# Genome-wide identification, transcriptome analysis and alternative splicing events of Hsf family genes in maize

**DOI:** 10.1038/s41598-020-65068-z

**Published:** 2020-05-15

**Authors:** Huaning Zhang, Guoliang Li, Cai Fu, Shuonan Duan, Dong Hu, Xiulin Guo

**Affiliations:** Plant Genetic Engineering Center of Hebei Province/Institute of Genetics and Physiology, Hebei Academy of Agriculture and Forestry Sciences, Shijiazhuang, 050051 P.R. China

**Keywords:** Transcriptional regulatory elements, Abiotic

## Abstract

Heat shock transcription factor (Hsf) plays a transcriptional regulatory role in plants during heat stress and other abiotic stresses. 31 non-redundant *ZmHsf* genes from maize were identified and clustered in the reference genome sequenced by Single Molecule Real Time (SMRT). The amino acid length, chromosome location, and presence of functional domains and motifs of all *ZmHsfs* sequences were analyzed and determined. Phylogenetics and collinearity analyses reveal gene duplication events in Hsf family and collinearity blocks shared by maize, rice and sorghum. The results of RNA-Seq analysis of anthesis and post-anthesis periods in maize show different expression patterns of *ZmHsf* family members. Specially, *ZmHsf26* of A2 subclass and *ZmHsf23* of *A6* subclass were distinctly up-regulated after heat shock (HS) at post-anthesis stage. Nanopore transcriptome sequencing of maize seedlings showed that alternative splicing (AS) events occur in *ZmHsf04* and *ZmHsf17* which belong to subclass A2 after heat shock. Through sequence alignment, semi-quantitative and quantitative RT-PCR, we found that intron retention events occur in response to heat shock, and newly splice isoforms, *ZmHsf04-II* and *ZmHsf17-II*, were transcribed. Both new isoforms contain several premature termination codons in their introns which may lead to early termination of translation. The *ZmHsf04* expression was highly increased than that of *ZmHsf17*, and the up-regulation of *ZmHsf04-I* transcription level were significantly higher than that of *ZmHsf04-II* after HS.

## Introduction

To address the agriculture and food needs, research has been focused on understanding abiotic stress responses in plants^1^. In particular, attention is being paid to heat stress in connection to global warming. During growth and development, especially the reproductive stage, crop is sensitive to extreme heat waves that consequently influence grain yield and quality^2,3^. Heat stress often reduces photosynthesis rates, primarily by changing the structure of thylakoid membranes^4^. The floral organs are sensitive to high temperature at anthesis, with poor pollination affecting seed development^5^. To survive under environmental stresses, plants have evolved intricate signal pathways and gene expression regulation. As a critical step in gene expression, transcriptional regulation has been extensively studied^6^. Transcription factors (TFs) can recognize and bind specific cis-elements so as to activate or repress target genes expression at specific times and locations^7^.

58 types of TFs, have been identified in plants currently, some of them are involved in heat shock (HS)^8^. The heat shock transcription factors (Hsfs) are important group of eukaryotic stress responsive TFs, which have been identified in several plant species after being firstly discovered in yeast^9–11^. The structure of Hsf protein is highly conserved, which indicating an important, conserved function of the family genes and providing a convenient opportunity for research^11,12^. Typical Hsf structure includes an N-terminal DNA binding domain (DBD), which has landmark central ‘wing’ helix-turn-helix (HTH) motif; and an oligomerization domain (OD), which has a bipartite heptad pattern of hydrophobic amino acid residues (HR-A/B)^13–15^. The DBD, which including three α helical bundles and four β stranded antiparallel sheets, can bind to heat shock elements (HSEs) with high selectivity^11,16^. The HSEs of most downstream target genes of eukaryotic cell contain repetitive palindromic motifs (5′-nGAAnnTTCn-3′) by which HSEs interact with Hsf trimers^17^. Hsf trimers are the active forms in plants. The HR-A and HR-B regions of the OD are responsible for forming specific homo-oligomeric or hetero-oligomeric combinations^18^. The number of amino acid between HR-A and HR-B regions is the basis to sort Hsf family into three classes: A, B and C. Members of the class A and C contain 21 and 7 inserted residues, respectively, and no inserted residues in class B^11^. Unlike the highly conserved N-terminal domain, C-terminal activation domains (CTAD) of different Hsf classes contain diversified motifs, in the class A, the aromatic and highly hydrophobic amino acid residues (AHA) motif is located in the CTAD to assist with transcriptional activation^19^. Some Hsf members contain nuclear localization signals (NLS) and nuclear export signals (NES) that dictating cellular localization.

Hsf can activate many target genes in response to several environment stresses, including high temperature, heavy metals, oxidants and drought^20,21^. Hsfs of subclass A1, constitutively-expressed genes, have been proved to be the major regulators and can specific bind affinity to Hsfs of subclass A2^22^. Typically, the Hsfs of subclass A2 are more dramatically induced by HS than other members^23,24^. *AtHsfA2* sustains the expression of Hsp genes to extend the duration of acquired thermotolerance in *Arabidopsis*^25^. The up-regulation of *HsfA4a* was proved to enhance Cd tolerance of wheat and rice^26^. The transcriptional activation activity of *HsfA6* has been connected to HS response in rice and wheat^27,28^. HsfB, such as *AtHsfB1* and *AtHsfB2b*, were considered to be transcriptional repressors that negatively regulate heat responsive genes^29^. But in both tomato and chickpea, HsfB1 and HsfB2 are co-activators which can interact with HsfA genes to produce positive HS response^30,31^. Lacking of AHA motifs, Hsfs both Class B and C are thought to have no transcriptional activation activity^31^. However, yeast one-hybrid assays of OsHsfC1a and OsHsfC1b, suggest that transcriptional activation of Hsfs may not require the AHA motif in rice^32^.

Using genomic techniques, Hsf family genes from various non-model plants have been characterized, such as maize, Chinese cabbage, pepper, grape and *Triticum aestivum*^24,33–36^. The Hsfs belong to typical multigene family. Previous studies have identified orthologous and paralogous Hsf genes in different species^23^, and gene functions have been assigned using homology comparison and gene collinearity analysis^37^. After several rounds of genome and segmental duplication, maize genome is diverse^38^. If based on second-generation sequencing technology, the maize reference genome may be fragmented and many complex repeat regions will be missed^39^. The latest released maize reference genome was assembled with PacBio Single Molecule Real Time (SMRT) sequencing and features a 52-fold increase in contig length compared to previous assemblies^39^. Our previous studies have cloned and characterized several *ZmHsfs* from maize and found variations in the expression profile, transcript activation and gene function of maize *Hsf* family^40–42^. In this work, we identified and classified putative *ZmHsfs* by blasting all Hsf genes of *Arabidopsis* and rice against the newly maize reference genome. Phylogenetics and collinearity analyses were carried out using data from *Arabidopsis*, rice, sorghum and maize. Further, transcriptome analyses of maize leaves at anthesis and post-anthesis stages were performed and possible alternative splicing events were identified by full-length transcriptome sequencing.

## Results

### Identification and sequence analysis of ZmHsfs

A total of 58 ZmHsf proteins were found by HMM and Blastp searches in the latest released maize genome. Among these, 31 non-redundant ZmHsf proteins containing DBD and HR-A/B regions were identified by the SMART program. 25 identified ZmHsfs were previously numbered according to their chromosomal locations^33^. Our newly identified Hsfs, ZmHsf26, 27, 28, 29, 30 and 31, were also named according to their chromosomal location too. The WoLFPSORT website analysis of their amino acid sequences predicts that most of the ZmHsfs are localized in the nucleus, ZmHsf23, ZmHsf10 and ZmHsf11 are located to the cytoplasm, chloroplast and ER, respectively. The length of ZmHsfs proteins varies from 250 to 622 amino acids, the molecular weight is between 27309.07~68553.79 Da and the pI value varies from 4.7 to 9.53 (Table 1).

**Table 1:** *ZmHsf* genes identified in maize.

Gene Name	Gene ID	Chromosome			Amino Acid Length (aa)	MW(Da)	pI	Extron	Intron	Localization
		No.	Position(bp)							
			From	To						
ZmHsf12	Zm00001d012823	5	925235	928518	556	60690.43	5.32	3	2	nucleus
ZmHsf06	Zm00001d034886	1	304715056	304719918	528	56724.50	5.11	2	1	nucleus
ZmHsf26	Zm00001d033987	1	280368696	280370932	372	41282.08	5.12	2	1	nucleus
ZmHsf17	Zm00001d018941	7	9991664	9995513	376	42043.73	4.70	2	1	nucleus
ZmHsf04	Zm00001d032923	1	243208029	243211663	358	40502.49	4.99	2	1	nucleus
ZmHsf01	Zm00001d027757	1	12714065	12716389	385	43268.46	5.30	2	1	nucleus
ZmHsf05	Zm00001d034433	1	292905093	292912832	622	68553.79	5.74	6	5	nucleus
ZmHsf15	Zm00001d016674	5	172180319	172183764	509	56061.66	4.96	2	1	nucleus
ZmHsf22	Zm00001d012749	8	179671303	179673854	434	48647.71	5.25	2	1	nucleus
ZmHsf20	Zm00001d010812	8	128602960	128604732	450	49977.79	5.15	2	1	nucleus
ZmHsf16	Zm00001d038746	6	163786743	163788401	470	51623.79	5.41	2	1	nucleus
ZmHsf14	Zm00001d016520	5	165944432	165950570	529	58138.65	5.57	2	1	nucleus
ZmHsf23	Zm00001d046204	9	71750080	71751360	366	39945.72	5.15	2	1	cytoplasm
ZmHsf10	Zm00001d044259	3	223146310	223151904	378	41038.95	5.82	2	1	chloroplast
ZmHsf24	Zm00001d048041	9	148611010	148615970	421	46799.35	5.04	4	3	nucleus
ZmHsf02	Zm00001d028269	1	28714876	28733871	409	45754.98	4.90	3	2	nucleus
ZmHsf08	Zm00001d005888	2	191778849	191782862	299	32270.31	9.13	2	1	nucleus
ZmHsf18	Zm00001d020714	7	129797898	129801599	299	32258.35	9.53	2	1	nucleus
ZmHsf25	Zm00001d026094	10	138304690	138305939	319	33947.51	5.70	2	1	nucleus
ZmHsf03	Zm00001d031736	1	200197812	200199489	415	44382.81	6.80	2	1	nucleus
ZmHsf11	Zm00001d052738	4	199404178	199405434	419	44863.40	6.80	1	0	ER
ZmHsf19	Zm00001d021263	7	147163459	147165213	395	41468.10	5.00	2	1	nucleus
ZmHsf28	Zm00001d022295	7	174697653	174700664	324	35560.65	6.57	2	1	nucleus
ZmHsf30	Zm00001d020704	7	129347595	129349243	406	42723.92	9.19	2	1	nucleus
ZmHsf07	Zm00001d005843	2	190605384	190606902	395	41742.83	7.81	2	1	nucleus
ZmHsf27	Zm00001d029270	1	64404103	64407052	333	37303.85	9.11	2	1	nucleus
ZmHsf09	Zm00001d044168	3	220964949	220966074	332	35883.69	5.94	2	1	nucleus
ZmHsf21	Zm00001d011406	8	149667369	149668532	388	41737.49	8.05	1	0	nucleus
ZmHsf31	Zm00001d043536	3	203125767	203126960	250	27309.07	9.44	2	1	nucleus
ZmHsf13	Zm00001d016255	5	153717773	153719275	258	27836.98	5.85	2	1	nucleus
ZmHsf29	Zm00001d046299	9	79124493	79126324	268	28328.77	6.99	2	1	nucleus

Thirty-one ZmHsfs were identified and their Gene ID, chromosome location, amino acid length, molecular weight (MW), isoelectric point (pI), protein localization, extrons and introns were listed.

**Figure 1:**
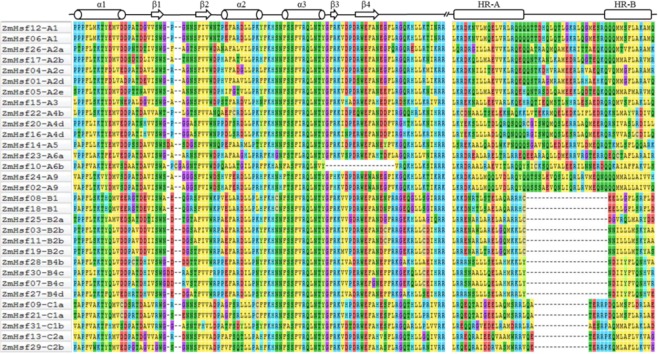
Multiple sequence alignment of DBD and HR-A/B domains of maize Hsfs. Arranged the ZmHsf proteins in the order according to the homology comparison with rice. The secondary structure elements of DBD are shown above the alignment.

Multiple sequence alignment illustrates the conservation of the DBD domain and HR-A/B regions of ZmHsfs (Fig. 1). The secondary structure of all ZmHsf proteins contains 3 α-helical structures and 4 β-fold structures in the DBD domain. Based on the length of flexible linkers between the HR-A and HR-B regions, 16, 10 and 5 members of ZmHsf were assigned to class A, B and C, respectively. There are 21 inserted residues in class A and 7 in class C between the HR-A and HR-B regions and no insertion in class B.

### Conserved protein motifs and gene structure of ZmHsfs

**Figure 2:**
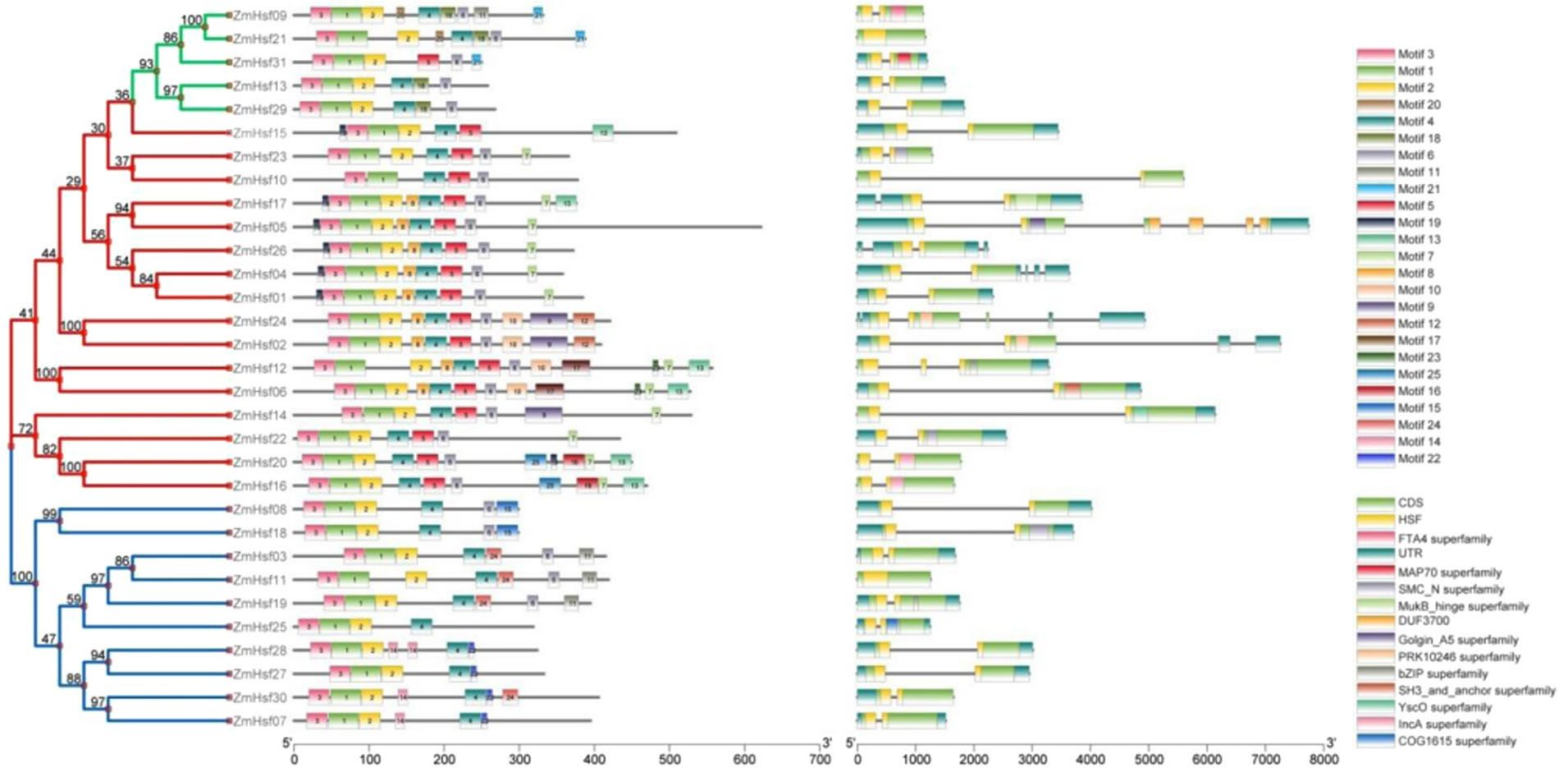
The conserved protein motifs and gene structures of maize Hsfs. ZmHsf proteins are displayed in order based on the phylogenetic analysis. Twenty-five motifs and thirteen domains identified in ZmHsf proteins marked by different colors.

The results of phylogenetic tree, motif identification and gene structure analysis of 31 ZmHsf proteins were merged using TBtools to assess conserved motifs and gene structure. All ZmHsfs contain motifs 1, 2, 3 and 4 except ZmHsf31, which is lack of motif 4. All members of class A include motif 5 and 6 except ZmHsf15, which does not have motif 6. All members of class C contain motif 6 (Fig. 2). Motifs 1, 2 and 3 function in the DBD domain, motif 4 functions in the HR-B domain, and motif 6 functions in the NLS domain. The Hsf domains were found in all ZmHsf proteins. Most class A members have longer introns and a larger number of exons than members of class B and C (Fig. 2).

### Analysis of phylogenetic tree and collinearity of AtHsfs, OsHsfs, SbHsfs and ZmHsfs

**Figure 3:**
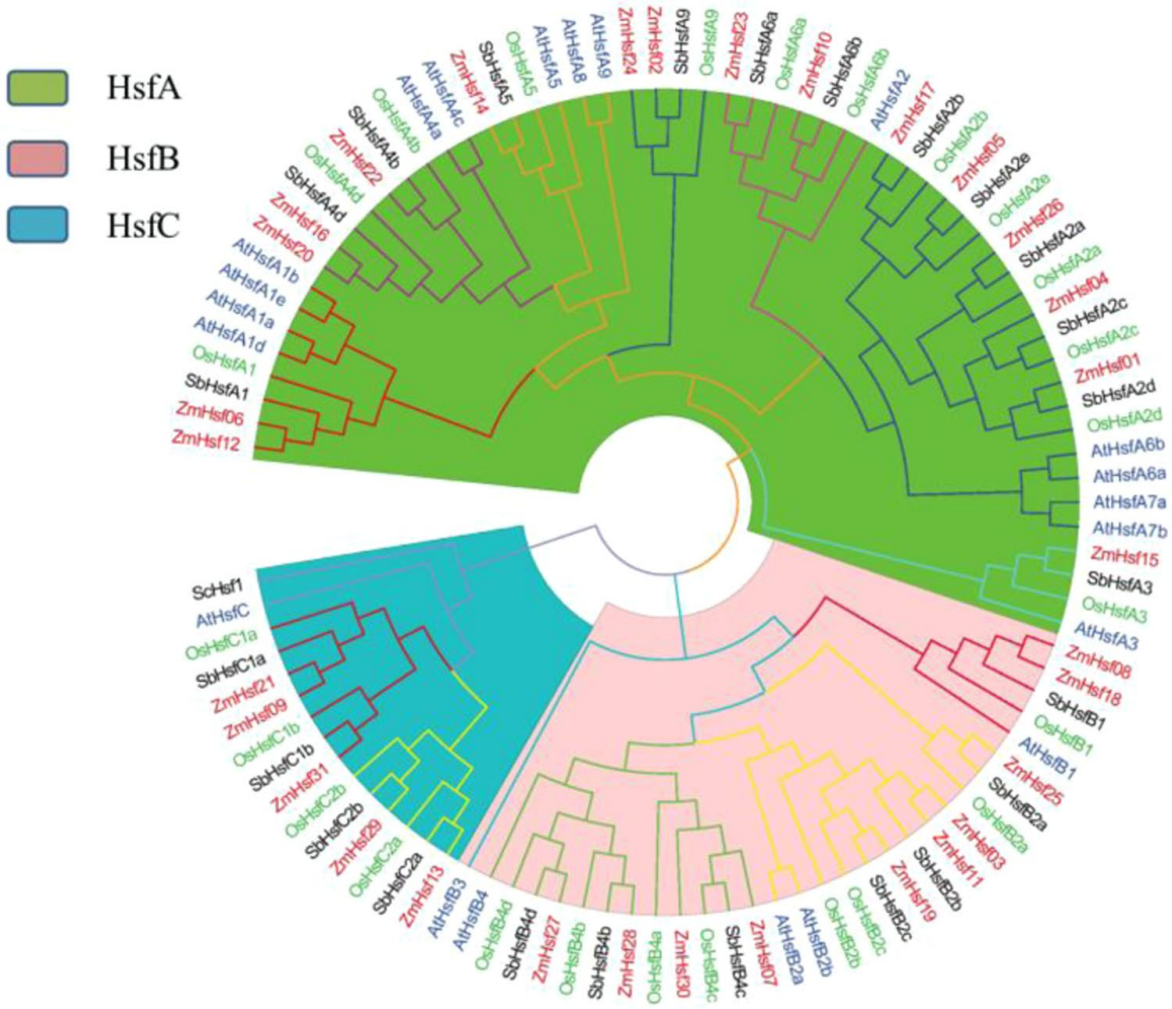
Phylogenetic tree of Hsf family proteins among Arabidopsis, rice, sorghum and maize. The Hsf family proteins are classified three classes (**A–C**) which marked by three underground colors. The font colors of Hsf ID from Arabidopsis, rice, sorghum and maize were blue, green, red and black respectively.

A phylogenetic tree was constructed from a multiple sequence alignment of full-length proteins from 31 maize ZmHsfs, 21 *Arabidopsis* AtHsfs, 25 rice OsHsfs and 25 sorghum SbHsfs (Fig. 3). The class and subclass of the ZmHsfs are highlighted with different background colors and different line colors, respectively (Fig. 3). There are 16, 10 and 5 ZmHsf proteins in class A, B and C, respectively. Same to the two Gramineae species, class A has more members than class B or C in maize, and the subclass A2 has the most members in class A. Compared with the dicot *Arabidopsis*, the three Gramineae genomes lack members of subclass A7, A8 and B3, and contain fewer members of subclass A1 and more subclass A2 and class C (Fig. 3).

**Figure 4:**
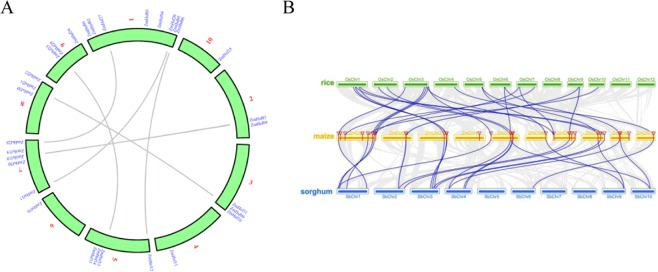
The collinearity analysis of maize itself and among maize, rice and sorghum. (**A**) The duplicated ZmHsf genes in maize based on the collinearity analysis of all the chromosomes from maize. The pairs of duplicated genes are connected with lines. (**B**) The collinearity analysis of the chromosomes from maize, rice and sorghum. The collinearity blocks include 10 successive homologous genes are connected with blue lines. The red flags represent the 31 ZmHsfs located on different chromosomes of maize.

Through homologous blast of maize amino acid sequences performed with MCScan toolkit, 7783 collinear genes, 1152 tandem repeat genes and 415 collinear blocks were identified (Table S3). Chromosome 1 contains the most ZmHsfs of class A and B. Chromosomes 2, 4 and 10 only contain class B genes. The majority of ZmHsfs were located at the end of chromosomes (Fig. 4a). Pairs of paralogous ZmHsf gene arose from whole genome duplication or segmental duplication in subclass A1, A2, B1, B2, C1 and C2, respectively (Fig. 4a). The most members might arise from transposition and have no tandem repeat genes. The results of homologous blast show the collinearity among maize, rice and sorghum (Fig. 4b). There are more collinear genes and blocks between maize and sorghum than between maize and rice (Table S4 and S5). Most ZmHsfs distribute in the regions in which there are more than 10 consecutive collinear genes (highlighted by blue lines), excepting ZmHsf03, ZmHsf11 and ZmHsf19 of subclass B2 and ZmHsf24 of subclass A9 (Fig. 4b). Maize chromosome 1 shares the most collinear blocks with chromosome 1 of sorghum bicolor and chromosome 3 of rice (Fig. 4b).

### Expression analysis of ZmHsfs under heat stress

**Figure 5:**
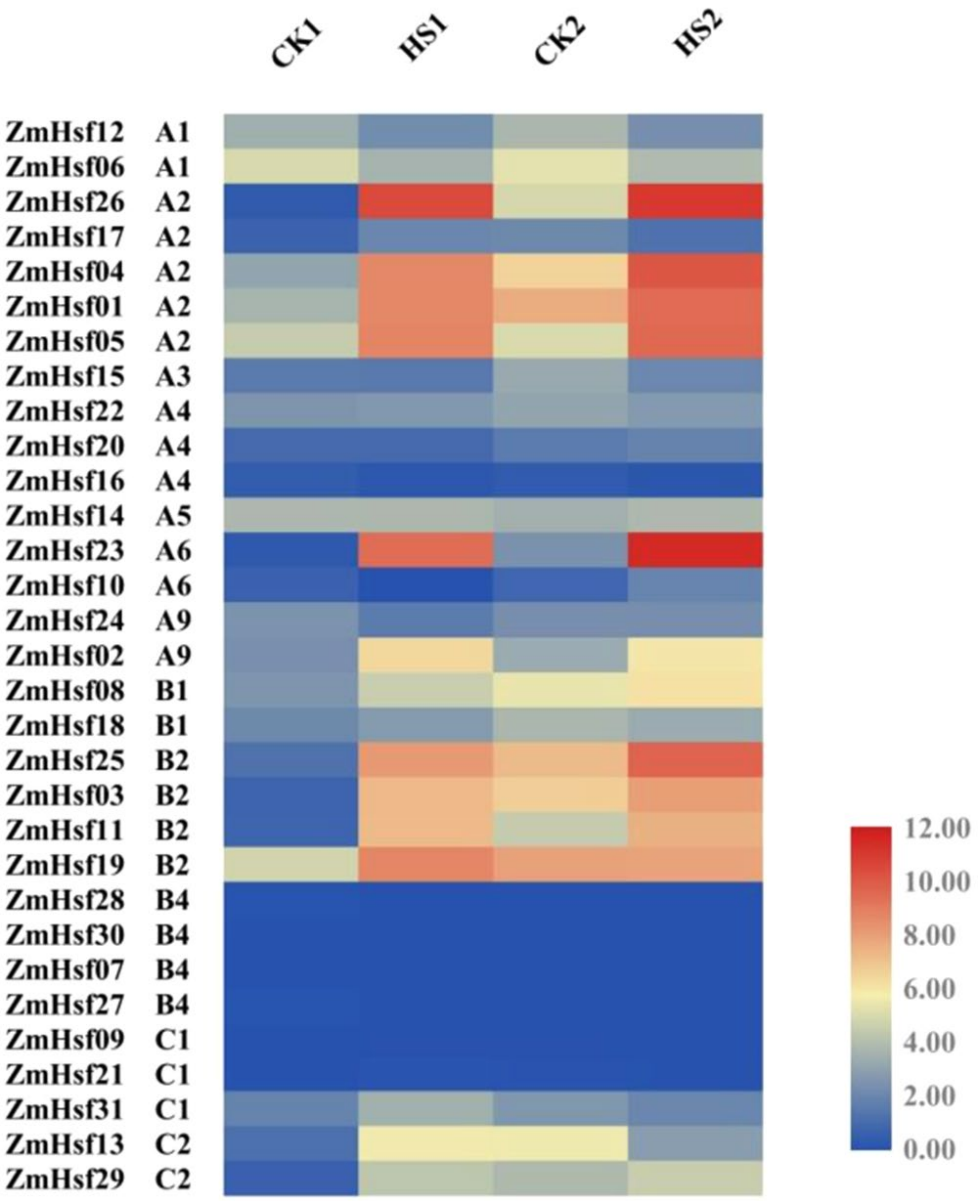
The transcription profiles of ZmHsf family genes in maize leaves at anthesis and post-anthesis stages after HS treatment. Different color correspond to the TPM levels from RNA-seq data, and number 0 to 12 represent the range of TPM levels (from the lowest to highest). Groups CK1/HS1 and CK2/HS2 represent anthesis and post-anthesis stages respectively.

Transcriptome sequencing analysis of the ZmHsf family was performed by measuring plants TPM levels both anthesis and post-anthesis stage under HS (Fig. 5). Most of ZmHsf members responded to HS, and expression patterns are similar in the same subclass. Total eight members of subclass A2 (*ZmHsf26*, *ZmHsf04*, *ZmHsf01*, *ZmHsf05*) and subclass B2 (*ZmHsf25*, *ZmHsf03*, *ZmHsf11*, *ZmHsf19*) were induced by both HS1 and HS2 treatments. In addition, the expressions of such genes were all improved after HS treatment: *ZmHsf23* of subclass A6, *ZmHsf02* of subclass A9, *ZmHsf08* of subclass B1 and *ZmHsf29* of subclass C2. The transcription levels of *ZmHsf17* of subclass A2 and *ZmHsf13* of subclass C2, were up-regulated in the anthesis stage but down-regulated in the post-anthesis stage after HS treatments. To further confirm transcription changes, qRT-PCR was done for 24 ZmHsfs responsive to HS, and the results of qRT-PCR were consistent with that of transcriptome sequencing analysis (Fig. S1).

### Alternative splicing (AS) analysis of ZmHsfs

**Figure 6:**
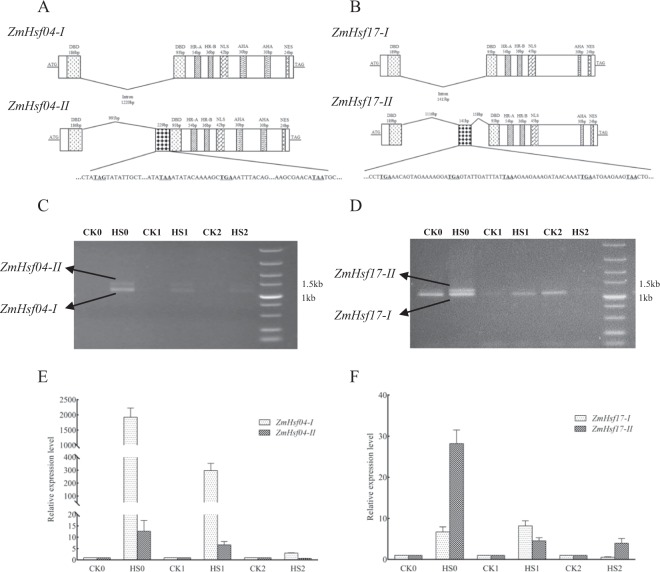
The structures of two transcripts from *ZmHsf04* and *ZmHsf17* are analyzed based on the alternative splice events after HS treatment. (**A,B**) sequence alignment of two transcripts from ZmHsf04 and ZmHsf17 respectively, the premature termination codons within the partial introns, (**C,D**) semi quantitative RT-PCR after HS treatment at three development stages, (**E,F**) quantitative RT-PCR after HS treatment at three development stages. The expression levels of CK0, CK1 and CK2 are set as ‘1’ respectively. CK0 and HS0: seedling stage, CK1 and HS1: anthesis stage, CK2 and HS2: post-anthesis stage.

The third generation full length transcriptome sequencing was used for AS analysis in the leaves of maize seedlings after HS treatment. The results showed that AS events occurred only in *ZmHsf04* and *ZmHsf17* in the form of intron retention, causing *ZmHsf04-II* and *ZmHsf17-II* with the retention lengths of intron 229 bp and 141 bp, respectively. New splicing sites were identified in the first intron both *ZmHsf04* and *ZmHsf17* genes. The sequences of retained regions contain several new ‘TAG’ and ‘TAA’ stop codons (Fig. 6A,B). The intron retention probably generated small peptides which retain only a truncated DBD domain, because the first intron was between the third α-helical and β-fold structures of the DBD domain. Compared the C-terminal amino acid sequences of ZmHsf04-II and ZmHsf17-II hypothetical small peptides, an additional Leucine-rich hydrophobic motif was found in ZmHsf04-II but not in ZmHsf17-II (Fig. S2)

The AS events in leaves were confirmed at seedling, anthesis and post-anthesis stages by semi-quantity RT-PCR and quantity RT-PCR experiments. Specific primers were designed in both ends of *ZmHsf04* and *ZmHsf17* genes and used with semi-quantitative PCR assays to assess the response of different isoforms to HS treatment. Two pairs of primers which can amplify two different isoforms respectively were designed and used for quantitative RT-PCR. Both isoforms of *ZmHsf04* and *ZmHsf17* were significantly induced by HS treatment (Fig. 6C,D), and the up-regulation were most significant at the seedling stages (Fig. 6E,F). The *ZmHsf04* expression have a far higher increase than *ZmHsf17* after HS treatment. The expression increasing in *ZmHsf04-I* were higher than that in *ZmHsf04-II* (Fig. 6C,E). Differently, the *ZmHsf17-I* expression decreased after HS treatment at post-anthesis stage, and the *ZmHsf17-II* expressions were up-regulated slightly after HS treatment at both anthesis and post-anthesis stages (Fig. 6D,F).

## Discussion

Since three Hsfs were identified from HS treated tomato cells, the Hsf family genes has been studied as one of key regulators of plant thermotolerance^43^. Sessile plants possess more Hsf family members than other eukaryotes. With the development of sequencing technology, most Hsfs have been identified, such as, 21 *AtHsfs* in *Arabidopsis*^11,44^, 26 *SlyHsfs* in tomato^23^, 25 *OsHsfs* in rice^45^, 25 *SbHsfs* in sorghum^46^ and 82 *TaHsfs* in wheat^24^. All genes identified by HMM research or BLASTP possess canonical DBD domain and OD domains. Recently the maize B73 inbred line was genome-sequenced and assembled using single-molecule PacBio sequencing technology, making it more precise for gene annotation and discovery of new genes^30^.

Base on homology comparison with rice, 31 *ZmHsfs* were identified from the maize ‘B73RefGen_V4′ and assigned into three class A, B and C, which contain 7, 3 and 2 subclasses, respectively. Similar to rice and sorghum bicolor, class A of maize has more members than the other classes, and subclass A2, with 5 members, is the most abundant subclass. In wheat, 18 members of *TaHsfA2* were recently identified^24^. Differently, *Arabidopsis* has only one *HsfA2* gene, but four *HsfA1* genes^44^. As the dominate Hsf, *HsfA2* is strongly induced by HS and has been improved to be a key regulator of abiotic stress response by recent research^24,45,46^. Speaking from structure (Fig. 2), all members of the subclass A2 possess the AHA motif (motif 7), which has a transcriptional activation function. Meanwhile the AHA motifs are different in subclass A1, A4 and A6. Subcellular localization predictions indicate that ZmHsf23 and ZmHsf10 of subclass A6 are not localized to the nucleus. Previously, AtHsfA6a has been simultaneously found in both cytoplasm and nucleus and appeared to be translocated into the nucleus after salt stress^47^.

Compared to the model dicot *Arabidopsis*, class B and C were relatively more abundant in monocots^24,33,45,46,48^, this was verified in maize in this study (Fig. 3). Curiously, in the maize B73 genome, two Hsfs are aimed to subclasses A1, A4d, A9, B1, B2b and C1a, respectively, but there is only one member of these subclasses in both rice and sorghum bicolor (Fig. 3). These perhaps because the maize genome underwent a whole genome duplication event about 5 to 12 mya and has continuously expanded over the last 3 million years via long terminal repeat retrotransposons^38^. Similarly, in the wheat genome, duplication events may be one important means of expanding Hsf family^37^. Gene loss and retention after duplication events are presumably the results of enhanced resistance to environmental stress^49^, redundant duplicates of ZmHsfs in subclasses A1 and A2 may be associated with the thermotolerance-regulating. Our previous research found that obvious differences existed in the regulating function and expression patterns of two highly homologous HsfA1 genes, *ZmHsf12*^50^ and *ZmHsf06*^40^.

The flowering, pollination and grain filling of maize are all highly susceptible to high temperature^51^. Transcriptome analysis of maize leaves under HS in both anthesis and post-anthesis stages showed that the expression levels of some ZmHsfs changed significantly. The three most distinctly up-regulated subclasses were A2, A6 and B2, similarly in wheat^24^. These can be proved by the close phylogenetic relationship of subclass A2 and A6 in our phylogenetic tree (Fig. 3). As one of key regulators for heat stress response, the up-regulation of *ZmHsfA2* was expected. Subclass A2 were also induced by HS in rice and sorghum^45,46^. The *ZmHsf05* of subclass A2 was induced by HS treatment in maize, and rescued the reduced thermotolerance of the *athsfa2* mutant in *Arabidopsis*. In *ZmHsf05*-overexpressing lines of *Arabidopsis*, the basal and acquired thermotolerance of plants were all enhanced^41^. Up-regulations of the four ZmHsfs of subclass B2 were observed in maize after heat treatment, similar to the expression of both *SlyHsf03* and *SlyHsf10* of subclass B2 in tomato, which increased dramatically after heat treatment for 1–2 h^23^. The *ZmHsf12* and *ZmHsf06* of subclass A1 were down-regulated in both anthesis and post-anthesis stages, differing from previous studies in maize seedlings^33^_._ The expression levels of *TaHsfA1a* was down-regulated after heat or drought stress in wheat seedlings too^24^. In *Arabidopsis*, HsfA1s, act as transcriptional activator, can be induced at the early HS response stage^25,52^. Those suggest that Hsf can demonstrate different expression responses during different developmental stages. In our experiment, *ZmHsf17* of subclass A2 and *ZmHsf13* of subclass C2 were observed to have different responses to HS at anthesis and post-anthesis stages, both transcriptional levels of two genes increased in anthesis stage and decreased in post-anthesis stage under HS treatment. Different express patterns suggest diversity and complexity of regulating roles.

Almost all Hsfs have the conserved DBD domain which containing two independent exons separated by one intron^53^. The conserved intron locates in the end of the central helix-turn-helix motif (H2-T-H3) which is necessary for specific recognition of the palindromic HSEs in plant^53^. AS of pre-messenger RNA is the important post-transcriptional regulation mechanism that often widely happen during various developmental stages of plants^54^. Previous studies verified that intron retention is the predominant form of AS in plants and exon skipping is the most frequent AS event in mammals^55^. More than 6000 *Arabidopsis* genes and 1000 grape genes have displayed AS patterns under salt and high-temperature stress, respectively^56,57^. In *Arabidopsis*, three spliced variants of *AtHsfA2* were identified after different HS treatment. Except for full spliced *AtHsfA2-I*, *AtHsfA2-II* and *AtHsfA2-III* have different C-terminal amino acid sequences due to different parts of the first intron were retained^58,59^. *AtHsfA2-II* without Leucine-rich hydrophobic motif (LRM) in C-terminal was degraded by nonsense-mediated mRNA decay (NMD)^58^. However, *AtHsfA2-III* with the LRM in C-terminal could be translated into a small truncated AtHsfA2 protein at HS recovered stage. This “small AtHsfA2” protein was shown to bind to the TATA box-proximal clusters of HSE in the HsfA2 promoter to activate the transcription of *HsfA2*^59^.

In our experiment, only *ZmHsf04* and *ZmHsf17* were found to have AS events after HS, in the form of intron retention. *ZmHsf04-II* and *ZmHsf17-II* retained different partial introns which containing several premature termination codons. The two truncated isoforms of *ZmHsf04* and *ZmHsf17* might be involved in different transcriptional regulation process. Based on the amino acid sequences, we speculated that *ZmHsf17-II* without the LRM may be degraded by NMD, like the *AtHsfA2-II of Arabidopsis*^58^. However, *ZmHsf04-II* with the LRM in C-terminal could be translated and activate the transcription of *ZmHsf04-I*, like the *AtHsfA2-III* of *Arabidopsi*s^59^. In previous reports, overexpression of *ZmHsf04-I* in *Arabidopsis* up-regulated the expression of heat and other related genes, the thermotolerance and salt tolerance of transgene plants were all enhanced^42^. At the same time, Quantitative RT-PCR showed that the expression increase of *ZmHsf04* were far higher than *ZmHsf17* after HS in three development periods, the expression of *ZmHsf17* were also observed in normal plants, these verify above speculation further. The full spliced HsfA2 protein contain typical complete functional domains and motifs and have been considered as the major transcriptional regulatory forms^57^. In rice, the full spliced OsHsfA2d-I could participated in the unfolded protein response by regulating expression of *OsBiP1*, but OsHsfA2d-II could not, because of its incomplete DBD domain and LRM motif^60^. The regulating functions of different isoforms both *ZmHsf04* and *ZmHsf17* need more deeply research.

## Conclusion

There is remarkable functional diversity in the members of plant Hsf family after the long-term adaptation to high temperature^11^. We identified and classified all possible ZmHsfs and found the orthologous and paralogous genes through amino acid sequence alignment, the results were valuable for the functional study of ZmHsfs. We analyzed the expression pattern of all the ZmHsfs responsive to heat stress at anthesis and post-anthesis stages through RNA-seq and quantitative RT-PCR, and found some up-regulated Hsf genes from subclass A2, A6 and B2. Intron retention events which often occurring within DBD domain were found in *ZmHsf04* and *ZmHsf17* of subclass A2 through full length transcriptome sequencing. The two truncated isoforms both *ZmHsf04* and *ZmHsf17* have different expression patterns after HS treatment in plants of seedlings, anthesis and post-anthesis stages. We infer that different spliced variants of Hsf genes in maize may involve in different transcriptional regulation process.

## Materials and Methods

### Plant culture and heat stress

Maize inbred variety ‘H21(♀ Huangzao 4 × H84 ♂)’ were selected for this study. Maize inbred variety ‘H21’ is one of the main inbred lines of China and it is widely used as a parent in breeding hybrid. Inbred H21 has typical characteristics like early maturing, compact plant type and strong combining ability, meanwhile it has good drought and disease resistance^61,62^. The plants were grown in large pots containing nutritive soil in a greenhouse and used for RNA sequencing experiments. When the seedlings grew to two-leaves-old, some plants were subjected to HS at 42 °C for 30 min (HS0). When pollens were released, some plants were subjected to same HS as seedlings (HS1). After 3 weeks, the other plants were subjected to HS either (HS2), and the plants growing under the normal conditions of different stages were performed as CK0, CK1 and CK2. The second leaves or the flag leaves were sampled quickly and frozed in liquid nitrogen and stored until RNA extraction.

### Identification and bioinformatic analysis of Hsfs in maize

The maize reference genome (Maize B73 RefGen_V4) was downloaded from the MaizeGDB database on the website ftp://ftp.ensemblgenomes.org/pub/release-41/plants/fasta/zea_mays/dna/. 47 non-redundant Hsfs amino acid sequences were downloaded from PlantTFDB and blasted against the amino acid sequences retrieved from the maize reference genome. Additionally, the Hsf-type DBD domain (Pfam: PF00447) was used as a query in BLASTP (P = 0.001) search for probable Hsf protein sequences in the maize genome reference data base. 58 maize Hsf proteins were collected after homologous comparison and HMM research. Results from the two searches described above were blasted, respectively, against the UniprotKB/Swiss-prot data base on the NCBI blastp suite and the NCBI batch CDD (https://www.ncbi.nlm.nih.gov/Structure/bwrpsb/bwrpsb.cgi). Redundant sequences were discarded. Using SMART, sequences without a DBD domain or HR-A/B domain were eliminated. Motif elicitation of Hsf proteins was completed using the MEME suite (http://meme-suite.org/tools/meme). The pI and MW of identified Hsf proteins were calculated using the Expasy website (https://web.expasy.org/compute_pi/). WoLFPSORT was used to predict subcellular localization motifs in amino acid sequences of the identified Hsf proteins (https://wolfpsort.hgc.jp/).

### Multiple sequence alignment and phylogenetic tree

The amino acid sequences of Hsf proteins from *Arabidopsis*, *Oryza sativa*, *Sorghum bicolor* and *Zea mays* were examined together. MEGA 7.0 was used for multiple sequence alignment and the phylogenetic tree was constructed using the Neighbor joining method with a bootstrap value of 1000. The phylogenetic tree was modified using Figtree software. The physical location and gene duplication events were assigned based on the maize genome annotation results from the MCScan toolkit in TBtools^63^. MCScan toolkit was also used to analyze the collinearity of rice, sorghum and maize Hsf proteins and plot collinear genes and blocks on the chromosomes^63^.

### RNA extraction and RNA sequencing analysis

Total RNA of leaves from different treatments were extracted and purified using the Total RNA Extractor kit and RNase-free DNase I (Sangon, China). The RNA quality and integrity were estimated with an Agilent 2100 Bioanalyzer (Agilent, USA), and the RNA quantity was measured with a NanoDrop ND-1000 spectrophotometer (Thermo scientific, USA). The cDNA libraries were constructed using 2 μg of fragmented RNA from different samples with slight modifications of previously published method^24^. Refer to the instruction manual of VAHTS mRNA-seq V3 library prep Kit for Illumina (Vazyme, China) for the specific construction process. The four cDNA libraries were sequenced using Hiseq XTen sequencers (Illumina, USA). A data quality assessment of the raw reads was attained with FastQC and Trimmomatic. The clean reads were mapped to the maize reference genome sequence (Maize B73 RefGen_V4), and the transcription-level expression was analyzed with HISAT, StringTie and Ballgown according to a previously reported standard process^64^. Gene expression levels were calculated in TPM (Transcripts per million) using StringTie and Ballgown, and the heat map of gene expression levels was plotted using TBtools.

### Quantitative RT-PCR for validation of RNA-Seq

The first strand cDNA was synthesized with 1 μg RNA by reverse transcription PCR. A SYBR Premix ExTaqTM kit (Takara, Japan) was used for quantitative RT-PCR assays in the ABI 7500 (Applied Biosystem, USA) according to the manufacturer instructions. The reaction procedure included pre-denaturation at 95 °C for 10 min, followed by 40 cycles of denaturation at 95 °C for 5 s and annealing/extension at 60 °C for 40 s. Three biological replicates were performed for each group. After the reaction, the data were analyzed using the 2^−ΔΔCt^ method^65,66^ and plotted with Microsoft Excel 2010. The expression level of CK1 was set as 1. For statistical analysis, each dataset was repeated at least three times. The *Actin2* gene was used as an endogenous control. All of the primers used in quantitative RT-PCR are listed in Table S1.

### Nanopore sequencing and alternative splicing analysis

The second leaves of two-leaf-old maize seedlings were sampled after HS for nanopore sequencing experiments. RNA extraction, cDNA library construction and long-read sequencing were performed according to the standard protocols of Oxford Nanopore Technologies (ONT)^67,68^. Ribosomal RNA and low-quality raw reads less than 500 bp in length were removed. Minmap2 software was used to map all full-length reads to the reference genome and remove the redundant transcript reads were removed. The alternative splicing events in both CK0 and HS0 treatments were identified using Astalavista software^69^. The structures of different isoforms were analyzed by sequence alignment in DNAman software. The RNA samples of maize leaves from seedling, anthesis and post-anthesis stages were used to analyze the transcriptional levels of different isoforms. The transcription abundances of the cDNA from different isoforms were tested by semi-quantity and quantity RT-PCR methods, respectively. According to the operation manual, the 2 × Taq Plus Master Mix II (Dye Plus) kits were used for semi-quantity RT-PCR experiments. The primers used in semi-quantity and quantity RT-PCR were listed in Table S2.

## Supplementary information

Supplementary Figure 1

Supplementary Figure 2

Supplementary Table 1

Supplementary Information.

Supplementary Information.

Supplementary Information.

Supplementary Information.
