# Supplementary material for: Genome-wide identification, transcriptome analysis and alternative splicing events of Hsf family genes in maize
Source: Sci Rep. 2020 May 15;10:8073. doi: 10.1038/s41598-020-65068-z (PMC7229205; doi:10.1038/s41598-020-65068-z)

Genome-wide identification, transcriptome analysis and alternative splicing events of Hsf family genes in maize

Huaning Zhang [1,2], Guoliang Li [1,2], Cai Fu[1], Shuonan Duan [1], Dong Hu[1, ✉] & Xiulin Guo [1, ✉]

[1] Plant Genetic Engineering Center of Hebei Province/Institute of Genetics and Physiology, Hebei Academy of Agriculture and Forestry Sciences, Shijiazhuang 050051, P.R. China

[2] These authors contributed equally: Huaning Zhang and Guoliang Li.

✉ e-mail: myhf2002@163.com, donghu1983@163.com.

Fig. S1 Relative expression level of partial ZmHsf genes analyzed by quantitative RT-PCR in response to HS treatment. The expression levels of CK1 are set as ‘1’. Groups CK1/HS1 and CK2/HS2 represent anthesis and post-anthesis stages respectively. Error bars indicate the Mean± SD of three replicates.

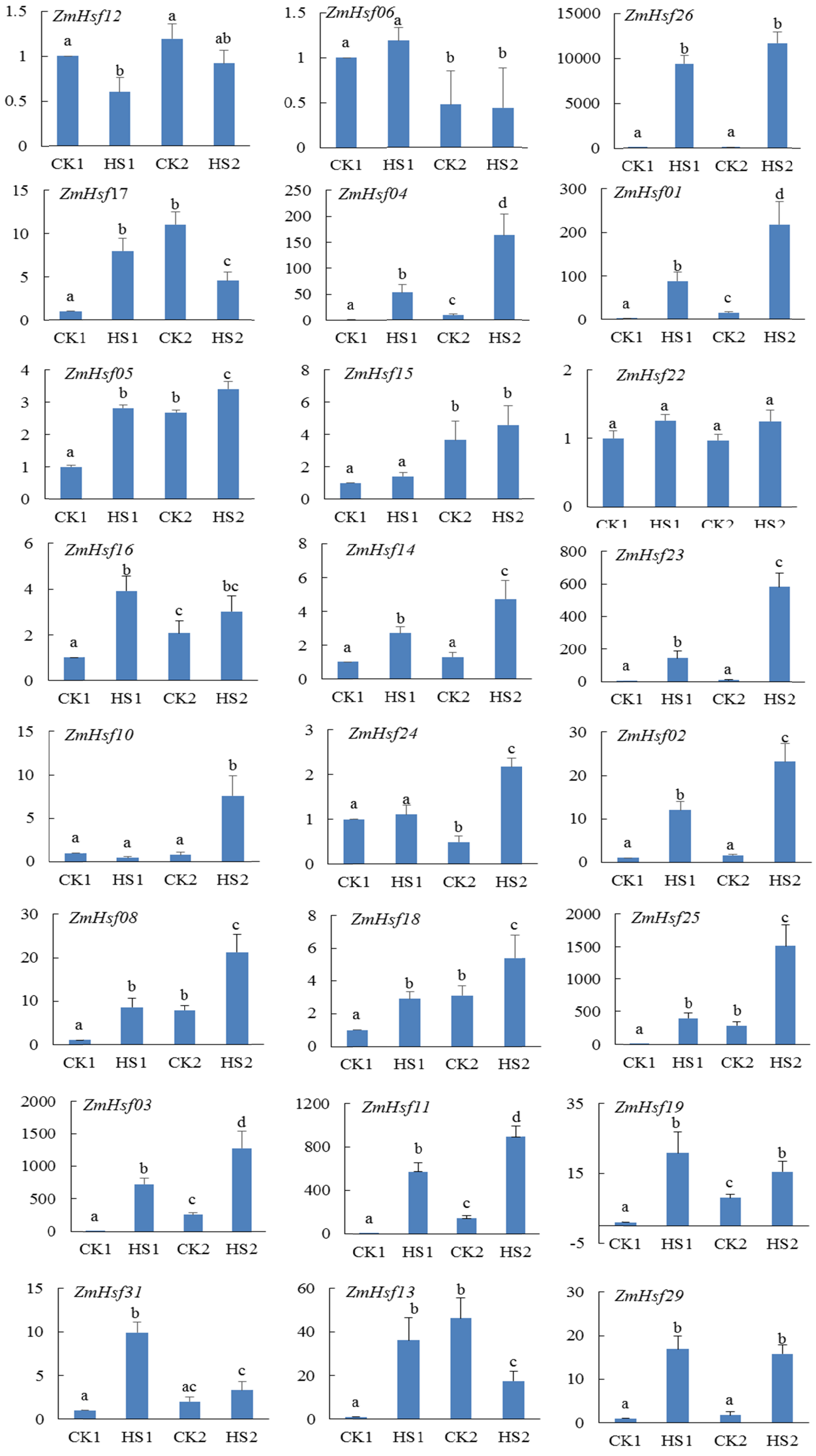

Supplement: Supplementary file 1 — Supplementary Figure 1 [file 41598_2020_65068_MOESM1_ESM.pdf]
