# Supplementary material for: Genome-wide identification, transcriptome analysis and alternative splicing events of Hsf family genes in maize
Source: Sci Rep. 2020 May 15;10:8073. doi: 10.1038/s41598-020-65068-z (PMC7229205; doi:10.1038/s41598-020-65068-z)
Supplement: Supplementary file 2 — Supplementary Figure 2 [file 41598_2020_65068_MOESM2_ESM.pdf]

Genome-wide identification, transcriptome analysis and alternative splicing events of Hsf family genes in maize

Huaning Zhang [1,2], Guoliang Li [1,2], Cai Fu[1], Shuonan Duan [1], Dong Hu[1, ✉] & Xiulin Guo [1, ✉]

[1] Plant Genetic Engineering Center of Hebei Province/Institute of Genetics and Physiology, Hebei Academy of Agriculture and Forestry Sciences, Shijiazhuang 050051, P.R. China

[2] These authors contributed equally: Huaning Zhang and Guoliang Li.

✉ e-mail: myhf2002@163.com, donghu1983@163.com.

Fig. S2 The amino acid sequences of ZmHsf04-II and ZmHsf17-II. A: ZmHsf04-II, B: ZmHsf17-II.

A

```
1      ATGGACAAGCCGGTGGCGCCGGGCATTATCAAGGAGGAGCTCCTGGAGCAGCAGCCGCCG
1       M  D  K  P  V  A  P  G  I  I  K  E  E  L  L  E  Q  Q  P  P

61     ACGCAGGACGGTGTGGGTGGCGGCGGGATGCGCCGCGCCCGATGGAGGGGCTGCACGAGG
21      T  Q  D  G  V  G  G  G  G  M  R  R  A  R  W  R  G  C  T  R

121    TGGGTCCCCGCCTTTCCTCACCAAGACGTTCGACCTGGTGGAGGACCCGGCCACCGACGC
41      W  V  P  A  F  P  H  Q  D  V  R  P  G  G  G  P  G  H  R  R

181    CGTCCTCTCCTGGAGCTGCGTTGGCAACAGCTTCATCGTCTGGGACCTGGCACTTCGCCG
61      R  P  L  L  E  L  R  W  Q  Q  L  H  R  L  G  P  G  T  S  P

241    ACGGGCTGCTCCCGCGCCTCTTCGAAGCACAGTAACTTCTCCAGCTTCGTGCGCCAGCTC
81      T  G  C  S  R  A  S  S  K  H  S  N  F  S  S  F  V  R  Q  L

301    AACACATATTCTAGATGGAACAAGATACTGATGAAGTACCAAGAATCAGATTGTTTTGTT
101     N  T  Y  S  R  W  N  K  I  L  M  K  Y  Q  E  S  D  C  F  V

361    CTAGAAGACTCTACTGACCACCTTCACATGCTATAG
121     L  E  D  S  T  D  H  L  H  M  L  *
```

B

```
1      ATGGACTCAACGCTGAACCAGGTGAAGGAGGAGAGCCATGGGGAGGGAGGAGATTTGATG
1       M  D  S  T  L  N  Q  V  K  E  E  S  H  G  E  G  G  D  L  M

61     GCAGGCACGGTGGAGGCCGCGGATGGGCCGTCTGCGGCCGTCGCCGCGGCACCAAAGCCG
21      A  G  T  V  E  A  A  D  G  P  S  A  A  V  A  A  A  P  K  P

121    ATGGAGGGTCTGCATGACCCTGGGCCGCCGCCGTTCCTCACCAAGACATATGACATGGTC
41      M  E  G  L  H  D  P  G  P  P  P  F  L  T  K  T  Y  D  M  V

181    GACGACTCGGACACCGACCTGATTGTGTCGTGGAGCGCCACCAACAACAGCTTCGTGGTG
61      D  D  S  D  T  D  L  I  V  S  W  S  A  T  N  N  S  F  V  V

241    TGGGATCCGCACGCCTTCGCCACGGTGCTGCTGCCCAGGCACTTCAAGCACAACAACTTC
81      W  D  P  H  A  F  A  T  V  L  L  P  R  H  F  K  H  N  N  F

301    TCCAGCTTCGTCCGGCAGCTCAACACCTATTACATGACAGGTCCTTGA
101     S  S  F  V  R  Q  L  N  T  Y  Y  M  T  G  P  *
```
