# Supplementary material for: Genome-wide identification, transcriptome analysis and alternative splicing events of Hsf family genes in maize
Source: Sci Rep. 2020 May 15;10:8073. doi: 10.1038/s41598-020-65068-z (PMC7229205; doi:10.1038/s41598-020-65068-z)
Supplement: Supplementary file 3 — Supplementary Table 1 [file 41598_2020_65068_MOESM3_ESM.pdf]

Genome-wide identification, transcriptome analysis and alternative splicing events of Hsf family genes in maize

Huaning Zhang [1,2], Guoliang Li [1,2], Cai Fu[1], Shuonan Duan [1], Dong Hu[1, ✉] & Xiulin Guo [1, ✉]

[1] Plant Genetic Engineering Center of Hebei Province/Institute of Genetics and Physiology, Hebei Academy of Agriculture and Forestry Sciences, Shijiazhuang 050051, P.R. China

[2] These authors contributed equally: Huaning Zhang and Guoliang Li.

✉ e-mail: myhf2002@163.com, donghu1983@163.com.

Table S1 The primers used in quantitative RT-PCR experiments of Fig. S1.

Gene	Forward Primer (5'-3')	Reverse Primer (5'-3')
ZmHsf23	GACGTTCGAGCTTGTGGAGGAC	GTAGGTGTTGAGCTGGCGTATG
ZmHsf10	GTCCTTCGTCAGGCAGCTCAAC	GCGATGCTTCTACCTGAGGCTTG
ZmHsf11	CACGCCGTTCCTCAGCAAGAC	CGCCACACGATGAAGGCAGAC
ZmHsf13	GCACTCCAACTTCTCCAGCTTCG	CACCGTTGCCGTCGTCCTTG
ZmHsf14	CACTCGCAGCAGGCAGCAG	CGCCGCCACCTCCTCCTC
ZmHsf15	CCACCGCGCACGCTAATGAC	AGCGGCAACTCCTCGTCCTC
ZmHsf16	TCGCCGACGCCGTTCCTC	GCTGCCTGATGAAGCTGGAGAAG
ZmHsf18	CGCGCACGCAGTGGCCAG	CTCGCGGCGTCCTTTAAGAG
ZmHsf19	GACGCCGTTCCTGACCAAGAC	GCCGCCACACCACGAACG
ZmHsf02	ATGGTTATGATCATCCACGGTTAGA	GGACATCAGATGCATGGCTTT
ZmHsf22	GCTTCGTCGTCGCCAACCAG	AAGCCGTAGGTGTTCAGTTGCC
ZmHsf24	TGGAGAAGTTGCCGTGCTTGAAG	TGGGACTCGCACGTTTTTG
ZmHsf25	ACTCGGCCACGGACGATACG	GTTGCTGTGCTTGAAGTGCTTGG
ZmHsf26	CGGCATTGTGAAGGAGGAGTTCG	TGTCTTGGTCAGGAACGGAGTGG
ZmHsf29	AGACGTACACGATGGTGGAGGAC	GCGGACGAAGCTGGAGAAGTTG
ZmHsf03	CACGCCGTTCCTCACCAAGAC	GGCCGCCACACGATGAAGG
ZmHsf31	GGCCACCTTCTCCGACTACCTG	TCCGTGTCCACCTTCCTGAACC
ZmHsf8	GCCGCAGCCATCCAAGTACG	TGCGCTCGTTGCCAGCGCCACC
ZmHsf06	ACATGTCGTCTAACCAAGTGCCCA	ACCAAGTCTGCCAGAGCTGAGAAT
ZmHsf12	ACTCAGGCTTGGCGTATGT	CATCATCACCTTCTTCTGGC
ZmHsf01	AGAACCTGGCGCTCAACA	TCAGCAGCTCCTCCCAAA
ZmHsf04	AGAACCTGGCGCTCAACA	TTCAGCAGCTCCTCCCAA
ZmHsf05	GCCACAGGGAGTGTTTGA	TCGCCTCAACAGACGAAC
ZmHsf17	CCACACCACGAAGCTGTT	GAGGAGATTTGATGGCAGG
