# Supplementary material for: Genome-wide identification, transcriptome analysis and alternative splicing events of Hsf family genes in maize
Source: Sci Rep. 2020 May 15;10:8073. doi: 10.1038/s41598-020-65068-z (PMC7229205; doi:10.1038/s41598-020-65068-z)
Supplement: Supplementary file 4 — Supplementary Information. [file 41598_2020_65068_MOESM4_ESM.pdf]

Genome-wide identification, transcriptome analysis and alternative splicing events of Hsf family genes in maize

Huaning Zhang [1,2], Guoliang Li [1,2], Cai Fu[1], Shuonan Duan [1], Dong Hu[1, ✉] & Xiulin Guo [1, ✉]

[1] Plant Genetic Engineering Center of Hebei Province/Institute of Genetics and Physiology, Hebei Academy of Agriculture and Forestry Sciences, Shijiazhuang 050051, P.R. China

[2] These authors contributed equally: Huaning Zhang and Guoliang Li.

✉ e-mail: myhf2002@163.com, donghu1983@163.com.

Table S2 The primers used in semi-quantity and quantity RT-PCR of Fig. 6.

	semi-quantitative RT-PCR(5'-3')		quantitative RT-PCR(5'-3')
ZmHsf04	CTCACCAAGACGTTC	ZmHsf04-I	TCAACACCTATGGCTTC
			CTCACCAACCTCCAGGC
	ACCGTCCACGAGATC	ZmHsf04-II	ACAGCTTCATCGTCTGGGACC
			CGCTTGGATGAGGTTCTC
ZmHsf17	TGACATTGTCCAACAGACTATTCC	ZmHsf17-I	TCAACACCTATGGTTTCAGG
			ATGTCAATCTCTGCATCATATC
	AGGAATTCAGAAGGGCATGGAAAC	ZmHsf17-II	GTTAACTGGAACGTATCC
			TGCTGGTTTGTGGCATTCTGAGC
Zmβ-actin	TGTCCATCACTTGTGAAGCCTCCT	Zmβ-actin	TGTCCATCACTTGTGAAGCCTCCT
	ACGACCTTAGCCAATATCGCA CCA		ACGACCTTAGCCAATATCGCACCA
