# Supplementary material for: Genome-wide identification, transcriptome analysis and alternative splicing events of Hsf family genes in maize
Source: Sci Rep. 2020 May 15;10:8073. doi: 10.1038/s41598-020-65068-z (PMC7229205; doi:10.1038/s41598-020-65068-z)
Supplement: Supplementary file 5 — Supplementary Information. [file 41598_2020_65068_MOESM5_ESM.pdf]

Genome-wide identification, transcriptome analysis and alternative splicing events of Hsf family genes in maize

Huaning Zhang [1,2], Guoliang Li [1,2], Cai Fu[1], Shuonan Duan [1], Dong Hu[1, ✉] & Xiulin Guo [1, ✉]

[1] Plant Genetic Engineering Center of Hebei Province/Institute of Genetics and Physiology, Hebei Academy of Agriculture and Forestry Sciences, Shijiazhuang 050051, P.R. China

[2] These authors contributed equally: Huaning Zhang and Guoliang Li.

✉ e-mail: myhf2002@163.com, donghu1983@163.com.

Table S3 Analysis of collinearity of ZmHsfs proteins.

```
############### Parameters ###############
# MATCH_SCORE: 50
# MATCH_SIZE: 5
# GAP_PENALTY: -1
# OVERLAP_WINDOW: 5
# E_VALUE: 1e-05
# MAX GAPS: 25
############### Statistics ###############
# Number of collinear genes: 7783, Percentage: 5.91
# Number of all genes: 131585
##########################################
## Alignment 0: score=384.0 e_value=1.3e-19 N=8 1&1 plus
  0-  0:transcript:Zm00001d031349_T002 transcript:Zm00001d031379_T001          0
  0-  1:transcript:Zm00001d031351_T001 transcript:Zm00001d031381_T001          0
  0-  2:transcript:Zm00001d031359_T001 transcript:Zm00001d031398_T001          0
  0-  3:transcript:Zm00001d031362_T001 transcript:Zm00001d031400_T001 6.00E-118
  0-  4:transcript:Zm00001d031364_T001 transcript:Zm00001d031402_T001  8.00E-39
  0-  5:transcript:Zm00001d031367_T001 transcript:Zm00001d031404_T001  3.00E-87
  0-  6:transcript:Zm00001d031372_T001 transcript:Zm00001d031407_T001          0
  0-  7:transcript:Zm00001d031373_T001 transcript:Zm00001d031408_T001  3.00E-37
## Alignment 1: score=305.0 e_value=1.1e-14 N=8 1&1 plus
  1-  0:transcript:Zm00001d027815_T002 transcript:Zm00001d032981_T001          0
  1-  1:transcript:Zm00001d027826_T001 transcript:Zm00001d032989_T001          0
  1-  2:transcript:Zm00001d027827_T001 transcript:Zm00001d032991_T001          0
  1-  3:transcript:Zm00001d027846_T001 transcript:Zm00001d032999_T001 3.00E-110
  1-  4:transcript:Zm00001d027851_T001 transcript:Zm00001d033003_T001  1.00E-41
  1-  5:transcript:Zm00001d027852_T001 transcript:Zm00001d033005_T001  2.00E-84
  1-  6:transcript:Zm00001d027854_T006 transcript:Zm00001d033011_T001          0
  1-  7:transcript:Zm00001d027856_T001 transcript:Zm00001d033012_T001          0
## Alignment 2: score=294.0 e_value=8.3e-15 N=7 1&1 plus
  2-  0:transcript:Zm00001d027514_T002 transcript:Zm00001d029782_T004          0
  2-  1:transcript:Zm00001d027520_T001 transcript:Zm00001d029783_T001 4.00E-126
  2-  2:transcript:Zm00001d027522_T001 transcript:Zm00001d029784_T001  8.00E-66
  2-  3:transcript:Zm00001d027523_T001 transcript:Zm00001d029785_T001          0
  2-  4:transcript:Zm00001d027524_T001 transcript:Zm00001d029794_T001  3.00E-82
  2-  5:transcript:Zm00001d027539_T001 transcript:Zm00001d029799_T002  2.00E-67
  2-  6:transcript:Zm00001d027540_T001 transcript:Zm00001d029801_T002  2.00E-61
## Alignment 3: score=277.0 e_value=1.3e-10 N=6 1&1 plus
  3-  0:transcript:Zm00001d031182_T001 transcript:Zm00001d033378_T019          0
  3-  1:transcript:Zm00001d031183_T001 transcript:Zm00001d033380_T001  1.00E-22
  3-  2:transcript:Zm00001d031184_T001 transcript:Zm00001d033386_T001 1.00E-101
  3-  3:transcript:Zm00001d031189_T001 transcript:Zm00001d033389_T001  1.00E-09
  3-  4:transcript:Zm00001d031195_T001 transcript:Zm00001d033391_T001  3.00E-08
  3-  5:transcript:Zm00001d031196_T001 transcript:Zm00001d033395_T001  5.00E-16
## Alignment 4: score=273.0 e_value=6.6e-09 N=6 1&1 plus
  4-  0:transcript:Zm00001d027355_T001 transcript:Zm00001d029667_T001 3.00E-160
  4-  1:transcript:Zm00001d027361_T001 transcript:Zm00001d029673_T001 6.00E-146
  4-  2:transcript:Zm00001d027365_T001 transcript:Zm00001d029675_T001          0
  4-  3:transcript:Zm00001d027369_T002 transcript:Zm00001d029679_T001  1.00E-38
  4-  4:transcript:Zm00001d027370_T001 transcript:Zm00001d029680_T001  4.00E-24
```

```
  4-  5:transcript:Zm00001d027375_T001 transcript:Zm00001d029681_T001  2.00E-37
## Alignment 5: score=443.0 e_value=2.4e-24 N=11 1&1 minus
  5-  0:transcript:Zm00001d031061_T007 transcript:Zm00001d033246_T002          0
  5-  1:transcript:Zm00001d031068_T001 transcript:Zm00001d033234_T001 5.00E-163
  5-  2:transcript:Zm00001d031072_T001 transcript:Zm00001d033233_T002 2.00E-121
  5-  3:transcript:Zm00001d031075_T001 transcript:Zm00001d033227_T001  2.00E-17
  5-  4:transcript:Zm00001d031081_T001 transcript:Zm00001d033223_T003 7.00E-122
  5-  5:transcript:Zm00001d031086_T001 transcript:Zm00001d033222_T001          0
  5-  6:transcript:Zm00001d031088_T001 transcript:Zm00001d033217_T004          0
  5-  7:transcript:Zm00001d031092_T001 transcript:Zm00001d033215_T003  7.00E-80
  5-  8:transcript:Zm00001d031094_T001 transcript:Zm00001d033213_T001 7.00E-146
  5-  9:transcript:Zm00001d031098_T004 transcript:Zm00001d033211_T002 9.00E-136
  5- 10:transcript:Zm00001d031101_T002 transcript:Zm00001d033204_T002  2.00E-46
## Alignment 6: score=293.0 e_value=1.4e-12 N=7 1&1 minus
  6-  0:transcript:Zm00001d031120_T003 transcript:Zm00001d033493_T002          0
  6-  1:transcript:Zm00001d031127_T001 transcript:Zm00001d033489_T001  7.00E-76
  6-  2:transcript:Zm00001d031128_T004 transcript:Zm00001d033480_T002          0
  6-  3:transcript:Zm00001d031129_T001 transcript:Zm00001d033478_T001  1.00E-24
  6-  4:transcript:Zm00001d031131_T001 transcript:Zm00001d033475_T001  2.00E-60
  6-  5:transcript:Zm00001d031146_T001 transcript:Zm00001d033464_T001  8.00E-36
  6-  6:transcript:Zm00001d031148_T001 transcript:Zm00001d033456_T001  3.00E-16
## Alignment 7: score=559.0 e_value=3.4e-30 N=13 1&2 plus
  7-  0:transcript:Zm00001d031790_T002 transcript:Zm00001d005884_T002  1.00E-73
  7-  1:transcript:Zm00001d031792_T001 transcript:Zm00001d005885_T001 1.00E-163
  7-  2:transcript:Zm00001d031794_T001 transcript:Zm00001d005890_T001          0
  7-  3:transcript:Zm00001d031796_T001 transcript:Zm00001d005892_T001  3.00E-65
  7-  4:transcript:Zm00001d031797_T001 transcript:Zm00001d005897_T001  4.00E-20
  7-  5:transcript:Zm00001d031801_T001 transcript:Zm00001d005905_T001 7.00E-139
  7-  6:transcript:Zm00001d031805_T001 transcript:Zm00001d005910_T001  2.00E-98
  7-  7:transcript:Zm00001d031807_T003 transcript:Zm00001d005917_T003 2.00E-104
  7-  8:transcript:Zm00001d031810_T001 transcript:Zm00001d005918_T001  2.00E-66
  7-  9:transcript:Zm00001d031818_T001 transcript:Zm00001d005919_T001  1.00E-63
  7- 10:transcript:Zm00001d031826_T001 transcript:Zm00001d005923_T001  4.00E-62
  7- 11:transcript:Zm00001d031837_T001 transcript:Zm00001d005928_T002  3.00E-98
  7- 12:transcript:Zm00001d031840_T001 transcript:Zm00001d005931_T001 6.00E-109
## Alignment 8: score=427.0 e_value=2e-23 N=10 1&2 plus
  8-  0:transcript:Zm00001d031545_T001 transcript:Zm00001d006059_T001  2.00E-49
  8-  1:transcript:Zm00001d031546_T001 transcript:Zm00001d006060_T001  1.00E-07
  8-  2:transcript:Zm00001d031554_T003 transcript:Zm00001d006063_T001          0
  8-  3:transcript:Zm00001d031560_T001 transcript:Zm00001d006064_T002  2.00E-91
  8-  4:transcript:Zm00001d031561_T002 transcript:Zm00001d006065_T002  1.00E-85
  8-  5:transcript:Zm00001d031570_T001 transcript:Zm00001d006066_T002  4.00E-19
  8-  6:transcript:Zm00001d031586_T001 transcript:Zm00001d006078_T001  4.00E-26
  8-  7:transcript:Zm00001d031587_T004 transcript:Zm00001d006079_T001  1.00E-09
  8-  8:transcript:Zm00001d031594_T001 transcript:Zm00001d006082_T001  8.00E-55
  8-  9:transcript:Zm00001d031602_T002 transcript:Zm00001d006084_T003          0
## Alignment 9: score=268.0 e_value=3.1e-08 N=6 1&2 minus
  9-  0:transcript:Zm00001d029734_T001 transcript:Zm00001d002579_T001          0
  9-  1:transcript:Zm00001d029740_T002 transcript:Zm00001d002570_T001  6.00E-68
  9-  2:transcript:Zm00001d029744_T001 transcript:Zm00001d002564_T001 9.00E-133
  9-  3:transcript:Zm00001d029749_T001 transcript:Zm00001d002562_T001  6.00E-55
  9-  4:transcript:Zm00001d029752_T001 transcript:Zm00001d002558_T001  1.00E-10
  9-  5:transcript:Zm00001d029754_T004 transcript:Zm00001d002553_T001 4.00E-168
## Alignment 10: score=516.0 e_value=4.8e-35 N=13 1&3 plus
```

```
 10-  0:transcript:Zm00001d030969_T001 transcript:Zm00001d041397_T001          0
 10-  1:transcript:Zm00001d030975_T001 transcript:Zm00001d041402_T001  1.00E-97
 10-  2:transcript:Zm00001d030985_T001 transcript:Zm00001d041405_T001          0
 10-  3:transcript:Zm00001d030987_T001 transcript:Zm00001d041407_T002  1.00E-23
 10-  4:transcript:Zm00001d030989_T001 transcript:Zm00001d041410_T001  3.00E-63
 10-  5:transcript:Zm00001d030990_T001 transcript:Zm00001d041414_T002          0
 10-  6:transcript:Zm00001d030993_T001 transcript:Zm00001d041418_T001  6.00E-67
 10-  7:transcript:Zm00001d030996_T008 transcript:Zm00001d041420_T025          0
 10-  8:transcript:Zm00001d030998_T007 transcript:Zm00001d041438_T001          0
 10-  9:transcript:Zm00001d030999_T001 transcript:Zm00001d041439_T001          0
 10- 10:transcript:Zm00001d031005_T004 transcript:Zm00001d041443_T012          0
 10- 11:transcript:Zm00001d031008_T001 transcript:Zm00001d041444_T001          0
 10- 12:transcript:Zm00001d031009_T004 transcript:Zm00001d041445_T006          0
## Alignment 11: score=300.0 e_value=6.5e-12 N=7 1&3 plus
 11-  0:transcript:Zm00001d031049_T001 transcript:Zm00001d041476_T001          0
 11-  1:transcript:Zm00001d031050_T001 transcript:Zm00001d041480_T001          0
 11-  2:transcript:Zm00001d031053_T001 transcript:Zm00001d041481_T001          0
 11-  3:transcript:Zm00001d031059_T006 transcript:Zm00001d041488_T002          0
 11-  4:transcript:Zm00001d031061_T007 transcript:Zm00001d041489_T009          0
 11-  5:transcript:Zm00001d031063_T001 transcript:Zm00001d041491_T001  5.00E-65
 11-  6:transcript:Zm00001d031064_T002 transcript:Zm00001d041497_T005          0
## Alignment 12: score=686.0 e_value=3.7e-50 N=17 1&3 minus
 12-  0:transcript:Zm00001d030677_T004 transcript:Zm00001d041575_T008          0
 12-  1:transcript:Zm00001d030678_T001 transcript:Zm00001d041576_T001 7.00E-157
 12-  2:transcript:Zm00001d030690_T001 transcript:Zm00001d041569_T001  3.00E-84
 12-  3:transcript:Zm00001d030691_T001 transcript:Zm00001d041568_T001          0
 12-  4:transcript:Zm00001d030694_T002 transcript:Zm00001d041553_T001  3.00E-63
 12-  5:transcript:Zm00001d030725_T003 transcript:Zm00001d041550_T006          0
 12-  6:transcript:Zm00001d030727_T002 transcript:Zm00001d041549_T001 1.00E-158
 12-  7:transcript:Zm00001d030732_T001 transcript:Zm00001d041548_T002          0
 12-  8:transcript:Zm00001d030735_T001 transcript:Zm00001d041538_T001  3.00E-11
 12-  9:transcript:Zm00001d030744_T001 transcript:Zm00001d041536_T001          0
 12- 10:transcript:Zm00001d030750_T001 transcript:Zm00001d041535_T001  7.00E-25
 12- 11:transcript:Zm00001d030751_T001 transcript:Zm00001d041534_T002          0
 12- 12:transcript:Zm00001d030760_T004 transcript:Zm00001d041530_T001          0
 12- 13:transcript:Zm00001d030766_T001 transcript:Zm00001d041525_T001          0
 12- 14:transcript:Zm00001d030773_T001 transcript:Zm00001d041523_T001 5.00E-172
 12- 15:transcript:Zm00001d030775_T001 transcript:Zm00001d041518_T001          0
 12- 16:transcript:Zm00001d030780_T001 transcript:Zm00001d041515_T006  2.00E-92
## Alignment 13: score=351.0 e_value=1.6e-13 N=8 1&3 minus
 13-  0:transcript:Zm00001d030613_T003 transcript:Zm00001d041608_T001          0
 13-  1:transcript:Zm00001d030614_T002 transcript:Zm00001d041606_T002          0
 13-  2:transcript:Zm00001d030617_T001 transcript:Zm00001d041604_T001  2.00E-87
 13-  3:transcript:Zm00001d030620_T002 transcript:Zm00001d041603_T001          0
 13-  4:transcript:Zm00001d030622_T001 transcript:Zm00001d041600_T001 8.00E-115
 13-  5:transcript:Zm00001d030626_T003 transcript:Zm00001d041599_T004          0
 13-  6:transcript:Zm00001d030627_T002 transcript:Zm00001d041597_T001 2.00E-129
 13-  7:transcript:Zm00001d030638_T001 transcript:Zm00001d041595_T001  4.00E-63
## Alignment 14: score=287.0 e_value=5.9e-14 N=7 1&3 minus
 14-  0:transcript:Zm00001d031074_T002 transcript:Zm00001d041323_T003          0
 14-  1:transcript:Zm00001d031086_T001 transcript:Zm00001d041319_T001          0
 14-  2:transcript:Zm00001d031088_T001 transcript:Zm00001d041307_T002          0
 14-  3:transcript:Zm00001d031094_T001 transcript:Zm00001d041305_T001 5.00E-180
 14-  4:transcript:Zm00001d031100_T001 transcript:Zm00001d041291_T001  7.00E-43
```

14- 5:transcript:Zm00001d031101_T002 transcript:Zm00001d041290_T002 3.00E-102
14- 6:transcript:Zm00001d031109_T002 transcript:Zm00001d041277_T001 2.00E-88
Alignment 15: score=649.0 e_value=2.7e-40 N=15 1&4 plus
15- 0:transcript:Zm00001d031530_T005 transcript:Zm00001d049762_T006 0
15- 1:transcript:Zm00001d031531_T002 transcript:Zm00001d049763_T002 0
15- 2:transcript:Zm00001d031532_T001 transcript:Zm00001d049764_T001 0
15- 3:transcript:Zm00001d031536_T001 transcript:Zm00001d049766_T001 0
15- 4:transcript:Zm00001d031542_T001 transcript:Zm00001d049767_T001 0
15- 5:transcript:Zm00001d031545_T001 transcript:Zm00001d049768_T001 2.00E-105
15- 6:transcript:Zm00001d031546_T001 transcript:Zm00001d049769_T001 3.00E-26
15- 7:transcript:Zm00001d031549_T002 transcript:Zm00001d049777_T001 2.00E-66
15- 8:transcript:Zm00001d031554_T003 transcript:Zm00001d049785_T003 0
15- 9:transcript:Zm00001d031555_T001 transcript:Zm00001d049786_T001 0
15- 10:transcript:Zm00001d031556_T001 transcript:Zm00001d049789_T001 1.00E-85
15- 11:transcript:Zm00001d031560_T001 transcript:Zm00001d049790_T007 1.00E-95
15- 12:transcript:Zm00001d031572_T001 transcript:Zm00001d049800_T001 1.00E-146
15- 13:transcript:Zm00001d031577_T001 transcript:Zm00001d049804_T001 0
15- 14:transcript:Zm00001d031587_T004 transcript:Zm00001d049806_T001 5.00E-15
Alignment 16: score=501.0 e_value=2.7e-30 N=12 1&4 plus
16- 0:transcript:Zm00001d031634_T001 transcript:Zm00001d049831_T001 0
16- 1:transcript:Zm00001d031636_T001 transcript:Zm00001d049834_T001 5.00E-93
16- 2:transcript:Zm00001d031637_T003 transcript:Zm00001d049835_T004 4.00E-155
16- 3:transcript:Zm00001d031638_T001 transcript:Zm00001d049851_T001 0
16- 4:transcript:Zm00001d031643_T001 transcript:Zm00001d049854_T001 7.00E-78
16- 5:transcript:Zm00001d031648_T004 transcript:Zm00001d049857_T002 0
16- 6:transcript:Zm00001d031651_T001 transcript:Zm00001d049858_T001 1.00E-41
16- 7:transcript:Zm00001d031655_T003 transcript:Zm00001d049860_T001 1.00E-113
16- 8:transcript:Zm00001d031661_T001 transcript:Zm00001d049865_T001 5.00E-10
16- 9:transcript:Zm00001d031665_T001 transcript:Zm00001d049870_T001 9.00E-128
16- 10:transcript:Zm00001d031667_T006 transcript:Zm00001d049871_T001 1.00E-120
16- 11:transcript:Zm00001d031668_T001 transcript:Zm00001d049873_T001 3.00E-57
Alignment 17: score=422.0 e_value=7.8e-19 N=9 1&4 plus
17- 0:transcript:Zm00001d031269_T002 transcript:Zm00001d052803_T004 6.00E-86
17- 1:transcript:Zm00001d031270_T001 transcript:Zm00001d052804_T001 1.00E-136
17- 2:transcript:Zm00001d031272_T001 transcript:Zm00001d052807_T001 2.00E-53
17- 3:transcript:Zm00001d031274_T001 transcript:Zm00001d052809_T002 9.00E-56
17- 4:transcript:Zm00001d031275_T001 transcript:Zm00001d052814_T001 5.00E-48
17- 5:transcript:Zm00001d031277_T001 transcript:Zm00001d052815_T001 6.00E-68
17- 6:transcript:Zm00001d031280_T001 transcript:Zm00001d052816_T001 0
17- 7:transcript:Zm00001d031282_T001 transcript:Zm00001d052817_T001 4.00E-52
17- 8:transcript:Zm00001d031288_T001 transcript:Zm00001d052820_T001 2.00E-49
Alignment 18: score=363.0 e_value=7e-16 N=8 1&4 plus
18- 0:transcript:Zm00001d031760_T001 transcript:Zm00001d049990_T001 0
18- 1:transcript:Zm00001d031766_T001 transcript:Zm00001d050000_T001 0
18- 2:transcript:Zm00001d031777_T001 transcript:Zm00001d050008_T001 0
18- 3:transcript:Zm00001d031778_T001 transcript:Zm00001d050011_T004 4.00E-115
18- 4:transcript:Zm00001d031781_T001 transcript:Zm00001d050016_T001 5.00E-100
18- 5:transcript:Zm00001d031790_T002 transcript:Zm00001d050018_T007 2.00E-166
18- 6:transcript:Zm00001d031792_T001 transcript:Zm00001d050019_T001 0
18- 7:transcript:Zm00001d031793_T002 transcript:Zm00001d050020_T003 0
Alignment 19: score=358.0 e_value=5.4e-18 N=8 1&4 plus
19- 0:transcript:Zm00001d031389_T001 transcript:Zm00001d052864_T001 9.00E-35
19- 1:transcript:Zm00001d031408_T001 transcript:Zm00001d052865_T001 3.00E-15
19- 2:transcript:Zm00001d031412_T001 transcript:Zm00001d052875_T001 0

```
 19-  3:transcript:Zm00001d031413_T001 transcript:Zm00001d052876_T001          0
 19-  4:transcript:Zm00001d031415_T001 transcript:Zm00001d052882_T001  1.00E-45
 19-  5:transcript:Zm00001d031416_T001 transcript:Zm00001d052883_T001          0
 19-  6:transcript:Zm00001d031420_T001 transcript:Zm00001d052886_T001          0
 19-  7:transcript:Zm00001d031422_T006 transcript:Zm00001d052888_T004          0
## Alignment 20: score=356.0 e_value=7.3e-18 N=9 1&4 plus
 20-  0:transcript:Zm00001d031674_T003 transcript:Zm00001d052689_T002          0
 20-  1:transcript:Zm00001d031678_T002 transcript:Zm00001d052695_T021          0
 20-  2:transcript:Zm00001d031684_T001 transcript:Zm00001d052697_T002          0
 20-  3:transcript:Zm00001d031688_T002 transcript:Zm00001d052698_T001 7.00E-168
 20-  4:transcript:Zm00001d031691_T001 transcript:Zm00001d052702_T001          0
 20-  5:transcript:Zm00001d031694_T003 transcript:Zm00001d052709_T002          0
 20-  6:transcript:Zm00001d031697_T001 transcript:Zm00001d052717_T001 8.00E-128
 20-  7:transcript:Zm00001d031701_T002 transcript:Zm00001d052718_T002  3.00E-37
 20-  8:transcript:Zm00001d031703_T001 transcript:Zm00001d052719_T002 7.00E-176
## Alignment 21: score=303.0 e_value=9.1e-12 N=7 1&4 plus
 21-  0:transcript:Zm00001d031953_T008 transcript:Zm00001d049939_T001          0
 21-  1:transcript:Zm00001d031957_T001 transcript:Zm00001d049944_T003  2.00E-59
 21-  2:transcript:Zm00001d031958_T001 transcript:Zm00001d049948_T001          0
 21-  3:transcript:Zm00001d031959_T001 transcript:Zm00001d049950_T001          0
 21-  4:transcript:Zm00001d031961_T002 transcript:Zm00001d049952_T001  4.00E-81
 21-  5:transcript:Zm00001d031967_T001 transcript:Zm00001d049959_T001          0
 21-  6:transcript:Zm00001d031969_T001 transcript:Zm00001d049965_T003 3.00E-165
## Alignment 22: score=301.0 e_value=3e-14 N=8 1&4 plus
 22-  0:transcript:Zm00001d031310_T001 transcript:Zm00001d052838_T001  7.00E-81
 22-  1:transcript:Zm00001d031316_T020 transcript:Zm00001d052840_T016          0
 22-  2:transcript:Zm00001d031318_T001 transcript:Zm00001d052845_T009 2.00E-166
 22-  3:transcript:Zm00001d031323_T001 transcript:Zm00001d052848_T004          0
 22-  4:transcript:Zm00001d031327_T009 transcript:Zm00001d052851_T002          0
 22-  5:transcript:Zm00001d031329_T002 transcript:Zm00001d052852_T001          0
 22-  6:transcript:Zm00001d031332_T007 transcript:Zm00001d052855_T001          0
 22-  7:transcript:Zm00001d031340_T001 transcript:Zm00001d052856_T001          0
## Alignment 23: score=269.0 e_value=9.4e-10 N=6 1&4 plus
 23-  0:transcript:Zm00001d032152_T001 transcript:Zm00001d050417_T001          0
 23-  1:transcript:Zm00001d032155_T001 transcript:Zm00001d050422_T001          0
 23-  2:transcript:Zm00001d032157_T001 transcript:Zm00001d050430_T003 1.00E-138
 23-  3:transcript:Zm00001d032158_T002 transcript:Zm00001d050433_T001 3.00E-177
 23-  4:transcript:Zm00001d032164_T001 transcript:Zm00001d050436_T001          0
 23-  5:transcript:Zm00001d032165_T003 transcript:Zm00001d050440_T004 3.00E-134
## Alignment 24: score=256.0 e_value=2.7e-09 N=6 1&4 plus
 24-  0:transcript:Zm00001d031848_T001 transcript:Zm00001d049880_T001          0
 24-  1:transcript:Zm00001d031850_T001 transcript:Zm00001d049883_T001  5.00E-71
 24-  2:transcript:Zm00001d031851_T002 transcript:Zm00001d049884_T006          0
 24-  3:transcript:Zm00001d031856_T002 transcript:Zm00001d049886_T001  1.00E-79
 24-  4:transcript:Zm00001d031861_T001 transcript:Zm00001d049889_T001 6.00E-133
 24-  5:transcript:Zm00001d031863_T013 transcript:Zm00001d049894_T011          0
## Alignment 25: score=650.0 e_value=4.9e-38 N=15 1&4 minus
 25-  0:transcript:Zm00001d032008_T001 transcript:Zm00001d053236_T001 1.00E-127
 25-  1:transcript:Zm00001d032010_T007 transcript:Zm00001d053232_T005          0
 25-  2:transcript:Zm00001d032012_T001 transcript:Zm00001d053230_T001  5.00E-29
 25-  3:transcript:Zm00001d032013_T001 transcript:Zm00001d053229_T001  3.00E-52
 25-  4:transcript:Zm00001d032019_T001 transcript:Zm00001d053228_T002  8.00E-86
 25-  5:transcript:Zm00001d032022_T003 transcript:Zm00001d053225_T005          0
 25-  6:transcript:Zm00001d032024_T001 transcript:Zm00001d053220_T001 1.00E-123
```

25- 7:transcript:Zm00001d032027_T002 transcript:Zm00001d053215_T001 0
25- 8:transcript:Zm00001d032028_T001 transcript:Zm00001d053214_T001 0
25- 9:transcript:Zm00001d032030_T001 transcript:Zm00001d053213_T001 3.00E-60
25- 10:transcript:Zm00001d032031_T001 transcript:Zm00001d053211_T009 6.00E-122
25- 11:transcript:Zm00001d032032_T001 transcript:Zm00001d053210_T001 3.00E-98
25- 12:transcript:Zm00001d032040_T001 transcript:Zm00001d053208_T001 2.00E-135
25- 13:transcript:Zm00001d032042_T001 transcript:Zm00001d053202_T001 0
25- 14:transcript:Zm00001d032044_T004 transcript:Zm00001d053200_T001 0
Alignment 26: score=521.0 e_value=1.3e-28 N=12 1&4 minus
26- 0:transcript:Zm00001d032181_T001 transcript:Zm00001d050411_T001 4.00E-79
26- 1:transcript:Zm00001d032182_T001 transcript:Zm00001d050410_T001 0
26- 2:transcript:Zm00001d032187_T001 transcript:Zm00001d050409_T001 0
26- 3:transcript:Zm00001d032188_T001 transcript:Zm00001d050406_T008 0
26- 4:transcript:Zm00001d032197_T001 transcript:Zm00001d050403_T001 1.00E-171
26- 5:transcript:Zm00001d032198_T002 transcript:Zm00001d050401_T003 0
26- 6:transcript:Zm00001d032206_T001 transcript:Zm00001d050400_T001 0
26- 7:transcript:Zm00001d032212_T005 transcript:Zm00001d050395_T002 5.00E-137
26- 8:transcript:Zm00001d032213_T001 transcript:Zm00001d050394_T001 1.00E-154
26- 9:transcript:Zm00001d032215_T001 transcript:Zm00001d050393_T001 1.00E-105
26- 10:transcript:Zm00001d032217_T001 transcript:Zm00001d050389_T001 3.00E-99
26- 11:transcript:Zm00001d032219_T001 transcript:Zm00001d050386_T001 2.00E-24
Alignment 27: score=487.0 e_value=2.5e-26 N=12 1&4 minus
27- 0:transcript:Zm00001d032224_T002 transcript:Zm00001d050383_T001 0
27- 1:transcript:Zm00001d032226_T002 transcript:Zm00001d050382_T001 0
27- 2:transcript:Zm00001d032229_T002 transcript:Zm00001d050380_T001 0
27- 3:transcript:Zm00001d032231_T001 transcript:Zm00001d050375_T001 0
27- 4:transcript:Zm00001d032239_T001 transcript:Zm00001d050368_T001 1.00E-97
27- 5:transcript:Zm00001d032242_T001 transcript:Zm00001d050357_T005 5.00E-60
27- 6:transcript:Zm00001d032244_T001 transcript:Zm00001d050355_T001 0
27- 7:transcript:Zm00001d032249_T002 transcript:Zm00001d050350_T001 2.00E-93
27- 8:transcript:Zm00001d032250_T001 transcript:Zm00001d050347_T002 4.00E-100
27- 9:transcript:Zm00001d032257_T001 transcript:Zm00001d050339_T001 0
27- 10:transcript:Zm00001d032262_T001 transcript:Zm00001d050335_T001 0
27- 11:transcript:Zm00001d032263_T001 transcript:Zm00001d050330_T003 4.00E-101
Alignment 28: score=290.0 e_value=2.2e-11 N=7 1&4 minus
28- 0:transcript:Zm00001d032109_T001 transcript:Zm00001d050081_T002 3.00E-173
28- 1:transcript:Zm00001d032115_T001 transcript:Zm00001d050079_T001 2.00E-48
28- 2:transcript:Zm00001d032116_T002 transcript:Zm00001d050076_T001 7.00E-175
28- 3:transcript:Zm00001d032118_T001 transcript:Zm00001d050069_T001 0
28- 4:transcript:Zm00001d032123_T001 transcript:Zm00001d050056_T002 4.00E-09
28- 5:transcript:Zm00001d032139_T002 transcript:Zm00001d050054_T002 2.00E-55
28- 6:transcript:Zm00001d032146_T001 transcript:Zm00001d050051_T001 1.00E-114
Alignment 29: score=1010.0 e_value=3.7e-70 N=22 1&5 plus
29- 0:transcript:Zm00001d033465_T001 transcript:Zm00001d013624_T001 0
29- 1:transcript:Zm00001d033466_T001 transcript:Zm00001d013625_T001 5.00E-82
29- 2:transcript:Zm00001d033467_T002 transcript:Zm00001d013627_T001 0
29- 3:transcript:Zm00001d033470_T001 transcript:Zm00001d013629_T001 3.00E-74
29- 4:transcript:Zm00001d033471_T001 transcript:Zm00001d013630_T001 4.00E-47
29- 5:transcript:Zm00001d033472_T004 transcript:Zm00001d013631_T001 1.00E-123
29- 6:transcript:Zm00001d033473_T001 transcript:Zm00001d013632_T001 9.00E-142
29- 7:transcript:Zm00001d033475_T001 transcript:Zm00001d013638_T001 0
29- 8:transcript:Zm00001d033477_T002 transcript:Zm00001d013639_T001 8.00E-87
29- 9:transcript:Zm00001d033478_T001 transcript:Zm00001d013641_T001 4.00E-82
29- 10:transcript:Zm00001d033479_T001 transcript:Zm00001d013642_T001 1.00E-53

29- 11:transcript:Zm00001d033480_T002 transcript:Zm00001d013644_T001 0
29- 12:transcript:Zm00001d033481_T001 transcript:Zm00001d013645_T001 2.00E-58
29- 13:transcript:Zm00001d033482_T001 transcript:Zm00001d013646_T001 0
29- 14:transcript:Zm00001d033483_T001 transcript:Zm00001d013647_T001 5.00E-17
29- 15:transcript:Zm00001d033493_T002 transcript:Zm00001d013652_T003 0
29- 16:transcript:Zm00001d033494_T011 transcript:Zm00001d013653_T010 0
29- 17:transcript:Zm00001d033496_T001 transcript:Zm00001d013655_T001 0
29- 18:transcript:Zm00001d033503_T002 transcript:Zm00001d013657_T001 9.00E-23
29- 19:transcript:Zm00001d033504_T001 transcript:Zm00001d013658_T001 0
29- 20:transcript:Zm00001d033507_T001 transcript:Zm00001d013660_T001 6.00E-79
29- 21:transcript:Zm00001d033508_T001 transcript:Zm00001d013661_T001 2.00E-31
Alignment 30: score=584.0 e_value=2.5e-36 N=14 1&5 plus
30- 0:transcript:Zm00001d032499_T001 transcript:Zm00001d013892_T004 2.00E-104
30- 1:transcript:Zm00001d032502_T001 transcript:Zm00001d013895_T002 1.00E-128
30- 2:transcript:Zm00001d032503_T001 transcript:Zm00001d013896_T001 0
30- 3:transcript:Zm00001d032519_T001 transcript:Zm00001d013900_T001 0
30- 4:transcript:Zm00001d032520_T018 transcript:Zm00001d013908_T003 0
30- 5:transcript:Zm00001d032522_T001 transcript:Zm00001d013909_T001 1.00E-18
30- 6:transcript:Zm00001d032526_T002 transcript:Zm00001d013910_T002 0
30- 7:transcript:Zm00001d032531_T001 transcript:Zm00001d013914_T001 5.00E-94
30- 8:transcript:Zm00001d032532_T001 transcript:Zm00001d013915_T001 0
30- 9:transcript:Zm00001d032535_T002 transcript:Zm00001d013919_T001 3.00E-170
30- 10:transcript:Zm00001d032539_T001 transcript:Zm00001d013920_T002 0
30- 11:transcript:Zm00001d032543_T001 transcript:Zm00001d013923_T001 3.00E-71
30- 12:transcript:Zm00001d032545_T001 transcript:Zm00001d013926_T001 0
30- 13:transcript:Zm00001d032546_T001 transcript:Zm00001d013927_T001 0
Alignment 31: score=500.0 e_value=8e-27 N=11 1&5 plus
31- 0:transcript:Zm00001d033204_T002 transcript:Zm00001d013667_T001 2.00E-159
31- 1:transcript:Zm00001d033205_T001 transcript:Zm00001d013668_T001 4.00E-156
31- 2:transcript:Zm00001d033210_T002 transcript:Zm00001d013669_T002 0
31- 3:transcript:Zm00001d033213_T001 transcript:Zm00001d013672_T001 0
31- 4:transcript:Zm00001d033214_T001 transcript:Zm00001d013673_T001 3.00E-174
31- 5:transcript:Zm00001d033215_T003 transcript:Zm00001d013676_T001 1.00E-169
31- 6:transcript:Zm00001d033216_T001 transcript:Zm00001d013677_T001 8.00E-59
31- 7:transcript:Zm00001d033217_T004 transcript:Zm00001d013680_T002 0
31- 8:transcript:Zm00001d033218_T002 transcript:Zm00001d013683_T003 0
31- 9:transcript:Zm00001d033222_T001 transcript:Zm00001d013689_T001 0
31- 10:transcript:Zm00001d033223_T003 transcript:Zm00001d013692_T001 2.00E-144
Alignment 32: score=448.0 e_value=1.4e-24 N=10 1&5 plus
32- 0:transcript:Zm00001d032661_T001 transcript:Zm00001d013990_T001 3.00E-95
32- 1:transcript:Zm00001d032662_T001 transcript:Zm00001d013991_T001 0
32- 2:transcript:Zm00001d032663_T001 transcript:Zm00001d013993_T001 4.00E-82
32- 3:transcript:Zm00001d032668_T001 transcript:Zm00001d013999_T001 1.00E-114
32- 4:transcript:Zm00001d032670_T004 transcript:Zm00001d014003_T004 2.00E-169
32- 5:transcript:Zm00001d032671_T001 transcript:Zm00001d014005_T001 1.00E-52
32- 6:transcript:Zm00001d032672_T001 transcript:Zm00001d014006_T001 2.00E-29
32- 7:transcript:Zm00001d032679_T001 transcript:Zm00001d014011_T001 0
32- 8:transcript:Zm00001d032683_T001 transcript:Zm00001d014013_T002 0
32- 9:transcript:Zm00001d032686_T001 transcript:Zm00001d014015_T001 3.00E-55
Alignment 33: score=425.0 e_value=1.3e-20 N=10 1&5 plus
33- 0:transcript:Zm00001d033378_T019 transcript:Zm00001d013547_T002 0
33- 1:transcript:Zm00001d033388_T001 transcript:Zm00001d013548_T002 0
33- 2:transcript:Zm00001d033389_T001 transcript:Zm00001d013550_T001 1.00E-11
33- 3:transcript:Zm00001d033391_T001 transcript:Zm00001d013554_T001 5.00E-144

```
 33-  4:transcript:Zm00001d033396_T003 transcript:Zm00001d013555_T003          0
 33-  5:transcript:Zm00001d033404_T002 transcript:Zm00001d013559_T001  5.00E-79
 33-  6:transcript:Zm00001d033415_T001 transcript:Zm00001d013569_T001          0
 33-  7:transcript:Zm00001d033419_T001 transcript:Zm00001d013571_T001  1.00E-77
 33-  8:transcript:Zm00001d033428_T001 transcript:Zm00001d013574_T001  7.00E-28
 33-  9:transcript:Zm00001d033429_T001 transcript:Zm00001d013575_T001          0
## Alignment 34: score=421.0 e_value=2.1e-16 N=9 1&5 plus
 34-  0:transcript:Zm00001d032465_T002 transcript:Zm00001d013860_T012          0
 34-  1:transcript:Zm00001d032467_T001 transcript:Zm00001d013862_T001 4.00E-169
 34-  2:transcript:Zm00001d032470_T001 transcript:Zm00001d013863_T007          0
 34-  3:transcript:Zm00001d032472_T001 transcript:Zm00001d013865_T001          0
 34-  4:transcript:Zm00001d032473_T001 transcript:Zm00001d013868_T001          0
 34-  5:transcript:Zm00001d032475_T001 transcript:Zm00001d013869_T001  1.00E-55
 34-  6:transcript:Zm00001d032480_T001 transcript:Zm00001d013873_T001          0
 34-  7:transcript:Zm00001d032481_T001 transcript:Zm00001d013879_T001  1.00E-40
 34-  8:transcript:Zm00001d032485_T001 transcript:Zm00001d013886_T002  3.00E-92
## Alignment 35: score=387.0 e_value=2.8e-19 N=9 1&5 plus
 35-  0:transcript:Zm00001d032993_T001 transcript:Zm00001d014193_T001  2.00E-70
 35-  1:transcript:Zm00001d032999_T001 transcript:Zm00001d014198_T001 6.00E-131
 35-  2:transcript:Zm00001d033003_T001 transcript:Zm00001d014200_T001  2.00E-37
 35-  3:transcript:Zm00001d033005_T001 transcript:Zm00001d014201_T001 8.00E-151
 35-  4:transcript:Zm00001d033012_T001 transcript:Zm00001d014203_T001          0
 35-  5:transcript:Zm00001d033018_T001 transcript:Zm00001d014204_T001          0
 35-  6:transcript:Zm00001d033019_T001 transcript:Zm00001d014205_T001  9.00E-67
 35-  7:transcript:Zm00001d033035_T001 transcript:Zm00001d014226_T001 3.00E-140
 35-  8:transcript:Zm00001d033037_T008 transcript:Zm00001d014232_T014          0
## Alignment 36: score=377.0 e_value=3.6e-17 N=9 1&5 plus
 36-  0:transcript:Zm00001d033324_T001 transcript:Zm00001d013722_T001  8.00E-19
 36-  1:transcript:Zm00001d033325_T001 transcript:Zm00001d013724_T001 4.00E-120
 36-  2:transcript:Zm00001d033327_T001 transcript:Zm00001d013725_T002 2.00E-113
 36-  3:transcript:Zm00001d033330_T001 transcript:Zm00001d013728_T001 3.00E-138
 36-  4:transcript:Zm00001d033335_T001 transcript:Zm00001d013732_T001 2.00E-117
 36-  5:transcript:Zm00001d033339_T002 transcript:Zm00001d013736_T001          0
 36-  6:transcript:Zm00001d033344_T001 transcript:Zm00001d013741_T006          0
 36-  7:transcript:Zm00001d033346_T001 transcript:Zm00001d013742_T001          0
 36-  8:transcript:Zm00001d033363_T001 transcript:Zm00001d013743_T001  1.00E-85
## Alignment 37: score=320.0 e_value=1.3e-14 N=8 1&5 plus
 37-  0:transcript:Zm00001d032859_T006 transcript:Zm00001d014124_T001          0
 37-  1:transcript:Zm00001d032866_T001 transcript:Zm00001d014126_T001          0
 37-  2:transcript:Zm00001d032873_T001 transcript:Zm00001d014128_T001 9.00E-139
 37-  3:transcript:Zm00001d032875_T009 transcript:Zm00001d014138_T004          0
 37-  4:transcript:Zm00001d032887_T001 transcript:Zm00001d014139_T001  2.00E-55
 37-  5:transcript:Zm00001d032889_T001 transcript:Zm00001d014141_T002 1.00E-166
 37-  6:transcript:Zm00001d032893_T002 transcript:Zm00001d014149_T001  5.00E-51
 37-  7:transcript:Zm00001d032896_T001 transcript:Zm00001d014153_T001  1.00E-82
## Alignment 38: score=286.0 e_value=4.3e-12 N=6 1&5 plus
 38-  0:transcript:Zm00001d031182_T001 transcript:Zm00001d013547_T002          0
 38-  1:transcript:Zm00001d031184_T001 transcript:Zm00001d013549_T001  6.00E-95
 38-  2:transcript:Zm00001d031189_T001 transcript:Zm00001d013550_T001  3.00E-08
 38-  3:transcript:Zm00001d031191_T001 transcript:Zm00001d013551_T001  1.00E-16
 38-  4:transcript:Zm00001d031192_T001 transcript:Zm00001d013553_T001  4.00E-08
 38-  5:transcript:Zm00001d031195_T001 transcript:Zm00001d013554_T001  1.00E-08
## Alignment 39: score=280.0 e_value=2.7e-10 N=7 1&5 plus
 39-  0:transcript:Zm00001d032822_T001 transcript:Zm00001d014100_T001  2.00E-24
```

```
 39-  1:transcript:Zm00001d032828_T001 transcript:Zm00001d014102_T001          0
 39-  2:transcript:Zm00001d032830_T003 transcript:Zm00001d014108_T002  2.00E-68
 39-  3:transcript:Zm00001d032836_T001 transcript:Zm00001d014110_T001  2.00E-41
 39-  4:transcript:Zm00001d032843_T002 transcript:Zm00001d014112_T003  5.00E-35
 39-  5:transcript:Zm00001d032850_T001 transcript:Zm00001d014116_T001 7.00E-180
 39-  6:transcript:Zm00001d032854_T001 transcript:Zm00001d014123_T001          0
## Alignment 40: score=276.0 e_value=4.1e-15 N=7 1&5 plus
 40-  0:transcript:Zm00001d027585_T001 transcript:Zm00001d014046_T001  8.00E-54
 40-  1:transcript:Zm00001d027593_T001 transcript:Zm00001d014060_T001          0
 40-  2:transcript:Zm00001d027595_T001 transcript:Zm00001d014073_T004  8.00E-23
 40-  3:transcript:Zm00001d027598_T001 transcript:Zm00001d014074_T001  5.00E-44
 40-  4:transcript:Zm00001d027618_T001 transcript:Zm00001d014082_T001 7.00E-114
 40-  5:transcript:Zm00001d027619_T002 transcript:Zm00001d014083_T001          0
 40-  6:transcript:Zm00001d027622_T001 transcript:Zm00001d014084_T001          0
## Alignment 41: score=263.0 e_value=2e-10 N=6 1&5 plus
 41-  0:transcript:Zm00001d033528_T005 transcript:Zm00001d013529_T002          0
 41-  1:transcript:Zm00001d033529_T001 transcript:Zm00001d013530_T001          0
 41-  2:transcript:Zm00001d033537_T002 transcript:Zm00001d013531_T002  2.00E-39
 41-  3:transcript:Zm00001d033539_T001 transcript:Zm00001d013532_T001 1.00E-163
 41-  4:transcript:Zm00001d033541_T002 transcript:Zm00001d013533_T001  1.00E-48
 41-  5:transcript:Zm00001d033543_T002 transcript:Zm00001d013536_T001 2.00E-145
## Alignment 42: score=1008.0 e_value=7.5e-69 N=22 1&5 minus
 42-  0:transcript:Zm00001d034167_T006 transcript:Zm00001d013195_T001          0
 42-  1:transcript:Zm00001d034175_T002 transcript:Zm00001d013193_T001 5.00E-111
 42-  2:transcript:Zm00001d034178_T001 transcript:Zm00001d013192_T001          0
 42-  3:transcript:Zm00001d034179_T001 transcript:Zm00001d013191_T001  5.00E-68
 42-  4:transcript:Zm00001d034180_T002 transcript:Zm00001d013188_T002 1.00E-152
 42-  5:transcript:Zm00001d034181_T001 transcript:Zm00001d013187_T001 1.00E-135
 42-  6:transcript:Zm00001d034183_T006 transcript:Zm00001d013186_T001  2.00E-38
 42-  7:transcript:Zm00001d034184_T001 transcript:Zm00001d013185_T001          0
 42-  8:transcript:Zm00001d034187_T001 transcript:Zm00001d013184_T001  4.00E-39
 42-  9:transcript:Zm00001d034188_T002 transcript:Zm00001d013179_T001 1.00E-117
 42- 10:transcript:Zm00001d034190_T001 transcript:Zm00001d013178_T001  4.00E-89
 42- 11:transcript:Zm00001d034191_T004 transcript:Zm00001d013177_T002          0
 42- 12:transcript:Zm00001d034197_T001 transcript:Zm00001d013175_T001  3.00E-76
 42- 13:transcript:Zm00001d034198_T001 transcript:Zm00001d013173_T001          0
 42- 14:transcript:Zm00001d034199_T001 transcript:Zm00001d013172_T001  8.00E-66
 42- 15:transcript:Zm00001d034204_T001 transcript:Zm00001d013170_T001 7.00E-151
 42- 16:transcript:Zm00001d034205_T001 transcript:Zm00001d013168_T001          0
 42- 17:transcript:Zm00001d034206_T001 transcript:Zm00001d013166_T001          0
 42- 18:transcript:Zm00001d034207_T001 transcript:Zm00001d013164_T002 1.00E-164
 42- 19:transcript:Zm00001d034221_T001 transcript:Zm00001d013163_T001          0
 42- 20:transcript:Zm00001d034240_T004 transcript:Zm00001d013162_T001          0
 42- 21:transcript:Zm00001d034244_T001 transcript:Zm00001d013156_T001 1.00E-162
## Alignment 43: score=935.0 e_value=7.6e-68 N=22 1&5 minus
 43-  0:transcript:Zm00001d034633_T057 transcript:Zm00001d012974_T003          0
 43-  1:transcript:Zm00001d034634_T001 transcript:Zm00001d012973_T001  6.00E-99
 43-  2:transcript:Zm00001d034635_T001 transcript:Zm00001d012972_T001 7.00E-133
 43-  3:transcript:Zm00001d034636_T001 transcript:Zm00001d012970_T001          0
 43-  4:transcript:Zm00001d034640_T003 transcript:Zm00001d012969_T008          0
 43-  5:transcript:Zm00001d034641_T001 transcript:Zm00001d012967_T001  5.00E-35
 43-  6:transcript:Zm00001d034643_T001 transcript:Zm00001d012966_T001  4.00E-47
 43-  7:transcript:Zm00001d034649_T001 transcript:Zm00001d012965_T001  1.00E-13
 43-  8:transcript:Zm00001d034650_T017 transcript:Zm00001d012964_T016          0
```

43- 9:transcript:Zm00001d034651_T001 transcript:Zm00001d012963_T001 2.00E-144
43- 10:transcript:Zm00001d034655_T001 transcript:Zm00001d012962_T001 0
43- 11:transcript:Zm00001d034659_T001 transcript:Zm00001d012960_T001 1.00E-23
43- 12:transcript:Zm00001d034662_T001 transcript:Zm00001d012958_T001 0
43- 13:transcript:Zm00001d034663_T001 transcript:Zm00001d012957_T002 7.00E-171
43- 14:transcript:Zm00001d034667_T003 transcript:Zm00001d012955_T001 1.00E-178
43- 15:transcript:Zm00001d034673_T001 transcript:Zm00001d012939_T001 2.00E-51
43- 16:transcript:Zm00001d034677_T001 transcript:Zm00001d012936_T001 1.00E-72
43- 17:transcript:Zm00001d034678_T001 transcript:Zm00001d012935_T004 0
43- 18:transcript:Zm00001d034679_T001 transcript:Zm00001d012934_T001 1.00E-11
43- 19:transcript:Zm00001d034680_T001 transcript:Zm00001d012933_T001 2.00E-46
43- 20:transcript:Zm00001d034689_T010 transcript:Zm00001d012931_T001 0
43- 21:transcript:Zm00001d034698_T001 transcript:Zm00001d012930_T001 6.00E-43
Alignment 44: score=771.0 e_value=2.3e-51 N=18 1&5 minus
44- 0:transcript:Zm00001d033554_T002 transcript:Zm00001d013526_T002 4.00E-136
44- 1:transcript:Zm00001d033556_T007 transcript:Zm00001d013524_T009 0
44- 2:transcript:Zm00001d033557_T001 transcript:Zm00001d013523_T002 0
44- 3:transcript:Zm00001d033563_T001 transcript:Zm00001d013521_T001 0
44- 4:transcript:Zm00001d033569_T001 transcript:Zm00001d013520_T001 3.00E-09
44- 5:transcript:Zm00001d033572_T004 transcript:Zm00001d013519_T001 0
44- 6:transcript:Zm00001d033574_T001 transcript:Zm00001d013517_T001 1.00E-100
44- 7:transcript:Zm00001d033575_T002 transcript:Zm00001d013512_T001 0
44- 8:transcript:Zm00001d033589_T001 transcript:Zm00001d013509_T001 3.00E-54
44- 9:transcript:Zm00001d033591_T001 transcript:Zm00001d013507_T001 0
44- 10:transcript:Zm00001d033594_T001 transcript:Zm00001d013506_T001 0
44- 11:transcript:Zm00001d033596_T001 transcript:Zm00001d013505_T001 3.00E-130
44- 12:transcript:Zm00001d033600_T003 transcript:Zm00001d013504_T001 6.00E-107
44- 13:transcript:Zm00001d033602_T002 transcript:Zm00001d013501_T002 3.00E-12
44- 14:transcript:Zm00001d033606_T001 transcript:Zm00001d013498_T001 8.00E-85
44- 15:transcript:Zm00001d033610_T001 transcript:Zm00001d013497_T001 0
44- 16:transcript:Zm00001d033611_T002 transcript:Zm00001d013496_T001 0
44- 17:transcript:Zm00001d033612_T001 transcript:Zm00001d013494_T001 3.00E-78
Alignment 45: score=740.0 e_value=2.1e-49 N=18 1&5 minus
45- 0:transcript:Zm00001d034823_T001 transcript:Zm00001d012861_T001 3.00E-98
45- 1:transcript:Zm00001d034827_T001 transcript:Zm00001d012860_T001 4.00E-83
45- 2:transcript:Zm00001d034830_T001 transcript:Zm00001d012859_T001 8.00E-127
45- 3:transcript:Zm00001d034833_T004 transcript:Zm00001d012858_T001 0
45- 4:transcript:Zm00001d034835_T001 transcript:Zm00001d012856_T001 7.00E-175
45- 5:transcript:Zm00001d034841_T001 transcript:Zm00001d012853_T001 1.00E-146
45- 6:transcript:Zm00001d034842_T001 transcript:Zm00001d012851_T001 0
45- 7:transcript:Zm00001d034844_T001 transcript:Zm00001d012850_T001 8.00E-100
45- 8:transcript:Zm00001d034849_T001 transcript:Zm00001d012848_T001 6.00E-98
45- 9:transcript:Zm00001d034856_T003 transcript:Zm00001d012846_T005 0
45- 10:transcript:Zm00001d034857_T001 transcript:Zm00001d012845_T001 4.00E-102
45- 11:transcript:Zm00001d034862_T002 transcript:Zm00001d012839_T002 7.00E-166
45- 12:transcript:Zm00001d034867_T001 transcript:Zm00001d012838_T001 2.00E-166
45- 13:transcript:Zm00001d034869_T001 transcript:Zm00001d012836_T001 4.00E-40
45- 14:transcript:Zm00001d034871_T001 transcript:Zm00001d012834_T003 0
45- 15:transcript:Zm00001d034876_T001 transcript:Zm00001d012831_T001 0
45- 16:transcript:Zm00001d034885_T004 transcript:Zm00001d012827_T001 0
45- 17:transcript:Zm00001d034886_T001 transcript:Zm00001d012823_T001 0
Alignment 46: score=719.0 e_value=4.4e-42 N=16 1&5 minus
46- 0:transcript:Zm00001d033669_T003 transcript:Zm00001d013470_T007 0
46- 1:transcript:Zm00001d033670_T001 transcript:Zm00001d013469_T002 0

```
 46-  2:transcript:Zm00001d033671_T001 transcript:Zm00001d013468_T002          0
 46-  3:transcript:Zm00001d033673_T001 transcript:Zm00001d013467_T001          0
 46-  4:transcript:Zm00001d033674_T001 transcript:Zm00001d013466_T005          0
 46-  5:transcript:Zm00001d033680_T001 transcript:Zm00001d013465_T001          0
 46-  6:transcript:Zm00001d033682_T001 transcript:Zm00001d013463_T001          0
 46-  7:transcript:Zm00001d033683_T001 transcript:Zm00001d013461_T002 3.00E-103
 46-  8:transcript:Zm00001d033684_T001 transcript:Zm00001d013459_T001  3.00E-47
 46-  9:transcript:Zm00001d033704_T007 transcript:Zm00001d013453_T001          0
 46- 10:transcript:Zm00001d033705_T001 transcript:Zm00001d013452_T002          0
 46- 11:transcript:Zm00001d033706_T001 transcript:Zm00001d013451_T001  3.00E-88
 46- 12:transcript:Zm00001d033709_T001 transcript:Zm00001d013448_T001  2.00E-34
 46- 13:transcript:Zm00001d033716_T001 transcript:Zm00001d013447_T001 1.00E-133
 46- 14:transcript:Zm00001d033717_T001 transcript:Zm00001d013446_T001 5.00E-117
 46- 15:transcript:Zm00001d033719_T001 transcript:Zm00001d013443_T001          0
## Alignment 47: score=689.0 e_value=5.4e-50 N=17 1&5 minus
 47-  0:transcript:Zm00001d034327_T001 transcript:Zm00001d013144_T001 2.00E-109
 47-  1:transcript:Zm00001d034330_T001 transcript:Zm00001d013143_T001  9.00E-12
 47-  2:transcript:Zm00001d034337_T005 transcript:Zm00001d013128_T004 2.00E-106
 47-  3:transcript:Zm00001d034341_T003 transcript:Zm00001d013126_T002  2.00E-16
 47-  4:transcript:Zm00001d034343_T001 transcript:Zm00001d013122_T008  8.00E-21
 47-  5:transcript:Zm00001d034353_T001 transcript:Zm00001d013119_T001          0
 47-  6:transcript:Zm00001d034359_T001 transcript:Zm00001d013118_T001  9.00E-49
 47-  7:transcript:Zm00001d034361_T001 transcript:Zm00001d013116_T002          0
 47-  8:transcript:Zm00001d034365_T001 transcript:Zm00001d013114_T001 1.00E-111
 47-  9:transcript:Zm00001d034366_T001 transcript:Zm00001d013112_T011          0
 47- 10:transcript:Zm00001d034368_T001 transcript:Zm00001d013111_T001          0
 47- 11:transcript:Zm00001d034369_T001 transcript:Zm00001d013110_T001  3.00E-93
 47- 12:transcript:Zm00001d034372_T001 transcript:Zm00001d013109_T002          0
 47- 13:transcript:Zm00001d034373_T001 transcript:Zm00001d013108_T001          0
 47- 14:transcript:Zm00001d034379_T001 transcript:Zm00001d013104_T001  2.00E-44
 47- 15:transcript:Zm00001d034380_T001 transcript:Zm00001d013100_T001 7.00E-167
 47- 16:transcript:Zm00001d034387_T002 transcript:Zm00001d013098_T002          0
## Alignment 48: score=684.0 e_value=3.8e-40 N=16 1&5 minus
 48-  0:transcript:Zm00001d034059_T006 transcript:Zm00001d013252_T001 2.00E-138
 48-  1:transcript:Zm00001d034062_T001 transcript:Zm00001d013250_T003  6.00E-72
 48-  2:transcript:Zm00001d034063_T001 transcript:Zm00001d013249_T001          0
 48-  3:transcript:Zm00001d034067_T001 transcript:Zm00001d013246_T001 1.00E-141
 48-  4:transcript:Zm00001d034072_T001 transcript:Zm00001d013245_T001          0
 48-  5:transcript:Zm00001d034076_T001 transcript:Zm00001d013243_T002          0
 48-  6:transcript:Zm00001d034080_T005 transcript:Zm00001d013241_T001          0
 48-  7:transcript:Zm00001d034082_T001 transcript:Zm00001d013240_T002          0
 48-  8:transcript:Zm00001d034085_T002 transcript:Zm00001d013239_T002          0
 48-  9:transcript:Zm00001d034087_T001 transcript:Zm00001d013238_T001  5.00E-31
 48- 10:transcript:Zm00001d034091_T001 transcript:Zm00001d013232_T001 1.00E-103
 48- 11:transcript:Zm00001d034097_T001 transcript:Zm00001d013230_T001 1.00E-145
 48- 12:transcript:Zm00001d034107_T001 transcript:Zm00001d013226_T001 3.00E-172
 48- 13:transcript:Zm00001d034109_T001 transcript:Zm00001d013220_T001          0
 48- 14:transcript:Zm00001d034110_T001 transcript:Zm00001d013219_T001  3.00E-26
 48- 15:transcript:Zm00001d034115_T001 transcript:Zm00001d013218_T003  1.00E-16
## Alignment 49: score=512.0 e_value=2.8e-27 N=12 1&5 minus
 49-  0:transcript:Zm00001d033870_T001 transcript:Zm00001d013357_T001          0
 49-  1:transcript:Zm00001d033874_T002 transcript:Zm00001d013348_T001  2.00E-62
 49-  2:transcript:Zm00001d033876_T004 transcript:Zm00001d013346_T001          0
 49-  3:transcript:Zm00001d033878_T002 transcript:Zm00001d013343_T001          0
```

```
 49-  4:transcript:Zm00001d033884_T001 transcript:Zm00001d013342_T001          0
 49-  5:transcript:Zm00001d033897_T002 transcript:Zm00001d013339_T001  3.00E-35
 49-  6:transcript:Zm00001d033903_T001 transcript:Zm00001d013336_T002          0
 49-  7:transcript:Zm00001d033906_T001 transcript:Zm00001d013335_T001          0
 49-  8:transcript:Zm00001d033912_T001 transcript:Zm00001d013330_T001          0
 49-  9:transcript:Zm00001d033913_T001 transcript:Zm00001d013329_T001  1.00E-98
 49- 10:transcript:Zm00001d033919_T001 transcript:Zm00001d013323_T001          0
 49- 11:transcript:Zm00001d033927_T004 transcript:Zm00001d013318_T035          0
## Alignment 50: score=474.0 e_value=3.7e-25 N=11 1&5 minus
 50-  0:transcript:Zm00001d034505_T001 transcript:Zm00001d013055_T002 7.00E-100
 50-  1:transcript:Zm00001d034507_T001 transcript:Zm00001d013054_T001  2.00E-22
 50-  2:transcript:Zm00001d034511_T001 transcript:Zm00001d013052_T001          0
 50-  3:transcript:Zm00001d034512_T002 transcript:Zm00001d013050_T001  2.00E-30
 50-  4:transcript:Zm00001d034516_T001 transcript:Zm00001d013048_T009          0
 50-  5:transcript:Zm00001d034517_T001 transcript:Zm00001d013047_T004          0
 50-  6:transcript:Zm00001d034518_T001 transcript:Zm00001d013046_T002          0
 50-  7:transcript:Zm00001d034520_T003 transcript:Zm00001d013045_T001  3.00E-75
 50-  8:transcript:Zm00001d034525_T001 transcript:Zm00001d013043_T001 4.00E-178
 50-  9:transcript:Zm00001d034527_T001 transcript:Zm00001d013042_T001          0
 50- 10:transcript:Zm00001d034528_T001 transcript:Zm00001d013041_T003  1.00E-55
## Alignment 51: score=439.0 e_value=8.1e-22 N=10 1&5 minus
 51-  0:transcript:Zm00001d033730_T001 transcript:Zm00001d013439_T007  2.00E-36
 51-  1:transcript:Zm00001d033741_T002 transcript:Zm00001d013432_T001  7.00E-17
 51-  2:transcript:Zm00001d033744_T001 transcript:Zm00001d013431_T002 4.00E-103
 51-  3:transcript:Zm00001d033745_T001 transcript:Zm00001d013430_T001          0
 51-  4:transcript:Zm00001d033746_T003 transcript:Zm00001d013428_T002          0
 51-  5:transcript:Zm00001d033750_T003 transcript:Zm00001d013427_T003          0
 51-  6:transcript:Zm00001d033751_T001 transcript:Zm00001d013426_T002          0
 51-  7:transcript:Zm00001d033753_T001 transcript:Zm00001d013424_T001          0
 51-  8:transcript:Zm00001d033754_T001 transcript:Zm00001d013423_T001          0
 51-  9:transcript:Zm00001d033765_T001 transcript:Zm00001d013418_T001          0
## Alignment 52: score=425.0 e_value=1.9e-22 N=10 1&5 minus
 52-  0:transcript:Zm00001d032600_T001 transcript:Zm00001d013962_T003          0
 52-  1:transcript:Zm00001d032601_T002 transcript:Zm00001d013960_T001          0
 52-  2:transcript:Zm00001d032603_T001 transcript:Zm00001d013958_T004          0
 52-  3:transcript:Zm00001d032609_T002 transcript:Zm00001d013957_T001          0
 52-  4:transcript:Zm00001d032610_T003 transcript:Zm00001d013956_T003 8.00E-163
 52-  5:transcript:Zm00001d032618_T001 transcript:Zm00001d013950_T001 9.00E-117
 52-  6:transcript:Zm00001d032620_T001 transcript:Zm00001d013947_T001 3.00E-122
 52-  7:transcript:Zm00001d032624_T001 transcript:Zm00001d013945_T001  2.00E-87
 52-  8:transcript:Zm00001d032633_T004 transcript:Zm00001d013944_T001  3.00E-45
 52-  9:transcript:Zm00001d032636_T001 transcript:Zm00001d013943_T001 2.00E-140
## Alignment 53: score=362.0 e_value=1.9e-16 N=9 1&5 minus
 53-  0:transcript:Zm00001d031081_T001 transcript:Zm00001d013692_T001          0
 53-  1:transcript:Zm00001d031086_T001 transcript:Zm00001d013689_T001          0
 53-  2:transcript:Zm00001d031088_T001 transcript:Zm00001d013680_T002          0
 53-  3:transcript:Zm00001d031092_T001 transcript:Zm00001d013676_T001  1.00E-74
 53-  4:transcript:Zm00001d031094_T001 transcript:Zm00001d013672_T001 3.00E-148
 53-  5:transcript:Zm00001d031101_T002 transcript:Zm00001d013667_T001  1.00E-38
 53-  6:transcript:Zm00001d031109_T002 transcript:Zm00001d013661_T001  8.00E-20
 53-  7:transcript:Zm00001d031113_T001 transcript:Zm00001d013660_T001  1.00E-15
 53-  8:transcript:Zm00001d031119_T001 transcript:Zm00001d013659_T001  6.00E-91
## Alignment 54: score=354.0 e_value=7.9e-15 N=8 1&5 minus
 54-  0:transcript:Zm00001d034425_T001 transcript:Zm00001d013082_T001  7.00E-49
```

54- 1:transcript:Zm00001d034427_T001 transcript:Zm00001d013080_T001 0
54- 2:transcript:Zm00001d034429_T001 transcript:Zm00001d013079_T001 2.00E-105
54- 3:transcript:Zm00001d034431_T001 transcript:Zm00001d013078_T004 3.00E-78
54- 4:transcript:Zm00001d034439_T001 transcript:Zm00001d013077_T004 3.00E-171
54- 5:transcript:Zm00001d034440_T001 transcript:Zm00001d013076_T001 0
54- 6:transcript:Zm00001d034446_T002 transcript:Zm00001d013075_T001 0
54- 7:transcript:Zm00001d034447_T001 transcript:Zm00001d013074_T003 4.00E-148
Alignment 55: score=351.0 e_value=3.7e-17 N=8 1&5 minus
55- 0:transcript:Zm00001d034456_T003 transcript:Zm00001d013075_T001 0
55- 1:transcript:Zm00001d034457_T003 transcript:Zm00001d013074_T003 0
55- 2:transcript:Zm00001d034462_T001 transcript:Zm00001d013072_T001 3.00E-44
55- 3:transcript:Zm00001d034463_T003 transcript:Zm00001d013071_T001 4.00E-75
55- 4:transcript:Zm00001d034467_T001 transcript:Zm00001d013070_T001 1.00E-149
55- 5:transcript:Zm00001d034469_T001 transcript:Zm00001d013069_T001 9.00E-78
55- 6:transcript:Zm00001d034479_T001 transcript:Zm00001d013066_T002 2.00E-43
55- 7:transcript:Zm00001d034480_T002 transcript:Zm00001d013065_T002 5.00E-176
Alignment 56: score=348.0 e_value=1.1e-16 N=9 1&5 minus
56- 0:transcript:Zm00001d033798_T030 transcript:Zm00001d013405_T004 0
56- 1:transcript:Zm00001d033799_T022 transcript:Zm00001d013402_T025 0
56- 2:transcript:Zm00001d033815_T001 transcript:Zm00001d013399_T001 2.00E-142
56- 3:transcript:Zm00001d033818_T001 transcript:Zm00001d013397_T002 8.00E-116
56- 4:transcript:Zm00001d033822_T001 transcript:Zm00001d013392_T001 2.00E-92
56- 5:transcript:Zm00001d033825_T001 transcript:Zm00001d013391_T004 0
56- 6:transcript:Zm00001d033830_T001 transcript:Zm00001d013389_T014 0
56- 7:transcript:Zm00001d033834_T001 transcript:Zm00001d013385_T001 0
56- 8:transcript:Zm00001d033835_T001 transcript:Zm00001d013381_T001 1.00E-40
Alignment 57: score=345.0 e_value=4.7e-18 N=9 1&5 minus
57- 0:transcript:Zm00001d034388_T010 transcript:Zm00001d013098_T002 0
57- 1:transcript:Zm00001d034399_T001 transcript:Zm00001d013095_T001 0
57- 2:transcript:Zm00001d034400_T003 transcript:Zm00001d013094_T001 0
57- 3:transcript:Zm00001d034401_T004 transcript:Zm00001d013093_T004 0
57- 4:transcript:Zm00001d034404_T001 transcript:Zm00001d013092_T002 5.00E-102
57- 5:transcript:Zm00001d034410_T001 transcript:Zm00001d013090_T002 0
57- 6:transcript:Zm00001d034414_T001 transcript:Zm00001d013089_T002 9.00E-15
57- 7:transcript:Zm00001d034417_T001 transcript:Zm00001d013087_T001 6.00E-81
57- 8:transcript:Zm00001d034422_T002 transcript:Zm00001d013086_T004 6.00E-85
Alignment 58: score=340.0 e_value=4.1e-16 N=8 1&5 minus
58- 0:transcript:Zm00001d033943_T001 transcript:Zm00001d013311_T001 3.00E-55
58- 1:transcript:Zm00001d033951_T001 transcript:Zm00001d013309_T001 3.00E-72
58- 2:transcript:Zm00001d033965_T001 transcript:Zm00001d013307_T003 0
58- 3:transcript:Zm00001d033966_T001 transcript:Zm00001d013306_T001 4.00E-136
58- 4:transcript:Zm00001d033967_T001 transcript:Zm00001d013303_T001 0
58- 5:transcript:Zm00001d033975_T002 transcript:Zm00001d013301_T004 0
58- 6:transcript:Zm00001d033979_T001 transcript:Zm00001d013300_T002 1.00E-93
58- 7:transcript:Zm00001d033981_T001 transcript:Zm00001d013296_T001 0
Alignment 59: score=319.0 e_value=1.4e-12 N=7 1&5 minus
59- 0:transcript:Zm00001d034125_T002 transcript:Zm00001d013217_T001 0
59- 1:transcript:Zm00001d034126_T001 transcript:Zm00001d013216_T001 1.00E-102
59- 2:transcript:Zm00001d034128_T001 transcript:Zm00001d013212_T001 0
59- 3:transcript:Zm00001d034130_T001 transcript:Zm00001d013210_T001 1.00E-45
59- 4:transcript:Zm00001d034131_T001 transcript:Zm00001d013209_T001 2.00E-148
59- 5:transcript:Zm00001d034145_T001 transcript:Zm00001d013208_T001 2.00E-111
59- 6:transcript:Zm00001d034152_T002 transcript:Zm00001d013206_T003 0
Alignment 60: score=307.0 e_value=1.9e-12 N=7 1&5 minus

60- 0:transcript:Zm00001d034601_T001 transcript:Zm00001d013003_T001 2.00E-174
60- 1:transcript:Zm00001d034602_T003 transcript:Zm00001d012999_T001 6.00E-146
60- 2:transcript:Zm00001d034604_T001 transcript:Zm00001d012996_T001 0
60- 3:transcript:Zm00001d034606_T001 transcript:Zm00001d012993_T001 0
60- 4:transcript:Zm00001d034609_T001 transcript:Zm00001d012992_T002 2.00E-64
60- 5:transcript:Zm00001d034618_T002 transcript:Zm00001d012984_T002 0
60- 6:transcript:Zm00001d034620_T001 transcript:Zm00001d012983_T001 1.00E-108
Alignment 61: score=286.0 e_value=2.7e-12 N=7 1&5 minus
61- 0:transcript:Zm00001d034740_T001 transcript:Zm00001d012907_T001 2.00E-56
61- 1:transcript:Zm00001d034745_T001 transcript:Zm00001d012906_T001 5.00E-59
61- 2:transcript:Zm00001d034752_T006 transcript:Zm00001d012896_T010 0
61- 3:transcript:Zm00001d034754_T001 transcript:Zm00001d012895_T003 0
61- 4:transcript:Zm00001d034755_T001 transcript:Zm00001d012893_T002 0
61- 5:transcript:Zm00001d034758_T003 transcript:Zm00001d012892_T002 0
61- 6:transcript:Zm00001d034763_T001 transcript:Zm00001d012889_T001 8.00E-31
Alignment 62: score=272.0 e_value=3.9e-09 N=6 1&5 minus
62- 0:transcript:Zm00001d033054_T001 transcript:Zm00001d013817_T001 6.00E-64
62- 1:transcript:Zm00001d033060_T001 transcript:Zm00001d013814_T001 0
62- 2:transcript:Zm00001d033061_T002 transcript:Zm00001d013809_T002 0
62- 3:transcript:Zm00001d033064_T001 transcript:Zm00001d013806_T001 8.00E-54
62- 4:transcript:Zm00001d033075_T001 transcript:Zm00001d013801_T001 7.00E-158
62- 5:transcript:Zm00001d033077_T001 transcript:Zm00001d013799_T001 0
Alignment 63: score=266.0 e_value=4.3e-08 N=6 1&5 minus
63- 0:transcript:Zm00001d034559_T001 transcript:Zm00001d013032_T001 0
63- 1:transcript:Zm00001d034561_T001 transcript:Zm00001d013031_T002 5.00E-50
63- 2:transcript:Zm00001d034562_T003 transcript:Zm00001d013030_T002 1.00E-144
63- 3:transcript:Zm00001d034572_T001 transcript:Zm00001d013028_T001 4.00E-177
63- 4:transcript:Zm00001d034575_T001 transcript:Zm00001d013027_T002 0
63- 5:transcript:Zm00001d034577_T003 transcript:Zm00001d013026_T001 4.00E-20
Alignment 64: score=261.0 e_value=5.4e-11 N=6 1&5 minus
64- 0:transcript:Zm00001d033128_T001 transcript:Zm00001d013777_T001 2.00E-154
64- 1:transcript:Zm00001d033129_T001 transcript:Zm00001d013768_T002 9.00E-58
64- 2:transcript:Zm00001d033130_T004 transcript:Zm00001d013767_T001 0
64- 3:transcript:Zm00001d033132_T001 transcript:Zm00001d013766_T001 4.00E-89
64- 4:transcript:Zm00001d033139_T001 transcript:Zm00001d013759_T001 0
64- 5:transcript:Zm00001d033147_T004 transcript:Zm00001d013758_T007 1.00E-84
Alignment 65: score=254.0 e_value=6.9e-11 N=6 1&5 minus
65- 0:transcript:Zm00001d033088_T001 transcript:Zm00001d013785_T001 2.00E-38
65- 1:transcript:Zm00001d033093_T001 transcript:Zm00001d013783_T001 7.00E-51
65- 2:transcript:Zm00001d033098_T001 transcript:Zm00001d013781_T001 4.00E-171
65- 3:transcript:Zm00001d033099_T001 transcript:Zm00001d013780_T002 1.00E-96
65- 4:transcript:Zm00001d033105_T001 transcript:Zm00001d013779_T001 7.00E-87
65- 5:transcript:Zm00001d033108_T007 transcript:Zm00001d013778_T001 0
Alignment 66: score=253.0 e_value=1.9e-09 N=6 1&5 minus
66- 0:transcript:Zm00001d033777_T001 transcript:Zm00001d013415_T001 0
66- 1:transcript:Zm00001d033786_T002 transcript:Zm00001d013412_T003 0
66- 2:transcript:Zm00001d033787_T001 transcript:Zm00001d013411_T003 0
66- 3:transcript:Zm00001d033790_T002 transcript:Zm00001d013410_T002 0
66- 4:transcript:Zm00001d033791_T001 transcript:Zm00001d013409_T001 2.00E-101
66- 5:transcript:Zm00001d033797_T003 transcript:Zm00001d013406_T002 0
Alignment 67: score=452.0 e_value=9.6e-23 N=10 1&7 plus
67- 0:transcript:Zm00001d031545_T001 transcript:Zm00001d021006_T001 2.00E-40
67- 1:transcript:Zm00001d031546_T001 transcript:Zm00001d021010_T001 3.00E-09
67- 2:transcript:Zm00001d031554_T003 transcript:Zm00001d021014_T001 0

67- 3:transcript:Zm00001d031555_T001 transcript:Zm00001d021016_T001 9.00E-161
67- 4:transcript:Zm00001d031560_T001 transcript:Zm00001d021018_T002 1.00E-88
67- 5:transcript:Zm00001d031561_T002 transcript:Zm00001d021019_T001 1.00E-91
67- 6:transcript:Zm00001d031569_T001 transcript:Zm00001d021021_T001 2.00E-28
67- 7:transcript:Zm00001d031570_T001 transcript:Zm00001d021023_T001 4.00E-21
67- 8:transcript:Zm00001d031571_T001 transcript:Zm00001d021024_T001 0
67- 9:transcript:Zm00001d031577_T001 transcript:Zm00001d021026_T001 0
Alignment 68: score=409.0 e_value=2.8e-20 N=10 1&7 plus
68- 0:transcript:Zm00001d034427_T001 transcript:Zm00001d018931_T001 8.00E-126
68- 1:transcript:Zm00001d034432_T001 transcript:Zm00001d018938_T001 1.00E-119
68- 2:transcript:Zm00001d034433_T003 transcript:Zm00001d018941_T001 9.00E-154
68- 3:transcript:Zm00001d034439_T001 transcript:Zm00001d018947_T001 5.00E-22
68- 4:transcript:Zm00001d034440_T001 transcript:Zm00001d018957_T001 0
68- 5:transcript:Zm00001d034443_T001 transcript:Zm00001d018961_T002 0
68- 6:transcript:Zm00001d034444_T002 transcript:Zm00001d018962_T001 3.00E-126
68- 7:transcript:Zm00001d034446_T002 transcript:Zm00001d018964_T003 0
68- 8:transcript:Zm00001d034452_T009 transcript:Zm00001d018969_T001 4.00E-143
68- 9:transcript:Zm00001d034455_T001 transcript:Zm00001d018971_T003 2.00E-41
Alignment 69: score=396.0 e_value=1.2e-16 N=9 1&7 plus
69- 0:transcript:Zm00001d032283_T004 transcript:Zm00001d020233_T001 2.00E-124
69- 1:transcript:Zm00001d032284_T001 transcript:Zm00001d020238_T002 0
69- 2:transcript:Zm00001d032286_T001 transcript:Zm00001d020243_T001 3.00E-33
69- 3:transcript:Zm00001d032287_T001 transcript:Zm00001d020251_T001 8.00E-08
69- 4:transcript:Zm00001d032291_T001 transcript:Zm00001d020257_T001 3.00E-61
69- 5:transcript:Zm00001d032292_T001 transcript:Zm00001d020264_T001 1.00E-42
69- 6:transcript:Zm00001d032295_T001 transcript:Zm00001d020267_T001 5.00E-84
69- 7:transcript:Zm00001d032298_T002 transcript:Zm00001d020272_T001 0
69- 8:transcript:Zm00001d032300_T001 transcript:Zm00001d020277_T001 3.00E-180
Alignment 70: score=346.0 e_value=1.4e-18 N=9 1&7 plus
70- 0:transcript:Zm00001d031640_T001 transcript:Zm00001d021061_T001 0
70- 1:transcript:Zm00001d031647_T001 transcript:Zm00001d021064_T001 2.00E-112
70- 2:transcript:Zm00001d031648_T004 transcript:Zm00001d021065_T001 0
70- 3:transcript:Zm00001d031659_T001 transcript:Zm00001d021070_T002 0
70- 4:transcript:Zm00001d031660_T002 transcript:Zm00001d021072_T001 0
70- 5:transcript:Zm00001d031665_T001 transcript:Zm00001d021086_T001 1.00E-82
70- 6:transcript:Zm00001d031667_T006 transcript:Zm00001d021087_T003 0
70- 7:transcript:Zm00001d031673_T001 transcript:Zm00001d021089_T001 5.00E-26
70- 8:transcript:Zm00001d031676_T002 transcript:Zm00001d021090_T001 0
Alignment 71: score=346.0 e_value=3.3e-15 N=8 1&7 plus
71- 0:transcript:Zm00001d031416_T001 transcript:Zm00001d020923_T001 4.00E-137
71- 1:transcript:Zm00001d031423_T001 transcript:Zm00001d020926_T001 8.00E-55
71- 2:transcript:Zm00001d031426_T001 transcript:Zm00001d020927_T001 5.00E-167
71- 3:transcript:Zm00001d031430_T005 transcript:Zm00001d020929_T002 0
71- 4:transcript:Zm00001d031431_T001 transcript:Zm00001d020930_T001 9.00E-68
71- 5:transcript:Zm00001d031441_T002 transcript:Zm00001d020932_T001 6.00E-118
71- 6:transcript:Zm00001d031444_T001 transcript:Zm00001d020937_T001 0
71- 7:transcript:Zm00001d031445_T001 transcript:Zm00001d020939_T001 0
Alignment 72: score=326.0 e_value=7.5e-16 N=8 1&7 plus
72- 0:transcript:Zm00001d034190_T001 transcript:Zm00001d018717_T001 4.00E-35
72- 1:transcript:Zm00001d034191_T004 transcript:Zm00001d018719_T001 0
72- 2:transcript:Zm00001d034199_T001 transcript:Zm00001d018729_T001 1.00E-14
72- 3:transcript:Zm00001d034200_T001 transcript:Zm00001d018730_T001 9.00E-34
72- 4:transcript:Zm00001d034204_T001 transcript:Zm00001d018731_T001 0
72- 5:transcript:Zm00001d034212_T001 transcript:Zm00001d018742_T001 3.00E-113

```
 72-  6:transcript:Zm00001d034221_T001 transcript:Zm00001d018749_T001 1.00E-154
 72-  7:transcript:Zm00001d034237_T001 transcript:Zm00001d018754_T001         0
## Alignment 73: score=273.0 e_value=2.5e-10 N=7 1&7 plus
 73-  0:transcript:Zm00001d031801_T001 transcript:Zm00001d020736_T001 4.00E-130
 73-  1:transcript:Zm00001d031806_T001 transcript:Zm00001d020746_T001  3.00E-09
 73-  2:transcript:Zm00001d031810_T001 transcript:Zm00001d020757_T001 7.00E-103
 73-  3:transcript:Zm00001d031818_T001 transcript:Zm00001d020763_T001  4.00E-58
 73-  4:transcript:Zm00001d031826_T001 transcript:Zm00001d020768_T001  1.00E-54
 73-  5:transcript:Zm00001d031840_T001 transcript:Zm00001d020774_T001 9.00E-108
 73-  6:transcript:Zm00001d031844_T001 transcript:Zm00001d020776_T002         0
## Alignment 74: score=262.0 e_value=5.1e-10 N=6 1&7 plus
 74-  0:transcript:Zm00001d031777_T001 transcript:Zm00001d020697_T001 4.00E-168
 74-  1:transcript:Zm00001d031781_T001 transcript:Zm00001d020705_T001  1.00E-53
 74-  2:transcript:Zm00001d031790_T002 transcript:Zm00001d020711_T001  9.00E-85
 74-  3:transcript:Zm00001d031792_T001 transcript:Zm00001d020713_T001 1.00E-155
 74-  4:transcript:Zm00001d031797_T001 transcript:Zm00001d020723_T001  3.00E-22
 74-  5:transcript:Zm00001d031798_T001 transcript:Zm00001d020725_T001  6.00E-21
## Alignment 75: score=349.0 e_value=8.5e-17 N=9 1&7 minus
 75-  0:transcript:Zm00001d029260_T004 transcript:Zm00001d022315_T001         0
 75-  1:transcript:Zm00001d029263_T001 transcript:Zm00001d022307_T003  2.00E-49
 75-  2:transcript:Zm00001d029264_T004 transcript:Zm00001d022305_T001         0
 75-  3:transcript:Zm00001d029270_T001 transcript:Zm00001d022295_T001  1.00E-94
 75-  4:transcript:Zm00001d029274_T001 transcript:Zm00001d022283_T001 2.00E-105
 75-  5:transcript:Zm00001d029279_T001 transcript:Zm00001d022280_T001 2.00E-114
 75-  6:transcript:Zm00001d029281_T007 transcript:Zm00001d022275_T001 3.00E-149
 75-  7:transcript:Zm00001d029285_T005 transcript:Zm00001d022274_T001         0
 75-  8:transcript:Zm00001d029286_T001 transcript:Zm00001d022273_T016  9.00E-59
## Alignment 76: score=289.0 e_value=2.4e-10 N=6 1&7 minus
 76-  0:transcript:Zm00001d032175_T001 transcript:Zm00001d020460_T001  3.00E-86
 76-  1:transcript:Zm00001d032177_T001 transcript:Zm00001d020459_T001  1.00E-31
 76-  2:transcript:Zm00001d032178_T001 transcript:Zm00001d020457_T001 3.00E-122
 76-  3:transcript:Zm00001d032181_T001 transcript:Zm00001d020450_T001  5.00E-77
 76-  4:transcript:Zm00001d032182_T001 transcript:Zm00001d020446_T001 3.00E-134
 76-  5:transcript:Zm00001d032183_T002 transcript:Zm00001d020445_T001 3.00E-107
## Alignment 77: score=984.0 e_value=7.6e-72 N=24 1&9 plus
 77-  0:transcript:Zm00001d027503_T036 transcript:Zm00001d048338_T035         0
 77-  1:transcript:Zm00001d027506_T003 transcript:Zm00001d048340_T002         0
 77-  2:transcript:Zm00001d027508_T001 transcript:Zm00001d048341_T001         0
 77-  3:transcript:Zm00001d027509_T002 transcript:Zm00001d048342_T001 3.00E-170
 77-  4:transcript:Zm00001d027514_T002 transcript:Zm00001d048343_T002         0
 77-  5:transcript:Zm00001d027520_T001 transcript:Zm00001d048344_T002 2.00E-153
 77-  6:transcript:Zm00001d027523_T001 transcript:Zm00001d048345_T003         0
 77-  7:transcript:Zm00001d027527_T001 transcript:Zm00001d048346_T001         0
 77-  8:transcript:Zm00001d027530_T001 transcript:Zm00001d048347_T001         0
 77-  9:transcript:Zm00001d027532_T002 transcript:Zm00001d048349_T002 2.00E-179
 77- 10:transcript:Zm00001d027540_T001 transcript:Zm00001d048353_T001 3.00E-166
 77- 11:transcript:Zm00001d027548_T001 transcript:Zm00001d048356_T001         0
 77- 12:transcript:Zm00001d027558_T004 transcript:Zm00001d048358_T004         0
 77- 13:transcript:Zm00001d027570_T005 transcript:Zm00001d048359_T004         0
 77- 14:transcript:Zm00001d027573_T001 transcript:Zm00001d048360_T001         0
 77- 15:transcript:Zm00001d027578_T001 transcript:Zm00001d048362_T002 2.00E-150
 77- 16:transcript:Zm00001d027585_T001 transcript:Zm00001d048363_T001 4.00E-122
 77- 17:transcript:Zm00001d027589_T001 transcript:Zm00001d048366_T001         0
 77- 18:transcript:Zm00001d027595_T001 transcript:Zm00001d048368_T007  9.00E-23
```

77- 19:transcript:Zm00001d027598_T001 transcript:Zm00001d048369_T002 1.00E-143
77- 20:transcript:Zm00001d027599_T001 transcript:Zm00001d048370_T001 0
77- 21:transcript:Zm00001d027612_T007 transcript:Zm00001d048372_T001 0
77- 22:transcript:Zm00001d027618_T001 transcript:Zm00001d048382_T001 2.00E-116
77- 23:transcript:Zm00001d027623_T001 transcript:Zm00001d048383_T001 6.00E-113
Alignment 78: score=528.0 e_value=2.1e-29 N=12 1&9 plus
78- 0:transcript:Zm00001d027674_T001 transcript:Zm00001d048396_T001 9.00E-89
78- 1:transcript:Zm00001d027675_T002 transcript:Zm00001d048397_T002 5.00E-116
78- 2:transcript:Zm00001d027677_T001 transcript:Zm00001d048400_T001 0
78- 3:transcript:Zm00001d027678_T001 transcript:Zm00001d048401_T001 1.00E-48
78- 4:transcript:Zm00001d027680_T003 transcript:Zm00001d048402_T001 1.00E-77
78- 5:transcript:Zm00001d027688_T002 transcript:Zm00001d048403_T003 4.00E-175
78- 6:transcript:Zm00001d027702_T001 transcript:Zm00001d048404_T005 0
78- 7:transcript:Zm00001d027703_T001 transcript:Zm00001d048407_T001 1.00E-133
78- 8:transcript:Zm00001d027707_T001 transcript:Zm00001d048409_T001 0
78- 9:transcript:Zm00001d027708_T002 transcript:Zm00001d048411_T001 2.00E-116
78- 10:transcript:Zm00001d027709_T001 transcript:Zm00001d048412_T001 0
78- 11:transcript:Zm00001d027710_T001 transcript:Zm00001d048413_T001 0
Alignment 79: score=498.0 e_value=5.8e-29 N=12 1&9 plus
79- 0:transcript:Zm00001d029553_T001 transcript:Zm00001d047298_T001 4.00E-129
79- 1:transcript:Zm00001d029557_T001 transcript:Zm00001d047302_T001 0
79- 2:transcript:Zm00001d029560_T001 transcript:Zm00001d047303_T001 3.00E-175
79- 3:transcript:Zm00001d029561_T002 transcript:Zm00001d047306_T007 0
79- 4:transcript:Zm00001d029564_T001 transcript:Zm00001d047309_T001 0
79- 5:transcript:Zm00001d029576_T002 transcript:Zm00001d047310_T001 2.00E-80
79- 6:transcript:Zm00001d029578_T001 transcript:Zm00001d047325_T001 7.00E-67
79- 7:transcript:Zm00001d029584_T001 transcript:Zm00001d047330_T001 6.00E-72
79- 8:transcript:Zm00001d029585_T001 transcript:Zm00001d047331_T001 0
79- 9:transcript:Zm00001d029589_T001 transcript:Zm00001d047333_T001 3.00E-18
79- 10:transcript:Zm00001d029590_T001 transcript:Zm00001d047335_T001 0
79- 11:transcript:Zm00001d029594_T001 transcript:Zm00001d047340_T001 4.00E-149
Alignment 80: score=498.0 e_value=1.2e-29 N=13 1&9 plus
80- 0:transcript:Zm00001d029329_T011 transcript:Zm00001d047174_T001 2.00E-46
80- 1:transcript:Zm00001d029331_T001 transcript:Zm00001d047178_T009 0
80- 2:transcript:Zm00001d029333_T008 transcript:Zm00001d047181_T005 0
80- 3:transcript:Zm00001d029336_T005 transcript:Zm00001d047183_T002 0
80- 4:transcript:Zm00001d029340_T003 transcript:Zm00001d047186_T001 3.00E-60
80- 5:transcript:Zm00001d029350_T001 transcript:Zm00001d047190_T001 0
80- 6:transcript:Zm00001d029352_T002 transcript:Zm00001d047191_T003 0
80- 7:transcript:Zm00001d029359_T001 transcript:Zm00001d047192_T001 0
80- 8:transcript:Zm00001d029372_T001 transcript:Zm00001d047201_T001 0
80- 9:transcript:Zm00001d029376_T001 transcript:Zm00001d047203_T001 5.00E-64
80- 10:transcript:Zm00001d029378_T012 transcript:Zm00001d047204_T003 0
80- 11:transcript:Zm00001d029383_T001 transcript:Zm00001d047208_T001 2.00E-131
80- 12:transcript:Zm00001d029387_T001 transcript:Zm00001d047210_T001 1.00E-161
Alignment 81: score=474.0 e_value=6.5e-24 N=11 1&9 plus
81- 0:transcript:Zm00001d028144_T001 transcript:Zm00001d048178_T001 0
81- 1:transcript:Zm00001d028153_T001 transcript:Zm00001d048181_T003 2.00E-68
81- 2:transcript:Zm00001d028159_T001 transcript:Zm00001d048185_T001 4.00E-61
81- 3:transcript:Zm00001d028161_T001 transcript:Zm00001d048188_T001 2.00E-38
81- 4:transcript:Zm00001d028164_T001 transcript:Zm00001d048189_T001 0
81- 5:transcript:Zm00001d028167_T001 transcript:Zm00001d048191_T001 0
81- 6:transcript:Zm00001d028171_T001 transcript:Zm00001d048195_T001 0
81- 7:transcript:Zm00001d028173_T002 transcript:Zm00001d048196_T001 1.00E-34

81- 8:transcript:Zm00001d028175_T001 transcript:Zm00001d048197_T001 2.00E-31
81- 9:transcript:Zm00001d028180_T002 transcript:Zm00001d048201_T002   0
81- 10:transcript:Zm00001d028181_T001 transcript:Zm00001d048204_T001 2.00E-98
Alignment 82: score=365.0 e_value=7.1e-18 N=9 1&9 plus
82- 0:transcript:Zm00001d028036_T001 transcript:Zm00001d048121_T001 1.00E-38
82- 1:transcript:Zm00001d028040_T002 transcript:Zm00001d048122_T003 3.00E-85
82- 2:transcript:Zm00001d028043_T001 transcript:Zm00001d048123_T001 2.00E-53
82- 3:transcript:Zm00001d028045_T007 transcript:Zm00001d048126_T003   0
82- 4:transcript:Zm00001d028053_T001 transcript:Zm00001d048132_T002 3.00E-83
82- 5:transcript:Zm00001d028055_T001 transcript:Zm00001d048134_T001 5.00E-148
82- 6:transcript:Zm00001d028064_T001 transcript:Zm00001d048135_T002 1.00E-48
82- 7:transcript:Zm00001d028073_T004 transcript:Zm00001d048137_T002   0
82- 8:transcript:Zm00001d028075_T011 transcript:Zm00001d048138_T004   0
Alignment 83: score=364.0 e_value=1.7e-15 N=8 1&9 plus
83- 0:transcript:Zm00001d029896_T001 transcript:Zm00001d047081_T001 1.00E-158
83- 1:transcript:Zm00001d029899_T001 transcript:Zm00001d047087_T001   0
83- 2:transcript:Zm00001d029903_T002 transcript:Zm00001d047089_T001   0
83- 3:transcript:Zm00001d029907_T001 transcript:Zm00001d047091_T001 5.00E-164
83- 4:transcript:Zm00001d029910_T001 transcript:Zm00001d047092_T001   0
83- 5:transcript:Zm00001d029917_T001 transcript:Zm00001d047102_T001 1.00E-144
83- 6:transcript:Zm00001d029918_T001 transcript:Zm00001d047103_T002   0
83- 7:transcript:Zm00001d029920_T001 transcript:Zm00001d047104_T001   0
Alignment 84: score=353.0 e_value=7.8e-19 N=9 1&9 plus
84- 0:transcript:Zm00001d028102_T001 transcript:Zm00001d048149_T002   0
84- 1:transcript:Zm00001d028103_T001 transcript:Zm00001d048151_T001   0
84- 2:transcript:Zm00001d028111_T004 transcript:Zm00001d048154_T001   0
84- 3:transcript:Zm00001d028112_T001 transcript:Zm00001d048155_T001 3.00E-132
84- 4:transcript:Zm00001d028117_T002 transcript:Zm00001d048157_T001   0
84- 5:transcript:Zm00001d028118_T001 transcript:Zm00001d048162_T001 2.00E-97
84- 6:transcript:Zm00001d028125_T001 transcript:Zm00001d048166_T001   0
84- 7:transcript:Zm00001d028129_T002 transcript:Zm00001d048172_T001 3.00E-105
84- 8:transcript:Zm00001d028130_T001 transcript:Zm00001d048173_T001 3.00E-176
Alignment 85: score=314.0 e_value=1.5e-10 N=7 1&9 plus
85- 0:transcript:Zm00001d029738_T001 transcript:Zm00001d047350_T002 4.00E-67
85- 1:transcript:Zm00001d029740_T002 transcript:Zm00001d047354_T001 3.00E-99
85- 2:transcript:Zm00001d029747_T001 transcript:Zm00001d047358_T001 1.00E-96
85- 3:transcript:Zm00001d029749_T001 transcript:Zm00001d047359_T001 4.00E-139
85- 4:transcript:Zm00001d029750_T001 transcript:Zm00001d047361_T002   0
85- 5:transcript:Zm00001d029753_T001 transcript:Zm00001d047362_T003   0
85- 6:transcript:Zm00001d029757_T001 transcript:Zm00001d047364_T001   0
Alignment 86: score=284.0 e_value=7.3e-12 N=7 1&9 plus
86- 0:transcript:Zm00001d027717_T001 transcript:Zm00001d048418_T001 1.00E-101
86- 1:transcript:Zm00001d027719_T001 transcript:Zm00001d048419_T004 3.00E-48
86- 2:transcript:Zm00001d027729_T001 transcript:Zm00001d048422_T001   0
86- 3:transcript:Zm00001d027731_T012 transcript:Zm00001d048424_T003   0
86- 4:transcript:Zm00001d027738_T001 transcript:Zm00001d048428_T001 3.00E-141
86- 5:transcript:Zm00001d027743_T002 transcript:Zm00001d048430_T005   0
86- 6:transcript:Zm00001d027748_T004 transcript:Zm00001d048432_T002   0
Alignment 87: score=262.0 e_value=2.5e-08 N=6 1&9 plus
87- 0:transcript:Zm00001d028930_T001 transcript:Zm00001d047579_T001 5.00E-166
87- 1:transcript:Zm00001d028931_T002 transcript:Zm00001d047582_T002   0
87- 2:transcript:Zm00001d028936_T002 transcript:Zm00001d047587_T005 5.00E-133
87- 3:transcript:Zm00001d028941_T001 transcript:Zm00001d047591_T001 7.00E-30
87- 4:transcript:Zm00001d028946_T001 transcript:Zm00001d047594_T001   0

87- 5:transcript:Zm00001d028948_T001 transcript:Zm00001d047597_T001 5.00E-86
Alignment 88: score=1429.0 e_value=6e-115 N=34 1&9 minus
88- 0:transcript:Zm00001d027826_T001 transcript:Zm00001d048336_T003 0
88- 1:transcript:Zm00001d027831_T001 transcript:Zm00001d048335_T001 9.00E-20
88- 2:transcript:Zm00001d027833_T001 transcript:Zm00001d048333_T001 3.00E-89
88- 3:transcript:Zm00001d027839_T001 transcript:Zm00001d048325_T001 3.00E-55
88- 4:transcript:Zm00001d027841_T002 transcript:Zm00001d048324_T001 1.00E-180
88- 5:transcript:Zm00001d027842_T003 transcript:Zm00001d048323_T001 9.00E-167
88- 6:transcript:Zm00001d027844_T001 transcript:Zm00001d048321_T001 1.00E-18
88- 7:transcript:Zm00001d027845_T001 transcript:Zm00001d048320_T001 0
88- 8:transcript:Zm00001d027848_T002 transcript:Zm00001d048318_T001 0
88- 9:transcript:Zm00001d027851_T001 transcript:Zm00001d048317_T001 4.00E-58
88- 10:transcript:Zm00001d027852_T001 transcript:Zm00001d048312_T001 0
88- 11:transcript:Zm00001d027854_T006 transcript:Zm00001d048311_T003 0
88- 12:transcript:Zm00001d027856_T001 transcript:Zm00001d048310_T005 0
88- 13:transcript:Zm00001d027859_T001 transcript:Zm00001d048309_T001 2.00E-52
88- 14:transcript:Zm00001d027866_T001 transcript:Zm00001d048303_T001 1.00E-08
88- 15:transcript:Zm00001d027868_T002 transcript:Zm00001d048301_T001 6.00E-134
88- 16:transcript:Zm00001d027869_T002 transcript:Zm00001d048299_T002 0
88- 17:transcript:Zm00001d027870_T001 transcript:Zm00001d048296_T001 7.00E-161
88- 18:transcript:Zm00001d027873_T001 transcript:Zm00001d048294_T001 1.00E-38
88- 19:transcript:Zm00001d027875_T001 transcript:Zm00001d048293_T001 0
88- 20:transcript:Zm00001d027876_T004 transcript:Zm00001d048292_T003 7.00E-154
88- 21:transcript:Zm00001d027877_T001 transcript:Zm00001d048288_T001 1.00E-105
88- 22:transcript:Zm00001d027885_T001 transcript:Zm00001d048284_T001 1.00E-94
88- 23:transcript:Zm00001d027894_T001 transcript:Zm00001d048273_T001 5.00E-69
88- 24:transcript:Zm00001d027895_T004 transcript:Zm00001d048272_T012 0
88- 25:transcript:Zm00001d027896_T001 transcript:Zm00001d048271_T001 0
88- 26:transcript:Zm00001d027898_T002 transcript:Zm00001d048270_T002 0
88- 27:transcript:Zm00001d027899_T001 transcript:Zm00001d048268_T001 4.00E-56
88- 28:transcript:Zm00001d027904_T001 transcript:Zm00001d048262_T001 5.00E-112
88- 29:transcript:Zm00001d027908_T001 transcript:Zm00001d048260_T001 0
88- 30:transcript:Zm00001d027916_T006 transcript:Zm00001d048258_T012 0
88- 31:transcript:Zm00001d027919_T001 transcript:Zm00001d048256_T002 0
88- 32:transcript:Zm00001d027922_T001 transcript:Zm00001d048252_T004 1.00E-129
88- 33:transcript:Zm00001d027934_T002 transcript:Zm00001d048247_T002 0
Alignment 89: score=1011.0 e_value=2.2e-64 N=22 1&9 minus
89- 0:transcript:Zm00001d027962_T001 transcript:Zm00001d048239_T001 3.00E-126
89- 1:transcript:Zm00001d027967_T001 transcript:Zm00001d048238_T001 4.00E-86
89- 2:transcript:Zm00001d027969_T006 transcript:Zm00001d048236_T004 0
89- 3:transcript:Zm00001d027971_T001 transcript:Zm00001d048235_T004 0
89- 4:transcript:Zm00001d027973_T001 transcript:Zm00001d048234_T001 6.00E-97
89- 5:transcript:Zm00001d027975_T001 transcript:Zm00001d048233_T001 0
89- 6:transcript:Zm00001d027978_T001 transcript:Zm00001d048232_T001 0
89- 7:transcript:Zm00001d027983_T001 transcript:Zm00001d048230_T001 5.00E-140
89- 8:transcript:Zm00001d027987_T001 transcript:Zm00001d048229_T001 6.00E-142
89- 9:transcript:Zm00001d027991_T001 transcript:Zm00001d048227_T001 2.00E-105
89- 10:transcript:Zm00001d027992_T001 transcript:Zm00001d048226_T001 2.00E-58
89- 11:transcript:Zm00001d027994_T001 transcript:Zm00001d048225_T001 0
89- 12:transcript:Zm00001d027998_T001 transcript:Zm00001d048224_T001 3.00E-54
89- 13:transcript:Zm00001d028002_T001 transcript:Zm00001d048222_T001 4.00E-162
89- 14:transcript:Zm00001d028004_T001 transcript:Zm00001d048220_T001 0
89- 15:transcript:Zm00001d028008_T001 transcript:Zm00001d048218_T001 5.00E-179
89- 16:transcript:Zm00001d028010_T001 transcript:Zm00001d048217_T001 0

```
 89- 17:transcript:Zm00001d028011_T001 transcript:Zm00001d048215_T001  2.00E-92
 89- 18:transcript:Zm00001d028013_T001 transcript:Zm00001d048210_T001  3.00E-24
 89- 19:transcript:Zm00001d028015_T001 transcript:Zm00001d048209_T001 3.00E-160
 89- 20:transcript:Zm00001d028017_T001 transcript:Zm00001d048208_T001 7.00E-166
 89- 21:transcript:Zm00001d028020_T001 transcript:Zm00001d048205_T003         0
## Alignment 90: score=914.0 e_value=4.7e-64 N=22 1&9 minus
 90-  0:transcript:Zm00001d028339_T018 transcript:Zm00001d047986_T029         0
 90-  1:transcript:Zm00001d028347_T001 transcript:Zm00001d047984_T002  3.00E-61
 90-  2:transcript:Zm00001d028352_T002 transcript:Zm00001d047983_T007 8.00E-160
 90-  3:transcript:Zm00001d028358_T003 transcript:Zm00001d047979_T005         0
 90-  4:transcript:Zm00001d028359_T001 transcript:Zm00001d047977_T004         0
 90-  5:transcript:Zm00001d028361_T001 transcript:Zm00001d047976_T001 1.00E-171
 90-  6:transcript:Zm00001d028366_T001 transcript:Zm00001d047972_T001         0
 90-  7:transcript:Zm00001d028370_T001 transcript:Zm00001d047969_T001         0
 90-  8:transcript:Zm00001d028371_T001 transcript:Zm00001d047968_T001 2.00E-102
 90-  9:transcript:Zm00001d028372_T005 transcript:Zm00001d047967_T003 1.00E-171
 90- 10:transcript:Zm00001d028373_T002 transcript:Zm00001d047965_T002         0
 90- 11:transcript:Zm00001d028377_T001 transcript:Zm00001d047962_T003  1.00E-78
 90- 12:transcript:Zm00001d028380_T002 transcript:Zm00001d047960_T001         0
 90- 13:transcript:Zm00001d028384_T006 transcript:Zm00001d047958_T001  1.00E-80
 90- 14:transcript:Zm00001d028385_T002 transcript:Zm00001d047955_T001         0
 90- 15:transcript:Zm00001d028389_T001 transcript:Zm00001d047944_T001  4.00E-61
 90- 16:transcript:Zm00001d028392_T001 transcript:Zm00001d047942_T001 6.00E-102
 90- 17:transcript:Zm00001d028396_T001 transcript:Zm00001d047941_T001         0
 90- 18:transcript:Zm00001d028399_T001 transcript:Zm00001d047939_T001 4.00E-149
 90- 19:transcript:Zm00001d028400_T001 transcript:Zm00001d047938_T001 1.00E-157
 90- 20:transcript:Zm00001d028405_T002 transcript:Zm00001d047933_T001  3.00E-23
 90- 21:transcript:Zm00001d028406_T001 transcript:Zm00001d047931_T001         0
## Alignment 91: score=829.0 e_value=2.3e-53 N=20 1&9 minus
 91-  0:transcript:Zm00001d028579_T027 transcript:Zm00001d047831_T001  2.00E-47
 91-  1:transcript:Zm00001d028586_T001 transcript:Zm00001d047828_T001         0
 91-  2:transcript:Zm00001d028590_T001 transcript:Zm00001d047824_T001         0
 91-  3:transcript:Zm00001d028593_T002 transcript:Zm00001d047823_T001 2.00E-110
 91-  4:transcript:Zm00001d028598_T001 transcript:Zm00001d047820_T001  4.00E-44
 91-  5:transcript:Zm00001d028601_T002 transcript:Zm00001d047814_T001         0
 91-  6:transcript:Zm00001d028603_T001 transcript:Zm00001d047813_T002         0
 91-  7:transcript:Zm00001d028606_T003 transcript:Zm00001d047812_T001         0
 91-  8:transcript:Zm00001d028608_T006 transcript:Zm00001d047808_T001         0
 91-  9:transcript:Zm00001d028615_T001 transcript:Zm00001d047807_T001         0
 91- 10:transcript:Zm00001d028616_T001 transcript:Zm00001d047806_T001         0
 91- 11:transcript:Zm00001d028619_T007 transcript:Zm00001d047805_T003 3.00E-173
 91- 12:transcript:Zm00001d028620_T001 transcript:Zm00001d047804_T001         0
 91- 13:transcript:Zm00001d028623_T001 transcript:Zm00001d047803_T001  2.00E-65
 91- 14:transcript:Zm00001d028625_T002 transcript:Zm00001d047802_T001         0
 91- 15:transcript:Zm00001d028642_T001 transcript:Zm00001d047801_T001 4.00E-122
 91- 16:transcript:Zm00001d028643_T001 transcript:Zm00001d047800_T001         0
 91- 17:transcript:Zm00001d028653_T005 transcript:Zm00001d047796_T001  3.00E-24
 91- 18:transcript:Zm00001d028655_T001 transcript:Zm00001d047794_T001 1.00E-101
 91- 19:transcript:Zm00001d028656_T001 transcript:Zm00001d047793_T001         0
## Alignment 92: score=695.0 e_value=2e-48 N=17 1&9 minus
 92-  0:transcript:Zm00001d028820_T001 transcript:Zm00001d047687_T002  3.00E-72
 92-  1:transcript:Zm00001d028824_T001 transcript:Zm00001d047685_T001         0
 92-  2:transcript:Zm00001d028825_T002 transcript:Zm00001d047683_T003         0
 92-  3:transcript:Zm00001d028826_T001 transcript:Zm00001d047681_T002  5.00E-52
```

92- 4:transcript:Zm00001d028829_T001 transcript:Zm00001d047680_T001 0
92- 5:transcript:Zm00001d028830_T013 transcript:Zm00001d047677_T016 0
92- 6:transcript:Zm00001d028834_T001 transcript:Zm00001d047676_T002 1.00E-33
92- 7:transcript:Zm00001d028838_T001 transcript:Zm00001d047674_T001 7.00E-87
92- 8:transcript:Zm00001d028840_T002 transcript:Zm00001d047673_T001 0
92- 9:transcript:Zm00001d028841_T001 transcript:Zm00001d047672_T001 7.00E-170
92- 10:transcript:Zm00001d028842_T001 transcript:Zm00001d047671_T001 4.00E-156
92- 11:transcript:Zm00001d028868_T002 transcript:Zm00001d047663_T001 3.00E-162
92- 12:transcript:Zm00001d028887_T001 transcript:Zm00001d047651_T001 0
92- 13:transcript:Zm00001d028889_T001 transcript:Zm00001d047638_T001 0
92- 14:transcript:Zm00001d028890_T002 transcript:Zm00001d047636_T002 0
92- 15:transcript:Zm00001d028894_T001 transcript:Zm00001d047635_T005 0
92- 16:transcript:Zm00001d028895_T001 transcript:Zm00001d047634_T001 4.00E-170
Alignment 93: score=664.0 e_value=1.5e-41 N=15 1&9 minus
93- 0:transcript:Zm00001d028272_T002 transcript:Zm00001d048028_T001 0
93- 1:transcript:Zm00001d028273_T001 transcript:Zm00001d048027_T001 0
93- 2:transcript:Zm00001d028274_T001 transcript:Zm00001d048026_T001 0
93- 3:transcript:Zm00001d028279_T001 transcript:Zm00001d048022_T001 1.00E-46
93- 4:transcript:Zm00001d028282_T001 transcript:Zm00001d048021_T001 0
93- 5:transcript:Zm00001d028284_T001 transcript:Zm00001d048019_T002 0
93- 6:transcript:Zm00001d028285_T003 transcript:Zm00001d048017_T003 0
93- 7:transcript:Zm00001d028286_T001 transcript:Zm00001d048016_T001 0
93- 8:transcript:Zm00001d028287_T001 transcript:Zm00001d048013_T001 0
93- 9:transcript:Zm00001d028294_T001 transcript:Zm00001d048008_T001 0
93- 10:transcript:Zm00001d028297_T001 transcript:Zm00001d047999_T001 2.00E-119
93- 11:transcript:Zm00001d028299_T001 transcript:Zm00001d047998_T001 0
93- 12:transcript:Zm00001d028304_T001 transcript:Zm00001d047995_T001 9.00E-110
93- 13:transcript:Zm00001d028307_T001 transcript:Zm00001d047993_T001 0
93- 14:transcript:Zm00001d028319_T002 transcript:Zm00001d047990_T001 0
Alignment 94: score=660.0 e_value=2e-39 N=15 1&9 minus
94- 0:transcript:Zm00001d027419_T001 transcript:Zm00001d048478_T001 3.00E-157
94- 1:transcript:Zm00001d027425_T001 transcript:Zm00001d048474_T001 8.00E-135
94- 2:transcript:Zm00001d027427_T002 transcript:Zm00001d048473_T003 3.00E-117
94- 3:transcript:Zm00001d027430_T001 transcript:Zm00001d048472_T001 0
94- 4:transcript:Zm00001d027431_T001 transcript:Zm00001d048471_T001 0
94- 5:transcript:Zm00001d027443_T001 transcript:Zm00001d048469_T001 3.00E-169
94- 6:transcript:Zm00001d027446_T001 transcript:Zm00001d048467_T001 0
94- 7:transcript:Zm00001d027447_T006 transcript:Zm00001d048466_T001 2.00E-28
94- 8:transcript:Zm00001d027448_T001 transcript:Zm00001d048463_T012 0
94- 9:transcript:Zm00001d027449_T001 transcript:Zm00001d048462_T001 3.00E-80
94- 10:transcript:Zm00001d027455_T001 transcript:Zm00001d048460_T001 0
94- 11:transcript:Zm00001d027459_T001 transcript:Zm00001d048456_T001 1.00E-98
94- 12:transcript:Zm00001d027461_T001 transcript:Zm00001d048455_T001 1.00E-166
94- 13:transcript:Zm00001d027462_T001 transcript:Zm00001d048454_T005 0
94- 14:transcript:Zm00001d027463_T001 transcript:Zm00001d048453_T001 0
Alignment 95: score=603.0 e_value=9.4e-32 N=13 1&9 minus
95- 0:transcript:Zm00001d028784_T001 transcript:Zm00001d047709_T001 4.00E-168
95- 1:transcript:Zm00001d028786_T001 transcript:Zm00001d047707_T002 2.00E-130
95- 2:transcript:Zm00001d028787_T001 transcript:Zm00001d047706_T001 2.00E-55
95- 3:transcript:Zm00001d028793_T001 transcript:Zm00001d047705_T001 8.00E-118
95- 4:transcript:Zm00001d028799_T001 transcript:Zm00001d047702_T001 1.00E-36
95- 5:transcript:Zm00001d028803_T002 transcript:Zm00001d047701_T002 0
95- 6:transcript:Zm00001d028804_T001 transcript:Zm00001d047699_T001 3.00E-168
95- 7:transcript:Zm00001d028806_T001 transcript:Zm00001d047698_T001 0

```
 95-  8:transcript:Zm00001d028808_T001 transcript:Zm00001d047696_T001  3.00E-85
 95-  9:transcript:Zm00001d028810_T002 transcript:Zm00001d047695_T001         0
 95- 10:transcript:Zm00001d028811_T001 transcript:Zm00001d047694_T001         0
 95- 11:transcript:Zm00001d028812_T005 transcript:Zm00001d047689_T002         0
 95- 12:transcript:Zm00001d028813_T001 transcript:Zm00001d047688_T001 2.00E-128
## Alignment 96: score=543.0 e_value=4.1e-34 N=13 1&9 minus
 96-  0:transcript:Zm00001d029980_T008 transcript:Zm00001d047078_T004         0
 96-  1:transcript:Zm00001d029983_T001 transcript:Zm00001d047077_T001         0
 96-  2:transcript:Zm00001d029988_T001 transcript:Zm00001d047069_T001 1.00E-139
 96-  3:transcript:Zm00001d029996_T001 transcript:Zm00001d047068_T001 2.00E-154
 96-  4:transcript:Zm00001d029997_T003 transcript:Zm00001d047067_T001  5.00E-79
 96-  5:transcript:Zm00001d030002_T002 transcript:Zm00001d047064_T001  1.00E-85
 96-  6:transcript:Zm00001d030004_T001 transcript:Zm00001d047060_T001  3.00E-54
 96-  7:transcript:Zm00001d030014_T006 transcript:Zm00001d047059_T003         0
 96-  8:transcript:Zm00001d030018_T001 transcript:Zm00001d047058_T002  3.00E-62
 96-  9:transcript:Zm00001d030019_T001 transcript:Zm00001d047057_T001 5.00E-101
 96- 10:transcript:Zm00001d030020_T001 transcript:Zm00001d047054_T001         0
 96- 11:transcript:Zm00001d030021_T004 transcript:Zm00001d047053_T004         0
 96- 12:transcript:Zm00001d030023_T001 transcript:Zm00001d047050_T001 4.00E-119
## Alignment 97: score=538.0 e_value=1.2e-31 N=13 1&9 minus
 97-  0:transcript:Zm00001d028667_T001 transcript:Zm00001d047786_T001  6.00E-94
 97-  1:transcript:Zm00001d028668_T001 transcript:Zm00001d047781_T001         0
 97-  2:transcript:Zm00001d028671_T002 transcript:Zm00001d047780_T002         0
 97-  3:transcript:Zm00001d028675_T001 transcript:Zm00001d047779_T001  4.00E-47
 97-  4:transcript:Zm00001d028676_T002 transcript:Zm00001d047777_T004  2.00E-94
 97-  5:transcript:Zm00001d028679_T002 transcript:Zm00001d047772_T002         0
 97-  6:transcript:Zm00001d028685_T001 transcript:Zm00001d047771_T001 4.00E-128
 97-  7:transcript:Zm00001d028687_T001 transcript:Zm00001d047769_T001         0
 97-  8:transcript:Zm00001d028691_T004 transcript:Zm00001d047767_T004 2.00E-138
 97-  9:transcript:Zm00001d028692_T004 transcript:Zm00001d047765_T001 3.00E-130
 97- 10:transcript:Zm00001d028696_T001 transcript:Zm00001d047764_T001  2.00E-97
 97- 11:transcript:Zm00001d028698_T002 transcript:Zm00001d047763_T001         0
 97- 12:transcript:Zm00001d028699_T026 transcript:Zm00001d047762_T045         0
## Alignment 98: score=481.0 e_value=6.4e-25 N=11 1&9 minus
 98-  0:transcript:Zm00001d029846_T001 transcript:Zm00001d047130_T001         0
 98-  1:transcript:Zm00001d029849_T006 transcript:Zm00001d047126_T003         0
 98-  2:transcript:Zm00001d029853_T001 transcript:Zm00001d047124_T001         0
 98-  3:transcript:Zm00001d029855_T001 transcript:Zm00001d047123_T001  1.00E-40
 98-  4:transcript:Zm00001d029856_T001 transcript:Zm00001d047119_T001  1.00E-53
 98-  5:transcript:Zm00001d029858_T001 transcript:Zm00001d047113_T001  1.00E-30
 98-  6:transcript:Zm00001d029859_T001 transcript:Zm00001d047111_T001  2.00E-47
 98-  7:transcript:Zm00001d029862_T001 transcript:Zm00001d047109_T001  4.00E-93
 98-  8:transcript:Zm00001d029868_T004 transcript:Zm00001d047107_T004         0
 98-  9:transcript:Zm00001d029869_T001 transcript:Zm00001d047106_T001  3.00E-86
 98- 10:transcript:Zm00001d029872_T001 transcript:Zm00001d047105_T007         0
## Alignment 99: score=464.0 e_value=9.3e-26 N=11 1&9 minus
 99-  0:transcript:Zm00001d028751_T001 transcript:Zm00001d047737_T001 3.00E-110
 99-  1:transcript:Zm00001d028752_T001 transcript:Zm00001d047732_T001         0
 99-  2:transcript:Zm00001d028754_T001 transcript:Zm00001d047728_T001         0
 99-  3:transcript:Zm00001d028755_T001 transcript:Zm00001d047727_T001 9.00E-128
 99-  4:transcript:Zm00001d028761_T002 transcript:Zm00001d047723_T001         0
 99-  5:transcript:Zm00001d028768_T001 transcript:Zm00001d047722_T001 2.00E-110
 99-  6:transcript:Zm00001d028769_T004 transcript:Zm00001d047721_T001         0
 99-  7:transcript:Zm00001d028773_T002 transcript:Zm00001d047720_T003  2.00E-76
```

```
 99-  8:transcript:Zm00001d028774_T001 transcript:Zm00001d047719_T001         0
 99-  9:transcript:Zm00001d028777_T001 transcript:Zm00001d047716_T001  9.00E-17
 99- 10:transcript:Zm00001d028782_T003 transcript:Zm00001d047713_T003         0
## Alignment 100: score=425.0 e_value=1.2e-23 N=10 1&9 minus
100-  0:transcript:Zm00001d028711_T006 transcript:Zm00001d047758_T004         0
100-  1:transcript:Zm00001d028712_T001 transcript:Zm00001d047755_T013         0
100-  2:transcript:Zm00001d028713_T001 transcript:Zm00001d047754_T002         0
100-  3:transcript:Zm00001d028714_T001 transcript:Zm00001d047753_T001 4.00E-121
100-  4:transcript:Zm00001d028721_T001 transcript:Zm00001d047752_T001 8.00E-109
100-  5:transcript:Zm00001d028725_T001 transcript:Zm00001d047750_T001         0
100-  6:transcript:Zm00001d028727_T001 transcript:Zm00001d047749_T001         0
100-  7:transcript:Zm00001d028730_T003 transcript:Zm00001d047747_T001 3.00E-174
100-  8:transcript:Zm00001d028736_T001 transcript:Zm00001d047745_T001 6.00E-148
100-  9:transcript:Zm00001d028742_T001 transcript:Zm00001d047743_T001         0
## Alignment 101: score=371.0 e_value=3.7e-15 N=8 1&9 minus
101-  0:transcript:Zm00001d028522_T001 transcript:Zm00001d047868_T001 9.00E-124
101-  1:transcript:Zm00001d028524_T001 transcript:Zm00001d047860_T001  1.00E-90
101-  2:transcript:Zm00001d028529_T001 transcript:Zm00001d047859_T001 2.00E-163
101-  3:transcript:Zm00001d028531_T001 transcript:Zm00001d047857_T001  4.00E-45
101-  4:transcript:Zm00001d028532_T001 transcript:Zm00001d047856_T001         0
101-  5:transcript:Zm00001d028533_T001 transcript:Zm00001d047855_T001         0
101-  6:transcript:Zm00001d028534_T005 transcript:Zm00001d047854_T002         0
101-  7:transcript:Zm00001d028535_T001 transcript:Zm00001d047853_T002         0
## Alignment 102: score=370.0 e_value=5.2e-18 N=9 1&9 minus
102-  0:transcript:Zm00001d028412_T029 transcript:Zm00001d047923_T001         0
102-  1:transcript:Zm00001d028413_T001 transcript:Zm00001d047921_T001 6.00E-110
102-  2:transcript:Zm00001d028414_T002 transcript:Zm00001d047920_T002 1.00E-117
102-  3:transcript:Zm00001d028415_T005 transcript:Zm00001d047918_T001         0
102-  4:transcript:Zm00001d028416_T001 transcript:Zm00001d047917_T001         0
102-  5:transcript:Zm00001d028423_T001 transcript:Zm00001d047916_T001         0
102-  6:transcript:Zm00001d028427_T001 transcript:Zm00001d047915_T001         0
102-  7:transcript:Zm00001d028429_T017 transcript:Zm00001d047911_T010         0
102-  8:transcript:Zm00001d028436_T001 transcript:Zm00001d047910_T001         0
## Alignment 103: score=363.0 e_value=4.7e-16 N=8 1&9 minus
103-  0:transcript:Zm00001d029285_T005 transcript:Zm00001d047422_T002         0
103-  1:transcript:Zm00001d029287_T001 transcript:Zm00001d047421_T001         0
103-  2:transcript:Zm00001d029288_T001 transcript:Zm00001d047418_T002         0
103-  3:transcript:Zm00001d029290_T001 transcript:Zm00001d047417_T001         0
103-  4:transcript:Zm00001d029297_T003 transcript:Zm00001d047412_T004         0
103-  5:transcript:Zm00001d029300_T001 transcript:Zm00001d047404_T001         0
103-  6:transcript:Zm00001d029305_T001 transcript:Zm00001d047401_T001  3.00E-42
103-  7:transcript:Zm00001d029313_T001 transcript:Zm00001d047399_T001         0
## Alignment 104: score=362.0 e_value=4.7e-14 N=8 1&9 minus
104-  0:transcript:Zm00001d028971_T002 transcript:Zm00001d047566_T002         0
104-  1:transcript:Zm00001d028974_T001 transcript:Zm00001d047563_T001         0
104-  2:transcript:Zm00001d028980_T001 transcript:Zm00001d047562_T002         0
104-  3:transcript:Zm00001d028982_T001 transcript:Zm00001d047560_T001  4.00E-28
104-  4:transcript:Zm00001d028986_T001 transcript:Zm00001d047559_T001 1.00E-131
104-  5:transcript:Zm00001d028989_T002 transcript:Zm00001d047558_T001         0
104-  6:transcript:Zm00001d028992_T001 transcript:Zm00001d047555_T001 1.00E-171
104-  7:transcript:Zm00001d028995_T001 transcript:Zm00001d047554_T001         0
## Alignment 105: score=347.0 e_value=3.4e-17 N=8 1&9 minus
105-  0:transcript:Zm00001d028560_T020 transcript:Zm00001d047842_T003         0
105-  1:transcript:Zm00001d028561_T001 transcript:Zm00001d047841_T001  2.00E-91
```

105- 2:transcript:Zm00001d028565_T002 transcript:Zm00001d047840_T001 0
105- 3:transcript:Zm00001d028566_T003 transcript:Zm00001d047839_T003 0
105- 4:transcript:Zm00001d028567_T001 transcript:Zm00001d047837_T003 0
105- 5:transcript:Zm00001d028568_T004 transcript:Zm00001d047835_T001 1.00E-179
105- 6:transcript:Zm00001d028569_T002 transcript:Zm00001d047834_T004 6.00E-154
105- 7:transcript:Zm00001d028570_T002 transcript:Zm00001d047833_T006 0
Alignment 106: score=341.0 e_value=3.6e-15 N=8 1&9 minus
106- 0:transcript:Zm00001d032989_T001 transcript:Zm00001d048336_T003 0
106- 1:transcript:Zm00001d032992_T001 transcript:Zm00001d048322_T001 4.00E-153
106- 2:transcript:Zm00001d032994_T002 transcript:Zm00001d048319_T003 0
106- 3:transcript:Zm00001d033003_T001 transcript:Zm00001d048317_T001 8.00E-42
106- 4:transcript:Zm00001d033004_T002 transcript:Zm00001d048314_T004 0
106- 5:transcript:Zm00001d033005_T001 transcript:Zm00001d048312_T001 1.00E-91
106- 6:transcript:Zm00001d033011_T001 transcript:Zm00001d048311_T003 0
106- 7:transcript:Zm00001d033012_T001 transcript:Zm00001d048310_T005 0
Alignment 107: score=325.0 e_value=2.9e-15 N=8 1&9 minus
107- 0:transcript:Zm00001d029143_T003 transcript:Zm00001d047482_T001 0
107- 1:transcript:Zm00001d029144_T003 transcript:Zm00001d047481_T003 0
107- 2:transcript:Zm00001d029154_T001 transcript:Zm00001d047480_T001 7.00E-165
107- 3:transcript:Zm00001d029170_T004 transcript:Zm00001d047479_T001 8.00E-95
107- 4:transcript:Zm00001d029172_T001 transcript:Zm00001d047478_T001 1.00E-53
107- 5:transcript:Zm00001d029173_T004 transcript:Zm00001d047477_T001 7.00E-119
107- 6:transcript:Zm00001d029174_T005 transcript:Zm00001d047472_T002 0
107- 7:transcript:Zm00001d029180_T001 transcript:Zm00001d047471_T002 0
Alignment 108: score=287.0 e_value=3.3e-12 N=7 1&9 minus
108- 0:transcript:Zm00001d029189_T003 transcript:Zm00001d047466_T001 0
108- 1:transcript:Zm00001d029196_T002 transcript:Zm00001d047464_T024 0
108- 2:transcript:Zm00001d029200_T001 transcript:Zm00001d047463_T001 0
108- 3:transcript:Zm00001d029201_T001 transcript:Zm00001d047462_T001 1.00E-141
108- 4:transcript:Zm00001d029208_T001 transcript:Zm00001d047460_T001 3.00E-40
108- 5:transcript:Zm00001d029209_T003 transcript:Zm00001d047457_T002 0
108- 6:transcript:Zm00001d029214_T001 transcript:Zm00001d047456_T002 1.00E-116
Alignment 109: score=266.0 e_value=6.7e-12 N=6 1&9 minus
109- 0:transcript:Zm00001d029044_T006 transcript:Zm00001d047534_T007 0
109- 1:transcript:Zm00001d029047_T001 transcript:Zm00001d047533_T001 0
109- 2:transcript:Zm00001d029049_T001 transcript:Zm00001d047532_T001 7.00E-74
109- 3:transcript:Zm00001d029050_T002 transcript:Zm00001d047531_T001 0
109- 4:transcript:Zm00001d029051_T001 transcript:Zm00001d047528_T001 9.00E-156
109- 5:transcript:Zm00001d029053_T005 transcript:Zm00001d047526_T006 0
Alignment 110: score=258.0 e_value=9.3e-13 N=6 1&9 minus
110- 0:transcript:Zm00001d027344_T001 transcript:Zm00001d048507_T007 0
110- 1:transcript:Zm00001d027345_T001 transcript:Zm00001d048506_T001 2.00E-112
110- 2:transcript:Zm00001d027350_T001 transcript:Zm00001d048505_T001 3.00E-20
110- 3:transcript:Zm00001d027359_T001 transcript:Zm00001d048502_T002 6.00E-86
110- 4:transcript:Zm00001d027373_T003 transcript:Zm00001d048497_T002 0
110- 5:transcript:Zm00001d027374_T001 transcript:Zm00001d048496_T002 8.00E-166
Alignment 111: score=252.0 e_value=7.6e-09 N=6 1&9 minus
111- 0:transcript:Zm00001d030290_T001 transcript:Zm00001d047941_T001 1.00E-119
111- 1:transcript:Zm00001d030303_T001 transcript:Zm00001d047939_T001 4.00E-115
111- 2:transcript:Zm00001d030304_T002 transcript:Zm00001d047938_T001 1.00E-126
111- 3:transcript:Zm00001d030308_T001 transcript:Zm00001d047937_T001 0
111- 4:transcript:Zm00001d030316_T001 transcript:Zm00001d047934_T001 1.00E-39
111- 5:transcript:Zm00001d030338_T001 transcript:Zm00001d047932_T001 3.00E-93
Alignment 112: score=285.0 e_value=2.9e-13 N=7 1&B73V4_ctg182 plus

112- 0:transcript:Zm00001d029934_T001 transcript:Zm00001d000025_T001 8.00E-15
112- 1:transcript:Zm00001d029937_T001 transcript:Zm00001d000028_T001 0
112- 2:transcript:Zm00001d029955_T001 transcript:Zm00001d000037_T001 0
112- 3:transcript:Zm00001d029974_T001 transcript:Zm00001d000045_T001 2.00E-179
112- 4:transcript:Zm00001d029976_T006 transcript:Zm00001d000046_T001 2.00E-85
112- 5:transcript:Zm00001d029978_T001 transcript:Zm00001d000052_T004 2.00E-140
112- 6:transcript:Zm00001d029979_T001 transcript:Zm00001d000053_T001 0
Alignment 113: score=273.0 e_value=1.3e-08 N=6 1&B73V4_ctg182 minus
113- 0:transcript:Zm00001d029938_T002 transcript:Zm00001d000042_T002 0
113- 1:transcript:Zm00001d029946_T003 transcript:Zm00001d000039_T001 0
113- 2:transcript:Zm00001d029948_T001 transcript:Zm00001d000038_T001 0
113- 3:transcript:Zm00001d029955_T001 transcript:Zm00001d000037_T001 0
113- 4:transcript:Zm00001d029964_T001 transcript:Zm00001d000034_T002 3.00E-161
113- 5:transcript:Zm00001d029973_T001 transcript:Zm00001d000030_T001 0
Alignment 114: score=549.0 e_value=2.8e-31 N=13 10&2 plus
114- 0:transcript:Zm00001d024924_T001 transcript:Zm00001d004526_T001 0
114- 1:transcript:Zm00001d024926_T001 transcript:Zm00001d004539_T001 1.00E-67
114- 2:transcript:Zm00001d024928_T003 transcript:Zm00001d004541_T009 0
114- 3:transcript:Zm00001d024933_T003 transcript:Zm00001d004543_T001 0
114- 4:transcript:Zm00001d024934_T001 transcript:Zm00001d004545_T001 0
114- 5:transcript:Zm00001d024935_T001 transcript:Zm00001d004546_T001 8.00E-161
114- 6:transcript:Zm00001d024937_T001 transcript:Zm00001d004547_T002 0
114- 7:transcript:Zm00001d024938_T005 transcript:Zm00001d004551_T007 2.00E-126
114- 8:transcript:Zm00001d024939_T002 transcript:Zm00001d004552_T003 2.00E-115
114- 9:transcript:Zm00001d024940_T001 transcript:Zm00001d004553_T001 0
114- 10:transcript:Zm00001d024941_T002 transcript:Zm00001d004554_T004 6.00E-169
114- 11:transcript:Zm00001d024943_T001 transcript:Zm00001d004555_T001 0
114- 12:transcript:Zm00001d024947_T005 transcript:Zm00001d004557_T002 0
Alignment 115: score=504.0 e_value=7.3e-30 N=12 10&2 plus
115- 0:transcript:Zm00001d026158_T001 transcript:Zm00001d002718_T001 0
115- 1:transcript:Zm00001d026159_T001 transcript:Zm00001d002720_T001 3.00E-18
115- 2:transcript:Zm00001d026160_T001 transcript:Zm00001d002721_T001 4.00E-55
115- 3:transcript:Zm00001d026164_T001 transcript:Zm00001d002723_T001 2.00E-68
115- 4:transcript:Zm00001d026165_T003 transcript:Zm00001d002725_T001 0
115- 5:transcript:Zm00001d026166_T002 transcript:Zm00001d002732_T001 0
115- 6:transcript:Zm00001d026176_T008 transcript:Zm00001d002739_T004 0
115- 7:transcript:Zm00001d026182_T001 transcript:Zm00001d002744_T001 9.00E-80
115- 8:transcript:Zm00001d026186_T001 transcript:Zm00001d002750_T001 4.00E-163
115- 9:transcript:Zm00001d026189_T001 transcript:Zm00001d002757_T001 0
115- 10:transcript:Zm00001d026190_T001 transcript:Zm00001d002758_T002 6.00E-133
115- 11:transcript:Zm00001d026191_T001 transcript:Zm00001d002760_T001 7.00E-28
Alignment 116: score=409.0 e_value=3.4e-17 N=9 10&2 plus
116- 0:transcript:Zm00001d026056_T002 transcript:Zm00001d002589_T001 5.00E-155
116- 1:transcript:Zm00001d026060_T003 transcript:Zm00001d002592_T002 0
116- 2:transcript:Zm00001d026061_T001 transcript:Zm00001d002594_T001 1.00E-117
116- 3:transcript:Zm00001d026063_T001 transcript:Zm00001d002599_T002 0
116- 4:transcript:Zm00001d026064_T005 transcript:Zm00001d002600_T005 0
116- 5:transcript:Zm00001d026066_T001 transcript:Zm00001d002609_T001 5.00E-170
116- 6:transcript:Zm00001d026067_T001 transcript:Zm00001d002610_T001 0
116- 7:transcript:Zm00001d026069_T001 transcript:Zm00001d002611_T002 5.00E-37
116- 8:transcript:Zm00001d026070_T002 transcript:Zm00001d002614_T001 0
Alignment 117: score=967.0 e_value=1.6e-66 N=22 10&2 minus
117- 0:transcript:Zm00001d025508_T001 transcript:Zm00001d003554_T001 9.00E-103
117- 1:transcript:Zm00001d025509_T001 transcript:Zm00001d003552_T001 5.00E-67

117- 2: transcript:Zm00001d025514_T001 transcript:Zm00001d003549_T001 4.00E-176
117- 3: transcript:Zm00001d025517_T003 transcript:Zm00001d003546_T001 5.00E-72
117- 4: transcript:Zm00001d025518_T001 transcript:Zm00001d003545_T001 5.00E-22
117- 5: transcript:Zm00001d025519_T003 transcript:Zm00001d003544_T003 2.00E-133
117- 6: transcript:Zm00001d025520_T001 transcript:Zm00001d003543_T001 0
117- 7: transcript:Zm00001d025522_T001 transcript:Zm00001d003535_T001 1.00E-46
117- 8: transcript:Zm00001d025524_T001 transcript:Zm00001d003533_T001 0
117- 9: transcript:Zm00001d025526_T003 transcript:Zm00001d003530_T001 1.00E-117
117- 10: transcript:Zm00001d025528_T002 transcript:Zm00001d003525_T010 0
117- 11: transcript:Zm00001d025533_T001 transcript:Zm00001d003524_T001 0
117- 12: transcript:Zm00001d025534_T001 transcript:Zm00001d003521_T001 1.00E-94
117- 13: transcript:Zm00001d025538_T001 transcript:Zm00001d003518_T003 0
117- 14: transcript:Zm00001d025541_T001 transcript:Zm00001d003515_T001 3.00E-65
117- 15: transcript:Zm00001d025544_T001 transcript:Zm00001d003512_T001 2.00E-42
117- 16: transcript:Zm00001d025547_T001 transcript:Zm00001d003510_T001 0
117- 17: transcript:Zm00001d025548_T001 transcript:Zm00001d003509_T001 0
117- 18: transcript:Zm00001d025552_T001 transcript:Zm00001d003505_T001 7.00E-24
117- 19: transcript:Zm00001d025559_T001 transcript:Zm00001d003502_T003 5.00E-25
117- 20: transcript:Zm00001d025566_T001 transcript:Zm00001d003494_T004 3.00E-60
117- 21: transcript:Zm00001d025568_T001 transcript:Zm00001d003492_T001 0
Alignment 118: score=963.0 e_value=3e-75 N=24 10&2 minus
118- 0: transcript:Zm00001d026257_T009 transcript:Zm00001d002388_T003 0
118- 1: transcript:Zm00001d026259_T001 transcript:Zm00001d002387_T002 5.00E-73
118- 2: transcript:Zm00001d026260_T003 transcript:Zm00001d002386_T003 8.00E-155
118- 3: transcript:Zm00001d026261_T001 transcript:Zm00001d002382_T002 0
118- 4: transcript:Zm00001d026262_T001 transcript:Zm00001d002374_T001 6.00E-101
118- 5: transcript:Zm00001d026265_T002 transcript:Zm00001d002373_T006 0
118- 6: transcript:Zm00001d026267_T001 transcript:Zm00001d002371_T002 0
118- 7: transcript:Zm00001d026268_T001 transcript:Zm00001d002370_T001 0
118- 8: transcript:Zm00001d026269_T001 transcript:Zm00001d002369_T003 2.00E-168
118- 9: transcript:Zm00001d026271_T001 transcript:Zm00001d002364_T001 2.00E-71
118- 10: transcript:Zm00001d026277_T002 transcript:Zm00001d002358_T001 0
118- 11: transcript:Zm00001d026278_T001 transcript:Zm00001d002352_T001 8.00E-40
118- 12: transcript:Zm00001d026281_T003 transcript:Zm00001d002348_T001 8.00E-62
118- 13: transcript:Zm00001d026282_T001 transcript:Zm00001d002347_T001 9.00E-52
118- 14: transcript:Zm00001d026284_T001 transcript:Zm00001d002344_T001 1.00E-65
118- 15: transcript:Zm00001d026285_T001 transcript:Zm00001d002341_T001 0
118- 16: transcript:Zm00001d026286_T001 transcript:Zm00001d002339_T001 2.00E-114
118- 17: transcript:Zm00001d026287_T005 transcript:Zm00001d002338_T001 0
118- 18: transcript:Zm00001d026289_T004 transcript:Zm00001d002333_T003 0
118- 19: transcript:Zm00001d026290_T001 transcript:Zm00001d002332_T026 0
118- 20: transcript:Zm00001d026291_T013 transcript:Zm00001d002330_T006 0
118- 21: transcript:Zm00001d026293_T001 transcript:Zm00001d002326_T003 0
118- 22: transcript:Zm00001d026295_T001 transcript:Zm00001d002325_T002 0
118- 23: transcript:Zm00001d026296_T014 transcript:Zm00001d002323_T024 0
Alignment 119: score=836.0 e_value=5.8e-54 N=19 10&2 minus
119- 0: transcript:Zm00001d025906_T001 transcript:Zm00001d002872_T001 4.00E-50
119- 1: transcript:Zm00001d025908_T001 transcript:Zm00001d002869_T001 0
119- 2: transcript:Zm00001d025910_T001 transcript:Zm00001d002867_T001 4.00E-168
119- 3: transcript:Zm00001d025911_T002 transcript:Zm00001d002865_T002 0
119- 4: transcript:Zm00001d025916_T001 transcript:Zm00001d002856_T001 0
119- 5: transcript:Zm00001d025917_T001 transcript:Zm00001d002851_T001 0
119- 6: transcript:Zm00001d025918_T001 transcript:Zm00001d002849_T001 1.00E-26
119- 7: transcript:Zm00001d025922_T001 transcript:Zm00001d002845_T001 4.00E-18

119- 8:transcript:Zm00001d025926_T001 transcript:Zm00001d002844_T003 4.00E-125
119- 9:transcript:Zm00001d025930_T001 transcript:Zm00001d002843_T001 2.00E-150
119- 10:transcript:Zm00001d025932_T003 transcript:Zm00001d002842_T001 7.00E-97
119- 11:transcript:Zm00001d025933_T001 transcript:Zm00001d002836_T006 2.00E-164
119- 12:transcript:Zm00001d025943_T001 transcript:Zm00001d002830_T001   0
119- 13:transcript:Zm00001d025944_T001 transcript:Zm00001d002829_T001 3.00E-123
119- 14:transcript:Zm00001d025946_T003 transcript:Zm00001d002827_T010   0
119- 15:transcript:Zm00001d025947_T001 transcript:Zm00001d002826_T001 2.00E-64
119- 16:transcript:Zm00001d025948_T001 transcript:Zm00001d002822_T001   0
119- 17:transcript:Zm00001d025949_T001 transcript:Zm00001d002820_T001 1.00E-114
119- 18:transcript:Zm00001d025950_T002 transcript:Zm00001d002819_T002   0
Alignment 120: score=702.0 e_value=1.6e-48 N=16 10&2 minus
120- 0:transcript:Zm00001d025866_T003 transcript:Zm00001d003014_T002 5.00E-72
120- 1:transcript:Zm00001d025868_T002 transcript:Zm00001d003013_T001 6.00E-131
120- 2:transcript:Zm00001d025869_T003 transcript:Zm00001d003012_T001 8.00E-116
120- 3:transcript:Zm00001d025871_T001 transcript:Zm00001d003011_T001 1.00E-50
120- 4:transcript:Zm00001d025872_T001 transcript:Zm00001d003009_T001 8.00E-72
120- 5:transcript:Zm00001d025874_T001 transcript:Zm00001d002996_T001 7.00E-48
120- 6:transcript:Zm00001d025885_T001 transcript:Zm00001d002989_T001   0
120- 7:transcript:Zm00001d025886_T001 transcript:Zm00001d002984_T001 1.00E-69
120- 8:transcript:Zm00001d025889_T001 transcript:Zm00001d002974_T001   0
120- 9:transcript:Zm00001d025891_T001 transcript:Zm00001d002969_T001 6.00E-178
120- 10:transcript:Zm00001d025892_T001 transcript:Zm00001d002967_T015   0
120- 11:transcript:Zm00001d025894_T002 transcript:Zm00001d002962_T001 2.00E-142
120- 12:transcript:Zm00001d025900_T001 transcript:Zm00001d002958_T001 4.00E-68
120- 13:transcript:Zm00001d025903_T004 transcript:Zm00001d002956_T005   0
120- 14:transcript:Zm00001d025904_T001 transcript:Zm00001d002955_T001 8.00E-47
120- 15:transcript:Zm00001d025905_T002 transcript:Zm00001d002954_T002 3.00E-61
Alignment 121: score=629.0 e_value=1.9e-38 N=15 10&2 minus
121- 0:transcript:Zm00001d026002_T001 transcript:Zm00001d002568_T001 5.00E-75
121- 1:transcript:Zm00001d026003_T001 transcript:Zm00001d002564_T001 5.00E-40
121- 2:transcript:Zm00001d026005_T001 transcript:Zm00001d002562_T001 4.00E-169
121- 3:transcript:Zm00001d026010_T001 transcript:Zm00001d002551_T001 3.00E-40
121- 4:transcript:Zm00001d026012_T001 transcript:Zm00001d002549_T002 1.00E-21
121- 5:transcript:Zm00001d026014_T001 transcript:Zm00001d002548_T001 2.00E-177
121- 6:transcript:Zm00001d026015_T001 transcript:Zm00001d002546_T001 6.00E-67
121- 7:transcript:Zm00001d026017_T001 transcript:Zm00001d002545_T002   0
121- 8:transcript:Zm00001d026018_T001 transcript:Zm00001d002540_T002   0
121- 9:transcript:Zm00001d026020_T001 transcript:Zm00001d002537_T002   0
121- 10:transcript:Zm00001d026021_T001 transcript:Zm00001d002535_T001 3.00E-89
121- 11:transcript:Zm00001d026026_T001 transcript:Zm00001d002523_T001 5.00E-19
121- 12:transcript:Zm00001d026028_T001 transcript:Zm00001d002520_T001 5.00E-25
121- 13:transcript:Zm00001d026032_T001 transcript:Zm00001d002519_T001 7.00E-152
121- 14:transcript:Zm00001d026042_T001 transcript:Zm00001d002514_T001 7.00E-118
Alignment 122: score=621.0 e_value=7.8e-40 N=15 10&2 minus
122- 0:transcript:Zm00001d026560_T001 transcript:Zm00001d001910_T002 8.00E-82
122- 1:transcript:Zm00001d026562_T001 transcript:Zm00001d001908_T001 1.00E-47
122- 2:transcript:Zm00001d026563_T001 transcript:Zm00001d001907_T001 9.00E-61
122- 3:transcript:Zm00001d026568_T003 transcript:Zm00001d001903_T003 2.00E-15
122- 4:transcript:Zm00001d026572_T001 transcript:Zm00001d001902_T002 7.00E-47
122- 5:transcript:Zm00001d026575_T001 transcript:Zm00001d001901_T002 5.00E-119
122- 6:transcript:Zm00001d026577_T002 transcript:Zm00001d001900_T002   0
122- 7:transcript:Zm00001d026578_T001 transcript:Zm00001d001899_T001 1.00E-43
122- 8:transcript:Zm00001d026579_T001 transcript:Zm00001d001898_T008 3.00E-179

```
122-  9:transcript:Zm00001d026582_T001 transcript:Zm00001d001895_T002          0
122- 10:transcript:Zm00001d026584_T001 transcript:Zm00001d001885_T001  6.00E-75
122- 11:transcript:Zm00001d026585_T001 transcript:Zm00001d001884_T001          0
122- 12:transcript:Zm00001d026586_T003 transcript:Zm00001d001883_T008          0
122- 13:transcript:Zm00001d026588_T004 transcript:Zm00001d001880_T001  3.00E-48
122- 14:transcript:Zm00001d026590_T003 transcript:Zm00001d001879_T035          0
## Alignment 123: score=598.0 e_value=5.9e-34 N=13 10&2 minus
123-  0:transcript:Zm00001d025752_T006 transcript:Zm00001d003193_T001  3.00E-28
123-  1:transcript:Zm00001d025753_T001 transcript:Zm00001d003188_T001 3.00E-128
123-  2:transcript:Zm00001d025754_T001 transcript:Zm00001d003185_T001 2.00E-129
123-  3:transcript:Zm00001d025756_T002 transcript:Zm00001d003179_T001  1.00E-33
123-  4:transcript:Zm00001d025757_T001 transcript:Zm00001d003176_T001          0
123-  5:transcript:Zm00001d025759_T001 transcript:Zm00001d003175_T001  2.00E-86
123-  6:transcript:Zm00001d025761_T002 transcript:Zm00001d003173_T001 1.00E-154
123-  7:transcript:Zm00001d025762_T001 transcript:Zm00001d003172_T001 2.00E-116
123-  8:transcript:Zm00001d025763_T003 transcript:Zm00001d003167_T002  2.00E-72
123-  9:transcript:Zm00001d025770_T001 transcript:Zm00001d003162_T001          0
123- 10:transcript:Zm00001d025771_T001 transcript:Zm00001d003160_T001          0
123- 11:transcript:Zm00001d025772_T001 transcript:Zm00001d003159_T001  8.00E-27
123- 12:transcript:Zm00001d025773_T002 transcript:Zm00001d003157_T001          0
## Alignment 124: score=554.0 e_value=1.7e-34 N=13 10&2 minus
124-  0:transcript:Zm00001d025645_T001 transcript:Zm00001d003370_T001  2.00E-33
124-  1:transcript:Zm00001d025646_T001 transcript:Zm00001d003369_T004 3.00E-101
124-  2:transcript:Zm00001d025654_T001 transcript:Zm00001d003355_T001  9.00E-17
124-  3:transcript:Zm00001d025658_T006 transcript:Zm00001d003352_T003          0
124-  4:transcript:Zm00001d025660_T001 transcript:Zm00001d003347_T002  5.00E-62
124-  5:transcript:Zm00001d025662_T001 transcript:Zm00001d003346_T001  2.00E-21
124-  6:transcript:Zm00001d025665_T002 transcript:Zm00001d003343_T001          0
124-  7:transcript:Zm00001d025666_T001 transcript:Zm00001d003335_T001  2.00E-75
124-  8:transcript:Zm00001d025667_T001 transcript:Zm00001d003334_T001          0
124-  9:transcript:Zm00001d025668_T005 transcript:Zm00001d003333_T005          0
124- 10:transcript:Zm00001d025669_T003 transcript:Zm00001d003331_T001          0
124- 11:transcript:Zm00001d025673_T005 transcript:Zm00001d003329_T003          0
124- 12:transcript:Zm00001d025675_T001 transcript:Zm00001d003328_T001          0
## Alignment 125: score=551.0 e_value=9.8e-35 N=13 10&2 minus
125-  0:transcript:Zm00001d026212_T001 transcript:Zm00001d002456_T002 7.00E-103
125-  1:transcript:Zm00001d026218_T001 transcript:Zm00001d002452_T001          0
125-  2:transcript:Zm00001d026223_T001 transcript:Zm00001d002451_T001  9.00E-61
125-  3:transcript:Zm00001d026231_T001 transcript:Zm00001d002449_T001          0
125-  4:transcript:Zm00001d026235_T001 transcript:Zm00001d002444_T003  2.00E-35
125-  5:transcript:Zm00001d026237_T001 transcript:Zm00001d002432_T001          0
125-  6:transcript:Zm00001d026239_T004 transcript:Zm00001d002430_T001  1.00E-28
125-  7:transcript:Zm00001d026240_T006 transcript:Zm00001d002429_T002 1.00E-164
125-  8:transcript:Zm00001d026241_T001 transcript:Zm00001d002428_T001  2.00E-19
125-  9:transcript:Zm00001d026242_T001 transcript:Zm00001d002427_T001          0
125- 10:transcript:Zm00001d026244_T001 transcript:Zm00001d002425_T001  8.00E-36
125- 11:transcript:Zm00001d026245_T001 transcript:Zm00001d002424_T002 4.00E-136
125- 12:transcript:Zm00001d026246_T001 transcript:Zm00001d002418_T030          0
## Alignment 126: score=516.0 e_value=6.3e-30 N=12 10&2 minus
126-  0:transcript:Zm00001d026440_T001 transcript:Zm00001d002088_T001  1.00E-45
126-  1:transcript:Zm00001d026442_T001 transcript:Zm00001d002087_T001 8.00E-175
126-  2:transcript:Zm00001d026444_T001 transcript:Zm00001d002083_T001  7.00E-89
126-  3:transcript:Zm00001d026445_T001 transcript:Zm00001d002080_T003          0
126-  4:transcript:Zm00001d026447_T001 transcript:Zm00001d002079_T001  9.00E-79
```

```
126-  5:transcript:Zm00001d026448_T001 transcript:Zm00001d002075_T001 5.00E-126
126-  6:transcript:Zm00001d026454_T001 transcript:Zm00001d002069_T001  1.00E-08
126-  7:transcript:Zm00001d026458_T002 transcript:Zm00001d002065_T006         0
126-  8:transcript:Zm00001d026460_T001 transcript:Zm00001d002064_T001         0
126-  9:transcript:Zm00001d026469_T001 transcript:Zm00001d002056_T006         0
126- 10:transcript:Zm00001d026470_T001 transcript:Zm00001d002055_T001 3.00E-128
126- 11:transcript:Zm00001d026472_T001 transcript:Zm00001d002051_T001         0
## Alignment 127: score=510.0 e_value=3.9e-26 N=12 10&2 minus
127-  0:transcript:Zm00001d025281_T001 transcript:Zm00001d003884_T001  2.00E-17
127-  1:transcript:Zm00001d025287_T001 transcript:Zm00001d003877_T001  8.00E-92
127-  2:transcript:Zm00001d025291_T001 transcript:Zm00001d003875_T001  8.00E-86
127-  3:transcript:Zm00001d025294_T002 transcript:Zm00001d003872_T002 5.00E-171
127-  4:transcript:Zm00001d025298_T001 transcript:Zm00001d003871_T001 1.00E-120
127-  5:transcript:Zm00001d025299_T001 transcript:Zm00001d003865_T001  2.00E-10
127-  6:transcript:Zm00001d025300_T007 transcript:Zm00001d003864_T002         0
127-  7:transcript:Zm00001d025303_T004 transcript:Zm00001d003859_T001         0
127-  8:transcript:Zm00001d025305_T001 transcript:Zm00001d003857_T001         0
127-  9:transcript:Zm00001d025307_T004 transcript:Zm00001d003855_T010  3.00E-28
127- 10:transcript:Zm00001d025319_T001 transcript:Zm00001d003850_T001 3.00E-123
127- 11:transcript:Zm00001d025326_T001 transcript:Zm00001d003848_T004         0
## Alignment 128: score=479.0 e_value=2.1e-23 N=11 10&2 minus
128-  0:transcript:Zm00001d025354_T001 transcript:Zm00001d003776_T001         0
128-  1:transcript:Zm00001d025359_T002 transcript:Zm00001d003773_T005         0
128-  2:transcript:Zm00001d025360_T001 transcript:Zm00001d003769_T001  1.00E-48
128-  3:transcript:Zm00001d025361_T001 transcript:Zm00001d003763_T001  1.00E-54
128-  4:transcript:Zm00001d025367_T001 transcript:Zm00001d003761_T001  7.00E-23
128-  5:transcript:Zm00001d025369_T002 transcript:Zm00001d003754_T003         0
128-  6:transcript:Zm00001d025371_T001 transcript:Zm00001d003751_T001 9.00E-139
128-  7:transcript:Zm00001d025373_T001 transcript:Zm00001d003749_T001         0
128-  8:transcript:Zm00001d025374_T001 transcript:Zm00001d003743_T001 3.00E-113
128-  9:transcript:Zm00001d025375_T001 transcript:Zm00001d003742_T001         0
128- 10:transcript:Zm00001d025382_T001 transcript:Zm00001d003730_T001  2.00E-94
## Alignment 129: score=437.0 e_value=1.8e-23 N=10 10&2 minus
129-  0:transcript:Zm00001d025205_T001 transcript:Zm00001d004007_T001 4.00E-151
129-  1:transcript:Zm00001d025208_T002 transcript:Zm00001d003996_T001         0
129-  2:transcript:Zm00001d025218_T001 transcript:Zm00001d003993_T001 4.00E-178
129-  3:transcript:Zm00001d025222_T001 transcript:Zm00001d003984_T001         0
129-  4:transcript:Zm00001d025225_T001 transcript:Zm00001d003981_T001  3.00E-61
129-  5:transcript:Zm00001d025228_T003 transcript:Zm00001d003975_T002  1.00E-97
129-  6:transcript:Zm00001d025235_T004 transcript:Zm00001d003958_T013         0
129-  7:transcript:Zm00001d025236_T001 transcript:Zm00001d003957_T002  8.00E-99
129-  8:transcript:Zm00001d025237_T001 transcript:Zm00001d003949_T001         0
129-  9:transcript:Zm00001d025238_T001 transcript:Zm00001d003948_T001         0
## Alignment 130: score=424.0 e_value=2.2e-21 N=10 10&2 minus
130-  0:transcript:Zm00001d026530_T001 transcript:Zm00001d001961_T001  1.00E-26
130-  1:transcript:Zm00001d026531_T002 transcript:Zm00001d001959_T001         0
130-  2:transcript:Zm00001d026535_T003 transcript:Zm00001d001953_T003         0
130-  3:transcript:Zm00001d026536_T002 transcript:Zm00001d001952_T001         0
130-  4:transcript:Zm00001d026537_T001 transcript:Zm00001d001948_T001 1.00E-128
130-  5:transcript:Zm00001d026540_T001 transcript:Zm00001d001945_T006         0
130-  6:transcript:Zm00001d026541_T001 transcript:Zm00001d001937_T003         0
130-  7:transcript:Zm00001d026542_T001 transcript:Zm00001d001936_T001         0
130-  8:transcript:Zm00001d026543_T007 transcript:Zm00001d001932_T001         0
130-  9:transcript:Zm00001d026547_T004 transcript:Zm00001d001929_T003 1.00E-114
```

Alignment 131: score=416.0 e_value=2.2e-16 N=9 10&2 minus
131- 0:transcript:Zm00001d025952_T002 transcript:Zm00001d002815_T001 2.00E-30
131- 1:transcript:Zm00001d025953_T003 transcript:Zm00001d002811_T002 0
131- 2:transcript:Zm00001d025955_T001 transcript:Zm00001d002810_T001 0
131- 3:transcript:Zm00001d025957_T001 transcript:Zm00001d002806_T001 2.00E-146
131- 4:transcript:Zm00001d025959_T001 transcript:Zm00001d002802_T001 0
131- 5:transcript:Zm00001d025960_T002 transcript:Zm00001d002801_T001 0
131- 6:transcript:Zm00001d025964_T001 transcript:Zm00001d002799_T001 7.00E-104
131- 7:transcript:Zm00001d025967_T001 transcript:Zm00001d002798_T001 6.00E-76
131- 8:transcript:Zm00001d025977_T001 transcript:Zm00001d002797_T001 0
Alignment 132: score=416.0 e_value=9.7e-23 N=10 10&2 minus
132- 0:transcript:Zm00001d025712_T003 transcript:Zm00001d003261_T003 0
132- 1:transcript:Zm00001d025713_T001 transcript:Zm00001d003258_T001 0
132- 2:transcript:Zm00001d025714_T001 transcript:Zm00001d003256_T001 0
132- 3:transcript:Zm00001d025715_T001 transcript:Zm00001d003254_T008 2.00E-126
132- 4:transcript:Zm00001d025716_T001 transcript:Zm00001d003251_T001 0
132- 5:transcript:Zm00001d025717_T001 transcript:Zm00001d003250_T001 0
132- 6:transcript:Zm00001d025721_T001 transcript:Zm00001d003249_T001 4.00E-103
132- 7:transcript:Zm00001d025724_T001 transcript:Zm00001d003248_T003 3.00E-82
132- 8:transcript:Zm00001d025726_T001 transcript:Zm00001d003245_T001 0
132- 9:transcript:Zm00001d025727_T006 transcript:Zm00001d003229_T002 0
Alignment 133: score=406.0 e_value=1.5e-21 N=10 10&2 minus
133- 0:transcript:Zm00001d025453_T006 transcript:Zm00001d003612_T001 2.00E-163
133- 1:transcript:Zm00001d025461_T006 transcript:Zm00001d003611_T003 0
133- 2:transcript:Zm00001d025462_T001 transcript:Zm00001d003604_T001 2.00E-147
133- 3:transcript:Zm00001d025467_T001 transcript:Zm00001d003600_T001 5.00E-51
133- 4:transcript:Zm00001d025470_T005 transcript:Zm00001d003599_T001 1.00E-35
133- 5:transcript:Zm00001d025472_T002 transcript:Zm00001d003598_T003 2.00E-66
133- 6:transcript:Zm00001d025474_T002 transcript:Zm00001d003594_T001 6.00E-45
133- 7:transcript:Zm00001d025483_T001 transcript:Zm00001d003583_T001 0
133- 8:transcript:Zm00001d025485_T001 transcript:Zm00001d003573_T001 8.00E-73
133- 9:transcript:Zm00001d025489_T001 transcript:Zm00001d003563_T001 1.00E-46
Alignment 134: score=400.0 e_value=4.5e-24 N=10 10&2 minus
134- 0:transcript:Zm00001d025247_T001 transcript:Zm00001d003930_T001 1.00E-50
134- 1:transcript:Zm00001d025251_T001 transcript:Zm00001d003929_T001 0
134- 2:transcript:Zm00001d025252_T001 transcript:Zm00001d003928_T001 5.00E-88
134- 3:transcript:Zm00001d025258_T009 transcript:Zm00001d003923_T002 0
134- 4:transcript:Zm00001d025265_T001 transcript:Zm00001d003913_T001 6.00E-13
134- 5:transcript:Zm00001d025268_T011 transcript:Zm00001d003911_T010 0
134- 6:transcript:Zm00001d025271_T001 transcript:Zm00001d003904_T003 2.00E-119
134- 7:transcript:Zm00001d025273_T019 transcript:Zm00001d003894_T004 0
134- 8:transcript:Zm00001d025274_T001 transcript:Zm00001d003890_T001 0
134- 9:transcript:Zm00001d025275_T001 transcript:Zm00001d003889_T001 0
Alignment 135: score=368.0 e_value=8.7e-17 N=8 10&2 minus
135- 0:transcript:Zm00001d026647_T005 transcript:Zm00001d001787_T002 1.00E-118
135- 1:transcript:Zm00001d026650_T001 transcript:Zm00001d001785_T001 0
135- 2:transcript:Zm00001d026652_T001 transcript:Zm00001d001784_T002 0
135- 3:transcript:Zm00001d026653_T004 transcript:Zm00001d001780_T002 0
135- 4:transcript:Zm00001d026657_T001 transcript:Zm00001d001774_T001 2.00E-164
135- 5:transcript:Zm00001d026658_T001 transcript:Zm00001d001773_T001 6.00E-127
135- 6:transcript:Zm00001d026662_T001 transcript:Zm00001d001772_T001 6.00E-118
135- 7:transcript:Zm00001d026665_T001 transcript:Zm00001d001766_T001 1.00E-101
Alignment 136: score=367.0 e_value=5.5e-19 N=9 10&2 minus
136- 0:transcript:Zm00001d025607_T001 transcript:Zm00001d003429_T001 0

```
136-  1:transcript:Zm00001d025612_T001 transcript:Zm00001d003419_T001  2.00E-22
136-  2:transcript:Zm00001d025613_T001 transcript:Zm00001d003418_T001 3.00E-115
136-  3:transcript:Zm00001d025616_T001 transcript:Zm00001d003411_T001  8.00E-94
136-  4:transcript:Zm00001d025617_T001 transcript:Zm00001d003401_T003         0
136-  5:transcript:Zm00001d025621_T001 transcript:Zm00001d003399_T001  7.00E-48
136-  6:transcript:Zm00001d025622_T001 transcript:Zm00001d003398_T001  9.00E-54
136-  7:transcript:Zm00001d025628_T002 transcript:Zm00001d003393_T002         0
136-  8:transcript:Zm00001d025639_T002 transcript:Zm00001d003382_T002         0
## Alignment 137: score=330.0 e_value=1.4e-15 N=8 10&2 minus
137-  0:transcript:Zm00001d026335_T001 transcript:Zm00001d002257_T006  5.00E-84
137-  1:transcript:Zm00001d026337_T011 transcript:Zm00001d002256_T022         0
137-  2:transcript:Zm00001d026341_T001 transcript:Zm00001d002255_T001  7.00E-40
137-  3:transcript:Zm00001d026343_T001 transcript:Zm00001d002253_T001  7.00E-96
137-  4:transcript:Zm00001d026344_T001 transcript:Zm00001d002252_T001         0
137-  5:transcript:Zm00001d026346_T002 transcript:Zm00001d002244_T003         0
137-  6:transcript:Zm00001d026347_T008 transcript:Zm00001d002243_T004         0
137-  7:transcript:Zm00001d026348_T004 transcript:Zm00001d002241_T002         0
## Alignment 138: score=329.0 e_value=9e-13 N=7 10&2 minus
138-  0:transcript:Zm00001d025412_T001 transcript:Zm00001d003674_T001 3.00E-178
138-  1:transcript:Zm00001d025413_T001 transcript:Zm00001d003672_T001 2.00E-118
138-  2:transcript:Zm00001d025414_T001 transcript:Zm00001d003669_T001 1.00E-144
138-  3:transcript:Zm00001d025416_T001 transcript:Zm00001d003666_T001         0
138-  4:transcript:Zm00001d025419_T001 transcript:Zm00001d003664_T001         0
138-  5:transcript:Zm00001d025420_T001 transcript:Zm00001d003663_T001 1.00E-117
138-  6:transcript:Zm00001d025421_T001 transcript:Zm00001d003661_T001 8.00E-120
## Alignment 139: score=315.0 e_value=9.1e-13 N=7 10&2 minus
139-  0:transcript:Zm00001d026311_T001 transcript:Zm00001d002292_T004         0
139-  1:transcript:Zm00001d026317_T002 transcript:Zm00001d002288_T002 1.00E-102
139-  2:transcript:Zm00001d026318_T001 transcript:Zm00001d002286_T001 1.00E-112
139-  3:transcript:Zm00001d026321_T001 transcript:Zm00001d002284_T003         0
139-  4:transcript:Zm00001d026322_T003 transcript:Zm00001d002283_T002         0
139-  5:transcript:Zm00001d026326_T001 transcript:Zm00001d002279_T001 4.00E-156
139-  6:transcript:Zm00001d026329_T001 transcript:Zm00001d002278_T001  2.00E-14
## Alignment 140: score=311.0 e_value=1e-16 N=7 10&2 minus
140-  0:transcript:Zm00001d026390_T001 transcript:Zm00001d002158_T001  1.00E-14
140-  1:transcript:Zm00001d026391_T004 transcript:Zm00001d002156_T001 2.00E-132
140-  2:transcript:Zm00001d026392_T002 transcript:Zm00001d002155_T001  8.00E-53
140-  3:transcript:Zm00001d026394_T001 transcript:Zm00001d002149_T007         0
140-  4:transcript:Zm00001d026395_T001 transcript:Zm00001d002150_T001         0
140-  5:transcript:Zm00001d026396_T004 transcript:Zm00001d002145_T001         0
140-  6:transcript:Zm00001d026398_T006 transcript:Zm00001d002143_T002         0
## Alignment 141: score=299.0 e_value=1.7e-15 N=7 10&2 minus
141-  0:transcript:Zm00001d025570_T006 transcript:Zm00001d003472_T002  2.00E-21
141-  1:transcript:Zm00001d025574_T001 transcript:Zm00001d003470_T001         0
141-  2:transcript:Zm00001d025576_T001 transcript:Zm00001d003463_T001  9.00E-75
141-  3:transcript:Zm00001d025577_T001 transcript:Zm00001d003462_T001  2.00E-36
141-  4:transcript:Zm00001d025581_T001 transcript:Zm00001d003438_T001         0
141-  5:transcript:Zm00001d025586_T001 transcript:Zm00001d003432_T001         0
141-  6:transcript:Zm00001d025588_T001 transcript:Zm00001d003431_T009         0
## Alignment 142: score=282.0 e_value=4e-10 N=6 10&2 minus
142-  0:transcript:Zm00001d026625_T001 transcript:Zm00001d001826_T002  3.00E-12
142-  1:transcript:Zm00001d026627_T001 transcript:Zm00001d001825_T001         0
142-  2:transcript:Zm00001d026628_T001 transcript:Zm00001d001824_T001  6.00E-72
142-  3:transcript:Zm00001d026629_T001 transcript:Zm00001d001819_T001 1.00E-173
```

142- 4:transcript:Zm00001d026630_T001 transcript:Zm00001d001814_T001 5.00E-148
142- 5:transcript:Zm00001d026634_T002 transcript:Zm00001d001811_T001 3.00E-104
Alignment 143: score=280.0 e_value=4.7e-09 N=6 10&2 minus
143- 0:transcript:Zm00001d026195_T002 transcript:Zm00001d002483_T007 0
143- 1:transcript:Zm00001d026197_T001 transcript:Zm00001d002482_T001 2.00E-110
143- 2:transcript:Zm00001d026200_T001 transcript:Zm00001d002479_T001 3.00E-75
143- 3:transcript:Zm00001d026202_T001 transcript:Zm00001d002477_T001 1.00E-14
143- 4:transcript:Zm00001d026203_T001 transcript:Zm00001d002476_T001 1.00E-175
143- 5:transcript:Zm00001d026206_T001 transcript:Zm00001d002475_T001 0
Alignment 144: score=268.0 e_value=6.1e-09 N=6 10&2 minus
144- 0:transcript:Zm00001d026492_T001 transcript:Zm00001d001997_T001 0
144- 1:transcript:Zm00001d026495_T001 transcript:Zm00001d001994_T001 0
144- 2:transcript:Zm00001d026498_T001 transcript:Zm00001d001990_T001 1.00E-25
144- 3:transcript:Zm00001d026500_T001 transcript:Zm00001d001989_T001 4.00E-34
144- 4:transcript:Zm00001d026506_T001 transcript:Zm00001d001988_T002 7.00E-18
144- 5:transcript:Zm00001d026510_T001 transcript:Zm00001d001982_T001 1.00E-68
Alignment 145: score=254.0 e_value=5e-08 N=6 10&2 minus
145- 0:transcript:Zm00001d026593_T002 transcript:Zm00001d001866_T012 1.00E-150
145- 1:transcript:Zm00001d026594_T001 transcript:Zm00001d001865_T001 8.00E-78
145- 2:transcript:Zm00001d026597_T001 transcript:Zm00001d001862_T001 1.00E-105
145- 3:transcript:Zm00001d026599_T001 transcript:Zm00001d001857_T001 1.00E-145
145- 4:transcript:Zm00001d026600_T001 transcript:Zm00001d001855_T002 0
145- 5:transcript:Zm00001d026603_T002 transcript:Zm00001d001850_T001 0
Alignment 146: score=254.0 e_value=1.2e-08 N=6 10&2 minus
146- 0:transcript:Zm00001d025684_T001 transcript:Zm00001d003293_T001 1.00E-171
146- 1:transcript:Zm00001d025689_T002 transcript:Zm00001d003291_T002 0
146- 2:transcript:Zm00001d025692_T002 transcript:Zm00001d003288_T001 0
146- 3:transcript:Zm00001d025694_T001 transcript:Zm00001d003284_T001 3.00E-72
146- 4:transcript:Zm00001d025696_T001 transcript:Zm00001d003281_T001 0
146- 5:transcript:Zm00001d025699_T001 transcript:Zm00001d003272_T001 0
Alignment 147: score=306.0 e_value=2e-12 N=7 10&3 plus
147- 0:transcript:Zm00001d023272_T009 transcript:Zm00001d044657_T003 0
147- 1:transcript:Zm00001d023280_T001 transcript:Zm00001d044660_T001 2.00E-30
147- 2:transcript:Zm00001d023283_T001 transcript:Zm00001d044661_T001 0
147- 3:transcript:Zm00001d023286_T001 transcript:Zm00001d044662_T001 2.00E-35
147- 4:transcript:Zm00001d023291_T001 transcript:Zm00001d044663_T002 0
147- 5:transcript:Zm00001d023293_T001 transcript:Zm00001d044664_T001 3.00E-98
147- 6:transcript:Zm00001d023299_T003 transcript:Zm00001d044667_T002 0
Alignment 148: score=255.0 e_value=2.8e-09 N=6 10&3 plus
148- 0:transcript:Zm00001d023815_T001 transcript:Zm00001d041624_T001 0
148- 1:transcript:Zm00001d023817_T001 transcript:Zm00001d041625_T008 0
148- 2:transcript:Zm00001d023820_T001 transcript:Zm00001d041626_T001 0
148- 3:transcript:Zm00001d023825_T005 transcript:Zm00001d041635_T002 0
148- 4:transcript:Zm00001d023830_T001 transcript:Zm00001d041638_T001 6.00E-79
148- 5:transcript:Zm00001d023838_T014 transcript:Zm00001d041647_T002 0
Alignment 149: score=317.0 e_value=1.3e-15 N=8 10&3 minus
149- 0:transcript:Zm00001d023539_T001 transcript:Zm00001d041883_T001 0
149- 1:transcript:Zm00001d023542_T001 transcript:Zm00001d041882_T002 3.00E-107
149- 2:transcript:Zm00001d023544_T011 transcript:Zm00001d041877_T002 0
149- 3:transcript:Zm00001d023560_T002 transcript:Zm00001d041871_T001 4.00E-116
149- 4:transcript:Zm00001d023565_T001 transcript:Zm00001d041866_T001 8.00E-150
149- 5:transcript:Zm00001d023569_T001 transcript:Zm00001d041856_T001 3.00E-31
149- 6:transcript:Zm00001d023576_T002 transcript:Zm00001d041852_T002 0
149- 7:transcript:Zm00001d023577_T001 transcript:Zm00001d041850_T001 3.00E-28

```
## Alignment 150: score=461.0 e_value=6.4e-26 N=12 10&4 plus
150-  0:transcript:Zm00001d024615_T001 transcript:Zm00001d049576_T001  3.00E-14
150-  1:transcript:Zm00001d024624_T004 transcript:Zm00001d049579_T003         0
150-  2:transcript:Zm00001d024627_T002 transcript:Zm00001d049581_T001         0
150-  3:transcript:Zm00001d024635_T001 transcript:Zm00001d049583_T003         0
150-  4:transcript:Zm00001d024648_T001 transcript:Zm00001d049596_T001  5.00E-15
150-  5:transcript:Zm00001d024661_T001 transcript:Zm00001d049598_T001 7.00E-159
150-  6:transcript:Zm00001d024664_T001 transcript:Zm00001d049601_T001         0
150-  7:transcript:Zm00001d024676_T001 transcript:Zm00001d049602_T001  2.00E-33
150-  8:transcript:Zm00001d024677_T001 transcript:Zm00001d049605_T036         0
150-  9:transcript:Zm00001d024687_T001 transcript:Zm00001d049607_T001         0
150- 10:transcript:Zm00001d024698_T002 transcript:Zm00001d049608_T001         0
150- 11:transcript:Zm00001d024702_T001 transcript:Zm00001d049610_T001         0
## Alignment 151: score=384.0 e_value=2.4e-16 N=9 10&4 plus
151-  0:transcript:Zm00001d024528_T001 transcript:Zm00001d049505_T001         0
151-  1:transcript:Zm00001d024531_T001 transcript:Zm00001d049510_T001         0
151-  2:transcript:Zm00001d024532_T001 transcript:Zm00001d049511_T001         0
151-  3:transcript:Zm00001d024534_T001 transcript:Zm00001d049516_T001 4.00E-100
151-  4:transcript:Zm00001d024537_T001 transcript:Zm00001d049521_T001 2.00E-117
151-  5:transcript:Zm00001d024540_T001 transcript:Zm00001d049533_T001         0
151-  6:transcript:Zm00001d024541_T001 transcript:Zm00001d049536_T001 2.00E-171
151-  7:transcript:Zm00001d024543_T001 transcript:Zm00001d049540_T002         0
151-  8:transcript:Zm00001d024546_T001 transcript:Zm00001d049543_T016         0
## Alignment 152: score=365.0 e_value=1.7e-18 N=9 10&4 plus
152-  0:transcript:Zm00001d025922_T001 transcript:Zm00001d051251_T003  5.00E-75
152-  1:transcript:Zm00001d025923_T001 transcript:Zm00001d051259_T001  1.00E-58
152-  2:transcript:Zm00001d025926_T001 transcript:Zm00001d051262_T001 6.00E-105
152-  3:transcript:Zm00001d025930_T001 transcript:Zm00001d051269_T001 2.00E-108
152-  4:transcript:Zm00001d025933_T001 transcript:Zm00001d051287_T001 2.00E-174
152-  5:transcript:Zm00001d025939_T003 transcript:Zm00001d051288_T001  6.00E-80
152-  6:transcript:Zm00001d025947_T001 transcript:Zm00001d051302_T001  2.00E-41
152-  7:transcript:Zm00001d025950_T002 transcript:Zm00001d051307_T001 2.00E-166
152-  8:transcript:Zm00001d025951_T002 transcript:Zm00001d051308_T009         0
## Alignment 153: score=314.0 e_value=1.5e-13 N=8 10&4 plus
153-  0:transcript:Zm00001d024357_T001 transcript:Zm00001d050190_T001  8.00E-63
153-  1:transcript:Zm00001d024364_T001 transcript:Zm00001d050191_T001         0
153-  2:transcript:Zm00001d024373_T001 transcript:Zm00001d050193_T001 5.00E-173
153-  3:transcript:Zm00001d024376_T001 transcript:Zm00001d050195_T001 2.00E-122
153-  4:transcript:Zm00001d024392_T001 transcript:Zm00001d050201_T001 7.00E-117
153-  5:transcript:Zm00001d024408_T005 transcript:Zm00001d050208_T003         0
153-  6:transcript:Zm00001d024412_T002 transcript:Zm00001d050218_T001         0
153-  7:transcript:Zm00001d024415_T001 transcript:Zm00001d050222_T001  6.00E-20
## Alignment 154: score=296.0 e_value=6e-12 N=7 10&4 plus
154-  0:transcript:Zm00001d023400_T002 transcript:Zm00001d052530_T001         0
154-  1:transcript:Zm00001d023405_T001 transcript:Zm00001d052534_T001  3.00E-20
154-  2:transcript:Zm00001d023420_T001 transcript:Zm00001d052537_T001 3.00E-109
154-  3:transcript:Zm00001d023422_T001 transcript:Zm00001d052540_T001         0
154-  4:transcript:Zm00001d023424_T001 transcript:Zm00001d052543_T001 7.00E-171
154-  5:transcript:Zm00001d023431_T002 transcript:Zm00001d052556_T001         0
154-  6:transcript:Zm00001d023443_T001 transcript:Zm00001d052562_T001  3.00E-42
## Alignment 155: score=270.0 e_value=4.2e-09 N=6 10&4 plus
155-  0:transcript:Zm00001d025896_T001 transcript:Zm00001d051216_T001  6.00E-48
155-  1:transcript:Zm00001d025900_T001 transcript:Zm00001d051223_T001 8.00E-116
155-  2:transcript:Zm00001d025901_T001 transcript:Zm00001d051225_T001  2.00E-80
```

155- 3:transcript:Zm00001d025908_T001 transcript:Zm00001d051235_T001 2.00E-124
155- 4:transcript:Zm00001d025910_T001 transcript:Zm00001d051239_T001 1.00E-74
155- 5:transcript:Zm00001d025911_T002 transcript:Zm00001d051241_T001 0
Alignment 156: score=258.0 e_value=6e-11 N=6 10&4 plus
156- 0:transcript:Zm00001d023538_T006 transcript:Zm00001d053009_T009 0
156- 1:transcript:Zm00001d023542_T001 transcript:Zm00001d053010_T001 3.00E-111
156- 2:transcript:Zm00001d023544_T011 transcript:Zm00001d053011_T010 0
156- 3:transcript:Zm00001d023559_T001 transcript:Zm00001d053015_T001 0
156- 4:transcript:Zm00001d023560_T002 transcript:Zm00001d053016_T002 2.00E-128
156- 5:transcript:Zm00001d023565_T001 transcript:Zm00001d053020_T001 4.00E-70
Alignment 157: score=584.0 e_value=1.3e-30 N=13 10&4 minus
157- 0:transcript:Zm00001d026152_T001 transcript:Zm00001d051394_T001 6.00E-10
157- 1:transcript:Zm00001d026153_T001 transcript:Zm00001d051393_T002 4.00E-25
157- 2:transcript:Zm00001d026154_T002 transcript:Zm00001d051392_T001 1.00E-108
157- 3:transcript:Zm00001d026158_T001 transcript:Zm00001d051387_T001 0
157- 4:transcript:Zm00001d026162_T001 transcript:Zm00001d051384_T001 8.00E-55
157- 5:transcript:Zm00001d026166_T002 transcript:Zm00001d051370_T001 1.00E-42
157- 6:transcript:Zm00001d026170_T001 transcript:Zm00001d051368_T001 5.00E-77
157- 7:transcript:Zm00001d026173_T001 transcript:Zm00001d051365_T001 4.00E-38
157- 8:transcript:Zm00001d026177_T001 transcript:Zm00001d051362_T001 9.00E-150
157- 9:transcript:Zm00001d026180_T001 transcript:Zm00001d051360_T001 2.00E-35
157- 10:transcript:Zm00001d026182_T001 transcript:Zm00001d051355_T001 1.00E-55
157- 11:transcript:Zm00001d026184_T001 transcript:Zm00001d051350_T001 4.00E-41
157- 12:transcript:Zm00001d026189_T001 transcript:Zm00001d051343_T001 5.00E-127
Alignment 158: score=390.0 e_value=8.4e-24 N=10 10&4 minus
158- 0:transcript:Zm00001d024305_T001 transcript:Zm00001d049376_T001 2.00E-44
158- 1:transcript:Zm00001d024310_T003 transcript:Zm00001d049372_T002 0
158- 2:transcript:Zm00001d024311_T001 transcript:Zm00001d049371_T002 5.00E-173
158- 3:transcript:Zm00001d024318_T009 transcript:Zm00001d049370_T002 0
158- 4:transcript:Zm00001d024321_T001 transcript:Zm00001d049369_T002 2.00E-109
158- 5:transcript:Zm00001d024324_T001 transcript:Zm00001d049364_T001 1.00E-30
158- 6:transcript:Zm00001d024338_T008 transcript:Zm00001d049361_T004 0
158- 7:transcript:Zm00001d024339_T001 transcript:Zm00001d049360_T004 0
158- 8:transcript:Zm00001d024342_T001 transcript:Zm00001d049357_T001 0
158- 9:transcript:Zm00001d024349_T001 transcript:Zm00001d049350_T001 4.00E-27
Alignment 159: score=357.0 e_value=3.2e-16 N=8 10&4 minus
159- 0:transcript:Zm00001d023238_T002 transcript:Zm00001d052485_T001 0
159- 1:transcript:Zm00001d023242_T001 transcript:Zm00001d052476_T001 3.00E-30
159- 2:transcript:Zm00001d023243_T002 transcript:Zm00001d052472_T012 0
159- 3:transcript:Zm00001d023246_T001 transcript:Zm00001d052468_T002 0
159- 4:transcript:Zm00001d023247_T001 transcript:Zm00001d052463_T001 1.00E-63
159- 5:transcript:Zm00001d023249_T001 transcript:Zm00001d052461_T001 2.00E-90
159- 6:transcript:Zm00001d023258_T002 transcript:Zm00001d052457_T001 0
159- 7:transcript:Zm00001d023260_T001 transcript:Zm00001d052453_T014 0
Alignment 160: score=466.0 e_value=1.9e-27 N=12 10&5 plus
160- 0:transcript:Zm00001d025910_T001 transcript:Zm00001d017366_T001 2.00E-79
160- 1:transcript:Zm00001d025911_T002 transcript:Zm00001d017371_T008 0
160- 2:transcript:Zm00001d025916_T001 transcript:Zm00001d017378_T001 2.00E-72
160- 3:transcript:Zm00001d025922_T001 transcript:Zm00001d017381_T001 8.00E-107
160- 4:transcript:Zm00001d025926_T001 transcript:Zm00001d017382_T001 4.00E-100
160- 5:transcript:Zm00001d025930_T001 transcript:Zm00001d017385_T001 7.00E-114
160- 6:transcript:Zm00001d025932_T003 transcript:Zm00001d017386_T001 9.00E-59
160- 7:transcript:Zm00001d025939_T003 transcript:Zm00001d017390_T001 4.00E-82
160- 8:transcript:Zm00001d025944_T001 transcript:Zm00001d017391_T002 2.00E-76

160- 9:transcript:Zm00001d025947_T001 transcript:Zm00001d017397_T001 1.00E-44
160- 10:transcript:Zm00001d025949_T001 transcript:Zm00001d017402_T001 5.00E-70
160- 11:transcript:Zm00001d025950_T002 transcript:Zm00001d017403_T001 3.00E-148
Alignment 161: score=265.0 e_value=9.5e-09 N=6 10&5 plus
161- 0:transcript:Zm00001d025953_T003 transcript:Zm00001d017409_T001 2.00E-92
161- 1:transcript:Zm00001d025957_T001 transcript:Zm00001d017412_T001 2.00E-58
161- 2:transcript:Zm00001d025958_T001 transcript:Zm00001d017418_T001   0
161- 3:transcript:Zm00001d025959_T001 transcript:Zm00001d017419_T001 9.00E-147
161- 4:transcript:Zm00001d025964_T001 transcript:Zm00001d017422_T001 9.00E-64
161- 5:transcript:Zm00001d025977_T001 transcript:Zm00001d017427_T002   0
Alignment 162: score=417.0 e_value=6.4e-21 N=10 10&5 minus
162- 0:transcript:Zm00001d026053_T001 transcript:Zm00001d017612_T001 8.00E-58
162- 1:transcript:Zm00001d026055_T001 transcript:Zm00001d017611_T001   0
162- 2:transcript:Zm00001d026061_T001 transcript:Zm00001d017606_T001 9.00E-28
162- 3:transcript:Zm00001d026070_T002 transcript:Zm00001d017597_T001   0
162- 4:transcript:Zm00001d026076_T001 transcript:Zm00001d017590_T001   0
162- 5:transcript:Zm00001d026079_T001 transcript:Zm00001d017583_T002 2.00E-130
162- 6:transcript:Zm00001d026083_T001 transcript:Zm00001d017582_T001   0
162- 7:transcript:Zm00001d026096_T001 transcript:Zm00001d017575_T001 2.00E-91
162- 8:transcript:Zm00001d026097_T005 transcript:Zm00001d017573_T003   0
162- 9:transcript:Zm00001d026102_T001 transcript:Zm00001d017570_T001   0
Alignment 163: score=392.0 e_value=5.8e-17 N=9 10&5 minus
163- 0:transcript:Zm00001d026169_T001 transcript:Zm00001d017493_T001 4.00E-107
163- 1:transcript:Zm00001d026173_T001 transcript:Zm00001d017486_T001 5.00E-34
163- 2:transcript:Zm00001d026177_T001 transcript:Zm00001d017485_T003 5.00E-146
163- 3:transcript:Zm00001d026180_T001 transcript:Zm00001d017482_T002 2.00E-43
163- 4:transcript:Zm00001d026182_T001 transcript:Zm00001d017478_T001 5.00E-42
163- 5:transcript:Zm00001d026184_T001 transcript:Zm00001d017477_T001 3.00E-56
163- 6:transcript:Zm00001d026189_T001 transcript:Zm00001d017473_T002 2.00E-148
163- 7:transcript:Zm00001d026190_T001 transcript:Zm00001d017468_T001 5.00E-99
163- 8:transcript:Zm00001d026191_T001 transcript:Zm00001d017462_T001 9.00E-95
Alignment 164: score=265.0 e_value=2.2e-10 N=6 10&5 minus
164- 0:transcript:Zm00001d025863_T001 transcript:Zm00001d014032_T001 3.00E-77
164- 1:transcript:Zm00001d025864_T001 transcript:Zm00001d014029_T001 4.00E-75
164- 2:transcript:Zm00001d025868_T002 transcript:Zm00001d014016_T001 6.00E-114
164- 3:transcript:Zm00001d025869_T003 transcript:Zm00001d014015_T001 6.00E-38
164- 4:transcript:Zm00001d025871_T001 transcript:Zm00001d014013_T002 2.00E-133
164- 5:transcript:Zm00001d025872_T001 transcript:Zm00001d014007_T001 6.00E-24
Alignment 165: score=322.0 e_value=5.6e-12 N=7 10&8 plus
165- 0:transcript:Zm00001d024767_T003 transcript:Zm00001d008189_T004   0
165- 1:transcript:Zm00001d024768_T001 transcript:Zm00001d008190_T001 1.00E-90
165- 2:transcript:Zm00001d024772_T001 transcript:Zm00001d008196_T001 1.00E-21
165- 3:transcript:Zm00001d024778_T001 transcript:Zm00001d008200_T001 8.00E-29
165- 4:transcript:Zm00001d024783_T001 transcript:Zm00001d008205_T001 6.00E-70
165- 5:transcript:Zm00001d024784_T001 transcript:Zm00001d008206_T001 7.00E-47
165- 6:transcript:Zm00001d024786_T001 transcript:Zm00001d008208_T001 1.00E-11
Alignment 166: score=495.0 e_value=7.4e-30 N=12 10&8 minus
166- 0:transcript:Zm00001d024722_T002 transcript:Zm00001d010940_T001 1.00E-98
166- 1:transcript:Zm00001d024725_T001 transcript:Zm00001d010933_T001 1.00E-144
166- 2:transcript:Zm00001d024733_T001 transcript:Zm00001d010927_T001 1.00E-131
166- 3:transcript:Zm00001d024734_T001 transcript:Zm00001d010926_T001 4.00E-107
166- 4:transcript:Zm00001d024738_T001 transcript:Zm00001d010924_T001 3.00E-90
166- 5:transcript:Zm00001d024754_T001 transcript:Zm00001d010920_T001   0
166- 6:transcript:Zm00001d024755_T004 transcript:Zm00001d010919_T001   0

166- 7:transcript:Zm00001d024756_T004 transcript:Zm00001d010918_T001 0
166- 8:transcript:Zm00001d024764_T001 transcript:Zm00001d010914_T001 2.00E-134
166- 9:transcript:Zm00001d024772_T001 transcript:Zm00001d010912_T001 1.00E-29
166- 10:transcript:Zm00001d024784_T001 transcript:Zm00001d010907_T001 3.00E-59
166- 11:transcript:Zm00001d024796_T001 transcript:Zm00001d010902_T002 0
Alignment 167: score=292.0 e_value=3.5e-09 N=6 10&9 plus
167- 0:transcript:Zm00001d025493_T001 transcript:Zm00001d048374_T001 3.00E-60
167- 1:transcript:Zm00001d025494_T001 transcript:Zm00001d048375_T001 2.00E-157
167- 2:transcript:Zm00001d025495_T002 transcript:Zm00001d048376_T001 6.00E-61
167- 3:transcript:Zm00001d025497_T003 transcript:Zm00001d048379_T002 0
167- 4:transcript:Zm00001d025502_T001 transcript:Zm00001d048380_T001 3.00E-91
167- 5:transcript:Zm00001d025503_T001 transcript:Zm00001d048381_T001 1.00E-82
Alignment 168: score=442.0 e_value=8.5e-19 N=9 2&2 plus
168- 0:transcript:Zm00001d002202_T001 transcript:Zm00001d002221_T001 9.00E-91
168- 1:transcript:Zm00001d002203_T001 transcript:Zm00001d002223_T001 2.00E-139
168- 2:transcript:Zm00001d002204_T001 transcript:Zm00001d002224_T001 2.00E-160
168- 3:transcript:Zm00001d002205_T001 transcript:Zm00001d002225_T001 0
168- 4:transcript:Zm00001d002206_T001 transcript:Zm00001d002226_T001 9.00E-172
168- 5:transcript:Zm00001d002207_T001 transcript:Zm00001d002227_T001 6.00E-103
168- 6:transcript:Zm00001d002214_T001 transcript:Zm00001d002228_T001 1.00E-89
168- 7:transcript:Zm00001d002216_T001 transcript:Zm00001d002230_T001 3.00E-86
168- 8:transcript:Zm00001d002217_T001 transcript:Zm00001d002232_T001 0
Alignment 169: score=258.0 e_value=1.4e-08 N=6 2&4 plus
169- 0:transcript:Zm00001d006052_T004 transcript:Zm00001d049764_T001 5.00E-169
169- 1:transcript:Zm00001d006054_T001 transcript:Zm00001d049765_T001 1.00E-110
169- 2:transcript:Zm00001d006059_T001 transcript:Zm00001d049768_T001 1.00E-49
169- 3:transcript:Zm00001d006060_T001 transcript:Zm00001d049769_T001 1.00E-11
169- 4:transcript:Zm00001d006063_T001 transcript:Zm00001d049785_T003 0
169- 5:transcript:Zm00001d006064_T002 transcript:Zm00001d049790_T007 7.00E-92
Alignment 170: score=250.0 e_value=4e-08 N=6 2&4 plus
170- 0:transcript:Zm00001d003041_T001 transcript:Zm00001d051128_T001 2.00E-23
170- 1:transcript:Zm00001d003049_T003 transcript:Zm00001d051135_T005 0
170- 2:transcript:Zm00001d003052_T001 transcript:Zm00001d051140_T001 6.00E-105
170- 3:transcript:Zm00001d003057_T001 transcript:Zm00001d051143_T001 3.00E-99
170- 4:transcript:Zm00001d003064_T001 transcript:Zm00001d051149_T001 1.00E-92
170- 5:transcript:Zm00001d003068_T001 transcript:Zm00001d051156_T001 6.00E-156
Alignment 171: score=748.0 e_value=8.5e-50 N=18 2&4 minus
171- 0:transcript:Zm00001d005003_T002 transcript:Zm00001d052415_T001 4.00E-97
171- 1:transcript:Zm00001d005005_T001 transcript:Zm00001d052412_T001 9.00E-35
171- 2:transcript:Zm00001d005006_T003 transcript:Zm00001d052411_T002 0
171- 3:transcript:Zm00001d005012_T002 transcript:Zm00001d052407_T004 0
171- 4:transcript:Zm00001d005016_T005 transcript:Zm00001d052405_T002 0
171- 5:transcript:Zm00001d005019_T001 transcript:Zm00001d052399_T009 0
171- 6:transcript:Zm00001d005026_T001 transcript:Zm00001d052392_T003 0
171- 7:transcript:Zm00001d005027_T002 transcript:Zm00001d052391_T001 5.00E-121
171- 8:transcript:Zm00001d005028_T001 transcript:Zm00001d052390_T001 2.00E-124
171- 9:transcript:Zm00001d005029_T001 transcript:Zm00001d052380_T001 0
171- 10:transcript:Zm00001d005035_T001 transcript:Zm00001d052378_T001 0
171- 11:transcript:Zm00001d005036_T003 transcript:Zm00001d052376_T006 0
171- 12:transcript:Zm00001d005037_T001 transcript:Zm00001d052375_T002 2.00E-48
171- 13:transcript:Zm00001d005038_T001 transcript:Zm00001d052373_T001 7.00E-86
171- 14:transcript:Zm00001d005053_T001 transcript:Zm00001d052363_T001 3.00E-99
171- 15:transcript:Zm00001d005056_T001 transcript:Zm00001d052358_T001 6.00E-23
171- 16:transcript:Zm00001d005057_T001 transcript:Zm00001d052355_T001 6.00E-129

```
171- 17:transcript:Zm00001d005060_T001 transcript:Zm00001d052354_T005  3.00E-46
## Alignment 172: score=624.0 e_value=1.9e-39 N=15 2&4 minus
172-  0:transcript:Zm00001d002819_T002 transcript:Zm00001d051307_T001 2.00E-164
172-  1:transcript:Zm00001d002823_T001 transcript:Zm00001d051306_T001         0
172-  2:transcript:Zm00001d002825_T001 transcript:Zm00001d051305_T001  1.00E-89
172-  3:transcript:Zm00001d002826_T001 transcript:Zm00001d051302_T001  3.00E-41
172-  4:transcript:Zm00001d002828_T001 transcript:Zm00001d051294_T001  5.00E-93
172-  5:transcript:Zm00001d002836_T006 transcript:Zm00001d051287_T001         0
172-  6:transcript:Zm00001d002837_T001 transcript:Zm00001d051272_T001 4.00E-103
172-  7:transcript:Zm00001d002843_T001 transcript:Zm00001d051269_T001 4.00E-104
172-  8:transcript:Zm00001d002844_T003 transcript:Zm00001d051262_T001 5.00E-104
172-  9:transcript:Zm00001d002845_T001 transcript:Zm00001d051251_T003  6.00E-19
172- 10:transcript:Zm00001d002849_T001 transcript:Zm00001d051248_T001  4.00E-08
172- 11:transcript:Zm00001d002865_T002 transcript:Zm00001d051241_T001         0
172- 12:transcript:Zm00001d002867_T001 transcript:Zm00001d051239_T001  2.00E-85
172- 13:transcript:Zm00001d002868_T001 transcript:Zm00001d051238_T001  2.00E-41
172- 14:transcript:Zm00001d002869_T001 transcript:Zm00001d051235_T001 1.00E-114
## Alignment 173: score=407.0 e_value=7.8e-19 N=10 2&4 minus
173-  0:transcript:Zm00001d004779_T001 transcript:Zm00001d053041_T002         0
173-  1:transcript:Zm00001d004782_T005 transcript:Zm00001d053038_T007         0
173-  2:transcript:Zm00001d004784_T001 transcript:Zm00001d053028_T001         0
173-  3:transcript:Zm00001d004790_T008 transcript:Zm00001d053022_T001         0
173-  4:transcript:Zm00001d004791_T001 transcript:Zm00001d053020_T001  3.00E-56
173-  5:transcript:Zm00001d004804_T001 transcript:Zm00001d053017_T001         0
173-  6:transcript:Zm00001d004812_T002 transcript:Zm00001d053016_T002         0
173-  7:transcript:Zm00001d004818_T001 transcript:Zm00001d053014_T001         0
173-  8:transcript:Zm00001d004821_T002 transcript:Zm00001d053010_T001 3.00E-111
173-  9:transcript:Zm00001d004822_T001 transcript:Zm00001d053006_T001 9.00E-101
## Alignment 174: score=395.0 e_value=3.2e-17 N=9 2&4 minus
174-  0:transcript:Zm00001d004895_T001 transcript:Zm00001d052578_T001 2.00E-151
174-  1:transcript:Zm00001d004898_T002 transcript:Zm00001d052565_T001  6.00E-54
174-  2:transcript:Zm00001d004909_T001 transcript:Zm00001d052551_T001 2.00E-170
174-  3:transcript:Zm00001d004910_T001 transcript:Zm00001d052545_T001  2.00E-33
174-  4:transcript:Zm00001d004913_T001 transcript:Zm00001d052542_T002 2.00E-161
174-  5:transcript:Zm00001d004914_T002 transcript:Zm00001d052538_T002 2.00E-165
174-  6:transcript:Zm00001d004915_T001 transcript:Zm00001d052533_T001 5.00E-160
174-  7:transcript:Zm00001d004916_T001 transcript:Zm00001d052525_T001         0
174-  8:transcript:Zm00001d004919_T001 transcript:Zm00001d052520_T001 2.00E-164
## Alignment 175: score=337.0 e_value=9.6e-16 N=8 2&4 minus
175-  0:transcript:Zm00001d002743_T001 transcript:Zm00001d051365_T001  5.00E-17
175-  1:transcript:Zm00001d002744_T001 transcript:Zm00001d051355_T001  8.00E-50
175-  2:transcript:Zm00001d002747_T001 transcript:Zm00001d051350_T001  3.00E-59
175-  3:transcript:Zm00001d002756_T001 transcript:Zm00001d051344_T001  1.00E-18
175-  4:transcript:Zm00001d002757_T001 transcript:Zm00001d051343_T001 3.00E-134
175-  5:transcript:Zm00001d002760_T001 transcript:Zm00001d051340_T001  5.00E-54
175-  6:transcript:Zm00001d002772_T001 transcript:Zm00001d051333_T001  7.00E-56
175-  7:transcript:Zm00001d002776_T001 transcript:Zm00001d051323_T003         0
## Alignment 176: score=328.0 e_value=4.5e-16 N=8 2&4 minus
176-  0:transcript:Zm00001d004846_T001 transcript:Zm00001d053192_T001         0
176-  1:transcript:Zm00001d004848_T001 transcript:Zm00001d053181_T001 7.00E-170
176-  2:transcript:Zm00001d004851_T001 transcript:Zm00001d053178_T001         0
176-  3:transcript:Zm00001d004855_T002 transcript:Zm00001d053177_T001         0
176-  4:transcript:Zm00001d004856_T001 transcript:Zm00001d053173_T006         0
176-  5:transcript:Zm00001d004857_T001 transcript:Zm00001d053162_T003         0
```

176- 6: transcript:Zm00001d004865_T001 transcript:Zm00001d053151_T003 3.00E-33
176- 7: transcript:Zm00001d004868_T001 transcript:Zm00001d053150_T007 0
Alignment 177: score=288.0 e_value=2.3e-10 N=7 2&4 minus
177- 0: transcript:Zm00001d002540_T002 transcript:Zm00001d051502_T001 0
177- 1: transcript:Zm00001d002545_T002 transcript:Zm00001d051480_T001 4.00E-57
177- 2: transcript:Zm00001d002549_T002 transcript:Zm00001d051475_T002 2.00E-06
177- 3: transcript:Zm00001d002562_T001 transcript:Zm00001d051471_T001 1.00E-104
177- 4: transcript:Zm00001d002568_T001 transcript:Zm00001d051468_T001 3.00E-40
177- 5: transcript:Zm00001d002573_T001 transcript:Zm00001d051461_T001 0
177- 6: transcript:Zm00001d002576_T001 transcript:Zm00001d051460_T001 5.00E-59
Alignment 178: score=269.0 e_value=9.4e-09 N=6 2&4 minus
178- 0: transcript:Zm00001d002992_T002 transcript:Zm00001d051180_T002 7.00E-108
178- 1: transcript:Zm00001d003006_T001 transcript:Zm00001d051174_T001 0
178- 2: transcript:Zm00001d003011_T001 transcript:Zm00001d051172_T001 8.00E-52
178- 3: transcript:Zm00001d003015_T001 transcript:Zm00001d051163_T001 0
178- 4: transcript:Zm00001d003016_T001 transcript:Zm00001d051161_T003 0
178- 5: transcript:Zm00001d003017_T002 transcript:Zm00001d051157_T001 0
Alignment 179: score=267.0 e_value=1.4e-10 N=7 2&4 minus
179- 0: transcript:Zm00001d003438_T001 transcript:Zm00001d050872_T001 0
179- 1: transcript:Zm00001d003447_T001 transcript:Zm00001d050864_T001 1.00E-12
179- 2: transcript:Zm00001d003451_T001 transcript:Zm00001d050861_T001 2.00E-150
179- 3: transcript:Zm00001d003468_T001 transcript:Zm00001d050860_T001 2.00E-139
179- 4: transcript:Zm00001d003477_T001 transcript:Zm00001d050850_T001 0
179- 5: transcript:Zm00001d003492_T001 transcript:Zm00001d050848_T001 7.00E-67
179- 6: transcript:Zm00001d003497_T001 transcript:Zm00001d050840_T001 1.00E-44
Alignment 180: score=250.0 e_value=5.1e-08 N=6 2&4 minus
180- 0: transcript:Zm00001d005841_T001 transcript:Zm00001d050016_T001 6.00E-51
180- 1: transcript:Zm00001d005844_T002 transcript:Zm00001d050011_T004 2.00E-53
180- 2: transcript:Zm00001d005849_T001 transcript:Zm00001d050008_T001 6.00E-160
180- 3: transcript:Zm00001d005859_T001 transcript:Zm00001d049996_T001 8.00E-76
180- 4: transcript:Zm00001d005865_T001 transcript:Zm00001d049991_T001 2.00E-94
180- 5: transcript:Zm00001d005869_T001 transcript:Zm00001d049990_T001 2.00E-113
Alignment 181: score=323.0 e_value=1e-10 N=7 2&5 plus
181- 0: transcript:Zm00001d003745_T001 transcript:Zm00001d016730_T001 2.00E-173
181- 1: transcript:Zm00001d003750_T001 transcript:Zm00001d016731_T001 0
181- 2: transcript:Zm00001d003751_T001 transcript:Zm00001d016733_T001 1.00E-87
181- 3: transcript:Zm00001d003754_T003 transcript:Zm00001d016735_T001 0
181- 4: transcript:Zm00001d003755_T001 transcript:Zm00001d016737_T002 8.00E-113
181- 5: transcript:Zm00001d003763_T001 transcript:Zm00001d016743_T001 6.00E-27
181- 6: transcript:Zm00001d003769_T001 transcript:Zm00001d016745_T001 6.00E-37
Alignment 182: score=715.0 e_value=3.8e-46 N=17 2&5 minus
182- 0: transcript:Zm00001d002819_T002 transcript:Zm00001d017403_T001 2.00E-158
182- 1: transcript:Zm00001d002820_T001 transcript:Zm00001d017402_T001 2.00E-71
182- 2: transcript:Zm00001d002823_T001 transcript:Zm00001d017401_T001 0
182- 3: transcript:Zm00001d002824_T001 transcript:Zm00001d017399_T001 0
182- 4: transcript:Zm00001d002826_T001 transcript:Zm00001d017397_T001 9.00E-43
182- 5: transcript:Zm00001d002828_T001 transcript:Zm00001d017396_T001 1.00E-92
182- 6: transcript:Zm00001d002829_T001 transcript:Zm00001d017391_T002 5.00E-89
182- 7: transcript:Zm00001d002842_T001 transcript:Zm00001d017386_T001 2.00E-61
182- 8: transcript:Zm00001d002843_T001 transcript:Zm00001d017385_T001 4.00E-103
182- 9: transcript:Zm00001d002844_T003 transcript:Zm00001d017382_T001 2.00E-102
182- 10: transcript:Zm00001d002848_T001 transcript:Zm00001d017381_T001 7.00E-104
182- 11: transcript:Zm00001d002854_T001 transcript:Zm00001d017379_T001 2.00E-71
182- 12: transcript:Zm00001d002856_T001 transcript:Zm00001d017378_T001 4.00E-74

182- 13:transcript:Zm00001d002860_T002 transcript:Zm00001d017377_T001 2.00E-142
182- 14:transcript:Zm00001d002865_T002 transcript:Zm00001d017371_T008 0
182- 15:transcript:Zm00001d002867_T001 transcript:Zm00001d017366_T001 6.00E-88
182- 16:transcript:Zm00001d002868_T001 transcript:Zm00001d017364_T001 7.00E-43
Alignment 183: score=600.0 e_value=2.2e-41 N=16 2&5 minus
183- 0:transcript:Zm00001d003432_T001 transcript:Zm00001d016934_T001 8.00E-155
183- 1:transcript:Zm00001d003438_T001 transcript:Zm00001d016933_T001 0
183- 2:transcript:Zm00001d003451_T001 transcript:Zm00001d016924_T001 2.00E-138
183- 3:transcript:Zm00001d003469_T001 transcript:Zm00001d016919_T001 1.00E-179
183- 4:transcript:Zm00001d003472_T002 transcript:Zm00001d016918_T001 2.00E-16
183- 5:transcript:Zm00001d003477_T001 transcript:Zm00001d016915_T002 0
183- 6:transcript:Zm00001d003483_T001 transcript:Zm00001d016910_T001 6.00E-96
183- 7:transcript:Zm00001d003492_T001 transcript:Zm00001d016909_T001 4.00E-59
183- 8:transcript:Zm00001d003493_T002 transcript:Zm00001d016908_T001 1.00E-94
183- 9:transcript:Zm00001d003499_T001 transcript:Zm00001d016901_T001 5.00E-47
183- 10:transcript:Zm00001d003505_T001 transcript:Zm00001d016899_T001 5.00E-10
183- 11:transcript:Zm00001d003509_T001 transcript:Zm00001d016894_T005 0
183- 12:transcript:Zm00001d003518_T003 transcript:Zm00001d016890_T001 4.00E-116
183- 13:transcript:Zm00001d003520_T001 transcript:Zm00001d016884_T002 4.00E-170
183- 14:transcript:Zm00001d003534_T001 transcript:Zm00001d016878_T001 2.00E-159
183- 15:transcript:Zm00001d003549_T001 transcript:Zm00001d016876_T001 2.00E-97
Alignment 184: score=489.0 e_value=8.2e-29 N=12 2&5 minus
184- 0:transcript:Zm00001d002512_T001 transcript:Zm00001d017657_T001 2.00E-70
184- 1:transcript:Zm00001d002519_T001 transcript:Zm00001d017653_T001 2.00E-93
184- 2:transcript:Zm00001d002520_T001 transcript:Zm00001d017648_T001 1.00E-17
184- 3:transcript:Zm00001d002523_T001 transcript:Zm00001d017646_T001 8.00E-19
184- 4:transcript:Zm00001d002535_T001 transcript:Zm00001d017645_T001 3.00E-60
184- 5:transcript:Zm00001d002542_T001 transcript:Zm00001d017643_T001 0
184- 6:transcript:Zm00001d002544_T004 transcript:Zm00001d017641_T004 0
184- 7:transcript:Zm00001d002549_T002 transcript:Zm00001d017622_T001 2.00E-08
184- 8:transcript:Zm00001d002562_T001 transcript:Zm00001d017618_T001 2.00E-120
184- 9:transcript:Zm00001d002568_T001 transcript:Zm00001d017615_T001 2.00E-38
184- 10:transcript:Zm00001d002576_T001 transcript:Zm00001d017612_T001 2.00E-58
184- 11:transcript:Zm00001d002579_T001 transcript:Zm00001d017611_T001 0
Alignment 185: score=389.0 e_value=3.2e-16 N=9 2&5 minus
185- 0:transcript:Zm00001d002992_T002 transcript:Zm00001d017298_T003 7.00E-109
185- 1:transcript:Zm00001d002999_T001 transcript:Zm00001d017294_T001 1.00E-158
185- 2:transcript:Zm00001d003006_T001 transcript:Zm00001d017288_T001 0
185- 3:transcript:Zm00001d003009_T001 transcript:Zm00001d017287_T001 3.00E-25
185- 4:transcript:Zm00001d003012_T001 transcript:Zm00001d017282_T001 1.00E-109
185- 5:transcript:Zm00001d003015_T001 transcript:Zm00001d017275_T001 0
185- 6:transcript:Zm00001d003016_T001 transcript:Zm00001d017274_T001 0
185- 7:transcript:Zm00001d003017_T002 transcript:Zm00001d017271_T005 0
185- 8:transcript:Zm00001d003021_T001 transcript:Zm00001d017264_T001 0
Alignment 186: score=362.0 e_value=1.6e-15 N=8 2&5 minus
186- 0:transcript:Zm00001d002743_T001 transcript:Zm00001d017486_T001 5.00E-19
186- 1:transcript:Zm00001d002744_T001 transcript:Zm00001d017478_T001 7.00E-47
186- 2:transcript:Zm00001d002748_T001 transcript:Zm00001d017477_T001 3.00E-25
186- 3:transcript:Zm00001d002756_T001 transcript:Zm00001d017474_T001 2.00E-28
186- 4:transcript:Zm00001d002757_T001 transcript:Zm00001d017473_T002 7.00E-154
186- 5:transcript:Zm00001d002758_T002 transcript:Zm00001d017468_T001 1.00E-101
186- 6:transcript:Zm00001d002760_T001 transcript:Zm00001d017466_T001 7.00E-71
186- 7:transcript:Zm00001d002762_T001 transcript:Zm00001d017462_T001 4.00E-97
Alignment 187: score=309.0 e_value=1.5e-11 N=7 2&5 minus

```
187-  0:transcript:Zm00001d002796_T004 transcript:Zm00001d017445_T001 4.00E-138
187-  1:transcript:Zm00001d002797_T001 transcript:Zm00001d017427_T002         0
187-  2:transcript:Zm00001d002799_T001 transcript:Zm00001d017422_T001  9.00E-63
187-  3:transcript:Zm00001d002801_T001 transcript:Zm00001d017420_T001  3.00E-85
187-  4:transcript:Zm00001d002802_T001 transcript:Zm00001d017419_T001 1.00E-148
187-  5:transcript:Zm00001d002806_T001 transcript:Zm00001d017412_T001  2.00E-59
187-  6:transcript:Zm00001d002811_T002 transcript:Zm00001d017409_T001  6.00E-95
## Alignment 188: score=295.0 e_value=1.3e-10 N=7 2&5 minus
188-  0:transcript:Zm00001d003347_T002 transcript:Zm00001d017026_T003  6.00E-56
188-  1:transcript:Zm00001d003353_T001 transcript:Zm00001d017019_T001  1.00E-16
188-  2:transcript:Zm00001d003357_T001 transcript:Zm00001d017014_T001  4.00E-18
188-  3:transcript:Zm00001d003358_T001 transcript:Zm00001d017009_T001  3.00E-21
188-  4:transcript:Zm00001d003363_T001 transcript:Zm00001d017008_T001  1.00E-08
188-  5:transcript:Zm00001d003369_T004 transcript:Zm00001d016998_T001  1.00E-46
188-  6:transcript:Zm00001d003375_T001 transcript:Zm00001d016992_T001  6.00E-17
## Alignment 189: score=265.0 e_value=2e-10 N=6 2&5 minus
189-  0:transcript:Zm00001d003287_T001 transcript:Zm00001d017095_T001  3.00E-73
189-  1:transcript:Zm00001d003288_T001 transcript:Zm00001d017094_T001 2.00E-175
189-  2:transcript:Zm00001d003292_T001 transcript:Zm00001d017085_T002         0
189-  3:transcript:Zm00001d003293_T001 transcript:Zm00001d017084_T001  7.00E-86
189-  4:transcript:Zm00001d003297_T001 transcript:Zm00001d017079_T001         0
189-  5:transcript:Zm00001d003300_T001 transcript:Zm00001d017072_T002  2.00E-66
## Alignment 190: score=263.0 e_value=4.1e-10 N=6 2&5 minus
190-  0:transcript:Zm00001d003833_T001 transcript:Zm00001d016671_T001  5.00E-58
190-  1:transcript:Zm00001d003840_T001 transcript:Zm00001d016669_T001  4.00E-61
190-  2:transcript:Zm00001d003846_T001 transcript:Zm00001d016665_T001 2.00E-131
190-  3:transcript:Zm00001d003847_T001 transcript:Zm00001d016664_T001         0
190-  4:transcript:Zm00001d003848_T004 transcript:Zm00001d016662_T003         0
190-  5:transcript:Zm00001d003850_T001 transcript:Zm00001d016648_T001  3.00E-67
## Alignment 191: score=263.0 e_value=7.1e-09 N=6 2&5 minus
191-  0:transcript:Zm00001d002478_T001 transcript:Zm00001d017692_T002  9.00E-40
191-  1:transcript:Zm00001d002488_T001 transcript:Zm00001d017684_T001  2.00E-21
191-  2:transcript:Zm00001d002489_T001 transcript:Zm00001d017682_T001 2.00E-121
191-  3:transcript:Zm00001d002491_T001 transcript:Zm00001d017678_T001 1.00E-104
191-  4:transcript:Zm00001d002492_T001 transcript:Zm00001d017677_T001  3.00E-40
191-  5:transcript:Zm00001d002498_T001 transcript:Zm00001d017671_T001 2.00E-170
## Alignment 192: score=1505.0 e_value=4.3e-118 N=34 2&7 plus
192-  0:transcript:Zm00001d006651_T001 transcript:Zm00001d021891_T005         0
192-  1:transcript:Zm00001d006653_T001 transcript:Zm00001d021892_T001 1.00E-133
192-  2:transcript:Zm00001d006654_T001 transcript:Zm00001d021893_T002 5.00E-112
192-  3:transcript:Zm00001d006656_T001 transcript:Zm00001d021898_T001  2.00E-51
192-  4:transcript:Zm00001d006658_T005 transcript:Zm00001d021903_T005         0
192-  5:transcript:Zm00001d006663_T001 transcript:Zm00001d021906_T001         0
192-  6:transcript:Zm00001d006667_T003 transcript:Zm00001d021908_T005         0
192-  7:transcript:Zm00001d006669_T001 transcript:Zm00001d021912_T001         0
192-  8:transcript:Zm00001d006670_T001 transcript:Zm00001d021913_T001  2.00E-78
192-  9:transcript:Zm00001d006673_T001 transcript:Zm00001d021915_T001         0
192- 10:transcript:Zm00001d006677_T001 transcript:Zm00001d021927_T001 7.00E-133
192- 11:transcript:Zm00001d006678_T001 transcript:Zm00001d021929_T001         0
192- 12:transcript:Zm00001d006679_T001 transcript:Zm00001d021930_T001         0
192- 13:transcript:Zm00001d006680_T001 transcript:Zm00001d021931_T001         0
192- 14:transcript:Zm00001d006682_T001 transcript:Zm00001d021932_T001         0
192- 15:transcript:Zm00001d006687_T001 transcript:Zm00001d021934_T001 6.00E-101
192- 16:transcript:Zm00001d006688_T001 transcript:Zm00001d021935_T001         0
```

192- 17:transcript:Zm00001d006699_T001 transcript:Zm00001d021943_T002 1.00E-27
192- 18:transcript:Zm00001d006701_T001 transcript:Zm00001d021946_T001 0
192- 19:transcript:Zm00001d006702_T001 transcript:Zm00001d021947_T002 0
192- 20:transcript:Zm00001d006704_T005 transcript:Zm00001d021950_T007 0
192- 21:transcript:Zm00001d006705_T001 transcript:Zm00001d021951_T001 0
192- 22:transcript:Zm00001d006708_T001 transcript:Zm00001d021952_T001 0
192- 23:transcript:Zm00001d006710_T004 transcript:Zm00001d021954_T001 0
192- 24:transcript:Zm00001d006711_T001 transcript:Zm00001d021955_T002 7.00E-34
192- 25:transcript:Zm00001d006713_T001 transcript:Zm00001d021957_T001 5.00E-164
192- 26:transcript:Zm00001d006714_T001 transcript:Zm00001d021958_T002 1.00E-47
192- 27:transcript:Zm00001d006715_T001 transcript:Zm00001d021959_T001 1.00E-42
192- 28:transcript:Zm00001d006717_T008 transcript:Zm00001d021966_T002 0
192- 29:transcript:Zm00001d006720_T001 transcript:Zm00001d021971_T001 2.00E-136
192- 30:transcript:Zm00001d006721_T001 transcript:Zm00001d021973_T001 0
192- 31:transcript:Zm00001d006722_T001 transcript:Zm00001d021974_T002 0
192- 32:transcript:Zm00001d006723_T001 transcript:Zm00001d021976_T001 8.00E-62
192- 33:transcript:Zm00001d006726_T001 transcript:Zm00001d021978_T001 0
Alignment 193: score=962.0 e_value=1.2e-65 N=22 2&7 plus
193- 0:transcript:Zm00001d006059_T001 transcript:Zm00001d021006_T001 9.00E-110
193- 1:transcript:Zm00001d006060_T001 transcript:Zm00001d021010_T001 9.00E-29
193- 2:transcript:Zm00001d006063_T001 transcript:Zm00001d021014_T001 0
193- 3:transcript:Zm00001d006064_T002 transcript:Zm00001d021018_T002 3.00E-89
193- 4:transcript:Zm00001d006065_T002 transcript:Zm00001d021019_T001 0
193- 5:transcript:Zm00001d006066_T002 transcript:Zm00001d021023_T001 3.00E-170
193- 6:transcript:Zm00001d006069_T001 transcript:Zm00001d021025_T001 5.00E-155
193- 7:transcript:Zm00001d006070_T004 transcript:Zm00001d021032_T001 5.00E-108
193- 8:transcript:Zm00001d006071_T001 transcript:Zm00001d021035_T001 2.00E-15
193- 9:transcript:Zm00001d006078_T001 transcript:Zm00001d021038_T001 2.00E-82
193- 10:transcript:Zm00001d006080_T004 transcript:Zm00001d021046_T003 0
193- 11:transcript:Zm00001d006085_T002 transcript:Zm00001d021051_T002 7.00E-162
193- 12:transcript:Zm00001d006090_T002 transcript:Zm00001d021054_T002 1.00E-121
193- 13:transcript:Zm00001d006091_T001 transcript:Zm00001d021056_T001 0
193- 14:transcript:Zm00001d006094_T001 transcript:Zm00001d021057_T001 2.00E-171
193- 15:transcript:Zm00001d006096_T001 transcript:Zm00001d021058_T001 2.00E-57
193- 16:transcript:Zm00001d006099_T001 transcript:Zm00001d021060_T001 2.00E-59
193- 17:transcript:Zm00001d006100_T001 transcript:Zm00001d021061_T001 0
193- 18:transcript:Zm00001d006101_T001 transcript:Zm00001d021062_T001 1.00E-71
193- 19:transcript:Zm00001d006102_T002 transcript:Zm00001d021065_T001 0
193- 20:transcript:Zm00001d006107_T001 transcript:Zm00001d021070_T002 0
193- 21:transcript:Zm00001d006108_T002 transcript:Zm00001d021071_T001 0
Alignment 194: score=834.0 e_value=1.9e-57 N=20 2&7 plus
194- 0:transcript:Zm00001d005970_T001 transcript:Zm00001d020837_T001 1.00E-91
194- 1:transcript:Zm00001d005971_T001 transcript:Zm00001d020840_T001 2.00E-111
194- 2:transcript:Zm00001d005973_T001 transcript:Zm00001d020857_T001 0
194- 3:transcript:Zm00001d005976_T012 transcript:Zm00001d020858_T001 0
194- 4:transcript:Zm00001d005978_T001 transcript:Zm00001d020861_T001 3.00E-81
194- 5:transcript:Zm00001d005984_T001 transcript:Zm00001d020871_T001 2.00E-63
194- 6:transcript:Zm00001d005989_T001 transcript:Zm00001d020872_T001 6.00E-35
194- 7:transcript:Zm00001d005993_T001 transcript:Zm00001d020874_T001 6.00E-60
194- 8:transcript:Zm00001d005996_T001 transcript:Zm00001d020877_T001 3.00E-87
194- 9:transcript:Zm00001d005997_T002 transcript:Zm00001d020879_T001 8.00E-123
194- 10:transcript:Zm00001d006001_T006 transcript:Zm00001d020881_T002 0
194- 11:transcript:Zm00001d006008_T001 transcript:Zm00001d020898_T002 0
194- 12:transcript:Zm00001d006009_T001 transcript:Zm00001d020903_T001 5.00E-126

194- 13:transcript:Zm00001d006011_T001 transcript:Zm00001d020913_T001 0
194- 14:transcript:Zm00001d006016_T001 transcript:Zm00001d020926_T001 2.00E-67
194- 15:transcript:Zm00001d006017_T001 transcript:Zm00001d020927_T001 0
194- 16:transcript:Zm00001d006022_T001 transcript:Zm00001d020932_T001 0
194- 17:transcript:Zm00001d006025_T001 transcript:Zm00001d020937_T001 0
194- 18:transcript:Zm00001d006027_T005 transcript:Zm00001d020938_T002 0
194- 19:transcript:Zm00001d006028_T001 transcript:Zm00001d020941_T001 1.00E-166
Alignment 195: score=735.0 e_value=1.1e-43 N=17 2&7 plus
195- 0:transcript:Zm00001d005802_T001 transcript:Zm00001d020605_T001 1.00E-94
195- 1:transcript:Zm00001d005803_T001 transcript:Zm00001d020606_T001 1.00E-88
195- 2:transcript:Zm00001d005804_T001 transcript:Zm00001d020607_T001 3.00E-21
195- 3:transcript:Zm00001d005807_T001 transcript:Zm00001d020610_T001 6.00E-46
195- 4:transcript:Zm00001d005808_T007 transcript:Zm00001d020612_T001 0
195- 5:transcript:Zm00001d005811_T001 transcript:Zm00001d020613_T001 5.00E-103
195- 6:transcript:Zm00001d005813_T002 transcript:Zm00001d020614_T001 3.00E-81
195- 7:transcript:Zm00001d005816_T001 transcript:Zm00001d020617_T001 0
195- 8:transcript:Zm00001d005817_T006 transcript:Zm00001d020618_T003 0
195- 9:transcript:Zm00001d005819_T001 transcript:Zm00001d020623_T001 2.00E-83
195- 10:transcript:Zm00001d005821_T002 transcript:Zm00001d020628_T001 0
195- 11:transcript:Zm00001d005824_T001 transcript:Zm00001d020629_T001 2.00E-98
195- 12:transcript:Zm00001d005828_T001 transcript:Zm00001d020636_T001 0
195- 13:transcript:Zm00001d005830_T001 transcript:Zm00001d020646_T001 1.00E-105
195- 14:transcript:Zm00001d005831_T003 transcript:Zm00001d020647_T002 0
195- 15:transcript:Zm00001d005833_T002 transcript:Zm00001d020650_T001 2.00E-98
195- 16:transcript:Zm00001d005837_T001 transcript:Zm00001d020653_T001 1.00E-133
Alignment 196: score=690.0 e_value=3.9e-41 N=15 2&7 plus
196- 0:transcript:Zm00001d005901_T001 transcript:Zm00001d020731_T001 4.00E-103
196- 1:transcript:Zm00001d005905_T001 transcript:Zm00001d020736_T001 0
196- 2:transcript:Zm00001d005910_T001 transcript:Zm00001d020742_T001 1.00E-69
196- 3:transcript:Zm00001d005912_T001 transcript:Zm00001d020748_T002 0
196- 4:transcript:Zm00001d005913_T001 transcript:Zm00001d020752_T001 0
196- 5:transcript:Zm00001d005917_T003 transcript:Zm00001d020755_T001 3.00E-10
196- 6:transcript:Zm00001d005918_T001 transcript:Zm00001d020757_T001 6.00E-76
196- 7:transcript:Zm00001d005919_T001 transcript:Zm00001d020763_T001 6.00E-109
196- 8:transcript:Zm00001d005920_T004 transcript:Zm00001d020764_T004 0
196- 9:transcript:Zm00001d005923_T001 transcript:Zm00001d020768_T001 2.00E-137
196- 10:transcript:Zm00001d005924_T002 transcript:Zm00001d020770_T002 9.00E-131
196- 11:transcript:Zm00001d005925_T002 transcript:Zm00001d020771_T003 0
196- 12:transcript:Zm00001d005928_T002 transcript:Zm00001d020772_T001 2.00E-104
196- 13:transcript:Zm00001d005931_T001 transcript:Zm00001d020774_T001 7.00E-113
196- 14:transcript:Zm00001d005933_T001 transcript:Zm00001d020781_T003 6.00E-113
Alignment 197: score=602.0 e_value=1.2e-37 N=14 2&7 plus
197- 0:transcript:Zm00001d006377_T001 transcript:Zm00001d021507_T002 0
197- 1:transcript:Zm00001d006388_T001 transcript:Zm00001d021508_T001 3.00E-147
197- 2:transcript:Zm00001d006389_T001 transcript:Zm00001d021512_T002 0
197- 3:transcript:Zm00001d006396_T001 transcript:Zm00001d021514_T001 3.00E-58
197- 4:transcript:Zm00001d006398_T001 transcript:Zm00001d021515_T001 7.00E-96
197- 5:transcript:Zm00001d006402_T004 transcript:Zm00001d021517_T001 6.00E-128
197- 6:transcript:Zm00001d006408_T001 transcript:Zm00001d021519_T001 0
197- 7:transcript:Zm00001d006409_T002 transcript:Zm00001d021520_T002 7.00E-164
197- 8:transcript:Zm00001d006410_T013 transcript:Zm00001d021522_T002 5.00E-169
197- 9:transcript:Zm00001d006415_T001 transcript:Zm00001d021524_T003 0
197- 10:transcript:Zm00001d006417_T002 transcript:Zm00001d021532_T001 0
197- 11:transcript:Zm00001d006419_T002 transcript:Zm00001d021534_T001 0

197- 12:transcript:Zm00001d006420_T002 transcript:Zm00001d021535_T001 0
197- 13:transcript:Zm00001d006421_T002 transcript:Zm00001d021536_T003 4.00E-46
Alignment 198: score=559.0 e_value=3.9e-34 N=13 2&7 plus
198- 0:transcript:Zm00001d006520_T001 transcript:Zm00001d021672_T001 0
198- 1:transcript:Zm00001d006521_T001 transcript:Zm00001d021673_T001 3.00E-84
198- 2:transcript:Zm00001d006524_T001 transcript:Zm00001d021674_T001 1.00E-126
198- 3:transcript:Zm00001d006525_T001 transcript:Zm00001d021677_T001 0
198- 4:transcript:Zm00001d006527_T001 transcript:Zm00001d021682_T001 2.00E-69
198- 5:transcript:Zm00001d006533_T007 transcript:Zm00001d021685_T022 0
198- 6:transcript:Zm00001d006534_T002 transcript:Zm00001d021688_T001 1.00E-09
198- 7:transcript:Zm00001d006535_T001 transcript:Zm00001d021692_T001 0
198- 8:transcript:Zm00001d006538_T001 transcript:Zm00001d021697_T001 0
198- 9:transcript:Zm00001d006539_T001 transcript:Zm00001d021702_T001 0
198- 10:transcript:Zm00001d006540_T001 transcript:Zm00001d021703_T001 2.00E-130
198- 11:transcript:Zm00001d006541_T002 transcript:Zm00001d021704_T001 4.00E-134
198- 12:transcript:Zm00001d006547_T001 transcript:Zm00001d021706_T001 2.00E-89
Alignment 199: score=485.0 e_value=3.4e-25 N=12 2&7 plus
199- 0:transcript:Zm00001d005456_T001 transcript:Zm00001d019265_T001 2.00E-119
199- 1:transcript:Zm00001d005459_T001 transcript:Zm00001d019279_T001 2.00E-131
199- 2:transcript:Zm00001d005460_T003 transcript:Zm00001d019283_T005 0
199- 3:transcript:Zm00001d005461_T001 transcript:Zm00001d019287_T001 0
199- 4:transcript:Zm00001d005462_T001 transcript:Zm00001d019288_T001 0
199- 5:transcript:Zm00001d005464_T001 transcript:Zm00001d019290_T001 2.00E-135
199- 6:transcript:Zm00001d005468_T001 transcript:Zm00001d019298_T002 2.00E-155
199- 7:transcript:Zm00001d005470_T001 transcript:Zm00001d019303_T001 2.00E-74
199- 8:transcript:Zm00001d005471_T001 transcript:Zm00001d019312_T002 1.00E-28
199- 9:transcript:Zm00001d005473_T001 transcript:Zm00001d019314_T003 0
199- 10:transcript:Zm00001d005478_T009 transcript:Zm00001d019317_T002 0
199- 11:transcript:Zm00001d005479_T001 transcript:Zm00001d019327_T001 0
Alignment 200: score=480.0 e_value=5.5e-25 N=11 2&7 plus
200- 0:transcript:Zm00001d005782_T001 transcript:Zm00001d020563_T001 0
200- 1:transcript:Zm00001d005784_T001 transcript:Zm00001d020569_T001 1.00E-164
200- 2:transcript:Zm00001d005785_T006 transcript:Zm00001d020572_T001 2.00E-30
200- 3:transcript:Zm00001d005786_T001 transcript:Zm00001d020573_T001 1.00E-56
200- 4:transcript:Zm00001d005788_T001 transcript:Zm00001d020577_T001 1.00E-44
200- 5:transcript:Zm00001d005789_T001 transcript:Zm00001d020580_T001 5.00E-96
200- 6:transcript:Zm00001d005790_T001 transcript:Zm00001d020583_T001 3.00E-138
200- 7:transcript:Zm00001d005792_T004 transcript:Zm00001d020586_T001 0
200- 8:transcript:Zm00001d005793_T001 transcript:Zm00001d020591_T001 3.00E-12
200- 9:transcript:Zm00001d005794_T009 transcript:Zm00001d020593_T020 0
200- 10:transcript:Zm00001d005798_T001 transcript:Zm00001d020595_T001 3.00E-112
Alignment 201: score=446.0 e_value=6.1e-20 N=10 2&7 plus
201- 0:transcript:Zm00001d005654_T001 transcript:Zm00001d020250_T001 3.00E-31
201- 1:transcript:Zm00001d005656_T001 transcript:Zm00001d020257_T001 8.00E-90
201- 2:transcript:Zm00001d005657_T002 transcript:Zm00001d020264_T001 3.00E-75
201- 3:transcript:Zm00001d005659_T001 transcript:Zm00001d020268_T002 9.00E-152
201- 4:transcript:Zm00001d005661_T001 transcript:Zm00001d020273_T001 4.00E-169
201- 5:transcript:Zm00001d005662_T001 transcript:Zm00001d020274_T001 4.00E-23
201- 6:transcript:Zm00001d005664_T001 transcript:Zm00001d020275_T001 2.00E-156
201- 7:transcript:Zm00001d005666_T001 transcript:Zm00001d020285_T001 0
201- 8:transcript:Zm00001d005671_T002 transcript:Zm00001d020296_T001 0
201- 9:transcript:Zm00001d005674_T001 transcript:Zm00001d020309_T001 0
Alignment 202: score=427.0 e_value=1.9e-21 N=10 2&7 plus
202- 0:transcript:Zm00001d007947_T001 transcript:Zm00001d018664_T004 0

202- 1:transcript:Zm00001d007949_T001 transcript:Zm00001d018667_T003 2.00E-148
202- 2:transcript:Zm00001d007951_T001 transcript:Zm00001d018669_T004 0
202- 3:transcript:Zm00001d007954_T001 transcript:Zm00001d018671_T001 7.00E-66
202- 4:transcript:Zm00001d007957_T001 transcript:Zm00001d018687_T002 2.00E-28
202- 5:transcript:Zm00001d007960_T001 transcript:Zm00001d018694_T002 0
202- 6:transcript:Zm00001d007962_T001 transcript:Zm00001d018698_T001 2.00E-176
202- 7:transcript:Zm00001d007971_T001 transcript:Zm00001d018707_T001 5.00E-12
202- 8:transcript:Zm00001d007973_T001 transcript:Zm00001d018716_T001 0
202- 9:transcript:Zm00001d007974_T001 transcript:Zm00001d018717_T001 4.00E-29
Alignment 203: score=420.0 e_value=3.5e-20 N=10 2&7 plus
203- 0:transcript:Zm00001d006906_T010 transcript:Zm00001d022189_T002 6.00E-85
203- 1:transcript:Zm00001d006910_T003 transcript:Zm00001d022190_T003 0
203- 2:transcript:Zm00001d006912_T001 transcript:Zm00001d022191_T001 7.00E-09
203- 3:transcript:Zm00001d006913_T002 transcript:Zm00001d022192_T002 0
203- 4:transcript:Zm00001d006915_T001 transcript:Zm00001d022195_T001 2.00E-51
203- 5:transcript:Zm00001d006916_T001 transcript:Zm00001d022199_T003 0
203- 6:transcript:Zm00001d006917_T001 transcript:Zm00001d022201_T002 0
203- 7:transcript:Zm00001d006918_T001 transcript:Zm00001d022202_T001 0
203- 8:transcript:Zm00001d006921_T003 transcript:Zm00001d022212_T002 1.00E-45
203- 9:transcript:Zm00001d006924_T001 transcript:Zm00001d022218_T001 2.00E-107
Alignment 204: score=410.0 e_value=2.2e-26 N=11 2&7 plus
204- 0:transcript:Zm00001d006472_T003 transcript:Zm00001d021609_T018 0
204- 1:transcript:Zm00001d006473_T001 transcript:Zm00001d021610_T003 0
204- 2:transcript:Zm00001d006475_T001 transcript:Zm00001d021620_T001 2.00E-122
204- 3:transcript:Zm00001d006479_T006 transcript:Zm00001d021623_T004 0
204- 4:transcript:Zm00001d006489_T001 transcript:Zm00001d021627_T001 2.00E-80
204- 5:transcript:Zm00001d006494_T004 transcript:Zm00001d021629_T003 2.00E-167
204- 6:transcript:Zm00001d006495_T001 transcript:Zm00001d021630_T001 4.00E-20
204- 7:transcript:Zm00001d006496_T004 transcript:Zm00001d021633_T006 0
204- 8:transcript:Zm00001d006499_T005 transcript:Zm00001d021635_T011 0
204- 9:transcript:Zm00001d006503_T001 transcript:Zm00001d021639_T001 0
204- 10:transcript:Zm00001d006504_T005 transcript:Zm00001d021641_T002 0
Alignment 205: score=362.0 e_value=5.5e-15 N=8 2&7 plus
205- 0:transcript:Zm00001d007202_T003 transcript:Zm00001d022560_T001 0
205- 1:transcript:Zm00001d007204_T002 transcript:Zm00001d022562_T002 1.00E-159
205- 2:transcript:Zm00001d007205_T001 transcript:Zm00001d022563_T001 0
205- 3:transcript:Zm00001d007207_T002 transcript:Zm00001d022565_T001 0
205- 4:transcript:Zm00001d007209_T001 transcript:Zm00001d022567_T001 1.00E-16
205- 5:transcript:Zm00001d007214_T001 transcript:Zm00001d022572_T001 1.00E-82
205- 6:transcript:Zm00001d007215_T001 transcript:Zm00001d022573_T001 0
205- 7:transcript:Zm00001d007216_T001 transcript:Zm00001d022575_T001 0
Alignment 206: score=348.0 e_value=4.6e-15 N=8 2&7 plus
206- 0:transcript:Zm00001d006213_T001 transcript:Zm00001d021300_T001 1.00E-97
206- 1:transcript:Zm00001d006214_T002 transcript:Zm00001d021301_T002 0
206- 2:transcript:Zm00001d006217_T004 transcript:Zm00001d021303_T003 0
206- 3:transcript:Zm00001d006219_T003 transcript:Zm00001d021306_T001 0
206- 4:transcript:Zm00001d006220_T002 transcript:Zm00001d021309_T003 4.00E-123
206- 5:transcript:Zm00001d006221_T001 transcript:Zm00001d021310_T001 0
206- 6:transcript:Zm00001d006226_T001 transcript:Zm00001d021314_T007 7.00E-128
206- 7:transcript:Zm00001d006231_T001 transcript:Zm00001d021318_T003 0
Alignment 207: score=329.0 e_value=1.3e-16 N=9 2&7 plus
207- 0:transcript:Zm00001d007037_T002 transcript:Zm00001d022314_T001 5.00E-156
207- 1:transcript:Zm00001d007038_T001 transcript:Zm00001d022316_T001 0
207- 2:transcript:Zm00001d007039_T001 transcript:Zm00001d022322_T001 0

207- 3:transcript:Zm00001d007043_T002 transcript:Zm00001d022335_T002 0
207- 4:transcript:Zm00001d007048_T001 transcript:Zm00001d022341_T001 0
207- 5:transcript:Zm00001d007050_T001 transcript:Zm00001d022347_T001 0
207- 6:transcript:Zm00001d007058_T002 transcript:Zm00001d022350_T001 2.00E-17
207- 7:transcript:Zm00001d007059_T001 transcript:Zm00001d022354_T001 1.00E-42
207- 8:transcript:Zm00001d007062_T001 transcript:Zm00001d022364_T002 2.00E-159
Alignment 208: score=327.0 e_value=1e-11 N=7 2&7 plus
208- 0:transcript:Zm00001d006746_T001 transcript:Zm00001d022001_T001 2.00E-88
208- 1:transcript:Zm00001d006749_T001 transcript:Zm00001d022006_T001 0
208- 2:transcript:Zm00001d006751_T001 transcript:Zm00001d022010_T004 0
208- 3:transcript:Zm00001d006752_T003 transcript:Zm00001d022016_T001 0
208- 4:transcript:Zm00001d006753_T001 transcript:Zm00001d022017_T001 0
208- 5:transcript:Zm00001d006754_T001 transcript:Zm00001d022022_T001 2.00E-104
208- 6:transcript:Zm00001d006756_T001 transcript:Zm00001d022025_T001 2.00E-53
Alignment 209: score=321.0 e_value=7e-12 N=7 2&7 plus
209- 0:transcript:Zm00001d005881_T011 transcript:Zm00001d020708_T002 0
209- 1:transcript:Zm00001d005884_T002 transcript:Zm00001d020711_T001 5.00E-121
209- 2:transcript:Zm00001d005885_T001 transcript:Zm00001d020713_T001 0
209- 3:transcript:Zm00001d005888_T002 transcript:Zm00001d020714_T001 3.00E-159
209- 4:transcript:Zm00001d005889_T001 transcript:Zm00001d020717_T001 0
209- 5:transcript:Zm00001d005893_T001 transcript:Zm00001d020719_T002 0
209- 6:transcript:Zm00001d005897_T001 transcript:Zm00001d020723_T001 1.00E-59
Alignment 210: score=319.0 e_value=3.2e-12 N=7 2&7 plus
210- 0:transcript:Zm00001d005936_T001 transcript:Zm00001d020789_T001 0
210- 1:transcript:Zm00001d005939_T001 transcript:Zm00001d020790_T001 4.00E-150
210- 2:transcript:Zm00001d005940_T001 transcript:Zm00001d020791_T001 1.00E-164
210- 3:transcript:Zm00001d005948_T001 transcript:Zm00001d020799_T003 2.00E-86
210- 4:transcript:Zm00001d005950_T001 transcript:Zm00001d020803_T001 9.00E-39
210- 5:transcript:Zm00001d005951_T001 transcript:Zm00001d020804_T001 6.00E-139
210- 6:transcript:Zm00001d005957_T001 transcript:Zm00001d020807_T003 3.00E-52
Alignment 211: score=313.0 e_value=3.2e-12 N=7 2&7 plus
211- 0:transcript:Zm00001d006167_T003 transcript:Zm00001d021203_T003 0
211- 1:transcript:Zm00001d006168_T002 transcript:Zm00001d021204_T005 0
211- 2:transcript:Zm00001d006169_T001 transcript:Zm00001d021205_T001 1.00E-93
211- 3:transcript:Zm00001d006171_T001 transcript:Zm00001d021216_T001 0
211- 4:transcript:Zm00001d006173_T001 transcript:Zm00001d021221_T001 6.00E-175
211- 5:transcript:Zm00001d006175_T001 transcript:Zm00001d021223_T001 1.00E-16
211- 6:transcript:Zm00001d006176_T001 transcript:Zm00001d021227_T003 0
Alignment 212: score=303.0 e_value=1.9e-13 N=7 2&7 plus
212- 0:transcript:Zm00001d006198_T001 transcript:Zm00001d021278_T001 4.00E-95
212- 1:transcript:Zm00001d006204_T001 transcript:Zm00001d021282_T001 4.00E-122
212- 2:transcript:Zm00001d006205_T001 transcript:Zm00001d021283_T001 0
212- 3:transcript:Zm00001d006209_T001 transcript:Zm00001d021285_T001 6.00E-150
212- 4:transcript:Zm00001d006210_T009 transcript:Zm00001d021287_T001 0
212- 5:transcript:Zm00001d006211_T001 transcript:Zm00001d021288_T001 1.00E-142
212- 6:transcript:Zm00001d006212_T002 transcript:Zm00001d021291_T002 0
Alignment 213: score=301.0 e_value=1.6e-12 N=7 2&7 plus
213- 0:transcript:Zm00001d006590_T002 transcript:Zm00001d021777_T001 0
213- 1:transcript:Zm00001d006591_T001 transcript:Zm00001d021779_T001 4.00E-45
213- 2:transcript:Zm00001d006592_T001 transcript:Zm00001d021784_T001 4.00E-53
213- 3:transcript:Zm00001d006593_T009 transcript:Zm00001d021785_T002 0
213- 4:transcript:Zm00001d006595_T001 transcript:Zm00001d021787_T003 0
213- 5:transcript:Zm00001d006596_T001 transcript:Zm00001d021788_T001 0
213- 6:transcript:Zm00001d006597_T003 transcript:Zm00001d021790_T010 0

Alignment 214: score=288.0 e_value=1.8e-13 N=7 2&7 plus
214- 0:transcript:Zm00001d006866_T003 transcript:Zm00001d022144_T003 0
214- 1:transcript:Zm00001d006869_T001 transcript:Zm00001d022150_T001 2.00E-107
214- 2:transcript:Zm00001d006874_T001 transcript:Zm00001d022151_T001 0
214- 3:transcript:Zm00001d006875_T001 transcript:Zm00001d022152_T001 0
214- 4:transcript:Zm00001d006879_T001 transcript:Zm00001d022154_T001 1.00E-96
214- 5:transcript:Zm00001d006883_T001 transcript:Zm00001d022163_T001 3.00E-103
214- 6:transcript:Zm00001d006885_T004 transcript:Zm00001d022166_T001 0
Alignment 215: score=284.0 e_value=4.2e-09 N=6 2&7 plus
215- 0:transcript:Zm00001d006608_T001 transcript:Zm00001d021812_T001 2.00E-60
215- 1:transcript:Zm00001d006612_T001 transcript:Zm00001d021813_T001 2.00E-49
215- 2:transcript:Zm00001d006613_T004 transcript:Zm00001d021815_T002 0
215- 3:transcript:Zm00001d006614_T001 transcript:Zm00001d021817_T002 0
215- 4:transcript:Zm00001d006616_T001 transcript:Zm00001d021818_T001 2.00E-13
215- 5:transcript:Zm00001d006617_T001 transcript:Zm00001d021820_T001 2.00E-130
Alignment 216: score=280.0 e_value=5.6e-10 N=6 2&7 plus
216- 0:transcript:Zm00001d006157_T001 transcript:Zm00001d021191_T001 0
216- 1:transcript:Zm00001d006158_T001 transcript:Zm00001d021192_T001 2.00E-31
216- 2:transcript:Zm00001d006160_T001 transcript:Zm00001d021196_T004 0
216- 3:transcript:Zm00001d006161_T001 transcript:Zm00001d021197_T001 0
216- 4:transcript:Zm00001d006162_T001 transcript:Zm00001d021199_T002 1.00E-146
216- 5:transcript:Zm00001d006166_T001 transcript:Zm00001d021201_T001 0
Alignment 217: score=279.0 e_value=3.2e-09 N=6 2&7 plus
217- 0:transcript:Zm00001d006619_T001 transcript:Zm00001d021831_T001 0
217- 1:transcript:Zm00001d006620_T001 transcript:Zm00001d021834_T001 1.00E-180
217- 2:transcript:Zm00001d006621_T004 transcript:Zm00001d021835_T007 0
217- 3:transcript:Zm00001d006623_T001 transcript:Zm00001d021836_T001 0
217- 4:transcript:Zm00001d006625_T002 transcript:Zm00001d021838_T001 0
217- 5:transcript:Zm00001d006626_T001 transcript:Zm00001d021839_T001 2.00E-116
Alignment 218: score=277.0 e_value=1.8e-10 N=6 2&7 plus
218- 0:transcript:Zm00001d006449_T001 transcript:Zm00001d021571_T001 0
218- 1:transcript:Zm00001d006451_T001 transcript:Zm00001d021573_T001 8.00E-46
218- 2:transcript:Zm00001d006453_T001 transcript:Zm00001d021574_T001 1.00E-33
218- 3:transcript:Zm00001d006454_T003 transcript:Zm00001d021579_T002 0
218- 4:transcript:Zm00001d006455_T001 transcript:Zm00001d021580_T001 1.00E-109
218- 5:transcript:Zm00001d006456_T001 transcript:Zm00001d021583_T001 0
Alignment 219: score=270.0 e_value=9.6e-11 N=6 2&7 plus
219- 0:transcript:Zm00001d006574_T001 transcript:Zm00001d021746_T001 8.00E-180
219- 1:transcript:Zm00001d006578_T001 transcript:Zm00001d021748_T002 0
219- 2:transcript:Zm00001d006580_T005 transcript:Zm00001d021754_T005 0
219- 3:transcript:Zm00001d006585_T001 transcript:Zm00001d021761_T001 6.00E-118
219- 4:transcript:Zm00001d006587_T001 transcript:Zm00001d021763_T001 0
219- 5:transcript:Zm00001d006588_T001 transcript:Zm00001d021764_T002 1.00E-144
Alignment 220: score=267.0 e_value=2.9e-08 N=6 2&7 plus
220- 0:transcript:Zm00001d006731_T003 transcript:Zm00001d021979_T001 0
220- 1:transcript:Zm00001d006733_T001 transcript:Zm00001d021988_T001 1.00E-139
220- 2:transcript:Zm00001d006735_T001 transcript:Zm00001d021990_T001 1.00E-31
220- 3:transcript:Zm00001d006737_T006 transcript:Zm00001d021991_T002 0
220- 4:transcript:Zm00001d006739_T001 transcript:Zm00001d021995_T001 5.00E-105
220- 5:transcript:Zm00001d006744_T001 transcript:Zm00001d021998_T001 0
Alignment 221: score=261.0 e_value=4.3e-10 N=6 2&7 plus
221- 0:transcript:Zm00001d006034_T003 transcript:Zm00001d020951_T002 5.00E-133
221- 1:transcript:Zm00001d006036_T001 transcript:Zm00001d020956_T001 8.00E-51
221- 2:transcript:Zm00001d006037_T001 transcript:Zm00001d020957_T001 9.00E-125

221- 3:transcript:Zm00001d006040_T001 transcript:Zm00001d020963_T001 0
221- 4:transcript:Zm00001d006041_T002 transcript:Zm00001d020965_T007 0
221- 5:transcript:Zm00001d006042_T001 transcript:Zm00001d020966_T001 9.00E-163
Alignment 222: score=260.0 e_value=2.8e-09 N=6 2&7 plus
222- 0:transcript:Zm00001d006628_T005 transcript:Zm00001d021858_T007 0
222- 1:transcript:Zm00001d006630_T001 transcript:Zm00001d021862_T001 3.00E-34
222- 2:transcript:Zm00001d006637_T001 transcript:Zm00001d021864_T001 2.00E-50
222- 3:transcript:Zm00001d006639_T001 transcript:Zm00001d021870_T001 7.00E-34
222- 4:transcript:Zm00001d006644_T002 transcript:Zm00001d021875_T002 0
222- 5:transcript:Zm00001d006645_T001 transcript:Zm00001d021877_T001 3.00E-144
Alignment 223: score=257.0 e_value=8.1e-12 N=6 2&7 plus
223- 0:transcript:Zm00001d005767_T001 transcript:Zm00001d020505_T001 4.00E-124
223- 1:transcript:Zm00001d005769_T001 transcript:Zm00001d020512_T001 0
223- 2:transcript:Zm00001d005772_T001 transcript:Zm00001d020519_T001 2.00E-91
223- 3:transcript:Zm00001d005773_T001 transcript:Zm00001d020521_T001 1.00E-118
223- 4:transcript:Zm00001d005775_T005 transcript:Zm00001d020531_T002 0
223- 5:transcript:Zm00001d005776_T002 transcript:Zm00001d020533_T001 0
Alignment 224: score=255.0 e_value=1.3e-08 N=6 2&7 plus
224- 0:transcript:Zm00001d005726_T001 transcript:Zm00001d020409_T002 8.00E-175
224- 1:transcript:Zm00001d005727_T001 transcript:Zm00001d020411_T001 0
224- 2:transcript:Zm00001d005729_T001 transcript:Zm00001d020418_T001 0
224- 3:transcript:Zm00001d005732_T001 transcript:Zm00001d020419_T003 4.00E-92
224- 4:transcript:Zm00001d005735_T001 transcript:Zm00001d020429_T001 0
224- 5:transcript:Zm00001d005736_T003 transcript:Zm00001d020432_T001 6.00E-49
Alignment 225: score=767.0 e_value=5.8e-55 N=19 2&7 minus
225- 0:transcript:Zm00001d005174_T001 transcript:Zm00001d019250_T002 2.00E-138
225- 1:transcript:Zm00001d005178_T001 transcript:Zm00001d019241_T002 2.00E-34
225- 2:transcript:Zm00001d005179_T001 transcript:Zm00001d019234_T001 1.00E-108
225- 3:transcript:Zm00001d005182_T001 transcript:Zm00001d019233_T001 1.00E-157
225- 4:transcript:Zm00001d005190_T001 transcript:Zm00001d019226_T003 0
225- 5:transcript:Zm00001d005193_T001 transcript:Zm00001d019223_T001 4.00E-88
225- 6:transcript:Zm00001d005196_T001 transcript:Zm00001d019222_T001 3.00E-51
225- 7:transcript:Zm00001d005200_T002 transcript:Zm00001d019219_T001 2.00E-97
225- 8:transcript:Zm00001d005203_T001 transcript:Zm00001d019216_T001 1.00E-47
225- 9:transcript:Zm00001d005205_T003 transcript:Zm00001d019215_T002 0
225- 10:transcript:Zm00001d005208_T001 transcript:Zm00001d019207_T001 0
225- 11:transcript:Zm00001d005229_T001 transcript:Zm00001d019194_T008 2.00E-159
225- 12:transcript:Zm00001d005230_T001 transcript:Zm00001d019192_T001 7.00E-17
225- 13:transcript:Zm00001d005231_T013 transcript:Zm00001d019191_T007 1.00E-131
225- 14:transcript:Zm00001d005238_T001 transcript:Zm00001d019182_T001 0
225- 15:transcript:Zm00001d005239_T001 transcript:Zm00001d019171_T001 1.00E-176
225- 16:transcript:Zm00001d005240_T004 transcript:Zm00001d019169_T004 0
225- 17:transcript:Zm00001d005244_T002 transcript:Zm00001d019166_T002 1.00E-177
225- 18:transcript:Zm00001d005248_T003 transcript:Zm00001d019164_T002 0
Alignment 226: score=490.0 e_value=6.7e-29 N=12 2&7 minus
226- 0:transcript:Zm00001d007135_T001 transcript:Zm00001d022469_T001 3.00E-33
226- 1:transcript:Zm00001d007143_T001 transcript:Zm00001d022467_T001 0
226- 2:transcript:Zm00001d007145_T001 transcript:Zm00001d022466_T006 0
226- 3:transcript:Zm00001d007146_T001 transcript:Zm00001d022465_T007 4.00E-158
226- 4:transcript:Zm00001d007154_T001 transcript:Zm00001d022463_T001 2.00E-73
226- 5:transcript:Zm00001d007160_T001 transcript:Zm00001d022456_T001 8.00E-147
226- 6:transcript:Zm00001d007166_T001 transcript:Zm00001d022450_T001 8.00E-106
226- 7:transcript:Zm00001d007168_T001 transcript:Zm00001d022446_T001 7.00E-78
226- 8:transcript:Zm00001d007169_T001 transcript:Zm00001d022444_T001 0

226- 9:transcript:Zm00001d007172_T001 transcript:Zm00001d022442_T002 0
226- 10:transcript:Zm00001d007173_T003 transcript:Zm00001d022440_T005 0
226- 11:transcript:Zm00001d007174_T001 transcript:Zm00001d022438_T001 2.00E-23
Alignment 227: score=433.0 e_value=1.1e-19 N=10 2&7 minus
227- 0:transcript:Zm00001d005841_T001 transcript:Zm00001d020705_T001 4.00E-82
227- 1:transcript:Zm00001d005843_T001 transcript:Zm00001d020704_T001 8.00E-152
227- 2:transcript:Zm00001d005849_T001 transcript:Zm00001d020697_T001 0
227- 3:transcript:Zm00001d005856_T003 transcript:Zm00001d020695_T001 0
227- 4:transcript:Zm00001d005865_T001 transcript:Zm00001d020688_T001 0
227- 5:transcript:Zm00001d005866_T003 transcript:Zm00001d020687_T002 0
227- 6:transcript:Zm00001d005871_T002 transcript:Zm00001d020683_T001 0
227- 7:transcript:Zm00001d005873_T001 transcript:Zm00001d020681_T001 3.00E-64
227- 8:transcript:Zm00001d005874_T001 transcript:Zm00001d020675_T001 6.00E-135
227- 9:transcript:Zm00001d005875_T001 transcript:Zm00001d020670_T001 2.00E-118
Alignment 228: score=419.0 e_value=6.6e-27 N=11 2&7 minus
228- 0:transcript:Zm00001d006267_T002 transcript:Zm00001d021473_T002 0
228- 1:transcript:Zm00001d006269_T001 transcript:Zm00001d021464_T005 0
228- 2:transcript:Zm00001d006270_T001 transcript:Zm00001d021462_T001 4.00E-78
228- 3:transcript:Zm00001d006276_T001 transcript:Zm00001d021457_T001 2.00E-57
228- 4:transcript:Zm00001d006277_T001 transcript:Zm00001d021456_T001 9.00E-46
228- 5:transcript:Zm00001d006282_T001 transcript:Zm00001d021454_T001 6.00E-68
228- 6:transcript:Zm00001d006286_T001 transcript:Zm00001d021452_T001 0
228- 7:transcript:Zm00001d006287_T001 transcript:Zm00001d021445_T001 2.00E-89
228- 8:transcript:Zm00001d006293_T024 transcript:Zm00001d021442_T013 0
228- 9:transcript:Zm00001d006294_T001 transcript:Zm00001d021441_T001 7.00E-118
228- 10:transcript:Zm00001d006295_T002 transcript:Zm00001d021438_T003 0
Alignment 229: score=406.0 e_value=1.4e-21 N=10 2&7 minus
229- 0:transcript:Zm00001d006297_T001 transcript:Zm00001d021434_T001 0
229- 1:transcript:Zm00001d006298_T001 transcript:Zm00001d021433_T002 2.00E-45
229- 2:transcript:Zm00001d006307_T002 transcript:Zm00001d021426_T002 3.00E-171
229- 3:transcript:Zm00001d006309_T007 transcript:Zm00001d021421_T003 0
229- 4:transcript:Zm00001d006310_T005 transcript:Zm00001d021420_T003 2.00E-166
229- 5:transcript:Zm00001d006311_T001 transcript:Zm00001d021419_T001 2.00E-13
229- 6:transcript:Zm00001d006312_T001 transcript:Zm00001d021418_T001 6.00E-105
229- 7:transcript:Zm00001d006317_T001 transcript:Zm00001d021404_T001 0
229- 8:transcript:Zm00001d006319_T001 transcript:Zm00001d021403_T001 0
229- 9:transcript:Zm00001d006320_T004 transcript:Zm00001d021401_T001 7.00E-89
Alignment 230: score=349.0 e_value=7.9e-20 N=9 2&7 minus
230- 0:transcript:Zm00001d006930_T001 transcript:Zm00001d022296_T001 3.00E-98
230- 1:transcript:Zm00001d006931_T002 transcript:Zm00001d022294_T002 6.00E-49
230- 2:transcript:Zm00001d006937_T001 transcript:Zm00001d022281_T001 2.00E-94
230- 3:transcript:Zm00001d006939_T001 transcript:Zm00001d022275_T001 2.00E-179
230- 4:transcript:Zm00001d006940_T001 transcript:Zm00001d022273_T016 9.00E-44
230- 5:transcript:Zm00001d006944_T001 transcript:Zm00001d022272_T002 0
230- 6:transcript:Zm00001d006945_T001 transcript:Zm00001d022266_T001 0
230- 7:transcript:Zm00001d006947_T001 transcript:Zm00001d022262_T001 0
230- 8:transcript:Zm00001d006950_T001 transcript:Zm00001d022249_T001 6.00E-27
Alignment 231: score=331.0 e_value=2.8e-14 N=8 2&7 minus
231- 0:transcript:Zm00001d005748_T001 transcript:Zm00001d020496_T001 0
231- 1:transcript:Zm00001d005749_T001 transcript:Zm00001d020492_T001 1.00E-120
231- 2:transcript:Zm00001d005750_T002 transcript:Zm00001d020490_T001 7.00E-103
231- 3:transcript:Zm00001d005751_T002 transcript:Zm00001d020485_T001 2.00E-152
231- 4:transcript:Zm00001d005754_T001 transcript:Zm00001d020472_T001 2.00E-13
231- 5:transcript:Zm00001d005757_T001 transcript:Zm00001d020460_T001 3.00E-161

231- 6:transcript:Zm00001d005760_T001 transcript:Zm00001d020457_T001 1.00E-179
231- 7:transcript:Zm00001d005764_T001 transcript:Zm00001d020454_T003 0
Alignment 232: score=303.0 e_value=3.3e-12 N=7 2&7 minus
232- 0:transcript:Zm00001d006810_T001 transcript:Zm00001d022102_T001 2.00E-92
232- 1:transcript:Zm00001d006813_T001 transcript:Zm00001d022099_T001 1.00E-98
232- 2:transcript:Zm00001d006816_T001 transcript:Zm00001d022090_T001 5.00E-06
232- 3:transcript:Zm00001d006820_T001 transcript:Zm00001d022085_T001 0
232- 4:transcript:Zm00001d006821_T001 transcript:Zm00001d022083_T001 4.00E-137
232- 5:transcript:Zm00001d006825_T003 transcript:Zm00001d022073_T001 0
232- 6:transcript:Zm00001d006828_T001 transcript:Zm00001d022072_T001 0
Alignment 233: score=298.0 e_value=4.8e-13 N=7 2&7 minus
233- 0:transcript:Zm00001d007758_T009 transcript:Zm00001d019106_T002 0
233- 1:transcript:Zm00001d007760_T001 transcript:Zm00001d019104_T001 0
233- 2:transcript:Zm00001d007764_T001 transcript:Zm00001d019094_T001 7.00E-116
233- 3:transcript:Zm00001d007767_T001 transcript:Zm00001d019091_T002 1.00E-161
233- 4:transcript:Zm00001d007769_T003 transcript:Zm00001d019090_T003 0
233- 5:transcript:Zm00001d007770_T001 transcript:Zm00001d019087_T015 0
233- 6:transcript:Zm00001d007772_T001 transcript:Zm00001d019084_T002 0
Alignment 234: score=293.0 e_value=8.9e-12 N=7 2&7 minus
234- 0:transcript:Zm00001d006243_T005 transcript:Zm00001d022621_T005 0
234- 1:transcript:Zm00001d006246_T001 transcript:Zm00001d022611_T001 0
234- 2:transcript:Zm00001d006247_T001 transcript:Zm00001d022605_T001 9.00E-46
234- 3:transcript:Zm00001d006249_T003 transcript:Zm00001d022604_T001 1.00E-80
234- 4:transcript:Zm00001d006256_T001 transcript:Zm00001d022596_T001 7.00E-73
234- 5:transcript:Zm00001d006257_T006 transcript:Zm00001d022594_T002 0
234- 6:transcript:Zm00001d006260_T001 transcript:Zm00001d022592_T001 1.00E-161
Alignment 235: score=279.0 e_value=1.3e-08 N=6 2&7 minus
235- 0:transcript:Zm00001d005434_T001 transcript:Zm00001d019534_T003 1.00E-140
235- 1:transcript:Zm00001d005439_T001 transcript:Zm00001d019527_T005 5.00E-133
235- 2:transcript:Zm00001d005442_T001 transcript:Zm00001d019522_T001 0
235- 3:transcript:Zm00001d005445_T001 transcript:Zm00001d019520_T001 0
235- 4:transcript:Zm00001d005446_T001 transcript:Zm00001d019518_T001 2.00E-83
235- 5:transcript:Zm00001d005450_T003 transcript:Zm00001d019515_T008 0
Alignment 236: score=278.0 e_value=9.5e-10 N=6 2&7 minus
236- 0:transcript:Zm00001d007908_T001 transcript:Zm00001d018853_T001 2.00E-99
236- 1:transcript:Zm00001d007909_T001 transcript:Zm00001d018848_T001 6.00E-88
236- 2:transcript:Zm00001d007910_T001 transcript:Zm00001d018846_T001 4.00E-88
236- 3:transcript:Zm00001d007911_T001 transcript:Zm00001d018844_T001 8.00E-24
236- 4:transcript:Zm00001d007918_T001 transcript:Zm00001d018839_T001 0
236- 5:transcript:Zm00001d007919_T001 transcript:Zm00001d018832_T001 2.00E-30
Alignment 237: score=266.0 e_value=9.2e-11 N=7 2&7 minus
237- 0:transcript:Zm00001d007820_T002 transcript:Zm00001d018962_T001 1.00E-175
237- 1:transcript:Zm00001d007823_T001 transcript:Zm00001d018959_T001 0
237- 2:transcript:Zm00001d007824_T001 transcript:Zm00001d018957_T001 0
237- 3:transcript:Zm00001d007826_T002 transcript:Zm00001d018944_T002 0
237- 4:transcript:Zm00001d007827_T001 transcript:Zm00001d018940_T001 6.00E-61
237- 5:transcript:Zm00001d007830_T001 transcript:Zm00001d018931_T001 9.00E-158
237- 6:transcript:Zm00001d007831_T001 transcript:Zm00001d018926_T001 3.00E-08
Alignment 238: score=260.0 e_value=3e-10 N=6 2&7 minus
238- 0:transcript:Zm00001d006232_T001 transcript:Zm00001d022647_T001 0
238- 1:transcript:Zm00001d006235_T001 transcript:Zm00001d022629_T001 0
238- 2:transcript:Zm00001d006236_T001 transcript:Zm00001d022628_T001 2.00E-132
238- 3:transcript:Zm00001d006237_T001 transcript:Zm00001d022627_T002 0
238- 4:transcript:Zm00001d006238_T003 transcript:Zm00001d022625_T006 0

238- 5:transcript:Zm00001d006242_T001 transcript:Zm00001d022622_T005 0
Alignment 239: score=268.0 e_value=4e-13 N=6 3&4 minus
239- 0:transcript:Zm00001d041912_T006 transcript:Zm00001d053182_T003 8.00E-128
239- 1:transcript:Zm00001d041913_T001 transcript:Zm00001d053181_T001 0
239- 2:transcript:Zm00001d041917_T001 transcript:Zm00001d053175_T002 2.00E-35
239- 3:transcript:Zm00001d041918_T001 transcript:Zm00001d053168_T001 0
239- 4:transcript:Zm00001d041919_T001 transcript:Zm00001d053163_T001 6.00E-110
239- 5:transcript:Zm00001d041920_T001 transcript:Zm00001d053162_T003 1.00E-142
Alignment 240: score=586.0 e_value=3.9e-36 N=14 3&6 plus
240- 0:transcript:Zm00001d043014_T001 transcript:Zm00001d038436_T001 1.00E-94
240- 1:transcript:Zm00001d043015_T001 transcript:Zm00001d038447_T001 0
240- 2:transcript:Zm00001d043022_T001 transcript:Zm00001d038449_T001 1.00E-179
240- 3:transcript:Zm00001d043024_T001 transcript:Zm00001d038450_T001 3.00E-56
240- 4:transcript:Zm00001d043025_T001 transcript:Zm00001d038451_T001 1.00E-144
240- 5:transcript:Zm00001d043029_T001 transcript:Zm00001d038459_T001 5.00E-178
240- 6:transcript:Zm00001d043031_T001 transcript:Zm00001d038465_T001 0
240- 7:transcript:Zm00001d043037_T001 transcript:Zm00001d038466_T001 4.00E-34
240- 8:transcript:Zm00001d043039_T001 transcript:Zm00001d038469_T001 6.00E-31
240- 9:transcript:Zm00001d043043_T001 transcript:Zm00001d038471_T001 0
240- 10:transcript:Zm00001d043046_T001 transcript:Zm00001d038473_T001 2.00E-83
240- 11:transcript:Zm00001d043047_T001 transcript:Zm00001d038476_T001 1.00E-154
240- 12:transcript:Zm00001d043050_T001 transcript:Zm00001d038478_T001 4.00E-34
240- 13:transcript:Zm00001d043058_T001 transcript:Zm00001d038481_T001 0
Alignment 241: score=510.0 e_value=2.3e-27 N=12 3&6 plus
241- 0:transcript:Zm00001d043921_T001 transcript:Zm00001d039004_T002 6.00E-143
241- 1:transcript:Zm00001d043922_T001 transcript:Zm00001d039006_T007 0
241- 2:transcript:Zm00001d043929_T001 transcript:Zm00001d039010_T001 2.00E-36
241- 3:transcript:Zm00001d043932_T001 transcript:Zm00001d039011_T001 3.00E-39
241- 4:transcript:Zm00001d043935_T001 transcript:Zm00001d039016_T001 2.00E-122
241- 5:transcript:Zm00001d043937_T001 transcript:Zm00001d039017_T001 2.00E-12
241- 6:transcript:Zm00001d043942_T001 transcript:Zm00001d039020_T001 4.00E-124
241- 7:transcript:Zm00001d043943_T001 transcript:Zm00001d039021_T001 5.00E-85
241- 8:transcript:Zm00001d043950_T001 transcript:Zm00001d039032_T001 3.00E-75
241- 9:transcript:Zm00001d043954_T003 transcript:Zm00001d039037_T001 0
241- 10:transcript:Zm00001d043955_T001 transcript:Zm00001d039038_T001 0
241- 11:transcript:Zm00001d043962_T001 transcript:Zm00001d039041_T007 0
Alignment 242: score=430.0 e_value=3.5e-21 N=10 3&6 plus
242- 0:transcript:Zm00001d042560_T001 transcript:Zm00001d038197_T001 2.00E-77
242- 1:transcript:Zm00001d042572_T001 transcript:Zm00001d038203_T001 1.00E-12
242- 2:transcript:Zm00001d042578_T001 transcript:Zm00001d038205_T002 0
242- 3:transcript:Zm00001d042580_T001 transcript:Zm00001d038207_T001 5.00E-113
242- 4:transcript:Zm00001d042582_T001 transcript:Zm00001d038208_T001 3.00E-127
242- 5:transcript:Zm00001d042585_T004 transcript:Zm00001d038209_T001 0
242- 6:transcript:Zm00001d042593_T001 transcript:Zm00001d038216_T001 7.00E-41
242- 7:transcript:Zm00001d042598_T003 transcript:Zm00001d038217_T002 1.00E-101
242- 8:transcript:Zm00001d042600_T001 transcript:Zm00001d038218_T001 2.00E-73
242- 9:transcript:Zm00001d042609_T001 transcript:Zm00001d038221_T001 3.00E-145
Alignment 243: score=381.0 e_value=8.5e-17 N=8 3&6 plus
243- 0:transcript:Zm00001d041725_T001 transcript:Zm00001d035304_T001 8.00E-140
243- 1:transcript:Zm00001d041726_T001 transcript:Zm00001d035308_T001 2.00E-160
243- 2:transcript:Zm00001d041727_T001 transcript:Zm00001d035312_T002 1.00E-20
243- 3:transcript:Zm00001d041730_T001 transcript:Zm00001d035313_T002 1.00E-62
243- 4:transcript:Zm00001d041732_T001 transcript:Zm00001d035317_T002 4.00E-68
243- 5:transcript:Zm00001d041733_T001 transcript:Zm00001d035318_T001 3.00E-72

243- 6:transcript:Zm00001d041735_T001 transcript:Zm00001d035320_T001 2.00E-64
243- 7:transcript:Zm00001d041740_T001 transcript:Zm00001d035323_T001 0
Alignment 244: score=327.0 e_value=1.1e-11 N=7 3&6 plus
244- 0:transcript:Zm00001d042736_T001 transcript:Zm00001d038281_T001 2.00E-74
244- 1:transcript:Zm00001d042747_T003 transcript:Zm00001d038282_T001 0
244- 2:transcript:Zm00001d042752_T001 transcript:Zm00001d038283_T001 8.00E-32
244- 3:transcript:Zm00001d042753_T001 transcript:Zm00001d038284_T001 4.00E-43
244- 4:transcript:Zm00001d042754_T001 transcript:Zm00001d038287_T001 1.00E-26
244- 5:transcript:Zm00001d042756_T001 transcript:Zm00001d038288_T001 3.00E-75
244- 6:transcript:Zm00001d042758_T001 transcript:Zm00001d038289_T001 5.00E-75
Alignment 245: score=297.0 e_value=5.4e-15 N=8 3&6 plus
245- 0:transcript:Zm00001d043889_T001 transcript:Zm00001d038989_T002 2.00E-178
245- 1:transcript:Zm00001d043890_T002 transcript:Zm00001d038991_T001 0
245- 2:transcript:Zm00001d043898_T001 transcript:Zm00001d038993_T001 2.00E-10
245- 3:transcript:Zm00001d043902_T001 transcript:Zm00001d038994_T001 6.00E-94
245- 4:transcript:Zm00001d043905_T003 transcript:Zm00001d038995_T001 3.00E-99
245- 5:transcript:Zm00001d043906_T002 transcript:Zm00001d038998_T001 2.00E-117
245- 6:transcript:Zm00001d043911_T001 transcript:Zm00001d038999_T001 1.00E-101
245- 7:transcript:Zm00001d043914_T001 transcript:Zm00001d039002_T019 2.00E-126
Alignment 246: score=289.0 e_value=2.9e-11 N=7 3&6 plus
246- 0:transcript:Zm00001d043709_T003 transcript:Zm00001d038870_T001 2.00E-22
246- 1:transcript:Zm00001d043713_T001 transcript:Zm00001d038873_T001 1.00E-22
246- 2:transcript:Zm00001d043725_T001 transcript:Zm00001d038876_T001 6.00E-26
246- 3:transcript:Zm00001d043729_T001 transcript:Zm00001d038878_T001 6.00E-33
246- 4:transcript:Zm00001d043733_T002 transcript:Zm00001d038880_T001 0
246- 5:transcript:Zm00001d043741_T001 transcript:Zm00001d038883_T001 3.00E-135
246- 6:transcript:Zm00001d043751_T001 transcript:Zm00001d038886_T001 2.00E-13
Alignment 247: score=283.0 e_value=2.3e-08 N=6 3&6 plus
247- 0:transcript:Zm00001d039453_T001 transcript:Zm00001d037745_T001 0
247- 1:transcript:Zm00001d039455_T001 transcript:Zm00001d037747_T001 5.00E-53
247- 2:transcript:Zm00001d039459_T001 transcript:Zm00001d037749_T001 3.00E-35
247- 3:transcript:Zm00001d039461_T001 transcript:Zm00001d037751_T001 5.00E-84
247- 4:transcript:Zm00001d039464_T001 transcript:Zm00001d037753_T001 1.00E-09
247- 5:transcript:Zm00001d039468_T001 transcript:Zm00001d037757_T001 2.00E-47
Alignment 248: score=275.0 e_value=1e-09 N=6 3&6 plus
248- 0:transcript:Zm00001d043165_T002 transcript:Zm00001d038553_T001 1.00E-149
248- 1:transcript:Zm00001d043171_T001 transcript:Zm00001d038554_T001 0
248- 2:transcript:Zm00001d043174_T001 transcript:Zm00001d038555_T001 0
248- 3:transcript:Zm00001d043175_T003 transcript:Zm00001d038558_T005 2.00E-33
248- 4:transcript:Zm00001d043178_T001 transcript:Zm00001d038562_T001 0
248- 5:transcript:Zm00001d043180_T001 transcript:Zm00001d038563_T001 8.00E-127
Alignment 249: score=272.0 e_value=5.3e-11 N=6 3&6 plus
249- 0:transcript:Zm00001d044027_T001 transcript:Zm00001d039082_T001 5.00E-06
249- 1:transcript:Zm00001d044034_T001 transcript:Zm00001d039083_T001 3.00E-72
249- 2:transcript:Zm00001d044035_T001 transcript:Zm00001d039084_T001 4.00E-23
249- 3:transcript:Zm00001d044038_T001 transcript:Zm00001d039086_T001 4.00E-145
249- 4:transcript:Zm00001d044042_T001 transcript:Zm00001d039089_T001 0
249- 5:transcript:Zm00001d044054_T001 transcript:Zm00001d039090_T001 3.00E-155
Alignment 250: score=422.0 e_value=5.8e-24 N=10 3&6 minus
250- 0:transcript:Zm00001d043504_T030 transcript:Zm00001d038797_T001 0
250- 1:transcript:Zm00001d043506_T004 transcript:Zm00001d038796_T001 1.00E-107
250- 2:transcript:Zm00001d043509_T002 transcript:Zm00001d038794_T002 0
250- 3:transcript:Zm00001d043510_T002 transcript:Zm00001d038793_T001 2.00E-63
250- 4:transcript:Zm00001d043511_T001 transcript:Zm00001d038792_T001 0

250- 5:transcript:Zm00001d043512_T001 transcript:Zm00001d038791_T001 0
250- 6:transcript:Zm00001d043515_T002 transcript:Zm00001d038784_T001 7.00E-78
250- 7:transcript:Zm00001d043523_T001 transcript:Zm00001d038783_T001 6.00E-81
250- 8:transcript:Zm00001d043525_T001 transcript:Zm00001d038780_T001 1.00E-21
250- 9:transcript:Zm00001d043527_T002 transcript:Zm00001d038779_T001 1.00E-140
Alignment 251: score=250.0 e_value=4.1e-10 N=6 3&6 minus
251- 0:transcript:Zm00001d042714_T001 transcript:Zm00001d038321_T001 1.00E-139
251- 1:transcript:Zm00001d042717_T001 transcript:Zm00001d038320_T001 3.00E-35
251- 2:transcript:Zm00001d042718_T001 transcript:Zm00001d038319_T003 1.00E-123
251- 3:transcript:Zm00001d042719_T001 transcript:Zm00001d038312_T001 4.00E-119
251- 4:transcript:Zm00001d042721_T001 transcript:Zm00001d038311_T001 3.00E-64
251- 5:transcript:Zm00001d042724_T001 transcript:Zm00001d038310_T003 0
Alignment 252: score=2472.0 e_value=2.9e-224 N=57 3&8 plus
252- 0:transcript:Zm00001d042910_T010 transcript:Zm00001d012404_T001 0
252- 1:transcript:Zm00001d042917_T001 transcript:Zm00001d012417_T001 4.00E-160
252- 2:transcript:Zm00001d042918_T001 transcript:Zm00001d012418_T001 3.00E-140
252- 3:transcript:Zm00001d042920_T001 transcript:Zm00001d012419_T001 3.00E-83
252- 4:transcript:Zm00001d042922_T007 transcript:Zm00001d012420_T001 0
252- 5:transcript:Zm00001d042929_T001 transcript:Zm00001d012421_T004 1.00E-117
252- 6:transcript:Zm00001d042930_T001 transcript:Zm00001d012423_T001 4.00E-91
252- 7:transcript:Zm00001d042931_T001 transcript:Zm00001d012424_T001 2.00E-54
252- 8:transcript:Zm00001d042932_T001 transcript:Zm00001d012426_T001 5.00E-163
252- 9:transcript:Zm00001d042933_T001 transcript:Zm00001d012427_T001 1.00E-52
252- 10:transcript:Zm00001d042940_T001 transcript:Zm00001d012431_T001 1.00E-41
252- 11:transcript:Zm00001d042943_T005 transcript:Zm00001d012433_T002 0
252- 12:transcript:Zm00001d042944_T001 transcript:Zm00001d012434_T001 2.00E-44
252- 13:transcript:Zm00001d042949_T001 transcript:Zm00001d012435_T001 1.00E-170
252- 14:transcript:Zm00001d042950_T001 transcript:Zm00001d012437_T001 0
252- 15:transcript:Zm00001d042953_T001 transcript:Zm00001d012438_T001 0
252- 16:transcript:Zm00001d042954_T001 transcript:Zm00001d012439_T001 6.00E-67
252- 17:transcript:Zm00001d042955_T001 transcript:Zm00001d012440_T002 0
252- 18:transcript:Zm00001d042958_T001 transcript:Zm00001d012441_T001 4.00E-139
252- 19:transcript:Zm00001d042963_T001 transcript:Zm00001d012443_T001 4.00E-21
252- 20:transcript:Zm00001d042964_T001 transcript:Zm00001d012444_T001 3.00E-34
252- 21:transcript:Zm00001d042968_T002 transcript:Zm00001d012445_T001 4.00E-63
252- 22:transcript:Zm00001d042969_T002 transcript:Zm00001d012446_T002 4.00E-145
252- 23:transcript:Zm00001d042972_T002 transcript:Zm00001d012447_T005 0
252- 24:transcript:Zm00001d042975_T003 transcript:Zm00001d012449_T001 2.00E-34
252- 25:transcript:Zm00001d042977_T001 transcript:Zm00001d012451_T004 0
252- 26:transcript:Zm00001d042979_T007 transcript:Zm00001d012452_T004 0
252- 27:transcript:Zm00001d042980_T002 transcript:Zm00001d012456_T004 0
252- 28:transcript:Zm00001d042985_T001 transcript:Zm00001d012460_T001 4.00E-160
252- 29:transcript:Zm00001d042998_T001 transcript:Zm00001d012465_T001 3.00E-112
252- 30:transcript:Zm00001d043006_T001 transcript:Zm00001d012471_T001 5.00E-66
252- 31:transcript:Zm00001d043011_T001 transcript:Zm00001d012473_T001 1.00E-106
252- 32:transcript:Zm00001d043014_T001 transcript:Zm00001d012475_T001 5.00E-129
252- 33:transcript:Zm00001d043019_T003 transcript:Zm00001d012477_T001 0
252- 34:transcript:Zm00001d043022_T001 transcript:Zm00001d012479_T004 1.00E-17
252- 35:transcript:Zm00001d043024_T001 transcript:Zm00001d012480_T001 3.00E-74
252- 36:transcript:Zm00001d043025_T001 transcript:Zm00001d012482_T001 0
252- 37:transcript:Zm00001d043026_T001 transcript:Zm00001d012484_T002 2.00E-27
252- 38:transcript:Zm00001d043031_T001 transcript:Zm00001d012488_T001 0
252- 39:transcript:Zm00001d043043_T001 transcript:Zm00001d012494_T001 0
252- 40:transcript:Zm00001d043046_T001 transcript:Zm00001d012498_T001 6.00E-154

252- 41:transcript:Zm00001d043050_T001 transcript:Zm00001d012500_T001 2.00E-79
252- 42:transcript:Zm00001d043052_T001 transcript:Zm00001d012501_T002 0
252- 43:transcript:Zm00001d043059_T002 transcript:Zm00001d012504_T001 4.00E-156
252- 44:transcript:Zm00001d043060_T001 transcript:Zm00001d012505_T001 4.00E-103
252- 45:transcript:Zm00001d043062_T001 transcript:Zm00001d012507_T001 4.00E-24
252- 46:transcript:Zm00001d043067_T002 transcript:Zm00001d012511_T010 0
252- 47:transcript:Zm00001d043069_T001 transcript:Zm00001d012512_T001 0
252- 48:transcript:Zm00001d043070_T001 transcript:Zm00001d012513_T001 6.00E-108
252- 49:transcript:Zm00001d043071_T002 transcript:Zm00001d012514_T001 0
252- 50:transcript:Zm00001d043074_T004 transcript:Zm00001d012515_T004 0
252- 51:transcript:Zm00001d043076_T001 transcript:Zm00001d012516_T001 3.00E-159
252- 52:transcript:Zm00001d043080_T001 transcript:Zm00001d012517_T001 0
252- 53:transcript:Zm00001d043086_T002 transcript:Zm00001d012518_T003 0
252- 54:transcript:Zm00001d043087_T001 transcript:Zm00001d012520_T001 5.00E-86
252- 55:transcript:Zm00001d043089_T001 transcript:Zm00001d012522_T001 0
252- 56:transcript:Zm00001d043090_T001 transcript:Zm00001d012524_T001 0
Alignment 253: score=2023.0 e_value=2e-172 N=46 3&8 plus
253- 0:transcript:Zm00001d043112_T002 transcript:Zm00001d012536_T002 0
253- 1:transcript:Zm00001d043113_T001 transcript:Zm00001d012537_T003 3.00E-95
253- 2:transcript:Zm00001d043117_T002 transcript:Zm00001d012538_T001 3.00E-117
253- 3:transcript:Zm00001d043119_T001 transcript:Zm00001d012539_T001 4.00E-164
253- 4:transcript:Zm00001d043131_T002 transcript:Zm00001d012544_T001 0
253- 5:transcript:Zm00001d043136_T001 transcript:Zm00001d012545_T001 9.00E-61
253- 6:transcript:Zm00001d043137_T004 transcript:Zm00001d012546_T002 3.00E-177
253- 7:transcript:Zm00001d043146_T002 transcript:Zm00001d012548_T001 2.00E-08
253- 8:transcript:Zm00001d043149_T002 transcript:Zm00001d012550_T001 2.00E-148
253- 9:transcript:Zm00001d043150_T002 transcript:Zm00001d012552_T005 0
253- 10:transcript:Zm00001d043153_T001 transcript:Zm00001d012553_T003 0
253- 11:transcript:Zm00001d043158_T002 transcript:Zm00001d012556_T001 0
253- 12:transcript:Zm00001d043160_T004 transcript:Zm00001d012559_T004 0
253- 13:transcript:Zm00001d043164_T002 transcript:Zm00001d012560_T002 0
253- 14:transcript:Zm00001d043175_T003 transcript:Zm00001d012561_T001 9.00E-74
253- 15:transcript:Zm00001d043178_T001 transcript:Zm00001d012562_T001 0
253- 16:transcript:Zm00001d043181_T001 transcript:Zm00001d012563_T001 1.00E-32
253- 17:transcript:Zm00001d043183_T001 transcript:Zm00001d012564_T001 0
253- 18:transcript:Zm00001d043185_T001 transcript:Zm00001d012566_T001 3.00E-52
253- 19:transcript:Zm00001d043187_T001 transcript:Zm00001d012569_T001 0
253- 20:transcript:Zm00001d043188_T001 transcript:Zm00001d012571_T002 3.00E-102
253- 21:transcript:Zm00001d043191_T005 transcript:Zm00001d012575_T001 0
253- 22:transcript:Zm00001d043193_T001 transcript:Zm00001d012576_T001 0
253- 23:transcript:Zm00001d043196_T001 transcript:Zm00001d012578_T002 0
253- 24:transcript:Zm00001d043200_T001 transcript:Zm00001d012580_T001 4.00E-06
253- 25:transcript:Zm00001d043202_T001 transcript:Zm00001d012581_T001 0
253- 26:transcript:Zm00001d043204_T001 transcript:Zm00001d012584_T001 2.00E-59
253- 27:transcript:Zm00001d043218_T001 transcript:Zm00001d012593_T001 0
253- 28:transcript:Zm00001d043220_T001 transcript:Zm00001d012594_T001 3.00E-38
253- 29:transcript:Zm00001d043222_T001 transcript:Zm00001d012595_T001 0
253- 30:transcript:Zm00001d043223_T001 transcript:Zm00001d012598_T001 2.00E-44
253- 31:transcript:Zm00001d043224_T001 transcript:Zm00001d012599_T001 2.00E-69
253- 32:transcript:Zm00001d043227_T002 transcript:Zm00001d012600_T001 0
253- 33:transcript:Zm00001d043229_T001 transcript:Zm00001d012601_T002 2.00E-156
253- 34:transcript:Zm00001d043232_T001 transcript:Zm00001d012602_T001 1.00E-80
253- 35:transcript:Zm00001d043235_T001 transcript:Zm00001d012603_T001 2.00E-148
253- 36:transcript:Zm00001d043243_T001 transcript:Zm00001d012605_T001 0

253- 37:transcript:Zm00001d043249_T001 transcript:Zm00001d012607_T001 1.00E-138
253- 38:transcript:Zm00001d043256_T001 transcript:Zm00001d012609_T001 0
253- 39:transcript:Zm00001d043261_T001 transcript:Zm00001d012610_T001 3.00E-176
253- 40:transcript:Zm00001d043262_T001 transcript:Zm00001d012611_T001 2.00E-24
253- 41:transcript:Zm00001d043264_T001 transcript:Zm00001d012612_T001 2.00E-71
253- 42:transcript:Zm00001d043265_T001 transcript:Zm00001d012613_T001 1.00E-20
253- 43:transcript:Zm00001d043266_T002 transcript:Zm00001d012615_T001 0
253- 44:transcript:Zm00001d043269_T001 transcript:Zm00001d012618_T001 1.00E-149
253- 45:transcript:Zm00001d043270_T001 transcript:Zm00001d012619_T001 2.00E-118
Alignment 254: score=1335.0 e_value=5.5e-111 N=32 3&8 plus
254- 0:transcript:Zm00001d042164_T002 transcript:Zm00001d011896_T001 0
254- 1:transcript:Zm00001d042168_T001 transcript:Zm00001d011898_T001 0
254- 2:transcript:Zm00001d042169_T001 transcript:Zm00001d011899_T001 1.00E-39
254- 3:transcript:Zm00001d042179_T001 transcript:Zm00001d011903_T001 7.00E-98
254- 4:transcript:Zm00001d042180_T003 transcript:Zm00001d011904_T001 0
254- 5:transcript:Zm00001d042185_T003 transcript:Zm00001d011908_T001 0
254- 6:transcript:Zm00001d042189_T004 transcript:Zm00001d011913_T002 0
254- 7:transcript:Zm00001d042196_T001 transcript:Zm00001d011915_T001 2.00E-71
254- 8:transcript:Zm00001d042204_T001 transcript:Zm00001d011917_T001 3.00E-36
254- 9:transcript:Zm00001d042213_T001 transcript:Zm00001d011918_T002 0
254- 10:transcript:Zm00001d042214_T001 transcript:Zm00001d011919_T001 2.00E-123
254- 11:transcript:Zm00001d042215_T002 transcript:Zm00001d011920_T003 0
254- 12:transcript:Zm00001d042234_T004 transcript:Zm00001d011923_T002 0
254- 13:transcript:Zm00001d042237_T001 transcript:Zm00001d011924_T001 4.00E-97
254- 14:transcript:Zm00001d042239_T001 transcript:Zm00001d011925_T001 6.00E-107
254- 15:transcript:Zm00001d042245_T001 transcript:Zm00001d011938_T001 4.00E-36
254- 16:transcript:Zm00001d042258_T001 transcript:Zm00001d011944_T001 2.00E-60
254- 17:transcript:Zm00001d042259_T002 transcript:Zm00001d011945_T001 2.00E-161
254- 18:transcript:Zm00001d042266_T001 transcript:Zm00001d011952_T001 3.00E-142
254- 19:transcript:Zm00001d042267_T003 transcript:Zm00001d011953_T008 0
254- 20:transcript:Zm00001d042270_T005 transcript:Zm00001d011955_T002 4.00E-73
254- 21:transcript:Zm00001d042272_T003 transcript:Zm00001d011956_T002 0
254- 22:transcript:Zm00001d042276_T001 transcript:Zm00001d011959_T001 0
254- 23:transcript:Zm00001d042279_T001 transcript:Zm00001d011964_T001 0
254- 24:transcript:Zm00001d042282_T003 transcript:Zm00001d011965_T001 0
254- 25:transcript:Zm00001d042285_T001 transcript:Zm00001d011967_T001 0
254- 26:transcript:Zm00001d042286_T001 transcript:Zm00001d011968_T001 2.00E-84
254- 27:transcript:Zm00001d042288_T002 transcript:Zm00001d011969_T001 2.00E-88
254- 28:transcript:Zm00001d042290_T001 transcript:Zm00001d011970_T001 0
254- 29:transcript:Zm00001d042291_T002 transcript:Zm00001d011975_T002 0
254- 30:transcript:Zm00001d042292_T001 transcript:Zm00001d011978_T001 5.00E-61
254- 31:transcript:Zm00001d042302_T002 transcript:Zm00001d011979_T001 0
Alignment 255: score=878.0 e_value=1.6e-63 N=20 3&8 plus
255- 0:transcript:Zm00001d042349_T001 transcript:Zm00001d012035_T001 0
255- 1:transcript:Zm00001d042353_T003 transcript:Zm00001d012036_T001 0
255- 2:transcript:Zm00001d042354_T001 transcript:Zm00001d012038_T001 1.00E-14
255- 3:transcript:Zm00001d042357_T002 transcript:Zm00001d012043_T002 6.00E-37
255- 4:transcript:Zm00001d042359_T001 transcript:Zm00001d012045_T001 5.00E-111
255- 5:transcript:Zm00001d042361_T002 transcript:Zm00001d012047_T001 2.00E-62
255- 6:transcript:Zm00001d042362_T001 transcript:Zm00001d012048_T001 0
255- 7:transcript:Zm00001d042363_T007 transcript:Zm00001d012049_T003 0
255- 8:transcript:Zm00001d042367_T002 transcript:Zm00001d012059_T001 2.00E-50
255- 9:transcript:Zm00001d042370_T003 transcript:Zm00001d012061_T005 0
255- 10:transcript:Zm00001d042371_T001 transcript:Zm00001d012063_T001 2.00E-41

```
255- 11:transcript:Zm00001d042372_T002 transcript:Zm00001d012064_T001  3.00E-81
255- 12:transcript:Zm00001d042373_T002 transcript:Zm00001d012067_T003         0
255- 13:transcript:Zm00001d042376_T001 transcript:Zm00001d012069_T001 4.00E-127
255- 14:transcript:Zm00001d042380_T001 transcript:Zm00001d012078_T001  6.00E-37
255- 15:transcript:Zm00001d042381_T001 transcript:Zm00001d012079_T001  3.00E-73
255- 16:transcript:Zm00001d042382_T001 transcript:Zm00001d012080_T001  6.00E-43
255- 17:transcript:Zm00001d042383_T001 transcript:Zm00001d012081_T001  4.00E-24
255- 18:transcript:Zm00001d042394_T001 transcript:Zm00001d012085_T005         0
255- 19:transcript:Zm00001d042398_T001 transcript:Zm00001d012086_T009         0
## Alignment 256: score=877.0 e_value=3.7e-55 N=20 3&8 plus
256-  0:transcript:Zm00001d042718_T001 transcript:Zm00001d012269_T003 3.00E-166
256-  1:transcript:Zm00001d042719_T001 transcript:Zm00001d012270_T001 5.00E-102
256-  2:transcript:Zm00001d042721_T001 transcript:Zm00001d012273_T001  1.00E-98
256-  3:transcript:Zm00001d042723_T001 transcript:Zm00001d012274_T001         0
256-  4:transcript:Zm00001d042729_T003 transcript:Zm00001d012275_T001 5.00E-134
256-  5:transcript:Zm00001d042731_T001 transcript:Zm00001d012277_T003         0
256-  6:transcript:Zm00001d042735_T001 transcript:Zm00001d012279_T001  1.00E-92
256-  7:transcript:Zm00001d042736_T001 transcript:Zm00001d012280_T001 1.00E-128
256-  8:transcript:Zm00001d042749_T001 transcript:Zm00001d012281_T001  4.00E-98
256-  9:transcript:Zm00001d042752_T001 transcript:Zm00001d012282_T001  6.00E-92
256- 10:transcript:Zm00001d042755_T001 transcript:Zm00001d012284_T001  1.00E-63
256- 11:transcript:Zm00001d042756_T001 transcript:Zm00001d012285_T001  2.00E-93
256- 12:transcript:Zm00001d042761_T001 transcript:Zm00001d012286_T001         0
256- 13:transcript:Zm00001d042764_T001 transcript:Zm00001d012290_T001 5.00E-108
256- 14:transcript:Zm00001d042766_T001 transcript:Zm00001d012291_T001 4.00E-111
256- 15:transcript:Zm00001d042767_T001 transcript:Zm00001d012292_T001         0
256- 16:transcript:Zm00001d042777_T006 transcript:Zm00001d012294_T009         0
256- 17:transcript:Zm00001d042778_T001 transcript:Zm00001d012295_T003 3.00E-171
256- 18:transcript:Zm00001d042779_T001 transcript:Zm00001d012296_T001 1.00E-162
256- 19:transcript:Zm00001d042786_T001 transcript:Zm00001d012302_T001         0
## Alignment 257: score=821.0 e_value=1.4e-55 N=19 3&8 plus
257-  0:transcript:Zm00001d042627_T005 transcript:Zm00001d012224_T014         0
257-  1:transcript:Zm00001d042633_T001 transcript:Zm00001d012227_T001  2.00E-19
257-  2:transcript:Zm00001d042634_T001 transcript:Zm00001d012228_T002         0
257-  3:transcript:Zm00001d042636_T002 transcript:Zm00001d012229_T001         0
257-  4:transcript:Zm00001d042639_T004 transcript:Zm00001d012234_T002 2.00E-163
257-  5:transcript:Zm00001d042640_T001 transcript:Zm00001d012237_T001 3.00E-114
257-  6:transcript:Zm00001d042641_T013 transcript:Zm00001d012238_T003         0
257-  7:transcript:Zm00001d042642_T002 transcript:Zm00001d012239_T003  2.00E-90
257-  8:transcript:Zm00001d042643_T001 transcript:Zm00001d012240_T001         0
257-  9:transcript:Zm00001d042646_T001 transcript:Zm00001d012241_T001         0
257- 10:transcript:Zm00001d042653_T001 transcript:Zm00001d012242_T001         0
257- 11:transcript:Zm00001d042657_T001 transcript:Zm00001d012244_T001  1.00E-47
257- 12:transcript:Zm00001d042658_T002 transcript:Zm00001d012245_T002 2.00E-116
257- 13:transcript:Zm00001d042660_T001 transcript:Zm00001d012246_T001 4.00E-154
257- 14:transcript:Zm00001d042661_T001 transcript:Zm00001d012247_T004         0
257- 15:transcript:Zm00001d042663_T001 transcript:Zm00001d012248_T001  4.00E-46
257- 16:transcript:Zm00001d042664_T001 transcript:Zm00001d012254_T002         0
257- 17:transcript:Zm00001d042665_T001 transcript:Zm00001d012255_T001 7.00E-132
257- 18:transcript:Zm00001d042669_T002 transcript:Zm00001d012257_T013         0
## Alignment 258: score=760.0 e_value=3.6e-46 N=17 3&8 plus
258-  0:transcript:Zm00001d042810_T001 transcript:Zm00001d012320_T005         0
258-  1:transcript:Zm00001d042811_T001 transcript:Zm00001d012321_T001         0
258-  2:transcript:Zm00001d042812_T001 transcript:Zm00001d012325_T001         0
```

258- 3:transcript:Zm00001d042814_T001 transcript:Zm00001d012326_T001 0
258- 4:transcript:Zm00001d042821_T001 transcript:Zm00001d012330_T001 4.00E-97
258- 5:transcript:Zm00001d042822_T001 transcript:Zm00001d012332_T001 7.00E-36
258- 6:transcript:Zm00001d042826_T001 transcript:Zm00001d012333_T001 0
258- 7:transcript:Zm00001d042830_T002 transcript:Zm00001d012337_T001 3.00E-121
258- 8:transcript:Zm00001d042833_T001 transcript:Zm00001d012338_T001 0
258- 9:transcript:Zm00001d042836_T001 transcript:Zm00001d012339_T001 8.00E-23
258- 10:transcript:Zm00001d042837_T001 transcript:Zm00001d012350_T001 0
258- 11:transcript:Zm00001d042845_T001 transcript:Zm00001d012361_T001 0
258- 12:transcript:Zm00001d042849_T001 transcript:Zm00001d012379_T001 7.00E-62
258- 13:transcript:Zm00001d042851_T003 transcript:Zm00001d012380_T002 0
258- 14:transcript:Zm00001d042853_T001 transcript:Zm00001d012381_T001 6.00E-177
258- 15:transcript:Zm00001d042856_T002 transcript:Zm00001d012382_T002 4.00E-08
258- 16:transcript:Zm00001d042863_T001 transcript:Zm00001d012383_T002 3.00E-73
Alignment 259: score=727.0 e_value=2.5e-45 N=17 3&8 plus
259- 0:transcript:Zm00001d044367_T001 transcript:Zm00001d011266_T002 4.00E-101
259- 1:transcript:Zm00001d044374_T001 transcript:Zm00001d011268_T001 4.00E-66
259- 2:transcript:Zm00001d044375_T001 transcript:Zm00001d011270_T001 4.00E-08
259- 3:transcript:Zm00001d044379_T006 transcript:Zm00001d011272_T007 0
259- 4:transcript:Zm00001d044385_T001 transcript:Zm00001d011276_T001 5.00E-120
259- 5:transcript:Zm00001d044391_T002 transcript:Zm00001d011278_T001 1.00E-79
259- 6:transcript:Zm00001d044393_T002 transcript:Zm00001d011282_T001 0
259- 7:transcript:Zm00001d044394_T002 transcript:Zm00001d011283_T002 0
259- 8:transcript:Zm00001d044395_T001 transcript:Zm00001d011284_T001 6.00E-97
259- 9:transcript:Zm00001d044396_T001 transcript:Zm00001d011285_T001 0
259- 10:transcript:Zm00001d044405_T002 transcript:Zm00001d011288_T006 0
259- 11:transcript:Zm00001d044409_T001 transcript:Zm00001d011297_T001 4.00E-130
259- 12:transcript:Zm00001d044421_T001 transcript:Zm00001d011299_T001 2.00E-169
259- 13:transcript:Zm00001d044425_T001 transcript:Zm00001d011302_T001 0
259- 14:transcript:Zm00001d044427_T001 transcript:Zm00001d011303_T001 7.00E-33
259- 15:transcript:Zm00001d044431_T001 transcript:Zm00001d011304_T001 0
259- 16:transcript:Zm00001d044432_T010 transcript:Zm00001d011309_T009 0
Alignment 260: score=631.0 e_value=1.3e-39 N=15 3&8 plus
260- 0:transcript:Zm00001d042303_T001 transcript:Zm00001d011984_T001 0
260- 1:transcript:Zm00001d042305_T001 transcript:Zm00001d011985_T001 7.00E-129
260- 2:transcript:Zm00001d042307_T001 transcript:Zm00001d011987_T001 1.00E-62
260- 3:transcript:Zm00001d042308_T001 transcript:Zm00001d011992_T001 1.00E-148
260- 4:transcript:Zm00001d042309_T001 transcript:Zm00001d011994_T001 0
260- 5:transcript:Zm00001d042310_T001 transcript:Zm00001d012003_T001 2.00E-52
260- 6:transcript:Zm00001d042312_T012 transcript:Zm00001d012005_T007 0
260- 7:transcript:Zm00001d042314_T004 transcript:Zm00001d012007_T006 0
260- 8:transcript:Zm00001d042319_T001 transcript:Zm00001d012015_T001 3.00E-179
260- 9:transcript:Zm00001d042328_T001 transcript:Zm00001d012016_T001 0
260- 10:transcript:Zm00001d042329_T001 transcript:Zm00001d012017_T001 1.00E-130
260- 11:transcript:Zm00001d042336_T001 transcript:Zm00001d012021_T001 6.00E-84
260- 12:transcript:Zm00001d042338_T002 transcript:Zm00001d012027_T002 0
260- 13:transcript:Zm00001d042340_T001 transcript:Zm00001d012030_T003 2.00E-68
260- 14:transcript:Zm00001d042341_T001 transcript:Zm00001d012031_T001 7.00E-120
Alignment 261: score=494.0 e_value=2.6e-25 N=12 3&8 plus
261- 0:transcript:Zm00001d043356_T004 transcript:Zm00001d012674_T010 6.00E-179
261- 1:transcript:Zm00001d043364_T001 transcript:Zm00001d012676_T001 4.00E-68
261- 2:transcript:Zm00001d043367_T001 transcript:Zm00001d012679_T004 4.00E-71
261- 3:transcript:Zm00001d043371_T001 transcript:Zm00001d012684_T001 7.00E-46
261- 4:transcript:Zm00001d043378_T001 transcript:Zm00001d012688_T001 0

261- 5:transcript:Zm00001d043380_T001 transcript:Zm00001d012689_T001 1.00E-11
261- 6:transcript:Zm00001d043383_T001 transcript:Zm00001d012691_T001 3.00E-143
261- 7:transcript:Zm00001d043387_T002 transcript:Zm00001d012694_T002   0
261- 8:transcript:Zm00001d043389_T003 transcript:Zm00001d012696_T001   0
261- 9:transcript:Zm00001d043392_T016 transcript:Zm00001d012699_T005 2.00E-178
261- 10:transcript:Zm00001d043399_T001 transcript:Zm00001d012700_T001 6.00E-84
261- 11:transcript:Zm00001d043401_T002 transcript:Zm00001d012701_T004   0
Alignment 262: score=490.0 e_value=3.5e-28 N=11 3&8 plus
262- 0:transcript:Zm00001d042574_T001 transcript:Zm00001d012194_T001 8.00E-12
262- 1:transcript:Zm00001d042575_T002 transcript:Zm00001d012195_T002   0
262- 2:transcript:Zm00001d042582_T001 transcript:Zm00001d012196_T005 3.00E-141
262- 3:transcript:Zm00001d042584_T001 transcript:Zm00001d012197_T002 2.00E-149
262- 4:transcript:Zm00001d042585_T004 transcript:Zm00001d012198_T001   0
262- 5:transcript:Zm00001d042599_T001 transcript:Zm00001d012204_T001   0
262- 6:transcript:Zm00001d042601_T001 transcript:Zm00001d012205_T001   0
262- 7:transcript:Zm00001d042603_T001 transcript:Zm00001d012206_T001 5.00E-124
262- 8:transcript:Zm00001d042610_T001 transcript:Zm00001d012211_T010 8.00E-54
262- 9:transcript:Zm00001d042611_T001 transcript:Zm00001d012212_T001   0
262- 10:transcript:Zm00001d042615_T003 transcript:Zm00001d012213_T003 1.00E-117
Alignment 263: score=442.0 e_value=4.8e-20 N=10 3&8 plus
263- 0:transcript:Zm00001d043001_T001 transcript:Zm00001d010591_T001 2.00E-23
263- 1:transcript:Zm00001d043006_T001 transcript:Zm00001d010594_T001 2.00E-96
263- 2:transcript:Zm00001d043011_T001 transcript:Zm00001d010596_T001 2.00E-87
263- 3:transcript:Zm00001d043012_T018 transcript:Zm00001d010599_T002   0
263- 4:transcript:Zm00001d043015_T001 transcript:Zm00001d010602_T001   0
263- 5:transcript:Zm00001d043018_T001 transcript:Zm00001d010603_T001 1.00E-105
263- 6:transcript:Zm00001d043022_T001 transcript:Zm00001d010604_T006   0
263- 7:transcript:Zm00001d043023_T002 transcript:Zm00001d010606_T004   0
263- 8:transcript:Zm00001d043026_T001 transcript:Zm00001d010607_T001 6.00E-106
263- 9:transcript:Zm00001d043039_T001 transcript:Zm00001d010613_T002 2.00E-17
Alignment 264: score=437.0 e_value=3.8e-21 N=10 3&8 plus
264- 0:transcript:Zm00001d039472_T001 transcript:Zm00001d008799_T001   0
264- 1:transcript:Zm00001d039475_T004 transcript:Zm00001d008800_T011   0
264- 2:transcript:Zm00001d039480_T001 transcript:Zm00001d008803_T002 2.00E-145
264- 3:transcript:Zm00001d039488_T002 transcript:Zm00001d008805_T001   0
264- 4:transcript:Zm00001d039492_T001 transcript:Zm00001d008808_T002 2.00E-130
264- 5:transcript:Zm00001d039495_T001 transcript:Zm00001d008812_T001   0
264- 6:transcript:Zm00001d039498_T001 transcript:Zm00001d008815_T001   0
264- 7:transcript:Zm00001d039499_T002 transcript:Zm00001d008816_T001   0
264- 8:transcript:Zm00001d039506_T001 transcript:Zm00001d008817_T001   0
264- 9:transcript:Zm00001d039510_T001 transcript:Zm00001d008819_T001   0
Alignment 265: score=390.0 e_value=5.5e-20 N=9 3&8 plus
265- 0:transcript:Zm00001d044316_T001 transcript:Zm00001d011241_T001 3.00E-10
265- 1:transcript:Zm00001d044318_T001 transcript:Zm00001d011242_T002   0
265- 2:transcript:Zm00001d044326_T001 transcript:Zm00001d011245_T001 8.00E-30
265- 3:transcript:Zm00001d044327_T001 transcript:Zm00001d011246_T001 2.00E-77
265- 4:transcript:Zm00001d044330_T002 transcript:Zm00001d011252_T001 3.00E-161
265- 5:transcript:Zm00001d044333_T001 transcript:Zm00001d011255_T001 3.00E-133
265- 6:transcript:Zm00001d044335_T001 transcript:Zm00001d011256_T001 3.00E-96
265- 7:transcript:Zm00001d044338_T003 transcript:Zm00001d011258_T001   0
265- 8:transcript:Zm00001d044339_T003 transcript:Zm00001d011259_T001 6.00E-173
Alignment 266: score=379.0 e_value=1.5e-19 N=10 3&8 plus
266- 0:transcript:Zm00001d043700_T006 transcript:Zm00001d009421_T001 5.00E-49
266- 1:transcript:Zm00001d043708_T001 transcript:Zm00001d009425_T001 3.00E-14

266- 2:transcript:Zm00001d043713_T001 transcript:Zm00001d009429_T001 8.00E-21
266- 3:transcript:Zm00001d043727_T001 transcript:Zm00001d009431_T001 0
266- 4:transcript:Zm00001d043729_T001 transcript:Zm00001d009435_T001 4.00E-52
266- 5:transcript:Zm00001d043733_T002 transcript:Zm00001d009439_T004 0
266- 6:transcript:Zm00001d043751_T001 transcript:Zm00001d009446_T001 5.00E-19
266- 7:transcript:Zm00001d043762_T001 transcript:Zm00001d009447_T002 0
266- 8:transcript:Zm00001d043766_T005 transcript:Zm00001d009448_T001 0
266- 9:transcript:Zm00001d043773_T003 transcript:Zm00001d009452_T007 2.00E-170
Alignment 267: score=364.0 e_value=1.9e-17 N=9 3&8 plus
267- 0:transcript:Zm00001d044442_T036 transcript:Zm00001d011319_T003 0
267- 1:transcript:Zm00001d044445_T001 transcript:Zm00001d011321_T001 4.00E-119
267- 2:transcript:Zm00001d044459_T002 transcript:Zm00001d011330_T001 2.00E-32
267- 3:transcript:Zm00001d044461_T001 transcript:Zm00001d011331_T001 2.00E-172
267- 4:transcript:Zm00001d044465_T001 transcript:Zm00001d011334_T003 0
267- 5:transcript:Zm00001d044469_T001 transcript:Zm00001d011336_T006 0
267- 6:transcript:Zm00001d044475_T001 transcript:Zm00001d011348_T001 0
267- 7:transcript:Zm00001d044476_T001 transcript:Zm00001d011350_T001 0
267- 8:transcript:Zm00001d044478_T022 transcript:Zm00001d011351_T001 0
Alignment 268: score=360.0 e_value=5.3e-14 N=8 3&8 plus
268- 0:transcript:Zm00001d044224_T001 transcript:Zm00001d011178_T002 0
268- 1:transcript:Zm00001d044226_T001 transcript:Zm00001d011180_T004 2.00E-95
268- 2:transcript:Zm00001d044228_T001 transcript:Zm00001d011183_T001 0
268- 3:transcript:Zm00001d044237_T001 transcript:Zm00001d011185_T001 1.00E-31
268- 4:transcript:Zm00001d044242_T001 transcript:Zm00001d011187_T001 8.00E-139
268- 5:transcript:Zm00001d044243_T001 transcript:Zm00001d011188_T001 0
268- 6:transcript:Zm00001d044244_T001 transcript:Zm00001d011189_T001 2.00E-53
268- 7:transcript:Zm00001d044250_T001 transcript:Zm00001d011192_T001 0
Alignment 269: score=359.0 e_value=2.5e-16 N=8 3&8 plus
269- 0:transcript:Zm00001d042040_T001 transcript:Zm00001d011828_T001 3.00E-97
269- 1:transcript:Zm00001d042049_T001 transcript:Zm00001d011833_T001 0
269- 2:transcript:Zm00001d042051_T001 transcript:Zm00001d011834_T001 0
269- 3:transcript:Zm00001d042052_T009 transcript:Zm00001d011835_T003 0
269- 4:transcript:Zm00001d042055_T001 transcript:Zm00001d011839_T001 9.00E-100
269- 5:transcript:Zm00001d042056_T001 transcript:Zm00001d011841_T001 5.00E-45
269- 6:transcript:Zm00001d042061_T001 transcript:Zm00001d011845_T001 2.00E-86
269- 7:transcript:Zm00001d042062_T001 transcript:Zm00001d011847_T001 6.00E-111
Alignment 270: score=350.0 e_value=1.4e-22 N=9 3&8 plus
270- 0:transcript:Zm00001d042084_T060 transcript:Zm00001d011854_T087 0
270- 1:transcript:Zm00001d042088_T001 transcript:Zm00001d011855_T002 0
270- 2:transcript:Zm00001d042093_T001 transcript:Zm00001d011873_T002 7.00E-101
270- 3:transcript:Zm00001d042094_T001 transcript:Zm00001d011874_T002 7.00E-100
270- 4:transcript:Zm00001d042111_T001 transcript:Zm00001d011878_T001 0
270- 5:transcript:Zm00001d042114_T001 transcript:Zm00001d011879_T001 3.00E-06
270- 6:transcript:Zm00001d042118_T001 transcript:Zm00001d011880_T002 3.00E-79
270- 7:transcript:Zm00001d042127_T001 transcript:Zm00001d011881_T001 0
270- 8:transcript:Zm00001d042133_T003 transcript:Zm00001d011885_T005 0
Alignment 271: score=336.0 e_value=1.4e-16 N=8 3&8 plus
271- 0:transcript:Zm00001d041984_T001 transcript:Zm00001d011793_T001 6.00E-120
271- 1:transcript:Zm00001d041993_T002 transcript:Zm00001d011795_T001 6.00E-44
271- 2:transcript:Zm00001d041995_T002 transcript:Zm00001d011796_T002 0
271- 3:transcript:Zm00001d042005_T001 transcript:Zm00001d011803_T003 0
271- 4:transcript:Zm00001d042014_T001 transcript:Zm00001d011810_T001 1.00E-19
271- 5:transcript:Zm00001d042016_T001 transcript:Zm00001d011812_T001 7.00E-65
271- 6:transcript:Zm00001d042023_T001 transcript:Zm00001d011816_T001 1.00E-101

271- 7:transcript:Zm00001d042026_T001 transcript:Zm00001d011819_T001 0
Alignment 272: score=324.0 e_value=7.6e-14 N=8 3&8 plus
272- 0:transcript:Zm00001d043854_T001 transcript:Zm00001d009506_T001 0
272- 1:transcript:Zm00001d043855_T001 transcript:Zm00001d009508_T001 1.00E-87
272- 2:transcript:Zm00001d043864_T001 transcript:Zm00001d009510_T001 0
272- 3:transcript:Zm00001d043870_T001 transcript:Zm00001d009511_T001 0
272- 4:transcript:Zm00001d043874_T001 transcript:Zm00001d009525_T002 1.00E-34
272- 5:transcript:Zm00001d043878_T001 transcript:Zm00001d009532_T001 3.00E-84
272- 6:transcript:Zm00001d043879_T002 transcript:Zm00001d009539_T002 0
272- 7:transcript:Zm00001d043889_T001 transcript:Zm00001d009549_T002 3.00E-173
Alignment 273: score=320.0 e_value=9.2e-15 N=7 3&8 plus
273- 0:transcript:Zm00001d040389_T002 transcript:Zm00001d008259_T001 4.00E-19
273- 1:transcript:Zm00001d040390_T003 transcript:Zm00001d008260_T001 7.00E-108
273- 2:transcript:Zm00001d040392_T001 transcript:Zm00001d008262_T001 9.00E-146
273- 3:transcript:Zm00001d040398_T001 transcript:Zm00001d008265_T001 0
273- 4:transcript:Zm00001d040399_T001 transcript:Zm00001d008266_T001 0
273- 5:transcript:Zm00001d040408_T003 transcript:Zm00001d008269_T001 0
273- 6:transcript:Zm00001d040415_T001 transcript:Zm00001d008272_T001 9.00E-150
Alignment 274: score=319.0 e_value=2.6e-11 N=7 3&8 plus
274- 0:transcript:Zm00001d043402_T001 transcript:Zm00001d012703_T001 5.00E-88
274- 1:transcript:Zm00001d043405_T001 transcript:Zm00001d012708_T001 0
274- 2:transcript:Zm00001d043406_T002 transcript:Zm00001d012709_T001 7.00E-167
274- 3:transcript:Zm00001d043407_T001 transcript:Zm00001d012710_T001 1.00E-128
274- 4:transcript:Zm00001d043411_T001 transcript:Zm00001d012712_T002 0
274- 5:transcript:Zm00001d043413_T001 transcript:Zm00001d012714_T001 1.00E-09
274- 6:transcript:Zm00001d043414_T001 transcript:Zm00001d012718_T004 0
Alignment 275: score=298.0 e_value=1.3e-12 N=7 3&8 plus
275- 0:transcript:Zm00001d042470_T003 transcript:Zm00001d012137_T010 0
275- 1:transcript:Zm00001d042473_T002 transcript:Zm00001d012138_T001 0
275- 2:transcript:Zm00001d042474_T001 transcript:Zm00001d012140_T001 0
275- 3:transcript:Zm00001d042476_T002 transcript:Zm00001d012142_T001 1.00E-109
275- 4:transcript:Zm00001d042479_T001 transcript:Zm00001d012145_T001 0
275- 5:transcript:Zm00001d042480_T002 transcript:Zm00001d012146_T003 0
275- 6:transcript:Zm00001d042486_T001 transcript:Zm00001d012147_T001 5.00E-158
Alignment 276: score=286.0 e_value=8e-13 N=7 3&8 plus
276- 0:transcript:Zm00001d039518_T002 transcript:Zm00001d008788_T003 0
276- 1:transcript:Zm00001d039522_T002 transcript:Zm00001d008789_T018 0
276- 2:transcript:Zm00001d039526_T002 transcript:Zm00001d008791_T001 0
276- 3:transcript:Zm00001d039527_T001 transcript:Zm00001d008792_T001 2.00E-57
276- 4:transcript:Zm00001d039531_T001 transcript:Zm00001d008793_T001 8.00E-80
276- 5:transcript:Zm00001d039532_T001 transcript:Zm00001d008794_T001 0
276- 6:transcript:Zm00001d039537_T002 transcript:Zm00001d008800_T011 6.00E-59
Alignment 277: score=273.0 e_value=4.9e-11 N=6 3&8 plus
277- 0:transcript:Zm00001d039425_T005 transcript:Zm00001d008874_T002 0
277- 1:transcript:Zm00001d039427_T002 transcript:Zm00001d008875_T001 0
277- 2:transcript:Zm00001d039428_T001 transcript:Zm00001d008878_T001 3.00E-91
277- 3:transcript:Zm00001d039429_T001 transcript:Zm00001d008881_T001 1.00E-51
277- 4:transcript:Zm00001d039434_T001 transcript:Zm00001d008882_T001 2.00E-139
277- 5:transcript:Zm00001d039446_T001 transcript:Zm00001d008890_T001 0
Alignment 278: score=272.0 e_value=3.3e-11 N=6 3&8 plus
278- 0:transcript:Zm00001d042525_T003 transcript:Zm00001d012161_T001 0
278- 1:transcript:Zm00001d042527_T001 transcript:Zm00001d012165_T001 6.00E-93
278- 2:transcript:Zm00001d042528_T007 transcript:Zm00001d012166_T002 0
278- 3:transcript:Zm00001d042530_T003 transcript:Zm00001d012168_T002 0

278- 4:transcript:Zm00001d042534_T002 transcript:Zm00001d012171_T001 9.00E-26
278- 5:transcript:Zm00001d042536_T001 transcript:Zm00001d012173_T001 0
Alignment 279: score=268.0 e_value=1.9e-10 N=6 3&8 plus
279- 0:transcript:Zm00001d044171_T001 transcript:Zm00001d009698_T001 7.00E-62
279- 1:transcript:Zm00001d044173_T002 transcript:Zm00001d009700_T001 4.00E-78
279- 2:transcript:Zm00001d044176_T001 transcript:Zm00001d009701_T001 4.00E-18
279- 3:transcript:Zm00001d044181_T002 transcript:Zm00001d009705_T001 0
279- 4:transcript:Zm00001d044184_T001 transcript:Zm00001d009707_T001 5.00E-115
279- 5:transcript:Zm00001d044185_T004 transcript:Zm00001d009708_T001 0
Alignment 280: score=262.0 e_value=3.1e-11 N=6 3&8 plus
280- 0:transcript:Zm00001d040717_T005 transcript:Zm00001d009161_T002 1.00E-145
280- 1:transcript:Zm00001d040721_T002 transcript:Zm00001d009163_T001 0
280- 2:transcript:Zm00001d040725_T004 transcript:Zm00001d009167_T001 0
280- 3:transcript:Zm00001d040726_T001 transcript:Zm00001d009171_T001 0
280- 4:transcript:Zm00001d040735_T001 transcript:Zm00001d009177_T001 0
280- 5:transcript:Zm00001d040741_T001 transcript:Zm00001d009178_T001 0
Alignment 281: score=261.0 e_value=8.2e-10 N=6 3&8 plus
281- 0:transcript:Zm00001d040323_T001 transcript:Zm00001d008278_T001 4.00E-77
281- 1:transcript:Zm00001d040324_T001 transcript:Zm00001d008279_T001 2.00E-172
281- 2:transcript:Zm00001d040333_T001 transcript:Zm00001d008283_T001 2.00E-120
281- 3:transcript:Zm00001d040334_T001 transcript:Zm00001d008284_T004 0
281- 4:transcript:Zm00001d040340_T005 transcript:Zm00001d008285_T005 0
281- 5:transcript:Zm00001d040343_T001 transcript:Zm00001d008293_T002 0
Alignment 282: score=1721.0 e_value=3.4e-144 N=40 3&8 minus
282- 0:transcript:Zm00001d039852_T001 transcript:Zm00001d008625_T001 0
282- 1:transcript:Zm00001d039854_T001 transcript:Zm00001d008624_T001 0
282- 2:transcript:Zm00001d039856_T002 transcript:Zm00001d008623_T001 4.00E-71
282- 3:transcript:Zm00001d039858_T001 transcript:Zm00001d008621_T001 0
282- 4:transcript:Zm00001d039859_T001 transcript:Zm00001d008620_T001 5.00E-22
282- 5:transcript:Zm00001d039865_T002 transcript:Zm00001d008619_T001 5.00E-173
282- 6:transcript:Zm00001d039867_T007 transcript:Zm00001d008618_T005 0
282- 7:transcript:Zm00001d039871_T002 transcript:Zm00001d008617_T001 0
282- 8:transcript:Zm00001d039879_T001 transcript:Zm00001d008613_T001 5.00E-140
282- 9:transcript:Zm00001d039881_T003 transcript:Zm00001d008611_T003 2.00E-178
282- 10:transcript:Zm00001d039882_T001 transcript:Zm00001d008610_T001 7.00E-31
282- 11:transcript:Zm00001d039893_T002 transcript:Zm00001d008601_T001 1.00E-50
282- 12:transcript:Zm00001d039901_T003 transcript:Zm00001d008599_T001 7.00E-76
282- 13:transcript:Zm00001d039902_T001 transcript:Zm00001d008598_T001 3.00E-126
282- 14:transcript:Zm00001d039904_T006 transcript:Zm00001d008596_T005 0
282- 15:transcript:Zm00001d039908_T001 transcript:Zm00001d008594_T001 9.00E-108
282- 16:transcript:Zm00001d039916_T001 transcript:Zm00001d008591_T001 0
282- 17:transcript:Zm00001d039918_T001 transcript:Zm00001d008588_T001 0
282- 18:transcript:Zm00001d039920_T001 transcript:Zm00001d008585_T001 0
282- 19:transcript:Zm00001d039930_T001 transcript:Zm00001d008582_T001 3.00E-48
282- 20:transcript:Zm00001d039932_T001 transcript:Zm00001d008579_T001 6.00E-31
282- 21:transcript:Zm00001d039933_T001 transcript:Zm00001d008577_T001 3.00E-74
282- 22:transcript:Zm00001d039944_T001 transcript:Zm00001d008573_T001 5.00E-86
282- 23:transcript:Zm00001d039963_T001 transcript:Zm00001d008570_T001 0
282- 24:transcript:Zm00001d039965_T001 transcript:Zm00001d008569_T001 0
282- 25:transcript:Zm00001d039969_T001 transcript:Zm00001d008568_T001 1.00E-146
282- 26:transcript:Zm00001d039973_T002 transcript:Zm00001d008567_T008 0
282- 27:transcript:Zm00001d039975_T001 transcript:Zm00001d008565_T001 0
282- 28:transcript:Zm00001d039977_T001 transcript:Zm00001d008562_T001 8.00E-105
282- 29:transcript:Zm00001d039982_T001 transcript:Zm00001d008552_T001 9.00E-62

282- 30:transcript:Zm00001d039989_T001 transcript:Zm00001d008545_T001 9.00E-19
282- 31:transcript:Zm00001d039991_T001 transcript:Zm00001d008540_T001 5.00E-65
282- 32:transcript:Zm00001d039993_T001 transcript:Zm00001d008539_T001 6.00E-39
282- 33:transcript:Zm00001d040005_T001 transcript:Zm00001d008535_T001 0
282- 34:transcript:Zm00001d040006_T001 transcript:Zm00001d008532_T001 2.00E-74
282- 35:transcript:Zm00001d040010_T003 transcript:Zm00001d008531_T001 0
282- 36:transcript:Zm00001d040011_T001 transcript:Zm00001d008530_T001 1.00E-121
282- 37:transcript:Zm00001d040014_T002 transcript:Zm00001d008529_T001 1.00E-102
282- 38:transcript:Zm00001d040019_T001 transcript:Zm00001d008528_T001 5.00E-134
282- 39:transcript:Zm00001d040020_T001 transcript:Zm00001d008524_T001 2.00E-97
Alignment 283: score=1143.0 e_value=1.4e-85 N=27 3&8 minus
283- 0:transcript:Zm00001d043674_T001 transcript:Zm00001d011710_T001 2.00E-161
283- 1:transcript:Zm00001d043675_T001 transcript:Zm00001d011708_T001 9.00E-155
283- 2:transcript:Zm00001d043682_T002 transcript:Zm00001d011705_T002 0
283- 3:transcript:Zm00001d043684_T001 transcript:Zm00001d011700_T014 0
283- 4:transcript:Zm00001d043686_T009 transcript:Zm00001d011699_T002 0
283- 5:transcript:Zm00001d043693_T002 transcript:Zm00001d011696_T002 0
283- 6:transcript:Zm00001d043695_T001 transcript:Zm00001d011692_T001 0
283- 7:transcript:Zm00001d043696_T004 transcript:Zm00001d011691_T002 1.00E-163
283- 8:transcript:Zm00001d043700_T006 transcript:Zm00001d011688_T004 1.00E-118
283- 9:transcript:Zm00001d043701_T001 transcript:Zm00001d011687_T001 0
283- 10:transcript:Zm00001d043703_T007 transcript:Zm00001d011685_T002 0
283- 11:transcript:Zm00001d043708_T001 transcript:Zm00001d011681_T001 2.00E-38
283- 12:transcript:Zm00001d043725_T001 transcript:Zm00001d011678_T002 3.00E-118
283- 13:transcript:Zm00001d043726_T001 transcript:Zm00001d011676_T002 0
283- 14:transcript:Zm00001d043727_T001 transcript:Zm00001d011673_T001 0
283- 15:transcript:Zm00001d043729_T001 transcript:Zm00001d011669_T001 1.00E-111
283- 16:transcript:Zm00001d043733_T002 transcript:Zm00001d011663_T003 0
283- 17:transcript:Zm00001d043736_T002 transcript:Zm00001d011658_T001 6.00E-138
283- 18:transcript:Zm00001d043737_T001 transcript:Zm00001d011657_T002 1.00E-98
283- 19:transcript:Zm00001d043738_T001 transcript:Zm00001d011656_T001 9.00E-133
283- 20:transcript:Zm00001d043740_T001 transcript:Zm00001d011654_T001 5.00E-114
283- 21:transcript:Zm00001d043745_T001 transcript:Zm00001d011652_T001 6.00E-71
283- 22:transcript:Zm00001d043751_T001 transcript:Zm00001d011650_T001 1.00E-66
283- 23:transcript:Zm00001d043752_T001 transcript:Zm00001d011649_T001 0
283- 24:transcript:Zm00001d043757_T001 transcript:Zm00001d011648_T001 0
283- 25:transcript:Zm00001d043766_T005 transcript:Zm00001d011644_T002 0
283- 26:transcript:Zm00001d043770_T001 transcript:Zm00001d011642_T002 4.00E-38
Alignment 284: score=835.0 e_value=2.3e-63 N=21 3&8 minus
284- 0:transcript:Zm00001d040232_T001 transcript:Zm00001d008381_T001 1.00E-147
284- 1:transcript:Zm00001d040233_T004 transcript:Zm00001d008380_T002 0
284- 2:transcript:Zm00001d040235_T005 transcript:Zm00001d008379_T001 0
284- 3:transcript:Zm00001d040238_T002 transcript:Zm00001d008376_T001 0
284- 4:transcript:Zm00001d040243_T002 transcript:Zm00001d008374_T001 0
284- 5:transcript:Zm00001d040257_T006 transcript:Zm00001d008369_T001 0
284- 6:transcript:Zm00001d040260_T001 transcript:Zm00001d008359_T001 7.00E-39
284- 7:transcript:Zm00001d040263_T010 transcript:Zm00001d008357_T002 0
284- 8:transcript:Zm00001d040269_T001 transcript:Zm00001d008347_T001 0
284- 9:transcript:Zm00001d040271_T001 transcript:Zm00001d008346_T001 1.00E-58
284- 10:transcript:Zm00001d040274_T001 transcript:Zm00001d008345_T003 0
284- 11:transcript:Zm00001d040279_T023 transcript:Zm00001d008338_T001 0
284- 12:transcript:Zm00001d040286_T003 transcript:Zm00001d008333_T003 2.00E-123
284- 13:transcript:Zm00001d040291_T001 transcript:Zm00001d008330_T001 2.00E-82
284- 14:transcript:Zm00001d040292_T001 transcript:Zm00001d008329_T001 3.00E-23

```
284- 15:transcript:Zm00001d040293_T001 transcript:Zm00001d008327_T001         0
284- 16:transcript:Zm00001d040298_T001 transcript:Zm00001d008325_T001  8.00E-70
284- 17:transcript:Zm00001d040301_T007 transcript:Zm00001d008323_T001         0
284- 18:transcript:Zm00001d040302_T005 transcript:Zm00001d008322_T002         0
284- 19:transcript:Zm00001d040305_T014 transcript:Zm00001d008321_T011         0
284- 20:transcript:Zm00001d040308_T001 transcript:Zm00001d008320_T004 4.00E-104
## Alignment 285: score=752.0 e_value=3.7e-48 N=18 3&8 minus
285-  0:transcript:Zm00001d044036_T001 transcript:Zm00001d011478_T002 2.00E-109
285-  1:transcript:Zm00001d044049_T001 transcript:Zm00001d011475_T001         0
285-  2:transcript:Zm00001d044054_T001 transcript:Zm00001d011474_T001 3.00E-166
285-  3:transcript:Zm00001d044056_T003 transcript:Zm00001d011473_T003         0
285-  4:transcript:Zm00001d044060_T001 transcript:Zm00001d011471_T001         0
285-  5:transcript:Zm00001d044074_T001 transcript:Zm00001d011470_T001 2.00E-146
285-  6:transcript:Zm00001d044076_T001 transcript:Zm00001d011468_T001  2.00E-30
285-  7:transcript:Zm00001d044079_T001 transcript:Zm00001d011467_T001  2.00E-51
285-  8:transcript:Zm00001d044087_T001 transcript:Zm00001d011463_T001 2.00E-180
285-  9:transcript:Zm00001d044091_T003 transcript:Zm00001d011458_T001         0
285- 10:transcript:Zm00001d044092_T001 transcript:Zm00001d011457_T001  3.00E-35
285- 11:transcript:Zm00001d044093_T001 transcript:Zm00001d011455_T001 3.00E-104
285- 12:transcript:Zm00001d044099_T002 transcript:Zm00001d011454_T001 7.00E-130
285- 13:transcript:Zm00001d044100_T001 transcript:Zm00001d011451_T001  1.00E-98
285- 14:transcript:Zm00001d044101_T003 transcript:Zm00001d011448_T001         0
285- 15:transcript:Zm00001d044104_T001 transcript:Zm00001d011446_T001         0
285- 16:transcript:Zm00001d044110_T001 transcript:Zm00001d011444_T002         0
285- 17:transcript:Zm00001d044122_T001 transcript:Zm00001d011438_T001         0
## Alignment 286: score=739.0 e_value=6.7e-45 N=17 3&8 minus
286-  0:transcript:Zm00001d043914_T001 transcript:Zm00001d011560_T012         0
286-  1:transcript:Zm00001d043921_T001 transcript:Zm00001d011555_T001 5.00E-173
286-  2:transcript:Zm00001d043923_T001 transcript:Zm00001d011550_T001         0
286-  3:transcript:Zm00001d043932_T001 transcript:Zm00001d011543_T001  6.00E-56
286-  4:transcript:Zm00001d043935_T001 transcript:Zm00001d011540_T001  3.00E-33
286-  5:transcript:Zm00001d043937_T001 transcript:Zm00001d011537_T001  4.00E-37
286-  6:transcript:Zm00001d043942_T001 transcript:Zm00001d011534_T004 3.00E-129
286-  7:transcript:Zm00001d043945_T001 transcript:Zm00001d011531_T001 1.00E-111
286-  8:transcript:Zm00001d043946_T001 transcript:Zm00001d011528_T001  2.00E-45
286-  9:transcript:Zm00001d043950_T001 transcript:Zm00001d011527_T001 4.00E-127
286- 10:transcript:Zm00001d043954_T003 transcript:Zm00001d011525_T002         0
286- 11:transcript:Zm00001d043959_T003 transcript:Zm00001d011520_T001 1.00E-164
286- 12:transcript:Zm00001d043962_T001 transcript:Zm00001d011517_T005         0
286- 13:transcript:Zm00001d043968_T001 transcript:Zm00001d011515_T001  2.00E-44
286- 14:transcript:Zm00001d043971_T001 transcript:Zm00001d011514_T001  3.00E-91
286- 15:transcript:Zm00001d043972_T001 transcript:Zm00001d011513_T001  4.00E-80
286- 16:transcript:Zm00001d043975_T001 transcript:Zm00001d011511_T001  1.00E-24
## Alignment 287: score=698.0 e_value=9.9e-40 N=16 3&8 minus
287-  0:transcript:Zm00001d044144_T008 transcript:Zm00001d011424_T008         0
287-  1:transcript:Zm00001d044146_T001 transcript:Zm00001d011422_T001         0
287-  2:transcript:Zm00001d044154_T001 transcript:Zm00001d011419_T002         0
287-  3:transcript:Zm00001d044159_T002 transcript:Zm00001d011417_T001         0
287-  4:transcript:Zm00001d044162_T001 transcript:Zm00001d011413_T001 4.00E-169
287-  5:transcript:Zm00001d044164_T002 transcript:Zm00001d011411_T002         0
287-  6:transcript:Zm00001d044167_T001 transcript:Zm00001d011410_T001 3.00E-121
287-  7:transcript:Zm00001d044168_T001 transcript:Zm00001d011406_T001 7.00E-143
287-  8:transcript:Zm00001d044170_T001 transcript:Zm00001d011405_T001         0
287-  9:transcript:Zm00001d044171_T001 transcript:Zm00001d011403_T001 1.00E-153
```

287- 10:transcript:Zm00001d044172_T002 transcript:Zm00001d011401_T001 0
287- 11:transcript:Zm00001d044173_T002 transcript:Zm00001d011399_T001 5.00E-114
287- 12:transcript:Zm00001d044176_T001 transcript:Zm00001d011398_T001 5.00E-98
287- 13:transcript:Zm00001d044179_T001 transcript:Zm00001d011396_T001 6.00E-103
287- 14:transcript:Zm00001d044181_T002 transcript:Zm00001d011393_T004 0
287- 15:transcript:Zm00001d044185_T004 transcript:Zm00001d011392_T003 0
Alignment 288: score=650.0 e_value=1.8e-35 N=15 3&8 minus
288- 0:transcript:Zm00001d040503_T005 transcript:Zm00001d008218_T002 0
288- 1:transcript:Zm00001d040509_T001 transcript:Zm00001d008216_T002 8.00E-166
288- 2:transcript:Zm00001d040515_T001 transcript:Zm00001d008215_T002 0
288- 3:transcript:Zm00001d040519_T002 transcript:Zm00001d008210_T002 1.00E-130
288- 4:transcript:Zm00001d040526_T001 transcript:Zm00001d008208_T001 4.00E-101
288- 5:transcript:Zm00001d040527_T001 transcript:Zm00001d008206_T001 2.00E-47
288- 6:transcript:Zm00001d040536_T005 transcript:Zm00001d008205_T001 0
288- 7:transcript:Zm00001d040539_T004 transcript:Zm00001d008203_T001 1.00E-35
288- 8:transcript:Zm00001d040541_T001 transcript:Zm00001d008201_T001 4.00E-41
288- 9:transcript:Zm00001d040544_T001 transcript:Zm00001d008200_T001 2.00E-33
288- 10:transcript:Zm00001d040545_T002 transcript:Zm00001d008196_T001 7.00E-15
288- 11:transcript:Zm00001d040548_T001 transcript:Zm00001d008194_T001 4.00E-28
288- 12:transcript:Zm00001d040554_T001 transcript:Zm00001d008190_T001 2.00E-165
288- 13:transcript:Zm00001d040555_T001 transcript:Zm00001d008187_T001 0
288- 14:transcript:Zm00001d040562_T002 transcript:Zm00001d008178_T012 0
Alignment 289: score=620.0 e_value=4.7e-36 N=14 3&8 minus
289- 0:transcript:Zm00001d039345_T001 transcript:Zm00001d008957_T001 0
289- 1:transcript:Zm00001d039349_T002 transcript:Zm00001d008954_T002 0
289- 2:transcript:Zm00001d039350_T001 transcript:Zm00001d008951_T002 6.00E-161
289- 3:transcript:Zm00001d039351_T001 transcript:Zm00001d008946_T001 2.00E-68
289- 4:transcript:Zm00001d039352_T001 transcript:Zm00001d008945_T001 5.00E-76
289- 5:transcript:Zm00001d039355_T010 transcript:Zm00001d008941_T002 0
289- 6:transcript:Zm00001d039362_T002 transcript:Zm00001d008930_T003 0
289- 7:transcript:Zm00001d039367_T001 transcript:Zm00001d008924_T001 2.00E-15
289- 8:transcript:Zm00001d039369_T004 transcript:Zm00001d008922_T001 0
289- 9:transcript:Zm00001d039371_T001 transcript:Zm00001d008919_T001 0
289- 10:transcript:Zm00001d039372_T002 transcript:Zm00001d008916_T001 3.00E-25
289- 11:transcript:Zm00001d039375_T001 transcript:Zm00001d008914_T001 1.00E-34
289- 12:transcript:Zm00001d039378_T001 transcript:Zm00001d008912_T001 5.00E-23
289- 13:transcript:Zm00001d039379_T001 transcript:Zm00001d008911_T001 7.00E-45
Alignment 290: score=598.0 e_value=6.5e-41 N=15 3&8 minus
290- 0:transcript:Zm00001d039238_T001 transcript:Zm00001d009032_T001 6.00E-63
290- 1:transcript:Zm00001d039249_T001 transcript:Zm00001d009031_T001 4.00E-55
290- 2:transcript:Zm00001d039254_T001 transcript:Zm00001d009030_T002 0
290- 3:transcript:Zm00001d039258_T001 transcript:Zm00001d009028_T001 0
290- 4:transcript:Zm00001d039259_T001 transcript:Zm00001d009027_T004 0
290- 5:transcript:Zm00001d039263_T001 transcript:Zm00001d009023_T001 3.00E-82
290- 6:transcript:Zm00001d039266_T004 transcript:Zm00001d009017_T002 0
290- 7:transcript:Zm00001d039271_T001 transcript:Zm00001d009013_T001 0
290- 8:transcript:Zm00001d039280_T007 transcript:Zm00001d009007_T001 3.00E-80
290- 9:transcript:Zm00001d039287_T002 transcript:Zm00001d009005_T002 0
290- 10:transcript:Zm00001d039292_T001 transcript:Zm00001d009003_T001 0
290- 11:transcript:Zm00001d039299_T001 transcript:Zm00001d009000_T001 2.00E-91
290- 12:transcript:Zm00001d039303_T001 transcript:Zm00001d008999_T001 7.00E-37
290- 13:transcript:Zm00001d039304_T001 transcript:Zm00001d008997_T002 0
290- 14:transcript:Zm00001d039305_T002 transcript:Zm00001d008996_T001 0
Alignment 291: score=526.0 e_value=8.3e-33 N=13 3&8 minus

291- 0:transcript:Zm00001d043874_T001 transcript:Zm00001d011592_T001 4.00E-81
291- 1:transcript:Zm00001d043877_T001 transcript:Zm00001d011589_T001 1.00E-99
291- 2:transcript:Zm00001d043878_T001 transcript:Zm00001d011588_T003 4.00E-134
291- 3:transcript:Zm00001d043880_T001 transcript:Zm00001d011585_T001 7.00E-126
291- 4:transcript:Zm00001d043890_T002 transcript:Zm00001d011580_T002   0
291- 5:transcript:Zm00001d043895_T003 transcript:Zm00001d011578_T003   0
291- 6:transcript:Zm00001d043898_T001 transcript:Zm00001d011576_T001 8.00E-41
291- 7:transcript:Zm00001d043900_T022 transcript:Zm00001d011572_T001 1.00E-179
291- 8:transcript:Zm00001d043902_T001 transcript:Zm00001d011571_T001 3.00E-168
291- 9:transcript:Zm00001d043903_T004 transcript:Zm00001d011570_T002   0
291- 10:transcript:Zm00001d043905_T003 transcript:Zm00001d011565_T002 2.00E-93
291- 11:transcript:Zm00001d043906_T002 transcript:Zm00001d011562_T003   0
291- 12:transcript:Zm00001d043911_T001 transcript:Zm00001d011561_T004 8.00E-173
Alignment 292: score=472.0 e_value=1e-22 N=11 3&8 minus
292- 0:transcript:Zm00001d043985_T001 transcript:Zm00001d011507_T001 2.00E-73
292- 1:transcript:Zm00001d043990_T001 transcript:Zm00001d011506_T001 2.00E-63
292- 2:transcript:Zm00001d043991_T001 transcript:Zm00001d011504_T003 4.00E-166
292- 3:transcript:Zm00001d043994_T001 transcript:Zm00001d011503_T001 2.00E-145
292- 4:transcript:Zm00001d043998_T001 transcript:Zm00001d011500_T014 7.00E-77
292- 5:transcript:Zm00001d044004_T001 transcript:Zm00001d011499_T001 5.00E-149
292- 6:transcript:Zm00001d044010_T001 transcript:Zm00001d011496_T002 3.00E-149
292- 7:transcript:Zm00001d044015_T001 transcript:Zm00001d011495_T001   0
292- 8:transcript:Zm00001d044019_T004 transcript:Zm00001d011492_T001   0
292- 9:transcript:Zm00001d044020_T001 transcript:Zm00001d011490_T003 5.00E-84
292- 10:transcript:Zm00001d044021_T001 transcript:Zm00001d011487_T001   0
Alignment 293: score=469.0 e_value=6.3e-25 N=11 3&8 minus
293- 0:transcript:Zm00001d040438_T004 transcript:Zm00001d008244_T003   0
293- 1:transcript:Zm00001d040440_T001 transcript:Zm00001d008242_T001 8.00E-105
293- 2:transcript:Zm00001d040442_T001 transcript:Zm00001d008239_T001   0
293- 3:transcript:Zm00001d040445_T001 transcript:Zm00001d008237_T001   0
293- 4:transcript:Zm00001d040448_T001 transcript:Zm00001d008236_T004   0
293- 5:transcript:Zm00001d040462_T002 transcript:Zm00001d008233_T001   0
293- 6:transcript:Zm00001d040463_T001 transcript:Zm00001d008230_T001   0
293- 7:transcript:Zm00001d040464_T001 transcript:Zm00001d008229_T001 6.00E-178
293- 8:transcript:Zm00001d040467_T003 transcript:Zm00001d008228_T001 6.00E-52
293- 9:transcript:Zm00001d040468_T001 transcript:Zm00001d008227_T001   0
293- 10:transcript:Zm00001d040475_T001 transcript:Zm00001d008224_T002   0
Alignment 294: score=415.0 e_value=3.8e-20 N=9 3&8 minus
294- 0:transcript:Zm00001d039701_T001 transcript:Zm00001d008693_T002 7.00E-120
294- 1:transcript:Zm00001d039702_T001 transcript:Zm00001d008692_T001   0
294- 2:transcript:Zm00001d039703_T001 transcript:Zm00001d008691_T001   0
294- 3:transcript:Zm00001d039706_T001 transcript:Zm00001d008690_T001   0
294- 4:transcript:Zm00001d039709_T001 transcript:Zm00001d008689_T003   0
294- 5:transcript:Zm00001d039715_T001 transcript:Zm00001d008681_T001 2.00E-42
294- 6:transcript:Zm00001d039716_T001 transcript:Zm00001d008680_T001 1.00E-65
294- 7:transcript:Zm00001d039717_T005 transcript:Zm00001d008679_T010   0
294- 8:transcript:Zm00001d039726_T001 transcript:Zm00001d008676_T001 7.00E-76
Alignment 295: score=390.0 e_value=3.8e-17 N=9 3&8 minus
295- 0:transcript:Zm00001d044253_T001 transcript:Zm00001d011228_T001   0
295- 1:transcript:Zm00001d044254_T001 transcript:Zm00001d011225_T003 3.00E-80
295- 2:transcript:Zm00001d044255_T001 transcript:Zm00001d011224_T001 1.00E-83
295- 3:transcript:Zm00001d044261_T001 transcript:Zm00001d011223_T002   0
295- 4:transcript:Zm00001d044266_T012 transcript:Zm00001d011220_T003   0
295- 5:transcript:Zm00001d044270_T001 transcript:Zm00001d011217_T001 3.00E-121

295- 6:transcript:Zm00001d044271_T011 transcript:Zm00001d011213_T005 0
295- 7:transcript:Zm00001d044272_T001 transcript:Zm00001d011212_T001 3.00E-143
295- 8:transcript:Zm00001d044276_T001 transcript:Zm00001d011211_T001 0
Alignment 296: score=351.0 e_value=1e-14 N=8 3&8 minus
296- 0:transcript:Zm00001d043566_T005 transcript:Zm00001d011762_T001 0
296- 1:transcript:Zm00001d043571_T001 transcript:Zm00001d011760_T001 0
296- 2:transcript:Zm00001d043573_T001 transcript:Zm00001d011758_T001 0
296- 3:transcript:Zm00001d043578_T008 transcript:Zm00001d011754_T001 0
296- 4:transcript:Zm00001d043586_T001 transcript:Zm00001d011752_T001 1.00E-37
296- 5:transcript:Zm00001d043589_T002 transcript:Zm00001d011748_T001 1.00E-111
296- 6:transcript:Zm00001d043590_T001 transcript:Zm00001d011747_T001 5.00E-18
296- 7:transcript:Zm00001d043592_T004 transcript:Zm00001d011746_T002 0
Alignment 297: score=346.0 e_value=4.1e-16 N=8 3&8 minus
297- 0:transcript:Zm00001d042853_T001 transcript:Zm00001d010066_T003 4.00E-90
297- 1:transcript:Zm00001d042861_T001 transcript:Zm00001d010062_T001 0
297- 2:transcript:Zm00001d042863_T001 transcript:Zm00001d010061_T001 5.00E-16
297- 3:transcript:Zm00001d042864_T003 transcript:Zm00001d010060_T001 1.00E-179
297- 4:transcript:Zm00001d042866_T001 transcript:Zm00001d010055_T003 2.00E-32
297- 5:transcript:Zm00001d042868_T001 transcript:Zm00001d010054_T001 3.00E-91
297- 6:transcript:Zm00001d042872_T001 transcript:Zm00001d010053_T001 8.00E-65
297- 7:transcript:Zm00001d042875_T001 transcript:Zm00001d010052_T001 2.00E-06
Alignment 298: score=341.0 e_value=4.4e-13 N=7 3&8 minus
298- 0:transcript:Zm00001d040169_T001 transcript:Zm00001d008409_T001 0
298- 1:transcript:Zm00001d040171_T001 transcript:Zm00001d008408_T001 5.00E-131
298- 2:transcript:Zm00001d040178_T001 transcript:Zm00001d008407_T001 8.00E-125
298- 3:transcript:Zm00001d040183_T001 transcript:Zm00001d008406_T002 0
298- 4:transcript:Zm00001d040185_T002 transcript:Zm00001d008405_T001 8.00E-78
298- 5:transcript:Zm00001d040186_T001 transcript:Zm00001d008404_T001 1.00E-109
298- 6:transcript:Zm00001d040189_T001 transcript:Zm00001d008403_T001 1.00E-129
Alignment 299: score=339.0 e_value=2.9e-13 N=8 3&8 minus
299- 0:transcript:Zm00001d044527_T002 transcript:Zm00001d009403_T002 1.00E-156
299- 1:transcript:Zm00001d044528_T001 transcript:Zm00001d009401_T001 0
299- 2:transcript:Zm00001d044529_T001 transcript:Zm00001d009399_T001 0
299- 3:transcript:Zm00001d044533_T003 transcript:Zm00001d009397_T001 4.00E-45
299- 4:transcript:Zm00001d044536_T001 transcript:Zm00001d009376_T001 0
299- 5:transcript:Zm00001d044540_T001 transcript:Zm00001d009374_T001 0
299- 6:transcript:Zm00001d044547_T003 transcript:Zm00001d009364_T001 2.00E-56
299- 7:transcript:Zm00001d044551_T003 transcript:Zm00001d009361_T001 1.00E-105
Alignment 300: score=315.0 e_value=5.1e-14 N=7 3&8 minus
300- 0:transcript:Zm00001d042444_T001 transcript:Zm00001d012126_T004 2.00E-51
300- 1:transcript:Zm00001d042446_T001 transcript:Zm00001d012120_T001 0
300- 2:transcript:Zm00001d042447_T002 transcript:Zm00001d012119_T001 0
300- 3:transcript:Zm00001d042448_T001 transcript:Zm00001d012118_T002 1.00E-43
300- 4:transcript:Zm00001d042449_T001 transcript:Zm00001d012109_T001 1.00E-92
300- 5:transcript:Zm00001d042452_T001 transcript:Zm00001d012108_T003 3.00E-39
300- 6:transcript:Zm00001d042453_T001 transcript:Zm00001d012103_T001 0
Alignment 301: score=280.0 e_value=5.1e-10 N=6 3&8 minus
301- 0:transcript:Zm00001d040094_T001 transcript:Zm00001d008462_T002 0
301- 1:transcript:Zm00001d040107_T001 transcript:Zm00001d008455_T001 0
301- 2:transcript:Zm00001d040109_T001 transcript:Zm00001d008454_T001 0
301- 3:transcript:Zm00001d040110_T001 transcript:Zm00001d008453_T001 1.00E-23
301- 4:transcript:Zm00001d040112_T001 transcript:Zm00001d008451_T001 2.00E-13
301- 5:transcript:Zm00001d040113_T003 transcript:Zm00001d008446_T006 2.00E-110
Alignment 302: score=274.0 e_value=2.9e-08 N=6 3&8 minus

302- 0:transcript:Zm00001d042656_T001 transcript:Zm00001d010212_T002 0
302- 1:transcript:Zm00001d042658_T002 transcript:Zm00001d010211_T002 4.00E-88
302- 2:transcript:Zm00001d042664_T001 transcript:Zm00001d010205_T002 0
302- 3:transcript:Zm00001d042665_T001 transcript:Zm00001d010201_T001 8.00E-83
302- 4:transcript:Zm00001d042667_T001 transcript:Zm00001d010200_T001 0
302- 5:transcript:Zm00001d042669_T002 transcript:Zm00001d010199_T002 0
Alignment 303: score=264.0 e_value=2.7e-11 N=7 3&8 minus
303- 0:transcript:Zm00001d043796_T001 transcript:Zm00001d011635_T001 7.00E-21
303- 1:transcript:Zm00001d043799_T001 transcript:Zm00001d011627_T001 2.00E-93
303- 2:transcript:Zm00001d043801_T002 transcript:Zm00001d011624_T001 0
303- 3:transcript:Zm00001d043803_T003 transcript:Zm00001d011623_T001 4.00E-30
303- 4:transcript:Zm00001d043805_T012 transcript:Zm00001d011622_T004 0
303- 5:transcript:Zm00001d043806_T001 transcript:Zm00001d011620_T001 1.00E-64
303- 6:transcript:Zm00001d043812_T001 transcript:Zm00001d011618_T001 0
Alignment 304: score=251.0 e_value=5e-12 N=6 3&8 minus
304- 0:transcript:Zm00001d040614_T002 transcript:Zm00001d009119_T001 3.00E-34
304- 1:transcript:Zm00001d040617_T001 transcript:Zm00001d009117_T001 2.00E-17
304- 2:transcript:Zm00001d040619_T001 transcript:Zm00001d009116_T001 5.00E-78
304- 3:transcript:Zm00001d040621_T002 transcript:Zm00001d009088_T001 8.00E-62
304- 4:transcript:Zm00001d040624_T001 transcript:Zm00001d009087_T001 3.00E-126
304- 5:transcript:Zm00001d040634_T001 transcript:Zm00001d009082_T001 2.00E-58
Alignment 305: score=993.0 e_value=1.9e-72 N=24 4&5 plus
305- 0:transcript:Zm00001d051214_T001 transcript:Zm00001d017352_T004 0
305- 1:transcript:Zm00001d051225_T001 transcript:Zm00001d017353_T001 1.00E-129
305- 2:transcript:Zm00001d051232_T009 transcript:Zm00001d017360_T013 0
305- 3:transcript:Zm00001d051234_T001 transcript:Zm00001d017361_T001 1.00E-176
305- 4:transcript:Zm00001d051238_T001 transcript:Zm00001d017364_T001 3.00E-78
305- 5:transcript:Zm00001d051239_T001 transcript:Zm00001d017366_T001 6.00E-173
305- 6:transcript:Zm00001d051241_T001 transcript:Zm00001d017371_T008 0
305- 7:transcript:Zm00001d051242_T002 transcript:Zm00001d017373_T001 0
305- 8:transcript:Zm00001d051250_T001 transcript:Zm00001d017379_T001 1.00E-109
305- 9:transcript:Zm00001d051251_T003 transcript:Zm00001d017381_T001 1.00E-84
305- 10:transcript:Zm00001d051262_T001 transcript:Zm00001d017382_T001 1.00E-121
305- 11:transcript:Zm00001d051267_T001 transcript:Zm00001d017383_T001 4.00E-118
305- 12:transcript:Zm00001d051268_T004 transcript:Zm00001d017384_T008 0
305- 13:transcript:Zm00001d051269_T001 transcript:Zm00001d017385_T001 7.00E-169
305- 14:transcript:Zm00001d051288_T001 transcript:Zm00001d017390_T001 7.00E-150
305- 15:transcript:Zm00001d051291_T003 transcript:Zm00001d017392_T009 0
305- 16:transcript:Zm00001d051294_T001 transcript:Zm00001d017396_T001 1.00E-59
305- 17:transcript:Zm00001d051302_T001 transcript:Zm00001d017397_T001 5.00E-73
305- 18:transcript:Zm00001d051306_T001 transcript:Zm00001d017401_T001 0
305- 19:transcript:Zm00001d051307_T001 transcript:Zm00001d017403_T001 0
305- 20:transcript:Zm00001d051309_T001 transcript:Zm00001d017412_T001 6.00E-63
305- 21:transcript:Zm00001d051314_T001 transcript:Zm00001d017419_T001 0
305- 22:transcript:Zm00001d051316_T001 transcript:Zm00001d017420_T001 0
305- 23:transcript:Zm00001d051321_T007 transcript:Zm00001d017424_T003 0
Alignment 306: score=917.0 e_value=1.8e-67 N=23 4&5 plus
306- 0:transcript:Zm00001d050833_T002 transcript:Zm00001d016879_T001 0
306- 1:transcript:Zm00001d050837_T001 transcript:Zm00001d016895_T001 0
306- 2:transcript:Zm00001d050838_T001 transcript:Zm00001d016897_T002 0
306- 3:transcript:Zm00001d050844_T005 transcript:Zm00001d016902_T002 0
306- 4:transcript:Zm00001d050848_T001 transcript:Zm00001d016909_T001 4.00E-148
306- 5:transcript:Zm00001d050850_T001 transcript:Zm00001d016915_T002 0
306- 6:transcript:Zm00001d050851_T009 transcript:Zm00001d016916_T004 0

306- 7:transcript:Zm00001d050860_T001 transcript:Zm00001d016919_T001 0
306- 8:transcript:Zm00001d050861_T001 transcript:Zm00001d016924_T001 0
306- 9:transcript:Zm00001d050868_T004 transcript:Zm00001d016928_T005 0
306- 10:transcript:Zm00001d050872_T001 transcript:Zm00001d016933_T001 0
306- 11:transcript:Zm00001d050874_T001 transcript:Zm00001d016935_T001 9.00E-146
306- 12:transcript:Zm00001d050884_T004 transcript:Zm00001d016941_T003 7.00E-171
306- 13:transcript:Zm00001d050889_T001 transcript:Zm00001d016943_T001 1.00E-93
306- 14:transcript:Zm00001d050893_T001 transcript:Zm00001d016950_T002 5.00E-164
306- 15:transcript:Zm00001d050897_T002 transcript:Zm00001d016957_T001 2.00E-84
306- 16:transcript:Zm00001d050903_T001 transcript:Zm00001d016973_T001 7.00E-118
306- 17:transcript:Zm00001d050905_T001 transcript:Zm00001d016977_T002 2.00E-116
306- 18:transcript:Zm00001d050907_T001 transcript:Zm00001d016980_T001 0
306- 19:transcript:Zm00001d050908_T001 transcript:Zm00001d016992_T001 3.00E-32
306- 20:transcript:Zm00001d050910_T004 transcript:Zm00001d016993_T007 0
306- 21:transcript:Zm00001d050911_T001 transcript:Zm00001d016998_T001 5.00E-41
306- 22:transcript:Zm00001d050913_T001 transcript:Zm00001d016999_T002 1.00E-166
Alignment 307: score=784.0 e_value=2.6e-50 N=18 4&5 plus
307- 0:transcript:Zm00001d051328_T002 transcript:Zm00001d017444_T001 2.00E-114
307- 1:transcript:Zm00001d051329_T002 transcript:Zm00001d017448_T001 2.00E-143
307- 2:transcript:Zm00001d051333_T001 transcript:Zm00001d017455_T001 5.00E-74
307- 3:transcript:Zm00001d051335_T001 transcript:Zm00001d017456_T003 2.00E-22
307- 4:transcript:Zm00001d051337_T002 transcript:Zm00001d017460_T003 6.00E-151
307- 5:transcript:Zm00001d051338_T002 transcript:Zm00001d017461_T001 0
307- 6:transcript:Zm00001d051340_T001 transcript:Zm00001d017466_T001 2.00E-144
307- 7:transcript:Zm00001d051343_T001 transcript:Zm00001d017473_T002 0
307- 8:transcript:Zm00001d051344_T001 transcript:Zm00001d017474_T001 7.00E-76
307- 9:transcript:Zm00001d051350_T001 transcript:Zm00001d017477_T001 3.00E-43
307- 10:transcript:Zm00001d051360_T001 transcript:Zm00001d017482_T002 1.00E-141
307- 11:transcript:Zm00001d051362_T001 transcript:Zm00001d017485_T003 2.00E-161
307- 12:transcript:Zm00001d051365_T001 transcript:Zm00001d017486_T001 2.00E-39
307- 13:transcript:Zm00001d051366_T002 transcript:Zm00001d017491_T001 7.00E-25
307- 14:transcript:Zm00001d051368_T001 transcript:Zm00001d017493_T001 2.00E-100
307- 15:transcript:Zm00001d051371_T001 transcript:Zm00001d017497_T001 6.00E-100
307- 16:transcript:Zm00001d051373_T001 transcript:Zm00001d017501_T002 5.00E-69
307- 17:transcript:Zm00001d051383_T001 transcript:Zm00001d017505_T003 3.00E-112
Alignment 308: score=684.0 e_value=9.3e-49 N=17 4&5 plus
308- 0:transcript:Zm00001d051439_T001 transcript:Zm00001d017575_T001 5.00E-143
308- 1:transcript:Zm00001d051442_T002 transcript:Zm00001d017583_T002 1.00E-142
308- 2:transcript:Zm00001d051451_T001 transcript:Zm00001d017591_T001 9.00E-89
308- 3:transcript:Zm00001d051456_T001 transcript:Zm00001d017602_T002 5.00E-08
308- 4:transcript:Zm00001d051457_T001 transcript:Zm00001d017607_T001 0
308- 5:transcript:Zm00001d051458_T002 transcript:Zm00001d017609_T011 7.00E-49
308- 6:transcript:Zm00001d051460_T001 transcript:Zm00001d017612_T001 1.00E-75
308- 7:transcript:Zm00001d051465_T002 transcript:Zm00001d017614_T003 1.00E-138
308- 8:transcript:Zm00001d051468_T001 transcript:Zm00001d017615_T001 6.00E-60
308- 9:transcript:Zm00001d051471_T001 transcript:Zm00001d017618_T001 0
308- 10:transcript:Zm00001d051475_T002 transcript:Zm00001d017622_T001 1.00E-83
308- 11:transcript:Zm00001d051479_T014 transcript:Zm00001d017641_T004 0
308- 12:transcript:Zm00001d051480_T001 transcript:Zm00001d017642_T001 6.00E-134
308- 13:transcript:Zm00001d051492_T001 transcript:Zm00001d017646_T001 3.00E-18
308- 14:transcript:Zm00001d051505_T001 transcript:Zm00001d017657_T001 3.00E-122
308- 15:transcript:Zm00001d051506_T001 transcript:Zm00001d017659_T001 1.00E-68
308- 16:transcript:Zm00001d051507_T007 transcript:Zm00001d017660_T021 0
Alignment 309: score=616.0 e_value=7e-45 N=15 4&5 plus

309- 0:transcript:Zm00001d053936_T001 transcript:Zm00001d015084_T001 7.00E-73
309- 1:transcript:Zm00001d053941_T001 transcript:Zm00001d015088_T002 5.00E-164
309- 2:transcript:Zm00001d053952_T001 transcript:Zm00001d015091_T003 9.00E-140
309- 3:transcript:Zm00001d053953_T001 transcript:Zm00001d015095_T002 2.00E-139
309- 4:transcript:Zm00001d053956_T001 transcript:Zm00001d015096_T002 0
309- 5:transcript:Zm00001d053957_T003 transcript:Zm00001d015103_T003 1.00E-97
309- 6:transcript:Zm00001d053966_T001 transcript:Zm00001d015116_T001 1.00E-68
309- 7:transcript:Zm00001d053967_T001 transcript:Zm00001d015118_T001 4.00E-127
309- 8:transcript:Zm00001d053968_T005 transcript:Zm00001d015120_T001 0
309- 9:transcript:Zm00001d053976_T001 transcript:Zm00001d015125_T001 6.00E-147
309- 10:transcript:Zm00001d053979_T001 transcript:Zm00001d015127_T001 1.00E-54
309- 11:transcript:Zm00001d053983_T002 transcript:Zm00001d015134_T001 8.00E-155
309- 12:transcript:Zm00001d053984_T002 transcript:Zm00001d015135_T002 0
309- 13:transcript:Zm00001d053985_T001 transcript:Zm00001d015141_T001 0
309- 14:transcript:Zm00001d053987_T003 transcript:Zm00001d015148_T002 0
Alignment 310: score=604.0 e_value=2.3e-34 N=14 4&5 plus
310- 0:transcript:Zm00001d051128_T001 transcript:Zm00001d017256_T001 8.00E-72
310- 1:transcript:Zm00001d051129_T002 transcript:Zm00001d017258_T010 0
310- 2:transcript:Zm00001d051135_T005 transcript:Zm00001d017261_T006 0
310- 3:transcript:Zm00001d051143_T001 transcript:Zm00001d017263_T001 4.00E-98
310- 4:transcript:Zm00001d051149_T001 transcript:Zm00001d017268_T001 6.00E-135
310- 5:transcript:Zm00001d051156_T001 transcript:Zm00001d017270_T001 4.00E-180
310- 6:transcript:Zm00001d051157_T001 transcript:Zm00001d017271_T005 0
310- 7:transcript:Zm00001d051161_T003 transcript:Zm00001d017274_T001 0
310- 8:transcript:Zm00001d051163_T001 transcript:Zm00001d017275_T001 0
310- 9:transcript:Zm00001d051166_T001 transcript:Zm00001d017276_T001 0
310- 10:transcript:Zm00001d051172_T001 transcript:Zm00001d017284_T001 0
310- 11:transcript:Zm00001d051174_T001 transcript:Zm00001d017288_T001 0
310- 12:transcript:Zm00001d051178_T001 transcript:Zm00001d017296_T001 0
310- 13:transcript:Zm00001d051180_T002 transcript:Zm00001d017298_T003 0
Alignment 311: score=583.0 e_value=4.1e-32 N=13 4&5 plus
311- 0:transcript:Zm00001d053410_T001 transcript:Zm00001d016120_T001 3.00E-27
311- 1:transcript:Zm00001d053425_T001 transcript:Zm00001d016130_T001 0
311- 2:transcript:Zm00001d053427_T004 transcript:Zm00001d016131_T006 0
311- 3:transcript:Zm00001d053429_T001 transcript:Zm00001d016132_T001 8.00E-157
311- 4:transcript:Zm00001d053432_T001 transcript:Zm00001d016134_T002 1.00E-159
311- 5:transcript:Zm00001d053433_T001 transcript:Zm00001d016136_T001 3.00E-108
311- 6:transcript:Zm00001d053434_T001 transcript:Zm00001d016137_T001 9.00E-64
311- 7:transcript:Zm00001d053435_T001 transcript:Zm00001d016142_T001 7.00E-26
311- 8:transcript:Zm00001d053438_T002 transcript:Zm00001d016149_T001 1.00E-145
311- 9:transcript:Zm00001d053442_T001 transcript:Zm00001d016154_T001 1.00E-108
311- 10:transcript:Zm00001d053446_T001 transcript:Zm00001d016160_T001 0
311- 11:transcript:Zm00001d053447_T001 transcript:Zm00001d016163_T001 6.00E-10
311- 12:transcript:Zm00001d053448_T001 transcript:Zm00001d016164_T003 0
Alignment 312: score=566.0 e_value=5.7e-36 N=14 4&5 plus
312- 0:transcript:Zm00001d051043_T001 transcript:Zm00001d017168_T002 0
312- 1:transcript:Zm00001d051047_T001 transcript:Zm00001d017176_T001 5.00E-144
312- 2:transcript:Zm00001d051052_T001 transcript:Zm00001d017177_T007 1.00E-63
312- 3:transcript:Zm00001d051053_T001 transcript:Zm00001d017178_T004 2.00E-72
312- 4:transcript:Zm00001d051054_T001 transcript:Zm00001d017180_T001 4.00E-10
312- 5:transcript:Zm00001d051062_T001 transcript:Zm00001d017187_T001 0
312- 6:transcript:Zm00001d051064_T001 transcript:Zm00001d017188_T001 5.00E-91
312- 7:transcript:Zm00001d051065_T001 transcript:Zm00001d017193_T002 2.00E-152
312- 8:transcript:Zm00001d051067_T003 transcript:Zm00001d017197_T015 0

312- 9:transcript:Zm00001d051069_T001 transcript:Zm00001d017201_T001 0
312- 10:transcript:Zm00001d051080_T004 transcript:Zm00001d017204_T003 1.00E-64
312- 11:transcript:Zm00001d051081_T001 transcript:Zm00001d017205_T001 0
312- 12:transcript:Zm00001d051082_T001 transcript:Zm00001d017206_T001 4.00E-46
312- 13:transcript:Zm00001d051083_T001 transcript:Zm00001d017212_T001 6.00E-145
Alignment 313: score=519.0 e_value=5.9e-29 N=12 4&5 plus
313- 0:transcript:Zm00001d051789_T002 transcript:Zm00001d017947_T001 3.00E-92
313- 1:transcript:Zm00001d051795_T001 transcript:Zm00001d017950_T001 1.00E-84
313- 2:transcript:Zm00001d051799_T001 transcript:Zm00001d017951_T001 4.00E-108
313- 3:transcript:Zm00001d051800_T002 transcript:Zm00001d017952_T001 4.00E-71
313- 4:transcript:Zm00001d051803_T001 transcript:Zm00001d017956_T001 1.00E-73
313- 5:transcript:Zm00001d051804_T001 transcript:Zm00001d017958_T002 0
313- 6:transcript:Zm00001d051806_T001 transcript:Zm00001d017959_T003 0
313- 7:transcript:Zm00001d051808_T002 transcript:Zm00001d017960_T001 0
313- 8:transcript:Zm00001d051810_T001 transcript:Zm00001d017969_T001 1.00E-15
313- 9:transcript:Zm00001d051812_T003 transcript:Zm00001d017977_T010 0
313- 10:transcript:Zm00001d051814_T001 transcript:Zm00001d017978_T001 0
313- 11:transcript:Zm00001d051815_T002 transcript:Zm00001d017979_T002 0
Alignment 314: score=511.0 e_value=2.6e-27 N=12 4&5 plus
314- 0:transcript:Zm00001d051610_T001 transcript:Zm00001d017811_T001 2.00E-95
314- 1:transcript:Zm00001d051615_T001 transcript:Zm00001d017821_T002 0
314- 2:transcript:Zm00001d051620_T001 transcript:Zm00001d017825_T001 7.00E-141
314- 3:transcript:Zm00001d051626_T003 transcript:Zm00001d017826_T001 0
314- 4:transcript:Zm00001d051627_T002 transcript:Zm00001d017829_T002 0
314- 5:transcript:Zm00001d051629_T003 transcript:Zm00001d017830_T001 0
314- 6:transcript:Zm00001d051632_T002 transcript:Zm00001d017831_T001 0
314- 7:transcript:Zm00001d051633_T003 transcript:Zm00001d017832_T006 0
314- 8:transcript:Zm00001d051634_T001 transcript:Zm00001d017833_T001 0
314- 9:transcript:Zm00001d051636_T001 transcript:Zm00001d017840_T001 0
314- 10:transcript:Zm00001d051637_T001 transcript:Zm00001d017842_T001 2.00E-110
314- 11:transcript:Zm00001d051644_T004 transcript:Zm00001d017848_T004 0
Alignment 315: score=460.0 e_value=1.7e-26 N=11 4&5 plus
315- 0:transcript:Zm00001d051403_T001 transcript:Zm00001d017526_T001 0
315- 1:transcript:Zm00001d051410_T001 transcript:Zm00001d017534_T001 1.00E-47
315- 2:transcript:Zm00001d051411_T001 transcript:Zm00001d017535_T001 3.00E-40
315- 3:transcript:Zm00001d051415_T001 transcript:Zm00001d017539_T002 0
315- 4:transcript:Zm00001d051416_T001 transcript:Zm00001d017540_T001 0
315- 5:transcript:Zm00001d051419_T002 transcript:Zm00001d017546_T001 9.00E-36
315- 6:transcript:Zm00001d051420_T001 transcript:Zm00001d017547_T001 4.00E-68
315- 7:transcript:Zm00001d051421_T002 transcript:Zm00001d017550_T009 1.00E-125
315- 8:transcript:Zm00001d051427_T002 transcript:Zm00001d017555_T001 5.00E-70
315- 9:transcript:Zm00001d051429_T001 transcript:Zm00001d017558_T001 0
315- 10:transcript:Zm00001d051431_T002 transcript:Zm00001d017568_T002 0
Alignment 316: score=453.0 e_value=1.8e-22 N=10 4&5 plus
316- 0:transcript:Zm00001d051568_T001 transcript:Zm00001d017773_T001 0
316- 1:transcript:Zm00001d051569_T001 transcript:Zm00001d017778_T001 1.00E-165
316- 2:transcript:Zm00001d051572_T001 transcript:Zm00001d017783_T001 0
316- 3:transcript:Zm00001d051573_T001 transcript:Zm00001d017784_T001 2.00E-141
316- 4:transcript:Zm00001d051577_T001 transcript:Zm00001d017788_T001 5.00E-160
316- 5:transcript:Zm00001d051585_T001 transcript:Zm00001d017789_T001 7.00E-83
316- 6:transcript:Zm00001d051586_T001 transcript:Zm00001d017790_T002 0
316- 7:transcript:Zm00001d051587_T001 transcript:Zm00001d017791_T002 3.00E-141
316- 8:transcript:Zm00001d051589_T004 transcript:Zm00001d017793_T002 0
316- 9:transcript:Zm00001d051590_T001 transcript:Zm00001d017798_T002 0

```
## Alignment 317: score=414.0 e_value=1e-19 N=10 4&5 plus
317-  0:transcript:Zm00001d050917_T002 transcript:Zm00001d017036_T002          0
317-  1:transcript:Zm00001d050918_T001 transcript:Zm00001d017041_T001  8.00E-26
317-  2:transcript:Zm00001d050920_T002 transcript:Zm00001d017047_T001 5.00E-151
317-  3:transcript:Zm00001d050923_T002 transcript:Zm00001d017049_T001          0
317-  4:transcript:Zm00001d050925_T007 transcript:Zm00001d017050_T002          0
317-  5:transcript:Zm00001d050935_T001 transcript:Zm00001d017060_T001  4.00E-66
317-  6:transcript:Zm00001d050942_T001 transcript:Zm00001d017061_T001  1.00E-44
317-  7:transcript:Zm00001d050943_T001 transcript:Zm00001d017067_T001          0
317-  8:transcript:Zm00001d050944_T001 transcript:Zm00001d017070_T001  3.00E-54
317-  9:transcript:Zm00001d050955_T002 transcript:Zm00001d017077_T001          0
## Alignment 318: score=386.0 e_value=1.8e-14 N=8 4&5 plus
318-  0:transcript:Zm00001d050959_T001 transcript:Zm00001d017079_T001          0
318-  1:transcript:Zm00001d050960_T001 transcript:Zm00001d017084_T001          0
318-  2:transcript:Zm00001d050961_T001 transcript:Zm00001d017085_T002          0
318-  3:transcript:Zm00001d050963_T001 transcript:Zm00001d017089_T001 3.00E-101
318-  4:transcript:Zm00001d050964_T001 transcript:Zm00001d017090_T001 3.00E-128
318-  5:transcript:Zm00001d050965_T001 transcript:Zm00001d017091_T001 3.00E-141
318-  6:transcript:Zm00001d050969_T001 transcript:Zm00001d017092_T001          0
318-  7:transcript:Zm00001d050970_T003 transcript:Zm00001d017096_T005          0
## Alignment 319: score=375.0 e_value=1.3e-16 N=9 4&5 plus
319-  0:transcript:Zm00001d051509_T001 transcript:Zm00001d017677_T001 2.00E-136
319-  1:transcript:Zm00001d051511_T001 transcript:Zm00001d017682_T001 2.00E-139
319-  2:transcript:Zm00001d051513_T001 transcript:Zm00001d017686_T003          0
319-  3:transcript:Zm00001d051525_T001 transcript:Zm00001d017698_T003          0
319-  4:transcript:Zm00001d051526_T002 transcript:Zm00001d017699_T001 1.00E-133
319-  5:transcript:Zm00001d051527_T001 transcript:Zm00001d017702_T001 2.00E-135
319-  6:transcript:Zm00001d051528_T001 transcript:Zm00001d017703_T002 7.00E-172
319-  7:transcript:Zm00001d051532_T002 transcript:Zm00001d017706_T002          0
319-  8:transcript:Zm00001d051535_T001 transcript:Zm00001d017707_T003          0
## Alignment 320: score=370.0 e_value=6.4e-19 N=9 4&5 plus
320-  0:transcript:Zm00001d050669_T004 transcript:Zm00001d016732_T006          0
320-  1:transcript:Zm00001d050698_T001 transcript:Zm00001d016755_T001          0
320-  2:transcript:Zm00001d050705_T001 transcript:Zm00001d016758_T001  4.00E-52
320-  3:transcript:Zm00001d050712_T001 transcript:Zm00001d016764_T001          0
320-  4:transcript:Zm00001d050715_T003 transcript:Zm00001d016766_T002          0
320-  5:transcript:Zm00001d050716_T002 transcript:Zm00001d016768_T001          0
320-  6:transcript:Zm00001d050723_T002 transcript:Zm00001d016783_T002          0
320-  7:transcript:Zm00001d050726_T001 transcript:Zm00001d016784_T001          0
320-  8:transcript:Zm00001d050730_T001 transcript:Zm00001d016793_T002          0
## Alignment 321: score=365.0 e_value=6.1e-14 N=8 4&5 plus
321-  0:transcript:Zm00001d053639_T001 transcript:Zm00001d015767_T002  3.00E-45
321-  1:transcript:Zm00001d053641_T001 transcript:Zm00001d015776_T001 1.00E-113
321-  2:transcript:Zm00001d053642_T001 transcript:Zm00001d015778_T001          0
321-  3:transcript:Zm00001d053643_T001 transcript:Zm00001d015780_T002          0
321-  4:transcript:Zm00001d053648_T005 transcript:Zm00001d015785_T005 3.00E-177
321-  5:transcript:Zm00001d053649_T002 transcript:Zm00001d015788_T001          0
321-  6:transcript:Zm00001d053654_T001 transcript:Zm00001d015796_T001 3.00E-115
321-  7:transcript:Zm00001d053655_T001 transcript:Zm00001d015798_T001  2.00E-64
## Alignment 322: score=342.0 e_value=5.5e-22 N=10 4&5 plus
322-  0:transcript:Zm00001d050737_T003 transcript:Zm00001d016822_T012          0
322-  1:transcript:Zm00001d050741_T004 transcript:Zm00001d016825_T008          0
322-  2:transcript:Zm00001d050748_T001 transcript:Zm00001d016826_T001 9.00E-109
322-  3:transcript:Zm00001d050753_T001 transcript:Zm00001d016827_T001          0
```

322- 4: transcript:Zm00001d050755_T003 transcript:Zm00001d016832_T001 0
322- 5: transcript:Zm00001d050775_T001 transcript:Zm00001d016834_T001 3.00E-104
322- 6: transcript:Zm00001d050781_T001 transcript:Zm00001d016838_T001 0
322- 7: transcript:Zm00001d050785_T001 transcript:Zm00001d016851_T001 7.00E-165
322- 8: transcript:Zm00001d050800_T001 transcript:Zm00001d016856_T009 0
322- 9: transcript:Zm00001d050816_T002 transcript:Zm00001d016861_T003 6.00E-172
Alignment 323: score=319.0 e_value=1e-16 N=8 4&5 plus
323- 0: transcript:Zm00001d051739_T001 transcript:Zm00001d017911_T001 4.00E-55
323- 1: transcript:Zm00001d051741_T001 transcript:Zm00001d017914_T001 2.00E-77
323- 2: transcript:Zm00001d051749_T001 transcript:Zm00001d017920_T001 4.00E-163
323- 3: transcript:Zm00001d051756_T004 transcript:Zm00001d017925_T004 0
323- 4: transcript:Zm00001d051759_T001 transcript:Zm00001d017926_T003 0
323- 5: transcript:Zm00001d051760_T001 transcript:Zm00001d017927_T001 4.00E-108
323- 6: transcript:Zm00001d051784_T001 transcript:Zm00001d017937_T003 1.00E-09
323- 7: transcript:Zm00001d051787_T001 transcript:Zm00001d017941_T001 0
Alignment 324: score=312.0 e_value=6.7e-14 N=8 4&5 plus
324- 0: transcript:Zm00001d053566_T002 transcript:Zm00001d016332_T001 1.00E-179
324- 1: transcript:Zm00001d053568_T001 transcript:Zm00001d016342_T001 4.00E-83
324- 2: transcript:Zm00001d053572_T002 transcript:Zm00001d016349_T002 7.00E-145
324- 3: transcript:Zm00001d053576_T001 transcript:Zm00001d016358_T006 0
324- 4: transcript:Zm00001d053580_T002 transcript:Zm00001d016365_T002 0
324- 5: transcript:Zm00001d053589_T001 transcript:Zm00001d016378_T002 7.00E-131
324- 6: transcript:Zm00001d053593_T001 transcript:Zm00001d016379_T001 2.00E-33
324- 7: transcript:Zm00001d053595_T004 transcript:Zm00001d016380_T001 1.00E-30
Alignment 325: score=308.0 e_value=1.2e-12 N=7 4&5 plus
325- 0: transcript:Zm00001d051553_T004 transcript:Zm00001d017755_T011 0
325- 1: transcript:Zm00001d051554_T002 transcript:Zm00001d017762_T003 0
325- 2: transcript:Zm00001d051556_T001 transcript:Zm00001d017766_T001 0
325- 3: transcript:Zm00001d051561_T001 transcript:Zm00001d017767_T001 1.00E-145
325- 4: transcript:Zm00001d051562_T001 transcript:Zm00001d017768_T006 0
325- 5: transcript:Zm00001d051563_T001 transcript:Zm00001d017769_T001 0
325- 6: transcript:Zm00001d051564_T001 transcript:Zm00001d017770_T001 2.00E-115
Alignment 326: score=294.0 e_value=3.6e-11 N=7 4&5 plus
326- 0: transcript:Zm00001d053377_T001 transcript:Zm00001d016072_T001 1.00E-110
326- 1: transcript:Zm00001d053378_T001 transcript:Zm00001d016075_T001 0
326- 2: transcript:Zm00001d053382_T001 transcript:Zm00001d016081_T002 0
326- 3: transcript:Zm00001d053391_T001 transcript:Zm00001d016095_T001 1.00E-163
326- 4: transcript:Zm00001d053393_T002 transcript:Zm00001d016103_T001 0
326- 5: transcript:Zm00001d053396_T001 transcript:Zm00001d016105_T001 1.00E-136
326- 6: transcript:Zm00001d053399_T001 transcript:Zm00001d016106_T001 0
Alignment 327: score=283.0 e_value=3.8e-08 N=6 4&5 plus
327- 0: transcript:Zm00001d051112_T001 transcript:Zm00001d017239_T001 0
327- 1: transcript:Zm00001d051114_T001 transcript:Zm00001d017241_T002 0
327- 2: transcript:Zm00001d051116_T001 transcript:Zm00001d017242_T001 5.00E-36
327- 3: transcript:Zm00001d051117_T001 transcript:Zm00001d017243_T001 5.00E-118
327- 4: transcript:Zm00001d051119_T001 transcript:Zm00001d017247_T003 0
327- 5: transcript:Zm00001d051121_T001 transcript:Zm00001d017248_T001 4.00E-71
Alignment 328: score=282.0 e_value=1.7e-10 N=6 4&5 plus
328- 0: transcript:Zm00001d053729_T001 transcript:Zm00001d015470_T001 6.00E-18
328- 1: transcript:Zm00001d053731_T001 transcript:Zm00001d015473_T003 2.00E-43
328- 2: transcript:Zm00001d053732_T001 transcript:Zm00001d015474_T001 0
328- 3: transcript:Zm00001d053734_T001 transcript:Zm00001d015475_T001 3.00E-53
328- 4: transcript:Zm00001d053739_T001 transcript:Zm00001d015490_T001 2.00E-56
328- 5: transcript:Zm00001d053741_T002 transcript:Zm00001d015493_T001 0

Alignment 329: score=275.0 e_value=7e-11 N=6 4&5 plus
329- 0:transcript:Zm00001d051193_T004 transcript:Zm00001d017323_T001 0
329- 1:transcript:Zm00001d051199_T001 transcript:Zm00001d017325_T001 3.00E-70
329- 2:transcript:Zm00001d051203_T001 transcript:Zm00001d017330_T001 1.00E-159
329- 3:transcript:Zm00001d051206_T001 transcript:Zm00001d017333_T002 0
329- 4:transcript:Zm00001d051207_T001 transcript:Zm00001d017334_T006 8.00E-39
329- 5:transcript:Zm00001d051211_T004 transcript:Zm00001d017336_T007 6.00E-157
Alignment 330: score=272.0 e_value=7.5e-09 N=6 4&5 plus
330- 0:transcript:Zm00001d051685_T001 transcript:Zm00001d017857_T002 0
330- 1:transcript:Zm00001d051687_T001 transcript:Zm00001d017859_T002 5.00E-134
330- 2:transcript:Zm00001d051688_T002 transcript:Zm00001d017860_T005 0
330- 3:transcript:Zm00001d051689_T001 transcript:Zm00001d017861_T001 4.00E-93
330- 4:transcript:Zm00001d051690_T001 transcript:Zm00001d017862_T001 0
330- 5:transcript:Zm00001d051692_T001 transcript:Zm00001d017864_T001 0
Alignment 331: score=256.0 e_value=2.5e-08 N=6 4&5 plus
331- 0:transcript:Zm00001d053664_T001 transcript:Zm00001d015837_T001 0
331- 1:transcript:Zm00001d053667_T001 transcript:Zm00001d015839_T001 0
331- 2:transcript:Zm00001d053671_T001 transcript:Zm00001d015842_T001 2.00E-90
331- 3:transcript:Zm00001d053674_T001 transcript:Zm00001d015851_T001 0
331- 4:transcript:Zm00001d053675_T001 transcript:Zm00001d015852_T004 0
331- 5:transcript:Zm00001d053684_T004 transcript:Zm00001d015856_T007 0
Alignment 332: score=1068.0 e_value=9.2e-73 N=24 4&5 minus
332- 0:transcript:Zm00001d052198_T002 transcript:Zm00001d018112_T004 7.00E-152
332- 1:transcript:Zm00001d052200_T005 transcript:Zm00001d018111_T002 0
332- 2:transcript:Zm00001d052205_T001 transcript:Zm00001d018105_T001 1.00E-98
332- 3:transcript:Zm00001d052208_T002 transcript:Zm00001d018104_T001 5.00E-63
332- 4:transcript:Zm00001d052212_T001 transcript:Zm00001d018103_T002 4.00E-79
332- 5:transcript:Zm00001d052215_T001 transcript:Zm00001d018099_T001 0
332- 6:transcript:Zm00001d052216_T001 transcript:Zm00001d018096_T001 1.00E-84
332- 7:transcript:Zm00001d052220_T001 transcript:Zm00001d018089_T001 8.00E-61
332- 8:transcript:Zm00001d052221_T001 transcript:Zm00001d018088_T001 0
332- 9:transcript:Zm00001d052223_T001 transcript:Zm00001d018087_T001 5.00E-162
332- 10:transcript:Zm00001d052225_T001 transcript:Zm00001d018085_T001 5.00E-51
332- 11:transcript:Zm00001d052226_T001 transcript:Zm00001d018082_T001 1.00E-08
332- 12:transcript:Zm00001d052229_T001 transcript:Zm00001d018081_T001 3.00E-164
332- 13:transcript:Zm00001d052232_T002 transcript:Zm00001d018078_T002 0
332- 14:transcript:Zm00001d052234_T001 transcript:Zm00001d018076_T001 3.00E-66
332- 15:transcript:Zm00001d052237_T001 transcript:Zm00001d018074_T001 0
332- 16:transcript:Zm00001d052238_T001 transcript:Zm00001d018073_T001 2.00E-28
332- 17:transcript:Zm00001d052239_T001 transcript:Zm00001d018072_T001 3.00E-77
332- 18:transcript:Zm00001d052242_T001 transcript:Zm00001d018069_T001 1.00E-136
332- 19:transcript:Zm00001d052243_T001 transcript:Zm00001d018064_T004 0
332- 20:transcript:Zm00001d052247_T001 transcript:Zm00001d018061_T002 0
332- 21:transcript:Zm00001d052252_T001 transcript:Zm00001d018058_T001 3.00E-164
332- 22:transcript:Zm00001d052254_T001 transcript:Zm00001d018056_T001 1.00E-49
332- 23:transcript:Zm00001d052255_T001 transcript:Zm00001d018050_T003 2.00E-78
Alignment 333: score=707.0 e_value=2.1e-51 N=18 4&5 minus
333- 0:transcript:Zm00001d051966_T001 transcript:Zm00001d018433_T001 5.00E-97
333- 1:transcript:Zm00001d051967_T001 transcript:Zm00001d018432_T001 0
333- 2:transcript:Zm00001d051976_T002 transcript:Zm00001d018430_T008 3.00E-21
333- 3:transcript:Zm00001d051977_T001 transcript:Zm00001d018428_T001 0
333- 4:transcript:Zm00001d051981_T005 transcript:Zm00001d018421_T004 0
333- 5:transcript:Zm00001d051986_T001 transcript:Zm00001d018419_T001 1.00E-61
333- 6:transcript:Zm00001d051987_T001 transcript:Zm00001d018418_T002 8.00E-180

333- 7:transcript:Zm00001d051989_T001 transcript:Zm00001d018417_T001 0
333- 8:transcript:Zm00001d051995_T001 transcript:Zm00001d018415_T002 0
333- 9:transcript:Zm00001d052001_T002 transcript:Zm00001d018413_T003 0
333- 10:transcript:Zm00001d052003_T002 transcript:Zm00001d018410_T001 7.00E-36
333- 11:transcript:Zm00001d052011_T003 transcript:Zm00001d018404_T002 2.00E-123
333- 12:transcript:Zm00001d052015_T001 transcript:Zm00001d018400_T001 1.00E-06
333- 13:transcript:Zm00001d052017_T001 transcript:Zm00001d018396_T001 8.00E-13
333- 14:transcript:Zm00001d052018_T001 transcript:Zm00001d018394_T003 2.00E-143
333- 15:transcript:Zm00001d052019_T001 transcript:Zm00001d018391_T005 0
333- 16:transcript:Zm00001d052021_T002 transcript:Zm00001d018384_T002 0
333- 17:transcript:Zm00001d052025_T001 transcript:Zm00001d018383_T002 1.00E-30
Alignment 334: score=632.0 e_value=8e-44 N=16 4&5 minus
334- 0:transcript:Zm00001d052031_T001 transcript:Zm00001d018378_T001 5.00E-33
334- 1:transcript:Zm00001d052036_T001 transcript:Zm00001d018375_T006 4.00E-46
334- 2:transcript:Zm00001d052038_T001 transcript:Zm00001d018374_T001 4.00E-146
334- 3:transcript:Zm00001d052043_T003 transcript:Zm00001d018365_T004 0
334- 4:transcript:Zm00001d052050_T001 transcript:Zm00001d018361_T001 1.00E-41
334- 5:transcript:Zm00001d052051_T001 transcript:Zm00001d018352_T001 0
334- 6:transcript:Zm00001d052056_T005 transcript:Zm00001d018349_T001 0
334- 7:transcript:Zm00001d052058_T001 transcript:Zm00001d018344_T001 2.00E-159
334- 8:transcript:Zm00001d052059_T001 transcript:Zm00001d018343_T001 0
334- 9:transcript:Zm00001d052060_T005 transcript:Zm00001d018342_T002 0
334- 10:transcript:Zm00001d052062_T002 transcript:Zm00001d018341_T007 4.00E-51
334- 11:transcript:Zm00001d052063_T003 transcript:Zm00001d018336_T002 0
334- 12:transcript:Zm00001d052064_T001 transcript:Zm00001d018329_T001 0
334- 13:transcript:Zm00001d052066_T001 transcript:Zm00001d018326_T001 0
334- 14:transcript:Zm00001d052068_T001 transcript:Zm00001d018324_T001 2.00E-83
334- 15:transcript:Zm00001d052069_T001 transcript:Zm00001d018319_T001 3.00E-58
Alignment 335: score=614.0 e_value=1.7e-38 N=14 4&5 minus
335- 0:transcript:Zm00001d052118_T001 transcript:Zm00001d018247_T001 7.00E-102
335- 1:transcript:Zm00001d052120_T001 transcript:Zm00001d018246_T015 5.00E-92
335- 2:transcript:Zm00001d052123_T001 transcript:Zm00001d018238_T001 7.00E-105
335- 3:transcript:Zm00001d052124_T002 transcript:Zm00001d018237_T001 0
335- 4:transcript:Zm00001d052125_T001 transcript:Zm00001d018235_T001 8.00E-60
335- 5:transcript:Zm00001d052130_T001 transcript:Zm00001d018227_T001 3.00E-114
335- 6:transcript:Zm00001d052131_T001 transcript:Zm00001d018226_T002 7.00E-74
335- 7:transcript:Zm00001d052133_T003 transcript:Zm00001d018225_T001 0
335- 8:transcript:Zm00001d052136_T001 transcript:Zm00001d018211_T001 0
335- 9:transcript:Zm00001d052137_T001 transcript:Zm00001d018209_T001 2.00E-88
335- 10:transcript:Zm00001d052138_T004 transcript:Zm00001d018207_T001 0
335- 11:transcript:Zm00001d052139_T001 transcript:Zm00001d018206_T001 0
335- 12:transcript:Zm00001d052144_T001 transcript:Zm00001d018204_T001 0
335- 13:transcript:Zm00001d052148_T001 transcript:Zm00001d018200_T001 1.00E-57
Alignment 336: score=477.0 e_value=1.8e-30 N=12 4&5 minus
336- 0:transcript:Zm00001d051881_T001 transcript:Zm00001d018514_T003 0
336- 1:transcript:Zm00001d051882_T001 transcript:Zm00001d018512_T001 3.00E-23
336- 2:transcript:Zm00001d051890_T001 transcript:Zm00001d018505_T001 3.00E-20
336- 3:transcript:Zm00001d051898_T001 transcript:Zm00001d018503_T002 0
336- 4:transcript:Zm00001d051899_T001 transcript:Zm00001d018502_T001 0
336- 5:transcript:Zm00001d051900_T001 transcript:Zm00001d018499_T003 0
336- 6:transcript:Zm00001d051901_T004 transcript:Zm00001d018497_T002 0
336- 7:transcript:Zm00001d051902_T003 transcript:Zm00001d018496_T007 0
336- 8:transcript:Zm00001d051906_T003 transcript:Zm00001d018489_T007 3.00E-172
336- 9:transcript:Zm00001d051912_T001 transcript:Zm00001d018485_T003 0

336- 10:transcript:Zm00001d051913_T005 transcript:Zm00001d018484_T003 0
336- 11:transcript:Zm00001d051914_T001 transcript:Zm00001d018483_T001 0
Alignment 337: score=392.0 e_value=5.7e-19 N=9 4&5 minus
337- 0:transcript:Zm00001d052152_T001 transcript:Zm00001d018191_T001 6.00E-92
337- 1:transcript:Zm00001d052154_T001 transcript:Zm00001d018190_T001 9.00E-144
337- 2:transcript:Zm00001d052155_T001 transcript:Zm00001d018183_T001 2.00E-88
337- 3:transcript:Zm00001d052157_T005 transcript:Zm00001d018179_T015 0
337- 4:transcript:Zm00001d052164_T002 transcript:Zm00001d018161_T001 0
337- 5:transcript:Zm00001d052167_T001 transcript:Zm00001d018158_T001 6.00E-80
337- 6:transcript:Zm00001d052168_T001 transcript:Zm00001d018155_T001 8.00E-176
337- 7:transcript:Zm00001d052173_T001 transcript:Zm00001d018150_T001 1.00E-87
337- 8:transcript:Zm00001d052174_T001 transcript:Zm00001d018149_T002 8.00E-95
Alignment 338: score=387.0 e_value=9.4e-21 N=9 4&5 minus
338- 0:transcript:Zm00001d052180_T001 transcript:Zm00001d018142_T001 4.00E-150
338- 1:transcript:Zm00001d052183_T001 transcript:Zm00001d018133_T013 2.00E-169
338- 2:transcript:Zm00001d052184_T001 transcript:Zm00001d018130_T001 5.00E-108
338- 3:transcript:Zm00001d052185_T001 transcript:Zm00001d018128_T001 0
338- 4:transcript:Zm00001d052186_T001 transcript:Zm00001d018127_T001 0
338- 5:transcript:Zm00001d052188_T001 transcript:Zm00001d018126_T001 0
338- 6:transcript:Zm00001d052189_T001 transcript:Zm00001d018122_T001 0
338- 7:transcript:Zm00001d052191_T001 transcript:Zm00001d018118_T001 4.00E-69
338- 8:transcript:Zm00001d052193_T012 transcript:Zm00001d018113_T006 0
Alignment 339: score=380.0 e_value=9.8e-19 N=9 4&5 minus
339- 0:transcript:Zm00001d051936_T001 transcript:Zm00001d018451_T001 2.00E-145
339- 1:transcript:Zm00001d051939_T001 transcript:Zm00001d018447_T001 0
339- 2:transcript:Zm00001d051945_T001 transcript:Zm00001d018445_T004 0
339- 3:transcript:Zm00001d051946_T001 transcript:Zm00001d018444_T001 0
339- 4:transcript:Zm00001d051948_T001 transcript:Zm00001d018443_T001 0
339- 5:transcript:Zm00001d051950_T003 transcript:Zm00001d018441_T008 0
339- 6:transcript:Zm00001d051951_T008 transcript:Zm00001d018440_T002 0
339- 7:transcript:Zm00001d051952_T001 transcript:Zm00001d018437_T001 1.00E-67
339- 8:transcript:Zm00001d051956_T001 transcript:Zm00001d018435_T001 5.00E-142
Alignment 340: score=372.0 e_value=9.2e-20 N=9 4&5 minus
340- 0:transcript:Zm00001d053699_T001 transcript:Zm00001d015686_T001 8.00E-20
340- 1:transcript:Zm00001d053702_T006 transcript:Zm00001d015665_T007 0
340- 2:transcript:Zm00001d053703_T001 transcript:Zm00001d015664_T001 9.00E-36
340- 3:transcript:Zm00001d053705_T001 transcript:Zm00001d015656_T001 1.00E-98
340- 4:transcript:Zm00001d053707_T001 transcript:Zm00001d015639_T001 5.00E-75
340- 5:transcript:Zm00001d053709_T001 transcript:Zm00001d015635_T002 9.00E-75
340- 6:transcript:Zm00001d053713_T001 transcript:Zm00001d015628_T001 3.00E-74
340- 7:transcript:Zm00001d053715_T001 transcript:Zm00001d015623_T001 0
340- 8:transcript:Zm00001d053716_T001 transcript:Zm00001d015613_T001 2.00E-143
Alignment 341: score=324.0 e_value=1.6e-11 N=7 4&5 minus
341- 0:transcript:Zm00001d052256_T001 transcript:Zm00001d018045_T001 2.00E-149
341- 1:transcript:Zm00001d052258_T001 transcript:Zm00001d018041_T001 0
341- 2:transcript:Zm00001d052259_T001 transcript:Zm00001d018040_T002 0
341- 3:transcript:Zm00001d052261_T001 transcript:Zm00001d018037_T001 3.00E-144
341- 4:transcript:Zm00001d052263_T001 transcript:Zm00001d018033_T001 0
341- 5:transcript:Zm00001d052268_T001 transcript:Zm00001d018028_T001 3.00E-123
341- 6:transcript:Zm00001d052269_T001 transcript:Zm00001d018024_T001 0
Alignment 342: score=298.0 e_value=2.8e-12 N=7 4&5 minus
342- 0:transcript:Zm00001d053718_T001 transcript:Zm00001d015603_T001 2.00E-99
342- 1:transcript:Zm00001d053719_T002 transcript:Zm00001d015600_T002 0
342- 2:transcript:Zm00001d053722_T001 transcript:Zm00001d015599_T001 6.00E-151

342- 3:transcript:Zm00001d053724_T001 transcript:Zm00001d015586_T003 6.00E-112
342- 4:transcript:Zm00001d053725_T002 transcript:Zm00001d015581_T001 0
342- 5:transcript:Zm00001d053727_T003 transcript:Zm00001d015569_T001 0
342- 6:transcript:Zm00001d053728_T001 transcript:Zm00001d015568_T001 4.00E-22
Alignment 343: score=293.0 e_value=1.3e-14 N=7 4&5 minus
343- 0:transcript:Zm00001d054014_T001 transcript:Zm00001d015012_T001 0
343- 1:transcript:Zm00001d054016_T004 transcript:Zm00001d015007_T004 0
343- 2:transcript:Zm00001d054017_T003 transcript:Zm00001d015005_T002 2.00E-81
343- 3:transcript:Zm00001d054038_T003 transcript:Zm00001d014995_T001 4.00E-154
343- 4:transcript:Zm00001d054039_T001 transcript:Zm00001d014994_T001 0
343- 5:transcript:Zm00001d054043_T003 transcript:Zm00001d014993_T003 0
343- 6:transcript:Zm00001d054047_T001 transcript:Zm00001d014988_T001 0
Alignment 344: score=271.0 e_value=8.6e-10 N=6 4&5 minus
344- 0:transcript:Zm00001d051542_T047 transcript:Zm00001d017720_T009 0
344- 1:transcript:Zm00001d051543_T001 transcript:Zm00001d017719_T001 2.00E-112
344- 2:transcript:Zm00001d051544_T004 transcript:Zm00001d017718_T004 0
344- 3:transcript:Zm00001d051546_T001 transcript:Zm00001d017717_T001 9.00E-132
344- 4:transcript:Zm00001d051548_T001 transcript:Zm00001d017713_T001 0
344- 5:transcript:Zm00001d051550_T001 transcript:Zm00001d017712_T001 0
Alignment 345: score=259.0 e_value=3.1e-12 N=6 4&5 minus
345- 0:transcript:Zm00001d054066_T001 transcript:Zm00001d014965_T001 0
345- 1:transcript:Zm00001d054069_T001 transcript:Zm00001d014961_T001 0
345- 2:transcript:Zm00001d054071_T001 transcript:Zm00001d014952_T001 0
345- 3:transcript:Zm00001d054072_T002 transcript:Zm00001d014950_T002 0
345- 4:transcript:Zm00001d054074_T001 transcript:Zm00001d014949_T001 3.00E-13
345- 5:transcript:Zm00001d054075_T001 transcript:Zm00001d014947_T001 9.00E-177
Alignment 346: score=413.0 e_value=2.6e-19 N=10 4&6 plus
346- 0:transcript:Zm00001d052252_T001 transcript:Zm00001d037218_T001 7.00E-107
346- 1:transcript:Zm00001d052256_T001 transcript:Zm00001d037221_T001 6.00E-116
346- 2:transcript:Zm00001d052258_T001 transcript:Zm00001d037225_T001 4.00E-156
346- 3:transcript:Zm00001d052259_T001 transcript:Zm00001d037227_T002 2.00E-73
346- 4:transcript:Zm00001d052261_T001 transcript:Zm00001d037228_T001 6.00E-123
346- 5:transcript:Zm00001d052263_T001 transcript:Zm00001d037234_T005 0
346- 6:transcript:Zm00001d052268_T001 transcript:Zm00001d037237_T002 3.00E-86
346- 7:transcript:Zm00001d052270_T005 transcript:Zm00001d037246_T002 3.00E-167
346- 8:transcript:Zm00001d052271_T003 transcript:Zm00001d037247_T007 0
346- 9:transcript:Zm00001d052277_T001 transcript:Zm00001d037251_T001 6.00E-44
Alignment 347: score=567.0 e_value=1.7e-40 N=14 4&6 minus
347- 0:transcript:Zm00001d049619_T003 transcript:Zm00001d035201_T001 0
347- 1:transcript:Zm00001d049621_T001 transcript:Zm00001d035194_T001 0
347- 2:transcript:Zm00001d049628_T001 transcript:Zm00001d035186_T001 0
347- 3:transcript:Zm00001d049636_T002 transcript:Zm00001d035170_T002 0
347- 4:transcript:Zm00001d049637_T001 transcript:Zm00001d035169_T001 0
347- 5:transcript:Zm00001d049638_T009 transcript:Zm00001d035167_T009 0
347- 6:transcript:Zm00001d049640_T002 transcript:Zm00001d035157_T001 0
347- 7:transcript:Zm00001d049641_T001 transcript:Zm00001d035156_T003 0
347- 8:transcript:Zm00001d049642_T001 transcript:Zm00001d035140_T001 0
347- 9:transcript:Zm00001d049649_T001 transcript:Zm00001d035136_T001 0
347- 10:transcript:Zm00001d049650_T001 transcript:Zm00001d035135_T001 4.00E-46
347- 11:transcript:Zm00001d049651_T001 transcript:Zm00001d035134_T001 1.00E-57
347- 12:transcript:Zm00001d049652_T008 transcript:Zm00001d035130_T001 0
347- 13:transcript:Zm00001d049660_T002 transcript:Zm00001d035119_T001 0
Alignment 348: score=427.0 e_value=1.2e-25 N=10 4&6 minus
348- 0:transcript:Zm00001d049707_T006 transcript:Zm00001d035041_T001 0

```
348-  1:transcript:Zm00001d049708_T007 transcript:Zm00001d035040_T007         0
348-  2:transcript:Zm00001d049713_T001 transcript:Zm00001d035035_T001         0
348-  3:transcript:Zm00001d049716_T001 transcript:Zm00001d035032_T002         0
348-  4:transcript:Zm00001d049717_T001 transcript:Zm00001d035031_T001         0
348-  5:transcript:Zm00001d049718_T008 transcript:Zm00001d035029_T006         0
348-  6:transcript:Zm00001d049721_T001 transcript:Zm00001d035027_T001  3.00E-86
348-  7:transcript:Zm00001d049722_T001 transcript:Zm00001d035020_T001  9.00E-71
348-  8:transcript:Zm00001d049723_T001 transcript:Zm00001d035019_T001  4.00E-97
348-  9:transcript:Zm00001d049725_T002 transcript:Zm00001d035015_T001         0
## Alignment 349: score=285.0 e_value=1.4e-11 N=7 4&6 minus
349-  0:transcript:Zm00001d049674_T002 transcript:Zm00001d035087_T001  9.00E-68
349-  1:transcript:Zm00001d049676_T001 transcript:Zm00001d035086_T001         0
349-  2:transcript:Zm00001d049678_T001 transcript:Zm00001d035084_T001         0
349-  3:transcript:Zm00001d049687_T001 transcript:Zm00001d035076_T001         0
349-  4:transcript:Zm00001d049690_T001 transcript:Zm00001d035069_T001         0
349-  5:transcript:Zm00001d049697_T001 transcript:Zm00001d035064_T001  1.00E-30
349-  6:transcript:Zm00001d049704_T001 transcript:Zm00001d035057_T001 9.00E-180
## Alignment 350: score=298.0 e_value=7.1e-12 N=7 4&7 plus
350-  0:transcript:Zm00001d049991_T001 transcript:Zm00001d020688_T001  2.00E-95
350-  1:transcript:Zm00001d050008_T001 transcript:Zm00001d020697_T001 2.00E-158
350-  2:transcript:Zm00001d050016_T001 transcript:Zm00001d020705_T001  1.00E-62
350-  3:transcript:Zm00001d050017_T001 transcript:Zm00001d020708_T002 4.00E-152
350-  4:transcript:Zm00001d050018_T007 transcript:Zm00001d020711_T001  3.00E-77
350-  5:transcript:Zm00001d050019_T001 transcript:Zm00001d020713_T001 1.00E-166
350-  6:transcript:Zm00001d050021_T001 transcript:Zm00001d020717_T001         0
## Alignment 351: score=274.0 e_value=2.8e-11 N=6 4&7 plus
351-  0:transcript:Zm00001d052892_T001 transcript:Zm00001d020944_T001  1.00E-83
351-  1:transcript:Zm00001d052893_T002 transcript:Zm00001d020946_T005  1.00E-63
351-  2:transcript:Zm00001d052895_T001 transcript:Zm00001d020948_T001 3.00E-102
351-  3:transcript:Zm00001d052898_T001 transcript:Zm00001d020949_T001  4.00E-06
351-  4:transcript:Zm00001d052901_T001 transcript:Zm00001d020951_T002  5.00E-94
351-  5:transcript:Zm00001d052903_T001 transcript:Zm00001d020963_T001         0
## Alignment 352: score=326.0 e_value=7.2e-18 N=9 4&9 minus
352-  0:transcript:Zm00001d052180_T001 transcript:Zm00001d044899_T001  3.00E-33
352-  1:transcript:Zm00001d052191_T001 transcript:Zm00001d044898_T001  5.00E-09
352-  2:transcript:Zm00001d052194_T001 transcript:Zm00001d044874_T001  9.00E-41
352-  3:transcript:Zm00001d052198_T002 transcript:Zm00001d044869_T001  7.00E-77
352-  4:transcript:Zm00001d052206_T001 transcript:Zm00001d044864_T001 1.00E-147
352-  5:transcript:Zm00001d052219_T002 transcript:Zm00001d044860_T001 3.00E-162
352-  6:transcript:Zm00001d052229_T001 transcript:Zm00001d044857_T001 9.00E-112
352-  7:transcript:Zm00001d052234_T001 transcript:Zm00001d044849_T001  1.00E-25
352-  8:transcript:Zm00001d052239_T001 transcript:Zm00001d044844_T001  1.00E-50
## Alignment 353: score=313.0 e_value=8.4e-12 N=7 4&9 minus
353-  0:transcript:Zm00001d052252_T001 transcript:Zm00001d044841_T001 2.00E-100
353-  1:transcript:Zm00001d052254_T001 transcript:Zm00001d044839_T001  8.00E-40
353-  2:transcript:Zm00001d052256_T001 transcript:Zm00001d044836_T001  2.00E-87
353-  3:transcript:Zm00001d052258_T001 transcript:Zm00001d044833_T001 8.00E-161
353-  4:transcript:Zm00001d052259_T001 transcript:Zm00001d044831_T001  3.00E-66
353-  5:transcript:Zm00001d052260_T001 transcript:Zm00001d044828_T001         0
353-  6:transcript:Zm00001d052261_T001 transcript:Zm00001d044826_T001 9.00E-119
## Alignment 354: score=378.0 e_value=6e-21 N=9 5&6 plus
354-  0:transcript:Zm00001d014355_T001 transcript:Zm00001d036570_T001  3.00E-93
354-  1:transcript:Zm00001d014358_T014 transcript:Zm00001d036571_T002         0
354-  2:transcript:Zm00001d014360_T001 transcript:Zm00001d036573_T002         0
```

354- 3: transcript:Zm00001d014364_T002 transcript:Zm00001d036577_T001 2.00E-144
354- 4: transcript:Zm00001d014368_T001 transcript:Zm00001d036588_T001 4.00E-172
354- 5: transcript:Zm00001d014377_T002 transcript:Zm00001d036593_T001 0
354- 6: transcript:Zm00001d014378_T001 transcript:Zm00001d036594_T001 0
354- 7: transcript:Zm00001d014381_T001 transcript:Zm00001d036597_T001 3.00E-71
354- 8: transcript:Zm00001d014382_T003 transcript:Zm00001d036598_T006 0
Alignment 355: score=296.0 e_value=4.1e-12 N=7 5&6 plus
355- 0: transcript:Zm00001d015504_T004 transcript:Zm00001d036710_T002 0
355- 1: transcript:Zm00001d015508_T001 transcript:Zm00001d036716_T001 2.00E-167
355- 2: transcript:Zm00001d015515_T001 transcript:Zm00001d036726_T001 4.00E-100
355- 3: transcript:Zm00001d015527_T001 transcript:Zm00001d036735_T002 0
355- 4: transcript:Zm00001d015546_T001 transcript:Zm00001d036736_T001 1.00E-63
355- 5: transcript:Zm00001d015550_T001 transcript:Zm00001d036737_T001 4.00E-14
355- 6: transcript:Zm00001d015559_T001 transcript:Zm00001d036739_T001 1.00E-159
Alignment 356: score=254.0 e_value=1.6e-09 N=6 5&6 plus
356- 0: transcript:Zm00001d015448_T001 transcript:Zm00001d036690_T001 1.00E-24
356- 1: transcript:Zm00001d015451_T001 transcript:Zm00001d036692_T001 2.00E-87
356- 2: transcript:Zm00001d015461_T001 transcript:Zm00001d036699_T001 2.00E-36
356- 3: transcript:Zm00001d015463_T001 transcript:Zm00001d036700_T001 8.00E-44
356- 4: transcript:Zm00001d015470_T001 transcript:Zm00001d036703_T001 9.00E-16
356- 5: transcript:Zm00001d015473_T003 transcript:Zm00001d036708_T001 1.00E-42
Alignment 357: score=935.0 e_value=2e-66 N=23 5&6 minus
357- 0: transcript:Zm00001d014743_T001 transcript:Zm00001d036436_T001 2.00E-28
357- 1: transcript:Zm00001d014753_T002 transcript:Zm00001d036432_T003 0
357- 2: transcript:Zm00001d014757_T001 transcript:Zm00001d036431_T001 2.00E-92
357- 3: transcript:Zm00001d014760_T002 transcript:Zm00001d036429_T006 0
357- 4: transcript:Zm00001d014761_T001 transcript:Zm00001d036428_T006 0
357- 5: transcript:Zm00001d014762_T001 transcript:Zm00001d036426_T001 2.00E-151
357- 6: transcript:Zm00001d014764_T006 transcript:Zm00001d036422_T003 0
357- 7: transcript:Zm00001d014765_T001 transcript:Zm00001d036418_T001 2.00E-112
357- 8: transcript:Zm00001d014773_T001 transcript:Zm00001d036416_T001 0
357- 9: transcript:Zm00001d014774_T001 transcript:Zm00001d036415_T001 3.00E-47
357- 10: transcript:Zm00001d014775_T002 transcript:Zm00001d036410_T002 0
357- 11: transcript:Zm00001d014785_T001 transcript:Zm00001d036403_T002 5.00E-171
357- 12: transcript:Zm00001d014789_T002 transcript:Zm00001d036402_T002 3.00E-136
357- 13: transcript:Zm00001d014792_T003 transcript:Zm00001d036401_T001 0
357- 14: transcript:Zm00001d014795_T001 transcript:Zm00001d036400_T001 9.00E-60
357- 15: transcript:Zm00001d014797_T006 transcript:Zm00001d036395_T016 0
357- 16: transcript:Zm00001d014804_T001 transcript:Zm00001d036394_T001 2.00E-19
357- 17: transcript:Zm00001d014809_T003 transcript:Zm00001d036387_T001 0
357- 18: transcript:Zm00001d014814_T002 transcript:Zm00001d036382_T001 1.00E-163
357- 19: transcript:Zm00001d014834_T001 transcript:Zm00001d036373_T001 1.00E-30
357- 20: transcript:Zm00001d014840_T001 transcript:Zm00001d036366_T001 2.00E-86
357- 21: transcript:Zm00001d014843_T001 transcript:Zm00001d036364_T001 7.00E-135
357- 22: transcript:Zm00001d014844_T007 transcript:Zm00001d036363_T001 2.00E-176
Alignment 358: score=550.0 e_value=5.4e-32 N=13 5&6 minus
358- 0: transcript:Zm00001d014640_T036 transcript:Zm00001d036507_T004 0
358- 1: transcript:Zm00001d014641_T001 transcript:Zm00001d036506_T001 0
358- 2: transcript:Zm00001d014648_T001 transcript:Zm00001d036499_T001 6.00E-44
358- 3: transcript:Zm00001d014655_T001 transcript:Zm00001d036495_T001 0
358- 4: transcript:Zm00001d014656_T003 transcript:Zm00001d036494_T001 2.00E-179
358- 5: transcript:Zm00001d014664_T001 transcript:Zm00001d036485_T003 9.00E-159
358- 6: transcript:Zm00001d014665_T002 transcript:Zm00001d036483_T006 0
358- 7: transcript:Zm00001d014668_T001 transcript:Zm00001d036482_T001 0

358- 8:transcript:Zm00001d014671_T001 transcript:Zm00001d036481_T001 4.00E-100
358- 9:transcript:Zm00001d014673_T001 transcript:Zm00001d036480_T001 0
358- 10:transcript:Zm00001d014674_T003 transcript:Zm00001d036477_T002 0
358- 11:transcript:Zm00001d014679_T001 transcript:Zm00001d036464_T001 3.00E-37
358- 12:transcript:Zm00001d014682_T001 transcript:Zm00001d036463_T001 3.00E-17
Alignment 359: score=427.0 e_value=1.4e-20 N=10 5&6 minus
359- 0:transcript:Zm00001d018016_T001 transcript:Zm00001d037244_T001 2.00E-45
359- 1:transcript:Zm00001d018017_T001 transcript:Zm00001d037243_T001 1.00E-43
359- 2:transcript:Zm00001d018027_T001 transcript:Zm00001d037239_T001 3.00E-19
359- 3:transcript:Zm00001d018028_T001 transcript:Zm00001d037237_T002 2.00E-88
359- 4:transcript:Zm00001d018033_T001 transcript:Zm00001d037234_T005 0
359- 5:transcript:Zm00001d018037_T001 transcript:Zm00001d037228_T001 2.00E-174
359- 6:transcript:Zm00001d018040_T002 transcript:Zm00001d037227_T002 4.00E-87
359- 7:transcript:Zm00001d018041_T001 transcript:Zm00001d037225_T001 3.00E-156
359- 8:transcript:Zm00001d018045_T001 transcript:Zm00001d037221_T001 4.00E-118
359- 9:transcript:Zm00001d018047_T002 transcript:Zm00001d037220_T001 2.00E-34
Alignment 360: score=372.0 e_value=6.9e-19 N=9 5&6 minus
360- 0:transcript:Zm00001d018090_T002 transcript:Zm00001d037211_T001 0
360- 1:transcript:Zm00001d018103_T002 transcript:Zm00001d037209_T001 1.00E-75
360- 2:transcript:Zm00001d018105_T001 transcript:Zm00001d037205_T001 7.00E-129
360- 3:transcript:Zm00001d018107_T001 transcript:Zm00001d037204_T001 3.00E-93
360- 4:transcript:Zm00001d018112_T004 transcript:Zm00001d037200_T004 9.00E-63
360- 5:transcript:Zm00001d018113_T006 transcript:Zm00001d037198_T001 0
360- 6:transcript:Zm00001d018117_T001 transcript:Zm00001d037197_T001 2.00E-08
360- 7:transcript:Zm00001d018118_T001 transcript:Zm00001d037194_T001 3.00E-37
360- 8:transcript:Zm00001d018122_T001 transcript:Zm00001d037192_T001 4.00E-86
Alignment 361: score=268.0 e_value=6.6e-11 N=7 5&7 minus
361- 0:transcript:Zm00001d013071_T001 transcript:Zm00001d018973_T001 1.00E-65
361- 1:transcript:Zm00001d013074_T003 transcript:Zm00001d018971_T003 2.00E-33
361- 2:transcript:Zm00001d013075_T001 transcript:Zm00001d018964_T003 0
361- 3:transcript:Zm00001d013076_T001 transcript:Zm00001d018957_T001 0
361- 4:transcript:Zm00001d013077_T004 transcript:Zm00001d018947_T001 5.00E-19
361- 5:transcript:Zm00001d013078_T004 transcript:Zm00001d018938_T001 1.00E-118
361- 6:transcript:Zm00001d013080_T001 transcript:Zm00001d018931_T001 2.00E-126
Alignment 362: score=365.0 e_value=4.2e-17 N=9 5&9 plus
362- 0:transcript:Zm00001d018016_T001 transcript:Zm00001d044808_T001 2.00E-155
362- 1:transcript:Zm00001d018024_T001 transcript:Zm00001d044812_T001 0
362- 2:transcript:Zm00001d018025_T001 transcript:Zm00001d044814_T002 0
362- 3:transcript:Zm00001d018029_T001 transcript:Zm00001d044816_T001 4.00E-79
362- 4:transcript:Zm00001d018035_T001 transcript:Zm00001d044825_T001 2.00E-31
362- 5:transcript:Zm00001d018037_T001 transcript:Zm00001d044826_T001 7.00E-169
362- 6:transcript:Zm00001d018040_T002 transcript:Zm00001d044831_T001 5.00E-79
362- 7:transcript:Zm00001d018041_T001 transcript:Zm00001d044833_T001 4.00E-160
362- 8:transcript:Zm00001d018045_T001 transcript:Zm00001d044836_T001 9.00E-91
Alignment 363: score=315.0 e_value=3.1e-16 N=8 5&9 plus
363- 0:transcript:Zm00001d015759_T001 transcript:Zm00001d046501_T001 1.00E-93
363- 1:transcript:Zm00001d015767_T002 transcript:Zm00001d046510_T001 4.00E-16
363- 2:transcript:Zm00001d015776_T001 transcript:Zm00001d046513_T001 6.00E-87
363- 3:transcript:Zm00001d015780_T002 transcript:Zm00001d046531_T007 1.00E-102
363- 4:transcript:Zm00001d015783_T001 transcript:Zm00001d046534_T001 0
363- 5:transcript:Zm00001d015785_T005 transcript:Zm00001d046538_T003 5.00E-179
363- 6:transcript:Zm00001d015788_T001 transcript:Zm00001d046539_T002 0
363- 7:transcript:Zm00001d015789_T002 transcript:Zm00001d046540_T003 0
Alignment 364: score=314.0 e_value=2.1e-11 N=7 5&9 plus

364- 0:transcript:Zm00001d015451_T001 transcript:Zm00001d046906_T001 3.00E-83
364- 1:transcript:Zm00001d015457_T001 transcript:Zm00001d046910_T001 5.00E-45
364- 2:transcript:Zm00001d015459_T003 transcript:Zm00001d046915_T001 0
364- 3:transcript:Zm00001d015463_T001 transcript:Zm00001d046916_T001 2.00E-45
364- 4:transcript:Zm00001d015468_T001 transcript:Zm00001d046925_T001 1.00E-111
364- 5:transcript:Zm00001d015470_T001 transcript:Zm00001d046927_T001 5.00E-18
364- 6:transcript:Zm00001d015473_T003 transcript:Zm00001d046929_T002 2.00E-42
Alignment 365: score=280.0 e_value=1.7e-10 N=7 5&9 plus
365- 0:transcript:Zm00001d015614_T001 transcript:Zm00001d046632_T001 3.00E-124
365- 1:transcript:Zm00001d015636_T001 transcript:Zm00001d046642_T001 4.00E-110
365- 2:transcript:Zm00001d015649_T003 transcript:Zm00001d046655_T001 0
365- 3:transcript:Zm00001d015656_T001 transcript:Zm00001d046660_T001 0
365- 4:transcript:Zm00001d015658_T003 transcript:Zm00001d046661_T003 0
365- 5:transcript:Zm00001d015664_T001 transcript:Zm00001d046664_T001 8.00E-14
365- 6:transcript:Zm00001d015670_T001 transcript:Zm00001d046667_T001 2.00E-17
Alignment 366: score=447.0 e_value=3.6e-21 N=10 5&9 minus
366- 0:transcript:Zm00001d016216_T001 transcript:Zm00001d046311_T001 1.00E-15
366- 1:transcript:Zm00001d016231_T001 transcript:Zm00001d046305_T001 7.00E-30
366- 2:transcript:Zm00001d016234_T001 transcript:Zm00001d046304_T001 0
366- 3:transcript:Zm00001d016237_T001 transcript:Zm00001d046303_T001 8.00E-84
366- 4:transcript:Zm00001d016253_T001 transcript:Zm00001d046300_T001 3.00E-90
366- 5:transcript:Zm00001d016255_T001 transcript:Zm00001d046299_T001 5.00E-90
366- 6:transcript:Zm00001d016256_T001 transcript:Zm00001d046297_T001 9.00E-44
366- 7:transcript:Zm00001d016260_T001 transcript:Zm00001d046292_T001 6.00E-44
366- 8:transcript:Zm00001d016269_T001 transcript:Zm00001d046288_T001 6.00E-45
366- 9:transcript:Zm00001d016271_T001 transcript:Zm00001d046281_T001 5.00E-42
Alignment 367: score=387.0 e_value=8.7e-20 N=10 5&9 minus
367- 0:transcript:Zm00001d015342_T001 transcript:Zm00001d046998_T001 5.00E-85
367- 1:transcript:Zm00001d015348_T002 transcript:Zm00001d046996_T001 9.00E-165
367- 2:transcript:Zm00001d015354_T001 transcript:Zm00001d046986_T001 7.00E-39
367- 3:transcript:Zm00001d015364_T003 transcript:Zm00001d046978_T002 4.00E-100
367- 4:transcript:Zm00001d015382_T001 transcript:Zm00001d046966_T001 1.00E-08
367- 5:transcript:Zm00001d015394_T001 transcript:Zm00001d046958_T001 2.00E-108
367- 6:transcript:Zm00001d015401_T001 transcript:Zm00001d046948_T002 0
367- 7:transcript:Zm00001d015407_T001 transcript:Zm00001d046947_T001 3.00E-115
367- 8:transcript:Zm00001d015414_T001 transcript:Zm00001d046938_T001 0
367- 9:transcript:Zm00001d015421_T001 transcript:Zm00001d046937_T001 5.00E-78
Alignment 368: score=1776.0 e_value=1.1e-152 N=42 6&8 plus
368- 0:transcript:Zm00001d038854_T006 transcript:Zm00001d009409_T002 0
368- 1:transcript:Zm00001d038856_T001 transcript:Zm00001d009410_T001 9.00E-34
368- 2:transcript:Zm00001d038859_T007 transcript:Zm00001d009411_T002 0
368- 3:transcript:Zm00001d038864_T001 transcript:Zm00001d009413_T001 7.00E-112
368- 4:transcript:Zm00001d038865_T003 transcript:Zm00001d009417_T006 4.00E-91
368- 5:transcript:Zm00001d038866_T002 transcript:Zm00001d009418_T001 0
368- 6:transcript:Zm00001d038868_T003 transcript:Zm00001d009424_T005 0
368- 7:transcript:Zm00001d038871_T001 transcript:Zm00001d009426_T005 2.00E-100
368- 8:transcript:Zm00001d038873_T001 transcript:Zm00001d009429_T001 4.00E-37
368- 9:transcript:Zm00001d038877_T001 transcript:Zm00001d009430_T002 0
368- 10:transcript:Zm00001d038878_T001 transcript:Zm00001d009435_T001 3.00E-113
368- 11:transcript:Zm00001d038879_T003 transcript:Zm00001d009436_T003 0
368- 12:transcript:Zm00001d038880_T001 transcript:Zm00001d009439_T004 0
368- 13:transcript:Zm00001d038881_T001 transcript:Zm00001d009440_T001 5.00E-127
368- 14:transcript:Zm00001d038886_T001 transcript:Zm00001d009446_T001 1.00E-98
368- 15:transcript:Zm00001d038890_T002 transcript:Zm00001d009450_T002 4.00E-79

368- 16:transcript:Zm00001d038892_T001 transcript:Zm00001d009452_T007 0
368- 17:transcript:Zm00001d038901_T001 transcript:Zm00001d009458_T001 1.00E-83
368- 18:transcript:Zm00001d038905_T001 transcript:Zm00001d009465_T001 0
368- 19:transcript:Zm00001d038907_T001 transcript:Zm00001d009468_T001 0
368- 20:transcript:Zm00001d038915_T001 transcript:Zm00001d009473_T001 0
368- 21:transcript:Zm00001d038918_T001 transcript:Zm00001d009475_T001 2.00E-77
368- 22:transcript:Zm00001d038923_T001 transcript:Zm00001d009480_T001 0
368- 23:transcript:Zm00001d038925_T003 transcript:Zm00001d009481_T001 0
368- 24:transcript:Zm00001d038929_T001 transcript:Zm00001d009488_T001 0
368- 25:transcript:Zm00001d038930_T001 transcript:Zm00001d009490_T001 9.00E-20
368- 26:transcript:Zm00001d038932_T001 transcript:Zm00001d009493_T001 2.00E-129
368- 27:transcript:Zm00001d038933_T001 transcript:Zm00001d009495_T001 2.00E-84
368- 28:transcript:Zm00001d038936_T001 transcript:Zm00001d009497_T004 0
368- 29:transcript:Zm00001d038944_T003 transcript:Zm00001d009500_T002 0
368- 30:transcript:Zm00001d038950_T001 transcript:Zm00001d009501_T001 3.00E-35
368- 31:transcript:Zm00001d038954_T001 transcript:Zm00001d009503_T001 2.00E-153
368- 32:transcript:Zm00001d038955_T001 transcript:Zm00001d009504_T002 0
368- 33:transcript:Zm00001d038963_T001 transcript:Zm00001d009508_T001 8.00E-157
368- 34:transcript:Zm00001d038968_T001 transcript:Zm00001d009510_T001 0
368- 35:transcript:Zm00001d038972_T002 transcript:Zm00001d009513_T003 0
368- 36:transcript:Zm00001d038973_T001 transcript:Zm00001d009517_T001 1.00E-123
368- 37:transcript:Zm00001d038978_T001 transcript:Zm00001d009520_T002 3.00E-55
368- 38:transcript:Zm00001d038979_T001 transcript:Zm00001d009530_T001 5.00E-122
368- 39:transcript:Zm00001d038980_T001 transcript:Zm00001d009532_T001 1.00E-130
368- 40:transcript:Zm00001d038981_T001 transcript:Zm00001d009539_T002 0
368- 41:transcript:Zm00001d038982_T007 transcript:Zm00001d009541_T002 0
Alignment 369: score=679.0 e_value=2e-42 N=16 6&8 plus
369- 0:transcript:Zm00001d039005_T003 transcript:Zm00001d009564_T001 0
369- 1:transcript:Zm00001d039010_T001 transcript:Zm00001d009566_T001 1.00E-32
369- 2:transcript:Zm00001d039011_T001 transcript:Zm00001d009568_T001 4.00E-47
369- 3:transcript:Zm00001d039014_T001 transcript:Zm00001d009570_T001 0
369- 4:transcript:Zm00001d039015_T001 transcript:Zm00001d009571_T002 0
369- 5:transcript:Zm00001d039016_T001 transcript:Zm00001d009572_T001 2.00E-154
369- 6:transcript:Zm00001d039019_T001 transcript:Zm00001d009573_T001 1.00E-104
369- 7:transcript:Zm00001d039020_T001 transcript:Zm00001d009578_T001 8.00E-88
369- 8:transcript:Zm00001d039031_T001 transcript:Zm00001d009583_T001 1.00E-48
369- 9:transcript:Zm00001d039037_T001 transcript:Zm00001d009587_T002 0
369- 10:transcript:Zm00001d039040_T001 transcript:Zm00001d009589_T001 0
369- 11:transcript:Zm00001d039041_T007 transcript:Zm00001d009591_T006 0
369- 12:transcript:Zm00001d039043_T011 transcript:Zm00001d009594_T011 0
369- 13:transcript:Zm00001d039044_T001 transcript:Zm00001d009595_T001 0
369- 14:transcript:Zm00001d039047_T001 transcript:Zm00001d009597_T002 0
369- 15:transcript:Zm00001d039049_T001 transcript:Zm00001d009599_T001 4.00E-46
Alignment 370: score=490.0 e_value=7.8e-32 N=13 6&8 plus
370- 0:transcript:Zm00001d038366_T001 transcript:Zm00001d010520_T001 0
370- 1:transcript:Zm00001d038373_T001 transcript:Zm00001d010526_T002 0
370- 2:transcript:Zm00001d038374_T007 transcript:Zm00001d010528_T001 2.00E-62
370- 3:transcript:Zm00001d038375_T003 transcript:Zm00001d010530_T002 0
370- 4:transcript:Zm00001d038376_T001 transcript:Zm00001d010534_T001 3.00E-90
370- 5:transcript:Zm00001d038378_T001 transcript:Zm00001d010537_T001 4.00E-115
370- 6:transcript:Zm00001d038380_T004 transcript:Zm00001d010538_T008 3.00E-159
370- 7:transcript:Zm00001d038388_T001 transcript:Zm00001d010543_T001 1.00E-137
370- 8:transcript:Zm00001d038392_T001 transcript:Zm00001d010545_T001 0
370- 9:transcript:Zm00001d038393_T012 transcript:Zm00001d010546_T003 0

370- 10:transcript:Zm00001d038394_T001 transcript:Zm00001d010548_T002 0
370- 11:transcript:Zm00001d038396_T011 transcript:Zm00001d010556_T001 0
370- 12:transcript:Zm00001d038404_T003 transcript:Zm00001d010564_T002 0
Alignment 371: score=482.0 e_value=1.6e-24 N=11 6&8 plus
371- 0:transcript:Zm00001d038311_T001 transcript:Zm00001d010168_T001 2.00E-62
371- 1:transcript:Zm00001d038318_T002 transcript:Zm00001d010173_T003 0
371- 2:transcript:Zm00001d038319_T003 transcript:Zm00001d010174_T004 5.00E-150
371- 3:transcript:Zm00001d038320_T001 transcript:Zm00001d010175_T002 4.00E-79
371- 4:transcript:Zm00001d038325_T001 transcript:Zm00001d010185_T001 6.00E-28
371- 5:transcript:Zm00001d038330_T001 transcript:Zm00001d010189_T001 0
371- 6:transcript:Zm00001d038331_T001 transcript:Zm00001d010191_T001 2.00E-46
371- 7:transcript:Zm00001d038333_T001 transcript:Zm00001d010195_T001 0
371- 8:transcript:Zm00001d038336_T001 transcript:Zm00001d010199_T002 0
371- 9:transcript:Zm00001d038338_T001 transcript:Zm00001d010201_T001 2.00E-96
371- 10:transcript:Zm00001d038340_T001 transcript:Zm00001d010202_T001 0
Alignment 372: score=476.0 e_value=1.5e-25 N=11 6&8 plus
372- 0:transcript:Zm00001d035439_T001 transcript:Zm00001d010948_T001 5.00E-170
372- 1:transcript:Zm00001d035445_T001 transcript:Zm00001d010950_T003 4.00E-164
372- 2:transcript:Zm00001d035447_T001 transcript:Zm00001d010954_T001 0
372- 3:transcript:Zm00001d035455_T001 transcript:Zm00001d010956_T001 0
372- 4:transcript:Zm00001d035467_T001 transcript:Zm00001d010961_T001 0
372- 5:transcript:Zm00001d035470_T004 transcript:Zm00001d010967_T001 0
372- 6:transcript:Zm00001d035473_T001 transcript:Zm00001d010968_T001 6.00E-60
372- 7:transcript:Zm00001d035474_T001 transcript:Zm00001d010969_T001 5.00E-170
372- 8:transcript:Zm00001d035475_T001 transcript:Zm00001d010970_T003 0
372- 9:transcript:Zm00001d035476_T002 transcript:Zm00001d010971_T003 0
372- 10:transcript:Zm00001d035487_T025 transcript:Zm00001d010974_T008 0
Alignment 373: score=451.0 e_value=1.1e-26 N=10 6&8 plus
373- 0:transcript:Zm00001d039077_T001 transcript:Zm00001d009622_T001 4.00E-107
373- 1:transcript:Zm00001d039080_T001 transcript:Zm00001d009628_T001 4.00E-152
373- 2:transcript:Zm00001d039081_T002 transcript:Zm00001d009631_T001 0
373- 3:transcript:Zm00001d039082_T001 transcript:Zm00001d009637_T001 6.00E-19
373- 4:transcript:Zm00001d039084_T001 transcript:Zm00001d009638_T001 5.00E-26
373- 5:transcript:Zm00001d039087_T001 transcript:Zm00001d009639_T001 0
373- 6:transcript:Zm00001d039089_T001 transcript:Zm00001d009640_T002 0
373- 7:transcript:Zm00001d039103_T001 transcript:Zm00001d009647_T001 3.00E-43
373- 8:transcript:Zm00001d039104_T001 transcript:Zm00001d009649_T001 0
373- 9:transcript:Zm00001d039108_T001 transcript:Zm00001d009650_T001 1.00E-59
Alignment 374: score=429.0 e_value=5.1e-27 N=11 6&8 plus
374- 0:transcript:Zm00001d035492_T003 transcript:Zm00001d010975_T003 0
374- 1:transcript:Zm00001d035494_T001 transcript:Zm00001d010976_T001 2.00E-135
374- 2:transcript:Zm00001d035498_T001 transcript:Zm00001d010978_T001 9.00E-87
374- 3:transcript:Zm00001d035501_T001 transcript:Zm00001d010982_T001 0
374- 4:transcript:Zm00001d035512_T001 transcript:Zm00001d010987_T001 2.00E-144
374- 5:transcript:Zm00001d035515_T001 transcript:Zm00001d010995_T001 0
374- 6:transcript:Zm00001d035527_T001 transcript:Zm00001d010997_T001 0
374- 7:transcript:Zm00001d035535_T001 transcript:Zm00001d010998_T001 3.00E-81
374- 8:transcript:Zm00001d035551_T007 transcript:Zm00001d011005_T005 2.00E-21
374- 9:transcript:Zm00001d035553_T001 transcript:Zm00001d011006_T001 0
374- 10:transcript:Zm00001d035559_T004 transcript:Zm00001d011012_T001 1.00E-148
Alignment 375: score=391.0 e_value=4.9e-17 N=9 6&8 plus
375- 0:transcript:Zm00001d038506_T001 transcript:Zm00001d010622_T002 0
375- 1:transcript:Zm00001d038509_T001 transcript:Zm00001d010623_T001 0
375- 2:transcript:Zm00001d038513_T002 transcript:Zm00001d010625_T001 0

375- 3:transcript:Zm00001d038514_T001 transcript:Zm00001d010627_T001 2.00E-114
375- 4:transcript:Zm00001d038517_T001 transcript:Zm00001d010629_T001 0
375- 5:transcript:Zm00001d038521_T001 transcript:Zm00001d010630_T003 0
375- 6:transcript:Zm00001d038523_T001 transcript:Zm00001d010632_T002 6.00E-35
375- 7:transcript:Zm00001d038527_T001 transcript:Zm00001d010634_T001 0
375- 8:transcript:Zm00001d038528_T001 transcript:Zm00001d010635_T001 3.00E-141
Alignment 376: score=356.0 e_value=1.6e-16 N=8 6&8 plus
376- 0:transcript:Zm00001d035634_T001 transcript:Zm00001d011051_T002 0
376- 1:transcript:Zm00001d035637_T001 transcript:Zm00001d011055_T001 7.00E-30
376- 2:transcript:Zm00001d035638_T001 transcript:Zm00001d011056_T001 1.00E-131
376- 3:transcript:Zm00001d035651_T001 transcript:Zm00001d011058_T001 4.00E-106
376- 4:transcript:Zm00001d035657_T001 transcript:Zm00001d011060_T001 2.00E-160
376- 5:transcript:Zm00001d035659_T001 transcript:Zm00001d011063_T001 3.00E-20
376- 6:transcript:Zm00001d035664_T005 transcript:Zm00001d011064_T003 1.00E-63
376- 7:transcript:Zm00001d035670_T001 transcript:Zm00001d011070_T001 0
Alignment 377: score=317.0 e_value=3.5e-14 N=8 6&8 plus
377- 0:transcript:Zm00001d038397_T005 transcript:Zm00001d010574_T002 7.00E-115
377- 1:transcript:Zm00001d038409_T001 transcript:Zm00001d010578_T003 0
377- 2:transcript:Zm00001d038411_T001 transcript:Zm00001d010584_T001 0
377- 3:transcript:Zm00001d038420_T001 transcript:Zm00001d010587_T001 7.00E-73
377- 4:transcript:Zm00001d038431_T001 transcript:Zm00001d010589_T001 0
377- 5:transcript:Zm00001d038442_T001 transcript:Zm00001d010596_T001 9.00E-92
377- 6:transcript:Zm00001d038447_T001 transcript:Zm00001d010602_T001 0
377- 7:transcript:Zm00001d038449_T001 transcript:Zm00001d010604_T006 0
Alignment 378: score=315.0 e_value=3.6e-13 N=7 6&8 plus
378- 0:transcript:Zm00001d039138_T012 transcript:Zm00001d009683_T004 0
378- 1:transcript:Zm00001d039139_T001 transcript:Zm00001d009686_T002 7.00E-113
378- 2:transcript:Zm00001d039140_T001 transcript:Zm00001d009687_T001 5.00E-81
378- 3:transcript:Zm00001d039141_T001 transcript:Zm00001d009688_T001 0
378- 4:transcript:Zm00001d039144_T002 transcript:Zm00001d009693_T001 0
378- 5:transcript:Zm00001d039145_T001 transcript:Zm00001d009695_T001 0
378- 6:transcript:Zm00001d039150_T001 transcript:Zm00001d009705_T001 0
Alignment 379: score=305.0 e_value=1.5e-14 N=7 6&8 plus
379- 0:transcript:Zm00001d038984_T001 transcript:Zm00001d009543_T001 1.00E-10
379- 1:transcript:Zm00001d038988_T005 transcript:Zm00001d009548_T005 0
379- 2:transcript:Zm00001d038989_T002 transcript:Zm00001d009549_T002 0
379- 3:transcript:Zm00001d038995_T001 transcript:Zm00001d009552_T002 5.00E-103
379- 4:transcript:Zm00001d038998_T001 transcript:Zm00001d009555_T003 0
379- 5:transcript:Zm00001d039000_T001 transcript:Zm00001d009556_T001 0
379- 6:transcript:Zm00001d039002_T019 transcript:Zm00001d009562_T007 0
Alignment 380: score=286.0 e_value=6.5e-12 N=6 6&8 plus
380- 0:transcript:Zm00001d035595_T003 transcript:Zm00001d011034_T004 6.00E-147
380- 1:transcript:Zm00001d035597_T001 transcript:Zm00001d011036_T001 0
380- 2:transcript:Zm00001d035599_T001 transcript:Zm00001d011037_T001 0
380- 3:transcript:Zm00001d035601_T001 transcript:Zm00001d011038_T002 0
380- 4:transcript:Zm00001d035603_T001 transcript:Zm00001d011041_T001 2.00E-122
380- 5:transcript:Zm00001d035610_T001 transcript:Zm00001d011050_T001 2.00E-32
Alignment 381: score=252.0 e_value=5.8e-09 N=6 6&8 plus
381- 0:transcript:Zm00001d038281_T001 transcript:Zm00001d012280_T001 1.00E-69
381- 1:transcript:Zm00001d038283_T001 transcript:Zm00001d012282_T001 1.00E-34
381- 2:transcript:Zm00001d038288_T001 transcript:Zm00001d012285_T001 1.00E-70
381- 3:transcript:Zm00001d038291_T001 transcript:Zm00001d012293_T001 2.00E-55
381- 4:transcript:Zm00001d038296_T001 transcript:Zm00001d012294_T009 0
381- 5:transcript:Zm00001d038300_T002 transcript:Zm00001d012304_T001 0

Alignment 382: score=251.0 e_value=3.7e-11 N=7 6&8 plus
382- 0:transcript:Zm00001d038682_T001 transcript:Zm00001d010728_T005 7.00E-67
382- 1:transcript:Zm00001d038683_T001 transcript:Zm00001d010730_T001 1.00E-148
382- 2:transcript:Zm00001d038688_T002 transcript:Zm00001d010732_T002 1.00E-151
382- 3:transcript:Zm00001d038699_T001 transcript:Zm00001d010736_T001 3.00E-154
382- 4:transcript:Zm00001d038717_T001 transcript:Zm00001d010749_T001 5.00E-112
382- 5:transcript:Zm00001d038725_T001 transcript:Zm00001d010752_T001 2.00E-81
382- 6:transcript:Zm00001d038728_T003 transcript:Zm00001d010755_T001   0
Alignment 383: score=455.0 e_value=1.5e-25 N=11 6&8 minus
383- 0:transcript:Zm00001d038907_T001 transcript:Zm00001d011639_T001 2.00E-159
383- 1:transcript:Zm00001d038908_T004 transcript:Zm00001d011638_T002 2.00E-155
383- 2:transcript:Zm00001d038909_T001 transcript:Zm00001d011636_T001 1.00E-89
383- 3:transcript:Zm00001d038910_T001 transcript:Zm00001d011631_T001 6.00E-95
383- 4:transcript:Zm00001d038911_T001 transcript:Zm00001d011630_T001 3.00E-18
383- 5:transcript:Zm00001d038915_T001 transcript:Zm00001d011625_T001   0
383- 6:transcript:Zm00001d038916_T001 transcript:Zm00001d011622_T004   0
383- 7:transcript:Zm00001d038918_T001 transcript:Zm00001d011620_T001 1.00E-27
383- 8:transcript:Zm00001d038921_T001 transcript:Zm00001d011618_T001 2.00E-126
383- 9:transcript:Zm00001d038926_T001 transcript:Zm00001d011616_T001 2.00E-63
383- 10:transcript:Zm00001d038930_T001 transcript:Zm00001d011614_T001 2.00E-83
Alignment 384: score=377.0 e_value=3.6e-18 N=9 6&8 minus
384- 0:transcript:Zm00001d039004_T002 transcript:Zm00001d011555_T001 1.00E-138
384- 1:transcript:Zm00001d039011_T001 transcript:Zm00001d011543_T001 4.00E-39
384- 2:transcript:Zm00001d039015_T001 transcript:Zm00001d011541_T004 1.00E-80
384- 3:transcript:Zm00001d039016_T001 transcript:Zm00001d011540_T001 6.00E-22
384- 4:transcript:Zm00001d039017_T001 transcript:Zm00001d011537_T001 8.00E-09
384- 5:transcript:Zm00001d039020_T001 transcript:Zm00001d011534_T004 3.00E-121
384- 6:transcript:Zm00001d039032_T001 transcript:Zm00001d011527_T001 2.00E-79
384- 7:transcript:Zm00001d039037_T001 transcript:Zm00001d011525_T002   0
384- 8:transcript:Zm00001d039041_T007 transcript:Zm00001d011517_T005   0
Alignment 385: score=336.0 e_value=2e-15 N=8 6&8 minus
385- 0:transcript:Zm00001d037725_T001 transcript:Zm00001d010020_T001 7.00E-83
385- 1:transcript:Zm00001d037728_T001 transcript:Zm00001d010018_T004   0
385- 2:transcript:Zm00001d037729_T001 transcript:Zm00001d010012_T001   0
385- 3:transcript:Zm00001d037730_T001 transcript:Zm00001d010010_T001 8.00E-08
385- 4:transcript:Zm00001d037732_T002 transcript:Zm00001d010009_T003 2.00E-112
385- 5:transcript:Zm00001d037743_T001 transcript:Zm00001d010001_T001 1.00E-114
385- 6:transcript:Zm00001d037748_T001 transcript:Zm00001d010000_T001 7.00E-72
385- 7:transcript:Zm00001d037749_T001 transcript:Zm00001d009999_T001 9.00E-25
Alignment 386: score=336.0 e_value=1.1e-14 N=8 6&8 minus
386- 0:transcript:Zm00001d038876_T001 transcript:Zm00001d011678_T002 1.00E-26
386- 1:transcript:Zm00001d038878_T001 transcript:Zm00001d011669_T001 2.00E-49
386- 2:transcript:Zm00001d038879_T003 transcript:Zm00001d011668_T001   0
386- 3:transcript:Zm00001d038880_T001 transcript:Zm00001d011663_T003   0
386- 4:transcript:Zm00001d038882_T002 transcript:Zm00001d011660_T003   0
386- 5:transcript:Zm00001d038883_T001 transcript:Zm00001d011655_T001 7.00E-131
386- 6:transcript:Zm00001d038886_T001 transcript:Zm00001d011650_T001 2.00E-21
386- 7:transcript:Zm00001d038891_T001 transcript:Zm00001d011642_T002   0
Alignment 387: score=312.0 e_value=7.3e-13 N=8 6&8 minus
387- 0:transcript:Zm00001d038190_T012 transcript:Zm00001d010282_T001   0
387- 1:transcript:Zm00001d038191_T001 transcript:Zm00001d010280_T001 6.00E-135
387- 2:transcript:Zm00001d038193_T005 transcript:Zm00001d010274_T001   0
387- 3:transcript:Zm00001d038197_T001 transcript:Zm00001d010264_T001 8.00E-91
387- 4:transcript:Zm00001d038200_T002 transcript:Zm00001d010255_T001   0

387- 5:transcript:Zm00001d038205_T002 transcript:Zm00001d010249_T001 0
387- 6:transcript:Zm00001d038209_T001 transcript:Zm00001d010243_T002 0
387- 7:transcript:Zm00001d038218_T001 transcript:Zm00001d010236_T001 8.00E-77
Alignment 388: score=291.0 e_value=7.5e-14 N=7 6&8 minus
388- 0:transcript:Zm00001d038311_T001 transcript:Zm00001d012273_T001 1.00E-37
388- 1:transcript:Zm00001d038312_T001 transcript:Zm00001d012270_T001 4.00E-56
388- 2:transcript:Zm00001d038319_T003 transcript:Zm00001d012269_T003 1.00E-140
388- 3:transcript:Zm00001d038326_T003 transcript:Zm00001d012263_T002 0
388- 4:transcript:Zm00001d038333_T001 transcript:Zm00001d012259_T001 0
388- 5:transcript:Zm00001d038336_T001 transcript:Zm00001d012257_T013 0
388- 6:transcript:Zm00001d038338_T001 transcript:Zm00001d012255_T001 8.00E-111
Alignment 389: score=282.0 e_value=1.5e-10 N=6 6&8 minus
389- 0:transcript:Zm00001d038780_T001 transcript:Zm00001d010798_T001 5.00E-44
389- 1:transcript:Zm00001d038783_T001 transcript:Zm00001d010797_T001 1.00E-86
389- 2:transcript:Zm00001d038792_T001 transcript:Zm00001d010796_T001 0
389- 3:transcript:Zm00001d038793_T001 transcript:Zm00001d010795_T001 1.00E-153
389- 4:transcript:Zm00001d038794_T002 transcript:Zm00001d010793_T002 0
389- 5:transcript:Zm00001d038795_T001 transcript:Zm00001d010792_T001 9.00E-38
Alignment 390: score=257.0 e_value=2.8e-09 N=6 6&8 minus
390- 0:transcript:Zm00001d038783_T001 transcript:Zm00001d012772_T001 4.00E-85
390- 1:transcript:Zm00001d038791_T001 transcript:Zm00001d012769_T001 0
390- 2:transcript:Zm00001d038793_T001 transcript:Zm00001d012767_T001 3.00E-43
390- 3:transcript:Zm00001d038794_T002 transcript:Zm00001d012766_T001 0
390- 4:transcript:Zm00001d038797_T001 transcript:Zm00001d012763_T002 0
390- 5:transcript:Zm00001d038801_T001 transcript:Zm00001d012757_T001 8.00E-97
Alignment 391: score=254.0 e_value=9.9e-09 N=6 6&8 minus
391- 0:transcript:Zm00001d039087_T001 transcript:Zm00001d011477_T001 3.00E-144
391- 1:transcript:Zm00001d039090_T001 transcript:Zm00001d011474_T001 3.00E-156
391- 2:transcript:Zm00001d039101_T001 transcript:Zm00001d011470_T001 6.00E-79
391- 3:transcript:Zm00001d039105_T001 transcript:Zm00001d011465_T001 0
391- 4:transcript:Zm00001d039108_T001 transcript:Zm00001d011463_T001 4.00E-140
391- 5:transcript:Zm00001d039112_T001 transcript:Zm00001d011451_T001 9.00E-44
Alignment 392: score=252.0 e_value=3.1e-10 N=6 6&8 minus
392- 0:transcript:Zm00001d038248_T002 transcript:Zm00001d010213_T001 2.00E-94
392- 1:transcript:Zm00001d038251_T001 transcript:Zm00001d010211_T002 3.00E-121
392- 2:transcript:Zm00001d038252_T001 transcript:Zm00001d010209_T001 2.00E-69
392- 3:transcript:Zm00001d038257_T018 transcript:Zm00001d010207_T001 2.00E-53
392- 4:transcript:Zm00001d038258_T001 transcript:Zm00001d010206_T001 7.00E-147
392- 5:transcript:Zm00001d038262_T001 transcript:Zm00001d010205_T002 0
Alignment 393: score=524.0 e_value=1e-30 N=12 6&9 plus
393- 0:transcript:Zm00001d036768_T001 transcript:Zm00001d046632_T001 9.00E-126
393- 1:transcript:Zm00001d036769_T001 transcript:Zm00001d046641_T001 2.00E-40
393- 2:transcript:Zm00001d036770_T001 transcript:Zm00001d046642_T001 0
393- 3:transcript:Zm00001d036771_T001 transcript:Zm00001d046643_T001 0
393- 4:transcript:Zm00001d036775_T001 transcript:Zm00001d046655_T001 0
393- 5:transcript:Zm00001d036777_T001 transcript:Zm00001d046656_T004 0
393- 6:transcript:Zm00001d036778_T001 transcript:Zm00001d046659_T001 3.00E-26
393- 7:transcript:Zm00001d036780_T001 transcript:Zm00001d046667_T001 2.00E-19
393- 8:transcript:Zm00001d036781_T001 transcript:Zm00001d046673_T001 2.00E-37
393- 9:transcript:Zm00001d036784_T002 transcript:Zm00001d046679_T002 0
393- 10:transcript:Zm00001d036785_T003 transcript:Zm00001d046683_T002 0
393- 11:transcript:Zm00001d036787_T001 transcript:Zm00001d046687_T001 5.00E-52
Alignment 394: score=500.0 e_value=1.9e-27 N=11 6&9 plus
394- 0:transcript:Zm00001d036676_T001 transcript:Zm00001d046888_T001 5.00E-68

394- 1:transcript:Zm00001d036678_T002 transcript:Zm00001d046889_T001 0
394- 2:transcript:Zm00001d036679_T001 transcript:Zm00001d046890_T001 9.00E-165
394- 3:transcript:Zm00001d036683_T001 transcript:Zm00001d046896_T001 6.00E-31
394- 4:transcript:Zm00001d036692_T001 transcript:Zm00001d046906_T001 3.00E-162
394- 5:transcript:Zm00001d036699_T001 transcript:Zm00001d046914_T001 1.00E-121
394- 6:transcript:Zm00001d036700_T001 transcript:Zm00001d046916_T001 7.00E-60
394- 7:transcript:Zm00001d036703_T001 transcript:Zm00001d046927_T001 2.00E-44
394- 8:transcript:Zm00001d036704_T002 transcript:Zm00001d046928_T002 0
394- 9:transcript:Zm00001d036708_T001 transcript:Zm00001d046929_T002 5.00E-43
394- 10:transcript:Zm00001d036709_T001 transcript:Zm00001d046930_T001 0
Alignment 395: score=424.0 e_value=3.8e-22 N=10 6&9 plus
395- 0:transcript:Zm00001d036742_T007 transcript:Zm00001d046590_T004 0
395- 1:transcript:Zm00001d036748_T001 transcript:Zm00001d046592_T001 1.00E-121
395- 2:transcript:Zm00001d036749_T001 transcript:Zm00001d046595_T002 0
395- 3:transcript:Zm00001d036752_T001 transcript:Zm00001d046599_T001 1.00E-29
395- 4:transcript:Zm00001d036756_T001 transcript:Zm00001d046600_T001 2.00E-67
395- 5:transcript:Zm00001d036759_T002 transcript:Zm00001d046601_T005 0
395- 6:transcript:Zm00001d036760_T001 transcript:Zm00001d046602_T001 2.00E-173
395- 7:transcript:Zm00001d036762_T001 transcript:Zm00001d046603_T001 3.00E-178
395- 8:transcript:Zm00001d036765_T009 transcript:Zm00001d046624_T005 0
395- 9:transcript:Zm00001d036766_T001 transcript:Zm00001d046626_T001 2.00E-87
Alignment 396: score=404.0 e_value=6.1e-21 N=10 6&9 plus
396- 0:transcript:Zm00001d036977_T001 transcript:Zm00001d046255_T035 0
396- 1:transcript:Zm00001d036984_T001 transcript:Zm00001d046263_T001 0
396- 2:transcript:Zm00001d036986_T002 transcript:Zm00001d046277_T008 0
396- 3:transcript:Zm00001d036989_T001 transcript:Zm00001d046286_T001 0
396- 4:transcript:Zm00001d036991_T001 transcript:Zm00001d046288_T001 2.00E-90
396- 5:transcript:Zm00001d036996_T001 transcript:Zm00001d046292_T001 3.00E-37
396- 6:transcript:Zm00001d037000_T002 transcript:Zm00001d046297_T001 2.00E-62
396- 7:transcript:Zm00001d037003_T001 transcript:Zm00001d046311_T001 7.00E-32
396- 8:transcript:Zm00001d037004_T002 transcript:Zm00001d046313_T001 2.00E-98
396- 9:transcript:Zm00001d037005_T001 transcript:Zm00001d046318_T001 0
Alignment 397: score=298.0 e_value=1.8e-14 N=8 6&9 plus
397- 0:transcript:Zm00001d036789_T003 transcript:Zm00001d046716_T001 1.00E-149
397- 1:transcript:Zm00001d036790_T009 transcript:Zm00001d046719_T006 0
397- 2:transcript:Zm00001d036791_T001 transcript:Zm00001d046728_T001 5.00E-20
397- 3:transcript:Zm00001d036796_T004 transcript:Zm00001d046740_T001 3.00E-151
397- 4:transcript:Zm00001d036801_T001 transcript:Zm00001d046749_T001 0
397- 5:transcript:Zm00001d036802_T001 transcript:Zm00001d046755_T001 2.00E-103
397- 6:transcript:Zm00001d036807_T001 transcript:Zm00001d046759_T001 7.00E-178
397- 7:transcript:Zm00001d036812_T001 transcript:Zm00001d046774_T001 0
Alignment 398: score=533.0 e_value=2.1e-35 N=13 6&9 minus
398- 0:transcript:Zm00001d036195_T004 transcript:Zm00001d045339_T002 1.00E-103
398- 1:transcript:Zm00001d036197_T001 transcript:Zm00001d045338_T001 1.00E-161
398- 2:transcript:Zm00001d036198_T001 transcript:Zm00001d045336_T001 3.00E-21
398- 3:transcript:Zm00001d036199_T001 transcript:Zm00001d045335_T003 1.00E-39
398- 4:transcript:Zm00001d036202_T001 transcript:Zm00001d045334_T001 8.00E-59
398- 5:transcript:Zm00001d036206_T001 transcript:Zm00001d045326_T004 0
398- 6:transcript:Zm00001d036214_T001 transcript:Zm00001d045323_T004 0
398- 7:transcript:Zm00001d036216_T001 transcript:Zm00001d045313_T001 0
398- 8:transcript:Zm00001d036228_T001 transcript:Zm00001d045309_T001 9.00E-131
398- 9:transcript:Zm00001d036237_T002 transcript:Zm00001d045298_T002 2.00E-58
398- 10:transcript:Zm00001d036240_T003 transcript:Zm00001d045297_T001 1.00E-167
398- 11:transcript:Zm00001d036244_T001 transcript:Zm00001d045283_T001 4.00E-124

398- 12:transcript:Zm00001d036246_T001 transcript:Zm00001d045270_T001 3.00E-120
Alignment 399: score=524.0 e_value=2.1e-29 N=13 6&9 minus
399- 0:transcript:Zm00001d037218_T001 transcript:Zm00001d044841_T001 9.00E-164
399- 1:transcript:Zm00001d037220_T001 transcript:Zm00001d044838_T002 7.00E-120
399- 2:transcript:Zm00001d037221_T001 transcript:Zm00001d044836_T001 6.00E-124
399- 3:transcript:Zm00001d037225_T001 transcript:Zm00001d044833_T001 0
399- 4:transcript:Zm00001d037227_T002 transcript:Zm00001d044831_T001 0
399- 5:transcript:Zm00001d037228_T001 transcript:Zm00001d044826_T001 0
399- 6:transcript:Zm00001d037232_T001 transcript:Zm00001d044824_T001 5.00E-68
399- 7:transcript:Zm00001d037233_T002 transcript:Zm00001d044823_T011 0
399- 8:transcript:Zm00001d037236_T001 transcript:Zm00001d044815_T002 2.00E-174
399- 9:transcript:Zm00001d037244_T001 transcript:Zm00001d044808_T001 3.00E-51
399- 10:transcript:Zm00001d037247_T007 transcript:Zm00001d044803_T001 2.00E-164
399- 11:transcript:Zm00001d037249_T001 transcript:Zm00001d044784_T001 0
399- 12:transcript:Zm00001d037251_T001 transcript:Zm00001d044783_T001 4.00E-58
Alignment 400: score=437.0 e_value=2.8e-22 N=10 6&9 minus
400- 0:transcript:Zm00001d036086_T002 transcript:Zm00001d045431_T001 0
400- 1:transcript:Zm00001d036088_T001 transcript:Zm00001d045427_T001 2.00E-91
400- 2:transcript:Zm00001d036090_T003 transcript:Zm00001d045425_T001 0
400- 3:transcript:Zm00001d036091_T001 transcript:Zm00001d045420_T001 0
400- 4:transcript:Zm00001d036103_T001 transcript:Zm00001d045406_T001 4.00E-108
400- 5:transcript:Zm00001d036107_T001 transcript:Zm00001d045404_T001 4.00E-157
400- 6:transcript:Zm00001d036108_T001 transcript:Zm00001d045403_T001 3.00E-177
400- 7:transcript:Zm00001d036118_T001 transcript:Zm00001d045398_T001 1.00E-51
400- 8:transcript:Zm00001d036125_T001 transcript:Zm00001d045390_T002 4.00E-53
400- 9:transcript:Zm00001d036135_T003 transcript:Zm00001d045384_T003 2.00E-118
Alignment 401: score=377.0 e_value=8.2e-19 N=9 6&9 minus
401- 0:transcript:Zm00001d036013_T002 transcript:Zm00001d045505_T003 0
401- 1:transcript:Zm00001d036014_T001 transcript:Zm00001d045499_T001 0
401- 2:transcript:Zm00001d036016_T001 transcript:Zm00001d045498_T001 9.00E-83
401- 3:transcript:Zm00001d036018_T001 transcript:Zm00001d045497_T001 9.00E-16
401- 4:transcript:Zm00001d036020_T002 transcript:Zm00001d045491_T003 0
401- 5:transcript:Zm00001d036022_T001 transcript:Zm00001d045487_T001 1.00E-70
401- 6:transcript:Zm00001d036023_T001 transcript:Zm00001d045486_T001 8.00E-37
401- 7:transcript:Zm00001d036025_T002 transcript:Zm00001d045483_T002 6.00E-37
401- 8:transcript:Zm00001d036031_T015 transcript:Zm00001d045482_T017 0
Alignment 402: score=334.0 e_value=6.9e-17 N=9 6&9 minus
402- 0:transcript:Zm00001d036264_T001 transcript:Zm00001d045238_T001 3.00E-17
402- 1:transcript:Zm00001d036279_T001 transcript:Zm00001d045231_T002 2.00E-27
402- 2:transcript:Zm00001d036283_T002 transcript:Zm00001d045220_T003 0
402- 3:transcript:Zm00001d036285_T001 transcript:Zm00001d045213_T002 0
402- 4:transcript:Zm00001d036293_T002 transcript:Zm00001d045206_T003 6.00E-164
402- 5:transcript:Zm00001d036298_T001 transcript:Zm00001d045204_T001 2.00E-68
402- 6:transcript:Zm00001d036300_T001 transcript:Zm00001d045202_T002 0
402- 7:transcript:Zm00001d036301_T007 transcript:Zm00001d045199_T003 0
402- 8:transcript:Zm00001d036305_T003 transcript:Zm00001d045195_T012 0
Alignment 403: score=320.0 e_value=2.7e-14 N=7 6&9 minus
403- 0:transcript:Zm00001d037158_T001 transcript:Zm00001d044953_T001 3.00E-162
403- 1:transcript:Zm00001d037160_T002 transcript:Zm00001d044951_T001 0
403- 2:transcript:Zm00001d037165_T001 transcript:Zm00001d044950_T001 1.00E-54
403- 3:transcript:Zm00001d037170_T001 transcript:Zm00001d044940_T004 0
403- 4:transcript:Zm00001d037171_T001 transcript:Zm00001d044934_T001 2.00E-25
403- 5:transcript:Zm00001d037173_T001 transcript:Zm00001d044930_T001 2.00E-55
403- 6:transcript:Zm00001d037174_T003 transcript:Zm00001d044929_T002 0

Alignment 404: score=314.0 e_value=7.8e-15 N=8 6&9 minus
404- 0:transcript:Zm00001d036632_T002 transcript:Zm00001d046974_T002 2.00E-160
404- 1:transcript:Zm00001d036646_T002 transcript:Zm00001d046968_T001 6.00E-88
404- 2:transcript:Zm00001d036648_T001 transcript:Zm00001d046967_T001 2.00E-140
404- 3:transcript:Zm00001d036650_T001 transcript:Zm00001d046961_T001 4.00E-67
404- 4:transcript:Zm00001d036653_T001 transcript:Zm00001d046950_T001 0
404- 5:transcript:Zm00001d036654_T002 transcript:Zm00001d046948_T002 0
404- 6:transcript:Zm00001d036656_T003 transcript:Zm00001d046939_T001 0
404- 7:transcript:Zm00001d036668_T002 transcript:Zm00001d046936_T002 0
Alignment 405: score=290.0 e_value=1.7e-10 N=7 6&9 minus
405- 0:transcript:Zm00001d036877_T001 transcript:Zm00001d046535_T001 4.00E-163
405- 1:transcript:Zm00001d036878_T007 transcript:Zm00001d046531_T007 0
405- 2:transcript:Zm00001d036883_T001 transcript:Zm00001d046517_T001 1.00E-72
405- 3:transcript:Zm00001d036889_T001 transcript:Zm00001d046501_T001 2.00E-88
405- 4:transcript:Zm00001d036892_T001 transcript:Zm00001d046499_T001 2.00E-165
405- 5:transcript:Zm00001d036893_T001 transcript:Zm00001d046496_T001 9.00E-79
405- 6:transcript:Zm00001d036894_T001 transcript:Zm00001d046492_T001 5.00E-174
Alignment 406: score=282.0 e_value=5.7e-11 N=7 6&9 minus
406- 0:transcript:Zm00001d037191_T002 transcript:Zm00001d044880_T001 5.00E-107
406- 1:transcript:Zm00001d037197_T001 transcript:Zm00001d044875_T001 4.00E-52
406- 2:transcript:Zm00001d037200_T004 transcript:Zm00001d044869_T001 1.00E-112
406- 3:transcript:Zm00001d037204_T001 transcript:Zm00001d044864_T001 4.00E-93
406- 4:transcript:Zm00001d037210_T001 transcript:Zm00001d044861_T001 4.00E-22
406- 5:transcript:Zm00001d037212_T001 transcript:Zm00001d044850_T001 1.00E-119
406- 6:transcript:Zm00001d037213_T001 transcript:Zm00001d044845_T001 6.00E-26
Alignment 407: score=264.0 e_value=1.6e-10 N=6 6&9 minus
407- 0:transcript:Zm00001d036710_T002 transcript:Zm00001d046828_T003 0
407- 1:transcript:Zm00001d036711_T001 transcript:Zm00001d046827_T002 1.00E-99
407- 2:transcript:Zm00001d036716_T001 transcript:Zm00001d046823_T001 0
407- 3:transcript:Zm00001d036723_T001 transcript:Zm00001d046810_T001 5.00E-39
407- 4:transcript:Zm00001d036726_T001 transcript:Zm00001d046805_T001 1.00E-140
407- 5:transcript:Zm00001d036727_T001 transcript:Zm00001d046794_T001 2.00E-76
Alignment 408: score=252.0 e_value=1.2e-10 N=6 6&9 minus
408- 0:transcript:Zm00001d037136_T001 transcript:Zm00001d044980_T001 3.00E-112
408- 1:transcript:Zm00001d037140_T004 transcript:Zm00001d044973_T001 0
408- 2:transcript:Zm00001d037141_T001 transcript:Zm00001d044972_T001 1.00E-51
408- 3:transcript:Zm00001d037142_T022 transcript:Zm00001d044971_T005 0
408- 4:transcript:Zm00001d037150_T001 transcript:Zm00001d044970_T001 2.00E-138
408- 5:transcript:Zm00001d037153_T002 transcript:Zm00001d044966_T001 1.00E-83
Alignment 409: score=252.0 e_value=6.3e-10 N=6 6&9 minus
409- 0:transcript:Zm00001d035987_T001 transcript:Zm00001d045522_T001 9.00E-120
409- 1:transcript:Zm00001d035988_T003 transcript:Zm00001d045521_T001 3.00E-24
409- 2:transcript:Zm00001d035989_T001 transcript:Zm00001d045517_T001 2.00E-69
409- 3:transcript:Zm00001d035990_T002 transcript:Zm00001d045516_T010 0
409- 4:transcript:Zm00001d035992_T001 transcript:Zm00001d045515_T001 0
409- 5:transcript:Zm00001d035999_T002 transcript:Zm00001d045513_T001 3.00E-147
Alignment 410: score=307.0 e_value=7.4e-14 N=7 7&9 plus
410- 0:transcript:Zm00001d022242_T001 transcript:Zm00001d047399_T001 3.00E-48
410- 1:transcript:Zm00001d022243_T002 transcript:Zm00001d047401_T001 3.00E-73
410- 2:transcript:Zm00001d022245_T001 transcript:Zm00001d047402_T001 0
410- 3:transcript:Zm00001d022250_T001 transcript:Zm00001d047403_T001 2.00E-95
410- 4:transcript:Zm00001d022252_T001 transcript:Zm00001d047404_T001 5.00E-62
410- 5:transcript:Zm00001d022264_T001 transcript:Zm00001d047419_T001 3.00E-20
410- 6:transcript:Zm00001d022266_T001 transcript:Zm00001d047421_T001 9.00E-96

Alignment 411: score=451.0 e_value=1.2e-22 N=10 8&8 plus
411- 0: transcript:Zm00001d010576_T002 transcript:Zm00001d012451_T004 6.00E-105
411- 1: transcript:Zm00001d010578_T003 transcript:Zm00001d012452_T004 0
411- 2: transcript:Zm00001d010579_T001 transcript:Zm00001d012457_T003 0
411- 3: transcript:Zm00001d010583_T001 transcript:Zm00001d012458_T001 7.00E-93
411- 4: transcript:Zm00001d010589_T001 transcript:Zm00001d012463_T001 1.00E-159
411- 5: transcript:Zm00001d010590_T001 transcript:Zm00001d012467_T001 0
411- 6: transcript:Zm00001d010592_T001 transcript:Zm00001d012468_T001 1.00E-08
411- 7: transcript:Zm00001d010594_T001 transcript:Zm00001d012471_T001 2.00E-47
411- 8: transcript:Zm00001d010596_T001 transcript:Zm00001d012473_T001 7.00E-89
411- 9: transcript:Zm00001d010604_T006 transcript:Zm00001d012479_T004 5.00E-12
Alignment 412: score=273.0 e_value=9.6e-09 N=6 8&8 plus
412- 0: transcript:Zm00001d010618_T003 transcript:Zm00001d012513_T001 8.00E-108
412- 1: transcript:Zm00001d010620_T001 transcript:Zm00001d012515_T004 0
412- 2: transcript:Zm00001d010622_T002 transcript:Zm00001d012517_T001 9.00E-126
412- 3: transcript:Zm00001d010627_T001 transcript:Zm00001d012520_T001 2.00E-65
412- 4: transcript:Zm00001d010629_T001 transcript:Zm00001d012521_T001 0
412- 5: transcript:Zm00001d010630_T003 transcript:Zm00001d012524_T001 0
Alignment 413: score=400.0 e_value=7.3e-20 N=10 8&8 minus
413- 0: transcript:Zm00001d010159_T002 transcript:Zm00001d012277_T003 0
413- 1: transcript:Zm00001d010168_T001 transcript:Zm00001d012273_T001 2.00E-14
413- 2: transcript:Zm00001d010174_T004 transcript:Zm00001d012269_T003 3.00E-144
413- 3: transcript:Zm00001d010179_T001 transcript:Zm00001d012268_T001 0
413- 4: transcript:Zm00001d010195_T001 transcript:Zm00001d012259_T001 0
413- 5: transcript:Zm00001d010199_T002 transcript:Zm00001d012257_T013 0
413- 6: transcript:Zm00001d010201_T001 transcript:Zm00001d012255_T001 7.00E-87
413- 7: transcript:Zm00001d010205_T002 transcript:Zm00001d012254_T002 0
413- 8: transcript:Zm00001d010211_T002 transcript:Zm00001d012245_T002 2.00E-89
413- 9: transcript:Zm00001d010213_T001 transcript:Zm00001d012239_T003 6.00E-53
Alignment 414: score=290.0 e_value=1.2e-13 N=7 8&8 minus
414- 0: transcript:Zm00001d009568_T001 transcript:Zm00001d011543_T001 3.00E-44
414- 1: transcript:Zm00001d009571_T002 transcript:Zm00001d011541_T004 1.00E-137
414- 2: transcript:Zm00001d009572_T001 transcript:Zm00001d011540_T001 7.00E-23
414- 3: transcript:Zm00001d009578_T001 transcript:Zm00001d011536_T001 3.00E-57
414- 4: transcript:Zm00001d009579_T001 transcript:Zm00001d011534_T004 5.00E-127
414- 5: transcript:Zm00001d009587_T002 transcript:Zm00001d011525_T002 0
414- 6: transcript:Zm00001d009591_T006 transcript:Zm00001d011517_T005 0
Alignment 415: score=257.0 e_value=2.8e-09 N=6 8&8 minus
415- 0: transcript:Zm00001d009421_T001 transcript:Zm00001d011688_T004 9.00E-50
415- 1: transcript:Zm00001d009425_T001 transcript:Zm00001d011681_T001 2.00E-14
415- 2: transcript:Zm00001d009431_T001 transcript:Zm00001d011673_T001 0
415- 3: transcript:Zm00001d009435_T001 transcript:Zm00001d011669_T001 1.00E-56
415- 4: transcript:Zm00001d009436_T003 transcript:Zm00001d011668_T001 0
415- 5: transcript:Zm00001d009439_T004 transcript:Zm00001d011663_T003 0
