# Supplementary material for: Genome-wide identification, transcriptome analysis and alternative splicing events of Hsf family genes in maize
Source: Sci Rep. 2020 May 15;10:8073. doi: 10.1038/s41598-020-65068-z (PMC7229205; doi:10.1038/s41598-020-65068-z)
Supplement: Supplementary file 6 — Supplementary Information. [file 41598_2020_65068_MOESM6_ESM.pdf]

Genome-wide identification, transcriptome analysis and alternative splicing events of Hsf family genes in maize

Huaning Zhang [1,2], Guoliang Li [1,2], Cai Fu[1], Shuonan Duan [1], Dong Hu[1, ✉] & Xiulin Guo [1, ✉]

[1] Plant Genetic Engineering Center of Hebei Province/Institute of Genetics and Physiology, Hebei Academy of Agriculture and Forestry Sciences, Shijiazhuang 050051, P.R. China

[2] These authors contributed equally: Huaning Zhang and Guoliang Li.

✉ e-mail: myhf2002@163.com, donghu1983@163.com.

Table S4 Analysis of collinearity of ZmHsfs and SbHsfs proteins.

```
############### Parameters ###############
# MATCH_SCORE: 50
# MATCH_SIZE: 5
# GAP_PENALTY: -1
# OVERLAP_WINDOW: 5
# E_VALUE: 1e-05
# MAX GAPS: 25
############### Statistics ###############
# Number of collinear genes: 33153, Percentage: 20.57
# Number of all genes: 161133
##########################################
## Alignment 0: score=324.0 e_value=2.9e-14 N=8 1&NC_008394.4 plus
  0-  0:  transcript:Zm00001d031561_T002  rna3837      1.00E-85
  0-  1:  transcript:Zm00001d031569_T001  rna3838      3.00E-29
  0-  2:  transcript:Zm00001d031586_T001  rna3854      2.00E-23
  0-  3:  transcript:Zm00001d031594_T001  rna3866      7.00E-81
  0-  4:  transcript:Zm00001d031600_T001  rna3868      5.00E-83
  0-  5:  transcript:Zm00001d031607_T001  rna3874      1.00E-88
  0-  6:  transcript:Zm00001d031614_T001  rna3879     5.00E-138
  0-  7:  transcript:Zm00001d031625_T001  rna3887      1.00E-25
## Alignment 1: score=253.0 e_value=2.1e-09 N=6 1&NC_008395.2 plus
  1-  0:  transcript:Zm00001d029734_T001  rna6704             0
  1-  1:  transcript:Zm00001d029749_T001  rna6714      4.00E-57
  1-  2:  transcript:Zm00001d029753_T001  rna6716     8.00E-129
  1-  3:  transcript:Zm00001d029754_T004  rna6719      5.00E-56
  1-  4:  transcript:Zm00001d029758_T004  rna6728             0
  1-  5:  transcript:Zm00001d029763_T001  rna6729      5.00E-35
## Alignment 2: score=304.0 e_value=5.1e-12 N=7 1&NC_008395.2 minus
  2-  0:  transcript:Zm00001d029917_T001  rna6597      5.00E-36
  2-  1:  transcript:Zm00001d029937_T001  rna6575      3.00E-07
  2-  2:  transcript:Zm00001d029940_T001  rna6567      2.00E-28
  2-  3:  transcript:Zm00001d029946_T003  rna6566     4.00E-104
  2-  4:  transcript:Zm00001d029948_T001  rna6563             0
  2-  5:  transcript:Zm00001d029955_T001  rna6560             0
  2-  6:  transcript:Zm00001d029970_T001  rna6549      5.00E-71
## Alignment 3: score=5919.0 e_value=0 N=131 1&NC_008396.2 plus
  3-  0:  transcript:Zm00001d028481_T017  rna8723             0
  3-  1:  transcript:Zm00001d028486_T001  rna8744      7.00E-51
  3-  2:  transcript:Zm00001d028490_T001  rna8745             0
  3-  3:  transcript:Zm00001d028493_T002  rna8749             0
  3-  4:  transcript:Zm00001d028495_T001  rna8751             0
  3-  5:  transcript:Zm00001d028503_T001  rna8753      3.00E-24
  3-  6:  transcript:Zm00001d028504_T001  rna8754     1.00E-140
  3-  7:  transcript:Zm00001d028505_T001  rna8755      9.00E-79
  3-  8:  transcript:Zm00001d028509_T001  rna8756     1.00E-167
  3-  9:  transcript:Zm00001d028510_T001  rna8757      2.00E-95
  3- 10:  transcript:Zm00001d028511_T028  rna8758      1.00E-57
  3- 11:  transcript:Zm00001d028512_T001  rna8759             0
  3- 12:  transcript:Zm00001d028513_T001  rna8760     2.00E-114
  3- 13:  transcript:Zm00001d028514_T002  rna8761      3.00E-28
  3- 14:  transcript:Zm00001d028515_T001  rna8763     2.00E-130
```

3- 15:	transcript:Zm00001d028522_T001	rna8765	3.00E-111
3- 16:	transcript:Zm00001d028524_T001	rna8767	1.00E-64
3- 17:	transcript:Zm00001d028528_T001	rna8768	4.00E-155
3- 18:	transcript:Zm00001d028529_T001	rna8769	8.00E-144
3- 19:	transcript:Zm00001d028531_T001	rna8771	4.00E-43
3- 20:	transcript:Zm00001d028532_T001	rna8772	0
3- 21:	transcript:Zm00001d028533_T001	rna8773	0
3- 22:	transcript:Zm00001d028534_T005	rna8774	4.00E-175
3- 23:	transcript:Zm00001d028535_T001	rna8775	6.00E-50
3- 24:	transcript:Zm00001d028536_T001	rna8776	0
3- 25:	transcript:Zm00001d028537_T001	rna8777	0
3- 26:	transcript:Zm00001d028538_T003	rna8779	0
3- 27:	transcript:Zm00001d028539_T001	rna8780	0
3- 28:	transcript:Zm00001d028540_T001	rna8781	3.00E-108
3- 29:	transcript:Zm00001d028542_T001	rna8782	0
3- 30:	transcript:Zm00001d028543_T001	rna8784	0
3- 31:	transcript:Zm00001d028546_T032	rna8786	0
3- 32:	transcript:Zm00001d028547_T001	rna8787	8.00E-65
3- 33:	transcript:Zm00001d028548_T001	rna8788	2.00E-85
3- 34:	transcript:Zm00001d028549_T005	rna8789	1.00E-72
3- 35:	transcript:Zm00001d028550_T001	rna8790	1.00E-123
3- 36:	transcript:Zm00001d028554_T003	rna8791	7.00E-37
3- 37:	transcript:Zm00001d028555_T001	rna8792	2.00E-92
3- 38:	transcript:Zm00001d028558_T003	rna8793	0
3- 39:	transcript:Zm00001d028560_T020	rna8794	0
3- 40:	transcript:Zm00001d028562_T001	rna8799	0
3- 41:	transcript:Zm00001d028565_T002	rna8800	0
3- 42:	transcript:Zm00001d028566_T003	rna8801	0
3- 43:	transcript:Zm00001d028567_T001	rna8803	0
3- 44:	transcript:Zm00001d028568_T004	rna8804	1.00E-180
3- 45:	transcript:Zm00001d028569_T002	rna8805	5.00E-114
3- 46:	transcript:Zm00001d028570_T002	rna8806	4.00E-75
3- 47:	transcript:Zm00001d028571_T001	rna8807	0
3- 48:	transcript:Zm00001d028572_T004	rna8808	0
3- 49:	transcript:Zm00001d028574_T001	rna8809	0
3- 50:	transcript:Zm00001d028575_T001	rna8810	2.00E-164
3- 51:	transcript:Zm00001d028576_T001	rna8815	1.00E-63
3- 52:	transcript:Zm00001d028577_T001	rna8818	1.00E-118
3- 53:	transcript:Zm00001d028579_T027	rna8819	2.00E-89
3- 54:	transcript:Zm00001d028580_T001	rna8820	1.00E-16
3- 55:	transcript:Zm00001d028584_T001	rna8824	1.00E-37
3- 56:	transcript:Zm00001d028585_T001	rna8825	0
3- 57:	transcript:Zm00001d028586_T001	rna8826	0
3- 58:	transcript:Zm00001d028587_T001	rna8827	0
3- 59:	transcript:Zm00001d028588_T001	rna8828	0
3- 60:	transcript:Zm00001d028590_T001	rna8830	2.00E-175
3- 61:	transcript:Zm00001d028591_T001	rna8832	0
3- 62:	transcript:Zm00001d028593_T002	rna8833	1.00E-136
3- 63:	transcript:Zm00001d028596_T001	rna8834	3.00E-83
3- 64:	transcript:Zm00001d028598_T001	rna8835	4.00E-38
3- 65:	transcript:Zm00001d028599_T001	rna8836	0
3- 66:	transcript:Zm00001d028601_T002	rna8837	0
3- 67:	transcript:Zm00001d028606_T003	rna8838	0
3- 68:	transcript:Zm00001d028608_T006	rna8839	4.00E-69

3- 69:	transcript:Zm00001d028612_T001	rna8841	5.00E-141
3- 70:	transcript:Zm00001d028613_T001	rna8842	0
3- 71:	transcript:Zm00001d028615_T001	rna8843	0
3- 72:	transcript:Zm00001d028616_T001	rna8844	1.00E-42
3- 73:	transcript:Zm00001d028619_T007	rna8845	1.00E-161
3- 74:	transcript:Zm00001d028620_T001	rna8846	0
3- 75:	transcript:Zm00001d028623_T001	rna8849	0
3- 76:	transcript:Zm00001d028630_T005	rna8850	0
3- 77:	transcript:Zm00001d028631_T001	rna8851	1.00E-149
3- 78:	transcript:Zm00001d028641_T003	rna8852	8.00E-46
3- 79:	transcript:Zm00001d028642_T001	rna8854	3.00E-111
3- 80:	transcript:Zm00001d028643_T001	rna8856	2.00E-164
3- 81:	transcript:Zm00001d028647_T002	rna8858	1.00E-67
3- 82:	transcript:Zm00001d028651_T001	rna8859	1.00E-141
3- 83:	transcript:Zm00001d028653_T005	rna8861	0
3- 84:	transcript:Zm00001d028655_T001	rna8863	2.00E-96
3- 85:	transcript:Zm00001d028656_T001	rna8864	0
3- 86:	transcript:Zm00001d028661_T001	rna8867	3.00E-84
3- 87:	transcript:Zm00001d028662_T001	rna8871	0
3- 88:	transcript:Zm00001d028664_T001	rna8872	4.00E-56
3- 89:	transcript:Zm00001d028665_T001	rna8873	2.00E-103
3- 90:	transcript:Zm00001d028667_T001	rna8874	7.00E-67
3- 91:	transcript:Zm00001d028668_T001	rna8876	0
3- 92:	transcript:Zm00001d028670_T001	rna8877	2.00E-140
3- 93:	transcript:Zm00001d028671_T002	rna8878	0
3- 94:	transcript:Zm00001d028675_T001	rna8879	1.00E-38
3- 95:	transcript:Zm00001d028679_T002	rna8884	0
3- 96:	transcript:Zm00001d028685_T001	rna8886	3.00E-69
3- 97:	transcript:Zm00001d028686_T001	rna8889	0
3- 98:	transcript:Zm00001d028687_T001	rna8890	0
3- 99:	transcript:Zm00001d028689_T001	rna8892	0
3-100:	transcript:Zm00001d028690_T007	rna8894	4.00E-152
3-101:	transcript:Zm00001d028691_T004	rna8896	2.00E-112
3-102:	transcript:Zm00001d028692_T004	rna8898	1.00E-75
3-103:	transcript:Zm00001d028695_T002	rna8901	4.00E-52
3-104:	transcript:Zm00001d028696_T001	rna8902	3.00E-56
3-105:	transcript:Zm00001d028697_T001	rna8903	0
3-106:	transcript:Zm00001d028698_T002	rna8904	8.00E-141
3-107:	transcript:Zm00001d028699_T026	rna8906	1.00E-89
3-108:	transcript:Zm00001d028701_T001	rna8907	8.00E-147
3-109:	transcript:Zm00001d028702_T001	rna8910	3.00E-134
3-110:	transcript:Zm00001d028704_T003	rna8912	3.00E-123
3-111:	transcript:Zm00001d028705_T001	rna8914	0
3-112:	transcript:Zm00001d028707_T003	rna8917	8.00E-68
3-113:	transcript:Zm00001d028709_T002	rna8920	1.00E-159
3-114:	transcript:Zm00001d028711_T006	rna8921	0
3-115:	transcript:Zm00001d028712_T001	rna8923	0
3-116:	transcript:Zm00001d028713_T001	rna8924	0
3-117:	transcript:Zm00001d028714_T001	rna8925	6.00E-145
3-118:	transcript:Zm00001d028715_T001	rna8926	0
3-119:	transcript:Zm00001d028718_T001	rna8927	5.00E-29
3-120:	transcript:Zm00001d028720_T001	rna8928	6.00E-114
3-121:	transcript:Zm00001d028721_T001	rna8929	3.00E-86
3-122:	transcript:Zm00001d028725_T001	rna8931	1.00E-178

3-123:	transcript:Zm00001d028726_T001	rna8932	4.00E-80
3-124:	transcript:Zm00001d028727_T001	rna8935	0
3-125:	transcript:Zm00001d028728_T001	rna8937	4.00E-57
3-126:	transcript:Zm00001d028730_T003	rna8938	0
3-127:	transcript:Zm00001d028731_T001	rna8939	2.00E-149
3-128:	transcript:Zm00001d028733_T002	rna8942	0
3-129:	transcript:Zm00001d028736_T001	rna8943	9.00E-145
3-130:	transcript:Zm00001d028742_T001	rna8948	0

Alignment 4: score=5545.0 e_value=0 N=119 1&NC_008396.2 plus

4- 0:	transcript:Zm00001d027415_T001	rna7863	0
4- 1:	transcript:Zm00001d027421_T001	rna7864	1.00E-113
4- 2:	transcript:Zm00001d027423_T002	rna7865	0
4- 3:	transcript:Zm00001d027425_T001	rna7867	3.00E-115
4- 4:	transcript:Zm00001d027427_T002	rna7869	3.00E-115
4- 5:	transcript:Zm00001d027429_T001	rna7870	4.00E-105
4- 6:	transcript:Zm00001d027430_T001	rna7871	0
4- 7:	transcript:Zm00001d027431_T001	rna7872	0
4- 8:	transcript:Zm00001d027434_T001	rna7874	4.00E-82
4- 9:	transcript:Zm00001d027435_T002	rna7875	0
4- 10:	transcript:Zm00001d027436_T001	rna7878	0
4- 11:	transcript:Zm00001d027439_T001	rna7879	0
4- 12:	transcript:Zm00001d027440_T001	rna7880	5.00E-43
4- 13:	transcript:Zm00001d027441_T001	rna7882	0
4- 14:	transcript:Zm00001d027442_T001	rna7883	2.00E-174
4- 15:	transcript:Zm00001d027443_T001	rna7884	6.00E-145
4- 16:	transcript:Zm00001d027444_T003	rna7886	0
4- 17:	transcript:Zm00001d027445_T002	rna7887	0
4- 18:	transcript:Zm00001d027446_T001	rna7888	4.00E-80
4- 19:	transcript:Zm00001d027447_T006	rna7889	3.00E-22
4- 20:	transcript:Zm00001d027448_T001	rna7890	0
4- 21:	transcript:Zm00001d027449_T001	rna7891	1.00E-72
4- 22:	transcript:Zm00001d027450_T001	rna7893	0
4- 23:	transcript:Zm00001d027451_T001	rna7894	9.00E-135
4- 24:	transcript:Zm00001d027454_T001	rna7895	2.00E-37
4- 25:	transcript:Zm00001d027455_T001	rna7896	5.00E-99
4- 26:	transcript:Zm00001d027457_T001	rna7898	2.00E-19
4- 27:	transcript:Zm00001d027458_T001	rna7899	1.00E-38
4- 28:	transcript:Zm00001d027459_T001	rna7901	2.00E-176
4- 29:	transcript:Zm00001d027461_T001	rna7902	0
4- 30:	transcript:Zm00001d027462_T001	rna7903	0
4- 31:	transcript:Zm00001d027463_T001	rna7904	0
4- 32:	transcript:Zm00001d027466_T001	rna7906	1.00E-139
4- 33:	transcript:Zm00001d027471_T001	rna7907	2.00E-29
4- 34:	transcript:Zm00001d027472_T002	rna7908	0
4- 35:	transcript:Zm00001d027474_T001	rna7909	4.00E-24
4- 36:	transcript:Zm00001d027477_T001	rna7910	2.00E-62
4- 37:	transcript:Zm00001d027478_T001	rna7911	5.00E-28
4- 38:	transcript:Zm00001d027479_T002	rna7912	0
4- 39:	transcript:Zm00001d027480_T002	rna7914	0
4- 40:	transcript:Zm00001d027481_T001	rna7915	8.00E-149
4- 41:	transcript:Zm00001d027485_T002	rna7917	9.00E-109
4- 42:	transcript:Zm00001d027486_T001	rna7918	0
4- 43:	transcript:Zm00001d027487_T017	rna7919	0
4- 44:	transcript:Zm00001d027488_T001	rna7920	0

4- 45:	transcript:Zm00001d027489_T001	rna7921	4.00E-167
4- 46:	transcript:Zm00001d027492_T001	rna7923	3.00E-60
4- 47:	transcript:Zm00001d027493_T001	rna7924	1.00E-60
4- 48:	transcript:Zm00001d027499_T001	rna7925	0
4- 49:	transcript:Zm00001d027500_T001	rna7926	2.00E-25
4- 50:	transcript:Zm00001d027502_T001	rna7927	0
4- 51:	transcript:Zm00001d027503_T036	rna7928	0
4- 52:	transcript:Zm00001d027504_T002	rna7929	0
4- 53:	transcript:Zm00001d027506_T003	rna7930	0
4- 54:	transcript:Zm00001d027508_T001	rna7931	0
4- 55:	transcript:Zm00001d027510_T001	rna7933	0
4- 56:	transcript:Zm00001d027511_T001	rna7934	0
4- 57:	transcript:Zm00001d027514_T002	rna7935	0
4- 58:	transcript:Zm00001d027515_T002	rna7936	0
4- 59:	transcript:Zm00001d027517_T001	rna7937	0
4- 60:	transcript:Zm00001d027518_T001	rna7938	0
4- 61:	transcript:Zm00001d027519_T001	rna7939	0
4- 62:	transcript:Zm00001d027520_T001	rna7940	3.00E-143
4- 63:	transcript:Zm00001d027522_T001	rna7942	7.00E-109
4- 64:	transcript:Zm00001d027523_T001	rna7943	0
4- 65:	transcript:Zm00001d027524_T001	rna7944	4.00E-139
4- 66:	transcript:Zm00001d027530_T001	rna7945	2.00E-145
4- 67:	transcript:Zm00001d027532_T002	rna7947	2.00E-172
4- 68:	transcript:Zm00001d027533_T001	rna7948	3.00E-131
4- 69:	transcript:Zm00001d027534_T001	rna7949	0
4- 70:	transcript:Zm00001d027536_T003	rna7952	0
4- 71:	transcript:Zm00001d027537_T001	rna7954	1.00E-109
4- 72:	transcript:Zm00001d027539_T001	rna7956	3.00E-152
4- 73:	transcript:Zm00001d027544_T002	rna7962	2.00E-171
4- 74:	transcript:Zm00001d027546_T001	rna7963	6.00E-148
4- 75:	transcript:Zm00001d027548_T001	rna7965	0
4- 76:	transcript:Zm00001d027549_T001	rna7966	0
4- 77:	transcript:Zm00001d027554_T001	rna7968	3.00E-66
4- 78:	transcript:Zm00001d027555_T001	rna7969	3.00E-41
4- 79:	transcript:Zm00001d027556_T001	rna7970	3.00E-139
4- 80:	transcript:Zm00001d027558_T004	rna7971	0
4- 81:	transcript:Zm00001d027570_T005	rna7972	0
4- 82:	transcript:Zm00001d027573_T001	rna7973	2.00E-139
4- 83:	transcript:Zm00001d027578_T001	rna7975	2.00E-129
4- 84:	transcript:Zm00001d027581_T001	rna7976	3.00E-103
4- 85:	transcript:Zm00001d027582_T001	rna7977	2.00E-145
4- 86:	transcript:Zm00001d027585_T001	rna7978	9.00E-84
4- 87:	transcript:Zm00001d027588_T003	rna7982	3.00E-30
4- 88:	transcript:Zm00001d027589_T001	rna7983	3.00E-133
4- 89:	transcript:Zm00001d027590_T001	rna7984	0
4- 90:	transcript:Zm00001d027593_T001	rna7985	0
4- 91:	transcript:Zm00001d027595_T001	rna7988	6.00E-23
4- 92:	transcript:Zm00001d027596_T009	rna7989	0
4- 93:	transcript:Zm00001d027597_T001	rna7990	9.00E-76
4- 94:	transcript:Zm00001d027598_T001	rna7991	6.00E-103
4- 95:	transcript:Zm00001d027599_T001	rna7992	0
4- 96:	transcript:Zm00001d027607_T001	rna7996	2.00E-141
4- 97:	transcript:Zm00001d027613_T002	rna7997	5.00E-128
4- 98:	transcript:Zm00001d027616_T001	rna7998	4.00E-61

4- 99:	transcript:Zm00001d027618_T001	rna7999	3.00E-112
4-100:	transcript:Zm00001d027619_T002	rna8001	0
4-101:	transcript:Zm00001d027620_T001	rna8003	3.00E-70
4-102:	transcript:Zm00001d027621_T001	rna8006	1.00E-156
4-103:	transcript:Zm00001d027623_T001	rna8007	2.00E-100
4-104:	transcript:Zm00001d027625_T001	rna8008	0
4-105:	transcript:Zm00001d027626_T004	rna8010	2.00E-95
4-106:	transcript:Zm00001d027630_T002	rna8015	0
4-107:	transcript:Zm00001d027636_T001	rna8018	2.00E-151
4-108:	transcript:Zm00001d027637_T002	rna8019	0
4-109:	transcript:Zm00001d027638_T001	rna8021	0
4-110:	transcript:Zm00001d027645_T001	rna8024	0
4-111:	transcript:Zm00001d027646_T001	rna8026	3.00E-74
4-112:	transcript:Zm00001d027648_T001	rna8031	1.00E-86
4-113:	transcript:Zm00001d027649_T003	rna8032	1.00E-71
4-114:	transcript:Zm00001d027652_T001	rna8034	7.00E-102
4-115:	transcript:Zm00001d027655_T001	rna8036	1.00E-43
4-116:	transcript:Zm00001d027656_T001	rna8037	0
4-117:	transcript:Zm00001d027659_T001	rna8038	1.00E-128
4-118:	transcript:Zm00001d027665_T004	rna8039	7.00E-17

Alignment 5: score=4970.0 e_value=0 N=108 1&NC_008396.2 plus

5- 0:	transcript:Zm00001d027799_T002	rna8153	0
5- 1:	transcript:Zm00001d027801_T010	rna8154	0
5- 2:	transcript:Zm00001d027802_T002	rna8157	0
5- 3:	transcript:Zm00001d027803_T002	rna8158	0
5- 4:	transcript:Zm00001d027807_T001	rna8159	0
5- 5:	transcript:Zm00001d027808_T001	rna8160	8.00E-151
5- 6:	transcript:Zm00001d027809_T001	rna8161	3.00E-157
5- 7:	transcript:Zm00001d027810_T001	rna8165	4.00E-81
5- 8:	transcript:Zm00001d027811_T001	rna8166	0
5- 9:	transcript:Zm00001d027813_T002	rna8168	3.00E-39
5- 10:	transcript:Zm00001d027815_T002	rna8169	0
5- 11:	transcript:Zm00001d027816_T001	rna8170	0
5- 12:	transcript:Zm00001d027826_T001	rna8173	0
5- 13:	transcript:Zm00001d027827_T001	rna8174	0
5- 14:	transcript:Zm00001d027831_T001	rna8175	5.00E-19
5- 15:	transcript:Zm00001d027832_T001	rna8176	4.00E-51
5- 16:	transcript:Zm00001d027833_T001	rna8178	8.00E-76
5- 17:	transcript:Zm00001d027835_T001	rna8179	0
5- 18:	transcript:Zm00001d027837_T001	rna8181	3.00E-111
5- 19:	transcript:Zm00001d027838_T001	rna8182	0
5- 20:	transcript:Zm00001d027839_T001	rna8184	1.00E-39
5- 21:	transcript:Zm00001d027841_T002	rna8185	2.00E-177
5- 22:	transcript:Zm00001d027842_T003	rna8186	4.00E-152
5- 23:	transcript:Zm00001d027843_T001	rna8187	0
5- 24:	transcript:Zm00001d027844_T001	rna8188	2.00E-82
5- 25:	transcript:Zm00001d027846_T001	rna8190	0
5- 26:	transcript:Zm00001d027847_T001	rna8192	3.00E-65
5- 27:	transcript:Zm00001d027851_T001	rna8193	8.00E-46
5- 28:	transcript:Zm00001d027852_T001	rna8199	2.00E-48
5- 29:	transcript:Zm00001d027854_T006	rna8200	0
5- 30:	transcript:Zm00001d027855_T001	rna8201	2.00E-44
5- 31:	transcript:Zm00001d027856_T001	rna8202	0
5- 32:	transcript:Zm00001d027861_T001	rna8204	0

5- 33:	transcript:Zm00001d027862_T001	rna8207	3.00E-180
5- 34:	transcript:Zm00001d027864_T001	rna8208	6.00E-17
5- 35:	transcript:Zm00001d027868_T002	rna8212	1.00E-162
5- 36:	transcript:Zm00001d027869_T002	rna8213	0
5- 37:	transcript:Zm00001d027870_T001	rna8216	3.00E-91
5- 38:	transcript:Zm00001d027871_T001	rna8217	0
5- 39:	transcript:Zm00001d027872_T001	rna8218	0
5- 40:	transcript:Zm00001d027874_T004	rna8220	9.00E-94
5- 41:	transcript:Zm00001d027875_T001	rna8221	2.00E-175
5- 42:	transcript:Zm00001d027876_T004	rna8222	0
5- 43:	transcript:Zm00001d027877_T001	rna8223	5.00E-98
5- 44:	transcript:Zm00001d027878_T002	rna8225	6.00E-53
5- 45:	transcript:Zm00001d027879_T002	rna8227	1.00E-110
5- 46:	transcript:Zm00001d027881_T001	rna8229	7.00E-38
5- 47:	transcript:Zm00001d027884_T003	rna8237	4.00E-25
5- 48:	transcript:Zm00001d027885_T001	rna8238	5.00E-107
5- 49:	transcript:Zm00001d027887_T001	rna8239	2.00E-117
5- 50:	transcript:Zm00001d027892_T001	rna8245	0
5- 51:	transcript:Zm00001d027893_T001	rna8247	0
5- 52:	transcript:Zm00001d027894_T001	rna8249	3.00E-45
5- 53:	transcript:Zm00001d027895_T004	rna8250	0
5- 54:	transcript:Zm00001d027896_T001	rna8251	6.00E-180
5- 55:	transcript:Zm00001d027897_T001	rna8253	2.00E-32
5- 56:	transcript:Zm00001d027898_T002	rna8254	0
5- 57:	transcript:Zm00001d027899_T001	rna8255	2.00E-31
5- 58:	transcript:Zm00001d027900_T001	rna8256	4.00E-56
5- 59:	transcript:Zm00001d027903_T001	rna8258	0
5- 60:	transcript:Zm00001d027904_T001	rna8259	0
5- 61:	transcript:Zm00001d027907_T002	rna8261	0
5- 62:	transcript:Zm00001d027916_T006	rna8263	0
5- 63:	transcript:Zm00001d027919_T001	rna8264	1.00E-36
5- 64:	transcript:Zm00001d027922_T001	rna8265	4.00E-145
5- 65:	transcript:Zm00001d027924_T001	rna8266	5.00E-45
5- 66:	transcript:Zm00001d027925_T001	rna8268	3.00E-81
5- 67:	transcript:Zm00001d027932_T001	rna8272	1.00E-38
5- 68:	transcript:Zm00001d027934_T002	rna8275	0
5- 69:	transcript:Zm00001d027936_T001	rna8277	0
5- 70:	transcript:Zm00001d027938_T001	rna8280	0
5- 71:	transcript:Zm00001d027939_T002	rna8282	2.00E-134
5- 72:	transcript:Zm00001d027941_T004	rna8283	7.00E-159
5- 73:	transcript:Zm00001d027943_T006	rna8290	2.00E-180
5- 74:	transcript:Zm00001d027944_T001	rna8291	7.00E-84
5- 75:	transcript:Zm00001d027946_T001	rna8292	0
5- 76:	transcript:Zm00001d027950_T002	rna8294	3.00E-152
5- 77:	transcript:Zm00001d027954_T001	rna8295	0
5- 78:	transcript:Zm00001d027955_T001	rna8296	9.00E-52
5- 79:	transcript:Zm00001d027962_T001	rna8297	3.00E-133
5- 80:	transcript:Zm00001d027965_T001	rna8298	0
5- 81:	transcript:Zm00001d027966_T001	rna8299	1.00E-41
5- 82:	transcript:Zm00001d027967_T001	rna8300	3.00E-71
5- 83:	transcript:Zm00001d027971_T001	rna8302	0
5- 84:	transcript:Zm00001d027973_T001	rna8304	4.00E-59
5- 85:	transcript:Zm00001d027975_T001	rna8305	5.00E-152
5- 86:	transcript:Zm00001d027976_T002	rna8306	2.00E-149

5- 87: transcript:Zm00001d027978_T001 rna8308 0
5- 88: transcript:Zm00001d027982_T002 rna8309 2.00E-37
5- 89: transcript:Zm00001d027983_T001 rna8310 4.00E-120
5- 90: transcript:Zm00001d027987_T001 rna8311 7.00E-118
5- 91: transcript:Zm00001d027991_T001 rna8314 7.00E-104
5- 92: transcript:Zm00001d027992_T001 rna8315 5.00E-61
5- 93: transcript:Zm00001d027994_T001 rna8316 0
5- 94: transcript:Zm00001d027995_T001 rna8317 0
5- 95: transcript:Zm00001d027998_T001 rna8319 7.00E-54
5- 96: transcript:Zm00001d027999_T001 rna8320 0
5- 97: transcript:Zm00001d028004_T001 rna8322 2.00E-74
5- 98: transcript:Zm00001d028005_T002 rna8325 0
5- 99: transcript:Zm00001d028006_T001 rna8326 9.00E-58
5-100: transcript:Zm00001d028007_T004 rna8327 0
5-101: transcript:Zm00001d028008_T001 rna8328 2.00E-174
5-102: transcript:Zm00001d028011_T001 rna8329 2.00E-60
5-103: transcript:Zm00001d028012_T001 rna8330 2.00E-61
5-104: transcript:Zm00001d028013_T001 rna8333 0
5-105: transcript:Zm00001d028017_T001 rna8334 6.00E-119
5-106: transcript:Zm00001d028018_T001 rna8335 9.00E-166
5-107: transcript:Zm00001d028019_T001 rna8336 0
Alignment 6: score=4864.0 e_value=0 N=110 1&NC_008396.2 plus
6- 0: transcript:Zm00001d029121_T005 rna9271 0
6- 1: transcript:Zm00001d029123_T005 rna9272 0
6- 2: transcript:Zm00001d029124_T001 rna9273 2.00E-51
6- 3: transcript:Zm00001d029125_T001 rna9274 9.00E-160
6- 4: transcript:Zm00001d029126_T001 rna9275 7.00E-28
6- 5: transcript:Zm00001d029129_T002 rna9279 0
6- 6: transcript:Zm00001d029130_T001 rna9280 0
6- 7: transcript:Zm00001d029133_T003 rna9281 0
6- 8: transcript:Zm00001d029134_T003 rna9282 8.00E-179
6- 9: transcript:Zm00001d029135_T001 rna9284 4.00E-109
6- 10: transcript:Zm00001d029138_T001 rna9287 2.00E-57
6- 11: transcript:Zm00001d029139_T001 rna9288 0
6- 12: transcript:Zm00001d029140_T002 rna9290 1.00E-52
6- 13: transcript:Zm00001d029141_T001 rna9291 4.00E-104
6- 14: transcript:Zm00001d029142_T007 rna9292 0
6- 15: transcript:Zm00001d029143_T003 rna9293 0
6- 16: transcript:Zm00001d029144_T003 rna9297 0
6- 17: transcript:Zm00001d029149_T001 rna9299 4.00E-169
6- 18: transcript:Zm00001d029150_T001 rna9300 0
6- 19: transcript:Zm00001d029164_T001 rna9301 0
6- 20: transcript:Zm00001d029165_T001 rna9302 0
6- 21: transcript:Zm00001d029170_T004 rna9303 8.00E-78
6- 22: transcript:Zm00001d029172_T001 rna9305 1.00E-06
6- 23: transcript:Zm00001d029174_T005 rna9308 0
6- 24: transcript:Zm00001d029177_T001 rna9309 0
6- 25: transcript:Zm00001d029180_T001 rna9310 5.00E-72
6- 26: transcript:Zm00001d029188_T006 rna9320 2.00E-77
6- 27: transcript:Zm00001d029196_T002 rna9323 0
6- 28: transcript:Zm00001d029197_T001 rna9324 4.00E-163
6- 29: transcript:Zm00001d029198_T003 rna9325 2.00E-141
6- 30: transcript:Zm00001d029200_T001 rna9326 1.00E-169
6- 31: transcript:Zm00001d029201_T001 rna9327 1.00E-138

6- 32:	transcript:Zm00001d029202_T001	rna9328	6.00E-102
6- 33:	transcript:Zm00001d029203_T001	rna9329	0
6- 34:	transcript:Zm00001d029206_T002	rna9331	5.00E-150
6- 35:	transcript:Zm00001d029208_T001	rna9333	3.00E-50
6- 36:	transcript:Zm00001d029209_T003	rna9335	0
6- 37:	transcript:Zm00001d029212_T003	rna9337	0
6- 38:	transcript:Zm00001d029214_T001	rna9338	1.00E-103
6- 39:	transcript:Zm00001d029215_T012	rna9339	0
6- 40:	transcript:Zm00001d029222_T001	rna9343	2.00E-32
6- 41:	transcript:Zm00001d029223_T004	rna9344	1.00E-32
6- 42:	transcript:Zm00001d029226_T001	rna9347	4.00E-27
6- 43:	transcript:Zm00001d029227_T001	rna9350	0
6- 44:	transcript:Zm00001d029235_T001	rna9352	0
6- 45:	transcript:Zm00001d029238_T001	rna9357	1.00E-89
6- 46:	transcript:Zm00001d029246_T001	rna9364	0
6- 47:	transcript:Zm00001d029248_T002	rna9367	0
6- 48:	transcript:Zm00001d029249_T001	rna9368	6.00E-85
6- 49:	transcript:Zm00001d029251_T001	rna9369	1.00E-59
6- 50:	transcript:Zm00001d029255_T001	rna9371	0
6- 51:	transcript:Zm00001d029256_T002	rna9372	1.00E-77
6- 52:	transcript:Zm00001d029258_T001	rna9376	0
6- 53:	transcript:Zm00001d029260_T004	rna9377	2.00E-155
6- 54:	transcript:Zm00001d029263_T001	rna9383	5.00E-57
6- 55:	transcript:Zm00001d029264_T004	rna9385	0
6- 56:	transcript:Zm00001d029266_T002	rna9388	2.00E-40
6- 57:	transcript:Zm00001d029272_T002	rna9390	0
6- 58:	transcript:Zm00001d029274_T001	rna9394	3.00E-147
6- 59:	transcript:Zm00001d029281_T007	rna9403	2.00E-174
6- 60:	transcript:Zm00001d029285_T005	rna9404	0
6- 61:	transcript:Zm00001d029286_T001	rna9405	2.00E-59
6- 62:	transcript:Zm00001d029287_T001	rna9406	2.00E-117
6- 63:	transcript:Zm00001d029288_T001	rna9412	0
6- 64:	transcript:Zm00001d029297_T003	rna9416	0
6- 65:	transcript:Zm00001d029300_T001	rna9417	0
6- 66:	transcript:Zm00001d029303_T001	rna9423	6.00E-132
6- 67:	transcript:Zm00001d029305_T001	rna9427	3.00E-25
6- 68:	transcript:Zm00001d029313_T001	rna9428	2.00E-153
6- 69:	transcript:Zm00001d029314_T001	rna9429	3.00E-50
6- 70:	transcript:Zm00001d029320_T001	rna9430	6.00E-119
6- 71:	transcript:Zm00001d029324_T001	rna9435	0
6- 72:	transcript:Zm00001d029325_T001	rna9436	1.00E-116
6- 73:	transcript:Zm00001d029328_T009	rna9438	0
6- 74:	transcript:Zm00001d029329_T011	rna9439	4.00E-177
6- 75:	transcript:Zm00001d029331_T001	rna9446	0
6- 76:	transcript:Zm00001d029333_T008	rna9448	0
6- 77:	transcript:Zm00001d029337_T001	rna9449	3.00E-56
6- 78:	transcript:Zm00001d029339_T001	rna9450	3.00E-116
6- 79:	transcript:Zm00001d029341_T001	rna9454	0
6- 80:	transcript:Zm00001d029342_T009	rna9455	5.00E-39
6- 81:	transcript:Zm00001d029343_T001	rna9456	1.00E-132
6- 82:	transcript:Zm00001d029349_T001	rna9458	1.00E-34
6- 83:	transcript:Zm00001d029350_T001	rna9459	0
6- 84:	transcript:Zm00001d029352_T002	rna9466	4.00E-134
6- 85:	transcript:Zm00001d029361_T001	rna9469	2.00E-24

```
  6- 86:  transcript:Zm00001d029365_T002  rna9470      6.00E-16
  6- 87:  transcript:Zm00001d029366_T003  rna9473     2.00E-180
  6- 88:  transcript:Zm00001d029371_T001  rna9474     3.00E-159
  6- 89:  transcript:Zm00001d029372_T001  rna9476             0
  6- 90:  transcript:Zm00001d029375_T002  rna9477             0
  6- 91:  transcript:Zm00001d029376_T001  rna9481     2.00E-133
  6- 92:  transcript:Zm00001d029378_T012  rna9482             0
  6- 93:  transcript:Zm00001d029380_T002  rna9483      6.00E-44
  6- 94:  transcript:Zm00001d029386_T006  rna9487             0
  6- 95:  transcript:Zm00001d029387_T001  rna9488     3.00E-110
  6- 96:  transcript:Zm00001d029391_T002  rna9493             0
  6- 97:  transcript:Zm00001d029392_T001  rna9494             0
  6- 98:  transcript:Zm00001d029393_T001  rna9498      3.00E-80
  6- 99:  transcript:Zm00001d029394_T003  rna9499      3.00E-95
  6-100:  transcript:Zm00001d029397_T001  rna9505             0
  6-101:  transcript:Zm00001d029402_T001  rna9506      5.00E-86
  6-102:  transcript:Zm00001d029408_T001  rna9508     1.00E-122
  6-103:  transcript:Zm00001d029409_T001  rna9509     2.00E-170
  6-104:  transcript:Zm00001d029410_T002  rna9510             0
  6-105:  transcript:Zm00001d029412_T001  rna9513             0
  6-106:  transcript:Zm00001d029417_T002  rna9515             0
  6-107:  transcript:Zm00001d029419_T005  rna9517      2.00E-20
  6-108:  transcript:Zm00001d029420_T001  rna9518             0
  6-109:  transcript:Zm00001d029422_T007  rna9519             0
## Alignment 7: score=4155.0 e_value=0 N=91 1&NC_008396.2 plus
  7-  0:  transcript:Zm00001d033734_T003  rna10385            0
  7-  1:  transcript:Zm00001d033738_T002  rna10391    2.00E-136
  7-  2:  transcript:Zm00001d033741_T002  rna10392     6.00E-15
  7-  3:  transcript:Zm00001d033744_T001  rna10393    3.00E-109
  7-  4:  transcript:Zm00001d033745_T001  rna10394    9.00E-106
  7-  5:  transcript:Zm00001d033746_T003  rna10396            0
  7-  6:  transcript:Zm00001d033747_T001  rna10397            0
  7-  7:  transcript:Zm00001d033750_T003  rna10399     6.00E-77
  7-  8:  transcript:Zm00001d033751_T001  rna10400     3.00E-63
  7-  9:  transcript:Zm00001d033753_T001  rna10402            0
  7- 10:  transcript:Zm00001d033766_T002  rna10403            0
  7- 11:  transcript:Zm00001d033776_T008  rna10409            0
  7- 12:  transcript:Zm00001d033777_T001  rna10413            0
  7- 13:  transcript:Zm00001d033778_T001  rna10414    7.00E-108
  7- 14:  transcript:Zm00001d033786_T002  rna10416    3.00E-120
  7- 15:  transcript:Zm00001d033787_T001  rna10417            0
  7- 16:  transcript:Zm00001d033788_T002  rna10418    3.00E-122
  7- 17:  transcript:Zm00001d033790_T002  rna10419            0
  7- 18:  transcript:Zm00001d033791_T001  rna10422     6.00E-92
  7- 19:  transcript:Zm00001d033792_T001  rna10423     8.00E-41
  7- 20:  transcript:Zm00001d033793_T001  rna10424     3.00E-47
  7- 21:  transcript:Zm00001d033794_T001  rna10425            0
  7- 22:  transcript:Zm00001d033795_T006  rna10427            0
  7- 23:  transcript:Zm00001d033797_T003  rna10428            0
  7- 24:  transcript:Zm00001d033798_T030  rna10429            0
  7- 25:  transcript:Zm00001d033799_T022  rna10431            0
  7- 26:  transcript:Zm00001d033800_T002  rna10433            0
  7- 27:  transcript:Zm00001d033805_T001  rna10434            0
  7- 28:  transcript:Zm00001d033815_T001  rna10436    2.00E-121
```

7- 29:	transcript:Zm00001d033817_T004	rna10438	0
7- 30:	transcript:Zm00001d033818_T001	rna10440	2.00E-108
7- 31:	transcript:Zm00001d033821_T001	rna10441	1.00E-65
7- 32:	transcript:Zm00001d033822_T001	rna10442	7.00E-83
7- 33:	transcript:Zm00001d033823_T001	rna10443	2.00E-169
7- 34:	transcript:Zm00001d033825_T001	rna10444	0
7- 35:	transcript:Zm00001d033827_T006	rna10446	2.00E-14
7- 36:	transcript:Zm00001d033830_T001	rna10448	2.00E-176
7- 37:	transcript:Zm00001d033834_T001	rna10451	0
7- 38:	transcript:Zm00001d033835_T001	rna10454	5.00E-29
7- 39:	transcript:Zm00001d033836_T001	rna10456	7.00E-165
7- 40:	transcript:Zm00001d033839_T001	rna10458	5.00E-122
7- 41:	transcript:Zm00001d033840_T001	rna10459	4.00E-52
7- 42:	transcript:Zm00001d033843_T007	rna10465	3.00E-63
7- 43:	transcript:Zm00001d033846_T001	rna10467	2.00E-12
7- 44:	transcript:Zm00001d033848_T004	rna10470	0
7- 45:	transcript:Zm00001d033850_T001	rna10471	0
7- 46:	transcript:Zm00001d033853_T002	rna10472	0
7- 47:	transcript:Zm00001d033854_T001	rna10474	1.00E-164
7- 48:	transcript:Zm00001d033855_T002	rna10475	0
7- 49:	transcript:Zm00001d033858_T003	rna10477	2.00E-06
7- 50:	transcript:Zm00001d033859_T002	rna10478	3.00E-121
7- 51:	transcript:Zm00001d033860_T001	rna10479	1.00E-109
7- 52:	transcript:Zm00001d033862_T001	rna10481	0
7- 53:	transcript:Zm00001d033863_T001	rna10482	4.00E-166
7- 54:	transcript:Zm00001d033866_T001	rna10483	0
7- 55:	transcript:Zm00001d033869_T001	rna10485	0
7- 56:	transcript:Zm00001d033870_T001	rna10490	4.00E-171
7- 57:	transcript:Zm00001d033872_T001	rna10491	0
7- 58:	transcript:Zm00001d033873_T001	rna10492	4.00E-111
7- 59:	transcript:Zm00001d033874_T002	rna10493	3.00E-63
7- 60:	transcript:Zm00001d033876_T004	rna10496	0
7- 61:	transcript:Zm00001d033877_T001	rna10497	8.00E-34
7- 62:	transcript:Zm00001d033878_T002	rna10499	0
7- 63:	transcript:Zm00001d033879_T001	rna10500	9.00E-122
7- 64:	transcript:Zm00001d033882_T001	rna10501	5.00E-114
7- 65:	transcript:Zm00001d033886_T001	rna10504	0
7- 66:	transcript:Zm00001d033890_T001	rna10505	6.00E-160
7- 67:	transcript:Zm00001d033893_T001	rna10506	4.00E-46
7- 68:	transcript:Zm00001d033895_T001	rna10508	0
7- 69:	transcript:Zm00001d033896_T001	rna10510	0
7- 70:	transcript:Zm00001d033898_T002	rna10513	0
7- 71:	transcript:Zm00001d033902_T001	rna10518	6.00E-35
7- 72:	transcript:Zm00001d033903_T001	rna10519	0
7- 73:	transcript:Zm00001d033905_T001	rna10520	1.00E-107
7- 74:	transcript:Zm00001d033906_T001	rna10521	6.00E-107
7- 75:	transcript:Zm00001d033909_T001	rna10522	5.00E-11
7- 76:	transcript:Zm00001d033910_T002	rna10532	0
7- 77:	transcript:Zm00001d033912_T001	rna10533	0
7- 78:	transcript:Zm00001d033913_T001	rna10535	6.00E-82
7- 79:	transcript:Zm00001d033915_T001	rna10538	0
7- 80:	transcript:Zm00001d033916_T001	rna10540	0
7- 81:	transcript:Zm00001d033917_T001	rna10541	0
7- 82:	transcript:Zm00001d033922_T002	rna10543	1.00E-11

```
  7- 83:  transcript:Zm00001d033924_T001  rna10545     2.00E-82
  7- 84:  transcript:Zm00001d033925_T001  rna10546    3.00E-115
  7- 85:  transcript:Zm00001d033928_T001  rna10550            0
  7- 86:  transcript:Zm00001d033929_T002  rna10551            0
  7- 87:  transcript:Zm00001d033930_T001  rna10552     4.00E-69
  7- 88:  transcript:Zm00001d033932_T002  rna10553     7.00E-21
  7- 89:  transcript:Zm00001d033933_T001  rna10554            0
  7- 90:  transcript:Zm00001d033935_T003  rna10555            0
## Alignment 8: score=3812.0 e_value=0 N=82 1&NC_008396.2 plus
  8-  0:  transcript:Zm00001d034167_T006  rna10728            0
  8-  1:  transcript:Zm00001d034175_T002  rna10729     2.00E-99
  8-  2:  transcript:Zm00001d034178_T001  rna10730            0
  8-  3:  transcript:Zm00001d034179_T001  rna10731     3.00E-58
  8-  4:  transcript:Zm00001d034180_T002  rna10732    3.00E-165
  8-  5:  transcript:Zm00001d034181_T001  rna10736    9.00E-105
  8-  6:  transcript:Zm00001d034182_T001  rna10738     3.00E-09
  8-  7:  transcript:Zm00001d034183_T006  rna10739            0
  8-  8:  transcript:Zm00001d034184_T001  rna10740            0
  8-  9:  transcript:Zm00001d034187_T001  rna10741     4.00E-39
  8- 10:  transcript:Zm00001d034188_T002  rna10744    4.00E-110
  8- 11:  transcript:Zm00001d034189_T001  rna10745     3.00E-74
  8- 12:  transcript:Zm00001d034190_T001  rna10750     2.00E-50
  8- 13:  transcript:Zm00001d034191_T004  rna10751            0
  8- 14:  transcript:Zm00001d034192_T001  rna10753    2.00E-109
  8- 15:  transcript:Zm00001d034194_T004  rna10755            0
  8- 16:  transcript:Zm00001d034195_T001  rna10756     1.00E-81
  8- 17:  transcript:Zm00001d034196_T003  rna10757            0
  8- 18:  transcript:Zm00001d034197_T001  rna10758     8.00E-55
  8- 19:  transcript:Zm00001d034198_T001  rna10760    2.00E-144
  8- 20:  transcript:Zm00001d034199_T001  rna10761     2.00E-39
  8- 21:  transcript:Zm00001d034201_T001  rna10762     6.00E-14
  8- 22:  transcript:Zm00001d034204_T001  rna10763     2.00E-22
  8- 23:  transcript:Zm00001d034205_T001  rna10764            0
  8- 24:  transcript:Zm00001d034206_T001  rna10765            0
  8- 25:  transcript:Zm00001d034207_T001  rna10766    3.00E-153
  8- 26:  transcript:Zm00001d034212_T001  rna10767    2.00E-106
  8- 27:  transcript:Zm00001d034217_T001  rna10770    2.00E-157
  8- 28:  transcript:Zm00001d034218_T001  rna10771     4.00E-56
  8- 29:  transcript:Zm00001d034219_T001  rna10772     1.00E-19
  8- 30:  transcript:Zm00001d034221_T001  rna10773            0
  8- 31:  transcript:Zm00001d034223_T002  rna10775     6.00E-35
  8- 32:  transcript:Zm00001d034232_T001  rna10777     5.00E-09
  8- 33:  transcript:Zm00001d034239_T001  rna10778            0
  8- 34:  transcript:Zm00001d034240_T004  rna10779            0
  8- 35:  transcript:Zm00001d034241_T001  rna10780            0
  8- 36:  transcript:Zm00001d034244_T001  rna10782    6.00E-143
  8- 37:  transcript:Zm00001d034245_T004  rna10783            0
  8- 38:  transcript:Zm00001d034248_T002  rna10784     3.00E-49
  8- 39:  transcript:Zm00001d034249_T001  rna10788            0
  8- 40:  transcript:Zm00001d034250_T002  rna10789            0
  8- 41:  transcript:Zm00001d034251_T001  rna10790            0
  8- 42:  transcript:Zm00001d034253_T001  rna10791            0
  8- 43:  transcript:Zm00001d034254_T002  rna10792     1.00E-68
  8- 44:  transcript:Zm00001d034255_T001  rna10793            0
```

```
  8- 45:  transcript:Zm00001d034256_T001  rna10795            0
  8- 46:  transcript:Zm00001d034257_T002  rna10796            0
  8- 47:  transcript:Zm00001d034270_T001  rna10798     2.00E-66
  8- 48:  transcript:Zm00001d034277_T001  rna10799    1.00E-127
  8- 49:  transcript:Zm00001d034278_T001  rna10800     2.00E-91
  8- 50:  transcript:Zm00001d034279_T001  rna10801     3.00E-96
  8- 51:  transcript:Zm00001d034282_T004  rna10803            0
  8- 52:  transcript:Zm00001d034283_T001  rna10805    1.00E-103
  8- 53:  transcript:Zm00001d034317_T001  rna10815    3.00E-177
  8- 54:  transcript:Zm00001d034318_T001  rna10816     6.00E-46
  8- 55:  transcript:Zm00001d034337_T005  rna10835    2.00E-113
  8- 56:  transcript:Zm00001d034338_T001  rna10837            0
  8- 57:  transcript:Zm00001d034340_T001  rna10838            0
  8- 58:  transcript:Zm00001d034341_T003  rna10839     9.00E-63
  8- 59:  transcript:Zm00001d034344_T001  rna10841    2.00E-120
  8- 60:  transcript:Zm00001d034345_T001  rna10842            0
  8- 61:  transcript:Zm00001d034346_T002  rna10844    7.00E-128
  8- 62:  transcript:Zm00001d034350_T012  rna10847     2.00E-69
  8- 63:  transcript:Zm00001d034351_T001  rna10848            0
  8- 64:  transcript:Zm00001d034353_T001  rna10849    9.00E-105
  8- 65:  transcript:Zm00001d034356_T001  rna10850    3.00E-103
  8- 66:  transcript:Zm00001d034358_T001  rna10851     5.00E-80
  8- 67:  transcript:Zm00001d034359_T001  rna10853     3.00E-44
  8- 68:  transcript:Zm00001d034360_T001  rna10854    7.00E-153
  8- 69:  transcript:Zm00001d034361_T001  rna10857     3.00E-99
  8- 70:  transcript:Zm00001d034364_T001  rna10858            0
  8- 71:  transcript:Zm00001d034365_T001  rna10859    5.00E-175
  8- 72:  transcript:Zm00001d034366_T001  rna10861    9.00E-172
  8- 73:  transcript:Zm00001d034368_T001  rna10862            0
  8- 74:  transcript:Zm00001d034369_T001  rna10866     1.00E-75
  8- 75:  transcript:Zm00001d034371_T002  rna10868            0
  8- 76:  transcript:Zm00001d034372_T001  rna10870            0
  8- 77:  transcript:Zm00001d034373_T001  rna10871            0
  8- 78:  transcript:Zm00001d034380_T001  rna10876    4.00E-149
  8- 79:  transcript:Zm00001d034383_T003  rna10883            0
  8- 80:  transcript:Zm00001d034385_T001  rna10884     1.00E-71
  8- 81:  transcript:Zm00001d034387_T002  rna10885            0
## Alignment 9: score=2916.0 e_value=3.3e-259 N=64 1&NC_008396.2 plus
  9-  0:  transcript:Zm00001d034813_T001  rna11265     2.00E-88
  9-  1:  transcript:Zm00001d034814_T001  rna11267    3.00E-153
  9-  2:  transcript:Zm00001d034817_T001  rna11272            0
  9-  3:  transcript:Zm00001d034818_T001  rna11273            0
  9-  4:  transcript:Zm00001d034823_T001  rna11274     2.00E-62
  9-  5:  transcript:Zm00001d034828_T002  rna11276    7.00E-158
  9-  6:  transcript:Zm00001d034829_T001  rna11277            0
  9-  7:  transcript:Zm00001d034830_T001  rna11279     1.00E-14
  9-  8:  transcript:Zm00001d034832_T006  rna11280            0
  9-  9:  transcript:Zm00001d034834_T001  rna11291            0
  9- 10:  transcript:Zm00001d034838_T001  rna11294            0
  9- 11:  transcript:Zm00001d034839_T003  rna11295            0
  9- 12:  transcript:Zm00001d034841_T001  rna11297    9.00E-134
  9- 13:  transcript:Zm00001d034842_T001  rna11303            0
  9- 14:  transcript:Zm00001d034844_T001  rna11306    1.00E-151
  9- 15:  transcript:Zm00001d034848_T001  rna11309            0
```

```
  9- 16:  transcript:Zm00001d034849_T001  rna11310     2.00E-94
  9- 17:  transcript:Zm00001d034851_T001  rna11311            0
  9- 18:  transcript:Zm00001d034852_T002  rna11312    9.00E-127
  9- 19:  transcript:Zm00001d034853_T001  rna11314     9.00E-91
  9- 20:  transcript:Zm00001d034855_T001  rna11316    2.00E-101
  9- 21:  transcript:Zm00001d034856_T003  rna11317            0
  9- 22:  transcript:Zm00001d034857_T001  rna11318     2.00E-80
  9- 23:  transcript:Zm00001d034858_T002  rna11320     2.00E-41
  9- 24:  transcript:Zm00001d034859_T001  rna11321     3.00E-95
  9- 25:  transcript:Zm00001d034862_T002  rna11322            0
  9- 26:  transcript:Zm00001d034865_T001  rna11323            0
  9- 27:  transcript:Zm00001d034866_T001  rna11324    1.00E-179
  9- 28:  transcript:Zm00001d034868_T002  rna11325    1.00E-124
  9- 29:  transcript:Zm00001d034869_T001  rna11326     4.00E-50
  9- 30:  transcript:Zm00001d034871_T001  rna11327            0
  9- 31:  transcript:Zm00001d034872_T001  rna11328     2.00E-58
  9- 32:  transcript:Zm00001d034874_T001  rna11329     2.00E-19
  9- 33:  transcript:Zm00001d034875_T001  rna11333            0
  9- 34:  transcript:Zm00001d034876_T001  rna11334            0
  9- 35:  transcript:Zm00001d034877_T001  rna11336    2.00E-158
  9- 36:  transcript:Zm00001d034878_T001  rna11337     4.00E-36
  9- 37:  transcript:Zm00001d034879_T001  rna11338            0
  9- 38:  transcript:Zm00001d034880_T001  rna11339    1.00E-126
  9- 39:  transcript:Zm00001d034884_T001  rna11341     2.00E-77
  9- 40:  transcript:Zm00001d034885_T004  rna11342            0
  9- 41:  transcript:Zm00001d034886_T001  rna11346            0
  9- 42:  transcript:Zm00001d034888_T001  rna11350     9.00E-81
  9- 43:  transcript:Zm00001d034890_T001  rna11352    7.00E-117
  9- 44:  transcript:Zm00001d034892_T001  rna11355            0
  9- 45:  transcript:Zm00001d034894_T001  rna11356     2.00E-46
  9- 46:  transcript:Zm00001d034896_T003  rna11358            0
  9- 47:  transcript:Zm00001d034897_T001  rna11359    5.00E-117
  9- 48:  transcript:Zm00001d034898_T001  rna11360            0
  9- 49:  transcript:Zm00001d034904_T001  rna11364    2.00E-128
  9- 50:  transcript:Zm00001d034914_T037  rna11369            0
  9- 51:  transcript:Zm00001d034915_T001  rna11371            0
  9- 52:  transcript:Zm00001d034916_T001  rna11374            0
  9- 53:  transcript:Zm00001d034917_T001  rna11375    1.00E-170
  9- 54:  transcript:Zm00001d034918_T003  rna11377     2.00E-75
  9- 55:  transcript:Zm00001d034919_T001  rna11380     6.00E-95
  9- 56:  transcript:Zm00001d034920_T001  rna11385     2.00E-51
  9- 57:  transcript:Zm00001d034927_T001  rna11387            0
  9- 58:  transcript:Zm00001d034929_T011  rna11388            0
  9- 59:  transcript:Zm00001d034931_T013  rna11390            0
  9- 60:  transcript:Zm00001d034932_T001  rna11394     2.00E-99
  9- 61:  transcript:Zm00001d034933_T001  rna11396            0
  9- 62:  transcript:Zm00001d034936_T001  rna11397     2.00E-29
  9- 63:  transcript:Zm00001d034935_T007  rna11398            0
## Alignment 10: score=2821.0 e_value=1.5e-244 N=62 1&NC_008396.2 plus
 10-  0:  transcript:Zm00001d028347_T001  rna8609      6.00E-54
 10-  1:  transcript:Zm00001d028349_T001  rna8611     1.00E-105
 10-  2:  transcript:Zm00001d028352_T002  rna8613     4.00E-154
 10-  3:  transcript:Zm00001d028354_T001  rna8615     6.00E-122
 10-  4:  transcript:Zm00001d028358_T003  rna8620      9.00E-49
```

10- 5:	transcript:Zm00001d028359_T001	rna8621	0
10- 6:	transcript:Zm00001d028360_T001	rna8622	0
10- 7:	transcript:Zm00001d028361_T001	rna8623	5.00E-25
10- 8:	transcript:Zm00001d028362_T001	rna8625	1.00E-41
10- 9:	transcript:Zm00001d028363_T001	rna8626	0
10- 10:	transcript:Zm00001d028365_T006	rna8627	1.00E-144
10- 11:	transcript:Zm00001d028366_T001	rna8630	0
10- 12:	transcript:Zm00001d028367_T002	rna8631	6.00E-118
10- 13:	transcript:Zm00001d028369_T001	rna8633	0
10- 14:	transcript:Zm00001d028370_T001	rna8635	0
10- 15:	transcript:Zm00001d028371_T001	rna8636	6.00E-76
10- 16:	transcript:Zm00001d028372_T005	rna8637	0
10- 17:	transcript:Zm00001d028373_T002	rna8640	0
10- 18:	transcript:Zm00001d028374_T001	rna8641	0
10- 19:	transcript:Zm00001d028375_T001	rna8642	8.00E-65
10- 20:	transcript:Zm00001d028377_T001	rna8643	4.00E-77
10- 21:	transcript:Zm00001d028380_T002	rna8645	0
10- 22:	transcript:Zm00001d028384_T006	rna8646	1.00E-65
10- 23:	transcript:Zm00001d028385_T002	rna8647	3.00E-88
10- 24:	transcript:Zm00001d028386_T001	rna8648	2.00E-140
10- 25:	transcript:Zm00001d028388_T001	rna8649	0
10- 26:	transcript:Zm00001d028389_T001	rna8653	1.00E-37
10- 27:	transcript:Zm00001d028391_T001	rna8655	0
10- 28:	transcript:Zm00001d028392_T001	rna8656	3.00E-101
10- 29:	transcript:Zm00001d028396_T001	rna8658	0
10- 30:	transcript:Zm00001d028397_T001	rna8661	2.00E-129
10- 31:	transcript:Zm00001d028398_T003	rna8662	0
10- 32:	transcript:Zm00001d028399_T001	rna8664	6.00E-147
10- 33:	transcript:Zm00001d028400_T001	rna8666	7.00E-150
10- 34:	transcript:Zm00001d028401_T005	rna8667	0
10- 35:	transcript:Zm00001d028404_T001	rna8669	1.00E-57
10- 36:	transcript:Zm00001d028405_T002	rna8671	3.00E-84
10- 37:	transcript:Zm00001d028406_T001	rna8674	0
10- 38:	transcript:Zm00001d028408_T001	rna8675	2.00E-118
10- 39:	transcript:Zm00001d028410_T001	rna8677	3.00E-176
10- 40:	transcript:Zm00001d028412_T029	rna8678	0
10- 41:	transcript:Zm00001d028413_T001	rna8681	3.00E-98
10- 42:	transcript:Zm00001d028414_T002	rna8682	5.00E-126
10- 43:	transcript:Zm00001d028415_T005	rna8683	0
10- 44:	transcript:Zm00001d028416_T001	rna8688	5.00E-67
10- 45:	transcript:Zm00001d028417_T001	rna8689	0
10- 46:	transcript:Zm00001d028419_T001	rna8690	0
10- 47:	transcript:Zm00001d028423_T001	rna8694	0
10- 48:	transcript:Zm00001d028424_T002	rna8695	2.00E-120
10- 49:	transcript:Zm00001d028425_T003	rna8696	4.00E-99
10- 50:	transcript:Zm00001d028426_T001	rna8697	6.00E-83
10- 51:	transcript:Zm00001d028427_T001	rna8698	0
10- 52:	transcript:Zm00001d028428_T001	rna8701	4.00E-40
10- 53:	transcript:Zm00001d028429_T017	rna8702	4.00E-103
10- 54:	transcript:Zm00001d028432_T001	rna8708	6.00E-119
10- 55:	transcript:Zm00001d028436_T001	rna8709	0
10- 56:	transcript:Zm00001d028439_T001	rna8710	0
10- 57:	transcript:Zm00001d028440_T001	rna8712	5.00E-168
10- 58:	transcript:Zm00001d028443_T001	rna8713	0

10- 59:	transcript:Zm00001d028447_T001	rna8715	0
10- 60:	transcript:Zm00001d028451_T001	rna8718	0
10- 61:	transcript:Zm00001d028463_T003	rna8735	2.00E-107

Alignment 11: score=2372.0 e_value=8.6e-192 N=51 1&NC_008396.2 plus

11- 0:	transcript:Zm00001d028909_T001	rna9082	6.00E-173
11- 1:	transcript:Zm00001d028910_T002	rna9083	0
11- 2:	transcript:Zm00001d028912_T001	rna9084	0
11- 3:	transcript:Zm00001d028915_T023	rna9089	2.00E-44
11- 4:	transcript:Zm00001d028916_T001	rna9091	2.00E-62
11- 5:	transcript:Zm00001d028918_T001	rna9095	1.00E-106
11- 6:	transcript:Zm00001d028919_T001	rna9096	7.00E-137
11- 7:	transcript:Zm00001d028920_T004	rna9097	0
11- 8:	transcript:Zm00001d028921_T004	rna9098	0
11- 9:	transcript:Zm00001d028922_T001	rna9099	4.00E-29
11- 10:	transcript:Zm00001d028923_T003	rna9101	0
11- 11:	transcript:Zm00001d028924_T001	rna9105	6.00E-120
11- 12:	transcript:Zm00001d028926_T006	rna9107	0
11- 13:	transcript:Zm00001d028930_T001	rna9108	4.00E-138
11- 14:	transcript:Zm00001d028931_T002	rna9110	0
11- 15:	transcript:Zm00001d028935_T001	rna9114	0
11- 16:	transcript:Zm00001d028936_T002	rna9120	0
11- 17:	transcript:Zm00001d028948_T001	rna9124	3.00E-86
11- 18:	transcript:Zm00001d028949_T002	rna9125	0
11- 19:	transcript:Zm00001d028951_T001	rna9126	5.00E-144
11- 20:	transcript:Zm00001d028952_T004	rna9127	4.00E-151
11- 21:	transcript:Zm00001d028953_T001	rna9130	5.00E-38
11- 22:	transcript:Zm00001d028954_T001	rna9132	6.00E-162
11- 23:	transcript:Zm00001d028955_T001	rna9133	2.00E-87
11- 24:	transcript:Zm00001d028957_T001	rna9135	7.00E-160
11- 25:	transcript:Zm00001d028958_T001	rna9136	2.00E-53
11- 26:	transcript:Zm00001d028960_T001	rna9137	0
11- 27:	transcript:Zm00001d028962_T001	rna9138	2.00E-104
11- 28:	transcript:Zm00001d028963_T001	rna9143	2.00E-86
11- 29:	transcript:Zm00001d028964_T002	rna9144	1.00E-166
11- 30:	transcript:Zm00001d028966_T005	rna9145	0
11- 31:	transcript:Zm00001d028967_T001	rna9150	0
11- 32:	transcript:Zm00001d028971_T002	rna9153	0
11- 33:	transcript:Zm00001d028973_T001	rna9154	9.00E-46
11- 34:	transcript:Zm00001d028974_T001	rna9155	0
11- 35:	transcript:Zm00001d028975_T001	rna9156	0
11- 36:	transcript:Zm00001d028980_T001	rna9157	0
11- 37:	transcript:Zm00001d028981_T002	rna9158	2.00E-133
11- 38:	transcript:Zm00001d028982_T001	rna9159	7.00E-24
11- 39:	transcript:Zm00001d028983_T001	rna9160	1.00E-34
11- 40:	transcript:Zm00001d028984_T001	rna9164	0
11- 41:	transcript:Zm00001d028986_T001	rna9165	1.00E-109
11- 42:	transcript:Zm00001d028987_T001	rna9167	1.00E-138
11- 43:	transcript:Zm00001d028988_T001	rna9168	0
11- 44:	transcript:Zm00001d028989_T002	rna9169	1.00E-162
11- 45:	transcript:Zm00001d028992_T001	rna9170	2.00E-47
11- 46:	transcript:Zm00001d028994_T001	rna9171	0
11- 47:	transcript:Zm00001d028995_T001	rna9172	4.00E-146
11- 48:	transcript:Zm00001d028998_T001	rna9173	0
11- 49:	transcript:Zm00001d028999_T001	rna9175	6.00E-95

11- 50: transcript:Zm00001d029004_T004 rna9176 0
Alignment 12: score=2267.0 e_value=2.3e-187 N=50 1&NC_008396.2 plus
12- 0: transcript:Zm00001d027308_T001 rna7748 2.00E-154
12- 1: transcript:Zm00001d027309_T007 rna7751 3.00E-29
12- 2: transcript:Zm00001d027311_T001 rna7752 0
12- 3: transcript:Zm00001d027312_T002 rna7755 1.00E-175
12- 4: transcript:Zm00001d027313_T001 rna7756 1.00E-165
12- 5: transcript:Zm00001d027314_T001 rna7757 2.00E-93
12- 6: transcript:Zm00001d027315_T002 rna7760 5.00E-22
12- 7: transcript:Zm00001d027317_T015 rna7766 0
12- 8: transcript:Zm00001d027318_T001 rna7768 2.00E-94
12- 9: transcript:Zm00001d027322_T001 rna7770 8.00E-26
12- 10: transcript:Zm00001d027323_T001 rna7773 2.00E-50
12- 11: transcript:Zm00001d027324_T002 rna7774 0
12- 12: transcript:Zm00001d027325_T002 rna7776 3.00E-41
12- 13: transcript:Zm00001d027329_T002 rna7778 0
12- 14: transcript:Zm00001d027330_T001 rna7782 1.00E-84
12- 15: transcript:Zm00001d027332_T001 rna7783 8.00E-30
12- 16: transcript:Zm00001d027333_T018 rna7789 6.00E-79
12- 17: transcript:Zm00001d027334_T001 rna7790 1.00E-73
12- 18: transcript:Zm00001d027335_T001 rna7797 3.00E-22
12- 19: transcript:Zm00001d027337_T001 rna7801 0
12- 20: transcript:Zm00001d027338_T001 rna7802 6.00E-13
12- 21: transcript:Zm00001d027339_T001 rna7803 1.00E-74
12- 22: transcript:Zm00001d027340_T001 rna7804 0
12- 23: transcript:Zm00001d027341_T001 rna7805 0
12- 24: transcript:Zm00001d027344_T001 rna7808 0
12- 25: transcript:Zm00001d027345_T001 rna7809 2.00E-111
12- 26: transcript:Zm00001d027346_T001 rna7810 3.00E-72
12- 27: transcript:Zm00001d027347_T002 rna7811 0
12- 28: transcript:Zm00001d027349_T001 rna7813 0
12- 29: transcript:Zm00001d027351_T001 rna7814 0
12- 30: transcript:Zm00001d027353_T001 rna7816 0
12- 31: transcript:Zm00001d027354_T001 rna7818 1.00E-168
12- 32: transcript:Zm00001d027355_T001 rna7820 0
12- 33: transcript:Zm00001d027359_T001 rna7821 0
12- 34: transcript:Zm00001d027361_T001 rna7823 7.00E-169
12- 35: transcript:Zm00001d027362_T001 rna7824 7.00E-176
12- 36: transcript:Zm00001d027365_T001 rna7825 0
12- 37: transcript:Zm00001d027366_T001 rna7827 1.00E-161
12- 38: transcript:Zm00001d027367_T002 rna7828 0
12- 39: transcript:Zm00001d027368_T001 rna7829 7.00E-125
12- 40: transcript:Zm00001d027369_T002 rna7830 9.00E-164
12- 41: transcript:Zm00001d027370_T001 rna7832 9.00E-57
12- 42: transcript:Zm00001d027373_T003 rna7834 2.00E-164
12- 43: transcript:Zm00001d027375_T001 rna7835 5.00E-45
12- 44: transcript:Zm00001d027383_T001 rna7839 0
12- 45: transcript:Zm00001d027384_T001 rna7840 0
12- 46: transcript:Zm00001d027385_T003 rna7841 2.00E-145
12- 47: transcript:Zm00001d027386_T002 rna7842 0
12- 48: transcript:Zm00001d027387_T001 rna7843 0
12- 49: transcript:Zm00001d027392_T003 rna7844 0
Alignment 13: score=2191.0 e_value=1.1e-175 N=48 1&NC_008396.2 plus
13- 0: transcript:Zm00001d034457_T003 rna10929 0

```
 13-  1:  transcript:Zm00001d034460_T010  rna10930    5.00E-163
 13-  2:  transcript:Zm00001d034462_T001  rna10935     2.00E-31
 13-  3:  transcript:Zm00001d034463_T003  rna10936     5.00E-56
 13-  4:  transcript:Zm00001d034468_T001  rna10939    3.00E-164
 13-  5:  transcript:Zm00001d034469_T001  rna10941     5.00E-74
 13-  6:  transcript:Zm00001d034475_T001  rna10942     2.00E-76
 13-  7:  transcript:Zm00001d034479_T001  rna10944     4.00E-37
 13-  8:  transcript:Zm00001d034480_T002  rna10945    1.00E-158
 13-  9:  transcript:Zm00001d034482_T001  rna10947     5.00E-10
 13- 10:  transcript:Zm00001d034485_T001  rna10948    9.00E-128
 13- 11:  transcript:Zm00001d034486_T003  rna10950            0
 13- 12:  transcript:Zm00001d034487_T002  rna10951     9.00E-16
 13- 13:  transcript:Zm00001d034488_T001  rna10952            0
 13- 14:  transcript:Zm00001d034490_T001  rna10953     2.00E-74
 13- 15:  transcript:Zm00001d034491_T001  rna10957     2.00E-70
 13- 16:  transcript:Zm00001d034492_T006  rna10964            0
 13- 17:  transcript:Zm00001d034493_T001  rna10965            0
 13- 18:  transcript:Zm00001d034494_T001  rna10967    3.00E-145
 13- 19:  transcript:Zm00001d034495_T001  rna10968            0
 13- 20:  transcript:Zm00001d034497_T001  rna10971     5.00E-16
 13- 21:  transcript:Zm00001d034498_T007  rna10972            0
 13- 22:  transcript:Zm00001d034501_T001  rna10974            0
 13- 23:  transcript:Zm00001d034502_T003  rna10977     1.00E-70
 13- 24:  transcript:Zm00001d034503_T001  rna10978    1.00E-126
 13- 25:  transcript:Zm00001d034505_T001  rna10979    8.00E-108
 13- 26:  transcript:Zm00001d034506_T001  rna10980     2.00E-07
 13- 27:  transcript:Zm00001d034508_T001  rna10984     2.00E-64
 13- 28:  transcript:Zm00001d034510_T001  rna10985            0
 13- 29:  transcript:Zm00001d034512_T002  rna10989     3.00E-93
 13- 30:  transcript:Zm00001d034513_T001  rna10990     5.00E-48
 13- 31:  transcript:Zm00001d034514_T001  rna10993    1.00E-106
 13- 32:  transcript:Zm00001d034516_T001  rna10999            0
 13- 33:  transcript:Zm00001d034517_T001  rna11001            0
 13- 34:  transcript:Zm00001d034518_T001  rna11002            0
 13- 35:  transcript:Zm00001d034519_T001  rna11003            0
 13- 36:  transcript:Zm00001d034523_T001  rna11004     2.00E-17
 13- 37:  transcript:Zm00001d034525_T001  rna11009    4.00E-153
 13- 38:  transcript:Zm00001d034526_T001  rna11010     1.00E-18
 13- 39:  transcript:Zm00001d034527_T001  rna11011            0
 13- 40:  transcript:Zm00001d034528_T001  rna11013     8.00E-69
 13- 41:  transcript:Zm00001d034533_T002  rna11015     2.00E-34
 13- 42:  transcript:Zm00001d034534_T001  rna11016    3.00E-104
 13- 43:  transcript:Zm00001d034546_T001  rna11018     4.00E-43
 13- 44:  transcript:Zm00001d034547_T002  rna11020            0
 13- 45:  transcript:Zm00001d034550_T001  rna11021    1.00E-160
 13- 46:  transcript:Zm00001d034551_T001  rna11023            0
 13- 47:  transcript:Zm00001d034553_T001  rna11024            0
## Alignment 14: score=2105.0 e_value=1.4e-169 N=46 1&NC_008396.2 plus
 14-  0:  transcript:Zm00001d029427_T001  rna9523     5.00E-148
 14-  1:  transcript:Zm00001d029428_T001  rna9524             0
 14-  2:  transcript:Zm00001d029429_T001  rna9527             0
 14-  3:  transcript:Zm00001d029432_T002  rna9529             0
 14-  4:  transcript:Zm00001d029435_T001  rna9530             0
 14-  5:  transcript:Zm00001d029438_T001  rna9535      1.00E-69
```

14- 6:	transcript:Zm00001d029441_T002	rna9537	0
14- 7:	transcript:Zm00001d029442_T001	rna9539	0
14- 8:	transcript:Zm00001d029443_T012	rna9540	0
14- 9:	transcript:Zm00001d029444_T001	rna9546	2.00E-15
14- 10:	transcript:Zm00001d029448_T001	rna9553	4.00E-107
14- 11:	transcript:Zm00001d029454_T010	rna9558	6.00E-111
14- 12:	transcript:Zm00001d029455_T001	rna9560	3.00E-170
14- 13:	transcript:Zm00001d029457_T001	rna9563	0
14- 14:	transcript:Zm00001d029460_T001	rna9566	2.00E-119
14- 15:	transcript:Zm00001d029461_T003	rna9568	3.00E-52
14- 16:	transcript:Zm00001d029462_T002	rna9569	0
14- 17:	transcript:Zm00001d029464_T001	rna9571	4.00E-17
14- 18:	transcript:Zm00001d029468_T002	rna9572	0
14- 19:	transcript:Zm00001d029473_T001	rna9574	8.00E-96
14- 20:	transcript:Zm00001d029475_T001	rna9575	1.00E-23
14- 21:	transcript:Zm00001d029476_T001	rna9576	2.00E-86
14- 22:	transcript:Zm00001d029482_T002	rna9582	4.00E-22
14- 23:	transcript:Zm00001d029483_T001	rna9583	3.00E-50
14- 24:	transcript:Zm00001d029487_T001	rna9584	0
14- 25:	transcript:Zm00001d029488_T001	rna9585	5.00E-14
14- 26:	transcript:Zm00001d029495_T001	rna9586	2.00E-48
14- 27:	transcript:Zm00001d029497_T001	rna9589	0
14- 28:	transcript:Zm00001d029498_T001	rna9592	4.00E-43
14- 29:	transcript:Zm00001d029499_T002	rna9593	8.00E-35
14- 30:	transcript:Zm00001d029501_T001	rna9594	5.00E-11
14- 31:	transcript:Zm00001d029502_T001	rna9595	0
14- 32:	transcript:Zm00001d029505_T001	rna9596	3.00E-85
14- 33:	transcript:Zm00001d029515_T002	rna9597	0
14- 34:	transcript:Zm00001d029516_T001	rna9598	0
14- 35:	transcript:Zm00001d029518_T001	rna9599	1.00E-19
14- 36:	transcript:Zm00001d029524_T001	rna9601	8.00E-13
14- 37:	transcript:Zm00001d029525_T001	rna9609	8.00E-160
14- 38:	transcript:Zm00001d029526_T001	rna9610	0
14- 39:	transcript:Zm00001d029527_T003	rna9611	2.00E-25
14- 40:	transcript:Zm00001d029529_T001	rna9618	5.00E-111
14- 41:	transcript:Zm00001d029531_T003	rna9620	3.00E-96
14- 42:	transcript:Zm00001d029534_T001	rna9622	1.00E-167
14- 43:	transcript:Zm00001d029539_T001	rna9625	2.00E-48
14- 44:	transcript:Zm00001d029540_T001	rna9630	6.00E-152
14- 45:	transcript:Zm00001d029543_T001	rna9637	3.00E-99

Alignment 15: score=2105.0 e_value=6.3e-167 N=45 1&NC_008396.2 plus

15- 0:	transcript:Zm00001d027667_T005	rna8044	0
15- 1:	transcript:Zm00001d027671_T001	rna8045	0
15- 2:	transcript:Zm00001d027672_T001	rna8046	4.00E-56
15- 3:	transcript:Zm00001d027673_T001	rna8047	0
15- 4:	transcript:Zm00001d027676_T001	rna8049	0
15- 5:	transcript:Zm00001d027677_T001	rna8050	3.00E-161
15- 6:	transcript:Zm00001d027678_T001	rna8051	3.00E-85
15- 7:	transcript:Zm00001d027680_T003	rna8053	2.00E-83
15- 8:	transcript:Zm00001d027682_T001	rna8054	9.00E-24
15- 9:	transcript:Zm00001d027683_T001	rna8055	6.00E-169
15- 10:	transcript:Zm00001d027684_T001	rna8056	4.00E-91
15- 11:	transcript:Zm00001d027686_T001	rna8058	2.00E-46
15- 12:	transcript:Zm00001d027700_T001	rna8059	0

15- 13: transcript:Zm00001d027701_T001 rna8064 0
15- 14: transcript:Zm00001d027703_T001 rna8067 4.00E-10
15- 15: transcript:Zm00001d027706_T004 rna8069 0
15- 16: transcript:Zm00001d027707_T001 rna8070 0
15- 17: transcript:Zm00001d027708_T002 rna8072 3.00E-89
15- 18: transcript:Zm00001d027709_T001 rna8073 0
15- 19: transcript:Zm00001d027710_T001 rna8074 2.00E-159
15- 20: transcript:Zm00001d027711_T001 rna8075 4.00E-28
15- 21: transcript:Zm00001d027714_T001 rna8077 0
15- 22: transcript:Zm00001d027715_T001 rna8079 0
15- 23: transcript:Zm00001d027717_T001 rna8088 4.00E-128
15- 24: transcript:Zm00001d027719_T001 rna8092 3.00E-40
15- 25: transcript:Zm00001d027720_T001 rna8093 3.00E-82
15- 26: transcript:Zm00001d027721_T001 rna8094 3.00E-165
15- 27: transcript:Zm00001d027722_T001 rna8096 0
15- 28: transcript:Zm00001d027723_T002 rna8097 0
15- 29: transcript:Zm00001d027726_T001 rna8102 0
15- 30: transcript:Zm00001d027728_T001 rna8105 2.00E-53
15- 31: transcript:Zm00001d027729_T001 rna8106 0
15- 32: transcript:Zm00001d027731_T012 rna8108 0
15- 33: transcript:Zm00001d027732_T001 rna8110 5.00E-144
15- 34: transcript:Zm00001d027733_T001 rna8111 7.00E-36
15- 35: transcript:Zm00001d027734_T004 rna8114 0
15- 36: transcript:Zm00001d027738_T001 rna8115 1.00E-122
15- 37: transcript:Zm00001d027739_T001 rna8116 1.00E-50
15- 38: transcript:Zm00001d027740_T001 rna8117 8.00E-86
15- 39: transcript:Zm00001d027741_T001 rna8118 0
15- 40: transcript:Zm00001d027742_T001 rna8120 4.00E-52
15- 41: transcript:Zm00001d027743_T002 rna8122 0
15- 42: transcript:Zm00001d027746_T001 rna8124 3.00E-40
15- 43: transcript:Zm00001d027748_T004 rna8125 0
15- 44: transcript:Zm00001d027749_T001 rna8130 0
Alignment 16: score=1995.0 e_value=7e-153 N=42 1&NC_008396.2 plus
16- 0: transcript:Zm00001d028750_T002 rna8952 1.00E-47
16- 1: transcript:Zm00001d028752_T001 rna8955 0
16- 2: transcript:Zm00001d028753_T003 rna8956 0
16- 3: transcript:Zm00001d028755_T001 rna8958 2.00E-103
16- 4: transcript:Zm00001d028756_T001 rna8961 7.00E-82
16- 5: transcript:Zm00001d028759_T001 rna8964 0
16- 6: transcript:Zm00001d028761_T002 rna8969 3.00E-64
16- 7: transcript:Zm00001d028762_T005 rna8970 0
16- 8: transcript:Zm00001d028767_T001 rna8971 8.00E-46
16- 9: transcript:Zm00001d028768_T001 rna8972 4.00E-97
16- 10: transcript:Zm00001d028769_T004 rna8974 0
16- 11: transcript:Zm00001d028770_T003 rna8975 0
16- 12: transcript:Zm00001d028771_T001 rna8976 0
16- 13: transcript:Zm00001d028773_T002 rna8979 8.00E-69
16- 14: transcript:Zm00001d028774_T001 rna8980 0
16- 15: transcript:Zm00001d028775_T001 rna8982 2.00E-74
16- 16: transcript:Zm00001d028777_T001 rna8983 4.00E-134
16- 17: transcript:Zm00001d028778_T002 rna8985 2.00E-34
16- 18: transcript:Zm00001d028779_T001 rna8986 1.00E-67
16- 19: transcript:Zm00001d028782_T003 rna8987 0
16- 20: transcript:Zm00001d028783_T003 rna8989 3.00E-104

16- 21: transcript:Zm00001d028784_T001 rna8990 0
16- 22: transcript:Zm00001d028785_T001 rna8992 2.00E-105
16- 23: transcript:Zm00001d028786_T001 rna8993 2.00E-131
16- 24: transcript:Zm00001d028787_T001 rna8995 0
16- 25: transcript:Zm00001d028793_T001 rna8996 1.00E-108
16- 26: transcript:Zm00001d028796_T001 rna8998 0
16- 27: transcript:Zm00001d028797_T001 rna8999 0
16- 28: transcript:Zm00001d028798_T001 rna9000 4.00E-75
16- 29: transcript:Zm00001d028799_T001 rna9001 9.00E-14
16- 30: transcript:Zm00001d028801_T001 rna9002 0
16- 31: transcript:Zm00001d028802_T001 rna9004 1.00E-71
16- 32: transcript:Zm00001d028803_T002 rna9005 0
16- 33: transcript:Zm00001d028804_T001 rna9006 2.00E-146
16- 34: transcript:Zm00001d028806_T001 rna9008 3.00E-175
16- 35: transcript:Zm00001d028808_T001 rna9009 9.00E-77
16- 36: transcript:Zm00001d028809_T001 rna9011 0
16- 37: transcript:Zm00001d028810_T002 rna9012 0
16- 38: transcript:Zm00001d028811_T001 rna9013 0
16- 39: transcript:Zm00001d028812_T005 rna9015 0
16- 40: transcript:Zm00001d028813_T001 rna9016 0
16- 41: transcript:Zm00001d028814_T001 rna9017 4.00E-100
Alignment 17: score=1836.0 e_value=6.6e-143 N=40 1&NC_008396.2 plus
17- 0: transcript:Zm00001d034634_T001 rna11096 4.00E-93
17- 1: transcript:Zm00001d034635_T001 rna11097 1.00E-121
17- 2: transcript:Zm00001d034636_T001 rna11098 2.00E-134
17- 3: transcript:Zm00001d034638_T001 rna11099 2.00E-155
17- 4: transcript:Zm00001d034640_T003 rna11100 0
17- 5: transcript:Zm00001d034641_T001 rna11101 5.00E-55
17- 6: transcript:Zm00001d034643_T001 rna11103 6.00E-74
17- 7: transcript:Zm00001d034649_T001 rna11105 7.00E-100
17- 8: transcript:Zm00001d034650_T017 rna11106 0
17- 9: transcript:Zm00001d034651_T001 rna11108 2.00E-135
17- 10: transcript:Zm00001d034655_T001 rna11110 0
17- 11: transcript:Zm00001d034659_T001 rna11115 0
17- 12: transcript:Zm00001d034662_T001 rna11116 0
17- 13: transcript:Zm00001d034663_T001 rna11117 8.00E-164
17- 14: transcript:Zm00001d034665_T001 rna11119 4.00E-176
17- 15: transcript:Zm00001d034666_T001 rna11120 2.00E-125
17- 16: transcript:Zm00001d034667_T003 rna11122 0
17- 17: transcript:Zm00001d034668_T001 rna11123 6.00E-48
17- 18: transcript:Zm00001d034670_T005 rna11124 0
17- 19: transcript:Zm00001d034671_T001 rna11125 0
17- 20: transcript:Zm00001d034673_T001 rna11127 3.00E-32
17- 21: transcript:Zm00001d034674_T001 rna11128 0
17- 22: transcript:Zm00001d034675_T002 rna11129 3.00E-153
17- 23: transcript:Zm00001d034678_T001 rna11130 0
17- 24: transcript:Zm00001d034680_T001 rna11132 6.00E-44
17- 25: transcript:Zm00001d034681_T001 rna11133 7.00E-16
17- 26: transcript:Zm00001d034682_T001 rna11137 5.00E-167
17- 27: transcript:Zm00001d034688_T001 rna11138 1.00E-26
17- 28: transcript:Zm00001d034692_T001 rna11139 0
17- 29: transcript:Zm00001d034698_T001 rna11142 2.00E-51
17- 30: transcript:Zm00001d034699_T001 rna11143 0
17- 31: transcript:Zm00001d034700_T001 rna11144 1.00E-175

```
 17- 32:  transcript:Zm00001d034701_T023  rna11145            0
 17- 33:  transcript:Zm00001d034710_T003  rna11151    2.00E-117
 17- 34:  transcript:Zm00001d034713_T002  rna11152            0
 17- 35:  transcript:Zm00001d034714_T001  rna11153            0
 17- 36:  transcript:Zm00001d034717_T002  rna11154    4.00E-123
 17- 37:  transcript:Zm00001d034718_T001  rna11157    2.00E-151
 17- 38:  transcript:Zm00001d034721_T002  rna11159            0
 17- 39:  transcript:Zm00001d034723_T001  rna11161     2.00E-75
## Alignment 18: score=1660.0 e_value=7.8e-126 N=36 1&NC_008396.2 plus
 18-  0:  transcript:Zm00001d033941_T001  rna10560            0
 18-  1:  transcript:Zm00001d033943_T001  rna10563     4.00E-55
 18-  2:  transcript:Zm00001d033954_T001  rna10565            0
 18-  3:  transcript:Zm00001d033955_T001  rna10566    1.00E-143
 18-  4:  transcript:Zm00001d033957_T002  rna10571     4.00E-89
 18-  5:  transcript:Zm00001d033965_T001  rna10572            0
 18-  6:  transcript:Zm00001d033966_T001  rna10574    1.00E-118
 18-  7:  transcript:Zm00001d033967_T001  rna10575     5.00E-40
 18-  8:  transcript:Zm00001d033968_T001  rna10576     8.00E-55
 18-  9:  transcript:Zm00001d033969_T002  rna10577    5.00E-136
 18- 10:  transcript:Zm00001d033975_T002  rna10578            0
 18- 11:  transcript:Zm00001d033976_T002  rna10579    1.00E-101
 18- 12:  transcript:Zm00001d033979_T001  rna10581     9.00E-94
 18- 13:  transcript:Zm00001d033980_T001  rna10582     1.00E-75
 18- 14:  transcript:Zm00001d033981_T001  rna10584            0
 18- 15:  transcript:Zm00001d033982_T001  rna10587            0
 18- 16:  transcript:Zm00001d033983_T001  rna10588    4.00E-116
 18- 17:  transcript:Zm00001d033984_T002  rna10589            0
 18- 18:  transcript:Zm00001d033985_T001  rna10590    1.00E-153
 18- 19:  transcript:Zm00001d033986_T003  rna10591    8.00E-140
 18- 20:  transcript:Zm00001d033987_T001  rna10592    1.00E-140
 18- 21:  transcript:Zm00001d033988_T001  rna10593    4.00E-142
 18- 22:  transcript:Zm00001d033989_T001  rna10594            0
 18- 23:  transcript:Zm00001d033990_T001  rna10597    2.00E-155
 18- 24:  transcript:Zm00001d033991_T001  rna10598            0
 18- 25:  transcript:Zm00001d033992_T009  rna10600            0
 18- 26:  transcript:Zm00001d033993_T002  rna10601            0
 18- 27:  transcript:Zm00001d033994_T002  rna10602            0
 18- 28:  transcript:Zm00001d033995_T002  rna10603     8.00E-79
 18- 29:  transcript:Zm00001d033998_T001  rna10605     3.00E-34
 18- 30:  transcript:Zm00001d033999_T001  rna10606     1.00E-56
 18- 31:  transcript:Zm00001d034000_T005  rna10607    5.00E-178
 18- 32:  transcript:Zm00001d034001_T001  rna10608            0
 18- 33:  transcript:Zm00001d034002_T001  rna10609     5.00E-93
 18- 34:  transcript:Zm00001d034003_T001  rna10610     2.00E-27
 18- 35:  transcript:Zm00001d034004_T004  rna10611    6.00E-152
## Alignment 19: score=1458.0 e_value=2.5e-111 N=33 1&NC_008396.2 plus
 19-  0:  transcript:Zm00001d028204_T002  rna8477      2.00E-34
 19-  1:  transcript:Zm00001d028205_T002  rna8479             0
 19-  2:  transcript:Zm00001d028208_T009  rna8480      1.00E-93
 19-  3:  transcript:Zm00001d028210_T001  rna8482             0
 19-  4:  transcript:Zm00001d028211_T002  rna8484             0
 19-  5:  transcript:Zm00001d028213_T001  rna8486             0
 19-  6:  transcript:Zm00001d028214_T001  rna8487             0
 19-  7:  transcript:Zm00001d028216_T001  rna8488     4.00E-115
```

```
19-  8:  transcript:Zm00001d028217_T001  rna8489     9.00E-120
19-  9:  transcript:Zm00001d028219_T001  rna8491             0
19- 10:  transcript:Zm00001d028220_T001  rna8492             0
19- 11:  transcript:Zm00001d028221_T001  rna8493             0
19- 12:  transcript:Zm00001d028222_T001  rna8494      4.00E-94
19- 13:  transcript:Zm00001d028225_T001  rna8495             0
19- 14:  transcript:Zm00001d028226_T001  rna8497     2.00E-153
19- 15:  transcript:Zm00001d028227_T009  rna8498             0
19- 16:  transcript:Zm00001d028228_T001  rna8499             0
19- 17:  transcript:Zm00001d028229_T001  rna8501      9.00E-81
19- 18:  transcript:Zm00001d028230_T001  rna8509             0
19- 19:  transcript:Zm00001d028231_T003  rna8511             0
19- 20:  transcript:Zm00001d028232_T012  rna8512      1.00E-60
19- 21:  transcript:Zm00001d028235_T001  rna8514             0
19- 22:  transcript:Zm00001d028240_T001  rna8519     3.00E-139
19- 23:  transcript:Zm00001d028241_T003  rna8520             0
19- 24:  transcript:Zm00001d028243_T001  rna8526             0
19- 25:  transcript:Zm00001d028245_T001  rna8528             0
19- 26:  transcript:Zm00001d028244_T010  rna8529      7.00E-92
19- 27:  transcript:Zm00001d028248_T001  rna8530             0
19- 28:  transcript:Zm00001d028249_T003  rna8532      7.00E-42
19- 29:  transcript:Zm00001d028256_T002  rna8533      2.00E-25
19- 30:  transcript:Zm00001d028258_T001  rna8535             0
19- 31:  transcript:Zm00001d028260_T005  rna8538             0
19- 32:  transcript:Zm00001d028261_T005  rna8539             0
## Alignment 20: score=1343.0 e_value=3.5e-93 N=29 1&NC_008396.2 plus
20-  0:  transcript:Zm00001d028819_T001  rna9018             0
20-  1:  transcript:Zm00001d028820_T001  rna9019      2.00E-65
20-  2:  transcript:Zm00001d028824_T001  rna9021             0
20-  3:  transcript:Zm00001d028826_T001  rna9023             0
20-  4:  transcript:Zm00001d028827_T006  rna9024     1.00E-168
20-  5:  transcript:Zm00001d028829_T001  rna9025      3.00E-15
20-  6:  transcript:Zm00001d028834_T001  rna9029     3.00E-104
20-  7:  transcript:Zm00001d028837_T001  rna9034      4.00E-57
20-  8:  transcript:Zm00001d028838_T001  rna9035      5.00E-68
20-  9:  transcript:Zm00001d028840_T002  rna9036             0
20- 10:  transcript:Zm00001d028841_T001  rna9037     2.00E-134
20- 11:  transcript:Zm00001d028862_T001  rna9038      2.00E-76
20- 12:  transcript:Zm00001d028863_T001  rna9040             0
20- 13:  transcript:Zm00001d028866_T001  rna9041             0
20- 14:  transcript:Zm00001d028867_T001  rna9042      1.00E-36
20- 15:  transcript:Zm00001d028868_T002  rna9044     6.00E-161
20- 16:  transcript:Zm00001d028870_T001  rna9045      1.00E-61
20- 17:  transcript:Zm00001d028873_T001  rna9048      1.00E-90
20- 18:  transcript:Zm00001d028874_T001  rna9050             0
20- 19:  transcript:Zm00001d028875_T001  rna9054             0
20- 20:  transcript:Zm00001d028879_T001  rna9055      2.00E-49
20- 21:  transcript:Zm00001d028880_T001  rna9056      8.00E-51
20- 22:  transcript:Zm00001d028885_T001  rna9058     5.00E-129
20- 23:  transcript:Zm00001d028887_T001  rna9060     5.00E-174
20- 24:  transcript:Zm00001d028889_T001  rna9063     4.00E-161
20- 25:  transcript:Zm00001d028890_T002  rna9066             0
20- 26:  transcript:Zm00001d028894_T001  rna9068     1.00E-180
20- 27:  transcript:Zm00001d028895_T001  rna9069     7.00E-168
```

```
 20- 28:  transcript:Zm00001d028896_T006  rna9070             0
## Alignment 21: score=1211.0 e_value=6.5e-81 N=26 1&NC_008396.2 plus
 21-  0:  transcript:Zm00001d033558_T001  rna10268            0
 21-  1:  transcript:Zm00001d033559_T001  rna10269     6.00E-47
 21-  2:  transcript:Zm00001d033563_T001  rna10274            0
 21-  3:  transcript:Zm00001d033567_T001  rna10275            0
 21-  4:  transcript:Zm00001d033568_T001  rna10276            0
 21-  5:  transcript:Zm00001d033572_T004  rna10277            0
 21-  6:  transcript:Zm00001d033573_T001  rna10278            0
 21-  7:  transcript:Zm00001d033575_T002  rna10280     3.00E-46
 21-  8:  transcript:Zm00001d033579_T002  rna10283     3.00E-77
 21-  9:  transcript:Zm00001d033580_T001  rna10284            0
 21- 10:  transcript:Zm00001d033583_T001  rna10285    3.00E-125
 21- 11:  transcript:Zm00001d033585_T002  rna10289            0
 21- 12:  transcript:Zm00001d033589_T001  rna10291     6.00E-41
 21- 13:  transcript:Zm00001d033590_T001  rna10292     4.00E-74
 21- 14:  transcript:Zm00001d033594_T001  rna10296    1.00E-178
 21- 15:  transcript:Zm00001d033596_T001  rna10297    7.00E-135
 21- 16:  transcript:Zm00001d033597_T004  rna10298    2.00E-149
 21- 17:  transcript:Zm00001d033600_T003  rna10299     1.00E-99
 21- 18:  transcript:Zm00001d033602_T002  rna10300    2.00E-121
 21- 19:  transcript:Zm00001d033605_T001  rna10301    5.00E-109
 21- 20:  transcript:Zm00001d033606_T001  rna10305     3.00E-80
 21- 21:  transcript:Zm00001d033610_T001  rna10306            0
 21- 22:  transcript:Zm00001d033611_T002  rna10307            0
 21- 23:  transcript:Zm00001d033612_T001  rna10308     3.00E-56
 21- 24:  transcript:Zm00001d033615_T002  rna10309     9.00E-66
 21- 25:  transcript:Zm00001d033616_T016  rna10310    1.00E-164
## Alignment 22: score=1116.0 e_value=6.6e-72 N=24 1&NC_008396.2 plus
 22-  0:  transcript:Zm00001d033664_T001  rna10345    6.00E-176
 22-  1:  transcript:Zm00001d033665_T002  rna10346     1.00E-75
 22-  2:  transcript:Zm00001d033666_T001  rna10347     6.00E-94
 22-  3:  transcript:Zm00001d033668_T006  rna10349            0
 22-  4:  transcript:Zm00001d033669_T003  rna10350            0
 22-  5:  transcript:Zm00001d033670_T001  rna10351            0
 22-  6:  transcript:Zm00001d033671_T001  rna10354    6.00E-134
 22-  7:  transcript:Zm00001d033673_T001  rna10355    1.00E-108
 22-  8:  transcript:Zm00001d033674_T001  rna10356            0
 22-  9:  transcript:Zm00001d033675_T001  rna10357     1.00E-98
 22- 10:  transcript:Zm00001d033680_T001  rna10359            0
 22- 11:  transcript:Zm00001d033682_T001  rna10360            0
 22- 12:  transcript:Zm00001d033683_T001  rna10361     2.00E-86
 22- 13:  transcript:Zm00001d033684_T001  rna10362     9.00E-76
 22- 14:  transcript:Zm00001d033685_T001  rna10363     7.00E-25
 22- 15:  transcript:Zm00001d033704_T007  rna10366            0
 22- 16:  transcript:Zm00001d033705_T001  rna10367            0
 22- 17:  transcript:Zm00001d033707_T001  rna10370     6.00E-92
 22- 18:  transcript:Zm00001d033709_T001  rna10371     4.00E-54
 22- 19:  transcript:Zm00001d033714_T001  rna10374    1.00E-114
 22- 20:  transcript:Zm00001d033717_T001  rna10378     9.00E-92
 22- 21:  transcript:Zm00001d033718_T001  rna10379    3.00E-138
 22- 22:  transcript:Zm00001d033719_T001  rna10382    3.00E-156
 22- 23:  transcript:Zm00001d033726_T002  rna10390            0
## Alignment 23: score=1116.0 e_value=1.3e-73 N=24 1&NC_008396.2 plus
```

```
 23-  0:  transcript:Zm00001d029008_T001  rna9177            0
 23-  1:  transcript:Zm00001d029009_T003  rna9180      1.00E-21
 23-  2:  transcript:Zm00001d029010_T001  rna9181            0
 23-  3:  transcript:Zm00001d029011_T001  rna9183            0
 23-  4:  transcript:Zm00001d029012_T002  rna9184      8.00E-97
 23-  5:  transcript:Zm00001d029020_T001  rna9185     1.00E-146
 23-  6:  transcript:Zm00001d029023_T007  rna9186            0
 23-  7:  transcript:Zm00001d029025_T001  rna9187            0
 23-  8:  transcript:Zm00001d029027_T001  rna9189      1.00E-66
 23-  9:  transcript:Zm00001d029028_T001  rna9190     5.00E-114
 23- 10:  transcript:Zm00001d029030_T002  rna9191      3.00E-44
 23- 11:  transcript:Zm00001d029031_T001  rna9192            0
 23- 12:  transcript:Zm00001d029036_T001  rna9194     3.00E-168
 23- 13:  transcript:Zm00001d029038_T001  rna9195      9.00E-69
 23- 14:  transcript:Zm00001d029039_T001  rna9196     2.00E-146
 23- 15:  transcript:Zm00001d029040_T001  rna9197            0
 23- 16:  transcript:Zm00001d029041_T002  rna9198     1.00E-167
 23- 17:  transcript:Zm00001d029046_T012  rna9199            0
 23- 18:  transcript:Zm00001d029047_T001  rna9200            0
 23- 19:  transcript:Zm00001d029048_T001  rna9201     3.00E-174
 23- 20:  transcript:Zm00001d029049_T001  rna9202      1.00E-69
 23- 21:  transcript:Zm00001d029050_T002  rna9205            0
 23- 22:  transcript:Zm00001d029051_T001  rna9207      3.00E-95
 23- 23:  transcript:Zm00001d029053_T005  rna9210            0
## Alignment 24: score=1012.0 e_value=1.5e-67 N=22 1&NC_008396.2 plus
 24-  0:  transcript:Zm00001d034584_T001  rna11038    2.00E-144
 24-  1:  transcript:Zm00001d034586_T002  rna11040     5.00E-37
 24-  2:  transcript:Zm00001d034590_T023  rna11042            0
 24-  3:  transcript:Zm00001d034591_T001  rna11044            0
 24-  4:  transcript:Zm00001d034594_T010  rna11046            0
 24-  5:  transcript:Zm00001d034595_T001  rna11047     4.00E-88
 24-  6:  transcript:Zm00001d034596_T001  rna11048     3.00E-75
 24-  7:  transcript:Zm00001d034597_T004  rna11049            0
 24-  8:  transcript:Zm00001d034598_T001  rna11051     6.00E-84
 24-  9:  transcript:Zm00001d034601_T001  rna11063    2.00E-169
 24- 10:  transcript:Zm00001d034602_T003  rna11064            0
 24- 11:  transcript:Zm00001d034604_T001  rna11067            0
 24- 12:  transcript:Zm00001d034605_T001  rna11068     4.00E-64
 24- 13:  transcript:Zm00001d034606_T001  rna11069            0
 24- 14:  transcript:Zm00001d034607_T001  rna11070    2.00E-150
 24- 15:  transcript:Zm00001d034608_T004  rna11071    5.00E-116
 24- 16:  transcript:Zm00001d034609_T001  rna11072     9.00E-21
 24- 17:  transcript:Zm00001d034610_T001  rna11074            0
 24- 18:  transcript:Zm00001d034611_T001  rna11075     4.00E-25
 24- 19:  transcript:Zm00001d034615_T001  rna11077     3.00E-50
 24- 20:  transcript:Zm00001d034616_T001  rna11079            0
 24- 21:  transcript:Zm00001d034618_T002  rna11087    1.00E-131
## Alignment 25: score=955.0 e_value=3e-59 N=21 1&NC_008396.2 plus
 25-  0:  transcript:Zm00001d034059_T006  rna10662     2.00E-75
 25-  1:  transcript:Zm00001d034062_T001  rna10667     2.00E-65
 25-  2:  transcript:Zm00001d034063_T001  rna10668    5.00E-173
 25-  3:  transcript:Zm00001d034064_T001  rna10670     3.00E-51
 25-  4:  transcript:Zm00001d034066_T001  rna10671    1.00E-168
 25-  5:  transcript:Zm00001d034069_T001  rna10672    6.00E-137
```

```
 25-  6:  transcript:Zm00001d034072_T001  rna10677            0
 25-  7:  transcript:Zm00001d034073_T001  rna10678     1.00E-76
 25-  8:  transcript:Zm00001d034074_T005  rna10679            0
 25-  9:  transcript:Zm00001d034076_T001  rna10680            0
 25- 10:  transcript:Zm00001d034080_T005  rna10684            0
 25- 11:  transcript:Zm00001d034081_T002  rna10685     6.00E-27
 25- 12:  transcript:Zm00001d034082_T001  rna10687            0
 25- 13:  transcript:Zm00001d034085_T002  rna10688     4.00E-46
 25- 14:  transcript:Zm00001d034087_T001  rna10689     1.00E-27
 25- 15:  transcript:Zm00001d034091_T001  rna10690     3.00E-95
 25- 16:  transcript:Zm00001d034095_T001  rna10691    8.00E-102
 25- 17:  transcript:Zm00001d034109_T001  rna10702     2.00E-59
 25- 18:  transcript:Zm00001d034110_T001  rna10703     8.00E-22
 25- 19:  transcript:Zm00001d034111_T001  rna10704            0
 25- 20:  transcript:Zm00001d034112_T001  rna10705            0
## Alignment 26: score=943.0 e_value=4e-60 N=21 1&NC_008396.2 plus
 26-  0:  transcript:Zm00001d034406_T002  rna10888     4.00E-36
 26-  1:  transcript:Zm00001d034410_T001  rna10902            0
 26-  2:  transcript:Zm00001d034413_T001  rna10903            0
 26-  3:  transcript:Zm00001d034415_T001  rna10905     6.00E-68
 26-  4:  transcript:Zm00001d034416_T001  rna10906     3.00E-42
 26-  5:  transcript:Zm00001d034417_T001  rna10907     1.00E-87
 26-  6:  transcript:Zm00001d034420_T009  rna10910            0
 26-  7:  transcript:Zm00001d034422_T002  rna10911     1.00E-88
 26-  8:  transcript:Zm00001d034424_T002  rna10914            0
 26-  9:  transcript:Zm00001d034425_T001  rna10915     1.00E-27
 26- 10:  transcript:Zm00001d034427_T001  rna10917            0
 26- 11:  transcript:Zm00001d034428_T001  rna10918            0
 26- 12:  transcript:Zm00001d034429_T001  rna10919     8.00E-94
 26- 13:  transcript:Zm00001d034431_T001  rna10920    6.00E-115
 26- 14:  transcript:Zm00001d034433_T003  rna10922    1.00E-180
 26- 15:  transcript:Zm00001d034439_T001  rna10925     2.00E-90
 26- 16:  transcript:Zm00001d034440_T001  rna10926            0
 26- 17:  transcript:Zm00001d034444_T002  rna10927    1.00E-177
 26- 18:  transcript:Zm00001d034446_T002  rna10928     1.00E-23
 26- 19:  transcript:Zm00001d034447_T001  rna10929    5.00E-130
 26- 20:  transcript:Zm00001d034452_T009  rna10930    1.00E-158
## Alignment 27: score=844.0 e_value=1.7e-56 N=19 1&NC_008396.2 plus
 27-  0:  transcript:Zm00001d034739_T001  rna11165    4.00E-140
 27-  1:  transcript:Zm00001d034740_T001  rna11166            0
 27-  2:  transcript:Zm00001d034741_T001  rna11168     5.00E-58
 27-  3:  transcript:Zm00001d034745_T001  rna11169     6.00E-34
 27-  4:  transcript:Zm00001d034746_T001  rna11174     2.00E-51
 27-  5:  transcript:Zm00001d034747_T001  rna11175     1.00E-42
 27-  6:  transcript:Zm00001d034750_T004  rna11177            0
 27-  7:  transcript:Zm00001d034751_T001  rna11178     8.00E-58
 27-  8:  transcript:Zm00001d034752_T006  rna11179     9.00E-99
 27-  9:  transcript:Zm00001d034753_T001  rna11180     1.00E-23
 27- 10:  transcript:Zm00001d034754_T001  rna11182            0
 27- 11:  transcript:Zm00001d034755_T001  rna11203            0
 27- 12:  transcript:Zm00001d034756_T001  rna11204     4.00E-26
 27- 13:  transcript:Zm00001d034757_T008  rna11207            0
 27- 14:  transcript:Zm00001d034758_T003  rna11208            0
 27- 15:  transcript:Zm00001d034760_T001  rna11210     1.00E-87
```

```
 27- 16:  transcript:Zm00001d034761_T002  rna11211            0
 27- 17:  transcript:Zm00001d034769_T001  rna11212            0
 27- 18:  transcript:Zm00001d034770_T017  rna11221            0
## Alignment 28: score=825.0 e_value=2.9e-52 N=18 1&NC_008396.2 plus
 28-  0:  transcript:Zm00001d034777_T005  rna11222            0
 28-  1:  transcript:Zm00001d034778_T001  rna11224    4.00E-105
 28-  2:  transcript:Zm00001d034779_T001  rna11225            0
 28-  3:  transcript:Zm00001d034781_T001  rna11227            0
 28-  4:  transcript:Zm00001d034782_T001  rna11228            0
 28-  5:  transcript:Zm00001d034783_T001  rna11230    3.00E-111
 28-  6:  transcript:Zm00001d034784_T001  rna11231     2.00E-89
 28-  7:  transcript:Zm00001d034787_T001  rna11238     1.00E-19
 28-  8:  transcript:Zm00001d034788_T001  rna11239            0
 28-  9:  transcript:Zm00001d034793_T001  rna11244     3.00E-55
 28- 10:  transcript:Zm00001d034795_T002  rna11248            0
 28- 11:  transcript:Zm00001d034796_T005  rna11255    1.00E-118
 28- 12:  transcript:Zm00001d034801_T001  rna11257    2.00E-103
 28- 13:  transcript:Zm00001d034802_T001  rna11259    3.00E-115
 28- 14:  transcript:Zm00001d034804_T002  rna11260     6.00E-62
 28- 15:  transcript:Zm00001d034807_T002  rna11261            0
 28- 16:  transcript:Zm00001d034808_T001  rna11262     4.00E-88
 28- 17:  transcript:Zm00001d034809_T001  rna11263    2.00E-104
## Alignment 29: score=823.0 e_value=1.6e-56 N=18 1&NC_008396.2 plus
 29-  0:  transcript:Zm00001d033154_T001  rna9857      1.00E-45
 29-  1:  transcript:Zm00001d033155_T001  rna9858             0
 29-  2:  transcript:Zm00001d033159_T002  rna9876     6.00E-108
 29-  3:  transcript:Zm00001d033160_T001  rna9880     8.00E-176
 29-  4:  transcript:Zm00001d033167_T001  rna9882             0
 29-  5:  transcript:Zm00001d033168_T003  rna9883             0
 29-  6:  transcript:Zm00001d033170_T001  rna9885      4.00E-55
 29-  7:  transcript:Zm00001d033171_T001  rna9887      7.00E-49
 29-  8:  transcript:Zm00001d033172_T009  rna9891      3.00E-43
 29-  9:  transcript:Zm00001d033174_T001  rna9893             0
 29- 10:  transcript:Zm00001d033180_T001  rna9899             0
 29- 11:  transcript:Zm00001d033181_T001  rna9902      3.00E-95
 29- 12:  transcript:Zm00001d033187_T001  rna9906             0
 29- 13:  transcript:Zm00001d033188_T001  rna9908             0
 29- 14:  transcript:Zm00001d033192_T001  rna9909      6.00E-24
 29- 15:  transcript:Zm00001d033193_T006  rna9911      2.00E-58
 29- 16:  transcript:Zm00001d033194_T001  rna9912             0
 29- 17:  transcript:Zm00001d033195_T001  rna9918      2.00E-20
## Alignment 30: score=775.0 e_value=5.9e-48 N=17 1&NC_008396.2 plus
 30-  0:  transcript:Zm00001d033054_T001  rna9762      7.00E-65
 30-  1:  transcript:Zm00001d033056_T001  rna9765             0
 30-  2:  transcript:Zm00001d033057_T001  rna9766      5.00E-53
 30-  3:  transcript:Zm00001d033060_T001  rna9767             0
 30-  4:  transcript:Zm00001d033061_T002  rna9773             0
 30-  5:  transcript:Zm00001d033065_T001  rna9784      5.00E-30
 30-  6:  transcript:Zm00001d033066_T001  rna9785             0
 30-  7:  transcript:Zm00001d033075_T001  rna9805     1.00E-138
 30-  8:  transcript:Zm00001d033077_T001  rna9806             0
 30-  9:  transcript:Zm00001d033079_T001  rna9811      3.00E-86
 30- 10:  transcript:Zm00001d033088_T001  rna9818      2.00E-33
 30- 11:  transcript:Zm00001d033090_T002  rna9821             0
```

30- 12: transcript:Zm00001d033091_T001 rna9826 0
30- 13: transcript:Zm00001d033094_T001 rna9831 0
30- 14: transcript:Zm00001d033098_T001 rna9833 6.00E-146
30- 15: transcript:Zm00001d033099_T001 rna9834 1.00E-73
30- 16: transcript:Zm00001d033104_T003 rna9835 0
Alignment 31: score=690.0 e_value=1.2e-44 N=17 1&NC_008396.2 plus
31- 0: transcript:Zm00001d031061_T007 rna10038 0
31- 1: transcript:Zm00001d031065_T001 rna10042 2.00E-63
31- 2: transcript:Zm00001d031068_T001 rna10043 3.00E-164
31- 3: transcript:Zm00001d031072_T001 rna10044 6.00E-117
31- 4: transcript:Zm00001d031081_T001 rna10058 5.00E-62
31- 5: transcript:Zm00001d031086_T001 rna10059 0
31- 6: transcript:Zm00001d031090_T001 rna10067 2.00E-136
31- 7: transcript:Zm00001d031092_T001 rna10068 2.00E-79
31- 8: transcript:Zm00001d031094_T001 rna10070 1.00E-153
31- 9: transcript:Zm00001d031098_T004 rna10073 9.00E-143
31- 10: transcript:Zm00001d031109_T002 rna10079 1.00E-62
31- 11: transcript:Zm00001d031119_T001 rna10081 8.00E-101
31- 12: transcript:Zm00001d031120_T003 rna10096 0
31- 13: transcript:Zm00001d031127_T001 rna10099 2.00E-45
31- 14: transcript:Zm00001d031129_T001 rna10109 1.00E-10
31- 15: transcript:Zm00001d031131_T001 rna10112 2.00E-70
31- 16: transcript:Zm00001d031148_T001 rna10130 4.00E-26
Alignment 32: score=633.0 e_value=1.3e-35 N=14 1&NC_008396.2 plus
32- 0: transcript:Zm00001d033632_T008 rna10322 0
32- 1: transcript:Zm00001d033633_T001 rna10324 9.00E-140
32- 2: transcript:Zm00001d033634_T001 rna10325 6.00E-20
32- 3: transcript:Zm00001d033636_T001 rna10326 4.00E-31
32- 4: transcript:Zm00001d033637_T001 rna10327 2.00E-137
32- 5: transcript:Zm00001d033640_T001 rna10328 2.00E-41
32- 6: transcript:Zm00001d033641_T002 rna10329 9.00E-123
32- 7: transcript:Zm00001d033642_T001 rna10330 0
32- 8: transcript:Zm00001d033645_T001 rna10331 0
32- 9: transcript:Zm00001d033646_T013 rna10332 2.00E-106
32- 10: transcript:Zm00001d033648_T002 rna10337 3.00E-138
32- 11: transcript:Zm00001d033651_T005 rna10339 0
32- 12: transcript:Zm00001d033652_T002 rna10341 0
32- 13: transcript:Zm00001d033654_T004 rna10342 1.00E-134
Alignment 33: score=590.0 e_value=3.9e-29 N=13 1&NC_008396.2 plus
33- 0: transcript:Zm00001d029091_T003 rna9249 0
33- 1: transcript:Zm00001d029093_T001 rna9251 0
33- 2: transcript:Zm00001d029095_T009 rna9252 0
33- 3: transcript:Zm00001d029096_T002 rna9255 0
33- 4: transcript:Zm00001d029098_T001 rna9256 9.00E-138
33- 5: transcript:Zm00001d029099_T001 rna9257 2.00E-26
33- 6: transcript:Zm00001d029102_T001 rna9260 3.00E-63
33- 7: transcript:Zm00001d029104_T002 rna9261 1.00E-72
33- 8: transcript:Zm00001d029105_T001 rna9262 0
33- 9: transcript:Zm00001d029106_T002 rna9264 0
33- 10: transcript:Zm00001d029107_T001 rna9265 1.00E-23
33- 11: transcript:Zm00001d029118_T001 rna9269 4.00E-90
33- 12: transcript:Zm00001d029120_T004 rna9270 0
Alignment 34: score=584.0 e_value=8.5e-32 N=13 1&NC_008396.2 plus
34- 0: transcript:Zm00001d029056_T003 rna9213 0

```
 34-  1:  transcript:Zm00001d029059_T001  rna9215             0
 34-  2:  transcript:Zm00001d029061_T001  rna9217             0
 34-  3:  transcript:Zm00001d029062_T001  rna9218      9.00E-87
 34-  4:  transcript:Zm00001d029064_T001  rna9219     7.00E-160
 34-  5:  transcript:Zm00001d029065_T001  rna9222     4.00E-125
 34-  6:  transcript:Zm00001d029066_T005  rna9224             0
 34-  7:  transcript:Zm00001d029067_T001  rna9225      8.00E-44
 34-  8:  transcript:Zm00001d029071_T001  rna9226      5.00E-20
 34-  9:  transcript:Zm00001d029072_T001  rna9228      2.00E-51
 34- 10:  transcript:Zm00001d029074_T001  rna9229             0
 34- 11:  transcript:Zm00001d029078_T002  rna9236      4.00E-82
 34- 12:  transcript:Zm00001d029087_T002  rna9249             0
## Alignment 35: score=529.0 e_value=9e-24 N=11 1&NC_008396.2 plus
 35-  0:  transcript:Zm00001d034124_T001  rna10707    6.00E-138
 35-  1:  transcript:Zm00001d034125_T002  rna10708     3.00E-41
 35-  2:  transcript:Zm00001d034126_T001  rna10709     2.00E-62
 35-  3:  transcript:Zm00001d034128_T001  rna10711    1.00E-169
 35-  4:  transcript:Zm00001d034130_T001  rna10713     1.00E-45
 35-  5:  transcript:Zm00001d034131_T001  rna10714    3.00E-164
 35-  6:  transcript:Zm00001d034133_T001  rna10717            0
 35-  7:  transcript:Zm00001d034137_T001  rna10718            0
 35-  8:  transcript:Zm00001d034143_T001  rna10719            0
 35-  9:  transcript:Zm00001d034145_T001  rna10720     1.00E-92
 35- 10:  transcript:Zm00001d034152_T002  rna10721            0
## Alignment 36: score=511.0 e_value=1.8e-24 N=11 1&NC_008396.2 plus
 36-  0:  transcript:Zm00001d029580_T001  rna9683             0
 36-  1:  transcript:Zm00001d029583_T001  rna9685      6.00E-82
 36-  2:  transcript:Zm00001d029584_T001  rna9687      7.00E-63
 36-  3:  transcript:Zm00001d029590_T001  rna9690             0
 36-  4:  transcript:Zm00001d029594_T001  rna9692     4.00E-136
 36-  5:  transcript:Zm00001d029595_T005  rna9701      5.00E-09
 36-  6:  transcript:Zm00001d029597_T002  rna9704     2.00E-129
 36-  7:  transcript:Zm00001d029598_T003  rna9705     2.00E-141
 36-  8:  transcript:Zm00001d029599_T001  rna9710      7.00E-23
 36-  9:  transcript:Zm00001d029601_T003  rna9713      6.00E-34
 36- 10:  transcript:Zm00001d029603_T004  rna9716             0
## Alignment 37: score=499.0 e_value=1.9e-23 N=11 1&NC_008396.2 plus
 37-  0:  transcript:Zm00001d029657_T001  rna7818     6.00E-142
 37-  1:  transcript:Zm00001d029662_T001  rna7819      5.00E-29
 37-  2:  transcript:Zm00001d029667_T001  rna7820     9.00E-177
 37-  3:  transcript:Zm00001d029673_T001  rna7823     7.00E-157
 37-  4:  transcript:Zm00001d029675_T001  rna7825             0
 37-  5:  transcript:Zm00001d029679_T001  rna7831      9.00E-51
 37-  6:  transcript:Zm00001d029680_T001  rna7832      7.00E-27
 37-  7:  transcript:Zm00001d029681_T001  rna7835      4.00E-37
 37-  8:  transcript:Zm00001d029683_T001  rna7838             0
 37-  9:  transcript:Zm00001d029684_T001  rna7842             0
 37- 10:  transcript:Zm00001d029686_T001  rna7843             0
## Alignment 38: score=475.0 e_value=4.4e-21 N=10 1&NC_008396.2 plus
 38-  0:  transcript:Zm00001d027755_T002  rna8132      2.00E-33
 38-  1:  transcript:Zm00001d027756_T001  rna8134     6.00E-138
 38-  2:  transcript:Zm00001d027757_T001  rna8135     7.00E-148
 38-  3:  transcript:Zm00001d027759_T001  rna8136             0
 38-  4:  transcript:Zm00001d027760_T001  rna8137      1.00E-92
```

38- 5: transcript:Zm00001d027763_T001 rna8140 1.00E-141
38- 6: transcript:Zm00001d027766_T001 rna8141 5.00E-146
38- 7: transcript:Zm00001d027767_T001 rna8142 0
38- 8: transcript:Zm00001d027768_T023 rna8144 0
38- 9: transcript:Zm00001d027769_T001 rna8146 0
Alignment 39: score=448.0 e_value=1.5e-20 N=10 1&NC_008396.2 plus
39- 0: transcript:Zm00001d028185_T002 rna8460 2.00E-23
39- 1: transcript:Zm00001d028186_T001 rna8461 0
39- 2: transcript:Zm00001d028187_T001 rna8462 0
39- 3: transcript:Zm00001d028188_T001 rna8463 1.00E-66
39- 4: transcript:Zm00001d028189_T001 rna8464 0
39- 5: transcript:Zm00001d028191_T002 rna8466 3.00E-70
39- 6: transcript:Zm00001d028195_T001 rna8469 4.00E-151
39- 7: transcript:Zm00001d028199_T004 rna8471 0
39- 8: transcript:Zm00001d028201_T004 rna8472 0
39- 9: transcript:Zm00001d028203_T002 rna8474 0
Alignment 40: score=431.0 e_value=1.3e-21 N=10 1&NC_008396.2 plus
40- 0: transcript:Zm00001d032859_T006 rna8070 0
40- 1: transcript:Zm00001d032873_T001 rna8072 6.00E-57
40- 2: transcript:Zm00001d032875_T009 rna8080 4.00E-40
40- 3: transcript:Zm00001d032876_T001 rna8088 2.00E-104
40- 4: transcript:Zm00001d032884_T001 rna8090 2.00E-108
40- 5: transcript:Zm00001d032893_T002 rna8100 9.00E-25
40- 6: transcript:Zm00001d032894_T001 rna8102 0
40- 7: transcript:Zm00001d032902_T001 rna8114 0
40- 8: transcript:Zm00001d032903_T001 rna8116 2.00E-14
40- 9: transcript:Zm00001d032904_T001 rna8117 6.00E-10
Alignment 41: score=430.0 e_value=4.8e-19 N=10 1&NC_008396.2 plus
41- 0: transcript:Zm00001d032981_T001 rna8169 0
41- 1: transcript:Zm00001d032989_T001 rna8173 0
41- 2: transcript:Zm00001d032991_T001 rna8174 0
41- 3: transcript:Zm00001d032994_T002 rna8189 0
41- 4: transcript:Zm00001d032999_T001 rna8190 1.00E-119
41- 5: transcript:Zm00001d033003_T001 rna8193 4.00E-40
41- 6: transcript:Zm00001d033004_T002 rna8197 3.00E-98
41- 7: transcript:Zm00001d033005_T001 rna8199 5.00E-14
41- 8: transcript:Zm00001d033011_T001 rna8200 0
41- 9: transcript:Zm00001d033012_T001 rna8202 0
Alignment 42: score=426.0 e_value=4.5e-28 N=10 1&NC_008396.2 plus
42- 0: transcript:Zm00001d033108_T007 rna9836 0
42- 1: transcript:Zm00001d033109_T003 rna9837 0
42- 2: transcript:Zm00001d033110_T001 rna9838 7.00E-175
42- 3: transcript:Zm00001d033111_T003 rna9839 1.00E-97
42- 4: transcript:Zm00001d033112_T001 rna9840 0
42- 5: transcript:Zm00001d033129_T001 rna9842 3.00E-17
42- 6: transcript:Zm00001d033130_T004 rna9843 6.00E-49
42- 7: transcript:Zm00001d033132_T001 rna9856 2.00E-179
42- 8: transcript:Zm00001d033139_T001 rna9859 0
42- 9: transcript:Zm00001d033148_T001 rna9872 3.00E-60
Alignment 43: score=414.0 e_value=1.7e-18 N=9 1&NC_008396.2 plus
43- 0: transcript:Zm00001d034005_T003 rna10615 0
43- 1: transcript:Zm00001d034006_T002 rna10617 2.00E-165
43- 2: transcript:Zm00001d034007_T001 rna10618 6.00E-141
43- 3: transcript:Zm00001d034010_T002 rna10619 0

```
 43-  4:  transcript:Zm00001d034011_T001  rna10621     2.00E-65
 43-  5:  transcript:Zm00001d034012_T001  rna10622            0
 43-  6:  transcript:Zm00001d034013_T002  rna10623     5.00E-40
 43-  7:  transcript:Zm00001d034015_T005  rna10625            0
 43-  8:  transcript:Zm00001d034018_T002  rna10629            0
## Alignment 44: score=407.0 e_value=5.5e-17 N=9 1&NC_008396.2 plus
 44-  0:  transcript:Zm00001d028264_T002  rna8540             0
 44-  1:  transcript:Zm00001d028265_T004  rna8541             0
 44-  2:  transcript:Zm00001d028267_T001  rna8542             0
 44-  3:  transcript:Zm00001d028273_T001  rna8546             0
 44-  4:  transcript:Zm00001d028274_T001  rna8547             0
 44-  5:  transcript:Zm00001d028275_T001  rna8548             0
 44-  6:  transcript:Zm00001d028279_T001  rna8550      5.00E-63
 44-  7:  transcript:Zm00001d028280_T001  rna8551             0
 44-  8:  transcript:Zm00001d028282_T001  rna8552             0
## Alignment 45: score=386.0 e_value=4.2e-15 N=8 1&NC_008396.2 plus
 45-  0:  transcript:Zm00001d029550_T008  rna9644             0
 45-  1:  transcript:Zm00001d029552_T001  rna9646     1.00E-145
 45-  2:  transcript:Zm00001d029553_T001  rna9647      3.00E-76
 45-  3:  transcript:Zm00001d029555_T002  rna9649      1.00E-72
 45-  4:  transcript:Zm00001d029557_T001  rna9650             0
 45-  5:  transcript:Zm00001d029559_T001  rna9651     7.00E-146
 45-  6:  transcript:Zm00001d029560_T001  rna9652     2.00E-157
 45-  7:  transcript:Zm00001d029561_T002  rna9654      3.00E-22
## Alignment 46: score=379.0 e_value=7e-19 N=9 1&NC_008396.2 plus
 46-  0:  transcript:Zm00001d034035_T002  rna10640            0
 46-  1:  transcript:Zm00001d034038_T001  rna10645            0
 46-  2:  transcript:Zm00001d034039_T001  rna10647            0
 46-  3:  transcript:Zm00001d034045_T003  rna10650    6.00E-112
 46-  4:  transcript:Zm00001d034047_T006  rna10651    4.00E-130
 46-  5:  transcript:Zm00001d034049_T002  rna10656    4.00E-124
 46-  6:  transcript:Zm00001d034050_T001  rna10657            0
 46-  7:  transcript:Zm00001d034054_T002  rna10658            0
 46-  8:  transcript:Zm00001d034055_T001  rna10659            0
## Alignment 47: score=378.0 e_value=1.2e-14 N=8 1&NC_008396.2 plus
 47-  0:  transcript:Zm00001d027281_T001  rna7713     7.00E-140
 47-  1:  transcript:Zm00001d027283_T001  rna7715     2.00E-153
 47-  2:  transcript:Zm00001d027285_T001  rna7716      8.00E-53
 47-  3:  transcript:Zm00001d027290_T001  rna7719      6.00E-59
 47-  4:  transcript:Zm00001d027292_T001  rna7720     2.00E-153
 47-  5:  transcript:Zm00001d027295_T002  rna7731      1.00E-92
 47-  6:  transcript:Zm00001d027296_T003  rna7733             0
 47-  7:  transcript:Zm00001d027298_T001  rna7734             0
## Alignment 48: score=369.0 e_value=1.6e-15 N=8 1&NC_008396.2 plus
 48-  0:  transcript:Zm00001d033511_T002  rna10224    9.00E-133
 48-  1:  transcript:Zm00001d033518_T001  rna10225            0
 48-  2:  transcript:Zm00001d033522_T001  rna10231            0
 48-  3:  transcript:Zm00001d033523_T001  rna10233    3.00E-122
 48-  4:  transcript:Zm00001d033525_T001  rna10234            0
 48-  5:  transcript:Zm00001d033526_T001  rna10235     3.00E-60
 48-  6:  transcript:Zm00001d033527_T001  rna10236            0
 48-  7:  transcript:Zm00001d033530_T001  rna10248     2.00E-55
## Alignment 49: score=369.0 e_value=1.6e-13 N=8 1&NC_008396.2 plus
 49-  0:  transcript:Zm00001d027393_T001  rna7846             0
```

49- 1: transcript:Zm00001d027395_T009 rna7849 0
49- 2: transcript:Zm00001d027401_T008 rna7851 0
49- 3: transcript:Zm00001d027403_T001 rna7852 0
49- 4: transcript:Zm00001d027405_T001 rna7854 3.00E-111
49- 5: transcript:Zm00001d027409_T001 rna7858 5.00E-114
49- 6: transcript:Zm00001d027411_T001 rna7859 2.00E-176
49- 7: transcript:Zm00001d027412_T001 rna7862 8.00E-96
Alignment 50: score=319.0 e_value=7.5e-14 N=7 1&NC_008396.2 plus
50- 0: transcript:Zm00001d034019_T001 rna10630 2.00E-134
50- 1: transcript:Zm00001d034027_T001 rna10631 5.00E-51
50- 2: transcript:Zm00001d034028_T003 rna10632 2.00E-77
50- 3: transcript:Zm00001d034030_T001 rna10633 0
50- 4: transcript:Zm00001d034031_T001 rna10635 7.00E-07
50- 5: transcript:Zm00001d034032_T001 rna10636 2.00E-116
50- 6: transcript:Zm00001d034033_T002 rna10637 0
Alignment 51: score=316.0 e_value=1.9e-12 N=7 1&NC_008396.2 plus
51- 0: transcript:Zm00001d034559_T001 rna11023 0
51- 1: transcript:Zm00001d034562_T003 rna11028 6.00E-137
51- 2: transcript:Zm00001d034563_T001 rna11030 3.00E-76
51- 3: transcript:Zm00001d034568_T001 rna11031 0
51- 4: transcript:Zm00001d034571_T001 rna11032 4.00E-27
51- 5: transcript:Zm00001d034575_T001 rna11033 0
51- 6: transcript:Zm00001d034578_T001 rna11037 5.00E-82
Alignment 52: score=309.0 e_value=9.2e-13 N=7 1&NC_008396.2 plus
52- 0: transcript:Zm00001d030834_T001 rna9909 1.00E-36
52- 1: transcript:Zm00001d030837_T001 rna9914 0
52- 2: transcript:Zm00001d030846_T001 rna9916 8.00E-11
52- 3: transcript:Zm00001d030849_T001 rna9922 3.00E-153
52- 4: transcript:Zm00001d030850_T001 rna9923 2.00E-34
52- 5: transcript:Zm00001d030858_T001 rna9934 1.00E-35
52- 6: transcript:Zm00001d030866_T001 rna9949 2.00E-51
Alignment 53: score=305.0 e_value=3.5e-13 N=7 1&NC_008396.2 plus
53- 0: transcript:Zm00001d029782_T004 rna7935 0
53- 1: transcript:Zm00001d029783_T001 rna7940 1.00E-129
53- 2: transcript:Zm00001d029784_T001 rna7942 3.00E-74
53- 3: transcript:Zm00001d029785_T001 rna7943 0
53- 4: transcript:Zm00001d029794_T001 rna7944 6.00E-87
53- 5: transcript:Zm00001d029799_T002 rna7956 8.00E-71
53- 6: transcript:Zm00001d029808_T001 rna7977 2.00E-140
Alignment 54: score=271.0 e_value=1.1e-10 N=6 1&NC_008396.2 plus
54- 0: transcript:Zm00001d027265_T007 rna7696 0
54- 1: transcript:Zm00001d027266_T001 rna7697 7.00E-122
54- 2: transcript:Zm00001d027267_T002 rna7699 2.00E-174
54- 3: transcript:Zm00001d027268_T001 rna7701 0
54- 4: transcript:Zm00001d027276_T001 rna7704 0
54- 5: transcript:Zm00001d027278_T001 rna7711 0
Alignment 55: score=257.0 e_value=2.9e-10 N=6 1&NC_008396.2 plus
55- 0: transcript:Zm00001d034159_T002 rna10701 1.00E-11
55- 1: transcript:Zm00001d034160_T001 rna10723 8.00E-116
55- 2: transcript:Zm00001d034161_T003 rna10724 0
55- 3: transcript:Zm00001d034163_T001 rna10725 7.00E-129
55- 4: transcript:Zm00001d034164_T002 rna10726 1.00E-171
55- 5: transcript:Zm00001d034165_T001 rna10728 8.00E-53
Alignment 56: score=2493.0 e_value=1.3e-215 N=56 1&NC_008396.2 minus

56- 0:	transcript:Zm00001d033205_T001	rna10076	4.00E-116
56- 1:	transcript:Zm00001d033209_T001	rna10075	3.00E-44
56- 2:	transcript:Zm00001d033210_T002	rna10074	0
56- 3:	transcript:Zm00001d033211_T002	rna10073	0
56- 4:	transcript:Zm00001d033213_T001	rna10070	0
56- 5:	transcript:Zm00001d033214_T001	rna10069	3.00E-142
56- 6:	transcript:Zm00001d033215_T003	rna10068	2.00E-111
56- 7:	transcript:Zm00001d033216_T001	rna10066	5.00E-38
56- 8:	transcript:Zm00001d033218_T002	rna10064	0
56- 9:	transcript:Zm00001d033221_T001	rna10061	0
56- 10:	transcript:Zm00001d033222_T001	rna10059	0
56- 11:	transcript:Zm00001d033223_T003	rna10058	2.00E-19
56- 12:	transcript:Zm00001d033225_T002	rna10057	0
56- 13:	transcript:Zm00001d033228_T002	rna10047	0
56- 14:	transcript:Zm00001d033233_T002	rna10044	7.00E-87
56- 15:	transcript:Zm00001d033234_T001	rna10043	0
56- 16:	transcript:Zm00001d033241_T001	rna10039	0
56- 17:	transcript:Zm00001d033246_T002	rna10038	0
56- 18:	transcript:Zm00001d033250_T001	rna10035	3.00E-27
56- 19:	transcript:Zm00001d033254_T001	rna10034	2.00E-177
56- 20:	transcript:Zm00001d033259_T002	rna10032	0
56- 21:	transcript:Zm00001d033262_T001	rna10031	8.00E-96
56- 22:	transcript:Zm00001d033267_T001	rna10029	1.00E-88
56- 23:	transcript:Zm00001d033271_T001	rna10026	5.00E-119
56- 24:	transcript:Zm00001d033274_T005	rna10023	9.00E-30
56- 25:	transcript:Zm00001d033278_T011	rna10020	0
56- 26:	transcript:Zm00001d033279_T001	rna10018	0
56- 27:	transcript:Zm00001d033280_T001	rna10016	5.00E-166
56- 28:	transcript:Zm00001d033283_T001	rna10012	1.00E-141
56- 29:	transcript:Zm00001d033287_T001	rna10011	9.00E-30
56- 30:	transcript:Zm00001d033292_T004	rna10000	0
56- 31:	transcript:Zm00001d033294_T002	rna9998	6.00E-71
56- 32:	transcript:Zm00001d033295_T001	rna9997	4.00E-157
56- 33:	transcript:Zm00001d033296_T002	rna9996	0
56- 34:	transcript:Zm00001d033297_T003	rna9995	0
56- 35:	transcript:Zm00001d033300_T001	rna9994	8.00E-77
56- 36:	transcript:Zm00001d033303_T004	rna9987	0
56- 37:	transcript:Zm00001d033304_T001	rna9985	5.00E-172
56- 38:	transcript:Zm00001d033308_T001	rna9982	3.00E-129
56- 39:	transcript:Zm00001d033310_T001	rna9980	3.00E-66
56- 40:	transcript:Zm00001d033312_T006	rna9976	3.00E-09
56- 41:	transcript:Zm00001d033324_T001	rna9963	4.00E-107
56- 42:	transcript:Zm00001d033325_T001	rna9961	6.00E-34
56- 43:	transcript:Zm00001d033327_T001	rna9958	6.00E-154
56- 44:	transcript:Zm00001d033328_T001	rna9957	0
56- 45:	transcript:Zm00001d033330_T001	rna9954	6.00E-88
56- 46:	transcript:Zm00001d033333_T001	rna9952	0
56- 47:	transcript:Zm00001d033334_T001	rna9950	0
56- 48:	transcript:Zm00001d033335_T001	rna9943	1.00E-80
56- 49:	transcript:Zm00001d033337_T002	rna9942	8.00E-44
56- 50:	transcript:Zm00001d033338_T002	rna9941	0
56- 51:	transcript:Zm00001d033339_T002	rna9938	0
56- 52:	transcript:Zm00001d033344_T001	rna9933	0
56- 53:	transcript:Zm00001d033347_T001	rna9932	1.00E-101

```
 56- 54:  transcript:Zm00001d033360_T001  rna9924      3.00E-20
 56- 55:  transcript:Zm00001d033363_T001  rna9923      8.00E-59
## Alignment 57: score=2298.0 e_value=8e-194 N=50 1&NC_008396.2 minus
 57-  0:  transcript:Zm00001d033378_T019  rna10218     7.00E-56
 57-  1:  transcript:Zm00001d033380_T001  rna10211     8.00E-31
 57-  2:  transcript:Zm00001d033383_T001  rna10210            0
 57-  3:  transcript:Zm00001d033385_T001  rna10206            0
 57-  4:  transcript:Zm00001d033386_T001  rna10205    1.00E-121
 57-  5:  transcript:Zm00001d033389_T001  rna10203     5.00E-15
 57-  6:  transcript:Zm00001d033391_T001  rna10198    2.00E-100
 57-  7:  transcript:Zm00001d033396_T003  rna10196            0
 57-  8:  transcript:Zm00001d033397_T004  rna10194     1.00E-70
 57-  9:  transcript:Zm00001d033401_T001  rna10193            0
 57- 10:  transcript:Zm00001d033402_T002  rna10190     1.00E-21
 57- 11:  transcript:Zm00001d033404_T002  rna10189     2.00E-48
 57- 12:  transcript:Zm00001d033405_T003  rna10185            0
 57- 13:  transcript:Zm00001d033412_T001  rna10183     3.00E-79
 57- 14:  transcript:Zm00001d033422_T001  rna10171    5.00E-116
 57- 15:  transcript:Zm00001d033446_T001  rna10159    5.00E-167
 57- 16:  transcript:Zm00001d033447_T005  rna10140    5.00E-175
 57- 17:  transcript:Zm00001d033451_T001  rna10139            0
 57- 18:  transcript:Zm00001d033455_T001  rna10131    2.00E-121
 57- 19:  transcript:Zm00001d033456_T001  rna10130     3.00E-73
 57- 20:  transcript:Zm00001d033457_T001  rna10129            0
 57- 21:  transcript:Zm00001d033459_T001  rna10128     2.00E-30
 57- 22:  transcript:Zm00001d033465_T001  rna10125     8.00E-87
 57- 23:  transcript:Zm00001d033466_T001  rna10124     2.00E-33
 57- 24:  transcript:Zm00001d033467_T002  rna10123            0
 57- 25:  transcript:Zm00001d033468_T001  rna10122     3.00E-84
 57- 26:  transcript:Zm00001d033469_T001  rna10121     2.00E-73
 57- 27:  transcript:Zm00001d033470_T001  rna10120     6.00E-58
 57- 28:  transcript:Zm00001d033471_T001  rna10118     6.00E-43
 57- 29:  transcript:Zm00001d033472_T004  rna10116    2.00E-139
 57- 30:  transcript:Zm00001d033473_T001  rna10115    3.00E-118
 57- 31:  transcript:Zm00001d033475_T001  rna10112            0
 57- 32:  transcript:Zm00001d033477_T002  rna10111     4.00E-73
 57- 33:  transcript:Zm00001d033478_T001  rna10109     1.00E-10
 57- 34:  transcript:Zm00001d033481_T001  rna10107     2.00E-41
 57- 35:  transcript:Zm00001d033482_T001  rna10106            0
 57- 36:  transcript:Zm00001d033483_T001  rna10105     4.00E-14
 57- 37:  transcript:Zm00001d033484_T002  rna10104     1.00E-31
 57- 38:  transcript:Zm00001d033488_T001  rna10103    2.00E-143
 57- 39:  transcript:Zm00001d033489_T001  rna10099     2.00E-46
 57- 40:  transcript:Zm00001d033492_T005  rna10097            0
 57- 41:  transcript:Zm00001d033493_T002  rna10096            0
 57- 42:  transcript:Zm00001d033494_T011  rna10093            0
 57- 43:  transcript:Zm00001d033497_T001  rna10088            0
 57- 44:  transcript:Zm00001d033502_T005  rna10087    3.00E-120
 57- 45:  transcript:Zm00001d033503_T002  rna10085            0
 57- 46:  transcript:Zm00001d033504_T001  rna10084    1.00E-132
 57- 47:  transcript:Zm00001d033505_T001  rna10083            0
 57- 48:  transcript:Zm00001d033508_T001  rna10079     8.00E-78
 57- 49:  transcript:Zm00001d033510_T001  rna10078     1.00E-63
## Alignment 58: score=1982.0 e_value=5e-159 N=44 1&NC_008396.2 minus
```

58- 0: transcript:Zm00001d028084_T002 rna8423 0
58- 1: transcript:Zm00001d028088_T003 rna8420 0
58- 2: transcript:Zm00001d028089_T001 rna8416 0
58- 3: transcript:Zm00001d028092_T001 rna8415 1.00E-43
58- 4: transcript:Zm00001d028093_T001 rna8414 0
58- 5: transcript:Zm00001d028094_T001 rna8413 0
58- 6: transcript:Zm00001d028096_T006 rna8412 0
58- 7: transcript:Zm00001d028097_T001 rna8411 3.00E-109
58- 8: transcript:Zm00001d028098_T001 rna8409 0
58- 9: transcript:Zm00001d028102_T001 rna8408 9.00E-175
58- 10: transcript:Zm00001d028104_T003 rna8404 0
58- 11: transcript:Zm00001d028105_T001 rna8403 2.00E-109
58- 12: transcript:Zm00001d028107_T001 rna8401 0
58- 13: transcript:Zm00001d028109_T006 rna8399 0
58- 14: transcript:Zm00001d028110_T001 rna8398 1.00E-95
58- 15: transcript:Zm00001d028112_T001 rna8397 1.00E-129
58- 16: transcript:Zm00001d028113_T001 rna8396 1.00E-161
58- 17: transcript:Zm00001d028114_T005 rna8395 5.00E-88
58- 18: transcript:Zm00001d028117_T002 rna8394 5.00E-170
58- 19: transcript:Zm00001d028118_T001 rna8392 3.00E-90
58- 20: transcript:Zm00001d028122_T001 rna8391 9.00E-39
58- 21: transcript:Zm00001d028125_T001 rna8388 0
58- 22: transcript:Zm00001d028128_T003 rna8386 3.00E-114
58- 23: transcript:Zm00001d028129_T002 rna8385 8.00E-90
58- 24: transcript:Zm00001d028130_T001 rna8384 5.00E-157
58- 25: transcript:Zm00001d028139_T001 rna8379 2.00E-95
58- 26: transcript:Zm00001d028143_T002 rna8378 0
58- 27: transcript:Zm00001d028144_T001 rna8375 0
58- 28: transcript:Zm00001d028152_T001 rna8373 4.00E-119
58- 29: transcript:Zm00001d028153_T001 rna8371 1.00E-82
58- 30: transcript:Zm00001d028154_T001 rna8370 1.00E-155
58- 31: transcript:Zm00001d028159_T001 rna8369 7.00E-61
58- 32: transcript:Zm00001d028160_T003 rna8368 2.00E-34
58- 33: transcript:Zm00001d028161_T001 rna8367 1.00E-38
58- 34: transcript:Zm00001d028162_T009 rna8360 0
58- 35: transcript:Zm00001d028165_T001 rna8358 0
58- 36: transcript:Zm00001d028167_T001 rna8353 3.00E-73
58- 37: transcript:Zm00001d028171_T001 rna8352 0
58- 38: transcript:Zm00001d028172_T001 rna8349 4.00E-151
58- 39: transcript:Zm00001d028173_T002 rna8347 0
58- 40: transcript:Zm00001d028177_T004 rna8343 0
58- 41: transcript:Zm00001d028180_T002 rna8342 0
58- 42: transcript:Zm00001d028181_T001 rna8338 1.00E-83
58- 43: transcript:Zm00001d028182_T001 rna8337 2.00E-148
Alignment 59: score=788.0 e_value=3.3e-45 N=17 1&NC_008396.2 minus
59- 0: transcript:Zm00001d028038_T008 rna8447 0
59- 1: transcript:Zm00001d028039_T004 rna8446 0
59- 2: transcript:Zm00001d028040_T002 rna8445 4.00E-91
59- 3: transcript:Zm00001d028043_T001 rna8444 5.00E-43
59- 4: transcript:Zm00001d028045_T007 rna8443 0
59- 5: transcript:Zm00001d028046_T001 rna8442 0
59- 6: transcript:Zm00001d028050_T001 rna8440 0
59- 7: transcript:Zm00001d028053_T001 rna8436 3.00E-83
59- 8: transcript:Zm00001d028054_T002 rna8435 2.00E-178

```
 59-  9:  transcript:Zm00001d028055_T001  rna8434     3.00E-132
 59- 10:  transcript:Zm00001d028056_T001  rna8432             0
 59- 11:  transcript:Zm00001d028062_T001  rna8431      7.00E-83
 59- 12:  transcript:Zm00001d028064_T001  rna8430      7.00E-40
 59- 13:  transcript:Zm00001d028071_T001  rna8429      3.00E-36
 59- 14:  transcript:Zm00001d028073_T004  rna8427             0
 59- 15:  transcript:Zm00001d028074_T001  rna8426     2.00E-175
 59- 16:  transcript:Zm00001d028075_T011  rna8424      1.00E-78
## Alignment 60: score=693.0 e_value=2.3e-38 N=15 1&NC_008396.2 minus
 60-  0:  transcript:Zm00001d028284_T001  rna8604             0
 60-  1:  transcript:Zm00001d028285_T003  rna8602             0
 60-  2:  transcript:Zm00001d028287_T001  rna8600     4.00E-157
 60-  3:  transcript:Zm00001d028293_T001  rna8599             0
 60-  4:  transcript:Zm00001d028294_T001  rna8597             0
 60-  5:  transcript:Zm00001d028296_T001  rna8592     1.00E-172
 60-  6:  transcript:Zm00001d028297_T001  rna8591      8.00E-47
 60-  7:  transcript:Zm00001d028298_T001  rna8590      1.00E-21
 60-  8:  transcript:Zm00001d028299_T001  rna8588             0
 60-  9:  transcript:Zm00001d028303_T003  rna8587             0
 60- 10:  transcript:Zm00001d028307_T001  rna8582     9.00E-168
 60- 11:  transcript:Zm00001d028317_T001  rna8571             0
 60- 12:  transcript:Zm00001d028319_T002  rna8568             0
 60- 13:  transcript:Zm00001d028325_T005  rna8566             0
 60- 14:  transcript:Zm00001d028328_T001  rna8565      1.00E-22
## Alignment 61: score=531.0 e_value=4.8e-23 N=11 1&NC_008396.2 minus
 61-  0:  transcript:Zm00001d029730_T001  rna7880      4.00E-25
 61-  1:  transcript:Zm00001d029734_T001  rna7876             0
 61-  2:  transcript:Zm00001d029736_T001  rna7875     3.00E-153
 61-  3:  transcript:Zm00001d029738_T001  rna7874      1.00E-64
 61-  4:  transcript:Zm00001d029740_T002  rna7869     4.00E-100
 61-  5:  transcript:Zm00001d029744_T001  rna7865             0
 61-  6:  transcript:Zm00001d029747_T001  rna7859     6.00E-162
 61-  7:  transcript:Zm00001d029749_T001  rna7858      3.00E-75
 61-  8:  transcript:Zm00001d029750_T001  rna7857      2.00E-28
 61-  9:  transcript:Zm00001d029752_T001  rna7854      3.00E-49
 61- 10:  transcript:Zm00001d029753_T001  rna7853     3.00E-176
## Alignment 62: score=462.0 e_value=1.3e-23 N=10 1&NC_008396.2 minus
 62-  0:  transcript:Zm00001d032396_T002  rna9755     2.00E-175
 62-  1:  transcript:Zm00001d032397_T001  rna9754     3.00E-156
 62-  2:  transcript:Zm00001d032398_T001  rna9750     4.00E-172
 62-  3:  transcript:Zm00001d032400_T001  rna9748             0
 62-  4:  transcript:Zm00001d032401_T001  rna9747      4.00E-90
 62-  5:  transcript:Zm00001d032402_T001  rna9746      1.00E-62
 62-  6:  transcript:Zm00001d032405_T001  rna9745     4.00E-176
 62-  7:  transcript:Zm00001d032407_T001  rna9741             0
 62-  8:  transcript:Zm00001d032408_T002  rna9725             0
 62-  9:  transcript:Zm00001d032420_T001  rna9718     4.00E-172
## Alignment 63: score=408.0 e_value=1.3e-17 N=9 1&NC_008396.2 minus
 63-  0:  transcript:Zm00001d034725_T001  rna11201     3.00E-68
 63-  1:  transcript:Zm00001d034726_T001  rna11199    6.00E-125
 63-  2:  transcript:Zm00001d034727_T003  rna11198            0
 63-  3:  transcript:Zm00001d034730_T002  rna11194            0
 63-  4:  transcript:Zm00001d034731_T001  rna11192    1.00E-149
 63-  5:  transcript:Zm00001d034732_T001  rna11190            0
```

63- 6: transcript:Zm00001d034733_T002 rna11189 0
63- 7: transcript:Zm00001d034734_T007 rna11188 0
63- 8: transcript:Zm00001d034738_T001 rna11187 0
Alignment 64: score=366.0 e_value=9.2e-15 N=8 1&NC_008396.2 minus
64- 0: transcript:Zm00001d032923_T002 rna8135 2.00E-145
64- 1: transcript:Zm00001d032925_T001 rna8134 2.00E-74
64- 2: transcript:Zm00001d032927_T001 rna8132 2.00E-12
64- 3: transcript:Zm00001d032932_T001 rna8131 2.00E-96
64- 4: transcript:Zm00001d032933_T020 rna8129 0
64- 5: transcript:Zm00001d032935_T006 rna8126 8.00E-18
64- 6: transcript:Zm00001d032938_T001 rna8124 8.00E-37
64- 7: transcript:Zm00001d032939_T001 rna8123 5.00E-60
Alignment 65: score=363.0 e_value=2.3e-15 N=8 1&NC_008396.2 minus
65- 0: transcript:Zm00001d033529_T001 rna10254 4.00E-179
65- 1: transcript:Zm00001d033532_T001 rna10246 0
65- 2: transcript:Zm00001d033533_T005 rna10245 0
65- 3: transcript:Zm00001d033537_T002 rna10243 2.00E-50
65- 4: transcript:Zm00001d033539_T001 rna10242 2.00E-146
65- 5: transcript:Zm00001d033541_T002 rna10241 2.00E-166
65- 6: transcript:Zm00001d033543_T002 rna10238 8.00E-107
65- 7: transcript:Zm00001d033544_T001 rna10237 0
Alignment 66: score=319.0 e_value=8.3e-16 N=7 1&NC_008396.2 minus
66- 0: transcript:Zm00001d031168_T001 rna10224 2.00E-25
66- 1: transcript:Zm00001d031182_T001 rna10218 4.00E-53
66- 2: transcript:Zm00001d031183_T001 rna10211 4.00E-32
66- 3: transcript:Zm00001d031184_T001 rna10205 2.00E-105
66- 4: transcript:Zm00001d031189_T001 rna10203 3.00E-07
66- 5: transcript:Zm00001d031191_T001 rna10202 4.00E-16
66- 6: transcript:Zm00001d031195_T001 rna10198 3.00E-06
Alignment 67: score=318.0 e_value=3.1e-11 N=7 1&NC_008396.2 minus
67- 0: transcript:Zm00001d028465_T001 rna8731 1.00E-49
67- 1: transcript:Zm00001d028471_T005 rna8730 0
67- 2: transcript:Zm00001d028472_T001 rna8729 0
67- 3: transcript:Zm00001d028473_T001 rna8728 5.00E-106
67- 4: transcript:Zm00001d028474_T002 rna8727 0
67- 5: transcript:Zm00001d028475_T002 rna8725 1.00E-178
67- 6: transcript:Zm00001d028477_T002 rna8724 0
Alignment 68: score=302.0 e_value=2e-12 N=7 1&NC_008396.2 minus
68- 0: transcript:Zm00001d029842_T001 rna7746 0
68- 1: transcript:Zm00001d029848_T002 rna7738 0
68- 2: transcript:Zm00001d029849_T006 rna7736 0
68- 3: transcript:Zm00001d029852_T001 rna7733 0
68- 4: transcript:Zm00001d029855_T001 rna7720 3.00E-131
68- 5: transcript:Zm00001d029857_T001 rna7719 2.00E-15
68- 6: transcript:Zm00001d029858_T001 rna7717 3.00E-39
Alignment 69: score=291.0 e_value=4.1e-09 N=6 1&NC_008396.2 minus
69- 0: transcript:Zm00001d033316_T001 rna10010 0
69- 1: transcript:Zm00001d033317_T001 rna10009 7.00E-54
69- 2: transcript:Zm00001d033318_T005 rna10008 1.00E-158
69- 3: transcript:Zm00001d033319_T001 rna10007 3.00E-77
69- 4: transcript:Zm00001d033321_T001 rna10005 0
69- 5: transcript:Zm00001d033322_T001 rna10004 1.00E-38
Alignment 70: score=277.0 e_value=1.1e-09 N=6 1&NC_008396.2 minus
70- 0: transcript:Zm00001d034389_T001 rna10897 6.00E-30

70- 1: transcript:Zm00001d034399_T001 rna10894 0
70- 2: transcript:Zm00001d034400_T003 rna10893 0
70- 3: transcript:Zm00001d034401_T004 rna10892 0
70- 4: transcript:Zm00001d034404_T001 rna10891 2.00E-105
70- 5: transcript:Zm00001d034405_T002 rna10890 0
Alignment 71: score=273.0 e_value=3.1e-08 N=6 1&NC_008396.2 minus
71- 0: transcript:Zm00001d034298_T001 rna10827 2.00E-153
71- 1: transcript:Zm00001d034313_T002 rna10822 1.00E-159
71- 2: transcript:Zm00001d034319_T001 rna10814 7.00E-163
71- 3: transcript:Zm00001d034320_T002 rna10812 6.00E-150
71- 4: transcript:Zm00001d034326_T001 rna10811 2.00E-86
71- 5: transcript:Zm00001d034330_T001 rna10810 7.00E-15
Alignment 72: score=267.0 e_value=6.7e-09 N=6 1&NC_008396.2 minus
72- 0: transcript:Zm00001d029604_T001 rna9676 1.00E-57
72- 1: transcript:Zm00001d029607_T001 rna9674 0
72- 2: transcript:Zm00001d029608_T011 rna9673 5.00E-108
72- 3: transcript:Zm00001d029625_T001 rna9665 3.00E-10
72- 4: transcript:Zm00001d029627_T002 rna9662 0
72- 5: transcript:Zm00001d029628_T001 rna9659 9.00E-44
Alignment 73: score=255.0 e_value=3.2e-09 N=6 1&NC_008396.2 minus
73- 0: transcript:Zm00001d030464_T001 rna8375 1.00E-169
73- 1: transcript:Zm00001d030496_T001 rna8355 2.00E-28
73- 2: transcript:Zm00001d030500_T001 rna8345 0
73- 3: transcript:Zm00001d030506_T001 rna8342 0
73- 4: transcript:Zm00001d030513_T001 rna8334 3.00E-62
73- 5: transcript:Zm00001d030518_T005 rna8327 0
Alignment 74: score=261.0 e_value=9.1e-08 N=6 1&NC_008397.2 plus
74- 0: transcript:Zm00001d029734_T001 rna13307 0
74- 1: transcript:Zm00001d029740_T002 rna13312 3.00E-35
74- 2: transcript:Zm00001d029744_T001 rna13316 2.00E-179
74- 3: transcript:Zm00001d029749_T001 rna13319 1.00E-56
74- 4: transcript:Zm00001d029754_T004 rna13323 4.00E-105
74- 5: transcript:Zm00001d029758_T004 rna13341 0
Alignment 75: score=250.0 e_value=1.3e-12 N=6 1&NC_008397.2 minus
75- 0: transcript:Zm00001d029848_T002 rna13196 0
75- 1: transcript:Zm00001d029849_T006 rna13195 5.00E-53
75- 2: transcript:Zm00001d029850_T001 rna13194 2.00E-179
75- 3: transcript:Zm00001d029855_T001 rna13169 3.00E-116
75- 4: transcript:Zm00001d029857_T001 rna13168 2.00E-15
75- 5: transcript:Zm00001d029858_T001 rna13166 9.00E-31
Alignment 76: score=478.0 e_value=5e-26 N=11 1&NC_008400.2 plus
76- 0: transcript:Zm00001d034190_T001 rna19074 1.00E-37
76- 1: transcript:Zm00001d034191_T004 rna19077 1.00E-40
76- 2: transcript:Zm00001d034194_T004 rna19079 0
76- 3: transcript:Zm00001d034197_T001 rna19082 2.00E-33
76- 4: transcript:Zm00001d034198_T001 rna19084 2.00E-117
76- 5: transcript:Zm00001d034200_T001 rna19088 1.00E-23
76- 6: transcript:Zm00001d034204_T001 rna19089 6.00E-127
76- 7: transcript:Zm00001d034212_T001 rna19102 2.00E-111
76- 8: transcript:Zm00001d034217_T001 rna19104 0
76- 9: transcript:Zm00001d034221_T001 rna19128 0
76- 10: transcript:Zm00001d034237_T001 rna19135 3.00E-17
Alignment 77: score=477.0 e_value=9.5e-25 N=11 1&NC_008400.2 plus
77- 0: transcript:Zm00001d034422_T002 rna19318 1.00E-88

77- 1: transcript:Zm00001d034427_T001 rna19337 1.00E-148
77- 2: transcript:Zm00001d034431_T001 rna19343 1.00E-89
77- 3: transcript:Zm00001d034433_T003 rna19348 1.00E-162
77- 4: transcript:Zm00001d034439_T001 rna19355 1.00E-21
77- 5: transcript:Zm00001d034440_T001 rna19361 0
77- 6: transcript:Zm00001d034443_T001 rna19362 0
77- 7: transcript:Zm00001d034444_T002 rna19365 3.00E-51
77- 8: transcript:Zm00001d034446_T002 rna19366 0
77- 9: transcript:Zm00001d034447_T001 rna19368 3.00E-59
77- 10: transcript:Zm00001d034452_T009 rna19369 2.00E-137
Alignment 78: score=382.0 e_value=2.9e-19 N=9 1&NC_008400.2 plus
78- 0: transcript:Zm00001d034497_T001 rna19419 2.00E-10
78- 1: transcript:Zm00001d034501_T001 rna19422 0
78- 2: transcript:Zm00001d034502_T003 rna19431 0
78- 3: transcript:Zm00001d034503_T001 rna19432 6.00E-22
78- 4: transcript:Zm00001d034505_T001 rna19433 1.00E-106
78- 5: transcript:Zm00001d034507_T001 rna19436 9.00E-22
78- 6: transcript:Zm00001d034508_T001 rna19441 0
78- 7: transcript:Zm00001d034513_T001 rna19452 3.00E-49
78- 8: transcript:Zm00001d034516_T001 rna19456 0
Alignment 79: score=328.0 e_value=9.6e-16 N=8 1&NC_008400.2 plus
79- 0: transcript:Zm00001d034456_T003 rna19366 0
79- 1: transcript:Zm00001d034457_T003 rna19368 5.00E-85
79- 2: transcript:Zm00001d034461_T001 rna19369 2.00E-116
79- 3: transcript:Zm00001d034463_T003 rna19371 3.00E-53
79- 4: transcript:Zm00001d034479_T001 rna19379 2.00E-22
79- 5: transcript:Zm00001d034484_T001 rna19388 3.00E-11
79- 6: transcript:Zm00001d034487_T002 rna19398 0
79- 7: transcript:Zm00001d034491_T001 rna19413 7.00E-58
Alignment 80: score=317.0 e_value=3.4e-13 N=7 1&NC_008400.2 plus
80- 0: transcript:Zm00001d034340_T001 rna19178 4.00E-135
80- 1: transcript:Zm00001d034344_T001 rna19187 0
80- 2: transcript:Zm00001d034345_T001 rna19188 0
80- 3: transcript:Zm00001d034350_T012 rna19200 0
80- 4: transcript:Zm00001d034353_T001 rna19208 1.00E-90
80- 5: transcript:Zm00001d034356_T001 rna19210 2.00E-56
80- 6: transcript:Zm00001d034358_T001 rna19211 3.00E-35
Alignment 81: score=250.0 e_value=6.7e-09 N=6 1&NC_008400.2 plus
81- 0: transcript:Zm00001d034528_T001 rna19477 5.00E-73
81- 1: transcript:Zm00001d034533_T002 rna19479 0
81- 2: transcript:Zm00001d034547_T002 rna19488 4.00E-20
81- 3: transcript:Zm00001d034550_T001 rna19490 1.00E-105
81- 4: transcript:Zm00001d034551_T001 rna19493 2.00E-98
81- 5: transcript:Zm00001d034553_T001 rna19494 0
Alignment 82: score=776.0 e_value=3.3e-49 N=18 1&NC_008400.2 minus
82- 0: transcript:Zm00001d029260_T004 rna20897 0
82- 1: transcript:Zm00001d029263_T001 rna20890 5.00E-52
82- 2: transcript:Zm00001d029264_T004 rna20886 6.00E-19
82- 3: transcript:Zm00001d029266_T002 rna20884 1.00E-151
82- 4: transcript:Zm00001d029270_T001 rna20883 5.00E-97
82- 5: transcript:Zm00001d029272_T002 rna20875 9.00E-113
82- 6: transcript:Zm00001d029274_T001 rna20872 1.00E-131
82- 7: transcript:Zm00001d029279_T001 rna20870 1.00E-123
82- 8: transcript:Zm00001d029281_T007 rna20861 2.00E-146

```
 82-  9:  transcript:Zm00001d029285_T005  rna20860           0
 82- 10:  transcript:Zm00001d029286_T001  rna20859     6.00E-61
 82- 11:  transcript:Zm00001d029287_T001  rna20851     7.00E-66
 82- 12:  transcript:Zm00001d029288_T001  rna20845           0
 82- 13:  transcript:Zm00001d029293_T001  rna20842     7.00E-09
 82- 14:  transcript:Zm00001d029300_T001  rna20838     2.00E-55
 82- 15:  transcript:Zm00001d029305_T001  rna20830     1.00E-25
 82- 16:  transcript:Zm00001d029313_T001  rna20829     5.00E-45
 82- 17:  transcript:Zm00001d029325_T001  rna20823    3.00E-100
## Alignment 83: score=465.0 e_value=6.3e-19 N=10 1&NC_008400.2 minus
 83-  0:  transcript:Zm00001d028930_T001  rna21189     2.00E-89
 83-  1:  transcript:Zm00001d028931_T002  rna21186           0
 83-  2:  transcript:Zm00001d028936_T002  rna21185    4.00E-161
 83-  3:  transcript:Zm00001d028941_T001  rna21182     2.00E-13
 83-  4:  transcript:Zm00001d028946_T001  rna21181    2.00E-107
 83-  5:  transcript:Zm00001d028948_T001  rna21180     3.00E-86
 83-  6:  transcript:Zm00001d028949_T002  rna21178     5.00E-50
 83-  7:  transcript:Zm00001d028952_T004  rna21177    7.00E-119
 83-  8:  transcript:Zm00001d028953_T001  rna21174     4.00E-16
 83-  9:  transcript:Zm00001d028960_T001  rna21173    1.00E-178
## Alignment 84: score=434.0 e_value=7e-24 N=11 1&NC_008400.2 minus
 84-  0:  transcript:Zm00001d029352_T002  rna20789    2.00E-128
 84-  1:  transcript:Zm00001d029365_T002  rna20787     6.00E-19
 84-  2:  transcript:Zm00001d029371_T001  rna20783    3.00E-139
 84-  3:  transcript:Zm00001d029376_T001  rna20781    2.00E-117
 84-  4:  transcript:Zm00001d029380_T002  rna20779     3.00E-47
 84-  5:  transcript:Zm00001d029386_T006  rna20774    5.00E-159
 84-  6:  transcript:Zm00001d029391_T002  rna20770           0
 84-  7:  transcript:Zm00001d029393_T001  rna20766     5.00E-15
 84-  8:  transcript:Zm00001d029407_T001  rna20760     4.00E-96
 84-  9:  transcript:Zm00001d029408_T001  rna20756    8.00E-110
 84- 10:  transcript:Zm00001d029409_T001  rna20754    1.00E-119
## Alignment 85: score=432.0 e_value=1.9e-24 N=11 1&NC_008400.2 minus
 85-  0:  transcript:Zm00001d029442_T001  rna20738           0
 85-  1:  transcript:Zm00001d029447_T001  rna20725     1.00E-07
 85-  2:  transcript:Zm00001d029448_T001  rna20723     3.00E-74
 85-  3:  transcript:Zm00001d029460_T001  rna20720     4.00E-19
 85-  4:  transcript:Zm00001d029462_T002  rna20716    7.00E-171
 85-  5:  transcript:Zm00001d029468_T002  rna20710           0
 85-  6:  transcript:Zm00001d029473_T001  rna20703     5.00E-41
 85-  7:  transcript:Zm00001d029483_T001  rna20692     7.00E-36
 85-  8:  transcript:Zm00001d029488_T001  rna20688     8.00E-09
 85-  9:  transcript:Zm00001d029499_T002  rna20680     7.00E-30
 85- 10:  transcript:Zm00001d029505_T001  rna20678     7.00E-75
## Alignment 86: score=318.0 e_value=4.2e-16 N=8 1&NC_008400.2 minus
 86-  0:  transcript:Zm00001d029140_T002  rna21057    3.00E-167
 86-  1:  transcript:Zm00001d029143_T003  rna21052     1.00E-37
 86-  2:  transcript:Zm00001d029149_T001  rna21050    2.00E-114
 86-  3:  transcript:Zm00001d029164_T001  rna21049    3.00E-161
 86-  4:  transcript:Zm00001d029165_T001  rna21048           0
 86-  5:  transcript:Zm00001d029170_T004  rna21040     4.00E-79
 86-  6:  transcript:Zm00001d029172_T001  rna21021     1.00E-05
 86-  7:  transcript:Zm00001d029174_T005  rna21014           0
## Alignment 87: score=287.0 e_value=1e-11 N=7 1&NC_008400.2 minus
```

87- 0: transcript:Zm00001d029056_T003 rna21129 0
87- 1: transcript:Zm00001d029064_T001 rna21127 2.00E-140
87- 2: transcript:Zm00001d029066_T005 rna21126 3.00E-17
87- 3: transcript:Zm00001d029067_T001 rna21124 4.00E-42
87- 4: transcript:Zm00001d029072_T001 rna21123 0
87- 5: transcript:Zm00001d029075_T001 rna21122 0
87- 6: transcript:Zm00001d029078_T002 rna21109 4.00E-67
Alignment 88: score=268.0 e_value=1.7e-09 N=6 1&NC_008400.2 minus
88- 0: transcript:Zm00001d029202_T001 rna21000 3.00E-91
88- 1: transcript:Zm00001d029203_T001 rna20999 0
88- 2: transcript:Zm00001d029208_T001 rna20996 7.00E-138
88- 3: transcript:Zm00001d029209_T003 rna20993 0
88- 4: transcript:Zm00001d029212_T003 rna20988 0
88- 5: transcript:Zm00001d029214_T001 rna20982 1.00E-74
Alignment 89: score=264.0 e_value=3.4e-09 N=6 1&NC_008400.2 minus
89- 0: transcript:Zm00001d028974_T001 rna21167 0
89- 1: transcript:Zm00001d028984_T001 rna21163 4.00E-121
89- 2: transcript:Zm00001d028986_T001 rna21161 3.00E-74
89- 3: transcript:Zm00001d028992_T001 rna21155 5.00E-108
89- 4: transcript:Zm00001d028998_T001 rna21151 7.00E-73
89- 5: transcript:Zm00001d028999_T001 rna21150 2.00E-24
Alignment 90: score=1735.0 e_value=3e-135 N=37 1&NC_008401.2 plus
90- 0: transcript:Zm00001d031738_T001 rna22677 5.00E-83
90- 1: transcript:Zm00001d031740_T001 rna22678 0
90- 2: transcript:Zm00001d031741_T001 rna22680 2.00E-73
90- 3: transcript:Zm00001d031745_T001 rna22681 1.00E-49
90- 4: transcript:Zm00001d031749_T005 rna22685 0
90- 5: transcript:Zm00001d031750_T001 rna22686 6.00E-18
90- 6: transcript:Zm00001d031751_T001 rna22687 6.00E-171
90- 7: transcript:Zm00001d031760_T001 rna22690 0
90- 8: transcript:Zm00001d031764_T002 rna22691 3.00E-177
90- 9: transcript:Zm00001d031769_T001 rna22694 0
90- 10: transcript:Zm00001d031777_T001 rna22703 9.00E-150
90- 11: transcript:Zm00001d031778_T001 rna22705 9.00E-25
90- 12: transcript:Zm00001d031790_T002 rna22710 5.00E-100
90- 13: transcript:Zm00001d031792_T001 rna22711 0
90- 14: transcript:Zm00001d031793_T002 rna22712 0
90- 15: transcript:Zm00001d031794_T001 rna22715 0
90- 16: transcript:Zm00001d031796_T001 rna22719 3.00E-53
90- 17: transcript:Zm00001d031797_T001 rna22720 1.00E-25
90- 18: transcript:Zm00001d031798_T001 rna22722 1.00E-26
90- 19: transcript:Zm00001d031801_T001 rna22726 8.00E-107
90- 20: transcript:Zm00001d031804_T001 rna22729 1.00E-147
90- 21: transcript:Zm00001d031807_T003 rna22731 8.00E-18
90- 22: transcript:Zm00001d031808_T001 rna22734 2.00E-157
90- 23: transcript:Zm00001d031809_T001 rna22735 7.00E-98
90- 24: transcript:Zm00001d031810_T001 rna22736 2.00E-120
90- 25: transcript:Zm00001d031811_T001 rna22738 0
90- 26: transcript:Zm00001d031816_T002 rna22741 1.00E-32
90- 27: transcript:Zm00001d031817_T003 rna22743 2.00E-84
90- 28: transcript:Zm00001d031818_T001 rna22744 6.00E-65
90- 29: transcript:Zm00001d031822_T001 rna22745 5.00E-24
90- 30: transcript:Zm00001d031824_T003 rna22746 1.00E-138
90- 31: transcript:Zm00001d031825_T001 rna22747 7.00E-99

90- 32: transcript:Zm00001d031826_T001 rna22748 4.00E-71
90- 33: transcript:Zm00001d031832_T003 rna22749 0
90- 34: transcript:Zm00001d031833_T001 rna22750 5.00E-30
90- 35: transcript:Zm00001d031837_T001 rna22752 2.00E-115
90- 36: transcript:Zm00001d031840_T001 rna22753 2.00E-133
Alignment 91: score=1591.0 e_value=9.5e-124 N=36 1&NC_008401.2 plus
91- 0: transcript:Zm00001d031634_T001 rna23063 0
91- 1: transcript:Zm00001d031635_T001 rna23066 1.00E-163
91- 2: transcript:Zm00001d031636_T001 rna23067 3.00E-67
91- 3: transcript:Zm00001d031637_T003 rna23068 7.00E-148
91- 4: transcript:Zm00001d031638_T001 rna23069 0
91- 5: transcript:Zm00001d031641_T002 rna23071 6.00E-137
91- 6: transcript:Zm00001d031647_T001 rna23079 8.00E-161
91- 7: transcript:Zm00001d031648_T004 rna23080 0
91- 8: transcript:Zm00001d031651_T001 rna23081 9.00E-37
91- 9: transcript:Zm00001d031653_T007 rna23082 0
91- 10: transcript:Zm00001d031655_T003 rna23083 4.00E-61
91- 11: transcript:Zm00001d031659_T001 rna23085 0
91- 12: transcript:Zm00001d031660_T002 rna23086 0
91- 13: transcript:Zm00001d031662_T001 rna23088 0
91- 14: transcript:Zm00001d031665_T001 rna23093 5.00E-75
91- 15: transcript:Zm00001d031667_T006 rna23096 0
91- 16: transcript:Zm00001d031668_T001 rna23097 1.00E-06
91- 17: transcript:Zm00001d031669_T001 rna23098 1.00E-102
91- 18: transcript:Zm00001d031674_T003 rna23099 0
91- 19: transcript:Zm00001d031676_T002 rna23101 0
91- 20: transcript:Zm00001d031677_T001 rna23103 0
91- 21: transcript:Zm00001d031678_T002 rna23104 0
91- 22: transcript:Zm00001d031683_T001 rna23107 3.00E-28
91- 23: transcript:Zm00001d031684_T001 rna23108 0
91- 24: transcript:Zm00001d031691_T001 rna23113 0
91- 25: transcript:Zm00001d031694_T003 rna23114 0
91- 26: transcript:Zm00001d031696_T002 rna23120 4.00E-109
91- 27: transcript:Zm00001d031700_T003 rna23121 0
91- 28: transcript:Zm00001d031703_T001 rna23125 6.00E-138
91- 29: transcript:Zm00001d031705_T001 rna23129 0
91- 30: transcript:Zm00001d031706_T001 rna23130 0
91- 31: transcript:Zm00001d031707_T001 rna23132 8.00E-146
91- 32: transcript:Zm00001d031711_T001 rna23133 0
91- 33: transcript:Zm00001d031712_T002 rna23138 0
91- 34: transcript:Zm00001d031723_T002 rna23139 0
91- 35: transcript:Zm00001d031724_T003 rna23141 0
Alignment 92: score=1494.0 e_value=1.3e-120 N=33 1&NC_008401.2 plus
92- 0: transcript:Zm00001d031372_T001 rna22869 1.00E-65
92- 1: transcript:Zm00001d031389_T001 rna22877 7.00E-24
92- 2: transcript:Zm00001d031404_T001 rna22878 1.00E-67
92- 3: transcript:Zm00001d031412_T001 rna22882 0
92- 4: transcript:Zm00001d031415_T001 rna22886 0
92- 5: transcript:Zm00001d031416_T001 rna22887 4.00E-85
92- 6: transcript:Zm00001d031421_T001 rna22889 5.00E-114
92- 7: transcript:Zm00001d031422_T006 rna22890 0
92- 8: transcript:Zm00001d031423_T001 rna22891 3.00E-60
92- 9: transcript:Zm00001d031430_T005 rna22894 0
92- 10: transcript:Zm00001d031434_T001 rna22897 0

```
 92- 11:  transcript:Zm00001d031436_T001  rna22898    3.00E-156
 92- 12:  transcript:Zm00001d031437_T001  rna22902            0
 92- 13:  transcript:Zm00001d031439_T001  rna22903    2.00E-110
 92- 14:  transcript:Zm00001d031441_T002  rna22904    2.00E-149
 92- 15:  transcript:Zm00001d031444_T001  rna22911     8.00E-07
 92- 16:  transcript:Zm00001d031445_T001  rna22914            0
 92- 17:  transcript:Zm00001d031449_T001  rna22915            0
 92- 18:  transcript:Zm00001d031450_T001  rna22919     7.00E-95
 92- 19:  transcript:Zm00001d031451_T001  rna22920    5.00E-124
 92- 20:  transcript:Zm00001d031453_T001  rna22922    2.00E-157
 92- 21:  transcript:Zm00001d031454_T001  rna22925    4.00E-162
 92- 22:  transcript:Zm00001d031463_T002  rna22929    4.00E-141
 92- 23:  transcript:Zm00001d031464_T001  rna22930     3.00E-52
 92- 24:  transcript:Zm00001d031470_T001  rna22932     4.00E-80
 92- 25:  transcript:Zm00001d031473_T001  rna22934     3.00E-62
 92- 26:  transcript:Zm00001d031480_T001  rna22935            0
 92- 27:  transcript:Zm00001d031481_T001  rna22936    2.00E-111
 92- 28:  transcript:Zm00001d031484_T001  rna22937    4.00E-110
 92- 29:  transcript:Zm00001d031485_T001  rna22938            0
 92- 30:  transcript:Zm00001d031487_T004  rna22945     6.00E-99
 92- 31:  transcript:Zm00001d031488_T002  rna22955     2.00E-24
 92- 32:  transcript:Zm00001d031489_T001  rna22956            0
## Alignment 93: score=1476.0 e_value=1.9e-112 N=33 1&NC_008401.2 plus
 93-  0:  transcript:Zm00001d031265_T029  rna22781            0
 93-  1:  transcript:Zm00001d031266_T001  rna22784     5.00E-92
 93-  2:  transcript:Zm00001d031267_T001  rna22787    6.00E-166
 93-  3:  transcript:Zm00001d031268_T010  rna22789            0
 93-  4:  transcript:Zm00001d031269_T002  rna22795     2.00E-66
 93-  5:  transcript:Zm00001d031270_T001  rna22796    2.00E-103
 93-  6:  transcript:Zm00001d031271_T003  rna22801     5.00E-29
 93-  7:  transcript:Zm00001d031272_T001  rna22802     1.00E-51
 93-  8:  transcript:Zm00001d031273_T001  rna22804     9.00E-94
 93-  9:  transcript:Zm00001d031274_T001  rna22806     6.00E-54
 93- 10:  transcript:Zm00001d031275_T001  rna22810            0
 93- 11:  transcript:Zm00001d031278_T001  rna22813     2.00E-67
 93- 12:  transcript:Zm00001d031280_T001  rna22814            0
 93- 13:  transcript:Zm00001d031287_T001  rna22822            0
 93- 14:  transcript:Zm00001d031288_T001  rna22823     3.00E-31
 93- 15:  transcript:Zm00001d031289_T007  rna22824            0
 93- 16:  transcript:Zm00001d031290_T001  rna22825     1.00E-50
 93- 17:  transcript:Zm00001d031291_T004  rna22828            0
 93- 18:  transcript:Zm00001d031292_T001  rna22829    8.00E-103
 93- 19:  transcript:Zm00001d031303_T006  rna22837            0
 93- 20:  transcript:Zm00001d031308_T001  rna22838     3.00E-71
 93- 21:  transcript:Zm00001d031311_T001  rna22840     3.00E-46
 93- 22:  transcript:Zm00001d031312_T001  rna22842    2.00E-136
 93- 23:  transcript:Zm00001d031315_T002  rna22845            0
 93- 24:  transcript:Zm00001d031318_T001  rna22852            0
 93- 25:  transcript:Zm00001d031321_T001  rna22857            0
 93- 26:  transcript:Zm00001d031323_T001  rna22859            0
 93- 27:  transcript:Zm00001d031327_T009  rna22860            0
 93- 28:  transcript:Zm00001d031328_T001  rna22861    6.00E-163
 93- 29:  transcript:Zm00001d031329_T002  rna22862            0
 93- 30:  transcript:Zm00001d031332_T007  rna22866            0
```

```
 93- 31:  transcript:Zm00001d031338_T002  rna22867            0
 93- 32:  transcript:Zm00001d031349_T002  rna22879            0
## Alignment 94: score=1390.0 e_value=1.4e-104 N=31 1&NC_008401.2 plus
 94-  0:  transcript:Zm00001d032265_T001  rna22335     7.00E-82
 94-  1:  transcript:Zm00001d032266_T003  rna22336     7.00E-55
 94-  2:  transcript:Zm00001d032267_T003  rna22337            0
 94-  3:  transcript:Zm00001d032268_T001  rna22338            0
 94-  4:  transcript:Zm00001d032270_T002  rna22339    3.00E-160
 94-  5:  transcript:Zm00001d032271_T001  rna22344            0
 94-  6:  transcript:Zm00001d032272_T001  rna22345            0
 94-  7:  transcript:Zm00001d032274_T001  rna22357            0
 94-  8:  transcript:Zm00001d032279_T005  rna22383            0
 94-  9:  transcript:Zm00001d032282_T012  rna22388            0
 94- 10:  transcript:Zm00001d032283_T004  rna22396            0
 94- 11:  transcript:Zm00001d032284_T001  rna22399            0
 94- 12:  transcript:Zm00001d032285_T001  rna22402     2.00E-17
 94- 13:  transcript:Zm00001d032291_T001  rna22407     4.00E-73
 94- 14:  transcript:Zm00001d032292_T001  rna22423     2.00E-51
 94- 15:  transcript:Zm00001d032293_T001  rna22424            0
 94- 16:  transcript:Zm00001d032295_T001  rna22426     9.00E-91
 94- 17:  transcript:Zm00001d032298_T002  rna22427     1.00E-29
 94- 18:  transcript:Zm00001d032300_T001  rna22429            0
 94- 19:  transcript:Zm00001d032301_T005  rna22436     1.00E-96
 94- 20:  transcript:Zm00001d032303_T002  rna22443     1.00E-65
 94- 21:  transcript:Zm00001d032304_T003  rna22452            0
 94- 22:  transcript:Zm00001d032306_T001  rna22453     2.00E-36
 94- 23:  transcript:Zm00001d032307_T001  rna22454     7.00E-20
 94- 24:  transcript:Zm00001d032308_T001  rna22455     3.00E-98
 94- 25:  transcript:Zm00001d032311_T001  rna22456            0
 94- 26:  transcript:Zm00001d032316_T001  rna22461    2.00E-111
 94- 27:  transcript:Zm00001d032317_T001  rna22462            0
 94- 28:  transcript:Zm00001d032318_T001  rna22465     9.00E-27
 94- 29:  transcript:Zm00001d032322_T001  rna22474            0
 94- 30:  transcript:Zm00001d032324_T001  rna22475     3.00E-60
## Alignment 95: score=1357.0 e_value=1.3e-95 N=29 1&NC_008401.2 plus
 95-  0:  transcript:Zm00001d031554_T003  rna23006            0
 95-  1:  transcript:Zm00001d031555_T001  rna23007            0
 95-  2:  transcript:Zm00001d031560_T001  rna23009     2.00E-93
 95-  3:  transcript:Zm00001d031561_T002  rna23010    8.00E-161
 95-  4:  transcript:Zm00001d031562_T001  rna23011     2.00E-72
 95-  5:  transcript:Zm00001d031569_T001  rna23013            0
 95-  6:  transcript:Zm00001d031570_T001  rna23014     3.00E-98
 95-  7:  transcript:Zm00001d031571_T001  rna23015            0
 95-  8:  transcript:Zm00001d031577_T001  rna23017            0
 95-  9:  transcript:Zm00001d031580_T001  rna23020     1.00E-75
 95- 10:  transcript:Zm00001d031581_T002  rna23021     8.00E-19
 95- 11:  transcript:Zm00001d031587_T004  rna23024     2.00E-13
 95- 12:  transcript:Zm00001d031588_T001  rna23033     3.00E-89
 95- 13:  transcript:Zm00001d031589_T001  rna23034    1.00E-149
 95- 14:  transcript:Zm00001d031593_T001  rna23038            0
 95- 15:  transcript:Zm00001d031594_T001  rna23039    2.00E-150
 95- 16:  transcript:Zm00001d031599_T001  rna23040    3.00E-148
 95- 17:  transcript:Zm00001d031600_T001  rna23041    1.00E-154
 95- 18:  transcript:Zm00001d031601_T001  rna23042    7.00E-120
```

```
 95- 19:  transcript:Zm00001d031602_T002  rna23046            0
 95- 20:  transcript:Zm00001d031607_T001  rna23047     2.00E-40
 95- 21:  transcript:Zm00001d031611_T006  rna23048    2.00E-113
 95- 22:  transcript:Zm00001d031614_T001  rna23049            0
 95- 23:  transcript:Zm00001d031617_T001  rna23050            0
 95- 24:  transcript:Zm00001d031618_T001  rna23051     4.00E-68
 95- 25:  transcript:Zm00001d031619_T001  rna23055            0
 95- 26:  transcript:Zm00001d031620_T001  rna23059    4.00E-131
 95- 27:  transcript:Zm00001d031625_T001  rna23060     3.00E-31
 95- 28:  transcript:Zm00001d031627_T003  rna23062     7.00E-56
## Alignment 96: score=435.0 e_value=7.8e-17 N=9 1&NC_008401.2 plus
 96-  0:  transcript:Zm00001d031725_T001  rna23145    8.00E-156
 96-  1:  transcript:Zm00001d031726_T001  rna23147            0
 96-  2:  transcript:Zm00001d031727_T004  rna23149            0
 96-  3:  transcript:Zm00001d031728_T001  rna23151     2.00E-63
 96-  4:  transcript:Zm00001d031729_T001  rna23154    6.00E-173
 96-  5:  transcript:Zm00001d031730_T001  rna23156            0
 96-  6:  transcript:Zm00001d031732_T001  rna23157    4.00E-142
 96-  7:  transcript:Zm00001d031736_T001  rna23164    2.00E-134
 96-  8:  transcript:Zm00001d031737_T001  rna23165    3.00E-143
## Alignment 97: score=384.0 e_value=5.9e-18 N=8 1&NC_008401.2 plus
 97-  0:  transcript:Zm00001d032185_T001  rna22547            0
 97-  1:  transcript:Zm00001d032186_T001  rna22548    2.00E-129
 97-  2:  transcript:Zm00001d032187_T001  rna22549            0
 97-  3:  transcript:Zm00001d032188_T001  rna22550     1.00E-24
 97-  4:  transcript:Zm00001d032190_T009  rna22551     1.00E-67
 97-  5:  transcript:Zm00001d032194_T002  rna22552    4.00E-113
 97-  6:  transcript:Zm00001d032197_T001  rna22554    6.00E-158
 97-  7:  transcript:Zm00001d032198_T002  rna22555            0
## Alignment 98: score=320.0 e_value=2.8e-12 N=7 1&NC_008401.2 plus
 98-  0:  transcript:Zm00001d031253_T001  rna22765    1.00E-156
 98-  1:  transcript:Zm00001d031256_T019  rna22767            0
 98-  2:  transcript:Zm00001d031257_T001  rna22770     4.00E-31
 98-  3:  transcript:Zm00001d031258_T003  rna22773     1.00E-96
 98-  4:  transcript:Zm00001d031259_T001  rna22774     8.00E-71
 98-  5:  transcript:Zm00001d031260_T001  rna22776     4.00E-34
 98-  6:  transcript:Zm00001d031261_T001  rna22779    5.00E-112
## Alignment 99: score=2833.0 e_value=2.4e-244 N=62 1&NC_008401.2 minus
 99-  0:  transcript:Zm00001d032046_T002  rna22660     1.00E-51
 99-  1:  transcript:Zm00001d032047_T001  rna22658     1.00E-80
 99-  2:  transcript:Zm00001d032049_T002  rna22657            0
 99-  3:  transcript:Zm00001d032055_T001  rna22655     8.00E-99
 99-  4:  transcript:Zm00001d032056_T001  rna22654            0
 99-  5:  transcript:Zm00001d032057_T002  rna22653     1.00E-22
 99-  6:  transcript:Zm00001d032058_T002  rna22652     9.00E-65
 99-  7:  transcript:Zm00001d032060_T001  rna22651            0
 99-  8:  transcript:Zm00001d032069_T001  rna22650            0
 99-  9:  transcript:Zm00001d032073_T001  rna22645     7.00E-21
 99- 10:  transcript:Zm00001d032075_T001  rna22644            0
 99- 11:  transcript:Zm00001d032076_T002  rna22643            0
 99- 12:  transcript:Zm00001d032078_T002  rna22637     2.00E-39
 99- 13:  transcript:Zm00001d032079_T001  rna22636            0
 99- 14:  transcript:Zm00001d032081_T001  rna22634     6.00E-13
 99- 15:  transcript:Zm00001d032088_T001  rna22632     3.00E-69
```

```
 99- 16:  transcript:Zm00001d032096_T005  rna22623            0
 99- 17:  transcript:Zm00001d032098_T001  rna22621     1.00E-24
 99- 18:  transcript:Zm00001d032099_T004  rna22620            0
 99- 19:  transcript:Zm00001d032100_T003  rna22617            0
 99- 20:  transcript:Zm00001d032102_T001  rna22610     7.00E-32
 99- 21:  transcript:Zm00001d032103_T001  rna22609            0
 99- 22:  transcript:Zm00001d032104_T001  rna22608            0
 99- 23:  transcript:Zm00001d032109_T001  rna22607     7.00E-17
 99- 24:  transcript:Zm00001d032111_T003  rna22606     8.00E-58
 99- 25:  transcript:Zm00001d032112_T001  rna22605            0
 99- 26:  transcript:Zm00001d032114_T001  rna22604    3.00E-104
 99- 27:  transcript:Zm00001d032115_T001  rna22603     3.00E-72
 99- 28:  transcript:Zm00001d032116_T002  rna22601            0
 99- 29:  transcript:Zm00001d032118_T001  rna22600            0
 99- 30:  transcript:Zm00001d032132_T001  rna22598     4.00E-29
 99- 31:  transcript:Zm00001d032142_T004  rna22593            0
 99- 32:  transcript:Zm00001d032144_T001  rna22591    6.00E-126
 99- 33:  transcript:Zm00001d032145_T001  rna22590     2.00E-73
 99- 34:  transcript:Zm00001d032146_T001  rna22589    4.00E-111
 99- 35:  transcript:Zm00001d032152_T001  rna22582            0
 99- 36:  transcript:Zm00001d032153_T002  rna22581     3.00E-63
 99- 37:  transcript:Zm00001d032155_T001  rna22578    2.00E-167
 99- 38:  transcript:Zm00001d032156_T003  rna22577    3.00E-127
 99- 39:  transcript:Zm00001d032157_T001  rna22575    6.00E-126
 99- 40:  transcript:Zm00001d032158_T002  rna22574     2.00E-28
 99- 41:  transcript:Zm00001d032162_T001  rna22572    3.00E-111
 99- 42:  transcript:Zm00001d032163_T001  rna22570     2.00E-96
 99- 43:  transcript:Zm00001d032164_T001  rna22569    3.00E-168
 99- 44:  transcript:Zm00001d032166_T003  rna22567            0
 99- 45:  transcript:Zm00001d032172_T002  rna22566            0
 99- 46:  transcript:Zm00001d032173_T001  rna22564            0
 99- 47:  transcript:Zm00001d032175_T001  rna22563     2.00E-77
 99- 48:  transcript:Zm00001d032177_T001  rna22562     2.00E-33
 99- 49:  transcript:Zm00001d032178_T001  rna22561    4.00E-126
 99- 50:  transcript:Zm00001d032181_T001  rna22560     1.00E-76
 99- 51:  transcript:Zm00001d032182_T001  rna22559            0
 99- 52:  transcript:Zm00001d032183_T002  rna22558     4.00E-22
 99- 53:  transcript:Zm00001d032184_T003  rna22556    4.00E-145
 99- 54:  transcript:Zm00001d032198_T002  rna22555            0
 99- 55:  transcript:Zm00001d032199_T001  rna22545     2.00E-63
 99- 56:  transcript:Zm00001d032206_T001  rna22543            0
 99- 57:  transcript:Zm00001d032209_T002  rna22541     6.00E-54
 99- 58:  transcript:Zm00001d032212_T005  rna22540     6.00E-99
 99- 59:  transcript:Zm00001d032213_T001  rna22538    6.00E-102
 99- 60:  transcript:Zm00001d032215_T001  rna22536     9.00E-96
 99- 61:  transcript:Zm00001d032218_T001  rna22535     6.00E-33
## Alignment 100: score=1632.0 e_value=4.2e-120 N=36 1&NC_008401.2 minus
100-  0:  transcript:Zm00001d031953_T008  rna23250    8.00E-174
100-  1:  transcript:Zm00001d031957_T001  rna23248     1.00E-54
100-  2:  transcript:Zm00001d031958_T001  rna23247            0
100-  3:  transcript:Zm00001d031959_T001  rna23246            0
100-  4:  transcript:Zm00001d031961_T002  rna23245     6.00E-73
100-  5:  transcript:Zm00001d031963_T001  rna23242    5.00E-119
100-  6:  transcript:Zm00001d031969_T001  rna23239    1.00E-103
```

100- 7:	transcript:Zm00001d031971_T002	rna23237	3.00E-176
100- 8:	transcript:Zm00001d031973_T001	rna23235	0
100- 9:	transcript:Zm00001d031975_T001	rna23234	6.00E-53
100- 10:	transcript:Zm00001d031977_T001	rna23232	0
100- 11:	transcript:Zm00001d031979_T001	rna23230	3.00E-65
100- 12:	transcript:Zm00001d031981_T002	rna23228	0
100- 13:	transcript:Zm00001d031985_T001	rna23227	2.00E-42
100- 14:	transcript:Zm00001d031988_T001	rna23221	0
100- 15:	transcript:Zm00001d031992_T001	rna23220	0
100- 16:	transcript:Zm00001d031993_T001	rna23218	0
100- 17:	transcript:Zm00001d031996_T006	rna23216	0
100- 18:	transcript:Zm00001d031997_T001	rna23215	0
100- 19:	transcript:Zm00001d032005_T001	rna23200	1.00E-14
100- 20:	transcript:Zm00001d032008_T001	rna23193	6.00E-96
100- 21:	transcript:Zm00001d032010_T007	rna23191	0
100- 22:	transcript:Zm00001d032012_T001	rna23190	5.00E-07
100- 23:	transcript:Zm00001d032013_T001	rna23189	3.00E-18
100- 24:	transcript:Zm00001d032019_T001	rna23187	1.00E-62
100- 25:	transcript:Zm00001d032022_T003	rna23186	0
100- 26:	transcript:Zm00001d032024_T001	rna23184	2.00E-121
100- 27:	transcript:Zm00001d032028_T001	rna23180	0
100- 28:	transcript:Zm00001d032030_T001	rna23179	6.00E-34
100- 29:	transcript:Zm00001d032031_T001	rna23177	0
100- 30:	transcript:Zm00001d032035_T001	rna23174	0
100- 31:	transcript:Zm00001d032036_T001	rna23173	1.00E-146
100- 32:	transcript:Zm00001d032040_T001	rna23172	3.00E-90
100- 33:	transcript:Zm00001d032042_T001	rna23170	0
100- 34:	transcript:Zm00001d032044_T004	rna23169	0
100- 35:	transcript:Zm00001d032045_T001	rna23168	1.00E-156

Alignment 101: score=1320.0 e_value=1.6e-93 N=29 1&NC_008401.2 minus

101- 0:	transcript:Zm00001d031848_T001	rna23310	3.00E-70
101- 1:	transcript:Zm00001d031849_T002	rna23309	8.00E-157
101- 2:	transcript:Zm00001d031850_T001	rna23306	7.00E-59
101- 3:	transcript:Zm00001d031853_T001	rna23304	0
101- 4:	transcript:Zm00001d031854_T001	rna23303	0
101- 5:	transcript:Zm00001d031855_T001	rna23302	0
101- 6:	transcript:Zm00001d031856_T002	rna23301	6.00E-75
101- 7:	transcript:Zm00001d031858_T001	rna23300	0
101- 8:	transcript:Zm00001d031861_T001	rna23299	2.00E-50
101- 9:	transcript:Zm00001d031863_T013	rna23296	0
101- 10:	transcript:Zm00001d031866_T001	rna23294	0
101- 11:	transcript:Zm00001d031868_T001	rna23293	9.00E-42
101- 12:	transcript:Zm00001d031871_T001	rna23291	0
101- 13:	transcript:Zm00001d031875_T001	rna23289	0
101- 14:	transcript:Zm00001d031882_T001	rna23288	1.00E-107
101- 15:	transcript:Zm00001d031883_T001	rna23287	2.00E-78
101- 16:	transcript:Zm00001d031887_T001	rna23286	2.00E-98
101- 17:	transcript:Zm00001d031891_T017	rna23284	0
101- 18:	transcript:Zm00001d031892_T001	rna23283	3.00E-91
101- 19:	transcript:Zm00001d031893_T001	rna23282	0
101- 20:	transcript:Zm00001d031895_T001	rna23281	2.00E-138
101- 21:	transcript:Zm00001d031898_T001	rna23280	0
101- 22:	transcript:Zm00001d031899_T002	rna23279	0
101- 23:	transcript:Zm00001d031904_T001	rna23278	5.00E-145

101- 24: transcript:Zm00001d031908_T001 rna23276 1.00E-113
101- 25: transcript:Zm00001d031911_T002 rna23275 0
101- 26: transcript:Zm00001d031921_T001 rna23272 5.00E-49
101- 27: transcript:Zm00001d031925_T001 rna23269 2.00E-94
101- 28: transcript:Zm00001d031926_T001 rna23268 0
Alignment 102: score=1127.0 e_value=3.1e-81 N=25 1&NC_008401.2 minus
102- 0: transcript:Zm00001d032326_T002 rna22328 7.00E-50
102- 1: transcript:Zm00001d032327_T001 rna22327 1.00E-70
102- 2: transcript:Zm00001d032328_T005 rna22326 2.00E-96
102- 3: transcript:Zm00001d032331_T001 rna22324 0
102- 4: transcript:Zm00001d032332_T001 rna22320 0
102- 5: transcript:Zm00001d032333_T004 rna22319 0
102- 6: transcript:Zm00001d032334_T002 rna22318 2.00E-139
102- 7: transcript:Zm00001d032335_T001 rna22317 3.00E-31
102- 8: transcript:Zm00001d032337_T001 rna22316 1.00E-82
102- 9: transcript:Zm00001d032339_T001 rna22312 8.00E-161
102- 10: transcript:Zm00001d032342_T001 rna22308 2.00E-134
102- 11: transcript:Zm00001d032344_T001 rna22306 0
102- 12: transcript:Zm00001d032345_T001 rna22303 2.00E-42
102- 13: transcript:Zm00001d032346_T002 rna22297 0
102- 14: transcript:Zm00001d032347_T001 rna22295 2.00E-161
102- 15: transcript:Zm00001d032350_T002 rna22294 1.00E-44
102- 16: transcript:Zm00001d032357_T001 rna22280 3.00E-44
102- 17: transcript:Zm00001d032359_T003 rna22270 4.00E-88
102- 18: transcript:Zm00001d032363_T001 rna22266 3.00E-150
102- 19: transcript:Zm00001d032367_T001 rna22258 4.00E-27
102- 20: transcript:Zm00001d032373_T004 rna22257 0
102- 21: transcript:Zm00001d032377_T002 rna22252 0
102- 22: transcript:Zm00001d032379_T002 rna22250 0
102- 23: transcript:Zm00001d032380_T001 rna22249 3.00E-134
102- 24: transcript:Zm00001d032381_T001 rna22248 3.00E-42
Alignment 103: score=960.0 e_value=3.6e-61 N=21 1&NC_008401.2 minus
103- 0: transcript:Zm00001d031509_T003 rna22993 0
103- 1: transcript:Zm00001d031510_T001 rna22992 6.00E-111
103- 2: transcript:Zm00001d031517_T001 rna22989 5.00E-20
103- 3: transcript:Zm00001d031522_T007 rna22988 0
103- 4: transcript:Zm00001d031523_T001 rna22987 0
103- 5: transcript:Zm00001d031525_T004 rna22986 0
103- 6: transcript:Zm00001d031527_T001 rna22984 0
103- 7: transcript:Zm00001d031528_T001 rna22983 0
103- 8: transcript:Zm00001d031529_T014 rna22982 0
103- 9: transcript:Zm00001d031530_T005 rna22981 2.00E-97
103- 10: transcript:Zm00001d031531_T002 rna22980 0
103- 11: transcript:Zm00001d031532_T001 rna22978 0
103- 12: transcript:Zm00001d031533_T001 rna22970 0
103- 13: transcript:Zm00001d031534_T001 rna22969 0
103- 14: transcript:Zm00001d031536_T001 rna22965 0
103- 15: transcript:Zm00001d031539_T004 rna22964 1.00E-108
103- 16: transcript:Zm00001d031540_T002 rna22963 0
103- 17: transcript:Zm00001d031542_T001 rna22961 3.00E-20
103- 18: transcript:Zm00001d031543_T005 rna22960 9.00E-47
103- 19: transcript:Zm00001d031545_T001 rna22958 1.00E-51
103- 20: transcript:Zm00001d031546_T001 rna22957 7.00E-16
Alignment 104: score=882.0 e_value=3.5e-52 N=19 1&NC_008401.2 minus

```
104-  0:  transcript:Zm00001d032224_T002  rna22530            0
104-  1:  transcript:Zm00001d032225_T001  rna22529            0
104-  2:  transcript:Zm00001d032226_T002  rna22528            0
104-  3:  transcript:Zm00001d032229_T002  rna22526            0
104-  4:  transcript:Zm00001d032231_T001  rna22524    1.00E-175
104-  5:  transcript:Zm00001d032233_T001  rna22519     1.00E-77
104-  6:  transcript:Zm00001d032238_T001  rna22517            0
104-  7:  transcript:Zm00001d032239_T001  rna22516     1.00E-83
104-  8:  transcript:Zm00001d032240_T001  rna22513     5.00E-55
104-  9:  transcript:Zm00001d032242_T001  rna22511     7.00E-35
104- 10:  transcript:Zm00001d032245_T003  rna22509            0
104- 11:  transcript:Zm00001d032249_T002  rna22504     2.00E-87
104- 12:  transcript:Zm00001d032250_T001  rna22503    1.00E-105
104- 13:  transcript:Zm00001d032253_T001  rna22501    3.00E-152
104- 14:  transcript:Zm00001d032256_T001  rna22499            0
104- 15:  transcript:Zm00001d032257_T001  rna22495            0
104- 16:  transcript:Zm00001d032262_T001  rna22485     1.00E-97
104- 17:  transcript:Zm00001d032263_T001  rna22483     9.00E-98
104- 18:  transcript:Zm00001d032264_T001  rna22482    4.00E-175
## Alignment 105: score=428.0 e_value=7e-18 N=9 1&NC_008401.2 minus
105-  0:  transcript:Zm00001d031937_T001  rna23261     2.00E-92
105-  1:  transcript:Zm00001d031938_T001  rna23260            0
105-  2:  transcript:Zm00001d031939_T001  rna23259     2.00E-56
105-  3:  transcript:Zm00001d031940_T001  rna23257     1.00E-35
105-  4:  transcript:Zm00001d031941_T001  rna23255    1.00E-119
105-  5:  transcript:Zm00001d031942_T002  rna23254    9.00E-167
105-  6:  transcript:Zm00001d031943_T002  rna23252            0
105-  7:  transcript:Zm00001d031944_T001  rna23251    8.00E-106
105-  8:  transcript:Zm00001d031945_T001  rna23250    9.00E-169
## Alignment 106: score=713.0 e_value=4.2e-43 N=16 1&NC_008402.2 plus
106-  0:  transcript:Zm00001d031545_T001  rna24511     1.00E-40
106-  1:  transcript:Zm00001d031554_T003  rna24518            0
106-  2:  transcript:Zm00001d031555_T001  rna24520     3.00E-47
106-  3:  transcript:Zm00001d031560_T001  rna24521     4.00E-89
106-  4:  transcript:Zm00001d031561_T002  rna24522    1.00E-102
106-  5:  transcript:Zm00001d031569_T001  rna24524     3.00E-30
106-  6:  transcript:Zm00001d031570_T001  rna24526     1.00E-25
106-  7:  transcript:Zm00001d031571_T001  rna24527            0
106-  8:  transcript:Zm00001d031577_T001  rna24536            0
106-  9:  transcript:Zm00001d031586_T001  rna24541     5.00E-28
106- 10:  transcript:Zm00001d031587_T004  rna24542     4.00E-12
106- 11:  transcript:Zm00001d031593_T001  rna24543     2.00E-68
106- 12:  transcript:Zm00001d031594_T001  rna24545     4.00E-51
106- 13:  transcript:Zm00001d031599_T001  rna24546    1.00E-141
106- 14:  transcript:Zm00001d031602_T002  rna24548            0
106- 15:  transcript:Zm00001d031620_T001  rna24556    4.00E-126
## Alignment 107: score=682.0 e_value=5.2e-42 N=16 1&NC_008402.2 plus
107-  0:  transcript:Zm00001d031745_T001  rna24204     5.00E-54
107-  1:  transcript:Zm00001d031753_T001  rna24211     2.00E-36
107-  2:  transcript:Zm00001d031759_T001  rna24215     1.00E-95
107-  3:  transcript:Zm00001d031773_T003  rna24223     4.00E-32
107-  4:  transcript:Zm00001d031777_T001  rna24245    5.00E-166
107-  5:  transcript:Zm00001d031778_T001  rna24249     4.00E-29
107-  6:  transcript:Zm00001d031781_T001  rna24254     1.00E-52
```

107- 7: transcript:Zm00001d031790_T002 rna24259 8.00E-96
107- 8: transcript:Zm00001d031792_T001 rna24260 0
107- 9: transcript:Zm00001d031794_T001 rna24264 0
107- 10: transcript:Zm00001d031796_T001 rna24269 1.00E-67
107- 11: transcript:Zm00001d031797_T001 rna24280 4.00E-20
107- 12: transcript:Zm00001d031798_T001 rna24281 4.00E-17
107- 13: transcript:Zm00001d031804_T001 rna24292 3.00E-84
107- 14: transcript:Zm00001d031807_T003 rna24304 2.00E-115
107- 15: transcript:Zm00001d031810_T001 rna24305 1.00E-109
Alignment 108: score=607.0 e_value=8.6e-34 N=14 1&NC_008402.2 plus
108- 0: transcript:Zm00001d032282_T012 rna23833 0
108- 1: transcript:Zm00001d032283_T004 rna23838 3.00E-142
108- 2: transcript:Zm00001d032284_T001 rna23842 0
108- 3: transcript:Zm00001d032286_T001 rna23844 2.00E-09
108- 4: transcript:Zm00001d032291_T001 rna23848 2.00E-33
108- 5: transcript:Zm00001d032295_T001 rna23861 7.00E-79
108- 6: transcript:Zm00001d032298_T002 rna23862 1.00E-171
108- 7: transcript:Zm00001d032300_T001 rna23868 0
108- 8: transcript:Zm00001d032306_T001 rna23891 3.00E-29
108- 9: transcript:Zm00001d032307_T001 rna23892 1.00E-12
108- 10: transcript:Zm00001d032311_T001 rna23895 0
108- 11: transcript:Zm00001d032316_T001 rna23902 8.00E-48
108- 12: transcript:Zm00001d032322_T001 rna23910 0
108- 13: transcript:Zm00001d032324_T001 rna23913 3.00E-56
Alignment 109: score=526.0 e_value=1.3e-28 N=12 1&NC_008402.2 plus
109- 0: transcript:Zm00001d031389_T001 rna24427 3.00E-23
109- 1: transcript:Zm00001d031404_T001 rna24428 7.00E-69
109- 2: transcript:Zm00001d031410_T001 rna24430 3.00E-33
109- 3: transcript:Zm00001d031416_T001 rna24444 7.00E-35
109- 4: transcript:Zm00001d031423_T001 rna24448 1.00E-62
109- 5: transcript:Zm00001d031426_T001 rna24449 5.00E-168
109- 6: transcript:Zm00001d031430_T005 rna24451 0
109- 7: transcript:Zm00001d031431_T001 rna24452 1.00E-65
109- 8: transcript:Zm00001d031436_T001 rna24454 2.00E-118
109- 9: transcript:Zm00001d031441_T002 rna24455 5.00E-119
109- 10: transcript:Zm00001d031444_T001 rna24459 0
109- 11: transcript:Zm00001d031445_T001 rna24463 0
Alignment 110: score=378.0 e_value=5.4e-17 N=9 1&NC_008402.2 plus
110- 0: transcript:Zm00001d031635_T001 rna24559 2.00E-152
110- 1: transcript:Zm00001d031637_T003 rna24560 3.00E-17
110- 2: transcript:Zm00001d031640_T001 rna24561 0
110- 3: transcript:Zm00001d031648_T004 rna24565 0
110- 4: transcript:Zm00001d031655_T003 rna24569 2.00E-39
110- 5: transcript:Zm00001d031659_T001 rna24570 0
110- 6: transcript:Zm00001d031660_T002 rna24572 0
110- 7: transcript:Zm00001d031665_T001 rna24579 6.00E-101
110- 8: transcript:Zm00001d031667_T006 rna24580 0
Alignment 111: score=365.0 e_value=2.3e-18 N=9 1&NC_008402.2 plus
111- 0: transcript:Zm00001d031683_T001 rna24584 1.00E-07
111- 1: transcript:Zm00001d031684_T001 rna24585 3.00E-101
111- 2: transcript:Zm00001d031689_T004 rna24600 8.00E-25
111- 3: transcript:Zm00001d031694_T003 rna24602 0
111- 4: transcript:Zm00001d031697_T001 rna24622 7.00E-72
111- 5: transcript:Zm00001d031700_T003 rna24626 0

111- 6: transcript:Zm00001d031706_T001 rna24639 0
111- 7: transcript:Zm00001d031708_T001 rna24640 5.00E-38
111- 8: transcript:Zm00001d031717_T001 rna24645 3.00E-71
Alignment 112: score=340.0 e_value=2.5e-15 N=8 1&NC_008402.2 plus
112- 0: transcript:Zm00001d031265_T029 rna24352 0
112- 1: transcript:Zm00001d031268_T010 rna24357 1.00E-45
112- 2: transcript:Zm00001d031271_T003 rna24362 5.00E-06
112- 3: transcript:Zm00001d031272_T001 rna24363 7.00E-46
112- 4: transcript:Zm00001d031274_T001 rna24365 9.00E-54
112- 5: transcript:Zm00001d031278_T001 rna24372 1.00E-50
112- 6: transcript:Zm00001d031280_T001 rna24374 1.00E-85
112- 7: transcript:Zm00001d031290_T001 rna24387 1.00E-36
Alignment 113: score=277.0 e_value=1.9e-08 N=6 1&NC_008402.2 plus
113- 0: transcript:Zm00001d027976_T002 rna24318 9.00E-80
113- 1: transcript:Zm00001d027978_T001 rna24324 1.00E-90
113- 2: transcript:Zm00001d027982_T002 rna24328 0
113- 3: transcript:Zm00001d027983_T001 rna24330 6.00E-32
113- 4: transcript:Zm00001d027987_T001 rna24332 3.00E-75
113- 5: transcript:Zm00001d027991_T001 rna24340 8.00E-47
Alignment 114: score=427.0 e_value=2.1e-21 N=10 1&NC_008402.2 minus
114- 0: transcript:Zm00001d031958_T001 rna24951 1.00E-173
114- 1: transcript:Zm00001d031959_T001 rna24947 0
114- 2: transcript:Zm00001d031961_T002 rna24946 5.00E-62
114- 3: transcript:Zm00001d031969_T001 rna24929 8.00E-101
114- 4: transcript:Zm00001d031971_T002 rna24925 1.00E-54
114- 5: transcript:Zm00001d031977_T001 rna24918 9.00E-178
114- 6: transcript:Zm00001d031979_T001 rna24905 4.00E-69
114- 7: transcript:Zm00001d031985_T001 rna24891 1.00E-19
114- 8: transcript:Zm00001d031988_T001 rna24883 0
114- 9: transcript:Zm00001d031992_T001 rna24869 0
Alignment 115: score=422.0 e_value=6.1e-20 N=10 1&NC_008402.2 minus
115- 0: transcript:Zm00001d032060_T001 rna24181 0
115- 1: transcript:Zm00001d032069_T001 rna24178 0
115- 2: transcript:Zm00001d032075_T001 rna24165 0
115- 3: transcript:Zm00001d032079_T001 rna24156 0
115- 4: transcript:Zm00001d032081_T001 rna24153 7.00E-36
115- 5: transcript:Zm00001d032088_T001 rna24151 1.00E-61
115- 6: transcript:Zm00001d032095_T001 rna24139 3.00E-38
115- 7: transcript:Zm00001d032096_T005 rna24137 1.00E-142
115- 8: transcript:Zm00001d032098_T001 rna24135 5.00E-36
115- 9: transcript:Zm00001d032100_T003 rna24134 0
Alignment 116: score=393.0 e_value=6.8e-20 N=9 1&NC_008402.2 minus
116- 0: transcript:Zm00001d032233_T001 rna23988 1.00E-16
116- 1: transcript:Zm00001d032238_T001 rna23987 2.00E-163
116- 2: transcript:Zm00001d032239_T001 rna23986 3.00E-65
116- 3: transcript:Zm00001d032240_T001 rna23980 4.00E-55
116- 4: transcript:Zm00001d032244_T001 rna23977 0
116- 5: transcript:Zm00001d032249_T002 rna23956 3.00E-64
116- 6: transcript:Zm00001d032253_T001 rna23953 1.00E-149
116- 7: transcript:Zm00001d032263_T001 rna23930 8.00E-98
116- 8: transcript:Zm00001d032264_T001 rna23928 5.00E-130
Alignment 117: score=375.0 e_value=1.9e-17 N=9 1&NC_008402.2 minus
117- 0: transcript:Zm00001d032019_T001 rna24771 3.00E-29
117- 1: transcript:Zm00001d032022_T003 rna24766 0

117- 2: transcript:Zm00001d032024_T001 rna24756   2.00E-109
117- 3: transcript:Zm00001d032027_T002 rna24743   5.00E-93
117- 4: transcript:Zm00001d032028_T001 rna24742   0
117- 5: transcript:Zm00001d032031_T001 rna24730   3.00E-51
117- 6: transcript:Zm00001d032032_T001 rna24721   9.00E-54
117- 7: transcript:Zm00001d032040_T001 rna24712   4.00E-38
117- 8: transcript:Zm00001d032045_T001 rna24696   2.00E-153
Alignment 118: score=324.0 e_value=1.8e-14 N=8 1&NC_008402.2 minus
118- 0: transcript:Zm00001d032118_T001 rna24109   0
118- 1: transcript:Zm00001d032132_T001 rna24107   2.00E-13
118- 2: transcript:Zm00001d032144_T001 rna24091   3.00E-116
118- 3: transcript:Zm00001d032148_T001 rna24081   0
118- 4: transcript:Zm00001d032152_T001 rna24063   0
118- 5: transcript:Zm00001d032155_T001 rna24061   0
118- 6: transcript:Zm00001d032158_T002 rna24054   4.00E-121
118- 7: transcript:Zm00001d032164_T001 rna24052   1.00E-73
Alignment 119: score=289.0 e_value=1.5e-08 N=6 1&NC_008402.2 minus
119- 0: transcript:Zm00001d032172_T002 rna24048   0
119- 1: transcript:Zm00001d032175_T001 rna24042   1.00E-35
119- 2: transcript:Zm00001d032177_T001 rna24041   3.00E-26
119- 3: transcript:Zm00001d032178_T001 rna24040   2.00E-115
119- 4: transcript:Zm00001d032181_T001 rna24037   2.00E-12
119- 5: transcript:Zm00001d032183_T002 rna24034   1.00E-37
Alignment 120: score=3130.0 e_value=7.8e-275 N=68 1&NC_008403.2 plus
120- 0: transcript:Zm00001d029706_T001 rna26324   4.00E-71
120- 1: transcript:Zm00001d029707_T001 rna26335   1.00E-81
120- 2: transcript:Zm00001d029708_T001 rna26339   2.00E-89
120- 3: transcript:Zm00001d029711_T001 rna26347   1.00E-170
120- 4: transcript:Zm00001d029714_T001 rna26349   0
120- 5: transcript:Zm00001d029715_T001 rna26350   0
120- 6: transcript:Zm00001d029716_T002 rna26351   4.00E-50
120- 7: transcript:Zm00001d029718_T001 rna26352   5.00E-77
120- 8: transcript:Zm00001d029719_T002 rna26354   0
120- 9: transcript:Zm00001d029720_T001 rna26355   6.00E-72
120- 10: transcript:Zm00001d029721_T001 rna26356   0
120- 11: transcript:Zm00001d029723_T001 rna26357   1.00E-178
120- 12: transcript:Zm00001d029725_T001 rna26362   1.00E-152
120- 13: transcript:Zm00001d029726_T004 rna26363   0
120- 14: transcript:Zm00001d029730_T001 rna26364   5.00E-48
120- 15: transcript:Zm00001d029734_T001 rna26367   0
120- 16: transcript:Zm00001d029736_T001 rna26369   3.00E-177
120- 17: transcript:Zm00001d029738_T001 rna26370   2.00E-57
120- 18: transcript:Zm00001d029740_T002 rna26372   5.00E-99
120- 19: transcript:Zm00001d029744_T001 rna26374   2.00E-97
120- 20: transcript:Zm00001d029745_T003 rna26375   2.00E-66
120- 21: transcript:Zm00001d029747_T001 rna26376   7.00E-89
120- 22: transcript:Zm00001d029750_T001 rna26378   0
120- 23: transcript:Zm00001d029753_T001 rna26379   0
120- 24: transcript:Zm00001d029754_T004 rna26380   0
120- 25: transcript:Zm00001d029758_T004 rna26386   0
120- 26: transcript:Zm00001d029761_T001 rna26388   0
120- 27: transcript:Zm00001d029762_T008 rna26389   0
120- 28: transcript:Zm00001d029763_T001 rna26390   1.00E-11
120- 29: transcript:Zm00001d029764_T001 rna26391   0

120- 30: transcript:Zm00001d029768_T001 rna26397 0
120- 31: transcript:Zm00001d029772_T002 rna26398 3.00E-119
120- 32: transcript:Zm00001d029778_T001 rna26403 4.00E-27
120- 33: transcript:Zm00001d029782_T004 rna26404 0
120- 34: transcript:Zm00001d029783_T001 rna26405 1.00E-146
120- 35: transcript:Zm00001d029784_T001 rna26406 1.00E-157
120- 36: transcript:Zm00001d029785_T001 rna26407 1.00E-124
120- 37: transcript:Zm00001d029794_T001 rna26408 5.00E-127
120- 38: transcript:Zm00001d029799_T002 rna26412 5.00E-80
120- 39: transcript:Zm00001d029806_T001 rna26416 5.00E-123
120- 40: transcript:Zm00001d029808_T001 rna26419 0
120- 41: transcript:Zm00001d029809_T002 rna26420 4.00E-43
120- 42: transcript:Zm00001d029810_T001 rna26421 0
120- 43: transcript:Zm00001d029811_T001 rna26422 1.00E-154
120- 44: transcript:Zm00001d029814_T001 rna26424 6.00E-171
120- 45: transcript:Zm00001d029815_T001 rna26427 6.00E-114
120- 46: transcript:Zm00001d029816_T001 rna26429 3.00E-53
120- 47: transcript:Zm00001d029818_T004 rna26431 0
120- 48: transcript:Zm00001d029820_T001 rna26432 9.00E-25
120- 49: transcript:Zm00001d029822_T001 rna26433 4.00E-146
120- 50: transcript:Zm00001d029825_T001 rna26434 8.00E-52
120- 51: transcript:Zm00001d029835_T001 rna26440 2.00E-117
120- 52: transcript:Zm00001d029846_T001 rna26446 0
120- 53: transcript:Zm00001d029848_T002 rna26451 7.00E-63
120- 54: transcript:Zm00001d029849_T006 rna26454 0
120- 55: transcript:Zm00001d029850_T001 rna26457 0
120- 56: transcript:Zm00001d029851_T001 rna26458 4.00E-100
120- 57: transcript:Zm00001d029852_T001 rna26460 5.00E-55
120- 58: transcript:Zm00001d029853_T001 rna26461 0
120- 59: transcript:Zm00001d029855_T001 rna26463 0
120- 60: transcript:Zm00001d029856_T001 rna26464 2.00E-71
120- 61: transcript:Zm00001d029857_T001 rna26467 2.00E-13
120- 62: transcript:Zm00001d029858_T001 rna26468 7.00E-34
120- 63: transcript:Zm00001d029860_T001 rna26475 0
120- 64: transcript:Zm00001d029862_T001 rna26477 0
120- 65: transcript:Zm00001d029865_T001 rna26478 0
120- 66: transcript:Zm00001d029868_T004 rna26479 9.00E-63
120- 67: transcript:Zm00001d029872_T001 rna26481 0
Alignment 121: score=1396.0 e_value=7e-110 N=31 1&NC_008403.2 plus
121- 0: transcript:Zm00001d029934_T001 rna26534 1.00E-91
121- 1: transcript:Zm00001d029937_T001 rna26536 3.00E-107
121- 2: transcript:Zm00001d029948_T001 rna26539 0
121- 3: transcript:Zm00001d029964_T001 rna26546 1.00E-37
121- 4: transcript:Zm00001d029965_T001 rna26547 3.00E-135
121- 5: transcript:Zm00001d029968_T001 rna26548 8.00E-94
121- 6: transcript:Zm00001d029969_T001 rna26549 3.00E-78
121- 7: transcript:Zm00001d029970_T001 rna26550 8.00E-103
121- 8: transcript:Zm00001d029973_T001 rna26551 3.00E-36
121- 9: transcript:Zm00001d029974_T001 rna26553 2.00E-144
121- 10: transcript:Zm00001d029975_T003 rna26556 0
121- 11: transcript:Zm00001d029976_T006 rna26557 8.00E-43
121- 12: transcript:Zm00001d029978_T001 rna26558 2.00E-145
121- 13: transcript:Zm00001d029979_T001 rna26559 0
121- 14: transcript:Zm00001d029980_T008 rna26560 1.00E-68

121- 15: transcript:Zm00001d029983_T001 rna26561 0
121- 16: transcript:Zm00001d029988_T001 rna26563 2.00E-09
121- 17: transcript:Zm00001d029997_T003 rna26573 8.00E-70
121- 18: transcript:Zm00001d030002_T002 rna26574 0
121- 19: transcript:Zm00001d030004_T001 rna26582 8.00E-78
121- 20: transcript:Zm00001d030005_T002 rna26583 0
121- 21: transcript:Zm00001d030007_T001 rna26586 3.00E-48
121- 22: transcript:Zm00001d030009_T001 rna26590 5.00E-79
121- 23: transcript:Zm00001d030010_T001 rna26591 4.00E-74
121- 24: transcript:Zm00001d030011_T001 rna26593 0
121- 25: transcript:Zm00001d030012_T001 rna26594 0
121- 26: transcript:Zm00001d030019_T001 rna26602 2.00E-61
121- 27: transcript:Zm00001d030020_T001 rna26603 0
121- 28: transcript:Zm00001d030021_T004 rna26604 0
121- 29: transcript:Zm00001d030023_T001 rna26606 2.00E-82
121- 30: transcript:Zm00001d030026_T006 rna26633 0
Alignment 122: score=1057.0 e_value=2.8e-72 N=23 1&NC_008403.2 plus
122- 0: transcript:Zm00001d029649_T002 rna26273 9.00E-148
122- 1: transcript:Zm00001d029650_T006 rna26274 0
122- 2: transcript:Zm00001d029654_T003 rna26275 0
122- 3: transcript:Zm00001d029656_T003 rna26276 2.00E-95
122- 4: transcript:Zm00001d029657_T001 rna26277 8.00E-18
122- 5: transcript:Zm00001d029662_T001 rna26278 1.00E-152
122- 6: transcript:Zm00001d029663_T001 rna26280 1.00E-148
122- 7: transcript:Zm00001d029664_T001 rna26281 0
122- 8: transcript:Zm00001d029667_T001 rna26282 8.00E-167
122- 9: transcript:Zm00001d029673_T001 rna26284 1.00E-67
122- 10: transcript:Zm00001d029674_T001 rna26285 1.00E-52
122- 11: transcript:Zm00001d029675_T001 rna26286 0
122- 12: transcript:Zm00001d029676_T001 rna26287 3.00E-20
122- 13: transcript:Zm00001d029680_T001 rna26293 2.00E-37
122- 14: transcript:Zm00001d029681_T001 rna26295 4.00E-38
122- 15: transcript:Zm00001d029683_T001 rna26296 0
122- 16: transcript:Zm00001d029684_T001 rna26297 0
122- 17: transcript:Zm00001d029686_T001 rna26298 0
122- 18: transcript:Zm00001d029688_T002 rna26299 9.00E-129
122- 19: transcript:Zm00001d029696_T003 rna26302 3.00E-109
122- 20: transcript:Zm00001d029699_T002 rna26304 3.00E-105
122- 21: transcript:Zm00001d029702_T001 rna26318 1.00E-49
122- 22: transcript:Zm00001d029704_T001 rna26324 3.00E-83
Alignment 123: score=561.0 e_value=3.7e-27 N=12 1&NC_008403.2 plus
123- 0: transcript:Zm00001d032921_T001 rna25748 6.00E-169
123- 1: transcript:Zm00001d032922_T001 rna25749 7.00E-134
123- 2: transcript:Zm00001d032923_T002 rna25750 0
123- 3: transcript:Zm00001d032925_T001 rna25751 7.00E-69
123- 4: transcript:Zm00001d032926_T001 rna25753 4.00E-139
123- 5: transcript:Zm00001d032931_T002 rna25754 1.00E-72
123- 6: transcript:Zm00001d032932_T001 rna25755 1.00E-58
123- 7: transcript:Zm00001d032933_T020 rna25761 0
123- 8: transcript:Zm00001d032935_T006 rna25764 0
123- 9: transcript:Zm00001d032937_T001 rna25767 6.00E-51
123- 10: transcript:Zm00001d032939_T001 rna25770 1.00E-71
123- 11: transcript:Zm00001d032942_T002 rna25771 3.00E-123
Alignment 124: score=546.0 e_value=4.6e-31 N=12 1&NC_008403.2 plus

```
124-  0:  transcript:Zm00001d030062_T001  rna26611            0
124-  1:  transcript:Zm00001d030069_T006  rna26635            0
124-  2:  transcript:Zm00001d030074_T001  rna26636    6.00E-154
124-  3:  transcript:Zm00001d030079_T001  rna26638     4.00E-48
124-  4:  transcript:Zm00001d030080_T002  rna26639     3.00E-36
124-  5:  transcript:Zm00001d030083_T001  rna26640            0
124-  6:  transcript:Zm00001d030086_T001  rna26641     3.00E-62
124-  7:  transcript:Zm00001d030090_T001  rna26642    7.00E-140
124-  8:  transcript:Zm00001d030091_T001  rna26643            0
124-  9:  transcript:Zm00001d030098_T001  rna26644     5.00E-67
124- 10:  transcript:Zm00001d030101_T001  rna26645     9.00E-94
124- 11:  transcript:Zm00001d030103_T001  rna26646    2.00E-147
## Alignment 125: score=510.0 e_value=5e-24 N=11 1&NC_008403.2 plus
125-  0:  transcript:Zm00001d032750_T001  rna25952            0
125-  1:  transcript:Zm00001d032753_T001  rna25953            0
125-  2:  transcript:Zm00001d032754_T002  rna25954            0
125-  3:  transcript:Zm00001d032760_T001  rna25956    3.00E-111
125-  4:  transcript:Zm00001d032761_T005  rna25959    1.00E-148
125-  5:  transcript:Zm00001d032763_T002  rna25962            0
125-  6:  transcript:Zm00001d032768_T005  rna25963     1.00E-70
125-  7:  transcript:Zm00001d032771_T001  rna25966     2.00E-07
125-  8:  transcript:Zm00001d032773_T003  rna25967            0
125-  9:  transcript:Zm00001d032775_T002  rna25968    1.00E-121
125- 10:  transcript:Zm00001d032776_T002  rna25969            0
## Alignment 126: score=493.0 e_value=1.7e-26 N=11 1&NC_008403.2 plus
126-  0:  transcript:Zm00001d030523_T001  rna25576    6.00E-115
126-  1:  transcript:Zm00001d030526_T003  rna25577            0
126-  2:  transcript:Zm00001d030529_T001  rna25580     5.00E-34
126-  3:  transcript:Zm00001d030532_T002  rna25581     1.00E-16
126-  4:  transcript:Zm00001d030533_T002  rna25583            0
126-  5:  transcript:Zm00001d030540_T001  rna25591     8.00E-76
126-  6:  transcript:Zm00001d030549_T001  rna25612     2.00E-57
126-  7:  transcript:Zm00001d030550_T001  rna25614     6.00E-32
126-  8:  transcript:Zm00001d030551_T001  rna25615            0
126-  9:  transcript:Zm00001d030554_T015  rna25617            0
126- 10:  transcript:Zm00001d030557_T001  rna25618            0
## Alignment 127: score=493.0 e_value=1.5e-30 N=13 1&NC_008403.2 plus
127-  0:  transcript:Zm00001d028406_T001  rna25214            0
127-  1:  transcript:Zm00001d028408_T001  rna25221     7.00E-08
127-  2:  transcript:Zm00001d028410_T001  rna25225    2.00E-166
127-  3:  transcript:Zm00001d028412_T029  rna25235            0
127-  4:  transcript:Zm00001d028413_T001  rna25236     1.00E-80
127-  5:  transcript:Zm00001d028419_T001  rna25239     6.00E-84
127-  6:  transcript:Zm00001d028425_T003  rna25254     2.00E-75
127-  7:  transcript:Zm00001d028426_T001  rna25255     2.00E-79
127-  8:  transcript:Zm00001d028428_T001  rna25269    2.00E-142
127-  9:  transcript:Zm00001d028429_T017  rna25271     6.00E-86
127- 10:  transcript:Zm00001d028436_T001  rna25287     2.00E-58
127- 11:  transcript:Zm00001d028439_T001  rna25294    3.00E-152
127- 12:  transcript:Zm00001d028443_T001  rna25304            0
## Alignment 128: score=475.0 e_value=3.4e-25 N=11 1&NC_008403.2 plus
128-  0:  transcript:Zm00001d027335_T001  rna26250     2.00E-09
128-  1:  transcript:Zm00001d027340_T001  rna26263            0
128-  2:  transcript:Zm00001d027341_T001  rna26265            0
```

128- 3: transcript:Zm00001d027344_T001 rna26267 0
128- 4: transcript:Zm00001d027345_T001 rna26268 2.00E-95
128- 5: transcript:Zm00001d027354_T001 rna26277 1.00E-16
128- 6: transcript:Zm00001d027355_T001 rna26282 2.00E-137
128- 7: transcript:Zm00001d027361_T001 rna26284 8.00E-62
128- 8: transcript:Zm00001d027365_T001 rna26286 0
128- 9: transcript:Zm00001d027374_T001 rna26294 4.00E-147
128- 10: transcript:Zm00001d027375_T001 rna26295 6.00E-35
Alignment 129: score=468.0 e_value=1.1e-25 N=11 1&NC_008403.2 plus
129- 0: transcript:Zm00001d030117_T006 rna26650 0
129- 1: transcript:Zm00001d030121_T001 rna26653 1.00E-176
129- 2: transcript:Zm00001d030129_T001 rna26655 0
129- 3: transcript:Zm00001d030131_T001 rna26658 0
129- 4: transcript:Zm00001d030132_T002 rna26659 0
129- 5: transcript:Zm00001d030133_T002 rna26660 0
129- 6: transcript:Zm00001d030138_T001 rna26662 7.00E-70
129- 7: transcript:Zm00001d030139_T001 rna26663 4.00E-54
129- 8: transcript:Zm00001d030146_T003 rna26664 1.00E-49
129- 9: transcript:Zm00001d030158_T001 rna26665 0
129- 10: transcript:Zm00001d030164_T026 rna26674 9.00E-24
Alignment 130: score=394.0 e_value=1.6e-18 N=9 1&NC_008403.2 plus
130- 0: transcript:Zm00001d030464_T001 rna25518 0
130- 1: transcript:Zm00001d030479_T001 rna25534 0
130- 2: transcript:Zm00001d030496_T001 rna25537 9.00E-15
130- 3: transcript:Zm00001d030498_T001 rna25539 2.00E-10
130- 4: transcript:Zm00001d030500_T001 rna25553 2.00E-122
130- 5: transcript:Zm00001d030506_T001 rna25554 0
130- 6: transcript:Zm00001d030509_T001 rna25562 2.00E-83
130- 7: transcript:Zm00001d030513_T001 rna25563 8.00E-18
130- 8: transcript:Zm00001d030518_T005 rna25575 0
Alignment 131: score=357.0 e_value=4e-19 N=9 1&NC_008403.2 plus
131- 0: transcript:Zm00001d028337_T005 rna25020 0
131- 1: transcript:Zm00001d028339_T018 rna25022 0
131- 2: transcript:Zm00001d028347_T001 rna25027 2.00E-50
131- 3: transcript:Zm00001d028349_T001 rna25032 3.00E-155
131- 4: transcript:Zm00001d028354_T001 rna25033 1.00E-67
131- 5: transcript:Zm00001d028359_T001 rna25042 0
131- 6: transcript:Zm00001d028360_T001 rna25045 5.00E-146
131- 7: transcript:Zm00001d028367_T002 rna25059 2.00E-109
131- 8: transcript:Zm00001d028369_T001 rna25060 1.00E-176
Alignment 132: score=303.0 e_value=2.4e-11 N=7 1&NC_008403.2 plus
132- 0: transcript:Zm00001d030285_T003 rna25146 0
132- 1: transcript:Zm00001d030290_T001 rna25149 3.00E-83
132- 2: transcript:Zm00001d030299_T001 rna25153 0
132- 3: transcript:Zm00001d030301_T002 rna25154 3.00E-21
132- 4: transcript:Zm00001d030303_T001 rna25167 2.00E-140
132- 5: transcript:Zm00001d030310_T001 rna25170 0
132- 6: transcript:Zm00001d030314_T001 rna25173 1.00E-70
Alignment 133: score=288.0 e_value=3.9e-11 N=7 1&NC_008403.2 plus
133- 0: transcript:Zm00001d030372_T001 rna25239 6.00E-110
133- 1: transcript:Zm00001d030373_T003 rna25245 0
133- 2: transcript:Zm00001d030379_T001 rna25247 2.00E-43
133- 3: transcript:Zm00001d030385_T002 rna25253 0
133- 4: transcript:Zm00001d030388_T001 rna25267 5.00E-43

133- 5: transcript:Zm00001d030398_T004 rna25279 0
133- 6: transcript:Zm00001d030409_T001 rna25299 2.00E-16
Alignment 134: score=288.0 e_value=1.3e-12 N=7 1&NC_008403.2 plus
134- 0: transcript:Zm00001d027514_T002 rna26404 0
134- 1: transcript:Zm00001d027520_T001 rna26405 2.00E-130
134- 2: transcript:Zm00001d027522_T001 rna26406 4.00E-79
134- 3: transcript:Zm00001d027523_T001 rna26407 3.00E-132
134- 4: transcript:Zm00001d027524_T001 rna26408 4.00E-94
134- 5: transcript:Zm00001d027539_T001 rna26412 1.00E-116
134- 6: transcript:Zm00001d027546_T001 rna26413 2.00E-81
Alignment 135: score=266.0 e_value=1e-08 N=6 1&NC_008403.2 plus
135- 0: transcript:Zm00001d030343_T001 rna25214 0
135- 1: transcript:Zm00001d030345_T001 rna25221 3.00E-46
135- 2: transcript:Zm00001d030347_T001 rna25223 0
135- 3: transcript:Zm00001d030357_T009 rna25224 0
135- 4: transcript:Zm00001d030361_T001 rna25225 0
135- 5: transcript:Zm00001d030364_T001 rna25236 4.00E-76
Alignment 136: score=250.0 e_value=3.9e-12 N=6 1&NC_008403.2 plus
136- 0: transcript:Zm00001d028039_T004 rna25426 0
136- 1: transcript:Zm00001d028040_T002 rna25427 5.00E-37
136- 2: transcript:Zm00001d028043_T001 rna25428 2.00E-26
136- 3: transcript:Zm00001d028054_T002 rna25444 3.00E-149
136- 4: transcript:Zm00001d028062_T001 rna25445 2.00E-69
136- 5: transcript:Zm00001d028073_T004 rna25454 1.00E-56
Alignment 137: score=1960.0 e_value=7.5e-157 N=43 1&NC_008403.2 minus
137- 0: transcript:Zm00001d032598_T023 rna26119 0
137- 1: transcript:Zm00001d032600_T001 rna26118 0
137- 2: transcript:Zm00001d032601_T002 rna26117 0
137- 3: transcript:Zm00001d032603_T001 rna26114 0
137- 4: transcript:Zm00001d032604_T001 rna26113 1.00E-99
137- 5: transcript:Zm00001d032608_T001 rna26108 3.00E-77
137- 6: transcript:Zm00001d032609_T002 rna26107 1.00E-179
137- 7: transcript:Zm00001d032610_T003 rna26106 9.00E-152
137- 8: transcript:Zm00001d032613_T001 rna26104 8.00E-102
137- 9: transcript:Zm00001d032615_T002 rna26101 1.00E-84
137- 10: transcript:Zm00001d032617_T002 rna26100 5.00E-55
137- 11: transcript:Zm00001d032618_T001 rna26095 5.00E-105
137- 12: transcript:Zm00001d032624_T001 rna26093 3.00E-82
137- 13: transcript:Zm00001d032632_T001 rna26088 0
137- 14: transcript:Zm00001d032633_T004 rna26087 0
137- 15: transcript:Zm00001d032636_T001 rna26086 1.00E-117
137- 16: transcript:Zm00001d032637_T004 rna26084 0
137- 17: transcript:Zm00001d032643_T001 rna26082 9.00E-22
137- 18: transcript:Zm00001d032644_T002 rna26081 0
137- 19: transcript:Zm00001d032646_T001 rna26075 3.00E-128
137- 20: transcript:Zm00001d032650_T001 rna26073 4.00E-31
137- 21: transcript:Zm00001d032651_T001 rna26069 0
137- 22: transcript:Zm00001d032652_T001 rna26068 2.00E-140
137- 23: transcript:Zm00001d032653_T001 rna26067 3.00E-18
137- 24: transcript:Zm00001d032655_T006 rna26065 0
137- 25: transcript:Zm00001d032656_T001 rna26064 1.00E-175
137- 26: transcript:Zm00001d032659_T004 rna26062 2.00E-150
137- 27: transcript:Zm00001d032661_T001 rna26059 0
137- 28: transcript:Zm00001d032662_T001 rna26058 0

137- 29: transcript:Zm00001d032663_T001 rna26054 1.00E-61
137- 30: transcript:Zm00001d032664_T001 rna26053 0
137- 31: transcript:Zm00001d032666_T002 rna26051 1.00E-60
137- 32: transcript:Zm00001d032668_T001 rna26050 1.00E-112
137- 33: transcript:Zm00001d032669_T001 rna26049 0
137- 34: transcript:Zm00001d032670_T004 rna26045 3.00E-48
137- 35: transcript:Zm00001d032671_T001 rna26044 1.00E-42
137- 36: transcript:Zm00001d032672_T001 rna26042 2.00E-24
137- 37: transcript:Zm00001d032679_T001 rna26039 0
137- 38: transcript:Zm00001d032681_T004 rna26038 0
137- 39: transcript:Zm00001d032683_T001 rna26037 0
137- 40: transcript:Zm00001d032685_T001 rna26036 3.00E-76
137- 41: transcript:Zm00001d032686_T001 rna26035 0
137- 42: transcript:Zm00001d032687_T001 rna26034 6.00E-75
Alignment 138: score=1806.0 e_value=1.5e-132 N=38 1&NC_008403.2 minus
138- 0: transcript:Zm00001d032465_T002 rna26232 0
138- 1: transcript:Zm00001d032467_T001 rna26230 2.00E-153
138- 2: transcript:Zm00001d032469_T001 rna26228 0
138- 3: transcript:Zm00001d032472_T001 rna26223 0
138- 4: transcript:Zm00001d032473_T001 rna26222 5.00E-149
138- 5: transcript:Zm00001d032475_T001 rna26220 3.00E-52
138- 6: transcript:Zm00001d032476_T001 rna26219 0
138- 7: transcript:Zm00001d032478_T001 rna26218 0
138- 8: transcript:Zm00001d032480_T001 rna26217 0
138- 9: transcript:Zm00001d032481_T001 rna26212 5.00E-30
138- 10: transcript:Zm00001d032485_T001 rna26210 1.00E-92
138- 11: transcript:Zm00001d032494_T001 rna26208 3.00E-13
138- 12: transcript:Zm00001d032495_T001 rna26207 6.00E-62
138- 13: transcript:Zm00001d032497_T001 rna26206 1.00E-61
138- 14: transcript:Zm00001d032498_T001 rna26205 5.00E-34
138- 15: transcript:Zm00001d032499_T001 rna26204 2.00E-102
138- 16: transcript:Zm00001d032502_T001 rna26203 3.00E-103
138- 17: transcript:Zm00001d032503_T001 rna26202 0
138- 18: transcript:Zm00001d032504_T001 rna26201 6.00E-58
138- 19: transcript:Zm00001d032505_T002 rna26200 0
138- 20: transcript:Zm00001d032507_T001 rna26199 3.00E-121
138- 21: transcript:Zm00001d032508_T001 rna26198 5.00E-104
138- 22: transcript:Zm00001d032510_T001 rna26197 7.00E-30
138- 23: transcript:Zm00001d032515_T001 rna26195 2.00E-69
138- 24: transcript:Zm00001d032517_T001 rna26193 1.00E-59
138- 25: transcript:Zm00001d032518_T001 rna26192 0
138- 26: transcript:Zm00001d032519_T001 rna26191 3.00E-69
138- 27: transcript:Zm00001d032520_T018 rna26186 0
138- 28: transcript:Zm00001d032521_T004 rna26184 2.00E-130
138- 29: transcript:Zm00001d032526_T002 rna26182 0
138- 30: transcript:Zm00001d032527_T001 rna26179 0
138- 31: transcript:Zm00001d032529_T001 rna26178 0
138- 32: transcript:Zm00001d032530_T001 rna26176 2.00E-175
138- 33: transcript:Zm00001d032531_T001 rna26174 1.00E-62
138- 34: transcript:Zm00001d032532_T001 rna26173 0
138- 35: transcript:Zm00001d032533_T001 rna26171 8.00E-26
138- 36: transcript:Zm00001d032535_T002 rna26169 1.00E-103
138- 37: transcript:Zm00001d032536_T001 rna26167 2.00E-156
Alignment 139: score=1198.0 e_value=1.9e-86 N=28 1&NC_008403.2 minus

139- 0:	transcript:Zm00001d032689_T036	rna26032	0
139- 1:	transcript:Zm00001d032691_T001	rna26027	1.00E-27
139- 2:	transcript:Zm00001d032693_T001	rna26026	0
139- 3:	transcript:Zm00001d032694_T001	rna26025	1.00E-96
139- 4:	transcript:Zm00001d032695_T001	rna26024	0
139- 5:	transcript:Zm00001d032696_T001	rna26022	9.00E-84
139- 6:	transcript:Zm00001d032699_T030	rna26021	0
139- 7:	transcript:Zm00001d032703_T020	rna26017	0
139- 8:	transcript:Zm00001d032704_T036	rna26016	0
139- 9:	transcript:Zm00001d032707_T001	rna26015	0
139- 10:	transcript:Zm00001d032708_T002	rna26012	0
139- 11:	transcript:Zm00001d032710_T003	rna26010	3.00E-127
139- 12:	transcript:Zm00001d032715_T002	rna26008	0
139- 13:	transcript:Zm00001d032716_T001	rna26006	0
139- 14:	transcript:Zm00001d032718_T001	rna26004	2.00E-123
139- 15:	transcript:Zm00001d032719_T001	rna25998	0
139- 16:	transcript:Zm00001d032722_T001	rna25996	6.00E-66
139- 17:	transcript:Zm00001d032724_T001	rna25995	0
139- 18:	transcript:Zm00001d032725_T001	rna25993	0
139- 19:	transcript:Zm00001d032728_T001	rna25991	0
139- 20:	transcript:Zm00001d032732_T002	rna25989	3.00E-24
139- 21:	transcript:Zm00001d032734_T007	rna25988	0
139- 22:	transcript:Zm00001d032735_T001	rna25987	3.00E-29
139- 23:	transcript:Zm00001d032736_T001	rna25986	8.00E-99
139- 24:	transcript:Zm00001d032737_T001	rna25984	1.00E-69
139- 25:	transcript:Zm00001d032739_T004	rna25983	7.00E-172
139- 26:	transcript:Zm00001d032740_T001	rna25980	0
139- 27:	transcript:Zm00001d032745_T001	rna25970	0

Alignment 140: score=1099.0 e_value=5.6e-76 N=25 1&NC_008403.2 minus

140- 0:	transcript:Zm00001d032828_T001	rna25875	1.00E-180
140- 1:	transcript:Zm00001d032830_T003	rna25869	1.00E-66
140- 2:	transcript:Zm00001d032832_T001	rna25866	3.00E-74
140- 3:	transcript:Zm00001d032836_T001	rna25864	4.00E-58
140- 4:	transcript:Zm00001d032849_T002	rna25860	7.00E-133
140- 5:	transcript:Zm00001d032850_T001	rna25857	0
140- 6:	transcript:Zm00001d032852_T001	rna25856	0
140- 7:	transcript:Zm00001d032855_T001	rna25851	0
140- 8:	transcript:Zm00001d032857_T001	rna25850	1.00E-95
140- 9:	transcript:Zm00001d032859_T006	rna25848	4.00E-118
140- 10:	transcript:Zm00001d032866_T001	rna25847	5.00E-176
140- 11:	transcript:Zm00001d032867_T002	rna25846	4.00E-101
140- 12:	transcript:Zm00001d032868_T001	rna25844	0
140- 13:	transcript:Zm00001d032873_T001	rna25842	1.00E-31
140- 14:	transcript:Zm00001d032875_T009	rna25837	3.00E-30
140- 15:	transcript:Zm00001d032876_T001	rna25836	2.00E-110
140- 16:	transcript:Zm00001d032887_T001	rna25835	2.00E-31
140- 17:	transcript:Zm00001d032888_T001	rna25833	0
140- 18:	transcript:Zm00001d032889_T001	rna25832	5.00E-138
140- 19:	transcript:Zm00001d032893_T002	rna25827	2.00E-43
140- 20:	transcript:Zm00001d032894_T001	rna25823	6.00E-29
140- 21:	transcript:Zm00001d032897_T001	rna25821	0
140- 22:	transcript:Zm00001d032899_T001	rna25820	0
140- 23:	transcript:Zm00001d032902_T001	rna25808	0
140- 24:	transcript:Zm00001d032905_T010	rna25806	0

```
## Alignment 141: score=875.0 e_value=1.4e-55 N=19 1&NC_008403.2 minus
141-  0:  transcript:Zm00001d032433_T001  rna26269    3.00E-114
141-  1:  transcript:Zm00001d032434_T001  rna26268    2.00E-102
141-  2:  transcript:Zm00001d032435_T001  rna26267            0
141-  3:  transcript:Zm00001d032438_T001  rna26265            0
141-  4:  transcript:Zm00001d032439_T002  rna26264            0
141-  5:  transcript:Zm00001d032440_T004  rna26263            0
141-  6:  transcript:Zm00001d032441_T001  rna26261            0
141-  7:  transcript:Zm00001d032442_T004  rna26260     6.00E-62
141-  8:  transcript:Zm00001d032443_T001  rna26258     3.00E-61
141-  9:  transcript:Zm00001d032447_T001  rna26253     2.00E-42
141- 10:  transcript:Zm00001d032449_T002  rna26252            0
141- 11:  transcript:Zm00001d032450_T001  rna26251     1.00E-72
141- 12:  transcript:Zm00001d032453_T001  rna26250     6.00E-33
141- 13:  transcript:Zm00001d032455_T002  rna26248            0
141- 14:  transcript:Zm00001d032457_T001  rna26247     1.00E-52
141- 15:  transcript:Zm00001d032458_T001  rna26237    2.00E-159
141- 16:  transcript:Zm00001d032460_T001  rna26235            0
141- 17:  transcript:Zm00001d032461_T001  rna26234     3.00E-94
141- 18:  transcript:Zm00001d032464_T008  rna26233     2.00E-63
## Alignment 142: score=748.0 e_value=8.7e-44 N=17 1&NC_008403.2 minus
142-  0:  transcript:Zm00001d032981_T001  rna25705            0
142-  1:  transcript:Zm00001d032989_T001  rna25701            0
142-  2:  transcript:Zm00001d032991_T001  rna25697            0
142-  3:  transcript:Zm00001d032992_T001  rna25688            0
142-  4:  transcript:Zm00001d032994_T002  rna25685    1.00E-172
142-  5:  transcript:Zm00001d032999_T001  rna25684     2.00E-47
142-  6:  transcript:Zm00001d033002_T001  rna25681            0
142-  7:  transcript:Zm00001d033003_T001  rna25679     6.00E-52
142-  8:  transcript:Zm00001d033004_T002  rna25678            0
142-  9:  transcript:Zm00001d033005_T001  rna25677     1.00E-97
142- 10:  transcript:Zm00001d033011_T001  rna25676            0
142- 11:  transcript:Zm00001d033018_T001  rna25675    8.00E-179
142- 12:  transcript:Zm00001d033020_T001  rna25673            0
142- 13:  transcript:Zm00001d033025_T001  rna25667    2.00E-133
142- 14:  transcript:Zm00001d033028_T002  rna25665            0
142- 15:  transcript:Zm00001d033029_T001  rna25655            0
142- 16:  transcript:Zm00001d033037_T008  rna25648            0
## Alignment 143: score=704.0 e_value=2.4e-43 N=16 1&NC_008403.2 minus
143-  0:  transcript:Zm00001d032777_T001  rna25947     5.00E-22
143-  1:  transcript:Zm00001d032781_T002  rna25946            0
143-  2:  transcript:Zm00001d032788_T001  rna25943            0
143-  3:  transcript:Zm00001d032789_T001  rna25941            0
143-  4:  transcript:Zm00001d032790_T002  rna25933     2.00E-25
143-  5:  transcript:Zm00001d032794_T001  rna25925    5.00E-144
143-  6:  transcript:Zm00001d032798_T001  rna25923    4.00E-163
143-  7:  transcript:Zm00001d032801_T005  rna25918            0
143-  8:  transcript:Zm00001d032804_T001  rna25916    4.00E-133
143-  9:  transcript:Zm00001d032805_T001  rna25915     1.00E-76
143- 10:  transcript:Zm00001d032806_T001  rna25913            0
143- 11:  transcript:Zm00001d032807_T001  rna25911            0
143- 12:  transcript:Zm00001d032810_T003  rna25909    9.00E-149
143- 13:  transcript:Zm00001d032811_T002  rna25908    5.00E-126
143- 14:  transcript:Zm00001d032815_T011  rna25907            0
```

143- 15: transcript:Zm00001d032819_T001 rna25906 0
Alignment 144: score=675.0 e_value=1e-34 N=15 1&NC_008403.2 minus
144- 0: transcript:Zm00001d027423_T002 rna26374 9.00E-86
144- 1: transcript:Zm00001d027425_T001 rna26373 1.00E-90
144- 2: transcript:Zm00001d027427_T002 rna26372 1.00E-111
144- 3: transcript:Zm00001d027434_T001 rna26370 6.00E-44
144- 4: transcript:Zm00001d027435_T002 rna26369 0
144- 5: transcript:Zm00001d027436_T001 rna26365 0
144- 6: transcript:Zm00001d027440_T001 rna26364 2.00E-27
144- 7: transcript:Zm00001d027443_T001 rna26359 9.00E-145
144- 8: transcript:Zm00001d027446_T001 rna26356 0
144- 9: transcript:Zm00001d027450_T001 rna26354 0
144- 10: transcript:Zm00001d027453_T001 rna26352 2.00E-36
144- 11: transcript:Zm00001d027454_T001 rna26351 6.00E-40
144- 12: transcript:Zm00001d027459_T001 rna26348 2.00E-135
144- 13: transcript:Zm00001d027461_T001 rna26347 2.00E-143
144- 14: transcript:Zm00001d027463_T001 rna26345 0
Alignment 145: score=588.0 e_value=8.6e-32 N=13 1&NC_008403.2 minus
145- 0: transcript:Zm00001d032555_T006 rna26161 0
145- 1: transcript:Zm00001d032556_T001 rna26159 3.00E-80
145- 2: transcript:Zm00001d032557_T001 rna26157 3.00E-168
145- 3: transcript:Zm00001d032560_T001 rna26152 3.00E-138
145- 4: transcript:Zm00001d032565_T001 rna26150 0
145- 5: transcript:Zm00001d032566_T002 rna26149 5.00E-31
145- 6: transcript:Zm00001d032567_T004 rna26148 0
145- 7: transcript:Zm00001d032570_T002 rna26142 1.00E-128
145- 8: transcript:Zm00001d032572_T001 rna26141 0
145- 9: transcript:Zm00001d032573_T001 rna26140 1.00E-122
145- 10: transcript:Zm00001d032575_T005 rna26132 0
145- 11: transcript:Zm00001d032576_T001 rna26131 0
145- 12: transcript:Zm00001d032578_T001 rna26129 8.00E-43
Alignment 146: score=564.0 e_value=3.9e-27 N=12 1&NC_008403.2 minus
146- 0: transcript:Zm00001d030028_T001 rna26631 0
146- 1: transcript:Zm00001d030031_T001 rna26629 0
146- 2: transcript:Zm00001d030032_T002 rna26628 4.00E-106
146- 3: transcript:Zm00001d030037_T001 rna26627 0
146- 4: transcript:Zm00001d030038_T001 rna26626 3.00E-53
146- 5: transcript:Zm00001d030040_T001 rna26625 0
146- 6: transcript:Zm00001d030045_T001 rna26619 1.00E-109
146- 7: transcript:Zm00001d030047_T001 rna26618 1.00E-09
146- 8: transcript:Zm00001d030048_T001 rna26616 0
146- 9: transcript:Zm00001d030053_T001 rna26615 2.00E-147
146- 10: transcript:Zm00001d030059_T001 rna26614 2.00E-11
146- 11: transcript:Zm00001d030061_T001 rna26612 0
Alignment 147: score=527.0 e_value=4.9e-29 N=12 1&NC_008403.2 minus
147- 0: transcript:Zm00001d027810_T001 rna25716 0
147- 1: transcript:Zm00001d027811_T001 rna25711 0
147- 2: transcript:Zm00001d027813_T002 rna25707 3.00E-39
147- 3: transcript:Zm00001d027815_T002 rna25705 0
147- 4: transcript:Zm00001d027816_T001 rna25704 6.00E-74
147- 5: transcript:Zm00001d027826_T001 rna25701 0
147- 6: transcript:Zm00001d027827_T001 rna25697 0
147- 7: transcript:Zm00001d027839_T001 rna25689 2.00E-32
147- 8: transcript:Zm00001d027846_T001 rna25684 3.00E-49

147- 9: transcript:Zm00001d027851_T001 rna25679 8.00E-43
147- 10: transcript:Zm00001d027852_T001 rna25677 8.00E-100
147- 11: transcript:Zm00001d027854_T006 rna25676 0
Alignment 148: score=466.0 e_value=1.3e-26 N=11 1&NC_008403.2 minus
148- 0: transcript:Zm00001d027585_T001 rna25989 9.00E-53
148- 1: transcript:Zm00001d027590_T001 rna25984 2.00E-50
148- 2: transcript:Zm00001d027593_T001 rna25980 0
148- 3: transcript:Zm00001d027595_T001 rna25964 6.00E-23
148- 4: transcript:Zm00001d027598_T001 rna25963 2.00E-52
148- 5: transcript:Zm00001d027616_T001 rna25959 9.00E-56
148- 6: transcript:Zm00001d027618_T001 rna25956 1.00E-111
148- 7: transcript:Zm00001d027619_T002 rna25955 0
148- 8: transcript:Zm00001d027621_T001 rna25953 0
148- 9: transcript:Zm00001d027622_T001 rna25952 0
148- 10: transcript:Zm00001d027628_T001 rna25942 1.00E-15
Alignment 149: score=456.0 e_value=4.1e-22 N=10 1&NC_008403.2 minus
149- 0: transcript:Zm00001d029892_T001 rna26502 1.00E-166
149- 1: transcript:Zm00001d029896_T001 rna26499 2.00E-89
149- 2: transcript:Zm00001d029897_T001 rna26495 2.00E-65
149- 3: transcript:Zm00001d029913_T001 rna26489 1.00E-133
149- 4: transcript:Zm00001d029917_T001 rna26488 1.00E-103
149- 5: transcript:Zm00001d029919_T002 rna26487 1.00E-105
149- 6: transcript:Zm00001d029920_T001 rna26486 0
149- 7: transcript:Zm00001d029921_T001 rna26485 9.00E-113
149- 8: transcript:Zm00001d029923_T001 rna26484 6.00E-40
149- 9: transcript:Zm00001d029932_T001 rna26482 0
Alignment 150: score=384.0 e_value=4.2e-15 N=8 1&NC_008403.2 minus
150- 0: transcript:Zm00001d029875_T001 rna26531 2.00E-132
150- 1: transcript:Zm00001d029878_T001 rna26529 0
150- 2: transcript:Zm00001d029884_T001 rna26525 2.00E-69
150- 3: transcript:Zm00001d029885_T001 rna26521 1.00E-76
150- 4: transcript:Zm00001d029886_T001 rna26519 9.00E-154
150- 5: transcript:Zm00001d029887_T004 rna26518 2.00E-113
150- 6: transcript:Zm00001d029888_T002 rna26517 0
150- 7: transcript:Zm00001d029889_T005 rna26515 3.00E-166
Alignment 151: score=356.0 e_value=2.5e-15 N=8 1&NC_008403.2 minus
151- 0: transcript:Zm00001d027281_T001 rna26489 1.00E-132
151- 1: transcript:Zm00001d027285_T001 rna26482 0
151- 2: transcript:Zm00001d027286_T001 rna26478 5.00E-156
151- 3: transcript:Zm00001d027287_T001 rna26477 0
151- 4: transcript:Zm00001d027290_T001 rna26465 7.00E-19
151- 5: transcript:Zm00001d027292_T001 rna26463 3.00E-170
151- 6: transcript:Zm00001d027296_T003 rna26460 5.00E-36
151- 7: transcript:Zm00001d027299_T014 rna26455 0
Alignment 152: score=337.0 e_value=6.9e-12 N=7 1&NC_008403.2 minus
152- 0: transcript:Zm00001d033039_T001 rna25638 2.00E-114
152- 1: transcript:Zm00001d033040_T001 rna25637 9.00E-122
152- 2: transcript:Zm00001d033044_T001 rna25632 7.00E-143
152- 3: transcript:Zm00001d033046_T005 rna25631 5.00E-99
152- 4: transcript:Zm00001d033047_T001 rna25630 5.00E-109
152- 5: transcript:Zm00001d033048_T001 rna25628 7.00E-16
152- 6: transcript:Zm00001d033049_T001 rna25626 1.00E-38
Alignment 153: score=301.0 e_value=1.9e-12 N=7 1&NC_008403.2 minus
153- 0: transcript:Zm00001d027700_T001 rna25857 0

153- 1: transcript:Zm00001d027701_T001 rna25856 0
153- 2: transcript:Zm00001d027707_T001 rna25848 2.00E-118
153- 3: transcript:Zm00001d027708_T002 rna25842 1.00E-20
153- 4: transcript:Zm00001d027709_T001 rna25841 6.00E-174
153- 5: transcript:Zm00001d027717_T001 rna25836 2.00E-103
153- 6: transcript:Zm00001d027726_T001 rna25823 3.00E-28
Alignment 154: score=280.0 e_value=5.6e-09 N=6 1&NC_008403.2 minus
154- 0: transcript:Zm00001d032543_T001 rna26163 7.00E-128
154- 1: transcript:Zm00001d032546_T001 rna26162 0
154- 2: transcript:Zm00001d032547_T010 rna26161 0
154- 3: transcript:Zm00001d032548_T001 rna26159 2.00E-78
154- 4: transcript:Zm00001d032550_T001 rna26157 2.00E-169
154- 5: transcript:Zm00001d032552_T002 rna26154 8.00E-42
Alignment 155: score=279.0 e_value=9.7e-08 N=6 1&NC_008403.2 minus
155- 0: transcript:Zm00001d029827_T001 rna26444 8.00E-74
155- 1: transcript:Zm00001d029829_T001 rna26442 0
155- 2: transcript:Zm00001d029833_T001 rna26441 0
155- 3: transcript:Zm00001d029840_T003 rna26438 2.00E-30
155- 4: transcript:Zm00001d029842_T001 rna26436 0
155- 5: transcript:Zm00001d029844_T001 rna26435 3.00E-63
Alignment 156: score=274.0 e_value=3.7e-15 N=7 1&NC_008404.2 plus
156- 0: transcript:Zm00001d030622_T001 rna28393 8.00E-36
156- 1: transcript:Zm00001d030626_T003 rna28395 2.00E-15
156- 2: transcript:Zm00001d030642_T001 rna28407 7.00E-37
156- 3: transcript:Zm00001d030643_T001 rna28408 3.00E-43
156- 4: transcript:Zm00001d030644_T001 rna28410 5.00E-105
156- 5: transcript:Zm00001d030652_T002 rna28432 0
156- 6: transcript:Zm00001d030659_T010 rna28434 1.00E-17
Alignment 157: score=2459.0 e_value=1.6e-217 N=55 1&NC_008405.2 plus
157- 0: transcript:Zm00001d031041_T002 rna30108 0
157- 1: transcript:Zm00001d031044_T001 rna30111 7.00E-103
157- 2: transcript:Zm00001d031047_T001 rna30117 5.00E-71
157- 3: transcript:Zm00001d031049_T001 rna30121 3.00E-141
157- 4: transcript:Zm00001d031050_T001 rna30122 5.00E-83
157- 5: transcript:Zm00001d031053_T001 rna30123 2.00E-106
157- 6: transcript:Zm00001d031059_T006 rna30128 0
157- 7: transcript:Zm00001d031060_T001 rna30129 4.00E-178
157- 8: transcript:Zm00001d031061_T007 rna30130 0
157- 9: transcript:Zm00001d031063_T001 rna30131 3.00E-48
157- 10: transcript:Zm00001d031064_T002 rna30137 0
157- 11: transcript:Zm00001d031065_T001 rna30138 4.00E-76
157- 12: transcript:Zm00001d031068_T001 rna30140 0
157- 13: transcript:Zm00001d031071_T001 rna30147 0
157- 14: transcript:Zm00001d031072_T001 rna30148 5.00E-154
157- 15: transcript:Zm00001d031074_T002 rna30152 0
157- 16: transcript:Zm00001d031077_T001 rna30155 0
157- 17: transcript:Zm00001d031079_T001 rna30159 9.00E-25
157- 18: transcript:Zm00001d031081_T001 rna30160 0
157- 19: transcript:Zm00001d031086_T001 rna30161 0
157- 20: transcript:Zm00001d031088_T001 rna30163 0
157- 21: transcript:Zm00001d031090_T001 rna30165 0
157- 22: transcript:Zm00001d031091_T001 rna30166 2.00E-08
157- 23: transcript:Zm00001d031092_T001 rna30167 2.00E-105
157- 24: transcript:Zm00001d031094_T001 rna30168 7.00E-163

157- 25: transcript:Zm00001d031098_T004 rna30169 1.00E-168
157- 26: transcript:Zm00001d031101_T002 rna30170 6.00E-62
157- 27: transcript:Zm00001d031109_T002 rna30174 7.00E-72
157- 28: transcript:Zm00001d031113_T001 rna30176 4.00E-27
157- 29: transcript:Zm00001d031114_T001 rna30178 3.00E-12
157- 30: transcript:Zm00001d031118_T001 rna30179 0
157- 31: transcript:Zm00001d031120_T003 rna30186 0
157- 32: transcript:Zm00001d031127_T001 rna30187 3.00E-108
157- 33: transcript:Zm00001d031128_T004 rna30189 0
157- 34: transcript:Zm00001d031131_T001 rna30192 7.00E-72
157- 35: transcript:Zm00001d031134_T001 rna30194 2.00E-134
157- 36: transcript:Zm00001d031135_T001 rna30195 7.00E-99
157- 37: transcript:Zm00001d031136_T003 rna30196 0
157- 38: transcript:Zm00001d031146_T001 rna30202 2.00E-33
157- 39: transcript:Zm00001d031148_T001 rna30207 1.00E-28
157- 40: transcript:Zm00001d031149_T002 rna30208 7.00E-145
157- 41: transcript:Zm00001d031152_T001 rna30210 3.00E-75
157- 42: transcript:Zm00001d031163_T004 rna30218 2.00E-155
157- 43: transcript:Zm00001d031168_T001 rna30227 1.00E-49
157- 44: transcript:Zm00001d031183_T001 rna30245 8.00E-71
157- 45: transcript:Zm00001d031189_T001 rna30247 7.00E-11
157- 46: transcript:Zm00001d031203_T001 rna30253 8.00E-177
157- 47: transcript:Zm00001d031205_T004 rna30255 3.00E-81
157- 48: transcript:Zm00001d031211_T002 rna30265 0
157- 49: transcript:Zm00001d031213_T001 rna30266 0
157- 50: transcript:Zm00001d031216_T002 rna30271 0
157- 51: transcript:Zm00001d031217_T005 rna30272 0
157- 52: transcript:Zm00001d031221_T001 rna30275 0
157- 53: transcript:Zm00001d031223_T001 rna30279 0
157- 54: transcript:Zm00001d031230_T001 rna30285 0
Alignment 158: score=1912.0 e_value=6.4e-171 N=43 1&NC_008405.2 plus
158- 0: transcript:Zm00001d030832_T001 rna29976 5.00E-156
158- 1: transcript:Zm00001d030833_T001 rna29977 2.00E-97
158- 2: transcript:Zm00001d030834_T001 rna29978 1.00E-51
158- 3: transcript:Zm00001d030843_T001 rna29979 1.00E-43
158- 4: transcript:Zm00001d030846_T001 rna29982 0
158- 5: transcript:Zm00001d030849_T001 rna29984 3.00E-164
158- 6: transcript:Zm00001d030850_T001 rna29985 3.00E-59
158- 7: transcript:Zm00001d030851_T001 rna29986 0
158- 8: transcript:Zm00001d030860_T003 rna29990 0
158- 9: transcript:Zm00001d030862_T001 rna29991 1.00E-90
158- 10: transcript:Zm00001d030863_T006 rna29992 9.00E-157
158- 11: transcript:Zm00001d030864_T001 rna29995 0
158- 12: transcript:Zm00001d030868_T003 rna29997 0
158- 13: transcript:Zm00001d030888_T001 rna30001 0
158- 14: transcript:Zm00001d030891_T001 rna30003 1.00E-105
158- 15: transcript:Zm00001d030892_T004 rna30004 1.00E-36
158- 16: transcript:Zm00001d030893_T004 rna30006 0
158- 17: transcript:Zm00001d030894_T002 rna30013 1.00E-133
158- 18: transcript:Zm00001d030895_T001 rna30015 9.00E-108
158- 19: transcript:Zm00001d030900_T001 rna30020 0
158- 20: transcript:Zm00001d030907_T001 rna30021 4.00E-09
158- 21: transcript:Zm00001d030910_T002 rna30023 2.00E-18
158- 22: transcript:Zm00001d030915_T001 rna30026 3.00E-20

158- 23: transcript:Zm00001d030916_T001 rna30027 0
158- 24: transcript:Zm00001d030935_T002 rna30029 0
158- 25: transcript:Zm00001d030937_T006 rna30030 0
158- 26: transcript:Zm00001d030938_T001 rna30033 1.00E-14
158- 27: transcript:Zm00001d030939_T004 rna30034 0
158- 28: transcript:Zm00001d030941_T001 rna30039 3.00E-66
158- 29: transcript:Zm00001d030944_T001 rna30040 4.00E-134
158- 30: transcript:Zm00001d030945_T001 rna30042 1.00E-106
158- 31: transcript:Zm00001d030947_T001 rna30045 8.00E-96
158- 32: transcript:Zm00001d030953_T001 rna30047 0
158- 33: transcript:Zm00001d030954_T001 rna30048 4.00E-135
158- 34: transcript:Zm00001d030955_T003 rna30049 9.00E-158
158- 35: transcript:Zm00001d030962_T001 rna30055 0
158- 36: transcript:Zm00001d030971_T001 rna30058 1.00E-10
158- 37: transcript:Zm00001d030982_T002 rna30061 9.00E-165
158- 38: transcript:Zm00001d030985_T001 rna30065 2.00E-174
158- 39: transcript:Zm00001d030987_T001 rna30066 1.00E-23
158- 40: transcript:Zm00001d030989_T001 rna30067 2.00E-52
158- 41: transcript:Zm00001d030990_T001 rna30068 0
158- 42: transcript:Zm00001d030993_T001 rna30071 4.00E-55
Alignment 159: score=1073.0 e_value=4.2e-74 N=25 1&NC_008405.2 plus
159- 0: transcript:Zm00001d030667_T001 rna29894 4.00E-48
159- 1: transcript:Zm00001d030675_T002 rna29896 4.00E-42
159- 2: transcript:Zm00001d030677_T004 rna29899 5.00E-38
159- 3: transcript:Zm00001d030678_T001 rna29900 1.00E-132
159- 4: transcript:Zm00001d030691_T001 rna29902 0
159- 5: transcript:Zm00001d030694_T002 rna29908 0
159- 6: transcript:Zm00001d030698_T001 rna29909 4.00E-06
159- 7: transcript:Zm00001d030725_T003 rna29914 5.00E-90
159- 8: transcript:Zm00001d030727_T002 rna29915 1.00E-111
159- 9: transcript:Zm00001d030732_T001 rna29916 0
159- 10: transcript:Zm00001d030733_T001 rna29919 0
159- 11: transcript:Zm00001d030737_T001 rna29932 2.00E-127
159- 12: transcript:Zm00001d030739_T008 rna29933 7.00E-107
159- 13: transcript:Zm00001d030741_T001 rna29936 0
159- 14: transcript:Zm00001d030742_T003 rna29937 2.00E-88
159- 15: transcript:Zm00001d030744_T001 rna29938 0
159- 16: transcript:Zm00001d030751_T001 rna29943 0
159- 17: transcript:Zm00001d030760_T004 rna29946 2.00E-168
159- 18: transcript:Zm00001d030762_T001 rna29948 2.00E-58
159- 19: transcript:Zm00001d030766_T001 rna29954 2.00E-153
159- 20: transcript:Zm00001d030770_T003 rna29959 0
159- 21: transcript:Zm00001d030774_T004 rna29960 1.00E-32
159- 22: transcript:Zm00001d030775_T001 rna29962 1.00E-130
159- 23: transcript:Zm00001d030784_T016 rna29965 0
159- 24: transcript:Zm00001d030790_T002 rna29970 0
Alignment 160: score=713.0 e_value=5.1e-41 N=16 1&NC_008405.2 plus
160- 0: transcript:Zm00001d030614_T002 rna29854 0
160- 1: transcript:Zm00001d030618_T001 rna29856 1.00E-38
160- 2: transcript:Zm00001d030622_T001 rna29860 6.00E-61
160- 3: transcript:Zm00001d030623_T006 rna29863 0
160- 4: transcript:Zm00001d030624_T001 rna29864 0
160- 5: transcript:Zm00001d030626_T003 rna29866 0
160- 6: transcript:Zm00001d030627_T002 rna29869 4.00E-124

160- 7: transcript:Zm00001d030639_T001 rna29873 0
160- 8: transcript:Zm00001d030640_T001 rna29874 0
160- 9: transcript:Zm00001d030642_T001 rna29875 1.00E-52
160- 10: transcript:Zm00001d030643_T001 rna29876 4.00E-59
160- 11: transcript:Zm00001d030644_T001 rna29877 3.00E-25
160- 12: transcript:Zm00001d030652_T002 rna29884 0
160- 13: transcript:Zm00001d030656_T001 rna29887 0
160- 14: transcript:Zm00001d030659_T010 rna29890 1.00E-41
160- 15: transcript:Zm00001d030665_T001 rna29892 2.00E-69
Alignment 161: score=509.0 e_value=5.9e-28 N=12 1&NC_008405.2 plus
161- 0: transcript:Zm00001d030998_T007 rna30081 0
161- 1: transcript:Zm00001d030999_T001 rna30082 0
161- 2: transcript:Zm00001d031002_T001 rna30083 9.00E-177
161- 3: transcript:Zm00001d031005_T004 rna30084 1.00E-134
161- 4: transcript:Zm00001d031008_T001 rna30085 3.00E-179
161- 5: transcript:Zm00001d031009_T004 rna30087 0
161- 6: transcript:Zm00001d031013_T002 rna30088 0
161- 7: transcript:Zm00001d031014_T001 rna30089 7.00E-175
161- 8: transcript:Zm00001d031019_T005 rna30094 9.00E-156
161- 9: transcript:Zm00001d031020_T001 rna30098 1.00E-49
161- 10: transcript:Zm00001d031024_T002 rna30099 0
161- 11: transcript:Zm00001d031026_T001 rna30106 1.00E-155
Alignment 162: score=875.0 e_value=1e-58 N=21 1&NC_008405.2 minus
162- 0: transcript:Zm00001d033204_T002 rna30170 1.00E-41
162- 1: transcript:Zm00001d033211_T002 rna30169 5.00E-158
162- 2: transcript:Zm00001d033213_T001 rna30168 0
162- 3: transcript:Zm00001d033215_T003 rna30167 2.00E-84
162- 4: transcript:Zm00001d033217_T004 rna30163 0
162- 5: transcript:Zm00001d033222_T001 rna30161 0
162- 6: transcript:Zm00001d033223_T003 rna30160 3.00E-123
162- 7: transcript:Zm00001d033225_T002 rna30158 0
162- 8: transcript:Zm00001d033229_T001 rna30157 5.00E-16
162- 9: transcript:Zm00001d033233_T002 rna30148 4.00E-81
162- 10: transcript:Zm00001d033234_T001 rna30140 2.00E-180
162- 11: transcript:Zm00001d033240_T001 rna30133 0
162- 12: transcript:Zm00001d033246_T002 rna30130 0
162- 13: transcript:Zm00001d033254_T001 rna30123 6.00E-95
162- 14: transcript:Zm00001d033262_T001 rna30120 4.00E-85
162- 15: transcript:Zm00001d033267_T001 rna30111 1.00E-98
162- 16: transcript:Zm00001d033271_T001 rna30109 1.00E-100
162- 17: transcript:Zm00001d033274_T005 rna30087 6.00E-141
162- 18: transcript:Zm00001d033278_T011 rna30084 8.00E-112
162- 19: transcript:Zm00001d033280_T001 rna30080 7.00E-71
162- 20: transcript:Zm00001d033283_T001 rna30079 4.00E-33
Alignment 163: score=446.0 e_value=7.9e-26 N=12 1&NC_008405.2 minus
163- 0: transcript:Zm00001d033291_T004 rna30051 6.00E-154
163- 1: transcript:Zm00001d033295_T001 rna30049 1.00E-153
163- 2: transcript:Zm00001d033297_T003 rna30047 0
163- 3: transcript:Zm00001d033304_T001 rna30028 1.00E-165
163- 4: transcript:Zm00001d033313_T001 rna30024 6.00E-23
163- 5: transcript:Zm00001d033324_T001 rna30021 2.00E-12
163- 6: transcript:Zm00001d033330_T001 rna30013 4.00E-81
163- 7: transcript:Zm00001d033334_T001 rna30006 0
163- 8: transcript:Zm00001d033339_T002 rna30002 0

163- 9: transcript:Zm00001d033344_T001 rna29995 0
163- 10: transcript:Zm00001d033353_T001 rna29991 4.00E-74
163- 11: transcript:Zm00001d033363_T001 rna29985 2.00E-49
Alignment 164: score=383.0 e_value=9.2e-14 N=8 1&NC_008405.2 minus
164- 0: transcript:Zm00001d031182_T001 rna30252 0
164- 1: transcript:Zm00001d031184_T001 rna30250 6.00E-114
164- 2: transcript:Zm00001d031187_T002 rna30248 2.00E-114
164- 3: transcript:Zm00001d031192_T001 rna30243 3.00E-10
164- 4: transcript:Zm00001d031194_T001 rna30242 5.00E-16
164- 5: transcript:Zm00001d031196_T001 rna30240 5.00E-27
164- 6: transcript:Zm00001d031200_T002 rna30235 3.00E-82
164- 7: transcript:Zm00001d031201_T001 rna30231 0
Alignment 165: score=288.0 e_value=4e-11 N=7 1&NC_008405.2 minus
165- 0: transcript:Zm00001d033455_T001 rna30215 3.00E-76
165- 1: transcript:Zm00001d033456_T001 rna30207 2.00E-21
165- 2: transcript:Zm00001d033460_T001 rna30202 5.00E-12
165- 3: transcript:Zm00001d033475_T001 rna30192 2.00E-157
165- 4: transcript:Zm00001d033480_T002 rna30189 0
165- 5: transcript:Zm00001d033489_T001 rna30187 5.00E-76
165- 6: transcript:Zm00001d033493_T002 rna30186 0
Alignment 166: score=455.0 e_value=4.9e-21 N=10 10&NC_008394.4 minus
166- 0: transcript:Zm00001d024754_T001 rna1166 0
166- 1: transcript:Zm00001d024756_T004 rna1164 2.00E-43
166- 2: transcript:Zm00001d024762_T001 rna1163 0
166- 3: transcript:Zm00001d024763_T001 rna1162 0
166- 4: transcript:Zm00001d024767_T003 rna1156 0
166- 5: transcript:Zm00001d024768_T001 rna1152 2.00E-41
166- 6: transcript:Zm00001d024772_T001 rna1148 8.00E-21
166- 7: transcript:Zm00001d024778_T001 rna1145 3.00E-10
166- 8: transcript:Zm00001d024783_T001 rna1140 1.00E-82
166- 9: transcript:Zm00001d024784_T001 rna1138 6.00E-59
Alignment 167: score=274.0 e_value=2.4e-08 N=6 10&NC_008394.4 minus
167- 0: transcript:Zm00001d024717_T001 rna1199 1.00E-62
167- 1: transcript:Zm00001d024722_T002 rna1187 3.00E-35
167- 2: transcript:Zm00001d024725_T001 rna1183 2.00E-75
167- 3: transcript:Zm00001d024729_T001 rna1176 0
167- 4: transcript:Zm00001d024732_T001 rna1173 1.00E-176
167- 5: transcript:Zm00001d024734_T001 rna1170 2.00E-151
Alignment 168: score=688.0 e_value=5.6e-40 N=16 10&NC_008395.2 plus
168- 0: transcript:Zm00001d025896_T001 rna6464 3.00E-50
168- 1: transcript:Zm00001d025900_T001 rna6470 1.00E-123
168- 2: transcript:Zm00001d025908_T001 rna6480 1.00E-47
168- 3: transcript:Zm00001d025910_T001 rna6483 2.00E-79
168- 4: transcript:Zm00001d025911_T002 rna6484 0
168- 5: transcript:Zm00001d025916_T001 rna6491 9.00E-85
168- 6: transcript:Zm00001d025920_T001 rna6496 0
168- 7: transcript:Zm00001d025926_T001 rna6499 2.00E-113
168- 8: transcript:Zm00001d025932_T003 rna6504 2.00E-67
168- 9: transcript:Zm00001d025933_T001 rna6508 2.00E-171
168- 10: transcript:Zm00001d025939_T003 rna6509 2.00E-85
168- 11: transcript:Zm00001d025944_T001 rna6510 2.00E-77
168- 12: transcript:Zm00001d025947_T001 rna6512 6.00E-34
168- 13: transcript:Zm00001d025949_T001 rna6515 5.00E-32
168- 14: transcript:Zm00001d025950_T002 rna6516 1.00E-167

```
168- 15:  transcript:Zm00001d025951_T002  rna6517     4.00E-100
## Alignment 169: score=602.0 e_value=1.1e-35 N=15 10&NC_008395.2 plus
169-  0:  transcript:Zm00001d025303_T004  rna5833             0
169-  1:  transcript:Zm00001d025319_T001  rna5857      1.00E-59
169-  2:  transcript:Zm00001d025326_T001  rna5864             0
169-  3:  transcript:Zm00001d025338_T001  rna5878      6.00E-78
169-  4:  transcript:Zm00001d025345_T001  rna5891      2.00E-18
169-  5:  transcript:Zm00001d025346_T001  rna5898     2.00E-106
169-  6:  transcript:Zm00001d025347_T001  rna5901      8.00E-31
169-  7:  transcript:Zm00001d025354_T001  rna5911             0
169-  8:  transcript:Zm00001d025360_T001  rna5918     1.00E-129
169-  9:  transcript:Zm00001d025361_T001  rna5921      3.00E-27
169- 10:  transcript:Zm00001d025369_T002  rna5928     7.00E-119
169- 11:  transcript:Zm00001d025371_T001  rna5932     3.00E-104
169- 12:  transcript:Zm00001d025374_T001  rna5936      1.00E-51
169- 13:  transcript:Zm00001d025375_T001  rna5938     3.00E-178
169- 14:  transcript:Zm00001d025379_T001  rna5941             0
## Alignment 170: score=483.0 e_value=4.9e-27 N=12 10&NC_008395.2 plus
170-  0:  transcript:Zm00001d026002_T001  rna6705      3.00E-15
170-  1:  transcript:Zm00001d026005_T001  rna6714     7.00E-119
170-  2:  transcript:Zm00001d026010_T001  rna6719      9.00E-67
170-  3:  transcript:Zm00001d026012_T001  rna6721      1.00E-34
170-  4:  transcript:Zm00001d026015_T001  rna6722      6.00E-67
170-  5:  transcript:Zm00001d026017_T001  rna6726      1.00E-71
170-  6:  transcript:Zm00001d026018_T001  rna6728             0
170-  7:  transcript:Zm00001d026021_T001  rna6729      2.00E-48
170-  8:  transcript:Zm00001d026026_T001  rna6734      4.00E-26
170-  9:  transcript:Zm00001d026032_T001  rna6743      5.00E-95
170- 10:  transcript:Zm00001d026042_T001  rna6748     1.00E-118
170- 11:  transcript:Zm00001d026047_T002  rna6765     8.00E-122
## Alignment 171: score=448.0 e_value=8.8e-24 N=10 10&NC_008395.2 plus
171-  0:  transcript:Zm00001d025857_T001  rna6406      7.00E-18
171-  1:  transcript:Zm00001d025863_T001  rna6410      3.00E-81
171-  2:  transcript:Zm00001d025864_T001  rna6413      3.00E-96
171-  3:  transcript:Zm00001d025865_T001  rna6414             0
171-  4:  transcript:Zm00001d025869_T003  rna6426             0
171-  5:  transcript:Zm00001d025871_T001  rna6427     1.00E-118
171-  6:  transcript:Zm00001d025872_T001  rna6430      8.00E-35
171-  7:  transcript:Zm00001d025873_T001  rna6433      8.00E-35
171-  8:  transcript:Zm00001d025874_T001  rna6439      5.00E-35
171-  9:  transcript:Zm00001d025891_T001  rna6454     4.00E-136
## Alignment 172: score=373.0 e_value=1.3e-17 N=9 10&NC_008395.2 plus
172-  0:  transcript:Zm00001d026194_T003  rna6769      2.00E-78
172-  1:  transcript:Zm00001d026203_T001  rna6777     3.00E-105
172-  2:  transcript:Zm00001d026205_T001  rna6779      2.00E-09
172-  3:  transcript:Zm00001d026218_T001  rna6795      2.00E-94
172-  4:  transcript:Zm00001d026221_T001  rna6796             0
172-  5:  transcript:Zm00001d026237_T001  rna6811     3.00E-133
172-  6:  transcript:Zm00001d026240_T006  rna6812     4.00E-137
172-  7:  transcript:Zm00001d026245_T001  rna6818      1.00E-17
172-  8:  transcript:Zm00001d026246_T001  rna6822             0
## Alignment 173: score=347.0 e_value=3.7e-17 N=8 10&NC_008395.2 plus
173-  0:  transcript:Zm00001d025548_T001  rna6073             0
173-  1:  transcript:Zm00001d025549_T001  rna6074     6.00E-120
```

173- 2: transcript:Zm00001d025551_T002 rna6075 2.00E-150
173- 3: transcript:Zm00001d025568_T001 rna6092 8.00E-95
173- 4: transcript:Zm00001d025570_T006 rna6097 0
173- 5: transcript:Zm00001d025572_T001 rna6098 0
173- 6: transcript:Zm00001d025581_T001 rna6111 0
173- 7: transcript:Zm00001d025586_T001 rna6112 9.00E-155
Alignment 174: score=337.0 e_value=1.1e-18 N=9 10&NC_008395.2 plus
174- 0: transcript:Zm00001d025753_T001 rna6297 1.00E-108
174- 1: transcript:Zm00001d025761_T002 rna6308 1.00E-159
174- 2: transcript:Zm00001d025767_T002 rna6326 0
174- 3: transcript:Zm00001d025770_T001 rna6327 2.00E-137
174- 4: transcript:Zm00001d025773_T002 rna6332 0
174- 5: transcript:Zm00001d025777_T007 rna6336 0
174- 6: transcript:Zm00001d025788_T001 rna6343 0
174- 7: transcript:Zm00001d025789_T014 rna6344 3.00E-99
174- 8: transcript:Zm00001d025798_T004 rna6346 0
Alignment 175: score=336.0 e_value=3.4e-15 N=8 10&NC_008395.2 plus
175- 0: transcript:Zm00001d025401_T001 rna5952 1.00E-16
175- 1: transcript:Zm00001d025409_T001 rna5965 1.00E-27
175- 2: transcript:Zm00001d025413_T001 rna5971 3.00E-107
175- 3: transcript:Zm00001d025419_T001 rna5973 2.00E-61
175- 4: transcript:Zm00001d025431_T002 rna5985 6.00E-56
175- 5: transcript:Zm00001d025444_T001 rna6002 1.00E-10
175- 6: transcript:Zm00001d025446_T006 rna6005 2.00E-111
175- 7: transcript:Zm00001d025449_T002 rna6012 0
Alignment 176: score=324.0 e_value=1e-10 N=7 10&NC_008395.2 plus
176- 0: transcript:Zm00001d025953_T003 rna6524 8.00E-108
176- 1: transcript:Zm00001d025957_T001 rna6525 5.00E-84
176- 2: transcript:Zm00001d025958_T001 rna6527 0
176- 3: transcript:Zm00001d025959_T001 rna6531 4.00E-151
176- 4: transcript:Zm00001d025960_T002 rna6532 3.00E-48
176- 5: transcript:Zm00001d025964_T001 rna6535 9.00E-68
176- 6: transcript:Zm00001d025977_T001 rna6539 0
Alignment 177: score=1027.0 e_value=3.9e-68 N=23 10&NC_008395.2 minus
177- 0: transcript:Zm00001d026120_T001 rna6661 3.00E-61
177- 1: transcript:Zm00001d026121_T001 rna6658 0
177- 2: transcript:Zm00001d026126_T001 rna6653 0
177- 3: transcript:Zm00001d026130_T002 rna6651 3.00E-169
177- 4: transcript:Zm00001d026133_T001 rna6647 1.00E-64
177- 5: transcript:Zm00001d026135_T002 rna6642 0
177- 6: transcript:Zm00001d026140_T001 rna6624 0
177- 7: transcript:Zm00001d026141_T001 rna6623 2.00E-17
177- 8: transcript:Zm00001d026153_T001 rna6619 3.00E-06
177- 9: transcript:Zm00001d026154_T002 rna6616 4.00E-108
177- 10: transcript:Zm00001d026158_T001 rna6611 0
177- 11: transcript:Zm00001d026159_T001 rna6610 4.00E-09
177- 12: transcript:Zm00001d026160_T001 rna6607 4.00E-37
177- 13: transcript:Zm00001d026166_T002 rna6595 3.00E-98
177- 14: transcript:Zm00001d026169_T001 rna6592 9.00E-104
177- 15: transcript:Zm00001d026173_T001 rna6588 1.00E-37
177- 16: transcript:Zm00001d026177_T001 rna6587 6.00E-156
177- 17: transcript:Zm00001d026180_T001 rna6584 2.00E-47
177- 18: transcript:Zm00001d026182_T001 rna6580 1.00E-42
177- 19: transcript:Zm00001d026185_T001 rna6579 3.00E-177

177- 20: transcript:Zm00001d026189_T001 rna6574 5.00E-161
177- 21: transcript:Zm00001d026190_T001 rna6571 1.00E-104
177- 22: transcript:Zm00001d026191_T001 rna6567 4.00E-90
Alignment 178: score=586.0 e_value=2e-30 N=13 10&NC_008395.2 minus
178- 0: transcript:Zm00001d026050_T001 rna6708 0
178- 1: transcript:Zm00001d026053_T001 rna6705 3.00E-57
178- 2: transcript:Zm00001d026055_T001 rna6704 0
178- 3: transcript:Zm00001d026061_T001 rna6698 2.00E-32
178- 4: transcript:Zm00001d026070_T002 rna6692 8.00E-29
178- 5: transcript:Zm00001d026076_T001 rna6683 0
178- 6: transcript:Zm00001d026078_T001 rna6681 9.00E-109
178- 7: transcript:Zm00001d026079_T001 rna6680 0
178- 8: transcript:Zm00001d026088_T002 rna6677 0
178- 9: transcript:Zm00001d026091_T001 rna6676 0
178- 10: transcript:Zm00001d026096_T001 rna6675 5.00E-84
178- 11: transcript:Zm00001d026097_T005 rna6673 0
178- 12: transcript:Zm00001d026102_T001 rna6670 5.00E-48
Alignment 179: score=4831.0 e_value=0 N=108 10&NC_008397.2 plus
179- 0: transcript:Zm00001d025281_T001 rna12216 3.00E-12
179- 1: transcript:Zm00001d025286_T001 rna12219 2.00E-178
179- 2: transcript:Zm00001d025287_T001 rna12221 6.00E-78
179- 3: transcript:Zm00001d025296_T001 rna12226 2.00E-84
179- 4: transcript:Zm00001d025298_T001 rna12227 2.00E-87
179- 5: transcript:Zm00001d025299_T001 rna12232 5.00E-07
179- 6: transcript:Zm00001d025300_T007 rna12234 3.00E-146
179- 7: transcript:Zm00001d025303_T004 rna12236 0
179- 8: transcript:Zm00001d025304_T002 rna12238 0
179- 9: transcript:Zm00001d025305_T001 rna12240 0
179- 10: transcript:Zm00001d025307_T004 rna12243 2.00E-39
179- 11: transcript:Zm00001d025310_T001 rna12244 2.00E-84
179- 12: transcript:Zm00001d025322_T001 rna12250 8.00E-23
179- 13: transcript:Zm00001d025323_T010 rna12251 0
179- 14: transcript:Zm00001d025325_T002 rna12254 3.00E-172
179- 15: transcript:Zm00001d025326_T001 rna12256 0
179- 16: transcript:Zm00001d025327_T001 rna12263 3.00E-166
179- 17: transcript:Zm00001d025333_T001 rna12272 0
179- 18: transcript:Zm00001d025338_T001 rna12275 1.00E-98
179- 19: transcript:Zm00001d025340_T002 rna12277 5.00E-100
179- 20: transcript:Zm00001d025342_T002 rna12280 0
179- 21: transcript:Zm00001d025343_T001 rna12284 0
179- 22: transcript:Zm00001d025345_T001 rna12286 1.00E-29
179- 23: transcript:Zm00001d025346_T001 rna12288 1.00E-111
179- 24: transcript:Zm00001d025347_T001 rna12291 4.00E-38
179- 25: transcript:Zm00001d025352_T001 rna12292 4.00E-60
179- 26: transcript:Zm00001d025353_T001 rna12299 0
179- 27: transcript:Zm00001d025354_T001 rna12302 0
179- 28: transcript:Zm00001d025359_T002 rna12308 0
179- 29: transcript:Zm00001d025360_T001 rna12310 1.00E-59
179- 30: transcript:Zm00001d025361_T001 rna12314 1.00E-48
179- 31: transcript:Zm00001d025362_T002 rna12317 2.00E-110
179- 32: transcript:Zm00001d025367_T001 rna12319 5.00E-15
179- 33: transcript:Zm00001d025369_T002 rna12325 0
179- 34: transcript:Zm00001d025371_T001 rna12326 4.00E-122
179- 35: transcript:Zm00001d025373_T001 rna12328 0

179- 36:	transcript:Zm00001d025374_T001	rna12330	2.00E-82
179- 37:	transcript:Zm00001d025375_T001	rna12331	0
179- 38:	transcript:Zm00001d025379_T001	rna12334	0
179- 39:	transcript:Zm00001d025380_T001	rna12341	5.00E-109
179- 40:	transcript:Zm00001d025382_T001	rna12342	2.00E-94
179- 41:	transcript:Zm00001d025383_T001	rna12343	0
179- 42:	transcript:Zm00001d025389_T001	rna12353	0
179- 43:	transcript:Zm00001d025398_T005	rna12354	0
179- 44:	transcript:Zm00001d025400_T001	rna12361	3.00E-117
179- 45:	transcript:Zm00001d025401_T001	rna12363	4.00E-10
179- 46:	transcript:Zm00001d025402_T002	rna12366	0
179- 47:	transcript:Zm00001d025407_T001	rna12372	3.00E-118
179- 48:	transcript:Zm00001d025412_T001	rna12381	0
179- 49:	transcript:Zm00001d025413_T001	rna12385	3.00E-107
179- 50:	transcript:Zm00001d025414_T001	rna12389	3.00E-129
179- 51:	transcript:Zm00001d025419_T001	rna12392	5.00E-96
179- 52:	transcript:Zm00001d025420_T001	rna12393	2.00E-98
179- 53:	transcript:Zm00001d025421_T001	rna12394	0
179- 54:	transcript:Zm00001d025425_T001	rna12397	2.00E-47
179- 55:	transcript:Zm00001d025430_T001	rna12410	2.00E-26
179- 56:	transcript:Zm00001d025431_T002	rna12411	2.00E-67
179- 57:	transcript:Zm00001d025433_T003	rna12414	0
179- 58:	transcript:Zm00001d025434_T001	rna12415	2.00E-56
179- 59:	transcript:Zm00001d025435_T002	rna12419	3.00E-60
179- 60:	transcript:Zm00001d025437_T001	rna12421	0
179- 61:	transcript:Zm00001d025444_T001	rna12426	3.00E-23
179- 62:	transcript:Zm00001d025445_T001	rna12427	4.00E-79
179- 63:	transcript:Zm00001d025446_T006	rna12429	0
179- 64:	transcript:Zm00001d025449_T002	rna12437	0
179- 65:	transcript:Zm00001d025450_T007	rna12446	2.00E-127
179- 66:	transcript:Zm00001d025453_T006	rna12453	0
179- 67:	transcript:Zm00001d025460_T001	rna12454	2.00E-125
179- 68:	transcript:Zm00001d025461_T006	rna12455	0
179- 69:	transcript:Zm00001d025469_T001	rna12461	4.00E-171
179- 70:	transcript:Zm00001d025470_T005	rna12462	0
179- 71:	transcript:Zm00001d025472_T002	rna12463	2.00E-65
179- 72:	transcript:Zm00001d025475_T001	rna12471	0
179- 73:	transcript:Zm00001d025476_T001	rna12474	5.00E-54
179- 74:	transcript:Zm00001d025485_T001	rna12475	4.00E-36
179- 75:	transcript:Zm00001d025489_T001	rna12480	1.00E-26
179- 76:	transcript:Zm00001d025504_T001	rna12482	2.00E-85
179- 77:	transcript:Zm00001d025508_T001	rna12485	3.00E-90
179- 78:	transcript:Zm00001d025509_T001	rna12486	7.00E-71
179- 79:	transcript:Zm00001d025513_T001	rna12488	8.00E-35
179- 80:	transcript:Zm00001d025514_T001	rna12490	9.00E-143
179- 81:	transcript:Zm00001d025517_T003	rna12492	3.00E-72
179- 82:	transcript:Zm00001d025519_T003	rna12493	1.00E-113
179- 83:	transcript:Zm00001d025520_T001	rna12494	0
179- 84:	transcript:Zm00001d025522_T001	rna12497	3.00E-180
179- 85:	transcript:Zm00001d025528_T002	rna12503	5.00E-179
179- 86:	transcript:Zm00001d025538_T001	rna12509	0
179- 87:	transcript:Zm00001d025541_T001	rna12510	0
179- 88:	transcript:Zm00001d025542_T001	rna12512	8.00E-94
179- 89:	transcript:Zm00001d025544_T001	rna12514	2.00E-58

179- 90: transcript:Zm00001d025547_T001 rna12516 0
179- 91: transcript:Zm00001d025548_T001 rna12519 3.00E-41
179- 92: transcript:Zm00001d025549_T001 rna12521 2.00E-151
179- 93: transcript:Zm00001d025551_T002 rna12522 0
179- 94: transcript:Zm00001d025552_T001 rna12523 1.00E-21
179- 95: transcript:Zm00001d025566_T001 rna12528 1.00E-65
179- 96: transcript:Zm00001d025568_T001 rna12531 0
179- 97: transcript:Zm00001d025570_T006 rna12538 0
179- 98: transcript:Zm00001d025572_T001 rna12540 0
179- 99: transcript:Zm00001d025576_T001 rna12552 1.00E-49
179-100: transcript:Zm00001d025577_T001 rna12553 3.00E-35
179-101: transcript:Zm00001d025579_T001 rna12565 3.00E-30
179-102: transcript:Zm00001d025580_T001 rna12566 0
179-103: transcript:Zm00001d025581_T001 rna12568 0
179-104: transcript:Zm00001d025586_T001 rna12569 2.00E-155
179-105: transcript:Zm00001d025587_T001 rna12573 5.00E-15
179-106: transcript:Zm00001d025588_T001 rna12574 0
179-107: transcript:Zm00001d025590_T002 rna12575 0
Alignment 180: score=3192.0 e_value=6.2e-294 N=71 10&NC_008397.2 plus
180- 0: transcript:Zm00001d026181_T001 rna13363 4.00E-22
180- 1: transcript:Zm00001d026193_T001 rna13390 0
180- 2: transcript:Zm00001d026192_T006 rna13391 2.00E-86
180- 3: transcript:Zm00001d026194_T003 rna13392 1.00E-80
180- 4: transcript:Zm00001d026195_T002 rna13393 0
180- 5: transcript:Zm00001d026197_T001 rna13394 1.00E-39
180- 6: transcript:Zm00001d026200_T001 rna13395 6.00E-54
180- 7: transcript:Zm00001d026203_T001 rna13401 6.00E-122
180- 8: transcript:Zm00001d026206_T001 rna13402 1.00E-180
180- 9: transcript:Zm00001d026207_T001 rna13404 0
180- 10: transcript:Zm00001d026211_T001 rna13406 0
180- 11: transcript:Zm00001d026212_T001 rna13407 4.00E-95
180- 12: transcript:Zm00001d026213_T001 rna13408 0
180- 13: transcript:Zm00001d026214_T002 rna13410 0
180- 14: transcript:Zm00001d026218_T001 rna13411 5.00E-127
180- 15: transcript:Zm00001d026220_T001 rna13412 3.00E-65
180- 16: transcript:Zm00001d026222_T001 rna13416 2.00E-25
180- 17: transcript:Zm00001d026223_T001 rna13417 2.00E-53
180- 18: transcript:Zm00001d026231_T001 rna13419 0
180- 19: transcript:Zm00001d026235_T001 rna13427 2.00E-73
180- 20: transcript:Zm00001d026237_T001 rna13437 9.00E-50
180- 21: transcript:Zm00001d026239_T004 rna13438 1.00E-97
180- 22: transcript:Zm00001d026240_T006 rna13439 5.00E-08
180- 23: transcript:Zm00001d026241_T001 rna13441 3.00E-18
180- 24: transcript:Zm00001d026243_T002 rna13444 4.00E-61
180- 25: transcript:Zm00001d026244_T001 rna13447 0
180- 26: transcript:Zm00001d026245_T001 rna13448 4.00E-84
180- 27: transcript:Zm00001d026246_T001 rna13453 0
180- 28: transcript:Zm00001d026248_T001 rna13457 8.00E-145
180- 29: transcript:Zm00001d026249_T003 rna13462 0
180- 30: transcript:Zm00001d026250_T001 rna13463 6.00E-97
180- 31: transcript:Zm00001d026252_T001 rna13470 3.00E-136
180- 32: transcript:Zm00001d026253_T002 rna13473 0
180- 33: transcript:Zm00001d026254_T001 rna13474 6.00E-166
180- 34: transcript:Zm00001d026255_T001 rna13476 2.00E-34

180- 35: transcript:Zm00001d026257_T009 rna13477 0
180- 36: transcript:Zm00001d026258_T004 rna13479 0
180- 37: transcript:Zm00001d026259_T001 rna13480 4.00E-39
180- 38: transcript:Zm00001d026260_T003 rna13481 4.00E-118
180- 39: transcript:Zm00001d026261_T001 rna13484 1.00E-111
180- 40: transcript:Zm00001d026262_T001 rna13492 5.00E-83
180- 41: transcript:Zm00001d026263_T013 rna13493 0
180- 42: transcript:Zm00001d026265_T002 rna13494 0
180- 43: transcript:Zm00001d026266_T001 rna13495 2.00E-103
180- 44: transcript:Zm00001d026267_T001 rna13497 0
180- 45: transcript:Zm00001d026268_T001 rna13500 0
180- 46: transcript:Zm00001d026269_T001 rna13502 6.00E-168
180- 47: transcript:Zm00001d026270_T013 rna13503 0
180- 48: transcript:Zm00001d026271_T001 rna13509 6.00E-66
180- 49: transcript:Zm00001d026277_T002 rna13513 0
180- 50: transcript:Zm00001d026278_T001 rna13519 3.00E-31
180- 51: transcript:Zm00001d026279_T001 rna13520 1.00E-15
180- 52: transcript:Zm00001d026280_T001 rna13521 0
180- 53: transcript:Zm00001d026281_T003 rna13525 1.00E-48
180- 54: transcript:Zm00001d026282_T001 rna13526 2.00E-47
180- 55: transcript:Zm00001d026283_T001 rna13529 0
180- 56: transcript:Zm00001d026284_T001 rna13532 3.00E-58
180- 57: transcript:Zm00001d026285_T001 rna13535 1.00E-142
180- 58: transcript:Zm00001d026286_T001 rna13537 4.00E-52
180- 59: transcript:Zm00001d026287_T005 rna13539 0
180- 60: transcript:Zm00001d026288_T003 rna13540 1.00E-112
180- 61: transcript:Zm00001d026289_T004 rna13541 0
180- 62: transcript:Zm00001d026290_T001 rna13542 0
180- 63: transcript:Zm00001d026293_T001 rna13544 0
180- 64: transcript:Zm00001d026295_T001 rna13546 1.00E-61
180- 65: transcript:Zm00001d026296_T014 rna13547 1.00E-135
180- 66: transcript:Zm00001d026298_T001 rna13551 3.00E-31
180- 67: transcript:Zm00001d026300_T001 rna13552 0
180- 68: transcript:Zm00001d026301_T003 rna13554 0
180- 69: transcript:Zm00001d026302_T003 rna13555 1.00E-172
180- 70: transcript:Zm00001d026303_T004 rna13557 2.00E-140
Alignment 181: score=2772.0 e_value=8.6e-257 N=63 10&NC_008397.2 plus
181- 0: transcript:Zm00001d026306_T009 rna13557 1.00E-161
181- 1: transcript:Zm00001d026308_T001 rna13566 1.00E-49
181- 2: transcript:Zm00001d026310_T001 rna13574 0
181- 3: transcript:Zm00001d026311_T001 rna13576 0
181- 4: transcript:Zm00001d026312_T002 rna13579 0
181- 5: transcript:Zm00001d026317_T002 rna13580 6.00E-88
181- 6: transcript:Zm00001d026318_T001 rna13582 2.00E-69
181- 7: transcript:Zm00001d026321_T001 rna13586 0
181- 8: transcript:Zm00001d026322_T003 rna13587 0
181- 9: transcript:Zm00001d026326_T001 rna13590 0
181- 10: transcript:Zm00001d026329_T001 rna13591 8.00E-10
181- 11: transcript:Zm00001d026331_T006 rna13595 0
181- 12: transcript:Zm00001d026333_T001 rna13606 9.00E-56
181- 13: transcript:Zm00001d026334_T001 rna13611 0
181- 14: transcript:Zm00001d026335_T001 rna13618 1.00E-76
181- 15: transcript:Zm00001d026336_T001 rna13619 4.00E-54
181- 16: transcript:Zm00001d026337_T011 rna13622 0

181- 17: transcript:Zm00001d026344_T001 rna13625 3.00E-102
181- 18: transcript:Zm00001d026345_T001 rna13626 2.00E-133
181- 19: transcript:Zm00001d026346_T002 rna13631 0
181- 20: transcript:Zm00001d026347_T008 rna13632 2.00E-154
181- 21: transcript:Zm00001d026348_T004 rna13633 3.00E-116
181- 22: transcript:Zm00001d026351_T003 rna13642 0
181- 23: transcript:Zm00001d026352_T001 rna13643 5.00E-138
181- 24: transcript:Zm00001d026354_T001 rna13644 7.00E-33
181- 25: transcript:Zm00001d026357_T001 rna13649 8.00E-148
181- 26: transcript:Zm00001d026358_T001 rna13651 0
181- 27: transcript:Zm00001d026359_T003 rna13652 0
181- 28: transcript:Zm00001d026360_T001 rna13653 6.00E-47
181- 29: transcript:Zm00001d026361_T001 rna13655 3.00E-157
181- 30: transcript:Zm00001d026366_T002 rna13658 0
181- 31: transcript:Zm00001d026367_T001 rna13659 4.00E-38
181- 32: transcript:Zm00001d026368_T001 rna13660 3.00E-74
181- 33: transcript:Zm00001d026370_T002 rna13664 1.00E-95
181- 34: transcript:Zm00001d026377_T001 rna13672 0
181- 35: transcript:Zm00001d026379_T001 rna13676 2.00E-151
181- 36: transcript:Zm00001d026382_T001 rna13677 0
181- 37: transcript:Zm00001d026383_T001 rna13685 0
181- 38: transcript:Zm00001d026390_T001 rna13699 8.00E-15
181- 39: transcript:Zm00001d026391_T004 rna13704 2.00E-120
181- 40: transcript:Zm00001d026394_T001 rna13707 0
181- 41: transcript:Zm00001d026395_T001 rna13708 5.00E-116
181- 42: transcript:Zm00001d026396_T004 rna13712 0
181- 43: transcript:Zm00001d026397_T001 rna13715 0
181- 44: transcript:Zm00001d026398_T006 rna13716 4.00E-55
181- 45: transcript:Zm00001d026402_T002 rna13718 0
181- 46: transcript:Zm00001d026404_T001 rna13723 5.00E-12
181- 47: transcript:Zm00001d026406_T001 rna13725 1.00E-16
181- 48: transcript:Zm00001d026414_T001 rna13727 4.00E-50
181- 49: transcript:Zm00001d026415_T003 rna13734 0
181- 50: transcript:Zm00001d026421_T010 rna13737 0
181- 51: transcript:Zm00001d026424_T001 rna13739 2.00E-59
181- 52: transcript:Zm00001d026425_T003 rna13740 2.00E-134
181- 53: transcript:Zm00001d026426_T005 rna13741 0
181- 54: transcript:Zm00001d026429_T002 rna13746 0
181- 55: transcript:Zm00001d026431_T001 rna13748 0
181- 56: transcript:Zm00001d026432_T001 rna13751 2.00E-27
181- 57: transcript:Zm00001d026434_T002 rna13752 0
181- 58: transcript:Zm00001d026435_T001 rna13754 0
181- 59: transcript:Zm00001d026436_T001 rna13756 4.00E-44
181- 60: transcript:Zm00001d026437_T001 rna13758 7.00E-105
181- 61: transcript:Zm00001d026438_T001 rna13760 1.00E-180
181- 62: transcript:Zm00001d026439_T009 rna13763 0
Alignment 182: score=2596.0 e_value=4.8e-221 N=57 10&NC_008397.2 plus
182- 0: transcript:Zm00001d026548_T049 rna13891 0
182- 1: transcript:Zm00001d026549_T006 rna13892 1.00E-83
182- 2: transcript:Zm00001d026553_T001 rna13897 1.00E-34
182- 3: transcript:Zm00001d026554_T001 rna13898 1.00E-17
182- 4: transcript:Zm00001d026555_T002 rna13901 7.00E-102
182- 5: transcript:Zm00001d026557_T001 rna13907 0
182- 6: transcript:Zm00001d026559_T001 rna13908 0

182- 7: transcript:Zm00001d026560_T001 rna13909   3.00E-121
182- 8: transcript:Zm00001d026562_T001 rna13911   2.00E-96
182- 9: transcript:Zm00001d026563_T001 rna13912   9.00E-45
182- 10: transcript:Zm00001d026572_T001 rna13915   2.00E-57
182- 11: transcript:Zm00001d026573_T002 rna13918   0
182- 12: transcript:Zm00001d026575_T001 rna13919   3.00E-111
182- 13: transcript:Zm00001d026576_T001 rna13920   0
182- 14: transcript:Zm00001d026577_T002 rna13921   0
182- 15: transcript:Zm00001d026580_T002 rna13924   3.00E-172
182- 16: transcript:Zm00001d026582_T001 rna13926   0
182- 17: transcript:Zm00001d026584_T001 rna13929   2.00E-21
182- 18: transcript:Zm00001d026586_T003 rna13930   0
182- 19: transcript:Zm00001d026587_T002 rna13933   0
182- 20: transcript:Zm00001d026590_T003 rna13934   0
182- 21: transcript:Zm00001d026591_T001 rna13937   6.00E-66
182- 22: transcript:Zm00001d026592_T001 rna13939   0
182- 23: transcript:Zm00001d026593_T002 rna13942   4.00E-105
182- 24: transcript:Zm00001d026594_T001 rna13943   4.00E-78
182- 25: transcript:Zm00001d026595_T001 rna13946   6.00E-57
182- 26: transcript:Zm00001d026597_T001 rna13947   5.00E-35
182- 27: transcript:Zm00001d026598_T001 rna13950   2.00E-160
182- 28: transcript:Zm00001d026604_T001 rna13956   0
182- 29: transcript:Zm00001d026605_T001 rna13958   0
182- 30: transcript:Zm00001d026606_T002 rna13962   0
182- 31: transcript:Zm00001d026607_T001 rna13963   0
182- 32: transcript:Zm00001d026608_T001 rna13967   3.00E-76
182- 33: transcript:Zm00001d026610_T001 rna13969   0
182- 34: transcript:Zm00001d026611_T001 rna13970   2.00E-22
182- 35: transcript:Zm00001d026613_T001 rna13972   1.00E-40
182- 36: transcript:Zm00001d026614_T001 rna13977   1.00E-63
182- 37: transcript:Zm00001d026617_T001 rna13979   8.00E-103
182- 38: transcript:Zm00001d026618_T002 rna13981   0
182- 39: transcript:Zm00001d026619_T001 rna13982   0
182- 40: transcript:Zm00001d026620_T001 rna13984   7.00E-26
182- 41: transcript:Zm00001d026621_T006 rna13985   5.00E-65
182- 42: transcript:Zm00001d026625_T001 rna13988   4.00E-91
182- 43: transcript:Zm00001d026627_T001 rna13989   0
182- 44: transcript:Zm00001d026628_T001 rna13990   3.00E-50
182- 45: transcript:Zm00001d026629_T001 rna13992   1.00E-149
182- 46: transcript:Zm00001d026630_T001 rna13995   2.00E-125
182- 47: transcript:Zm00001d026632_T001 rna13997   3.00E-70
182- 48: transcript:Zm00001d026633_T001 rna13999   1.00E-58
182- 49: transcript:Zm00001d026634_T002 rna14000   1.00E-109
182- 50: transcript:Zm00001d026635_T002 rna14003   1.00E-124
182- 51: transcript:Zm00001d026638_T009 rna14007   7.00E-104
182- 52: transcript:Zm00001d026639_T002 rna14010   2.00E-175
182- 53: transcript:Zm00001d026641_T001 rna14012   0
182- 54: transcript:Zm00001d026642_T001 rna14014   1.00E-80
182- 55: transcript:Zm00001d026643_T002 rna14017   0
182- 56: transcript:Zm00001d026645_T001 rna14019   0
Alignment 183: score=2032.0 e_value=1.3e-164 N=45 10&NC_008397.2 plus
183- 0: transcript:Zm00001d025607_T001 rna12577   0
183- 1: transcript:Zm00001d025613_T001 rna12584   3.00E-105
183- 2: transcript:Zm00001d025616_T001 rna12590   7.00E-81

183- 3:	transcript:Zm00001d025617_T001	rna12600	0
183- 4:	transcript:Zm00001d025619_T001	rna12603	2.00E-165
183- 5:	transcript:Zm00001d025621_T001	rna12604	1.00E-42
183- 6:	transcript:Zm00001d025622_T001	rna12606	3.00E-21
183- 7:	transcript:Zm00001d025623_T001	rna12608	2.00E-148
183- 8:	transcript:Zm00001d025624_T001	rna12610	5.00E-165
183- 9:	transcript:Zm00001d025625_T001	rna12612	0
183- 10:	transcript:Zm00001d025626_T002	rna12614	0
183- 11:	transcript:Zm00001d025628_T002	rna12617	1.00E-142
183- 12:	transcript:Zm00001d025633_T007	rna12620	0
183- 13:	transcript:Zm00001d025639_T002	rna12622	8.00E-137
183- 14:	transcript:Zm00001d025640_T002	rna12626	3.00E-124
183- 15:	transcript:Zm00001d025644_T002	rna12634	2.00E-25
183- 16:	transcript:Zm00001d025645_T001	rna12636	2.00E-13
183- 17:	transcript:Zm00001d025646_T001	rna12637	7.00E-42
183- 18:	transcript:Zm00001d025651_T001	rna12638	4.00E-78
183- 19:	transcript:Zm00001d025652_T005	rna12639	4.00E-158
183- 20:	transcript:Zm00001d025653_T001	rna12640	0
183- 21:	transcript:Zm00001d025654_T001	rna12642	6.00E-16
183- 22:	transcript:Zm00001d025656_T001	rna12644	0
183- 23:	transcript:Zm00001d025659_T001	rna12645	0
183- 24:	transcript:Zm00001d025660_T001	rna12649	4.00E-137
183- 25:	transcript:Zm00001d025663_T004	rna12652	3.00E-109
183- 26:	transcript:Zm00001d025665_T002	rna12655	0
183- 27:	transcript:Zm00001d025667_T001	rna12657	6.00E-106
183- 28:	transcript:Zm00001d025668_T005	rna12658	0
183- 29:	transcript:Zm00001d025669_T003	rna12659	0
183- 30:	transcript:Zm00001d025672_T001	rna12661	5.00E-41
183- 31:	transcript:Zm00001d025673_T005	rna12663	7.00E-39
183- 32:	transcript:Zm00001d025674_T001	rna12664	0
183- 33:	transcript:Zm00001d025679_T001	rna12686	5.00E-164
183- 34:	transcript:Zm00001d025681_T001	rna12691	2.00E-158
183- 35:	transcript:Zm00001d025684_T001	rna12695	1.00E-51
183- 36:	transcript:Zm00001d025687_T003	rna12701	0
183- 37:	transcript:Zm00001d025689_T002	rna12702	2.00E-164
183- 38:	transcript:Zm00001d025690_T003	rna12703	0
183- 39:	transcript:Zm00001d025692_T002	rna12709	0
183- 40:	transcript:Zm00001d025694_T001	rna12712	1.00E-102
183- 41:	transcript:Zm00001d025696_T001	rna12717	0
183- 42:	transcript:Zm00001d025699_T001	rna12724	3.00E-179
183- 43:	transcript:Zm00001d025703_T002	rna12725	0
183- 44:	transcript:Zm00001d025704_T001	rna12726	1.00E-138
## Alignment 184: score=1916.0 e_value=9.5e-153 N=42 10&NC_008397.2 plus			
184- 0:	transcript:Zm00001d025745_T001	rna12781	1.00E-153
184- 1:	transcript:Zm00001d025746_T001	rna12784	0
184- 2:	transcript:Zm00001d025747_T002	rna12785	1.00E-121
184- 3:	transcript:Zm00001d025748_T001	rna12788	2.00E-164
184- 4:	transcript:Zm00001d025749_T002	rna12790	0
184- 5:	transcript:Zm00001d025750_T011	rna12791	0
184- 6:	transcript:Zm00001d025751_T002	rna12793	0
184- 7:	transcript:Zm00001d025752_T006	rna12799	2.00E-30
184- 8:	transcript:Zm00001d025753_T001	rna12801	2.00E-101
184- 9:	transcript:Zm00001d025754_T001	rna12805	4.00E-145
184- 10:	transcript:Zm00001d025757_T001	rna12814	0

184- 11: transcript:Zm00001d025759_T001 rna12815 5.00E-80
184- 12: transcript:Zm00001d025761_T002 rna12816 6.00E-77
184- 13: transcript:Zm00001d025762_T001 rna12817 3.00E-110
184- 14: transcript:Zm00001d025763_T003 rna12820 3.00E-44
184- 15: transcript:Zm00001d025764_T001 rna12824 1.00E-58
184- 16: transcript:Zm00001d025765_T002 rna12826 2.00E-25
184- 17: transcript:Zm00001d025767_T002 rna12828 0
184- 18: transcript:Zm00001d025770_T001 rna12829 3.00E-168
184- 19: transcript:Zm00001d025771_T001 rna12831 7.00E-22
184- 20: transcript:Zm00001d025773_T002 rna12835 0
184- 21: transcript:Zm00001d025774_T003 rna12836 5.00E-177
184- 22: transcript:Zm00001d025776_T002 rna12837 0
184- 23: transcript:Zm00001d025777_T007 rna12842 0
184- 24: transcript:Zm00001d025780_T001 rna12849 4.00E-100
184- 25: transcript:Zm00001d025784_T002 rna12850 1.00E-85
184- 26: transcript:Zm00001d025788_T001 rna12853 0
184- 27: transcript:Zm00001d025789_T014 rna12854 0
184- 28: transcript:Zm00001d025793_T001 rna12855 0
184- 29: transcript:Zm00001d025794_T002 rna12856 2.00E-92
184- 30: transcript:Zm00001d025795_T010 rna12859 0
184- 31: transcript:Zm00001d025797_T001 rna12861 6.00E-52
184- 32: transcript:Zm00001d025798_T004 rna12862 0
184- 33: transcript:Zm00001d025799_T001 rna12866 1.00E-45
184- 34: transcript:Zm00001d025801_T007 rna12874 0
184- 35: transcript:Zm00001d025803_T001 rna12875 0
184- 36: transcript:Zm00001d025804_T001 rna12876 6.00E-123
184- 37: transcript:Zm00001d025807_T001 rna12878 0
184- 38: transcript:Zm00001d025808_T002 rna12880 0
184- 39: transcript:Zm00001d025814_T001 rna12884 2.00E-168
184- 40: transcript:Zm00001d025815_T001 rna12885 2.00E-58
184- 41: transcript:Zm00001d025816_T002 rna12886 0
Alignment 185: score=1906.0 e_value=6.1e-157 N=42 10&NC_008397.2 plus
185- 0: transcript:Zm00001d025857_T001 rna12941 8.00E-90
185- 1: transcript:Zm00001d025859_T001 rna12942 3.00E-67
185- 2: transcript:Zm00001d025861_T001 rna12944 1.00E-123
185- 3: transcript:Zm00001d025862_T002 rna12946 0
185- 4: transcript:Zm00001d025864_T001 rna12951 6.00E-108
185- 5: transcript:Zm00001d025865_T001 rna12953 2.00E-131
185- 6: transcript:Zm00001d025867_T003 rna12962 4.00E-06
185- 7: transcript:Zm00001d025868_T002 rna12963 9.00E-129
185- 8: transcript:Zm00001d025871_T001 rna12965 1.00E-123
185- 9: transcript:Zm00001d025872_T001 rna12968 9.00E-62
185- 10: transcript:Zm00001d025874_T001 rna12978 5.00E-49
185- 11: transcript:Zm00001d025886_T001 rna12983 2.00E-98
185- 12: transcript:Zm00001d025887_T001 rna12988 0
185- 13: transcript:Zm00001d025891_T001 rna12996 1.00E-175
185- 14: transcript:Zm00001d025894_T002 rna12998 0
185- 15: transcript:Zm00001d025896_T001 rna12999 2.00E-73
185- 16: transcript:Zm00001d025900_T001 rna13003 3.00E-133
185- 17: transcript:Zm00001d025903_T004 rna13007 0
185- 18: transcript:Zm00001d025904_T001 rna13008 2.00E-65
185- 19: transcript:Zm00001d025905_T002 rna13009 2.00E-48
185- 20: transcript:Zm00001d025906_T001 rna13014 1.00E-23
185- 21: transcript:Zm00001d025907_T001 rna13016 0

185- 22: transcript:Zm00001d025908_T001 rna13017 2.00E-134
185- 23: transcript:Zm00001d025910_T001 rna13019 4.00E-100
185- 24: transcript:Zm00001d025911_T002 rna13020 3.00E-75
185- 25: transcript:Zm00001d025912_T001 rna13023 2.00E-176
185- 26: transcript:Zm00001d025915_T002 rna13030 0
185- 27: transcript:Zm00001d025916_T001 rna13031 2.00E-126
185- 28: transcript:Zm00001d025917_T001 rna13034 0
185- 29: transcript:Zm00001d025919_T001 rna13036 0
185- 30: transcript:Zm00001d025920_T001 rna13037 0
185- 31: transcript:Zm00001d025921_T001 rna13038 0
185- 32: transcript:Zm00001d025922_T001 rna13040 7.00E-29
185- 33: transcript:Zm00001d025926_T001 rna13047 6.00E-116
185- 34: transcript:Zm00001d025932_T003 rna13051 9.00E-92
185- 35: transcript:Zm00001d025933_T001 rna13054 0
185- 36: transcript:Zm00001d025941_T001 rna13058 3.00E-87
185- 37: transcript:Zm00001d025947_T001 rna13067 3.00E-52
185- 38: transcript:Zm00001d025948_T001 rna13071 0
185- 39: transcript:Zm00001d025949_T001 rna13073 4.00E-74
185- 40: transcript:Zm00001d025950_T002 rna13074 0
185- 41: transcript:Zm00001d025951_T002 rna13076 0
Alignment 186: score=1241.0 e_value=6e-92 N=28 10&NC_008397.2 plus
186- 0: transcript:Zm00001d026647_T005 rna14020 0
186- 1: transcript:Zm00001d026649_T001 rna14024 0
186- 2: transcript:Zm00001d026650_T001 rna14026 0
186- 3: transcript:Zm00001d026652_T001 rna14027 1.00E-145
186- 4: transcript:Zm00001d026653_T004 rna14030 0
186- 5: transcript:Zm00001d026654_T001 rna14033 0
186- 6: transcript:Zm00001d026657_T001 rna14036 1.00E-112
186- 7: transcript:Zm00001d026661_T001 rna14037 2.00E-66
186- 8: transcript:Zm00001d026662_T001 rna14040 4.00E-109
186- 9: transcript:Zm00001d026664_T001 rna14041 8.00E-125
186- 10: transcript:Zm00001d026668_T001 rna14046 0
186- 11: transcript:Zm00001d026676_T003 rna14053 5.00E-71
186- 12: transcript:Zm00001d026677_T001 rna14054 6.00E-30
186- 13: transcript:Zm00001d026678_T001 rna14055 1.00E-145
186- 14: transcript:Zm00001d026679_T001 rna14056 7.00E-79
186- 15: transcript:Zm00001d026680_T002 rna14057 2.00E-151
186- 16: transcript:Zm00001d026681_T001 rna14063 5.00E-62
186- 17: transcript:Zm00001d026683_T001 rna14065 3.00E-81
186- 18: transcript:Zm00001d026684_T001 rna14076 1.00E-131
186- 19: transcript:Zm00001d026690_T001 rna14086 0
186- 20: transcript:Zm00001d026691_T004 rna14087 3.00E-131
186- 21: transcript:Zm00001d026695_T001 rna14088 0
186- 22: transcript:Zm00001d026696_T019 rna14089 0
186- 23: transcript:Zm00001d026697_T001 rna14090 1.00E-57
186- 24: transcript:Zm00001d026698_T026 rna14091 0
186- 25: transcript:Zm00001d026700_T002 rna14096 1.00E-133
186- 26: transcript:Zm00001d026701_T001 rna14097 1.00E-107
186- 27: transcript:Zm00001d026702_T002 rna14103 1.00E-70
Alignment 187: score=890.0 e_value=3.4e-59 N=20 10&NC_008397.2 plus
187- 0: transcript:Zm00001d026002_T001 rna13315 5.00E-71
187- 1: transcript:Zm00001d026003_T001 rna13316 2.00E-61
187- 2: transcript:Zm00001d026005_T001 rna13319 1.00E-87
187- 3: transcript:Zm00001d026010_T001 rna13323 5.00E-175

187- 4: transcript:Zm00001d026012_T001 rna13331   4.00E-38
187- 5: transcript:Zm00001d026014_T001 rna13332   4.00E-162
187- 6: transcript:Zm00001d026015_T001 rna13333   6.00E-67
187- 7: transcript:Zm00001d026016_T001 rna13335   3.00E-18
187- 8: transcript:Zm00001d026017_T001 rna13336   8.00E-131
187- 9: transcript:Zm00001d026018_T001 rna13341   0
187- 10: transcript:Zm00001d026020_T001 rna13344   5.00E-140
187- 11: transcript:Zm00001d026021_T001 rna13345   5.00E-18
187- 12: transcript:Zm00001d026022_T004 rna13348   0
187- 13: transcript:Zm00001d026025_T002 rna13350   2.00E-98
187- 14: transcript:Zm00001d026026_T001 rna13351   2.00E-54
187- 15: transcript:Zm00001d026028_T001 rna13353   3.00E-15
187- 16: transcript:Zm00001d026032_T001 rna13358   5.00E-117
187- 17: transcript:Zm00001d026041_T005 rna13368   0
187- 18: transcript:Zm00001d026046_T001 rna13381   4.00E-12
187- 19: transcript:Zm00001d026047_T002 rna13385   2.00E-139
Alignment 188: score=683.0 e_value=5.7e-43 N=15 10&NC_008397.2 plus
188- 0: transcript:Zm00001d025193_T003 rna12117   0
188- 1: transcript:Zm00001d025203_T001 rna12141   0
188- 2: transcript:Zm00001d025206_T001 rna12143   0
188- 3: transcript:Zm00001d025208_T002 rna12147   5.00E-15
188- 4: transcript:Zm00001d025218_T001 rna12157   2.00E-160
188- 5: transcript:Zm00001d025222_T001 rna12158   3.00E-151
188- 6: transcript:Zm00001d025225_T001 rna12159   3.00E-34
188- 7: transcript:Zm00001d025228_T003 rna12161   4.00E-60
188- 8: transcript:Zm00001d025229_T001 rna12163   1.00E-36
188- 9: transcript:Zm00001d025233_T001 rna12164   7.00E-67
188- 10: transcript:Zm00001d025235_T004 rna12170   0
188- 11: transcript:Zm00001d025236_T001 rna12171   2.00E-57
188- 12: transcript:Zm00001d025237_T001 rna12172   1.00E-43
188- 13: transcript:Zm00001d025238_T001 rna12173   0
188- 14: transcript:Zm00001d025239_T001 rna12174   1.00E-56
Alignment 189: score=653.0 e_value=2e-39 N=15 10&NC_008397.2 plus
189- 0: transcript:Zm00001d026514_T002 rna13855   0
189- 1: transcript:Zm00001d026515_T003 rna13857   2.00E-30
189- 2: transcript:Zm00001d026516_T002 rna13859   3.00E-88
189- 3: transcript:Zm00001d026517_T001 rna13860   1.00E-75
189- 4: transcript:Zm00001d026518_T001 rna13861   3.00E-85
189- 5: transcript:Zm00001d026530_T001 rna13863   2.00E-63
189- 6: transcript:Zm00001d026531_T002 rna13866   0
189- 7: transcript:Zm00001d026532_T007 rna13867   0
189- 8: transcript:Zm00001d026535_T003 rna13870   0
189- 9: transcript:Zm00001d026536_T002 rna13871   8.00E-122
189- 10: transcript:Zm00001d026540_T001 rna13877   0
189- 11: transcript:Zm00001d026541_T001 rna13885   1.00E-155
189- 12: transcript:Zm00001d026542_T001 rna13886   4.00E-148
189- 13: transcript:Zm00001d026543_T007 rna13889   3.00E-173
189- 14: transcript:Zm00001d026546_T001 rna13890   2.00E-27
Alignment 190: score=641.0 e_value=1.6e-36 N=14 10&NC_008397.2 plus
190- 0: transcript:Zm00001d025707_T001 rna12729   2.00E-143
190- 1: transcript:Zm00001d025711_T001 rna12733   1.00E-53
190- 2: transcript:Zm00001d025712_T003 rna12737   6.00E-146
190- 3: transcript:Zm00001d025713_T001 rna12743   0
190- 4: transcript:Zm00001d025714_T001 rna12745   0

```
190-  5:  transcript:Zm00001d025715_T001  rna12746    6.00E-114
190-  6:  transcript:Zm00001d025716_T001  rna12749     2.00E-28
190-  7:  transcript:Zm00001d025717_T001  rna12750    2.00E-157
190-  8:  transcript:Zm00001d025720_T001  rna12752            0
190-  9:  transcript:Zm00001d025721_T001  rna12753    5.00E-123
190- 10:  transcript:Zm00001d025722_T002  rna12755            0
190- 11:  transcript:Zm00001d025724_T001  rna12756     4.00E-75
190- 12:  transcript:Zm00001d025726_T001  rna12760            0
190- 13:  transcript:Zm00001d025727_T006  rna12776            0
## Alignment 191: score=597.0 e_value=1.6e-38 N=15 10&NC_008397.2 plus
191-  0:  transcript:Zm00001d025020_T001  rna11623     2.00E-72
191-  1:  transcript:Zm00001d025023_T001  rna11624            0
191-  2:  transcript:Zm00001d025024_T018  rna11626    2.00E-159
191-  3:  transcript:Zm00001d025025_T001  rna11627    1.00E-163
191-  4:  transcript:Zm00001d025027_T009  rna11638            0
191-  5:  transcript:Zm00001d025028_T001  rna11639            0
191-  6:  transcript:Zm00001d025031_T001  rna11661            0
191-  7:  transcript:Zm00001d025033_T001  rna11662     1.00E-59
191-  8:  transcript:Zm00001d025034_T002  rna11677            0
191-  9:  transcript:Zm00001d025035_T001  rna11680            0
191- 10:  transcript:Zm00001d025037_T001  rna11689            0
191- 11:  transcript:Zm00001d025038_T001  rna11702    2.00E-110
191- 12:  transcript:Zm00001d025043_T004  rna11709            0
191- 13:  transcript:Zm00001d025044_T029  rna11714            0
191- 14:  transcript:Zm00001d025047_T001  rna11733    4.00E-162
## Alignment 192: score=579.0 e_value=1e-30 N=12 10&NC_008397.2 plus
192-  0:  transcript:Zm00001d026440_T001  rna13764     7.00E-42
192-  1:  transcript:Zm00001d026441_T001  rna13765     2.00E-96
192-  2:  transcript:Zm00001d026442_T001  rna13766    4.00E-164
192-  3:  transcript:Zm00001d026444_T001  rna13778     1.00E-63
192-  4:  transcript:Zm00001d026445_T001  rna13780    3.00E-169
192-  5:  transcript:Zm00001d026447_T001  rna13781     3.00E-69
192-  6:  transcript:Zm00001d026448_T001  rna13784     1.00E-20
192-  7:  transcript:Zm00001d026452_T001  rna13785            0
192-  8:  transcript:Zm00001d026453_T001  rna13786     2.00E-81
192-  9:  transcript:Zm00001d026454_T001  rna13787     9.00E-20
192- 10:  transcript:Zm00001d026457_T001  rna13788     7.00E-16
192- 11:  transcript:Zm00001d026458_T002  rna13791     6.00E-71
## Alignment 193: score=525.0 e_value=1.4e-21 N=11 10&NC_008397.2 plus
193-  0:  transcript:Zm00001d025952_T002  rna13078     4.00E-29
193-  1:  transcript:Zm00001d025953_T003  rna13080     8.00E-57
193-  2:  transcript:Zm00001d025955_T001  rna13082     3.00E-67
193-  3:  transcript:Zm00001d025957_T001  rna13083    9.00E-114
193-  4:  transcript:Zm00001d025958_T001  rna13087            0
193-  5:  transcript:Zm00001d025959_T001  rna13088            0
193-  6:  transcript:Zm00001d025960_T002  rna13089            0
193-  7:  transcript:Zm00001d025963_T002  rna13090            0
193-  8:  transcript:Zm00001d025964_T001  rna13091     8.00E-90
193-  9:  transcript:Zm00001d025966_T001  rna13092    9.00E-105
193- 10:  transcript:Zm00001d025977_T001  rna13095            0
## Alignment 194: score=511.0 e_value=4e-23 N=11 10&NC_008397.2 plus
194-  0:  transcript:Zm00001d026488_T001  rna13821    2.00E-156
194-  1:  transcript:Zm00001d026490_T010  rna13824            0
194-  2:  transcript:Zm00001d026491_T001  rna13825    5.00E-176
```

194- 3: transcript:Zm00001d026492_T001 rna13833 0
194- 4: transcript:Zm00001d026495_T001 rna13836 0
194- 5: transcript:Zm00001d026498_T001 rna13838 8.00E-18
194- 6: transcript:Zm00001d026500_T001 rna13839 2.00E-09
194- 7: transcript:Zm00001d026501_T009 rna13841 0
194- 8: transcript:Zm00001d026506_T001 rna13845 7.00E-17
194- 9: transcript:Zm00001d026509_T001 rna13848 4.00E-29
194- 10: transcript:Zm00001d026510_T001 rna13849 1.00E-48
Alignment 195: score=489.0 e_value=6.3e-22 N=10 10&NC_008397.2 plus
195- 0: transcript:Zm00001d025817_T041 rna12888 0
195- 1: transcript:Zm00001d025818_T001 rna12889 0
195- 2: transcript:Zm00001d025819_T001 rna12890 8.00E-50
195- 3: transcript:Zm00001d025820_T001 rna12891 0
195- 4: transcript:Zm00001d025821_T001 rna12893 0
195- 5: transcript:Zm00001d025822_T001 rna12894 2.00E-30
195- 6: transcript:Zm00001d025823_T001 rna12899 9.00E-94
195- 7: transcript:Zm00001d025824_T001 rna12900 0
195- 8: transcript:Zm00001d025825_T001 rna12901 0
195- 9: transcript:Zm00001d025827_T002 rna12904 0
Alignment 196: score=459.0 e_value=5.7e-23 N=10 10&NC_008397.2 plus
196- 0: transcript:Zm00001d025981_T004 rna13102 0
196- 1: transcript:Zm00001d025982_T001 rna13103 6.00E-66
196- 2: transcript:Zm00001d025983_T002 rna13104 0
196- 3: transcript:Zm00001d025984_T003 rna13106 3.00E-117
196- 4: transcript:Zm00001d025988_T001 rna13111 2.00E-133
196- 5: transcript:Zm00001d025991_T004 rna13114 4.00E-132
196- 6: transcript:Zm00001d025992_T001 rna13115 0
196- 7: transcript:Zm00001d025996_T001 rna13121 6.00E-92
196- 8: transcript:Zm00001d025997_T002 rna13122 0
196- 9: transcript:Zm00001d025998_T001 rna13123 1.00E-63
Alignment 197: score=417.0 e_value=1.5e-17 N=9 10&NC_008397.2 plus
197- 0: transcript:Zm00001d025831_T001 rna12909 0
197- 1: transcript:Zm00001d025833_T001 rna12920 7.00E-158
197- 2: transcript:Zm00001d025834_T002 rna12922 0
197- 3: transcript:Zm00001d025837_T001 rna12924 0
197- 4: transcript:Zm00001d025842_T001 rna12929 1.00E-38
197- 5: transcript:Zm00001d025845_T001 rna12931 1.00E-47
197- 6: transcript:Zm00001d025846_T001 rna12932 0
197- 7: transcript:Zm00001d025848_T001 rna12938 5.00E-119
197- 8: transcript:Zm00001d025853_T001 rna12940 0
Alignment 198: score=413.0 e_value=4.1e-22 N=10 10&NC_008397.2 plus
198- 0: transcript:Zm00001d025240_T036 rna12179 0
198- 1: transcript:Zm00001d025241_T002 rna12180 4.00E-11
198- 2: transcript:Zm00001d025249_T001 rna12188 0
198- 3: transcript:Zm00001d025252_T001 rna12193 5.00E-61
198- 4: transcript:Zm00001d025258_T009 rna12197 0
198- 5: transcript:Zm00001d025263_T002 rna12200 0
198- 6: transcript:Zm00001d025268_T011 rna12203 0
198- 7: transcript:Zm00001d025273_T019 rna12210 0
198- 8: transcript:Zm00001d025274_T001 rna12211 7.00E-10
198- 9: transcript:Zm00001d025275_T001 rna12212 1.00E-123
Alignment 199: score=368.0 e_value=3.9e-16 N=8 10&NC_008397.2 plus
199- 0: transcript:Zm00001d026471_T001 rna13794 0
199- 1: transcript:Zm00001d026472_T001 rna13798 0

199- 2: transcript:Zm00001d026475_T001 rna13807 1.00E-127
199- 3: transcript:Zm00001d026477_T002 rna13808 2.00E-60
199- 4: transcript:Zm00001d026478_T001 rna13809 8.00E-29
199- 5: transcript:Zm00001d026479_T006 rna13810 0
199- 6: transcript:Zm00001d026480_T001 rna13811 8.00E-49
199- 7: transcript:Zm00001d026483_T001 rna13812 7.00E-79
Alignment 200: score=276.0 e_value=9.1e-09 N=6 10&NC_008397.2 plus
200- 0: transcript:Zm00001d023440_T001 rna12306 5.00E-16
200- 1: transcript:Zm00001d023453_T001 rna12314 8.00E-06
200- 2: transcript:Zm00001d023455_T001 rna12316 2.00E-23
200- 3: transcript:Zm00001d023459_T001 rna12317 1.00E-57
200- 4: transcript:Zm00001d023465_T002 rna12323 2.00E-102
200- 5: transcript:Zm00001d023468_T001 rna12326 2.00E-73
Alignment 201: score=1786.0 e_value=5.7e-131 N=38 10&NC_008397.2 minus
201- 0: transcript:Zm00001d026119_T002 rna13237 9.00E-30
201- 1: transcript:Zm00001d026120_T001 rna13236 5.00E-68
201- 2: transcript:Zm00001d026121_T001 rna13235 0
201- 3: transcript:Zm00001d026124_T001 rna13228 5.00E-35
201- 4: transcript:Zm00001d026126_T001 rna13223 0
201- 5: transcript:Zm00001d026127_T001 rna13222 3.00E-178
201- 6: transcript:Zm00001d026129_T001 rna13221 0
201- 7: transcript:Zm00001d026130_T002 rna13215 0
201- 8: transcript:Zm00001d026131_T001 rna13213 0
201- 9: transcript:Zm00001d026132_T001 rna13211 0
201- 10: transcript:Zm00001d026135_T002 rna13206 0
201- 11: transcript:Zm00001d026137_T002 rna13205 2.00E-49
201- 12: transcript:Zm00001d026139_T001 rna13204 0
201- 13: transcript:Zm00001d026140_T001 rna13192 0
201- 14: transcript:Zm00001d026141_T001 rna13191 1.00E-33
201- 15: transcript:Zm00001d026143_T001 rna13190 1.00E-10
201- 16: transcript:Zm00001d026144_T004 rna13189 6.00E-108
201- 17: transcript:Zm00001d026147_T002 rna13186 7.00E-169
201- 18: transcript:Zm00001d026148_T002 rna13185 5.00E-175
201- 19: transcript:Zm00001d026152_T001 rna13184 8.00E-41
201- 20: transcript:Zm00001d026154_T002 rna13179 7.00E-110
201- 21: transcript:Zm00001d026156_T002 rna13170 0
201- 22: transcript:Zm00001d026158_T001 rna13169 0
201- 23: transcript:Zm00001d026160_T001 rna13166 2.00E-35
201- 24: transcript:Zm00001d026165_T003 rna13164 0
201- 25: transcript:Zm00001d026166_T002 rna13157 7.00E-131
201- 26: transcript:Zm00001d026169_T001 rna13154 1.00E-105
201- 27: transcript:Zm00001d026173_T001 rna13151 2.00E-44
201- 28: transcript:Zm00001d026175_T001 rna13150 3.00E-92
201- 29: transcript:Zm00001d026177_T001 rna13146 1.00E-62
201- 30: transcript:Zm00001d026180_T001 rna13145 6.00E-61
201- 31: transcript:Zm00001d026182_T001 rna13139 1.00E-39
201- 32: transcript:Zm00001d026185_T001 rna13138 1.00E-169
201- 33: transcript:Zm00001d026186_T001 rna13137 3.00E-98
201- 34: transcript:Zm00001d026187_T001 rna13136 7.00E-35
201- 35: transcript:Zm00001d026189_T001 rna13132 2.00E-80
201- 36: transcript:Zm00001d026190_T001 rna13129 8.00E-114
201- 37: transcript:Zm00001d026191_T001 rna13125 3.00E-112
Alignment 202: score=1348.0 e_value=4.7e-95 N=29 10&NC_008397.2 minus
202- 0: transcript:Zm00001d026050_T001 rna13311 0

```
202-  1:  transcript:Zm00001d026051_T002  rna13310           0
202-  2:  transcript:Zm00001d026053_T001  rna13309     1.00E-70
202-  3:  transcript:Zm00001d026055_T001  rna13307           0
202-  4:  transcript:Zm00001d026056_T002  rna13304    7.00E-140
202-  5:  transcript:Zm00001d026060_T003  rna13302           0
202-  6:  transcript:Zm00001d026061_T001  rna13300     1.00E-72
202-  7:  transcript:Zm00001d026062_T001  rna13297           0
202-  8:  transcript:Zm00001d026063_T001  rna13294           0
202-  9:  transcript:Zm00001d026064_T005  rna13293           0
202- 10:  transcript:Zm00001d026066_T001  rna13282    7.00E-174
202- 11:  transcript:Zm00001d026067_T001  rna13281           0
202- 12:  transcript:Zm00001d026068_T001  rna13280           0
202- 13:  transcript:Zm00001d026069_T001  rna13279     9.00E-35
202- 14:  transcript:Zm00001d026070_T002  rna13277           0
202- 15:  transcript:Zm00001d026076_T001  rna13270           0
202- 16:  transcript:Zm00001d026078_T001  rna13269    6.00E-125
202- 17:  transcript:Zm00001d026079_T001  rna13267           0
202- 18:  transcript:Zm00001d026083_T001  rna13262           0
202- 19:  transcript:Zm00001d026084_T001  rna13261           0
202- 20:  transcript:Zm00001d026088_T002  rna13257           0
202- 21:  transcript:Zm00001d026089_T001  rna13256           0
202- 22:  transcript:Zm00001d026091_T001  rna13255    1.00E-144
202- 23:  transcript:Zm00001d026094_T001  rna13252    2.00E-127
202- 24:  transcript:Zm00001d026095_T001  rna13251    3.00E-105
202- 25:  transcript:Zm00001d026102_T001  rna13246           0
202- 26:  transcript:Zm00001d026104_T002  rna13244     4.00E-47
202- 27:  transcript:Zm00001d026106_T001  rna13242     2.00E-84
202- 28:  transcript:Zm00001d026111_T008  rna13241           0
## Alignment 203: score=800.0 e_value=4.9e-49 N=18 10&NC_008397.2 minus
203-  0:  transcript:Zm00001d024898_T001  rna11433     1.00E-93
203-  1:  transcript:Zm00001d024904_T001  rna11428    1.00E-125
203-  2:  transcript:Zm00001d024906_T001  rna11427    2.00E-151
203-  3:  transcript:Zm00001d024908_T003  rna11426           0
203-  4:  transcript:Zm00001d024913_T001  rna11425     1.00E-66
203-  5:  transcript:Zm00001d024919_T001  rna11423     2.00E-22
203-  6:  transcript:Zm00001d024925_T001  rna11421    6.00E-144
203-  7:  transcript:Zm00001d024926_T001  rna11420     3.00E-41
203-  8:  transcript:Zm00001d024927_T002  rna11419    3.00E-169
203-  9:  transcript:Zm00001d024928_T003  rna11418    3.00E-172
203- 10:  transcript:Zm00001d024933_T003  rna11410           0
203- 11:  transcript:Zm00001d024934_T001  rna11408    7.00E-167
203- 12:  transcript:Zm00001d024936_T002  rna11407           0
203- 13:  transcript:Zm00001d024939_T002  rna11405     5.00E-30
203- 14:  transcript:Zm00001d024940_T001  rna11404           0
203- 15:  transcript:Zm00001d024941_T002  rna11403    2.00E-145
203- 16:  transcript:Zm00001d024943_T001  rna11402           0
203- 17:  transcript:Zm00001d024947_T005  rna11400    5.00E-175
## Alignment 204: score=783.0 e_value=2.7e-47 N=18 10&NC_008397.2 minus
204-  0:  transcript:Zm00001d024314_T001  rna13530    2.00E-177
204-  1:  transcript:Zm00001d024317_T001  rna13518    4.00E-120
204-  2:  transcript:Zm00001d024324_T001  rna13509     4.00E-27
204-  3:  transcript:Zm00001d024327_T001  rna13503           0
204-  4:  transcript:Zm00001d024338_T008  rna13497           0
204-  5:  transcript:Zm00001d024339_T001  rna13496           0
```

204- 6: transcript:Zm00001d024342_T001 rna13494 0
204- 7: transcript:Zm00001d024348_T002 rna13484 5.00E-106
204- 8: transcript:Zm00001d024357_T001 rna13480 2.00E-37
204- 9: transcript:Zm00001d024364_T001 rna13479 0
204- 10: transcript:Zm00001d024371_T001 rna13476 5.00E-27
204- 11: transcript:Zm00001d024373_T001 rna13474 2.00E-159
204- 12: transcript:Zm00001d024376_T001 rna13470 7.00E-75
204- 13: transcript:Zm00001d024379_T001 rna13469 5.00E-90
204- 14: transcript:Zm00001d024392_T001 rna13463 2.00E-126
204- 15: transcript:Zm00001d024403_T001 rna13459 3.00E-25
204- 16: transcript:Zm00001d024406_T003 rna13457 6.00E-83
204- 17: transcript:Zm00001d024408_T005 rna13455 5.00E-37
Alignment 205: score=400.0 e_value=3e-16 N=9 10&NC_008397.2 minus
205- 0: transcript:Zm00001d025170_T001 rna12131 0
205- 1: transcript:Zm00001d025171_T001 rna12128 2.00E-85
205- 2: transcript:Zm00001d025174_T001 rna12125 1.00E-135
205- 3: transcript:Zm00001d025178_T001 rna12122 3.00E-109
205- 4: transcript:Zm00001d025180_T001 rna12120 0
205- 5: transcript:Zm00001d025182_T007 rna12118 5.00E-66
205- 6: transcript:Zm00001d025185_T001 rna12117 0
205- 7: transcript:Zm00001d025199_T001 rna12116 0
205- 8: transcript:Zm00001d025201_T002 rna12115 2.00E-114
Alignment 206: score=2521.0 e_value=1.7e-219 N=56 10&NC_008398.2 plus
206- 0: transcript:Zm00001d024717_T001 rna14308 2.00E-41
206- 1: transcript:Zm00001d024718_T004 rna14312 5.00E-140
206- 2: transcript:Zm00001d024722_T002 rna14314 1.00E-44
206- 3: transcript:Zm00001d024723_T001 rna14316 0
206- 4: transcript:Zm00001d024726_T001 rna14320 1.00E-14
206- 5: transcript:Zm00001d024729_T001 rna14325 0
206- 6: transcript:Zm00001d024732_T001 rna14326 0
206- 7: transcript:Zm00001d024733_T001 rna14327 1.00E-25
206- 8: transcript:Zm00001d024734_T001 rna14328 6.00E-111
206- 9: transcript:Zm00001d024735_T001 rna14329 2.00E-96
206- 10: transcript:Zm00001d024738_T001 rna14331 4.00E-146
206- 11: transcript:Zm00001d024750_T001 rna14333 3.00E-72
206- 12: transcript:Zm00001d024752_T001 rna14335 4.00E-123
206- 13: transcript:Zm00001d024754_T001 rna14336 0
206- 14: transcript:Zm00001d024755_T004 rna14337 0
206- 15: transcript:Zm00001d024756_T004 rna14338 5.00E-177
206- 16: transcript:Zm00001d024757_T001 rna14339 8.00E-59
206- 17: transcript:Zm00001d024762_T001 rna14340 0
206- 18: transcript:Zm00001d024763_T001 rna14343 0
206- 19: transcript:Zm00001d024765_T001 rna14347 2.00E-114
206- 20: transcript:Zm00001d024767_T003 rna14348 0
206- 21: transcript:Zm00001d024770_T001 rna14350 1.00E-73
206- 22: transcript:Zm00001d024772_T001 rna14352 2.00E-18
206- 23: transcript:Zm00001d024778_T001 rna14354 6.00E-31
206- 24: transcript:Zm00001d024783_T001 rna14356 8.00E-110
206- 25: transcript:Zm00001d024784_T001 rna14360 2.00E-58
206- 26: transcript:Zm00001d024786_T001 rna14362 5.00E-52
206- 27: transcript:Zm00001d024787_T002 rna14363 2.00E-48
206- 28: transcript:Zm00001d024788_T002 rna14364 2.00E-61
206- 29: transcript:Zm00001d024789_T001 rna14369 1.00E-170
206- 30: transcript:Zm00001d024796_T001 rna14372 2.00E-172

206- 31: transcript:Zm00001d024799_T001 rna14374 2.00E-15
206- 32: transcript:Zm00001d024800_T001 rna14375 1.00E-11
206- 33: transcript:Zm00001d024802_T001 rna14376 7.00E-13
206- 34: transcript:Zm00001d024803_T001 rna14380 4.00E-18
206- 35: transcript:Zm00001d024804_T001 rna14381 5.00E-169
206- 36: transcript:Zm00001d024805_T001 rna14382 2.00E-20
206- 37: transcript:Zm00001d024807_T008 rna14383 2.00E-88
206- 38: transcript:Zm00001d024815_T005 rna14390 3.00E-37
206- 39: transcript:Zm00001d024816_T007 rna14391 0
206- 40: transcript:Zm00001d024819_T002 rna14394 0
206- 41: transcript:Zm00001d024821_T002 rna14395 2.00E-93
206- 42: transcript:Zm00001d024823_T001 rna14398 9.00E-53
206- 43: transcript:Zm00001d024824_T001 rna14399 0
206- 44: transcript:Zm00001d024828_T003 rna14407 0
206- 45: transcript:Zm00001d024830_T001 rna14409 4.00E-167
206- 46: transcript:Zm00001d024831_T001 rna14410 8.00E-121
206- 47: transcript:Zm00001d024833_T001 rna14417 1.00E-86
206- 48: transcript:Zm00001d024839_T001 rna14420 4.00E-70
206- 49: transcript:Zm00001d024841_T001 rna14423 0
206- 50: transcript:Zm00001d024843_T001 rna14424 2.00E-126
206- 51: transcript:Zm00001d024854_T001 rna14426 3.00E-92
206- 52: transcript:Zm00001d024855_T006 rna14427 0
206- 53: transcript:Zm00001d024857_T002 rna14428 0
206- 54: transcript:Zm00001d024858_T004 rna14429 0
206- 55: transcript:Zm00001d024861_T007 rna14433 0
Alignment 207: score=1264.0 e_value=1e-91 N=28 10&NC_008401.2 plus
207- 0: transcript:Zm00001d024318_T009 rna21825 0
207- 1: transcript:Zm00001d024327_T001 rna21829 0
207- 2: transcript:Zm00001d024337_T001 rna21831 0
207- 3: transcript:Zm00001d024338_T008 rna21832 0
207- 4: transcript:Zm00001d024339_T001 rna21833 0
207- 5: transcript:Zm00001d024342_T001 rna21836 0
207- 6: transcript:Zm00001d024343_T001 rna21837 1.00E-52
207- 7: transcript:Zm00001d024344_T001 rna21838 6.00E-11
207- 8: transcript:Zm00001d024347_T001 rna21840 7.00E-15
207- 9: transcript:Zm00001d024348_T002 rna21841 0
207- 10: transcript:Zm00001d024349_T001 rna21844 3.00E-73
207- 11: transcript:Zm00001d024357_T001 rna21854 3.00E-60
207- 12: transcript:Zm00001d024364_T001 rna21855 0
207- 13: transcript:Zm00001d024365_T001 rna21857 0
207- 14: transcript:Zm00001d024371_T001 rna21860 8.00E-26
207- 15: transcript:Zm00001d024373_T001 rna21873 8.00E-164
207- 16: transcript:Zm00001d024376_T001 rna21881 5.00E-78
207- 17: transcript:Zm00001d024379_T001 rna21887 1.00E-126
207- 18: transcript:Zm00001d024392_T001 rna21890 4.00E-94
207- 19: transcript:Zm00001d024403_T001 rna21905 8.00E-19
207- 20: transcript:Zm00001d024406_T003 rna21906 5.00E-123
207- 21: transcript:Zm00001d024408_T005 rna21907 5.00E-25
207- 22: transcript:Zm00001d024410_T001 rna21910 6.00E-83
207- 23: transcript:Zm00001d024412_T002 rna21915 0
207- 24: transcript:Zm00001d024413_T001 rna21916 4.00E-155
207- 25: transcript:Zm00001d024416_T001 rna21917 0
207- 26: transcript:Zm00001d024418_T004 rna21918 1.00E-163
207- 27: transcript:Zm00001d024420_T007 rna21919 5.00E-94

Alignment 208: score=602.0 e_value=4.1e-37 N=14 10&NC_008401.2 plus
208- 0: transcript:Zm00001d024239_T002 rna21720 2.00E-73
208- 1: transcript:Zm00001d024245_T001 rna21721 8.00E-174
208- 2: transcript:Zm00001d024248_T001 rna21723 0
208- 3: transcript:Zm00001d024251_T001 rna21726 1.00E-178
208- 4: transcript:Zm00001d024265_T001 rna21729 1.00E-23
208- 5: transcript:Zm00001d024275_T013 rna21751 0
208- 6: transcript:Zm00001d024291_T001 rna21771 3.00E-26
208- 7: transcript:Zm00001d024292_T002 rna21787 7.00E-170
208- 8: transcript:Zm00001d024294_T001 rna21791 0
208- 9: transcript:Zm00001d024298_T001 rna21792 8.00E-142
208- 10: transcript:Zm00001d024300_T003 rna21796 3.00E-81
208- 11: transcript:Zm00001d024301_T002 rna21798 0
208- 12: transcript:Zm00001d024305_T001 rna21799 2.00E-24
208- 13: transcript:Zm00001d024307_T007 rna21800 0
Alignment 209: score=493.0 e_value=9.7e-24 N=11 10&NC_008401.2 plus
209- 0: transcript:Zm00001d024066_T001 rna21983 5.00E-115
209- 1: transcript:Zm00001d024068_T001 rna21984 0
209- 2: transcript:Zm00001d024082_T001 rna21995 2.00E-47
209- 3: transcript:Zm00001d024094_T003 rna21999 0
209- 4: transcript:Zm00001d024102_T002 rna22005 2.00E-41
209- 5: transcript:Zm00001d024105_T001 rna22006 2.00E-14
209- 6: transcript:Zm00001d024126_T001 rna22014 1.00E-82
209- 7: transcript:Zm00001d024128_T003 rna22024 7.00E-127
209- 8: transcript:Zm00001d024134_T001 rna22026 0
209- 9: transcript:Zm00001d024141_T001 rna22027 2.00E-46
209- 10: transcript:Zm00001d024148_T001 rna22028 0
Alignment 210: score=329.0 e_value=1.8e-11 N=7 10&NC_008401.2 plus
210- 0: transcript:Zm00001d024621_T001 rna21515 8.00E-112
210- 1: transcript:Zm00001d024624_T004 rna21516 0
210- 2: transcript:Zm00001d024627_T002 rna21517 2.00E-97
210- 3: transcript:Zm00001d024630_T002 rna21518 6.00E-123
210- 4: transcript:Zm00001d024631_T001 rna21521 8.00E-90
210- 5: transcript:Zm00001d024632_T002 rna21522 0
210- 6: transcript:Zm00001d024633_T001 rna21523 0
Alignment 211: score=321.0 e_value=8.8e-14 N=7 10&NC_008401.2 plus
211- 0: transcript:Zm00001d024462_T006 rna21628 1.00E-41
211- 1: transcript:Zm00001d024463_T009 rna21629 0
211- 2: transcript:Zm00001d024464_T002 rna21630 3.00E-136
211- 3: transcript:Zm00001d024466_T001 rna21636 0
211- 4: transcript:Zm00001d024467_T004 rna21637 6.00E-144
211- 5: transcript:Zm00001d024468_T003 rna21638 3.00E-15
211- 6: transcript:Zm00001d024470_T003 rna21639 0
Alignment 212: score=943.0 e_value=6.4e-66 N=21 10&NC_008401.2 minus
212- 0: transcript:Zm00001d024633_T001 rna21523 0
212- 1: transcript:Zm00001d024640_T003 rna21504 0
212- 2: transcript:Zm00001d024644_T001 rna21499 2.00E-138
212- 3: transcript:Zm00001d024645_T001 rna21496 0
212- 4: transcript:Zm00001d024646_T001 rna21494 0
212- 5: transcript:Zm00001d024647_T001 rna21493 0
212- 6: transcript:Zm00001d024660_T001 rna21490 2.00E-14
212- 7: transcript:Zm00001d024661_T001 rna21487 9.00E-120
212- 8: transcript:Zm00001d024664_T001 rna21483 0
212- 9: transcript:Zm00001d024674_T001 rna21479 3.00E-100

212- 10: transcript:Zm00001d024675_T001 rna21478 2.00E-75
212- 11: transcript:Zm00001d024676_T001 rna21477 1.00E-24
212- 12: transcript:Zm00001d024678_T001 rna21476 7.00E-27
212- 13: transcript:Zm00001d024679_T002 rna21474 7.00E-144
212- 14: transcript:Zm00001d024691_T001 rna21469 8.00E-62
212- 15: transcript:Zm00001d024698_T002 rna21465 0
212- 16: transcript:Zm00001d024701_T001 rna21458 1.00E-78
212- 17: transcript:Zm00001d024702_T001 rna21457 0
212- 18: transcript:Zm00001d024703_T010 rna21456 0
212- 19: transcript:Zm00001d024705_T001 rna21452 3.00E-32
212- 20: transcript:Zm00001d024708_T001 rna21450 0
Alignment 213: score=765.0 e_value=4.9e-42 N=16 10&NC_008401.2 minus
213- 0: transcript:Zm00001d024525_T001 rna21585 2.00E-31
213- 1: transcript:Zm00001d024527_T001 rna21584 4.00E-95
213- 2: transcript:Zm00001d024528_T001 rna21583 0
213- 3: transcript:Zm00001d024530_T007 rna21581 2.00E-169
213- 4: transcript:Zm00001d024531_T001 rna21579 0
213- 5: transcript:Zm00001d024532_T001 rna21577 4.00E-131
213- 6: transcript:Zm00001d024533_T001 rna21575 0
213- 7: transcript:Zm00001d024534_T001 rna21574 0
213- 8: transcript:Zm00001d024537_T001 rna21573 6.00E-119
213- 9: transcript:Zm00001d024538_T001 rna21569 8.00E-125
213- 10: transcript:Zm00001d024539_T001 rna21568 0
213- 11: transcript:Zm00001d024540_T001 rna21565 2.00E-172
213- 12: transcript:Zm00001d024541_T001 rna21564 2.00E-163
213- 13: transcript:Zm00001d024543_T001 rna21563 0
213- 14: transcript:Zm00001d024544_T002 rna21562 2.00E-142
213- 15: transcript:Zm00001d024546_T001 rna21561 0
Alignment 214: score=691.0 e_value=2.1e-38 N=15 10&NC_008401.2 minus
214- 0: transcript:Zm00001d024567_T004 rna21557 0
214- 1: transcript:Zm00001d024568_T001 rna21556 0
214- 2: transcript:Zm00001d024571_T001 rna21554 6.00E-79
214- 3: transcript:Zm00001d024572_T002 rna21552 0
214- 4: transcript:Zm00001d024583_T003 rna21546 8.00E-15
214- 5: transcript:Zm00001d024587_T001 rna21545 0
214- 6: transcript:Zm00001d024588_T001 rna21544 2.00E-132
214- 7: transcript:Zm00001d024591_T001 rna21541 2.00E-23
214- 8: transcript:Zm00001d024594_T001 rna21538 0
214- 9: transcript:Zm00001d024596_T002 rna21536 2.00E-129
214- 10: transcript:Zm00001d024597_T001 rna21534 2.00E-107
214- 11: transcript:Zm00001d024601_T003 rna21529 0
214- 12: transcript:Zm00001d024602_T001 rna21528 6.00E-35
214- 13: transcript:Zm00001d024605_T001 rna21526 8.00E-179
214- 14: transcript:Zm00001d024606_T001 rna21525 3.00E-128
Alignment 215: score=492.0 e_value=8.3e-30 N=12 10&NC_008401.2 minus
215- 0: transcript:Zm00001d024468_T003 rna21638 3.00E-15
215- 1: transcript:Zm00001d024476_T001 rna21622 1.00E-146
215- 2: transcript:Zm00001d024479_T001 rna21619 7.00E-29
215- 3: transcript:Zm00001d024489_T001 rna21615 0
215- 4: transcript:Zm00001d024497_T001 rna21614 5.00E-152
215- 5: transcript:Zm00001d024500_T001 rna21599 2.00E-64
215- 6: transcript:Zm00001d024507_T001 rna21597 0
215- 7: transcript:Zm00001d024509_T002 rna21596 1.00E-33
215- 8: transcript:Zm00001d024518_T001 rna21594 4.00E-49

215- 9: transcript:Zm00001d024519_T005 rna21592 2.00E-149
215- 10: transcript:Zm00001d024520_T001 rna21589 1.00E-70
215- 11: transcript:Zm00001d024523_T003 rna21586 0
Alignment 216: score=337.0 e_value=1.3e-17 N=8 10&NC_008401.2 minus
216- 0: transcript:Zm00001d024421_T001 rna21662 7.00E-162
216- 1: transcript:Zm00001d024423_T002 rna21657 0
216- 2: transcript:Zm00001d024425_T003 rna21656 0
216- 3: transcript:Zm00001d024429_T001 rna21655 0
216- 4: transcript:Zm00001d024430_T002 rna21652 0
216- 5: transcript:Zm00001d024432_T001 rna21648 3.00E-85
216- 6: transcript:Zm00001d024436_T001 rna21647 1.00E-37
216- 7: transcript:Zm00001d024462_T006 rna21628 1.00E-41
Alignment 217: score=361.0 e_value=7e-17 N=8 10&NC_008403.2 plus
217- 0: transcript:Zm00001d025861_T001 rna26019 1.00E-84
217- 1: transcript:Zm00001d025863_T001 rna26022 6.00E-81
217- 2: transcript:Zm00001d025864_T001 rna26025 2.00E-74
217- 3: transcript:Zm00001d025868_T002 rna26033 6.00E-111
217- 4: transcript:Zm00001d025869_T003 rna26035 2.00E-167
217- 5: transcript:Zm00001d025871_T001 rna26037 1.00E-100
217- 6: transcript:Zm00001d025872_T001 rna26040 3.00E-22
217- 7: transcript:Zm00001d025885_T001 rna26053 0
Alignment 218: score=1203.0 e_value=1.4e-89 N=27 10&NC_008404.2 plus
218- 0: transcript:Zm00001d023372_T001 rna26944 7.00E-47
218- 1: transcript:Zm00001d023376_T002 rna26947 1.00E-156
218- 2: transcript:Zm00001d023377_T001 rna26948 7.00E-83
218- 3: transcript:Zm00001d023378_T001 rna26949 4.00E-78
218- 4: transcript:Zm00001d023379_T001 rna26952 0
218- 5: transcript:Zm00001d023391_T001 rna26954 0
218- 6: transcript:Zm00001d023394_T001 rna26960 0
218- 7: transcript:Zm00001d023395_T001 rna26963 4.00E-47
218- 8: transcript:Zm00001d023396_T001 rna26964 9.00E-28
218- 9: transcript:Zm00001d023400_T002 rna26972 0
218- 10: transcript:Zm00001d023404_T001 rna26975 8.00E-175
218- 11: transcript:Zm00001d023420_T001 rna26978 9.00E-119
218- 12: transcript:Zm00001d023424_T001 rna26980 2.00E-165
218- 13: transcript:Zm00001d023431_T002 rna26987 4.00E-93
218- 14: transcript:Zm00001d023439_T001 rna26990 0
218- 15: transcript:Zm00001d023440_T001 rna26992 2.00E-48
218- 16: transcript:Zm00001d023443_T001 rna26994 3.00E-46
218- 17: transcript:Zm00001d023445_T001 rna26995 4.00E-31
218- 18: transcript:Zm00001d023450_T001 rna26997 0
218- 19: transcript:Zm00001d023455_T001 rna27003 1.00E-55
218- 20: transcript:Zm00001d023456_T001 rna27004 1.00E-31
218- 21: transcript:Zm00001d023459_T001 rna27005 3.00E-95
218- 22: transcript:Zm00001d023461_T001 rna27006 1.00E-33
218- 23: transcript:Zm00001d023468_T001 rna27010 1.00E-124
218- 24: transcript:Zm00001d023469_T001 rna27012 0
218- 25: transcript:Zm00001d023479_T006 rna27018 0
218- 26: transcript:Zm00001d023492_T004 rna27024 8.00E-39
Alignment 219: score=862.0 e_value=4.5e-71 N=22 10&NC_008404.2 plus
219- 0: transcript:Zm00001d023507_T001 rna27027 2.00E-97
219- 1: transcript:Zm00001d023514_T003 rna27048 4.00E-120
219- 2: transcript:Zm00001d023521_T003 rna27051 2.00E-36
219- 3: transcript:Zm00001d023529_T001 rna27053 4.00E-28

219- 4: transcript:Zm00001d023535_T001 rna27058 2.00E-24
219- 5: transcript:Zm00001d023538_T006 rna27065 0
219- 6: transcript:Zm00001d023542_T001 rna27066 9.00E-111
219- 7: transcript:Zm00001d023543_T002 rna27067 0
219- 8: transcript:Zm00001d023544_T011 rna27069 0
219- 9: transcript:Zm00001d023559_T001 rna27074 0
219- 10: transcript:Zm00001d023560_T002 rna27076 4.00E-131
219- 11: transcript:Zm00001d023565_T001 rna27098 4.00E-64
219- 12: transcript:Zm00001d023566_T001 rna27104 0
219- 13: transcript:Zm00001d023570_T001 rna27107 2.00E-52
219- 14: transcript:Zm00001d023576_T002 rna27118 0
219- 15: transcript:Zm00001d023579_T002 rna27134 1.00E-179
219- 16: transcript:Zm00001d023580_T001 rna27141 0
219- 17: transcript:Zm00001d023581_T001 rna27146 0
219- 18: transcript:Zm00001d023587_T002 rna27164 2.00E-43
219- 19: transcript:Zm00001d023588_T001 rna27165 0
219- 20: transcript:Zm00001d023603_T001 rna27168 0
219- 21: transcript:Zm00001d023605_T001 rna27169 3.00E-27
Alignment 220: score=309.0 e_value=5.5e-13 N=7 10&NC_008404.2 plus
220- 0: transcript:Zm00001d023220_T001 rna26711 0
220- 1: transcript:Zm00001d023222_T024 rna26713 0
220- 2: transcript:Zm00001d023223_T001 rna26714 1.00E-35
220- 3: transcript:Zm00001d023225_T001 rna26717 0
220- 4: transcript:Zm00001d023227_T001 rna26732 0
220- 5: transcript:Zm00001d023229_T001 rna26734 0
220- 6: transcript:Zm00001d023230_T006 rna26736 3.00E-57
Alignment 221: score=282.0 e_value=7.6e-13 N=7 10&NC_008404.2 plus
221- 0: transcript:Zm00001d023945_T003 rna27364 0
221- 1: transcript:Zm00001d023950_T001 rna27365 5.00E-74
221- 2: transcript:Zm00001d023957_T005 rna27372 3.00E-47
221- 3: transcript:Zm00001d023962_T007 rna27374 4.00E-126
221- 4: transcript:Zm00001d023968_T005 rna27376 5.00E-20
221- 5: transcript:Zm00001d023987_T001 rna27394 1.00E-78
221- 6: transcript:Zm00001d023990_T001 rna27410 4.00E-162
Alignment 222: score=774.0 e_value=1.1e-46 N=17 10&NC_008404.2 minus
222- 0: transcript:Zm00001d023266_T001 rna26858 1.00E-62
222- 1: transcript:Zm00001d023269_T001 rna26849 2.00E-17
222- 2: transcript:Zm00001d023270_T001 rna26845 4.00E-93
222- 3: transcript:Zm00001d023271_T003 rna26844 5.00E-175
222- 4: transcript:Zm00001d023272_T009 rna26842 1.00E-112
222- 5: transcript:Zm00001d023277_T001 rna26834 2.00E-85
222- 6: transcript:Zm00001d023278_T002 rna26833 0
222- 7: transcript:Zm00001d023279_T001 rna26832 5.00E-23
222- 8: transcript:Zm00001d023282_T001 rna26830 5.00E-93
222- 9: transcript:Zm00001d023283_T001 rna26829 0
222- 10: transcript:Zm00001d023286_T001 rna26828 1.00E-32
222- 11: transcript:Zm00001d023291_T001 rna26827 5.00E-107
222- 12: transcript:Zm00001d023293_T001 rna26826 1.00E-93
222- 13: transcript:Zm00001d023294_T001 rna26821 8.00E-83
222- 14: transcript:Zm00001d023298_T001 rna26816 1.00E-72
222- 15: transcript:Zm00001d023300_T002 rna26814 4.00E-106
222- 16: transcript:Zm00001d023301_T002 rna26813 0
Alignment 223: score=547.0 e_value=1.1e-29 N=12 10&NC_008404.2 minus
223- 0: transcript:Zm00001d023238_T002 rna26940 0

223- 1: transcript:Zm00001d023239_T001 rna26938 0
223- 2: transcript:Zm00001d023241_T001 rna26937 1.00E-47
223- 3: transcript:Zm00001d023242_T001 rna26920 2.00E-48
223- 4: transcript:Zm00001d023243_T002 rna26918 2.00E-42
223- 5: transcript:Zm00001d023246_T001 rna26915 0
223- 6: transcript:Zm00001d023247_T001 rna26913 1.00E-70
223- 7: transcript:Zm00001d023249_T001 rna26909 7.00E-69
223- 8: transcript:Zm00001d023258_T002 rna26905 2.00E-66
223- 9: transcript:Zm00001d023259_T001 rna26903 1.00E-84
223- 10: transcript:Zm00001d023260_T001 rna26899 2.00E-85
223- 11: transcript:Zm00001d023261_T001 rna26898 0
Alignment 224: score=383.0 e_value=2.1e-17 N=9 10&NC_008404.2 minus
224- 0: transcript:Zm00001d023336_T001 rna26779 2.00E-86
224- 1: transcript:Zm00001d023340_T001 rna26775 9.00E-108
224- 2: transcript:Zm00001d023343_T002 rna26766 4.00E-21
224- 3: transcript:Zm00001d023347_T001 rna26761 4.00E-80
224- 4: transcript:Zm00001d023353_T001 rna26760 0
224- 5: transcript:Zm00001d023366_T001 rna26759 2.00E-44
224- 6: transcript:Zm00001d023367_T002 rna26758 0
224- 7: transcript:Zm00001d023368_T001 rna26755 3.00E-18
224- 8: transcript:Zm00001d023371_T001 rna26754 1.00E-36
Alignment 225: score=1405.0 e_value=8.3e-106 N=31 10&NC_008405.2 plus
225- 0: transcript:Zm00001d023372_T001 rna28790 1.00E-42
225- 1: transcript:Zm00001d023376_T002 rna28792 8.00E-151
225- 2: transcript:Zm00001d023378_T001 rna28794 0
225- 3: transcript:Zm00001d023379_T001 rna28798 0
225- 4: transcript:Zm00001d023390_T001 rna28801 1.00E-58
225- 5: transcript:Zm00001d023398_T001 rna28813 5.00E-174
225- 6: transcript:Zm00001d023400_T002 rna28818 0
225- 7: transcript:Zm00001d023404_T001 rna28820 8.00E-90
225- 8: transcript:Zm00001d023420_T001 rna28824 2.00E-117
225- 9: transcript:Zm00001d023422_T001 rna28828 0
225- 10: transcript:Zm00001d023423_T002 rna28829 1.00E-150
225- 11: transcript:Zm00001d023424_T001 rna28831 7.00E-167
225- 12: transcript:Zm00001d023434_T002 rna28844 8.00E-88
225- 13: transcript:Zm00001d023440_T001 rna28847 8.00E-47
225- 14: transcript:Zm00001d023443_T001 rna28849 2.00E-38
225- 15: transcript:Zm00001d023445_T001 rna28850 0
225- 16: transcript:Zm00001d023446_T001 rna28852 6.00E-55
225- 17: transcript:Zm00001d023452_T001 rna28854 0
225- 18: transcript:Zm00001d023453_T001 rna28855 9.00E-32
225- 19: transcript:Zm00001d023455_T001 rna28856 2.00E-53
225- 20: transcript:Zm00001d023456_T001 rna28857 9.00E-45
225- 21: transcript:Zm00001d023459_T001 rna28858 2.00E-94
225- 22: transcript:Zm00001d023461_T001 rna28859 7.00E-50
225- 23: transcript:Zm00001d023465_T002 rna28860 0
225- 24: transcript:Zm00001d023468_T001 rna28865 2.00E-135
225- 25: transcript:Zm00001d023469_T001 rna28866 0
225- 26: transcript:Zm00001d023477_T001 rna28867 7.00E-10
225- 27: transcript:Zm00001d023478_T001 rna28868 8.00E-21
225- 28: transcript:Zm00001d023479_T006 rna28870 0
225- 29: transcript:Zm00001d023480_T001 rna28873 5.00E-57
225- 30: transcript:Zm00001d023492_T004 rna28875 2.00E-73
Alignment 226: score=1242.0 e_value=1.7e-92 N=28 10&NC_008405.2 plus

226- 0: transcript:Zm00001d023525_T001 rna28888 0
226- 1: transcript:Zm00001d023530_T011 rna28907 3.00E-11
226- 2: transcript:Zm00001d023534_T002 rna28909 0
226- 3: transcript:Zm00001d023535_T001 rna28910 3.00E-45
226- 4: transcript:Zm00001d023537_T002 rna28912 0
226- 5: transcript:Zm00001d023538_T006 rna28914 0
226- 6: transcript:Zm00001d023539_T001 rna28915 9.00E-71
226- 7: transcript:Zm00001d023542_T001 rna28916 5.00E-111
226- 8: transcript:Zm00001d023543_T002 rna28917 1.00E-46
226- 9: transcript:Zm00001d023544_T011 rna28918 0
226- 10: transcript:Zm00001d023558_T001 rna28926 1.00E-154
226- 11: transcript:Zm00001d023564_T002 rna28931 0
226- 12: transcript:Zm00001d023565_T001 rna28939 7.00E-123
226- 13: transcript:Zm00001d023566_T001 rna28943 0
226- 14: transcript:Zm00001d023568_T001 rna28944 1.00E-09
226- 15: transcript:Zm00001d023569_T001 rna28945 6.00E-15
226- 16: transcript:Zm00001d023570_T001 rna28946 2.00E-85
226- 17: transcript:Zm00001d023576_T002 rna28950 0
226- 18: transcript:Zm00001d023578_T001 rna28956 0
226- 19: transcript:Zm00001d023579_T002 rna28960 0
226- 20: transcript:Zm00001d023580_T001 rna28962 9.00E-113
226- 21: transcript:Zm00001d023582_T001 rna28963 0
226- 22: transcript:Zm00001d023583_T005 rna28967 0
226- 23: transcript:Zm00001d023585_T001 rna28971 0
226- 24: transcript:Zm00001d023587_T002 rna28974 2.00E-43
226- 25: transcript:Zm00001d023603_T001 rna28980 0
226- 26: transcript:Zm00001d023606_T005 rna28983 0
226- 27: transcript:Zm00001d023608_T001 rna28984 0
Alignment 227: score=476.0 e_value=2.5e-30 N=11 10&NC_008405.2 plus
227- 0: transcript:Zm00001d024003_T005 rna29148 0
227- 1: transcript:Zm00001d024004_T001 rna29150 9.00E-62
227- 2: transcript:Zm00001d024030_T001 rna29154 5.00E-42
227- 3: transcript:Zm00001d024034_T001 rna29156 5.00E-89
227- 4: transcript:Zm00001d024036_T005 rna29158 2.00E-120
227- 5: transcript:Zm00001d024041_T002 rna29160 9.00E-151
227- 6: transcript:Zm00001d024042_T001 rna29161 1.00E-24
227- 7: transcript:Zm00001d024047_T001 rna29164 2.00E-112
227- 8: transcript:Zm00001d024048_T004 rna29165 0
227- 9: transcript:Zm00001d024049_T013 rna29166 2.00E-113
227- 10: transcript:Zm00001d024051_T001 rna29169 1.00E-156
Alignment 228: score=355.0 e_value=5.5e-16 N=8 10&NC_008405.2 plus
228- 0: transcript:Zm00001d023945_T003 rna29050 2.00E-108
228- 1: transcript:Zm00001d023946_T001 rna29051 3.00E-13
228- 2: transcript:Zm00001d023950_T001 rna29055 9.00E-143
228- 3: transcript:Zm00001d023953_T001 rna29060 5.00E-26
228- 4: transcript:Zm00001d023955_T003 rna29062 3.00E-127
228- 5: transcript:Zm00001d023957_T005 rna29078 0
228- 6: transcript:Zm00001d023968_T005 rna29082 2.00E-132
228- 7: transcript:Zm00001d023969_T002 rna29089 0
Alignment 229: score=324.0 e_value=6.3e-11 N=7 10&NC_008405.2 plus
229- 0: transcript:Zm00001d023899_T001 rna29720 9.00E-165
229- 1: transcript:Zm00001d023901_T001 rna29722 3.00E-83
229- 2: transcript:Zm00001d023903_T001 rna29723 9.00E-49
229- 3: transcript:Zm00001d023908_T001 rna29724 4.00E-112

229- 4: transcript:Zm00001d023910_T001 rna29728 0
229- 5: transcript:Zm00001d023912_T001 rna29731 9.00E-98
229- 6: transcript:Zm00001d023915_T001 rna29732 2.00E-126
Alignment 230: score=302.0 e_value=1e-13 N=7 10&NC_008405.2 plus
230- 0: transcript:Zm00001d023220_T001 rna28546 0
230- 1: transcript:Zm00001d023222_T024 rna28550 0
230- 2: transcript:Zm00001d023223_T001 rna28551 8.00E-136
230- 3: transcript:Zm00001d023225_T001 rna28554 0
230- 4: transcript:Zm00001d023227_T001 rna28576 5.00E-22
230- 5: transcript:Zm00001d023229_T001 rna28577 0
230- 6: transcript:Zm00001d023230_T006 rna28579 0
Alignment 231: score=270.0 e_value=2.7e-08 N=6 10&NC_008405.2 plus
231- 0: transcript:Zm00001d023625_T001 rna29269 9.00E-112
231- 1: transcript:Zm00001d023629_T002 rna29278 0
231- 2: transcript:Zm00001d023630_T005 rna29287 4.00E-63
231- 3: transcript:Zm00001d023634_T001 rna29301 4.00E-71
231- 4: transcript:Zm00001d023635_T001 rna29304 4.00E-170
231- 5: transcript:Zm00001d023636_T001 rna29305 0
Alignment 232: score=913.0 e_value=1.3e-66 N=21 10&NC_008405.2 minus
232- 0: transcript:Zm00001d023859_T001 rna29774 1.00E-22
232- 1: transcript:Zm00001d023863_T001 rna29770 8.00E-148
232- 2: transcript:Zm00001d023865_T001 rna29767 5.00E-58
232- 3: transcript:Zm00001d023867_T005 rna29762 0
232- 4: transcript:Zm00001d023877_T001 rna29752 1.00E-58
232- 5: transcript:Zm00001d023881_T007 rna29751 3.00E-72
232- 6: transcript:Zm00001d023885_T002 rna29749 0
232- 7: transcript:Zm00001d023887_T001 rna29743 1.00E-69
232- 8: transcript:Zm00001d023888_T001 rna29742 5.00E-61
232- 9: transcript:Zm00001d023892_T001 rna29741 9.00E-90
232- 10: transcript:Zm00001d023895_T002 rna29739 1.00E-109
232- 11: transcript:Zm00001d023896_T003 rna29737 7.00E-55
232- 12: transcript:Zm00001d023908_T001 rna29724 4.00E-112
232- 13: transcript:Zm00001d023914_T003 rna29717 0
232- 14: transcript:Zm00001d023922_T004 rna29681 0
232- 15: transcript:Zm00001d023927_T001 rna29680 0
232- 16: transcript:Zm00001d023929_T001 rna29679 0
232- 17: transcript:Zm00001d023931_T001 rna29678 8.00E-51
232- 18: transcript:Zm00001d023934_T001 rna29677 2.00E-66
232- 19: transcript:Zm00001d023936_T001 rna29673 5.00E-24
232- 20: transcript:Zm00001d023939_T001 rna29671 0
Alignment 233: score=807.0 e_value=3.4e-51 N=18 10&NC_008405.2 minus
233- 0: transcript:Zm00001d023264_T004 rna28712 2.00E-33
233- 1: transcript:Zm00001d023266_T001 rna28701 6.00E-57
233- 2: transcript:Zm00001d023269_T001 rna28692 1.00E-17
233- 3: transcript:Zm00001d023270_T001 rna28687 5.00E-96
233- 4: transcript:Zm00001d023271_T003 rna28686 3.00E-174
233- 5: transcript:Zm00001d023272_T009 rna28685 0
233- 6: transcript:Zm00001d023277_T001 rna28677 1.00E-43
233- 7: transcript:Zm00001d023278_T002 rna28676 0
233- 8: transcript:Zm00001d023279_T001 rna28675 0
233- 9: transcript:Zm00001d023282_T001 rna28673 4.00E-93
233- 10: transcript:Zm00001d023283_T001 rna28671 0
233- 11: transcript:Zm00001d023286_T001 rna28670 1.00E-32
233- 12: transcript:Zm00001d023291_T001 rna28669 0

233- 13: transcript:Zm00001d023293_T001 rna28667 1.00E-93
233- 14: transcript:Zm00001d023294_T001 rna28662 5.00E-83
233- 15: transcript:Zm00001d023298_T001 rna28660 6.00E-92
233- 16: transcript:Zm00001d023300_T002 rna28658 2.00E-104
233- 17: transcript:Zm00001d023301_T002 rna28657 2.00E-08
Alignment 234: score=597.0 e_value=2.1e-34 N=13 10&NC_008405.2 minus
234- 0: transcript:Zm00001d023646_T002 rna29574 0
234- 1: transcript:Zm00001d023648_T003 rna29572 8.00E-179
234- 2: transcript:Zm00001d023650_T002 rna29558 7.00E-74
234- 3: transcript:Zm00001d023652_T002 rna29554 0
234- 4: transcript:Zm00001d023653_T001 rna29553 9.00E-68
234- 5: transcript:Zm00001d023654_T001 rna29552 2.00E-92
234- 6: transcript:Zm00001d023658_T002 rna29544 0
234- 7: transcript:Zm00001d023659_T007 rna29542 0
234- 8: transcript:Zm00001d023661_T001 rna29540 2.00E-06
234- 9: transcript:Zm00001d023664_T001 rna29536 1.00E-76
234- 10: transcript:Zm00001d023671_T001 rna29534 8.00E-25
234- 11: transcript:Zm00001d023673_T001 rna29529 6.00E-61
234- 12: transcript:Zm00001d023681_T001 rna29526 7.00E-58
Alignment 235: score=555.0 e_value=7.4e-34 N=13 10&NC_008405.2 minus
235- 0: transcript:Zm00001d023808_T001 rna29832 3.00E-48
235- 1: transcript:Zm00001d023810_T002 rna29830 0
235- 2: transcript:Zm00001d023811_T001 rna29824 1.00E-52
235- 3: transcript:Zm00001d023813_T001 rna29823 0
235- 4: transcript:Zm00001d023815_T001 rna29822 0
235- 5: transcript:Zm00001d023824_T002 rna29818 2.00E-39
235- 6: transcript:Zm00001d023825_T005 rna29810 0
235- 7: transcript:Zm00001d023830_T001 rna29808 2.00E-78
235- 8: transcript:Zm00001d023833_T001 rna29794 1.00E-108
235- 9: transcript:Zm00001d023838_T014 rna29793 0
235- 10: transcript:Zm00001d023839_T001 rna29792 0
235- 11: transcript:Zm00001d023843_T002 rna29791 2.00E-123
235- 12: transcript:Zm00001d023856_T001 rna29778 2.00E-33
Alignment 236: score=446.0 e_value=2.5e-24 N=10 10&NC_008405.2 minus
236- 0: transcript:Zm00001d023238_T002 rna28787 0
236- 1: transcript:Zm00001d023239_T001 rna28784 0
236- 2: transcript:Zm00001d023241_T001 rna28783 3.00E-49
236- 3: transcript:Zm00001d023242_T001 rna28762 8.00E-50
236- 4: transcript:Zm00001d023243_T002 rna28760 0
236- 5: transcript:Zm00001d023246_T001 rna28757 0
236- 6: transcript:Zm00001d023258_T002 rna28748 0
236- 7: transcript:Zm00001d023259_T001 rna28747 3.00E-39
236- 8: transcript:Zm00001d023260_T001 rna28744 1.00E-170
236- 9: transcript:Zm00001d023261_T001 rna28743 0
Alignment 237: score=406.0 e_value=3.9e-18 N=9 10&NC_008405.2 minus
237- 0: transcript:Zm00001d023720_T001 rna29618 0
237- 1: transcript:Zm00001d023728_T001 rna29617 4.00E-180
237- 2: transcript:Zm00001d023729_T005 rna29615 0
237- 3: transcript:Zm00001d023732_T002 rna29613 0
237- 4: transcript:Zm00001d023734_T003 rna29612 2.00E-58
237- 5: transcript:Zm00001d023735_T001 rna29610 2.00E-27
237- 6: transcript:Zm00001d023736_T001 rna29608 1.00E-71
237- 7: transcript:Zm00001d023737_T001 rna29607 4.00E-89
237- 8: transcript:Zm00001d023738_T001 rna29604 0

Alignment 238: score=385.0 e_value=3.9e-17 N=9 10&NC_008405.2 minus
238- 0: transcript:Zm00001d023336_T001 rna28619 2.00E-71
238- 1: transcript:Zm00001d023340_T001 rna28615 2.00E-107
238- 2: transcript:Zm00001d023343_T002 rna28607 7.00E-22
238- 3: transcript:Zm00001d023347_T001 rna28602 1.00E-81
238- 4: transcript:Zm00001d023353_T001 rna28599 0
238- 5: transcript:Zm00001d023366_T001 rna28598 1.00E-42
238- 6: transcript:Zm00001d023367_T002 rna28597 0
238- 7: transcript:Zm00001d023368_T001 rna28595 0
238- 8: transcript:Zm00001d023371_T001 rna28594 4.00E-103
Alignment 239: score=325.0 e_value=1.3e-13 N=7 10&NC_008405.2 minus
239- 0: transcript:Zm00001d023753_T001 rna29599 8.00E-41
239- 1: transcript:Zm00001d023756_T001 rna29598 1.00E-64
239- 2: transcript:Zm00001d023757_T001 rna29595 9.00E-45
239- 3: transcript:Zm00001d023760_T001 rna29594 1.00E-123
239- 4: transcript:Zm00001d023762_T001 rna29593 2.00E-23
239- 5: transcript:Zm00001d023767_T001 rna29589 1.00E-73
239- 6: transcript:Zm00001d023781_T001 rna29586 0
Alignment 240: score=270.0 e_value=3.1e-11 N=7 2&NC_008394.4 minus
240- 0: transcript:Zm00001d003745_T001 rna3520 2.00E-83
240- 1: transcript:Zm00001d003762_T002 rna3509 1.00E-26
240- 2: transcript:Zm00001d003763_T001 rna3508 2.00E-13
240- 3: transcript:Zm00001d003775_T001 rna3502 3.00E-19
240- 4: transcript:Zm00001d003784_T001 rna3488 1.00E-150
240- 5: transcript:Zm00001d003797_T001 rna3486 4.00E-46
240- 6: transcript:Zm00001d003800_T011 rna3477 8.00E-108
Alignment 241: score=927.0 e_value=8.2e-69 N=23 2&NC_008395.2 minus
241- 0: transcript:Zm00001d003432_T001 rna6112 2.00E-151
241- 1: transcript:Zm00001d003438_T001 rna6111 0
241- 2: transcript:Zm00001d003447_T001 rna6106 2.00E-10
241- 3: transcript:Zm00001d003451_T001 rna6104 3.00E-16
241- 4: transcript:Zm00001d003464_T001 rna6102 5.00E-152
241- 5: transcript:Zm00001d003468_T001 rna6098 2.00E-140
241- 6: transcript:Zm00001d003472_T002 rna6097 3.00E-17
241- 7: transcript:Zm00001d003477_T001 rna6095 0
241- 8: transcript:Zm00001d003492_T001 rna6092 7.00E-111
241- 9: transcript:Zm00001d003497_T001 rna6090 1.00E-47
241- 10: transcript:Zm00001d003499_T001 rna6089 8.00E-89
241- 11: transcript:Zm00001d003509_T001 rna6073 0
241- 12: transcript:Zm00001d003518_T003 rna6068 5.00E-112
241- 13: transcript:Zm00001d003520_T001 rna6066 1.00E-175
241- 14: transcript:Zm00001d003534_T001 rna6060 8.00E-118
241- 15: transcript:Zm00001d003545_T001 rna6058 1.00E-07
241- 16: transcript:Zm00001d003549_T001 rna6056 3.00E-103
241- 17: transcript:Zm00001d003572_T001 rna6041 0
241- 18: transcript:Zm00001d003575_T001 rna6039 4.00E-35
241- 19: transcript:Zm00001d003584_T001 rna6037 0
241- 20: transcript:Zm00001d003594_T001 rna6031 2.00E-41
241- 21: transcript:Zm00001d003598_T003 rna6030 8.00E-56
241- 22: transcript:Zm00001d003601_T006 rna6025 0
Alignment 242: score=873.0 e_value=5.6e-52 N=19 2&NC_008395.2 minus
242- 0: transcript:Zm00001d002819_T002 rna6516 5.00E-166
242- 1: transcript:Zm00001d002820_T001 rna6515 3.00E-34
242- 2: transcript:Zm00001d002823_T001 rna6514 0

242- 3: transcript:Zm00001d002824_T001 rna6513 0
242- 4: transcript:Zm00001d002826_T001 rna6512 2.00E-35
242- 5: transcript:Zm00001d002829_T001 rna6510 3.00E-84
242- 6: transcript:Zm00001d002836_T006 rna6508 0
242- 7: transcript:Zm00001d002837_T001 rna6505 4.00E-50
242- 8: transcript:Zm00001d002842_T001 rna6504 2.00E-64
242- 9: transcript:Zm00001d002844_T003 rna6499 3.00E-115
242- 10: transcript:Zm00001d002850_T001 rna6493 1.00E-72
242- 11: transcript:Zm00001d002854_T001 rna6492 8.00E-75
242- 12: transcript:Zm00001d002856_T001 rna6491 8.00E-85
242- 13: transcript:Zm00001d002859_T001 rna6489 0
242- 14: transcript:Zm00001d002860_T002 rna6488 6.00E-23
242- 15: transcript:Zm00001d002865_T002 rna6484 0
242- 16: transcript:Zm00001d002867_T001 rna6483 4.00E-93
242- 17: transcript:Zm00001d002868_T001 rna6481 4.00E-48
242- 18: transcript:Zm00001d002869_T001 rna6480 2.00E-36
Alignment 243: score=840.0 e_value=8.4e-52 N=19 2&NC_008395.2 minus
243- 0: transcript:Zm00001d003742_T001 rna5938 0
243- 1: transcript:Zm00001d003743_T001 rna5936 3.00E-49
243- 2: transcript:Zm00001d003745_T001 rna5935 8.00E-171
243- 3: transcript:Zm00001d003750_T001 rna5934 0
243- 4: transcript:Zm00001d003751_T001 rna5932 5.00E-107
243- 5: transcript:Zm00001d003754_T003 rna5928 7.00E-108
243- 6: transcript:Zm00001d003755_T001 rna5926 1.00E-110
243- 7: transcript:Zm00001d003763_T001 rna5921 4.00E-30
243- 8: transcript:Zm00001d003769_T001 rna5918 4.00E-31
243- 9: transcript:Zm00001d003776_T001 rna5911 0
243- 10: transcript:Zm00001d003777_T001 rna5910 3.00E-20
243- 11: transcript:Zm00001d003780_T001 rna5907 8.00E-13
243- 12: transcript:Zm00001d003781_T002 rna5905 1.00E-110
243- 13: transcript:Zm00001d003784_T001 rna5902 0
243- 14: transcript:Zm00001d003799_T001 rna5901 1.00E-33
243- 15: transcript:Zm00001d003800_T011 rna5899 1.00E-177
243- 16: transcript:Zm00001d003804_T001 rna5898 3.00E-105
243- 17: transcript:Zm00001d003806_T001 rna5891 2.00E-19
243- 18: transcript:Zm00001d003817_T002 rna5879 0
Alignment 244: score=789.0 e_value=7.2e-48 N=18 2&NC_008395.2 minus
244- 0: transcript:Zm00001d002958_T001 rna6470 1.00E-67
244- 1: transcript:Zm00001d002966_T001 rna6456 6.00E-153
244- 2: transcript:Zm00001d002969_T001 rna6454 2.00E-134
244- 3: transcript:Zm00001d002972_T001 rna6451 7.00E-33
244- 4: transcript:Zm00001d002979_T001 rna6448 0
244- 5: transcript:Zm00001d002982_T001 rna6442 2.00E-77
244- 6: transcript:Zm00001d002992_T002 rna6440 3.00E-104
244- 7: transcript:Zm00001d002996_T001 rna6439 6.00E-19
244- 8: transcript:Zm00001d002999_T001 rna6436 6.00E-38
244- 9: transcript:Zm00001d003006_T001 rna6431 0
244- 10: transcript:Zm00001d003009_T001 rna6430 1.00E-33
244- 11: transcript:Zm00001d003011_T001 rna6427 2.00E-54
244- 12: transcript:Zm00001d003012_T001 rna6426 1.00E-110
244- 13: transcript:Zm00001d003015_T001 rna6421 0
244- 14: transcript:Zm00001d003016_T001 rna6420 0
244- 15: transcript:Zm00001d003017_T002 rna6418 0
244- 16: transcript:Zm00001d003021_T001 rna6412 4.00E-149

```
244- 17:  transcript:Zm00001d003025_T001  rna6393            0
## Alignment 245: score=708.0 e_value=1.9e-41 N=16 2&NC_008395.2 minus
245-  0:  transcript:Zm00001d002512_T001  rna6749      4.00E-81
245-  1:  transcript:Zm00001d002514_T001  rna6748     7.00E-118
245-  2:  transcript:Zm00001d002517_T001  rna6746      8.00E-43
245-  3:  transcript:Zm00001d002519_T001  rna6743      2.00E-98
245-  4:  transcript:Zm00001d002520_T001  rna6734      2.00E-17
245-  5:  transcript:Zm00001d002531_T001  rna6730     1.00E-178
245-  6:  transcript:Zm00001d002535_T001  rna6729      2.00E-53
245-  7:  transcript:Zm00001d002540_T002  rna6728            0
245-  8:  transcript:Zm00001d002542_T001  rna6727            0
245-  9:  transcript:Zm00001d002545_T002  rna6726      1.00E-98
245- 10:  transcript:Zm00001d002551_T001  rna6719      1.00E-42
245- 11:  transcript:Zm00001d002562_T001  rna6714     3.00E-130
245- 12:  transcript:Zm00001d002573_T001  rna6708            0
245- 13:  transcript:Zm00001d002576_T001  rna6705      4.00E-76
245- 14:  transcript:Zm00001d002579_T001  rna6704            0
245- 15:  transcript:Zm00001d002580_T004  rna6703            0
## Alignment 246: score=436.0 e_value=2.1e-21 N=10 2&NC_008395.2 minus
246-  0:  transcript:Zm00001d003281_T001  rna6237     8.00E-151
246-  1:  transcript:Zm00001d003287_T001  rna6234      9.00E-77
246-  2:  transcript:Zm00001d003288_T001  rna6233     1.00E-177
246-  3:  transcript:Zm00001d003292_T001  rna6226            0
246-  4:  transcript:Zm00001d003293_T001  rna6225      3.00E-87
246-  5:  transcript:Zm00001d003297_T001  rna6224      3.00E-54
246-  6:  transcript:Zm00001d003300_T001  rna6219      2.00E-66
246-  7:  transcript:Zm00001d003309_T002  rna6215      1.00E-83
246-  8:  transcript:Zm00001d003311_T001  rna6207      1.00E-94
246-  9:  transcript:Zm00001d003322_T002  rna6201      9.00E-21
## Alignment 247: score=430.0 e_value=3.1e-20 N=10 2&NC_008395.2 minus
247-  0:  transcript:Zm00001d002418_T030  rna6822            0
247-  1:  transcript:Zm00001d002422_T004  rna6820     1.00E-157
247-  2:  transcript:Zm00001d002424_T002  rna6818      1.00E-16
247-  3:  transcript:Zm00001d002429_T002  rna6812     6.00E-124
247-  4:  transcript:Zm00001d002432_T001  rna6811     1.00E-125
247-  5:  transcript:Zm00001d002434_T001  rna6808     3.00E-148
247-  6:  transcript:Zm00001d002439_T002  rna6807      1.00E-53
247-  7:  transcript:Zm00001d002450_T001  rna6800     8.00E-111
247-  8:  transcript:Zm00001d002452_T001  rna6795     6.00E-135
247-  9:  transcript:Zm00001d002454_T002  rna6793            0
## Alignment 248: score=397.0 e_value=3.5e-17 N=9 2&NC_008395.2 minus
248-  0:  transcript:Zm00001d002679_T001  rna6642            0
248-  1:  transcript:Zm00001d002687_T001  rna6632            0
248-  2:  transcript:Zm00001d002688_T002  rna6631            0
248-  3:  transcript:Zm00001d002690_T001  rna6630            0
248-  4:  transcript:Zm00001d002693_T001  rna6626            0
248-  5:  transcript:Zm00001d002699_T001  rna6624            0
248-  6:  transcript:Zm00001d002700_T001  rna6623      3.00E-16
248-  7:  transcript:Zm00001d002703_T001  rna6619      2.00E-19
248-  8:  transcript:Zm00001d002704_T002  rna6616     2.00E-114
## Alignment 249: score=392.0 e_value=4.7e-18 N=9 2&NC_008395.2 minus
249-  0:  transcript:Zm00001d002743_T001  rna6588      5.00E-22
249-  1:  transcript:Zm00001d002744_T001  rna6580      5.00E-43
249-  2:  transcript:Zm00001d002756_T001  rna6575      3.00E-18
```

249- 3: transcript:Zm00001d002757_T001 rna6574 1.00E-174
249- 4: transcript:Zm00001d002758_T002 rna6571 1.00E-106
249- 5: transcript:Zm00001d002760_T001 rna6569 6.00E-56
249- 6: transcript:Zm00001d002762_T001 rna6567 7.00E-92
249- 7: transcript:Zm00001d002772_T001 rna6556 6.00E-52
249- 8: transcript:Zm00001d002776_T001 rna6541 0
Alignment 250: score=357.0 e_value=1.5e-13 N=8 2&NC_008395.2 minus
250- 0: transcript:Zm00001d002796_T004 rna6551 3.00E-146
250- 1: transcript:Zm00001d002797_T001 rna6539 0
250- 2: transcript:Zm00001d002799_T001 rna6535 3.00E-64
250- 3: transcript:Zm00001d002801_T001 rna6532 4.00E-54
250- 4: transcript:Zm00001d002802_T001 rna6531 2.00E-153
250- 5: transcript:Zm00001d002806_T001 rna6525 5.00E-90
250- 6: transcript:Zm00001d002811_T002 rna6524 5.00E-110
250- 7: transcript:Zm00001d002817_T004 rna6517 2.00E-100
Alignment 251: score=311.0 e_value=2.6e-12 N=7 2&NC_008395.2 minus
251- 0: transcript:Zm00001d003917_T007 rna5813 1.00E-07
251- 1: transcript:Zm00001d003923_T002 rna5812 0
251- 2: transcript:Zm00001d003924_T002 rna5811 2.00E-59
251- 3: transcript:Zm00001d003925_T001 rna5807 0
251- 4: transcript:Zm00001d003927_T001 rna5806 9.00E-71
251- 5: transcript:Zm00001d003930_T001 rna5800 2.00E-34
251- 6: transcript:Zm00001d003938_T001 rna5798 2.00E-83
Alignment 252: score=310.0 e_value=9.1e-12 N=7 2&NC_008395.2 minus
252- 0: transcript:Zm00001d002476_T001 rna6777 9.00E-106
252- 1: transcript:Zm00001d002478_T001 rna6775 1.00E-82
252- 2: transcript:Zm00001d002488_T001 rna6766 9.00E-25
252- 3: transcript:Zm00001d002489_T001 rna6765 4.00E-124
252- 4: transcript:Zm00001d002491_T001 rna6763 4.00E-124
252- 5: transcript:Zm00001d002498_T001 rna6757 0
252- 6: transcript:Zm00001d002500_T001 rna6754 8.00E-17
Alignment 253: score=300.0 e_value=2.2e-13 N=7 2&NC_008395.2 minus
253- 0: transcript:Zm00001d003833_T001 rna5871 6.00E-76
253- 1: transcript:Zm00001d003840_T001 rna5869 5.00E-85
253- 2: transcript:Zm00001d003846_T001 rna5867 4.00E-42
253- 3: transcript:Zm00001d003847_T001 rna5866 0
253- 4: transcript:Zm00001d003848_T004 rna5864 0
253- 5: transcript:Zm00001d003850_T001 rna5857 2.00E-63
253- 6: transcript:Zm00001d003859_T001 rna5833 0
Alignment 254: score=276.0 e_value=5.9e-08 N=6 2&NC_008395.2 minus
254- 0: transcript:Zm00001d005881_T011 rna7225 3.00E-78
254- 1: transcript:Zm00001d005884_T002 rna7223 3.00E-31
254- 2: transcript:Zm00001d005890_T001 rna7216 9.00E-52
254- 3: transcript:Zm00001d005892_T001 rna7215 1.00E-46
254- 4: transcript:Zm00001d005895_T001 rna7206 1.00E-17
254- 5: transcript:Zm00001d005897_T001 rna7204 6.00E-36
Alignment 255: score=268.0 e_value=6e-10 N=6 2&NC_008395.2 minus
255- 0: transcript:Zm00001d002618_T001 rna6688 4.00E-86
255- 1: transcript:Zm00001d002620_T001 rna6687 8.00E-58
255- 2: transcript:Zm00001d002621_T002 rna6685 7.00E-62
255- 3: transcript:Zm00001d002623_T001 rna6683 0
255- 4: transcript:Zm00001d002631_T003 rna6680 1.00E-164
255- 5: transcript:Zm00001d002639_T001 rna6676 0
Alignment 256: score=262.0 e_value=2.1e-09 N=6 2&NC_008395.2 minus

256- 0: transcript:Zm00001d003068_T001 rna6417 3.00E-154
256- 1: transcript:Zm00001d003069_T001 rna6412 3.00E-152
256- 2: transcript:Zm00001d003079_T001 rna6388 0
256- 3: transcript:Zm00001d003081_T003 rna6384 6.00E-44
256- 4: transcript:Zm00001d003085_T001 rna6377 0
256- 5: transcript:Zm00001d003087_T002 rna6376 0
Alignment 257: score=261.0 e_value=1.3e-09 N=6 2&NC_008395.2 minus
257- 0: transcript:Zm00001d002718_T001 rna6611 0
257- 1: transcript:Zm00001d002720_T001 rna6610 8.00E-08
257- 2: transcript:Zm00001d002721_T001 rna6607 8.00E-36
257- 3: transcript:Zm00001d002731_T001 rna6597 5.00E-78
257- 4: transcript:Zm00001d002732_T001 rna6595 4.00E-93
257- 5: transcript:Zm00001d002734_T001 rna6583 2.00E-171
Alignment 258: score=257.0 e_value=1.4e-09 N=6 2&NC_008395.2 minus
258- 0: transcript:Zm00001d003345_T001 rna6177 2.00E-60
258- 1: transcript:Zm00001d003347_T002 rna6176 6.00E-63
258- 2: transcript:Zm00001d003353_T001 rna6168 1.00E-12
258- 3: transcript:Zm00001d003363_T001 rna6159 1.00E-12
258- 4: transcript:Zm00001d003369_T004 rna6154 5.00E-06
258- 5: transcript:Zm00001d003375_T001 rna6146 4.00E-18
Alignment 259: score=364.0 e_value=6.5e-19 N=9 2&NC_008396.2 plus
259- 0: transcript:Zm00001d006931_T002 rna9388 3.00E-33
259- 1: transcript:Zm00001d006933_T001 rna9394 9.00E-132
259- 2: transcript:Zm00001d006939_T001 rna9403 8.00E-150
259- 3: transcript:Zm00001d006940_T001 rna9405 1.00E-21
259- 4: transcript:Zm00001d006945_T001 rna9406 3.00E-104
259- 5: transcript:Zm00001d006947_T001 rna9412 0
259- 6: transcript:Zm00001d006951_T001 rna9434 4.00E-119
259- 7: transcript:Zm00001d007012_T001 rna9436 4.00E-111
259- 8: transcript:Zm00001d007027_T001 rna9444 5.00E-22
Alignment 260: score=343.0 e_value=2.5e-16 N=8 2&NC_008396.2 plus
260- 0: transcript:Zm00001d007951_T001 rna10708 1.00E-59
260- 1: transcript:Zm00001d007952_T001 rna10711 5.00E-116
260- 2: transcript:Zm00001d007953_T001 rna10717 5.00E-97
260- 3: transcript:Zm00001d007962_T001 rna10723 8.00E-105
260- 4: transcript:Zm00001d007969_T001 rna10744 1.00E-60
260- 5: transcript:Zm00001d007974_T001 rna10750 2.00E-22
260- 6: transcript:Zm00001d007975_T001 rna10755 0
260- 7: transcript:Zm00001d007977_T001 rna10758 8.00E-36
Alignment 261: score=289.0 e_value=1.1e-14 N=8 2&NC_008396.2 plus
261- 0: transcript:Zm00001d005459_T001 rna11199 5.00E-125
261- 1: transcript:Zm00001d005470_T001 rna11214 2.00E-29
261- 2: transcript:Zm00001d005473_T001 rna11220 3.00E-43
261- 3: transcript:Zm00001d005478_T009 rna11221 0
261- 4: transcript:Zm00001d005484_T003 rna11243 0
261- 5: transcript:Zm00001d005485_T001 rna11248 2.00E-179
261- 6: transcript:Zm00001d005495_T001 rna11259 5.00E-141
261- 7: transcript:Zm00001d005501_T001 rna11272 0
Alignment 262: score=266.0 e_value=1.2e-08 N=6 2&NC_008396.2 plus
262- 0: transcript:Zm00001d007839_T001 rna10762 2.00E-15
262- 1: transcript:Zm00001d007840_T001 rna10763 9.00E-13
262- 2: transcript:Zm00001d007842_T002 rna10767 2.00E-92
262- 3: transcript:Zm00001d007845_T001 rna10773 4.00E-161
262- 4: transcript:Zm00001d007848_T001 rna10796 2.00E-161

262- 5: transcript:Zm00001d007850_T001 rna10799   9.00E-98
Alignment 263: score=504.0 e_value=1.8e-27 N=12 2&NC_008396.2 minus
263- 0: transcript:Zm00001d007164_T001 rna9244   0
263- 1: transcript:Zm00001d007168_T001 rna9225   2.00E-20
263- 2: transcript:Zm00001d007169_T001 rna9224   0
263- 3: transcript:Zm00001d007172_T001 rna9219   9.00E-142
263- 4: transcript:Zm00001d007175_T001 rna9207   3.00E-84
263- 5: transcript:Zm00001d007179_T001 rna9201   1.00E-163
263- 6: transcript:Zm00001d007180_T001 rna9192   0
263- 7: transcript:Zm00001d007181_T001 rna9190   1.00E-92
263- 8: transcript:Zm00001d007184_T001 rna9186   0
263- 9: transcript:Zm00001d007186_T004 rna9180   1.00E-17
263- 10: transcript:Zm00001d007187_T002 rna9166   1.00E-31
263- 11: transcript:Zm00001d007188_T001 rna9155   7.00E-169
Alignment 264: score=404.0 e_value=1.6e-18 N=9 2&NC_008396.2 minus
264- 0: transcript:Zm00001d007757_T004 rna11000   0
264- 1: transcript:Zm00001d007760_T001 rna10992   0
264- 2: transcript:Zm00001d007762_T001 rna10987   0
264- 3: transcript:Zm00001d007764_T001 rna10984   2.00E-68
264- 4: transcript:Zm00001d007765_T001 rna10983   6.00E-113
264- 5: transcript:Zm00001d007767_T001 rna10979   1.00E-136
264- 6: transcript:Zm00001d007768_T001 rna10978   5.00E-97
264- 7: transcript:Zm00001d007769_T003 rna10977   3.00E-73
264- 8: transcript:Zm00001d007773_T001 rna10974   6.00E-150
Alignment 265: score=322.0 e_value=5.4e-15 N=8 2&NC_008396.2 minus
265- 0: transcript:Zm00001d006899_T001 rna9495   9.00E-173
265- 1: transcript:Zm00001d006900_T004 rna9493   0
265- 2: transcript:Zm00001d006906_T010 rna9483   2.00E-75
265- 3: transcript:Zm00001d006910_T003 rna9481   2.00E-124
265- 4: transcript:Zm00001d006912_T001 rna9479   6.00E-14
265- 5: transcript:Zm00001d006913_T002 rna9474   3.00E-136
265- 6: transcript:Zm00001d006915_T001 rna9470   6.00E-13
265- 7: transcript:Zm00001d006924_T001 rna9452   1.00E-59
Alignment 266: score=304.0 e_value=3.1e-15 N=8 2&NC_008396.2 minus
266- 0: transcript:Zm00001d005193_T001 rna11077   1.00E-38
266- 1: transcript:Zm00001d005196_T001 rna11075   3.00E-18
266- 2: transcript:Zm00001d005222_T001 rna11071   5.00E-31
266- 3: transcript:Zm00001d005231_T013 rna11053   5.00E-131
266- 4: transcript:Zm00001d005239_T001 rna11038   7.00E-146
266- 5: transcript:Zm00001d005240_T004 rna11035   0
266- 6: transcript:Zm00001d005244_T002 rna11032   1.00E-15
266- 7: transcript:Zm00001d005250_T001 rna11024   0
Alignment 267: score=299.0 e_value=2.8e-12 N=7 2&NC_008396.2 minus
267- 0: transcript:Zm00001d007202_T003 rna9054   0
267- 1: transcript:Zm00001d007205_T001 rna9041   0
267- 2: transcript:Zm00001d007206_T001 rna9033   1.00E-15
267- 3: transcript:Zm00001d007207_T002 rna9026   0
267- 4: transcript:Zm00001d007208_T001 rna9023   3.00E-135
267- 5: transcript:Zm00001d007209_T001 rna9018   2.00E-12
267- 6: transcript:Zm00001d007215_T001 rna9002   0
Alignment 268: score=255.0 e_value=9.9e-09 N=6 2&NC_008396.2 minus
268- 0: transcript:Zm00001d007819_T001 rna10943   1.00E-98
268- 1: transcript:Zm00001d007820_T002 rna10927   2.00E-135
268- 2: transcript:Zm00001d007824_T001 rna10926   0

268- 3: transcript:Zm00001d007825_T003 rna10925   2.00E-22
268- 4: transcript:Zm00001d007830_T001 rna10917   2.00E-167
268- 5: transcript:Zm00001d007835_T002 rna10913   0
Alignment 269: score=251.0 e_value=1.3e-08 N=6 2&NC_008396.2 minus
269- 0: transcript:Zm00001d007225_T019 rna8959   9.00E-73
269- 1: transcript:Zm00001d007228_T001 rna8948   2.00E-161
269- 2: transcript:Zm00001d007229_T001 rna8944   0
269- 3: transcript:Zm00001d007231_T002 rna8931   4.00E-75
269- 4: transcript:Zm00001d007234_T001 rna8920   3.00E-155
269- 5: transcript:Zm00001d007240_T001 rna8909   1.00E-179
Alignment 270: score=1040.0 e_value=1.1e-62 N=22 2&NC_008397.2 plus
270- 0: transcript:Zm00001d003024_T001 rna12907   3.00E-62
270- 1: transcript:Zm00001d003025_T001 rna12909   0
270- 2: transcript:Zm00001d003031_T002 rna12915   0
270- 3: transcript:Zm00001d003033_T005 rna12916   5.00E-69
270- 4: transcript:Zm00001d003034_T001 rna12918   6.00E-117
270- 5: transcript:Zm00001d003038_T001 rna12925   4.00E-137
270- 6: transcript:Zm00001d003039_T001 rna12927   2.00E-149
270- 7: transcript:Zm00001d003040_T001 rna12928   0
270- 8: transcript:Zm00001d003041_T001 rna12929   9.00E-42
270- 9: transcript:Zm00001d003044_T001 rna12932   0
270- 10: transcript:Zm00001d003047_T001 rna12939   0
270- 11: transcript:Zm00001d003049_T003 rna12940   0
270- 12: transcript:Zm00001d003051_T001 rna12942   4.00E-63
270- 13: transcript:Zm00001d003052_T001 rna12944   5.00E-133
270- 14: transcript:Zm00001d003057_T001 rna12945   3.00E-107
270- 15: transcript:Zm00001d003059_T005 rna12946   0
270- 16: transcript:Zm00001d003060_T001 rna12949   1.00E-43
270- 17: transcript:Zm00001d003062_T001 rna12950   7.00E-63
270- 18: transcript:Zm00001d003064_T001 rna12951   3.00E-110
270- 19: transcript:Zm00001d003067_T002 rna12952   1.00E-170
270- 20: transcript:Zm00001d003068_T001 rna12954   1.00E-120
270- 21: transcript:Zm00001d003069_T001 rna12955   2.00E-106
Alignment 271: score=920.0 e_value=3.3e-58 N=20 2&NC_008397.2 plus
271- 0: transcript:Zm00001d002876_T001 rna14049   0
271- 1: transcript:Zm00001d002878_T001 rna14050   1.00E-64
271- 2: transcript:Zm00001d002881_T001 rna14052   0
271- 3: transcript:Zm00001d002882_T001 rna14055   3.00E-149
271- 4: transcript:Zm00001d002884_T001 rna14056   1.00E-74
271- 5: transcript:Zm00001d002889_T002 rna14057   1.00E-142
271- 6: transcript:Zm00001d002890_T001 rna14059   5.00E-80
271- 7: transcript:Zm00001d002891_T001 rna14060   2.00E-62
271- 8: transcript:Zm00001d002893_T001 rna14061   7.00E-170
271- 9: transcript:Zm00001d002896_T001 rna14062   0
271- 10: transcript:Zm00001d002897_T001 rna14064   9.00E-179
271- 11: transcript:Zm00001d002902_T001 rna14065   4.00E-42
271- 12: transcript:Zm00001d002904_T008 rna14070   7.00E-180
271- 13: transcript:Zm00001d002905_T001 rna14071   0
271- 14: transcript:Zm00001d002906_T001 rna14072   7.00E-112
271- 15: transcript:Zm00001d002917_T001 rna14073   3.00E-101
271- 16: transcript:Zm00001d002919_T001 rna14077   1.00E-53
271- 17: transcript:Zm00001d002925_T002 rna14078   5.00E-76
271- 18: transcript:Zm00001d002928_T001 rna14079   4.00E-60
271- 19: transcript:Zm00001d002933_T014 rna14087   3.00E-129

Alignment 272: score=699.0 e_value=1.3e-47 N=17 2&NC_008397.2 plus
272- 0: transcript:Zm00001d004132_T001 rna11751 0
272- 1: transcript:Zm00001d004133_T001 rna11752 8.00E-140
272- 2: transcript:Zm00001d004136_T001 rna11798 2.00E-159
272- 3: transcript:Zm00001d004138_T001 rna11800 5.00E-105
272- 4: transcript:Zm00001d004143_T001 rna11809 1.00E-121
272- 5: transcript:Zm00001d004153_T002 rna11811 4.00E-153
272- 6: transcript:Zm00001d004159_T003 rna11813 0
272- 7: transcript:Zm00001d004165_T001 rna11820 0
272- 8: transcript:Zm00001d004171_T001 rna11827 0
272- 9: transcript:Zm00001d004172_T005 rna11840 0
272- 10: transcript:Zm00001d004176_T001 rna11841 5.00E-66
272- 11: transcript:Zm00001d004187_T001 rna11842 0
272- 12: transcript:Zm00001d004193_T001 rna11860 4.00E-132
272- 13: transcript:Zm00001d004196_T002 rna11867 0
272- 14: transcript:Zm00001d004198_T001 rna11874 5.00E-112
272- 15: transcript:Zm00001d004209_T001 rna11876 4.00E-89
272- 16: transcript:Zm00001d004222_T001 rna11882 8.00E-55
Alignment 273: score=550.0 e_value=4.5e-28 N=12 2&NC_008397.2 plus
273- 0: transcript:Zm00001d002775_T002 rna13099 0
273- 1: transcript:Zm00001d002776_T001 rna13100 0
273- 2: transcript:Zm00001d002781_T003 rna13102 0
273- 3: transcript:Zm00001d002782_T001 rna13103 8.00E-70
273- 4: transcript:Zm00001d002783_T001 rna13105 0
273- 5: transcript:Zm00001d002787_T001 rna13108 2.00E-60
273- 6: transcript:Zm00001d002788_T001 rna13109 5.00E-97
273- 7: transcript:Zm00001d002789_T001 rna13110 0
273- 8: transcript:Zm00001d002790_T001 rna13111 1.00E-129
273- 9: transcript:Zm00001d002791_T002 rna13112 0
273- 10: transcript:Zm00001d002794_T001 rna13113 7.00E-98
273- 11: transcript:Zm00001d002796_T004 rna13114 1.00E-147
Alignment 274: score=549.0 e_value=2.1e-34 N=13 2&NC_008397.2 plus
274- 0: transcript:Zm00001d004259_T002 rna11614 8.00E-105
274- 1: transcript:Zm00001d004293_T001 rna11627 1.00E-166
274- 2: transcript:Zm00001d004300_T006 rna11638 0
274- 3: transcript:Zm00001d004301_T001 rna11639 0
274- 4: transcript:Zm00001d004309_T001 rna11644 6.00E-31
274- 5: transcript:Zm00001d004310_T001 rna11645 2.00E-11
274- 6: transcript:Zm00001d004322_T001 rna11646 9.00E-21
274- 7: transcript:Zm00001d004328_T001 rna11651 2.00E-28
274- 8: transcript:Zm00001d004335_T001 rna11661 0
274- 9: transcript:Zm00001d004339_T003 rna11677 0
274- 10: transcript:Zm00001d004340_T002 rna11678 3.00E-123
274- 11: transcript:Zm00001d004355_T001 rna11688 2.00E-170
274- 12: transcript:Zm00001d004366_T001 rna11689 0
Alignment 275: score=313.0 e_value=1.9e-12 N=7 2&NC_008397.2 plus
275- 0: transcript:Zm00001d002184_T003 rna13655 9.00E-83
275- 1: transcript:Zm00001d002185_T003 rna13656 0
275- 2: transcript:Zm00001d002186_T001 rna13662 0
275- 3: transcript:Zm00001d002190_T001 rna13663 0
275- 4: transcript:Zm00001d002191_T001 rna13672 0
275- 5: transcript:Zm00001d002193_T003 rna13673 5.00E-145
275- 6: transcript:Zm00001d002198_T002 rna13685 6.00E-87
Alignment 276: score=294.0 e_value=9.1e-10 N=6 2&NC_008397.2 plus

```
276-  0:  transcript:Zm00001d002828_T001  rna13055     6.00E-99
276-  1:  transcript:Zm00001d002829_T001  rna13056    1.00E-109
276-  2:  transcript:Zm00001d002830_T001  rna13057            0
276-  3:  transcript:Zm00001d002833_T001  rna13059    3.00E-102
276-  4:  transcript:Zm00001d002834_T001  rna13062    9.00E-135
276-  5:  transcript:Zm00001d002835_T003  rna13066     3.00E-89
## Alignment 277: score=262.0 e_value=2.5e-10 N=6 2&NC_008397.2 plus
277-  0:  transcript:Zm00001d002734_T001  rna13143            0
277-  1:  transcript:Zm00001d002736_T001  rna13144            0
277-  2:  transcript:Zm00001d002738_T001  rna13146     5.00E-63
277-  3:  transcript:Zm00001d002741_T001  rna13148            0
277-  4:  transcript:Zm00001d002742_T002  rna13149            0
277-  5:  transcript:Zm00001d002743_T001  rna13151     5.00E-35
## Alignment 278: score=256.0 e_value=1e-08 N=6 2&NC_008397.2 plus
278-  0:  transcript:Zm00001d004079_T001  rna11891    3.00E-103
278-  1:  transcript:Zm00001d004086_T001  rna11893    2.00E-153
278-  2:  transcript:Zm00001d004089_T001  rna11895     1.00E-70
278-  3:  transcript:Zm00001d004091_T003  rna11918     6.00E-19
278-  4:  transcript:Zm00001d004094_T003  rna11920            0
278-  5:  transcript:Zm00001d004095_T001  rna11924     8.00E-45
## Alignment 279: score=6899.0 e_value=0 N=150 2&NC_008397.2 minus
279-  0:  transcript:Zm00001d003268_T001  rna12731     5.00E-93
279-  1:  transcript:Zm00001d003271_T001  rna12728    1.00E-118
279-  2:  transcript:Zm00001d003272_T001  rna12724    2.00E-172
279-  3:  transcript:Zm00001d003274_T002  rna12723     2.00E-26
279-  4:  transcript:Zm00001d003275_T001  rna12722     5.00E-92
279-  5:  transcript:Zm00001d003276_T001  rna12721    2.00E-149
279-  6:  transcript:Zm00001d003279_T001  rna12720     5.00E-56
279-  7:  transcript:Zm00001d003281_T001  rna12717            0
279-  8:  transcript:Zm00001d003283_T001  rna12714     1.00E-79
279-  9:  transcript:Zm00001d003284_T001  rna12711            0
279- 10:  transcript:Zm00001d003287_T001  rna12710     1.00E-81
279- 11:  transcript:Zm00001d003288_T001  rna12709    1.00E-169
279- 12:  transcript:Zm00001d003291_T002  rna12702    2.00E-127
279- 13:  transcript:Zm00001d003292_T001  rna12696            0
279- 14:  transcript:Zm00001d003293_T001  rna12695     6.00E-51
279- 15:  transcript:Zm00001d003294_T001  rna12694            0
279- 16:  transcript:Zm00001d003297_T001  rna12693            0
279- 17:  transcript:Zm00001d003300_T001  rna12692     1.00E-76
279- 18:  transcript:Zm00001d003301_T001  rna12690            0
279- 19:  transcript:Zm00001d003302_T001  rna12689            0
279- 20:  transcript:Zm00001d003308_T001  rna12688     1.00E-48
279- 21:  transcript:Zm00001d003309_T002  rna12687            0
279- 22:  transcript:Zm00001d003311_T001  rna12683    3.00E-169
279- 23:  transcript:Zm00001d003313_T001  rna12682    2.00E-121
279- 24:  transcript:Zm00001d003314_T001  rna12681     3.00E-81
279- 25:  transcript:Zm00001d003316_T001  rna12673     6.00E-28
279- 26:  transcript:Zm00001d003319_T001  rna12672            0
279- 27:  transcript:Zm00001d003321_T001  rna12668            0
279- 28:  transcript:Zm00001d003322_T002  rna12666     7.00E-77
279- 29:  transcript:Zm00001d003329_T003  rna12663     3.00E-36
279- 30:  transcript:Zm00001d003331_T001  rna12659            0
279- 31:  transcript:Zm00001d003333_T005  rna12658            0
279- 32:  transcript:Zm00001d003334_T001  rna12657    7.00E-107
```

279- 33:	transcript:Zm00001d003343_T001	rna12655	0
279- 34:	transcript:Zm00001d003345_T001	rna12651	2.00E-72
279- 35:	transcript:Zm00001d003347_T002	rna12649	8.00E-60
279- 36:	transcript:Zm00001d003349_T001	rna12648	0
279- 37:	transcript:Zm00001d003353_T001	rna12642	9.00E-20
279- 38:	transcript:Zm00001d003364_T001	rna12641	5.00E-10
279- 39:	transcript:Zm00001d003369_T004	rna12637	6.00E-104
279- 40:	transcript:Zm00001d003370_T001	rna12636	2.00E-20
279- 41:	transcript:Zm00001d003372_T001	rna12635	0
279- 42:	transcript:Zm00001d003373_T002	rna12633	6.00E-46
279- 43:	transcript:Zm00001d003375_T001	rna12632	2.00E-33
279- 44:	transcript:Zm00001d003376_T002	rna12630	5.00E-129
279- 45:	transcript:Zm00001d003378_T001	rna12628	0
279- 46:	transcript:Zm00001d003379_T001	rna12627	1.00E-90
279- 47:	transcript:Zm00001d003380_T001	rna12624	2.00E-34
279- 48:	transcript:Zm00001d003381_T001	rna12623	2.00E-170
279- 49:	transcript:Zm00001d003382_T002	rna12622	2.00E-143
279- 50:	transcript:Zm00001d003392_T001	rna12618	7.00E-158
279- 51:	transcript:Zm00001d003393_T002	rna12617	2.00E-145
279- 52:	transcript:Zm00001d003394_T001	rna12611	1.00E-94
279- 53:	transcript:Zm00001d003395_T001	rna12609	2.00E-161
279- 54:	transcript:Zm00001d003396_T001	rna12607	1.00E-32
279- 55:	transcript:Zm00001d003398_T001	rna12606	5.00E-88
279- 56:	transcript:Zm00001d003399_T001	rna12604	0
279- 57:	transcript:Zm00001d003400_T001	rna12601	2.00E-71
279- 58:	transcript:Zm00001d003401_T003	rna12600	0
279- 59:	transcript:Zm00001d003403_T001	rna12599	3.00E-164
279- 60:	transcript:Zm00001d003404_T001	rna12598	2.00E-148
279- 61:	transcript:Zm00001d003405_T001	rna12597	3.00E-43
279- 62:	transcript:Zm00001d003406_T002	rna12595	0
279- 63:	transcript:Zm00001d003409_T001	rna12591	2.00E-53
279- 64:	transcript:Zm00001d003411_T001	rna12590	1.00E-79
279- 65:	transcript:Zm00001d003412_T001	rna12589	4.00E-160
279- 66:	transcript:Zm00001d003414_T001	rna12586	1.00E-140
279- 67:	transcript:Zm00001d003415_T002	rna12585	5.00E-124
279- 68:	transcript:Zm00001d003418_T001	rna12584	4.00E-102
279- 69:	transcript:Zm00001d003422_T001	rna12582	0
279- 70:	transcript:Zm00001d003426_T002	rna12581	0
279- 71:	transcript:Zm00001d003427_T001	rna12580	0
279- 72:	transcript:Zm00001d003428_T001	rna12578	9.00E-116
279- 73:	transcript:Zm00001d003429_T001	rna12577	0
279- 74:	transcript:Zm00001d003431_T009	rna12574	0
279- 75:	transcript:Zm00001d003432_T001	rna12569	5.00E-155
279- 76:	transcript:Zm00001d003438_T001	rna12568	0
279- 77:	transcript:Zm00001d003446_T002	rna12563	2.00E-44
279- 78:	transcript:Zm00001d003447_T001	rna12562	2.00E-09
279- 79:	transcript:Zm00001d003448_T002	rna12560	2.00E-145
279- 80:	transcript:Zm00001d003457_T001	rna12556	5.00E-58
279- 81:	transcript:Zm00001d003460_T001	rna12554	3.00E-174
279- 82:	transcript:Zm00001d003462_T001	rna12553	0
279- 83:	transcript:Zm00001d003463_T001	rna12552	2.00E-55
279- 84:	transcript:Zm00001d003464_T001	rna12551	4.00E-179
279- 85:	transcript:Zm00001d003468_T001	rna12541	7.00E-146
279- 86:	transcript:Zm00001d003469_T001	rna12540	0

279- 87:	transcript:Zm00001d003472_T002	rna12538	1.00E-21
279- 88:	transcript:Zm00001d003477_T001	rna12537	0
279- 89:	transcript:Zm00001d003482_T001	rna12535	1.00E-06
279- 90:	transcript:Zm00001d003483_T001	rna12534	0
279- 91:	transcript:Zm00001d003491_T005	rna12533	5.00E-166
279- 92:	transcript:Zm00001d003492_T001	rna12531	0
279- 93:	transcript:Zm00001d003493_T002	rna12529	2.00E-37
279- 94:	transcript:Zm00001d003494_T004	rna12528	8.00E-66
279- 95:	transcript:Zm00001d003495_T008	rna12527	1.00E-100
279- 96:	transcript:Zm00001d003497_T001	rna12526	4.00E-87
279- 97:	transcript:Zm00001d003500_T001	rna12525	4.00E-163
279- 98:	transcript:Zm00001d003504_T002	rna12524	9.00E-145
279- 99:	transcript:Zm00001d003505_T001	rna12523	3.00E-30
279-100:	transcript:Zm00001d003508_T001	rna12520	0
279-101:	transcript:Zm00001d003509_T001	rna12519	3.00E-41
279-102:	transcript:Zm00001d003510_T001	rna12516	0
279-103:	transcript:Zm00001d003511_T001	rna12515	2.00E-118
279-104:	transcript:Zm00001d003512_T001	rna12514	0
279-105:	transcript:Zm00001d003515_T001	rna12510	1.00E-70
279-106:	transcript:Zm00001d003518_T003	rna12509	0
279-107:	transcript:Zm00001d003525_T010	rna12503	1.00E-170
279-108:	transcript:Zm00001d003531_T002	rna12502	0
279-109:	transcript:Zm00001d003535_T001	rna12497	9.00E-29
279-110:	transcript:Zm00001d003540_T003	rna12495	4.00E-94
279-111:	transcript:Zm00001d003543_T001	rna12494	0
279-112:	transcript:Zm00001d003544_T003	rna12493	8.00E-119
279-113:	transcript:Zm00001d003546_T001	rna12492	3.00E-83
279-114:	transcript:Zm00001d003549_T001	rna12490	9.00E-134
279-115:	transcript:Zm00001d003550_T001	rna12487	1.00E-71
279-116:	transcript:Zm00001d003552_T001	rna12486	3.00E-64
279-117:	transcript:Zm00001d003554_T001	rna12485	3.00E-97
279-118:	transcript:Zm00001d003555_T001	rna12483	0
279-119:	transcript:Zm00001d003559_T001	rna12481	0
279-120:	transcript:Zm00001d003563_T001	rna12480	0
279-121:	transcript:Zm00001d003566_T001	rna12479	0
279-122:	transcript:Zm00001d003569_T001	rna12478	0
279-123:	transcript:Zm00001d003572_T001	rna12476	0
279-124:	transcript:Zm00001d003573_T001	rna12475	1.00E-29
279-125:	transcript:Zm00001d003590_T001	rna12473	0
279-126:	transcript:Zm00001d003593_T001	rna12470	4.00E-70
279-127:	transcript:Zm00001d003598_T003	rna12463	1.00E-83
279-128:	transcript:Zm00001d003599_T001	rna12462	4.00E-30
279-129:	transcript:Zm00001d003601_T006	rna12460	0
279-130:	transcript:Zm00001d003602_T002	rna12459	0
279-131:	transcript:Zm00001d003603_T001	rna12458	0
279-132:	transcript:Zm00001d003605_T001	rna12456	3.00E-39
279-133:	transcript:Zm00001d003611_T003	rna12455	0
279-134:	transcript:Zm00001d003612_T001	rna12453	7.00E-141
279-135:	transcript:Zm00001d003614_T001	rna12452	0
279-136:	transcript:Zm00001d003615_T002	rna12447	0
279-137:	transcript:Zm00001d003616_T024	rna12446	1.00E-129
279-138:	transcript:Zm00001d003617_T001	rna12442	0
279-139:	transcript:Zm00001d003618_T001	rna12441	2.00E-139
279-140:	transcript:Zm00001d003622_T001	rna12437	1.00E-49

279-141: transcript:Zm00001d003624_T001 rna12434 2.00E-175
279-142: transcript:Zm00001d003626_T002 rna12433 3.00E-81
279-143: transcript:Zm00001d003630_T001 rna12427 1.00E-79
279-144: transcript:Zm00001d003631_T001 rna12426 3.00E-20
279-145: transcript:Zm00001d003632_T003 rna12425 0
279-146: transcript:Zm00001d003640_T001 rna12424 0
279-147: transcript:Zm00001d003643_T005 rna12418 0
279-148: transcript:Zm00001d003645_T001 rna12416 2.00E-83
279-149: transcript:Zm00001d003646_T002 rna12414 1.00E-96
Alignment 280: score=3512.0 e_value=9.4e-321 N=77 2&NC_008397.2 minus
280- 0: transcript:Zm00001d003742_T001 rna12331 0
280- 1: transcript:Zm00001d003743_T001 rna12330 4.00E-89
280- 2: transcript:Zm00001d003745_T001 rna12329 3.00E-175
280- 3: transcript:Zm00001d003749_T001 rna12328 0
280- 4: transcript:Zm00001d003750_T001 rna12327 1.00E-76
280- 5: transcript:Zm00001d003751_T001 rna12326 9.00E-124
280- 6: transcript:Zm00001d003754_T003 rna12325 0
280- 7: transcript:Zm00001d003755_T001 rna12323 4.00E-99
280- 8: transcript:Zm00001d003756_T001 rna12322 4.00E-81
280- 9: transcript:Zm00001d003757_T001 rna12320 5.00E-53
280- 10: transcript:Zm00001d003761_T001 rna12319 1.00E-34
280- 11: transcript:Zm00001d003762_T002 rna12316 5.00E-64
280- 12: transcript:Zm00001d003763_T001 rna12314 3.00E-50
280- 13: transcript:Zm00001d003765_T001 rna12312 0
280- 14: transcript:Zm00001d003767_T001 rna12311 5.00E-85
280- 15: transcript:Zm00001d003773_T005 rna12308 0
280- 16: transcript:Zm00001d003775_T001 rna12306 1.00E-79
280- 17: transcript:Zm00001d003776_T001 rna12302 0
280- 18: transcript:Zm00001d003777_T001 rna12301 1.00E-44
280- 19: transcript:Zm00001d003778_T001 rna12300 0
280- 20: transcript:Zm00001d003779_T001 rna12298 0
280- 21: transcript:Zm00001d003780_T001 rna12297 7.00E-40
280- 22: transcript:Zm00001d003784_T001 rna12293 0
280- 23: transcript:Zm00001d003797_T001 rna12292 8.00E-67
280- 24: transcript:Zm00001d003799_T001 rna12291 6.00E-36
280- 25: transcript:Zm00001d003800_T011 rna12289 7.00E-176
280- 26: transcript:Zm00001d003804_T001 rna12288 2.00E-110
280- 27: transcript:Zm00001d003806_T001 rna12286 4.00E-28
280- 28: transcript:Zm00001d003807_T001 rna12283 0
280- 29: transcript:Zm00001d003812_T004 rna12280 0
280- 30: transcript:Zm00001d003813_T001 rna12278 0
280- 31: transcript:Zm00001d003817_T002 rna12276 1.00E-151
280- 32: transcript:Zm00001d003822_T001 rna12273 1.00E-25
280- 33: transcript:Zm00001d003823_T001 rna12271 0
280- 34: transcript:Zm00001d003829_T001 rna12268 1.00E-71
280- 35: transcript:Zm00001d003833_T001 rna12265 6.00E-14
280- 36: transcript:Zm00001d003836_T005 rna12264 3.00E-158
280- 37: transcript:Zm00001d003839_T004 rna12262 0
280- 38: transcript:Zm00001d003840_T001 rna12261 2.00E-77
280- 39: transcript:Zm00001d003841_T002 rna12259 1.00E-144
280- 40: transcript:Zm00001d003846_T001 rna12258 3.00E-138
280- 41: transcript:Zm00001d003847_T001 rna12257 1.00E-18
280- 42: transcript:Zm00001d003848_T004 rna12256 0
280- 43: transcript:Zm00001d003851_T001 rna12247 0

280- 44: transcript:Zm00001d003855_T010 rna12243 5.00E-21
280- 45: transcript:Zm00001d003857_T001 rna12240 0
280- 46: transcript:Zm00001d003859_T001 rna12236 0
280- 47: transcript:Zm00001d003864_T002 rna12234 0
280- 48: transcript:Zm00001d003865_T001 rna12229 1.00E-08
280- 49: transcript:Zm00001d003867_T001 rna12228 5.00E-21
280- 50: transcript:Zm00001d003871_T001 rna12227 4.00E-84
280- 51: transcript:Zm00001d003877_T001 rna12221 1.00E-83
280- 52: transcript:Zm00001d003878_T001 rna12220 0
280- 53: transcript:Zm00001d003879_T010 rna12217 0
280- 54: transcript:Zm00001d003884_T001 rna12216 2.00E-31
280- 55: transcript:Zm00001d003888_T002 rna12213 8.00E-55
280- 56: transcript:Zm00001d003889_T001 rna12212 2.00E-114
280- 57: transcript:Zm00001d003890_T001 rna12211 7.00E-09
280- 58: transcript:Zm00001d003894_T004 rna12210 0
280- 59: transcript:Zm00001d003895_T001 rna12208 0
280- 60: transcript:Zm00001d003902_T002 rna12206 0
280- 61: transcript:Zm00001d003903_T001 rna12205 3.00E-38
280- 62: transcript:Zm00001d003905_T001 rna12204 4.00E-13
280- 63: transcript:Zm00001d003911_T010 rna12203 0
280- 64: transcript:Zm00001d003917_T007 rna12199 3.00E-115
280- 65: transcript:Zm00001d003922_T002 rna12198 0
280- 66: transcript:Zm00001d003923_T002 rna12197 0
280- 67: transcript:Zm00001d003924_T002 rna12196 8.00E-151
280- 68: transcript:Zm00001d003925_T001 rna12195 0
280- 69: transcript:Zm00001d003927_T001 rna12194 2.00E-68
280- 70: transcript:Zm00001d003928_T001 rna12193 5.00E-60
280- 71: transcript:Zm00001d003931_T001 rna12187 0
280- 72: transcript:Zm00001d003937_T002 rna12185 0
280- 73: transcript:Zm00001d003938_T001 rna12183 3.00E-107
280- 74: transcript:Zm00001d003941_T003 rna12181 1.00E-150
280- 75: transcript:Zm00001d003945_T002 rna12180 4.00E-10
280- 76: transcript:Zm00001d003947_T005 rna12179 0
Alignment 281: score=3135.0 e_value=4.7e-270 N=67 2&NC_008397.2 minus
281- 0: transcript:Zm00001d002473_T001 rna13403 8.00E-137
281- 1: transcript:Zm00001d002475_T001 rna13402 2.00E-180
281- 2: transcript:Zm00001d002476_T001 rna13401 4.00E-135
281- 3: transcript:Zm00001d002478_T001 rna13397 4.00E-116
281- 4: transcript:Zm00001d002479_T001 rna13395 1.00E-52
281- 5: transcript:Zm00001d002482_T001 rna13394 2.00E-46
281- 6: transcript:Zm00001d002483_T007 rna13393 0
281- 7: transcript:Zm00001d002485_T001 rna13390 0
281- 8: transcript:Zm00001d002488_T001 rna13386 5.00E-38
281- 9: transcript:Zm00001d002489_T001 rna13385 3.00E-143
281- 10: transcript:Zm00001d002490_T001 rna13383 3.00E-115
281- 11: transcript:Zm00001d002491_T001 rna13382 9.00E-148
281- 12: transcript:Zm00001d002495_T001 rna13378 4.00E-153
281- 13: transcript:Zm00001d002496_T001 rna13377 1.00E-94
281- 14: transcript:Zm00001d002498_T001 rna13374 0
281- 15: transcript:Zm00001d002499_T003 rna13373 0
281- 16: transcript:Zm00001d002500_T001 rna13372 1.00E-19
281- 17: transcript:Zm00001d002501_T001 rna13370 1.00E-29
281- 18: transcript:Zm00001d002503_T001 rna13368 0
281- 19: transcript:Zm00001d002510_T001 rna13366 0

281- 20: transcript:Zm00001d002512_T001 rna13365 2.00E-44
281- 21: transcript:Zm00001d002514_T001 rna13361 5.00E-108
281- 22: transcript:Zm00001d002517_T001 rna13359 8.00E-32
281- 23: transcript:Zm00001d002519_T001 rna13358 6.00E-116
281- 24: transcript:Zm00001d002522_T001 rna13352 1.00E-12
281- 25: transcript:Zm00001d002531_T001 rna13349 0
281- 26: transcript:Zm00001d002532_T003 rna13347 2.00E-161
281- 27: transcript:Zm00001d002534_T001 rna13346 0
281- 28: transcript:Zm00001d002535_T001 rna13345 8.00E-19
281- 29: transcript:Zm00001d002537_T002 rna13344 5.00E-142
281- 30: transcript:Zm00001d002538_T001 rna13343 0
281- 31: transcript:Zm00001d002539_T001 rna13342 0
281- 32: transcript:Zm00001d002540_T002 rna13341 0
281- 33: transcript:Zm00001d002541_T001 rna13340 2.00E-104
281- 34: transcript:Zm00001d002542_T001 rna13339 4.00E-78
281- 35: transcript:Zm00001d002545_T002 rna13336 5.00E-156
281- 36: transcript:Zm00001d002548_T001 rna13332 5.00E-164
281- 37: transcript:Zm00001d002550_T001 rna13330 0
281- 38: transcript:Zm00001d002551_T001 rna13323 4.00E-77
281- 39: transcript:Zm00001d002558_T001 rna13322 4.00E-62
281- 40: transcript:Zm00001d002562_T001 rna13319 1.00E-95
281- 41: transcript:Zm00001d002564_T001 rna13316 3.00E-129
281- 42: transcript:Zm00001d002565_T001 rna13315 8.00E-11
281- 43: transcript:Zm00001d002569_T001 rna13313 3.00E-89
281- 44: transcript:Zm00001d002570_T001 rna13312 1.00E-55
281- 45: transcript:Zm00001d002573_T001 rna13311 0
281- 46: transcript:Zm00001d002576_T001 rna13309 2.00E-91
281- 47: transcript:Zm00001d002579_T001 rna13307 0
281- 48: transcript:Zm00001d002580_T004 rna13306 0
281- 49: transcript:Zm00001d002589_T001 rna13304 5.00E-159
281- 50: transcript:Zm00001d002591_T001 rna13303 4.00E-33
281- 51: transcript:Zm00001d002592_T002 rna13302 0
281- 52: transcript:Zm00001d002593_T001 rna13301 3.00E-56
281- 53: transcript:Zm00001d002594_T001 rna13300 3.00E-83
281- 54: transcript:Zm00001d002595_T002 rna13298 6.00E-146
281- 55: transcript:Zm00001d002597_T002 rna13296 0
281- 56: transcript:Zm00001d002598_T001 rna13295 0
281- 57: transcript:Zm00001d002599_T002 rna13294 0
281- 58: transcript:Zm00001d002600_T005 rna13293 4.00E-174
281- 59: transcript:Zm00001d002602_T002 rna13288 0
281- 60: transcript:Zm00001d002603_T001 rna13286 0
281- 61: transcript:Zm00001d002607_T001 rna13284 0
281- 62: transcript:Zm00001d002609_T001 rna13282 0
281- 63: transcript:Zm00001d002610_T001 rna13281 0
281- 64: transcript:Zm00001d002611_T002 rna13279 1.00E-163
281- 65: transcript:Zm00001d002613_T002 rna13278 0
281- 66: transcript:Zm00001d002614_T001 rna13277 0
Alignment 282: score=2365.0 e_value=2.7e-205 N=54 2&NC_008397.2 minus
282- 0: transcript:Zm00001d003088_T001 rna12887 2.00E-26
282- 1: transcript:Zm00001d003089_T001 rna12885 0
282- 2: transcript:Zm00001d003091_T001 rna12884 4.00E-173
282- 3: transcript:Zm00001d003099_T003 rna12883 0
282- 4: transcript:Zm00001d003101_T013 rna12882 2.00E-84
282- 5: transcript:Zm00001d003102_T001 rna12881 0

282- 6: transcript:Zm00001d003103_T001 rna12880 0
282- 7: transcript:Zm00001d003104_T001 rna12876 9.00E-126
282- 8: transcript:Zm00001d003106_T003 rna12874 0
282- 9: transcript:Zm00001d003107_T001 rna12870 3.00E-54
282- 10: transcript:Zm00001d003108_T007 rna12869 3.00E-102
282- 11: transcript:Zm00001d003109_T002 rna12867 0
282- 12: transcript:Zm00001d003110_T002 rna12866 8.00E-47
282- 13: transcript:Zm00001d003112_T004 rna12865 0
282- 14: transcript:Zm00001d003114_T006 rna12862 0
282- 15: transcript:Zm00001d003116_T001 rna12861 0
282- 16: transcript:Zm00001d003118_T001 rna12860 4.00E-105
282- 17: transcript:Zm00001d003119_T001 rna12859 0
282- 18: transcript:Zm00001d003123_T001 rna12854 0
282- 19: transcript:Zm00001d003125_T001 rna12851 0
282- 20: transcript:Zm00001d003127_T003 rna12850 6.00E-85
282- 21: transcript:Zm00001d003129_T001 rna12848 3.00E-83
282- 22: transcript:Zm00001d003139_T001 rna12845 6.00E-25
282- 23: transcript:Zm00001d003147_T001 rna12842 0
282- 24: transcript:Zm00001d003148_T002 rna12841 6.00E-35
282- 25: transcript:Zm00001d003149_T001 rna12839 0
282- 26: transcript:Zm00001d003153_T004 rna12838 6.00E-93
282- 27: transcript:Zm00001d003157_T001 rna12835 0
282- 28: transcript:Zm00001d003160_T001 rna12831 3.00E-21
282- 29: transcript:Zm00001d003161_T001 rna12830 0
282- 30: transcript:Zm00001d003162_T001 rna12829 3.00E-162
282- 31: transcript:Zm00001d003166_T006 rna12822 0
282- 32: transcript:Zm00001d003167_T002 rna12820 2.00E-36
282- 33: transcript:Zm00001d003172_T001 rna12817 3.00E-115
282- 34: transcript:Zm00001d003173_T001 rna12816 4.00E-45
282- 35: transcript:Zm00001d003175_T001 rna12815 4.00E-59
282- 36: transcript:Zm00001d003176_T001 rna12814 0
282- 37: transcript:Zm00001d003180_T001 rna12812 1.00E-40
282- 38: transcript:Zm00001d003181_T005 rna12811 1.00E-35
282- 39: transcript:Zm00001d003182_T001 rna12810 5.00E-68
282- 40: transcript:Zm00001d003183_T001 rna12805 8.00E-165
282- 41: transcript:Zm00001d003188_T001 rna12801 2.00E-108
282- 42: transcript:Zm00001d003193_T001 rna12799 2.00E-82
282- 43: transcript:Zm00001d003195_T001 rna12798 2.00E-31
282- 44: transcript:Zm00001d003196_T001 rna12797 0
282- 45: transcript:Zm00001d003197_T001 rna12796 6.00E-88
282- 46: transcript:Zm00001d003198_T010 rna12794 3.00E-138
282- 47: transcript:Zm00001d003200_T002 rna12793 0
282- 48: transcript:Zm00001d003203_T014 rna12792 0
282- 49: transcript:Zm00001d003207_T010 rna12791 0
282- 50: transcript:Zm00001d003208_T001 rna12788 0
282- 51: transcript:Zm00001d003212_T001 rna12782 2.00E-156
282- 52: transcript:Zm00001d003213_T001 rna12779 9.00E-51
282- 53: transcript:Zm00001d003214_T002 rna12778 2.00E-37
Alignment 283: score=1686.0 e_value=2.1e-128 N=36 2&NC_008397.2 minus
283- 0: transcript:Zm00001d002332_T026 rna13542 0
283- 1: transcript:Zm00001d002333_T003 rna13541 0
283- 2: transcript:Zm00001d002338_T001 rna13539 0
283- 3: transcript:Zm00001d002339_T001 rna13537 4.00E-52
283- 4: transcript:Zm00001d002340_T006 rna13536 0

283- 5: transcript:Zm00001d002341_T001 rna13535 6.00E-148
283- 6: transcript:Zm00001d002342_T001 rna13534 0
283- 7: transcript:Zm00001d002343_T001 rna13533 0
283- 8: transcript:Zm00001d002344_T001 rna13532 8.00E-61
283- 9: transcript:Zm00001d002345_T001 rna13531 0
283- 10: transcript:Zm00001d002346_T002 rna13530 0
283- 11: transcript:Zm00001d002347_T001 rna13526 5.00E-47
283- 12: transcript:Zm00001d002348_T001 rna13525 2.00E-46
283- 13: transcript:Zm00001d002350_T001 rna13522 0
283- 14: transcript:Zm00001d002352_T001 rna13519 2.00E-33
283- 15: transcript:Zm00001d002353_T001 rna13518 0
283- 16: transcript:Zm00001d002357_T007 rna13514 0
283- 17: transcript:Zm00001d002358_T001 rna13513 0
283- 18: transcript:Zm00001d002359_T001 rna13512 0
283- 19: transcript:Zm00001d002360_T001 rna13511 5.00E-93
283- 20: transcript:Zm00001d002362_T001 rna13510 9.00E-62
283- 21: transcript:Zm00001d002364_T001 rna13509 8.00E-64
283- 22: transcript:Zm00001d002369_T003 rna13502 0
283- 23: transcript:Zm00001d002370_T001 rna13500 0
283- 24: transcript:Zm00001d002371_T002 rna13497 0
283- 25: transcript:Zm00001d002372_T002 rna13496 0
283- 26: transcript:Zm00001d002373_T006 rna13494 0
283- 27: transcript:Zm00001d002374_T001 rna13492 3.00E-86
283- 28: transcript:Zm00001d002377_T001 rna13491 0
283- 29: transcript:Zm00001d002378_T001 rna13489 1.00E-107
283- 30: transcript:Zm00001d002382_T002 rna13484 9.00E-115
283- 31: transcript:Zm00001d002384_T001 rna13483 7.00E-129
283- 32: transcript:Zm00001d002385_T002 rna13482 1.00E-110
283- 33: transcript:Zm00001d002386_T003 rna13481 2.00E-114
283- 34: transcript:Zm00001d002387_T002 rna13480 1.00E-35
283- 35: transcript:Zm00001d002388_T003 rna13477 0
Alignment 284: score=1511.0 e_value=4.1e-106 N=32 2&NC_008397.2 minus
284- 0: transcript:Zm00001d002952_T001 rna13011 5.00E-125
284- 1: transcript:Zm00001d002953_T001 rna13010 0
284- 2: transcript:Zm00001d002954_T002 rna13009 2.00E-63
284- 3: transcript:Zm00001d002956_T005 rna13007 0
284- 4: transcript:Zm00001d002958_T001 rna13003 2.00E-67
284- 5: transcript:Zm00001d002960_T001 rna13002 3.00E-31
284- 6: transcript:Zm00001d002962_T001 rna12998 6.00E-158
284- 7: transcript:Zm00001d002966_T001 rna12997 0
284- 8: transcript:Zm00001d002969_T001 rna12996 1.00E-168
284- 9: transcript:Zm00001d002970_T001 rna12994 2.00E-150
284- 10: transcript:Zm00001d002971_T001 rna12993 1.00E-119
284- 11: transcript:Zm00001d002972_T001 rna12988 0
284- 12: transcript:Zm00001d002980_T001 rna12987 0
284- 13: transcript:Zm00001d002982_T001 rna12986 2.00E-80
284- 14: transcript:Zm00001d002984_T001 rna12983 1.00E-65
284- 15: transcript:Zm00001d002992_T002 rna12981 2.00E-144
284- 16: transcript:Zm00001d002993_T001 rna12980 5.00E-64
284- 17: transcript:Zm00001d002996_T001 rna12978 9.00E-32
284- 18: transcript:Zm00001d002999_T001 rna12977 0
284- 19: transcript:Zm00001d003002_T001 rna12975 0
284- 20: transcript:Zm00001d003003_T001 rna12974 3.00E-34
284- 21: transcript:Zm00001d003004_T001 rna12973 0

284- 22: transcript:Zm00001d003005_T001 rna12972 1.00E-27
284- 23: transcript:Zm00001d003006_T001 rna12971 0
284- 24: transcript:Zm00001d003007_T001 rna12969 0
284- 25: transcript:Zm00001d003009_T001 rna12968 3.00E-69
284- 26: transcript:Zm00001d003011_T001 rna12965 1.00E-62
284- 27: transcript:Zm00001d003013_T001 rna12963 1.00E-150
284- 28: transcript:Zm00001d003015_T001 rna12958 0
284- 29: transcript:Zm00001d003017_T002 rna12957 0
284- 30: transcript:Zm00001d003018_T001 rna12956 5.00E-49
284- 31: transcript:Zm00001d003019_T002 rna12955 8.00E-115
Alignment 285: score=1423.0 e_value=3.7e-100 N=30 2&NC_008397.2 minus
285- 0: transcript:Zm00001d002819_T002 rna13074 0
285- 1: transcript:Zm00001d002820_T001 rna13073 8.00E-80
285- 2: transcript:Zm00001d002821_T001 rna13072 2.00E-69
285- 3: transcript:Zm00001d002822_T001 rna13071 0
285- 4: transcript:Zm00001d002823_T001 rna13070 0
285- 5: transcript:Zm00001d002824_T001 rna13069 0
285- 6: transcript:Zm00001d002825_T001 rna13068 4.00E-151
285- 7: transcript:Zm00001d002826_T001 rna13067 1.00E-56
285- 8: transcript:Zm00001d002834_T001 rna13062 9.00E-135
285- 9: transcript:Zm00001d002836_T006 rna13054 7.00E-172
285- 10: transcript:Zm00001d002837_T001 rna13052 7.00E-116
285- 11: transcript:Zm00001d002842_T001 rna13051 1.00E-96
285- 12: transcript:Zm00001d002844_T003 rna13047 4.00E-118
285- 13: transcript:Zm00001d002845_T001 rna13041 9.00E-14
285- 14: transcript:Zm00001d002850_T001 rna13035 6.00E-75
285- 15: transcript:Zm00001d002851_T001 rna13034 0
285- 16: transcript:Zm00001d002853_T001 rna13033 0
285- 17: transcript:Zm00001d002854_T001 rna13032 5.00E-85
285- 18: transcript:Zm00001d002856_T001 rna13031 1.00E-124
285- 19: transcript:Zm00001d002857_T001 rna13029 2.00E-96
285- 20: transcript:Zm00001d002860_T002 rna13026 7.00E-67
285- 21: transcript:Zm00001d002864_T001 rna13024 0
285- 22: transcript:Zm00001d002865_T002 rna13020 1.00E-74
285- 23: transcript:Zm00001d002867_T001 rna13019 2.00E-114
285- 24: transcript:Zm00001d002868_T001 rna13018 7.00E-61
285- 25: transcript:Zm00001d002869_T001 rna13017 6.00E-108
285- 26: transcript:Zm00001d002871_T001 rna13015 0
285- 27: transcript:Zm00001d002872_T001 rna13014 9.00E-19
285- 28: transcript:Zm00001d002873_T004 rna13013 4.00E-45
285- 29: transcript:Zm00001d002874_T001 rna13012 2.00E-87
Alignment 286: score=1393.0 e_value=9.7e-131 N=34 2&NC_008397.2 minus
286- 0: transcript:Zm00001d004413_T002 rna11569 0
286- 1: transcript:Zm00001d004417_T001 rna11568 8.00E-37
286- 2: transcript:Zm00001d004437_T001 rna11563 2.00E-131
286- 3: transcript:Zm00001d004438_T005 rna11562 0
286- 4: transcript:Zm00001d004439_T001 rna11554 4.00E-64
286- 5: transcript:Zm00001d004446_T001 rna11529 3.00E-14
286- 6: transcript:Zm00001d004448_T001 rna11528 0
286- 7: transcript:Zm00001d004452_T001 rna11526 7.00E-55
286- 8: transcript:Zm00001d004460_T004 rna11519 0
286- 9: transcript:Zm00001d004463_T006 rna11517 0
286- 10: transcript:Zm00001d004465_T001 rna11515 1.00E-67
286- 11: transcript:Zm00001d004466_T010 rna11514 0

286- 12: transcript:Zm00001d004467_T001 rna11510 0
286- 13: transcript:Zm00001d004472_T002 rna11487 0
286- 14: transcript:Zm00001d004473_T001 rna11486 0
286- 15: transcript:Zm00001d004477_T005 rna11482 2.00E-96
286- 16: transcript:Zm00001d004495_T001 rna11468 2.00E-62
286- 17: transcript:Zm00001d004497_T001 rna11463 3.00E-158
286- 18: transcript:Zm00001d004498_T001 rna11462 2.00E-27
286- 19: transcript:Zm00001d004512_T001 rna11454 5.00E-147
286- 20: transcript:Zm00001d004513_T001 rna11447 2.00E-157
286- 21: transcript:Zm00001d004517_T005 rna11445 6.00E-31
286- 22: transcript:Zm00001d004521_T001 rna11441 4.00E-79
286- 23: transcript:Zm00001d004523_T001 rna11440 2.00E-115
286- 24: transcript:Zm00001d004524_T001 rna11437 3.00E-58
286- 25: transcript:Zm00001d004539_T001 rna11420 5.00E-42
286- 26: transcript:Zm00001d004541_T009 rna11418 0
286- 27: transcript:Zm00001d004543_T001 rna11410 0
286- 28: transcript:Zm00001d004545_T001 rna11408 1.00E-168
286- 29: transcript:Zm00001d004552_T003 rna11405 3.00E-42
286- 30: transcript:Zm00001d004553_T001 rna11404 0
286- 31: transcript:Zm00001d004554_T004 rna11403 3.00E-147
286- 32: transcript:Zm00001d004555_T001 rna11402 0
286- 33: transcript:Zm00001d004557_T002 rna11400 4.00E-179
Alignment 287: score=1369.0 e_value=3.8e-100 N=30 2&NC_008397.2 minus
287- 0: transcript:Zm00001d002679_T001 rna13206 0
287- 1: transcript:Zm00001d002680_T003 rna13203 4.00E-35
287- 2: transcript:Zm00001d002684_T001 rna13202 0
287- 3: transcript:Zm00001d002685_T001 rna13201 1.00E-81
287- 4: transcript:Zm00001d002687_T001 rna13200 0
287- 5: transcript:Zm00001d002688_T002 rna13199 0
287- 6: transcript:Zm00001d002690_T001 rna13198 6.00E-40
287- 7: transcript:Zm00001d002694_T004 rna13196 0
287- 8: transcript:Zm00001d002695_T009 rna13195 0
287- 9: transcript:Zm00001d002696_T001 rna13194 0
287- 10: transcript:Zm00001d002698_T001 rna13193 0
287- 11: transcript:Zm00001d002699_T001 rna13192 0
287- 12: transcript:Zm00001d002700_T001 rna13191 3.00E-21
287- 13: transcript:Zm00001d002707_T001 rna13180 6.00E-146
287- 14: transcript:Zm00001d002708_T001 rna13177 0
287- 15: transcript:Zm00001d002711_T001 rna13176 0
287- 16: transcript:Zm00001d002713_T001 rna13175 4.00E-140
287- 17: transcript:Zm00001d002714_T003 rna13174 6.00E-38
287- 18: transcript:Zm00001d002715_T001 rna13172 0
287- 19: transcript:Zm00001d002716_T001 rna13171 0
287- 20: transcript:Zm00001d002718_T001 rna13169 0
287- 21: transcript:Zm00001d002721_T001 rna13166 5.00E-34
287- 22: transcript:Zm00001d002725_T001 rna13164 0
287- 23: transcript:Zm00001d002726_T002 rna13163 8.00E-135
287- 24: transcript:Zm00001d002727_T002 rna13161 2.00E-11
287- 25: transcript:Zm00001d002730_T001 rna13160 0
287- 26: transcript:Zm00001d002731_T001 rna13159 2.00E-107
287- 27: transcript:Zm00001d002732_T001 rna13157 2.00E-118
287- 28: transcript:Zm00001d002733_T001 rna13156 2.00E-103
287- 29: transcript:Zm00001d002736_T001 rna13144 0
Alignment 288: score=1327.0 e_value=1.5e-91 N=28 2&NC_008397.2 minus

288-	0:	transcript:Zm00001d002418_T030	rna13453	0
288-	1:	transcript:Zm00001d002420_T001	rna13452	0
288-	2:	transcript:Zm00001d002421_T001	rna13451	0
288-	3:	transcript:Zm00001d002422_T004	rna13450	0
288-	4:	transcript:Zm00001d002423_T001	rna13449	0
288-	5:	transcript:Zm00001d002424_T002	rna13448	2.00E-84
288-	6:	transcript:Zm00001d002425_T001	rna13447	5.00E-29
288-	7:	transcript:Zm00001d002426_T003	rna13446	0
288-	8:	transcript:Zm00001d002427_T001	rna13445	4.00E-15
288-	9:	transcript:Zm00001d002428_T001	rna13441	2.00E-110
288-	10:	transcript:Zm00001d002429_T002	rna13439	3.00E-08
288-	11:	transcript:Zm00001d002430_T001	rna13438	2.00E-27
288-	12:	transcript:Zm00001d002432_T001	rna13437	1.00E-44
288-	13:	transcript:Zm00001d002433_T002	rna13436	2.00E-159
288-	14:	transcript:Zm00001d002434_T001	rna13434	2.00E-151
288-	15:	transcript:Zm00001d002437_T001	rna13433	2.00E-08
288-	16:	transcript:Zm00001d002439_T002	rna13432	2.00E-41
288-	17:	transcript:Zm00001d002440_T002	rna13431	0
288-	18:	transcript:Zm00001d002441_T002	rna13428	0
288-	19:	transcript:Zm00001d002444_T003	rna13427	1.00E-111
288-	20:	transcript:Zm00001d002446_T001	rna13420	4.00E-107
288-	21:	transcript:Zm00001d002449_T001	rna13419	0
288-	22:	transcript:Zm00001d002450_T001	rna13418	4.00E-112
288-	23:	transcript:Zm00001d002451_T001	rna13417	2.00E-55
288-	24:	transcript:Zm00001d002452_T001	rna13411	6.00E-127
288-	25:	transcript:Zm00001d002454_T002	rna13409	0
288-	26:	transcript:Zm00001d002456_T002	rna13407	1.00E-92
288-	27:	transcript:Zm00001d002457_T002	rna13403	2.00E-65
## Alignment 289: score=1214.0 e_value=9.7e-89 N=27 2&NC_008397.2 minus				
289-	0:	transcript:Zm00001d002115_T001	rna13742	1.00E-83
289-	1:	transcript:Zm00001d002119_T006	rna13741	0
289-	2:	transcript:Zm00001d002122_T007	rna13740	6.00E-131
289-	3:	transcript:Zm00001d002123_T001	rna13739	0
289-	4:	transcript:Zm00001d002124_T001	rna13738	4.00E-80
289-	5:	transcript:Zm00001d002125_T019	rna13737	0
289-	6:	transcript:Zm00001d002126_T001	rna13736	0
289-	7:	transcript:Zm00001d002127_T001	rna13735	1.00E-34
289-	8:	transcript:Zm00001d002128_T001	rna13733	3.00E-151
289-	9:	transcript:Zm00001d002129_T001	rna13732	0
289-	10:	transcript:Zm00001d002130_T002	rna13728	2.00E-117
289-	11:	transcript:Zm00001d002131_T001	rna13727	5.00E-82
289-	12:	transcript:Zm00001d002132_T001	rna13725	4.00E-16
289-	13:	transcript:Zm00001d002136_T001	rna13724	1.00E-109
289-	14:	transcript:Zm00001d002137_T001	rna13723	6.00E-13
289-	15:	transcript:Zm00001d002139_T001	rna13720	0
289-	16:	transcript:Zm00001d002141_T022	rna13715	0
289-	17:	transcript:Zm00001d002145_T001	rna13712	9.00E-142
289-	18:	transcript:Zm00001d002146_T001	rna13711	0
289-	19:	transcript:Zm00001d002147_T002	rna13709	6.00E-84
289-	20:	transcript:Zm00001d002150_T001	rna13708	3.00E-123
289-	21:	transcript:Zm00001d002149_T007	rna13707	0
289-	22:	transcript:Zm00001d002153_T001	rna13706	0
289-	23:	transcript:Zm00001d002154_T003	rna13705	0
289-	24:	transcript:Zm00001d002156_T001	rna13704	4.00E-130

```
289- 25:  transcript:Zm00001d002158_T001  rna13698     1.00E-20
289- 26:  transcript:Zm00001d002163_T001  rna13695            0
## Alignment 290: score=1152.0 e_value=5.8e-77 N=25 2&NC_008397.2 minus
290-  0:  transcript:Zm00001d002035_T001  rna13806    8.00E-178
290-  1:  transcript:Zm00001d002036_T001  rna13804            0
290-  2:  transcript:Zm00001d002038_T003  rna13803            0
290-  3:  transcript:Zm00001d002039_T001  rna13801            0
290-  4:  transcript:Zm00001d002042_T001  rna13800            0
290-  5:  transcript:Zm00001d002046_T003  rna13799     8.00E-74
290-  6:  transcript:Zm00001d002051_T001  rna13798            0
290-  7:  transcript:Zm00001d002053_T001  rna13796            0
290-  8:  transcript:Zm00001d002054_T001  rna13795    6.00E-151
290-  9:  transcript:Zm00001d002063_T001  rna13794            0
290- 10:  transcript:Zm00001d002065_T006  rna13791     2.00E-70
290- 11:  transcript:Zm00001d002066_T001  rna13790    8.00E-176
290- 12:  transcript:Zm00001d002068_T001  rna13789            0
290- 13:  transcript:Zm00001d002071_T001  rna13788     7.00E-16
290- 14:  transcript:Zm00001d002075_T001  rna13784     1.00E-23
290- 15:  transcript:Zm00001d002079_T001  rna13781     1.00E-67
290- 16:  transcript:Zm00001d002080_T003  rna13780    5.00E-172
290- 17:  transcript:Zm00001d002082_T004  rna13779    2.00E-155
290- 18:  transcript:Zm00001d002083_T001  rna13778     7.00E-57
290- 19:  transcript:Zm00001d002084_T007  rna13777            0
290- 20:  transcript:Zm00001d002085_T001  rna13772            0
290- 21:  transcript:Zm00001d002086_T001  rna13770            0
290- 22:  transcript:Zm00001d002087_T001  rna13766            0
290- 23:  transcript:Zm00001d002088_T001  rna13764     2.00E-45
290- 24:  transcript:Zm00001d002089_T003  rna13763            0
## Alignment 291: score=1150.0 e_value=4.4e-76 N=25 2&NC_008397.2 minus
291-  0:  transcript:Zm00001d002277_T001  rna13592    3.00E-127
291-  1:  transcript:Zm00001d002278_T001  rna13591     5.00E-13
291-  2:  transcript:Zm00001d002279_T001  rna13590    9.00E-135
291-  3:  transcript:Zm00001d002282_T001  rna13589            0
291-  4:  transcript:Zm00001d002283_T002  rna13587            0
291-  5:  transcript:Zm00001d002284_T003  rna13586            0
291-  6:  transcript:Zm00001d002285_T001  rna13583     1.00E-91
291-  7:  transcript:Zm00001d002286_T001  rna13582     2.00E-79
291-  8:  transcript:Zm00001d002287_T001  rna13581            0
291-  9:  transcript:Zm00001d002288_T002  rna13580     3.00E-99
291- 10:  transcript:Zm00001d002292_T004  rna13576            0
291- 11:  transcript:Zm00001d002295_T001  rna13572     3.00E-68
291- 12:  transcript:Zm00001d002296_T001  rna13571    1.00E-135
291- 13:  transcript:Zm00001d002301_T001  rna13570     2.00E-23
291- 14:  transcript:Zm00001d002304_T001  rna13566     3.00E-11
291- 15:  transcript:Zm00001d002309_T001  rna13563     3.00E-54
291- 16:  transcript:Zm00001d002313_T003  rna13561     4.00E-50
291- 17:  transcript:Zm00001d002314_T001  rna13556    1.00E-108
291- 18:  transcript:Zm00001d002315_T002  rna13555    2.00E-173
291- 19:  transcript:Zm00001d002316_T001  rna13552            0
291- 20:  transcript:Zm00001d002317_T002  rna13550            0
291- 21:  transcript:Zm00001d002319_T001  rna13548            0
291- 22:  transcript:Zm00001d002323_T024  rna13547    1.00E-134
291- 23:  transcript:Zm00001d002325_T002  rna13546     1.00E-54
291- 24:  transcript:Zm00001d002326_T003  rna13544            0
```

Alignment 292: score=1120.0 e_value=2.5e-77 N=24 2&NC_008397.2 minus
292- 0: transcript:Zm00001d003649_T005 rna12411 1.00E-74
292- 1: transcript:Zm00001d003650_T001 rna12410 2.00E-25
292- 2: transcript:Zm00001d003652_T003 rna12409 4.00E-47
292- 3: transcript:Zm00001d003653_T002 rna12405 2.00E-51
292- 4: transcript:Zm00001d003654_T001 rna12404 0
292- 5: transcript:Zm00001d003655_T006 rna12400 7.00E-123
292- 6: transcript:Zm00001d003657_T001 rna12399 7.00E-111
292- 7: transcript:Zm00001d003659_T002 rna12398 0
292- 8: transcript:Zm00001d003660_T001 rna12395 1.00E-34
292- 9: transcript:Zm00001d003661_T001 rna12394 3.00E-104
292- 10: transcript:Zm00001d003663_T001 rna12393 2.00E-128
292- 11: transcript:Zm00001d003664_T001 rna12392 6.00E-100
292- 12: transcript:Zm00001d003667_T002 rna12391 2.00E-51
292- 13: transcript:Zm00001d003668_T001 rna12390 0
292- 14: transcript:Zm00001d003669_T001 rna12389 6.00E-125
292- 15: transcript:Zm00001d003670_T005 rna12387 0
292- 16: transcript:Zm00001d003671_T001 rna12386 0
292- 17: transcript:Zm00001d003672_T001 rna12385 1.00E-106
292- 18: transcript:Zm00001d003673_T001 rna12384 0
292- 19: transcript:Zm00001d003674_T001 rna12381 3.00E-178
292- 20: transcript:Zm00001d003676_T001 rna12379 0
292- 21: transcript:Zm00001d003677_T003 rna12376 2.00E-29
292- 22: transcript:Zm00001d003678_T003 rna12375 7.00E-43
292- 23: transcript:Zm00001d003679_T006 rna12374 0
Alignment 293: score=1040.0 e_value=1.2e-68 N=23 2&NC_008397.2 minus
293- 0: transcript:Zm00001d001979_T002 rna13851 0
293- 1: transcript:Zm00001d001980_T001 rna13850 0
293- 2: transcript:Zm00001d001982_T001 rna13849 2.00E-51
293- 3: transcript:Zm00001d001983_T003 rna13847 1.00E-47
293- 4: transcript:Zm00001d001984_T001 rna13846 8.00E-36
293- 5: transcript:Zm00001d001988_T002 rna13845 5.00E-143
293- 6: transcript:Zm00001d001989_T001 rna13839 3.00E-08
293- 7: transcript:Zm00001d001990_T001 rna13838 9.00E-19
293- 8: transcript:Zm00001d001993_T001 rna13837 0
293- 9: transcript:Zm00001d001994_T001 rna13836 0
293- 10: transcript:Zm00001d001995_T003 rna13835 0
293- 11: transcript:Zm00001d001996_T001 rna13834 7.00E-36
293- 12: transcript:Zm00001d001997_T001 rna13833 0
293- 13: transcript:Zm00001d001999_T009 rna13832 0
293- 14: transcript:Zm00001d002001_T001 rna13831 3.00E-21
293- 15: transcript:Zm00001d002004_T003 rna13826 0
293- 16: transcript:Zm00001d002005_T001 rna13825 1.00E-178
293- 17: transcript:Zm00001d002006_T013 rna13824 0
293- 18: transcript:Zm00001d002012_T002 rna13823 0
293- 19: transcript:Zm00001d002019_T001 rna13821 0
293- 20: transcript:Zm00001d002024_T002 rna13812 4.00E-80
293- 21: transcript:Zm00001d002028_T001 rna13809 1.00E-56
293- 22: transcript:Zm00001d002029_T003 rna13808 2.00E-62
Alignment 294: score=1040.0 e_value=4.1e-70 N=23 2&NC_008397.2 minus
294- 0: transcript:Zm00001d001789_T002 rna14015 1.00E-173
294- 1: transcript:Zm00001d001790_T004 rna14012 0
294- 2: transcript:Zm00001d001791_T001 rna14011 1.00E-74
294- 3: transcript:Zm00001d001798_T002 rna14010 0

294- 4: transcript:Zm00001d001803_T001 rna14006 0
294- 5: transcript:Zm00001d001804_T005 rna14005 3.00E-142
294- 6: transcript:Zm00001d001806_T001 rna14004 0
294- 7: transcript:Zm00001d001807_T001 rna14002 5.00E-78
294- 8: transcript:Zm00001d001809_T002 rna14001 0
294- 9: transcript:Zm00001d001811_T001 rna14000 8.00E-117
294- 10: transcript:Zm00001d001813_T001 rna13996 0
294- 11: transcript:Zm00001d001814_T001 rna13995 6.00E-123
294- 12: transcript:Zm00001d001817_T001 rna13993 0
294- 13: transcript:Zm00001d001819_T001 rna13992 1.00E-145
294- 14: transcript:Zm00001d001820_T001 rna13991 0
294- 15: transcript:Zm00001d001824_T001 rna13990 1.00E-54
294- 16: transcript:Zm00001d001825_T001 rna13989 0
294- 17: transcript:Zm00001d001827_T005 rna13986 0
294- 18: transcript:Zm00001d001828_T033 rna13985 2.00E-63
294- 19: transcript:Zm00001d001829_T001 rna13984 7.00E-18
294- 20: transcript:Zm00001d001831_T001 rna13982 0
294- 21: transcript:Zm00001d001832_T001 rna13980 1.00E-105
294- 22: transcript:Zm00001d001833_T001 rna13978 0
Alignment 295: score=964.0 e_value=4.3e-60 N=21 2&NC_008397.2 minus
295- 0: transcript:Zm00001d001835_T001 rna13978 0
295- 1: transcript:Zm00001d001837_T001 rna13972 3.00E-149
295- 2: transcript:Zm00001d001838_T001 rna13971 2.00E-67
295- 3: transcript:Zm00001d001839_T001 rna13970 2.00E-27
295- 4: transcript:Zm00001d001842_T001 rna13968 0
295- 5: transcript:Zm00001d001843_T001 rna13967 2.00E-81
295- 6: transcript:Zm00001d001844_T001 rna13966 2.00E-24
295- 7: transcript:Zm00001d001845_T001 rna13964 0
295- 8: transcript:Zm00001d001846_T001 rna13963 0
295- 9: transcript:Zm00001d001848_T003 rna13960 0
295- 10: transcript:Zm00001d001849_T001 rna13957 0
295- 11: transcript:Zm00001d001856_T003 rna13953 2.00E-143
295- 12: transcript:Zm00001d001858_T001 rna13951 0
295- 13: transcript:Zm00001d001861_T002 rna13949 3.00E-54
295- 14: transcript:Zm00001d001862_T001 rna13947 1.00E-35
295- 15: transcript:Zm00001d001864_T002 rna13944 6.00E-159
295- 16: transcript:Zm00001d001865_T001 rna13943 1.00E-71
295- 17: transcript:Zm00001d001866_T012 rna13942 3.00E-105
295- 18: transcript:Zm00001d001868_T001 rna13941 0
295- 19: transcript:Zm00001d001870_T001 rna13940 4.00E-137
295- 20: transcript:Zm00001d001877_T001 rna13935 0
Alignment 296: score=903.0 e_value=1.1e-61 N=20 2&NC_008397.2 minus
296- 0: transcript:Zm00001d003224_T005 rna12768 4.00E-86
296- 1: transcript:Zm00001d003227_T002 rna12767 0
296- 2: transcript:Zm00001d003242_T001 rna12761 7.00E-22
296- 3: transcript:Zm00001d003245_T001 rna12760 0
296- 4: transcript:Zm00001d003246_T001 rna12759 0
296- 5: transcript:Zm00001d003247_T002 rna12757 2.00E-66
296- 6: transcript:Zm00001d003248_T003 rna12756 1.00E-76
296- 7: transcript:Zm00001d003249_T001 rna12753 5.00E-109
296- 8: transcript:Zm00001d003250_T001 rna12750 1.00E-159
296- 9: transcript:Zm00001d003251_T001 rna12749 1.00E-32
296- 10: transcript:Zm00001d003252_T002 rna12747 0
296- 11: transcript:Zm00001d003254_T008 rna12746 6.00E-89

296- 12: transcript:Zm00001d003256_T001 rna12745 0
296- 13: transcript:Zm00001d003257_T002 rna12744 0
296- 14: transcript:Zm00001d003258_T001 rna12743 0
296- 15: transcript:Zm00001d003259_T006 rna12739 0
296- 16: transcript:Zm00001d003261_T003 rna12737 2.00E-139
296- 17: transcript:Zm00001d003262_T004 rna12735 0
296- 18: transcript:Zm00001d003264_T001 rna12734 4.00E-32
296- 19: transcript:Zm00001d003265_T003 rna12732 0
Alignment 297: score=814.0 e_value=4.2e-48 N=17 2&NC_008397.2 minus
297- 0: transcript:Zm00001d001932_T001 rna13889 2.00E-177
297- 1: transcript:Zm00001d001933_T001 rna13888 1.00E-168
297- 2: transcript:Zm00001d001934_T001 rna13887 5.00E-20
297- 3: transcript:Zm00001d001936_T001 rna13886 3.00E-141
297- 4: transcript:Zm00001d001937_T003 rna13885 2.00E-155
297- 5: transcript:Zm00001d001939_T003 rna13884 0
297- 6: transcript:Zm00001d001940_T001 rna13882 2.00E-38
297- 7: transcript:Zm00001d001941_T001 rna13880 0
297- 8: transcript:Zm00001d001945_T006 rna13877 0
297- 9: transcript:Zm00001d001947_T001 rna13874 0
297- 10: transcript:Zm00001d001949_T001 rna13873 5.00E-62
297- 11: transcript:Zm00001d001951_T001 rna13872 6.00E-76
297- 12: transcript:Zm00001d001952_T001 rna13871 1.00E-119
297- 13: transcript:Zm00001d001953_T003 rna13870 0
297- 14: transcript:Zm00001d001959_T001 rna13866 0
297- 15: transcript:Zm00001d001960_T001 rna13865 4.00E-21
297- 16: transcript:Zm00001d001961_T001 rna13863 1.00E-24
Alignment 298: score=787.0 e_value=1e-48 N=18 2&NC_008397.2 minus
298- 0: transcript:Zm00001d001879_T035 rna13934 0
298- 1: transcript:Zm00001d001881_T001 rna13932 0
298- 2: transcript:Zm00001d001883_T008 rna13930 0
298- 3: transcript:Zm00001d001885_T001 rna13929 3.00E-16
298- 4: transcript:Zm00001d001894_T001 rna13927 6.00E-78
298- 5: transcript:Zm00001d001895_T002 rna13926 0
298- 6: transcript:Zm00001d001896_T002 rna13925 4.00E-52
298- 7: transcript:Zm00001d001897_T001 rna13922 7.00E-142
298- 8: transcript:Zm00001d001900_T002 rna13921 0
298- 9: transcript:Zm00001d001901_T002 rna13919 9.00E-100
298- 10: transcript:Zm00001d001902_T002 rna13915 1.00E-73
298- 11: transcript:Zm00001d001905_T001 rna13914 2.00E-29
298- 12: transcript:Zm00001d001906_T004 rna13913 0
298- 13: transcript:Zm00001d001907_T001 rna13912 4.00E-50
298- 14: transcript:Zm00001d001908_T001 rna13910 1.00E-143
298- 15: transcript:Zm00001d001910_T002 rna13909 3.00E-73
298- 16: transcript:Zm00001d001913_T004 rna13900 1.00E-108
298- 17: transcript:Zm00001d001914_T001 rna13899 7.00E-32
Alignment 299: score=763.0 e_value=7e-49 N=18 2&NC_008397.2 minus
299- 0: transcript:Zm00001d002240_T001 rna13636 0
299- 1: transcript:Zm00001d002241_T002 rna13633 2.00E-110
299- 2: transcript:Zm00001d002243_T004 rna13632 3.00E-166
299- 3: transcript:Zm00001d002244_T003 rna13631 0
299- 4: transcript:Zm00001d002247_T001 rna13629 1.00E-41
299- 5: transcript:Zm00001d002250_T001 rna13628 4.00E-142
299- 6: transcript:Zm00001d002252_T001 rna13625 3.00E-105
299- 7: transcript:Zm00001d002256_T022 rna13622 0

299- 8: transcript:Zm00001d002257_T006 rna13618 1.00E-76
299- 9: transcript:Zm00001d002258_T001 rna13616 0
299- 10: transcript:Zm00001d002259_T003 rna13615 5.00E-50
299- 11: transcript:Zm00001d002261_T003 rna13613 0
299- 12: transcript:Zm00001d002266_T002 rna13611 0
299- 13: transcript:Zm00001d002267_T001 rna13606 1.00E-57
299- 14: transcript:Zm00001d002268_T001 rna13600 2.00E-20
299- 15: transcript:Zm00001d002272_T007 rna13595 0
299- 16: transcript:Zm00001d002274_T001 rna13594 1.00E-150
299- 17: transcript:Zm00001d002275_T001 rna13593 3.00E-132
Alignment 300: score=679.0 e_value=2.7e-40 N=16 2&NC_008397.2 minus
300- 0: transcript:Zm00001d002618_T001 rna13274 4.00E-108
300- 1: transcript:Zm00001d002621_T002 rna13272 1.00E-79
300- 2: transcript:Zm00001d002623_T001 rna13270 0
300- 3: transcript:Zm00001d002624_T003 rna13268 6.00E-115
300- 4: transcript:Zm00001d002625_T001 rna13267 0
300- 5: transcript:Zm00001d002626_T001 rna13262 0
300- 6: transcript:Zm00001d002630_T002 rna13261 0
300- 7: transcript:Zm00001d002631_T003 rna13259 0
300- 8: transcript:Zm00001d002632_T001 rna13258 0
300- 9: transcript:Zm00001d002639_T001 rna13255 3.00E-150
300- 10: transcript:Zm00001d002640_T011 rna13253 7.00E-20
300- 11: transcript:Zm00001d002641_T001 rna13250 0
300- 12: transcript:Zm00001d002644_T030 rna13244 2.00E-39
300- 13: transcript:Zm00001d002649_T001 rna13242 2.00E-91
300- 14: transcript:Zm00001d002650_T003 rna13241 0
300- 15: transcript:Zm00001d002654_T001 rna13240 0
Alignment 301: score=658.0 e_value=3.2e-34 N=14 2&NC_008397.2 minus
301- 0: transcript:Zm00001d002741_T001 rna13148 0
301- 1: transcript:Zm00001d002744_T001 rna13139 7.00E-40
301- 2: transcript:Zm00001d002750_T001 rna13137 3.00E-108
301- 3: transcript:Zm00001d002751_T001 rna13135 0
301- 4: transcript:Zm00001d002754_T001 rna13134 4.00E-112
301- 5: transcript:Zm00001d002756_T001 rna13133 9.00E-11
301- 6: transcript:Zm00001d002757_T001 rna13132 7.00E-91
301- 7: transcript:Zm00001d002758_T002 rna13129 1.00E-113
301- 8: transcript:Zm00001d002759_T001 rna13128 0
301- 9: transcript:Zm00001d002760_T001 rna13127 1.00E-71
301- 10: transcript:Zm00001d002761_T001 rna13125 6.00E-28
301- 11: transcript:Zm00001d002768_T001 rna13124 2.00E-71
301- 12: transcript:Zm00001d002772_T001 rna13119 1.00E-51
301- 13: transcript:Zm00001d002773_T001 rna13118 2.00E-77
Alignment 302: score=650.0 e_value=8.8e-38 N=14 2&NC_008397.2 minus
302- 0: transcript:Zm00001d003948_T001 rna12173 0
302- 1: transcript:Zm00001d003949_T001 rna12172 3.00E-43
302- 2: transcript:Zm00001d003957_T002 rna12171 1.00E-48
302- 3: transcript:Zm00001d003958_T013 rna12170 0
302- 4: transcript:Zm00001d003961_T001 rna12169 3.00E-31
302- 5: transcript:Zm00001d003962_T001 rna12168 6.00E-39
302- 6: transcript:Zm00001d003974_T001 rna12162 3.00E-67
302- 7: transcript:Zm00001d003975_T002 rna12161 1.00E-63
302- 8: transcript:Zm00001d003981_T001 rna12159 1.00E-35
302- 9: transcript:Zm00001d003984_T001 rna12158 2.00E-153
302- 10: transcript:Zm00001d003993_T001 rna12157 3.00E-168

302- 11: transcript:Zm00001d003996_T001 rna12147 2.00E-11
302- 12: transcript:Zm00001d003999_T001 rna12145 4.00E-80
302- 13: transcript:Zm00001d004006_T001 rna12144 0
Alignment 303: score=627.0 e_value=1.6e-31 N=13 2&NC_008397.2 minus
303- 0: transcript:Zm00001d002090_T001 rna13762 1.00E-49
303- 1: transcript:Zm00001d002091_T002 rna13761 4.00E-113
303- 2: transcript:Zm00001d002093_T001 rna13757 2.00E-42
303- 3: transcript:Zm00001d002094_T001 rna13756 7.00E-45
303- 4: transcript:Zm00001d002095_T001 rna13755 3.00E-42
303- 5: transcript:Zm00001d002096_T001 rna13753 4.00E-52
303- 6: transcript:Zm00001d002097_T001 rna13751 6.00E-09
303- 7: transcript:Zm00001d002098_T001 rna13750 0
303- 8: transcript:Zm00001d002099_T003 rna13749 3.00E-167
303- 9: transcript:Zm00001d002100_T001 rna13748 5.00E-107
303- 10: transcript:Zm00001d002103_T002 rna13746 0
303- 11: transcript:Zm00001d002104_T001 rna13745 2.00E-34
303- 12: transcript:Zm00001d002105_T001 rna13744 0
Alignment 304: score=588.0 e_value=4.5e-30 N=13 2&NC_008397.2 minus
304- 0: transcript:Zm00001d002656_T002 rna13238 0
304- 1: transcript:Zm00001d002658_T003 rna13230 0
304- 2: transcript:Zm00001d002659_T001 rna13229 2.00E-45
304- 3: transcript:Zm00001d002661_T001 rna13224 1.00E-125
304- 4: transcript:Zm00001d002662_T010 rna13221 0
304- 5: transcript:Zm00001d002663_T004 rna13217 2.00E-53
304- 6: transcript:Zm00001d002668_T004 rna13216 2.00E-18
304- 7: transcript:Zm00001d002669_T002 rna13215 0
304- 8: transcript:Zm00001d002671_T001 rna13214 8.00E-95
304- 9: transcript:Zm00001d002673_T001 rna13213 0
304- 10: transcript:Zm00001d002676_T001 rna13210 0
304- 11: transcript:Zm00001d002677_T001 rna13209 1.00E-97
304- 12: transcript:Zm00001d002678_T004 rna13208 0
Alignment 305: score=573.0 e_value=3.6e-27 N=12 2&NC_008397.2 minus
305- 0: transcript:Zm00001d002797_T001 rna13095 0
305- 1: transcript:Zm00001d002799_T001 rna13091 1.00E-93
305- 2: transcript:Zm00001d002801_T001 rna13089 0
305- 3: transcript:Zm00001d002802_T001 rna13088 0
305- 4: transcript:Zm00001d002803_T001 rna13084 1.00E-82
305- 5: transcript:Zm00001d002806_T001 rna13083 5.00E-116
305- 6: transcript:Zm00001d002810_T001 rna13082 4.00E-67
305- 7: transcript:Zm00001d002811_T002 rna13080 3.00E-53
305- 8: transcript:Zm00001d002812_T002 rna13079 2.00E-119
305- 9: transcript:Zm00001d002815_T001 rna13078 6.00E-102
305- 10: transcript:Zm00001d002816_T001 rna13077 3.00E-46
305- 11: transcript:Zm00001d002817_T004 rna13076 0
Alignment 306: score=548.0 e_value=4.9e-31 N=12 2&NC_008397.2 minus
306- 0: transcript:Zm00001d004012_T001 rna12137 0
306- 1: transcript:Zm00001d004015_T001 rna12133 2.00E-54
306- 2: transcript:Zm00001d004019_T001 rna12131 0
306- 3: transcript:Zm00001d004020_T003 rna12130 9.00E-118
306- 4: transcript:Zm00001d004021_T001 rna12129 0
306- 5: transcript:Zm00001d004022_T001 rna12128 2.00E-92
306- 6: transcript:Zm00001d004023_T001 rna12122 8.00E-110
306- 7: transcript:Zm00001d004025_T001 rna12121 2.00E-129
306- 8: transcript:Zm00001d004036_T002 rna12118 7.00E-58

306- 9: transcript:Zm00001d004042_T002 rna12115 2.00E-54
306- 10: transcript:Zm00001d004051_T001 rna12113 3.00E-100
306- 11: transcript:Zm00001d004052_T001 rna12112 1.00E-69
Alignment 307: score=545.0 e_value=5.6e-26 N=12 2&NC_008397.2 minus
307- 0: transcript:Zm00001d002391_T005 rna13479 0
307- 1: transcript:Zm00001d002393_T001 rna13476 1.00E-32
307- 2: transcript:Zm00001d002395_T002 rna13474 2.00E-166
307- 3: transcript:Zm00001d002396_T001 rna13473 0
307- 4: transcript:Zm00001d002399_T001 rna13472 2.00E-123
307- 5: transcript:Zm00001d002403_T001 rna13471 8.00E-82
307- 6: transcript:Zm00001d002405_T001 rna13470 2.00E-150
307- 7: transcript:Zm00001d002409_T002 rna13468 6.00E-10
307- 8: transcript:Zm00001d002410_T001 rna13463 3.00E-100
307- 9: transcript:Zm00001d002413_T005 rna13462 0
307- 10: transcript:Zm00001d002415_T001 rna13459 6.00E-33
307- 11: transcript:Zm00001d002416_T002 rna13457 2.00E-145
Alignment 308: score=518.0 e_value=3.2e-25 N=11 2&NC_008397.2 minus
308- 0: transcript:Zm00001d001765_T001 rna14042 3.00E-73
308- 1: transcript:Zm00001d001771_T001 rna14039 6.00E-133
308- 2: transcript:Zm00001d001774_T001 rna14036 4.00E-113
308- 3: transcript:Zm00001d001776_T001 rna14034 5.00E-30
308- 4: transcript:Zm00001d001779_T001 rna14031 3.00E-27
308- 5: transcript:Zm00001d001780_T002 rna14030 0
308- 6: transcript:Zm00001d001784_T002 rna14027 5.00E-164
308- 7: transcript:Zm00001d001785_T001 rna14026 0
308- 8: transcript:Zm00001d001786_T005 rna14023 5.00E-141
308- 9: transcript:Zm00001d001787_T002 rna14021 1.00E-149
308- 10: transcript:Zm00001d001788_T004 rna14019 0
Alignment 309: score=480.0 e_value=2.5e-20 N=10 2&NC_008397.2 minus
309- 0: transcript:Zm00001d003071_T003 rna12905 2.00E-93
309- 1: transcript:Zm00001d003072_T001 rna12903 5.00E-158
309- 2: transcript:Zm00001d003076_T001 rna12902 8.00E-98
309- 3: transcript:Zm00001d003079_T001 rna12900 0
309- 4: transcript:Zm00001d003080_T001 rna12898 2.00E-105
309- 5: transcript:Zm00001d003081_T003 rna12897 1.00E-66
309- 6: transcript:Zm00001d003083_T002 rna12896 0
309- 7: transcript:Zm00001d003084_T001 rna12894 2.00E-34
309- 8: transcript:Zm00001d003085_T001 rna12889 0
309- 9: transcript:Zm00001d003087_T002 rna12888 0
Alignment 310: score=368.0 e_value=7.5e-15 N=8 2&NC_008397.2 minus
310- 0: transcript:Zm00001d001963_T001 rna13863 3.00E-27
310- 1: transcript:Zm00001d001965_T001 rna13862 3.00E-22
310- 2: transcript:Zm00001d001966_T001 rna13860 1.00E-90
310- 3: transcript:Zm00001d001967_T001 rna13859 6.00E-77
310- 4: transcript:Zm00001d001968_T001 rna13858 0
310- 5: transcript:Zm00001d001974_T001 rna13855 0
310- 6: transcript:Zm00001d001976_T001 rna13854 0
310- 7: transcript:Zm00001d001978_T012 rna13852 0
Alignment 311: score=309.0 e_value=1.7e-16 N=7 2&NC_008397.2 minus
311- 0: transcript:Zm00001d002200_T001 rna13676 2.00E-150
311- 1: transcript:Zm00001d002227_T001 rna13650 7.00E-51
311- 2: transcript:Zm00001d002228_T001 rna13645 3.00E-14
311- 3: transcript:Zm00001d002229_T001 rna13644 4.00E-43
311- 4: transcript:Zm00001d002230_T001 rna13643 2.00E-73

311- 5: transcript:Zm00001d002234_T001 rna13642 0
311- 6: transcript:Zm00001d002235_T002 rna13641 0
Alignment 312: score=295.0 e_value=8.5e-14 N=7 2&NC_008397.2 minus
312- 0: transcript:Zm00001d003684_T001 rna12373 2.00E-60
312- 1: transcript:Zm00001d003685_T001 rna12372 5.00E-120
312- 2: transcript:Zm00001d003707_T002 rna12366 0
312- 3: transcript:Zm00001d003710_T002 rna12364 0
312- 4: transcript:Zm00001d003712_T001 rna12363 2.00E-22
312- 5: transcript:Zm00001d003713_T006 rna12354 0
312- 6: transcript:Zm00001d003723_T001 rna12343 0
Alignment 313: score=3282.0 e_value=0 N=76 2&NC_008400.2 plus
313- 0: transcript:Zm00001d006439_T001 rna20091 3.00E-124
313- 1: transcript:Zm00001d006447_T001 rna20117 6.00E-59
313- 2: transcript:Zm00001d006449_T001 rna20127 0
313- 3: transcript:Zm00001d006451_T001 rna20133 3.00E-42
313- 4: transcript:Zm00001d006453_T001 rna20134 2.00E-32
313- 5: transcript:Zm00001d006454_T003 rna20136 0
313- 6: transcript:Zm00001d006455_T001 rna20137 2.00E-161
313- 7: transcript:Zm00001d006456_T001 rna20139 3.00E-94
313- 8: transcript:Zm00001d006457_T002 rna20141 1.00E-117
313- 9: transcript:Zm00001d006459_T003 rna20146 0
313- 10: transcript:Zm00001d006460_T002 rna20147 2.00E-49
313- 11: transcript:Zm00001d006461_T001 rna20148 0
313- 12: transcript:Zm00001d006463_T001 rna20150 8.00E-99
313- 13: transcript:Zm00001d006464_T001 rna20152 2.00E-176
313- 14: transcript:Zm00001d006467_T002 rna20154 0
313- 15: transcript:Zm00001d006470_T003 rna20155 0
313- 16: transcript:Zm00001d006472_T003 rna20168 2.00E-133
313- 17: transcript:Zm00001d006474_T002 rna20171 2.00E-69
313- 18: transcript:Zm00001d006475_T001 rna20172 4.00E-118
313- 19: transcript:Zm00001d006478_T001 rna20173 0
313- 20: transcript:Zm00001d006479_T006 rna20180 0
313- 21: transcript:Zm00001d006481_T001 rna20183 2.00E-145
313- 22: transcript:Zm00001d006489_T001 rna20185 1.00E-43
313- 23: transcript:Zm00001d006494_T004 rna20188 0
313- 24: transcript:Zm00001d006495_T001 rna20189 1.00E-21
313- 25: transcript:Zm00001d006496_T004 rna20190 4.00E-39
313- 26: transcript:Zm00001d006497_T002 rna20191 0
313- 27: transcript:Zm00001d006499_T005 rna20193 0
313- 28: transcript:Zm00001d006503_T001 rna20197 6.00E-95
313- 29: transcript:Zm00001d006504_T005 rna20207 0
313- 30: transcript:Zm00001d006505_T002 rna20208 7.00E-34
313- 31: transcript:Zm00001d006507_T021 rna20210 5.00E-108
313- 32: transcript:Zm00001d006508_T011 rna20221 0
313- 33: transcript:Zm00001d006509_T001 rna20222 2.00E-113
313- 34: transcript:Zm00001d006510_T001 rna20223 7.00E-69
313- 35: transcript:Zm00001d006511_T002 rna20228 0
313- 36: transcript:Zm00001d006512_T001 rna20231 0
313- 37: transcript:Zm00001d006515_T001 rna20232 1.00E-30
313- 38: transcript:Zm00001d006517_T001 rna20237 0
313- 39: transcript:Zm00001d006520_T001 rna20258 0
313- 40: transcript:Zm00001d006521_T001 rna20259 2.00E-56
313- 41: transcript:Zm00001d006524_T001 rna20262 5.00E-96
313- 42: transcript:Zm00001d006525_T001 rna20268 7.00E-93

313- 43: transcript:Zm00001d006527_T001 rna20276 8.00E-85
313- 44: transcript:Zm00001d006531_T001 rna20282 1.00E-28
313- 45: transcript:Zm00001d006534_T002 rna20303 7.00E-14
313- 46: transcript:Zm00001d006536_T001 rna20310 5.00E-17
313- 47: transcript:Zm00001d006539_T001 rna20322 0
313- 48: transcript:Zm00001d006540_T001 rna20329 2.00E-90
313- 49: transcript:Zm00001d006541_T002 rna20330 3.00E-121
313- 50: transcript:Zm00001d006547_T001 rna20332 4.00E-93
313- 51: transcript:Zm00001d006549_T002 rna20334 0
313- 52: transcript:Zm00001d006551_T001 rna20335 0
313- 53: transcript:Zm00001d006553_T001 rna20340 0
313- 54: transcript:Zm00001d006561_T001 rna20349 4.00E-76
313- 55: transcript:Zm00001d006562_T001 rna20352 2.00E-146
313- 56: transcript:Zm00001d006564_T002 rna20353 2.00E-45
313- 57: transcript:Zm00001d006565_T002 rna20354 1.00E-64
313- 58: transcript:Zm00001d006566_T002 rna20355 1.00E-41
313- 59: transcript:Zm00001d006568_T001 rna20361 7.00E-111
313- 60: transcript:Zm00001d006571_T001 rna20362 0
313- 61: transcript:Zm00001d006573_T023 rna20377 0
313- 62: transcript:Zm00001d006574_T001 rna20382 2.00E-115
313- 63: transcript:Zm00001d006580_T005 rna20388 0
313- 64: transcript:Zm00001d006581_T001 rna20389 1.00E-151
313- 65: transcript:Zm00001d006582_T002 rna20390 0
313- 66: transcript:Zm00001d006585_T001 rna20391 1.00E-110
313- 67: transcript:Zm00001d006587_T001 rna20394 0
313- 68: transcript:Zm00001d006588_T001 rna20395 0
313- 69: transcript:Zm00001d006590_T002 rna20403 0
313- 70: transcript:Zm00001d006591_T001 rna20407 2.00E-46
313- 71: transcript:Zm00001d006592_T001 rna20413 2.00E-49
313- 72: transcript:Zm00001d006593_T009 rna20414 0
313- 73: transcript:Zm00001d006594_T001 rna20415 5.00E-44
313- 74: transcript:Zm00001d006595_T001 rna20416 2.00E-67
313- 75: transcript:Zm00001d006597_T003 rna20417 1.00E-148
Alignment 314: score=1781.0 e_value=3.5e-144 N=39 2&NC_008400.2 plus
314- 0: transcript:Zm00001d007123_T001 rna21075 1.00E-50
314- 1: transcript:Zm00001d007124_T001 rna21076 1.00E-36
314- 2: transcript:Zm00001d007125_T001 rna21077 0
314- 3: transcript:Zm00001d007130_T001 rna21078 2.00E-23
314- 4: transcript:Zm00001d007133_T002 rna21080 0
314- 5: transcript:Zm00001d007135_T001 rna21081 1.00E-31
314- 6: transcript:Zm00001d007139_T007 rna21084 0
314- 7: transcript:Zm00001d007143_T001 rna21085 0
314- 8: transcript:Zm00001d007144_T001 rna21086 5.00E-152
314- 9: transcript:Zm00001d007145_T001 rna21087 0
314- 10: transcript:Zm00001d007152_T001 rna21093 0
314- 11: transcript:Zm00001d007153_T001 rna21095 2.00E-42
314- 12: transcript:Zm00001d007154_T001 rna21097 4.00E-66
314- 13: transcript:Zm00001d007155_T001 rna21098 0
314- 14: transcript:Zm00001d007157_T001 rna21099 2.00E-67
314- 15: transcript:Zm00001d007158_T014 rna21103 0
314- 16: transcript:Zm00001d007160_T001 rna21112 1.00E-130
314- 17: transcript:Zm00001d007161_T001 rna21113 5.00E-129
314- 18: transcript:Zm00001d007164_T001 rna21119 0
314- 19: transcript:Zm00001d007166_T001 rna21121 0

314- 20: transcript:Zm00001d007167_T001 rna21122 0
314- 21: transcript:Zm00001d007168_T001 rna21124 5.00E-60
314- 22: transcript:Zm00001d007169_T001 rna21126 2.00E-31
314- 23: transcript:Zm00001d007172_T001 rna21127 2.00E-173
314- 24: transcript:Zm00001d007173_T003 rna21128 0
314- 25: transcript:Zm00001d007174_T001 rna21130 3.00E-20
314- 26: transcript:Zm00001d007175_T001 rna21133 2.00E-145
314- 27: transcript:Zm00001d007179_T001 rna21135 3.00E-170
314- 28: transcript:Zm00001d007180_T001 rna21138 0
314- 29: transcript:Zm00001d007181_T001 rna21139 1.00E-100
314- 30: transcript:Zm00001d007183_T002 rna21140 0
314- 31: transcript:Zm00001d007184_T001 rna21142 0
314- 32: transcript:Zm00001d007185_T001 rna21148 7.00E-133
314- 33: transcript:Zm00001d007186_T004 rna21149 0
314- 34: transcript:Zm00001d007187_T002 rna21151 7.00E-80
314- 35: transcript:Zm00001d007188_T001 rna21167 0
314- 36: transcript:Zm00001d007189_T003 rna21168 3.00E-52
314- 37: transcript:Zm00001d007195_T003 rna21177 2.00E-134
314- 38: transcript:Zm00001d007196_T001 rna21179 1.00E-139
Alignment 315: score=1558.0 e_value=1.2e-113 N=34 2&NC_008400.2 plus
315- 0: transcript:Zm00001d006646_T001 rna20490 2.00E-23
315- 1: transcript:Zm00001d006651_T001 rna20502 0
315- 2: transcript:Zm00001d006654_T001 rna20504 5.00E-111
315- 3: transcript:Zm00001d006657_T001 rna20511 0
315- 4: transcript:Zm00001d006658_T005 rna20516 0
315- 5: transcript:Zm00001d006659_T001 rna20518 4.00E-70
315- 6: transcript:Zm00001d006663_T001 rna20519 2.00E-165
315- 7: transcript:Zm00001d006667_T003 rna20520 7.00E-24
315- 8: transcript:Zm00001d006670_T001 rna20521 1.00E-74
315- 9: transcript:Zm00001d006673_T001 rna20522 0
315- 10: transcript:Zm00001d006676_T001 rna20525 9.00E-134
315- 11: transcript:Zm00001d006677_T001 rna20532 1.00E-126
315- 12: transcript:Zm00001d006678_T001 rna20535 0
315- 13: transcript:Zm00001d006679_T001 rna20536 0
315- 14: transcript:Zm00001d006681_T004 rna20537 0
315- 15: transcript:Zm00001d006687_T001 rna20539 9.00E-82
315- 16: transcript:Zm00001d006688_T001 rna20541 0
315- 17: transcript:Zm00001d006699_T001 rna20547 1.00E-37
315- 18: transcript:Zm00001d006700_T003 rna20548 0
315- 19: transcript:Zm00001d006701_T001 rna20550 0
315- 20: transcript:Zm00001d006702_T001 rna20551 0
315- 21: transcript:Zm00001d006704_T005 rna20555 0
315- 22: transcript:Zm00001d006705_T001 rna20556 0
315- 23: transcript:Zm00001d006708_T001 rna20557 0
315- 24: transcript:Zm00001d006710_T004 rna20558 0
315- 25: transcript:Zm00001d006713_T001 rna20562 8.00E-101
315- 26: transcript:Zm00001d006714_T001 rna20563 5.00E-104
315- 27: transcript:Zm00001d006717_T008 rna20567 0
315- 28: transcript:Zm00001d006720_T001 rna20570 4.00E-129
315- 29: transcript:Zm00001d006721_T001 rna20571 0
315- 30: transcript:Zm00001d006722_T001 rna20573 0
315- 31: transcript:Zm00001d006723_T001 rna20574 1.00E-24
315- 32: transcript:Zm00001d006725_T001 rna20576 0
315- 33: transcript:Zm00001d006726_T001 rna20577 0

```
## Alignment 316: score=1318.0 e_value=6.1e-103 N=30 2&NC_008400.2 plus
316-  0:  transcript:Zm00001d006866_T003  rna20730            0
316-  1:  transcript:Zm00001d006868_T007  rna20731            0
316-  2:  transcript:Zm00001d006871_T001  rna20732    4.00E-166
316-  3:  transcript:Zm00001d006873_T001  rna20736     1.00E-26
316-  4:  transcript:Zm00001d006874_T001  rna20738    4.00E-170
316-  5:  transcript:Zm00001d006875_T001  rna20739            0
316-  6:  transcript:Zm00001d006879_T001  rna20740     4.00E-12
316-  7:  transcript:Zm00001d006882_T001  rna20747     2.00E-40
316-  8:  transcript:Zm00001d006883_T001  rna20750    4.00E-106
316-  9:  transcript:Zm00001d006884_T001  rna20752     4.00E-25
316- 10:  transcript:Zm00001d006885_T004  rna20753            0
316- 11:  transcript:Zm00001d006894_T001  rna20761            0
316- 12:  transcript:Zm00001d006896_T001  rna20763     5.00E-83
316- 13:  transcript:Zm00001d006899_T001  rna20769    2.00E-164
316- 14:  transcript:Zm00001d006900_T004  rna20770            0
316- 15:  transcript:Zm00001d006903_T001  rna20775     1.00E-60
316- 16:  transcript:Zm00001d006904_T001  rna20776     3.00E-66
316- 17:  transcript:Zm00001d006906_T010  rna20779     3.00E-86
316- 18:  transcript:Zm00001d006907_T002  rna20780     5.00E-46
316- 19:  transcript:Zm00001d006910_T003  rna20781    6.00E-167
316- 20:  transcript:Zm00001d006913_T002  rna20783    4.00E-148
316- 21:  transcript:Zm00001d006914_T001  rna20786     3.00E-24
316- 22:  transcript:Zm00001d006915_T001  rna20787     2.00E-32
316- 23:  transcript:Zm00001d006916_T001  rna20790            0
316- 24:  transcript:Zm00001d006917_T001  rna20792            0
316- 25:  transcript:Zm00001d006918_T001  rna20793            0
316- 26:  transcript:Zm00001d006922_T001  rna20800            0
316- 27:  transcript:Zm00001d006924_T001  rna20805     4.00E-19
316- 28:  transcript:Zm00001d006931_T002  rna20833     4.00E-13
316- 29:  transcript:Zm00001d006940_T001  rna20859     1.00E-34
## Alignment 317: score=1081.0 e_value=4.6e-71 N=23 2&NC_008400.2 plus
317-  0:  transcript:Zm00001d006610_T001  rna20436            0
317-  1:  transcript:Zm00001d006611_T001  rna20437            0
317-  2:  transcript:Zm00001d006612_T001  rna20438     3.00E-42
317-  3:  transcript:Zm00001d006614_T001  rna20439            0
317-  4:  transcript:Zm00001d006616_T001  rna20440     3.00E-13
317-  5:  transcript:Zm00001d006617_T001  rna20441     7.00E-63
317-  6:  transcript:Zm00001d006619_T001  rna20451            0
317-  7:  transcript:Zm00001d006620_T001  rna20454    2.00E-144
317-  8:  transcript:Zm00001d006621_T004  rna20455            0
317-  9:  transcript:Zm00001d006623_T001  rna20456            0
317- 10:  transcript:Zm00001d006624_T001  rna20458     1.00E-40
317- 11:  transcript:Zm00001d006625_T002  rna20459            0
317- 12:  transcript:Zm00001d006626_T001  rna20460    2.00E-101
317- 13:  transcript:Zm00001d006627_T001  rna20465    1.00E-170
317- 14:  transcript:Zm00001d006628_T005  rna20473            0
317- 15:  transcript:Zm00001d006630_T001  rna20476    4.00E-131
317- 16:  transcript:Zm00001d006631_T001  rna20477            0
317- 17:  transcript:Zm00001d006637_T001  rna20479     7.00E-52
317- 18:  transcript:Zm00001d006638_T001  rna20481            0
317- 19:  transcript:Zm00001d006639_T001  rna20483     2.00E-28
317- 20:  transcript:Zm00001d006640_T001  rna20485            0
317- 21:  transcript:Zm00001d006644_T002  rna20486            0
```

317- 22: transcript:Zm00001d006645_T001 rna20489 5.00E-143
Alignment 318: score=1074.0 e_value=4.4e-71 N=24 2&NC_008400.2 plus
318- 0: transcript:Zm00001d006731_T003 rna20578 0
318- 1: transcript:Zm00001d006732_T001 rna20580 0
318- 2: transcript:Zm00001d006735_T001 rna20582 1.00E-31
318- 3: transcript:Zm00001d006738_T001 rna20584 0
318- 4: transcript:Zm00001d006739_T001 rna20586 2.00E-94
318- 5: transcript:Zm00001d006744_T001 rna20587 0
318- 6: transcript:Zm00001d006746_T001 rna20589 5.00E-66
318- 7: transcript:Zm00001d006749_T001 rna20595 1.00E-118
318- 8: transcript:Zm00001d006750_T001 rna20599 3.00E-35
318- 9: transcript:Zm00001d006751_T001 rna20601 0
318- 10: transcript:Zm00001d006752_T003 rna20602 0
318- 11: transcript:Zm00001d006753_T001 rna20603 0
318- 12: transcript:Zm00001d006754_T001 rna20604 5.00E-61
318- 13: transcript:Zm00001d006756_T001 rna20606 3.00E-41
318- 14: transcript:Zm00001d006757_T003 rna20610 4.00E-133
318- 15: transcript:Zm00001d006758_T003 rna20612 0
318- 16: transcript:Zm00001d006759_T012 rna20613 0
318- 17: transcript:Zm00001d006760_T001 rna20615 2.00E-108
318- 18: transcript:Zm00001d006761_T001 rna20623 1.00E-171
318- 19: transcript:Zm00001d006763_T001 rna20627 0
318- 20: transcript:Zm00001d006769_T003 rna20629 0
318- 21: transcript:Zm00001d006774_T001 rna20631 9.00E-14
318- 22: transcript:Zm00001d006775_T001 rna20632 6.00E-21
318- 23: transcript:Zm00001d006776_T001 rna20640 3.00E-52
Alignment 319: score=1020.0 e_value=3.7e-76 N=24 2&NC_008400.2 plus
319- 0: transcript:Zm00001d006345_T001 rna19978 1.00E-49
319- 1: transcript:Zm00001d006350_T001 rna19994 0
319- 2: transcript:Zm00001d006351_T001 rna19997 0
319- 3: transcript:Zm00001d006353_T001 rna19999 2.00E-154
319- 4: transcript:Zm00001d006354_T002 rna20001 0
319- 5: transcript:Zm00001d006355_T011 rna20002 0
319- 6: transcript:Zm00001d006357_T001 rna20003 0
319- 7: transcript:Zm00001d006358_T001 rna20005 7.00E-50
319- 8: transcript:Zm00001d006360_T001 rna20010 3.00E-134
319- 9: transcript:Zm00001d006361_T001 rna20012 5.00E-164
319- 10: transcript:Zm00001d006368_T003 rna20019 2.00E-131
319- 11: transcript:Zm00001d006371_T001 rna20036 0
319- 12: transcript:Zm00001d006373_T002 rna20038 0
319- 13: transcript:Zm00001d006375_T001 rna20044 0
319- 14: transcript:Zm00001d006377_T001 rna20046 0
319- 15: transcript:Zm00001d006382_T004 rna20051 1.00E-134
319- 16: transcript:Zm00001d006384_T005 rna20052 0
319- 17: transcript:Zm00001d006389_T001 rna20054 0
319- 18: transcript:Zm00001d006397_T001 rna20055 0
319- 19: transcript:Zm00001d006398_T001 rna20062 1.00E-75
319- 20: transcript:Zm00001d006408_T001 rna20068 0
319- 21: transcript:Zm00001d006409_T002 rna20069 6.00E-130
319- 22: transcript:Zm00001d006410_T013 rna20071 9.00E-65
319- 23: transcript:Zm00001d006415_T001 rna20073 9.00E-109
Alignment 320: score=966.0 e_value=5.8e-70 N=22 2&NC_008400.2 plus
320- 0: transcript:Zm00001d005456_T001 rna19608 7.00E-144
320- 1: transcript:Zm00001d005457_T001 rna19621 3.00E-21

```
320-  2:  transcript:Zm00001d005459_T001  rna19628    2.00E-128
320-  3:  transcript:Zm00001d005460_T003  rna19630            0
320-  4:  transcript:Zm00001d005461_T001  rna19631            0
320-  5:  transcript:Zm00001d005462_T001  rna19632     1.00E-97
320-  6:  transcript:Zm00001d005464_T001  rna19633     5.00E-91
320-  7:  transcript:Zm00001d005466_T001  rna19634    3.00E-150
320-  8:  transcript:Zm00001d005468_T001  rna19640    2.00E-143
320-  9:  transcript:Zm00001d005473_T001  rna19641            0
320- 10:  transcript:Zm00001d005478_T009  rna19657            0
320- 11:  transcript:Zm00001d005479_T001  rna19666            0
320- 12:  transcript:Zm00001d005480_T001  rna19676            0
320- 13:  transcript:Zm00001d005481_T001  rna19677            0
320- 14:  transcript:Zm00001d005482_T001  rna19678    9.00E-133
320- 15:  transcript:Zm00001d005484_T003  rna19679            0
320- 16:  transcript:Zm00001d005485_T001  rna19681            0
320- 17:  transcript:Zm00001d005489_T006  rna19686            0
320- 18:  transcript:Zm00001d005497_T001  rna19700     7.00E-21
320- 19:  transcript:Zm00001d005498_T001  rna19701            0
320- 20:  transcript:Zm00001d005500_T001  rna19716     2.00E-51
320- 21:  transcript:Zm00001d005501_T001  rna19718            0
## Alignment 321: score=901.0 e_value=1.6e-62 N=21 2&NC_008400.2 plus
321-  0:  transcript:Zm00001d007839_T001  rna19088    6.00E-143
321-  1:  transcript:Zm00001d007840_T001  rna19089    7.00E-137
321-  2:  transcript:Zm00001d007842_T002  rna19102    4.00E-171
321-  3:  transcript:Zm00001d007843_T001  rna19111            0
321-  4:  transcript:Zm00001d007844_T001  rna19124     6.00E-41
321-  5:  transcript:Zm00001d007845_T001  rna19128            0
321-  6:  transcript:Zm00001d007848_T001  rna19149    4.00E-169
321-  7:  transcript:Zm00001d007849_T002  rna19151            0
321-  8:  transcript:Zm00001d007857_T001  rna19158    1.00E-129
321-  9:  transcript:Zm00001d007858_T001  rna19169            0
321- 10:  transcript:Zm00001d007862_T002  rna19180            0
321- 11:  transcript:Zm00001d007864_T001  rna19195     5.00E-67
321- 12:  transcript:Zm00001d007865_T001  rna19197            0
321- 13:  transcript:Zm00001d007868_T001  rna19208    5.00E-141
321- 14:  transcript:Zm00001d007870_T001  rna19211    1.00E-108
321- 15:  transcript:Zm00001d007872_T001  rna19219     7.00E-37
321- 16:  transcript:Zm00001d007877_T001  rna19224     3.00E-98
321- 17:  transcript:Zm00001d007881_T002  rna19229     1.00E-32
321- 18:  transcript:Zm00001d007882_T001  rna19234     1.00E-32
321- 19:  transcript:Zm00001d007884_T001  rna19240     4.00E-70
321- 20:  transcript:Zm00001d007885_T001  rna19241     3.00E-74
## Alignment 322: score=800.0 e_value=9.2e-50 N=18 2&NC_008400.2 plus
322-  0:  transcript:Zm00001d007197_T001  rna21175            0
322-  1:  transcript:Zm00001d007202_T003  rna21194     2.00E-11
322-  2:  transcript:Zm00001d007205_T001  rna21198            0
322-  3:  transcript:Zm00001d007206_T001  rna21200     1.00E-19
322-  4:  transcript:Zm00001d007207_T002  rna21202            0
322-  5:  transcript:Zm00001d007208_T001  rna21203            0
322-  6:  transcript:Zm00001d007209_T001  rna21204     1.00E-15
322-  7:  transcript:Zm00001d007213_T001  rna21205            0
322-  8:  transcript:Zm00001d007215_T001  rna21209            0
322-  9:  transcript:Zm00001d007216_T001  rna21213            0
322- 10:  transcript:Zm00001d007225_T019  rna21223     5.00E-28
```

```
322- 11:  transcript:Zm00001d007228_T001  rna21227            0
322- 12:  transcript:Zm00001d007229_T001  rna21229            0
322- 13:  transcript:Zm00001d007231_T002  rna21232     7.00E-73
322- 14:  transcript:Zm00001d007232_T001  rna21233    6.00E-143
322- 15:  transcript:Zm00001d007234_T001  rna21235    8.00E-168
322- 16:  transcript:Zm00001d007240_T001  rna21237            0
322- 17:  transcript:Zm00001d007241_T001  rna21239            0
## Alignment 323: score=578.0 e_value=2e-32 N=13 2&NC_008400.2 plus
323-  0:  transcript:Zm00001d007031_T002  rna20890     7.00E-55
323-  1:  transcript:Zm00001d007032_T001  rna20891     2.00E-22
323-  2:  transcript:Zm00001d007037_T002  rna20896    3.00E-167
323-  3:  transcript:Zm00001d007042_T001  rna20907            0
323-  4:  transcript:Zm00001d007043_T002  rna20909            0
323-  5:  transcript:Zm00001d007044_T001  rna20910            0
323-  6:  transcript:Zm00001d007045_T001  rna20912    4.00E-115
323-  7:  transcript:Zm00001d007047_T001  rna20915     5.00E-82
323-  8:  transcript:Zm00001d007048_T001  rna20916            0
323-  9:  transcript:Zm00001d007050_T001  rna20921    2.00E-148
323- 10:  transcript:Zm00001d007058_T002  rna20928            0
323- 11:  transcript:Zm00001d007059_T001  rna20932     7.00E-40
323- 12:  transcript:Zm00001d007060_T003  rna20934     1.00E-75
## Alignment 324: score=554.0 e_value=3.4e-34 N=13 2&NC_008400.2 plus
324-  0:  transcript:Zm00001d006813_T001  rna20678     4.00E-85
324-  1:  transcript:Zm00001d006834_T002  rna20687    2.00E-122
324-  2:  transcript:Zm00001d006835_T001  rna20688     1.00E-96
324-  3:  transcript:Zm00001d006836_T002  rna20689            0
324-  4:  transcript:Zm00001d006838_T002  rna20691     3.00E-57
324-  5:  transcript:Zm00001d006839_T002  rna20693            0
324-  6:  transcript:Zm00001d006840_T001  rna20695     2.00E-29
324-  7:  transcript:Zm00001d006841_T001  rna20702    7.00E-177
324-  8:  transcript:Zm00001d006845_T002  rna20709            0
324-  9:  transcript:Zm00001d006853_T001  rna20714    4.00E-176
324- 10:  transcript:Zm00001d006855_T001  rna20716    2.00E-144
324- 11:  transcript:Zm00001d006856_T001  rna20721    5.00E-111
324- 12:  transcript:Zm00001d006860_T001  rna20723    2.00E-104
## Alignment 325: score=493.0 e_value=6.4e-25 N=11 2&NC_008400.2 plus
325-  0:  transcript:Zm00001d007951_T001  rna19026            0
325-  1:  transcript:Zm00001d007952_T001  rna19030    1.00E-174
325-  2:  transcript:Zm00001d007956_T001  rna19034     3.00E-28
325-  3:  transcript:Zm00001d007957_T001  rna19036     4.00E-21
325-  4:  transcript:Zm00001d007961_T001  rna19042     2.00E-82
325-  5:  transcript:Zm00001d007965_T002  rna19045            0
325-  6:  transcript:Zm00001d007971_T001  rna19058     3.00E-30
325-  7:  transcript:Zm00001d007972_T002  rna19071            0
325-  8:  transcript:Zm00001d007974_T001  rna19074     1.00E-24
325-  9:  transcript:Zm00001d007975_T001  rna19079            0
325- 10:  transcript:Zm00001d007977_T001  rna19082     1.00E-42
## Alignment 326: score=416.0 e_value=6.7e-17 N=9 2&NC_008400.2 plus
326-  0:  transcript:Zm00001d007076_T001  rna20981            0
326-  1:  transcript:Zm00001d007077_T001  rna20982     4.00E-69
326-  2:  transcript:Zm00001d007079_T001  rna20985     1.00E-32
326-  3:  transcript:Zm00001d007081_T002  rna20988            0
326-  4:  transcript:Zm00001d007082_T001  rna20993            0
326-  5:  transcript:Zm00001d007083_T001  rna20995     8.00E-65
```

326- 6: transcript:Zm00001d007085_T001 rna20996   3.00E-151
326- 7: transcript:Zm00001d007086_T001 rna20998   4.00E-25
326- 8: transcript:Zm00001d007089_T003 rna21002   0
Alignment 327: score=372.0 e_value=9.3e-16 N=8 2&NC_008400.2 plus
327- 0: transcript:Zm00001d007105_T001 rna21046   5.00E-114
327- 1: transcript:Zm00001d007106_T001 rna21048   2.00E-121
327- 2: transcript:Zm00001d007107_T001 rna21050   3.00E-113
327- 3: transcript:Zm00001d007108_T001 rna21051   0
327- 4: transcript:Zm00001d007111_T001 rna21052   6.00E-45
327- 5: transcript:Zm00001d007113_T001 rna21061   0
327- 6: transcript:Zm00001d007117_T001 rna21062   0
327- 7: transcript:Zm00001d007119_T001 rna21063   6.00E-100
Alignment 328: score=350.0 e_value=5.3e-14 N=8 2&NC_008400.2 plus
328- 0: transcript:Zm00001d007090_T001 rna21003   0
328- 1: transcript:Zm00001d007091_T001 rna21004   0
328- 2: transcript:Zm00001d007094_T004 rna21007   0
328- 3: transcript:Zm00001d007095_T008 rna21008   0
328- 4: transcript:Zm00001d007096_T007 rna21009   0
328- 5: transcript:Zm00001d007097_T001 rna21010   3.00E-45
328- 6: transcript:Zm00001d007102_T001 rna21012   1.00E-128
328- 7: transcript:Zm00001d007103_T001 rna21017   2.00E-130
Alignment 329: score=291.0 e_value=1.3e-11 N=6 2&NC_008400.2 plus
329- 0: transcript:Zm00001d006601_T001 rna20418   9.00E-93
329- 1: transcript:Zm00001d006602_T001 rna20421   0
329- 2: transcript:Zm00001d006603_T001 rna20424   2.00E-46
329- 3: transcript:Zm00001d006604_T001 rna20425   1.00E-74
329- 4: transcript:Zm00001d006605_T002 rna20427   0
329- 5: transcript:Zm00001d006606_T001 rna20429   0
Alignment 330: score=262.0 e_value=4.8e-09 N=6 2&NC_008400.2 plus
330- 0: transcript:Zm00001d007927_T001 rna18945   0
330- 1: transcript:Zm00001d007930_T001 rna18953   7.00E-171
330- 2: transcript:Zm00001d007931_T001 rna18971   0
330- 3: transcript:Zm00001d007932_T001 rna18972   4.00E-101
330- 4: transcript:Zm00001d007935_T001 rna18979   9.00E-38
330- 5: transcript:Zm00001d007937_T001 rna18988   0
Alignment 331: score=704.0 e_value=3e-46 N=17 2&NC_008400.2 minus
331- 0: transcript:Zm00001d007788_T001 rna19403   5.00E-92
331- 1: transcript:Zm00001d007799_T001 rna19396   1.00E-82
331- 2: transcript:Zm00001d007800_T001 rna19389   4.00E-56
331- 3: transcript:Zm00001d007807_T006 rna19380   0
331- 4: transcript:Zm00001d007810_T001 rna19376   1.00E-53
331- 5: transcript:Zm00001d007820_T002 rna19365   1.00E-65
331- 6: transcript:Zm00001d007824_T001 rna19361   0
331- 7: transcript:Zm00001d007825_T003 rna19355   1.00E-67
331- 8: transcript:Zm00001d007826_T002 rna19353   1.00E-53
331- 9: transcript:Zm00001d007827_T001 rna19349   6.00E-67
331- 10: transcript:Zm00001d007830_T001 rna19337   1.00E-156
331- 11: transcript:Zm00001d007831_T001 rna19331   4.00E-41
331- 12: transcript:Zm00001d007832_T001 rna19325   3.00E-06
331- 13: transcript:Zm00001d007835_T002 rna19315   0
331- 14: transcript:Zm00001d007836_T001 rna19314   4.00E-57
331- 15: transcript:Zm00001d007837_T002 rna19313   0
331- 16: transcript:Zm00001d007838_T001 rna19312   0
Alignment 332: score=698.0 e_value=9.4e-42 N=16 2&NC_008400.2 minus

```
332-  0: transcript:Zm00001d007889_T009  rna19309            0
332-  1: transcript:Zm00001d007890_T002  rna19303            0
332-  2: transcript:Zm00001d007892_T001  rna19301            0
332-  3: transcript:Zm00001d007894_T001  rna19298            0
332-  4: transcript:Zm00001d007896_T001  rna19294            0
332-  5: transcript:Zm00001d007897_T002  rna19282     1.00E-80
332-  6: transcript:Zm00001d007898_T001  rna19280     6.00E-81
332-  7: transcript:Zm00001d007901_T001  rna19272     3.00E-17
332-  8: transcript:Zm00001d007902_T001  rna19268            0
332-  9: transcript:Zm00001d007904_T003  rna19263     1.00E-70
332- 10: transcript:Zm00001d007905_T001  rna19262            0
332- 11: transcript:Zm00001d007907_T003  rna19260     4.00E-52
332- 12: transcript:Zm00001d007908_T001  rna19254     2.00E-93
332- 13: transcript:Zm00001d007910_T001  rna19252     4.00E-91
332- 14: transcript:Zm00001d007911_T001  rna19251     7.00E-16
332- 15: transcript:Zm00001d007912_T009  rna19250            0
## Alignment 333: score=636.0 e_value=8.8e-39 N=15 2&NC_008400.2 minus
333-  0: transcript:Zm00001d005174_T001  rna19608            0
333-  1: transcript:Zm00001d005177_T001  rna19607     8.00E-90
333-  2: transcript:Zm00001d005178_T001  rna19603     2.00E-29
333-  3: transcript:Zm00001d005179_T001  rna19593     4.00E-54
333-  4: transcript:Zm00001d005182_T001  rna19592    7.00E-139
333-  5: transcript:Zm00001d005185_T001  rna19584    1.00E-125
333-  6: transcript:Zm00001d005190_T001  rna19583            0
333-  7: transcript:Zm00001d005193_T001  rna19574    2.00E-103
333-  8: transcript:Zm00001d005196_T001  rna19573     2.00E-14
333-  9: transcript:Zm00001d005200_T002  rna19570     9.00E-88
333- 10: transcript:Zm00001d005205_T003  rna19559            0
333- 11: transcript:Zm00001d005208_T001  rna19555    5.00E-119
333- 12: transcript:Zm00001d005229_T001  rna19547    2.00E-142
333- 13: transcript:Zm00001d005231_T013  rna19546    9.00E-133
333- 14: transcript:Zm00001d005238_T001  rna19541            0
## Alignment 334: score=619.0 e_value=8.2e-36 N=14 2&NC_008400.2 minus
334-  0: transcript:Zm00001d006928_T001  rna20877     6.00E-55
334-  1: transcript:Zm00001d006933_T001  rna20870    2.00E-123
334-  2: transcript:Zm00001d006936_T002  rna20869     1.00E-72
334-  3: transcript:Zm00001d006938_T001  rna20862            0
334-  4: transcript:Zm00001d006939_T001  rna20861    2.00E-167
334-  5: transcript:Zm00001d006942_T007  rna20858     2.00E-28
334-  6: transcript:Zm00001d006943_T001  rna20856            0
334-  7: transcript:Zm00001d006945_T001  rna20851     6.00E-90
334-  8: transcript:Zm00001d006947_T001  rna20843            0
334-  9: transcript:Zm00001d006951_T001  rna20826    7.00E-135
334- 10: transcript:Zm00001d007009_T001  rna20825            0
334- 11: transcript:Zm00001d007012_T001  rna20823    4.00E-124
334- 12: transcript:Zm00001d007015_T001  rna20821     2.00E-58
334- 13: transcript:Zm00001d007027_T001  rna20818     2.00E-64
## Alignment 335: score=580.0 e_value=9.1e-33 N=13 2&NC_008400.2 minus
335-  0: transcript:Zm00001d007754_T001  rna19466            0
335-  1: transcript:Zm00001d007757_T004  rna19463            0
335-  2: transcript:Zm00001d007758_T009  rna19458            0
335-  3: transcript:Zm00001d007760_T001  rna19454            0
335-  4: transcript:Zm00001d007762_T001  rna19441            0
335-  5: transcript:Zm00001d007765_T001  rna19438    1.00E-148
```

```
335-  6:  transcript:Zm00001d007767_T001  rna19433    5.00E-163
335-  7:  transcript:Zm00001d007768_T001  rna19432     4.00E-18
335-  8:  transcript:Zm00001d007769_T003  rna19431            0
335-  9:  transcript:Zm00001d007770_T001  rna19428    1.00E-168
335- 10:  transcript:Zm00001d007773_T001  rna19425            0
335- 11:  transcript:Zm00001d007774_T001  rna19417            0
335- 12:  transcript:Zm00001d007775_T001  rna19416            0
## Alignment 336: score=440.0 e_value=2.4e-21 N=10 2&NC_008400.2 minus
336-  0:  transcript:Zm00001d005424_T004  rna19927            0
336-  1:  transcript:Zm00001d005425_T001  rna19909     2.00E-07
336-  2:  transcript:Zm00001d005426_T003  rna19906     6.00E-49
336-  3:  transcript:Zm00001d005429_T004  rna19905            0
336-  4:  transcript:Zm00001d005431_T006  rna19903     2.00E-43
336-  5:  transcript:Zm00001d005437_T001  rna19889     2.00E-25
336-  6:  transcript:Zm00001d005439_T001  rna19884            0
336-  7:  transcript:Zm00001d005442_T001  rna19880            0
336-  8:  transcript:Zm00001d005445_T001  rna19879    6.00E-160
336-  9:  transcript:Zm00001d005446_T001  rna19877     3.00E-46
## Alignment 337: score=407.0 e_value=9.8e-20 N=9 2&NC_008400.2 minus
337-  0:  transcript:Zm00001d006416_T001  rna20114     2.00E-73
337-  1:  transcript:Zm00001d006417_T002  rna20113    8.00E-112
337-  2:  transcript:Zm00001d006419_T002  rna20110            0
337-  3:  transcript:Zm00001d006420_T002  rna20108            0
337-  4:  transcript:Zm00001d006421_T002  rna20107     1.00E-45
337-  5:  transcript:Zm00001d006422_T006  rna20105            0
337-  6:  transcript:Zm00001d006428_T026  rna20103            0
337-  7:  transcript:Zm00001d006430_T001  rna20102            0
337-  8:  transcript:Zm00001d006433_T001  rna20096    2.00E-151
## Alignment 338: score=379.0 e_value=1.3e-14 N=8 2&NC_008400.2 minus
338-  0:  transcript:Zm00001d006808_T001  rna20684     2.00E-72
338-  1:  transcript:Zm00001d006810_T001  rna20680     1.00E-62
338-  2:  transcript:Zm00001d006820_T001  rna20669            0
338-  3:  transcript:Zm00001d006821_T001  rna20666    1.00E-113
338-  4:  transcript:Zm00001d006822_T001  rna20661            0
338-  5:  transcript:Zm00001d006823_T001  rna20660            0
338-  6:  transcript:Zm00001d006825_T003  rna20658            0
338-  7:  transcript:Zm00001d006828_T001  rna20656            0
## Alignment 339: score=293.0 e_value=3.1e-12 N=7 2&NC_008400.2 minus
339-  0:  transcript:Zm00001d005230_T001  rna19516     6.00E-08
339-  1:  transcript:Zm00001d005240_T004  rna19507            0
339-  2:  transcript:Zm00001d005241_T001  rna19506            0
339-  3:  transcript:Zm00001d005244_T002  rna19502    9.00E-107
339-  4:  transcript:Zm00001d005248_T003  rna19498            0
339-  5:  transcript:Zm00001d005250_T001  rna19494            0
339-  6:  transcript:Zm00001d005252_T001  rna19489            0
## Alignment 340: score=1236.0 e_value=4.9e-90 N=28 2&NC_008401.2 plus
340-  0:  transcript:Zm00001d005869_T001  rna22690    1.00E-112
340-  1:  transcript:Zm00001d005881_T011  rna22708    5.00E-137
340-  2:  transcript:Zm00001d005884_T002  rna22710     2.00E-58
340-  3:  transcript:Zm00001d005885_T001  rna22711    4.00E-173
340-  4:  transcript:Zm00001d005889_T001  rna22713            0
340-  5:  transcript:Zm00001d005890_T001  rna22715            0
340-  6:  transcript:Zm00001d005892_T001  rna22719     2.00E-44
340-  7:  transcript:Zm00001d005897_T001  rna22720     3.00E-31
```

340- 8: transcript:Zm00001d005899_T001 rna22723 3.00E-71
340- 9: transcript:Zm00001d005902_T003 rna22724 0
340- 10: transcript:Zm00001d005905_T001 rna22726 6.00E-120
340- 11: transcript:Zm00001d005910_T001 rna22729 4.00E-85
340- 12: transcript:Zm00001d005917_T003 rna22731 2.00E-21
340- 13: transcript:Zm00001d005918_T001 rna22736 3.00E-69
340- 14: transcript:Zm00001d005919_T001 rna22744 7.00E-72
340- 15: transcript:Zm00001d005923_T001 rna22748 1.00E-81
340- 16: transcript:Zm00001d005925_T002 rna22751 0
340- 17: transcript:Zm00001d005928_T002 rna22752 9.00E-116
340- 18: transcript:Zm00001d005931_T001 rna22753 4.00E-100
340- 19: transcript:Zm00001d005944_T001 rna22774 3.00E-53
340- 20: transcript:Zm00001d005958_T002 rna22789 1.00E-94
340- 21: transcript:Zm00001d005961_T001 rna22801 3.00E-22
340- 22: transcript:Zm00001d005962_T001 rna22802 5.00E-29
340- 23: transcript:Zm00001d005964_T001 rna22805 1.00E-77
340- 24: transcript:Zm00001d005966_T001 rna22811 0
340- 25: transcript:Zm00001d005969_T004 rna22812 3.00E-138
340- 26: transcript:Zm00001d005970_T001 rna22813 4.00E-46
340- 27: transcript:Zm00001d005971_T001 rna22815 1.00E-56
Alignment 341: score=866.0 e_value=3.4e-55 N=20 2&NC_008401.2 plus
341- 0: transcript:Zm00001d005976_T012 rna22820 0
341- 1: transcript:Zm00001d005978_T001 rna22825 3.00E-41
341- 2: transcript:Zm00001d005980_T001 rna22826 3.00E-14
341- 3: transcript:Zm00001d005981_T006 rna22839 1.00E-46
341- 4: transcript:Zm00001d005993_T001 rna22841 2.00E-44
341- 5: transcript:Zm00001d005995_T008 rna22844 0
341- 6: transcript:Zm00001d005997_T002 rna22846 6.00E-93
341- 7: transcript:Zm00001d005998_T003 rna22847 6.00E-98
341- 8: transcript:Zm00001d006001_T006 rna22853 5.00E-94
341- 9: transcript:Zm00001d006008_T001 rna22866 0
341- 10: transcript:Zm00001d006009_T001 rna22877 1.00E-73
341- 11: transcript:Zm00001d006016_T001 rna22891 1.00E-58
341- 12: transcript:Zm00001d006019_T001 rna22898 6.00E-25
341- 13: transcript:Zm00001d006022_T001 rna22904 5.00E-121
341- 14: transcript:Zm00001d006025_T001 rna22911 3.00E-13
341- 15: transcript:Zm00001d006026_T001 rna22912 2.00E-41
341- 16: transcript:Zm00001d006028_T001 rna22920 4.00E-106
341- 17: transcript:Zm00001d006030_T001 rna22923 7.00E-40
341- 18: transcript:Zm00001d006031_T001 rna22926 1.00E-104
341- 19: transcript:Zm00001d006032_T001 rna22933 1.00E-30
Alignment 342: score=712.0 e_value=1.6e-48 N=18 2&NC_008401.2 plus
342- 0: transcript:Zm00001d006063_T001 rna23006 0
342- 1: transcript:Zm00001d006064_T002 rna23009 3.00E-90
342- 2: transcript:Zm00001d006065_T002 rna23010 5.00E-92
342- 3: transcript:Zm00001d006066_T002 rna23014 8.00E-19
342- 4: transcript:Zm00001d006079_T001 rna23024 2.00E-56
342- 5: transcript:Zm00001d006082_T001 rna23039 9.00E-145
342- 6: transcript:Zm00001d006085_T002 rna23040 9.00E-144
342- 7: transcript:Zm00001d006091_T001 rna23058 2.00E-147
342- 8: transcript:Zm00001d006094_T001 rna23059 7.00E-131
342- 9: transcript:Zm00001d006097_T001 rna23066 9.00E-151
342- 10: transcript:Zm00001d006102_T002 rna23080 0
342- 11: transcript:Zm00001d006106_T001 rna23083 2.00E-37

342- 12: transcript:Zm00001d006107_T001 rna23085 0
342- 13: transcript:Zm00001d006110_T002 rna23093 1.00E-50
342- 14: transcript:Zm00001d006115_T002 rna23112 0
342- 15: transcript:Zm00001d006117_T001 rna23119 5.00E-125
342- 16: transcript:Zm00001d006130_T001 rna23121 0
342- 17: transcript:Zm00001d006142_T002 rna23130 0
Alignment 343: score=505.0 e_value=1.4e-26 N=12 2&NC_008401.2 plus
343- 0: transcript:Zm00001d005622_T001 rna22335 9.00E-58
343- 1: transcript:Zm00001d005629_T001 rna22353 0
343- 2: transcript:Zm00001d005636_T003 rna22356 0
343- 3: transcript:Zm00001d005638_T001 rna22357 0
343- 4: transcript:Zm00001d005645_T001 rna22382 2.00E-84
343- 5: transcript:Zm00001d005648_T002 rna22386 0
343- 6: transcript:Zm00001d005650_T004 rna22390 1.00E-52
343- 7: transcript:Zm00001d005653_T004 rna22396 2.00E-143
343- 8: transcript:Zm00001d005656_T001 rna22407 8.00E-65
343- 9: transcript:Zm00001d005657_T002 rna22423 3.00E-40
343- 10: transcript:Zm00001d005658_T001 rna22427 4.00E-28
343- 11: transcript:Zm00001d005664_T001 rna22428 4.00E-62
Alignment 344: score=429.0 e_value=4e-23 N=10 2&NC_008401.2 plus
344- 0: transcript:Zm00001d005684_T001 rna22453 2.00E-38
344- 1: transcript:Zm00001d005687_T001 rna22456 0
344- 2: transcript:Zm00001d005692_T001 rna22461 7.00E-48
344- 3: transcript:Zm00001d005696_T001 rna22481 0
344- 4: transcript:Zm00001d005699_T001 rna22482 5.00E-95
344- 5: transcript:Zm00001d005705_T001 rna22483 4.00E-88
344- 6: transcript:Zm00001d005713_T001 rna22502 7.00E-166
344- 7: transcript:Zm00001d005714_T001 rna22503 8.00E-46
344- 8: transcript:Zm00001d005726_T001 rna22515 3.00E-83
344- 9: transcript:Zm00001d005732_T001 rna22522 6.00E-159
Alignment 345: score=343.0 e_value=1.4e-15 N=8 2&NC_008401.2 plus
345- 0: transcript:Zm00001d006147_T006 rna23130 0
345- 1: transcript:Zm00001d006157_T001 rna23139 7.00E-85
345- 2: transcript:Zm00001d006165_T001 rna23145 1.00E-73
345- 3: transcript:Zm00001d006166_T001 rna23146 0
345- 4: transcript:Zm00001d006169_T001 rna23151 1.00E-48
345- 5: transcript:Zm00001d006171_T001 rna23156 0
345- 6: transcript:Zm00001d006173_T001 rna23157 2.00E-127
345- 7: transcript:Zm00001d006176_T001 rna23159 0
Alignment 346: score=327.0 e_value=2.6e-10 N=7 2&NC_008401.2 plus
346- 0: transcript:Zm00001d006179_T005 rna23163 0
346- 1: transcript:Zm00001d006180_T001 rna23165 7.00E-146
346- 2: transcript:Zm00001d006181_T003 rna23166 4.00E-21
346- 3: transcript:Zm00001d006185_T001 rna23168 2.00E-149
346- 4: transcript:Zm00001d006192_T001 rna23169 0
346- 5: transcript:Zm00001d006193_T001 rna23170 0
346- 6: transcript:Zm00001d006197_T003 rna23171 0
Alignment 347: score=275.0 e_value=6e-12 N=7 2&NC_008401.2 plus
347- 0: transcript:Zm00001d005507_T012 rna22181 0
347- 1: transcript:Zm00001d005513_T001 rna22200 6.00E-120
347- 2: transcript:Zm00001d005516_T001 rna22201 0
347- 3: transcript:Zm00001d005535_T002 rna22207 0
347- 4: transcript:Zm00001d005542_T001 rna22213 0
347- 5: transcript:Zm00001d005546_T002 rna22219 0

347- 6: transcript:Zm00001d005547_T001 rna22220 5.00E-112
Alignment 348: score=273.0 e_value=2.8e-09 N=6 2&NC_008401.2 plus
348- 0: transcript:Zm00001d002405_T001 rna21881 9.00E-101
348- 1: transcript:Zm00001d002409_T002 rna21890 1.00E-17
348- 2: transcript:Zm00001d002410_T001 rna21902 5.00E-96
348- 3: transcript:Zm00001d002413_T005 rna21904 0
348- 4: transcript:Zm00001d002415_T001 rna21905 2.00E-22
348- 5: transcript:Zm00001d002416_T002 rna21906 5.00E-90
Alignment 349: score=266.0 e_value=7e-12 N=7 2&NC_008401.2 plus
349- 0: transcript:Zm00001d005816_T001 rna22644 0
349- 1: transcript:Zm00001d005821_T002 rna22646 0
349- 2: transcript:Zm00001d005826_T004 rna22651 0
349- 3: transcript:Zm00001d005833_T002 rna22672 0
349- 4: transcript:Zm00001d005837_T001 rna22676 2.00E-59
349- 5: transcript:Zm00001d005846_T002 rna22701 0
349- 6: transcript:Zm00001d005849_T001 rna22703 4.00E-153
Alignment 350: score=260.0 e_value=2.2e-10 N=6 2&NC_008401.2 minus
350- 0: transcript:Zm00001d005849_T001 rna22703 4.00E-153
350- 1: transcript:Zm00001d005859_T001 rna22700 2.00E-74
350- 2: transcript:Zm00001d005860_T001 rna22699 1.00E-179
350- 3: transcript:Zm00001d005865_T001 rna22693 7.00E-111
350- 4: transcript:Zm00001d005867_T002 rna22692 1.00E-19
350- 5: transcript:Zm00001d005875_T001 rna22681 6.00E-21
Alignment 351: score=253.0 e_value=1.7e-09 N=6 2&NC_008401.2 minus
351- 0: transcript:Zm00001d005751_T002 rna22571 1.00E-141
351- 1: transcript:Zm00001d005752_T001 rna22569 1.00E-126
351- 2: transcript:Zm00001d005756_T002 rna22566 0
351- 3: transcript:Zm00001d005757_T001 rna22563 3.00E-83
351- 4: transcript:Zm00001d005760_T001 rna22561 7.00E-140
351- 5: transcript:Zm00001d005765_T004 rna22557 3.00E-140
Alignment 352: score=3320.0 e_value=2e-292 N=72 2&NC_008402.2 plus
352- 0: transcript:Zm00001d005881_T011 rna24258 3.00E-157
352- 1: transcript:Zm00001d005884_T002 rna24259 2.00E-85
352- 2: transcript:Zm00001d005885_T001 rna24260 0
352- 3: transcript:Zm00001d005888_T002 rna24261 7.00E-134
352- 4: transcript:Zm00001d005889_T001 rna24263 0
352- 5: transcript:Zm00001d005890_T001 rna24264 0
352- 6: transcript:Zm00001d005892_T001 rna24269 1.00E-76
352- 7: transcript:Zm00001d005893_T001 rna24270 0
352- 8: transcript:Zm00001d005894_T001 rna24273 0
352- 9: transcript:Zm00001d005895_T001 rna24278 1.00E-32
352- 10: transcript:Zm00001d005897_T001 rna24280 4.00E-60
352- 11: transcript:Zm00001d005899_T001 rna24285 5.00E-79
352- 12: transcript:Zm00001d005901_T001 rna24286 3.00E-90
352- 13: transcript:Zm00001d005902_T003 rna24287 3.00E-29
352- 14: transcript:Zm00001d005905_T001 rna24294 0
352- 15: transcript:Zm00001d005911_T001 rna24301 0
352- 16: transcript:Zm00001d005912_T001 rna24302 0
352- 17: transcript:Zm00001d005913_T001 rna24303 3.00E-128
352- 18: transcript:Zm00001d005917_T003 rna24304 0
352- 19: transcript:Zm00001d005918_T001 rna24305 3.00E-81
352- 20: transcript:Zm00001d005920_T004 rna24308 3.00E-166
352- 21: transcript:Zm00001d005924_T002 rna24313 2.00E-77
352- 22: transcript:Zm00001d005925_T002 rna24314 0

352- 23: transcript:Zm00001d005926_T001 rna24315 1.00E-76
352- 24: transcript:Zm00001d005928_T002 rna24316 2.00E-139
352- 25: transcript:Zm00001d005931_T001 rna24319 7.00E-107
352- 26: transcript:Zm00001d005933_T001 rna24324 3.00E-179
352- 27: transcript:Zm00001d005936_T001 rna24330 5.00E-143
352- 28: transcript:Zm00001d005939_T001 rna24332 4.00E-99
352- 29: transcript:Zm00001d005944_T001 rna24334 2.00E-73
352- 30: transcript:Zm00001d005948_T001 rna24337 1.00E-85
352- 31: transcript:Zm00001d005950_T001 rna24339 5.00E-58
352- 32: transcript:Zm00001d005951_T001 rna24340 5.00E-110
352- 33: transcript:Zm00001d005957_T001 rna24341 2.00E-48
352- 34: transcript:Zm00001d005958_T002 rna24357 0
352- 35: transcript:Zm00001d005959_T001 rna24358 0
352- 36: transcript:Zm00001d005961_T001 rna24362 2.00E-14
352- 37: transcript:Zm00001d005962_T001 rna24363 2.00E-46
352- 38: transcript:Zm00001d005964_T001 rna24364 0
352- 39: transcript:Zm00001d005966_T001 rna24368 0
352- 40: transcript:Zm00001d005969_T004 rna24369 0
352- 41: transcript:Zm00001d005970_T001 rna24372 4.00E-71
352- 42: transcript:Zm00001d005971_T001 rna24377 7.00E-90
352- 43: transcript:Zm00001d005975_T001 rna24385 2.00E-83
352- 44: transcript:Zm00001d005976_T012 rna24386 0
352- 45: transcript:Zm00001d005978_T001 rna24387 2.00E-22
352- 46: transcript:Zm00001d005980_T001 rna24390 2.00E-78
352- 47: transcript:Zm00001d005984_T001 rna24400 4.00E-42
352- 48: transcript:Zm00001d005989_T001 rna24403 1.00E-22
352- 49: transcript:Zm00001d005993_T001 rna24404 4.00E-52
352- 50: transcript:Zm00001d005995_T008 rna24406 0
352- 51: transcript:Zm00001d005996_T001 rna24407 2.00E-80
352- 52: transcript:Zm00001d005997_T002 rna24408 3.00E-58
352- 53: transcript:Zm00001d005998_T003 rna24409 1.00E-144
352- 54: transcript:Zm00001d006001_T006 rna24411 0
352- 55: transcript:Zm00001d006002_T001 rna24412 9.00E-65
352- 56: transcript:Zm00001d006008_T001 rna24414 0
352- 57: transcript:Zm00001d006009_T001 rna24427 6.00E-99
352- 58: transcript:Zm00001d006011_T001 rna24441 0
352- 59: transcript:Zm00001d006013_T001 rna24446 1.00E-49
352- 60: transcript:Zm00001d006016_T001 rna24448 2.00E-66
352- 61: transcript:Zm00001d006017_T001 rna24449 0
352- 62: transcript:Zm00001d006019_T001 rna24454 4.00E-34
352- 63: transcript:Zm00001d006022_T001 rna24455 3.00E-127
352- 64: transcript:Zm00001d006024_T001 rna24457 0
352- 65: transcript:Zm00001d006025_T001 rna24459 0
352- 66: transcript:Zm00001d006026_T001 rna24460 5.00E-55
352- 67: transcript:Zm00001d006027_T005 rna24461 0
352- 68: transcript:Zm00001d006028_T001 rna24466 4.00E-108
352- 69: transcript:Zm00001d006029_T001 rna24467 5.00E-09
352- 70: transcript:Zm00001d006031_T001 rna24471 6.00E-164
352- 71: transcript:Zm00001d006032_T001 rna24472 1.00E-30
Alignment 353: score=3181.0 e_value=4.1e-290 N=70 2&NC_008402.2 plus
353- 0: transcript:Zm00001d006034_T003 rna24473 3.00E-113
353- 1: transcript:Zm00001d006036_T001 rna24476 0
353- 2: transcript:Zm00001d006037_T001 rna24477 6.00E-136
353- 3: transcript:Zm00001d006040_T001 rna24483 0

353-	4:	transcript:Zm00001d006046_T002	rna24492	9.00E-147
353-	5:	transcript:Zm00001d006049_T001	rna24499	2.00E-173
353-	6:	transcript:Zm00001d006050_T001	rna24500	4.00E-180
353-	7:	transcript:Zm00001d006051_T001	rna24501	2.00E-41
353-	8:	transcript:Zm00001d006052_T004	rna24502	0
353-	9:	transcript:Zm00001d006053_T001	rna24503	2.00E-170
353-	10:	transcript:Zm00001d006055_T002	rna24508	0
353-	11:	transcript:Zm00001d006057_T001	rna24510	0
353-	12:	transcript:Zm00001d006059_T001	rna24511	4.00E-82
353-	13:	transcript:Zm00001d006061_T001	rna24517	1.00E-61
353-	14:	transcript:Zm00001d006063_T001	rna24518	0
353-	15:	transcript:Zm00001d006064_T002	rna24521	7.00E-88
353-	16:	transcript:Zm00001d006065_T002	rna24522	5.00E-134
353-	17:	transcript:Zm00001d006066_T002	rna24526	1.00E-45
353-	18:	transcript:Zm00001d006069_T001	rna24528	2.00E-166
353-	19:	transcript:Zm00001d006070_T004	rna24538	2.00E-73
353-	20:	transcript:Zm00001d006071_T001	rna24539	4.00E-87
353-	21:	transcript:Zm00001d006078_T001	rna24541	1.00E-39
353-	22:	transcript:Zm00001d006079_T001	rna24542	0
353-	23:	transcript:Zm00001d006080_T004	rna24544	0
353-	24:	transcript:Zm00001d006082_T001	rna24545	2.00E-161
353-	25:	transcript:Zm00001d006085_T002	rna24546	1.00E-159
353-	26:	transcript:Zm00001d006089_T002	rna24553	0
353-	27:	transcript:Zm00001d006090_T002	rna24554	2.00E-55
353-	28:	transcript:Zm00001d006091_T001	rna24555	1.00E-173
353-	29:	transcript:Zm00001d006094_T001	rna24556	4.00E-157
353-	30:	transcript:Zm00001d006096_T001	rna24558	4.00E-42
353-	31:	transcript:Zm00001d006097_T001	rna24559	0
353-	32:	transcript:Zm00001d006100_T001	rna24561	0
353-	33:	transcript:Zm00001d006101_T001	rna24562	2.00E-32
353-	34:	transcript:Zm00001d006102_T002	rna24565	0
353-	35:	transcript:Zm00001d006106_T001	rna24569	7.00E-61
353-	36:	transcript:Zm00001d006107_T001	rna24570	0
353-	37:	transcript:Zm00001d006108_T002	rna24571	0
353-	38:	transcript:Zm00001d006110_T002	rna24579	1.00E-99
353-	39:	transcript:Zm00001d006112_T002	rna24594	1.00E-141
353-	40:	transcript:Zm00001d006115_T002	rna24597	0
353-	41:	transcript:Zm00001d006116_T001	rna24598	8.00E-50
353-	42:	transcript:Zm00001d006118_T005	rna24607	0
353-	43:	transcript:Zm00001d006119_T001	rna24609	1.00E-92
353-	44:	transcript:Zm00001d006124_T001	rna24612	0
353-	45:	transcript:Zm00001d006125_T001	rna24614	4.00E-169
353-	46:	transcript:Zm00001d006127_T003	rna24622	5.00E-159
353-	47:	transcript:Zm00001d006128_T002	rna24624	5.00E-84
353-	48:	transcript:Zm00001d006130_T001	rna24626	0
353-	49:	transcript:Zm00001d006131_T003	rna24628	0
353-	50:	transcript:Zm00001d006132_T001	rna24632	0
353-	51:	transcript:Zm00001d006133_T001	rna24633	0
353-	52:	transcript:Zm00001d006145_T001	rna24635	5.00E-91
353-	53:	transcript:Zm00001d006147_T006	rna24639	0
353-	54:	transcript:Zm00001d006148_T001	rna24642	2.00E-37
353-	55:	transcript:Zm00001d006149_T001	rna24643	0
353-	56:	transcript:Zm00001d006150_T001	rna24645	4.00E-96
353-	57:	transcript:Zm00001d006153_T003	rna24649	0

353- 58: transcript:Zm00001d006154_T001 rna24650 0
353- 59: transcript:Zm00001d006158_T001 rna24652 1.00E-15
353- 60: transcript:Zm00001d006160_T001 rna24655 0
353- 61: transcript:Zm00001d006161_T001 rna24656 0
353- 62: transcript:Zm00001d006166_T001 rna24658 0
353- 63: transcript:Zm00001d006167_T003 rna24659 0
353- 64: transcript:Zm00001d006169_T001 rna24662 1.00E-42
353- 65: transcript:Zm00001d006171_T001 rna24667 0
353- 66: transcript:Zm00001d006173_T001 rna24669 5.00E-155
353- 67: transcript:Zm00001d006175_T001 rna24670 3.00E-124
353- 68: transcript:Zm00001d006176_T001 rna24674 0
353- 69: transcript:Zm00001d006177_T002 rna24675 8.00E-81
Alignment 354: score=2044.0 e_value=2.5e-173 N=46 2&NC_008402.2 plus
354- 0: transcript:Zm00001d006198_T001 rna24689 1.00E-89
354- 1: transcript:Zm00001d006199_T001 rna24704 0
354- 2: transcript:Zm00001d006204_T001 rna24707 4.00E-89
354- 3: transcript:Zm00001d006205_T001 rna24708 0
354- 4: transcript:Zm00001d006206_T001 rna24710 2.00E-16
354- 5: transcript:Zm00001d006207_T001 rna24711 1.00E-45
354- 6: transcript:Zm00001d006209_T001 rna24712 2.00E-65
354- 7: transcript:Zm00001d006210_T009 rna24713 0
354- 8: transcript:Zm00001d006211_T001 rna24715 9.00E-134
354- 9: transcript:Zm00001d006212_T002 rna24717 0
354- 10: transcript:Zm00001d006214_T002 rna24725 0
354- 11: transcript:Zm00001d006217_T004 rna24726 0
354- 12: transcript:Zm00001d006219_T003 rna24730 9.00E-54
354- 13: transcript:Zm00001d006220_T002 rna24732 2.00E-126
354- 14: transcript:Zm00001d006221_T001 rna24734 0
354- 15: transcript:Zm00001d006226_T001 rna24735 1.00E-101
354- 16: transcript:Zm00001d006227_T003 rna24737 0
354- 17: transcript:Zm00001d006231_T001 rna24740 0
354- 18: transcript:Zm00001d006232_T001 rna24742 0
354- 19: transcript:Zm00001d006235_T001 rna24755 9.00E-179
354- 20: transcript:Zm00001d006236_T001 rna24756 2.00E-138
354- 21: transcript:Zm00001d006237_T001 rna24758 0
354- 22: transcript:Zm00001d006238_T003 rna24762 0
354- 23: transcript:Zm00001d006242_T001 rna24765 0
354- 24: transcript:Zm00001d006243_T005 rna24766 0
354- 25: transcript:Zm00001d006244_T001 rna24775 5.00E-152
354- 26: transcript:Zm00001d006246_T001 rna24778 1.00E-101
354- 27: transcript:Zm00001d006249_T003 rna24781 1.00E-87
354- 28: transcript:Zm00001d006250_T001 rna24786 4.00E-118
354- 29: transcript:Zm00001d006251_T001 rna24788 9.00E-142
354- 30: transcript:Zm00001d006254_T001 rna24791 7.00E-30
354- 31: transcript:Zm00001d006255_T001 rna24792 0
354- 32: transcript:Zm00001d006256_T001 rna24793 1.00E-76
354- 33: transcript:Zm00001d006257_T006 rna24794 0
354- 34: transcript:Zm00001d006260_T001 rna24796 3.00E-171
354- 35: transcript:Zm00001d006267_T002 rna24798 0
354- 36: transcript:Zm00001d006269_T001 rna24800 0
354- 37: transcript:Zm00001d006270_T001 rna24801 0
354- 38: transcript:Zm00001d006274_T002 rna24807 3.00E-53
354- 39: transcript:Zm00001d006276_T001 rna24808 2.00E-55
354- 40: transcript:Zm00001d006279_T001 rna24809 3.00E-57

354- 41: transcript:Zm00001d006286_T001 rna24819 0
354- 42: transcript:Zm00001d006291_T002 rna24829 3.00E-56
354- 43: transcript:Zm00001d006293_T024 rna24830 2.00E-116
354- 44: transcript:Zm00001d006294_T001 rna24831 4.00E-120
354- 45: transcript:Zm00001d006295_T002 rna24834 0
Alignment 355: score=1961.0 e_value=2.1e-157 N=43 2&NC_008402.2 plus
355- 0: transcript:Zm00001d005641_T001 rna23823 1.00E-151
355- 1: transcript:Zm00001d005645_T001 rna23824 3.00E-121
355- 2: transcript:Zm00001d005647_T002 rna23827 0
355- 3: transcript:Zm00001d005648_T002 rna23828 0
355- 4: transcript:Zm00001d005649_T001 rna23830 2.00E-61
355- 5: transcript:Zm00001d005650_T004 rna23835 0
355- 6: transcript:Zm00001d005653_T004 rna23838 0
355- 7: transcript:Zm00001d005654_T001 rna23846 4.00E-24
355- 8: transcript:Zm00001d005656_T001 rna23848 3.00E-51
355- 9: transcript:Zm00001d005657_T002 rna23856 3.00E-08
355- 10: transcript:Zm00001d005658_T001 rna23862 8.00E-177
355- 11: transcript:Zm00001d005659_T001 rna23863 1.00E-136
355- 12: transcript:Zm00001d005661_T001 rna23864 7.00E-127
355- 13: transcript:Zm00001d005662_T001 rna23865 2.00E-162
355- 14: transcript:Zm00001d005663_T001 rna23866 9.00E-161
355- 15: transcript:Zm00001d005664_T001 rna23867 1.00E-100
355- 16: transcript:Zm00001d005671_T002 rna23874 2.00E-41
355- 17: transcript:Zm00001d005674_T001 rna23883 4.00E-54
355- 18: transcript:Zm00001d005680_T001 rna23885 0
355- 19: transcript:Zm00001d005681_T001 rna23886 3.00E-106
355- 20: transcript:Zm00001d005682_T001 rna23887 2.00E-118
355- 21: transcript:Zm00001d005684_T001 rna23891 2.00E-42
355- 22: transcript:Zm00001d005685_T001 rna23894 1.00E-81
355- 23: transcript:Zm00001d005687_T001 rna23895 0
355- 24: transcript:Zm00001d005688_T001 rna23900 0
355- 25: transcript:Zm00001d005692_T001 rna23902 4.00E-69
355- 26: transcript:Zm00001d005694_T001 rna23918 5.00E-80
355- 27: transcript:Zm00001d005695_T001 rna23919 8.00E-38
355- 28: transcript:Zm00001d005696_T001 rna23921 0
355- 29: transcript:Zm00001d005698_T001 rna23926 2.00E-81
355- 30: transcript:Zm00001d005699_T001 rna23928 0
355- 31: transcript:Zm00001d005705_T001 rna23930 4.00E-88
355- 32: transcript:Zm00001d005707_T007 rna23931 0
355- 33: transcript:Zm00001d005708_T001 rna23945 0
355- 34: transcript:Zm00001d005711_T005 rna23950 0
355- 35: transcript:Zm00001d005713_T001 rna23955 0
355- 36: transcript:Zm00001d005715_T002 rna23961 8.00E-78
355- 37: transcript:Zm00001d005716_T001 rna23966 5.00E-75
355- 38: transcript:Zm00001d005726_T001 rna23983 2.00E-119
355- 39: transcript:Zm00001d005727_T001 rna23984 4.00E-180
355- 40: transcript:Zm00001d005729_T001 rna23989 0
355- 41: transcript:Zm00001d005732_T001 rna24001 0
355- 42: transcript:Zm00001d005735_T001 rna24010 0
Alignment 356: score=1337.0 e_value=5.1e-97 N=30 2&NC_008402.2 plus
356- 0: transcript:Zm00001d005780_T001 rna24092 5.00E-125
356- 1: transcript:Zm00001d005782_T001 rna24112 9.00E-130
356- 2: transcript:Zm00001d005785_T006 rna24125 3.00E-55
356- 3: transcript:Zm00001d005786_T001 rna24126 3.00E-25

356- 4: transcript:Zm00001d005790_T001 rna24129 9.00E-158
356- 5: transcript:Zm00001d005791_T001 rna24132 4.00E-67
356- 6: transcript:Zm00001d005792_T004 rna24134 0
356- 7: transcript:Zm00001d005794_T009 rna24137 2.00E-175
356- 8: transcript:Zm00001d005795_T002 rna24138 7.00E-173
356- 9: transcript:Zm00001d005798_T001 rna24139 2.00E-93
356- 10: transcript:Zm00001d005802_T001 rna24151 2.00E-73
356- 11: transcript:Zm00001d005803_T001 rna24152 2.00E-59
356- 12: transcript:Zm00001d005804_T001 rna24153 1.00E-20
356- 13: transcript:Zm00001d005807_T001 rna24157 1.00E-27
356- 14: transcript:Zm00001d005808_T007 rna24158 9.00E-131
356- 15: transcript:Zm00001d005812_T002 rna24159 2.00E-81
356- 16: transcript:Zm00001d005813_T002 rna24162 6.00E-56
356- 17: transcript:Zm00001d005814_T001 rna24164 3.00E-153
356- 18: transcript:Zm00001d005816_T001 rna24165 0
356- 19: transcript:Zm00001d005817_T006 rna24166 0
356- 20: transcript:Zm00001d005818_T001 rna24170 0
356- 21: transcript:Zm00001d005819_T001 rna24172 7.00E-73
356- 22: transcript:Zm00001d005821_T002 rna24174 0
356- 23: transcript:Zm00001d005824_T001 rna24177 5.00E-43
356- 24: transcript:Zm00001d005826_T004 rna24181 0
356- 25: transcript:Zm00001d005830_T001 rna24189 2.00E-25
356- 26: transcript:Zm00001d005831_T003 rna24190 0
356- 27: transcript:Zm00001d005833_T002 rna24191 3.00E-14
356- 28: transcript:Zm00001d005834_T001 rna24193 5.00E-176
356- 29: transcript:Zm00001d005837_T001 rna24197 4.00E-75
Alignment 357: score=963.0 e_value=1.2e-73 N=22 2&NC_008402.2 plus
357- 0: transcript:Zm00001d005578_T001 rna23630 6.00E-55
357- 1: transcript:Zm00001d005579_T003 rna23639 0
357- 2: transcript:Zm00001d005581_T001 rna23643 4.00E-51
357- 3: transcript:Zm00001d005586_T001 rna23653 0
357- 4: transcript:Zm00001d005590_T001 rna23673 5.00E-42
357- 5: transcript:Zm00001d005594_T001 rna23680 0
357- 6: transcript:Zm00001d005598_T001 rna23689 0
357- 7: transcript:Zm00001d005599_T001 rna23690 0
357- 8: transcript:Zm00001d005609_T001 rna23705 3.00E-156
357- 9: transcript:Zm00001d005612_T001 rna23714 4.00E-46
357- 10: transcript:Zm00001d005614_T001 rna23716 0
357- 11: transcript:Zm00001d005615_T001 rna23718 5.00E-33
357- 12: transcript:Zm00001d005616_T001 rna23719 7.00E-25
357- 13: transcript:Zm00001d005622_T001 rna23738 3.00E-91
357- 14: transcript:Zm00001d005624_T001 rna23748 0
357- 15: transcript:Zm00001d005628_T001 rna23770 5.00E-36
357- 16: transcript:Zm00001d005629_T001 rna23771 0
357- 17: transcript:Zm00001d005630_T001 rna23772 1.00E-70
357- 18: transcript:Zm00001d005631_T001 rna23773 1.00E-65
357- 19: transcript:Zm00001d005632_T001 rna23776 0
357- 20: transcript:Zm00001d005636_T003 rna23777 0
357- 21: transcript:Zm00001d005638_T001 rna23781 0
Alignment 358: score=748.0 e_value=2.1e-51 N=17 2&NC_008402.2 plus
358- 0: transcript:Zm00001d005504_T001 rna23570 2.00E-66
358- 1: transcript:Zm00001d005506_T001 rna23571 5.00E-174
358- 2: transcript:Zm00001d005507_T012 rna23572 0
358- 3: transcript:Zm00001d005513_T001 rna23578 2.00E-137

```
358-  4:  transcript:Zm00001d005516_T001  rna23579            0
358-  5:  transcript:Zm00001d005520_T001  rna23585    2.00E-111
358-  6:  transcript:Zm00001d005532_T003  rna23593    5.00E-106
358-  7:  transcript:Zm00001d005534_T001  rna23596    9.00E-131
358-  8:  transcript:Zm00001d005535_T002  rna23597            0
358-  9:  transcript:Zm00001d005538_T001  rna23606    3.00E-110
358- 10:  transcript:Zm00001d005539_T003  rna23607            0
358- 11:  transcript:Zm00001d005542_T001  rna23609            0
358- 12:  transcript:Zm00001d005545_T001  rna23611     2.00E-62
358- 13:  transcript:Zm00001d005546_T002  rna23613            0
358- 14:  transcript:Zm00001d005547_T001  rna23617     8.00E-60
358- 15:  transcript:Zm00001d005554_T001  rna23619            0
358- 16:  transcript:Zm00001d005563_T006  rna23636    1.00E-150
## Alignment 359: score=537.0 e_value=5.6e-28 N=12 2&NC_008402.2 plus
359-  0:  transcript:Zm00001d006297_T001  rna24836    2.00E-129
359-  1:  transcript:Zm00001d006306_T006  rna24842     4.00E-70
359-  2:  transcript:Zm00001d006307_T002  rna24845     1.00E-09
359-  3:  transcript:Zm00001d006308_T001  rna24847            0
359-  4:  transcript:Zm00001d006309_T007  rna24853            0
359-  5:  transcript:Zm00001d006310_T005  rna24854     5.00E-82
359-  6:  transcript:Zm00001d006311_T001  rna24855     5.00E-83
359-  7:  transcript:Zm00001d006312_T001  rna24856     4.00E-95
359-  8:  transcript:Zm00001d006315_T001  rna24859    2.00E-106
359-  9:  transcript:Zm00001d006319_T001  rna24869    3.00E-136
359- 10:  transcript:Zm00001d006320_T004  rna24871     5.00E-48
359- 11:  transcript:Zm00001d006322_T001  rna24876            0
## Alignment 360: score=513.0 e_value=7.2e-25 N=11 2&NC_008402.2 plus
360-  0:  transcript:Zm00001d006324_T005  rna24878    1.00E-160
360-  1:  transcript:Zm00001d006328_T003  rna24883            0
360-  2:  transcript:Zm00001d006329_T001  rna24886     5.00E-41
360-  3:  transcript:Zm00001d006331_T001  rna24887    3.00E-140
360-  4:  transcript:Zm00001d006332_T001  rna24895     4.00E-88
360-  5:  transcript:Zm00001d006333_T001  rna24898            0
360-  6:  transcript:Zm00001d006335_T003  rna24899            0
360-  7:  transcript:Zm00001d006336_T001  rna24902     2.00E-52
360-  8:  transcript:Zm00001d006337_T001  rna24903     2.00E-94
360-  9:  transcript:Zm00001d006340_T002  rna24907    3.00E-159
360- 10:  transcript:Zm00001d006344_T007  rna24909            0
## Alignment 361: score=335.0 e_value=6.7e-13 N=7 2&NC_008402.2 plus
361-  0:  transcript:Zm00001d005767_T001  rna24069    5.00E-155
361-  1:  transcript:Zm00001d005769_T001  rna24073            0
361-  2:  transcript:Zm00001d005770_T001  rna24076            0
361-  3:  transcript:Zm00001d005772_T001  rna24077     7.00E-28
361-  4:  transcript:Zm00001d005773_T001  rna24078     2.00E-26
361-  5:  transcript:Zm00001d005775_T005  rna24082            0
361-  6:  transcript:Zm00001d005776_T002  rna24084            0
## Alignment 362: score=968.0 e_value=1.9e-60 N=21 2&NC_008402.2 minus
362-  0:  transcript:Zm00001d005306_T002  rna24998            0
362-  1:  transcript:Zm00001d005308_T002  rna24990            0
362-  2:  transcript:Zm00001d005313_T001  rna24986            0
362-  3:  transcript:Zm00001d005315_T001  rna24984     3.00E-98
362-  4:  transcript:Zm00001d005317_T001  rna24980            0
362-  5:  transcript:Zm00001d005322_T001  rna24979     8.00E-47
362-  6:  transcript:Zm00001d005323_T002  rna24973            0
```

362- 7: transcript:Zm00001d005324_T004 rna24972 0
362- 8: transcript:Zm00001d005328_T002 rna24964 4.00E-119
362- 9: transcript:Zm00001d005329_T001 rna24961 0
362- 10: transcript:Zm00001d005333_T001 rna24956 2.00E-56
362- 11: transcript:Zm00001d005334_T001 rna24955 2.00E-15
362- 12: transcript:Zm00001d005338_T005 rna24952 1.00E-63
362- 13: transcript:Zm00001d005343_T003 rna24951 0
362- 14: transcript:Zm00001d005344_T002 rna24946 1.00E-90
362- 15: transcript:Zm00001d005346_T001 rna24945 0
362- 16: transcript:Zm00001d005347_T001 rna24944 0
362- 17: transcript:Zm00001d005348_T001 rna24940 5.00E-27
362- 18: transcript:Zm00001d005350_T005 rna24937 0
362- 19: transcript:Zm00001d005351_T001 rna24934 0
362- 20: transcript:Zm00001d005356_T001 rna24932 2.00E-41
Alignment 363: score=893.0 e_value=1.1e-53 N=19 2&NC_008402.2 minus
363- 0: transcript:Zm00001d005841_T001 rna24254 1.00E-54
363- 1: transcript:Zm00001d005843_T001 rna24253 1.00E-142
363- 2: transcript:Zm00001d005844_T002 rna24249 8.00E-51
363- 3: transcript:Zm00001d005846_T002 rna24248 0
363- 4: transcript:Zm00001d005847_T001 rna24247 3.00E-179
363- 5: transcript:Zm00001d005848_T003 rna24246 7.00E-169
363- 6: transcript:Zm00001d005849_T001 rna24245 0
363- 7: transcript:Zm00001d005851_T002 rna24243 3.00E-41
363- 8: transcript:Zm00001d005856_T003 rna24242 0
363- 9: transcript:Zm00001d005857_T001 rna24239 4.00E-83
363- 10: transcript:Zm00001d005858_T001 rna24237 9.00E-95
363- 11: transcript:Zm00001d005859_T001 rna24233 8.00E-103
363- 12: transcript:Zm00001d005864_T001 rna24230 3.00E-92
363- 13: transcript:Zm00001d005865_T001 rna24229 1.00E-42
363- 14: transcript:Zm00001d005866_T003 rna24228 0
363- 15: transcript:Zm00001d005867_T002 rna24223 2.00E-37
363- 16: transcript:Zm00001d005871_T002 rna24215 8.00E-94
363- 17: transcript:Zm00001d005874_T001 rna24211 1.00E-72
363- 18: transcript:Zm00001d005875_T001 rna24204 1.00E-48
Alignment 364: score=599.0 e_value=9.6e-29 N=13 2&NC_008402.2 minus
364- 0: transcript:Zm00001d005748_T001 rna24060 0
364- 1: transcript:Zm00001d005749_T001 rna24058 7.00E-95
364- 2: transcript:Zm00001d005750_T002 rna24056 2.00E-124
364- 3: transcript:Zm00001d005751_T002 rna24053 3.00E-143
364- 4: transcript:Zm00001d005752_T001 rna24052 5.00E-75
364- 5: transcript:Zm00001d005754_T001 rna24050 2.00E-20
364- 6: transcript:Zm00001d005756_T002 rna24048 0
364- 7: transcript:Zm00001d005757_T001 rna24042 9.00E-39
364- 8: transcript:Zm00001d005760_T001 rna24040 4.00E-139
364- 9: transcript:Zm00001d005762_T002 rna24039 2.00E-130
364- 10: transcript:Zm00001d005764_T001 rna24038 0
364- 11: transcript:Zm00001d005765_T004 rna24032 3.00E-42
364- 12: transcript:Zm00001d005766_T001 rna24027 1.00E-30
Alignment 365: score=486.0 e_value=1.3e-20 N=10 2&NC_008402.2 minus
365- 0: transcript:Zm00001d006182_T001 rna24700 3.00E-171
365- 1: transcript:Zm00001d006183_T001 rna24699 1.00E-98
365- 2: transcript:Zm00001d006184_T001 rna24697 4.00E-111
365- 3: transcript:Zm00001d006185_T001 rna24696 1.00E-170
365- 4: transcript:Zm00001d006192_T001 rna24695 0

365- 5: transcript:Zm00001d006193_T001 rna24694 0
365- 6: transcript:Zm00001d006194_T001 rna24693 9.00E-106
365- 7: transcript:Zm00001d006195_T001 rna24692 4.00E-70
365- 8: transcript:Zm00001d006196_T001 rna24691 6.00E-40
365- 9: transcript:Zm00001d006197_T003 rna24690 0
Alignment 366: score=258.0 e_value=6.5e-09 N=6 2&NC_008402.2 minus
366- 0: transcript:Zm00001d005293_T001 rna23344 2.00E-107
366- 1: transcript:Zm00001d005295_T001 rna23342 0
366- 2: transcript:Zm00001d005298_T001 rna23339 0
366- 3: transcript:Zm00001d005300_T001 rna23331 2.00E-98
366- 4: transcript:Zm00001d005302_T001 rna23326 1.00E-66
366- 5: transcript:Zm00001d005303_T001 rna23325 4.00E-143
Alignment 367: score=274.0 e_value=2.4e-10 N=6 2&NC_008403.2 minus
367- 0: transcript:Zm00001d002989_T001 rna26053 0
367- 1: transcript:Zm00001d003006_T001 rna26041 2.00E-91
367- 2: transcript:Zm00001d003009_T001 rna26040 1.00E-20
367- 3: transcript:Zm00001d003011_T001 rna26037 9.00E-50
367- 4: transcript:Zm00001d003012_T001 rna26035 1.00E-69
367- 5: transcript:Zm00001d003013_T001 rna26033 5.00E-116
Alignment 368: score=1665.0 e_value=3.6e-153 N=41 2&NC_008404.2 plus
368- 0: transcript:Zm00001d007411_T001 rna27853 2.00E-63
368- 1: transcript:Zm00001d007419_T001 rna27857 0
368- 2: transcript:Zm00001d007420_T001 rna27862 3.00E-67
368- 3: transcript:Zm00001d007422_T008 rna27877 0
368- 4: transcript:Zm00001d007433_T001 rna27880 2.00E-114
368- 5: transcript:Zm00001d007434_T002 rna27894 6.00E-70
368- 6: transcript:Zm00001d007435_T008 rna27897 2.00E-169
368- 7: transcript:Zm00001d007436_T002 rna27898 5.00E-137
368- 8: transcript:Zm00001d007437_T002 rna27903 4.00E-100
368- 9: transcript:Zm00001d007441_T001 rna27906 0
368- 10: transcript:Zm00001d007442_T001 rna27908 0
368- 11: transcript:Zm00001d007446_T003 rna27910 2.00E-169
368- 12: transcript:Zm00001d007454_T003 rna27920 1.00E-91
368- 13: transcript:Zm00001d007455_T003 rna27927 0
368- 14: transcript:Zm00001d007457_T003 rna27929 6.00E-58
368- 15: transcript:Zm00001d007465_T003 rna27938 6.00E-132
368- 16: transcript:Zm00001d007468_T001 rna27940 1.00E-76
368- 17: transcript:Zm00001d007473_T001 rna27941 3.00E-47
368- 18: transcript:Zm00001d007474_T001 rna27943 1.00E-161
368- 19: transcript:Zm00001d007478_T001 rna27951 2.00E-07
368- 20: transcript:Zm00001d007479_T001 rna27952 2.00E-136
368- 21: transcript:Zm00001d007485_T001 rna27971 0
368- 22: transcript:Zm00001d007489_T001 rna27980 9.00E-161
368- 23: transcript:Zm00001d007490_T001 rna27981 4.00E-90
368- 24: transcript:Zm00001d007496_T002 rna27991 2.00E-162
368- 25: transcript:Zm00001d007500_T034 rna27992 0
368- 26: transcript:Zm00001d007502_T001 rna27997 0
368- 27: transcript:Zm00001d007507_T001 rna28001 2.00E-44
368- 28: transcript:Zm00001d007509_T001 rna28012 0
368- 29: transcript:Zm00001d007514_T001 rna28023 3.00E-69
368- 30: transcript:Zm00001d007518_T001 rna28035 0
368- 31: transcript:Zm00001d007522_T023 rna28040 0
368- 32: transcript:Zm00001d007530_T001 rna28041 7.00E-107
368- 33: transcript:Zm00001d007550_T001 rna28045 5.00E-89

368- 34: transcript:Zm00001d007571_T001 rna28067 0
368- 35: transcript:Zm00001d007572_T001 rna28068 2.00E-55
368- 36: transcript:Zm00001d007573_T001 rna28073 0
368- 37: transcript:Zm00001d007577_T001 rna28082 4.00E-21
368- 38: transcript:Zm00001d007581_T001 rna28090 1.00E-126
368- 39: transcript:Zm00001d007592_T001 rna28115 2.00E-137
368- 40: transcript:Zm00001d007594_T002 rna28125 2.00E-102
Alignment 369: score=504.0 e_value=5.7e-27 N=12 2&NC_008404.2 plus
369- 0: transcript:Zm00001d007255_T001 rna27338 0
369- 1: transcript:Zm00001d007257_T001 rna27351 6.00E-178
369- 2: transcript:Zm00001d007259_T001 rna27356 3.00E-133
369- 3: transcript:Zm00001d007264_T001 rna27362 4.00E-126
369- 4: transcript:Zm00001d007267_T002 rna27367 0
369- 5: transcript:Zm00001d007271_T001 rna27371 5.00E-74
369- 6: transcript:Zm00001d007272_T001 rna27373 2.00E-134
369- 7: transcript:Zm00001d007274_T001 rna27385 0
369- 8: transcript:Zm00001d007280_T001 rna27389 3.00E-15
369- 9: transcript:Zm00001d007285_T002 rna27410 6.00E-175
369- 10: transcript:Zm00001d007288_T005 rna27411 2.00E-50
369- 11: transcript:Zm00001d007294_T001 rna27433 3.00E-159
Alignment 370: score=435.0 e_value=4.9e-22 N=10 2&NC_008404.2 plus
370- 0: transcript:Zm00001d007318_T007 rna27729 3.00E-23
370- 1: transcript:Zm00001d007320_T002 rna27740 8.00E-84
370- 2: transcript:Zm00001d007327_T001 rna27743 2.00E-126
370- 3: transcript:Zm00001d007328_T003 rna27747 3.00E-39
370- 4: transcript:Zm00001d007329_T001 rna27748 3.00E-77
370- 5: transcript:Zm00001d007331_T001 rna27760 3.00E-74
370- 6: transcript:Zm00001d007339_T001 rna27761 4.00E-161
370- 7: transcript:Zm00001d007341_T001 rna27762 3.00E-73
370- 8: transcript:Zm00001d007345_T001 rna27763 0
370- 9: transcript:Zm00001d007365_T001 rna27778 3.00E-141
Alignment 371: score=421.0 e_value=5.2e-24 N=10 2&NC_008404.2 plus
371- 0: transcript:Zm00001d007372_T002 rna27815 0
371- 1: transcript:Zm00001d007382_T001 rna27817 4.00E-176
371- 2: transcript:Zm00001d007383_T002 rna27823 0
371- 3: transcript:Zm00001d007387_T005 rna27825 6.00E-126
371- 4: transcript:Zm00001d007388_T005 rna27834 1.00E-153
371- 5: transcript:Zm00001d007390_T001 rna27835 5.00E-72
371- 6: transcript:Zm00001d007391_T005 rna27836 0
371- 7: transcript:Zm00001d007394_T001 rna27837 2.00E-60
371- 8: transcript:Zm00001d007395_T001 rna27838 4.00E-111
371- 9: transcript:Zm00001d007400_T001 rna27840 2.00E-125
Alignment 372: score=316.0 e_value=4.6e-15 N=8 2&NC_008404.2 plus
372- 0: transcript:Zm00001d007629_T001 rna28221 3.00E-30
372- 1: transcript:Zm00001d007632_T003 rna28247 0
372- 2: transcript:Zm00001d007633_T001 rna28251 5.00E-41
372- 3: transcript:Zm00001d007638_T001 rna28253 4.00E-59
372- 4: transcript:Zm00001d007639_T003 rna28254 0
372- 5: transcript:Zm00001d007645_T002 rna28259 9.00E-61
372- 6: transcript:Zm00001d007653_T003 rna28271 0
372- 7: transcript:Zm00001d007657_T001 rna28278 3.00E-20
Alignment 373: score=284.0 e_value=9e-12 N=7 2&NC_008404.2 plus
373- 0: transcript:Zm00001d004646_T001 rna27650 3.00E-161
373- 1: transcript:Zm00001d004649_T001 rna27659 7.00E-54

373- 2: transcript:Zm00001d004650_T001 rna27678   3.00E-44
373- 3: transcript:Zm00001d004651_T001 rna27693   0
373- 4: transcript:Zm00001d004655_T002 rna27695   3.00E-105
373- 5: transcript:Zm00001d004659_T002 rna27697   5.00E-171
373- 6: transcript:Zm00001d004667_T003 rna27719   0
Alignment 374: score=282.0 e_value=1.1e-10 N=7 2&NC_008404.2 plus
374- 0: transcript:Zm00001d007161_T001 rna26750   1.00E-131
374- 1: transcript:Zm00001d007166_T001 rna26760   5.00E-105
374- 2: transcript:Zm00001d007169_T001 rna26764   1.00E-127
374- 3: transcript:Zm00001d007175_T001 rna26782   2.00E-11
374- 4: transcript:Zm00001d007180_T001 rna26793   7.00E-93
374- 5: transcript:Zm00001d007184_T001 rna26813   0
374- 6: transcript:Zm00001d007186_T004 rna26816   4.00E-38
Alignment 375: score=265.0 e_value=3e-10 N=6 2&NC_008404.2 plus
375- 0: transcript:Zm00001d007596_T001 rna28124   0
375- 1: transcript:Zm00001d007597_T001 rna28128   0
375- 2: transcript:Zm00001d007599_T002 rna28132   0
375- 3: transcript:Zm00001d007600_T001 rna28133   2.00E-107
375- 4: transcript:Zm00001d007602_T001 rna28138   7.00E-16
375- 5: transcript:Zm00001d007604_T001 rna28164   0
Alignment 376: score=3125.0 e_value=5.4e-296 N=71 2&NC_008404.2 minus
376- 0: transcript:Zm00001d004705_T001 rna27168   0
376- 1: transcript:Zm00001d004706_T002 rna27167   0
376- 2: transcript:Zm00001d004707_T002 rna27166   0
376- 3: transcript:Zm00001d004709_T002 rna27154   8.00E-120
376- 4: transcript:Zm00001d004716_T002 rna27152   5.00E-14
376- 5: transcript:Zm00001d004718_T001 rna27145   5.00E-177
376- 6: transcript:Zm00001d004720_T001 rna27142   0
376- 7: transcript:Zm00001d004731_T001 rna27141   0
376- 8: transcript:Zm00001d004734_T001 rna27134   0
376- 9: transcript:Zm00001d004738_T001 rna27129   7.00E-44
376- 10: transcript:Zm00001d004739_T001 rna27128   0
376- 11: transcript:Zm00001d004740_T002 rna27118   0
376- 12: transcript:Zm00001d004744_T001 rna27117   2.00E-110
376- 13: transcript:Zm00001d004751_T001 rna27113   6.00E-31
376- 14: transcript:Zm00001d004753_T001 rna27107   2.00E-40
376- 15: transcript:Zm00001d004762_T001 rna27106   0
376- 16: transcript:Zm00001d004775_T004 rna27104   0
376- 17: transcript:Zm00001d004779_T001 rna27102   0
376- 18: transcript:Zm00001d004782_T005 rna27101   0
376- 19: transcript:Zm00001d004785_T001 rna27100   0
376- 20: transcript:Zm00001d004791_T001 rna27098   1.00E-37
376- 21: transcript:Zm00001d004804_T001 rna27096   0
376- 22: transcript:Zm00001d004807_T001 rna27092   0
376- 23: transcript:Zm00001d004810_T002 rna27078   0
376- 24: transcript:Zm00001d004812_T002 rna27076   0
376- 25: transcript:Zm00001d004814_T001 rna27075   1.00E-135
376- 26: transcript:Zm00001d004818_T001 rna27070   1.00E-27
376- 27: transcript:Zm00001d004821_T002 rna27066   9.00E-111
376- 28: transcript:Zm00001d004822_T001 rna27062   3.00E-83
376- 29: transcript:Zm00001d004837_T001 rna27057   2.00E-103
376- 30: transcript:Zm00001d004839_T007 rna27056   0
376- 31: transcript:Zm00001d004843_T003 rna27053   1.00E-21
376- 32: transcript:Zm00001d004846_T001 rna27052   0

376- 33: transcript:Zm00001d004847_T001 rna27047 2.00E-10
376- 34: transcript:Zm00001d004848_T001 rna27043 2.00E-143
376- 35: transcript:Zm00001d004851_T001 rna27041 0
376- 36: transcript:Zm00001d004855_T002 rna27040 0
376- 37: transcript:Zm00001d004857_T001 rna27027 1.00E-103
376- 38: transcript:Zm00001d004861_T002 rna27024 3.00E-49
376- 39: transcript:Zm00001d004862_T001 rna27023 7.00E-76
376- 40: transcript:Zm00001d004865_T001 rna27019 3.00E-17
376- 41: transcript:Zm00001d004868_T001 rna27018 0
376- 42: transcript:Zm00001d004875_T002 rna27013 0
376- 43: transcript:Zm00001d004876_T001 rna27011 2.00E-91
376- 44: transcript:Zm00001d004881_T001 rna27004 5.00E-51
376- 45: transcript:Zm00001d004882_T001 rna27003 2.00E-58
376- 46: transcript:Zm00001d004895_T001 rna26996 6.00E-132
376- 47: transcript:Zm00001d004896_T002 rna26995 3.00E-30
376- 48: transcript:Zm00001d004897_T001 rna26994 2.00E-41
376- 49: transcript:Zm00001d004903_T001 rna26986 1.00E-84
376- 50: transcript:Zm00001d004908_T001 rna26984 2.00E-91
376- 51: transcript:Zm00001d004909_T001 rna26983 1.00E-93
376- 52: transcript:Zm00001d004910_T001 rna26981 7.00E-27
376- 53: transcript:Zm00001d004917_T001 rna26964 3.00E-42
376- 54: transcript:Zm00001d004919_T001 rna26962 2.00E-119
376- 55: transcript:Zm00001d004924_T001 rna26956 3.00E-135
376- 56: transcript:Zm00001d004925_T001 rna26954 0
376- 57: transcript:Zm00001d004927_T001 rna26953 1.00E-82
376- 58: transcript:Zm00001d004930_T001 rna26950 3.00E-32
376- 59: transcript:Zm00001d004931_T001 rna26944 6.00E-35
376- 60: transcript:Zm00001d004934_T001 rna26930 2.00E-61
376- 61: transcript:Zm00001d004936_T001 rna26929 2.00E-18
376- 62: transcript:Zm00001d004955_T001 rna26926 3.00E-34
376- 63: transcript:Zm00001d004956_T001 rna26921 8.00E-106
376- 64: transcript:Zm00001d004957_T001 rna26919 0
376- 65: transcript:Zm00001d004960_T002 rna26918 2.00E-42
376- 66: transcript:Zm00001d004966_T001 rna26915 0
376- 67: transcript:Zm00001d004972_T001 rna26911 5.00E-36
376- 68: transcript:Zm00001d004976_T002 rna26910 0
376- 69: transcript:Zm00001d004977_T002 rna26908 9.00E-47
376- 70: transcript:Zm00001d004978_T001 rna26907 2.00E-13
Alignment 377: score=1267.0 e_value=7.3e-97 N=29 2&NC_008404.2 minus
377- 0: transcript:Zm00001d004986_T014 rna26899 9.00E-95
377- 1: transcript:Zm00001d004992_T001 rna26883 0
377- 2: transcript:Zm00001d004998_T001 rna26881 0
377- 3: transcript:Zm00001d005001_T001 rna26874 0
377- 4: transcript:Zm00001d005003_T002 rna26870 2.00E-62
377- 5: transcript:Zm00001d005007_T001 rna26849 1.00E-12
377- 6: transcript:Zm00001d005008_T002 rna26848 6.00E-78
377- 7: transcript:Zm00001d005010_T001 rna26845 2.00E-102
377- 8: transcript:Zm00001d005011_T001 rna26844 1.00E-30
377- 9: transcript:Zm00001d005013_T001 rna26842 1.00E-154
377- 10: transcript:Zm00001d005015_T001 rna26840 1.00E-165
377- 11: transcript:Zm00001d005018_T001 rna26834 3.00E-92
377- 12: transcript:Zm00001d005023_T001 rna26826 2.00E-95
377- 13: transcript:Zm00001d005027_T002 rna26824 8.00E-158
377- 14: transcript:Zm00001d005028_T001 rna26822 9.00E-78

377- 15: transcript:Zm00001d005029_T001 rna26811 0
377- 16: transcript:Zm00001d005032_T001 rna26810 0
377- 17: transcript:Zm00001d005035_T001 rna26808 1.00E-48
377- 18: transcript:Zm00001d005036_T003 rna26802 2.00E-114
377- 19: transcript:Zm00001d005037_T001 rna26801 3.00E-30
377- 20: transcript:Zm00001d005038_T001 rna26798 6.00E-26
377- 21: transcript:Zm00001d005039_T001 rna26796 4.00E-96
377- 22: transcript:Zm00001d005047_T001 rna26789 3.00E-163
377- 23: transcript:Zm00001d005051_T001 rna26786 0
377- 24: transcript:Zm00001d005052_T002 rna26785 4.00E-97
377- 25: transcript:Zm00001d005053_T001 rna26783 1.00E-50
377- 26: transcript:Zm00001d005056_T001 rna26779 6.00E-22
377- 27: transcript:Zm00001d005057_T001 rna26778 4.00E-27
377- 28: transcript:Zm00001d005062_T003 rna26777 2.00E-158
Alignment 378: score=401.0 e_value=2.7e-21 N=9 2&NC_008404.2 minus
378- 0: transcript:Zm00001d005063_T001 rna26736 7.00E-44
378- 1: transcript:Zm00001d005065_T001 rna26735 3.00E-142
378- 2: transcript:Zm00001d005071_T001 rna26727 0
378- 3: transcript:Zm00001d005072_T003 rna26726 6.00E-46
378- 4: transcript:Zm00001d005081_T001 rna26719 1.00E-39
378- 5: transcript:Zm00001d005082_T001 rna26718 0
378- 6: transcript:Zm00001d005085_T002 rna26713 0
378- 7: transcript:Zm00001d005087_T001 rna26710 6.00E-26
378- 8: transcript:Zm00001d005089_T005 rna26703 3.00E-126
Alignment 379: score=351.0 e_value=2.7e-13 N=8 2&NC_008404.2 minus
379- 0: transcript:Zm00001d003750_T001 rna27017 0
379- 1: transcript:Zm00001d003751_T001 rna27010 4.00E-68
379- 2: transcript:Zm00001d003754_T003 rna27007 4.00E-58
379- 3: transcript:Zm00001d003762_T002 rna27003 1.00E-30
379- 4: transcript:Zm00001d003769_T001 rna26996 2.00E-15
379- 5: transcript:Zm00001d003775_T001 rna26992 3.00E-16
379- 6: transcript:Zm00001d003777_T001 rna26986 3.00E-11
379- 7: transcript:Zm00001d003781_T002 rna26983 6.00E-52
Alignment 380: score=318.0 e_value=3.2e-12 N=7 2&NC_008404.2 minus
380- 0: transcript:Zm00001d007300_T005 rna27184 0
380- 1: transcript:Zm00001d007301_T001 rna27182 0
380- 2: transcript:Zm00001d007302_T003 rna27177 2.00E-171
380- 3: transcript:Zm00001d007305_T001 rna27175 7.00E-26
380- 4: transcript:Zm00001d007307_T003 rna27174 3.00E-72
380- 5: transcript:Zm00001d007308_T001 rna27171 2.00E-42
380- 6: transcript:Zm00001d007309_T001 rna27169 3.00E-28
Alignment 381: score=312.0 e_value=1.2e-12 N=7 2&NC_008404.2 minus
381- 0: transcript:Zm00001d007351_T001 rna27813 0
381- 1: transcript:Zm00001d007354_T001 rna27798 0
381- 2: transcript:Zm00001d007357_T003 rna27796 0
381- 3: transcript:Zm00001d007360_T001 rna27795 9.00E-112
381- 4: transcript:Zm00001d007361_T001 rna27793 1.00E-52
381- 5: transcript:Zm00001d007363_T001 rna27789 6.00E-58
381- 6: transcript:Zm00001d007367_T001 rna27774 2.00E-31
Alignment 382: score=1314.0 e_value=4.3e-101 N=30 2&NC_008405.2 minus
382- 0: transcript:Zm00001d004986_T014 rna28744 0
382- 1: transcript:Zm00001d004992_T001 rna28730 0
382- 2: transcript:Zm00001d004998_T001 rna28728 0
382- 3: transcript:Zm00001d005001_T001 rna28720 3.00E-128

382- 4: transcript:Zm00001d005003_T002 rna28718 6.00E-78
382- 5: transcript:Zm00001d005007_T001 rna28692 3.00E-10
382- 6: transcript:Zm00001d005008_T002 rna28691 6.00E-87
382- 7: transcript:Zm00001d005010_T001 rna28687 4.00E-106
382- 8: transcript:Zm00001d005011_T001 rna28686 6.00E-31
382- 9: transcript:Zm00001d005013_T001 rna28685 1.00E-153
382- 10: transcript:Zm00001d005015_T001 rna28683 7.00E-72
382- 11: transcript:Zm00001d005016_T005 rna28682 4.00E-147
382- 12: transcript:Zm00001d005018_T001 rna28677 5.00E-47
382- 13: transcript:Zm00001d005023_T001 rna28667 2.00E-95
382- 14: transcript:Zm00001d005027_T002 rna28664 3.00E-152
382- 15: transcript:Zm00001d005028_T001 rna28663 3.00E-96
382- 16: transcript:Zm00001d005029_T001 rna28655 0
382- 17: transcript:Zm00001d005032_T001 rna28654 0
382- 18: transcript:Zm00001d005035_T001 rna28652 0
382- 19: transcript:Zm00001d005036_T003 rna28650 2.00E-115
382- 20: transcript:Zm00001d005037_T001 rna28649 2.00E-32
382- 21: transcript:Zm00001d005038_T001 rna28644 6.00E-26
382- 22: transcript:Zm00001d005039_T001 rna28641 1.00E-94
382- 23: transcript:Zm00001d005047_T001 rna28631 3.00E-163
382- 24: transcript:Zm00001d005051_T001 rna28626 0
382- 25: transcript:Zm00001d005052_T002 rna28624 0
382- 26: transcript:Zm00001d005053_T001 rna28622 3.00E-111
382- 27: transcript:Zm00001d005056_T001 rna28620 4.00E-20
382- 28: transcript:Zm00001d005057_T001 rna28618 1.00E-26
382- 29: transcript:Zm00001d005060_T001 rna28616 4.00E-45
Alignment 383: score=1211.0 e_value=4.5e-88 N=27 2&NC_008405.2 minus
383- 0: transcript:Zm00001d004881_T001 rna28857 2.00E-45
383- 1: transcript:Zm00001d004882_T001 rna28856 3.00E-54
383- 2: transcript:Zm00001d004895_T001 rna28851 9.00E-111
383- 3: transcript:Zm00001d004896_T002 rna28850 7.00E-172
383- 4: transcript:Zm00001d004897_T001 rna28849 2.00E-40
383- 5: transcript:Zm00001d004903_T001 rna28843 5.00E-82
383- 6: transcript:Zm00001d004908_T001 rna28835 3.00E-75
383- 7: transcript:Zm00001d004909_T001 rna28833 1.00E-125
383- 8: transcript:Zm00001d004910_T001 rna28832 8.00E-19
383- 9: transcript:Zm00001d004915_T001 rna28822 3.00E-150
383- 10: transcript:Zm00001d004916_T001 rna28816 0
383- 11: transcript:Zm00001d004917_T001 rna28811 6.00E-08
383- 12: transcript:Zm00001d004919_T001 rna28810 2.00E-28
383- 13: transcript:Zm00001d004924_T001 rna28802 5.00E-96
383- 14: transcript:Zm00001d004927_T001 rna28800 3.00E-27
383- 15: transcript:Zm00001d004930_T001 rna28795 0
383- 16: transcript:Zm00001d004931_T001 rna28790 7.00E-32
383- 17: transcript:Zm00001d004934_T001 rna28782 3.00E-166
383- 18: transcript:Zm00001d004936_T001 rna28781 6.00E-48
383- 19: transcript:Zm00001d004955_T001 rna28772 0
383- 20: transcript:Zm00001d004956_T001 rna28763 5.00E-105
383- 21: transcript:Zm00001d004957_T001 rna28761 0
383- 22: transcript:Zm00001d004960_T002 rna28760 0
383- 23: transcript:Zm00001d004966_T001 rna28757 7.00E-169
383- 24: transcript:Zm00001d004972_T001 rna28756 8.00E-35
383- 25: transcript:Zm00001d004976_T002 rna28752 0
383- 26: transcript:Zm00001d004977_T002 rna28750 2.00E-31

```
## Alignment 384: score=448.0 e_value=5e-26 N=10 2&NC_008405.2 minus
384-  0:  transcript:Zm00001d005063_T001  rna28579            0
384-  1:  transcript:Zm00001d005065_T001  rna28578    1.00E-143
384-  2:  transcript:Zm00001d005071_T001  rna28568            0
384-  3:  transcript:Zm00001d005072_T003  rna28567            0
384-  4:  transcript:Zm00001d005080_T002  rna28557            0
384-  5:  transcript:Zm00001d005081_T001  rna28556     2.00E-40
384-  6:  transcript:Zm00001d005082_T001  rna28555            0
384-  7:  transcript:Zm00001d005085_T002  rna28550            0
384-  8:  transcript:Zm00001d005087_T001  rna28545     8.00E-52
384-  9:  transcript:Zm00001d005089_T005  rna28542     2.00E-33
## Alignment 385: score=440.0 e_value=2.8e-23 N=11 2&NC_008405.2 minus
385-  0:  transcript:Zm00001d004791_T001  rna28939     4.00E-29
385-  1:  transcript:Zm00001d004812_T002  rna28923            0
385-  2:  transcript:Zm00001d004818_T001  rna28920            0
385-  3:  transcript:Zm00001d004821_T002  rna28916    5.00E-111
385-  4:  transcript:Zm00001d004839_T007  rna28908            0
385-  5:  transcript:Zm00001d004846_T001  rna28898            0
385-  6:  transcript:Zm00001d004848_T001  rna28892    1.00E-162
385-  7:  transcript:Zm00001d004851_T001  rna28887            0
385-  8:  transcript:Zm00001d004857_T001  rna28880    4.00E-145
385-  9:  transcript:Zm00001d004861_T002  rna28875     1.00E-38
385- 10:  transcript:Zm00001d004868_T001  rna28870            0
## Alignment 386: score=349.0 e_value=9.6e-13 N=8 2&NC_008405.2 minus
386-  0:  transcript:Zm00001d003745_T001  rna28873     8.00E-54
386-  1:  transcript:Zm00001d003751_T001  rna28865     6.00E-72
386-  2:  transcript:Zm00001d003755_T001  rna28860     1.00E-90
386-  3:  transcript:Zm00001d003762_T002  rna28856     4.00E-25
386-  4:  transcript:Zm00001d003769_T001  rna28851     3.00E-15
386-  5:  transcript:Zm00001d003775_T001  rna28847     2.00E-16
386-  6:  transcript:Zm00001d003777_T001  rna28843     1.00E-12
386-  7:  transcript:Zm00001d003781_T002  rna28833     3.00E-71
## Alignment 387: score=2496.0 e_value=4.6e-219 N=56 3&NC_008394.4 plus
387-  0:  transcript:Zm00001d040581_T001  rna1170     5.00E-174
387-  1:  transcript:Zm00001d040583_T001  rna1174      2.00E-61
387-  2:  transcript:Zm00001d040589_T001  rna1177             0
387-  3:  transcript:Zm00001d040593_T001  rna1179     2.00E-123
387-  4:  transcript:Zm00001d040594_T001  rna1183      2.00E-93
387-  5:  transcript:Zm00001d040596_T001  rna1186      8.00E-58
387-  6:  transcript:Zm00001d040603_T001  rna1190             0
387-  7:  transcript:Zm00001d040606_T004  rna1193             0
387-  8:  transcript:Zm00001d040611_T002  rna1202     1.00E-166
387-  9:  transcript:Zm00001d040612_T003  rna1207             0
387- 10:  transcript:Zm00001d040613_T001  rna1208      3.00E-25
387- 11:  transcript:Zm00001d040614_T002  rna1209     3.00E-116
387- 12:  transcript:Zm00001d040617_T001  rna1211      1.00E-34
387- 13:  transcript:Zm00001d040619_T001  rna1214      9.00E-51
387- 14:  transcript:Zm00001d040621_T002  rna1218     3.00E-101
387- 15:  transcript:Zm00001d040622_T001  rna1219      2.00E-26
387- 16:  transcript:Zm00001d040623_T001  rna1220      3.00E-20
387- 17:  transcript:Zm00001d040624_T001  rna1221     5.00E-109
387- 18:  transcript:Zm00001d040625_T002  rna1223     7.00E-173
387- 19:  transcript:Zm00001d040627_T001  rna1224             0
387- 20:  transcript:Zm00001d040634_T001  rna1227      3.00E-54
```

387- 21: transcript:Zm00001d040637_T001 rna1229 2.00E-44
387- 22: transcript:Zm00001d040639_T001 rna1239 9.00E-51
387- 23: transcript:Zm00001d040641_T002 rna1240 0
387- 24: transcript:Zm00001d040649_T001 rna1243 0
387- 25: transcript:Zm00001d040651_T001 rna1246 7.00E-75
387- 26: transcript:Zm00001d040659_T001 rna1251 6.00E-35
387- 27: transcript:Zm00001d040666_T003 rna1252 4.00E-62
387- 28: transcript:Zm00001d040667_T001 rna1253 0
387- 29: transcript:Zm00001d040672_T013 rna1256 7.00E-84
387- 30: transcript:Zm00001d040678_T003 rna1258 2.00E-53
387- 31: transcript:Zm00001d040681_T001 rna1259 4.00E-42
387- 32: transcript:Zm00001d040682_T001 rna1262 4.00E-54
387- 33: transcript:Zm00001d040684_T001 rna1265 0
387- 34: transcript:Zm00001d040686_T001 rna1270 6.00E-32
387- 35: transcript:Zm00001d040689_T001 rna1276 0
387- 36: transcript:Zm00001d040691_T005 rna1277 0
387- 37: transcript:Zm00001d040695_T001 rna1278 0
387- 38: transcript:Zm00001d040696_T001 rna1279 0
387- 39: transcript:Zm00001d040702_T001 rna1284 4.00E-158
387- 40: transcript:Zm00001d040710_T001 rna1289 7.00E-29
387- 41: transcript:Zm00001d040712_T002 rna1290 1.00E-17
387- 42: transcript:Zm00001d040715_T001 rna1295 1.00E-08
387- 43: transcript:Zm00001d040717_T005 rna1296 8.00E-129
387- 44: transcript:Zm00001d040720_T001 rna1297 9.00E-16
387- 45: transcript:Zm00001d040721_T002 rna1298 0
387- 46: transcript:Zm00001d040724_T001 rna1299 0
387- 47: transcript:Zm00001d040725_T004 rna1301 0
387- 48: transcript:Zm00001d040726_T001 rna1302 0
387- 49: transcript:Zm00001d040730_T002 rna1304 0
387- 50: transcript:Zm00001d040734_T001 rna1310 2.00E-115
387- 51: transcript:Zm00001d040735_T001 rna1311 0
387- 52: transcript:Zm00001d040737_T001 rna1312 1.00E-35
387- 53: transcript:Zm00001d040741_T001 rna1313 0
387- 54: transcript:Zm00001d040745_T001 rna1316 3.00E-71
387- 55: transcript:Zm00001d040748_T008 rna1323 0
Alignment 388: score=1658.0 e_value=5.7e-128 N=36 3&NC_008394.4 plus
388- 0: transcript:Zm00001d044356_T001 rna1905 1.00E-15
388- 1: transcript:Zm00001d044358_T002 rna1907 0
388- 2: transcript:Zm00001d044364_T002 rna1908 0
388- 3: transcript:Zm00001d044365_T001 rna1910 7.00E-119
388- 4: transcript:Zm00001d044367_T001 rna1912 5.00E-96
388- 5: transcript:Zm00001d044373_T001 rna1914 0
388- 6: transcript:Zm00001d044374_T001 rna1915 4.00E-27
388- 7: transcript:Zm00001d044376_T001 rna1917 4.00E-15
388- 8: transcript:Zm00001d044379_T006 rna1918 1.00E-49
388- 9: transcript:Zm00001d044382_T001 rna1921 2.00E-138
388- 10: transcript:Zm00001d044385_T001 rna1922 3.00E-77
388- 11: transcript:Zm00001d044387_T001 rna1931 0
388- 12: transcript:Zm00001d044389_T001 rna1934 9.00E-125
388- 13: transcript:Zm00001d044390_T001 rna1936 0
388- 14: transcript:Zm00001d044391_T002 rna1939 4.00E-75
388- 15: transcript:Zm00001d044393_T002 rna1940 6.00E-09
388- 16: transcript:Zm00001d044394_T002 rna1941 5.00E-136
388- 17: transcript:Zm00001d044395_T001 rna1943 2.00E-50

388- 18: transcript:Zm00001d044396_T001 rna1944 3.00E-180
388- 19: transcript:Zm00001d044405_T002 rna1953 1.00E-12
388- 20: transcript:Zm00001d044407_T001 rna1954 2.00E-29
388- 21: transcript:Zm00001d044409_T001 rna1957 5.00E-110
388- 22: transcript:Zm00001d044410_T001 rna1963 3.00E-09
388- 23: transcript:Zm00001d044411_T001 rna1964 9.00E-104
388- 24: transcript:Zm00001d044412_T001 rna1967 0
388- 25: transcript:Zm00001d044416_T001 rna1970 0
388- 26: transcript:Zm00001d044415_T002 rna1971 2.00E-56
388- 27: transcript:Zm00001d044418_T001 rna1972 0
388- 28: transcript:Zm00001d044419_T001 rna1973 0
388- 29: transcript:Zm00001d044420_T001 rna1974 0
388- 30: transcript:Zm00001d044421_T001 rna1976 1.00E-134
388- 31: transcript:Zm00001d044422_T002 rna1979 2.00E-42
388- 32: transcript:Zm00001d044425_T001 rna1980 4.00E-136
388- 33: transcript:Zm00001d044426_T001 rna1983 3.00E-71
388- 34: transcript:Zm00001d044430_T001 rna1985 1.00E-65
388- 35: transcript:Zm00001d044432_T010 rna1987 0
Alignment 389: score=1295.0 e_value=2.9e-93 N=29 3&NC_008394.4 plus
389- 0: transcript:Zm00001d040244_T001 rna935 1.00E-134
389- 1: transcript:Zm00001d040246_T001 rna940 5.00E-17
389- 2: transcript:Zm00001d040247_T001 rna941 8.00E-134
389- 3: transcript:Zm00001d040254_T001 rna945 1.00E-105
389- 4: transcript:Zm00001d040259_T001 rna950 0
389- 5: transcript:Zm00001d040263_T010 rna954 0
389- 6: transcript:Zm00001d040264_T001 rna959 5.00E-61
389- 7: transcript:Zm00001d040265_T001 rna960 0
389- 8: transcript:Zm00001d040269_T001 rna962 0
389- 9: transcript:Zm00001d040270_T011 rna963 0
389- 10: transcript:Zm00001d040271_T001 rna964 3.00E-50
389- 11: transcript:Zm00001d040274_T001 rna965 0
389- 12: transcript:Zm00001d040275_T001 rna966 1.00E-132
389- 13: transcript:Zm00001d040276_T001 rna968 2.00E-37
389- 14: transcript:Zm00001d040277_T001 rna969 3.00E-52
389- 15: transcript:Zm00001d040278_T001 rna971 3.00E-140
389- 16: transcript:Zm00001d040279_T023 rna972 0
389- 17: transcript:Zm00001d040281_T001 rna973 3.00E-18
389- 18: transcript:Zm00001d040286_T003 rna976 2.00E-146
389- 19: transcript:Zm00001d040290_T001 rna977 5.00E-175
389- 20: transcript:Zm00001d040291_T001 rna980 4.00E-66
389- 21: transcript:Zm00001d040294_T002 rna985 1.00E-71
389- 22: transcript:Zm00001d040297_T001 rna986 2.00E-49
389- 23: transcript:Zm00001d040298_T001 rna987 9.00E-60
389- 24: transcript:Zm00001d040301_T007 rna988 0
389- 25: transcript:Zm00001d040303_T001 rna994 0
389- 26: transcript:Zm00001d040305_T014 rna996 0
389- 27: transcript:Zm00001d040308_T001 rna997 6.00E-37
389- 28: transcript:Zm00001d040309_T003 rna998 5.00E-103
Alignment 390: score=1271.0 e_value=5.5e-98 N=30 3&NC_008394.4 plus
390- 0: transcript:Zm00001d040752_T001 rna1326 1.00E-139
390- 1: transcript:Zm00001d040760_T001 rna1328 0
390- 2: transcript:Zm00001d040766_T001 rna1336 0
390- 3: transcript:Zm00001d040771_T001 rna1340 2.00E-36
390- 4: transcript:Zm00001d040774_T001 rna1348 5.00E-06

390- 5: transcript:Zm00001d040775_T001 rna1349 2.00E-46
390- 6: transcript:Zm00001d040777_T001 rna1353 1.00E-85
390- 7: transcript:Zm00001d040780_T001 rna1354 3.00E-28
390- 8: transcript:Zm00001d040783_T001 rna1355 2.00E-47
390- 9: transcript:Zm00001d040784_T003 rna1358 0
390- 10: transcript:Zm00001d040789_T001 rna1375 6.00E-94
390- 11: transcript:Zm00001d040795_T002 rna1390 0
390- 12: transcript:Zm00001d040796_T002 rna1393 0
390- 13: transcript:Zm00001d040798_T001 rna1395 1.00E-131
390- 14: transcript:Zm00001d040802_T001 rna1397 0
390- 15: transcript:Zm00001d040803_T001 rna1404 4.00E-36
390- 16: transcript:Zm00001d040804_T001 rna1406 0
390- 17: transcript:Zm00001d040805_T003 rna1413 0
390- 18: transcript:Zm00001d040807_T001 rna1425 8.00E-82
390- 19: transcript:Zm00001d040823_T001 rna1436 7.00E-150
390- 20: transcript:Zm00001d040827_T003 rna1438 7.00E-174
390- 21: transcript:Zm00001d040829_T003 rna1441 0
390- 22: transcript:Zm00001d040831_T021 rna1442 0
390- 23: transcript:Zm00001d040836_T001 rna1445 3.00E-59
390- 24: transcript:Zm00001d040838_T001 rna1449 1.00E-95
390- 25: transcript:Zm00001d040842_T001 rna1452 3.00E-134
390- 26: transcript:Zm00001d040846_T001 rna1475 0
390- 27: transcript:Zm00001d040850_T003 rna1476 0
390- 28: transcript:Zm00001d040855_T001 rna1495 3.00E-112
390- 29: transcript:Zm00001d040864_T003 rna1514 0
Alignment 391: score=1090.0 e_value=6.9e-81 N=24 3&NC_008394.4 plus
391- 0: transcript:Zm00001d044225_T001 rna1784 0
391- 1: transcript:Zm00001d044226_T001 rna1797 1.00E-90
391- 2: transcript:Zm00001d044227_T001 rna1798 6.00E-50
391- 3: transcript:Zm00001d044230_T001 rna1799 3.00E-105
391- 4: transcript:Zm00001d044231_T001 rna1800 2.00E-25
391- 5: transcript:Zm00001d044232_T003 rna1802 4.00E-105
391- 6: transcript:Zm00001d044242_T001 rna1805 2.00E-89
391- 7: transcript:Zm00001d044243_T001 rna1806 0
391- 8: transcript:Zm00001d044244_T001 rna1808 2.00E-44
391- 9: transcript:Zm00001d044245_T001 rna1809 0
391- 10: transcript:Zm00001d044250_T001 rna1814 0
391- 11: transcript:Zm00001d044253_T001 rna1815 0
391- 12: transcript:Zm00001d044254_T001 rna1817 2.00E-36
391- 13: transcript:Zm00001d044255_T001 rna1818 4.00E-68
391- 14: transcript:Zm00001d044259_T001 rna1819 2.00E-117
391- 15: transcript:Zm00001d044260_T002 rna1822 7.00E-68
391- 16: transcript:Zm00001d044261_T001 rna1825 2.00E-158
391- 17: transcript:Zm00001d044266_T012 rna1828 0
391- 18: transcript:Zm00001d044267_T004 rna1830 0
391- 19: transcript:Zm00001d044270_T001 rna1831 1.00E-85
391- 20: transcript:Zm00001d044271_T011 rna1832 0
391- 21: transcript:Zm00001d044273_T002 rna1834 0
391- 22: transcript:Zm00001d044276_T001 rna1838 0
391- 23: transcript:Zm00001d044278_T001 rna1839 0
Alignment 392: score=1082.0 e_value=1.8e-69 N=24 3&NC_008394.4 plus
392- 0: transcript:Zm00001d044442_T036 rna1994 0
392- 1: transcript:Zm00001d044445_T001 rna2000 3.00E-115
392- 2: transcript:Zm00001d044446_T001 rna2002 5.00E-30

392- 3: transcript:Zm00001d044447_T007 rna2004 0
392- 4: transcript:Zm00001d044451_T002 rna2005 3.00E-142
392- 5: transcript:Zm00001d044452_T001 rna2006 2.00E-51
392- 6: transcript:Zm00001d044455_T001 rna2007 0
392- 7: transcript:Zm00001d044457_T001 rna2010 0
392- 8: transcript:Zm00001d044459_T002 rna2013 3.00E-32
392- 9: transcript:Zm00001d044460_T001 rna2014 2.00E-128
392- 10: transcript:Zm00001d044461_T001 rna2015 1.00E-118
392- 11: transcript:Zm00001d044463_T001 rna2016 5.00E-71
392- 12: transcript:Zm00001d044464_T001 rna2017 1.00E-61
392- 13: transcript:Zm00001d044465_T001 rna2018 2.00E-30
392- 14: transcript:Zm00001d044467_T001 rna2019 5.00E-91
392- 15: transcript:Zm00001d044468_T002 rna2023 0
392- 16: transcript:Zm00001d044469_T001 rna2026 3.00E-173
392- 17: transcript:Zm00001d044470_T006 rna2028 7.00E-117
392- 18: transcript:Zm00001d044475_T001 rna2033 0
392- 19: transcript:Zm00001d044476_T001 rna2035 0
392- 20: transcript:Zm00001d044478_T022 rna2037 0
392- 21: transcript:Zm00001d044479_T001 rna2038 2.00E-30
392- 22: transcript:Zm00001d044481_T002 rna2039 2.00E-159
392- 23: transcript:Zm00001d044488_T003 rna2043 6.00E-17
Alignment 393: score=888.0 e_value=5.3e-51 N=19 3&NC_008394.4 plus
393- 0: transcript:Zm00001d040503_T005 rna1123 1.00E-109
393- 1: transcript:Zm00001d040504_T001 rna1124 7.00E-98
393- 2: transcript:Zm00001d040508_T001 rna1125 4.00E-48
393- 3: transcript:Zm00001d040513_T001 rna1126 0
393- 4: transcript:Zm00001d040515_T001 rna1127 5.00E-169
393- 5: transcript:Zm00001d040518_T001 rna1129 4.00E-08
393- 6: transcript:Zm00001d040519_T002 rna1134 3.00E-121
393- 7: transcript:Zm00001d040526_T001 rna1136 1.00E-74
393- 8: transcript:Zm00001d040527_T001 rna1138 1.00E-89
393- 9: transcript:Zm00001d040535_T005 rna1139 8.00E-168
393- 10: transcript:Zm00001d040536_T005 rna1140 0
393- 11: transcript:Zm00001d040539_T004 rna1142 4.00E-35
393- 12: transcript:Zm00001d040541_T001 rna1143 3.00E-49
393- 13: transcript:Zm00001d040544_T001 rna1145 1.00E-10
393- 14: transcript:Zm00001d040545_T002 rna1148 8.00E-07
393- 15: transcript:Zm00001d040548_T001 rna1150 1.00E-13
393- 16: transcript:Zm00001d040554_T001 rna1152 5.00E-63
393- 17: transcript:Zm00001d040555_T001 rna1157 0
393- 18: transcript:Zm00001d040562_T002 rna1160 4.00E-84
Alignment 394: score=886.0 e_value=5.8e-55 N=19 3&NC_008394.4 plus
394- 0: transcript:Zm00001d043439_T006 rna2764 0
394- 1: transcript:Zm00001d043442_T001 rna2765 0
394- 2: transcript:Zm00001d043443_T001 rna2766 0
394- 3: transcript:Zm00001d043444_T001 rna2767 1.00E-73
394- 4: transcript:Zm00001d043445_T003 rna2769 0
394- 5: transcript:Zm00001d043446_T001 rna2770 0
394- 6: transcript:Zm00001d043449_T001 rna2771 1.00E-162
394- 7: transcript:Zm00001d043450_T001 rna2772 2.00E-79
394- 8: transcript:Zm00001d043451_T001 rna2773 0
394- 9: transcript:Zm00001d043453_T001 rna2775 3.00E-66
394- 10: transcript:Zm00001d043454_T001 rna2776 3.00E-169
394- 11: transcript:Zm00001d043455_T001 rna2777 1.00E-30

394- 12: transcript:Zm00001d043458_T001 rna2778 4.00E-57
394- 13: transcript:Zm00001d043459_T005 rna2779 1.00E-114
394- 14: transcript:Zm00001d043461_T001 rna2780 1.00E-93
394- 15: transcript:Zm00001d043462_T004 rna2781 0
394- 16: transcript:Zm00001d043463_T001 rna2782 5.00E-156
394- 17: transcript:Zm00001d043464_T001 rna2783 2.00E-148
394- 18: transcript:Zm00001d043465_T004 rna2784 6.00E-134
Alignment 395: score=875.0 e_value=1.2e-52 N=19 3&NC_008394.4 plus
395- 0: transcript:Zm00001d043528_T001 rna2677 7.00E-139
395- 1: transcript:Zm00001d043534_T002 rna2681 2.00E-165
395- 2: transcript:Zm00001d043535_T001 rna2682 2.00E-106
395- 3: transcript:Zm00001d043536_T001 rna2686 2.00E-121
395- 4: transcript:Zm00001d043538_T001 rna2687 0
395- 5: transcript:Zm00001d043539_T002 rna2688 0
395- 6: transcript:Zm00001d043541_T001 rna2690 7.00E-47
395- 7: transcript:Zm00001d043543_T002 rna2691 0
395- 8: transcript:Zm00001d043544_T002 rna2693 9.00E-130
395- 9: transcript:Zm00001d043549_T001 rna2704 2.00E-122
395- 10: transcript:Zm00001d043550_T001 rna2705 7.00E-81
395- 11: transcript:Zm00001d043551_T003 rna2707 8.00E-158
395- 12: transcript:Zm00001d043552_T006 rna2709 1.00E-17
395- 13: transcript:Zm00001d043554_T001 rna2711 2.00E-72
395- 14: transcript:Zm00001d043555_T002 rna2713 0
395- 15: transcript:Zm00001d043556_T001 rna2714 0
395- 16: transcript:Zm00001d043558_T001 rna2715 1.00E-32
395- 17: transcript:Zm00001d043560_T006 rna2716 1.00E-43
395- 18: transcript:Zm00001d043562_T008 rna2717 0
Alignment 396: score=748.0 e_value=1.7e-43 N=16 3&NC_008394.4 plus
396- 0: transcript:Zm00001d044315_T009 rna1876 0
396- 1: transcript:Zm00001d044316_T001 rna1880 2.00E-21
396- 2: transcript:Zm00001d044318_T001 rna1882 0
396- 3: transcript:Zm00001d044323_T001 rna1883 9.00E-53
396- 4: transcript:Zm00001d044324_T001 rna1884 0
396- 5: transcript:Zm00001d044326_T001 rna1887 5.00E-30
396- 6: transcript:Zm00001d044327_T001 rna1888 8.00E-77
396- 7: transcript:Zm00001d044328_T001 rna1889 0
396- 8: transcript:Zm00001d044329_T003 rna1891 2.00E-101
396- 9: transcript:Zm00001d044330_T002 rna1892 6.00E-127
396- 10: transcript:Zm00001d044331_T001 rna1893 6.00E-62
396- 11: transcript:Zm00001d044332_T001 rna1894 2.00E-112
396- 12: transcript:Zm00001d044333_T001 rna1895 4.00E-97
396- 13: transcript:Zm00001d044335_T001 rna1896 6.00E-82
396- 14: transcript:Zm00001d044338_T003 rna1901 0
396- 15: transcript:Zm00001d044339_T003 rna1902 0
Alignment 397: score=746.0 e_value=4.9e-42 N=16 3&NC_008394.4 plus
397- 0: transcript:Zm00001d040438_T004 rna1088 0
397- 1: transcript:Zm00001d040440_T001 rna1089 1.00E-71
397- 2: transcript:Zm00001d040442_T001 rna1091 0
397- 3: transcript:Zm00001d040445_T001 rna1093 1.00E-85
397- 4: transcript:Zm00001d040446_T001 rna1094 0
397- 5: transcript:Zm00001d040448_T001 rna1095 2.00E-147
397- 6: transcript:Zm00001d040450_T002 rna1096 1.00E-22
397- 7: transcript:Zm00001d040455_T001 rna1098 8.00E-154
397- 8: transcript:Zm00001d040456_T002 rna1099 0

397- 9: transcript:Zm00001d040459_T006 rna1100 2.00E-137
397- 10: transcript:Zm00001d040461_T001 rna1102 1.00E-24
397- 11: transcript:Zm00001d040465_T001 rna1107 1.00E-165
397- 12: transcript:Zm00001d040468_T001 rna1111 0
397- 13: transcript:Zm00001d040470_T001 rna1115 0
397- 14: transcript:Zm00001d040475_T001 rna1117 0
397- 15: transcript:Zm00001d040477_T004 rna1120 0
Alignment 398: score=717.0 e_value=2.5e-37 N=15 3&NC_008394.4 plus
398- 0: transcript:Zm00001d039422_T003 rna647 0
398- 1: transcript:Zm00001d039424_T001 rna654 3.00E-21
398- 2: transcript:Zm00001d039425_T005 rna656 2.00E-57
398- 3: transcript:Zm00001d039426_T001 rna657 1.00E-115
398- 4: transcript:Zm00001d039427_T002 rna658 0
398- 5: transcript:Zm00001d039429_T001 rna659 1.00E-32
398- 6: transcript:Zm00001d039432_T004 rna660 6.00E-61
398- 7: transcript:Zm00001d039434_T001 rna661 2.00E-81
398- 8: transcript:Zm00001d039435_T001 rna662 8.00E-123
398- 9: transcript:Zm00001d039437_T001 rna663 7.00E-128
398- 10: transcript:Zm00001d039439_T001 rna667 5.00E-169
398- 11: transcript:Zm00001d039441_T001 rna669 9.00E-95
398- 12: transcript:Zm00001d039444_T001 rna671 0
398- 13: transcript:Zm00001d039446_T001 rna672 0
398- 14: transcript:Zm00001d039448_T002 rna674 0
Alignment 399: score=648.0 e_value=6.9e-35 N=14 3&NC_008394.4 plus
399- 0: transcript:Zm00001d044511_T001 rna1729 0
399- 1: transcript:Zm00001d044514_T004 rna1732 0
399- 2: transcript:Zm00001d044515_T001 rna1733 4.00E-142
399- 3: transcript:Zm00001d044516_T001 rna1734 1.00E-138
399- 4: transcript:Zm00001d044517_T001 rna1735 0
399- 5: transcript:Zm00001d044518_T001 rna1738 9.00E-34
399- 6: transcript:Zm00001d044520_T001 rna1741 6.00E-37
399- 7: transcript:Zm00001d044525_T001 rna1745 0
399- 8: transcript:Zm00001d044526_T001 rna1754 6.00E-67
399- 9: transcript:Zm00001d044527_T002 rna1756 2.00E-133
399- 10: transcript:Zm00001d044528_T001 rna1760 0
399- 11: transcript:Zm00001d044529_T001 rna1762 0
399- 12: transcript:Zm00001d044532_T001 rna1763 5.00E-24
399- 13: transcript:Zm00001d044533_T003 rna1767 0
Alignment 400: score=422.0 e_value=3.8e-18 N=9 3&NC_008394.4 plus
400- 0: transcript:Zm00001d044297_T002 rna1859 0
400- 1: transcript:Zm00001d044300_T001 rna1860 0
400- 2: transcript:Zm00001d044301_T001 rna1861 3.00E-87
400- 3: transcript:Zm00001d044302_T001 rna1864 4.00E-44
400- 4: transcript:Zm00001d044303_T003 rna1865 1.00E-23
400- 5: transcript:Zm00001d044307_T001 rna1870 0
400- 6: transcript:Zm00001d044310_T001 rna1872 9.00E-81
400- 7: transcript:Zm00001d044312_T003 rna1874 0
400- 8: transcript:Zm00001d044313_T004 rna1875 0
Alignment 401: score=372.0 e_value=2e-16 N=8 3&NC_008394.4 plus
401- 0: transcript:Zm00001d043486_T001 rna2798 5.00E-30
401- 1: transcript:Zm00001d043489_T001 rna2800 0
401- 2: transcript:Zm00001d043490_T001 rna2803 0
401- 3: transcript:Zm00001d043491_T001 rna2806 2.00E-42
401- 4: transcript:Zm00001d043492_T001 rna2807 0

401- 5: transcript:Zm00001d043494_T005 rna2808 0
401- 6: transcript:Zm00001d043497_T001 rna2809 3.00E-85
401- 7: transcript:Zm00001d043500_T008 rna2810 4.00E-131
Alignment 402: score=366.0 e_value=1.4e-14 N=8 3&NC_008394.4 plus
402- 0: transcript:Zm00001d043469_T022 rna2787 0
402- 1: transcript:Zm00001d043473_T004 rna2788 0
402- 2: transcript:Zm00001d043474_T001 rna2790 4.00E-60
402- 3: transcript:Zm00001d043477_T001 rna2791 0
402- 4: transcript:Zm00001d043478_T001 rna2792 0
402- 5: transcript:Zm00001d043479_T001 rna2793 2.00E-126
402- 6: transcript:Zm00001d043480_T001 rna2794 0
402- 7: transcript:Zm00001d043483_T001 rna2797 0
Alignment 403: score=322.0 e_value=7.8e-12 N=7 3&NC_008394.4 plus
403- 0: transcript:Zm00001d043612_T002 rna2593 1.00E-60
403- 1: transcript:Zm00001d043613_T004 rna2594 0
403- 2: transcript:Zm00001d043614_T001 rna2597 0
403- 3: transcript:Zm00001d043615_T001 rna2599 1.00E-76
403- 4: transcript:Zm00001d043621_T001 rna2603 4.00E-141
403- 5: transcript:Zm00001d043624_T001 rna2607 8.00E-37
403- 6: transcript:Zm00001d043625_T002 rna2615 1.00E-121
Alignment 404: score=288.0 e_value=1.8e-08 N=6 3&NC_008394.4 plus
404- 0: transcript:Zm00001d040124_T001 rna68 4.00E-06
404- 1: transcript:Zm00001d040126_T001 rna72 2.00E-07
404- 2: transcript:Zm00001d040127_T001 rna74 0
404- 3: transcript:Zm00001d040129_T001 rna75 0
404- 4: transcript:Zm00001d040130_T001 rna76 0
404- 5: transcript:Zm00001d040142_T001 rna78 0
Alignment 405: score=252.0 e_value=1.2e-08 N=6 3&NC_008394.4 plus
405- 0: transcript:Zm00001d043612_T002 rna1910 6.00E-21
405- 1: transcript:Zm00001d043614_T001 rna1913 0
405- 2: transcript:Zm00001d043617_T001 rna1936 2.00E-129
405- 3: transcript:Zm00001d043618_T002 rna1939 2.00E-49
405- 4: transcript:Zm00001d043621_T001 rna1943 2.00E-27
405- 5: transcript:Zm00001d043625_T002 rna1954 8.00E-82
Alignment 406: score=12533.0 e_value=0 N=270 3&NC_008394.4 minus
406- 0: transcript:Zm00001d042543_T002 rna3707 0
406- 1: transcript:Zm00001d042549_T001 rna3704 0
406- 2: transcript:Zm00001d042551_T009 rna3702 0
406- 3: transcript:Zm00001d042553_T001 rna3699 4.00E-45
406- 4: transcript:Zm00001d042555_T001 rna3693 0
406- 5: transcript:Zm00001d042556_T001 rna3692 0
406- 6: transcript:Zm00001d042558_T002 rna3689 3.00E-163
406- 7: transcript:Zm00001d042560_T001 rna3682 1.00E-135
406- 8: transcript:Zm00001d042567_T005 rna3681 3.00E-60
406- 9: transcript:Zm00001d042569_T001 rna3680 0
406- 10: transcript:Zm00001d042572_T001 rna3679 2.00E-30
406- 11: transcript:Zm00001d042575_T002 rna3677 0
406- 12: transcript:Zm00001d042578_T001 rna3676 0
406- 13: transcript:Zm00001d042580_T001 rna3674 3.00E-156
406- 14: transcript:Zm00001d042582_T001 rna3672 9.00E-141
406- 15: transcript:Zm00001d042583_T001 rna3671 8.00E-23
406- 16: transcript:Zm00001d042584_T001 rna3670 1.00E-22
406- 17: transcript:Zm00001d042589_T001 rna3666 2.00E-43
406- 18: transcript:Zm00001d042590_T001 rna3664 0

406- 19:	transcript:Zm00001d042591_T002	rna3663	1.00E-128
406- 20:	transcript:Zm00001d042596_T001	rna3659	0
406- 21:	transcript:Zm00001d042598_T003	rna3657	0
406- 22:	transcript:Zm00001d042599_T001	rna3656	0
406- 23:	transcript:Zm00001d042600_T001	rna3655	2.00E-58
406- 24:	transcript:Zm00001d042601_T001	rna3654	1.00E-30
406- 25:	transcript:Zm00001d042602_T001	rna3653	0
406- 26:	transcript:Zm00001d042603_T001	rna3651	4.00E-93
406- 27:	transcript:Zm00001d042605_T001	rna3650	2.00E-96
406- 28:	transcript:Zm00001d042608_T002	rna3649	0
406- 29:	transcript:Zm00001d042609_T001	rna3648	1.00E-170
406- 30:	transcript:Zm00001d042610_T001	rna3646	3.00E-50
406- 31:	transcript:Zm00001d042611_T001	rna3645	0
406- 32:	transcript:Zm00001d042615_T003	rna3642	0
406- 33:	transcript:Zm00001d042618_T001	rna3641	3.00E-139
406- 34:	transcript:Zm00001d042619_T002	rna3639	0
406- 35:	transcript:Zm00001d042620_T001	rna3638	3.00E-57
406- 36:	transcript:Zm00001d042621_T005	rna3636	0
406- 37:	transcript:Zm00001d042624_T001	rna3618	1.00E-149
406- 38:	transcript:Zm00001d042627_T005	rna3617	0
406- 39:	transcript:Zm00001d042632_T002	rna3613	0
406- 40:	transcript:Zm00001d042633_T001	rna3612	4.00E-89
406- 41:	transcript:Zm00001d042634_T001	rna3608	3.00E-157
406- 42:	transcript:Zm00001d042636_T002	rna3605	0
406- 43:	transcript:Zm00001d042637_T002	rna3604	0
406- 44:	transcript:Zm00001d042638_T002	rna3601	1.00E-52
406- 45:	transcript:Zm00001d042639_T004	rna3596	0
406- 46:	transcript:Zm00001d042640_T001	rna3594	1.00E-107
406- 47:	transcript:Zm00001d042641_T013	rna3593	4.00E-38
406- 48:	transcript:Zm00001d042642_T002	rna3592	2.00E-46
406- 49:	transcript:Zm00001d042643_T001	rna3591	0
406- 50:	transcript:Zm00001d042646_T001	rna3590	1.00E-155
406- 51:	transcript:Zm00001d042651_T001	rna3588	6.00E-50
406- 52:	transcript:Zm00001d042652_T001	rna3587	0
406- 53:	transcript:Zm00001d042653_T001	rna3586	5.00E-176
406- 54:	transcript:Zm00001d042656_T001	rna3585	2.00E-87
406- 55:	transcript:Zm00001d042657_T001	rna3584	9.00E-37
406- 56:	transcript:Zm00001d042658_T002	rna3583	4.00E-108
406- 57:	transcript:Zm00001d042660_T001	rna3582	2.00E-139
406- 58:	transcript:Zm00001d042661_T001	rna3581	0
406- 59:	transcript:Zm00001d042663_T001	rna3580	0
406- 60:	transcript:Zm00001d042664_T001	rna3579	0
406- 61:	transcript:Zm00001d042665_T001	rna3578	1.00E-136
406- 62:	transcript:Zm00001d042667_T001	rna3577	0
406- 63:	transcript:Zm00001d042669_T002	rna3576	2.00E-57
406- 64:	transcript:Zm00001d042670_T025	rna3575	0
406- 65:	transcript:Zm00001d042672_T001	rna3574	2.00E-64
406- 66:	transcript:Zm00001d042673_T001	rna3572	7.00E-66
406- 67:	transcript:Zm00001d042676_T001	rna3569	0
406- 68:	transcript:Zm00001d042680_T001	rna3560	1.00E-128
406- 69:	transcript:Zm00001d042683_T001	rna3559	2.00E-159
406- 70:	transcript:Zm00001d042685_T001	rna3556	0
406- 71:	transcript:Zm00001d042686_T001	rna3555	0
406- 72:	transcript:Zm00001d042689_T002	rna3554	1.00E-31

406- 73:	transcript:Zm00001d042694_T002	rna3551	0
406- 74:	transcript:Zm00001d042695_T002	rna3550	0
406- 75:	transcript:Zm00001d042697_T002	rna3549	1.00E-130
406- 76:	transcript:Zm00001d042699_T001	rna3547	3.00E-164
406- 77:	transcript:Zm00001d042708_T001	rna3544	3.00E-51
406- 78:	transcript:Zm00001d042712_T001	rna3542	0
406- 79:	transcript:Zm00001d042713_T005	rna3540	0
406- 80:	transcript:Zm00001d042714_T001	rna3539	1.00E-145
406- 81:	transcript:Zm00001d042717_T001	rna3538	3.00E-82
406- 82:	transcript:Zm00001d042718_T001	rna3537	8.00E-167
406- 83:	transcript:Zm00001d042719_T001	rna3535	5.00E-150
406- 84:	transcript:Zm00001d042720_T005	rna3534	0
406- 85:	transcript:Zm00001d042721_T001	rna3533	1.00E-93
406- 86:	transcript:Zm00001d042722_T001	rna3532	3.00E-168
406- 87:	transcript:Zm00001d042724_T001	rna3530	0
406- 88:	transcript:Zm00001d042725_T001	rna3529	2.00E-37
406- 89:	transcript:Zm00001d042726_T001	rna3528	4.00E-50
406- 90:	transcript:Zm00001d042727_T001	rna3526	0
406- 91:	transcript:Zm00001d042729_T003	rna3525	8.00E-127
406- 92:	transcript:Zm00001d042731_T001	rna3523	3.00E-97
406- 93:	transcript:Zm00001d042735_T001	rna3522	1.00E-41
406- 94:	transcript:Zm00001d042736_T001	rna3521	3.00E-158
406- 95:	transcript:Zm00001d042738_T001	rna3520	0
406- 96:	transcript:Zm00001d042739_T001	rna3518	0
406- 97:	transcript:Zm00001d042746_T003	rna3517	0
406- 98:	transcript:Zm00001d042747_T003	rna3516	7.00E-156
406- 99:	transcript:Zm00001d042748_T001	rna3513	7.00E-17
406-100:	transcript:Zm00001d042751_T001	rna3512	5.00E-25
406-101:	transcript:Zm00001d042753_T001	rna3509	2.00E-75
406-102:	transcript:Zm00001d042754_T001	rna3508	9.00E-39
406-103:	transcript:Zm00001d042755_T001	rna3507	2.00E-08
406-104:	transcript:Zm00001d042756_T001	rna3506	2.00E-63
406-105:	transcript:Zm00001d042758_T001	rna3503	7.00E-101
406-106:	transcript:Zm00001d042760_T001	rna3502	1.00E-88
406-107:	transcript:Zm00001d042761_T001	rna3499	0
406-108:	transcript:Zm00001d042762_T001	rna3496	4.00E-90
406-109:	transcript:Zm00001d042764_T001	rna3493	0
406-110:	transcript:Zm00001d042765_T001	rna3491	5.00E-34
406-111:	transcript:Zm00001d042766_T001	rna3489	3.00E-62
406-112:	transcript:Zm00001d042767_T001	rna3488	0
406-113:	transcript:Zm00001d042768_T001	rna3483	2.00E-132
406-114:	transcript:Zm00001d042772_T001	rna3482	5.00E-42
406-115:	transcript:Zm00001d042774_T001	rna3481	0
406-116:	transcript:Zm00001d042777_T006	rna3480	0
406-117:	transcript:Zm00001d042778_T001	rna3479	2.00E-164
406-118:	transcript:Zm00001d042779_T001	rna3478	2.00E-94
406-119:	transcript:Zm00001d042780_T001	rna3477	0
406-120:	transcript:Zm00001d042781_T001	rna3476	2.00E-137
406-121:	transcript:Zm00001d042786_T001	rna3475	0
406-122:	transcript:Zm00001d042787_T001	rna3474	0
406-123:	transcript:Zm00001d042792_T002	rna3473	7.00E-82
406-124:	transcript:Zm00001d042793_T002	rna3470	0
406-125:	transcript:Zm00001d042794_T005	rna3469	4.00E-86
406-126:	transcript:Zm00001d042796_T001	rna3468	7.00E-102

406-127:	transcript:Zm00001d042800_T001	rna3467	5.00E-111
406-128:	transcript:Zm00001d042801_T001	rna3465	0
406-129:	transcript:Zm00001d042802_T001	rna3464	9.00E-66
406-130:	transcript:Zm00001d042804_T001	rna3460	0
406-131:	transcript:Zm00001d042808_T001	rna3458	3.00E-74
406-132:	transcript:Zm00001d042809_T003	rna3456	0
406-133:	transcript:Zm00001d042810_T001	rna3455	0
406-134:	transcript:Zm00001d042811_T001	rna3452	0
406-135:	transcript:Zm00001d042812_T001	rna3450	0
406-136:	transcript:Zm00001d042813_T001	rna3449	0
406-137:	transcript:Zm00001d042814_T001	rna3448	0
406-138:	transcript:Zm00001d042821_T001	rna3447	1.00E-118
406-139:	transcript:Zm00001d042822_T001	rna3446	2.00E-47
406-140:	transcript:Zm00001d042830_T002	rna3443	3.00E-117
406-141:	transcript:Zm00001d042833_T001	rna3441	0
406-142:	transcript:Zm00001d042836_T001	rna3440	4.00E-22
406-143:	transcript:Zm00001d042837_T001	rna3436	0
406-144:	transcript:Zm00001d042841_T002	rna3433	4.00E-45
406-145:	transcript:Zm00001d042842_T003	rna3432	0
406-146:	transcript:Zm00001d042843_T001	rna3431	2.00E-121
406-147:	transcript:Zm00001d042845_T001	rna3430	2.00E-174
406-148:	transcript:Zm00001d042846_T001	rna3429	1.00E-92
406-149:	transcript:Zm00001d042847_T001	rna3427	0
406-150:	transcript:Zm00001d042848_T001	rna3424	0
406-151:	transcript:Zm00001d042849_T001	rna3423	9.00E-36
406-152:	transcript:Zm00001d042850_T001	rna3422	5.00E-109
406-153:	transcript:Zm00001d042851_T003	rna3421	0
406-154:	transcript:Zm00001d042853_T001	rna3420	2.00E-129
406-155:	transcript:Zm00001d042856_T002	rna3419	1.00E-06
406-156:	transcript:Zm00001d042857_T001	rna3418	1.00E-28
406-157:	transcript:Zm00001d042861_T001	rna3417	0
406-158:	transcript:Zm00001d042862_T001	rna3416	8.00E-63
406-159:	transcript:Zm00001d042863_T001	rna3415	2.00E-49
406-160:	transcript:Zm00001d042864_T003	rna3412	0
406-161:	transcript:Zm00001d042866_T001	rna3407	9.00E-36
406-162:	transcript:Zm00001d042868_T001	rna3404	6.00E-39
406-163:	transcript:Zm00001d042872_T001	rna3403	2.00E-73
406-164:	transcript:Zm00001d042874_T001	rna3402	0
406-165:	transcript:Zm00001d042875_T001	rna3401	3.00E-22
406-166:	transcript:Zm00001d042879_T003	rna3400	0
406-167:	transcript:Zm00001d042880_T001	rna3399	2.00E-123
406-168:	transcript:Zm00001d042883_T001	rna3398	0
406-169:	transcript:Zm00001d042885_T006	rna3396	1.00E-27
406-170:	transcript:Zm00001d042886_T001	rna3394	0
406-171:	transcript:Zm00001d042887_T001	rna3391	7.00E-178
406-172:	transcript:Zm00001d042892_T001	rna3390	3.00E-50
406-173:	transcript:Zm00001d042898_T001	rna3387	0
406-174:	transcript:Zm00001d042899_T001	rna3386	7.00E-179
406-175:	transcript:Zm00001d042900_T001	rna3384	2.00E-21
406-176:	transcript:Zm00001d042902_T001	rna3382	7.00E-40
406-177:	transcript:Zm00001d042907_T001	rna3376	0
406-178:	transcript:Zm00001d042908_T002	rna3375	0
406-179:	transcript:Zm00001d042909_T001	rna3374	1.00E-78
406-180:	transcript:Zm00001d042910_T010	rna3372	0

406-181:	transcript:Zm00001d042911_T001	rna3371	3.00E-21
406-182:	transcript:Zm00001d042916_T003	rna3369	0
406-183:	transcript:Zm00001d042917_T001	rna3368	6.00E-141
406-184:	transcript:Zm00001d042918_T001	rna3366	7.00E-125
406-185:	transcript:Zm00001d042920_T001	rna3363	3.00E-49
406-186:	transcript:Zm00001d042921_T001	rna3362	0
406-187:	transcript:Zm00001d042922_T007	rna3361	0
406-188:	transcript:Zm00001d042926_T001	rna3360	4.00E-83
406-189:	transcript:Zm00001d042929_T001	rna3359	9.00E-116
406-190:	transcript:Zm00001d042930_T001	rna3356	4.00E-60
406-191:	transcript:Zm00001d042931_T001	rna3355	0
406-192:	transcript:Zm00001d042932_T001	rna3353	2.00E-151
406-193:	transcript:Zm00001d042933_T001	rna3348	2.00E-19
406-194:	transcript:Zm00001d042936_T001	rna3344	5.00E-37
406-195:	transcript:Zm00001d042938_T004	rna3342	0
406-196:	transcript:Zm00001d042939_T002	rna3341	0
406-197:	transcript:Zm00001d042940_T001	rna3339	8.00E-36
406-198:	transcript:Zm00001d042941_T001	rna3338	1.00E-15
406-199:	transcript:Zm00001d042943_T005	rna3336	0
406-200:	transcript:Zm00001d042944_T001	rna3335	1.00E-64
406-201:	transcript:Zm00001d042948_T001	rna3334	0
406-202:	transcript:Zm00001d042949_T001	rna3333	0
406-203:	transcript:Zm00001d042950_T001	rna3332	0
406-204:	transcript:Zm00001d042953_T001	rna3331	0
406-205:	transcript:Zm00001d042955_T001	rna3328	0
406-206:	transcript:Zm00001d042958_T001	rna3327	7.00E-108
406-207:	transcript:Zm00001d042960_T001	rna3326	5.00E-59
406-208:	transcript:Zm00001d042961_T002	rna3325	0
406-209:	transcript:Zm00001d042962_T002	rna3322	2.00E-21
406-210:	transcript:Zm00001d042965_T001	rna3319	0
406-211:	transcript:Zm00001d042966_T001	rna3317	6.00E-100
406-212:	transcript:Zm00001d042969_T002	rna3314	2.00E-148
406-213:	transcript:Zm00001d042972_T002	rna3313	0
406-214:	transcript:Zm00001d042973_T003	rna3312	0
406-215:	transcript:Zm00001d042975_T003	rna3309	0
406-216:	transcript:Zm00001d042976_T001	rna3307	5.00E-91
406-217:	transcript:Zm00001d042977_T001	rna3306	0
406-218:	transcript:Zm00001d042978_T001	rna3305	0
406-219:	transcript:Zm00001d042979_T007	rna3304	0
406-220:	transcript:Zm00001d042980_T002	rna3303	0
406-221:	transcript:Zm00001d042985_T001	rna3296	1.00E-163
406-222:	transcript:Zm00001d042988_T001	rna3295	4.00E-67
406-223:	transcript:Zm00001d042993_T001	rna3294	0
406-224:	transcript:Zm00001d042996_T001	rna3293	2.00E-171
406-225:	transcript:Zm00001d042998_T001	rna3289	4.00E-101
406-226:	transcript:Zm00001d043001_T001	rna3285	3.00E-92
406-227:	transcript:Zm00001d043006_T001	rna3284	1.00E-129
406-228:	transcript:Zm00001d043009_T002	rna3278	3.00E-151
406-229:	transcript:Zm00001d043012_T018	rna3277	0
406-230:	transcript:Zm00001d043015_T001	rna3273	0
406-231:	transcript:Zm00001d043018_T001	rna3272	3.00E-85
406-232:	transcript:Zm00001d043019_T003	rna3271	0
406-233:	transcript:Zm00001d043022_T001	rna3270	0
406-234:	transcript:Zm00001d043023_T002	rna3269	0

```
406-235: transcript:Zm00001d043024_T001 rna3268      2.00E-71
406-236: transcript:Zm00001d043025_T001 rna3267             0
406-237: transcript:Zm00001d043026_T001 rna3265             0
406-238: transcript:Zm00001d043029_T001 rna3263             0
406-239: transcript:Zm00001d043031_T001 rna3262             0
406-240: transcript:Zm00001d043036_T001 rna3260     5.00E-108
406-241: transcript:Zm00001d043037_T001 rna3259      1.00E-58
406-242: transcript:Zm00001d043038_T001 rna3255             0
406-243: transcript:Zm00001d043043_T001 rna3252             0
406-244: transcript:Zm00001d043044_T001 rna3251             0
406-245: transcript:Zm00001d043045_T001 rna3250      5.00E-96
406-246: transcript:Zm00001d043046_T001 rna3249     1.00E-131
406-247: transcript:Zm00001d043047_T001 rna3245     1.00E-163
406-248: transcript:Zm00001d043049_T001 rna3243      1.00E-50
406-249: transcript:Zm00001d043050_T001 rna3242      5.00E-65
406-250: transcript:Zm00001d043056_T002 rna3241             0
406-251: transcript:Zm00001d043058_T001 rna3240             0
406-252: transcript:Zm00001d043060_T001 rna3237      3.00E-64
406-253: transcript:Zm00001d043062_T001 rna3236      4.00E-77
406-254: transcript:Zm00001d043067_T002 rna3233             0
406-255: transcript:Zm00001d043068_T001 rna3232             0
406-256: transcript:Zm00001d043069_T001 rna3231     4.00E-124
406-257: transcript:Zm00001d043070_T001 rna3228     2.00E-107
406-258: transcript:Zm00001d043071_T002 rna3225      5.00E-91
406-259: transcript:Zm00001d043074_T004 rna3224             0
406-260: transcript:Zm00001d043075_T002 rna3221             0
406-261: transcript:Zm00001d043076_T001 rna3220     7.00E-145
406-262: transcript:Zm00001d043080_T001 rna3219     3.00E-161
406-263: transcript:Zm00001d043083_T003 rna3217             0
406-264: transcript:Zm00001d043086_T002 rna3215             0
406-265: transcript:Zm00001d043087_T001 rna3214      5.00E-86
406-266: transcript:Zm00001d043088_T002 rna3212     4.00E-147
406-267: transcript:Zm00001d043089_T001 rna3211     2.00E-153
406-268: transcript:Zm00001d043090_T001 rna3210             0
406-269: transcript:Zm00001d043091_T002 rna3209      7.00E-21
## Alignment 407: score=6393.0 e_value=0 N=139 3&NC_008394.4 minus
407-  0: transcript:Zm00001d043094_T001 rna3203             0
407-  1: transcript:Zm00001d043095_T004 rna3201             0
407-  2: transcript:Zm00001d043096_T001 rna3200      2.00E-12
407-  3: transcript:Zm00001d043097_T001 rna3199      2.00E-87
407-  4: transcript:Zm00001d043098_T002 rna3198     3.00E-176
407-  5: transcript:Zm00001d043101_T001 rna3197      2.00E-63
407-  6: transcript:Zm00001d043102_T001 rna3196      5.00E-73
407-  7: transcript:Zm00001d043104_T001 rna3195      5.00E-92
407-  8: transcript:Zm00001d043107_T001 rna3194             0
407-  9: transcript:Zm00001d043108_T001 rna3190     2.00E-146
407- 10: transcript:Zm00001d043109_T004 rna3189             0
407- 11: transcript:Zm00001d043110_T012 rna3187      8.00E-14
407- 12: transcript:Zm00001d043112_T002 rna3186             0
407- 13: transcript:Zm00001d043113_T001 rna3185     5.00E-131
407- 14: transcript:Zm00001d043117_T002 rna3184     1.00E-139
407- 15: transcript:Zm00001d043118_T002 rna3183      1.00E-15
407- 16: transcript:Zm00001d043119_T001 rna3182     2.00E-149
407- 17: transcript:Zm00001d043125_T001 rna3179             0
```

407- 18:	transcript:Zm00001d043128_T001	rna3178	3.00E-104
407- 19:	transcript:Zm00001d043131_T002	rna3176	0
407- 20:	transcript:Zm00001d043134_T004	rna3175	0
407- 21:	transcript:Zm00001d043135_T003	rna3174	0
407- 22:	transcript:Zm00001d043136_T001	rna3173	3.00E-55
407- 23:	transcript:Zm00001d043137_T004	rna3172	9.00E-175
407- 24:	transcript:Zm00001d043144_T001	rna3164	6.00E-93
407- 25:	transcript:Zm00001d043145_T001	rna3163	4.00E-139
407- 26:	transcript:Zm00001d043146_T002	rna3162	0
407- 27:	transcript:Zm00001d043147_T002	rna3160	0
407- 28:	transcript:Zm00001d043149_T002	rna3159	5.00E-133
407- 29:	transcript:Zm00001d043150_T002	rna3156	0
407- 30:	transcript:Zm00001d043152_T002	rna3154	0
407- 31:	transcript:Zm00001d043155_T001	rna3153	3.00E-34
407- 32:	transcript:Zm00001d043156_T001	rna3148	0
407- 33:	transcript:Zm00001d043157_T002	rna3147	1.00E-58
407- 34:	transcript:Zm00001d043158_T002	rna3146	0
407- 35:	transcript:Zm00001d043160_T004	rna3144	0
407- 36:	transcript:Zm00001d043164_T002	rna3143	0
407- 37:	transcript:Zm00001d043165_T002	rna3141	2.00E-116
407- 38:	transcript:Zm00001d043167_T001	rna3140	1.00E-148
407- 39:	transcript:Zm00001d043168_T001	rna3139	4.00E-74
407- 40:	transcript:Zm00001d043170_T001	rna3137	1.00E-44
407- 41:	transcript:Zm00001d043171_T001	rna3131	0
407- 42:	transcript:Zm00001d043175_T003	rna3130	1.00E-51
407- 43:	transcript:Zm00001d043178_T001	rna3129	0
407- 44:	transcript:Zm00001d043179_T001	rna3128	0
407- 45:	transcript:Zm00001d043180_T001	rna3127	2.00E-144
407- 46:	transcript:Zm00001d043181_T001	rna3125	6.00E-25
407- 47:	transcript:Zm00001d043182_T001	rna3124	0
407- 48:	transcript:Zm00001d043183_T001	rna3123	1.00E-147
407- 49:	transcript:Zm00001d043185_T001	rna3121	0
407- 50:	transcript:Zm00001d043187_T001	rna3118	3.00E-145
407- 51:	transcript:Zm00001d043188_T001	rna3116	4.00E-137
407- 52:	transcript:Zm00001d043190_T001	rna3114	3.00E-16
407- 53:	transcript:Zm00001d043193_T001	rna3111	0
407- 54:	transcript:Zm00001d043194_T001	rna3110	0
407- 55:	transcript:Zm00001d043195_T001	rna3106	0
407- 56:	transcript:Zm00001d043196_T001	rna3104	0
407- 57:	transcript:Zm00001d043197_T001	rna3103	3.00E-110
407- 58:	transcript:Zm00001d043198_T001	rna3102	0
407- 59:	transcript:Zm00001d043200_T001	rna3101	0
407- 60:	transcript:Zm00001d043201_T001	rna3100	7.00E-95
407- 61:	transcript:Zm00001d043205_T001	rna3094	8.00E-91
407- 62:	transcript:Zm00001d043206_T002	rna3092	7.00E-82
407- 63:	transcript:Zm00001d043207_T004	rna3090	3.00E-119
407- 64:	transcript:Zm00001d043209_T001	rna3086	2.00E-08
407- 65:	transcript:Zm00001d043211_T001	rna3078	0
407- 66:	transcript:Zm00001d043217_T002	rna3077	6.00E-70
407- 67:	transcript:Zm00001d043220_T001	rna3073	1.00E-33
407- 68:	transcript:Zm00001d043225_T001	rna3070	7.00E-153
407- 69:	transcript:Zm00001d043226_T001	rna3069	2.00E-135
407- 70:	transcript:Zm00001d043227_T002	rna3046	0
407- 71:	transcript:Zm00001d043228_T001	rna3045	1.00E-162

407- 72:	transcript:Zm00001d043230_T001	rna3043	0
407- 73:	transcript:Zm00001d043231_T001	rna3042	0
407- 74:	transcript:Zm00001d043232_T001	rna3041	1.00E-68
407- 75:	transcript:Zm00001d043233_T001	rna3040	0
407- 76:	transcript:Zm00001d043234_T001	rna3037	1.00E-76
407- 77:	transcript:Zm00001d043235_T001	rna3036	5.00E-147
407- 78:	transcript:Zm00001d043238_T001	rna3033	3.00E-166
407- 79:	transcript:Zm00001d043239_T004	rna3032	0
407- 80:	transcript:Zm00001d043240_T002	rna3030	5.00E-51
407- 81:	transcript:Zm00001d043242_T001	rna3029	8.00E-37
407- 82:	transcript:Zm00001d043243_T001	rna3027	0
407- 83:	transcript:Zm00001d043244_T001	rna3024	0
407- 84:	transcript:Zm00001d043248_T001	rna3022	3.00E-148
407- 85:	transcript:Zm00001d043249_T001	rna3021	1.00E-135
407- 86:	transcript:Zm00001d043253_T002	rna3017	0
407- 87:	transcript:Zm00001d043257_T004	rna3015	0
407- 88:	transcript:Zm00001d043258_T001	rna3014	5.00E-44
407- 89:	transcript:Zm00001d043259_T001	rna3013	6.00E-45
407- 90:	transcript:Zm00001d043261_T001	rna3012	3.00E-123
407- 91:	transcript:Zm00001d043262_T001	rna3011	1.00E-22
407- 92:	transcript:Zm00001d043263_T006	rna3010	0
407- 93:	transcript:Zm00001d043264_T001	rna3009	1.00E-66
407- 94:	transcript:Zm00001d043267_T002	rna3007	0
407- 95:	transcript:Zm00001d043269_T001	rna2998	4.00E-126
407- 96:	transcript:Zm00001d043270_T001	rna2996	2.00E-142
407- 97:	transcript:Zm00001d043272_T002	rna2994	0
407- 98:	transcript:Zm00001d043273_T001	rna2992	3.00E-67
407- 99:	transcript:Zm00001d043274_T008	rna2987	0
407-100:	transcript:Zm00001d043275_T002	rna2986	0
407-101:	transcript:Zm00001d043276_T001	rna2985	1.00E-15
407-102:	transcript:Zm00001d043278_T001	rna2982	4.00E-52
407-103:	transcript:Zm00001d043280_T001	rna2980	6.00E-96
407-104:	transcript:Zm00001d043286_T002	rna2979	1.00E-26
407-105:	transcript:Zm00001d043287_T002	rna2976	0
407-106:	transcript:Zm00001d043288_T001	rna2973	0
407-107:	transcript:Zm00001d043289_T002	rna2971	1.00E-21
407-108:	transcript:Zm00001d043291_T001	rna2970	0
407-109:	transcript:Zm00001d043292_T001	rna2968	2.00E-46
407-110:	transcript:Zm00001d043293_T001	rna2964	0
407-111:	transcript:Zm00001d043294_T006	rna2963	0
407-112:	transcript:Zm00001d043295_T003	rna2962	0
407-113:	transcript:Zm00001d043296_T002	rna2961	0
407-114:	transcript:Zm00001d043298_T001	rna2960	0
407-115:	transcript:Zm00001d043299_T001	rna2955	1.00E-40
407-116:	transcript:Zm00001d043301_T006	rna2950	0
407-117:	transcript:Zm00001d043302_T005	rna2948	5.00E-165
407-118:	transcript:Zm00001d043303_T003	rna2947	0
407-119:	transcript:Zm00001d043305_T001	rna2946	0
407-120:	transcript:Zm00001d043307_T001	rna2943	9.00E-128
407-121:	transcript:Zm00001d043309_T004	rna2938	0
407-122:	transcript:Zm00001d043312_T001	rna2937	9.00E-135
407-123:	transcript:Zm00001d043314_T001	rna2935	2.00E-94
407-124:	transcript:Zm00001d043317_T001	rna2934	2.00E-109
407-125:	transcript:Zm00001d043318_T001	rna2933	1.00E-105

407-126: transcript:Zm00001d043323_T001 rna2931 2.00E-57
407-127: transcript:Zm00001d043324_T001 rna2929 0
407-128: transcript:Zm00001d043325_T001 rna2925 0
407-129: transcript:Zm00001d043327_T002 rna2920 0
407-130: transcript:Zm00001d043328_T003 rna2919 0
407-131: transcript:Zm00001d043329_T001 rna2918 0
407-132: transcript:Zm00001d043330_T001 rna2917 5.00E-131
407-133: transcript:Zm00001d043335_T006 rna2905 0
407-134: transcript:Zm00001d043336_T001 rna2904 1.00E-103
407-135: transcript:Zm00001d043338_T001 rna2901 1.00E-35
407-136: transcript:Zm00001d043339_T001 rna2900 2.00E-110
407-137: transcript:Zm00001d043346_T001 rna2898 5.00E-75
407-138: transcript:Zm00001d043347_T006 rna2894 2.00E-130
Alignment 408: score=4880.0 e_value=0 N=107 3&NC_008394.4 minus
408- 0: transcript:Zm00001d042164_T002 rna4016 0
408- 1: transcript:Zm00001d042168_T001 rna4013 0
408- 2: transcript:Zm00001d042169_T001 rna4012 4.00E-17
408- 3: transcript:Zm00001d042172_T001 rna4010 1.00E-64
408- 4: transcript:Zm00001d042179_T001 rna3998 2.00E-92
408- 5: transcript:Zm00001d042180_T003 rna3997 2.00E-165
408- 6: transcript:Zm00001d042182_T001 rna3995 8.00E-08
408- 7: transcript:Zm00001d042183_T001 rna3992 3.00E-102
408- 8: transcript:Zm00001d042185_T003 rna3991 0
408- 9: transcript:Zm00001d042187_T001 rna3990 1.00E-58
408- 10: transcript:Zm00001d042191_T002 rna3986 1.00E-67
408- 11: transcript:Zm00001d042192_T001 rna3984 0
408- 12: transcript:Zm00001d042193_T001 rna3983 2.00E-124
408- 13: transcript:Zm00001d042196_T001 rna3982 5.00E-74
408- 14: transcript:Zm00001d042202_T001 rna3981 7.00E-158
408- 15: transcript:Zm00001d042204_T001 rna3977 2.00E-40
408- 16: transcript:Zm00001d042211_T001 rna3975 3.00E-106
408- 17: transcript:Zm00001d042212_T007 rna3974 0
408- 18: transcript:Zm00001d042213_T001 rna3973 0
408- 19: transcript:Zm00001d042214_T001 rna3972 2.00E-100
408- 20: transcript:Zm00001d042215_T002 rna3971 0
408- 21: transcript:Zm00001d042216_T001 rna3970 2.00E-111
408- 22: transcript:Zm00001d042217_T001 rna3968 1.00E-54
408- 23: transcript:Zm00001d042225_T002 rna3965 2.00E-75
408- 24: transcript:Zm00001d042229_T001 rna3963 8.00E-07
408- 25: transcript:Zm00001d042234_T004 rna3961 0
408- 26: transcript:Zm00001d042241_T001 rna3957 1.00E-158
408- 27: transcript:Zm00001d042243_T001 rna3950 1.00E-36
408- 28: transcript:Zm00001d042244_T001 rna3949 0
408- 29: transcript:Zm00001d042245_T001 rna3947 8.00E-39
408- 30: transcript:Zm00001d042250_T001 rna3942 2.00E-164
408- 31: transcript:Zm00001d042256_T010 rna3940 4.00E-71
408- 32: transcript:Zm00001d042257_T001 rna3939 7.00E-111
408- 33: transcript:Zm00001d042258_T001 rna3934 4.00E-34
408- 34: transcript:Zm00001d042259_T002 rna3933 2.00E-44
408- 35: transcript:Zm00001d042262_T003 rna3932 2.00E-64
408- 36: transcript:Zm00001d042263_T001 rna3931 4.00E-152
408- 37: transcript:Zm00001d042264_T006 rna3930 0
408- 38: transcript:Zm00001d042267_T003 rna3929 0
408- 39: transcript:Zm00001d042268_T001 rna3928 0

408- 40:	transcript:Zm00001d042269_T001	rna3927	0
408- 41:	transcript:Zm00001d042270_T005	rna3926	2.00E-47
408- 42:	transcript:Zm00001d042271_T001	rna3925	6.00E-101
408- 43:	transcript:Zm00001d042272_T003	rna3924	0
408- 44:	transcript:Zm00001d042274_T001	rna3922	3.00E-136
408- 45:	transcript:Zm00001d042276_T001	rna3917	0
408- 46:	transcript:Zm00001d042282_T003	rna3916	0
408- 47:	transcript:Zm00001d042284_T003	rna3915	5.00E-135
408- 48:	transcript:Zm00001d042285_T001	rna3913	1.00E-100
408- 49:	transcript:Zm00001d042286_T001	rna3912	2.00E-27
408- 50:	transcript:Zm00001d042288_T002	rna3911	3.00E-54
408- 51:	transcript:Zm00001d042290_T001	rna3910	0
408- 52:	transcript:Zm00001d042291_T002	rna3907	1.00E-80
408- 53:	transcript:Zm00001d042292_T001	rna3906	5.00E-48
408- 54:	transcript:Zm00001d042302_T002	rna3904	2.00E-151
408- 55:	transcript:Zm00001d042305_T001	rna3900	5.00E-102
408- 56:	transcript:Zm00001d042306_T004	rna3899	0
408- 57:	transcript:Zm00001d042307_T001	rna3898	3.00E-61
408- 58:	transcript:Zm00001d042309_T001	rna3895	0
408- 59:	transcript:Zm00001d042312_T012	rna3893	2.00E-39
408- 60:	transcript:Zm00001d042313_T004	rna3892	0
408- 61:	transcript:Zm00001d042314_T004	rna3890	4.00E-124
408- 62:	transcript:Zm00001d042315_T001	rna3887	8.00E-34
408- 63:	transcript:Zm00001d042317_T001	rna3886	2.00E-15
408- 64:	transcript:Zm00001d042319_T001	rna3885	5.00E-99
408- 65:	transcript:Zm00001d042325_T001	rna3884	3.00E-82
408- 66:	transcript:Zm00001d042328_T001	rna3882	2.00E-172
408- 67:	transcript:Zm00001d042329_T001	rna3881	1.00E-113
408- 68:	transcript:Zm00001d042333_T001	rna3878	0
408- 69:	transcript:Zm00001d042335_T006	rna3877	0
408- 70:	transcript:Zm00001d042336_T001	rna3874	3.00E-155
408- 71:	transcript:Zm00001d042337_T001	rna3873	2.00E-83
408- 72:	transcript:Zm00001d042338_T002	rna3872	0
408- 73:	transcript:Zm00001d042340_T001	rna3870	4.00E-54
408- 74:	transcript:Zm00001d042341_T001	rna3869	1.00E-82
408- 75:	transcript:Zm00001d042343_T001	rna3868	0
408- 76:	transcript:Zm00001d042344_T002	rna3867	0
408- 77:	transcript:Zm00001d042345_T001	rna3866	0
408- 78:	transcript:Zm00001d042346_T001	rna3865	5.00E-151
408- 79:	transcript:Zm00001d042349_T001	rna3864	0
408- 80:	transcript:Zm00001d042350_T002	rna3863	2.00E-66
408- 81:	transcript:Zm00001d042353_T003	rna3862	3.00E-44
408- 82:	transcript:Zm00001d042357_T002	rna3855	3.00E-141
408- 83:	transcript:Zm00001d042359_T001	rna3854	1.00E-79
408- 84:	transcript:Zm00001d042361_T002	rna3853	1.00E-34
408- 85:	transcript:Zm00001d042362_T001	rna3852	0
408- 86:	transcript:Zm00001d042363_T007	rna3851	0
408- 87:	transcript:Zm00001d042365_T001	rna3850	1.00E-31
408- 88:	transcript:Zm00001d042366_T002	rna3849	0
408- 89:	transcript:Zm00001d042367_T002	rna3844	0
408- 90:	transcript:Zm00001d042370_T003	rna3842	0
408- 91:	transcript:Zm00001d042371_T001	rna3841	2.00E-10
408- 92:	transcript:Zm00001d042372_T002	rna3838	3.00E-91
408- 93:	transcript:Zm00001d042373_T002	rna3837	0

408- 94: transcript:Zm00001d042376_T001 rna3833 2.00E-125
408- 95: transcript:Zm00001d042378_T001 rna3831 0
408- 96: transcript:Zm00001d042380_T001 rna3830 0
408- 97: transcript:Zm00001d042381_T001 rna3829 2.00E-57
408- 98: transcript:Zm00001d042382_T001 rna3826 4.00E-12
408- 99: transcript:Zm00001d042386_T001 rna3825 2.00E-59
408-100: transcript:Zm00001d042388_T001 rna3818 1.00E-61
408-101: transcript:Zm00001d042389_T001 rna3816 0
408-102: transcript:Zm00001d042393_T002 rna3813 6.00E-22
408-103: transcript:Zm00001d042394_T001 rna3812 0
408-104: transcript:Zm00001d042396_T003 rna3811 0
408-105: transcript:Zm00001d042397_T001 rna3809 0
408-106: transcript:Zm00001d042398_T001 rna3808 0
Alignment 409: score=3659.0 e_value=0 N=81 3&NC_008394.4 minus
409- 0: transcript:Zm00001d043669_T004 rna2564 0
409- 1: transcript:Zm00001d043674_T001 rna2561 4.00E-155
409- 2: transcript:Zm00001d043675_T001 rna2557 2.00E-142
409- 3: transcript:Zm00001d043680_T001 rna2550 0
409- 4: transcript:Zm00001d043681_T001 rna2548 0
409- 5: transcript:Zm00001d043683_T001 rna2546 2.00E-97
409- 6: transcript:Zm00001d043684_T001 rna2545 0
409- 7: transcript:Zm00001d043686_T009 rna2544 0
409- 8: transcript:Zm00001d043687_T001 rna2542 2.00E-24
409- 9: transcript:Zm00001d043691_T001 rna2540 0
409- 10: transcript:Zm00001d043692_T001 rna2538 3.00E-131
409- 11: transcript:Zm00001d043693_T002 rna2537 4.00E-22
409- 12: transcript:Zm00001d043694_T001 rna2536 9.00E-128
409- 13: transcript:Zm00001d043695_T001 rna2534 0
409- 14: transcript:Zm00001d043696_T004 rna2533 6.00E-105
409- 15: transcript:Zm00001d043699_T001 rna2532 2.00E-108
409- 16: transcript:Zm00001d043700_T006 rna2531 1.00E-112
409- 17: transcript:Zm00001d043701_T001 rna2530 0
409- 18: transcript:Zm00001d043703_T007 rna2527 0
409- 19: transcript:Zm00001d043704_T001 rna2526 1.00E-30
409- 20: transcript:Zm00001d043705_T005 rna2521 1.00E-56
409- 21: transcript:Zm00001d043706_T001 rna2519 0
409- 22: transcript:Zm00001d043707_T002 rna2517 3.00E-94
409- 23: transcript:Zm00001d043708_T001 rna2516 1.00E-23
409- 24: transcript:Zm00001d043709_T003 rna2515 2.00E-56
409- 25: transcript:Zm00001d043721_T001 rna2514 6.00E-133
409- 26: transcript:Zm00001d043723_T001 rna2512 4.00E-83
409- 27: transcript:Zm00001d043724_T001 rna2511 3.00E-124
409- 28: transcript:Zm00001d043725_T001 rna2509 2.00E-73
409- 29: transcript:Zm00001d043726_T001 rna2508 0
409- 30: transcript:Zm00001d043727_T001 rna2506 0
409- 31: transcript:Zm00001d043729_T001 rna2502 2.00E-87
409- 32: transcript:Zm00001d043730_T001 rna2501 2.00E-35
409- 33: transcript:Zm00001d043731_T001 rna2498 0
409- 34: transcript:Zm00001d043733_T002 rna2496 0
409- 35: transcript:Zm00001d043734_T001 rna2495 0
409- 36: transcript:Zm00001d043735_T001 rna2488 2.00E-136
409- 37: transcript:Zm00001d043736_T002 rna2487 5.00E-62
409- 38: transcript:Zm00001d043737_T001 rna2486 7.00E-93
409- 39: transcript:Zm00001d043738_T001 rna2482 3.00E-120

409- 40: transcript:Zm00001d043743_T001 rna2478 0
409- 41: transcript:Zm00001d043745_T001 rna2476 2.00E-48
409- 42: transcript:Zm00001d043751_T001 rna2475 1.00E-15
409- 43: transcript:Zm00001d043752_T001 rna2473 0
409- 44: transcript:Zm00001d043765_T001 rna2469 0
409- 45: transcript:Zm00001d043766_T005 rna2465 0
409- 46: transcript:Zm00001d043767_T002 rna2464 3.00E-53
409- 47: transcript:Zm00001d043768_T001 rna2462 0
409- 48: transcript:Zm00001d043770_T001 rna2461 9.00E-39
409- 49: transcript:Zm00001d043773_T003 rna2460 0
409- 50: transcript:Zm00001d043775_T006 rna2455 4.00E-75
409- 51: transcript:Zm00001d043776_T001 rna2454 9.00E-165
409- 52: transcript:Zm00001d043779_T001 rna2451 2.00E-145
409- 53: transcript:Zm00001d043780_T001 rna2450 0
409- 54: transcript:Zm00001d043782_T001 rna2448 0
409- 55: transcript:Zm00001d043784_T002 rna2446 3.00E-126
409- 56: transcript:Zm00001d043785_T002 rna2445 0
409- 57: transcript:Zm00001d043786_T001 rna2443 0
409- 58: transcript:Zm00001d043795_T001 rna2440 4.00E-52
409- 59: transcript:Zm00001d043797_T020 rna2438 0
409- 60: transcript:Zm00001d043798_T001 rna2427 1.00E-29
409- 61: transcript:Zm00001d043801_T002 rna2426 0
409- 62: transcript:Zm00001d043803_T003 rna2425 4.00E-33
409- 63: transcript:Zm00001d043805_T012 rna2424 3.00E-50
409- 64: transcript:Zm00001d043806_T001 rna2423 2.00E-53
409- 65: transcript:Zm00001d043808_T001 rna2420 7.00E-57
409- 66: transcript:Zm00001d043813_T004 rna2415 5.00E-171
409- 67: transcript:Zm00001d043814_T002 rna2414 1.00E-90
409- 68: transcript:Zm00001d043815_T005 rna2413 0
409- 69: transcript:Zm00001d043827_T001 rna2406 1.00E-42
409- 70: transcript:Zm00001d043829_T001 rna2404 4.00E-169
409- 71: transcript:Zm00001d043830_T001 rna2402 2.00E-57
409- 72: transcript:Zm00001d043831_T001 rna2401 0
409- 73: transcript:Zm00001d043834_T001 rna2400 0
409- 74: transcript:Zm00001d043835_T006 rna2398 2.00E-159
409- 75: transcript:Zm00001d043838_T002 rna2393 0
409- 76: transcript:Zm00001d043839_T001 rna2389 2.00E-22
409- 77: transcript:Zm00001d043840_T001 rna2386 0
409- 78: transcript:Zm00001d043841_T003 rna2380 1.00E-158
409- 79: transcript:Zm00001d043842_T002 rna2379 2.00E-152
409- 80: transcript:Zm00001d043843_T001 rna2375 2.00E-13
Alignment 410: score=3416.0 e_value=3.3e-316 N=75 3&NC_008394.4 minus
410- 0: transcript:Zm00001d039848_T002 rna356 0
410- 1: transcript:Zm00001d039851_T002 rna355 0
410- 2: transcript:Zm00001d039852_T001 rna352 0
410- 3: transcript:Zm00001d039854_T001 rna351 0
410- 4: transcript:Zm00001d039856_T002 rna345 2.00E-74
410- 5: transcript:Zm00001d039858_T001 rna343 0
410- 6: transcript:Zm00001d039859_T001 rna336 2.00E-10
410- 7: transcript:Zm00001d039860_T003 rna330 2.00E-79
410- 8: transcript:Zm00001d039865_T002 rna329 3.00E-164
410- 9: transcript:Zm00001d039866_T001 rna328 4.00E-152
410- 10: transcript:Zm00001d039867_T007 rna325 6.00E-65
410- 11: transcript:Zm00001d039870_T002 rna324 1.00E-68

410- 12:	transcript:Zm00001d039871_T002	rna323	0
410- 13:	transcript:Zm00001d039873_T002	rna311	7.00E-14
410- 14:	transcript:Zm00001d039874_T001	rna310	3.00E-85
410- 15:	transcript:Zm00001d039878_T003	rna308	1.00E-126
410- 16:	transcript:Zm00001d039879_T001	rna307	4.00E-152
410- 17:	transcript:Zm00001d039881_T003	rna305	1.00E-174
410- 18:	transcript:Zm00001d039882_T001	rna299	1.00E-11
410- 19:	transcript:Zm00001d039884_T002	rna298	0
410- 20:	transcript:Zm00001d039893_T002	rna295	1.00E-128
410- 21:	transcript:Zm00001d039899_T002	rna292	8.00E-99
410- 22:	transcript:Zm00001d039900_T002	rna291	0
410- 23:	transcript:Zm00001d039902_T001	rna290	8.00E-143
410- 24:	transcript:Zm00001d039903_T003	rna287	1.00E-133
410- 25:	transcript:Zm00001d039904_T006	rna286	0
410- 26:	transcript:Zm00001d039907_T001	rna285	2.00E-153
410- 27:	transcript:Zm00001d039908_T001	rna284	3.00E-38
410- 28:	transcript:Zm00001d039911_T002	rna283	0
410- 29:	transcript:Zm00001d039916_T001	rna278	0
410- 30:	transcript:Zm00001d039918_T001	rna276	0
410- 31:	transcript:Zm00001d039919_T002	rna273	1.00E-128
410- 32:	transcript:Zm00001d039922_T001	rna268	1.00E-13
410- 33:	transcript:Zm00001d039930_T001	rna260	6.00E-08
410- 34:	transcript:Zm00001d039932_T001	rna255	3.00E-20
410- 35:	transcript:Zm00001d039933_T001	rna252	4.00E-75
410- 36:	transcript:Zm00001d039941_T001	rna251	2.00E-35
410- 37:	transcript:Zm00001d039944_T001	rna249	2.00E-80
410- 38:	transcript:Zm00001d039958_T001	rna245	3.00E-162
410- 39:	transcript:Zm00001d039963_T001	rna244	0
410- 40:	transcript:Zm00001d039965_T001	rna243	9.00E-175
410- 41:	transcript:Zm00001d039967_T001	rna241	0
410- 42:	transcript:Zm00001d039969_T001	rna239	3.00E-155
410- 43:	transcript:Zm00001d039971_T002	rna238	0
410- 44:	transcript:Zm00001d039973_T002	rna237	0
410- 45:	transcript:Zm00001d039974_T001	rna236	1.00E-59
410- 46:	transcript:Zm00001d039975_T001	rna235	0
410- 47:	transcript:Zm00001d039977_T001	rna233	1.00E-85
410- 48:	transcript:Zm00001d039982_T001	rna231	2.00E-41
410- 49:	transcript:Zm00001d039983_T001	rna230	5.00E-14
410- 50:	transcript:Zm00001d039988_T001	rna228	8.00E-38
410- 51:	transcript:Zm00001d039991_T001	rna221	3.00E-42
410- 52:	transcript:Zm00001d039993_T001	rna220	2.00E-35
410- 53:	transcript:Zm00001d039994_T001	rna218	0
410- 54:	transcript:Zm00001d040002_T001	rna216	0
410- 55:	transcript:Zm00001d040003_T001	rna214	1.00E-10
410- 56:	transcript:Zm00001d040004_T001	rna213	5.00E-125
410- 57:	transcript:Zm00001d040005_T001	rna212	0
410- 58:	transcript:Zm00001d040006_T001	rna211	9.00E-38
410- 59:	transcript:Zm00001d040008_T005	rna210	0
410- 60:	transcript:Zm00001d040010_T003	rna208	0
410- 61:	transcript:Zm00001d040011_T001	rna207	4.00E-100
410- 62:	transcript:Zm00001d040014_T002	rna204	4.00E-157
410- 63:	transcript:Zm00001d040019_T001	rna203	9.00E-128
410- 64:	transcript:Zm00001d040020_T001	rna202	0
410- 65:	transcript:Zm00001d040023_T001	rna201	4.00E-40

410- 66: transcript:Zm00001d040026_T002 rna200 2.00E-23
410- 67: transcript:Zm00001d040033_T002 rna192 0
410- 68: transcript:Zm00001d040034_T001 rna189 0
410- 69: transcript:Zm00001d040036_T021 rna187 0
410- 70: transcript:Zm00001d040038_T001 rna186 0
410- 71: transcript:Zm00001d040040_T001 rna184 2.00E-98
410- 72: transcript:Zm00001d040044_T001 rna170 5.00E-08
410- 73: transcript:Zm00001d040047_T001 rna169 0
410- 74: transcript:Zm00001d040048_T001 rna166 2.00E-23
Alignment 411: score=3075.0 e_value=1.9e-273 N=67 3&NC_008394.4 minus
411- 0: transcript:Zm00001d044030_T001 rna2220 0
411- 1: transcript:Zm00001d044031_T001 rna2218 1.00E-148
411- 2: transcript:Zm00001d044034_T001 rna2217 1.00E-76
411- 3: transcript:Zm00001d044035_T001 rna2216 0
411- 4: transcript:Zm00001d044037_T001 rna2214 0
411- 5: transcript:Zm00001d044038_T001 rna2213 9.00E-48
411- 6: transcript:Zm00001d044039_T001 rna2211 3.00E-168
411- 7: transcript:Zm00001d044043_T001 rna2209 4.00E-17
411- 8: transcript:Zm00001d044049_T001 rna2200 2.00E-91
411- 9: transcript:Zm00001d044056_T003 rna2192 0
411- 10: transcript:Zm00001d044059_T001 rna2190 0
411- 11: transcript:Zm00001d044060_T001 rna2188 0
411- 12: transcript:Zm00001d044061_T001 rna2187 0
411- 13: transcript:Zm00001d044065_T001 rna2184 0
411- 14: transcript:Zm00001d044068_T001 rna2181 0
411- 15: transcript:Zm00001d044069_T001 rna2180 3.00E-24
411- 16: transcript:Zm00001d044074_T001 rna2178 1.00E-113
411- 17: transcript:Zm00001d044075_T001 rna2177 2.00E-57
411- 18: transcript:Zm00001d044076_T001 rna2176 7.00E-22
411- 19: transcript:Zm00001d044078_T001 rna2174 6.00E-43
411- 20: transcript:Zm00001d044079_T001 rna2173 5.00E-33
411- 21: transcript:Zm00001d044080_T001 rna2172 6.00E-29
411- 22: transcript:Zm00001d044081_T001 rna2170 3.00E-123
411- 23: transcript:Zm00001d044083_T001 rna2169 0
411- 24: transcript:Zm00001d044086_T001 rna2168 1.00E-113
411- 25: transcript:Zm00001d044087_T001 rna2167 2.00E-156
411- 26: transcript:Zm00001d044088_T001 rna2164 0
411- 27: transcript:Zm00001d044089_T002 rna2162 5.00E-162
411- 28: transcript:Zm00001d044091_T003 rna2158 0
411- 29: transcript:Zm00001d044092_T001 rna2157 6.00E-34
411- 30: transcript:Zm00001d044093_T001 rna2156 9.00E-23
411- 31: transcript:Zm00001d044094_T001 rna2155 0
411- 32: transcript:Zm00001d044099_T002 rna2151 5.00E-117
411- 33: transcript:Zm00001d044100_T001 rna2150 4.00E-67
411- 34: transcript:Zm00001d044104_T001 rna2149 0
411- 35: transcript:Zm00001d044106_T001 rna2146 0
411- 36: transcript:Zm00001d044107_T001 rna2145 5.00E-75
411- 37: transcript:Zm00001d044110_T001 rna2140 0
411- 38: transcript:Zm00001d044111_T001 rna2139 3.00E-40
411- 39: transcript:Zm00001d044116_T001 rna2136 0
411- 40: transcript:Zm00001d044117_T001 rna2135 1.00E-40
411- 41: transcript:Zm00001d044119_T001 rna2134 1.00E-124
411- 42: transcript:Zm00001d044121_T002 rna2132 0
411- 43: transcript:Zm00001d044124_T001 rna2128 0

411- 44:	transcript:Zm00001d044127_T003	rna2127	0
411- 45:	transcript:Zm00001d044129_T001	rna2126	0
411- 46:	transcript:Zm00001d044130_T002	rna2125	1.00E-84
411- 47:	transcript:Zm00001d044131_T001	rna2120	7.00E-32
411- 48:	transcript:Zm00001d044132_T001	rna2118	0
411- 49:	transcript:Zm00001d044136_T001	rna2114	1.00E-144
411- 50:	transcript:Zm00001d044138_T001	rna2113	3.00E-135
411- 51:	transcript:Zm00001d044142_T001	rna2110	4.00E-28
411- 52:	transcript:Zm00001d044144_T008	rna2107	2.00E-100
411- 53:	transcript:Zm00001d044147_T001	rna2096	0
411- 54:	transcript:Zm00001d044162_T001	rna2092	9.00E-138
411- 55:	transcript:Zm00001d044164_T002	rna2090	2.00E-124
411- 56:	transcript:Zm00001d044167_T001	rna2089	1.00E-98
411- 57:	transcript:Zm00001d044168_T001	rna2088	3.00E-119
411- 58:	transcript:Zm00001d044170_T001	rna2087	3.00E-132
411- 59:	transcript:Zm00001d044171_T001	rna2086	6.00E-102
411- 60:	transcript:Zm00001d044172_T002	rna2085	0
411- 61:	transcript:Zm00001d044173_T002	rna2084	1.00E-91
411- 62:	transcript:Zm00001d044175_T002	rna2083	0
411- 63:	transcript:Zm00001d044176_T001	rna2082	3.00E-61
411- 64:	transcript:Zm00001d044181_T002	rna2079	0
411- 65:	transcript:Zm00001d044184_T001	rna2077	6.00E-155
411- 66:	transcript:Zm00001d044185_T004	rna2076	2.00E-26

Alignment 412: score=2972.0 e_value=3.9e-258 N=64 3&NC_008394.4 minus

412- 0:	transcript:Zm00001d039238_T001	rna934	7.00E-40
412- 1:	transcript:Zm00001d039240_T001	rna933	3.00E-85
412- 2:	transcript:Zm00001d039241_T001	rna932	6.00E-35
412- 3:	transcript:Zm00001d039243_T001	rna930	0
412- 4:	transcript:Zm00001d039245_T001	rna929	5.00E-142
412- 5:	transcript:Zm00001d039249_T001	rna928	1.00E-43
412- 6:	transcript:Zm00001d039250_T001	rna927	4.00E-128
412- 7:	transcript:Zm00001d039251_T001	rna916	1.00E-50
412- 8:	transcript:Zm00001d039253_T001	rna905	6.00E-100
412- 9:	transcript:Zm00001d039254_T001	rna904	2.00E-150
412- 10:	transcript:Zm00001d039255_T003	rna901	4.00E-44
412- 11:	transcript:Zm00001d039256_T001	rna900	1.00E-30
412- 12:	transcript:Zm00001d039258_T001	rna892	0
412- 13:	transcript:Zm00001d039259_T001	rna891	1.00E-171
412- 14:	transcript:Zm00001d039260_T002	rna890	1.00E-132
412- 15:	transcript:Zm00001d039262_T002	rna888	1.00E-29
412- 16:	transcript:Zm00001d039263_T001	rna886	1.00E-84
412- 17:	transcript:Zm00001d039264_T001	rna885	0
412- 18:	transcript:Zm00001d039265_T001	rna877	0
412- 19:	transcript:Zm00001d039266_T004	rna875	8.00E-75
412- 20:	transcript:Zm00001d039267_T005	rna874	0
412- 21:	transcript:Zm00001d039268_T001	rna873	4.00E-55
412- 22:	transcript:Zm00001d039270_T001	rna872	1.00E-141
412- 23:	transcript:Zm00001d039271_T001	rna870	0
412- 24:	transcript:Zm00001d039273_T001	rna867	3.00E-121
412- 25:	transcript:Zm00001d039274_T001	rna866	0
412- 26:	transcript:Zm00001d039279_T001	rna860	1.00E-84
412- 27:	transcript:Zm00001d039280_T007	rna855	6.00E-177
412- 28:	transcript:Zm00001d039282_T001	rna854	0
412- 29:	transcript:Zm00001d039283_T004	rna850	2.00E-62

```
412- 30:  transcript:Zm00001d039284_T001  rna849       2.00E-28
412- 31:  transcript:Zm00001d039285_T001  rna846              0
412- 32:  transcript:Zm00001d039287_T002  rna844              0
412- 33:  transcript:Zm00001d039293_T001  rna841      7.00E-131
412- 34:  transcript:Zm00001d039294_T007  rna839       1.00E-75
412- 35:  transcript:Zm00001d039296_T005  rna837              0
412- 36:  transcript:Zm00001d039300_T003  rna834       2.00E-95
412- 37:  transcript:Zm00001d039301_T001  rna832       2.00E-93
412- 38:  transcript:Zm00001d039302_T001  rna831              0
412- 39:  transcript:Zm00001d039303_T001  rna829       2.00E-42
412- 40:  transcript:Zm00001d039304_T001  rna826              0
412- 41:  transcript:Zm00001d039305_T002  rna825              0
412- 42:  transcript:Zm00001d039306_T002  rna824       2.00E-67
412- 43:  transcript:Zm00001d039310_T001  rna814       2.00E-65
412- 44:  transcript:Zm00001d039311_T001  rna812              0
412- 45:  transcript:Zm00001d039312_T001  rna811              0
412- 46:  transcript:Zm00001d039313_T001  rna810       4.00E-53
412- 47:  transcript:Zm00001d039314_T001  rna809              0
412- 48:  transcript:Zm00001d039315_T003  rna807              0
412- 49:  transcript:Zm00001d039316_T001  rna806      1.00E-120
412- 50:  transcript:Zm00001d039318_T001  rna805      3.00E-110
412- 51:  transcript:Zm00001d039319_T002  rna799       1.00E-88
412- 52:  transcript:Zm00001d039321_T008  rna798      2.00E-115
412- 53:  transcript:Zm00001d039323_T001  rna796              0
412- 54:  transcript:Zm00001d039324_T001  rna791      3.00E-115
412- 55:  transcript:Zm00001d039325_T001  rna787              0
412- 56:  transcript:Zm00001d039326_T001  rna782      3.00E-148
412- 57:  transcript:Zm00001d039327_T001  rna780       1.00E-85
412- 58:  transcript:Zm00001d039328_T006  rna779              0
412- 59:  transcript:Zm00001d039330_T001  rna777      9.00E-119
412- 60:  transcript:Zm00001d039331_T001  rna776      2.00E-135
412- 61:  transcript:Zm00001d039337_T001  rna774       3.00E-79
412- 62:  transcript:Zm00001d039338_T001  rna771       6.00E-19
412- 63:  transcript:Zm00001d039339_T001  rna770       2.00E-29
## Alignment 413: score=2785.0 e_value=2.2e-233 N=60 3&NC_008394.4 minus
413-  0:  transcript:Zm00001d043849_T001  rna2376      3.00E-07
413-  1:  transcript:Zm00001d043850_T001  rna2375             0
413-  2:  transcript:Zm00001d043851_T001  rna2373             0
413-  3:  transcript:Zm00001d043853_T001  rna2371             0
413-  4:  transcript:Zm00001d043854_T001  rna2369     8.00E-179
413-  5:  transcript:Zm00001d043855_T001  rna2368     1.00E-157
413-  6:  transcript:Zm00001d043857_T001  rna2365      1.00E-54
413-  7:  transcript:Zm00001d043858_T001  rna2364             0
413-  8:  transcript:Zm00001d043860_T001  rna2362             0
413-  9:  transcript:Zm00001d043863_T001  rna2360      5.00E-60
413- 10:  transcript:Zm00001d043864_T001  rna2359      9.00E-55
413- 11:  transcript:Zm00001d043868_T001  rna2354      1.00E-21
413- 12:  transcript:Zm00001d043870_T001  rna2347     3.00E-143
413- 13:  transcript:Zm00001d043873_T001  rna2344             0
413- 14:  transcript:Zm00001d043874_T001  rna2343      2.00E-42
413- 15:  transcript:Zm00001d043875_T001  rna2341     2.00E-126
413- 16:  transcript:Zm00001d043877_T001  rna2340      7.00E-35
413- 17:  transcript:Zm00001d043878_T001  rna2339     3.00E-113
413- 18:  transcript:Zm00001d043879_T002  rna2338             0
```

413- 19: transcript:Zm00001d043889_T001 rna2334 0
413- 20: transcript:Zm00001d043890_T002 rna2333 0
413- 21: transcript:Zm00001d043898_T001 rna2330 7.00E-23
413- 22: transcript:Zm00001d043900_T022 rna2327 0
413- 23: transcript:Zm00001d043902_T001 rna2326 2.00E-135
413- 24: transcript:Zm00001d043903_T004 rna2325 2.00E-26
413- 25: transcript:Zm00001d043905_T003 rna2323 5.00E-103
413- 26: transcript:Zm00001d043906_T002 rna2320 9.00E-165
413- 27: transcript:Zm00001d043907_T003 rna2319 0
413- 28: transcript:Zm00001d043909_T010 rna2318 0
413- 29: transcript:Zm00001d043911_T001 rna2317 3.00E-129
413- 30: transcript:Zm00001d043914_T001 rna2316 3.00E-128
413- 31: transcript:Zm00001d043921_T001 rna2315 5.00E-67
413- 32: transcript:Zm00001d043922_T001 rna2313 0
413- 33: transcript:Zm00001d043923_T001 rna2305 0
413- 34: transcript:Zm00001d043928_T001 rna2303 2.00E-07
413- 35: transcript:Zm00001d043929_T001 rna2302 2.00E-122
413- 36: transcript:Zm00001d043932_T001 rna2300 2.00E-56
413- 37: transcript:Zm00001d043935_T001 rna2297 4.00E-142
413- 38: transcript:Zm00001d043937_T001 rna2296 4.00E-09
413- 39: transcript:Zm00001d043941_T001 rna2294 7.00E-150
413- 40: transcript:Zm00001d043942_T001 rna2292 3.00E-129
413- 41: transcript:Zm00001d043943_T001 rna2290 2.00E-89
413- 42: transcript:Zm00001d043944_T001 rna2289 9.00E-80
413- 43: transcript:Zm00001d043945_T001 rna2288 4.00E-149
413- 44: transcript:Zm00001d043946_T001 rna2285 2.00E-47
413- 45: transcript:Zm00001d043947_T001 rna2284 4.00E-120
413- 46: transcript:Zm00001d043950_T001 rna2282 3.00E-99
413- 47: transcript:Zm00001d043953_T001 rna2280 0
413- 48: transcript:Zm00001d043954_T003 rna2279 0
413- 49: transcript:Zm00001d043959_T003 rna2277 0
413- 50: transcript:Zm00001d043962_T001 rna2274 0
413- 51: transcript:Zm00001d043963_T001 rna2273 1.00E-82
413- 52: transcript:Zm00001d043965_T001 rna2271 0
413- 53: transcript:Zm00001d043969_T001 rna2270 1.00E-92
413- 54: transcript:Zm00001d043970_T001 rna2269 0
413- 55: transcript:Zm00001d043971_T001 rna2268 5.00E-99
413- 56: transcript:Zm00001d043972_T001 rna2267 9.00E-67
413- 57: transcript:Zm00001d043973_T001 rna2263 3.00E-31
413- 58: transcript:Zm00001d043974_T001 rna2262 1.00E-09
413- 59: transcript:Zm00001d043975_T001 rna2260 3.00E-153
Alignment 414: score=2060.0 e_value=1.7e-172 N=46 3&NC_008394.4 minus
414- 0: transcript:Zm00001d042433_T001 rna3797 5.00E-08
414- 1: transcript:Zm00001d042437_T005 rna3791 0
414- 2: transcript:Zm00001d042438_T001 rna3790 8.00E-76
414- 3: transcript:Zm00001d042441_T001 rna3789 0
414- 4: transcript:Zm00001d042443_T001 rna3787 1.00E-149
414- 5: transcript:Zm00001d042444_T001 rna3786 3.00E-47
414- 6: transcript:Zm00001d042445_T001 rna3785 0
414- 7: transcript:Zm00001d042446_T001 rna3783 0
414- 8: transcript:Zm00001d042447_T002 rna3782 4.00E-131
414- 9: transcript:Zm00001d042448_T001 rna3781 1.00E-33
414- 10: transcript:Zm00001d042449_T001 rna3780 8.00E-90
414- 11: transcript:Zm00001d042450_T002 rna3779 0

414- 12:	transcript:Zm00001d042451_T003	rna3777	0
414- 13:	transcript:Zm00001d042453_T001	rna3775	0
414- 14:	transcript:Zm00001d042455_T001	rna3774	1.00E-141
414- 15:	transcript:Zm00001d042460_T002	rna3772	5.00E-170
414- 16:	transcript:Zm00001d042461_T001	rna3770	5.00E-164
414- 17:	transcript:Zm00001d042463_T001	rna3769	2.00E-172
414- 18:	transcript:Zm00001d042464_T002	rna3768	0
414- 19:	transcript:Zm00001d042468_T004	rna3767	0
414- 20:	transcript:Zm00001d042469_T002	rna3766	2.00E-69
414- 21:	transcript:Zm00001d042470_T003	rna3765	0
414- 22:	transcript:Zm00001d042473_T002	rna3763	9.00E-168
414- 23:	transcript:Zm00001d042474_T001	rna3762	0
414- 24:	transcript:Zm00001d042475_T001	rna3761	0
414- 25:	transcript:Zm00001d042476_T002	rna3757	9.00E-100
414- 26:	transcript:Zm00001d042478_T003	rna3752	6.00E-141
414- 27:	transcript:Zm00001d042479_T001	rna3751	0
414- 28:	transcript:Zm00001d042480_T002	rna3750	0
414- 29:	transcript:Zm00001d042481_T004	rna3749	7.00E-98
414- 30:	transcript:Zm00001d042482_T001	rna3748	1.00E-142
414- 31:	transcript:Zm00001d042486_T001	rna3747	0
414- 32:	transcript:Zm00001d042487_T002	rna3746	2.00E-99
414- 33:	transcript:Zm00001d042492_T001	rna3745	3.00E-140
414- 34:	transcript:Zm00001d042493_T001	rna3744	1.00E-106
414- 35:	transcript:Zm00001d042494_T004	rna3743	0
414- 36:	transcript:Zm00001d042499_T001	rna3742	1.00E-77
414- 37:	transcript:Zm00001d042500_T001	rna3740	0
414- 38:	transcript:Zm00001d042503_T001	rna3738	3.00E-63
414- 39:	transcript:Zm00001d042504_T001	rna3736	0
414- 40:	transcript:Zm00001d042505_T005	rna3733	0
414- 41:	transcript:Zm00001d042506_T002	rna3732	2.00E-92
414- 42:	transcript:Zm00001d042507_T001	rna3731	2.00E-56
414- 43:	transcript:Zm00001d042508_T001	rna3730	3.00E-52
414- 44:	transcript:Zm00001d042512_T006	rna3728	1.00E-46
414- 45:	transcript:Zm00001d042520_T001	rna3727	0

Alignment 415: score=1936.0 e_value=3.7e-153 N=42 3&NC_008394.4 minus

415- 0:	transcript:Zm00001d039449_T001	rna637	0
415- 1:	transcript:Zm00001d039452_T002	rna634	4.00E-32
415- 2:	transcript:Zm00001d039453_T001	rna633	0
415- 3:	transcript:Zm00001d039454_T001	rna631	0
415- 4:	transcript:Zm00001d039455_T001	rna630	3.00E-82
415- 5:	transcript:Zm00001d039459_T001	rna627	3.00E-50
415- 6:	transcript:Zm00001d039460_T003	rna626	1.00E-116
415- 7:	transcript:Zm00001d039465_T001	rna624	1.00E-128
415- 8:	transcript:Zm00001d039467_T001	rna622	0
415- 9:	transcript:Zm00001d039468_T001	rna621	1.00E-51
415- 10:	transcript:Zm00001d039469_T001	rna620	0
415- 11:	transcript:Zm00001d039471_T001	rna619	0
415- 12:	transcript:Zm00001d039472_T001	rna618	1.00E-73
415- 13:	transcript:Zm00001d039475_T004	rna617	0
415- 14:	transcript:Zm00001d039477_T001	rna616	0
415- 15:	transcript:Zm00001d039481_T001	rna615	0
415- 16:	transcript:Zm00001d039480_T001	rna614	1.00E-37
415- 17:	transcript:Zm00001d039487_T001	rna612	0
415- 18:	transcript:Zm00001d039488_T002	rna611	0

```
415- 19:  transcript:Zm00001d039492_T001  rna609       1.00E-94
415- 20:  transcript:Zm00001d039495_T001  rna608      1.00E-175
415- 21:  transcript:Zm00001d039496_T001  rna607       2.00E-84
415- 22:  transcript:Zm00001d039498_T001  rna606              0
415- 23:  transcript:Zm00001d039499_T002  rna605              0
415- 24:  transcript:Zm00001d039505_T001  rna604              0
415- 25:  transcript:Zm00001d039506_T001  rna602              0
415- 26:  transcript:Zm00001d039510_T001  rna597      3.00E-155
415- 27:  transcript:Zm00001d039512_T001  rna596              0
415- 28:  transcript:Zm00001d039513_T001  rna595       2.00E-75
415- 29:  transcript:Zm00001d039514_T002  rna594              0
415- 30:  transcript:Zm00001d039520_T001  rna582      2.00E-109
415- 31:  transcript:Zm00001d039521_T002  rna581       2.00E-27
415- 32:  transcript:Zm00001d039522_T002  rna580              0
415- 33:  transcript:Zm00001d039524_T001  rna576       2.00E-98
415- 34:  transcript:Zm00001d039526_T002  rna575              0
415- 35:  transcript:Zm00001d039527_T001  rna574       5.00E-26
415- 36:  transcript:Zm00001d039530_T001  rna573              0
415- 37:  transcript:Zm00001d039531_T001  rna572       3.00E-45
415- 38:  transcript:Zm00001d039532_T001  rna571      4.00E-160
415- 39:  transcript:Zm00001d039533_T001  rna570       2.00E-53
415- 40:  transcript:Zm00001d039534_T001  rna569              0
415- 41:  transcript:Zm00001d039535_T001  rna567              0
## Alignment 416: score=1740.0 e_value=7.7e-140 N=37 3&NC_008394.4 minus
416-  0:  transcript:Zm00001d041954_T002  rna4247             0
416-  1:  transcript:Zm00001d041955_T001  rna4243     2.00E-161
416-  2:  transcript:Zm00001d041956_T002  rna4239      1.00E-86
416-  3:  transcript:Zm00001d041957_T004  rna4236             0
416-  4:  transcript:Zm00001d041958_T001  rna4235      2.00E-77
416-  5:  transcript:Zm00001d041959_T001  rna4233     1.00E-161
416-  6:  transcript:Zm00001d041960_T001  rna4230      9.00E-95
416-  7:  transcript:Zm00001d041961_T001  rna4229      1.00E-92
416-  8:  transcript:Zm00001d041962_T002  rna4228             0
416-  9:  transcript:Zm00001d041963_T001  rna4227      3.00E-91
416- 10:  transcript:Zm00001d041964_T001  rna4225             0
416- 11:  transcript:Zm00001d041965_T001  rna4224      7.00E-78
416- 12:  transcript:Zm00001d041968_T001  rna4222             0
416- 13:  transcript:Zm00001d041969_T001  rna4219      1.00E-63
416- 14:  transcript:Zm00001d041973_T001  rna4218     5.00E-135
416- 15:  transcript:Zm00001d041979_T002  rna4214      3.00E-38
416- 16:  transcript:Zm00001d041981_T001  rna4212      2.00E-81
416- 17:  transcript:Zm00001d041982_T001  rna4211             0
416- 18:  transcript:Zm00001d041983_T006  rna4210     6.00E-137
416- 19:  transcript:Zm00001d041984_T001  rna4206     6.00E-139
416- 20:  transcript:Zm00001d041988_T001  rna4205             0
416- 21:  transcript:Zm00001d041989_T001  rna4204             0
416- 22:  transcript:Zm00001d041990_T001  rna4199     2.00E-125
416- 23:  transcript:Zm00001d041991_T001  rna4194             0
416- 24:  transcript:Zm00001d041993_T002  rna4192      6.00E-75
416- 25:  transcript:Zm00001d041994_T002  rna4191             0
416- 26:  transcript:Zm00001d041995_T002  rna4190             0
416- 27:  transcript:Zm00001d041996_T001  rna4188             0
416- 28:  transcript:Zm00001d042005_T001  rna4182             0
416- 29:  transcript:Zm00001d042007_T001  rna4180             0
```

416- 30: transcript:Zm00001d042017_T002 rna4174 7.00E-75
416- 31: transcript:Zm00001d042018_T003 rna4173 1.00E-142
416- 32: transcript:Zm00001d042019_T002 rna4169 1.00E-117
416- 33: transcript:Zm00001d042023_T001 rna4168 3.00E-100
416- 34: transcript:Zm00001d042024_T001 rna4167 0
416- 35: transcript:Zm00001d042025_T001 rna4166 2.00E-57
416- 36: transcript:Zm00001d042027_T001 rna4163 3.00E-62
Alignment 417: score=1648.0 e_value=9.9e-126 N=36 3&NC_008394.4 minus
417- 0: transcript:Zm00001d039343_T001 rna769 3.00E-98
417- 1: transcript:Zm00001d039345_T001 rna768 0
417- 2: transcript:Zm00001d039347_T001 rna767 2.00E-28
417- 3: transcript:Zm00001d039348_T001 rna764 1.00E-65
417- 4: transcript:Zm00001d039349_T002 rna763 1.00E-81
417- 5: transcript:Zm00001d039351_T001 rna757 1.00E-24
417- 6: transcript:Zm00001d039352_T001 rna756 2.00E-113
417- 7: transcript:Zm00001d039354_T001 rna754 3.00E-96
417- 8: transcript:Zm00001d039355_T010 rna752 0
417- 9: transcript:Zm00001d039362_T002 rna749 0
417- 10: transcript:Zm00001d039363_T001 rna745 1.00E-143
417- 11: transcript:Zm00001d039366_T001 rna742 0
417- 12: transcript:Zm00001d039368_T003 rna731 0
417- 13: transcript:Zm00001d039369_T004 rna728 3.00E-07
417- 14: transcript:Zm00001d039370_T002 rna727 6.00E-111
417- 15: transcript:Zm00001d039371_T001 rna726 0
417- 16: transcript:Zm00001d039374_T001 rna724 3.00E-46
417- 17: transcript:Zm00001d039375_T001 rna722 9.00E-18
417- 18: transcript:Zm00001d039378_T001 rna721 1.00E-08
417- 19: transcript:Zm00001d039379_T001 rna720 2.00E-30
417- 20: transcript:Zm00001d039380_T001 rna719 0
417- 21: transcript:Zm00001d039383_T001 rna717 1.00E-108
417- 22: transcript:Zm00001d039384_T001 rna713 0
417- 23: transcript:Zm00001d039385_T003 rna712 1.00E-106
417- 24: transcript:Zm00001d039386_T001 rna711 0
417- 25: transcript:Zm00001d039387_T002 rna709 1.00E-127
417- 26: transcript:Zm00001d039390_T001 rna708 0
417- 27: transcript:Zm00001d039391_T001 rna707 8.00E-120
417- 28: transcript:Zm00001d039392_T001 rna706 0
417- 29: transcript:Zm00001d039394_T001 rna705 2.00E-131
417- 30: transcript:Zm00001d039400_T002 rna704 3.00E-81
417- 31: transcript:Zm00001d039401_T001 rna703 6.00E-172
417- 32: transcript:Zm00001d039403_T003 rna700 0
417- 33: transcript:Zm00001d039406_T002 rna686 0
417- 34: transcript:Zm00001d039407_T001 rna680 3.00E-154
417- 35: transcript:Zm00001d039408_T001 rna679 3.00E-80
Alignment 418: score=1586.0 e_value=2.8e-110 N=34 3&NC_008394.4 minus
418- 0: transcript:Zm00001d043348_T020 rna2891 0
418- 1: transcript:Zm00001d043350_T001 rna2890 0
418- 2: transcript:Zm00001d043352_T002 rna2889 1.00E-88
418- 3: transcript:Zm00001d043353_T001 rna2888 1.00E-51
418- 4: transcript:Zm00001d043356_T004 rna2886 1.00E-159
418- 5: transcript:Zm00001d043358_T001 rna2885 2.00E-146
418- 6: transcript:Zm00001d043361_T001 rna2882 3.00E-62
418- 7: transcript:Zm00001d043365_T001 rna2880 0
418- 8: transcript:Zm00001d043366_T001 rna2879 2.00E-17

418- 9: transcript:Zm00001d043367_T001 rna2877 2.00E-61
418- 10: transcript:Zm00001d043368_T001 rna2876 1.00E-108
418- 11: transcript:Zm00001d043370_T002 rna2874 3.00E-113
418- 12: transcript:Zm00001d043371_T001 rna2873 4.00E-43
418- 13: transcript:Zm00001d043374_T001 rna2865 4.00E-149
418- 14: transcript:Zm00001d043376_T006 rna2864 0
418- 15: transcript:Zm00001d043378_T001 rna2863 0
418- 16: transcript:Zm00001d043382_T001 rna2859 0
418- 17: transcript:Zm00001d043383_T001 rna2857 0
418- 18: transcript:Zm00001d043386_T001 rna2855 1.00E-24
418- 19: transcript:Zm00001d043387_T002 rna2853 2.00E-114
418- 20: transcript:Zm00001d043389_T003 rna2852 0
418- 21: transcript:Zm00001d043391_T001 rna2849 8.00E-53
418- 22: transcript:Zm00001d043395_T002 rna2848 0
418- 23: transcript:Zm00001d043399_T001 rna2845 1.00E-23
418- 24: transcript:Zm00001d043401_T002 rna2844 0
418- 25: transcript:Zm00001d043402_T001 rna2842 1.00E-52
418- 26: transcript:Zm00001d043403_T001 rna2841 6.00E-79
418- 27: transcript:Zm00001d043404_T001 rna2839 0
418- 28: transcript:Zm00001d043405_T001 rna2838 3.00E-99
418- 29: transcript:Zm00001d043406_T002 rna2837 4.00E-90
418- 30: transcript:Zm00001d043407_T001 rna2836 6.00E-120
418- 31: transcript:Zm00001d043410_T002 rna2835 0
418- 32: transcript:Zm00001d043411_T001 rna2834 0
418- 33: transcript:Zm00001d043414_T001 rna2831 0
Alignment 419: score=1582.0 e_value=8.2e-120 N=35 3&NC_008394.4 minus
419- 0: transcript:Zm00001d039623_T001 rna518 0
419- 1: transcript:Zm00001d039624_T001 rna516 1.00E-104
419- 2: transcript:Zm00001d039625_T001 rna515 2.00E-108
419- 3: transcript:Zm00001d039626_T002 rna513 5.00E-165
419- 4: transcript:Zm00001d039628_T001 rna511 1.00E-133
419- 5: transcript:Zm00001d039631_T002 rna510 0
419- 6: transcript:Zm00001d039634_T001 rna509 0
419- 7: transcript:Zm00001d039636_T001 rna506 0
419- 8: transcript:Zm00001d039637_T001 rna505 5.00E-103
419- 9: transcript:Zm00001d039638_T001 rna504 0
419- 10: transcript:Zm00001d039639_T001 rna503 1.00E-78
419- 11: transcript:Zm00001d039642_T001 rna498 3.00E-96
419- 12: transcript:Zm00001d039647_T001 rna497 0
419- 13: transcript:Zm00001d039648_T001 rna492 6.00E-44
419- 14: transcript:Zm00001d039653_T005 rna491 7.00E-157
419- 15: transcript:Zm00001d039654_T001 rna489 0
419- 16: transcript:Zm00001d039656_T003 rna485 0
419- 17: transcript:Zm00001d039657_T001 rna483 5.00E-38
419- 18: transcript:Zm00001d039658_T001 rna482 2.00E-54
419- 19: transcript:Zm00001d039661_T001 rna480 3.00E-36
419- 20: transcript:Zm00001d039663_T005 rna479 2.00E-47
419- 21: transcript:Zm00001d039667_T001 rna478 3.00E-93
419- 22: transcript:Zm00001d039670_T007 rna474 0
419- 23: transcript:Zm00001d039673_T002 rna472 0
419- 24: transcript:Zm00001d039674_T001 rna471 0
419- 25: transcript:Zm00001d039675_T002 rna470 2.00E-101
419- 26: transcript:Zm00001d039677_T004 rna469 6.00E-121
419- 27: transcript:Zm00001d039678_T002 rna467 1.00E-86

419- 28: transcript:Zm00001d039679_T001 rna465 2.00E-173
419- 29: transcript:Zm00001d039682_T003 rna462 0
419- 30: transcript:Zm00001d039683_T009 rna459 3.00E-52
419- 31: transcript:Zm00001d039685_T001 rna458 0
419- 32: transcript:Zm00001d039686_T001 rna457 0
419- 33: transcript:Zm00001d039691_T001 rna456 6.00E-117
419- 34: transcript:Zm00001d039693_T013 rna455 0
Alignment 420: score=1316.0 e_value=1.3e-96 N=29 3&NC_008394.4 minus
420- 0: transcript:Zm00001d043565_T001 rna2676 4.00E-68
420- 1: transcript:Zm00001d043566_T005 rna2675 0
420- 2: transcript:Zm00001d043569_T001 rna2673 5.00E-95
420- 3: transcript:Zm00001d043570_T001 rna2672 9.00E-16
420- 4: transcript:Zm00001d043571_T001 rna2671 2.00E-171
420- 5: transcript:Zm00001d043573_T001 rna2666 0
420- 6: transcript:Zm00001d043574_T001 rna2664 0
420- 7: transcript:Zm00001d043578_T008 rna2653 1.00E-100
420- 8: transcript:Zm00001d043579_T001 rna2652 9.00E-39
420- 9: transcript:Zm00001d043580_T001 rna2650 8.00E-82
420- 10: transcript:Zm00001d043581_T001 rna2649 0
420- 11: transcript:Zm00001d043586_T001 rna2646 2.00E-10
420- 12: transcript:Zm00001d043587_T001 rna2644 0
420- 13: transcript:Zm00001d043588_T001 rna2642 7.00E-81
420- 14: transcript:Zm00001d043589_T002 rna2641 5.00E-102
420- 15: transcript:Zm00001d043590_T001 rna2638 9.00E-16
420- 16: transcript:Zm00001d043592_T004 rna2637 0
420- 17: transcript:Zm00001d043595_T001 rna2636 0
420- 18: transcript:Zm00001d043596_T001 rna2633 1.00E-74
420- 19: transcript:Zm00001d043598_T003 rna2631 0
420- 20: transcript:Zm00001d043599_T001 rna2629 0
420- 21: transcript:Zm00001d043600_T001 rna2627 3.00E-136
420- 22: transcript:Zm00001d043601_T003 rna2625 0
420- 23: transcript:Zm00001d043606_T001 rna2624 1.00E-86
420- 24: transcript:Zm00001d043607_T001 rna2620 6.00E-140
420- 25: transcript:Zm00001d043611_T001 rna2618 1.00E-99
420- 26: transcript:Zm00001d043622_T001 rna2608 3.00E-118
420- 27: transcript:Zm00001d043634_T001 rna2590 0
420- 28: transcript:Zm00001d043639_T001 rna2583 3.00E-20
Alignment 421: score=1080.0 e_value=4.2e-79 N=24 3&NC_008394.4 minus
421- 0: transcript:Zm00001d042087_T001 rna4091 0
421- 1: transcript:Zm00001d042088_T001 rna4089 0
421- 2: transcript:Zm00001d042091_T002 rna4087 0
421- 3: transcript:Zm00001d042093_T001 rna4085 3.00E-77
421- 4: transcript:Zm00001d042094_T001 rna4084 5.00E-61
421- 5: transcript:Zm00001d042095_T001 rna4079 7.00E-93
421- 6: transcript:Zm00001d042098_T001 rna4077 8.00E-89
421- 7: transcript:Zm00001d042107_T001 rna4074 4.00E-17
421- 8: transcript:Zm00001d042111_T001 rna4072 0
421- 9: transcript:Zm00001d042116_T001 rna4070 0
421- 10: transcript:Zm00001d042117_T002 rna4069 1.00E-169
421- 11: transcript:Zm00001d042118_T001 rna4068 1.00E-79
421- 12: transcript:Zm00001d042127_T001 rna4067 0
421- 13: transcript:Zm00001d042128_T005 rna4066 0
421- 14: transcript:Zm00001d042132_T001 rna4054 8.00E-173
421- 15: transcript:Zm00001d042133_T003 rna4053 9.00E-169

421- 16: transcript:Zm00001d042134_T001 rna4048 3.00E-57
421- 17: transcript:Zm00001d042138_T001 rna4028 3.00E-104
421- 18: transcript:Zm00001d042140_T001 rna4027 3.00E-89
421- 19: transcript:Zm00001d042144_T001 rna4026 7.00E-38
421- 20: transcript:Zm00001d042146_T002 rna4025 0
421- 21: transcript:Zm00001d042148_T001 rna4024 0
421- 22: transcript:Zm00001d042150_T001 rna4022 5.00E-70
421- 23: transcript:Zm00001d042152_T005 rna4021 0
Alignment 422: score=1035.0 e_value=3.8e-69 N=23 3&NC_008394.4 minus
422- 0: transcript:Zm00001d042030_T001 rna4157 9.00E-12
422- 1: transcript:Zm00001d042034_T002 rna4151 0
422- 2: transcript:Zm00001d042039_T001 rna4143 0
422- 3: transcript:Zm00001d042042_T001 rna4136 2.00E-101
422- 4: transcript:Zm00001d042043_T001 rna4135 0
422- 5: transcript:Zm00001d042044_T001 rna4133 0
422- 6: transcript:Zm00001d042045_T001 rna4131 1.00E-153
422- 7: transcript:Zm00001d042047_T001 rna4129 3.00E-87
422- 8: transcript:Zm00001d042050_T001 rna4128 2.00E-165
422- 9: transcript:Zm00001d042051_T001 rna4127 0
422- 10: transcript:Zm00001d042052_T009 rna4126 0
422- 11: transcript:Zm00001d042054_T001 rna4119 1.00E-151
422- 12: transcript:Zm00001d042055_T001 rna4118 1.00E-27
422- 13: transcript:Zm00001d042056_T001 rna4116 5.00E-53
422- 14: transcript:Zm00001d042057_T001 rna4115 0
422- 15: transcript:Zm00001d042060_T001 rna4107 4.00E-68
422- 16: transcript:Zm00001d042061_T001 rna4106 7.00E-116
422- 17: transcript:Zm00001d042063_T001 rna4101 3.00E-67
422- 18: transcript:Zm00001d042064_T010 rna4099 0
422- 19: transcript:Zm00001d042066_T016 rna4098 0
422- 20: transcript:Zm00001d042074_T001 rna4096 0
422- 21: transcript:Zm00001d042078_T001 rna4095 1.00E-68
422- 22: transcript:Zm00001d042082_T001 rna4093 3.00E-92
Alignment 423: score=1016.0 e_value=1.6e-64 N=22 3&NC_008394.4 minus
423- 0: transcript:Zm00001d043984_T001 rna2259 2.00E-52
423- 1: transcript:Zm00001d043985_T001 rna2257 6.00E-70
423- 2: transcript:Zm00001d043986_T005 rna2253 2.00E-153
423- 3: transcript:Zm00001d043988_T001 rna2252 6.00E-155
423- 4: transcript:Zm00001d043989_T001 rna2249 0
423- 5: transcript:Zm00001d043990_T001 rna2248 3.00E-136
423- 6: transcript:Zm00001d043991_T001 rna2246 2.00E-150
423- 7: transcript:Zm00001d043992_T001 rna2245 2.00E-152
423- 8: transcript:Zm00001d043993_T003 rna2244 0
423- 9: transcript:Zm00001d043998_T001 rna2242 2.00E-103
423- 10: transcript:Zm00001d044004_T001 rna2240 1.00E-131
423- 11: transcript:Zm00001d044005_T001 rna2237 1.00E-21
423- 12: transcript:Zm00001d044008_T010 rna2236 0
423- 13: transcript:Zm00001d044010_T001 rna2235 5.00E-71
423- 14: transcript:Zm00001d044015_T001 rna2234 4.00E-161
423- 15: transcript:Zm00001d044016_T001 rna2233 0
423- 16: transcript:Zm00001d044017_T001 rna2232 0
423- 17: transcript:Zm00001d044019_T004 rna2231 0
423- 18: transcript:Zm00001d044020_T001 rna2230 2.00E-28
423- 19: transcript:Zm00001d044021_T001 rna2228 0
423- 20: transcript:Zm00001d044022_T001 rna2227 5.00E-32

423- 21: transcript:Zm00001d044023_T017 rna2225 2.00E-149
Alignment 424: score=935.0 e_value=2.7e-65 N=21 3&NC_008394.4 minus
424- 0: transcript:Zm00001d039732_T001 rna431 2.00E-98
424- 1: transcript:Zm00001d039740_T001 rna423 1.00E-35
424- 2: transcript:Zm00001d039745_T001 rna422 0
424- 3: transcript:Zm00001d039746_T001 rna421 0
424- 4: transcript:Zm00001d039747_T002 rna420 2.00E-109
424- 5: transcript:Zm00001d039754_T001 rna406 0
424- 6: transcript:Zm00001d039764_T001 rna404 8.00E-105
424- 7: transcript:Zm00001d039768_T003 rna401 0
424- 8: transcript:Zm00001d039769_T002 rna400 7.00E-168
424- 9: transcript:Zm00001d039771_T001 rna398 1.00E-110
424- 10: transcript:Zm00001d039772_T001 rna393 0
424- 11: transcript:Zm00001d039776_T001 rna390 7.00E-110
424- 12: transcript:Zm00001d039780_T001 rna389 1.00E-65
424- 13: transcript:Zm00001d039783_T001 rna387 7.00E-34
424- 14: transcript:Zm00001d039785_T001 rna384 1.00E-108
424- 15: transcript:Zm00001d039787_T001 rna380 0
424- 16: transcript:Zm00001d039795_T001 rna373 1.00E-74
424- 17: transcript:Zm00001d039796_T001 rna371 5.00E-69
424- 18: transcript:Zm00001d039808_T001 rna368 0
424- 19: transcript:Zm00001d039821_T001 rna359 8.00E-67
424- 20: transcript:Zm00001d039822_T001 rna358 0
Alignment 425: score=891.0 e_value=8.8e-58 N=20 3&NC_008394.4 minus
425- 0: transcript:Zm00001d040320_T004 rna1075 2.00E-148
425- 1: transcript:Zm00001d040322_T002 rna1067 7.00E-162
425- 2: transcript:Zm00001d040323_T001 rna1049 7.00E-77
425- 3: transcript:Zm00001d040324_T001 rna1048 1.00E-113
425- 4: transcript:Zm00001d040331_T001 rna1047 0
425- 5: transcript:Zm00001d040333_T001 rna1045 1.00E-117
425- 6: transcript:Zm00001d040334_T001 rna1044 0
425- 7: transcript:Zm00001d040340_T005 rna1041 0
425- 8: transcript:Zm00001d040341_T001 rna1040 6.00E-116
425- 9: transcript:Zm00001d040344_T001 rna1032 3.00E-165
425- 10: transcript:Zm00001d040348_T002 rna1031 3.00E-101
425- 11: transcript:Zm00001d040351_T005 rna1028 0
425- 12: transcript:Zm00001d040356_T001 rna1026 0
425- 13: transcript:Zm00001d040360_T001 rna1024 0
425- 14: transcript:Zm00001d040362_T001 rna1019 0
425- 15: transcript:Zm00001d040364_T001 rna1015 2.00E-134
425- 16: transcript:Zm00001d040365_T001 rna1014 6.00E-25
425- 17: transcript:Zm00001d040371_T001 rna1013 0
425- 18: transcript:Zm00001d040372_T002 rna1008 2.00E-62
425- 19: transcript:Zm00001d040380_T001 rna997 4.00E-34
Alignment 426: score=857.0 e_value=1.4e-53 N=19 3&NC_008394.4 minus
426- 0: transcript:Zm00001d043502_T001 rna2759 0
426- 1: transcript:Zm00001d043503_T001 rna2757 7.00E-150
426- 2: transcript:Zm00001d043504_T030 rna2755 0
426- 3: transcript:Zm00001d043505_T001 rna2754 7.00E-85
426- 4: transcript:Zm00001d043506_T004 rna2753 2.00E-141
426- 5: transcript:Zm00001d043508_T002 rna2751 0
426- 6: transcript:Zm00001d043509_T002 rna2748 0
426- 7: transcript:Zm00001d043510_T002 rna2747 7.00E-102
426- 8: transcript:Zm00001d043511_T001 rna2746 0

426- 9: transcript:Zm00001d043512_T001 rna2745 0
426- 10: transcript:Zm00001d043514_T001 rna2743 0
426- 11: transcript:Zm00001d043515_T002 rna2739 9.00E-119
426- 12: transcript:Zm00001d043516_T001 rna2737 0
426- 13: transcript:Zm00001d043517_T002 rna2733 0
426- 14: transcript:Zm00001d043520_T001 rna2732 5.00E-130
426- 15: transcript:Zm00001d043524_T001 rna2729 0
426- 16: transcript:Zm00001d043525_T001 rna2728 1.00E-61
426- 17: transcript:Zm00001d043526_T004 rna2727 1.00E-101
426- 18: transcript:Zm00001d043527_T002 rna2725 5.00E-180
Alignment 427: score=710.0 e_value=3.3e-39 N=15 3&NC_008394.4 minus
427- 0: transcript:Zm00001d039694_T001 rna454 6.00E-130
427- 1: transcript:Zm00001d039701_T001 rna453 6.00E-100
427- 2: transcript:Zm00001d039702_T001 rna451 0
427- 3: transcript:Zm00001d039703_T001 rna450 0
427- 4: transcript:Zm00001d039706_T001 rna449 0
427- 5: transcript:Zm00001d039709_T001 rna448 1.00E-164
427- 6: transcript:Zm00001d039710_T004 rna447 1.00E-77
427- 7: transcript:Zm00001d039711_T001 rna444 4.00E-88
427- 8: transcript:Zm00001d039714_T001 rna442 0
427- 9: transcript:Zm00001d039717_T005 rna440 0
427- 10: transcript:Zm00001d039718_T001 rna437 0
427- 11: transcript:Zm00001d039719_T002 rna436 1.00E-78
427- 12: transcript:Zm00001d039726_T001 rna434 7.00E-40
427- 13: transcript:Zm00001d039727_T001 rna433 2.00E-19
427- 14: transcript:Zm00001d039728_T001 rna431 4.00E-27
Alignment 428: score=628.0 e_value=3.6e-32 N=13 3&NC_008394.4 minus
428- 0: transcript:Zm00001d040171_T001 rna49 2.00E-20
428- 1: transcript:Zm00001d040172_T001 rna48 4.00E-17
428- 2: transcript:Zm00001d040173_T001 rna46 3.00E-145
428- 3: transcript:Zm00001d040178_T001 rna45 7.00E-117
428- 4: transcript:Zm00001d040183_T001 rna44 0
428- 5: transcript:Zm00001d040185_T002 rna43 5.00E-75
428- 6: transcript:Zm00001d040186_T001 rna42 2.00E-76
428- 7: transcript:Zm00001d040188_T001 rna38 0
428- 8: transcript:Zm00001d040189_T001 rna35 2.00E-75
428- 9: transcript:Zm00001d040190_T001 rna34 4.00E-112
428- 10: transcript:Zm00001d040191_T002 rna32 2.00E-161
428- 11: transcript:Zm00001d040192_T001 rna31 0
428- 12: transcript:Zm00001d040193_T005 rna28 1.00E-157
Alignment 429: score=610.0 e_value=1.2e-30 N=13 3&NC_008394.4 minus
429- 0: transcript:Zm00001d043650_T001 rna2585 0
429- 1: transcript:Zm00001d043652_T003 rna2584 0
429- 2: transcript:Zm00001d043653_T003 rna2583 0
429- 3: transcript:Zm00001d043654_T001 rna2582 8.00E-154
429- 4: transcript:Zm00001d043655_T002 rna2579 0
429- 5: transcript:Zm00001d043656_T001 rna2576 0
429- 6: transcript:Zm00001d043660_T001 rna2575 2.00E-176
429- 7: transcript:Zm00001d043661_T001 rna2574 5.00E-110
429- 8: transcript:Zm00001d043662_T001 rna2573 0
429- 9: transcript:Zm00001d043663_T001 rna2569 1.00E-74
429- 10: transcript:Zm00001d043665_T001 rna2568 2.00E-41
429- 11: transcript:Zm00001d043666_T001 rna2567 0
429- 12: transcript:Zm00001d043667_T003 rna2566 0

```
## Alignment 430: score=547.0 e_value=2.4e-27 N=12 3&NC_008394.4 minus
430-  0:  transcript:Zm00001d039589_T136  rna544              0
430-  1:  transcript:Zm00001d039590_T001  rna542      2.00E-165
430-  2:  transcript:Zm00001d039591_T001  rna541       1.00E-45
430-  3:  transcript:Zm00001d039594_T001  rna536              0
430-  4:  transcript:Zm00001d039595_T002  rna533       5.00E-46
430-  5:  transcript:Zm00001d039596_T004  rna532              0
430-  6:  transcript:Zm00001d039597_T001  rna531       1.00E-31
430-  7:  transcript:Zm00001d039598_T001  rna530              0
430-  8:  transcript:Zm00001d039600_T001  rna529      5.00E-176
430-  9:  transcript:Zm00001d039606_T002  rna527              0
430- 10:  transcript:Zm00001d039607_T001  rna525              0
430- 11:  transcript:Zm00001d039608_T007  rna524              0
## Alignment 431: score=536.0 e_value=1.5e-30 N=13 3&NC_008394.4 minus
431-  0:  transcript:Zm00001d044189_T001  rna2071             0
431-  1:  transcript:Zm00001d044191_T004  rna2067      3.00E-41
431-  2:  transcript:Zm00001d044192_T001  rna2066             0
431-  3:  transcript:Zm00001d044193_T002  rna2061             0
431-  4:  transcript:Zm00001d044195_T018  rna2058             0
431-  5:  transcript:Zm00001d044201_T005  rna2056             0
431-  6:  transcript:Zm00001d044202_T001  rna2055     1.00E-146
431-  7:  transcript:Zm00001d044208_T001  rna2053             0
431-  8:  transcript:Zm00001d044212_T001  rna2050             0
431-  9:  transcript:Zm00001d044217_T001  rna2048     7.00E-133
431- 10:  transcript:Zm00001d044219_T004  rna2047      2.00E-76
431- 11:  transcript:Zm00001d044221_T001  rna2046      7.00E-19
431- 12:  transcript:Zm00001d044222_T032  rna2044             0
## Alignment 432: score=480.0 e_value=4.8e-25 N=11 3&NC_008394.4 minus
432-  0:  transcript:Zm00001d040196_T001  rna27       1.00E-108
432-  1:  transcript:Zm00001d040201_T001  rna23               0
432-  2:  transcript:Zm00001d040202_T002  rna21       5.00E-149
432-  3:  transcript:Zm00001d040203_T003  rna19               0
432-  4:  transcript:Zm00001d040204_T001  rna16               0
432-  5:  transcript:Zm00001d040205_T001  rna13               0
432-  6:  transcript:Zm00001d040213_T002  rna12       3.00E-146
432-  7:  transcript:Zm00001d040215_T001  rna11        1.00E-54
432-  8:  transcript:Zm00001d040218_T016  rna10               0
432-  9:  transcript:Zm00001d040220_T002  rna8        1.00E-122
432- 10:  transcript:Zm00001d040234_T001  rna0        1.00E-173
## Alignment 433: score=447.0 e_value=6.8e-20 N=10 3&NC_008394.4 minus
433-  0:  transcript:Zm00001d040144_T002  rna66        1.00E-93
433-  1:  transcript:Zm00001d040147_T001  rna64       2.00E-112
433-  2:  transcript:Zm00001d040148_T001  rna60        5.00E-38
433-  3:  transcript:Zm00001d040154_T001  rna58               0
433-  4:  transcript:Zm00001d040155_T003  rna57       3.00E-172
433-  5:  transcript:Zm00001d040160_T001  rna54        2.00E-09
433-  6:  transcript:Zm00001d040161_T002  rna53       2.00E-153
433-  7:  transcript:Zm00001d040163_T001  rna52               0
433-  8:  transcript:Zm00001d040164_T002  rna51        9.00E-83
433-  9:  transcript:Zm00001d040166_T014  rna50               0
## Alignment 434: score=433.0 e_value=8.6e-21 N=10 3&NC_008394.4 minus
434-  0:  transcript:Zm00001d040056_T005  rna165              0
434-  1:  transcript:Zm00001d040057_T001  rna162       3.00E-65
434-  2:  transcript:Zm00001d040059_T002  rna153              0
```

434- 3: transcript:Zm00001d040060_T001 rna151 1.00E-126
434- 4: transcript:Zm00001d040071_T001 rna149 8.00E-175
434- 5: transcript:Zm00001d040076_T008 rna148 7.00E-163
434- 6: transcript:Zm00001d040082_T003 rna147 2.00E-39
434- 7: transcript:Zm00001d040084_T001 rna146 0
434- 8: transcript:Zm00001d040089_T001 rna145 0
434- 9: transcript:Zm00001d040094_T001 rna134 0
Alignment 435: score=426.0 e_value=7e-19 N=9 3&NC_008394.4 minus
435- 0: transcript:Zm00001d039565_T001 rna562 2.00E-43
435- 1: transcript:Zm00001d039566_T001 rna558 3.00E-73
435- 2: transcript:Zm00001d039568_T001 rna557 0
435- 3: transcript:Zm00001d039576_T001 rna556 4.00E-154
435- 4: transcript:Zm00001d039579_T001 rna555 2.00E-168
435- 5: transcript:Zm00001d039580_T001 rna554 2.00E-49
435- 6: transcript:Zm00001d039581_T001 rna551 1.00E-49
435- 7: transcript:Zm00001d039582_T002 rna550 0
435- 8: transcript:Zm00001d039584_T001 rna545 2.00E-63
Alignment 436: score=424.0 e_value=1.2e-19 N=9 3&NC_008394.4 minus
436- 0: transcript:Zm00001d042525_T003 rna3724 5.00E-78
436- 1: transcript:Zm00001d042526_T001 rna3723 0
436- 2: transcript:Zm00001d042527_T001 rna3722 1.00E-85
436- 3: transcript:Zm00001d042528_T007 rna3721 0
436- 4: transcript:Zm00001d042530_T003 rna3715 0
436- 5: transcript:Zm00001d042533_T002 rna3713 4.00E-94
436- 6: transcript:Zm00001d042534_T002 rna3711 7.00E-93
436- 7: transcript:Zm00001d042535_T001 rna3710 2.00E-74
436- 8: transcript:Zm00001d042536_T001 rna3709 0
Alignment 437: score=420.0 e_value=5.4e-20 N=9 3&NC_008394.4 minus
437- 0: transcript:Zm00001d041935_T001 rna4271 1.00E-60
437- 1: transcript:Zm00001d041941_T001 rna4268 0
437- 2: transcript:Zm00001d041944_T001 rna4264 0
437- 3: transcript:Zm00001d041947_T001 rna4260 0
437- 4: transcript:Zm00001d041948_T002 rna4259 0
437- 5: transcript:Zm00001d041949_T001 rna4253 2.00E-29
437- 6: transcript:Zm00001d041950_T001 rna4252 0
437- 7: transcript:Zm00001d041951_T001 rna4251 0
437- 8: transcript:Zm00001d041953_T010 rna4250 0
Alignment 438: score=397.0 e_value=1.1e-18 N=9 3&NC_008394.4 minus
438- 0: transcript:Zm00001d040379_T001 rna1080 3.00E-129
438- 1: transcript:Zm00001d040389_T002 rna1072 1.00E-07
438- 2: transcript:Zm00001d040398_T001 rna1064 0
438- 3: transcript:Zm00001d040399_T001 rna1063 0
438- 4: transcript:Zm00001d040408_T003 rna1057 2.00E-170
438- 5: transcript:Zm00001d040409_T001 rna1055 3.00E-128
438- 6: transcript:Zm00001d040411_T001 rna1054 2.00E-168
438- 7: transcript:Zm00001d040416_T004 rna1052 0
438- 8: transcript:Zm00001d040422_T007 rna1051 0
Alignment 439: score=386.0 e_value=2.4e-14 N=8 3&NC_008394.4 minus
439- 0: transcript:Zm00001d043418_T136 rna2830 2.00E-35
439- 1: transcript:Zm00001d043419_T001 rna2829 1.00E-16
439- 2: transcript:Zm00001d043420_T001 rna2828 2.00E-118
439- 3: transcript:Zm00001d043422_T001 rna2827 2.00E-74
439- 4: transcript:Zm00001d043423_T001 rna2825 6.00E-166
439- 5: transcript:Zm00001d043425_T003 rna2824 0

439- 6: transcript:Zm00001d043426_T001 rna2822 0
439- 7: transcript:Zm00001d043428_T005 rna2819 0
Alignment 440: score=316.0 e_value=6.9e-13 N=7 3&NC_008394.4 minus
440- 0: transcript:Zm00001d044495_T005 rna1781 0
440- 1: transcript:Zm00001d044496_T013 rna1780 0
440- 2: transcript:Zm00001d044497_T002 rna1776 6.00E-145
440- 3: transcript:Zm00001d044498_T003 rna1775 1.00E-32
440- 4: transcript:Zm00001d044500_T001 rna1771 0
440- 5: transcript:Zm00001d044502_T001 rna1770 2.00E-107
440- 6: transcript:Zm00001d044503_T004 rna1769 0
Alignment 441: score=314.0 e_value=1e-14 N=7 3&NC_008394.4 minus
441- 0: transcript:Zm00001d040094_T001 rna98 0
441- 1: transcript:Zm00001d040108_T001 rna91 2.00E-118
441- 2: transcript:Zm00001d040109_T001 rna88 2.00E-153
441- 3: transcript:Zm00001d040111_T001 rna86 5.00E-12
441- 4: transcript:Zm00001d040112_T001 rna85 5.00E-18
441- 5: transcript:Zm00001d040113_T003 rna83 4.00E-96
441- 6: transcript:Zm00001d040123_T007 rna68 0
Alignment 442: score=279.0 e_value=6e-11 N=6 3&NC_008394.4 minus
442- 0: transcript:Zm00001d044534_T001 rna1718 0
442- 1: transcript:Zm00001d044535_T001 rna1711 5.00E-62
442- 2: transcript:Zm00001d044536_T001 rna1710 2.00E-71
442- 3: transcript:Zm00001d044537_T001 rna1709 0
442- 4: transcript:Zm00001d044546_T001 rna1701 4.00E-31
442- 5: transcript:Zm00001d044547_T003 rna1699 3.00E-43
Alignment 443: score=337.0 e_value=3.4e-16 N=8 3&NC_008396.2 plus
443- 0: transcript:Zm00001d042922_T007 rna8850 0
443- 1: transcript:Zm00001d042933_T001 rna8874 1.00E-23
443- 2: transcript:Zm00001d042940_T001 rna8879 2.00E-26
443- 3: transcript:Zm00001d042943_T005 rna8882 4.00E-147
443- 4: transcript:Zm00001d042948_T001 rna8884 0
443- 5: transcript:Zm00001d042949_T001 rna8886 2.00E-06
443- 6: transcript:Zm00001d042953_T001 rna8892 0
443- 7: transcript:Zm00001d042962_T002 rna8906 5.00E-82
Alignment 444: score=1425.0 e_value=2.3e-108 N=33 3&NC_008398.2 plus
444- 0: transcript:Zm00001d043853_T001 rna16230 4.00E-23
444- 1: transcript:Zm00001d043854_T001 rna16231 0
444- 2: transcript:Zm00001d043855_T001 rna16232 7.00E-66
444- 3: transcript:Zm00001d043858_T001 rna16234 1.00E-150
444- 4: transcript:Zm00001d043864_T001 rna16237 0
444- 5: transcript:Zm00001d043870_T001 rna16240 2.00E-107
444- 6: transcript:Zm00001d043873_T001 rna16249 0
444- 7: transcript:Zm00001d043874_T001 rna16250 7.00E-39
444- 8: transcript:Zm00001d043878_T001 rna16251 1.00E-94
444- 9: transcript:Zm00001d043879_T002 rna16252 0
444- 10: transcript:Zm00001d043889_T001 rna16257 0
444- 11: transcript:Zm00001d043890_T002 rna16259 2.00E-180
444- 12: transcript:Zm00001d043895_T003 rna16260 3.00E-173
444- 13: transcript:Zm00001d043898_T001 rna16261 1.00E-15
444- 14: transcript:Zm00001d043902_T001 rna16262 1.00E-95
444- 15: transcript:Zm00001d043906_T002 rna16266 9.00E-81
444- 16: transcript:Zm00001d043911_T001 rna16270 4.00E-115
444- 17: transcript:Zm00001d043914_T001 rna16272 2.00E-170
444- 18: transcript:Zm00001d043921_T001 rna16274 5.00E-145

444- 19: transcript:Zm00001d043922_T001 rna16277 0
444- 20: transcript:Zm00001d043929_T001 rna16279 3.00E-83
444- 21: transcript:Zm00001d043935_T001 rna16287 8.00E-127
444- 22: transcript:Zm00001d043937_T001 rna16288 2.00E-15
444- 23: transcript:Zm00001d043942_T001 rna16290 3.00E-129
444- 24: transcript:Zm00001d043943_T001 rna16291 6.00E-89
444- 25: transcript:Zm00001d043950_T001 rna16297 4.00E-49
444- 26: transcript:Zm00001d043953_T001 rna16298 8.00E-28
444- 27: transcript:Zm00001d043954_T003 rna16300 0
444- 28: transcript:Zm00001d043955_T001 rna16301 0
444- 29: transcript:Zm00001d043962_T001 rna16305 0
444- 30: transcript:Zm00001d043971_T001 rna16313 4.00E-87
444- 31: transcript:Zm00001d043972_T001 rna16315 1.00E-59
444- 32: transcript:Zm00001d043974_T001 rna16316 7.00E-51
Alignment 445: score=1069.0 e_value=3.7e-73 N=24 3&NC_008398.2 plus
445- 0: transcript:Zm00001d042973_T003 rna15678 2.00E-48
445- 1: transcript:Zm00001d042977_T001 rna15697 7.00E-19
445- 2: transcript:Zm00001d042979_T007 rna15698 0
445- 3: transcript:Zm00001d042993_T001 rna15717 0
445- 4: transcript:Zm00001d043001_T001 rna15722 3.00E-27
445- 5: transcript:Zm00001d043006_T001 rna15724 1.00E-95
445- 6: transcript:Zm00001d043012_T018 rna15727 0
445- 7: transcript:Zm00001d043014_T001 rna15731 8.00E-90
445- 8: transcript:Zm00001d043015_T001 rna15734 0
445- 9: transcript:Zm00001d043018_T001 rna15736 3.00E-110
445- 10: transcript:Zm00001d043022_T001 rna15737 2.00E-27
445- 11: transcript:Zm00001d043024_T001 rna15739 1.00E-50
445- 12: transcript:Zm00001d043025_T001 rna15740 1.00E-147
445- 13: transcript:Zm00001d043026_T001 rna15741 6.00E-122
445- 14: transcript:Zm00001d043029_T001 rna15742 0
445- 15: transcript:Zm00001d043031_T001 rna15745 0
445- 16: transcript:Zm00001d043037_T001 rna15747 5.00E-37
445- 17: transcript:Zm00001d043039_T001 rna15750 6.00E-32
445- 18: transcript:Zm00001d043043_T001 rna15751 0
445- 19: transcript:Zm00001d043046_T001 rna15753 4.00E-61
445- 20: transcript:Zm00001d043047_T001 rna15756 1.00E-157
445- 21: transcript:Zm00001d043049_T001 rna15758 1.00E-38
445- 22: transcript:Zm00001d043050_T001 rna15759 2.00E-39
445- 23: transcript:Zm00001d043058_T001 rna15761 0
Alignment 446: score=943.0 e_value=3.7e-67 N=22 3&NC_008398.2 plus
446- 0: transcript:Zm00001d043164_T002 rna15835 6.00E-170
446- 1: transcript:Zm00001d043165_T002 rna15836 6.00E-154
446- 2: transcript:Zm00001d043171_T001 rna15837 0
446- 3: transcript:Zm00001d043174_T001 rna15838 0
446- 4: transcript:Zm00001d043175_T003 rna15840 3.00E-37
446- 5: transcript:Zm00001d043178_T001 rna15841 0
446- 6: transcript:Zm00001d043180_T001 rna15842 5.00E-122
446- 7: transcript:Zm00001d043194_T001 rna15853 2.00E-57
446- 8: transcript:Zm00001d043195_T001 rna15854 9.00E-164
446- 9: transcript:Zm00001d043204_T001 rna15857 5.00E-37
446- 10: transcript:Zm00001d043206_T002 rna15861 1.00E-30
446- 11: transcript:Zm00001d043217_T002 rna15867 1.00E-68
446- 12: transcript:Zm00001d043218_T001 rna15868 2.00E-53
446- 13: transcript:Zm00001d043227_T002 rna15871 0

446- 14: transcript:Zm00001d043238_T001 rna15873 6.00E-92
446- 15: transcript:Zm00001d043244_T001 rna15885 0
446- 16: transcript:Zm00001d043248_T001 rna15886 9.00E-68
446- 17: transcript:Zm00001d043249_T001 rna15887 7.00E-132
446- 18: transcript:Zm00001d043253_T002 rna15889 0
446- 19: transcript:Zm00001d043261_T001 rna15891 3.00E-107
446- 20: transcript:Zm00001d043267_T002 rna15894 0
446- 21: transcript:Zm00001d043272_T002 rna15899 0
Alignment 447: score=876.0 e_value=7e-57 N=20 3&NC_008398.2 plus
447- 0: transcript:Zm00001d042714_T001 rna15546 1.00E-135
447- 1: transcript:Zm00001d042717_T001 rna15547 4.00E-37
447- 2: transcript:Zm00001d042718_T001 rna15548 3.00E-148
447- 3: transcript:Zm00001d042719_T001 rna15551 4.00E-132
447- 4: transcript:Zm00001d042721_T001 rna15552 4.00E-54
447- 5: transcript:Zm00001d042724_T001 rna15554 0
447- 6: transcript:Zm00001d042727_T001 rna15556 9.00E-91
447- 7: transcript:Zm00001d042730_T001 rna15557 5.00E-93
447- 8: transcript:Zm00001d042731_T001 rna15558 0
447- 9: transcript:Zm00001d042735_T001 rna15559 9.00E-36
447- 10: transcript:Zm00001d042736_T001 rna15564 3.00E-90
447- 11: transcript:Zm00001d042747_T003 rna15568 0
447- 12: transcript:Zm00001d042752_T001 rna15569 2.00E-38
447- 13: transcript:Zm00001d042753_T001 rna15570 3.00E-54
447- 14: transcript:Zm00001d042754_T001 rna15572 2.00E-22
447- 15: transcript:Zm00001d042756_T001 rna15573 1.00E-70
447- 16: transcript:Zm00001d042758_T001 rna15574 9.00E-79
447- 17: transcript:Zm00001d042767_T001 rna15577 3.00E-161
447- 18: transcript:Zm00001d042777_T006 rna15581 0
447- 19: transcript:Zm00001d042781_T001 rna15582 5.00E-119
Alignment 448: score=841.0 e_value=4.3e-53 N=19 3&NC_008398.2 plus
448- 0: transcript:Zm00001d042800_T001 rna15587 1.00E-72
448- 1: transcript:Zm00001d042808_T001 rna15595 8.00E-86
448- 2: transcript:Zm00001d042809_T003 rna15596 0
448- 3: transcript:Zm00001d042812_T001 rna15598 1.00E-178
448- 4: transcript:Zm00001d042813_T001 rna15599 1.00E-113
448- 5: transcript:Zm00001d042826_T001 rna15603 0
448- 6: transcript:Zm00001d042833_T001 rna15604 0
448- 7: transcript:Zm00001d042837_T001 rna15608 1.00E-179
448- 8: transcript:Zm00001d042841_T002 rna15609 5.00E-127
448- 9: transcript:Zm00001d042850_T001 rna15616 7.00E-98
448- 10: transcript:Zm00001d042851_T003 rna15617 0
448- 11: transcript:Zm00001d042853_T001 rna15619 2.00E-100
448- 12: transcript:Zm00001d042861_T001 rna15623 0
448- 13: transcript:Zm00001d042863_T001 rna15625 1.00E-14
448- 14: transcript:Zm00001d042864_T003 rna15626 0
448- 15: transcript:Zm00001d042866_T001 rna15629 7.00E-33
448- 16: transcript:Zm00001d042868_T001 rna15635 4.00E-71
448- 17: transcript:Zm00001d042872_T001 rna15637 1.00E-68
448- 18: transcript:Zm00001d042875_T001 rna15638 9.00E-12
Alignment 449: score=816.0 e_value=1.1e-54 N=19 3&NC_008398.2 plus
449- 0: transcript:Zm00001d042560_T001 rna15448 2.00E-107
449- 1: transcript:Zm00001d042572_T001 rna15453 7.00E-14
449- 2: transcript:Zm00001d042573_T001 rna15454 8.00E-06
449- 3: transcript:Zm00001d042578_T001 rna15456 2.00E-167

449- 4: transcript:Zm00001d042580_T001 rna15457   1.00E-121
449- 5: transcript:Zm00001d042582_T001 rna15459   4.00E-135
449- 6: transcript:Zm00001d042585_T004 rna15460   0
449- 7: transcript:Zm00001d042590_T001 rna15461   5.00E-180
449- 8: transcript:Zm00001d042598_T003 rna15467   4.00E-104
449- 9: transcript:Zm00001d042600_T001 rna15468   4.00E-77
449- 10: transcript:Zm00001d042609_T001 rna15474   9.00E-55
449- 11: transcript:Zm00001d042611_T001 rna15475   4.00E-38
449- 12: transcript:Zm00001d042615_T003 rna15479   2.00E-115
449- 13: transcript:Zm00001d042618_T001 rna15483   4.00E-116
449- 14: transcript:Zm00001d042619_T002 rna15486   0
449- 15: transcript:Zm00001d042621_T005 rna15489   0
449- 16: transcript:Zm00001d042627_T005 rna15497   5.00E-148
449- 17: transcript:Zm00001d042638_T002 rna15499   5.00E-29
449- 18: transcript:Zm00001d042639_T004 rna15502   2.00E-131
Alignment 450: score=662.0 e_value=4.4e-43 N=16 3&NC_008398.2 plus
450- 0: transcript:Zm00001d043691_T001 rna16127   5.00E-151
450- 1: transcript:Zm00001d043692_T001 rna16128   1.00E-114
450- 2: transcript:Zm00001d043699_T001 rna16129   1.00E-62
450- 3: transcript:Zm00001d043704_T001 rna16134   1.00E-16
450- 4: transcript:Zm00001d043708_T001 rna16137   1.00E-12
450- 5: transcript:Zm00001d043709_T003 rna16138   5.00E-35
450- 6: transcript:Zm00001d043713_T001 rna16140   3.00E-13
450- 7: transcript:Zm00001d043725_T001 rna16143   1.00E-24
450- 8: transcript:Zm00001d043726_T001 rna16144   0
450- 9: transcript:Zm00001d043727_T001 rna16147   0
450- 10: transcript:Zm00001d043733_T002 rna16152   0
450- 11: transcript:Zm00001d043738_T001 rna16155   2.00E-130
450- 12: transcript:Zm00001d043751_T001 rna16170   7.00E-18
450- 13: transcript:Zm00001d043766_T005 rna16172   0
450- 14: transcript:Zm00001d043770_T001 rna16177   1.00E-35
450- 15: transcript:Zm00001d043773_T003 rna16178   0
Alignment 451: score=619.0 e_value=2.1e-37 N=15 3&NC_008398.2 plus
451- 0: transcript:Zm00001d044065_T001 rna16356   4.00E-140
451- 1: transcript:Zm00001d044074_T001 rna16362   2.00E-101
451- 2: transcript:Zm00001d044075_T001 rna16364   1.00E-25
451- 3: transcript:Zm00001d044080_T001 rna16365   6.00E-29
451- 4: transcript:Zm00001d044083_T001 rna16367   7.00E-19
451- 5: transcript:Zm00001d044087_T001 rna16369   4.00E-152
451- 6: transcript:Zm00001d044088_T001 rna16370   0
451- 7: transcript:Zm00001d044091_T003 rna16372   0
451- 8: transcript:Zm00001d044100_T001 rna16376   1.00E-50
451- 9: transcript:Zm00001d044104_T001 rna16377   0
451- 10: transcript:Zm00001d044110_T001 rna16378   0
451- 11: transcript:Zm00001d044117_T001 rna16386   1.00E-39
451- 12: transcript:Zm00001d044121_T002 rna16388   6.00E-128
451- 13: transcript:Zm00001d044129_T001 rna16389   0
451- 14: transcript:Zm00001d044131_T001 rna16400   3.00E-22
Alignment 452: score=546.0 e_value=3.5e-31 N=13 3&NC_008398.2 plus
452- 0: transcript:Zm00001d042899_T001 rna15645   4.00E-166
452- 1: transcript:Zm00001d042901_T001 rna15647   0
452- 2: transcript:Zm00001d042910_T010 rna15651   1.00E-156
452- 3: transcript:Zm00001d042916_T003 rna15655   0
452- 4: transcript:Zm00001d042917_T001 rna15656   3.00E-138

452- 5: transcript:Zm00001d042922_T007 rna15658 0
452- 6: transcript:Zm00001d042929_T001 rna15659 1.00E-114
452- 7: transcript:Zm00001d042936_T001 rna15669 3.00E-25
452- 8: transcript:Zm00001d042939_T002 rna15670 0
452- 9: transcript:Zm00001d042940_T001 rna15671 7.00E-36
452- 10: transcript:Zm00001d042950_T001 rna15673 0
452- 11: transcript:Zm00001d042954_T001 rna15675 4.00E-67
452- 12: transcript:Zm00001d042962_T002 rna15688 0
Alignment 453: score=543.0 e_value=2.8e-32 N=13 3&NC_008398.2 plus
453- 0: transcript:Zm00001d040614_T002 rna15157 4.00E-78
453- 1: transcript:Zm00001d040617_T001 rna15160 1.00E-34
453- 2: transcript:Zm00001d040623_T001 rna15163 6.00E-15
453- 3: transcript:Zm00001d040629_T002 rna15168 0
453- 4: transcript:Zm00001d040633_T001 rna15170 8.00E-10
453- 5: transcript:Zm00001d040634_T001 rna15171 3.00E-28
453- 6: transcript:Zm00001d040639_T001 rna15177 9.00E-31
453- 7: transcript:Zm00001d040649_T001 rna15183 0
453- 8: transcript:Zm00001d040659_T001 rna15189 3.00E-12
453- 9: transcript:Zm00001d040666_T003 rna15190 0
453- 10: transcript:Zm00001d040667_T001 rna15191 0
453- 11: transcript:Zm00001d040672_T013 rna15196 7.00E-113
453- 12: transcript:Zm00001d040678_T003 rna15198 1.00E-60
Alignment 454: score=476.0 e_value=1.8e-22 N=11 3&NC_008398.2 plus
454- 0: transcript:Zm00001d043069_T001 rna15764 2.00E-34
454- 1: transcript:Zm00001d043071_T002 rna15769 0
454- 2: transcript:Zm00001d043074_T004 rna15771 0
454- 3: transcript:Zm00001d043075_T002 rna15775 0
454- 4: transcript:Zm00001d043080_T001 rna15777 2.00E-137
454- 5: transcript:Zm00001d043083_T003 rna15781 0
454- 6: transcript:Zm00001d043086_T002 rna15784 0
454- 7: transcript:Zm00001d043087_T001 rna15787 2.00E-77
454- 8: transcript:Zm00001d043088_T002 rna15789 3.00E-138
454- 9: transcript:Zm00001d043090_T001 rna15791 8.00E-55
454- 10: transcript:Zm00001d043091_T002 rna15792 0
Alignment 455: score=435.0 e_value=1e-19 N=10 3&NC_008398.2 plus
455- 0: transcript:Zm00001d043113_T001 rna15807 1.00E-131
455- 1: transcript:Zm00001d043117_T002 rna15808 1.00E-124
455- 2: transcript:Zm00001d043125_T001 rna15814 0
455- 3: transcript:Zm00001d043126_T007 rna15815 4.00E-77
455- 4: transcript:Zm00001d043131_T002 rna15817 1.00E-78
455- 5: transcript:Zm00001d043135_T003 rna15818 0
455- 6: transcript:Zm00001d043137_T004 rna15819 8.00E-78
455- 7: transcript:Zm00001d043144_T001 rna15821 2.00E-34
455- 8: transcript:Zm00001d043149_T002 rna15824 9.00E-80
455- 9: transcript:Zm00001d043150_T002 rna15827 0
Alignment 456: score=374.0 e_value=1.6e-20 N=9 3&NC_008398.2 plus
456- 0: transcript:Zm00001d043504_T030 rna16009 0
456- 1: transcript:Zm00001d043506_T004 rna16011 1.00E-115
456- 2: transcript:Zm00001d043509_T002 rna16012 0
456- 3: transcript:Zm00001d043510_T002 rna16015 3.00E-72
456- 4: transcript:Zm00001d043511_T001 rna16017 0
456- 5: transcript:Zm00001d043512_T001 rna16018 1.00E-49
456- 6: transcript:Zm00001d043515_T002 rna16022 5.00E-94
456- 7: transcript:Zm00001d043525_T001 rna16023 7.00E-24

456- 8: transcript:Zm00001d043527_T002 rna16024 5.00E-142
Alignment 457: score=330.0 e_value=2.7e-10 N=7 3&NC_008398.2 plus
457- 0: transcript:Zm00001d042656_T001 rna15508 0
457- 1: transcript:Zm00001d042658_T002 rna15509 6.00E-95
457- 2: transcript:Zm00001d042661_T001 rna15513 0
457- 3: transcript:Zm00001d042664_T001 rna15515 0
457- 4: transcript:Zm00001d042665_T001 rna15518 4.00E-116
457- 5: transcript:Zm00001d042667_T001 rna15519 0
457- 6: transcript:Zm00001d042669_T002 rna15520 0
Alignment 458: score=285.0 e_value=7e-09 N=6 3&NC_008398.2 plus
458- 0: transcript:Zm00001d043829_T001 rna16207 4.00E-146
458- 1: transcript:Zm00001d043830_T001 rna16208 7.00E-43
458- 2: transcript:Zm00001d043831_T001 rna16209 0
458- 3: transcript:Zm00001d043834_T001 rna16210 0
458- 4: transcript:Zm00001d043837_T001 rna16212 2.00E-93
458- 5: transcript:Zm00001d043838_T002 rna16215 4.00E-34
Alignment 459: score=261.0 e_value=2.1e-09 N=6 3&NC_008398.2 plus
459- 0: transcript:Zm00001d043652_T003 rna16094 0
459- 1: transcript:Zm00001d043653_T003 rna16096 0
459- 2: transcript:Zm00001d043655_T002 rna16099 6.00E-173
459- 3: transcript:Zm00001d043661_T001 rna16101 7.00E-106
459- 4: transcript:Zm00001d043663_T001 rna16106 2.00E-34
459- 5: transcript:Zm00001d043667_T003 rna16107 0
Alignment 460: score=394.0 e_value=2.5e-18 N=9 3&NC_008398.2 minus
460- 0: transcript:Zm00001d039480_T001 rna14670 2.00E-08
460- 1: transcript:Zm00001d039488_T002 rna14667 4.00E-67
460- 2: transcript:Zm00001d039492_T001 rna14665 8.00E-83
460- 3: transcript:Zm00001d039495_T001 rna14663 4.00E-151
460- 4: transcript:Zm00001d039499_T002 rna14661 6.00E-166
460- 5: transcript:Zm00001d039502_T001 rna14660 4.00E-120
460- 6: transcript:Zm00001d039506_T001 rna14658 1.00E-30
460- 7: transcript:Zm00001d039510_T001 rna14633 1.00E-126
460- 8: transcript:Zm00001d039512_T001 rna14632 0
Alignment 461: score=366.0 e_value=1.9e-14 N=8 3&NC_008398.2 minus
461- 0: transcript:Zm00001d039623_T001 rna14594 1.00E-158
461- 1: transcript:Zm00001d039624_T001 rna14592 5.00E-98
461- 2: transcript:Zm00001d039625_T001 rna14591 0
461- 3: transcript:Zm00001d039634_T001 rna14589 2.00E-137
461- 4: transcript:Zm00001d039639_T001 rna14588 6.00E-149
461- 5: transcript:Zm00001d039642_T001 rna14587 6.00E-135
461- 6: transcript:Zm00001d039647_T001 rna14583 0
461- 7: transcript:Zm00001d039648_T001 rna14582 3.00E-31
Alignment 462: score=355.0 e_value=1e-17 N=9 3&NC_008398.2 minus
462- 0: transcript:Zm00001d039272_T002 rna14821 0
462- 1: transcript:Zm00001d039279_T001 rna14812 7.00E-73
462- 2: transcript:Zm00001d039282_T001 rna14809 0
462- 3: transcript:Zm00001d039285_T001 rna14797 1.00E-179
462- 4: transcript:Zm00001d039293_T001 rna14794 2.00E-97
462- 5: transcript:Zm00001d039300_T003 rna14785 8.00E-80
462- 6: transcript:Zm00001d039301_T001 rna14784 7.00E-89
462- 7: transcript:Zm00001d039305_T002 rna14782 0
462- 8: transcript:Zm00001d039314_T001 rna14760 0
Alignment 463: score=334.0 e_value=3.6e-15 N=8 3&NC_008398.2 minus
463- 0: transcript:Zm00001d040702_T001 rna14508 5.00E-118

463- 1: transcript:Zm00001d040715_T001 rna14496 1.00E-08
463- 2: transcript:Zm00001d040721_T002 rna14494 3.00E-40
463- 3: transcript:Zm00001d040730_T002 rna14492 6.00E-131
463- 4: transcript:Zm00001d040734_T001 rna14491 3.00E-101
463- 5: transcript:Zm00001d040737_T001 rna14488 3.00E-130
463- 6: transcript:Zm00001d040741_T001 rna14487 0
463- 7: transcript:Zm00001d040748_T008 rna14477 1.00E-105
Alignment 464: score=284.0 e_value=1.2e-08 N=6 3&NC_008398.2 minus
464- 0: transcript:Zm00001d043541_T001 rna16052 1.00E-48
464- 1: transcript:Zm00001d043543_T002 rna16051 0
464- 2: transcript:Zm00001d043544_T002 rna16048 6.00E-36
464- 3: transcript:Zm00001d043549_T001 rna16040 3.00E-94
464- 4: transcript:Zm00001d043550_T001 rna16039 7.00E-75
464- 5: transcript:Zm00001d043551_T003 rna16037 2.00E-146
Alignment 465: score=272.0 e_value=5e-08 N=6 3&NC_008398.2 minus
465- 0: transcript:Zm00001d044405_T002 rna16457 6.00E-33
465- 1: transcript:Zm00001d044407_T001 rna16456 5.00E-22
465- 2: transcript:Zm00001d044409_T001 rna16455 4.00E-69
465- 3: transcript:Zm00001d044412_T001 rna16450 1.00E-168
465- 4: transcript:Zm00001d044416_T001 rna16448 0
465- 5: transcript:Zm00001d044421_T001 rna16445 4.00E-109
Alignment 466: score=266.0 e_value=1.7e-11 N=6 3&NC_008398.2 minus
466- 0: transcript:Zm00001d039703_T001 rna14546 6.00E-174
466- 1: transcript:Zm00001d039710_T004 rna14544 5.00E-76
466- 2: transcript:Zm00001d039711_T001 rna14543 6.00E-180
466- 3: transcript:Zm00001d039717_T005 rna14542 0
466- 4: transcript:Zm00001d039726_T001 rna14529 8.00E-23
466- 5: transcript:Zm00001d039727_T001 rna14527 8.00E-81
Alignment 467: score=259.0 e_value=1.2e-09 N=6 3&NC_008398.2 minus
467- 0: transcript:Zm00001d039764_T001 rna14521 6.00E-54
467- 1: transcript:Zm00001d039768_T003 rna14520 0
467- 2: transcript:Zm00001d039769_T002 rna14518 5.00E-112
467- 3: transcript:Zm00001d039770_T001 rna14517 3.00E-64
467- 4: transcript:Zm00001d039785_T001 rna14513 8.00E-28
467- 5: transcript:Zm00001d039787_T001 rna14510 3.00E-98
Alignment 468: score=272.0 e_value=1.3e-09 N=6 3&NC_008404.2 plus
468- 0: transcript:Zm00001d044615_T001 rna26703 6.00E-149
468- 1: transcript:Zm00001d044618_T001 rna26718 0
468- 2: transcript:Zm00001d044619_T001 rna26719 1.00E-40
468- 3: transcript:Zm00001d044622_T002 rna26726 4.00E-45
468- 4: transcript:Zm00001d044623_T001 rna26730 0
468- 5: transcript:Zm00001d044624_T004 rna26737 6.00E-60
Alignment 469: score=846.0 e_value=4.3e-54 N=19 3&NC_008404.2 minus
469- 0: transcript:Zm00001d044650_T001 rna26852 3.00E-27
469- 1: transcript:Zm00001d044657_T003 rna26842 2.00E-138
469- 2: transcript:Zm00001d044659_T001 rna26831 1.00E-62
469- 3: transcript:Zm00001d044661_T001 rna26829 5.00E-176
469- 4: transcript:Zm00001d044662_T001 rna26828 7.00E-35
469- 5: transcript:Zm00001d044663_T002 rna26827 1.00E-103
469- 6: transcript:Zm00001d044664_T001 rna26826 3.00E-97
469- 7: transcript:Zm00001d044666_T001 rna26825 5.00E-68
469- 8: transcript:Zm00001d044669_T001 rna26812 9.00E-89
469- 9: transcript:Zm00001d044670_T001 rna26798 3.00E-22
469- 10: transcript:Zm00001d044674_T003 rna26790 1.00E-162

```
469- 11:  transcript:Zm00001d044680_T001  rna26779     2.00E-20
469- 12:  transcript:Zm00001d044683_T001  rna26774    3.00E-100
469- 13:  transcript:Zm00001d044685_T001  rna26766     7.00E-25
469- 14:  transcript:Zm00001d044689_T002  rna26764            0
469- 15:  transcript:Zm00001d044691_T001  rna26759     5.00E-39
469- 16:  transcript:Zm00001d044692_T001  rna26758            0
469- 17:  transcript:Zm00001d044693_T001  rna26756    2.00E-156
469- 18:  transcript:Zm00001d044694_T002  rna26754     2.00E-37
## Alignment 470: score=432.0 e_value=1.3e-23 N=11 3&NC_008404.2 minus
470-  0:  transcript:Zm00001d041842_T002  rna27168            0
470-  1:  transcript:Zm00001d041844_T021  rna27142            0
470-  2:  transcript:Zm00001d041847_T002  rna27132            0
470-  3:  transcript:Zm00001d041852_T002  rna27118            0
470-  4:  transcript:Zm00001d041853_T001  rna27117     8.00E-90
470-  5:  transcript:Zm00001d041854_T002  rna27113     2.00E-28
470-  6:  transcript:Zm00001d041864_T007  rna27101            0
470-  7:  transcript:Zm00001d041866_T001  rna27098     3.00E-80
470-  8:  transcript:Zm00001d041871_T001  rna27076    3.00E-103
470-  9:  transcript:Zm00001d041877_T002  rna27069            0
470- 10:  transcript:Zm00001d041882_T002  rna27066    8.00E-107
## Alignment 471: score=260.0 e_value=5.9e-10 N=6 3&NC_008404.2 minus
471-  0:  transcript:Zm00001d041912_T006  rna27051     9.00E-38
471-  1:  transcript:Zm00001d041913_T001  rna27043            0
471-  2:  transcript:Zm00001d041917_T001  rna27036     2.00E-37
471-  3:  transcript:Zm00001d041920_T001  rna27027    8.00E-103
471-  4:  transcript:Zm00001d041926_T001  rna27018            0
471-  5:  transcript:Zm00001d041927_T001  rna27010    4.00E-122
## Alignment 472: score=254.0 e_value=1.9e-08 N=6 3&NC_008404.2 minus
472-  0:  transcript:Zm00001d042748_T001  rna27010     5.00E-15
472-  1:  transcript:Zm00001d042753_T001  rna27003     7.00E-22
472-  2:  transcript:Zm00001d042758_T001  rna26993     4.00E-62
472-  3:  transcript:Zm00001d042760_T001  rna26992     1.00E-15
472-  4:  transcript:Zm00001d042766_T001  rna26984     5.00E-15
472-  5:  transcript:Zm00001d042777_T006  rna26980    4.00E-139
## Alignment 473: score=1117.0 e_value=9.3e-80 N=25 3&NC_008405.2 plus
473-  0:  transcript:Zm00001d041422_T001  rna30076            0
473-  1:  transcript:Zm00001d041430_T001  rna30077     4.00E-73
473-  2:  transcript:Zm00001d041437_T001  rna30080     5.00E-73
473-  3:  transcript:Zm00001d041438_T001  rna30081            0
473-  4:  transcript:Zm00001d041439_T001  rna30082            0
473-  5:  transcript:Zm00001d041443_T012  rna30084    3.00E-137
473-  6:  transcript:Zm00001d041444_T001  rna30085    3.00E-157
473-  7:  transcript:Zm00001d041445_T006  rna30087            0
473-  8:  transcript:Zm00001d041455_T002  rna30094            0
473-  9:  transcript:Zm00001d041462_T001  rna30107     7.00E-37
473- 10:  transcript:Zm00001d041465_T001  rna30110     1.00E-22
473- 11:  transcript:Zm00001d041467_T001  rna30113     2.00E-73
473- 12:  transcript:Zm00001d041471_T001  rna30114            0
473- 13:  transcript:Zm00001d041472_T001  rna30115    8.00E-128
473- 14:  transcript:Zm00001d041474_T001  rna30120     5.00E-90
473- 15:  transcript:Zm00001d041476_T001  rna30121    1.00E-141
473- 16:  transcript:Zm00001d041480_T001  rna30122     6.00E-82
473- 17:  transcript:Zm00001d041481_T001  rna30123    3.00E-124
473- 18:  transcript:Zm00001d041484_T001  rna30127            0
```

473- 19: transcript:Zm00001d041488_T002 rna30128 0
473- 20: transcript:Zm00001d041489_T009 rna30130 0
473- 21: transcript:Zm00001d041491_T001 rna30131 2.00E-111
473- 22: transcript:Zm00001d041495_T005 rna30135 3.00E-67
473- 23: transcript:Zm00001d041496_T001 rna30136 2.00E-125
473- 24: transcript:Zm00001d041497_T005 rna30137 0
Alignment 474: score=1105.0 e_value=4.9e-83 N=25 3&NC_008405.2 plus
474- 0: transcript:Zm00001d041329_T001 rna29977 2.00E-112
474- 1: transcript:Zm00001d041330_T001 rna29978 8.00E-65
474- 2: transcript:Zm00001d041334_T001 rna29984 9.00E-160
474- 3: transcript:Zm00001d041343_T001 rna30001 0
474- 4: transcript:Zm00001d041345_T001 rna30013 2.00E-136
474- 5: transcript:Zm00001d041346_T001 rna30014 0
474- 6: transcript:Zm00001d041352_T001 rna30015 9.00E-108
474- 7: transcript:Zm00001d041353_T002 rna30019 0
474- 8: transcript:Zm00001d041375_T001 rna30023 9.00E-23
474- 9: transcript:Zm00001d041381_T001 rna30028 0
474- 10: transcript:Zm00001d041382_T001 rna30033 5.00E-13
474- 11: transcript:Zm00001d041383_T002 rna30034 0
474- 12: transcript:Zm00001d041384_T001 rna30041 7.00E-32
474- 13: transcript:Zm00001d041387_T001 rna30042 4.00E-107
474- 14: transcript:Zm00001d041390_T001 rna30043 1.00E-70
474- 15: transcript:Zm00001d041392_T001 rna30049 9.00E-113
474- 16: transcript:Zm00001d041397_T001 rna30051 1.00E-178
474- 17: transcript:Zm00001d041403_T001 rna30060 3.00E-48
474- 18: transcript:Zm00001d041405_T001 rna30065 2.00E-177
474- 19: transcript:Zm00001d041407_T002 rna30066 0
474- 20: transcript:Zm00001d041410_T001 rna30067 1.00E-54
474- 21: transcript:Zm00001d041414_T002 rna30068 0
474- 22: transcript:Zm00001d041415_T001 rna30069 3.00E-161
474- 23: transcript:Zm00001d041416_T001 rna30070 1.00E-105
474- 24: transcript:Zm00001d041418_T001 rna30071 6.00E-78
Alignment 475: score=637.0 e_value=4.5e-40 N=15 3&NC_008405.2 plus
475- 0: transcript:Zm00001d041792_T001 rna28985 1.00E-25
475- 1: transcript:Zm00001d041796_T001 rna28997 3.00E-22
475- 2: transcript:Zm00001d041803_T037 rna28998 3.00E-09
475- 3: transcript:Zm00001d041804_T001 rna29000 5.00E-79
475- 4: transcript:Zm00001d041816_T001 rna29001 4.00E-73
475- 5: transcript:Zm00001d041819_T001 rna29003 1.00E-76
475- 6: transcript:Zm00001d041823_T003 rna29008 0
475- 7: transcript:Zm00001d041824_T001 rna29009 3.00E-74
475- 8: transcript:Zm00001d041827_T001 rna29012 7.00E-179
475- 9: transcript:Zm00001d041829_T001 rna29017 0
475- 10: transcript:Zm00001d041830_T001 rna29018 3.00E-28
475- 11: transcript:Zm00001d041831_T002 rna29023 6.00E-98
475- 12: transcript:Zm00001d041833_T005 rna29026 0
475- 13: transcript:Zm00001d041836_T001 rna29027 7.00E-120
475- 14: transcript:Zm00001d041842_T002 rna29028 0
Alignment 476: score=353.0 e_value=2.2e-16 N=8 3&NC_008405.2 plus
476- 0: transcript:Zm00001d044695_T001 rna28794 0
476- 1: transcript:Zm00001d044696_T002 rna28797 3.00E-71
476- 2: transcript:Zm00001d044698_T003 rna28804 0
476- 3: transcript:Zm00001d044703_T001 rna28818 0
476- 4: transcript:Zm00001d044705_T001 rna28824 3.00E-118

```
476-  5:  transcript:Zm00001d044704_T018  rna28825           0
476-  6:  transcript:Zm00001d044707_T001  rna28828           0
476-  7:  transcript:Zm00001d044713_T005  rna28829    3.00E-154
## Alignment 477: score=307.0 e_value=5.6e-12 N=7 3&NC_008405.2 plus
477-  0:  transcript:Zm00001d041578_T001  rna29875     3.00E-92
477-  1:  transcript:Zm00001d041582_T005  rna29879           0
477-  2:  transcript:Zm00001d041584_T001  rna29880           0
477-  3:  transcript:Zm00001d041590_T001  rna29887           0
477-  4:  transcript:Zm00001d041592_T002  rna29890     5.00E-37
477-  5:  transcript:Zm00001d041593_T001  rna29891           0
477-  6:  transcript:Zm00001d041594_T001  rna29892     4.00E-67
## Alignment 478: score=268.0 e_value=9.4e-10 N=6 3&NC_008405.2 plus
478-  0:  transcript:Zm00001d044615_T001  rna28542     2.00E-34
478-  1:  transcript:Zm00001d044618_T001  rna28555           0
478-  2:  transcript:Zm00001d044619_T001  rna28556     3.00E-41
478-  3:  transcript:Zm00001d044622_T002  rna28567           0
478-  4:  transcript:Zm00001d044623_T001  rna28572           0
478-  5:  transcript:Zm00001d044624_T004  rna28580           0
## Alignment 479: score=258.0 e_value=1.7e-09 N=6 3&NC_008405.2 plus
479-  0:  transcript:Zm00001d043018_T001  rna28562     9.00E-68
479-  1:  transcript:Zm00001d043019_T003  rna28564           0
479-  2:  transcript:Zm00001d043026_T001  rna28576     5.00E-29
479-  3:  transcript:Zm00001d043038_T001  rna28599           0
479-  4:  transcript:Zm00001d043044_T001  rna28602     3.00E-65
479-  5:  transcript:Zm00001d043049_T001  rna28608     7.00E-20
## Alignment 480: score=897.0 e_value=3.1e-56 N=20 3&NC_008405.2 minus
480-  0:  transcript:Zm00001d044650_T001  rna28696     8.00E-37
480-  1:  transcript:Zm00001d044657_T003  rna28685           0
480-  2:  transcript:Zm00001d044659_T001  rna28674     7.00E-62
480-  3:  transcript:Zm00001d044661_T001  rna28671    2.00E-174
480-  4:  transcript:Zm00001d044662_T001  rna28670     7.00E-35
480-  5:  transcript:Zm00001d044663_T002  rna28669           0
480-  6:  transcript:Zm00001d044664_T001  rna28667     3.00E-97
480-  7:  transcript:Zm00001d044666_T001  rna28665     8.00E-68
480-  8:  transcript:Zm00001d044669_T001  rna28656     1.00E-91
480-  9:  transcript:Zm00001d044670_T001  rna28644     2.00E-22
480- 10:  transcript:Zm00001d044674_T003  rna28632    8.00E-112
480- 11:  transcript:Zm00001d044680_T001  rna28619     6.00E-19
480- 12:  transcript:Zm00001d044682_T001  rna28618     5.00E-16
480- 13:  transcript:Zm00001d044683_T001  rna28614    4.00E-101
480- 14:  transcript:Zm00001d044685_T001  rna28607     5.00E-26
480- 15:  transcript:Zm00001d044689_T002  rna28605     9.00E-88
480- 16:  transcript:Zm00001d044691_T001  rna28598     6.00E-40
480- 17:  transcript:Zm00001d044692_T001  rna28597           0
480- 18:  transcript:Zm00001d044693_T001  rna28596     3.00E-43
480- 19:  transcript:Zm00001d044694_T002  rna28594    1.00E-104
## Alignment 481: score=874.0 e_value=1.8e-56 N=20 3&NC_008405.2 minus
481-  0:  transcript:Zm00001d041506_T001  rna29973     2.00E-51
481-  1:  transcript:Zm00001d041510_T003  rna29970           0
481-  2:  transcript:Zm00001d041511_T003  rna29969           0
481-  3:  transcript:Zm00001d041514_T002  rna29964     2.00E-86
481-  4:  transcript:Zm00001d041518_T001  rna29962    2.00E-153
481-  5:  transcript:Zm00001d041525_T001  rna29954    3.00E-172
481-  6:  transcript:Zm00001d041530_T001  rna29946    3.00E-176
```

481- 7: transcript:Zm00001d041534_T002 rna29943   0
481- 8: transcript:Zm00001d041536_T001 rna29938   0
481- 9: transcript:Zm00001d041539_T001 rna29925   6.00E-107
481- 10: transcript:Zm00001d041548_T002 rna29916   0
481- 11: transcript:Zm00001d041549_T001 rna29915   3.00E-106
481- 12: transcript:Zm00001d041550_T006 rna29914   3.00E-90
481- 13: transcript:Zm00001d041553_T001 rna29909   2.00E-58
481- 14: transcript:Zm00001d041556_T004 rna29908   0
481- 15: transcript:Zm00001d041567_T001 rna29907   3.00E-29
481- 16: transcript:Zm00001d041568_T001 rna29902   0
481- 17: transcript:Zm00001d041573_T002 rna29901   3.00E-46
481- 18: transcript:Zm00001d041576_T001 rna29900   4.00E-126
481- 19: transcript:Zm00001d041575_T008 rna29899   4.00E-31
Alignment 482: score=764.0 e_value=1.7e-45 N=17 3&NC_008405.2 minus
482- 0: transcript:Zm00001d041191_T001 rna30291   2.00E-143
482- 1: transcript:Zm00001d041198_T001 rna30282   2.00E-72
482- 2: transcript:Zm00001d041201_T001 rna30278   0
482- 3: transcript:Zm00001d041203_T001 rna30274   0
482- 4: transcript:Zm00001d041212_T001 rna30272   1.00E-56
482- 5: transcript:Zm00001d041214_T003 rna30270   0
482- 6: transcript:Zm00001d041215_T002 rna30265   0
482- 7: transcript:Zm00001d041216_T001 rna30255   3.00E-116
482- 8: transcript:Zm00001d041217_T002 rna30253   1.00E-173
482- 9: transcript:Zm00001d041220_T001 rna30249   3.00E-151
482- 10: transcript:Zm00001d041221_T001 rna30248   7.00E-115
482- 11: transcript:Zm00001d041229_T002 rna30238   0
482- 12: transcript:Zm00001d041230_T001 rna30237   6.00E-68
482- 13: transcript:Zm00001d041232_T001 rna30231   0
482- 14: transcript:Zm00001d041243_T001 rna30230   0
482- 15: transcript:Zm00001d041246_T001 rna30227   1.00E-48
482- 16: transcript:Zm00001d041258_T001 rna30223   7.00E-103
Alignment 483: score=751.0 e_value=1.9e-50 N=17 3&NC_008405.2 minus
483- 0: transcript:Zm00001d041595_T001 rna29872   1.00E-56
483- 1: transcript:Zm00001d041596_T001 rna29870   0
483- 2: transcript:Zm00001d041597_T001 rna29869   3.00E-126
483- 3: transcript:Zm00001d041599_T004 rna29866   0
483- 4: transcript:Zm00001d041600_T001 rna29860   3.00E-55
483- 5: transcript:Zm00001d041601_T001 rna29858   2.00E-157
483- 6: transcript:Zm00001d041606_T002 rna29854   0
483- 7: transcript:Zm00001d041607_T001 rna29853   2.00E-99
483- 8: transcript:Zm00001d041609_T001 rna29851   5.00E-72
483- 9: transcript:Zm00001d041612_T001 rna29840   4.00E-79
483- 10: transcript:Zm00001d041620_T001 rna29837   0
483- 11: transcript:Zm00001d041624_T001 rna29822   0
483- 12: transcript:Zm00001d041632_T002 rna29821   0
483- 13: transcript:Zm00001d041634_T001 rna29811   4.00E-30
483- 14: transcript:Zm00001d041635_T002 rna29810   0
483- 15: transcript:Zm00001d041638_T001 rna29808   7.00E-85
483- 16: transcript:Zm00001d041647_T002 rna29793   0
Alignment 484: score=654.0 e_value=4.5e-37 N=15 3&NC_008405.2 minus
484- 0: transcript:Zm00001d041839_T001 rna28983   0
484- 1: transcript:Zm00001d041842_T002 rna28980   0
484- 2: transcript:Zm00001d041844_T021 rna28965   7.00E-76
484- 3: transcript:Zm00001d041847_T002 rna28957   0

484- 4: transcript:Zm00001d041852_T002 rna28950 0
484- 5: transcript:Zm00001d041854_T002 rna28947 6.00E-71
484- 6: transcript:Zm00001d041856_T001 rna28945 4.00E-13
484- 7: transcript:Zm00001d041857_T001 rna28942 4.00E-144
484- 8: transcript:Zm00001d041864_T007 rna28938 0
484- 9: transcript:Zm00001d041870_T001 rna28925 1.00E-159
484- 10: transcript:Zm00001d041871_T001 rna28923 1.00E-102
484- 11: transcript:Zm00001d041877_T002 rna28918 0
484- 12: transcript:Zm00001d041882_T002 rna28916 4.00E-107
484- 13: transcript:Zm00001d041883_T001 rna28915 1.00E-71
484- 14: transcript:Zm00001d041884_T001 rna28911 1.00E-119
Alignment 485: score=610.0 e_value=3e-39 N=14 3&NC_008405.2 minus
485- 0: transcript:Zm00001d041719_T001 rna29675 0
485- 1: transcript:Zm00001d041725_T001 rna29658 8.00E-63
485- 2: transcript:Zm00001d041727_T001 rna29643 5.00E-36
485- 3: transcript:Zm00001d041730_T001 rna29642 2.00E-85
485- 4: transcript:Zm00001d041732_T001 rna29641 1.00E-51
485- 5: transcript:Zm00001d041733_T001 rna29640 2.00E-66
485- 6: transcript:Zm00001d041735_T001 rna29638 1.00E-51
485- 7: transcript:Zm00001d041740_T001 rna29634 0
485- 8: transcript:Zm00001d041744_T001 rna29626 8.00E-74
485- 9: transcript:Zm00001d041748_T003 rna29618 0
485- 10: transcript:Zm00001d041750_T001 rna29612 1.00E-53
485- 11: transcript:Zm00001d041751_T001 rna29611 1.00E-52
485- 12: transcript:Zm00001d041759_T002 rna29593 3.00E-23
485- 13: transcript:Zm00001d041762_T001 rna29580 0
Alignment 486: score=562.0 e_value=4.6e-32 N=13 3&NC_008405.2 minus
486- 0: transcript:Zm00001d041900_T011 rna28904 3.00E-42
486- 1: transcript:Zm00001d041908_T001 rna28898 0
486- 2: transcript:Zm00001d041910_T001 rna28895 4.00E-62
486- 3: transcript:Zm00001d041911_T011 rna28894 0
486- 4: transcript:Zm00001d041912_T006 rna28893 5.00E-43
486- 5: transcript:Zm00001d041913_T001 rna28892 0
486- 6: transcript:Zm00001d041918_T001 rna28890 8.00E-33
486- 7: transcript:Zm00001d041920_T001 rna28880 2.00E-151
486- 8: transcript:Zm00001d041921_T001 rna28879 7.00E-35
486- 9: transcript:Zm00001d041923_T002 rna28872 4.00E-158
486- 10: transcript:Zm00001d041926_T001 rna28870 0
486- 11: transcript:Zm00001d041927_T001 rna28865 9.00E-136
486- 12: transcript:Zm00001d041929_T001 rna28861 4.00E-48
Alignment 487: score=542.0 e_value=9.7e-28 N=12 3&NC_008405.2 minus
487- 0: transcript:Zm00001d041268_T004 rna30185 2.00E-105
487- 1: transcript:Zm00001d041269_T010 rna30177 0
487- 2: transcript:Zm00001d041277_T001 rna30174 1.00E-82
487- 3: transcript:Zm00001d041280_T001 rna30173 0
487- 4: transcript:Zm00001d041287_T001 rna30171 0
487- 5: transcript:Zm00001d041290_T002 rna30170 2.00E-54
487- 6: transcript:Zm00001d041305_T001 rna30168 0
487- 7: transcript:Zm00001d041307_T002 rna30163 0
487- 8: transcript:Zm00001d041308_T001 rna30162 0
487- 9: transcript:Zm00001d041319_T001 rna30161 0
487- 10: transcript:Zm00001d041322_T002 rna30153 9.00E-111
487- 11: transcript:Zm00001d041323_T003 rna30152 0
Alignment 488: score=530.0 e_value=1.7e-29 N=12 3&NC_008405.2 minus

```
488-  0:  transcript:Zm00001d041685_T001  rna29778     2.00E-34
488-  1:  transcript:Zm00001d041691_T002  rna29775     8.00E-73
488-  2:  transcript:Zm00001d041692_T001  rna29763     2.00E-65
488-  3:  transcript:Zm00001d041693_T004  rna29762            0
488-  4:  transcript:Zm00001d041696_T001  rna29752     2.00E-64
488-  5:  transcript:Zm00001d041698_T001  rna29749            0
488-  6:  transcript:Zm00001d041701_T002  rna29743     2.00E-69
488-  7:  transcript:Zm00001d041707_T002  rna29731    2.00E-105
488-  8:  transcript:Zm00001d041709_T001  rna29730            0
488-  9:  transcript:Zm00001d041710_T001  rna29727     5.00E-78
488- 10:  transcript:Zm00001d041711_T003  rna29724     1.00E-92
488- 11:  transcript:Zm00001d041712_T001  rna29723     3.00E-11
## Alignment 489: score=509.0 e_value=2.3e-28 N=12 3&NC_008405.2 minus
489-  0:  transcript:Zm00001d042724_T001  rna28886            0
489-  1:  transcript:Zm00001d042738_T001  rna28873     7.00E-46
489-  2:  transcript:Zm00001d042748_T001  rna28862     1.00E-15
489-  3:  transcript:Zm00001d042750_T001  rna28861     2.00E-08
489-  4:  transcript:Zm00001d042752_T001  rna28857     2.00E-17
489-  5:  transcript:Zm00001d042753_T001  rna28856     1.00E-19
489-  6:  transcript:Zm00001d042758_T001  rna28848     3.00E-60
489-  7:  transcript:Zm00001d042760_T001  rna28847     3.00E-15
489-  8:  transcript:Zm00001d042766_T001  rna28835     4.00E-09
489-  9:  transcript:Zm00001d042777_T006  rna28831    2.00E-137
489- 10:  transcript:Zm00001d042780_T001  rna28825    3.00E-133
489- 11:  transcript:Zm00001d042786_T001  rna28822     2.00E-98
## Alignment 490: score=352.0 e_value=2.9e-14 N=8 3&NC_008405.2 minus
490-  0:  transcript:Zm00001d041142_T030  rna29160            0
490-  1:  transcript:Zm00001d041154_T001  rna29154     7.00E-41
490-  2:  transcript:Zm00001d041163_T001  rna29151            0
490-  3:  transcript:Zm00001d041165_T004  rna29148            0
490-  4:  transcript:Zm00001d041175_T002  rna29143    6.00E-132
490-  5:  transcript:Zm00001d041179_T004  rna29128    2.00E-154
490-  6:  transcript:Zm00001d041180_T003  rna29126     5.00E-80
490-  7:  transcript:Zm00001d041181_T001  rna29125            0
## Alignment 491: score=4783.0 e_value=0 N=105 4&NC_008395.2 plus
491-  0:  transcript:Zm00001d051214_T001  rna6459             0
491-  1:  transcript:Zm00001d051216_T001  rna6464      3.00E-58
491-  2:  transcript:Zm00001d051219_T001  rna6468     2.00E-107
491-  3:  transcript:Zm00001d051223_T001  rna6470     1.00E-138
491-  4:  transcript:Zm00001d051227_T001  rna6475             0
491-  5:  transcript:Zm00001d051229_T002  rna6476             0
491-  6:  transcript:Zm00001d051233_T001  rna6477      2.00E-61
491-  7:  transcript:Zm00001d051234_T001  rna6478      4.00E-60
491-  8:  transcript:Zm00001d051235_T001  rna6480      4.00E-74
491-  9:  transcript:Zm00001d051238_T001  rna6481      9.00E-58
491- 10:  transcript:Zm00001d051239_T001  rna6483     6.00E-147
491- 11:  transcript:Zm00001d051241_T001  rna6484             0
491- 12:  transcript:Zm00001d051242_T002  rna6485             0
491- 13:  transcript:Zm00001d051245_T001  rna6490             0
491- 14:  transcript:Zm00001d051250_T001  rna6492      2.00E-80
491- 15:  transcript:Zm00001d051262_T001  rna6499     1.00E-118
491- 16:  transcript:Zm00001d051267_T001  rna6500      2.00E-24
491- 17:  transcript:Zm00001d051268_T004  rna6501             0
491- 18:  transcript:Zm00001d051272_T001  rna6505      7.00E-55
```

491- 19:	transcript:Zm00001d051287_T001	rna6508	0
491- 20:	transcript:Zm00001d051288_T001	rna6509	1.00E-112
491- 21:	transcript:Zm00001d051302_T001	rna6512	1.00E-54
491- 22:	transcript:Zm00001d051306_T001	rna6514	0
491- 23:	transcript:Zm00001d051307_T001	rna6516	0
491- 24:	transcript:Zm00001d051308_T009	rna6523	0
491- 25:	transcript:Zm00001d051309_T001	rna6525	2.00E-92
491- 26:	transcript:Zm00001d051313_T001	rna6529	0
491- 27:	transcript:Zm00001d051314_T001	rna6531	4.00E-179
491- 28:	transcript:Zm00001d051316_T001	rna6532	1.00E-108
491- 29:	transcript:Zm00001d051320_T001	rna6536	0
491- 30:	transcript:Zm00001d051321_T007	rna6537	0
491- 31:	transcript:Zm00001d051323_T003	rna6541	0
491- 32:	transcript:Zm00001d051329_T002	rna6552	4.00E-68
491- 33:	transcript:Zm00001d051333_T001	rna6556	1.00E-62
491- 34:	transcript:Zm00001d051334_T001	rna6558	1.00E-129
491- 35:	transcript:Zm00001d051335_T001	rna6559	2.00E-22
491- 36:	transcript:Zm00001d051337_T002	rna6565	3.00E-149
491- 37:	transcript:Zm00001d051338_T002	rna6566	0
491- 38:	transcript:Zm00001d051339_T001	rna6568	1.00E-139
491- 39:	transcript:Zm00001d051340_T001	rna6569	6.00E-89
491- 40:	transcript:Zm00001d051343_T001	rna6574	6.00E-162
491- 41:	transcript:Zm00001d051344_T001	rna6575	7.00E-35
491- 42:	transcript:Zm00001d051345_T003	rna6576	1.00E-25
491- 43:	transcript:Zm00001d051350_T001	rna6580	6.00E-39
491- 44:	transcript:Zm00001d051359_T001	rna6583	2.00E-174
491- 45:	transcript:Zm00001d051360_T001	rna6584	3.00E-100
491- 46:	transcript:Zm00001d051361_T001	rna6585	8.00E-43
491- 47:	transcript:Zm00001d051362_T001	rna6587	4.00E-154
491- 48:	transcript:Zm00001d051365_T001	rna6588	1.00E-24
491- 49:	transcript:Zm00001d051366_T002	rna6589	5.00E-23
491- 50:	transcript:Zm00001d051367_T001	rna6590	4.00E-173
491- 51:	transcript:Zm00001d051368_T001	rna6592	7.00E-74
491- 52:	transcript:Zm00001d051370_T001	rna6595	2.00E-64
491- 53:	transcript:Zm00001d051371_T001	rna6597	1.00E-77
491- 54:	transcript:Zm00001d051373_T001	rna6600	4.00E-65
491- 55:	transcript:Zm00001d051380_T001	rna6605	2.00E-102
491- 56:	transcript:Zm00001d051383_T001	rna6606	1.00E-24
491- 57:	transcript:Zm00001d051384_T001	rna6607	1.00E-65
491- 58:	transcript:Zm00001d051387_T001	rna6611	0
491- 59:	transcript:Zm00001d051388_T001	rna6614	2.00E-94
491- 60:	transcript:Zm00001d051389_T001	rna6615	0
491- 61:	transcript:Zm00001d051392_T001	rna6616	2.00E-123
491- 62:	transcript:Zm00001d051393_T002	rna6619	1.00E-14
491- 63:	transcript:Zm00001d051396_T004	rna6622	0
491- 64:	transcript:Zm00001d051397_T001	rna6626	0
491- 65:	transcript:Zm00001d051403_T001	rna6630	0
491- 66:	transcript:Zm00001d051404_T004	rna6631	0
491- 67:	transcript:Zm00001d051405_T001	rna6635	1.00E-86
491- 68:	transcript:Zm00001d051406_T001	rna6636	1.00E-45
491- 69:	transcript:Zm00001d051410_T001	rna6637	6.00E-39
491- 70:	transcript:Zm00001d051411_T001	rna6638	3.00E-34
491- 71:	transcript:Zm00001d051415_T001	rna6642	0
491- 72:	transcript:Zm00001d051416_T001	rna6643	0

491- 73: transcript:Zm00001d051418_T001 rna6647 2.00E-80
491- 74: transcript:Zm00001d051420_T001 rna6648 1.00E-54
491- 75: transcript:Zm00001d051424_T001 rna6653 0
491- 76: transcript:Zm00001d051427_T002 rna6654 3.00E-50
491- 77: transcript:Zm00001d051429_T001 rna6659 7.00E-15
491- 78: transcript:Zm00001d051430_T001 rna6668 2.00E-59
491- 79: transcript:Zm00001d051431_T002 rna6669 0
491- 80: transcript:Zm00001d051439_T001 rna6675 3.00E-108
491- 81: transcript:Zm00001d051441_T001 rna6676 0
491- 82: transcript:Zm00001d051442_T002 rna6680 0
491- 83: transcript:Zm00001d051447_T001 rna6681 4.00E-171
491- 84: transcript:Zm00001d051448_T001 rna6685 3.00E-83
491- 85: transcript:Zm00001d051451_T001 rna6687 3.00E-86
491- 86: transcript:Zm00001d051453_T001 rna6689 4.00E-114
491- 87: transcript:Zm00001d051456_T001 rna6695 3.00E-98
491- 88: transcript:Zm00001d051457_T001 rna6699 0
491- 89: transcript:Zm00001d051458_T002 rna6701 1.00E-135
491- 90: transcript:Zm00001d051460_T001 rna6705 1.00E-58
491- 91: transcript:Zm00001d051461_T001 rna6708 0
491- 92: transcript:Zm00001d051465_T002 rna6709 4.00E-136
491- 93: transcript:Zm00001d051471_T001 rna6714 1.00E-131
491- 94: transcript:Zm00001d051473_T001 rna6718 3.00E-172
491- 95: transcript:Zm00001d051474_T001 rna6720 4.00E-142
491- 96: transcript:Zm00001d051475_T002 rna6721 2.00E-51
491- 97: transcript:Zm00001d051479_T014 rna6725 2.00E-152
491- 98: transcript:Zm00001d051480_T001 rna6726 6.00E-53
491- 99: transcript:Zm00001d051500_T001 rna6730 0
491-100: transcript:Zm00001d051503_T001 rna6740 8.00E-06
491-101: transcript:Zm00001d051504_T001 rna6748 0
491-102: transcript:Zm00001d051505_T001 rna6749 6.00E-113
491-103: transcript:Zm00001d051506_T001 rna6750 2.00E-19
491-104: transcript:Zm00001d051507_T007 rna6751 0
Alignment 492: score=2646.0 e_value=4.7e-235 N=59 4&NC_008395.2 plus
492- 0: transcript:Zm00001d050747_T007 rna6019 0
492- 1: transcript:Zm00001d050748_T001 rna6021 5.00E-78
492- 2: transcript:Zm00001d050753_T001 rna6022 0
492- 3: transcript:Zm00001d050755_T003 rna6024 0
492- 4: transcript:Zm00001d050775_T001 rna6029 5.00E-98
492- 5: transcript:Zm00001d050783_T001 rna6033 5.00E-38
492- 6: transcript:Zm00001d050785_T001 rna6034 7.00E-82
492- 7: transcript:Zm00001d050798_T001 rna6039 2.00E-64
492- 8: transcript:Zm00001d050800_T001 rna6041 0
492- 9: transcript:Zm00001d050805_T001 rna6043 1.00E-27
492- 10: transcript:Zm00001d050810_T001 rna6044 0
492- 11: transcript:Zm00001d050822_T008 rna6051 1.00E-110
492- 12: transcript:Zm00001d050823_T002 rna6052 4.00E-75
492- 13: transcript:Zm00001d050824_T001 rna6053 6.00E-21
492- 14: transcript:Zm00001d050827_T003 rna6057 9.00E-90
492- 15: transcript:Zm00001d050830_T001 rna6058 1.00E-07
492- 16: transcript:Zm00001d050833_T002 rna6061 0
492- 17: transcript:Zm00001d050834_T001 rna6062 1.00E-93
492- 18: transcript:Zm00001d050837_T001 rna6074 2.00E-159
492- 19: transcript:Zm00001d050838_T001 rna6075 4.00E-165
492- 20: transcript:Zm00001d050840_T001 rna6090 3.00E-59

492- 21: transcript:Zm00001d050844_T005 rna6091 0
492- 22: transcript:Zm00001d050848_T001 rna6092 5.00E-150
492- 23: transcript:Zm00001d050850_T001 rna6095 0
492- 24: transcript:Zm00001d050851_T009 rna6096 1.00E-104
492- 25: transcript:Zm00001d050860_T001 rna6098 0
492- 26: transcript:Zm00001d050861_T001 rna6104 2.00E-22
492- 27: transcript:Zm00001d050862_T001 rna6105 0
492- 28: transcript:Zm00001d050864_T001 rna6106 4.00E-16
492- 29: transcript:Zm00001d050865_T001 rna6107 0
492- 30: transcript:Zm00001d050872_T001 rna6111 0
492- 31: transcript:Zm00001d050873_T001 rna6115 0
492- 32: transcript:Zm00001d050874_T001 rna6116 2.00E-136
492- 33: transcript:Zm00001d050884_T004 rna6118 2.00E-152
492- 34: transcript:Zm00001d050885_T001 rna6119 0
492- 35: transcript:Zm00001d050886_T001 rna6121 2.00E-130
492- 36: transcript:Zm00001d050889_T001 rna6128 8.00E-78
492- 37: transcript:Zm00001d050893_T001 rna6130 3.00E-138
492- 38: transcript:Zm00001d050897_T002 rna6133 5.00E-73
492- 39: transcript:Zm00001d050899_T001 rna6134 1.00E-85
492- 40: transcript:Zm00001d050903_T001 rna6137 4.00E-21
492- 41: transcript:Zm00001d050905_T001 rna6138 1.00E-80
492- 42: transcript:Zm00001d050907_T001 rna6141 0
492- 43: transcript:Zm00001d050908_T001 rna6146 2.00E-28
492- 44: transcript:Zm00001d050910_T004 rna6147 0
492- 45: transcript:Zm00001d050914_T001 rna6156 0
492- 46: transcript:Zm00001d050915_T004 rna6176 1.00E-133
492- 47: transcript:Zm00001d050916_T002 rna6177 9.00E-61
492- 48: transcript:Zm00001d050917_T002 rna6185 0
492- 49: transcript:Zm00001d050918_T001 rna6187 6.00E-19
492- 50: transcript:Zm00001d050920_T002 rna6203 2.00E-139
492- 51: transcript:Zm00001d050923_T002 rna6204 0
492- 52: transcript:Zm00001d050925_T007 rna6206 2.00E-164
492- 53: transcript:Zm00001d050929_T002 rna6209 0
492- 54: transcript:Zm00001d050935_T001 rna6210 8.00E-64
492- 55: transcript:Zm00001d050942_T001 rna6211 6.00E-163
492- 56: transcript:Zm00001d050944_T001 rna6216 2.00E-53
492- 57: transcript:Zm00001d050947_T007 rna6221 0
492- 58: transcript:Zm00001d050948_T001 rna6223 1.00E-76
Alignment 493: score=1595.0 e_value=3.2e-120 N=35 4&NC_008395.2 plus
493- 0: transcript:Zm00001d051567_T001 rna6838 0
493- 1: transcript:Zm00001d051568_T001 rna6839 0
493- 2: transcript:Zm00001d051569_T001 rna6841 8.00E-123
493- 3: transcript:Zm00001d051572_T001 rna6845 0
493- 4: transcript:Zm00001d051573_T001 rna6846 1.00E-87
493- 5: transcript:Zm00001d051577_T001 rna6850 3.00E-104
493- 6: transcript:Zm00001d051584_T001 rna6853 4.00E-116
493- 7: transcript:Zm00001d051586_T001 rna6854 0
493- 8: transcript:Zm00001d051587_T001 rna6855 2.00E-134
493- 9: transcript:Zm00001d051588_T002 rna6856 2.00E-24
493- 10: transcript:Zm00001d051589_T004 rna6857 0
493- 11: transcript:Zm00001d051590_T001 rna6858 8.00E-159
493- 12: transcript:Zm00001d051592_T001 rna6861 5.00E-47
493- 13: transcript:Zm00001d051594_T001 rna6863 2.00E-30
493- 14: transcript:Zm00001d051598_T001 rna6865 8.00E-93

493- 15: transcript:Zm00001d051599_T004 rna6867 0
493- 16: transcript:Zm00001d051600_T004 rna6868 0
493- 17: transcript:Zm00001d051606_T001 rna6871 9.00E-110
493- 18: transcript:Zm00001d051609_T001 rna6873 0
493- 19: transcript:Zm00001d051610_T001 rna6875 2.00E-72
493- 20: transcript:Zm00001d051611_T001 rna6879 4.00E-66
493- 21: transcript:Zm00001d051615_T001 rna6884 0
493- 22: transcript:Zm00001d051620_T001 rna6890 8.00E-129
493- 23: transcript:Zm00001d051626_T003 rna6891 8.00E-143
493- 24: transcript:Zm00001d051627_T002 rna6892 2.00E-136
493- 25: transcript:Zm00001d051628_T002 rna6893 0
493- 26: transcript:Zm00001d051629_T003 rna6894 0
493- 27: transcript:Zm00001d051632_T002 rna6895 3.00E-161
493- 28: transcript:Zm00001d051633_T003 rna6896 0
493- 29: transcript:Zm00001d051634_T001 rna6897 0
493- 30: transcript:Zm00001d051635_T001 rna6903 6.00E-21
493- 31: transcript:Zm00001d051636_T001 rna6905 0
493- 32: transcript:Zm00001d051637_T001 rna6907 0
493- 33: transcript:Zm00001d051643_T001 rna6909 9.00E-66
493- 34: transcript:Zm00001d051644_T004 rna6911 0
Alignment 494: score=1287.0 e_value=1.7e-96 N=28 4&NC_008395.2 plus
494- 0: transcript:Zm00001d051736_T001 rna6981 1.00E-103
494- 1: transcript:Zm00001d051740_T005 rna6983 0
494- 2: transcript:Zm00001d051741_T001 rna6985 2.00E-115
494- 3: transcript:Zm00001d051746_T001 rna6991 6.00E-178
494- 4: transcript:Zm00001d051749_T001 rna6993 5.00E-136
494- 5: transcript:Zm00001d051754_T003 rna6995 0
494- 6: transcript:Zm00001d051756_T004 rna6997 0
494- 7: transcript:Zm00001d051759_T001 rna6998 0
494- 8: transcript:Zm00001d051760_T001 rna6999 2.00E-89
494- 9: transcript:Zm00001d051761_T001 rna7000 0
494- 10: transcript:Zm00001d051785_T001 rna7007 7.00E-57
494- 11: transcript:Zm00001d051787_T001 rna7009 0
494- 12: transcript:Zm00001d051788_T001 rna7014 1.00E-111
494- 13: transcript:Zm00001d051790_T001 rna7017 0
494- 14: transcript:Zm00001d051793_T001 rna7020 1.00E-49
494- 15: transcript:Zm00001d051795_T001 rna7021 2.00E-57
494- 16: transcript:Zm00001d051796_T003 rna7023 0
494- 17: transcript:Zm00001d051798_T001 rna7024 5.00E-24
494- 18: transcript:Zm00001d051799_T001 rna7025 9.00E-91
494- 19: transcript:Zm00001d051800_T002 rna7026 2.00E-52
494- 20: transcript:Zm00001d051803_T001 rna7031 3.00E-72
494- 21: transcript:Zm00001d051804_T001 rna7033 0
494- 22: transcript:Zm00001d051806_T001 rna7034 0
494- 23: transcript:Zm00001d051807_T001 rna7035 0
494- 24: transcript:Zm00001d051808_T002 rna7036 1.00E-94
494- 25: transcript:Zm00001d051809_T001 rna7041 0
494- 26: transcript:Zm00001d051814_T001 rna7052 0
494- 27: transcript:Zm00001d051815_T002 rna7053 6.00E-172
Alignment 495: score=1210.0 e_value=1.8e-84 N=26 4&NC_008395.2 plus
495- 0: transcript:Zm00001d051110_T001 rna6377 0
495- 1: transcript:Zm00001d051111_T001 rna6378 0
495- 2: transcript:Zm00001d051114_T001 rna6385 0
495- 3: transcript:Zm00001d051116_T001 rna6386 2.00E-37

495- 4: transcript:Zm00001d051117_T001 rna6387 5.00E-84
495- 5: transcript:Zm00001d051119_T001 rna6389 0
495- 6: transcript:Zm00001d051120_T001 rna6391 4.00E-22
495- 7: transcript:Zm00001d051121_T001 rna6392 4.00E-69
495- 8: transcript:Zm00001d051126_T004 rna6396 0
495- 9: transcript:Zm00001d051128_T001 rna6401 2.00E-30
495- 10: transcript:Zm00001d051129_T002 rna6402 0
495- 11: transcript:Zm00001d051130_T001 rna6403 0
495- 12: transcript:Zm00001d051135_T005 rna6404 0
495- 13: transcript:Zm00001d051136_T001 rna6405 5.00E-180
495- 14: transcript:Zm00001d051138_T001 rna6407 2.00E-132
495- 15: transcript:Zm00001d051139_T002 rna6408 4.00E-68
495- 16: transcript:Zm00001d051143_T001 rna6410 9.00E-90
495- 17: transcript:Zm00001d051149_T001 rna6413 1.00E-123
495- 18: transcript:Zm00001d051156_T001 rna6417 7.00E-158
495- 19: transcript:Zm00001d051157_T001 rna6418 0
495- 20: transcript:Zm00001d051161_T003 rna6420 0
495- 21: transcript:Zm00001d051164_T001 rna6421 1.00E-58
495- 22: transcript:Zm00001d051172_T001 rna6427 0
495- 23: transcript:Zm00001d051174_T001 rna6431 0
495- 24: transcript:Zm00001d051178_T001 rna6438 0
495- 25: transcript:Zm00001d051180_T002 rna6440 3.00E-139
Alignment 496: score=969.0 e_value=6.8e-66 N=21 4&NC_008395.2 plus
496- 0: transcript:Zm00001d053941_T001 rna4462 1.00E-160
496- 1: transcript:Zm00001d053952_T001 rna4464 1.00E-134
496- 2: transcript:Zm00001d053954_T001 rna4469 2.00E-167
496- 3: transcript:Zm00001d053956_T001 rna4471 0
496- 4: transcript:Zm00001d053957_T003 rna4477 6.00E-151
496- 5: transcript:Zm00001d053961_T002 rna4486 2.00E-38
496- 6: transcript:Zm00001d053964_T001 rna4487 2.00E-22
496- 7: transcript:Zm00001d053965_T001 rna4488 2.00E-64
496- 8: transcript:Zm00001d053966_T001 rna4489 1.00E-51
496- 9: transcript:Zm00001d053967_T001 rna4490 4.00E-133
496- 10: transcript:Zm00001d053969_T002 rna4491 4.00E-110
496- 11: transcript:Zm00001d053972_T001 rna4492 3.00E-169
496- 12: transcript:Zm00001d053974_T001 rna4498 1.00E-84
496- 13: transcript:Zm00001d053975_T003 rna4499 1.00E-178
496- 14: transcript:Zm00001d053978_T001 rna4502 8.00E-171
496- 15: transcript:Zm00001d053979_T001 rna4503 3.00E-44
496- 16: transcript:Zm00001d053981_T002 rna4504 6.00E-160
496- 17: transcript:Zm00001d053983_T002 rna4513 1.00E-137
496- 18: transcript:Zm00001d053986_T001 rna4518 0
496- 19: transcript:Zm00001d053987_T003 rna4519 0
496- 20: transcript:Zm00001d053988_T001 rna4521 1.00E-61
Alignment 497: score=958.0 e_value=2.6e-63 N=22 4&NC_008395.2 plus
497- 0: transcript:Zm00001d050637_T002 rna5867 9.00E-42
497- 1: transcript:Zm00001d050642_T001 rna5870 0
497- 2: transcript:Zm00001d050645_T005 rna5883 0
497- 3: transcript:Zm00001d050649_T001 rna5898 7.00E-116
497- 4: transcript:Zm00001d050660_T001 rna5917 2.00E-62
497- 5: transcript:Zm00001d050664_T002 rna5926 0
497- 6: transcript:Zm00001d050666_T001 rna5927 4.00E-157
497- 7: transcript:Zm00001d050669_T004 rna5933 2.00E-173
497- 8: transcript:Zm00001d050689_T001 rna5941 0

497- 9: transcript:Zm00001d050694_T001 rna5944 2.00E-139
497- 10: transcript:Zm00001d050696_T005 rna5945 2.00E-40
497- 11: transcript:Zm00001d050698_T001 rna5948 6.00E-156
497- 12: transcript:Zm00001d050705_T001 rna5951 1.00E-27
497- 13: transcript:Zm00001d050708_T002 rna5956 0
497- 14: transcript:Zm00001d050712_T001 rna5968 0
497- 15: transcript:Zm00001d050715_T003 rna5973 0
497- 16: transcript:Zm00001d050716_T002 rna5977 0
497- 17: transcript:Zm00001d050723_T002 rna5981 0
497- 18: transcript:Zm00001d050726_T001 rna5982 3.00E-112
497- 19: transcript:Zm00001d050730_T001 rna5986 2.00E-160
497- 20: transcript:Zm00001d050735_T003 rna5995 2.00E-52
497- 21: transcript:Zm00001d050737_T003 rna6000 0
Alignment 498: score=895.0 e_value=6.3e-58 N=20 4&NC_008395.2 plus
498- 0: transcript:Zm00001d051041_T001 rna6308 5.00E-159
498- 1: transcript:Zm00001d051043_T001 rna6314 0
498- 2: transcript:Zm00001d051047_T001 rna6327 8.00E-116
498- 3: transcript:Zm00001d051052_T001 rna6328 6.00E-69
498- 4: transcript:Zm00001d051053_T001 rna6329 2.00E-69
498- 5: transcript:Zm00001d051056_T001 rna6332 0
498- 6: transcript:Zm00001d051057_T002 rna6333 7.00E-89
498- 7: transcript:Zm00001d051061_T001 rna6335 3.00E-72
498- 8: transcript:Zm00001d051062_T001 rna6338 3.00E-36
498- 9: transcript:Zm00001d051063_T001 rna6339 8.00E-98
498- 10: transcript:Zm00001d051065_T001 rna6341 1.00E-134
498- 11: transcript:Zm00001d051067_T003 rna6344 0
498- 12: transcript:Zm00001d051069_T001 rna6346 0
498- 13: transcript:Zm00001d051075_T002 rna6348 6.00E-139
498- 14: transcript:Zm00001d051080_T004 rna6350 4.00E-53
498- 15: transcript:Zm00001d051081_T001 rna6351 0
498- 16: transcript:Zm00001d051082_T001 rna6352 1.00E-44
498- 17: transcript:Zm00001d051093_T001 rna6360 1.00E-72
498- 18: transcript:Zm00001d051096_T001 rna6361 0
498- 19: transcript:Zm00001d051102_T001 rna6366 9.00E-06
Alignment 499: score=748.0 e_value=5.6e-45 N=17 4&NC_008395.2 plus
499- 0: transcript:Zm00001d050544_T001 rna5763 1.00E-67
499- 1: transcript:Zm00001d050550_T001 rna5771 8.00E-46
499- 2: transcript:Zm00001d050551_T007 rna5772 0
499- 3: transcript:Zm00001d050552_T001 rna5774 2.00E-123
499- 4: transcript:Zm00001d050553_T001 rna5777 2.00E-69
499- 5: transcript:Zm00001d050557_T001 rna5781 2.00E-51
499- 6: transcript:Zm00001d050558_T001 rna5784 1.00E-56
499- 7: transcript:Zm00001d050563_T001 rna5790 7.00E-62
499- 8: transcript:Zm00001d050565_T001 rna5791 0
499- 9: transcript:Zm00001d050567_T001 rna5793 0
499- 10: transcript:Zm00001d050575_T002 rna5807 0
499- 11: transcript:Zm00001d050577_T001 rna5808 6.00E-140
499- 12: transcript:Zm00001d050580_T001 rna5817 0
499- 13: transcript:Zm00001d050583_T001 rna5821 2.00E-111
499- 14: transcript:Zm00001d050584_T034 rna5826 0
499- 15: transcript:Zm00001d050600_T002 rna5839 9.00E-91
499- 16: transcript:Zm00001d050603_T001 rna5841 2.00E-16
Alignment 500: score=722.0 e_value=1.3e-39 N=15 4&NC_008395.2 plus
500- 0: transcript:Zm00001d053729_T001 rna4819 2.00E-16

500- 1: transcript:Zm00001d053731_T001 rna4820 2.00E-43
500- 2: transcript:Zm00001d053732_T001 rna4821 0
500- 3: transcript:Zm00001d053734_T001 rna4822 2.00E-17
500- 4: transcript:Zm00001d053736_T001 rna4827 1.00E-116
500- 5: transcript:Zm00001d053738_T001 rna4828 0
500- 6: transcript:Zm00001d053739_T001 rna4829 2.00E-129
500- 7: transcript:Zm00001d053740_T001 rna4830 0
500- 8: transcript:Zm00001d053741_T002 rna4831 0
500- 9: transcript:Zm00001d053743_T002 rna4835 3.00E-113
500- 10: transcript:Zm00001d053746_T001 rna4840 2.00E-131
500- 11: transcript:Zm00001d053747_T002 rna4842 2.00E-175
500- 12: transcript:Zm00001d053748_T001 rna4847 9.00E-57
500- 13: transcript:Zm00001d053749_T002 rna4848 2.00E-140
500- 14: transcript:Zm00001d053750_T001 rna4849 4.00E-09
Alignment 501: score=675.0 e_value=2.2e-38 N=15 4&NC_008395.2 plus
501- 0: transcript:Zm00001d050974_T001 rna6237 6.00E-172
501- 1: transcript:Zm00001d050977_T001 rna6238 0
501- 2: transcript:Zm00001d050978_T001 rna6239 3.00E-146
501- 3: transcript:Zm00001d050981_T001 rna6240 0
501- 4: transcript:Zm00001d050985_T002 rna6241 0
501- 5: transcript:Zm00001d050988_T001 rna6243 1.00E-67
501- 6: transcript:Zm00001d050992_T001 rna6246 1.00E-89
501- 7: transcript:Zm00001d050993_T001 rna6267 2.00E-66
501- 8: transcript:Zm00001d051001_T001 rna6268 0
501- 9: transcript:Zm00001d051005_T001 rna6274 4.00E-69
501- 10: transcript:Zm00001d051007_T001 rna6277 9.00E-47
501- 11: transcript:Zm00001d051009_T002 rna6282 7.00E-85
501- 12: transcript:Zm00001d051012_T001 rna6285 0
501- 13: transcript:Zm00001d051014_T001 rna6291 0
501- 14: transcript:Zm00001d051016_T002 rna6305 1.00E-53
Alignment 502: score=534.0 e_value=6.6e-28 N=12 4&NC_008395.2 plus
502- 0: transcript:Zm00001d051511_T001 rna6765 8.00E-125
502- 1: transcript:Zm00001d051513_T001 rna6768 2.00E-45
502- 2: transcript:Zm00001d051514_T002 rna6770 0
502- 3: transcript:Zm00001d051516_T001 rna6771 3.00E-176
502- 4: transcript:Zm00001d051518_T001 rna6776 2.00E-174
502- 5: transcript:Zm00001d051520_T001 rna6777 5.00E-133
502- 6: transcript:Zm00001d051526_T002 rna6782 7.00E-124
502- 7: transcript:Zm00001d051528_T001 rna6785 6.00E-106
502- 8: transcript:Zm00001d051529_T001 rna6787 0
502- 9: transcript:Zm00001d051532_T002 rna6788 2.00E-33
502- 10: transcript:Zm00001d051535_T001 rna6789 0
502- 11: transcript:Zm00001d051540_T001 rna6815 3.00E-88
Alignment 503: score=430.0 e_value=9.7e-19 N=9 4&NC_008395.2 plus
503- 0: transcript:Zm00001d053352_T001 rna5282 6.00E-77
503- 1: transcript:Zm00001d053359_T001 rna5292 6.00E-109
503- 2: transcript:Zm00001d053367_T001 rna5295 2.00E-19
503- 3: transcript:Zm00001d053369_T001 rna5297 2.00E-106
503- 4: transcript:Zm00001d053371_T001 rna5299 4.00E-135
503- 5: transcript:Zm00001d053372_T001 rna5301 0
503- 6: transcript:Zm00001d053374_T001 rna5303 4.00E-39
503- 7: transcript:Zm00001d053375_T001 rna5304 1.00E-103
503- 8: transcript:Zm00001d053376_T003 rna5306 0
Alignment 504: score=418.0 e_value=1.3e-20 N=9 4&NC_008395.2 plus

504- 0: transcript:Zm00001d051543_T001 rna6800 2.00E-111
504- 1: transcript:Zm00001d051552_T001 rna6816 0
504- 2: transcript:Zm00001d051553_T004 rna6823 0
504- 3: transcript:Zm00001d051554_T002 rna6827 4.00E-40
504- 4: transcript:Zm00001d051556_T001 rna6830 0
504- 5: transcript:Zm00001d051561_T001 rna6832 1.00E-84
504- 6: transcript:Zm00001d051562_T001 rna6833 0
504- 7: transcript:Zm00001d051563_T001 rna6835 6.00E-34
504- 8: transcript:Zm00001d051564_T001 rna6836 5.00E-121
Alignment 505: score=400.0 e_value=9.4e-17 N=9 4&NC_008395.2 plus
505- 0: transcript:Zm00001d053918_T001 rna4437 3.00E-127
505- 1: transcript:Zm00001d053919_T001 rna4440 2.00E-76
505- 2: transcript:Zm00001d053923_T003 rna4443 1.00E-100
505- 3: transcript:Zm00001d053925_T001 rna4445 2.00E-29
505- 4: transcript:Zm00001d053926_T001 rna4446 8.00E-41
505- 5: transcript:Zm00001d053927_T021 rna4448 4.00E-44
505- 6: transcript:Zm00001d053930_T001 rna4449 0
505- 7: transcript:Zm00001d053931_T003 rna4450 1.00E-139
505- 8: transcript:Zm00001d053932_T002 rna4453 0
Alignment 506: score=393.0 e_value=2.7e-19 N=9 4&NC_008395.2 plus
506- 0: transcript:Zm00001d051938_T002 rna7488 0
506- 1: transcript:Zm00001d051939_T001 rna7489 2.00E-168
506- 2: transcript:Zm00001d051944_T001 rna7490 3.00E-11
506- 3: transcript:Zm00001d051945_T001 rna7494 0
506- 4: transcript:Zm00001d051946_T001 rna7495 0
506- 5: transcript:Zm00001d051950_T003 rna7497 1.00E-94
506- 6: transcript:Zm00001d051951_T008 rna7499 0
506- 7: transcript:Zm00001d051956_T001 rna7502 2.00E-111
506- 8: transcript:Zm00001d051960_T001 rna7517 4.00E-41
Alignment 507: score=389.0 e_value=2.6e-15 N=8 4&NC_008395.2 plus
507- 0: transcript:Zm00001d051696_T001 rna6965 2.00E-35
507- 1: transcript:Zm00001d051697_T001 rna6967 7.00E-85
507- 2: transcript:Zm00001d051699_T001 rna6969 1.00E-88
507- 3: transcript:Zm00001d051700_T001 rna6970 4.00E-59
507- 4: transcript:Zm00001d051702_T002 rna6971 6.00E-126
507- 5: transcript:Zm00001d051703_T001 rna6976 0
507- 6: transcript:Zm00001d051704_T002 rna6978 0
507- 7: transcript:Zm00001d051706_T001 rna6980 1.00E-54
Alignment 508: score=374.0 e_value=2.7e-15 N=8 4&NC_008395.2 plus
508- 0: transcript:Zm00001d053895_T001 rna4426 3.00E-168
508- 1: transcript:Zm00001d053897_T001 rna4427 6.00E-105
508- 2: transcript:Zm00001d053899_T001 rna4429 1.00E-120
508- 3: transcript:Zm00001d053900_T001 rna4430 0
508- 4: transcript:Zm00001d053901_T001 rna4431 0
508- 5: transcript:Zm00001d053909_T002 rna4433 0
508- 6: transcript:Zm00001d053911_T002 rna4435 3.00E-133
508- 7: transcript:Zm00001d053916_T002 rna4437 0
Alignment 509: score=374.0 e_value=1.3e-14 N=8 4&NC_008395.2 plus
509- 0: transcript:Zm00001d051685_T001 rna6920 0
509- 1: transcript:Zm00001d051687_T001 rna6921 9.00E-115
509- 2: transcript:Zm00001d051688_T002 rna6922 0
509- 3: transcript:Zm00001d051689_T001 rna6924 1.00E-72
509- 4: transcript:Zm00001d051690_T001 rna6926 5.00E-23
509- 5: transcript:Zm00001d051691_T001 rna6930 2.00E-82

509- 6: transcript:Zm00001d051692_T001 rna6931 2.00E-70
509- 7: transcript:Zm00001d051695_T001 rna6932 0
Alignment 510: score=353.0 e_value=3.3e-16 N=8 4&NC_008395.2 plus
510- 0: transcript:Zm00001d051674_T037 rna6941 0
510- 1: transcript:Zm00001d051675_T001 rna6944 0
510- 2: transcript:Zm00001d051676_T007 rna6946 3.00E-172
510- 3: transcript:Zm00001d051677_T003 rna6948 4.00E-24
510- 4: transcript:Zm00001d051678_T001 rna6949 0
510- 5: transcript:Zm00001d051681_T001 rna6950 0
510- 6: transcript:Zm00001d051682_T001 rna6951 1.00E-63
510- 7: transcript:Zm00001d051684_T004 rna6955 0
Alignment 511: score=352.0 e_value=5.3e-17 N=8 4&NC_008395.2 plus
511- 0: transcript:Zm00001d053522_T001 rna5146 3.00E-179
511- 1: transcript:Zm00001d053541_T002 rna5156 0
511- 2: transcript:Zm00001d053543_T001 rna5157 5.00E-18
511- 3: transcript:Zm00001d053544_T001 rna5161 0
511- 4: transcript:Zm00001d053545_T003 rna5163 0
511- 5: transcript:Zm00001d053547_T003 rna5169 1.00E-129
511- 6: transcript:Zm00001d053548_T001 rna5173 0
511- 7: transcript:Zm00001d053554_T001 rna5181 0
Alignment 512: score=346.0 e_value=9.6e-12 N=7 4&NC_008395.2 plus
512- 0: transcript:Zm00001d050959_T001 rna6224 8.00E-69
512- 1: transcript:Zm00001d050960_T001 rna6225 0
512- 2: transcript:Zm00001d050961_T001 rna6226 0
512- 3: transcript:Zm00001d050963_T001 rna6229 5.00E-20
512- 4: transcript:Zm00001d050964_T001 rna6230 6.00E-118
512- 5: transcript:Zm00001d050965_T001 rna6231 2.00E-135
512- 6: transcript:Zm00001d050969_T001 rna6232 0
Alignment 513: score=322.0 e_value=5.4e-13 N=7 4&NC_008395.2 plus
513- 0: transcript:Zm00001d051183_T031 rna6441 1.00E-36
513- 1: transcript:Zm00001d051187_T009 rna6444 0
513- 2: transcript:Zm00001d051193_T004 rna6451 5.00E-39
513- 3: transcript:Zm00001d051203_T001 rna6454 2.00E-160
513- 4: transcript:Zm00001d051206_T001 rna6456 0
513- 5: transcript:Zm00001d051207_T001 rna6457 5.00E-38
513- 6: transcript:Zm00001d051211_T004 rna6458 7.00E-156
Alignment 514: score=253.0 e_value=1.7e-09 N=6 4&NC_008395.2 plus
514- 0: transcript:Zm00001d053994_T008 rna4524 0
514- 1: transcript:Zm00001d053995_T001 rna4533 7.00E-45
514- 2: transcript:Zm00001d053997_T004 rna4539 0
514- 3: transcript:Zm00001d053998_T001 rna4540 4.00E-74
514- 4: transcript:Zm00001d054000_T001 rna4541 0
514- 5: transcript:Zm00001d054001_T007 rna4566 0
Alignment 515: score=3323.0 e_value=3.7e-291 N=71 4&NC_008395.2 minus
515- 0: transcript:Zm00001d052153_T001 rna7231 2.00E-174
515- 1: transcript:Zm00001d052155_T001 rna7230 1.00E-82
515- 2: transcript:Zm00001d052157_T005 rna7225 0
515- 3: transcript:Zm00001d052158_T001 rna7224 0
515- 4: transcript:Zm00001d052162_T001 rna7222 4.00E-62
515- 5: transcript:Zm00001d052163_T001 rna7221 5.00E-70
515- 6: transcript:Zm00001d052164_T002 rna7220 8.00E-144
515- 7: transcript:Zm00001d052167_T001 rna7215 1.00E-77
515- 8: transcript:Zm00001d052168_T001 rna7208 2.00E-153
515- 9: transcript:Zm00001d052171_T001 rna7207 1.00E-92

515- 10:	transcript:Zm00001d052172_T001	rna7206	3.00E-54
515- 11:	transcript:Zm00001d052173_T001	rna7204	1.00E-65
515- 12:	transcript:Zm00001d052174_T001	rna7203	8.00E-74
515- 13:	transcript:Zm00001d052176_T001	rna7197	1.00E-17
515- 14:	transcript:Zm00001d052177_T001	rna7196	2.00E-39
515- 15:	transcript:Zm00001d052179_T001	rna7194	5.00E-121
515- 16:	transcript:Zm00001d052180_T001	rna7191	1.00E-133
515- 17:	transcript:Zm00001d052182_T001	rna7187	2.00E-59
515- 18:	transcript:Zm00001d052183_T001	rna7185	4.00E-163
515- 19:	transcript:Zm00001d052184_T001	rna7183	5.00E-66
515- 20:	transcript:Zm00001d052186_T001	rna7182	0
515- 21:	transcript:Zm00001d052188_T001	rna7181	0
515- 22:	transcript:Zm00001d052189_T001	rna7179	9.00E-123
515- 23:	transcript:Zm00001d052192_T001	rna7178	2.00E-66
515- 24:	transcript:Zm00001d052193_T012	rna7175	0
515- 25:	transcript:Zm00001d052194_T001	rna7174	3.00E-104
515- 26:	transcript:Zm00001d052198_T002	rna7173	1.00E-105
515- 27:	transcript:Zm00001d052200_T005	rna7171	0
515- 28:	transcript:Zm00001d052205_T001	rna7165	2.00E-100
515- 29:	transcript:Zm00001d052208_T002	rna7163	1.00E-39
515- 30:	transcript:Zm00001d052209_T001	rna7160	2.00E-179
515- 31:	transcript:Zm00001d052212_T001	rna7159	3.00E-54
515- 32:	transcript:Zm00001d052213_T002	rna7157	5.00E-51
515- 33:	transcript:Zm00001d052215_T001	rna7156	2.00E-178
515- 34:	transcript:Zm00001d052216_T001	rna7150	2.00E-77
515- 35:	transcript:Zm00001d052218_T001	rna7149	0
515- 36:	transcript:Zm00001d052219_T002	rna7148	0
515- 37:	transcript:Zm00001d052220_T001	rna7145	5.00E-24
515- 38:	transcript:Zm00001d052221_T001	rna7144	3.00E-125
515- 39:	transcript:Zm00001d052223_T001	rna7143	6.00E-122
515- 40:	transcript:Zm00001d052225_T001	rna7141	4.00E-120
515- 41:	transcript:Zm00001d052226_T001	rna7140	5.00E-08
515- 42:	transcript:Zm00001d052229_T001	rna7139	7.00E-145
515- 43:	transcript:Zm00001d052230_T001	rna7137	2.00E-119
515- 44:	transcript:Zm00001d052231_T003	rna7135	7.00E-179
515- 45:	transcript:Zm00001d052232_T002	rna7134	0
515- 46:	transcript:Zm00001d052233_T001	rna7133	0
515- 47:	transcript:Zm00001d052234_T001	rna7131	4.00E-41
515- 48:	transcript:Zm00001d052237_T001	rna7130	0
515- 49:	transcript:Zm00001d052239_T001	rna7129	5.00E-64
515- 50:	transcript:Zm00001d052240_T001	rna7127	1.00E-118
515- 51:	transcript:Zm00001d052242_T001	rna7124	6.00E-120
515- 52:	transcript:Zm00001d052244_T001	rna7121	3.00E-18
515- 53:	transcript:Zm00001d052247_T001	rna7118	6.00E-110
515- 54:	transcript:Zm00001d052248_T009	rna7116	2.00E-72
515- 55:	transcript:Zm00001d052252_T001	rna7113	1.00E-168
515- 56:	transcript:Zm00001d052254_T001	rna7110	3.00E-27
515- 57:	transcript:Zm00001d052255_T001	rna7109	2.00E-30
515- 58:	transcript:Zm00001d052256_T001	rna7107	2.00E-146
515- 59:	transcript:Zm00001d052258_T001	rna7103	7.00E-171
515- 60:	transcript:Zm00001d052259_T001	rna7102	2.00E-101
515- 61:	transcript:Zm00001d052260_T001	rna7101	0
515- 62:	transcript:Zm00001d052261_T001	rna7098	1.00E-126
515- 63:	transcript:Zm00001d052263_T001	rna7094	0

515- 64: transcript:Zm00001d052266_T001 rna7090 3.00E-153
515- 65: transcript:Zm00001d052268_T001 rna7087 2.00E-113
515- 66: transcript:Zm00001d052269_T001 rna7085 0
515- 67: transcript:Zm00001d052271_T003 rna7079 2.00E-81
515- 68: transcript:Zm00001d052273_T004 rna7078 4.00E-120
515- 69: transcript:Zm00001d052276_T005 rna7074 1.00E-110
515- 70: transcript:Zm00001d052277_T001 rna7072 1.00E-64
Alignment 516: score=2510.0 e_value=4.1e-221 N=55 4&NC_008395.2 minus
516- 0: transcript:Zm00001d051849_T023 rna7644 0
516- 1: transcript:Zm00001d051850_T001 rna7635 1.00E-74
516- 2: transcript:Zm00001d051851_T005 rna7634 1.00E-161
516- 3: transcript:Zm00001d051854_T001 rna7633 1.00E-36
516- 4: transcript:Zm00001d051859_T001 rna7625 4.00E-09
516- 5: transcript:Zm00001d051860_T001 rna7613 5.00E-16
516- 6: transcript:Zm00001d051861_T001 rna7612 0
516- 7: transcript:Zm00001d051863_T001 rna7610 0
516- 8: transcript:Zm00001d051864_T003 rna7609 0
516- 9: transcript:Zm00001d051866_T004 rna7608 0
516- 10: transcript:Zm00001d051869_T001 rna7607 3.00E-40
516- 11: transcript:Zm00001d051870_T001 rna7606 0
516- 12: transcript:Zm00001d051871_T002 rna7605 0
516- 13: transcript:Zm00001d051872_T001 rna7603 0
516- 14: transcript:Zm00001d051873_T002 rna7602 0
516- 15: transcript:Zm00001d051876_T002 rna7600 0
516- 16: transcript:Zm00001d051877_T001 rna7599 3.00E-71
516- 17: transcript:Zm00001d051879_T001 rna7596 0
516- 18: transcript:Zm00001d051881_T001 rna7595 1.00E-96
516- 19: transcript:Zm00001d051882_T001 rna7594 3.00E-22
516- 20: transcript:Zm00001d051883_T001 rna7593 0
516- 21: transcript:Zm00001d051884_T002 rna7592 0
516- 22: transcript:Zm00001d051885_T001 rna7590 7.00E-30
516- 23: transcript:Zm00001d051886_T001 rna7588 0
516- 24: transcript:Zm00001d051887_T001 rna7587 0
516- 25: transcript:Zm00001d051889_T002 rna7583 0
516- 26: transcript:Zm00001d051891_T001 rna7579 1.00E-102
516- 27: transcript:Zm00001d051892_T001 rna7578 0
516- 28: transcript:Zm00001d051893_T001 rna7577 0
516- 29: transcript:Zm00001d051894_T001 rna7576 1.00E-61
516- 30: transcript:Zm00001d051898_T001 rna7575 2.00E-177
516- 31: transcript:Zm00001d051899_T001 rna7574 3.00E-176
516- 32: transcript:Zm00001d051900_T001 rna7572 0
516- 33: transcript:Zm00001d051901_T004 rna7570 0
516- 34: transcript:Zm00001d051902_T003 rna7569 1.00E-22
516- 35: transcript:Zm00001d051905_T001 rna7566 8.00E-74
516- 36: transcript:Zm00001d051906_T003 rna7563 1.00E-97
516- 37: transcript:Zm00001d051908_T001 rna7561 8.00E-108
516- 38: transcript:Zm00001d051911_T002 rna7557 8.00E-124
516- 39: transcript:Zm00001d051912_T001 rna7556 0
516- 40: transcript:Zm00001d051913_T005 rna7554 0
516- 41: transcript:Zm00001d051915_T001 rna7549 0
516- 42: transcript:Zm00001d051917_T007 rna7547 0
516- 43: transcript:Zm00001d051923_T001 rna7545 0
516- 44: transcript:Zm00001d051924_T002 rna7544 2.00E-151
516- 45: transcript:Zm00001d051925_T001 rna7542 0

516- 46: transcript:Zm00001d051926_T001 rna7534 3.00E-80
516- 47: transcript:Zm00001d051927_T001 rna7532 5.00E-103
516- 48: transcript:Zm00001d051928_T001 rna7531 0
516- 49: transcript:Zm00001d051929_T001 rna7530 3.00E-98
516- 50: transcript:Zm00001d051930_T002 rna7529 0
516- 51: transcript:Zm00001d051932_T002 rna7525 0
516- 52: transcript:Zm00001d051936_T001 rna7511 1.00E-121
516- 53: transcript:Zm00001d051938_T002 rna7506 0
516- 54: transcript:Zm00001d051946_T001 rna7495 0
Alignment 517: score=1631.0 e_value=1e-124 N=36 4&NC_008395.2 minus
517- 0: transcript:Zm00001d053664_T001 rna4952 0
517- 1: transcript:Zm00001d053665_T002 rna4950 5.00E-160
517- 2: transcript:Zm00001d053667_T001 rna4948 7.00E-161
517- 3: transcript:Zm00001d053671_T001 rna4946 1.00E-78
517- 4: transcript:Zm00001d053672_T007 rna4945 0
517- 5: transcript:Zm00001d053674_T001 rna4940 0
517- 6: transcript:Zm00001d053675_T001 rna4939 0
517- 7: transcript:Zm00001d053676_T002 rna4938 0
517- 8: transcript:Zm00001d053680_T001 rna4936 2.00E-101
517- 9: transcript:Zm00001d053682_T001 rna4935 1.00E-60
517- 10: transcript:Zm00001d053685_T005 rna4934 0
517- 11: transcript:Zm00001d053687_T001 rna4933 7.00E-169
517- 12: transcript:Zm00001d053688_T002 rna4931 9.00E-114
517- 13: transcript:Zm00001d053694_T001 rna4929 1.00E-23
517- 14: transcript:Zm00001d053695_T001 rna4924 2.00E-19
517- 15: transcript:Zm00001d053696_T003 rna4922 0
517- 16: transcript:Zm00001d053697_T007 rna4920 9.00E-36
517- 17: transcript:Zm00001d053698_T004 rna4918 3.00E-63
517- 18: transcript:Zm00001d053702_T006 rna4916 2.00E-89
517- 19: transcript:Zm00001d053703_T001 rna4915 1.00E-24
517- 20: transcript:Zm00001d053704_T001 rna4914 3.00E-43
517- 21: transcript:Zm00001d053705_T001 rna4908 2.00E-85
517- 22: transcript:Zm00001d053706_T002 rna4899 0
517- 23: transcript:Zm00001d053707_T001 rna4897 5.00E-49
517- 24: transcript:Zm00001d053709_T001 rna4892 7.00E-64
517- 25: transcript:Zm00001d053713_T001 rna4891 3.00E-58
517- 26: transcript:Zm00001d053714_T001 rna4889 7.00E-10
517- 27: transcript:Zm00001d053715_T001 rna4886 0
517- 28: transcript:Zm00001d053716_T001 rna4883 3.00E-126
517- 29: transcript:Zm00001d053719_T002 rna4879 0
517- 30: transcript:Zm00001d053722_T001 rna4878 2.00E-43
517- 31: transcript:Zm00001d053724_T001 rna4874 2.00E-92
517- 32: transcript:Zm00001d053725_T002 rna4873 0
517- 33: transcript:Zm00001d053726_T001 rna4872 0
517- 34: transcript:Zm00001d053727_T003 rna4858 0
517- 35: transcript:Zm00001d053728_T001 rna4857 4.00E-06
Alignment 518: score=1238.0 e_value=9.8e-92 N=28 4&NC_008395.2 minus
518- 0: transcript:Zm00001d053816_T012 rna4737 5.00E-180
518- 1: transcript:Zm00001d053817_T001 rna4730 5.00E-101
518- 2: transcript:Zm00001d053818_T003 rna4728 0
518- 3: transcript:Zm00001d053819_T002 rna4726 0
518- 4: transcript:Zm00001d053822_T001 rna4725 2.00E-35
518- 5: transcript:Zm00001d053825_T002 rna4723 3.00E-132
518- 6: transcript:Zm00001d053828_T001 rna4713 5.00E-18

```
518-  7:  transcript:Zm00001d053829_T001  rna4710      6.00E-64
518-  8:  transcript:Zm00001d053838_T005  rna4708             0
518-  9:  transcript:Zm00001d053839_T001  rna4707      4.00E-77
518- 10:  transcript:Zm00001d053841_T014  rna4703             0
518- 11:  transcript:Zm00001d053843_T001  rna4700      1.00E-24
518- 12:  transcript:Zm00001d053846_T001  rna4697             0
518- 13:  transcript:Zm00001d053848_T001  rna4695             0
518- 14:  transcript:Zm00001d053850_T001  rna4694      5.00E-36
518- 15:  transcript:Zm00001d053852_T001  rna4688             0
518- 16:  transcript:Zm00001d053855_T001  rna4683     5.00E-134
518- 17:  transcript:Zm00001d053856_T001  rna4682             0
518- 18:  transcript:Zm00001d053858_T001  rna4681             0
518- 19:  transcript:Zm00001d053859_T001  rna4680      4.00E-48
518- 20:  transcript:Zm00001d053862_T002  rna4678      5.00E-55
518- 21:  transcript:Zm00001d053865_T007  rna4655             0
518- 22:  transcript:Zm00001d053866_T001  rna4651             0
518- 23:  transcript:Zm00001d053868_T001  rna4649      7.00E-51
518- 24:  transcript:Zm00001d053872_T021  rna4648             0
518- 25:  transcript:Zm00001d053873_T001  rna4646     2.00E-153
518- 26:  transcript:Zm00001d053875_T001  rna4645             0
518- 27:  transcript:Zm00001d053876_T011  rna4643             0
## Alignment 519: score=1189.0 e_value=6e-92 N=27 4&NC_008395.2 minus
519-  0:  transcript:Zm00001d051966_T001  rna7503      1.00E-92
519-  1:  transcript:Zm00001d051976_T002  rna7478     4.00E-163
519-  2:  transcript:Zm00001d051977_T001  rna7474             0
519-  3:  transcript:Zm00001d051980_T004  rna7473             0
519-  4:  transcript:Zm00001d051986_T001  rna7467      3.00E-51
519-  5:  transcript:Zm00001d051987_T001  rna7466     3.00E-171
519-  6:  transcript:Zm00001d051988_T001  rna7465             0
519-  7:  transcript:Zm00001d051989_T001  rna7464             0
519-  8:  transcript:Zm00001d051995_T001  rna7461             0
519-  9:  transcript:Zm00001d051998_T003  rna7459             0
519- 10:  transcript:Zm00001d052001_T002  rna7455             0
519- 11:  transcript:Zm00001d052002_T001  rna7454      7.00E-29
519- 12:  transcript:Zm00001d052003_T002  rna7452      2.00E-36
519- 13:  transcript:Zm00001d052007_T001  rna7446      2.00E-84
519- 14:  transcript:Zm00001d052008_T003  rna7445             0
519- 15:  transcript:Zm00001d052009_T002  rna7444     2.00E-123
519- 16:  transcript:Zm00001d052010_T001  rna7439      4.00E-59
519- 17:  transcript:Zm00001d052011_T003  rna7437     1.00E-117
519- 18:  transcript:Zm00001d052015_T001  rna7428      2.00E-39
519- 19:  transcript:Zm00001d052016_T001  rna7427      2.00E-11
519- 20:  transcript:Zm00001d052018_T001  rna7423     6.00E-150
519- 21:  transcript:Zm00001d052019_T001  rna7420      2.00E-92
519- 22:  transcript:Zm00001d052020_T001  rna7416             0
519- 23:  transcript:Zm00001d052021_T002  rna7411             0
519- 24:  transcript:Zm00001d052022_T002  rna7410             0
519- 25:  transcript:Zm00001d052026_T001  rna7408      5.00E-71
519- 26:  transcript:Zm00001d052028_T007  rna7406             0
## Alignment 520: score=1075.0 e_value=3.1e-73 N=23 4&NC_008395.2 minus
520-  0:  transcript:Zm00001d052110_T044  rna7295             0
520-  1:  transcript:Zm00001d052111_T007  rna7293             0
520-  2:  transcript:Zm00001d052112_T001  rna7292     1.00E-157
520-  3:  transcript:Zm00001d052113_T002  rna7291             0
```

520- 4: transcript:Zm00001d052118_T001 rna7286   2.00E-102
520- 5: transcript:Zm00001d052120_T001 rna7285   3.00E-84
520- 6: transcript:Zm00001d052122_T001 rna7276   6.00E-36
520- 7: transcript:Zm00001d052123_T001 rna7275   3.00E-82
520- 8: transcript:Zm00001d052124_T002 rna7273   5.00E-35
520- 9: transcript:Zm00001d052125_T001 rna7272   2.00E-40
520- 10: transcript:Zm00001d052130_T001 rna7267   5.00E-41
520- 11: transcript:Zm00001d052131_T001 rna7266   3.00E-51
520- 12: transcript:Zm00001d052135_T001 rna7264   5.00E-09
520- 13: transcript:Zm00001d052136_T001 rna7258   0
520- 14: transcript:Zm00001d052137_T001 rna7256   6.00E-98
520- 15: transcript:Zm00001d052138_T004 rna7254   0
520- 16: transcript:Zm00001d052139_T001 rna7253   0
520- 17: transcript:Zm00001d052143_T009 rna7252   0
520- 18: transcript:Zm00001d052144_T001 rna7251   0
520- 19: transcript:Zm00001d052145_T001 rna7248   0
520- 20: transcript:Zm00001d052146_T001 rna7246   0
520- 21: transcript:Zm00001d052147_T001 rna7244   6.00E-30
520- 22: transcript:Zm00001d052148_T001 rna7241   1.00E-46
Alignment 521: score=1071.0 e_value=4.6e-73 N=24 4&NC_008395.2 minus
521- 0: transcript:Zm00001d053753_T003 rna4815   0
521- 1: transcript:Zm00001d053754_T001 rna4812   4.00E-57
521- 2: transcript:Zm00001d053756_T001 rna4810   5.00E-68
521- 3: transcript:Zm00001d053757_T002 rna4809   2.00E-157
521- 4: transcript:Zm00001d053759_T001 rna4806   8.00E-54
521- 5: transcript:Zm00001d053761_T001 rna4804   0
521- 6: transcript:Zm00001d053762_T001 rna4802   3.00E-156
521- 7: transcript:Zm00001d053763_T001 rna4798   0
521- 8: transcript:Zm00001d053765_T001 rna4797   0
521- 9: transcript:Zm00001d053770_T001 rna4789   0
521- 10: transcript:Zm00001d053775_T001 rna4788   1.00E-87
521- 11: transcript:Zm00001d053776_T015 rna4786   5.00E-125
521- 12: transcript:Zm00001d053778_T001 rna4785   0
521- 13: transcript:Zm00001d053779_T001 rna4783   4.00E-28
521- 14: transcript:Zm00001d053781_T001 rna4778   3.00E-21
521- 15: transcript:Zm00001d053782_T006 rna4777   3.00E-178
521- 16: transcript:Zm00001d053783_T001 rna4773   0
521- 17: transcript:Zm00001d053784_T001 rna4771   8.00E-15
521- 18: transcript:Zm00001d053798_T004 rna4768   0
521- 19: transcript:Zm00001d053799_T005 rna4767   0
521- 20: transcript:Zm00001d053800_T001 rna4758   6.00E-59
521- 21: transcript:Zm00001d053805_T003 rna4752   1.00E-104
521- 22: transcript:Zm00001d053808_T001 rna4748   1.00E-122
521- 23: transcript:Zm00001d053813_T002 rna4743   0
Alignment 522: score=1061.0 e_value=7.8e-72 N=24 4&NC_008395.2 minus
522- 0: transcript:Zm00001d052030_T001 rna7403   0
522- 1: transcript:Zm00001d052031_T001 rna7402   0
522- 2: transcript:Zm00001d052036_T001 rna7401   2.00E-41
522- 3: transcript:Zm00001d052039_T001 rna7398   6.00E-65
522- 4: transcript:Zm00001d052040_T001 rna7393   6.00E-32
522- 5: transcript:Zm00001d052043_T003 rna7390   3.00E-92
522- 6: transcript:Zm00001d052044_T002 rna7389   2.00E-63
522- 7: transcript:Zm00001d052047_T001 rna7387   2.00E-125
522- 8: transcript:Zm00001d052050_T001 rna7383   0

522- 9: transcript:Zm00001d052051_T001 rna7372 0
522- 10: transcript:Zm00001d052054_T001 rna7368 8.00E-21
522- 11: transcript:Zm00001d052057_T013 rna7367 0
522- 12: transcript:Zm00001d052059_T001 rna7366 4.00E-173
522- 13: transcript:Zm00001d052060_T005 rna7365 0
522- 14: transcript:Zm00001d052061_T001 rna7363 0
522- 15: transcript:Zm00001d052062_T002 rna7362 2.00E-58
522- 16: transcript:Zm00001d052063_T003 rna7360 1.00E-163
522- 17: transcript:Zm00001d052064_T001 rna7353 0
522- 18: transcript:Zm00001d052066_T001 rna7351 0
522- 19: transcript:Zm00001d052067_T004 rna7349 0
522- 20: transcript:Zm00001d052068_T001 rna7346 6.00E-29
522- 21: transcript:Zm00001d052069_T001 rna7343 2.00E-131
522- 22: transcript:Zm00001d052070_T032 rna7341 3.00E-36
522- 23: transcript:Zm00001d052072_T003 rna7340 0
Alignment 523: score=1059.0 e_value=2e-77 N=25 4&NC_008395.2 minus
523- 0: transcript:Zm00001d053547_T003 rna5169 1.00E-129
523- 1: transcript:Zm00001d053555_T001 rna5145 0
523- 2: transcript:Zm00001d053556_T004 rna5144 1.00E-38
523- 3: transcript:Zm00001d053559_T005 rna5132 0
523- 4: transcript:Zm00001d053560_T001 rna5131 1.00E-55
523- 5: transcript:Zm00001d053561_T001 rna5128 0
523- 6: transcript:Zm00001d053562_T001 rna5117 2.00E-132
523- 7: transcript:Zm00001d053563_T002 rna5114 0
523- 8: transcript:Zm00001d053564_T020 rna5112 4.00E-140
523- 9: transcript:Zm00001d053565_T014 rna5111 0
523- 10: transcript:Zm00001d053566_T002 rna5110 9.00E-144
523- 11: transcript:Zm00001d053568_T001 rna5109 3.00E-37
523- 12: transcript:Zm00001d053569_T001 rna5104 0
523- 13: transcript:Zm00001d053572_T002 rna5101 5.00E-136
523- 14: transcript:Zm00001d053575_T005 rna5096 6.00E-70
523- 15: transcript:Zm00001d053576_T001 rna5094 0
523- 16: transcript:Zm00001d053585_T002 rna5089 0
523- 17: transcript:Zm00001d053586_T001 rna5084 0
523- 18: transcript:Zm00001d053587_T001 rna5083 0
523- 19: transcript:Zm00001d053588_T001 rna5081 0
523- 20: transcript:Zm00001d053589_T001 rna5076 3.00E-126
523- 21: transcript:Zm00001d053591_T002 rna5075 0
523- 22: transcript:Zm00001d053593_T001 rna5070 2.00E-56
523- 23: transcript:Zm00001d053595_T004 rna5057 0
523- 24: transcript:Zm00001d053597_T006 rna5054 3.00E-13
Alignment 524: score=974.0 e_value=5.1e-66 N=21 4&NC_008395.2 minus
524- 0: transcript:Zm00001d053620_T043 rna5004 1.00E-78
524- 1: transcript:Zm00001d053622_T003 rna5001 1.00E-75
524- 2: transcript:Zm00001d053623_T001 rna5000 0
524- 3: transcript:Zm00001d053624_T001 rna4994 2.00E-176
524- 4: transcript:Zm00001d053625_T001 rna4993 4.00E-23
524- 5: transcript:Zm00001d053626_T001 rna4990 8.00E-102
524- 6: transcript:Zm00001d053639_T001 rna4985 2.00E-28
524- 7: transcript:Zm00001d053641_T001 rna4984 2.00E-56
524- 8: transcript:Zm00001d053642_T001 rna4983 0
524- 9: transcript:Zm00001d053643_T001 rna4982 3.00E-119
524- 10: transcript:Zm00001d053648_T005 rna4979 3.00E-166
524- 11: transcript:Zm00001d053649_T002 rna4978 0

524- 12: transcript:Zm00001d053650_T001 rna4975 3.00E-160
524- 13: transcript:Zm00001d053652_T001 rna4973 3.00E-89
524- 14: transcript:Zm00001d053654_T001 rna4971 2.00E-117
524- 15: transcript:Zm00001d053655_T001 rna4970 5.00E-34
524- 16: transcript:Zm00001d053658_T002 rna4969 0
524- 17: transcript:Zm00001d053659_T001 rna4965 1.00E-142
524- 18: transcript:Zm00001d053660_T001 rna4959 2.00E-89
524- 19: transcript:Zm00001d053661_T003 rna4958 1.00E-29
524- 20: transcript:Zm00001d053663_T009 rna4955 0
Alignment 525: score=909.0 e_value=8.7e-62 N=21 4&NC_008395.2 minus
525- 0: transcript:Zm00001d053369_T001 rna5297 2.00E-106
525- 1: transcript:Zm00001d053377_T001 rna5275 1.00E-75
525- 2: transcript:Zm00001d053378_T001 rna5274 2.00E-125
525- 3: transcript:Zm00001d053382_T001 rna5271 0
525- 4: transcript:Zm00001d053395_T001 rna5261 0
525- 5: transcript:Zm00001d053396_T001 rna5257 4.00E-131
525- 6: transcript:Zm00001d053399_T001 rna5255 0
525- 7: transcript:Zm00001d053401_T014 rna5254 0
525- 8: transcript:Zm00001d053404_T001 rna5250 3.00E-123
525- 9: transcript:Zm00001d053406_T001 rna5249 2.00E-14
525- 10: transcript:Zm00001d053409_T001 rna5244 4.00E-64
525- 11: transcript:Zm00001d053415_T001 rna5235 1.00E-17
525- 12: transcript:Zm00001d053416_T001 rna5234 2.00E-60
525- 13: transcript:Zm00001d053425_T001 rna5222 0
525- 14: transcript:Zm00001d053427_T004 rna5221 0
525- 15: transcript:Zm00001d053433_T001 rna5215 1.00E-67
525- 16: transcript:Zm00001d053435_T001 rna5212 3.00E-27
525- 17: transcript:Zm00001d053442_T001 rna5207 7.00E-89
525- 18: transcript:Zm00001d053445_T001 rna5205 9.00E-09
525- 19: transcript:Zm00001d053446_T001 rna5201 0
525- 20: transcript:Zm00001d053448_T001 rna5194 0
Alignment 526: score=692.0 e_value=1.8e-41 N=15 4&NC_008395.2 minus
526- 0: transcript:Zm00001d054009_T001 rna4419 7.00E-152
526- 1: transcript:Zm00001d054010_T001 rna4418 5.00E-21
526- 2: transcript:Zm00001d054011_T002 rna4415 0
526- 3: transcript:Zm00001d054014_T001 rna4408 0
526- 4: transcript:Zm00001d054015_T001 rna4403 5.00E-111
526- 5: transcript:Zm00001d054016_T004 rna4402 0
526- 6: transcript:Zm00001d054017_T003 rna4400 1.00E-39
526- 7: transcript:Zm00001d054033_T001 rna4397 3.00E-93
526- 8: transcript:Zm00001d054034_T001 rna4396 0
526- 9: transcript:Zm00001d054038_T003 rna4394 9.00E-128
526- 10: transcript:Zm00001d054039_T001 rna4393 0
526- 11: transcript:Zm00001d054042_T008 rna4391 0
526- 12: transcript:Zm00001d054043_T003 rna4388 0
526- 13: transcript:Zm00001d054044_T001 rna4387 0
526- 14: transcript:Zm00001d054047_T001 rna4381 0
Alignment 527: score=551.0 e_value=2.7e-30 N=12 4&NC_008395.2 minus
527- 0: transcript:Zm00001d054077_T003 rna4320 9.00E-113
527- 1: transcript:Zm00001d054078_T002 rna4319 3.00E-126
527- 2: transcript:Zm00001d054079_T002 rna4318 0
527- 3: transcript:Zm00001d054080_T001 rna4317 3.00E-133
527- 4: transcript:Zm00001d054084_T001 rna4311 7.00E-123
527- 5: transcript:Zm00001d054086_T001 rna4308 8.00E-87

```
527-  6:  transcript:Zm00001d054089_T002  rna4305            0
527-  7:  transcript:Zm00001d054090_T008  rna4303            0
527-  8:  transcript:Zm00001d054093_T002  rna4299      8.00E-26
527-  9:  transcript:Zm00001d054094_T001  rna4297      1.00E-94
527- 10:  transcript:Zm00001d054095_T001  rna4296      4.00E-85
527- 11:  transcript:Zm00001d054104_T002  rna4295            0
## Alignment 528: score=550.0 e_value=5.1e-30 N=12 4&NC_008395.2 minus
528-  0:  transcript:Zm00001d054055_T001  rna4375            0
528-  1:  transcript:Zm00001d054056_T001  rna4374      2.00E-20
528-  2:  transcript:Zm00001d054057_T001  rna4366            0
528-  3:  transcript:Zm00001d054065_T001  rna4362      7.00E-61
528-  4:  transcript:Zm00001d054067_T001  rna4355            0
528-  5:  transcript:Zm00001d054069_T001  rna4352            0
528-  6:  transcript:Zm00001d054070_T002  rna4351            0
528-  7:  transcript:Zm00001d054071_T001  rna4336     4.00E-149
528-  8:  transcript:Zm00001d054072_T002  rna4334            0
528-  9:  transcript:Zm00001d054074_T001  rna4332      9.00E-14
528- 10:  transcript:Zm00001d054075_T001  rna4330            0
528- 11:  transcript:Zm00001d054076_T010  rna4321            0
## Alignment 529: score=510.0 e_value=7.3e-27 N=11 4&NC_008395.2 minus
529-  0:  transcript:Zm00001d051823_T001  rna7681     2.00E-172
529-  1:  transcript:Zm00001d051830_T001  rna7674      1.00E-60
529-  2:  transcript:Zm00001d051832_T001  rna7673     3.00E-126
529-  3:  transcript:Zm00001d051833_T001  rna7668      4.00E-51
529-  4:  transcript:Zm00001d051835_T001  rna7667      8.00E-67
529-  5:  transcript:Zm00001d051836_T001  rna7664      2.00E-34
529-  6:  transcript:Zm00001d051837_T002  rna7662            0
529-  7:  transcript:Zm00001d051838_T001  rna7658      1.00E-81
529-  8:  transcript:Zm00001d051839_T004  rna7657            0
529-  9:  transcript:Zm00001d051842_T003  rna7649     3.00E-157
529- 10:  transcript:Zm00001d051846_T001  rna7647            0
## Alignment 530: score=456.0 e_value=6.3e-24 N=11 4&NC_008395.2 minus
530-  0:  transcript:Zm00001d052079_T039  rna7332            0
530-  1:  transcript:Zm00001d052081_T002  rna7331            0
530-  2:  transcript:Zm00001d052082_T001  rna7328     9.00E-146
530-  3:  transcript:Zm00001d052087_T001  rna7327     1.00E-174
530-  4:  transcript:Zm00001d052093_T002  rna7324      1.00E-39
530-  5:  transcript:Zm00001d052098_T011  rna7323            0
530-  6:  transcript:Zm00001d052101_T001  rna7317     7.00E-138
530-  7:  transcript:Zm00001d052103_T001  rna7316            0
530-  8:  transcript:Zm00001d052104_T001  rna7305      3.00E-64
530-  9:  transcript:Zm00001d052107_T001  rna7301            0
530- 10:  transcript:Zm00001d052108_T003  rna7300     3.00E-135
## Alignment 531: score=391.0 e_value=2.4e-18 N=9 4&NC_008395.2 minus
531-  0:  transcript:Zm00001d053880_T001  rna4616            0
531-  1:  transcript:Zm00001d053881_T005  rna4607            0
531-  2:  transcript:Zm00001d053882_T001  rna4604            0
531-  3:  transcript:Zm00001d053884_T001  rna4595      2.00E-17
531-  4:  transcript:Zm00001d053885_T001  rna4583      1.00E-91
531-  5:  transcript:Zm00001d053886_T001  rna4581            0
531-  6:  transcript:Zm00001d053887_T001  rna4575     6.00E-100
531-  7:  transcript:Zm00001d053889_T004  rna4569      1.00E-47
531-  8:  transcript:Zm00001d053890_T003  rna4568      3.00E-49
## Alignment 532: score=322.0 e_value=5.8e-11 N=7 4&NC_008395.2 minus
```

532- 0: transcript:Zm00001d051542_T047 rna6801 0
532- 1: transcript:Zm00001d051543_T001 rna6800 2.00E-111
532- 2: transcript:Zm00001d051545_T003 rna6798 0
532- 3: transcript:Zm00001d051546_T001 rna6797 4.00E-58
532- 4: transcript:Zm00001d051548_T001 rna6796 0
532- 5: transcript:Zm00001d051550_T001 rna6795 2.00E-132
532- 6: transcript:Zm00001d051551_T001 rna6794 4.00E-117
Alignment 533: score=298.0 e_value=6.1e-11 N=6 4&NC_008395.2 minus
533- 0: transcript:Zm00001d051658_T001 rna6918 7.00E-12
533- 1: transcript:Zm00001d051660_T001 rna6917 5.00E-99
533- 2: transcript:Zm00001d051661_T001 rna6916 9.00E-128
533- 3: transcript:Zm00001d051662_T001 rna6915 3.00E-41
533- 4: transcript:Zm00001d051663_T001 rna6914 4.00E-88
533- 5: transcript:Zm00001d051664_T001 rna6912 0
Alignment 534: score=288.0 e_value=1.1e-09 N=6 4&NC_008395.2 minus
534- 0: transcript:Zm00001d051484_T001 rna6737 6.00E-42
534- 1: transcript:Zm00001d051492_T001 rna6734 3.00E-20
534- 2: transcript:Zm00001d051498_T001 rna6733 0
534- 3: transcript:Zm00001d051499_T001 rna6732 1.00E-30
534- 4: transcript:Zm00001d051501_T001 rna6729 9.00E-77
534- 5: transcript:Zm00001d051502_T001 rna6728 0
Alignment 535: score=273.0 e_value=2.8e-09 N=6 4&NC_008395.2 minus
535- 0: transcript:Zm00001d053610_T002 rna5029 0
535- 1: transcript:Zm00001d053611_T001 rna5027 0
535- 2: transcript:Zm00001d053612_T004 rna5025 6.00E-113
535- 3: transcript:Zm00001d053613_T001 rna5023 1.00E-52
535- 4: transcript:Zm00001d053616_T002 rna5011 0
535- 5: transcript:Zm00001d053617_T001 rna5007 0
Alignment 536: score=253.0 e_value=6.1e-10 N=6 4&NC_008396.2 plus
536- 0: transcript:Zm00001d051883_T001 rna8044 0
536- 1: transcript:Zm00001d051887_T001 rna8049 7.00E-123
536- 2: transcript:Zm00001d051891_T001 rna8051 1.00E-49
536- 3: transcript:Zm00001d051893_T001 rna8055 3.00E-107
536- 4: transcript:Zm00001d051894_T001 rna8056 1.00E-24
536- 5: transcript:Zm00001d051905_T001 rna8072 6.00E-18
Alignment 537: score=1896.0 e_value=2.1e-155 N=43 4&NC_008397.2 plus
537- 0: transcript:Zm00001d051235_T001 rna13017 8.00E-72
537- 1: transcript:Zm00001d051238_T001 rna13018 6.00E-59
537- 2: transcript:Zm00001d051239_T001 rna13019 6.00E-88
537- 3: transcript:Zm00001d051241_T001 rna13020 2.00E-73
537- 4: transcript:Zm00001d051242_T002 rna13021 0
537- 5: transcript:Zm00001d051249_T003 rna13035 1.00E-71
537- 6: transcript:Zm00001d051251_T003 rna13041 1.00E-60
537- 7: transcript:Zm00001d051262_T001 rna13047 3.00E-105
537- 8: transcript:Zm00001d051267_T001 rna13049 1.00E-91
537- 9: transcript:Zm00001d051268_T004 rna13050 0
537- 10: transcript:Zm00001d051272_T001 rna13052 1.00E-125
537- 11: transcript:Zm00001d051287_T001 rna13054 0
537- 12: transcript:Zm00001d051294_T001 rna13055 2.00E-95
537- 13: transcript:Zm00001d051302_T001 rna13067 3.00E-51
537- 14: transcript:Zm00001d051305_T001 rna13068 1.00E-91
537- 15: transcript:Zm00001d051306_T001 rna13070 0
537- 16: transcript:Zm00001d051307_T001 rna13074 0
537- 17: transcript:Zm00001d051308_T009 rna13076 0

537- 18: transcript:Zm00001d051309_T001 rna13083 1.00E-88
537- 19: transcript:Zm00001d051314_T001 rna13088 1.00E-161
537- 20: transcript:Zm00001d051316_T001 rna13089 3.00E-114
537- 21: transcript:Zm00001d051322_T001 rna13099 9.00E-18
537- 22: transcript:Zm00001d051323_T003 rna13100 0
537- 23: transcript:Zm00001d051328_T002 rna13113 2.00E-72
537- 24: transcript:Zm00001d051333_T001 rna13118 3.00E-32
537- 25: transcript:Zm00001d051334_T001 rna13120 1.00E-83
537- 26: transcript:Zm00001d051337_T002 rna13123 2.00E-60
537- 27: transcript:Zm00001d051340_T001 rna13125 6.00E-28
537- 28: transcript:Zm00001d051343_T001 rna13132 1.00E-53
537- 29: transcript:Zm00001d051350_T001 rna13139 3.00E-42
537- 30: transcript:Zm00001d051359_T001 rna13143 0
537- 31: transcript:Zm00001d051360_T001 rna13145 1.00E-68
537- 32: transcript:Zm00001d051362_T001 rna13146 2.00E-57
537- 33: transcript:Zm00001d051365_T001 rna13151 9.00E-26
537- 34: transcript:Zm00001d051368_T001 rna13154 2.00E-76
537- 35: transcript:Zm00001d051370_T001 rna13157 4.00E-39
537- 36: transcript:Zm00001d051371_T001 rna13159 1.00E-66
537- 37: transcript:Zm00001d051383_T001 rna13165 4.00E-73
537- 38: transcript:Zm00001d051384_T001 rna13166 1.00E-58
537- 39: transcript:Zm00001d051387_T001 rna13169 0
537- 40: transcript:Zm00001d051388_T001 rna13174 6.00E-88
537- 41: transcript:Zm00001d051392_T001 rna13179 3.00E-112
537- 42: transcript:Zm00001d051394_T001 rna13184 2.00E-09
Alignment 538: score=684.0 e_value=2.9e-40 N=16 4&NC_008397.2 plus
538- 0: transcript:Zm00001d051110_T001 rna12889 4.00E-156
538- 1: transcript:Zm00001d051117_T001 rna12899 2.00E-60
538- 2: transcript:Zm00001d051119_T001 rna12901 0
538- 3: transcript:Zm00001d051121_T001 rna12907 1.00E-54
538- 4: transcript:Zm00001d051128_T001 rna12929 6.00E-21
538- 5: transcript:Zm00001d051135_T005 rna12940 0
538- 6: transcript:Zm00001d051140_T001 rna12944 2.00E-107
538- 7: transcript:Zm00001d051143_T001 rna12945 5.00E-89
538- 8: transcript:Zm00001d051149_T001 rna12951 1.00E-101
538- 9: transcript:Zm00001d051156_T001 rna12954 1.00E-119
538- 10: transcript:Zm00001d051157_T001 rna12957 0
538- 11: transcript:Zm00001d051161_T003 rna12958 0
538- 12: transcript:Zm00001d051163_T001 rna12960 0
538- 13: transcript:Zm00001d051172_T001 rna12965 9.00E-172
538- 14: transcript:Zm00001d051174_T001 rna12971 0
538- 15: transcript:Zm00001d051180_T002 rna12981 3.00E-107
Alignment 539: score=640.0 e_value=9.9e-37 N=15 4&NC_008397.2 plus
539- 0: transcript:Zm00001d050837_T001 rna12521 9.00E-130
539- 1: transcript:Zm00001d050838_T001 rna12522 0
539- 2: transcript:Zm00001d050840_T001 rna12526 4.00E-45
539- 3: transcript:Zm00001d050848_T001 rna12531 2.00E-73
539- 4: transcript:Zm00001d050850_T001 rna12537 0
539- 5: transcript:Zm00001d050860_T001 rna12540 0
539- 6: transcript:Zm00001d050864_T001 rna12562 4.00E-09
539- 7: transcript:Zm00001d050868_T004 rna12566 0
539- 8: transcript:Zm00001d050872_T001 rna12568 0
539- 9: transcript:Zm00001d050874_T001 rna12579 4.00E-104
539- 10: transcript:Zm00001d050893_T001 rna12586 8.00E-121

539- 11: transcript:Zm00001d050897_T002 rna12591 7.00E-51
539- 12: transcript:Zm00001d050899_T001 rna12593 7.00E-62
539- 13: transcript:Zm00001d050903_T001 rna12600 6.00E-22
539- 14: transcript:Zm00001d050905_T001 rna12601 1.00E-76
Alignment 540: score=531.0 e_value=3.8e-28 N=12 4&NC_008397.2 plus
540- 0: transcript:Zm00001d051460_T001 rna13309 2.00E-55
540- 1: transcript:Zm00001d051461_T001 rna13311 0
540- 2: transcript:Zm00001d051465_T002 rna13314 3.00E-107
540- 3: transcript:Zm00001d051468_T001 rna13315 3.00E-38
540- 4: transcript:Zm00001d051471_T001 rna13319 2.00E-67
540- 5: transcript:Zm00001d051475_T002 rna13331 1.00E-34
540- 6: transcript:Zm00001d051479_T014 rna13334 0
540- 7: transcript:Zm00001d051480_T001 rna13336 1.00E-75
540- 8: transcript:Zm00001d051500_T001 rna13349 0
540- 9: transcript:Zm00001d051503_T001 rna13359 3.00E-17
540- 10: transcript:Zm00001d051504_T001 rna13361 7.00E-173
540- 11: transcript:Zm00001d051505_T001 rna13365 2.00E-41
Alignment 541: score=345.0 e_value=5.1e-18 N=9 4&NC_008397.2 plus
541- 0: transcript:Zm00001d051041_T001 rna12816 1.00E-66
541- 1: transcript:Zm00001d051047_T001 rna12829 5.00E-100
541- 2: transcript:Zm00001d051055_T003 rna12833 6.00E-34
541- 3: transcript:Zm00001d051056_T001 rna12835 0
541- 4: transcript:Zm00001d051061_T001 rna12841 1.00E-38
541- 5: transcript:Zm00001d051067_T003 rna12854 0
541- 6: transcript:Zm00001d051069_T001 rna12862 0
541- 7: transcript:Zm00001d051080_T004 rna12869 2.00E-50
541- 8: transcript:Zm00001d051102_T001 rna12876 5.00E-57
Alignment 542: score=313.0 e_value=6.6e-14 N=8 4&NC_008397.2 plus
542- 0: transcript:Zm00001d050637_T002 rna12258 1.00E-136
542- 1: transcript:Zm00001d050645_T005 rna12279 8.00E-62
542- 2: transcript:Zm00001d050649_T001 rna12288 1.00E-112
542- 3: transcript:Zm00001d050658_T001 rna12307 5.00E-49
542- 4: transcript:Zm00001d050664_T002 rna12323 3.00E-134
542- 5: transcript:Zm00001d050666_T001 rna12324 2.00E-49
542- 6: transcript:Zm00001d050689_T001 rna12334 8.00E-161
542- 7: transcript:Zm00001d050697_T001 rna12342 2.00E-94
Alignment 543: score=276.0 e_value=1.3e-10 N=7 4&NC_008397.2 plus
543- 0: transcript:Zm00001d050552_T001 rna12131 0
543- 1: transcript:Zm00001d050553_T001 rna12145 8.00E-41
543- 2: transcript:Zm00001d050557_T001 rna12159 7.00E-26
543- 3: transcript:Zm00001d050565_T001 rna12173 0
543- 4: transcript:Zm00001d050575_T002 rna12195 0
543- 5: transcript:Zm00001d050580_T001 rna12200 0
543- 6: transcript:Zm00001d050583_T001 rna12212 2.00E-93
Alignment 544: score=269.0 e_value=4.5e-10 N=6 4&NC_008397.2 plus
544- 0: transcript:Zm00001d050908_T001 rna12632 8.00E-20
544- 1: transcript:Zm00001d050911_T001 rna12637 6.00E-10
544- 2: transcript:Zm00001d050915_T004 rna12649 2.00E-128
544- 3: transcript:Zm00001d050916_T002 rna12651 1.00E-73
544- 4: transcript:Zm00001d050917_T002 rna12662 0
544- 5: transcript:Zm00001d050920_T002 rna12671 3.00E-139
Alignment 545: score=277.0 e_value=2.2e-08 N=6 4&NC_008397.2 minus
545- 0: transcript:Zm00001d050190_T001 rna13480 3.00E-33
545- 1: transcript:Zm00001d050191_T001 rna13479 0

545- 2: transcript:Zm00001d050193_T001 rna13474 2.00E-160
545- 3: transcript:Zm00001d050195_T001 rna13470 3.00E-68
545- 4: transcript:Zm00001d050201_T001 rna13463 1.00E-104
545- 5: transcript:Zm00001d050208_T003 rna13455 6.00E-32
Alignment 546: score=1574.0 e_value=7.5e-121 N=36 4&NC_008399.2 plus
546- 0: transcript:Zm00001d052168_T001 rna17164 1.00E-169
546- 1: transcript:Zm00001d052170_T001 rna17169 0
546- 2: transcript:Zm00001d052176_T001 rna17174 4.00E-06
546- 3: transcript:Zm00001d052179_T001 rna17180 5.00E-61
546- 4: transcript:Zm00001d052180_T001 rna17197 1.00E-125
546- 5: transcript:Zm00001d052184_T001 rna17200 3.00E-29
546- 6: transcript:Zm00001d052185_T001 rna17201 1.00E-94
546- 7: transcript:Zm00001d052191_T001 rna17207 2.00E-42
546- 8: transcript:Zm00001d052193_T012 rna17209 8.00E-179
546- 9: transcript:Zm00001d052194_T001 rna17211 2.00E-64
546- 10: transcript:Zm00001d052198_T002 rna17212 6.00E-81
546- 11: transcript:Zm00001d052200_T005 rna17213 4.00E-172
546- 12: transcript:Zm00001d052206_T001 rna17215 2.00E-99
546- 13: transcript:Zm00001d052211_T001 rna17219 1.00E-160
546- 14: transcript:Zm00001d052215_T001 rna17221 8.00E-113
546- 15: transcript:Zm00001d052219_T002 rna17224 6.00E-172
546- 16: transcript:Zm00001d052226_T001 rna17227 3.00E-07
546- 17: transcript:Zm00001d052229_T001 rna17228 5.00E-24
546- 18: transcript:Zm00001d052232_T002 rna17230 0
546- 19: transcript:Zm00001d052234_T001 rna17233 5.00E-28
546- 20: transcript:Zm00001d052239_T001 rna17236 1.00E-38
546- 21: transcript:Zm00001d052247_T001 rna17251 5.00E-159
546- 22: transcript:Zm00001d052248_T009 rna17252 3.00E-174
546- 23: transcript:Zm00001d052252_T001 rna17255 1.00E-101
546- 24: transcript:Zm00001d052254_T001 rna17256 5.00E-41
546- 25: transcript:Zm00001d052256_T001 rna17258 4.00E-108
546- 26: transcript:Zm00001d052258_T001 rna17259 4.00E-160
546- 27: transcript:Zm00001d052259_T001 rna17260 8.00E-58
546- 28: transcript:Zm00001d052261_T001 rna17262 2.00E-120
546- 29: transcript:Zm00001d052263_T001 rna17272 0
546- 30: transcript:Zm00001d052268_T001 rna17281 5.00E-91
546- 31: transcript:Zm00001d052269_T001 rna17285 0
546- 32: transcript:Zm00001d052270_T005 rna17292 4.00E-169
546- 33: transcript:Zm00001d052271_T003 rna17294 0
546- 34: transcript:Zm00001d052273_T004 rna17295 2.00E-105
546- 35: transcript:Zm00001d052277_T001 rna17305 5.00E-51
Alignment 547: score=707.0 e_value=4.5e-47 N=17 4&NC_008399.2 plus
547- 0: transcript:Zm00001d053815_T001 rna18557 1.00E-37
547- 1: transcript:Zm00001d053817_T001 rna18573 4.00E-65
547- 2: transcript:Zm00001d053818_T003 rna18582 0
547- 3: transcript:Zm00001d053819_T002 rna18591 0
547- 4: transcript:Zm00001d053825_T002 rna18603 0
547- 5: transcript:Zm00001d053829_T001 rna18611 6.00E-44
547- 6: transcript:Zm00001d053831_T001 rna18612 1.00E-35
547- 7: transcript:Zm00001d053838_T005 rna18613 0
547- 8: transcript:Zm00001d053839_T001 rna18614 4.00E-64
547- 9: transcript:Zm00001d053841_T014 rna18619 1.00E-19
547- 10: transcript:Zm00001d053843_T001 rna18627 2.00E-25
547- 11: transcript:Zm00001d053848_T001 rna18634 3.00E-35

547- 12: transcript:Zm00001d053852_T001 rna18643 0
547- 13: transcript:Zm00001d053857_T007 rna18650 0
547- 14: transcript:Zm00001d053858_T001 rna18651 0
547- 15: transcript:Zm00001d053859_T001 rna18662 4.00E-49
547- 16: transcript:Zm00001d053865_T007 rna18670 0
Alignment 548: score=490.0 e_value=3.7e-26 N=12 4&NC_008399.2 plus
548- 0: transcript:Zm00001d053378_T001 rna17908 0
548- 1: transcript:Zm00001d053382_T001 rna17913 0
548- 2: transcript:Zm00001d053391_T001 rna17920 6.00E-105
548- 3: transcript:Zm00001d053396_T001 rna17922 2.00E-12
548- 4: transcript:Zm00001d053399_T001 rna17925 3.00E-75
548- 5: transcript:Zm00001d053401_T014 rna17928 0
548- 6: transcript:Zm00001d053404_T001 rna17934 0
548- 7: transcript:Zm00001d053406_T001 rna17937 8.00E-14
548- 8: transcript:Zm00001d053415_T001 rna17941 5.00E-12
548- 9: transcript:Zm00001d053425_T001 rna17946 0
548- 10: transcript:Zm00001d053434_T001 rna17957 2.00E-19
548- 11: transcript:Zm00001d053445_T001 rna17961 1.00E-08
Alignment 549: score=460.0 e_value=7.2e-23 N=11 4&NC_008399.2 plus
549- 0: transcript:Zm00001d053565_T014 rna18049 0
549- 1: transcript:Zm00001d053568_T001 rna18051 9.00E-10
549- 2: transcript:Zm00001d053569_T001 rna18057 0
549- 3: transcript:Zm00001d053572_T002 rna18061 2.00E-88
549- 4: transcript:Zm00001d053576_T001 rna18083 0
549- 5: transcript:Zm00001d053585_T002 rna18088 0
549- 6: transcript:Zm00001d053587_T001 rna18091 0
549- 7: transcript:Zm00001d053589_T001 rna18093 1.00E-127
549- 8: transcript:Zm00001d053593_T001 rna18110 8.00E-52
549- 9: transcript:Zm00001d053595_T004 rna18118 0
549- 10: transcript:Zm00001d053597_T006 rna18120 2.00E-13
Alignment 550: score=434.0 e_value=2.7e-22 N=10 4&NC_008399.2 plus
550- 0: transcript:Zm00001d053755_T001 rna18471 1.00E-40
550- 1: transcript:Zm00001d053756_T001 rna18478 3.00E-59
550- 2: transcript:Zm00001d053761_T001 rna18489 3.00E-73
550- 3: transcript:Zm00001d053763_T001 rna18499 0
550- 4: transcript:Zm00001d053765_T001 rna18500 0
550- 5: transcript:Zm00001d053770_T001 rna18514 0
550- 6: transcript:Zm00001d053775_T001 rna18515 2.00E-18
550- 7: transcript:Zm00001d053778_T001 rna18525 0
550- 8: transcript:Zm00001d053779_T001 rna18526 1.00E-26
550- 9: transcript:Zm00001d053781_T001 rna18531 1.00E-16
Alignment 551: score=368.0 e_value=3.7e-20 N=9 4&NC_008399.2 plus
551- 0: transcript:Zm00001d053671_T001 rna18266 5.00E-80
551- 1: transcript:Zm00001d053672_T007 rna18267 0
551- 2: transcript:Zm00001d053674_T001 rna18277 0
551- 3: transcript:Zm00001d053680_T001 rna18284 6.00E-144
551- 4: transcript:Zm00001d053682_T001 rna18285 1.00E-54
551- 5: transcript:Zm00001d053686_T001 rna18294 1.00E-167
551- 6: transcript:Zm00001d053688_T002 rna18306 0
551- 7: transcript:Zm00001d053695_T001 rna18318 0
551- 8: transcript:Zm00001d053696_T003 rna18322 1.00E-101
Alignment 552: score=341.0 e_value=1.4e-15 N=8 4&NC_008399.2 plus
552- 0: transcript:Zm00001d053625_T001 rna18197 3.00E-66
552- 1: transcript:Zm00001d053626_T001 rna18205 1.00E-114

552- 2: transcript:Zm00001d053639_T001 rna18212 2.00E-24
552- 3: transcript:Zm00001d053642_T001 rna18217 8.00E-58
552- 4: transcript:Zm00001d053643_T001 rna18221 4.00E-112
552- 5: transcript:Zm00001d053648_T005 rna18225 8.00E-37
552- 6: transcript:Zm00001d053649_T002 rna18229 0
552- 7: transcript:Zm00001d053659_T001 rna18244 1.00E-119
Alignment 553: score=280.0 e_value=3.4e-12 N=7 4&NC_008399.2 plus
553- 0: transcript:Zm00001d052003_T002 rna16965 6.00E-36
553- 1: transcript:Zm00001d052008_T003 rna16977 0
553- 2: transcript:Zm00001d052011_T003 rna16995 4.00E-107
553- 3: transcript:Zm00001d052018_T001 rna16999 2.00E-132
553- 4: transcript:Zm00001d052022_T002 rna17009 0
553- 5: transcript:Zm00001d052025_T001 rna17010 2.00E-30
553- 6: transcript:Zm00001d052026_T001 rna17011 5.00E-68
Alignment 554: score=497.0 e_value=2e-30 N=12 4&NC_008399.2 minus
554- 0: transcript:Zm00001d051741_T001 rna17447 2.00E-73
554- 1: transcript:Zm00001d051754_T003 rna17427 0
554- 2: transcript:Zm00001d051756_T004 rna17426 0
554- 3: transcript:Zm00001d051759_T001 rna17425 0
554- 4: transcript:Zm00001d051761_T001 rna17421 0
554- 5: transcript:Zm00001d051785_T001 rna17405 1.00E-76
554- 6: transcript:Zm00001d051787_T001 rna17394 0
554- 7: transcript:Zm00001d051788_T001 rna17389 8.00E-102
554- 8: transcript:Zm00001d051791_T002 rna17388 0
554- 9: transcript:Zm00001d051793_T001 rna17384 5.00E-32
554- 10: transcript:Zm00001d051799_T001 rna17377 5.00E-76
554- 11: transcript:Zm00001d051800_T002 rna17370 1.00E-35
Alignment 555: score=341.0 e_value=7e-17 N=8 4&NC_008399.2 minus
555- 0: transcript:Zm00001d053725_T002 rna18461 5.00E-128
555- 1: transcript:Zm00001d053731_T001 rna18458 9.00E-42
555- 2: transcript:Zm00001d053739_T001 rna18448 2.00E-93
555- 3: transcript:Zm00001d053740_T001 rna18447 0
555- 4: transcript:Zm00001d053746_T001 rna18429 7.00E-101
555- 5: transcript:Zm00001d053748_T001 rna18415 1.00E-15
555- 6: transcript:Zm00001d053749_T002 rna18414 2.00E-162
555- 7: transcript:Zm00001d053750_T001 rna18411 1.00E-19
Alignment 556: score=2361.0 e_value=4.7e-202 N=53 4&NC_008401.2 plus
556- 0: transcript:Zm00001d050256_T001 rna22372 1.00E-65
556- 1: transcript:Zm00001d050259_T002 rna22383 2.00E-83
556- 2: transcript:Zm00001d050265_T001 rna22386 0
556- 3: transcript:Zm00001d050266_T001 rna22389 2.00E-119
556- 4: transcript:Zm00001d050267_T001 rna22390 3.00E-51
556- 5: transcript:Zm00001d050269_T001 rna22401 5.00E-74
556- 6: transcript:Zm00001d050273_T001 rna22403 0
556- 7: transcript:Zm00001d050277_T001 rna22413 5.00E-54
556- 8: transcript:Zm00001d050283_T001 rna22414 0
556- 9: transcript:Zm00001d050284_T005 rna22419 1.00E-25
556- 10: transcript:Zm00001d050285_T001 rna22423 5.00E-60
556- 11: transcript:Zm00001d050294_T001 rna22428 2.00E-63
556- 12: transcript:Zm00001d050300_T001 rna22442 5.00E-130
556- 13: transcript:Zm00001d050302_T001 rna22446 0
556- 14: transcript:Zm00001d050303_T001 rna22448 0
556- 15: transcript:Zm00001d050304_T002 rna22452 0
556- 16: transcript:Zm00001d050307_T001 rna22453 5.00E-37

```
556- 17:  transcript:Zm00001d050308_T001  rna22459            0
556- 18:  transcript:Zm00001d050310_T001  rna22461    2.00E-114
556- 19:  transcript:Zm00001d050315_T001  rna22462            0
556- 20:  transcript:Zm00001d050316_T035  rna22464    6.00E-164
556- 21:  transcript:Zm00001d050319_T001  rna22466     1.00E-61
556- 22:  transcript:Zm00001d050329_T003  rna22481            0
556- 23:  transcript:Zm00001d050330_T003  rna22483    5.00E-144
556- 24:  transcript:Zm00001d050333_T001  rna22484    5.00E-114
556- 25:  transcript:Zm00001d050335_T001  rna22485     3.00E-97
556- 26:  transcript:Zm00001d050336_T003  rna22494            0
556- 27:  transcript:Zm00001d050339_T001  rna22495            0
556- 28:  transcript:Zm00001d050340_T001  rna22498            0
556- 29:  transcript:Zm00001d050341_T001  rna22500     7.00E-50
556- 30:  transcript:Zm00001d050346_T001  rna22502    4.00E-176
556- 31:  transcript:Zm00001d050347_T002  rna22503     8.00E-79
556- 32:  transcript:Zm00001d050350_T001  rna22504     2.00E-50
556- 33:  transcript:Zm00001d050352_T001  rna22507     4.00E-94
556- 34:  transcript:Zm00001d050357_T005  rna22511    2.00E-147
556- 35:  transcript:Zm00001d050359_T001  rna22512    5.00E-133
556- 36:  transcript:Zm00001d050365_T006  rna22515     2.00E-78
556- 37:  transcript:Zm00001d050368_T001  rna22516     2.00E-81
556- 38:  transcript:Zm00001d050372_T002  rna22520            0
556- 39:  transcript:Zm00001d050375_T001  rna22524    8.00E-179
556- 40:  transcript:Zm00001d050380_T001  rna22526            0
556- 41:  transcript:Zm00001d050382_T001  rna22528            0
556- 42:  transcript:Zm00001d050383_T001  rna22530            0
556- 43:  transcript:Zm00001d050384_T001  rna22531     1.00E-66
556- 44:  transcript:Zm00001d050388_T001  rna22535     2.00E-16
556- 45:  transcript:Zm00001d050393_T001  rna22536     3.00E-94
556- 46:  transcript:Zm00001d050394_T001  rna22538     2.00E-89
556- 47:  transcript:Zm00001d050395_T002  rna22540    8.00E-106
556- 48:  transcript:Zm00001d050400_T001  rna22543            0
556- 49:  transcript:Zm00001d050403_T001  rna22554    2.00E-157
556- 50:  transcript:Zm00001d050410_T001  rna22559            0
556- 51:  transcript:Zm00001d050411_T001  rna22560     2.00E-78
556- 52:  transcript:Zm00001d050428_T003  rna22576            0
## Alignment 557: score=2078.0 e_value=2.3e-174 N=47 4&NC_008401.2 plus
557-  0:  transcript:Zm00001d052794_T001  rna22760            0
557-  1:  transcript:Zm00001d052795_T001  rna22761    1.00E-176
557-  2:  transcript:Zm00001d052797_T002  rna22782            0
557-  3:  transcript:Zm00001d052798_T001  rna22784    4.00E-100
557-  4:  transcript:Zm00001d052799_T001  rna22786     2.00E-80
557-  5:  transcript:Zm00001d052800_T002  rna22789    2.00E-172
557-  6:  transcript:Zm00001d052801_T004  rna22794    2.00E-107
557-  7:  transcript:Zm00001d052803_T004  rna22795     4.00E-66
557-  8:  transcript:Zm00001d052804_T001  rna22796    6.00E-106
557-  9:  transcript:Zm00001d052807_T001  rna22802     2.00E-43
557- 10:  transcript:Zm00001d052809_T002  rna22806            0
557- 11:  transcript:Zm00001d052810_T001  rna22807            0
557- 12:  transcript:Zm00001d052813_T001  rna22809     2.00E-57
557- 13:  transcript:Zm00001d052814_T001  rna22810     7.00E-45
557- 14:  transcript:Zm00001d052816_T001  rna22814    2.00E-153
557- 15:  transcript:Zm00001d052818_T001  rna22815     7.00E-63
557- 16:  transcript:Zm00001d052820_T001  rna22823    8.00E-149
```

557- 17: transcript:Zm00001d052835_T001 rna22827 0
557- 18: transcript:Zm00001d052837_T001 rna22835 0
557- 19: transcript:Zm00001d052838_T001 rna22841 7.00E-76
557- 20: transcript:Zm00001d052840_T016 rna22844 0
557- 21: transcript:Zm00001d052842_T001 rna22847 4.00E-137
557- 22: transcript:Zm00001d052843_T001 rna22850 1.00E-67
557- 23: transcript:Zm00001d052845_T009 rna22851 0
557- 24: transcript:Zm00001d052847_T001 rna22853 0
557- 25: transcript:Zm00001d052850_T001 rna22858 0
557- 26: transcript:Zm00001d052851_T002 rna22860 0
557- 27: transcript:Zm00001d052852_T001 rna22862 0
557- 28: transcript:Zm00001d052853_T001 rna22864 5.00E-138
557- 29: transcript:Zm00001d052854_T001 rna22865 0
557- 30: transcript:Zm00001d052855_T001 rna22866 0
557- 31: transcript:Zm00001d052859_T001 rna22875 0
557- 32: transcript:Zm00001d052864_T001 rna22877 7.00E-86
557- 33: transcript:Zm00001d052873_T001 rna22880 3.00E-146
557- 34: transcript:Zm00001d052875_T001 rna22882 0
557- 35: transcript:Zm00001d052882_T001 rna22886 1.00E-43
557- 36: transcript:Zm00001d052883_T001 rna22887 3.00E-80
557- 37: transcript:Zm00001d052888_T004 rna22890 0
557- 38: transcript:Zm00001d052889_T001 rna22911 3.00E-13
557- 39: transcript:Zm00001d052890_T001 rna22920 1.00E-125
557- 40: transcript:Zm00001d052893_T002 rna22923 4.00E-75
557- 41: transcript:Zm00001d052895_T001 rna22926 3.00E-119
557- 42: transcript:Zm00001d052898_T001 rna22930 1.00E-43
557- 43: transcript:Zm00001d052901_T001 rna22936 2.00E-116
557- 44: transcript:Zm00001d052903_T001 rna22939 2.00E-150
557- 45: transcript:Zm00001d052905_T002 rna22945 3.00E-98
557- 46: transcript:Zm00001d052906_T001 rna22946 0
Alignment 558: score=1345.0 e_value=2.7e-101 N=30 4&NC_008401.2 plus
558- 0: transcript:Zm00001d049763_T002 rna22980 0
558- 1: transcript:Zm00001d049785_T003 rna23006 0
558- 2: transcript:Zm00001d049789_T001 rna23007 2.00E-42
558- 3: transcript:Zm00001d049790_T007 rna23009 8.00E-93
558- 4: transcript:Zm00001d049796_T001 rna23012 1.00E-119
558- 5: transcript:Zm00001d049804_T001 rna23017 0
558- 6: transcript:Zm00001d049805_T001 rna23023 2.00E-166
558- 7: transcript:Zm00001d049806_T001 rna23024 2.00E-102
558- 8: transcript:Zm00001d049807_T001 rna23035 1.00E-31
558- 9: transcript:Zm00001d049809_T001 rna23037 0
558- 10: transcript:Zm00001d049813_T001 rna23045 5.00E-72
558- 11: transcript:Zm00001d049815_T005 rna23048 2.00E-114
558- 12: transcript:Zm00001d049816_T001 rna23055 1.00E-77
558- 13: transcript:Zm00001d049817_T003 rna23056 4.00E-06
558- 14: transcript:Zm00001d049821_T001 rna23057 3.00E-117
558- 15: transcript:Zm00001d049822_T001 rna23058 2.00E-138
558- 16: transcript:Zm00001d049826_T001 rna23062 3.00E-70
558- 17: transcript:Zm00001d049831_T001 rna23063 0
558- 18: transcript:Zm00001d049832_T001 rna23064 1.00E-52
558- 19: transcript:Zm00001d049834_T001 rna23067 9.00E-69
558- 20: transcript:Zm00001d049835_T004 rna23068 1.00E-147
558- 21: transcript:Zm00001d049851_T001 rna23069 0
558- 22: transcript:Zm00001d049853_T001 rna23072 3.00E-124

558- 23: transcript:Zm00001d049857_T002 rna23080 0
558- 24: transcript:Zm00001d049858_T001 rna23081 3.00E-41
558- 25: transcript:Zm00001d049860_T001 rna23083 5.00E-58
558- 26: transcript:Zm00001d049865_T001 rna23087 3.00E-38
558- 27: transcript:Zm00001d049870_T001 rna23093 1.00E-56
558- 28: transcript:Zm00001d049871_T001 rna23096 2.00E-85
558- 29: transcript:Zm00001d049873_T001 rna23097 1.00E-138
Alignment 559: score=952.0 e_value=1.3e-59 N=21 4&NC_008401.2 plus
559- 0: transcript:Zm00001d050048_T001 rna22587 5.00E-47
559- 1: transcript:Zm00001d050051_T001 rna22589 4.00E-122
559- 2: transcript:Zm00001d050056_T002 rna22596 8.00E-48
559- 3: transcript:Zm00001d050063_T001 rna22599 0
559- 4: transcript:Zm00001d050069_T001 rna22600 0
559- 5: transcript:Zm00001d050076_T001 rna22601 1.00E-147
559- 6: transcript:Zm00001d050079_T001 rna22603 5.00E-60
559- 7: transcript:Zm00001d050081_T002 rna22607 3.00E-14
559- 8: transcript:Zm00001d050082_T001 rna22616 5.00E-108
559- 9: transcript:Zm00001d050089_T003 rna22618 0
559- 10: transcript:Zm00001d050090_T001 rna22628 8.00E-133
559- 11: transcript:Zm00001d050092_T001 rna22630 0
559- 12: transcript:Zm00001d050093_T001 rna22632 8.00E-60
559- 13: transcript:Zm00001d050095_T001 rna22644 0
559- 14: transcript:Zm00001d050097_T001 rna22646 0
559- 15: transcript:Zm00001d050099_T001 rna22649 1.00E-12
559- 16: transcript:Zm00001d050104_T002 rna22654 0
559- 17: transcript:Zm00001d050106_T001 rna22655 1.00E-99
559- 18: transcript:Zm00001d050107_T001 rna22657 0
559- 19: transcript:Zm00001d050109_T003 rna22672 7.00E-173
559- 20: transcript:Zm00001d050111_T001 rna22676 1.00E-72
Alignment 560: score=876.0 e_value=5.8e-51 N=19 4&NC_008401.2 plus
560- 0: transcript:Zm00001d053197_T006 rna23166 2.00E-106
560- 1: transcript:Zm00001d053200_T001 rna23169 0
560- 2: transcript:Zm00001d053202_T001 rna23170 0
560- 3: transcript:Zm00001d053208_T001 rna23172 5.00E-103
560- 4: transcript:Zm00001d053210_T001 rna23176 2.00E-51
560- 5: transcript:Zm00001d053211_T009 rna23177 7.00E-124
560- 6: transcript:Zm00001d053212_T002 rna23178 1.00E-109
560- 7: transcript:Zm00001d053213_T001 rna23179 3.00E-37
560- 8: transcript:Zm00001d053214_T001 rna23180 0
560- 9: transcript:Zm00001d053217_T001 rna23183 3.00E-179
560- 10: transcript:Zm00001d053220_T001 rna23184 9.00E-118
560- 11: transcript:Zm00001d053223_T001 rna23185 2.00E-160
560- 12: transcript:Zm00001d053225_T005 rna23186 0
560- 13: transcript:Zm00001d053228_T002 rna23187 1.00E-63
560- 14: transcript:Zm00001d053229_T001 rna23189 7.00E-20
560- 15: transcript:Zm00001d053232_T005 rna23191 0
560- 16: transcript:Zm00001d053234_T001 rna23192 0
560- 17: transcript:Zm00001d053236_T001 rna23193 6.00E-105
560- 18: transcript:Zm00001d053237_T001 rna23195 0
Alignment 561: score=610.0 e_value=8.4e-30 N=13 4&NC_008401.2 plus
561- 0: transcript:Zm00001d049987_T001 rna22688 0
561- 1: transcript:Zm00001d049990_T001 rna22690 0
561- 2: transcript:Zm00001d049991_T001 rna22693 7.00E-122
561- 3: transcript:Zm00001d049995_T001 rna22694 0

```
561-  4:  transcript:Zm00001d049996_T001  rna22699    3.00E-175
561-  5:  transcript:Zm00001d050000_T001  rna22701            0
561-  6:  transcript:Zm00001d050008_T001  rna22703    4.00E-147
561-  7:  transcript:Zm00001d050011_T004  rna22706     3.00E-62
561-  8:  transcript:Zm00001d050017_T001  rna22708            0
561-  9:  transcript:Zm00001d050018_T007  rna22710    3.00E-109
561- 10:  transcript:Zm00001d050019_T001  rna22711            0
561- 11:  transcript:Zm00001d050020_T003  rna22712            0
561- 12:  transcript:Zm00001d050021_T001  rna22713            0
## Alignment 562: score=567.0 e_value=2.3e-33 N=13 4&NC_008401.2 plus
562-  0:  transcript:Zm00001d050190_T001  rna21854     3.00E-46
562-  1:  transcript:Zm00001d050191_T001  rna21855            0
562-  2:  transcript:Zm00001d050193_T001  rna21873    1.00E-163
562-  3:  transcript:Zm00001d050195_T001  rna21881     3.00E-68
562-  4:  transcript:Zm00001d050196_T001  rna21892    4.00E-111
562-  5:  transcript:Zm00001d050201_T001  rna21902    1.00E-134
562-  6:  transcript:Zm00001d050202_T002  rna21903    4.00E-135
562-  7:  transcript:Zm00001d050208_T003  rna21907     4.00E-26
562-  8:  transcript:Zm00001d050214_T001  rna21908            0
562-  9:  transcript:Zm00001d050215_T001  rna21909     4.00E-55
562- 10:  transcript:Zm00001d050216_T003  rna21913            0
562- 11:  transcript:Zm00001d050218_T001  rna21915            0
562- 12:  transcript:Zm00001d050224_T002  rna21923            0
## Alignment 563: score=530.0 e_value=1.1e-26 N=12 4&NC_008401.2 plus
563-  0:  transcript:Zm00001d052689_T002  rna23099            0
563-  1:  transcript:Zm00001d052690_T001  rna23100    6.00E-113
563-  2:  transcript:Zm00001d052695_T021  rna23104            0
563-  3:  transcript:Zm00001d052696_T001  rna23106            0
563-  4:  transcript:Zm00001d052697_T002  rna23108            0
563-  5:  transcript:Zm00001d052700_T001  rna23110     6.00E-72
563-  6:  transcript:Zm00001d052701_T001  rna23112            0
563-  7:  transcript:Zm00001d052702_T001  rna23113            0
563-  8:  transcript:Zm00001d052709_T002  rna23114            0
563-  9:  transcript:Zm00001d052713_T002  rna23115    8.00E-128
563- 10:  transcript:Zm00001d052715_T001  rna23116    2.00E-154
563- 11:  transcript:Zm00001d052719_T002  rna23125    1.00E-139
## Alignment 564: score=321.0 e_value=2.6e-13 N=7 4&NC_008401.2 plus
564-  0:  transcript:Zm00001d052753_T002  rna21250     9.00E-72
564-  1:  transcript:Zm00001d052758_T001  rna21262            0
564-  2:  transcript:Zm00001d052763_T001  rna21265     6.00E-53
564-  3:  transcript:Zm00001d052765_T002  rna21267            0
564-  4:  transcript:Zm00001d052766_T001  rna21268    6.00E-116
564-  5:  transcript:Zm00001d052767_T002  rna21269            0
564-  6:  transcript:Zm00001d052768_T001  rna21270    2.00E-112
## Alignment 565: score=300.0 e_value=8e-12 N=7 4&NC_008401.2 plus
565-  0:  transcript:Zm00001d049305_T002  rna22253     2.00E-78
565-  1:  transcript:Zm00001d049308_T001  rna22255     3.00E-40
565-  2:  transcript:Zm00001d049313_T004  rna22268            0
565-  3:  transcript:Zm00001d049320_T001  rna22270     1.00E-96
565-  4:  transcript:Zm00001d049324_T001  rna22274            0
565-  5:  transcript:Zm00001d049325_T002  rna22280     5.00E-84
565-  6:  transcript:Zm00001d049331_T001  rna22303     1.00E-59
## Alignment 566: score=269.0 e_value=4.4e-08 N=6 4&NC_008401.2 plus
566-  0:  transcript:Zm00001d052731_T001  rna23147            0
```

566- 1: transcript:Zm00001d052732_T001 rna23151 5.00E-39
566- 2: transcript:Zm00001d052733_T001 rna23157 2.00E-144
566- 3: transcript:Zm00001d052735_T013 rna23159 0
566- 4: transcript:Zm00001d052738_T001 rna23164 1.00E-134
566- 5: transcript:Zm00001d052741_T001 rna23165 1.00E-160
Alignment 567: score=269.0 e_value=3.9e-09 N=6 4&NC_008401.2 plus
567- 0: transcript:Zm00001d050241_T001 rna22321 0
567- 1: transcript:Zm00001d050242_T002 rna22326 2.00E-102
567- 2: transcript:Zm00001d050245_T001 rna22334 8.00E-73
567- 3: transcript:Zm00001d050247_T001 rna22335 9.00E-76
567- 4: transcript:Zm00001d050251_T002 rna22337 0
567- 5: transcript:Zm00001d050253_T002 rna22338 0
Alignment 568: score=1995.0 e_value=7.3e-169 N=44 4&NC_008401.2 minus
568- 0: transcript:Zm00001d049606_T003 rna21475 0
568- 1: transcript:Zm00001d049607_T001 rna21468 0
568- 2: transcript:Zm00001d049608_T001 rna21465 0
568- 3: transcript:Zm00001d049610_T001 rna21457 0
568- 4: transcript:Zm00001d049612_T001 rna21454 5.00E-30
568- 5: transcript:Zm00001d049614_T001 rna21453 3.00E-51
568- 6: transcript:Zm00001d049615_T001 rna21451 3.00E-47
568- 7: transcript:Zm00001d049617_T007 rna21435 2.00E-23
568- 8: transcript:Zm00001d049619_T003 rna21432 0
568- 9: transcript:Zm00001d049625_T001 rna21428 2.00E-47
568- 10: transcript:Zm00001d049628_T001 rna21422 5.00E-151
568- 11: transcript:Zm00001d049629_T002 rna21418 1.00E-171
568- 12: transcript:Zm00001d049630_T001 rna21416 0
568- 13: transcript:Zm00001d049636_T002 rna21412 0
568- 14: transcript:Zm00001d049637_T001 rna21411 2.00E-151
568- 15: transcript:Zm00001d049640_T002 rna21408 0
568- 16: transcript:Zm00001d049641_T001 rna21404 0
568- 17: transcript:Zm00001d049642_T001 rna21388 0
568- 18: transcript:Zm00001d049643_T001 rna21387 0
568- 19: transcript:Zm00001d049647_T001 rna21386 8.00E-50
568- 20: transcript:Zm00001d049648_T001 rna21381 0
568- 21: transcript:Zm00001d049649_T001 rna21380 3.00E-137
568- 22: transcript:Zm00001d049650_T001 rna21378 1.00E-44
568- 23: transcript:Zm00001d049651_T001 rna21377 2.00E-38
568- 24: transcript:Zm00001d049652_T008 rna21374 0
568- 25: transcript:Zm00001d049659_T001 rna21370 3.00E-43
568- 26: transcript:Zm00001d049660_T002 rna21369 0
568- 27: transcript:Zm00001d049661_T001 rna21368 3.00E-150
568- 28: transcript:Zm00001d049662_T001 rna21367 1.00E-148
568- 29: transcript:Zm00001d049663_T001 rna21366 0
568- 30: transcript:Zm00001d049665_T001 rna21364 1.00E-98
568- 31: transcript:Zm00001d049666_T001 rna21362 5.00E-104
568- 32: transcript:Zm00001d049674_T002 rna21356 4.00E-34
568- 33: transcript:Zm00001d049676_T001 rna21355 3.00E-139
568- 34: transcript:Zm00001d049677_T001 rna21354 2.00E-15
568- 35: transcript:Zm00001d049678_T001 rna21353 2.00E-122
568- 36: transcript:Zm00001d049683_T001 rna21350 0
568- 37: transcript:Zm00001d049685_T001 rna21345 2.00E-54
568- 38: transcript:Zm00001d049687_T001 rna21342 1.00E-115
568- 39: transcript:Zm00001d049688_T001 rna21341 1.00E-156
568- 40: transcript:Zm00001d049691_T002 rna21335 5.00E-85

568- 41: transcript:Zm00001d049692_T001 rna21334 9.00E-117
568- 42: transcript:Zm00001d049698_T003 rna21332 0
568- 43: transcript:Zm00001d049704_T001 rna21325 0
Alignment 569: score=1881.0 e_value=9.7e-161 N=43 4&NC_008401.2 minus
569- 0: transcript:Zm00001d049479_T003 rna21656 0
569- 1: transcript:Zm00001d049480_T005 rna21652 0
569- 2: transcript:Zm00001d049485_T001 rna21651 8.00E-77
569- 3: transcript:Zm00001d049489_T001 rna21641 4.00E-109
569- 4: transcript:Zm00001d049490_T001 rna21616 4.00E-121
569- 5: transcript:Zm00001d049491_T001 rna21615 0
569- 6: transcript:Zm00001d049493_T001 rna21611 2.00E-148
569- 7: transcript:Zm00001d049494_T001 rna21597 0
569- 8: transcript:Zm00001d049503_T001 rna21593 3.00E-35
569- 9: transcript:Zm00001d049505_T001 rna21583 0
569- 10: transcript:Zm00001d049507_T001 rna21582 4.00E-54
569- 11: transcript:Zm00001d049510_T001 rna21579 0
569- 12: transcript:Zm00001d049511_T001 rna21577 2.00E-128
569- 13: transcript:Zm00001d049516_T001 rna21574 8.00E-89
569- 14: transcript:Zm00001d049521_T001 rna21573 1.00E-111
569- 15: transcript:Zm00001d049525_T004 rna21572 5.00E-48
569- 16: transcript:Zm00001d049526_T002 rna21570 3.00E-123
569- 17: transcript:Zm00001d049533_T001 rna21565 2.00E-152
569- 18: transcript:Zm00001d049536_T001 rna21564 0
569- 19: transcript:Zm00001d049540_T002 rna21563 0
569- 20: transcript:Zm00001d049543_T016 rna21561 0
569- 21: transcript:Zm00001d049545_T001 rna21558 1.00E-57
569- 22: transcript:Zm00001d049548_T002 rna21557 0
569- 23: transcript:Zm00001d049550_T001 rna21555 0
569- 24: transcript:Zm00001d049552_T007 rna21554 4.00E-74
569- 25: transcript:Zm00001d049553_T001 rna21553 1.00E-146
569- 26: transcript:Zm00001d049554_T001 rna21552 0
569- 27: transcript:Zm00001d049556_T011 rna21542 0
569- 28: transcript:Zm00001d049557_T002 rna21541 3.00E-64
569- 29: transcript:Zm00001d049558_T002 rna21540 0
569- 30: transcript:Zm00001d049559_T001 rna21539 5.00E-60
569- 31: transcript:Zm00001d049564_T001 rna21534 6.00E-93
569- 32: transcript:Zm00001d049571_T001 rna21528 2.00E-33
569- 33: transcript:Zm00001d049572_T004 rna21527 0
569- 34: transcript:Zm00001d049573_T001 rna21524 1.00E-170
569- 35: transcript:Zm00001d049581_T001 rna21517 3.00E-95
569- 36: transcript:Zm00001d049588_T001 rna21506 3.00E-98
569- 37: transcript:Zm00001d049594_T001 rna21495 0
569- 38: transcript:Zm00001d049596_T001 rna21489 1.00E-11
569- 39: transcript:Zm00001d049597_T001 rna21488 2.00E-138
569- 40: transcript:Zm00001d049598_T001 rna21487 2.00E-118
569- 41: transcript:Zm00001d049601_T001 rna21483 0
569- 42: transcript:Zm00001d049602_T001 rna21477 3.00E-37
Alignment 570: score=1443.0 e_value=8.2e-105 N=32 4&NC_008401.2 minus
570- 0: transcript:Zm00001d049880_T001 rna23310 3.00E-67
570- 1: transcript:Zm00001d049881_T001 rna23308 4.00E-129
570- 2: transcript:Zm00001d049883_T001 rna23306 7.00E-62
570- 3: transcript:Zm00001d049886_T001 rna23301 3.00E-73
570- 4: transcript:Zm00001d049889_T001 rna23299 2.00E-51
570- 5: transcript:Zm00001d049894_T011 rna23296 0

570- 6: transcript:Zm00001d049902_T001 rna23295 6.00E-13
570- 7: transcript:Zm00001d049905_T001 rna23290 0
570- 8: transcript:Zm00001d049906_T001 rna23286 7.00E-96
570- 9: transcript:Zm00001d049910_T001 rna23285 0
570- 10: transcript:Zm00001d049913_T001 rna23280 0
570- 11: transcript:Zm00001d049914_T002 rna23275 0
570- 12: transcript:Zm00001d049920_T001 rna23272 1.00E-47
570- 13: transcript:Zm00001d049921_T001 rna23271 4.00E-118
570- 14: transcript:Zm00001d049922_T002 rna23270 0
570- 15: transcript:Zm00001d049926_T001 rna23268 0
570- 16: transcript:Zm00001d049928_T005 rna23267 0
570- 17: transcript:Zm00001d049929_T002 rna23264 0
570- 18: transcript:Zm00001d049932_T001 rna23262 5.00E-07
570- 19: transcript:Zm00001d049938_T011 rna23252 0
570- 20: transcript:Zm00001d049939_T001 rna23250 2.00E-148
570- 21: transcript:Zm00001d049940_T002 rna23249 3.00E-112
570- 22: transcript:Zm00001d049944_T003 rna23248 1.00E-22
570- 23: transcript:Zm00001d049948_T001 rna23247 0
570- 24: transcript:Zm00001d049950_T001 rna23246 0
570- 25: transcript:Zm00001d049952_T001 rna23245 3.00E-72
570- 26: transcript:Zm00001d049954_T001 rna23244 0
570- 27: transcript:Zm00001d049958_T004 rna23243 5.00E-107
570- 28: transcript:Zm00001d049960_T001 rna23240 0
570- 29: transcript:Zm00001d049965_T003 rna23239 8.00E-102
570- 30: transcript:Zm00001d049966_T001 rna23238 3.00E-48
570- 31: transcript:Zm00001d049974_T004 rna23233 4.00E-65
Alignment 571: score=677.0 e_value=1.1e-42 N=15 4&NC_008401.2 minus
571- 0: transcript:Zm00001d049707_T006 rna21322 0
571- 1: transcript:Zm00001d049708_T007 rna21320 0
571- 2: transcript:Zm00001d049709_T001 rna21315 0
571- 3: transcript:Zm00001d049712_T006 rna21314 9.00E-180
571- 4: transcript:Zm00001d049713_T001 rna21313 0
571- 5: transcript:Zm00001d049714_T003 rna21312 0
571- 6: transcript:Zm00001d049715_T001 rna21311 8.00E-71
571- 7: transcript:Zm00001d049716_T001 rna21309 0
571- 8: transcript:Zm00001d049718_T008 rna21307 0
571- 9: transcript:Zm00001d049721_T001 rna21305 3.00E-73
571- 10: transcript:Zm00001d049722_T001 rna21302 2.00E-68
571- 11: transcript:Zm00001d049723_T001 rna21301 1.00E-105
571- 12: transcript:Zm00001d049724_T001 rna21298 0
571- 13: transcript:Zm00001d049725_T002 rna21296 0
571- 14: transcript:Zm00001d049732_T001 rna21277 5.00E-59
Alignment 572: score=513.0 e_value=4.1e-31 N=12 4&NC_008401.2 minus
572- 0: transcript:Zm00001d049404_T001 rna21721 3.00E-38
572- 1: transcript:Zm00001d049405_T001 rna21720 6.00E-82
572- 2: transcript:Zm00001d049413_T001 rna21714 4.00E-64
572- 3: transcript:Zm00001d049416_T001 rna21711 5.00E-30
572- 4: transcript:Zm00001d049426_T001 rna21710 7.00E-88
572- 5: transcript:Zm00001d049437_T001 rna21701 6.00E-120
572- 6: transcript:Zm00001d049440_T004 rna21699 0
572- 7: transcript:Zm00001d049441_T002 rna21698 0
572- 8: transcript:Zm00001d049442_T009 rna21697 0
572- 9: transcript:Zm00001d049443_T003 rna21696 3.00E-92
572- 10: transcript:Zm00001d049450_T007 rna21687 0

572- 11: transcript:Zm00001d049455_T008 rna21686 0
Alignment 573: score=418.0 e_value=1.7e-21 N=10 4&NC_008401.2 minus
573- 0: transcript:Zm00001d050146_T001 rna22032 7.00E-156
573- 1: transcript:Zm00001d050148_T001 rna22030 0
573- 2: transcript:Zm00001d050157_T001 rna22015 5.00E-07
573- 3: transcript:Zm00001d050163_T001 rna22014 1.00E-22
573- 4: transcript:Zm00001d050165_T001 rna22010 4.00E-78
573- 5: transcript:Zm00001d050166_T001 rna22009 1.00E-105
573- 6: transcript:Zm00001d050173_T001 rna21996 6.00E-126
573- 7: transcript:Zm00001d050182_T001 rna21983 6.00E-119
573- 8: transcript:Zm00001d050184_T010 rna21974 0
573- 9: transcript:Zm00001d050187_T001 rna21940 0
Alignment 574: score=407.0 e_value=1e-16 N=9 4&NC_008401.2 minus
574- 0: transcript:Zm00001d050412_T002 rna22583 2.00E-139
574- 1: transcript:Zm00001d050417_T001 rna22582 0
574- 2: transcript:Zm00001d050422_T001 rna22578 1.00E-155
574- 3: transcript:Zm00001d050430_T003 rna22575 9.00E-129
574- 4: transcript:Zm00001d050433_T001 rna22574 4.00E-27
574- 5: transcript:Zm00001d050434_T002 rna22573 3.00E-148
574- 6: transcript:Zm00001d050436_T001 rna22569 2.00E-170
574- 7: transcript:Zm00001d050443_T001 rna22563 2.00E-85
574- 8: transcript:Zm00001d050452_T001 rna22562 2.00E-28
Alignment 575: score=316.0 e_value=1.4e-12 N=7 4&NC_008401.2 minus
575- 0: transcript:Zm00001d049746_T005 rna22995 3.00E-158
575- 1: transcript:Zm00001d049748_T001 rna22994 2.00E-169
575- 2: transcript:Zm00001d049750_T002 rna22993 0
575- 3: transcript:Zm00001d049753_T001 rna22991 0
575- 4: transcript:Zm00001d049759_T003 rna22986 0
575- 5: transcript:Zm00001d049760_T001 rna22985 0
575- 6: transcript:Zm00001d049761_T009 rna22982 0
Alignment 576: score=305.0 e_value=4.4e-13 N=7 4&NC_008401.2 minus
576- 0: transcript:Zm00001d049350_T001 rna21844 2.00E-37
576- 1: transcript:Zm00001d049351_T002 rna21838 5.00E-49
576- 2: transcript:Zm00001d049357_T001 rna21836 0
576- 3: transcript:Zm00001d049359_T001 rna21835 0
576- 4: transcript:Zm00001d049360_T004 rna21833 0
576- 5: transcript:Zm00001d049361_T004 rna21832 0
576- 6: transcript:Zm00001d049370_T002 rna21825 0
Alignment 577: score=291.0 e_value=1.7e-10 N=6 4&NC_008401.2 minus
577- 0: transcript:Zm00001d052744_T001 rna21260 0
577- 1: transcript:Zm00001d052746_T001 rna21259 4.00E-92
577- 2: transcript:Zm00001d052747_T001 rna21256 4.00E-94
577- 3: transcript:Zm00001d052749_T001 rna21255 2.00E-78
577- 4: transcript:Zm00001d052750_T001 rna21254 3.00E-126
577- 5: transcript:Zm00001d052751_T005 rna21249 0
Alignment 578: score=273.0 e_value=1.1e-08 N=6 4&NC_008401.2 minus
578- 0: transcript:Zm00001d049762_T006 rna22981 1.00E-100
578- 1: transcript:Zm00001d049764_T001 rna22978 0
578- 2: transcript:Zm00001d049766_T001 rna22965 0
578- 3: transcript:Zm00001d049767_T001 rna22961 8.00E-19
578- 4: transcript:Zm00001d049768_T001 rna22958 3.00E-69
578- 5: transcript:Zm00001d049769_T001 rna22957 3.00E-14
Alignment 579: score=676.0 e_value=4.4e-41 N=16 4&NC_008402.2 plus
579- 0: transcript:Zm00001d053197_T006 rna24685 4.00E-28

579- 1: transcript:Zm00001d053200_T001 rna24695 0
579- 2: transcript:Zm00001d053208_T001 rna24712 1.00E-37
579- 3: transcript:Zm00001d053210_T001 rna24721 2.00E-115
579- 4: transcript:Zm00001d053211_T009 rna24730 2.00E-31
579- 5: transcript:Zm00001d053212_T002 rna24735 1.00E-85
579- 6: transcript:Zm00001d053214_T001 rna24742 0
579- 7: transcript:Zm00001d053215_T001 rna24743 5.00E-93
579- 8: transcript:Zm00001d053217_T001 rna24755 3.00E-177
579- 9: transcript:Zm00001d053220_T001 rna24756 6.00E-106
579- 10: transcript:Zm00001d053223_T001 rna24759 2.00E-30
579- 11: transcript:Zm00001d053225_T005 rna24766 0
579- 12: transcript:Zm00001d053228_T002 rna24771 4.00E-30
579- 13: transcript:Zm00001d053232_T005 rna24785 0
579- 14: transcript:Zm00001d053236_T001 rna24790 8.00E-48
579- 15: transcript:Zm00001d053237_T001 rna24802 0
Alignment 580: score=553.0 e_value=4.6e-33 N=14 4&NC_008402.2 plus
580- 0: transcript:Zm00001d050319_T001 rna23905 6.00E-80
580- 1: transcript:Zm00001d050329_T003 rna23921 0
580- 2: transcript:Zm00001d050330_T003 rna23930 5.00E-137
580- 3: transcript:Zm00001d050340_T001 rna23944 0
580- 4: transcript:Zm00001d050346_T001 rna23955 0
580- 5: transcript:Zm00001d050350_T001 rna23956 3.00E-29
580- 6: transcript:Zm00001d050355_T001 rna23977 0
580- 7: transcript:Zm00001d050365_T006 rna23983 3.00E-48
580- 8: transcript:Zm00001d050368_T001 rna23986 3.00E-70
580- 9: transcript:Zm00001d050371_T001 rna23989 0
580- 10: transcript:Zm00001d050382_T001 rna24008 2.00E-27
580- 11: transcript:Zm00001d050383_T001 rna24009 0
580- 12: transcript:Zm00001d050389_T001 rna24018 7.00E-38
580- 13: transcript:Zm00001d050394_T001 rna24019 3.00E-40
Alignment 581: score=512.0 e_value=1.5e-30 N=13 4&NC_008402.2 plus
581- 0: transcript:Zm00001d049764_T001 rna24502 0
581- 1: transcript:Zm00001d049768_T001 rna24511 2.00E-48
581- 2: transcript:Zm00001d049785_T003 rna24518 0
581- 3: transcript:Zm00001d049786_T001 rna24520 2.00E-46
581- 4: transcript:Zm00001d049790_T007 rna24521 2.00E-89
581- 5: transcript:Zm00001d049804_T001 rna24536 0
581- 6: transcript:Zm00001d049806_T001 rna24542 3.00E-57
581- 7: transcript:Zm00001d049822_T001 rna24555 2.00E-121
581- 8: transcript:Zm00001d049835_T004 rna24560 2.00E-17
581- 9: transcript:Zm00001d049857_T002 rna24565 0
581- 10: transcript:Zm00001d049860_T001 rna24569 3.00E-38
581- 11: transcript:Zm00001d049870_T001 rna24579 2.00E-87
581- 12: transcript:Zm00001d049871_T001 rna24580 7.00E-74
Alignment 582: score=442.0 e_value=4.3e-20 N=10 4&NC_008402.2 plus
582- 0: transcript:Zm00001d049987_T001 rna24210 0
582- 1: transcript:Zm00001d049991_T001 rna24229 2.00E-35
582- 2: transcript:Zm00001d049996_T001 rna24233 2.00E-145
582- 3: transcript:Zm00001d050008_T001 rna24245 3.00E-153
582- 4: transcript:Zm00001d050011_T004 rna24249 8.00E-30
582- 5: transcript:Zm00001d050016_T001 rna24254 9.00E-65
582- 6: transcript:Zm00001d050017_T001 rna24258 1.00E-150
582- 7: transcript:Zm00001d050018_T007 rna24259 2.00E-102
582- 8: transcript:Zm00001d050019_T001 rna24260 0

582- 9: transcript:Zm00001d050021_T001 rna24263 0
Alignment 583: score=428.0 e_value=2.3e-21 N=10 4&NC_008402.2 plus
583- 0: transcript:Zm00001d052807_T001 rna24363 2.00E-28
583- 1: transcript:Zm00001d052809_T002 rna24365 0
583- 2: transcript:Zm00001d052816_T001 rna24374 1.00E-132
583- 3: transcript:Zm00001d052818_T001 rna24377 5.00E-72
583- 4: transcript:Zm00001d052835_T001 rna24391 5.00E-113
583- 5: transcript:Zm00001d052838_T001 rna24404 2.00E-73
583- 6: transcript:Zm00001d052840_T016 rna24406 0
583- 7: transcript:Zm00001d052842_T001 rna24409 6.00E-96
583- 8: transcript:Zm00001d052845_T009 rna24410 0
583- 9: transcript:Zm00001d052847_T001 rna24411 5.00E-76
Alignment 584: score=310.0 e_value=9.3e-13 N=7 4&NC_008402.2 plus
584- 0: transcript:Zm00001d052155_T001 rna24250 8.00E-69
584- 1: transcript:Zm00001d052157_T005 rna24258 2.00E-101
584- 2: transcript:Zm00001d052167_T001 rna24269 7.00E-34
584- 3: transcript:Zm00001d052168_T001 rna24271 9.00E-147
584- 4: transcript:Zm00001d052172_T001 rna24278 3.00E-15
584- 5: transcript:Zm00001d052173_T001 rna24280 3.00E-30
584- 6: transcript:Zm00001d052174_T001 rna24284 5.00E-11
Alignment 585: score=287.0 e_value=4.4e-11 N=7 4&NC_008402.2 plus
585- 0: transcript:Zm00001d052727_T002 rna24628 7.00E-12
585- 1: transcript:Zm00001d052728_T001 rna24645 2.00E-85
585- 2: transcript:Zm00001d052732_T001 rna24663 3.00E-36
585- 3: transcript:Zm00001d052733_T001 rna24669 3.00E-134
585- 4: transcript:Zm00001d052735_T013 rna24674 0
585- 5: transcript:Zm00001d052738_T001 rna24682 1.00E-122
585- 6: transcript:Zm00001d052741_T001 rna24684 1.00E-129
Alignment 586: score=286.0 e_value=1.8e-11 N=7 4&NC_008402.2 plus
586- 0: transcript:Zm00001d052696_T001 rna24565 7.00E-139
586- 1: transcript:Zm00001d052697_T002 rna24585 4.00E-94
586- 2: transcript:Zm00001d052700_T001 rna24595 0
586- 3: transcript:Zm00001d052701_T001 rna24597 4.00E-176
586- 4: transcript:Zm00001d052709_T002 rna24602 0
586- 5: transcript:Zm00001d052713_T002 rna24605 9.00E-112
586- 6: transcript:Zm00001d052717_T001 rna24622 3.00E-114
Alignment 587: score=292.0 e_value=5.3e-11 N=7 4&NC_008402.2 minus
587- 0: transcript:Zm00001d050413_T001 rna24082 2.00E-32
587- 1: transcript:Zm00001d050417_T001 rna24063 0
587- 2: transcript:Zm00001d050422_T001 rna24061 0
587- 3: transcript:Zm00001d050433_T001 rna24054 1.00E-109
587- 4: transcript:Zm00001d050436_T001 rna24052 4.00E-76
587- 5: transcript:Zm00001d050443_T001 rna24042 2.00E-35
587- 6: transcript:Zm00001d050452_T001 rna24041 1.00E-22
Alignment 588: score=268.0 e_value=5.8e-10 N=6 4&NC_008402.2 minus
588- 0: transcript:Zm00001d049948_T001 rna24951 2.00E-173
588- 1: transcript:Zm00001d049950_T001 rna24947 0
588- 2: transcript:Zm00001d049952_T001 rna24946 5.00E-60
588- 3: transcript:Zm00001d049954_T001 rna24944 0
588- 4: transcript:Zm00001d049965_T003 rna24929 2.00E-103
588- 5: transcript:Zm00001d049974_T004 rna24921 8.00E-06
Alignment 589: score=302.0 e_value=2.4e-11 N=7 4&NC_008403.2 plus
589- 0: transcript:Zm00001d051130_T001 rna25998 0
589- 1: transcript:Zm00001d051135_T005 rna26008 0

589- 2: transcript:Zm00001d051140_T001 rna26019 1.00E-90
589- 3: transcript:Zm00001d051143_T001 rna26022 6.00E-83
589- 4: transcript:Zm00001d051149_T001 rna26025 5.00E-80
589- 5: transcript:Zm00001d051172_T001 rna26037 3.00E-140
589- 6: transcript:Zm00001d051174_T001 rna26041 1.00E-96
Alignment 590: score=1730.0 e_value=2.8e-143 N=39 4&NC_008404.2 plus
590- 0: transcript:Zm00001d052329_T002 rna26739 8.00E-148
590- 1: transcript:Zm00001d052331_T001 rna26746 9.00E-44
590- 2: transcript:Zm00001d052334_T001 rna26749 3.00E-29
590- 3: transcript:Zm00001d052335_T003 rna26750 2.00E-140
590- 4: transcript:Zm00001d052339_T001 rna26758 0
590- 5: transcript:Zm00001d052340_T001 rna26760 0
590- 6: transcript:Zm00001d052344_T003 rna26764 0
590- 7: transcript:Zm00001d052354_T005 rna26777 1.00E-159
590- 8: transcript:Zm00001d052355_T001 rna26778 6.00E-24
590- 9: transcript:Zm00001d052358_T001 rna26779 6.00E-64
590- 10: transcript:Zm00001d052361_T001 rna26782 2.00E-129
590- 11: transcript:Zm00001d052363_T001 rna26783 4.00E-62
590- 12: transcript:Zm00001d052367_T003 rna26792 9.00E-166
590- 13: transcript:Zm00001d052368_T001 rna26797 0
590- 14: transcript:Zm00001d052371_T001 rna26798 3.00E-80
590- 15: transcript:Zm00001d052375_T002 rna26801 5.00E-33
590- 16: transcript:Zm00001d052376_T006 rna26802 0
590- 17: transcript:Zm00001d052378_T001 rna26808 2.00E-48
590- 18: transcript:Zm00001d052379_T001 rna26809 2.00E-95
590- 19: transcript:Zm00001d052380_T001 rna26811 0
590- 20: transcript:Zm00001d052384_T001 rna26815 7.00E-30
590- 21: transcript:Zm00001d052386_T001 rna26816 3.00E-65
590- 22: transcript:Zm00001d052390_T001 rna26822 4.00E-72
590- 23: transcript:Zm00001d052391_T001 rna26824 2.00E-98
590- 24: transcript:Zm00001d052395_T001 rna26828 2.00E-33
590- 25: transcript:Zm00001d052397_T001 rna26830 3.00E-109
590- 26: transcript:Zm00001d052403_T001 rna26837 2.00E-60
590- 27: transcript:Zm00001d052404_T002 rna26838 2.00E-21
590- 28: transcript:Zm00001d052407_T004 rna26842 8.00E-134
590- 29: transcript:Zm00001d052409_T001 rna26843 2.00E-98
590- 30: transcript:Zm00001d052410_T005 rna26850 2.00E-22
590- 31: transcript:Zm00001d052424_T001 rna26855 1.00E-19
590- 32: transcript:Zm00001d052433_T001 rna26873 2.00E-148
590- 33: transcript:Zm00001d052434_T001 rna26874 0
590- 34: transcript:Zm00001d052437_T001 rna26880 1.00E-148
590- 35: transcript:Zm00001d052438_T001 rna26881 0
590- 36: transcript:Zm00001d052442_T001 rna26884 0
590- 37: transcript:Zm00001d052444_T002 rna26886 2.00E-32
590- 38: transcript:Zm00001d052445_T003 rna26887 0
Alignment 591: score=1634.0 e_value=6.4e-122 N=36 4&NC_008404.2 plus
591- 0: transcript:Zm00001d052450_T001 rna26891 4.00E-35
591- 1: transcript:Zm00001d052451_T001 rna26894 3.00E-162
591- 2: transcript:Zm00001d052452_T001 rna26897 3.00E-106
591- 3: transcript:Zm00001d052453_T014 rna26899 1.00E-89
591- 4: transcript:Zm00001d052457_T001 rna26905 1.00E-67
591- 5: transcript:Zm00001d052458_T001 rna26906 3.00E-149
591- 6: transcript:Zm00001d052461_T001 rna26909 3.00E-83
591- 7: transcript:Zm00001d052462_T001 rna26910 0

591- 8:	transcript:Zm00001d052463_T001	rna26913	1.00E-79
591- 9:	transcript:Zm00001d052468_T002	rna26915	0
591- 10:	transcript:Zm00001d052471_T001	rna26916	2.00E-17
591- 11:	transcript:Zm00001d052472_T012	rna26918	4.00E-44
591- 12:	transcript:Zm00001d052475_T001	rna26919	0
591- 13:	transcript:Zm00001d052476_T001	rna26920	5.00E-34
591- 14:	transcript:Zm00001d052478_T001	rna26925	0
591- 15:	transcript:Zm00001d052479_T001	rna26928	2.00E-133
591- 16:	transcript:Zm00001d052485_T001	rna26940	0
591- 17:	transcript:Zm00001d052488_T001	rna26941	2.00E-133
591- 18:	transcript:Zm00001d052493_T001	rna26947	4.00E-131
591- 19:	transcript:Zm00001d052494_T001	rna26952	0
591- 20:	transcript:Zm00001d052495_T001	rna26956	5.00E-145
591- 21:	transcript:Zm00001d052500_T001	rna26959	7.00E-15
591- 22:	transcript:Zm00001d052509_T001	rna26960	0
591- 23:	transcript:Zm00001d052519_T004	rna26961	0
591- 24:	transcript:Zm00001d052520_T001	rna26962	1.00E-107
591- 25:	transcript:Zm00001d052523_T001	rna26967	0
591- 26:	transcript:Zm00001d052527_T001	rna26969	1.00E-72
591- 27:	transcript:Zm00001d052530_T001	rna26972	0
591- 28:	transcript:Zm00001d052532_T001	rna26973	0
591- 29:	transcript:Zm00001d052537_T001	rna26978	3.00E-110
591- 30:	transcript:Zm00001d052543_T001	rna26980	0
591- 31:	transcript:Zm00001d052545_T001	rna26981	8.00E-27
591- 32:	transcript:Zm00001d052551_T001	rna26983	3.00E-94
591- 33:	transcript:Zm00001d052556_T001	rna26987	8.00E-89
591- 34:	transcript:Zm00001d052564_T001	rna26993	8.00E-89
591- 35:	transcript:Zm00001d052578_T001	rna26996	4.00E-117
## Alignment 592: score=1152.0 e_value=5e-84 N=26 4&NC_008404.2 plus			
592- 0:	transcript:Zm00001d053004_T001	rna27061	0
592- 1:	transcript:Zm00001d053006_T001	rna27062	9.00E-131
592- 2:	transcript:Zm00001d053008_T001	rna27064	0
592- 3:	transcript:Zm00001d053009_T009	rna27065	0
592- 4:	transcript:Zm00001d053010_T001	rna27066	9.00E-111
592- 5:	transcript:Zm00001d053011_T010	rna27069	0
592- 6:	transcript:Zm00001d053014_T001	rna27070	4.00E-30
592- 7:	transcript:Zm00001d053015_T001	rna27074	0
592- 8:	transcript:Zm00001d053016_T002	rna27076	0
592- 9:	transcript:Zm00001d053017_T001	rna27096	0
592- 10:	transcript:Zm00001d053018_T004	rna27097	0
592- 11:	transcript:Zm00001d053020_T001	rna27098	1.00E-96
592- 12:	transcript:Zm00001d053028_T001	rna27100	0
592- 13:	transcript:Zm00001d053038_T007	rna27101	0
592- 14:	transcript:Zm00001d053041_T002	rna27102	0
592- 15:	transcript:Zm00001d053049_T001	rna27106	0
592- 16:	transcript:Zm00001d053055_T001	rna27107	5.00E-78
592- 17:	transcript:Zm00001d053059_T007	rna27115	1.00E-25
592- 18:	transcript:Zm00001d053060_T001	rna27117	4.00E-119
592- 19:	transcript:Zm00001d053065_T002	rna27120	9.00E-25
592- 20:	transcript:Zm00001d053066_T001	rna27121	3.00E-124
592- 21:	transcript:Zm00001d053067_T001	rna27123	0
592- 22:	transcript:Zm00001d053068_T001	rna27126	5.00E-33
592- 23:	transcript:Zm00001d053070_T001	rna27143	0
592- 24:	transcript:Zm00001d053076_T001	rna27152	4.00E-15

592- 25: transcript:Zm00001d053080_T002 rna27165 0
Alignment 593: score=692.0 e_value=3.8e-39 N=15 4&NC_008404.2 plus
593- 0: transcript:Zm00001d053094_T001 rna27916 5.00E-113
593- 1: transcript:Zm00001d053100_T004 rna27918 8.00E-87
593- 2: transcript:Zm00001d053101_T001 rna27926 6.00E-55
593- 3: transcript:Zm00001d053102_T001 rna27928 1.00E-46
593- 4: transcript:Zm00001d053103_T001 rna27932 4.00E-35
593- 5: transcript:Zm00001d053104_T002 rna27933 8.00E-80
593- 6: transcript:Zm00001d053107_T001 rna27939 0
593- 7: transcript:Zm00001d053109_T002 rna27940 2.00E-141
593- 8: transcript:Zm00001d053117_T001 rna27941 1.00E-61
593- 9: transcript:Zm00001d053119_T002 rna27943 2.00E-169
593- 10: transcript:Zm00001d053120_T001 rna27954 0
593- 11: transcript:Zm00001d053122_T001 rna27956 2.00E-154
593- 12: transcript:Zm00001d053123_T001 rna27958 2.00E-58
593- 13: transcript:Zm00001d053124_T001 rna27959 9.00E-120
593- 14: transcript:Zm00001d053127_T005 rna27960 0
Alignment 594: score=672.0 e_value=1.4e-39 N=15 4&NC_008404.2 plus
594- 0: transcript:Zm00001d053146_T001 rna27016 6.00E-87
594- 1: transcript:Zm00001d053149_T002 rna27017 0
594- 2: transcript:Zm00001d053150_T007 rna27018 0
594- 3: transcript:Zm00001d053151_T003 rna27019 0
594- 4: transcript:Zm00001d053155_T002 rna27020 3.00E-147
594- 5: transcript:Zm00001d053158_T001 rna27025 0
594- 6: transcript:Zm00001d053162_T003 rna27027 2.00E-113
594- 7: transcript:Zm00001d053175_T002 rna27036 4.00E-38
594- 8: transcript:Zm00001d053177_T001 rna27040 0
594- 9: transcript:Zm00001d053178_T001 rna27041 0
594- 10: transcript:Zm00001d053179_T002 rna27042 7.00E-147
594- 11: transcript:Zm00001d053181_T001 rna27043 1.00E-162
594- 12: transcript:Zm00001d053189_T004 rna27048 6.00E-123
594- 13: transcript:Zm00001d053192_T001 rna27052 0
594- 14: transcript:Zm00001d053195_T002 rna27058 1.00E-31
Alignment 595: score=532.0 e_value=3.1e-26 N=12 4&NC_008404.2 plus
595- 0: transcript:Zm00001d049252_T001 rna27778 1.00E-140
595- 1: transcript:Zm00001d049258_T001 rna27795 1.00E-119
595- 2: transcript:Zm00001d049259_T001 rna27798 0
595- 3: transcript:Zm00001d049266_T001 rna27808 3.00E-14
595- 4: transcript:Zm00001d049269_T004 rna27811 0
595- 5: transcript:Zm00001d049273_T001 rna27814 0
595- 6: transcript:Zm00001d049276_T003 rna27815 0
595- 7: transcript:Zm00001d049277_T001 rna27816 1.00E-133
595- 8: transcript:Zm00001d049294_T001 rna27817 0
595- 9: transcript:Zm00001d049295_T001 rna27818 0
595- 10: transcript:Zm00001d049297_T003 rna27819 3.00E-162
595- 11: transcript:Zm00001d049299_T001 rna27820 0
Alignment 596: score=514.0 e_value=4.7e-23 N=11 4&NC_008404.2 plus
596- 0: transcript:Zm00001d048989_T001 rna27362 2.00E-114
596- 1: transcript:Zm00001d048994_T003 rna27364 0
596- 2: transcript:Zm00001d048997_T001 rna27366 6.00E-54
596- 3: transcript:Zm00001d048998_T001 rna27367 0
596- 4: transcript:Zm00001d049000_T001 rna27368 2.00E-93
596- 5: transcript:Zm00001d049002_T001 rna27373 5.00E-149
596- 6: transcript:Zm00001d049006_T001 rna27374 1.00E-71

596- 7: transcript:Zm00001d049011_T001 rna27380 1.00E-113
596- 8: transcript:Zm00001d049016_T001 rna27385 0
596- 9: transcript:Zm00001d049018_T001 rna27387 0
596- 10: transcript:Zm00001d049019_T003 rna27388 0
Alignment 597: score=451.0 e_value=1.8e-25 N=10 4&NC_008404.2 plus
597- 0: transcript:Zm00001d052911_T002 rna27973 2.00E-113
597- 1: transcript:Zm00001d052918_T001 rna27980 5.00E-166
597- 2: transcript:Zm00001d052921_T002 rna27982 0
597- 3: transcript:Zm00001d052922_T003 rna27983 0
597- 4: transcript:Zm00001d052928_T001 rna27991 2.00E-125
597- 5: transcript:Zm00001d052929_T002 rna27995 0
597- 6: transcript:Zm00001d052930_T003 rna27996 9.00E-53
597- 7: transcript:Zm00001d052932_T001 rna27997 0
597- 8: transcript:Zm00001d052933_T001 rna27998 0
597- 9: transcript:Zm00001d052941_T001 rna28001 9.00E-47
Alignment 598: score=424.0 e_value=2.7e-22 N=10 4&NC_008404.2 plus
598- 0: transcript:Zm00001d052943_T023 rna27999 4.00E-165
598- 1: transcript:Zm00001d052947_T001 rna28013 0
598- 2: transcript:Zm00001d052952_T001 rna28023 3.00E-68
598- 3: transcript:Zm00001d052953_T001 rna28024 0
598- 4: transcript:Zm00001d052955_T001 rna28025 0
598- 5: transcript:Zm00001d052964_T002 rna28029 4.00E-99
598- 6: transcript:Zm00001d052974_T002 rna28040 0
598- 7: transcript:Zm00001d052977_T001 rna28042 0
598- 8: transcript:Zm00001d052978_T001 rna28044 1.00E-130
598- 9: transcript:Zm00001d052981_T001 rna28046 0
Alignment 599: score=384.0 e_value=4.8e-14 N=8 4&NC_008404.2 plus
599- 0: transcript:Zm00001d052586_T002 rna26997 0
599- 1: transcript:Zm00001d052591_T001 rna27000 7.00E-103
599- 2: transcript:Zm00001d052592_T001 rna27002 0
599- 3: transcript:Zm00001d052605_T001 rna27003 2.00E-57
599- 4: transcript:Zm00001d052606_T001 rna27004 1.00E-36
599- 5: transcript:Zm00001d052609_T001 rna27006 1.00E-38
599- 6: transcript:Zm00001d052612_T001 rna27007 3.00E-129
599- 7: transcript:Zm00001d052613_T001 rna27008 7.00E-107
Alignment 600: score=376.0 e_value=1.6e-15 N=8 4&NC_008404.2 plus
600- 0: transcript:Zm00001d052316_T001 rna26714 1.00E-40
600- 1: transcript:Zm00001d052317_T003 rna26715 1.00E-80
600- 2: transcript:Zm00001d052318_T002 rna26717 0
600- 3: transcript:Zm00001d052321_T002 rna26720 6.00E-80
600- 4: transcript:Zm00001d052322_T001 rna26721 4.00E-80
600- 5: transcript:Zm00001d052323_T001 rna26722 0
600- 6: transcript:Zm00001d052324_T001 rna26724 0
600- 7: transcript:Zm00001d052327_T006 rna26736 2.00E-47
Alignment 601: score=351.0 e_value=5e-16 N=8 4&NC_008404.2 plus
601- 0: transcript:Zm00001d048962_T001 rna27332 0
601- 1: transcript:Zm00001d048969_T004 rna27337 0
601- 2: transcript:Zm00001d048970_T003 rna27343 3.00E-50
601- 3: transcript:Zm00001d048979_T001 rna27344 1.00E-156
601- 4: transcript:Zm00001d048982_T001 rna27356 6.00E-46
601- 5: transcript:Zm00001d048984_T001 rna27357 2.00E-135
601- 6: transcript:Zm00001d048985_T001 rna27358 1.00E-59
601- 7: transcript:Zm00001d048988_T001 rna27361 0
Alignment 602: score=316.0 e_value=2.2e-12 N=7 4&NC_008404.2 plus

```
602-  0:  transcript:Zm00001d049092_T002  rna27174     4.00E-81
602-  1:  transcript:Zm00001d049093_T001  rna27175     1.00E-14
602-  2:  transcript:Zm00001d049094_T001  rna27176    4.00E-104
602-  3:  transcript:Zm00001d049095_T002  rna27177    1.00E-169
602-  4:  transcript:Zm00001d049096_T001  rna27178    1.00E-118
602-  5:  transcript:Zm00001d049099_T005  rna27182            0
602-  6:  transcript:Zm00001d049100_T003  rna27184            0
## Alignment 603: score=256.0 e_value=5.4e-10 N=6 4&NC_008404.2 plus
603-  0:  transcript:Zm00001d052618_T001  rna27893    6.00E-145
603-  1:  transcript:Zm00001d052620_T002  rna27894     1.00E-67
603-  2:  transcript:Zm00001d052621_T002  rna27898    2.00E-136
603-  3:  transcript:Zm00001d052622_T001  rna27899            0
603-  4:  transcript:Zm00001d052624_T007  rna27904            0
603-  5:  transcript:Zm00001d052625_T001  rna27909            0
## Alignment 604: score=1259.0 e_value=1.4e-100 N=28 4&NC_008404.2 minus
604-  0:  transcript:Zm00001d048582_T001  rna28511     4.00E-56
604-  1:  transcript:Zm00001d048583_T001  rna28510            0
604-  2:  transcript:Zm00001d048585_T002  rna28508            0
604-  3:  transcript:Zm00001d048590_T001  rna28507     9.00E-28
604-  4:  transcript:Zm00001d048592_T001  rna28505            0
604-  5:  transcript:Zm00001d048595_T001  rna28503    6.00E-132
604-  6:  transcript:Zm00001d048599_T002  rna28501     8.00E-64
604-  7:  transcript:Zm00001d048600_T001  rna28498    2.00E-174
604-  8:  transcript:Zm00001d048606_T001  rna28497            0
604-  9:  transcript:Zm00001d048609_T001  rna28496     5.00E-51
604- 10:  transcript:Zm00001d048611_T001  rna28493     4.00E-23
604- 11:  transcript:Zm00001d048614_T001  rna28491            0
604- 12:  transcript:Zm00001d048615_T001  rna28487     2.00E-73
604- 13:  transcript:Zm00001d048616_T001  rna28485    3.00E-129
604- 14:  transcript:Zm00001d048618_T001  rna28483     6.00E-50
604- 15:  transcript:Zm00001d048620_T001  rna28481            0
604- 16:  transcript:Zm00001d048623_T002  rna28465    2.00E-125
604- 17:  transcript:Zm00001d048627_T001  rna28456     4.00E-19
604- 18:  transcript:Zm00001d048628_T001  rna28455     3.00E-31
604- 19:  transcript:Zm00001d048631_T001  rna28437     4.00E-62
604- 20:  transcript:Zm00001d048632_T001  rna28433            0
604- 21:  transcript:Zm00001d048635_T001  rna28421     4.00E-72
604- 22:  transcript:Zm00001d048636_T001  rna28414     9.00E-06
604- 23:  transcript:Zm00001d048637_T001  rna28411    4.00E-151
604- 24:  transcript:Zm00001d048647_T001  rna28410    7.00E-102
604- 25:  transcript:Zm00001d048648_T006  rna28408            0
604- 26:  transcript:Zm00001d048649_T001  rna28407     2.00E-76
604- 27:  transcript:Zm00001d048655_T012  rna28403            0
## Alignment 605: score=938.0 e_value=2e-66 N=21 4&NC_008404.2 minus
605-  0:  transcript:Zm00001d048877_T002  rna28148            0
605-  1:  transcript:Zm00001d048880_T001  rna28147            0
605-  2:  transcript:Zm00001d048890_T002  rna28136     6.00E-24
605-  3:  transcript:Zm00001d048892_T001  rna28133    5.00E-105
605-  4:  transcript:Zm00001d048893_T002  rna28132            0
605-  5:  transcript:Zm00001d048898_T001  rna28124            0
605-  6:  transcript:Zm00001d048899_T001  rna28119    7.00E-168
605-  7:  transcript:Zm00001d048901_T001  rna28116     1.00E-89
605-  8:  transcript:Zm00001d048907_T001  rna28113    4.00E-111
605-  9:  transcript:Zm00001d048908_T002  rna28112    2.00E-133
```

605- 10: transcript:Zm00001d048913_T001 rna28110 9.00E-53
605- 11: transcript:Zm00001d048924_T001 rna28108 0
605- 12: transcript:Zm00001d048925_T001 rna28106 0
605- 13: transcript:Zm00001d048926_T001 rna28104 3.00E-174
605- 14: transcript:Zm00001d048930_T001 rna28085 0
605- 15: transcript:Zm00001d048943_T001 rna28074 1.00E-100
605- 16: transcript:Zm00001d048944_T007 rna28072 2.00E-25
605- 17: transcript:Zm00001d048945_T001 rna28070 5.00E-111
605- 18: transcript:Zm00001d048947_T001 rna28062 5.00E-40
605- 19: transcript:Zm00001d048953_T001 rna28059 0
605- 20: transcript:Zm00001d048954_T001 rna28058 0
Alignment 606: score=919.0 e_value=8.7e-60 N=20 4&NC_008404.2 minus
606- 0: transcript:Zm00001d049161_T001 rna27763 0
606- 1: transcript:Zm00001d049162_T001 rna27760 4.00E-80
606- 2: transcript:Zm00001d049163_T001 rna27759 0
606- 3: transcript:Zm00001d049166_T001 rna27758 3.00E-38
606- 4: transcript:Zm00001d049168_T003 rna27757 6.00E-97
606- 5: transcript:Zm00001d049169_T001 rna27756 0
606- 6: transcript:Zm00001d049171_T001 rna27749 0
606- 7: transcript:Zm00001d049173_T001 rna27748 5.00E-76
606- 8: transcript:Zm00001d049174_T001 rna27746 2.00E-119
606- 9: transcript:Zm00001d049175_T001 rna27745 2.00E-26
606- 10: transcript:Zm00001d049183_T001 rna27739 2.00E-87
606- 11: transcript:Zm00001d049187_T001 rna27730 0
606- 12: transcript:Zm00001d049188_T008 rna27729 5.00E-23
606- 13: transcript:Zm00001d049190_T002 rna27728 0
606- 14: transcript:Zm00001d049191_T001 rna27727 0
606- 15: transcript:Zm00001d049196_T001 rna27726 0
606- 16: transcript:Zm00001d049197_T002 rna27725 4.00E-173
606- 17: transcript:Zm00001d049201_T001 rna27723 0
606- 18: transcript:Zm00001d049203_T001 rna27721 0
606- 19: transcript:Zm00001d049208_T003 rna27719 0
Alignment 607: score=802.0 e_value=3.3e-57 N=18 4&NC_008404.2 minus
607- 0: transcript:Zm00001d048785_T003 rna28242 1.00E-53
607- 1: transcript:Zm00001d048787_T001 rna28241 7.00E-35
607- 2: transcript:Zm00001d048789_T001 rna28239 1.00E-28
607- 3: transcript:Zm00001d048794_T001 rna28226 4.00E-159
607- 4: transcript:Zm00001d048795_T001 rna28225 4.00E-34
607- 5: transcript:Zm00001d048796_T003 rna28224 2.00E-132
607- 6: transcript:Zm00001d048799_T001 rna28222 8.00E-35
607- 7: transcript:Zm00001d048801_T001 rna28205 1.00E-59
607- 8: transcript:Zm00001d048802_T003 rna28203 4.00E-73
607- 9: transcript:Zm00001d048804_T001 rna28202 1.00E-125
607- 10: transcript:Zm00001d048822_T001 rna28199 0
607- 11: transcript:Zm00001d048824_T004 rna28196 0
607- 12: transcript:Zm00001d048825_T001 rna28195 1.00E-89
607- 13: transcript:Zm00001d048829_T001 rna28179 2.00E-154
607- 14: transcript:Zm00001d048831_T001 rna28178 4.00E-63
607- 15: transcript:Zm00001d048833_T001 rna28170 1.00E-86
607- 16: transcript:Zm00001d048834_T001 rna28169 0
607- 17: transcript:Zm00001d048835_T004 rna28168 4.00E-142
Alignment 608: score=615.0 e_value=1.1e-39 N=14 4&NC_008404.2 minus
608- 0: transcript:Zm00001d048715_T004 rna28327 0
608- 1: transcript:Zm00001d048717_T004 rna28324 0

608- 2: transcript:Zm00001d048718_T010 rna28323 0
608- 3: transcript:Zm00001d048719_T001 rna28318 4.00E-52
608- 4: transcript:Zm00001d048721_T001 rna28317 0
608- 5: transcript:Zm00001d048723_T001 rna28306 7.00E-137
608- 6: transcript:Zm00001d048727_T001 rna28301 3.00E-32
608- 7: transcript:Zm00001d048729_T001 rna28300 3.00E-49
608- 8: transcript:Zm00001d048731_T011 rna28295 2.00E-133
608- 9: transcript:Zm00001d048732_T005 rna28294 0
608- 10: transcript:Zm00001d048736_T001 rna28286 2.00E-42
608- 11: transcript:Zm00001d048737_T001 rna28283 3.00E-38
608- 12: transcript:Zm00001d048740_T001 rna28281 0
608- 13: transcript:Zm00001d048741_T004 rna28279 1.00E-54
Alignment 609: score=538.0 e_value=3.1e-33 N=12 4&NC_008404.2 minus
609- 0: transcript:Zm00001d049210_T003 rna27714 0
609- 1: transcript:Zm00001d049211_T001 rna27711 0
609- 2: transcript:Zm00001d049222_T002 rna27694 7.00E-168
609- 3: transcript:Zm00001d049223_T001 rna27693 0
609- 4: transcript:Zm00001d049224_T001 rna27678 1.00E-44
609- 5: transcript:Zm00001d049225_T001 rna27677 5.00E-71
609- 6: transcript:Zm00001d049226_T001 rna27672 0
609- 7: transcript:Zm00001d049230_T002 rna27655 9.00E-93
609- 8: transcript:Zm00001d049232_T002 rna27653 0
609- 9: transcript:Zm00001d049233_T001 rna27652 3.00E-40
609- 10: transcript:Zm00001d049234_T001 rna27651 0
609- 11: transcript:Zm00001d049239_T001 rna27650 0
Alignment 610: score=456.0 e_value=1.8e-23 N=11 4&NC_008404.2 minus
610- 0: transcript:Zm00001d052624_T007 rna27904 0
610- 1: transcript:Zm00001d052630_T001 rna27892 4.00E-83
610- 2: transcript:Zm00001d052632_T005 rna27890 6.00E-39
610- 3: transcript:Zm00001d052636_T001 rna27880 4.00E-118
610- 4: transcript:Zm00001d052651_T001 rna27873 0
610- 5: transcript:Zm00001d052654_T001 rna27865 2.00E-90
610- 6: transcript:Zm00001d052660_T002 rna27863 0
610- 7: transcript:Zm00001d052661_T001 rna27862 1.00E-75
610- 8: transcript:Zm00001d052662_T001 rna27861 2.00E-49
610- 9: transcript:Zm00001d052663_T001 rna27857 0
610- 10: transcript:Zm00001d052666_T015 rna27854 0
Alignment 611: score=359.0 e_value=7.6e-18 N=8 4&NC_008404.2 minus
611- 0: transcript:Zm00001d048669_T001 rna28380 0
611- 1: transcript:Zm00001d048670_T001 rna28378 0
611- 2: transcript:Zm00001d048671_T001 rna28377 3.00E-76
611- 3: transcript:Zm00001d048672_T001 rna28375 0
611- 4: transcript:Zm00001d048673_T003 rna28373 1.00E-16
611- 5: transcript:Zm00001d048680_T001 rna28358 5.00E-25
611- 6: transcript:Zm00001d048684_T001 rna28355 7.00E-07
611- 7: transcript:Zm00001d048689_T004 rna28345 8.00E-163
Alignment 612: score=322.0 e_value=4.6e-11 N=7 4&NC_008404.2 minus
612- 0: transcript:Zm00001d048759_T001 rna28271 0
612- 1: transcript:Zm00001d048760_T001 rna28270 2.00E-31
612- 2: transcript:Zm00001d048763_T001 rna28257 3.00E-10
612- 3: transcript:Zm00001d048766_T001 rna28253 3.00E-59
612- 4: transcript:Zm00001d048770_T001 rna28251 3.00E-35
612- 5: transcript:Zm00001d048775_T001 rna28249 0
612- 6: transcript:Zm00001d048777_T004 rna28247 0

```
## Alignment 613: score=307.0 e_value=4.6e-12 N=7 4&NC_008404.2 minus
613-  0:  transcript:Zm00001d049055_T001  rna27258            0
613-  1:  transcript:Zm00001d049059_T001  rna27244            0
613-  2:  transcript:Zm00001d049071_T001  rna27242     3.00E-82
613-  3:  transcript:Zm00001d049073_T001  rna27240            0
613-  4:  transcript:Zm00001d049076_T001  rna27239    4.00E-101
613-  5:  transcript:Zm00001d049079_T001  rna27224     2.00E-87
613-  6:  transcript:Zm00001d049080_T001  rna27221            0
## Alignment 614: score=288.0 e_value=1e-09 N=6 4&NC_008404.2 minus
614-  0:  transcript:Zm00001d049038_T014  rna27277            0
614-  1:  transcript:Zm00001d049042_T001  rna27274            0
614-  2:  transcript:Zm00001d049043_T001  rna27273    9.00E-164
614-  3:  transcript:Zm00001d049046_T002  rna27272            0
614-  4:  transcript:Zm00001d049048_T001  rna27270    3.00E-120
614-  5:  transcript:Zm00001d049049_T001  rna27269            0
## Alignment 615: score=267.0 e_value=2.3e-08 N=6 4&NC_008404.2 minus
615-  0:  transcript:Zm00001d052667_T001  rna27845    4.00E-116
615-  1:  transcript:Zm00001d052668_T001  rna27844     7.00E-60
615-  2:  transcript:Zm00001d052673_T001  rna27843            0
615-  3:  transcript:Zm00001d052675_T002  rna27840    2.00E-140
615-  4:  transcript:Zm00001d052678_T002  rna27835     2.00E-87
615-  5:  transcript:Zm00001d052680_T002  rna27825     4.00E-59
## Alignment 616: score=258.0 e_value=1.3e-09 N=6 4&NC_008404.2 minus
616-  0:  transcript:Zm00001d048836_T001  rna28167    4.00E-103
616-  1:  transcript:Zm00001d048837_T001  rna28166     4.00E-18
616-  2:  transcript:Zm00001d048841_T001  rna28164            0
616-  3:  transcript:Zm00001d048856_T001  rna28159            0
616-  4:  transcript:Zm00001d048857_T006  rna28158            0
616-  5:  transcript:Zm00001d048868_T002  rna28153     3.00E-31
## Alignment 617: score=1373.0 e_value=1.9e-103 N=31 4&NC_008405.2 plus
617-  0:  transcript:Zm00001d052449_T001  rna28735            0
617-  1:  transcript:Zm00001d052451_T001  rna28738    6.00E-174
617-  2:  transcript:Zm00001d052452_T001  rna28742     1.00E-68
617-  3:  transcript:Zm00001d052453_T014  rna28744            0
617-  4:  transcript:Zm00001d052457_T001  rna28748            0
617-  5:  transcript:Zm00001d052458_T001  rna28749    9.00E-149
617-  6:  transcript:Zm00001d052462_T001  rna28752            0
617-  7:  transcript:Zm00001d052468_T002  rna28757            0
617-  8:  transcript:Zm00001d052472_T012  rna28760            0
617-  9:  transcript:Zm00001d052475_T001  rna28761            0
617- 10:  transcript:Zm00001d052476_T001  rna28762     2.00E-34
617- 11:  transcript:Zm00001d052478_T001  rna28771            0
617- 12:  transcript:Zm00001d052485_T001  rna28787            0
617- 13:  transcript:Zm00001d052488_T001  rna28788     3.00E-42
617- 14:  transcript:Zm00001d052493_T001  rna28792    1.00E-126
617- 15:  transcript:Zm00001d052494_T001  rna28798            0
617- 16:  transcript:Zm00001d052495_T001  rna28802    6.00E-100
617- 17:  transcript:Zm00001d052500_T001  rna28805     4.00E-10
617- 18:  transcript:Zm00001d052520_T001  rna28810     9.00E-26
617- 19:  transcript:Zm00001d052523_T001  rna28814            0
617- 20:  transcript:Zm00001d052525_T001  rna28816            0
617- 21:  transcript:Zm00001d052530_T001  rna28818            0
617- 22:  transcript:Zm00001d052533_T001  rna28822            0
617- 23:  transcript:Zm00001d052537_T001  rna28824    3.00E-110
```

617- 24: transcript:Zm00001d052539_T001 rna28825 2.00E-28
617- 25: transcript:Zm00001d052540_T001 rna28828 0
617- 26: transcript:Zm00001d052543_T001 rna28831 0
617- 27: transcript:Zm00001d052545_T001 rna28832 5.00E-18
617- 28: transcript:Zm00001d052551_T001 rna28833 2.00E-124
617- 29: transcript:Zm00001d052564_T001 rna28848 5.00E-82
617- 30: transcript:Zm00001d052578_T001 rna28851 8.00E-96
Alignment 618: score=1354.0 e_value=1.8e-99 N=30 4&NC_008405.2 plus
618- 0: transcript:Zm00001d052329_T002 rna28583 6.00E-159
618- 1: transcript:Zm00001d052331_T001 rna28588 4.00E-44
618- 2: transcript:Zm00001d052333_T002 rna28589 5.00E-51
618- 3: transcript:Zm00001d052334_T001 rna28590 7.00E-22
618- 4: transcript:Zm00001d052335_T003 rna28592 0
618- 5: transcript:Zm00001d052339_T001 rna28597 0
618- 6: transcript:Zm00001d052340_T001 rna28599 0
618- 7: transcript:Zm00001d052343_T001 rna28604 0
618- 8: transcript:Zm00001d052344_T003 rna28605 5.00E-51
618- 9: transcript:Zm00001d052354_T005 rna28616 0
618- 10: transcript:Zm00001d052355_T001 rna28618 2.00E-24
618- 11: transcript:Zm00001d052358_T001 rna28619 2.00E-46
618- 12: transcript:Zm00001d052363_T001 rna28622 7.00E-129
618- 13: transcript:Zm00001d052367_T003 rna28634 1.00E-173
618- 14: transcript:Zm00001d052368_T001 rna28643 0
618- 15: transcript:Zm00001d052371_T001 rna28644 3.00E-80
618- 16: transcript:Zm00001d052375_T002 rna28649 3.00E-36
618- 17: transcript:Zm00001d052376_T006 rna28650 0
618- 18: transcript:Zm00001d052378_T001 rna28652 0
618- 19: transcript:Zm00001d052379_T001 rna28653 3.00E-92
618- 20: transcript:Zm00001d052380_T001 rna28655 0
618- 21: transcript:Zm00001d052386_T001 rna28660 0
618- 22: transcript:Zm00001d052390_T001 rna28663 4.00E-90
618- 23: transcript:Zm00001d052391_T001 rna28664 1.00E-93
618- 24: transcript:Zm00001d052393_T001 rna28666 3.00E-64
618- 25: transcript:Zm00001d052395_T001 rna28670 3.00E-32
618- 26: transcript:Zm00001d052397_T001 rna28673 6.00E-109
618- 27: transcript:Zm00001d052404_T002 rna28681 2.00E-154
618- 28: transcript:Zm00001d052405_T002 rna28682 7.00E-111
618- 29: transcript:Zm00001d052407_T004 rna28685 0
Alignment 619: score=373.0 e_value=2.5e-16 N=8 4&NC_008405.2 plus
619- 0: transcript:Zm00001d052316_T001 rna28551 3.00E-138
619- 1: transcript:Zm00001d052317_T003 rna28552 4.00E-78
619- 2: transcript:Zm00001d052318_T002 rna28554 0
619- 3: transcript:Zm00001d052321_T002 rna28561 2.00E-66
619- 4: transcript:Zm00001d052322_T001 rna28562 1.00E-77
619- 5: transcript:Zm00001d052323_T001 rna28563 0
619- 6: transcript:Zm00001d052324_T001 rna28564 0
619- 7: transcript:Zm00001d052327_T006 rna28579 0
Alignment 620: score=326.0 e_value=3.9e-15 N=8 4&NC_008405.2 plus
620- 0: transcript:Zm00001d053150_T007 rna28870 0
620- 1: transcript:Zm00001d053151_T003 rna28871 2.00E-78
620- 2: transcript:Zm00001d053158_T001 rna28878 3.00E-157
620- 3: transcript:Zm00001d053162_T003 rna28880 5.00E-147
620- 4: transcript:Zm00001d053178_T001 rna28887 0
620- 5: transcript:Zm00001d053181_T001 rna28892 6.00E-179

620- 6: transcript:Zm00001d053189_T004 rna28899 2.00E-141
620- 7: transcript:Zm00001d053195_T002 rna28910 2.00E-44
Alignment 621: score=305.0 e_value=1.4e-15 N=7 4&NC_008405.2 plus
621- 0: transcript:Zm00001d052425_T006 rna28697 0
621- 1: transcript:Zm00001d052434_T001 rna28720 2.00E-145
621- 2: transcript:Zm00001d052437_T001 rna28727 4.00E-140
621- 3: transcript:Zm00001d052438_T001 rna28728 0
621- 4: transcript:Zm00001d052442_T001 rna28731 5.00E-156
621- 5: transcript:Zm00001d052444_T002 rna28733 0
621- 6: transcript:Zm00001d052445_T003 rna28734 0
Alignment 622: score=272.0 e_value=7.2e-10 N=6 4&NC_008405.2 plus
622- 0: transcript:Zm00001d053009_T009 rna28914 0
622- 1: transcript:Zm00001d053010_T001 rna28916 5.00E-111
622- 2: transcript:Zm00001d053011_T010 rna28918 0
622- 3: transcript:Zm00001d053014_T001 rna28920 0
622- 4: transcript:Zm00001d053016_T002 rna28923 0
622- 5: transcript:Zm00001d053020_T001 rna28939 6.00E-79
Alignment 623: score=259.0 e_value=3e-08 N=6 4&NC_008405.2 plus
623- 0: transcript:Zm00001d053038_T007 rna28938 0
623- 1: transcript:Zm00001d053042_T001 rna28944 1.00E-09
623- 2: transcript:Zm00001d053046_T001 rna28945 6.00E-14
623- 3: transcript:Zm00001d053055_T001 rna28946 5.00E-52
623- 4: transcript:Zm00001d053060_T001 rna28948 3.00E-96
623- 5: transcript:Zm00001d053066_T001 rna28953 2.00E-85
Alignment 624: score=258.0 e_value=1.4e-09 N=6 5&NC_008394.4 minus
624- 0: transcript:Zm00001d016231_T001 rna667 6.00E-17
624- 1: transcript:Zm00001d016237_T001 rna665 6.00E-107
624- 2: transcript:Zm00001d016256_T001 rna660 1.00E-31
624- 3: transcript:Zm00001d016260_T001 rna654 3.00E-39
624- 4: transcript:Zm00001d016269_T001 rna646 9.00E-23
624- 5: transcript:Zm00001d016271_T001 rna643 3.00E-19
Alignment 625: score=4187.0 e_value=0 N=90 5&NC_008395.2 plus
625- 0: transcript:Zm00001d018014_T030 rna7081 0
625- 1: transcript:Zm00001d018016_T001 rna7083 0
625- 2: transcript:Zm00001d018017_T001 rna7084 1.00E-78
625- 3: transcript:Zm00001d018024_T001 rna7085 0
625- 4: transcript:Zm00001d018025_T001 rna7086 0
625- 5: transcript:Zm00001d018028_T001 rna7087 1.00E-118
625- 6: transcript:Zm00001d018030_T001 rna7089 1.00E-46
625- 7: transcript:Zm00001d018031_T001 rna7091 5.00E-161
625- 8: transcript:Zm00001d018032_T001 rna7093 3.00E-52
625- 9: transcript:Zm00001d018033_T001 rna7094 0
625- 10: transcript:Zm00001d018034_T001 rna7096 0
625- 11: transcript:Zm00001d018036_T002 rna7097 2.00E-170
625- 12: transcript:Zm00001d018037_T001 rna7098 0
625- 13: transcript:Zm00001d018038_T001 rna7100 9.00E-33
625- 14: transcript:Zm00001d018040_T002 rna7102 3.00E-119
625- 15: transcript:Zm00001d018041_T001 rna7103 1.00E-170
625- 16: transcript:Zm00001d018042_T001 rna7104 2.00E-50
625- 17: transcript:Zm00001d018043_T001 rna7105 0
625- 18: transcript:Zm00001d018045_T001 rna7107 4.00E-147
625- 19: transcript:Zm00001d018047_T002 rna7108 2.00E-37
625- 20: transcript:Zm00001d018050_T003 rna7109 1.00E-34
625- 21: transcript:Zm00001d018056_T001 rna7110 7.00E-31

625- 22:	transcript:Zm00001d018057_T001	rna7111	0
625- 23:	transcript:Zm00001d018058_T001	rna7113	3.00E-156
625- 24:	transcript:Zm00001d018061_T002	rna7118	7.00E-107
625- 25:	transcript:Zm00001d018066_T001	rna7122	2.00E-100
625- 26:	transcript:Zm00001d018069_T001	rna7124	2.00E-111
625- 27:	transcript:Zm00001d018070_T001	rna7128	0
625- 28:	transcript:Zm00001d018072_T001	rna7129	2.00E-66
625- 29:	transcript:Zm00001d018074_T001	rna7130	0
625- 30:	transcript:Zm00001d018076_T001	rna7131	1.00E-42
625- 31:	transcript:Zm00001d018077_T001	rna7132	7.00E-115
625- 32:	transcript:Zm00001d018078_T002	rna7134	0
625- 33:	transcript:Zm00001d018080_T001	rna7136	0
625- 34:	transcript:Zm00001d018081_T001	rna7139	4.00E-121
625- 35:	transcript:Zm00001d018082_T001	rna7140	0
625- 36:	transcript:Zm00001d018085_T001	rna7141	2.00E-40
625- 37:	transcript:Zm00001d018087_T001	rna7143	6.00E-140
625- 38:	transcript:Zm00001d018088_T001	rna7144	2.00E-122
625- 39:	transcript:Zm00001d018089_T001	rna7145	2.00E-27
625- 40:	transcript:Zm00001d018090_T002	rna7146	0
625- 41:	transcript:Zm00001d018093_T001	rna7147	1.00E-76
625- 42:	transcript:Zm00001d018096_T001	rna7150	6.00E-73
625- 43:	transcript:Zm00001d018097_T001	rna7151	1.00E-53
625- 44:	transcript:Zm00001d018098_T005	rna7152	0
625- 45:	transcript:Zm00001d018099_T001	rna7156	6.00E-176
625- 46:	transcript:Zm00001d018102_T001	rna7158	0
625- 47:	transcript:Zm00001d018103_T002	rna7159	4.00E-55
625- 48:	transcript:Zm00001d018104_T001	rna7163	4.00E-45
625- 49:	transcript:Zm00001d018105_T001	rna7165	4.00E-132
625- 50:	transcript:Zm00001d018111_T002	rna7171	0
625- 51:	transcript:Zm00001d018112_T004	rna7173	2.00E-86
625- 52:	transcript:Zm00001d018113_T006	rna7175	0
625- 53:	transcript:Zm00001d018117_T001	rna7176	1.00E-32
625- 54:	transcript:Zm00001d018118_T001	rna7177	2.00E-47
625- 55:	transcript:Zm00001d018119_T001	rna7178	8.00E-69
625- 56:	transcript:Zm00001d018122_T001	rna7179	2.00E-124
625- 57:	transcript:Zm00001d018125_T001	rna7180	0
625- 58:	transcript:Zm00001d018126_T001	rna7181	0
625- 59:	transcript:Zm00001d018127_T001	rna7182	0
625- 60:	transcript:Zm00001d018130_T001	rna7183	1.00E-70
625- 61:	transcript:Zm00001d018131_T004	rna7184	1.00E-58
625- 62:	transcript:Zm00001d018133_T013	rna7185	0
625- 63:	transcript:Zm00001d018134_T001	rna7186	4.00E-72
625- 64:	transcript:Zm00001d018135_T001	rna7188	2.00E-104
625- 65:	transcript:Zm00001d018142_T001	rna7191	3.00E-137
625- 66:	transcript:Zm00001d018144_T003	rna7193	0
625- 67:	transcript:Zm00001d018145_T001	rna7195	2.00E-53
625- 68:	transcript:Zm00001d018146_T008	rna7198	0
625- 69:	transcript:Zm00001d018148_T003	rna7202	0
625- 70:	transcript:Zm00001d018149_T002	rna7203	6.00E-60
625- 71:	transcript:Zm00001d018150_T001	rna7204	2.00E-65
625- 72:	transcript:Zm00001d018151_T003	rna7205	0
625- 73:	transcript:Zm00001d018155_T001	rna7210	0
625- 74:	transcript:Zm00001d018157_T001	rna7214	6.00E-150
625- 75:	transcript:Zm00001d018158_T001	rna7215	1.00E-80

625- 76: transcript:Zm00001d018159_T001 rna7216 2.00E-59
625- 77: transcript:Zm00001d018161_T001 rna7220 6.00E-142
625- 78: transcript:Zm00001d018178_T001 rna7223 7.00E-154
625- 79: transcript:Zm00001d018179_T015 rna7225 0
625- 80: transcript:Zm00001d018180_T003 rna7226 0
625- 81: transcript:Zm00001d018181_T001 rna7228 2.00E-171
625- 82: transcript:Zm00001d018182_T001 rna7229 0
625- 83: transcript:Zm00001d018183_T001 rna7230 0
625- 84: transcript:Zm00001d018192_T001 rna7232 8.00E-21
625- 85: transcript:Zm00001d018193_T001 rna7233 3.00E-85
625- 86: transcript:Zm00001d018194_T001 rna7235 6.00E-95
625- 87: transcript:Zm00001d018195_T001 rna7236 0
625- 88: transcript:Zm00001d018197_T001 rna7238 3.00E-179
625- 89: transcript:Zm00001d018198_T001 rna7239 1.00E-79
Alignment 626: score=3821.0 e_value=0 N=85 5&NC_008395.2 plus
626- 0: transcript:Zm00001d016838_T001 rna6025 0
626- 1: transcript:Zm00001d016850_T002 rna6033 4.00E-39
626- 2: transcript:Zm00001d016856_T009 rna6041 0
626- 3: transcript:Zm00001d016858_T001 rna6042 4.00E-123
626- 4: transcript:Zm00001d016860_T001 rna6047 1.00E-101
626- 5: transcript:Zm00001d016864_T001 rna6049 2.00E-39
626- 6: transcript:Zm00001d016865_T001 rna6050 5.00E-128
626- 7: transcript:Zm00001d016873_T001 rna6054 7.00E-28
626- 8: transcript:Zm00001d016876_T001 rna6056 4.00E-136
626- 9: transcript:Zm00001d016878_T001 rna6060 0
626- 10: transcript:Zm00001d016879_T001 rna6061 0
626- 11: transcript:Zm00001d016884_T002 rna6066 0
626- 12: transcript:Zm00001d016890_T001 rna6068 0
626- 13: transcript:Zm00001d016891_T001 rna6069 6.00E-23
626- 14: transcript:Zm00001d016892_T001 rna6071 1.00E-58
626- 15: transcript:Zm00001d016893_T001 rna6072 1.00E-74
626- 16: transcript:Zm00001d016894_T005 rna6073 0
626- 17: transcript:Zm00001d016895_T001 rna6074 5.00E-160
626- 18: transcript:Zm00001d016897_T002 rna6075 2.00E-134
626- 19: transcript:Zm00001d016898_T001 rna6076 0
626- 20: transcript:Zm00001d016901_T001 rna6089 1.00E-47
626- 21: transcript:Zm00001d016902_T002 rna6091 0
626- 22: transcript:Zm00001d016909_T001 rna6092 7.00E-143
626- 23: transcript:Zm00001d016911_T001 rna6093 2.00E-177
626- 24: transcript:Zm00001d016915_T002 rna6095 0
626- 25: transcript:Zm00001d016916_T004 rna6096 2.00E-107
626- 26: transcript:Zm00001d016918_T001 rna6097 0
626- 27: transcript:Zm00001d016919_T001 rna6098 0
626- 28: transcript:Zm00001d016922_T009 rna6101 1.00E-123
626- 29: transcript:Zm00001d016924_T001 rna6104 3.00E-20
626- 30: transcript:Zm00001d016926_T001 rna6109 0
626- 31: transcript:Zm00001d016933_T001 rna6111 0
626- 32: transcript:Zm00001d016934_T001 rna6112 0
626- 33: transcript:Zm00001d016938_T001 rna6115 0
626- 34: transcript:Zm00001d016941_T003 rna6118 9.00E-165
626- 35: transcript:Zm00001d016942_T001 rna6122 0
626- 36: transcript:Zm00001d016943_T001 rna6128 1.00E-81
626- 37: transcript:Zm00001d016947_T001 rna6129 4.00E-09
626- 38: transcript:Zm00001d016950_T002 rna6130 9.00E-128

626- 39: transcript:Zm00001d016952_T001 rna6132 9.00E-149
626- 40: transcript:Zm00001d016957_T001 rna6133 3.00E-151
626- 41: transcript:Zm00001d016972_T001 rna6136 0
626- 42: transcript:Zm00001d016977_T002 rna6138 1.00E-74
626- 43: transcript:Zm00001d016979_T001 rna6139 6.00E-178
626- 44: transcript:Zm00001d016980_T001 rna6141 5.00E-178
626- 45: transcript:Zm00001d016982_T001 rna6143 0
626- 46: transcript:Zm00001d016990_T007 rna6144 0
626- 47: transcript:Zm00001d016991_T001 rna6145 1.00E-13
626- 48: transcript:Zm00001d016992_T001 rna6146 1.00E-23
626- 49: transcript:Zm00001d016993_T007 rna6147 0
626- 50: transcript:Zm00001d016995_T001 rna6148 3.00E-77
626- 51: transcript:Zm00001d016996_T001 rna6149 9.00E-133
626- 52: transcript:Zm00001d016997_T001 rna6152 8.00E-10
626- 53: transcript:Zm00001d017000_T001 rna6157 4.00E-125
626- 54: transcript:Zm00001d017001_T001 rna6158 2.00E-09
626- 55: transcript:Zm00001d017014_T001 rna6168 2.00E-29
626- 56: transcript:Zm00001d017026_T003 rna6176 2.00E-149
626- 57: transcript:Zm00001d017030_T001 rna6178 2.00E-152
626- 58: transcript:Zm00001d017034_T007 rna6184 0
626- 59: transcript:Zm00001d017036_T002 rna6185 0
626- 60: transcript:Zm00001d017037_T001 rna6186 1.00E-59
626- 61: transcript:Zm00001d017041_T001 rna6187 5.00E-20
626- 62: transcript:Zm00001d017042_T002 rna6199 1.00E-62
626- 63: transcript:Zm00001d017043_T002 rna6201 3.00E-40
626- 64: transcript:Zm00001d017046_T001 rna6202 0
626- 65: transcript:Zm00001d017047_T001 rna6203 3.00E-131
626- 66: transcript:Zm00001d017049_T001 rna6204 0
626- 67: transcript:Zm00001d017050_T002 rna6206 3.00E-116
626- 68: transcript:Zm00001d017053_T003 rna6208 0
626- 69: transcript:Zm00001d017060_T001 rna6210 8.00E-64
626- 70: transcript:Zm00001d017061_T001 rna6211 2.00E-44
626- 71: transcript:Zm00001d017065_T001 rna6214 8.00E-31
626- 72: transcript:Zm00001d017070_T001 rna6216 1.00E-52
626- 73: transcript:Zm00001d017071_T002 rna6218 0
626- 74: transcript:Zm00001d017072_T002 rna6219 0
626- 75: transcript:Zm00001d017079_T001 rna6224 2.00E-81
626- 76: transcript:Zm00001d017084_T001 rna6225 0
626- 77: transcript:Zm00001d017085_T002 rna6226 0
626- 78: transcript:Zm00001d017086_T001 rna6228 1.00E-32
626- 79: transcript:Zm00001d017089_T001 rna6229 2.00E-16
626- 80: transcript:Zm00001d017090_T001 rna6230 8.00E-118
626- 81: transcript:Zm00001d017091_T001 rna6231 0
626- 82: transcript:Zm00001d017092_T001 rna6232 0
626- 83: transcript:Zm00001d017094_T001 rna6233 0
626- 84: transcript:Zm00001d017095_T001 rna6234 8.00E-87
Alignment 627: score=3741.0 e_value=0 N=81 5&NC_008395.2 plus
627- 0: transcript:Zm00001d017317_T001 rna6450 0
627- 1: transcript:Zm00001d017323_T001 rna6451 5.00E-35
627- 2: transcript:Zm00001d017324_T001 rna6452 4.00E-94
627- 3: transcript:Zm00001d017330_T001 rna6454 1.00E-155
627- 4: transcript:Zm00001d017331_T002 rna6455 0
627- 5: transcript:Zm00001d017333_T002 rna6456 0
627- 6: transcript:Zm00001d017334_T006 rna6457 3.00E-115

627- 7:	transcript:Zm00001d017338_T006	rna6458	4.00E-168
627- 8:	transcript:Zm00001d017352_T004	rna6459	0
627- 9:	transcript:Zm00001d017353_T001	rna6470	2.00E-72
627- 10:	transcript:Zm00001d017357_T001	rna6474	1.00E-15
627- 11:	transcript:Zm00001d017361_T001	rna6478	2.00E-57
627- 12:	transcript:Zm00001d017364_T001	rna6481	1.00E-61
627- 13:	transcript:Zm00001d017365_T001	rna6482	3.00E-174
627- 14:	transcript:Zm00001d017366_T001	rna6483	6.00E-148
627- 15:	transcript:Zm00001d017371_T008	rna6484	0
627- 16:	transcript:Zm00001d017373_T001	rna6485	0
627- 17:	transcript:Zm00001d017374_T002	rna6486	8.00E-175
627- 18:	transcript:Zm00001d017375_T001	rna6487	2.00E-111
627- 19:	transcript:Zm00001d017377_T001	rna6488	7.00E-25
627- 20:	transcript:Zm00001d017378_T001	rna6491	6.00E-76
627- 21:	transcript:Zm00001d017379_T001	rna6492	1.00E-79
627- 22:	transcript:Zm00001d017380_T001	rna6495	3.00E-110
627- 23:	transcript:Zm00001d017382_T001	rna6499	2.00E-115
627- 24:	transcript:Zm00001d017383_T001	rna6500	2.00E-55
627- 25:	transcript:Zm00001d017384_T008	rna6501	0
627- 26:	transcript:Zm00001d017386_T001	rna6504	3.00E-51
627- 27:	transcript:Zm00001d017387_T006	rna6507	0
627- 28:	transcript:Zm00001d017390_T001	rna6509	1.00E-116
627- 29:	transcript:Zm00001d017391_T002	rna6510	8.00E-124
627- 30:	transcript:Zm00001d017395_T002	rna6511	5.00E-128
627- 31:	transcript:Zm00001d017397_T001	rna6512	2.00E-65
627- 32:	transcript:Zm00001d017399_T001	rna6513	0
627- 33:	transcript:Zm00001d017401_T001	rna6514	0
627- 34:	transcript:Zm00001d017402_T001	rna6515	2.00E-37
627- 35:	transcript:Zm00001d017403_T001	rna6516	0
627- 36:	transcript:Zm00001d017404_T001	rna6522	9.00E-62
627- 37:	transcript:Zm00001d017409_T001	rna6524	6.00E-116
627- 38:	transcript:Zm00001d017412_T001	rna6525	1.00E-62
627- 39:	transcript:Zm00001d017415_T001	rna6526	3.00E-15
627- 40:	transcript:Zm00001d017418_T001	rna6527	0
627- 41:	transcript:Zm00001d017419_T001	rna6531	0
627- 42:	transcript:Zm00001d017420_T001	rna6532	8.00E-102
627- 43:	transcript:Zm00001d017422_T001	rna6535	3.00E-96
627- 44:	transcript:Zm00001d017424_T003	rna6537	0
627- 45:	transcript:Zm00001d017425_T001	rna6538	3.00E-67
627- 46:	transcript:Zm00001d017427_T002	rna6539	0
627- 47:	transcript:Zm00001d017432_T001	rna6543	2.00E-112
627- 48:	transcript:Zm00001d017435_T001	rna6546	0
627- 49:	transcript:Zm00001d017441_T001	rna6549	6.00E-94
627- 50:	transcript:Zm00001d017445_T001	rna6551	2.00E-176
627- 51:	transcript:Zm00001d017448_T001	rna6552	9.00E-72
627- 52:	transcript:Zm00001d017449_T001	rna6553	1.00E-109
627- 53:	transcript:Zm00001d017453_T001	rna6554	0
627- 54:	transcript:Zm00001d017455_T001	rna6556	4.00E-58
627- 55:	transcript:Zm00001d017456_T003	rna6559	1.00E-142
627- 56:	transcript:Zm00001d017457_T001	rna6560	0
627- 57:	transcript:Zm00001d017458_T003	rna6561	7.00E-66
627- 58:	transcript:Zm00001d017460_T003	rna6565	4.00E-142
627- 59:	transcript:Zm00001d017461_T001	rna6566	0
627- 60:	transcript:Zm00001d017462_T001	rna6567	5.00E-103

627- 61: transcript:Zm00001d017467_T001 rna6570 5.00E-130
627- 62: transcript:Zm00001d017468_T001 rna6571 7.00E-121
627- 63: transcript:Zm00001d017470_T001 rna6572 1.00E-35
627- 64: transcript:Zm00001d017472_T002 rna6573 0
627- 65: transcript:Zm00001d017473_T002 rna6574 0
627- 66: transcript:Zm00001d017474_T001 rna6575 4.00E-63
627- 67: transcript:Zm00001d017475_T001 rna6577 0
627- 68: transcript:Zm00001d017476_T001 rna6579 0
627- 69: transcript:Zm00001d017477_T001 rna6580 4.00E-79
627- 70: transcript:Zm00001d017482_T002 rna6584 2.00E-109
627- 71: transcript:Zm00001d017485_T003 rna6587 2.00E-158
627- 72: transcript:Zm00001d017486_T001 rna6588 2.00E-27
627- 73: transcript:Zm00001d017491_T001 rna6589 2.00E-16
627- 74: transcript:Zm00001d017492_T001 rna6591 0
627- 75: transcript:Zm00001d017493_T001 rna6592 1.00E-127
627- 76: transcript:Zm00001d017497_T001 rna6597 1.00E-67
627- 77: transcript:Zm00001d017501_T002 rna6600 4.00E-124
627- 78: transcript:Zm00001d017502_T001 rna6601 0
627- 79: transcript:Zm00001d017503_T001 rna6603 2.00E-97
627- 80: transcript:Zm00001d017505_T003 rna6606 6.00E-21
Alignment 628: score=3305.0 e_value=3.3e-297 N=72 5&NC_008395.2 plus
628- 0: transcript:Zm00001d017508_T001 rna6607 1.00E-63
628- 1: transcript:Zm00001d017512_T003 rna6609 4.00E-148
628- 2: transcript:Zm00001d017513_T001 rna6611 0
628- 3: transcript:Zm00001d017514_T002 rna6613 2.00E-17
628- 4: transcript:Zm00001d017516_T001 rna6614 7.00E-93
628- 5: transcript:Zm00001d017522_T003 rna6618 0
628- 6: transcript:Zm00001d017523_T001 rna6620 2.00E-15
628- 7: transcript:Zm00001d017524_T001 rna6624 0
628- 8: transcript:Zm00001d017526_T001 rna6630 0
628- 9: transcript:Zm00001d017528_T001 rna6632 0
628- 10: transcript:Zm00001d017530_T001 rna6633 2.00E-64
628- 11: transcript:Zm00001d017531_T001 rna6634 0
628- 12: transcript:Zm00001d017534_T001 rna6637 2.00E-43
628- 13: transcript:Zm00001d017535_T001 rna6638 4.00E-38
628- 14: transcript:Zm00001d017536_T001 rna6639 1.00E-122
628- 15: transcript:Zm00001d017537_T001 rna6640 7.00E-169
628- 16: transcript:Zm00001d017539_T002 rna6642 0
628- 17: transcript:Zm00001d017540_T001 rna6643 0
628- 18: transcript:Zm00001d017544_T002 rna6645 0
628- 19: transcript:Zm00001d017545_T001 rna6646 8.00E-118
628- 20: transcript:Zm00001d017546_T001 rna6647 1.00E-37
628- 21: transcript:Zm00001d017547_T001 rna6648 1.00E-59
628- 22: transcript:Zm00001d017553_T001 rna6651 0
628- 23: transcript:Zm00001d017555_T001 rna6654 8.00E-54
628- 24: transcript:Zm00001d017557_T001 rna6658 0
628- 25: transcript:Zm00001d017558_T001 rna6659 4.00E-18
628- 26: transcript:Zm00001d017559_T001 rna6661 2.00E-149
628- 27: transcript:Zm00001d017560_T002 rna6663 0
628- 28: transcript:Zm00001d017564_T001 rna6665 7.00E-132
628- 29: transcript:Zm00001d017566_T001 rna6667 0
628- 30: transcript:Zm00001d017568_T002 rna6669 0
628- 31: transcript:Zm00001d017570_T001 rna6670 2.00E-49
628- 32: transcript:Zm00001d017573_T003 rna6673 0

628- 33: transcript:Zm00001d017574_T001 rna6674 7.00E-106
628- 34: transcript:Zm00001d017575_T001 rna6675 1.00E-132
628- 35: transcript:Zm00001d017583_T002 rna6680 1.00E-142
628- 36: transcript:Zm00001d017584_T002 rna6682 0
628- 37: transcript:Zm00001d017590_T001 rna6683 0
628- 38: transcript:Zm00001d017591_T001 rna6687 9.00E-82
628- 39: transcript:Zm00001d017592_T001 rna6688 5.00E-97
628- 40: transcript:Zm00001d017595_T002 rna6690 1.00E-42
628- 41: transcript:Zm00001d017597_T001 rna6692 1.00E-134
628- 42: transcript:Zm00001d017598_T001 rna6693 5.00E-129
628- 43: transcript:Zm00001d017601_T001 rna6694 0
628- 44: transcript:Zm00001d017603_T001 rna6696 0
628- 45: transcript:Zm00001d017604_T001 rna6697 0
628- 46: transcript:Zm00001d017606_T001 rna6698 6.00E-73
628- 47: transcript:Zm00001d017607_T001 rna6699 0
628- 48: transcript:Zm00001d017608_T001 rna6701 1.00E-134
628- 49: transcript:Zm00001d017611_T001 rna6704 0
628- 50: transcript:Zm00001d017612_T001 rna6705 3.00E-63
628- 51: transcript:Zm00001d017613_T001 rna6706 1.00E-126
628- 52: transcript:Zm00001d017614_T003 rna6709 2.00E-105
628- 53: transcript:Zm00001d017616_T001 rna6711 0
628- 54: transcript:Zm00001d017617_T001 rna6712 1.00E-33
628- 55: transcript:Zm00001d017618_T001 rna6714 3.00E-144
628- 56: transcript:Zm00001d017620_T005 rna6716 9.00E-159
628- 57: transcript:Zm00001d017621_T001 rna6717 2.00E-56
628- 58: transcript:Zm00001d017625_T001 rna6720 4.00E-140
628- 59: transcript:Zm00001d017640_T006 rna6723 4.00E-26
628- 60: transcript:Zm00001d017641_T004 rna6725 2.00E-99
628- 61: transcript:Zm00001d017642_T001 rna6726 5.00E-122
628- 62: transcript:Zm00001d017643_T001 rna6727 0
628- 63: transcript:Zm00001d017645_T001 rna6729 1.00E-80
628- 64: transcript:Zm00001d017646_T001 rna6734 2.00E-31
628- 65: transcript:Zm00001d017649_T001 rna6737 8.00E-43
628- 66: transcript:Zm00001d017651_T001 rna6741 0
628- 67: transcript:Zm00001d017653_T001 rna6743 4.00E-142
628- 68: transcript:Zm00001d017654_T002 rna6745 0
628- 69: transcript:Zm00001d017657_T001 rna6749 2.00E-128
628- 70: transcript:Zm00001d017659_T001 rna6750 1.00E-36
628- 71: transcript:Zm00001d017660_T021 rna6751 0
Alignment 629: score=2875.0 e_value=3e-265 N=64 5&NC_008395.2 plus
629- 0: transcript:Zm00001d015248_T018 rna4604 0
629- 1: transcript:Zm00001d015249_T002 rna4605 0
629- 2: transcript:Zm00001d015251_T001 rna4606 0
629- 3: transcript:Zm00001d015259_T001 rna4607 0
629- 4: transcript:Zm00001d015260_T001 rna4609 4.00E-61
629- 5: transcript:Zm00001d015265_T003 rna4615 1.00E-166
629- 6: transcript:Zm00001d015268_T001 rna4616 0
629- 7: transcript:Zm00001d015269_T001 rna4618 0
629- 8: transcript:Zm00001d015272_T004 rna4619 1.00E-160
629- 9: transcript:Zm00001d015274_T001 rna4620 0
629- 10: transcript:Zm00001d015275_T001 rna4622 1.00E-133
629- 11: transcript:Zm00001d015277_T001 rna4627 0
629- 12: transcript:Zm00001d015279_T003 rna4628 4.00E-168
629- 13: transcript:Zm00001d015284_T001 rna4631 8.00E-11

629- 14: transcript:Zm00001d015289_T002 rna4634 0
629- 15: transcript:Zm00001d015290_T001 rna4635 0
629- 16: transcript:Zm00001d015291_T003 rna4638 2.00E-24
629- 17: transcript:Zm00001d015292_T001 rna4639 0
629- 18: transcript:Zm00001d015293_T015 rna4645 0
629- 19: transcript:Zm00001d015297_T003 rna4650 0
629- 20: transcript:Zm00001d015298_T001 rna4674 4.00E-106
629- 21: transcript:Zm00001d015306_T001 rna4682 0
629- 22: transcript:Zm00001d015307_T001 rna4684 4.00E-103
629- 23: transcript:Zm00001d015308_T001 rna4685 4.00E-94
629- 24: transcript:Zm00001d015309_T002 rna4686 0
629- 25: transcript:Zm00001d015310_T001 rna4687 0
629- 26: transcript:Zm00001d015313_T001 rna4691 3.00E-117
629- 27: transcript:Zm00001d015314_T003 rna4693 5.00E-21
629- 28: transcript:Zm00001d015319_T001 rna4695 0
629- 29: transcript:Zm00001d015325_T001 rna4701 0
629- 30: transcript:Zm00001d015326_T003 rna4702 0
629- 31: transcript:Zm00001d015327_T001 rna4708 0
629- 32: transcript:Zm00001d015330_T001 rna4713 7.00E-18
629- 33: transcript:Zm00001d015338_T001 rna4730 2.00E-114
629- 34: transcript:Zm00001d015342_T001 rna4735 6.00E-83
629- 35: transcript:Zm00001d015344_T001 rna4736 1.00E-27
629- 36: transcript:Zm00001d015346_T001 rna4738 0
629- 37: transcript:Zm00001d015348_T002 rna4739 0
629- 38: transcript:Zm00001d015355_T001 rna4745 2.00E-141
629- 39: transcript:Zm00001d015356_T001 rna4746 7.00E-143
629- 40: transcript:Zm00001d015361_T001 rna4749 2.00E-22
629- 41: transcript:Zm00001d015362_T001 rna4750 0
629- 42: transcript:Zm00001d015364_T003 rna4751 2.00E-145
629- 43: transcript:Zm00001d015366_T001 rna4753 0
629- 44: transcript:Zm00001d015367_T001 rna4754 7.00E-82
629- 45: transcript:Zm00001d015374_T001 rna4755 0
629- 46: transcript:Zm00001d015376_T002 rna4756 0
629- 47: transcript:Zm00001d015377_T003 rna4761 0
629- 48: transcript:Zm00001d015379_T001 rna4763 3.00E-60
629- 49: transcript:Zm00001d015381_T001 rna4764 9.00E-136
629- 50: transcript:Zm00001d015382_T001 rna4766 4.00E-08
629- 51: transcript:Zm00001d015383_T001 rna4768 0
629- 52: transcript:Zm00001d015394_T001 rna4774 2.00E-111
629- 53: transcript:Zm00001d015397_T001 rna4778 3.00E-47
629- 54: transcript:Zm00001d015399_T002 rna4782 0
629- 55: transcript:Zm00001d015400_T004 rna4784 0
629- 56: transcript:Zm00001d015401_T001 rna4785 0
629- 57: transcript:Zm00001d015406_T002 rna4786 2.00E-113
629- 58: transcript:Zm00001d015407_T001 rna4787 5.00E-159
629- 59: transcript:Zm00001d015410_T002 rna4788 2.00E-92
629- 60: transcript:Zm00001d015412_T001 rna4790 1.00E-69
629- 61: transcript:Zm00001d015420_T001 rna4793 1.00E-74
629- 62: transcript:Zm00001d015421_T001 rna4796 1.00E-133
629- 63: transcript:Zm00001d015426_T001 rna4797 0
Alignment 630: score=2528.0 e_value=6.1e-236 N=57 5&NC_008395.2 plus
630- 0: transcript:Zm00001d015085_T002 rna4461 2.00E-47
630- 1: transcript:Zm00001d015088_T002 rna4462 0
630- 2: transcript:Zm00001d015090_T001 rna4463 3.00E-127

630- 3:	transcript:Zm00001d015091_T003	rna4464	7.00E-135
630- 4:	transcript:Zm00001d015092_T001	rna4465	4.00E-38
630- 5:	transcript:Zm00001d015094_T002	rna4467	8.00E-132
630- 6:	transcript:Zm00001d015096_T002	rna4471	0
630- 7:	transcript:Zm00001d015100_T002	rna4472	0
630- 8:	transcript:Zm00001d015101_T001	rna4473	0
630- 9:	transcript:Zm00001d015102_T001	rna4476	6.00E-145
630- 10:	transcript:Zm00001d015103_T003	rna4477	6.00E-73
630- 11:	transcript:Zm00001d015114_T001	rna4484	0
630- 12:	transcript:Zm00001d015115_T002	rna4485	0
630- 13:	transcript:Zm00001d015116_T001	rna4489	3.00E-56
630- 14:	transcript:Zm00001d015118_T001	rna4490	2.00E-127
630- 15:	transcript:Zm00001d015121_T001	rna4493	7.00E-138
630- 16:	transcript:Zm00001d015123_T001	rna4495	0
630- 17:	transcript:Zm00001d015126_T001	rna4501	2.00E-18
630- 18:	transcript:Zm00001d015127_T001	rna4503	7.00E-41
630- 19:	transcript:Zm00001d015129_T001	rna4505	0
630- 20:	transcript:Zm00001d015130_T001	rna4506	3.00E-130
630- 21:	transcript:Zm00001d015131_T003	rna4507	4.00E-162
630- 22:	transcript:Zm00001d015140_T001	rna4510	2.00E-24
630- 23:	transcript:Zm00001d015148_T002	rna4519	0
630- 24:	transcript:Zm00001d015152_T001	rna4520	8.00E-169
630- 25:	transcript:Zm00001d015153_T001	rna4521	4.00E-65
630- 26:	transcript:Zm00001d015156_T001	rna4522	1.00E-179
630- 27:	transcript:Zm00001d015164_T001	rna4523	3.00E-17
630- 28:	transcript:Zm00001d015174_T010	rna4524	0
630- 29:	transcript:Zm00001d015175_T002	rna4525	0
630- 30:	transcript:Zm00001d015180_T001	rna4529	2.00E-172
630- 31:	transcript:Zm00001d015181_T001	rna4530	0
630- 32:	transcript:Zm00001d015182_T001	rna4531	3.00E-85
630- 33:	transcript:Zm00001d015183_T002	rna4535	0
630- 34:	transcript:Zm00001d015186_T001	rna4541	0
630- 35:	transcript:Zm00001d015195_T001	rna4542	0
630- 36:	transcript:Zm00001d015196_T003	rna4543	0
630- 37:	transcript:Zm00001d015200_T003	rna4545	2.00E-153
630- 38:	transcript:Zm00001d015201_T001	rna4546	3.00E-64
630- 39:	transcript:Zm00001d015202_T001	rna4550	0
630- 40:	transcript:Zm00001d015203_T005	rna4551	0
630- 41:	transcript:Zm00001d015204_T001	rna4553	9.00E-154
630- 42:	transcript:Zm00001d015208_T003	rna4554	0
630- 43:	transcript:Zm00001d015209_T022	rna4555	2.00E-45
630- 44:	transcript:Zm00001d015210_T001	rna4556	0
630- 45:	transcript:Zm00001d015211_T001	rna4558	2.00E-51
630- 46:	transcript:Zm00001d015213_T001	rna4559	0
630- 47:	transcript:Zm00001d015215_T001	rna4560	1.00E-45
630- 48:	transcript:Zm00001d015226_T001	rna4564	4.00E-138
630- 49:	transcript:Zm00001d015227_T004	rna4565	1.00E-86
630- 50:	transcript:Zm00001d015228_T004	rna4566	2.00E-176
630- 51:	transcript:Zm00001d015231_T002	rna4567	0
630- 52:	transcript:Zm00001d015233_T001	rna4568	7.00E-78
630- 53:	transcript:Zm00001d015234_T002	rna4569	9.00E-51
630- 54:	transcript:Zm00001d015242_T002	rna4579	9.00E-76
630- 55:	transcript:Zm00001d015243_T001	rna4580	7.00E-163
630- 56:	transcript:Zm00001d015245_T002	rna4589	1.00E-29

Alignment 631: score=2412.0 e_value=1.1e-202 N=53 5&NC_008395.2 plus

631- 0:	transcript:Zm00001d018200_T001	rna7241	3.00E-42
631- 1:	transcript:Zm00001d018201_T001	rna7242	4.00E-121
631- 2:	transcript:Zm00001d018203_T001	rna7250	8.00E-176
631- 3:	transcript:Zm00001d018204_T001	rna7251	0
631- 4:	transcript:Zm00001d018206_T001	rna7253	0
631- 5:	transcript:Zm00001d018207_T001	rna7254	0
631- 6:	transcript:Zm00001d018208_T001	rna7255	9.00E-57
631- 7:	transcript:Zm00001d018209_T001	rna7256	1.00E-72
631- 8:	transcript:Zm00001d018211_T001	rna7258	0
631- 9:	transcript:Zm00001d018214_T001	rna7259	0
631- 10:	transcript:Zm00001d018217_T003	rna7260	1.00E-176
631- 11:	transcript:Zm00001d018218_T001	rna7265	2.00E-102
631- 12:	transcript:Zm00001d018226_T002	rna7266	0
631- 13:	transcript:Zm00001d018227_T001	rna7267	2.00E-61
631- 14:	transcript:Zm00001d018229_T001	rna7268	4.00E-107
631- 15:	transcript:Zm00001d018230_T001	rna7269	0
631- 16:	transcript:Zm00001d018233_T011	rna7270	0
631- 17:	transcript:Zm00001d018234_T001	rna7271	1.00E-36
631- 18:	transcript:Zm00001d018235_T001	rna7272	3.00E-31
631- 19:	transcript:Zm00001d018237_T001	rna7273	5.00E-35
631- 20:	transcript:Zm00001d018238_T001	rna7275	9.00E-89
631- 21:	transcript:Zm00001d018241_T001	rna7279	2.00E-145
631- 22:	transcript:Zm00001d018242_T001	rna7282	3.00E-131
631- 23:	transcript:Zm00001d018243_T001	rna7283	0
631- 24:	transcript:Zm00001d018244_T001	rna7284	2.00E-32
631- 25:	transcript:Zm00001d018246_T015	rna7285	0
631- 26:	transcript:Zm00001d018247_T001	rna7286	1.00E-91
631- 27:	transcript:Zm00001d018255_T001	rna7288	2.00E-48
631- 28:	transcript:Zm00001d018256_T001	rna7289	2.00E-68
631- 29:	transcript:Zm00001d018258_T012	rna7290	0
631- 30:	transcript:Zm00001d018260_T001	rna7292	3.00E-133
631- 31:	transcript:Zm00001d018261_T002	rna7294	0
631- 32:	transcript:Zm00001d018262_T001	rna7296	4.00E-23
631- 33:	transcript:Zm00001d018274_T003	rna7298	0
631- 34:	transcript:Zm00001d018275_T001	rna7299	0
631- 35:	transcript:Zm00001d018277_T002	rna7300	4.00E-138
631- 36:	transcript:Zm00001d018278_T001	rna7301	0
631- 37:	transcript:Zm00001d018279_T011	rna7302	4.00E-58
631- 38:	transcript:Zm00001d018281_T001	rna7305	2.00E-58
631- 39:	transcript:Zm00001d018284_T001	rna7308	2.00E-27
631- 40:	transcript:Zm00001d018286_T002	rna7314	0
631- 41:	transcript:Zm00001d018287_T001	rna7315	1.00E-36
631- 42:	transcript:Zm00001d018289_T001	rna7318	0
631- 43:	transcript:Zm00001d018290_T001	rna7319	4.00E-132
631- 44:	transcript:Zm00001d018291_T002	rna7320	4.00E-139
631- 45:	transcript:Zm00001d018292_T001	rna7321	8.00E-114
631- 46:	transcript:Zm00001d018295_T001	rna7322	0
631- 47:	transcript:Zm00001d018297_T002	rna7324	9.00E-14
631- 48:	transcript:Zm00001d018298_T001	rna7325	5.00E-82
631- 49:	transcript:Zm00001d018303_T010	rna7326	0
631- 50:	transcript:Zm00001d018305_T001	rna7327	3.00E-73
631- 51:	transcript:Zm00001d018309_T003	rna7336	1.00E-171
631- 52:	transcript:Zm00001d018312_T001	rna7338	7.00E-85

Alignment 632: score=1972.0 e_value=5.8e-164 N=43 5&NC_008395.2 plus

632- 0:	transcript:Zm00001d017158_T001	rna6294	5.00E-89
632- 1:	transcript:Zm00001d017165_T002	rna6309	1.00E-178
632- 2:	transcript:Zm00001d017166_T001	rna6312	0
632- 3:	transcript:Zm00001d017167_T001	rna6313	1.00E-179
632- 4:	transcript:Zm00001d017168_T002	rna6314	0
632- 5:	transcript:Zm00001d017171_T001	rna6317	0
632- 6:	transcript:Zm00001d017174_T001	rna6320	2.00E-58
632- 7:	transcript:Zm00001d017175_T003	rna6326	0
632- 8:	transcript:Zm00001d017176_T001	rna6327	2.00E-134
632- 9:	transcript:Zm00001d017177_T007	rna6328	7.00E-73
632- 10:	transcript:Zm00001d017178_T004	rna6329	3.00E-55
632- 11:	transcript:Zm00001d017179_T001	rna6330	0
632- 12:	transcript:Zm00001d017185_T001	rna6334	0
632- 13:	transcript:Zm00001d017186_T001	rna6337	0
632- 14:	transcript:Zm00001d017187_T001	rna6338	5.00E-39
632- 15:	transcript:Zm00001d017193_T002	rna6341	4.00E-141
632- 16:	transcript:Zm00001d017195_T001	rna6343	0
632- 17:	transcript:Zm00001d017197_T015	rna6344	0
632- 18:	transcript:Zm00001d017199_T001	rna6345	3.00E-116
632- 19:	transcript:Zm00001d017201_T001	rna6346	0
632- 20:	transcript:Zm00001d017203_T021	rna6347	0
632- 21:	transcript:Zm00001d017204_T003	rna6350	9.00E-118
632- 22:	transcript:Zm00001d017205_T001	rna6351	0
632- 23:	transcript:Zm00001d017206_T001	rna6352	1.00E-40
632- 24:	transcript:Zm00001d017207_T001	rna6353	2.00E-165
632- 25:	transcript:Zm00001d017208_T001	rna6354	4.00E-117
632- 26:	transcript:Zm00001d017209_T001	rna6357	0
632- 27:	transcript:Zm00001d017210_T001	rna6358	0
632- 28:	transcript:Zm00001d017212_T001	rna6361	0
632- 29:	transcript:Zm00001d017213_T004	rna6373	1.00E-76
632- 30:	transcript:Zm00001d017214_T002	rna6375	1.00E-86
632- 31:	transcript:Zm00001d017215_T001	rna6379	3.00E-10
632- 32:	transcript:Zm00001d017216_T001	rna6380	8.00E-135
632- 33:	transcript:Zm00001d017240_T001	rna6384	4.00E-48
632- 34:	transcript:Zm00001d017241_T002	rna6385	0
632- 35:	transcript:Zm00001d017242_T001	rna6386	7.00E-40
632- 36:	transcript:Zm00001d017243_T001	rna6387	2.00E-108
632- 37:	transcript:Zm00001d017246_T001	rna6388	0
632- 38:	transcript:Zm00001d017247_T003	rna6389	0
632- 39:	transcript:Zm00001d017248_T001	rna6392	7.00E-70
632- 40:	transcript:Zm00001d017249_T001	rna6393	0
632- 41:	transcript:Zm00001d017250_T001	rna6395	0
632- 42:	transcript:Zm00001d017251_T003	rna6398	0

Alignment 633: score=1796.0 e_value=8.4e-136 N=39 5&NC_008395.2 plus

633- 0:	transcript:Zm00001d017949_T001	rna7019	0
633- 1:	transcript:Zm00001d017950_T001	rna7021	6.00E-61
633- 2:	transcript:Zm00001d017951_T001	rna7025	4.00E-94
633- 3:	transcript:Zm00001d017952_T001	rna7026	3.00E-47
633- 4:	transcript:Zm00001d017955_T001	rna7030	4.00E-114
633- 5:	transcript:Zm00001d017956_T001	rna7031	7.00E-73
633- 6:	transcript:Zm00001d017957_T002	rna7032	0
633- 7:	transcript:Zm00001d017958_T002	rna7033	0
633- 8:	transcript:Zm00001d017959_T003	rna7034	0

633- 9: transcript:Zm00001d017960_T001 rna7036 8.00E-102
633- 10: transcript:Zm00001d017961_T003 rna7037 0
633- 11: transcript:Zm00001d017964_T001 rna7038 0
633- 12: transcript:Zm00001d017965_T001 rna7039 0
633- 13: transcript:Zm00001d017966_T001 rna7040 0
633- 14: transcript:Zm00001d017967_T006 rna7042 0
633- 15: transcript:Zm00001d017968_T001 rna7043 3.00E-11
633- 16: transcript:Zm00001d017976_T001 rna7049 0
633- 17: transcript:Zm00001d017978_T001 rna7052 0
633- 18: transcript:Zm00001d017979_T002 rna7053 0
633- 19: transcript:Zm00001d017982_T001 rna7054 0
633- 20: transcript:Zm00001d017983_T001 rna7055 0
633- 21: transcript:Zm00001d017984_T001 rna7056 0
633- 22: transcript:Zm00001d017985_T002 rna7057 0
633- 23: transcript:Zm00001d017986_T005 rna7058 0
633- 24: transcript:Zm00001d017988_T002 rna7060 0
633- 25: transcript:Zm00001d017989_T001 rna7061 0
633- 26: transcript:Zm00001d017990_T001 rna7062 4.00E-43
633- 27: transcript:Zm00001d017991_T001 rna7063 9.00E-38
633- 28: transcript:Zm00001d017992_T001 rna7064 2.00E-142
633- 29: transcript:Zm00001d017994_T001 rna7066 4.00E-151
633- 30: transcript:Zm00001d017995_T003 rna7067 0
633- 31: transcript:Zm00001d017996_T001 rna7068 0
633- 32: transcript:Zm00001d017997_T010 rna7069 0
633- 33: transcript:Zm00001d017998_T001 rna7072 6.00E-67
633- 34: transcript:Zm00001d018001_T001 rna7073 0
633- 35: transcript:Zm00001d018004_T002 rna7074 2.00E-100
633- 36: transcript:Zm00001d018005_T001 rna7079 2.00E-77
633- 37: transcript:Zm00001d018006_T003 rna7080 3.00E-168
633- 38: transcript:Zm00001d018009_T026 rna7081 0
Alignment 634: score=1770.0 e_value=2e-137 N=39 5&NC_008395.2 plus
634- 0: transcript:Zm00001d017874_T001 rna6940 3.00E-62
634- 1: transcript:Zm00001d017876_T018 rna6941 0
634- 2: transcript:Zm00001d017877_T001 rna6944 0
634- 3: transcript:Zm00001d017880_T006 rna6945 0
634- 4: transcript:Zm00001d017881_T002 rna6946 0
634- 5: transcript:Zm00001d017882_T001 rna6948 4.00E-25
634- 6: transcript:Zm00001d017883_T002 rna6949 0
634- 7: transcript:Zm00001d017885_T001 rna6955 1.00E-176
634- 8: transcript:Zm00001d017886_T001 rna6958 0
634- 9: transcript:Zm00001d017887_T002 rna6959 9.00E-17
634- 10: transcript:Zm00001d017889_T001 rna6960 2.00E-152
634- 11: transcript:Zm00001d017895_T001 rna6963 0
634- 12: transcript:Zm00001d017896_T002 rna6964 3.00E-32
634- 13: transcript:Zm00001d017897_T002 rna6966 4.00E-84
634- 14: transcript:Zm00001d017898_T001 rna6967 9.00E-86
634- 15: transcript:Zm00001d017900_T001 rna6970 1.00E-86
634- 16: transcript:Zm00001d017905_T001 rna6971 9.00E-144
634- 17: transcript:Zm00001d017906_T003 rna6972 9.00E-53
634- 18: transcript:Zm00001d017907_T001 rna6973 3.00E-70
634- 19: transcript:Zm00001d017908_T001 rna6974 1.00E-51
634- 20: transcript:Zm00001d017912_T001 rna6984 2.00E-58
634- 21: transcript:Zm00001d017913_T001 rna6985 3.00E-163
634- 22: transcript:Zm00001d017914_T001 rna6986 1.00E-93

634- 23: transcript:Zm00001d017915_T001 rna6987 7.00E-126
634- 24: transcript:Zm00001d017918_T001 rna6989 0
634- 25: transcript:Zm00001d017920_T001 rna6993 4.00E-147
634- 26: transcript:Zm00001d017923_T001 rna6994 3.00E-38
634- 27: transcript:Zm00001d017925_T004 rna6997 0
634- 28: transcript:Zm00001d017926_T003 rna6998 0
634- 29: transcript:Zm00001d017927_T001 rna6999 3.00E-117
634- 30: transcript:Zm00001d017930_T003 rna7001 9.00E-177
634- 31: transcript:Zm00001d017931_T001 rna7002 0
634- 32: transcript:Zm00001d017932_T002 rna7003 6.00E-116
634- 33: transcript:Zm00001d017936_T001 rna7005 8.00E-46
634- 34: transcript:Zm00001d017939_T001 rna7008 2.00E-117
634- 35: transcript:Zm00001d017941_T001 rna7009 0
634- 36: transcript:Zm00001d017943_T001 rna7011 3.00E-46
634- 37: transcript:Zm00001d017945_T002 rna7013 2.00E-105
634- 38: transcript:Zm00001d017946_T001 rna7015 0
Alignment 635: score=1704.0 e_value=8.7e-144 N=38 5&NC_008395.2 plus
635- 0: transcript:Zm00001d014969_T001 rna4362 9.00E-62
635- 1: transcript:Zm00001d014971_T001 rna4363 0
635- 2: transcript:Zm00001d014976_T001 rna4366 0
635- 3: transcript:Zm00001d014977_T007 rna4374 3.00E-131
635- 4: transcript:Zm00001d014983_T001 rna4377 0
635- 5: transcript:Zm00001d014984_T001 rna4378 0
635- 6: transcript:Zm00001d014986_T002 rna4380 0
635- 7: transcript:Zm00001d014988_T001 rna4381 0
635- 8: transcript:Zm00001d014989_T001 rna4382 4.00E-134
635- 9: transcript:Zm00001d014991_T004 rna4386 0
635- 10: transcript:Zm00001d014993_T003 rna4388 0
635- 11: transcript:Zm00001d014994_T001 rna4393 0
635- 12: transcript:Zm00001d014995_T001 rna4394 3.00E-116
635- 13: transcript:Zm00001d014996_T001 rna4395 0
635- 14: transcript:Zm00001d015004_T001 rna4399 0
635- 15: transcript:Zm00001d015005_T002 rna4401 2.00E-80
635- 16: transcript:Zm00001d015007_T004 rna4402 0
635- 17: transcript:Zm00001d015008_T001 rna4404 0
635- 18: transcript:Zm00001d015009_T002 rna4405 3.00E-174
635- 19: transcript:Zm00001d015010_T001 rna4406 0
635- 20: transcript:Zm00001d015012_T001 rna4408 0
635- 21: transcript:Zm00001d015014_T001 rna4409 0
635- 22: transcript:Zm00001d015020_T001 rna4413 4.00E-123
635- 23: transcript:Zm00001d015025_T001 rna4414 0
635- 24: transcript:Zm00001d015029_T002 rna4420 0
635- 25: transcript:Zm00001d015030_T001 rna4421 0
635- 26: transcript:Zm00001d015032_T003 rna4422 0
635- 27: transcript:Zm00001d015033_T001 rna4423 3.00E-130
635- 28: transcript:Zm00001d015036_T007 rna4429 4.00E-146
635- 29: transcript:Zm00001d015037_T002 rna4431 0
635- 30: transcript:Zm00001d015053_T001 rna4437 0
635- 31: transcript:Zm00001d015058_T003 rna4440 5.00E-100
635- 32: transcript:Zm00001d015059_T001 rna4441 0
635- 33: transcript:Zm00001d015060_T001 rna4442 1.00E-23
635- 34: transcript:Zm00001d015061_T001 rna4443 2.00E-109
635- 35: transcript:Zm00001d015065_T005 rna4452 0
635- 36: transcript:Zm00001d015067_T001 rna4455 0

635- 37: transcript:Zm00001d015082_T001 rna4459 0
Alignment 636: score=1600.0 e_value=4.2e-118 N=35 5&NC_008395.2 plus
636- 0: transcript:Zm00001d017729_T029 rna6810 0
636- 1: transcript:Zm00001d017730_T001 rna6811 6.00E-165
636- 2: transcript:Zm00001d017742_T001 rna6812 3.00E-165
636- 3: transcript:Zm00001d017746_T001 rna6813 0
636- 4: transcript:Zm00001d017748_T001 rna6816 0
636- 5: transcript:Zm00001d017751_T001 rna6817 0
636- 6: transcript:Zm00001d017752_T001 rna6818 8.00E-20
636- 7: transcript:Zm00001d017753_T002 rna6820 0
636- 8: transcript:Zm00001d017754_T031 rna6822 0
636- 9: transcript:Zm00001d017755_T011 rna6823 0
636- 10: transcript:Zm00001d017756_T001 rna6825 7.00E-119
636- 11: transcript:Zm00001d017761_T001 rna6826 5.00E-103
636- 12: transcript:Zm00001d017762_T003 rna6827 2.00E-40
636- 13: transcript:Zm00001d017764_T001 rna6829 1.00E-12
636- 14: transcript:Zm00001d017766_T001 rna6830 0
636- 15: transcript:Zm00001d017767_T001 rna6832 8.00E-88
636- 16: transcript:Zm00001d017768_T006 rna6833 0
636- 17: transcript:Zm00001d017769_T001 rna6835 7.00E-34
636- 18: transcript:Zm00001d017770_T001 rna6836 1.00E-128
636- 19: transcript:Zm00001d017771_T001 rna6837 0
636- 20: transcript:Zm00001d017773_T001 rna6839 0
636- 21: transcript:Zm00001d017777_T003 rna6840 9.00E-97
636- 22: transcript:Zm00001d017778_T001 rna6841 3.00E-122
636- 23: transcript:Zm00001d017780_T002 rna6843 0
636- 24: transcript:Zm00001d017782_T001 rna6844 2.00E-25
636- 25: transcript:Zm00001d017783_T001 rna6845 0
636- 26: transcript:Zm00001d017784_T001 rna6846 5.00E-95
636- 27: transcript:Zm00001d017785_T002 rna6847 2.00E-132
636- 28: transcript:Zm00001d017786_T003 rna6848 4.00E-91
636- 29: transcript:Zm00001d017788_T001 rna6850 9.00E-122
636- 30: transcript:Zm00001d017789_T001 rna6852 6.00E-68
636- 31: transcript:Zm00001d017790_T002 rna6854 0
636- 32: transcript:Zm00001d017791_T002 rna6855 0
636- 33: transcript:Zm00001d017793_T002 rna6857 0
636- 34: transcript:Zm00001d017798_T002 rna6858 1.00E-161
Alignment 637: score=1545.0 e_value=1.9e-122 N=34 5&NC_008395.2 plus
637- 0: transcript:Zm00001d018383_T002 rna7407 3.00E-30
637- 1: transcript:Zm00001d018384_T002 rna7411 0
637- 2: transcript:Zm00001d018386_T005 rna7412 0
637- 3: transcript:Zm00001d018388_T001 rna7413 4.00E-38
637- 4: transcript:Zm00001d018389_T001 rna7414 0
637- 5: transcript:Zm00001d018390_T001 rna7417 1.00E-35
637- 6: transcript:Zm00001d018391_T005 rna7420 0
637- 7: transcript:Zm00001d018392_T004 rna7421 0
637- 8: transcript:Zm00001d018394_T003 rna7423 0
637- 9: transcript:Zm00001d018399_T002 rna7425 0
637- 10: transcript:Zm00001d018400_T001 rna7427 3.00E-10
637- 11: transcript:Zm00001d018401_T001 rna7429 2.00E-65
637- 12: transcript:Zm00001d018403_T001 rna7430 1.00E-76
637- 13: transcript:Zm00001d018404_T002 rna7437 0
637- 14: transcript:Zm00001d018406_T001 rna7442 1.00E-171
637- 15: transcript:Zm00001d018407_T001 rna7443 5.00E-155

637- 16: transcript:Zm00001d018408_T001 rna7447 1.00E-132
637- 17: transcript:Zm00001d018409_T007 rna7448 0
637- 18: transcript:Zm00001d018410_T001 rna7452 1.00E-37
637- 19: transcript:Zm00001d018411_T001 rna7453 0
637- 20: transcript:Zm00001d018413_T003 rna7455 0
637- 21: transcript:Zm00001d018414_T001 rna7460 4.00E-62
637- 22: transcript:Zm00001d018415_T002 rna7461 0
637- 23: transcript:Zm00001d018416_T001 rna7462 3.00E-22
637- 24: transcript:Zm00001d018417_T001 rna7464 0
637- 25: transcript:Zm00001d018418_T002 rna7466 0
637- 26: transcript:Zm00001d018419_T001 rna7467 2.00E-60
637- 27: transcript:Zm00001d018420_T008 rna7468 0
637- 28: transcript:Zm00001d018422_T001 rna7472 0
637- 29: transcript:Zm00001d018428_T001 rna7474 0
637- 30: transcript:Zm00001d018429_T001 rna7477 1.00E-157
637- 31: transcript:Zm00001d018430_T008 rna7478 2.00E-19
637- 32: transcript:Zm00001d018431_T001 rna7485 0
637- 33: transcript:Zm00001d018439_T003 rna7500 0
Alignment 638: score=1527.0 e_value=2.9e-112 N=33 5&NC_008395.2 plus
638- 0: transcript:Zm00001d017799_T001 rna6860 0
638- 1: transcript:Zm00001d017800_T001 rna6861 4.00E-45
638- 2: transcript:Zm00001d017802_T001 rna6862 0
638- 3: transcript:Zm00001d017803_T001 rna6863 9.00E-25
638- 4: transcript:Zm00001d017804_T001 rna6869 5.00E-102
638- 5: transcript:Zm00001d017806_T002 rna6870 0
638- 6: transcript:Zm00001d017807_T001 rna6871 1.00E-133
638- 7: transcript:Zm00001d017808_T001 rna6872 0
638- 8: transcript:Zm00001d017809_T002 rna6873 0
638- 9: transcript:Zm00001d017810_T010 rna6874 5.00E-115
638- 10: transcript:Zm00001d017811_T001 rna6875 0
638- 11: transcript:Zm00001d017812_T002 rna6876 4.00E-96
638- 12: transcript:Zm00001d017813_T001 rna6877 2.00E-43
638- 13: transcript:Zm00001d017814_T001 rna6878 1.00E-50
638- 14: transcript:Zm00001d017818_T001 rna6880 0
638- 15: transcript:Zm00001d017820_T001 rna6882 0
638- 16: transcript:Zm00001d017821_T002 rna6884 0
638- 17: transcript:Zm00001d017823_T001 rna6887 0
638- 18: transcript:Zm00001d017825_T001 rna6890 1.00E-138
638- 19: transcript:Zm00001d017826_T001 rna6891 3.00E-144
638- 20: transcript:Zm00001d017829_T002 rna6892 5.00E-136
638- 21: transcript:Zm00001d017830_T001 rna6894 0
638- 22: transcript:Zm00001d017831_T001 rna6895 0
638- 23: transcript:Zm00001d017832_T006 rna6896 0
638- 24: transcript:Zm00001d017833_T001 rna6897 0
638- 25: transcript:Zm00001d017834_T001 rna6899 3.00E-161
638- 26: transcript:Zm00001d017836_T002 rna6901 0
638- 27: transcript:Zm00001d017837_T001 rna6902 1.00E-85
638- 28: transcript:Zm00001d017840_T001 rna6905 0
638- 29: transcript:Zm00001d017842_T001 rna6907 4.00E-100
638- 30: transcript:Zm00001d017845_T001 rna6908 7.00E-141
638- 31: transcript:Zm00001d017847_T003 rna6910 4.00E-88
638- 32: transcript:Zm00001d017848_T004 rna6911 0
Alignment 639: score=1261.0 e_value=5.7e-83 N=27 5&NC_008395.2 plus
639- 0: transcript:Zm00001d017666_T001 rna6752 0

639- 1: transcript:Zm00001d017670_T001 rna6756   0
639- 2: transcript:Zm00001d017671_T001 rna6757   0
639- 3: transcript:Zm00001d017675_T003 rna6758   7.00E-169
639- 4: transcript:Zm00001d017676_T001 rna6759   0
639- 5: transcript:Zm00001d017678_T001 rna6763   4.00E-180
639- 6: transcript:Zm00001d017682_T001 rna6765   1.00E-141
639- 7: transcript:Zm00001d017684_T001 rna6766   3.00E-63
639- 8: transcript:Zm00001d017685_T003 rna6767   2.00E-71
639- 9: transcript:Zm00001d017686_T003 rna6768   1.00E-71
639- 10: transcript:Zm00001d017689_T002 rna6769   1.00E-142
639- 11: transcript:Zm00001d017691_T001 rna6772   4.00E-78
639- 12: transcript:Zm00001d017692_T002 rna6774   8.00E-12
639- 13: transcript:Zm00001d017697_T001 rna6780   3.00E-51
639- 14: transcript:Zm00001d017699_T001 rna6782   0
639- 15: transcript:Zm00001d017700_T020 rna6784   0
639- 16: transcript:Zm00001d017703_T002 rna6785   1.00E-109
639- 17: transcript:Zm00001d017704_T001 rna6786   0
639- 18: transcript:Zm00001d017706_T002 rna6788   6.00E-33
639- 19: transcript:Zm00001d017707_T003 rna6789   0
639- 20: transcript:Zm00001d017708_T001 rna6790   2.00E-83
639- 21: transcript:Zm00001d017711_T001 rna6793   0
639- 22: transcript:Zm00001d017712_T001 rna6795   1.00E-136
639- 23: transcript:Zm00001d017713_T001 rna6796   0
639- 24: transcript:Zm00001d017717_T001 rna6797   3.00E-53
639- 25: transcript:Zm00001d017719_T001 rna6800   1.00E-111
639- 26: transcript:Zm00001d017720_T009 rna6801   0
Alignment 640: score=1242.0 e_value=1.1e-91 N=27 5&NC_008395.2 plus
640- 0: transcript:Zm00001d017097_T001 rna6235   7.00E-113
640- 1: transcript:Zm00001d017100_T006 rna6239   1.00E-145
640- 2: transcript:Zm00001d017106_T001 rna6240   0
640- 3: transcript:Zm00001d017107_T001 rna6243   5.00E-75
640- 4: transcript:Zm00001d017110_T002 rna6244   0
640- 5: transcript:Zm00001d017111_T001 rna6246   1.00E-90
640- 6: transcript:Zm00001d017112_T001 rna6247   2.00E-133
640- 7: transcript:Zm00001d017113_T002 rna6248   4.00E-134
640- 8: transcript:Zm00001d017114_T001 rna6249   0
640- 9: transcript:Zm00001d017117_T001 rna6262   0
640- 10: transcript:Zm00001d017118_T001 rna6263   6.00E-119
640- 11: transcript:Zm00001d017119_T003 rna6264   0
640- 12: transcript:Zm00001d017120_T002 rna6267   5.00E-67
640- 13: transcript:Zm00001d017121_T001 rna6268   0
640- 14: transcript:Zm00001d017124_T001 rna6271   0
640- 15: transcript:Zm00001d017125_T005 rna6272   0
640- 16: transcript:Zm00001d017126_T001 rna6273   0
640- 17: transcript:Zm00001d017128_T002 rna6275   0
640- 18: transcript:Zm00001d017130_T001 rna6278   4.00E-163
640- 19: transcript:Zm00001d017131_T001 rna6279   8.00E-110
640- 20: transcript:Zm00001d017135_T001 rna6284   0
640- 21: transcript:Zm00001d017138_T001 rna6287   8.00E-33
640- 22: transcript:Zm00001d017139_T003 rna6288   0
640- 23: transcript:Zm00001d017140_T001 rna6289   6.00E-123
640- 24: transcript:Zm00001d017141_T001 rna6290   1.00E-60
640- 25: transcript:Zm00001d017144_T007 rna6291   0
640- 26: transcript:Zm00001d017150_T001 rna6299   4.00E-64

Alignment 641: score=1236.0 e_value=5.7e-92 N=27 5&NC_008395.2 plus
641- 0: transcript:Zm00001d016737_T002 rna5926 0
641- 1: transcript:Zm00001d016746_T003 rna5938 0
641- 2: transcript:Zm00001d016755_T001 rna5948 3.00E-159
641- 3: transcript:Zm00001d016756_T001 rna5949 4.00E-11
641- 4: transcript:Zm00001d016758_T001 rna5951 5.00E-37
641- 5: transcript:Zm00001d016760_T001 rna5952 1.00E-14
641- 6: transcript:Zm00001d016762_T002 rna5953 0
641- 7: transcript:Zm00001d016764_T001 rna5968 0
641- 8: transcript:Zm00001d016765_T001 rna5970 0
641- 9: transcript:Zm00001d016766_T002 rna5973 7.00E-165
641- 10: transcript:Zm00001d016767_T001 rna5976 5.00E-146
641- 11: transcript:Zm00001d016768_T001 rna5977 0
641- 12: transcript:Zm00001d016770_T002 rna5978 0
641- 13: transcript:Zm00001d016771_T002 rna5979 0
641- 14: transcript:Zm00001d016772_T001 rna5980 2.00E-161
641- 15: transcript:Zm00001d016783_T002 rna5981 0
641- 16: transcript:Zm00001d016784_T001 rna5982 6.00E-136
641- 17: transcript:Zm00001d016792_T004 rna5985 8.00E-125
641- 18: transcript:Zm00001d016793_T002 rna5986 9.00E-163
641- 19: transcript:Zm00001d016797_T004 rna5987 0
641- 20: transcript:Zm00001d016798_T001 rna5988 0
641- 21: transcript:Zm00001d016799_T001 rna5989 3.00E-119
641- 22: transcript:Zm00001d016801_T001 rna5990 5.00E-39
641- 23: transcript:Zm00001d016802_T001 rna5993 0
641- 24: transcript:Zm00001d016803_T001 rna5996 1.00E-31
641- 25: transcript:Zm00001d016804_T002 rna5997 0
641- 26: transcript:Zm00001d016811_T001 rna6009 0
Alignment 642: score=1221.0 e_value=3.8e-88 N=27 5&NC_008395.2 plus
642- 0: transcript:Zm00001d018482_T004 rna7553 0
642- 1: transcript:Zm00001d018484_T003 rna7554 0
642- 2: transcript:Zm00001d018485_T003 rna7556 0
642- 3: transcript:Zm00001d018487_T001 rna7558 0
642- 4: transcript:Zm00001d018488_T001 rna7560 0
642- 5: transcript:Zm00001d018489_T007 rna7563 2.00E-154
642- 6: transcript:Zm00001d018490_T001 rna7564 0
642- 7: transcript:Zm00001d018491_T001 rna7565 1.00E-18
642- 8: transcript:Zm00001d018494_T002 rna7567 9.00E-146
642- 9: transcript:Zm00001d018495_T001 rna7568 4.00E-43
642- 10: transcript:Zm00001d018496_T007 rna7569 1.00E-23
642- 11: transcript:Zm00001d018497_T002 rna7570 0
642- 12: transcript:Zm00001d018498_T002 rna7571 1.00E-119
642- 13: transcript:Zm00001d018499_T003 rna7572 0
642- 14: transcript:Zm00001d018501_T001 rna7573 8.00E-92
642- 15: transcript:Zm00001d018502_T001 rna7574 3.00E-169
642- 16: transcript:Zm00001d018503_T002 rna7575 9.00E-179
642- 17: transcript:Zm00001d018504_T001 rna7580 0
642- 18: transcript:Zm00001d018507_T002 rna7581 2.00E-74
642- 19: transcript:Zm00001d018508_T001 rna7582 2.00E-118
642- 20: transcript:Zm00001d018512_T001 rna7594 0
642- 21: transcript:Zm00001d018514_T003 rna7595 3.00E-92
642- 22: transcript:Zm00001d018515_T003 rna7612 0
642- 23: transcript:Zm00001d018516_T002 rna7623 0
642- 24: transcript:Zm00001d018517_T001 rna7624 0

```
642- 25:  transcript:Zm00001d018521_T001  rna7626      5.00E-24
642- 26:  transcript:Zm00001d018522_T001  rna7627      1.00E-20
## Alignment 643: score=1083.0 e_value=2.3e-72 N=24 5&NC_008395.2 plus
643-  0:  transcript:Zm00001d015499_T003  rna4832      5.00E-68
643-  1:  transcript:Zm00001d015504_T004  rna4833             0
643-  2:  transcript:Zm00001d015505_T001  rna4835     5.00E-120
643-  3:  transcript:Zm00001d015509_T001  rna4836     1.00E-132
643-  4:  transcript:Zm00001d015513_T001  rna4837             0
643-  5:  transcript:Zm00001d015515_T001  rna4840     2.00E-146
643-  6:  transcript:Zm00001d015520_T001  rna4843     6.00E-111
643-  7:  transcript:Zm00001d015521_T001  rna4844             0
643-  8:  transcript:Zm00001d015526_T002  rna4845      2.00E-25
643-  9:  transcript:Zm00001d015527_T001  rna4846      1.00E-53
643- 10:  transcript:Zm00001d015546_T001  rna4847      2.00E-76
643- 11:  transcript:Zm00001d015549_T001  rna4848     2.00E-141
643- 12:  transcript:Zm00001d015550_T001  rna4849      3.00E-11
643- 13:  transcript:Zm00001d015560_T001  rna4852     9.00E-168
643- 14:  transcript:Zm00001d015569_T001  rna4858             0
643- 15:  transcript:Zm00001d015570_T002  rna4859             0
643- 16:  transcript:Zm00001d015571_T001  rna4860     6.00E-115
643- 17:  transcript:Zm00001d015578_T001  rna4861     1.00E-162
643- 18:  transcript:Zm00001d015581_T001  rna4873             0
643- 19:  transcript:Zm00001d015586_T003  rna4874      2.00E-99
643- 20:  transcript:Zm00001d015589_T001  rna4875             0
643- 21:  transcript:Zm00001d015599_T001  rna4878      3.00E-48
643- 22:  transcript:Zm00001d015600_T002  rna4879             0
643- 23:  transcript:Zm00001d015609_T003  rna4881             0
## Alignment 644: score=1081.0 e_value=3e-74 N=24 5&NC_008395.2 plus
644-  0:  transcript:Zm00001d018525_T004  rna7628             0
644-  1:  transcript:Zm00001d018526_T001  rna7629      1.00E-22
644-  2:  transcript:Zm00001d018527_T002  rna7630             0
644-  3:  transcript:Zm00001d018529_T001  rna7639     7.00E-175
644-  4:  transcript:Zm00001d018530_T001  rna7641      1.00E-77
644-  5:  transcript:Zm00001d018531_T004  rna7646     1.00E-134
644-  6:  transcript:Zm00001d018532_T001  rna7648             0
644-  7:  transcript:Zm00001d018534_T001  rna7651             0
644-  8:  transcript:Zm00001d018537_T001  rna7653             0
644-  9:  transcript:Zm00001d018535_T003  rna7654             0
644- 10:  transcript:Zm00001d018541_T002  rna7656      1.00E-50
644- 11:  transcript:Zm00001d018544_T001  rna7659      7.00E-85
644- 12:  transcript:Zm00001d018545_T001  rna7660             0
644- 13:  transcript:Zm00001d018552_T002  rna7661      4.00E-78
644- 14:  transcript:Zm00001d018554_T001  rna7664      5.00E-26
644- 15:  transcript:Zm00001d018555_T001  rna7665             0
644- 16:  transcript:Zm00001d018557_T001  rna7669      1.00E-50
644- 17:  transcript:Zm00001d018562_T006  rna7671     2.00E-131
644- 18:  transcript:Zm00001d018565_T003  rna7672             0
644- 19:  transcript:Zm00001d018567_T001  rna7680             0
644- 20:  transcript:Zm00001d018571_T003  rna7683      3.00E-32
644- 21:  transcript:Zm00001d018573_T002  rna7685     3.00E-140
644- 22:  transcript:Zm00001d018574_T001  rna7687      6.00E-87
644- 23:  transcript:Zm00001d018575_T001  rna7688     8.00E-114
## Alignment 645: score=1050.0 e_value=3.4e-74 N=23 5&NC_008395.2 plus
645-  0:  transcript:Zm00001d018445_T004  rna7494             0
```

645- 1: transcript:Zm00001d018450_T009 rna7510 0
645- 2: transcript:Zm00001d018451_T001 rna7518 2.00E-58
645- 3: transcript:Zm00001d018454_T004 rna7520 0
645- 4: transcript:Zm00001d018455_T001 rna7521 0
645- 5: transcript:Zm00001d018460_T003 rna7522 0
645- 6: transcript:Zm00001d018461_T001 rna7523 1.00E-144
645- 7: transcript:Zm00001d018462_T009 rna7524 0
645- 8: transcript:Zm00001d018464_T001 rna7526 4.00E-20
645- 9: transcript:Zm00001d018465_T003 rna7528 1.00E-08
645- 10: transcript:Zm00001d018466_T001 rna7535 8.00E-121
645- 11: transcript:Zm00001d018467_T001 rna7536 6.00E-18
645- 12: transcript:Zm00001d018468_T001 rna7537 9.00E-42
645- 13: transcript:Zm00001d018469_T001 rna7539 0
645- 14: transcript:Zm00001d018470_T001 rna7540 9.00E-84
645- 15: transcript:Zm00001d018471_T002 rna7541 8.00E-80
645- 16: transcript:Zm00001d018472_T002 rna7542 0
645- 17: transcript:Zm00001d018473_T001 rna7543 0
645- 18: transcript:Zm00001d018474_T001 rna7545 0
645- 19: transcript:Zm00001d018475_T001 rna7546 0
645- 20: transcript:Zm00001d018477_T006 rna7547 0
645- 21: transcript:Zm00001d018478_T001 rna7548 1.00E-44
645- 22: transcript:Zm00001d018479_T001 rna7551 0
Alignment 646: score=1012.0 e_value=1.8e-64 N=22 5&NC_008395.2 plus
646- 0: transcript:Zm00001d017252_T002 rna6400 4.00E-175
646- 1: transcript:Zm00001d017256_T001 rna6401 5.00E-34
646- 2: transcript:Zm00001d017258_T010 rna6402 0
646- 3: transcript:Zm00001d017261_T006 rna6404 0
646- 4: transcript:Zm00001d017263_T001 rna6410 9.00E-91
646- 5: transcript:Zm00001d017264_T001 rna6412 0
646- 6: transcript:Zm00001d017268_T001 rna6413 6.00E-124
646- 7: transcript:Zm00001d017270_T001 rna6417 4.00E-156
646- 8: transcript:Zm00001d017271_T005 rna6418 0
646- 9: transcript:Zm00001d017274_T001 rna6420 0
646- 10: transcript:Zm00001d017275_T001 rna6421 0
646- 11: transcript:Zm00001d017281_T001 rna6424 0
646- 12: transcript:Zm00001d017282_T001 rna6426 0
646- 13: transcript:Zm00001d017284_T001 rna6427 0
646- 14: transcript:Zm00001d017287_T001 rna6430 1.00E-36
646- 15: transcript:Zm00001d017288_T001 rna6431 0
646- 16: transcript:Zm00001d017291_T001 rna6432 0
646- 17: transcript:Zm00001d017292_T003 rna6433 2.00E-40
646- 18: transcript:Zm00001d017294_T001 rna6436 1.00E-42
646- 19: transcript:Zm00001d017296_T001 rna6438 0
646- 20: transcript:Zm00001d017297_T001 rna6439 6.00E-16
646- 21: transcript:Zm00001d017298_T003 rna6440 2.00E-140
Alignment 647: score=998.0 e_value=9.8e-65 N=21 5&NC_008395.2 plus
647- 0: transcript:Zm00001d015612_T001 rna4882 5.00E-98
647- 1: transcript:Zm00001d015613_T001 rna4883 4.00E-123
647- 2: transcript:Zm00001d015614_T001 rna4884 2.00E-146
647- 3: transcript:Zm00001d015618_T001 rna4885 0
647- 4: transcript:Zm00001d015623_T001 rna4886 0
647- 5: transcript:Zm00001d015626_T001 rna4887 9.00E-06
647- 6: transcript:Zm00001d015627_T002 rna4888 4.00E-86
647- 7: transcript:Zm00001d015628_T001 rna4891 1.00E-56

647- 8: transcript:Zm00001d015635_T002 rna4892 6.00E-64
647- 9: transcript:Zm00001d015636_T001 rna4894 1.00E-110
647- 10: transcript:Zm00001d015638_T004 rna4895 1.00E-52
647- 11: transcript:Zm00001d015639_T001 rna4897 9.00E-50
647- 12: transcript:Zm00001d015649_T003 rna4905 0
647- 13: transcript:Zm00001d015651_T001 rna4906 1.00E-178
647- 14: transcript:Zm00001d015654_T001 rna4907 3.00E-12
647- 15: transcript:Zm00001d015656_T001 rna4908 0
647- 16: transcript:Zm00001d015657_T001 rna4909 2.00E-67
647- 17: transcript:Zm00001d015658_T003 rna4913 0
647- 18: transcript:Zm00001d015664_T001 rna4915 6.00E-40
647- 19: transcript:Zm00001d015665_T007 rna4916 6.00E-90
647- 20: transcript:Zm00001d015670_T001 rna4917 3.00E-34
Alignment 648: score=989.0 e_value=4.3e-76 N=24 5&NC_008395.2 plus
648- 0: transcript:Zm00001d016545_T001 rna5763 5.00E-67
648- 1: transcript:Zm00001d016549_T001 rna5764 3.00E-75
648- 2: transcript:Zm00001d016550_T002 rna5765 6.00E-60
648- 3: transcript:Zm00001d016552_T002 rna5772 0
648- 4: transcript:Zm00001d016553_T001 rna5775 0
648- 5: transcript:Zm00001d016559_T001 rna5780 8.00E-164
648- 6: transcript:Zm00001d016561_T001 rna5781 9.00E-53
648- 7: transcript:Zm00001d016566_T001 rna5786 2.00E-84
648- 8: transcript:Zm00001d016567_T001 rna5787 2.00E-40
648- 9: transcript:Zm00001d016570_T001 rna5790 9.00E-50
648- 10: transcript:Zm00001d016572_T001 rna5793 0
648- 11: transcript:Zm00001d016577_T002 rna5796 9.00E-109
648- 12: transcript:Zm00001d016580_T001 rna5798 1.00E-61
648- 13: transcript:Zm00001d016581_T004 rna5802 0
648- 14: transcript:Zm00001d016582_T001 rna5803 1.00E-54
648- 15: transcript:Zm00001d016593_T001 rna5806 1.00E-67
648- 16: transcript:Zm00001d016599_T002 rna5819 6.00E-69
648- 17: transcript:Zm00001d016602_T001 rna5820 0
648- 18: transcript:Zm00001d016604_T001 rna5821 1.00E-96
648- 19: transcript:Zm00001d016606_T022 rna5826 0
648- 20: transcript:Zm00001d016607_T001 rna5827 1.00E-36
648- 21: transcript:Zm00001d016625_T001 rna5837 6.00E-17
648- 22: transcript:Zm00001d016640_T003 rna5854 0
648- 23: transcript:Zm00001d016644_T001 rna5880 1.00E-19
Alignment 649: score=984.0 e_value=4.9e-66 N=22 5&NC_008395.2 plus
649- 0: transcript:Zm00001d016457_T001 rna5639 3.00E-53
649- 1: transcript:Zm00001d016458_T002 rna5640 6.00E-36
649- 2: transcript:Zm00001d016459_T001 rna5642 4.00E-49
649- 3: transcript:Zm00001d016461_T001 rna5648 4.00E-83
649- 4: transcript:Zm00001d016463_T001 rna5654 0
649- 5: transcript:Zm00001d016465_T001 rna5655 7.00E-133
649- 6: transcript:Zm00001d016469_T001 rna5658 1.00E-141
649- 7: transcript:Zm00001d016470_T001 rna5659 2.00E-106
649- 8: transcript:Zm00001d016471_T001 rna5661 0
649- 9: transcript:Zm00001d016472_T001 rna5669 0
649- 10: transcript:Zm00001d016473_T001 rna5672 0
649- 11: transcript:Zm00001d016474_T001 rna5684 0
649- 12: transcript:Zm00001d016475_T001 rna5687 0
649- 13: transcript:Zm00001d016476_T001 rna5688 0
649- 14: transcript:Zm00001d016479_T001 rna5691 0

649- 15: transcript:Zm00001d016483_T002 rna5692 0
649- 16: transcript:Zm00001d016486_T001 rna5697 1.00E-80
649- 17: transcript:Zm00001d016490_T001 rna5700 6.00E-145
649- 18: transcript:Zm00001d016491_T001 rna5702 7.00E-58
649- 19: transcript:Zm00001d016493_T001 rna5704 4.00E-78
649- 20: transcript:Zm00001d016496_T004 rna5706 0
649- 21: transcript:Zm00001d016501_T001 rna5721 0
Alignment 650: score=901.0 e_value=7.5e-63 N=20 5&NC_008395.2 plus
650- 0: transcript:Zm00001d014924_T001 rna4300 1.00E-34
650- 1: transcript:Zm00001d014925_T001 rna4301 5.00E-105
650- 2: transcript:Zm00001d014926_T001 rna4302 1.00E-91
650- 3: transcript:Zm00001d014928_T002 rna4303 0
650- 4: transcript:Zm00001d014932_T001 rna4308 1.00E-83
650- 5: transcript:Zm00001d014937_T001 rna4315 4.00E-97
650- 6: transcript:Zm00001d014938_T001 rna4317 2.00E-123
650- 7: transcript:Zm00001d014943_T002 rna4321 0
650- 8: transcript:Zm00001d014944_T002 rna4324 0
650- 9: transcript:Zm00001d014946_T001 rna4326 3.00E-152
650- 10: transcript:Zm00001d014947_T001 rna4330 0
650- 11: transcript:Zm00001d014949_T001 rna4332 2.00E-60
650- 12: transcript:Zm00001d014950_T002 rna4334 0
650- 13: transcript:Zm00001d014951_T002 rna4335 1.00E-176
650- 14: transcript:Zm00001d014952_T001 rna4336 5.00E-148
650- 15: transcript:Zm00001d014960_T001 rna4350 1.00E-26
650- 16: transcript:Zm00001d014961_T001 rna4352 0
650- 17: transcript:Zm00001d014962_T003 rna4353 3.00E-20
650- 18: transcript:Zm00001d014966_T001 rna4356 0
650- 19: transcript:Zm00001d014967_T019 rna4359 8.00E-53
Alignment 651: score=825.0 e_value=1.1e-51 N=18 5&NC_008395.2 plus
651- 0: transcript:Zm00001d018350_T001 rna7370 0
651- 1: transcript:Zm00001d018352_T001 rna7372 0
651- 2: transcript:Zm00001d018355_T005 rna7373 0
651- 3: transcript:Zm00001d018358_T001 rna7377 0
651- 4: transcript:Zm00001d018359_T002 rna7378 0
651- 5: transcript:Zm00001d018360_T001 rna7379 0
651- 6: transcript:Zm00001d018361_T001 rna7383 4.00E-21
651- 7: transcript:Zm00001d018363_T001 rna7386 6.00E-64
651- 8: transcript:Zm00001d018364_T001 rna7388 1.00E-118
651- 9: transcript:Zm00001d018365_T004 rna7390 4.00E-93
651- 10: transcript:Zm00001d018366_T001 rna7391 5.00E-108
651- 11: transcript:Zm00001d018369_T001 rna7392 0
651- 12: transcript:Zm00001d018370_T003 rna7394 0
651- 13: transcript:Zm00001d018371_T006 rna7395 0
651- 14: transcript:Zm00001d018375_T006 rna7401 0
651- 15: transcript:Zm00001d018378_T001 rna7402 2.00E-27
651- 16: transcript:Zm00001d018380_T001 rna7404 0
651- 17: transcript:Zm00001d018382_T011 rna7406 0
Alignment 652: score=797.0 e_value=3.6e-57 N=18 5&NC_008395.2 plus
652- 0: transcript:Zm00001d016686_T002 rna5884 1.00E-133
652- 1: transcript:Zm00001d016687_T001 rna5885 0
652- 2: transcript:Zm00001d016690_T001 rna5888 0
652- 3: transcript:Zm00001d016691_T001 rna5889 1.00E-35
652- 4: transcript:Zm00001d016693_T001 rna5891 1.00E-22
652- 5: transcript:Zm00001d016694_T003 rna5892 0

652- 6: transcript:Zm00001d016695_T004 rna5895   2.00E-159
652- 7: transcript:Zm00001d016696_T002 rna5899   0
652- 8: transcript:Zm00001d016697_T001 rna5900   3.00E-128
652- 9: transcript:Zm00001d016698_T001 rna5901   1.00E-42
652- 10: transcript:Zm00001d016702_T006 rna5903   1.00E-52
652- 11: transcript:Zm00001d016705_T001 rna5908   6.00E-23
652- 12: transcript:Zm00001d016708_T002 rna5911   0
652- 13: transcript:Zm00001d016714_T003 rna5914   3.00E-168
652- 14: transcript:Zm00001d016719_T001 rna5941   0
652- 15: transcript:Zm00001d016720_T001 rna5944   3.00E-144
652- 16: transcript:Zm00001d016722_T003 rna5945   3.00E-41
652- 17: transcript:Zm00001d016730_T001 rna5968   1.00E-44
Alignment 653: score=779.0 e_value=2.9e-44 N=17 5&NC_008395.2 plus
653- 0: transcript:Zm00001d015446_T016 rna4808   0
653- 1: transcript:Zm00001d015451_T001 rna4810   1.00E-102
653- 2: transcript:Zm00001d015457_T001 rna4811   3.00E-90
653- 3: transcript:Zm00001d015460_T001 rna4812   2.00E-55
653- 4: transcript:Zm00001d015463_T001 rna4814   1.00E-70
653- 5: transcript:Zm00001d015464_T004 rna4815   0
653- 6: transcript:Zm00001d015468_T001 rna4818   6.00E-72
653- 7: transcript:Zm00001d015470_T001 rna4819   2.00E-20
653- 8: transcript:Zm00001d015473_T003 rna4820   2.00E-43
653- 9: transcript:Zm00001d015474_T001 rna4821   0
653- 10: transcript:Zm00001d015475_T001 rna4822   4.00E-19
653- 11: transcript:Zm00001d015476_T001 rna4823   3.00E-38
653- 12: transcript:Zm00001d015477_T001 rna4824   0
653- 13: transcript:Zm00001d015484_T001 rna4826   2.00E-125
653- 14: transcript:Zm00001d015491_T001 rna4829   2.00E-116
653- 15: transcript:Zm00001d015493_T001 rna4831   0
653- 16: transcript:Zm00001d015495_T013 rna4832   1.00E-120
Alignment 654: score=769.0 e_value=4.6e-42 N=17 5&NC_008395.2 plus
654- 0: transcript:Zm00001d018316_T028 rna7341   4.00E-36
654- 1: transcript:Zm00001d018318_T006 rna7342   0
654- 2: transcript:Zm00001d018319_T001 rna7343   2.00E-57
654- 3: transcript:Zm00001d018321_T001 rna7344   5.00E-62
654- 4: transcript:Zm00001d018323_T001 rna7346   3.00E-94
654- 5: transcript:Zm00001d018325_T001 rna7348   0
654- 6: transcript:Zm00001d018326_T001 rna7351   0
654- 7: transcript:Zm00001d018328_T003 rna7352   0
654- 8: transcript:Zm00001d018332_T001 rna7353   0
654- 9: transcript:Zm00001d018333_T001 rna7355   0
654- 10: transcript:Zm00001d018334_T001 rna7358   7.00E-114
654- 11: transcript:Zm00001d018335_T001 rna7359   0
654- 12: transcript:Zm00001d018336_T002 rna7360   1.00E-157
654- 13: transcript:Zm00001d018339_T001 rna7361   6.00E-135
654- 14: transcript:Zm00001d018341_T007 rna7362   2.00E-66
654- 15: transcript:Zm00001d018342_T002 rna7365   0
654- 16: transcript:Zm00001d018343_T001 rna7366   0
Alignment 655: score=617.0 e_value=9e-31 N=13 5&NC_008395.2 plus
655- 0: transcript:Zm00001d016647_T001 rna5855   5.00E-147
655- 1: transcript:Zm00001d016648_T001 rna5857   7.00E-69
655- 2: transcript:Zm00001d016653_T001 rna5858   0
655- 3: transcript:Zm00001d016655_T001 rna5862   9.00E-35
655- 4: transcript:Zm00001d016659_T001 rna5863   0

655- 5: transcript:Zm00001d016662_T003 rna5864 0
655- 6: transcript:Zm00001d016664_T001 rna5866 0
655- 7: transcript:Zm00001d016665_T001 rna5867 1.00E-44
655- 8: transcript:Zm00001d016666_T001 rna5868 0
655- 9: transcript:Zm00001d016669_T001 rna5869 2.00E-126
655- 10: transcript:Zm00001d016671_T001 rna5871 3.00E-59
655- 11: transcript:Zm00001d016674_T001 rna5872 0
655- 12: transcript:Zm00001d016675_T013 rna5874 0
Alignment 656: score=540.0 e_value=3e-28 N=12 5&NC_008395.2 plus
656- 0: transcript:Zm00001d017849_T001 rna6913 1.00E-131
656- 1: transcript:Zm00001d017850_T001 rna6915 4.00E-44
656- 2: transcript:Zm00001d017857_T002 rna6920 0
656- 3: transcript:Zm00001d017859_T002 rna6921 1.00E-124
656- 4: transcript:Zm00001d017860_T005 rna6922 0
656- 5: transcript:Zm00001d017861_T001 rna6924 1.00E-70
656- 6: transcript:Zm00001d017862_T001 rna6926 2.00E-20
656- 7: transcript:Zm00001d017863_T001 rna6929 3.00E-129
656- 8: transcript:Zm00001d017867_T001 rna6931 2.00E-66
656- 9: transcript:Zm00001d017868_T010 rna6933 0
656- 10: transcript:Zm00001d017869_T001 rna6934 0
656- 11: transcript:Zm00001d017872_T001 rna6939 0
Alignment 657: score=332.0 e_value=1.6e-14 N=8 5&NC_008395.2 plus
657- 0: transcript:Zm00001d015921_T001 rna5380 1.00E-68
657- 1: transcript:Zm00001d015929_T002 rna5382 0
657- 2: transcript:Zm00001d015934_T001 rna5390 2.00E-80
657- 3: transcript:Zm00001d015948_T010 rna5413 2.00E-108
657- 4: transcript:Zm00001d015955_T004 rna5418 0
657- 5: transcript:Zm00001d015968_T003 rna5425 0
657- 6: transcript:Zm00001d015969_T001 rna5430 7.00E-31
657- 7: transcript:Zm00001d015970_T002 rna5432 4.00E-43
Alignment 658: score=280.0 e_value=1.7e-13 N=7 5&NC_008395.2 plus
658- 0: transcript:Zm00001d016823_T001 rna5999 9.00E-54
658- 1: transcript:Zm00001d016824_T001 rna6015 9.00E-65
658- 2: transcript:Zm00001d016826_T001 rna6021 6.00E-81
658- 3: transcript:Zm00001d016827_T001 rna6022 3.00E-152
658- 4: transcript:Zm00001d016832_T001 rna6024 0
658- 5: transcript:Zm00001d016836_T002 rna6027 2.00E-118
658- 6: transcript:Zm00001d016839_T001 rna6040 4.00E-136
Alignment 659: score=276.0 e_value=1.7e-08 N=6 5&NC_008395.2 plus
659- 0: transcript:Zm00001d015429_T006 rna4798 0
659- 1: transcript:Zm00001d015430_T001 rna4800 0
659- 2: transcript:Zm00001d015433_T001 rna4802 2.00E-156
659- 3: transcript:Zm00001d015434_T001 rna4803 9.00E-53
659- 4: transcript:Zm00001d015435_T016 rna4804 0
659- 5: transcript:Zm00001d015440_T001 rna4806 2.00E-33
Alignment 660: score=2262.0 e_value=4e-197 N=52 5&NC_008395.2 minus
660- 0: transcript:Zm00001d016177_T001 rna5181 3.00E-157
660- 1: transcript:Zm00001d016182_T001 rna5176 6.00E-168
660- 2: transcript:Zm00001d016185_T001 rna5175 1.00E-70
660- 3: transcript:Zm00001d016188_T002 rna5173 0
660- 4: transcript:Zm00001d016197_T001 rna5172 0
660- 5: transcript:Zm00001d016198_T003 rna5171 0
660- 6: transcript:Zm00001d016207_T002 rna5167 2.00E-81
660- 7: transcript:Zm00001d016216_T001 rna5165 4.00E-23

660- 8: transcript:Zm00001d016225_T001 rna5161 0
660- 9: transcript:Zm00001d016228_T001 rna5160 0
660- 10: transcript:Zm00001d016231_T001 rna5157 3.00E-26
660- 11: transcript:Zm00001d016234_T001 rna5156 0
660- 12: transcript:Zm00001d016237_T001 rna5155 6.00E-161
660- 13: transcript:Zm00001d016242_T002 rna5154 3.00E-116
660- 14: transcript:Zm00001d016248_T001 rna5153 0
660- 15: transcript:Zm00001d016254_T001 rna5152 0
660- 16: transcript:Zm00001d016255_T001 rna5151 3.00E-110
660- 17: transcript:Zm00001d016256_T001 rna5147 2.00E-97
660- 18: transcript:Zm00001d016271_T001 rna5143 1.00E-64
660- 19: transcript:Zm00001d016273_T001 rna5141 4.00E-169
660- 20: transcript:Zm00001d016274_T006 rna5140 0
660- 21: transcript:Zm00001d016276_T001 rna5139 6.00E-90
660- 22: transcript:Zm00001d016277_T002 rna5138 7.00E-105
660- 23: transcript:Zm00001d016285_T001 rna5133 2.00E-101
660- 24: transcript:Zm00001d016287_T001 rna5130 1.00E-55
660- 25: transcript:Zm00001d016294_T001 rna5127 7.00E-113
660- 26: transcript:Zm00001d016298_T001 rna5126 1.00E-144
660- 27: transcript:Zm00001d016299_T001 rna5123 1.00E-35
660- 28: transcript:Zm00001d016301_T001 rna5122 1.00E-103
660- 29: transcript:Zm00001d016305_T001 rna5121 3.00E-127
660- 30: transcript:Zm00001d016306_T001 rna5117 1.00E-132
660- 31: transcript:Zm00001d016308_T003 rna5114 0
660- 32: transcript:Zm00001d016311_T002 rna5113 0
660- 33: transcript:Zm00001d016326_T001 rna5112 1.00E-142
660- 34: transcript:Zm00001d016332_T001 rna5110 0
660- 35: transcript:Zm00001d016342_T001 rna5109 1.00E-54
660- 36: transcript:Zm00001d016347_T002 rna5103 0
660- 37: transcript:Zm00001d016349_T002 rna5101 2.00E-148
660- 38: transcript:Zm00001d016350_T001 rna5099 2.00E-52
660- 39: transcript:Zm00001d016354_T001 rna5098 1.00E-143
660- 40: transcript:Zm00001d016358_T006 rna5094 0
660- 41: transcript:Zm00001d016361_T001 rna5093 2.00E-111
660- 42: transcript:Zm00001d016364_T001 rna5091 1.00E-146
660- 43: transcript:Zm00001d016373_T003 rna5079 0
660- 44: transcript:Zm00001d016374_T002 rna5077 0
660- 45: transcript:Zm00001d016378_T002 rna5076 0
660- 46: transcript:Zm00001d016379_T001 rna5070 4.00E-30
660- 47: transcript:Zm00001d016382_T004 rna5058 0
660- 48: transcript:Zm00001d016395_T001 rna5040 0
660- 49: transcript:Zm00001d016397_T008 rna5039 3.00E-84
660- 50: transcript:Zm00001d016400_T001 rna5033 7.00E-20
660- 51: transcript:Zm00001d016402_T012 rna5030 2.00E-114
Alignment 661: score=1495.0 e_value=3.5e-116 N=34 5&NC_008395.2 minus
661- 0: transcript:Zm00001d016016_T001 rna5319 2.00E-54
661- 1: transcript:Zm00001d016021_T001 rna5318 3.00E-169
661- 2: transcript:Zm00001d016035_T001 rna5311 2.00E-103
661- 3: transcript:Zm00001d016036_T001 rna5310 1.00E-140
661- 4: transcript:Zm00001d016037_T001 rna5307 3.00E-50
661- 5: transcript:Zm00001d016041_T001 rna5302 2.00E-124
661- 6: transcript:Zm00001d016044_T002 rna5301 2.00E-169
661- 7: transcript:Zm00001d016049_T003 rna5299 8.00E-91
661- 8: transcript:Zm00001d016052_T001 rna5297 2.00E-105

661- 9: transcript:Zm00001d016061_T001 rna5292 1.00E-118
661- 10: transcript:Zm00001d016062_T001 rna5282 6.00E-73
661- 11: transcript:Zm00001d016063_T006 rna5281 0
661- 12: transcript:Zm00001d016066_T001 rna5278 0
661- 13: transcript:Zm00001d016070_T004 rna5276 9.00E-43
661- 14: transcript:Zm00001d016072_T001 rna5275 4.00E-88
661- 15: transcript:Zm00001d016075_T001 rna5274 5.00E-136
661- 16: transcript:Zm00001d016076_T001 rna5272 2.00E-43
661- 17: transcript:Zm00001d016081_T002 rna5271 0
661- 18: transcript:Zm00001d016082_T001 rna5270 1.00E-90
661- 19: transcript:Zm00001d016100_T001 rna5268 2.00E-167
661- 20: transcript:Zm00001d016105_T001 rna5257 4.00E-136
661- 21: transcript:Zm00001d016106_T001 rna5255 0
661- 22: transcript:Zm00001d016119_T001 rna5238 5.00E-145
661- 23: transcript:Zm00001d016128_T001 rna5223 3.00E-67
661- 24: transcript:Zm00001d016130_T001 rna5222 8.00E-173
661- 25: transcript:Zm00001d016131_T006 rna5221 0
661- 26: transcript:Zm00001d016136_T001 rna5215 5.00E-66
661- 27: transcript:Zm00001d016142_T001 rna5212 2.00E-33
661- 28: transcript:Zm00001d016152_T002 rna5211 6.00E-45
661- 29: transcript:Zm00001d016154_T001 rna5207 2.00E-104
661- 30: transcript:Zm00001d016156_T003 rna5206 6.00E-146
661- 31: transcript:Zm00001d016158_T001 rna5203 5.00E-76
661- 32: transcript:Zm00001d016160_T001 rna5201 0
661- 33: transcript:Zm00001d016164_T003 rna5194 0
Alignment 662: score=948.0 e_value=7.1e-64 N=22 5&NC_008395.2 minus
662- 0: transcript:Zm00001d015893_T001 rna5439 0
662- 1: transcript:Zm00001d015905_T001 rna5437 3.00E-163
662- 2: transcript:Zm00001d015917_T001 rna5435 2.00E-94
662- 3: transcript:Zm00001d015945_T001 rna5416 6.00E-67
662- 4: transcript:Zm00001d015952_T001 rna5412 0
662- 5: transcript:Zm00001d015960_T001 rna5404 0
662- 6: transcript:Zm00001d015962_T001 rna5403 0
662- 7: transcript:Zm00001d015965_T001 rna5391 2.00E-76
662- 8: transcript:Zm00001d015971_T004 rna5376 0
662- 9: transcript:Zm00001d015973_T001 rna5371 1.00E-73
662- 10: transcript:Zm00001d015974_T003 rna5366 2.00E-132
662- 11: transcript:Zm00001d015975_T001 rna5363 1.00E-44
662- 12: transcript:Zm00001d015977_T001 rna5360 3.00E-152
662- 13: transcript:Zm00001d015984_T002 rna5350 2.00E-112
662- 14: transcript:Zm00001d015985_T003 rna5349 0
662- 15: transcript:Zm00001d015986_T002 rna5347 7.00E-63
662- 16: transcript:Zm00001d015988_T003 rna5343 6.00E-102
662- 17: transcript:Zm00001d015990_T001 rna5340 2.00E-35
662- 18: transcript:Zm00001d015992_T001 rna5338 0
662- 19: transcript:Zm00001d015997_T001 rna5337 2.00E-32
662- 20: transcript:Zm00001d015999_T001 rna5335 0
662- 21: transcript:Zm00001d016000_T006 rna5326 2.00E-55
Alignment 663: score=862.0 e_value=7.9e-54 N=19 5&NC_008395.2 minus
663- 0: transcript:Zm00001d015759_T001 rna4989 4.00E-116
663- 1: transcript:Zm00001d015767_T002 rna4985 4.00E-18
663- 2: transcript:Zm00001d015776_T001 rna4984 9.00E-58
663- 3: transcript:Zm00001d015778_T001 rna4983 0
663- 4: transcript:Zm00001d015780_T002 rna4982 7.00E-125

663- 5: transcript:Zm00001d015783_T001 rna4981 0
663- 6: transcript:Zm00001d015785_T005 rna4979 0
663- 7: transcript:Zm00001d015788_T001 rna4978 0
663- 8: transcript:Zm00001d015789_T002 rna4977 0
663- 9: transcript:Zm00001d015791_T001 rna4976 2.00E-169
663- 10: transcript:Zm00001d015795_T001 rna4972 2.00E-110
663- 11: transcript:Zm00001d015796_T001 rna4971 4.00E-78
663- 12: transcript:Zm00001d015798_T001 rna4970 0
663- 13: transcript:Zm00001d015799_T001 rna4968 7.00E-11
663- 14: transcript:Zm00001d015802_T001 rna4967 0
663- 15: transcript:Zm00001d015804_T013 rna4966 0
663- 16: transcript:Zm00001d015811_T002 rna4963 2.00E-23
663- 17: transcript:Zm00001d015816_T003 rna4956 4.00E-151
663- 18: transcript:Zm00001d015820_T002 rna4954 3.00E-69
Alignment 664: score=714.0 e_value=1.3e-41 N=16 5&NC_008395.2 minus
664- 0: transcript:Zm00001d015825_T001 rna4954 0
664- 1: transcript:Zm00001d015829_T001 rna4953 0
664- 2: transcript:Zm00001d015837_T001 rna4952 0
664- 3: transcript:Zm00001d015839_T001 rna4948 2.00E-163
664- 4: transcript:Zm00001d015842_T001 rna4946 1.00E-103
664- 5: transcript:Zm00001d015844_T001 rna4943 0
664- 6: transcript:Zm00001d015845_T001 rna4942 6.00E-52
664- 7: transcript:Zm00001d015846_T001 rna4941 6.00E-43
664- 8: transcript:Zm00001d015851_T001 rna4940 0
664- 9: transcript:Zm00001d015852_T004 rna4939 0
664- 10: transcript:Zm00001d015853_T001 rna4937 1.00E-155
664- 11: transcript:Zm00001d015856_T007 rna4934 0
664- 12: transcript:Zm00001d015861_T002 rna4932 4.00E-141
664- 13: transcript:Zm00001d015863_T001 rna4929 0
664- 14: transcript:Zm00001d015867_T001 rna4925 2.00E-47
664- 15: transcript:Zm00001d015884_T001 rna4924 7.00E-19
Alignment 665: score=482.0 e_value=1.6e-20 N=10 5&NC_008395.2 minus
665- 0: transcript:Zm00001d016730_T001 rna5935 0
665- 1: transcript:Zm00001d016731_T001 rna5934 0
665- 2: transcript:Zm00001d016732_T006 rna5933 7.00E-168
665- 3: transcript:Zm00001d016733_T001 rna5932 2.00E-105
665- 4: transcript:Zm00001d016735_T001 rna5928 2.00E-127
665- 5: transcript:Zm00001d016736_T001 rna5927 1.00E-160
665- 6: transcript:Zm00001d016738_T001 rna5922 6.00E-43
665- 7: transcript:Zm00001d016743_T001 rna5921 7.00E-26
665- 8: transcript:Zm00001d016744_T001 rna5919 8.00E-42
665- 9: transcript:Zm00001d016745_T001 rna5918 2.00E-143
Alignment 666: score=455.0 e_value=7.9e-25 N=10 5&NC_008395.2 minus
666- 0: transcript:Zm00001d016406_T015 rna5029 0
666- 1: transcript:Zm00001d016407_T007 rna5027 0
666- 2: transcript:Zm00001d016408_T001 rna5026 2.00E-15
666- 3: transcript:Zm00001d016409_T001 rna5025 7.00E-66
666- 4: transcript:Zm00001d016410_T006 rna5023 9.00E-51
666- 5: transcript:Zm00001d016411_T001 rna5022 3.00E-65
666- 6: transcript:Zm00001d016414_T001 rna5015 4.00E-46
666- 7: transcript:Zm00001d016417_T001 rna5014 1.00E-131
666- 8: transcript:Zm00001d016438_T001 rna5013 0
666- 9: transcript:Zm00001d016439_T003 rna5011 0
Alignment 667: score=411.0 e_value=1.4e-16 N=9 5&NC_008395.2 minus

667- 0: transcript:Zm00001d018431_T001 rna7505 0
667- 1: transcript:Zm00001d018432_T001 rna7504 4.00E-15
667- 2: transcript:Zm00001d018433_T001 rna7503 3.00E-95
667- 3: transcript:Zm00001d018435_T001 rna7502 1.00E-115
667- 4: transcript:Zm00001d018438_T003 rna7501 0
667- 5: transcript:Zm00001d018440_T002 rna7499 0
667- 6: transcript:Zm00001d018441_T008 rna7497 5.00E-84
667- 7: transcript:Zm00001d018444_T001 rna7495 0
667- 8: transcript:Zm00001d018447_T001 rna7489 0
Alignment 668: score=374.0 e_value=3.2e-15 N=8 5&NC_008395.2 minus
668- 0: transcript:Zm00001d016806_T001 rna6013 0
668- 1: transcript:Zm00001d016807_T001 rna6012 0
668- 2: transcript:Zm00001d016810_T001 rna6010 0
668- 3: transcript:Zm00001d016814_T001 rna6008 3.00E-96
668- 4: transcript:Zm00001d016815_T001 rna6005 0
668- 5: transcript:Zm00001d016816_T002 rna6004 0
668- 6: transcript:Zm00001d016817_T001 rna6002 2.00E-10
668- 7: transcript:Zm00001d016822_T012 rna6000 0
Alignment 669: score=364.0 e_value=2.4e-16 N=8 5&NC_008395.2 minus
669- 0: transcript:Zm00001d016607_T001 rna5851 7.00E-06
669- 1: transcript:Zm00001d016615_T002 rna5845 0
669- 2: transcript:Zm00001d016619_T001 rna5843 3.00E-164
669- 3: transcript:Zm00001d016620_T001 rna5841 1.00E-16
669- 4: transcript:Zm00001d016621_T001 rna5839 3.00E-104
669- 5: transcript:Zm00001d016634_T003 rna5831 0
669- 6: transcript:Zm00001d016635_T001 rna5830 3.00E-85
669- 7: transcript:Zm00001d016636_T001 rna5828 2.00E-36
Alignment 670: score=353.0 e_value=9.5e-17 N=8 5&NC_008395.2 minus
670- 0: transcript:Zm00001d017140_T001 rna6316 1.00E-132
670- 1: transcript:Zm00001d017145_T003 rna6306 5.00E-126
670- 2: transcript:Zm00001d017146_T001 rna6305 2.00E-59
670- 3: transcript:Zm00001d017151_T002 rna6298 2.00E-113
670- 4: transcript:Zm00001d017152_T001 rna6297 2.00E-153
670- 5: transcript:Zm00001d017153_T018 rna6296 0
670- 6: transcript:Zm00001d017157_T001 rna6295 0
670- 7: transcript:Zm00001d017160_T001 rna6292 2.00E-56
Alignment 671: score=321.0 e_value=1.5e-14 N=8 5&NC_008395.2 minus
671- 0: transcript:Zm00001d014689_T006 rna4581 0
671- 1: transcript:Zm00001d014690_T014 rna4580 3.00E-152
671- 2: transcript:Zm00001d014692_T001 rna4579 1.00E-64
671- 3: transcript:Zm00001d014695_T001 rna4571 0
671- 4: transcript:Zm00001d014698_T006 rna4568 3.00E-67
671- 5: transcript:Zm00001d014701_T002 rna4564 1.00E-148
671- 6: transcript:Zm00001d014704_T002 rna4560 1.00E-28
671- 7: transcript:Zm00001d014716_T002 rna4557 2.00E-17
Alignment 672: score=287.0 e_value=2.3e-09 N=6 5&NC_008395.2 minus
672- 0: transcript:Zm00001d016584_T001 rna5818 8.00E-39
672- 1: transcript:Zm00001d016586_T002 rna5817 0
672- 2: transcript:Zm00001d016587_T001 rna5814 4.00E-165
672- 3: transcript:Zm00001d016588_T002 rna5811 3.00E-87
672- 4: transcript:Zm00001d016590_T001 rna5808 1.00E-137
672- 5: transcript:Zm00001d016591_T002 rna5807 0
Alignment 673: score=406.0 e_value=1.4e-22 N=10 5&NC_008396.2 plus
673- 0: transcript:Zm00001d014046_T001 rna7978 4.00E-54

673- 1: transcript:Zm00001d014060_T001 rna7985 0
673- 2: transcript:Zm00001d014073_T004 rna7988 2.00E-96
673- 3: transcript:Zm00001d014074_T001 rna7991 3.00E-78
673- 4: transcript:Zm00001d014082_T001 rna7999 3.00E-111
673- 5: transcript:Zm00001d014083_T001 rna8001 0
673- 6: transcript:Zm00001d014084_T001 rna8006 0
673- 7: transcript:Zm00001d014090_T002 rna8013 0
673- 8: transcript:Zm00001d014097_T002 rna8033 5.00E-117
673- 9: transcript:Zm00001d014102_T001 rna8044 0
Alignment 674: score=316.0 e_value=5.8e-12 N=7 5&NC_008396.2 plus
674- 0: transcript:Zm00001d014198_T001 rna8190 4.00E-92
674- 1: transcript:Zm00001d014200_T001 rna8193 3.00E-27
674- 2: transcript:Zm00001d014201_T001 rna8199 1.00E-23
674- 3: transcript:Zm00001d014203_T001 rna8202 0
674- 4: transcript:Zm00001d014219_T001 rna8208 2.00E-07
674- 5: transcript:Zm00001d014224_T001 rna8221 2.00E-144
674- 6: transcript:Zm00001d014226_T001 rna8223 1.00E-55
Alignment 675: score=280.0 e_value=1.5e-12 N=7 5&NC_008396.2 plus
675- 0: transcript:Zm00001d014113_T001 rna8058 2.00E-45
675- 1: transcript:Zm00001d014116_T001 rna8059 4.00E-157
675- 2: transcript:Zm00001d014124_T001 rna8070 0
675- 3: transcript:Zm00001d014128_T001 rna8072 2.00E-51
675- 4: transcript:Zm00001d014132_T001 rna8073 5.00E-60
675- 5: transcript:Zm00001d014138_T004 rna8080 5.00E-40
675- 6: transcript:Zm00001d014146_T001 rna8100 1.00E-06
Alignment 676: score=7679.0 e_value=0 N=168 5&NC_008396.2 minus
676- 0: transcript:Zm00001d012980_T001 rna11089 2.00E-170
676- 1: transcript:Zm00001d012983_T001 rna11084 0
676- 2: transcript:Zm00001d012988_T002 rna11082 2.00E-76
676- 3: transcript:Zm00001d012991_T001 rna11075 6.00E-140
676- 4: transcript:Zm00001d012992_T002 rna11072 1.00E-21
676- 5: transcript:Zm00001d012993_T001 rna11069 0
676- 6: transcript:Zm00001d012996_T001 rna11067 0
676- 7: transcript:Zm00001d012998_T001 rna11065 6.00E-123
676- 8: transcript:Zm00001d012999_T001 rna11064 2.00E-137
676- 9: transcript:Zm00001d013003_T001 rna11063 9.00E-160
676- 10: transcript:Zm00001d013004_T001 rna11057 6.00E-45
676- 11: transcript:Zm00001d013005_T002 rna11055 4.00E-139
676- 12: transcript:Zm00001d013006_T007 rna11054 0
676- 13: transcript:Zm00001d013007_T001 rna11053 1.00E-132
676- 14: transcript:Zm00001d013008_T001 rna11052 2.00E-39
676- 15: transcript:Zm00001d013009_T001 rna11051 1.00E-83
676- 16: transcript:Zm00001d013010_T002 rna11050 2.00E-110
676- 17: transcript:Zm00001d013012_T003 rna11049 2.00E-178
676- 18: transcript:Zm00001d013013_T003 rna11045 0
676- 19: transcript:Zm00001d013014_T001 rna11044 2.00E-87
676- 20: transcript:Zm00001d013015_T001 rna11043 1.00E-161
676- 21: transcript:Zm00001d013019_T012 rna11042 0
676- 22: transcript:Zm00001d013021_T001 rna11038 2.00E-136
676- 23: transcript:Zm00001d013025_T001 rna11036 1.00E-42
676- 24: transcript:Zm00001d013027_T002 rna11033 0
676- 25: transcript:Zm00001d013030_T002 rna11028 0
676- 26: transcript:Zm00001d013032_T001 rna11023 0
676- 27: transcript:Zm00001d013033_T001 rna11022 3.00E-93

676- 28:	transcript:Zm00001d013034_T001	rna11021	1.00E-160
676- 29:	transcript:Zm00001d013037_T003	rna11019	2.00E-77
676- 30:	transcript:Zm00001d013038_T002	rna11018	2.00E-63
676- 31:	transcript:Zm00001d013041_T003	rna11013	1.00E-44
676- 32:	transcript:Zm00001d013042_T001	rna11011	0
676- 33:	transcript:Zm00001d013043_T001	rna11009	1.00E-143
676- 34:	transcript:Zm00001d013046_T002	rna11002	0
676- 35:	transcript:Zm00001d013047_T004	rna11001	0
676- 36:	transcript:Zm00001d013048_T009	rna10999	0
676- 37:	transcript:Zm00001d013049_T002	rna10992	0
676- 38:	transcript:Zm00001d013050_T001	rna10989	4.00E-26
676- 39:	transcript:Zm00001d013052_T001	rna10984	5.00E-60
676- 40:	transcript:Zm00001d013053_T002	rna10983	2.00E-165
676- 41:	transcript:Zm00001d013055_T002	rna10979	2.00E-113
676- 42:	transcript:Zm00001d013056_T001	rna10976	2.00E-62
676- 43:	transcript:Zm00001d013057_T001	rna10967	2.00E-159
676- 44:	transcript:Zm00001d013058_T001	rna10966	0
676- 45:	transcript:Zm00001d013059_T003	rna10964	0
676- 46:	transcript:Zm00001d013060_T001	rna10958	0
676- 47:	transcript:Zm00001d013061_T003	rna10956	1.00E-131
676- 48:	transcript:Zm00001d013063_T001	rna10954	1.00E-27
676- 49:	transcript:Zm00001d013065_T002	rna10945	5.00E-131
676- 50:	transcript:Zm00001d013066_T002	rna10944	7.00E-36
676- 51:	transcript:Zm00001d013069_T001	rna10941	4.00E-77
676- 52:	transcript:Zm00001d013071_T001	rna10936	5.00E-61
676- 53:	transcript:Zm00001d013072_T001	rna10935	1.00E-144
676- 54:	transcript:Zm00001d013073_T002	rna10934	3.00E-161
676- 55:	transcript:Zm00001d013074_T003	rna10929	2.00E-155
676- 56:	transcript:Zm00001d013075_T001	rna10928	2.00E-20
676- 57:	transcript:Zm00001d013076_T001	rna10926	0
676- 58:	transcript:Zm00001d013077_T004	rna10925	7.00E-94
676- 59:	transcript:Zm00001d013078_T004	rna10920	5.00E-78
676- 60:	transcript:Zm00001d013079_T001	rna10919	2.00E-108
676- 61:	transcript:Zm00001d013080_T001	rna10917	0
676- 62:	transcript:Zm00001d013081_T001	rna10916	0
676- 63:	transcript:Zm00001d013082_T001	rna10915	1.00E-31
676- 64:	transcript:Zm00001d013083_T005	rna10912	0
676- 65:	transcript:Zm00001d013086_T004	rna10911	4.00E-104
676- 66:	transcript:Zm00001d013087_T001	rna10907	3.00E-75
676- 67:	transcript:Zm00001d013089_T002	rna10904	3.00E-23
676- 68:	transcript:Zm00001d013090_T002	rna10902	0
676- 69:	transcript:Zm00001d013094_T001	rna10893	0
676- 70:	transcript:Zm00001d013098_T002	rna10884	2.00E-74
676- 71:	transcript:Zm00001d013099_T001	rna10882	8.00E-150
676- 72:	transcript:Zm00001d013100_T001	rna10876	3.00E-126
676- 73:	transcript:Zm00001d013107_T001	rna10873	5.00E-132
676- 74:	transcript:Zm00001d013108_T001	rna10871	0
676- 75:	transcript:Zm00001d013109_T002	rna10870	0
676- 76:	transcript:Zm00001d013110_T001	rna10866	2.00E-86
676- 77:	transcript:Zm00001d013111_T001	rna10862	0
676- 78:	transcript:Zm00001d013112_T011	rna10861	9.00E-161
676- 79:	transcript:Zm00001d013113_T001	rna10860	2.00E-36
676- 80:	transcript:Zm00001d013114_T001	rna10859	1.00E-103
676- 81:	transcript:Zm00001d013116_T002	rna10857	2.00E-99

676- 82:	transcript:Zm00001d013117_T001	rna10855	7.00E-38
676- 83:	transcript:Zm00001d013118_T001	rna10853	2.00E-43
676- 84:	transcript:Zm00001d013119_T001	rna10849	2.00E-117
676- 85:	transcript:Zm00001d013120_T001	rna10843	4.00E-12
676- 86:	transcript:Zm00001d013122_T008	rna10841	8.00E-17
676- 87:	transcript:Zm00001d013126_T002	rna10839	1.00E-12
676- 88:	transcript:Zm00001d013128_T004	rna10835	8.00E-107
676- 89:	transcript:Zm00001d013130_T001	rna10827	7.00E-134
676- 90:	transcript:Zm00001d013133_T001	rna10824	1.00E-07
676- 91:	transcript:Zm00001d013135_T002	rna10819	3.00E-142
676- 92:	transcript:Zm00001d013139_T001	rna10813	1.00E-152
676- 93:	transcript:Zm00001d013140_T002	rna10812	3.00E-109
676- 94:	transcript:Zm00001d013141_T001	rna10811	1.00E-101
676- 95:	transcript:Zm00001d013143_T001	rna10810	5.00E-32
676- 96:	transcript:Zm00001d013144_T001	rna10806	5.00E-107
676- 97:	transcript:Zm00001d013146_T001	rna10805	1.00E-107
676- 98:	transcript:Zm00001d013147_T001	rna10802	2.00E-132
676- 99:	transcript:Zm00001d013150_T001	rna10800	1.00E-101
676-100:	transcript:Zm00001d013151_T001	rna10799	6.00E-136
676-101:	transcript:Zm00001d013153_T001	rna10797	8.00E-150
676-102:	transcript:Zm00001d013154_T001	rna10794	0
676-103:	transcript:Zm00001d013156_T001	rna10782	2.00E-143
676-104:	transcript:Zm00001d013162_T001	rna10779	0
676-105:	transcript:Zm00001d013163_T001	rna10773	0
676-106:	transcript:Zm00001d013164_T002	rna10766	3.00E-137
676-107:	transcript:Zm00001d013166_T001	rna10765	0
676-108:	transcript:Zm00001d013168_T001	rna10764	0
676-109:	transcript:Zm00001d013170_T001	rna10763	1.00E-16
676-110:	transcript:Zm00001d013172_T001	rna10761	2.00E-21
676-111:	transcript:Zm00001d013173_T001	rna10760	2.00E-167
676-112:	transcript:Zm00001d013175_T001	rna10758	3.00E-53
676-113:	transcript:Zm00001d013176_T002	rna10752	2.00E-18
676-114:	transcript:Zm00001d013177_T002	rna10751	0
676-115:	transcript:Zm00001d013178_T001	rna10750	3.00E-39
676-116:	transcript:Zm00001d013179_T001	rna10744	1.00E-112
676-117:	transcript:Zm00001d013181_T001	rna10743	2.00E-122
676-118:	transcript:Zm00001d013182_T003	rna10742	0
676-119:	transcript:Zm00001d013184_T001	rna10741	5.00E-140
676-120:	transcript:Zm00001d013185_T001	rna10740	0
676-121:	transcript:Zm00001d013186_T001	rna10739	1.00E-33
676-122:	transcript:Zm00001d013187_T001	rna10736	5.00E-105
676-123:	transcript:Zm00001d013188_T002	rna10732	2.00E-149
676-124:	transcript:Zm00001d013191_T001	rna10731	2.00E-57
676-125:	transcript:Zm00001d013192_T001	rna10730	0
676-126:	transcript:Zm00001d013193_T001	rna10729	9.00E-104
676-127:	transcript:Zm00001d013195_T001	rna10728	0
676-128:	transcript:Zm00001d013196_T001	rna10727	4.00E-107
676-129:	transcript:Zm00001d013200_T001	rna10725	1.00E-113
676-130:	transcript:Zm00001d013201_T003	rna10724	0
676-131:	transcript:Zm00001d013202_T001	rna10723	2.00E-102
676-132:	transcript:Zm00001d013206_T003	rna10721	0
676-133:	transcript:Zm00001d013208_T001	rna10720	8.00E-102
676-134:	transcript:Zm00001d013209_T001	rna10714	3.00E-123
676-135:	transcript:Zm00001d013210_T001	rna10713	1.00E-40

676-136: transcript:Zm00001d013212_T001 rna10711 0
676-137: transcript:Zm00001d013216_T001 rna10709 2.00E-56
676-138: transcript:Zm00001d013217_T001 rna10708 1.00E-43
676-139: transcript:Zm00001d013218_T003 rna10706 0
676-140: transcript:Zm00001d013219_T001 rna10703 0
676-141: transcript:Zm00001d013220_T001 rna10702 6.00E-57
676-142: transcript:Zm00001d013221_T001 rna10701 5.00E-15
676-143: transcript:Zm00001d013222_T001 rna10699 1.00E-42
676-144: transcript:Zm00001d013223_T002 rna10698 3.00E-131
676-145: transcript:Zm00001d013228_T001 rna10697 0
676-146: transcript:Zm00001d013229_T001 rna10691 7.00E-131
676-147: transcript:Zm00001d013232_T001 rna10690 2.00E-83
676-148: transcript:Zm00001d013238_T001 rna10689 0
676-149: transcript:Zm00001d013239_T002 rna10688 2.00E-45
676-150: transcript:Zm00001d013240_T002 rna10687 0
676-151: transcript:Zm00001d013241_T001 rna10684 0
676-152: transcript:Zm00001d013242_T001 rna10681 3.00E-43
676-153: transcript:Zm00001d013243_T002 rna10680 0
676-154: transcript:Zm00001d013245_T001 rna10677 0
676-155: transcript:Zm00001d013247_T002 rna10669 1.00E-39
676-156: transcript:Zm00001d013249_T001 rna10668 0
676-157: transcript:Zm00001d013250_T003 rna10667 3.00E-55
676-158: transcript:Zm00001d013251_T001 rna10666 0
676-159: transcript:Zm00001d013252_T001 rna10662 2.00E-81
676-160: transcript:Zm00001d013253_T001 rna10661 3.00E-31
676-161: transcript:Zm00001d013254_T002 rna10660 0
676-162: transcript:Zm00001d013256_T001 rna10656 4.00E-126
676-163: transcript:Zm00001d013258_T001 rna10651 6.00E-55
676-164: transcript:Zm00001d013259_T001 rna10650 6.00E-128
676-165: transcript:Zm00001d013262_T001 rna10645 0
676-166: transcript:Zm00001d013263_T001 rna10642 1.00E-31
676-167: transcript:Zm00001d013265_T001 rna10637 0
Alignment 677: score=3465.0 e_value=0 N=77 5&NC_008396.2 minus
677- 0: transcript:Zm00001d013443_T001 rna10382 3.00E-167
677- 1: transcript:Zm00001d013444_T002 rna10381 0
677- 2: transcript:Zm00001d013446_T001 rna10378 5.00E-95
677- 3: transcript:Zm00001d013448_T001 rna10371 2.00E-31
677- 4: transcript:Zm00001d013449_T001 rna10369 0
677- 5: transcript:Zm00001d013452_T002 rna10367 0
677- 6: transcript:Zm00001d013453_T001 rna10366 0
677- 7: transcript:Zm00001d013455_T001 rna10365 9.00E-134
677- 8: transcript:Zm00001d013456_T001 rna10364 0
677- 9: transcript:Zm00001d013459_T001 rna10362 3.00E-48
677- 10: transcript:Zm00001d013461_T002 rna10361 4.00E-85
677- 11: transcript:Zm00001d013463_T001 rna10360 0
677- 12: transcript:Zm00001d013465_T001 rna10359 0
677- 13: transcript:Zm00001d013466_T005 rna10356 0
677- 14: transcript:Zm00001d013467_T001 rna10355 3.00E-102
677- 15: transcript:Zm00001d013468_T002 rna10354 2.00E-145
677- 16: transcript:Zm00001d013469_T002 rna10351 0
677- 17: transcript:Zm00001d013470_T007 rna10350 0
677- 18: transcript:Zm00001d013471_T001 rna10348 1.00E-77
677- 19: transcript:Zm00001d013477_T008 rna10343 0
677- 20: transcript:Zm00001d013481_T001 rna10338 0

677- 21:	transcript:Zm00001d013485_T007	rna10332	2.00E-102
677- 22:	transcript:Zm00001d013486_T001	rna10330	0
677- 23:	transcript:Zm00001d013487_T001	rna10326	4.00E-31
677- 24:	transcript:Zm00001d013489_T001	rna10323	2.00E-75
677- 25:	transcript:Zm00001d013492_T001	rna10321	0
677- 26:	transcript:Zm00001d013493_T001	rna10314	0
677- 27:	transcript:Zm00001d013495_T002	rna10309	1.00E-108
677- 28:	transcript:Zm00001d013496_T001	rna10307	6.00E-179
677- 29:	transcript:Zm00001d013497_T001	rna10306	0
677- 30:	transcript:Zm00001d013498_T001	rna10305	9.00E-78
677- 31:	transcript:Zm00001d013499_T002	rna10303	0
677- 32:	transcript:Zm00001d013501_T002	rna10300	2.00E-12
677- 33:	transcript:Zm00001d013504_T001	rna10299	1.00E-76
677- 34:	transcript:Zm00001d013505_T001	rna10297	7.00E-127
677- 35:	transcript:Zm00001d013506_T001	rna10296	0
677- 36:	transcript:Zm00001d013509_T001	rna10291	8.00E-43
677- 37:	transcript:Zm00001d013517_T001	rna10283	2.00E-18
677- 38:	transcript:Zm00001d013519_T001	rna10277	0
677- 39:	transcript:Zm00001d013521_T001	rna10274	0
677- 40:	transcript:Zm00001d013522_T001	rna10273	1.00E-53
677- 41:	transcript:Zm00001d013527_T002	rna10263	0
677- 42:	transcript:Zm00001d013528_T001	rna10262	5.00E-141
677- 43:	transcript:Zm00001d013530_T001	rna10254	4.00E-158
677- 44:	transcript:Zm00001d013531_T002	rna10243	6.00E-40
677- 45:	transcript:Zm00001d013532_T001	rna10242	5.00E-150
677- 46:	transcript:Zm00001d013533_T001	rna10241	2.00E-40
677- 47:	transcript:Zm00001d013534_T002	rna10240	3.00E-90
677- 48:	transcript:Zm00001d013536_T001	rna10238	3.00E-118
677- 49:	transcript:Zm00001d013540_T020	rna10232	5.00E-38
677- 50:	transcript:Zm00001d013542_T003	rna10225	0
677- 51:	transcript:Zm00001d013543_T009	rna10224	4.00E-142
677- 52:	transcript:Zm00001d013544_T003	rna10223	9.00E-62
677- 53:	transcript:Zm00001d013546_T003	rna10221	6.00E-90
677- 54:	transcript:Zm00001d013547_T002	rna10218	5.00E-55
677- 55:	transcript:Zm00001d013549_T001	rna10205	2.00E-114
677- 56:	transcript:Zm00001d013550_T001	rna10203	2.00E-18
677- 57:	transcript:Zm00001d013551_T001	rna10202	2.00E-26
677- 58:	transcript:Zm00001d013552_T001	rna10201	1.00E-84
677- 59:	transcript:Zm00001d013554_T001	rna10198	3.00E-100
677- 60:	transcript:Zm00001d013555_T003	rna10196	0
677- 61:	transcript:Zm00001d013561_T001	rna10188	0
677- 62:	transcript:Zm00001d013566_T001	rna10186	8.00E-153
677- 63:	transcript:Zm00001d013568_T001	rna10182	5.00E-45
677- 64:	transcript:Zm00001d013569_T001	rna10160	1.00E-53
677- 65:	transcript:Zm00001d013570_T001	rna10159	2.00E-32
677- 66:	transcript:Zm00001d013581_T001	rna10158	8.00E-86
677- 67:	transcript:Zm00001d013582_T006	rna10155	3.00E-180
677- 68:	transcript:Zm00001d013583_T002	rna10153	2.00E-137
677- 69:	transcript:Zm00001d013589_T006	rna10152	0
677- 70:	transcript:Zm00001d013590_T001	rna10150	8.00E-101
677- 71:	transcript:Zm00001d013593_T001	rna10148	1.00E-62
677- 72:	transcript:Zm00001d013595_T001	rna10147	2.00E-128
677- 73:	transcript:Zm00001d013596_T001	rna10144	2.00E-41
677- 74:	transcript:Zm00001d013597_T001	rna10143	0

677- 75: transcript:Zm00001d013598_T001 rna10142 8.00E-20
677- 76: transcript:Zm00001d013603_T006 rna10139 7.00E-179
Alignment 678: score=3288.0 e_value=3.7e-309 N=73 5&NC_008396.2 minus
678- 0: transcript:Zm00001d013624_T001 rna10125 2.00E-81
678- 1: transcript:Zm00001d013625_T001 rna10124 1.00E-39
678- 2: transcript:Zm00001d013627_T001 rna10123 0
678- 3: transcript:Zm00001d013629_T001 rna10119 1.00E-81
678- 4: transcript:Zm00001d013630_T001 rna10118 2.00E-41
678- 5: transcript:Zm00001d013631_T001 rna10116 1.00E-122
678- 6: transcript:Zm00001d013632_T001 rna10115 2.00E-91
678- 7: transcript:Zm00001d013635_T001 rna10114 0
678- 8: transcript:Zm00001d013638_T001 rna10112 0
678- 9: transcript:Zm00001d013639_T001 rna10111 3.00E-68
678- 10: transcript:Zm00001d013641_T001 rna10109 6.00E-08
678- 11: transcript:Zm00001d013645_T001 rna10107 3.00E-37
678- 12: transcript:Zm00001d013646_T001 rna10106 0
678- 13: transcript:Zm00001d013647_T001 rna10105 2.00E-12
678- 14: transcript:Zm00001d013651_T001 rna10098 1.00E-17
678- 15: transcript:Zm00001d013652_T003 rna10096 0
678- 16: transcript:Zm00001d013653_T010 rna10093 0
678- 17: transcript:Zm00001d013654_T001 rna10092 2.00E-179
678- 18: transcript:Zm00001d013656_T001 rna10086 2.00E-67
678- 19: transcript:Zm00001d013657_T001 rna10085 1.00E-22
678- 20: transcript:Zm00001d013658_T001 rna10084 2.00E-144
678- 21: transcript:Zm00001d013659_T001 rna10081 0
678- 22: transcript:Zm00001d013661_T001 rna10079 3.00E-29
678- 23: transcript:Zm00001d013664_T006 rna10077 0
678- 24: transcript:Zm00001d013668_T001 rna10076 6.00E-121
678- 25: transcript:Zm00001d013669_T002 rna10074 0
678- 26: transcript:Zm00001d013672_T001 rna10070 0
678- 27: transcript:Zm00001d013673_T001 rna10069 9.00E-138
678- 28: transcript:Zm00001d013676_T001 rna10068 3.00E-103
678- 29: transcript:Zm00001d013677_T001 rna10066 1.00E-34
678- 30: transcript:Zm00001d013683_T003 rna10064 0
678- 31: transcript:Zm00001d013688_T001 rna10062 0
678- 32: transcript:Zm00001d013689_T001 rna10059 0
678- 33: transcript:Zm00001d013692_T001 rna10058 1.00E-97
678- 34: transcript:Zm00001d013693_T001 rna10049 1.00E-105
678- 35: transcript:Zm00001d013699_T001 rna10038 0
678- 36: transcript:Zm00001d013700_T001 rna10036 2.00E-50
678- 37: transcript:Zm00001d013702_T001 rna10035 0
678- 38: transcript:Zm00001d013703_T001 rna10017 0
678- 39: transcript:Zm00001d013705_T001 rna10012 2.00E-136
678- 40: transcript:Zm00001d013706_T001 rna10008 8.00E-106
678- 41: transcript:Zm00001d013707_T001 rna10007 2.00E-76
678- 42: transcript:Zm00001d013708_T001 rna10001 5.00E-83
678- 43: transcript:Zm00001d013712_T003 rna9999 0
678- 44: transcript:Zm00001d013713_T002 rna9998 7.00E-70
678- 45: transcript:Zm00001d013716_T002 rna9988 0
678- 46: transcript:Zm00001d013717_T001 rna9982 8.00E-130
678- 47: transcript:Zm00001d013722_T001 rna9963 3.00E-29
678- 48: transcript:Zm00001d013724_T001 rna9961 8.00E-40
678- 49: transcript:Zm00001d013725_T002 rna9958 1.00E-156
678- 50: transcript:Zm00001d013728_T001 rna9954 5.00E-91

```
678- 51:  transcript:Zm00001d013729_T003  rna9949            0
678- 52:  transcript:Zm00001d013730_T001  rna9944      2.00E-35
678- 53:  transcript:Zm00001d013732_T001  rna9943      9.00E-76
678- 54:  transcript:Zm00001d013736_T001  rna9938            0
678- 55:  transcript:Zm00001d013737_T001  rna9935     9.00E-154
678- 56:  transcript:Zm00001d013741_T006  rna9933            0
678- 57:  transcript:Zm00001d013743_T001  rna9923      2.00E-53
678- 58:  transcript:Zm00001d013744_T001  rna9922     2.00E-157
678- 59:  transcript:Zm00001d013745_T002  rna9921     8.00E-117
678- 60:  transcript:Zm00001d013746_T001  rna9914            0
678- 61:  transcript:Zm00001d013747_T001  rna9907            0
678- 62:  transcript:Zm00001d013750_T001  rna9894      7.00E-18
678- 63:  transcript:Zm00001d013751_T005  rna9893            0
678- 64:  transcript:Zm00001d013755_T001  rna9887      5.00E-24
678- 65:  transcript:Zm00001d013757_T007  rna9876            0
678- 66:  transcript:Zm00001d013759_T001  rna9859            0
678- 67:  transcript:Zm00001d013766_T001  rna9856      1.00E-80
678- 68:  transcript:Zm00001d013768_T002  rna9842      3.00E-17
678- 69:  transcript:Zm00001d013778_T001  rna9836            0
678- 70:  transcript:Zm00001d013780_T002  rna9834      1.00E-72
678- 71:  transcript:Zm00001d013781_T001  rna9833     1.00E-139
678- 72:  transcript:Zm00001d013787_T003  rna9821     2.00E-149
## Alignment 679: score=1911.0 e_value=1.5e-153 N=42 5&NC_008396.2 minus
679-  0:  transcript:Zm00001d012808_T001  rna11382           0
679-  1:  transcript:Zm00001d012809_T001  rna11381    4.00E-140
679-  2:  transcript:Zm00001d012811_T003  rna11377     1.00E-92
679-  3:  transcript:Zm00001d012812_T004  rna11373     1.00E-92
679-  4:  transcript:Zm00001d012813_T001  rna11372           0
679-  5:  transcript:Zm00001d012814_T001  rna11370     3.00E-47
679-  6:  transcript:Zm00001d012815_T022  rna11369           0
679-  7:  transcript:Zm00001d012816_T004  rna11365    3.00E-139
679-  8:  transcript:Zm00001d012817_T002  rna11358           0
679-  9:  transcript:Zm00001d012818_T001  rna11357     2.00E-52
679- 10:  transcript:Zm00001d012819_T001  rna11354    2.00E-160
679- 11:  transcript:Zm00001d012820_T001  rna11352    5.00E-119
679- 12:  transcript:Zm00001d012821_T003  rna11349    1.00E-137
679- 13:  transcript:Zm00001d012823_T001  rna11346           0
679- 14:  transcript:Zm00001d012824_T001  rna11345     4.00E-46
679- 15:  transcript:Zm00001d012825_T001  rna11344           0
679- 16:  transcript:Zm00001d012826_T003  rna11343    9.00E-113
679- 17:  transcript:Zm00001d012827_T001  rna11342           0
679- 18:  transcript:Zm00001d012830_T002  rna11340    2.00E-168
679- 19:  transcript:Zm00001d012831_T001  rna11334           0
679- 20:  transcript:Zm00001d012832_T003  rna11331     4.00E-83
679- 21:  transcript:Zm00001d012833_T002  rna11330     2.00E-66
679- 22:  transcript:Zm00001d012834_T003  rna11327           0
679- 23:  transcript:Zm00001d012836_T001  rna11326     6.00E-48
679- 24:  transcript:Zm00001d012839_T002  rna11322    1.00E-147
679- 25:  transcript:Zm00001d012844_T002  rna11319           0
679- 26:  transcript:Zm00001d012845_T001  rna11318     2.00E-78
679- 27:  transcript:Zm00001d012846_T005  rna11317           0
679- 28:  transcript:Zm00001d012847_T001  rna11313    8.00E-122
679- 29:  transcript:Zm00001d012848_T001  rna11310     1.00E-82
679- 30:  transcript:Zm00001d012849_T001  rna11307    1.00E-155
```

```
679- 31:  transcript:Zm00001d012850_T001  rna11305    2.00E-108
679- 32:  transcript:Zm00001d012851_T001  rna11303            0
679- 33:  transcript:Zm00001d012852_T003  rna11298     3.00E-85
679- 34:  transcript:Zm00001d012853_T001  rna11297    6.00E-129
679- 35:  transcript:Zm00001d012854_T001  rna11296     1.00E-83
679- 36:  transcript:Zm00001d012857_T006  rna11282     6.00E-84
679- 37:  transcript:Zm00001d012859_T001  rna11279     3.00E-08
679- 38:  transcript:Zm00001d012861_T001  rna11274     2.00E-91
679- 39:  transcript:Zm00001d012862_T001  rna11268            0
679- 40:  transcript:Zm00001d012863_T003  rna11264            0
679- 41:  transcript:Zm00001d012865_T004  rna11263            0
## Alignment 680: score=1766.0 e_value=1e-140 N=40 5&NC_008396.2 minus
680-  0:  transcript:Zm00001d013281_T001  rna10605     1.00E-36
680-  1:  transcript:Zm00001d013283_T001  rna10604            0
680-  2:  transcript:Zm00001d013287_T001  rna10603     1.00E-71
680-  3:  transcript:Zm00001d013288_T003  rna10598    3.00E-175
680-  4:  transcript:Zm00001d013289_T001  rna10597    2.00E-155
680-  5:  transcript:Zm00001d013290_T004  rna10591    2.00E-141
680-  6:  transcript:Zm00001d013296_T001  rna10584            0
680-  7:  transcript:Zm00001d013300_T002  rna10581     9.00E-94
680-  8:  transcript:Zm00001d013302_T002  rna10579     8.00E-97
680-  9:  transcript:Zm00001d013303_T001  rna10575     2.00E-30
680- 10:  transcript:Zm00001d013306_T001  rna10574    3.00E-105
680- 11:  transcript:Zm00001d013307_T003  rna10572            0
680- 12:  transcript:Zm00001d013310_T001  rna10564            0
680- 13:  transcript:Zm00001d013311_T001  rna10563     9.00E-49
680- 14:  transcript:Zm00001d013314_T005  rna10552            0
680- 15:  transcript:Zm00001d013317_T001  rna10548     5.00E-92
680- 16:  transcript:Zm00001d013319_T001  rna10541            0
680- 17:  transcript:Zm00001d013329_T001  rna10535     1.00E-89
680- 18:  transcript:Zm00001d013330_T001  rna10533            0
680- 19:  transcript:Zm00001d013331_T001  rna10531    8.00E-136
680- 20:  transcript:Zm00001d013333_T001  rna10528     1.00E-21
680- 21:  transcript:Zm00001d013335_T001  rna10521    1.00E-108
680- 22:  transcript:Zm00001d013336_T002  rna10519            0
680- 23:  transcript:Zm00001d013339_T001  rna10516     9.00E-26
680- 24:  transcript:Zm00001d013340_T001  rna10509    4.00E-162
680- 25:  transcript:Zm00001d013343_T001  rna10499            0
680- 26:  transcript:Zm00001d013344_T002  rna10498    3.00E-120
680- 27:  transcript:Zm00001d013346_T001  rna10496            0
680- 28:  transcript:Zm00001d013348_T001  rna10493    9.00E-128
680- 29:  transcript:Zm00001d013357_T001  rna10490    4.00E-142
680- 30:  transcript:Zm00001d013358_T005  rna10489            0
680- 31:  transcript:Zm00001d013359_T002  rna10487     9.00E-76
680- 32:  transcript:Zm00001d013360_T002  rna10484    1.00E-126
680- 33:  transcript:Zm00001d013361_T001  rna10477    1.00E-168
680- 34:  transcript:Zm00001d013362_T001  rna10474    2.00E-158
680- 35:  transcript:Zm00001d013364_T007  rna10473            0
680- 36:  transcript:Zm00001d013367_T001  rna10471            0
680- 37:  transcript:Zm00001d013370_T001  rna10470    1.00E-151
680- 38:  transcript:Zm00001d013375_T003  rna10468    2.00E-170
680- 39:  transcript:Zm00001d013378_T001  rna10461     1.00E-96
## Alignment 681: score=1177.0 e_value=3e-80 N=26 5&NC_008396.2 minus
681-  0:  transcript:Zm00001d012922_T003  rna11159            0
```

681- 1: transcript:Zm00001d012924_T001 rna11153 0
681- 2: transcript:Zm00001d012925_T001 rna11152 0
681- 3: transcript:Zm00001d012929_T002 rna11146 7.00E-161
681- 4: transcript:Zm00001d012930_T001 rna11142 5.00E-40
681- 5: transcript:Zm00001d012931_T001 rna11141 0
681- 6: transcript:Zm00001d012932_T001 rna11134 8.00E-161
681- 7: transcript:Zm00001d012933_T001 rna11132 6.00E-32
681- 8: transcript:Zm00001d012934_T001 rna11131 2.00E-50
681- 9: transcript:Zm00001d012935_T004 rna11130 0
681- 10: transcript:Zm00001d012939_T001 rna11127 1.00E-35
681- 11: transcript:Zm00001d012955_T001 rna11122 9.00E-164
681- 12: transcript:Zm00001d012956_T001 rna11121 2.00E-27
681- 13: transcript:Zm00001d012957_T002 rna11117 2.00E-167
681- 14: transcript:Zm00001d012958_T001 rna11116 0
681- 15: transcript:Zm00001d012960_T001 rna11115 1.00E-22
681- 16: transcript:Zm00001d012961_T001 rna11114 0
681- 17: transcript:Zm00001d012962_T001 rna11110 0
681- 18: transcript:Zm00001d012963_T001 rna11108 2.00E-111
681- 19: transcript:Zm00001d012964_T016 rna11106 0
681- 20: transcript:Zm00001d012966_T001 rna11103 9.00E-51
681- 21: transcript:Zm00001d012967_T001 rna11101 6.00E-32
681- 22: transcript:Zm00001d012969_T008 rna11100 0
681- 23: transcript:Zm00001d012970_T001 rna11098 4.00E-141
681- 24: transcript:Zm00001d012972_T001 rna11097 5.00E-116
681- 25: transcript:Zm00001d012973_T001 rna11096 3.00E-86
Alignment 682: score=895.0 e_value=1.2e-58 N=20 5&NC_008396.2 minus
682- 0: transcript:Zm00001d013406_T002 rna10428 0
682- 1: transcript:Zm00001d013409_T001 rna10422 3.00E-86
682- 2: transcript:Zm00001d013410_T002 rna10419 0
682- 3: transcript:Zm00001d013411_T003 rna10417 0
682- 4: transcript:Zm00001d013412_T003 rna10416 5.00E-122
682- 5: transcript:Zm00001d013415_T001 rna10413 0
682- 6: transcript:Zm00001d013416_T001 rna10411 0
682- 7: transcript:Zm00001d013421_T002 rna10408 0
682- 8: transcript:Zm00001d013424_T001 rna10402 0
682- 9: transcript:Zm00001d013425_T001 rna10401 9.00E-45
682- 10: transcript:Zm00001d013426_T002 rna10400 3.00E-63
682- 11: transcript:Zm00001d013427_T003 rna10399 4.00E-75
682- 12: transcript:Zm00001d013428_T002 rna10396 0
682- 13: transcript:Zm00001d013430_T001 rna10394 8.00E-107
682- 14: transcript:Zm00001d013431_T002 rna10393 1.00E-95
682- 15: transcript:Zm00001d013432_T001 rna10392 0
682- 16: transcript:Zm00001d013439_T007 rna10390 0
682- 17: transcript:Zm00001d013440_T001 rna10387 6.00E-16
682- 18: transcript:Zm00001d013441_T003 rna10385 0
682- 19: transcript:Zm00001d013442_T041 rna10383 0
Alignment 683: score=717.0 e_value=1.2e-41 N=16 5&NC_008396.2 minus
683- 0: transcript:Zm00001d012875_T001 rna11244 2.00E-53
683- 1: transcript:Zm00001d012881_T001 rna11240 0
683- 2: transcript:Zm00001d012882_T001 rna11239 1.00E-174
683- 3: transcript:Zm00001d012883_T001 rna11234 5.00E-82
683- 4: transcript:Zm00001d012884_T003 rna11233 0
683- 5: transcript:Zm00001d012885_T001 rna11229 6.00E-44
683- 6: transcript:Zm00001d012887_T001 rna11218 8.00E-126

683- 7: transcript:Zm00001d012888_T002 rna11216   2.00E-37
683- 8: transcript:Zm00001d012889_T001 rna11214   2.00E-31
683- 9: transcript:Zm00001d012892_T002 rna11208   0
683- 10: transcript:Zm00001d012893_T002 rna11203   0
683- 11: transcript:Zm00001d012895_T003 rna11182   0
683- 12: transcript:Zm00001d012896_T010 rna11179   6.00E-100
683- 13: transcript:Zm00001d012898_T001 rna11176   0
683- 14: transcript:Zm00001d012906_T001 rna11169   2.00E-34
683- 15: transcript:Zm00001d012907_T001 rna11166   3.00E-54
Alignment 684: score=625.0 e_value=5.1e-34 N=14 5&NC_008396.2 minus
684- 0: transcript:Zm00001d013381_T001 rna10454   2.00E-11
684- 1: transcript:Zm00001d013383_T001 rna10453   0
684- 2: transcript:Zm00001d013384_T001 rna10452   1.00E-52
684- 3: transcript:Zm00001d013385_T001 rna10451   0
684- 4: transcript:Zm00001d013389_T014 rna10448   2.00E-170
684- 5: transcript:Zm00001d013390_T001 rna10447   0
684- 6: transcript:Zm00001d013391_T004 rna10444   0
684- 7: transcript:Zm00001d013392_T001 rna10442   6.00E-84
684- 8: transcript:Zm00001d013397_T002 rna10440   2.00E-111
684- 9: transcript:Zm00001d013398_T001 rna10439   0
684- 10: transcript:Zm00001d013399_T001 rna10436   1.00E-123
684- 11: transcript:Zm00001d013400_T001 rna10432   0
684- 12: transcript:Zm00001d013402_T025 rna10431   0
684- 13: transcript:Zm00001d013405_T004 rna10429   0
Alignment 685: score=463.0 e_value=7.1e-24 N=10 5&NC_008396.2 minus
685- 0: transcript:Zm00001d013809_T002 rna9773   0
685- 1: transcript:Zm00001d013812_T001 rna9768   0
685- 2: transcript:Zm00001d013814_T001 rna9767   0
685- 3: transcript:Zm00001d013816_T001 rna9763   0
685- 4: transcript:Zm00001d013817_T001 rna9762   4.00E-62
685- 5: transcript:Zm00001d013820_T001 rna9758   8.00E-22
685- 6: transcript:Zm00001d013821_T003 rna9755   1.00E-47
685- 7: transcript:Zm00001d013823_T001 rna9741   0
685- 8: transcript:Zm00001d013824_T001 rna9739   3.00E-166
685- 9: transcript:Zm00001d013825_T001 rna9731   2.00E-43
Alignment 686: score=1884.0 e_value=2.7e-152 N=43 5&NC_008397.2 plus
686- 0: transcript:Zm00001d017364_T001 rna13018   2.00E-59
686- 1: transcript:Zm00001d017366_T001 rna13019   3.00E-86
686- 2: transcript:Zm00001d017371_T008 rna13020   2.00E-76
686- 3: transcript:Zm00001d017373_T001 rna13021   0
686- 4: transcript:Zm00001d017377_T001 rna13026   7.00E-61
686- 5: transcript:Zm00001d017378_T001 rna13031   1.00E-78
686- 6: transcript:Zm00001d017379_T001 rna13032   7.00E-74
686- 7: transcript:Zm00001d017381_T001 rna13040   6.00E-28
686- 8: transcript:Zm00001d017382_T001 rna13047   5.00E-99
686- 9: transcript:Zm00001d017383_T001 rna13049   4.00E-115
686- 10: transcript:Zm00001d017384_T008 rna13050   0
686- 11: transcript:Zm00001d017386_T001 rna13051   9.00E-62
686- 12: transcript:Zm00001d017391_T002 rna13056   7.00E-106
686- 13: transcript:Zm00001d017397_T001 rna13067   4.00E-53
686- 14: transcript:Zm00001d017399_T001 rna13069   0
686- 15: transcript:Zm00001d017401_T001 rna13070   0
686- 16: transcript:Zm00001d017402_T001 rna13073   2.00E-66
686- 17: transcript:Zm00001d017403_T001 rna13074   0

686- 18: transcript:Zm00001d017409_T001 rna13080 3.00E-51
686- 19: transcript:Zm00001d017412_T001 rna13083 1.00E-63
686- 20: transcript:Zm00001d017418_T001 rna13087 0
686- 21: transcript:Zm00001d017419_T001 rna13088 6.00E-169
686- 22: transcript:Zm00001d017420_T001 rna13089 8.00E-122
686- 23: transcript:Zm00001d017422_T001 rna13091 2.00E-68
686- 24: transcript:Zm00001d017427_T002 rna13095 0
686- 25: transcript:Zm00001d017441_T001 rna13109 2.00E-87
686- 26: transcript:Zm00001d017444_T001 rna13113 3.00E-71
686- 27: transcript:Zm00001d017445_T001 rna13114 3.00E-151
686- 28: transcript:Zm00001d017455_T001 rna13118 1.00E-32
686- 29: transcript:Zm00001d017460_T003 rna13123 1.00E-55
686- 30: transcript:Zm00001d017462_T001 rna13125 2.00E-94
686- 31: transcript:Zm00001d017466_T001 rna13127 2.00E-84
686- 32: transcript:Zm00001d017468_T001 rna13129 1.00E-86
686- 33: transcript:Zm00001d017473_T002 rna13132 6.00E-74
686- 34: transcript:Zm00001d017476_T001 rna13138 0
686- 35: transcript:Zm00001d017477_T001 rna13139 1.00E-61
686- 36: transcript:Zm00001d017482_T002 rna13145 3.00E-66
686- 37: transcript:Zm00001d017485_T003 rna13146 5.00E-60
686- 38: transcript:Zm00001d017486_T001 rna13151 6.00E-30
686- 39: transcript:Zm00001d017493_T001 rna13154 2.00E-136
686- 40: transcript:Zm00001d017497_T001 rna13157 6.00E-06
686- 41: transcript:Zm00001d017499_T001 rna13161 7.00E-08
686- 42: transcript:Zm00001d017505_T003 rna13165 1.00E-70
Alignment 687: score=1127.0 e_value=1.4e-82 N=26 5&NC_008397.2 plus
687- 0: transcript:Zm00001d017570_T001 rna13246 0
687- 1: transcript:Zm00001d017574_T001 rna13247 3.00E-102
687- 2: transcript:Zm00001d017582_T001 rna13262 0
687- 3: transcript:Zm00001d017583_T002 rna13267 5.00E-135
687- 4: transcript:Zm00001d017590_T001 rna13270 0
687- 5: transcript:Zm00001d017592_T001 rna13274 8.00E-95
687- 6: transcript:Zm00001d017595_T002 rna13276 7.00E-14
687- 7: transcript:Zm00001d017597_T001 rna13277 0
687- 8: transcript:Zm00001d017598_T001 rna13278 0
687- 9: transcript:Zm00001d017602_T002 rna13285 3.00E-30
687- 10: transcript:Zm00001d017604_T001 rna13296 0
687- 11: transcript:Zm00001d017606_T001 rna13300 1.00E-30
687- 12: transcript:Zm00001d017611_T001 rna13307 0
687- 13: transcript:Zm00001d017612_T001 rna13309 1.00E-55
687- 14: transcript:Zm00001d017614_T003 rna13314 1.00E-80
687- 15: transcript:Zm00001d017615_T001 rna13315 2.00E-37
687- 16: transcript:Zm00001d017617_T001 rna13318 4.00E-42
687- 17: transcript:Zm00001d017618_T001 rna13319 4.00E-76
687- 18: transcript:Zm00001d017622_T001 rna13331 3.00E-33
687- 19: transcript:Zm00001d017641_T004 rna13334 0
687- 20: transcript:Zm00001d017642_T001 rna13336 6.00E-90
687- 21: transcript:Zm00001d017643_T001 rna13339 1.00E-68
687- 22: transcript:Zm00001d017645_T001 rna13345 1.00E-18
687- 23: transcript:Zm00001d017646_T001 rna13351 2.00E-22
687- 24: transcript:Zm00001d017653_T001 rna13358 2.00E-89
687- 25: transcript:Zm00001d017657_T001 rna13365 4.00E-44
Alignment 688: score=843.0 e_value=3.8e-66 N=21 5&NC_008397.2 plus
688- 0: transcript:Zm00001d016856_T009 rna12476 0

688- 1: transcript:Zm00001d016861_T003 rna12482   1.00E-122
688- 2: transcript:Zm00001d016876_T001 rna12490   4.00E-100
688- 3: transcript:Zm00001d016890_T001 rna12509   2.00E-126
688- 4: transcript:Zm00001d016894_T005 rna12519   2.00E-35
688- 5: transcript:Zm00001d016895_T001 rna12521   2.00E-129
688- 6: transcript:Zm00001d016897_T002 rna12522   0
688- 7: transcript:Zm00001d016899_T001 rna12523   3.00E-10
688- 8: transcript:Zm00001d016908_T001 rna12529   2.00E-37
688- 9: transcript:Zm00001d016909_T001 rna12531   1.00E-67
688- 10: transcript:Zm00001d016910_T001 rna12534   2.00E-93
688- 11: transcript:Zm00001d016915_T002 rna12537   0
688- 12: transcript:Zm00001d016918_T001 rna12538   0
688- 13: transcript:Zm00001d016919_T001 rna12542   3.00E-178
688- 14: transcript:Zm00001d016928_T005 rna12566   0
688- 15: transcript:Zm00001d016933_T001 rna12568   0
688- 16: transcript:Zm00001d016934_T001 rna12569   3.00E-175
688- 17: transcript:Zm00001d016935_T001 rna12579   3.00E-105
688- 18: transcript:Zm00001d016950_T002 rna12586   1.00E-110
688- 19: transcript:Zm00001d016952_T001 rna12589   1.00E-125
688- 20: transcript:Zm00001d016957_T001 rna12591   7.00E-55
Alignment 689: score=615.0 e_value=1.6e-33 N=14 5&NC_008397.2 plus
689- 0: transcript:Zm00001d017256_T001 rna12929   9.00E-24
689- 1: transcript:Zm00001d017261_T006 rna12940   0
689- 2: transcript:Zm00001d017263_T001 rna12945   3.00E-87
689- 3: transcript:Zm00001d017268_T001 rna12951   2.00E-106
689- 4: transcript:Zm00001d017270_T001 rna12954   3.00E-119
689- 5: transcript:Zm00001d017271_T005 rna12957   0
689- 6: transcript:Zm00001d017274_T001 rna12958   0
689- 7: transcript:Zm00001d017275_T001 rna12960   0
689- 8: transcript:Zm00001d017284_T001 rna12965   5.00E-166
689- 9: transcript:Zm00001d017287_T001 rna12968   5.00E-30
689- 10: transcript:Zm00001d017288_T001 rna12971   0
689- 11: transcript:Zm00001d017294_T001 rna12977   5.00E-156
689- 12: transcript:Zm00001d017297_T001 rna12978   2.00E-18
689- 13: transcript:Zm00001d017298_T003 rna12981   2.00E-111
Alignment 690: score=603.0 e_value=4.9e-33 N=14 5&NC_008397.2 plus
690- 0: transcript:Zm00001d017523_T001 rna13184   2.00E-18
690- 1: transcript:Zm00001d017524_T001 rna13192   0
690- 2: transcript:Zm00001d017526_T001 rna13198   4.00E-38
690- 3: transcript:Zm00001d017528_T001 rna13200   0
690- 4: transcript:Zm00001d017539_T002 rna13206   0
690- 5: transcript:Zm00001d017545_T001 rna13207   4.00E-98
690- 6: transcript:Zm00001d017550_T009 rna13212   1.00E-100
690- 7: transcript:Zm00001d017551_T001 rna13214   1.00E-74
690- 8: transcript:Zm00001d017553_T001 rna13215   0
690- 9: transcript:Zm00001d017555_T001 rna13229   3.00E-11
690- 10: transcript:Zm00001d017557_T001 rna13235   0
690- 11: transcript:Zm00001d017559_T001 rna13236   7.00E-61
690- 12: transcript:Zm00001d017560_T002 rna13240   9.00E-165
690- 13: transcript:Zm00001d017563_T002 rna13242   2.00E-43
Alignment 691: score=474.0 e_value=1.5e-27 N=12 5&NC_008397.2 plus
691- 0: transcript:Zm00001d017166_T001 rna12822   0
691- 1: transcript:Zm00001d017175_T003 rna12828   8.00E-176
691- 2: transcript:Zm00001d017176_T001 rna12829   6.00E-118

691- 3: transcript:Zm00001d017186_T001 rna12848   0
691- 4: transcript:Zm00001d017195_T001 rna12853   0
691- 5: transcript:Zm00001d017197_T015 rna12854   0
691- 6: transcript:Zm00001d017201_T001 rna12862   0
691- 7: transcript:Zm00001d017203_T021 rna12865   0
691- 8: transcript:Zm00001d017204_T003 rna12869   4.00E-113
691- 9: transcript:Zm00001d017207_T001 rna12871   2.00E-15
691- 10: transcript:Zm00001d017209_T001 rna12875   5.00E-149
691- 11: transcript:Zm00001d017213_T004 rna12885   0
Alignment 692: score=421.0 e_value=4.3e-20 N=10 5&NC_008397.2 plus
692- 0: transcript:Zm00001d017117_T001 rna12744   0
692- 1: transcript:Zm00001d017119_T003 rna12747   0
692- 2: transcript:Zm00001d017121_T001 rna12754   0
692- 3: transcript:Zm00001d017128_T002 rna12762   0
692- 4: transcript:Zm00001d017134_T001 rna12766   4.00E-114
692- 5: transcript:Zm00001d017135_T001 rna12770   3.00E-161
692- 6: transcript:Zm00001d017138_T001 rna12774   1.00E-70
692- 7: transcript:Zm00001d017144_T007 rna12783   7.00E-176
692- 8: transcript:Zm00001d017151_T002 rna12800   3.00E-78
692- 9: transcript:Zm00001d017152_T001 rna12801   1.00E-92
Alignment 693: score=384.0 e_value=8e-20 N=9 5&NC_008397.2 plus
693- 0: transcript:Zm00001d016760_T001 rna12363   6.00E-08
693- 1: transcript:Zm00001d016762_T002 rna12365   0
693- 2: transcript:Zm00001d016765_T001 rna12384   0
693- 3: transcript:Zm00001d016766_T002 rna12392   2.00E-33
693- 4: transcript:Zm00001d016783_T002 rna12398   0
693- 5: transcript:Zm00001d016786_T001 rna12404   0
693- 6: transcript:Zm00001d016787_T001 rna12405   4.00E-46
693- 7: transcript:Zm00001d016788_T001 rna12410   2.00E-16
693- 8: transcript:Zm00001d016792_T004 rna12411   2.00E-33
Alignment 694: score=374.0 e_value=2.3e-16 N=8 5&NC_008397.2 plus
694- 0: transcript:Zm00001d017071_T002 rna12691   3.00E-125
694- 1: transcript:Zm00001d017072_T002 rna12692   6.00E-119
694- 2: transcript:Zm00001d017079_T001 rna12693   0
694- 3: transcript:Zm00001d017084_T001 rna12695   5.00E-61
694- 4: transcript:Zm00001d017085_T002 rna12696   0
694- 5: transcript:Zm00001d017091_T001 rna12703   0
694- 6: transcript:Zm00001d017094_T001 rna12709   3.00E-131
694- 7: transcript:Zm00001d017095_T001 rna12710   7.00E-79
Alignment 695: score=363.0 e_value=2.1e-14 N=8 5&NC_008397.2 plus
695- 0: transcript:Zm00001d017671_T001 rna13374   0
695- 1: transcript:Zm00001d017677_T001 rna13381   5.00E-13
695- 2: transcript:Zm00001d017678_T001 rna13382   9.00E-137
695- 3: transcript:Zm00001d017682_T001 rna13385   3.00E-124
695- 4: transcript:Zm00001d017684_T001 rna13386   3.00E-34
695- 5: transcript:Zm00001d017689_T002 rna13392   9.00E-138
695- 6: transcript:Zm00001d017692_T002 rna13397   9.00E-41
695- 7: transcript:Zm00001d017698_T003 rna13404   0
Alignment 696: score=333.0 e_value=1.1e-15 N=8 5&NC_008397.2 plus
696- 0: transcript:Zm00001d016549_T001 rna12112   1.00E-76
696- 1: transcript:Zm00001d016550_T002 rna12115   5.00E-111
696- 2: transcript:Zm00001d016553_T001 rna12134   0
696- 3: transcript:Zm00001d016561_T001 rna12159   9.00E-27
696- 4: transcript:Zm00001d016567_T001 rna12168   1.00E-32

696- 5: transcript:Zm00001d016569_T001 rna12174 9.00E-21
696- 6: transcript:Zm00001d016577_T002 rna12180 5.00E-09
696- 7: transcript:Zm00001d016580_T001 rna12183 6.00E-33
Alignment 697: score=329.0 e_value=2.7e-13 N=7 5&NC_008397.2 plus
697- 0: transcript:Zm00001d016648_T001 rna12244 1.00E-68
697- 1: transcript:Zm00001d016655_T001 rna12252 1.00E-24
697- 2: transcript:Zm00001d016659_T001 rna12255 6.00E-53
697- 3: transcript:Zm00001d016662_T003 rna12256 0
697- 4: transcript:Zm00001d016664_T001 rna12257 3.00E-13
697- 5: transcript:Zm00001d016665_T001 rna12258 3.00E-125
697- 6: transcript:Zm00001d016669_T001 rna12261 1.00E-57
Alignment 698: score=325.0 e_value=1.6e-14 N=8 5&NC_008397.2 plus
698- 0: transcript:Zm00001d016977_T002 rna12601 1.00E-68
698- 1: transcript:Zm00001d016979_T001 rna12603 6.00E-152
698- 2: transcript:Zm00001d016982_T001 rna12616 0
698- 3: transcript:Zm00001d016992_T001 rna12632 1.00E-16
698- 4: transcript:Zm00001d016998_T001 rna12637 3.00E-21
698- 5: transcript:Zm00001d017008_T001 rna12641 8.00E-25
698- 6: transcript:Zm00001d017009_T001 rna12642 5.00E-19
698- 7: transcript:Zm00001d017026_T003 rna12649 2.00E-133
Alignment 699: score=316.0 e_value=1.3e-12 N=7 5&NC_008397.2 plus
699- 0: transcript:Zm00001d017240_T001 rna12897 1.00E-55
699- 1: transcript:Zm00001d017243_T001 rna12899 2.00E-91
699- 2: transcript:Zm00001d017246_T001 rna12900 0
699- 3: transcript:Zm00001d017247_T003 rna12901 0
699- 4: transcript:Zm00001d017248_T001 rna12907 1.00E-56
699- 5: transcript:Zm00001d017249_T001 rna12909 0
699- 6: transcript:Zm00001d017251_T003 rna12926 7.00E-65
Alignment 700: score=416.0 e_value=2.5e-18 N=9 5&NC_008397.2 minus
700- 0: transcript:Zm00001d016721_T001 rna12341 8.00E-82
700- 1: transcript:Zm00001d016730_T001 rna12329 9.00E-147
700- 2: transcript:Zm00001d016731_T001 rna12327 2.00E-67
700- 3: transcript:Zm00001d016733_T001 rna12326 5.00E-86
700- 4: transcript:Zm00001d016735_T001 rna12325 8.00E-155
700- 5: transcript:Zm00001d016736_T001 rna12324 4.00E-49
700- 6: transcript:Zm00001d016737_T002 rna12323 2.00E-127
700- 7: transcript:Zm00001d016743_T001 rna12314 2.00E-25
700- 8: transcript:Zm00001d016745_T001 rna12310 4.00E-49
Alignment 701: score=2276.0 e_value=2.9e-189 N=49 5&NC_008399.2 plus
701- 0: transcript:Zm00001d014742_T001 rna18794 7.00E-176
701- 1: transcript:Zm00001d014744_T001 rna18796 8.00E-113
701- 2: transcript:Zm00001d014745_T001 rna18797 1.00E-84
701- 3: transcript:Zm00001d014748_T001 rna18798 0
701- 4: transcript:Zm00001d014751_T003 rna18799 0
701- 5: transcript:Zm00001d014752_T001 rna18800 7.00E-90
701- 6: transcript:Zm00001d014753_T002 rna18802 6.00E-94
701- 7: transcript:Zm00001d014755_T001 rna18805 3.00E-123
701- 8: transcript:Zm00001d014757_T001 rna18806 8.00E-87
701- 9: transcript:Zm00001d014758_T001 rna18807 1.00E-34
701- 10: transcript:Zm00001d014760_T002 rna18809 0
701- 11: transcript:Zm00001d014761_T001 rna18810 0
701- 12: transcript:Zm00001d014762_T001 rna18812 5.00E-139
701- 13: transcript:Zm00001d014763_T001 rna18816 0
701- 14: transcript:Zm00001d014765_T001 rna18817 4.00E-80

```
701- 15:  transcript:Zm00001d014770_T001  rna18823     1.00E-36
701- 16:  transcript:Zm00001d014771_T001  rna18824            0
701- 17:  transcript:Zm00001d014772_T001  rna18825     7.00E-75
701- 18:  transcript:Zm00001d014774_T001  rna18827     2.00E-38
701- 19:  transcript:Zm00001d014775_T002  rna18829            0
701- 20:  transcript:Zm00001d014778_T001  rna18831     8.00E-15
701- 21:  transcript:Zm00001d014780_T001  rna18833    2.00E-110
701- 22:  transcript:Zm00001d014781_T001  rna18837     8.00E-52
701- 23:  transcript:Zm00001d014782_T001  rna18838            0
701- 24:  transcript:Zm00001d014783_T001  rna18839            0
701- 25:  transcript:Zm00001d014785_T001  rna18840    2.00E-145
701- 26:  transcript:Zm00001d014786_T001  rna18841            0
701- 27:  transcript:Zm00001d014788_T002  rna18842            0
701- 28:  transcript:Zm00001d014789_T002  rna18843    7.00E-136
701- 29:  transcript:Zm00001d014790_T001  rna18844     2.00E-41
701- 30:  transcript:Zm00001d014791_T001  rna18845    2.00E-103
701- 31:  transcript:Zm00001d014792_T003  rna18846            0
701- 32:  transcript:Zm00001d014793_T001  rna18849            0
701- 33:  transcript:Zm00001d014796_T001  rna18852    2.00E-140
701- 34:  transcript:Zm00001d014797_T006  rna18854            0
701- 35:  transcript:Zm00001d014804_T001  rna18855    3.00E-100
701- 36:  transcript:Zm00001d014807_T001  rna18867     2.00E-37
701- 37:  transcript:Zm00001d014808_T001  rna18872    1.00E-161
701- 38:  transcript:Zm00001d014809_T003  rna18874            0
701- 39:  transcript:Zm00001d014811_T001  rna18875    8.00E-116
701- 40:  transcript:Zm00001d014812_T005  rna18877     2.00E-78
701- 41:  transcript:Zm00001d014813_T024  rna18878            0
701- 42:  transcript:Zm00001d014816_T001  rna18881     6.00E-71
701- 43:  transcript:Zm00001d014817_T001  rna18882    3.00E-168
701- 44:  transcript:Zm00001d014826_T001  rna18884     2.00E-73
701- 45:  transcript:Zm00001d014834_T001  rna18886     1.00E-46
701- 46:  transcript:Zm00001d014840_T001  rna18889     3.00E-78
701- 47:  transcript:Zm00001d014843_T001  rna18891    4.00E-120
701- 48:  transcript:Zm00001d014844_T007  rna18892            0
## Alignment 702: score=833.0 e_value=1.1e-51 N=18 5&NC_008399.2 plus
702-  0:  transcript:Zm00001d014641_T001  rna18710     2.00E-60
702-  1:  transcript:Zm00001d014642_T001  rna18711            0
702-  2:  transcript:Zm00001d014648_T001  rna18714     1.00E-34
702-  3:  transcript:Zm00001d014655_T001  rna18717            0
702-  4:  transcript:Zm00001d014656_T003  rna18718     3.00E-21
702-  5:  transcript:Zm00001d014658_T001  rna18721            0
702-  6:  transcript:Zm00001d014659_T001  rna18722     3.00E-70
702-  7:  transcript:Zm00001d014664_T001  rna18723    9.00E-121
702-  8:  transcript:Zm00001d014665_T002  rna18726            0
702-  9:  transcript:Zm00001d014666_T001  rna18727            0
702- 10:  transcript:Zm00001d014667_T001  rna18728     3.00E-61
702- 11:  transcript:Zm00001d014668_T001  rna18729            0
702- 12:  transcript:Zm00001d014669_T001  rna18730            0
702- 13:  transcript:Zm00001d014673_T001  rna18732            0
702- 14:  transcript:Zm00001d014674_T003  rna18733            0
702- 15:  transcript:Zm00001d014679_T001  rna18735     3.00E-35
702- 16:  transcript:Zm00001d014680_T001  rna18736            0
702- 17:  transcript:Zm00001d014682_T001  rna18738     6.00E-16
## Alignment 703: score=763.0 e_value=1.3e-48 N=17 5&NC_008399.2 plus
```

703- 0: transcript:Zm00001d014451_T001 rna18606 0
703- 1: transcript:Zm00001d014459_T001 rna18607 0
703- 2: transcript:Zm00001d014463_T001 rna18611 8.00E-61
703- 3: transcript:Zm00001d014464_T001 rna18613 3.00E-13
703- 4: transcript:Zm00001d014467_T002 rna18614 2.00E-172
703- 5: transcript:Zm00001d014468_T001 rna18615 0
703- 6: transcript:Zm00001d014485_T001 rna18621 2.00E-135
703- 7: transcript:Zm00001d014488_T001 rna18623 2.00E-116
703- 8: transcript:Zm00001d014489_T010 rna18624 4.00E-164
703- 9: transcript:Zm00001d014491_T001 rna18625 2.00E-66
703- 10: transcript:Zm00001d014492_T001 rna18626 1.00E-78
703- 11: transcript:Zm00001d014493_T001 rna18627 1.00E-40
703- 12: transcript:Zm00001d014494_T001 rna18628 1.00E-13
703- 13: transcript:Zm00001d014495_T001 rna18629 8.00E-143
703- 14: transcript:Zm00001d014496_T001 rna18630 0
703- 15: transcript:Zm00001d014499_T004 rna18635 4.00E-17
703- 16: transcript:Zm00001d014507_T001 rna18637 0
Alignment 704: score=752.0 e_value=1.8e-46 N=16 5&NC_008399.2 plus
704- 0: transcript:Zm00001d014575_T002 rna18653 0
704- 1: transcript:Zm00001d014583_T001 rna18674 0
704- 2: transcript:Zm00001d014585_T002 rna18677 0
704- 3: transcript:Zm00001d014587_T001 rna18678 0
704- 4: transcript:Zm00001d014594_T001 rna18680 0
704- 5: transcript:Zm00001d014595_T001 rna18681 0
704- 6: transcript:Zm00001d014596_T001 rna18682 0
704- 7: transcript:Zm00001d014597_T001 rna18684 4.00E-83
704- 8: transcript:Zm00001d014599_T001 rna18686 0
704- 9: transcript:Zm00001d014600_T001 rna18687 3.00E-81
704- 10: transcript:Zm00001d014601_T001 rna18689 2.00E-155
704- 11: transcript:Zm00001d014609_T002 rna18693 0
704- 12: transcript:Zm00001d014610_T001 rna18694 5.00E-42
704- 13: transcript:Zm00001d014611_T001 rna18695 3.00E-116
704- 14: transcript:Zm00001d014612_T001 rna18696 0
704- 15: transcript:Zm00001d014613_T001 rna18697 1.00E-167
Alignment 705: score=682.0 e_value=1.9e-42 N=16 5&NC_008399.2 plus
705- 0: transcript:Zm00001d016216_T001 rna18005 6.00E-18
705- 1: transcript:Zm00001d016223_T001 rna18007 5.00E-165
705- 2: transcript:Zm00001d016231_T001 rna18008 1.00E-25
705- 3: transcript:Zm00001d016234_T001 rna18009 0
705- 4: transcript:Zm00001d016253_T001 rna18010 3.00E-86
705- 5: transcript:Zm00001d016255_T001 rna18011 1.00E-29
705- 6: transcript:Zm00001d016256_T001 rna18012 7.00E-39
705- 7: transcript:Zm00001d016269_T001 rna18014 5.00E-37
705- 8: transcript:Zm00001d016271_T001 rna18016 1.00E-38
705- 9: transcript:Zm00001d016273_T001 rna18017 8.00E-166
705- 10: transcript:Zm00001d016276_T001 rna18020 3.00E-73
705- 11: transcript:Zm00001d016285_T001 rna18027 0
705- 12: transcript:Zm00001d016287_T001 rna18034 3.00E-18
705- 13: transcript:Zm00001d016294_T001 rna18044 9.00E-101
705- 14: transcript:Zm00001d016298_T001 rna18045 3.00E-88
705- 15: transcript:Zm00001d016308_T003 rna18048 0
Alignment 706: score=629.0 e_value=3.9e-39 N=14 5&NC_008399.2 plus
706- 0: transcript:Zm00001d014689_T006 rna18743 0
706- 1: transcript:Zm00001d014690_T014 rna18744 0

706- 2: transcript:Zm00001d014692_T001 rna18745 3.00E-99
706- 3: transcript:Zm00001d014696_T002 rna18746 2.00E-177
706- 4: transcript:Zm00001d014697_T002 rna18748 1.00E-123
706- 5: transcript:Zm00001d014698_T006 rna18752 2.00E-153
706- 6: transcript:Zm00001d014701_T002 rna18756 0
706- 7: transcript:Zm00001d014703_T001 rna18758 0
706- 8: transcript:Zm00001d014704_T002 rna18759 7.00E-33
706- 9: transcript:Zm00001d014705_T002 rna18760 0
706- 10: transcript:Zm00001d014715_T002 rna18763 0
706- 11: transcript:Zm00001d014716_T002 rna18764 0
706- 12: transcript:Zm00001d014717_T001 rna18767 6.00E-93
706- 13: transcript:Zm00001d014718_T001 rna18768 4.00E-99
Alignment 707: score=555.0 e_value=3.9e-28 N=12 5&NC_008399.2 plus
707- 0: transcript:Zm00001d014845_T001 rna18895 0
707- 1: transcript:Zm00001d014846_T001 rna18896 4.00E-36
707- 2: transcript:Zm00001d014848_T001 rna18897 0
707- 3: transcript:Zm00001d014849_T003 rna18898 0
707- 4: transcript:Zm00001d014850_T002 rna18899 0
707- 5: transcript:Zm00001d014858_T001 rna18902 6.00E-71
707- 6: transcript:Zm00001d014861_T001 rna18906 3.00E-93
707- 7: transcript:Zm00001d014862_T002 rna18907 0
707- 8: transcript:Zm00001d014863_T002 rna18908 4.00E-160
707- 9: transcript:Zm00001d014865_T001 rna18909 2.00E-145
707- 10: transcript:Zm00001d014867_T001 rna18910 3.00E-50
707- 11: transcript:Zm00001d014875_T001 rna18912 0
Alignment 708: score=503.0 e_value=4.1e-29 N=12 5&NC_008399.2 plus
708- 0: transcript:Zm00001d015759_T001 rna18206 2.00E-47
708- 1: transcript:Zm00001d015767_T002 rna18212 4.00E-24
708- 2: transcript:Zm00001d015778_T001 rna18217 4.00E-62
708- 3: transcript:Zm00001d015780_T002 rna18221 6.00E-114
708- 4: transcript:Zm00001d015783_T001 rna18223 0
708- 5: transcript:Zm00001d015785_T005 rna18225 1.00E-34
708- 6: transcript:Zm00001d015788_T001 rna18229 0
708- 7: transcript:Zm00001d015789_T002 rna18230 0
708- 8: transcript:Zm00001d015798_T001 rna18241 8.00E-50
708- 9: transcript:Zm00001d015804_T013 rna18242 1.00E-27
708- 10: transcript:Zm00001d015810_T001 rna18245 2.00E-98
708- 11: transcript:Zm00001d015820_T002 rna18260 3.00E-50
Alignment 709: score=405.0 e_value=5e-20 N=10 5&NC_008399.2 plus
709- 0: transcript:Zm00001d016075_T001 rna17908 0
709- 1: transcript:Zm00001d016076_T001 rna17909 1.00E-34
709- 2: transcript:Zm00001d016081_T002 rna17913 0
709- 3: transcript:Zm00001d016083_T001 rna17919 2.00E-10
709- 4: transcript:Zm00001d016095_T001 rna17920 2.00E-99
709- 5: transcript:Zm00001d016105_T001 rna17922 1.00E-14
709- 6: transcript:Zm00001d016106_T001 rna17925 1.00E-73
709- 7: transcript:Zm00001d016119_T001 rna17940 1.00E-110
709- 8: transcript:Zm00001d016130_T001 rna17946 4.00E-157
709- 9: transcript:Zm00001d016137_T001 rna17950 4.00E-21
Alignment 710: score=397.0 e_value=9.1e-20 N=9 5&NC_008399.2 plus
710- 0: transcript:Zm00001d014721_T001 rna18778 0
710- 1: transcript:Zm00001d014722_T001 rna18780 0
710- 2: transcript:Zm00001d014726_T003 rna18785 0
710- 3: transcript:Zm00001d014727_T001 rna18786 0

710- 4: transcript:Zm00001d014728_T006 rna18787 1.00E-96
710- 5: transcript:Zm00001d014729_T001 rna18788 2.00E-35
710- 6: transcript:Zm00001d014731_T017 rna18789 3.00E-56
710- 7: transcript:Zm00001d014733_T001 rna18790 1.00E-58
710- 8: transcript:Zm00001d014735_T001 rna18792 2.00E-47
Alignment 711: score=258.0 e_value=3.7e-09 N=6 5&NC_008399.2 plus
711- 0: transcript:Zm00001d016342_T001 rna18051 2.00E-12
711- 1: transcript:Zm00001d016349_T002 rna18061 3.00E-90
711- 2: transcript:Zm00001d016354_T001 rna18079 4.00E-98
711- 3: transcript:Zm00001d016358_T006 rna18083 0
711- 4: transcript:Zm00001d016361_T001 rna18084 2.00E-47
711- 5: transcript:Zm00001d016364_T001 rna18086 5.00E-121
Alignment 712: score=1030.0 e_value=7.1e-76 N=24 5&NC_008399.2 minus
712- 0: transcript:Zm00001d017949_T001 rna17385 0
712- 1: transcript:Zm00001d017951_T001 rna17377 9.00E-73
712- 2: transcript:Zm00001d017952_T001 rna17371 5.00E-54
712- 3: transcript:Zm00001d017953_T002 rna17370 8.00E-36
712- 4: transcript:Zm00001d017955_T001 rna17369 1.00E-91
712- 5: transcript:Zm00001d017961_T003 rna17365 0
712- 6: transcript:Zm00001d017965_T001 rna17363 0
712- 7: transcript:Zm00001d017969_T001 rna17359 9.00E-07
712- 8: transcript:Zm00001d017976_T001 rna17357 0
712- 9: transcript:Zm00001d017978_T001 rna17354 0
712- 10: transcript:Zm00001d017979_T002 rna17353 0
712- 11: transcript:Zm00001d017984_T001 rna17350 6.00E-48
712- 12: transcript:Zm00001d017985_T002 rna17347 0
712- 13: transcript:Zm00001d017986_T005 rna17345 3.00E-168
712- 14: transcript:Zm00001d017987_T003 rna17341 0
712- 15: transcript:Zm00001d017991_T001 rna17322 2.00E-20
712- 16: transcript:Zm00001d017992_T001 rna17321 3.00E-115
712- 17: transcript:Zm00001d017994_T001 rna17318 6.00E-110
712- 18: transcript:Zm00001d017995_T003 rna17314 0
712- 19: transcript:Zm00001d017996_T001 rna17313 3.00E-161
712- 20: transcript:Zm00001d017998_T001 rna17305 3.00E-60
712- 21: transcript:Zm00001d018004_T002 rna17295 9.00E-128
712- 22: transcript:Zm00001d018005_T001 rna17294 0
712- 23: transcript:Zm00001d018006_T003 rna17293 1.00E-154
Alignment 713: score=869.0 e_value=1.7e-55 N=20 5&NC_008399.2 minus
713- 0: transcript:Zm00001d018056_T001 rna17256 2.00E-48
713- 1: transcript:Zm00001d018058_T001 rna17255 8.00E-94
713- 2: transcript:Zm00001d018061_T002 rna17251 5.00E-157
713- 3: transcript:Zm00001d018065_T001 rna17239 2.00E-58
713- 4: transcript:Zm00001d018072_T001 rna17236 1.00E-39
713- 5: transcript:Zm00001d018076_T001 rna17233 1.00E-25
713- 6: transcript:Zm00001d018078_T002 rna17230 0
713- 7: transcript:Zm00001d018081_T001 rna17228 6.00E-15
713- 8: transcript:Zm00001d018082_T001 rna17227 2.00E-149
713- 9: transcript:Zm00001d018090_T002 rna17225 0
713- 10: transcript:Zm00001d018097_T001 rna17223 8.00E-124
713- 11: transcript:Zm00001d018099_T001 rna17221 3.00E-111
713- 12: transcript:Zm00001d018105_T001 rna17216 2.00E-117
713- 13: transcript:Zm00001d018111_T002 rna17213 1.00E-171
713- 14: transcript:Zm00001d018112_T004 rna17212 1.00E-59
713- 15: transcript:Zm00001d018113_T006 rna17209 0

713- 16: transcript:Zm00001d018117_T001 rna17208 2.00E-06
713- 17: transcript:Zm00001d018118_T001 rna17207 4.00E-39
713- 18: transcript:Zm00001d018128_T001 rna17201 4.00E-94
713- 19: transcript:Zm00001d018130_T001 rna17200 5.00E-31
Alignment 714: score=722.0 e_value=1.3e-43 N=16 5&NC_008399.2 minus
714- 0: transcript:Zm00001d014358_T014 rna18605 0
714- 1: transcript:Zm00001d014360_T001 rna18604 5.00E-24
714- 2: transcript:Zm00001d014361_T001 rna18603 0
714- 3: transcript:Zm00001d014364_T002 rna18602 2.00E-144
714- 4: transcript:Zm00001d014366_T002 rna18598 0
714- 5: transcript:Zm00001d014367_T004 rna18594 0
714- 6: transcript:Zm00001d014368_T001 rna18593 7.00E-134
714- 7: transcript:Zm00001d014377_T002 rna18591 0
714- 8: transcript:Zm00001d014378_T001 rna18590 0
714- 9: transcript:Zm00001d014381_T001 rna18589 2.00E-44
714- 10: transcript:Zm00001d014382_T003 rna18588 0
714- 11: transcript:Zm00001d014383_T006 rna18587 0
714- 12: transcript:Zm00001d014385_T001 rna18586 0
714- 13: transcript:Zm00001d014386_T001 rna18585 0
714- 14: transcript:Zm00001d014405_T001 rna18575 1.00E-168
714- 15: transcript:Zm00001d014412_T001 rna18573 1.00E-134
Alignment 715: score=678.0 e_value=2.7e-40 N=16 5&NC_008399.2 minus
715- 0: transcript:Zm00001d015338_T001 rna18573 9.00E-84
715- 1: transcript:Zm00001d015342_T001 rna18563 1.00E-91
715- 2: transcript:Zm00001d015348_T002 rna18562 0
715- 3: transcript:Zm00001d015354_T001 rna18557 8.00E-39
715- 4: transcript:Zm00001d015364_T003 rna18551 2.00E-129
715- 5: transcript:Zm00001d015376_T002 rna18543 0
715- 6: transcript:Zm00001d015378_T001 rna18541 5.00E-94
715- 7: transcript:Zm00001d015381_T001 rna18537 6.00E-63
715- 8: transcript:Zm00001d015382_T001 rna18534 1.00E-13
715- 9: transcript:Zm00001d015383_T001 rna18533 0
715- 10: transcript:Zm00001d015397_T001 rna18531 6.00E-32
715- 11: transcript:Zm00001d015401_T001 rna18525 0
715- 12: transcript:Zm00001d015407_T001 rna18524 1.00E-113
715- 13: transcript:Zm00001d015410_T002 rna18515 3.00E-18
715- 14: transcript:Zm00001d015421_T001 rna18503 2.00E-75
715- 15: transcript:Zm00001d015426_T001 rna18500 0
Alignment 716: score=645.0 e_value=6.4e-38 N=15 5&NC_008399.2 minus
716- 0: transcript:Zm00001d015504_T004 rna18444 0
716- 1: transcript:Zm00001d015515_T001 rna18429 3.00E-111
716- 2: transcript:Zm00001d015517_T001 rna18426 9.00E-31
716- 3: transcript:Zm00001d015521_T001 rna18418 6.00E-113
716- 4: transcript:Zm00001d015546_T001 rna18415 1.00E-22
716- 5: transcript:Zm00001d015549_T001 rna18414 7.00E-165
716- 6: transcript:Zm00001d015550_T001 rna18411 2.00E-18
716- 7: transcript:Zm00001d015559_T001 rna18407 2.00E-177
716- 8: transcript:Zm00001d015569_T001 rna18398 0
716- 9: transcript:Zm00001d015570_T002 rna18395 2.00E-104
716- 10: transcript:Zm00001d015571_T001 rna18388 1.00E-120
716- 11: transcript:Zm00001d015589_T001 rna18384 6.00E-38
716- 12: transcript:Zm00001d015592_T001 rna18380 3.00E-90
716- 13: transcript:Zm00001d015599_T001 rna18368 9.00E-108
716- 14: transcript:Zm00001d015600_T002 rna18365 2.00E-140

Alignment 717: score=612.0 e_value=6.7e-40 N=15 5&NC_008399.2 minus
717- 0: transcript:Zm00001d017883_T002 rna17539 0
717- 1: transcript:Zm00001d017885_T001 rna17526 1.00E-166
717- 2: transcript:Zm00001d017886_T001 rna17521 5.00E-31
717- 3: transcript:Zm00001d017889_T001 rna17502 3.00E-144
717- 4: transcript:Zm00001d017896_T002 rna17483 5.00E-40
717- 5: transcript:Zm00001d017898_T001 rna17478 1.00E-46
717- 6: transcript:Zm00001d017900_T001 rna17475 5.00E-45
717- 7: transcript:Zm00001d017906_T003 rna17474 0
717- 8: transcript:Zm00001d017913_T001 rna17448 2.00E-58
717- 9: transcript:Zm00001d017914_T001 rna17447 3.00E-94
717- 10: transcript:Zm00001d017919_T001 rna17438 1.00E-116
717- 11: transcript:Zm00001d017923_T001 rna17432 9.00E-40
717- 12: transcript:Zm00001d017925_T004 rna17426 0
717- 13: transcript:Zm00001d017926_T003 rna17425 0
717- 14: transcript:Zm00001d017928_T001 rna17422 2.00E-174
Alignment 718: score=595.0 e_value=3.5e-31 N=13 5&NC_008399.2 minus
718- 0: transcript:Zm00001d018016_T001 rna17291 7.00E-158
718- 1: transcript:Zm00001d018017_T001 rna17290 3.00E-07
718- 2: transcript:Zm00001d018024_T001 rna17285 0
718- 3: transcript:Zm00001d018025_T001 rna17283 1.00E-171
718- 4: transcript:Zm00001d018027_T001 rna17282 3.00E-18
718- 5: transcript:Zm00001d018028_T001 rna17281 7.00E-88
718- 6: transcript:Zm00001d018029_T001 rna17278 8.00E-93
718- 7: transcript:Zm00001d018033_T001 rna17272 0
718- 8: transcript:Zm00001d018037_T001 rna17262 2.00E-171
718- 9: transcript:Zm00001d018040_T002 rna17260 1.00E-71
718- 10: transcript:Zm00001d018041_T001 rna17259 2.00E-160
718- 11: transcript:Zm00001d018045_T001 rna17258 5.00E-109
718- 12: transcript:Zm00001d018047_T002 rna17257 7.00E-35
Alignment 719: score=453.0 e_value=9.1e-27 N=12 5&NC_008399.2 minus
719- 0: transcript:Zm00001d015277_T001 rna18711 0
719- 1: transcript:Zm00001d015279_T003 rna18708 3.00E-155
719- 2: transcript:Zm00001d015284_T001 rna18694 4.00E-18
719- 3: transcript:Zm00001d015289_T002 rna18693 4.00E-163
719- 4: transcript:Zm00001d015290_T001 rna18680 0
719- 5: transcript:Zm00001d015297_T003 rna18671 0
719- 6: transcript:Zm00001d015298_T001 rna18667 0
719- 7: transcript:Zm00001d015307_T001 rna18649 2.00E-148
719- 8: transcript:Zm00001d015309_T002 rna18646 6.00E-20
719- 9: transcript:Zm00001d015319_T001 rna18634 4.00E-34
719- 10: transcript:Zm00001d015327_T001 rna18613 0
719- 11: transcript:Zm00001d015331_T001 rna18611 1.00E-25
Alignment 720: score=366.0 e_value=5.1e-14 N=8 5&NC_008399.2 minus
720- 0: transcript:Zm00001d014553_T001 rna18671 0
720- 1: transcript:Zm00001d014555_T001 rna18667 0
720- 2: transcript:Zm00001d014559_T001 rna18665 4.00E-77
720- 3: transcript:Zm00001d014562_T001 rna18664 0
720- 4: transcript:Zm00001d014563_T001 rna18663 2.00E-148
720- 5: transcript:Zm00001d014565_T001 rna18662 2.00E-68
720- 6: transcript:Zm00001d014568_T023 rna18661 0
720- 7: transcript:Zm00001d014569_T002 rna18659 0
Alignment 721: score=349.0 e_value=1.9e-13 N=8 5&NC_008399.2 minus
721- 0: transcript:Zm00001d015451_T001 rna18478 1.00E-78

721- 1: transcript:Zm00001d015457_T001 rna18476 3.00E-52
721- 2: transcript:Zm00001d015461_T001 rna18471 8.00E-35
721- 3: transcript:Zm00001d015463_T001 rna18467 6.00E-49
721- 4: transcript:Zm00001d015473_T003 rna18458 6.00E-42
721- 5: transcript:Zm00001d015476_T001 rna18455 2.00E-34
721- 6: transcript:Zm00001d015477_T001 rna18453 0
721- 7: transcript:Zm00001d015491_T001 rna18448 6.00E-95
Alignment 722: score=341.0 e_value=6.5e-16 N=8 5&NC_008399.2 minus
722- 0: transcript:Zm00001d015612_T001 rna18364 7.00E-62
722- 1: transcript:Zm00001d015613_T001 rna18363 2.00E-107
722- 2: transcript:Zm00001d015614_T001 rna18360 7.00E-127
722- 3: transcript:Zm00001d015636_T001 rna18357 2.00E-111
722- 4: transcript:Zm00001d015649_T003 rna18348 2.00E-57
722- 5: transcript:Zm00001d015656_T001 rna18343 0
722- 6: transcript:Zm00001d015658_T003 rna18342 0
722- 7: transcript:Zm00001d015670_T001 rna18339 3.00E-28
Alignment 723: score=262.0 e_value=3e-12 N=7 5&NC_008399.2 minus
723- 0: transcript:Zm00001d018383_T002 rna17010 1.00E-30
723- 1: transcript:Zm00001d018390_T001 rna17003 2.00E-16
723- 2: transcript:Zm00001d018394_T003 rna16999 0
723- 3: transcript:Zm00001d018404_T002 rna16995 0
723- 4: transcript:Zm00001d018408_T001 rna16973 8.00E-123
723- 5: transcript:Zm00001d018409_T007 rna16969 0
723- 6: transcript:Zm00001d018410_T001 rna16965 5.00E-38
Alignment 724: score=471.0 e_value=2e-24 N=11 5&NC_008400.2 minus
724- 0: transcript:Zm00001d013060_T001 rna19414 0
724- 1: transcript:Zm00001d013063_T001 rna19400 0
724- 2: transcript:Zm00001d013067_T002 rna19379 2.00E-24
724- 3: transcript:Zm00001d013071_T001 rna19371 4.00E-61
724- 4: transcript:Zm00001d013073_T002 rna19370 1.00E-92
724- 5: transcript:Zm00001d013074_T003 rna19368 4.00E-71
724- 6: transcript:Zm00001d013075_T001 rna19366 0
724- 7: transcript:Zm00001d013076_T001 rna19361 0
724- 8: transcript:Zm00001d013077_T004 rna19355 7.00E-28
724- 9: transcript:Zm00001d013078_T004 rna19343 5.00E-75
724- 10: transcript:Zm00001d013080_T001 rna19337 1.00E-152
Alignment 725: score=279.0 e_value=5.4e-11 N=6 5&NC_008400.2 minus
725- 0: transcript:Zm00001d013048_T009 rna19456 0
725- 1: transcript:Zm00001d013049_T002 rna19454 0
725- 2: transcript:Zm00001d013052_T001 rna19441 0
725- 3: transcript:Zm00001d013053_T002 rna19438 5.00E-115
725- 4: transcript:Zm00001d013054_T001 rna19436 2.00E-16
725- 5: transcript:Zm00001d013055_T002 rna19433 6.00E-114
Alignment 726: score=262.0 e_value=7.9e-13 N=7 5&NC_008400.2 minus
726- 0: transcript:Zm00001d013089_T002 rna19320 6.00E-17
726- 1: transcript:Zm00001d013097_T001 rna19294 0
726- 2: transcript:Zm00001d013098_T002 rna19273 2.00E-61
726- 3: transcript:Zm00001d013099_T001 rna19254 8.00E-92
726- 4: transcript:Zm00001d013104_T001 rna19251 4.00E-18
726- 5: transcript:Zm00001d013105_T001 rna19250 0
726- 6: transcript:Zm00001d013108_T001 rna19247 0
Alignment 727: score=260.0 e_value=5e-09 N=6 5&NC_008400.2 minus
727- 0: transcript:Zm00001d013202_T001 rna19047 2.00E-68
727- 1: transcript:Zm00001d013206_T003 rna19045 0

```
727-  2:  transcript:Zm00001d013210_T001  rna19033     8.00E-25
727-  3:  transcript:Zm00001d013212_T001  rna19030    2.00E-119
727-  4:  transcript:Zm00001d013217_T001  rna19026            0
727-  5:  transcript:Zm00001d013220_T001  rna19024    7.00E-155
## Alignment 728: score=253.0 e_value=3.4e-10 N=6 5&NC_008400.2 minus
728-  0:  transcript:Zm00001d013662_T001  rna19243     4.00E-29
728-  1:  transcript:Zm00001d013668_T001  rna19241     2.00E-42
728-  2:  transcript:Zm00001d013672_T001  rna19235    6.00E-121
728-  3:  transcript:Zm00001d013676_T001  rna19234     3.00E-38
728-  4:  transcript:Zm00001d013677_T001  rna19233     7.00E-24
728-  5:  transcript:Zm00001d013689_T001  rna19218            0
## Alignment 729: score=431.0 e_value=8.8e-23 N=10 5&NC_008402.2 minus
729-  0:  transcript:Zm00001d018149_T002  rna24284     9.00E-11
729-  1:  transcript:Zm00001d018150_T001  rna24280     3.00E-31
729-  2:  transcript:Zm00001d018158_T001  rna24269     8.00E-35
729-  3:  transcript:Zm00001d018159_T001  rna24264            0
729-  4:  transcript:Zm00001d018178_T001  rna24259     8.00E-85
729-  5:  transcript:Zm00001d018179_T015  rna24258     5.00E-84
729-  6:  transcript:Zm00001d018182_T001  rna24251            0
729-  7:  transcript:Zm00001d018183_T001  rna24250    3.00E-178
729-  8:  transcript:Zm00001d018193_T001  rna24247     6.00E-55
729-  9:  transcript:Zm00001d018194_T001  rna24239     1.00E-54
## Alignment 730: score=290.0 e_value=1.7e-11 N=7 5&NC_008402.2 minus
730-  0:  transcript:Zm00001d017889_T001  rna24386    1.00E-131
730-  1:  transcript:Zm00001d017895_T001  rna24382     2.00E-44
730-  2:  transcript:Zm00001d017900_T001  rna24372     9.00E-29
730-  3:  transcript:Zm00001d017906_T003  rna24370     3.00E-69
730-  4:  transcript:Zm00001d017908_T001  rna24369     8.00E-26
730-  5:  transcript:Zm00001d017911_T001  rna24363     1.00E-35
730-  6:  transcript:Zm00001d017920_T001  rna24340     2.00E-38
## Alignment 731: score=553.0 e_value=1.1e-30 N=12 5&NC_008403.2 plus
731-  0:  transcript:Zm00001d013941_T001  rna26085    2.00E-141
731-  1:  transcript:Zm00001d013943_T001  rna26086    9.00E-124
731-  2:  transcript:Zm00001d013944_T001  rna26087     2.00E-39
731-  3:  transcript:Zm00001d013945_T001  rna26093     3.00E-79
731-  4:  transcript:Zm00001d013950_T001  rna26095     4.00E-84
731-  5:  transcript:Zm00001d013956_T003  rna26106    6.00E-155
731-  6:  transcript:Zm00001d013957_T001  rna26107            0
731-  7:  transcript:Zm00001d013958_T004  rna26114            0
731-  8:  transcript:Zm00001d013960_T001  rna26117            0
731-  9:  transcript:Zm00001d013962_T003  rna26118            0
731- 10:  transcript:Zm00001d013963_T002  rna26121            0
731- 11:  transcript:Zm00001d013964_T003  rna26124            0
## Alignment 732: score=286.0 e_value=1e-11 N=7 5&NC_008403.2 plus
732-  0:  transcript:Zm00001d017375_T001  rna26152     5.00E-64
732-  1:  transcript:Zm00001d017380_T001  rna26174     6.00E-55
732-  2:  transcript:Zm00001d017382_T001  rna26177     2.00E-87
732-  3:  transcript:Zm00001d017384_T008  rna26182            0
732-  4:  transcript:Zm00001d017386_T001  rna26183     2.00E-09
732-  5:  transcript:Zm00001d017390_T001  rna26199     3.00E-54
732-  6:  transcript:Zm00001d017391_T002  rna26203     4.00E-67
## Alignment 733: score=260.0 e_value=9.7e-09 N=6 5&NC_008403.2 plus
733-  0:  transcript:Zm00001d017699_T001  rna26646    7.00E-121
733-  1:  transcript:Zm00001d017700_T020  rna26647            0
```

733-	2:	transcript:Zm00001d017704_T001	rna26651	9.00E-111
733-	3:	transcript:Zm00001d017712_T001	rna26663	2.00E-52
733-	4:	transcript:Zm00001d017713_T001	rna26665	7.00E-179
733-	5:	transcript:Zm00001d017717_T001	rna26667	4.00E-122

Alignment 734: score=2500.0 e_value=3.8e-219 N=56 5&NC_008403.2 minus

734-	0:	transcript:Zm00001d013967_T004	rna26084	0
734-	1:	transcript:Zm00001d013971_T001	rna26075	2.00E-134
734-	2:	transcript:Zm00001d013976_T002	rna26074	0
734-	3:	transcript:Zm00001d013977_T001	rna26073	4.00E-34
734-	4:	transcript:Zm00001d013979_T001	rna26069	0
734-	5:	transcript:Zm00001d013983_T001	rna26064	1.00E-179
734-	6:	transcript:Zm00001d013984_T012	rna26062	2.00E-161
734-	7:	transcript:Zm00001d013985_T004	rna26061	0
734-	8:	transcript:Zm00001d013989_T028	rna26060	0
734-	9:	transcript:Zm00001d013990_T001	rna26059	3.00E-83
734-	10:	transcript:Zm00001d013991_T001	rna26058	0
734-	11:	transcript:Zm00001d013992_T001	rna26057	0
734-	12:	transcript:Zm00001d013993_T001	rna26054	4.00E-59
734-	13:	transcript:Zm00001d013997_T001	rna26052	8.00E-24
734-	14:	transcript:Zm00001d013999_T001	rna26050	2.00E-93
734-	15:	transcript:Zm00001d014001_T001	rna26046	0
734-	16:	transcript:Zm00001d014003_T004	rna26045	3.00E-42
734-	17:	transcript:Zm00001d014005_T001	rna26044	2.00E-45
734-	18:	transcript:Zm00001d014006_T001	rna26042	5.00E-20
734-	19:	transcript:Zm00001d014007_T001	rna26040	1.00E-41
734-	20:	transcript:Zm00001d014011_T001	rna26039	0
734-	21:	transcript:Zm00001d014013_T002	rna26037	0
734-	22:	transcript:Zm00001d014015_T001	rna26035	6.00E-49
734-	23:	transcript:Zm00001d014016_T001	rna26033	5.00E-142
734-	24:	transcript:Zm00001d014021_T001	rna26026	0
734-	25:	transcript:Zm00001d014029_T001	rna26025	3.00E-100
734-	26:	transcript:Zm00001d014030_T001	rna26024	0
734-	27:	transcript:Zm00001d014032_T001	rna26022	1.00E-85
734-	28:	transcript:Zm00001d014033_T004	rna26021	0
734-	29:	transcript:Zm00001d014035_T001	rna26018	0
734-	30:	transcript:Zm00001d014036_T002	rna26017	0
734-	31:	transcript:Zm00001d014037_T004	rna26012	0
734-	32:	transcript:Zm00001d014038_T011	rna26008	0
734-	33:	transcript:Zm00001d014039_T001	rna26005	7.00E-169
734-	34:	transcript:Zm00001d014043_T001	rna25990	2.00E-121
734-	35:	transcript:Zm00001d014046_T001	rna25989	9.00E-62
734-	36:	transcript:Zm00001d014055_T002	rna25985	0
734-	37:	transcript:Zm00001d014058_T002	rna25983	5.00E-173
734-	38:	transcript:Zm00001d014060_T001	rna25980	0
734-	39:	transcript:Zm00001d014062_T001	rna25975	1.00E-123
734-	40:	transcript:Zm00001d014063_T001	rna25970	0
734-	41:	transcript:Zm00001d014065_T001	rna25968	3.00E-121
734-	42:	transcript:Zm00001d014073_T004	rna25964	2.00E-96
734-	43:	transcript:Zm00001d014074_T001	rna25963	3.00E-72
734-	44:	transcript:Zm00001d014078_T001	rna25962	0
734-	45:	transcript:Zm00001d014079_T001	rna25961	2.00E-92
734-	46:	transcript:Zm00001d014080_T001	rna25960	1.00E-34
734-	47:	transcript:Zm00001d014082_T001	rna25956	6.00E-111
734-	48:	transcript:Zm00001d014083_T001	rna25955	0

734- 49: transcript:Zm00001d014084_T001 rna25952 0
734- 50: transcript:Zm00001d014085_T001 rna25949 0
734- 51: transcript:Zm00001d014089_T001 rna25944 1.00E-109
734- 52: transcript:Zm00001d014090_T002 rna25941 0
734- 53: transcript:Zm00001d014093_T002 rna25917 0
734- 54: transcript:Zm00001d014094_T002 rna25911 0
734- 55: transcript:Zm00001d014097_T002 rna25908 2.00E-124
Alignment 735: score=900.0 e_value=1.8e-60 N=21 5&NC_008403.2 minus
735- 0: transcript:Zm00001d014187_T011 rna25711 0
735- 1: transcript:Zm00001d014188_T010 rna25710 0
735- 2: transcript:Zm00001d014189_T003 rna25708 2.00E-12
735- 3: transcript:Zm00001d014191_T001 rna25691 2.00E-19
735- 4: transcript:Zm00001d014194_T001 rna25687 2.00E-53
735- 5: transcript:Zm00001d014198_T001 rna25684 4.00E-42
735- 6: transcript:Zm00001d014199_T003 rna25682 3.00E-102
735- 7: transcript:Zm00001d014200_T001 rna25679 2.00E-36
735- 8: transcript:Zm00001d014201_T001 rna25677 1.00E-109
735- 9: transcript:Zm00001d014204_T001 rna25675 3.00E-165
735- 10: transcript:Zm00001d014222_T001 rna25661 0
735- 11: transcript:Zm00001d014224_T001 rna25651 2.00E-148
735- 12: transcript:Zm00001d014232_T014 rna25648 0
735- 13: transcript:Zm00001d014235_T002 rna25647 2.00E-159
735- 14: transcript:Zm00001d014241_T001 rna25642 1.00E-104
735- 15: transcript:Zm00001d014243_T001 rna25641 0
735- 16: transcript:Zm00001d014244_T001 rna25632 6.00E-133
735- 17: transcript:Zm00001d014246_T002 rna25631 2.00E-93
735- 18: transcript:Zm00001d014249_T001 rna25628 6.00E-14
735- 19: transcript:Zm00001d014253_T001 rna25626 4.00E-29
735- 20: transcript:Zm00001d014258_T002 rna25618 0
Alignment 736: score=871.0 e_value=1.1e-59 N=20 5&NC_008403.2 minus
736- 0: transcript:Zm00001d013892_T004 rna26204 0
736- 1: transcript:Zm00001d013895_T002 rna26203 2.00E-96
736- 2: transcript:Zm00001d013896_T001 rna26202 0
736- 3: transcript:Zm00001d013900_T001 rna26191 1.00E-72
736- 4: transcript:Zm00001d013907_T001 rna26187 5.00E-21
736- 5: transcript:Zm00001d013908_T003 rna26186 0
736- 6: transcript:Zm00001d013910_T002 rna26182 0
736- 7: transcript:Zm00001d013911_T001 rna26180 0
736- 8: transcript:Zm00001d013914_T001 rna26174 3.00E-60
736- 9: transcript:Zm00001d013915_T001 rna26173 0
736- 10: transcript:Zm00001d013918_T001 rna26170 4.00E-128
736- 11: transcript:Zm00001d013919_T001 rna26169 2.00E-103
736- 12: transcript:Zm00001d013923_T001 rna26163 8.00E-41
736- 13: transcript:Zm00001d013927_T001 rna26162 0
736- 14: transcript:Zm00001d013933_T001 rna26152 3.00E-132
736- 15: transcript:Zm00001d013934_T001 rna26142 2.00E-133
736- 16: transcript:Zm00001d013935_T001 rna26132 0
736- 17: transcript:Zm00001d013937_T001 rna26131 0
736- 18: transcript:Zm00001d013940_T004 rna26126 1.00E-97
736- 19: transcript:Zm00001d013956_T003 rna26106 6.00E-155
Alignment 737: score=853.0 e_value=4.4e-54 N=19 5&NC_008403.2 minus
737- 0: transcript:Zm00001d014116_T001 rna25858 7.00E-164
737- 1: transcript:Zm00001d014117_T001 rna25854 4.00E-28
737- 2: transcript:Zm00001d014122_T002 rna25853 1.00E-155

737- 3: transcript:Zm00001d014124_T001 rna25848   2.00E-116
737- 4: transcript:Zm00001d014126_T001 rna25847   6.00E-180
737- 5: transcript:Zm00001d014128_T001 rna25842   1.00E-27
737- 6: transcript:Zm00001d014132_T001 rna25841   8.00E-70
737- 7: transcript:Zm00001d014134_T001 rna25838   0
737- 8: transcript:Zm00001d014138_T004 rna25837   5.00E-49
737- 9: transcript:Zm00001d014139_T001 rna25835   5.00E-38
737- 10: transcript:Zm00001d014140_T001 rna25834   7.00E-30
737- 11: transcript:Zm00001d014141_T002 rna25832   1.00E-135
737- 12: transcript:Zm00001d014145_T001 rna25831   2.00E-117
737- 13: transcript:Zm00001d014146_T001 rna25828   4.00E-38
737- 14: transcript:Zm00001d014149_T001 rna25827   3.00E-19
737- 15: transcript:Zm00001d014150_T005 rna25826   0
737- 16: transcript:Zm00001d014152_T003 rna25822   0
737- 17: transcript:Zm00001d014157_T001 rna25819   3.00E-44
737- 18: transcript:Zm00001d014158_T002 rna25801   4.00E-49
Alignment 738: score=523.0 e_value=4.4e-23 N=11 5&NC_008403.2 minus
738- 0: transcript:Zm00001d013860_T012 rna26232   0
738- 1: transcript:Zm00001d013861_T001 rna26231   1.00E-89
738- 2: transcript:Zm00001d013862_T001 rna26230   0
738- 3: transcript:Zm00001d013865_T001 rna26223   0
738- 4: transcript:Zm00001d013868_T001 rna26222   2.00E-139
738- 5: transcript:Zm00001d013869_T001 rna26220   2.00E-52
738- 6: transcript:Zm00001d013873_T001 rna26217   0
738- 7: transcript:Zm00001d013879_T001 rna26212   5.00E-25
738- 8: transcript:Zm00001d013885_T001 rna26211   8.00E-46
738- 9: transcript:Zm00001d013886_T002 rna26210   5.00E-87
738- 10: transcript:Zm00001d013887_T002 rna26209   0
Alignment 739: score=419.0 e_value=2.6e-22 N=10 5&NC_008403.2 minus
739- 0: transcript:Zm00001d013834_T001 rna26265   0
739- 1: transcript:Zm00001d013836_T001 rna26263   0
739- 2: transcript:Zm00001d013838_T002 rna26261   0
739- 3: transcript:Zm00001d013839_T001 rna26260   4.00E-69
739- 4: transcript:Zm00001d013840_T002 rna26259   0
739- 5: transcript:Zm00001d013844_T001 rna26256   0
739- 6: transcript:Zm00001d013847_T001 rna26251   0
739- 7: transcript:Zm00001d013849_T003 rna26250   3.00E-35
739- 8: transcript:Zm00001d013858_T001 rna26247   2.00E-47
739- 9: transcript:Zm00001d013859_T005 rna26233   9.00E-65
Alignment 740: score=324.0 e_value=1.3e-14 N=8 5&NC_008403.2 minus
740- 0: transcript:Zm00001d014160_T001 rna25761   2.00E-177
740- 1: transcript:Zm00001d014166_T001 rna25745   0
740- 2: transcript:Zm00001d014168_T001 rna25739   1.00E-171
740- 3: transcript:Zm00001d014175_T004 rna25736   0
740- 4: transcript:Zm00001d014178_T013 rna25734   0
740- 5: transcript:Zm00001d014179_T001 rna25731   0
740- 6: transcript:Zm00001d014180_T004 rna25722   1.00E-168
740- 7: transcript:Zm00001d014183_T008 rna25717   0
Alignment 741: score=422.0 e_value=3.6e-20 N=10 5&NC_008405.2 minus
741- 0: transcript:Zm00001d013660_T001 rna30176   2.00E-18
741- 1: transcript:Zm00001d013661_T001 rna30174   4.00E-20
741- 2: transcript:Zm00001d013667_T001 rna30170   1.00E-41
741- 3: transcript:Zm00001d013672_T001 rna30168   0
741- 4: transcript:Zm00001d013676_T001 rna30167   6.00E-78

741- 5: transcript:Zm00001d013680_T002 rna30163   0
741- 6: transcript:Zm00001d013689_T001 rna30161   0
741- 7: transcript:Zm00001d013692_T001 rna30160   0
741- 8: transcript:Zm00001d013694_T001 rna30134   7.00E-107
741- 9: transcript:Zm00001d013699_T001 rna30130   0
Alignment 742: score=400.0 e_value=1e-18 N=9 5&NC_008405.2 minus
742- 0: transcript:Zm00001d013722_T001 rna30024   3.00E-18
742- 1: transcript:Zm00001d013723_T001 rna30021   1.00E-13
742- 2: transcript:Zm00001d013728_T001 rna30013   7.00E-87
742- 3: transcript:Zm00001d013736_T001 rna30002   0
742- 4: transcript:Zm00001d013741_T006 rna29995   0
742- 5: transcript:Zm00001d013743_T001 rna29985   1.00E-48
742- 6: transcript:Zm00001d013744_T001 rna29984   4.00E-143
742- 7: transcript:Zm00001d013745_T002 rna29983   7.00E-147
742- 8: transcript:Zm00001d013746_T001 rna29981   3.00E-79
Alignment 743: score=1041.0 e_value=1.1e-71 N=24 6&NC_008394.4 plus
743- 0: transcript:Zm00001d038740_T002 rna2638   7.00E-57
743- 1: transcript:Zm00001d038742_T001 rna2662   1.00E-180
743- 2: transcript:Zm00001d038747_T003 rna2666   3.00E-174
743- 3: transcript:Zm00001d038751_T001 rna2671   3.00E-150
743- 4: transcript:Zm00001d038752_T001 rna2675   0
743- 5: transcript:Zm00001d038753_T002 rna2676   6.00E-55
743- 6: transcript:Zm00001d038761_T001 rna2689   4.00E-68
743- 7: transcript:Zm00001d038762_T001 rna2691   0
743- 8: transcript:Zm00001d038763_T001 rna2693   6.00E-107
743- 9: transcript:Zm00001d038764_T001 rna2699   5.00E-141
743- 10: transcript:Zm00001d038766_T001 rna2705   8.00E-82
743- 11: transcript:Zm00001d038768_T001 rna2707   3.00E-134
743- 12: transcript:Zm00001d038772_T001 rna2718   4.00E-67
743- 13: transcript:Zm00001d038776_T001 rna2724   0
743- 14: transcript:Zm00001d038779_T001 rna2725   7.00E-156
743- 15: transcript:Zm00001d038780_T001 rna2728   5.00E-32
743- 16: transcript:Zm00001d038784_T001 rna2739   1.00E-81
743- 17: transcript:Zm00001d038791_T001 rna2745   0
743- 18: transcript:Zm00001d038792_T001 rna2746   0
743- 19: transcript:Zm00001d038793_T001 rna2747   1.00E-78
743- 20: transcript:Zm00001d038794_T002 rna2748   0
743- 21: transcript:Zm00001d038796_T001 rna2753   0
743- 22: transcript:Zm00001d038797_T001 rna2755   0
743- 23: transcript:Zm00001d038801_T001 rna2763   9.00E-115
Alignment 744: score=593.0 e_value=2.9e-32 N=13 6&NC_008394.4 plus
744- 0: transcript:Zm00001d038310_T003 rna3530   0
744- 1: transcript:Zm00001d038311_T001 rna3533   4.00E-62
744- 2: transcript:Zm00001d038312_T001 rna3535   3.00E-122
744- 3: transcript:Zm00001d038318_T002 rna3536   0
744- 4: transcript:Zm00001d038319_T003 rna3537   2.00E-142
744- 5: transcript:Zm00001d038320_T001 rna3538   4.00E-38
744- 6: transcript:Zm00001d038321_T001 rna3539   0
744- 7: transcript:Zm00001d038326_T003 rna3550   0
744- 8: transcript:Zm00001d038328_T001 rna3554   0
744- 9: transcript:Zm00001d038331_T001 rna3557   3.00E-43
744- 10: transcript:Zm00001d038333_T001 rna3559   0
744- 11: transcript:Zm00001d038336_T001 rna3576   3.00E-36
744- 12: transcript:Zm00001d038338_T001 rna3578   3.00E-102

Alignment 745: score=328.0 e_value=1.1e-14 N=8 6&NC_008394.4 plus
745- 0: transcript:Zm00001d038049_T001 rna4064 8.00E-112
745- 1: transcript:Zm00001d038061_T001 rna4074 3.00E-16
745- 2: transcript:Zm00001d038062_T001 rna4084 1.00E-43
745- 3: transcript:Zm00001d038063_T001 rna4088 4.00E-170
745- 4: transcript:Zm00001d038064_T001 rna4091 0
745- 5: transcript:Zm00001d038070_T001 rna4101 8.00E-71
745- 6: transcript:Zm00001d038081_T001 rna4111 1.00E-77
745- 7: transcript:Zm00001d038085_T001 rna4128 0
Alignment 746: score=294.0 e_value=5.4e-13 N=7 6&NC_008394.4 plus
746- 0: transcript:Zm00001d037010_T003 rna680 3.00E-149
746- 1: transcript:Zm00001d037017_T001 rna706 0
746- 2: transcript:Zm00001d037018_T001 rna710 6.00E-50
746- 3: transcript:Zm00001d037019_T001 rna722 2.00E-22
746- 4: transcript:Zm00001d037024_T001 rna731 2.00E-15
746- 5: transcript:Zm00001d037025_T001 rna732 2.00E-178
746- 6: transcript:Zm00001d037032_T001 rna742 7.00E-123
Alignment 747: score=261.0 e_value=6.6e-09 N=6 6&NC_008394.4 plus
747- 0: transcript:Zm00001d038263_T001 rna3428 0
747- 1: transcript:Zm00001d038268_T001 rna3435 6.00E-118
747- 2: transcript:Zm00001d038269_T001 rna3436 0
747- 3: transcript:Zm00001d038273_T001 rna3441 0
747- 4: transcript:Zm00001d038274_T002 rna3450 8.00E-175
747- 5: transcript:Zm00001d038275_T002 rna3456 0
Alignment 748: score=1603.0 e_value=6.3e-127 N=37 6&NC_008394.4 minus
748- 0: transcript:Zm00001d038860_T001 rna2547 3.00E-32
748- 1: transcript:Zm00001d038861_T001 rna2540 5.00E-135
748- 2: transcript:Zm00001d038862_T001 rna2538 3.00E-121
748- 3: transcript:Zm00001d038863_T001 rna2532 1.00E-56
748- 4: transcript:Zm00001d038865_T003 rna2525 1.00E-87
748- 5: transcript:Zm00001d038870_T001 rna2515 2.00E-37
748- 6: transcript:Zm00001d038876_T001 rna2509 3.00E-27
748- 7: transcript:Zm00001d038878_T001 rna2502 6.00E-109
748- 8: transcript:Zm00001d038879_T003 rna2500 6.00E-172
748- 9: transcript:Zm00001d038880_T001 rna2496 0
748- 10: transcript:Zm00001d038882_T002 rna2489 0
748- 11: transcript:Zm00001d038883_T001 rna2482 1.00E-131
748- 12: transcript:Zm00001d038886_T001 rna2475 7.00E-07
748- 13: transcript:Zm00001d038891_T001 rna2461 0
748- 14: transcript:Zm00001d038892_T001 rna2460 0
748- 15: transcript:Zm00001d038903_T001 rna2451 1.00E-112
748- 16: transcript:Zm00001d038904_T001 rna2450 0
748- 17: transcript:Zm00001d038907_T001 rna2448 7.00E-167
748- 18: transcript:Zm00001d038908_T004 rna2447 3.00E-150
748- 19: transcript:Zm00001d038909_T001 rna2446 1.00E-81
748- 20: transcript:Zm00001d038910_T001 rna2436 1.00E-76
748- 21: transcript:Zm00001d038911_T001 rna2433 6.00E-18
748- 22: transcript:Zm00001d038915_T001 rna2427 0
748- 23: transcript:Zm00001d038916_T001 rna2424 2.00E-43
748- 24: transcript:Zm00001d038918_T001 rna2420 6.00E-09
748- 25: transcript:Zm00001d038925_T003 rna2411 2.00E-159
748- 26: transcript:Zm00001d038926_T001 rna2409 2.00E-24
748- 27: transcript:Zm00001d038929_T001 rna2400 0
748- 28: transcript:Zm00001d038932_T001 rna2395 2.00E-56

748- 29: transcript:Zm00001d038936_T001 rna2387 1.00E-15
748- 30: transcript:Zm00001d038948_T001 rna2378 0
748- 31: transcript:Zm00001d038950_T001 rna2377 4.00E-07
748- 32: transcript:Zm00001d038955_T001 rna2372 1.00E-14
748- 33: transcript:Zm00001d038959_T001 rna2371 0
748- 34: transcript:Zm00001d038963_T001 rna2368 3.00E-73
748- 35: transcript:Zm00001d038965_T001 rna2364 2.00E-154
748- 36: transcript:Zm00001d038968_T001 rna2359 2.00E-47
Alignment 749: score=1548.0 e_value=3.5e-129 N=37 6&NC_008394.4 minus
749- 0: transcript:Zm00001d038445_T001 rna3284 4.00E-90
749- 1: transcript:Zm00001d038447_T001 rna3273 0
749- 2: transcript:Zm00001d038449_T001 rna3270 2.00E-178
749- 3: transcript:Zm00001d038450_T001 rna3268 2.00E-68
749- 4: transcript:Zm00001d038451_T001 rna3267 2.00E-138
749- 5: transcript:Zm00001d038459_T001 rna3263 2.00E-165
749- 6: transcript:Zm00001d038465_T001 rna3262 0
749- 7: transcript:Zm00001d038466_T001 rna3259 3.00E-32
749- 8: transcript:Zm00001d038471_T001 rna3252 0
749- 9: transcript:Zm00001d038473_T001 rna3249 8.00E-94
749- 10: transcript:Zm00001d038476_T001 rna3245 2.00E-155
749- 11: transcript:Zm00001d038478_T001 rna3242 5.00E-35
749- 12: transcript:Zm00001d038481_T001 rna3240 0
749- 13: transcript:Zm00001d038483_T001 rna3231 5.00E-61
749- 14: transcript:Zm00001d038487_T003 rna3225 2.00E-67
749- 15: transcript:Zm00001d038489_T001 rna3224 0
749- 16: transcript:Zm00001d038494_T011 rna3221 7.00E-176
749- 17: transcript:Zm00001d038506_T001 rna3219 3.00E-86
749- 18: transcript:Zm00001d038513_T002 rna3216 0
749- 19: transcript:Zm00001d038514_T001 rna3214 8.00E-81
749- 20: transcript:Zm00001d038517_T001 rna3213 0
749- 21: transcript:Zm00001d038521_T001 rna3210 0
749- 22: transcript:Zm00001d038522_T002 rna3209 8.00E-22
749- 23: transcript:Zm00001d038523_T001 rna3200 2.00E-10
749- 24: transcript:Zm00001d038529_T003 rna3198 5.00E-169
749- 25: transcript:Zm00001d038530_T003 rna3193 9.00E-55
749- 26: transcript:Zm00001d038533_T004 rna3185 2.00E-131
749- 27: transcript:Zm00001d038535_T001 rna3181 4.00E-62
749- 28: transcript:Zm00001d038537_T001 rna3179 0
749- 29: transcript:Zm00001d038538_T001 rna3177 9.00E-104
749- 30: transcript:Zm00001d038541_T002 rna3174 0
749- 31: transcript:Zm00001d038543_T004 rna3164 8.00E-72
749- 32: transcript:Zm00001d038553_T001 rna3141 1.00E-125
749- 33: transcript:Zm00001d038554_T001 rna3131 2.00E-164
749- 34: transcript:Zm00001d038558_T005 rna3130 3.00E-35
749- 35: transcript:Zm00001d038562_T001 rna3129 0
749- 36: transcript:Zm00001d038563_T001 rna3127 6.00E-125
Alignment 750: score=870.0 e_value=3.2e-56 N=20 6&NC_008394.4 minus
750- 0: transcript:Zm00001d038195_T001 rna3695 3.00E-68
750- 1: transcript:Zm00001d038196_T001 rna3691 0
750- 2: transcript:Zm00001d038197_T001 rna3682 3.00E-74
750- 3: transcript:Zm00001d038203_T001 rna3679 2.00E-14
750- 4: transcript:Zm00001d038205_T002 rna3676 0
750- 5: transcript:Zm00001d038207_T001 rna3674 1.00E-119
750- 6: transcript:Zm00001d038208_T001 rna3672 8.00E-139

750- 7: transcript:Zm00001d038217_T002 rna3657   6.00E-109
750- 8: transcript:Zm00001d038218_T001 rna3655   5.00E-55
750- 9: transcript:Zm00001d038221_T001 rna3648   3.00E-145
750- 10: transcript:Zm00001d038222_T003 rna3636   0
750- 11: transcript:Zm00001d038224_T003 rna3632   0
750- 12: transcript:Zm00001d038225_T003 rna3627   0
750- 13: transcript:Zm00001d038226_T001 rna3625   1.00E-124
750- 14: transcript:Zm00001d038228_T001 rna3619   5.00E-164
750- 15: transcript:Zm00001d038229_T001 rna3617   0
750- 16: transcript:Zm00001d038239_T001 rna3601   3.00E-29
750- 17: transcript:Zm00001d038248_T002 rna3592   1.00E-51
750- 18: transcript:Zm00001d038250_T001 rna3586   1.00E-135
750- 19: transcript:Zm00001d038251_T001 rna3583   5.00E-93
Alignment 751: score=680.0 e_value=2.3e-43 N=16 6&NC_008394.4 minus
751- 0: transcript:Zm00001d038363_T001 rna3405   1.00E-31
751- 1: transcript:Zm00001d038365_T002 rna3404   5.00E-38
751- 2: transcript:Zm00001d038367_T002 rna3386   3.00E-174
751- 3: transcript:Zm00001d038368_T002 rna3385   1.00E-91
751- 4: transcript:Zm00001d038371_T002 rna3377   0
751- 5: transcript:Zm00001d038373_T001 rna3369   0
751- 6: transcript:Zm00001d038374_T007 rna3364   1.00E-60
751- 7: transcript:Zm00001d038376_T001 rna3359   3.00E-108
751- 8: transcript:Zm00001d038377_T002 rna3351   0
751- 9: transcript:Zm00001d038378_T001 rna3350   3.00E-95
751- 10: transcript:Zm00001d038379_T001 rna3348   3.00E-42
751- 11: transcript:Zm00001d038387_T001 rna3339   3.00E-35
751- 12: transcript:Zm00001d038388_T001 rna3333   1.00E-111
751- 13: transcript:Zm00001d038392_T001 rna3332   0
751- 14: transcript:Zm00001d038393_T012 rna3330   0
751- 15: transcript:Zm00001d038395_T005 rna3312   1.00E-57
Alignment 752: score=674.0 e_value=3.6e-38 N=15 6&NC_008394.4 minus
752- 0: transcript:Zm00001d039004_T002 rna2315   2.00E-55
752- 1: transcript:Zm00001d039006_T007 rna2313   0
752- 2: transcript:Zm00001d039010_T001 rna2302   2.00E-38
752- 3: transcript:Zm00001d039011_T001 rna2300   7.00E-50
752- 4: transcript:Zm00001d039014_T001 rna2299   1.00E-122
752- 5: transcript:Zm00001d039015_T001 rna2298   5.00E-96
752- 6: transcript:Zm00001d039016_T001 rna2297   1.00E-128
752- 7: transcript:Zm00001d039017_T001 rna2296   2.00E-06
752- 8: transcript:Zm00001d039020_T001 rna2292   4.00E-122
752- 9: transcript:Zm00001d039021_T001 rna2290   5.00E-50
752- 10: transcript:Zm00001d039031_T001 rna2283   6.00E-22
752- 11: transcript:Zm00001d039032_T001 rna2282   3.00E-74
752- 12: transcript:Zm00001d039037_T001 rna2279   0
752- 13: transcript:Zm00001d039041_T007 rna2274   0
752- 14: transcript:Zm00001d039043_T011 rna2272   0
Alignment 753: score=663.0 e_value=7.6e-40 N=15 6&NC_008394.4 minus
753- 0: transcript:Zm00001d039079_T002 rna2228   0
753- 1: transcript:Zm00001d039081_T002 rna2224   0
753- 2: transcript:Zm00001d039083_T001 rna2217   7.00E-73
753- 3: transcript:Zm00001d039084_T001 rna2216   9.00E-24
753- 4: transcript:Zm00001d039086_T001 rna2213   5.00E-37
753- 5: transcript:Zm00001d039087_T001 rna2208   5.00E-136
753- 6: transcript:Zm00001d039089_T001 rna2196   0

```
753-  7:  transcript:Zm00001d039100_T001  rna2177      1.00E-06
753-  8:  transcript:Zm00001d039105_T001  rna2171             0
753-  9:  transcript:Zm00001d039108_T001  rna2167     7.00E-150
753- 10:  transcript:Zm00001d039109_T001  rna2164      1.00E-35
753- 11:  transcript:Zm00001d039112_T001  rna2150      1.00E-51
753- 12:  transcript:Zm00001d039116_T001  rna2137      6.00E-52
753- 13:  transcript:Zm00001d039118_T001  rna2135      8.00E-33
753- 14:  transcript:Zm00001d039120_T002  rna2132             0
## Alignment 754: score=613.0 e_value=4.1e-32 N=14 6&NC_008394.4 minus
754-  0:  transcript:Zm00001d037729_T001  rna671      6.00E-160
754-  1:  transcript:Zm00001d037733_T001  rna665      1.00E-117
754-  2:  transcript:Zm00001d037735_T001  rna663      5.00E-104
754-  3:  transcript:Zm00001d037737_T001  rna661       6.00E-57
754-  4:  transcript:Zm00001d037738_T001  rna659       7.00E-38
754-  5:  transcript:Zm00001d037743_T001  rna643       1.00E-59
754-  6:  transcript:Zm00001d037744_T001  rna642              0
754-  7:  transcript:Zm00001d037745_T001  rna633              0
754-  8:  transcript:Zm00001d037747_T001  rna630       1.00E-56
754-  9:  transcript:Zm00001d037749_T001  rna627       1.00E-38
754- 10:  transcript:Zm00001d037757_T001  rna621       1.00E-45
754- 11:  transcript:Zm00001d037762_T001  rna614       1.00E-06
754- 12:  transcript:Zm00001d037766_T001  rna611      2.00E-165
754- 13:  transcript:Zm00001d037769_T001  rna608      4.00E-159
## Alignment 755: score=498.0 e_value=6.2e-22 N=11 6&NC_008394.4 minus
755-  0:  transcript:Zm00001d038281_T001  rna3521      2.00E-78
755-  1:  transcript:Zm00001d038282_T001  rna3516      2.00E-86
755-  2:  transcript:Zm00001d038284_T001  rna3509      2.00E-59
755-  3:  transcript:Zm00001d038287_T001  rna3508      3.00E-32
755-  4:  transcript:Zm00001d038288_T001  rna3506      3.00E-68
755-  5:  transcript:Zm00001d038289_T001  rna3503      3.00E-79
755-  6:  transcript:Zm00001d038291_T001  rna3486      5.00E-54
755-  7:  transcript:Zm00001d038296_T001  rna3480             0
755-  8:  transcript:Zm00001d038297_T001  rna3476     1.00E-117
755-  9:  transcript:Zm00001d038300_T002  rna3472             0
755- 10:  transcript:Zm00001d038301_T002  rna3467      5.00E-65
## Alignment 756: score=478.0 e_value=5.3e-24 N=11 6&NC_008394.4 minus
756-  0:  transcript:Zm00001d038594_T002  rna3077      5.00E-67
756-  1:  transcript:Zm00001d038596_T001  rna3073      7.00E-11
756-  2:  transcript:Zm00001d038599_T001  rna3033     1.00E-127
756-  3:  transcript:Zm00001d038610_T001  rna3025      2.00E-87
756-  4:  transcript:Zm00001d038612_T001  rna3022      7.00E-37
756-  5:  transcript:Zm00001d038613_T001  rna3021      8.00E-22
756-  6:  transcript:Zm00001d038616_T001  rna3012     9.00E-118
756-  7:  transcript:Zm00001d038618_T001  rna3008             0
756-  8:  transcript:Zm00001d038620_T006  rna3007             0
756-  9:  transcript:Zm00001d038623_T001  rna2994     6.00E-165
756- 10:  transcript:Zm00001d038624_T001  rna2992      2.00E-34
## Alignment 757: score=408.0 e_value=2.1e-17 N=9 6&NC_008394.4 minus
757-  0:  transcript:Zm00001d038987_T001  rna2339      2.00E-28
757-  1:  transcript:Zm00001d038989_T002  rna2334             0
757-  2:  transcript:Zm00001d038991_T001  rna2333     1.00E-177
757-  3:  transcript:Zm00001d038993_T001  rna2330      2.00E-08
757-  4:  transcript:Zm00001d038994_T001  rna2326      3.00E-89
757-  5:  transcript:Zm00001d038995_T001  rna2323     6.00E-106
```

757- 6: transcript:Zm00001d038998_T001 rna2320 2.00E-90
757- 7: transcript:Zm00001d038999_T001 rna2317 7.00E-103
757- 8: transcript:Zm00001d039002_T019 rna2316 2.00E-88
Alignment 758: score=343.0 e_value=2.4e-13 N=8 6&NC_008394.4 minus
758- 0: transcript:Zm00001d037619_T001 rna520 0
758- 1: transcript:Zm00001d037624_T001 rna518 1.00E-155
758- 2: transcript:Zm00001d037626_T001 rna515 2.00E-110
758- 3: transcript:Zm00001d037627_T001 rna509 3.00E-138
758- 4: transcript:Zm00001d037630_T001 rna498 1.00E-94
758- 5: transcript:Zm00001d037631_T001 rna492 8.00E-25
758- 6: transcript:Zm00001d037637_T001 rna480 6.00E-29
758- 7: transcript:Zm00001d037643_T001 rna474 0
Alignment 759: score=337.0 e_value=2.9e-17 N=8 6&NC_008394.4 minus
759- 0: transcript:Zm00001d038690_T001 rna2852 0
759- 1: transcript:Zm00001d038691_T001 rna2850 1.00E-154
759- 2: transcript:Zm00001d038692_T001 rna2848 7.00E-13
759- 3: transcript:Zm00001d038693_T001 rna2842 3.00E-34
759- 4: transcript:Zm00001d038695_T001 rna2834 2.00E-154
759- 5: transcript:Zm00001d038698_T001 rna2815 0
759- 6: transcript:Zm00001d038704_T002 rna2810 5.00E-93
759- 7: transcript:Zm00001d038708_T001 rna2794 8.00E-173
Alignment 760: score=327.0 e_value=7.9e-12 N=7 6&NC_008394.4 minus
760- 0: transcript:Zm00001d038342_T002 rna3422 1.00E-96
760- 1: transcript:Zm00001d038343_T001 rna3421 0
760- 2: transcript:Zm00001d038346_T002 rna3420 7.00E-113
760- 3: transcript:Zm00001d038351_T004 rna3419 1.00E-46
760- 4: transcript:Zm00001d038352_T001 rna3415 5.00E-10
760- 5: transcript:Zm00001d038355_T001 rna3412 0
760- 6: transcript:Zm00001d038358_T002 rna3409 0
Alignment 761: score=279.0 e_value=8.9e-10 N=6 6&NC_008394.4 minus
761- 0: transcript:Zm00001d038842_T001 rna2570 4.00E-109
761- 1: transcript:Zm00001d038843_T001 rna2569 1.00E-58
761- 2: transcript:Zm00001d038844_T001 rna2566 0
761- 3: transcript:Zm00001d038845_T002 rna2565 3.00E-50
761- 4: transcript:Zm00001d038851_T001 rna2558 8.00E-139
761- 5: transcript:Zm00001d038852_T001 rna2557 2.00E-124
Alignment 762: score=256.0 e_value=2.3e-08 N=6 6&NC_008394.4 minus
762- 0: transcript:Zm00001d037934_T011 rna1253 0
762- 1: transcript:Zm00001d037936_T001 rna1251 2.00E-16
762- 2: transcript:Zm00001d037941_T001 rna1246 1.00E-31
762- 3: transcript:Zm00001d037943_T001 rna1239 2.00E-40
762- 4: transcript:Zm00001d037946_T001 rna1231 0
762- 5: transcript:Zm00001d037958_T001 rna1227 3.00E-42
Alignment 763: score=596.0 e_value=5.7e-36 N=14 6&NC_008395.2 plus
763- 0: transcript:Zm00001d036672_T005 rna4800 0
763- 1: transcript:Zm00001d036676_T001 rna4803 3.00E-40
763- 2: transcript:Zm00001d036678_T002 rna4804 0
763- 3: transcript:Zm00001d036692_T001 rna4810 1.00E-76
763- 4: transcript:Zm00001d036700_T001 rna4814 3.00E-58
763- 5: transcript:Zm00001d036703_T001 rna4819 4.00E-18
763- 6: transcript:Zm00001d036708_T001 rna4820 6.00E-43
763- 7: transcript:Zm00001d036710_T002 rna4833 0
763- 8: transcript:Zm00001d036726_T001 rna4840 1.00E-102
763- 9: transcript:Zm00001d036735_T002 rna4846 6.00E-49

```
763- 10:  transcript:Zm00001d036736_T001  rna4847      3.00E-50
763- 11:  transcript:Zm00001d036737_T001  rna4849      3.00E-08
763- 12:  transcript:Zm00001d036739_T001  rna4853     2.00E-124
763- 13:  transcript:Zm00001d036741_T009  rna4854             0
## Alignment 764: score=487.0 e_value=2e-27 N=12 6&NC_008395.2 plus
764-  0:  transcript:Zm00001d036750_T001  rna4829      2.00E-64
764-  1:  transcript:Zm00001d036758_T002  rna4845      2.00E-23
764-  2:  transcript:Zm00001d036760_T001  rna4860     4.00E-102
764-  3:  transcript:Zm00001d036762_T001  rna4870      5.00E-84
764-  4:  transcript:Zm00001d036763_T001  rna4871      5.00E-37
764-  5:  transcript:Zm00001d036768_T001  rna4884      5.00E-92
764-  6:  transcript:Zm00001d036770_T001  rna4894     1.00E-178
764-  7:  transcript:Zm00001d036772_T002  rna4895      3.00E-29
764-  8:  transcript:Zm00001d036775_T001  rna4905             0
764-  9:  transcript:Zm00001d036778_T001  rna4907      3.00E-08
764- 10:  transcript:Zm00001d036780_T001  rna4917      9.00E-22
764- 11:  transcript:Zm00001d036787_T001  rna4925      2.00E-27
## Alignment 765: score=468.0 e_value=8.8e-25 N=11 6&NC_008395.2 plus
765-  0:  transcript:Zm00001d037023_T001  rna5215      6.00E-18
765-  1:  transcript:Zm00001d037025_T001  rna5222             0
765-  2:  transcript:Zm00001d037029_T001  rna5238     2.00E-143
765-  3:  transcript:Zm00001d037033_T001  rna5249      2.00E-08
765-  4:  transcript:Zm00001d037035_T002  rna5254             0
765-  5:  transcript:Zm00001d037042_T001  rna5272      8.00E-35
765-  6:  transcript:Zm00001d037044_T001  rna5274      3.00E-15
765-  7:  transcript:Zm00001d037050_T001  rna5280     2.00E-160
765-  8:  transcript:Zm00001d037051_T001  rna5281             0
765-  9:  transcript:Zm00001d037052_T001  rna5283      1.00E-70
765- 10:  transcript:Zm00001d037054_T001  rna5297      5.00E-44
## Alignment 766: score=319.0 e_value=4.7e-13 N=8 6&NC_008395.2 plus
766-  0:  transcript:Zm00001d036925_T001  rna5028     4.00E-118
766-  1:  transcript:Zm00001d036931_T001  rna5036     3.00E-160
766-  2:  transcript:Zm00001d036945_T001  rna5054     5.00E-167
766-  3:  transcript:Zm00001d036947_T001  rna5063             0
766-  4:  transcript:Zm00001d036949_T004  rna5076             0
766-  5:  transcript:Zm00001d036956_T001  rna5091     2.00E-112
766-  6:  transcript:Zm00001d036959_T003  rna5094             0
766-  7:  transcript:Zm00001d036966_T001  rna5101     8.00E-122
## Alignment 767: score=610.0 e_value=3e-33 N=14 6&NC_008395.2 minus
767-  0:  transcript:Zm00001d037218_T001  rna7113     1.00E-110
767-  1:  transcript:Zm00001d037220_T001  rna7108     7.00E-100
767-  2:  transcript:Zm00001d037221_T001  rna7107     2.00E-123
767-  3:  transcript:Zm00001d037225_T001  rna7103     1.00E-161
767-  4:  transcript:Zm00001d037227_T002  rna7102     2.00E-102
767-  5:  transcript:Zm00001d037228_T001  rna7098     3.00E-172
767-  6:  transcript:Zm00001d037234_T005  rna7094             0
767-  7:  transcript:Zm00001d037236_T001  rna7088      1.00E-81
767-  8:  transcript:Zm00001d037237_T002  rna7087      6.00E-91
767-  9:  transcript:Zm00001d037243_T001  rna7084      9.00E-47
767- 10:  transcript:Zm00001d037244_T001  rna7083      2.00E-42
767- 11:  transcript:Zm00001d037247_T007  rna7079      1.00E-80
767- 12:  transcript:Zm00001d037251_T001  rna7072      8.00E-46
767- 13:  transcript:Zm00001d037257_T001  rna7067             0
## Alignment 768: score=497.0 e_value=6.7e-24 N=11 6&NC_008395.2 minus
```

768- 0: transcript:Zm00001d037181_T001 rna7194 4.00E-27
768- 1: transcript:Zm00001d037189_T004 rna7192 0
768- 2: transcript:Zm00001d037192_T001 rna7179 5.00E-83
768- 3: transcript:Zm00001d037194_T001 rna7177 6.00E-38
768- 4: transcript:Zm00001d037198_T001 rna7175 0
768- 5: transcript:Zm00001d037200_T004 rna7173 2.00E-114
768- 6: transcript:Zm00001d037204_T001 rna7166 5.00E-92
768- 7: transcript:Zm00001d037205_T001 rna7165 2.00E-135
768- 8: transcript:Zm00001d037207_T001 rna7160 1.00E-155
768- 9: transcript:Zm00001d037209_T001 rna7159 3.00E-52
768- 10: transcript:Zm00001d037211_T001 rna7146 0
Alignment 769: score=273.0 e_value=2.8e-10 N=6 6&NC_008395.2 minus
769- 0: transcript:Zm00001d037479_T001 rna7452 1.00E-37
769- 1: transcript:Zm00001d037481_T003 rna7448 0
769- 2: transcript:Zm00001d037483_T002 rna7447 3.00E-122
769- 3: transcript:Zm00001d037484_T001 rna7445 0
769- 4: transcript:Zm00001d037492_T001 rna7440 0
769- 5: transcript:Zm00001d037493_T001 rna7437 0
Alignment 770: score=271.0 e_value=8.9e-10 N=6 6&NC_008395.2 minus
770- 0: transcript:Zm00001d037158_T001 rna7239 7.00E-79
770- 1: transcript:Zm00001d037160_T002 rna7236 2.00E-180
770- 2: transcript:Zm00001d037163_T002 rna7235 5.00E-126
770- 3: transcript:Zm00001d037170_T001 rna7223 2.00E-116
770- 4: transcript:Zm00001d037171_T001 rna7210 2.00E-24
770- 5: transcript:Zm00001d037173_T001 rna7206 3.00E-36
Alignment 771: score=348.0 e_value=6.9e-18 N=9 6&NC_008396.2 minus
771- 0: transcript:Zm00001d038511_T001 rna9192 7.00E-133
771- 1: transcript:Zm00001d038522_T002 rna9184 9.00E-61
771- 2: transcript:Zm00001d038527_T001 rna9164 3.00E-106
771- 3: transcript:Zm00001d038528_T001 rna9159 4.00E-50
771- 4: transcript:Zm00001d038532_T001 rna9154 5.00E-21
771- 5: transcript:Zm00001d038536_T003 rna9136 2.00E-38
771- 6: transcript:Zm00001d038537_T001 rna9135 2.00E-143
771- 7: transcript:Zm00001d038541_T002 rna9129 1.00E-128
771- 8: transcript:Zm00001d038543_T004 rna9124 2.00E-86
Alignment 772: score=6934.0 e_value=0 N=152 6&NC_008398.2 plus
772- 0: transcript:Zm00001d038342_T002 rna15616 1.00E-109
772- 1: transcript:Zm00001d038343_T001 rna15617 0
772- 2: transcript:Zm00001d038346_T002 rna15619 1.00E-149
772- 3: transcript:Zm00001d038348_T002 rna15620 1.00E-42
772- 4: transcript:Zm00001d038351_T004 rna15621 2.00E-64
772- 5: transcript:Zm00001d038352_T001 rna15625 4.00E-46
772- 6: transcript:Zm00001d038355_T001 rna15626 0
772- 7: transcript:Zm00001d038357_T001 rna15627 2.00E-18
772- 8: transcript:Zm00001d038358_T002 rna15628 0
772- 9: transcript:Zm00001d038361_T001 rna15631 3.00E-87
772- 10: transcript:Zm00001d038364_T001 rna15634 2.00E-16
772- 11: transcript:Zm00001d038366_T001 rna15643 0
772- 12: transcript:Zm00001d038367_T002 rna15645 2.00E-164
772- 13: transcript:Zm00001d038368_T002 rna15646 3.00E-102
772- 14: transcript:Zm00001d038371_T002 rna15647 0
772- 15: transcript:Zm00001d038373_T001 rna15655 0
772- 16: transcript:Zm00001d038374_T007 rna15657 2.00E-60
772- 17: transcript:Zm00001d038376_T001 rna15659 8.00E-122

772- 18:	transcript:Zm00001d038378_T001	rna15666	3.00E-108
772- 19:	transcript:Zm00001d038380_T004	rna15667	8.00E-152
772- 20:	transcript:Zm00001d038381_T001	rna15668	2.00E-76
772- 21:	transcript:Zm00001d038387_T001	rna15671	3.00E-56
772- 22:	transcript:Zm00001d038389_T008	rna15672	0
772- 23:	transcript:Zm00001d038392_T001	rna15673	0
772- 24:	transcript:Zm00001d038393_T012	rna15674	0
772- 25:	transcript:Zm00001d038394_T001	rna15676	0
772- 26:	transcript:Zm00001d038395_T005	rna15678	6.00E-53
772- 27:	transcript:Zm00001d038396_T011	rna15679	6.00E-109
772- 28:	transcript:Zm00001d038397_T005	rna15682	6.00E-28
772- 29:	transcript:Zm00001d038401_T001	rna15685	2.00E-12
772- 30:	transcript:Zm00001d038403_T001	rna15686	4.00E-11
772- 31:	transcript:Zm00001d038404_T003	rna15688	0
772- 32:	transcript:Zm00001d038407_T004	rna15694	0
772- 33:	transcript:Zm00001d038408_T001	rna15696	5.00E-70
772- 34:	transcript:Zm00001d038409_T001	rna15698	0
772- 35:	transcript:Zm00001d038411_T001	rna15709	3.00E-175
772- 36:	transcript:Zm00001d038412_T001	rna15711	0
772- 37:	transcript:Zm00001d038420_T001	rna15713	3.00E-64
772- 38:	transcript:Zm00001d038431_T001	rna15720	0
772- 39:	transcript:Zm00001d038432_T006	rna15723	0
772- 40:	transcript:Zm00001d038440_T001	rna15728	5.00E-164
772- 41:	transcript:Zm00001d038446_T001	rna15732	2.00E-23
772- 42:	transcript:Zm00001d038447_T001	rna15734	0
772- 43:	transcript:Zm00001d038449_T001	rna15737	5.00E-28
772- 44:	transcript:Zm00001d038450_T001	rna15739	3.00E-74
772- 45:	transcript:Zm00001d038451_T001	rna15740	9.00E-151
772- 46:	transcript:Zm00001d038459_T001	rna15742	0
772- 47:	transcript:Zm00001d038460_T007	rna15743	0
772- 48:	transcript:Zm00001d038465_T001	rna15745	0
772- 49:	transcript:Zm00001d038466_T001	rna15747	8.00E-59
772- 50:	transcript:Zm00001d038467_T001	rna15749	0
772- 51:	transcript:Zm00001d038469_T001	rna15750	9.00E-37
772- 52:	transcript:Zm00001d038471_T001	rna15751	0
772- 53:	transcript:Zm00001d038473_T001	rna15753	5.00E-94
772- 54:	transcript:Zm00001d038476_T001	rna15756	7.00E-159
772- 55:	transcript:Zm00001d038478_T001	rna15759	1.00E-42
772- 56:	transcript:Zm00001d038480_T001	rna15760	1.00E-41
772- 57:	transcript:Zm00001d038481_T001	rna15761	0
772- 58:	transcript:Zm00001d038483_T001	rna15764	3.00E-62
772- 59:	transcript:Zm00001d038485_T001	rna15765	3.00E-130
772- 60:	transcript:Zm00001d038486_T002	rna15767	2.00E-39
772- 61:	transcript:Zm00001d038487_T003	rna15769	0
772- 62:	transcript:Zm00001d038489_T001	rna15771	0
772- 63:	transcript:Zm00001d038490_T002	rna15772	3.00E-141
772- 64:	transcript:Zm00001d038491_T002	rna15774	2.00E-119
772- 65:	transcript:Zm00001d038503_T001	rna15775	0
772- 66:	transcript:Zm00001d038504_T001	rna15776	3.00E-91
772- 67:	transcript:Zm00001d038506_T001	rna15777	2.00E-101
772- 68:	transcript:Zm00001d038508_T001	rna15778	3.00E-34
772- 69:	transcript:Zm00001d038509_T001	rna15779	4.00E-21
772- 70:	transcript:Zm00001d038510_T001	rna15780	0
772- 71:	transcript:Zm00001d038511_T001	rna15782	0

772- 72:	transcript:Zm00001d038513_T002	rna15783	0
772- 73:	transcript:Zm00001d038514_T001	rna15787	3.00E-96
772- 74:	transcript:Zm00001d038517_T001	rna15788	0
772- 75:	transcript:Zm00001d038521_T001	rna15791	9.00E-59
772- 76:	transcript:Zm00001d038522_T002	rna15792	0
772- 77:	transcript:Zm00001d038525_T004	rna15794	6.00E-113
772- 78:	transcript:Zm00001d038526_T002	rna15796	4.00E-19
772- 79:	transcript:Zm00001d038527_T001	rna15801	0
772- 80:	transcript:Zm00001d038528_T001	rna15802	3.00E-150
772- 81:	transcript:Zm00001d038529_T003	rna15803	3.00E-172
772- 82:	transcript:Zm00001d038530_T003	rna15804	0
772- 83:	transcript:Zm00001d038531_T002	rna15805	3.00E-168
772- 84:	transcript:Zm00001d038532_T001	rna15806	3.00E-62
772- 85:	transcript:Zm00001d038533_T004	rna15807	3.00E-132
772- 86:	transcript:Zm00001d038535_T001	rna15812	4.00E-66
772- 87:	transcript:Zm00001d038536_T003	rna15813	0
772- 88:	transcript:Zm00001d038537_T001	rna15814	0
772- 89:	transcript:Zm00001d038538_T001	rna15815	1.00E-72
772- 90:	transcript:Zm00001d038540_T001	rna15816	1.00E-92
772- 91:	transcript:Zm00001d038541_T002	rna15818	0
772- 92:	transcript:Zm00001d038542_T001	rna15820	1.00E-67
772- 93:	transcript:Zm00001d038543_T004	rna15822	3.00E-86
772- 94:	transcript:Zm00001d038546_T001	rna15825	1.00E-162
772- 95:	transcript:Zm00001d038548_T001	rna15829	0
772- 96:	transcript:Zm00001d038549_T001	rna15833	4.00E-122
772- 97:	transcript:Zm00001d038553_T001	rna15836	0
772- 98:	transcript:Zm00001d038554_T001	rna15837	0
772- 99:	transcript:Zm00001d038555_T001	rna15838	0
772-100:	transcript:Zm00001d038557_T005	rna15839	3.00E-98
772-101:	transcript:Zm00001d038558_T005	rna15840	5.00E-44
772-102:	transcript:Zm00001d038562_T001	rna15841	0
772-103:	transcript:Zm00001d038563_T001	rna15842	3.00E-132
772-104:	transcript:Zm00001d038570_T005	rna15844	2.00E-131
772-105:	transcript:Zm00001d038572_T001	rna15845	1.00E-159
772-106:	transcript:Zm00001d038574_T004	rna15846	8.00E-104
772-107:	transcript:Zm00001d038576_T001	rna15848	0
772-108:	transcript:Zm00001d038577_T001	rna15850	0
772-109:	transcript:Zm00001d038578_T001	rna15851	2.00E-46
772-110:	transcript:Zm00001d038579_T001	rna15853	6.00E-113
772-111:	transcript:Zm00001d038583_T001	rna15856	0
772-112:	transcript:Zm00001d038584_T001	rna15857	2.00E-56
772-113:	transcript:Zm00001d038587_T033	rna15859	2.00E-136
772-114:	transcript:Zm00001d038589_T005	rna15863	0
772-115:	transcript:Zm00001d038594_T002	rna15867	4.00E-69
772-116:	transcript:Zm00001d038595_T001	rna15868	3.00E-60
772-117:	transcript:Zm00001d038597_T001	rna15872	3.00E-42
772-118:	transcript:Zm00001d038598_T001	rna15873	0
772-119:	transcript:Zm00001d038600_T001	rna15875	7.00E-46
772-120:	transcript:Zm00001d038604_T001	rna15876	3.00E-177
772-121:	transcript:Zm00001d038606_T001	rna15880	3.00E-140
772-122:	transcript:Zm00001d038607_T001	rna15881	8.00E-42
772-123:	transcript:Zm00001d038608_T001	rna15882	8.00E-96
772-124:	transcript:Zm00001d038609_T001	rna15883	0
772-125:	transcript:Zm00001d038610_T001	rna15884	1.00E-97

772-126: transcript:Zm00001d038612_T001 rna15886 7.00E-31
772-127: transcript:Zm00001d038613_T001 rna15887 9.00E-23
772-128: transcript:Zm00001d038616_T001 rna15891 9.00E-117
772-129: transcript:Zm00001d038618_T001 rna15892 0
772-130: transcript:Zm00001d038620_T006 rna15894 0
772-131: transcript:Zm00001d038622_T001 rna15898 0
772-132: transcript:Zm00001d038623_T001 rna15899 9.00E-175
772-133: transcript:Zm00001d038625_T001 rna15900 0
772-134: transcript:Zm00001d038641_T001 rna15902 6.00E-13
772-135: transcript:Zm00001d038642_T001 rna15903 0
772-136: transcript:Zm00001d038643_T001 rna15904 3.00E-115
772-137: transcript:Zm00001d038644_T004 rna15905 4.00E-176
772-138: transcript:Zm00001d038645_T001 rna15908 6.00E-52
772-139: transcript:Zm00001d038646_T002 rna15912 2.00E-117
772-140: transcript:Zm00001d038647_T001 rna15913 4.00E-64
772-141: transcript:Zm00001d038648_T005 rna15915 0
772-142: transcript:Zm00001d038650_T001 rna15917 0
772-143: transcript:Zm00001d038651_T004 rna15918 0
772-144: transcript:Zm00001d038652_T003 rna15919 5.00E-65
772-145: transcript:Zm00001d038655_T003 rna15920 0
772-146: transcript:Zm00001d038657_T005 rna15921 0
772-147: transcript:Zm00001d038658_T001 rna15923 3.00E-172
772-148: transcript:Zm00001d038660_T003 rna15925 0
772-149: transcript:Zm00001d038661_T002 rna15926 0
772-150: transcript:Zm00001d038665_T001 rna15934 0
772-151: transcript:Zm00001d038668_T001 rna15945 8.00E-120
Alignment 773: score=4215.0 e_value=0 N=91 6&NC_008398.2 plus
773- 0: transcript:Zm00001d038820_T001 rna16089 2.00E-69
773- 1: transcript:Zm00001d038823_T001 rna16091 0
773- 2: transcript:Zm00001d038824_T002 rna16094 0
773- 3: transcript:Zm00001d038829_T001 rna16095 0
773- 4: transcript:Zm00001d038830_T002 rna16096 4.00E-100
773- 5: transcript:Zm00001d038835_T002 rna16098 0
773- 6: transcript:Zm00001d038838_T001 rna16099 0
773- 7: transcript:Zm00001d038840_T001 rna16101 3.00E-159
773- 8: transcript:Zm00001d038841_T007 rna16103 5.00E-51
773- 9: transcript:Zm00001d038842_T001 rna16105 2.00E-116
773- 10: transcript:Zm00001d038843_T001 rna16106 9.00E-44
773- 11: transcript:Zm00001d038844_T001 rna16107 0
773- 12: transcript:Zm00001d038845_T002 rna16108 0
773- 13: transcript:Zm00001d038846_T001 rna16109 0
773- 14: transcript:Zm00001d038847_T001 rna16113 1.00E-163
773- 15: transcript:Zm00001d038849_T005 rna16114 5.00E-142
773- 16: transcript:Zm00001d038850_T001 rna16115 2.00E-58
773- 17: transcript:Zm00001d038851_T001 rna16116 0
773- 18: transcript:Zm00001d038852_T001 rna16117 4.00E-142
773- 19: transcript:Zm00001d038854_T006 rna16119 0
773- 20: transcript:Zm00001d038855_T001 rna16120 0
773- 21: transcript:Zm00001d038856_T001 rna16121 5.00E-30
773- 22: transcript:Zm00001d038857_T001 rna16122 0
773- 23: transcript:Zm00001d038860_T001 rna16125 2.00E-70
773- 24: transcript:Zm00001d038861_T001 rna16127 3.00E-141
773- 25: transcript:Zm00001d038862_T001 rna16128 1.00E-129
773- 26: transcript:Zm00001d038863_T001 rna16129 3.00E-66

773- 27:	transcript:Zm00001d038864_T001	rna16131	6.00E-09
773- 28:	transcript:Zm00001d038865_T003	rna16133	2.00E-19
773- 29:	transcript:Zm00001d038866_T002	rna16136	0
773- 30:	transcript:Zm00001d038870_T001	rna16138	2.00E-54
773- 31:	transcript:Zm00001d038871_T001	rna16139	0
773- 32:	transcript:Zm00001d038873_T001	rna16140	2.00E-12
773- 33:	transcript:Zm00001d038874_T002	rna16141	0
773- 34:	transcript:Zm00001d038876_T001	rna16143	2.00E-21
773- 35:	transcript:Zm00001d038877_T001	rna16146	0
773- 36:	transcript:Zm00001d038878_T001	rna16148	2.00E-102
773- 37:	transcript:Zm00001d038879_T003	rna16149	0
773- 38:	transcript:Zm00001d038880_T001	rna16152	0
773- 39:	transcript:Zm00001d038882_T002	rna16153	3.00E-48
773- 40:	transcript:Zm00001d038883_T001	rna16154	0
773- 41:	transcript:Zm00001d038884_T001	rna16155	1.00E-151
773- 42:	transcript:Zm00001d038886_T001	rna16170	9.00E-37
773- 43:	transcript:Zm00001d038887_T001	rna16171	2.00E-67
773- 44:	transcript:Zm00001d038890_T002	rna16176	5.00E-26
773- 45:	transcript:Zm00001d038891_T001	rna16177	1.00E-130
773- 46:	transcript:Zm00001d038892_T001	rna16178	0
773- 47:	transcript:Zm00001d038894_T002	rna16179	0
773- 48:	transcript:Zm00001d038901_T001	rna16180	1.00E-56
773- 49:	transcript:Zm00001d038903_T001	rna16181	1.00E-65
773- 50:	transcript:Zm00001d038904_T001	rna16182	0
773- 51:	transcript:Zm00001d038907_T001	rna16183	0
773- 52:	transcript:Zm00001d038908_T004	rna16184	1.00E-139
773- 53:	transcript:Zm00001d038909_T001	rna16185	4.00E-24
773- 54:	transcript:Zm00001d038911_T001	rna16187	2.00E-31
773- 55:	transcript:Zm00001d038915_T001	rna16190	4.00E-97
773- 56:	transcript:Zm00001d038916_T001	rna16191	0
773- 57:	transcript:Zm00001d038918_T001	rna16193	2.00E-49
773- 58:	transcript:Zm00001d038919_T001	rna16194	2.00E-54
773- 59:	transcript:Zm00001d038921_T001	rna16198	2.00E-140
773- 60:	transcript:Zm00001d038922_T001	rna16199	5.00E-22
773- 61:	transcript:Zm00001d038924_T001	rna16202	0
773- 62:	transcript:Zm00001d038926_T001	rna16204	3.00E-106
773- 63:	transcript:Zm00001d038927_T001	rna16205	3.00E-178
773- 64:	transcript:Zm00001d038929_T001	rna16210	0
773- 65:	transcript:Zm00001d038930_T001	rna16212	5.00E-96
773- 66:	transcript:Zm00001d038931_T002	rna16213	2.00E-180
773- 67:	transcript:Zm00001d038932_T001	rna16214	9.00E-131
773- 68:	transcript:Zm00001d038933_T001	rna16216	3.00E-75
773- 69:	transcript:Zm00001d038936_T001	rna16217	0
773- 70:	transcript:Zm00001d038937_T001	rna16218	0
773- 71:	transcript:Zm00001d038944_T003	rna16222	0
773- 72:	transcript:Zm00001d038946_T001	rna16224	2.00E-62
773- 73:	transcript:Zm00001d038948_T001	rna16225	0
773- 74:	transcript:Zm00001d038950_T001	rna16226	8.00E-19
773- 75:	transcript:Zm00001d038954_T001	rna16228	0
773- 76:	transcript:Zm00001d038955_T001	rna16229	0
773- 77:	transcript:Zm00001d038959_T001	rna16230	4.00E-36
773- 78:	transcript:Zm00001d038963_T001	rna16232	7.00E-61
773- 79:	transcript:Zm00001d038965_T001	rna16234	5.00E-174
773- 80:	transcript:Zm00001d038968_T001	rna16237	0

773- 81: transcript:Zm00001d038970_T004 rna16238 0
773- 82: transcript:Zm00001d038971_T001 rna16239 0
773- 83: transcript:Zm00001d038972_T002 rna16241 0
773- 84: transcript:Zm00001d038973_T001 rna16243 1.00E-109
773- 85: transcript:Zm00001d038974_T001 rna16244 0
773- 86: transcript:Zm00001d038975_T001 rna16245 8.00E-143
773- 87: transcript:Zm00001d038978_T001 rna16247 2.00E-44
773- 88: transcript:Zm00001d038980_T001 rna16251 9.00E-119
773- 89: transcript:Zm00001d038981_T001 rna16252 0
773- 90: transcript:Zm00001d038982_T007 rna16255 0
Alignment 774: score=2719.0 e_value=3.5e-229 N=59 6&NC_008398.2 plus
774- 0: transcript:Zm00001d039004_T002 rna16274 1.00E-162
774- 1: transcript:Zm00001d039005_T003 rna16275 1.00E-75
774- 2: transcript:Zm00001d039006_T007 rna16277 0
774- 3: transcript:Zm00001d039007_T001 rna16278 1.00E-28
774- 4: transcript:Zm00001d039010_T001 rna16279 2.00E-36
774- 5: transcript:Zm00001d039012_T001 rna16283 7.00E-46
774- 6: transcript:Zm00001d039013_T001 rna16284 4.00E-139
774- 7: transcript:Zm00001d039014_T001 rna16285 2.00E-160
774- 8: transcript:Zm00001d039015_T001 rna16286 6.00E-111
774- 9: transcript:Zm00001d039016_T001 rna16287 4.00E-152
774- 10: transcript:Zm00001d039017_T001 rna16288 4.00E-116
774- 11: transcript:Zm00001d039020_T001 rna16289 9.00E-58
774- 12: transcript:Zm00001d039021_T001 rna16291 2.00E-128
774- 13: transcript:Zm00001d039029_T001 rna16294 1.00E-153
774- 14: transcript:Zm00001d039031_T001 rna16296 1.00E-41
774- 15: transcript:Zm00001d039032_T001 rna16297 1.00E-82
774- 16: transcript:Zm00001d039036_T003 rna16299 4.00E-139
774- 17: transcript:Zm00001d039037_T001 rna16300 0
774- 18: transcript:Zm00001d039038_T001 rna16301 0
774- 19: transcript:Zm00001d039039_T002 rna16304 2.00E-107
774- 20: transcript:Zm00001d039041_T007 rna16305 0
774- 21: transcript:Zm00001d039043_T011 rna16306 0
774- 22: transcript:Zm00001d039047_T001 rna16308 0
774- 23: transcript:Zm00001d039050_T001 rna16309 2.00E-117
774- 24: transcript:Zm00001d039051_T002 rna16310 6.00E-87
774- 25: transcript:Zm00001d039053_T001 rna16312 0
774- 26: transcript:Zm00001d039057_T001 rna16316 2.00E-33
774- 27: transcript:Zm00001d039059_T001 rna16318 0
774- 28: transcript:Zm00001d039064_T001 rna16320 1.00E-70
774- 29: transcript:Zm00001d039065_T011 rna16321 0
774- 30: transcript:Zm00001d039066_T001 rna16322 8.00E-100
774- 31: transcript:Zm00001d039071_T001 rna16330 1.00E-74
774- 32: transcript:Zm00001d039072_T005 rna16331 0
774- 33: transcript:Zm00001d039076_T001 rna16335 7.00E-80
774- 34: transcript:Zm00001d039077_T001 rna16339 2.00E-21
774- 35: transcript:Zm00001d039078_T001 rna16341 0
774- 36: transcript:Zm00001d039079_T002 rna16343 0
774- 37: transcript:Zm00001d039081_T002 rna16344 0
774- 38: transcript:Zm00001d039084_T001 rna16346 2.00E-24
774- 39: transcript:Zm00001d039086_T001 rna16347 5.00E-77
774- 40: transcript:Zm00001d039087_T001 rna16348 2.00E-92
774- 41: transcript:Zm00001d039089_T001 rna16351 0
774- 42: transcript:Zm00001d039090_T001 rna16352 1.00E-158

774- 43:	transcript:Zm00001d039094_T001	rna16353	0
774- 44:	transcript:Zm00001d039096_T001	rna16355	0
774- 45:	transcript:Zm00001d039098_T001	rna16357	2.00E-22
774- 46:	transcript:Zm00001d039101_T001	rna16362	6.00E-102
774- 47:	transcript:Zm00001d039102_T001	rna16363	2.00E-20
774- 48:	transcript:Zm00001d039105_T001	rna16366	0
774- 49:	transcript:Zm00001d039108_T001	rna16369	3.00E-165
774- 50:	transcript:Zm00001d039109_T001	rna16370	2.00E-35
774- 51:	transcript:Zm00001d039110_T001	rna16371	2.00E-98
774- 52:	transcript:Zm00001d039111_T002	rna16375	4.00E-69
774- 53:	transcript:Zm00001d039112_T001	rna16376	3.00E-63
774- 54:	transcript:Zm00001d039113_T001	rna16380	4.00E-42
774- 55:	transcript:Zm00001d039115_T001	rna16382	2.00E-152
774- 56:	transcript:Zm00001d039116_T001	rna16383	0
774- 57:	transcript:Zm00001d039118_T001	rna16386	3.00E-45
774- 58:	transcript:Zm00001d039120_T002	rna16388	0

Alignment 775: score=2063.0 e_value=1.2e-168 N=46 6&NC_008398.2 plus

775- 0:	transcript:Zm00001d038049_T001	rna15252	0
775- 1:	transcript:Zm00001d038051_T001	rna15253	0
775- 2:	transcript:Zm00001d038054_T002	rna15259	0
775- 3:	transcript:Zm00001d038056_T001	rna15260	2.00E-44
775- 4:	transcript:Zm00001d038057_T002	rna15261	5.00E-62
775- 5:	transcript:Zm00001d038059_T001	rna15263	0
775- 6:	transcript:Zm00001d038061_T001	rna15272	2.00E-82
775- 7:	transcript:Zm00001d038062_T001	rna15275	2.00E-97
775- 8:	transcript:Zm00001d038063_T001	rna15277	0
775- 9:	transcript:Zm00001d038065_T001	rna15282	6.00E-41
775- 10:	transcript:Zm00001d038068_T001	rna15289	0
775- 11:	transcript:Zm00001d038070_T001	rna15292	7.00E-95
775- 12:	transcript:Zm00001d038076_T002	rna15297	0
775- 13:	transcript:Zm00001d038084_T001	rna15304	0
775- 14:	transcript:Zm00001d038085_T001	rna15305	0
775- 15:	transcript:Zm00001d038087_T002	rna15306	7.00E-155
775- 16:	transcript:Zm00001d038088_T002	rna15307	8.00E-60
775- 17:	transcript:Zm00001d038089_T001	rna15308	3.00E-113
775- 18:	transcript:Zm00001d038091_T006	rna15309	0
775- 19:	transcript:Zm00001d038092_T005	rna15310	0
775- 20:	transcript:Zm00001d038097_T002	rna15316	0
775- 21:	transcript:Zm00001d038098_T001	rna15317	2.00E-61
775- 22:	transcript:Zm00001d038100_T002	rna15318	0
775- 23:	transcript:Zm00001d038101_T002	rna15323	0
775- 24:	transcript:Zm00001d038104_T003	rna15324	0
775- 25:	transcript:Zm00001d038105_T002	rna15325	0
775- 26:	transcript:Zm00001d038107_T002	rna15327	2.00E-57
775- 27:	transcript:Zm00001d038108_T001	rna15328	6.00E-71
775- 28:	transcript:Zm00001d038109_T002	rna15329	0
775- 29:	transcript:Zm00001d038113_T001	rna15330	0
775- 30:	transcript:Zm00001d038114_T001	rna15331	0
775- 31:	transcript:Zm00001d038117_T001	rna15334	2.00E-75
775- 32:	transcript:Zm00001d038118_T001	rna15335	7.00E-164
775- 33:	transcript:Zm00001d038121_T001	rna15336	3.00E-137
775- 34:	transcript:Zm00001d038122_T001	rna15342	0
775- 35:	transcript:Zm00001d038123_T003	rna15343	0
775- 36:	transcript:Zm00001d038125_T001	rna15346	0

775- 37: transcript:Zm00001d038126_T001 rna15347 1.00E-44
775- 38: transcript:Zm00001d038127_T001 rna15349 5.00E-130
775- 39: transcript:Zm00001d038128_T004 rna15350 0
775- 40: transcript:Zm00001d038131_T004 rna15352 0
775- 41: transcript:Zm00001d038133_T001 rna15353 6.00E-41
775- 42: transcript:Zm00001d038134_T001 rna15354 0
775- 43: transcript:Zm00001d038135_T001 rna15355 0
775- 44: transcript:Zm00001d038136_T002 rna15356 2.00E-140
775- 45: transcript:Zm00001d038139_T001 rna15367 4.00E-127
Alignment 776: score=1838.0 e_value=4.1e-143 N=41 6&NC_008398.2 plus
776- 0: transcript:Zm00001d038164_T001 rna15402 7.00E-21
776- 1: transcript:Zm00001d038165_T003 rna15404 0
776- 2: transcript:Zm00001d038166_T002 rna15406 0
776- 3: transcript:Zm00001d038167_T001 rna15408 0
776- 4: transcript:Zm00001d038170_T001 rna15409 0
776- 5: transcript:Zm00001d038171_T001 rna15410 0
776- 6: transcript:Zm00001d038173_T001 rna15412 5.00E-57
776- 7: transcript:Zm00001d038174_T002 rna15413 0
776- 8: transcript:Zm00001d038176_T001 rna15415 0
776- 9: transcript:Zm00001d038177_T001 rna15417 3.00E-151
776- 10: transcript:Zm00001d038179_T001 rna15418 4.00E-144
776- 11: transcript:Zm00001d038189_T010 rna15421 0
776- 12: transcript:Zm00001d038190_T012 rna15423 3.00E-73
776- 13: transcript:Zm00001d038191_T001 rna15425 1.00E-113
776- 14: transcript:Zm00001d038192_T001 rna15429 1.00E-26
776- 15: transcript:Zm00001d038193_T005 rna15433 1.00E-166
776- 16: transcript:Zm00001d038195_T001 rna15439 0
776- 17: transcript:Zm00001d038196_T001 rna15444 0
776- 18: transcript:Zm00001d038197_T001 rna15448 1.00E-78
776- 19: transcript:Zm00001d038198_T001 rna15449 0
776- 20: transcript:Zm00001d038204_T001 rna15452 9.00E-109
776- 21: transcript:Zm00001d038205_T002 rna15456 1.00E-175
776- 22: transcript:Zm00001d038207_T001 rna15457 2.00E-132
776- 23: transcript:Zm00001d038208_T001 rna15459 9.00E-150
776- 24: transcript:Zm00001d038209_T001 rna15460 0
776- 25: transcript:Zm00001d038211_T001 rna15463 0
776- 26: transcript:Zm00001d038217_T002 rna15467 0
776- 27: transcript:Zm00001d038218_T001 rna15468 1.00E-74
776- 28: transcript:Zm00001d038220_T001 rna15472 8.00E-58
776- 29: transcript:Zm00001d038221_T001 rna15474 7.00E-71
776- 30: transcript:Zm00001d038222_T003 rna15489 0
776- 31: transcript:Zm00001d038223_T001 rna15490 0
776- 32: transcript:Zm00001d038224_T003 rna15492 0
776- 33: transcript:Zm00001d038226_T001 rna15493 9.00E-136
776- 34: transcript:Zm00001d038227_T002 rna15495 1.00E-160
776- 35: transcript:Zm00001d038228_T001 rna15496 0
776- 36: transcript:Zm00001d038229_T001 rna15497 6.00E-162
776- 37: transcript:Zm00001d038239_T001 rna15499 6.00E-37
776- 38: transcript:Zm00001d038248_T002 rna15503 2.00E-82
776- 39: transcript:Zm00001d038251_T001 rna15509 8.00E-105
776- 40: transcript:Zm00001d038252_T001 rna15511 0
Alignment 777: score=1200.0 e_value=3e-83 N=26 6&NC_008398.2 plus
777- 0: transcript:Zm00001d038676_T001 rna15936 4.00E-152
777- 1: transcript:Zm00001d038678_T001 rna15949 0

```
777-  2:  transcript:Zm00001d038681_T002  rna15951     9.00E-23
777-  3:  transcript:Zm00001d038682_T001  rna15952     3.00E-37
777-  4:  transcript:Zm00001d038683_T001  rna15953    2.00E-104
777-  5:  transcript:Zm00001d038684_T003  rna15955    8.00E-144
777-  6:  transcript:Zm00001d038685_T002  rna15956            0
777-  7:  transcript:Zm00001d038687_T001  rna15958            0
777-  8:  transcript:Zm00001d038688_T002  rna15959    2.00E-139
777-  9:  transcript:Zm00001d038689_T001  rna15960    7.00E-167
777- 10:  transcript:Zm00001d038690_T001  rna15961            0
777- 11:  transcript:Zm00001d038691_T001  rna15962            0
777- 12:  transcript:Zm00001d038692_T001  rna15963     2.00E-17
777- 13:  transcript:Zm00001d038695_T001  rna15965     2.00E-46
777- 14:  transcript:Zm00001d038698_T001  rna15967            0
777- 15:  transcript:Zm00001d038699_T001  rna15968    1.00E-165
777- 16:  transcript:Zm00001d038704_T002  rna15970            0
777- 17:  transcript:Zm00001d038706_T001  rna15973     2.00E-30
777- 18:  transcript:Zm00001d038708_T001  rna15975    3.00E-179
777- 19:  transcript:Zm00001d038709_T001  rna15977    2.00E-150
777- 20:  transcript:Zm00001d038712_T001  rna15978     5.00E-30
777- 21:  transcript:Zm00001d038718_T001  rna15985     1.00E-85
777- 22:  transcript:Zm00001d038719_T001  rna15986     4.00E-24
777- 23:  transcript:Zm00001d038724_T001  rna15987     7.00E-49
777- 24:  transcript:Zm00001d038726_T001  rna15989    3.00E-172
777- 25:  transcript:Zm00001d038728_T003  rna15990    3.00E-128
## Alignment 778: score=818.0 e_value=9.9e-48 N=18 6&NC_008398.2 plus
778-  0:  transcript:Zm00001d038280_T001  rna15563            0
778-  1:  transcript:Zm00001d038281_T001  rna15564    2.00E-103
778-  2:  transcript:Zm00001d038282_T001  rna15568            0
778-  3:  transcript:Zm00001d038283_T001  rna15569     5.00E-38
778-  4:  transcript:Zm00001d038284_T001  rna15570     1.00E-77
778-  5:  transcript:Zm00001d038287_T001  rna15572     6.00E-29
778-  6:  transcript:Zm00001d038288_T001  rna15573     1.00E-90
778-  7:  transcript:Zm00001d038289_T001  rna15574    1.00E-106
778-  8:  transcript:Zm00001d038290_T004  rna15576            0
778-  9:  transcript:Zm00001d038291_T001  rna15578     4.00E-76
778- 10:  transcript:Zm00001d038292_T004  rna15579    1.00E-170
778- 11:  transcript:Zm00001d038296_T001  rna15581            0
778- 12:  transcript:Zm00001d038297_T001  rna15582    2.00E-130
778- 13:  transcript:Zm00001d038299_T003  rna15583            0
778- 14:  transcript:Zm00001d038300_T002  rna15585            0
778- 15:  transcript:Zm00001d038301_T002  rna15587            0
778- 16:  transcript:Zm00001d038302_T041  rna15588            0
778- 17:  transcript:Zm00001d038304_T001  rna15594    3.00E-105
## Alignment 779: score=512.0 e_value=5.1e-26 N=11 6&NC_008398.2 plus
779-  0:  transcript:Zm00001d038984_T001  rna16256     3.00E-67
779-  1:  transcript:Zm00001d038989_T002  rna16257            0
779-  2:  transcript:Zm00001d038990_T001  rna16258    5.00E-122
779-  3:  transcript:Zm00001d038991_T001  rna16259    8.00E-180
779-  4:  transcript:Zm00001d038993_T001  rna16261     2.00E-18
779-  5:  transcript:Zm00001d038994_T001  rna16262    3.00E-131
779-  6:  transcript:Zm00001d038996_T001  rna16263    2.00E-169
779-  7:  transcript:Zm00001d038998_T001  rna16266    1.00E-149
779-  8:  transcript:Zm00001d038999_T001  rna16270    2.00E-138
779-  9:  transcript:Zm00001d039000_T001  rna16271            0
```

779- 10: transcript:Zm00001d039002_T019 rna16272 0
Alignment 780: score=421.0 e_value=2.8e-18 N=9 6&NC_008398.2 plus
780- 0: transcript:Zm00001d038003_T001 rna15118 1.00E-35
780- 1: transcript:Zm00001d038004_T001 rna15119 2.00E-103
780- 2: transcript:Zm00001d038005_T001 rna15120 5.00E-45
780- 3: transcript:Zm00001d038006_T001 rna15121 0
780- 4: transcript:Zm00001d038007_T001 rna15124 0
780- 5: transcript:Zm00001d038012_T001 rna15127 1.00E-116
780- 6: transcript:Zm00001d038013_T001 rna15129 9.00E-89
780- 7: transcript:Zm00001d038016_T001 rna15132 1.00E-91
780- 8: transcript:Zm00001d038022_T001 rna15149 0
Alignment 781: score=405.0 e_value=4.1e-18 N=9 6&NC_008398.2 plus
781- 0: transcript:Zm00001d037982_T006 rna15136 0
781- 1: transcript:Zm00001d037984_T004 rna15139 0
781- 2: transcript:Zm00001d037985_T001 rna15140 4.00E-40
781- 3: transcript:Zm00001d037992_T001 rna15141 0
781- 4: transcript:Zm00001d037993_T001 rna15142 7.00E-96
781- 5: transcript:Zm00001d037994_T001 rna15144 7.00E-60
781- 6: transcript:Zm00001d038001_T001 rna15145 4.00E-56
781- 7: transcript:Zm00001d038002_T001 rna15146 7.00E-80
781- 8: transcript:Zm00001d038018_T001 rna15152 3.00E-108
Alignment 782: score=397.0 e_value=8.1e-19 N=9 6&NC_008398.2 plus
782- 0: transcript:Zm00001d039131_T007 rna16389 0
782- 1: transcript:Zm00001d039132_T001 rna16391 5.00E-117
782- 2: transcript:Zm00001d039135_T001 rna16400 7.00E-53
782- 3: transcript:Zm00001d039138_T012 rna16401 0
782- 4: transcript:Zm00001d039139_T001 rna16402 7.00E-72
782- 5: transcript:Zm00001d039140_T001 rna16403 4.00E-78
782- 6: transcript:Zm00001d039144_T002 rna16404 0
782- 7: transcript:Zm00001d039147_T003 rna16411 0
782- 8: transcript:Zm00001d039150_T001 rna16421 0
Alignment 783: score=391.0 e_value=8.1e-19 N=9 6&NC_008398.2 plus
783- 0: transcript:Zm00001d037839_T001 rna15037 0
783- 1: transcript:Zm00001d037843_T001 rna15049 5.00E-33
783- 2: transcript:Zm00001d037849_T001 rna15050 0
783- 3: transcript:Zm00001d037854_T001 rna15054 8.00E-88
783- 4: transcript:Zm00001d037856_T001 rna15056 0
783- 5: transcript:Zm00001d037857_T001 rna15057 2.00E-23
783- 6: transcript:Zm00001d037858_T005 rna15058 0
783- 7: transcript:Zm00001d037859_T002 rna15059 3.00E-149
783- 8: transcript:Zm00001d037860_T001 rna15061 2.00E-48
Alignment 784: score=374.0 e_value=1.2e-15 N=8 6&NC_008398.2 plus
784- 0: transcript:Zm00001d037890_T001 rna15217 6.00E-140
784- 1: transcript:Zm00001d037891_T001 rna15221 1.00E-07
784- 2: transcript:Zm00001d037892_T001 rna15225 2.00E-66
784- 3: transcript:Zm00001d037897_T002 rna15233 4.00E-23
784- 4: transcript:Zm00001d037898_T012 rna15234 5.00E-31
784- 5: transcript:Zm00001d037899_T001 rna15235 6.00E-83
784- 6: transcript:Zm00001d037900_T001 rna15239 0
784- 7: transcript:Zm00001d037902_T001 rna15240 4.00E-116
Alignment 785: score=370.0 e_value=2.8e-14 N=8 6&NC_008398.2 plus
785- 0: transcript:Zm00001d037593_T004 rna14456 0
785- 1: transcript:Zm00001d037596_T001 rna14457 0
785- 2: transcript:Zm00001d037599_T001 rna14458 0

785- 3: transcript:Zm00001d037602_T001 rna14459 4.00E-163
785- 4: transcript:Zm00001d037603_T002 rna14462 7.00E-120
785- 5: transcript:Zm00001d037604_T001 rna14463 0
785- 6: transcript:Zm00001d037605_T001 rna14465 3.00E-41
785- 7: transcript:Zm00001d037606_T001 rna14466 0
Alignment 786: score=344.0 e_value=2.9e-11 N=7 6&NC_008398.2 plus
786- 0: transcript:Zm00001d037704_T001 rna14598 1.00E-112
786- 1: transcript:Zm00001d037711_T001 rna14599 0
786- 2: transcript:Zm00001d037712_T001 rna14600 0
786- 3: transcript:Zm00001d037713_T001 rna14601 4.00E-64
786- 4: transcript:Zm00001d037714_T001 rna14603 1.00E-37
786- 5: transcript:Zm00001d037715_T001 rna14604 1.00E-08
786- 6: transcript:Zm00001d037717_T002 rna14605 0
Alignment 787: score=336.0 e_value=1e-13 N=8 6&NC_008398.2 plus
787- 0: transcript:Zm00001d038142_T034 rna15368 2.00E-49
787- 1: transcript:Zm00001d038143_T002 rna15369 1.00E-70
787- 2: transcript:Zm00001d038147_T001 rna15374 1.00E-173
787- 3: transcript:Zm00001d038150_T001 rna15376 1.00E-19
787- 4: transcript:Zm00001d038151_T001 rna15378 0
787- 5: transcript:Zm00001d038158_T001 rna15381 4.00E-108
787- 6: transcript:Zm00001d038161_T001 rna15386 2.00E-109
787- 7: transcript:Zm00001d038163_T001 rna15390 0
Alignment 788: score=312.0 e_value=7.2e-12 N=7 6&NC_008398.2 plus
788- 0: transcript:Zm00001d035701_T001 rna14149 0
788- 1: transcript:Zm00001d035702_T001 rna14150 3.00E-79
788- 2: transcript:Zm00001d035717_T001 rna14151 1.00E-147
788- 3: transcript:Zm00001d035721_T002 rna14153 3.00E-96
788- 4: transcript:Zm00001d035722_T007 rna14154 0
788- 5: transcript:Zm00001d035723_T001 rna14155 0
788- 6: transcript:Zm00001d035731_T002 rna14159 0
Alignment 789: score=290.0 e_value=3.6e-11 N=7 6&NC_008398.2 plus
789- 0: transcript:Zm00001d037771_T001 rna14785 3.00E-127
789- 1: transcript:Zm00001d037772_T001 rna14790 0
789- 2: transcript:Zm00001d037775_T001 rna14792 3.00E-121
789- 3: transcript:Zm00001d037779_T001 rna14794 4.00E-135
789- 4: transcript:Zm00001d037781_T001 rna14805 0
789- 5: transcript:Zm00001d037782_T001 rna14809 0
789- 6: transcript:Zm00001d037790_T001 rna14811 0
Alignment 790: score=259.0 e_value=5.2e-10 N=6 6&NC_008398.2 plus
790- 0: transcript:Zm00001d035526_T010 rna14227 0
790- 1: transcript:Zm00001d035527_T001 rna14228 0
790- 2: transcript:Zm00001d035540_T001 rna14230 4.00E-27
790- 3: transcript:Zm00001d035543_T001 rna14235 2.00E-15
790- 4: transcript:Zm00001d035551_T007 rna14242 2.00E-21
790- 5: transcript:Zm00001d035556_T001 rna14244 5.00E-76
Alignment 791: score=2147.0 e_value=4.3e-177 N=47 6&NC_008398.2 minus
791- 0: transcript:Zm00001d038730_T001 rna16086 9.00E-108
791- 1: transcript:Zm00001d038732_T001 rna16085 1.00E-93
791- 2: transcript:Zm00001d038733_T001 rna16084 4.00E-135
791- 3: transcript:Zm00001d038734_T003 rna16083 0
791- 4: transcript:Zm00001d038737_T001 rna16079 0
791- 5: transcript:Zm00001d038739_T001 rna16069 0
791- 6: transcript:Zm00001d038740_T002 rna16067 4.00E-14
791- 7: transcript:Zm00001d038742_T001 rna16064 0

791- 8:	transcript:Zm00001d038745_T002	rna16063	0
791- 9:	transcript:Zm00001d038746_T001	rna16062	1.00E-156
791- 10:	transcript:Zm00001d038747_T003	rna16061	1.00E-27
791- 11:	transcript:Zm00001d038750_T003	rna16060	0
791- 12:	transcript:Zm00001d038751_T001	rna16059	1.00E-160
791- 13:	transcript:Zm00001d038752_T001	rna16058	0
791- 14:	transcript:Zm00001d038753_T002	rna16057	3.00E-55
791- 15:	transcript:Zm00001d038754_T002	rna16056	3.00E-106
791- 16:	transcript:Zm00001d038755_T001	rna16055	1.00E-131
791- 17:	transcript:Zm00001d038756_T001	rna16054	3.00E-177
791- 18:	transcript:Zm00001d038762_T001	rna16051	0
791- 19:	transcript:Zm00001d038763_T001	rna16043	2.00E-115
791- 20:	transcript:Zm00001d038764_T001	rna16041	7.00E-175
791- 21:	transcript:Zm00001d038766_T001	rna16039	9.00E-97
791- 22:	transcript:Zm00001d038768_T001	rna16037	2.00E-153
791- 23:	transcript:Zm00001d038769_T003	rna16036	3.00E-55
791- 24:	transcript:Zm00001d038772_T001	rna16034	3.00E-125
791- 25:	transcript:Zm00001d038775_T002	rna16026	0
791- 26:	transcript:Zm00001d038776_T001	rna16025	0
791- 27:	transcript:Zm00001d038779_T001	rna16024	0
791- 28:	transcript:Zm00001d038780_T001	rna16023	1.00E-26
791- 29:	transcript:Zm00001d038784_T001	rna16022	2.00E-101
791- 30:	transcript:Zm00001d038791_T001	rna16018	1.00E-64
791- 31:	transcript:Zm00001d038792_T001	rna16017	0
791- 32:	transcript:Zm00001d038793_T001	rna16015	3.00E-116
791- 33:	transcript:Zm00001d038794_T002	rna16012	0
791- 34:	transcript:Zm00001d038796_T001	rna16011	0
791- 35:	transcript:Zm00001d038797_T001	rna16009	0
791- 36:	transcript:Zm00001d038799_T001	rna16005	2.00E-61
791- 37:	transcript:Zm00001d038801_T001	rna16004	3.00E-123
791- 38:	transcript:Zm00001d038803_T001	rna16002	6.00E-126
791- 39:	transcript:Zm00001d038804_T002	rna16001	0
791- 40:	transcript:Zm00001d038805_T002	rna16000	7.00E-135
791- 41:	transcript:Zm00001d038806_T001	rna15999	0
791- 42:	transcript:Zm00001d038807_T005	rna15997	0
791- 43:	transcript:Zm00001d038808_T001	rna15996	0
791- 44:	transcript:Zm00001d038809_T008	rna15993	0
791- 45:	transcript:Zm00001d038810_T001	rna15992	8.00E-67
791- 46:	transcript:Zm00001d038814_T001	rna15991	2.00E-21
## Alignment 792: score=1827.0 e_value=1e-147 N=40 6&NC_008398.2 minus			
792- 0:	transcript:Zm00001d035568_T001	rna14247	1.00E-66
792- 1:	transcript:Zm00001d035569_T001	rna14246	1.00E-09
792- 2:	transcript:Zm00001d035574_T001	rna14224	0
792- 3:	transcript:Zm00001d035579_T007	rna14223	1.00E-180
792- 4:	transcript:Zm00001d035593_T001	rna14219	1.00E-161
792- 5:	transcript:Zm00001d035595_T003	rna14215	2.00E-130
792- 6:	transcript:Zm00001d035597_T001	rna14214	0
792- 7:	transcript:Zm00001d035598_T001	rna14213	0
792- 8:	transcript:Zm00001d035599_T001	rna14212	0
792- 9:	transcript:Zm00001d035601_T001	rna14211	0
792- 10:	transcript:Zm00001d035603_T001	rna14208	2.00E-81
792- 11:	transcript:Zm00001d035604_T001	rna14206	3.00E-19
792- 12:	transcript:Zm00001d035608_T001	rna14204	0
792- 13:	transcript:Zm00001d035609_T001	rna14203	2.00E-80

792- 14: transcript:Zm00001d035616_T001 rna14202 3.00E-149
792- 15: transcript:Zm00001d035617_T001 rna14201 3.00E-25
792- 16: transcript:Zm00001d035628_T002 rna14200 8.00E-74
792- 17: transcript:Zm00001d035629_T001 rna14199 6.00E-89
792- 18: transcript:Zm00001d035631_T001 rna14198 0
792- 19: transcript:Zm00001d035634_T001 rna14196 0
792- 20: transcript:Zm00001d035646_T001 rna14194 7.00E-123
792- 21: transcript:Zm00001d035649_T001 rna14193 8.00E-75
792- 22: transcript:Zm00001d035651_T001 rna14192 3.00E-90
792- 23: transcript:Zm00001d035652_T001 rna14191 0
792- 24: transcript:Zm00001d035657_T001 rna14190 4.00E-161
792- 25: transcript:Zm00001d035659_T001 rna14188 2.00E-08
792- 26: transcript:Zm00001d035664_T005 rna14187 2.00E-61
792- 27: transcript:Zm00001d035666_T002 rna14185 0
792- 28: transcript:Zm00001d035669_T002 rna14181 9.00E-68
792- 29: transcript:Zm00001d035670_T001 rna14179 0
792- 30: transcript:Zm00001d035672_T001 rna14178 2.00E-74
792- 31: transcript:Zm00001d035677_T001 rna14175 5.00E-148
792- 32: transcript:Zm00001d035679_T006 rna14174 6.00E-33
792- 33: transcript:Zm00001d035685_T001 rna14172 7.00E-26
792- 34: transcript:Zm00001d035689_T001 rna14171 0
792- 35: transcript:Zm00001d035693_T001 rna14170 4.00E-25
792- 36: transcript:Zm00001d035694_T001 rna14169 1.00E-151
792- 37: transcript:Zm00001d035695_T001 rna14168 0
792- 38: transcript:Zm00001d035696_T001 rna14167 0
792- 39: transcript:Zm00001d035698_T001 rna14165 0
Alignment 793: score=1053.0 e_value=1.6e-69 N=23 6&NC_008398.2 minus
793- 0: transcript:Zm00001d037719_T001 rna14738 9.00E-115
793- 1: transcript:Zm00001d037722_T001 rna14723 3.00E-42
793- 2: transcript:Zm00001d037724_T002 rna14715 3.00E-22
793- 3: transcript:Zm00001d037725_T001 rna14713 0
793- 4: transcript:Zm00001d037728_T001 rna14711 0
793- 5: transcript:Zm00001d037729_T001 rna14706 7.00E-160
793- 6: transcript:Zm00001d037733_T001 rna14700 6.00E-30
793- 7: transcript:Zm00001d037734_T011 rna14699 0
793- 8: transcript:Zm00001d037735_T001 rna14698 5.00E-133
793- 9: transcript:Zm00001d037737_T001 rna14695 7.00E-30
793- 10: transcript:Zm00001d037743_T001 rna14687 4.00E-87
793- 11: transcript:Zm00001d037744_T001 rna14686 0
793- 12: transcript:Zm00001d037745_T001 rna14685 3.00E-64
793- 13: transcript:Zm00001d037748_T001 rna14684 3.00E-103
793- 14: transcript:Zm00001d037749_T001 rna14683 3.00E-36
793- 15: transcript:Zm00001d037751_T001 rna14680 8.00E-38
793- 16: transcript:Zm00001d037756_T004 rna14678 0
793- 17: transcript:Zm00001d037757_T001 rna14677 4.00E-50
793- 18: transcript:Zm00001d037759_T002 rna14673 0
793- 19: transcript:Zm00001d037760_T001 rna14671 0
793- 20: transcript:Zm00001d037762_T001 rna14670 2.00E-28
793- 21: transcript:Zm00001d037766_T001 rna14667 9.00E-60
793- 22: transcript:Zm00001d037769_T001 rna14663 2.00E-178
Alignment 794: score=856.0 e_value=4.4e-51 N=18 6&NC_008398.2 minus
794- 0: transcript:Zm00001d038310_T003 rna15554 0
794- 1: transcript:Zm00001d038311_T001 rna15552 1.00E-61
794- 2: transcript:Zm00001d038312_T001 rna15551 3.00E-119

794- 3: transcript:Zm00001d038316_T001 rna15550 3.00E-06
794- 4: transcript:Zm00001d038318_T002 rna15549 0
794- 5: transcript:Zm00001d038319_T003 rna15548 2.00E-148
794- 6: transcript:Zm00001d038320_T001 rna15547 3.00E-58
794- 7: transcript:Zm00001d038321_T001 rna15546 0
794- 8: transcript:Zm00001d038325_T001 rna15533 1.00E-54
794- 9: transcript:Zm00001d038326_T003 rna15532 3.00E-118
794- 10: transcript:Zm00001d038327_T001 rna15531 9.00E-57
794- 11: transcript:Zm00001d038328_T001 rna15530 0
794- 12: transcript:Zm00001d038329_T001 rna15529 7.00E-97
794- 13: transcript:Zm00001d038330_T001 rna15528 0
794- 14: transcript:Zm00001d038333_T001 rna15524 0
794- 15: transcript:Zm00001d038337_T001 rna15521 0
794- 16: transcript:Zm00001d038338_T001 rna15518 1.00E-117
794- 17: transcript:Zm00001d038340_T001 rna15517 0
Alignment 795: score=834.0 e_value=1.5e-52 N=18 6&NC_008398.2 minus
795- 0: transcript:Zm00001d035439_T001 rna14302 6.00E-157
795- 1: transcript:Zm00001d035440_T001 rna14300 4.00E-46
795- 2: transcript:Zm00001d035442_T001 rna14299 0
795- 3: transcript:Zm00001d035443_T001 rna14298 6.00E-102
795- 4: transcript:Zm00001d035445_T001 rna14297 2.00E-164
795- 5: transcript:Zm00001d035447_T001 rna14294 0
795- 6: transcript:Zm00001d035454_T001 rna14293 3.00E-34
795- 7: transcript:Zm00001d035455_T001 rna14292 3.00E-139
795- 8: transcript:Zm00001d035456_T001 rna14291 2.00E-27
795- 9: transcript:Zm00001d035457_T001 rna14290 2.00E-106
795- 10: transcript:Zm00001d035462_T001 rna14289 0
795- 11: transcript:Zm00001d035467_T001 rna14287 0
795- 12: transcript:Zm00001d035473_T001 rna14281 2.00E-70
795- 13: transcript:Zm00001d035474_T001 rna14280 5.00E-98
795- 14: transcript:Zm00001d035475_T001 rna14276 0
795- 15: transcript:Zm00001d035476_T002 rna14272 0
795- 16: transcript:Zm00001d035486_T001 rna14271 3.00E-37
795- 17: transcript:Zm00001d035487_T025 rna14270 0
Alignment 796: score=772.0 e_value=1.5e-46 N=17 6&NC_008398.2 minus
796- 0: transcript:Zm00001d037607_T001 rna14620 5.00E-67
796- 1: transcript:Zm00001d037612_T001 rna14619 1.00E-165
796- 2: transcript:Zm00001d037613_T001 rna14617 3.00E-161
796- 3: transcript:Zm00001d037615_T002 rna14615 0
796- 4: transcript:Zm00001d037616_T001 rna14613 3.00E-156
796- 5: transcript:Zm00001d037619_T001 rna14596 0
796- 6: transcript:Zm00001d037623_T001 rna14595 3.00E-20
796- 7: transcript:Zm00001d037624_T001 rna14594 0
796- 8: transcript:Zm00001d037625_T001 rna14593 1.00E-64
796- 9: transcript:Zm00001d037626_T001 rna14591 0
796- 10: transcript:Zm00001d037627_T001 rna14589 2.00E-148
796- 11: transcript:Zm00001d037630_T001 rna14587 1.00E-141
796- 12: transcript:Zm00001d037631_T001 rna14582 9.00E-37
796- 13: transcript:Zm00001d037636_T001 rna14577 0
796- 14: transcript:Zm00001d037637_T001 rna14565 2.00E-43
796- 15: transcript:Zm00001d037640_T001 rna14564 0
796- 16: transcript:Zm00001d037643_T001 rna14560 0
Alignment 797: score=750.0 e_value=2.7e-44 N=17 6&NC_008398.2 minus
797- 0: transcript:Zm00001d037914_T001 rna15213 6.00E-21

797- 1: transcript:Zm00001d037915_T001 rna15212 3.00E-89
797- 2: transcript:Zm00001d037916_T001 rna15210 2.00E-130
797- 3: transcript:Zm00001d037917_T001 rna15208 9.00E-102
797- 4: transcript:Zm00001d037918_T001 rna15201 0
797- 5: transcript:Zm00001d037920_T001 rna15198 2.00E-46
797- 6: transcript:Zm00001d037925_T006 rna15196 2.00E-111
797- 7: transcript:Zm00001d037932_T003 rna15192 0
797- 8: transcript:Zm00001d037934_T011 rna15191 0
797- 9: transcript:Zm00001d037936_T001 rna15189 1.00E-41
797- 10: transcript:Zm00001d037937_T001 rna15187 3.00E-67
797- 11: transcript:Zm00001d037939_T003 rna15186 0
797- 12: transcript:Zm00001d037941_T001 rna15184 1.00E-58
797- 13: transcript:Zm00001d037943_T001 rna15177 9.00E-30
797- 14: transcript:Zm00001d037946_T001 rna15175 3.00E-32
797- 15: transcript:Zm00001d037949_T001 rna15174 0
797- 16: transcript:Zm00001d037958_T001 rna15171 7.00E-44
Alignment 798: score=729.0 e_value=1.1e-42 N=16 6&NC_008398.2 minus
798- 0: transcript:Zm00001d035751_T002 rna14139 1.00E-47
798- 1: transcript:Zm00001d035752_T002 rna14138 1.00E-177
798- 2: transcript:Zm00001d035753_T001 rna14134 7.00E-133
798- 3: transcript:Zm00001d035757_T003 rna14132 8.00E-40
798- 4: transcript:Zm00001d035759_T001 rna14126 3.00E-24
798- 5: transcript:Zm00001d035761_T001 rna14125 7.00E-156
798- 6: transcript:Zm00001d035762_T001 rna14122 2.00E-150
798- 7: transcript:Zm00001d035763_T005 rna14121 0
798- 8: transcript:Zm00001d035764_T003 rna14120 0
798- 9: transcript:Zm00001d035765_T001 rna14119 4.00E-62
798- 10: transcript:Zm00001d035767_T001 rna14116 3.00E-125
798- 11: transcript:Zm00001d035772_T001 rna14112 4.00E-156
798- 12: transcript:Zm00001d035773_T003 rna14111 7.00E-17
798- 13: transcript:Zm00001d035774_T001 rna14109 2.00E-156
798- 14: transcript:Zm00001d035775_T001 rna14108 0
798- 15: transcript:Zm00001d035776_T006 rna14106 5.00E-71
Alignment 799: score=691.0 e_value=3.7e-39 N=15 6&NC_008398.2 minus
799- 0: transcript:Zm00001d039165_T046 rna16468 0
799- 1: transcript:Zm00001d039166_T002 rna16467 5.00E-133
799- 2: transcript:Zm00001d039167_T001 rna16466 0
799- 3: transcript:Zm00001d039173_T002 rna16465 0
799- 4: transcript:Zm00001d039174_T001 rna16464 8.00E-137
799- 5: transcript:Zm00001d039175_T001 rna16460 4.00E-108
799- 6: transcript:Zm00001d039176_T001 rna16457 1.00E-32
799- 7: transcript:Zm00001d039181_T001 rna16452 1.00E-171
799- 8: transcript:Zm00001d039182_T001 rna16450 0
799- 9: transcript:Zm00001d039183_T001 rna16448 0
799- 10: transcript:Zm00001d039188_T001 rna16443 2.00E-145
799- 11: transcript:Zm00001d039192_T001 rna16435 0
799- 12: transcript:Zm00001d039193_T001 rna16433 5.00E-90
799- 13: transcript:Zm00001d039194_T001 rna16431 0
799- 14: transcript:Zm00001d039200_T001 rna16427 3.00E-28
Alignment 800: score=518.0 e_value=9.7e-25 N=11 6&NC_008398.2 minus
800- 0: transcript:Zm00001d037675_T001 rna14660 0
800- 1: transcript:Zm00001d037680_T001 rna14643 5.00E-40
800- 2: transcript:Zm00001d037682_T001 rna14641 0
800- 3: transcript:Zm00001d037684_T001 rna14637 9.00E-29

800- 4: transcript:Zm00001d037687_T001 rna14635   4.00E-13
800- 5: transcript:Zm00001d037688_T001 rna14633   3.00E-159
800- 6: transcript:Zm00001d037689_T001 rna14632   0
800- 7: transcript:Zm00001d037691_T003 rna14630   2.00E-06
800- 8: transcript:Zm00001d037693_T003 rna14629   0
800- 9: transcript:Zm00001d037694_T001 rna14628   5.00E-78
800- 10: transcript:Zm00001d037695_T003 rna14626   0
Alignment 801: score=480.0 e_value=7.4e-27 N=11 6&NC_008398.2 minus
801- 0: transcript:Zm00001d035492_T003 rna14264   1.00E-169
801- 1: transcript:Zm00001d035494_T001 rna14263   2.00E-119
801- 2: transcript:Zm00001d035501_T001 rna14261   1.00E-60
801- 3: transcript:Zm00001d035505_T001 rna14260   6.00E-27
801- 4: transcript:Zm00001d035506_T003 rna14259   0
801- 5: transcript:Zm00001d035509_T001 rna14257   7.00E-59
801- 6: transcript:Zm00001d035512_T001 rna14253   7.00E-89
801- 7: transcript:Zm00001d035514_T001 rna14251   6.00E-162
801- 8: transcript:Zm00001d035515_T001 rna14250   0
801- 9: transcript:Zm00001d035519_T001 rna14248   7.00E-102
801- 10: transcript:Zm00001d035527_T001 rna14228   0
Alignment 802: score=438.0 e_value=1.8e-25 N=11 6&NC_008398.2 minus
802- 0: transcript:Zm00001d037509_T001 rna14540   5.00E-141
802- 1: transcript:Zm00001d037512_T001 rna14539   0
802- 2: transcript:Zm00001d037515_T001 rna14529   4.00E-51
802- 3: transcript:Zm00001d037519_T004 rna14525   5.00E-157
802- 4: transcript:Zm00001d037528_T001 rna14521   9.00E-71
802- 5: transcript:Zm00001d037536_T006 rna14518   3.00E-143
802- 6: transcript:Zm00001d037537_T001 rna14515   3.00E-148
802- 7: transcript:Zm00001d037538_T001 rna14514   0
802- 8: transcript:Zm00001d037543_T001 rna14512   1.00E-37
802- 9: transcript:Zm00001d037544_T001 rna14511   2.00E-61
802- 10: transcript:Zm00001d037546_T011 rna14510   6.00E-119
Alignment 803: score=421.0 e_value=2.4e-19 N=9 6&NC_008398.2 minus
803- 0: transcript:Zm00001d037557_T065 rna14490   0
803- 1: transcript:Zm00001d037565_T002 rna14488   4.00E-177
803- 2: transcript:Zm00001d037573_T001 rna14481   2.00E-96
803- 3: transcript:Zm00001d037574_T001 rna14480   5.00E-111
803- 4: transcript:Zm00001d037576_T001 rna14478   0
803- 5: transcript:Zm00001d037577_T001 rna14477   3.00E-52
803- 6: transcript:Zm00001d037582_T001 rna14475   0
803- 7: transcript:Zm00001d037586_T001 rna14474   5.00E-86
803- 8: transcript:Zm00001d037590_T002 rna14472   0
Alignment 804: score=419.0 e_value=1.7e-17 N=9 6&NC_008398.2 minus
804- 0: transcript:Zm00001d038263_T001 rna15612   0
804- 1: transcript:Zm00001d038267_T001 rna15611   4.00E-84
804- 2: transcript:Zm00001d038268_T001 rna15609   0
804- 3: transcript:Zm00001d038269_T001 rna15608   0
804- 4: transcript:Zm00001d038270_T001 rna15606   4.00E-150
804- 5: transcript:Zm00001d038272_T006 rna15605   0
804- 6: transcript:Zm00001d038273_T001 rna15604   0
804- 7: transcript:Zm00001d038274_T002 rna15598   0
804- 8: transcript:Zm00001d038275_T002 rna15596   0
Alignment 805: score=355.0 e_value=7.1e-14 N=8 6&NC_008398.2 minus
805- 0: transcript:Zm00001d038023_T001 rna15116   0
805- 1: transcript:Zm00001d038027_T002 rna15115   2.00E-48

805- 2: transcript:Zm00001d038029_T001 rna15113 1.00E-11
805- 3: transcript:Zm00001d038032_T001 rna15112 3.00E-27
805- 4: transcript:Zm00001d038038_T001 rna15107 6.00E-70
805- 5: transcript:Zm00001d038039_T001 rna15106 2.00E-51
805- 6: transcript:Zm00001d038047_T001 rna15102 2.00E-58
805- 7: transcript:Zm00001d038048_T001 rna15088 0
Alignment 806: score=298.0 e_value=7.3e-10 N=6 6&NC_008398.2 minus
806- 0: transcript:Zm00001d039216_T001 rna16494 6.00E-106
806- 1: transcript:Zm00001d039217_T001 rna16492 0
806- 2: transcript:Zm00001d039218_T002 rna16491 0
806- 3: transcript:Zm00001d039219_T001 rna16490 2.00E-150
806- 4: transcript:Zm00001d039221_T001 rna16489 3.00E-125
806- 5: transcript:Zm00001d039222_T001 rna16488 1.00E-126
Alignment 807: score=291.0 e_value=6.4e-09 N=6 6&NC_008398.2 minus
807- 0: transcript:Zm00001d037659_T001 rna14559 2.00E-26
807- 1: transcript:Zm00001d037664_T001 rna14556 7.00E-10
807- 2: transcript:Zm00001d037666_T001 rna14555 0
807- 3: transcript:Zm00001d037668_T001 rna14551 1.00E-30
807- 4: transcript:Zm00001d037672_T001 rna14550 3.00E-120
807- 5: transcript:Zm00001d037674_T001 rna14547 9.00E-161
Alignment 808: score=268.0 e_value=5.3e-09 N=6 6&NC_008398.2 minus
808- 0: transcript:Zm00001d037972_T001 rna15160 6.00E-35
808- 1: transcript:Zm00001d037974_T001 rna15157 5.00E-83
808- 2: transcript:Zm00001d037975_T001 rna15156 0
808- 3: transcript:Zm00001d037976_T001 rna15155 2.00E-21
808- 4: transcript:Zm00001d037978_T001 rna15154 0
808- 5: transcript:Zm00001d037979_T001 rna15153 0
Alignment 809: score=2733.0 e_value=8.5e-232 N=60 6&NC_008399.2 plus
809- 0: transcript:Zm00001d037180_T001 rna17176 2.00E-100
809- 1: transcript:Zm00001d037181_T001 rna17180 1.00E-50
809- 2: transcript:Zm00001d037182_T001 rna17184 0
809- 3: transcript:Zm00001d037189_T004 rna17195 0
809- 4: transcript:Zm00001d037191_T002 rna17202 2.00E-33
809- 5: transcript:Zm00001d037195_T003 rna17206 0
809- 6: transcript:Zm00001d037197_T001 rna17208 1.00E-36
809- 7: transcript:Zm00001d037198_T001 rna17209 0
809- 8: transcript:Zm00001d037199_T001 rna17210 0
809- 9: transcript:Zm00001d037200_T004 rna17212 5.00E-117
809- 10: transcript:Zm00001d037204_T001 rna17215 7.00E-165
809- 11: transcript:Zm00001d037207_T001 rna17219 0
809- 12: transcript:Zm00001d037210_T001 rna17222 6.00E-17
809- 13: transcript:Zm00001d037211_T001 rna17225 0
809- 14: transcript:Zm00001d037212_T001 rna17232 2.00E-118
809- 15: transcript:Zm00001d037213_T001 rna17234 5.00E-25
809- 16: transcript:Zm00001d037215_T011 rna17237 0
809- 17: transcript:Zm00001d037218_T001 rna17255 8.00E-101
809- 18: transcript:Zm00001d037220_T001 rna17257 2.00E-121
809- 19: transcript:Zm00001d037221_T001 rna17258 2.00E-130
809- 20: transcript:Zm00001d037225_T001 rna17259 0
809- 21: transcript:Zm00001d037227_T002 rna17260 6.00E-111
809- 22: transcript:Zm00001d037228_T001 rna17262 0
809- 23: transcript:Zm00001d037229_T001 rna17263 0
809- 24: transcript:Zm00001d037232_T001 rna17267 4.00E-103
809- 25: transcript:Zm00001d037233_T002 rna17269 0

809- 26: transcript:Zm00001d037234_T005 rna17272 0
809- 27: transcript:Zm00001d037235_T001 rna17275 2.00E-98
809- 28: transcript:Zm00001d037236_T001 rna17279 4.00E-174
809- 29: transcript:Zm00001d037237_T002 rna17281 7.00E-97
809- 30: transcript:Zm00001d037239_T001 rna17282 9.00E-106
809- 31: transcript:Zm00001d037240_T001 rna17287 4.00E-151
809- 32: transcript:Zm00001d037243_T001 rna17290 3.00E-06
809- 33: transcript:Zm00001d037244_T001 rna17291 6.00E-39
809- 34: transcript:Zm00001d037246_T002 rna17292 8.00E-174
809- 35: transcript:Zm00001d037247_T007 rna17294 0
809- 36: transcript:Zm00001d037248_T001 rna17297 0
809- 37: transcript:Zm00001d037249_T001 rna17299 7.00E-43
809- 38: transcript:Zm00001d037251_T001 rna17305 3.00E-61
809- 39: transcript:Zm00001d037252_T002 rna17311 0
809- 40: transcript:Zm00001d037257_T001 rna17314 0
809- 41: transcript:Zm00001d037258_T002 rna17315 5.00E-86
809- 42: transcript:Zm00001d037261_T001 rna17317 8.00E-35
809- 43: transcript:Zm00001d037263_T001 rna17319 2.00E-127
809- 44: transcript:Zm00001d037264_T002 rna17320 5.00E-60
809- 45: transcript:Zm00001d037265_T001 rna17324 2.00E-54
809- 46: transcript:Zm00001d037266_T003 rna17325 6.00E-151
809- 47: transcript:Zm00001d037268_T003 rna17339 0
809- 48: transcript:Zm00001d037270_T001 rna17340 1.00E-65
809- 49: transcript:Zm00001d037271_T001 rna17341 0
809- 50: transcript:Zm00001d037272_T001 rna17343 0
809- 51: transcript:Zm00001d037274_T002 rna17345 0
809- 52: transcript:Zm00001d037275_T001 rna17346 4.00E-53
809- 53: transcript:Zm00001d037278_T001 rna17352 0
809- 54: transcript:Zm00001d037279_T011 rna17353 0
809- 55: transcript:Zm00001d037280_T001 rna17359 5.00E-10
809- 56: transcript:Zm00001d037284_T001 rna17360 2.00E-136
809- 57: transcript:Zm00001d037289_T002 rna17361 3.00E-78
809- 58: transcript:Zm00001d037290_T002 rna17362 8.00E-40
809- 59: transcript:Zm00001d037291_T001 rna17365 0
Alignment 810: score=1379.0 e_value=9.7e-105 N=32 6&NC_008399.2 plus
810- 0: transcript:Zm00001d036175_T001 rna16821 1.00E-74
810- 1: transcript:Zm00001d036177_T001 rna16823 3.00E-167
810- 2: transcript:Zm00001d036197_T001 rna16832 7.00E-162
810- 3: transcript:Zm00001d036206_T001 rna16839 1.00E-86
810- 4: transcript:Zm00001d036213_T002 rna16841 1.00E-50
810- 5: transcript:Zm00001d036214_T001 rna16842 4.00E-176
810- 6: transcript:Zm00001d036222_T002 rna16852 2.00E-168
810- 7: transcript:Zm00001d036230_T001 rna16866 0
810- 8: transcript:Zm00001d036234_T001 rna16868 2.00E-91
810- 9: transcript:Zm00001d036237_T002 rna16869 0
810- 10: transcript:Zm00001d036240_T003 rna16871 0
810- 11: transcript:Zm00001d036242_T001 rna16873 1.00E-111
810- 12: transcript:Zm00001d036244_T001 rna16877 2.00E-66
810- 13: transcript:Zm00001d036245_T005 rna16878 2.00E-113
810- 14: transcript:Zm00001d036246_T001 rna16884 2.00E-75
810- 15: transcript:Zm00001d036250_T001 rna16887 1.00E-94
810- 16: transcript:Zm00001d036255_T001 rna16895 3.00E-113
810- 17: transcript:Zm00001d036267_T001 rna16900 4.00E-20
810- 18: transcript:Zm00001d036274_T001 rna16904 4.00E-31

810- 19: transcript:Zm00001d036279_T001 rna16906 1.00E-21
810- 20: transcript:Zm00001d036283_T002 rna16909 0
810- 21: transcript:Zm00001d036285_T001 rna16916 0
810- 22: transcript:Zm00001d036293_T002 rna16928 2.00E-161
810- 23: transcript:Zm00001d036297_T001 rna16932 0
810- 24: transcript:Zm00001d036298_T001 rna16933 1.00E-58
810- 25: transcript:Zm00001d036300_T001 rna16936 0
810- 26: transcript:Zm00001d036301_T007 rna16937 0
810- 27: transcript:Zm00001d036305_T003 rna16938 0
810- 28: transcript:Zm00001d036306_T001 rna16940 4.00E-134
810- 29: transcript:Zm00001d036313_T001 rna16942 2.00E-18
810- 30: transcript:Zm00001d036319_T001 rna16943 8.00E-180
810- 31: transcript:Zm00001d036322_T002 rna16947 0
Alignment 811: score=1051.0 e_value=2.1e-86 N=25 6&NC_008399.2 plus
811- 0: transcript:Zm00001d035889_T001 rna16538 0
811- 1: transcript:Zm00001d035898_T001 rna16542 0
811- 2: transcript:Zm00001d035903_T001 rna16552 7.00E-25
811- 3: transcript:Zm00001d035907_T001 rna16554 2.00E-156
811- 4: transcript:Zm00001d035913_T002 rna16559 0
811- 5: transcript:Zm00001d035916_T001 rna16561 9.00E-97
811- 6: transcript:Zm00001d035925_T001 rna16569 0
811- 7: transcript:Zm00001d035934_T002 rna16585 0
811- 8: transcript:Zm00001d035935_T001 rna16587 1.00E-10
811- 9: transcript:Zm00001d035945_T001 rna16591 2.00E-22
811- 10: transcript:Zm00001d035947_T002 rna16593 0
811- 11: transcript:Zm00001d035948_T001 rna16598 9.00E-46
811- 12: transcript:Zm00001d035957_T001 rna16599 0
811- 13: transcript:Zm00001d035962_T001 rna16603 0
811- 14: transcript:Zm00001d035963_T001 rna16604 4.00E-39
811- 15: transcript:Zm00001d035964_T003 rna16606 0
811- 16: transcript:Zm00001d035965_T004 rna16607 4.00E-149
811- 17: transcript:Zm00001d035973_T005 rna16610 0
811- 18: transcript:Zm00001d035974_T001 rna16612 0
811- 19: transcript:Zm00001d035980_T001 rna16620 7.00E-06
811- 20: transcript:Zm00001d035981_T001 rna16631 3.00E-102
811- 21: transcript:Zm00001d035987_T001 rna16640 0
811- 22: transcript:Zm00001d035988_T003 rna16641 4.00E-10
811- 23: transcript:Zm00001d035989_T001 rna16646 0
811- 24: transcript:Zm00001d035990_T002 rna16652 0
Alignment 812: score=740.0 e_value=1.8e-46 N=17 6&NC_008399.2 plus
812- 0: transcript:Zm00001d036073_T002 rna16719 6.00E-08
812- 1: transcript:Zm00001d036086_T002 rna16726 0
812- 2: transcript:Zm00001d036088_T001 rna16729 3.00E-72
812- 3: transcript:Zm00001d036090_T003 rna16730 8.00E-27
812- 4: transcript:Zm00001d036091_T001 rna16734 0
812- 5: transcript:Zm00001d036092_T004 rna16735 8.00E-68
812- 6: transcript:Zm00001d036103_T001 rna16739 0
812- 7: transcript:Zm00001d036107_T001 rna16746 2.00E-143
812- 8: transcript:Zm00001d036108_T001 rna16747 8.00E-166
812- 9: transcript:Zm00001d036110_T002 rna16749 0
812- 10: transcript:Zm00001d036118_T001 rna16752 9.00E-63
812- 11: transcript:Zm00001d036125_T001 rna16760 4.00E-13
812- 12: transcript:Zm00001d036131_T001 rna16775 0
812- 13: transcript:Zm00001d036135_T003 rna16777 1.00E-139

812- 14: transcript:Zm00001d036136_T001 rna16778 6.00E-18
812- 15: transcript:Zm00001d036137_T001 rna16779 0
812- 16: transcript:Zm00001d036145_T001 rna16800 5.00E-89
Alignment 813: score=610.0 e_value=5.8e-30 N=13 6&NC_008399.2 plus
813- 0: transcript:Zm00001d037473_T004 rna16962 1.00E-156
813- 1: transcript:Zm00001d037476_T001 rna16963 0
813- 2: transcript:Zm00001d037477_T001 rna16964 2.00E-83
813- 3: transcript:Zm00001d037479_T001 rna16965 5.00E-38
813- 4: transcript:Zm00001d037480_T001 rna16967 0
813- 5: transcript:Zm00001d037481_T003 rna16969 0
813- 6: transcript:Zm00001d037483_T002 rna16973 8.00E-139
813- 7: transcript:Zm00001d037484_T001 rna16977 0
813- 8: transcript:Zm00001d037485_T001 rna16979 5.00E-46
813- 9: transcript:Zm00001d037487_T001 rna16982 8.00E-137
813- 10: transcript:Zm00001d037492_T001 rna16994 0
813- 11: transcript:Zm00001d037493_T001 rna16995 0
813- 12: transcript:Zm00001d037495_T002 rna16996 0
Alignment 814: score=567.0 e_value=9.6e-34 N=13 6&NC_008399.2 plus
814- 0: transcript:Zm00001d037073_T001 rna17031 1.00E-97
814- 1: transcript:Zm00001d037079_T001 rna17038 3.00E-40
814- 2: transcript:Zm00001d037080_T001 rna17040 0
814- 3: transcript:Zm00001d037084_T004 rna17042 8.00E-99
814- 4: transcript:Zm00001d037085_T005 rna17043 0
814- 5: transcript:Zm00001d037086_T006 rna17046 0
814- 6: transcript:Zm00001d037087_T001 rna17047 0
814- 7: transcript:Zm00001d037089_T001 rna17068 0
814- 8: transcript:Zm00001d037095_T001 rna17077 9.00E-99
814- 9: transcript:Zm00001d037096_T004 rna17079 1.00E-131
814- 10: transcript:Zm00001d037097_T001 rna17081 5.00E-49
814- 11: transcript:Zm00001d037098_T003 rna17082 0
814- 12: transcript:Zm00001d037099_T012 rna17087 0
Alignment 815: score=546.0 e_value=1.2e-30 N=12 6&NC_008399.2 plus
815- 0: transcript:Zm00001d035999_T002 rna16655 4.00E-134
815- 1: transcript:Zm00001d036001_T006 rna16656 7.00E-149
815- 2: transcript:Zm00001d036003_T001 rna16657 5.00E-63
815- 3: transcript:Zm00001d036004_T017 rna16658 0
815- 4: transcript:Zm00001d036008_T001 rna16659 5.00E-74
815- 5: transcript:Zm00001d036010_T003 rna16662 0
815- 6: transcript:Zm00001d036013_T002 rna16666 0
815- 7: transcript:Zm00001d036014_T001 rna16670 4.00E-28
815- 8: transcript:Zm00001d036020_T002 rna16676 0
815- 9: transcript:Zm00001d036022_T001 rna16679 5.00E-50
815- 10: transcript:Zm00001d036023_T001 rna16680 0
815- 11: transcript:Zm00001d036025_T002 rna16681 5.00E-41
Alignment 816: score=351.0 e_value=1.1e-16 N=8 6&NC_008399.2 plus
816- 0: transcript:Zm00001d035859_T001 rna16508 1.00E-70
816- 1: transcript:Zm00001d035865_T001 rna16519 0
816- 2: transcript:Zm00001d035872_T001 rna16521 4.00E-53
816- 3: transcript:Zm00001d035874_T003 rna16524 4.00E-163
816- 4: transcript:Zm00001d035875_T002 rna16525 0
816- 5: transcript:Zm00001d035876_T002 rna16527 0
816- 6: transcript:Zm00001d035878_T003 rna16528 0
816- 7: transcript:Zm00001d035879_T001 rna16529 2.00E-143
Alignment 817: score=336.0 e_value=4.2e-13 N=7 6&NC_008399.2 plus

817- 0: transcript:Zm00001d037160_T002 rna17154 6.00E-32
817- 1: transcript:Zm00001d037163_T002 rna17155 4.00E-133
817- 2: transcript:Zm00001d037164_T001 rna17156 0
817- 3: transcript:Zm00001d037165_T001 rna17157 3.00E-53
817- 4: transcript:Zm00001d037170_T001 rna17162 7.00E-146
817- 5: transcript:Zm00001d037172_T001 rna17164 0
817- 6: transcript:Zm00001d037174_T003 rna17172 0
Alignment 818: score=314.0 e_value=2.8e-12 N=7 6&NC_008399.2 plus
818- 0: transcript:Zm00001d037105_T001 rna17089 3.00E-133
818- 1: transcript:Zm00001d037107_T001 rna17092 7.00E-49
818- 2: transcript:Zm00001d037108_T003 rna17093 0
818- 3: transcript:Zm00001d037109_T008 rna17104 0
818- 4: transcript:Zm00001d037110_T002 rna17107 0
818- 5: transcript:Zm00001d037112_T001 rna17121 0
818- 6: transcript:Zm00001d037114_T002 rna17126 0
Alignment 819: score=304.0 e_value=3.2e-13 N=7 6&NC_008399.2 plus
819- 0: transcript:Zm00001d037140_T004 rna17143 9.00E-86
819- 1: transcript:Zm00001d037141_T001 rna17144 8.00E-31
819- 2: transcript:Zm00001d037142_T022 rna17145 0
819- 3: transcript:Zm00001d037150_T001 rna17146 2.00E-130
819- 4: transcript:Zm00001d037151_T001 rna17147 1.00E-65
819- 5: transcript:Zm00001d037155_T001 rna17148 3.00E-180
819- 6: transcript:Zm00001d037156_T034 rna17149 0
Alignment 820: score=292.0 e_value=9.6e-09 N=6 6&NC_008399.2 plus
820- 0: transcript:Zm00001d037367_T002 rna17474 0
820- 1: transcript:Zm00001d037369_T001 rna17476 6.00E-152
820- 2: transcript:Zm00001d037371_T001 rna17479 0
820- 3: transcript:Zm00001d037375_T001 rna17481 2.00E-92
820- 4: transcript:Zm00001d037376_T001 rna17483 1.00E-52
820- 5: transcript:Zm00001d037377_T002 rna17485 0
Alignment 821: score=289.0 e_value=5.3e-14 N=7 6&NC_008399.2 plus
821- 0: transcript:Zm00001d036613_T001 rna18535 0
821- 1: transcript:Zm00001d036629_T010 rna18547 0
821- 2: transcript:Zm00001d036630_T001 rna18548 6.00E-170
821- 3: transcript:Zm00001d036632_T002 rna18550 1.00E-116
821- 4: transcript:Zm00001d036635_T001 rna18552 5.00E-18
821- 5: transcript:Zm00001d036637_T001 rna18553 2.00E-98
821- 6: transcript:Zm00001d036638_T001 rna18554 1.00E-76
Alignment 822: score=286.0 e_value=5.4e-09 N=6 6&NC_008399.2 plus
822- 0: transcript:Zm00001d036927_T001 rna18137 0
822- 1: transcript:Zm00001d036929_T001 rna18138 1.00E-168
822- 2: transcript:Zm00001d036930_T001 rna18141 7.00E-27
822- 3: transcript:Zm00001d036931_T001 rna18145 0
822- 4: transcript:Zm00001d036933_T001 rna18154 2.00E-47
822- 5: transcript:Zm00001d036936_T002 rna18156 9.00E-163
Alignment 823: score=264.0 e_value=6.2e-08 N=6 6&NC_008399.2 plus
823- 0: transcript:Zm00001d035820_T002 rna17001 0
823- 1: transcript:Zm00001d035826_T001 rna17009 2.00E-90
823- 2: transcript:Zm00001d035835_T001 rna17011 4.00E-74
823- 3: transcript:Zm00001d035841_T002 rna17014 0
823- 4: transcript:Zm00001d035844_T004 rna17016 0
823- 5: transcript:Zm00001d035845_T001 rna17017 7.00E-61
Alignment 824: score=2267.0 e_value=1.4e-192 N=50 6&NC_008399.2 minus
824- 0: transcript:Zm00001d036355_T001 rna18902 7.00E-34

824- 1: transcript:Zm00001d036357_T001 rna18901   2.00E-177
824- 2: transcript:Zm00001d036360_T002 rna18894   0
824- 3: transcript:Zm00001d036361_T018 rna18893   0
824- 4: transcript:Zm00001d036363_T001 rna18892   1.00E-168
824- 5: transcript:Zm00001d036364_T001 rna18891   1.00E-128
824- 6: transcript:Zm00001d036366_T001 rna18889   4.00E-152
824- 7: transcript:Zm00001d036371_T001 rna18888   0
824- 8: transcript:Zm00001d036373_T001 rna18886   5.00E-32
824- 9: transcript:Zm00001d036382_T001 rna18879   2.00E-55
824- 10: transcript:Zm00001d036386_T001 rna18876   0
824- 11: transcript:Zm00001d036387_T001 rna18874   0
824- 12: transcript:Zm00001d036388_T002 rna18873   0
824- 13: transcript:Zm00001d036392_T001 rna18858   7.00E-62
824- 14: transcript:Zm00001d036394_T001 rna18855   7.00E-16
824- 15: transcript:Zm00001d036395_T016 rna18854   0
824- 16: transcript:Zm00001d036396_T001 rna18851   0
824- 17: transcript:Zm00001d036398_T001 rna18849   2.00E-46
824- 18: transcript:Zm00001d036400_T001 rna18847   2.00E-52
824- 19: transcript:Zm00001d036401_T001 rna18846   0
824- 20: transcript:Zm00001d036402_T002 rna18843   0
824- 21: transcript:Zm00001d036403_T002 rna18840   2.00E-154
824- 22: transcript:Zm00001d036406_T001 rna18836   4.00E-17
824- 23: transcript:Zm00001d036409_T001 rna18832   8.00E-41
824- 24: transcript:Zm00001d036410_T002 rna18829   0
824- 25: transcript:Zm00001d036415_T001 rna18827   7.00E-46
824- 26: transcript:Zm00001d036416_T001 rna18826   4.00E-65
824- 27: transcript:Zm00001d036417_T004 rna18818   9.00E-84
824- 28: transcript:Zm00001d036418_T001 rna18817   5.00E-77
824- 29: transcript:Zm00001d036423_T002 rna18815   1.00E-90
824- 30: transcript:Zm00001d036425_T002 rna18813   2.00E-143
824- 31: transcript:Zm00001d036426_T001 rna18812   7.00E-132
824- 32: transcript:Zm00001d036428_T006 rna18810   0
824- 33: transcript:Zm00001d036429_T006 rna18809   0
824- 34: transcript:Zm00001d036430_T001 rna18808   1.00E-93
824- 35: transcript:Zm00001d036431_T001 rna18806   2.00E-82
824- 36: transcript:Zm00001d036432_T003 rna18802   2.00E-09
824- 37: transcript:Zm00001d036439_T001 rna18793   4.00E-171
824- 38: transcript:Zm00001d036440_T003 rna18791   0
824- 39: transcript:Zm00001d036441_T001 rna18790   2.00E-59
824- 40: transcript:Zm00001d036442_T001 rna18789   3.00E-122
824- 41: transcript:Zm00001d036443_T002 rna18788   1.00E-36
824- 42: transcript:Zm00001d036446_T001 rna18787   9.00E-27
824- 43: transcript:Zm00001d036448_T019 rna18785   0
824- 44: transcript:Zm00001d036450_T001 rna18781   0
824- 45: transcript:Zm00001d036451_T002 rna18771   1.00E-39
824- 46: transcript:Zm00001d036452_T001 rna18770   3.00E-18
824- 47: transcript:Zm00001d036454_T001 rna18769   5.00E-113
824- 48: transcript:Zm00001d036455_T001 rna18762   2.00E-129
824- 49: transcript:Zm00001d036459_T005 rna18748   4.00E-122
Alignment 825: score=1732.0 e_value=5.3e-139 N=38 6&NC_008399.2 minus
825- 0: transcript:Zm00001d036742_T007 rna18401   0
825- 1: transcript:Zm00001d036748_T001 rna18397   9.00E-116
825- 2: transcript:Zm00001d036749_T001 rna18395   3.00E-106
825- 3: transcript:Zm00001d036750_T001 rna18394   0

825- 4: transcript:Zm00001d036751_T001 rna18393 0
825- 5: transcript:Zm00001d036752_T001 rna18391 4.00E-27
825- 6: transcript:Zm00001d036756_T001 rna18390 5.00E-67
825- 7: transcript:Zm00001d036759_T002 rna18389 0
825- 8: transcript:Zm00001d036760_T001 rna18388 4.00E-139
825- 9: transcript:Zm00001d036761_T001 rna18387 3.00E-24
825- 10: transcript:Zm00001d036762_T001 rna18384 7.00E-48
825- 11: transcript:Zm00001d036763_T001 rna18375 0
825- 12: transcript:Zm00001d036764_T004 rna18372 0
825- 13: transcript:Zm00001d036765_T009 rna18370 2.00E-178
825- 14: transcript:Zm00001d036768_T001 rna18360 2.00E-126
825- 15: transcript:Zm00001d036769_T001 rna18358 1.00E-14
825- 16: transcript:Zm00001d036770_T001 rna18357 0
825- 17: transcript:Zm00001d036771_T001 rna18356 0
825- 18: transcript:Zm00001d036773_T001 rna18352 4.00E-62
825- 19: transcript:Zm00001d036775_T001 rna18348 2.00E-67
825- 20: transcript:Zm00001d036777_T001 rna18347 0
825- 21: transcript:Zm00001d036780_T001 rna18339 1.00E-29
825- 22: transcript:Zm00001d036782_T001 rna18332 8.00E-64
825- 23: transcript:Zm00001d036784_T002 rna18324 0
825- 24: transcript:Zm00001d036785_T003 rna18320 0
825- 25: transcript:Zm00001d036787_T001 rna18316 5.00E-45
825- 26: transcript:Zm00001d036788_T003 rna18312 0
825- 27: transcript:Zm00001d036789_T003 rna18307 3.00E-151
825- 28: transcript:Zm00001d036790_T009 rna18301 0
825- 29: transcript:Zm00001d036791_T001 rna18290 1.00E-12
825- 30: transcript:Zm00001d036795_T002 rna18286 5.00E-31
825- 31: transcript:Zm00001d036796_T004 rna18285 4.00E-148
825- 32: transcript:Zm00001d036797_T002 rna18284 4.00E-157
825- 33: transcript:Zm00001d036798_T002 rna18277 0
825- 34: transcript:Zm00001d036801_T001 rna18275 0
825- 35: transcript:Zm00001d036807_T001 rna18272 2.00E-105
825- 36: transcript:Zm00001d036812_T001 rna18264 8.00E-146
825- 37: transcript:Zm00001d036815_T001 rna18259 8.00E-104
Alignment 826: score=1490.0 e_value=4.1e-115 N=32 6&NC_008399.2 minus
826- 0: transcript:Zm00001d036671_T001 rna18494 5.00E-39
826- 1: transcript:Zm00001d036672_T005 rna18493 7.00E-159
826- 2: transcript:Zm00001d036678_T002 rna18489 2.00E-83
826- 3: transcript:Zm00001d036679_T001 rna18488 2.00E-144
826- 4: transcript:Zm00001d036683_T001 rna18484 1.00E-13
826- 5: transcript:Zm00001d036688_T001 rna18481 0
826- 6: transcript:Zm00001d036689_T001 rna18479 2.00E-102
826- 7: transcript:Zm00001d036692_T001 rna18478 2.00E-119
826- 8: transcript:Zm00001d036699_T001 rna18471 4.00E-88
826- 9: transcript:Zm00001d036700_T001 rna18467 9.00E-60
826- 10: transcript:Zm00001d036701_T001 rna18466 0
826- 11: transcript:Zm00001d036704_T002 rna18460 0
826- 12: transcript:Zm00001d036707_T006 rna18459 0
826- 13: transcript:Zm00001d036708_T001 rna18458 2.00E-41
826- 14: transcript:Zm00001d036709_T001 rna18457 0
826- 15: transcript:Zm00001d036710_T002 rna18444 0
826- 16: transcript:Zm00001d036711_T001 rna18443 6.00E-61
826- 17: transcript:Zm00001d036714_T002 rna18438 0
826- 18: transcript:Zm00001d036715_T001 rna18436 0

826- 19: transcript:Zm00001d036717_T001 rna18435 1.00E-95
826- 20: transcript:Zm00001d036718_T002 rna18434 0
826- 21: transcript:Zm00001d036719_T001 rna18433 2.00E-170
826- 22: transcript:Zm00001d036720_T001 rna18432 0
826- 23: transcript:Zm00001d036723_T001 rna18430 7.00E-34
826- 24: transcript:Zm00001d036726_T001 rna18429 3.00E-130
826- 25: transcript:Zm00001d036727_T001 rna18419 0
826- 26: transcript:Zm00001d036736_T001 rna18415 4.00E-15
826- 27: transcript:Zm00001d036737_T001 rna18411 2.00E-26
826- 28: transcript:Zm00001d036738_T001 rna18410 0
826- 29: transcript:Zm00001d036739_T001 rna18407 5.00E-165
826- 30: transcript:Zm00001d036740_T001 rna18406 5.00E-62
826- 31: transcript:Zm00001d036741_T009 rna18405 0
Alignment 827: score=951.0 e_value=1.9e-60 N=21 6&NC_008399.2 minus
827- 0: transcript:Zm00001d036877_T001 rna18224 1.00E-136
827- 1: transcript:Zm00001d036878_T007 rna18221 8.00E-176
827- 2: transcript:Zm00001d036879_T001 rna18218 0
827- 3: transcript:Zm00001d036880_T003 rna18217 8.00E-69
827- 4: transcript:Zm00001d036883_T001 rna18216 9.00E-13
827- 5: transcript:Zm00001d036889_T001 rna18206 2.00E-38
827- 6: transcript:Zm00001d036892_T001 rna18204 4.00E-25
827- 7: transcript:Zm00001d036893_T001 rna18203 1.00E-60
827- 8: transcript:Zm00001d036894_T001 rna18201 7.00E-149
827- 9: transcript:Zm00001d036895_T001 rna18199 7.00E-113
827- 10: transcript:Zm00001d036896_T001 rna18197 7.00E-14
827- 11: transcript:Zm00001d036897_T001 rna18194 0
827- 12: transcript:Zm00001d036902_T001 rna18191 3.00E-44
827- 13: transcript:Zm00001d036903_T001 rna18188 7.00E-88
827- 14: transcript:Zm00001d036905_T002 rna18183 0
827- 15: transcript:Zm00001d036912_T001 rna18171 0
827- 16: transcript:Zm00001d036917_T009 rna18170 0
827- 17: transcript:Zm00001d036918_T001 rna18168 9.00E-83
827- 18: transcript:Zm00001d036919_T002 rna18167 0
827- 19: transcript:Zm00001d036922_T001 rna18163 4.00E-166
827- 20: transcript:Zm00001d036925_T001 rna18162 0
Alignment 828: score=687.0 e_value=2.6e-39 N=15 6&NC_008399.2 minus
828- 0: transcript:Zm00001d036463_T001 rna18738 1.00E-61
828- 1: transcript:Zm00001d036464_T001 rna18735 7.00E-33
828- 2: transcript:Zm00001d036475_T001 rna18734 0
828- 3: transcript:Zm00001d036477_T002 rna18733 0
828- 4: transcript:Zm00001d036480_T001 rna18732 0
828- 5: transcript:Zm00001d036482_T001 rna18729 0
828- 6: transcript:Zm00001d036483_T006 rna18726 0
828- 7: transcript:Zm00001d036484_T001 rna18724 0
828- 8: transcript:Zm00001d036485_T003 rna18723 1.00E-84
828- 9: transcript:Zm00001d036490_T001 rna18719 0
828- 10: transcript:Zm00001d036494_T001 rna18718 3.00E-22
828- 11: transcript:Zm00001d036495_T001 rna18717 0
828- 12: transcript:Zm00001d036496_T001 rna18715 0
828- 13: transcript:Zm00001d036499_T001 rna18714 1.00E-27
828- 14: transcript:Zm00001d036506_T001 rna18710 2.00E-58
Alignment 829: score=674.0 e_value=2.1e-37 N=15 6&NC_008399.2 minus
829- 0: transcript:Zm00001d037008_T001 rna17996 5.00E-176
829- 1: transcript:Zm00001d037009_T001 rna17993 0

829- 2: transcript:Zm00001d037010_T003 rna17991 0
829- 3: transcript:Zm00001d037013_T001 rna17981 9.00E-103
829- 4: transcript:Zm00001d037015_T001 rna17977 7.00E-145
829- 5: transcript:Zm00001d037017_T001 rna17966 0
829- 6: transcript:Zm00001d037018_T001 rna17963 1.00E-73
829- 7: transcript:Zm00001d037019_T001 rna17952 1.00E-21
829- 8: transcript:Zm00001d037024_T001 rna17947 1.00E-21
829- 9: transcript:Zm00001d037025_T001 rna17946 0
829- 10: transcript:Zm00001d037029_T001 rna17940 1.00E-129
829- 11: transcript:Zm00001d037033_T001 rna17937 1.00E-15
829- 12: transcript:Zm00001d037034_T002 rna17936 0
829- 13: transcript:Zm00001d037035_T002 rna17928 0
829- 14: transcript:Zm00001d037042_T001 rna17909 2.00E-36
Alignment 830: score=550.0 e_value=4.5e-32 N=13 6&NC_008399.2 minus
830- 0: transcript:Zm00001d036945_T001 rna18120 0
830- 1: transcript:Zm00001d036947_T001 rna18114 0
830- 2: transcript:Zm00001d036949_T004 rna18093 0
830- 3: transcript:Zm00001d036956_T001 rna18086 1.00E-126
830- 4: transcript:Zm00001d036959_T003 rna18083 0
830- 5: transcript:Zm00001d036961_T004 rna18069 0
830- 6: transcript:Zm00001d036962_T001 rna18068 1.00E-115
830- 7: transcript:Zm00001d036964_T001 rna18065 8.00E-43
830- 8: transcript:Zm00001d036965_T001 rna18062 4.00E-26
830- 9: transcript:Zm00001d036966_T001 rna18061 5.00E-108
830- 10: transcript:Zm00001d036968_T015 rna18060 0
830- 11: transcript:Zm00001d036970_T001 rna18059 1.00E-81
830- 12: transcript:Zm00001d036971_T004 rna18047 3.00E-150
Alignment 831: score=543.0 e_value=3.9e-28 N=12 6&NC_008399.2 minus
831- 0: transcript:Zm00001d036571_T002 rna18605 0
831- 1: transcript:Zm00001d036573_T002 rna18604 1.00E-21
831- 2: transcript:Zm00001d036577_T001 rna18602 7.00E-100
831- 3: transcript:Zm00001d036579_T003 rna18597 0
831- 4: transcript:Zm00001d036583_T001 rna18596 1.00E-140
831- 5: transcript:Zm00001d036588_T001 rna18593 9.00E-101
831- 6: transcript:Zm00001d036593_T001 rna18591 0
831- 7: transcript:Zm00001d036594_T001 rna18590 0
831- 8: transcript:Zm00001d036597_T001 rna18589 2.00E-46
831- 9: transcript:Zm00001d036598_T006 rna18588 0
831- 10: transcript:Zm00001d036602_T003 rna18582 0
831- 11: transcript:Zm00001d036608_T003 rna18576 0
Alignment 832: score=470.0 e_value=1.4e-26 N=11 6&NC_008399.2 minus
832- 0: transcript:Zm00001d036981_T006 rna18042 0
832- 1: transcript:Zm00001d036984_T001 rna18023 0
832- 2: transcript:Zm00001d036985_T001 rna18020 3.00E-71
832- 3: transcript:Zm00001d036986_T002 rna18018 0
832- 4: transcript:Zm00001d036987_T001 rna18017 1.00E-103
832- 5: transcript:Zm00001d036989_T001 rna18015 6.00E-110
832- 6: transcript:Zm00001d036991_T001 rna18014 4.00E-77
832- 7: transcript:Zm00001d037000_T002 rna18012 8.00E-65
832- 8: transcript:Zm00001d037003_T001 rna18005 2.00E-26
832- 9: transcript:Zm00001d037004_T002 rna18004 7.00E-98
832- 10: transcript:Zm00001d037005_T001 rna17993 0
Alignment 833: score=462.0 e_value=3e-22 N=10 6&NC_008399.2 minus
833- 0: transcript:Zm00001d036543_T001 rna18652 9.00E-171

833- 1: transcript:Zm00001d036545_T001 rna18647 4.00E-52
833- 2: transcript:Zm00001d036546_T007 rna18646 8.00E-20
833- 3: transcript:Zm00001d036549_T001 rna18640 9.00E-28
833- 4: transcript:Zm00001d036550_T001 rna18638 2.00E-35
833- 5: transcript:Zm00001d036557_T002 rna18631 0
833- 6: transcript:Zm00001d036558_T001 rna18629 9.00E-140
833- 7: transcript:Zm00001d036560_T001 rna18619 2.00E-15
833- 8: transcript:Zm00001d036563_T001 rna18618 0
833- 9: transcript:Zm00001d036564_T001 rna18615 0
Alignment 834: score=394.0 e_value=1e-19 N=9 6&NC_008399.2 minus
834- 0: transcript:Zm00001d036641_T001 rna18544 3.00E-105
834- 1: transcript:Zm00001d036642_T001 rna18540 1.00E-56
834- 2: transcript:Zm00001d036648_T001 rna18536 3.00E-119
834- 3: transcript:Zm00001d036650_T001 rna18531 1.00E-37
834- 4: transcript:Zm00001d036654_T002 rna18525 0
834- 5: transcript:Zm00001d036655_T003 rna18516 4.00E-81
834- 6: transcript:Zm00001d036656_T003 rna18512 0
834- 7: transcript:Zm00001d036657_T003 rna18511 1.00E-59
834- 8: transcript:Zm00001d036668_T002 rna18499 0
Alignment 835: score=362.0 e_value=2.6e-14 N=8 6&NC_008399.2 minus
835- 0: transcript:Zm00001d037321_T001 rna17418 0
835- 1: transcript:Zm00001d037323_T001 rna17405 2.00E-80
835- 2: transcript:Zm00001d037326_T002 rna17401 0
835- 3: transcript:Zm00001d037327_T001 rna17400 1.00E-164
835- 4: transcript:Zm00001d037328_T001 rna17394 0
835- 5: transcript:Zm00001d037329_T001 rna17393 7.00E-134
835- 6: transcript:Zm00001d037332_T001 rna17392 2.00E-117
835- 7: transcript:Zm00001d037333_T002 rna17391 0
Alignment 836: score=286.0 e_value=1.6e-10 N=6 6&NC_008399.2 minus
836- 0: transcript:Zm00001d037334_T001 rna17389 3.00E-104
836- 1: transcript:Zm00001d037336_T001 rna17388 0
836- 2: transcript:Zm00001d037337_T001 rna17387 8.00E-53
836- 3: transcript:Zm00001d037338_T001 rna17385 0
836- 4: transcript:Zm00001d037340_T001 rna17384 2.00E-54
836- 5: transcript:Zm00001d037346_T007 rna17375 0
Alignment 837: score=286.0 e_value=5.9e-13 N=6 6&NC_008399.2 minus
837- 0: transcript:Zm00001d036181_T001 rna16836 0
837- 1: transcript:Zm00001d036195_T004 rna16835 2.00E-95
837- 2: transcript:Zm00001d036196_T001 rna16834 2.00E-149
837- 3: transcript:Zm00001d036198_T001 rna16830 5.00E-16
837- 4: transcript:Zm00001d036199_T001 rna16829 5.00E-62
837- 5: transcript:Zm00001d036201_T001 rna16828 7.00E-140
Alignment 838: score=274.0 e_value=9e-11 N=6 6&NC_008399.2 minus
838- 0: transcript:Zm00001d036515_T001 rna18703 3.00E-07
838- 1: transcript:Zm00001d036517_T001 rna18687 3.00E-17
838- 2: transcript:Zm00001d036521_T001 rna18685 1.00E-110
838- 3: transcript:Zm00001d036522_T001 rna18684 2.00E-81
838- 4: transcript:Zm00001d036524_T001 rna18680 0
838- 5: transcript:Zm00001d036529_T001 rna18678 0
Alignment 839: score=1348.0 e_value=8.3e-105 N=30 6&NC_008401.2 plus
839- 0: transcript:Zm00001d034964_T083 rna21249 0
839- 1: transcript:Zm00001d034965_T002 rna21254 3.00E-117
839- 2: transcript:Zm00001d034968_T001 rna21258 0
839- 3: transcript:Zm00001d034969_T001 rna21261 2.00E-79

839- 4: transcript:Zm00001d034970_T002 rna21262 0
839- 5: transcript:Zm00001d034977_T001 rna21263 5.00E-110
839- 6: transcript:Zm00001d034984_T001 rna21273 3.00E-102
839- 7: transcript:Zm00001d034998_T004 rna21275 0
839- 8: transcript:Zm00001d035000_T001 rna21276 2.00E-25
839- 9: transcript:Zm00001d035001_T001 rna21277 8.00E-67
839- 10: transcript:Zm00001d035004_T002 rna21279 0
839- 11: transcript:Zm00001d035005_T001 rna21280 4.00E-102
839- 12: transcript:Zm00001d035007_T001 rna21281 0
839- 13: transcript:Zm00001d035008_T006 rna21282 3.00E-170
839- 14: transcript:Zm00001d035013_T001 rna21284 0
839- 15: transcript:Zm00001d035015_T001 rna21296 6.00E-175
839- 16: transcript:Zm00001d035019_T001 rna21301 3.00E-85
839- 17: transcript:Zm00001d035020_T001 rna21302 1.00E-61
839- 18: transcript:Zm00001d035023_T001 rna21303 0
839- 19: transcript:Zm00001d035024_T001 rna21304 6.00E-37
839- 20: transcript:Zm00001d035027_T001 rna21305 1.00E-81
839- 21: transcript:Zm00001d035028_T001 rna21306 4.00E-100
839- 22: transcript:Zm00001d035029_T006 rna21307 0
839- 23: transcript:Zm00001d035030_T002 rna21308 0
839- 24: transcript:Zm00001d035032_T002 rna21309 0
839- 25: transcript:Zm00001d035034_T002 rna21310 2.00E-152
839- 26: transcript:Zm00001d035035_T001 rna21313 0
839- 27: transcript:Zm00001d035039_T001 rna21319 0
839- 28: transcript:Zm00001d035040_T007 rna21320 0
839- 29: transcript:Zm00001d035041_T001 rna21322 0
Alignment 840: score=1126.0 e_value=1.8e-80 N=25 6&NC_008401.2 plus
840- 0: transcript:Zm00001d035046_T001 rna21324 0
840- 1: transcript:Zm00001d035048_T003 rna21330 0
840- 2: transcript:Zm00001d035050_T002 rna21332 0
840- 3: transcript:Zm00001d035052_T001 rna21333 4.00E-93
840- 4: transcript:Zm00001d035053_T002 rna21334 1.00E-106
840- 5: transcript:Zm00001d035054_T002 rna21335 1.00E-83
840- 6: transcript:Zm00001d035063_T002 rna21336 0
840- 7: transcript:Zm00001d035065_T002 rna21339 0
840- 8: transcript:Zm00001d035066_T001 rna21340 3.00E-50
840- 9: transcript:Zm00001d035067_T001 rna21341 8.00E-138
840- 10: transcript:Zm00001d035076_T001 rna21342 3.00E-117
840- 11: transcript:Zm00001d035080_T001 rna21346 6.00E-18
840- 12: transcript:Zm00001d035081_T001 rna21348 1.00E-136
840- 13: transcript:Zm00001d035082_T001 rna21349 0
840- 14: transcript:Zm00001d035084_T001 rna21353 1.00E-130
840- 15: transcript:Zm00001d035086_T001 rna21355 3.00E-147
840- 16: transcript:Zm00001d035087_T001 rna21356 1.00E-33
840- 17: transcript:Zm00001d035089_T003 rna21357 0
840- 18: transcript:Zm00001d035090_T002 rna21359 0
840- 19: transcript:Zm00001d035091_T001 rna21360 7.00E-153
840- 20: transcript:Zm00001d035092_T001 rna21361 1.00E-110
840- 21: transcript:Zm00001d035094_T001 rna21362 5.00E-102
840- 22: transcript:Zm00001d035095_T001 rna21364 1.00E-98
840- 23: transcript:Zm00001d035097_T001 rna21365 0
840- 24: transcript:Zm00001d035098_T002 rna21367 8.00E-148
Alignment 841: score=1110.0 e_value=2.2e-90 N=25 6&NC_008401.2 plus
841- 0: transcript:Zm00001d035119_T001 rna21369 0

```
841-  1:  transcript:Zm00001d035124_T001  rna21373    1.00E-151
841-  2:  transcript:Zm00001d035130_T001  rna21374            0
841-  3:  transcript:Zm00001d035132_T001  rna21376     4.00E-70
841-  4:  transcript:Zm00001d035134_T001  rna21377     2.00E-33
841-  5:  transcript:Zm00001d035135_T001  rna21378     5.00E-39
841-  6:  transcript:Zm00001d035136_T001  rna21380    1.00E-148
841-  7:  transcript:Zm00001d035139_T002  rna21383            0
841-  8:  transcript:Zm00001d035140_T001  rna21388            0
841-  9:  transcript:Zm00001d035156_T003  rna21404            0
841- 10:  transcript:Zm00001d035157_T001  rna21408            0
841- 11:  transcript:Zm00001d035167_T009  rna21409     9.00E-12
841- 12:  transcript:Zm00001d035168_T003  rna21410     3.00E-93
841- 13:  transcript:Zm00001d035169_T001  rna21411    8.00E-163
841- 14:  transcript:Zm00001d035170_T002  rna21412            0
841- 15:  transcript:Zm00001d035171_T001  rna21414     2.00E-18
841- 16:  transcript:Zm00001d035184_T001  rna21417            0
841- 17:  transcript:Zm00001d035186_T001  rna21422    2.00E-145
841- 18:  transcript:Zm00001d035191_T004  rna21423            0
841- 19:  transcript:Zm00001d035195_T001  rna21428     2.00E-70
841- 20:  transcript:Zm00001d035197_T001  rna21430            0
841- 21:  transcript:Zm00001d035200_T001  rna21431    4.00E-130
841- 22:  transcript:Zm00001d035201_T001  rna21432            0
841- 23:  transcript:Zm00001d035208_T001  rna21433            0
841- 24:  transcript:Zm00001d035211_T001  rna21452            0
## Alignment 842: score=917.0 e_value=2.6e-64 N=20 6&NC_008405.2 minus
842-  0:  transcript:Zm00001d035267_T003  rna29677    3.00E-105
842-  1:  transcript:Zm00001d035270_T011  rna29676     2.00E-71
842-  2:  transcript:Zm00001d035285_T003  rna29675            0
842-  3:  transcript:Zm00001d035288_T002  rna29672     9.00E-42
842-  4:  transcript:Zm00001d035294_T001  rna29664            0
842-  5:  transcript:Zm00001d035297_T001  rna29661            0
842-  6:  transcript:Zm00001d035300_T001  rna29660    1.00E-165
842-  7:  transcript:Zm00001d035303_T001  rna29659     1.00E-76
842-  8:  transcript:Zm00001d035304_T001  rna29658     9.00E-64
842-  9:  transcript:Zm00001d035306_T001  rna29657     1.00E-06
842- 10:  transcript:Zm00001d035309_T001  rna29655     3.00E-19
842- 11:  transcript:Zm00001d035312_T002  rna29643     4.00E-20
842- 12:  transcript:Zm00001d035313_T002  rna29642     1.00E-44
842- 13:  transcript:Zm00001d035317_T002  rna29641     2.00E-63
842- 14:  transcript:Zm00001d035318_T001  rna29640     1.00E-99
842- 15:  transcript:Zm00001d035320_T001  rna29638     3.00E-49
842- 16:  transcript:Zm00001d035319_T005  rna29637     1.00E-06
842- 17:  transcript:Zm00001d035321_T001  rna29636     2.00E-23
842- 18:  transcript:Zm00001d035322_T001  rna29635     2.00E-88
842- 19:  transcript:Zm00001d035323_T001  rna29634            0
## Alignment 843: score=297.0 e_value=6.9e-12 N=7 7&NC_008394.4 plus
843-  0:  transcript:Zm00001d021038_T001  rna3854      5.00E-22
843-  1:  transcript:Zm00001d021048_T001  rna3874      2.00E-79
843-  2:  transcript:Zm00001d021056_T001  rna3885      6.00E-46
843-  3:  transcript:Zm00001d021058_T001  rna3888      1.00E-15
843-  4:  transcript:Zm00001d021061_T001  rna3903     4.00E-157
843-  5:  transcript:Zm00001d021062_T001  rna3906      4.00E-28
843-  6:  transcript:Zm00001d021065_T001  rna3910             0
## Alignment 844: score=410.0 e_value=7.7e-21 N=10 7&NC_008395.2 minus
```

844- 0: transcript:Zm00001d020675_T001 rna7267 3.00E-29
844- 1: transcript:Zm00001d020686_T001 rna7258 6.00E-170
844- 2: transcript:Zm00001d020688_T001 rna7255 8.00E-14
844- 3: transcript:Zm00001d020691_T001 rna7242 4.00E-61
844- 4: transcript:Zm00001d020702_T001 rna7230 1.00E-176
844- 5: transcript:Zm00001d020703_T001 rna7229 0
844- 6: transcript:Zm00001d020708_T002 rna7225 3.00E-104
844- 7: transcript:Zm00001d020711_T001 rna7223 7.00E-65
844- 8: transcript:Zm00001d020721_T001 rna7208 3.00E-160
844- 9: transcript:Zm00001d020723_T001 rna7204 6.00E-38
Alignment 845: score=539.0 e_value=2.1e-33 N=13 7&NC_008396.2 plus
845- 0: transcript:Zm00001d019165_T002 rna11031 0
845- 1: transcript:Zm00001d019166_T002 rna11032 5.00E-17
845- 2: transcript:Zm00001d019169_T004 rna11035 7.00E-145
845- 3: transcript:Zm00001d019171_T001 rna11038 2.00E-138
845- 4: transcript:Zm00001d019180_T001 rna11040 3.00E-33
845- 5: transcript:Zm00001d019191_T007 rna11053 3.00E-131
845- 6: transcript:Zm00001d019207_T001 rna11063 3.00E-125
845- 7: transcript:Zm00001d019215_T002 rna11067 7.00E-165
845- 8: transcript:Zm00001d019216_T001 rna11068 1.00E-31
845- 9: transcript:Zm00001d019222_T001 rna11075 4.00E-18
845- 10: transcript:Zm00001d019223_T001 rna11077 2.00E-31
845- 11: transcript:Zm00001d019225_T001 rna11083 8.00E-174
845- 12: transcript:Zm00001d019230_T002 rna11092 1.00E-130
Alignment 846: score=476.0 e_value=3.7e-26 N=12 7&NC_008396.2 plus
846- 0: transcript:Zm00001d018915_T007 rna10904 2.00E-19
846- 1: transcript:Zm00001d018926_T001 rna10905 3.00E-30
846- 2: transcript:Zm00001d018931_T001 rna10917 2.00E-128
846- 3: transcript:Zm00001d018938_T001 rna10920 7.00E-81
846- 4: transcript:Zm00001d018941_T001 rna10922 8.00E-163
846- 5: transcript:Zm00001d018947_T001 rna10925 5.00E-21
846- 6: transcript:Zm00001d018957_T001 rna10926 0
846- 7: transcript:Zm00001d018962_T001 rna10927 2.00E-131
846- 8: transcript:Zm00001d018964_T003 rna10928 6.00E-15
846- 9: transcript:Zm00001d018971_T003 rna10929 8.00E-63
846- 10: transcript:Zm00001d018973_T001 rna10936 3.00E-60
846- 11: transcript:Zm00001d018976_T003 rna10940 0
Alignment 847: score=444.0 e_value=3.5e-20 N=10 7&NC_008396.2 plus
847- 0: transcript:Zm00001d022437_T001 rna9212 3.00E-33
847- 1: transcript:Zm00001d022439_T001 rna9213 0
847- 2: transcript:Zm00001d022442_T002 rna9219 7.00E-145
847- 3: transcript:Zm00001d022444_T001 rna9224 5.00E-179
847- 4: transcript:Zm00001d022446_T001 rna9225 2.00E-30
847- 5: transcript:Zm00001d022449_T002 rna9228 1.00E-50
847- 6: transcript:Zm00001d022457_T002 rna9242 3.00E-122
847- 7: transcript:Zm00001d022462_T001 rna9254 6.00E-125
847- 8: transcript:Zm00001d022463_T001 rna9263 1.00E-69
847- 9: transcript:Zm00001d022464_T001 rna9266 7.00E-09
Alignment 848: score=386.0 e_value=3.6e-21 N=10 7&NC_008396.2 plus
848- 0: transcript:Zm00001d018669_T004 rna10718 2.00E-158
848- 1: transcript:Zm00001d018677_T001 rna10719 6.00E-180
848- 2: transcript:Zm00001d018698_T001 rna10723 2.00E-93
848- 3: transcript:Zm00001d018717_T001 rna10750 1.00E-26
848- 4: transcript:Zm00001d018719_T001 rna10751 0

848- 5: transcript:Zm00001d018730_T001 rna10762 1.00E-15
848- 6: transcript:Zm00001d018731_T001 rna10763 3.00E-14
848- 7: transcript:Zm00001d018742_T001 rna10767 3.00E-101
848- 8: transcript:Zm00001d018749_T001 rna10773 5.00E-149
848- 9: transcript:Zm00001d018752_T001 rna10779 0
Alignment 849: score=312.0 e_value=7.5e-13 N=7 7&NC_008396.2 plus
849- 0: transcript:Zm00001d018798_T002 rna10847 2.00E-55
849- 1: transcript:Zm00001d018806_T001 rna10849 1.00E-71
849- 2: transcript:Zm00001d018809_T001 rna10850 5.00E-98
849- 3: transcript:Zm00001d018810_T002 rna10852 0
849- 4: transcript:Zm00001d018816_T001 rna10853 2.00E-20
849- 5: transcript:Zm00001d018823_T001 rna10866 3.00E-57
849- 6: transcript:Zm00001d018826_T001 rna10867 4.00E-66
Alignment 850: score=288.0 e_value=1.5e-08 N=6 7&NC_008396.2 plus
850- 0: transcript:Zm00001d022430_T001 rna9190 9.00E-94
850- 1: transcript:Zm00001d022431_T001 rna9195 2.00E-36
850- 2: transcript:Zm00001d022432_T001 rna9197 0
850- 3: transcript:Zm00001d022433_T001 rna9201 9.00E-164
850- 4: transcript:Zm00001d022434_T001 rna9205 0
850- 5: transcript:Zm00001d022435_T001 rna9207 7.00E-22
Alignment 851: score=268.0 e_value=2.8e-08 N=6 7&NC_008396.2 plus
851- 0: transcript:Zm00001d019090_T003 rna10977 6.00E-74
851- 1: transcript:Zm00001d019091_T002 rna10979 3.00E-133
851- 2: transcript:Zm00001d019094_T001 rna10987 3.00E-140
851- 3: transcript:Zm00001d019100_T001 rna10990 6.00E-37
851- 4: transcript:Zm00001d019104_T001 rna10992 0
851- 5: transcript:Zm00001d019107_T001 rna10998 5.00E-25
Alignment 852: score=255.0 e_value=9.9e-09 N=6 7&NC_008396.2 plus
852- 0: transcript:Zm00001d019303_T001 rna11214 9.00E-28
852- 1: transcript:Zm00001d019305_T001 rna11216 2.00E-33
852- 2: transcript:Zm00001d019311_T001 rna11218 1.00E-109
852- 3: transcript:Zm00001d019314_T003 rna11220 2.00E-41
852- 4: transcript:Zm00001d019317_T002 rna11221 0
852- 5: transcript:Zm00001d019326_T001 rna11224 3.00E-44
Alignment 853: score=251.0 e_value=1.6e-09 N=6 7&NC_008396.2 plus
853- 0: transcript:Zm00001d022117_T002 rna8239 3.00E-37
853- 1: transcript:Zm00001d022126_T001 rna8245 3.00E-176
853- 2: transcript:Zm00001d022130_T001 rna8249 7.00E-19
853- 3: transcript:Zm00001d022131_T001 rna8251 3.00E-83
853- 4: transcript:Zm00001d022139_T001 rna8256 1.00E-25
853- 5: transcript:Zm00001d022142_T001 rna8260 0
Alignment 854: score=823.0 e_value=6.1e-57 N=20 7&NC_008396.2 minus
854- 0: transcript:Zm00001d022149_T001 rna9548 3.00E-31
854- 1: transcript:Zm00001d022151_T001 rna9539 4.00E-166
854- 2: transcript:Zm00001d022154_T001 rna9535 1.00E-63
854- 3: transcript:Zm00001d022155_T001 rna9532 8.00E-115
854- 4: transcript:Zm00001d022161_T001 rna9518 0
854- 5: transcript:Zm00001d022168_T001 rna9509 1.00E-71
854- 6: transcript:Zm00001d022172_T001 rna9508 7.00E-112
854- 7: transcript:Zm00001d022174_T001 rna9507 8.00E-19
854- 8: transcript:Zm00001d022179_T001 rna9497 0
854- 9: transcript:Zm00001d022180_T009 rna9495 4.00E-171
854- 10: transcript:Zm00001d022181_T004 rna9493 0
854- 11: transcript:Zm00001d022182_T001 rna9487 2.00E-149

854- 12: transcript:Zm00001d022185_T003 rna9485 2.00E-154
854- 13: transcript:Zm00001d022188_T002 rna9484 3.00E-35
854- 14: transcript:Zm00001d022189_T002 rna9483 3.00E-74
854- 15: transcript:Zm00001d022190_T003 rna9481 1.00E-115
854- 16: transcript:Zm00001d022191_T001 rna9479 6.00E-15
854- 17: transcript:Zm00001d022192_T002 rna9474 4.00E-131
854- 18: transcript:Zm00001d022195_T001 rna9470 8.00E-15
854- 19: transcript:Zm00001d022198_T002 rna9466 5.00E-94
Alignment 855: score=614.0 e_value=1.3e-36 N=14 7&NC_008396.2 minus
855- 0: transcript:Zm00001d022211_T001 rna9458 7.00E-17
855- 1: transcript:Zm00001d022218_T001 rna9452 2.00E-53
855- 2: transcript:Zm00001d022225_T001 rna9450 8.00E-81
855- 3: transcript:Zm00001d022230_T001 rna9445 0
855- 4: transcript:Zm00001d022237_T001 rna9436 5.00E-110
855- 5: transcript:Zm00001d022238_T001 rna9434 7.00E-120
855- 6: transcript:Zm00001d022242_T001 rna9428 2.00E-54
855- 7: transcript:Zm00001d022243_T002 rna9427 7.00E-82
855- 8: transcript:Zm00001d022245_T001 rna9426 3.00E-48
855- 9: transcript:Zm00001d022250_T001 rna9424 9.00E-98
855- 10: transcript:Zm00001d022252_T001 rna9417 9.00E-77
855- 11: transcript:Zm00001d022264_T001 rna9409 7.00E-24
855- 12: transcript:Zm00001d022265_T001 rna9408 0
855- 13: transcript:Zm00001d022266_T001 rna9406 1.00E-111
Alignment 856: score=552.0 e_value=9e-32 N=13 7&NC_008396.2 minus
856- 0: transcript:Zm00001d022557_T001 rna9060 1.00E-140
856- 1: transcript:Zm00001d022560_T001 rna9054 0
856- 2: transcript:Zm00001d022561_T003 rna9042 5.00E-38
856- 3: transcript:Zm00001d022563_T001 rna9041 0
856- 4: transcript:Zm00001d022564_T001 rna9040 6.00E-174
856- 5: transcript:Zm00001d022565_T001 rna9026 0
856- 6: transcript:Zm00001d022567_T001 rna9018 0
856- 7: transcript:Zm00001d022569_T001 rna9009 2.00E-56
856- 8: transcript:Zm00001d022573_T001 rna9002 0
856- 9: transcript:Zm00001d022574_T001 rna9001 5.00E-35
856- 10: transcript:Zm00001d022578_T001 rna8976 0
856- 11: transcript:Zm00001d022579_T001 rna8964 0
856- 12: transcript:Zm00001d022582_T002 rna8951 5.00E-100
Alignment 857: score=477.0 e_value=1.4e-23 N=11 7&NC_008396.2 minus
857- 0: transcript:Zm00001d022273_T016 rna9405 8.00E-53
857- 1: transcript:Zm00001d022274_T001 rna9404 0
857- 2: transcript:Zm00001d022275_T001 rna9403 2.00E-147
857- 3: transcript:Zm00001d022279_T001 rna9400 8.00E-118
857- 4: transcript:Zm00001d022283_T001 rna9396 1.00E-110
857- 5: transcript:Zm00001d022294_T002 rna9388 2.00E-13
857- 6: transcript:Zm00001d022305_T001 rna9385 0
857- 7: transcript:Zm00001d022307_T003 rna9383 2.00E-51
857- 8: transcript:Zm00001d022309_T002 rna9381 2.00E-81
857- 9: transcript:Zm00001d022314_T001 rna9378 4.00E-17
857- 10: transcript:Zm00001d022315_T001 rna9377 6.00E-124
Alignment 858: score=374.0 e_value=1.1e-14 N=8 7&NC_008396.2 minus
858- 0: transcript:Zm00001d022059_T001 rna9625 2.00E-46
858- 1: transcript:Zm00001d022069_T001 rna9618 1.00E-82
858- 2: transcript:Zm00001d022071_T001 rna9617 9.00E-58
858- 3: transcript:Zm00001d022072_T001 rna9616 0

858- 4: transcript:Zm00001d022075_T001 rna9610 0
858- 5: transcript:Zm00001d022077_T003 rna9609 1.00E-171
858- 6: transcript:Zm00001d022081_T001 rna9606 0
858- 7: transcript:Zm00001d022083_T001 rna9604 4.00E-84
Alignment 859: score=273.0 e_value=4.4e-10 N=6 7&NC_008396.2 minus
859- 0: transcript:Zm00001d019279_T001 rna11199 8.00E-126
859- 1: transcript:Zm00001d019282_T001 rna11198 0
859- 2: transcript:Zm00001d019283_T005 rna11185 0
859- 3: transcript:Zm00001d019287_T001 rna11183 1.00E-171
859- 4: transcript:Zm00001d019288_T001 rna11179 4.00E-91
859- 5: transcript:Zm00001d019290_T001 rna11174 1.00E-27
Alignment 860: score=260.0 e_value=1e-08 N=6 7&NC_008396.2 minus
860- 0: transcript:Zm00001d022124_T001 rna9573 6.00E-93
860- 1: transcript:Zm00001d022126_T001 rna9572 0
860- 2: transcript:Zm00001d022130_T001 rna9570 5.00E-46
860- 3: transcript:Zm00001d022132_T002 rna9569 2.00E-163
860- 4: transcript:Zm00001d022134_T002 rna9567 1.00E-110
860- 5: transcript:Zm00001d022139_T001 rna9553 5.00E-76
Alignment 861: score=262.0 e_value=4.8e-09 N=6 7&NC_008397.2 minus
861- 0: transcript:Zm00001d021207_T001 rna13274 7.00E-33
861- 1: transcript:Zm00001d021216_T001 rna13270 1.00E-165
861- 2: transcript:Zm00001d021224_T001 rna13269 2.00E-124
861- 3: transcript:Zm00001d021225_T005 rna13268 3.00E-48
861- 4: transcript:Zm00001d021231_T005 rna13258 5.00E-130
861- 5: transcript:Zm00001d021236_T004 rna13257 0
Alignment 862: score=5268.0 e_value=0 N=116 7&NC_008400.2 plus
862- 0: transcript:Zm00001d022117_T002 rna20702 1.00E-83
862- 1: transcript:Zm00001d022119_T001 rna20707 2.00E-40
862- 2: transcript:Zm00001d022124_T001 rna20708 2.00E-90
862- 3: transcript:Zm00001d022125_T002 rna20709 0
862- 4: transcript:Zm00001d022126_T001 rna20710 0
862- 5: transcript:Zm00001d022130_T001 rna20712 1.00E-49
862- 6: transcript:Zm00001d022131_T001 rna20715 1.00E-68
862- 7: transcript:Zm00001d022132_T002 rna20716 0
862- 8: transcript:Zm00001d022133_T001 rna20717 6.00E-82
862- 9: transcript:Zm00001d022134_T002 rna20718 7.00E-124
862- 10: transcript:Zm00001d022139_T001 rna20723 2.00E-99
862- 11: transcript:Zm00001d022141_T007 rna20726 2.00E-61
862- 12: transcript:Zm00001d022142_T001 rna20727 0
862- 13: transcript:Zm00001d022143_T010 rna20728 0
862- 14: transcript:Zm00001d022144_T003 rna20730 0
862- 15: transcript:Zm00001d022148_T001 rna20734 0
862- 16: transcript:Zm00001d022151_T001 rna20738 2.00E-169
862- 17: transcript:Zm00001d022152_T001 rna20739 0
862- 18: transcript:Zm00001d022155_T001 rna20741 0
862- 19: transcript:Zm00001d022159_T001 rna20743 4.00E-161
862- 20: transcript:Zm00001d022160_T001 rna20745 3.00E-105
862- 21: transcript:Zm00001d022161_T001 rna20746 0
862- 22: transcript:Zm00001d022163_T001 rna20750 1.00E-82
862- 23: transcript:Zm00001d022166_T001 rna20753 0
862- 24: transcript:Zm00001d022168_T001 rna20754 3.00E-177
862- 25: transcript:Zm00001d022171_T001 rna20755 9.00E-18
862- 26: transcript:Zm00001d022172_T001 rna20756 7.00E-125
862- 27: transcript:Zm00001d022174_T001 rna20757 1.00E-20

862- 28:	transcript:Zm00001d022175_T001	rna20760	5.00E-143
862- 29:	transcript:Zm00001d022176_T002	rna20765	0
862- 30:	transcript:Zm00001d022179_T001	rna20768	0
862- 31:	transcript:Zm00001d022180_T009	rna20769	1.00E-168
862- 32:	transcript:Zm00001d022181_T004	rna20770	0
862- 33:	transcript:Zm00001d022182_T001	rna20774	3.00E-179
862- 34:	transcript:Zm00001d022185_T003	rna20777	1.00E-140
862- 35:	transcript:Zm00001d022188_T002	rna20778	1.00E-46
862- 36:	transcript:Zm00001d022189_T002	rna20779	4.00E-81
862- 37:	transcript:Zm00001d022190_T003	rna20781	6.00E-158
862- 38:	transcript:Zm00001d022192_T002	rna20783	2.00E-149
862- 39:	transcript:Zm00001d022195_T001	rna20787	1.00E-39
862- 40:	transcript:Zm00001d022197_T001	rna20788	6.00E-51
862- 41:	transcript:Zm00001d022198_T002	rna20789	1.00E-123
862- 42:	transcript:Zm00001d022199_T003	rna20790	0
862- 43:	transcript:Zm00001d022200_T003	rna20791	1.00E-150
862- 44:	transcript:Zm00001d022201_T002	rna20792	0
862- 45:	transcript:Zm00001d022202_T001	rna20793	0
862- 46:	transcript:Zm00001d022203_T001	rna20796	0
862- 47:	transcript:Zm00001d022205_T001	rna20797	1.00E-138
862- 48:	transcript:Zm00001d022206_T004	rna20798	3.00E-76
862- 49:	transcript:Zm00001d022212_T002	rna20801	1.00E-37
862- 50:	transcript:Zm00001d022225_T001	rna20806	4.00E-112
862- 51:	transcript:Zm00001d022226_T002	rna20807	0
862- 52:	transcript:Zm00001d022227_T001	rna20810	4.00E-84
862- 53:	transcript:Zm00001d022228_T001	rna20813	3.00E-111
862- 54:	transcript:Zm00001d022229_T001	rna20814	0
862- 55:	transcript:Zm00001d022230_T001	rna20815	0
862- 56:	transcript:Zm00001d022231_T001	rna20816	1.00E-138
862- 57:	transcript:Zm00001d022233_T001	rna20819	7.00E-15
862- 58:	transcript:Zm00001d022234_T001	rna20821	2.00E-65
862- 59:	transcript:Zm00001d022237_T001	rna20823	2.00E-135
862- 60:	transcript:Zm00001d022238_T001	rna20826	8.00E-137
862- 61:	transcript:Zm00001d022239_T001	rna20827	1.00E-21
862- 62:	transcript:Zm00001d022241_T001	rna20828	2.00E-103
862- 63:	transcript:Zm00001d022242_T001	rna20829	2.00E-69
862- 64:	transcript:Zm00001d022243_T002	rna20830	1.00E-90
862- 65:	transcript:Zm00001d022245_T001	rna20831	0
862- 66:	transcript:Zm00001d022246_T001	rna20832	0
862- 67:	transcript:Zm00001d022247_T001	rna20833	0
862- 68:	transcript:Zm00001d022248_T001	rna20834	4.00E-25
862- 69:	transcript:Zm00001d022250_T001	rna20835	4.00E-111
862- 70:	transcript:Zm00001d022251_T002	rna20837	7.00E-145
862- 71:	transcript:Zm00001d022252_T001	rna20838	7.00E-71
862- 72:	transcript:Zm00001d022254_T002	rna20839	9.00E-150
862- 73:	transcript:Zm00001d022258_T002	rna20841	0
862- 74:	transcript:Zm00001d022259_T002	rna20842	1.00E-86
862- 75:	transcript:Zm00001d022262_T001	rna20843	0
862- 76:	transcript:Zm00001d022263_T001	rna20848	0
862- 77:	transcript:Zm00001d022264_T001	rna20849	2.00E-30
862- 78:	transcript:Zm00001d022265_T001	rna20850	0
862- 79:	transcript:Zm00001d022266_T001	rna20851	1.00E-91
862- 80:	transcript:Zm00001d022268_T001	rna20852	5.00E-34
862- 81:	transcript:Zm00001d022269_T001	rna20853	1.00E-85

862- 82: transcript:Zm00001d022270_T002 rna20854 2.00E-68
862- 83: transcript:Zm00001d022272_T002 rna20857 0
862- 84: transcript:Zm00001d022273_T016 rna20859 0
862- 85: transcript:Zm00001d022274_T001 rna20860 0
862- 86: transcript:Zm00001d022275_T001 rna20861 7.00E-169
862- 87: transcript:Zm00001d022276_T002 rna20864 2.00E-166
862- 88: transcript:Zm00001d022277_T001 rna20865 0
862- 89: transcript:Zm00001d022278_T001 rna20866 3.00E-127
862- 90: transcript:Zm00001d022279_T001 rna20869 9.00E-143
862- 91: transcript:Zm00001d022283_T001 rna20870 7.00E-120
862- 92: transcript:Zm00001d022302_T001 rna20875 1.00E-149
862- 93: transcript:Zm00001d022306_T001 rna20889 4.00E-91
862- 94: transcript:Zm00001d022307_T003 rna20890 1.00E-56
862- 95: transcript:Zm00001d022309_T002 rna20893 6.00E-126
862- 96: transcript:Zm00001d022313_T001 rna20895 0
862- 97: transcript:Zm00001d022314_T001 rna20896 5.00E-112
862- 98: transcript:Zm00001d022315_T001 rna20897 0
862- 99: transcript:Zm00001d022317_T001 rna20899 5.00E-137
862-100: transcript:Zm00001d022330_T001 rna20906 2.00E-99
862-101: transcript:Zm00001d022334_T002 rna20908 0
862-102: transcript:Zm00001d022335_T002 rna20909 0
862-103: transcript:Zm00001d022338_T002 rna20913 0
862-104: transcript:Zm00001d022341_T001 rna20916 0
862-105: transcript:Zm00001d022342_T001 rna20917 0
862-106: transcript:Zm00001d022344_T001 rna20920 6.00E-27
862-107: transcript:Zm00001d022347_T001 rna20921 3.00E-153
862-108: transcript:Zm00001d022349_T001 rna20927 0
862-109: transcript:Zm00001d022350_T001 rna20928 1.00E-19
862-110: transcript:Zm00001d022351_T001 rna20929 0
862-111: transcript:Zm00001d022352_T001 rna20930 5.00E-15
862-112: transcript:Zm00001d022353_T001 rna20931 5.00E-154
862-113: transcript:Zm00001d022354_T001 rna20932 1.00E-41
862-114: transcript:Zm00001d022355_T001 rna20933 2.00E-83
862-115: transcript:Zm00001d022356_T006 rna20935 0
Alignment 863: score=2007.0 e_value=4e-160 N=44 7&NC_008400.2 plus
863- 0: transcript:Zm00001d021891_T005 rna20502 0
863- 1: transcript:Zm00001d021893_T002 rna20504 0
863- 2: transcript:Zm00001d021895_T001 rna20506 0
863- 3: transcript:Zm00001d021896_T001 rna20507 0
863- 4: transcript:Zm00001d021899_T002 rna20509 5.00E-102
863- 5: transcript:Zm00001d021900_T001 rna20510 0
863- 6: transcript:Zm00001d021903_T005 rna20516 0
863- 7: transcript:Zm00001d021904_T001 rna20517 1.00E-91
863- 8: transcript:Zm00001d021906_T001 rna20519 3.00E-166
863- 9: transcript:Zm00001d021908_T005 rna20520 1.00E-24
863- 10: transcript:Zm00001d021913_T001 rna20521 5.00E-73
863- 11: transcript:Zm00001d021915_T001 rna20522 0
863- 12: transcript:Zm00001d021927_T001 rna20532 2.00E-126
863- 13: transcript:Zm00001d021929_T001 rna20535 0
863- 14: transcript:Zm00001d021930_T001 rna20536 0
863- 15: transcript:Zm00001d021934_T001 rna20539 7.00E-86
863- 16: transcript:Zm00001d021935_T001 rna20541 0
863- 17: transcript:Zm00001d021946_T001 rna20550 0
863- 18: transcript:Zm00001d021947_T002 rna20551 0

863- 19:	transcript:Zm00001d021948_T001	rna20553	0
863- 20:	transcript:Zm00001d021949_T002	rna20554	0
863- 21:	transcript:Zm00001d021950_T007	rna20555	0
863- 22:	transcript:Zm00001d021951_T001	rna20556	0
863- 23:	transcript:Zm00001d021952_T001	rna20557	0
863- 24:	transcript:Zm00001d021954_T001	rna20558	0
863- 25:	transcript:Zm00001d021956_T002	rna20561	2.00E-46
863- 26:	transcript:Zm00001d021957_T001	rna20562	2.00E-106
863- 27:	transcript:Zm00001d021958_T002	rna20563	9.00E-47
863- 28:	transcript:Zm00001d021960_T001	rna20564	2.00E-26
863- 29:	transcript:Zm00001d021961_T001	rna20565	0
863- 30:	transcript:Zm00001d021966_T002	rna20567	0
863- 31:	transcript:Zm00001d021967_T001	rna20568	3.00E-32
863- 32:	transcript:Zm00001d021968_T001	rna20569	0
863- 33:	transcript:Zm00001d021971_T001	rna20570	3.00E-133
863- 34:	transcript:Zm00001d021973_T001	rna20571	0
863- 35:	transcript:Zm00001d021974_T002	rna20573	0
863- 36:	transcript:Zm00001d021976_T001	rna20574	1.00E-69
863- 37:	transcript:Zm00001d021977_T001	rna20575	3.00E-09
863- 38:	transcript:Zm00001d021978_T001	rna20577	0
863- 39:	transcript:Zm00001d021979_T001	rna20578	0
863- 40:	transcript:Zm00001d021985_T001	rna20579	0
863- 41:	transcript:Zm00001d021990_T001	rna20582	3.00E-45
863- 42:	transcript:Zm00001d021995_T001	rna20586	4.00E-100
863- 43:	transcript:Zm00001d021998_T001	rna20587	0

Alignment 864: score=1995.0 e_value=1.6e-155 N=43 7&NC_008400.2 plus

864- 0:	transcript:Zm00001d022038_T007	rna20610	2.00E-131
864- 1:	transcript:Zm00001d022040_T002	rna20612	0
864- 2:	transcript:Zm00001d022041_T001	rna20613	0
864- 3:	transcript:Zm00001d022042_T006	rna20615	2.00E-108
864- 4:	transcript:Zm00001d022043_T001	rna20616	9.00E-45
864- 5:	transcript:Zm00001d022044_T001	rna20617	0
864- 6:	transcript:Zm00001d022046_T004	rna20624	0
864- 7:	transcript:Zm00001d022048_T002	rna20628	0
864- 8:	transcript:Zm00001d022049_T003	rna20629	0
864- 9:	transcript:Zm00001d022052_T001	rna20630	0
864- 10:	transcript:Zm00001d022053_T001	rna20631	9.00E-09
864- 11:	transcript:Zm00001d022057_T001	rna20634	1.00E-07
864- 12:	transcript:Zm00001d022058_T001	rna20639	0
864- 13:	transcript:Zm00001d022059_T001	rna20640	2.00E-53
864- 14:	transcript:Zm00001d022060_T001	rna20641	2.00E-173
864- 15:	transcript:Zm00001d022065_T001	rna20649	2.00E-49
864- 16:	transcript:Zm00001d022066_T001	rna20650	0
864- 17:	transcript:Zm00001d022067_T001	rna20651	7.00E-171
864- 18:	transcript:Zm00001d022069_T001	rna20652	4.00E-48
864- 19:	transcript:Zm00001d022071_T001	rna20653	4.00E-106
864- 20:	transcript:Zm00001d022072_T001	rna20656	0
864- 21:	transcript:Zm00001d022073_T001	rna20658	5.00E-139
864- 22:	transcript:Zm00001d022075_T001	rna20659	0
864- 23:	transcript:Zm00001d022077_T003	rna20662	1.00E-137
864- 24:	transcript:Zm00001d022081_T001	rna20664	0
864- 25:	transcript:Zm00001d022083_T001	rna20666	3.00E-116
864- 26:	transcript:Zm00001d022084_T001	rna20667	1.00E-47
864- 27:	transcript:Zm00001d022085_T001	rna20669	0

864- 28: transcript:Zm00001d022088_T004 rna20671 3.00E-131
864- 29: transcript:Zm00001d022089_T001 rna20672 3.00E-12
864- 30: transcript:Zm00001d022092_T001 rna20673 2.00E-162
864- 31: transcript:Zm00001d022097_T004 rna20677 5.00E-68
864- 32: transcript:Zm00001d022099_T001 rna20678 8.00E-83
864- 33: transcript:Zm00001d022101_T001 rna20679 7.00E-143
864- 34: transcript:Zm00001d022102_T001 rna20680 1.00E-74
864- 35: transcript:Zm00001d022103_T002 rna20682 9.00E-142
864- 36: transcript:Zm00001d022104_T001 rna20683 6.00E-178
864- 37: transcript:Zm00001d022106_T001 rna20686 2.00E-35
864- 38: transcript:Zm00001d022108_T004 rna20687 2.00E-124
864- 39: transcript:Zm00001d022109_T011 rna20688 1.00E-98
864- 40: transcript:Zm00001d022110_T002 rna20690 2.00E-124
864- 41: transcript:Zm00001d022111_T001 rna20691 9.00E-160
864- 42: transcript:Zm00001d022112_T001 rna20695 2.00E-99
Alignment 865: score=1810.0 e_value=2.9e-150 N=42 7&NC_008400.2 plus
865- 0: transcript:Zm00001d021562_T001 rna20118 6.00E-156
865- 1: transcript:Zm00001d021565_T001 rna20120 1.00E-126
865- 2: transcript:Zm00001d021566_T005 rna20122 0
865- 3: transcript:Zm00001d021567_T001 rna20123 9.00E-156
865- 4: transcript:Zm00001d021569_T001 rna20124 0
865- 5: transcript:Zm00001d021571_T001 rna20125 0
865- 6: transcript:Zm00001d021573_T001 rna20133 4.00E-39
865- 7: transcript:Zm00001d021574_T001 rna20134 0
865- 8: transcript:Zm00001d021579_T002 rna20136 0
865- 9: transcript:Zm00001d021580_T001 rna20137 2.00E-80
865- 10: transcript:Zm00001d021582_T001 rna20138 1.00E-58
865- 11: transcript:Zm00001d021583_T001 rna20139 1.00E-89
865- 12: transcript:Zm00001d021584_T003 rna20140 0
865- 13: transcript:Zm00001d021587_T002 rna20147 4.00E-162
865- 14: transcript:Zm00001d021588_T002 rna20149 0
865- 15: transcript:Zm00001d021591_T001 rna20150 2.00E-116
865- 16: transcript:Zm00001d021592_T001 rna20151 0
865- 17: transcript:Zm00001d021596_T004 rna20154 0
865- 18: transcript:Zm00001d021598_T005 rna20156 0
865- 19: transcript:Zm00001d021599_T002 rna20162 5.00E-43
865- 20: transcript:Zm00001d021600_T002 rna20163 0
865- 21: transcript:Zm00001d021607_T001 rna20167 0
865- 22: transcript:Zm00001d021609_T018 rna20168 8.00E-137
865- 23: transcript:Zm00001d021611_T001 rna20169 4.00E-19
865- 24: transcript:Zm00001d021613_T001 rna20170 0
865- 25: transcript:Zm00001d021620_T001 rna20172 0
865- 26: transcript:Zm00001d021623_T004 rna20180 0
865- 27: transcript:Zm00001d021626_T002 rna20184 6.00E-84
865- 28: transcript:Zm00001d021627_T001 rna20185 3.00E-39
865- 29: transcript:Zm00001d021628_T001 rna20186 2.00E-23
865- 30: transcript:Zm00001d021629_T003 rna20188 0
865- 31: transcript:Zm00001d021630_T001 rna20189 2.00E-12
865- 32: transcript:Zm00001d021633_T006 rna20190 1.00E-37
865- 33: transcript:Zm00001d021634_T001 rna20192 1.00E-57
865- 34: transcript:Zm00001d021635_T011 rna20193 0
865- 35: transcript:Zm00001d021636_T004 rna20194 0
865- 36: transcript:Zm00001d021638_T001 rna20195 2.00E-176
865- 37: transcript:Zm00001d021639_T001 rna20197 7.00E-98

865- 38: transcript:Zm00001d021641_T002 rna20207 0
865- 39: transcript:Zm00001d021645_T001 rna20209 8.00E-46
865- 40: transcript:Zm00001d021646_T024 rna20210 2.00E-116
865- 41: transcript:Zm00001d021647_T003 rna20220 0
Alignment 866: score=1746.0 e_value=3.2e-137 N=39 7&NC_008400.2 plus
866- 0: transcript:Zm00001d018882_T001 rna19280 4.00E-82
866- 1: transcript:Zm00001d018883_T001 rna19281 0
866- 2: transcript:Zm00001d018884_T002 rna19282 8.00E-69
866- 3: transcript:Zm00001d018887_T002 rna19285 0
866- 4: transcript:Zm00001d018891_T001 rna19290 0
866- 5: transcript:Zm00001d018895_T001 rna19302 1.00E-133
866- 6: transcript:Zm00001d018896_T002 rna19303 0
866- 7: transcript:Zm00001d018901_T002 rna19307 7.00E-135
866- 8: transcript:Zm00001d018902_T001 rna19308 6.00E-124
866- 9: transcript:Zm00001d018904_T004 rna19309 0
866- 10: transcript:Zm00001d018907_T003 rna19313 0
866- 11: transcript:Zm00001d018908_T001 rna19314 5.00E-61
866- 12: transcript:Zm00001d018911_T001 rna19315 0
866- 13: transcript:Zm00001d018912_T010 rna19316 8.00E-117
866- 14: transcript:Zm00001d018913_T001 rna19318 2.00E-30
866- 15: transcript:Zm00001d018915_T007 rna19320 2.00E-22
866- 16: transcript:Zm00001d018916_T001 rna19326 0
866- 17: transcript:Zm00001d018917_T001 rna19328 2.00E-34
866- 18: transcript:Zm00001d018918_T001 rna19329 0
866- 19: transcript:Zm00001d018929_T002 rna19334 0
866- 20: transcript:Zm00001d018930_T003 rna19335 0
866- 21: transcript:Zm00001d018931_T001 rna19337 1.00E-106
866- 22: transcript:Zm00001d018935_T003 rna19339 0
866- 23: transcript:Zm00001d018936_T001 rna19340 1.00E-153
866- 24: transcript:Zm00001d018937_T012 rna19342 0
866- 25: transcript:Zm00001d018938_T001 rna19344 1.00E-142
866- 26: transcript:Zm00001d018939_T001 rna19347 0
866- 27: transcript:Zm00001d018941_T001 rna19348 0
866- 28: transcript:Zm00001d018943_T003 rna19352 0
866- 29: transcript:Zm00001d018944_T002 rna19353 2.00E-52
866- 30: transcript:Zm00001d018946_T001 rna19354 3.00E-103
866- 31: transcript:Zm00001d018950_T001 rna19360 1.00E-19
866- 32: transcript:Zm00001d018957_T001 rna19361 0
866- 33: transcript:Zm00001d018961_T002 rna19362 0
866- 34: transcript:Zm00001d018962_T001 rna19365 1.00E-66
866- 35: transcript:Zm00001d018964_T003 rna19366 0
866- 36: transcript:Zm00001d018971_T003 rna19368 3.00E-41
866- 37: transcript:Zm00001d018973_T001 rna19371 3.00E-75
866- 38: transcript:Zm00001d018976_T003 rna19372 0
Alignment 867: score=1724.0 e_value=0 N=37 7&NC_008400.2 plus
867- 0: transcript:Zm00001d018797_T001 rna19193 3.00E-76
867- 1: transcript:Zm00001d018798_T002 rna19200 0
867- 2: transcript:Zm00001d018799_T001 rna19201 0
867- 3: transcript:Zm00001d018803_T001 rna19203 1.00E-128
867- 4: transcript:Zm00001d018804_T002 rna19204 2.00E-134
867- 5: transcript:Zm00001d018806_T001 rna19208 2.00E-151
867- 6: transcript:Zm00001d018807_T001 rna19209 1.00E-68
867- 7: transcript:Zm00001d018809_T001 rna19210 3.00E-57
867- 8: transcript:Zm00001d018810_T002 rna19212 0

867- 9: transcript:Zm00001d018811_T001 rna19213 4.00E-22
867- 10: transcript:Zm00001d018812_T001 rna19215 7.00E-104
867- 11: transcript:Zm00001d018813_T001 rna19216 0
867- 12: transcript:Zm00001d018819_T001 rna19218 0
867- 13: transcript:Zm00001d018820_T004 rna19223 0
867- 14: transcript:Zm00001d018821_T001 rna19232 7.00E-132
867- 15: transcript:Zm00001d018822_T001 rna19235 4.00E-138
867- 16: transcript:Zm00001d018825_T001 rna19239 2.00E-83
867- 17: transcript:Zm00001d018826_T001 rna19240 4.00E-73
867- 18: transcript:Zm00001d018827_T001 rna19241 3.00E-77
867- 19: transcript:Zm00001d018828_T002 rna19242 0
867- 20: transcript:Zm00001d018829_T002 rna19243 2.00E-96
867- 21: transcript:Zm00001d018831_T008 rna19244 3.00E-96
867- 22: transcript:Zm00001d018842_T001 rna19249 0
867- 23: transcript:Zm00001d018844_T001 rna19251 4.00E-18
867- 24: transcript:Zm00001d018846_T001 rna19252 2.00E-105
867- 25: transcript:Zm00001d018848_T001 rna19254 1.00E-176
867- 26: transcript:Zm00001d018854_T001 rna19258 8.00E-157
867- 27: transcript:Zm00001d018859_T010 rna19263 2.00E-71
867- 28: transcript:Zm00001d018862_T001 rna19264 0
867- 29: transcript:Zm00001d018863_T003 rna19265 0
867- 30: transcript:Zm00001d018864_T002 rna19266 0
867- 31: transcript:Zm00001d018867_T002 rna19268 0
867- 32: transcript:Zm00001d018868_T001 rna19272 3.00E-14
867- 33: transcript:Zm00001d018869_T001 rna19273 2.00E-73
867- 34: transcript:Zm00001d018870_T001 rna19274 0
867- 35: transcript:Zm00001d018871_T001 rna19275 0
867- 36: transcript:Zm00001d018872_T009 rna19276 0
Alignment 868: score=1703.0 e_value=1.6e-139 N=38 7&NC_008400.2 plus
868- 0: transcript:Zm00001d018668_T002 rna19025 4.00E-29
868- 1: transcript:Zm00001d018669_T004 rna19026 0
868- 2: transcript:Zm00001d018671_T001 rna19034 1.00E-64
868- 3: transcript:Zm00001d018680_T001 rna19036 3.00E-83
868- 4: transcript:Zm00001d018682_T001 rna19040 5.00E-85
868- 5: transcript:Zm00001d018698_T001 rna19047 3.00E-118
868- 6: transcript:Zm00001d018703_T001 rna19054 1.00E-37
868- 7: transcript:Zm00001d018707_T001 rna19059 2.00E-12
868- 8: transcript:Zm00001d018714_T001 rna19073 4.00E-18
868- 9: transcript:Zm00001d018717_T001 rna19074 2.00E-29
868- 10: transcript:Zm00001d018718_T001 rna19076 3.00E-105
868- 11: transcript:Zm00001d018719_T001 rna19077 1.00E-41
868- 12: transcript:Zm00001d018724_T003 rna19081 5.00E-50
868- 13: transcript:Zm00001d018725_T001 rna19083 2.00E-12
868- 14: transcript:Zm00001d018727_T001 rna19085 4.00E-11
868- 15: transcript:Zm00001d018730_T001 rna19088 2.00E-150
868- 16: transcript:Zm00001d018731_T001 rna19089 1.00E-144
868- 17: transcript:Zm00001d018732_T003 rna19090 3.00E-125
868- 18: transcript:Zm00001d018734_T001 rna19091 4.00E-63
868- 19: transcript:Zm00001d018737_T001 rna19092 6.00E-52
868- 20: transcript:Zm00001d018742_T001 rna19102 2.00E-176
868- 21: transcript:Zm00001d018748_T001 rna19124 2.00E-29
868- 22: transcript:Zm00001d018749_T001 rna19128 0
868- 23: transcript:Zm00001d018751_T001 rna19130 0
868- 24: transcript:Zm00001d018752_T001 rna19132 0

868- 25: transcript:Zm00001d018756_T001 rna19136 0
868- 26: transcript:Zm00001d018758_T001 rna19137 0
868- 27: transcript:Zm00001d018770_T003 rna19150 0
868- 28: transcript:Zm00001d018772_T001 rna19151 1.00E-25
868- 29: transcript:Zm00001d018776_T004 rna19154 0
868- 30: transcript:Zm00001d018779_T001 rna19158 2.00E-141
868- 31: transcript:Zm00001d018781_T001 rna19162 6.00E-127
868- 32: transcript:Zm00001d018787_T001 rna19175 1.00E-125
868- 33: transcript:Zm00001d018788_T001 rna19178 1.00E-155
868- 34: transcript:Zm00001d018789_T001 rna19185 0
868- 35: transcript:Zm00001d018793_T001 rna19186 4.00E-39
868- 36: transcript:Zm00001d018792_T004 rna19187 0
868- 37: transcript:Zm00001d018794_T003 rna19188 0
Alignment 869: score=1184.0 e_value=3.5e-87 N=26 7&NC_008400.2 plus
869- 0: transcript:Zm00001d021686_T002 rna20282 3.00E-159
869- 1: transcript:Zm00001d021688_T001 rna20283 0
869- 2: transcript:Zm00001d021690_T001 rna20291 0
869- 3: transcript:Zm00001d021692_T001 rna20303 0
869- 4: transcript:Zm00001d021693_T001 rna20311 3.00E-52
869- 5: transcript:Zm00001d021698_T001 rna20316 1.00E-138
869- 6: transcript:Zm00001d021700_T001 rna20320 5.00E-53
869- 7: transcript:Zm00001d021701_T002 rna20321 3.00E-124
869- 8: transcript:Zm00001d021702_T001 rna20322 0
869- 9: transcript:Zm00001d021703_T001 rna20329 1.00E-101
869- 10: transcript:Zm00001d021704_T001 rna20330 5.00E-124
869- 11: transcript:Zm00001d021705_T002 rna20331 8.00E-57
869- 12: transcript:Zm00001d021706_T001 rna20332 1.00E-89
869- 13: transcript:Zm00001d021708_T003 rna20336 9.00E-31
869- 14: transcript:Zm00001d021709_T001 rna20337 7.00E-167
869- 15: transcript:Zm00001d021710_T004 rna20338 0
869- 16: transcript:Zm00001d021711_T001 rna20339 8.00E-19
869- 17: transcript:Zm00001d021714_T002 rna20340 0
869- 18: transcript:Zm00001d021715_T001 rna20342 0
869- 19: transcript:Zm00001d021716_T001 rna20347 0
869- 20: transcript:Zm00001d021718_T001 rna20349 2.00E-83
869- 21: transcript:Zm00001d021719_T001 rna20350 3.00E-112
869- 22: transcript:Zm00001d021720_T001 rna20351 3.00E-35
869- 23: transcript:Zm00001d021721_T001 rna20354 2.00E-42
869- 24: transcript:Zm00001d021726_T003 rna20357 2.00E-45
869- 25: transcript:Zm00001d021727_T001 rna20359 0
Alignment 870: score=1182.0 e_value=2e-86 N=27 7&NC_008400.2 plus
870- 0: transcript:Zm00001d019337_T003 rna19676 0
870- 1: transcript:Zm00001d019341_T001 rna19681 3.00E-29
870- 2: transcript:Zm00001d019343_T001 rna19687 2.00E-112
870- 3: transcript:Zm00001d019344_T001 rna19690 4.00E-30
870- 4: transcript:Zm00001d019345_T001 rna19704 1.00E-84
870- 5: transcript:Zm00001d019348_T001 rna19705 0
870- 6: transcript:Zm00001d019354_T001 rna19711 5.00E-58
870- 7: transcript:Zm00001d019358_T001 rna19716 9.00E-33
870- 8: transcript:Zm00001d019363_T001 rna19718 0
870- 9: transcript:Zm00001d019372_T001 rna19720 4.00E-172
870- 10: transcript:Zm00001d019374_T001 rna19721 4.00E-07
870- 11: transcript:Zm00001d019376_T004 rna19732 4.00E-13
870- 12: transcript:Zm00001d019394_T001 rna19741 1.00E-84

```
870- 13: transcript:Zm00001d019397_T006 rna19745     9.00E-64
870- 14: transcript:Zm00001d019398_T001 rna19748            0
870- 15: transcript:Zm00001d019399_T001 rna19752     6.00E-41
870- 16: transcript:Zm00001d019404_T001 rna19753    8.00E-137
870- 17: transcript:Zm00001d019414_T001 rna19757    1.00E-106
870- 18: transcript:Zm00001d019420_T001 rna19761     3.00E-90
870- 19: transcript:Zm00001d019422_T001 rna19764            0
870- 20: transcript:Zm00001d019430_T001 rna19765    2.00E-168
870- 21: transcript:Zm00001d019432_T002 rna19768            0
870- 22: transcript:Zm00001d019434_T001 rna19778     6.00E-71
870- 23: transcript:Zm00001d019445_T001 rna19779     5.00E-44
870- 24: transcript:Zm00001d019446_T001 rna19781     9.00E-83
870- 25: transcript:Zm00001d019447_T001 rna19782     2.00E-64
870- 26: transcript:Zm00001d019449_T004 rna19786            0
## Alignment 871: score=1094.0 e_value=3.7e-74 N=24 7&NC_008400.2 plus
871-  0: transcript:Zm00001d019182_T001 rna19541            0
871-  1: transcript:Zm00001d019185_T001 rna19542    8.00E-145
871-  2: transcript:Zm00001d019186_T001 rna19543     6.00E-75
871-  3: transcript:Zm00001d019191_T007 rna19545     4.00E-29
871-  4: transcript:Zm00001d019194_T008 rna19547    9.00E-156
871-  5: transcript:Zm00001d019200_T001 rna19552            0
871-  6: transcript:Zm00001d019206_T001 rna19554     3.00E-99
871-  7: transcript:Zm00001d019207_T001 rna19555    1.00E-130
871-  8: transcript:Zm00001d019215_T002 rna19559    1.00E-178
871-  9: transcript:Zm00001d019217_T002 rna19562     4.00E-67
871- 10: transcript:Zm00001d019218_T001 rna19569    9.00E-167
871- 11: transcript:Zm00001d019219_T001 rna19570     8.00E-88
871- 12: transcript:Zm00001d019220_T001 rna19572    2.00E-109
871- 13: transcript:Zm00001d019222_T001 rna19573     6.00E-14
871- 14: transcript:Zm00001d019223_T001 rna19574     2.00E-81
871- 15: transcript:Zm00001d019225_T001 rna19575            0
871- 16: transcript:Zm00001d019227_T001 rna19582     2.00E-06
871- 17: transcript:Zm00001d019230_T002 rna19587            0
871- 18: transcript:Zm00001d019233_T001 rna19592    9.00E-140
871- 19: transcript:Zm00001d019234_T001 rna19593     1.00E-67
871- 20: transcript:Zm00001d019241_T002 rna19603     2.00E-30
871- 21: transcript:Zm00001d019248_T001 rna19605            0
871- 22: transcript:Zm00001d019249_T001 rna19606            0
871- 23: transcript:Zm00001d019250_T002 rna19608    7.00E-139
## Alignment 872: score=1019.0 e_value=7.9e-72 N=23 7&NC_008400.2 plus
872-  0: transcript:Zm00001d019254_T001 rna19608    1.00E-150
872-  1: transcript:Zm00001d019256_T001 rna19609    1.00E-127
872-  2: transcript:Zm00001d019265_T001 rna19610    6.00E-116
872-  3: transcript:Zm00001d019266_T002 rna19622            0
872-  4: transcript:Zm00001d019268_T001 rna19624            0
872-  5: transcript:Zm00001d019269_T001 rna19625            0
872-  6: transcript:Zm00001d019279_T001 rna19628    8.00E-130
872-  7: transcript:Zm00001d019282_T001 rna19629            0
872-  8: transcript:Zm00001d019283_T005 rna19630            0
872-  9: transcript:Zm00001d019287_T001 rna19631            0
872- 10: transcript:Zm00001d019288_T001 rna19632     2.00E-97
872- 11: transcript:Zm00001d019290_T001 rna19633     5.00E-93
872- 12: transcript:Zm00001d019297_T001 rna19639            0
872- 13: transcript:Zm00001d019298_T002 rna19640    3.00E-138
```

872- 14: transcript:Zm00001d019311_T001 rna19642 6.00E-22
872- 15: transcript:Zm00001d019317_T002 rna19657 0
872- 16: transcript:Zm00001d019320_T001 rna19659 6.00E-06
872- 17: transcript:Zm00001d019325_T002 rna19664 4.00E-102
872- 18: transcript:Zm00001d019327_T001 rna19666 0
872- 19: transcript:Zm00001d019328_T003 rna19670 5.00E-138
872- 20: transcript:Zm00001d019329_T001 rna19671 1.00E-42
872- 21: transcript:Zm00001d019333_T001 rna19682 6.00E-172
872- 22: transcript:Zm00001d019334_T001 rna19683 6.00E-126
Alignment 873: score=1008.0 e_value=8.9e-61 N=21 7&NC_008400.2 plus
873- 0: transcript:Zm00001d021813_T001 rna20438 2.00E-42
873- 1: transcript:Zm00001d021817_T002 rna20439 0
873- 2: transcript:Zm00001d021818_T001 rna20440 7.00E-171
873- 3: transcript:Zm00001d021820_T001 rna20441 2.00E-70
873- 4: transcript:Zm00001d021821_T001 rna20442 0
873- 5: transcript:Zm00001d021823_T002 rna20443 0
873- 6: transcript:Zm00001d021825_T001 rna20444 1.00E-46
873- 7: transcript:Zm00001d021826_T001 rna20445 6.00E-19
873- 8: transcript:Zm00001d021827_T001 rna20447 1.00E-06
873- 9: transcript:Zm00001d021828_T004 rna20448 0
873- 10: transcript:Zm00001d021829_T001 rna20449 0
873- 11: transcript:Zm00001d021830_T002 rna20450 0
873- 12: transcript:Zm00001d021831_T001 rna20451 0
873- 13: transcript:Zm00001d021834_T001 rna20454 1.00E-148
873- 14: transcript:Zm00001d021835_T007 rna20455 0
873- 15: transcript:Zm00001d021836_T001 rna20456 0
873- 16: transcript:Zm00001d021838_T001 rna20459 0
873- 17: transcript:Zm00001d021839_T001 rna20460 3.00E-112
873- 18: transcript:Zm00001d021840_T001 rna20462 5.00E-109
873- 19: transcript:Zm00001d021841_T001 rna20463 9.00E-74
873- 20: transcript:Zm00001d021842_T001 rna20464 2.00E-50
Alignment 874: score=1002.0 e_value=5.7e-75 N=23 7&NC_008400.2 plus
874- 0: transcript:Zm00001d021473_T002 rna20013 0
874- 1: transcript:Zm00001d021484_T004 rna20023 6.00E-62
874- 2: transcript:Zm00001d021486_T001 rna20025 3.00E-28
874- 3: transcript:Zm00001d021488_T003 rna20028 6.00E-91
874- 4: transcript:Zm00001d021489_T001 rna20029 0
874- 5: transcript:Zm00001d021490_T002 rna20030 3.00E-66
874- 6: transcript:Zm00001d021491_T001 rna20031 0
874- 7: transcript:Zm00001d021494_T001 rna20036 0
874- 8: transcript:Zm00001d021498_T008 rna20038 0
874- 9: transcript:Zm00001d021504_T001 rna20039 1.00E-26
874- 10: transcript:Zm00001d021506_T001 rna20045 0
874- 11: transcript:Zm00001d021507_T002 rna20046 0
874- 12: transcript:Zm00001d021512_T002 rna20054 0
874- 13: transcript:Zm00001d021515_T001 rna20062 1.00E-89
874- 14: transcript:Zm00001d021516_T003 rna20065 0
874- 15: transcript:Zm00001d021518_T001 rna20067 3.00E-135
874- 16: transcript:Zm00001d021519_T001 rna20068 0
874- 17: transcript:Zm00001d021520_T002 rna20069 1.00E-133
874- 18: transcript:Zm00001d021521_T001 rna20070 4.00E-60
874- 19: transcript:Zm00001d021522_T002 rna20071 7.00E-65
874- 20: transcript:Zm00001d021524_T003 rna20073 6.00E-106
874- 21: transcript:Zm00001d021525_T002 rna20075 0

874- 22: transcript:Zm00001d021526_T001 rna20076 1.00E-100
Alignment 875: score=909.0 e_value=2e-61 N=20 7&NC_008400.2 plus
875- 0: transcript:Zm00001d021649_T001 rna20222 7.00E-113
875- 1: transcript:Zm00001d021652_T001 rna20223 7.00E-69
875- 2: transcript:Zm00001d021653_T001 rna20228 0
875- 3: transcript:Zm00001d021654_T001 rna20230 2.00E-65
875- 4: transcript:Zm00001d021655_T001 rna20234 0
875- 5: transcript:Zm00001d021659_T010 rna20237 0
875- 6: transcript:Zm00001d021662_T002 rna20245 0
875- 7: transcript:Zm00001d021664_T001 rna20248 1.00E-73
875- 8: transcript:Zm00001d021666_T001 rna20249 0
875- 9: transcript:Zm00001d021667_T001 rna20253 3.00E-93
875- 10: transcript:Zm00001d021668_T008 rna20254 7.00E-81
875- 11: transcript:Zm00001d021669_T007 rna20255 0
875- 12: transcript:Zm00001d021672_T001 rna20258 0
875- 13: transcript:Zm00001d021673_T001 rna20259 7.00E-52
875- 14: transcript:Zm00001d021674_T001 rna20262 2.00E-95
875- 15: transcript:Zm00001d021675_T001 rna20263 0
875- 16: transcript:Zm00001d021676_T001 rna20264 0
875- 17: transcript:Zm00001d021677_T001 rna20268 4.00E-80
875- 18: transcript:Zm00001d021680_T001 rna20276 4.00E-11
875- 19: transcript:Zm00001d021682_T001 rna20282 8.00E-170
Alignment 876: score=845.0 e_value=4e-50 N=18 7&NC_008400.2 plus
876- 0: transcript:Zm00001d022547_T001 rna21178 1.00E-55
876- 1: transcript:Zm00001d022554_T003 rna21193 0
876- 2: transcript:Zm00001d022560_T001 rna21194 2.00E-11
876- 3: transcript:Zm00001d022563_T001 rna21198 0
876- 4: transcript:Zm00001d022564_T001 rna21199 2.00E-133
876- 5: transcript:Zm00001d022565_T001 rna21202 0
876- 6: transcript:Zm00001d022567_T001 rna21204 0
876- 7: transcript:Zm00001d022569_T001 rna21206 3.00E-24
876- 8: transcript:Zm00001d022570_T001 rna21208 2.00E-14
876- 9: transcript:Zm00001d022573_T001 rna21209 0
876- 10: transcript:Zm00001d022574_T001 rna21211 7.00E-48
876- 11: transcript:Zm00001d022575_T001 rna21213 0
876- 12: transcript:Zm00001d022576_T001 rna21214 1.00E-144
876- 13: transcript:Zm00001d022578_T001 rna21218 0
876- 14: transcript:Zm00001d022579_T001 rna21220 0
876- 15: transcript:Zm00001d022581_T001 rna21224 0
876- 16: transcript:Zm00001d022582_T002 rna21225 0
876- 17: transcript:Zm00001d022584_T001 rna21226 0
Alignment 877: score=837.0 e_value=1.1e-52 N=18 7&NC_008400.2 plus
877- 0: transcript:Zm00001d018596_T002 rna18939 0
877- 1: transcript:Zm00001d018598_T001 rna18941 0
877- 2: transcript:Zm00001d018599_T001 rna18942 3.00E-50
877- 3: transcript:Zm00001d018601_T001 rna18943 0
877- 4: transcript:Zm00001d018603_T003 rna18946 4.00E-151
877- 5: transcript:Zm00001d018609_T001 rna18951 2.00E-68
877- 6: transcript:Zm00001d018610_T001 rna18952 0
877- 7: transcript:Zm00001d018613_T004 rna18954 0
877- 8: transcript:Zm00001d018614_T003 rna18957 0
877- 9: transcript:Zm00001d018616_T001 rna18958 1.00E-35
877- 10: transcript:Zm00001d018617_T001 rna18959 6.00E-143
877- 11: transcript:Zm00001d018618_T001 rna18961 2.00E-93

877- 12: transcript:Zm00001d018619_T001 rna18963 4.00E-156
877- 13: transcript:Zm00001d018620_T001 rna18964 4.00E-159
877- 14: transcript:Zm00001d018621_T001 rna18965 1.00E-51
877- 15: transcript:Zm00001d018622_T001 rna18968 6.00E-17
877- 16: transcript:Zm00001d018623_T001 rna18969 1.00E-86
877- 17: transcript:Zm00001d018624_T001 rna18970 0
Alignment 878: score=814.0 e_value=1.5e-51 N=18 7&NC_008400.2 plus
878- 0: transcript:Zm00001d021846_T016 rna20466 1.00E-17
878- 1: transcript:Zm00001d021850_T001 rna20469 1.00E-28
878- 2: transcript:Zm00001d021858_T007 rna20473 0
878- 3: transcript:Zm00001d021861_T007 rna20475 2.00E-19
878- 4: transcript:Zm00001d021862_T001 rna20476 2.00E-25
878- 5: transcript:Zm00001d021864_T001 rna20479 0
878- 6: transcript:Zm00001d021866_T001 rna20480 0
878- 7: transcript:Zm00001d021869_T001 rna20482 8.00E-166
878- 8: transcript:Zm00001d021870_T001 rna20483 2.00E-28
878- 9: transcript:Zm00001d021875_T002 rna20486 0
878- 10: transcript:Zm00001d021876_T001 rna20488 2.00E-23
878- 11: transcript:Zm00001d021877_T001 rna20489 3.00E-142
878- 12: transcript:Zm00001d021878_T003 rna20491 2.00E-173
878- 13: transcript:Zm00001d021879_T001 rna20492 0
878- 14: transcript:Zm00001d021880_T001 rna20495 0
878- 15: transcript:Zm00001d021881_T001 rna20496 1.00E-141
878- 16: transcript:Zm00001d021883_T001 rna20498 0
878- 17: transcript:Zm00001d021886_T001 rna20500 2.00E-14
Alignment 879: score=687.0 e_value=3.3e-41 N=16 7&NC_008400.2 plus
879- 0: transcript:Zm00001d021774_T001 rna20399 8.00E-156
879- 1: transcript:Zm00001d021775_T001 rna20400 0
879- 2: transcript:Zm00001d021777_T001 rna20403 0
879- 3: transcript:Zm00001d021779_T001 rna20407 3.00E-34
879- 4: transcript:Zm00001d021781_T001 rna20409 3.00E-34
879- 5: transcript:Zm00001d021784_T001 rna20413 2.00E-176
879- 6: transcript:Zm00001d021785_T002 rna20414 0
879- 7: transcript:Zm00001d021787_T003 rna20416 8.00E-74
879- 8: transcript:Zm00001d021790_T010 rna20417 2.00E-147
879- 9: transcript:Zm00001d021791_T001 rna20418 3.00E-104
879- 10: transcript:Zm00001d021799_T010 rna20419 0
879- 11: transcript:Zm00001d021802_T001 rna20421 0
879- 12: transcript:Zm00001d021803_T001 rna20424 3.00E-46
879- 13: transcript:Zm00001d021804_T001 rna20426 0
879- 14: transcript:Zm00001d021805_T006 rna20427 0
879- 15: transcript:Zm00001d021806_T019 rna20428 9.00E-154
Alignment 880: score=591.0 e_value=8.4e-30 N=13 7&NC_008400.2 plus
880- 0: transcript:Zm00001d019115_T048 rna19469 0
880- 1: transcript:Zm00001d019117_T002 rna19472 1.00E-31
880- 2: transcript:Zm00001d019120_T001 rna19473 5.00E-31
880- 3: transcript:Zm00001d019123_T002 rna19474 5.00E-177
880- 4: transcript:Zm00001d019124_T003 rna19476 0
880- 5: transcript:Zm00001d019125_T003 rna19479 0
880- 6: transcript:Zm00001d019130_T005 rna19480 0
880- 7: transcript:Zm00001d019138_T001 rna19485 0
880- 8: transcript:Zm00001d019139_T012 rna19486 0
880- 9: transcript:Zm00001d019142_T001 rna19487 2.00E-62
880- 10: transcript:Zm00001d019145_T001 rna19489 0

880- 11: transcript:Zm00001d019147_T001 rna19490 4.00E-107
880- 12: transcript:Zm00001d019149_T006 rna19494 0
Alignment 881: score=564.0 e_value=3e-34 N=13 7&NC_008400.2 plus
881- 0: transcript:Zm00001d019510_T001 rna19859 0
881- 1: transcript:Zm00001d019518_T001 rna19877 7.00E-43
881- 2: transcript:Zm00001d019520_T001 rna19879 2.00E-161
881- 3: transcript:Zm00001d019522_T001 rna19880 0
881- 4: transcript:Zm00001d019525_T001 rna19882 0
881- 5: transcript:Zm00001d019527_T005 rna19884 9.00E-126
881- 6: transcript:Zm00001d019536_T001 rna19891 0
881- 7: transcript:Zm00001d019538_T002 rna19893 1.00E-50
881- 8: transcript:Zm00001d019542_T003 rna19905 0
881- 9: transcript:Zm00001d019546_T001 rna19906 1.00E-34
881- 10: transcript:Zm00001d019547_T002 rna19907 0
881- 11: transcript:Zm00001d019549_T002 rna19909 2.00E-14
881- 12: transcript:Zm00001d019552_T002 rna19926 1.00E-07
Alignment 882: score=556.0 e_value=2e-29 N=13 7&NC_008400.2 plus
882- 0: transcript:Zm00001d022380_T001 rna20981 0
882- 1: transcript:Zm00001d022381_T016 rna20984 0
882- 2: transcript:Zm00001d022384_T002 rna20986 3.00E-27
882- 3: transcript:Zm00001d022386_T001 rna20987 5.00E-130
882- 4: transcript:Zm00001d022387_T010 rna20988 0
882- 5: transcript:Zm00001d022388_T004 rna20989 0
882- 6: transcript:Zm00001d022390_T001 rna20992 0
882- 7: transcript:Zm00001d022391_T001 rna20993 0
882- 8: transcript:Zm00001d022395_T001 rna20996 4.00E-151
882- 9: transcript:Zm00001d022396_T003 rna20997 0
882- 10: transcript:Zm00001d022400_T003 rna20998 3.00E-131
882- 11: transcript:Zm00001d022401_T002 rna20999 0
882- 12: transcript:Zm00001d022403_T001 rna21000 5.00E-108
Alignment 883: score=549.0 e_value=1.6e-30 N=12 7&NC_008400.2 plus
883- 0: transcript:Zm00001d019087_T015 rna19428 4.00E-123
883- 1: transcript:Zm00001d019089_T001 rna19429 0
883- 2: transcript:Zm00001d019090_T003 rna19431 0
883- 3: transcript:Zm00001d019091_T002 rna19433 6.00E-159
883- 4: transcript:Zm00001d019092_T001 rna19436 1.00E-18
883- 5: transcript:Zm00001d019093_T001 rna19440 6.00E-75
883- 6: transcript:Zm00001d019094_T001 rna19441 5.00E-151
883- 7: transcript:Zm00001d019100_T001 rna19452 6.00E-60
883- 8: transcript:Zm00001d019104_T001 rna19454 0
883- 9: transcript:Zm00001d019106_T002 rna19458 0
883- 10: transcript:Zm00001d019107_T001 rna19459 1.00E-34
883- 11: transcript:Zm00001d019108_T002 rna19467 0
Alignment 884: score=523.0 e_value=2.4e-25 N=11 7&NC_008400.2 plus
884- 0: transcript:Zm00001d022001_T001 rna20589 1.00E-68
884- 1: transcript:Zm00001d022002_T001 rna20591 8.00E-39
884- 2: transcript:Zm00001d022003_T001 rna20593 0
884- 3: transcript:Zm00001d022006_T001 rna20595 2.00E-124
884- 4: transcript:Zm00001d022009_T001 rna20600 5.00E-151
884- 5: transcript:Zm00001d022010_T004 rna20601 0
884- 6: transcript:Zm00001d022016_T001 rna20602 0
884- 7: transcript:Zm00001d022017_T001 rna20603 0
884- 8: transcript:Zm00001d022022_T001 rna20604 9.00E-66
884- 9: transcript:Zm00001d022025_T001 rna20606 6.00E-44

884- 10: transcript:Zm00001d022027_T001 rna20608 4.00E-52
Alignment 885: score=521.0 e_value=7.5e-27 N=12 7&NC_008400.2 plus
885- 0: transcript:Zm00001d019462_T001 rna19802 0
885- 1: transcript:Zm00001d019467_T001 rna19803 0
885- 2: transcript:Zm00001d019472_T005 rna19804 0
885- 3: transcript:Zm00001d019475_T001 rna19811 3.00E-43
885- 4: transcript:Zm00001d019478_T001 rna19814 0
885- 5: transcript:Zm00001d019479_T004 rna19815 0
885- 6: transcript:Zm00001d019481_T001 rna19816 6.00E-107
885- 7: transcript:Zm00001d019483_T001 rna19821 2.00E-166
885- 8: transcript:Zm00001d019490_T001 rna19838 5.00E-89
885- 9: transcript:Zm00001d019492_T001 rna19845 2.00E-140
885- 10: transcript:Zm00001d019493_T001 rna19848 3.00E-101
885- 11: transcript:Zm00001d019497_T004 rna19850 2.00E-33
Alignment 886: score=463.0 e_value=3.5e-22 N=10 7&NC_008400.2 plus
886- 0: transcript:Zm00001d019039_T002 rna19391 2.00E-157
886- 1: transcript:Zm00001d019040_T001 rna19395 9.00E-47
886- 2: transcript:Zm00001d019041_T001 rna19396 2.00E-112
886- 3: transcript:Zm00001d019048_T001 rna19398 0
886- 4: transcript:Zm00001d019052_T001 rna19402 4.00E-109
886- 5: transcript:Zm00001d019053_T001 rna19404 0
886- 6: transcript:Zm00001d019054_T001 rna19405 0
886- 7: transcript:Zm00001d019057_T001 rna19407 2.00E-56
886- 8: transcript:Zm00001d019060_T001 rna19409 0
886- 9: transcript:Zm00001d019061_T003 rna19414 6.00E-114
Alignment 887: score=443.0 e_value=1.4e-21 N=10 7&NC_008400.2 plus
887- 0: transcript:Zm00001d022516_T001 rna21151 8.00E-75
887- 1: transcript:Zm00001d022518_T002 rna21155 3.00E-137
887- 2: transcript:Zm00001d022524_T001 rna21161 5.00E-103
887- 3: transcript:Zm00001d022525_T001 rna21162 0
887- 4: transcript:Zm00001d022526_T001 rna21163 3.00E-114
887- 5: transcript:Zm00001d022527_T005 rna21164 6.00E-139
887- 6: transcript:Zm00001d022529_T001 rna21165 0
887- 7: transcript:Zm00001d022530_T001 rna21167 8.00E-166
887- 8: transcript:Zm00001d022533_T001 rna21169 0
887- 9: transcript:Zm00001d022537_T001 rna21188 0
Alignment 888: score=424.0 e_value=3e-18 N=9 7&NC_008400.2 plus
888- 0: transcript:Zm00001d018625_T106 rna18973 0
888- 1: transcript:Zm00001d018626_T001 rna18974 0
888- 2: transcript:Zm00001d018627_T001 rna18975 0
888- 3: transcript:Zm00001d018628_T001 rna18977 3.00E-39
888- 4: transcript:Zm00001d018631_T001 rna18979 3.00E-38
888- 5: transcript:Zm00001d018635_T001 rna18983 0
888- 6: transcript:Zm00001d018636_T001 rna18984 4.00E-100
888- 7: transcript:Zm00001d018638_T001 rna18987 0
888- 8: transcript:Zm00001d018639_T002 rna18988 0
Alignment 889: score=412.0 e_value=1.1e-18 N=9 7&NC_008400.2 plus
889- 0: transcript:Zm00001d021744_T001 rna20371 5.00E-63
889- 1: transcript:Zm00001d021745_T001 rna20380 2.00E-148
889- 2: transcript:Zm00001d021746_T001 rna20382 4.00E-118
889- 3: transcript:Zm00001d021747_T001 rna20385 7.00E-171
889- 4: transcript:Zm00001d021754_T005 rna20388 0
889- 5: transcript:Zm00001d021761_T001 rna20391 1.00E-138
889- 6: transcript:Zm00001d021762_T001 rna20392 5.00E-121

889- 7: transcript:Zm00001d021763_T001 rna20394 0
889- 8: transcript:Zm00001d021764_T002 rna20395 4.00E-133
Alignment 890: score=386.0 e_value=4.4e-16 N=8 7&NC_008400.2 plus
890- 0: transcript:Zm00001d022366_T001 rna20964 0
890- 1: transcript:Zm00001d022367_T002 rna20971 0
890- 2: transcript:Zm00001d022369_T001 rna20972 0
890- 3: transcript:Zm00001d022371_T001 rna20974 2.00E-108
890- 4: transcript:Zm00001d022373_T001 rna20976 7.00E-57
890- 5: transcript:Zm00001d022374_T001 rna20977 0
890- 6: transcript:Zm00001d022376_T001 rna20978 7.00E-114
890- 7: transcript:Zm00001d022378_T003 rna20979 0
Alignment 891: score=381.0 e_value=1.1e-15 N=8 7&NC_008400.2 plus
891- 0: transcript:Zm00001d021729_T001 rna20359 0
891- 1: transcript:Zm00001d021730_T001 rna20361 5.00E-130
891- 2: transcript:Zm00001d021731_T001 rna20362 0
891- 3: transcript:Zm00001d021736_T001 rna20363 0
891- 4: transcript:Zm00001d021737_T001 rna20367 0
891- 5: transcript:Zm00001d021739_T001 rna20370 2.00E-54
891- 6: transcript:Zm00001d021740_T001 rna20371 2.00E-82
891- 7: transcript:Zm00001d021741_T001 rna20377 0
Alignment 892: score=375.0 e_value=1.9e-14 N=8 7&NC_008400.2 plus
892- 0: transcript:Zm00001d019165_T002 rna19497 0
892- 1: transcript:Zm00001d019166_T002 rna19502 2.00E-99
892- 2: transcript:Zm00001d019169_T004 rna19507 1.00E-171
892- 3: transcript:Zm00001d019172_T001 rna19508 0
892- 4: transcript:Zm00001d019173_T001 rna19509 2.00E-30
892- 5: transcript:Zm00001d019177_T001 rna19510 0
892- 6: transcript:Zm00001d019180_T001 rna19511 2.00E-42
892- 7: transcript:Zm00001d019181_T001 rna19514 1.00E-107
Alignment 893: score=330.0 e_value=1.1e-11 N=7 7&NC_008400.2 plus
893- 0: transcript:Zm00001d018979_T001 rna19376 4.00E-81
893- 1: transcript:Zm00001d018981_T002 rna19379 2.00E-27
893- 2: transcript:Zm00001d018983_T001 rna19381 0
893- 3: transcript:Zm00001d018984_T001 rna19384 1.00E-79
893- 4: transcript:Zm00001d018987_T001 rna19386 0
893- 5: transcript:Zm00001d018988_T002 rna19387 1.00E-57
893- 6: transcript:Zm00001d019002_T001 rna19388 2.00E-18
Alignment 894: score=308.0 e_value=3.4e-11 N=7 7&NC_008400.2 plus
894- 0: transcript:Zm00001d019595_T001 rna19954 0
894- 1: transcript:Zm00001d019596_T001 rna19957 2.00E-104
894- 2: transcript:Zm00001d019597_T001 rna19963 5.00E-94
894- 3: transcript:Zm00001d019599_T002 rna19964 9.00E-76
894- 4: transcript:Zm00001d019600_T006 rna19966 0
894- 5: transcript:Zm00001d019613_T001 rna19968 4.00E-114
894- 6: transcript:Zm00001d019627_T002 rna19970 0
Alignment 895: score=272.0 e_value=2.2e-12 N=7 7&NC_008400.2 plus
895- 0: transcript:Zm00001d019565_T001 rna19914 0
895- 1: transcript:Zm00001d019576_T001 rna19928 1.00E-110
895- 2: transcript:Zm00001d019579_T007 rna19939 0
895- 3: transcript:Zm00001d019582_T001 rna19940 3.00E-54
895- 4: transcript:Zm00001d019586_T016 rna19941 0
895- 5: transcript:Zm00001d019587_T002 rna19944 0
895- 6: transcript:Zm00001d019591_T006 rna19953 1.00E-105
Alignment 896: score=264.0 e_value=4.1e-09 N=6 7&NC_008400.2 plus

896- 0: transcript:Zm00001d022405_T051 rna21002 0
896- 1: transcript:Zm00001d022407_T007 rna21008 0
896- 2: transcript:Zm00001d022414_T001 rna21009 0
896- 3: transcript:Zm00001d022416_T001 rna21010 2.00E-37
896- 4: transcript:Zm00001d022417_T003 rna21011 0
896- 5: transcript:Zm00001d022418_T001 rna21013 4.00E-25
Alignment 897: score=1451.0 e_value=2.4e-105 N=32 7&NC_008400.2 minus
897- 0: transcript:Zm00001d022425_T002 rna21149 0
897- 1: transcript:Zm00001d022427_T003 rna21146 0
897- 2: transcript:Zm00001d022428_T005 rna21142 0
897- 3: transcript:Zm00001d022429_T009 rna21140 0
897- 4: transcript:Zm00001d022430_T001 rna21139 2.00E-100
897- 5: transcript:Zm00001d022431_T001 rna21137 9.00E-81
897- 6: transcript:Zm00001d022432_T001 rna21136 0
897- 7: transcript:Zm00001d022433_T001 rna21135 7.00E-171
897- 8: transcript:Zm00001d022434_T001 rna21134 0
897- 9: transcript:Zm00001d022435_T001 rna21133 8.00E-29
897- 10: transcript:Zm00001d022436_T005 rna21132 8.00E-89
897- 11: transcript:Zm00001d022437_T001 rna21131 6.00E-58
897- 12: transcript:Zm00001d022438_T001 rna21130 5.00E-35
897- 13: transcript:Zm00001d022439_T001 rna21129 0
897- 14: transcript:Zm00001d022440_T005 rna21128 0
897- 15: transcript:Zm00001d022442_T002 rna21127 0
897- 16: transcript:Zm00001d022444_T001 rna21126 2.00E-32
897- 17: transcript:Zm00001d022445_T002 rna21125 0
897- 18: transcript:Zm00001d022446_T001 rna21124 1.00E-61
897- 19: transcript:Zm00001d022449_T002 rna21123 0
897- 20: transcript:Zm00001d022450_T001 rna21121 5.00E-110
897- 21: transcript:Zm00001d022453_T001 rna21118 0
897- 22: transcript:Zm00001d022456_T001 rna21112 2.00E-97
897- 23: transcript:Zm00001d022459_T001 rna21106 5.00E-76
897- 24: transcript:Zm00001d022460_T001 rna21104 0
897- 25: transcript:Zm00001d022461_T001 rna21102 2.00E-64
897- 26: transcript:Zm00001d022462_T001 rna21101 2.00E-125
897- 27: transcript:Zm00001d022463_T001 rna21097 1.00E-70
897- 28: transcript:Zm00001d022464_T001 rna21091 8.00E-21
897- 29: transcript:Zm00001d022466_T006 rna21087 0
897- 30: transcript:Zm00001d022467_T001 rna21085 0
897- 31: transcript:Zm00001d022469_T001 rna21080 0
Alignment 898: score=943.0 e_value=3.4e-60 N=21 7&NC_008400.2 minus
898- 0: transcript:Zm00001d022485_T001 rna21065 0
898- 1: transcript:Zm00001d022487_T001 rna21064 1.00E-55
898- 2: transcript:Zm00001d022488_T001 rna21063 2.00E-103
898- 3: transcript:Zm00001d022490_T002 rna21061 0
898- 4: transcript:Zm00001d022491_T005 rna21060 1.00E-72
898- 5: transcript:Zm00001d022492_T001 rna21059 0
898- 6: transcript:Zm00001d022493_T001 rna21057 0
898- 7: transcript:Zm00001d022494_T001 rna21056 3.00E-59
898- 8: transcript:Zm00001d022496_T001 rna21055 2.00E-115
898- 9: transcript:Zm00001d022498_T007 rna21054 0
898- 10: transcript:Zm00001d022499_T001 rna21052 2.00E-45
898- 11: transcript:Zm00001d022500_T001 rna21050 2.00E-40
898- 12: transcript:Zm00001d022501_T001 rna21049 0
898- 13: transcript:Zm00001d022504_T001 rna21047 0

898- 14: transcript:Zm00001d022505_T002 rna21040 7.00E-84
898- 15: transcript:Zm00001d022506_T001 rna21027 5.00E-111
898- 16: transcript:Zm00001d022509_T001 rna21021 9.00E-21
898- 17: transcript:Zm00001d022510_T016 rna21020 0
898- 18: transcript:Zm00001d022512_T001 rna21019 2.00E-163
898- 19: transcript:Zm00001d022513_T001 rna21016 4.00E-112
898- 20: transcript:Zm00001d022514_T001 rna21015 0
Alignment 899: score=646.0 e_value=1.6e-34 N=14 7&NC_008400.2 minus
899- 0: transcript:Zm00001d021529_T002 rna20117 8.00E-89
899- 1: transcript:Zm00001d021532_T001 rna20113 0
899- 2: transcript:Zm00001d021533_T001 rna20112 6.00E-124
899- 3: transcript:Zm00001d021534_T001 rna20110 0
899- 4: transcript:Zm00001d021535_T001 rna20108 0
899- 5: transcript:Zm00001d021536_T003 rna20107 5.00E-47
899- 6: transcript:Zm00001d021537_T001 rna20106 4.00E-56
899- 7: transcript:Zm00001d021541_T008 rna20103 0
899- 8: transcript:Zm00001d021542_T001 rna20102 5.00E-66
899- 9: transcript:Zm00001d021544_T001 rna20101 0
899- 10: transcript:Zm00001d021545_T005 rna20099 0
899- 11: transcript:Zm00001d021546_T003 rna20097 0
899- 12: transcript:Zm00001d021551_T002 rna20096 9.00E-141
899- 13: transcript:Zm00001d021553_T004 rna20095 0
Alignment 900: score=613.0 e_value=3.8e-35 N=14 7&NC_008400.2 minus
900- 0: transcript:Zm00001d021335_T003 rna20017 2.00E-112
900- 1: transcript:Zm00001d021336_T004 rna20013 0
900- 2: transcript:Zm00001d021337_T001 rna20011 1.00E-45
900- 3: transcript:Zm00001d021340_T001 rna20009 6.00E-90
900- 4: transcript:Zm00001d021341_T001 rna20005 2.00E-40
900- 5: transcript:Zm00001d021342_T017 rna20002 0
900- 6: transcript:Zm00001d021344_T002 rna19999 6.00E-170
900- 7: transcript:Zm00001d021345_T001 rna19997 0
900- 8: transcript:Zm00001d021346_T002 rna19995 0
900- 9: transcript:Zm00001d021347_T004 rna19994 0
900- 10: transcript:Zm00001d021350_T003 rna19992 9.00E-135
900- 11: transcript:Zm00001d021351_T003 rna19991 3.00E-177
900- 12: transcript:Zm00001d021356_T001 rna19990 1.00E-28
900- 13: transcript:Zm00001d021362_T001 rna19976 2.00E-133
Alignment 901: score=412.0 e_value=3.3e-17 N=9 7&NC_008400.2 minus
901- 0: transcript:Zm00001d022535_T004 rna21191 0
901- 1: transcript:Zm00001d022536_T008 rna21190 0
901- 2: transcript:Zm00001d022538_T001 rna21186 9.00E-136
901- 3: transcript:Zm00001d022542_T002 rna21185 0
901- 4: transcript:Zm00001d022544_T002 rna21183 5.00E-158
901- 5: transcript:Zm00001d022545_T001 rna21181 6.00E-119
901- 6: transcript:Zm00001d022546_T001 rna21179 1.00E-142
901- 7: transcript:Zm00001d022549_T001 rna21176 3.00E-165
901- 8: transcript:Zm00001d022550_T001 rna21170 5.00E-76
Alignment 902: score=308.0 e_value=1.1e-11 N=7 7&NC_008400.2 minus
902- 0: transcript:Zm00001d018639_T002 rna19022 0
902- 1: transcript:Zm00001d018641_T001 rna19018 1.00E-70
902- 2: transcript:Zm00001d018645_T001 rna19016 3.00E-51
902- 3: transcript:Zm00001d018648_T001 rna19012 0
902- 4: transcript:Zm00001d018653_T001 rna19011 0
902- 5: transcript:Zm00001d018656_T001 rna19010 9.00E-69

```
902-  6:  transcript:Zm00001d018657_T004  rna19004            0
## Alignment 903: score=289.0 e_value=1.5e-09 N=6 7&NC_008400.2 minus
903-  0:  transcript:Zm00001d022473_T010  rna21079    3.00E-141
903-  1:  transcript:Zm00001d022474_T001  rna21078     7.00E-58
903-  2:  transcript:Zm00001d022477_T001  rna21075     1.00E-51
903-  3:  transcript:Zm00001d022478_T001  rna21070     2.00E-47
903-  4:  transcript:Zm00001d022479_T001  rna21069     6.00E-08
903-  5:  transcript:Zm00001d022480_T001  rna21068            0
## Alignment 904: score=274.0 e_value=6.8e-10 N=6 7&NC_008400.2 minus
904-  0:  transcript:Zm00001d019299_T001  rna19654            0
904-  1:  transcript:Zm00001d019300_T001  rna19651     2.00E-11
904-  2:  transcript:Zm00001d019303_T001  rna19648     4.00E-69
904-  3:  transcript:Zm00001d019305_T001  rna19647     1.00E-34
904-  4:  transcript:Zm00001d019306_T001  rna19642     1.00E-19
904-  5:  transcript:Zm00001d019314_T003  rna19641            0
## Alignment 905: score=869.0 e_value=7.5e-56 N=20 7&NC_008401.2 plus
905-  0:  transcript:Zm00001d020837_T001  rna22813     2.00E-48
905-  1:  transcript:Zm00001d020840_T001  rna22815     2.00E-54
905-  2:  transcript:Zm00001d020851_T001  rna22819     2.00E-17
905-  3:  transcript:Zm00001d020858_T001  rna22820            0
905-  4:  transcript:Zm00001d020861_T001  rna22825     6.00E-38
905-  5:  transcript:Zm00001d020862_T001  rna22827            0
905-  6:  transcript:Zm00001d020874_T001  rna22841     2.00E-78
905-  7:  transcript:Zm00001d020875_T001  rna22842     8.00E-17
905-  8:  transcript:Zm00001d020879_T001  rna22846     6.00E-42
905-  9:  transcript:Zm00001d020881_T002  rna22853     1.00E-92
905- 10:  transcript:Zm00001d020898_T002  rna22866            0
905- 11:  transcript:Zm00001d020903_T001  rna22877     6.00E-76
905- 12:  transcript:Zm00001d020921_T001  rna22885            0
905- 13:  transcript:Zm00001d020923_T001  rna22887     6.00E-81
905- 14:  transcript:Zm00001d020926_T001  rna22891     9.00E-57
905- 15:  transcript:Zm00001d020929_T002  rna22894            0
905- 16:  transcript:Zm00001d020932_T001  rna22904    9.00E-129
905- 17:  transcript:Zm00001d020937_T001  rna22911     6.00E-16
905- 18:  transcript:Zm00001d020939_T001  rna22914            0
905- 19:  transcript:Zm00001d020941_T001  rna22920    2.00E-120
## Alignment 906: score=569.0 e_value=2.1e-34 N=14 7&NC_008401.2 plus
906-  0:  transcript:Zm00001d020315_T001  rna22453     1.00E-41
906-  1:  transcript:Zm00001d020317_T001  rna22454     2.00E-16
906-  2:  transcript:Zm00001d020332_T001  rna22459            0
906-  3:  transcript:Zm00001d020344_T001  rna22466     3.00E-59
906-  4:  transcript:Zm00001d020345_T001  rna22474            0
906-  5:  transcript:Zm00001d020348_T001  rna22475     3.00E-59
906-  6:  transcript:Zm00001d020353_T001  rna22478     5.00E-53
906-  7:  transcript:Zm00001d020355_T002  rna22481            0
906-  8:  transcript:Zm00001d020358_T001  rna22482    8.00E-139
906-  9:  transcript:Zm00001d020361_T001  rna22483    3.00E-136
906- 10:  transcript:Zm00001d020365_T001  rna22498            0
906- 11:  transcript:Zm00001d020378_T001  rna22501    3.00E-153
906- 12:  transcript:Zm00001d020381_T008  rna22502    1.00E-162
906- 13:  transcript:Zm00001d020382_T001  rna22505     3.00E-13
## Alignment 907: score=469.0 e_value=1.8e-25 N=11 7&NC_008401.2 plus
907-  0:  transcript:Zm00001d021051_T002  rna23040    4.00E-143
907-  1:  transcript:Zm00001d021056_T001  rna23058    6.00E-144
```

907- 2: transcript:Zm00001d021057_T001 rna23059   6.00E-124
907- 3: transcript:Zm00001d021064_T001 rna23079   3.00E-116
907- 4: transcript:Zm00001d021065_T001 rna23080   0
907- 5: transcript:Zm00001d021070_T002 rna23085   0
907- 6: transcript:Zm00001d021072_T001 rna23086   0
907- 7: transcript:Zm00001d021086_T001 rna23093   3.00E-55
907- 8: transcript:Zm00001d021087_T003 rna23096   0
907- 9: transcript:Zm00001d021090_T001 rna23101   2.00E-167
907- 10: transcript:Zm00001d021093_T001 rna23107   2.00E-19
Alignment 908: score=454.0 e_value=2.2e-21 N=10 7&NC_008401.2 plus
908- 0: transcript:Zm00001d020736_T001 rna22726   3.00E-121
908- 1: transcript:Zm00001d020744_T001 rna22729   3.00E-61
908- 2: transcript:Zm00001d020757_T001 rna22736   6.00E-111
908- 3: transcript:Zm00001d020762_T001 rna22742   3.00E-105
908- 4: transcript:Zm00001d020763_T001 rna22744   9.00E-66
908- 5: transcript:Zm00001d020768_T001 rna22748   1.00E-69
908- 6: transcript:Zm00001d020769_T005 rna22749   0
908- 7: transcript:Zm00001d020771_T003 rna22751   0
908- 8: transcript:Zm00001d020772_T001 rna22752   2.00E-49
908- 9: transcript:Zm00001d020774_T001 rna22753   2.00E-98
Alignment 909: score=445.0 e_value=2.4e-22 N=10 7&NC_008401.2 plus
909- 0: transcript:Zm00001d020445_T001 rna22558   2.00E-21
909- 1: transcript:Zm00001d020446_T001 rna22559   3.00E-140
909- 2: transcript:Zm00001d020450_T001 rna22560   2.00E-78
909- 3: transcript:Zm00001d020457_T001 rna22561   1.00E-139
909- 4: transcript:Zm00001d020459_T001 rna22562   6.00E-28
909- 5: transcript:Zm00001d020460_T001 rna22563   8.00E-86
909- 6: transcript:Zm00001d020470_T001 rna22569   2.00E-123
909- 7: transcript:Zm00001d020485_T001 rna22571   9.00E-146
909- 8: transcript:Zm00001d020486_T001 rna22574   7.00E-35
909- 9: transcript:Zm00001d020497_T001 rna22578   1.00E-136
Alignment 910: score=418.0 e_value=3.9e-21 N=10 7&NC_008401.2 plus
910- 0: transcript:Zm00001d020605_T001 rna22632   1.00E-54
910- 1: transcript:Zm00001d020607_T001 rna22634   1.00E-11
910- 2: transcript:Zm00001d020609_T001 rna22636   0
910- 3: transcript:Zm00001d020617_T001 rna22644   0
910- 4: transcript:Zm00001d020628_T001 rna22646   0
910- 5: transcript:Zm00001d020631_T001 rna22650   0
910- 6: transcript:Zm00001d020639_T002 rna22651   0
910- 7: transcript:Zm00001d020650_T001 rna22672   2.00E-82
910- 8: transcript:Zm00001d020651_T001 rna22673   1.00E-100
910- 9: transcript:Zm00001d020653_T001 rna22676   9.00E-47
Alignment 911: score=393.0 e_value=2.3e-16 N=9 7&NC_008401.2 plus
911- 0: transcript:Zm00001d020233_T001 rna22396   3.00E-149
911- 1: transcript:Zm00001d020238_T002 rna22399   0
911- 2: transcript:Zm00001d020243_T001 rna22401   2.00E-33
911- 3: transcript:Zm00001d020257_T001 rna22407   8.00E-73
911- 4: transcript:Zm00001d020264_T001 rna22423   9.00E-43
911- 5: transcript:Zm00001d020267_T001 rna22426   1.00E-88
911- 6: transcript:Zm00001d020272_T001 rna22427   5.00E-28
911- 7: transcript:Zm00001d020275_T001 rna22428   3.00E-39
911- 8: transcript:Zm00001d020277_T001 rna22429   0
Alignment 912: score=390.0 e_value=8.4e-23 N=9 7&NC_008401.2 plus
912- 0: transcript:Zm00001d020946_T005 rna22923   1.00E-41

912- 1: transcript:Zm00001d020947_T001 rna22925 1.00E-109
912- 2: transcript:Zm00001d020948_T001 rna22926 5.00E-106
912- 3: transcript:Zm00001d020950_T001 rna22933 2.00E-65
912- 4: transcript:Zm00001d020951_T002 rna22936 5.00E-77
912- 5: transcript:Zm00001d020963_T001 rna22939 0
912- 6: transcript:Zm00001d020965_T007 rna22944 0
912- 7: transcript:Zm00001d020969_T013 rna22953 0
912- 8: transcript:Zm00001d020970_T001 rna22955 5.00E-26
Alignment 913: score=373.0 e_value=9.2e-14 N=8 7&NC_008401.2 plus
913- 0: transcript:Zm00001d021014_T001 rna23006 0
913- 1: transcript:Zm00001d021016_T001 rna23007 5.00E-165
913- 2: transcript:Zm00001d021018_T002 rna23009 2.00E-87
913- 3: transcript:Zm00001d021019_T001 rna23010 2.00E-109
913- 4: transcript:Zm00001d021021_T001 rna23013 2.00E-26
913- 5: transcript:Zm00001d021023_T001 rna23014 3.00E-19
913- 6: transcript:Zm00001d021024_T001 rna23015 0
913- 7: transcript:Zm00001d021026_T001 rna23017 0
Alignment 914: score=348.0 e_value=4.7e-14 N=8 7&NC_008401.2 plus
914- 0: transcript:Zm00001d020688_T001 rna22693 6.00E-112
914- 1: transcript:Zm00001d020697_T001 rna22703 3.00E-155
914- 2: transcript:Zm00001d020708_T002 rna22708 0
914- 3: transcript:Zm00001d020711_T001 rna22710 1.00E-86
914- 4: transcript:Zm00001d020713_T001 rna22711 2.00E-169
914- 5: transcript:Zm00001d020717_T001 rna22713 0
914- 6: transcript:Zm00001d020723_T001 rna22720 2.00E-28
914- 7: transcript:Zm00001d020725_T001 rna22722 2.00E-29
Alignment 915: score=341.0 e_value=5.4e-16 N=8 7&NC_008401.2 plus
915- 0: transcript:Zm00001d020804_T001 rna22763 2.00E-101
915- 1: transcript:Zm00001d020810_T002 rna22781 0
915- 2: transcript:Zm00001d020820_T001 rna22798 1.00E-22
915- 3: transcript:Zm00001d020823_T001 rna22802 3.00E-35
915- 4: transcript:Zm00001d020826_T001 rna22805 1.00E-97
915- 5: transcript:Zm00001d020827_T001 rna22806 0
915- 6: transcript:Zm00001d020828_T001 rna22811 0
915- 7: transcript:Zm00001d020834_T004 rna22812 1.00E-17
Alignment 916: score=310.0 e_value=1.6e-10 N=7 7&NC_008401.2 plus
916- 0: transcript:Zm00001d020405_T001 rna22513 1.00E-88
916- 1: transcript:Zm00001d020409_T002 rna22515 6.00E-103
916- 2: transcript:Zm00001d020412_T001 rna22516 1.00E-21
916- 3: transcript:Zm00001d020414_T002 rna22517 0
916- 4: transcript:Zm00001d020417_T001 rna22519 1.00E-72
916- 5: transcript:Zm00001d020419_T003 rna22522 2.00E-115
916- 6: transcript:Zm00001d020426_T001 rna22530 0
Alignment 917: score=250.0 e_value=1.9e-08 N=6 7&NC_008401.2 plus
917- 0: transcript:Zm00001d021205_T001 rna23151 3.00E-50
917- 1: transcript:Zm00001d021216_T001 rna23156 0
917- 2: transcript:Zm00001d021221_T001 rna23157 3.00E-127
917- 3: transcript:Zm00001d021227_T003 rna23159 0
917- 4: transcript:Zm00001d021231_T005 rna23163 0
917- 5: transcript:Zm00001d021240_T001 rna23169 0
Alignment 918: score=6980.0 e_value=0 N=150 7&NC_008402.2 plus
918- 0: transcript:Zm00001d020603_T004 rna24150 2.00E-111
918- 1: transcript:Zm00001d020605_T001 rna24151 4.00E-70
918- 2: transcript:Zm00001d020606_T001 rna24152 1.00E-73

918-	3:	transcript:Zm00001d020607_T001	rna24153	4.00E-44
918-	4:	transcript:Zm00001d020609_T001	rna24156	0
918-	5:	transcript:Zm00001d020610_T001	rna24157	7.00E-50
918-	6:	transcript:Zm00001d020612_T001	rna24158	5.00E-136
918-	7:	transcript:Zm00001d020614_T001	rna24162	5.00E-55
918-	8:	transcript:Zm00001d020615_T001	rna24163	0
918-	9:	transcript:Zm00001d020617_T001	rna24165	0
918-	10:	transcript:Zm00001d020618_T003	rna24166	0
918-	11:	transcript:Zm00001d020620_T001	rna24167	7.00E-55
918-	12:	transcript:Zm00001d020622_T002	rna24169	0
918-	13:	transcript:Zm00001d020623_T001	rna24172	4.00E-62
918-	14:	transcript:Zm00001d020627_T001	rna24173	0
918-	15:	transcript:Zm00001d020628_T001	rna24174	0
918-	16:	transcript:Zm00001d020629_T001	rna24177	2.00E-17
918-	17:	transcript:Zm00001d020631_T001	rna24178	0
918-	18:	transcript:Zm00001d020636_T001	rna24179	0
918-	19:	transcript:Zm00001d020638_T002	rna24180	0
918-	20:	transcript:Zm00001d020639_T002	rna24181	0
918-	21:	transcript:Zm00001d020640_T002	rna24183	2.00E-57
918-	22:	transcript:Zm00001d020643_T002	rna24184	0
918-	23:	transcript:Zm00001d020644_T001	rna24187	7.00E-144
918-	24:	transcript:Zm00001d020646_T001	rna24189	2.00E-28
918-	25:	transcript:Zm00001d020647_T002	rna24190	0
918-	26:	transcript:Zm00001d020651_T001	rna24194	6.00E-156
918-	27:	transcript:Zm00001d020653_T001	rna24197	6.00E-73
918-	28:	transcript:Zm00001d020655_T001	rna24198	8.00E-42
918-	29:	transcript:Zm00001d020656_T001	rna24199	0
918-	30:	transcript:Zm00001d020658_T001	rna24200	4.00E-23
918-	31:	transcript:Zm00001d020664_T016	rna24201	0
918-	32:	transcript:Zm00001d020669_T001	rna24203	0
918-	33:	transcript:Zm00001d020670_T001	rna24204	2.00E-57
918-	34:	transcript:Zm00001d020673_T001	rna24206	1.00E-113
918-	35:	transcript:Zm00001d020674_T001	rna24210	0
918-	36:	transcript:Zm00001d020675_T001	rna24211	4.00E-76
918-	37:	transcript:Zm00001d020679_T001	rna24212	3.00E-11
918-	38:	transcript:Zm00001d020680_T001	rna24213	0
918-	39:	transcript:Zm00001d020683_T001	rna24215	2.00E-94
918-	40:	transcript:Zm00001d020684_T001	rna24221	6.00E-52
918-	41:	transcript:Zm00001d020685_T002	rna24222	0
918-	42:	transcript:Zm00001d020686_T001	rna24226	0
918-	43:	transcript:Zm00001d020687_T002	rna24228	0
918-	44:	transcript:Zm00001d020688_T001	rna24229	9.00E-41
918-	45:	transcript:Zm00001d020691_T001	rna24231	0
918-	46:	transcript:Zm00001d020692_T002	rna24236	0
918-	47:	transcript:Zm00001d020694_T001	rna24241	0
918-	48:	transcript:Zm00001d020695_T001	rna24242	0
918-	49:	transcript:Zm00001d020696_T001	rna24244	0
918-	50:	transcript:Zm00001d020697_T001	rna24245	0
918-	51:	transcript:Zm00001d020702_T001	rna24250	0
918-	52:	transcript:Zm00001d020703_T001	rna24251	0
918-	53:	transcript:Zm00001d020704_T001	rna24253	2.00E-140
918-	54:	transcript:Zm00001d020705_T001	rna24254	1.00E-77
918-	55:	transcript:Zm00001d020706_T001	rna24255	5.00E-54
918-	56:	transcript:Zm00001d020707_T001	rna24256	8.00E-134

918- 57:	transcript:Zm00001d020708_T002	rna24258	0
918- 58:	transcript:Zm00001d020711_T001	rna24259	5.00E-131
918- 59:	transcript:Zm00001d020713_T001	rna24260	0
918- 60:	transcript:Zm00001d020714_T001	rna24261	2.00E-131
918- 61:	transcript:Zm00001d020717_T001	rna24263	0
918- 62:	transcript:Zm00001d020719_T002	rna24270	0
918- 63:	transcript:Zm00001d020721_T001	rna24271	0
918- 64:	transcript:Zm00001d020722_T001	rna24277	2.00E-92
918- 65:	transcript:Zm00001d020723_T001	rna24280	2.00E-55
918- 66:	transcript:Zm00001d020725_T001	rna24281	3.00E-34
918- 67:	transcript:Zm00001d020726_T001	rna24282	0
918- 68:	transcript:Zm00001d020728_T005	rna24283	0
918- 69:	transcript:Zm00001d020731_T001	rna24286	2.00E-78
918- 70:	transcript:Zm00001d020732_T001	rna24288	3.00E-177
918- 71:	transcript:Zm00001d020736_T001	rna24294	0
918- 72:	transcript:Zm00001d020742_T001	rna24298	8.00E-46
918- 73:	transcript:Zm00001d020744_T001	rna24299	3.00E-91
918- 74:	transcript:Zm00001d020748_T002	rna24302	0
918- 75:	transcript:Zm00001d020750_T001	rna24303	7.00E-149
918- 76:	transcript:Zm00001d020755_T001	rna24304	3.00E-07
918- 77:	transcript:Zm00001d020757_T001	rna24305	7.00E-151
918- 78:	transcript:Zm00001d020762_T001	rna24306	4.00E-155
918- 79:	transcript:Zm00001d020764_T004	rna24308	2.00E-175
918- 80:	transcript:Zm00001d020769_T005	rna24310	0
918- 81:	transcript:Zm00001d020770_T002	rna24313	2.00E-61
918- 82:	transcript:Zm00001d020771_T003	rna24314	0
918- 83:	transcript:Zm00001d020772_T001	rna24316	2.00E-68
918- 84:	transcript:Zm00001d020773_T001	rna24318	3.00E-119
918- 85:	transcript:Zm00001d020774_T001	rna24319	1.00E-113
918- 86:	transcript:Zm00001d020776_T002	rna24321	0
918- 87:	transcript:Zm00001d020779_T001	rna24322	8.00E-27
918- 88:	transcript:Zm00001d020780_T001	rna24323	9.00E-136
918- 89:	transcript:Zm00001d020781_T003	rna24324	1.00E-118
918- 90:	transcript:Zm00001d020787_T001	rna24329	2.00E-142
918- 91:	transcript:Zm00001d020789_T001	rna24330	1.00E-154
918- 92:	transcript:Zm00001d020790_T001	rna24332	1.00E-110
918- 93:	transcript:Zm00001d020793_T001	rna24335	3.00E-36
918- 94:	transcript:Zm00001d020799_T003	rna24337	0
918- 95:	transcript:Zm00001d020804_T001	rna24340	4.00E-131
918- 96:	transcript:Zm00001d020807_T003	rna24341	0
918- 97:	transcript:Zm00001d020808_T001	rna24343	2.00E-109
918- 98:	transcript:Zm00001d020810_T002	rna24352	0
918- 99:	transcript:Zm00001d020811_T001	rna24353	0
918-100:	transcript:Zm00001d020820_T001	rna24359	5.00E-39
918-101:	transcript:Zm00001d020823_T001	rna24363	9.00E-49
918-102:	transcript:Zm00001d020826_T001	rna24364	0
918-103:	transcript:Zm00001d020827_T001	rna24365	0
918-104:	transcript:Zm00001d020828_T001	rna24368	0
918-105:	transcript:Zm00001d020834_T004	rna24369	3.00E-31
918-106:	transcript:Zm00001d020835_T022	rna24371	0
918-107:	transcript:Zm00001d020837_T001	rna24372	3.00E-64
918-108:	transcript:Zm00001d020838_T001	rna24373	4.00E-127
918-109:	transcript:Zm00001d020840_T001	rna24377	2.00E-83
918-110:	transcript:Zm00001d020843_T001	rna24378	6.00E-09

918-111:	transcript:Zm00001d020852_T001	rna24382	3.00E-58
918-112:	transcript:Zm00001d020858_T001	rna24386	0
918-113:	transcript:Zm00001d020861_T001	rna24387	2.00E-24
918-114:	transcript:Zm00001d020862_T001	rna24391	2.00E-133
918-115:	transcript:Zm00001d020863_T001	rna24392	3.00E-131
918-116:	transcript:Zm00001d020866_T001	rna24396	1.00E-31
918-117:	transcript:Zm00001d020869_T001	rna24399	7.00E-26
918-118:	transcript:Zm00001d020871_T001	rna24400	6.00E-40
918-119:	transcript:Zm00001d020872_T001	rna24403	3.00E-23
918-120:	transcript:Zm00001d020874_T001	rna24404	6.00E-86
918-121:	transcript:Zm00001d020875_T001	rna24405	3.00E-08
918-122:	transcript:Zm00001d020877_T001	rna24407	8.00E-75
918-123:	transcript:Zm00001d020879_T001	rna24408	6.00E-58
918-124:	transcript:Zm00001d020881_T002	rna24411	0
918-125:	transcript:Zm00001d020882_T001	rna24413	2.00E-171
918-126:	transcript:Zm00001d020886_T001	rna24415	0
918-127:	transcript:Zm00001d020888_T001	rna24416	4.00E-34
918-128:	transcript:Zm00001d020892_T003	rna24419	1.00E-31
918-129:	transcript:Zm00001d020899_T001	rna24422	2.00E-67
918-130:	transcript:Zm00001d020900_T001	rna24423	0
918-131:	transcript:Zm00001d020901_T001	rna24424	3.00E-44
918-132:	transcript:Zm00001d020902_T002	rna24425	1.00E-178
918-133:	transcript:Zm00001d020903_T001	rna24427	2.00E-103
918-134:	transcript:Zm00001d020909_T001	rna24438	0
918-135:	transcript:Zm00001d020913_T001	rna24441	0
918-136:	transcript:Zm00001d020915_T002	rna24442	0
918-137:	transcript:Zm00001d020921_T001	rna24443	0
918-138:	transcript:Zm00001d020923_T001	rna24444	3.00E-35
918-139:	transcript:Zm00001d020926_T001	rna24448	2.00E-62
918-140:	transcript:Zm00001d020927_T001	rna24449	0
918-141:	transcript:Zm00001d020929_T002	rna24451	0
918-142:	transcript:Zm00001d020930_T001	rna24452	8.00E-60
918-143:	transcript:Zm00001d020931_T001	rna24453	0
918-144:	transcript:Zm00001d020932_T001	rna24455	1.00E-143
918-145:	transcript:Zm00001d020936_T001	rna24458	3.00E-169
918-146:	transcript:Zm00001d020937_T001	rna24459	0
918-147:	transcript:Zm00001d020938_T002	rna24461	0
918-148:	transcript:Zm00001d020939_T001	rna24463	0
918-149:	transcript:Zm00001d020941_T001	rna24466	2.00E-117

Alignment 919: score=6349.0 e_value=0 N=138 7&NC_008402.2 plus

919- 0:	transcript:Zm00001d020228_T002	rna23835	6.00E-34
919- 1:	transcript:Zm00001d020230_T006	rna23836	0
919- 2:	transcript:Zm00001d020233_T001	rna23838	0
919- 3:	transcript:Zm00001d020234_T001	rna23839	1.00E-163
919- 4:	transcript:Zm00001d020236_T001	rna23840	7.00E-58
919- 5:	transcript:Zm00001d020237_T001	rna23841	1.00E-163
919- 6:	transcript:Zm00001d020238_T002	rna23842	0
919- 7:	transcript:Zm00001d020242_T002	rna23843	0
919- 8:	transcript:Zm00001d020250_T001	rna23846	5.00E-22
919- 9:	transcript:Zm00001d020256_T001	rna23847	0
919- 10:	transcript:Zm00001d020257_T001	rna23848	2.00E-51
919- 11:	transcript:Zm00001d020260_T001	rna23853	2.00E-140
919- 12:	transcript:Zm00001d020264_T001	rna23856	2.00E-09
919- 13:	transcript:Zm00001d020267_T001	rna23861	1.00E-130

919- 14:	transcript:Zm00001d020268_T002	rna23863	1.00E-105
919- 15:	transcript:Zm00001d020273_T001	rna23864	2.00E-108
919- 16:	transcript:Zm00001d020274_T001	rna23865	2.00E-23
919- 17:	transcript:Zm00001d020275_T001	rna23867	3.00E-80
919- 18:	transcript:Zm00001d020277_T001	rna23868	0
919- 19:	transcript:Zm00001d020283_T001	rna23873	7.00E-70
919- 20:	transcript:Zm00001d020296_T001	rna23874	2.00E-41
919- 21:	transcript:Zm00001d020297_T002	rna23875	0
919- 22:	transcript:Zm00001d020303_T001	rna23876	0
919- 23:	transcript:Zm00001d020306_T001	rna23879	8.00E-170
919- 24:	transcript:Zm00001d020309_T001	rna23883	2.00E-55
919- 25:	transcript:Zm00001d020311_T001	rna23889	2.00E-55
919- 26:	transcript:Zm00001d020312_T017	rna23890	3.00E-159
919- 27:	transcript:Zm00001d020315_T001	rna23891	2.00E-43
919- 28:	transcript:Zm00001d020317_T001	rna23892	1.00E-14
919- 29:	transcript:Zm00001d020322_T001	rna23893	0
919- 30:	transcript:Zm00001d020323_T001	rna23894	2.00E-84
919- 31:	transcript:Zm00001d020325_T001	rna23896	0
919- 32:	transcript:Zm00001d020332_T001	rna23898	0
919- 33:	transcript:Zm00001d020337_T001	rna23900	3.00E-148
919- 34:	transcript:Zm00001d020339_T001	rna23903	0
919- 35:	transcript:Zm00001d020343_T002	rna23904	4.00E-89
919- 36:	transcript:Zm00001d020344_T001	rna23905	2.00E-95
919- 37:	transcript:Zm00001d020345_T001	rna23910	0
919- 38:	transcript:Zm00001d020348_T001	rna23913	1.00E-70
919- 39:	transcript:Zm00001d020353_T001	rna23917	6.00E-77
919- 40:	transcript:Zm00001d020354_T001	rna23919	2.00E-31
919- 41:	transcript:Zm00001d020355_T002	rna23921	0
919- 42:	transcript:Zm00001d020357_T003	rna23922	0
919- 43:	transcript:Zm00001d020358_T001	rna23928	0
919- 44:	transcript:Zm00001d020359_T003	rna23929	0
919- 45:	transcript:Zm00001d020361_T001	rna23930	4.00E-141
919- 46:	transcript:Zm00001d020363_T002	rna23931	0
919- 47:	transcript:Zm00001d020365_T001	rna23944	0
919- 48:	transcript:Zm00001d020367_T003	rna23949	0
919- 49:	transcript:Zm00001d020371_T001	rna23951	0
919- 50:	transcript:Zm00001d020374_T003	rna23952	3.00E-20
919- 51:	transcript:Zm00001d020378_T001	rna23953	0
919- 52:	transcript:Zm00001d020380_T002	rna23954	0
919- 53:	transcript:Zm00001d020381_T008	rna23955	0
919- 54:	transcript:Zm00001d020382_T001	rna23956	1.00E-21
919- 55:	transcript:Zm00001d020392_T002	rna23958	9.00E-94
919- 56:	transcript:Zm00001d020395_T002	rna23961	1.00E-80
919- 57:	transcript:Zm00001d020396_T004	rna23964	0
919- 58:	transcript:Zm00001d020399_T007	rna23965	0
919- 59:	transcript:Zm00001d020400_T001	rna23971	0
919- 60:	transcript:Zm00001d020403_T001	rna23977	0
919- 61:	transcript:Zm00001d020405_T001	rna23980	1.00E-108
919- 62:	transcript:Zm00001d020409_T002	rna23983	3.00E-145
919- 63:	transcript:Zm00001d020411_T001	rna23984	1.00E-177
919- 64:	transcript:Zm00001d020412_T001	rna23986	7.00E-29
919- 65:	transcript:Zm00001d020414_T002	rna23987	0
919- 66:	transcript:Zm00001d020417_T001	rna23988	4.00E-38
919- 67:	transcript:Zm00001d020418_T001	rna23989	0

919- 68:	transcript:Zm00001d020419_T003	rna24001	1.00E-85
919- 69:	transcript:Zm00001d020420_T001	rna24002	3.00E-158
919- 70:	transcript:Zm00001d020424_T002	rna24003	2.00E-27
919- 71:	transcript:Zm00001d020425_T001	rna24008	2.00E-39
919- 72:	transcript:Zm00001d020426_T001	rna24009	0
919- 73:	transcript:Zm00001d020429_T001	rna24010	0
919- 74:	transcript:Zm00001d020432_T001	rna24016	3.00E-63
919- 75:	transcript:Zm00001d020433_T001	rna24018	2.00E-59
919- 76:	transcript:Zm00001d020435_T001	rna24021	0
919- 77:	transcript:Zm00001d020437_T001	rna24022	2.00E-125
919- 78:	transcript:Zm00001d020438_T001	rna24023	5.00E-75
919- 79:	transcript:Zm00001d020441_T001	rna24026	0
919- 80:	transcript:Zm00001d020443_T001	rna24030	5.00E-30
919- 81:	transcript:Zm00001d020444_T003	rna24033	4.00E-87
919- 82:	transcript:Zm00001d020445_T001	rna24034	2.00E-76
919- 83:	transcript:Zm00001d020448_T001	rna24035	0
919- 84:	transcript:Zm00001d020450_T001	rna24037	2.00E-12
919- 85:	transcript:Zm00001d020454_T003	rna24038	0
919- 86:	transcript:Zm00001d020457_T001	rna24040	3.00E-152
919- 87:	transcript:Zm00001d020459_T001	rna24041	3.00E-40
919- 88:	transcript:Zm00001d020460_T001	rna24042	8.00E-43
919- 89:	transcript:Zm00001d020461_T001	rna24047	0
919- 90:	transcript:Zm00001d020463_T002	rna24049	0
919- 91:	transcript:Zm00001d020472_T001	rna24050	3.00E-08
919- 92:	transcript:Zm00001d020485_T001	rna24053	3.00E-149
919- 93:	transcript:Zm00001d020486_T001	rna24054	0
919- 94:	transcript:Zm00001d020487_T001	rna24055	0
919- 95:	transcript:Zm00001d020490_T001	rna24056	4.00E-80
919- 96:	transcript:Zm00001d020492_T001	rna24058	1.00E-121
919- 97:	transcript:Zm00001d020496_T001	rna24060	0
919- 98:	transcript:Zm00001d020497_T001	rna24061	0
919- 99:	transcript:Zm00001d020501_T001	rna24064	1.00E-127
919-100:	transcript:Zm00001d020505_T001	rna24069	4.00E-105
919-101:	transcript:Zm00001d020506_T001	rna24070	2.00E-118
919-102:	transcript:Zm00001d020510_T003	rna24071	4.00E-30
919-103:	transcript:Zm00001d020511_T001	rna24072	2.00E-78
919-104:	transcript:Zm00001d020512_T001	rna24073	0
919-105:	transcript:Zm00001d020515_T002	rna24075	8.00E-137
919-106:	transcript:Zm00001d020519_T001	rna24077	2.00E-50
919-107:	transcript:Zm00001d020521_T001	rna24078	1.00E-44
919-108:	transcript:Zm00001d020528_T001	rna24081	2.00E-101
919-109:	transcript:Zm00001d020531_T002	rna24082	0
919-110:	transcript:Zm00001d020532_T002	rna24083	0
919-111:	transcript:Zm00001d020533_T001	rna24084	0
919-112:	transcript:Zm00001d020535_T001	rna24086	0
919-113:	transcript:Zm00001d020536_T001	rna24088	0
919-114:	transcript:Zm00001d020537_T001	rna24089	2.00E-118
919-115:	transcript:Zm00001d020538_T001	rna24090	7.00E-45
919-116:	transcript:Zm00001d020540_T002	rna24091	2.00E-179
919-117:	transcript:Zm00001d020543_T001	rna24093	0
919-118:	transcript:Zm00001d020549_T005	rna24094	0
919-119:	transcript:Zm00001d020552_T001	rna24101	1.00E-160
919-120:	transcript:Zm00001d020555_T001	rna24102	1.00E-178
919-121:	transcript:Zm00001d020557_T001	rna24105	0

919-122: transcript:Zm00001d020558_T001 rna24107 1.00E-20
919-123: transcript:Zm00001d020560_T001 rna24110 0
919-124: transcript:Zm00001d020561_T002 rna24115 0
919-125: transcript:Zm00001d020563_T001 rna24119 0
919-126: transcript:Zm00001d020571_T003 rna24124 3.00E-86
919-127: transcript:Zm00001d020572_T001 rna24125 2.00E-24
919-128: transcript:Zm00001d020573_T001 rna24126 2.00E-22
919-129: transcript:Zm00001d020578_T001 rna24127 4.00E-70
919-130: transcript:Zm00001d020583_T001 rna24129 1.00E-129
919-131: transcript:Zm00001d020584_T001 rna24132 4.00E-67
919-132: transcript:Zm00001d020586_T001 rna24134 5.00E-172
919-133: transcript:Zm00001d020589_T001 rna24135 6.00E-36
919-134: transcript:Zm00001d020590_T009 rna24136 0
919-135: transcript:Zm00001d020593_T020 rna24137 3.00E-176
919-136: transcript:Zm00001d020595_T001 rna24139 3.00E-78
919-137: transcript:Zm00001d020602_T009 rna24149 2.00E-169
Alignment 920: score=2374.0 e_value=9.7e-207 N=53 7&NC_008402.2 plus
920- 0: transcript:Zm00001d021006_T001 rna24511 6.00E-79
920- 1: transcript:Zm00001d021014_T001 rna24518 0
920- 2: transcript:Zm00001d021015_T002 rna24519 0
920- 3: transcript:Zm00001d021016_T001 rna24520 2.00E-45
920- 4: transcript:Zm00001d021018_T002 rna24521 5.00E-85
920- 5: transcript:Zm00001d021019_T001 rna24522 1.00E-151
920- 6: transcript:Zm00001d021021_T001 rna24524 9.00E-86
920- 7: transcript:Zm00001d021023_T001 rna24526 2.00E-54
920- 8: transcript:Zm00001d021024_T001 rna24527 0
920- 9: transcript:Zm00001d021025_T001 rna24528 9.00E-157
920- 10: transcript:Zm00001d021026_T001 rna24536 0
920- 11: transcript:Zm00001d021031_T002 rna24537 2.00E-172
920- 12: transcript:Zm00001d021032_T001 rna24538 6.00E-78
920- 13: transcript:Zm00001d021035_T001 rna24539 2.00E-09
920- 14: transcript:Zm00001d021036_T001 rna24540 5.00E-155
920- 15: transcript:Zm00001d021038_T001 rna24541 7.00E-50
920- 16: transcript:Zm00001d021046_T003 rna24544 0
920- 17: transcript:Zm00001d021050_T002 rna24547 2.00E-151
920- 18: transcript:Zm00001d021054_T002 rna24554 7.00E-44
920- 19: transcript:Zm00001d021056_T001 rna24555 4.00E-167
920- 20: transcript:Zm00001d021057_T001 rna24556 1.00E-149
920- 21: transcript:Zm00001d021058_T001 rna24558 3.00E-44
920- 22: transcript:Zm00001d021061_T001 rna24561 0
920- 23: transcript:Zm00001d021062_T001 rna24562 2.00E-37
920- 24: transcript:Zm00001d021063_T001 rna24564 5.00E-129
920- 25: transcript:Zm00001d021065_T001 rna24565 0
920- 26: transcript:Zm00001d021070_T002 rna24570 0
920- 27: transcript:Zm00001d021071_T001 rna24571 0
920- 28: transcript:Zm00001d021072_T001 rna24572 0
920- 29: transcript:Zm00001d021073_T002 rna24573 0
920- 30: transcript:Zm00001d021085_T001 rna24578 2.00E-49
920- 31: transcript:Zm00001d021086_T001 rna24579 3.00E-109
920- 32: transcript:Zm00001d021087_T003 rna24580 0
920- 33: transcript:Zm00001d021093_T001 rna24584 7.00E-12
920- 34: transcript:Zm00001d021119_T008 rna24586 0
920- 35: transcript:Zm00001d021120_T001 rna24592 0
920- 36: transcript:Zm00001d021122_T001 rna24593 3.00E-38

920- 37: transcript:Zm00001d021123_T002 rna24594 6.00E-144
920- 38: transcript:Zm00001d021126_T002 rna24595 0
920- 39: transcript:Zm00001d021131_T002 rna24596 1.00E-75
920- 40: transcript:Zm00001d021136_T001 rna24600 6.00E-26
920- 41: transcript:Zm00001d021137_T002 rna24601 5.00E-127
920- 42: transcript:Zm00001d021138_T003 rna24604 0
920- 43: transcript:Zm00001d021139_T008 rna24605 0
920- 44: transcript:Zm00001d021140_T002 rna24608 2.00E-50
920- 45: transcript:Zm00001d021141_T001 rna24609 4.00E-88
920- 46: transcript:Zm00001d021142_T006 rna24610 6.00E-109
920- 47: transcript:Zm00001d021145_T003 rna24613 0
920- 48: transcript:Zm00001d021147_T002 rna24614 4.00E-160
920- 49: transcript:Zm00001d021160_T001 rna24618 3.00E-08
920- 50: transcript:Zm00001d021168_T001 rna24635 3.00E-148
920- 51: transcript:Zm00001d021169_T002 rna24639 0
920- 52: transcript:Zm00001d021170_T016 rna24641 0
Alignment 921: score=1686.0 e_value=2.6e-144 N=38 7&NC_008402.2 plus
921- 0: transcript:Zm00001d019950_T001 rna23562 0
921- 1: transcript:Zm00001d019967_T001 rna23579 5.00E-107
921- 2: transcript:Zm00001d019978_T001 rna23582 0
921- 3: transcript:Zm00001d019980_T001 rna23585 2.00E-106
921- 4: transcript:Zm00001d019981_T002 rna23593 3.00E-106
921- 5: transcript:Zm00001d019983_T001 rna23594 0
921- 6: transcript:Zm00001d019987_T001 rna23596 2.00E-149
921- 7: transcript:Zm00001d019988_T001 rna23597 0
921- 8: transcript:Zm00001d019989_T001 rna23602 2.00E-121
921- 9: transcript:Zm00001d019993_T001 rna23604 0
921- 10: transcript:Zm00001d020002_T002 rna23605 0
921- 11: transcript:Zm00001d020003_T002 rna23608 1.00E-94
921- 12: transcript:Zm00001d020004_T003 rna23609 1.00E-172
921- 13: transcript:Zm00001d020006_T001 rna23612 1.00E-11
921- 14: transcript:Zm00001d020011_T001 rna23614 0
921- 15: transcript:Zm00001d020013_T001 rna23615 0
921- 16: transcript:Zm00001d020014_T001 rna23616 0
921- 17: transcript:Zm00001d020019_T002 rna23617 6.00E-57
921- 18: transcript:Zm00001d020020_T003 rna23618 6.00E-171
921- 19: transcript:Zm00001d020023_T002 rna23619 0
921- 20: transcript:Zm00001d020025_T001 rna23630 8.00E-26
921- 21: transcript:Zm00001d020028_T001 rna23631 4.00E-08
921- 22: transcript:Zm00001d020030_T001 rna23632 1.00E-49
921- 23: transcript:Zm00001d020036_T002 rna23634 0
921- 24: transcript:Zm00001d020038_T001 rna23636 3.00E-149
921- 25: transcript:Zm00001d020040_T003 rna23639 0
921- 26: transcript:Zm00001d020042_T001 rna23642 2.00E-38
921- 27: transcript:Zm00001d020043_T001 rna23644 1.00E-93
921- 28: transcript:Zm00001d020051_T001 rna23653 1.00E-161
921- 29: transcript:Zm00001d020052_T002 rna23654 0
921- 30: transcript:Zm00001d020053_T002 rna23655 0
921- 31: transcript:Zm00001d020057_T004 rna23664 3.00E-18
921- 32: transcript:Zm00001d020063_T001 rna23665 0
921- 33: transcript:Zm00001d020064_T001 rna23666 4.00E-21
921- 34: transcript:Zm00001d020069_T002 rna23676 0
921- 35: transcript:Zm00001d020071_T001 rna23680 0
921- 36: transcript:Zm00001d020072_T001 rna23681 3.00E-55

921- 37: transcript:Zm00001d020086_T001 rna23682 1.00E-20
Alignment 922: score=1231.0 e_value=3e-85 N=27 7&NC_008402.2 plus
922- 0: transcript:Zm00001d021278_T001 rna24689 3.00E-131
922- 1: transcript:Zm00001d021280_T004 rna24702 0
922- 2: transcript:Zm00001d021282_T001 rna24707 9.00E-95
922- 3: transcript:Zm00001d021283_T001 rna24708 0
922- 4: transcript:Zm00001d021284_T001 rna24709 2.00E-22
922- 5: transcript:Zm00001d021285_T001 rna24712 4.00E-64
922- 6: transcript:Zm00001d021287_T001 rna24713 0
922- 7: transcript:Zm00001d021288_T001 rna24715 2.00E-129
922- 8: transcript:Zm00001d021289_T001 rna24716 1.00E-73
922- 9: transcript:Zm00001d021291_T002 rna24717 0
922- 10: transcript:Zm00001d021294_T001 rna24718 3.00E-15
922- 11: transcript:Zm00001d021295_T006 rna24719 0
922- 12: transcript:Zm00001d021296_T004 rna24721 2.00E-137
922- 13: transcript:Zm00001d021297_T006 rna24722 0
922- 14: transcript:Zm00001d021298_T001 rna24723 0
922- 15: transcript:Zm00001d021299_T001 rna24724 0
922- 16: transcript:Zm00001d021301_T002 rna24725 0
922- 17: transcript:Zm00001d021303_T003 rna24726 0
922- 18: transcript:Zm00001d021304_T001 rna24728 0
922- 19: transcript:Zm00001d021305_T001 rna24729 0
922- 20: transcript:Zm00001d021306_T001 rna24730 4.00E-55
922- 21: transcript:Zm00001d021309_T003 rna24732 0
922- 22: transcript:Zm00001d021310_T001 rna24734 0
922- 23: transcript:Zm00001d021314_T007 rna24735 3.00E-118
922- 24: transcript:Zm00001d021315_T001 rna24738 7.00E-118
922- 25: transcript:Zm00001d021316_T001 rna24739 7.00E-57
922- 26: transcript:Zm00001d021318_T003 rna24740 0
Alignment 923: score=948.0 e_value=4.8e-66 N=21 7&NC_008402.2 plus
923- 0: transcript:Zm00001d020947_T001 rna24469 2.00E-18
923- 1: transcript:Zm00001d020948_T001 rna24471 3.00E-166
923- 2: transcript:Zm00001d020950_T001 rna24472 1.00E-76
923- 3: transcript:Zm00001d020951_T002 rna24473 1.00E-102
923- 4: transcript:Zm00001d020953_T001 rna24474 0
923- 5: transcript:Zm00001d020954_T001 rna24475 5.00E-151
923- 6: transcript:Zm00001d020956_T001 rna24476 6.00E-49
923- 7: transcript:Zm00001d020957_T001 rna24477 2.00E-121
923- 8: transcript:Zm00001d020963_T001 rna24483 0
923- 9: transcript:Zm00001d020967_T001 rna24484 5.00E-21
923- 10: transcript:Zm00001d020969_T013 rna24486 0
923- 11: transcript:Zm00001d020970_T001 rna24487 0
923- 12: transcript:Zm00001d020971_T002 rna24488 1.00E-95
923- 13: transcript:Zm00001d020973_T002 rna24490 0
923- 14: transcript:Zm00001d020974_T001 rna24491 0
923- 15: transcript:Zm00001d020975_T001 rna24492 2.00E-150
923- 16: transcript:Zm00001d020977_T001 rna24493 2.00E-120
923- 17: transcript:Zm00001d020980_T001 rna24497 0
923- 18: transcript:Zm00001d020982_T001 rna24503 2.00E-172
923- 19: transcript:Zm00001d020983_T001 rna24505 7.00E-87
923- 20: transcript:Zm00001d020986_T002 rna24510 0
Alignment 924: score=722.0 e_value=1.8e-47 N=16 7&NC_008402.2 plus
924- 0: transcript:Zm00001d021203_T003 rna24659 0
924- 1: transcript:Zm00001d021205_T001 rna24662 2.00E-43

924- 2: transcript:Zm00001d021216_T001 rna24667 0
924- 3: transcript:Zm00001d021221_T001 rna24669 4.00E-157
924- 4: transcript:Zm00001d021223_T001 rna24670 1.00E-17
924- 5: transcript:Zm00001d021224_T001 rna24671 1.00E-167
924- 6: transcript:Zm00001d021225_T005 rna24672 3.00E-109
924- 7: transcript:Zm00001d021226_T001 rna24673 2.00E-28
924- 8: transcript:Zm00001d021227_T003 rna24674 0
924- 9: transcript:Zm00001d021231_T005 rna24677 0
924- 10: transcript:Zm00001d021232_T001 rna24678 3.00E-24
924- 11: transcript:Zm00001d021236_T004 rna24679 2.00E-140
924- 12: transcript:Zm00001d021240_T001 rna24695 0
924- 13: transcript:Zm00001d021241_T009 rna24697 1.00E-31
924- 14: transcript:Zm00001d021243_T001 rna24698 6.00E-178
924- 15: transcript:Zm00001d021245_T001 rna24701 7.00E-150
Alignment 925: score=716.0 e_value=2.4e-44 N=16 7&NC_008402.2 plus
925- 0: transcript:Zm00001d020093_T002 rna23689 9.00E-172
925- 1: transcript:Zm00001d020097_T003 rna23690 0
925- 2: transcript:Zm00001d020100_T001 rna23705 2.00E-153
925- 3: transcript:Zm00001d020101_T001 rna23709 0
925- 4: transcript:Zm00001d020102_T001 rna23714 3.00E-45
925- 5: transcript:Zm00001d020103_T003 rna23715 0
925- 6: transcript:Zm00001d020108_T002 rna23717 0
925- 7: transcript:Zm00001d020109_T001 rna23718 2.00E-109
925- 8: transcript:Zm00001d020112_T001 rna23721 7.00E-93
925- 9: transcript:Zm00001d020122_T001 rna23723 0
925- 10: transcript:Zm00001d020133_T001 rna23732 3.00E-19
925- 11: transcript:Zm00001d020134_T003 rna23733 0
925- 12: transcript:Zm00001d020136_T001 rna23738 8.00E-78
925- 13: transcript:Zm00001d020138_T001 rna23740 0
925- 14: transcript:Zm00001d020139_T001 rna23747 0
925- 15: transcript:Zm00001d020147_T001 rna23748 0
Alignment 926: score=587.0 e_value=1.2e-35 N=13 7&NC_008402.2 plus
926- 0: transcript:Zm00001d020170_T001 rna23771 0
926- 1: transcript:Zm00001d020172_T001 rna23774 0
926- 2: transcript:Zm00001d020175_T002 rna23777 0
926- 3: transcript:Zm00001d020176_T001 rna23780 4.00E-173
926- 4: transcript:Zm00001d020178_T002 rna23781 0
926- 5: transcript:Zm00001d020180_T002 rna23783 4.00E-64
926- 6: transcript:Zm00001d020182_T002 rna23788 0
926- 7: transcript:Zm00001d020183_T002 rna23791 0
926- 8: transcript:Zm00001d020184_T001 rna23802 1.00E-58
926- 9: transcript:Zm00001d020185_T003 rna23809 0
926- 10: transcript:Zm00001d020187_T001 rna23821 4.00E-60
926- 11: transcript:Zm00001d020188_T001 rna23822 0
926- 12: transcript:Zm00001d020193_T001 rna23823 7.00E-30
Alignment 927: score=369.0 e_value=1.5e-14 N=8 7&NC_008402.2 plus
927- 0: transcript:Zm00001d020205_T001 rna23824 1.00E-117
927- 1: transcript:Zm00001d020206_T001 rna23825 0
927- 2: transcript:Zm00001d020208_T003 rna23826 1.00E-127
927- 3: transcript:Zm00001d020213_T003 rna23827 0
927- 4: transcript:Zm00001d020219_T004 rna23830 4.00E-163
927- 5: transcript:Zm00001d020220_T001 rna23831 9.00E-139
927- 6: transcript:Zm00001d020224_T002 rna23832 6.00E-41
927- 7: transcript:Zm00001d020225_T002 rna23833 0

Alignment 928: score=306.0 e_value=7.5e-15 N=7 7&NC_008402.2 plus

928- 0:	transcript:Zm00001d021181_T005	rna24626	0
928- 1:	transcript:Zm00001d021192_T001	rna24652	4.00E-18
928- 2:	transcript:Zm00001d021195_T002	rna24654	0
928- 3:	transcript:Zm00001d021196_T004	rna24655	0
928- 4:	transcript:Zm00001d021197_T001	rna24656	0
928- 5:	transcript:Zm00001d021200_T001	rna24657	0
928- 6:	transcript:Zm00001d021201_T001	rna24658	0

Alignment 929: score=2320.0 e_value=6e-202 N=51 7&NC_008402.2 minus

929- 0:	transcript:Zm00001d021371_T001	rna24906	3.00E-165
929- 1:	transcript:Zm00001d021372_T001	rna24904	9.00E-37
929- 2:	transcript:Zm00001d021375_T003	rna24902	3.00E-35
929- 3:	transcript:Zm00001d021378_T007	rna24900	0
929- 4:	transcript:Zm00001d021385_T001	rna24892	0
929- 5:	transcript:Zm00001d021386_T001	rna24891	5.00E-24
929- 6:	transcript:Zm00001d021388_T001	rna24888	0
929- 7:	transcript:Zm00001d021389_T001	rna24887	2.00E-148
929- 8:	transcript:Zm00001d021390_T001	rna24885	0
929- 9:	transcript:Zm00001d021391_T001	rna24884	1.00E-77
929- 10:	transcript:Zm00001d021392_T002	rna24883	0
929- 11:	transcript:Zm00001d021395_T004	rna24878	0
929- 12:	transcript:Zm00001d021396_T001	rna24875	8.00E-125
929- 13:	transcript:Zm00001d021400_T001	rna24872	0
929- 14:	transcript:Zm00001d021401_T001	rna24871	6.00E-25
929- 15:	transcript:Zm00001d021403_T001	rna24869	5.00E-176
929- 16:	transcript:Zm00001d021407_T001	rna24868	0
929- 17:	transcript:Zm00001d021410_T001	rna24867	0
929- 18:	transcript:Zm00001d021411_T001	rna24866	0
929- 19:	transcript:Zm00001d021412_T001	rna24865	0
929- 20:	transcript:Zm00001d021413_T001	rna24861	0
929- 21:	transcript:Zm00001d021414_T001	rna24860	3.00E-64
929- 22:	transcript:Zm00001d021416_T001	rna24858	0
929- 23:	transcript:Zm00001d021417_T001	rna24857	4.00E-40
929- 24:	transcript:Zm00001d021418_T001	rna24856	9.00E-107
929- 25:	transcript:Zm00001d021419_T001	rna24855	1.00E-117
929- 26:	transcript:Zm00001d021420_T003	rna24854	2.00E-83
929- 27:	transcript:Zm00001d021421_T003	rna24853	0
929- 28:	transcript:Zm00001d021422_T001	rna24852	2.00E-67
929- 29:	transcript:Zm00001d021423_T001	rna24851	3.00E-137
929- 30:	transcript:Zm00001d021430_T013	rna24844	0
929- 31:	transcript:Zm00001d021433_T002	rna24842	1.00E-70
929- 32:	transcript:Zm00001d021434_T001	rna24836	6.00E-124
929- 33:	transcript:Zm00001d021438_T003	rna24834	0
929- 34:	transcript:Zm00001d021439_T001	rna24833	3.00E-76
929- 35:	transcript:Zm00001d021440_T001	rna24832	3.00E-79
929- 36:	transcript:Zm00001d021441_T001	rna24831	2.00E-118
929- 37:	transcript:Zm00001d021442_T013	rna24830	1.00E-117
929- 38:	transcript:Zm00001d021443_T001	rna24826	1.00E-20
929- 39:	transcript:Zm00001d021446_T002	rna24825	3.00E-51
929- 40:	transcript:Zm00001d021447_T001	rna24824	0
929- 41:	transcript:Zm00001d021448_T002	rna24823	8.00E-06
929- 42:	transcript:Zm00001d021452_T001	rna24819	0
929- 43:	transcript:Zm00001d021453_T002	rna24818	0
929- 44:	transcript:Zm00001d021454_T001	rna24809	6.00E-52

929- 45: transcript:Zm00001d021456_T001 rna24803 7.00E-48
929- 46: transcript:Zm00001d021461_T002 rna24802 0
929- 47: transcript:Zm00001d021462_T001 rna24801 3.00E-80
929- 48: transcript:Zm00001d021464_T005 rna24800 0
929- 49: transcript:Zm00001d021469_T002 rna24799 9.00E-130
929- 50: transcript:Zm00001d021473_T002 rna24798 0
Alignment 930: score=1009.0 e_value=3.6e-61 N=21 7&NC_008402.2 minus
930- 0: transcript:Zm00001d022592_T001 rna24796 0
930- 1: transcript:Zm00001d022593_T001 rna24795 0
930- 2: transcript:Zm00001d022594_T002 rna24794 0
930- 3: transcript:Zm00001d022596_T001 rna24793 3.00E-48
930- 4: transcript:Zm00001d022597_T001 rna24790 2.00E-44
930- 5: transcript:Zm00001d022598_T001 rna24789 3.00E-168
930- 6: transcript:Zm00001d022600_T001 rna24787 0
930- 7: transcript:Zm00001d022602_T001 rna24783 6.00E-102
930- 8: transcript:Zm00001d022603_T002 rna24782 1.00E-54
930- 9: transcript:Zm00001d022604_T001 rna24781 2.00E-94
930- 10: transcript:Zm00001d022607_T001 rna24780 3.00E-44
930- 11: transcript:Zm00001d022608_T001 rna24779 5.00E-150
930- 12: transcript:Zm00001d022611_T001 rna24778 5.00E-98
930- 13: transcript:Zm00001d022613_T001 rna24777 3.00E-25
930- 14: transcript:Zm00001d022614_T002 rna24776 0
930- 15: transcript:Zm00001d022615_T001 rna24774 0
930- 16: transcript:Zm00001d022616_T001 rna24771 5.00E-32
930- 17: transcript:Zm00001d022617_T001 rna24770 2.00E-28
930- 18: transcript:Zm00001d022618_T001 rna24769 2.00E-30
930- 19: transcript:Zm00001d022619_T001 rna24768 0
930- 20: transcript:Zm00001d022621_T005 rna24766 0
Alignment 931: score=521.0 e_value=5e-27 N=12 7&NC_008402.2 minus
931- 0: transcript:Zm00001d019670_T021 rna23353 0
931- 1: transcript:Zm00001d019677_T001 rna23352 1.00E-31
931- 2: transcript:Zm00001d019696_T001 rna23344 3.00E-114
931- 3: transcript:Zm00001d019698_T001 rna23341 1.00E-64
931- 4: transcript:Zm00001d019701_T001 rna23339 0
931- 5: transcript:Zm00001d019703_T001 rna23338 0
931- 6: transcript:Zm00001d019704_T001 rna23337 3.00E-08
931- 7: transcript:Zm00001d019706_T002 rna23336 2.00E-178
931- 8: transcript:Zm00001d019712_T001 rna23331 2.00E-119
931- 9: transcript:Zm00001d019718_T001 rna23326 1.00E-90
931- 10: transcript:Zm00001d019719_T001 rna23324 5.00E-171
931- 11: transcript:Zm00001d019721_T001 rna23320 7.00E-67
Alignment 932: score=519.0 e_value=5.2e-25 N=11 7&NC_008402.2 minus
932- 0: transcript:Zm00001d022622_T005 rna24765 0
932- 1: transcript:Zm00001d022625_T006 rna24762 0
932- 2: transcript:Zm00001d022626_T001 rna24760 2.00E-68
932- 3: transcript:Zm00001d022627_T002 rna24758 0
932- 4: transcript:Zm00001d022628_T001 rna24756 4.00E-137
932- 5: transcript:Zm00001d022629_T001 rna24755 7.00E-167
932- 6: transcript:Zm00001d022630_T001 rna24752 4.00E-58
932- 7: transcript:Zm00001d022631_T002 rna24751 9.00E-136
932- 8: transcript:Zm00001d022636_T002 rna24745 0
932- 9: transcript:Zm00001d022637_T001 rna24743 4.00E-86
932- 10: transcript:Zm00001d022647_T001 rna24742 0
Alignment 933: score=428.0 e_value=8.8e-23 N=10 7&NC_008402.2 minus

933- 0: transcript:Zm00001d019725_T001 rna24996 3.00E-59
933- 1: transcript:Zm00001d019727_T001 rna24995 0
933- 2: transcript:Zm00001d019733_T007 rna24990 3.00E-113
933- 3: transcript:Zm00001d019734_T001 rna24987 7.00E-48
933- 4: transcript:Zm00001d019750_T001 rna24984 2.00E-103
933- 5: transcript:Zm00001d019753_T001 rna24983 1.00E-176
933- 6: transcript:Zm00001d019759_T001 rna24980 0
933- 7: transcript:Zm00001d019765_T001 rna24976 9.00E-162
933- 8: transcript:Zm00001d019775_T001 rna24966 5.00E-39
933- 9: transcript:Zm00001d019789_T002 rna24949 0
Alignment 934: score=371.0 e_value=5.1e-14 N=8 7&NC_008402.2 minus
934- 0: transcript:Zm00001d021173_T001 rna24632 0
934- 1: transcript:Zm00001d021176_T001 rna24630 7.00E-145
934- 2: transcript:Zm00001d021177_T001 rna24629 9.00E-20
934- 3: transcript:Zm00001d021180_T005 rna24628 0
934- 4: transcript:Zm00001d021181_T005 rna24626 0
934- 5: transcript:Zm00001d021187_T002 rna24625 0
934- 6: transcript:Zm00001d021188_T006 rna24623 0
934- 7: transcript:Zm00001d021189_T001 rna24622 5.00E-160
Alignment 935: score=340.0 e_value=4.3e-19 N=8 7&NC_008402.2 minus
935- 0: transcript:Zm00001d019922_T003 rna23556 0
935- 1: transcript:Zm00001d019925_T001 rna23553 2.00E-19
935- 2: transcript:Zm00001d019926_T001 rna23552 0
935- 3: transcript:Zm00001d019933_T001 rna23538 1.00E-06
935- 4: transcript:Zm00001d019934_T001 rna23536 0
935- 5: transcript:Zm00001d019936_T001 rna23533 2.00E-48
935- 6: transcript:Zm00001d019944_T004 rna23518 0
935- 7: transcript:Zm00001d019947_T025 rna23517 0
Alignment 936: score=3194.0 e_value=1.1e-287 N=71 8&NC_008394.4 plus
936- 0: transcript:Zm00001d011610_T063 rna2378 0
936- 1: transcript:Zm00001d011611_T005 rna2383 0
936- 2: transcript:Zm00001d011615_T004 rna2401 0
936- 3: transcript:Zm00001d011616_T001 rna2409 5.00E-54
936- 4: transcript:Zm00001d011619_T002 rna2419 0
936- 5: transcript:Zm00001d011620_T001 rna2423 5.00E-49
936- 6: transcript:Zm00001d011622_T004 rna2424 3.00E-50
936- 7: transcript:Zm00001d011623_T001 rna2425 5.00E-22
936- 8: transcript:Zm00001d011624_T001 rna2426 0
936- 9: transcript:Zm00001d011625_T001 rna2427 0
936- 10: transcript:Zm00001d011628_T001 rna2429 0
936- 11: transcript:Zm00001d011630_T001 rna2433 6.00E-11
936- 12: transcript:Zm00001d011631_T001 rna2436 6.00E-88
936- 13: transcript:Zm00001d011632_T002 rna2437 3.00E-178
936- 14: transcript:Zm00001d011634_T002 rna2439 0
936- 15: transcript:Zm00001d011636_T001 rna2446 2.00E-119
936- 16: transcript:Zm00001d011638_T002 rna2447 0
936- 17: transcript:Zm00001d011639_T001 rna2448 7.00E-178
936- 18: transcript:Zm00001d011641_T001 rna2452 0
936- 19: transcript:Zm00001d011642_T002 rna2461 0
936- 20: transcript:Zm00001d011644_T002 rna2465 0
936- 21: transcript:Zm00001d011645_T001 rna2467 6.00E-78
936- 22: transcript:Zm00001d011649_T001 rna2473 0
936- 23: transcript:Zm00001d011650_T001 rna2475 2.00E-30
936- 24: transcript:Zm00001d011652_T001 rna2476 0

```
936- 25: transcript:Zm00001d011654_T001 rna2482     5.00E-141
936- 26: transcript:Zm00001d011657_T002 rna2486      2.00E-90
936- 27: transcript:Zm00001d011658_T001 rna2487      6.00E-56
936- 28: transcript:Zm00001d011660_T003 rna2489             0
936- 29: transcript:Zm00001d011663_T003 rna2496             0
936- 30: transcript:Zm00001d011668_T001 rna2500             0
936- 31: transcript:Zm00001d011669_T001 rna2502      6.00E-97
936- 32: transcript:Zm00001d011673_T001 rna2506             0
936- 33: transcript:Zm00001d011676_T002 rna2508             0
936- 34: transcript:Zm00001d011678_T002 rna2509      3.00E-72
936- 35: transcript:Zm00001d011679_T001 rna2510             0
936- 36: transcript:Zm00001d011681_T001 rna2516      8.00E-26
936- 37: transcript:Zm00001d011684_T002 rna2524             0
936- 38: transcript:Zm00001d011685_T002 rna2527             0
936- 39: transcript:Zm00001d011687_T001 rna2530             0
936- 40: transcript:Zm00001d011688_T004 rna2531     1.00E-150
936- 41: transcript:Zm00001d011691_T002 rna2533     1.00E-108
936- 42: transcript:Zm00001d011692_T001 rna2534             0
936- 43: transcript:Zm00001d011696_T002 rna2537      3.00E-22
936- 44: transcript:Zm00001d011697_T001 rna2541             0
936- 45: transcript:Zm00001d011699_T002 rna2544             0
936- 46: transcript:Zm00001d011700_T014 rna2545             0
936- 47: transcript:Zm00001d011705_T002 rna2546             0
936- 48: transcript:Zm00001d011706_T003 rna2553             0
936- 49: transcript:Zm00001d011707_T001 rna2554             0
936- 50: transcript:Zm00001d011708_T001 rna2557     7.00E-140
936- 51: transcript:Zm00001d011709_T003 rna2560             0
936- 52: transcript:Zm00001d011710_T001 rna2561     5.00E-150
936- 53: transcript:Zm00001d011712_T001 rna2563     6.00E-116
936- 54: transcript:Zm00001d011713_T003 rna2565      5.00E-81
936- 55: transcript:Zm00001d011716_T001 rna2567             0
936- 56: transcript:Zm00001d011717_T001 rna2570     1.00E-125
936- 57: transcript:Zm00001d011718_T002 rna2571      3.00E-99
936- 58: transcript:Zm00001d011719_T008 rna2580      8.00E-62
936- 59: transcript:Zm00001d011720_T002 rna2581             0
936- 60: transcript:Zm00001d011721_T001 rna2590             0
936- 61: transcript:Zm00001d011722_T001 rna2591      3.00E-10
936- 62: transcript:Zm00001d011730_T001 rna2592      1.00E-87
936- 63: transcript:Zm00001d011731_T001 rna2599      3.00E-64
936- 64: transcript:Zm00001d011734_T001 rna2603     3.00E-134
936- 65: transcript:Zm00001d011735_T001 rna2606             0
936- 66: transcript:Zm00001d011736_T001 rna2607     2.00E-110
936- 67: transcript:Zm00001d011738_T002 rna2616     1.00E-167
936- 68: transcript:Zm00001d011739_T001 rna2618      4.00E-87
936- 69: transcript:Zm00001d011740_T001 rna2623             0
936- 70: transcript:Zm00001d011741_T002 rna2624      9.00E-87
## Alignment 937: score=3067.0 e_value=4.2e-277 N=67 8&NC_008394.4 plus
937-  0: transcript:Zm00001d008512_T002 rna168              0
937-  1: transcript:Zm00001d008513_T001 rna169              0
937-  2: transcript:Zm00001d008514_T001 rna170       2.00E-85
937-  3: transcript:Zm00001d008515_T001 rna184       2.00E-92
937-  4: transcript:Zm00001d008516_T001 rna185      1.00E-173
937-  5: transcript:Zm00001d008517_T001 rna186              0
937-  6: transcript:Zm00001d008520_T001 rna187              0
```

937-	7:	transcript:Zm00001d008524_T001	rna202	1.00E-81
937-	8:	transcript:Zm00001d008528_T001	rna203	9.00E-131
937-	9:	transcript:Zm00001d008529_T001	rna204	3.00E-103
937-	10:	transcript:Zm00001d008530_T001	rna207	8.00E-92
937-	11:	transcript:Zm00001d008531_T001	rna208	0
937-	12:	transcript:Zm00001d008532_T001	rna211	2.00E-42
937-	13:	transcript:Zm00001d008535_T001	rna212	0
937-	14:	transcript:Zm00001d008539_T001	rna220	2.00E-28
937-	15:	transcript:Zm00001d008540_T001	rna221	6.00E-36
937-	16:	transcript:Zm00001d008546_T001	rna222	6.00E-87
937-	17:	transcript:Zm00001d008548_T001	rna224	2.00E-38
937-	18:	transcript:Zm00001d008552_T001	rna231	2.00E-33
937-	19:	transcript:Zm00001d008562_T001	rna233	1.00E-81
937-	20:	transcript:Zm00001d008565_T001	rna235	2.00E-148
937-	21:	transcript:Zm00001d008567_T008	rna237	0
937-	22:	transcript:Zm00001d008568_T001	rna239	5.00E-151
937-	23:	transcript:Zm00001d008569_T001	rna243	1.00E-167
937-	24:	transcript:Zm00001d008570_T001	rna244	4.00E-172
937-	25:	transcript:Zm00001d008573_T001	rna249	1.00E-76
937-	26:	transcript:Zm00001d008577_T001	rna250	2.00E-51
937-	27:	transcript:Zm00001d008579_T001	rna255	3.00E-21
937-	28:	transcript:Zm00001d008581_T001	rna256	0
937-	29:	transcript:Zm00001d008582_T001	rna258	2.00E-16
937-	30:	transcript:Zm00001d008585_T001	rna263	6.00E-161
937-	31:	transcript:Zm00001d008587_T001	rna275	0
937-	32:	transcript:Zm00001d008588_T001	rna276	0
937-	33:	transcript:Zm00001d008590_T001	rna277	3.00E-30
937-	34:	transcript:Zm00001d008591_T001	rna278	0
937-	35:	transcript:Zm00001d008592_T001	rna279	2.00E-178
937-	36:	transcript:Zm00001d008594_T001	rna284	1.00E-38
937-	37:	transcript:Zm00001d008596_T005	rna286	0
937-	38:	transcript:Zm00001d008597_T001	rna288	0
937-	39:	transcript:Zm00001d008598_T001	rna290	2.00E-94
937-	40:	transcript:Zm00001d008600_T001	rna293	0
937-	41:	transcript:Zm00001d008601_T001	rna295	6.00E-55
937-	42:	transcript:Zm00001d008604_T011	rna297	0
937-	43:	transcript:Zm00001d008610_T001	rna299	2.00E-47
937-	44:	transcript:Zm00001d008611_T003	rna305	3.00E-173
937-	45:	transcript:Zm00001d008612_T002	rna306	5.00E-99
937-	46:	transcript:Zm00001d008613_T001	rna307	1.00E-133
937-	47:	transcript:Zm00001d008617_T001	rna323	0
937-	48:	transcript:Zm00001d008618_T005	rna325	2.00E-65
937-	49:	transcript:Zm00001d008619_T001	rna329	3.00E-162
937-	50:	transcript:Zm00001d008620_T001	rna336	2.00E-20
937-	51:	transcript:Zm00001d008621_T001	rna343	0
937-	52:	transcript:Zm00001d008622_T002	rna344	0
937-	53:	transcript:Zm00001d008623_T001	rna346	6.00E-90
937-	54:	transcript:Zm00001d008624_T001	rna351	0
937-	55:	transcript:Zm00001d008625_T001	rna352	0
937-	56:	transcript:Zm00001d008628_T001	rna353	1.00E-166
937-	57:	transcript:Zm00001d008631_T001	rna357	0
937-	58:	transcript:Zm00001d008632_T001	rna358	0
937-	59:	transcript:Zm00001d008634_T001	rna373	9.00E-131
937-	60:	transcript:Zm00001d008640_T002	rna391	0

937- 61: transcript:Zm00001d008641_T001 rna392 2.00E-130
937- 62: transcript:Zm00001d008642_T002 rna393 0
937- 63: transcript:Zm00001d008645_T002 rna394 3.00E-47
937- 64: transcript:Zm00001d008648_T003 rna397 0
937- 65: transcript:Zm00001d008649_T003 rna400 2.00E-168
937- 66: transcript:Zm00001d008651_T001 rna405 0
Alignment 938: score=2892.0 e_value=5.2e-258 N=63 8&NC_008394.4 plus
938- 0: transcript:Zm00001d008843_T001 rna621 6.00E-51
938- 1: transcript:Zm00001d008850_T001 rna625 8.00E-06
938- 2: transcript:Zm00001d008851_T002 rna626 2.00E-111
938- 3: transcript:Zm00001d008853_T001 rna627 9.00E-56
938- 4: transcript:Zm00001d008858_T001 rna635 8.00E-15
938- 5: transcript:Zm00001d008859_T001 rna637 2.00E-153
938- 6: transcript:Zm00001d008862_T001 rna638 1.00E-33
938- 7: transcript:Zm00001d008863_T004 rna642 0
938- 8: transcript:Zm00001d008866_T001 rna643 5.00E-56
938- 9: transcript:Zm00001d008869_T001 rna651 0
938- 10: transcript:Zm00001d008870_T009 rna652 1.00E-149
938- 11: transcript:Zm00001d008871_T001 rna653 1.00E-172
938- 12: transcript:Zm00001d008872_T001 rna654 7.00E-58
938- 13: transcript:Zm00001d008874_T002 rna656 1.00E-59
938- 14: transcript:Zm00001d008875_T001 rna658 0
938- 15: transcript:Zm00001d008881_T001 rna659 7.00E-37
938- 16: transcript:Zm00001d008882_T001 rna661 4.00E-65
938- 17: transcript:Zm00001d008890_T001 rna672 0
938- 18: transcript:Zm00001d008892_T001 rna679 5.00E-86
938- 19: transcript:Zm00001d008893_T001 rna680 6.00E-164
938- 20: transcript:Zm00001d008898_T001 rna681 8.00E-154
938- 21: transcript:Zm00001d008899_T001 rna683 0
938- 22: transcript:Zm00001d008900_T001 rna684 1.00E-16
938- 23: transcript:Zm00001d008903_T001 rna687 0
938- 24: transcript:Zm00001d008906_T003 rna697 0
938- 25: transcript:Zm00001d008907_T001 rna703 2.00E-166
938- 26: transcript:Zm00001d008909_T002 rna705 2.00E-136
938- 27: transcript:Zm00001d008910_T001 rna710 3.00E-48
938- 28: transcript:Zm00001d008911_T001 rna720 6.00E-33
938- 29: transcript:Zm00001d008912_T001 rna721 6.00E-15
938- 30: transcript:Zm00001d008914_T001 rna722 3.00E-18
938- 31: transcript:Zm00001d008919_T001 rna726 4.00E-162
938- 32: transcript:Zm00001d008922_T001 rna728 4.00E-26
938- 33: transcript:Zm00001d008923_T001 rna730 3.00E-16
938- 34: transcript:Zm00001d008925_T002 rna732 0
938- 35: transcript:Zm00001d008927_T001 rna733 2.00E-59
938- 36: transcript:Zm00001d008930_T003 rna749 0
938- 37: transcript:Zm00001d008939_T001 rna750 8.00E-118
938- 38: transcript:Zm00001d008940_T002 rna751 0
938- 39: transcript:Zm00001d008941_T002 rna752 0
938- 40: transcript:Zm00001d008944_T002 rna755 1.00E-14
938- 41: transcript:Zm00001d008945_T001 rna756 5.00E-33
938- 42: transcript:Zm00001d008946_T001 rna757 2.00E-35
938- 43: transcript:Zm00001d008950_T001 rna758 4.00E-46
938- 44: transcript:Zm00001d008952_T001 rna759 0
938- 45: transcript:Zm00001d008954_T002 rna763 5.00E-82
938- 46: transcript:Zm00001d008956_T001 rna766 2.00E-57

938- 47: transcript:Zm00001d008957_T001 rna768 0
938- 48: transcript:Zm00001d008961_T001 rna770 3.00E-31
938- 49: transcript:Zm00001d008962_T001 rna771 7.00E-38
938- 50: transcript:Zm00001d008965_T001 rna787 0
938- 51: transcript:Zm00001d008966_T001 rna788 0
938- 52: transcript:Zm00001d008968_T001 rna791 1.00E-100
938- 53: transcript:Zm00001d008970_T001 rna792 2.00E-81
938- 54: transcript:Zm00001d008971_T001 rna795 8.00E-21
938- 55: transcript:Zm00001d008974_T001 rna797 5.00E-157
938- 56: transcript:Zm00001d008976_T001 rna801 2.00E-67
938- 57: transcript:Zm00001d008980_T001 rna805 1.00E-104
938- 58: transcript:Zm00001d008982_T001 rna806 4.00E-120
938- 59: transcript:Zm00001d008983_T001 rna807 0
938- 60: transcript:Zm00001d008984_T001 rna808 2.00E-173
938- 61: transcript:Zm00001d008985_T001 rna814 3.00E-64
938- 62: transcript:Zm00001d008992_T001 rna819 2.00E-136
Alignment 939: score=1713.0 e_value=2.9e-136 N=38 8&NC_008394.4 plus
939- 0: transcript:Zm00001d008656_T002 rna420 2.00E-125
939- 1: transcript:Zm00001d008662_T001 rna423 4.00E-39
939- 2: transcript:Zm00001d008665_T001 rna426 2.00E-117
939- 3: transcript:Zm00001d008666_T003 rna428 0
939- 4: transcript:Zm00001d008676_T001 rna434 1.00E-37
939- 5: transcript:Zm00001d008679_T010 rna440 0
939- 6: transcript:Zm00001d008689_T003 rna448 5.00E-166
939- 7: transcript:Zm00001d008690_T001 rna449 0
939- 8: transcript:Zm00001d008691_T001 rna450 0
939- 9: transcript:Zm00001d008692_T001 rna451 0
939- 10: transcript:Zm00001d008693_T002 rna453 3.00E-141
939- 11: transcript:Zm00001d008700_T002 rna456 4.00E-119
939- 12: transcript:Zm00001d008708_T001 rna457 0
939- 13: transcript:Zm00001d008715_T001 rna460 0
939- 14: transcript:Zm00001d008716_T002 rna462 0
939- 15: transcript:Zm00001d008721_T001 rna463 2.00E-91
939- 16: transcript:Zm00001d008722_T001 rna466 3.00E-13
939- 17: transcript:Zm00001d008724_T005 rna467 1.00E-83
939- 18: transcript:Zm00001d008725_T004 rna472 0
939- 19: transcript:Zm00001d008726_T001 rna473 2.00E-50
939- 20: transcript:Zm00001d008727_T006 rna474 0
939- 21: transcript:Zm00001d008730_T001 rna477 4.00E-62
939- 22: transcript:Zm00001d008731_T001 rna479 8.00E-13
939- 23: transcript:Zm00001d008733_T001 rna481 0
939- 24: transcript:Zm00001d008734_T001 rna482 3.00E-68
939- 25: transcript:Zm00001d008735_T001 rna488 3.00E-26
939- 26: transcript:Zm00001d008736_T001 rna490 1.00E-90
939- 27: transcript:Zm00001d008737_T001 rna491 4.00E-73
939- 28: transcript:Zm00001d008738_T001 rna502 6.00E-170
939- 29: transcript:Zm00001d008742_T010 rna507 0
939- 30: transcript:Zm00001d008743_T004 rna508 0
939- 31: transcript:Zm00001d008744_T007 rna511 0
939- 32: transcript:Zm00001d008748_T001 rna512 0
939- 33: transcript:Zm00001d008749_T002 rna516 6.00E-100
939- 34: transcript:Zm00001d008750_T001 rna517 9.00E-165
939- 35: transcript:Zm00001d008756_T001 rna520 0
939- 36: transcript:Zm00001d008758_T001 rna521 0

939- 37: transcript:Zm00001d008759_T002 rna522 3.00E-78
Alignment 940: score=1663.0 e_value=2.3e-125 N=36 8&NC_008394.4 plus
940- 0: transcript:Zm00001d011486_T003 rna2224 0
940- 1: transcript:Zm00001d011487_T001 rna2228 0
940- 2: transcript:Zm00001d011490_T003 rna2230 2.00E-27
940- 3: transcript:Zm00001d011492_T001 rna2231 0
940- 4: transcript:Zm00001d011495_T001 rna2234 2.00E-148
940- 5: transcript:Zm00001d011496_T002 rna2235 6.00E-89
940- 6: transcript:Zm00001d011497_T001 rna2238 6.00E-27
940- 7: transcript:Zm00001d011498_T001 rna2239 0
940- 8: transcript:Zm00001d011499_T001 rna2240 9.00E-122
940- 9: transcript:Zm00001d011500_T014 rna2242 8.00E-78
940- 10: transcript:Zm00001d011504_T003 rna2246 4.00E-149
940- 11: transcript:Zm00001d011506_T001 rna2248 2.00E-53
940- 12: transcript:Zm00001d011507_T001 rna2257 5.00E-92
940- 13: transcript:Zm00001d011510_T001 rna2258 3.00E-152
940- 14: transcript:Zm00001d011511_T001 rna2260 1.00E-19
940- 15: transcript:Zm00001d011512_T004 rna2261 8.00E-103
940- 16: transcript:Zm00001d011513_T001 rna2267 1.00E-61
940- 17: transcript:Zm00001d011514_T001 rna2268 3.00E-77
940- 18: transcript:Zm00001d011517_T005 rna2274 0
940- 19: transcript:Zm00001d011518_T001 rna2275 0
940- 20: transcript:Zm00001d011519_T003 rna2276 0
940- 21: transcript:Zm00001d011520_T001 rna2277 5.00E-147
940- 22: transcript:Zm00001d011525_T002 rna2279 0
940- 23: transcript:Zm00001d011526_T002 rna2281 0
940- 24: transcript:Zm00001d011527_T001 rna2282 3.00E-57
940- 25: transcript:Zm00001d011528_T001 rna2285 2.00E-28
940- 26: transcript:Zm00001d011531_T001 rna2287 1.00E-139
940- 27: transcript:Zm00001d011532_T002 rna2291 8.00E-160
940- 28: transcript:Zm00001d011534_T004 rna2292 2.00E-125
940- 29: transcript:Zm00001d011536_T001 rna2293 1.00E-89
940- 30: transcript:Zm00001d011540_T001 rna2297 1.00E-24
940- 31: transcript:Zm00001d011541_T004 rna2298 0
940- 32: transcript:Zm00001d011543_T001 rna2300 7.00E-62
940- 33: transcript:Zm00001d011550_T001 rna2305 0
940- 34: transcript:Zm00001d011555_T001 rna2315 5.00E-67
940- 35: transcript:Zm00001d011560_T012 rna2316 2.00E-118
Alignment 941: score=1655.0 e_value=6.9e-130 N=37 8&NC_008394.4 plus
941- 0: transcript:Zm00001d011238_T001 rna1877 2.00E-90
941- 1: transcript:Zm00001d011239_T001 rna1878 0
941- 2: transcript:Zm00001d011241_T001 rna1880 6.00E-26
941- 3: transcript:Zm00001d011242_T002 rna1882 1.00E-178
941- 4: transcript:Zm00001d011243_T001 rna1885 3.00E-34
941- 5: transcript:Zm00001d011245_T001 rna1887 0
941- 6: transcript:Zm00001d011246_T001 rna1888 2.00E-137
941- 7: transcript:Zm00001d011252_T001 rna1892 2.00E-122
941- 8: transcript:Zm00001d011255_T001 rna1895 9.00E-102
941- 9: transcript:Zm00001d011256_T001 rna1896 4.00E-82
941- 10: transcript:Zm00001d011258_T001 rna1901 0
941- 11: transcript:Zm00001d011259_T001 rna1902 0
941- 12: transcript:Zm00001d011260_T001 rna1905 3.00E-11
941- 13: transcript:Zm00001d011263_T001 rna1911 7.00E-156
941- 14: transcript:Zm00001d011266_T002 rna1912 5.00E-96

941- 15: transcript:Zm00001d011268_T001 rna1915 1.00E-24
941- 16: transcript:Zm00001d011270_T001 rna1917 1.00E-13
941- 17: transcript:Zm00001d011272_T007 rna1918 1.00E-52
941- 18: transcript:Zm00001d011275_T001 rna1920 7.00E-74
941- 19: transcript:Zm00001d011276_T001 rna1935 1.00E-75
941- 20: transcript:Zm00001d011277_T001 rna1938 0
941- 21: transcript:Zm00001d011278_T001 rna1939 2.00E-77
941- 22: transcript:Zm00001d011282_T001 rna1940 6.00E-06
941- 23: transcript:Zm00001d011283_T002 rna1941 1.00E-148
941- 24: transcript:Zm00001d011285_T001 rna1944 1.00E-180
941- 25: transcript:Zm00001d011288_T006 rna1953 1.00E-12
941- 26: transcript:Zm00001d011297_T001 rna1957 3.00E-100
941- 27: transcript:Zm00001d011298_T001 rna1975 1.00E-172
941- 28: transcript:Zm00001d011299_T001 rna1976 8.00E-134
941- 29: transcript:Zm00001d011302_T001 rna1980 4.00E-142
941- 30: transcript:Zm00001d011303_T001 rna1984 4.00E-06
941- 31: transcript:Zm00001d011304_T001 rna1985 0
941- 32: transcript:Zm00001d011308_T001 rna1986 7.00E-71
941- 33: transcript:Zm00001d011309_T009 rna1987 7.00E-178
941- 34: transcript:Zm00001d011311_T001 rna1989 4.00E-169
941- 35: transcript:Zm00001d011312_T001 rna1990 3.00E-90
941- 36: transcript:Zm00001d011315_T002 rna1994 0
Alignment 942: score=1376.0 e_value=2.8e-99 N=30 8&NC_008394.4 plus
942- 0: transcript:Zm00001d008430_T006 rna69 8.00E-54
942- 1: transcript:Zm00001d008432_T003 rna73 0
942- 2: transcript:Zm00001d008433_T007 rna74 0
942- 3: transcript:Zm00001d008439_T001 rna78 0
942- 4: transcript:Zm00001d008443_T002 rna81 0
942- 5: transcript:Zm00001d008444_T001 rna82 1.00E-55
942- 6: transcript:Zm00001d008446_T006 rna83 5.00E-91
942- 7: transcript:Zm00001d008449_T001 rna84 8.00E-39
942- 8: transcript:Zm00001d008451_T001 rna85 9.00E-24
942- 9: transcript:Zm00001d008453_T001 rna87 4.00E-07
942- 10: transcript:Zm00001d008454_T001 rna88 1.00E-170
942- 11: transcript:Zm00001d008457_T001 rna97 0
942- 12: transcript:Zm00001d008462_T002 rna98 0
942- 13: transcript:Zm00001d008466_T001 rna100 2.00E-172
942- 14: transcript:Zm00001d008477_T003 rna106 2.00E-40
942- 15: transcript:Zm00001d008478_T001 rna114 1.00E-79
942- 16: transcript:Zm00001d008479_T001 rna116 2.00E-109
942- 17: transcript:Zm00001d008485_T001 rna121 0
942- 18: transcript:Zm00001d008487_T001 rna126 2.00E-88
942- 19: transcript:Zm00001d008491_T001 rna146 0
942- 20: transcript:Zm00001d008493_T001 rna148 5.00E-163
942- 21: transcript:Zm00001d008494_T001 rna151 2.00E-130
942- 22: transcript:Zm00001d008495_T001 rna153 0
942- 23: transcript:Zm00001d008497_T002 rna156 7.00E-80
942- 24: transcript:Zm00001d008498_T001 rna157 1.00E-132
942- 25: transcript:Zm00001d008499_T001 rna158 8.00E-132
942- 26: transcript:Zm00001d008500_T001 rna160 9.00E-48
942- 27: transcript:Zm00001d008501_T001 rna162 1.00E-63
942- 28: transcript:Zm00001d008502_T001 rna163 1.00E-140
942- 29: transcript:Zm00001d008503_T002 rna164 7.00E-154
Alignment 943: score=1279.0 e_value=8.4e-93 N=28 8&NC_008394.4 plus

943- 0: transcript:Zm00001d011428_T003 rna2113 2.00E-137
943- 1: transcript:Zm00001d011430_T001 rna2114 3.00E-144
943- 2: transcript:Zm00001d011431_T001 rna2115 1.00E-78
943- 3: transcript:Zm00001d011433_T002 rna2116 0
943- 4: transcript:Zm00001d011434_T001 rna2117 6.00E-30
943- 5: transcript:Zm00001d011435_T001 rna2120 9.00E-13
943- 6: transcript:Zm00001d011442_T001 rna2133 6.00E-172
943- 7: transcript:Zm00001d011444_T002 rna2140 0
943- 8: transcript:Zm00001d011445_T009 rna2141 1.00E-75
943- 9: transcript:Zm00001d011446_T001 rna2149 0
943- 10: transcript:Zm00001d011451_T001 rna2150 3.00E-64
943- 11: transcript:Zm00001d011454_T001 rna2151 8.00E-127
943- 12: transcript:Zm00001d011455_T001 rna2156 1.00E-19
943- 13: transcript:Zm00001d011457_T001 rna2157 0
943- 14: transcript:Zm00001d011458_T001 rna2158 0
943- 15: transcript:Zm00001d011459_T001 rna2163 0
943- 16: transcript:Zm00001d011461_T001 rna2166 2.00E-15
943- 17: transcript:Zm00001d011463_T001 rna2167 2.00E-135
943- 18: transcript:Zm00001d011465_T001 rna2171 0
943- 19: transcript:Zm00001d011467_T001 rna2173 2.00E-23
943- 20: transcript:Zm00001d011468_T001 rna2176 4.00E-19
943- 21: transcript:Zm00001d011470_T001 rna2178 2.00E-110
943- 22: transcript:Zm00001d011471_T001 rna2188 0
943- 23: transcript:Zm00001d011473_T003 rna2192 0
943- 24: transcript:Zm00001d011475_T001 rna2200 2.00E-93
943- 25: transcript:Zm00001d011476_T001 rna2207 0
943- 26: transcript:Zm00001d011477_T001 rna2208 2.00E-139
943- 27: transcript:Zm00001d011483_T004 rna2223 0
Alignment 944: score=1223.0 e_value=5.1e-93 N=29 8&NC_008394.4 plus
944- 0: transcript:Zm00001d010159_T002 rna3523 2.00E-97
944- 1: transcript:Zm00001d010171_T002 rna3527 3.00E-131
944- 2: transcript:Zm00001d010173_T003 rna3536 0
944- 3: transcript:Zm00001d010174_T004 rna3537 5.00E-145
944- 4: transcript:Zm00001d010175_T002 rna3538 8.00E-45
944- 5: transcript:Zm00001d010179_T001 rna3541 0
944- 6: transcript:Zm00001d010190_T002 rna3556 0
944- 7: transcript:Zm00001d010191_T001 rna3557 4.00E-39
944- 8: transcript:Zm00001d010195_T001 rna3559 0
944- 9: transcript:Zm00001d010197_T001 rna3572 5.00E-51
944- 10: transcript:Zm00001d010198_T001 rna3574 0
944- 11: transcript:Zm00001d010199_T002 rna3576 7.00E-51
944- 12: transcript:Zm00001d010200_T001 rna3577 0
944- 13: transcript:Zm00001d010201_T001 rna3578 4.00E-77
944- 14: transcript:Zm00001d010205_T002 rna3579 0
944- 15: transcript:Zm00001d010211_T002 rna3583 5.00E-86
944- 16: transcript:Zm00001d010213_T001 rna3592 5.00E-51
944- 17: transcript:Zm00001d010217_T001 rna3600 5.00E-16
944- 18: transcript:Zm00001d010222_T001 rna3619 0
944- 19: transcript:Zm00001d010228_T001 rna3624 6.00E-144
944- 20: transcript:Zm00001d010230_T001 rna3636 0
944- 21: transcript:Zm00001d010231_T001 rna3639 0
944- 22: transcript:Zm00001d010234_T001 rna3640 0
944- 23: transcript:Zm00001d010235_T022 rna3650 0
944- 24: transcript:Zm00001d010236_T001 rna3655 4.00E-56

944- 25: transcript:Zm00001d010241_T002 rna3672 5.00E-83
944- 26: transcript:Zm00001d010249_T001 rna3676 0
944- 27: transcript:Zm00001d010256_T001 rna3679 3.00E-14
944- 28: transcript:Zm00001d010264_T001 rna3682 2.00E-83
Alignment 945: score=1039.0 e_value=5.4e-74 N=23 8&NC_008394.4 plus
945- 0: transcript:Zm00001d008993_T030 rna823 0
945- 1: transcript:Zm00001d008996_T001 rna825 0
945- 2: transcript:Zm00001d008997_T002 rna826 0
945- 3: transcript:Zm00001d008999_T001 rna829 3.00E-72
945- 4: transcript:Zm00001d009004_T002 rna843 0
945- 5: transcript:Zm00001d009005_T002 rna844 0
945- 6: transcript:Zm00001d009007_T001 rna846 2.00E-119
945- 7: transcript:Zm00001d009009_T001 rna849 6.00E-25
945- 8: transcript:Zm00001d009010_T003 rna850 7.00E-62
945- 9: transcript:Zm00001d009011_T001 rna851 1.00E-117
945- 10: transcript:Zm00001d009013_T001 rna870 0
945- 11: transcript:Zm00001d009014_T004 rna871 0
945- 12: transcript:Zm00001d009017_T002 rna876 0
945- 13: transcript:Zm00001d009020_T001 rna879 2.00E-64
945- 14: transcript:Zm00001d009022_T001 rna881 2.00E-32
945- 15: transcript:Zm00001d009024_T001 rna883 8.00E-54
945- 16: transcript:Zm00001d009025_T001 rna887 0
945- 17: transcript:Zm00001d009027_T004 rna891 0
945- 18: transcript:Zm00001d009028_T001 rna892 0
945- 19: transcript:Zm00001d009029_T001 rna894 0
945- 20: transcript:Zm00001d009030_T002 rna904 2.00E-163
945- 21: transcript:Zm00001d009031_T001 rna928 1.00E-51
945- 22: transcript:Zm00001d009032_T001 rna934 1.00E-39
Alignment 946: score=813.0 e_value=2.1e-53 N=18 8&NC_008394.4 plus
946- 0: transcript:Zm00001d008386_T002 rna3 0
946- 1: transcript:Zm00001d008387_T002 rna5 5.00E-132
946- 2: transcript:Zm00001d008388_T002 rna6 4.00E-179
946- 3: transcript:Zm00001d008389_T003 rna7 0
946- 4: transcript:Zm00001d008390_T001 rna10 0
946- 5: transcript:Zm00001d008392_T001 rna11 7.00E-45
946- 6: transcript:Zm00001d008393_T001 rna12 1.00E-146
946- 7: transcript:Zm00001d008394_T001 rna14 1.00E-63
946- 8: transcript:Zm00001d008395_T001 rna27 4.00E-127
946- 9: transcript:Zm00001d008398_T001 rna30 0
946- 10: transcript:Zm00001d008399_T001 rna33 1.00E-74
946- 11: transcript:Zm00001d008403_T001 rna35 3.00E-55
946- 12: transcript:Zm00001d008404_T001 rna42 3.00E-45
946- 13: transcript:Zm00001d008405_T001 rna43 6.00E-74
946- 14: transcript:Zm00001d008406_T002 rna44 0
946- 15: transcript:Zm00001d008407_T001 rna45 1.00E-119
946- 16: transcript:Zm00001d008408_T001 rna49 3.00E-23
946- 17: transcript:Zm00001d008417_T003 rna66 2.00E-91
Alignment 947: score=804.0 e_value=7.8e-44 N=17 8&NC_008394.4 plus
947- 0: transcript:Zm00001d011745_T001 rna2626 4.00E-17
947- 1: transcript:Zm00001d011746_T002 rna2637 0
947- 2: transcript:Zm00001d011747_T001 rna2638 6.00E-68
947- 3: transcript:Zm00001d011748_T001 rna2641 2.00E-110
947- 4: transcript:Zm00001d011750_T004 rna2645 3.00E-149
947- 5: transcript:Zm00001d011751_T001 rna2646 3.00E-18

947- 6: transcript:Zm00001d011754_T001 rna2653 7.00E-78
947- 7: transcript:Zm00001d011755_T001 rna2657 2.00E-158
947- 8: transcript:Zm00001d011756_T001 rna2660 2.00E-166
947- 9: transcript:Zm00001d011757_T001 rna2662 0
947- 10: transcript:Zm00001d011758_T001 rna2666 0
947- 11: transcript:Zm00001d011759_T001 rna2669 0
947- 12: transcript:Zm00001d011760_T001 rna2671 0
947- 13: transcript:Zm00001d011761_T001 rna2674 4.00E-110
947- 14: transcript:Zm00001d011762_T001 rna2675 0
947- 15: transcript:Zm00001d011763_T005 rna2678 2.00E-56
947- 16: transcript:Zm00001d011764_T001 rna2683 1.00E-142
Alignment 948: score=764.0 e_value=2.4e-45 N=17 8&NC_008394.4 plus
948- 0: transcript:Zm00001d011386_T004 rna2073 5.00E-134
948- 1: transcript:Zm00001d011392_T003 rna2076 9.00E-27
948- 2: transcript:Zm00001d011393_T004 rna2079 0
948- 3: transcript:Zm00001d011398_T001 rna2082 1.00E-85
948- 4: transcript:Zm00001d011399_T001 rna2084 1.00E-89
948- 5: transcript:Zm00001d011401_T001 rna2085 0
948- 6: transcript:Zm00001d011403_T001 rna2086 1.00E-102
948- 7: transcript:Zm00001d011405_T001 rna2087 2.00E-120
948- 8: transcript:Zm00001d011406_T001 rna2088 1.00E-113
948- 9: transcript:Zm00001d011410_T001 rna2089 1.00E-91
948- 10: transcript:Zm00001d011411_T002 rna2090 3.00E-124
948- 11: transcript:Zm00001d011412_T001 rna2091 1.00E-34
948- 12: transcript:Zm00001d011413_T001 rna2092 6.00E-134
948- 13: transcript:Zm00001d011417_T001 rna2095 0
948- 14: transcript:Zm00001d011424_T008 rna2107 2.00E-107
948- 15: transcript:Zm00001d011425_T001 rna2108 0
948- 16: transcript:Zm00001d011426_T002 rna2109 0
Alignment 949: score=712.0 e_value=3.4e-42 N=16 8&NC_008394.4 plus
949- 0: transcript:Zm00001d011561_T004 rna2317 7.00E-138
949- 1: transcript:Zm00001d011562_T003 rna2320 7.00E-137
949- 2: transcript:Zm00001d011563_T019 rna2322 0
949- 3: transcript:Zm00001d011565_T002 rna2323 8.00E-100
949- 4: transcript:Zm00001d011569_T001 rna2324 7.00E-156
949- 5: transcript:Zm00001d011571_T001 rna2326 1.00E-129
949- 6: transcript:Zm00001d011572_T001 rna2327 0
949- 7: transcript:Zm00001d011576_T001 rna2330 6.00E-25
949- 8: transcript:Zm00001d011579_T001 rna2332 2.00E-158
949- 9: transcript:Zm00001d011580_T002 rna2333 0
949- 10: transcript:Zm00001d011581_T001 rna2335 1.00E-103
949- 11: transcript:Zm00001d011588_T003 rna2339 2.00E-111
949- 12: transcript:Zm00001d011589_T001 rna2340 6.00E-45
949- 13: transcript:Zm00001d011592_T001 rna2343 2.00E-43
949- 14: transcript:Zm00001d011594_T001 rna2345 2.00E-24
949- 15: transcript:Zm00001d011595_T007 rna2351 0
Alignment 950: score=672.0 e_value=4e-41 N=15 8&NC_008394.4 plus
950- 0: transcript:Zm00001d011159_T001 rna1770 2.00E-133
950- 1: transcript:Zm00001d011160_T002 rna1771 0
950- 2: transcript:Zm00001d011162_T002 rna1772 8.00E-147
950- 3: transcript:Zm00001d011164_T001 rna1773 2.00E-20
950- 4: transcript:Zm00001d011166_T001 rna1774 2.00E-136
950- 5: transcript:Zm00001d011167_T001 rna1778 1.00E-108
950- 6: transcript:Zm00001d011168_T007 rna1779 6.00E-79

950- 7: transcript:Zm00001d011174_T001 rna1781 0
950- 8: transcript:Zm00001d011179_T001 rna1785 2.00E-132
950- 9: transcript:Zm00001d011180_T004 rna1797 1.00E-74
950- 10: transcript:Zm00001d011185_T001 rna1800 6.00E-25
950- 11: transcript:Zm00001d011187_T001 rna1805 2.00E-129
950- 12: transcript:Zm00001d011188_T001 rna1806 0
950- 13: transcript:Zm00001d011189_T001 rna1808 7.00E-47
950- 14: transcript:Zm00001d011192_T001 rna1814 0
Alignment 951: score=649.0 e_value=2.5e-33 N=14 8&NC_008394.4 plus
951- 0: transcript:Zm00001d011319_T003 rna1994 0
951- 1: transcript:Zm00001d011321_T001 rna2000 3.00E-115
951- 2: transcript:Zm00001d011325_T001 rna2003 7.00E-180
951- 3: transcript:Zm00001d011328_T001 rna2008 2.00E-79
951- 4: transcript:Zm00001d011329_T002 rna2011 1.00E-54
951- 5: transcript:Zm00001d011330_T001 rna2013 7.00E-74
951- 6: transcript:Zm00001d011331_T001 rna2015 4.00E-120
951- 7: transcript:Zm00001d011334_T003 rna2018 2.00E-30
951- 8: transcript:Zm00001d011335_T001 rna2024 0
951- 9: transcript:Zm00001d011336_T006 rna2026 1.00E-170
951- 10: transcript:Zm00001d011347_T005 rna2029 0
951- 11: transcript:Zm00001d011348_T001 rna2033 0
951- 12: transcript:Zm00001d011350_T001 rna2035 0
951- 13: transcript:Zm00001d011351_T001 rna2037 0
Alignment 952: score=622.0 e_value=3.5e-33 N=14 8&NC_008394.4 plus
952- 0: transcript:Zm00001d011352_T001 rna2038 4.00E-29
952- 1: transcript:Zm00001d011355_T003 rna2039 6.00E-172
952- 2: transcript:Zm00001d011357_T003 rna2043 4.00E-17
952- 3: transcript:Zm00001d011359_T001 rna2044 3.00E-42
952- 4: transcript:Zm00001d011360_T004 rna2046 2.00E-89
952- 5: transcript:Zm00001d011362_T005 rna2048 9.00E-111
952- 6: transcript:Zm00001d011363_T002 rna2050 2.00E-164
952- 7: transcript:Zm00001d011364_T001 rna2051 5.00E-48
952- 8: transcript:Zm00001d011365_T001 rna2055 1.00E-138
952- 9: transcript:Zm00001d011366_T002 rna2056 0
952- 10: transcript:Zm00001d011369_T001 rna2057 4.00E-180
952- 11: transcript:Zm00001d011370_T001 rna2059 1.00E-128
952- 12: transcript:Zm00001d011373_T009 rna2067 7.00E-41
952- 13: transcript:Zm00001d011378_T001 rna2069 0
Alignment 953: score=484.0 e_value=1.9e-27 N=11 8&NC_008394.4 plus
953- 0: transcript:Zm00001d010052_T001 rna3401 2.00E-12
953- 1: transcript:Zm00001d010053_T001 rna3403 7.00E-71
953- 2: transcript:Zm00001d010054_T001 rna3404 3.00E-41
953- 3: transcript:Zm00001d010055_T003 rna3407 4.00E-137
953- 4: transcript:Zm00001d010056_T002 rna3409 0
953- 5: transcript:Zm00001d010060_T001 rna3412 0
953- 6: transcript:Zm00001d010061_T001 rna3415 5.00E-13
953- 7: transcript:Zm00001d010062_T001 rna3417 0
953- 8: transcript:Zm00001d010066_T003 rna3420 2.00E-105
953- 9: transcript:Zm00001d010082_T002 rna3441 0
953- 10: transcript:Zm00001d010087_T001 rna3443 1.00E-80
Alignment 954: score=408.0 e_value=1.2e-20 N=10 8&NC_008394.4 plus
954- 0: transcript:Zm00001d010899_T001 rna1136 2.00E-23
954- 1: transcript:Zm00001d010907_T001 rna1138 0
954- 2: transcript:Zm00001d010911_T001 rna1146 1.00E-131

954- 3: transcript:Zm00001d010912_T001 rna1148 1.00E-20
954- 4: transcript:Zm00001d010918_T001 rna1164 1.00E-48
954- 5: transcript:Zm00001d010920_T001 rna1166 0
954- 6: transcript:Zm00001d010926_T001 rna1170 3.00E-78
954- 7: transcript:Zm00001d010933_T001 rna1183 3.00E-74
954- 8: transcript:Zm00001d010940_T001 rna1187 3.00E-40
954- 9: transcript:Zm00001d010948_T001 rna1202 1.00E-154
Alignment 955: score=397.0 e_value=3.1e-19 N=10 8&NC_008394.4 plus
955- 0: transcript:Zm00001d008431_T001 rna68 8.00E-08
955- 1: transcript:Zm00001d008440_T001 rna77 4.00E-129
955- 2: transcript:Zm00001d008450_T001 rna86 7.00E-06
955- 3: transcript:Zm00001d008458_T001 rna100 0
955- 4: transcript:Zm00001d008462_T002 rna106 1.00E-40
955- 5: transcript:Zm00001d008466_T001 rna113 2.00E-178
955- 6: transcript:Zm00001d008477_T003 rna121 0
955- 7: transcript:Zm00001d008479_T001 rna125 2.00E-111
955- 8: transcript:Zm00001d008485_T001 rna128 0
955- 9: transcript:Zm00001d008487_T001 rna139 2.00E-87
Alignment 956: score=367.0 e_value=1.1e-16 N=8 8&NC_008394.4 plus
956- 0: transcript:Zm00001d012644_T001 rna2929 0
956- 1: transcript:Zm00001d012645_T001 rna2930 1.00E-153
956- 2: transcript:Zm00001d012647_T001 rna2943 1.00E-133
956- 3: transcript:Zm00001d012648_T001 rna2944 0
956- 4: transcript:Zm00001d012651_T004 rna2945 0
956- 5: transcript:Zm00001d012652_T003 rna2947 0
956- 6: transcript:Zm00001d012653_T002 rna2950 0
956- 7: transcript:Zm00001d012654_T004 rna2951 1.00E-85
Alignment 957: score=363.0 e_value=2e-14 N=8 8&NC_008394.4 plus
957- 0: transcript:Zm00001d012103_T001 rna3775 0
957- 1: transcript:Zm00001d012108_T003 rna3776 4.00E-46
957- 2: transcript:Zm00001d012109_T001 rna3780 5.00E-89
957- 3: transcript:Zm00001d012118_T002 rna3781 0
957- 4: transcript:Zm00001d012119_T001 rna3782 0
957- 5: transcript:Zm00001d012120_T001 rna3783 0
957- 6: transcript:Zm00001d012126_T004 rna3786 0
957- 7: transcript:Zm00001d012127_T001 rna3788 2.00E-36
Alignment 958: score=323.0 e_value=2.8e-12 N=7 8&NC_008394.4 plus
958- 0: transcript:Zm00001d010651_T001 rna3125 3.00E-10
958- 1: transcript:Zm00001d010652_T001 rna3127 1.00E-125
958- 2: transcript:Zm00001d010654_T001 rna3129 0
958- 3: transcript:Zm00001d010655_T001 rna3131 2.00E-155
958- 4: transcript:Zm00001d010656_T001 rna3143 2.00E-147
958- 5: transcript:Zm00001d010659_T001 rna3156 0
958- 6: transcript:Zm00001d010661_T001 rna3157 4.00E-16
Alignment 959: score=320.0 e_value=1.1e-14 N=8 8&NC_008394.4 plus
959- 0: transcript:Zm00001d009352_T001 rna1648 0
959- 1: transcript:Zm00001d009354_T001 rna1656 0
959- 2: transcript:Zm00001d009359_T004 rna1680 0
959- 3: transcript:Zm00001d009364_T001 rna1699 0
959- 4: transcript:Zm00001d009373_T001 rna1703 0
959- 5: transcript:Zm00001d009376_T001 rna1710 2.00E-68
959- 6: transcript:Zm00001d009387_T001 rna1713 2.00E-147
959- 7: transcript:Zm00001d009395_T001 rna1716 2.00E-09
Alignment 960: score=319.0 e_value=1.1e-10 N=7 8&NC_008394.4 plus

960- 0: transcript:Zm00001d010356_T001 rna576 3.00E-67
960- 1: transcript:Zm00001d010358_T001 rna587 0
960- 2: transcript:Zm00001d010360_T001 rna595 6.00E-64
960- 3: transcript:Zm00001d010366_T001 rna597 9.00E-139
960- 4: transcript:Zm00001d010373_T001 rna601 7.00E-63
960- 5: transcript:Zm00001d010375_T001 rna605 0
960- 6: transcript:Zm00001d010380_T001 rna608 7.00E-154
Alignment 961: score=313.0 e_value=2.4e-14 N=7 8&NC_008394.4 plus
961- 0: transcript:Zm00001d009150_T002 rna1272 0
961- 1: transcript:Zm00001d009161_T002 rna1296 2.00E-104
961- 2: transcript:Zm00001d009163_T001 rna1298 0
961- 3: transcript:Zm00001d009167_T001 rna1301 0
961- 4: transcript:Zm00001d009171_T001 rna1302 0
961- 5: transcript:Zm00001d009177_T001 rna1311 0
961- 6: transcript:Zm00001d009178_T001 rna1313 0
Alignment 962: score=284.0 e_value=1.1e-11 N=7 8&NC_008394.4 plus
962- 0: transcript:Zm00001d009230_T001 rna1376 8.00E-101
962- 1: transcript:Zm00001d009240_T002 rna1393 1.00E-06
962- 2: transcript:Zm00001d009243_T001 rna1394 8.00E-154
962- 3: transcript:Zm00001d009247_T001 rna1404 2.00E-34
962- 4: transcript:Zm00001d009248_T022 rna1413 0
962- 5: transcript:Zm00001d009260_T001 rna1414 8.00E-97
962- 6: transcript:Zm00001d009270_T001 rna1429 0
Alignment 963: score=259.0 e_value=2.2e-08 N=6 8&NC_008394.4 plus
963- 0: transcript:Zm00001d012702_T008 rna698 0
963- 1: transcript:Zm00001d012712_T002 rna705 2.00E-104
963- 2: transcript:Zm00001d012717_T002 rna711 0
963- 3: transcript:Zm00001d012719_T001 rna717 9.00E-63
963- 4: transcript:Zm00001d012720_T001 rna722 4.00E-22
963- 5: transcript:Zm00001d012725_T002 rna726 2.00E-38
Alignment 964: score=259.0 e_value=2.6e-09 N=6 8&NC_008394.4 plus
964- 0: transcript:Zm00001d011625_T001 rna256 3.00E-166
964- 1: transcript:Zm00001d011626_T001 rna268 1.00E-06
964- 2: transcript:Zm00001d011628_T001 rna271 5.00E-140
964- 3: transcript:Zm00001d011631_T001 rna277 3.00E-67
964- 4: transcript:Zm00001d011638_T002 rna279 2.00E-111
964- 5: transcript:Zm00001d011639_T001 rna285 8.00E-107
Alignment 965: score=257.0 e_value=1.7e-11 N=6 8&NC_008394.4 plus
965- 0: transcript:Zm00001d008465_T001 rna97 0
965- 1: transcript:Zm00001d008477_T003 rna98 0
965- 2: transcript:Zm00001d008478_T001 rna105 5.00E-50
965- 3: transcript:Zm00001d008485_T001 rna107 0
965- 4: transcript:Zm00001d008487_T001 rna110 8.00E-87
965- 5: transcript:Zm00001d008488_T006 rna121 0
Alignment 966: score=256.0 e_value=1.6e-10 N=6 8&NC_008394.4 plus
966- 0: transcript:Zm00001d008458_T001 rna107 0
966- 1: transcript:Zm00001d008461_T001 rna113 2.00E-38
966- 2: transcript:Zm00001d008462_T002 rna121 0
966- 3: transcript:Zm00001d008466_T001 rna123 4.00E-86
966- 4: transcript:Zm00001d008467_T002 rna128 0
966- 5: transcript:Zm00001d008479_T001 rna133 1.00E-110
Alignment 967: score=8131.0 e_value=0 N=177 8&NC_008394.4 minus
967- 0: transcript:Zm00001d012158_T001 rna3732 2.00E-84
967- 1: transcript:Zm00001d012159_T006 rna3731 2.00E-56

967-	2:	transcript:Zm00001d012161_T001	rna3724	5.00E-79
967-	3:	transcript:Zm00001d012165_T001	rna3722	7.00E-83
967-	4:	transcript:Zm00001d012166_T002	rna3721	0
967-	5:	transcript:Zm00001d012167_T001	rna3720	4.00E-125
967-	6:	transcript:Zm00001d012168_T002	rna3715	0
967-	7:	transcript:Zm00001d012171_T001	rna3711	1.00E-16
967-	8:	transcript:Zm00001d012173_T001	rna3709	0
967-	9:	transcript:Zm00001d012175_T004	rna3699	4.00E-57
967-	10:	transcript:Zm00001d012176_T001	rna3691	0
967-	11:	transcript:Zm00001d012177_T001	rna3684	0
967-	12:	transcript:Zm00001d012178_T001	rna3683	9.00E-143
967-	13:	transcript:Zm00001d012195_T002	rna3677	0
967-	14:	transcript:Zm00001d012196_T005	rna3672	2.00E-129
967-	15:	transcript:Zm00001d012197_T002	rna3670	3.00E-25
967-	16:	transcript:Zm00001d012199_T002	rna3667	0
967-	17:	transcript:Zm00001d012203_T001	rna3661	6.00E-148
967-	18:	transcript:Zm00001d012204_T001	rna3656	0
967-	19:	transcript:Zm00001d012205_T001	rna3654	4.00E-33
967-	20:	transcript:Zm00001d012206_T001	rna3651	2.00E-96
967-	21:	transcript:Zm00001d012211_T010	rna3646	0
967-	22:	transcript:Zm00001d012212_T001	rna3645	0
967-	23:	transcript:Zm00001d012213_T003	rna3642	3.00E-09
967-	24:	transcript:Zm00001d012216_T001	rna3640	0
967-	25:	transcript:Zm00001d012217_T001	rna3637	1.00E-118
967-	26:	transcript:Zm00001d012220_T001	rna3624	0
967-	27:	transcript:Zm00001d012221_T001	rna3621	0
967-	28:	transcript:Zm00001d012223_T001	rna3619	0
967-	29:	transcript:Zm00001d012224_T014	rna3617	0
967-	30:	transcript:Zm00001d012226_T001	rna3614	2.00E-120
967-	31:	transcript:Zm00001d012227_T001	rna3612	1.00E-15
967-	32:	transcript:Zm00001d012228_T002	rna3608	4.00E-161
967-	33:	transcript:Zm00001d012229_T001	rna3605	1.00E-180
967-	34:	transcript:Zm00001d012233_T001	rna3598	0
967-	35:	transcript:Zm00001d012234_T002	rna3597	0
967-	36:	transcript:Zm00001d012237_T001	rna3594	6.00E-107
967-	37:	transcript:Zm00001d012238_T003	rna3593	4.00E-37
967-	38:	transcript:Zm00001d012239_T003	rna3592	6.00E-44
967-	39:	transcript:Zm00001d012240_T001	rna3591	0
967-	40:	transcript:Zm00001d012241_T001	rna3590	3.00E-152
967-	41:	transcript:Zm00001d012242_T001	rna3586	2.00E-179
967-	42:	transcript:Zm00001d012244_T001	rna3584	0
967-	43:	transcript:Zm00001d012245_T002	rna3583	1.00E-101
967-	44:	transcript:Zm00001d012246_T001	rna3582	4.00E-140
967-	45:	transcript:Zm00001d012247_T004	rna3581	0
967-	46:	transcript:Zm00001d012248_T001	rna3580	6.00E-46
967-	47:	transcript:Zm00001d012254_T002	rna3579	0
967-	48:	transcript:Zm00001d012255_T001	rna3578	4.00E-128
967-	49:	transcript:Zm00001d012257_T013	rna3576	1.00E-58
967-	50:	transcript:Zm00001d012259_T001	rna3559	0
967-	51:	transcript:Zm00001d012260_T001	rna3555	0
967-	52:	transcript:Zm00001d012261_T001	rna3552	0
967-	53:	transcript:Zm00001d012263_T002	rna3550	0
967-	54:	transcript:Zm00001d012265_T001	rna3547	6.00E-165
967-	55:	transcript:Zm00001d012266_T001	rna3543	2.00E-45

967- 56:	transcript:Zm00001d012268_T001	rna3541	0
967- 57:	transcript:Zm00001d012269_T003	rna3537	0
967- 58:	transcript:Zm00001d012270_T001	rna3535	1.00E-90
967- 59:	transcript:Zm00001d012273_T001	rna3533	6.00E-65
967- 60:	transcript:Zm00001d012275_T001	rna3525	1.00E-132
967- 61:	transcript:Zm00001d012277_T003	rna3523	2.00E-98
967- 62:	transcript:Zm00001d012279_T001	rna3522	5.00E-50
967- 63:	transcript:Zm00001d012280_T001	rna3521	4.00E-119
967- 64:	transcript:Zm00001d012281_T001	rna3513	2.00E-24
967- 65:	transcript:Zm00001d012284_T001	rna3507	1.00E-117
967- 66:	transcript:Zm00001d012285_T001	rna3506	2.00E-70
967- 67:	transcript:Zm00001d012286_T001	rna3499	0
967- 68:	transcript:Zm00001d012287_T001	rna3498	7.00E-76
967- 69:	transcript:Zm00001d012289_T001	rna3495	0
967- 70:	transcript:Zm00001d012290_T001	rna3493	5.00E-93
967- 71:	transcript:Zm00001d012291_T001	rna3489	3.00E-55
967- 72:	transcript:Zm00001d012292_T001	rna3488	0
967- 73:	transcript:Zm00001d012293_T001	rna3486	6.00E-63
967- 74:	transcript:Zm00001d012294_T009	rna3480	0
967- 75:	transcript:Zm00001d012295_T003	rna3479	4.00E-158
967- 76:	transcript:Zm00001d012296_T001	rna3478	9.00E-130
967- 77:	transcript:Zm00001d012302_T001	rna3475	0
967- 78:	transcript:Zm00001d012304_T001	rna3472	0
967- 79:	transcript:Zm00001d012307_T001	rna3466	5.00E-156
967- 80:	transcript:Zm00001d012312_T001	rna3462	7.00E-160
967- 81:	transcript:Zm00001d012313_T001	rna3459	4.00E-71
967- 82:	transcript:Zm00001d012314_T005	rna3457	6.00E-64
967- 83:	transcript:Zm00001d012320_T005	rna3455	0
967- 84:	transcript:Zm00001d012321_T001	rna3452	0
967- 85:	transcript:Zm00001d012325_T001	rna3450	0
967- 86:	transcript:Zm00001d012326_T001	rna3448	0
967- 87:	transcript:Zm00001d012330_T001	rna3447	2.00E-97
967- 88:	transcript:Zm00001d012332_T001	rna3446	9.00E-32
967- 89:	transcript:Zm00001d012336_T001	rna3444	4.00E-135
967- 90:	transcript:Zm00001d012337_T001	rna3443	8.00E-93
967- 91:	transcript:Zm00001d012338_T001	rna3441	0
967- 92:	transcript:Zm00001d012339_T001	rna3440	1.00E-147
967- 93:	transcript:Zm00001d012340_T001	rna3439	0
967- 94:	transcript:Zm00001d012349_T001	rna3437	2.00E-158
967- 95:	transcript:Zm00001d012350_T001	rna3436	0
967- 96:	transcript:Zm00001d012361_T001	rna3430	1.00E-176
967- 97:	transcript:Zm00001d012362_T001	rna3428	5.00E-170
967- 98:	transcript:Zm00001d012378_T001	rna3426	6.00E-115
967- 99:	transcript:Zm00001d012379_T001	rna3423	6.00E-159
967-100:	transcript:Zm00001d012380_T002	rna3421	0
967-101:	transcript:Zm00001d012381_T001	rna3420	1.00E-137
967-102:	transcript:Zm00001d012382_T002	rna3419	1.00E-87
967-103:	transcript:Zm00001d012383_T002	rna3415	6.00E-46
967-104:	transcript:Zm00001d012391_T003	rna3409	0
967-105:	transcript:Zm00001d012395_T005	rna3402	0
967-106:	transcript:Zm00001d012396_T007	rna3400	0
967-107:	transcript:Zm00001d012398_T002	rna3399	2.00E-122
967-108:	transcript:Zm00001d012399_T003	rna3398	2.00E-156
967-109:	transcript:Zm00001d012401_T001	rna3394	0

967-110:	transcript:Zm00001d012402_T001	rna3391	1.00E-171
967-111:	transcript:Zm00001d012408_T001	rna3377	0
967-112:	transcript:Zm00001d012417_T001	rna3368	0
967-113:	transcript:Zm00001d012418_T001	rna3366	9.00E-109
967-114:	transcript:Zm00001d012419_T001	rna3363	8.00E-44
967-115:	transcript:Zm00001d012420_T001	rna3361	0
967-116:	transcript:Zm00001d012421_T004	rna3359	5.00E-119
967-117:	transcript:Zm00001d012423_T001	rna3356	4.00E-60
967-118:	transcript:Zm00001d012424_T001	rna3355	1.00E-50
967-119:	transcript:Zm00001d012425_T002	rna3354	8.00E-101
967-120:	transcript:Zm00001d012426_T001	rna3353	2.00E-123
967-121:	transcript:Zm00001d012427_T001	rna3348	3.00E-19
967-122:	transcript:Zm00001d012431_T001	rna3339	1.00E-33
967-123:	transcript:Zm00001d012433_T002	rna3336	0
967-124:	transcript:Zm00001d012434_T001	rna3335	7.00E-41
967-125:	transcript:Zm00001d012435_T001	rna3333	6.00E-164
967-126:	transcript:Zm00001d012437_T001	rna3332	0
967-127:	transcript:Zm00001d012438_T001	rna3331	0
967-128:	transcript:Zm00001d012439_T001	rna3329	6.00E-67
967-129:	transcript:Zm00001d012440_T002	rna3328	0
967-130:	transcript:Zm00001d012441_T001	rna3327	1.00E-103
967-131:	transcript:Zm00001d012446_T002	rna3314	0
967-132:	transcript:Zm00001d012447_T005	rna3313	2.00E-171
967-133:	transcript:Zm00001d012449_T001	rna3309	6.00E-34
967-134:	transcript:Zm00001d012450_T001	rna3308	2.00E-67
967-135:	transcript:Zm00001d012451_T004	rna3306	0
967-136:	transcript:Zm00001d012452_T004	rna3304	0
967-137:	transcript:Zm00001d012456_T004	rna3303	0
967-138:	transcript:Zm00001d012457_T003	rna3302	4.00E-30
967-139:	transcript:Zm00001d012458_T001	rna3301	2.00E-26
967-140:	transcript:Zm00001d012459_T001	rna3299	2.00E-153
967-141:	transcript:Zm00001d012460_T001	rna3296	6.00E-143
967-142:	transcript:Zm00001d012463_T001	rna3291	0
967-143:	transcript:Zm00001d012464_T001	rna3290	0
967-144:	transcript:Zm00001d012465_T001	rna3289	5.00E-150
967-145:	transcript:Zm00001d012466_T001	rna3288	0
967-146:	transcript:Zm00001d012471_T001	rna3284	4.00E-55
967-147:	transcript:Zm00001d012472_T001	rna3279	0
967-148:	transcript:Zm00001d012476_T001	rna3275	4.00E-163
967-149:	transcript:Zm00001d012477_T001	rna3271	0
967-150:	transcript:Zm00001d012479_T004	rna3270	1.00E-11
967-151:	transcript:Zm00001d012480_T001	rna3268	3.00E-75
967-152:	transcript:Zm00001d012482_T001	rna3267	0
967-153:	transcript:Zm00001d012484_T002	rna3265	2.00E-12
967-154:	transcript:Zm00001d012485_T001	rna3264	0
967-155:	transcript:Zm00001d012488_T001	rna3262	0
967-156:	transcript:Zm00001d012494_T001	rna3252	0
967-157:	transcript:Zm00001d012498_T001	rna3249	8.00E-135
967-158:	transcript:Zm00001d012499_T001	rna3248	2.00E-117
967-159:	transcript:Zm00001d012500_T001	rna3242	4.00E-66
967-160:	transcript:Zm00001d012503_T003	rna3239	0
967-161:	transcript:Zm00001d012505_T001	rna3237	9.00E-58
967-162:	transcript:Zm00001d012507_T001	rna3236	5.00E-26
967-163:	transcript:Zm00001d012510_T001	rna3234	1.00E-48

967-164: transcript:Zm00001d012511_T010 rna3233 0
967-165: transcript:Zm00001d012512_T001 rna3231 3.00E-110
967-166: transcript:Zm00001d012513_T001 rna3228 2.00E-107
967-167: transcript:Zm00001d012514_T001 rna3225 2.00E-84
967-168: transcript:Zm00001d012515_T004 rna3224 0
967-169: transcript:Zm00001d012516_T001 rna3220 1.00E-109
967-170: transcript:Zm00001d012517_T001 rna3219 1.00E-162
967-171: transcript:Zm00001d012518_T003 rna3215 0
967-172: transcript:Zm00001d012520_T001 rna3214 3.00E-81
967-173: transcript:Zm00001d012521_T001 rna3213 0
967-174: transcript:Zm00001d012522_T001 rna3211 3.00E-159
967-175: transcript:Zm00001d012524_T001 rna3210 0
967-176: transcript:Zm00001d012525_T012 rna3206 0
Alignment 968: score=2972.0 e_value=1.4e-264 N=65 8&NC_008394.4 minus
968- 0: transcript:Zm00001d008171_T001 rna1173 0
968- 1: transcript:Zm00001d008173_T001 rna1170 1.00E-170
968- 2: transcript:Zm00001d008174_T002 rna1167 2.00E-147
968- 3: transcript:Zm00001d008175_T001 rna1166 5.00E-35
968- 4: transcript:Zm00001d008176_T002 rna1165 0
968- 5: transcript:Zm00001d008178_T012 rna1160 5.00E-85
968- 6: transcript:Zm00001d008187_T001 rna1157 0
968- 7: transcript:Zm00001d008189_T004 rna1156 0
968- 8: transcript:Zm00001d008190_T001 rna1152 1.00E-59
968- 9: transcript:Zm00001d008194_T001 rna1150 3.00E-07
968- 10: transcript:Zm00001d008196_T001 rna1148 2.00E-32
968- 11: transcript:Zm00001d008199_T001 rna1146 1.00E-151
968- 12: transcript:Zm00001d008200_T001 rna1145 5.00E-10
968- 13: transcript:Zm00001d008201_T001 rna1143 5.00E-26
968- 14: transcript:Zm00001d008203_T001 rna1142 0
968- 15: transcript:Zm00001d008205_T001 rna1140 0
968- 16: transcript:Zm00001d008206_T001 rna1138 4.00E-47
968- 17: transcript:Zm00001d008208_T001 rna1136 1.00E-73
968- 18: transcript:Zm00001d008209_T001 rna1135 3.00E-30
968- 19: transcript:Zm00001d008210_T002 rna1134 2.00E-117
968- 20: transcript:Zm00001d008214_T001 rna1130 5.00E-160
968- 21: transcript:Zm00001d008215_T002 rna1127 2.00E-173
968- 22: transcript:Zm00001d008216_T002 rna1125 2.00E-164
968- 23: transcript:Zm00001d008218_T002 rna1123 2.00E-114
968- 24: transcript:Zm00001d008221_T001 rna1122 8.00E-177
968- 25: transcript:Zm00001d008222_T002 rna1121 7.00E-39
968- 26: transcript:Zm00001d008223_T001 rna1118 6.00E-26
968- 27: transcript:Zm00001d008224_T002 rna1117 0
968- 28: transcript:Zm00001d008225_T001 rna1114 0
968- 29: transcript:Zm00001d008226_T001 rna1112 0
968- 30: transcript:Zm00001d008227_T001 rna1111 0
968- 31: transcript:Zm00001d008230_T001 rna1108 0
968- 32: transcript:Zm00001d008236_T004 rna1095 0
968- 33: transcript:Zm00001d008237_T001 rna1093 1.00E-80
968- 34: transcript:Zm00001d008238_T001 rna1092 1.00E-164
968- 35: transcript:Zm00001d008239_T001 rna1091 0
968- 36: transcript:Zm00001d008241_T001 rna1090 2.00E-13
968- 37: transcript:Zm00001d008242_T001 rna1089 0
968- 38: transcript:Zm00001d008244_T003 rna1088 0
968- 39: transcript:Zm00001d008245_T001 rna1087 4.00E-75

```
968- 40:  transcript:Zm00001d008248_T002  rna1086            0
968- 41:  transcript:Zm00001d008249_T012  rna1082            0
968- 42:  transcript:Zm00001d008255_T001  rna1076            0
968- 43:  transcript:Zm00001d008260_T001  rna1073     2.00E-103
968- 44:  transcript:Zm00001d008264_T003  rna1065     4.00E-113
968- 45:  transcript:Zm00001d008265_T001  rna1064     4.00E-180
968- 46:  transcript:Zm00001d008266_T001  rna1063     4.00E-169
968- 47:  transcript:Zm00001d008268_T001  rna1062     5.00E-129
968- 48:  transcript:Zm00001d008269_T001  rna1057     4.00E-164
968- 49:  transcript:Zm00001d008271_T002  rna1056            0
968- 50:  transcript:Zm00001d008273_T001  rna1053            0
968- 51:  transcript:Zm00001d008278_T001  rna1049      2.00E-86
968- 52:  transcript:Zm00001d008279_T001  rna1048     2.00E-119
968- 53:  transcript:Zm00001d008282_T002  rna1046      1.00E-77
968- 54:  transcript:Zm00001d008283_T001  rna1045     2.00E-150
968- 55:  transcript:Zm00001d008284_T004  rna1044            0
968- 56:  transcript:Zm00001d008285_T005  rna1041            0
968- 57:  transcript:Zm00001d008287_T001  rna1039     3.00E-168
968- 58:  transcript:Zm00001d008288_T001  rna1038      1.00E-14
968- 59:  transcript:Zm00001d008289_T007  rna1034            0
968- 60:  transcript:Zm00001d008294_T001  rna1033      4.00E-97
968- 61:  transcript:Zm00001d008295_T002  rna1027     9.00E-133
968- 62:  transcript:Zm00001d008296_T001  rna1023     3.00E-130
968- 63:  transcript:Zm00001d008297_T001  rna1022      1.00E-69
968- 64:  transcript:Zm00001d008299_T002  rna1015     2.00E-130
## Alignment 969: score=2720.0 e_value=2.9e-233 N=61 8&NC_008394.4 minus
969-  0:  transcript:Zm00001d012655_T001  rna2920            0
969-  1:  transcript:Zm00001d012656_T002  rna2919            0
969-  2:  transcript:Zm00001d012659_T009  rna2913            0
969-  3:  transcript:Zm00001d012660_T002  rna2911            0
969-  4:  transcript:Zm00001d012661_T002  rna2910            0
969-  5:  transcript:Zm00001d012662_T001  rna2909            0
969-  6:  transcript:Zm00001d012667_T009  rna2906            0
969-  7:  transcript:Zm00001d012670_T002  rna2897     2.00E-117
969-  8:  transcript:Zm00001d012671_T001  rna2893     2.00E-133
969-  9:  transcript:Zm00001d012672_T001  rna2892            0
969- 10:  transcript:Zm00001d012673_T002  rna2887      3.00E-42
969- 11:  transcript:Zm00001d012674_T010  rna2886     2.00E-165
969- 12:  transcript:Zm00001d012675_T001  rna2883     2.00E-123
969- 13:  transcript:Zm00001d012676_T001  rna2881      8.00E-10
969- 14:  transcript:Zm00001d012677_T012  rna2878            0
969- 15:  transcript:Zm00001d012679_T004  rna2877      3.00E-56
969- 16:  transcript:Zm00001d012681_T003  rna2875      4.00E-87
969- 17:  transcript:Zm00001d012684_T001  rna2873      9.00E-46
969- 18:  transcript:Zm00001d012685_T004  rna2870      2.00E-49
969- 19:  transcript:Zm00001d012686_T001  rna2869      1.00E-33
969- 20:  transcript:Zm00001d012687_T001  rna2868            0
969- 21:  transcript:Zm00001d012688_T001  rna2863            0
969- 22:  transcript:Zm00001d012689_T001  rna2862      5.00E-60
969- 23:  transcript:Zm00001d012690_T002  rna2861      5.00E-75
969- 24:  transcript:Zm00001d012691_T001  rna2857     2.00E-133
969- 25:  transcript:Zm00001d012693_T002  rna2854            0
969- 26:  transcript:Zm00001d012694_T002  rna2853     8.00E-132
969- 27:  transcript:Zm00001d012696_T001  rna2852            0
```

```
969- 28:  transcript:Zm00001d012697_T009  rna2851            0
969- 29:  transcript:Zm00001d012700_T001  rna2845      3.00E-23
969- 30:  transcript:Zm00001d012701_T004  rna2844            0
969- 31:  transcript:Zm00001d012702_T008  rna2843            0
969- 32:  transcript:Zm00001d012703_T001  rna2842      1.00E-61
969- 33:  transcript:Zm00001d012708_T001  rna2838     3.00E-101
969- 34:  transcript:Zm00001d012709_T001  rna2837      4.00E-86
969- 35:  transcript:Zm00001d012710_T001  rna2836     3.00E-115
969- 36:  transcript:Zm00001d012712_T002  rna2834            0
969- 37:  transcript:Zm00001d012717_T002  rna2832            0
969- 38:  transcript:Zm00001d012718_T004  rna2831            0
969- 39:  transcript:Zm00001d012719_T001  rna2828     1.00E-105
969- 40:  transcript:Zm00001d012720_T001  rna2827      5.00E-69
969- 41:  transcript:Zm00001d012725_T002  rna2825     1.00E-166
969- 42:  transcript:Zm00001d012726_T001  rna2823      1.00E-08
969- 43:  transcript:Zm00001d012728_T001  rna2821            0
969- 44:  transcript:Zm00001d012729_T009  rna2817      6.00E-33
969- 45:  transcript:Zm00001d012731_T010  rna2815            0
969- 46:  transcript:Zm00001d012732_T002  rna2812      2.00E-79
969- 47:  transcript:Zm00001d012734_T002  rna2810     3.00E-132
969- 48:  transcript:Zm00001d012735_T003  rna2809      1.00E-85
969- 49:  transcript:Zm00001d012736_T001  rna2806      1.00E-07
969- 50:  transcript:Zm00001d012737_T001  rna2805      3.00E-73
969- 51:  transcript:Zm00001d012738_T001  rna2800            0
969- 52:  transcript:Zm00001d012740_T001  rna2798      3.00E-26
969- 53:  transcript:Zm00001d012741_T001  rna2795      2.00E-44
969- 54:  transcript:Zm00001d012743_T002  rna2794            0
969- 55:  transcript:Zm00001d012744_T002  rna2791            0
969- 56:  transcript:Zm00001d012746_T001  rna2790      9.00E-55
969- 57:  transcript:Zm00001d012747_T007  rna2788            0
969- 58:  transcript:Zm00001d012748_T002  rna2787            0
969- 59:  transcript:Zm00001d012749_T001  rna2786            0
969- 60:  transcript:Zm00001d012750_T002  rna2781            0
## Alignment 970: score=2670.0 e_value=2e-226 N=58 8&NC_008394.4 minus
970-  0:  transcript:Zm00001d012527_T001  rna3205     9.00E-154
970-  1:  transcript:Zm00001d012528_T001  rna3204            0
970-  2:  transcript:Zm00001d012529_T001  rna3201            0
970-  3:  transcript:Zm00001d012530_T001  rna3199      3.00E-95
970-  4:  transcript:Zm00001d012532_T001  rna3195      2.00E-77
970-  5:  transcript:Zm00001d012534_T001  rna3193      3.00E-80
970-  6:  transcript:Zm00001d012536_T002  rna3186            0
970-  7:  transcript:Zm00001d012537_T003  rna3185      7.00E-95
970-  8:  transcript:Zm00001d012538_T001  rna3184      3.00E-85
970-  9:  transcript:Zm00001d012539_T001  rna3182     4.00E-140
970- 10:  transcript:Zm00001d012544_T001  rna3176            0
970- 11:  transcript:Zm00001d012545_T001  rna3173      4.00E-54
970- 12:  transcript:Zm00001d012546_T002  rna3172     5.00E-176
970- 13:  transcript:Zm00001d012548_T001  rna3162      2.00E-09
970- 14:  transcript:Zm00001d012549_T001  rna3161     2.00E-133
970- 15:  transcript:Zm00001d012550_T001  rna3159     3.00E-133
970- 16:  transcript:Zm00001d012551_T001  rna3157      5.00E-38
970- 17:  transcript:Zm00001d012552_T005  rna3156            0
970- 18:  transcript:Zm00001d012555_T001  rna3150     1.00E-131
970- 19:  transcript:Zm00001d012556_T001  rna3146            0
```

970- 20:	transcript:Zm00001d012557_T001	rna3145	8.00E-143
970- 21:	transcript:Zm00001d012559_T004	rna3144	0
970- 22:	transcript:Zm00001d012560_T002	rna3143	0
970- 23:	transcript:Zm00001d012561_T001	rna3130	5.00E-55
970- 24:	transcript:Zm00001d012562_T001	rna3129	0
970- 25:	transcript:Zm00001d012563_T001	rna3125	5.00E-19
970- 26:	transcript:Zm00001d012564_T001	rna3123	4.00E-141
970- 27:	transcript:Zm00001d012566_T001	rna3121	1.00E-51
970- 28:	transcript:Zm00001d012567_T002	rna3120	0
970- 29:	transcript:Zm00001d012568_T001	rna3119	5.00E-176
970- 30:	transcript:Zm00001d012569_T001	rna3118	2.00E-127
970- 31:	transcript:Zm00001d012571_T002	rna3116	0
970- 32:	transcript:Zm00001d012573_T001	rna3113	0
970- 33:	transcript:Zm00001d012576_T001	rna3111	7.00E-162
970- 34:	transcript:Zm00001d012577_T001	rna3107	2.00E-14
970- 35:	transcript:Zm00001d012578_T002	rna3104	0
970- 36:	transcript:Zm00001d012585_T001	rna3094	1.00E-81
970- 37:	transcript:Zm00001d012590_T001	rna3089	0
970- 38:	transcript:Zm00001d012591_T001	rna3076	0
970- 39:	transcript:Zm00001d012594_T001	rna3073	4.00E-26
970- 40:	transcript:Zm00001d012596_T001	rna3072	2.00E-14
970- 41:	transcript:Zm00001d012600_T001	rna3046	0
970- 42:	transcript:Zm00001d012602_T001	rna3041	1.00E-69
970- 43:	transcript:Zm00001d012603_T001	rna3036	3.00E-125
970- 44:	transcript:Zm00001d012604_T001	rna3035	7.00E-60
970- 45:	transcript:Zm00001d012605_T001	rna3027	0
970- 46:	transcript:Zm00001d012606_T001	rna3026	2.00E-176
970- 47:	transcript:Zm00001d012607_T001	rna3021	3.00E-134
970- 48:	transcript:Zm00001d012609_T001	rna3015	0
970- 49:	transcript:Zm00001d012610_T001	rna3012	3.00E-124
970- 50:	transcript:Zm00001d012611_T001	rna3011	7.00E-104
970- 51:	transcript:Zm00001d012612_T001	rna3009	0
970- 52:	transcript:Zm00001d012614_T001	rna3008	0
970- 53:	transcript:Zm00001d012618_T001	rna2998	7.00E-125
970- 54:	transcript:Zm00001d012619_T001	rna2996	4.00E-106
970- 55:	transcript:Zm00001d012620_T025	rna2993	0
970- 56:	transcript:Zm00001d012621_T001	rna2992	1.00E-71
970- 57:	transcript:Zm00001d012624_T006	rna2991	0
## Alignment 971: score=2661.0 e_value=4.9e-236 N=58 8&NC_008394.4 minus			
971- 0:	transcript:Zm00001d011855_T002	rna4088	0
971- 1:	transcript:Zm00001d011872_T003	rna4086	0
971- 2:	transcript:Zm00001d011873_T002	rna4085	6.00E-84
971- 3:	transcript:Zm00001d011874_T002	rna4084	8.00E-91
971- 4:	transcript:Zm00001d011876_T001	rna4075	2.00E-166
971- 5:	transcript:Zm00001d011877_T003	rna4073	4.00E-59
971- 6:	transcript:Zm00001d011878_T001	rna4072	0
971- 7:	transcript:Zm00001d011880_T002	rna4068	2.00E-143
971- 8:	transcript:Zm00001d011881_T001	rna4067	0
971- 9:	transcript:Zm00001d011885_T005	rna4053	0
971- 10:	transcript:Zm00001d011886_T001	rna4029	8.00E-118
971- 11:	transcript:Zm00001d011887_T001	rna4026	1.00E-34
971- 12:	transcript:Zm00001d011889_T001	rna4025	0
971- 13:	transcript:Zm00001d011890_T001	rna4024	0
971- 14:	transcript:Zm00001d011891_T001	rna4022	0

971- 15: transcript:Zm00001d011895_T001 rna4018 0
971- 16: transcript:Zm00001d011896_T001 rna4016 0
971- 17: transcript:Zm00001d011898_T001 rna4013 0
971- 18: transcript:Zm00001d011899_T001 rna4012 4.00E-20
971- 19: transcript:Zm00001d011900_T001 rna4011 0
971- 20: transcript:Zm00001d011902_T001 rna4009 7.00E-10
971- 21: transcript:Zm00001d011903_T001 rna3998 5.00E-80
971- 22: transcript:Zm00001d011904_T001 rna3997 2.00E-170
971- 23: transcript:Zm00001d011908_T001 rna3991 0
971- 24: transcript:Zm00001d011912_T002 rna3988 2.00E-114
971- 25: transcript:Zm00001d011913_T002 rna3985 0
971- 26: transcript:Zm00001d011915_T001 rna3982 1.00E-69
971- 27: transcript:Zm00001d011917_T001 rna3977 1.00E-61
971- 28: transcript:Zm00001d011918_T002 rna3973 0
971- 29: transcript:Zm00001d011919_T001 rna3972 4.00E-100
971- 30: transcript:Zm00001d011920_T003 rna3971 0
971- 31: transcript:Zm00001d011923_T002 rna3961 0
971- 32: transcript:Zm00001d011927_T001 rna3956 0
971- 33: transcript:Zm00001d011928_T001 rna3955 1.00E-137
971- 34: transcript:Zm00001d011929_T002 rna3954 0
971- 35: transcript:Zm00001d011930_T001 rna3952 2.00E-123
971- 36: transcript:Zm00001d011938_T001 rna3947 6.00E-36
971- 37: transcript:Zm00001d011940_T001 rna3944 3.00E-10
971- 38: transcript:Zm00001d011941_T003 rna3938 0
971- 39: transcript:Zm00001d011943_T001 rna3935 2.00E-31
971- 40: transcript:Zm00001d011944_T001 rna3934 2.00E-28
971- 41: transcript:Zm00001d011945_T001 rna3933 7.00E-48
971- 42: transcript:Zm00001d011953_T008 rna3929 0
971- 43: transcript:Zm00001d011955_T002 rna3926 1.00E-47
971- 44: transcript:Zm00001d011956_T002 rna3924 0
971- 45: transcript:Zm00001d011964_T001 rna3917 0
971- 46: transcript:Zm00001d011965_T001 rna3916 0
971- 47: transcript:Zm00001d011966_T003 rna3914 2.00E-124
971- 48: transcript:Zm00001d011967_T001 rna3913 4.00E-97
971- 49: transcript:Zm00001d011968_T001 rna3912 1.00E-13
971- 50: transcript:Zm00001d011969_T001 rna3911 2.00E-46
971- 51: transcript:Zm00001d011970_T001 rna3910 0
971- 52: transcript:Zm00001d011971_T001 rna3909 3.00E-179
971- 53: transcript:Zm00001d011972_T001 rna3908 7.00E-60
971- 54: transcript:Zm00001d011975_T002 rna3907 2.00E-145
971- 55: transcript:Zm00001d011978_T001 rna3906 8.00E-47
971- 56: transcript:Zm00001d011979_T001 rna3904 0
971- 57: transcript:Zm00001d011981_T007 rna3901 8.00E-165
Alignment 972: score=2461.0 e_value=1e-204 N=53 8&NC_008394.4 minus
972- 0: transcript:Zm00001d011767_T003 rna4270 2.00E-63
972- 1: transcript:Zm00001d011769_T001 rna4269 2.00E-113
972- 2: transcript:Zm00001d011771_T001 rna4267 6.00E-41
972- 3: transcript:Zm00001d011773_T003 rna4265 0
972- 4: transcript:Zm00001d011774_T001 rna4264 0
972- 5: transcript:Zm00001d011778_T001 rna4258 4.00E-155
972- 6: transcript:Zm00001d011780_T002 rna4241 0
972- 7: transcript:Zm00001d011781_T001 rna4239 1.00E-75
972- 8: transcript:Zm00001d011782_T003 rna4237 4.00E-166
972- 9: transcript:Zm00001d011784_T001 rna4234 8.00E-22

972- 10: transcript:Zm00001d011785_T001 rna4230 4.00E-90
972- 11: transcript:Zm00001d011786_T001 rna4227 9.00E-90
972- 12: transcript:Zm00001d011787_T001 rna4226 0
972- 13: transcript:Zm00001d011789_T001 rna4223 4.00E-23
972- 14: transcript:Zm00001d011790_T001 rna4220 7.00E-128
972- 15: transcript:Zm00001d011792_T003 rna4215 0
972- 16: transcript:Zm00001d011793_T001 rna4206 3.00E-155
972- 17: transcript:Zm00001d011795_T001 rna4192 2.00E-41
972- 18: transcript:Zm00001d011796_T002 rna4190 0
972- 19: transcript:Zm00001d011797_T002 rna4189 3.00E-70
972- 20: transcript:Zm00001d011799_T001 rna4186 3.00E-89
972- 21: transcript:Zm00001d011800_T001 rna4185 2.00E-11
972- 22: transcript:Zm00001d011802_T001 rna4183 8.00E-42
972- 23: transcript:Zm00001d011803_T003 rna4182 1.00E-178
972- 24: transcript:Zm00001d011805_T001 rna4179 0
972- 25: transcript:Zm00001d011811_T002 rna4177 9.00E-79
972- 26: transcript:Zm00001d011813_T001 rna4175 6.00E-30
972- 27: transcript:Zm00001d011816_T001 rna4168 5.00E-97
972- 28: transcript:Zm00001d011817_T003 rna4164 0
972- 29: transcript:Zm00001d011821_T007 rna4153 0
972- 30: transcript:Zm00001d011823_T002 rna4150 0
972- 31: transcript:Zm00001d011825_T001 rna4148 1.00E-91
972- 32: transcript:Zm00001d011826_T001 rna4147 4.00E-70
972- 33: transcript:Zm00001d011827_T002 rna4146 2.00E-27
972- 34: transcript:Zm00001d011828_T001 rna4140 7.00E-81
972- 35: transcript:Zm00001d011829_T001 rna4139 0
972- 36: transcript:Zm00001d011830_T001 rna4138 2.00E-113
972- 37: transcript:Zm00001d011831_T001 rna4137 0
972- 38: transcript:Zm00001d011832_T001 rna4134 0
972- 39: transcript:Zm00001d011834_T001 rna4127 0
972- 40: transcript:Zm00001d011835_T003 rna4126 0
972- 41: transcript:Zm00001d011838_T001 rna4120 0
972- 42: transcript:Zm00001d011839_T001 rna4118 1.00E-27
972- 43: transcript:Zm00001d011841_T001 rna4116 6.00E-59
972- 44: transcript:Zm00001d011842_T001 rna4114 0
972- 45: transcript:Zm00001d011843_T001 rna4111 2.00E-136
972- 46: transcript:Zm00001d011844_T001 rna4109 2.00E-08
972- 47: transcript:Zm00001d011845_T001 rna4106 4.00E-87
972- 48: transcript:Zm00001d011846_T001 rna4105 2.00E-92
972- 49: transcript:Zm00001d011847_T001 rna4100 6.00E-88
972- 50: transcript:Zm00001d011849_T002 rna4097 8.00E-96
972- 51: transcript:Zm00001d011851_T001 rna4096 0
972- 52: transcript:Zm00001d011853_T001 rna4093 1.00E-114
Alignment 973: score=1541.0 e_value=6.7e-112 N=33 8&NC_008394.4 minus
973- 0: transcript:Zm00001d012033_T002 rna3867 0
973- 1: transcript:Zm00001d012035_T001 rna3864 0
973- 2: transcript:Zm00001d012036_T001 rna3862 3.00E-47
973- 3: transcript:Zm00001d012040_T003 rna3858 0
973- 4: transcript:Zm00001d012042_T002 rna3856 2.00E-39
973- 5: transcript:Zm00001d012043_T002 rna3855 3.00E-28
973- 6: transcript:Zm00001d012045_T001 rna3854 1.00E-90
973- 7: transcript:Zm00001d012047_T001 rna3853 1.00E-43
973- 8: transcript:Zm00001d012048_T001 rna3852 0
973- 9: transcript:Zm00001d012049_T003 rna3851 0

973- 10: transcript:Zm00001d012050_T001 rna3846 1.00E-16
973- 11: transcript:Zm00001d012052_T001 rna3845 1.00E-55
973- 12: transcript:Zm00001d012059_T001 rna3844 6.00E-48
973- 13: transcript:Zm00001d012060_T003 rna3843 0
973- 14: transcript:Zm00001d012061_T005 rna3842 0
973- 15: transcript:Zm00001d012063_T001 rna3841 1.00E-08
973- 16: transcript:Zm00001d012064_T001 rna3838 2.00E-74
973- 17: transcript:Zm00001d012067_T003 rna3837 0
973- 18: transcript:Zm00001d012068_T001 rna3834 1.00E-42
973- 19: transcript:Zm00001d012069_T001 rna3833 2.00E-87
973- 20: transcript:Zm00001d012074_T001 rna3832 0
973- 21: transcript:Zm00001d012078_T001 rna3830 2.00E-29
973- 22: transcript:Zm00001d012079_T001 rna3829 1.00E-61
973- 23: transcript:Zm00001d012080_T001 rna3826 8.00E-13
973- 24: transcript:Zm00001d012083_T001 rna3815 3.00E-151
973- 25: transcript:Zm00001d012084_T001 rna3814 7.00E-119
973- 26: transcript:Zm00001d012085_T005 rna3812 0
973- 27: transcript:Zm00001d012086_T009 rna3808 0
973- 28: transcript:Zm00001d012087_T005 rna3806 1.00E-159
973- 29: transcript:Zm00001d012088_T001 rna3805 2.00E-27
973- 30: transcript:Zm00001d012091_T001 rna3803 4.00E-07
973- 31: transcript:Zm00001d012092_T001 rna3797 5.00E-29
973- 32: transcript:Zm00001d012097_T001 rna3792 0
Alignment 974: score=1357.0 e_value=4.2e-105 N=31 8&NC_008394.4 minus
974- 0: transcript:Zm00001d010575_T001 rna3329 6.00E-67
974- 1: transcript:Zm00001d010576_T002 rna3306 1.00E-114
974- 2: transcript:Zm00001d010578_T003 rna3304 0
974- 3: transcript:Zm00001d010579_T001 rna3302 4.00E-30
974- 4: transcript:Zm00001d010583_T001 rna3301 1.00E-16
974- 5: transcript:Zm00001d010584_T001 rna3300 4.00E-143
974- 6: transcript:Zm00001d010589_T001 rna3291 0
974- 7: transcript:Zm00001d010591_T001 rna3285 7.00E-40
974- 8: transcript:Zm00001d010594_T001 rna3284 1.00E-98
974- 9: transcript:Zm00001d010599_T002 rna3277 0
974- 10: transcript:Zm00001d010602_T001 rna3273 0
974- 11: transcript:Zm00001d010603_T001 rna3272 6.00E-73
974- 12: transcript:Zm00001d010604_T006 rna3270 0
974- 13: transcript:Zm00001d010606_T004 rna3269 0
974- 14: transcript:Zm00001d010607_T001 rna3265 7.00E-108
974- 15: transcript:Zm00001d010614_T007 rna3247 6.00E-70
974- 16: transcript:Zm00001d010617_T001 rna3237 3.00E-36
974- 17: transcript:Zm00001d010618_T003 rna3228 6.00E-108
974- 18: transcript:Zm00001d010620_T001 rna3224 0
974- 19: transcript:Zm00001d010622_T002 rna3219 3.00E-121
974- 20: transcript:Zm00001d010625_T001 rna3216 0
974- 21: transcript:Zm00001d010627_T001 rna3214 6.00E-79
974- 22: transcript:Zm00001d010629_T001 rna3213 0
974- 23: transcript:Zm00001d010630_T003 rna3210 0
974- 24: transcript:Zm00001d010631_T003 rna3202 3.00E-88
974- 25: transcript:Zm00001d010632_T002 rna3200 2.00E-07
974- 26: transcript:Zm00001d010633_T001 rna3199 4.00E-19
974- 27: transcript:Zm00001d010638_T001 rna3184 5.00E-114
974- 28: transcript:Zm00001d010640_T001 rna3179 0
974- 29: transcript:Zm00001d010646_T001 rna3174 0

974- 30: transcript:Zm00001d010661_T001 rna3157 4.00E-16
Alignment 975: score=1080.0 e_value=8.2e-77 N=25 8&NC_008394.4 minus
975- 0: transcript:Zm00001d009468_T001 rna2448 3.00E-156
975- 1: transcript:Zm00001d009469_T001 rna2443 3.00E-33
975- 2: transcript:Zm00001d009473_T001 rna2427 0
975- 3: transcript:Zm00001d009475_T001 rna2420 8.00E-09
975- 4: transcript:Zm00001d009481_T001 rna2411 5.00E-140
975- 5: transcript:Zm00001d009484_T001 rna2404 2.00E-137
975- 6: transcript:Zm00001d009487_T001 rna2402 9.00E-36
975- 7: transcript:Zm00001d009488_T001 rna2400 0
975- 8: transcript:Zm00001d009493_T001 rna2395 6.00E-57
975- 9: transcript:Zm00001d009494_T001 rna2393 0
975- 10: transcript:Zm00001d009496_T001 rna2388 1.00E-32
975- 11: transcript:Zm00001d009497_T004 rna2387 5.00E-16
975- 12: transcript:Zm00001d009504_T002 rna2372 1.00E-14
975- 13: transcript:Zm00001d009506_T001 rna2369 7.00E-168
975- 14: transcript:Zm00001d009508_T001 rna2368 8.00E-84
975- 15: transcript:Zm00001d009509_T001 rna2361 1.00E-61
975- 16: transcript:Zm00001d009510_T001 rna2359 1.00E-52
975- 17: transcript:Zm00001d009511_T001 rna2347 0
975- 18: transcript:Zm00001d009525_T002 rna2343 5.00E-25
975- 19: transcript:Zm00001d009532_T001 rna2339 3.00E-93
975- 20: transcript:Zm00001d009539_T002 rna2338 0
975- 21: transcript:Zm00001d009549_T002 rna2334 0
975- 22: transcript:Zm00001d009552_T002 rna2323 1.00E-106
975- 23: transcript:Zm00001d009555_T003 rna2320 2.00E-102
975- 24: transcript:Zm00001d009562_T007 rna2316 2.00E-83
Alignment 976: score=1025.0 e_value=2e-70 N=24 8&NC_008394.4 minus
976- 0: transcript:Zm00001d008305_T001 rna1012 1.00E-90
976- 1: transcript:Zm00001d008307_T001 rna1007 2.00E-27
976- 2: transcript:Zm00001d008312_T001 rna1003 5.00E-25
976- 3: transcript:Zm00001d008314_T002 rna1002 5.00E-116
976- 4: transcript:Zm00001d008316_T001 rna999 1.00E-109
976- 5: transcript:Zm00001d008320_T004 rna997 0
976- 6: transcript:Zm00001d008321_T011 rna996 0
976- 7: transcript:Zm00001d008322_T002 rna988 8.00E-167
976- 8: transcript:Zm00001d008325_T001 rna987 3.00E-58
976- 9: transcript:Zm00001d008330_T001 rna980 1.00E-68
976- 10: transcript:Zm00001d008333_T003 rna976 4.00E-106
976- 11: transcript:Zm00001d008334_T001 rna974 1.00E-134
976- 12: transcript:Zm00001d008338_T001 rna972 0
976- 13: transcript:Zm00001d008340_T003 rna970 0
976- 14: transcript:Zm00001d008343_T001 rna967 5.00E-52
976- 15: transcript:Zm00001d008345_T003 rna965 0
976- 16: transcript:Zm00001d008346_T001 rna964 7.00E-62
976- 17: transcript:Zm00001d008347_T001 rna962 0
976- 18: transcript:Zm00001d008354_T006 rna961 8.00E-153
976- 19: transcript:Zm00001d008355_T001 rna956 3.00E-13
976- 20: transcript:Zm00001d008357_T002 rna954 0
976- 21: transcript:Zm00001d008360_T001 rna951 0
976- 22: transcript:Zm00001d008363_T007 rna949 0
976- 23: transcript:Zm00001d008370_T001 rna939 5.00E-148
Alignment 977: score=901.0 e_value=4.2e-57 N=20 8&NC_008394.4 minus
977- 0: transcript:Zm00001d011983_T031 rna3901 5.00E-175

977- 1: transcript:Zm00001d011985_T001 rna3900 5.00E-102
977- 2: transcript:Zm00001d011987_T001 rna3898 3.00E-54
977- 3: transcript:Zm00001d011993_T001 rna3896 2.00E-108
977- 4: transcript:Zm00001d011994_T001 rna3895 0
977- 5: transcript:Zm00001d011997_T002 rna3894 0
977- 6: transcript:Zm00001d012005_T007 rna3893 1.00E-42
977- 7: transcript:Zm00001d012007_T006 rna3890 2.00E-131
977- 8: transcript:Zm00001d012010_T001 rna3888 2.00E-33
977- 9: transcript:Zm00001d012015_T001 rna3885 5.00E-90
977- 10: transcript:Zm00001d012016_T001 rna3882 3.00E-166
977- 11: transcript:Zm00001d012017_T001 rna3881 1.00E-111
977- 12: transcript:Zm00001d012019_T001 rna3880 0
977- 13: transcript:Zm00001d012020_T001 rna3879 0
977- 14: transcript:Zm00001d012021_T001 rna3874 1.00E-90
977- 15: transcript:Zm00001d012023_T001 rna3873 5.00E-44
977- 16: transcript:Zm00001d012027_T002 rna3872 0
977- 17: transcript:Zm00001d012029_T001 rna3871 3.00E-38
977- 18: transcript:Zm00001d012030_T003 rna3870 3.00E-64
977- 19: transcript:Zm00001d012031_T001 rna3869 3.00E-85
Alignment 978: score=862.0 e_value=9.8e-52 N=19 8&NC_008394.4 minus
978- 0: transcript:Zm00001d012757_T001 rna2763 2.00E-124
978- 1: transcript:Zm00001d012760_T002 rna2759 0
978- 2: transcript:Zm00001d012761_T002 rna2758 0
978- 3: transcript:Zm00001d012763_T002 rna2755 0
978- 4: transcript:Zm00001d012764_T001 rna2752 0
978- 5: transcript:Zm00001d012765_T005 rna2750 0
978- 6: transcript:Zm00001d012766_T001 rna2748 0
978- 7: transcript:Zm00001d012767_T001 rna2747 9.00E-83
978- 8: transcript:Zm00001d012769_T001 rna2745 0
978- 9: transcript:Zm00001d012770_T001 rna2742 0
978- 10: transcript:Zm00001d012771_T001 rna2740 5.00E-84
978- 11: transcript:Zm00001d012774_T001 rna2717 0
978- 12: transcript:Zm00001d012775_T001 rna2715 2.00E-26
978- 13: transcript:Zm00001d012776_T001 rna2707 3.00E-157
978- 14: transcript:Zm00001d012779_T001 rna2705 3.00E-84
978- 15: transcript:Zm00001d012782_T001 rna2699 0
978- 16: transcript:Zm00001d012788_T001 rna2693 3.00E-165
978- 17: transcript:Zm00001d012789_T001 rna2689 5.00E-76
978- 18: transcript:Zm00001d012791_T001 rna2687 8.00E-154
Alignment 979: score=841.0 e_value=3.8e-61 N=19 8&NC_008394.4 minus
979- 0: transcript:Zm00001d009074_T002 rna1250 3.00E-129
979- 1: transcript:Zm00001d009075_T001 rna1231 0
979- 2: transcript:Zm00001d009077_T001 rna1230 0
979- 3: transcript:Zm00001d009082_T001 rna1227 4.00E-58
979- 4: transcript:Zm00001d009087_T001 rna1221 7.00E-102
979- 5: transcript:Zm00001d009088_T001 rna1218 4.00E-25
979- 6: transcript:Zm00001d009112_T001 rna1216 2.00E-39
979- 7: transcript:Zm00001d009116_T001 rna1214 1.00E-45
979- 8: transcript:Zm00001d009117_T001 rna1211 7.00E-18
979- 9: transcript:Zm00001d009118_T001 rna1210 2.00E-94
979- 10: transcript:Zm00001d009119_T001 rna1209 6.00E-27
979- 11: transcript:Zm00001d009122_T001 rna1194 0
979- 12: transcript:Zm00001d009123_T001 rna1193 0
979- 13: transcript:Zm00001d009125_T008 rna1192 0

979- 14: transcript:Zm00001d009127_T001 rna1190 0
979- 15: transcript:Zm00001d009130_T001 rna1188 4.00E-67
979- 16: transcript:Zm00001d009131_T001 rna1187 3.00E-71
979- 17: transcript:Zm00001d009132_T001 rna1186 7.00E-56
979- 18: transcript:Zm00001d009146_T002 rna1166 0
Alignment 980: score=754.0 e_value=9.7e-48 N=17 8&NC_008394.4 minus
980- 0: transcript:Zm00001d009686_T002 rna2115 3.00E-51
980- 1: transcript:Zm00001d009688_T001 rna2109 2.00E-162
980- 2: transcript:Zm00001d009698_T001 rna2086 3.00E-62
980- 3: transcript:Zm00001d009700_T001 rna2084 8.00E-80
980- 4: transcript:Zm00001d009701_T001 rna2082 8.00E-21
980- 5: transcript:Zm00001d009702_T001 rna2080 0
980- 6: transcript:Zm00001d009705_T001 rna2079 0
980- 7: transcript:Zm00001d009707_T001 rna2077 3.00E-132
980- 8: transcript:Zm00001d009708_T001 rna2076 1.00E-26
980- 9: transcript:Zm00001d009709_T001 rna2071 0
980- 10: transcript:Zm00001d009710_T001 rna2069 0
980- 11: transcript:Zm00001d009716_T001 rna2067 5.00E-36
980- 12: transcript:Zm00001d009717_T001 rna2057 4.00E-173
980- 13: transcript:Zm00001d009719_T003 rna2051 1.00E-42
980- 14: transcript:Zm00001d009724_T009 rna2038 3.00E-22
980- 15: transcript:Zm00001d009726_T001 rna2014 6.00E-135
980- 16: transcript:Zm00001d009727_T006 rna2013 6.00E-28
Alignment 981: score=633.0 e_value=2.4e-35 N=14 8&NC_008394.4 minus
981- 0: transcript:Zm00001d012128_T001 rna3769 2.00E-179
981- 1: transcript:Zm00001d012137_T010 rna3765 0
981- 2: transcript:Zm00001d012138_T001 rna3763 4.00E-138
981- 3: transcript:Zm00001d012140_T001 rna3762 0
981- 4: transcript:Zm00001d012141_T001 rna3758 0
981- 5: transcript:Zm00001d012142_T001 rna3757 3.00E-101
981- 6: transcript:Zm00001d012144_T001 rna3754 3.00E-35
981- 7: transcript:Zm00001d012145_T001 rna3751 0
981- 8: transcript:Zm00001d012146_T003 rna3750 0
981- 9: transcript:Zm00001d012147_T001 rna3747 6.00E-141
981- 10: transcript:Zm00001d012153_T002 rna3739 0
981- 11: transcript:Zm00001d012154_T001 rna3738 5.00E-55
981- 12: transcript:Zm00001d012155_T021 rna3734 0
981- 13: transcript:Zm00001d012156_T007 rna3733 0
Alignment 982: score=623.0 e_value=1e-38 N=15 8&NC_008394.4 minus
982- 0: transcript:Zm00001d009619_T002 rna2235 9.00E-38
982- 1: transcript:Zm00001d009626_T001 rna2234 1.00E-147
982- 2: transcript:Zm00001d009631_T001 rna2224 0
982- 3: transcript:Zm00001d009638_T001 rna2216 0
982- 4: transcript:Zm00001d009639_T001 rna2208 3.00E-101
982- 5: transcript:Zm00001d009640_T002 rna2196 0
982- 6: transcript:Zm00001d009646_T001 rna2184 8.00E-128
982- 7: transcript:Zm00001d009647_T001 rna2177 2.00E-08
982- 8: transcript:Zm00001d009648_T001 rna2172 1.00E-24
982- 9: transcript:Zm00001d009657_T001 rna2167 1.00E-29
982- 10: transcript:Zm00001d009658_T001 rna2164 1.00E-169
982- 11: transcript:Zm00001d009660_T001 rna2158 0
982- 12: transcript:Zm00001d009666_T001 rna2149 0
982- 13: transcript:Zm00001d009667_T001 rna2140 0
982- 14: transcript:Zm00001d009674_T001 rna2137 1.00E-56

Alignment 983: score=586.0 e_value=1e-32 N=13 8&NC_008394.4 minus
983- 0: transcript:Zm00001d009181_T005 rna1370 5.00E-138
983- 1: transcript:Zm00001d009182_T009 rna1365 0
983- 2: transcript:Zm00001d009183_T008 rna1357 0
983- 3: transcript:Zm00001d009186_T001 rna1354 6.00E-14
983- 4: transcript:Zm00001d009187_T001 rna1353 9.00E-15
983- 5: transcript:Zm00001d009189_T001 rna1350 5.00E-27
983- 6: transcript:Zm00001d009193_T001 rna1349 2.00E-51
983- 7: transcript:Zm00001d009202_T001 rna1332 9.00E-11
983- 8: transcript:Zm00001d009205_T001 rna1328 0
983- 9: transcript:Zm00001d009211_T001 rna1326 8.00E-133
983- 10: transcript:Zm00001d009212_T004 rna1325 0
983- 11: transcript:Zm00001d009220_T002 rna1323 0
983- 12: transcript:Zm00001d009222_T001 rna1322 0
Alignment 984: score=528.0 e_value=1.1e-28 N=12 8&NC_008394.4 minus
984- 0: transcript:Zm00001d012625_T001 rna2990 0
984- 1: transcript:Zm00001d012626_T001 rna2988 6.00E-19
984- 2: transcript:Zm00001d012627_T001 rna2987 6.00E-153
984- 3: transcript:Zm00001d012632_T001 rna2981 1.00E-09
984- 4: transcript:Zm00001d012633_T001 rna2974 0
984- 5: transcript:Zm00001d012634_T002 rna2973 0
984- 6: transcript:Zm00001d012635_T001 rna2971 7.00E-21
984- 7: transcript:Zm00001d012636_T001 rna2967 3.00E-09
984- 8: transcript:Zm00001d012640_T010 rna2965 6.00E-58
984- 9: transcript:Zm00001d012641_T001 rna2964 0
984- 10: transcript:Zm00001d012642_T001 rna2956 2.00E-40
984- 11: transcript:Zm00001d012643_T007 rna2954 0
Alignment 985: score=510.0 e_value=8e-25 N=11 8&NC_008394.4 minus
985- 0: transcript:Zm00001d008799_T001 rna618 3.00E-65
985- 1: transcript:Zm00001d008800_T011 rna617 0
985- 2: transcript:Zm00001d008803_T002 rna614 6.00E-47
985- 3: transcript:Zm00001d008805_T001 rna611 0
985- 4: transcript:Zm00001d008808_T002 rna609 8.00E-96
985- 5: transcript:Zm00001d008812_T001 rna608 0
985- 6: transcript:Zm00001d008815_T001 rna606 0
985- 7: transcript:Zm00001d008816_T001 rna605 0
985- 8: transcript:Zm00001d008817_T001 rna602 0
985- 9: transcript:Zm00001d008819_T001 rna597 3.00E-132
985- 10: transcript:Zm00001d008820_T002 rna585 4.00E-31
Alignment 986: score=496.0 e_value=5.8e-25 N=11 8&NC_008394.4 minus
986- 0: transcript:Zm00001d009566_T001 rna2302 1.00E-86
986- 1: transcript:Zm00001d009568_T001 rna2300 5.00E-45
986- 2: transcript:Zm00001d009570_T001 rna2299 1.00E-122
986- 3: transcript:Zm00001d009571_T002 rna2298 2.00E-155
986- 4: transcript:Zm00001d009572_T001 rna2297 4.00E-129
986- 5: transcript:Zm00001d009578_T001 rna2293 9.00E-49
986- 6: transcript:Zm00001d009579_T001 rna2292 3.00E-127
986- 7: transcript:Zm00001d009583_T001 rna2283 2.00E-21
986- 8: transcript:Zm00001d009587_T002 rna2279 0
986- 9: transcript:Zm00001d009591_T006 rna2274 0
986- 10: transcript:Zm00001d009594_T011 rna2272 0
Alignment 987: score=468.0 e_value=1.2e-23 N=11 8&NC_008394.4 minus
987- 0: transcript:Zm00001d010522_T001 rna3379 0
987- 1: transcript:Zm00001d010526_T002 rna3369 0

987- 2: transcript:Zm00001d010527_T001 rna3368 0
987- 3: transcript:Zm00001d010528_T001 rna3364 4.00E-64
987- 4: transcript:Zm00001d010529_T001 rna3361 0
987- 5: transcript:Zm00001d010534_T001 rna3359 5.00E-88
987- 6: transcript:Zm00001d010537_T001 rna3350 4.00E-87
987- 7: transcript:Zm00001d010540_T001 rna3344 3.00E-23
987- 8: transcript:Zm00001d010543_T001 rna3333 4.00E-101
987- 9: transcript:Zm00001d010545_T001 rna3332 0
987- 10: transcript:Zm00001d010546_T003 rna3330 0
Alignment 988: score=462.0 e_value=2.3e-20 N=10 8&NC_008394.4 minus
988- 0: transcript:Zm00001d011211_T001 rna1838 0
988- 1: transcript:Zm00001d011213_T005 rna1832 0
988- 2: transcript:Zm00001d011217_T001 rna1831 1.00E-100
988- 3: transcript:Zm00001d011220_T003 rna1828 0
988- 4: transcript:Zm00001d011222_T001 rna1826 8.00E-132
988- 5: transcript:Zm00001d011223_T002 rna1825 7.00E-161
988- 6: transcript:Zm00001d011224_T001 rna1818 6.00E-68
988- 7: transcript:Zm00001d011225_T003 rna1817 4.00E-47
988- 8: transcript:Zm00001d011226_T002 rna1816 0
988- 9: transcript:Zm00001d011228_T001 rna1815 0
Alignment 989: score=391.0 e_value=1.9e-20 N=9 8&NC_008394.4 minus
989- 0: transcript:Zm00001d008461_T001 rna139 8.00E-40
989- 1: transcript:Zm00001d008465_T001 rna133 0
989- 2: transcript:Zm00001d008466_T001 rna130 8.00E-177
989- 3: transcript:Zm00001d008467_T002 rna128 0
989- 4: transcript:Zm00001d008478_T001 rna120 2.00E-97
989- 5: transcript:Zm00001d008479_T001 rna109 3.00E-113
989- 6: transcript:Zm00001d008485_T001 rna107 0
989- 7: transcript:Zm00001d008488_T006 rna98 0
989- 8: transcript:Zm00001d008489_T001 rna97 0
Alignment 990: score=387.0 e_value=4.2e-18 N=9 8&NC_008394.4 minus
990- 0: transcript:Zm00001d010791_T001 rna2754 2.00E-74
990- 1: transcript:Zm00001d010793_T002 rna2748 0
990- 2: transcript:Zm00001d010795_T001 rna2747 4.00E-83
990- 3: transcript:Zm00001d010796_T001 rna2746 0
990- 4: transcript:Zm00001d010798_T001 rna2728 4.00E-33
990- 5: transcript:Zm00001d010800_T001 rna2704 1.00E-67
990- 6: transcript:Zm00001d010802_T001 rna2694 1.00E-147
990- 7: transcript:Zm00001d010803_T001 rna2690 3.00E-81
990- 8: transcript:Zm00001d010805_T001 rna2689 8.00E-65
Alignment 991: score=368.0 e_value=1.2e-17 N=9 8&NC_008394.4 minus
991- 0: transcript:Zm00001d009421_T001 rna2531 4.00E-47
991- 1: transcript:Zm00001d009425_T001 rna2516 9.00E-10
991- 2: transcript:Zm00001d009431_T001 rna2506 0
991- 3: transcript:Zm00001d009435_T001 rna2502 5.00E-97
991- 4: transcript:Zm00001d009436_T003 rna2500 1.00E-178
991- 5: transcript:Zm00001d009439_T004 rna2496 0
991- 6: transcript:Zm00001d009446_T001 rna2475 4.00E-07
991- 7: transcript:Zm00001d009447_T002 rna2465 0
991- 8: transcript:Zm00001d009452_T007 rna2460 0
Alignment 992: score=343.0 e_value=1.9e-11 N=7 8&NC_008394.4 minus
992- 0: transcript:Zm00001d009397_T001 rna1767 1.00E-26
992- 1: transcript:Zm00001d009398_T003 rna1764 1.00E-140
992- 2: transcript:Zm00001d009399_T001 rna1762 0

992- 3: transcript:Zm00001d009401_T001 rna1760 0
992- 4: transcript:Zm00001d009402_T001 rna1758 1.00E-99
992- 5: transcript:Zm00001d009403_T002 rna1756 3.00E-130
992- 6: transcript:Zm00001d009404_T001 rna1755 5.00E-164
Alignment 993: score=313.0 e_value=2.1e-13 N=8 8&NC_008394.4 minus
993- 0: transcript:Zm00001d010402_T001 rna860 2.00E-68
993- 1: transcript:Zm00001d010404_T001 rna853 7.00E-108
993- 2: transcript:Zm00001d010407_T001 rna847 2.00E-16
993- 3: transcript:Zm00001d010410_T001 rna841 5.00E-84
993- 4: transcript:Zm00001d010411_T001 rna836 2.00E-118
993- 5: transcript:Zm00001d010416_T002 rna834 6.00E-87
993- 6: transcript:Zm00001d010421_T001 rna812 0
993- 7: transcript:Zm00001d010430_T001 rna809 1.00E-180
Alignment 994: score=294.0 e_value=1.8e-11 N=7 8&NC_008394.4 minus
994- 0: transcript:Zm00001d010723_T001 rna2913 3.00E-144
994- 1: transcript:Zm00001d010724_T001 rna2903 2.00E-117
994- 2: transcript:Zm00001d010726_T001 rna2901 2.00E-15
994- 3: transcript:Zm00001d010728_T005 rna2882 3.00E-25
994- 4: transcript:Zm00001d010730_T001 rna2876 1.00E-65
994- 5: transcript:Zm00001d010731_T001 rna2870 3.00E-46
994- 6: transcript:Zm00001d010743_T001 rna2852 0
Alignment 995: score=282.0 e_value=1.1e-09 N=6 8&NC_008394.4 minus
995- 0: transcript:Zm00001d011193_T001 rna1870 0
995- 1: transcript:Zm00001d011197_T007 rna1865 1.00E-54
995- 2: transcript:Zm00001d011198_T001 rna1864 2.00E-59
995- 3: transcript:Zm00001d011199_T002 rna1862 5.00E-137
995- 4: transcript:Zm00001d011202_T001 rna1861 8.00E-79
995- 5: transcript:Zm00001d011210_T001 rna1855 4.00E-129
Alignment 996: score=265.0 e_value=2e-12 N=6 8&NC_008394.4 minus
996- 0: transcript:Zm00001d010655_T001 rna3131 2.00E-155
996- 1: transcript:Zm00001d010670_T001 rna3120 0
996- 2: transcript:Zm00001d010671_T001 rna3119 1.00E-104
996- 3: transcript:Zm00001d010672_T001 rna3110 8.00E-59
996- 4: transcript:Zm00001d010673_T001 rna3106 5.00E-163
996- 5: transcript:Zm00001d010678_T003 rna3092 2.00E-176
Alignment 997: score=252.0 e_value=2e-10 N=6 8&NC_008394.4 minus
997- 0: transcript:Zm00001d009138_T001 rna1284 2.00E-120
997- 1: transcript:Zm00001d009147_T002 rna1273 2.00E-104
997- 2: transcript:Zm00001d009150_T002 rna1272 0
997- 3: transcript:Zm00001d009152_T001 rna1265 2.00E-153
997- 4: transcript:Zm00001d009156_T001 rna1263 1.00E-108
997- 5: transcript:Zm00001d009160_T001 rna1262 1.00E-50
Alignment 998: score=372.0 e_value=4.2e-20 N=9 8&NC_008396.2 plus
998- 0: transcript:Zm00001d012401_T001 rna8809 2.00E-118
998- 1: transcript:Zm00001d012402_T001 rna8821 7.00E-56
998- 2: transcript:Zm00001d012408_T001 rna8836 0
998- 3: transcript:Zm00001d012420_T001 rna8850 0
998- 4: transcript:Zm00001d012427_T001 rna8874 3.00E-24
998- 5: transcript:Zm00001d012431_T001 rna8879 1.00E-23
998- 6: transcript:Zm00001d012432_T001 rna8881 2.00E-17
998- 7: transcript:Zm00001d012433_T002 rna8882 0
998- 8: transcript:Zm00001d012438_T001 rna8892 0
Alignment 999: score=306.0 e_value=5e-12 N=7 8&NC_008396.2 minus
999- 0: transcript:Zm00001d010634_T001 rna9164 2.00E-100

999- 1: transcript:Zm00001d010635_T001 rna9159 3.00E-39
999- 2: transcript:Zm00001d010639_T002 rna9136 4.00E-38
999- 3: transcript:Zm00001d010640_T001 rna9135 2.00E-136
999- 4: transcript:Zm00001d010646_T001 rna9129 6.00E-131
999- 5: transcript:Zm00001d010649_T001 rna9124 7.00E-30
999- 6: transcript:Zm00001d010658_T001 rna9120 3.00E-130
Alignment 1000: score=252.0 e_value=3.4e-08 N=6 8&NC_008396.2 minus
1000- 0: transcript:Zm00001d010976_T001 rna8931 9.00E-108
1000- 1: transcript:Zm00001d010978_T001 rna8929 2.00E-63
1000- 2: transcript:Zm00001d010985_T001 rna8924 2.00E-149
1000- 3: transcript:Zm00001d010994_T001 rna8910 8.00E-57
1000- 4: transcript:Zm00001d011006_T001 rna8892 0
1000- 5: transcript:Zm00001d011013_T001 rna8890 0
Alignment 1001: score=2863.0 e_value=1.4e-252 N=62 8&NC_008398.2 plus
1001- 0: transcript:Zm00001d010574_T002 rna15682 4.00E-19
1001- 1: transcript:Zm00001d010575_T001 rna15695 6.00E-67
1001- 2: transcript:Zm00001d010576_T002 rna15697 8.00E-21
1001- 3: transcript:Zm00001d010578_T003 rna15698 0
1001- 4: transcript:Zm00001d010579_T001 rna15699 0
1001- 5: transcript:Zm00001d010583_T001 rna15700 3.00E-107
1001- 6: transcript:Zm00001d010584_T001 rna15709 3.00E-178
1001- 7: transcript:Zm00001d010585_T002 rna15710 0
1001- 8: transcript:Zm00001d010586_T001 rna15712 2.00E-89
1001- 9: transcript:Zm00001d010587_T001 rna15713 5.00E-63
1001- 10: transcript:Zm00001d010588_T001 rna15714 0
1001- 11: transcript:Zm00001d010589_T001 rna15720 0
1001- 12: transcript:Zm00001d010590_T001 rna15721 0
1001- 13: transcript:Zm00001d010591_T001 rna15722 6.00E-96
1001- 14: transcript:Zm00001d010594_T001 rna15724 3.00E-107
1001- 15: transcript:Zm00001d010599_T002 rna15727 0
1001- 16: transcript:Zm00001d010601_T001 rna15733 0
1001- 17: transcript:Zm00001d010602_T001 rna15734 0
1001- 18: transcript:Zm00001d010603_T001 rna15736 4.00E-109
1001- 19: transcript:Zm00001d010604_T006 rna15737 1.00E-30
1001- 20: transcript:Zm00001d010607_T001 rna15741 0
1001- 21: transcript:Zm00001d010610_T001 rna15748 0
1001- 22: transcript:Zm00001d010611_T002 rna15749 0
1001- 23: transcript:Zm00001d010613_T002 rna15750 9.00E-20
1001- 24: transcript:Zm00001d010614_T007 rna15754 6.00E-70
1001- 25: transcript:Zm00001d010615_T001 rna15760 2.00E-55
1001- 26: transcript:Zm00001d010620_T001 rna15771 0
1001- 27: transcript:Zm00001d010622_T002 rna15777 2.00E-147
1001- 28: transcript:Zm00001d010623_T001 rna15779 5.00E-23
1001- 29: transcript:Zm00001d010625_T001 rna15783 0
1001- 30: transcript:Zm00001d010627_T001 rna15787 1.00E-90
1001- 31: transcript:Zm00001d010629_T001 rna15788 0
1001- 32: transcript:Zm00001d010630_T003 rna15791 2.00E-58
1001- 33: transcript:Zm00001d010631_T003 rna15795 1.00E-88
1001- 34: transcript:Zm00001d010632_T002 rna15796 2.00E-17
1001- 35: transcript:Zm00001d010633_T001 rna15800 1.00E-20
1001- 36: transcript:Zm00001d010634_T001 rna15801 0
1001- 37: transcript:Zm00001d010635_T001 rna15802 4.00E-121
1001- 38: transcript:Zm00001d010638_T001 rna15808 3.00E-139
1001- 39: transcript:Zm00001d010639_T002 rna15813 0

1001- 40:	transcript:Zm00001d010640_T001	rna15814	0
1001- 41:	transcript:Zm00001d010646_T001	rna15818	0
1001- 42:	transcript:Zm00001d010649_T001	rna15821	1.00E-58
1001- 43:	transcript:Zm00001d010650_T001	rna15822	4.00E-77
1001- 44:	transcript:Zm00001d010656_T001	rna15835	0
1001- 45:	transcript:Zm00001d010667_T001	rna15846	4.00E-73
1001- 46:	transcript:Zm00001d010670_T001	rna15849	0
1001- 47:	transcript:Zm00001d010671_T001	rna15850	1.00E-112
1001- 48:	transcript:Zm00001d010672_T001	rna15853	3.00E-115
1001- 49:	transcript:Zm00001d010673_T001	rna15854	3.00E-170
1001- 50:	transcript:Zm00001d010676_T001	rna15857	7.00E-61
1001- 51:	transcript:Zm00001d010678_T003	rna15861	1.00E-159
1001- 52:	transcript:Zm00001d010679_T001	rna15862	3.00E-179
1001- 53:	transcript:Zm00001d010684_T003	rna15866	2.00E-57
1001- 54:	transcript:Zm00001d010687_T004	rna15871	0
1001- 55:	transcript:Zm00001d010689_T001	rna15876	0
1001- 56:	transcript:Zm00001d010693_T001	rna15882	2.00E-98
1001- 57:	transcript:Zm00001d010697_T001	rna15885	0
1001- 58:	transcript:Zm00001d010698_T002	rna15887	3.00E-135
1001- 59:	transcript:Zm00001d010700_T002	rna15889	0
1001- 60:	transcript:Zm00001d010702_T001	rna15897	2.00E-95
1001- 61:	transcript:Zm00001d010703_T002	rna15899	2.00E-170

Alignment 1002: score=2476.0 e_value=7.9e-219 N=55 8&NC_008398.2 plus

1002- 0:	transcript:Zm00001d009603_T002	rna16310	3.00E-61
1002- 1:	transcript:Zm00001d009608_T001	rna16312	0
1002- 2:	transcript:Zm00001d009612_T001	rna16316	5.00E-52
1002- 3:	transcript:Zm00001d009613_T001	rna16318	0
1002- 4:	transcript:Zm00001d009617_T017	rna16331	0
1002- 5:	transcript:Zm00001d009618_T001	rna16332	0
1002- 6:	transcript:Zm00001d009619_T002	rna16333	3.00E-63
1002- 7:	transcript:Zm00001d009622_T001	rna16339	9.00E-28
1002- 8:	transcript:Zm00001d009626_T001	rna16340	4.00E-150
1002- 9:	transcript:Zm00001d009627_T001	rna16342	2.00E-23
1002- 10:	transcript:Zm00001d009631_T001	rna16344	0
1002- 11:	transcript:Zm00001d009636_T001	rna16345	1.00E-65
1002- 12:	transcript:Zm00001d009638_T001	rna16346	0
1002- 13:	transcript:Zm00001d009639_T001	rna16348	5.00E-91
1002- 14:	transcript:Zm00001d009640_T002	rna16351	0
1002- 15:	transcript:Zm00001d009645_T001	rna16354	3.00E-113
1002- 16:	transcript:Zm00001d009646_T001	rna16356	3.00E-145
1002- 17:	transcript:Zm00001d009647_T001	rna16364	1.00E-28
1002- 18:	transcript:Zm00001d009648_T001	rna16365	2.00E-64
1002- 19:	transcript:Zm00001d009657_T001	rna16369	6.00E-42
1002- 20:	transcript:Zm00001d009658_T001	rna16370	0
1002- 21:	transcript:Zm00001d009660_T001	rna16372	0
1002- 22:	transcript:Zm00001d009661_T002	rna16373	0
1002- 23:	transcript:Zm00001d009666_T001	rna16377	0
1002- 24:	transcript:Zm00001d009667_T001	rna16378	0
1002- 25:	transcript:Zm00001d009668_T001	rna16380	3.00E-42
1002- 26:	transcript:Zm00001d009669_T005	rna16381	0
1002- 27:	transcript:Zm00001d009673_T001	rna16382	2.00E-139
1002- 28:	transcript:Zm00001d009674_T001	rna16384	4.00E-81
1002- 29:	transcript:Zm00001d009677_T001	rna16397	0
1002- 30:	transcript:Zm00001d009683_T004	rna16401	0

1002- 31: transcript:Zm00001d009686_T002 rna16402 2.00E-76
1002- 32: transcript:Zm00001d009687_T001 rna16403 4.00E-74
1002- 33: transcript:Zm00001d009693_T001 rna16404 0
1002- 34: transcript:Zm00001d009698_T001 rna16408 6.00E-81
1002- 35: transcript:Zm00001d009702_T001 rna16420 0
1002- 36: transcript:Zm00001d009705_T001 rna16421 0
1002- 37: transcript:Zm00001d009707_T001 rna16422 4.00E-137
1002- 38: transcript:Zm00001d009708_T001 rna16423 0
1002- 39: transcript:Zm00001d009710_T001 rna16426 0
1002- 40: transcript:Zm00001d009711_T001 rna16427 3.00E-31
1002- 41: transcript:Zm00001d009714_T002 rna16428 0
1002- 42: transcript:Zm00001d009716_T001 rna16429 0
1002- 43: transcript:Zm00001d009718_T001 rna16432 4.00E-94
1002- 44: transcript:Zm00001d009719_T003 rna16435 0
1002- 45: transcript:Zm00001d009723_T001 rna16436 2.00E-73
1002- 46: transcript:Zm00001d009724_T009 rna16437 0
1002- 47: transcript:Zm00001d009726_T001 rna16441 5.00E-14
1002- 48: transcript:Zm00001d009728_T001 rna16442 3.00E-89
1002- 49: transcript:Zm00001d009729_T001 rna16446 6.00E-65
1002- 50: transcript:Zm00001d009734_T001 rna16448 0
1002- 51: transcript:Zm00001d009740_T004 rna16456 6.00E-116
1002- 52: transcript:Zm00001d009741_T001 rna16459 0
1002- 53: transcript:Zm00001d009745_T023 rna16468 0
1002- 54: transcript:Zm00001d009746_T001 rna16473 0
Alignment 1003: score=2312.0 e_value=1.1e-195 N=50 8&NC_008398.2 plus
1003- 0: transcript:Zm00001d009425_T001 rna16137 8.00E-21
1003- 1: transcript:Zm00001d009426_T005 rna16139 4.00E-107
1003- 2: transcript:Zm00001d009429_T001 rna16140 9.00E-14
1003- 3: transcript:Zm00001d009430_T002 rna16146 0
1003- 4: transcript:Zm00001d009431_T001 rna16147 0
1003- 5: transcript:Zm00001d009435_T001 rna16148 9.00E-100
1003- 6: transcript:Zm00001d009436_T003 rna16149 0
1003- 7: transcript:Zm00001d009439_T004 rna16152 0
1003- 8: transcript:Zm00001d009445_T001 rna16162 2.00E-40
1003- 9: transcript:Zm00001d009446_T001 rna16170 3.00E-41
1003- 10: transcript:Zm00001d009447_T002 rna16172 0
1003- 11: transcript:Zm00001d009449_T001 rna16174 0
1003- 12: transcript:Zm00001d009450_T002 rna16176 6.00E-37
1003- 13: transcript:Zm00001d009452_T007 rna16178 0
1003- 14: transcript:Zm00001d009458_T001 rna16180 3.00E-60
1003- 15: transcript:Zm00001d009468_T001 rna16183 1.00E-172
1003- 16: transcript:Zm00001d009473_T001 rna16190 7.00E-105
1003- 17: transcript:Zm00001d009474_T001 rna16192 9.00E-139
1003- 18: transcript:Zm00001d009475_T001 rna16193 5.00E-60
1003- 19: transcript:Zm00001d009481_T001 rna16201 3.00E-152
1003- 20: transcript:Zm00001d009484_T001 rna16207 0
1003- 21: transcript:Zm00001d009487_T001 rna16208 2.00E-62
1003- 22: transcript:Zm00001d009488_T001 rna16210 0
1003- 23: transcript:Zm00001d009493_T001 rna16214 6.00E-128
1003- 24: transcript:Zm00001d009494_T001 rna16215 8.00E-35
1003- 25: transcript:Zm00001d009495_T001 rna16216 9.00E-73
1003- 26: transcript:Zm00001d009497_T004 rna16217 0
1003- 27: transcript:Zm00001d009500_T002 rna16222 0
1003- 28: transcript:Zm00001d009501_T001 rna16226 1.00E-23

1003- 29: transcript:Zm00001d009503_T001 rna16228 4.00E-151
1003- 30: transcript:Zm00001d009504_T002 rna16229 0
1003- 31: transcript:Zm00001d009506_T001 rna16231 0
1003- 32: transcript:Zm00001d009508_T001 rna16232 3.00E-79
1003- 33: transcript:Zm00001d009509_T001 rna16236 1.00E-61
1003- 34: transcript:Zm00001d009510_T001 rna16237 0
1003- 35: transcript:Zm00001d009511_T001 rna16240 0
1003- 36: transcript:Zm00001d009513_T003 rna16241 0
1003- 37: transcript:Zm00001d009517_T001 rna16243 8.00E-90
1003- 38: transcript:Zm00001d009520_T002 rna16247 0
1003- 39: transcript:Zm00001d009525_T002 rna16250 2.00E-43
1003- 40: transcript:Zm00001d009532_T001 rna16251 8.00E-117
1003- 41: transcript:Zm00001d009539_T002 rna16252 0
1003- 42: transcript:Zm00001d009541_T002 rna16255 0
1003- 43: transcript:Zm00001d009543_T001 rna16256 1.00E-08
1003- 44: transcript:Zm00001d009549_T002 rna16257 0
1003- 45: transcript:Zm00001d009553_T001 rna16264 5.00E-06
1003- 46: transcript:Zm00001d009554_T001 rna16265 0
1003- 47: transcript:Zm00001d009555_T003 rna16266 1.00E-165
1003- 48: transcript:Zm00001d009556_T001 rna16271 0
1003- 49: transcript:Zm00001d009557_T001 rna16273 2.00E-29
Alignment 1004: score=1880.0 e_value=1e-164 N=45 8&NC_008398.2 plus
1004- 0: transcript:Zm00001d012176_T001 rna15444 0
1004- 1: transcript:Zm00001d012196_T005 rna15459 2.00E-124
1004- 2: transcript:Zm00001d012198_T001 rna15460 0
1004- 3: transcript:Zm00001d012203_T001 rna15466 8.00E-111
1004- 4: transcript:Zm00001d012212_T001 rna15475 7.00E-37
1004- 5: transcript:Zm00001d012213_T003 rna15479 2.00E-29
1004- 6: transcript:Zm00001d012216_T001 rna15484 0
1004- 7: transcript:Zm00001d012220_T001 rna15494 1.00E-172
1004- 8: transcript:Zm00001d012223_T001 rna15496 0
1004- 9: transcript:Zm00001d012224_T014 rna15497 7.00E-155
1004- 10: transcript:Zm00001d012234_T002 rna15502 0
1004- 11: transcript:Zm00001d012239_T003 rna15503 3.00E-60
1004- 12: transcript:Zm00001d012245_T002 rna15509 2.00E-89
1004- 13: transcript:Zm00001d012247_T004 rna15513 0
1004- 14: transcript:Zm00001d012254_T002 rna15515 0
1004- 15: transcript:Zm00001d012255_T001 rna15518 4.00E-119
1004- 16: transcript:Zm00001d012257_T013 rna15520 0
1004- 17: transcript:Zm00001d012259_T001 rna15524 0
1004- 18: transcript:Zm00001d012263_T002 rna15532 1.00E-102
1004- 19: transcript:Zm00001d012268_T001 rna15543 0
1004- 20: transcript:Zm00001d012269_T003 rna15548 3.00E-165
1004- 21: transcript:Zm00001d012270_T001 rna15551 9.00E-80
1004- 22: transcript:Zm00001d012273_T001 rna15552 1.00E-31
1004- 23: transcript:Zm00001d012276_T001 rna15557 3.00E-94
1004- 24: transcript:Zm00001d012277_T003 rna15558 0
1004- 25: transcript:Zm00001d012279_T001 rna15559 2.00E-43
1004- 26: transcript:Zm00001d012280_T001 rna15564 3.00E-75
1004- 27: transcript:Zm00001d012282_T001 rna15569 9.00E-44
1004- 28: transcript:Zm00001d012285_T001 rna15573 2.00E-76
1004- 29: transcript:Zm00001d012292_T001 rna15577 1.00E-149
1004- 30: transcript:Zm00001d012293_T001 rna15578 2.00E-55
1004- 31: transcript:Zm00001d012294_T009 rna15581 0

1004- 32: transcript:Zm00001d012304_T001 rna15585 0
1004- 33: transcript:Zm00001d012313_T001 rna15594 2.00E-56
1004- 34: transcript:Zm00001d012325_T001 rna15598 0
1004- 35: transcript:Zm00001d012333_T001 rna15603 0
1004- 36: transcript:Zm00001d012337_T001 rna15606 6.00E-84
1004- 37: transcript:Zm00001d012339_T001 rna15607 2.00E-07
1004- 38: transcript:Zm00001d012350_T001 rna15608 3.00E-168
1004- 39: transcript:Zm00001d012362_T001 rna15612 0
1004- 40: transcript:Zm00001d012380_T002 rna15617 0
1004- 41: transcript:Zm00001d012381_T001 rna15619 2.00E-108
1004- 42: transcript:Zm00001d012382_T002 rna15621 2.00E-70
1004- 43: transcript:Zm00001d012383_T002 rna15625 3.00E-14
1004- 44: transcript:Zm00001d012391_T003 rna15628 0
Alignment 1005: score=881.0 e_value=1.8e-52 N=19 8&NC_008398.2 plus
1005- 0: transcript:Zm00001d010793_T002 rna16012 0
1005- 1: transcript:Zm00001d010795_T001 rna16015 1.00E-119
1005- 2: transcript:Zm00001d010796_T001 rna16017 0
1005- 3: transcript:Zm00001d010798_T001 rna16023 6.00E-18
1005- 4: transcript:Zm00001d010800_T001 rna16034 9.00E-123
1005- 5: transcript:Zm00001d010802_T001 rna16046 1.00E-88
1005- 6: transcript:Zm00001d010803_T001 rna16052 7.00E-82
1005- 7: transcript:Zm00001d010806_T001 rna16054 4.00E-175
1005- 8: transcript:Zm00001d010808_T001 rna16056 4.00E-154
1005- 9: transcript:Zm00001d010812_T001 rna16062 6.00E-169
1005- 10: transcript:Zm00001d010811_T007 rna16063 0
1005- 11: transcript:Zm00001d010813_T001 rna16064 0
1005- 12: transcript:Zm00001d010814_T001 rna16066 6.00E-33
1005- 13: transcript:Zm00001d010816_T001 rna16069 0
1005- 14: transcript:Zm00001d010818_T001 rna16072 2.00E-130
1005- 15: transcript:Zm00001d010819_T001 rna16075 4.00E-25
1005- 16: transcript:Zm00001d010821_T002 rna16080 0
1005- 17: transcript:Zm00001d010822_T001 rna16085 1.00E-91
1005- 18: transcript:Zm00001d010823_T002 rna16088 0
Alignment 1006: score=838.0 e_value=5.2e-55 N=19 8&NC_008398.2 plus
1006- 0: transcript:Zm00001d010519_T002 rna15642 0
1006- 1: transcript:Zm00001d010520_T001 rna15643 0
1006- 2: transcript:Zm00001d010522_T001 rna15648 0
1006- 3: transcript:Zm00001d010525_T001 rna15653 0
1006- 4: transcript:Zm00001d010526_T002 rna15655 0
1006- 5: transcript:Zm00001d010527_T001 rna15656 0
1006- 6: transcript:Zm00001d010528_T001 rna15657 8.00E-62
1006- 7: transcript:Zm00001d010529_T001 rna15658 0
1006- 8: transcript:Zm00001d010534_T001 rna15659 3.00E-99
1006- 9: transcript:Zm00001d010535_T001 rna15663 0
1006- 10: transcript:Zm00001d010536_T001 rna15665 2.00E-130
1006- 11: transcript:Zm00001d010537_T001 rna15666 4.00E-101
1006- 12: transcript:Zm00001d010538_T008 rna15667 8.00E-152
1006- 13: transcript:Zm00001d010540_T001 rna15669 1.00E-26
1006- 14: transcript:Zm00001d010545_T001 rna15673 0
1006- 15: transcript:Zm00001d010546_T003 rna15674 0
1006- 16: transcript:Zm00001d010548_T002 rna15676 0
1006- 17: transcript:Zm00001d010556_T001 rna15679 9.00E-114
1006- 18: transcript:Zm00001d010558_T001 rna15694 0
Alignment 1007: score=715.0 e_value=7.4e-43 N=16 8&NC_008398.2 plus

1007- 0: transcript:Zm00001d012450_T001 rna15696 4.00E-56
1007- 1: transcript:Zm00001d012451_T004 rna15697 5.00E-19
1007- 2: transcript:Zm00001d012452_T004 rna15698 0
1007- 3: transcript:Zm00001d012457_T003 rna15699 0
1007- 4: transcript:Zm00001d012458_T001 rna15700 9.00E-97
1007- 5: transcript:Zm00001d012463_T001 rna15720 4.00E-164
1007- 6: transcript:Zm00001d012467_T001 rna15721 0
1007- 7: transcript:Zm00001d012471_T001 rna15724 4.00E-50
1007- 8: transcript:Zm00001d012475_T001 rna15731 6.00E-86
1007- 9: transcript:Zm00001d012479_T004 rna15737 3.00E-09
1007- 10: transcript:Zm00001d012480_T001 rna15739 3.00E-57
1007- 11: transcript:Zm00001d012482_T001 rna15740 1.00E-136
1007- 12: transcript:Zm00001d012488_T001 rna15745 0
1007- 13: transcript:Zm00001d012494_T001 rna15751 0
1007- 14: transcript:Zm00001d012498_T001 rna15753 4.00E-72
1007- 15: transcript:Zm00001d012500_T001 rna15759 4.00E-39
Alignment 1008: score=688.0 e_value=3.3e-42 N=15 8&NC_008398.2 plus
1008- 0: transcript:Zm00001d009564_T001 rna16275 4.00E-75
1008- 1: transcript:Zm00001d009566_T001 rna16279 1.00E-81
1008- 2: transcript:Zm00001d009570_T001 rna16285 2.00E-168
1008- 3: transcript:Zm00001d009571_T002 rna16286 2.00E-179
1008- 4: transcript:Zm00001d009572_T001 rna16287 1.00E-155
1008- 5: transcript:Zm00001d009578_T001 rna16289 3.00E-55
1008- 6: transcript:Zm00001d009579_T001 rna16290 3.00E-128
1008- 7: transcript:Zm00001d009580_T001 rna16292 5.00E-174
1008- 8: transcript:Zm00001d009582_T001 rna16295 2.00E-161
1008- 9: transcript:Zm00001d009583_T001 rna16296 2.00E-43
1008- 10: transcript:Zm00001d009587_T002 rna16300 0
1008- 11: transcript:Zm00001d009591_T006 rna16305 0
1008- 12: transcript:Zm00001d009594_T011 rna16306 0
1008- 13: transcript:Zm00001d009596_T001 rna16307 3.00E-63
1008- 14: transcript:Zm00001d009597_T002 rna16308 0
Alignment 1009: score=517.0 e_value=1.8e-27 N=12 8&NC_008398.2 plus
1009- 0: transcript:Zm00001d012560_T002 rna15835 5.00E-179
1009- 1: transcript:Zm00001d012561_T001 rna15840 2.00E-35
1009- 2: transcript:Zm00001d012562_T001 rna15841 0
1009- 3: transcript:Zm00001d012567_T002 rna15849 0
1009- 4: transcript:Zm00001d012568_T001 rna15850 2.00E-166
1009- 5: transcript:Zm00001d012584_T001 rna15857 1.00E-44
1009- 6: transcript:Zm00001d012593_T001 rna15868 1.00E-45
1009- 7: transcript:Zm00001d012600_T001 rna15871 0
1009- 8: transcript:Zm00001d012607_T001 rna15887 1.00E-129
1009- 9: transcript:Zm00001d012609_T001 rna15889 0
1009- 10: transcript:Zm00001d012610_T001 rna15891 2.00E-109
1009- 11: transcript:Zm00001d012614_T001 rna15892 0
Alignment 1010: score=465.0 e_value=4.2e-24 N=11 8&NC_008398.2 plus
1010- 0: transcript:Zm00001d010718_T001 rna15915 3.00E-15
1010- 1: transcript:Zm00001d010720_T001 rna15942 0
1010- 2: transcript:Zm00001d010726_T001 rna15948 6.00E-49
1010- 3: transcript:Zm00001d010728_T005 rna15952 5.00E-32
1010- 4: transcript:Zm00001d010730_T001 rna15953 4.00E-93
1010- 5: transcript:Zm00001d010731_T001 rna15954 8.00E-124
1010- 6: transcript:Zm00001d010732_T002 rna15959 3.00E-141
1010- 7: transcript:Zm00001d010736_T001 rna15968 2.00E-154

1010- 8: transcript:Zm00001d010744_T001 rna15984 0
1010- 9: transcript:Zm00001d010754_T001 rna15988 4.00E-141
1010- 10: transcript:Zm00001d010755_T001 rna15990 2.00E-125
Alignment 1011: score=461.0 e_value=1.8e-19 N=10 8&NC_008398.2 plus
1011- 0: transcript:Zm00001d009997_T001 rna14681 1.00E-176
1011- 1: transcript:Zm00001d009999_T001 rna14683 2.00E-24
1011- 2: transcript:Zm00001d010000_T001 rna14684 8.00E-58
1011- 3: transcript:Zm00001d010001_T001 rna14687 3.00E-84
1011- 4: transcript:Zm00001d010007_T001 rna14689 7.00E-59
1011- 5: transcript:Zm00001d010008_T001 rna14701 2.00E-117
1011- 6: transcript:Zm00001d010012_T001 rna14706 3.00E-152
1011- 7: transcript:Zm00001d010016_T002 rna14708 6.00E-130
1011- 8: transcript:Zm00001d010018_T004 rna14711 0
1011- 9: transcript:Zm00001d010020_T001 rna14713 2.00E-81
Alignment 1012: score=460.0 e_value=7e-20 N=10 8&NC_008398.2 plus
1012- 0: transcript:Zm00001d012530_T001 rna15800 7.00E-71
1012- 1: transcript:Zm00001d012534_T001 rna15804 0
1012- 2: transcript:Zm00001d012535_T001 rna15806 2.00E-48
1012- 3: transcript:Zm00001d012537_T003 rna15807 3.00E-94
1012- 4: transcript:Zm00001d012538_T001 rna15808 3.00E-77
1012- 5: transcript:Zm00001d012544_T001 rna15817 6.00E-67
1012- 6: transcript:Zm00001d012546_T002 rna15819 4.00E-71
1012- 7: transcript:Zm00001d012550_T001 rna15824 7.00E-78
1012- 8: transcript:Zm00001d012551_T001 rna15826 2.00E-17
1012- 9: transcript:Zm00001d012552_T005 rna15827 0
Alignment 1013: score=457.0 e_value=6e-21 N=10 8&NC_008398.2 plus
1013- 0: transcript:Zm00001d009888_T001 rna15172 4.00E-46
1013- 1: transcript:Zm00001d009890_T001 rna15173 3.00E-164
1013- 2: transcript:Zm00001d009894_T001 rna15177 6.00E-42
1013- 3: transcript:Zm00001d009899_T001 rna15183 0
1013- 4: transcript:Zm00001d009902_T001 rna15187 7.00E-70
1013- 5: transcript:Zm00001d009907_T001 rna15190 0
1013- 6: transcript:Zm00001d009908_T005 rna15191 0
1013- 7: transcript:Zm00001d009910_T001 rna15193 8.00E-57
1013- 8: transcript:Zm00001d009912_T001 rna15195 0
1013- 9: transcript:Zm00001d009918_T001 rna15197 1.00E-170
Alignment 1014: score=447.0 e_value=2e-20 N=10 8&NC_008398.2 plus
1014- 0: transcript:Zm00001d010461_T001 rna15307 8.00E-60
1014- 1: transcript:Zm00001d010462_T002 rna15310 0
1014- 2: transcript:Zm00001d010463_T001 rna15314 2.00E-35
1014- 3: transcript:Zm00001d010465_T001 rna15319 1.00E-131
1014- 4: transcript:Zm00001d010466_T001 rna15325 0
1014- 5: transcript:Zm00001d010476_T002 rna15329 0
1014- 6: transcript:Zm00001d010478_T003 rna15331 0
1014- 7: transcript:Zm00001d010480_T001 rna15333 0
1014- 8: transcript:Zm00001d010481_T001 rna15334 1.00E-75
1014- 9: transcript:Zm00001d010488_T008 rna15340 1.00E-42
Alignment 1015: score=436.0 e_value=1.3e-22 N=10 8&NC_008398.2 plus
1015- 0: transcript:Zm00001d008666_T003 rna14525 3.00E-110
1015- 1: transcript:Zm00001d008676_T001 rna14529 4.00E-19
1015- 2: transcript:Zm00001d008679_T010 rna14542 0
1015- 3: transcript:Zm00001d008691_T001 rna14546 2.00E-161
1015- 4: transcript:Zm00001d008700_T002 rna14547 0
1015- 5: transcript:Zm00001d008708_T001 rna14548 4.00E-171

1015- 6: transcript:Zm00001d008715_T001 rna14550 2.00E-95
1015- 7: transcript:Zm00001d008716_T002 rna14551 4.00E-30
1015- 8: transcript:Zm00001d008722_T001 rna14556 7.00E-06
1015- 9: transcript:Zm00001d008724_T005 rna14557 6.00E-31
Alignment 1016: score=407.0 e_value=2.1e-16 N=9 8&NC_008398.2 plus
1016- 0: transcript:Zm00001d012408_T001 rna15649 0
1016- 1: transcript:Zm00001d012417_T001 rna15656 0
1016- 2: transcript:Zm00001d012420_T001 rna15658 0
1016- 3: transcript:Zm00001d012421_T004 rna15659 2.00E-119
1016- 4: transcript:Zm00001d012425_T002 rna15664 4.00E-65
1016- 5: transcript:Zm00001d012431_T001 rna15671 1.00E-31
1016- 6: transcript:Zm00001d012437_T001 rna15673 0
1016- 7: transcript:Zm00001d012439_T001 rna15675 4.00E-67
1016- 8: transcript:Zm00001d012445_T001 rna15682 2.00E-06
Alignment 1017: score=382.0 e_value=8.6e-18 N=9 8&NC_008398.2 plus
1017- 0: transcript:Zm00001d012757_T001 rna16004 8.00E-104
1017- 1: transcript:Zm00001d012763_T002 rna16009 0
1017- 2: transcript:Zm00001d012766_T001 rna16012 0
1017- 3: transcript:Zm00001d012767_T001 rna16015 1.00E-57
1017- 4: transcript:Zm00001d012769_T001 rna16018 8.00E-53
1017- 5: transcript:Zm00001d012776_T001 rna16037 1.00E-144
1017- 6: transcript:Zm00001d012779_T001 rna16039 7.00E-71
1017- 7: transcript:Zm00001d012781_T001 rna16041 2.00E-25
1017- 8: transcript:Zm00001d012788_T001 rna16050 5.00E-147
Alignment 1018: score=369.0 e_value=5.9e-14 N=8 8&NC_008398.2 plus
1018- 0: transcript:Zm00001d012512_T001 rna15764 1.00E-32
1018- 1: transcript:Zm00001d012514_T001 rna15769 0
1018- 2: transcript:Zm00001d012515_T004 rna15771 0
1018- 3: transcript:Zm00001d012517_T001 rna15777 8.00E-140
1018- 4: transcript:Zm00001d012518_T003 rna15784 0
1018- 5: transcript:Zm00001d012520_T001 rna15787 6.00E-65
1018- 6: transcript:Zm00001d012521_T001 rna15788 0
1018- 7: transcript:Zm00001d012524_T001 rna15791 4.00E-55
Alignment 1019: score=341.0 e_value=9e-13 N=7 8&NC_008398.2 plus
1019- 0: transcript:Zm00001d009771_T003 rna16482 0
1019- 1: transcript:Zm00001d009772_T001 rna16485 5.00E-64
1019- 2: transcript:Zm00001d009773_T001 rna16488 2.00E-144
1019- 3: transcript:Zm00001d009774_T001 rna16489 3.00E-136
1019- 4: transcript:Zm00001d009775_T001 rna16492 0
1019- 5: transcript:Zm00001d009776_T001 rna16493 5.00E-37
1019- 6: transcript:Zm00001d009777_T001 rna16494 2.00E-110
Alignment 1020: score=330.0 e_value=1.7e-13 N=7 8&NC_008398.2 plus
1020- 0: transcript:Zm00001d009407_T001 rna16118 7.00E-70
1020- 1: transcript:Zm00001d009409_T002 rna16119 0
1020- 2: transcript:Zm00001d009410_T001 rna16121 1.00E-65
1020- 3: transcript:Zm00001d009413_T001 rna16131 2.00E-07
1020- 4: transcript:Zm00001d009416_T001 rna16132 8.00E-48
1020- 5: transcript:Zm00001d009417_T006 rna16133 2.00E-18
1020- 6: transcript:Zm00001d009418_T001 rna16136 0
Alignment 1021: score=322.0 e_value=5.9e-12 N=7 8&NC_008398.2 plus
1021- 0: transcript:Zm00001d008189_T004 rna14348 0
1021- 1: transcript:Zm00001d008196_T001 rna14352 9.00E-14
1021- 2: transcript:Zm00001d008199_T001 rna14353 8.00E-123
1021- 3: transcript:Zm00001d008200_T001 rna14354 4.00E-27

1021- 4: transcript:Zm00001d008205_T001 rna14356 6.00E-78
1021- 5: transcript:Zm00001d008206_T001 rna14360 3.00E-46
1021- 6: transcript:Zm00001d008208_T001 rna14362 7.00E-23
Alignment 1022: score=313.0 e_value=1.7e-10 N=7 8&NC_008398.2 plus
1022- 0: transcript:Zm00001d009837_T001 rna14457 0
1022- 1: transcript:Zm00001d009840_T001 rna14458 0
1022- 2: transcript:Zm00001d009847_T001 rna14460 3.00E-72
1022- 3: transcript:Zm00001d009848_T001 rna14462 9.00E-129
1022- 4: transcript:Zm00001d009849_T001 rna14464 8.00E-153
1022- 5: transcript:Zm00001d009850_T002 rna14466 0
1022- 6: transcript:Zm00001d009856_T005 rna14468 0
Alignment 1023: score=311.0 e_value=6.3e-11 N=7 8&NC_008398.2 plus
1023- 0: transcript:Zm00001d010356_T001 rna14623 7.00E-130
1023- 1: transcript:Zm00001d010358_T001 rna14629 0
1023- 2: transcript:Zm00001d010366_T001 rna14633 8.00E-156
1023- 3: transcript:Zm00001d010368_T003 rna14635 3.00E-15
1023- 4: transcript:Zm00001d010373_T001 rna14643 7.00E-32
1023- 5: transcript:Zm00001d010375_T001 rna14661 0
1023- 6: transcript:Zm00001d010380_T001 rna14663 1.00E-167
Alignment 1024: score=304.0 e_value=1.8e-11 N=7 8&NC_008398.2 plus
1024- 0: transcript:Zm00001d009182_T009 rna14461 0
1024- 1: transcript:Zm00001d009193_T001 rna14465 8.00E-45
1024- 2: transcript:Zm00001d009196_T001 rna14471 7.00E-86
1024- 3: transcript:Zm00001d009210_T001 rna14474 4.00E-69
1024- 4: transcript:Zm00001d009212_T004 rna14476 5.00E-68
1024- 5: transcript:Zm00001d009220_T002 rna14477 2.00E-10
1024- 6: transcript:Zm00001d009222_T001 rna14478 0
Alignment 1025: score=290.0 e_value=8e-09 N=6 8&NC_008398.2 plus
1025- 0: transcript:Zm00001d010239_T001 rna15457 2.00E-132
1025- 1: transcript:Zm00001d010240_T001 rna15458 0
1025- 2: transcript:Zm00001d010241_T002 rna15459 7.00E-94
1025- 3: transcript:Zm00001d010243_T002 rna15460 0
1025- 4: transcript:Zm00001d010244_T001 rna15462 1.00E-40
1025- 5: transcript:Zm00001d010248_T001 rna15466 2.00E-170
Alignment 1026: score=284.0 e_value=8.4e-08 N=6 8&NC_008398.2 plus
1026- 0: transcript:Zm00001d010067_T001 rna15595 2.00E-97
1026- 1: transcript:Zm00001d010075_T001 rna15598 0
1026- 2: transcript:Zm00001d010078_T001 rna15599 3.00E-115
1026- 3: transcript:Zm00001d010079_T001 rna15603 0
1026- 4: transcript:Zm00001d010082_T002 rna15604 0
1026- 5: transcript:Zm00001d010087_T001 rna15606 4.00E-86
Alignment 1027: score=273.0 e_value=2e-08 N=6 8&NC_008398.2 plus
1027- 0: transcript:Zm00001d010858_T007 rna14427 0
1027- 1: transcript:Zm00001d010862_T002 rna14428 0
1027- 2: transcript:Zm00001d010863_T001 rna14434 1.00E-165
1027- 3: transcript:Zm00001d010866_T004 rna14435 0
1027- 4: transcript:Zm00001d010867_T001 rna14437 0
1027- 5: transcript:Zm00001d010868_T001 rna14438 0
Alignment 1028: score=2612.0 e_value=6.2e-230 N=58 8&NC_008398.2 minus
1028- 0: transcript:Zm00001d010152_T002 rna15565 1.00E-142
1028- 1: transcript:Zm00001d010159_T002 rna15558 0
1028- 2: transcript:Zm00001d010171_T002 rna15555 8.00E-142
1028- 3: transcript:Zm00001d010173_T003 rna15549 0
1028- 4: transcript:Zm00001d010174_T004 rna15548 6.00E-149

```
1028-  5: transcript:Zm00001d010175_T002  rna15547     2.00E-49
1028-  6: transcript:Zm00001d010178_T001  rna15544            0
1028-  7: transcript:Zm00001d010179_T001  rna15543            0
1028-  8: transcript:Zm00001d010180_T002  rna15542    6.00E-120
1028-  9: transcript:Zm00001d010183_T001  rna15540     2.00E-58
1028- 10: transcript:Zm00001d010185_T001  rna15533     3.00E-26
1028- 11: transcript:Zm00001d010189_T001  rna15528            0
1028- 12: transcript:Zm00001d010193_T001  rna15527     4.00E-48
1028- 13: transcript:Zm00001d010195_T001  rna15524            0
1028- 14: transcript:Zm00001d010197_T001  rna15523            0
1028- 15: transcript:Zm00001d010198_T001  rna15522            0
1028- 16: transcript:Zm00001d010199_T002  rna15520            0
1028- 17: transcript:Zm00001d010200_T001  rna15519            0
1028- 18: transcript:Zm00001d010201_T001  rna15518     5.00E-93
1028- 19: transcript:Zm00001d010202_T001  rna15517            0
1028- 20: transcript:Zm00001d010204_T001  rna15516     4.00E-44
1028- 21: transcript:Zm00001d010205_T002  rna15515            0
1028- 22: transcript:Zm00001d010210_T001  rna15511            0
1028- 23: transcript:Zm00001d010211_T002  rna15509    3.00E-100
1028- 24: transcript:Zm00001d010212_T002  rna15508            0
1028- 25: transcript:Zm00001d010213_T001  rna15503    5.00E-105
1028- 26: transcript:Zm00001d010214_T003  rna15501    5.00E-104
1028- 27: transcript:Zm00001d010217_T001  rna15500     2.00E-34
1028- 28: transcript:Zm00001d010222_T001  rna15496            0
1028- 29: transcript:Zm00001d010227_T004  rna15495    3.00E-161
1028- 30: transcript:Zm00001d010228_T001  rna15494            0
1028- 31: transcript:Zm00001d010230_T001  rna15489            0
1028- 32: transcript:Zm00001d010231_T001  rna15486            0
1028- 33: transcript:Zm00001d010234_T001  rna15484            0
1028- 34: transcript:Zm00001d010236_T001  rna15468     3.00E-75
1028- 35: transcript:Zm00001d010243_T002  rna15460            0
1028- 36: transcript:Zm00001d010249_T001  rna15456    1.00E-137
1028- 37: transcript:Zm00001d010251_T003  rna15455            0
1028- 38: transcript:Zm00001d010256_T001  rna15453     8.00E-30
1028- 39: transcript:Zm00001d010259_T001  rna15450    2.00E-114
1028- 40: transcript:Zm00001d010264_T001  rna15448     2.00E-69
1028- 41: transcript:Zm00001d010265_T001  rna15445     1.00E-88
1028- 42: transcript:Zm00001d010266_T001  rna15435     1.00E-81
1028- 43: transcript:Zm00001d010271_T003  rna15434     1.00E-61
1028- 44: transcript:Zm00001d010274_T001  rna15433    3.00E-165
1028- 45: transcript:Zm00001d010275_T001  rna15432    4.00E-145
1028- 46: transcript:Zm00001d010280_T001  rna15425    2.00E-120
1028- 47: transcript:Zm00001d010282_T001  rna15423     4.00E-73
1028- 48: transcript:Zm00001d010284_T001  rna15417            0
1028- 49: transcript:Zm00001d010287_T002  rna15414            0
1028- 50: transcript:Zm00001d010290_T002  rna15412     2.00E-54
1028- 51: transcript:Zm00001d010294_T001  rna15410            0
1028- 52: transcript:Zm00001d010308_T001  rna15404            0
1028- 53: transcript:Zm00001d010313_T001  rna15396            0
1028- 54: transcript:Zm00001d010314_T001  rna15395     5.00E-95
1028- 55: transcript:Zm00001d010315_T012  rna15394            0
1028- 56: transcript:Zm00001d010319_T001  rna15392    3.00E-119
1028- 57: transcript:Zm00001d010321_T001  rna15390            0
## Alignment 1029: score=1804.0 e_value=4.5e-147 N=41 8&NC_008398.2 minus
```

1029- 0: transcript:Zm00001d010851_T001 rna14446 2.00E-174
1029- 1: transcript:Zm00001d010852_T002 rna14443 0
1029- 2: transcript:Zm00001d010855_T001 rna14442 5.00E-126
1029- 3: transcript:Zm00001d010856_T001 rna14441 4.00E-54
1029- 4: transcript:Zm00001d010859_T001 rna14430 7.00E-146
1029- 5: transcript:Zm00001d010862_T002 rna14428 0
1029- 6: transcript:Zm00001d010869_T001 rna14423 0
1029- 7: transcript:Zm00001d010870_T001 rna14420 3.00E-65
1029- 8: transcript:Zm00001d010873_T001 rna14417 7.00E-33
1029- 9: transcript:Zm00001d010880_T001 rna14400 0
1029- 10: transcript:Zm00001d010884_T001 rna14398 2.00E-54
1029- 11: transcript:Zm00001d010885_T001 rna14397 2.00E-09
1029- 12: transcript:Zm00001d010887_T002 rna14392 0
1029- 13: transcript:Zm00001d010892_T013 rna14388 0
1029- 14: transcript:Zm00001d010901_T001 rna14363 2.00E-43
1029- 15: transcript:Zm00001d010907_T001 rna14360 0
1029- 16: transcript:Zm00001d010911_T001 rna14353 6.00E-127
1029- 17: transcript:Zm00001d010912_T001 rna14352 1.00E-17
1029- 18: transcript:Zm00001d010918_T001 rna14338 0
1029- 19: transcript:Zm00001d010919_T001 rna14337 0
1029- 20: transcript:Zm00001d010920_T001 rna14336 0
1029- 21: transcript:Zm00001d010924_T001 rna14331 1.00E-97
1029- 22: transcript:Zm00001d010926_T001 rna14328 2.00E-47
1029- 23: transcript:Zm00001d010927_T001 rna14327 2.00E-74
1029- 24: transcript:Zm00001d010928_T001 rna14324 0
1029- 25: transcript:Zm00001d010933_T001 rna14320 1.00E-14
1029- 26: transcript:Zm00001d010940_T001 rna14314 3.00E-57
1029- 27: transcript:Zm00001d010945_T002 rna14305 9.00E-47
1029- 28: transcript:Zm00001d010946_T004 rna14304 0
1029- 29: transcript:Zm00001d010948_T001 rna14302 4.00E-162
1029- 30: transcript:Zm00001d010950_T003 rna14297 0
1029- 31: transcript:Zm00001d010954_T001 rna14294 0
1029- 32: transcript:Zm00001d010956_T001 rna14292 2.00E-136
1029- 33: transcript:Zm00001d010958_T001 rna14288 9.00E-11
1029- 34: transcript:Zm00001d010961_T001 rna14287 0
1029- 35: transcript:Zm00001d010965_T001 rna14284 4.00E-37
1029- 36: transcript:Zm00001d010968_T001 rna14281 2.00E-57
1029- 37: transcript:Zm00001d010969_T001 rna14280 1.00E-95
1029- 38: transcript:Zm00001d010970_T003 rna14276 0
1029- 39: transcript:Zm00001d010971_T003 rna14272 0
1029- 40: transcript:Zm00001d010974_T008 rna14270 0
Alignment 1030: score=1073.0 e_value=5.2e-73 N=25 8&NC_008398.2 minus
1030- 0: transcript:Zm00001d011610_T063 rna16225 0
1030- 1: transcript:Zm00001d011614_T001 rna16212 1.00E-97
1030- 2: transcript:Zm00001d011615_T004 rna16209 0
1030- 3: transcript:Zm00001d011616_T001 rna16204 3.00E-80
1030- 4: transcript:Zm00001d011618_T001 rna16198 4.00E-130
1030- 5: transcript:Zm00001d011620_T001 rna16193 2.00E-34
1030- 6: transcript:Zm00001d011622_T004 rna16191 0
1030- 7: transcript:Zm00001d011625_T001 rna16190 5.00E-105
1030- 8: transcript:Zm00001d011628_T001 rna16189 5.00E-41
1030- 9: transcript:Zm00001d011630_T001 rna16187 4.00E-21
1030- 10: transcript:Zm00001d011636_T001 rna16185 2.00E-16
1030- 11: transcript:Zm00001d011638_T002 rna16184 3.00E-121

1030- 12: transcript:Zm00001d011639_T001 rna16183 4.00E-158
1030- 13: transcript:Zm00001d011642_T002 rna16177 2.00E-131
1030- 14: transcript:Zm00001d011644_T002 rna16172 0
1030- 15: transcript:Zm00001d011650_T001 rna16170 1.00E-33
1030- 16: transcript:Zm00001d011654_T001 rna16155 2.00E-117
1030- 17: transcript:Zm00001d011655_T001 rna16154 4.00E-122
1030- 18: transcript:Zm00001d011660_T003 rna16153 6.00E-55
1030- 19: transcript:Zm00001d011663_T003 rna16152 0
1030- 20: transcript:Zm00001d011668_T001 rna16149 0
1030- 21: transcript:Zm00001d011673_T001 rna16147 0
1030- 22: transcript:Zm00001d011676_T002 rna16144 0
1030- 23: transcript:Zm00001d011678_T002 rna16143 1.00E-21
1030- 24: transcript:Zm00001d011681_T001 rna16137 7.00E-12
Alignment 1031: score=899.0 e_value=8e-58 N=20 8&NC_008398.2 minus
1031- 0: transcript:Zm00001d010402_T001 rna14812 9.00E-92
1031- 1: transcript:Zm00001d010403_T001 rna14811 0
1031- 2: transcript:Zm00001d010404_T001 rna14806 5.00E-53
1031- 3: transcript:Zm00001d010406_T004 rna14805 0
1031- 4: transcript:Zm00001d010407_T001 rna14800 9.00E-25
1031- 5: transcript:Zm00001d010409_T001 rna14795 2.00E-145
1031- 6: transcript:Zm00001d010410_T001 rna14794 9.00E-132
1031- 7: transcript:Zm00001d010413_T004 rna14790 0
1031- 8: transcript:Zm00001d010416_T002 rna14785 2.00E-125
1031- 9: transcript:Zm00001d010426_T001 rna14767 0
1031- 10: transcript:Zm00001d010430_T001 rna14760 0
1031- 11: transcript:Zm00001d010434_T001 rna14752 2.00E-18
1031- 12: transcript:Zm00001d010443_T001 rna14744 1.00E-119
1031- 13: transcript:Zm00001d010445_T001 rna14738 6.00E-113
1031- 14: transcript:Zm00001d010446_T001 rna14735 0
1031- 15: transcript:Zm00001d010447_T001 rna14734 1.00E-46
1031- 16: transcript:Zm00001d010450_T001 rna14726 9.00E-100
1031- 17: transcript:Zm00001d010451_T001 rna14724 0
1031- 18: transcript:Zm00001d010452_T003 rna14722 1.00E-69
1031- 19: transcript:Zm00001d010454_T001 rna14719 0
Alignment 1032: score=806.0 e_value=3.9e-50 N=17 8&NC_008398.2 minus
1032- 0: transcript:Zm00001d011034_T004 rna14215 7.00E-130
1032- 1: transcript:Zm00001d011036_T001 rna14214 0
1032- 2: transcript:Zm00001d011037_T001 rna14212 0
1032- 3: transcript:Zm00001d011038_T002 rna14211 0
1032- 4: transcript:Zm00001d011041_T001 rna14208 7.00E-84
1032- 5: transcript:Zm00001d011051_T002 rna14196 0
1032- 6: transcript:Zm00001d011058_T001 rna14192 2.00E-81
1032- 7: transcript:Zm00001d011060_T001 rna14190 0
1032- 8: transcript:Zm00001d011061_T005 rna14189 3.00E-23
1032- 9: transcript:Zm00001d011063_T001 rna14188 5.00E-13
1032- 10: transcript:Zm00001d011064_T003 rna14187 5.00E-90
1032- 11: transcript:Zm00001d011066_T004 rna14184 0
1032- 12: transcript:Zm00001d011067_T001 rna14183 4.00E-94
1032- 13: transcript:Zm00001d011068_T001 rna14182 7.00E-75
1032- 14: transcript:Zm00001d011070_T001 rna14179 0
1032- 15: transcript:Zm00001d011072_T001 rna14177 2.00E-11
1032- 16: transcript:Zm00001d011073_T001 rna14176 4.00E-51
Alignment 1033: score=580.0 e_value=6.4e-32 N=13 8&NC_008398.2 minus
1033- 0: transcript:Zm00001d009789_T006 rna14583 0

1033- 1: transcript:Zm00001d009792_T001 rna14580   5.00E-120
1033- 2: transcript:Zm00001d009795_T001 rna14577   0
1033- 3: transcript:Zm00001d009797_T001 rna14565   2.00E-44
1033- 4: transcript:Zm00001d009799_T001 rna14564   0
1033- 5: transcript:Zm00001d009800_T001 rna14558   8.00E-147
1033- 6: transcript:Zm00001d009801_T001 rna14557   8.00E-34
1033- 7: transcript:Zm00001d009810_T003 rna14553   4.00E-119
1033- 8: transcript:Zm00001d009811_T001 rna14543   0
1033- 9: transcript:Zm00001d009813_T001 rna14542   0
1033- 10: transcript:Zm00001d009820_T001 rna14529   1.00E-53
1033- 11: transcript:Zm00001d009823_T001 rna14528   3.00E-86
1033- 12: transcript:Zm00001d009826_T001 rna14526   1.00E-61
Alignment 1034: score=577.0 e_value=1.1e-33 N=13 8&NC_008398.2 minus
1034- 0: transcript:Zm00001d011513_T001 rna16315   2.00E-52
1034- 1: transcript:Zm00001d011514_T001 rna16313   1.00E-70
1034- 2: transcript:Zm00001d011517_T005 rna16305   0
1034- 3: transcript:Zm00001d011525_T002 rna16300   0
1034- 4: transcript:Zm00001d011527_T001 rna16297   3.00E-53
1034- 5: transcript:Zm00001d011534_T004 rna16290   2.00E-125
1034- 6: transcript:Zm00001d011536_T001 rna16289   2.00E-51
1034- 7: transcript:Zm00001d011537_T001 rna16288   9.00E-08
1034- 8: transcript:Zm00001d011540_T001 rna16287   4.00E-24
1034- 9: transcript:Zm00001d011541_T004 rna16286   1.00E-143
1034- 10: transcript:Zm00001d011555_T001 rna16274   3.00E-138
1034- 11: transcript:Zm00001d011558_T001 rna16273   9.00E-26
1034- 12: transcript:Zm00001d011560_T012 rna16272   0
Alignment 1035: score=549.0 e_value=8.1e-29 N=12 8&NC_008398.2 minus
1035- 0: transcript:Zm00001d010112_T001 rna15612   0
1035- 1: transcript:Zm00001d010115_T001 rna15609   0
1035- 2: transcript:Zm00001d010116_T001 rna15608   0
1035- 3: transcript:Zm00001d010126_T004 rna15593   0
1035- 4: transcript:Zm00001d010127_T001 rna15588   2.00E-53
1035- 5: transcript:Zm00001d010128_T003 rna15586   3.00E-34
1035- 6: transcript:Zm00001d010131_T001 rna15582   2.00E-123
1035- 7: transcript:Zm00001d010135_T003 rna15580   0
1035- 8: transcript:Zm00001d010136_T001 rna15578   7.00E-71
1035- 9: transcript:Zm00001d010137_T001 rna15577   0
1035- 10: transcript:Zm00001d010141_T005 rna15576   0
1035- 11: transcript:Zm00001d010144_T001 rna15574   6.00E-96
Alignment 1036: score=518.0 e_value=4.2e-27 N=11 8&NC_008398.2 minus
1036- 0: transcript:Zm00001d011099_T012 rna14140   0
1036- 1: transcript:Zm00001d011100_T001 rna14138   0
1036- 2: transcript:Zm00001d011101_T002 rna14130   1.00E-110
1036- 3: transcript:Zm00001d011102_T001 rna14129   0
1036- 4: transcript:Zm00001d011103_T001 rna14128   1.00E-56
1036- 5: transcript:Zm00001d011104_T001 rna14125   2.00E-153
1036- 6: transcript:Zm00001d011108_T004 rna14120   0
1036- 7: transcript:Zm00001d011111_T001 rna14119   2.00E-67
1036- 8: transcript:Zm00001d011117_T001 rna14115   1.00E-157
1036- 9: transcript:Zm00001d011118_T001 rna14113   6.00E-06
1036- 10: transcript:Zm00001d011119_T004 rna14112   0
Alignment 1037: score=516.0 e_value=2.4e-26 N=12 8&NC_008398.2 minus
1037- 0: transcript:Zm00001d011431_T001 rna16402   4.00E-46
1037- 1: transcript:Zm00001d011435_T001 rna16400   5.00E-18

1037- 2: transcript:Zm00001d011443_T001 rna16383 0
1037- 3: transcript:Zm00001d011444_T002 rna16378 0
1037- 4: transcript:Zm00001d011446_T001 rna16377 0
1037- 5: transcript:Zm00001d011451_T001 rna16376 9.00E-53
1037- 6: transcript:Zm00001d011458_T001 rna16372 0
1037- 7: transcript:Zm00001d011463_T001 rna16369 1.00E-137
1037- 8: transcript:Zm00001d011465_T001 rna16366 0
1037- 9: transcript:Zm00001d011470_T001 rna16362 1.00E-92
1037- 10: transcript:Zm00001d011474_T001 rna16352 3.00E-163
1037- 11: transcript:Zm00001d011477_T001 rna16348 4.00E-66
Alignment 1038: score=515.0 e_value=3.1e-27 N=11 8&NC_008398.2 minus
1038- 0: transcript:Zm00001d010975_T003 rna14264 1.00E-166
1038- 1: transcript:Zm00001d010976_T001 rna14263 2.00E-176
1038- 2: transcript:Zm00001d010982_T001 rna14261 5.00E-59
1038- 3: transcript:Zm00001d010984_T001 rna14258 3.00E-109
1038- 4: transcript:Zm00001d010985_T001 rna14256 2.00E-176
1038- 5: transcript:Zm00001d010986_T001 rna14254 0
1038- 6: transcript:Zm00001d010987_T001 rna14253 6.00E-66
1038- 7: transcript:Zm00001d010994_T001 rna14252 9.00E-106
1038- 8: transcript:Zm00001d010995_T001 rna14250 0
1038- 9: transcript:Zm00001d011001_T001 rna14232 1.00E-162
1038- 10: transcript:Zm00001d011002_T001 rna14231 1.00E-54
Alignment 1039: score=498.0 e_value=4.2e-26 N=11 8&NC_008398.2 minus
1039- 0: transcript:Zm00001d010052_T001 rna15638 2.00E-29
1039- 1: transcript:Zm00001d010053_T001 rna15637 2.00E-74
1039- 2: transcript:Zm00001d010054_T001 rna15635 3.00E-94
1039- 3: transcript:Zm00001d010055_T003 rna15629 2.00E-146
1039- 4: transcript:Zm00001d010056_T002 rna15628 0
1039- 5: transcript:Zm00001d010060_T001 rna15626 0
1039- 6: transcript:Zm00001d010061_T001 rna15625 1.00E-43
1039- 7: transcript:Zm00001d010062_T001 rna15623 0
1039- 8: transcript:Zm00001d010063_T001 rna15620 4.00E-11
1039- 9: transcript:Zm00001d010066_T003 rna15619 2.00E-142
1039- 10: transcript:Zm00001d010082_T002 rna15604 0
Alignment 1040: score=440.0 e_value=6.6e-25 N=11 8&NC_008398.2 minus
1040- 0: transcript:Zm00001d011855_T002 rna15277 3.00E-158
1040- 1: transcript:Zm00001d011874_T002 rna15275 8.00E-25
1040- 2: transcript:Zm00001d011885_T005 rna15254 6.00E-17
1040- 3: transcript:Zm00001d011890_T001 rna15249 0
1040- 4: transcript:Zm00001d011898_T001 rna15245 1.00E-99
1040- 5: transcript:Zm00001d011900_T001 rna15244 0
1040- 6: transcript:Zm00001d011904_T001 rna15242 2.00E-109
1040- 7: transcript:Zm00001d011908_T001 rna15236 0
1040- 8: transcript:Zm00001d011912_T002 rna15234 2.00E-22
1040- 9: transcript:Zm00001d011917_T001 rna15227 1.00E-95
1040- 10: transcript:Zm00001d011919_T001 rna15225 2.00E-48
Alignment 1041: score=396.0 e_value=1.4e-15 N=9 8&NC_008398.2 minus
1041- 0: transcript:Zm00001d009939_T001 rna14620 6.00E-69
1041- 1: transcript:Zm00001d009941_T001 rna14617 2.00E-160
1041- 2: transcript:Zm00001d009943_T001 rna14612 8.00E-39
1041- 3: transcript:Zm00001d009945_T001 rna14609 5.00E-11
1041- 4: transcript:Zm00001d009950_T002 rna14605 0
1041- 5: transcript:Zm00001d009952_T001 rna14599 0
1041- 6: transcript:Zm00001d009954_T001 rna14597 9.00E-76

1041- 7: transcript:Zm00001d009960_T001 rna14596 0
1041- 8: transcript:Zm00001d009961_T001 rna14595 4.00E-20
Alignment 1042: score=378.0 e_value=2e-14 N=8 8&NC_008398.2 minus
1042- 0: transcript:Zm00001d009965_T002 rna15300 0
1042- 1: transcript:Zm00001d009967_T001 rna15298 0
1042- 2: transcript:Zm00001d009971_T001 rna15294 9.00E-85
1042- 3: transcript:Zm00001d009975_T001 rna15292 7.00E-98
1042- 4: transcript:Zm00001d009978_T001 rna15285 1.00E-90
1042- 5: transcript:Zm00001d009979_T002 rna15284 1.00E-168
1042- 6: transcript:Zm00001d009983_T003 rna15274 5.00E-63
1042- 7: transcript:Zm00001d009985_T001 rna15272 5.00E-82
Alignment 1043: score=317.0 e_value=1.5e-13 N=7 8&NC_008398.2 minus
1043- 0: transcript:Zm00001d010034_T002 rna14858 2.00E-164
1043- 1: transcript:Zm00001d010038_T001 rna14837 1.00E-63
1043- 2: transcript:Zm00001d010039_T003 rna14834 0
1043- 3: transcript:Zm00001d010040_T001 rna14825 5.00E-25
1043- 4: transcript:Zm00001d010042_T001 rna14824 5.00E-17
1043- 5: transcript:Zm00001d010043_T001 rna14823 7.00E-94
1043- 6: transcript:Zm00001d010047_T001 rna14821 0
Alignment 1044: score=313.0 e_value=7.3e-12 N=7 8&NC_008398.2 minus
1044- 0: transcript:Zm00001d008803_T002 rna14670 2.00E-06
1044- 1: transcript:Zm00001d008805_T001 rna14667 7.00E-58
1044- 2: transcript:Zm00001d008808_T002 rna14665 5.00E-80
1044- 3: transcript:Zm00001d008812_T001 rna14663 1.00E-157
1044- 4: transcript:Zm00001d008816_T001 rna14661 2.00E-168
1044- 5: transcript:Zm00001d008817_T001 rna14658 1.00E-30
1044- 6: transcript:Zm00001d008819_T001 rna14633 7.00E-110
Alignment 1045: score=285.0 e_value=7.4e-09 N=6 8&NC_008398.2 minus
1045- 0: transcript:Zm00001d010652_T001 rna15842 5.00E-135
1045- 1: transcript:Zm00001d010654_T001 rna15841 0
1045- 2: transcript:Zm00001d010655_T001 rna15837 0
1045- 3: transcript:Zm00001d010659_T001 rna15827 0
1045- 4: transcript:Zm00001d010661_T001 rna15826 3.00E-23
1045- 5: transcript:Zm00001d010662_T001 rna15823 7.00E-138
Alignment 1046: score=284.0 e_value=6.9e-08 N=6 8&NC_008398.2 minus
1046- 0: transcript:Zm00001d011747_T001 rna16067 6.00E-13
1046- 1: transcript:Zm00001d011752_T001 rna16066 2.00E-14
1046- 2: transcript:Zm00001d011757_T001 rna16064 0
1046- 3: transcript:Zm00001d011758_T001 rna16061 4.00E-23
1046- 4: transcript:Zm00001d011760_T001 rna16059 7.00E-163
1046- 5: transcript:Zm00001d011762_T001 rna16058 0
Alignment 1047: score=283.0 e_value=1.5e-08 N=6 8&NC_008398.2 minus
1047- 0: transcript:Zm00001d009928_T001 rna15372 1.00E-43
1047- 1: transcript:Zm00001d009929_T001 rna15370 8.00E-159
1047- 2: transcript:Zm00001d009930_T001 rna15366 0
1047- 3: transcript:Zm00001d009932_T001 rna15365 2.00E-09
1047- 4: transcript:Zm00001d009936_T001 rna15358 7.00E-161
1047- 5: transcript:Zm00001d009938_T006 rna15356 2.00E-123
Alignment 1048: score=278.0 e_value=4.7e-12 N=6 8&NC_008398.2 minus
1048- 0: transcript:Zm00001d009103_T001 rna15184 4.00E-30
1048- 1: transcript:Zm00001d009106_T001 rna15179 1.00E-20
1048- 2: transcript:Zm00001d009109_T001 rna15174 6.00E-17
1048- 3: transcript:Zm00001d009117_T001 rna15160 7.00E-18
1048- 4: transcript:Zm00001d009118_T001 rna15158 1.00E-31

1048- 5: transcript:Zm00001d009119_T001 rna15157 6.00E-23
Alignment 1049: score=265.0 e_value=2.8e-09 N=6 8&NC_008398.2 minus
1049- 0: transcript:Zm00001d011571_T001 rna16262 3.00E-95
1049- 1: transcript:Zm00001d011576_T001 rna16261 1.00E-14
1049- 2: transcript:Zm00001d011578_T003 rna16260 0
1049- 3: transcript:Zm00001d011580_T002 rna16259 1.00E-179
1049- 4: transcript:Zm00001d011588_T003 rna16251 6.00E-94
1049- 5: transcript:Zm00001d011592_T001 rna16250 4.00E-40
Alignment 1050: score=259.0 e_value=2.8e-09 N=6 8&NC_008398.2 minus
1050- 0: transcript:Zm00001d010705_T001 rna15932 0
1050- 1: transcript:Zm00001d010710_T002 rna15931 0
1050- 2: transcript:Zm00001d010711_T003 rna15928 2.00E-158
1050- 3: transcript:Zm00001d010713_T006 rna15925 2.00E-170
1050- 4: transcript:Zm00001d010714_T005 rna15924 3.00E-105
1050- 5: transcript:Zm00001d010716_T002 rna15920 0
Alignment 1051: score=320.0 e_value=2.4e-11 N=7 9&NC_008394.4 plus
1051- 0: transcript:Zm00001d046281_T001 rna643 3.00E-44
1051- 1: transcript:Zm00001d046288_T001 rna646 1.00E-43
1051- 2: transcript:Zm00001d046292_T001 rna654 2.00E-38
1051- 3: transcript:Zm00001d046297_T001 rna660 5.00E-21
1051- 4: transcript:Zm00001d046303_T001 rna665 2.00E-69
1051- 5: transcript:Zm00001d046305_T001 rna667 4.00E-127
1051- 6: transcript:Zm00001d046317_T001 rna678 2.00E-131
Alignment 1052: score=286.0 e_value=1e-11 N=7 9&NC_008394.4 minus
1052- 0: transcript:Zm00001d046135_T001 rna745 4.00E-132
1052- 1: transcript:Zm00001d046136_T001 rna731 7.00E-138
1052- 2: transcript:Zm00001d046142_T001 rna722 2.00E-23
1052- 3: transcript:Zm00001d046166_T004 rna703 1.00E-115
1052- 4: transcript:Zm00001d046170_T001 rna698 0
1052- 5: transcript:Zm00001d046175_T001 rna685 1.00E-117
1052- 6: transcript:Zm00001d046184_T001 rna681 4.00E-136
Alignment 1053: score=673.0 e_value=1e-41 N=16 9&NC_008395.2 plus
1053- 0: transcript:Zm00001d044826_T001 rna7098 2.00E-167
1053- 1: transcript:Zm00001d044828_T001 rna7101 0
1053- 2: transcript:Zm00001d044831_T001 rna7102 6.00E-95
1053- 3: transcript:Zm00001d044833_T001 rna7103 5.00E-167
1053- 4: transcript:Zm00001d044836_T001 rna7107 4.00E-100
1053- 5: transcript:Zm00001d044838_T002 rna7108 8.00E-98
1053- 6: transcript:Zm00001d044839_T001 rna7110 2.00E-29
1053- 7: transcript:Zm00001d044841_T001 rna7113 8.00E-107
1053- 8: transcript:Zm00001d044844_T001 rna7129 2.00E-53
1053- 9: transcript:Zm00001d044849_T001 rna7131 6.00E-21
1053- 10: transcript:Zm00001d044857_T001 rna7139 3.00E-101
1053- 11: transcript:Zm00001d044860_T001 rna7148 1.00E-160
1053- 12: transcript:Zm00001d044864_T001 rna7165 5.00E-126
1053- 13: transcript:Zm00001d044869_T001 rna7173 4.00E-95
1053- 14: transcript:Zm00001d044874_T001 rna7174 4.00E-37
1053- 15: transcript:Zm00001d044899_T001 rna7191 3.00E-32
Alignment 1054: score=582.0 e_value=2.9e-29 N=13 9&NC_008395.2 plus
1054- 0: transcript:Zm00001d044918_T001 rna7194 3.00E-54
1054- 1: transcript:Zm00001d044922_T001 rna7197 9.00E-163
1054- 2: transcript:Zm00001d044927_T001 rna7203 6.00E-19
1054- 3: transcript:Zm00001d044930_T001 rna7206 2.00E-40
1054- 4: transcript:Zm00001d044933_T001 rna7208 0

1054- 5: transcript:Zm00001d044934_T001 rna7210   3.00E-173
1054- 6: transcript:Zm00001d044937_T001 rna7212   2.00E-178
1054- 7: transcript:Zm00001d044940_T004 rna7223   2.00E-114
1054- 8: transcript:Zm00001d044947_T001 rna7227   0
1054- 9: transcript:Zm00001d044951_T001 rna7236   0
1054- 10: transcript:Zm00001d044953_T001 rna7239   5.00E-74
1054- 11: transcript:Zm00001d044970_T001 rna7256   4.00E-94
1054- 12: transcript:Zm00001d044971_T005 rna7261   0
Alignment 1055: score=524.0 e_value=1.5e-26 N=12 9&NC_008395.2 plus
1055- 0: transcript:Zm00001d046632_T001 rna4884   3.00E-108
1055- 1: transcript:Zm00001d046642_T001 rna4894   1.00E-175
1055- 2: transcript:Zm00001d046655_T001 rna4905   0
1055- 3: transcript:Zm00001d046660_T001 rna4908   0
1055- 4: transcript:Zm00001d046661_T003 rna4913   0
1055- 5: transcript:Zm00001d046663_T001 rna4914   2.00E-23
1055- 6: transcript:Zm00001d046664_T001 rna4915   5.00E-17
1055- 7: transcript:Zm00001d046667_T001 rna4917   8.00E-15
1055- 8: transcript:Zm00001d046672_T001 rna4919   0
1055- 9: transcript:Zm00001d046681_T001 rna4922   0
1055- 10: transcript:Zm00001d046682_T001 rna4923   1.00E-95
1055- 11: transcript:Zm00001d046687_T001 rna4925   8.00E-31
Alignment 1056: score=466.0 e_value=1.3e-23 N=11 9&NC_008395.2 plus
1056- 0: transcript:Zm00001d044757_T010 rna7053   0
1056- 1: transcript:Zm00001d044769_T001 rna7063   1.00E-35
1056- 2: transcript:Zm00001d044771_T001 rna7064   1.00E-152
1056- 3: transcript:Zm00001d044783_T001 rna7072   2.00E-46
1056- 4: transcript:Zm00001d044802_T001 rna7078   6.00E-135
1056- 5: transcript:Zm00001d044803_T001 rna7079   6.00E-29
1056- 6: transcript:Zm00001d044806_T001 rna7080   8.00E-137
1056- 7: transcript:Zm00001d044808_T001 rna7083   4.00E-171
1056- 8: transcript:Zm00001d044812_T001 rna7085   0
1056- 9: transcript:Zm00001d044814_T002 rna7086   0
1056- 10: transcript:Zm00001d044815_T002 rna7088   4.00E-63
Alignment 1057: score=458.0 e_value=4.5e-21 N=10 9&NC_008395.2 plus
1057- 0: transcript:Zm00001d046888_T001 rna4803   2.00E-35
1057- 1: transcript:Zm00001d046889_T001 rna4804   0
1057- 2: transcript:Zm00001d046897_T001 rna4806   6.00E-51
1057- 3: transcript:Zm00001d046906_T001 rna4810   3.00E-69
1057- 4: transcript:Zm00001d046910_T001 rna4811   5.00E-71
1057- 5: transcript:Zm00001d046915_T001 rna4813   0
1057- 6: transcript:Zm00001d046916_T001 rna4814   2.00E-57
1057- 7: transcript:Zm00001d046925_T001 rna4818   4.00E-47
1057- 8: transcript:Zm00001d046927_T001 rna4819   2.00E-20
1057- 9: transcript:Zm00001d046929_T002 rna4820   8.00E-43
Alignment 1058: score=386.0 e_value=1.3e-20 N=9 9&NC_008395.2 plus
1058- 0: transcript:Zm00001d045075_T001 rna7361   9.00E-125
1058- 1: transcript:Zm00001d045076_T003 rna7362   5.00E-77
1058- 2: transcript:Zm00001d045077_T001 rna7366   1.00E-105
1058- 3: transcript:Zm00001d045078_T001 rna7370   0
1058- 4: transcript:Zm00001d045084_T002 rna7387   6.00E-154
1058- 5: transcript:Zm00001d045085_T001 rna7392   2.00E-128
1058- 6: transcript:Zm00001d045102_T001 rna7395   1.00E-96
1058- 7: transcript:Zm00001d045104_T005 rna7396   0
1058- 8: transcript:Zm00001d045105_T001 rna7398   6.00E-47

Alignment 1059: score=384.0 e_value=1.8e-17 N=9 9&NC_008395.2 plus
1059- 0: transcript:Zm00001d046740_T001 rna4935 3.00E-126
1059- 1: transcript:Zm00001d046751_T001 rna4941 5.00E-83
1059- 2: transcript:Zm00001d046765_T001 rna4945 0
1059- 3: transcript:Zm00001d046767_T002 rna4946 8.00E-97
1059- 4: transcript:Zm00001d046774_T001 rna4951 5.00E-93
1059- 5: transcript:Zm00001d046781_T001 rna4957 3.00E-157
1059- 6: transcript:Zm00001d046786_T001 rna4960 3.00E-68
1059- 7: transcript:Zm00001d046792_T001 rna4965 4.00E-124
1059- 8: transcript:Zm00001d046793_T001 rna4970 0
Alignment 1060: score=371.0 e_value=3.3e-15 N=8 9&NC_008395.2 plus
1060- 0: transcript:Zm00001d046281_T001 rna5143 2.00E-51
1060- 1: transcript:Zm00001d046297_T001 rna5147 2.00E-41
1060- 2: transcript:Zm00001d046299_T001 rna5151 7.00E-88
1060- 3: transcript:Zm00001d046303_T001 rna5155 1.00E-84
1060- 4: transcript:Zm00001d046304_T001 rna5156 0
1060- 5: transcript:Zm00001d046305_T001 rna5157 2.00E-112
1060- 6: transcript:Zm00001d046311_T001 rna5165 4.00E-17
1060- 7: transcript:Zm00001d046317_T001 rna5169 6.00E-132
Alignment 1061: score=305.0 e_value=4e-13 N=7 9&NC_008395.2 plus
1061- 0: transcript:Zm00001d045112_T001 rna7404 0
1061- 1: transcript:Zm00001d045120_T001 rna7408 5.00E-69
1061- 2: transcript:Zm00001d045122_T001 rna7410 0
1061- 3: transcript:Zm00001d045125_T001 rna7417 8.00E-49
1061- 4: transcript:Zm00001d045128_T001 rna7423 0
1061- 5: transcript:Zm00001d045138_T001 rna7437 0
1061- 6: transcript:Zm00001d045139_T002 rna7440 0
Alignment 1062: score=490.0 e_value=4.6e-28 N=12 9&NC_008395.2 minus
1062- 0: transcript:Zm00001d046925_T001 rna4818 4.00E-47
1062- 1: transcript:Zm00001d046936_T002 rna4798 0
1062- 2: transcript:Zm00001d046937_T001 rna4796 1.00E-70
1062- 3: transcript:Zm00001d046947_T001 rna4787 2.00E-101
1062- 4: transcript:Zm00001d046948_T002 rna4785 4.00E-175
1062- 5: transcript:Zm00001d046949_T001 rna4783 1.00E-48
1062- 6: transcript:Zm00001d046958_T001 rna4774 3.00E-105
1062- 7: transcript:Zm00001d046967_T001 rna4765 2.00E-115
1062- 8: transcript:Zm00001d046978_T002 rna4751 2.00E-107
1062- 9: transcript:Zm00001d046981_T001 rna4747 5.00E-34
1062- 10: transcript:Zm00001d046996_T001 rna4739 1.00E-165
1062- 11: transcript:Zm00001d046998_T001 rna4735 4.00E-83
Alignment 1063: score=490.0 e_value=2.5e-26 N=12 9&NC_008395.2 minus
1063- 0: transcript:Zm00001d046055_T001 rna5272 1.00E-33
1063- 1: transcript:Zm00001d046072_T001 rna5271 0
1063- 2: transcript:Zm00001d046112_T002 rna5250 2.00E-94
1063- 3: transcript:Zm00001d046118_T001 rna5249 1.00E-10
1063- 4: transcript:Zm00001d046126_T001 rna5238 5.00E-141
1063- 5: transcript:Zm00001d046132_T001 rna5235 1.00E-14
1063- 6: transcript:Zm00001d046136_T001 rna5222 3.00E-127
1063- 7: transcript:Zm00001d046142_T001 rna5215 7.00E-27
1063- 8: transcript:Zm00001d046143_T001 rna5212 6.00E-28
1063- 9: transcript:Zm00001d046166_T004 rna5196 6.00E-128
1063- 10: transcript:Zm00001d046178_T001 rna5184 0
1063- 11: transcript:Zm00001d046184_T001 rna5175 4.00E-52
Alignment 1064: score=433.0 e_value=2.1e-20 N=10 9&NC_008395.2 minus

1064- 0: transcript:Zm00001d046500_T001 rna4990 9.00E-121
1064- 1: transcript:Zm00001d046501_T001 rna4989 3.00E-96
1064- 2: transcript:Zm00001d046509_T002 rna4986 3.00E-64
1064- 3: transcript:Zm00001d046510_T001 rna4985 1.00E-14
1064- 4: transcript:Zm00001d046513_T001 rna4984 1.00E-60
1064- 5: transcript:Zm00001d046531_T007 rna4982 5.00E-103
1064- 6: transcript:Zm00001d046534_T001 rna4981 3.00E-173
1064- 7: transcript:Zm00001d046538_T003 rna4979 0
1064- 8: transcript:Zm00001d046539_T002 rna4978 0
1064- 9: transcript:Zm00001d046540_T003 rna4977 0
Alignment 1065: score=341.0 e_value=9.1e-13 N=8 9&NC_008395.2 minus
1065- 0: transcript:Zm00001d046346_T001 rna5098 1.00E-48
1065- 1: transcript:Zm00001d046352_T001 rna5094 0
1065- 2: transcript:Zm00001d046354_T001 rna5093 9.00E-77
1065- 3: transcript:Zm00001d046357_T001 rna5089 0
1065- 4: transcript:Zm00001d046364_T001 rna5081 5.00E-117
1065- 5: transcript:Zm00001d046369_T002 rna5076 0
1065- 6: transcript:Zm00001d046374_T001 rna5074 0
1065- 7: transcript:Zm00001d046378_T001 rna5070 2.00E-43
Alignment 1066: score=251.0 e_value=2e-09 N=6 9&NC_008395.2 minus
1066- 0: transcript:Zm00001d045919_T001 rna6894 0
1066- 1: transcript:Zm00001d045927_T002 rna6893 6.00E-123
1066- 2: transcript:Zm00001d045928_T001 rna6890 2.00E-109
1066- 3: transcript:Zm00001d045940_T001 rna6871 1.00E-79
1066- 4: transcript:Zm00001d045945_T002 rna6870 0
1066- 5: transcript:Zm00001d045946_T001 rna6865 8.00E-69
Alignment 1067: score=2900.0 e_value=1.5e-259 N=65 9&NC_008396.2 plus
1067- 0: transcript:Zm00001d048338_T035 rna7928 0
1067- 1: transcript:Zm00001d048340_T002 rna7930 0
1067- 2: transcript:Zm00001d048341_T001 rna7931 0
1067- 3: transcript:Zm00001d048342_T001 rna7932 2.00E-44
1067- 4: transcript:Zm00001d048343_T002 rna7935 0
1067- 5: transcript:Zm00001d048344_T002 rna7940 9.00E-135
1067- 6: transcript:Zm00001d048345_T003 rna7943 0
1067- 7: transcript:Zm00001d048347_T001 rna7945 5.00E-139
1067- 8: transcript:Zm00001d048348_T001 rna7946 7.00E-51
1067- 9: transcript:Zm00001d048349_T002 rna7947 0
1067- 10: transcript:Zm00001d048353_T001 rna7956 2.00E-56
1067- 11: transcript:Zm00001d048355_T001 rna7960 0
1067- 12: transcript:Zm00001d048356_T001 rna7965 0
1067- 13: transcript:Zm00001d048358_T004 rna7971 0
1067- 14: transcript:Zm00001d048359_T004 rna7972 0
1067- 15: transcript:Zm00001d048360_T001 rna7973 6.00E-138
1067- 16: transcript:Zm00001d048361_T001 rna7974 0
1067- 17: transcript:Zm00001d048362_T002 rna7975 8.00E-155
1067- 18: transcript:Zm00001d048363_T001 rna7978 5.00E-95
1067- 19: transcript:Zm00001d048364_T002 rna7980 8.00E-127
1067- 20: transcript:Zm00001d048366_T001 rna7983 1.00E-129
1067- 21: transcript:Zm00001d048368_T007 rna7988 3.00E-98
1067- 22: transcript:Zm00001d048369_T002 rna7991 4.00E-156
1067- 23: transcript:Zm00001d048370_T001 rna7992 0
1067- 24: transcript:Zm00001d048382_T001 rna7999 3.00E-113
1067- 25: transcript:Zm00001d048383_T001 rna8007 3.00E-94
1067- 26: transcript:Zm00001d048385_T001 rna8014 1.00E-147

1067- 27:	transcript:Zm00001d048387_T001	rna8021	0
1067- 28:	transcript:Zm00001d048390_T001	rna8024	0
1067- 29:	transcript:Zm00001d048391_T001	rna8025	9.00E-64
1067- 30:	transcript:Zm00001d048392_T006	rna8027	0
1067- 31:	transcript:Zm00001d048393_T001	rna8033	2.00E-139
1067- 32:	transcript:Zm00001d048394_T002	rna8036	2.00E-49
1067- 33:	transcript:Zm00001d048398_T001	rna8048	7.00E-170
1067- 34:	transcript:Zm00001d048400_T001	rna8050	2.00E-152
1067- 35:	transcript:Zm00001d048401_T001	rna8051	1.00E-48
1067- 36:	transcript:Zm00001d048402_T001	rna8053	2.00E-84
1067- 37:	transcript:Zm00001d048404_T005	rna8065	3.00E-65
1067- 38:	transcript:Zm00001d048407_T001	rna8068	3.00E-89
1067- 39:	transcript:Zm00001d048409_T001	rna8070	0
1067- 40:	transcript:Zm00001d048410_T004	rna8071	3.00E-125
1067- 41:	transcript:Zm00001d048411_T001	rna8072	2.00E-88
1067- 42:	transcript:Zm00001d048412_T001	rna8073	0
1067- 43:	transcript:Zm00001d048413_T001	rna8074	5.00E-158
1067- 44:	transcript:Zm00001d048414_T001	rna8076	3.00E-142
1067- 45:	transcript:Zm00001d048415_T005	rna8080	1.00E-31
1067- 46:	transcript:Zm00001d048416_T001	rna8082	0
1067- 47:	transcript:Zm00001d048418_T001	rna8088	9.00E-110
1067- 48:	transcript:Zm00001d048419_T004	rna8092	8.00E-97
1067- 49:	transcript:Zm00001d048420_T001	rna8099	2.00E-164
1067- 50:	transcript:Zm00001d048421_T001	rna8101	0
1067- 51:	transcript:Zm00001d048422_T001	rna8106	0
1067- 52:	transcript:Zm00001d048424_T003	rna8108	0
1067- 53:	transcript:Zm00001d048428_T001	rna8115	0
1067- 54:	transcript:Zm00001d048430_T005	rna8122	0
1067- 55:	transcript:Zm00001d048431_T001	rna8123	3.00E-78
1067- 56:	transcript:Zm00001d048432_T002	rna8125	0
1067- 57:	transcript:Zm00001d048434_T002	rna8126	6.00E-19
1067- 58:	transcript:Zm00001d048437_T002	rna8131	4.00E-139
1067- 59:	transcript:Zm00001d048438_T001	rna8133	2.00E-37
1067- 60:	transcript:Zm00001d048440_T003	rna8158	0
1067- 61:	transcript:Zm00001d048442_T004	rna8159	0
1067- 62:	transcript:Zm00001d048443_T002	rna8160	2.00E-114
1067- 63:	transcript:Zm00001d048444_T001	rna8161	7.00E-162
1067- 64:	transcript:Zm00001d048445_T002	rna8162	1.00E-78

Alignment 1068: score=1290.0 e_value=2.4e-101 N=30 9&NC_008396.2 plus

1068- 0:	transcript:Zm00001d047163_T001	rna9434	4.00E-85
1068- 1:	transcript:Zm00001d047166_T001	rna9435	0
1068- 2:	transcript:Zm00001d047174_T001	rna9439	2.00E-138
1068- 3:	transcript:Zm00001d047178_T009	rna9446	0
1068- 4:	transcript:Zm00001d047181_T005	rna9448	0
1068- 5:	transcript:Zm00001d047183_T002	rna9449	0
1068- 6:	transcript:Zm00001d047184_T001	rna9451	2.00E-78
1068- 7:	transcript:Zm00001d047186_T001	rna9452	3.00E-60
1068- 8:	transcript:Zm00001d047187_T001	rna9457	2.00E-80
1068- 9:	transcript:Zm00001d047190_T001	rna9459	0
1068- 10:	transcript:Zm00001d047191_T003	rna9466	3.00E-133
1068- 11:	transcript:Zm00001d047201_T001	rna9476	0
1068- 12:	transcript:Zm00001d047202_T001	rna9479	3.00E-36
1068- 13:	transcript:Zm00001d047203_T001	rna9481	1.00E-76
1068- 14:	transcript:Zm00001d047204_T003	rna9482	0

1068- 15: transcript:Zm00001d047207_T001 rna9484 9.00E-43
1068- 16: transcript:Zm00001d047209_T003 rna9485 4.00E-168
1068- 17: transcript:Zm00001d047210_T001 rna9488 2.00E-104
1068- 18: transcript:Zm00001d047220_T003 rna9497 0
1068- 19: transcript:Zm00001d047235_T001 rna9510 9.00E-174
1068- 20: transcript:Zm00001d047236_T003 rna9515 0
1068- 21: transcript:Zm00001d047238_T001 rna9516 1.00E-139
1068- 22: transcript:Zm00001d047240_T001 rna9518 0
1068- 23: transcript:Zm00001d047241_T003 rna9528 0
1068- 24: transcript:Zm00001d047242_T001 rna9529 0
1068- 25: transcript:Zm00001d047245_T001 rna9530 0
1068- 26: transcript:Zm00001d047247_T001 rna9533 3.00E-24
1068- 27: transcript:Zm00001d047251_T003 rna9537 0
1068- 28: transcript:Zm00001d047252_T001 rna9542 6.00E-36
1068- 29: transcript:Zm00001d047253_T018 rna9550 0
Alignment 1069: score=817.0 e_value=5.5e-49 N=18 9&NC_008396.2 plus
1069- 0: transcript:Zm00001d047986_T029 rna8557 0
1069- 1: transcript:Zm00001d047988_T009 rna8563 8.00E-175
1069- 2: transcript:Zm00001d047990_T001 rna8568 0
1069- 3: transcript:Zm00001d047992_T006 rna8573 9.00E-106
1069- 4: transcript:Zm00001d047993_T001 rna8582 1.00E-157
1069- 5: transcript:Zm00001d047994_T002 rna8583 8.00E-157
1069- 6: transcript:Zm00001d047998_T001 rna8588 0
1069- 7: transcript:Zm00001d047999_T001 rna8591 2.00E-43
1069- 8: transcript:Zm00001d048004_T001 rna8594 0
1069- 9: transcript:Zm00001d048006_T001 rna8595 9.00E-30
1069- 10: transcript:Zm00001d048007_T003 rna8596 2.00E-175
1069- 11: transcript:Zm00001d048008_T001 rna8597 0
1069- 12: transcript:Zm00001d048009_T001 rna8598 3.00E-177
1069- 13: transcript:Zm00001d048013_T001 rna8600 9.00E-165
1069- 14: transcript:Zm00001d048014_T001 rna8601 4.00E-142
1069- 15: transcript:Zm00001d048017_T003 rna8602 0
1069- 16: transcript:Zm00001d048019_T002 rna8604 0
1069- 17: transcript:Zm00001d048020_T002 rna8605 4.00E-84
Alignment 1070: score=553.0 e_value=9.9e-27 N=12 9&NC_008396.2 plus
1070- 0: transcript:Zm00001d047261_T012 rna9558 7.00E-125
1070- 1: transcript:Zm00001d047265_T001 rna9574 3.00E-97
1070- 2: transcript:Zm00001d047269_T001 rna9581 1.00E-49
1070- 3: transcript:Zm00001d047271_T002 rna9583 8.00E-51
1070- 4: transcript:Zm00001d047272_T001 rna9588 6.00E-69
1070- 5: transcript:Zm00001d047273_T001 rna9598 0
1070- 6: transcript:Zm00001d047274_T001 rna9599 5.00E-35
1070- 7: transcript:Zm00001d047275_T003 rna9603 0
1070- 8: transcript:Zm00001d047276_T001 rna9606 0
1070- 9: transcript:Zm00001d047279_T001 rna9610 5.00E-98
1070- 10: transcript:Zm00001d047282_T001 rna9611 1.00E-31
1070- 11: transcript:Zm00001d047283_T006 rna9612 0
Alignment 1071: score=392.0 e_value=2.4e-17 N=9 9&NC_008396.2 plus
1071- 0: transcript:Zm00001d047289_T004 rna9617 2.00E-176
1071- 1: transcript:Zm00001d047290_T001 rna9618 1.00E-108
1071- 2: transcript:Zm00001d047292_T001 rna9622 7.00E-176
1071- 3: transcript:Zm00001d047293_T001 rna9625 3.00E-53
1071- 4: transcript:Zm00001d047296_T001 rna9637 3.00E-99
1071- 5: transcript:Zm00001d047298_T001 rna9647 2.00E-91

1071- 6: transcript:Zm00001d047302_T001 rna9650 0
1071- 7: transcript:Zm00001d047303_T001 rna9652 5.00E-170
1071- 8: transcript:Zm00001d047306_T007 rna9654 3.00E-22
Alignment 1072: score=318.0 e_value=1.5e-12 N=7 9&NC_008396.2 plus
1072- 0: transcript:Zm00001d047579_T001 rna9108 7.00E-147
1072- 1: transcript:Zm00001d047581_T001 rna9109 0
1072- 2: transcript:Zm00001d047582_T002 rna9110 0
1072- 3: transcript:Zm00001d047583_T001 rna9112 0
1072- 4: transcript:Zm00001d047584_T001 rna9116 0
1072- 5: transcript:Zm00001d047587_T005 rna9119 0
1072- 6: transcript:Zm00001d047597_T001 rna9124 3.00E-86
Alignment 1073: score=5574.0 e_value=0 N=123 9&NC_008396.2 minus
1073- 0: transcript:Zm00001d048096_T023 rna8474 0
1073- 1: transcript:Zm00001d048098_T004 rna8473 1.00E-106
1073- 2: transcript:Zm00001d048100_T004 rna8472 0
1073- 3: transcript:Zm00001d048102_T003 rna8470 0
1073- 4: transcript:Zm00001d048104_T001 rna8469 1.00E-86
1073- 5: transcript:Zm00001d048106_T001 rna8465 0
1073- 6: transcript:Zm00001d048109_T002 rna8460 7.00E-28
1073- 7: transcript:Zm00001d048111_T001 rna8459 0
1073- 8: transcript:Zm00001d048112_T001 rna8457 1.00E-154
1073- 9: transcript:Zm00001d048115_T026 rna8455 0
1073- 10: transcript:Zm00001d048116_T001 rna8454 1.00E-10
1073- 11: transcript:Zm00001d048117_T001 rna8452 4.00E-50
1073- 12: transcript:Zm00001d048119_T022 rna8451 0
1073- 13: transcript:Zm00001d048122_T003 rna8445 3.00E-85
1073- 14: transcript:Zm00001d048123_T001 rna8444 2.00E-42
1073- 15: transcript:Zm00001d048126_T003 rna8443 0
1073- 16: transcript:Zm00001d048129_T004 rna8441 0
1073- 17: transcript:Zm00001d048131_T001 rna8438 0
1073- 18: transcript:Zm00001d048132_T002 rna8436 4.00E-82
1073- 19: transcript:Zm00001d048134_T001 rna8434 3.00E-130
1073- 20: transcript:Zm00001d048135_T002 rna8430 9.00E-36
1073- 21: transcript:Zm00001d048137_T002 rna8427 0
1073- 22: transcript:Zm00001d048138_T004 rna8425 0
1073- 23: transcript:Zm00001d048139_T002 rna8424 1.00E-86
1073- 24: transcript:Zm00001d048142_T001 rna8416 0
1073- 25: transcript:Zm00001d048143_T003 rna8415 2.00E-117
1073- 26: transcript:Zm00001d048146_T001 rna8413 0
1073- 27: transcript:Zm00001d048149_T002 rna8408 7.00E-175
1073- 28: transcript:Zm00001d048150_T007 rna8407 7.00E-32
1073- 29: transcript:Zm00001d048151_T001 rna8402 1.00E-156
1073- 30: transcript:Zm00001d048153_T001 rna8400 2.00E-101
1073- 31: transcript:Zm00001d048155_T001 rna8397 1.00E-131
1073- 32: transcript:Zm00001d048157_T001 rna8394 5.00E-170
1073- 33: transcript:Zm00001d048162_T001 rna8392 6.00E-78
1073- 34: transcript:Zm00001d048166_T001 rna8388 0
1073- 35: transcript:Zm00001d048171_T003 rna8387 8.00E-69
1073- 36: transcript:Zm00001d048172_T001 rna8385 5.00E-77
1073- 37: transcript:Zm00001d048173_T001 rna8384 8.00E-153
1073- 38: transcript:Zm00001d048174_T001 rna8383 0
1073- 39: transcript:Zm00001d048175_T002 rna8382 0
1073- 40: transcript:Zm00001d048176_T010 rna8377 0
1073- 41: transcript:Zm00001d048178_T001 rna8375 0

1073- 42:	transcript:Zm00001d048180_T001	rna8372	5.00E-147
1073- 43:	transcript:Zm00001d048181_T003	rna8371	2.00E-69
1073- 44:	transcript:Zm00001d048185_T001	rna8369	1.00E-59
1073- 45:	transcript:Zm00001d048188_T001	rna8367	1.00E-36
1073- 46:	transcript:Zm00001d048189_T001	rna8360	0
1073- 47:	transcript:Zm00001d048190_T001	rna8359	2.00E-151
1073- 48:	transcript:Zm00001d048192_T001	rna8356	9.00E-68
1073- 49:	transcript:Zm00001d048193_T002	rna8355	4.00E-37
1073- 50:	transcript:Zm00001d048195_T001	rna8352	0
1073- 51:	transcript:Zm00001d048198_T002	rna8346	1.00E-69
1073- 52:	transcript:Zm00001d048199_T001	rna8345	0
1073- 53:	transcript:Zm00001d048201_T002	rna8342	0
1073- 54:	transcript:Zm00001d048202_T002	rna8341	1.00E-44
1073- 55:	transcript:Zm00001d048203_T002	rna8339	0
1073- 56:	transcript:Zm00001d048204_T001	rna8338	1.00E-83
1073- 57:	transcript:Zm00001d048208_T001	rna8334	3.00E-125
1073- 58:	transcript:Zm00001d048210_T001	rna8333	3.00E-21
1073- 59:	transcript:Zm00001d048215_T001	rna8329	3.00E-62
1073- 60:	transcript:Zm00001d048218_T001	rna8328	0
1073- 61:	transcript:Zm00001d048220_T001	rna8322	1.00E-78
1073- 62:	transcript:Zm00001d048224_T001	rna8319	1.00E-45
1073- 63:	transcript:Zm00001d048225_T001	rna8316	0
1073- 64:	transcript:Zm00001d048226_T001	rna8315	4.00E-53
1073- 65:	transcript:Zm00001d048227_T001	rna8314	8.00E-73
1073- 66:	transcript:Zm00001d048229_T001	rna8311	2.00E-126
1073- 67:	transcript:Zm00001d048230_T001	rna8310	3.00E-116
1073- 68:	transcript:Zm00001d048232_T001	rna8308	0
1073- 69:	transcript:Zm00001d048233_T001	rna8305	7.00E-149
1073- 70:	transcript:Zm00001d048234_T001	rna8304	8.00E-63
1073- 71:	transcript:Zm00001d048235_T004	rna8302	0
1073- 72:	transcript:Zm00001d048238_T001	rna8300	2.00E-76
1073- 73:	transcript:Zm00001d048239_T001	rna8297	7.00E-129
1073- 74:	transcript:Zm00001d048242_T001	rna8291	5.00E-82
1073- 75:	transcript:Zm00001d048243_T001	rna8287	0
1073- 76:	transcript:Zm00001d048244_T001	rna8282	4.00E-128
1073- 77:	transcript:Zm00001d048245_T003	rna8281	0
1073- 78:	transcript:Zm00001d048246_T001	rna8278	8.00E-61
1073- 79:	transcript:Zm00001d048247_T002	rna8275	0
1073- 80:	transcript:Zm00001d048255_T002	rna8265	1.00E-99
1073- 81:	transcript:Zm00001d048256_T002	rna8264	2.00E-41
1073- 82:	transcript:Zm00001d048258_T012	rna8263	0
1073- 83:	transcript:Zm00001d048262_T001	rna8259	1.00E-99
1073- 84:	transcript:Zm00001d048268_T001	rna8255	5.00E-32
1073- 85:	transcript:Zm00001d048270_T002	rna8254	0
1073- 86:	transcript:Zm00001d048271_T001	rna8251	1.00E-177
1073- 87:	transcript:Zm00001d048272_T012	rna8250	0
1073- 88:	transcript:Zm00001d048273_T001	rna8249	9.00E-48
1073- 89:	transcript:Zm00001d048281_T001	rna8246	2.00E-117
1073- 90:	transcript:Zm00001d048282_T002	rna8243	0
1073- 91:	transcript:Zm00001d048284_T001	rna8238	1.00E-70
1073- 92:	transcript:Zm00001d048285_T006	rna8232	0
1073- 93:	transcript:Zm00001d048286_T002	rna8230	1.00E-173
1073- 94:	transcript:Zm00001d048288_T001	rna8223	8.00E-76
1073- 95:	transcript:Zm00001d048292_T003	rna8222	1.00E-148

1073- 96:	transcript:Zm00001d048293_T001	rna8221	1.00E-169
1073- 97:	transcript:Zm00001d048296_T001	rna8216	4.00E-89
1073- 98:	transcript:Zm00001d048298_T003	rna8215	0
1073- 99:	transcript:Zm00001d048299_T002	rna8214	0
1073-100:	transcript:Zm00001d048301_T001	rna8212	1.00E-130
1073-101:	transcript:Zm00001d048302_T001	rna8209	5.00E-20
1073-102:	transcript:Zm00001d048303_T001	rna8208	3.00E-30
1073-103:	transcript:Zm00001d048307_T001	rna8206	1.00E-18
1073-104:	transcript:Zm00001d048308_T002	rna8205	0
1073-105:	transcript:Zm00001d048310_T005	rna8202	0
1073-106:	transcript:Zm00001d048311_T003	rna8200	0
1073-107:	transcript:Zm00001d048312_T001	rna8199	1.00E-42
1073-108:	transcript:Zm00001d048313_T001	rna8198	8.00E-59
1073-109:	transcript:Zm00001d048314_T004	rna8197	5.00E-104
1073-110:	transcript:Zm00001d048315_T001	rna8196	0
1073-111:	transcript:Zm00001d048316_T001	rna8194	5.00E-53
1073-112:	transcript:Zm00001d048317_T001	rna8193	9.00E-48
1073-113:	transcript:Zm00001d048319_T003	rna8189	0
1073-114:	transcript:Zm00001d048321_T001	rna8188	8.00E-13
1073-115:	transcript:Zm00001d048323_T001	rna8186	0
1073-116:	transcript:Zm00001d048324_T001	rna8185	7.00E-160
1073-117:	transcript:Zm00001d048325_T001	rna8184	2.00E-46
1073-118:	transcript:Zm00001d048333_T001	rna8178	6.00E-128
1073-119:	transcript:Zm00001d048334_T001	rna8177	2.00E-135
1073-120:	transcript:Zm00001d048335_T001	rna8175	0
1073-121:	transcript:Zm00001d048336_T003	rna8173	0
1073-122:	transcript:Zm00001d048337_T001	rna8172	0

Alignment 1074: score=4280.0 e_value=0 N=95 9&NC_008396.2 minus

1074- 0:	transcript:Zm00001d047762_T045	rna8906	5.00E-89
1074- 1:	transcript:Zm00001d047763_T001	rna8904	1.00E-147
1074- 2:	transcript:Zm00001d047764_T001	rna8902	2.00E-83
1074- 3:	transcript:Zm00001d047765_T001	rna8898	1.00E-85
1074- 4:	transcript:Zm00001d047766_T001	rna8897	1.00E-107
1074- 5:	transcript:Zm00001d047767_T004	rna8896	2.00E-114
1074- 6:	transcript:Zm00001d047769_T001	rna8890	0
1074- 7:	transcript:Zm00001d047770_T003	rna8887	0
1074- 8:	transcript:Zm00001d047771_T001	rna8886	1.00E-77
1074- 9:	transcript:Zm00001d047772_T002	rna8884	0
1074- 10:	transcript:Zm00001d047774_T002	rna8882	5.00E-81
1074- 11:	transcript:Zm00001d047775_T001	rna8881	8.00E-137
1074- 12:	transcript:Zm00001d047776_T001	rna8880	0
1074- 13:	transcript:Zm00001d047779_T001	rna8879	6.00E-41
1074- 14:	transcript:Zm00001d047780_T002	rna8878	0
1074- 15:	transcript:Zm00001d047781_T001	rna8876	0
1074- 16:	transcript:Zm00001d047786_T001	rna8874	5.00E-54
1074- 17:	transcript:Zm00001d047787_T001	rna8870	3.00E-78
1074- 18:	transcript:Zm00001d047789_T001	rna8868	5.00E-82
1074- 19:	transcript:Zm00001d047790_T001	rna8865	0
1074- 20:	transcript:Zm00001d047793_T001	rna8864	0
1074- 21:	transcript:Zm00001d047794_T001	rna8863	2.00E-61
1074- 22:	transcript:Zm00001d047797_T010	rna8861	0
1074- 23:	transcript:Zm00001d047800_T001	rna8856	3.00E-159
1074- 24:	transcript:Zm00001d047801_T001	rna8854	2.00E-127
1074- 25:	transcript:Zm00001d047803_T001	rna8849	2.00E-54

1074- 26:	transcript:Zm00001d047804_T001	rna8846	0
1074- 27:	transcript:Zm00001d047805_T003	rna8845	1.00E-115
1074- 28:	transcript:Zm00001d047806_T001	rna8844	6.00E-42
1074- 29:	transcript:Zm00001d047807_T001	rna8843	0
1074- 30:	transcript:Zm00001d047808_T001	rna8839	2.00E-102
1074- 31:	transcript:Zm00001d047812_T001	rna8838	0
1074- 32:	transcript:Zm00001d047814_T001	rna8837	0
1074- 33:	transcript:Zm00001d047820_T001	rna8835	1.00E-35
1074- 34:	transcript:Zm00001d047823_T001	rna8833	6.00E-96
1074- 35:	transcript:Zm00001d047824_T001	rna8830	9.00E-176
1074- 36:	transcript:Zm00001d047827_T001	rna8829	5.00E-23
1074- 37:	transcript:Zm00001d047828_T001	rna8826	0
1074- 38:	transcript:Zm00001d047829_T001	rna8821	1.00E-118
1074- 39:	transcript:Zm00001d047831_T001	rna8819	1.00E-28
1074- 40:	transcript:Zm00001d047833_T006	rna8806	8.00E-74
1074- 41:	transcript:Zm00001d047834_T004	rna8805	0
1074- 42:	transcript:Zm00001d047835_T001	rna8804	0
1074- 43:	transcript:Zm00001d047837_T003	rna8803	0
1074- 44:	transcript:Zm00001d047838_T001	rna8802	0
1074- 45:	transcript:Zm00001d047839_T003	rna8801	0
1074- 46:	transcript:Zm00001d047840_T001	rna8800	0
1074- 47:	transcript:Zm00001d047842_T003	rna8794	0
1074- 48:	transcript:Zm00001d047843_T002	rna8791	6.00E-173
1074- 49:	transcript:Zm00001d047847_T003	rna8785	4.00E-45
1074- 50:	transcript:Zm00001d047848_T001	rna8784	0
1074- 51:	transcript:Zm00001d047849_T001	rna8780	0
1074- 52:	transcript:Zm00001d047852_T001	rna8778	0
1074- 53:	transcript:Zm00001d047853_T002	rna8775	3.00E-46
1074- 54:	transcript:Zm00001d047854_T002	rna8774	1.00E-178
1074- 55:	transcript:Zm00001d047855_T001	rna8773	0
1074- 56:	transcript:Zm00001d047856_T001	rna8772	0
1074- 57:	transcript:Zm00001d047857_T001	rna8771	1.00E-39
1074- 58:	transcript:Zm00001d047858_T001	rna8770	0
1074- 59:	transcript:Zm00001d047859_T001	rna8769	6.00E-145
1074- 60:	transcript:Zm00001d047860_T001	rna8767	2.00E-60
1074- 61:	transcript:Zm00001d047863_T004	rna8766	0
1074- 62:	transcript:Zm00001d047868_T001	rna8765	6.00E-112
1074- 63:	transcript:Zm00001d047873_T002	rna8762	1.00E-146
1074- 64:	transcript:Zm00001d047875_T003	rna8761	2.00E-28
1074- 65:	transcript:Zm00001d047876_T001	rna8760	2.00E-112
1074- 66:	transcript:Zm00001d047877_T001	rna8757	4.00E-94
1074- 67:	transcript:Zm00001d047878_T001	rna8754	3.00E-118
1074- 68:	transcript:Zm00001d047879_T001	rna8753	4.00E-22
1074- 69:	transcript:Zm00001d047880_T001	rna8752	3.00E-78
1074- 70:	transcript:Zm00001d047881_T002	rna8750	0
1074- 71:	transcript:Zm00001d047882_T010	rna8745	0
1074- 72:	transcript:Zm00001d047883_T001	rna8741	0
1074- 73:	transcript:Zm00001d047884_T001	rna8736	1.00E-32
1074- 74:	transcript:Zm00001d047889_T013	rna8734	0
1074- 75:	transcript:Zm00001d047893_T003	rna8730	0
1074- 76:	transcript:Zm00001d047894_T002	rna8728	2.00E-172
1074- 77:	transcript:Zm00001d047896_T009	rna8724	0
1074- 78:	transcript:Zm00001d047897_T013	rna8723	0
1074- 79:	transcript:Zm00001d047900_T001	rna8720	6.00E-126

1074- 80: transcript:Zm00001d047901_T001 rna8718 0
1074- 81: transcript:Zm00001d047904_T001 rna8717 3.00E-175
1074- 82: transcript:Zm00001d047906_T009 rna8714 0
1074- 83: transcript:Zm00001d047908_T002 rna8713 0
1074- 84: transcript:Zm00001d047910_T001 rna8709 0
1074- 85: transcript:Zm00001d047911_T010 rna8702 1.00E-105
1074- 86: transcript:Zm00001d047912_T002 rna8700 6.00E-79
1074- 87: transcript:Zm00001d047913_T001 rna8699 2.00E-50
1074- 88: transcript:Zm00001d047915_T001 rna8698 0
1074- 89: transcript:Zm00001d047916_T001 rna8694 0
1074- 90: transcript:Zm00001d047917_T001 rna8688 3.00E-63
1074- 91: transcript:Zm00001d047918_T001 rna8683 0
1074- 92: transcript:Zm00001d047920_T002 rna8682 3.00E-116
1074- 93: transcript:Zm00001d047921_T001 rna8681 2.00E-97
1074- 94: transcript:Zm00001d047923_T001 rna8678 0
Alignment 1075: score=2325.0 e_value=5.5e-209 N=53 9&NC_008396.2 minus
1075- 0: transcript:Zm00001d047554_T001 rna9172 5.00E-153
1075- 1: transcript:Zm00001d047555_T001 rna9170 6.00E-51
1075- 2: transcript:Zm00001d047558_T001 rna9169 0
1075- 3: transcript:Zm00001d047559_T001 rna9165 6.00E-114
1075- 4: transcript:Zm00001d047560_T001 rna9159 6.00E-116
1075- 5: transcript:Zm00001d047562_T002 rna9157 0
1075- 6: transcript:Zm00001d047563_T001 rna9155 0
1075- 7: transcript:Zm00001d047566_T002 rna9153 0
1075- 8: transcript:Zm00001d047567_T001 rna9146 4.00E-85
1075- 9: transcript:Zm00001d047573_T001 rna9135 0
1075- 10: transcript:Zm00001d047587_T005 rna9119 0
1075- 11: transcript:Zm00001d047599_T001 rna9103 0
1075- 12: transcript:Zm00001d047600_T001 rna9102 0
1075- 13: transcript:Zm00001d047601_T006 rna9101 0
1075- 14: transcript:Zm00001d047603_T002 rna9098 0
1075- 15: transcript:Zm00001d047607_T001 rna9097 0
1075- 16: transcript:Zm00001d047612_T001 rna9094 0
1075- 17: transcript:Zm00001d047614_T001 rna9091 1.00E-63
1075- 18: transcript:Zm00001d047618_T001 rna9086 2.00E-60
1075- 19: transcript:Zm00001d047620_T002 rna9081 0
1075- 20: transcript:Zm00001d047632_T001 rna9077 0
1075- 21: transcript:Zm00001d047633_T002 rna9071 0
1075- 22: transcript:Zm00001d047634_T001 rna9069 1.00E-161
1075- 23: transcript:Zm00001d047635_T005 rna9068 6.00E-166
1075- 24: transcript:Zm00001d047636_T002 rna9066 0
1075- 25: transcript:Zm00001d047637_T001 rna9065 0
1075- 26: transcript:Zm00001d047638_T001 rna9063 1.00E-154
1075- 27: transcript:Zm00001d047656_T001 rna9060 8.00E-178
1075- 28: transcript:Zm00001d047664_T001 rna9045 9.00E-74
1075- 29: transcript:Zm00001d047672_T001 rna9037 3.00E-136
1075- 30: transcript:Zm00001d047673_T001 rna9036 0
1075- 31: transcript:Zm00001d047674_T001 rna9035 2.00E-63
1075- 32: transcript:Zm00001d047675_T001 rna9033 1.00E-42
1075- 33: transcript:Zm00001d047676_T002 rna9029 6.00E-25
1075- 34: transcript:Zm00001d047677_T016 rna9027 0
1075- 35: transcript:Zm00001d047679_T001 rna9026 0
1075- 36: transcript:Zm00001d047680_T001 rna9025 4.00E-21
1075- 37: transcript:Zm00001d047681_T002 rna9023 1.00E-53

```
1075- 38: transcript:Zm00001d047685_T001  rna9021           0
1075- 39: transcript:Zm00001d047687_T002  rna9019      5.00E-62
1075- 40: transcript:Zm00001d047688_T001  rna9016     3.00E-105
1075- 41: transcript:Zm00001d047689_T002  rna9015           0
1075- 42: transcript:Zm00001d047694_T001  rna9013           0
1075- 43: transcript:Zm00001d047695_T001  rna9012           0
1075- 44: transcript:Zm00001d047696_T001  rna9009      6.00E-75
1075- 45: transcript:Zm00001d047698_T001  rna9008     4.00E-175
1075- 46: transcript:Zm00001d047699_T001  rna9006     4.00E-150
1075- 47: transcript:Zm00001d047701_T002  rna9005           0
1075- 48: transcript:Zm00001d047702_T001  rna9001      2.00E-75
1075- 49: transcript:Zm00001d047705_T001  rna8996     1.00E-111
1075- 50: transcript:Zm00001d047706_T001  rna8995      4.00E-53
1075- 51: transcript:Zm00001d047707_T002  rna8993     3.00E-129
1075- 52: transcript:Zm00001d047709_T001  rna8990     7.00E-140
## Alignment 1076: score=2104.0 e_value=9.5e-186 N=48 9&NC_008396.2 minus
1076-  0: transcript:Zm00001d047456_T002  rna9338      2.00E-98
1076-  1: transcript:Zm00001d047457_T002  rna9335           0
1076-  2: transcript:Zm00001d047460_T001  rna9333      5.00E-39
1076-  3: transcript:Zm00001d047461_T001  rna9332     2.00E-107
1076-  4: transcript:Zm00001d047462_T001  rna9327     3.00E-132
1076-  5: transcript:Zm00001d047463_T001  rna9326     7.00E-153
1076-  6: transcript:Zm00001d047464_T024  rna9323           0
1076-  7: transcript:Zm00001d047465_T001  rna9322      3.00E-59
1076-  8: transcript:Zm00001d047468_T003  rna9316      1.00E-71
1076-  9: transcript:Zm00001d047469_T001  rna9315      3.00E-82
1076- 10: transcript:Zm00001d047470_T005  rna9314      1.00E-46
1076- 11: transcript:Zm00001d047471_T002  rna9310      7.00E-69
1076- 12: transcript:Zm00001d047472_T002  rna9308           0
1076- 13: transcript:Zm00001d047474_T003  rna9307     1.00E-166
1076- 14: transcript:Zm00001d047476_T001  rna9306     2.00E-179
1076- 15: transcript:Zm00001d047478_T001  rna9305      4.00E-18
1076- 16: transcript:Zm00001d047479_T001  rna9303      9.00E-79
1076- 17: transcript:Zm00001d047480_T001  rna9301     3.00E-134
1076- 18: transcript:Zm00001d047481_T003  rna9297           0
1076- 19: transcript:Zm00001d047482_T001  rna9293           0
1076- 20: transcript:Zm00001d047483_T002  rna9286           0
1076- 21: transcript:Zm00001d047487_T001  rna9284     3.00E-105
1076- 22: transcript:Zm00001d047489_T002  rna9280     8.00E-170
1076- 23: transcript:Zm00001d047491_T004  rna9278      3.00E-34
1076- 24: transcript:Zm00001d047492_T001  rna9275      9.00E-26
1076- 25: transcript:Zm00001d047493_T001  rna9273      3.00E-42
1076- 26: transcript:Zm00001d047494_T042  rna9270           0
1076- 27: transcript:Zm00001d047496_T001  rna9269      8.00E-09
1076- 28: transcript:Zm00001d047498_T010  rna9267     1.00E-115
1076- 29: transcript:Zm00001d047499_T001  rna9265      3.00E-28
1076- 30: transcript:Zm00001d047500_T001  rna9263      4.00E-56
1076- 31: transcript:Zm00001d047501_T001  rna9262           0
1076- 32: transcript:Zm00001d047503_T001  rna9260      2.00E-64
1076- 33: transcript:Zm00001d047506_T006  rna9252           0
1076- 34: transcript:Zm00001d047512_T002  rna9236      3.00E-80
1076- 35: transcript:Zm00001d047517_T001  rna9223     2.00E-135
1076- 36: transcript:Zm00001d047518_T004  rna9220           0
1076- 37: transcript:Zm00001d047519_T001  rna9219     3.00E-167
```

1076- 38: transcript:Zm00001d047522_T002 rna9213 0
1076- 39: transcript:Zm00001d047523_T001 rna9212 8.00E-53
1076- 40: transcript:Zm00001d047526_T006 rna9210 0
1076- 41: transcript:Zm00001d047527_T004 rna9209 0
1076- 42: transcript:Zm00001d047528_T001 rna9206 2.00E-71
1076- 43: transcript:Zm00001d047531_T001 rna9205 0
1076- 44: transcript:Zm00001d047532_T001 rna9202 5.00E-66
1076- 45: transcript:Zm00001d047533_T001 rna9200 0
1076- 46: transcript:Zm00001d047535_T001 rna9190 6.00E-102
1076- 47: transcript:Zm00001d047536_T006 rna9188 0
Alignment 1077: score=1549.0 e_value=7.1e-113 N=33 9&NC_008396.2 minus
1077- 0: transcript:Zm00001d047931_T001 rna8674 0
1077- 1: transcript:Zm00001d047932_T001 rna8673 1.00E-164
1077- 2: transcript:Zm00001d047933_T001 rna8672 6.00E-57
1077- 3: transcript:Zm00001d047934_T001 rna8671 3.00E-87
1077- 4: transcript:Zm00001d047935_T003 rna8670 0
1077- 5: transcript:Zm00001d047937_T001 rna8668 0
1077- 6: transcript:Zm00001d047938_T001 rna8664 3.00E-91
1077- 7: transcript:Zm00001d047940_T008 rna8663 2.00E-34
1077- 8: transcript:Zm00001d047941_T001 rna8658 0
1077- 9: transcript:Zm00001d047942_T001 rna8656 1.00E-98
1077- 10: transcript:Zm00001d047944_T001 rna8653 3.00E-36
1077- 11: transcript:Zm00001d047945_T002 rna8652 0
1077- 12: transcript:Zm00001d047946_T001 rna8651 0
1077- 13: transcript:Zm00001d047951_T002 rna8650 0
1077- 14: transcript:Zm00001d047955_T001 rna8647 2.00E-89
1077- 15: transcript:Zm00001d047958_T001 rna8646 1.00E-72
1077- 16: transcript:Zm00001d047960_T001 rna8645 0
1077- 17: transcript:Zm00001d047962_T003 rna8643 3.00E-77
1077- 18: transcript:Zm00001d047965_T002 rna8640 0
1077- 19: transcript:Zm00001d047966_T001 rna8639 0
1077- 20: transcript:Zm00001d047967_T003 rna8637 2.00E-179
1077- 21: transcript:Zm00001d047968_T001 rna8636 1.00E-58
1077- 22: transcript:Zm00001d047969_T001 rna8635 0
1077- 23: transcript:Zm00001d047970_T001 rna8634 4.00E-172
1077- 24: transcript:Zm00001d047971_T002 rna8632 1.00E-169
1077- 25: transcript:Zm00001d047972_T001 rna8630 0
1077- 26: transcript:Zm00001d047976_T001 rna8623 4.00E-26
1077- 27: transcript:Zm00001d047977_T004 rna8621 0
1077- 28: transcript:Zm00001d047979_T005 rna8620 5.00E-52
1077- 29: transcript:Zm00001d047981_T001 rna8619 0
1077- 30: transcript:Zm00001d047982_T001 rna8614 0
1077- 31: transcript:Zm00001d047983_T007 rna8613 8.00E-172
1077- 32: transcript:Zm00001d047984_T002 rna8609 3.00E-47
Alignment 1078: score=1494.0 e_value=1.4e-115 N=34 9&NC_008396.2 minus
1078- 0: transcript:Zm00001d048446_T001 rna7925 0
1078- 1: transcript:Zm00001d048448_T001 rna7923 2.00E-44
1078- 2: transcript:Zm00001d048450_T003 rna7917 1.00E-112
1078- 3: transcript:Zm00001d048453_T001 rna7904 0
1078- 4: transcript:Zm00001d048454_T005 rna7903 0
1078- 5: transcript:Zm00001d048455_T001 rna7902 6.00E-148
1078- 6: transcript:Zm00001d048456_T001 rna7901 3.00E-101
1078- 7: transcript:Zm00001d048460_T001 rna7896 3.00E-97
1078- 8: transcript:Zm00001d048461_T001 rna7892 0

1078- 9: transcript:Zm00001d048462_T001 rna7891 1.00E-59
1078- 10: transcript:Zm00001d048463_T012 rna7890 0
1078- 11: transcript:Zm00001d048466_T001 rna7889 1.00E-50
1078- 12: transcript:Zm00001d048467_T001 rna7888 7.00E-70
1078- 13: transcript:Zm00001d048469_T001 rna7884 4.00E-140
1078- 14: transcript:Zm00001d048470_T001 rna7873 2.00E-161
1078- 15: transcript:Zm00001d048471_T001 rna7872 0
1078- 16: transcript:Zm00001d048472_T001 rna7871 0
1078- 17: transcript:Zm00001d048473_T003 rna7869 1.00E-115
1078- 18: transcript:Zm00001d048474_T001 rna7867 5.00E-119
1078- 19: transcript:Zm00001d048479_T002 rna7861 9.00E-85
1078- 20: transcript:Zm00001d048480_T005 rna7853 6.00E-161
1078- 21: transcript:Zm00001d048481_T001 rna7852 0
1078- 22: transcript:Zm00001d048493_T003 rna7849 0
1078- 23: transcript:Zm00001d048495_T001 rna7843 0
1078- 24: transcript:Zm00001d048497_T002 rna7834 0
1078- 25: transcript:Zm00001d048499_T001 rna7833 0
1078- 26: transcript:Zm00001d048502_T002 rna7821 5.00E-81
1078- 27: transcript:Zm00001d048503_T001 rna7819 4.00E-42
1078- 28: transcript:Zm00001d048506_T001 rna7809 2.00E-111
1078- 29: transcript:Zm00001d048507_T007 rna7808 0
1078- 30: transcript:Zm00001d048508_T001 rna7800 7.00E-50
1078- 31: transcript:Zm00001d048509_T001 rna7791 0
1078- 32: transcript:Zm00001d048510_T003 rna7790 5.00E-71
1078- 33: transcript:Zm00001d048511_T008 rna7789 4.00E-77
Alignment 1079: score=1276.0 e_value=1.4e-94 N=28 9&NC_008396.2 minus
1079- 0: transcript:Zm00001d047399_T001 rna9428 1.00E-160
1079- 1: transcript:Zm00001d047401_T001 rna9427 2.00E-83
1079- 2: transcript:Zm00001d047402_T001 rna9426 1.00E-68
1079- 3: transcript:Zm00001d047403_T001 rna9424 0
1079- 4: transcript:Zm00001d047404_T001 rna9417 0
1079- 5: transcript:Zm00001d047412_T004 rna9416 0
1079- 6: transcript:Zm00001d047414_T001 rna9415 9.00E-22
1079- 7: transcript:Zm00001d047417_T001 rna9412 5.00E-144
1079- 8: transcript:Zm00001d047419_T001 rna9409 2.00E-22
1079- 9: transcript:Zm00001d047420_T001 rna9407 3.00E-108
1079- 10: transcript:Zm00001d047421_T001 rna9406 1.00E-118
1079- 11: transcript:Zm00001d047422_T002 rna9404 0
1079- 12: transcript:Zm00001d047423_T002 rna9393 3.00E-124
1079- 13: transcript:Zm00001d047424_T001 rna9391 1.00E-20
1079- 14: transcript:Zm00001d047426_T003 rna9390 0
1079- 15: transcript:Zm00001d047427_T002 rna9389 4.00E-89
1079- 16: transcript:Zm00001d047428_T001 rna9385 0
1079- 17: transcript:Zm00001d047434_T006 rna9378 2.00E-32
1079- 18: transcript:Zm00001d047435_T006 rna9377 4.00E-140
1079- 19: transcript:Zm00001d047437_T001 rna9376 0
1079- 20: transcript:Zm00001d047438_T001 rna9374 4.00E-169
1079- 21: transcript:Zm00001d047439_T002 rna9373 0
1079- 22: transcript:Zm00001d047440_T001 rna9359 2.00E-63
1079- 23: transcript:Zm00001d047442_T001 rna9356 3.00E-128
1079- 24: transcript:Zm00001d047443_T002 rna9355 8.00E-93
1079- 25: transcript:Zm00001d047446_T002 rna9347 3.00E-26
1079- 26: transcript:Zm00001d047448_T002 rna9346 5.00E-113
1079- 27: transcript:Zm00001d047449_T006 rna9344 1.00E-33

Alignment 1080: score=825.0 e_value=1.6e-50 N=18 9&NC_008396.2 minus
1080- 0: transcript:Zm00001d048050_T004 rna8538 0
1080- 1: transcript:Zm00001d048053_T001 rna8534 0
1080- 2: transcript:Zm00001d048055_T001 rna8526 0
1080- 3: transcript:Zm00001d048059_T002 rna8525 1.00E-160
1080- 4: transcript:Zm00001d048060_T001 rna8524 0
1080- 5: transcript:Zm00001d048061_T001 rna8520 0
1080- 6: transcript:Zm00001d048065_T002 rna8513 0
1080- 7: transcript:Zm00001d048066_T001 rna8511 0
1080- 8: transcript:Zm00001d048073_T001 rna8510 0
1080- 9: transcript:Zm00001d048075_T001 rna8502 4.00E-41
1080- 10: transcript:Zm00001d048076_T001 rna8501 5.00E-137
1080- 11: transcript:Zm00001d048079_T001 rna8500 0
1080- 12: transcript:Zm00001d048080_T002 rna8498 0
1080- 13: transcript:Zm00001d048082_T001 rna8489 4.00E-123
1080- 14: transcript:Zm00001d048083_T001 rna8488 4.00E-117
1080- 15: transcript:Zm00001d048084_T001 rna8487 0
1080- 16: transcript:Zm00001d048085_T002 rna8483 2.00E-129
1080- 17: transcript:Zm00001d048086_T001 rna8482 0
Alignment 1081: score=741.0 e_value=1.2e-41 N=16 9&NC_008396.2 minus
1081- 0: transcript:Zm00001d047711_T001 rna8988 2.00E-46
1081- 1: transcript:Zm00001d047713_T003 rna8987 0
1081- 2: transcript:Zm00001d047716_T001 rna8983 2.00E-17
1081- 3: transcript:Zm00001d047717_T014 rna8981 0
1081- 4: transcript:Zm00001d047719_T001 rna8980 0
1081- 5: transcript:Zm00001d047720_T003 rna8979 4.00E-67
1081- 6: transcript:Zm00001d047721_T001 rna8974 0
1081- 7: transcript:Zm00001d047722_T001 rna8972 4.00E-83
1081- 8: transcript:Zm00001d047723_T001 rna8969 1.00E-66
1081- 9: transcript:Zm00001d047726_T001 rna8960 0
1081- 10: transcript:Zm00001d047727_T001 rna8958 4.00E-109
1081- 11: transcript:Zm00001d047732_T001 rna8955 0
1081- 12: transcript:Zm00001d047736_T001 rna8952 2.00E-46
1081- 13: transcript:Zm00001d047737_T001 rna8951 0
1081- 14: transcript:Zm00001d047738_T001 rna8950 0
1081- 15: transcript:Zm00001d047739_T004 rna8949 0
Alignment 1082: score=684.0 e_value=2.4e-36 N=15 9&NC_008396.2 minus
1082- 0: transcript:Zm00001d047743_T001 rna8948 4.00E-179
1082- 1: transcript:Zm00001d047744_T001 rna8946 0
1082- 2: transcript:Zm00001d047745_T001 rna8943 6.00E-146
1082- 3: transcript:Zm00001d047746_T007 rna8940 0
1082- 4: transcript:Zm00001d047747_T001 rna8938 7.00E-103
1082- 5: transcript:Zm00001d047749_T001 rna8935 0
1082- 6: transcript:Zm00001d047750_T001 rna8931 5.00E-169
1082- 7: transcript:Zm00001d047752_T001 rna8929 3.00E-87
1082- 8: transcript:Zm00001d047753_T001 rna8925 2.00E-121
1082- 9: transcript:Zm00001d047754_T002 rna8924 0
1082- 10: transcript:Zm00001d047755_T013 rna8923 0
1082- 11: transcript:Zm00001d047758_T004 rna8921 0
1082- 12: transcript:Zm00001d047759_T003 rna8915 0
1082- 13: transcript:Zm00001d047760_T001 rna8911 0
1082- 14: transcript:Zm00001d047761_T008 rna8909 0
Alignment 1083: score=527.0 e_value=6.5e-31 N=13 9&NC_008396.2 minus
1083- 0: transcript:Zm00001d048512_T004 rna7788 0

```
1083-  1: transcript:Zm00001d048513_T001  rna7784      3.00E-34
1083-  2: transcript:Zm00001d048514_T001  rna7781             0
1083-  3: transcript:Zm00001d048516_T001  rna7778             0
1083-  4: transcript:Zm00001d048521_T001  rna7776      8.00E-64
1083-  5: transcript:Zm00001d048522_T001  rna7775     4.00E-125
1083-  6: transcript:Zm00001d048523_T001  rna7770      5.00E-21
1083-  7: transcript:Zm00001d048526_T001  rna7768      2.00E-88
1083-  8: transcript:Zm00001d048527_T028  rna7766             0
1083-  9: transcript:Zm00001d048531_T001  rna7759             0
1083- 10: transcript:Zm00001d048532_T001  rna7752             0
1083- 11: transcript:Zm00001d048533_T001  rna7750      2.00E-23
1083- 12: transcript:Zm00001d048535_T020  rna7747             0
## Alignment 1084: score=327.0 e_value=1.1e-10 N=7 9&NC_008396.2 minus
1084-  0: transcript:Zm00001d047350_T002  rna7874      1.00E-52
1084-  1: transcript:Zm00001d047351_T001  rna7873     3.00E-110
1084-  2: transcript:Zm00001d047354_T001  rna7869     6.00E-112
1084-  3: transcript:Zm00001d047355_T001  rna7867      3.00E-34
1084-  4: transcript:Zm00001d047358_T001  rna7859      1.00E-94
1084-  5: transcript:Zm00001d047359_T001  rna7858      6.00E-75
1084-  6: transcript:Zm00001d047362_T003  rna7853             0
## Alignment 1085: score=290.0 e_value=2.5e-09 N=6 9&NC_008396.2 minus
1085-  0: transcript:Zm00001d048020_T002  rna8553      2.00E-88
1085-  1: transcript:Zm00001d048021_T001  rna8552             0
1085-  2: transcript:Zm00001d048022_T001  rna8550      4.00E-25
1085-  3: transcript:Zm00001d048023_T002  rna8549             0
1085-  4: transcript:Zm00001d048026_T001  rna8547             0
1085-  5: transcript:Zm00001d048027_T001  rna8546             0
## Alignment 1086: score=1458.0 e_value=1.4e-121 N=34 9&NC_008399.2 plus
1086-  0: transcript:Zm00001d045639_T001  rna17345     1.00E-88
1086-  1: transcript:Zm00001d045643_T001  rna17359     6.00E-06
1086-  2: transcript:Zm00001d045655_T013  rna17380            0
1086-  3: transcript:Zm00001d045660_T001  rna17394            0
1086-  4: transcript:Zm00001d045661_T001  rna17400    2.00E-172
1086-  5: transcript:Zm00001d045665_T003  rna17401            0
1086-  6: transcript:Zm00001d045667_T001  rna17402            0
1086-  7: transcript:Zm00001d045668_T001  rna17403     1.00E-52
1086-  8: transcript:Zm00001d045673_T003  rna17406    2.00E-137
1086-  9: transcript:Zm00001d045675_T003  rna17407            0
1086- 10: transcript:Zm00001d045692_T010  rna17418            0
1086- 11: transcript:Zm00001d045695_T001  rna17421            0
1086- 12: transcript:Zm00001d045696_T001  rna17422            0
1086- 13: transcript:Zm00001d045703_T002  rna17425            0
1086- 14: transcript:Zm00001d045706_T001  rna17427            0
1086- 15: transcript:Zm00001d045720_T001  rna17436            0
1086- 16: transcript:Zm00001d045722_T001  rna17438     4.00E-99
1086- 17: transcript:Zm00001d045724_T001  rna17445     1.00E-20
1086- 18: transcript:Zm00001d045725_T002  rna17446     4.00E-18
1086- 19: transcript:Zm00001d045728_T003  rna17448     2.00E-56
1086- 20: transcript:Zm00001d045731_T001  rna17450     9.00E-59
1086- 21: transcript:Zm00001d045735_T002  rna17451    1.00E-143
1086- 22: transcript:Zm00001d045739_T001  rna17455            0
1086- 23: transcript:Zm00001d045740_T001  rna17456            0
1086- 24: transcript:Zm00001d045744_T002  rna17457            0
1086- 25: transcript:Zm00001d045747_T004  rna17466    5.00E-110
```

1086- 26: transcript:Zm00001d045755_T001 rna17471 0
1086- 27: transcript:Zm00001d045756_T001 rna17472 0
1086- 28: transcript:Zm00001d045757_T001 rna17473 0
1086- 29: transcript:Zm00001d045763_T001 rna17474 0
1086- 30: transcript:Zm00001d045764_T002 rna17476 1.00E-155
1086- 31: transcript:Zm00001d045772_T001 rna17478 1.00E-52
1086- 32: transcript:Zm00001d045778_T001 rna17481 3.00E-38
1086- 33: transcript:Zm00001d045782_T001 rna17484 6.00E-107
Alignment 1087: score=1454.0 e_value=3.5e-98 N=31 9&NC_008399.2 plus
1087- 0: transcript:Zm00001d046055_T001 rna17909 1.00E-35
1087- 1: transcript:Zm00001d046060_T001 rna17910 0
1087- 2: transcript:Zm00001d046072_T001 rna17913 0
1087- 3: transcript:Zm00001d046073_T002 rna17914 0
1087- 4: transcript:Zm00001d046089_T001 rna17919 1.00E-39
1087- 5: transcript:Zm00001d046096_T001 rna17920 8.00E-117
1087- 6: transcript:Zm00001d046097_T001 rna17924 2.00E-35
1087- 7: transcript:Zm00001d046112_T002 rna17934 0
1087- 8: transcript:Zm00001d046118_T001 rna17937 6.00E-22
1087- 9: transcript:Zm00001d046120_T001 rna17938 4.00E-44
1087- 10: transcript:Zm00001d046126_T001 rna17940 1.00E-127
1087- 11: transcript:Zm00001d046132_T001 rna17941 3.00E-29
1087- 12: transcript:Zm00001d046134_T001 rna17943 0
1087- 13: transcript:Zm00001d046135_T001 rna17944 0
1087- 14: transcript:Zm00001d046136_T001 rna17946 3.00E-143
1087- 15: transcript:Zm00001d046138_T001 rna17948 3.00E-20
1087- 16: transcript:Zm00001d046143_T001 rna17950 5.00E-27
1087- 17: transcript:Zm00001d046148_T001 rna17954 1.00E-98
1087- 18: transcript:Zm00001d046152_T001 rna17957 4.00E-22
1087- 19: transcript:Zm00001d046155_T001 rna17958 1.00E-66
1087- 20: transcript:Zm00001d046157_T001 rna17961 1.00E-28
1087- 21: transcript:Zm00001d046161_T001 rna17967 8.00E-21
1087- 22: transcript:Zm00001d046166_T004 rna17972 1.00E-20
1087- 23: transcript:Zm00001d046168_T001 rna17973 1.00E-154
1087- 24: transcript:Zm00001d046169_T001 rna17974 1.00E-61
1087- 25: transcript:Zm00001d046174_T001 rna17977 3.00E-149
1087- 26: transcript:Zm00001d046175_T001 rna17978 5.00E-150
1087- 27: transcript:Zm00001d046178_T001 rna17980 6.00E-119
1087- 28: transcript:Zm00001d046182_T001 rna17984 0
1087- 29: transcript:Zm00001d046183_T001 rna17986 7.00E-128
1087- 30: transcript:Zm00001d046184_T001 rna17987 9.00E-147
Alignment 1088: score=1426.0 e_value=3.6e-109 N=31 9&NC_008399.2 plus
1088- 0: transcript:Zm00001d046914_T001 rna18471 2.00E-82
1088- 1: transcript:Zm00001d046934_T002 rna18497 7.00E-20
1088- 2: transcript:Zm00001d046935_T001 rna18498 0
1088- 3: transcript:Zm00001d046936_T002 rna18499 0
1088- 4: transcript:Zm00001d046937_T001 rna18503 1.00E-98
1088- 5: transcript:Zm00001d046939_T001 rna18512 0
1088- 6: transcript:Zm00001d046940_T001 rna18517 5.00E-100
1088- 7: transcript:Zm00001d046941_T001 rna18518 0
1088- 8: transcript:Zm00001d046942_T001 rna18521 0
1088- 9: transcript:Zm00001d046945_T001 rna18522 0
1088- 10: transcript:Zm00001d046947_T001 rna18524 1.00E-124
1088- 11: transcript:Zm00001d046948_T002 rna18525 0
1088- 12: transcript:Zm00001d046949_T001 rna18526 6.00E-174

1088- 13: transcript:Zm00001d046952_T001 rna18527 0
1088- 14: transcript:Zm00001d046953_T002 rna18528 1.00E-89
1088- 15: transcript:Zm00001d046959_T002 rna18529 8.00E-33
1088- 16: transcript:Zm00001d046961_T001 rna18531 2.00E-46
1088- 17: transcript:Zm00001d046966_T001 rna18534 2.00E-15
1088- 18: transcript:Zm00001d046967_T001 rna18536 9.00E-124
1088- 19: transcript:Zm00001d046968_T001 rna18544 1.00E-85
1088- 20: transcript:Zm00001d046971_T001 rna18548 3.00E-170
1088- 21: transcript:Zm00001d046973_T001 rna18549 5.00E-70
1088- 22: transcript:Zm00001d046974_T002 rna18550 1.00E-120
1088- 23: transcript:Zm00001d046978_T002 rna18551 3.00E-110
1088- 24: transcript:Zm00001d046979_T001 rna18552 7.00E-19
1088- 25: transcript:Zm00001d046981_T001 rna18554 3.00E-68
1088- 26: transcript:Zm00001d046986_T001 rna18557 3.00E-39
1088- 27: transcript:Zm00001d046995_T001 rna18561 0
1088- 28: transcript:Zm00001d046996_T001 rna18562 8.00E-165
1088- 29: transcript:Zm00001d046998_T001 rna18563 6.00E-95
1088- 30: transcript:Zm00001d047000_T001 rna18569 1.00E-06
Alignment 1089: score=1257.0 e_value=1.9e-97 N=29 9&NC_008399.2 plus
1089- 0: transcript:Zm00001d045808_T025 rna17528 0
1089- 1: transcript:Zm00001d045812_T001 rna17529 1.00E-121
1089- 2: transcript:Zm00001d045817_T001 rna17530 6.00E-90
1089- 3: transcript:Zm00001d045819_T001 rna17537 6.00E-145
1089- 4: transcript:Zm00001d045825_T001 rna17538 3.00E-115
1089- 5: transcript:Zm00001d045836_T001 rna17553 2.00E-36
1089- 6: transcript:Zm00001d045845_T001 rna17556 1.00E-138
1089- 7: transcript:Zm00001d045853_T001 rna17559 4.00E-38
1089- 8: transcript:Zm00001d045864_T004 rna17563 0
1089- 9: transcript:Zm00001d045870_T001 rna17564 0
1089- 10: transcript:Zm00001d045872_T002 rna17567 0
1089- 11: transcript:Zm00001d045873_T002 rna17573 7.00E-38
1089- 12: transcript:Zm00001d045877_T001 rna17590 6.00E-06
1089- 13: transcript:Zm00001d045883_T001 rna17596 0
1089- 14: transcript:Zm00001d045885_T002 rna17598 0
1089- 15: transcript:Zm00001d045887_T004 rna17599 0
1089- 16: transcript:Zm00001d045888_T001 rna17602 8.00E-11
1089- 17: transcript:Zm00001d045889_T002 rna17604 9.00E-71
1089- 18: transcript:Zm00001d045893_T001 rna17606 5.00E-62
1089- 19: transcript:Zm00001d045894_T001 rna17607 0
1089- 20: transcript:Zm00001d045896_T001 rna17609 8.00E-55
1089- 21: transcript:Zm00001d045897_T003 rna17611 0
1089- 22: transcript:Zm00001d045906_T001 rna17622 1.00E-26
1089- 23: transcript:Zm00001d045911_T002 rna17626 0
1089- 24: transcript:Zm00001d045919_T001 rna17630 0
1089- 25: transcript:Zm00001d045926_T002 rna17631 1.00E-106
1089- 26: transcript:Zm00001d045927_T002 rna17632 5.00E-148
1089- 27: transcript:Zm00001d045928_T001 rna17633 1.00E-100
1089- 28: transcript:Zm00001d045935_T001 rna17640 8.00E-40
Alignment 1090: score=1006.0 e_value=4.5e-68 N=22 9&NC_008399.2 plus
1090- 0: transcript:Zm00001d046490_T001 rna18200 4.00E-91
1090- 1: transcript:Zm00001d046492_T001 rna18201 4.00E-152
1090- 2: transcript:Zm00001d046496_T001 rna18203 2.00E-60
1090- 3: transcript:Zm00001d046499_T001 rna18204 1.00E-28
1090- 4: transcript:Zm00001d046500_T001 rna18205 7.00E-164

1090- 5: transcript:Zm00001d046501_T001 rna18206 6.00E-47
1090- 6: transcript:Zm00001d046508_T001 rna18208 0
1090- 7: transcript:Zm00001d046509_T002 rna18211 0
1090- 8: transcript:Zm00001d046510_T001 rna18212 2.00E-36
1090- 9: transcript:Zm00001d046530_T001 rna18220 7.00E-53
1090- 10: transcript:Zm00001d046531_T007 rna18221 0
1090- 11: transcript:Zm00001d046534_T001 rna18223 0
1090- 12: transcript:Zm00001d046535_T001 rna18224 2.00E-136
1090- 13: transcript:Zm00001d046538_T003 rna18225 4.00E-35
1090- 14: transcript:Zm00001d046539_T002 rna18229 0
1090- 15: transcript:Zm00001d046540_T003 rna18230 0
1090- 16: transcript:Zm00001d046542_T001 rna18231 2.00E-15
1090- 17: transcript:Zm00001d046545_T010 rna18234 2.00E-93
1090- 18: transcript:Zm00001d046549_T002 rna18235 0
1090- 19: transcript:Zm00001d046552_T001 rna18237 9.00E-120
1090- 20: transcript:Zm00001d046553_T003 rna18238 1.00E-92
1090- 21: transcript:Zm00001d046554_T001 rna18240 0
Alignment 1091: score=775.0 e_value=2.6e-48 N=17 9&NC_008399.2 plus
1091- 0: transcript:Zm00001d046330_T002 rna18066 1.00E-43
1091- 1: transcript:Zm00001d046335_T001 rna18068 0
1091- 2: transcript:Zm00001d046336_T001 rna18069 0
1091- 3: transcript:Zm00001d046342_T002 rna18070 0
1091- 4: transcript:Zm00001d046346_T001 rna18079 1.00E-91
1091- 5: transcript:Zm00001d046348_T003 rna18081 0
1091- 6: transcript:Zm00001d046350_T001 rna18082 5.00E-19
1091- 7: transcript:Zm00001d046352_T001 rna18083 0
1091- 8: transcript:Zm00001d046354_T001 rna18084 1.00E-85
1091- 9: transcript:Zm00001d046357_T001 rna18088 0
1091- 10: transcript:Zm00001d046358_T001 rna18089 0
1091- 11: transcript:Zm00001d046363_T001 rna18090 1.00E-36
1091- 12: transcript:Zm00001d046364_T001 rna18091 0
1091- 13: transcript:Zm00001d046366_T001 rna18092 0
1091- 14: transcript:Zm00001d046369_T002 rna18093 0
1091- 15: transcript:Zm00001d046370_T001 rna18095 0
1091- 16: transcript:Zm00001d046378_T001 rna18110 3.00E-49
Alignment 1092: score=438.0 e_value=5.4e-22 N=10 9&NC_008399.2 plus
1092- 0: transcript:Zm00001d046794_T001 rna18419 9.00E-69
1092- 1: transcript:Zm00001d046803_T001 rna18426 7.00E-59
1092- 2: transcript:Zm00001d046805_T001 rna18429 2.00E-137
1092- 3: transcript:Zm00001d046810_T001 rna18430 0
1092- 4: transcript:Zm00001d046812_T001 rna18439 4.00E-57
1092- 5: transcript:Zm00001d046824_T001 rna18442 2.00E-30
1092- 6: transcript:Zm00001d046827_T002 rna18443 8.00E-93
1092- 7: transcript:Zm00001d046831_T002 rna18444 0
1092- 8: transcript:Zm00001d046834_T001 rna18445 1.00E-33
1092- 9: transcript:Zm00001d046835_T004 rna18446 0
Alignment 1093: score=335.0 e_value=1.5e-12 N=7 9&NC_008399.2 plus
1093- 0: transcript:Zm00001d044717_T001 rna17361 3.00E-78
1093- 1: transcript:Zm00001d044723_T001 rna17363 0
1093- 2: transcript:Zm00001d044724_T001 rna17365 0
1093- 3: transcript:Zm00001d044726_T001 rna17366 0
1093- 4: transcript:Zm00001d044728_T001 rna17368 5.00E-66
1093- 5: transcript:Zm00001d044729_T001 rna17369 3.00E-140
1093- 6: transcript:Zm00001d044730_T001 rna17370 4.00E-62

Alignment 1094: score=317.0 e_value=1.4e-10 N=7 9&NC_008399.2 plus
1094- 0: transcript:Zm00001d044745_T006 rna17344 1.00E-165
1094- 1: transcript:Zm00001d044746_T001 rna17345 2.00E-11
1094- 2: transcript:Zm00001d044747_T001 rna17346 8.00E-43
1094- 3: transcript:Zm00001d044748_T002 rna17347 0
1094- 4: transcript:Zm00001d044752_T001 rna17350 2.00E-51
1094- 5: transcript:Zm00001d044754_T001 rna17352 0
1094- 6: transcript:Zm00001d044757_T010 rna17353 0
Alignment 1095: score=303.0 e_value=1.7e-10 N=7 9&NC_008399.2 plus
1095- 0: transcript:Zm00001d044975_T001 rna17129 7.00E-94
1095- 1: transcript:Zm00001d044976_T001 rna17131 0
1095- 2: transcript:Zm00001d044979_T001 rna17133 0
1095- 3: transcript:Zm00001d044982_T009 rna17136 1.00E-55
1095- 4: transcript:Zm00001d044985_T014 rna17138 0
1095- 5: transcript:Zm00001d044986_T001 rna17140 2.00E-27
1095- 6: transcript:Zm00001d044987_T001 rna17141 0
Alignment 1096: score=301.0 e_value=1.4e-12 N=7 9&NC_008399.2 plus
1096- 0: transcript:Zm00001d045783_T064 rna17485 0
1096- 1: transcript:Zm00001d045785_T001 rna17492 0
1096- 2: transcript:Zm00001d045788_T001 rna17493 0
1096- 3: transcript:Zm00001d045793_T001 rna17508 0
1096- 4: transcript:Zm00001d045798_T001 rna17522 0
1096- 5: transcript:Zm00001d045800_T001 rna17523 0
1096- 6: transcript:Zm00001d045804_T003 rna17526 0
Alignment 1097: score=274.0 e_value=5.7e-09 N=6 9&NC_008399.2 plus
1097- 0: transcript:Zm00001d046235_T002 rna18023 0
1097- 1: transcript:Zm00001d046236_T001 rna18027 0
1097- 2: transcript:Zm00001d046242_T001 rna18037 2.00E-65
1097- 3: transcript:Zm00001d046246_T001 rna18038 3.00E-53
1097- 4: transcript:Zm00001d046249_T001 rna18039 1.00E-117
1097- 5: transcript:Zm00001d046254_T001 rna18040 9.00E-173
Alignment 1098: score=273.0 e_value=1e-10 N=6 9&NC_008399.2 plus
1098- 0: transcript:Zm00001d046438_T003 rna18170 0
1098- 1: transcript:Zm00001d046440_T001 rna18172 0
1098- 2: transcript:Zm00001d046441_T002 rna18173 0
1098- 3: transcript:Zm00001d046442_T002 rna18174 2.00E-157
1098- 4: transcript:Zm00001d046444_T003 rna18182 0
1098- 5: transcript:Zm00001d046445_T004 rna18183 0
Alignment 1099: score=265.0 e_value=1.2e-08 N=6 9&NC_008399.2 plus
1099- 0: transcript:Zm00001d045551_T032 rna16570 0
1099- 1: transcript:Zm00001d045553_T001 rna16571 3.00E-174
1099- 2: transcript:Zm00001d045557_T001 rna16572 0
1099- 3: transcript:Zm00001d045558_T003 rna16574 0
1099- 4: transcript:Zm00001d045559_T002 rna16578 1.00E-160
1099- 5: transcript:Zm00001d045560_T001 rna16580 2.00E-108
Alignment 1100: score=5217.0 e_value=0 N=116 9&NC_008399.2 minus
1100- 0: transcript:Zm00001d045256_T001 rna16900 2.00E-21
1100- 1: transcript:Zm00001d045263_T002 rna16894 7.00E-84
1100- 2: transcript:Zm00001d045264_T001 rna16893 0
1100- 3: transcript:Zm00001d045268_T001 rna16887 1.00E-94
1100- 4: transcript:Zm00001d045269_T021 rna16885 3.00E-48
1100- 5: transcript:Zm00001d045270_T001 rna16884 4.00E-63
1100- 6: transcript:Zm00001d045271_T001 rna16883 4.00E-80
1100- 7: transcript:Zm00001d045272_T001 rna16882 4.00E-137

1100- 8:	transcript:Zm00001d045283_T001	rna16877	2.00E-87
1100- 9:	transcript:Zm00001d045295_T001	rna16876	0
1100- 10:	transcript:Zm00001d045296_T001	rna16875	3.00E-15
1100- 11:	transcript:Zm00001d045297_T001	rna16871	3.00E-143
1100- 12:	transcript:Zm00001d045298_T002	rna16869	8.00E-60
1100- 13:	transcript:Zm00001d045304_T001	rna16865	0
1100- 14:	transcript:Zm00001d045305_T001	rna16863	3.00E-175
1100- 15:	transcript:Zm00001d045310_T001	rna16859	0
1100- 16:	transcript:Zm00001d045311_T015	rna16858	5.00E-76
1100- 17:	transcript:Zm00001d045313_T001	rna16857	0
1100- 18:	transcript:Zm00001d045314_T001	rna16856	5.00E-138
1100- 19:	transcript:Zm00001d045315_T003	rna16855	5.00E-169
1100- 20:	transcript:Zm00001d045316_T002	rna16852	4.00E-174
1100- 21:	transcript:Zm00001d045318_T001	rna16849	2.00E-50
1100- 22:	transcript:Zm00001d045319_T001	rna16846	0
1100- 23:	transcript:Zm00001d045322_T001	rna16844	1.00E-167
1100- 24:	transcript:Zm00001d045323_T004	rna16842	2.00E-169
1100- 25:	transcript:Zm00001d045324_T001	rna16840	1.00E-49
1100- 26:	transcript:Zm00001d045326_T004	rna16839	2.00E-84
1100- 27:	transcript:Zm00001d045339_T002	rna16835	2.00E-95
1100- 28:	transcript:Zm00001d045341_T003	rna16826	2.00E-99
1100- 29:	transcript:Zm00001d045351_T001	rna16824	4.00E-43
1100- 30:	transcript:Zm00001d045352_T002	rna16823	0
1100- 31:	transcript:Zm00001d045358_T001	rna16813	4.00E-37
1100- 32:	transcript:Zm00001d045359_T002	rna16812	0
1100- 33:	transcript:Zm00001d045360_T001	rna16806	1.00E-19
1100- 34:	transcript:Zm00001d045361_T001	rna16803	1.00E-31
1100- 35:	transcript:Zm00001d045369_T001	rna16801	6.00E-27
1100- 36:	transcript:Zm00001d045372_T002	rna16800	4.00E-98
1100- 37:	transcript:Zm00001d045373_T001	rna16799	2.00E-148
1100- 38:	transcript:Zm00001d045381_T001	rna16784	2.00E-173
1100- 39:	transcript:Zm00001d045385_T001	rna16778	7.00E-20
1100- 40:	transcript:Zm00001d045388_T001	rna16773	0
1100- 41:	transcript:Zm00001d045390_T002	rna16760	2.00E-16
1100- 42:	transcript:Zm00001d045393_T001	rna16759	3.00E-84
1100- 43:	transcript:Zm00001d045394_T001	rna16758	0
1100- 44:	transcript:Zm00001d045395_T001	rna16757	0
1100- 45:	transcript:Zm00001d045396_T001	rna16755	1.00E-21
1100- 46:	transcript:Zm00001d045397_T001	rna16754	0
1100- 47:	transcript:Zm00001d045398_T001	rna16752	1.00E-54
1100- 48:	transcript:Zm00001d045403_T001	rna16747	2.00E-166
1100- 49:	transcript:Zm00001d045404_T001	rna16746	2.00E-136
1100- 50:	transcript:Zm00001d045405_T001	rna16745	1.00E-144
1100- 51:	transcript:Zm00001d045406_T001	rna16739	1.00E-100
1100- 52:	transcript:Zm00001d045417_T002	rna16737	0
1100- 53:	transcript:Zm00001d045418_T001	rna16736	1.00E-140
1100- 54:	transcript:Zm00001d045420_T001	rna16734	0
1100- 55:	transcript:Zm00001d045421_T001	rna16733	1.00E-112
1100- 56:	transcript:Zm00001d045425_T001	rna16730	2.00E-27
1100- 57:	transcript:Zm00001d045427_T001	rna16729	1.00E-94
1100- 58:	transcript:Zm00001d045430_T002	rna16728	0
1100- 59:	transcript:Zm00001d045431_T001	rna16726	0
1100- 60:	transcript:Zm00001d045432_T001	rna16724	4.00E-37
1100- 61:	transcript:Zm00001d045434_T004	rna16721	0

1100- 62:	transcript:Zm00001d045435_T009	rna16718	3.00E-123
1100- 63:	transcript:Zm00001d045436_T001	rna16717	0
1100- 64:	transcript:Zm00001d045445_T001	rna16716	0
1100- 65:	transcript:Zm00001d045447_T002	rna16709	2.00E-102
1100- 66:	transcript:Zm00001d045448_T001	rna16708	3.00E-69
1100- 67:	transcript:Zm00001d045450_T001	rna16707	0
1100- 68:	transcript:Zm00001d045451_T001	rna16706	0
1100- 69:	transcript:Zm00001d045458_T001	rna16705	0
1100- 70:	transcript:Zm00001d045459_T001	rna16703	2.00E-14
1100- 71:	transcript:Zm00001d045461_T001	rna16701	4.00E-12
1100- 72:	transcript:Zm00001d045462_T001	rna16699	0
1100- 73:	transcript:Zm00001d045463_T001	rna16692	5.00E-98
1100- 74:	transcript:Zm00001d045470_T001	rna16690	0
1100- 75:	transcript:Zm00001d045472_T002	rna16689	5.00E-176
1100- 76:	transcript:Zm00001d045475_T001	rna16688	3.00E-95
1100- 77:	transcript:Zm00001d045477_T001	rna16686	0
1100- 78:	transcript:Zm00001d045479_T001	rna16684	0
1100- 79:	transcript:Zm00001d045480_T003	rna16683	3.00E-159
1100- 80:	transcript:Zm00001d045481_T004	rna16682	0
1100- 81:	transcript:Zm00001d045483_T002	rna16681	0
1100- 82:	transcript:Zm00001d045486_T001	rna16680	4.00E-33
1100- 83:	transcript:Zm00001d045487_T001	rna16679	1.00E-83
1100- 84:	transcript:Zm00001d045488_T003	rna16678	3.00E-164
1100- 85:	transcript:Zm00001d045491_T003	rna16676	0
1100- 86:	transcript:Zm00001d045493_T002	rna16675	1.00E-179
1100- 87:	transcript:Zm00001d045494_T001	rna16674	0
1100- 88:	transcript:Zm00001d045495_T001	rna16673	2.00E-49
1100- 89:	transcript:Zm00001d045496_T001	rna16672	1.00E-141
1100- 90:	transcript:Zm00001d045499_T001	rna16670	9.00E-31
1100- 91:	transcript:Zm00001d045500_T004	rna16669	6.00E-84
1100- 92:	transcript:Zm00001d045501_T001	rna16668	0
1100- 93:	transcript:Zm00001d045505_T003	rna16666	0
1100- 94:	transcript:Zm00001d045507_T001	rna16663	0
1100- 95:	transcript:Zm00001d045509_T002	rna16660	1.00E-114
1100- 96:	transcript:Zm00001d045513_T001	rna16655	2.00E-140
1100- 97:	transcript:Zm00001d045514_T001	rna16654	8.00E-47
1100- 98:	transcript:Zm00001d045516_T010	rna16652	0
1100- 99:	transcript:Zm00001d045517_T001	rna16651	5.00E-92
1100-100:	transcript:Zm00001d045518_T001	rna16649	4.00E-137
1100-101:	transcript:Zm00001d045519_T002	rna16647	3.00E-131
1100-102:	transcript:Zm00001d045520_T001	rna16642	6.00E-16
1100-103:	transcript:Zm00001d045521_T001	rna16641	1.00E-08
1100-104:	transcript:Zm00001d045522_T001	rna16621	2.00E-88
1100-105:	transcript:Zm00001d045528_T001	rna16611	1.00E-106
1100-106:	transcript:Zm00001d045530_T001	rna16608	4.00E-22
1100-107:	transcript:Zm00001d045533_T001	rna16607	3.00E-176
1100-108:	transcript:Zm00001d045535_T001	rna16604	2.00E-36
1100-109:	transcript:Zm00001d045537_T001	rna16603	0
1100-110:	transcript:Zm00001d045538_T001	rna16601	2.00E-177
1100-111:	transcript:Zm00001d045539_T001	rna16600	1.00E-50
1100-112:	transcript:Zm00001d045540_T001	rna16595	9.00E-120
1100-113:	transcript:Zm00001d045544_T002	rna16587	0
1100-114:	transcript:Zm00001d045547_T001	rna16582	3.00E-116
1100-115:	transcript:Zm00001d045550_T002	rna16567	0

Alignment 1101: score=3335.0 e_value=5.3e-311 N=75 9&NC_008399.2 minus

1101- 0:	transcript:Zm00001d044744_T001	rna17354	0
1101- 1:	transcript:Zm00001d044754_T001	rna17352	0
1101- 2:	transcript:Zm00001d044759_T001	rna17343	0
1101- 3:	transcript:Zm00001d044760_T001	rna17341	0
1101- 4:	transcript:Zm00001d044762_T001	rna17330	8.00E-142
1101- 5:	transcript:Zm00001d044766_T001	rna17324	9.00E-55
1101- 6:	transcript:Zm00001d044768_T001	rna17323	0
1101- 7:	transcript:Zm00001d044769_T001	rna17322	6.00E-26
1101- 8:	transcript:Zm00001d044771_T001	rna17321	8.00E-163
1101- 9:	transcript:Zm00001d044773_T001	rna17316	0
1101- 10:	transcript:Zm00001d044775_T001	rna17312	4.00E-30
1101- 11:	transcript:Zm00001d044780_T001	rna17310	0
1101- 12:	transcript:Zm00001d044781_T001	rna17309	4.00E-105
1101- 13:	transcript:Zm00001d044783_T001	rna17305	1.00E-48
1101- 14:	transcript:Zm00001d044784_T001	rna17300	1.00E-98
1101- 15:	transcript:Zm00001d044802_T001	rna17295	2.00E-134
1101- 16:	transcript:Zm00001d044803_T001	rna17294	1.00E-151
1101- 17:	transcript:Zm00001d044806_T001	rna17293	9.00E-150
1101- 18:	transcript:Zm00001d044808_T001	rna17291	0
1101- 19:	transcript:Zm00001d044812_T001	rna17285	0
1101- 20:	transcript:Zm00001d044813_T001	rna17284	0
1101- 21:	transcript:Zm00001d044814_T002	rna17283	0
1101- 22:	transcript:Zm00001d044815_T002	rna17279	2.00E-144
1101- 23:	transcript:Zm00001d044816_T001	rna17278	2.00E-109
1101- 24:	transcript:Zm00001d044821_T001	rna17271	0
1101- 25:	transcript:Zm00001d044823_T011	rna17269	0
1101- 26:	transcript:Zm00001d044824_T001	rna17267	5.00E-64
1101- 27:	transcript:Zm00001d044825_T001	rna17266	0
1101- 28:	transcript:Zm00001d044826_T001	rna17262	0
1101- 29:	transcript:Zm00001d044831_T001	rna17260	1.00E-129
1101- 30:	transcript:Zm00001d044833_T001	rna17259	0
1101- 31:	transcript:Zm00001d044836_T001	rna17258	1.00E-105
1101- 32:	transcript:Zm00001d044838_T002	rna17257	1.00E-118
1101- 33:	transcript:Zm00001d044839_T001	rna17256	9.00E-50
1101- 34:	transcript:Zm00001d044841_T001	rna17255	8.00E-109
1101- 35:	transcript:Zm00001d044844_T001	rna17236	8.00E-36
1101- 36:	transcript:Zm00001d044845_T001	rna17234	0
1101- 37:	transcript:Zm00001d044849_T001	rna17233	3.00E-42
1101- 38:	transcript:Zm00001d044850_T001	rna17232	6.00E-93
1101- 39:	transcript:Zm00001d044851_T001	rna17231	3.00E-30
1101- 40:	transcript:Zm00001d044857_T001	rna17228	4.00E-19
1101- 41:	transcript:Zm00001d044860_T001	rna17224	0
1101- 42:	transcript:Zm00001d044861_T001	rna17222	2.00E-14
1101- 43:	transcript:Zm00001d044864_T001	rna17215	3.00E-99
1101- 44:	transcript:Zm00001d044866_T001	rna17214	0
1101- 45:	transcript:Zm00001d044869_T001	rna17212	3.00E-96
1101- 46:	transcript:Zm00001d044874_T001	rna17211	5.00E-49
1101- 47:	transcript:Zm00001d044875_T001	rna17208	4.00E-30
1101- 48:	transcript:Zm00001d044878_T002	rna17203	8.00E-09
1101- 49:	transcript:Zm00001d044880_T001	rna17202	2.00E-36
1101- 50:	transcript:Zm00001d044903_T026	rna17199	8.00E-139
1101- 51:	transcript:Zm00001d044904_T001	rna17193	0
1101- 52:	transcript:Zm00001d044908_T001	rna17184	0

```
1101- 53: transcript:Zm00001d044916_T001  rna17182           0
1101- 54: transcript:Zm00001d044917_T001  rna17181    2.00E-148
1101- 55: transcript:Zm00001d044918_T001  rna17180     3.00E-93
1101- 56: transcript:Zm00001d044920_T001  rna17178           0
1101- 57: transcript:Zm00001d044921_T001  rna17176    7.00E-180
1101- 58: transcript:Zm00001d044922_T001  rna17174           0
1101- 59: transcript:Zm00001d044923_T001  rna17173     2.00E-40
1101- 60: transcript:Zm00001d044929_T002  rna17172           0
1101- 61: transcript:Zm00001d044931_T001  rna17171     5.00E-32
1101- 62: transcript:Zm00001d044933_T001  rna17167    4.00E-142
1101- 63: transcript:Zm00001d044934_T001  rna17166           0
1101- 64: transcript:Zm00001d044936_T001  rna17164    3.00E-167
1101- 65: transcript:Zm00001d044940_T004  rna17162    5.00E-141
1101- 66: transcript:Zm00001d044947_T001  rna17160           0
1101- 67: transcript:Zm00001d044950_T001  rna17157     3.00E-45
1101- 68: transcript:Zm00001d044951_T001  rna17154     3.00E-31
1101- 69: transcript:Zm00001d044966_T001  rna17148           0
1101- 70: transcript:Zm00001d044970_T001  rna17146    1.00E-124
1101- 71: transcript:Zm00001d044971_T005  rna17145           0
1101- 72: transcript:Zm00001d044972_T001  rna17144     1.00E-35
1101- 73: transcript:Zm00001d044973_T001  rna17143     2.00E-85
1101- 74: transcript:Zm00001d044979_T001  rna17133           0
## Alignment 1102: score=3300.0 e_value=8.2e-312 N=73 9&NC_008399.2 minus
1102-  0: transcript:Zm00001d046586_T001  rna18403           0
1102-  1: transcript:Zm00001d046587_T003  rna18402     1.00E-45
1102-  2: transcript:Zm00001d046590_T004  rna18401           0
1102-  3: transcript:Zm00001d046591_T001  rna18398           0
1102-  4: transcript:Zm00001d046592_T001  rna18397    3.00E-102
1102-  5: transcript:Zm00001d046593_T001  rna18396           0
1102-  6: transcript:Zm00001d046595_T002  rna18395    1.00E-109
1102-  7: transcript:Zm00001d046596_T001  rna18392     5.00E-39
1102-  8: transcript:Zm00001d046599_T001  rna18391    3.00E-144
1102-  9: transcript:Zm00001d046600_T001  rna18390    2.00E-138
1102- 10: transcript:Zm00001d046601_T005  rna18389           0
1102- 11: transcript:Zm00001d046602_T001  rna18388    1.00E-141
1102- 12: transcript:Zm00001d046603_T001  rna18375    2.00E-172
1102- 13: transcript:Zm00001d046613_T007  rna18374    5.00E-127
1102- 14: transcript:Zm00001d046616_T001  rna18373           0
1102- 15: transcript:Zm00001d046621_T001  rna18371     2.00E-13
1102- 16: transcript:Zm00001d046624_T005  rna18370           0
1102- 17: transcript:Zm00001d046626_T001  rna18367    1.00E-101
1102- 18: transcript:Zm00001d046628_T001  rna18364     1.00E-34
1102- 19: transcript:Zm00001d046629_T001  rna18363    2.00E-107
1102- 20: transcript:Zm00001d046630_T003  rna18362     3.00E-70
1102- 21: transcript:Zm00001d046632_T001  rna18360    1.00E-134
1102- 22: transcript:Zm00001d046634_T001  rna18359     2.00E-07
1102- 23: transcript:Zm00001d046641_T001  rna18358     5.00E-20
1102- 24: transcript:Zm00001d046642_T001  rna18357           0
1102- 25: transcript:Zm00001d046643_T001  rna18356           0
1102- 26: transcript:Zm00001d046644_T001  rna18355    3.00E-135
1102- 27: transcript:Zm00001d046652_T001  rna18351           0
1102- 28: transcript:Zm00001d046654_T002  rna18349    2.00E-128
1102- 29: transcript:Zm00001d046655_T001  rna18348     3.00E-65
1102- 30: transcript:Zm00001d046656_T004  rna18347    1.00E-178
```

1102- 31: transcript:Zm00001d046658_T001 rna18345 0
1102- 32: transcript:Zm00001d046659_T001 rna18344 4.00E-51
1102- 33: transcript:Zm00001d046660_T001 rna18343 0
1102- 34: transcript:Zm00001d046661_T003 rna18342 0
1102- 35: transcript:Zm00001d046663_T001 rna18341 4.00E-54
1102- 36: transcript:Zm00001d046665_T001 rna18340 1.00E-14
1102- 37: transcript:Zm00001d046667_T001 rna18339 2.00E-21
1102- 38: transcript:Zm00001d046672_T001 rna18338 0
1102- 39: transcript:Zm00001d046679_T002 rna18324 0
1102- 40: transcript:Zm00001d046680_T001 rna18323 0
1102- 41: transcript:Zm00001d046681_T001 rna18322 1.00E-102
1102- 42: transcript:Zm00001d046682_T001 rna18321 2.00E-107
1102- 43: transcript:Zm00001d046683_T002 rna18320 0
1102- 44: transcript:Zm00001d046687_T001 rna18316 8.00E-42
1102- 45: transcript:Zm00001d046688_T002 rna18315 3.00E-65
1102- 46: transcript:Zm00001d046690_T001 rna18314 0
1102- 47: transcript:Zm00001d046705_T001 rna18310 2.00E-76
1102- 48: transcript:Zm00001d046711_T001 rna18309 2.00E-34
1102- 49: transcript:Zm00001d046716_T001 rna18307 6.00E-141
1102- 50: transcript:Zm00001d046717_T009 rna18306 0
1102- 51: transcript:Zm00001d046718_T001 rna18305 3.00E-118
1102- 52: transcript:Zm00001d046719_T006 rna18301 0
1102- 53: transcript:Zm00001d046722_T007 rna18300 0
1102- 54: transcript:Zm00001d046723_T001 rna18299 2.00E-156
1102- 55: transcript:Zm00001d046725_T001 rna18293 7.00E-152
1102- 56: transcript:Zm00001d046728_T001 rna18290 4.00E-39
1102- 57: transcript:Zm00001d046740_T001 rna18285 3.00E-146
1102- 58: transcript:Zm00001d046742_T002 rna18283 0
1102- 59: transcript:Zm00001d046751_T001 rna18276 4.00E-179
1102- 60: transcript:Zm00001d046758_T001 rna18273 0
1102- 61: transcript:Zm00001d046759_T001 rna18272 8.00E-108
1102- 62: transcript:Zm00001d046761_T001 rna18271 0
1102- 63: transcript:Zm00001d046764_T001 rna18270 0
1102- 64: transcript:Zm00001d046765_T001 rna18267 0
1102- 65: transcript:Zm00001d046767_T002 rna18266 7.00E-101
1102- 66: transcript:Zm00001d046774_T001 rna18264 4.00E-148
1102- 67: transcript:Zm00001d046778_T001 rna18256 5.00E-28
1102- 68: transcript:Zm00001d046781_T001 rna18252 6.00E-13
1102- 69: transcript:Zm00001d046784_T001 rna18249 3.00E-32
1102- 70: transcript:Zm00001d046788_T001 rna18246 0
1102- 71: transcript:Zm00001d046792_T001 rna18244 3.00E-149
1102- 72: transcript:Zm00001d046793_T001 rna18241 2.00E-63
Alignment 1103: score=1914.0 e_value=5.8e-149 N=41 9&NC_008399.2 minus
1103- 0: transcript:Zm00001d045074_T001 rna17052 0
1103- 1: transcript:Zm00001d045075_T001 rna17051 2.00E-141
1103- 2: transcript:Zm00001d045076_T003 rna17050 8.00E-69
1103- 3: transcript:Zm00001d045078_T001 rna17047 0
1103- 4: transcript:Zm00001d045080_T001 rna17046 0
1103- 5: transcript:Zm00001d045082_T002 rna17044 0
1103- 6: transcript:Zm00001d045084_T002 rna17043 0
1103- 7: transcript:Zm00001d045085_T001 rna17041 0
1103- 8: transcript:Zm00001d045086_T001 rna17040 0
1103- 9: transcript:Zm00001d045089_T001 rna17037 0
1103- 10: transcript:Zm00001d045090_T001 rna17036 0

1103- 11: transcript:Zm00001d045091_T001 rna17031 1.00E-105
1103- 12: transcript:Zm00001d045094_T001 rna17030 0
1103- 13: transcript:Zm00001d045096_T001 rna17028 2.00E-36
1103- 14: transcript:Zm00001d045097_T001 rna17027 3.00E-31
1103- 15: transcript:Zm00001d045098_T001 rna17025 0
1103- 16: transcript:Zm00001d045102_T001 rna17024 0
1103- 17: transcript:Zm00001d045104_T005 rna17023 0
1103- 18: transcript:Zm00001d045105_T001 rna17022 4.00E-79
1103- 19: transcript:Zm00001d045106_T001 rna17021 2.00E-28
1103- 20: transcript:Zm00001d045108_T001 rna17020 0
1103- 21: transcript:Zm00001d045109_T016 rna17019 3.00E-20
1103- 22: transcript:Zm00001d045111_T001 rna17018 2.00E-34
1103- 23: transcript:Zm00001d045112_T001 rna17017 0
1103- 24: transcript:Zm00001d045113_T001 rna17015 1.00E-179
1103- 25: transcript:Zm00001d045118_T001 rna17013 7.00E-91
1103- 26: transcript:Zm00001d045120_T001 rna17011 6.00E-70
1103- 27: transcript:Zm00001d045121_T001 rna17010 1.00E-30
1103- 28: transcript:Zm00001d045122_T001 rna17009 0
1103- 29: transcript:Zm00001d045123_T001 rna17006 3.00E-161
1103- 30: transcript:Zm00001d045124_T012 rna17004 0
1103- 31: transcript:Zm00001d045125_T001 rna17003 3.00E-49
1103- 32: transcript:Zm00001d045127_T002 rna17002 1.00E-174
1103- 33: transcript:Zm00001d045128_T001 rna16999 0
1103- 34: transcript:Zm00001d045130_T001 rna16998 0
1103- 35: transcript:Zm00001d045136_T001 rna16996 0
1103- 36: transcript:Zm00001d045138_T001 rna16995 0
1103- 37: transcript:Zm00001d045139_T002 rna16994 0
1103- 38: transcript:Zm00001d045141_T001 rna16986 2.00E-67
1103- 39: transcript:Zm00001d045145_T001 rna16982 2.00E-90
1103- 40: transcript:Zm00001d045146_T002 rna16981 0
Alignment 1104: score=1722.0 e_value=4.4e-136 N=38 9&NC_008399.2 minus
1104- 0: transcript:Zm00001d045153_T002 rna16978 0
1104- 1: transcript:Zm00001d045154_T002 rna16977 0
1104- 2: transcript:Zm00001d045155_T001 rna16976 2.00E-179
1104- 3: transcript:Zm00001d045157_T002 rna16975 7.00E-38
1104- 4: transcript:Zm00001d045160_T002 rna16970 2.00E-46
1104- 5: transcript:Zm00001d045163_T001 rna16969 2.00E-103
1104- 6: transcript:Zm00001d045171_T007 rna16968 1.00E-112
1104- 7: transcript:Zm00001d045174_T001 rna16965 3.00E-37
1104- 8: transcript:Zm00001d045176_T001 rna16962 0
1104- 9: transcript:Zm00001d045179_T016 rna16961 0
1104- 10: transcript:Zm00001d045180_T001 rna16960 1.00E-113
1104- 11: transcript:Zm00001d045181_T001 rna16959 2.00E-155
1104- 12: transcript:Zm00001d045183_T001 rna16954 0
1104- 13: transcript:Zm00001d045184_T001 rna16953 5.00E-144
1104- 14: transcript:Zm00001d045191_T001 rna16949 0
1104- 15: transcript:Zm00001d045192_T017 rna16947 0
1104- 16: transcript:Zm00001d045193_T002 rna16944 2.00E-172
1104- 17: transcript:Zm00001d045194_T001 rna16943 0
1104- 18: transcript:Zm00001d045195_T012 rna16938 0
1104- 19: transcript:Zm00001d045199_T003 rna16937 0
1104- 20: transcript:Zm00001d045202_T002 rna16936 0
1104- 21: transcript:Zm00001d045203_T001 rna16934 7.00E-38
1104- 22: transcript:Zm00001d045204_T001 rna16933 2.00E-55

1104- 23: transcript:Zm00001d045205_T001 rna16930 2.00E-79
1104- 24: transcript:Zm00001d045206_T003 rna16928 2.00E-153
1104- 25: transcript:Zm00001d045207_T001 rna16921 0
1104- 26: transcript:Zm00001d045208_T001 rna16918 2.00E-138
1104- 27: transcript:Zm00001d045212_T001 rna16917 7.00E-86
1104- 28: transcript:Zm00001d045213_T002 rna16916 0
1104- 29: transcript:Zm00001d045215_T001 rna16915 0
1104- 30: transcript:Zm00001d045216_T002 rna16912 2.00E-80
1104- 31: transcript:Zm00001d045217_T011 rna16911 0
1104- 32: transcript:Zm00001d045218_T003 rna16910 0
1104- 33: transcript:Zm00001d045220_T003 rna16909 0
1104- 34: transcript:Zm00001d045230_T001 rna16907 0
1104- 35: transcript:Zm00001d045231_T002 rna16906 1.00E-129
1104- 36: transcript:Zm00001d045233_T002 rna16903 0
1104- 37: transcript:Zm00001d045235_T001 rna16900 4.00E-43
Alignment 1105: score=955.0 e_value=7.3e-54 N=20 9&NC_008399.2 minus
1105- 0: transcript:Zm00001d046882_T001 rna18496 0
1105- 1: transcript:Zm00001d046883_T001 rna18495 4.00E-50
1105- 2: transcript:Zm00001d046889_T001 rna18489 8.00E-88
1105- 3: transcript:Zm00001d046890_T001 rna18488 1.00E-158
1105- 4: transcript:Zm00001d046891_T001 rna18486 2.00E-35
1105- 5: transcript:Zm00001d046893_T001 rna18485 0
1105- 6: transcript:Zm00001d046896_T001 rna18484 3.00E-20
1105- 7: transcript:Zm00001d046900_T003 rna18482 1.00E-43
1105- 8: transcript:Zm00001d046905_T001 rna18480 0
1105- 9: transcript:Zm00001d046906_T001 rna18478 7.00E-110
1105- 10: transcript:Zm00001d046910_T001 rna18476 1.00E-139
1105- 11: transcript:Zm00001d046913_T001 rna18472 0
1105- 12: transcript:Zm00001d046915_T001 rna18469 0
1105- 13: transcript:Zm00001d046916_T001 rna18467 3.00E-63
1105- 14: transcript:Zm00001d046921_T001 rna18465 0
1105- 15: transcript:Zm00001d046923_T001 rna18463 0
1105- 16: transcript:Zm00001d046926_T001 rna18461 0
1105- 17: transcript:Zm00001d046928_T002 rna18460 0
1105- 18: transcript:Zm00001d046929_T002 rna18458 1.00E-41
1105- 19: transcript:Zm00001d046930_T001 rna18457 0
Alignment 1106: score=838.0 e_value=5.9e-55 N=20 9&NC_008399.2 minus
1106- 0: transcript:Zm00001d045938_T005 rna17842 1.00E-96
1106- 1: transcript:Zm00001d045940_T001 rna17834 9.00E-79
1106- 2: transcript:Zm00001d045945_T002 rna17831 0
1106- 3: transcript:Zm00001d045946_T001 rna17829 8.00E-38
1106- 4: transcript:Zm00001d045948_T002 rna17824 0
1106- 5: transcript:Zm00001d045950_T003 rna17823 0
1106- 6: transcript:Zm00001d045951_T001 rna17822 3.00E-52
1106- 7: transcript:Zm00001d045953_T016 rna17803 0
1106- 8: transcript:Zm00001d045954_T004 rna17802 0
1106- 9: transcript:Zm00001d045956_T017 rna17801 0
1106- 10: transcript:Zm00001d045962_T001 rna17799 4.00E-48
1106- 11: transcript:Zm00001d045965_T001 rna17793 0
1106- 12: transcript:Zm00001d045967_T002 rna17792 0
1106- 13: transcript:Zm00001d045968_T001 rna17783 1.00E-38
1106- 14: transcript:Zm00001d045975_T003 rna17776 0
1106- 15: transcript:Zm00001d045978_T002 rna17758 4.00E-23
1106- 16: transcript:Zm00001d045987_T002 rna17756 0

1106- 17: transcript:Zm00001d045988_T001 rna17753 2.00E-42
1106- 18: transcript:Zm00001d045993_T002 rna17747 4.00E-07
1106- 19: transcript:Zm00001d046004_T001 rna17737 0
Alignment 1107: score=814.0 e_value=3.9e-48 N=18 9&NC_008399.2 minus
1107- 0: transcript:Zm00001d046254_T001 rna18040 9.00E-173
1107- 1: transcript:Zm00001d046255_T035 rna18032 0
1107- 2: transcript:Zm00001d046263_T001 rna18023 0
1107- 3: transcript:Zm00001d046272_T001 rna18021 3.00E-107
1107- 4: transcript:Zm00001d046277_T008 rna18018 0
1107- 5: transcript:Zm00001d046281_T001 rna18016 9.00E-99
1107- 6: transcript:Zm00001d046286_T001 rna18015 2.00E-98
1107- 7: transcript:Zm00001d046288_T001 rna18014 2.00E-90
1107- 8: transcript:Zm00001d046297_T001 rna18012 5.00E-61
1107- 9: transcript:Zm00001d046299_T001 rna18011 4.00E-32
1107- 10: transcript:Zm00001d046300_T001 rna18010 3.00E-110
1107- 11: transcript:Zm00001d046304_T001 rna18009 0
1107- 12: transcript:Zm00001d046305_T001 rna18008 0
1107- 13: transcript:Zm00001d046311_T001 rna18005 2.00E-25
1107- 14: transcript:Zm00001d046313_T001 rna18004 3.00E-155
1107- 15: transcript:Zm00001d046314_T001 rna18002 1.00E-73
1107- 16: transcript:Zm00001d046317_T001 rna18001 1.00E-131
1107- 17: transcript:Zm00001d046318_T001 rna17993 0
Alignment 1108: score=765.0 e_value=6.9e-46 N=17 9&NC_008399.2 minus
1108- 0: transcript:Zm00001d046445_T004 rna18183 0
1108- 1: transcript:Zm00001d046450_T001 rna18169 0
1108- 2: transcript:Zm00001d046454_T003 rna18167 0
1108- 3: transcript:Zm00001d046455_T001 rna18163 7.00E-76
1108- 4: transcript:Zm00001d046456_T009 rna18159 0
1108- 5: transcript:Zm00001d046460_T001 rna18157 4.00E-124
1108- 6: transcript:Zm00001d046467_T001 rna18156 0
1108- 7: transcript:Zm00001d046471_T001 rna18155 2.00E-60
1108- 8: transcript:Zm00001d046472_T001 rna18152 6.00E-106
1108- 9: transcript:Zm00001d046479_T001 rna18147 0
1108- 10: transcript:Zm00001d046480_T001 rna18146 0
1108- 11: transcript:Zm00001d046482_T001 rna18145 0
1108- 12: transcript:Zm00001d046483_T001 rna18143 2.00E-76
1108- 13: transcript:Zm00001d046485_T001 rna18141 0
1108- 14: transcript:Zm00001d046487_T001 rna18140 2.00E-103
1108- 15: transcript:Zm00001d046488_T001 rna18139 3.00E-55
1108- 16: transcript:Zm00001d046489_T001 rna18138 8.00E-171
Alignment 1109: score=611.0 e_value=9.2e-32 N=13 9&NC_008399.2 minus
1109- 0: transcript:Zm00001d045042_T014 rna17086 0
1109- 1: transcript:Zm00001d045044_T001 rna17083 1.00E-159
1109- 2: transcript:Zm00001d045046_T001 rna17082 0
1109- 3: transcript:Zm00001d045048_T001 rna17080 0
1109- 4: transcript:Zm00001d045049_T001 rna17079 5.00E-143
1109- 5: transcript:Zm00001d045050_T001 rna17078 0
1109- 6: transcript:Zm00001d045051_T001 rna17077 9.00E-95
1109- 7: transcript:Zm00001d045052_T001 rna17073 2.00E-143
1109- 8: transcript:Zm00001d045055_T001 rna17072 0
1109- 9: transcript:Zm00001d045056_T003 rna17070 4.00E-23
1109- 10: transcript:Zm00001d045063_T001 rna17068 0
1109- 11: transcript:Zm00001d045064_T001 rna17066 0
1109- 12: transcript:Zm00001d045065_T001 rna17065 0

Alignment 1110: score=419.0 e_value=1.2e-20 N=9 9&NC_008399.2 minus
1110- 0: transcript:Zm00001d046399_T001 rna18199 2.00E-114
1110- 1: transcript:Zm00001d046400_T001 rna18198 1.00E-110
1110- 2: transcript:Zm00001d046401_T001 rna18197 2.00E-35
1110- 3: transcript:Zm00001d046402_T001 rna18192 6.00E-27
1110- 4: transcript:Zm00001d046404_T001 rna18191 1.00E-37
1110- 5: transcript:Zm00001d046405_T001 rna18190 9.00E-140
1110- 6: transcript:Zm00001d046420_T001 rna18188 2.00E-85
1110- 7: transcript:Zm00001d046422_T001 rna18187 0
1110- 8: transcript:Zm00001d046438_T003 rna18170 0
Alignment 1111: score=390.0 e_value=4.7e-17 N=9 9&NC_008399.2 minus
1111- 0: transcript:Zm00001d046555_T001 rna18417 1.00E-62
1111- 1: transcript:Zm00001d046558_T001 rna18416 1.00E-158
1111- 2: transcript:Zm00001d046564_T001 rna18415 8.00E-16
1111- 3: transcript:Zm00001d046568_T001 rna18414 6.00E-173
1111- 4: transcript:Zm00001d046569_T002 rna18412 7.00E-29
1111- 5: transcript:Zm00001d046574_T001 rna18411 1.00E-25
1111- 6: transcript:Zm00001d046579_T019 rna18408 4.00E-29
1111- 7: transcript:Zm00001d046580_T001 rna18406 1.00E-58
1111- 8: transcript:Zm00001d046581_T003 rna18405 0
Alignment 1112: score=373.0 e_value=1.1e-16 N=8 9&NC_008399.2 minus
1112- 0: transcript:Zm00001d045558_T003 rna16574 0
1112- 1: transcript:Zm00001d045563_T001 rna16562 0
1112- 2: transcript:Zm00001d045564_T001 rna16560 5.00E-82
1112- 3: transcript:Zm00001d045566_T001 rna16559 0
1112- 4: transcript:Zm00001d045567_T001 rna16557 0
1112- 5: transcript:Zm00001d045568_T001 rna16556 7.00E-30
1112- 6: transcript:Zm00001d045570_T001 rna16555 2.00E-162
1112- 7: transcript:Zm00001d045574_T003 rna16551 1.00E-23
Alignment 1113: score=313.0 e_value=2.5e-12 N=7 9&NC_008399.2 minus
1113- 0: transcript:Zm00001d045021_T004 rna17106 1.00E-131
1113- 1: transcript:Zm00001d045024_T001 rna17099 6.00E-95
1113- 2: transcript:Zm00001d045025_T005 rna17095 5.00E-127
1113- 3: transcript:Zm00001d045026_T002 rna17094 3.00E-170
1113- 4: transcript:Zm00001d045027_T001 rna17093 0
1113- 5: transcript:Zm00001d045030_T001 rna17090 8.00E-24
1113- 6: transcript:Zm00001d045041_T015 rna17087 0
Alignment 1114: score=312.0 e_value=2.3e-12 N=7 9&NC_008399.2 minus
1114- 0: transcript:Zm00001d045604_T003 rna16523 0
1114- 1: transcript:Zm00001d045606_T002 rna16522 0
1114- 2: transcript:Zm00001d045607_T001 rna16521 8.00E-54
1114- 3: transcript:Zm00001d045610_T003 rna16520 0
1114- 4: transcript:Zm00001d045617_T001 rna16509 2.00E-132
1114- 5: transcript:Zm00001d045620_T001 rna16508 3.00E-70
1114- 6: transcript:Zm00001d045625_T001 rna16505 2.00E-133
Alignment 1115: score=282.0 e_value=6.6e-10 N=6 9&NC_008399.2 minus
1115- 0: transcript:Zm00001d045575_T001 rna16551 0
1115- 1: transcript:Zm00001d045579_T001 rna16548 0
1115- 2: transcript:Zm00001d045580_T001 rna16547 1.00E-82
1115- 3: transcript:Zm00001d045581_T002 rna16540 3.00E-116
1115- 4: transcript:Zm00001d045582_T002 rna16539 4.00E-146
1115- 5: transcript:Zm00001d045583_T004 rna16537 0
Alignment 1116: score=596.0 e_value=1.2e-34 N=14 9&NC_008400.2 plus
1116- 0: transcript:Zm00001d047399_T001 rna20829 1.00E-49

1116- 1: transcript:Zm00001d047401_T001 rna20830 1.00E-68
1116- 2: transcript:Zm00001d047402_T001 rna20831 0
1116- 3: transcript:Zm00001d047403_T001 rna20835 3.00E-106
1116- 4: transcript:Zm00001d047404_T001 rna20838 2.00E-53
1116- 5: transcript:Zm00001d047417_T001 rna20844 4.00E-150
1116- 6: transcript:Zm00001d047419_T001 rna20849 6.00E-23
1116- 7: transcript:Zm00001d047421_T001 rna20851 5.00E-64
1116- 8: transcript:Zm00001d047422_T002 rna20860 0
1116- 9: transcript:Zm00001d047423_T002 rna20875 6.00E-121
1116- 10: transcript:Zm00001d047428_T001 rna20886 4.00E-22
1116- 11: transcript:Zm00001d047434_T006 rna20896 3.00E-132
1116- 12: transcript:Zm00001d047435_T006 rna20897 0
1116- 13: transcript:Zm00001d047438_T001 rna20908 8.00E-153
Alignment 1117: score=498.0 e_value=1.2e-28 N=12 9&NC_008400.2 plus
1117- 0: transcript:Zm00001d047498_T010 rna21090 1.00E-126
1117- 1: transcript:Zm00001d047499_T001 rna21094 3.00E-20
1117- 2: transcript:Zm00001d047500_T001 rna21097 1.00E-55
1117- 3: transcript:Zm00001d047512_T002 rna21109 3.00E-65
1117- 4: transcript:Zm00001d047514_T001 rna21118 7.00E-125
1117- 5: transcript:Zm00001d047516_T001 rna21119 3.00E-172
1117- 6: transcript:Zm00001d047519_T001 rna21127 4.00E-148
1117- 7: transcript:Zm00001d047522_T002 rna21129 0
1117- 8: transcript:Zm00001d047523_T001 rna21131 2.00E-42
1117- 9: transcript:Zm00001d047528_T001 rna21133 5.00E-17
1117- 10: transcript:Zm00001d047531_T001 rna21134 0
1117- 11: transcript:Zm00001d047535_T001 rna21139 3.00E-82
Alignment 1118: score=280.0 e_value=6.4e-15 N=8 9&NC_008400.2 plus
1118- 0: transcript:Zm00001d047470_T005 rna21010 1.00E-21
1118- 1: transcript:Zm00001d047472_T002 rna21014 0
1118- 2: transcript:Zm00001d047478_T001 rna21021 3.00E-07
1118- 3: transcript:Zm00001d047479_T001 rna21040 2.00E-80
1118- 4: transcript:Zm00001d047480_T001 rna21049 3.00E-87
1118- 5: transcript:Zm00001d047482_T001 rna21052 9.00E-42
1118- 6: transcript:Zm00001d047492_T001 rna21078 5.00E-14
1118- 7: transcript:Zm00001d047494_T042 rna21084 0
Alignment 1119: score=374.0 e_value=1.2e-16 N=9 9&NC_008400.2 minus
1119- 0: transcript:Zm00001d047253_T018 rna20733 0
1119- 1: transcript:Zm00001d047255_T001 rna20730 0
1119- 2: transcript:Zm00001d047260_T001 rna20725 3.00E-13
1119- 3: transcript:Zm00001d047263_T001 rna20717 4.00E-69
1119- 4: transcript:Zm00001d047265_T001 rna20703 2.00E-38
1119- 5: transcript:Zm00001d047271_T002 rna20692 4.00E-36
1119- 6: transcript:Zm00001d047272_T001 rna20684 1.00E-62
1119- 7: transcript:Zm00001d047276_T001 rna20664 0
1119- 8: transcript:Zm00001d047279_T001 rna20659 4.00E-132
Alignment 1120: score=269.0 e_value=2.3e-08 N=6 9&NC_008400.2 minus
1120- 0: transcript:Zm00001d047579_T001 rna21189 7.00E-93
1120- 1: transcript:Zm00001d047582_T002 rna21186 0
1120- 2: transcript:Zm00001d047587_T005 rna21185 9.00E-145
1120- 3: transcript:Zm00001d047591_T001 rna21182 4.00E-14
1120- 4: transcript:Zm00001d047594_T001 rna21181 3.00E-97
1120- 5: transcript:Zm00001d047597_T001 rna21180 3.00E-86
Alignment 1121: score=264.0 e_value=4.9e-11 N=7 9&NC_008400.2 minus
1121- 0: transcript:Zm00001d047182_T001 rna20810 1.00E-77

1121- 1: transcript:Zm00001d047186_T001 rna20805 4.00E-19
1121- 2: transcript:Zm00001d047191_T003 rna20789 9.00E-156
1121- 3: transcript:Zm00001d047203_T001 rna20781 2.00E-71
1121- 4: transcript:Zm00001d047207_T001 rna20778 2.00E-31
1121- 5: transcript:Zm00001d047209_T003 rna20777 4.00E-121
1121- 6: transcript:Zm00001d047214_T001 rna20754 6.00E-124
Alignment 1122: score=444.0 e_value=3.6e-20 N=10 9&NC_008403.2 plus
1122- 0: transcript:Zm00001d047373_T001 rna26404 0
1122- 1: transcript:Zm00001d047378_T001 rna26406 6.00E-97
1122- 2: transcript:Zm00001d047379_T001 rna26412 1.00E-17
1122- 3: transcript:Zm00001d047382_T001 rna26415 0
1122- 4: transcript:Zm00001d047385_T002 rna26418 0
1122- 5: transcript:Zm00001d047388_T001 rna26419 0
1122- 6: transcript:Zm00001d047389_T006 rna26422 4.00E-151
1122- 7: transcript:Zm00001d047395_T005 rna26425 8.00E-118
1122- 8: transcript:Zm00001d047396_T001 rna26426 2.00E-79
1122- 9: transcript:Zm00001d047397_T001 rna26427 2.00E-114
Alignment 1123: score=388.0 e_value=3.1e-20 N=9 9&NC_008403.2 plus
1123- 0: transcript:Zm00001d048307_T001 rna25666 2.00E-06
1123- 1: transcript:Zm00001d048311_T003 rna25676 0
1123- 2: transcript:Zm00001d048312_T001 rna25677 6.00E-107
1123- 3: transcript:Zm00001d048314_T004 rna25678 0
1123- 4: transcript:Zm00001d048317_T001 rna25679 1.00E-45
1123- 5: transcript:Zm00001d048319_T003 rna25685 1.00E-143
1123- 6: transcript:Zm00001d048322_T001 rna25688 1.00E-150
1123- 7: transcript:Zm00001d048325_T001 rna25689 7.00E-34
1123- 8: transcript:Zm00001d048336_T003 rna25701 0
Alignment 1124: score=383.0 e_value=1.3e-18 N=9 9&NC_008403.2 plus
1124- 0: transcript:Zm00001d048188_T001 rna25530 1.00E-18
1124- 1: transcript:Zm00001d048193_T002 rna25537 4.00E-09
1124- 2: transcript:Zm00001d048198_T002 rna25551 5.00E-10
1124- 3: transcript:Zm00001d048199_T001 rna25553 3.00E-112
1124- 4: transcript:Zm00001d048201_T002 rna25554 0
1124- 5: transcript:Zm00001d048203_T002 rna25555 4.00E-163
1124- 6: transcript:Zm00001d048208_T001 rna25563 1.00E-15
1124- 7: transcript:Zm00001d048220_T001 rna25577 3.00E-166
1124- 8: transcript:Zm00001d048222_T001 rna25582 4.00E-148
Alignment 1125: score=374.0 e_value=1.2e-14 N=8 9&NC_008403.2 plus
1125- 0: transcript:Zm00001d047349_T001 rna26360 0
1125- 1: transcript:Zm00001d047350_T002 rna26370 2.00E-56
1125- 2: transcript:Zm00001d047351_T001 rna26371 2.00E-75
1125- 3: transcript:Zm00001d047354_T001 rna26372 8.00E-111
1125- 4: transcript:Zm00001d047355_T001 rna26373 5.00E-34
1125- 5: transcript:Zm00001d047358_T001 rna26376 5.00E-150
1125- 6: transcript:Zm00001d047361_T002 rna26378 2.00E-81
1125- 7: transcript:Zm00001d047362_T003 rna26379 0
Alignment 1126: score=268.0 e_value=1.1e-08 N=6 9&NC_008403.2 plus
1126- 0: transcript:Zm00001d048467_T001 rna26356 0
1126- 1: transcript:Zm00001d048469_T001 rna26359 2.00E-131
1126- 2: transcript:Zm00001d048470_T001 rna26371 4.00E-78
1126- 3: transcript:Zm00001d048473_T003 rna26372 3.00E-112
1126- 4: transcript:Zm00001d048474_T001 rna26373 1.00E-89
1126- 5: transcript:Zm00001d048480_T005 rna26379 3.00E-155
Alignment 1127: score=895.0 e_value=4.9e-56 N=20 9&NC_008403.2 minus

1127- 0: transcript:Zm00001d047079_T001 rna26515   1.00E-157
1127- 1: transcript:Zm00001d047081_T001 rna26499   9.00E-90
1127- 2: transcript:Zm00001d047086_T001 rna26497   1.00E-110
1127- 3: transcript:Zm00001d047089_T001 rna26489   3.00E-146
1127- 4: transcript:Zm00001d047102_T001 rna26488   2.00E-96
1127- 5: transcript:Zm00001d047104_T001 rna26486   0
1127- 6: transcript:Zm00001d047105_T007 rna26481   1.00E-178
1127- 7: transcript:Zm00001d047107_T004 rna26479   9.00E-64
1127- 8: transcript:Zm00001d047109_T001 rna26477   7.00E-78
1127- 9: transcript:Zm00001d047110_T001 rna26476   0
1127- 10: transcript:Zm00001d047111_T001 rna26472   3.00E-38
1127- 11: transcript:Zm00001d047115_T003 rna26468   5.00E-27
1127- 12: transcript:Zm00001d047119_T001 rna26464   1.00E-57
1127- 13: transcript:Zm00001d047123_T001 rna26463   1.00E-29
1127- 14: transcript:Zm00001d047124_T001 rna26461   0
1127- 15: transcript:Zm00001d047125_T001 rna26459   1.00E-91
1127- 16: transcript:Zm00001d047126_T003 rna26454   0
1127- 17: transcript:Zm00001d047128_T002 rna26448   0
1127- 18: transcript:Zm00001d047130_T001 rna26446   0
1127- 19: transcript:Zm00001d047135_T009 rna26444   1.00E-152
Alignment 1128: score=702.0 e_value=3.8e-43 N=16 9&NC_008403.2 minus
1128- 0: transcript:Zm00001d047037_T001 rna26619   1.00E-107
1128- 1: transcript:Zm00001d047042_T004 rna26615   2.00E-146
1128- 2: transcript:Zm00001d047044_T005 rna26612   0
1128- 3: transcript:Zm00001d047045_T001 rna26611   0
1128- 4: transcript:Zm00001d047046_T003 rna26607   7.00E-98
1128- 5: transcript:Zm00001d047050_T001 rna26606   2.00E-61
1128- 6: transcript:Zm00001d047053_T004 rna26604   0
1128- 7: transcript:Zm00001d047054_T001 rna26603   0
1128- 8: transcript:Zm00001d047057_T001 rna26602   5.00E-60
1128- 9: transcript:Zm00001d047060_T001 rna26582   1.00E-54
1128- 10: transcript:Zm00001d047063_T002 rna26576   0
1128- 11: transcript:Zm00001d047064_T001 rna26574   5.00E-90
1128- 12: transcript:Zm00001d047067_T001 rna26573   9.00E-71
1128- 13: transcript:Zm00001d047074_T002 rna26562   0
1128- 14: transcript:Zm00001d047077_T001 rna26561   0
1128- 15: transcript:Zm00001d047078_T004 rna26560   9.00E-73
Alignment 1129: score=366.0 e_value=9.6e-14 N=8 9&NC_008403.2 minus
1129- 0: transcript:Zm00001d048558_T001 rna26316   2.00E-80
1129- 1: transcript:Zm00001d048559_T001 rna26304   6.00E-96
1129- 2: transcript:Zm00001d048561_T001 rna26292   0
1129- 3: transcript:Zm00001d048562_T002 rna26289   5.00E-106
1129- 4: transcript:Zm00001d048563_T001 rna26286   0
1129- 5: transcript:Zm00001d048564_T003 rna26281   0
1129- 6: transcript:Zm00001d048565_T001 rna26278   0
1129- 7: transcript:Zm00001d048566_T001 rna26273   2.00E-144
Alignment 1130: score=363.0 e_value=3.4e-17 N=9 9&NC_008403.2 minus
1130- 0: transcript:Zm00001d048397_T002 rna25869   7.00E-99
1130- 1: transcript:Zm00001d048402_T001 rna25861   3.00E-80
1130- 2: transcript:Zm00001d048409_T001 rna25848   1.00E-123
1130- 3: transcript:Zm00001d048410_T004 rna25843   5.00E-117
1130- 4: transcript:Zm00001d048411_T001 rna25842   3.00E-25
1130- 5: transcript:Zm00001d048412_T001 rna25841   9.00E-153
1130- 6: transcript:Zm00001d048415_T005 rna25837   6.00E-29

1130- 7: transcript:Zm00001d048418_T001 rna25836 1.00E-118
1130- 8: transcript:Zm00001d048420_T001 rna25829 7.00E-148
Alignment 1131: score=285.0 e_value=5.9e-12 N=7 9&NC_008403.2 minus
1131- 0: transcript:Zm00001d047931_T001 rna25214 0
1131- 1: transcript:Zm00001d047932_T001 rna25202 4.00E-90
1131- 2: transcript:Zm00001d047933_T001 rna25185 8.00E-29
1131- 3: transcript:Zm00001d047937_T001 rna25171 0
1131- 4: transcript:Zm00001d047938_T001 rna25167 1.00E-92
1131- 5: transcript:Zm00001d047941_T001 rna25149 5.00E-94
1131- 6: transcript:Zm00001d047944_T001 rna25148 3.00E-13
Alignment 1132: score=266.0 e_value=6.3e-09 N=6 9&NC_008403.2 minus
1132- 0: transcript:Zm00001d047147_T001 rna26444 8.00E-152
1132- 1: transcript:Zm00001d047150_T003 rna26438 3.00E-30
1132- 2: transcript:Zm00001d047153_T004 rna26434 0
1132- 3: transcript:Zm00001d047159_T001 rna26432 2.00E-25
1132- 4: transcript:Zm00001d047160_T001 rna26431 2.00E-48
1132- 5: transcript:Zm00001d047162_T005 rna26430 0
Alignment 1133: score=282.0 e_value=3.4e-08 N=6 B73V4_ctg107&NC_008395.2 plus
1133- 0: transcript:Zm00001d000301_T001 rna4627 0
1133- 1: transcript:Zm00001d000302_T002 rna4628 8.00E-165
1133- 2: transcript:Zm00001d000304_T005 rna4631 3.00E-08
1133- 3: transcript:Zm00001d000305_T003 rna4632 0
1133- 4: transcript:Zm00001d000306_T001 rna4635 1.00E-61
1133- 5: transcript:Zm00001d000307_T001 rna4636 8.00E-130
Alignment 1134: score=268.0 e_value=1.6e-09 N=6 B73V4_ctg107&NC_008399.2 minus
1134- 0: transcript:Zm00001d000300_T001 rna18712 0
1134- 1: transcript:Zm00001d000301_T001 rna18711 0
1134- 2: transcript:Zm00001d000302_T002 rna18708 2.00E-156
1134- 3: transcript:Zm00001d000304_T005 rna18694 1.00E-19
1134- 4: transcript:Zm00001d000306_T001 rna18680 4.00E-60
1134- 5: transcript:Zm00001d000307_T001 rna18678 4.00E-128
Alignment 1135: score=344.0 e_value=1.9e-11 N=7 B73V4_ctg181&NC_008403.2 plus
1135- 0: transcript:Zm00001d000175_T001 rna26519 2.00E-161
1135- 1: transcript:Zm00001d000176_T001 rna26521 8.00E-56
1135- 2: transcript:Zm00001d000178_T001 rna26522 2.00E-117
1135- 3: transcript:Zm00001d000179_T001 rna26525 3.00E-76
1135- 4: transcript:Zm00001d000180_T001 rna26526 6.00E-55
1135- 5: transcript:Zm00001d000183_T001 rna26530 0
1135- 6: transcript:Zm00001d000184_T001 rna26531 4.00E-131
Alignment 1136: score=470.0 e_value=4.9e-20 N=10 B73V4_ctg182&NC_008403.2 plus
1136- 0: transcript:Zm00001d000029_T003 rna26535 0
1136- 1: transcript:Zm00001d000037_T001 rna26538 0
1136- 2: transcript:Zm00001d000038_T001 rna26539 0
1136- 3: transcript:Zm00001d000039_T001 rna26540 0
1136- 4: transcript:Zm00001d000040_T001 rna26542 4.00E-54
1136- 5: transcript:Zm00001d000042_T002 rna26543 0
1136- 6: transcript:Zm00001d000045_T001 rna26553 2.00E-146
1136- 7: transcript:Zm00001d000046_T001 rna26557 3.00E-28
1136- 8: transcript:Zm00001d000052_T004 rna26558 4.00E-132
1136- 9: transcript:Zm00001d000053_T001 rna26559 0
Alignment 1137: score=271.0 e_value=8.2e-09 N=6 B73V4_ctg56&NC_008397.2 plus
1137- 0: transcript:Zm00001d000122_T006 rna12248 0
1137- 1: transcript:Zm00001d000123_T003 rna12249 0
1137- 2: transcript:Zm00001d000124_T010 rna12251 0

1137- 3:	transcript:Zm00001d000126_T002	rna12252	2.00E-22
1137- 4:	transcript:Zm00001d000128_T002	rna12254	5.00E-65
1137- 5:	transcript:Zm00001d000133_T009	rna12255	1.00E-54
