# Supplementary material for: Genome-wide identification, transcriptome analysis and alternative splicing events of Hsf family genes in maize
Source: Sci Rep. 2020 May 15;10:8073. doi: 10.1038/s41598-020-65068-z (PMC7229205; doi:10.1038/s41598-020-65068-z)
Supplement: Supplementary file 7 — Supplementary Information. [file 41598_2020_65068_MOESM7_ESM.pdf]

Genome-wide identification, transcriptome analysis and alternative splicing events of Hsf family genes in maize

Huaning Zhang [1,2], Guoliang Li [1,2], Cai Fu[1], Shuonan Duan [1], Dong Hu[1, ✉] & Xiulin Guo [1, ✉]

[1] Plant Genetic Engineering Center of Hebei Province/Institute of Genetics and Physiology, Hebei Academy of Agriculture and Forestry Sciences, Shijiazhuang 050051, P.R. China

[2] These authors contributed equally: Huaning Zhang and Guoliang Li.

✉ e-mail: myhf2002@163.com, donghu1983@163.com.

Table S5 Analysis of collinearity of ZmHsfs and OsHsfs proteins.

```
################ Parameters ################
# MATCH_SCORE: 50
# MATCH_SIZE: 5
# GAP_PENALTY: -1
# OVERLAP_WINDOW: 5
# E_VALUE: 1e-05
# MAX GAPS: 25
################ Statistics ################
# Number of collinear genes: 37332, Percentage: 20.89
# Number of all genes: 178695
##########################################
## Alignment 0: score=2161.0 e_value=2.9e-181 N=48 1&1 plus
  0-  0: transcript:OQU91573            transcript:Zm00001d032598_T023          0
  0-  1: transcript:OQU91575            transcript:Zm00001d032600_T001 2.00E-165
  0-  2: transcript:KXG38261            transcript:Zm00001d032601_T002          0
  0-  3: transcript:EER91435            transcript:Zm00001d032603_T001          0
  0-  4: transcript:EER94017            transcript:Zm00001d032604_T001          0
  0-  5: transcript:EER94018            transcript:Zm00001d032605_T002          0
  0-  6: transcript:EER91436            transcript:Zm00001d032609_T002          0
  0-  7: transcript:EER94020            transcript:Zm00001d032610_T003 1.00E-157
  0-  8: transcript:EER94021            transcript:Zm00001d032613_T001          0
  0-  9: transcript:OQU91582            transcript:Zm00001d032615_T002          0
  0- 10: transcript:KXG38267            transcript:Zm00001d032616_T001  3.00E-81
  0- 11: transcript:EER91438            transcript:Zm00001d032617_T002  4.00E-63
  0- 12: transcript:EER94023            transcript:Zm00001d032618_T001 5.00E-102
  0- 13: transcript:KXG38271            transcript:Zm00001d032620_T001 1.00E-124
  0- 14: transcript:EER91446            transcript:Zm00001d032621_T002          0
  0- 15: transcript:EER91447            transcript:Zm00001d032624_T001  2.00E-84
  0- 16: transcript:EER91450            transcript:Zm00001d032626_T001  3.00E-55
  0- 17: transcript:KXG38280            transcript:Zm00001d032632_T001          0
  0- 18: transcript:OQU91600            transcript:Zm00001d032633_T004          0
  0- 19: transcript:EER91454            transcript:Zm00001d032636_T001 1.00E-126
  0- 20: transcript:EER94044            transcript:Zm00001d032637_T004          0
  0- 21: transcript:OQU91605            transcript:Zm00001d032643_T001  2.00E-96
  0- 22: transcript:KXG38287            transcript:Zm00001d032644_T002          0
  0- 23: transcript:EER91456            transcript:Zm00001d032649_T001  1.00E-56
  0- 24: transcript:OQU91607            transcript:Zm00001d032650_T001  1.00E-56
  0- 25: transcript:KXG38289            transcript:Zm00001d032651_T001          0
  0- 26: transcript:EER94051            transcript:Zm00001d032652_T001 1.00E-155
  0- 27: transcript:EER91460            transcript:Zm00001d032653_T001  4.00E-74
  0- 28: transcript:OQU91610            transcript:Zm00001d032654_T001  9.00E-76
  0- 29: transcript:OQU91611            transcript:Zm00001d032655_T006          0
  0- 30: transcript:EER94055            transcript:Zm00001d032656_T001          0
  0- 31: transcript:KXG38297            transcript:Zm00001d032659_T004          0
  0- 32: transcript:EER91465            transcript:Zm00001d032661_T001          0
  0- 33: transcript:EER91459            transcript:Zm00001d032662_T001          0
  0- 34: transcript:EER94059            transcript:Zm00001d032663_T001  2.00E-85
  0- 35: transcript:KXG38303            transcript:Zm00001d032664_T001          0
  0- 36: transcript:EER91467            transcript:Zm00001d032666_T002          0
  0- 37: transcript:EER91468            transcript:Zm00001d032668_T001 5.00E-115
  0- 38: transcript:EER91469            transcript:Zm00001d032669_T001          0
```

```
  0- 39: transcript:EER91473           transcript:Zm00001d032670_T004 6.00E-130
  0- 40: transcript:OQU91620           transcript:Zm00001d032671_T001  2.00E-54
  0- 41: transcript:KXG38312           transcript:Zm00001d032672_T001  4.00E-44
  0- 42: transcript:KXG38314           transcript:Zm00001d032679_T001         0
  0- 43: transcript:EER94065           transcript:Zm00001d032681_T004         0
  0- 44: transcript:EER94066           transcript:Zm00001d032683_T001         0
  0- 45: transcript:EER91476           transcript:Zm00001d032685_T001         0
  0- 46: transcript:OQU91622           transcript:Zm00001d032686_T001         0
  0- 47: transcript:OQU91624           transcript:Zm00001d032687_T001  1.00E-82
## Alignment 1: score=1994.0 e_value=1.3e-158 N=43 1&1 plus
  1-  0: transcript:OQU91232           transcript:Zm00001d033447_T005         0
  1-  1: transcript:EER93704           transcript:Zm00001d033448_T003         0
  1-  2: transcript:KXG37885           transcript:Zm00001d033451_T001         0
  1-  3: transcript:EER91118           transcript:Zm00001d033454_T001 2.00E-117
  1-  4: transcript:EER93707           transcript:Zm00001d033455_T001  4.00E-60
  1-  5: transcript:EER93711           transcript:Zm00001d033456_T001 6.00E-122
  1-  6: transcript:EER93713           transcript:Zm00001d033457_T001         0
  1-  7: transcript:EER91121           transcript:Zm00001d033459_T001  5.00E-81
  1-  8: transcript:KXG37888           transcript:Zm00001d033460_T001  8.00E-22
  1-  9: transcript:KXG37892           transcript:Zm00001d033464_T001  5.00E-48
  1- 10: transcript:EER91123           transcript:Zm00001d033465_T001         0
  1- 11: transcript:EER93717           transcript:Zm00001d033466_T001  2.00E-82
  1- 12: transcript:EER91124           transcript:Zm00001d033467_T002         0
  1- 13: transcript:OQU91238           transcript:Zm00001d033468_T001  8.00E-89
  1- 14: transcript:EER93719           transcript:Zm00001d033469_T001  3.00E-88
  1- 15: transcript:KXG37894           transcript:Zm00001d033470_T001 5.00E-128
  1- 16: transcript:KXG37899           transcript:Zm00001d033471_T001  5.00E-46
  1- 17: transcript:OQU91240           transcript:Zm00001d033472_T004 8.00E-118
  1- 18: transcript:KXG37901           transcript:Zm00001d033473_T001 6.00E-153
  1- 19: transcript:EER93724           transcript:Zm00001d033475_T001         0
  1- 20: transcript:EER91129           transcript:Zm00001d033477_T002  4.00E-96
  1- 21: transcript:EER91130           transcript:Zm00001d033478_T001  2.00E-82
  1- 22: transcript:EER91131           transcript:Zm00001d033479_T001  2.00E-51
  1- 23: transcript:KXG37902           transcript:Zm00001d033480_T002         0
  1- 24: transcript:OQU91243           transcript:Zm00001d033481_T001  1.00E-71
  1- 25: transcript:EER93727           transcript:Zm00001d033482_T001         0
  1- 26: transcript:EER93728           transcript:Zm00001d033483_T001  2.00E-13
  1- 27: transcript:KXG37909           transcript:Zm00001d033484_T002  2.00E-93
  1- 28: transcript:EER91135           transcript:Zm00001d033488_T001 2.00E-167
  1- 29: transcript:OQU91248           transcript:Zm00001d033489_T001 4.00E-121
  1- 30: transcript:EER91145           transcript:Zm00001d033492_T005         0
  1- 31: transcript:EER91146           transcript:Zm00001d033493_T002         0
  1- 32: transcript:KXG37918           transcript:Zm00001d033494_T011         0
  1- 33: transcript:KXG37920           transcript:Zm00001d033495_T001         0
  1- 34: transcript:EER93732           transcript:Zm00001d033496_T001         0
  1- 35: transcript:EER91149           transcript:Zm00001d033497_T001         0
  1- 36: transcript:EER91150           transcript:Zm00001d033502_T005         0
  1- 37: transcript:KXG37921           transcript:Zm00001d033503_T002         0
  1- 38: transcript:OQU91257           transcript:Zm00001d033504_T001         0
  1- 39: transcript:KXG37924           transcript:Zm00001d033505_T001         0
  1- 40: transcript:KXG37931           transcript:Zm00001d033507_T001  5.00E-85
  1- 41: transcript:OQU91263           transcript:Zm00001d033508_T001  2.00E-48
  1- 42: transcript:KXG37933           transcript:Zm00001d033510_T001         0
## Alignment 2: score=1892.0 e_value=6.3e-159 N=43 1&1 plus
```

```
  2-  0: transcript:EER91558            transcript:Zm00001d032854_T001          0
  2-  1: transcript:OQU91718            transcript:Zm00001d032855_T001          0
  2-  2: transcript:OQU91721            transcript:Zm00001d032857_T001          0
  2-  3: transcript:EER94171            transcript:Zm00001d032859_T006          0
  2-  4: transcript:EER91563            transcript:Zm00001d032866_T001          0
  2-  5: transcript:EER91565            transcript:Zm00001d032867_T002          0
  2-  6: transcript:EER94172            transcript:Zm00001d032868_T001          0
  2-  7: transcript:KXG38456            transcript:Zm00001d032870_T001          0
  2-  8: transcript:EER94175            transcript:Zm00001d032873_T001 6.00E-145
  2-  9: transcript:EER94178            transcript:Zm00001d032875_T009          0
  2- 10: transcript:EER91568            transcript:Zm00001d032876_T001 1.00E-152
  2- 11: transcript:EER91569            transcript:Zm00001d032881_T001 1.00E-156
  2- 12: transcript:KXG38462            transcript:Zm00001d032882_T001 7.00E-139
  2- 13: transcript:KXG38463            transcript:Zm00001d032887_T001  4.00E-57
  2- 14: transcript:KXG38464            transcript:Zm00001d032888_T001          0
  2- 15: transcript:EER94184            transcript:Zm00001d032889_T001 2.00E-175
  2- 16: transcript:KXG38466            transcript:Zm00001d032893_T002  8.00E-86
  2- 17: transcript:KXG38469            transcript:Zm00001d032894_T001          0
  2- 18: transcript:EER91580            transcript:Zm00001d032896_T001  7.00E-83
  2- 19: transcript:OQU91733            transcript:Zm00001d032897_T001          0
  2- 20: transcript:KXG38473            transcript:Zm00001d032899_T001          0
  2- 21: transcript:EER94188            transcript:Zm00001d032901_T001 4.00E-167
  2- 22: transcript:EER91583            transcript:Zm00001d032902_T001          0
  2- 23: transcript:EER94197            transcript:Zm00001d032903_T001  2.00E-97
  2- 24: transcript:EER94198            transcript:Zm00001d032904_T001  3.00E-19
  2- 25: transcript:EER94199            transcript:Zm00001d032905_T010          0
  2- 26: transcript:KXG38485            transcript:Zm00001d032909_T001          0
  2- 27: transcript:EER94210            transcript:Zm00001d032911_T001          0
  2- 28: transcript:OQU91746            transcript:Zm00001d032916_T001          0
  2- 29: transcript:EER91587            transcript:Zm00001d032919_T001 2.00E-102
  2- 30: transcript:EER91589            transcript:Zm00001d032921_T001          0
  2- 31: transcript:KXG38489            transcript:Zm00001d032922_T001          0
  2- 32: transcript:EER94213            transcript:Zm00001d032923_T002          0
  2- 33: transcript:KXG38490            transcript:Zm00001d032925_T001  1.00E-73
  2- 34: transcript:OQU91749            transcript:Zm00001d032926_T001          0
  2- 35: transcript:OQU91752            transcript:Zm00001d032927_T001  3.00E-38
  2- 36: transcript:KXG38499            transcript:Zm00001d032929_T001  5.00E-08
  2- 37: transcript:EER94226            transcript:Zm00001d032931_T002 8.00E-126
  2- 38: transcript:KXG38501            transcript:Zm00001d032932_T001          0
  2- 39: transcript:EER94227            transcript:Zm00001d032933_T020          0
  2- 40: transcript:KXG38503            transcript:Zm00001d032934_T001          0
  2- 41: transcript:EER91601            transcript:Zm00001d032935_T006          0
  2- 42: transcript:EER94230            transcript:Zm00001d032937_T001 1.00E-124
## Alignment 3: score=1525.0 e_value=3.2e-116 N=35 1&1 plus
  3-  0: transcript:EER91173            transcript:Zm00001d033227_T001  9.00E-60
  3-  1: transcript:OQU91276            transcript:Zm00001d033232_T001  2.00E-81
  3-  2: transcript:EER93759            transcript:Zm00001d033234_T001          0
  3-  3: transcript:KXG37951            transcript:Zm00001d033237_T001 2.00E-158
  3-  4: transcript:EER91175            transcript:Zm00001d033240_T001          0
  3-  5: transcript:EER91176            transcript:Zm00001d033241_T001          0
  3-  6: transcript:EER91177            transcript:Zm00001d033244_T001          0
  3-  7: transcript:EER91178            transcript:Zm00001d033246_T002          0
  3-  8: transcript:EER91180            transcript:Zm00001d033250_T001  4.00E-27
  3-  9: transcript:EER91181            transcript:Zm00001d033254_T001          0
```

```
  3- 10: transcript:OQU91279          transcript:Zm00001d033258_T001          0
  3- 11: transcript:KXG37959          transcript:Zm00001d033259_T002          0
  3- 12: transcript:EER91186          transcript:Zm00001d033262_T001 3.00E-116
  3- 13: transcript:OQU91281          transcript:Zm00001d033265_T001 5.00E-141
  3- 14: transcript:KXG37960          transcript:Zm00001d033266_T001  1.00E-87
  3- 15: transcript:EER93765          transcript:Zm00001d033267_T001          0
  3- 16: transcript:OQU91286          transcript:Zm00001d033268_T001  4.00E-67
  3- 17: transcript:EER91191          transcript:Zm00001d033271_T001 3.00E-169
  3- 18: transcript:KXG37967          transcript:Zm00001d033274_T005          0
  3- 19: transcript:OQU91301          transcript:Zm00001d033278_T011          0
  3- 20: transcript:OQU91304          transcript:Zm00001d033279_T001          0
  3- 21: transcript:EER93770          transcript:Zm00001d033280_T001          0
  3- 22: transcript:KXG37970          transcript:Zm00001d033283_T001          0
  3- 23: transcript:EER93772          transcript:Zm00001d033284_T001          0
  3- 24: transcript:EER93773          transcript:Zm00001d033286_T001          0
  3- 25: transcript:EER91202          transcript:Zm00001d033287_T001  4.00E-36
  3- 26: transcript:OQU91310          transcript:Zm00001d033288_T001          0
  3- 27: transcript:EER91209          transcript:Zm00001d033291_T004          0
  3- 28: transcript:EER93781          transcript:Zm00001d033292_T004          0
  3- 29: transcript:EER91211          transcript:Zm00001d033296_T002          0
  3- 30: transcript:OQU91318          transcript:Zm00001d033297_T003          0
  3- 31: transcript:EER93785          transcript:Zm00001d033300_T001          0
  3- 32: transcript:EER93786          transcript:Zm00001d033301_T001  2.00E-31
  3- 33: transcript:EER91212          transcript:Zm00001d033303_T004          0
  3- 34: transcript:OQU91330          transcript:Zm00001d033304_T001          0
## Alignment 4: score=1444.0 e_value=1.9e-104 N=32 1&1 plus
  4-  0: transcript:EER91397          transcript:Zm00001d032502_T001 7.00E-137
  4-  1: transcript:EER91391          transcript:Zm00001d032503_T001          0
  4-  2: transcript:EER93966          transcript:Zm00001d032504_T001          0
  4-  3: transcript:EER91398          transcript:Zm00001d032505_T002          0
  4-  4: transcript:OQU91531          transcript:Zm00001d032506_T001  3.00E-31
  4-  5: transcript:EER91402          transcript:Zm00001d032507_T001 8.00E-180
  4-  6: transcript:EER93968          transcript:Zm00001d032508_T001 1.00E-142
  4-  7: transcript:KXG38211          transcript:Zm00001d032510_T001          0
  4-  8: transcript:OQU91535          transcript:Zm00001d032515_T001  9.00E-81
  4-  9: transcript:EER91404          transcript:Zm00001d032517_T001  5.00E-60
  4- 10: transcript:KXG38215          transcript:Zm00001d032518_T001          0
  4- 11: transcript:KXG38216          transcript:Zm00001d032519_T001          0
  4- 12: transcript:KXG38217          transcript:Zm00001d032520_T018          0
  4- 13: transcript:EER91407          transcript:Zm00001d032521_T004 3.00E-156
  4- 14: transcript:KXG38218          transcript:Zm00001d032522_T001  3.00E-25
  4- 15: transcript:EER91410          transcript:Zm00001d032526_T002          0
  4- 16: transcript:EER93977          transcript:Zm00001d032527_T001          0
  4- 17: transcript:EER91414          transcript:Zm00001d032529_T001          0
  4- 18: transcript:OQU91545          transcript:Zm00001d032530_T001          0
  4- 19: transcript:EER91417          transcript:Zm00001d032531_T001  1.00E-98
  4- 20: transcript:EER91419          transcript:Zm00001d032532_T001          0
  4- 21: transcript:EER93980          transcript:Zm00001d032533_T001  6.00E-35
  4- 22: transcript:EER93981          transcript:Zm00001d032535_T002 4.00E-164
  4- 23: transcript:EER93983          transcript:Zm00001d032536_T001 3.00E-179
  4- 24: transcript:KXG38228          transcript:Zm00001d032539_T001          0
  4- 25: transcript:OQU91552          transcript:Zm00001d032543_T001          0
  4- 26: transcript:KXG38231          transcript:Zm00001d032545_T001          0
  4- 27: transcript:KXG38234          transcript:Zm00001d032546_T001          0
```

```
  4- 28: transcript:KXG38235           transcript:Zm00001d032547_T010          0
  4- 29: transcript:EER91423           transcript:Zm00001d032548_T001  2.00E-98
  4- 30: transcript:EER93992           transcript:Zm00001d032550_T001 8.00E-178
  4- 31: transcript:KXG38238           transcript:Zm00001d032552_T002  7.00E-93
## Alignment 5: score=1310.0 e_value=1.1e-96 N=30 1&1 plus
  5-  0: transcript:Zm00001d028085_T001 transcript:KXG39918           8.00E-150
  5-  1: transcript:Zm00001d028086_T001 transcript:KXG39922            1.00E-31
  5-  2: transcript:Zm00001d028088_T003 transcript:EER92676                   0
  5-  3: transcript:Zm00001d028089_T001 transcript:KXG39923                   0
  5-  4: transcript:Zm00001d028092_T001 transcript:EER92677            3.00E-77
  5-  5: transcript:Zm00001d028093_T001 transcript:EER95313                   0
  5-  6: transcript:Zm00001d028094_T001 transcript:EER95314                   0
  5-  7: transcript:Zm00001d028096_T006 transcript:KXG39927                   0
  5-  8: transcript:Zm00001d028097_T001 transcript:EER92678                   0
  5-  9: transcript:Zm00001d028098_T001 transcript:EER92679                   0
  5- 10: transcript:Zm00001d028102_T001 transcript:EER92681                   0
  5- 11: transcript:Zm00001d028103_T001 transcript:EER92683                   0
  5- 12: transcript:Zm00001d028104_T003 transcript:OQU93046                   0
  5- 13: transcript:Zm00001d028105_T001 transcript:EER92685                   0
  5- 14: transcript:Zm00001d028107_T001 transcript:KXG39937                   0
  5- 15: transcript:Zm00001d028109_T006 transcript:EER92686                   0
  5- 16: transcript:Zm00001d028110_T001 transcript:KXG39939           7.00E-158
  5- 17: transcript:Zm00001d028111_T004 transcript:OQU93049                   0
  5- 18: transcript:Zm00001d028112_T001 transcript:EER92687           4.00E-131
  5- 19: transcript:Zm00001d028113_T001 transcript:EER92688                   0
  5- 20: transcript:Zm00001d028114_T005 transcript:EER92689                   0
  5- 21: transcript:Zm00001d028117_T002 transcript:KXG39941                   0
  5- 22: transcript:Zm00001d028118_T001 transcript:KXG39943           1.00E-105
  5- 23: transcript:Zm00001d028120_T001 transcript:EER95325                   0
  5- 24: transcript:Zm00001d028122_T001 transcript:EER92691           1.00E-104
  5- 25: transcript:Zm00001d028125_T001 transcript:EER95327                   0
  5- 26: transcript:Zm00001d028128_T003 transcript:EER95324                   0
  5- 27: transcript:Zm00001d028129_T002 transcript:EER92693           4.00E-117
  5- 28: transcript:Zm00001d028130_T001 transcript:EER95329                   0
  5- 29: transcript:Zm00001d028139_T001 transcript:EER95332                   0
## Alignment 6: score=1188.0 e_value=2.3e-78 N=26 1&1 plus
  6-  0: transcript:EER91079           transcript:Zm00001d033378_T019          0
  6-  1: transcript:KXG37850           transcript:Zm00001d033380_T001  9.00E-34
  6-  2: transcript:EER93671           transcript:Zm00001d033383_T001          0
  6-  3: transcript:EER93672           transcript:Zm00001d033385_T001          0
  6-  4: transcript:EER93673           transcript:Zm00001d033386_T001 1.00E-164
  6-  5: transcript:EER93674           transcript:Zm00001d033388_T001          0
  6-  6: transcript:EER91085           transcript:Zm00001d033389_T001  2.00E-36
  6-  7: transcript:OQU91206           transcript:Zm00001d033390_T001  8.00E-30
  6-  8: transcript:EER91088           transcript:Zm00001d033391_T001 2.00E-135
  6-  9: transcript:EER93678           transcript:Zm00001d033395_T001  7.00E-60
  6- 10: transcript:EER93679           transcript:Zm00001d033396_T003          0
  6- 11: transcript:OQU91209           transcript:Zm00001d033397_T004          0
  6- 12: transcript:KXG37863           transcript:Zm00001d033401_T001          0
  6- 13: transcript:EER91090           transcript:Zm00001d033404_T002          0
  6- 14: transcript:EER91093           transcript:Zm00001d033405_T003          0
  6- 15: transcript:EER93684           transcript:Zm00001d033407_T001 4.00E-175
  6- 16: transcript:OQU91213           transcript:Zm00001d033412_T001 2.00E-105
  6- 17: transcript:EER91096           transcript:Zm00001d033414_T001  3.00E-95
```

```
  6- 18: transcript:OQU91218           transcript:Zm00001d033415_T001         0
  6- 19: transcript:EER91098           transcript:Zm00001d033419_T001  2.00E-72
  6- 20: transcript:EER91099           transcript:Zm00001d033420_T001  4.00E-96
  6- 21: transcript:EER93687           transcript:Zm00001d033421_T001 5.00E-123
  6- 22: transcript:EER93689           transcript:Zm00001d033422_T001         0
  6- 23: transcript:EER91103           transcript:Zm00001d033428_T001  4.00E-38
  6- 24: transcript:EER91105           transcript:Zm00001d033429_T001         0
  6- 25: transcript:EER91106           transcript:Zm00001d033446_T001         0
## Alignment 7: score=1037.0 e_value=1.3e-70 N=23 1&1 plus
  7-  0: transcript:EER93926           transcript:Zm00001d032428_T004         0
  7-  1: transcript:EER93927           transcript:Zm00001d032429_T001 1.00E-110
  7-  2: transcript:KXG38173           transcript:Zm00001d032430_T002         0
  7-  3: transcript:EER91356           transcript:Zm00001d032432_T001 2.00E-160
  7-  4: transcript:EER93931           transcript:Zm00001d032433_T001         0
  7-  5: transcript:EER91359           transcript:Zm00001d032434_T001 7.00E-130
  7-  6: transcript:KXG38177           transcript:Zm00001d032435_T001         0
  7-  7: transcript:OQU91505           transcript:Zm00001d032438_T001         0
  7-  8: transcript:EER91361           transcript:Zm00001d032439_T002         0
  7-  9: transcript:EER93933           transcript:Zm00001d032440_T004         0
  7- 10: transcript:OQU91506           transcript:Zm00001d032441_T001         0
  7- 11: transcript:EER91363           transcript:Zm00001d032442_T004  2.00E-73
  7- 12: transcript:EER91364           transcript:Zm00001d032443_T001 4.00E-104
  7- 13: transcript:KXG38181           transcript:Zm00001d032447_T001  3.00E-63
  7- 14: transcript:EER93937           transcript:Zm00001d032449_T002         0
  7- 15: transcript:KXG38182           transcript:Zm00001d032450_T001  3.00E-75
  7- 16: transcript:EER91374           transcript:Zm00001d032453_T001         0
  7- 17: transcript:KXG38189           transcript:Zm00001d032455_T002         0
  7- 18: transcript:EER91375           transcript:Zm00001d032457_T001         0
  7- 19: transcript:EER93943           transcript:Zm00001d032458_T001         0
  7- 20: transcript:EER93946           transcript:Zm00001d032460_T001         0
  7- 21: transcript:EER91378           transcript:Zm00001d032461_T001         0
  7- 22: transcript:KXG38192           transcript:Zm00001d032464_T008         0
## Alignment 8: score=894.0 e_value=3.8e-55 N=19 1&1 plus
  8-  0: transcript:EER91379           transcript:Zm00001d032465_T002         0
  8-  1: transcript:EER91381           transcript:Zm00001d032467_T001 9.00E-170
  8-  2: transcript:KXG38193           transcript:Zm00001d032469_T001         0
  8-  3: transcript:OQU91520           transcript:Zm00001d032470_T001         0
  8-  4: transcript:KXG38194           transcript:Zm00001d032472_T001         0
  8-  5: transcript:KXG38195           transcript:Zm00001d032473_T001         0
  8-  6: transcript:EER91385           transcript:Zm00001d032475_T001  4.00E-54
  8-  7: transcript:EER93951           transcript:Zm00001d032476_T001         0
  8-  8: transcript:EER91386           transcript:Zm00001d032478_T001         0
  8-  9: transcript:OQU91523           transcript:Zm00001d032479_T001  1.00E-98
  8- 10: transcript:EER93954           transcript:Zm00001d032480_T001         0
  8- 11: transcript:EER91389           transcript:Zm00001d032481_T001  2.00E-40
  8- 12: transcript:OQU91527           transcript:Zm00001d032485_T001  7.00E-97
  8- 13: transcript:KXG38204           transcript:Zm00001d032491_T001         0
  8- 14: transcript:EER93958           transcript:Zm00001d032494_T001  1.00E-49
  8- 15: transcript:EER91393           transcript:Zm00001d032495_T001  2.00E-75
  8- 16: transcript:EER93964           transcript:Zm00001d032497_T001  4.00E-67
  8- 17: transcript:OQU91529           transcript:Zm00001d032498_T001  7.00E-38
  8- 18: transcript:KXG38207           transcript:Zm00001d032499_T001 2.00E-107
## Alignment 9: score=862.0 e_value=0 N=21 1&1 plus
  9-  0: transcript:Zm00001d028143_T002 transcript:EER92695                 0
```

```
  9-  1: transcript:Zm00001d028151_T001 transcript:KXG39951                  0
  9-  2: transcript:Zm00001d028152_T001 transcript:OQU93065          4.00E-175
  9-  3: transcript:Zm00001d028153_T001 transcript:KXG39955           1.00E-55
  9-  4: transcript:Zm00001d028154_T001 transcript:EER92698          2.00E-176
  9-  5: transcript:Zm00001d028159_T001 transcript:OQU93068           9.00E-65
  9-  6: transcript:Zm00001d028160_T003 transcript:OQU93069          4.00E-103
  9-  7: transcript:Zm00001d028161_T001 transcript:EER92700           8.00E-60
  9-  8: transcript:Zm00001d028162_T009 transcript:EER95337                  0
  9-  9: transcript:Zm00001d028164_T001 transcript:OQU93070                  0
  9- 10: transcript:Zm00001d028165_T001 transcript:KXG39963                  0
  9- 11: transcript:Zm00001d028167_T001 transcript:EER95339                  0
  9- 12: transcript:Zm00001d028170_T001 transcript:EER92705                  0
  9- 13: transcript:Zm00001d028171_T001 transcript:KXG39967                  0
  9- 14: transcript:Zm00001d028172_T001 transcript:KXG39969                  0
  9- 15: transcript:Zm00001d028173_T002 transcript:EER92706                  0
  9- 16: transcript:Zm00001d028175_T001 transcript:OQU93078                  0
  9- 17: transcript:Zm00001d028177_T004 transcript:OQU93081           5.00E-19
  9- 18: transcript:Zm00001d028180_T002 transcript:KXG39973                  0
  9- 19: transcript:Zm00001d028181_T001 transcript:EER92709          7.00E-100
  9- 20: transcript:Zm00001d028182_T001 transcript:EER92710                  0
## Alignment 10: score=839.0 e_value=3.3e-51 N=19 1&1 plus
 10-  0: transcript:EER91220            transcript:Zm00001d033325_T001  7.00E-95
 10-  1: transcript:EER91221            transcript:Zm00001d033327_T001 1.00E-145
 10-  2: transcript:KXG38015            transcript:Zm00001d033328_T001         0
 10-  3: transcript:EER91226            transcript:Zm00001d033330_T001 3.00E-136
 10-  4: transcript:EER91229            transcript:Zm00001d033333_T001         0
 10-  5: transcript:EER93815            transcript:Zm00001d033334_T001         0
 10-  6: transcript:EER93817            transcript:Zm00001d033335_T001 2.00E-128
 10-  7: transcript:KXG38028            transcript:Zm00001d033337_T002 5.00E-153
 10-  8: transcript:OQU91377            transcript:Zm00001d033338_T002 4.00E-140
 10-  9: transcript:EER91234            transcript:Zm00001d033339_T002         0
 10- 10: transcript:OQU91378            transcript:Zm00001d033340_T001 2.00E-156
 10- 11: transcript:EER91236            transcript:Zm00001d033344_T001         0
 10- 12: transcript:EER91237            transcript:Zm00001d033345_T007         0
 10- 13: transcript:KXG38038            transcript:Zm00001d033346_T001         0
 10- 14: transcript:KXG38039            transcript:Zm00001d033347_T001 2.00E-109
 10- 15: transcript:OQU91384            transcript:Zm00001d033353_T001 5.00E-169
 10- 16: transcript:KXG38041            transcript:Zm00001d033358_T001  2.00E-13
 10- 17: transcript:OQU91385            transcript:Zm00001d033360_T001 3.00E-100
 10- 18: transcript:EER91243            transcript:Zm00001d033363_T001 8.00E-105
## Alignment 11: score=832.0 e_value=1.1e-51 N=18 1&1 plus
 11-  0: transcript:EER93910            transcript:Zm00001d032396_T002         0
 11-  1: transcript:KXG38156            transcript:Zm00001d032397_T001         0
 11-  2: transcript:EER91338            transcript:Zm00001d032399_T001         0
 11-  3: transcript:EER91339            transcript:Zm00001d032400_T001         0
 11-  4: transcript:EER91340            transcript:Zm00001d032401_T001 6.00E-111
 11-  5: transcript:OQU91486            transcript:Zm00001d032402_T001  2.00E-80
 11-  6: transcript:KXG38160            transcript:Zm00001d032405_T001         0
 11-  7: transcript:EER93914            transcript:Zm00001d032406_T001         0
 11-  8: transcript:OQU91487            transcript:Zm00001d032407_T001         0
 11-  9: transcript:EER93919            transcript:Zm00001d032408_T002         0
 11- 10: transcript:EER93920            transcript:Zm00001d032409_T001         0
 11- 11: transcript:OQU91492            transcript:Zm00001d032410_T001  1.00E-27
 11- 12: transcript:EER91347            transcript:Zm00001d032418_T001         0
```

11- 13: transcript:EER91348 transcript:Zm00001d032419_T001 2.00E-104
11- 14: transcript:EER93922 transcript:Zm00001d032420_T001 0
11- 15: transcript:KXG38169 transcript:Zm00001d032422_T004 1.00E-51
11- 16: transcript:EER91353 transcript:Zm00001d032424_T001 0
11- 17: transcript:OQU91499 transcript:Zm00001d032427_T001 0
Alignment 12: score=812.0 e_value=3.1e-53 N=18 1&1 plus
12- 0: transcript:EER91158 transcript:Zm00001d033204_T002 3.00E-161
12- 1: transcript:EER91159 transcript:Zm00001d033205_T001 2.00E-165
12- 2: transcript:EER91156 transcript:Zm00001d033206_T001 0
12- 3: transcript:EER93744 transcript:Zm00001d033207_T001 5.00E-35
12- 4: transcript:EER91162 transcript:Zm00001d033209_T001 1.00E-69
12- 5: transcript:EER93745 transcript:Zm00001d033210_T002 0
12- 6: transcript:EER91163 transcript:Zm00001d033211_T002 0
12- 7: transcript:KXG37936 transcript:Zm00001d033213_T001 0
12- 8: transcript:EER91165 transcript:Zm00001d033214_T001 2.00E-173
12- 9: transcript:KXG37939 transcript:Zm00001d033215_T003 2.00E-175
12- 10: transcript:KXG37940 transcript:Zm00001d033216_T001 2.00E-82
12- 11: transcript:EER91167 transcript:Zm00001d033217_T004 0
12- 12: transcript:KXG37943 transcript:Zm00001d033218_T002 0
12- 13: transcript:KXG37944 transcript:Zm00001d033221_T001 0
12- 14: transcript:EER93751 transcript:Zm00001d033222_T001 0
12- 15: transcript:EER91168 transcript:Zm00001d033223_T003 4.00E-143
12- 16: transcript:EER93752 transcript:Zm00001d033225_T002 0
12- 17: transcript:EER91170 transcript:Zm00001d033229_T001 2.00E-97
Alignment 13: score=727.0 e_value=3.1e-42 N=16 1&1 plus
13- 0: transcript:EER91936 transcript:Zm00001d029892_T001 0
13- 1: transcript:EER91937 transcript:Zm00001d029896_T001 4.00E-168
13- 2: transcript:OQU92150 transcript:Zm00001d029898_T001 0
13- 3: transcript:EER94500 transcript:Zm00001d029899_T001 0
13- 4: transcript:KXG38933 transcript:Zm00001d029903_T002 0
13- 5: transcript:EER91945 transcript:Zm00001d029907_T001 2.00E-163
13- 6: transcript:EER91946 transcript:Zm00001d029910_T001 0
13- 7: transcript:EER91948 transcript:Zm00001d029913_T001 7.00E-106
13- 8: transcript:EER91950 transcript:Zm00001d029917_T001 7.00E-133
13- 9: transcript:OQU92159 transcript:Zm00001d029918_T001 2.00E-121
13- 10: transcript:EER91951 transcript:Zm00001d029919_T002 1.00E-109
13- 11: transcript:EER94513 transcript:Zm00001d029920_T001 0
13- 12: transcript:EER91952 transcript:Zm00001d029921_T001 3.00E-178
13- 13: transcript:KXG38941 transcript:Zm00001d029922_T001 0
13- 14: transcript:OQU92162 transcript:Zm00001d029923_T001 5.00E-57
13- 15: transcript:EER91957 transcript:Zm00001d029932_T001 0
Alignment 14: score=636.0 e_value=2.2e-38 N=15 1&1 plus
14- 0: transcript:KXG38235 transcript:Zm00001d032555_T006 0
14- 1: transcript:EER91423 transcript:Zm00001d032556_T001 4.00E-100
14- 2: transcript:EER93992 transcript:Zm00001d032557_T001 9.00E-177
14- 3: transcript:KXG38242 transcript:Zm00001d032560_T001 0
14- 4: transcript:EER91426 transcript:Zm00001d032565_T001 0
14- 5: transcript:EER93997 transcript:Zm00001d032566_T002 0
14- 6: transcript:EER93998 transcript:Zm00001d032567_T004 0
14- 7: transcript:OQU91561 transcript:Zm00001d032570_T002 0
14- 8: transcript:OQU91563 transcript:Zm00001d032572_T001 0
14- 9: transcript:EER94006 transcript:Zm00001d032573_T001 2.00E-146
14- 10: transcript:KXG38249 transcript:Zm00001d032575_T005 0
14- 11: transcript:EER94008 transcript:Zm00001d032576_T001 0

```
 14- 12: transcript:EER91427            transcript:Zm00001d032578_T001  3.00E-64
 14- 13: transcript:KXG38254            transcript:Zm00001d032588_T001  2.00E-67
 14- 14: transcript:KXG38256            transcript:Zm00001d032597_T006         0
## Alignment 15: score=635.0 e_value=1.1e-39 N=16 1&1 plus
 15-  0: transcript:EER94071            transcript:Zm00001d032689_T036         0
 15-  1: transcript:OQU91629            transcript:Zm00001d032691_T001 4.00E-112
 15-  2: transcript:OQU91633            transcript:Zm00001d032693_T001         0
 15-  3: transcript:EER91482            transcript:Zm00001d032694_T001 3.00E-142
 15-  4: transcript:EER94077            transcript:Zm00001d032695_T001         0
 15-  5: transcript:KXG38327            transcript:Zm00001d032696_T001 3.00E-110
 15-  6: transcript:KXG38329            transcript:Zm00001d032699_T030         0
 15-  7: transcript:KXG38331            transcript:Zm00001d032703_T020         0
 15-  8: transcript:EER94081            transcript:Zm00001d032704_T036         0
 15-  9: transcript:EER91487            transcript:Zm00001d032707_T001         0
 15- 10: transcript:EER94083            transcript:Zm00001d032708_T002         0
 15- 11: transcript:EER91490            transcript:Zm00001d032710_T003 1.00E-138
 15- 12: transcript:KXG38336            transcript:Zm00001d032715_T002         0
 15- 13: transcript:KXG38339            transcript:Zm00001d032716_T001         0
 15- 14: transcript:KXG38341            transcript:Zm00001d032718_T001 3.00E-132
 15- 15: transcript:EER91488            transcript:Zm00001d032719_T001         0
## Alignment 16: score=609.0 e_value=1.3e-33 N=14 1&1 plus
 16-  0: transcript:KXG39194            transcript:Zm00001d030028_T001         0
 16-  1: transcript:EER91883            transcript:Zm00001d030031_T001         0
 16-  2: transcript:EER91885            transcript:Zm00001d030032_T002 1.00E-138
 16-  3: transcript:EER94449            transcript:Zm00001d030035_T001  4.00E-95
 16-  4: transcript:OQU92086            transcript:Zm00001d030037_T001         0
 16-  5: transcript:OQU92087            transcript:Zm00001d030038_T001 3.00E-120
 16-  6: transcript:EER91888            transcript:Zm00001d030040_T001         0
 16-  7: transcript:OQU92088            transcript:Zm00001d030045_T001 3.00E-133
 16-  8: transcript:EER94451            transcript:Zm00001d030048_T001         0
 16-  9: transcript:KXG38864            transcript:Zm00001d030051_T002         0
 16- 10: transcript:KXG38867            transcript:Zm00001d030053_T001         0
 16- 11: transcript:EER91893            transcript:Zm00001d030059_T001  4.00E-14
 16- 12: transcript:KXG39219            transcript:Zm00001d030061_T001         0
 16- 13: transcript:KXG38871            transcript:Zm00001d030062_T001         0
## Alignment 17: score=591.0 e_value=1.1e-36 N=14 1&1 plus
 17-  0: transcript:KXG38419            transcript:Zm00001d032801_T005         0
 17-  1: transcript:EER91526            transcript:Zm00001d032804_T001 1.00E-141
 17-  2: transcript:KXG38420            transcript:Zm00001d032805_T001 3.00E-162
 17-  3: transcript:KXG38421            transcript:Zm00001d032806_T001         0
 17-  4: transcript:EER94133            transcript:Zm00001d032807_T001         0
 17-  5: transcript:EER91529            transcript:Zm00001d032810_T003 5.00E-174
 17-  6: transcript:EER91530            transcript:Zm00001d032811_T002 5.00E-136
 17-  7: transcript:EER91525            transcript:Zm00001d032815_T011         0
 17-  8: transcript:EER94138            transcript:Zm00001d032819_T001         0
 17-  9: transcript:EER94142            transcript:Zm00001d032821_T001  1.00E-22
 17- 10: transcript:EER91535            transcript:Zm00001d032822_T001  6.00E-25
 17- 11: transcript:EER91545            transcript:Zm00001d032823_T001  2.00E-07
 17- 12: transcript:OQU91699            transcript:Zm00001d032828_T001         0
 17- 13: transcript:OQU91701            transcript:Zm00001d032830_T003  2.00E-98
## Alignment 18: score=584.0 e_value=9e-31 N=13 1&1 plus
 18-  0: transcript:Zm00001d028050_T001 transcript:EER92666                   0
 18-  1: transcript:Zm00001d028051_T001 transcript:OQU93033                   0
 18-  2: transcript:Zm00001d028053_T001 transcript:EER92667            1.00E-83
```

18- 3: transcript:Zm00001d028054_T002 transcript:EER92668 0
18- 4: transcript:Zm00001d028055_T001 transcript:EER95307 3.00E-153
18- 5: transcript:Zm00001d028056_T001 transcript:KXG39906 0
18- 6: transcript:Zm00001d028062_T001 transcript:OQU93034 4.00E-104
18- 7: transcript:Zm00001d028064_T001 transcript:EER92670 4.00E-49
18- 8: transcript:Zm00001d028071_T001 transcript:KXG39908 3.00E-116
18- 9: transcript:Zm00001d028072_T001 transcript:KXG39910 3.00E-55
18- 10: transcript:Zm00001d028073_T004 transcript:EER92673 0
18- 11: transcript:Zm00001d028074_T001 transcript:EER92674 0
18- 12: transcript:Zm00001d028075_T011 transcript:OQU93039 0

Alignment 19: score=576.0 e_value=2.4e-30 N=13 1&1 plus

19- 0: transcript:OQU91644 transcript:Zm00001d032722_T001 1.00E-70
19- 1: transcript:KXG38346 transcript:Zm00001d032724_T001 0
19- 2: transcript:KXG38347 transcript:Zm00001d032725_T001 0
19- 3: transcript:EER94093 transcript:Zm00001d032728_T001 0
19- 4: transcript:EER91497 transcript:Zm00001d032732_T002 4.00E-42
19- 5: transcript:KXG38355 transcript:Zm00001d032734_T007 0
19- 6: transcript:KXG38358 transcript:Zm00001d032735_T001 0
19- 7: transcript:EER94096 transcript:Zm00001d032736_T001 2.00E-113
19- 8: transcript:OQU91659 transcript:Zm00001d032737_T001 0
19- 9: transcript:KXG38359 transcript:Zm00001d032739_T004 0
19- 10: transcript:EER94099 transcript:Zm00001d032740_T001 0
19- 11: transcript:OQU91665 transcript:Zm00001d032745_T001 0
19- 12: transcript:EER94106 transcript:Zm00001d032748_T001 3.00E-156

Alignment 20: score=480.0 e_value=1.5e-22 N=11 1&1 plus

20- 0: transcript:Zm00001d028465_T001 transcript:EER92532 1.00E-125
20- 1: transcript:Zm00001d028471_T005 transcript:KXG39751 0
20- 2: transcript:Zm00001d028472_T001 transcript:OQU92895 0
20- 3: transcript:Zm00001d028473_T001 transcript:OQU92897 3.00E-113
20- 4: transcript:Zm00001d028474_T002 transcript:EER92534 0
20- 5: transcript:Zm00001d028475_T002 transcript:EER92535 0
20- 6: transcript:Zm00001d028477_T002 transcript:EER92537 0
20- 7: transcript:Zm00001d028481_T017 transcript:EER92538 0
20- 8: transcript:Zm00001d028482_T001 transcript:EER95163 0
20- 9: transcript:Zm00001d028484_T002 transcript:EER92539 0
20- 10: transcript:Zm00001d028485_T001 transcript:EER92540 3.00E-155

Alignment 21: score=451.0 e_value=9.3e-19 N=10 1&1 plus

21- 0: transcript:OQU93348 transcript:Zm00001d029730_T001 2.00E-32
21- 1: transcript:EER95603 transcript:Zm00001d029734_T001 0
21- 2: transcript:KXG40303 transcript:Zm00001d029736_T001 4.00E-134
21- 3: transcript:EER95608 transcript:Zm00001d029738_T001 4.00E-58
21- 4: transcript:EER95612 transcript:Zm00001d029740_T002 2.00E-100
21- 5: transcript:EER92960 transcript:Zm00001d029744_T001 0
21- 6: transcript:EER92966 transcript:Zm00001d029747_T001 1.00E-154
21- 7: transcript:OQU93364 transcript:Zm00001d029749_T001 1.00E-63
21- 8: transcript:EER92967 transcript:Zm00001d029752_T001 2.00E-53
21- 9: transcript:KXG40316 transcript:Zm00001d029753_T001 3.00E-123

Alignment 22: score=451.0 e_value=6.5e-22 N=10 1&1 plus

22- 0: transcript:EER91626 transcript:Zm00001d032968_T003 0
22- 1: transcript:KXG38539 transcript:Zm00001d032969_T001 3.00E-148
22- 2: transcript:KXG38540 transcript:Zm00001d032970_T006 2.00E-29
22- 3: transcript:EER94252 transcript:Zm00001d032972_T001 5.00E-175
22- 4: transcript:EER91628 transcript:Zm00001d032973_T001 0
22- 5: transcript:EER94254 transcript:Zm00001d032974_T003 0

```
 22-  6: transcript:EER94255           transcript:Zm00001d032976_T001 4.00E-105
 22-  7: transcript:KXG38547           transcript:Zm00001d032978_T001  3.00E-81
 22-  8: transcript:OQU91798           transcript:Zm00001d032979_T001         0
 22-  9: transcript:KXG38553           transcript:Zm00001d032980_T001         0
## Alignment 23: score=408.0 e_value=2.8e-17 N=9 1&1 plus
 23-  0: transcript:KXG38970           transcript:Zm00001d029827_T001  8.00E-75
 23-  1: transcript:OQU92186           transcript:Zm00001d029829_T001  7.00E-87
 23-  2: transcript:EER91982           transcript:Zm00001d029832_T002         0
 23-  3: transcript:KXG38976           transcript:Zm00001d029833_T001         0
 23-  4: transcript:EER91984           transcript:Zm00001d029835_T001 8.00E-142
 23-  5: transcript:EER91985           transcript:Zm00001d029839_T010 2.00E-176
 23-  6: transcript:OQU92191           transcript:Zm00001d029840_T003 6.00E-179
 23-  7: transcript:EER94541           transcript:Zm00001d029842_T001         0
 23-  8: transcript:EER91990           transcript:Zm00001d029844_T001 8.00E-121
## Alignment 24: score=404.0 e_value=2.3e-18 N=9 1&1 plus
 24-  0: transcript:KXG38400           transcript:Zm00001d032777_T001  1.00E-47
 24-  1: transcript:EER91517           transcript:Zm00001d032781_T002         0
 24-  2: transcript:KXG38401           transcript:Zm00001d032784_T001         0
 24-  3: transcript:KXG38403           transcript:Zm00001d032788_T001         0
 24-  4: transcript:EER94121           transcript:Zm00001d032789_T001         0
 24-  5: transcript:EER94118           transcript:Zm00001d032790_T002         0
 24-  6: transcript:EER91520           transcript:Zm00001d032791_T001         0
 24-  7: transcript:EER91521           transcript:Zm00001d032794_T001         0
 24-  8: transcript:KXG38410           transcript:Zm00001d032798_T001         0
## Alignment 25: score=401.0 e_value=1.3e-17 N=9 1&1 plus
 25-  0: transcript:EER91066           transcript:Zm00001d033528_T005         0
 25-  1: transcript:OQU91183           transcript:Zm00001d033529_T001         0
 25-  2: transcript:EER93654           transcript:Zm00001d033530_T001  4.00E-63
 25-  3: transcript:OQU91185           transcript:Zm00001d033532_T001         0
 25-  4: transcript:EER91067           transcript:Zm00001d033537_T002  4.00E-53
 25-  5: transcript:EER93656           transcript:Zm00001d033539_T001 4.00E-164
 25-  6: transcript:OQU91186           transcript:Zm00001d033541_T002         0
 25-  7: transcript:EER91068           transcript:Zm00001d033543_T002 9.00E-140
 25-  8: transcript:KXG37831           transcript:Zm00001d033544_T001         0
## Alignment 26: score=397.0 e_value=5.3e-21 N=9 1&1 plus
 26-  0: transcript:EER91079           transcript:Zm00001d031182_T001         0
 26-  1: transcript:EER93669           transcript:Zm00001d031183_T001  2.00E-52
 26-  2: transcript:EER93673           transcript:Zm00001d031184_T001 2.00E-102
 26-  3: transcript:EER91085           transcript:Zm00001d031189_T001  6.00E-06
 26-  4: transcript:EER93676           transcript:Zm00001d031191_T001  6.00E-18
 26-  5: transcript:KXG37858           transcript:Zm00001d031192_T001  6.00E-07
 26-  6: transcript:EER91088           transcript:Zm00001d031195_T001  1.00E-06
 26-  7: transcript:EER93678           transcript:Zm00001d031196_T001  3.00E-11
 26-  8: transcript:EER91091           transcript:Zm00001d031197_T001  3.00E-11
## Alignment 27: score=372.0 e_value=1.8e-15 N=8 1&1 plus
 27-  0: transcript:EER91928           transcript:Zm00001d029875_T001         0
 27-  1: transcript:EER91929           transcript:Zm00001d029879_T001 1.00E-169
 27-  2: transcript:KXG38915           transcript:Zm00001d029884_T001  8.00E-73
 27-  3: transcript:OQU92139           transcript:Zm00001d029885_T001 1.00E-111
 27-  4: transcript:EER94492           transcript:Zm00001d029886_T001         0
 27-  5: transcript:EER94493           transcript:Zm00001d029887_T004 3.00E-123
 27-  6: transcript:EER91932           transcript:Zm00001d029888_T002         0
 27-  7: transcript:KXG38921           transcript:Zm00001d029889_T005         0
## Alignment 28: score=342.0 e_value=1.7e-16 N=8 1&1 plus
```

```
 28-  0: transcript:EER94148           transcript:Zm00001d032832_T001          0
 28-  1: transcript:EER91549           transcript:Zm00001d032836_T001 6.00E-165
 28-  2: transcript:KXG38436           transcript:Zm00001d032838_T001  4.00E-61
 28-  3: transcript:KXG38438           transcript:Zm00001d032839_T001  2.00E-20
 28-  4: transcript:EER91551           transcript:Zm00001d032847_T002  5.00E-35
 28-  5: transcript:EER91552           transcript:Zm00001d032849_T002 9.00E-162
 28-  6: transcript:EER91556           transcript:Zm00001d032850_T001          0
 28-  7: transcript:OQU91706           transcript:Zm00001d032852_T001          0
## Alignment 29: score=330.0 e_value=2.5e-12 N=7 1&1 plus
 29-  0: transcript:EER91108           transcript:Zm00001d034944_T004          0
 29-  1: transcript:OQU91224           transcript:Zm00001d034945_T001  4.00E-87
 29-  2: transcript:EER91109           transcript:Zm00001d034948_T009          0
 29-  3: transcript:EER93694           transcript:Zm00001d034949_T001 1.00E-142
 29-  4: transcript:EER93695           transcript:Zm00001d034953_T001  4.00E-99
 29-  5: transcript:KXG37876           transcript:Zm00001d034954_T001          0
 29-  6: transcript:OQU91227           transcript:Zm00001d034956_T001  5.00E-50
## Alignment 30: score=330.0 e_value=3.7e-13 N=7 1&1 plus
 30-  0: transcript:EER91264           transcript:Zm00001d033147_T004  2.00E-83
 30-  1: transcript:KXG38074           transcript:Zm00001d033148_T001          0
 30-  2: transcript:EER91268           transcript:Zm00001d033149_T001  7.00E-78
 30-  3: transcript:EER93849           transcript:Zm00001d033150_T001  3.00E-64
 30-  4: transcript:EER93852           transcript:Zm00001d033152_T001          0
 30-  5: transcript:EER93854           transcript:Zm00001d033153_T001          0
 30-  6: transcript:EER93859           transcript:Zm00001d033154_T001  5.00E-69
## Alignment 31: score=323.0 e_value=1.5e-11 N=7 1&1 plus
 31-  0: transcript:KXG37432           transcript:Zm00001d034298_T001 1.00E-105
 31-  1: transcript:KXG37434           transcript:Zm00001d034312_T001  3.00E-66
 31-  2: transcript:EER90792           transcript:Zm00001d034318_T001          0
 31-  3: transcript:EER93353           transcript:Zm00001d034319_T001 2.00E-168
 31-  4: transcript:EER93355           transcript:Zm00001d034320_T002          0
 31-  5: transcript:EER90794           transcript:Zm00001d034326_T001  7.00E-88
 31-  6: transcript:OQU90896           transcript:Zm00001d034330_T001  5.00E-08
## Alignment 32: score=315.0 e_value=3.6e-12 N=7 1&1 plus
 32-  0: transcript:EER95463           transcript:Zm00001d032923_T002 5.00E-124
 32-  1: transcript:OQU93187           transcript:Zm00001d032925_T001  9.00E-73
 32-  2: transcript:KXG40132           transcript:Zm00001d032927_T001  3.00E-22
 32-  3: transcript:KXG40135           transcript:Zm00001d032932_T001  1.00E-97
 32-  4: transcript:EER92819           transcript:Zm00001d032935_T006          0
 32-  5: transcript:EER92825           transcript:Zm00001d032938_T001  7.00E-35
 32-  6: transcript:EER95466           transcript:Zm00001d032939_T001  4.00E-53
## Alignment 33: score=294.0 e_value=4.8e-14 N=6 1&1 plus
 33-  0: transcript:EER91853           transcript:Zm00001d030148_T001          0
 33-  1: transcript:EER94414           transcript:Zm00001d030149_T001          0
 33-  2: transcript:EER91855           transcript:Zm00001d030152_T001 6.00E-134
 33-  3: transcript:EER94415           transcript:Zm00001d030153_T001  6.00E-90
 33-  4: transcript:EER94416           transcript:Zm00001d030157_T001 3.00E-144
 33-  5: transcript:EER94417           transcript:Zm00001d030158_T001          0
## Alignment 34: score=289.0 e_value=2.1e-09 N=6 1&1 plus
 34-  0: transcript:KXG37976           transcript:Zm00001d033316_T001          0
 34-  1: transcript:EER91204           transcript:Zm00001d033317_T001  5.00E-71
 34-  2: transcript:EER91205           transcript:Zm00001d033318_T005          0
 34-  3: transcript:EER93778           transcript:Zm00001d033319_T001  6.00E-98
 34-  4: transcript:EER93780           transcript:Zm00001d033321_T001          0
 34-  5: transcript:KXG37982           transcript:Zm00001d033322_T001          0
```

Alignment 35: score=281.0 e_value=2.5e-11 N=7 1&1 plus
35- 0: transcript:Zm00001d028036_T001 transcript:EER92660 2.00E-39
35- 1: transcript:Zm00001d028038_T008 transcript:EER92662 0
35- 2: transcript:Zm00001d028039_T004 transcript:KXG39903 0
35- 3: transcript:Zm00001d028040_T002 transcript:EER95301 4.00E-92
35- 4: transcript:Zm00001d028043_T001 transcript:KXG39905 6.00E-58
35- 5: transcript:Zm00001d028045_T007 transcript:EER92664 0
35- 6: transcript:Zm00001d028046_T001 transcript:OQU93031 0
Alignment 36: score=273.0 e_value=1.3e-08 N=6 1&1 plus
36- 0: transcript:EER91824 transcript:Zm00001d030199_T002 4.00E-173
36- 1: transcript:KXG38771 transcript:Zm00001d030207_T001 0
36- 2: transcript:EER91823 transcript:Zm00001d030212_T001 0
36- 3: transcript:EER91830 transcript:Zm00001d030214_T001 0
36- 4: transcript:EER91831 transcript:Zm00001d030217_T002 0
36- 5: transcript:EER91832 transcript:Zm00001d030218_T003 0
Alignment 37: score=271.0 e_value=2.7e-10 N=6 1&1 plus
37- 0: transcript:Zm00001d028452_T001 transcript:EER95152 0
37- 1: transcript:Zm00001d028454_T001 transcript:OQU92890 0
37- 2: transcript:Zm00001d028456_T001 transcript:EER92527 0
37- 3: transcript:Zm00001d028458_T001 transcript:EER95158 9.00E-51
37- 4: transcript:Zm00001d028463_T003 transcript:KXG39746 5.00E-142
37- 5: transcript:Zm00001d028464_T017 transcript:KXG39749 0
Alignment 38: score=270.0 e_value=8.4e-10 N=6 1&1 plus
38- 0: transcript:EER94293 transcript:Zm00001d033039_T001 0
38- 1: transcript:EER91677 transcript:Zm00001d033040_T001 0
38- 2: transcript:OQU91857 transcript:Zm00001d033044_T001 2.00E-156
38- 3: transcript:KXG38620 transcript:Zm00001d033046_T005 0
38- 4: transcript:KXG38624 transcript:Zm00001d033047_T001 1.00E-131
38- 5: transcript:EER91683 transcript:Zm00001d033048_T001 1.00E-95
Alignment 39: score=254.0 e_value=6.8e-10 N=6 1&1 plus
39- 0: transcript:OQU91335 transcript:Zm00001d033308_T001 2.00E-167
39- 1: transcript:EER91215 transcript:Zm00001d033310_T001 7.00E-148
39- 2: transcript:EER91218 transcript:Zm00001d033312_T006 5.00E-34
39- 3: transcript:KXG37996 transcript:Zm00001d033313_T001 1.00E-22
39- 4: transcript:KXG38006 transcript:Zm00001d033314_T001 1.00E-39
39- 5: transcript:OQU91353 transcript:Zm00001d033315_T002 3.00E-98
Alignment 40: score=4664.0 e_value=0 N=105 1&1 minus
40- 0: transcript:OQU91030 transcript:Zm00001d033935_T003 0
40- 1: transcript:KXG37604 transcript:Zm00001d033933_T001 0
40- 2: transcript:KXG37605 transcript:Zm00001d033932_T002 0
40- 3: transcript:KXG37606 transcript:Zm00001d033931_T001 0
40- 4: transcript:EER93494 transcript:Zm00001d033930_T001 6.00E-70
40- 5: transcript:KXG37613 transcript:Zm00001d033929_T002 0
40- 6: transcript:KXG37611 transcript:Zm00001d033928_T001 0
40- 7: transcript:KXG37618 transcript:Zm00001d033927_T004 0
40- 8: transcript:KXG37619 transcript:Zm00001d033925_T001 9.00E-126
40- 9: transcript:EER93496 transcript:Zm00001d033924_T001 1.00E-136
40- 10: transcript:KXG37622 transcript:Zm00001d033919_T001 0
40- 11: transcript:EER90913 transcript:Zm00001d033917_T001 0
40- 12: transcript:EER93499 transcript:Zm00001d033916_T001 0
40- 13: transcript:EER90914 transcript:Zm00001d033915_T001 0
40- 14: transcript:EER90918 transcript:Zm00001d033913_T001 3.00E-103
40- 15: transcript:KXG37626 transcript:Zm00001d033912_T001 0
40- 16: transcript:EER90919 transcript:Zm00001d033910_T002 0

40- 17:	transcript:OQU91043	transcript:Zm00001d033909_T001	2.00E-08
40- 18:	transcript:EER90924	transcript:Zm00001d033906_T001	0
40- 19:	transcript:EER90925	transcript:Zm00001d033905_T001	2.00E-151
40- 20:	transcript:KXG37635	transcript:Zm00001d033902_T001	8.00E-106
40- 21:	transcript:EER93512	transcript:Zm00001d033898_T002	0
40- 22:	transcript:KXG37637	transcript:Zm00001d033896_T001	3.00E-15
40- 23:	transcript:OQU91049	transcript:Zm00001d033895_T001	0
40- 24:	transcript:EER93515	transcript:Zm00001d033893_T001	8.00E-54
40- 25:	transcript:EER93516	transcript:Zm00001d033890_T001	0
40- 26:	transcript:KXG37640	transcript:Zm00001d033886_T001	0
40- 27:	transcript:OQU91053	transcript:Zm00001d033884_T001	0
40- 28:	transcript:OQU91054	transcript:Zm00001d033882_T001	2.00E-124
40- 29:	transcript:KXG37647	transcript:Zm00001d033879_T001	0
40- 30:	transcript:EER93521	transcript:Zm00001d033878_T002	0
40- 31:	transcript:EER93523	transcript:Zm00001d033877_T001	1.00E-104
40- 32:	transcript:EER90936	transcript:Zm00001d033876_T004	0
40- 33:	transcript:EER90939	transcript:Zm00001d033874_T002	1.00E-108
40- 34:	transcript:EER90941	transcript:Zm00001d033873_T001	2.00E-176
40- 35:	transcript:EER90942	transcript:Zm00001d033872_T001	0
40- 36:	transcript:OQU91059	transcript:Zm00001d033870_T001	0
40- 37:	transcript:OQU91062	transcript:Zm00001d033869_T001	0
40- 38:	transcript:KXG37655	transcript:Zm00001d033866_T001	0
40- 39:	transcript:EER93529	transcript:Zm00001d033863_T001	0
40- 40:	transcript:EER90948	transcript:Zm00001d033862_T001	0
40- 41:	transcript:OQU91064	transcript:Zm00001d033861_T001	0
40- 42:	transcript:KXG37658	transcript:Zm00001d033860_T001	5.00E-134
40- 43:	transcript:EER90950	transcript:Zm00001d033859_T002	0
40- 44:	transcript:KXG37660	transcript:Zm00001d033858_T003	4.00E-12
40- 45:	transcript:EER90952	transcript:Zm00001d033857_T001	4.00E-51
40- 46:	transcript:EER90953	transcript:Zm00001d033855_T002	0
40- 47:	transcript:EER93531	transcript:Zm00001d033854_T001	0
40- 48:	transcript:EER93532	transcript:Zm00001d033853_T002	0
40- 49:	transcript:KXG37666	transcript:Zm00001d033850_T001	0
40- 50:	transcript:EER90956	transcript:Zm00001d033849_T002	0
40- 51:	transcript:KXG37671	transcript:Zm00001d033848_T004	0
40- 52:	transcript:EER93534	transcript:Zm00001d033847_T003	0
40- 53:	transcript:EER93536	transcript:Zm00001d033846_T001	5.00E-33
40- 54:	transcript:KXG37677	transcript:Zm00001d033843_T007	0
40- 55:	transcript:EER90959	transcript:Zm00001d033840_T001	1.00E-58
40- 56:	transcript:EER90960	transcript:Zm00001d033839_T001	0
40- 57:	transcript:EER93541	transcript:Zm00001d033836_T001	5.00E-174
40- 58:	transcript:EER90961	transcript:Zm00001d033835_T001	6.00E-64
40- 59:	transcript:KXG37683	transcript:Zm00001d033834_T001	0
40- 60:	transcript:KXG37688	transcript:Zm00001d033830_T001	0
40- 61:	transcript:EER93548	transcript:Zm00001d033829_T001	4.00E-07
40- 62:	transcript:EER93549	transcript:Zm00001d033827_T006	0
40- 63:	transcript:EER93546	transcript:Zm00001d033825_T001	0
40- 64:	transcript:EER90964	transcript:Zm00001d033823_T001	0
40- 65:	transcript:EER93551	transcript:Zm00001d033822_T001	1.00E-91
40- 66:	transcript:EER93547	transcript:Zm00001d033821_T001	0
40- 67:	transcript:EER90966	transcript:Zm00001d033818_T001	8.00E-136
40- 68:	transcript:KXG37692	transcript:Zm00001d033817_T004	0
40- 69:	transcript:EER93554	transcript:Zm00001d033815_T001	2.00E-140
40- 70:	transcript:EER93555	transcript:Zm00001d033805_T001	0

40- 71:	transcript:EER90971	transcript:Zm00001d033800_T002	0
40- 72:	transcript:OQU91089	transcript:Zm00001d033799_T022	6.00E-65
40- 73:	transcript:KXG37701	transcript:Zm00001d033798_T030	0
40- 74:	transcript:EER90977	transcript:Zm00001d033797_T003	0
40- 75:	transcript:EER90978	transcript:Zm00001d033795_T006	0
40- 76:	transcript:EER90980	transcript:Zm00001d033794_T001	0
40- 77:	transcript:EER93557	transcript:Zm00001d033793_T001	2.00E-60
40- 78:	transcript:EER90982	transcript:Zm00001d033792_T001	8.00E-136
40- 79:	transcript:EER93559	transcript:Zm00001d033791_T001	9.00E-111
40- 80:	transcript:KXG37705	transcript:Zm00001d033790_T002	0
40- 81:	transcript:EER90983	transcript:Zm00001d033788_T002	8.00E-137
40- 82:	transcript:KXG37709	transcript:Zm00001d033787_T001	0
40- 83:	transcript:EER93561	transcript:Zm00001d033786_T002	0
40- 84:	transcript:EER90986	transcript:Zm00001d033782_T001	1.00E-36
40- 85:	transcript:EER93562	transcript:Zm00001d033780_T006	0
40- 86:	transcript:EER93563	transcript:Zm00001d033778_T001	1.00E-144
40- 87:	transcript:EER90988	transcript:Zm00001d033777_T001	0
40- 88:	transcript:EER90990	transcript:Zm00001d033776_T008	0
40- 89:	transcript:KXG37716	transcript:Zm00001d033775_T001	0
40- 90:	transcript:OQU91105	transcript:Zm00001d033766_T002	0
40- 91:	transcript:OQU91108	transcript:Zm00001d033765_T001	0
40- 92:	transcript:EER93570	transcript:Zm00001d033764_T016	0
40- 93:	transcript:EER90993	transcript:Zm00001d033753_T001	0
40- 94:	transcript:EER90995	transcript:Zm00001d033752_T001	2.00E-81
40- 95:	transcript:KXG37728	transcript:Zm00001d033751_T001	0
40- 96:	transcript:EER93572	transcript:Zm00001d033750_T003	0
40- 97:	transcript:KXG37730	transcript:Zm00001d033749_T001	0
40- 98:	transcript:OQU91109	transcript:Zm00001d033747_T001	0
40- 99:	transcript:EER93574	transcript:Zm00001d033746_T003	0
40-100:	transcript:EER90996	transcript:Zm00001d033745_T001	0
40-101:	transcript:EER93575	transcript:Zm00001d033744_T001	1.00E-114
40-102:	transcript:KXG37731	transcript:Zm00001d033741_T002	8.00E-16
40-103:	transcript:EER93577	transcript:Zm00001d033738_T002	4.00E-175
40-104:	transcript:EER93583	transcript:Zm00001d033731_T001	0

Alignment 41: score=4300.0 e_value=0 N=97 1&1 minus

41- 0:	transcript:Zm00001d028486_T001	transcript:EER95151	3.00E-75
41- 1:	transcript:Zm00001d028490_T001	transcript:EER95150	0
41- 2:	transcript:Zm00001d028493_T002	transcript:EER92523	0
41- 3:	transcript:Zm00001d028495_T001	transcript:KXG39737	0
41- 4:	transcript:Zm00001d028503_T001	transcript:OQU92885	3.00E-29
41- 5:	transcript:Zm00001d028504_T001	transcript:OQU92884	8.00E-154
41- 6:	transcript:Zm00001d028505_T001	transcript:EER95144	9.00E-93
41- 7:	transcript:Zm00001d028509_T001	transcript:EER95143	1.00E-176
41- 8:	transcript:Zm00001d028510_T001	transcript:EER92517	4.00E-175
41- 9:	transcript:Zm00001d028511_T028	transcript:EER92516	0
41- 10:	transcript:Zm00001d028512_T001	transcript:OQU92882	0
41- 11:	transcript:Zm00001d028513_T001	transcript:OQU92881	3.00E-145
41- 12:	transcript:Zm00001d028514_T002	transcript:EER92515	0
41- 13:	transcript:Zm00001d028515_T001	transcript:EER92511	2.00E-146
41- 14:	transcript:Zm00001d028522_T001	transcript:EER95140	6.00E-129
41- 15:	transcript:Zm00001d028524_T001	transcript:EER92509	3.00E-96
41- 16:	transcript:Zm00001d028526_T001	transcript:OQU92876	0
41- 17:	transcript:Zm00001d028528_T001	transcript:EER95137	0
41- 18:	transcript:Zm00001d028529_T001	transcript:EER92508	6.00E-167

41- 19:	transcript:Zm00001d028531_T001	transcript:EER95136	1.00E-69
41- 20:	transcript:Zm00001d028532_T001	transcript:OQU92875	6.00E-176
41- 21:	transcript:Zm00001d028533_T001	transcript:EER95134	0
41- 22:	transcript:Zm00001d028534_T005	transcript:EER92506	0
41- 23:	transcript:Zm00001d028535_T001	transcript:EER95133	0
41- 24:	transcript:Zm00001d028536_T001	transcript:KXG39728	0
41- 25:	transcript:Zm00001d028537_T001	transcript:EER92505	0
41- 26:	transcript:Zm00001d028538_T003	transcript:KXG39727	0
41- 27:	transcript:Zm00001d028539_T001	transcript:EER95132	0
41- 28:	transcript:Zm00001d028540_T001	transcript:EER92502	1.00E-123
41- 29:	transcript:Zm00001d028542_T001	transcript:EER95540	0
41- 30:	transcript:Zm00001d028543_T001	transcript:EER95129	0
41- 31:	transcript:Zm00001d028546_T032	transcript:OQU92867	0
41- 32:	transcript:Zm00001d028547_T001	transcript:KXG39720	2.00E-82
41- 33:	transcript:Zm00001d028548_T001	transcript:EER92501	3.00E-103
41- 34:	transcript:Zm00001d028549_T005	transcript:EER95530	5.00E-70
41- 35:	transcript:Zm00001d028550_T001	transcript:EER95125	1.00E-142
41- 36:	transcript:Zm00001d028554_T003	transcript:EER92500	3.00E-136
41- 37:	transcript:Zm00001d028555_T001	transcript:EER95124	3.00E-109
41- 38:	transcript:Zm00001d028558_T003	transcript:OQU92865	0
41- 39:	transcript:Zm00001d028560_T020	transcript:EER95121	0
41- 40:	transcript:Zm00001d028561_T001	transcript:EER95120	1.00E-101
41- 41:	transcript:Zm00001d028562_T001	transcript:KXG39716	0
41- 42:	transcript:Zm00001d028565_T002	transcript:EER95119	0
41- 43:	transcript:Zm00001d028566_T003	transcript:EER92498	0
41- 44:	transcript:Zm00001d028567_T001	transcript:EER92497	0
41- 45:	transcript:Zm00001d028568_T004	transcript:EER95117	0
41- 46:	transcript:Zm00001d028569_T002	transcript:EER92496	2.00E-153
41- 47:	transcript:Zm00001d028570_T002	transcript:EER92495	0
41- 48:	transcript:Zm00001d028571_T001	transcript:EER92494	0
41- 49:	transcript:Zm00001d028572_T004	transcript:KXG39711	0
41- 50:	transcript:Zm00001d028574_T001	transcript:EER92493	0
41- 51:	transcript:Zm00001d028575_T001	transcript:OQU92858	4.00E-180
41- 52:	transcript:Zm00001d028576_T001	transcript:OQU92853	0
41- 53:	transcript:Zm00001d028577_T001	transcript:EER95114	9.00E-138
41- 54:	transcript:Zm00001d028579_T027	transcript:OQU92852	0
41- 55:	transcript:Zm00001d028584_T001	transcript:EER92487	7.00E-84
41- 56:	transcript:Zm00001d028585_T001	transcript:EER95113	0
41- 57:	transcript:Zm00001d028586_T001	transcript:EER95112	0
41- 58:	transcript:Zm00001d028587_T001	transcript:KXG39704	0
41- 59:	transcript:Zm00001d028588_T001	transcript:EER92486	0
41- 60:	transcript:Zm00001d028590_T001	transcript:EER95109	0
41- 61:	transcript:Zm00001d028591_T001	transcript:KXG40203	0
41- 62:	transcript:Zm00001d028593_T002	transcript:EER95107	0
41- 63:	transcript:Zm00001d028596_T001	transcript:OQU92834	6.00E-141
41- 64:	transcript:Zm00001d028597_T001	transcript:EER95105	0
41- 65:	transcript:Zm00001d028598_T001	transcript:OQU92832	2.00E-50
41- 66:	transcript:Zm00001d028599_T001	transcript:EER92483	0
41- 67:	transcript:Zm00001d028601_T002	transcript:EER95104	0
41- 68:	transcript:Zm00001d028603_T001	transcript:OQU92829	0
41- 69:	transcript:Zm00001d028606_T003	transcript:OQU92826	0
41- 70:	transcript:Zm00001d028608_T006	transcript:KXG39690	0
41- 71:	transcript:Zm00001d028612_T001	transcript:EER95101	0
41- 72:	transcript:Zm00001d028613_T001	transcript:EER92482	0

```
 41- 73: transcript:Zm00001d028615_T001 transcript:EER95099                0
 41- 74: transcript:Zm00001d028616_T001 transcript:EER92481                0
 41- 75: transcript:Zm00001d028619_T007 transcript:KXG39683                0
 41- 76: transcript:Zm00001d028620_T001 transcript:EER92480                0
 41- 77: transcript:Zm00001d028623_T001 transcript:OQU92820                0
 41- 78: transcript:Zm00001d028625_T002 transcript:KXG39680        5.00E-178
 41- 79: transcript:Zm00001d028631_T001 transcript:EER92474                0
 41- 80: transcript:Zm00001d028639_T001 transcript:KXG39675                0
 41- 81: transcript:Zm00001d028641_T003 transcript:EER92468         1.00E-51
 41- 82: transcript:Zm00001d028642_T001 transcript:EER92467        2.00E-119
 41- 83: transcript:Zm00001d028643_T001 transcript:KXG39674                0
 41- 84: transcript:Zm00001d028647_T002 transcript:EER92465         4.00E-85
 41- 85: transcript:Zm00001d028649_T001 transcript:OQU92813                0
 41- 86: transcript:Zm00001d028651_T001 transcript:EER92464        1.00E-156
 41- 87: transcript:Zm00001d028653_T005 transcript:EER95086         6.00E-25
 41- 88: transcript:Zm00001d028655_T001 transcript:EER92463        3.00E-145
 41- 89: transcript:Zm00001d028656_T001 transcript:EER92462                0
 41- 90: transcript:Zm00001d028660_T013 transcript:OQU92811                0
 41- 91: transcript:Zm00001d028661_T001 transcript:EER92461        1.00E-100
 41- 92: transcript:Zm00001d028662_T001 transcript:EER92456                0
 41- 93: transcript:Zm00001d028664_T001 transcript:OQU92806        1.00E-124
 41- 94: transcript:Zm00001d028665_T001 transcript:KXG39660        1.00E-118
 41- 95: transcript:Zm00001d028667_T001 transcript:EER92453        6.00E-104
 41- 96: transcript:Zm00001d028669_T010 transcript:EER95074                0
## Alignment 42: score=2931.0 e_value=4.1e-257 N=66 1&1 minus
 42-  0: transcript:Zm00001d028344_T002 transcript:EER92593                0
 42-  1: transcript:Zm00001d028347_T001 transcript:EER92588        8.00E-108
 42-  2: transcript:Zm00001d028352_T002 transcript:EER95208        3.00E-161
 42-  3: transcript:Zm00001d028354_T001 transcript:EER92586                0
 42-  4: transcript:Zm00001d028356_T001 transcript:EER95203         2.00E-16
 42-  5: transcript:Zm00001d028358_T003 transcript:EER95202                0
 42-  6: transcript:Zm00001d028359_T001 transcript:EER95201                0
 42-  7: transcript:Zm00001d028360_T001 transcript:EER95200                0
 42-  8: transcript:Zm00001d028361_T001 transcript:KXG39813        5.00E-164
 42-  9: transcript:Zm00001d028362_T001 transcript:OQU92943        1.00E-122
 42- 10: transcript:Zm00001d028363_T001 transcript:EER92583                0
 42- 11: transcript:Zm00001d028365_T006 transcript:OQU92938        2.00E-159
 42- 12: transcript:Zm00001d028366_T001 transcript:KXG39809                0
 42- 13: transcript:Zm00001d028367_T002 transcript:KXG39805         3.00E-28
 42- 14: transcript:Zm00001d028368_T001 transcript:KXG39806         1.00E-45
 42- 15: transcript:Zm00001d028369_T001 transcript:KXG39803                0
 42- 16: transcript:Zm00001d028370_T001 transcript:EER95194                0
 42- 17: transcript:Zm00001d028371_T001 transcript:EER92577        3.00E-123
 42- 18: transcript:Zm00001d028372_T005 transcript:KXG39801                0
 42- 19: transcript:Zm00001d028373_T002 transcript:KXG39798                0
 42- 20: transcript:Zm00001d028374_T001 transcript:EER92576                0
 42- 21: transcript:Zm00001d028375_T001 transcript:EER92575         3.00E-72
 42- 22: transcript:Zm00001d028377_T001 transcript:EER95192         2.00E-77
 42- 23: transcript:Zm00001d028380_T002 transcript:KXG39796                0
 42- 24: transcript:Zm00001d028384_T006 transcript:EER95190         1.00E-80
 42- 25: transcript:Zm00001d028385_T002 transcript:EER95189                0
 42- 26: transcript:Zm00001d028386_T001 transcript:EER92574                0
 42- 27: transcript:Zm00001d028388_T001 transcript:KXG39793                0
 42- 28: transcript:Zm00001d028389_T001 transcript:EER92572         4.00E-73
```

42- 29:	transcript:Zm00001d028391_T001	transcript:EER92571	0
42- 30:	transcript:Zm00001d028392_T001	transcript:OQU92927	4.00E-64
42- 31:	transcript:Zm00001d028396_T001	transcript:EER92569	0
42- 32:	transcript:Zm00001d028397_T001	transcript:OQU92926	0
42- 33:	transcript:Zm00001d028398_T003	transcript:OQU92925	0
42- 34:	transcript:Zm00001d028399_T001	transcript:KXG39786	2.00E-171
42- 35:	transcript:Zm00001d028400_T001	transcript:KXG39784	9.00E-174
42- 36:	transcript:Zm00001d028401_T005	transcript:EER95182	0
42- 37:	transcript:Zm00001d028404_T001	transcript:EER92566	2.00E-70
42- 38:	transcript:Zm00001d028405_T002	transcript:EER92564	2.00E-23
42- 39:	transcript:Zm00001d028406_T001	transcript:KXG39781	0
42- 40:	transcript:Zm00001d028408_T001	transcript:EER95179	1.00E-148
42- 41:	transcript:Zm00001d028409_T001	transcript:OQU92920	0
42- 42:	transcript:Zm00001d028412_T029	transcript:EER95178	0
42- 43:	transcript:Zm00001d028413_T001	transcript:EER92557	9.00E-115
42- 44:	transcript:Zm00001d028414_T002	transcript:EER92556	4.00E-138
42- 45:	transcript:Zm00001d028415_T005	transcript:EER95177	0
42- 46:	transcript:Zm00001d028416_T001	transcript:EER95176	0
42- 47:	transcript:Zm00001d028417_T001	transcript:OQU92911	0
42- 48:	transcript:Zm00001d028422_T001	transcript:EER95175	0
42- 49:	transcript:Zm00001d028423_T001	transcript:KXG39773	0
42- 50:	transcript:Zm00001d028424_T002	transcript:KXG39770	3.00E-135
42- 51:	transcript:Zm00001d028425_T003	transcript:EER95173	3.00E-128
42- 52:	transcript:Zm00001d028426_T001	transcript:EER95172	2.00E-86
42- 53:	transcript:Zm00001d028427_T001	transcript:EER95171	0
42- 54:	transcript:Zm00001d028428_T001	transcript:KXG39767	0
42- 55:	transcript:Zm00001d028429_T017	transcript:KXG39766	0
42- 56:	transcript:Zm00001d028432_T001	transcript:KXG39763	1.00E-132
42- 57:	transcript:Zm00001d028436_T001	transcript:EER95168	0
42- 58:	transcript:Zm00001d028437_T001	transcript:EER92549	0
42- 59:	transcript:Zm00001d028439_T001	transcript:OQU92904	0
42- 60:	transcript:Zm00001d028440_T001	transcript:EER92547	0
42- 61:	transcript:Zm00001d028443_T001	transcript:EER92546	0
42- 62:	transcript:Zm00001d028445_T001	transcript:EER92545	2.00E-36
42- 63:	transcript:Zm00001d028447_T001	transcript:EER95166	0
42- 64:	transcript:Zm00001d028450_T001	transcript:EER92542	5.00E-131
42- 65:	transcript:Zm00001d028451_T001	transcript:EER92541	0

Alignment 43: score=2346.0 e_value=3.5e-200 N=53 1&1 minus

43- 0:	transcript:Zm00001d028915_T023	transcript:KXG39515	3.00E-108
43- 1:	transcript:Zm00001d028916_T001	transcript:OQU92704	4.00E-77
43- 2:	transcript:Zm00001d028917_T001	transcript:KXG39511	2.00E-44
43- 3:	transcript:Zm00001d028918_T001	transcript:EER94961	5.00E-98
43- 4:	transcript:Zm00001d028919_T001	transcript:OQU92701	0
43- 5:	transcript:Zm00001d028920_T004	transcript:OQU92698	0
43- 6:	transcript:Zm00001d028921_T004	transcript:KXG39506	2.00E-162
43- 7:	transcript:Zm00001d028922_T001	transcript:EER92359	2.00E-48
43- 8:	transcript:Zm00001d028923_T003	transcript:KXG39505	0
43- 9:	transcript:Zm00001d028924_T001	transcript:EER94953	9.00E-146
43- 10:	transcript:Zm00001d028926_T006	transcript:EER92357	0
43- 11:	transcript:Zm00001d028930_T001	transcript:KXG39500	3.00E-174
43- 12:	transcript:Zm00001d028931_T002	transcript:EER94952	0
43- 13:	transcript:Zm00001d028935_T001	transcript:OQU92696	0
43- 14:	transcript:Zm00001d028936_T002	transcript:KXG39494	0
43- 15:	transcript:Zm00001d028941_T001	transcript:EER94949	2.00E-41

43- 16: transcript:Zm00001d028946_T001 transcript:EER94948 0
43- 17: transcript:Zm00001d028947_T001 transcript:EER94947 0
43- 18: transcript:Zm00001d028948_T001 transcript:EER94946 3.00E-86
43- 19: transcript:Zm00001d028949_T002 transcript:EER92350 0
43- 20: transcript:Zm00001d028950_T001 transcript:OQU92692 3.00E-80
43- 21: transcript:Zm00001d028951_T001 transcript:EER92349 1.00E-170
43- 22: transcript:Zm00001d028952_T004 transcript:KXG39489 0
43- 23: transcript:Zm00001d028953_T001 transcript:EER92346 1.00E-71
43- 24: transcript:Zm00001d028954_T001 transcript:OQU92690 0
43- 25: transcript:Zm00001d028955_T001 transcript:EER94942 1.00E-115
43- 26: transcript:Zm00001d028957_T001 transcript:EER94941 2.00E-172
43- 27: transcript:Zm00001d028958_T001 transcript:EER92345 0
43- 28: transcript:Zm00001d028960_T001 transcript:OQU92687 0
43- 29: transcript:Zm00001d028962_T001 transcript:EER94940 6.00E-130
43- 30: transcript:Zm00001d028963_T001 transcript:EER94938 3.00E-89
43- 31: transcript:Zm00001d028964_T002 transcript:EER94937 0
43- 32: transcript:Zm00001d028966_T005 transcript:KXG39479 0
43- 33: transcript:Zm00001d028967_T001 transcript:OQU92671 0
43- 34: transcript:Zm00001d028968_T001 transcript:EER92337 8.00E-70
43- 35: transcript:Zm00001d028971_T002 transcript:EER94935 0
43- 36: transcript:Zm00001d028973_T001 transcript:EER94934 9.00E-57
43- 37: transcript:Zm00001d028974_T001 transcript:EER94933 0
43- 38: transcript:Zm00001d028975_T001 transcript:EER94930 0
43- 39: transcript:Zm00001d028980_T001 transcript:EER94932 0
43- 40: transcript:Zm00001d028981_T002 transcript:EER92335 1.00E-149
43- 41: transcript:Zm00001d028982_T001 transcript:KXG39475 8.00E-28
43- 42: transcript:Zm00001d028983_T001 transcript:EER92334 4.00E-56
43- 43: transcript:Zm00001d028984_T001 transcript:EER94931 0
43- 44: transcript:Zm00001d028986_T001 transcript:KXG39473 9.00E-138
43- 45: transcript:Zm00001d028987_T001 transcript:OQU92666 0
43- 46: transcript:Zm00001d028989_T002 transcript:EER92330 0
43- 47: transcript:Zm00001d028992_T001 transcript:KXG39469 8.00E-175
43- 48: transcript:Zm00001d028994_T001 transcript:EER92329 0
43- 49: transcript:Zm00001d028995_T001 transcript:EER92328 0
43- 50: transcript:Zm00001d028998_T001 transcript:EER92327 0
43- 51: transcript:Zm00001d028999_T001 transcript:EER92326 0
43- 52: transcript:Zm00001d029004_T004 transcript:KXG39465 0
Alignment 44: score=2172.0 e_value=3.8e-168 N=46 1&1 minus
44- 0: transcript:EER93368 transcript:Zm00001d034255_T001 0
44- 1: transcript:EER93369 transcript:Zm00001d034254_T002 2.00E-70
44- 2: transcript:OQU90916 transcript:Zm00001d034253_T001 0
44- 3: transcript:EER93370 transcript:Zm00001d034252_T003 1.00E-57
44- 4: transcript:EER93372 transcript:Zm00001d034250_T002 0
44- 5: transcript:EER90809 transcript:Zm00001d034249_T001 0
44- 6: transcript:EER93373 transcript:Zm00001d034248_T002 5.00E-91
44- 7: transcript:OQU90918 transcript:Zm00001d034245_T004 0
44- 8: transcript:KXG37457 transcript:Zm00001d034244_T001 5.00E-143
44- 9: transcript:KXG37460 transcript:Zm00001d034241_T001 0
44- 10: transcript:EER93376 transcript:Zm00001d034240_T004 0
44- 11: transcript:EER93377 transcript:Zm00001d034239_T001 0
44- 12: transcript:EER90813 transcript:Zm00001d034232_T001 6.00E-23
44- 13: transcript:KXG37462 transcript:Zm00001d034223_T002 5.00E-45
44- 14: transcript:EER90815 transcript:Zm00001d034221_T001 0
44- 15: transcript:OQU90920 transcript:Zm00001d034219_T001 2.00E-49

44- 16:	transcript:EER93378	transcript:Zm00001d034218_T001	6.00E-64
44- 17:	transcript:EER90817	transcript:Zm00001d034217_T001	0
44- 18:	transcript:OQU90922	transcript:Zm00001d034212_T001	3.00E-161
44- 19:	transcript:EER90818	transcript:Zm00001d034207_T001	0
44- 20:	transcript:EER90819	transcript:Zm00001d034206_T001	0
44- 21:	transcript:EER90820	transcript:Zm00001d034205_T001	0
44- 22:	transcript:EER93382	transcript:Zm00001d034204_T001	0
44- 23:	transcript:KXG37469	transcript:Zm00001d034200_T001	2.00E-78
44- 24:	transcript:EER93384	transcript:Zm00001d034199_T001	1.00E-78
44- 25:	transcript:KXG37470	transcript:Zm00001d034198_T001	0
44- 26:	transcript:EER93386	transcript:Zm00001d034197_T001	1.00E-82
44- 27:	transcript:EER90821	transcript:Zm00001d034196_T003	0
44- 28:	transcript:EER90822	transcript:Zm00001d034195_T001	2.00E-98
44- 29:	transcript:KXG37471	transcript:Zm00001d034194_T004	0
44- 30:	transcript:EER90823	transcript:Zm00001d034192_T001	1.00E-156
44- 31:	transcript:EER90825	transcript:Zm00001d034191_T004	0
44- 32:	transcript:EER93387	transcript:Zm00001d034190_T001	6.00E-92
44- 33:	transcript:KXG37475	transcript:Zm00001d034189_T001	3.00E-99
44- 34:	transcript:EER93389	transcript:Zm00001d034188_T002	7.00E-119
44- 35:	transcript:KXG37477	transcript:Zm00001d034187_T001	9.00E-38
44- 36:	transcript:EER90827	transcript:Zm00001d034184_T001	0
44- 37:	transcript:OQU90932	transcript:Zm00001d034183_T006	0
44- 38:	transcript:KXG37479	transcript:Zm00001d034182_T001	2.00E-16
44- 39:	transcript:EER93393	transcript:Zm00001d034181_T001	6.00E-127
44- 40:	transcript:EER90828	transcript:Zm00001d034180_T002	3.00E-168
44- 41:	transcript:KXG37482	transcript:Zm00001d034179_T001	4.00E-67
44- 42:	transcript:EER93395	transcript:Zm00001d034178_T001	0
44- 43:	transcript:EER90829	transcript:Zm00001d034175_T002	1.00E-112
44- 44:	transcript:OQU90933	transcript:Zm00001d034173_T001	1.00E-12
44- 45:	transcript:EER90831	transcript:Zm00001d034167_T006	0
## Alignment 45: score=2120.0 e_value=4.3e-168 N=46 1&1 minus			
45- 0:	transcript:EER94543	transcript:Zm00001d029826_T004	1.00E-54
45- 1:	transcript:EER94540	transcript:Zm00001d029825_T001	2.00E-74
45- 2:	transcript:EER94546	transcript:Zm00001d029823_T001	4.00E-114
45- 3:	transcript:KXG38982	transcript:Zm00001d029822_T001	3.00E-154
45- 4:	transcript:EER91992	transcript:Zm00001d029820_T001	2.00E-54
45- 5:	transcript:EER94548	transcript:Zm00001d029818_T004	0
45- 6:	transcript:EER94552	transcript:Zm00001d029816_T001	2.00E-78
45- 7:	transcript:KXG38983	transcript:Zm00001d029815_T001	3.00E-151
45- 8:	transcript:EER94556	transcript:Zm00001d029814_T001	0
45- 9:	transcript:EER91995	transcript:Zm00001d029811_T001	0
45- 10:	transcript:EER94557	transcript:Zm00001d029810_T001	0
45- 11:	transcript:EER94558	transcript:Zm00001d029809_T002	2.00E-81
45- 12:	transcript:EER91996	transcript:Zm00001d029808_T001	0
45- 13:	transcript:EER94560	transcript:Zm00001d029806_T001	8.00E-136
45- 14:	transcript:EER91998	transcript:Zm00001d029803_T001	0
45- 15:	transcript:OQU92206	transcript:Zm00001d029802_T001	3.00E-24
45- 16:	transcript:OQU92208	transcript:Zm00001d029801_T002	5.00E-62
45- 17:	transcript:KXG38989	transcript:Zm00001d029793_T001	2.00E-63
45- 18:	transcript:OQU92209	transcript:Zm00001d029792_T001	8.00E-118
45- 19:	transcript:EER94565	transcript:Zm00001d029785_T001	0
45- 20:	transcript:EER94567	transcript:Zm00001d029784_T001	3.00E-158
45- 21:	transcript:KXG38992	transcript:Zm00001d029783_T001	8.00E-163
45- 22:	transcript:EER92003	transcript:Zm00001d029782_T004	0

```
 45- 23: transcript:EER94573          transcript:Zm00001d029778_T001  3.00E-42
 45- 24: transcript:EER94575          transcript:Zm00001d029777_T001         0
 45- 25: transcript:OQU92218          transcript:Zm00001d029776_T002         0
 45- 26: transcript:OQU92219          transcript:Zm00001d029775_T001  1.00E-06
 45- 27: transcript:KXG39004          transcript:Zm00001d029773_T001  8.00E-27
 45- 28: transcript:OQU92223          transcript:Zm00001d029772_T002         0
 45- 29: transcript:EER92006          transcript:Zm00001d029768_T001         0
 45- 30: transcript:KXG39007          transcript:Zm00001d029764_T001         0
 45- 31: transcript:EER94578          transcript:Zm00001d029762_T008         0
 45- 32: transcript:EER94579          transcript:Zm00001d029761_T001         0
 45- 33: transcript:EER94580          transcript:Zm00001d029758_T004         0
 45- 34: transcript:OQU92227          transcript:Zm00001d029757_T001         0
 45- 35: transcript:KXG39009          transcript:Zm00001d029754_T004         0
 45- 36: transcript:EER94581          transcript:Zm00001d029753_T001         0
 45- 37: transcript:KXG39011          transcript:Zm00001d029752_T001  1.00E-88
 45- 38: transcript:OQU92229          transcript:Zm00001d029750_T001         0
 45- 39: transcript:EER92015          transcript:Zm00001d029749_T001 6.00E-143
 45- 40: transcript:EER94583          transcript:Zm00001d029747_T001         0
 45- 41: transcript:KXG39014          transcript:Zm00001d029745_T003  4.00E-66
 45- 42: transcript:EER94585          transcript:Zm00001d029744_T001         0
 45- 43: transcript:EER92016          transcript:Zm00001d029740_T002 5.00E-103
 45- 44: transcript:EER92017          transcript:Zm00001d029738_T001  1.00E-85
 45- 45: transcript:EER92018          transcript:Zm00001d029736_T001         0
## Alignment 46: score=2069.0 e_value=7.1e-169 N=45 1&1 minus
 46-  0: transcript:Zm00001d028749_T002 transcript:KXG39596                 0
 46-  1: transcript:Zm00001d028750_T002 transcript:KXG39586                 0
 46-  2: transcript:Zm00001d028751_T001 transcript:EER95036                 0
 46-  3: transcript:Zm00001d028752_T001 transcript:EER95033                 0
 46-  4: transcript:Zm00001d028753_T003 transcript:EER95032                 0
 46-  5: transcript:Zm00001d028754_T001 transcript:OQU92769                 0
 46-  6: transcript:Zm00001d028755_T001 transcript:EER92413         3.00E-115
 46-  7: transcript:Zm00001d028756_T001 transcript:KXG39581         7.00E-157
 46-  8: transcript:Zm00001d028757_T001 transcript:KXG39580                 0
 46-  9: transcript:Zm00001d028759_T001 transcript:EER92412                 0
 46- 10: transcript:Zm00001d028761_T002 transcript:KXG39578                 0
 46- 11: transcript:Zm00001d028762_T005 transcript:EER95027                 0
 46- 12: transcript:Zm00001d028767_T001 transcript:OQU92764          2.00E-09
 46- 13: transcript:Zm00001d028769_T004 transcript:EER95025                 0
 46- 14: transcript:Zm00001d028770_T003 transcript:KXG39575                 0
 46- 15: transcript:Zm00001d028771_T001 transcript:OQU92762                 0
 46- 16: transcript:Zm00001d028773_T002 transcript:OQU92761          3.00E-74
 46- 17: transcript:Zm00001d028774_T001 transcript:KXG39573                 0
 46- 18: transcript:Zm00001d028775_T001 transcript:KXG39571         6.00E-104
 46- 19: transcript:Zm00001d028777_T001 transcript:OQU92760         6.00E-171
 46- 20: transcript:Zm00001d028778_T002 transcript:KXG39569         2.00E-123
 46- 21: transcript:Zm00001d028779_T001 transcript:EER92408          1.00E-96
 46- 22: transcript:Zm00001d028782_T003 transcript:EER95021                 0
 46- 23: transcript:Zm00001d028783_T003 transcript:KXG39567         3.00E-124
 46- 24: transcript:Zm00001d028784_T001 transcript:OQU92753                 0
 46- 25: transcript:Zm00001d028785_T001 transcript:EER92407         9.00E-119
 46- 26: transcript:Zm00001d028786_T001 transcript:KXG39564         5.00E-135
 46- 27: transcript:Zm00001d028787_T001 transcript:EER92406                 0
 46- 28: transcript:Zm00001d028793_T001 transcript:EER92405         1.00E-124
 46- 29: transcript:Zm00001d028796_T001 transcript:EER95015                 0
```

```
 46- 30: transcript:Zm00001d028797_T001 transcript:EER95014                  0
 46- 31: transcript:Zm00001d028798_T001 transcript:EER95013           1.00E-95
 46- 32: transcript:Zm00001d028799_T001 transcript:OQU92750           3.00E-18
 46- 33: transcript:Zm00001d028802_T001 transcript:OQU92749          2.00E-100
 46- 34: transcript:Zm00001d028803_T002 transcript:EER92402                  0
 46- 35: transcript:Zm00001d028804_T001 transcript:EER92401          2.00E-167
 46- 36: transcript:Zm00001d028806_T001 transcript:EER95011                  0
 46- 37: transcript:Zm00001d028808_T001 transcript:OQU92744          8.00E-103
 46- 38: transcript:Zm00001d028809_T001 transcript:EER92399                  0
 46- 39: transcript:Zm00001d028810_T002 transcript:EER95009                  0
 46- 40: transcript:Zm00001d028811_T001 transcript:EER92398                  0
 46- 41: transcript:Zm00001d028812_T005 transcript:EER92397                  0
 46- 42: transcript:Zm00001d028813_T001 transcript:EER95008                  0
 46- 43: transcript:Zm00001d028814_T001 transcript:EER95005          4.00E-101
 46- 44: transcript:Zm00001d028818_T015 transcript:EER95003                  0
## Alignment 47: score=2031.0 e_value=2.6e-173 N=47 1&1 minus
 47-  0: transcript:Zm00001d027427_T002 transcript:EER95612          2.00E-117
 47-  1: transcript:Zm00001d027428_T001 transcript:EER92958                  0
 47-  2: transcript:Zm00001d027429_T001 transcript:OQU93351           2.00E-53
 47-  3: transcript:Zm00001d027431_T001 transcript:KXG40306                  0
 47-  4: transcript:Zm00001d027434_T001 transcript:EER95608           2.00E-98
 47-  5: transcript:Zm00001d027435_T002 transcript:KXG40303                  0
 47-  6: transcript:Zm00001d027436_T001 transcript:EER95602                  0
 47-  7: transcript:Zm00001d027439_T001 transcript:EER95601                  0
 47-  8: transcript:Zm00001d027440_T001 transcript:OQU93348           1.00E-76
 47-  9: transcript:Zm00001d027441_T001 transcript:EER95599                  0
 47- 10: transcript:Zm00001d027442_T001 transcript:KXG40295          3.00E-110
 47- 11: transcript:Zm00001d027444_T003 transcript:EER95598                  0
 47- 12: transcript:Zm00001d027445_T002 transcript:EER95597                  0
 47- 13: transcript:Zm00001d027446_T001 transcript:EER95596                  0
 47- 14: transcript:Zm00001d027447_T006 transcript:EER92954           8.00E-29
 47- 15: transcript:Zm00001d027448_T001 transcript:KXG40290                  0
 47- 16: transcript:Zm00001d027449_T001 transcript:OQU93342           1.00E-38
 47- 17: transcript:Zm00001d027451_T001 transcript:OQU93339                  0
 47- 18: transcript:Zm00001d027453_T001 transcript:EER92948           3.00E-94
 47- 19: transcript:Zm00001d027454_T001 transcript:EER95593           1.00E-55
 47- 20: transcript:Zm00001d027455_T001 transcript:EER92947                  0
 47- 21: transcript:Zm00001d027457_T001 transcript:KXG40284           2.00E-38
 47- 22: transcript:Zm00001d027458_T001 transcript:EER92946           2.00E-61
 47- 23: transcript:Zm00001d027459_T001 transcript:OQU93335                  0
 47- 24: transcript:Zm00001d027460_T002 transcript:KXG40279                  0
 47- 25: transcript:Zm00001d027461_T001 transcript:KXG40277                  0
 47- 26: transcript:Zm00001d027462_T001 transcript:EER92944                  0
 47- 27: transcript:Zm00001d027463_T001 transcript:EER95591                  0
 47- 28: transcript:Zm00001d027464_T001 transcript:EER95590          4.00E-157
 47- 29: transcript:Zm00001d027466_T001 transcript:EER92943                  0
 47- 30: transcript:Zm00001d027468_T001 transcript:EER95589                  0
 47- 31: transcript:Zm00001d027469_T002 transcript:EER95588           6.00E-72
 47- 32: transcript:Zm00001d027471_T001 transcript:OQU93332                  0
 47- 33: transcript:Zm00001d027474_T001 transcript:OQU93328                  0
 47- 34: transcript:Zm00001d027477_T001 transcript:EER92940           9.00E-61
 47- 35: transcript:Zm00001d027478_T001 transcript:EER92939           1.00E-59
 47- 36: transcript:Zm00001d027479_T002 transcript:KXG40270                  0
 47- 37: transcript:Zm00001d027480_T002 transcript:EER95585                  0
```

```
 47- 38: transcript:Zm00001d027481_T001 transcript:KXG40268                     0
 47- 39: transcript:Zm00001d027483_T001 transcript:EER92934             2.00E-100
 47- 40: transcript:Zm00001d027484_T002 transcript:EER92933                     0
 47- 41: transcript:Zm00001d027485_T002 transcript:OQU93320             2.00E-107
 47- 42: transcript:Zm00001d027486_T001 transcript:EER92932                     0
 47- 43: transcript:Zm00001d027487_T017 transcript:EER92931                     0
 47- 44: transcript:Zm00001d027488_T001 transcript:KXG40264                     0
 47- 45: transcript:Zm00001d027489_T001 transcript:OQU93316                     0
 47- 46: transcript:Zm00001d027490_T002 transcript:EER92929                     0
## Alignment 48: score=1969.0 e_value=4.9e-154 N=43 1&1 minus
 48-  0: transcript:EER90684            transcript:Zm00001d034553_T001          0
 48-  1: transcript:EER93240            transcript:Zm00001d034551_T001          0
 48-  2: transcript:EER93242            transcript:Zm00001d034550_T001          0
 48-  3: transcript:EER93244            transcript:Zm00001d034547_T002          0
 48-  4: transcript:OQU90794            transcript:Zm00001d034546_T001  1.00E-60
 48-  5: transcript:EER93246            transcript:Zm00001d034543_T001 9.00E-125
 48-  6: transcript:EER90692            transcript:Zm00001d034534_T001 1.00E-126
 48-  7: transcript:EER93247            transcript:Zm00001d034533_T002          0
 48-  8: transcript:OQU90796            transcript:Zm00001d034532_T001 3.00E-115
 48-  9: transcript:KXG37311            transcript:Zm00001d034531_T001          0
 48- 10: transcript:KXG37312            transcript:Zm00001d034528_T001  3.00E-82
 48- 11: transcript:EER93250            transcript:Zm00001d034527_T001          0
 48- 12: transcript:OQU90797            transcript:Zm00001d034526_T001  2.00E-15
 48- 13: transcript:KXG37314            transcript:Zm00001d034525_T001          0
 48- 14: transcript:KXG37316            transcript:Zm00001d034524_T002  2.00E-58
 48- 15: transcript:EER93253            transcript:Zm00001d034523_T001          0
 48- 16: transcript:EER90695            transcript:Zm00001d034522_T003          0
 48- 17: transcript:OQU90799            transcript:Zm00001d034520_T003  8.00E-54
 48- 18: transcript:EER90699            transcript:Zm00001d034519_T001          0
 48- 19: transcript:KXG37324            transcript:Zm00001d034518_T001          0
 48- 20: transcript:KXG37326            transcript:Zm00001d034517_T001          0
 48- 21: transcript:EER93257            transcript:Zm00001d034516_T001          0
 48- 22: transcript:EER90701            transcript:Zm00001d034515_T002          0
 48- 23: transcript:EER93258            transcript:Zm00001d034514_T001 3.00E-131
 48- 24: transcript:EER93260            transcript:Zm00001d034513_T001 4.00E-105
 48- 25: transcript:EER93261            transcript:Zm00001d034512_T002 4.00E-114
 48- 26: transcript:EER90705            transcript:Zm00001d034511_T001          0
 48- 27: transcript:OQU90807            transcript:Zm00001d034510_T001          0
 48- 28: transcript:OQU90810            transcript:Zm00001d034509_T002          0
 48- 29: transcript:EER90708            transcript:Zm00001d034508_T001          0
 48- 30: transcript:OQU90814            transcript:Zm00001d034506_T001  7.00E-17
 48- 31: transcript:OQU90815            transcript:Zm00001d034505_T001 8.00E-119
 48- 32: transcript:EER90712            transcript:Zm00001d034503_T001 1.00E-159
 48- 33: transcript:KXG37334            transcript:Zm00001d034502_T003          0
 48- 34: transcript:EER93263            transcript:Zm00001d034501_T001          0
 48- 35: transcript:EER90715            transcript:Zm00001d034498_T007          0
 48- 36: transcript:EER93264            transcript:Zm00001d034497_T001  2.00E-16
 48- 37: transcript:OQU90818            transcript:Zm00001d034496_T001          0
 48- 38: transcript:EER93266            transcript:Zm00001d034495_T001          0
 48- 39: transcript:OQU90821            transcript:Zm00001d034494_T001          0
 48- 40: transcript:KXG37340            transcript:Zm00001d034493_T001          0
 48- 41: transcript:EER93269            transcript:Zm00001d034492_T006          0
 48- 42: transcript:EER90722            transcript:Zm00001d034491_T001  2.00E-87
## Alignment 49: score=1895.0 e_value=2.1e-174 N=47 1&1 minus
```

```
 49-  0: transcript:Zm00001d027572_T006 transcript:EER95550                   0
 49-  1: transcript:Zm00001d027573_T001 transcript:KXG40228                   0
 49-  2: transcript:Zm00001d027578_T001 transcript:EER95548          7.00E-149
 49-  3: transcript:Zm00001d027581_T001 transcript:OQU93275          6.00E-135
 49-  4: transcript:Zm00001d027582_T001 transcript:OQU93276                   0
 49-  5: transcript:Zm00001d027585_T001 transcript:EER95545          3.00E-122
 49-  6: transcript:Zm00001d027587_T001 transcript:EER95543          4.00E-176
 49-  7: transcript:Zm00001d027588_T003 transcript:KXG40223           1.00E-49
 49-  8: transcript:Zm00001d027589_T001 transcript:OQU93273                   0
 49-  9: transcript:Zm00001d027590_T001 transcript:EER92892                   0
 49- 10: transcript:Zm00001d027593_T001 transcript:OQU93272                   0
 49- 11: transcript:Zm00001d027595_T001 transcript:EER91503           1.00E-22
 49- 12: transcript:Zm00001d027596_T009 transcript:KXG40218                   0
 49- 13: transcript:Zm00001d027597_T001 transcript:OQU93267          7.00E-117
 49- 14: transcript:Zm00001d027598_T001 transcript:OQU93266          6.00E-153
 49- 15: transcript:Zm00001d027604_T001 transcript:EER95538           7.00E-43
 49- 16: transcript:Zm00001d027605_T001 transcript:OQU93265           6.00E-24
 49- 17: transcript:Zm00001d027607_T001 transcript:EER92884          3.00E-166
 49- 18: transcript:Zm00001d027610_T001 transcript:OQU93263                   0
 49- 19: transcript:Zm00001d027611_T001 transcript:KXG40213                   0
 49- 20: transcript:Zm00001d027612_T007 transcript:EER92882                   0
 49- 21: transcript:Zm00001d027613_T002 transcript:EER92881          6.00E-172
 49- 22: transcript:Zm00001d027616_T001 transcript:OQU93260          9.00E-145
 49- 23: transcript:Zm00001d027618_T001 transcript:EER95532          4.00E-115
 49- 24: transcript:Zm00001d027619_T002 transcript:EER95531                   0
 49- 25: transcript:Zm00001d027620_T001 transcript:OQU93259          4.00E-159
 49- 26: transcript:Zm00001d027622_T001 transcript:OQU93257                   0
 49- 27: transcript:Zm00001d027623_T001 transcript:EER92876          1.00E-120
 49- 28: transcript:Zm00001d027625_T001 transcript:OQU93255                   0
 49- 29: transcript:Zm00001d027626_T004 transcript:EER92872                   0
 49- 30: transcript:Zm00001d027627_T001 transcript:OQU93252                   0
 49- 31: transcript:Zm00001d027628_T001 transcript:OQU93251           7.00E-47
 49- 32: transcript:Zm00001d027630_T002 transcript:KXG40198                   0
 49- 33: transcript:Zm00001d027633_T011 transcript:OQU93248                   0
 49- 34: transcript:Zm00001d027636_T001 transcript:EER95520                   0
 49- 35: transcript:Zm00001d027637_T002 transcript:KXG40196                   0
 49- 36: transcript:Zm00001d027638_T001 transcript:KXG40193                   0
 49- 37: transcript:Zm00001d027642_T001 transcript:KXG40192          8.00E-176
 49- 38: transcript:Zm00001d027645_T001 transcript:EER92863                   0
 49- 39: transcript:Zm00001d027646_T001 transcript:EER95514                   0
 49- 40: transcript:Zm00001d027647_T001 transcript:EER92861                   0
 49- 41: transcript:Zm00001d027648_T001 transcript:EER92859          5.00E-142
 49- 42: transcript:Zm00001d027649_T003 transcript:EER95510           2.00E-79
 49- 43: transcript:Zm00001d027652_T001 transcript:EER92857          1.00E-158
 49- 44: transcript:Zm00001d027655_T001 transcript:KXG40187           7.00E-44
 49- 45: transcript:Zm00001d027656_T001 transcript:EER95507                   0
 49- 46: transcript:Zm00001d027659_T001 transcript:OQU93236          5.00E-157
## Alignment 50: score=1883.0 e_value=2.5e-152 N=42 1&1 minus
 50-  0: transcript:Zm00001d028668_T001 transcript:EER95073                   0
 50-  1: transcript:Zm00001d028670_T001 transcript:KXG39655          6.00E-157
 50-  2: transcript:Zm00001d028671_T002 transcript:EER92449                   0
 50-  3: transcript:Zm00001d028675_T001 transcript:EER95072           2.00E-50
 50-  4: transcript:Zm00001d028676_T002 transcript:EER95070          5.00E-128
 50-  5: transcript:Zm00001d028679_T002 transcript:EER95067                   0
```

50- 6: transcript:Zm00001d028680_T003 transcript:EER95066 0
50- 7: transcript:Zm00001d028685_T001 transcript:KXG39648 7.00E-138
50- 8: transcript:Zm00001d028686_T001 transcript:EER95065 0
50- 9: transcript:Zm00001d028687_T001 transcript:EER92445 0
50- 10: transcript:Zm00001d028689_T001 transcript:EER95064 0
50- 11: transcript:Zm00001d028690_T007 transcript:KXG39644 0
50- 12: transcript:Zm00001d028691_T004 transcript:EER92441 8.00E-137
50- 13: transcript:Zm00001d028692_T004 transcript:EER92440 7.00E-102
50- 14: transcript:Zm00001d028695_T002 transcript:EER95058 3.00E-71
50- 15: transcript:Zm00001d028696_T001 transcript:EER92438 6.00E-129
50- 16: transcript:Zm00001d028697_T001 transcript:EER92437 0
50- 17: transcript:Zm00001d028698_T002 transcript:EER92436 9.00E-174
50- 18: transcript:Zm00001d028699_T026 transcript:OQU92785 0
50- 19: transcript:Zm00001d028701_T001 transcript:KXG39633 0
50- 20: transcript:Zm00001d028702_T001 transcript:EER92433 4.00E-151
50- 21: transcript:Zm00001d028704_T003 transcript:EER95054 1.00E-160
50- 22: transcript:Zm00001d028705_T001 transcript:EER95053 0
50- 23: transcript:Zm00001d028707_T003 transcript:KXG39630 4.00E-53
50- 24: transcript:Zm00001d028709_T002 transcript:EER95051 2.00E-169
50- 25: transcript:Zm00001d028711_T006 transcript:KXG39626 0
50- 26: transcript:Zm00001d028712_T001 transcript:EER95050 0
50- 27: transcript:Zm00001d028713_T001 transcript:KXG39624 0
50- 28: transcript:Zm00001d028714_T001 transcript:EER95048 3.00E-155
50- 29: transcript:Zm00001d028715_T001 transcript:EER95047 0
50- 30: transcript:Zm00001d028718_T001 transcript:EER92424 4.00E-30
50- 31: transcript:Zm00001d028720_T001 transcript:OQU92781 1.00E-162
50- 32: transcript:Zm00001d028721_T001 transcript:EER92422 6.00E-118
50- 33: transcript:Zm00001d028725_T001 transcript:EER95046 0
50- 34: transcript:Zm00001d028726_T001 transcript:EER92421 9.00E-98
50- 35: transcript:Zm00001d028727_T001 transcript:EER95045 0
50- 36: transcript:Zm00001d028728_T001 transcript:OQU92778 0
50- 37: transcript:Zm00001d028730_T003 transcript:OQU92777 0
50- 38: transcript:Zm00001d028731_T001 transcript:OQU92775 3.00E-154
50- 39: transcript:Zm00001d028733_T002 transcript:KXG39614 0
50- 40: transcript:Zm00001d028736_T001 transcript:KXG39609 3.00E-178
50- 41: transcript:Zm00001d028742_T001 transcript:EER92416 0
Alignment 51: score=1817.0 e_value=4.1e-137 N=39 1&1 minus
51- 0: transcript:EER93615 transcript:Zm00001d033616_T016 0
51- 1: transcript:EER91037 transcript:Zm00001d033615_T002 0
51- 2: transcript:KXG37789 transcript:Zm00001d033612_T001 1.00E-75
51- 3: transcript:OQU91156 transcript:Zm00001d033611_T002 0
51- 4: transcript:EER93617 transcript:Zm00001d033610_T001 0
51- 5: transcript:OQU91159 transcript:Zm00001d033607_T001 2.00E-11
51- 6: transcript:EER93618 transcript:Zm00001d033606_T001 7.00E-86
51- 7: transcript:EER93620 transcript:Zm00001d033605_T001 2.00E-131
51- 8: transcript:KXG37797 transcript:Zm00001d033602_T002 3.00E-150
51- 9: transcript:EER93624 transcript:Zm00001d033600_T003 2.00E-118
51- 10: transcript:KXG37801 transcript:Zm00001d033597_T004 0
51- 11: transcript:EER91045 transcript:Zm00001d033596_T001 2.00E-148
51- 12: transcript:EER93625 transcript:Zm00001d033595_T001 5.00E-63
51- 13: transcript:EER93626 transcript:Zm00001d033594_T001 0
51- 14: transcript:EER91047 transcript:Zm00001d033592_T001 0
51- 15: transcript:KXG37805 transcript:Zm00001d033591_T001 0
51- 16: transcript:EER93630 transcript:Zm00001d033590_T001 2.00E-75

```
 51- 17: transcript:OQU91164            transcript:Zm00001d033589_T001  5.00E-53
 51- 18: transcript:EER91049            transcript:Zm00001d033586_T001 2.00E-109
 51- 19: transcript:EER93631            transcript:Zm00001d033585_T002         0
 51- 20: transcript:EER91050            transcript:Zm00001d033583_T001 3.00E-149
 51- 21: transcript:EER93634            transcript:Zm00001d033580_T001         0
 51- 22: transcript:EER91051            transcript:Zm00001d033579_T002  2.00E-90
 51- 23: transcript:EER93635            transcript:Zm00001d033578_T001 3.00E-175
 51- 24: transcript:EER91052            transcript:Zm00001d033575_T002         0
 51- 25: transcript:KXG37808            transcript:Zm00001d033573_T001         0
 51- 26: transcript:EER91056            transcript:Zm00001d033572_T004         0
 51- 27: transcript:KXG37809            transcript:Zm00001d033571_T001  6.00E-68
 51- 28: transcript:KXG37810            transcript:Zm00001d033569_T001  1.00E-28
 51- 29: transcript:KXG37813            transcript:Zm00001d033568_T001         0
 51- 30: transcript:EER93638            transcript:Zm00001d033567_T001         0
 51- 31: transcript:EER93639            transcript:Zm00001d033563_T001         0
 51- 32: transcript:EER93640            transcript:Zm00001d033559_T001  3.00E-50
 51- 33: transcript:KXG37815            transcript:Zm00001d033558_T001         0
 51- 34: transcript:EER93641            transcript:Zm00001d033557_T001         0
 51- 35: transcript:OQU91172            transcript:Zm00001d033556_T007         0
 51- 36: transcript:EER91060            transcript:Zm00001d033555_T002 3.00E-158
 51- 37: transcript:EER93643            transcript:Zm00001d033554_T002 7.00E-156
 51- 38: transcript:OQU91173            transcript:Zm00001d033553_T073         0
## Alignment 52: score=1726.0 e_value=3.3e-132 N=38 1&1 minus
 52-  0: transcript:OQU90997            transcript:Zm00001d034004_T004         0
 52-  1: transcript:OQU90998            transcript:Zm00001d034002_T001  1.00E-19
 52-  2: transcript:OQU90999            transcript:Zm00001d034001_T001         0
 52-  3: transcript:EER90880            transcript:Zm00001d034000_T005         0
 52-  4: transcript:EER93464            transcript:Zm00001d033999_T001         0
 52-  5: transcript:EER93465            transcript:Zm00001d033998_T001  1.00E-38
 52-  6: transcript:EER90883            transcript:Zm00001d033995_T002 3.00E-117
 52-  7: transcript:KXG37566            transcript:Zm00001d033994_T002         0
 52-  8: transcript:KXG37567            transcript:Zm00001d033993_T002         0
 52-  9: transcript:KXG37569            transcript:Zm00001d033992_T009         0
 52- 10: transcript:EER93470            transcript:Zm00001d033991_T001         0
 52- 11: transcript:EER93471            transcript:Zm00001d033990_T001 2.00E-175
 52- 12: transcript:EER90887            transcript:Zm00001d033989_T001         0
 52- 13: transcript:KXG37575            transcript:Zm00001d033988_T001         0
 52- 14: transcript:EER90889            transcript:Zm00001d033987_T001         0
 52- 15: transcript:EER90890            transcript:Zm00001d033986_T003 2.00E-154
 52- 16: transcript:KXG37578            transcript:Zm00001d033985_T001         0
 52- 17: transcript:KXG37582            transcript:Zm00001d033984_T002         0
 52- 18: transcript:KXG37583            transcript:Zm00001d033983_T001 2.00E-131
 52- 19: transcript:KXG37585            transcript:Zm00001d033982_T001         0
 52- 20: transcript:EER90894            transcript:Zm00001d033981_T001         0
 52- 21: transcript:KXG37587            transcript:Zm00001d033980_T001  7.00E-78
 52- 22: transcript:KXG37588            transcript:Zm00001d033979_T001  1.00E-93
 52- 23: transcript:KXG37589            transcript:Zm00001d033977_T001         0
 52- 24: transcript:EER90896            transcript:Zm00001d033976_T002 4.00E-162
 52- 25: transcript:EER93475            transcript:Zm00001d033975_T002         0
 52- 26: transcript:EER90897            transcript:Zm00001d033969_T002         0
 52- 27: transcript:EER93476            transcript:Zm00001d033968_T001  8.00E-79
 52- 28: transcript:KXG37592            transcript:Zm00001d033967_T001         0
 52- 29: transcript:EER93477            transcript:Zm00001d033966_T001 7.00E-165
 52- 30: transcript:EER93478            transcript:Zm00001d033965_T001         0
```

52- 31: transcript:EER93480 transcript:Zm00001d033957_T002 7.00E-120
52- 32: transcript:EER93482 transcript:Zm00001d033955_T001 3.00E-172
52- 33: transcript:EER93483 transcript:Zm00001d033954_T001 0
52- 34: transcript:EER90901 transcript:Zm00001d033951_T001 9.00E-73
52- 35: transcript:KXG37596 transcript:Zm00001d033943_T001 2.00E-70
52- 36: transcript:KXG37598 transcript:Zm00001d033942_T001 9.00E-78
52- 37: transcript:OQU91026 transcript:Zm00001d033941_T001 0
Alignment 53: score=1657.0 e_value=6.1e-138 N=39 1&1 minus
53- 0: transcript:Zm00001d027808_T001 transcript:KXG40114 9.00E-157
53- 1: transcript:Zm00001d027809_T001 transcript:EER92802 0
53- 2: transcript:Zm00001d027810_T001 transcript:KXG40111 0
53- 3: transcript:Zm00001d027811_T001 transcript:KXG40109 0
53- 4: transcript:Zm00001d027812_T001 transcript:EER95447 2.00E-99
53- 5: transcript:Zm00001d027813_T002 transcript:OQU93167 3.00E-43
53- 6: transcript:Zm00001d027815_T002 transcript:OQU93166 0
53- 7: transcript:Zm00001d027816_T001 transcript:EER95445 0
53- 8: transcript:Zm00001d027819_T001 transcript:KXG40104 3.00E-26
53- 9: transcript:Zm00001d027824_T001 transcript:OQU93163 1.00E-62
53- 10: transcript:Zm00001d027826_T001 transcript:EER95434 0
53- 11: transcript:Zm00001d027827_T001 transcript:OQU93161 0
53- 12: transcript:Zm00001d027831_T001 transcript:EER95441 7.00E-20
53- 13: transcript:Zm00001d027832_T001 transcript:EER95431 5.00E-136
53- 14: transcript:Zm00001d027833_T001 transcript:EER92797 1.00E-90
53- 15: transcript:Zm00001d027835_T001 transcript:KXG40098 0
53- 16: transcript:Zm00001d027837_T001 transcript:EER92794 0
53- 17: transcript:Zm00001d027838_T001 transcript:EER92793 0
53- 18: transcript:Zm00001d027839_T001 transcript:EER95435 4.00E-50
53- 19: transcript:Zm00001d027841_T002 transcript:EER92786 1.00E-169
53- 20: transcript:Zm00001d027842_T003 transcript:EER92792 2.00E-166
53- 21: transcript:Zm00001d027843_T001 transcript:EER95433 0
53- 22: transcript:Zm00001d027844_T001 transcript:EER92791 7.00E-120
53- 23: transcript:Zm00001d027845_T001 transcript:EER95430 0
53- 24: transcript:Zm00001d027846_T001 transcript:EER95426 0
53- 25: transcript:Zm00001d027847_T001 transcript:OQU93153 1.00E-83
53- 26: transcript:Zm00001d027848_T002 transcript:KXG40087 0
53- 27: transcript:Zm00001d027851_T001 transcript:EER92785 7.00E-59
53- 28: transcript:Zm00001d027852_T001 transcript:EER92780 0
53- 29: transcript:Zm00001d027853_T001 transcript:EER95421 2.00E-171
53- 30: transcript:Zm00001d027854_T006 transcript:EER92779 0
53- 31: transcript:Zm00001d027855_T001 transcript:OQU93146 1.00E-47
53- 32: transcript:Zm00001d027856_T001 transcript:KXG40080 0
53- 33: transcript:Zm00001d027859_T001 transcript:EER95420 1.00E-54
53- 34: transcript:Zm00001d027861_T001 transcript:KXG40078 0
53- 35: transcript:Zm00001d027862_T001 transcript:EER92777 0
53- 36: transcript:Zm00001d027863_T001 transcript:EER92773 0
53- 37: transcript:Zm00001d027864_T001 transcript:EER92770 1.00E-11
53- 38: transcript:Zm00001d027868_T002 transcript:KXG40075 2.00E-174
Alignment 54: score=1611.0 e_value=3.7e-123 N=35 1&1 minus
54- 0: transcript:KXG37218 transcript:Zm00001d034722_T001 1.00E-34
54- 1: transcript:KXG37220 transcript:Zm00001d034721_T002 0
54- 2: transcript:EER90596 transcript:Zm00001d034719_T002 0
54- 3: transcript:EER93162 transcript:Zm00001d034718_T001 0
54- 4: transcript:EER93163 transcript:Zm00001d034717_T002 1.00E-149
54- 5: transcript:EER93165 transcript:Zm00001d034715_T001 5.00E-134

54- 6: transcript:EER93166 transcript:Zm00001d034714_T001 0
54- 7: transcript:KXG37226 transcript:Zm00001d034713_T002 0
54- 8: transcript:EER93167 transcript:Zm00001d034710_T003 4.00E-177
54- 9: transcript:KXG37228 transcript:Zm00001d034706_T001 0
54- 10: transcript:KXG37229 transcript:Zm00001d034705_T001 1.00E-48
54- 11: transcript:KXG37230 transcript:Zm00001d034703_T001 2.00E-112
54- 12: transcript:OQU90696 transcript:Zm00001d034702_T003 6.00E-117
54- 13: transcript:EER93170 transcript:Zm00001d034701_T023 0
54- 14: transcript:EER90605 transcript:Zm00001d034700_T001 0
54- 15: transcript:EER93171 transcript:Zm00001d034699_T001 0
54- 16: transcript:EER90606 transcript:Zm00001d034698_T001 7.00E-56
54- 17: transcript:EER93172 transcript:Zm00001d034689_T010 0
54- 18: transcript:OQU90701 transcript:Zm00001d034686_T001 8.00E-112
54- 19: transcript:EER93175 transcript:Zm00001d034682_T001 5.00E-178
54- 20: transcript:OQU90703 transcript:Zm00001d034681_T001 3.00E-15
54- 21: transcript:EER90611 transcript:Zm00001d034680_T001 7.00E-46
54- 22: transcript:EER90613 transcript:Zm00001d034678_T001 0
54- 23: transcript:EER90614 transcript:Zm00001d034677_T001 6.00E-68
54- 24: transcript:KXG37238 transcript:Zm00001d034675_T002 1.00E-157
54- 25: transcript:EER90615 transcript:Zm00001d034674_T001 0
54- 26: transcript:KXG37239 transcript:Zm00001d034673_T001 1.00E-59
54- 27: transcript:KXG37240 transcript:Zm00001d034671_T001 0
54- 28: transcript:OQU90709 transcript:Zm00001d034669_T001 5.00E-84
54- 29: transcript:EER93178 transcript:Zm00001d034668_T001 1.00E-148
54- 30: transcript:KXG37246 transcript:Zm00001d034667_T003 0
54- 31: transcript:OQU90713 transcript:Zm00001d034666_T001 6.00E-130
54- 32: transcript:KXG37247 transcript:Zm00001d034665_T001 0
54- 33: transcript:KXG37250 transcript:Zm00001d034664_T001 0
54- 34: transcript:EER90621 transcript:Zm00001d034663_T001 5.00E-171
Alignment 55: score=1530.0 e_value=3.8e-121 N=34 1&1 minus
55- 0: transcript:EER94455 transcript:Zm00001d030023_T001 9.00E-153
55- 1: transcript:EER94456 transcript:Zm00001d030021_T004 0
55- 2: transcript:EER94457 transcript:Zm00001d030020_T001 0
55- 3: transcript:EER94458 transcript:Zm00001d030019_T001 2.00E-122
55- 4: transcript:OQU92106 transcript:Zm00001d030018_T001 1.00E-65
55- 5: transcript:EER94459 transcript:Zm00001d030016_T001 0
55- 6: transcript:KXG38876 transcript:Zm00001d030014_T006 0
55- 7: transcript:KXG38880 transcript:Zm00001d030012_T001 0
55- 8: transcript:EER91898 transcript:Zm00001d030011_T001 0
55- 9: transcript:EER94461 transcript:Zm00001d030010_T001 1.00E-126
55- 10: transcript:OQU92108 transcript:Zm00001d030009_T001 2.00E-178
55- 11: transcript:OQU92111 transcript:Zm00001d030007_T001 0
55- 12: transcript:OQU92117 transcript:Zm00001d030005_T002 0
55- 13: transcript:EER91903 transcript:Zm00001d030004_T001 7.00E-97
55- 14: transcript:EER94465 transcript:Zm00001d030002_T002 4.00E-86
55- 15: transcript:EER94466 transcript:Zm00001d029997_T003 2.00E-81
55- 16: transcript:EER94467 transcript:Zm00001d029996_T001 8.00E-155
55- 17: transcript:KXG38894 transcript:Zm00001d029988_T001 9.00E-161
55- 18: transcript:EER91913 transcript:Zm00001d029983_T001 0
55- 19: transcript:KXG38897 transcript:Zm00001d029981_T001 5.00E-168
55- 20: transcript:EER94468 transcript:Zm00001d029980_T008 0
55- 21: transcript:EER94469 transcript:Zm00001d029979_T001 0
55- 22: transcript:EER91910 transcript:Zm00001d029978_T001 3.00E-149
55- 23: transcript:EER94470 transcript:Zm00001d029976_T006 6.00E-141

```
 55- 24: transcript:KXG38898            transcript:Zm00001d029975_T003          0
 55- 25: transcript:EER94471            transcript:Zm00001d029974_T001 1.00E-177
 55- 26: transcript:EER94472            transcript:Zm00001d029973_T001          0
 55- 27: transcript:EER94473            transcript:Zm00001d029970_T001 3.00E-122
 55- 28: transcript:EER91918            transcript:Zm00001d029969_T001 1.00E-134
 55- 29: transcript:EER91919            transcript:Zm00001d029968_T001 5.00E-105
 55- 30: transcript:EER94474            transcript:Zm00001d029965_T001 2.00E-156
 55- 31: transcript:KXG38901            transcript:Zm00001d029964_T001 4.00E-172
 55- 32: transcript:EER94477            transcript:Zm00001d029963_T001 2.00E-143
 55- 33: transcript:EER91920            transcript:Zm00001d029955_T001          0
## Alignment 56: score=1512.0 e_value=3e-114 N=34 1&1 minus
 56-  0: transcript:KXG37182            transcript:Zm00001d034771_T008  2.00E-18
 56-  1: transcript:EER93135            transcript:Zm00001d034770_T017          0
 56-  2: transcript:KXG37189            transcript:Zm00001d034768_T002 3.00E-126
 56-  3: transcript:EER90564            transcript:Zm00001d034761_T002          0
 56-  4: transcript:EER93144            transcript:Zm00001d034760_T001 5.00E-102
 56-  5: transcript:KXG37190            transcript:Zm00001d034759_T001          0
 56-  6: transcript:EER90565            transcript:Zm00001d034758_T003          0
 56-  7: transcript:KXG37193            transcript:Zm00001d034757_T008 9.00E-127
 56-  8: transcript:EER90566            transcript:Zm00001d034756_T001  1.00E-35
 56-  9: transcript:OQU90673            transcript:Zm00001d034755_T001          0
 56- 10: transcript:EER93146            transcript:Zm00001d034754_T001          0
 56- 11: transcript:KXG37195            transcript:Zm00001d034753_T001  2.00E-39
 56- 12: transcript:OQU90677            transcript:Zm00001d034752_T006 8.00E-146
 56- 13: transcript:KXG37198            transcript:Zm00001d034751_T001  9.00E-73
 56- 14: transcript:EER90567            transcript:Zm00001d034750_T004          0
 56- 15: transcript:KXG37199            transcript:Zm00001d034749_T001 5.00E-119
 56- 16: transcript:EER90570            transcript:Zm00001d034747_T001  5.00E-92
 56- 17: transcript:EER90571            transcript:Zm00001d034746_T001  3.00E-92
 56- 18: transcript:EER90573            transcript:Zm00001d034745_T001  1.00E-85
 56- 19: transcript:EER90577            transcript:Zm00001d034741_T001 4.00E-113
 56- 20: transcript:EER90578            transcript:Zm00001d034740_T001  3.00E-76
 56- 21: transcript:EER93154            transcript:Zm00001d034739_T001 2.00E-166
 56- 22: transcript:EER90579            transcript:Zm00001d034738_T001          0
 56- 23: transcript:EER90581            transcript:Zm00001d034734_T007          0
 56- 24: transcript:EER90582            transcript:Zm00001d034733_T002          0
 56- 25: transcript:KXG37209            transcript:Zm00001d034732_T001          0
 56- 26: transcript:EER90584            transcript:Zm00001d034731_T001 7.00E-172
 56- 27: transcript:EER93157            transcript:Zm00001d034730_T002          0
 56- 28: transcript:EER90586            transcript:Zm00001d034729_T002          0
 56- 29: transcript:EER90587            transcript:Zm00001d034727_T003          0
 56- 30: transcript:KXG37214            transcript:Zm00001d034726_T001 2.00E-134
 56- 31: transcript:EER90590            transcript:Zm00001d034725_T001          0
 56- 32: transcript:EER90592            transcript:Zm00001d034724_T001 3.00E-107
 56- 33: transcript:OQU90687            transcript:Zm00001d034723_T001          0
## Alignment 57: score=1506.0 e_value=2.3e-128 N=35 1&1 minus
 57-  0: transcript:KXG38076            transcript:Zm00001d033139_T001  2.00E-82
 57-  1: transcript:EER93860            transcript:Zm00001d033138_T001          0
 57-  2: transcript:EER93861            transcript:Zm00001d033132_T001          0
 57-  3: transcript:EER91272            transcript:Zm00001d033130_T004          0
 57-  4: transcript:EER91273            transcript:Zm00001d033129_T001  4.00E-44
 57-  5: transcript:EER93866            transcript:Zm00001d033128_T001 7.00E-163
 57-  6: transcript:EER91274            transcript:Zm00001d033112_T001          0
 57-  7: transcript:EER93868            transcript:Zm00001d033111_T003          0
```

```
 57-  8: transcript:KXG38083           transcript:Zm00001d033110_T001          0
 57-  9: transcript:EER93867           transcript:Zm00001d033109_T003          0
 57- 10: transcript:EER91275           transcript:Zm00001d033108_T007          0
 57- 11: transcript:KXG38088           transcript:Zm00001d033105_T001  8.00E-79
 57- 12: transcript:OQU91425           transcript:Zm00001d033104_T003          0
 57- 13: transcript:KXG38091           transcript:Zm00001d033099_T001  2.00E-76
 57- 14: transcript:EER93872           transcript:Zm00001d033098_T001 4.00E-169
 57- 15: transcript:OQU91426           transcript:Zm00001d033094_T001          0
 57- 16: transcript:EER91281           transcript:Zm00001d033093_T001 1.00E-110
 57- 17: transcript:EER93873           transcript:Zm00001d033092_T001          0
 57- 18: transcript:EER93874           transcript:Zm00001d033091_T001          0
 57- 19: transcript:OQU91444           transcript:Zm00001d033090_T002 1.00E-131
 57- 20: transcript:OQU91446           transcript:Zm00001d033088_T001  1.00E-43
 57- 21: transcript:KXG38108           transcript:Zm00001d033087_T001          0
 57- 22: transcript:KXG38109           transcript:Zm00001d033084_T001  5.00E-33
 57- 23: transcript:OQU91447           transcript:Zm00001d033082_T001 5.00E-170
 57- 24: transcript:EER91295           transcript:Zm00001d033080_T001          0
 57- 25: transcript:KXG38115           transcript:Zm00001d033079_T001 1.00E-129
 57- 26: transcript:EER91306           transcript:Zm00001d033077_T001          0
 57- 27: transcript:KXG38121           transcript:Zm00001d033075_T001          0
 57- 28: transcript:KXG38122           transcript:Zm00001d033074_T001          0
 57- 29: transcript:EER91312           transcript:Zm00001d033066_T001          0
 57- 30: transcript:EER91315           transcript:Zm00001d033065_T001  5.00E-37
 57- 31: transcript:EER91316           transcript:Zm00001d033064_T001  1.00E-55
 57- 32: transcript:KXG38134           transcript:Zm00001d033062_T001          0
 57- 33: transcript:OQU91467           transcript:Zm00001d033060_T001          0
 57- 34: transcript:EER91323           transcript:Zm00001d033058_T002          0
## Alignment 58: score=1489.0 e_value=2.1e-110 N=33 1&1 minus
 58-  0: transcript:Zm00001d028819_T001 transcript:EER92394                  0
 58-  1: transcript:Zm00001d028820_T001 transcript:KXG39549           1.00E-79
 58-  2: transcript:Zm00001d028821_T001 transcript:OQU92740                  0
 58-  3: transcript:Zm00001d028825_T002 transcript:EER95001                  0
 58-  4: transcript:Zm00001d028826_T001 transcript:EER95000                  0
 58-  5: transcript:Zm00001d028827_T006 transcript:KXG39547                  0
 58-  6: transcript:Zm00001d028828_T001 transcript:OQU92737                  0
 58-  7: transcript:Zm00001d028829_T001 transcript:OQU92735                  0
 58-  8: transcript:Zm00001d028830_T013 transcript:EER94990                  0
 58-  9: transcript:Zm00001d028834_T001 transcript:EER94995          1.00E-155
 58- 10: transcript:Zm00001d028837_T001 transcript:OQU92732           5.00E-58
 58- 11: transcript:Zm00001d028838_T001 transcript:KXG39539           3.00E-89
 58- 12: transcript:Zm00001d028840_T002 transcript:EER94989                  0
 58- 13: transcript:Zm00001d028841_T001 transcript:EER94986          9.00E-165
 58- 14: transcript:Zm00001d028842_T001 transcript:OQU92728           3.00E-83
 58- 15: transcript:Zm00001d028862_T001 transcript:OQU92725           2.00E-27
 58- 16: transcript:Zm00001d028863_T001 transcript:KXG39536                  0
 58- 17: transcript:Zm00001d028866_T001 transcript:EER92383                  0
 58- 18: transcript:Zm00001d028867_T001 transcript:EER92382           1.00E-37
 58- 19: transcript:Zm00001d028868_T002 transcript:EER92380          8.00E-165
 58- 20: transcript:Zm00001d028870_T001 transcript:EER92379           1.00E-80
 58- 21: transcript:Zm00001d028873_T001 transcript:KXG39534          2.00E-110
 58- 22: transcript:Zm00001d028874_T001 transcript:EER92376                  0
 58- 23: transcript:Zm00001d028875_T001 transcript:KXG39532                  0
 58- 24: transcript:Zm00001d028879_T001 transcript:EER94981           2.00E-82
 58- 25: transcript:Zm00001d028880_T001 transcript:EER94980           8.00E-67
```

58- 26: transcript:Zm00001d028885_T001 transcript:KXG39530 5.00E-147
58- 27: transcript:Zm00001d028887_T001 transcript:KXG39529 0
58- 28: transcript:Zm00001d028889_T001 transcript:EER94977 0
58- 29: transcript:Zm00001d028890_T002 transcript:KXG40039 0
58- 30: transcript:Zm00001d028894_T001 transcript:KXG39527 0
58- 31: transcript:Zm00001d028895_T001 transcript:EER92371 0
58- 32: transcript:Zm00001d028896_T006 transcript:EER92370 0
Alignment 59: score=1477.0 e_value=5.5e-108 N=33 1&1 minus
59- 0: transcript:Zm00001d028263_T001 transcript:KXG39842 0
59- 1: transcript:Zm00001d028264_T002 transcript:EER95244 0
59- 2: transcript:Zm00001d028265_T004 transcript:OQU92974 0
59- 3: transcript:Zm00001d028266_T001 transcript:EER92619 0
59- 4: transcript:Zm00001d028267_T001 transcript:EER92618 0
59- 5: transcript:Zm00001d028272_T002 transcript:EER95241 0
59- 6: transcript:Zm00001d028273_T001 transcript:EER92617 0
59- 7: transcript:Zm00001d028274_T001 transcript:OQU92973 0
59- 8: transcript:Zm00001d028275_T001 transcript:KXG39840 0
59- 9: transcript:Zm00001d028278_T001 transcript:EER95239 0
59- 10: transcript:Zm00001d028279_T001 transcript:OQU92971 0
59- 11: transcript:Zm00001d028280_T001 transcript:EER95236 0
59- 12: transcript:Zm00001d028282_T001 transcript:OQU92970 0
59- 13: transcript:Zm00001d028284_T001 transcript:EER92614 0
59- 14: transcript:Zm00001d028285_T003 transcript:EER92613 0
59- 15: transcript:Zm00001d028286_T001 transcript:OQU92969 0
59- 16: transcript:Zm00001d028287_T001 transcript:EER92612 0
59- 17: transcript:Zm00001d028293_T001 transcript:EER95232 0
59- 18: transcript:Zm00001d028294_T001 transcript:EER95229 0
59- 19: transcript:Zm00001d028296_T001 transcript:EER95223 0
59- 20: transcript:Zm00001d028297_T001 transcript:EER95218 1.00E-172
59- 21: transcript:Zm00001d028298_T001 transcript:EER95221 5.00E-99
59- 22: transcript:Zm00001d028299_T001 transcript:EER95220 0
59- 23: transcript:Zm00001d028303_T003 transcript:OQU92960 0
59- 24: transcript:Zm00001d028304_T001 transcript:EER92607 3.00E-127
59- 25: transcript:Zm00001d028307_T001 transcript:EER92605 0
59- 26: transcript:Zm00001d028308_T001 transcript:EER95216 4.00E-14
59- 27: transcript:Zm00001d028311_T001 transcript:KXG39827 0
59- 28: transcript:Zm00001d028312_T001 transcript:KXG39824 2.00E-162
59- 29: transcript:Zm00001d028317_T001 transcript:KXG39823 0
59- 30: transcript:Zm00001d028319_T002 transcript:EER95212 0
59- 31: transcript:Zm00001d028325_T005 transcript:EER92600 0
59- 32: transcript:Zm00001d028328_T001 transcript:EER92599 5.00E-25
Alignment 60: score=1475.0 e_value=1.5e-111 N=34 1&1 minus
60- 0: transcript:EER90735 transcript:Zm00001d034460_T010 3.00E-127
60- 1: transcript:EER90736 transcript:Zm00001d034453_T001 0
60- 2: transcript:OQU90833 transcript:Zm00001d034452_T009 4.00E-59
60- 3: transcript:EER90738 transcript:Zm00001d034447_T001 6.00E-155
60- 4: transcript:EER93294 transcript:Zm00001d034446_T002 0
60- 5: transcript:EER93295 transcript:Zm00001d034444_T002 0
60- 6: transcript:KXG37365 transcript:Zm00001d034443_T001 0
60- 7: transcript:EER90739 transcript:Zm00001d034440_T001 0
60- 8: transcript:EER90740 transcript:Zm00001d034439_T001 0
60- 9: transcript:OQU90835 transcript:Zm00001d034435_T001 1.00E-49
60- 10: transcript:EER93297 transcript:Zm00001d034433_T003 2.00E-158
60- 11: transcript:EER90742 transcript:Zm00001d034431_T001 2.00E-82

```
 60- 12: transcript:EER93299          transcript:Zm00001d034429_T001 1.00E-107
 60- 13: transcript:KXG37369          transcript:Zm00001d034428_T001         0
 60- 14: transcript:EER90744          transcript:Zm00001d034427_T001         0
 60- 15: transcript:EER90745          transcript:Zm00001d034425_T001  1.00E-50
 60- 16: transcript:EER90746          transcript:Zm00001d034424_T002         0
 60- 17: transcript:EER90748          transcript:Zm00001d034422_T002  2.00E-89
 60- 18: transcript:OQU90838          transcript:Zm00001d034420_T009         0
 60- 19: transcript:OQU90843          transcript:Zm00001d034417_T001  9.00E-53
 60- 20: transcript:EER93307          transcript:Zm00001d034416_T001  5.00E-89
 60- 21: transcript:EER90749          transcript:Zm00001d034415_T001  4.00E-88
 60- 22: transcript:OQU90844          transcript:Zm00001d034413_T001         0
 60- 23: transcript:EER93309          transcript:Zm00001d034410_T001         0
 60- 24: transcript:EER93311          transcript:Zm00001d034409_T001         0
 60- 25: transcript:EER93318          transcript:Zm00001d034406_T002 3.00E-162
 60- 26: transcript:KXG37385          transcript:Zm00001d034405_T002         0
 60- 27: transcript:KXG37383          transcript:Zm00001d034404_T001  2.00E-70
 60- 28: transcript:EER90754          transcript:Zm00001d034401_T004         0
 60- 29: transcript:EER90755          transcript:Zm00001d034400_T003         0
 60- 30: transcript:KXG37386          transcript:Zm00001d034399_T001         0
 60- 31: transcript:OQU90853          transcript:Zm00001d034396_T001  5.00E-61
 60- 32: transcript:OQU90855          transcript:Zm00001d034389_T001  3.00E-42
 60- 33: transcript:EER90757          transcript:Zm00001d034388_T010         0
## Alignment 61: score=1446.0 e_value=3.9e-112 N=33 1&1 minus
 61-  0: transcript:Zm00001d027335_T001 transcript:KXG40355                 0
 61-  1: transcript:Zm00001d027337_T001 transcript:KXG40352                 0
 61-  2: transcript:Zm00001d027338_T001 transcript:EER95650          6.00E-94
 61-  3: transcript:Zm00001d027339_T001 transcript:EER92990          1.00E-89
 61-  4: transcript:Zm00001d027340_T001 transcript:OQU93388                 0
 61-  5: transcript:Zm00001d027341_T001 transcript:EER92989                 0
 61-  6: transcript:Zm00001d027342_T002 transcript:EER92988          1.00E-62
 61-  7: transcript:Zm00001d027344_T001 transcript:KXG40347                 0
 61-  8: transcript:Zm00001d027345_T001 transcript:EER92987         1.00E-113
 61-  9: transcript:Zm00001d027346_T001 transcript:EER95648          2.00E-82
 61- 10: transcript:Zm00001d027347_T002 transcript:EER92986                 0
 61- 11: transcript:Zm00001d027348_T001 transcript:EER95647                 0
 61- 12: transcript:Zm00001d027349_T001 transcript:KXG40345                 0
 61- 13: transcript:Zm00001d027350_T001 transcript:EER92984          3.00E-24
 61- 14: transcript:Zm00001d027351_T001 transcript:EER95646                 0
 61- 15: transcript:Zm00001d027352_T001 transcript:OQU93382          3.00E-76
 61- 16: transcript:Zm00001d027353_T001 transcript:OQU93381                 0
 61- 17: transcript:Zm00001d027354_T001 transcript:EER95644                 0
 61- 18: transcript:Zm00001d027355_T001 transcript:EER95642                 0
 61- 19: transcript:Zm00001d027359_T001 transcript:EER92981                 0
 61- 20: transcript:Zm00001d027361_T001 transcript:EER95641                 0
 61- 21: transcript:Zm00001d027362_T001 transcript:OQU93380                 0
 61- 22: transcript:Zm00001d027363_T001 transcript:KXG40341                 0
 61- 23: transcript:Zm00001d027365_T001 transcript:KXG40340                 0
 61- 24: transcript:Zm00001d027366_T001 transcript:EER92978         6.00E-168
 61- 25: transcript:Zm00001d027367_T002 transcript:EER95637                 0
 61- 26: transcript:Zm00001d027368_T001 transcript:EER95636         6.00E-145
 61- 27: transcript:Zm00001d027369_T002 transcript:KXG40338          9.00E-78
 61- 28: transcript:Zm00001d027370_T001 transcript:EER95633         9.00E-166
 61- 29: transcript:Zm00001d027371_T001 transcript:EER95631                 0
 61- 30: transcript:Zm00001d027373_T003 transcript:KXG40336         5.00E-165
```

```
 61- 31: transcript:Zm00001d027374_T001 transcript:EER92977          2.00E-165
 61- 32: transcript:Zm00001d027375_T001 transcript:OQU93375           8.00E-52
## Alignment 62: score=1441.0 e_value=0 N=33 1&1 minus
 62-  0: transcript:EER90848            transcript:Zm00001d034108_T006         0
 62-  1: transcript:EER93412            transcript:Zm00001d034107_T001 2.00E-157
 62-  2: transcript:OQU90942            transcript:Zm00001d034095_T001 4.00E-107
 62-  3: transcript:EER93415            transcript:Zm00001d034091_T001 6.00E-122
 62-  4: transcript:KXG37500            transcript:Zm00001d034089_T001         0
 62-  5: transcript:OQU90947            transcript:Zm00001d034087_T001  2.00E-35
 62-  6: transcript:EER90852            transcript:Zm00001d034085_T002         0
 62-  7: transcript:EER93417            transcript:Zm00001d034084_T001         0
 62-  8: transcript:KXG37501            transcript:Zm00001d034082_T001         0
 62-  9: transcript:OQU90948            transcript:Zm00001d034081_T002 1.00E-176
 62- 10: transcript:KXG37510            transcript:Zm00001d034080_T005         0
 62- 11: transcript:OQU90956            transcript:Zm00001d034076_T001         0
 62- 12: transcript:KXG37513            transcript:Zm00001d034074_T005         0
 62- 13: transcript:EER90854            transcript:Zm00001d034073_T001 2.00E-140
 62- 14: transcript:KXG37517            transcript:Zm00001d034072_T001         0
 62- 15: transcript:OQU90961            transcript:Zm00001d034066_T001 9.00E-146
 62- 16: transcript:EER93428            transcript:Zm00001d034064_T001  3.00E-59
 62- 17: transcript:EER90856            transcript:Zm00001d034063_T001         0
 62- 18: transcript:KXG37528            transcript:Zm00001d034062_T001  4.00E-98
 62- 19: transcript:EER93429            transcript:Zm00001d034059_T006 1.00E-140
 62- 20: transcript:OQU90964            transcript:Zm00001d034057_T001  3.00E-59
 62- 21: transcript:OQU90965            transcript:Zm00001d034055_T001         0
 62- 22: transcript:KXG37532            transcript:Zm00001d034054_T002         0
 62- 23: transcript:OQU90966            transcript:Zm00001d034050_T001         0
 62- 24: transcript:EER90859            transcript:Zm00001d034049_T002         0
 62- 25: transcript:EER93435            transcript:Zm00001d034048_T003  1.00E-88
 62- 26: transcript:KXG37536            transcript:Zm00001d034047_T006 2.00E-167
 62- 27: transcript:EER90862            transcript:Zm00001d034045_T003 5.00E-154
 62- 28: transcript:EER90863            transcript:Zm00001d034039_T001         0
 62- 29: transcript:EER93439            transcript:Zm00001d034038_T001         0
 62- 30: transcript:EER93441            transcript:Zm00001d034037_T001  1.00E-43
 62- 31: transcript:KXG37542            transcript:Zm00001d034036_T003         0
 62- 32: transcript:EER90865            transcript:Zm00001d034035_T002         0
## Alignment 63: score=1365.0 e_value=7.8e-106 N=32 1&1 minus
 63-  0: transcript:Zm00001d027869_T002 transcript:KXG40074                  0
 63-  1: transcript:Zm00001d027870_T001 transcript:KXG40072                  0
 63-  2: transcript:Zm00001d027871_T001 transcript:EER95407                  0
 63-  3: transcript:Zm00001d027872_T001 transcript:OQU93141                  0
 63-  4: transcript:Zm00001d027873_T001 transcript:KXG40067           1.00E-37
 63-  5: transcript:Zm00001d027874_T004 transcript:EER92762          8.00E-113
 63-  6: transcript:Zm00001d027876_T004 transcript:OQU93138           8.00E-10
 63-  7: transcript:Zm00001d027877_T001 transcript:KXG40062          4.00E-128
 63-  8: transcript:Zm00001d027878_T002 transcript:KXG40060                  0
 63-  9: transcript:Zm00001d027879_T002 transcript:EER92763          3.00E-127
 63- 10: transcript:Zm00001d027880_T002 transcript:OQU93136          5.00E-151
 63- 11: transcript:Zm00001d027881_T001 transcript:EER92760           1.00E-49
 63- 12: transcript:Zm00001d027883_T001 transcript:EER95403           2.00E-73
 63- 13: transcript:Zm00001d027884_T003 transcript:KXG40056                  0
 63- 14: transcript:Zm00001d027885_T001 transcript:EER95402          6.00E-138
 63- 15: transcript:Zm00001d027886_T002 transcript:OQU93134          1.00E-137
 63- 16: transcript:Zm00001d027887_T001 transcript:EER92755                  0
```

```
 63- 17: transcript:Zm00001d027888_T002 transcript:KXG40055                0
 63- 18: transcript:Zm00001d027890_T001 transcript:EER95401       3.00E-136
 63- 19: transcript:Zm00001d027892_T001 transcript:KXG40053                0
 63- 20: transcript:Zm00001d027893_T001 transcript:EER95400                0
 63- 21: transcript:Zm00001d027894_T001 transcript:EER92752         2.00E-67
 63- 22: transcript:Zm00001d027895_T004 transcript:KXG40051                0
 63- 23: transcript:Zm00001d027896_T001 transcript:EER92751                0
 63- 24: transcript:Zm00001d027897_T001 transcript:OQU93129         3.00E-72
 63- 25: transcript:Zm00001d027898_T002 transcript:OQU93127                0
 63- 26: transcript:Zm00001d027899_T001 transcript:KXG40045         1.00E-21
 63- 27: transcript:Zm00001d027903_T001 transcript:EER95396                0
 63- 28: transcript:Zm00001d027904_T001 transcript:EER95395                0
 63- 29: transcript:Zm00001d027907_T002 transcript:EER95393                0
 63- 30: transcript:Zm00001d027908_T001 transcript:EER95392                0
 63- 31: transcript:Zm00001d027916_T006 transcript:EER95391                0
## Alignment 64: score=1217.0 e_value=2.2e-85 N=27 1&1 minus
 64-  0: transcript:OQU91111            transcript:Zm00001d033726_T002        0
 64-  1: transcript:EER93585            transcript:Zm00001d033719_T001        0
 64-  2: transcript:EER91001            transcript:Zm00001d033718_T001 5.00E-165
 64-  3: transcript:EER91002            transcript:Zm00001d033717_T001 1.00E-120
 64-  4: transcript:EER91004            transcript:Zm00001d033716_T001 6.00E-136
 64-  5: transcript:EER93588            transcript:Zm00001d033714_T001        0
 64-  6: transcript:EER91005            transcript:Zm00001d033709_T001  2.00E-33
 64-  7: transcript:EER91008            transcript:Zm00001d033708_T001        0
 64-  8: transcript:EER91009            transcript:Zm00001d033707_T001  7.00E-96
 64-  9: transcript:EER91011            transcript:Zm00001d033706_T001 5.00E-126
 64- 10: transcript:OQU91119            transcript:Zm00001d033705_T001        0
 64- 11: transcript:KXG37746            transcript:Zm00001d033704_T007        0
 64- 12: transcript:EER93590            transcript:Zm00001d033685_T001  5.00E-44
 64- 13: transcript:EER91016            transcript:Zm00001d033684_T001        0
 64- 14: transcript:EER93591            transcript:Zm00001d033683_T001 1.00E-103
 64- 15: transcript:EER91017            transcript:Zm00001d033682_T001        0
 64- 16: transcript:EER93592            transcript:Zm00001d033680_T001        0
 64- 17: transcript:KXG37753            transcript:Zm00001d033675_T001        0
 64- 18: transcript:EER91018            transcript:Zm00001d033674_T001        0
 64- 19: transcript:EER93595            transcript:Zm00001d033673_T001 1.00E-178
 64- 20: transcript:EER93596            transcript:Zm00001d033671_T001        0
 64- 21: transcript:EER91019            transcript:Zm00001d033670_T001        0
 64- 22: transcript:OQU91126            transcript:Zm00001d033669_T003        0
 64- 23: transcript:OQU91127            transcript:Zm00001d033668_T006        0
 64- 24: transcript:EER93599            transcript:Zm00001d033666_T001 4.00E-110
 64- 25: transcript:KXG37760            transcript:Zm00001d033665_T002 6.00E-114
 64- 26: transcript:EER93600            transcript:Zm00001d033664_T001        0
## Alignment 65: score=1187.0 e_value=6.7e-81 N=26 1&1 minus
 65-  0: transcript:EER92034            transcript:Zm00001d029696_T003 4.00E-129
 65-  1: transcript:EER92037            transcript:Zm00001d029688_T002        0
 65-  2: transcript:EER92040            transcript:Zm00001d029686_T001        0
 65-  3: transcript:EER92041            transcript:Zm00001d029684_T001        0
 65-  4: transcript:EER94621            transcript:Zm00001d029683_T001        0
 65-  5: transcript:EER94622            transcript:Zm00001d029681_T001  5.00E-56
 65-  6: transcript:EER94623            transcript:Zm00001d029680_T001 1.00E-109
 65-  7: transcript:OQU92275            transcript:Zm00001d029677_T001        0
 65-  8: transcript:KXG39044            transcript:Zm00001d029676_T001  7.00E-28
 65-  9: transcript:EER94627            transcript:Zm00001d029675_T001        0
```

```
 65- 10: transcript:KXG39046           transcript:Zm00001d029674_T001          0
 65- 11: transcript:EER94629           transcript:Zm00001d029673_T001          0
 65- 12: transcript:OQU92278           transcript:Zm00001d029669_T001  1.00E-23
 65- 13: transcript:EER94630           transcript:Zm00001d029667_T001          0
 65- 14: transcript:EER92044           transcript:Zm00001d029664_T001          0
 65- 15: transcript:EER92045           transcript:Zm00001d029663_T001          0
 65- 16: transcript:EER94631           transcript:Zm00001d029662_T001          0
 65- 17: transcript:EER94633           transcript:Zm00001d029657_T001          0
 65- 18: transcript:EER92046           transcript:Zm00001d029656_T003  7.00E-99
 65- 19: transcript:OQU92283           transcript:Zm00001d029654_T003          0
 65- 20: transcript:EER92048           transcript:Zm00001d029653_T001          0
 65- 21: transcript:EER92049           transcript:Zm00001d029652_T001          0
 65- 22: transcript:OQU92286           transcript:Zm00001d029651_T001  4.00E-52
 65- 23: transcript:EER92050           transcript:Zm00001d029650_T006          0
 65- 24: transcript:EER92051           transcript:Zm00001d029649_T002          0
 65- 25: transcript:EER92053           transcript:Zm00001d029647_T001          0
## Alignment 66: score=1140.0 e_value=1.1e-77 N=25 1&1 minus
 66-  0: transcript:EER90766           transcript:Zm00001d034373_T001          0
 66-  1: transcript:EER93328           transcript:Zm00001d034372_T001          0
 66-  2: transcript:EER93329           transcript:Zm00001d034371_T002          0
 66-  3: transcript:EER93331           transcript:Zm00001d034369_T001  4.00E-87
 66-  4: transcript:KXG37403           transcript:Zm00001d034368_T001          0
 66-  5: transcript:EER90769           transcript:Zm00001d034366_T001          0
 66-  6: transcript:EER93336           transcript:Zm00001d034365_T001          0
 66-  7: transcript:EER93337           transcript:Zm00001d034364_T001          0
 66-  8: transcript:EER93338           transcript:Zm00001d034361_T001          0
 66-  9: transcript:EER90772           transcript:Zm00001d034360_T001 4.00E-177
 66- 10: transcript:EER90773           transcript:Zm00001d034359_T001  2.00E-78
 66- 11: transcript:OQU90873           transcript:Zm00001d034358_T001  4.00E-89
 66- 12: transcript:EER90776           transcript:Zm00001d034356_T001 3.00E-145
 66- 13: transcript:EER93342           transcript:Zm00001d034353_T001          0
 66- 14: transcript:KXG37410           transcript:Zm00001d034351_T001          0
 66- 15: transcript:KXG37412           transcript:Zm00001d034350_T012  5.00E-12
 66- 16: transcript:EER90780           transcript:Zm00001d034346_T002 4.00E-145
 66- 17: transcript:EER90782           transcript:Zm00001d034345_T001          0
 66- 18: transcript:KXG37420           transcript:Zm00001d034344_T001          0
 66- 19: transcript:OQU90882           transcript:Zm00001d034343_T001          0
 66- 20: transcript:EER90784           transcript:Zm00001d034341_T003 2.00E-124
 66- 21: transcript:EER90785           transcript:Zm00001d034340_T001          0
 66- 22: transcript:OQU90885           transcript:Zm00001d034339_T001  8.00E-55
 66- 23: transcript:KXG37426           transcript:Zm00001d034338_T001          0
 66- 24: transcript:KXG37427           transcript:Zm00001d034337_T005 2.00E-113
## Alignment 67: score=1012.0 e_value=6.7e-69 N=23 1&1 minus
 67-  0: transcript:Zm00001d027718_T001 transcript:OQU93208           1.00E-158
 67-  1: transcript:Zm00001d027719_T001 transcript:EER95479            7.00E-54
 67-  2: transcript:Zm00001d027720_T001 transcript:EER92834           7.00E-104
 67-  3: transcript:Zm00001d027721_T001 transcript:EER92833                   0
 67-  4: transcript:Zm00001d027722_T001 transcript:EER95478                   0
 67-  5: transcript:Zm00001d027723_T002 transcript:EER92832                   0
 67-  6: transcript:Zm00001d027726_T001 transcript:KXG40142                   0
 67-  7: transcript:Zm00001d027727_T001 transcript:EER92823                   0
 67-  8: transcript:Zm00001d027728_T001 transcript:EER95473                   0
 67-  9: transcript:Zm00001d027729_T001 transcript:KXG40141                   0
 67- 10: transcript:Zm00001d027731_T012 transcript:KXG40138                   0
```

67- 11: transcript:Zm00001d027732_T001 transcript:KXG40137 0
67- 12: transcript:Zm00001d027734_T004 transcript:EER95470 0
67- 13: transcript:Zm00001d027738_T001 transcript:OQU93195 1.00E-146
67- 14: transcript:Zm00001d027739_T001 transcript:EER92828 3.00E-57
67- 15: transcript:Zm00001d027740_T001 transcript:EER92827 1.00E-122
67- 16: transcript:Zm00001d027741_T001 transcript:OQU93192 7.00E-169
67- 17: transcript:Zm00001d027742_T001 transcript:EER92826 3.00E-78
67- 18: transcript:Zm00001d027743_T002 transcript:EER95467 0
67- 19: transcript:Zm00001d027746_T001 transcript:EER92825 2.00E-57
67- 20: transcript:Zm00001d027748_T004 transcript:EER92824 0
67- 21: transcript:Zm00001d027749_T001 transcript:EER92818 0
67- 22: transcript:Zm00001d027751_T001 transcript:KXG40133 5.00E-18
Alignment 68: score=998.0 e_value=1.5e-68 N=23 1&1 minus
68- 0: transcript:Zm00001d028225_T001 transcript:EER95269 1.00E-49
68- 1: transcript:Zm00001d028226_T001 transcript:EER92637 0
68- 2: transcript:Zm00001d028227_T009 transcript:EER95268 0
68- 3: transcript:Zm00001d028228_T001 transcript:EER92636 0
68- 4: transcript:Zm00001d028229_T001 transcript:EER95266 0
68- 5: transcript:Zm00001d028230_T001 transcript:EER92634 0
68- 6: transcript:Zm00001d028231_T003 transcript:EER95261 0
68- 7: transcript:Zm00001d028232_T012 transcript:EER95258 2.00E-66
68- 8: transcript:Zm00001d028235_T001 transcript:OQU92989 0
68- 9: transcript:Zm00001d028237_T002 transcript:EER95260 1.00E-124
68- 10: transcript:Zm00001d028238_T001 transcript:KXG39851 0
68- 11: transcript:Zm00001d028240_T001 transcript:EER95259 3.00E-158
68- 12: transcript:Zm00001d028241_T003 transcript:EER92628 0
68- 13: transcript:Zm00001d028243_T001 transcript:EER95256 0
68- 14: transcript:Zm00001d028245_T001 transcript:KXG39847 0
68- 15: transcript:Zm00001d028244_T010 transcript:EER92626 0
68- 16: transcript:Zm00001d028248_T001 transcript:EER92625 0
68- 17: transcript:Zm00001d028249_T003 transcript:EER95252 4.00E-160
68- 18: transcript:Zm00001d028256_T002 transcript:OQU92981 0
68- 19: transcript:Zm00001d028257_T004 transcript:OQU92980 7.00E-59
68- 20: transcript:Zm00001d028258_T001 transcript:EER95246 0
68- 21: transcript:Zm00001d028260_T005 transcript:KXG39844 3.00E-137
68- 22: transcript:Zm00001d028261_T005 transcript:EER92621 0
Alignment 69: score=986.0 e_value=2.7e-67 N=22 1&1 minus
69- 0: transcript:OQU91388 transcript:Zm00001d033195_T001 7.00E-29
69- 1: transcript:KXG38048 transcript:Zm00001d033194_T001 0
69- 2: transcript:EER91249 transcript:Zm00001d033193_T006 0
69- 3: transcript:EER93832 transcript:Zm00001d033192_T001 1.00E-58
69- 4: transcript:OQU91390 transcript:Zm00001d033189_T003 6.00E-10
69- 5: transcript:EER91252 transcript:Zm00001d033188_T001 0
69- 6: transcript:EER93836 transcript:Zm00001d033187_T001 0
69- 7: transcript:EER93837 transcript:Zm00001d033186_T002 3.00E-25
69- 8: transcript:EER93838 transcript:Zm00001d033181_T001 0
69- 9: transcript:EER93839 transcript:Zm00001d033180_T001 0
69- 10: transcript:EER93841 transcript:Zm00001d033175_T001 0
69- 11: transcript:EER91258 transcript:Zm00001d033174_T001 0
69- 12: transcript:OQU91404 transcript:Zm00001d033172_T009 0
69- 13: transcript:EER91259 transcript:Zm00001d033171_T001 4.00E-87
69- 14: transcript:EER93844 transcript:Zm00001d033170_T001 6.00E-82
69- 15: transcript:EER91261 transcript:Zm00001d033169_T001 6.00E-113
69- 16: transcript:EER91262 transcript:Zm00001d033168_T003 0

```
 69- 17: transcript:EER93845           transcript:Zm00001d033167_T001          0
 69- 18: transcript:EER91260           transcript:Zm00001d033166_T001          0
 69- 19: transcript:OQU91408           transcript:Zm00001d033160_T001          0
 69- 20: transcript:EER91265           transcript:Zm00001d033159_T002 2.00E-166
 69- 21: transcript:OQU91411           transcript:Zm00001d033158_T002          0
## Alignment 70: score=886.0 e_value=2e-59 N=21 1&1 minus
 70-  0: transcript:Zm00001d027506_T003 transcript:EER95577                    0
 70-  1: transcript:Zm00001d027508_T001 transcript:EER95576                    0
 70-  2: transcript:Zm00001d027509_T002 transcript:EER95575                    0
 70-  3: transcript:Zm00001d027510_T001 transcript:EER92921                    0
 70-  4: transcript:Zm00001d027511_T001 transcript:KXG40255                    0
 70-  5: transcript:Zm00001d027512_T002 transcript:EER92920                    0
 70-  6: transcript:Zm00001d027514_T002 transcript:EER92919                    0
 70-  7: transcript:Zm00001d027515_T002 transcript:OQU93308                    0
 70-  8: transcript:Zm00001d027516_T002 transcript:EER95572            3.00E-97
 70-  9: transcript:Zm00001d027517_T001 transcript:OQU93304                    0
 70- 10: transcript:Zm00001d027518_T001 transcript:OQU93303                    0
 70- 11: transcript:Zm00001d027519_T001 transcript:EER95569                    0
 70- 12: transcript:Zm00001d027520_T001 transcript:EER95568           7.00E-167
 70- 13: transcript:Zm00001d027522_T001 transcript:KXG40248           2.00E-137
 70- 14: transcript:Zm00001d027523_T001 transcript:EER95565                    0
 70- 15: transcript:Zm00001d027524_T001 transcript:EER95563           6.00E-154
 70- 16: transcript:Zm00001d027530_T001 transcript:EER92917                    0
 70- 17: transcript:Zm00001d027532_T002 transcript:OQU93301           1.00E-179
 70- 18: transcript:Zm00001d027533_T001 transcript:KXG40244                    0
 70- 19: transcript:Zm00001d027534_T001 transcript:KXG40242                    0
 70- 20: transcript:Zm00001d027535_T001 transcript:KXG40238                    0
## Alignment 71: score=866.0 e_value=8.4e-56 N=20 1&1 minus
 71-  0: transcript:Zm00001d029008_T001 transcript:EER92325                    0
 71-  1: transcript:Zm00001d029009_T003 transcript:EER92323                    0
 71-  2: transcript:Zm00001d029010_T001 transcript:EER94923                    0
 71-  3: transcript:Zm00001d029011_T001 transcript:EER92322                    0
 71-  4: transcript:Zm00001d029012_T002 transcript:KXG39452                    0
 71-  5: transcript:Zm00001d029014_T002 transcript:KXG39450                    0
 71-  6: transcript:Zm00001d029020_T001 transcript:KXG39447                    0
 71-  7: transcript:Zm00001d029023_T007 transcript:EER92321                    0
 71-  8: transcript:Zm00001d029025_T001 transcript:EER94920                    0
 71-  9: transcript:Zm00001d029027_T001 transcript:EER94919           5.00E-131
 71- 10: transcript:Zm00001d029028_T001 transcript:KXG39446           9.00E-122
 71- 11: transcript:Zm00001d029030_T002 transcript:EER94918            3.00E-48
 71- 12: transcript:Zm00001d029031_T001 transcript:EER92317                    0
 71- 13: transcript:Zm00001d029034_T002 transcript:EER94914                    0
 71- 14: transcript:Zm00001d029035_T001 transcript:OQU92656                    0
 71- 15: transcript:Zm00001d029036_T001 transcript:EER92316                    0
 71- 16: transcript:Zm00001d029038_T001 transcript:KXG39440           3.00E-157
 71- 17: transcript:Zm00001d029039_T001 transcript:KXG39438                    0
 71- 18: transcript:Zm00001d029040_T001 transcript:KXG39436                    0
 71- 19: transcript:Zm00001d029041_T002 transcript:KXG39435                    0
## Alignment 72: score=856.0 e_value=2.5e-54 N=19 1&1 minus
 72-  0: transcript:EER90644           transcript:Zm00001d034620_T001          0
 72-  1: transcript:EER93209           transcript:Zm00001d034616_T001          0
 72-  2: transcript:EER90648           transcript:Zm00001d034615_T001 3.00E-128
 72-  3: transcript:EER93211           transcript:Zm00001d034611_T001  1.00E-48
 72-  4: transcript:EER90650           transcript:Zm00001d034610_T001          0
```

```
 72-  5: transcript:OQU90753           transcript:Zm00001d034609_T001  6.00E-65
 72-  6: transcript:KXG37273           transcript:Zm00001d034608_T004 2.00E-149
 72-  7: transcript:EER90651           transcript:Zm00001d034607_T001         0
 72-  8: transcript:EER90652           transcript:Zm00001d034606_T001         0
 72-  9: transcript:EER90653           transcript:Zm00001d034605_T001 4.00E-117
 72- 10: transcript:EER93213           transcript:Zm00001d034604_T001         0
 72- 11: transcript:EER90654           transcript:Zm00001d034602_T003         0
 72- 12: transcript:EER93215           transcript:Zm00001d034601_T001         0
 72- 13: transcript:EER93216           transcript:Zm00001d034600_T001         0
 72- 14: transcript:EER93219           transcript:Zm00001d034598_T001  1.00E-91
 72- 15: transcript:EER90662           transcript:Zm00001d034597_T004         0
 72- 16: transcript:OQU90763           transcript:Zm00001d034596_T001  2.00E-94
 72- 17: transcript:EER93221           transcript:Zm00001d034595_T001  5.00E-94
 72- 18: transcript:OQU90765           transcript:Zm00001d034594_T010         0
## Alignment 73: score=778.0 e_value=6e-51 N=19 1&1 minus
 73-  0: transcript:Zm00001d027311_T001 transcript:EER95677                  0
 73-  1: transcript:Zm00001d027312_T002 transcript:OQU93429                  0
 73-  2: transcript:Zm00001d027313_T001 transcript:EER93011                  0
 73-  3: transcript:Zm00001d027314_T001 transcript:EER95671          5.00E-145
 73-  4: transcript:Zm00001d027315_T002 transcript:EER93009           7.00E-49
 73-  5: transcript:Zm00001d027317_T015 transcript:EER95667                  0
 73-  6: transcript:Zm00001d027318_T001 transcript:EER93008           2.00E-93
 73-  7: transcript:Zm00001d027319_T001 transcript:OQU93424          4.00E-162
 73-  8: transcript:Zm00001d027320_T002 transcript:OQU93422                  0
 73-  9: transcript:Zm00001d027322_T001 transcript:OQU93419                  0
 73- 10: transcript:Zm00001d027323_T001 transcript:EER95663           2.00E-77
 73- 11: transcript:Zm00001d027324_T002 transcript:OQU93418                  0
 73- 12: transcript:Zm00001d027325_T002 transcript:EER95660          1.00E-120
 73- 13: transcript:Zm00001d027326_T001 transcript:EER93001                  0
 73- 14: transcript:Zm00001d027329_T002 transcript:OQU93414                  0
 73- 15: transcript:Zm00001d027330_T001 transcript:EER92997           4.00E-97
 73- 16: transcript:Zm00001d027332_T001 transcript:OQU93402           9.00E-39
 73- 17: transcript:Zm00001d027333_T018 transcript:KXG40365                  0
 73- 18: transcript:Zm00001d027334_T001 transcript:EER95651                  0
## Alignment 74: score=771.0 e_value=7.4e-45 N=17 1&1 minus
 74-  0: transcript:Zm00001d027998_T001 transcript:OQU93090           2.00E-56
 74-  1: transcript:Zm00001d027999_T001 transcript:KXG39997                  0
 74-  2: transcript:Zm00001d028002_T001 transcript:EER92725          3.00E-160
 74-  3: transcript:Zm00001d028004_T001 transcript:EER92724                  0
 74-  4: transcript:Zm00001d028005_T002 transcript:EER92723                  0
 74-  5: transcript:Zm00001d028006_T001 transcript:EER92718                  0
 74-  6: transcript:Zm00001d028007_T004 transcript:OQU93088                  0
 74-  7: transcript:Zm00001d028009_T001 transcript:KXG39986                  0
 74-  8: transcript:Zm00001d028010_T001 transcript:EER92716                  0
 74-  9: transcript:Zm00001d028011_T001 transcript:EER92715          7.00E-104
 74- 10: transcript:Zm00001d028012_T001 transcript:KXG39985          4.00E-110
 74- 11: transcript:Zm00001d028013_T001 transcript:OQU93087                  0
 74- 12: transcript:Zm00001d028015_T001 transcript:OQU93086          1.00E-155
 74- 13: transcript:Zm00001d028017_T001 transcript:EER92713          4.00E-158
 74- 14: transcript:Zm00001d028018_T001 transcript:EER92712                  0
 74- 15: transcript:Zm00001d028019_T001 transcript:EER95353                  0
 74- 16: transcript:Zm00001d028020_T001 transcript:OQU93085                  0
## Alignment 75: score=740.0 e_value=8.5e-44 N=16 1&1 minus
 75-  0: transcript:EER92019           transcript:Zm00001d029734_T001         0
```

```
 75-  1: transcript:OQU92244           transcript:Zm00001d029733_T001 4.00E-106
 75-  2: transcript:EER92020           transcript:Zm00001d029730_T001  3.00E-76
 75-  3: transcript:OQU92247           transcript:Zm00001d029726_T004         0
 75-  4: transcript:EER94588           transcript:Zm00001d029725_T001 9.00E-174
 75-  5: transcript:EER94590           transcript:Zm00001d029723_T001         0
 75-  6: transcript:OQU92249           transcript:Zm00001d029721_T001         0
 75-  7: transcript:KXG39024           transcript:Zm00001d029720_T001 1.00E-106
 75-  8: transcript:OQU92250           transcript:Zm00001d029719_T002         0
 75-  9: transcript:EER94592           transcript:Zm00001d029718_T001 6.00E-107
 75- 10: transcript:OQU92251           transcript:Zm00001d029716_T002  7.00E-66
 75- 11: transcript:KXG39026           transcript:Zm00001d029715_T001         0
 75- 12: transcript:EER94594           transcript:Zm00001d029714_T001         0
 75- 13: transcript:OQU92255           transcript:Zm00001d029711_T001 4.00E-163
 75- 14: transcript:EER94596           transcript:Zm00001d029707_T001  5.00E-95
 75- 15: transcript:EER92030           transcript:Zm00001d029706_T001 1.00E-101
## Alignment 76: score=737.0 e_value=7e-48 N=17 1&1 minus
 76-  0: transcript:Zm00001d029061_T001 transcript:EER94902                 0
 76-  1: transcript:Zm00001d029062_T001 transcript:EER92308                 0
 76-  2: transcript:Zm00001d029064_T001 transcript:OQU92641         1.00E-180
 76-  3: transcript:Zm00001d029065_T001 transcript:KXG39424         3.00E-156
 76-  4: transcript:Zm00001d029066_T005 transcript:KXG39422                 0
 76-  5: transcript:Zm00001d029067_T001 transcript:OQU92638         1.00E-105
 76-  6: transcript:Zm00001d029071_T001 transcript:EER94896          9.00E-30
 76-  7: transcript:Zm00001d029072_T001 transcript:EER94895                 0
 76-  8: transcript:Zm00001d029074_T001 transcript:EER94893                 0
 76-  9: transcript:Zm00001d029075_T001 transcript:EER94892                 0
 76- 10: transcript:Zm00001d029077_T002 transcript:EER94890                 0
 76- 11: transcript:Zm00001d029078_T002 transcript:EER92303         3.00E-106
 76- 12: transcript:Zm00001d029083_T001 transcript:EER94889                 0
 76- 13: transcript:Zm00001d029084_T001 transcript:KXG39416                 0
 76- 14: transcript:Zm00001d029085_T001 transcript:KXG39415          4.00E-61
 76- 15: transcript:Zm00001d029086_T001 transcript:EER94885          5.00E-60
 76- 16: transcript:Zm00001d029087_T002 transcript:EER92301                 0
## Alignment 77: score=735.0 e_value=5.7e-41 N=16 1&1 minus
 77-  0: transcript:EER90833           transcript:Zm00001d034152_T002         0
 77-  1: transcript:EER90834           transcript:Zm00001d034145_T001 5.00E-125
 77-  2: transcript:EER93401           transcript:Zm00001d034143_T001         0
 77-  3: transcript:KXG37490           transcript:Zm00001d034137_T001         0
 77-  4: transcript:OQU90937           transcript:Zm00001d034133_T001         0
 77-  5: transcript:EER90837           transcript:Zm00001d034131_T001         0
 77-  6: transcript:EER93404           transcript:Zm00001d034130_T001  6.00E-49
 77-  7: transcript:EER93405           transcript:Zm00001d034128_T001 4.00E-106
 77-  8: transcript:EER90839           transcript:Zm00001d034126_T001 6.00E-124
 77-  9: transcript:EER90840           transcript:Zm00001d034125_T002         0
 77- 10: transcript:EER93408           transcript:Zm00001d034124_T001 9.00E-147
 77- 11: transcript:EER93409           transcript:Zm00001d034115_T001  5.00E-14
 77- 12: transcript:EER90842           transcript:Zm00001d034112_T001         0
 77- 13: transcript:EER93771           transcript:Zm00001d034111_T001         0
 77- 14: transcript:KXG37496           transcript:Zm00001d034110_T001  4.00E-26
 77- 15: transcript:KXG37497           transcript:Zm00001d034109_T001         0
## Alignment 78: score=715.0 e_value=3.4e-43 N=16 1&1 minus
 78-  0: transcript:EER93184           transcript:Zm00001d034662_T001         0
 78-  1: transcript:KXG37252           transcript:Zm00001d034659_T001         0
 78-  2: transcript:EER90623           transcript:Zm00001d034655_T001         0
```

```
 78-  3: transcript:KXG37254           transcript:Zm00001d034651_T001 1.00E-168
 78-  4: transcript:EER90625           transcript:Zm00001d034650_T017         0
 78-  5: transcript:EER90626           transcript:Zm00001d034649_T001 1.00E-134
 78-  6: transcript:KXG37255           transcript:Zm00001d034644_T001  1.00E-97
 78-  7: transcript:EER93192           transcript:Zm00001d034643_T001  9.00E-87
 78-  8: transcript:EER93193           transcript:Zm00001d034641_T001  6.00E-32
 78-  9: transcript:KXG37259           transcript:Zm00001d034640_T003         0
 78- 10: transcript:EER90627           transcript:Zm00001d034639_T001  6.00E-65
 78- 11: transcript:EER93195           transcript:Zm00001d034638_T001 7.00E-155
 78- 12: transcript:OQU90728           transcript:Zm00001d034636_T001         0
 78- 13: transcript:EER90629           transcript:Zm00001d034635_T001 4.00E-141
 78- 14: transcript:EER90630           transcript:Zm00001d034634_T001  1.00E-95
 78- 15: transcript:OQU90735           transcript:Zm00001d034633_T057         0
## Alignment 79: score=684.0 e_value=1.4e-47 N=18 1&1 minus
 79-  0: transcript:Zm00001d029138_T001 transcript:KXG39391           8.00E-111
 79-  1: transcript:Zm00001d029139_T001 transcript:KXG39389                   0
 79-  2: transcript:Zm00001d029140_T002 transcript:KXG39388                   0
 79-  3: transcript:Zm00001d029141_T001 transcript:EER92275           1.00E-118
 79-  4: transcript:Zm00001d029142_T007 transcript:EER92274                   0
 79-  5: transcript:Zm00001d029143_T003 transcript:KXG39386                   0
 79-  6: transcript:Zm00001d029144_T003 transcript:KXG39385                   0
 79-  7: transcript:Zm00001d029149_T001 transcript:EER94860                   0
 79-  8: transcript:Zm00001d029150_T001 transcript:EER92272                   0
 79-  9: transcript:Zm00001d029151_T002 transcript:EER94859                   0
 79- 10: transcript:Zm00001d029153_T001 transcript:OQU92598            6.00E-31
 79- 11: transcript:Zm00001d029154_T001 transcript:EER94858                   0
 79- 12: transcript:Zm00001d029165_T001 transcript:KXG39376                   0
 79- 13: transcript:Zm00001d029170_T004 transcript:KXG39375            2.00E-85
 79- 14: transcript:Zm00001d029172_T001 transcript:EER94857            6.00E-36
 79- 15: transcript:Zm00001d029173_T004 transcript:EER94856           4.00E-117
 79- 16: transcript:Zm00001d029174_T005 transcript:EER92268                   0
 79- 17: transcript:Zm00001d029177_T001 transcript:EER94854                   0
## Alignment 80: score=674.0 e_value=1e-39 N=15 1&1 minus
 80-  0: transcript:EER91964           transcript:Zm00001d029860_T001         0
 80-  1: transcript:EER91965           transcript:Zm00001d029859_T001  2.00E-56
 80-  2: transcript:EER91967           transcript:Zm00001d029857_T001  3.00E-29
 80-  3: transcript:EER94525           transcript:Zm00001d029856_T001  2.00E-66
 80-  4: transcript:EER91968           transcript:Zm00001d029855_T001         0
 80-  5: transcript:EER94526           transcript:Zm00001d029853_T001         0
 80-  6: transcript:KXG38956           transcript:Zm00001d029852_T001         0
 80-  7: transcript:EER94531           transcript:Zm00001d029851_T001         0
 80-  8: transcript:EER91970           transcript:Zm00001d029850_T001         0
 80-  9: transcript:KXG38962           transcript:Zm00001d029849_T006         0
 80- 10: transcript:KXG38963           transcript:Zm00001d029848_T002         0
 80- 11: transcript:EER91973           transcript:Zm00001d029847_T001         0
 80- 12: transcript:EER91974           transcript:Zm00001d029846_T001         0
 80- 13: transcript:OQU92183           transcript:Zm00001d029829_T001  6.00E-73
 80- 14: transcript:EER94537           transcript:Zm00001d029827_T001  9.00E-77
## Alignment 81: score=668.0 e_value=2e-38 N=15 1&1 minus
 81-  0: transcript:EER91868           transcript:Zm00001d030103_T001         0
 81-  1: transcript:EER91870           transcript:Zm00001d030101_T001         0
 81-  2: transcript:EER91871           transcript:Zm00001d030098_T001 3.00E-122
 81-  3: transcript:KXG38826           transcript:Zm00001d030094_T002  6.00E-97
 81-  4: transcript:EER91872           transcript:Zm00001d030091_T001         0
```

81- 5: transcript:EER94430 transcript:Zm00001d030090_T001 0
81- 6: transcript:EER91873 transcript:Zm00001d030087_T001 5.00E-80
81- 7: transcript:EER91874 transcript:Zm00001d030086_T001 5.00E-122
81- 8: transcript:EER91875 transcript:Zm00001d030083_T001 0
81- 9: transcript:EER94437 transcript:Zm00001d030080_T002 6.00E-71
81- 10: transcript:EER91876 transcript:Zm00001d030079_T001 7.00E-55
81- 11: transcript:KXG38834 transcript:Zm00001d030076_T002 1.00E-39
81- 12: transcript:EER94440 transcript:Zm00001d030074_T001 0
81- 13: transcript:KXG38837 transcript:Zm00001d030069_T006 0
81- 14: transcript:OQU92070 transcript:Zm00001d030068_T001 4.00E-83
Alignment 82: score=658.0 e_value=7.1e-41 N=16 1&1 minus
82- 0: transcript:Zm00001d027921_T003 transcript:EER95390 0
82- 1: transcript:Zm00001d027922_T001 transcript:EER92748 1.00E-157
82- 2: transcript:Zm00001d027924_T001 transcript:OQU93122 1.00E-99
82- 3: transcript:Zm00001d027928_T001 transcript:KXG40035 3.00E-94
82- 4: transcript:Zm00001d027932_T001 transcript:EER92744 2.00E-72
82- 5: transcript:Zm00001d027934_T002 transcript:EER95387 0
82- 6: transcript:Zm00001d027936_T001 transcript:OQU93120 6.00E-58
82- 7: transcript:Zm00001d027937_T013 transcript:EER95370 0
82- 8: transcript:Zm00001d027938_T001 transcript:EER95378 0
82- 9: transcript:Zm00001d027939_T002 transcript:EER92740 6.00E-153
82- 10: transcript:Zm00001d027941_T004 transcript:OQU93112 3.00E-140
82- 11: transcript:Zm00001d027943_T006 transcript:EER95373 0
82- 12: transcript:Zm00001d027944_T001 transcript:EER95372 4.00E-87
82- 13: transcript:Zm00001d027950_T002 transcript:EER95371 0
82- 14: transcript:Zm00001d027954_T001 transcript:KXG40021 0
82- 15: transcript:Zm00001d027955_T001 transcript:KXG40017 1.00E-65
Alignment 83: score=652.0 e_value=1.5e-39 N=15 1&1 minus
83- 0: transcript:EER92111 transcript:Zm00001d030164_T026 0
83- 1: transcript:EER94413 transcript:Zm00001d030163_T001 2.00E-54
83- 2: transcript:EER94415 transcript:Zm00001d030153_T001 6.00E-90
83- 3: transcript:OQU92053 transcript:Zm00001d030146_T003 1.00E-90
83- 4: transcript:KXG38806 transcript:Zm00001d030143_T001 0
83- 5: transcript:OQU92054 transcript:Zm00001d030139_T001 0
83- 6: transcript:KXG38808 transcript:Zm00001d030138_T001 4.00E-73
83- 7: transcript:EER91861 transcript:Zm00001d030133_T002 0
83- 8: transcript:KXG38810 transcript:Zm00001d030132_T002 0
83- 9: transcript:EER91863 transcript:Zm00001d030131_T001 0
83- 10: transcript:EER91864 transcript:Zm00001d030127_T001 0
83- 11: transcript:KXG38813 transcript:Zm00001d030126_T002 0
83- 12: transcript:KXG38815 transcript:Zm00001d030123_T001 0
83- 13: transcript:EER94423 transcript:Zm00001d030121_T001 0
83- 14: transcript:EER91862 transcript:Zm00001d030117_T006 0
Alignment 84: score=625.0 e_value=8.5e-35 N=14 1&1 minus
84- 0: transcript:Zm00001d027671_T001 transcript:EER92855 0
84- 1: transcript:Zm00001d027672_T001 transcript:OQU93234 2.00E-37
84- 2: transcript:Zm00001d027673_T001 transcript:EER92854 0
84- 3: transcript:Zm00001d027674_T001 transcript:OQU93232 1.00E-99
84- 4: transcript:Zm00001d027675_T002 transcript:EER92852 3.00E-129
84- 5: transcript:Zm00001d027676_T001 transcript:EER95499 0
84- 6: transcript:Zm00001d027677_T001 transcript:OQU93230 0
84- 7: transcript:Zm00001d027678_T001 transcript:EER92849 2.00E-50
84- 8: transcript:Zm00001d027680_T003 transcript:EER92848 2.00E-74
84- 9: transcript:Zm00001d027682_T001 transcript:EER92847 0

84- 10: transcript:Zm00001d027683_T001 transcript:EER95495 0
84- 11: transcript:Zm00001d027684_T001 transcript:EER92845 2.00E-22
84- 12: transcript:Zm00001d027686_T001 transcript:KXG40163 1.00E-62
84- 13: transcript:Zm00001d027688_T002 transcript:OQU93221 2.00E-176
Alignment 85: score=625.0 e_value=5.1e-36 N=14 1&1 minus
85- 0: transcript:EER91023 transcript:Zm00001d033654_T004 0
85- 1: transcript:OQU91137 transcript:Zm00001d033652_T002 0
85- 2: transcript:EER91024 transcript:Zm00001d033650_T001 0
85- 3: transcript:EER93603 transcript:Zm00001d033648_T002 0
85- 4: transcript:EER91029 transcript:Zm00001d033646_T013 0
85- 5: transcript:KXG37770 transcript:Zm00001d033645_T001 0
85- 6: transcript:EER91030 transcript:Zm00001d033642_T001 0
85- 7: transcript:EER91031 transcript:Zm00001d033641_T002 0
85- 8: transcript:EER91032 transcript:Zm00001d033640_T001 6.00E-74
85- 9: transcript:EER91033 transcript:Zm00001d033637_T001 1.00E-164
85- 10: transcript:OQU91144 transcript:Zm00001d033636_T001 5.00E-31
85- 11: transcript:OQU91145 transcript:Zm00001d033634_T001 1.00E-87
85- 12: transcript:KXG37775 transcript:Zm00001d033633_T001 2.00E-153
85- 13: transcript:KXG37780 transcript:Zm00001d033632_T008 0
Alignment 86: score=594.0 e_value=3.4e-38 N=15 1&1 minus
86- 0: transcript:Zm00001d029093_T001 transcript:EER94884 0
86- 1: transcript:Zm00001d029095_T009 transcript:OQU92635 0
86- 2: transcript:Zm00001d029096_T002 transcript:EER92299 0
86- 3: transcript:Zm00001d029098_T001 transcript:EER94881 5.00E-164
86- 4: transcript:Zm00001d029100_T001 transcript:OQU92624 1.00E-59
86- 5: transcript:Zm00001d029101_T001 transcript:EER92297 2.00E-15
86- 6: transcript:Zm00001d029102_T001 transcript:EER92294 1.00E-81
86- 7: transcript:Zm00001d029104_T002 transcript:OQU92618 0
86- 8: transcript:Zm00001d029105_T001 transcript:EER94877 0
86- 9: transcript:Zm00001d029106_T002 transcript:OQU92612 4.00E-24
86- 10: transcript:Zm00001d029107_T001 transcript:EER94871 6.00E-78
86- 11: transcript:Zm00001d029115_T001 transcript:KXG39404 7.00E-158
86- 12: transcript:Zm00001d029118_T001 transcript:EER92289 0
86- 13: transcript:Zm00001d029119_T001 transcript:KXG39402 2.00E-131
86- 14: transcript:Zm00001d029120_T004 transcript:EER92288 0
Alignment 87: score=593.0 e_value=9.6e-30 N=13 1&1 minus
87- 0: transcript:EER93230 transcript:Zm00001d034581_T001 0
87- 1: transcript:OQU90773 transcript:Zm00001d034578_T001 2.00E-111
87- 2: transcript:EER90673 transcript:Zm00001d034577_T003 0
87- 3: transcript:EER90674 transcript:Zm00001d034575_T001 0
87- 4: transcript:EER93233 transcript:Zm00001d034572_T001 2.00E-180
87- 5: transcript:OQU90776 transcript:Zm00001d034571_T001 6.00E-162
87- 6: transcript:EER90677 transcript:Zm00001d034569_T001 0
87- 7: transcript:OQU90781 transcript:Zm00001d034568_T001 0
87- 8: transcript:KXG37302 transcript:Zm00001d034565_T002 0
87- 9: transcript:EER93237 transcript:Zm00001d034562_T003 4.00E-149
87- 10: transcript:KXG37304 transcript:Zm00001d034560_T001 0
87- 11: transcript:EER90681 transcript:Zm00001d034559_T001 0
87- 12: transcript:OQU90788 transcript:Zm00001d034558_T001 1.00E-142
Alignment 88: score=558.0 e_value=1.9e-32 N=13 1&1 minus
88- 0: transcript:Zm00001d027536_T003 transcript:EER92913 0
88- 1: transcript:Zm00001d027537_T001 transcript:EER92912 0
88- 2: transcript:Zm00001d027539_T001 transcript:OQU93289 9.00E-59
88- 3: transcript:Zm00001d027540_T001 transcript:EER92907 2.00E-64

```
 88-  4: transcript:Zm00001d027544_T002 transcript:OQU93284                   0
 88-  5: transcript:Zm00001d027546_T001 transcript:EER92904          1.00E-167
 88-  6: transcript:Zm00001d027548_T001 transcript:EER95554                   0
 88-  7: transcript:Zm00001d027549_T001 transcript:KXG40235                   0
 88-  8: transcript:Zm00001d027550_T001 transcript:EER95552            8.00E-81
 88-  9: transcript:Zm00001d027554_T001 transcript:EER95551            9.00E-92
 88- 10: transcript:Zm00001d027555_T001 transcript:KXG40233                   0
 88- 11: transcript:Zm00001d027556_T001 transcript:EER92900                   0
 88- 12: transcript:Zm00001d027558_T004 transcript:EER92899                   0
## Alignment 89: score=534.0 e_value=3.9e-28 N=12 1&1 minus
 89-  0: transcript:Zm00001d029188_T006 transcript:EER94850                   0
 89-  1: transcript:Zm00001d029189_T003 transcript:EER92266                   0
 89-  2: transcript:Zm00001d029190_T001 transcript:KXG39360          2.00E-174
 89-  3: transcript:Zm00001d029191_T002 transcript:OQU92586                   0
 89-  4: transcript:Zm00001d029193_T001 transcript:EER92264                   0
 89-  5: transcript:Zm00001d029194_T001 transcript:KXG39356                   0
 89-  6: transcript:Zm00001d029195_T001 transcript:OQU92578                   0
 89-  7: transcript:Zm00001d029196_T002 transcript:EER94839                   0
 89-  8: transcript:Zm00001d029197_T001 transcript:EER94844                   0
 89-  9: transcript:Zm00001d029198_T003 transcript:KXG39349            5.00E-15
 89- 10: transcript:Zm00001d029200_T001 transcript:EER92259                   0
 89- 11: transcript:Zm00001d029201_T001 transcript:EER92258          1.00E-158
## Alignment 90: score=531.0 e_value=1.1e-30 N=12 1&1 minus
 90-  0: transcript:EER90788            transcript:Zm00001d034313_T002 1.00E-176
 90-  1: transcript:EER90791            transcript:Zm00001d034305_T001  1.00E-43
 90-  2: transcript:EER90796            transcript:Zm00001d034283_T001 6.00E-153
 90-  3: transcript:OQU90898            transcript:Zm00001d034282_T004         0
 90-  4: transcript:EER90798            transcript:Zm00001d034279_T001  2.00E-94
 90-  5: transcript:KXG37443            transcript:Zm00001d034278_T001         0
 90-  6: transcript:EER93360            transcript:Zm00001d034277_T001 1.00E-179
 90-  7: transcript:EER93361            transcript:Zm00001d034270_T001  6.00E-86
 90-  8: transcript:OQU90901            transcript:Zm00001d034265_T001  4.00E-28
 90-  9: transcript:OQU90902            transcript:Zm00001d034263_T004         0
 90- 10: transcript:EER90803            transcript:Zm00001d034257_T002         0
 90- 11: transcript:KXG37448            transcript:Zm00001d034256_T001         0
## Alignment 91: score=526.0 e_value=1.6e-27 N=12 1&1 minus
 91-  0: transcript:Zm00001d029226_T001 transcript:EER92238                   0
 91-  1: transcript:Zm00001d029227_T001 transcript:KXG39328                   0
 91-  2: transcript:Zm00001d029231_T001 transcript:KXG39323          2.00E-163
 91-  3: transcript:Zm00001d029232_T001 transcript:EER92236                   0
 91-  4: transcript:Zm00001d029233_T001 transcript:EER94828            3.00E-32
 91-  5: transcript:Zm00001d029234_T016 transcript:EER92235          3.00E-174
 91-  6: transcript:Zm00001d029235_T001 transcript:OQU92556                   0
 91-  7: transcript:Zm00001d029238_T001 transcript:EER94826            3.00E-92
 91-  8: transcript:Zm00001d029241_T001 transcript:EER94824                   0
 91-  9: transcript:Zm00001d029242_T001 transcript:KXG39318          3.00E-136
 91- 10: transcript:Zm00001d029243_T001 transcript:EER94822                   0
 91- 11: transcript:Zm00001d029246_T001 transcript:EER94818                   0
## Alignment 92: score=504.0 e_value=4.1e-33 N=14 1&1 minus
 92-  0: transcript:EER92180            transcript:Zm00001d029402_T001 2.00E-112
 92-  1: transcript:KXG39221            transcript:Zm00001d029397_T001         0
 92-  2: transcript:EER94753            transcript:Zm00001d029396_T001 1.00E-121
 92-  3: transcript:KXG39223            transcript:Zm00001d029395_T001 3.00E-128
 92-  4: transcript:OQU92483            transcript:Zm00001d029394_T003  2.00E-93
```

```
 92-  5: transcript:KXG39228           transcript:Zm00001d029393_T001  5.00E-72
 92-  6: transcript:OQU92485           transcript:Zm00001d029392_T001         0
 92-  7: transcript:EER94760           transcript:Zm00001d029391_T002         0
 92-  8: transcript:EER92183           transcript:Zm00001d029388_T001 6.00E-106
 92-  9: transcript:KXG39234           transcript:Zm00001d029386_T006         0
 92- 10: transcript:EER92186           transcript:Zm00001d029383_T001 1.00E-167
 92- 11: transcript:KXG39239           transcript:Zm00001d029380_T002  1.00E-58
 92- 12: transcript:KXG39241           transcript:Zm00001d029378_T012         0
 92- 13: transcript:EER94763           transcript:Zm00001d029376_T001  3.00E-70
## Alignment 93: score=498.0 e_value=2.7e-27 N=12 1&1 minus
 93-  0: transcript:Zm00001d027700_T001 transcript:EER92843                  0
 93-  1: transcript:Zm00001d027701_T001 transcript:EER92842                  0
 93-  2: transcript:Zm00001d027702_T001 transcript:KXG40161                  0
 93-  3: transcript:Zm00001d027703_T001 transcript:EER92841          1.00E-139
 93-  4: transcript:Zm00001d027706_T004 transcript:EER95491                  0
 93-  5: transcript:Zm00001d027707_T001 transcript:EER92840                  0
 93-  6: transcript:Zm00001d027708_T002 transcript:KXG40156          2.00E-133
 93-  7: transcript:Zm00001d027709_T001 transcript:EER95490                  0
 93-  8: transcript:Zm00001d027710_T001 transcript:EER95489                  0
 93-  9: transcript:Zm00001d027711_T001 transcript:KXG40154                  0
 93- 10: transcript:Zm00001d027714_T001 transcript:OQU93211                  0
 93- 11: transcript:Zm00001d027715_T001 transcript:OQU93210                  0
## Alignment 94: score=482.0 e_value=4e-26 N=11 1&1 minus
 94-  0: transcript:OQU92037           transcript:Zm00001d030184_T001         0
 94-  1: transcript:EER91839           transcript:Zm00001d030181_T001         0
 94-  2: transcript:OQU92040           transcript:Zm00001d030180_T001  7.00E-31
 94-  3: transcript:OQU92046           transcript:Zm00001d030176_T001  1.00E-23
 94-  4: transcript:KXG38789           transcript:Zm00001d030174_T001  1.00E-75
 94-  5: transcript:OQU92047           transcript:Zm00001d030173_T007         0
 94-  6: transcript:KXG38791           transcript:Zm00001d030172_T004 2.00E-163
 94-  7: transcript:EER94408           transcript:Zm00001d030171_T001         0
 94-  8: transcript:EER91850           transcript:Zm00001d030167_T002 2.00E-148
 94-  9: transcript:EER91851           transcript:Zm00001d030166_T005         0
 94- 10: transcript:KXG38794           transcript:Zm00001d030165_T001         0
## Alignment 95: score=473.0 e_value=1e-24 N=11 1&1 minus
 95-  0: transcript:EER94107           transcript:Zm00001d032776_T002         0
 95-  1: transcript:EER94108           transcript:Zm00001d032775_T002 4.00E-134
 95-  2: transcript:EER94110           transcript:Zm00001d032773_T003         0
 95-  3: transcript:KXG38374           transcript:Zm00001d032771_T001  6.00E-22
 95-  4: transcript:OQU91666           transcript:Zm00001d032768_T005 3.00E-143
 95-  5: transcript:KXG38386           transcript:Zm00001d032763_T002         0
 95-  6: transcript:OQU91671           transcript:Zm00001d032761_T005         0
 95-  7: transcript:EER91506           transcript:Zm00001d032760_T001 5.00E-117
 95-  8: transcript:EER91507           transcript:Zm00001d032754_T002         0
 95-  9: transcript:EER91508           transcript:Zm00001d032753_T001         0
 95- 10: transcript:EER91513           transcript:Zm00001d032750_T001         0
## Alignment 96: score=469.0 e_value=1.9e-20 N=10 1&1 minus
 96-  0: transcript:OQU91191           transcript:Zm00001d033527_T001         0
 96-  1: transcript:KXG37833           transcript:Zm00001d033526_T001  1.00E-89
 96-  2: transcript:OQU91192           transcript:Zm00001d033525_T001         0
 96-  3: transcript:EER91071           transcript:Zm00001d033523_T001 3.00E-157
 96-  4: transcript:EER93662           transcript:Zm00001d033522_T001         0
 96-  5: transcript:OQU91195           transcript:Zm00001d033521_T001         0
 96-  6: transcript:OQU91197           transcript:Zm00001d033518_T001         0
```

```
 96-  7: transcript:EER91075           transcript:Zm00001d033517_T001          0
 96-  8: transcript:EER91076           transcript:Zm00001d033514_T001 1.00E-125
 96-  9: transcript:KXG37841           transcript:Zm00001d033511_T002 2.00E-149
## Alignment 97: score=462.0 e_value=1.9e-22 N=11 1&1 minus
 97-  0: transcript:KXG40080           transcript:Zm00001d033012_T001          0
 97-  1: transcript:EER92779           transcript:Zm00001d033011_T001          0
 97-  2: transcript:EER92780           transcript:Zm00001d033005_T001  2.00E-88
 97-  3: transcript:EER92783           transcript:Zm00001d033004_T002          0
 97-  4: transcript:EER92785           transcript:Zm00001d033003_T001  2.00E-45
 97-  5: transcript:EER95426           transcript:Zm00001d032999_T001 1.00E-128
 97-  6: transcript:EER95427           transcript:Zm00001d032994_T002          0
 97-  7: transcript:OQU93156           transcript:Zm00001d032992_T001 6.00E-130
 97-  8: transcript:OQU93161           transcript:Zm00001d032991_T001          0
 97-  9: transcript:EER95434           transcript:Zm00001d032989_T001          0
 97- 10: transcript:OQU93166           transcript:Zm00001d032981_T001          0
## Alignment 98: score=442.0 e_value=9.8e-25 N=11 1&1 minus
 98-  0: transcript:Zm00001d027755_T002 transcript:KXG40132           3.00E-108
 98-  1: transcript:Zm00001d027756_T001 transcript:OQU93187           4.00E-138
 98-  2: transcript:Zm00001d027757_T001 transcript:EER95463                   0
 98-  3: transcript:Zm00001d027759_T001 transcript:OQU93186                   0
 98-  4: transcript:Zm00001d027760_T001 transcript:KXG40126            2.00E-78
 98-  5: transcript:Zm00001d027763_T001 transcript:EER95459           3.00E-156
 98-  6: transcript:Zm00001d027766_T001 transcript:EER95458                   0
 98-  7: transcript:Zm00001d027767_T001 transcript:EER95457                   0
 98-  8: transcript:Zm00001d027768_T023 transcript:EER92811                   0
 98-  9: transcript:Zm00001d027769_T001 transcript:EER95455                   0
 98- 10: transcript:Zm00001d027772_T001 transcript:KXG40120            2.00E-10
## Alignment 99: score=440.0 e_value=7.6e-21 N=10 1&1 minus
 99-  0: transcript:EER92974           transcript:Zm00001d029686_T001          0
 99-  1: transcript:KXG40331           transcript:Zm00001d029684_T001          0
 99-  2: transcript:EER95627           transcript:Zm00001d029683_T001          0
 99-  3: transcript:OQU93375           transcript:Zm00001d029681_T001  9.00E-40
 99-  4: transcript:KXG40338           transcript:Zm00001d029679_T001  3.00E-48
 99-  5: transcript:KXG40340           transcript:Zm00001d029675_T001          0
 99-  6: transcript:EER95641           transcript:Zm00001d029673_T001 7.00E-154
 99-  7: transcript:EER95642           transcript:Zm00001d029667_T001 1.00E-165
 99-  8: transcript:EER95643           transcript:Zm00001d029662_T001 6.00E-152
 99-  9: transcript:EER95644           transcript:Zm00001d029657_T001 5.00E-139
## Alignment 100: score=438.0 e_value=8.3e-18 N=9 1&1 minus
100-  0: transcript:EER93274           transcript:Zm00001d034490_T001          0
100-  1: transcript:OQU90828           transcript:Zm00001d034488_T001          0
100-  2: transcript:EER93276           transcript:Zm00001d034487_T002          0
100-  3: transcript:EER93277           transcript:Zm00001d034486_T003          0
100-  4: transcript:EER93278           transcript:Zm00001d034485_T001 6.00E-146
100-  5: transcript:EER90727           transcript:Zm00001d034484_T001  7.00E-52
100-  6: transcript:KXG37349           transcript:Zm00001d034482_T001  7.00E-14
100-  7: transcript:EER93281           transcript:Zm00001d034480_T002          0
100-  8: transcript:OQU90831           transcript:Zm00001d034479_T001  3.00E-39
## Alignment 101: score=437.0 e_value=1.1e-24 N=11 1&1 minus
101-  0: transcript:Zm00001d027393_T001 transcript:EER95623                   0
101-  1: transcript:Zm00001d027395_T009 transcript:EER95621                   0
101-  2: transcript:Zm00001d027399_T002 transcript:EER95620            2.00E-74
101-  3: transcript:Zm00001d027400_T001 transcript:OQU93368           2.00E-151
101-  4: transcript:Zm00001d027402_T004 transcript:OQU93366           2.00E-117
```

```
101-  5: transcript:Zm00001d027401_T008 transcript:KXG40322                 0
101-  6: transcript:Zm00001d027403_T001 transcript:KXG40319                 0
101-  7: transcript:Zm00001d027405_T001 transcript:EER92967        2.00E-131
101-  8: transcript:Zm00001d027409_T001 transcript:OQU93364        9.00E-159
101-  9: transcript:Zm00001d027411_T001 transcript:EER92966                 0
101- 10: transcript:Zm00001d027412_T001 transcript:KXG40313                 0
## Alignment 102: score=422.0 e_value=5.5e-21 N=10 1&1 minus
102-  0: transcript:Zm00001d028210_T001 transcript:EER95276                 0
102-  1: transcript:Zm00001d028211_T002 transcript:EER92641                 0
102-  2: transcript:Zm00001d028214_T001 transcript:KXG39871                 0
102-  3: transcript:Zm00001d028216_T001 transcript:EER95274        2.00E-144
102-  4: transcript:Zm00001d028217_T001 transcript:OQU93000        9.00E-152
102-  5: transcript:Zm00001d028218_T005 transcript:KXG40410                 0
102-  6: transcript:Zm00001d028219_T001 transcript:KXG39865                 0
102-  7: transcript:Zm00001d028220_T001 transcript:KXG39858                 0
102-  8: transcript:Zm00001d028221_T001 transcript:EER92639                 0
102-  9: transcript:Zm00001d028222_T001 transcript:EER92638        2.00E-112
## Alignment 103: score=416.0 e_value=2.9e-24 N=10 1&1 minus
103-  0: transcript:OQU90570            transcript:Zm00001d034940_T003         0
103-  1: transcript:EER90454            transcript:Zm00001d034937_T001 4.00E-126
103-  2: transcript:EER93049            transcript:Zm00001d034935_T007         0
103-  3: transcript:EER90455            transcript:Zm00001d034936_T001 5.00E-131
103-  4: transcript:KXG37063            transcript:Zm00001d034934_T001 3.00E-115
103-  5: transcript:EER93051            transcript:Zm00001d034933_T001         0
103-  6: transcript:EER90457            transcript:Zm00001d034932_T001 4.00E-132
103-  7: transcript:OQU90580            transcript:Zm00001d034931_T013         0
103-  8: transcript:KXG37070            transcript:Zm00001d034929_T011         0
103-  9: transcript:EER90459            transcript:Zm00001d034927_T001         0
## Alignment 104: score=408.0 e_value=1.8e-17 N=9 1&1 minus
104-  0: transcript:EER90872            transcript:Zm00001d034018_T002         0
104-  1: transcript:KXG37545            transcript:Zm00001d034015_T005         0
104-  2: transcript:KXG37551            transcript:Zm00001d034013_T002         0
104-  3: transcript:KXG37554            transcript:Zm00001d034012_T001         0
104-  4: transcript:EER93457            transcript:Zm00001d034011_T001 5.00E-105
104-  5: transcript:EER93458            transcript:Zm00001d034010_T002         0
104-  6: transcript:EER93455            transcript:Zm00001d034007_T001         0
104-  7: transcript:EER93460            transcript:Zm00001d034006_T002         0
104-  8: transcript:KXG37556            transcript:Zm00001d034005_T003         0
## Alignment 105: score=405.0 e_value=1.7e-24 N=11 1&1 minus
105-  0: transcript:KXG39256            transcript:Zm00001d029353_T002  7.00E-70
105-  1: transcript:KXG39259            transcript:Zm00001d029352_T002         0
105-  2: transcript:KXG39261            transcript:Zm00001d029350_T001         0
105-  3: transcript:EER92197            transcript:Zm00001d029349_T001  2.00E-65
105-  4: transcript:KXG39263            transcript:Zm00001d029347_T001  8.00E-62
105-  5: transcript:KXG39267            transcript:Zm00001d029343_T001 7.00E-136
105-  6: transcript:EER94780            transcript:Zm00001d029342_T009         0
105-  7: transcript:EER92199            transcript:Zm00001d029341_T001         0
105-  8: transcript:OQU92521            transcript:Zm00001d029340_T003  2.00E-76
105-  9: transcript:OQU92522            transcript:Zm00001d029339_T001 1.00E-173
105- 10: transcript:EER94782            transcript:Zm00001d029337_T001  1.00E-57
## Alignment 106: score=394.0 e_value=1.8e-19 N=9 1&1 minus
106-  0: transcript:OQU90979            transcript:Zm00001d034034_T011         0
106-  1: transcript:EER90868            transcript:Zm00001d034033_T002         0
106-  2: transcript:EER90869            transcript:Zm00001d034032_T001 2.00E-133
```

106- 3: transcript:OQU90983 transcript:Zm00001d034031_T001 7.00E-28
106- 4: transcript:OQU90984 transcript:Zm00001d034030_T001 0
106- 5: transcript:OQU90987 transcript:Zm00001d034028_T003 0
106- 6: transcript:EER93447 transcript:Zm00001d034027_T001 4.00E-102
106- 7: transcript:OQU90988 transcript:Zm00001d034020_T001 9.00E-78
106- 8: transcript:EER93453 transcript:Zm00001d034019_T001 3.00E-168
Alignment 107: score=370.0 e_value=2.8e-15 N=8 1&1 minus
107- 0: transcript:Zm00001d029046_T012 transcript:EER92309 0
107- 1: transcript:Zm00001d029047_T001 transcript:EER94913 0
107- 2: transcript:Zm00001d029048_T001 transcript:EER94912 0
107- 3: transcript:Zm00001d029049_T001 transcript:EER94911 8.00E-97
107- 4: transcript:Zm00001d029050_T002 transcript:KXG39433 0
107- 5: transcript:Zm00001d029051_T001 transcript:OQU92647 3.00E-162
107- 6: transcript:Zm00001d029052_T001 transcript:KXG39430 6.00E-36
107- 7: transcript:Zm00001d029053_T005 transcript:OQU92645 0
Alignment 108: score=370.0 e_value=4e-19 N=9 1&1 minus
108- 0: transcript:EER92827 transcript:Zm00001d032904_T001 2.00E-10
108- 1: transcript:EER92828 transcript:Zm00001d032903_T001 4.00E-14
108- 2: transcript:EER95470 transcript:Zm00001d032902_T001 0
108- 3: transcript:KXG40142 transcript:Zm00001d032894_T001 0
108- 4: transcript:OQU93207 transcript:Zm00001d032881_T001 1.00E-115
108- 5: transcript:OQU93208 transcript:Zm00001d032876_T001 3.00E-114
108- 6: transcript:EER92836 transcript:Zm00001d032875_T009 0
108- 7: transcript:KXG40156 transcript:Zm00001d032873_T001 1.00E-61
108- 8: transcript:EER92840 transcript:Zm00001d032859_T006 0
Alignment 109: score=369.0 e_value=2.4e-18 N=9 1&1 minus
109- 0: transcript:EER94677 transcript:Zm00001d029561_T002 0
109- 1: transcript:EER92108 transcript:Zm00001d029560_T001 2.00E-175
109- 2: transcript:EER92109 transcript:Zm00001d029559_T001 3.00E-176
109- 3: transcript:EER92110 transcript:Zm00001d029558_T001 2.00E-106
109- 4: transcript:EER94678 transcript:Zm00001d029557_T001 0
109- 5: transcript:EER94679 transcript:Zm00001d029555_T002 4.00E-81
109- 6: transcript:OQU92376 transcript:Zm00001d029553_T001 2.00E-154
109- 7: transcript:OQU92377 transcript:Zm00001d029552_T001 0
109- 8: transcript:EER94680 transcript:Zm00001d029550_T008 0
Alignment 110: score=367.0 e_value=3.5e-19 N=9 1&1 minus
110- 0: transcript:Zm00001d029121_T005 transcript:OQU92609 0
110- 1: transcript:Zm00001d029122_T001 transcript:EER92286 5.00E-67
110- 2: transcript:Zm00001d029123_T005 transcript:EER92285 0
110- 3: transcript:Zm00001d029124_T001 transcript:OQU92608 4.00E-55
110- 4: transcript:Zm00001d029125_T001 transcript:OQU92607 0
110- 5: transcript:Zm00001d029126_T001 transcript:KXG39397 1.00E-132
110- 6: transcript:Zm00001d029127_T001 transcript:EER94862 6.00E-111
110- 7: transcript:Zm00001d029129_T002 transcript:KXG39395 0
110- 8: transcript:Zm00001d029130_T001 transcript:EER92281 0
Alignment 111: score=356.0 e_value=1.9e-17 N=9 1&1 minus
111- 0: transcript:EER91737 transcript:Zm00001d030314_T001 4.00E-60
111- 1: transcript:EER91740 transcript:Zm00001d030308_T001 0
111- 2: transcript:OQU91969 transcript:Zm00001d030304_T002 0
111- 3: transcript:EER91743 transcript:Zm00001d030303_T001 0
111- 4: transcript:EER94357 transcript:Zm00001d030301_T002 2.00E-51
111- 5: transcript:EER94358 transcript:Zm00001d030299_T001 0
111- 6: transcript:EER91744 transcript:Zm00001d030290_T001 2.00E-163
111- 7: transcript:EER91750 transcript:Zm00001d030285_T003 0

111- 8: transcript:OQU91980 transcript:Zm00001d030282_T007 0
Alignment 112: score=354.0 e_value=2.7e-15 N=8 1&1 minus
112- 0: transcript:Zm00001d028898_T011 transcript:EER94974 0
112- 1: transcript:Zm00001d028899_T001 transcript:KXG39525 2.00E-17
112- 2: transcript:Zm00001d028901_T001 transcript:OQU92709 0
112- 3: transcript:Zm00001d028902_T003 transcript:KXG39524 5.00E-63
112- 4: transcript:Zm00001d028903_T002 transcript:EER92366 0
112- 5: transcript:Zm00001d028905_T001 transcript:EER94971 0
112- 6: transcript:Zm00001d028906_T001 transcript:KXG39520 4.00E-08
112- 7: transcript:Zm00001d028907_T016 transcript:KXG39519 0
Alignment 113: score=350.0 e_value=4e-15 N=8 1&1 minus
113- 0: transcript:EER93328 transcript:Zm00001d029609_T001 6.00E-45
113- 1: transcript:Zm00001d027415_T001 transcript:OQU93361 0
113- 2: transcript:Zm00001d027416_T001 transcript:OQU93359 0
113- 3: transcript:Zm00001d027419_T001 transcript:OQU93358 3.00E-157
113- 4: transcript:Zm00001d027421_T001 transcript:EER92962 7.00E-149
113- 5: transcript:Zm00001d027422_T001 transcript:EER92961 1.00E-170
113- 6: transcript:Zm00001d027423_T002 transcript:EER92960 0
113- 7: transcript:Zm00001d027425_T001 transcript:OQU93352 2.00E-130
Alignment 114: score=348.0 e_value=1.6e-15 N=8 1&1 minus
114- 0: transcript:EER94478 transcript:Zm00001d029950_T001 0
114- 1: transcript:EER91921 transcript:Zm00001d029948_T001 0
114- 2: transcript:EER94479 transcript:Zm00001d029947_T001 4.00E-131
114- 3: transcript:EER94480 transcript:Zm00001d029946_T003 0
114- 4: transcript:OQU92128 transcript:Zm00001d029940_T001 5.00E-105
114- 5: transcript:EER94481 transcript:Zm00001d029938_T002 0
114- 6: transcript:KXG38908 transcript:Zm00001d029937_T001 0
114- 7: transcript:EER94486 transcript:Zm00001d029934_T001 1.00E-141
Alignment 115: score=338.0 e_value=4.6e-15 N=8 1&1 minus
115- 0: transcript:Zm00001d027978_T001 transcript:OQU93095 1.00E-153
115- 1: transcript:Zm00001d027982_T002 transcript:KXG40007 0
115- 2: transcript:Zm00001d027983_T001 transcript:EER92732 4.00E-152
115- 3: transcript:Zm00001d027987_T001 transcript:EER95359 2.00E-141
115- 4: transcript:Zm00001d027991_T001 transcript:EER92730 8.00E-168
115- 5: transcript:Zm00001d027992_T001 transcript:KXG40003 1.00E-64
115- 6: transcript:Zm00001d027994_T001 transcript:KXG40002 0
115- 7: transcript:Zm00001d027995_T001 transcript:KXG39999 2.00E-174
Alignment 116: score=335.0 e_value=2.9e-14 N=8 1&1 minus
116- 0: transcript:Zm00001d029248_T002 transcript:EER94813 0
116- 1: transcript:Zm00001d029249_T001 transcript:EER94816 5.00E-141
116- 2: transcript:Zm00001d029251_T001 transcript:KXG39308 4.00E-52
116- 3: transcript:Zm00001d029255_T001 transcript:OQU92553 0
116- 4: transcript:Zm00001d029256_T002 transcript:KXG39307 7.00E-92
116- 5: transcript:Zm00001d029258_T001 transcript:OQU92552 0
116- 6: transcript:Zm00001d029260_T004 transcript:OQU92551 0
116- 7: transcript:Zm00001d029263_T001 transcript:EER92223 8.00E-60
Alignment 117: score=333.0 e_value=6.6e-14 N=8 1&1 minus
117- 0: transcript:KXG37156 transcript:Zm00001d034809_T001 2.00E-98
117- 1: transcript:EER93115 transcript:Zm00001d034808_T001 4.00E-141
117- 2: transcript:KXG37162 transcript:Zm00001d034807_T002 0
117- 3: transcript:EER90539 transcript:Zm00001d034805_T002 0
117- 4: transcript:EER93116 transcript:Zm00001d034804_T002 0
117- 5: transcript:EER90540 transcript:Zm00001d034802_T001 2.00E-130
117- 6: transcript:KXG37163 transcript:Zm00001d034801_T001 1.00E-125

117- 7: transcript:OQU90646 transcript:Zm00001d034799_T001 1.00E-126
Alignment 118: score=316.0 e_value=1.2e-12 N=7 1&1 minus
118- 0: transcript:EER91236 transcript:Zm00001d030864_T001 0
118- 1: transcript:OQU91384 transcript:Zm00001d030862_T001 2.00E-69
118- 2: transcript:EER91243 transcript:Zm00001d030850_T001 3.00E-46
118- 3: transcript:OQU91386 transcript:Zm00001d030849_T001 1.00E-132
118- 4: transcript:KXG38042 transcript:Zm00001d030846_T001 0
118- 5: transcript:EER91248 transcript:Zm00001d030837_T001 0
118- 6: transcript:EER93832 transcript:Zm00001d030834_T001 4.00E-24
Alignment 119: score=301.0 e_value=7.4e-16 N=8 1&1 minus
119- 0: transcript:EER93724 transcript:Zm00001d031131_T001 2.00E-55
119- 1: transcript:EER91130 transcript:Zm00001d031129_T001 4.00E-30
119- 2: transcript:KXG37902 transcript:Zm00001d031128_T004 0
119- 3: transcript:KXG37912 transcript:Zm00001d031127_T001 9.00E-45
119- 4: transcript:EER91146 transcript:Zm00001d031120_T003 0
119- 5: transcript:KXG37926 transcript:Zm00001d031119_T001 2.00E-89
119- 6: transcript:KXG37931 transcript:Zm00001d031113_T001 8.00E-15
119- 7: transcript:EER91158 transcript:Zm00001d031101_T002 8.00E-53
Alignment 120: score=293.0 e_value=4.5e-10 N=6 1&1 minus
120- 0: transcript:EER91955 transcript:Zm00001d029872_T001 0
120- 1: transcript:KXG38947 transcript:Zm00001d029869_T001 1.00E-107
120- 2: transcript:KXG38949 transcript:Zm00001d029868_T004 0
120- 3: transcript:EER91959 transcript:Zm00001d029865_T001 0
120- 4: transcript:EER94520 transcript:Zm00001d029862_T001 0
120- 5: transcript:OQU92169 transcript:Zm00001d029861_T002 0
Alignment 121: score=291.0 e_value=1.7e-09 N=6 1&1 minus
121- 0: transcript:KXG37354 transcript:Zm00001d034475_T001 3.00E-142
121- 1: transcript:EER90731 transcript:Zm00001d034469_T001 1.00E-100
121- 2: transcript:EER93290 transcript:Zm00001d034468_T001 0
121- 3: transcript:KXG37358 transcript:Zm00001d034467_T001 0
121- 4: transcript:EER90734 transcript:Zm00001d034463_T003 7.00E-97
121- 5: transcript:EER93291 transcript:Zm00001d034462_T001 3.00E-45
Alignment 122: score=287.0 e_value=2.1e-12 N=7 1&1 minus
122- 0: transcript:EER92206 transcript:Zm00001d029314_T001 0
122- 1: transcript:KXG39284 transcript:Zm00001d029313_T001 0
122- 2: transcript:EER92211 transcript:Zm00001d029305_T001 3.00E-44
122- 3: transcript:EER92214 transcript:Zm00001d029303_T001 2.00E-171
122- 4: transcript:OQU92532 transcript:Zm00001d029300_T001 0
122- 5: transcript:EER92215 transcript:Zm00001d029299_T001 1.00E-59
122- 6: transcript:OQU92535 transcript:Zm00001d029297_T003 0
Alignment 123: score=284.0 e_value=8e-15 N=8 1&1 minus
123- 0: transcript:Zm00001d027295_T002 transcript:EER95686 0
123- 1: transcript:Zm00001d027296_T003 transcript:EER93025 0
123- 2: transcript:Zm00001d027298_T001 transcript:OQU93465 0
123- 3: transcript:Zm00001d027299_T014 transcript:EER93022 0
123- 4: transcript:Zm00001d027300_T001 transcript:KXG40406 0
123- 5: transcript:Zm00001d027302_T002 transcript:EER93012 0
123- 6: transcript:Zm00001d027304_T026 transcript:OQU93436 0
123- 7: transcript:Zm00001d027305_T002 transcript:EER93014 0
Alignment 124: score=281.0 e_value=3.7e-08 N=6 1&1 minus
124- 0: transcript:Zm00001d028183_T002 transcript:EER92655 0
124- 1: transcript:Zm00001d028185_T002 transcript:KXG39897 4.00E-176
124- 2: transcript:Zm00001d028186_T001 transcript:EER95295 0
124- 3: transcript:Zm00001d028188_T001 transcript:KXG39896 0

```
124-  4: transcript:Zm00001d028189_T001 transcript:EER92652                    0
124-  5: transcript:Zm00001d028191_T002 transcript:EER95291            3.00E-133
## Alignment 125: score=276.0 e_value=6.4e-11 N=6 1&1 minus
125-  0: transcript:EER90757            transcript:Zm00001d034385_T001          0
125-  1: transcript:EER93325            transcript:Zm00001d034384_T001   3.00E-73
125-  2: transcript:EER93326            transcript:Zm00001d034383_T003          0
125-  3: transcript:EER90763            transcript:Zm00001d034380_T001          0
125-  4: transcript:EER93327            transcript:Zm00001d034379_T001   2.00E-56
125-  5: transcript:EER90764            transcript:Zm00001d034372_T001          0
## Alignment 126: score=271.0 e_value=7.4e-12 N=7 1&1 minus
126-  0: transcript:KXG39207            transcript:Zm00001d029424_T001   1.00E-24
126-  1: transcript:OQU92464            transcript:Zm00001d029422_T007          0
126-  2: transcript:EER92167            transcript:Zm00001d029420_T001          0
126-  3: transcript:KXG39208            transcript:Zm00001d029419_T005          0
126-  4: transcript:OQU92466            transcript:Zm00001d029418_T001   9.00E-44
126-  5: transcript:EER92170            transcript:Zm00001d029417_T002          0
126-  6: transcript:KXG39211            transcript:Zm00001d029416_T001          0
## Alignment 127: score=270.0 e_value=4.1e-09 N=6 1&1 minus
127-  0: transcript:KXG38738            transcript:Zm00001d030251_T001          0
127-  1: transcript:OQU92000            transcript:Zm00001d030250_T001          0
127-  2: transcript:EER91787            transcript:Zm00001d030248_T001          0
127-  3: transcript:EER91792            transcript:Zm00001d030247_T001   4.00E-58
127-  4: transcript:KXG38746            transcript:Zm00001d030239_T007          0
127-  5: transcript:KXG38749            transcript:Zm00001d030236_T001   1.00E-53
## Alignment 128: score=267.0 e_value=1.4e-10 N=6 1&1 minus
128-  0: transcript:Zm00001d027383_T001 transcript:OQU93373                    0
128-  1: transcript:Zm00001d027384_T001 transcript:OQU93372                    0
128-  2: transcript:Zm00001d027385_T003 transcript:OQU93371            7.00E-164
128-  3: transcript:Zm00001d027386_T002 transcript:KXG40331                    0
128-  4: transcript:Zm00001d027387_T001 transcript:EER92974                    0
128-  5: transcript:Zm00001d027392_T003 transcript:KXG40330                    0
## Alignment 129: score=267.0 e_value=2.4e-08 N=6 1&1 minus
129-  0: transcript:EER90831            transcript:Zm00001d034165_T001   6.00E-58
129-  1: transcript:EER93397            transcript:Zm00001d034164_T002          0
129-  2: transcript:KXG37485            transcript:Zm00001d034163_T001  6.00E-153
129-  3: transcript:EER93398            transcript:Zm00001d034161_T003          0
129-  4: transcript:KXG37487            transcript:Zm00001d034160_T001  6.00E-150
129-  5: transcript:EER93399            transcript:Zm00001d034159_T002  2.00E-143
## Alignment 130: score=265.0 e_value=3.6e-08 N=6 1&1 minus
130-  0: transcript:EER95179            transcript:Zm00001d030317_T001   2.00E-08
130-  1: transcript:EER92564            transcript:Zm00001d030314_T001   5.00E-19
130-  2: transcript:EER95182            transcript:Zm00001d030310_T001          0
130-  3: transcript:KXG39784            transcript:Zm00001d030304_T002  1.00E-128
130-  4: transcript:KXG39786            transcript:Zm00001d030303_T001  4.00E-118
130-  5: transcript:EER92569            transcript:Zm00001d030290_T001  2.00E-123
## Alignment 131: score=257.0 e_value=6.5e-10 N=6 1&1 minus
131-  0: transcript:EER92097            transcript:Zm00001d029570_T001          0
131-  1: transcript:EER94674            transcript:Zm00001d029568_T001          0
131-  2: transcript:EER92102            transcript:Zm00001d029567_T001   1.00E-78
131-  3: transcript:EER92103            transcript:Zm00001d029566_T005          0
131-  4: transcript:EER94675            transcript:Zm00001d029564_T001          0
131-  5: transcript:OQU92361            transcript:Zm00001d029563_T002          0
## Alignment 132: score=252.0 e_value=1.7e-08 N=6 1&1 minus
132-  0: transcript:EER94734            transcript:Zm00001d029435_T001          0
```

132- 1:	transcript:EER94736	transcript:Zm00001d029432_T002	0
132- 2:	transcript:KXG39199	transcript:Zm00001d029429_T001	0
132- 3:	transcript:OQU92455	transcript:Zm00001d029428_T001	0
132- 4:	transcript:OQU92460	transcript:Zm00001d029427_T001	0
132- 5:	transcript:KXG39205	transcript:Zm00001d029426_T024	0
## Alignment 133: score=250.0 e_value=1.4e-09 N=6 1&1 minus			
133- 0:	transcript:Zm00001d029279_T001	transcript:EER94803	0
133- 1:	transcript:Zm00001d029281_T007	transcript:EER92219	0
133- 2:	transcript:Zm00001d029285_T005	transcript:EER94801	0
133- 3:	transcript:Zm00001d029286_T001	transcript:OQU92540	0
133- 4:	transcript:Zm00001d029287_T001	transcript:EER92218	0
133- 5:	transcript:Zm00001d029289_T001	transcript:KXG39295	0
## Alignment 134: score=1033.0 e_value=1.9e-76 N=25 1&2 plus			
134- 0:	transcript:Zm00001d031745_T001	transcript:EER98990	2.00E-106
134- 1:	transcript:Zm00001d031753_T001	transcript:KXG35739	1.00E-32
134- 2:	transcript:Zm00001d031759_T001	transcript:EER96827	0
134- 3:	transcript:Zm00001d031760_T001	transcript:EER96828	1.00E-104
134- 4:	transcript:Zm00001d031773_T003	transcript:OQU89603	3.00E-53
134- 5:	transcript:Zm00001d031777_T001	transcript:KXG35752	5.00E-167
134- 6:	transcript:Zm00001d031778_T001	transcript:KXG35754	2.00E-63
134- 7:	transcript:Zm00001d031781_T001	transcript:EER96848	2.00E-54
134- 8:	transcript:Zm00001d031790_T002	transcript:EER96850	6.00E-78
134- 9:	transcript:Zm00001d031792_T001	transcript:EER99009	9.00E-165
134- 10:	transcript:Zm00001d031794_T001	transcript:EER96852	0
134- 11:	transcript:Zm00001d031796_T001	transcript:EER96855	1.00E-60
134- 12:	transcript:Zm00001d031797_T001	transcript:EER96862	8.00E-20
134- 13:	transcript:Zm00001d031798_T001	transcript:OQU89614	2.00E-25
134- 14:	transcript:Zm00001d031801_T001	transcript:KXG35780	6.00E-136
134- 15:	transcript:Zm00001d031804_T001	transcript:EER99019	4.00E-84
134- 16:	transcript:Zm00001d031806_T001	transcript:KXG35788	2.00E-09
134- 17:	transcript:Zm00001d031807_T003	transcript:KXG35796	2.00E-96
134- 18:	transcript:Zm00001d031810_T001	transcript:OQU89629	2.00E-100
134- 19:	transcript:Zm00001d031818_T001	transcript:EER96881	3.00E-61
134- 20:	transcript:Zm00001d031826_T001	transcript:OQU89633	5.00E-52
134- 21:	transcript:Zm00001d031832_T003	transcript:KXG35804	1.00E-81
134- 22:	transcript:Zm00001d031837_T001	transcript:EER96886	1.00E-103
134- 23:	transcript:Zm00001d031840_T001	transcript:EER96888	9.00E-106
134- 24:	transcript:Zm00001d031844_T001	transcript:OQU89636	3.00E-153
## Alignment 135: score=709.0 e_value=6.2e-46 N=17 1&2 plus			
135- 0:	transcript:Zm00001d031527_T001	transcript:EER96987	0
135- 1:	transcript:Zm00001d031532_T001	transcript:OQU89720	0
135- 2:	transcript:Zm00001d031543_T005	transcript:EER99121	0
135- 3:	transcript:Zm00001d031545_T001	transcript:EER99130	5.00E-47
135- 4:	transcript:Zm00001d031546_T001	transcript:EER96997	4.00E-12
135- 5:	transcript:Zm00001d031554_T003	transcript:KXG35959	0
135- 6:	transcript:Zm00001d031555_T001	transcript:EER99135	2.00E-161
135- 7:	transcript:Zm00001d031560_T001	transcript:EER97002	7.00E-94
135- 8:	transcript:Zm00001d031561_T002	transcript:OQU89735	5.00E-92
135- 9:	transcript:Zm00001d031569_T001	transcript:EER99137	2.00E-28
135- 10:	transcript:Zm00001d031570_T001	transcript:KXG35961	6.00E-21
135- 11:	transcript:Zm00001d031571_T001	transcript:EER99139	0
135- 12:	transcript:Zm00001d031577_T001	transcript:EER99141	0
135- 13:	transcript:Zm00001d031586_T001	transcript:OQU89743	2.00E-35
135- 14:	transcript:Zm00001d031594_T001	transcript:KXG35972	2.00E-54

135- 15: transcript:Zm00001d031602_T002 transcript:EER99151 0
135- 16: transcript:Zm00001d031620_T001 transcript:EER97012 5.00E-120
Alignment 136: score=547.0 e_value=5e-29 N=13 1&2 plus
136- 0: transcript:Zm00001d031635_T001 transcript:OQU89751 1.00E-139
136- 1: transcript:Zm00001d031637_T003 transcript:KXG35978 3.00E-104
136- 2: transcript:Zm00001d031640_T001 transcript:EER99160 0
136- 3: transcript:Zm00001d031647_T001 transcript:OQU89754 4.00E-112
136- 4: transcript:Zm00001d031648_T004 transcript:EER99164 0
136- 5: transcript:Zm00001d031651_T001 transcript:OQU89758 7.00E-12
136- 6: transcript:Zm00001d031655_T003 transcript:EER97018 2.00E-43
136- 7: transcript:Zm00001d031659_T001 transcript:EER97019 0
136- 8: transcript:Zm00001d031660_T002 transcript:KXG35983 0
136- 9: transcript:Zm00001d031665_T001 transcript:EER99171 3.00E-96
136- 10: transcript:Zm00001d031667_T006 transcript:EER99172 0
136- 11: transcript:Zm00001d031673_T001 transcript:EER97023 2.00E-26
136- 12: transcript:Zm00001d031676_T002 transcript:EER99165 0
Alignment 137: score=463.0 e_value=8.4e-25 N=11 1&2 plus
137- 0: transcript:Zm00001d031389_T001 transcript:KXG35889 3.00E-21
137- 1: transcript:Zm00001d031404_T001 transcript:EER96959 7.00E-63
137- 2: transcript:Zm00001d031416_T001 transcript:EER96965 7.00E-126
137- 3: transcript:Zm00001d031423_T001 transcript:OQU89687 1.00E-56
137- 4: transcript:Zm00001d031426_T001 transcript:EER99080 5.00E-162
137- 5: transcript:Zm00001d031430_T005 transcript:EER99082 0
137- 6: transcript:Zm00001d031431_T001 transcript:EER99083 2.00E-67
137- 7: transcript:Zm00001d031436_T001 transcript:KXG35901 3.00E-126
137- 8: transcript:Zm00001d031441_T002 transcript:KXG35903 5.00E-113
137- 9: transcript:Zm00001d031444_T001 transcript:EER99087 0
137- 10: transcript:Zm00001d031445_T001 transcript:EER99089 0
Alignment 138: score=382.0 e_value=1.3e-17 N=9 1&2 plus
138- 0: transcript:Zm00001d034190_T001 transcript:EER95817 1.00E-31
138- 1: transcript:Zm00001d034191_T004 transcript:KXG34315 0
138- 2: transcript:Zm00001d034194_T004 transcript:EER97908 0
138- 3: transcript:Zm00001d034197_T001 transcript:EER97910 2.00E-39
138- 4: transcript:Zm00001d034198_T001 transcript:OQU88381 5.00E-69
138- 5: transcript:Zm00001d034199_T001 transcript:EER95819 3.00E-18
138- 6: transcript:Zm00001d034200_T001 transcript:EER95824 5.00E-29
138- 7: transcript:Zm00001d034204_T001 transcript:KXG34322 0
138- 8: transcript:Zm00001d034205_T001 transcript:KXG34323 0
Alignment 139: score=380.0 e_value=3e-21 N=9 1&2 plus
139- 0: transcript:Zm00001d034494_T001 transcript:KXG34607 5.00E-108
139- 1: transcript:Zm00001d034497_T001 transcript:EER96015 2.00E-10
139- 2: transcript:Zm00001d034501_T001 transcript:KXG34614 3.00E-16
139- 3: transcript:Zm00001d034502_T003 transcript:EER98148 0
139- 4: transcript:Zm00001d034503_T001 transcript:OQU88647 3.00E-48
139- 5: transcript:Zm00001d034505_T001 transcript:EER96027 7.00E-106
139- 6: transcript:Zm00001d034507_T001 transcript:OQU88648 5.00E-13
139- 7: transcript:Zm00001d034508_T001 transcript:EER98156 0
139- 8: transcript:Zm00001d034511_T001 transcript:KXG34620 0
Alignment 140: score=360.0 e_value=1.1e-16 N=9 1&2 plus
140- 0: transcript:Zm00001d028930_T001 transcript:KXG36991 3.00E-91
140- 1: transcript:Zm00001d028931_T002 transcript:EER97763 0
140- 2: transcript:Zm00001d028936_T002 transcript:KXG36997 1.00E-161
140- 3: transcript:Zm00001d028946_T001 transcript:OQU90515 1.00E-130
140- 4: transcript:Zm00001d028948_T001 transcript:KXG37006 4.00E-82

140- 5: transcript:Zm00001d028949_T002 transcript:KXG37001 0
140- 6: transcript:Zm00001d028952_T004 transcript:OQU90522 0
140- 7: transcript:Zm00001d028953_T001 transcript:OQU90528 3.00E-22
140- 8: transcript:Zm00001d028960_T001 transcript:OQU90529 2.00E-57
Alignment 141: score=346.0 e_value=3.8e-15 N=8 1&2 plus
141- 0: transcript:EER94885 transcript:Zm00001d007159_T001 1.00E-21
141- 1: transcript:EER92305 transcript:Zm00001d007164_T001 0
141- 2: transcript:EER94892 transcript:Zm00001d007167_T001 0
141- 3: transcript:OQU92638 transcript:Zm00001d007168_T001 3.00E-26
141- 4: transcript:KXG39422 transcript:Zm00001d007169_T001 0
141- 5: transcript:OQU92641 transcript:Zm00001d007172_T001 1.00E-138
141- 6: transcript:OQU92647 transcript:Zm00001d007175_T001 3.00E-85
141- 7: transcript:EER94912 transcript:Zm00001d007179_T001 3.00E-159
Alignment 142: score=346.0 e_value=2.2e-15 N=8 1&2 plus
142- 0: transcript:KXG37329 transcript:Zm00001d007757_T004 0
142- 1: transcript:OQU90803 transcript:Zm00001d007760_T001 0
142- 2: transcript:EER90705 transcript:Zm00001d007762_T001 0
142- 3: transcript:EER90709 transcript:Zm00001d007765_T001 5.00E-109
142- 4: transcript:OQU90815 transcript:Zm00001d007767_T001 1.00E-159
142- 5: transcript:EER90712 transcript:Zm00001d007768_T001 1.00E-95
142- 6: transcript:KXG37334 transcript:Zm00001d007769_T003 0
142- 7: transcript:EER93263 transcript:Zm00001d007773_T001 8.00E-154
Alignment 143: score=291.0 e_value=2.2e-11 N=7 1&2 plus
143- 0: transcript:Zm00001d032306_T001 transcript:KXG35533 6.00E-36
143- 1: transcript:Zm00001d032307_T001 transcript:OQU89431 1.00E-13
143- 2: transcript:Zm00001d032311_T001 transcript:EER98826 0
143- 3: transcript:Zm00001d032316_T001 transcript:EER98832 5.00E-52
143- 4: transcript:Zm00001d032318_T001 transcript:EER96684 6.00E-19
143- 5: transcript:Zm00001d032322_T001 transcript:EER96683 0
143- 6: transcript:Zm00001d032324_T001 transcript:EER96686 2.00E-52
Alignment 144: score=286.0 e_value=2e-12 N=7 1&2 plus
144- 0: transcript:Zm00001d034461_T001 transcript:EER95952 1.00E-120
144- 1: transcript:Zm00001d034468_T001 transcript:KXG34543 3.00E-16
144- 2: transcript:Zm00001d034479_T001 transcript:EER95966 3.00E-30
144- 3: transcript:Zm00001d034480_T002 transcript:EER95968 2.00E-105
144- 4: transcript:Zm00001d034482_T001 transcript:KXG34552 2.00E-06
144- 5: transcript:Zm00001d034484_T001 transcript:EER98088 3.00E-12
144- 6: transcript:Zm00001d034491_T001 transcript:OQU88584 1.00E-37
Alignment 145: score=260.0 e_value=8.9e-13 N=7 1&2 plus
145- 0: transcript:EER92317 transcript:Zm00001d007180_T001 0
145- 1: transcript:KXG39446 transcript:Zm00001d007181_T001 2.00E-89
145- 2: transcript:EER92321 transcript:Zm00001d007184_T001 0
145- 3: transcript:KXG39454 transcript:Zm00001d007185_T001 8.00E-07
145- 4: transcript:EER92323 transcript:Zm00001d007186_T004 0
145- 5: transcript:EER92327 transcript:Zm00001d007187_T002 4.00E-90
145- 6: transcript:EER94933 transcript:Zm00001d007188_T001 0
Alignment 146: score=491.0 e_value=1.5e-29 N=13 1&2 minus
146- 0: transcript:Zm00001d032008_T001 transcript:KXG36092 7.00E-84
146- 1: transcript:Zm00001d032013_T001 transcript:EER97111 1.00E-11
146- 2: transcript:Zm00001d032019_T001 transcript:EER99269 4.00E-56
146- 3: transcript:Zm00001d032022_T003 transcript:KXG36088 0
146- 4: transcript:Zm00001d032024_T001 transcript:EER99264 3.00E-105
146- 5: transcript:Zm00001d032027_T002 transcript:EER97098 6.00E-137
146- 6: transcript:Zm00001d032028_T001 transcript:OQU89832 0

```
146-  7: transcript:Zm00001d032031_T001 transcript:EER97092                  0
146-  8: transcript:Zm00001d032032_T001 transcript:KXG36070           3.00E-48
146-  9: transcript:Zm00001d032040_T001 transcript:KXG36062           5.00E-83
146- 10: transcript:Zm00001d032042_T001 transcript:EER99230                  0
146- 11: transcript:Zm00001d032044_T004 transcript:EER97077                  0
146- 12: transcript:Zm00001d032045_T001 transcript:EER97076          9.00E-153
## Alignment 147: score=332.0 e_value=7.4e-12 N=7 1&2 minus
147-  0: transcript:Zm00001d032172_T002 transcript:EER98900                  0
147-  1: transcript:Zm00001d032175_T001 transcript:KXG35632           6.00E-85
147-  2: transcript:Zm00001d032177_T001 transcript:KXG35630           2.00E-28
147-  3: transcript:Zm00001d032178_T001 transcript:KXG35628          2.00E-121
147-  4: transcript:Zm00001d032181_T001 transcript:EER98897           4.00E-77
147-  5: transcript:Zm00001d032182_T001 transcript:OQU89481          3.00E-147
147-  6: transcript:Zm00001d032183_T002 transcript:EER96727           4.00E-83
## Alignment 148: score=268.0 e_value=3.8e-11 N=7 1&2 minus
148-  0: transcript:Zm00001d028974_T001 transcript:OQU90496                  0
148-  1: transcript:Zm00001d028984_T001 transcript:EER99928          2.00E-126
148-  2: transcript:Zm00001d028986_T001 transcript:EER97739           5.00E-71
148-  3: transcript:Zm00001d028992_T001 transcript:KXG36973          4.00E-104
148-  4: transcript:Zm00001d028995_T001 transcript:EER97754          6.00E-101
148-  5: transcript:Zm00001d028998_T001 transcript:KXG36963           2.00E-91
148-  6: transcript:Zm00001d028999_T001 transcript:EER99924           4.00E-97
## Alignment 149: score=252.0 e_value=9.6e-09 N=6 1&3 minus
149-  0: transcript:OQU92829            transcript:Zm00001d042907_T001        0
149-  1: transcript:EER92483            transcript:Zm00001d042901_T001        0
149-  2: transcript:OQU92832            transcript:Zm00001d042900_T001 2.00E-14
149-  3: transcript:KXG40203            transcript:Zm00001d042898_T001 3.00E-152
149-  4: transcript:EER92488            transcript:Zm00001d042887_T001 8.00E-97
149-  5: transcript:EER92493            transcript:Zm00001d042886_T001 1.00E-119
## Alignment 150: score=3103.0 e_value=7e-289 N=71 1&5 plus
150-  0: transcript:EER93367            transcript:Zm00001d013154_T001        0
150-  1: transcript:KXG37457            transcript:Zm00001d013156_T001 7.00E-158
150-  2: transcript:EER93374            transcript:Zm00001d013159_T001        0
150-  3: transcript:EER93376            transcript:Zm00001d013162_T001        0
150-  4: transcript:EER90815            transcript:Zm00001d013163_T001        0
150-  5: transcript:EER90818            transcript:Zm00001d013164_T002        0
150-  6: transcript:EER90819            transcript:Zm00001d013166_T001        0
150-  7: transcript:EER90820            transcript:Zm00001d013168_T001        0
150-  8: transcript:EER93382            transcript:Zm00001d013170_T001 7.00E-123
150-  9: transcript:EER93384            transcript:Zm00001d013172_T001 4.00E-60
150- 10: transcript:KXG37470            transcript:Zm00001d013173_T001        0
150- 11: transcript:EER93386            transcript:Zm00001d013175_T001 7.00E-83
150- 12: transcript:KXG37474            transcript:Zm00001d013176_T002        0
150- 13: transcript:EER90825            transcript:Zm00001d013177_T002        0
150- 14: transcript:EER93387            transcript:Zm00001d013178_T001 1.00E-77
150- 15: transcript:EER93389            transcript:Zm00001d013179_T001 7.00E-120
150- 16: transcript:EER90826            transcript:Zm00001d013181_T001        0
150- 17: transcript:EER93390            transcript:Zm00001d013182_T003        0
150- 18: transcript:KXG37476            transcript:Zm00001d013184_T001 3.00E-101
150- 19: transcript:EER90827            transcript:Zm00001d013185_T001        0
150- 20: transcript:OQU90932            transcript:Zm00001d013186_T001 2.00E-36
150- 21: transcript:EER93393            transcript:Zm00001d013187_T001 2.00E-141
150- 22: transcript:EER90828            transcript:Zm00001d013188_T002 1.00E-152
150- 23: transcript:KXG37482            transcript:Zm00001d013191_T001 4.00E-60
```

```
150- 24: transcript:EER93395            transcript:Zm00001d013192_T001          0
150- 25: transcript:EER90829            transcript:Zm00001d013193_T001 4.00E-115
150- 26: transcript:EER90831            transcript:Zm00001d013195_T001          0
150- 27: transcript:EER93396            transcript:Zm00001d013196_T001          0
150- 28: transcript:KXG37485            transcript:Zm00001d013200_T001 2.00E-149
150- 29: transcript:EER93398            transcript:Zm00001d013201_T003          0
150- 30: transcript:KXG37487            transcript:Zm00001d013202_T001 7.00E-135
150- 31: transcript:EER93399            transcript:Zm00001d013204_T001 7.00E-154
150- 32: transcript:EER90833            transcript:Zm00001d013206_T003          0
150- 33: transcript:EER90834            transcript:Zm00001d013208_T001 3.00E-115
150- 34: transcript:EER90837            transcript:Zm00001d013209_T001 7.00E-154
150- 35: transcript:EER93404            transcript:Zm00001d013210_T001  3.00E-46
150- 36: transcript:EER93405            transcript:Zm00001d013212_T001 2.00E-106
150- 37: transcript:EER90839            transcript:Zm00001d013216_T001 1.00E-110
150- 38: transcript:EER90840            transcript:Zm00001d013217_T001          0
150- 39: transcript:EER93409            transcript:Zm00001d013218_T003          0
150- 40: transcript:KXG37496            transcript:Zm00001d013219_T001          0
150- 41: transcript:KXG37497            transcript:Zm00001d013220_T001          0
150- 42: transcript:EER90846            transcript:Zm00001d013221_T001  6.00E-66
150- 43: transcript:EER90847            transcript:Zm00001d013222_T001  3.00E-46
150- 44: transcript:EER93410            transcript:Zm00001d013223_T002 3.00E-154
150- 45: transcript:EER90849            transcript:Zm00001d013228_T001          0
150- 46: transcript:EER93412            transcript:Zm00001d013229_T001 6.00E-123
150- 47: transcript:OQU90944            transcript:Zm00001d013230_T001 3.00E-123
150- 48: transcript:EER93415            transcript:Zm00001d013232_T001 1.00E-121
150- 49: transcript:OQU90947            transcript:Zm00001d013238_T001          0
150- 50: transcript:EER90852            transcript:Zm00001d013239_T002          0
150- 51: transcript:KXG37501            transcript:Zm00001d013240_T002          0
150- 52: transcript:KXG37510            transcript:Zm00001d013241_T001          0
150- 53: transcript:EER93420            transcript:Zm00001d013242_T001 2.00E-148
150- 54: transcript:OQU90956            transcript:Zm00001d013243_T002          0
150- 55: transcript:KXG37517            transcript:Zm00001d013245_T001          0
150- 56: transcript:EER93423            transcript:Zm00001d013246_T001 5.00E-132
150- 57: transcript:KXG37527            transcript:Zm00001d013247_T002          0
150- 58: transcript:EER90856            transcript:Zm00001d013249_T001          0
150- 59: transcript:KXG37528            transcript:Zm00001d013250_T003  1.00E-76
150- 60: transcript:EER90858            transcript:Zm00001d013251_T001          0
150- 61: transcript:EER93429            transcript:Zm00001d013252_T001 1.00E-146
150- 62: transcript:KXG37529            transcript:Zm00001d013253_T001          0
150- 63: transcript:EER93433            transcript:Zm00001d013254_T002          0
150- 64: transcript:EER90859            transcript:Zm00001d013256_T001          0
150- 65: transcript:EER93435            transcript:Zm00001d013257_T002  1.00E-86
150- 66: transcript:KXG37536            transcript:Zm00001d013258_T001  2.00E-83
150- 67: transcript:EER90862            transcript:Zm00001d013259_T001 4.00E-142
150- 68: transcript:EER93437            transcript:Zm00001d013261_T001          0
150- 69: transcript:OQU90969            transcript:Zm00001d013262_T001 3.00E-149
150- 70: transcript:EER93441            transcript:Zm00001d013263_T001  3.00E-42
## Alignment 151: score=2318.0 e_value=1e-209 N=54 1&5 plus
151-  0: transcript:EER93585            transcript:Zm00001d013443_T001          0
151-  1: transcript:EER93586            transcript:Zm00001d013444_T002          0
151-  2: transcript:EER91002            transcript:Zm00001d013446_T001 1.00E-118
151-  3: transcript:EER91004            transcript:Zm00001d013447_T001 5.00E-156
151-  4: transcript:EER91005            transcript:Zm00001d013448_T001  5.00E-66
151-  5: transcript:EER93589            transcript:Zm00001d013449_T001          0
```

151- 6:	transcript:EER91011	transcript:Zm00001d013451_T001	1.00E-86
151- 7:	transcript:OQU91119	transcript:Zm00001d013452_T002	0
151- 8:	transcript:KXG37746	transcript:Zm00001d013453_T001	0
151- 9:	transcript:EER91015	transcript:Zm00001d013455_T001	8.00E-159
151- 10:	transcript:KXG37747	transcript:Zm00001d013456_T001	0
151- 11:	transcript:EER91016	transcript:Zm00001d013459_T001	7.00E-58
151- 12:	transcript:EER93591	transcript:Zm00001d013461_T002	7.00E-100
151- 13:	transcript:EER91017	transcript:Zm00001d013463_T001	0
151- 14:	transcript:EER93592	transcript:Zm00001d013465_T001	0
151- 15:	transcript:EER91018	transcript:Zm00001d013466_T005	0
151- 16:	transcript:EER93595	transcript:Zm00001d013467_T001	1.00E-180
151- 17:	transcript:EER93596	transcript:Zm00001d013468_T002	0
151- 18:	transcript:EER91019	transcript:Zm00001d013469_T002	0
151- 19:	transcript:OQU91126	transcript:Zm00001d013470_T007	0
151- 20:	transcript:EER91020	transcript:Zm00001d013471_T001	0
151- 21:	transcript:OQU91136	transcript:Zm00001d013477_T008	0
151- 22:	transcript:OQU91138	transcript:Zm00001d013480_T001	1.00E-149
151- 23:	transcript:EER93602	transcript:Zm00001d013481_T001	0
151- 24:	transcript:EER91027	transcript:Zm00001d013482_T001	4.00E-84
151- 25:	transcript:EER91029	transcript:Zm00001d013485_T007	0
151- 26:	transcript:EER91030	transcript:Zm00001d013486_T001	0
151- 27:	transcript:OQU91144	transcript:Zm00001d013487_T001	5.00E-31
151- 28:	transcript:KXG37778	transcript:Zm00001d013489_T001	0
151- 29:	transcript:KXG37779	transcript:Zm00001d013492_T001	0
151- 30:	transcript:KXG37784	transcript:Zm00001d013493_T001	0
151- 31:	transcript:EER91037	transcript:Zm00001d013495_T002	0
151- 32:	transcript:OQU91156	transcript:Zm00001d013496_T001	0
151- 33:	transcript:EER93617	transcript:Zm00001d013497_T001	0
151- 34:	transcript:EER93618	transcript:Zm00001d013498_T001	3.00E-83
151- 35:	transcript:EER91041	transcript:Zm00001d013499_T002	0
151- 36:	transcript:EER93619	transcript:Zm00001d013500_T001	1.00E-77
151- 37:	transcript:KXG37797	transcript:Zm00001d013501_T002	4.00E-12
151- 38:	transcript:KXG37800	transcript:Zm00001d013502_T001	5.00E-50
151- 39:	transcript:EER93624	transcript:Zm00001d013504_T001	3.00E-97
151- 40:	transcript:EER91045	transcript:Zm00001d013505_T001	2.00E-138
151- 41:	transcript:EER93626	transcript:Zm00001d013506_T001	0
151- 42:	transcript:KXG37805	transcript:Zm00001d013507_T001	0
151- 43:	transcript:OQU91164	transcript:Zm00001d013509_T001	8.00E-51
151- 44:	transcript:EER91052	transcript:Zm00001d013514_T001	3.00E-56
151- 45:	transcript:EER91056	transcript:Zm00001d013519_T001	0
151- 46:	transcript:KXG37810	transcript:Zm00001d013520_T001	1.00E-11
151- 47:	transcript:EER93639	transcript:Zm00001d013521_T001	0
151- 48:	transcript:OQU91171	transcript:Zm00001d013522_T001	3.00E-75
151- 49:	transcript:EER93641	transcript:Zm00001d013523_T002	0
151- 50:	transcript:OQU91172	transcript:Zm00001d013524_T009	0
151- 51:	transcript:EER93643	transcript:Zm00001d013526_T002	2.00E-133
151- 52:	transcript:EER93646	transcript:Zm00001d013527_T002	0
151- 53:	transcript:EER93647	transcript:Zm00001d013528_T001	0

Alignment 152: score=1854.0 e_value=2.8e-154 N=42 1&5 plus

152- 0:	transcript:EER91123	transcript:Zm00001d013624_T001	0
152- 1:	transcript:EER93717	transcript:Zm00001d013625_T001	1.00E-96
152- 2:	transcript:EER93718	transcript:Zm00001d013626_T002	2.00E-98
152- 3:	transcript:EER91124	transcript:Zm00001d013627_T001	0
152- 4:	transcript:EER91122	transcript:Zm00001d013629_T001	0

152- 5:	transcript:KXG37899	transcript:Zm00001d013630_T001	9.00E-54
152- 6:	transcript:OQU91240	transcript:Zm00001d013631_T001	9.00E-115
152- 7:	transcript:KXG37901	transcript:Zm00001d013632_T001	7.00E-120
152- 8:	transcript:OQU91241	transcript:Zm00001d013635_T001	0
152- 9:	transcript:EER93724	transcript:Zm00001d013638_T001	0
152- 10:	transcript:EER91129	transcript:Zm00001d013639_T001	3.00E-87
152- 11:	transcript:EER91130	transcript:Zm00001d013641_T001	2.00E-86
152- 12:	transcript:EER91131	transcript:Zm00001d013642_T001	3.00E-51
152- 13:	transcript:KXG37902	transcript:Zm00001d013644_T001	0
152- 14:	transcript:OQU91243	transcript:Zm00001d013645_T001	9.00E-63
152- 15:	transcript:EER93727	transcript:Zm00001d013646_T001	0
152- 16:	transcript:EER93728	transcript:Zm00001d013647_T001	5.00E-12
152- 17:	transcript:KXG37915	transcript:Zm00001d013651_T001	0
152- 18:	transcript:EER91146	transcript:Zm00001d013652_T003	0
152- 19:	transcript:KXG37918	transcript:Zm00001d013653_T010	0
152- 20:	transcript:OQU91253	transcript:Zm00001d013654_T001	0
152- 21:	transcript:EER93732	transcript:Zm00001d013655_T001	0
152- 22:	transcript:EER93733	transcript:Zm00001d013656_T001	2.00E-88
152- 23:	transcript:KXG37921	transcript:Zm00001d013657_T001	1.00E-24
152- 24:	transcript:OQU91257	transcript:Zm00001d013658_T001	0
152- 25:	transcript:KXG37926	transcript:Zm00001d013659_T001	0
152- 26:	transcript:KXG37931	transcript:Zm00001d013660_T001	3.00E-76
152- 27:	transcript:OQU91263	transcript:Zm00001d013661_T001	3.00E-36
152- 28:	transcript:EER93742	transcript:Zm00001d013664_T006	0
152- 29:	transcript:EER91158	transcript:Zm00001d013667_T001	8.00E-157
152- 30:	transcript:EER91159	transcript:Zm00001d013668_T001	4.00E-161
152- 31:	transcript:EER93745	transcript:Zm00001d013669_T002	0
152- 32:	transcript:KXG37936	transcript:Zm00001d013672_T001	0
152- 33:	transcript:EER91165	transcript:Zm00001d013673_T001	2.00E-171
152- 34:	transcript:KXG37939	transcript:Zm00001d013676_T001	4.00E-179
152- 35:	transcript:KXG37940	transcript:Zm00001d013677_T001	3.00E-55
152- 36:	transcript:EER91167	transcript:Zm00001d013680_T002	0
152- 37:	transcript:KXG37943	transcript:Zm00001d013683_T003	0
152- 38:	transcript:OQU91272	transcript:Zm00001d013688_T001	0
152- 39:	transcript:EER93751	transcript:Zm00001d013689_T001	0
152- 40:	transcript:EER91168	transcript:Zm00001d013692_T001	0
152- 41:	transcript:EER91171	transcript:Zm00001d013693_T001	2.00E-138

Alignment 153: score=1467.0 e_value=6.5e-117 N=35 1&5 plus

153- 0:	transcript:OQU90772	transcript:Zm00001d013021_T001	2.00E-167
153- 1:	transcript:EER93229	transcript:Zm00001d013022_T001	0
153- 2:	transcript:EER90667	transcript:Zm00001d013023_T004	0
153- 3:	transcript:OQU90774	transcript:Zm00001d013025_T001	2.00E-153
153- 4:	transcript:EER90673	transcript:Zm00001d013026_T001	4.00E-21
153- 5:	transcript:EER90674	transcript:Zm00001d013027_T002	0
153- 6:	transcript:EER93233	transcript:Zm00001d013028_T001	5.00E-176
153- 7:	transcript:EER90679	transcript:Zm00001d013029_T001	0
153- 8:	transcript:EER93237	transcript:Zm00001d013030_T002	0
153- 9:	transcript:EER93240	transcript:Zm00001d013032_T001	0
153- 10:	transcript:EER93241	transcript:Zm00001d013033_T001	2.00E-121
153- 11:	transcript:EER93242	transcript:Zm00001d013034_T001	0
153- 12:	transcript:KXG37308	transcript:Zm00001d013035_T004	0
153- 13:	transcript:OQU90794	transcript:Zm00001d013038_T002	6.00E-103
153- 14:	transcript:EER93246	transcript:Zm00001d013039_T001	8.00E-124
153- 15:	transcript:EER90693	transcript:Zm00001d013040_T008	0

153- 16: transcript:KXG37312 transcript:Zm00001d013041_T003 1.00E-53
153- 17: transcript:EER93250 transcript:Zm00001d013042_T001 0
153- 18: transcript:KXG37314 transcript:Zm00001d013043_T001 1.00E-172
153- 19: transcript:OQU90799 transcript:Zm00001d013045_T001 4.00E-54
153- 20: transcript:KXG37324 transcript:Zm00001d013046_T002 0
153- 21: transcript:KXG37326 transcript:Zm00001d013047_T004 0
153- 22: transcript:EER93257 transcript:Zm00001d013048_T009 0
153- 23: transcript:OQU90803 transcript:Zm00001d013049_T002 0
153- 24: transcript:EER93261 transcript:Zm00001d013050_T001 4.00E-28
153- 25: transcript:EER90705 transcript:Zm00001d013052_T001 0
153- 26: transcript:EER90709 transcript:Zm00001d013053_T002 0
153- 27: transcript:OQU90815 transcript:Zm00001d013055_T002 1.00E-118
153- 28: transcript:EER90714 transcript:Zm00001d013056_T001 0
153- 29: transcript:OQU90821 transcript:Zm00001d013057_T001 0
153- 30: transcript:OQU90823 transcript:Zm00001d013058_T001 0
153- 31: transcript:EER93269 transcript:Zm00001d013059_T003 0
153- 32: transcript:KXG37341 transcript:Zm00001d013060_T001 0
153- 33: transcript:EER90723 transcript:Zm00001d013061_T003 1.00E-160
153- 34: transcript:EER93271 transcript:Zm00001d013063_T001 0
Alignment 154: score=1398.0 e_value=1.4e-105 N=32 1&5 plus
154- 0: transcript:EER90765 transcript:Zm00001d013107_T001 0
154- 1: transcript:EER90766 transcript:Zm00001d013108_T001 0
154- 2: transcript:EER93328 transcript:Zm00001d013109_T002 0
154- 3: transcript:EER93331 transcript:Zm00001d013110_T001 1.00E-97
154- 4: transcript:KXG37403 transcript:Zm00001d013111_T001 0
154- 5: transcript:EER90769 transcript:Zm00001d013112_T011 0
154- 6: transcript:OQU90868 transcript:Zm00001d013113_T001 9.00E-137
154- 7: transcript:EER93336 transcript:Zm00001d013114_T001 2.00E-108
154- 8: transcript:EER93338 transcript:Zm00001d013116_T002 0
154- 9: transcript:EER90771 transcript:Zm00001d013117_T001 2.00E-49
154- 10: transcript:EER90773 transcript:Zm00001d013118_T001 2.00E-90
154- 11: transcript:EER93342 transcript:Zm00001d013119_T001 0
154- 12: transcript:EER90781 transcript:Zm00001d013120_T001 0
154- 13: transcript:OQU90882 transcript:Zm00001d013122_T008 2.00E-26
154- 14: transcript:EER90784 transcript:Zm00001d013126_T002 1.00E-17
154- 15: transcript:EER90787 transcript:Zm00001d013127_T002 5.00E-103
154- 16: transcript:KXG37427 transcript:Zm00001d013128_T004 3.00E-110
154- 17: transcript:KXG37432 transcript:Zm00001d013130_T001 4.00E-109
154- 18: transcript:OQU90890 transcript:Zm00001d013133_T001 8.00E-06
154- 19: transcript:KXG37436 transcript:Zm00001d013135_T002 0
154- 20: transcript:OQU90893 transcript:Zm00001d013136_T001 0
154- 21: transcript:KXG37439 transcript:Zm00001d013138_T001 7.00E-130
154- 22: transcript:EER93354 transcript:Zm00001d013139_T001 8.00E-153
154- 23: transcript:EER93355 transcript:Zm00001d013140_T002 0
154- 24: transcript:EER90794 transcript:Zm00001d013141_T001 1.00E-103
154- 25: transcript:EER93356 transcript:Zm00001d013142_T001 4.00E-46
154- 26: transcript:EER93357 transcript:Zm00001d013144_T001 8.00E-121
154- 27: transcript:EER90796 transcript:Zm00001d013146_T001 6.00E-159
154- 28: transcript:EER93358 transcript:Zm00001d013147_T001 0
154- 29: transcript:KXG37443 transcript:Zm00001d013150_T001 0
154- 30: transcript:EER93360 transcript:Zm00001d013151_T001 0
154- 31: transcript:EER90800 transcript:Zm00001d013153_T001 0
Alignment 155: score=1024.0 e_value=2.8e-67 N=23 1&5 plus
155- 0: transcript:EER94048 transcript:Zm00001d013976_T002 0

155- 1:	transcript:OQU91607	transcript:Zm00001d013977_T001	6.00E-57
155- 2:	transcript:KXG38289	transcript:Zm00001d013979_T001	0
155- 3:	transcript:KXG38295	transcript:Zm00001d013982_T002	3.00E-56
155- 4:	transcript:EER94055	transcript:Zm00001d013983_T001	0
155- 5:	transcript:KXG38297	transcript:Zm00001d013984_T012	0
155- 6:	transcript:EER91463	transcript:Zm00001d013985_T004	0
155- 7:	transcript:OQU91613	transcript:Zm00001d013989_T028	0
155- 8:	transcript:EER91465	transcript:Zm00001d013990_T001	7.00E-103
155- 9:	transcript:EER91459	transcript:Zm00001d013991_T001	0
155- 10:	transcript:EER91466	transcript:Zm00001d013992_T001	0
155- 11:	transcript:EER94059	transcript:Zm00001d013993_T001	6.00E-86
155- 12:	transcript:OQU91617	transcript:Zm00001d013997_T001	7.00E-135
155- 13:	transcript:EER91468	transcript:Zm00001d013999_T001	2.00E-93
155- 14:	transcript:EER94063	transcript:Zm00001d014001_T001	0
155- 15:	transcript:EER91473	transcript:Zm00001d014003_T004	2.00E-105
155- 16:	transcript:OQU91620	transcript:Zm00001d014005_T001	9.00E-72
155- 17:	transcript:KXG38312	transcript:Zm00001d014006_T001	3.00E-32
155- 18:	transcript:EER91475	transcript:Zm00001d014007_T001	2.00E-72
155- 19:	transcript:KXG38314	transcript:Zm00001d014011_T001	0
155- 20:	transcript:EER94066	transcript:Zm00001d014013_T002	0
155- 21:	transcript:OQU91622	transcript:Zm00001d014015_T001	3.00E-53
155- 22:	transcript:OQU91625	transcript:Zm00001d014016_T001	4.00E-108

Alignment 156: score=1004.0 e_value=2e-71 N=23 1&5 plus

156- 0:	transcript:EER91090	transcript:Zm00001d013559_T001	3.00E-80
156- 1:	transcript:EER93683	transcript:Zm00001d013561_T001	0
156- 2:	transcript:EER91092	transcript:Zm00001d013566_T001	0
156- 3:	transcript:KXG37870	transcript:Zm00001d013568_T001	2.00E-52
156- 4:	transcript:OQU91218	transcript:Zm00001d013569_T001	0
156- 5:	transcript:EER91098	transcript:Zm00001d013571_T001	9.00E-115
156- 6:	transcript:EER91100	transcript:Zm00001d013572_T001	0
156- 7:	transcript:KXG37872	transcript:Zm00001d013573_T001	0
156- 8:	transcript:EER91105	transcript:Zm00001d013575_T001	0
156- 9:	transcript:EER91108	transcript:Zm00001d013581_T001	4.00E-111
156- 10:	transcript:EER91109	transcript:Zm00001d013582_T006	0
156- 11:	transcript:EER93694	transcript:Zm00001d013583_T002	1.00E-146
156- 12:	transcript:EER93695	transcript:Zm00001d013588_T001	0
156- 13:	transcript:KXG37876	transcript:Zm00001d013589_T006	0
156- 14:	transcript:KXG37878	transcript:Zm00001d013590_T001	0
156- 15:	transcript:OQU91228	transcript:Zm00001d013592_T005	4.00E-08
156- 16:	transcript:OQU91229	transcript:Zm00001d013593_T001	1.00E-66
156- 17:	transcript:EER91113	transcript:Zm00001d013595_T001	2.00E-162
156- 18:	transcript:EER93700	transcript:Zm00001d013596_T001	7.00E-103
156- 19:	transcript:EER91115	transcript:Zm00001d013597_T001	0
156- 20:	transcript:EER93702	transcript:Zm00001d013598_T001	5.00E-101
156- 21:	transcript:EER93704	transcript:Zm00001d013599_T006	0
156- 22:	transcript:KXG37885	transcript:Zm00001d013603_T006	0

Alignment 157: score=984.0 e_value=9.6e-68 N=22 1&5 plus

157- 0:	transcript:EER91499	transcript:Zm00001d014055_T002	0
157- 1:	transcript:KXG38359	transcript:Zm00001d014058_T002	0
157- 2:	transcript:EER94099	transcript:Zm00001d014060_T001	0
157- 3:	transcript:OQU91665	transcript:Zm00001d014062_T001	0
157- 4:	transcript:KXG38367	transcript:Zm00001d014063_T001	0
157- 5:	transcript:EER94108	transcript:Zm00001d014065_T001	2.00E-135
157- 6:	transcript:KXG38374	transcript:Zm00001d014066_T001	7.00E-29

```
157-  7: transcript:KXG38377           transcript:Zm00001d014070_T001  4.00E-07
157-  8: transcript:EER95541           transcript:Zm00001d014073_T004  6.00E-97
157-  9: transcript:OQU91666           transcript:Zm00001d014074_T001 6.00E-137
157- 10: transcript:KXG38386           transcript:Zm00001d014078_T001         0
157- 11: transcript:KXG38387           transcript:Zm00001d014079_T001  9.00E-98
157- 12: transcript:KXG38389           transcript:Zm00001d014080_T001 3.00E-153
157- 13: transcript:OQU91673           transcript:Zm00001d014081_T001  2.00E-60
157- 14: transcript:EER91506           transcript:Zm00001d014082_T001 3.00E-115
157- 15: transcript:OQU91674           transcript:Zm00001d014083_T001         0
157- 16: transcript:EER91513           transcript:Zm00001d014084_T001         0
157- 17: transcript:EER91514           transcript:Zm00001d014085_T001         0
157- 18: transcript:OQU91677           transcript:Zm00001d014088_T001         0
157- 19: transcript:EER94119           transcript:Zm00001d014089_T001 2.00E-100
157- 20: transcript:EER94121           transcript:Zm00001d014090_T002         0
157- 21: transcript:OQU91684           transcript:Zm00001d014091_T001 4.00E-122
## Alignment 158: score=963.0 e_value=2.3e-69 N=23 1&5 plus
158-  0: transcript:EER91397           transcript:Zm00001d013895_T002 4.00E-134
158-  1: transcript:EER91391           transcript:Zm00001d013896_T001         0
158-  2: transcript:KXG38216           transcript:Zm00001d013900_T001         0
158-  3: transcript:EER91405           transcript:Zm00001d013907_T001  6.00E-38
158-  4: transcript:KXG38217           transcript:Zm00001d013908_T003         0
158-  5: transcript:KXG38218           transcript:Zm00001d013909_T001  7.00E-16
158-  6: transcript:EER91410           transcript:Zm00001d013910_T002         0
158-  7: transcript:KXG38219           transcript:Zm00001d013911_T001         0
158-  8: transcript:EER91417           transcript:Zm00001d013914_T001 2.00E-107
158-  9: transcript:EER91419           transcript:Zm00001d013915_T001         0
158- 10: transcript:KXG38225           transcript:Zm00001d013917_T001  7.00E-30
158- 11: transcript:EER91420           transcript:Zm00001d013918_T001 9.00E-152
158- 12: transcript:EER93981           transcript:Zm00001d013919_T001 1.00E-177
158- 13: transcript:KXG38228           transcript:Zm00001d013920_T002         0
158- 14: transcript:OQU91552           transcript:Zm00001d013923_T001  3.00E-67
158- 15: transcript:KXG38231           transcript:Zm00001d013926_T001         0
158- 16: transcript:KXG38234           transcript:Zm00001d013927_T001         0
158- 17: transcript:KXG38237           transcript:Zm00001d013931_T001 1.00E-111
158- 18: transcript:KXG38242           transcript:Zm00001d013933_T001         0
158- 19: transcript:OQU91561           transcript:Zm00001d013934_T001         0
158- 20: transcript:OQU91568           transcript:Zm00001d013935_T001 4.00E-168
158- 21: transcript:EER94008           transcript:Zm00001d013937_T001         0
158- 22: transcript:KXG38251           transcript:Zm00001d013940_T004         0
## Alignment 159: score=955.0 e_value=1.9e-61 N=21 1&5 plus
159-  0: transcript:KXG37096           transcript:Zm00001d012822_T001         0
159-  1: transcript:EER93074           transcript:Zm00001d012823_T001         0
159-  2: transcript:KXG37098           transcript:Zm00001d012824_T001  1.00E-65
159-  3: transcript:EER93075           transcript:Zm00001d012825_T001         0
159-  4: transcript:EER90492           transcript:Zm00001d012826_T003 2.00E-131
159-  5: transcript:EER90493           transcript:Zm00001d012827_T001         0
159-  6: transcript:EER90494           transcript:Zm00001d012830_T002         0
159-  7: transcript:EER90498           transcript:Zm00001d012831_T001         0
159-  8: transcript:OQU90603           transcript:Zm00001d012832_T003  7.00E-98
159-  9: transcript:EER90499           transcript:Zm00001d012833_T002  1.00E-65
159- 10: transcript:KXG37105           transcript:Zm00001d012834_T003 3.00E-131
159- 11: transcript:EER90503           transcript:Zm00001d012836_T001  6.00E-66
159- 12: transcript:EER90505           transcript:Zm00001d012837_T001  2.00E-74
159- 13: transcript:KXG37109           transcript:Zm00001d012838_T001 9.00E-176
```

```
159- 14: transcript:KXG37112          transcript:Zm00001d012839_T002 1.00E-169
159- 15: transcript:EER90506          transcript:Zm00001d012844_T002         0
159- 16: transcript:EER93091          transcript:Zm00001d012845_T001 5.00E-102
159- 17: transcript:EER90508          transcript:Zm00001d012846_T005         0
159- 18: transcript:EER90511          transcript:Zm00001d012847_T001 3.00E-157
159- 19: transcript:EER90512          transcript:Zm00001d012848_T001  3.00E-99
159- 20: transcript:EER90514          transcript:Zm00001d012849_T001 6.00E-177
## Alignment 160: score=883.0 e_value=1.3e-58 N=20 1&5 plus
160-  0: transcript:EER91066          transcript:Zm00001d013529_T002         0
160-  1: transcript:OQU91183          transcript:Zm00001d013530_T001         0
160-  2: transcript:EER91067          transcript:Zm00001d013531_T002  1.00E-41
160-  3: transcript:EER93656          transcript:Zm00001d013532_T001 4.00E-158
160-  4: transcript:OQU91186          transcript:Zm00001d013533_T001  9.00E-34
160-  5: transcript:EER93655          transcript:Zm00001d013534_T002 5.00E-121
160-  6: transcript:EER91068          transcript:Zm00001d013536_T001 2.00E-144
160-  7: transcript:EER93658          transcript:Zm00001d013540_T020         0
160-  8: transcript:OQU91197          transcript:Zm00001d013542_T003         0
160-  9: transcript:KXG37841          transcript:Zm00001d013543_T009 6.00E-167
160- 10: transcript:KXG37844          transcript:Zm00001d013544_T003         0
160- 11: transcript:EER91078          transcript:Zm00001d013546_T003  3.00E-91
160- 12: transcript:EER91079          transcript:Zm00001d013547_T002         0
160- 13: transcript:EER93674          transcript:Zm00001d013548_T002         0
160- 14: transcript:EER91085          transcript:Zm00001d013550_T001  4.00E-33
160- 15: transcript:EER93676          transcript:Zm00001d013551_T001  1.00E-33
160- 16: transcript:OQU91204          transcript:Zm00001d013552_T001 6.00E-106
160- 17: transcript:KXG37858          transcript:Zm00001d013553_T001  1.00E-59
160- 18: transcript:EER91088          transcript:Zm00001d013554_T001 2.00E-151
160- 19: transcript:EER93679          transcript:Zm00001d013555_T003         0
## Alignment 161: score=878.0 e_value=1.2e-61 N=21 1&5 plus
161-  0: transcript:KXG37609          transcript:Zm00001d013313_T001  9.00E-26
161-  1: transcript:EER93494          transcript:Zm00001d013314_T005         0
161-  2: transcript:KXG37616          transcript:Zm00001d013317_T001 1.00E-118
161-  3: transcript:KXG37618          transcript:Zm00001d013318_T035         0
161-  4: transcript:KXG37622          transcript:Zm00001d013323_T001         0
161-  5: transcript:EER90916          transcript:Zm00001d013327_T001         0
161-  6: transcript:EER90918          transcript:Zm00001d013329_T001 2.00E-104
161-  7: transcript:KXG37626          transcript:Zm00001d013330_T001         0
161-  8: transcript:EER90915          transcript:Zm00001d013331_T001 1.00E-164
161-  9: transcript:EER93504          transcript:Zm00001d013332_T001  7.00E-89
161- 10: transcript:OQU91042          transcript:Zm00001d013333_T001  5.00E-31
161- 11: transcript:KXG37629          transcript:Zm00001d013334_T001         0
161- 12: transcript:EER90924          transcript:Zm00001d013335_T001         0
161- 13: transcript:EER93511          transcript:Zm00001d013337_T001 3.00E-119
161- 14: transcript:EER90929          transcript:Zm00001d013339_T001  3.00E-47
161- 15: transcript:EER93513          transcript:Zm00001d013340_T001         0
161- 16: transcript:OQU91053          transcript:Zm00001d013342_T001         0
161- 17: transcript:EER93521          transcript:Zm00001d013343_T001         0
161- 18: transcript:KXG37649          transcript:Zm00001d013344_T002 7.00E-152
161- 19: transcript:EER90936          transcript:Zm00001d013346_T001         0
161- 20: transcript:EER90939          transcript:Zm00001d013348_T001  5.00E-99
## Alignment 162: score=769.0 e_value=1.4e-49 N=18 1&5 plus
162-  0: transcript:EER90977          transcript:Zm00001d013406_T002         0
162-  1: transcript:EER93559          transcript:Zm00001d013409_T001 7.00E-109
162-  2: transcript:KXG37705          transcript:Zm00001d013410_T002         0
```

162- 3: transcript:KXG37709 transcript:Zm00001d013411_T003 0
162- 4: transcript:EER93561 transcript:Zm00001d013412_T003 0
162- 5: transcript:EER90988 transcript:Zm00001d013415_T001 0
162- 6: transcript:EER90989 transcript:Zm00001d013416_T001 0
162- 7: transcript:EER93566 transcript:Zm00001d013420_T002 0
162- 8: transcript:OQU91108 transcript:Zm00001d013423_T001 3.00E-150
162- 9: transcript:EER90993 transcript:Zm00001d013424_T001 0
162- 10: transcript:KXG37726 transcript:Zm00001d013425_T001 8.00E-86
162- 11: transcript:KXG37728 transcript:Zm00001d013426_T002 0
162- 12: transcript:EER93572 transcript:Zm00001d013427_T003 0
162- 13: transcript:EER93574 transcript:Zm00001d013428_T002 0
162- 14: transcript:EER90996 transcript:Zm00001d013430_T001 0
162- 15: transcript:EER93575 transcript:Zm00001d013431_T002 1.00E-101
162- 16: transcript:KXG37731 transcript:Zm00001d013432_T001 0
162- 17: transcript:OQU91111 transcript:Zm00001d013433_T001 6.00E-60
Alignment 163: score=747.0 e_value=8.4e-47 N=17 1&5 plus
163- 0: transcript:EER93465 transcript:Zm00001d013281_T001 2.00E-47
163- 1: transcript:OQU91002 transcript:Zm00001d013282_T002 6.00E-09
163- 2: transcript:EER90882 transcript:Zm00001d013283_T001 0
163- 3: transcript:EER90883 transcript:Zm00001d013287_T001 2.00E-102
163- 4: transcript:EER93470 transcript:Zm00001d013288_T003 0
163- 5: transcript:EER93471 transcript:Zm00001d013289_T001 3.00E-176
163- 6: transcript:EER90890 transcript:Zm00001d013290_T004 6.00E-158
163- 7: transcript:EER93472 transcript:Zm00001d013295_T001 0
163- 8: transcript:EER90894 transcript:Zm00001d013296_T001 0
163- 9: transcript:KXG37588 transcript:Zm00001d013300_T002 1.00E-93
163- 10: transcript:EER90896 transcript:Zm00001d013302_T002 2.00E-106
163- 11: transcript:KXG37592 transcript:Zm00001d013303_T001 0
163- 12: transcript:EER93477 transcript:Zm00001d013306_T001 6.00E-139
163- 13: transcript:EER93478 transcript:Zm00001d013307_T003 0
163- 14: transcript:EER90901 transcript:Zm00001d013309_T001 4.00E-72
163- 15: transcript:EER93485 transcript:Zm00001d013310_T001 0
163- 16: transcript:KXG37596 transcript:Zm00001d013311_T001 4.00E-55
Alignment 164: score=641.0 e_value=3.3e-39 N=15 1&5 plus
164- 0: transcript:EER90748 transcript:Zm00001d013086_T004 5.00E-105
164- 1: transcript:OQU90843 transcript:Zm00001d013087_T001 4.00E-38
164- 2: transcript:EER93307 transcript:Zm00001d013089_T002 1.00E-12
164- 3: transcript:EER93309 transcript:Zm00001d013090_T002 0
164- 4: transcript:KXG37379 transcript:Zm00001d013091_T001 2.00E-151
164- 5: transcript:KXG37383 transcript:Zm00001d013092_T002 2.00E-72
164- 6: transcript:EER90754 transcript:Zm00001d013093_T004 0
164- 7: transcript:EER90755 transcript:Zm00001d013094_T001 0
164- 8: transcript:KXG37386 transcript:Zm00001d013095_T001 0
164- 9: transcript:EER90756 transcript:Zm00001d013097_T001 0
164- 10: transcript:EER90757 transcript:Zm00001d013098_T002 0
164- 11: transcript:EER90762 transcript:Zm00001d013099_T001 0
164- 12: transcript:EER90763 transcript:Zm00001d013100_T001 2.00E-167
164- 13: transcript:EER93327 transcript:Zm00001d013104_T001 3.00E-48
164- 14: transcript:EER90764 transcript:Zm00001d013105_T001 0
Alignment 165: score=622.0 e_value=6.2e-38 N=14 1&5 plus
165- 0: transcript:OQU91727 transcript:Zm00001d014128_T001 1.00E-12
165- 1: transcript:KXG38461 transcript:Zm00001d014129_T004 0
165- 2: transcript:EER91566 transcript:Zm00001d014132_T001 4.00E-87
165- 3: transcript:EER94178 transcript:Zm00001d014138_T004 0

```
165-  4: transcript:KXG38463           transcript:Zm00001d014139_T001  4.00E-55
165-  5: transcript:EER94183           transcript:Zm00001d014140_T001  2.00E-50
165-  6: transcript:EER94184           transcript:Zm00001d014141_T002 2.00E-170
165-  7: transcript:EER91576           transcript:Zm00001d014145_T001         0
165-  8: transcript:EER94186           transcript:Zm00001d014146_T001  2.00E-71
165-  9: transcript:KXG38466           transcript:Zm00001d014149_T001  6.00E-78
165- 10: transcript:OQU91730           transcript:Zm00001d014150_T005         0
165- 11: transcript:OQU91732           transcript:Zm00001d014152_T003         0
165- 12: transcript:EER91580           transcript:Zm00001d014153_T001  2.00E-83
165- 13: transcript:KXG38474           transcript:Zm00001d014157_T001 4.00E-169
## Alignment 166: score=593.0 e_value=6.1e-34 N=14 1&5 plus
166-  0: transcript:EER93098           transcript:Zm00001d012850_T001 4.00E-105
166-  1: transcript:OQU90613           transcript:Zm00001d012851_T001         0
166-  2: transcript:KXG37123           transcript:Zm00001d012852_T003 6.00E-120
166-  3: transcript:EER90518           transcript:Zm00001d012853_T001 3.00E-148
166-  4: transcript:EER93100           transcript:Zm00001d012854_T001  1.00E-83
166-  5: transcript:EER93101           transcript:Zm00001d012855_T001         0
166-  6: transcript:OQU90624           transcript:Zm00001d012856_T001         0
166-  7: transcript:EER90523           transcript:Zm00001d012857_T006  6.00E-96
166-  8: transcript:EER90525           transcript:Zm00001d012858_T001         0
166-  9: transcript:EER90527           transcript:Zm00001d012859_T001 1.00E-132
166- 10: transcript:OQU90635           transcript:Zm00001d012860_T001  9.00E-92
166- 11: transcript:KXG37151           transcript:Zm00001d012861_T001         0
166- 12: transcript:OQU90639           transcript:Zm00001d012862_T001         0
166- 13: transcript:EER93114           transcript:Zm00001d012863_T003         0
## Alignment 167: score=585.0 e_value=5.2e-34 N=14 1&5 plus
167-  0: transcript:EER90961           transcript:Zm00001d013381_T001  2.00E-49
167-  1: transcript:EER93543           transcript:Zm00001d013383_T001         0
167-  2: transcript:EER93545           transcript:Zm00001d013384_T001  2.00E-82
167-  3: transcript:KXG37683           transcript:Zm00001d013385_T001         0
167-  4: transcript:KXG37688           transcript:Zm00001d013389_T014         0
167-  5: transcript:EER93548           transcript:Zm00001d013390_T001         0
167-  6: transcript:EER93546           transcript:Zm00001d013391_T004         0
167-  7: transcript:EER93551           transcript:Zm00001d013392_T001  5.00E-94
167-  8: transcript:EER90966           transcript:Zm00001d013397_T002 2.00E-116
167-  9: transcript:KXG37691           transcript:Zm00001d013398_T001         0
167- 10: transcript:EER93554           transcript:Zm00001d013399_T001 7.00E-157
167- 11: transcript:KXG37697           transcript:Zm00001d013400_T001         0
167- 12: transcript:OQU91089           transcript:Zm00001d013402_T025  2.00E-66
167- 13: transcript:KXG37701           transcript:Zm00001d013405_T004         0
## Alignment 168: score=582.0 e_value=4.4e-35 N=14 1&5 plus
168-  0: transcript:OQU90586           transcript:Zm00001d012808_T001         0
168-  1: transcript:EER90465           transcript:Zm00001d012809_T001         0
168-  2: transcript:KXG37075           transcript:Zm00001d012810_T003 2.00E-161
168-  3: transcript:EER90468           transcript:Zm00001d012811_T003         0
168-  4: transcript:KXG37080           transcript:Zm00001d012812_T004         0
168-  5: transcript:KXG37081           transcript:Zm00001d012813_T001         0
168-  6: transcript:OQU90591           transcript:Zm00001d012814_T001  8.00E-72
168-  7: transcript:EER90476           transcript:Zm00001d012815_T022         0
168-  8: transcript:EER93060           transcript:Zm00001d012816_T004  5.00E-95
168-  9: transcript:EER93064           transcript:Zm00001d012817_T002         0
168- 10: transcript:EER90483           transcript:Zm00001d012818_T001  2.00E-67
168- 11: transcript:KXG37089           transcript:Zm00001d012819_T001         0
168- 12: transcript:EER93070           transcript:Zm00001d012820_T001 5.00E-138
```

```
168- 13: transcript:EER90487           transcript:Zm00001d012821_T003        0
## Alignment 169: score=579.0 e_value=1.2e-34 N=14 1&5 plus
169-  0: transcript:EER91354           transcript:Zm00001d013828_T002        0
169-  1: transcript:EER93923           transcript:Zm00001d013829_T001        0
169-  2: transcript:EER91356           transcript:Zm00001d013830_T001        0
169-  3: transcript:OQU91505           transcript:Zm00001d013834_T001        0
169-  4: transcript:EER93933           transcript:Zm00001d013836_T001        0
169-  5: transcript:OQU91506           transcript:Zm00001d013838_T002        0
169-  6: transcript:EER91363           transcript:Zm00001d013839_T001  6.00E-84
169-  7: transcript:EER93935           transcript:Zm00001d013840_T002        0
169-  8: transcript:EER91365           transcript:Zm00001d013842_T003        0
169-  9: transcript:EER91366           transcript:Zm00001d013844_T001        0
169- 10: transcript:KXG38182           transcript:Zm00001d013847_T001        0
169- 11: transcript:EER91374           transcript:Zm00001d013849_T003        0
169- 12: transcript:KXG38188           transcript:Zm00001d013856_T001  3.00E-55
169- 13: transcript:EER91375           transcript:Zm00001d013858_T001        0
## Alignment 170: score=559.0 e_value=3.1e-26 N=12 1&5 plus
170-  0: transcript:EER91379           transcript:Zm00001d013860_T012        0
170-  1: transcript:EER91380           transcript:Zm00001d013861_T001 8.00E-132
170-  2: transcript:EER91381           transcript:Zm00001d013862_T001        0
170-  3: transcript:OQU91520           transcript:Zm00001d013863_T007        0
170-  4: transcript:KXG38194           transcript:Zm00001d013865_T001        0
170-  5: transcript:KXG38195           transcript:Zm00001d013868_T001 1.00E-166
170-  6: transcript:EER91385           transcript:Zm00001d013869_T001  5.00E-58
170-  7: transcript:EER93954           transcript:Zm00001d013873_T001        0
170-  8: transcript:EER91389           transcript:Zm00001d013879_T001  8.00E-36
170-  9: transcript:EER93960           transcript:Zm00001d013885_T001 2.00E-178
170- 10: transcript:OQU91527           transcript:Zm00001d013886_T002  5.00E-97
170- 11: transcript:OQU91528           transcript:Zm00001d013887_T002        0
## Alignment 171: score=558.0 e_value=2.1e-32 N=13 1&5 plus
171-  0: transcript:KXG37270           transcript:Zm00001d012980_T001        0
171-  1: transcript:EER93204           transcript:Zm00001d012982_T001        0
171-  2: transcript:EER90646           transcript:Zm00001d012983_T001        0
171-  3: transcript:EER90647           transcript:Zm00001d012988_T002  7.00E-99
171-  4: transcript:EER93210           transcript:Zm00001d012991_T001        0
171-  5: transcript:OQU90753           transcript:Zm00001d012992_T002  5.00E-91
171-  6: transcript:EER90652           transcript:Zm00001d012993_T001        0
171-  7: transcript:EER93213           transcript:Zm00001d012996_T001        0
171-  8: transcript:OQU90755           transcript:Zm00001d012998_T001 2.00E-146
171-  9: transcript:EER90654           transcript:Zm00001d013002_T001  5.00E-35
171- 10: transcript:EER93215           transcript:Zm00001d013003_T001 2.00E-177
171- 11: transcript:OQU90756           transcript:Zm00001d013004_T001  2.00E-67
171- 12: transcript:KXG37275           transcript:Zm00001d013005_T002        0
## Alignment 172: score=557.0 e_value=1.4e-33 N=14 1&5 plus
172-  0: transcript:EER93184           transcript:Zm00001d012958_T001        0
172-  1: transcript:KXG37252           transcript:Zm00001d012960_T001  7.00E-22
172-  2: transcript:OQU90715           transcript:Zm00001d012961_T001        0
172-  3: transcript:EER90623           transcript:Zm00001d012962_T001        0
172-  4: transcript:KXG37254           transcript:Zm00001d012963_T001 2.00E-144
172-  5: transcript:EER90625           transcript:Zm00001d012964_T016        0
172-  6: transcript:EER90626           transcript:Zm00001d012965_T001  3.00E-16
172-  7: transcript:KXG37255           transcript:Zm00001d012966_T001  5.00E-98
172-  8: transcript:EER93193           transcript:Zm00001d012967_T001  4.00E-47
172-  9: transcript:KXG37259           transcript:Zm00001d012969_T008        0
```

172- 10: transcript:OQU90728 transcript:Zm00001d012970_T001 0
172- 11: transcript:EER90629 transcript:Zm00001d012972_T001 1.00E-131
172- 12: transcript:EER90630 transcript:Zm00001d012973_T001 1.00E-102
172- 13: transcript:OQU90735 transcript:Zm00001d012974_T003 0
Alignment 173: score=534.0 e_value=2.1e-33 N=13 1&5 plus
173- 0: transcript:OQU91413 transcript:Zm00001d013757_T007 5.00E-129
173- 1: transcript:EER91264 transcript:Zm00001d013758_T007 3.00E-111
173- 2: transcript:KXG38076 transcript:Zm00001d013759_T001 7.00E-61
173- 3: transcript:EER93854 transcript:Zm00001d013765_T002 0
173- 4: transcript:EER93861 transcript:Zm00001d013766_T001 1.00E-84
173- 5: transcript:EER91272 transcript:Zm00001d013767_T001 0
173- 6: transcript:EER91273 transcript:Zm00001d013768_T002 1.00E-44
173- 7: transcript:EER93866 transcript:Zm00001d013777_T001 2.00E-153
173- 8: transcript:EER91275 transcript:Zm00001d013778_T001 0
173- 9: transcript:KXG38088 transcript:Zm00001d013779_T001 1.00E-84
173- 10: transcript:KXG38091 transcript:Zm00001d013780_T002 2.00E-74
173- 11: transcript:EER93872 transcript:Zm00001d013781_T001 3.00E-167
173- 12: transcript:EER91281 transcript:Zm00001d013783_T001 5.00E-51
Alignment 174: score=508.0 e_value=2.6e-28 N=12 1&5 plus
174- 0: transcript:OQU90668 transcript:Zm00001d012887_T001 0
174- 1: transcript:OQU90670 transcript:Zm00001d012888_T002 1.00E-39
174- 2: transcript:EER93139 transcript:Zm00001d012889_T001 9.00E-121
174- 3: transcript:EER93140 transcript:Zm00001d012890_T001 2.00E-65
174- 4: transcript:EER90565 transcript:Zm00001d012892_T002 0
174- 5: transcript:OQU90673 transcript:Zm00001d012893_T002 0
174- 6: transcript:EER93146 transcript:Zm00001d012895_T003 0
174- 7: transcript:OQU90677 transcript:Zm00001d012896_T010 2.00E-138
174- 8: transcript:EER90568 transcript:Zm00001d012898_T001 0
174- 9: transcript:EER90573 transcript:Zm00001d012906_T001 1.00E-58
174- 10: transcript:EER90578 transcript:Zm00001d012908_T001 3.00E-57
174- 11: transcript:OQU90680 transcript:Zm00001d012910_T001 4.00E-42
Alignment 175: score=499.0 e_value=1.6e-29 N=12 1&5 plus
175- 0: transcript:EER91234 transcript:Zm00001d013736_T001 0
175- 1: transcript:EER93821 transcript:Zm00001d013737_T001 0
175- 2: transcript:EER91236 transcript:Zm00001d013741_T006 0
175- 3: transcript:KXG38038 transcript:Zm00001d013742_T001 0
175- 4: transcript:EER91243 transcript:Zm00001d013743_T001 3.00E-86
175- 5: transcript:OQU91386 transcript:Zm00001d013744_T001 2.00E-167
175- 6: transcript:EER93827 transcript:Zm00001d013745_T002 0
175- 7: transcript:EER91248 transcript:Zm00001d013746_T001 0
175- 8: transcript:KXG38050 transcript:Zm00001d013747_T001 0
175- 9: transcript:EER91257 transcript:Zm00001d013750_T001 1.00E-116
175- 10: transcript:EER91258 transcript:Zm00001d013751_T005 0
175- 11: transcript:EER91259 transcript:Zm00001d013755_T001 5.00E-146
Alignment 176: score=475.0 e_value=1.1e-23 N=11 1&5 plus
176- 0: transcript:EER93292 transcript:Zm00001d013073_T002 0
176- 1: transcript:EER90738 transcript:Zm00001d013074_T003 0
176- 2: transcript:EER93294 transcript:Zm00001d013075_T001 0
176- 3: transcript:EER90739 transcript:Zm00001d013076_T001 0
176- 4: transcript:EER90740 transcript:Zm00001d013077_T004 0
176- 5: transcript:EER90742 transcript:Zm00001d013078_T004 5.00E-158
176- 6: transcript:EER93299 transcript:Zm00001d013079_T001 6.00E-122
176- 7: transcript:EER90744 transcript:Zm00001d013080_T001 0
176- 8: transcript:EER93301 transcript:Zm00001d013081_T001 0

```
176-  9: transcript:EER90745           transcript:Zm00001d013082_T001  7.00E-50
176- 10: transcript:EER93302           transcript:Zm00001d013083_T005         0
## Alignment 177: score=458.0 e_value=1e-19 N=10 1&5 plus
177-  0: transcript:KXG37230           transcript:Zm00001d012928_T001 4.00E-145
177-  1: transcript:EER90604           transcript:Zm00001d012929_T002         0
177-  2: transcript:EER90606           transcript:Zm00001d012930_T001  3.00E-44
177-  3: transcript:EER93172           transcript:Zm00001d012931_T001         0
177-  4: transcript:EER90610           transcript:Zm00001d012932_T001 1.00E-141
177-  5: transcript:EER90611           transcript:Zm00001d012933_T001  2.00E-41
177-  6: transcript:EER90612           transcript:Zm00001d012934_T001         0
177-  7: transcript:EER90613           transcript:Zm00001d012935_T004         0
177-  8: transcript:EER90614           transcript:Zm00001d012936_T001  6.00E-75
177-  9: transcript:EER90617           transcript:Zm00001d012938_T002         0
## Alignment 178: score=419.0 e_value=1.3e-20 N=10 1&5 plus
178-  0: transcript:OQU91633           transcript:Zm00001d014021_T001         0
178-  1: transcript:EER91482           transcript:Zm00001d014029_T001 6.00E-130
178-  2: transcript:EER94077           transcript:Zm00001d014030_T001         0
178-  3: transcript:KXG38327           transcript:Zm00001d014032_T001 6.00E-139
178-  4: transcript:KXG38329           transcript:Zm00001d014033_T004         0
178-  5: transcript:EER94078           transcript:Zm00001d014035_T001         0
178-  6: transcript:KXG38331           transcript:Zm00001d014036_T002         0
178-  7: transcript:EER94083           transcript:Zm00001d014037_T004         0
178-  8: transcript:KXG38336           transcript:Zm00001d014038_T011         0
178-  9: transcript:EER94087           transcript:Zm00001d014039_T001         0
## Alignment 179: score=377.0 e_value=6.9e-14 N=8 1&5 plus
179-  0: transcript:OQU90651           transcript:Zm00001d012873_T001  2.00E-42
179-  1: transcript:EER93119           transcript:Zm00001d012875_T001  5.00E-55
179-  2: transcript:EER90550           transcript:Zm00001d012878_T001         0
179-  3: transcript:OQU90655           transcript:Zm00001d012880_T001         0
179-  4: transcript:EER93123           transcript:Zm00001d012881_T001         0
179-  5: transcript:EER90554           transcript:Zm00001d012882_T001         0
179-  6: transcript:EER93126           transcript:Zm00001d012883_T001         0
179-  7: transcript:EER90555           transcript:Zm00001d012884_T003         0
## Alignment 180: score=321.0 e_value=1.6e-12 N=7 1&5 plus
180-  0: transcript:KXG37278           transcript:Zm00001d013006_T007         0
180-  1: transcript:EER93218           transcript:Zm00001d013007_T001 6.00E-133
180-  2: transcript:EER90659           transcript:Zm00001d013008_T001  7.00E-47
180-  3: transcript:EER93219           transcript:Zm00001d013009_T001  1.00E-91
180-  4: transcript:EER90660           transcript:Zm00001d013010_T002 1.00E-120
180-  5: transcript:EER90661           transcript:Zm00001d013011_T001         0
180-  6: transcript:EER90662           transcript:Zm00001d013012_T003         0
## Alignment 181: score=305.0 e_value=7.4e-11 N=7 1&5 plus
181-  0: transcript:EER90872           transcript:Zm00001d013267_T034         0
181-  1: transcript:KXG37547           transcript:Zm00001d013268_T009         0
181-  2: transcript:KXG37550           transcript:Zm00001d013270_T001         0
181-  3: transcript:EER93456           transcript:Zm00001d013271_T003         0
181-  4: transcript:KXG37554           transcript:Zm00001d013272_T001         0
181-  5: transcript:EER93458           transcript:Zm00001d013273_T003         0
181-  6: transcript:EER93459           transcript:Zm00001d013274_T016         0
## Alignment 182: score=302.0 e_value=3e-11 N=7 1&5 plus
182-  0: transcript:EER91117           transcript:Zm00001d013607_T003         0
182-  1: transcript:EER93707           transcript:Zm00001d013610_T001 3.00E-129
182-  2: transcript:EER93711           transcript:Zm00001d013611_T001 2.00E-124
182-  3: transcript:EER93713           transcript:Zm00001d013612_T003         0
```

182- 4: transcript:EER91121   transcript:Zm00001d013614_T001 1.00E-32
182- 5: transcript:KXG37888   transcript:Zm00001d013616_T001 4.00E-43
182- 6: transcript:KXG37893   transcript:Zm00001d013620_T003   0
Alignment 183: score=286.0 e_value=2.8e-11 N=7 1&5 plus
183- 0: transcript:EER94131   transcript:Zm00001d014093_T002   0
183- 1: transcript:EER94133   transcript:Zm00001d014094_T002   0
183- 2: transcript:EER91530   transcript:Zm00001d014097_T002 3.00E-133
183- 3: transcript:EER91535   transcript:Zm00001d014100_T001 7.00E-19
183- 4: transcript:EER91540   transcript:Zm00001d014101_T001 8.00E-26
183- 5: transcript:OQU91699   transcript:Zm00001d014102_T001   0
183- 6: transcript:OQU91701   transcript:Zm00001d014108_T002 2.00E-99
Alignment 184: score=280.0 e_value=1.2e-08 N=6 1&5 plus
184- 0: transcript:KXG38448   transcript:Zm00001d014117_T001 3.00E-15
184- 1: transcript:EER94164   transcript:Zm00001d014121_T001   0
184- 2: transcript:EER94165   transcript:Zm00001d014122_T002   0
184- 3: transcript:EER91558   transcript:Zm00001d014123_T001   0
184- 4: transcript:EER94171   transcript:Zm00001d014124_T001   0
184- 5: transcript:EER91563   transcript:Zm00001d014126_T001   0
Alignment 185: score=261.0 e_value=7.1e-09 N=6 1&5 plus
185- 0: transcript:EER91204   transcript:Zm00001d013706_T001 9.00E-57
185- 1: transcript:EER93778   transcript:Zm00001d013707_T001 7.00E-95
185- 2: transcript:EER91208   transcript:Zm00001d013708_T001 1.00E-85
185- 3: transcript:EER91209   transcript:Zm00001d013709_T003   0
185- 4: transcript:OQU91315   transcript:Zm00001d013712_T003   0
185- 5: transcript:EER93787   transcript:Zm00001d013716_T002 7.00E-172
Alignment 186: score=260.0 e_value=1.3e-08 N=6 1&5 plus
186- 0: transcript:KXG37210   transcript:Zm00001d012913_T005   0
186- 1: transcript:EER90585   transcript:Zm00001d012915_T001   0
186- 2: transcript:EER93157   transcript:Zm00001d012916_T001   0
186- 3: transcript:EER90586   transcript:Zm00001d012917_T001   0
186- 4: transcript:EER90588   transcript:Zm00001d012918_T003   0
186- 5: transcript:EER90589   transcript:Zm00001d012919_T001 3.00E-25
Alignment 187: score=259.0 e_value=3.9e-10 N=6 1&5 plus
187- 0: transcript:EER91220   transcript:Zm00001d013724_T001 3.00E-123
187- 1: transcript:EER91221   transcript:Zm00001d013725_T002 3.00E-156
187- 2: transcript:EER91226   transcript:Zm00001d013728_T001 8.00E-149
187- 3: transcript:KXG38022   transcript:Zm00001d013729_T003   0
187- 4: transcript:EER91230   transcript:Zm00001d013730_T001 2.00E-158
187- 5: transcript:EER93817   transcript:Zm00001d013732_T001 1.00E-119
Alignment 188: score=254.0 e_value=2.1e-08 N=6 1&5 plus
188- 0: transcript:EER91670   transcript:Zm00001d014235_T002 1.00E-161
188- 1: transcript:OQU91853   transcript:Zm00001d014239_T001   0
188- 2: transcript:OQU91855   transcript:Zm00001d014243_T001   0
188- 3: transcript:OQU91857   transcript:Zm00001d014244_T001 2.00E-114
188- 4: transcript:KXG38620   transcript:Zm00001d014246_T002   0
188- 5: transcript:EER91683   transcript:Zm00001d014249_T001 1.00E-90
Alignment 189: score=253.0 e_value=5.2e-12 N=7 1&5 plus
189- 0: transcript:EER91618   transcript:Zm00001d014168_T001   0
189- 1: transcript:OQU91781   transcript:Zm00001d014175_T004   0
189- 2: transcript:EER94244   transcript:Zm00001d014178_T013   0
189- 3: transcript:EER91624   transcript:Zm00001d014179_T001   0
189- 4: transcript:EER94248   transcript:Zm00001d014180_T004   0
189- 5: transcript:OQU91790   transcript:Zm00001d014181_T001   0
189- 6: transcript:EER94254   transcript:Zm00001d014183_T008   0

```
## Alignment 190: score=596.0 e_value=6.9e-36 N=14 1&5 minus
190-  0: transcript:KXG38253           transcript:Zm00001d013964_T003  4.00E-35
190-  1: transcript:KXG38256           transcript:Zm00001d013963_T002         0
190-  2: transcript:OQU91575           transcript:Zm00001d013962_T003 3.00E-166
190-  3: transcript:KXG38261           transcript:Zm00001d013960_T001         0
190-  4: transcript:EER91435           transcript:Zm00001d013958_T004         0
190-  5: transcript:EER91436           transcript:Zm00001d013957_T001         0
190-  6: transcript:EER94020           transcript:Zm00001d013956_T003 1.00E-158
190-  7: transcript:EER94023           transcript:Zm00001d013955_T001 4.00E-135
190-  8: transcript:EER91441           transcript:Zm00001d013949_T001  6.00E-99
190-  9: transcript:KXG38271           transcript:Zm00001d013947_T001 1.00E-108
190- 10: transcript:EER91449           transcript:Zm00001d013945_T001  1.00E-85
190- 11: transcript:OQU91600           transcript:Zm00001d013944_T001  2.00E-45
190- 12: transcript:EER91454           transcript:Zm00001d013943_T001 1.00E-128
190- 13: transcript:OQU91601           transcript:Zm00001d013941_T001         0
## Alignment 191: score=321.0 e_value=3.1e-13 N=7 1&5 minus
191-  0: transcript:KXG38096           transcript:Zm00001d013796_T005         0
191-  1: transcript:EER93874           transcript:Zm00001d013795_T002         0
191-  2: transcript:EER93875           transcript:Zm00001d013794_T001  3.00E-69
191-  3: transcript:KXG38099           transcript:Zm00001d013792_T001 2.00E-135
191-  4: transcript:OQU91444           transcript:Zm00001d013787_T003 2.00E-117
191-  5: transcript:EER93876           transcript:Zm00001d013786_T001         0
191-  6: transcript:OQU91446           transcript:Zm00001d013785_T001  2.00E-41
## Alignment 192: score=292.0 e_value=4.1e-14 N=7 1&5 minus
192-  0: transcript:KXG40202           transcript:Zm00001d014090_T002         0
192-  1: transcript:OQU93257           transcript:Zm00001d014084_T001         0
192-  2: transcript:EER95531           transcript:Zm00001d014083_T001         0
192-  3: transcript:EER95532           transcript:Zm00001d014082_T001 1.00E-112
192-  4: transcript:OQU93266           transcript:Zm00001d014074_T001  1.00E-67
192-  5: transcript:EER91503           transcript:Zm00001d014073_T004  6.00E-97
192-  6: transcript:OQU93272           transcript:Zm00001d014060_T001         0
## Alignment 193: score=315.0 e_value=2.5e-11 N=7 1&6 plus
193-  0: transcript:Zm00001d029667_T001 transcript:EES11282          4.00E-136
193-  1: transcript:Zm00001d029673_T001 transcript:EES11284          2.00E-122
193-  2: transcript:Zm00001d029677_T001 transcript:EES11285                  0
193-  3: transcript:Zm00001d029679_T001 transcript:EES11287           2.00E-36
193-  4: transcript:Zm00001d029681_T001 transcript:KXG26952           3.00E-33
193-  5: transcript:Zm00001d029683_T001 transcript:KXG26955                  0
193-  6: transcript:Zm00001d029688_T002 transcript:EES11294                  0
## Alignment 194: score=297.0 e_value=1.3e-10 N=7 1&6 plus
194-  0: transcript:Zm00001d029734_T001 transcript:EES12701                  0
194-  1: transcript:Zm00001d029740_T002 transcript:OQU82197           4.00E-60
194-  2: transcript:Zm00001d029744_T001 transcript:KXG26978          7.00E-172
194-  3: transcript:Zm00001d029749_T001 transcript:EES12712           2.00E-54
194-  4: transcript:Zm00001d029752_T001 transcript:OQU82200           5.00E-10
194-  5: transcript:Zm00001d029754_T004 transcript:EES11273          1.00E-146
194-  6: transcript:Zm00001d029758_T004 transcript:EES11322                  0
## Alignment 195: score=282.0 e_value=3.7e-11 N=7 1&6 minus
195-  0: transcript:Zm00001d029934_T001 transcript:EES12613           1.00E-69
195-  1: transcript:Zm00001d029937_T001 transcript:OQU82082           1.00E-09
195-  2: transcript:Zm00001d029940_T001 transcript:OQU82076           7.00E-29
195-  3: transcript:Zm00001d029948_T001 transcript:EES11209                  0
195-  4: transcript:Zm00001d029970_T001 transcript:EES12591           3.00E-73
195-  5: transcript:Zm00001d029974_T001 transcript:OQU82063           3.00E-26
```

```
195-  6: transcript:Zm00001d029978_T001 transcript:OQU82059            4.00E-108
## Alignment 196: score=3947.0 e_value=0 N=85 1&7 plus
196-  0: transcript:Zm00001d031738_T001 transcript:OQU80611            6.00E-119
196-  1: transcript:Zm00001d031740_T001 transcript:EES15108                    0
196-  2: transcript:Zm00001d031741_T001 transcript:OQU80613            8.00E-108
196-  3: transcript:Zm00001d031745_T001 transcript:KXG25294            2.00E-149
196-  4: transcript:Zm00001d031747_T001 transcript:EES13977                    0
196-  5: transcript:Zm00001d031749_T005 transcript:OQU80616                    0
196-  6: transcript:Zm00001d031750_T001 transcript:EES15109            2.00E-143
196-  7: transcript:Zm00001d031753_T001 transcript:KXG25300            1.00E-101
196-  8: transcript:Zm00001d031759_T001 transcript:EES13982                    0
196-  9: transcript:Zm00001d031760_T001 transcript:EES13983                    0
196- 10: transcript:Zm00001d031764_T002 transcript:EES15112                    0
196- 11: transcript:Zm00001d031769_T001 transcript:EES13985                    0
196- 12: transcript:Zm00001d031777_T001 transcript:OQU80624                    0
196- 13: transcript:Zm00001d031778_T001 transcript:EES15117            3.00E-133
196- 14: transcript:Zm00001d031781_T001 transcript:EES15124             3.00E-90
196- 15: transcript:Zm00001d031782_T001 transcript:OQU80628                    0
196- 16: transcript:Zm00001d031784_T002 transcript:KXG25314            2.00E-130
196- 17: transcript:Zm00001d031790_T002 transcript:OQU80630            3.00E-148
196- 18: transcript:Zm00001d031792_T001 transcript:OQU80634                    0
196- 19: transcript:Zm00001d031793_T002 transcript:KXG25318                    0
196- 20: transcript:Zm00001d031794_T001 transcript:EES14002                    0
196- 21: transcript:Zm00001d031796_T001 transcript:OQU80635             3.00E-97
196- 22: transcript:Zm00001d031797_T001 transcript:EES14004             6.00E-39
196- 23: transcript:Zm00001d031798_T001 transcript:EES15129             5.00E-64
196- 24: transcript:Zm00001d031801_T001 transcript:OQU80637            2.00E-106
196- 25: transcript:Zm00001d031804_T001 transcript:OQU80638             9.00E-33
196- 26: transcript:Zm00001d031805_T001 transcript:KXG25323            1.00E-155
196- 27: transcript:Zm00001d031806_T001 transcript:EES14016             9.00E-45
196- 28: transcript:Zm00001d031807_T003 transcript:KXG25324                    0
196- 29: transcript:Zm00001d031808_T001 transcript:EES14019            5.00E-165
196- 30: transcript:Zm00001d031809_T001 transcript:EES15132            2.00E-174
196- 31: transcript:Zm00001d031810_T001 transcript:EES14020            2.00E-154
196- 32: transcript:Zm00001d031811_T001 transcript:KXG25325                    0
196- 33: transcript:Zm00001d031816_T002 transcript:KXG25327             2.00E-38
196- 34: transcript:Zm00001d031817_T003 transcript:KXG25328            3.00E-167
196- 35: transcript:Zm00001d031818_T001 transcript:EES14023            8.00E-106
196- 36: transcript:Zm00001d031822_T001 transcript:OQU80646             7.00E-49
196- 37: transcript:Zm00001d031824_T003 transcript:OQU80648            2.00E-104
196- 38: transcript:Zm00001d031825_T001 transcript:EES15140            4.00E-101
196- 39: transcript:Zm00001d031826_T001 transcript:KXG25333            6.00E-106
196- 40: transcript:Zm00001d031832_T003 transcript:OQU80649                    0
196- 41: transcript:Zm00001d031833_T001 transcript:EES15143                    0
196- 42: transcript:Zm00001d031837_T001 transcript:EES14027            3.00E-160
196- 43: transcript:Zm00001d031840_T001 transcript:EES15144            5.00E-156
196- 44: transcript:Zm00001d031844_T001 transcript:OQU80661                    0
196- 45: transcript:Zm00001d031847_T031 transcript:KXG25342                    0
196- 46: transcript:Zm00001d031848_T001 transcript:EES15148                    0
196- 47: transcript:Zm00001d031849_T002 transcript:KXG25343                    0
196- 48: transcript:Zm00001d031850_T001 transcript:EES15149             7.00E-74
196- 49: transcript:Zm00001d031851_T002 transcript:EES15150                    0
196- 50: transcript:Zm00001d031852_T001 transcript:EES14032             6.00E-50
196- 51: transcript:Zm00001d031853_T001 transcript:EES14033                    0
```

196- 52: transcript:Zm00001d031854_T001 transcript:OQU80666 0
196- 53: transcript:Zm00001d031855_T001 transcript:OQU80667 0
196- 54: transcript:Zm00001d031856_T002 transcript:OQU80669 8.00E-70
196- 55: transcript:Zm00001d031858_T001 transcript:EES14036 0
196- 56: transcript:Zm00001d031860_T003 transcript:OQU80670 3.00E-174
196- 57: transcript:Zm00001d031861_T001 transcript:OQU80673 1.00E-104
196- 58: transcript:Zm00001d031863_T013 transcript:KXG25352 4.00E-29
196- 59: transcript:Zm00001d031866_T001 transcript:EES15157 0
196- 60: transcript:Zm00001d031867_T001 transcript:KXG25355 0
196- 61: transcript:Zm00001d031868_T001 transcript:EES14048 2.00E-43
196- 62: transcript:Zm00001d031871_T001 transcript:OQU80682 0
196- 63: transcript:Zm00001d031875_T001 transcript:OQU80695 0
196- 64: transcript:Zm00001d031877_T001 transcript:EES14054 1.00E-27
196- 65: transcript:Zm00001d031878_T001 transcript:OQU80696 5.00E-18
196- 66: transcript:Zm00001d031880_T001 transcript:KXG25363 7.00E-128
196- 67: transcript:Zm00001d031882_T001 transcript:EES14059 5.00E-145
196- 68: transcript:Zm00001d031883_T001 transcript:EES14052 3.00E-85
196- 69: transcript:Zm00001d031887_T001 transcript:KXG25365 4.00E-136
196- 70: transcript:Zm00001d031891_T017 transcript:EES14057 0
196- 71: transcript:Zm00001d031892_T001 transcript:EES15243 1.00E-130
196- 72: transcript:Zm00001d031893_T001 transcript:EES14067 0
196- 73: transcript:Zm00001d031894_T005 transcript:EES14068 0
196- 74: transcript:Zm00001d031895_T001 transcript:OQU80698 0
196- 75: transcript:Zm00001d031898_T001 transcript:OQU80699 0
196- 76: transcript:Zm00001d031899_T002 transcript:KXG25370 0
196- 77: transcript:Zm00001d031902_T002 transcript:EES14072 5.00E-121
196- 78: transcript:Zm00001d031904_T001 transcript:KXG25373 3.00E-162
196- 79: transcript:Zm00001d031908_T001 transcript:EES15166 5.00E-126
196- 80: transcript:Zm00001d031911_T002 transcript:OQU80701 0
196- 81: transcript:Zm00001d031913_T003 transcript:EES15167 0
196- 82: transcript:Zm00001d031921_T001 transcript:EES15168 8.00E-52
196- 83: transcript:Zm00001d031925_T001 transcript:KXG25377 6.00E-119
196- 84: transcript:Zm00001d031926_T001 transcript:EES15170 0
Alignment 197: score=2332.0 e_value=8.6e-196 N=52 1&7 plus
197- 0: transcript:Zm00001d031933_T011 transcript:KXG25380 0
197- 1: transcript:Zm00001d031934_T001 transcript:EES14083 3.00E-174
197- 2: transcript:Zm00001d031937_T001 transcript:EES14084 5.00E-99
197- 3: transcript:Zm00001d031938_T001 transcript:EES15174 0
197- 4: transcript:Zm00001d031940_T001 transcript:EES15175 4.00E-40
197- 5: transcript:Zm00001d031941_T001 transcript:EES14085 4.00E-145
197- 6: transcript:Zm00001d031942_T002 transcript:KXG25383 7.00E-180
197- 7: transcript:Zm00001d031943_T002 transcript:EES14087 0
197- 8: transcript:Zm00001d031944_T001 transcript:EES15176 2.00E-129
197- 9: transcript:Zm00001d031948_T002 transcript:EES15177 0
197- 10: transcript:Zm00001d031952_T002 transcript:EES14089 4.00E-51
197- 11: transcript:Zm00001d031957_T001 transcript:EES15178 5.00E-156
197- 12: transcript:Zm00001d031958_T001 transcript:OQU80713 0
197- 13: transcript:Zm00001d031959_T001 transcript:KXG25401 0
197- 14: transcript:Zm00001d031961_T002 transcript:EES15189 2.00E-87
197- 15: transcript:Zm00001d031963_T001 transcript:EES15192 4.00E-158
197- 16: transcript:Zm00001d031967_T001 transcript:KXG25407 1.00E-85
197- 17: transcript:Zm00001d031969_T001 transcript:EES14094 6.00E-169
197- 18: transcript:Zm00001d031970_T001 transcript:OQU80724 5.00E-131
197- 19: transcript:Zm00001d031971_T002 transcript:EES15197 0

197- 20:	transcript:Zm00001d031973_T001	transcript:KXG25410	0
197- 21:	transcript:Zm00001d031975_T001	transcript:EES15199	3.00E-59
197- 22:	transcript:Zm00001d031977_T001	transcript:KXG25411	0
197- 23:	transcript:Zm00001d031979_T001	transcript:EES15202	5.00E-82
197- 24:	transcript:Zm00001d031981_T002	transcript:EES14097	0
197- 25:	transcript:Zm00001d031985_T001	transcript:OQU80725	4.00E-61
197- 26:	transcript:Zm00001d031988_T001	transcript:EES15204	0
197- 27:	transcript:Zm00001d031992_T001	transcript:KXG25416	0
197- 28:	transcript:Zm00001d031993_T001	transcript:EES15206	0
197- 29:	transcript:Zm00001d031996_T006	transcript:EES15208	0
197- 30:	transcript:Zm00001d031997_T001	transcript:EES14101	0
197- 31:	transcript:Zm00001d032000_T001	transcript:OQU80731	1.00E-101
197- 32:	transcript:Zm00001d032005_T001	transcript:OQU80736	4.00E-82
197- 33:	transcript:Zm00001d032008_T001	transcript:OQU80738	1.00E-109
197- 34:	transcript:Zm00001d032010_T007	transcript:EES14113	0
197- 35:	transcript:Zm00001d032011_T001	transcript:EES15224	1.00E-59
197- 36:	transcript:Zm00001d032012_T001	transcript:EES15226	9.00E-33
197- 37:	transcript:Zm00001d032019_T001	transcript:EES14114	1.00E-86
197- 38:	transcript:Zm00001d032022_T003	transcript:KXG25431	0
197- 39:	transcript:Zm00001d032024_T001	transcript:EES14116	9.00E-143
197- 40:	transcript:Zm00001d032027_T002	transcript:EES15233	0
197- 41:	transcript:Zm00001d032028_T001	transcript:EES15234	0
197- 42:	transcript:Zm00001d032029_T003	transcript:OQU80745	5.00E-71
197- 43:	transcript:Zm00001d032030_T001	transcript:KXG25435	6.00E-56
197- 44:	transcript:Zm00001d032031_T001	transcript:EES15240	0
197- 45:	transcript:Zm00001d032032_T001	transcript:OQU80746	1.00E-98
197- 46:	transcript:Zm00001d032035_T001	transcript:EES14120	0
197- 47:	transcript:Zm00001d032036_T001	transcript:EES14122	0
197- 48:	transcript:Zm00001d032040_T001	transcript:KXG25440	6.00E-123
197- 49:	transcript:Zm00001d032042_T001	transcript:OQU80753	0
197- 50:	transcript:Zm00001d032044_T004	transcript:EES15245	0
197- 51:	transcript:Zm00001d032045_T001	transcript:OQU80754	0

Alignment 198: score=822.0 e_value=2.1e-49 N=18 1&7 plus

198- 0:	transcript:Zm00001d032282_T012	transcript:KXG25106	0
198- 1:	transcript:Zm00001d032283_T004	transcript:EES13800	0
198- 2:	transcript:Zm00001d032284_T001	transcript:OQU80404	0
198- 3:	transcript:Zm00001d032285_T001	transcript:EES14962	8.00E-76
198- 4:	transcript:Zm00001d032286_T001	transcript:EES13801	0
198- 5:	transcript:Zm00001d032291_T001	transcript:EES13808	2.00E-102
198- 6:	transcript:Zm00001d032292_T001	transcript:EES14968	2.00E-81
198- 7:	transcript:Zm00001d032293_T001	transcript:EES13813	0
198- 8:	transcript:Zm00001d032295_T001	transcript:EES13814	8.00E-128
198- 9:	transcript:Zm00001d032298_T002	transcript:EES13815	0
198- 10:	transcript:Zm00001d032300_T001	transcript:EES13816	0
198- 11:	transcript:Zm00001d032301_T005	transcript:EES14971	1.00E-140
198- 12:	transcript:Zm00001d032303_T002	transcript:EES13819	2.00E-150
198- 13:	transcript:Zm00001d032304_T003	transcript:EES13821	0
198- 14:	transcript:Zm00001d032306_T001	transcript:EES13822	2.00E-75
198- 15:	transcript:Zm00001d032307_T001	transcript:EES14978	2.00E-30
198- 16:	transcript:Zm00001d032308_T001	transcript:EES13823	0
198- 17:	transcript:Zm00001d032310_T001	transcript:OQU80422	0

Alignment 199: score=597.0 e_value=2.3e-34 N=14 1&7 plus

199- 0:	transcript:EER92197	transcript:Zm00001d022211_T001	4.00E-17
199- 1:	transcript:OQU92521	transcript:Zm00001d022218_T001	1.00E-81

199- 2: transcript:OQU92522 transcript:Zm00001d022225_T001 1.00E-83
199- 3: transcript:KXG39272 transcript:Zm00001d022227_T001 4.00E-93
199- 4: transcript:EER92203 transcript:Zm00001d022237_T001 3.00E-115
199- 5: transcript:EER92205 transcript:Zm00001d022238_T001 2.00E-119
199- 6: transcript:KXG39284 transcript:Zm00001d022242_T001 6.00E-53
199- 7: transcript:EER92211 transcript:Zm00001d022243_T002 1.00E-77
199- 8: transcript:EER92212 transcript:Zm00001d022245_T001 0
199- 9: transcript:EER94791 transcript:Zm00001d022250_T001 8.00E-103
199- 10: transcript:OQU92532 transcript:Zm00001d022252_T001 1.00E-57
199- 11: transcript:EER92216 transcript:Zm00001d022259_T002 1.00E-89
199- 12: transcript:OQU92539 transcript:Zm00001d022264_T001 6.00E-21
199- 13: transcript:EER92218 transcript:Zm00001d022266_T001 3.00E-100
Alignment 200: score=554.0 e_value=1e-34 N=13 1&7 plus
200- 0: transcript:Zm00001d032361_T001 transcript:KXG24984 1.00E-140
200- 1: transcript:Zm00001d032362_T002 transcript:EES13704 0
200- 2: transcript:Zm00001d032363_T001 transcript:EES14882 0
200- 3: transcript:Zm00001d032366_T001 transcript:EES14884 2.00E-75
200- 4: transcript:Zm00001d032367_T001 transcript:EES14886 2.00E-80
200- 5: transcript:Zm00001d032368_T001 transcript:EES14887 0
200- 6: transcript:Zm00001d032373_T004 transcript:EES13710 0
200- 7: transcript:Zm00001d032377_T002 transcript:OQU80310 0
200- 8: transcript:Zm00001d032379_T002 transcript:KXG24994 0
200- 9: transcript:Zm00001d032380_T001 transcript:EES14893 2.00E-173
200- 10: transcript:Zm00001d032381_T001 transcript:OQU80318 6.00E-20
200- 11: transcript:Zm00001d032382_T001 transcript:KXG25005 1.00E-15
200- 12: transcript:Zm00001d032388_T001 transcript:EES14900 3.00E-149
Alignment 201: score=521.0 e_value=1.7e-27 N=11 1&7 plus
201- 0: transcript:Zm00001d032265_T001 transcript:OQU80381 8.00E-168
201- 1: transcript:Zm00001d032266_T003 transcript:OQU80382 2.00E-70
201- 2: transcript:Zm00001d032267_T003 transcript:EES13774 0
201- 3: transcript:Zm00001d032268_T001 transcript:EES13775 0
201- 4: transcript:Zm00001d032270_T002 transcript:OQU80384 0
201- 5: transcript:Zm00001d032271_T001 transcript:EES13778 0
201- 6: transcript:Zm00001d032272_T001 transcript:EES14948 0
201- 7: transcript:Zm00001d032274_T001 transcript:OQU80385 0
201- 8: transcript:Zm00001d032275_T001 transcript:KXG25095 0
201- 9: transcript:Zm00001d032279_T005 transcript:EES14950 0
201- 10: transcript:Zm00001d032280_T001 transcript:EES14951 2.00E-180
Alignment 202: score=417.0 e_value=2.9e-21 N=10 1&7 plus
202- 0: transcript:OQU92540 transcript:Zm00001d022273_T016 7.00E-60
202- 1: transcript:EER94801 transcript:Zm00001d022274_T001 0
202- 2: transcript:EER92219 transcript:Zm00001d022275_T001 5.00E-147
202- 3: transcript:EER94803 transcript:Zm00001d022279_T001 3.00E-115
202- 4: transcript:KXG39300 transcript:Zm00001d022280_T001 2.00E-111
202- 5: transcript:EER94809 transcript:Zm00001d022294_T002 6.00E-34
202- 6: transcript:EER94810 transcript:Zm00001d022305_T001 0
202- 7: transcript:EER92223 transcript:Zm00001d022307_T003 3.00E-49
202- 8: transcript:KXG39304 transcript:Zm00001d022314_T001 2.00E-63
202- 9: transcript:OQU92551 transcript:Zm00001d022315_T001 0
Alignment 203: score=340.0 e_value=2.3e-14 N=8 1&7 plus
203- 0: transcript:EER92116 transcript:Zm00001d022059_T001 1.00E-40
203- 1: transcript:KXG39123 transcript:Zm00001d022069_T001 1.00E-78
203- 2: transcript:EER94684 transcript:Zm00001d022071_T001 5.00E-44
203- 3: transcript:EER94686 transcript:Zm00001d022072_T001 0

```
203-  4: transcript:KXG39130           transcript:Zm00001d022075_T001         0
203-  5: transcript:EER92124           transcript:Zm00001d022077_T003 6.00E-157
203-  6: transcript:OQU92393           transcript:Zm00001d022081_T001         0
203-  7: transcript:KXG39136           transcript:Zm00001d022083_T001  2.00E-08
## Alignment 204: score=280.0 e_value=1.9e-11 N=7 1&7 plus
204-  0: transcript:EER92182           transcript:Zm00001d022179_T001         0
204-  1: transcript:EER94760           transcript:Zm00001d022181_T004         0
204-  2: transcript:KXG39234           transcript:Zm00001d022182_T001 1.00E-156
204-  3: transcript:EER94762           transcript:Zm00001d022185_T003         0
204-  4: transcript:OQU92492           transcript:Zm00001d022188_T002  2.00E-31
204-  5: transcript:KXG39239           transcript:Zm00001d022189_T002  1.00E-61
204-  6: transcript:EER94763           transcript:Zm00001d022190_T003  8.00E-62
## Alignment 205: score=276.0 e_value=1e-10 N=6 1&7 plus
205-  0: transcript:Zm00001d031253_T001 transcript:EES14336                    0
205-  1: transcript:Zm00001d031254_T004 transcript:OQU81037             4.00E-86
205-  2: transcript:Zm00001d031256_T019 transcript:EES14342                    0
205-  3: transcript:Zm00001d031257_T001 transcript:KXG25739             1.00E-71
205-  4: transcript:Zm00001d031258_T003 transcript:EES15487            1.00E-140
205-  5: transcript:Zm00001d031259_T001 transcript:EES15488            6.00E-121
## Alignment 206: score=265.0 e_value=1.6e-08 N=6 1&7 plus
206-  0: transcript:Zm00001d032311_T001 transcript:EES14981                    0
206-  1: transcript:Zm00001d032313_T001 transcript:EES13825            2.00E-104
206-  2: transcript:Zm00001d032316_T001 transcript:KXG25139            1.00E-113
206-  3: transcript:Zm00001d032317_T001 transcript:OQU80427                    0
206-  4: transcript:Zm00001d032318_T001 transcript:EES13830             2.00E-39
206-  5: transcript:Zm00001d032321_T001 transcript:KXG25151             7.00E-59
## Alignment 207: score=4345.0 e_value=0 N=96 1&7 minus
207-  0: transcript:Zm00001d031265_T029 transcript:KXG25732                    0
207-  1: transcript:Zm00001d031266_T001 transcript:EES15479            3.00E-153
207-  2: transcript:Zm00001d031267_T001 transcript:OQU81028                    0
207-  3: transcript:Zm00001d031268_T010 transcript:EES15476                    0
207-  4: transcript:Zm00001d031269_T002 transcript:KXG25727             5.00E-88
207-  5: transcript:Zm00001d031270_T001 transcript:EES14333            4.00E-122
207-  6: transcript:Zm00001d031271_T003 transcript:EES15473             7.00E-71
207-  7: transcript:Zm00001d031272_T001 transcript:KXG25724             4.00E-71
207-  8: transcript:Zm00001d031273_T001 transcript:EES14331            3.00E-156
207-  9: transcript:Zm00001d031274_T001 transcript:EES14330             7.00E-54
207- 10: transcript:Zm00001d031275_T001 transcript:EES15471                    0
207- 11: transcript:Zm00001d031277_T001 transcript:EES14325             8.00E-79
207- 12: transcript:Zm00001d031278_T001 transcript:OQU81009             1.00E-90
207- 13: transcript:Zm00001d031279_T001 transcript:EES15464                    0
207- 14: transcript:Zm00001d031280_T001 transcript:EES15461                    0
207- 15: transcript:Zm00001d031282_T001 transcript:EES15463             2.00E-48
207- 16: transcript:Zm00001d031283_T001 transcript:KXG25712             2.00E-11
207- 17: transcript:Zm00001d031287_T001 transcript:EES15457                    0
207- 18: transcript:Zm00001d031288_T001 transcript:OQU81002             5.00E-40
207- 19: transcript:Zm00001d031289_T007 transcript:EES15456                    0
207- 20: transcript:Zm00001d031290_T001 transcript:EES14320             7.00E-65
207- 21: transcript:Zm00001d031291_T004 transcript:EES15450                    0
207- 22: transcript:Zm00001d031292_T001 transcript:EES14318            3.00E-156
207- 23: transcript:Zm00001d031303_T006 transcript:EES15448                    0
207- 24: transcript:Zm00001d031308_T001 transcript:EES15446             2.00E-95
207- 25: transcript:Zm00001d031310_T001 transcript:EES15444             4.00E-93
207- 26: transcript:Zm00001d031311_T001 transcript:OQU80993            6.00E-114
```

207- 27:	transcript:Zm00001d031312_T001	transcript:OQU80992	3.00E-139
207- 28:	transcript:Zm00001d031315_T002	transcript:EES15441	0
207- 29:	transcript:Zm00001d031318_T001	transcript:EES15437	0
207- 30:	transcript:Zm00001d031321_T001	transcript:EES15434	0
207- 31:	transcript:Zm00001d031323_T001	transcript:EES14305	0
207- 32:	transcript:Zm00001d031325_T001	transcript:KXG25677	1.00E-101
207- 33:	transcript:Zm00001d031326_T001	transcript:KXG25675	0
207- 34:	transcript:Zm00001d031327_T009	transcript:EES15427	0
207- 35:	transcript:Zm00001d031328_T001	transcript:EES14298	0
207- 36:	transcript:Zm00001d031329_T002	transcript:KXG25674	0
207- 37:	transcript:Zm00001d031332_T007	transcript:EES14299	0
207- 38:	transcript:Zm00001d031338_T002	transcript:EES15426	0
207- 39:	transcript:Zm00001d031339_T001	transcript:EES15423	5.00E-43
207- 40:	transcript:Zm00001d031340_T001	transcript:EES15422	0
207- 41:	transcript:Zm00001d031367_T001	transcript:EES14285	3.00E-85
207- 42:	transcript:Zm00001d031381_T001	transcript:OQU80962	0
207- 43:	transcript:Zm00001d031396_T001	transcript:EES15417	4.00E-56
207- 44:	transcript:Zm00001d031402_T001	transcript:EES14284	5.00E-50
207- 45:	transcript:Zm00001d031412_T001	transcript:OQU80956	0
207- 46:	transcript:Zm00001d031413_T001	transcript:EES14282	0
207- 47:	transcript:Zm00001d031415_T001	transcript:EES15413	0
207- 48:	transcript:Zm00001d031416_T001	transcript:EES15412	0
207- 49:	transcript:Zm00001d031418_T001	transcript:OQU80953	3.00E-128
207- 50:	transcript:Zm00001d031419_T001	transcript:KXG25648	4.00E-65
207- 51:	transcript:Zm00001d031420_T001	transcript:KXG25647	0
207- 52:	transcript:Zm00001d031421_T001	transcript:OQU80952	6.00E-161
207- 53:	transcript:Zm00001d031422_T006	transcript:EES15409	0
207- 54:	transcript:Zm00001d031423_T001	transcript:OQU80949	1.00E-73
207- 55:	transcript:Zm00001d031426_T001	transcript:EES15407	0
207- 56:	transcript:Zm00001d031427_T001	transcript:OQU80948	0
207- 57:	transcript:Zm00001d031428_T001	transcript:EES14277	0
207- 58:	transcript:Zm00001d031429_T001	transcript:EES15400	0
207- 59:	transcript:Zm00001d031430_T005	transcript:EES14276	0
207- 60:	transcript:Zm00001d031431_T001	transcript:EES14275	8.00E-75
207- 61:	transcript:Zm00001d031434_T001	transcript:EES14271	0
207- 62:	transcript:Zm00001d031436_T001	transcript:OQU80944	0
207- 63:	transcript:Zm00001d031437_T001	transcript:KXG25642	0
207- 64:	transcript:Zm00001d031439_T001	transcript:OQU80941	1.00E-129
207- 65:	transcript:Zm00001d031441_T002	transcript:OQU80938	4.00E-177
207- 66:	transcript:Zm00001d031444_T001	transcript:EES14272	0
207- 67:	transcript:Zm00001d031445_T001	transcript:EES15398	0
207- 68:	transcript:Zm00001d031447_T003	transcript:OQU80926	0
207- 69:	transcript:Zm00001d031449_T001	transcript:OQU80925	0
207- 70:	transcript:Zm00001d031450_T001	transcript:KXG25635	2.00E-128
207- 71:	transcript:Zm00001d031451_T001	transcript:EES14266	0
207- 72:	transcript:Zm00001d031453_T001	transcript:EES14264	2.00E-174
207- 73:	transcript:Zm00001d031454_T001	transcript:EES14263	0
207- 74:	transcript:Zm00001d031463_T002	transcript:EES14261	0
207- 75:	transcript:Zm00001d031464_T001	transcript:EES14260	2.00E-70
207- 76:	transcript:Zm00001d031465_T001	transcript:EES14259	6.00E-61
207- 77:	transcript:Zm00001d031466_T001	transcript:OQU80911	0
207- 78:	transcript:Zm00001d031470_T001	transcript:EES14257	0
207- 79:	transcript:Zm00001d031471_T002	transcript:EES15379	0
207- 80:	transcript:Zm00001d031473_T001	transcript:EES14254	0

207- 81:	transcript:Zm00001d031475_T001	transcript:EES15387	6.00E-147
207- 82:	transcript:Zm00001d031476_T001	transcript:KXG25627	2.00E-57
207- 83:	transcript:Zm00001d031480_T001	transcript:EES15385	0
207- 84:	transcript:Zm00001d031481_T001	transcript:KXG25625	0
207- 85:	transcript:Zm00001d031484_T001	transcript:EES15384	2.00E-157
207- 86:	transcript:Zm00001d031485_T001	transcript:OQU80908	0
207- 87:	transcript:Zm00001d031486_T001	transcript:OQU80907	0
207- 88:	transcript:Zm00001d031487_T004	transcript:KXG25620	0
207- 89:	transcript:Zm00001d031488_T002	transcript:EES14250	1.00E-143
207- 90:	transcript:Zm00001d031489_T001	transcript:EES14249	0
207- 91:	transcript:Zm00001d031490_T003	transcript:KXG25610	0
207- 92:	transcript:Zm00001d031494_T001	transcript:EES15371	1.00E-171
207- 93:	transcript:Zm00001d031496_T001	transcript:OQU80891	7.00E-115
207- 94:	transcript:Zm00001d031498_T001	transcript:EES14245	3.00E-33
207- 95:	transcript:Zm00001d031502_T008	transcript:OQU80887	0
## Alignment 208: score=3901.0 e_value=0 N=85 1&7 minus			
208- 0:	transcript:Zm00001d032035_T001	transcript:OQU80614	2.00E-65
208- 1:	transcript:Zm00001d032046_T002	transcript:KXG25291	0
208- 2:	transcript:Zm00001d032047_T001	transcript:EES15103	3.00E-102
208- 3:	transcript:Zm00001d032049_T002	transcript:EES13971	0
208- 4:	transcript:Zm00001d032053_T001	transcript:KXG25341	3.00E-85
208- 5:	transcript:Zm00001d032055_T001	transcript:KXG25288	7.00E-125
208- 6:	transcript:Zm00001d032056_T001	transcript:KXG25287	0
208- 7:	transcript:Zm00001d032057_T002	transcript:OQU80606	4.00E-73
208- 8:	transcript:Zm00001d032058_T002	transcript:EES13970	6.00E-99
208- 9:	transcript:Zm00001d032060_T001	transcript:KXG25280	0
208- 10:	transcript:Zm00001d032069_T001	transcript:KXG25279	0
208- 11:	transcript:Zm00001d032071_T001	transcript:EES13965	7.00E-70
208- 12:	transcript:Zm00001d032073_T001	transcript:EES13961	2.00E-23
208- 13:	transcript:Zm00001d032075_T001	transcript:EES13957	0
208- 14:	transcript:Zm00001d032076_T002	transcript:EES13960	0
208- 15:	transcript:Zm00001d032077_T001	transcript:KXG25274	2.00E-118
208- 16:	transcript:Zm00001d032078_T002	transcript:EES13956	0
208- 17:	transcript:Zm00001d032079_T001	transcript:KXG25273	0
208- 18:	transcript:Zm00001d032081_T001	transcript:KXG25271	1.00E-48
208- 19:	transcript:Zm00001d032084_T001	transcript:KXG25268	2.00E-67
208- 20:	transcript:Zm00001d032088_T001	transcript:EES13953	3.00E-70
208- 21:	transcript:Zm00001d032095_T001	transcript:EES13952	0
208- 22:	transcript:Zm00001d032096_T005	transcript:KXG25261	0
208- 23:	transcript:Zm00001d032098_T001	transcript:EES13947	6.00E-85
208- 24:	transcript:Zm00001d032099_T004	transcript:EES13942	0
208- 25:	transcript:Zm00001d032100_T003	transcript:EES15086	0
208- 26:	transcript:Zm00001d032101_T001	transcript:KXG25260	0
208- 27:	transcript:Zm00001d032102_T001	transcript:EES13938	9.00E-62
208- 28:	transcript:Zm00001d032103_T001	transcript:EES15085	0
208- 29:	transcript:Zm00001d032104_T001	transcript:OQU80571	0
208- 30:	transcript:Zm00001d032109_T001	transcript:EES15084	0
208- 31:	transcript:Zm00001d032111_T003	transcript:OQU80569	4.00E-103
208- 32:	transcript:Zm00001d032112_T001	transcript:OQU80568	0
208- 33:	transcript:Zm00001d032114_T001	transcript:EES15079	0
208- 34:	transcript:Zm00001d032115_T001	transcript:EES13936	6.00E-73
208- 35:	transcript:Zm00001d032116_T002	transcript:OQU80565	8.00E-113
208- 36:	transcript:Zm00001d032118_T001	transcript:EES13934	0
208- 37:	transcript:Zm00001d032127_T002	transcript:KXG25248	3.00E-142

208- 38: transcript:Zm00001d032138_T001 transcript:EES15070 5.00E-06
208- 39: transcript:Zm00001d032139_T002 transcript:EES13926 5.00E-65
208- 40: transcript:Zm00001d032142_T004 transcript:OQU80552 0
208- 41: transcript:Zm00001d032143_T001 transcript:OQU80550 2.00E-178
208- 42: transcript:Zm00001d032144_T001 transcript:OQU80549 0
208- 43: transcript:Zm00001d032145_T001 transcript:OQU80548 0
208- 44: transcript:Zm00001d032146_T001 transcript:EES13922 1.00E-113
208- 45: transcript:Zm00001d032148_T001 transcript:EES15064 0
208- 46: transcript:Zm00001d032152_T001 transcript:EES15061 0
208- 47: transcript:Zm00001d032153_T002 transcript:KXG25234 1.00E-73
208- 48: transcript:Zm00001d032155_T001 transcript:EES13913 0
208- 49: transcript:Zm00001d032156_T003 transcript:EES15058 0
208- 50: transcript:Zm00001d032157_T001 transcript:EES15057 1.00E-139
208- 51: transcript:Zm00001d032158_T002 transcript:EES13910 0
208- 52: transcript:Zm00001d032160_T001 transcript:EES15056 3.00E-156
208- 53: transcript:Zm00001d032162_T001 transcript:OQU80533 3.00E-119
208- 54: transcript:Zm00001d032163_T001 transcript:EES13907 1.00E-143
208- 55: transcript:Zm00001d032164_T001 transcript:EES13906 0
208- 56: transcript:Zm00001d032165_T003 transcript:EES13905 4.00E-124
208- 57: transcript:Zm00001d032166_T003 transcript:EES13904 0
208- 58: transcript:Zm00001d032172_T002 transcript:KXG25278 0
208- 59: transcript:Zm00001d032173_T001 transcript:KXG25228 0
208- 60: transcript:Zm00001d032175_T001 transcript:EES15050 5.00E-146
208- 61: transcript:Zm00001d032177_T001 transcript:KXG25227 1.00E-44
208- 62: transcript:Zm00001d032178_T001 transcript:EES13898 3.00E-136
208- 63: transcript:Zm00001d032181_T001 transcript:EES15047 4.00E-79
208- 64: transcript:Zm00001d032182_T001 transcript:EES13897 0
208- 65: transcript:Zm00001d032183_T002 transcript:KXG25224 9.00E-178
208- 66: transcript:Zm00001d032184_T003 transcript:EES13893 3.00E-170
208- 67: transcript:Zm00001d032185_T001 transcript:EES15045 0
208- 68: transcript:Zm00001d032186_T001 transcript:EES15044 3.00E-151
208- 69: transcript:Zm00001d032187_T001 transcript:OQU80513 0
208- 70: transcript:Zm00001d032188_T001 transcript:KXG25219 0
208- 71: transcript:Zm00001d032190_T009 transcript:OQU80510 8.00E-98
208- 72: transcript:Zm00001d032194_T002 transcript:OQU80507 2.00E-163
208- 73: transcript:Zm00001d032197_T001 transcript:KXG25214 5.00E-174
208- 74: transcript:Zm00001d032198_T002 transcript:EES13890 0
208- 75: transcript:Zm00001d032199_T001 transcript:EES15041 9.00E-81
208- 76: transcript:Zm00001d032204_T001 transcript:EES15040 0
208- 77: transcript:Zm00001d032206_T001 transcript:OQU80505 0
208- 78: transcript:Zm00001d032208_T001 transcript:OQU80504 2.00E-179
208- 79: transcript:Zm00001d032209_T002 transcript:EES13884 1.00E-64
208- 80: transcript:Zm00001d032212_T005 transcript:EES15039 6.00E-150
208- 81: transcript:Zm00001d032213_T001 transcript:EES15038 3.00E-154
208- 82: transcript:Zm00001d032215_T001 transcript:EES15037 2.00E-110
208- 83: transcript:Zm00001d032217_T001 transcript:EES13882 2.00E-126
208- 84: transcript:Zm00001d032218_T001 transcript:OQU80503 6.00E-33
Alignment 209: score=2826.0 e_value=5e-242 N=61 1&7 minus
209- 0: transcript:Zm00001d031509_T003 transcript:EES15369 0
209- 1: transcript:Zm00001d031510_T001 transcript:EES14244 1.00E-167
209- 2: transcript:Zm00001d031514_T001 transcript:EES14316 1.00E-46
209- 3: transcript:Zm00001d031515_T001 transcript:EES15365 1.00E-128
209- 4: transcript:Zm00001d031517_T001 transcript:EES15363 1.00E-54
209- 5: transcript:Zm00001d031520_T001 transcript:EES15360 1.00E-73

209- 6:	transcript:Zm00001d031522_T007	transcript:EES15359	0
209- 7:	transcript:Zm00001d031523_T001	transcript:EES15358	0
209- 8:	transcript:Zm00001d031525_T004	transcript:EES15356	0
209- 9:	transcript:Zm00001d031526_T001	transcript:KXG25603	0
209- 10:	transcript:Zm00001d031527_T001	transcript:EES15354	0
209- 11:	transcript:Zm00001d031528_T001	transcript:KXG25601	0
209- 12:	transcript:Zm00001d031529_T014	transcript:KXG25600	0
209- 13:	transcript:Zm00001d031530_T005	transcript:OQU80872	0
209- 14:	transcript:Zm00001d031531_T002	transcript:EES14236	0
209- 15:	transcript:Zm00001d031532_T001	transcript:KXG25593	0
209- 16:	transcript:Zm00001d031533_T001	transcript:EES15347	0
209- 17:	transcript:Zm00001d031534_T001	transcript:KXG25590	0
209- 18:	transcript:Zm00001d031535_T001	transcript:EES15346	0
209- 19:	transcript:Zm00001d031536_T001	transcript:EES14235	0
209- 20:	transcript:Zm00001d031539_T004	transcript:KXG25672	4.00E-168
209- 21:	transcript:Zm00001d031540_T002	transcript:KXG25583	1.00E-25
209- 22:	transcript:Zm00001d031542_T001	transcript:EES15338	0
209- 23:	transcript:Zm00001d031543_T005	transcript:EES15337	0
209- 24:	transcript:Zm00001d031544_T001	transcript:OQU80868	5.00E-84
209- 25:	transcript:Zm00001d031545_T001	transcript:KXG25581	3.00E-89
209- 26:	transcript:Zm00001d031546_T001	transcript:EES15334	6.00E-34
209- 27:	transcript:Zm00001d031549_T002	transcript:OQU80861	3.00E-50
209- 28:	transcript:Zm00001d031554_T003	transcript:EES15333	0
209- 29:	transcript:Zm00001d031555_T001	transcript:EES14216	0
209- 30:	transcript:Zm00001d031556_T001	transcript:EES15332	2.00E-121
209- 31:	transcript:Zm00001d031560_T001	transcript:EES15331	5.00E-95
209- 32:	transcript:Zm00001d031561_T002	transcript:EES15330	0
209- 33:	transcript:Zm00001d031562_T001	transcript:OQU80857	1.00E-73
209- 34:	transcript:Zm00001d031569_T001	transcript:KXG25559	0
209- 35:	transcript:Zm00001d031570_T001	transcript:OQU80851	0
209- 36:	transcript:Zm00001d031571_T001	transcript:EES14212	0
209- 37:	transcript:Zm00001d031573_T001	transcript:KXG25555	7.00E-140
209- 38:	transcript:Zm00001d031577_T001	transcript:EES14210	0
209- 39:	transcript:Zm00001d031580_T001	transcript:KXG25553	2.00E-107
209- 40:	transcript:Zm00001d031581_T002	transcript:EES14200	1.00E-07
209- 41:	transcript:Zm00001d031586_T001	transcript:OQU80845	2.00E-89
209- 42:	transcript:Zm00001d031587_T004	transcript:EES14205	1.00E-93
209- 43:	transcript:Zm00001d031588_T001	transcript:KXG25545	2.00E-146
209- 44:	transcript:Zm00001d031589_T001	transcript:EES14204	3.00E-163
209- 45:	transcript:Zm00001d031593_T001	transcript:EES15320	3.00E-89
209- 46:	transcript:Zm00001d031594_T001	transcript:OQU80833	3.00E-92
209- 47:	transcript:Zm00001d031599_T001	transcript:EES15319	8.00E-158
209- 48:	transcript:Zm00001d031600_T001	transcript:EES14199	0
209- 49:	transcript:Zm00001d031601_T001	transcript:OQU80832	0
209- 50:	transcript:Zm00001d031602_T002	transcript:EES15317	0
209- 51:	transcript:Zm00001d031607_T001	transcript:EES15316	0
209- 52:	transcript:Zm00001d031611_T006	transcript:OQU80827	4.00E-89
209- 53:	transcript:Zm00001d031614_T001	transcript:EES15315	0
209- 54:	transcript:Zm00001d031617_T001	transcript:EES15314	0
209- 55:	transcript:Zm00001d031618_T001	transcript:EES14193	2.00E-112
209- 56:	transcript:Zm00001d031619_T001	transcript:EES15312	0
209- 57:	transcript:Zm00001d031620_T001	transcript:EES15309	2.00E-169
209- 58:	transcript:Zm00001d031621_T003	transcript:EES15308	1.00E-157
209- 59:	transcript:Zm00001d031625_T001	transcript:KXG25532	2.00E-29

209- 60: transcript:Zm00001d031627_T003 transcript:EES14191 2.00E-61
Alignment 210: score=2181.0 e_value=2.2e-176 N=48 1&7 minus
210- 0: transcript:Zm00001d031634_T001 transcript:EES15304 0
210- 1: transcript:Zm00001d031635_T001 transcript:EES14190 0
210- 2: transcript:Zm00001d031636_T001 transcript:EES14189 1.00E-99
210- 3: transcript:Zm00001d031637_T003 transcript:EES14188 1.00E-154
210- 4: transcript:Zm00001d031638_T001 transcript:EES15302 0
210- 5: transcript:Zm00001d031639_T001 transcript:EES15301 0
210- 6: transcript:Zm00001d031641_T002 transcript:EES15300 1.00E-160
210- 7: transcript:Zm00001d031643_T001 transcript:EES15299 0
210- 8: transcript:Zm00001d031647_T001 transcript:EES14183 0
210- 9: transcript:Zm00001d031648_T004 transcript:EES14182 0
210- 10: transcript:Zm00001d031651_T001 transcript:EES14180 1.00E-42
210- 11: transcript:Zm00001d031653_T007 transcript:KXG25516 0
210- 12: transcript:Zm00001d031655_T003 transcript:OQU80816 1.00E-123
210- 13: transcript:Zm00001d031657_T001 transcript:OQU80814 0
210- 14: transcript:Zm00001d031659_T001 transcript:KXG25507 0
210- 15: transcript:Zm00001d031660_T002 transcript:KXG25506 0
210- 16: transcript:Zm00001d031661_T001 transcript:EES15294 5.00E-10
210- 17: transcript:Zm00001d031662_T001 transcript:EES14176 0
210- 18: transcript:Zm00001d031665_T001 transcript:EES14175 2.00E-166
210- 19: transcript:Zm00001d031667_T006 transcript:OQU80812 0
210- 20: transcript:Zm00001d031668_T001 transcript:EES15282 6.00E-54
210- 21: transcript:Zm00001d031669_T001 transcript:OQU80808 0
210- 22: transcript:Zm00001d031673_T001 transcript:OQU80805 6.00E-39
210- 23: transcript:Zm00001d031674_T003 transcript:OQU80804 0
210- 24: transcript:Zm00001d031676_T002 transcript:OQU80802 0
210- 25: transcript:Zm00001d031677_T001 transcript:EES15280 0
210- 26: transcript:Zm00001d031678_T002 transcript:EES14166 0
210- 27: transcript:Zm00001d031683_T001 transcript:EES15278 1.00E-64
210- 28: transcript:Zm00001d031684_T001 transcript:OQU80800 0
210- 29: transcript:Zm00001d031688_T002 transcript:EES15274 8.00E-174
210- 30: transcript:Zm00001d031689_T004 transcript:KXG25484 0
210- 31: transcript:Zm00001d031691_T001 transcript:KXG25483 3.00E-180
210- 32: transcript:Zm00001d031694_T003 transcript:EES15269 0
210- 33: transcript:Zm00001d031696_T002 transcript:EES15264 2.00E-135
210- 34: transcript:Zm00001d031697_T001 transcript:EES14151 1.00E-136
210- 35: transcript:Zm00001d031700_T003 transcript:EES14150 0
210- 36: transcript:Zm00001d031701_T002 transcript:OQU80786 2.00E-42
210- 37: transcript:Zm00001d031703_T001 transcript:EES15262 3.00E-178
210- 38: transcript:Zm00001d031705_T001 transcript:KXG25470 0
210- 39: transcript:Zm00001d031706_T001 transcript:EES15259 0
210- 40: transcript:Zm00001d031707_T001 transcript:EES15258 0
210- 41: transcript:Zm00001d031708_T001 transcript:EES14144 6.00E-153
210- 42: transcript:Zm00001d031711_T001 transcript:OQU80781 0
210- 43: transcript:Zm00001d031712_T002 transcript:EES14142 0
210- 44: transcript:Zm00001d031717_T001 transcript:KXG25462 1.00E-87
210- 45: transcript:Zm00001d031720_T001 transcript:OQU80773 0
210- 46: transcript:Zm00001d031723_T002 transcript:EES15257 0
210- 47: transcript:Zm00001d031724_T003 transcript:OQU80771 0
Alignment 211: score=1042.0 e_value=6.4e-70 N=23 1&7 minus
211- 0: transcript:Zm00001d032221_T001 transcript:KXG25199 4.00E-32
211- 1: transcript:Zm00001d032222_T001 transcript:EES15032 0
211- 2: transcript:Zm00001d032224_T002 transcript:EES15031 0

211- 3: transcript:Zm00001d032225_T001 transcript:OQU80500 0
211- 4: transcript:Zm00001d032226_T002 transcript:EES13877 0
211- 5: transcript:Zm00001d032229_T002 transcript:KXG25196 0
211- 6: transcript:Zm00001d032231_T001 transcript:OQU80487 0
211- 7: transcript:Zm00001d032233_T001 transcript:OQU80478 9.00E-81
211- 8: transcript:Zm00001d032234_T001 transcript:KXG25183 2.00E-29
211- 9: transcript:Zm00001d032238_T001 transcript:EES15018 0
211- 10: transcript:Zm00001d032239_T001 transcript:EES15017 3.00E-100
211- 11: transcript:Zm00001d032240_T001 transcript:KXG25181 1.00E-57
211- 12: transcript:Zm00001d032242_T001 transcript:EES15014 8.00E-58
211- 13: transcript:Zm00001d032244_T001 transcript:EES15012 0
211- 14: transcript:Zm00001d032245_T003 transcript:EES13866 0
211- 15: transcript:Zm00001d032249_T002 transcript:KXG25180 2.00E-153
211- 16: transcript:Zm00001d032250_T001 transcript:EES15006 4.00E-123
211- 17: transcript:Zm00001d032253_T001 transcript:EES15004 0
211- 18: transcript:Zm00001d032256_T001 transcript:KXG25176 0
211- 19: transcript:Zm00001d032257_T001 transcript:EES13852 0
211- 20: transcript:Zm00001d032262_T001 transcript:EES15000 0
211- 21: transcript:Zm00001d032263_T001 transcript:KXG25172 1.00E-105
211- 22: transcript:Zm00001d032264_T001 transcript:OQU80448 0
Alignment 212: score=640.0 e_value=5.5e-40 N=14 1&7 minus
212- 0: transcript:Zm00001d032326_T002 transcript:EES14943 6.00E-157
212- 1: transcript:Zm00001d032327_T001 transcript:EES13771 2.00E-164
212- 2: transcript:Zm00001d032328_T005 transcript:EES13767 2.00E-108
212- 3: transcript:Zm00001d032331_T001 transcript:OQU80374 0
212- 4: transcript:Zm00001d032332_T001 transcript:KXG25077 8.00E-166
212- 5: transcript:Zm00001d032333_T004 transcript:OQU80369 0
212- 6: transcript:Zm00001d032334_T002 transcript:EES13762 0
212- 7: transcript:Zm00001d032335_T001 transcript:KXG25075 3.00E-71
212- 8: transcript:Zm00001d032337_T001 transcript:EES14934 1.00E-115
212- 9: transcript:Zm00001d032338_T002 transcript:OQU80359 4.00E-171
212- 10: transcript:Zm00001d032339_T001 transcript:OQU80358 6.00E-166
212- 11: transcript:Zm00001d032342_T001 transcript:EES13760 0
212- 12: transcript:Zm00001d032344_T001 transcript:OQU80357 0
212- 13: transcript:Zm00001d032345_T001 transcript:EES13753 2.00E-36
Alignment 213: score=431.0 e_value=2.8e-17 N=9 1&7 minus
213- 0: transcript:Zm00001d031725_T001 transcript:OQU80770 0
213- 1: transcript:Zm00001d031726_T001 transcript:OQU80769 0
213- 2: transcript:Zm00001d031727_T004 transcript:EES15252 0
213- 3: transcript:Zm00001d031728_T001 transcript:EES14131 1.00E-79
213- 4: transcript:Zm00001d031729_T001 transcript:OQU80761 7.00E-160
213- 5: transcript:Zm00001d031730_T001 transcript:EES14130 0
213- 6: transcript:Zm00001d031732_T001 transcript:EES14129 0
213- 7: transcript:Zm00001d031736_T001 transcript:OQU80758 0
213- 8: transcript:Zm00001d031737_T001 transcript:EES14126 1.00E-143
Alignment 214: score=409.0 e_value=6.6e-17 N=9 1&7 minus
214- 0: transcript:KXG39419 transcript:Zm00001d022457_T002 2.00E-141
214- 1: transcript:KXG39418 transcript:Zm00001d022453_T001 1.00E-119
214- 2: transcript:EER94892 transcript:Zm00001d022450_T001 0
214- 3: transcript:EER94895 transcript:Zm00001d022449_T002 0
214- 4: transcript:OQU92638 transcript:Zm00001d022446_T001 4.00E-33
214- 5: transcript:KXG39422 transcript:Zm00001d022444_T001 0
214- 6: transcript:OQU92641 transcript:Zm00001d022442_T002 9.00E-142
214- 7: transcript:EER94904 transcript:Zm00001d022439_T001 0

```
214-  8: transcript:EER94905           transcript:Zm00001d022437_T001  6.00E-45
## Alignment 215: score=300.0 e_value=6.1e-12 N=7 1&7 minus
215-  0: transcript:KXG40041           transcript:Zm00001d022142_T001         0
215-  1: transcript:EER95398           transcript:Zm00001d022139_T001  6.00E-33
215-  2: transcript:EER92751           transcript:Zm00001d022131_T001  2.00E-81
215-  3: transcript:EER92752           transcript:Zm00001d022130_T001  1.00E-20
215-  4: transcript:KXG40053           transcript:Zm00001d022126_T001 3.00E-171
215-  5: transcript:KXG40055           transcript:Zm00001d022120_T001  8.00E-49
215-  6: transcript:EER92755           transcript:Zm00001d022117_T002  7.00E-40
## Alignment 216: score=287.0 e_value=3.7e-14 N=7 1&7 minus
216-  0: transcript:EER93207           transcript:Zm00001d019225_T001 8.00E-176
216-  1: transcript:EER90648           transcript:Zm00001d019223_T001  5.00E-48
216-  2: transcript:EER93211           transcript:Zm00001d019222_T001  5.00E-12
216-  3: transcript:EER90653           transcript:Zm00001d019216_T001  3.00E-24
216-  4: transcript:EER93213           transcript:Zm00001d019215_T002 7.00E-164
216-  5: transcript:EER93215           transcript:Zm00001d019207_T001 8.00E-125
216-  6: transcript:EER93218           transcript:Zm00001d019191_T007 1.00E-130
## Alignment 217: score=263.0 e_value=1.7e-09 N=6 1&7 minus
217-  0: transcript:EER92350           transcript:Zm00001d022547_T001         0
217-  1: transcript:EER94946           transcript:Zm00001d022546_T001  5.00E-74
217-  2: transcript:EER94948           transcript:Zm00001d022545_T001 4.00E-131
217-  3: transcript:KXG39494           transcript:Zm00001d022542_T002         0
217-  4: transcript:EER94952           transcript:Zm00001d022538_T001 2.00E-133
217-  5: transcript:OQU92698           transcript:Zm00001d022535_T004         0
## Alignment 218: score=261.0 e_value=5e-09 N=6 1&7 minus
218-  0: transcript:OQU92647           transcript:Zm00001d022435_T001  5.00E-19
218-  1: transcript:KXG39433           transcript:Zm00001d022434_T001         0
218-  2: transcript:EER94912           transcript:Zm00001d022433_T001 5.00E-160
218-  3: transcript:KXG39436           transcript:Zm00001d022432_T001         0
218-  4: transcript:KXG39440           transcript:Zm00001d022431_T001  1.00E-71
218-  5: transcript:KXG39446           transcript:Zm00001d022430_T001  1.00E-91
## Alignment 219: score=255.0 e_value=1.1e-08 N=6 1&7 minus
219-  0: transcript:EER90700           transcript:Zm00001d019107_T001  6.00E-21
219-  1: transcript:OQU90803           transcript:Zm00001d019104_T001         0
219-  2: transcript:EER93260           transcript:Zm00001d019100_T001  4.00E-45
219-  3: transcript:EER90708           transcript:Zm00001d019094_T001 2.00E-111
219-  4: transcript:OQU90815           transcript:Zm00001d019091_T002 2.00E-157
219-  5: transcript:KXG37334           transcript:Zm00001d019090_T003         0
## Alignment 220: score=3717.0 e_value=0 N=86 1&8 plus
220-  0: transcript:Zm00001d030998_T007 transcript:OQU79536                  0
220-  1: transcript:Zm00001d030999_T001 transcript:OQU79537                  0
220-  2: transcript:Zm00001d031002_T001 transcript:EES17348                  0
220-  3: transcript:Zm00001d031005_T004 transcript:EES17351                  0
220-  4: transcript:Zm00001d031008_T001 transcript:EES17352                  0
220-  5: transcript:Zm00001d031013_T002 transcript:OQU79541                  0
220-  6: transcript:Zm00001d031014_T001 transcript:KXG23933                  0
220-  7: transcript:Zm00001d031017_T001 transcript:KXG23935           2.00E-07
220-  8: transcript:Zm00001d031018_T002 transcript:OQU79545          2.00E-174
220-  9: transcript:Zm00001d031019_T005 transcript:OQU79550          1.00E-174
220- 10: transcript:Zm00001d031020_T001 transcript:OQU79553           6.00E-48
220- 11: transcript:Zm00001d031024_T002 transcript:EES16326                  0
220- 12: transcript:Zm00001d031026_T001 transcript:OQU79556           2.00E-51
220- 13: transcript:Zm00001d031028_T006 transcript:EES16331                  0
220- 14: transcript:Zm00001d031040_T005 transcript:OQU79568                  0
```

220- 15:	transcript:Zm00001d031041_T002	transcript:EES16340	0
220- 16:	transcript:Zm00001d031044_T001	transcript:KXG23955	3.00E-114
220- 17:	transcript:Zm00001d031047_T001	transcript:EES17365	2.00E-76
220- 18:	transcript:Zm00001d031049_T001	transcript:OQU79583	3.00E-24
220- 19:	transcript:Zm00001d031050_T001	transcript:EES17375	0
220- 20:	transcript:Zm00001d031053_T001	transcript:OQU79585	1.00E-173
220- 21:	transcript:Zm00001d031057_T001	transcript:KXG23974	8.00E-24
220- 22:	transcript:Zm00001d031059_T006	transcript:EES17384	0
220- 23:	transcript:Zm00001d031060_T001	transcript:EES16357	0
220- 24:	transcript:Zm00001d031061_T007	transcript:EES17386	0
220- 25:	transcript:Zm00001d031062_T001	transcript:EES16363	2.00E-104
220- 26:	transcript:Zm00001d031063_T001	transcript:EES17387	3.00E-64
220- 27:	transcript:Zm00001d031064_T002	transcript:EES17392	0
220- 28:	transcript:Zm00001d031065_T001	transcript:KXG23999	1.00E-116
220- 29:	transcript:Zm00001d031066_T001	transcript:OQU79610	4.00E-123
220- 30:	transcript:Zm00001d031068_T001	transcript:OQU79615	0
220- 31:	transcript:Zm00001d031071_T001	transcript:EES17401	0
220- 32:	transcript:Zm00001d031072_T001	transcript:EES17402	0
220- 33:	transcript:Zm00001d031073_T001	transcript:EES17403	0
220- 34:	transcript:Zm00001d031074_T002	transcript:KXG24011	0
220- 35:	transcript:Zm00001d031075_T001	transcript:OQU79627	3.00E-71
220- 36:	transcript:Zm00001d031077_T001	transcript:OQU79628	0
220- 37:	transcript:Zm00001d031079_T001	transcript:EES17410	2.00E-14
220- 38:	transcript:Zm00001d031081_T001	transcript:KXG24018	0
220- 39:	transcript:Zm00001d031088_T001	transcript:KXG24021	0
220- 40:	transcript:Zm00001d031090_T001	transcript:EES17418	0
220- 41:	transcript:Zm00001d031091_T001	transcript:KXG24025	0
220- 42:	transcript:Zm00001d031094_T001	transcript:EES17420	4.00E-179
220- 43:	transcript:Zm00001d031098_T004	transcript:KXG24027	0
220- 44:	transcript:Zm00001d031099_T001	transcript:EES16399	6.00E-41
220- 45:	transcript:Zm00001d031100_T001	transcript:OQU79657	8.00E-46
220- 46:	transcript:Zm00001d031101_T002	transcript:EES17423	2.00E-132
220- 47:	transcript:Zm00001d031109_T002	transcript:EES16403	2.00E-106
220- 48:	transcript:Zm00001d031114_T001	transcript:EES17426	2.00E-24
220- 49:	transcript:Zm00001d031118_T001	transcript:KXG24039	0
220- 50:	transcript:Zm00001d031119_T001	transcript:OQU79668	0
220- 51:	transcript:Zm00001d031120_T003	transcript:KXG24043	0
220- 52:	transcript:Zm00001d031127_T001	transcript:EES17430	8.00E-148
220- 53:	transcript:Zm00001d031128_T004	transcript:KXG24060	0
220- 54:	transcript:Zm00001d031129_T001	transcript:OQU79684	8.00E-59
220- 55:	transcript:Zm00001d031130_T001	transcript:OQU79685	0
220- 56:	transcript:Zm00001d031131_T001	transcript:EES16410	3.00E-93
220- 57:	transcript:Zm00001d031132_T002	transcript:EES17440	2.00E-45
220- 58:	transcript:Zm00001d031134_T001	transcript:EES17441	0
220- 59:	transcript:Zm00001d031135_T001	transcript:KXG24063	2.00E-164
220- 60:	transcript:Zm00001d031136_T003	transcript:KXG24064	0
220- 61:	transcript:Zm00001d031146_T001	transcript:EES17445	2.00E-57
220- 62:	transcript:Zm00001d031148_T001	transcript:EES16418	1.00E-75
220- 63:	transcript:Zm00001d031149_T002	transcript:EES16419	2.00E-157
220- 64:	transcript:Zm00001d031152_T001	transcript:EES17455	8.00E-77
220- 65:	transcript:Zm00001d031161_T001	transcript:EES16427	3.00E-17
220- 66:	transcript:Zm00001d031163_T004	transcript:EES17465	2.00E-179
220- 67:	transcript:Zm00001d031164_T001	transcript:EES17466	2.00E-145
220- 68:	transcript:Zm00001d031178_T001	transcript:OQU79715	0

220- 69: transcript:Zm00001d031196_T001 transcript:KXG24118 9.00E-53
220- 70: transcript:Zm00001d031202_T001 transcript:KXG24131 2.00E-42
220- 71: transcript:Zm00001d031203_T001 transcript:EES17493 0
220- 72: transcript:Zm00001d031205_T004 transcript:EES17494 4.00E-91
220- 73: transcript:Zm00001d031210_T002 transcript:KXG24139 0
220- 74: transcript:Zm00001d031211_T002 transcript:EES17498 0
220- 75: transcript:Zm00001d031212_T004 transcript:KXG24143 0
220- 76: transcript:Zm00001d031213_T001 transcript:KXG24141 0
220- 77: transcript:Zm00001d031216_T002 transcript:KXG24146 0
220- 78: transcript:Zm00001d031217_T005 transcript:EES17501 0
220- 79: transcript:Zm00001d031220_T001 transcript:EES17503 5.00E-99
220- 80: transcript:Zm00001d031221_T001 transcript:KXG24149 0
220- 81: transcript:Zm00001d031223_T001 transcript:EES16461 0
220- 82: transcript:Zm00001d031225_T001 transcript:EES17508 6.00E-126
220- 83: transcript:Zm00001d031227_T001 transcript:EES16464 1.00E-74
220- 84: transcript:Zm00001d031228_T001 transcript:KXG24152 4.00E-45
220- 85: transcript:Zm00001d031230_T001 transcript:EES17510 0
Alignment 221: score=3452.0 e_value=0 N=78 1&8 plus
221- 0: transcript:Zm00001d030750_T001 transcript:KXG23779 4.00E-27
221- 1: transcript:Zm00001d030751_T001 transcript:EES17245 0
221- 2: transcript:Zm00001d030759_T001 transcript:EES17246 0
221- 3: transcript:Zm00001d030760_T004 transcript:EES17247 0
221- 4: transcript:Zm00001d030762_T001 transcript:KXG23781 2.00E-79
221- 5: transcript:Zm00001d030765_T004 transcript:EES16213 0
221- 6: transcript:Zm00001d030766_T001 transcript:KXG23785 0
221- 7: transcript:Zm00001d030770_T003 transcript:KXG23787 0
221- 8: transcript:Zm00001d030773_T001 transcript:KXG23790 5.00E-172
221- 9: transcript:Zm00001d030774_T004 transcript:KXG23791 0
221- 10: transcript:Zm00001d030775_T001 transcript:EES16218 0
221- 11: transcript:Zm00001d030780_T001 transcript:KXG23793 4.00E-104
221- 12: transcript:Zm00001d030784_T016 transcript:EES17262 0
221- 13: transcript:Zm00001d030795_T001 transcript:EES17258 0
221- 14: transcript:Zm00001d030814_T001 transcript:OQU79423 2.00E-165
221- 15: transcript:Zm00001d030819_T005 transcript:KXG23805 0
221- 16: transcript:Zm00001d030832_T001 transcript:EES16229 0
221- 17: transcript:Zm00001d030833_T001 transcript:EES16230 6.00E-178
221- 18: transcript:Zm00001d030834_T001 transcript:EES16231 1.00E-78
221- 19: transcript:Zm00001d030843_T001 transcript:KXG23810 8.00E-64
221- 20: transcript:Zm00001d030846_T001 transcript:EES17269 0
221- 21: transcript:Zm00001d030849_T001 transcript:EES16237 0
221- 22: transcript:Zm00001d030851_T001 transcript:EES17271 0
221- 23: transcript:Zm00001d030855_T001 transcript:OQU79437 3.00E-12
221- 24: transcript:Zm00001d030858_T001 transcript:EES16240 1.00E-83
221- 25: transcript:Zm00001d030859_T001 transcript:EES16245 0
221- 26: transcript:Zm00001d030860_T003 transcript:OQU79441 0
221- 27: transcript:Zm00001d030862_T001 transcript:OQU79442 5.00E-173
221- 28: transcript:Zm00001d030863_T006 transcript:EES17279 0
221- 29: transcript:Zm00001d030864_T001 transcript:KXG23824 0
221- 30: transcript:Zm00001d030868_T003 transcript:KXG23827 0
221- 31: transcript:Zm00001d030877_T001 transcript:EES16250 0
221- 32: transcript:Zm00001d030888_T001 transcript:EES16256 0
221- 33: transcript:Zm00001d030891_T001 transcript:OQU79454 8.00E-130
221- 34: transcript:Zm00001d030892_T004 transcript:EES16259 0
221- 35: transcript:Zm00001d030893_T004 transcript:KXG23838 0

221- 36:	transcript:Zm00001d030894_T002	transcript:EES17294	0
221- 37:	transcript:Zm00001d030895_T001	transcript:EES17297	8.00E-125
221- 38:	transcript:Zm00001d030897_T001	transcript:EES17301	6.00E-16
221- 39:	transcript:Zm00001d030900_T001	transcript:EES16260	0
221- 40:	transcript:Zm00001d030901_T001	transcript:OQU79472	0
221- 41:	transcript:Zm00001d030907_T001	transcript:EES17302	0
221- 42:	transcript:Zm00001d030910_T002	transcript:OQU79477	1.00E-31
221- 43:	transcript:Zm00001d030911_T001	transcript:OQU79478	5.00E-61
221- 44:	transcript:Zm00001d030914_T002	transcript:KXG23863	0
221- 45:	transcript:Zm00001d030915_T001	transcript:EES16269	4.00E-28
221- 46:	transcript:Zm00001d030916_T001	transcript:EES17308	0
221- 47:	transcript:Zm00001d030934_T002	transcript:EES16271	2.00E-71
221- 48:	transcript:Zm00001d030935_T002	transcript:OQU79485	0
221- 49:	transcript:Zm00001d030937_T006	transcript:OQU79487	0
221- 50:	transcript:Zm00001d030938_T001	transcript:EES16277	1.00E-28
221- 51:	transcript:Zm00001d030939_T004	transcript:OQU79488	2.00E-145
221- 52:	transcript:Zm00001d030940_T001	transcript:EES16278	0
221- 53:	transcript:Zm00001d030944_T001	transcript:EES17314	0
221- 54:	transcript:Zm00001d030945_T001	transcript:OQU79494	1.00E-158
221- 55:	transcript:Zm00001d030947_T001	transcript:OQU79497	0
221- 56:	transcript:Zm00001d030951_T002	transcript:OQU79498	0
221- 57:	transcript:Zm00001d030953_T001	transcript:EES16286	0
221- 58:	transcript:Zm00001d030954_T001	transcript:EES16287	1.00E-177
221- 59:	transcript:Zm00001d030955_T003	transcript:OQU79501	5.00E-162
221- 60:	transcript:Zm00001d030958_T001	transcript:OQU79502	0
221- 61:	transcript:Zm00001d030962_T001	transcript:EES16280	0
221- 62:	transcript:Zm00001d030967_T002	transcript:EES17320	0
221- 63:	transcript:Zm00001d030968_T006	transcript:EES16283	0
221- 64:	transcript:Zm00001d030969_T001	transcript:EES17321	0
221- 65:	transcript:Zm00001d030971_T001	transcript:OQU79506	4.00E-06
221- 66:	transcript:Zm00001d030972_T001	transcript:OQU79507	2.00E-06
221- 67:	transcript:Zm00001d030975_T001	transcript:OQU79511	1.00E-98
221- 68:	transcript:Zm00001d030976_T001	transcript:OQU79512	0
221- 69:	transcript:Zm00001d030982_T002	transcript:EES17326	2.00E-39
221- 70:	transcript:Zm00001d030985_T001	transcript:OQU79520	0
221- 71:	transcript:Zm00001d030987_T001	transcript:KXG23899	2.00E-23
221- 72:	transcript:Zm00001d030989_T001	transcript:EES17331	4.00E-68
221- 73:	transcript:Zm00001d030990_T001	transcript:OQU79521	0
221- 74:	transcript:Zm00001d030993_T001	transcript:EES16315	2.00E-65
221- 75:	transcript:Zm00001d030995_T002	transcript:EES16316	1.00E-172
221- 76:	transcript:Zm00001d030996_T008	transcript:EES17335	0
221- 77:	transcript:Zm00001d030997_T001	transcript:KXG23907	2.00E-83
## Alignment 222: score=1241.0 e_value=3.2e-88 N=28 1&8 plus			
222- 0:	transcript:Zm00001d030667_T001	transcript:EES16178	9.00E-62
222- 1:	transcript:Zm00001d030675_T002	transcript:KXG23727	0
222- 2:	transcript:Zm00001d030676_T001	transcript:OQU79348	8.00E-33
222- 3:	transcript:Zm00001d030677_T004	transcript:KXG23730	3.00E-144
222- 4:	transcript:Zm00001d030678_T001	transcript:KXG23733	5.00E-135
222- 5:	transcript:Zm00001d030682_T001	transcript:KXG23738	0
222- 6:	transcript:Zm00001d030683_T002	transcript:KXG23740	0
222- 7:	transcript:Zm00001d030688_T005	transcript:EES16183	3.00E-169
222- 8:	transcript:Zm00001d030690_T001	transcript:OQU79368	3.00E-82
222- 9:	transcript:Zm00001d030691_T001	transcript:KXG23748	0
222- 10:	transcript:Zm00001d030694_T002	transcript:EES16185	0

222- 11: transcript:Zm00001d030696_T001 transcript:OQU79369 2.00E-33
222- 12: transcript:Zm00001d030698_T001 transcript:EES16188 3.00E-96
222- 13: transcript:Zm00001d030701_T001 transcript:OQU79375 1.00E-81
222- 14: transcript:Zm00001d030707_T001 transcript:EES16190 0
222- 15: transcript:Zm00001d030712_T001 transcript:OQU79376 1.00E-119
222- 16: transcript:Zm00001d030725_T003 transcript:EES16191 0
222- 17: transcript:Zm00001d030727_T002 transcript:OQU79380 1.00E-164
222- 18: transcript:Zm00001d030732_T001 transcript:OQU79382 0
222- 19: transcript:Zm00001d030733_T001 transcript:KXG23759 0
222- 20: transcript:Zm00001d030735_T001 transcript:KXG23762 3.00E-19
222- 21: transcript:Zm00001d030737_T001 transcript:EES17234 0
222- 22: transcript:Zm00001d030739_T008 transcript:EES16196 2.00E-127
222- 23: transcript:Zm00001d030741_T001 transcript:KXG23772 0
222- 24: transcript:Zm00001d030742_T003 transcript:EES16205 1.00E-140
222- 25: transcript:Zm00001d030744_T001 transcript:EES16206 0
222- 26: transcript:Zm00001d030745_T001 transcript:EES17243 0
222- 27: transcript:Zm00001d030750_T001 transcript:KXG23778 1.00E-16
Alignment 223: score=909.0 e_value=2.3e-59 N=20 1&8 plus
223- 0: transcript:Zm00001d030613_T003 transcript:KXG23698 0
223- 1: transcript:Zm00001d030614_T002 transcript:KXG23699 0
223- 2: transcript:Zm00001d030617_T001 transcript:EES16161 3.00E-90
223- 3: transcript:Zm00001d030618_T001 transcript:EES17196 9.00E-41
223- 4: transcript:Zm00001d030620_T002 transcript:KXG23702 0
223- 5: transcript:Zm00001d030622_T001 transcript:EES17198 7.00E-102
223- 6: transcript:Zm00001d030623_T006 transcript:EES17199 0
223- 7: transcript:Zm00001d030624_T001 transcript:EES16159 0
223- 8: transcript:Zm00001d030626_T003 transcript:EES16163 0
223- 9: transcript:Zm00001d030627_T002 transcript:EES16167 1.00E-129
223- 10: transcript:Zm00001d030638_T001 transcript:EES17205 4.00E-62
223- 11: transcript:Zm00001d030639_T001 transcript:EES16169 0
223- 12: transcript:Zm00001d030640_T001 transcript:EES16170 0
223- 13: transcript:Zm00001d030642_T001 transcript:EES16171 1.00E-57
223- 14: transcript:Zm00001d030643_T001 transcript:EES17206 2.00E-92
223- 15: transcript:Zm00001d030644_T001 transcript:EES17207 2.00E-130
223- 16: transcript:Zm00001d030652_T002 transcript:EES17213 0
223- 17: transcript:Zm00001d030656_T001 transcript:KXG23716 0
223- 18: transcript:Zm00001d030659_T010 transcript:KXG23720 0
223- 19: transcript:Zm00001d030665_T001 transcript:EES16176 1.00E-71
Alignment 224: score=272.0 e_value=5e-12 N=7 1&8 plus
224- 0: transcript:EER94931 transcript:Zm00001d010634_T001 2.00E-101
224- 1: transcript:KXG39475 transcript:Zm00001d010635_T001 4.00E-52
224- 2: transcript:KXG39483 transcript:Zm00001d010638_T001 5.00E-30
224- 3: transcript:EER92345 transcript:Zm00001d010639_T002 0
224- 4: transcript:EER94941 transcript:Zm00001d010640_T001 7.00E-138
224- 5: transcript:EER94946 transcript:Zm00001d010649_T001 1.00E-29
224- 6: transcript:EER94949 transcript:Zm00001d010661_T001 9.00E-08
Alignment 225: score=621.0 e_value=7.5e-33 N=13 1&8 minus
225- 0: transcript:Zm00001d031182_T001 transcript:KXG24130 0
225- 1: transcript:Zm00001d031183_T001 transcript:EES16450 8.00E-115
225- 2: transcript:Zm00001d031184_T001 transcript:EES16449 7.00E-126
225- 3: transcript:Zm00001d031186_T001 transcript:EES16448 9.00E-116
225- 4: transcript:Zm00001d031187_T002 transcript:KXG24124 0
225- 5: transcript:Zm00001d031189_T001 transcript:OQU79728 2.00E-34
225- 6: transcript:Zm00001d031191_T001 transcript:KXG24122 1.00E-28

225- 7: transcript:Zm00001d031192_T001 transcript:OQU79727 7.00E-24
225- 8: transcript:Zm00001d031194_T001 transcript:KXG24120 1.00E-38
225- 9: transcript:Zm00001d031195_T001 transcript:OQU79723 3.00E-54
225- 10: transcript:Zm00001d031197_T001 transcript:KXG24114 3.00E-18
225- 11: transcript:Zm00001d031200_T002 transcript:OQU79719 5.00E-98
225- 12: transcript:Zm00001d031201_T001 transcript:EES16439 0
Alignment 226: score=467.0 e_value=5.6e-27 N=12 1&8 minus
226- 0: transcript:Zm00001d033291_T004 transcript:EES17321 5.00E-148
226- 1: transcript:Zm00001d033295_T001 transcript:OQU79501 3.00E-154
226- 2: transcript:Zm00001d033297_T003 transcript:EES16286 0
226- 3: transcript:Zm00001d033304_T001 transcript:OQU79483 5.00E-139
226- 4: transcript:Zm00001d033310_T001 transcript:OQU79478 2.00E-38
226- 5: transcript:Zm00001d033312_T006 transcript:OQU79477 3.00E-14
226- 6: transcript:Zm00001d033313_T001 transcript:EES17302 2.00E-20
226- 7: transcript:Zm00001d033324_T001 transcript:OQU79468 8.00E-39
226- 8: transcript:Zm00001d033325_T001 transcript:EES17301 4.00E-34
226- 9: transcript:Zm00001d033330_T001 transcript:EES17294 7.00E-73
226- 10: transcript:Zm00001d033334_T001 transcript:KXG23838 0
226- 11: transcript:Zm00001d033339_T002 transcript:EES17292 0
Alignment 227: score=402.0 e_value=5e-21 N=10 1&8 minus
227- 0: transcript:Zm00001d033204_T002 transcript:EES17423 9.00E-48
227- 1: transcript:Zm00001d033211_T002 transcript:KXG24027 5.00E-145
227- 2: transcript:Zm00001d033213_T001 transcript:EES17420 4.00E-180
227- 3: transcript:Zm00001d033215_T003 transcript:OQU79642 7.00E-80
227- 4: transcript:Zm00001d033217_T004 transcript:KXG24021 0
227- 5: transcript:Zm00001d033223_T003 transcript:KXG24018 4.00E-128
227- 6: transcript:Zm00001d033225_T002 transcript:EES16391 0
227- 7: transcript:Zm00001d033227_T001 transcript:OQU79627 2.00E-18
227- 8: transcript:Zm00001d033233_T002 transcript:EES17402 5.00E-123
227- 9: transcript:Zm00001d033234_T001 transcript:OQU79615 2.00E-168
Alignment 228: score=386.0 e_value=1e-16 N=9 1&8 minus
228- 0: transcript:Zm00001d033456_T001 transcript:EES16418 1.00E-28
228- 1: transcript:Zm00001d033464_T001 transcript:EES17445 2.00E-37
228- 2: transcript:Zm00001d033467_T002 transcript:EES16411 1.00E-119
228- 3: transcript:Zm00001d033472_T004 transcript:EES16412 5.00E-142
228- 4: transcript:Zm00001d033475_T001 transcript:EES16410 4.00E-175
228- 5: transcript:Zm00001d033478_T001 transcript:OQU79684 3.00E-28
228- 6: transcript:Zm00001d033480_T002 transcript:KXG24060 0
228- 7: transcript:Zm00001d033489_T001 transcript:EES17430 7.00E-79
228- 8: transcript:Zm00001d033493_T002 transcript:KXG24043 0
Alignment 229: score=337.0 e_value=0 N=8 1&8 minus
229- 0: transcript:Zm00001d033378_T019 transcript:KXG24130 0
229- 1: transcript:Zm00001d033386_T001 transcript:EES16449 1.00E-110
229- 2: transcript:Zm00001d033389_T001 transcript:OQU79728 2.00E-06
229- 3: transcript:Zm00001d033391_T001 transcript:OQU79723 1.00E-26
229- 4: transcript:Zm00001d033395_T001 transcript:KXG24118 1.00E-12
229- 5: transcript:Zm00001d033401_T001 transcript:OQU79722 0
229- 6: transcript:Zm00001d033407_T001 transcript:OQU79717 3.00E-55
229- 7: transcript:Zm00001d033412_T001 transcript:KXG24110 8.00E-76
Alignment 230: score=6488.0 e_value=0 N=145 1&9 plus
230- 0: transcript:EER95280 transcript:Zm00001d048096_T023 0
230- 1: transcript:EER95282 transcript:Zm00001d048098_T004 5.00E-170
230- 2: transcript:KXG39887 transcript:Zm00001d048099_T003 6.00E-151
230- 3: transcript:EER95286 transcript:Zm00001d048102_T003 0

230- 4:	transcript:EER95287	transcript:Zm00001d048104_T001	5.00E-171
230- 5:	transcript:OQU93020	transcript:Zm00001d048105_T011	0
230- 6:	transcript:EER95292	transcript:Zm00001d048106_T001	0
230- 7:	transcript:KXG39896	transcript:Zm00001d048108_T001	7.00E-111
230- 8:	transcript:KXG39897	transcript:Zm00001d048109_T002	1.00E-174
230- 9:	transcript:KXG39899	transcript:Zm00001d048111_T001	0
230- 10:	transcript:EER95297	transcript:Zm00001d048112_T001	0
230- 11:	transcript:KXG39900	transcript:Zm00001d048113_T001	1.00E-50
230- 12:	transcript:EER92656	transcript:Zm00001d048115_T026	0
230- 13:	transcript:OQU93025	transcript:Zm00001d048116_T001	5.00E-87
230- 14:	transcript:EER95300	transcript:Zm00001d048117_T001	5.00E-108
230- 15:	transcript:EER92659	transcript:Zm00001d048119_T022	0
230- 16:	transcript:EER92660	transcript:Zm00001d048121_T001	0
230- 17:	transcript:EER95301	transcript:Zm00001d048122_T003	1.00E-85
230- 18:	transcript:KXG39905	transcript:Zm00001d048123_T001	1.00E-49
230- 19:	transcript:EER92664	transcript:Zm00001d048126_T003	0
230- 20:	transcript:EER95303	transcript:Zm00001d048127_T001	1.00E-149
230- 21:	transcript:EER92665	transcript:Zm00001d048129_T004	0
230- 22:	transcript:EER95306	transcript:Zm00001d048131_T001	0
230- 23:	transcript:EER92667	transcript:Zm00001d048132_T002	3.00E-82
230- 24:	transcript:EER95307	transcript:Zm00001d048134_T001	2.00E-154
230- 25:	transcript:EER92670	transcript:Zm00001d048135_T002	5.00E-44
230- 26:	transcript:EER92673	transcript:Zm00001d048137_T002	0
230- 27:	transcript:OQU93039	transcript:Zm00001d048138_T004	0
230- 28:	transcript:EER95310	transcript:Zm00001d048139_T002	4.00E-102
230- 29:	transcript:KXG39918	transcript:Zm00001d048141_T001	3.00E-134
230- 30:	transcript:KXG39923	transcript:Zm00001d048142_T001	0
230- 31:	transcript:EER92677	transcript:Zm00001d048143_T003	1.00E-168
230- 32:	transcript:EER95314	transcript:Zm00001d048146_T001	0
230- 33:	transcript:EER92681	transcript:Zm00001d048149_T002	0
230- 34:	transcript:EER92682	transcript:Zm00001d048150_T007	4.00E-173
230- 35:	transcript:EER92683	transcript:Zm00001d048151_T001	0
230- 36:	transcript:EER95320	transcript:Zm00001d048153_T001	2.00E-125
230- 37:	transcript:OQU93049	transcript:Zm00001d048154_T001	0
230- 38:	transcript:EER92687	transcript:Zm00001d048155_T001	1.00E-133
230- 39:	transcript:KXG39941	transcript:Zm00001d048157_T001	0
230- 40:	transcript:KXG39942	transcript:Zm00001d048161_T002	2.00E-17
230- 41:	transcript:KXG39943	transcript:Zm00001d048162_T001	3.00E-98
230- 42:	transcript:EER95327	transcript:Zm00001d048166_T001	0
230- 43:	transcript:OQU93055	transcript:Zm00001d048171_T003	2.00E-144
230- 44:	transcript:EER92693	transcript:Zm00001d048172_T001	1.00E-101
230- 45:	transcript:EER95329	transcript:Zm00001d048173_T001	1.00E-177
230- 46:	transcript:EER95330	transcript:Zm00001d048174_T001	0
230- 47:	transcript:EER95331	transcript:Zm00001d048175_T002	0
230- 48:	transcript:EER95333	transcript:Zm00001d048176_T010	0
230- 49:	transcript:KXG39951	transcript:Zm00001d048178_T001	0
230- 50:	transcript:EER92696	transcript:Zm00001d048180_T001	0
230- 51:	transcript:KXG39955	transcript:Zm00001d048181_T003	0
230- 52:	transcript:OQU93068	transcript:Zm00001d048185_T001	3.00E-62
230- 53:	transcript:EER95337	transcript:Zm00001d048187_T001	6.00E-96
230- 54:	transcript:EER92701	transcript:Zm00001d048189_T001	0
230- 55:	transcript:EER92703	transcript:Zm00001d048190_T001	5.00E-168
230- 56:	transcript:EER95339	transcript:Zm00001d048191_T001	0
230- 57:	transcript:OQU93076	transcript:Zm00001d048193_T002	8.00E-123

230- 58:	transcript:EER95344	transcript:Zm00001d048194_T002	4.00E-142
230- 59:	transcript:KXG39967	transcript:Zm00001d048195_T001	0
230- 60:	transcript:EER92706	transcript:Zm00001d048196_T001	7.00E-40
230- 61:	transcript:OQU93078	transcript:Zm00001d048197_T001	1.00E-37
230- 62:	transcript:EER95347	transcript:Zm00001d048198_T002	7.00E-77
230- 63:	transcript:EER95348	transcript:Zm00001d048199_T001	0
230- 64:	transcript:KXG39973	transcript:Zm00001d048201_T002	0
230- 65:	transcript:EER92708	transcript:Zm00001d048202_T002	1.00E-48
230- 66:	transcript:KXG39979	transcript:Zm00001d048203_T002	0
230- 67:	transcript:EER92709	transcript:Zm00001d048204_T001	1.00E-99
230- 68:	transcript:OQU93085	transcript:Zm00001d048205_T003	0
230- 69:	transcript:EER92713	transcript:Zm00001d048208_T001	1.00E-175
230- 70:	transcript:OQU93086	transcript:Zm00001d048209_T001	7.00E-156
230- 71:	transcript:OQU93087	transcript:Zm00001d048210_T001	6.00E-29
230- 72:	transcript:EER92715	transcript:Zm00001d048215_T001	3.00E-99
230- 73:	transcript:EER92716	transcript:Zm00001d048217_T001	0
230- 74:	transcript:KXG39986	transcript:Zm00001d048218_T001	0
230- 75:	transcript:EER92724	transcript:Zm00001d048220_T001	0
230- 76:	transcript:EER92725	transcript:Zm00001d048222_T001	1.00E-160
230- 77:	transcript:OQU93090	transcript:Zm00001d048224_T001	1.00E-49
230- 78:	transcript:KXG40002	transcript:Zm00001d048225_T001	0
230- 79:	transcript:KXG40003	transcript:Zm00001d048226_T001	8.00E-58
230- 80:	transcript:EER92730	transcript:Zm00001d048227_T001	2.00E-125
230- 81:	transcript:EER95359	transcript:Zm00001d048229_T001	4.00E-159
230- 82:	transcript:EER92732	transcript:Zm00001d048230_T001	2.00E-152
230- 83:	transcript:OQU93095	transcript:Zm00001d048232_T001	5.00E-150
230- 84:	transcript:EER92735	transcript:Zm00001d048233_T001	0
230- 85:	transcript:EER95363	transcript:Zm00001d048234_T001	6.00E-99
230- 86:	transcript:KXG40011	transcript:Zm00001d048235_T004	0
230- 87:	transcript:EER95365	transcript:Zm00001d048236_T004	0
230- 88:	transcript:EER95366	transcript:Zm00001d048238_T001	1.00E-101
230- 89:	transcript:KXG40015	transcript:Zm00001d048239_T001	5.00E-136
230- 90:	transcript:EER95372	transcript:Zm00001d048242_T001	1.00E-84
230- 91:	transcript:OQU93104	transcript:Zm00001d048243_T001	0
230- 92:	transcript:EER92740	transcript:Zm00001d048244_T001	3.00E-154
230- 93:	transcript:KXG40025	transcript:Zm00001d048245_T003	0
230- 94:	transcript:KXG40032	transcript:Zm00001d048246_T001	6.00E-95
230- 95:	transcript:EER95387	transcript:Zm00001d048247_T002	0
230- 96:	transcript:EER92748	transcript:Zm00001d048255_T002	9.00E-111
230- 97:	transcript:EER95391	transcript:Zm00001d048258_T012	0
230- 98:	transcript:EER95392	transcript:Zm00001d048260_T001	0
230- 99:	transcript:EER95395	transcript:Zm00001d048262_T001	5.00E-106
230-100:	transcript:KXG40045	transcript:Zm00001d048268_T001	5.00E-20
230-101:	transcript:OQU93127	transcript:Zm00001d048270_T002	0
230-102:	transcript:EER92751	transcript:Zm00001d048271_T001	0
230-103:	transcript:KXG40051	transcript:Zm00001d048272_T012	0
230-104:	transcript:EER92752	transcript:Zm00001d048273_T001	7.00E-65
230-105:	transcript:KXG40052	transcript:Zm00001d048281_T001	5.00E-125
230-106:	transcript:OQU93130	transcript:Zm00001d048282_T002	0
230-107:	transcript:EER95402	transcript:Zm00001d048284_T001	1.00E-101
230-108:	transcript:EER95404	transcript:Zm00001d048285_T006	0
230-109:	transcript:KXG40058	transcript:Zm00001d048286_T002	0
230-110:	transcript:KXG40062	transcript:Zm00001d048288_T001	3.00E-119
230-111:	transcript:EER92766	transcript:Zm00001d048291_T001	0

230-112:	transcript:KXG40065	transcript:Zm00001d048292_T003	3.00E-166
230-113:	transcript:KXG40067	transcript:Zm00001d048294_T001	1.00E-38
230-114:	transcript:KXG40072	transcript:Zm00001d048296_T001	2.00E-168
230-115:	transcript:OQU93143	transcript:Zm00001d048298_T003	0
230-116:	transcript:KXG40074	transcript:Zm00001d048299_T002	0
230-117:	transcript:KXG40075	transcript:Zm00001d048301_T001	4.00E-141
230-118:	transcript:EER92771	transcript:Zm00001d048302_T001	2.00E-61
230-119:	transcript:EER95417	transcript:Zm00001d048307_T001	1.00E-28
230-120:	transcript:EER95418	transcript:Zm00001d048308_T002	0
230-121:	transcript:EER95420	transcript:Zm00001d048309_T001	2.00E-52
230-122:	transcript:KXG40080	transcript:Zm00001d048310_T005	0
230-123:	transcript:EER92779	transcript:Zm00001d048311_T003	0
230-124:	transcript:EER92780	transcript:Zm00001d048312_T001	3.00E-179
230-125:	transcript:EER92781	transcript:Zm00001d048313_T001	2.00E-114
230-126:	transcript:EER92783	transcript:Zm00001d048314_T004	0
230-127:	transcript:KXG40086	transcript:Zm00001d048315_T001	0
230-128:	transcript:EER92784	transcript:Zm00001d048316_T001	4.00E-67
230-129:	transcript:EER92785	transcript:Zm00001d048317_T001	1.00E-71
230-130:	transcript:KXG40087	transcript:Zm00001d048318_T001	0
230-131:	transcript:EER95427	transcript:Zm00001d048319_T003	0
230-132:	transcript:EER95430	transcript:Zm00001d048320_T001	0
230-133:	transcript:EER92791	transcript:Zm00001d048321_T001	9.00E-19
230-134:	transcript:OQU93156	transcript:Zm00001d048322_T001	1.00E-173
230-135:	transcript:EER92792	transcript:Zm00001d048323_T001	0
230-136:	transcript:EER92786	transcript:Zm00001d048324_T001	1.00E-152
230-137:	transcript:EER95435	transcript:Zm00001d048325_T001	1.00E-57
230-138:	transcript:KXG40096	transcript:Zm00001d048327_T001	9.00E-18
230-139:	transcript:EER92795	transcript:Zm00001d048332_T001	0
230-140:	transcript:EER92797	transcript:Zm00001d048333_T001	5.00E-165
230-141:	transcript:EER95440	transcript:Zm00001d048334_T001	1.00E-148
230-142:	transcript:EER95441	transcript:Zm00001d048335_T001	0
230-143:	transcript:EER95434	transcript:Zm00001d048336_T003	0
230-144:	transcript:KXG40100	transcript:Zm00001d048337_T001	0

Alignment 231: score=3480.0 e_value=0 N=79 1&9 plus

231- 0:	transcript:KXG39680	transcript:Zm00001d047802_T001	0
231- 1:	transcript:OQU92820	transcript:Zm00001d047803_T001	7.00E-64
231- 2:	transcript:EER92480	transcript:Zm00001d047804_T001	0
231- 3:	transcript:KXG39683	transcript:Zm00001d047805_T003	5.00E-169
231- 4:	transcript:EER92481	transcript:Zm00001d047806_T001	0
231- 5:	transcript:EER95099	transcript:Zm00001d047807_T001	0
231- 6:	transcript:KXG39690	transcript:Zm00001d047808_T001	0
231- 7:	transcript:OQU92826	transcript:Zm00001d047812_T001	0
231- 8:	transcript:OQU92829	transcript:Zm00001d047813_T002	0
231- 9:	transcript:EER95104	transcript:Zm00001d047814_T001	0
231- 10:	transcript:OQU92832	transcript:Zm00001d047820_T001	2.00E-42
231- 11:	transcript:OQU92834	transcript:Zm00001d047821_T001	1.00E-06
231- 12:	transcript:EER95107	transcript:Zm00001d047823_T001	3.00E-122
231- 13:	transcript:EER95109	transcript:Zm00001d047824_T001	0
231- 14:	transcript:EER95110	transcript:Zm00001d047827_T001	1.00E-36
231- 15:	transcript:EER95112	transcript:Zm00001d047828_T001	0
231- 16:	transcript:EER92488	transcript:Zm00001d047829_T001	0
231- 17:	transcript:OQU92850	transcript:Zm00001d047830_T002	6.00E-28
231- 18:	transcript:OQU92852	transcript:Zm00001d047831_T001	1.00E-49
231- 19:	transcript:EER92495	transcript:Zm00001d047833_T006	0

231- 20:	transcript:EER92496	transcript:Zm00001d047834_T004	0
231- 21:	transcript:EER95117	transcript:Zm00001d047835_T001	0
231- 22:	transcript:EER92497	transcript:Zm00001d047837_T003	0
231- 23:	transcript:OQU92863	transcript:Zm00001d047838_T001	0
231- 24:	transcript:EER92498	transcript:Zm00001d047839_T003	0
231- 25:	transcript:EER95119	transcript:Zm00001d047840_T001	0
231- 26:	transcript:EER95120	transcript:Zm00001d047841_T001	3.00E-85
231- 27:	transcript:EER95121	transcript:Zm00001d047842_T003	0
231- 28:	transcript:EER92500	transcript:Zm00001d047843_T002	0
231- 29:	transcript:OQU92870	transcript:Zm00001d047847_T003	4.00E-110
231- 30:	transcript:EER95129	transcript:Zm00001d047848_T001	0
231- 31:	transcript:EER95132	transcript:Zm00001d047849_T001	0
231- 32:	transcript:OQU92873	transcript:Zm00001d047852_T001	0
231- 33:	transcript:EER95133	transcript:Zm00001d047853_T002	0
231- 34:	transcript:EER92506	transcript:Zm00001d047854_T002	0
231- 35:	transcript:EER95134	transcript:Zm00001d047855_T001	0
231- 36:	transcript:OQU92875	transcript:Zm00001d047856_T001	0
231- 37:	transcript:EER95136	transcript:Zm00001d047857_T001	3.00E-45
231- 38:	transcript:EER92507	transcript:Zm00001d047858_T001	0
231- 39:	transcript:EER92508	transcript:Zm00001d047859_T001	0
231- 40:	transcript:EER92509	transcript:Zm00001d047860_T001	1.00E-105
231- 41:	transcript:OQU92877	transcript:Zm00001d047863_T004	0
231- 42:	transcript:EER95140	transcript:Zm00001d047868_T001	2.00E-157
231- 43:	transcript:KXG39732	transcript:Zm00001d047873_T002	0
231- 44:	transcript:EER92515	transcript:Zm00001d047875_T003	0
231- 45:	transcript:OQU92881	transcript:Zm00001d047876_T001	1.00E-143
231- 46:	transcript:EER92517	transcript:Zm00001d047877_T001	2.00E-155
231- 47:	transcript:OQU92884	transcript:Zm00001d047878_T001	7.00E-117
231- 48:	transcript:OQU92885	transcript:Zm00001d047879_T001	6.00E-28
231- 49:	transcript:KXG39739	transcript:Zm00001d047880_T001	0
231- 50:	transcript:EER95149	transcript:Zm00001d047881_T002	0
231- 51:	transcript:EER95150	transcript:Zm00001d047882_T010	0
231- 52:	transcript:EER95156	transcript:Zm00001d047883_T001	0
231- 53:	transcript:EER92527	transcript:Zm00001d047884_T001	6.00E-24
231- 54:	transcript:EER95158	transcript:Zm00001d047888_T001	1.00E-46
231- 55:	transcript:KXG39749	transcript:Zm00001d047889_T013	0
231- 56:	transcript:EER95159	transcript:Zm00001d047890_T001	3.00E-87
231- 57:	transcript:KXG39751	transcript:Zm00001d047893_T003	0
231- 58:	transcript:OQU92897	transcript:Zm00001d047894_T002	0
231- 59:	transcript:EER92537	transcript:Zm00001d047896_T009	0
231- 60:	transcript:EER92538	transcript:Zm00001d047897_T013	0
231- 61:	transcript:EER92539	transcript:Zm00001d047900_T001	3.00E-144
231- 62:	transcript:EER92541	transcript:Zm00001d047901_T001	0
231- 63:	transcript:OQU92901	transcript:Zm00001d047904_T001	2.00E-102
231- 64:	transcript:EER92544	transcript:Zm00001d047906_T009	0
231- 65:	transcript:EER92545	transcript:Zm00001d047907_T001	2.00E-35
231- 66:	transcript:EER92546	transcript:Zm00001d047908_T002	0
231- 67:	transcript:EER92548	transcript:Zm00001d047909_T001	0
231- 68:	transcript:EER95168	transcript:Zm00001d047910_T001	0
231- 69:	transcript:KXG39766	transcript:Zm00001d047911_T010	0
231- 70:	transcript:EER95169	transcript:Zm00001d047912_T002	2.00E-92
231- 71:	transcript:EER92551	transcript:Zm00001d047913_T001	1.00E-67
231- 72:	transcript:EER95171	transcript:Zm00001d047915_T001	0
231- 73:	transcript:KXG39773	transcript:Zm00001d047916_T001	0

231- 74:	transcript:EER95176	transcript:Zm00001d047917_T001	0
231- 75:	transcript:EER95177	transcript:Zm00001d047918_T001	0
231- 76:	transcript:EER92556	transcript:Zm00001d047920_T002	5.00E-130
231- 77:	transcript:EER92557	transcript:Zm00001d047921_T001	2.00E-111
231- 78:	transcript:EER95178	transcript:Zm00001d047923_T001	0

Alignment 232: score=2734.0 e_value=9.9e-235 N=60 1&9 plus

232- 0:	transcript:KXG39781	transcript:Zm00001d047931_T001	0
232- 1:	transcript:EER92562	transcript:Zm00001d047932_T001	0
232- 2:	transcript:EER92564	transcript:Zm00001d047933_T001	2.00E-72
232- 3:	transcript:EER95181	transcript:Zm00001d047935_T003	0
232- 4:	transcript:EER92567	transcript:Zm00001d047937_T001	0
232- 5:	transcript:KXG39784	transcript:Zm00001d047938_T001	1.00E-170
232- 6:	transcript:KXG39786	transcript:Zm00001d047939_T001	1.00E-156
232- 7:	transcript:KXG39788	transcript:Zm00001d047940_T008	0
232- 8:	transcript:EER92569	transcript:Zm00001d047941_T001	0
232- 9:	transcript:OQU92927	transcript:Zm00001d047942_T001	6.00E-66
232- 10:	transcript:EER92572	transcript:Zm00001d047944_T001	3.00E-71
232- 11:	transcript:OQU92931	transcript:Zm00001d047945_T002	1.00E-128
232- 12:	transcript:EER95187	transcript:Zm00001d047946_T001	0
232- 13:	transcript:KXG39792	transcript:Zm00001d047951_T002	0
232- 14:	transcript:EER95189	transcript:Zm00001d047955_T001	0
232- 15:	transcript:EER95190	transcript:Zm00001d047958_T001	3.00E-89
232- 16:	transcript:KXG39796	transcript:Zm00001d047960_T001	0
232- 17:	transcript:EER95192	transcript:Zm00001d047962_T003	9.00E-78
232- 18:	transcript:KXG39798	transcript:Zm00001d047965_T002	0
232- 19:	transcript:EER95193	transcript:Zm00001d047966_T001	0
232- 20:	transcript:KXG39801	transcript:Zm00001d047967_T003	0
232- 21:	transcript:EER92577	transcript:Zm00001d047968_T001	4.00E-98
232- 22:	transcript:EER95194	transcript:Zm00001d047969_T001	0
232- 23:	transcript:KXG39804	transcript:Zm00001d047970_T001	0
232- 24:	transcript:KXG39807	transcript:Zm00001d047971_T002	0
232- 25:	transcript:KXG39809	transcript:Zm00001d047972_T001	0
232- 26:	transcript:KXG39813	transcript:Zm00001d047976_T001	2.00E-173
232- 27:	transcript:EER95201	transcript:Zm00001d047977_T004	0
232- 28:	transcript:EER95202	transcript:Zm00001d047979_T005	0
232- 29:	transcript:EER95206	transcript:Zm00001d047981_T001	0
232- 30:	transcript:EER95207	transcript:Zm00001d047982_T001	0
232- 31:	transcript:EER95208	transcript:Zm00001d047983_T007	1.00E-178
232- 32:	transcript:EER92589	transcript:Zm00001d047984_T002	8.00E-114
232- 33:	transcript:EER92590	transcript:Zm00001d047985_T002	3.00E-155
232- 34:	transcript:EER92594	transcript:Zm00001d047986_T029	0
232- 35:	transcript:EER92598	transcript:Zm00001d047988_T009	0
232- 36:	transcript:EER95213	transcript:Zm00001d047989_T003	5.00E-162
232- 37:	transcript:EER92601	transcript:Zm00001d047992_T006	8.00E-112
232- 38:	transcript:EER92605	transcript:Zm00001d047993_T001	0
232- 39:	transcript:EER92606	transcript:Zm00001d047994_T002	4.00E-170
232- 40:	transcript:EER92607	transcript:Zm00001d047995_T001	1.00E-112
232- 41:	transcript:EER95220	transcript:Zm00001d047998_T001	0
232- 42:	transcript:EER95218	transcript:Zm00001d047999_T001	6.00E-106
232- 43:	transcript:KXG39831	transcript:Zm00001d048004_T001	0
232- 44:	transcript:EER92608	transcript:Zm00001d048006_T001	3.00E-31
232- 45:	transcript:EER92611	transcript:Zm00001d048007_T003	0
232- 46:	transcript:EER95229	transcript:Zm00001d048008_T001	0
232- 47:	transcript:EER95230	transcript:Zm00001d048009_T001	0

232- 48: transcript:EER92612 transcript:Zm00001d048013_T001 0
232- 49: transcript:EER95233 transcript:Zm00001d048014_T001 1.00E-156
232- 50: transcript:OQU92969 transcript:Zm00001d048016_T001 0
232- 51: transcript:EER92613 transcript:Zm00001d048017_T003 0
232- 52: transcript:EER92614 transcript:Zm00001d048019_T002 0
232- 53: transcript:KXG39834 transcript:Zm00001d048020_T002 2.00E-102
232- 54: transcript:OQU92970 transcript:Zm00001d048021_T001 0
232- 55: transcript:OQU92971 transcript:Zm00001d048022_T001 1.00E-49
232- 56: transcript:EER95238 transcript:Zm00001d048023_T002 0
232- 57: transcript:OQU92973 transcript:Zm00001d048026_T001 0
232- 58: transcript:EER92617 transcript:Zm00001d048027_T001 3.00E-179
232- 59: transcript:EER95241 transcript:Zm00001d048028_T001 0
Alignment 233: score=2425.0 e_value=6.8e-227 N=58 1&9 plus
233- 0: transcript:OQU92567 transcript:Zm00001d047451_T005 0
233- 1: transcript:EER92245 transcript:Zm00001d047452_T001 0
233- 2: transcript:EER94835 transcript:Zm00001d047453_T001 0
233- 3: transcript:KXG39337 transcript:Zm00001d047455_T002 0
233- 4: transcript:EER92249 transcript:Zm00001d047456_T002 6.00E-118
233- 5: transcript:OQU92571 transcript:Zm00001d047457_T002 0
233- 6: transcript:EER92252 transcript:Zm00001d047458_T001 4.00E-111
233- 7: transcript:KXG39344 transcript:Zm00001d047460_T001 1.00E-39
233- 8: transcript:OQU92576 transcript:Zm00001d047461_T001 4.00E-112
233- 9: transcript:EER92258 transcript:Zm00001d047462_T001 2.00E-143
233- 10: transcript:EER92259 transcript:Zm00001d047463_T001 0
233- 11: transcript:EER94839 transcript:Zm00001d047464_T024 0
233- 12: transcript:EER92266 transcript:Zm00001d047466_T001 0
233- 13: transcript:EER94850 transcript:Zm00001d047467_T001 1.00E-06
233- 14: transcript:EER94848 transcript:Zm00001d047468_T003 0
233- 15: transcript:EER94852 transcript:Zm00001d047469_T001 8.00E-118
233- 16: transcript:KXG39364 transcript:Zm00001d047470_T005 6.00E-63
233- 17: transcript:OQU92590 transcript:Zm00001d047471_T002 0
233- 18: transcript:EER92268 transcript:Zm00001d047472_T002 0
233- 19: transcript:KXG39369 transcript:Zm00001d047474_T003 0
233- 20: transcript:EER92269 transcript:Zm00001d047476_T001 0
233- 21: transcript:EER94856 transcript:Zm00001d047477_T001 8.00E-114
233- 22: transcript:EER94857 transcript:Zm00001d047478_T001 1.00E-59
233- 23: transcript:KXG39375 transcript:Zm00001d047479_T001 4.00E-86
233- 24: transcript:EER94858 transcript:Zm00001d047480_T001 1.00E-172
233- 25: transcript:KXG39385 transcript:Zm00001d047481_T003 0
233- 26: transcript:KXG39386 transcript:Zm00001d047482_T001 0
233- 27: transcript:KXG39392 transcript:Zm00001d047483_T002 0
233- 28: transcript:EER92278 transcript:Zm00001d047487_T001 4.00E-138
233- 29: transcript:EER92281 transcript:Zm00001d047489_T002 0
233- 30: transcript:EER92280 transcript:Zm00001d047491_T004 0
233- 31: transcript:KXG39397 transcript:Zm00001d047492_T001 1.00E-128
233- 32: transcript:OQU92608 transcript:Zm00001d047493_T001 8.00E-45
233- 33: transcript:EER92288 transcript:Zm00001d047494_T042 0
233- 34: transcript:EER94869 transcript:Zm00001d047497_T003 0
233- 35: transcript:EER94870 transcript:Zm00001d047498_T010 0
233- 36: transcript:EER94871 transcript:Zm00001d047499_T001 3.00E-76
233- 37: transcript:EER92292 transcript:Zm00001d047500_T001 2.00E-60
233- 38: transcript:EER94877 transcript:Zm00001d047501_T001 0
233- 39: transcript:EER92293 transcript:Zm00001d047502_T001 0
233- 40: transcript:EER92294 transcript:Zm00001d047503_T001 4.00E-82

233- 41:	transcript:OQU92623	transcript:Zm00001d047504_T001	0
233- 42:	transcript:OQU92635	transcript:Zm00001d047506_T006	0
233- 43:	transcript:EER94885	transcript:Zm00001d047507_T001	3.00E-20
233- 44:	transcript:EER92303	transcript:Zm00001d047512_T002	3.00E-102
233- 45:	transcript:KXG39418	transcript:Zm00001d047514_T001	0
233- 46:	transcript:EER92305	transcript:Zm00001d047516_T001	0
233- 47:	transcript:EER92307	transcript:Zm00001d047517_T001	5.00E-158
233- 48:	transcript:KXG39425	transcript:Zm00001d047518_T004	0
233- 49:	transcript:OQU92641	transcript:Zm00001d047519_T001	5.00E-177
233- 50:	transcript:EER94904	transcript:Zm00001d047522_T002	0
233- 51:	transcript:EER94905	transcript:Zm00001d047523_T001	5.00E-130
233- 52:	transcript:OQU92645	transcript:Zm00001d047526_T006	0
233- 53:	transcript:EER94908	transcript:Zm00001d047527_T004	0
233- 54:	transcript:OQU92647	transcript:Zm00001d047528_T001	3.00E-127
233- 55:	transcript:KXG39433	transcript:Zm00001d047531_T001	0
233- 56:	transcript:EER94911	transcript:Zm00001d047532_T001	2.00E-85
233- 57:	transcript:EER94913	transcript:Zm00001d047533_T001	0

Alignment 234: score=1556.0 e_value=4.3e-127 N=36 1&9 plus

234- 0:	transcript:EER95591	transcript:Zm00001d048453_T001	0
234- 1:	transcript:EER92944	transcript:Zm00001d048454_T005	0
234- 2:	transcript:KXG40277	transcript:Zm00001d048455_T001	2.00E-160
234- 3:	transcript:OQU93335	transcript:Zm00001d048456_T001	1.00E-101
234- 4:	transcript:EER92947	transcript:Zm00001d048460_T001	0
234- 5:	transcript:KXG40289	transcript:Zm00001d048461_T001	0
234- 6:	transcript:OQU93342	transcript:Zm00001d048462_T001	7.00E-43
234- 7:	transcript:KXG40290	transcript:Zm00001d048463_T012	0
234- 8:	transcript:EER92954	transcript:Zm00001d048466_T001	2.00E-64
234- 9:	transcript:EER95596	transcript:Zm00001d048467_T001	0
234- 10:	transcript:KXG40304	transcript:Zm00001d048470_T001	6.00E-156
234- 11:	transcript:KXG40306	transcript:Zm00001d048471_T001	0
234- 12:	transcript:OQU93351	transcript:Zm00001d048472_T001	0
234- 13:	transcript:EER95612	transcript:Zm00001d048473_T003	7.00E-118
234- 14:	transcript:OQU93352	transcript:Zm00001d048474_T001	2.00E-146
234- 15:	transcript:EER95613	transcript:Zm00001d048476_T001	0
234- 16:	transcript:OQU93356	transcript:Zm00001d048477_T001	6.00E-125
234- 17:	transcript:OQU93358	transcript:Zm00001d048478_T001	3.00E-164
234- 18:	transcript:OQU93363	transcript:Zm00001d048479_T002	1.00E-90
234- 19:	transcript:KXG40316	transcript:Zm00001d048480_T005	3.00E-136
234- 20:	transcript:KXG40319	transcript:Zm00001d048481_T001	0
234- 21:	transcript:OQU93368	transcript:Zm00001d048483_T001	7.00E-153
234- 22:	transcript:EER95620	transcript:Zm00001d048492_T001	2.00E-79
234- 23:	transcript:EER95621	transcript:Zm00001d048493_T003	0
234- 24:	transcript:EER92974	transcript:Zm00001d048495_T001	0
234- 25:	transcript:EER92977	transcript:Zm00001d048496_T002	0
234- 26:	transcript:KXG40336	transcript:Zm00001d048497_T002	0
234- 27:	transcript:EER95630	transcript:Zm00001d048499_T001	0
234- 28:	transcript:EER92981	transcript:Zm00001d048502_T002	2.00E-90
234- 29:	transcript:EER95643	transcript:Zm00001d048503_T001	0
234- 30:	transcript:EER92984	transcript:Zm00001d048505_T001	5.00E-154
234- 31:	transcript:EER92987	transcript:Zm00001d048506_T001	3.00E-134
234- 32:	transcript:KXG40347	transcript:Zm00001d048507_T007	0
234- 33:	transcript:OQU93397	transcript:Zm00001d048509_T001	1.00E-180
234- 34:	transcript:EER95651	transcript:Zm00001d048510_T003	0
234- 35:	transcript:KXG40365	transcript:Zm00001d048511_T008	0

Alignment 235: score=1289.0 e_value=3.3e-98 N=29 1&9 plus
235- 0: transcript:KXG39284 transcript:Zm00001d047399_T001 0
235- 1: transcript:EER92211 transcript:Zm00001d047401_T001 1.00E-103
235- 2: transcript:EER92212 transcript:Zm00001d047402_T001 0
235- 3: transcript:EER94791 transcript:Zm00001d047403_T001 0
235- 4: transcript:OQU92532 transcript:Zm00001d047404_T001 0
235- 5: transcript:OQU92535 transcript:Zm00001d047412_T004 0
235- 6: transcript:EER94794 transcript:Zm00001d047414_T001 3.00E-24
235- 7: transcript:KXG39291 transcript:Zm00001d047417_T001 0
235- 8: transcript:KXG39293 transcript:Zm00001d047418_T002 0
235- 9: transcript:OQU92539 transcript:Zm00001d047419_T001 7.00E-31
235- 10: transcript:EER94799 transcript:Zm00001d047420_T001 2.00E-116
235- 11: transcript:EER92218 transcript:Zm00001d047421_T001 0
235- 12: transcript:EER94801 transcript:Zm00001d047422_T002 0
235- 13: transcript:EER94804 transcript:Zm00001d047423_T002 4.00E-142
235- 14: transcript:EER94806 transcript:Zm00001d047424_T001 0
235- 15: transcript:EER94808 transcript:Zm00001d047426_T003 0
235- 16: transcript:EER92221 transcript:Zm00001d047427_T002 3.00E-100
235- 17: transcript:EER94810 transcript:Zm00001d047428_T001 0
235- 18: transcript:KXG39304 transcript:Zm00001d047434_T006 0
235- 19: transcript:OQU92551 transcript:Zm00001d047435_T006 0
235- 20: transcript:EER94812 transcript:Zm00001d047436_T001 0
235- 21: transcript:OQU92552 transcript:Zm00001d047437_T001 0
235- 22: transcript:EER94811 transcript:Zm00001d047438_T001 0
235- 23: transcript:EER94814 transcript:Zm00001d047439_T002 0
235- 24: transcript:KXG39318 transcript:Zm00001d047442_T001 1.00E-136
235- 25: transcript:EER92233 transcript:Zm00001d047443_T002 8.00E-118
235- 26: transcript:EER92238 transcript:Zm00001d047446_T002 0
235- 27: transcript:KXG39329 transcript:Zm00001d047448_T002 1.00E-119
235- 28: transcript:KXG39331 transcript:Zm00001d047449_T006 0
Alignment 236: score=929.0 e_value=2.7e-67 N=22 1&9 plus
236- 0: transcript:EER94941 transcript:Zm00001d047573_T001 0
236- 1: transcript:KXG39494 transcript:Zm00001d047587_T005 6.00E-177
236- 2: transcript:EER94955 transcript:Zm00001d047599_T001 0
236- 3: transcript:EER92358 transcript:Zm00001d047600_T001 0
236- 4: transcript:KXG39505 transcript:Zm00001d047601_T006 0
236- 5: transcript:KXG39506 transcript:Zm00001d047603_T002 4.00E-163
236- 6: transcript:OQU92698 transcript:Zm00001d047607_T001 0
236- 7: transcript:OQU92704 transcript:Zm00001d047614_T001 2.00E-79
236- 8: transcript:OQU92705 transcript:Zm00001d047617_T001 0
236- 9: transcript:OQU92706 transcript:Zm00001d047618_T001 1.00E-59
236- 10: transcript:EER94970 transcript:Zm00001d047620_T002 0
236- 11: transcript:EER94971 transcript:Zm00001d047632_T001 0
236- 12: transcript:EER94974 transcript:Zm00001d047633_T002 0
236- 13: transcript:EER92371 transcript:Zm00001d047634_T001 1.00E-161
236- 14: transcript:KXG39527 transcript:Zm00001d047635_T005 0
236- 15: transcript:KXG40039 transcript:Zm00001d047636_T002 0
236- 16: transcript:OQU92716 transcript:Zm00001d047637_T001 0
236- 17: transcript:EER94977 transcript:Zm00001d047638_T001 0
236- 18: transcript:KXG39529 transcript:Zm00001d047639_T004 0
236- 19: transcript:KXG39531 transcript:Zm00001d047640_T001 3.00E-153
236- 20: transcript:EER94981 transcript:Zm00001d047644_T001 1.00E-61
236- 21: transcript:EER92376 transcript:Zm00001d047647_T001 0
Alignment 237: score=867.0 e_value=1.9e-53 N=19 1&9 plus

237- 0:	transcript:EER95256	transcript:Zm00001d048055_T001	0
237- 1:	transcript:OQU92983	transcript:Zm00001d048059_T002	0
237- 2:	transcript:EER95257	transcript:Zm00001d048060_T001	0
237- 3:	transcript:EER92628	transcript:Zm00001d048061_T001	0
237- 4:	transcript:EER92631	transcript:Zm00001d048065_T002	0
237- 5:	transcript:EER95261	transcript:Zm00001d048066_T001	0
237- 6:	transcript:EER92633	transcript:Zm00001d048073_T001	0
237- 7:	transcript:OQU92994	transcript:Zm00001d048074_T001	0
237- 8:	transcript:OQU92992	transcript:Zm00001d048075_T001	2.00E-67
237- 9:	transcript:EER95266	transcript:Zm00001d048076_T001	0
237- 10:	transcript:KXG39856	transcript:Zm00001d048079_T001	0
237- 11:	transcript:EER95268	transcript:Zm00001d048080_T002	0
237- 12:	transcript:KXG40410	transcript:Zm00001d048081_T001	0
237- 13:	transcript:OQU93000	transcript:Zm00001d048082_T001	1.00E-151
237- 14:	transcript:EER95274	transcript:Zm00001d048083_T001	5.00E-142
237- 15:	transcript:KXG39871	transcript:Zm00001d048084_T001	0
237- 16:	transcript:OQU93003	transcript:Zm00001d048085_T002	1.00E-135
237- 17:	transcript:EER95276	transcript:Zm00001d048086_T001	0
237- 18:	transcript:OQU93005	transcript:Zm00001d048087_T001	0

Alignment 238: score=831.0 e_value=3.8e-48 N=18 1&9 plus

238- 0:	transcript:OQU92785	transcript:Zm00001d047762_T045	0
238- 1:	transcript:EER92436	transcript:Zm00001d047763_T001	4.00E-178
238- 2:	transcript:EER92438	transcript:Zm00001d047764_T001	3.00E-143
238- 3:	transcript:EER92440	transcript:Zm00001d047765_T001	1.00E-116
238- 4:	transcript:EER95061	transcript:Zm00001d047766_T001	1.00E-133
238- 5:	transcript:EER92441	transcript:Zm00001d047767_T004	1.00E-144
238- 6:	transcript:KXG39645	transcript:Zm00001d047768_T001	4.00E-74
238- 7:	transcript:EER92445	transcript:Zm00001d047769_T001	0
238- 8:	transcript:KXG39647	transcript:Zm00001d047770_T003	0
238- 9:	transcript:KXG39648	transcript:Zm00001d047771_T001	7.00E-159
238- 10:	transcript:EER95067	transcript:Zm00001d047772_T002	0
238- 11:	transcript:OQU92799	transcript:Zm00001d047774_T002	3.00E-90
238- 12:	transcript:EER95068	transcript:Zm00001d047775_T001	2.00E-162
238- 13:	transcript:KXG39653	transcript:Zm00001d047776_T001	0
238- 14:	transcript:EER95070	transcript:Zm00001d047777_T004	4.00E-134
238- 15:	transcript:EER95072	transcript:Zm00001d047779_T001	2.00E-51
238- 16:	transcript:EER92449	transcript:Zm00001d047780_T002	0
238- 17:	transcript:EER95073	transcript:Zm00001d047781_T001	0

Alignment 239: score=782.0 e_value=6.6e-46 N=17 1&9 plus

239- 0:	transcript:EER95019	transcript:Zm00001d047711_T001	2.00E-72
239- 1:	transcript:EER95020	transcript:Zm00001d047712_T001	0
239- 2:	transcript:EER95021	transcript:Zm00001d047713_T003	0
239- 3:	transcript:OQU92760	transcript:Zm00001d047716_T001	7.00E-17
239- 4:	transcript:KXG40097	transcript:Zm00001d047717_T014	0
239- 5:	transcript:KXG39573	transcript:Zm00001d047719_T001	0
239- 6:	transcript:OQU92761	transcript:Zm00001d047720_T003	2.00E-72
239- 7:	transcript:EER95025	transcript:Zm00001d047721_T001	0
239- 8:	transcript:OQU92764	transcript:Zm00001d047722_T001	2.00E-109
239- 9:	transcript:KXG39578	transcript:Zm00001d047723_T001	0
239- 10:	transcript:KXG40108	transcript:Zm00001d047726_T001	0
239- 11:	transcript:EER92413	transcript:Zm00001d047727_T001	6.00E-117
239- 12:	transcript:OQU92769	transcript:Zm00001d047728_T001	0
239- 13:	transcript:EER95033	transcript:Zm00001d047732_T001	0
239- 14:	transcript:KXG39586	transcript:Zm00001d047736_T001	0

```
239- 15: transcript:EER95038           transcript:Zm00001d047738_T001         0
239- 16: transcript:KXG39597           transcript:Zm00001d047739_T004         0
## Alignment 240: score=716.0 e_value=3.4e-45 N=17 1&9 plus
240-  0: transcript:OQU93400           transcript:Zm00001d048512_T004         0
240-  1: transcript:OQU93401           transcript:Zm00001d048513_T001  1.00E-38
240-  2: transcript:OQU93403           transcript:Zm00001d048514_T001         0
240-  3: transcript:OQU93406           transcript:Zm00001d048515_T003 1.00E-158
240-  4: transcript:OQU93414           transcript:Zm00001d048516_T001         0
240-  5: transcript:EER95659           transcript:Zm00001d048520_T014  2.00E-59
240-  6: transcript:EER95660           transcript:Zm00001d048521_T001 2.00E-174
240-  7: transcript:KXG40377           transcript:Zm00001d048522_T001 2.00E-140
240-  8: transcript:OQU93419           transcript:Zm00001d048523_T001         0
240-  9: transcript:OQU93422           transcript:Zm00001d048524_T004         0
240- 10: transcript:EER93008           transcript:Zm00001d048526_T001  3.00E-92
240- 11: transcript:EER95667           transcript:Zm00001d048527_T028         0
240- 12: transcript:EER95669           transcript:Zm00001d048529_T002         0
240- 13: transcript:EER95670           transcript:Zm00001d048531_T001         0
240- 14: transcript:EER95677           transcript:Zm00001d048532_T001         0
240- 15: transcript:OQU93434           transcript:Zm00001d048533_T001  3.00E-39
240- 16: transcript:KXG40395           transcript:Zm00001d048535_T020         0
## Alignment 241: score=705.0 e_value=5.4e-42 N=16 1&9 plus
241-  0: transcript:KXG38921           transcript:Zm00001d047079_T001         0
241-  1: transcript:EER91937           transcript:Zm00001d047081_T001 3.00E-165
241-  2: transcript:KXG38927           transcript:Zm00001d047086_T001 1.00E-134
241-  3: transcript:EER94500           transcript:Zm00001d047087_T001         0
241-  4: transcript:KXG38933           transcript:Zm00001d047089_T001         0
241-  5: transcript:EER91945           transcript:Zm00001d047091_T001 3.00E-162
241-  6: transcript:EER91946           transcript:Zm00001d047092_T001         0
241-  7: transcript:EER91948           transcript:Zm00001d047093_T001 3.00E-104
241-  8: transcript:OQU92158           transcript:Zm00001d047097_T001 5.00E-131
241-  9: transcript:EER91950           transcript:Zm00001d047102_T001 1.00E-118
241- 10: transcript:OQU92159           transcript:Zm00001d047103_T002 1.00E-131
241- 11: transcript:EER94513           transcript:Zm00001d047104_T001         0
241- 12: transcript:EER91955           transcript:Zm00001d047105_T007         0
241- 13: transcript:KXG38947           transcript:Zm00001d047106_T001  2.00E-98
241- 14: transcript:KXG38949           transcript:Zm00001d047107_T004         0
241- 15: transcript:EER94520           transcript:Zm00001d047109_T001  8.00E-95
## Alignment 242: score=670.0 e_value=8.9e-36 N=15 1&9 plus
242-  0: transcript:EER92416           transcript:Zm00001d047743_T001         0
242-  1: transcript:EER92419           transcript:Zm00001d047744_T001         0
242-  2: transcript:KXG39609           transcript:Zm00001d047745_T001 2.00E-178
242-  3: transcript:EER95042           transcript:Zm00001d047746_T007         0
242-  4: transcript:OQU92777           transcript:Zm00001d047747_T001         0
242-  5: transcript:EER95045           transcript:Zm00001d047749_T001         0
242-  6: transcript:EER95046           transcript:Zm00001d047750_T001         0
242-  7: transcript:EER92422           transcript:Zm00001d047752_T001 7.00E-116
242-  8: transcript:EER95048           transcript:Zm00001d047753_T001 2.00E-132
242-  9: transcript:KXG39624           transcript:Zm00001d047754_T002         0
242- 10: transcript:EER95050           transcript:Zm00001d047755_T013         0
242- 11: transcript:EER95051           transcript:Zm00001d047757_T002 8.00E-172
242- 12: transcript:EER92428           transcript:Zm00001d047759_T003         0
242- 13: transcript:EER92432           transcript:Zm00001d047760_T001         0
242- 14: transcript:EER92434           transcript:Zm00001d047761_T008         0
## Alignment 243: score=668.0 e_value=2.4e-33 N=14 1&9 plus
```

```
243-  0: transcript:EER95008           transcript:Zm00001d047688_T001 3.00E-130
243-  1: transcript:EER92397           transcript:Zm00001d047689_T002         0
243-  2: transcript:EER92398           transcript:Zm00001d047694_T001         0
243-  3: transcript:EER95009           transcript:Zm00001d047695_T001         0
243-  4: transcript:OQU92744           transcript:Zm00001d047696_T001  8.00E-92
243-  5: transcript:OQU92746           transcript:Zm00001d047697_T002  1.00E-39
243-  6: transcript:EER95011           transcript:Zm00001d047698_T001         0
243-  7: transcript:EER92401           transcript:Zm00001d047699_T001 1.00E-170
243-  8: transcript:EER92402           transcript:Zm00001d047701_T002         0
243-  9: transcript:OQU92750           transcript:Zm00001d047702_T001  3.00E-54
243- 10: transcript:EER92405           transcript:Zm00001d047705_T001 2.00E-125
243- 11: transcript:EER92406           transcript:Zm00001d047706_T001  4.00E-56
243- 12: transcript:KXG39564           transcript:Zm00001d047707_T002 1.00E-133
243- 13: transcript:OQU92753           transcript:Zm00001d047709_T001 3.00E-168
## Alignment 244: score=573.0 e_value=1.3e-31 N=13 1&9 plus
244-  0: transcript:EER92379           transcript:Zm00001d047664_T001  2.00E-95
244-  1: transcript:OQU92728           transcript:Zm00001d047671_T001  2.00E-92
244-  2: transcript:EER94986           transcript:Zm00001d047672_T001 2.00E-169
244-  3: transcript:EER94989           transcript:Zm00001d047673_T001         0
244-  4: transcript:KXG39539           transcript:Zm00001d047674_T001  2.00E-91
244-  5: transcript:EER94992           transcript:Zm00001d047675_T001  2.00E-62
244-  6: transcript:OQU92733           transcript:Zm00001d047676_T002         0
244-  7: transcript:EER94990           transcript:Zm00001d047677_T016         0
244-  8: transcript:EER92392           transcript:Zm00001d047679_T001         0
244-  9: transcript:OQU92735           transcript:Zm00001d047680_T001         0
244- 10: transcript:OQU92738           transcript:Zm00001d047681_T002         0
244- 11: transcript:EER95001           transcript:Zm00001d047683_T003         0
244- 12: transcript:KXG39549           transcript:Zm00001d047687_T002  3.00E-83
## Alignment 245: score=496.0 e_value=1.9e-25 N=11 1&9 plus
245-  0: transcript:EER94451           transcript:Zm00001d047032_T001  2.00E-86
245-  1: transcript:KXG38867           transcript:Zm00001d047042_T004         0
245-  2: transcript:KXG39219           transcript:Zm00001d047044_T005         0
245-  3: transcript:KXG38871           transcript:Zm00001d047045_T001         0
245-  4: transcript:OQU92099           transcript:Zm00001d047046_T003 2.00E-139
245-  5: transcript:EER94455           transcript:Zm00001d047050_T001 2.00E-137
245-  6: transcript:EER94456           transcript:Zm00001d047053_T004         0
245-  7: transcript:EER94457           transcript:Zm00001d047054_T001         0
245-  8: transcript:EER94458           transcript:Zm00001d047057_T001 8.00E-113
245-  9: transcript:OQU92106           transcript:Zm00001d047058_T002  2.00E-64
245- 10: transcript:KXG38876           transcript:Zm00001d047059_T003         0
## Alignment 246: score=495.0 e_value=1.9e-23 N=11 1&9 plus
246-  0: transcript:EER92453           transcript:Zm00001d047786_T001  8.00E-88
246-  1: transcript:EER92457           transcript:Zm00001d047787_T001  1.00E-82
246-  2: transcript:EER92459           transcript:Zm00001d047789_T001 3.00E-101
246-  3: transcript:KXG40160           transcript:Zm00001d047790_T001         0
246-  4: transcript:EER92462           transcript:Zm00001d047793_T001         0
246-  5: transcript:EER92463           transcript:Zm00001d047794_T001 4.00E-101
246-  6: transcript:EER95086           transcript:Zm00001d047796_T001         0
246-  7: transcript:KXG39670           transcript:Zm00001d047797_T010         0
246-  8: transcript:EER92466           transcript:Zm00001d047799_T002         0
246-  9: transcript:KXG39674           transcript:Zm00001d047800_T001         0
246- 10: transcript:EER92467           transcript:Zm00001d047801_T001         0
## Alignment 247: score=490.0 e_value=3.1e-23 N=11 1&9 plus
247-  0: transcript:EER91963           transcript:Zm00001d047110_T001         0
```

247- 1: transcript:EER91965 transcript:Zm00001d047111_T001 7.00E-51
247- 2: transcript:OQU92173 transcript:Zm00001d047113_T001 4.00E-48
247- 3: transcript:EER94525 transcript:Zm00001d047119_T001 9.00E-55
247- 4: transcript:EER91968 transcript:Zm00001d047123_T001 4.00E-43
247- 5: transcript:EER94526 transcript:Zm00001d047124_T001 0
247- 6: transcript:KXG38959 transcript:Zm00001d047125_T001 0
247- 7: transcript:KXG38962 transcript:Zm00001d047126_T003 0
247- 8: transcript:EER91972 transcript:Zm00001d047128_T002 0
247- 9: transcript:EER91974 transcript:Zm00001d047130_T001 0
247- 10: transcript:KXG38970 transcript:Zm00001d047135_T009 5.00E-158
Alignment 248: score=439.0 e_value=1.2e-19 N=10 1&9 plus
248- 0: transcript:EER92328 transcript:Zm00001d047554_T001 0
248- 1: transcript:KXG39469 transcript:Zm00001d047555_T001 0
248- 2: transcript:EER92330 transcript:Zm00001d047558_T001 0
248- 3: transcript:KXG39473 transcript:Zm00001d047559_T001 3.00E-139
248- 4: transcript:KXG39475 transcript:Zm00001d047560_T001 0
248- 5: transcript:EER94932 transcript:Zm00001d047562_T002 0
248- 6: transcript:EER94933 transcript:Zm00001d047563_T001 0
248- 7: transcript:EER94935 transcript:Zm00001d047566_T002 0
248- 8: transcript:EER92338 transcript:Zm00001d047567_T001 6.00E-132
248- 9: transcript:EER94937 transcript:Zm00001d047574_T005 0
Alignment 249: score=384.0 e_value=5.5e-18 N=9 1&9 plus
249- 0: transcript:EER91903 transcript:Zm00001d047060_T001 3.00E-68
249- 1: transcript:EER91904 transcript:Zm00001d047063_T002 0
249- 2: transcript:EER94465 transcript:Zm00001d047064_T001 0
249- 3: transcript:EER94466 transcript:Zm00001d047067_T001 9.00E-84
249- 4: transcript:EER94467 transcript:Zm00001d047068_T001 2.00E-155
249- 5: transcript:KXG38894 transcript:Zm00001d047069_T001 3.00E-133
249- 6: transcript:KXG38896 transcript:Zm00001d047074_T002 0
249- 7: transcript:EER91913 transcript:Zm00001d047077_T001 0
249- 8: transcript:EER94468 transcript:Zm00001d047078_T004 0
Alignment 250: score=338.0 e_value=2.2e-15 N=8 1&9 plus
250- 0: transcript:KXG38970 transcript:Zm00001d047146_T001 1.00E-157
250- 1: transcript:EER94537 transcript:Zm00001d047147_T001 4.00E-162
250- 2: transcript:KXG38975 transcript:Zm00001d047149_T001 0
250- 3: transcript:OQU92191 transcript:Zm00001d047150_T003 0
250- 4: transcript:EER94540 transcript:Zm00001d047153_T004 0
250- 5: transcript:EER91992 transcript:Zm00001d047159_T001 6.00E-46
250- 6: transcript:EER94548 transcript:Zm00001d047160_T001 6.00E-49
250- 7: transcript:EER94547 transcript:Zm00001d047162_T005 0
Alignment 251: score=314.0 e_value=4.8e-13 N=7 1&9 plus
251- 0: transcript:EER95699 transcript:Zm00001d048547_T001 0
251- 1: transcript:EER95701 transcript:Zm00001d048550_T001 0
251- 2: transcript:KXG40426 transcript:Zm00001d048551_T005 3.00E-106
251- 3: transcript:OQU93482 transcript:Zm00001d048552_T005 0
251- 4: transcript:EER93042 transcript:Zm00001d048553_T001 1.00E-103
251- 5: transcript:EER95704 transcript:Zm00001d048555_T001 4.00E-170
251- 6: transcript:OQU93496 transcript:Zm00001d048556_T002 0
Alignment 252: score=313.0 e_value=1.7e-14 N=8 1&9 plus
252- 0: transcript:EER93012 transcript:Zm00001d048536_T009 0
252- 1: transcript:OQU93460 transcript:Zm00001d048539_T001 0
252- 2: transcript:KXG40406 transcript:Zm00001d048540_T002 5.00E-34
252- 3: transcript:EER93022 transcript:Zm00001d048541_T004 0
252- 4: transcript:KXG40411 transcript:Zm00001d048542_T001 0

252- 5: transcript:KXG40415 transcript:Zm00001d048544_T003 0
252- 6: transcript:KXG40418 transcript:Zm00001d048545_T001 0
252- 7: transcript:OQU93476 transcript:Zm00001d048546_T008 0
Alignment 253: score=280.0 e_value=2.8e-11 N=7 1&9 plus
253- 0: transcript:EER92051 transcript:Zm00001d048566_T001 0
253- 1: transcript:KXG39054 transcript:Zm00001d048567_T002 1.00E-166
253- 2: transcript:EER92053 transcript:Zm00001d048568_T003 0
253- 3: transcript:OQU92290 transcript:Zm00001d048569_T001 7.00E-159
253- 4: transcript:EER92060 transcript:Zm00001d048570_T001 5.00E-148
253- 5: transcript:EER94644 transcript:Zm00001d048574_T001 0
253- 6: transcript:OQU92307 transcript:Zm00001d048575_T016 0
Alignment 254: score=2869.0 e_value=7.5e-261 N=66 1&9 minus
254- 0: transcript:EER92814 transcript:Zm00001d048438_T001 6.00E-47
254- 1: transcript:KXG40135 transcript:Zm00001d048437_T002 0
254- 2: transcript:EER92819 transcript:Zm00001d048434_T002 0
254- 3: transcript:EER92824 transcript:Zm00001d048432_T002 0
254- 4: transcript:EER95466 transcript:Zm00001d048431_T001 4.00E-108
254- 5: transcript:EER95467 transcript:Zm00001d048430_T005 0
254- 6: transcript:OQU93195 transcript:Zm00001d048428_T001 0
254- 7: transcript:KXG40138 transcript:Zm00001d048424_T003 0
254- 8: transcript:KXG40141 transcript:Zm00001d048422_T001 0
254- 9: transcript:KXG40145 transcript:Zm00001d048421_T001 0
254- 10: transcript:KXG40147 transcript:Zm00001d048420_T001 0
254- 11: transcript:EER95479 transcript:Zm00001d048419_T004 1.00E-132
254- 12: transcript:OQU93207 transcript:Zm00001d048418_T001 2.00E-160
254- 13: transcript:EER95483 transcript:Zm00001d048416_T001 0
254- 14: transcript:EER92836 transcript:Zm00001d048415_T005 0
254- 15: transcript:EER92837 transcript:Zm00001d048414_T001 6.00E-162
254- 16: transcript:EER95489 transcript:Zm00001d048413_T001 0
254- 17: transcript:EER95490 transcript:Zm00001d048412_T001 0
254- 18: transcript:KXG40156 transcript:Zm00001d048411_T001 6.00E-126
254- 19: transcript:OQU93218 transcript:Zm00001d048410_T004 6.00E-135
254- 20: transcript:EER92840 transcript:Zm00001d048409_T001 0
254- 21: transcript:EER92841 transcript:Zm00001d048407_T001 2.00E-139
254- 22: transcript:KXG40161 transcript:Zm00001d048404_T005 0
254- 23: transcript:OQU93221 transcript:Zm00001d048403_T003 2.00E-175
254- 24: transcript:EER92848 transcript:Zm00001d048402_T001 6.00E-82
254- 25: transcript:EER92849 transcript:Zm00001d048401_T001 1.00E-122
254- 26: transcript:OQU93230 transcript:Zm00001d048400_T001 0
254- 27: transcript:OQU93231 transcript:Zm00001d048398_T001 0
254- 28: transcript:EER92852 transcript:Zm00001d048397_T002 9.00E-129
254- 29: transcript:OQU93232 transcript:Zm00001d048396_T001 3.00E-96
254- 30: transcript:KXG40187 transcript:Zm00001d048394_T002 2.00E-59
254- 31: transcript:EER95509 transcript:Zm00001d048393_T001 3.00E-148
254- 32: transcript:EER95503 transcript:Zm00001d048392_T006 0
254- 33: transcript:OQU93244 transcript:Zm00001d048391_T001 1.00E-74
254- 34: transcript:EER92863 transcript:Zm00001d048390_T001 0
254- 35: transcript:OQU93246 transcript:Zm00001d048389_T001 0
254- 36: transcript:KXG40201 transcript:Zm00001d048385_T001 5.00E-127
254- 37: transcript:EER92876 transcript:Zm00001d048383_T001 3.00E-114
254- 38: transcript:EER95532 transcript:Zm00001d048382_T001 4.00E-116
254- 39: transcript:OQU93261 transcript:Zm00001d048373_T002 0
254- 40: transcript:EER92882 transcript:Zm00001d048372_T001 0
254- 41: transcript:OQU93265 transcript:Zm00001d048370_T001 1.00E-36

254- 42: transcript:OQU93266 transcript:Zm00001d048369_T002 0
254- 43: transcript:EER91503 transcript:Zm00001d048368_T007 1.00E-97
254- 44: transcript:OQU93273 transcript:Zm00001d048366_T001 0
254- 45: transcript:OQU93274 transcript:Zm00001d048364_T002 0
254- 46: transcript:EER95545 transcript:Zm00001d048363_T001 9.00E-131
254- 47: transcript:EER95548 transcript:Zm00001d048362_T002 1.00E-172
254- 48: transcript:EER95549 transcript:Zm00001d048361_T001 0
254- 49: transcript:KXG40228 transcript:Zm00001d048360_T001 0
254- 50: transcript:EER95550 transcript:Zm00001d048359_T004 0
254- 51: transcript:EER92899 transcript:Zm00001d048358_T004 0
254- 52: transcript:EER95554 transcript:Zm00001d048356_T001 0
254- 53: transcript:EER92906 transcript:Zm00001d048355_T001 0
254- 54: transcript:EER92911 transcript:Zm00001d048354_T001 1.00E-58
254- 55: transcript:EER95556 transcript:Zm00001d048352_T001 1.00E-132
254- 56: transcript:OQU93301 transcript:Zm00001d048349_T002 0
254- 57: transcript:EER95562 transcript:Zm00001d048348_T001 9.00E-59
254- 58: transcript:EER92917 transcript:Zm00001d048347_T001 0
254- 59: transcript:EER95565 transcript:Zm00001d048345_T003 0
254- 60: transcript:EER95568 transcript:Zm00001d048344_T002 1.00E-155
254- 61: transcript:EER92919 transcript:Zm00001d048343_T002 0
254- 62: transcript:EER95575 transcript:Zm00001d048342_T001 0
254- 63: transcript:EER95576 transcript:Zm00001d048341_T001 0
254- 64: transcript:EER95577 transcript:Zm00001d048340_T002 0
254- 65: transcript:EER92924 transcript:Zm00001d048338_T035 0
Alignment 255: score=889.0 e_value=2.6e-60 N=21 1&9 minus
255- 0: transcript:KXG38983 transcript:Zm00001d047397_T001 2.00E-150
255- 1: transcript:EER94555 transcript:Zm00001d047396_T001 4.00E-89
255- 2: transcript:OQU92199 transcript:Zm00001d047392_T002 0
255- 3: transcript:EER91995 transcript:Zm00001d047389_T006 0
255- 4: transcript:EER91996 transcript:Zm00001d047388_T001 0
255- 5: transcript:EER94559 transcript:Zm00001d047385_T002 0
255- 6: transcript:KXG38988 transcript:Zm00001d047382_T001 0
255- 7: transcript:OQU92208 transcript:Zm00001d047379_T001 7.00E-46
255- 8: transcript:EER94567 transcript:Zm00001d047378_T001 1.00E-104
255- 9: transcript:EER92003 transcript:Zm00001d047373_T001 0
255- 10: transcript:OQU92219 transcript:Zm00001d047372_T001 7.00E-38
255- 11: transcript:OQU92227 transcript:Zm00001d047364_T001 0
255- 12: transcript:EER94581 transcript:Zm00001d047362_T003 0
255- 13: transcript:OQU92229 transcript:Zm00001d047361_T002 0
255- 14: transcript:EER92015 transcript:Zm00001d047359_T001 4.00E-146
255- 15: transcript:EER94583 transcript:Zm00001d047358_T001 6.00E-88
255- 16: transcript:KXG39017 transcript:Zm00001d047355_T001 5.00E-36
255- 17: transcript:EER92016 transcript:Zm00001d047354_T001 6.00E-115
255- 18: transcript:EER94586 transcript:Zm00001d047353_T001 2.00E-176
255- 19: transcript:EER94587 transcript:Zm00001d047351_T001 7.00E-143
255- 20: transcript:EER92017 transcript:Zm00001d047350_T002 8.00E-65
Alignment 256: score=802.0 e_value=1.1e-52 N=19 1&9 minus
256- 0: transcript:EER92084 transcript:Zm00001d047345_T001 0
256- 1: transcript:OQU92340 transcript:Zm00001d047342_T001 7.00E-63
256- 2: transcript:EER92085 transcript:Zm00001d047340_T001 1.00E-141
256- 3: transcript:EER92086 transcript:Zm00001d047335_T001 0
256- 4: transcript:EER94663 transcript:Zm00001d047331_T001 0
256- 5: transcript:OQU92346 transcript:Zm00001d047325_T001 4.00E-99
256- 6: transcript:OQU92347 transcript:Zm00001d047324_T001 0

```
256-  7: transcript:EER92101          transcript:Zm00001d047310_T001  3.00E-82
256-  8: transcript:EER94675          transcript:Zm00001d047309_T001         0
256-  9: transcript:OQU92373          transcript:Zm00001d047307_T001         0
256- 10: transcript:EER94677          transcript:Zm00001d047306_T007         0
256- 11: transcript:EER92108          transcript:Zm00001d047303_T001         0
256- 12: transcript:EER94678          transcript:Zm00001d047302_T001         0
256- 13: transcript:OQU92376          transcript:Zm00001d047298_T001 8.00E-169
256- 14: transcript:KXG39114          transcript:Zm00001d047296_T001  2.00E-78
256- 15: transcript:EER92116          transcript:Zm00001d047293_T001  5.00E-84
256- 16: transcript:EER92117          transcript:Zm00001d047292_T001         0
256- 17: transcript:KXG39123          transcript:Zm00001d047290_T001 2.00E-144
256- 18: transcript:EER94684          transcript:Zm00001d047289_T004         0
## Alignment 257: score=677.0 e_value=1.1e-42 N=16 1&9 minus
257-  0: transcript:OQU92441          transcript:Zm00001d047253_T018         0
257-  1: transcript:KXG39188          transcript:Zm00001d047252_T001  9.00E-52
257-  2: transcript:EER94725          transcript:Zm00001d047251_T003         0
257-  3: transcript:OQU92445          transcript:Zm00001d047250_T001  1.00E-94
257-  4: transcript:OQU92448          transcript:Zm00001d047247_T001  4.00E-40
257-  5: transcript:EER94734          transcript:Zm00001d047245_T001         0
257-  6: transcript:EER94736          transcript:Zm00001d047242_T001         0
257-  7: transcript:OQU92453          transcript:Zm00001d047241_T003         0
257-  8: transcript:EER92167          transcript:Zm00001d047240_T001         0
257-  9: transcript:KXG39208          transcript:Zm00001d047239_T005         0
257- 10: transcript:EER92168          transcript:Zm00001d047238_T001 1.00E-176
257- 11: transcript:EER92170          transcript:Zm00001d047236_T003         0
257- 12: transcript:EER94745          transcript:Zm00001d047231_T001  2.00E-09
257- 13: transcript:EER92172          transcript:Zm00001d047223_T001         0
257- 14: transcript:KXG39745          transcript:Zm00001d047222_T001         0
257- 15: transcript:OQU92474          transcript:Zm00001d047216_T001  1.00E-25
## Alignment 258: score=613.0 e_value=1.3e-34 N=14 1&9 minus
258-  0: transcript:KXG39256          transcript:Zm00001d047192_T001         0
258-  1: transcript:KXG39259          transcript:Zm00001d047191_T003         0
258-  2: transcript:KXG39261          transcript:Zm00001d047190_T001         0
258-  3: transcript:KXG39265          transcript:Zm00001d047187_T001  6.00E-86
258-  4: transcript:OQU92521          transcript:Zm00001d047186_T001 3.00E-113
258-  5: transcript:EER92200          transcript:Zm00001d047184_T001  1.00E-94
258-  6: transcript:EER94782          transcript:Zm00001d047183_T002         0
258-  7: transcript:KXG39272          transcript:Zm00001d047182_T001 2.00E-154
258-  8: transcript:EER94783          transcript:Zm00001d047181_T005         0
258-  9: transcript:EER94784          transcript:Zm00001d047178_T009         0
258- 10: transcript:KXG39276          transcript:Zm00001d047174_T001 2.00E-139
258- 11: transcript:EER92201          transcript:Zm00001d047169_T001  1.00E-11
258- 12: transcript:OQU92526          transcript:Zm00001d047166_T001         0
258- 13: transcript:EER92205          transcript:Zm00001d047163_T001  3.00E-93
## Alignment 259: score=371.0 e_value=4.1e-14 N=8 1&9 minus
259-  0: transcript:EER94946          transcript:Zm00001d047597_T001  6.00E-86
259-  1: transcript:EER94948          transcript:Zm00001d047594_T001         0
259-  2: transcript:EER94949          transcript:Zm00001d047591_T001  9.00E-37
259-  3: transcript:OQU92695          transcript:Zm00001d047584_T001         0
259-  4: transcript:EER94951          transcript:Zm00001d047583_T001         0
259-  5: transcript:EER94952          transcript:Zm00001d047582_T002         0
259-  6: transcript:EER92355          transcript:Zm00001d047581_T001         0
259-  7: transcript:KXG39500          transcript:Zm00001d047579_T001         0
## Alignment 260: score=365.0 e_value=2.4e-14 N=8 1&9 minus
```

260- 0: transcript:EER94698 transcript:Zm00001d047272_T001 2.00E-90
260- 1: transcript:EER94701 transcript:Zm00001d047271_T002 3.00E-52
260- 2: transcript:EER92142 transcript:Zm00001d047269_T001 8.00E-65
260- 3: transcript:EER92143 transcript:Zm00001d047268_T001 0
260- 4: transcript:OQU92409 transcript:Zm00001d047266_T001 0
260- 5: transcript:EER94702 transcript:Zm00001d047265_T001 1.00E-112
260- 6: transcript:EER92152 transcript:Zm00001d047263_T001 4.00E-144
260- 7: transcript:KXG39171 transcript:Zm00001d047262_T001 6.00E-85
Alignment 261: score=355.0 e_value=5.5e-15 N=8 1&9 minus
261- 0: transcript:OQU92482 transcript:Zm00001d047217_T001 0
261- 1: transcript:EER92183 transcript:Zm00001d047210_T001 6.00E-89
261- 2: transcript:EER94762 transcript:Zm00001d047209_T003 0
261- 3: transcript:EER92186 transcript:Zm00001d047208_T001 0
261- 4: transcript:OQU92492 transcript:Zm00001d047207_T001 5.00E-72
261- 5: transcript:KXG39241 transcript:Zm00001d047204_T003 0
261- 6: transcript:EER94763 transcript:Zm00001d047203_T001 2.00E-162
261- 7: transcript:OQU92495 transcript:Zm00001d047202_T001 6.00E-59
Alignment 262: score=292.0 e_value=7.1e-13 N=7 1&9 minus
262- 0: transcript:KXG40112 transcript:Zm00001d048445_T002 2.00E-83
262- 1: transcript:EER92802 transcript:Zm00001d048444_T001 0
262- 2: transcript:KXG40114 transcript:Zm00001d048443_T002 4.00E-119
262- 3: transcript:OQU93169 transcript:Zm00001d048442_T004 0
262- 4: transcript:EER92804 transcript:Zm00001d048441_T001 4.00E-116
262- 5: transcript:EER92805 transcript:Zm00001d048440_T003 0
262- 6: transcript:EER92810 transcript:Zm00001d048439_T008 0
Alignment 263: score=386.0 e_value=1e-15 N=8 1&B73V4_ctg181 minus
263- 0: transcript:EER91928 transcript:Zm00001d000184_T001 0
263- 1: transcript:EER94488 transcript:Zm00001d000183_T001 0
263- 2: transcript:EER91929 transcript:Zm00001d000181_T001 3.00E-155
263- 3: transcript:OQU92136 transcript:Zm00001d000180_T001 3.00E-64
263- 4: transcript:KXG38915 transcript:Zm00001d000179_T001 2.00E-84
263- 5: transcript:KXG38917 transcript:Zm00001d000178_T001 0
263- 6: transcript:OQU92139 transcript:Zm00001d000176_T001 9.00E-95
263- 7: transcript:EER94492 transcript:Zm00001d000175_T001 0
Alignment 264: score=620.0 e_value=8.6e-42 N=15 10&3 minus
264- 0: transcript:Zm00001d024729_T001 transcript:KXG32344 0
264- 1: transcript:Zm00001d024732_T001 transcript:EES02832 6.00E-168
264- 2: transcript:Zm00001d024735_T001 transcript:OQU86757 1.00E-60
264- 3: transcript:Zm00001d024745_T001 transcript:KXG32338 5.00E-107
264- 4: transcript:Zm00001d024754_T001 transcript:EES02825 0
264- 5: transcript:Zm00001d024755_T004 transcript:OQU86753 0
264- 6: transcript:Zm00001d024756_T004 transcript:EES00611 3.00E-94
264- 7: transcript:Zm00001d024757_T001 transcript:KXG32332 2.00E-32
264- 8: transcript:Zm00001d024762_T001 transcript:EES00612 0
264- 9: transcript:Zm00001d024767_T003 transcript:KXG32321 0
264- 10: transcript:Zm00001d024768_T001 transcript:EES00609 5.00E-104
264- 11: transcript:Zm00001d024772_T001 transcript:EES02810 2.00E-20
264- 12: transcript:Zm00001d024777_T001 transcript:KXG32310 3.00E-14
264- 13: transcript:Zm00001d024778_T001 transcript:KXG32309 1.00E-29
264- 14: transcript:Zm00001d024783_T001 transcript:KXG32305 2.00E-79
Alignment 265: score=1354.0 e_value=2.5e-105 N=32 10&4 plus
265- 0: transcript:KXG19603 transcript:Zm00001d052185_T001 7.00E-115
265- 1: transcript:OQU76058 transcript:Zm00001d052186_T001 6.00E-162
265- 2: transcript:OQU76061 transcript:Zm00001d052189_T001 2.00E-89

```
265-  3: transcript:OQU76064           transcript:Zm00001d052191_T001  8.00E-39
265-  4: transcript:OQU76066           transcript:Zm00001d052193_T012 2.00E-126
265-  5: transcript:EER89430           transcript:Zm00001d052194_T001  3.00E-36
265-  6: transcript:EER88092           transcript:Zm00001d052198_T002  4.00E-93
265-  7: transcript:KXG19617           transcript:Zm00001d052205_T001  2.00E-93
265-  8: transcript:EER89439           transcript:Zm00001d052206_T001 4.00E-127
265-  9: transcript:EER89441           transcript:Zm00001d052211_T001 2.00E-164
265- 10: transcript:OQU76075           transcript:Zm00001d052212_T001  2.00E-50
265- 11: transcript:KXG19622           transcript:Zm00001d052219_T002 4.00E-163
265- 12: transcript:KXG19623           transcript:Zm00001d052227_T001 8.00E-169
265- 13: transcript:EER89448           transcript:Zm00001d052229_T001 3.00E-109
265- 14: transcript:KXG19625           transcript:Zm00001d052232_T002         0
265- 15: transcript:EER89450           transcript:Zm00001d052233_T001 1.00E-167
265- 16: transcript:OQU76080           transcript:Zm00001d052234_T001  8.00E-25
265- 17: transcript:EER88105           transcript:Zm00001d052239_T001  8.00E-59
265- 18: transcript:OQU76097           transcript:Zm00001d052248_T009         0
265- 19: transcript:EER88108           transcript:Zm00001d052252_T001 2.00E-107
265- 20: transcript:EER88111           transcript:Zm00001d052254_T001  5.00E-40
265- 21: transcript:EER88112           transcript:Zm00001d052256_T001 8.00E-125
265- 22: transcript:EER89469           transcript:Zm00001d052258_T001 5.00E-156
265- 23: transcript:EER89470           transcript:Zm00001d052259_T001  2.00E-71
265- 24: transcript:EER89471           transcript:Zm00001d052260_T001         0
265- 25: transcript:EER89472           transcript:Zm00001d052261_T001 2.00E-123
265- 26: transcript:KXG19644           transcript:Zm00001d052263_T001         0
265- 27: transcript:EER89480           transcript:Zm00001d052268_T001  3.00E-87
265- 28: transcript:EER88128           transcript:Zm00001d052269_T001         0
265- 29: transcript:EER89486           transcript:Zm00001d052270_T005 1.00E-166
265- 30: transcript:KXG19670           transcript:Zm00001d052271_T003         0
265- 31: transcript:EER88130           transcript:Zm00001d052273_T004 2.00E-105
## Alignment 266: score=553.0 e_value=1.7e-28 N=13 10&4 plus
266-  0: transcript:Zm00001d026050_T001 transcript:EES05727                  0
266-  1: transcript:Zm00001d026055_T001 transcript:EES05729                  0
266-  2: transcript:Zm00001d026061_T001 transcript:OQU85631           1.00E-29
266-  3: transcript:Zm00001d026070_T002 transcript:EES07458                  0
266-  4: transcript:Zm00001d026076_T001 transcript:EES07463                  0
266-  5: transcript:Zm00001d026078_T001 transcript:KXG31015          3.00E-114
266-  6: transcript:Zm00001d026079_T001 transcript:EES05735                  0
266-  7: transcript:Zm00001d026083_T001 transcript:EES05736                  0
266-  8: transcript:Zm00001d026091_T001 transcript:KXG31018           7.00E-54
266-  9: transcript:Zm00001d026096_T001 transcript:KXG31020           8.00E-98
266- 10: transcript:Zm00001d026097_T005 transcript:EES07467                  0
266- 11: transcript:Zm00001d026102_T001 transcript:EES05741                  0
266- 12: transcript:Zm00001d026106_T001 transcript:KXG31025           2.00E-47
## Alignment 267: score=546.0 e_value=9.3e-32 N=13 10&4 plus
267-  0: transcript:Zm00001d026120_T001 transcript:EES07470           6.00E-60
267-  1: transcript:Zm00001d026121_T001 transcript:EES07473                  0
267-  2: transcript:Zm00001d026126_T001 transcript:EES05745                  0
267-  3: transcript:Zm00001d026130_T002 transcript:OQU85656                  0
267-  4: transcript:Zm00001d026133_T001 transcript:KXG31039           8.00E-63
267-  5: transcript:Zm00001d026135_T002 transcript:EES07479                  0
267-  6: transcript:Zm00001d026140_T001 transcript:KXG31053                  0
267-  7: transcript:Zm00001d026141_T001 transcript:KXG31055           1.00E-19
267-  8: transcript:Zm00001d026152_T001 transcript:EES05761           7.00E-08
267-  9: transcript:Zm00001d026153_T001 transcript:EES07487           8.00E-25
```

```
267- 10: transcript:Zm00001d026154_T002 transcript:KXG31059             2.00E-67
267- 11: transcript:Zm00001d026158_T001 transcript:EES05768                    0
267- 12: transcript:Zm00001d026160_T001 transcript:EES07497             2.00E-36
## Alignment 268: score=507.0 e_value=1.2e-26 N=12 10&4 plus
268-  0: transcript:Zm00001d026160_T001 transcript:KXG31076             8.00E-37
268-  1: transcript:Zm00001d026166_T002 transcript:OQU85697             8.00E-76
268-  2: transcript:Zm00001d026169_T001 transcript:EES05780            5.00E-121
268-  3: transcript:Zm00001d026170_T001 transcript:EES07509            4.00E-131
268-  4: transcript:Zm00001d026173_T001 transcript:EES05783             4.00E-39
268-  5: transcript:Zm00001d026177_T001 transcript:KXG31086            1.00E-150
268-  6: transcript:Zm00001d026180_T001 transcript:KXG31088             1.00E-44
268-  7: transcript:Zm00001d026182_T001 transcript:EES07513             7.00E-53
268-  8: transcript:Zm00001d026185_T001 transcript:EES07516            3.00E-175
268-  9: transcript:Zm00001d026189_T001 transcript:EES05790            1.00E-150
268- 10: transcript:Zm00001d026190_T001 transcript:EES07522            3.00E-104
268- 11: transcript:Zm00001d026191_T001 transcript:EES05793             5.00E-96
## Alignment 269: score=475.0 e_value=1.2e-27 N=11 10&4 plus
269-  0: transcript:Zm00001d025861_T001 transcript:EES05451            9.00E-104
269-  1: transcript:Zm00001d025863_T001 transcript:KXG30661             2.00E-80
269-  2: transcript:Zm00001d025864_T001 transcript:EES07173            1.00E-103
269-  3: transcript:Zm00001d025868_T002 transcript:EES05457            8.00E-119
269-  4: transcript:Zm00001d025869_T003 transcript:KXG30670                    0
269-  5: transcript:Zm00001d025871_T001 transcript:EES05458            3.00E-155
269-  6: transcript:Zm00001d025872_T001 transcript:EES07181             5.00E-20
269-  7: transcript:Zm00001d025873_T001 transcript:EES05460             4.00E-34
269-  8: transcript:Zm00001d025874_T001 transcript:OQU85352             2.00E-35
269-  9: transcript:Zm00001d025887_T001 transcript:OQU85362                    0
269- 10: transcript:Zm00001d025891_T001 transcript:EES05469            2.00E-141
## Alignment 270: score=414.0 e_value=1.3e-20 N=10 10&4 plus
270-  0: transcript:Zm00001d025548_T001 transcript:EES07012                    0
270-  1: transcript:Zm00001d025549_T001 transcript:EES05288            7.00E-122
270-  2: transcript:Zm00001d025551_T002 transcript:EES05289                    0
270-  3: transcript:Zm00001d025552_T001 transcript:EES05291             4.00E-14
270-  4: transcript:Zm00001d025568_T001 transcript:KXG30480             7.00E-70
270-  5: transcript:Zm00001d025570_T006 transcript:OQU85190            5.00E-140
270-  6: transcript:Zm00001d025572_T001 transcript:OQU85191             3.00E-34
270-  7: transcript:Zm00001d025580_T001 transcript:EES07030                    0
270-  8: transcript:Zm00001d025581_T001 transcript:EES05307                    0
270-  9: transcript:Zm00001d025586_T001 transcript:EES05308            4.00E-149
## Alignment 271: score=413.0 e_value=2.8e-21 N=10 10&4 plus
271-  0: transcript:EER90151            transcript:Zm00001d053755_T001  2.00E-44
271-  1: transcript:EER88765            transcript:Zm00001d053756_T001  5.00E-62
271-  2: transcript:EER90156            transcript:Zm00001d053759_T001  1.00E-50
271-  3: transcript:OQU76823            transcript:Zm00001d053761_T001         0
271-  4: transcript:KXG20537            transcript:Zm00001d053763_T001         0
271-  5: transcript:EER90164            transcript:Zm00001d053765_T001         0
271-  6: transcript:EER90166            transcript:Zm00001d053769_T001         0
271-  7: transcript:EER88783            transcript:Zm00001d053778_T001         0
271-  8: transcript:EER90176            transcript:Zm00001d053779_T001  7.00E-28
271-  9: transcript:KXG20553            transcript:Zm00001d053783_T001         0
## Alignment 272: score=375.0 e_value=2.6e-20 N=9 10&4 plus
272-  0: transcript:EER88613            transcript:Zm00001d053625_T001  4.00E-64
272-  1: transcript:EER90017            transcript:Zm00001d053626_T001 5.00E-117
272-  2: transcript:EER88620            transcript:Zm00001d053639_T001  3.00E-25
```

```
272-  3: transcript:KXG20322           transcript:Zm00001d053641_T001  5.00E-77
272-  4: transcript:EER90022           transcript:Zm00001d053642_T001         0
272-  5: transcript:KXG20324           transcript:Zm00001d053643_T001 2.00E-104
272-  6: transcript:EER90026           transcript:Zm00001d053648_T005 3.00E-146
272-  7: transcript:EER88627           transcript:Zm00001d053649_T002         0
272-  8: transcript:EER90030           transcript:Zm00001d053659_T001 4.00E-118
## Alignment 273: score=372.0 e_value=2.1e-19 N=9 10&4 plus
273-  0: transcript:EER90210           transcript:Zm00001d053817_T001  4.00E-86
273-  1: transcript:EER90214           transcript:Zm00001d053818_T003         0
273-  2: transcript:EER90218           transcript:Zm00001d053819_T002         0
273-  3: transcript:OQU76888           transcript:Zm00001d053824_T001  2.00E-12
273-  4: transcript:EER90231           transcript:Zm00001d053829_T001  2.00E-51
273-  5: transcript:KXG20643           transcript:Zm00001d053831_T001  4.00E-32
273-  6: transcript:KXG20644           transcript:Zm00001d053834_T001         0
273-  7: transcript:EER88826           transcript:Zm00001d053839_T001  2.00E-63
273-  8: transcript:EER88830           transcript:Zm00001d053841_T014  4.00E-97
## Alignment 274: score=360.0 e_value=2.1e-16 N=8 10&4 plus
274-  0: transcript:OQU76020           transcript:Zm00001d052112_T001  4.00E-48
274-  1: transcript:OQU76029           transcript:Zm00001d052118_T001  6.00E-49
274-  2: transcript:KXG19565           transcript:Zm00001d052120_T001  1.00E-44
274-  3: transcript:EER88035           transcript:Zm00001d052122_T001  4.00E-24
274-  4: transcript:EER88036           transcript:Zm00001d052124_T002         0
274-  5: transcript:EER88037           transcript:Zm00001d052125_T001  5.00E-26
274-  6: transcript:EER89393           transcript:Zm00001d052133_T003         0
274-  7: transcript:EER89396           transcript:Zm00001d052137_T001  1.00E-94
## Alignment 275: score=346.0 e_value=3.6e-17 N=9 10&4 plus
275-  0: transcript:EER88428           transcript:Zm00001d053391_T001 3.00E-110
275-  1: transcript:KXG20091           transcript:Zm00001d053399_T001 7.00E-144
275-  2: transcript:OQU76494           transcript:Zm00001d053401_T014         0
275-  3: transcript:EER88432           transcript:Zm00001d053404_T001         0
275-  4: transcript:OQU76497           transcript:Zm00001d053406_T001  5.00E-16
275-  5: transcript:EER88443           transcript:Zm00001d053415_T001  7.00E-16
275-  6: transcript:EER89867           transcript:Zm00001d053425_T001         0
275-  7: transcript:KXG20113           transcript:Zm00001d053433_T001  1.00E-16
275-  8: transcript:EER89870           transcript:Zm00001d053434_T001  2.00E-20
## Alignment 276: score=324.0 e_value=2.6e-14 N=8 10&4 plus
276-  0: transcript:EER89969           transcript:Zm00001d053576_T001         0
276-  1: transcript:KXG20232           transcript:Zm00001d053578_T001         0
276-  2: transcript:EER88554           transcript:Zm00001d053585_T002         0
276-  3: transcript:EER89974           transcript:Zm00001d053587_T001         0
276-  4: transcript:EER88560           transcript:Zm00001d053589_T001 5.00E-129
276-  5: transcript:EER88566           transcript:Zm00001d053593_T001  1.00E-47
276-  6: transcript:KXG20269           transcript:Zm00001d053595_T004         0
276-  7: transcript:EER88571           transcript:Zm00001d053597_T006  3.00E-13
## Alignment 277: score=317.0 e_value=4e-11 N=7 10&4 plus
277-  0: transcript:Zm00001d025369_T002 transcript:EES05200                  0
277-  1: transcript:Zm00001d025371_T001 transcript:EES06921           4.00E-87
277-  2: transcript:Zm00001d025375_T001 transcript:KXG30352                  0
277-  3: transcript:Zm00001d025377_T001 transcript:KXG30353           2.00E-09
277-  4: transcript:Zm00001d025379_T001 transcript:EES05205          2.00E-172
277-  5: transcript:Zm00001d025380_T001 transcript:OQU85090           1.00E-63
277-  6: transcript:Zm00001d025382_T001 transcript:EES05210           2.00E-94
## Alignment 278: score=298.0 e_value=4e-10 N=7 10&4 plus
278-  0: transcript:Zm00001d025896_T001 transcript:EES05476           6.00E-59
```

```
278-  1: transcript:Zm00001d025900_T001 transcript:EES07199           7.00E-126
278-  2: transcript:Zm00001d025903_T004 transcript:EES05482                   0
278-  3: transcript:Zm00001d025908_T001 transcript:EES07205           5.00E-144
278-  4: transcript:Zm00001d025910_T001 transcript:OQU85375            9.00E-82
278-  5: transcript:Zm00001d025911_T002 transcript:EES05484                   0
278-  6: transcript:Zm00001d025916_T001 transcript:EES07211            5.00E-74
## Alignment 279: score=280.0 e_value=1e-10 N=7 10&4 plus
279-  0: transcript:Zm00001d025338_T001 transcript:EES06901            3.00E-72
279-  1: transcript:Zm00001d025345_T001 transcript:OQU85055            2.00E-20
279-  2: transcript:Zm00001d025346_T001 transcript:EES06907           7.00E-106
279-  3: transcript:Zm00001d025347_T001 transcript:EES05177            2.00E-30
279-  4: transcript:Zm00001d025354_T001 transcript:KXG30339                   0
279-  5: transcript:Zm00001d025360_T001 transcript:OQU85064           2.00E-130
279-  6: transcript:Zm00001d025361_T001 transcript:EES05192            2.00E-25
## Alignment 280: score=253.0 e_value=8.6e-09 N=6 10&4 plus
280-  0: transcript:EER89404            transcript:Zm00001d052168_T001 3.00E-156
280-  1: transcript:OQU76045            transcript:Zm00001d052170_T001         0
280-  2: transcript:KXG19588            transcript:Zm00001d052172_T001  1.00E-44
280-  3: transcript:EER88065            transcript:Zm00001d052176_T001  8.00E-07
280-  4: transcript:EER88070            transcript:Zm00001d052179_T001  1.00E-61
280-  5: transcript:EER88077            transcript:Zm00001d052180_T001 9.00E-114
## Alignment 281: score=516.0 e_value=1.2e-27 N=12 10&4 minus
281-  0: transcript:Zm00001d025920_T001 transcript:EES07549                   0
281-  1: transcript:Zm00001d025922_T001 transcript:KXG31133            6.00E-98
281-  2: transcript:Zm00001d025926_T001 transcript:EES05836           6.00E-105
281-  3: transcript:Zm00001d025930_T001 transcript:OQU85763            1.00E-47
281-  4: transcript:Zm00001d025932_T003 transcript:KXG31130            4.00E-68
281-  5: transcript:Zm00001d025933_T001 transcript:EES07545           5.00E-177
281-  6: transcript:Zm00001d025939_T003 transcript:KXG31127            9.00E-81
281-  7: transcript:Zm00001d025944_T001 transcript:EES05828            3.00E-83
281-  8: transcript:Zm00001d025947_T001 transcript:EES07542            3.00E-40
281-  9: transcript:Zm00001d025949_T001 transcript:OQU85754            2.00E-60
281- 10: transcript:Zm00001d025950_T002 transcript:EES05821           2.00E-171
281- 11: transcript:Zm00001d025951_T002 transcript:KXG31119                   0
## Alignment 282: score=497.0 e_value=2.6e-26 N=12 10&4 minus
282-  0: transcript:Zm00001d026002_T001 transcript:EES05726            3.00E-38
282-  1: transcript:Zm00001d026005_T001 transcript:EES07445           2.00E-106
282-  2: transcript:Zm00001d026010_T001 transcript:EES05721           2.00E-171
282-  3: transcript:Zm00001d026012_T001 transcript:EES05720            7.00E-30
282-  4: transcript:Zm00001d026015_T001 transcript:OQU85624            6.00E-67
282-  5: transcript:Zm00001d026017_T001 transcript:EES05718            2.00E-69
282-  6: transcript:Zm00001d026018_T001 transcript:OQU85618                   0
282-  7: transcript:Zm00001d026021_T001 transcript:EES05717            5.00E-57
282-  8: transcript:Zm00001d026026_T001 transcript:EES05711            5.00E-18
282-  9: transcript:Zm00001d026028_T001 transcript:KXG30991            4.00E-15
282- 10: transcript:Zm00001d026032_T001 transcript:KXG30986            5.00E-97
282- 11: transcript:Zm00001d026042_T001 transcript:OQU85596            1.00E-48
## Alignment 283: score=469.0 e_value=9.4e-27 N=12 10&4 minus
283-  0: transcript:Zm00001d026193_T001 transcript:EES07420                   0
283-  1: transcript:Zm00001d026194_T003 transcript:EES05689            7.00E-75
283-  2: transcript:Zm00001d026203_T001 transcript:EES05685            4.00E-99
283-  3: transcript:Zm00001d026206_T001 transcript:OQU85574           5.00E-110
283-  4: transcript:Zm00001d026207_T001 transcript:EES05682                   0
283-  5: transcript:Zm00001d026214_T002 transcript:KXG30949           1.00E-132
```

```
283-  6: transcript:Zm00001d026218_T001 transcript:EES07413            5.00E-80
283-  7: transcript:Zm00001d026222_T001 transcript:KXG30941            1.00E-23
283-  8: transcript:Zm00001d026237_T001 transcript:OQU85560           2.00E-125
283-  9: transcript:Zm00001d026240_T006 transcript:EES07405           9.00E-126
283- 10: transcript:Zm00001d026245_T001 transcript:OQU85554            2.00E-19
283- 11: transcript:Zm00001d026246_T001 transcript:EES05664                   0
## Alignment 284: score=401.0 e_value=7.5e-23 N=10 10&4 minus
284-  0: transcript:OQU76779            transcript:Zm00001d053750_T001  1.00E-18
284-  1: transcript:KXG20488            transcript:Zm00001d053749_T002 3.00E-164
284-  2: transcript:EER90114            transcript:Zm00001d053748_T001  1.00E-46
284-  3: transcript:EER88729            transcript:Zm00001d053746_T001 2.00E-104
284-  4: transcript:EER90127            transcript:Zm00001d053745_T001 5.00E-174
284-  5: transcript:EER88742            transcript:Zm00001d053740_T001         0
284-  6: transcript:EER90136            transcript:Zm00001d053739_T001 3.00E-136
284-  7: transcript:EER90142            transcript:Zm00001d053731_T001  3.00E-42
284-  8: transcript:EER88756            transcript:Zm00001d053729_T001  7.00E-17
284-  9: transcript:EER88757            transcript:Zm00001d053725_T002 2.00E-137
## Alignment 285: score=325.0 e_value=1.5e-10 N=7 10&4 minus
285-  0: transcript:Zm00001d025953_T003 transcript:KXG31117            3.00E-90
285-  1: transcript:Zm00001d025957_T001 transcript:EES07539            3.00E-79
285-  2: transcript:Zm00001d025958_T001 transcript:EES05817                   0
285-  3: transcript:Zm00001d025959_T001 transcript:EES05816           1.00E-145
285-  4: transcript:Zm00001d025960_T002 transcript:EES05815            3.00E-62
285-  5: transcript:Zm00001d025964_T001 transcript:EES05814            5.00E-63
285-  6: transcript:Zm00001d025977_T001 transcript:EES05808                   0
## Alignment 286: score=1834.0 e_value=4.2e-160 N=43 10&5 plus
286-  0: transcript:KXG20829            transcript:Zm00001d014742_T001         0
286-  1: transcript:OQU77072            transcript:Zm00001d014743_T001  2.00E-32
286-  2: transcript:OQU77073            transcript:Zm00001d014744_T001 7.00E-135
286-  3: transcript:EER90359            transcript:Zm00001d014745_T001 1.00E-135
286-  4: transcript:EER88939            transcript:Zm00001d014748_T001         0
286-  5: transcript:EER90361            transcript:Zm00001d014749_T001  2.00E-79
286-  6: transcript:OQU77077            transcript:Zm00001d014751_T003         0
286-  7: transcript:OQU77081            transcript:Zm00001d014752_T001 3.00E-131
286-  8: transcript:EER88944            transcript:Zm00001d014753_T002         0
286-  9: transcript:EER88947            transcript:Zm00001d014755_T001 2.00E-169
286- 10: transcript:KXG20841            transcript:Zm00001d014756_T006         0
286- 11: transcript:KXG20844            transcript:Zm00001d014757_T001 4.00E-107
286- 12: transcript:EER88948            transcript:Zm00001d014758_T001  1.00E-49
286- 13: transcript:OQU77085            transcript:Zm00001d014759_T001         0
286- 14: transcript:EER88950            transcript:Zm00001d014760_T002         0
286- 15: transcript:EER90369            transcript:Zm00001d014761_T001         0
286- 16: transcript:EER88951            transcript:Zm00001d014762_T001 1.00E-160
286- 17: transcript:EER90371            transcript:Zm00001d014763_T001         0
286- 18: transcript:KXG20848            transcript:Zm00001d014764_T006         0
286- 19: transcript:KXG20850            transcript:Zm00001d014765_T001 3.00E-133
286- 20: transcript:OQU77093            transcript:Zm00001d014766_T001         0
286- 21: transcript:EER90378            transcript:Zm00001d014770_T001 2.00E-105
286- 22: transcript:EER90386            transcript:Zm00001d014771_T001         0
286- 23: transcript:OQU77107            transcript:Zm00001d014772_T001  1.00E-79
286- 24: transcript:KXG20873            transcript:Zm00001d014773_T001         0
286- 25: transcript:EER90388            transcript:Zm00001d014774_T001  1.00E-43
286- 26: transcript:KXG20882            transcript:Zm00001d014775_T002         0
286- 27: transcript:EER90390            transcript:Zm00001d014778_T001  2.00E-32
```

```
286- 28: transcript:EER90391           transcript:Zm00001d014779_T002  3.00E-34
286- 29: transcript:EER90392           transcript:Zm00001d014780_T001 4.00E-116
286- 30: transcript:EER88960           transcript:Zm00001d014781_T001  4.00E-84
286- 31: transcript:EER90394           transcript:Zm00001d014782_T001         0
286- 32: transcript:KXG20884           transcript:Zm00001d014783_T001         0
286- 33: transcript:EER88961           transcript:Zm00001d014785_T001 4.00E-170
286- 34: transcript:KXG20887           transcript:Zm00001d014786_T001         0
286- 35: transcript:EER90395           transcript:Zm00001d014788_T002         0
286- 36: transcript:KXG20892           transcript:Zm00001d014789_T002 1.00E-146
286- 37: transcript:EER88964           transcript:Zm00001d014790_T001  1.00E-61
286- 38: transcript:EER88966           transcript:Zm00001d014791_T001 3.00E-131
286- 39: transcript:KXG20895           transcript:Zm00001d014792_T003         0
286- 40: transcript:KXG20897           transcript:Zm00001d014793_T001         0
286- 41: transcript:EER88969           transcript:Zm00001d014796_T001 7.00E-153
286- 42: transcript:EER90402           transcript:Zm00001d014797_T006         0
## Alignment 287: score=1076.0 e_value=3.6e-76 N=25 10&5 plus
287-  0: transcript:EER88892           transcript:Zm00001d014640_T036         0
287-  1: transcript:OQU76985           transcript:Zm00001d014641_T001         0
287-  2: transcript:EER88890           transcript:Zm00001d014642_T001         0
287-  3: transcript:EER88891           transcript:Zm00001d014648_T001  3.00E-48
287-  4: transcript:KXG20754           transcript:Zm00001d014650_T001         0
287-  5: transcript:EER90312           transcript:Zm00001d014655_T001         0
287-  6: transcript:EER88899           transcript:Zm00001d014656_T003         0
287-  7: transcript:EER88894           transcript:Zm00001d014657_T001         0
287-  8: transcript:EER88895           transcript:Zm00001d014658_T001         0
287-  9: transcript:EER90314           transcript:Zm00001d014659_T001  7.00E-73
287- 10: transcript:EER88897           transcript:Zm00001d014663_T001         0
287- 11: transcript:KXG20763           transcript:Zm00001d014664_T001         0
287- 12: transcript:EER88901           transcript:Zm00001d014665_T002         0
287- 13: transcript:KXG20766           transcript:Zm00001d014666_T001         0
287- 14: transcript:EER90318           transcript:Zm00001d014667_T001  1.00E-94
287- 15: transcript:KXG20769           transcript:Zm00001d014668_T001         0
287- 16: transcript:EER90320           transcript:Zm00001d014669_T001         0
287- 17: transcript:EER88903           transcript:Zm00001d014671_T001 8.00E-101
287- 18: transcript:OQU76995           transcript:Zm00001d014673_T001         0
287- 19: transcript:KXG20771           transcript:Zm00001d014674_T003         0
287- 20: transcript:EER90326           transcript:Zm00001d014679_T001  5.00E-44
287- 21: transcript:EER90327           transcript:Zm00001d014680_T001         0
287- 22: transcript:EER88905           transcript:Zm00001d014681_T001 4.00E-144
287- 23: transcript:EER90331           transcript:Zm00001d014682_T001  5.00E-61
287- 24: transcript:KXG20779           transcript:Zm00001d014683_T011         0
## Alignment 288: score=1048.0 e_value=3e-72 N=23 10&5 plus
288-  0: transcript:KXG20638           transcript:Zm00001d014451_T001         0
288-  1: transcript:EER90227           transcript:Zm00001d014455_T001 2.00E-160
288-  2: transcript:EER88824           transcript:Zm00001d014459_T001         0
288-  3: transcript:KXG20642           transcript:Zm00001d014462_T002  2.00E-59
288-  4: transcript:EER90231           transcript:Zm00001d014463_T001  8.00E-74
288-  5: transcript:KXG20645           transcript:Zm00001d014464_T001  4.00E-13
288-  6: transcript:EER88826           transcript:Zm00001d014467_T002         0
288-  7: transcript:KXG20650           transcript:Zm00001d014468_T001         0
288-  8: transcript:OQU76897           transcript:Zm00001d014471_T001         0
288-  9: transcript:EER88830           transcript:Zm00001d014472_T004 1.00E-152
288- 10: transcript:OQU76902           transcript:Zm00001d014481_T001         0
288- 11: transcript:OQU76903           transcript:Zm00001d014487_T001         0
```

```
288- 12: transcript:EER90241          transcript:Zm00001d014488_T001 7.00E-129
288- 13: transcript:EER88831          transcript:Zm00001d014489_T010         0
288- 14: transcript:EER88832          transcript:Zm00001d014491_T001  4.00E-73
288- 15: transcript:EER90246          transcript:Zm00001d014493_T001  2.00E-43
288- 16: transcript:KXG20663          transcript:Zm00001d014494_T001  2.00E-30
288- 17: transcript:OQU76916          transcript:Zm00001d014495_T001         0
288- 18: transcript:EER90247          transcript:Zm00001d014496_T001         0
288- 19: transcript:EER88837          transcript:Zm00001d014497_T001  7.00E-97
288- 20: transcript:EER88838          transcript:Zm00001d014498_T001         0
288- 21: transcript:EER88839          transcript:Zm00001d014499_T004         0
288- 22: transcript:EER90260          transcript:Zm00001d014500_T003         0
## Alignment 289: score=812.0 e_value=3.1e-54 N=19 10&5 plus
289-  0: transcript:OQU76549          transcript:Zm00001d016216_T001  5.00E-14
289-  1: transcript:EER88498          transcript:Zm00001d016223_T001         0
289-  2: transcript:EER88499          transcript:Zm00001d016231_T001  2.00E-29
289-  3: transcript:EER89914          transcript:Zm00001d016234_T001         0
289-  4: transcript:KXG20157          transcript:Zm00001d016237_T001 3.00E-104
289-  5: transcript:EER89918          transcript:Zm00001d016253_T001  2.00E-90
289-  6: transcript:KXG20158          transcript:Zm00001d016254_T001  3.00E-52
289-  7: transcript:EER89919          transcript:Zm00001d016255_T001  6.00E-90
289-  8: transcript:EER89920          transcript:Zm00001d016256_T001  2.00E-41
289-  9: transcript:KXG20160          transcript:Zm00001d016260_T001  5.00E-39
289- 10: transcript:KXG20161          transcript:Zm00001d016269_T001  3.00E-39
289- 11: transcript:EER89925          transcript:Zm00001d016271_T001  2.00E-45
289- 12: transcript:KXG20162          transcript:Zm00001d016273_T001         0
289- 13: transcript:EER88512          transcript:Zm00001d016276_T001  5.00E-90
289- 14: transcript:OQU76569          transcript:Zm00001d016285_T001  2.00E-30
289- 15: transcript:KXG20189          transcript:Zm00001d016287_T001  1.00E-13
289- 16: transcript:EER88529          transcript:Zm00001d016294_T001 4.00E-105
289- 17: transcript:EER89946          transcript:Zm00001d016298_T001  2.00E-79
289- 18: transcript:KXG20202          transcript:Zm00001d016308_T003         0
## Alignment 290: score=735.0 e_value=3.5e-52 N=18 10&5 plus
290-  0: transcript:KXG20781          transcript:Zm00001d014685_T009         0
290-  1: transcript:EER88910          transcript:Zm00001d014688_T001         0
290-  2: transcript:OQU77002          transcript:Zm00001d014689_T006         0
290-  3: transcript:EER90336          transcript:Zm00001d014690_T014         0
290-  4: transcript:EER90333          transcript:Zm00001d014692_T001 3.00E-113
290-  5: transcript:OQU77004          transcript:Zm00001d014695_T001         0
290-  6: transcript:EER88914          transcript:Zm00001d014696_T002         0
290-  7: transcript:KXG20794          transcript:Zm00001d014697_T002 3.00E-169
290-  8: transcript:OQU77017          transcript:Zm00001d014698_T006         0
290-  9: transcript:OQU77022          transcript:Zm00001d014701_T002         0
290- 10: transcript:OQU77024          transcript:Zm00001d014702_T003  2.00E-46
290- 11: transcript:OQU77029          transcript:Zm00001d014703_T001         0
290- 12: transcript:EER88920          transcript:Zm00001d014705_T002         0
290- 13: transcript:KXG20799          transcript:Zm00001d014715_T002         0
290- 14: transcript:OQU77030          transcript:Zm00001d014716_T002         0
290- 15: transcript:EER88922          transcript:Zm00001d014717_T001         0
290- 16: transcript:EER88923          transcript:Zm00001d014718_T001 6.00E-125
290- 17: transcript:EER90345          transcript:Zm00001d014719_T005  3.00E-85
## Alignment 291: score=659.0 e_value=2.9e-39 N=15 10&5 plus
291-  0: transcript:EER88873          transcript:Zm00001d014583_T001         0
291-  1: transcript:KXG20719          transcript:Zm00001d014584_T004 3.00E-133
291-  2: transcript:OQU76963          transcript:Zm00001d014585_T002 3.00E-139
```

```
291-  3: transcript:KXG20722          transcript:Zm00001d014587_T001          0
291-  4: transcript:EER90290          transcript:Zm00001d014594_T001          0
291-  5: transcript:EER88876          transcript:Zm00001d014595_T001          0
291-  6: transcript:KXG20728          transcript:Zm00001d014596_T001          0
291-  7: transcript:EER90291          transcript:Zm00001d014597_T001 6.00E-148
291-  8: transcript:EER90296          transcript:Zm00001d014600_T001 2.00E-117
291-  9: transcript:EER90297          transcript:Zm00001d014606_T001 6.00E-137
291- 10: transcript:KXG20741          transcript:Zm00001d014609_T002          0
291- 11: transcript:OQU76979          transcript:Zm00001d014610_T001 6.00E-165
291- 12: transcript:EER90300          transcript:Zm00001d014611_T001 2.00E-122
291- 13: transcript:EER90301          transcript:Zm00001d014612_T001          0
291- 14: transcript:EER88885          transcript:Zm00001d014613_T001          0
## Alignment 292: score=641.0 e_value=4.6e-39 N=15 10&5 plus
292-  0: transcript:EER90018          transcript:Zm00001d015759_T001  1.00E-98
292-  1: transcript:EER88620          transcript:Zm00001d015767_T002  2.00E-23
292-  2: transcript:KXG20322          transcript:Zm00001d015776_T001  9.00E-79
292-  3: transcript:EER90022          transcript:Zm00001d015778_T001          0
292-  4: transcript:KXG20324          transcript:Zm00001d015780_T002 1.00E-106
292-  5: transcript:EER88625          transcript:Zm00001d015783_T001          0
292-  6: transcript:EER90026          transcript:Zm00001d015785_T005          0
292-  7: transcript:EER88627          transcript:Zm00001d015788_T001          0
292-  8: transcript:OQU76685          transcript:Zm00001d015789_T002          0
292-  9: transcript:EER88633          transcript:Zm00001d015798_T001          0
292- 10: transcript:EER88634          transcript:Zm00001d015804_T013 5.00E-176
292- 11: transcript:KXG20343          transcript:Zm00001d015810_T001  6.00E-95
292- 12: transcript:OQU76691          transcript:Zm00001d015815_T001 6.00E-154
292- 13: transcript:EER88642          transcript:Zm00001d015820_T002  6.00E-52
292- 14: transcript:EER88643          transcript:Zm00001d015821_T001 4.00E-106
## Alignment 293: score=542.0 e_value=7.3e-33 N=13 10&5 plus
293-  0: transcript:KXG20980          transcript:Zm00001d014876_T037          0
293-  1: transcript:EER90438          transcript:Zm00001d014879_T002          0
293-  2: transcript:EER90439          transcript:Zm00001d014882_T001 5.00E-166
293-  3: transcript:KXG20969          transcript:Zm00001d014883_T001 1.00E-120
293-  4: transcript:EER90440          transcript:Zm00001d014885_T001 4.00E-150
293-  5: transcript:KXG20971          transcript:Zm00001d014887_T005          0
293-  6: transcript:KXG20973          transcript:Zm00001d014888_T001  1.00E-34
293-  7: transcript:EER89014          transcript:Zm00001d014889_T002  5.00E-61
293-  8: transcript:KXG20978          transcript:Zm00001d014890_T001          0
293-  9: transcript:EER89015          transcript:Zm00001d014894_T001 1.00E-102
293- 10: transcript:EER90446          transcript:Zm00001d014895_T006          0
293- 11: transcript:EER90447          transcript:Zm00001d014896_T001          0
293- 12: transcript:EER90448          transcript:Zm00001d014897_T005          0
## Alignment 294: score=445.0 e_value=2.5e-28 N=13 10&5 plus
294-  0: transcript:EER89811          transcript:Zm00001d016041_T001 6.00E-109
294-  1: transcript:OQU76459          transcript:Zm00001d016052_T001  1.00E-23
294-  2: transcript:EER89830          transcript:Zm00001d016063_T006          0
294-  3: transcript:EER88424          transcript:Zm00001d016075_T001          0
294-  4: transcript:EER89844          transcript:Zm00001d016076_T001  5.00E-36
294-  5: transcript:EER88427          transcript:Zm00001d016081_T002          0
294-  6: transcript:OQU76485          transcript:Zm00001d016083_T001  7.00E-09
294-  7: transcript:EER88428          transcript:Zm00001d016095_T001 2.00E-101
294-  8: transcript:KXG20091          transcript:Zm00001d016106_T001 3.00E-136
294-  9: transcript:OQU76499          transcript:Zm00001d016119_T001  2.00E-81
294- 10: transcript:EER89867          transcript:Zm00001d016130_T001 4.00E-155
```

294- 11: transcript:OQU76505 transcript:Zm00001d016140_T001 3.00E-18
294- 12: transcript:KXG20125 transcript:Zm00001d016145_T001 1.00E-05
Alignment 295: score=411.0 e_value=1.9e-21 N=9 10&5 plus
295- 0: transcript:EER88841 transcript:Zm00001d014512_T001 0
295- 1: transcript:KXG20670 transcript:Zm00001d014513_T001 3.00E-49
295- 2: transcript:EER88842 transcript:Zm00001d014516_T001 0
295- 3: transcript:OQU76929 transcript:Zm00001d014517_T001 2.00E-117
295- 4: transcript:EER90263 transcript:Zm00001d014518_T001 3.00E-62
295- 5: transcript:KXG20671 transcript:Zm00001d014519_T001 3.00E-17
295- 6: transcript:KXG20672 transcript:Zm00001d014520_T001 5.00E-38
295- 7: transcript:KXG20673 transcript:Zm00001d014521_T001 0
295- 8: transcript:OQU76934 transcript:Zm00001d014525_T001 0
Alignment 296: score=389.0 e_value=3.3e-18 N=9 10&5 plus
296- 0: transcript:EER88970 transcript:Zm00001d014804_T001 0
296- 1: transcript:KXG20903 transcript:Zm00001d014806_T001 7.00E-120
296- 2: transcript:EER88973 transcript:Zm00001d014807_T001 2.00E-66
296- 3: transcript:OQU77119 transcript:Zm00001d014808_T001 0
296- 4: transcript:OQU77122 transcript:Zm00001d014809_T003 0
296- 5: transcript:OQU77123 transcript:Zm00001d014811_T001 5.00E-138
296- 6: transcript:KXG20906 transcript:Zm00001d014812_T005 1.00E-85
296- 7: transcript:EER88977 transcript:Zm00001d014814_T002 0
296- 8: transcript:OQU77126 transcript:Zm00001d014813_T024 0
Alignment 297: score=369.0 e_value=5.4e-15 N=8 10&5 plus
297- 0: transcript:EER90437 transcript:Zm00001d014845_T001 0
297- 1: transcript:KXG20947 transcript:Zm00001d014846_T001 1.00E-81
297- 2: transcript:EER88998 transcript:Zm00001d014848_T001 0
297- 3: transcript:EER90432 transcript:Zm00001d014849_T003 0
297- 4: transcript:EER90433 transcript:Zm00001d014850_T002 0
297- 5: transcript:KXG20954 transcript:Zm00001d014852_T001 0
297- 6: transcript:OQU77165 transcript:Zm00001d014853_T002 0
297- 7: transcript:KXG20955 transcript:Zm00001d014858_T001 5.00E-59
Alignment 298: score=271.0 e_value=3.2e-10 N=6 10&5 plus
298- 0: transcript:EER88885 transcript:Zm00001d014617_T001 6.00E-143
298- 1: transcript:KXG20746 transcript:Zm00001d014620_T001 4.00E-30
298- 2: transcript:EER90304 transcript:Zm00001d014621_T001 2.00E-35
298- 3: transcript:KXG20747 transcript:Zm00001d014623_T001 6.00E-178
298- 4: transcript:EER90306 transcript:Zm00001d014626_T002 0
298- 5: transcript:EER90307 transcript:Zm00001d014628_T001 4.00E-84
Alignment 299: score=253.0 e_value=5.3e-09 N=6 10&5 plus
299- 0: transcript:KXG20822 transcript:Zm00001d014728_T006 0
299- 1: transcript:EER88930 transcript:Zm00001d014729_T001 4.00E-133
299- 2: transcript:EER88931 transcript:Zm00001d014731_T017 5.00E-67
299- 3: transcript:EER88935 transcript:Zm00001d014733_T001 5.00E-74
299- 4: transcript:KXG20827 transcript:Zm00001d014734_T001 0
299- 5: transcript:OQU77071 transcript:Zm00001d014736_T001 0
Alignment 300: score=797.0 e_value=2.7e-49 N=18 10&5 minus
300- 0: transcript:EER90210 transcript:Zm00001d014412_T001 9.00E-148
300- 1: transcript:EER88807 transcript:Zm00001d014405_T001 0
300- 2: transcript:EER90213 transcript:Zm00001d014391_T004 0
300- 3: transcript:EER90215 transcript:Zm00001d014386_T001 0
300- 4: transcript:EER88810 transcript:Zm00001d014385_T001 0
300- 5: transcript:EER88811 transcript:Zm00001d014382_T003 0
300- 6: transcript:EER90217 transcript:Zm00001d014381_T001 2.00E-70
300- 7: transcript:EER88812 transcript:Zm00001d014378_T001 0

```
300-  8: transcript:EER90218           transcript:Zm00001d014377_T002          0
300-  9: transcript:EER88814           transcript:Zm00001d014368_T001          0
300- 10: transcript:EER90219           transcript:Zm00001d014367_T004          0
300- 11: transcript:EER90222           transcript:Zm00001d014366_T002          0
300- 12: transcript:KXG20634           transcript:Zm00001d014365_T001  2.00E-21
300- 13: transcript:EER90224           transcript:Zm00001d014364_T002          0
300- 14: transcript:EER88815           transcript:Zm00001d014361_T001          0
300- 15: transcript:EER90225           transcript:Zm00001d014360_T001          0
300- 16: transcript:KXG20635           transcript:Zm00001d014358_T014          0
300- 17: transcript:KXG20637           transcript:Zm00001d014355_T001 2.00E-100
## Alignment 301: score=741.0 e_value=3.1e-48 N=18 10&5 minus
301-  0: transcript:KXG19603           transcript:Zm00001d018128_T001 2.00E-114
301-  1: transcript:OQU76058           transcript:Zm00001d018127_T001 6.00E-166
301-  2: transcript:OQU76061           transcript:Zm00001d018122_T001  1.00E-87
301-  3: transcript:OQU76064           transcript:Zm00001d018118_T001  2.00E-36
301-  4: transcript:KXG19612           transcript:Zm00001d018117_T001  3.00E-06
301-  5: transcript:OQU76066           transcript:Zm00001d018113_T006          0
301-  6: transcript:EER88092           transcript:Zm00001d018112_T004  1.00E-62
301-  7: transcript:EER89439           transcript:Zm00001d018105_T001 2.00E-104
301-  8: transcript:OQU76075           transcript:Zm00001d018103_T002  2.00E-74
301-  9: transcript:EER88097           transcript:Zm00001d018090_T002          0
301- 10: transcript:KXG19623           transcript:Zm00001d018082_T001 1.00E-159
301- 11: transcript:EER89448           transcript:Zm00001d018081_T001 2.00E-102
301- 12: transcript:KXG19625           transcript:Zm00001d018078_T002          0
301- 13: transcript:OQU76080           transcript:Zm00001d018076_T001  1.00E-24
301- 14: transcript:EER88105           transcript:Zm00001d018072_T001  1.00E-60
301- 15: transcript:OQU76090           transcript:Zm00001d018065_T001  2.00E-65
301- 16: transcript:EER88108           transcript:Zm00001d018058_T001  3.00E-94
301- 17: transcript:EER88111           transcript:Zm00001d018056_T001  6.00E-47
## Alignment 302: score=621.0 e_value=2.7e-34 N=14 10&5 minus
302-  0: transcript:EER89468           transcript:Zm00001d018047_T002  2.00E-34
302-  1: transcript:EER88112           transcript:Zm00001d018045_T001 5.00E-123
302-  2: transcript:EER89469           transcript:Zm00001d018041_T001 8.00E-157
302-  3: transcript:EER89470           transcript:Zm00001d018040_T002  7.00E-84
302-  4: transcript:EER89472           transcript:Zm00001d018037_T001 2.00E-176
302-  5: transcript:KXG19645           transcript:Zm00001d018035_T001  4.00E-31
302-  6: transcript:KXG19644           transcript:Zm00001d018033_T001          0
302-  7: transcript:KXG19653           transcript:Zm00001d018029_T001  8.00E-83
302-  8: transcript:EER89480           transcript:Zm00001d018028_T001  7.00E-91
302-  9: transcript:EER89482           transcript:Zm00001d018027_T001  3.00E-19
302- 10: transcript:KXG19659           transcript:Zm00001d018025_T001          0
302- 11: transcript:EER88128           transcript:Zm00001d018024_T001          0
302- 12: transcript:KXG19666           transcript:Zm00001d018017_T001  4.00E-40
302- 13: transcript:OQU76123           transcript:Zm00001d018016_T001 4.00E-157
## Alignment 303: score=525.0 e_value=1.3e-26 N=12 10&5 minus
303-  0: transcript:OQU76944           transcript:Zm00001d014571_T001          0
303-  1: transcript:KXG20697           transcript:Zm00001d014569_T002          0
303-  2: transcript:OQU76948           transcript:Zm00001d014568_T023          0
303-  3: transcript:EER88863           transcript:Zm00001d014565_T001  2.00E-87
303-  4: transcript:KXG20703           transcript:Zm00001d014564_T001          0
303-  5: transcript:EER88862           transcript:Zm00001d014563_T001 6.00E-154
303-  6: transcript:EER90283           transcript:Zm00001d014562_T001          0
303-  7: transcript:EER90284           transcript:Zm00001d014559_T001  4.00E-91
303-  8: transcript:OQU76953           transcript:Zm00001d014556_T001  1.00E-25
```

303- 9: transcript:EER88869   transcript:Zm00001d014555_T001   0
303- 10: transcript:EER88867   transcript:Zm00001d014554_T001   0
303- 11: transcript:OQU76954   transcript:Zm00001d014553_T001   0
Alignment 304: score=488.0 e_value=1.4e-22 N=11 10&5 minus
304- 0: transcript:EER90136   transcript:Zm00001d015490_T001 4.00E-29
304- 1: transcript:EER88750   transcript:Zm00001d015477_T001   0
304- 2: transcript:KXG20510   transcript:Zm00001d015476_T001 2.00E-30
304- 3: transcript:EER90142   transcript:Zm00001d015473_T003 2.00E-42
304- 4: transcript:EER88756   transcript:Zm00001d015470_T001 9.00E-26
304- 5: transcript:EER90145   transcript:Zm00001d015468_T001 3.00E-103
304- 6: transcript:EER88760   transcript:Zm00001d015463_T001 4.00E-45
304- 7: transcript:EER90151   transcript:Zm00001d015461_T001 4.00E-39
304- 8: transcript:KXG20523   transcript:Zm00001d015457_T001 3.00E-49
304- 9: transcript:EER88765   transcript:Zm00001d015451_T001 5.00E-86
304- 10: transcript:EER90153   transcript:Zm00001d015448_T001 1.00E-20
Alignment 305: score=470.0 e_value=1.7e-25 N=11 10&5 minus
305- 0: transcript:EER88680   transcript:Zm00001d015686_T001 8.00E-06
305- 1: transcript:EER88683   transcript:Zm00001d015670_T001 4.00E-20
305- 2: transcript:KXG20422   transcript:Zm00001d015664_T001 6.00E-13
305- 3: transcript:EER88687   transcript:Zm00001d015658_T003   0
305- 4: transcript:OQU76743   transcript:Zm00001d015656_T001   0
305- 5: transcript:KXG20428   transcript:Zm00001d015649_T003   0
305- 6: transcript:KXG20463   transcript:Zm00001d015638_T004 2.00E-20
305- 7: transcript:EER90082   transcript:Zm00001d015636_T001 6.00E-115
305- 8: transcript:EER90085   transcript:Zm00001d015614_T001 3.00E-123
305- 9: transcript:EER90086   transcript:Zm00001d015613_T001 5.00E-101
305- 10: transcript:EER88696   transcript:Zm00001d015612_T001 2.00E-90
Alignment 306: score=376.0 e_value=1e-16 N=9 10&5 minus
306- 0: transcript:OQU76779   transcript:Zm00001d015550_T001 2.00E-15
306- 1: transcript:KXG20488   transcript:Zm00001d015549_T001 1.00E-163
306- 2: transcript:EER90114   transcript:Zm00001d015546_T001 4.00E-64
306- 3: transcript:EER88722   transcript:Zm00001d015527_T001   0
306- 4: transcript:OQU76785   transcript:Zm00001d015521_T001 3.00E-68
306- 5: transcript:OQU76787   transcript:Zm00001d015517_T001 7.00E-16
306- 6: transcript:EER88729   transcript:Zm00001d015515_T001 4.00E-113
306- 7: transcript:EER90127   transcript:Zm00001d015508_T001 9.00E-174
306- 8: transcript:EER90135   transcript:Zm00001d015504_T004   0
Alignment 307: score=360.0 e_value=2.1e-17 N=9 10&5 minus
307- 0: transcript:EER90164   transcript:Zm00001d015426_T001   0
307- 1: transcript:EER90165   transcript:Zm00001d015421_T001 1.00E-76
307- 2: transcript:EER90166   transcript:Zm00001d015414_T001   0
307- 3: transcript:KXG20552   transcript:Zm00001d015407_T001 9.00E-114
307- 4: transcript:EER88783   transcript:Zm00001d015401_T001   0
307- 5: transcript:EER90181   transcript:Zm00001d015394_T001 1.00E-107
307- 6: transcript:EER88786   transcript:Zm00001d015382_T001 9.00E-12
307- 7: transcript:KXG20559   transcript:Zm00001d015381_T001 1.00E-47
307- 8: transcript:KXG20562   transcript:Zm00001d015376_T002   0
Alignment 308: score=350.0 e_value=1.2e-15 N=8 10&5 minus
308- 0: transcript:KXG19701   transcript:Zm00001d017987_T003   0
308- 1: transcript:EER88156   transcript:Zm00001d017986_T005 3.00E-175
308- 2: transcript:OQU76159   transcript:Zm00001d017985_T002   0
308- 3: transcript:EER88158   transcript:Zm00001d017984_T001   0
308- 4: transcript:EER88160   transcript:Zm00001d017979_T002   0
308- 5: transcript:OQU76161   transcript:Zm00001d017978_T001   0

308- 6: transcript:KXG19704 transcript:Zm00001d017976_T001 0
308- 7: transcript:EER89526 transcript:Zm00001d017969_T001 8.00E-11
Alignment 309: score=343.0 e_value=2.2e-16 N=8 10&5 minus
309- 0: transcript:EER90336 transcript:Zm00001d015243_T001 0
309- 1: transcript:EER90333 transcript:Zm00001d015242_T002 8.00E-61
309- 2: transcript:OQU77004 transcript:Zm00001d015239_T007 7.00E-168
309- 3: transcript:OQU77011 transcript:Zm00001d015234_T002 0
309- 4: transcript:OQU77017 transcript:Zm00001d015233_T001 5.00E-126
309- 5: transcript:OQU77022 transcript:Zm00001d015226_T001 8.00E-123
309- 6: transcript:OQU77025 transcript:Zm00001d015215_T001 5.00E-36
309- 7: transcript:EER88921 transcript:Zm00001d015213_T001 0
Alignment 310: score=323.0 e_value=2.1e-11 N=7 10&5 minus
310- 0: transcript:OQU76034 transcript:Zm00001d018199_T001 0
310- 1: transcript:KXG19576 transcript:Zm00001d018198_T001 2.00E-69
310- 2: transcript:EER88045 transcript:Zm00001d018195_T001 0
310- 3: transcript:EER88046 transcript:Zm00001d018194_T001 3.00E-88
310- 4: transcript:EER88048 transcript:Zm00001d018191_T001 8.00E-58
310- 5: transcript:EER89401 transcript:Zm00001d018190_T001 1.00E-79
310- 6: transcript:EER88051 transcript:Zm00001d018183_T001 0
Alignment 311: score=263.0 e_value=9.7e-10 N=6 10&5 minus
311- 0: transcript:EER88701 transcript:Zm00001d015579_T001 5.00E-180
311- 1: transcript:EER90097 transcript:Zm00001d015578_T001 1.00E-103
311- 2: transcript:EER88702 transcript:Zm00001d015571_T001 3.00E-35
311- 3: transcript:OQU76768 transcript:Zm00001d015570_T002 0
311- 4: transcript:OQU76769 transcript:Zm00001d015569_T001 0
311- 5: transcript:EER88713 transcript:Zm00001d015556_T001 6.00E-144
Alignment 312: score=260.0 e_value=1.5e-09 N=6 10&5 minus
312- 0: transcript:KXG19779 transcript:Zm00001d017923_T001 8.00E-40
312- 1: transcript:EER89594 transcript:Zm00001d017918_T001 9.00E-115
312- 2: transcript:EER88218 transcript:Zm00001d017917_T001 1.00E-71
312- 3: transcript:EER88221 transcript:Zm00001d017914_T001 2.00E-93
312- 4: transcript:EER88222 transcript:Zm00001d017913_T001 0
312- 5: transcript:KXG19789 transcript:Zm00001d017911_T001 2.00E-33
Alignment 313: score=3117.0 e_value=5.5e-279 N=69 10&6 plus
313- 0: transcript:Zm00001d026548_T049 transcript:EES13049 0
313- 1: transcript:Zm00001d026549_T006 transcript:KXG27373 0
313- 2: transcript:Zm00001d026551_T002 transcript:OQU82533 1.00E-87
313- 3: transcript:Zm00001d026554_T001 transcript:EES11614 3.00E-59
313- 4: transcript:Zm00001d026555_T002 transcript:EES11616 1.00E-170
313- 5: transcript:Zm00001d026556_T001 transcript:KXG27378 3.00E-155
313- 6: transcript:Zm00001d026557_T001 transcript:EES11618 0
313- 7: transcript:Zm00001d026559_T001 transcript:EES13055 0
313- 8: transcript:Zm00001d026560_T001 transcript:EES11619 2.00E-142
313- 9: transcript:Zm00001d026562_T001 transcript:EES13057 5.00E-129
313- 10: transcript:Zm00001d026563_T001 transcript:OQU82539 7.00E-61
313- 11: transcript:Zm00001d026569_T001 transcript:KXG27385 0
313- 12: transcript:Zm00001d026572_T001 transcript:OQU82541 4.00E-54
313- 13: transcript:Zm00001d026573_T002 transcript:KXG27389 0
313- 14: transcript:Zm00001d026575_T001 transcript:EES11624 9.00E-124
313- 15: transcript:Zm00001d026576_T001 transcript:EES11626 0
313- 16: transcript:Zm00001d026577_T002 transcript:EES13064 0
313- 17: transcript:Zm00001d026578_T001 transcript:EES13065 4.00E-41
313- 18: transcript:Zm00001d026579_T001 transcript:EES11627 0
313- 19: transcript:Zm00001d026580_T002 transcript:OQU82544 0

313- 20: transcript:Zm00001d026581_T001 transcript:EES11630 0
313- 21: transcript:Zm00001d026582_T001 transcript:EES11631 0
313- 22: transcript:Zm00001d026584_T001 transcript:OQU82545 5.00E-168
313- 23: transcript:Zm00001d026585_T001 transcript:KXG27402 0
313- 24: transcript:Zm00001d026586_T003 transcript:EES13070 0
313- 25: transcript:Zm00001d026587_T002 transcript:EES13074 0
313- 26: transcript:Zm00001d026588_T004 transcript:EES13075 8.00E-41
313- 27: transcript:Zm00001d026590_T003 transcript:KXG27404 0
313- 28: transcript:Zm00001d026591_T001 transcript:EES13078 8.00E-78
313- 29: transcript:Zm00001d026592_T001 transcript:EES13077 0
313- 30: transcript:Zm00001d026593_T002 transcript:KXG27408 2.00E-180
313- 31: transcript:Zm00001d026594_T001 transcript:EES11637 1.00E-86
313- 32: transcript:Zm00001d026595_T001 transcript:EES13083 2.00E-69
313- 33: transcript:Zm00001d026597_T001 transcript:EES13084 7.00E-106
313- 34: transcript:Zm00001d026598_T001 transcript:KXG27416 1.00E-165
313- 35: transcript:Zm00001d026599_T001 transcript:OQU82564 1.00E-146
313- 36: transcript:Zm00001d026600_T001 transcript:EES13087 0
313- 37: transcript:Zm00001d026603_T002 transcript:EES11642 0
313- 38: transcript:Zm00001d026605_T001 transcript:EES13091 0
313- 39: transcript:Zm00001d026606_T002 transcript:EES11644 0
313- 40: transcript:Zm00001d026607_T001 transcript:EES13093 0
313- 41: transcript:Zm00001d026608_T001 transcript:EES11645 3.00E-108
313- 42: transcript:Zm00001d026609_T001 transcript:KXG27428 2.00E-82
313- 43: transcript:Zm00001d026610_T001 transcript:OQU82568 0
313- 44: transcript:Zm00001d026611_T001 transcript:OQU82569 8.00E-55
313- 45: transcript:Zm00001d026613_T001 transcript:EES13099 2.00E-49
313- 46: transcript:Zm00001d026614_T001 transcript:EES11648 0
313- 47: transcript:Zm00001d026617_T001 transcript:KXG27431 0
313- 48: transcript:Zm00001d026618_T002 transcript:KXG27432 0
313- 49: transcript:Zm00001d026619_T001 transcript:EES13103 0
313- 50: transcript:Zm00001d026620_T001 transcript:EES11651 7.00E-68
313- 51: transcript:Zm00001d026621_T006 transcript:OQU82573 0
313- 52: transcript:Zm00001d026625_T001 transcript:EES11652 1.00E-98
313- 53: transcript:Zm00001d026627_T001 transcript:EES11653 0
313- 54: transcript:Zm00001d026628_T001 transcript:EES13107 5.00E-91
313- 55: transcript:Zm00001d026629_T001 transcript:OQU82575 4.00E-177
313- 56: transcript:Zm00001d026630_T001 transcript:EES11661 1.00E-162
313- 57: transcript:Zm00001d026632_T001 transcript:EES13111 4.00E-167
313- 58: transcript:Zm00001d026633_T001 transcript:KXG27451 7.00E-157
313- 59: transcript:Zm00001d026634_T002 transcript:EES13114 4.00E-123
313- 60: transcript:Zm00001d026635_T002 transcript:EES11665 0
313- 61: transcript:Zm00001d026636_T002 transcript:KXG27455 2.00E-129
313- 62: transcript:Zm00001d026638_T009 transcript:EES11668 0
313- 63: transcript:Zm00001d026639_T002 transcript:EES13117 0
313- 64: transcript:Zm00001d026640_T001 transcript:EES13118 9.00E-83
313- 65: transcript:Zm00001d026641_T001 transcript:KXG27461 0
313- 66: transcript:Zm00001d026642_T001 transcript:EES11669 6.00E-90
313- 67: transcript:Zm00001d026643_T002 transcript:EES13120 0
313- 68: transcript:Zm00001d026645_T001 transcript:OQU82588 0
Alignment 314: score=2617.0 e_value=9.1e-225 N=57 10&6 plus
314- 0: transcript:Zm00001d025857_T001 transcript:EES12512 5.00E-93
314- 1: transcript:Zm00001d025859_T001 transcript:EES11113 2.00E-75
314- 2: transcript:Zm00001d025860_T002 transcript:KXG26718 0
314- 3: transcript:Zm00001d025861_T001 transcript:KXG26719 3.00E-145

314- 4:	transcript:Zm00001d025862_T002	transcript:OQU81978	7.00E-30
314- 5:	transcript:Zm00001d025863_T001	transcript:EES11114	7.00E-110
314- 6:	transcript:Zm00001d025864_T001	transcript:EES12516	8.00E-154
314- 7:	transcript:Zm00001d025865_T001	transcript:EES11117	0
314- 8:	transcript:Zm00001d025867_T003	transcript:OQU81985	6.00E-09
314- 9:	transcript:Zm00001d025868_T002	transcript:EES11120	2.00E-138
314- 10:	transcript:Zm00001d025869_T003	transcript:EES11121	0
314- 11:	transcript:Zm00001d025870_T001	transcript:OQU81991	0
314- 12:	transcript:Zm00001d025871_T001	transcript:KXG26729	0
314- 13:	transcript:Zm00001d025872_T001	transcript:KXG26730	8.00E-65
314- 14:	transcript:Zm00001d025873_T001	transcript:EES11129	2.00E-46
314- 15:	transcript:Zm00001d025874_T001	transcript:OQU81997	8.00E-64
314- 16:	transcript:Zm00001d025885_T001	transcript:EES11132	0
314- 17:	transcript:Zm00001d025886_T001	transcript:KXG26678	5.00E-86
314- 18:	transcript:Zm00001d025887_T001	transcript:KXG26749	0
314- 19:	transcript:Zm00001d025891_T001	transcript:KXG26752	6.00E-175
314- 20:	transcript:Zm00001d025892_T001	transcript:KXG26754	0
314- 21:	transcript:Zm00001d025894_T002	transcript:EES11144	0
314- 22:	transcript:Zm00001d025896_T001	transcript:EES11145	4.00E-86
314- 23:	transcript:Zm00001d025900_T001	transcript:KXG26758	4.00E-143
314- 24:	transcript:Zm00001d025903_T004	transcript:KXG26761	0
314- 25:	transcript:Zm00001d025904_T001	transcript:EES12541	0
314- 26:	transcript:Zm00001d025905_T002	transcript:EES11149	2.00E-60
314- 27:	transcript:Zm00001d025906_T001	transcript:EES11154	2.00E-56
314- 28:	transcript:Zm00001d025907_T001	transcript:EES12543	0
314- 29:	transcript:Zm00001d025908_T001	transcript:EES12544	0
314- 30:	transcript:Zm00001d025910_T001	transcript:KXG26694	5.00E-169
314- 31:	transcript:Zm00001d025911_T002	transcript:EES11157	0
314- 32:	transcript:Zm00001d025912_T001	transcript:OQU82011	0
314- 33:	transcript:Zm00001d025915_T002	transcript:EES11165	0
314- 34:	transcript:Zm00001d025916_T001	transcript:EES12550	2.00E-168
314- 35:	transcript:Zm00001d025917_T001	transcript:EES11166	0
314- 36:	transcript:Zm00001d025918_T001	transcript:EES12554	6.00E-28
314- 37:	transcript:Zm00001d025919_T001	transcript:OQU82015	0
314- 38:	transcript:Zm00001d025920_T001	transcript:EES12555	0
314- 39:	transcript:Zm00001d025922_T001	transcript:OQU82017	5.00E-65
314- 40:	transcript:Zm00001d025924_T001	transcript:EES11171	7.00E-134
314- 41:	transcript:Zm00001d025926_T001	transcript:EES12556	1.00E-131
314- 42:	transcript:Zm00001d025930_T001	transcript:EES11178	4.00E-162
314- 43:	transcript:Zm00001d025932_T003	transcript:EES12557	3.00E-102
314- 44:	transcript:Zm00001d025933_T001	transcript:KXG26784	0
314- 45:	transcript:Zm00001d025938_T002	transcript:EES11183	1.00E-160
314- 46:	transcript:Zm00001d025939_T003	transcript:EES12558	3.00E-141
314- 47:	transcript:Zm00001d025940_T001	transcript:OQU82019	2.00E-134
314- 48:	transcript:Zm00001d025941_T001	transcript:EES12562	3.00E-110
314- 49:	transcript:Zm00001d025943_T001	transcript:OQU82023	0
314- 50:	transcript:Zm00001d025944_T001	transcript:EES12563	4.00E-110
314- 51:	transcript:Zm00001d025946_T003	transcript:OQU82027	0
314- 52:	transcript:Zm00001d025947_T001	transcript:EES11187	4.00E-70
314- 53:	transcript:Zm00001d025948_T001	transcript:EES12566	0
314- 54:	transcript:Zm00001d025949_T001	transcript:EES12569	9.00E-115
314- 55:	transcript:Zm00001d025950_T002	transcript:EES12570	0
314- 56:	transcript:Zm00001d025951_T002	transcript:KXG26789	0

Alignment 315: score=2484.0 e_value=1.6e-226 N=57 10&6 plus

315- 0:	transcript:Zm00001d026306_T009	transcript:KXG27116	0
315- 1:	transcript:Zm00001d026307_T001	transcript:EES12861	1.00E-156
315- 2:	transcript:Zm00001d026308_T001	transcript:EES12863	3.00E-67
315- 3:	transcript:Zm00001d026310_T001	transcript:OQU82336	0
315- 4:	transcript:Zm00001d026311_T001	transcript:KXG27129	0
315- 5:	transcript:Zm00001d026312_T002	transcript:EES12869	0
315- 6:	transcript:Zm00001d026317_T002	transcript:EES11436	1.00E-102
315- 7:	transcript:Zm00001d026318_T001	transcript:OQU82343	2.00E-122
315- 8:	transcript:Zm00001d026321_T001	transcript:KXG27134	0
315- 9:	transcript:Zm00001d026322_T003	transcript:EES12875	0
315- 10:	transcript:Zm00001d026326_T001	transcript:OQU82350	0
315- 11:	transcript:Zm00001d026329_T001	transcript:EES11440	4.00E-17
315- 12:	transcript:Zm00001d026331_T006	transcript:OQU82356	0
315- 13:	transcript:Zm00001d026333_T001	transcript:OQU82367	2.00E-163
315- 14:	transcript:Zm00001d026334_T001	transcript:EES12883	0
315- 15:	transcript:Zm00001d026335_T001	transcript:KXG27145	5.00E-84
315- 16:	transcript:Zm00001d026337_T011	transcript:KXG27148	0
315- 17:	transcript:Zm00001d026341_T001	transcript:OQU82369	4.00E-39
315- 18:	transcript:Zm00001d026343_T001	transcript:EES11452	3.00E-93
315- 19:	transcript:Zm00001d026344_T001	transcript:EES12886	0
315- 20:	transcript:Zm00001d026345_T001	transcript:EES11453	0
315- 21:	transcript:Zm00001d026346_T002	transcript:KXG27152	0
315- 22:	transcript:Zm00001d026347_T008	transcript:EES11463	0
315- 23:	transcript:Zm00001d026348_T004	transcript:KXG27155	0
315- 24:	transcript:Zm00001d026351_T003	transcript:EES11464	0
315- 25:	transcript:Zm00001d026352_T001	transcript:EES12892	6.00E-162
315- 26:	transcript:Zm00001d026354_T001	transcript:EES12893	2.00E-62
315- 27:	transcript:Zm00001d026357_T001	transcript:EES12895	0
315- 28:	transcript:Zm00001d026359_T003	transcript:EES12901	0
315- 29:	transcript:Zm00001d026360_T001	transcript:OQU82384	1.00E-89
315- 30:	transcript:Zm00001d026361_T001	transcript:OQU82385	1.00E-156
315- 31:	transcript:Zm00001d026363_T001	transcript:OQU82386	2.00E-175
315- 32:	transcript:Zm00001d026366_T002	transcript:EES12902	0
315- 33:	transcript:Zm00001d026367_T001	transcript:EES12909	4.00E-56
315- 34:	transcript:Zm00001d026368_T001	transcript:OQU82388	5.00E-86
315- 35:	transcript:Zm00001d026370_T002	transcript:KXG27175	4.00E-164
315- 36:	transcript:Zm00001d026377_T001	transcript:EES11491	0
315- 37:	transcript:Zm00001d026379_T001	transcript:EES11495	0
315- 38:	transcript:Zm00001d026381_T001	transcript:EES11496	0
315- 39:	transcript:Zm00001d026382_T001	transcript:OQU82401	0
315- 40:	transcript:Zm00001d026383_T001	transcript:KXG27204	0
315- 41:	transcript:Zm00001d026390_T001	transcript:EES12925	8.00E-16
315- 42:	transcript:Zm00001d026391_T004	transcript:EES12930	1.00E-127
315- 43:	transcript:Zm00001d026392_T002	transcript:EES12932	4.00E-54
315- 44:	transcript:Zm00001d026394_T001	transcript:EES11508	0
315- 45:	transcript:Zm00001d026395_T001	transcript:EES12873	0
315- 46:	transcript:Zm00001d026396_T004	transcript:EES12936	0
315- 47:	transcript:Zm00001d026397_T001	transcript:EES12939	0
315- 48:	transcript:Zm00001d026398_T006	transcript:KXG27222	0
315- 49:	transcript:Zm00001d026401_T001	transcript:KXG27223	6.00E-51
315- 50:	transcript:Zm00001d026402_T002	transcript:EES11511	0
315- 51:	transcript:Zm00001d026404_T001	transcript:OQU82415	3.00E-58
315- 52:	transcript:Zm00001d026405_T001	transcript:EES12942	0
315- 53:	transcript:Zm00001d026406_T001	transcript:OQU82417	5.00E-06

315- 54: transcript:Zm00001d026413_T001 transcript:KXG27233 2.00E-06
315- 55: transcript:Zm00001d026414_T001 transcript:EES11521 5.00E-66
315- 56: transcript:Zm00001d026415_T003 transcript:EES12947 0
Alignment 316: score=2442.0 e_value=1.3e-210 N=55 10&6 plus
316- 0: transcript:Zm00001d026237_T001 transcript:OQU82255 0
316- 1: transcript:Zm00001d026239_T004 transcript:KXG27043 0
316- 2: transcript:Zm00001d026240_T006 transcript:EES12787 0
316- 3: transcript:Zm00001d026241_T001 transcript:EES12788 9.00E-18
316- 4: transcript:Zm00001d026242_T001 transcript:OQU82256 2.00E-172
316- 5: transcript:Zm00001d026243_T002 transcript:EES11377 1.00E-134
316- 6: transcript:Zm00001d026244_T001 transcript:EES12790 0
316- 7: transcript:Zm00001d026245_T001 transcript:EES12791 3.00E-145
316- 8: transcript:Zm00001d026246_T001 transcript:KXG27048 0
316- 9: transcript:Zm00001d026248_T001 transcript:EES12797 0
316- 10: transcript:Zm00001d026249_T003 transcript:EES11381 0
316- 11: transcript:Zm00001d026250_T001 transcript:EES12801 1.00E-98
316- 12: transcript:Zm00001d026252_T001 transcript:EES11384 7.00E-147
316- 13: transcript:Zm00001d026253_T002 transcript:EES12809 0
316- 14: transcript:Zm00001d026254_T001 transcript:KXG27062 3.00E-153
316- 15: transcript:Zm00001d026255_T001 transcript:EES12810 2.00E-39
316- 16: transcript:Zm00001d026257_T009 transcript:EES12812 0
316- 17: transcript:Zm00001d026258_T004 transcript:EES12814 0
316- 18: transcript:Zm00001d026259_T001 transcript:KXG27066 3.00E-71
316- 19: transcript:Zm00001d026260_T003 transcript:EES11389 1.00E-159
316- 20: transcript:Zm00001d026261_T001 transcript:EES12818 0
316- 21: transcript:Zm00001d026262_T001 transcript:OQU82277 9.00E-103
316- 22: transcript:Zm00001d026263_T013 transcript:EES11391 0
316- 23: transcript:Zm00001d026265_T002 transcript:KXG27072 0
316- 24: transcript:Zm00001d026266_T001 transcript:EES12823 0
316- 25: transcript:Zm00001d026267_T001 transcript:EES12824 0
316- 26: transcript:Zm00001d026268_T001 transcript:EES11393 0
316- 27: transcript:Zm00001d026269_T001 transcript:EES12828 2.00E-173
316- 28: transcript:Zm00001d026270_T013 transcript:OQU82282 0
316- 29: transcript:Zm00001d026271_T001 transcript:EES11400 8.00E-85
316- 30: transcript:Zm00001d026273_T003 transcript:EES12834 0
316- 31: transcript:Zm00001d026277_T002 transcript:EES11403 0
316- 32: transcript:Zm00001d026278_T001 transcript:EES11406 2.00E-39
316- 33: transcript:Zm00001d026279_T001 transcript:KXG27024 2.00E-06
316- 34: transcript:Zm00001d026280_T001 transcript:EES11404 0
316- 35: transcript:Zm00001d026281_T003 transcript:KXG27096 4.00E-58
316- 36: transcript:Zm00001d026282_T001 transcript:OQU82307 3.00E-48
316- 37: transcript:Zm00001d026283_T001 transcript:EES12840 0
316- 38: transcript:Zm00001d026284_T001 transcript:EES11411 5.00E-68
316- 39: transcript:Zm00001d026285_T001 transcript:OQU82312 2.00E-124
316- 40: transcript:Zm00001d026286_T001 transcript:EES11414 2.00E-114
316- 41: transcript:Zm00001d026287_T005 transcript:KXG27103 0
316- 42: transcript:Zm00001d026288_T003 transcript:OQU82315 7.00E-168
316- 43: transcript:Zm00001d026289_T004 transcript:EES12852 0
316- 44: transcript:Zm00001d026290_T001 transcript:EES11416 0
316- 45: transcript:Zm00001d026291_T013 transcript:EES12853 0
316- 46: transcript:Zm00001d026293_T001 transcript:EES11419 0
316- 47: transcript:Zm00001d026295_T001 transcript:EES11421 0
316- 48: transcript:Zm00001d026296_T014 transcript:KXG27107 0
316- 49: transcript:Zm00001d026297_T001 transcript:EES12854 8.00E-06

316- 50: transcript:Zm00001d026298_T001 transcript:OQU82320 2.00E-101
316- 51: transcript:Zm00001d026300_T001 transcript:KXG27113 0
316- 52: transcript:Zm00001d026301_T003 transcript:OQU82321 0
316- 53: transcript:Zm00001d026302_T003 transcript:EES12856 0
316- 54: transcript:Zm00001d026303_T004 transcript:KXG27116 0
Alignment 317: score=2133.0 e_value=2.3e-175 N=47 10&6 plus
317- 0: transcript:Zm00001d025740_T001 transcript:EES11033 0
317- 1: transcript:Zm00001d025741_T001 transcript:OQU81845 5.00E-41
317- 2: transcript:Zm00001d025744_T001 transcript:KXG26611 1.00E-11
317- 3: transcript:Zm00001d025745_T001 transcript:EES12421 8.00E-166
317- 4: transcript:Zm00001d025746_T001 transcript:KXG26616 0
317- 5: transcript:Zm00001d025747_T002 transcript:EES12423 5.00E-130
317- 6: transcript:Zm00001d025748_T001 transcript:KXG26617 3.00E-135
317- 7: transcript:Zm00001d025749_T002 transcript:EES12429 0
317- 8: transcript:Zm00001d025750_T011 transcript:EES11042 0
317- 9: transcript:Zm00001d025751_T002 transcript:OQU81856 0
317- 10: transcript:Zm00001d025752_T006 transcript:KXG26623 7.00E-31
317- 11: transcript:Zm00001d025753_T001 transcript:OQU81860 5.00E-101
317- 12: transcript:Zm00001d025754_T001 transcript:KXG26629 0
317- 13: transcript:Zm00001d025756_T002 transcript:KXG26632 2.00E-33
317- 14: transcript:Zm00001d025757_T001 transcript:KXG26633 0
317- 15: transcript:Zm00001d025759_T001 transcript:OQU81865 3.00E-100
317- 16: transcript:Zm00001d025761_T002 transcript:OQU81866 0
317- 17: transcript:Zm00001d025762_T001 transcript:OQU81867 2.00E-115
317- 18: transcript:Zm00001d025763_T003 transcript:KXG26636 4.00E-89
317- 19: transcript:Zm00001d025764_T001 transcript:EES11061 0
317- 20: transcript:Zm00001d025765_T002 transcript:EES12387 4.00E-85
317- 21: transcript:Zm00001d025767_T002 transcript:EES12447 0
317- 22: transcript:Zm00001d025770_T001 transcript:EES12446 0
317- 23: transcript:Zm00001d025771_T001 transcript:OQU81872 0
317- 24: transcript:Zm00001d025773_T002 transcript:OQU81878 0
317- 25: transcript:Zm00001d025774_T003 transcript:OQU81880 0
317- 26: transcript:Zm00001d025776_T002 transcript:KXG26645 0
317- 27: transcript:Zm00001d025777_T007 transcript:EES12452 0
317- 28: transcript:Zm00001d025778_T001 transcript:OQU81889 4.00E-30
317- 29: transcript:Zm00001d025780_T001 transcript:KXG26648 0
317- 30: transcript:Zm00001d025784_T002 transcript:KXG26651 2.00E-103
317- 31: transcript:Zm00001d025786_T003 transcript:EES11076 0
317- 32: transcript:Zm00001d025788_T001 transcript:OQU81891 0
317- 33: transcript:Zm00001d025789_T014 transcript:OQU81892 0
317- 34: transcript:Zm00001d025793_T001 transcript:OQU81893 0
317- 35: transcript:Zm00001d025794_T002 transcript:EES11077 7.00E-132
317- 36: transcript:Zm00001d025795_T010 transcript:KXG26655 0
317- 37: transcript:Zm00001d025797_T001 transcript:EES11079 2.00E-71
317- 38: transcript:Zm00001d025798_T004 transcript:KXG26657 0
317- 39: transcript:Zm00001d025799_T001 transcript:OQU81903 0
317- 40: transcript:Zm00001d025801_T007 transcript:EES12460 0
317- 41: transcript:Zm00001d025803_T001 transcript:EES11085 0
317- 42: transcript:Zm00001d025807_T001 transcript:EES12461 0
317- 43: transcript:Zm00001d025808_T002 transcript:KXG26667 0
317- 44: transcript:Zm00001d025814_T001 transcript:EES11087 0
317- 45: transcript:Zm00001d025815_T001 transcript:EES12465 3.00E-143
317- 46: transcript:Zm00001d025816_T002 transcript:KXG26672 0
Alignment 318: score=1969.0 e_value=5.4e-165 N=44 10&6 plus

318- 0: transcript:Zm00001d025407_T001 transcript:OQU81583 1.00E-133
318- 1: transcript:Zm00001d025409_T001 transcript:KXG26312 2.00E-85
318- 2: transcript:Zm00001d025412_T001 transcript:KXG26318 0
318- 3: transcript:Zm00001d025413_T001 transcript:EES10818 2.00E-122
318- 4: transcript:Zm00001d025414_T001 transcript:EES10820 2.00E-151
318- 5: transcript:Zm00001d025416_T001 transcript:EES10822 0
318- 6: transcript:Zm00001d025418_T001 transcript:OQU81595 1.00E-26
318- 7: transcript:Zm00001d025419_T001 transcript:OQU81597 0
318- 8: transcript:Zm00001d025420_T001 transcript:EES12177 2.00E-117
318- 9: transcript:Zm00001d025421_T001 transcript:EES10826 0
318- 10: transcript:Zm00001d025425_T001 transcript:EES12179 9.00E-59
318- 11: transcript:Zm00001d025430_T001 transcript:KXG26333 9.00E-35
318- 12: transcript:Zm00001d025431_T002 transcript:KXG26334 0
318- 13: transcript:Zm00001d025433_T003 transcript:EES12187 0
318- 14: transcript:Zm00001d025434_T001 transcript:EES10831 0
318- 15: transcript:Zm00001d025435_T002 transcript:EES10833 1.00E-145
318- 16: transcript:Zm00001d025437_T001 transcript:EES10834 0
318- 17: transcript:Zm00001d025444_T001 transcript:KXG26342 1.00E-38
318- 18: transcript:Zm00001d025445_T001 transcript:OQU81612 3.00E-105
318- 19: transcript:Zm00001d025446_T006 transcript:EES12195 0
318- 20: transcript:Zm00001d025447_T001 transcript:KXG26348 2.00E-38
318- 21: transcript:Zm00001d025449_T002 transcript:OQU81615 0
318- 22: transcript:Zm00001d025450_T007 transcript:OQU81622 1.00E-177
318- 23: transcript:Zm00001d025451_T001 transcript:EES10843 7.00E-49
318- 24: transcript:Zm00001d025452_T001 transcript:EES10844 0
318- 25: transcript:Zm00001d025453_T006 transcript:EES12206 0
318- 26: transcript:Zm00001d025460_T001 transcript:EES12207 1.00E-166
318- 27: transcript:Zm00001d025461_T006 transcript:KXG26361 0
318- 28: transcript:Zm00001d025462_T001 transcript:EES10849 3.00E-151
318- 29: transcript:Zm00001d025467_T001 transcript:EES12221 7.00E-45
318- 30: transcript:Zm00001d025468_T001 transcript:EES10853 0
318- 31: transcript:Zm00001d025469_T001 transcript:EES10854 0
318- 32: transcript:Zm00001d025470_T005 transcript:EES10855 0
318- 33: transcript:Zm00001d025472_T002 transcript:EES10856 2.00E-92
318- 34: transcript:Zm00001d025474_T002 transcript:EES12223 0
318- 35: transcript:Zm00001d025475_T001 transcript:EES12225 0
318- 36: transcript:Zm00001d025476_T001 transcript:OQU81645 2.00E-66
318- 37: transcript:Zm00001d025477_T001 transcript:EES12227 1.00E-53
318- 38: transcript:Zm00001d025479_T001 transcript:KXG26379 6.00E-128
318- 39: transcript:Zm00001d025483_T001 transcript:EES12234 0
318- 40: transcript:Zm00001d025485_T001 transcript:KXG26384 5.00E-71
318- 41: transcript:Zm00001d025490_T001 transcript:EES10865 4.00E-32
318- 42: transcript:Zm00001d025504_T001 transcript:KXG26388 1.00E-122
318- 43: transcript:Zm00001d025508_T001 transcript:KXG26390 3.00E-105
Alignment 319: score=1898.0 e_value=4.4e-159 N=43 10&6 plus
319- 0: transcript:Zm00001d026647_T005 transcript:EES11671 0
319- 1: transcript:Zm00001d026648_T001 transcript:EES13127 8.00E-73
319- 2: transcript:Zm00001d026649_T001 transcript:KXG27467 0
319- 3: transcript:Zm00001d026650_T001 transcript:KXG27468 0
319- 4: transcript:Zm00001d026652_T001 transcript:KXG27470 0
319- 5: transcript:Zm00001d026653_T004 transcript:EES11678 0
319- 6: transcript:Zm00001d026654_T001 transcript:EES11680 0
319- 7: transcript:Zm00001d026655_T001 transcript:OQU82593 0
319- 8: transcript:Zm00001d026657_T001 transcript:EES11684 5.00E-170

```
319-  9: transcript:Zm00001d026658_T001 transcript:KXG27475          7.00E-131
319- 10: transcript:Zm00001d026661_T001 transcript:KXG27476          2.00E-105
319- 11: transcript:Zm00001d026662_T001 transcript:OQU82595          2.00E-127
319- 12: transcript:Zm00001d026664_T001 transcript:EES13132          4.00E-137
319- 13: transcript:Zm00001d026665_T001 transcript:EES13135           1.00E-98
319- 14: transcript:Zm00001d026668_T001 transcript:OQU82599                  0
319- 15: transcript:Zm00001d026669_T001 transcript:EES11689                  0
319- 16: transcript:Zm00001d026670_T001 transcript:OQU82602           3.00E-85
319- 17: transcript:Zm00001d026671_T001 transcript:EES13138           6.00E-40
319- 18: transcript:Zm00001d026673_T001 transcript:OQU82618           1.00E-42
319- 19: transcript:Zm00001d026675_T002 transcript:EES11691                  0
319- 20: transcript:Zm00001d026676_T003 transcript:EES13140                  0
319- 21: transcript:Zm00001d026677_T001 transcript:OQU82623                  0
319- 22: transcript:Zm00001d026678_T001 transcript:EES13145                  0
319- 23: transcript:Zm00001d026679_T001 transcript:EES11692          4.00E-108
319- 24: transcript:Zm00001d026680_T002 transcript:KXG27493          2.00E-156
319- 25: transcript:Zm00001d026681_T001 transcript:EES11697                  0
319- 26: transcript:Zm00001d026683_T001 transcript:EES13148           1.00E-70
319- 27: transcript:Zm00001d026684_T001 transcript:EES11701                  0
319- 28: transcript:Zm00001d026685_T002 transcript:OQU82636                  0
319- 29: transcript:Zm00001d026687_T001 transcript:EES13156                  0
319- 30: transcript:Zm00001d026690_T001 transcript:EES11705                  0
319- 31: transcript:Zm00001d026691_T004 transcript:EES13157                  0
319- 32: transcript:Zm00001d026695_T001 transcript:OQU82640                  0
319- 33: transcript:Zm00001d026696_T019 transcript:EES11709                  0
319- 34: transcript:Zm00001d026697_T001 transcript:OQU82643           7.00E-61
319- 35: transcript:Zm00001d026698_T026 transcript:OQU82644                  0
319- 36: transcript:Zm00001d026700_T002 transcript:EES11712          1.00E-150
319- 37: transcript:Zm00001d026701_T001 transcript:KXG27521          8.00E-160
319- 38: transcript:Zm00001d026702_T002 transcript:EES11715                  0
319- 39: transcript:Zm00001d026703_T005 transcript:KXG27528          3.00E-133
319- 40: transcript:Zm00001d026709_T001 transcript:KXG27531           1.00E-76
319- 41: transcript:Zm00001d026711_T001 transcript:EES11719                  0
319- 42: transcript:Zm00001d026712_T001 transcript:OQU82665                  0
## Alignment 320: score=1823.0 e_value=3.2e-143 N=41 10&6 plus
320-  0: transcript:EER88080            transcript:Zm00001d037189_T004         0
320-  1: transcript:EER89424            transcript:Zm00001d037190_T001         0
320-  2: transcript:OQU76060            transcript:Zm00001d037191_T002 4.00E-122
320-  3: transcript:OQU76061            transcript:Zm00001d037192_T001         0
320-  4: transcript:OQU76064            transcript:Zm00001d037194_T001  1.00E-75
320-  5: transcript:KXG19611            transcript:Zm00001d037195_T003 3.00E-177
320-  6: transcript:KXG19612            transcript:Zm00001d037197_T001  3.00E-59
320-  7: transcript:OQU76066            transcript:Zm00001d037198_T001         0
320-  8: transcript:OQU76069            transcript:Zm00001d037199_T001         0
320-  9: transcript:EER88092            transcript:Zm00001d037200_T004 6.00E-150
320- 10: transcript:OQU76072            transcript:Zm00001d037203_T001         0
320- 11: transcript:KXG19617            transcript:Zm00001d037204_T001         0
320- 12: transcript:EER89439            transcript:Zm00001d037205_T001 9.00E-134
320- 13: transcript:EER89441            transcript:Zm00001d037207_T001         0
320- 14: transcript:OQU76075            transcript:Zm00001d037209_T001 7.00E-127
320- 15: transcript:EER88096            transcript:Zm00001d037210_T001  2.00E-30
320- 16: transcript:EER88097            transcript:Zm00001d037211_T001         0
320- 17: transcript:KXG19627            transcript:Zm00001d037212_T001         0
320- 18: transcript:KXG19632            transcript:Zm00001d037213_T001  1.00E-24
```

320- 19: transcript:KXG19635 transcript:Zm00001d037215_T011 0
320- 20: transcript:EER89460 transcript:Zm00001d037216_T001 0
320- 21: transcript:EER88108 transcript:Zm00001d037218_T001 5.00E-157
320- 22: transcript:EER89468 transcript:Zm00001d037220_T001 7.00E-135
320- 23: transcript:EER88112 transcript:Zm00001d037221_T001 7.00E-164
320- 24: transcript:EER89469 transcript:Zm00001d037225_T001 0
320- 25: transcript:EER89470 transcript:Zm00001d037227_T002 0
320- 26: transcript:EER89472 transcript:Zm00001d037228_T001 0
320- 27: transcript:OQU76109 transcript:Zm00001d037229_T001 0
320- 28: transcript:EER89473 transcript:Zm00001d037232_T001 0
320- 29: transcript:OQU76111 transcript:Zm00001d037233_T002 0
320- 30: transcript:KXG19644 transcript:Zm00001d037234_T005 0
320- 31: transcript:OQU76113 transcript:Zm00001d037235_T001 0
320- 32: transcript:KXG19654 transcript:Zm00001d037236_T001 2.00E-172
320- 33: transcript:EER89480 transcript:Zm00001d037237_T002 6.00E-137
320- 34: transcript:EER89482 transcript:Zm00001d037239_T001 4.00E-162
320- 35: transcript:KXG19661 transcript:Zm00001d037240_T001 0
320- 36: transcript:EER89485 transcript:Zm00001d037242_T001 0
320- 37: transcript:KXG19666 transcript:Zm00001d037243_T001 2.00E-71
320- 38: transcript:OQU76123 transcript:Zm00001d037244_T001 8.00E-43
320- 39: transcript:EER89486 transcript:Zm00001d037246_T002 0
320- 40: transcript:KXG19670 transcript:Zm00001d037247_T007 0
Alignment 321: score=1786.0 e_value=4e-144 N=40 10&6 plus
321- 0: transcript:Zm00001d025607_T001 transcript:KXG26463 0
321- 1: transcript:Zm00001d025613_T001 transcript:EES10944 1.00E-114
321- 2: transcript:Zm00001d025616_T001 transcript:EES10949 2.00E-97
321- 3: transcript:Zm00001d025617_T001 transcript:EES10954 0
321- 4: transcript:Zm00001d025619_T001 transcript:EES12301 0
321- 5: transcript:Zm00001d025621_T001 transcript:KXG26481 2.00E-49
321- 6: transcript:Zm00001d025622_T001 transcript:OQU81717 2.00E-47
321- 7: transcript:Zm00001d025623_T001 transcript:EES12303 1.00E-164
321- 8: transcript:Zm00001d025624_T001 transcript:EES10957 0
321- 9: transcript:Zm00001d025625_T001 transcript:EES12305 0
321- 10: transcript:Zm00001d025626_T002 transcript:KXG26485 0
321- 11: transcript:Zm00001d025628_T002 transcript:OQU81720 0
321- 12: transcript:Zm00001d025633_T007 transcript:KXG26492 0
321- 13: transcript:Zm00001d025639_T002 transcript:EES10959 0
321- 14: transcript:Zm00001d025640_T002 transcript:EES12315 8.00E-169
321- 15: transcript:Zm00001d025644_T002 transcript:EES10966 0
321- 16: transcript:Zm00001d025645_T001 transcript:KXG26505 2.00E-38
321- 17: transcript:Zm00001d025646_T001 transcript:OQU81742 9.00E-117
321- 18: transcript:Zm00001d025650_T001 transcript:EES12319 1.00E-177
321- 19: transcript:Zm00001d025651_T001 transcript:EES12320 0
321- 20: transcript:Zm00001d025652_T005 transcript:KXG26508 0
321- 21: transcript:Zm00001d025653_T001 transcript:KXG26509 0
321- 22: transcript:Zm00001d025654_T001 transcript:EES12328 5.00E-33
321- 23: transcript:Zm00001d025656_T001 transcript:OQU81758 0
321- 24: transcript:Zm00001d025657_T001 transcript:EES12336 1.00E-125
321- 25: transcript:Zm00001d025658_T006 transcript:EES12337 0
321- 26: transcript:Zm00001d025659_T001 transcript:EES12338 0
321- 27: transcript:Zm00001d025660_T001 transcript:EES10970 8.00E-156
321- 28: transcript:Zm00001d025662_T001 transcript:EES12339 1.00E-23
321- 29: transcript:Zm00001d025663_T004 transcript:EES12340 6.00E-139
321- 30: transcript:Zm00001d025664_T001 transcript:EES12341 0

321- 31: transcript:Zm00001d025665_T002 transcript:KXG26523 0
321- 32: transcript:Zm00001d025666_T001 transcript:KXG26524 3.00E-82
321- 33: transcript:Zm00001d025667_T001 transcript:OQU81760 0
321- 34: transcript:Zm00001d025668_T005 transcript:OQU81762 0
321- 35: transcript:Zm00001d025669_T003 transcript:KXG26525 0
321- 36: transcript:Zm00001d025672_T001 transcript:EES12346 7.00E-129
321- 37: transcript:Zm00001d025673_T005 transcript:EES10975 0
321- 38: transcript:Zm00001d025674_T001 transcript:EES10976 0
321- 39: transcript:Zm00001d025675_T001 transcript:EES12349 0
Alignment 322: score=1763.0 e_value=2.8e-153 N=42 10&6 plus
322- 0: transcript:Zm00001d025303_T004 transcript:KXG26179 0
322- 1: transcript:Zm00001d025304_T002 transcript:OQU81461 0
322- 2: transcript:Zm00001d025305_T001 transcript:KXG26188 0
322- 3: transcript:Zm00001d025307_T004 transcript:OQU81468 2.00E-156
322- 4: transcript:Zm00001d025310_T001 transcript:OQU81469 9.00E-96
322- 5: transcript:Zm00001d025322_T001 transcript:EES12086 6.00E-28
322- 6: transcript:Zm00001d025323_T010 transcript:EES12087 0
322- 7: transcript:Zm00001d025325_T002 transcript:EES12089 0
322- 8: transcript:Zm00001d025326_T001 transcript:EES12091 0
322- 9: transcript:Zm00001d025327_T001 transcript:EES10723 0
322- 10: transcript:Zm00001d025333_T001 transcript:EES10725 0
322- 11: transcript:Zm00001d025337_T001 transcript:KXG26216 7.00E-180
322- 12: transcript:Zm00001d025338_T001 transcript:EES12098 2.00E-111
322- 13: transcript:Zm00001d025340_T002 transcript:KXG26222 1.00E-116
322- 14: transcript:Zm00001d025342_T002 transcript:EES12107 0
322- 15: transcript:Zm00001d025343_T001 transcript:KXG26223 0
322- 16: transcript:Zm00001d025345_T001 transcript:EES10732 2.00E-40
322- 17: transcript:Zm00001d025346_T001 transcript:EES12110 8.00E-117
322- 18: transcript:Zm00001d025347_T001 transcript:KXG26229 1.00E-64
322- 19: transcript:Zm00001d025352_T001 transcript:EES10739 6.00E-88
322- 20: transcript:Zm00001d025353_T001 transcript:OQU81524 0
322- 21: transcript:Zm00001d025359_T002 transcript:KXG26266 0
322- 22: transcript:Zm00001d025360_T001 transcript:EES12134 4.00E-172
322- 23: transcript:Zm00001d025361_T001 transcript:EES10769 2.00E-51
322- 24: transcript:Zm00001d025362_T002 transcript:EES10771 9.00E-110
322- 25: transcript:Zm00001d025367_T001 transcript:KXG26271 3.00E-19
322- 26: transcript:Zm00001d025369_T002 transcript:EES10778 0
322- 27: transcript:Zm00001d025371_T001 transcript:EES12138 5.00E-132
322- 28: transcript:Zm00001d025373_T001 transcript:OQU81544 0
322- 29: transcript:Zm00001d025374_T001 transcript:OQU81546 5.00E-94
322- 30: transcript:Zm00001d025375_T001 transcript:EES10782 0
322- 31: transcript:Zm00001d025377_T001 transcript:KXG26281 3.00E-18
322- 32: transcript:Zm00001d025379_T001 transcript:OQU81548 0
322- 33: transcript:Zm00001d025380_T001 transcript:OQU81550 9.00E-121
322- 34: transcript:Zm00001d025381_T001 transcript:EES10786 8.00E-94
322- 35: transcript:Zm00001d025383_T001 transcript:OQU81562 0
322- 36: transcript:Zm00001d025389_T001 transcript:EES12148 0
322- 37: transcript:Zm00001d025398_T005 transcript:OQU81566 0
322- 38: transcript:Zm00001d025399_T001 transcript:KXG26294 2.00E-53
322- 39: transcript:Zm00001d025400_T001 transcript:OQU81571 0
322- 40: transcript:Zm00001d025401_T001 transcript:EES12153 2.00E-43
322- 41: transcript:Zm00001d025402_T002 transcript:EES10805 0
Alignment 323: score=1692.0 e_value=3.9e-132 N=38 10&6 plus
323- 0: transcript:Zm00001d025675_T001 transcript:OQU81789 2.00E-26

323- 1: transcript:Zm00001d025679_T001 transcript:EES10985 0
323- 2: transcript:Zm00001d025680_T002 transcript:OQU81793 2.00E-178
323- 3: transcript:Zm00001d025681_T001 transcript:EES10994 0
323- 4: transcript:Zm00001d025684_T001 transcript:OQU81795 4.00E-118
323- 5: transcript:Zm00001d025687_T003 transcript:KXG26558 0
323- 6: transcript:Zm00001d025689_T002 transcript:OQU81802 0
323- 7: transcript:Zm00001d025690_T003 transcript:KXG26562 0
323- 8: transcript:Zm00001d025692_T002 transcript:EES10998 0
323- 9: transcript:Zm00001d025694_T001 transcript:KXG26564 2.00E-134
323- 10: transcript:Zm00001d025695_T001 transcript:EES12381 0
323- 11: transcript:Zm00001d025696_T001 transcript:EES11002 0
323- 12: transcript:Zm00001d025699_T001 transcript:KXG26570 0
323- 13: transcript:Zm00001d025703_T002 transcript:OQU81809 0
323- 14: transcript:Zm00001d025704_T001 transcript:EES12388 0
323- 15: transcript:Zm00001d025705_T008 transcript:KXG26572 0
323- 16: transcript:Zm00001d025706_T012 transcript:EES12393 0
323- 17: transcript:Zm00001d025707_T001 transcript:EES11006 0
323- 18: transcript:Zm00001d025709_T001 transcript:EES12396 2.00E-28
323- 19: transcript:Zm00001d025710_T001 transcript:KXG26579 0
323- 20: transcript:Zm00001d025711_T001 transcript:EES12401 4.00E-67
323- 21: transcript:Zm00001d025712_T003 transcript:OQU81824 0
323- 22: transcript:Zm00001d025713_T001 transcript:EES12405 0
323- 23: transcript:Zm00001d025714_T001 transcript:KXG26588 0
323- 24: transcript:Zm00001d025715_T001 transcript:EES11019 2.00E-174
323- 25: transcript:Zm00001d025716_T001 transcript:EES11020 0
323- 26: transcript:Zm00001d025717_T001 transcript:EES12408 0
323- 27: transcript:Zm00001d025720_T001 transcript:EES11021 0
323- 28: transcript:Zm00001d025721_T001 transcript:EES12410 2.00E-133
323- 29: transcript:Zm00001d025722_T002 transcript:EES12342 0
323- 30: transcript:Zm00001d025724_T001 transcript:EES11024 3.00E-83
323- 31: transcript:Zm00001d025726_T001 transcript:EES11026 0
323- 32: transcript:Zm00001d025727_T006 transcript:EES12415 0
323- 33: transcript:Zm00001d025734_T011 transcript:KXG26602 0
323- 34: transcript:Zm00001d025735_T001 transcript:EES11030 0
323- 35: transcript:Zm00001d025737_T001 transcript:EES11032 5.00E-124
323- 36: transcript:Zm00001d025738_T001 transcript:KXG26609 0
323- 37: transcript:Zm00001d025739_T006 transcript:EES12419 0
Alignment 324: score=1589.0 e_value=2.5e-127 N=36 10&6 plus
324- 0: transcript:Zm00001d025509_T001 transcript:KXG26394 1.00E-91
324- 1: transcript:Zm00001d025513_T001 transcript:KXG26395 3.00E-49
324- 2: transcript:Zm00001d025514_T001 transcript:EES12241 0
324- 3: transcript:Zm00001d025517_T003 transcript:EES10876 5.00E-74
324- 4: transcript:Zm00001d025518_T001 transcript:OQU81655 5.00E-14
324- 5: transcript:Zm00001d025519_T003 transcript:EES10878 2.00E-136
324- 6: transcript:Zm00001d025520_T001 transcript:OQU81656 0
324- 7: transcript:Zm00001d025522_T001 transcript:EES10880 0
324- 8: transcript:Zm00001d025524_T001 transcript:EES10882 0
324- 9: transcript:Zm00001d025526_T003 transcript:EES10885 0
324- 10: transcript:Zm00001d025534_T001 transcript:KXG26409 3.00E-89
324- 11: transcript:Zm00001d025538_T001 transcript:EES10893 0
324- 12: transcript:Zm00001d025541_T001 transcript:EES10894 0
324- 13: transcript:Zm00001d025542_T001 transcript:EES12252 3.00E-110
324- 14: transcript:Zm00001d025544_T001 transcript:EES12253 8.00E-79
324- 15: transcript:Zm00001d025547_T001 transcript:EES12255 0

324- 16: transcript:Zm00001d025548_T001 transcript:KXG26417 0
324- 17: transcript:Zm00001d025549_T001 transcript:KXG26418 7.00E-180
324- 18: transcript:Zm00001d025551_T002 transcript:EES10898 0
324- 19: transcript:Zm00001d025552_T001 transcript:KXG26420 6.00E-30
324- 20: transcript:Zm00001d025559_T001 transcript:EES10907 5.00E-26
324- 21: transcript:Zm00001d025564_T001 transcript:EES12260 1.00E-70
324- 22: transcript:Zm00001d025566_T001 transcript:OQU81673 1.00E-92
324- 23: transcript:Zm00001d025568_T001 transcript:KXG26433 0
324- 24: transcript:Zm00001d025570_T006 transcript:EES12269 0
324- 25: transcript:Zm00001d025572_T001 transcript:KXG26442 0
324- 26: transcript:Zm00001d025574_T001 transcript:EES12270 0
324- 27: transcript:Zm00001d025576_T001 transcript:EES12277 4.00E-80
324- 28: transcript:Zm00001d025577_T001 transcript:EES12278 4.00E-38
324- 29: transcript:Zm00001d025579_T001 transcript:KXG26456 1.00E-41
324- 30: transcript:Zm00001d025580_T001 transcript:EES12285 0
324- 31: transcript:Zm00001d025581_T001 transcript:EES10931 0
324- 32: transcript:Zm00001d025584_T001 transcript:EES10933 0
324- 33: transcript:Zm00001d025587_T001 transcript:KXG26458 3.00E-41
324- 34: transcript:Zm00001d025588_T001 transcript:EES12286 0
324- 35: transcript:Zm00001d025590_T002 transcript:EES12287 0
Alignment 325: score=1312.0 e_value=1e-100 N=30 10&6 plus
325- 0: transcript:EER88133 transcript:Zm00001d037248_T001 0
325- 1: transcript:EER89492 transcript:Zm00001d037249_T001 0
325- 2: transcript:KXG19675 transcript:Zm00001d037250_T001 2.00E-92
325- 3: transcript:KXG19676 transcript:Zm00001d037251_T001 3.00E-82
325- 4: transcript:EER89500 transcript:Zm00001d037252_T002 0
325- 5: transcript:OQU76128 transcript:Zm00001d037254_T001 0
325- 6: transcript:EER89501 transcript:Zm00001d037257_T001 0
325- 7: transcript:EER89502 transcript:Zm00001d037258_T002 2.00E-113
325- 8: transcript:EER89504 transcript:Zm00001d037261_T001 2.00E-40
325- 9: transcript:OQU76145 transcript:Zm00001d037263_T001 7.00E-174
325- 10: transcript:KXG19690 transcript:Zm00001d037264_T002 5.00E-142
325- 11: transcript:EER88149 transcript:Zm00001d037265_T001 0
325- 12: transcript:EER88150 transcript:Zm00001d037266_T003 7.00E-168
325- 13: transcript:KXG19695 transcript:Zm00001d037267_T005 6.00E-105
325- 14: transcript:EER88152 transcript:Zm00001d037268_T003 0
325- 15: transcript:KXG19700 transcript:Zm00001d037270_T001 1.00E-90
325- 16: transcript:KXG19701 transcript:Zm00001d037271_T001 0
325- 17: transcript:EER89512 transcript:Zm00001d037272_T001 0
325- 18: transcript:EER88155 transcript:Zm00001d037273_T001 1.00E-110
325- 19: transcript:EER88156 transcript:Zm00001d037274_T002 0
325- 20: transcript:EER89513 transcript:Zm00001d037275_T001 3.00E-58
325- 21: transcript:EER88159 transcript:Zm00001d037277_T001 2.00E-57
325- 22: transcript:EER89514 transcript:Zm00001d037278_T001 0
325- 23: transcript:EER88160 transcript:Zm00001d037279_T011 0
325- 24: transcript:OQU76164 transcript:Zm00001d037280_T001 3.00E-27
325- 25: transcript:KXG19707 transcript:Zm00001d037284_T001 2.00E-167
325- 26: transcript:EER88163 transcript:Zm00001d037288_T001 6.00E-162
325- 27: transcript:KXG19708 transcript:Zm00001d037289_T002 0
325- 28: transcript:KXG19709 transcript:Zm00001d037290_T002 0
325- 29: transcript:EER89530 transcript:Zm00001d037291_T001 0
Alignment 326: score=1250.0 e_value=2.7e-102 N=29 10&6 plus
326- 0: transcript:EER89074 transcript:Zm00001d035934_T002 0
326- 1: transcript:KXG19148 transcript:Zm00001d035935_T001 4.00E-16

326- 2:	transcript:EER89078	transcript:Zm00001d035945_T001	3.00E-39
326- 3:	transcript:OQU75721	transcript:Zm00001d035947_T002	0
326- 4:	transcript:EER87739	transcript:Zm00001d035948_T001	7.00E-49
326- 5:	transcript:EER89081	transcript:Zm00001d035957_T001	0
326- 6:	transcript:EER87740	transcript:Zm00001d035962_T001	0
326- 7:	transcript:EER89085	transcript:Zm00001d035963_T001	6.00E-103
326- 8:	transcript:EER87741	transcript:Zm00001d035964_T003	0
326- 9:	transcript:EER89089	transcript:Zm00001d035965_T004	6.00E-151
326- 10:	transcript:EER89092	transcript:Zm00001d035973_T005	0
326- 11:	transcript:EER89093	transcript:Zm00001d035974_T001	0
326- 12:	transcript:EER89095	transcript:Zm00001d035978_T001	8.00E-09
326- 13:	transcript:EER89096	transcript:Zm00001d035981_T001	2.00E-145
326- 14:	transcript:KXG19160	transcript:Zm00001d035987_T001	0
326- 15:	transcript:EER87745	transcript:Zm00001d035988_T003	6.00E-29
326- 16:	transcript:KXG19162	transcript:Zm00001d035989_T001	0
326- 17:	transcript:KXG19168	transcript:Zm00001d035990_T002	0
326- 18:	transcript:EER89103	transcript:Zm00001d035992_T001	0
326- 19:	transcript:EER87751	transcript:Zm00001d035999_T002	3.00E-140
326- 20:	transcript:EER87752	transcript:Zm00001d036001_T006	1.00E-156
326- 21:	transcript:EER87753	transcript:Zm00001d036003_T001	9.00E-75
326- 22:	transcript:OQU75728	transcript:Zm00001d036004_T017	0
326- 23:	transcript:EER89106	transcript:Zm00001d036008_T001	0
326- 24:	transcript:OQU75731	transcript:Zm00001d036010_T003	0
326- 25:	transcript:KXG19179	transcript:Zm00001d036013_T002	0
326- 26:	transcript:OQU75732	transcript:Zm00001d036014_T001	2.00E-177
326- 27:	transcript:KXG19182	transcript:Zm00001d036016_T001	8.00E-94
326- 28:	transcript:EER87763	transcript:Zm00001d036018_T001	2.00E-43

Alignment 327: score=1155.0 e_value=1.6e-84 N=26 10&6 plus

327- 0:	transcript:Zm00001d026440_T001	transcript:OQU82436	9.00E-43
327- 1:	transcript:Zm00001d026441_T001	transcript:EES11543	1.00E-126
327- 2:	transcript:Zm00001d026442_T001	transcript:EES12961	5.00E-179
327- 3:	transcript:Zm00001d026444_T001	transcript:EES11545	2.00E-103
327- 4:	transcript:Zm00001d026445_T001	transcript:EES11547	0
327- 5:	transcript:Zm00001d026447_T001	transcript:KXG27254	8.00E-84
327- 6:	transcript:Zm00001d026448_T001	transcript:OQU82447	7.00E-131
327- 7:	transcript:Zm00001d026451_T001	transcript:OQU82448	4.00E-100
327- 8:	transcript:Zm00001d026452_T001	transcript:KXG27258	0
327- 9:	transcript:Zm00001d026453_T001	transcript:OQU82449	1.00E-122
327- 10:	transcript:Zm00001d026454_T001	transcript:OQU82450	5.00E-40
327- 11:	transcript:Zm00001d026457_T001	transcript:OQU82451	6.00E-48
327- 12:	transcript:Zm00001d026458_T002	transcript:KXG27268	0
327- 13:	transcript:Zm00001d026460_T001	transcript:EES12978	0
327- 14:	transcript:Zm00001d026463_T002	transcript:EES11552	9.00E-20
327- 15:	transcript:Zm00001d026469_T001	transcript:EES11553	0
327- 16:	transcript:Zm00001d026470_T001	transcript:KXG27271	3.00E-121
327- 17:	transcript:Zm00001d026471_T001	transcript:KXG27273	0
327- 18:	transcript:Zm00001d026472_T001	transcript:EES12983	0
327- 19:	transcript:Zm00001d026475_T001	transcript:EES12992	2.00E-138
327- 20:	transcript:Zm00001d026476_T002	transcript:EES11563	2.00E-139
327- 21:	transcript:Zm00001d026477_T002	transcript:OQU82468	4.00E-86
327- 22:	transcript:Zm00001d026478_T001	transcript:EES12994	3.00E-27
327- 23:	transcript:Zm00001d026479_T006	transcript:KXG27283	0
327- 24:	transcript:Zm00001d026480_T001	transcript:EES11567	3.00E-63
327- 25:	transcript:Zm00001d026483_T001	transcript:EES11568	0

Alignment 328: score=1023.0 e_value=8.7e-72 N=23 10&6 plus
328- 0: transcript:Zm00001d026002_T001 transcript:EES12708 4.00E-82
328- 1: transcript:Zm00001d026003_T001 transcript:KXG26978 3.00E-66
328- 2: transcript:Zm00001d026004_T001 transcript:EES11314 0
328- 3: transcript:Zm00001d026005_T001 transcript:EES12712 5.00E-172
328- 4: transcript:Zm00001d026010_T001 transcript:EES11273 0
328- 5: transcript:Zm00001d026012_T001 transcript:EES12722 3.00E-77
328- 6: transcript:Zm00001d026014_T001 transcript:EES11318 0
328- 7: transcript:Zm00001d026015_T001 transcript:EES12723 6.00E-67
328- 8: transcript:Zm00001d026016_T001 transcript:KXG26990 1.00E-80
328- 9: transcript:Zm00001d026017_T001 transcript:OQU82207 0
328- 10: transcript:Zm00001d026018_T001 transcript:EES11322 0
328- 11: transcript:Zm00001d026020_T001 transcript:EES12727 0
328- 12: transcript:Zm00001d026021_T001 transcript:EES12728 2.00E-89
328- 13: transcript:Zm00001d026022_T004 transcript:KXG26998 0
328- 14: transcript:Zm00001d026025_T002 transcript:EES12735 4.00E-122
328- 15: transcript:Zm00001d026026_T001 transcript:KXG27001 1.00E-62
328- 16: transcript:Zm00001d026028_T001 transcript:EES12739 2.00E-42
328- 17: transcript:Zm00001d026030_T014 transcript:EES11326 0
328- 18: transcript:Zm00001d026031_T003 transcript:EES11327 3.00E-58
328- 19: transcript:Zm00001d026032_T001 transcript:KXG27003 6.00E-154
328- 20: transcript:Zm00001d026041_T005 transcript:KXG27008 0
328- 21: transcript:Zm00001d026046_T001 transcript:KXG27015 8.00E-149
328- 22: transcript:Zm00001d026047_T002 transcript:EES12755 1.00E-140
Alignment 329: score=978.0 e_value=2.8e-60 N=21 10&6 plus
329- 0: transcript:Zm00001d026193_T001 transcript:EES11350 0
329- 1: transcript:Zm00001d026192_T006 transcript:KXG27019 0
329- 2: transcript:Zm00001d026194_T003 transcript:EES12761 4.00E-86
329- 3: transcript:Zm00001d026195_T002 transcript:EES12762 0
329- 4: transcript:Zm00001d026197_T001 transcript:KXG27021 3.00E-163
329- 5: transcript:Zm00001d026199_T001 transcript:EES12764 0
329- 6: transcript:Zm00001d026202_T001 transcript:KXG27022 4.00E-15
329- 7: transcript:Zm00001d026203_T001 transcript:EES12768 3.00E-162
329- 8: transcript:Zm00001d026205_T001 transcript:EES11357 8.00E-41
329- 9: transcript:Zm00001d026206_T001 transcript:EES12769 0
329- 10: transcript:Zm00001d026207_T001 transcript:EES11358 0
329- 11: transcript:Zm00001d026211_T001 transcript:OQU82237 0
329- 12: transcript:Zm00001d026212_T001 transcript:EES12775 6.00E-104
329- 13: transcript:Zm00001d026213_T001 transcript:OQU82239 0
329- 14: transcript:Zm00001d026214_T002 transcript:OQU82240 8.00E-148
329- 15: transcript:Zm00001d026218_T001 transcript:KXG27029 0
329- 16: transcript:Zm00001d026220_T001 transcript:OQU82243 6.00E-94
329- 17: transcript:Zm00001d026222_T001 transcript:KXG27031 0
329- 18: transcript:Zm00001d026223_T001 transcript:EES12779 3.00E-62
329- 19: transcript:Zm00001d026231_T001 transcript:EES11362 0
329- 20: transcript:Zm00001d026235_T001 transcript:KXG27033 5.00E-79
Alignment 330: score=858.0 e_value=4.8e-58 N=21 10&6 plus
330- 0: transcript:EER89146 transcript:Zm00001d036073_T002 0
330- 1: transcript:KXG19225 transcript:Zm00001d036077_T001 3.00E-98
330- 2: transcript:OQU75768 transcript:Zm00001d036086_T002 0
330- 3: transcript:EER89149 transcript:Zm00001d036088_T001 4.00E-86
330- 4: transcript:EER89151 transcript:Zm00001d036090_T003 0
330- 5: transcript:EER87803 transcript:Zm00001d036091_T001 0
330- 6: transcript:EER87804 transcript:Zm00001d036092_T004 0

330- 7: transcript:KXG19244   transcript:Zm00001d036097_T001 4.00E-161
330- 8: transcript:KXG19245   transcript:Zm00001d036098_T001 4.00E-164
330- 9: transcript:EER89158   transcript:Zm00001d036103_T001 0
330- 10: transcript:EER87808   transcript:Zm00001d036107_T001 1.00E-171
330- 11: transcript:EER89160   transcript:Zm00001d036108_T001 3.00E-174
330- 12: transcript:EER89162   transcript:Zm00001d036110_T002 0
330- 13: transcript:EER87812   transcript:Zm00001d036118_T001 8.00E-98
330- 14: transcript:EER89168   transcript:Zm00001d036122_T001 0
330- 15: transcript:KXG19264   transcript:Zm00001d036123_T001 0
330- 16: transcript:EER89174   transcript:Zm00001d036125_T001 5.00E-53
330- 17: transcript:KXG19267   transcript:Zm00001d036127_T001 3.00E-63
330- 18: transcript:EER87820   transcript:Zm00001d036131_T001 0
330- 19: transcript:EER87823   transcript:Zm00001d036135_T003 5.00E-133
330- 20: transcript:EER87824   transcript:Zm00001d036137_T001 0
Alignment 331: score=829.0 e_value=3e-55 N=19 10&6 plus
331- 0: transcript:Zm00001d026512_T001 transcript:OQU82497 3.00E-146
331- 1: transcript:Zm00001d026513_T001 transcript:EES13021 4.00E-82
331- 2: transcript:Zm00001d026514_T002 transcript:KXG27325 0
331- 3: transcript:Zm00001d026515_T003 transcript:EES11599 0
331- 4: transcript:Zm00001d026516_T002 transcript:EES13023 7.00E-110
331- 5: transcript:Zm00001d026517_T001 transcript:EES11600 0
331- 6: transcript:Zm00001d026518_T001 transcript:EES11601 4.00E-109
331- 7: transcript:Zm00001d026530_T001 transcript:OQU82508 2.00E-28
331- 8: transcript:Zm00001d026531_T002 transcript:KXG27351 0
331- 9: transcript:Zm00001d026532_T007 transcript:EES11604 0
331- 10: transcript:Zm00001d026535_T003 transcript:EES11606 0
331- 11: transcript:Zm00001d026536_T002 transcript:EES13033 0
331- 12: transcript:Zm00001d026537_T001 transcript:OQU82514 3.00E-129
331- 13: transcript:Zm00001d026540_T001 transcript:OQU82517 0
331- 14: transcript:Zm00001d026541_T001 transcript:KXG27277 0
331- 15: transcript:Zm00001d026542_T001 transcript:OQU82518 0
331- 16: transcript:Zm00001d026543_T007 transcript:EES11611 0
331- 17: transcript:Zm00001d026546_T001 transcript:OQU82525 3.00E-67
331- 18: transcript:Zm00001d026547_T004 transcript:OQU82526 3.00E-134
Alignment 332: score=700.0 e_value=1.8e-41 N=16 10&6 plus
332- 0: transcript:EER87933   transcript:Zm00001d037473_T004 0
332- 1: transcript:EER87934   transcript:Zm00001d037476_T001 0
332- 2: transcript:EER87935   transcript:Zm00001d037477_T001 3.00E-114
332- 3: transcript:EER87936   transcript:Zm00001d037479_T001 7.00E-38
332- 4: transcript:KXG19424   transcript:Zm00001d037480_T001 0
332- 5: transcript:EER87942   transcript:Zm00001d037481_T003 0
332- 6: transcript:OQU75934   transcript:Zm00001d037482_T001 2.00E-60
332- 7: transcript:OQU75937   transcript:Zm00001d037483_T002 5.00E-134
332- 8: transcript:KXG19435   transcript:Zm00001d037484_T001 0
332- 9: transcript:EER87950   transcript:Zm00001d037485_T001 5.00E-47
332- 10: transcript:EER87955   transcript:Zm00001d037487_T001 0
332- 11: transcript:KXG19444   transcript:Zm00001d037489_T002 1.00E-100
332- 12: transcript:KXG19445   transcript:Zm00001d037492_T001 0
332- 13: transcript:KXG19454   transcript:Zm00001d037493_T001 0
332- 14: transcript:OQU75953   transcript:Zm00001d037494_T001 1.00E-102
332- 15: transcript:EER89314   transcript:Zm00001d037495_T002 0
Alignment 333: score=658.0 e_value=3.2e-42 N=16 10&6 plus
333- 0: transcript:OQU75869   transcript:Zm00001d036246_T001 4.00E-127
333- 1: transcript:EER87888   transcript:Zm00001d036247_T003 7.00E-141

333- 2: transcript:EER89236   transcript:Zm00001d036250_T001 2.00E-94
333- 3: transcript:EER89242   transcript:Zm00001d036251_T001 2.00E-112
333- 4: transcript:OQU75871   transcript:Zm00001d036255_T001   0
333- 5: transcript:KXG19355   transcript:Zm00001d036256_T001   0
333- 6: transcript:KXG19373   transcript:Zm00001d036270_T001 2.00E-51
333- 7: transcript:EER89249   transcript:Zm00001d036274_T001 2.00E-36
333- 8: transcript:EER89250   transcript:Zm00001d036279_T001 1.00E-26
333- 9: transcript:OQU75886   transcript:Zm00001d036283_T002 3.00E-138
333- 10: transcript:EER89259   transcript:Zm00001d036285_T001   0
333- 11: transcript:EER87917   transcript:Zm00001d036293_T002   0
333- 12: transcript:EER89265   transcript:Zm00001d036296_T004   0
333- 13: transcript:EER87918   transcript:Zm00001d036297_T001   0
333- 14: transcript:EER87919   transcript:Zm00001d036298_T001 1.00E-73
333- 15: transcript:EER87920   transcript:Zm00001d036300_T001   0
Alignment 334: score=654.0 e_value=8.9e-40 N=15 10&6 plus
334- 0: transcript:Zm00001d026484_T001 transcript:KXG27288   2.00E-113
334- 1: transcript:Zm00001d026485_T017 transcript:OQU82469   0
334- 2: transcript:Zm00001d026487_T001 transcript:OQU82476   0
334- 3: transcript:Zm00001d026490_T010 transcript:EES11577   0
334- 4: transcript:Zm00001d026491_T001 transcript:EES11579   0
334- 5: transcript:Zm00001d026492_T001 transcript:EES11583   0
334- 6: transcript:Zm00001d026494_T001 transcript:OQU82489   2.00E-90
334- 7: transcript:Zm00001d026495_T001 transcript:OQU82490   0
334- 8: transcript:Zm00001d026498_T001 transcript:EES13012   5.00E-24
334- 9: transcript:Zm00001d026500_T001 transcript:EES11587   1.00E-38
334- 10: transcript:Zm00001d026501_T009 transcript:EES13013   0
334- 11: transcript:Zm00001d026505_T001 transcript:EES11588   1.00E-91
334- 12: transcript:Zm00001d026506_T001 transcript:EES13014   9.00E-18
334- 13: transcript:Zm00001d026509_T001 transcript:EES11592   3.00E-50
334- 14: transcript:Zm00001d026510_T001 transcript:KXG27318   8.00E-72
Alignment 335: score=606.0 e_value=3.6e-29 N=13 10&6 plus
335- 0: transcript:Zm00001d025952_T002 transcript:EES11192   6.00E-30
335- 1: transcript:Zm00001d025953_T003 transcript:EES11194   0
335- 2: transcript:Zm00001d025955_T001 transcript:OQU82036   0
335- 3: transcript:Zm00001d025957_T001 transcript:EES11195   4.00E-144
335- 4: transcript:Zm00001d025958_T001 transcript:OQU82042   0
335- 5: transcript:Zm00001d025959_T001 transcript:OQU82043   0
335- 6: transcript:Zm00001d025960_T002 transcript:OQU82045   0
335- 7: transcript:Zm00001d025963_T002 transcript:OQU82050   0
335- 8: transcript:Zm00001d025964_T001 transcript:EES11198   3.00E-118
335- 9: transcript:Zm00001d025966_T001 transcript:EES11199   1.00E-155
335- 10: transcript:Zm00001d025967_T001 transcript:EES11200   3.00E-76
335- 11: transcript:Zm00001d025977_T001 transcript:OQU82052   0
335- 12: transcript:Zm00001d025979_T001 transcript:KXG26815   0
Alignment 336: score=552.0 e_value=4.5e-29 N=12 10&6 plus
336- 0: transcript:KXG19576   transcript:Zm00001d037158_T001 4.00E-160
336- 1: transcript:EER88044   transcript:Zm00001d037159_T001   0
336- 2: transcript:EER88045   transcript:Zm00001d037160_T002   0
336- 3: transcript:EER88046   transcript:Zm00001d037163_T002 2.00E-149
336- 4: transcript:EER88047   transcript:Zm00001d037164_T001   0
336- 5: transcript:EER88048   transcript:Zm00001d037165_T001 1.00E-77
336- 6: transcript:EER88049   transcript:Zm00001d037166_T001 1.00E-49
336- 7: transcript:EER89401   transcript:Zm00001d037167_T001 2.00E-133
336- 8: transcript:KXG19586   transcript:Zm00001d037170_T001   0

```
336-  9: transcript:EER89405           transcript:Zm00001d037171_T001  2.00E-28
336- 10: transcript:KXG19588           transcript:Zm00001d037173_T001  9.00E-52
336- 11: transcript:OQU76046           transcript:Zm00001d037174_T003         0
## Alignment 337: score=549.0 e_value=1.6e-32 N=13 10&6 plus
337-  0: transcript:KXG19557           transcript:Zm00001d037115_T024         0
337-  1: transcript:OQU76020           transcript:Zm00001d037117_T001 4.00E-138
337-  2: transcript:EER89392           transcript:Zm00001d037120_T001         0
337-  3: transcript:EER88029           transcript:Zm00001d037122_T009         0
337-  4: transcript:OQU76027           transcript:Zm00001d037124_T001  9.00E-78
337-  5: transcript:OQU76029           transcript:Zm00001d037136_T001 3.00E-120
337-  6: transcript:EER89393           transcript:Zm00001d037140_T004         0
337-  7: transcript:KXG19571           transcript:Zm00001d037141_T001  5.00E-54
337-  8: transcript:EER88041           transcript:Zm00001d037142_T022         0
337-  9: transcript:EER89396           transcript:Zm00001d037150_T001 8.00E-141
337- 10: transcript:EER88039           transcript:Zm00001d037151_T001         0
337- 11: transcript:KXG19573           transcript:Zm00001d037153_T002  2.00E-79
337- 12: transcript:OQU76034           transcript:Zm00001d037156_T034         0
## Alignment 338: score=540.0 e_value=9.8e-32 N=13 10&6 plus
338-  0: transcript:KXG19184           transcript:Zm00001d036020_T002         0
338-  1: transcript:EER87768           transcript:Zm00001d036022_T001  4.00E-76
338-  2: transcript:EER89116           transcript:Zm00001d036023_T001         0
338-  3: transcript:KXG19188           transcript:Zm00001d036024_T001         0
338-  4: transcript:EER87773           transcript:Zm00001d036025_T002  4.00E-36
338-  5: transcript:KXG19189           transcript:Zm00001d036031_T015         0
338-  6: transcript:EER87777           transcript:Zm00001d036035_T001 7.00E-116
338-  7: transcript:EER89124           transcript:Zm00001d036037_T001         0
338-  8: transcript:EER87779           transcript:Zm00001d036044_T001         0
338-  9: transcript:EER87780           transcript:Zm00001d036045_T001         0
338- 10: transcript:EER87781           transcript:Zm00001d036046_T001         0
338- 11: transcript:EER87784           transcript:Zm00001d036050_T001 1.00E-170
338- 12: transcript:KXG19201           transcript:Zm00001d036051_T001         0
## Alignment 339: score=539.0 e_value=2.9e-27 N=12 10&6 plus
339-  0: transcript:Zm00001d026421_T010 transcript:EES11527                   0
339-  1: transcript:Zm00001d026422_T011 transcript:EES11528                   0
339-  2: transcript:Zm00001d026424_T001 transcript:OQU82428            7.00E-66
339-  3: transcript:Zm00001d026425_T003 transcript:EES12950           1.00E-157
339-  4: transcript:Zm00001d026426_T005 transcript:EES12952                   0
339-  5: transcript:Zm00001d026429_T002 transcript:EES12953                   0
339-  6: transcript:Zm00001d026432_T001 transcript:EES11535            6.00E-65
339-  7: transcript:Zm00001d026434_T002 transcript:EES11536                   0
339-  8: transcript:Zm00001d026436_T001 transcript:EES12957           1.00E-122
339-  9: transcript:Zm00001d026437_T001 transcript:KXG27241           5.00E-115
339- 10: transcript:Zm00001d026438_T001 transcript:OQU82434                   0
339- 11: transcript:Zm00001d026439_T009 transcript:KXG27246                   0
## Alignment 340: score=495.0 e_value=5.7e-27 N=11 10&6 plus
340-  0: transcript:Zm00001d025981_T004 transcript:KXG26818                   0
340-  1: transcript:Zm00001d025982_T001 transcript:OQU82063            1.00E-92
340-  2: transcript:Zm00001d025983_T002 transcript:OQU82062                   0
340-  3: transcript:Zm00001d025985_T001 transcript:OQU82069           1.00E-114
340-  4: transcript:Zm00001d025988_T001 transcript:KXG26822                   0
340-  5: transcript:Zm00001d025991_T004 transcript:EES12597           1.00E-159
340-  6: transcript:Zm00001d025992_T001 transcript:OQU82072                   0
340-  7: transcript:Zm00001d025996_T001 transcript:EES11208                   0
340-  8: transcript:Zm00001d025997_T002 transcript:EES11209                   0
```

```
340-  9: transcript:Zm00001d025998_T001 transcript:EES11210           1.00E-163
340- 10: transcript:Zm00001d026000_T004 transcript:EES12606                   0
## Alignment 341: score=487.0 e_value=8.5e-27 N=11 10&6 plus
341-  0: transcript:EER88006           transcript:Zm00001d037103_T001 4.00E-135
341-  1: transcript:OQU76001           transcript:Zm00001d037104_T002 2.00E-121
341-  2: transcript:KXG19532           transcript:Zm00001d037105_T001         0
341-  3: transcript:OQU76003           transcript:Zm00001d037107_T001  4.00E-98
341-  4: transcript:EER89376           transcript:Zm00001d037108_T003         0
341-  5: transcript:EER89379           transcript:Zm00001d037109_T008         0
341-  6: transcript:EER88012           transcript:Zm00001d037110_T002         0
341-  7: transcript:EER88019           transcript:Zm00001d037111_T001  9.00E-98
341-  8: transcript:EER89389           transcript:Zm00001d037112_T001         0
341-  9: transcript:EER88023           transcript:Zm00001d037113_T001 3.00E-101
341- 10: transcript:OQU76019           transcript:Zm00001d037114_T002         0
## Alignment 342: score=471.0 e_value=1.1e-21 N=10 10&6 plus
342-  0: transcript:Zm00001d025817_T041 transcript:KXG26674                   0
342-  1: transcript:Zm00001d025818_T001 transcript:OQU81918                   0
342-  2: transcript:Zm00001d025819_T001 transcript:OQU81917                   0
342-  3: transcript:Zm00001d025820_T001 transcript:EES11089                   0
342-  4: transcript:Zm00001d025821_T001 transcript:EES12472                   0
342-  5: transcript:Zm00001d025822_T001 transcript:EES11092            2.00E-35
342-  6: transcript:Zm00001d025823_T001 transcript:KXG26684           3.00E-147
342-  7: transcript:Zm00001d025824_T001 transcript:EES11097                   0
342-  8: transcript:Zm00001d025825_T001 transcript:OQU81932                   0
342-  9: transcript:Zm00001d025827_T002 transcript:KXG26688                   0
## Alignment 343: score=464.0 e_value=7.6e-25 N=11 10&6 plus
343-  0: transcript:Zm00001d025240_T036 transcript:EES10634                   0
343-  1: transcript:Zm00001d025241_T002 transcript:KXG26117            6.00E-93
343-  2: transcript:Zm00001d025247_T001 transcript:KXG26124            1.00E-66
343-  3: transcript:Zm00001d025249_T001 transcript:EES10648                   0
343-  4: transcript:Zm00001d025251_T001 transcript:KXG26128           4.00E-177
343-  5: transcript:Zm00001d025252_T001 transcript:EES12040            1.00E-82
343-  6: transcript:Zm00001d025255_T001 transcript:KXG26129                   0
343-  7: transcript:Zm00001d025263_T002 transcript:EES10655                   0
343-  8: transcript:Zm00001d025265_T001 transcript:EES10658            6.00E-14
343-  9: transcript:Zm00001d025268_T011 transcript:KXG26136                   0
343- 10: transcript:Zm00001d025271_T001 transcript:KXG26137           7.00E-129
## Alignment 344: score=409.0 e_value=1.6e-23 N=10 10&6 plus
344-  0: transcript:KXG19297           transcript:Zm00001d036164_T001         0
344-  1: transcript:EER87840           transcript:Zm00001d036175_T001 1.00E-147
344-  2: transcript:OQU75822           transcript:Zm00001d036176_T003  8.00E-64
344-  3: transcript:OQU75826           transcript:Zm00001d036177_T001         0
344-  4: transcript:EER89205           transcript:Zm00001d036178_T001         0
344-  5: transcript:EER87855           transcript:Zm00001d036197_T001 4.00E-167
344-  6: transcript:EER89214           transcript:Zm00001d036206_T001         0
344-  7: transcript:EER87860           transcript:Zm00001d036213_T002  2.00E-49
344-  8: transcript:KXG19322           transcript:Zm00001d036214_T001         0
344-  9: transcript:EER87865           transcript:Zm00001d036224_T001 1.00E-118
## Alignment 345: score=397.0 e_value=6.4e-20 N=9 10&6 plus
345-  0: transcript:Zm00001d025228_T003 transcript:OQU81387            1.00E-97
345-  1: transcript:Zm00001d025229_T001 transcript:OQU81391           3.00E-163
345-  2: transcript:Zm00001d025233_T001 transcript:KXG26102            1.00E-89
345-  3: transcript:Zm00001d025234_T001 transcript:KXG26103                   0
345-  4: transcript:Zm00001d025235_T004 transcript:OQU81398                   0
```

345- 5: transcript:Zm00001d025236_T001 transcript:EES12034 3.00E-101
345- 6: transcript:Zm00001d025237_T001 transcript:EES10631 0
345- 7: transcript:Zm00001d025238_T001 transcript:EES10633 0
345- 8: transcript:Zm00001d025239_T001 transcript:EES12036 4.00E-61
Alignment 346: score=394.0 e_value=4.2e-18 N=9 10&6 plus
346- 0: transcript:EER89342 transcript:Zm00001d037072_T001 4.00E-28
346- 1: transcript:EER87979 transcript:Zm00001d037073_T001 0
346- 2: transcript:EER87981 transcript:Zm00001d037079_T001 4.00E-153
346- 3: transcript:OQU75978 transcript:Zm00001d037080_T001 0
346- 4: transcript:KXG19494 transcript:Zm00001d037084_T004 0
346- 5: transcript:KXG19495 transcript:Zm00001d037085_T005 0
346- 6: transcript:OQU75979 transcript:Zm00001d037086_T006 0
346- 7: transcript:OQU75981 transcript:Zm00001d037087_T001 0
346- 8: transcript:OQU75985 transcript:Zm00001d037089_T001 0
Alignment 347: score=381.0 e_value=5.5e-17 N=9 10&6 plus
347- 0: transcript:Zm00001d025281_T001 transcript:OQU81440 3.00E-17
347- 1: transcript:Zm00001d025286_T001 transcript:EES10690 0
347- 2: transcript:Zm00001d025287_T001 transcript:EES12056 2.00E-96
347- 3: transcript:Zm00001d025291_T001 transcript:EES12060 1.00E-136
347- 4: transcript:Zm00001d025296_T001 transcript:EES10692 3.00E-117
347- 5: transcript:Zm00001d025298_T001 transcript:EES10693 1.00E-110
347- 6: transcript:Zm00001d025299_T001 transcript:KXG26165 7.00E-06
347- 7: transcript:Zm00001d025300_T007 transcript:OQU81455 0
347- 8: transcript:Zm00001d025303_T004 transcript:EES10698 0
Alignment 348: score=375.0 e_value=2.4e-15 N=8 10&6 plus
348- 0: transcript:Zm00001d025831_T001 transcript:KXG26692 0
348- 1: transcript:Zm00001d025833_T001 transcript:EES12484 0
348- 2: transcript:Zm00001d025834_T002 transcript:EES12485 0
348- 3: transcript:Zm00001d025837_T001 transcript:KXG26693 0
348- 4: transcript:Zm00001d025842_T001 transcript:EES12491 7.00E-61
348- 5: transcript:Zm00001d025844_T001 transcript:KXG26699 0
348- 6: transcript:Zm00001d025845_T001 transcript:EES11110 1.00E-93
348- 7: transcript:Zm00001d025846_T001 transcript:OQU81951 0
Alignment 349: score=306.0 e_value=2.9e-11 N=7 10&6 plus
349- 0: transcript:OQU76854 transcript:Zm00001d036629_T010 0
349- 1: transcript:KXG20578 transcript:Zm00001d036630_T001 0
349- 2: transcript:EER90198 transcript:Zm00001d036631_T002 1.00E-151
349- 3: transcript:KXG20581 transcript:Zm00001d036632_T002 9.00E-105
349- 4: transcript:KXG20587 transcript:Zm00001d036635_T001 0
349- 5: transcript:KXG20586 transcript:Zm00001d036637_T001 0
349- 6: transcript:OQU76857 transcript:Zm00001d036638_T001 3.00E-105
Alignment 350: score=282.0 e_value=1.3e-09 N=6 10&6 plus
350- 0: transcript:EER89710 transcript:Zm00001d037429_T001 0
350- 1: transcript:EER88342 transcript:Zm00001d037434_T001 0
350- 2: transcript:EER88345 transcript:Zm00001d037435_T001 1.00E-47
350- 3: transcript:OQU76359 transcript:Zm00001d037436_T001 1.00E-17
350- 4: transcript:KXG19937 transcript:Zm00001d037438_T002 0
350- 5: transcript:KXG19938 transcript:Zm00001d037439_T001 5.00E-107
Alignment 351: score=276.0 e_value=1.1e-08 N=6 10&6 plus
351- 0: transcript:OQU76632 transcript:Zm00001d036927_T001 0
351- 1: transcript:EER88574 transcript:Zm00001d036929_T001 0
351- 2: transcript:EER89985 transcript:Zm00001d036930_T001 6.00E-39
351- 3: transcript:EER89986 transcript:Zm00001d036931_T001 0
351- 4: transcript:KXG20268 transcript:Zm00001d036933_T001 0

```
351-  5: transcript:EER88586           transcript:Zm00001d036936_T002 1.00E-175
## Alignment 352: score=275.0 e_value=4.2e-10 N=6 10&6 plus
352-  0: transcript:OQU75991           transcript:Zm00001d037094_T001 1.00E-114
352-  1: transcript:EER87996           transcript:Zm00001d037095_T001 3.00E-128
352-  2: transcript:EER89368           transcript:Zm00001d037096_T004 3.00E-149
352-  3: transcript:EER89369           transcript:Zm00001d037097_T001  3.00E-72
352-  4: transcript:EER88000           transcript:Zm00001d037098_T003         0
352-  5: transcript:EER89373           transcript:Zm00001d037099_T012         0
## Alignment 353: score=2016.0 e_value=2.1e-158 N=44 10&6 minus
353-  0: transcript:Zm00001d026119_T002 transcript:EES12673           3.00E-46
353-  1: transcript:Zm00001d026120_T001 transcript:OQU82140          3.00E-113
353-  2: transcript:Zm00001d026121_T001 transcript:OQU82139                  0
353-  3: transcript:Zm00001d026122_T004 transcript:OQU82137                  0
353-  4: transcript:Zm00001d026124_T001 transcript:KXG26904                  0
353-  5: transcript:Zm00001d026126_T001 transcript:KXG26903                  0
353-  6: transcript:Zm00001d026127_T001 transcript:OQU82135                  0
353-  7: transcript:Zm00001d026129_T001 transcript:EES12664                  0
353-  8: transcript:Zm00001d026130_T002 transcript:KXG26898                  0
353-  9: transcript:Zm00001d026131_T001 transcript:EES12656                  0
353- 10: transcript:Zm00001d026132_T001 transcript:EES12655                  0
353- 11: transcript:Zm00001d026133_T001 transcript:KXG26897          7.00E-122
353- 12: transcript:Zm00001d026135_T002 transcript:KXG26894                  0
353- 13: transcript:Zm00001d026137_T002 transcript:EES11260           7.00E-71
353- 14: transcript:Zm00001d026139_T001 transcript:EES12651                  0
353- 15: transcript:Zm00001d026140_T001 transcript:EES11255                  0
353- 16: transcript:Zm00001d026143_T001 transcript:KXG26889           3.00E-58
353- 17: transcript:Zm00001d026144_T004 transcript:KXG26887          3.00E-117
353- 18: transcript:Zm00001d026147_T002 transcript:EES11251          1.00E-174
353- 19: transcript:Zm00001d026148_T002 transcript:KXG26871           3.00E-49
353- 20: transcript:Zm00001d026152_T001 transcript:OQU82115           5.00E-82
353- 21: transcript:Zm00001d026154_T002 transcript:EES11249          2.00E-114
353- 22: transcript:Zm00001d026156_T002 transcript:OQU82104                  0
353- 23: transcript:Zm00001d026158_T001 transcript:EES11245                  0
353- 24: transcript:Zm00001d026159_T001 transcript:EES12624           1.00E-17
353- 25: transcript:Zm00001d026160_T001 transcript:EES11244           8.00E-55
353- 26: transcript:Zm00001d026162_T001 transcript:EES11241           4.00E-62
353- 27: transcript:Zm00001d026164_T001 transcript:EES12623           9.00E-71
353- 28: transcript:Zm00001d026165_T003 transcript:OQU82099                  0
353- 29: transcript:Zm00001d026166_T002 transcript:EES11237                  0
353- 30: transcript:Zm00001d026169_T001 transcript:EES11233          9.00E-138
353- 31: transcript:Zm00001d026173_T001 transcript:OQU82096           1.00E-57
353- 32: transcript:Zm00001d026175_T001 transcript:EES11232          1.00E-137
353- 33: transcript:Zm00001d026176_T008 transcript:KXG26851                  0
353- 34: transcript:Zm00001d026177_T001 transcript:OQU82093           1.00E-60
353- 35: transcript:Zm00001d026180_T001 transcript:KXG26850           7.00E-98
353- 36: transcript:Zm00001d026182_T001 transcript:OQU82089           4.00E-75
353- 37: transcript:Zm00001d026184_T001 transcript:OQU82086           2.00E-93
353- 38: transcript:Zm00001d026185_T001 transcript:OQU82085          2.00E-180
353- 39: transcript:Zm00001d026186_T001 transcript:EES11224          4.00E-163
353- 40: transcript:Zm00001d026187_T001 transcript:EES11223           9.00E-36
353- 41: transcript:Zm00001d026189_T001 transcript:EES12610                  0
353- 42: transcript:Zm00001d026190_T001 transcript:EES11216          2.00E-140
353- 43: transcript:Zm00001d026191_T001 transcript:OQU82076          3.00E-138
## Alignment 354: score=1850.0 e_value=1.3e-153 N=42 10&6 minus
```

354- 0: transcript:EER88642 transcript:Zm00001d036815_T001 0
354- 1: transcript:EER88644 transcript:Zm00001d036812_T001 0
354- 2: transcript:KXG20357 transcript:Zm00001d036807_T001 2.00E-161
354- 3: transcript:KXG20365 transcript:Zm00001d036803_T001 1.00E-41
354- 4: transcript:EER90047 transcript:Zm00001d036801_T001 0
354- 5: transcript:KXG20366 transcript:Zm00001d036798_T002 0
354- 6: transcript:EER88659 transcript:Zm00001d036797_T002 0
354- 7: transcript:KXG20371 transcript:Zm00001d036796_T004 0
354- 8: transcript:OQU76714 transcript:Zm00001d036795_T002 0
354- 9: transcript:EER90053 transcript:Zm00001d036791_T001 5.00E-18
354- 10: transcript:KXG20383 transcript:Zm00001d036790_T009 0
354- 11: transcript:EER90058 transcript:Zm00001d036789_T003 3.00E-178
354- 12: transcript:KXG20396 transcript:Zm00001d036788_T003 0
354- 13: transcript:EER88673 transcript:Zm00001d036787_T001 1.00E-58
354- 14: transcript:OQU76732 transcript:Zm00001d036785_T003 0
354- 15: transcript:EER90068 transcript:Zm00001d036784_T002 0
354- 16: transcript:KXG20413 transcript:Zm00001d036782_T001 4.00E-70
354- 17: transcript:EER88680 transcript:Zm00001d036781_T001 1.00E-31
354- 18: transcript:EER88683 transcript:Zm00001d036780_T001 1.00E-23
354- 19: transcript:KXG20425 transcript:Zm00001d036778_T001 7.00E-26
354- 20: transcript:OQU76744 transcript:Zm00001d036777_T001 0
354- 21: transcript:KXG20428 transcript:Zm00001d036775_T001 0
354- 22: transcript:EER90080 transcript:Zm00001d036773_T001 0
354- 23: transcript:KXG20463 transcript:Zm00001d036772_T002 4.00E-109
354- 24: transcript:EER90083 transcript:Zm00001d036771_T001 0
354- 25: transcript:EER90082 transcript:Zm00001d036770_T001 0
354- 26: transcript:EER88692 transcript:Zm00001d036769_T001 5.00E-44
354- 27: transcript:EER90085 transcript:Zm00001d036768_T001 2.00E-125
354- 28: transcript:EER90087 transcript:Zm00001d036766_T001 1.00E-86
354- 29: transcript:KXG20452 transcript:Zm00001d036765_T009 0
354- 30: transcript:KXG20456 transcript:Zm00001d036764_T004 0
354- 31: transcript:EER88700 transcript:Zm00001d036763_T001 0
354- 32: transcript:EER88701 transcript:Zm00001d036762_T001 0
354- 33: transcript:EER88702 transcript:Zm00001d036760_T001 7.00E-34
354- 34: transcript:EER90100 transcript:Zm00001d036759_T002 0
354- 35: transcript:EER90101 transcript:Zm00001d036756_T001 2.00E-67
354- 36: transcript:EER88704 transcript:Zm00001d036752_T001 1.00E-29
354- 37: transcript:OQU76766 transcript:Zm00001d036751_T001 0
354- 38: transcript:EER90104 transcript:Zm00001d036750_T001 0
354- 39: transcript:OQU76768 transcript:Zm00001d036749_T001 0
354- 40: transcript:EER88706 transcript:Zm00001d036748_T001 1.00E-139
354- 41: transcript:EER88713 transcript:Zm00001d036742_T007 0
Alignment 355: score=1741.0 e_value=6.6e-137 N=38 10&6 minus
355- 0: transcript:EER90112 transcript:Zm00001d036738_T001 0
355- 1: transcript:OQU76779 transcript:Zm00001d036737_T001 2.00E-26
355- 2: transcript:EER90114 transcript:Zm00001d036736_T001 6.00E-90
355- 3: transcript:EER88722 transcript:Zm00001d036735_T002 0
355- 4: transcript:EER88725 transcript:Zm00001d036728_T001 0
355- 5: transcript:EER90117 transcript:Zm00001d036727_T001 0
355- 6: transcript:EER88729 transcript:Zm00001d036726_T001 9.00E-139
355- 7: transcript:KXG20497 transcript:Zm00001d036724_T001 5.00E-10
355- 8: transcript:KXG20499 transcript:Zm00001d036723_T001 9.00E-39
355- 9: transcript:EER88731 transcript:Zm00001d036720_T001 0
355- 10: transcript:EER90125 transcript:Zm00001d036719_T001 0

355- 11:	transcript:OQU76791	transcript:Zm00001d036718_T002	0
355- 12:	transcript:EER88734	transcript:Zm00001d036717_T001	8.00E-96
355- 13:	transcript:EER90127	transcript:Zm00001d036716_T001	0
355- 14:	transcript:EER88736	transcript:Zm00001d036715_T001	0
355- 15:	transcript:OQU76793	transcript:Zm00001d036714_T002	0
355- 16:	transcript:OQU76796	transcript:Zm00001d036711_T001	5.00E-108
355- 17:	transcript:EER90135	transcript:Zm00001d036710_T002	0
355- 18:	transcript:EER90141	transcript:Zm00001d036709_T001	0
355- 19:	transcript:EER90142	transcript:Zm00001d036708_T001	5.00E-43
355- 20:	transcript:KXG20513	transcript:Zm00001d036707_T006	0
355- 21:	transcript:EER88755	transcript:Zm00001d036704_T002	0
355- 22:	transcript:EER88756	transcript:Zm00001d036703_T001	2.00E-44
355- 23:	transcript:EER88759	transcript:Zm00001d036701_T001	0
355- 24:	transcript:EER88760	transcript:Zm00001d036700_T001	3.00E-76
355- 25:	transcript:EER90151	transcript:Zm00001d036699_T001	4.00E-120
355- 26:	transcript:KXG20531	transcript:Zm00001d036698_T001	0
355- 27:	transcript:EER88765	transcript:Zm00001d036692_T001	1.00E-174
355- 28:	transcript:EER90153	transcript:Zm00001d036690_T001	5.00E-34
355- 29:	transcript:KXG20524	transcript:Zm00001d036689_T001	0
355- 30:	transcript:EER88766	transcript:Zm00001d036688_T001	0
355- 31:	transcript:EER90158	transcript:Zm00001d036683_T001	5.00E-39
355- 32:	transcript:EER88770	transcript:Zm00001d036679_T001	4.00E-167
355- 33:	transcript:OQU76823	transcript:Zm00001d036678_T002	0
355- 34:	transcript:OQU76824	transcript:Zm00001d036676_T001	2.00E-67
355- 35:	transcript:KXG20532	transcript:Zm00001d036673_T001	0
355- 36:	transcript:KXG20534	transcript:Zm00001d036672_T005	0
355- 37:	transcript:EER90161	transcript:Zm00001d036671_T001	4.00E-76

Alignment 356: score=1519.0 e_value=2e-107 N=33 10&6 minus

356- 0:	transcript:Zm00001d026050_T001	transcript:OQU82196	0
356- 1:	transcript:Zm00001d026051_T002	transcript:KXG26972	0
356- 2:	transcript:Zm00001d026053_T001	transcript:KXG26971	3.00E-73
356- 3:	transcript:Zm00001d026055_T001	transcript:EES12701	0
356- 4:	transcript:Zm00001d026056_T002	transcript:EES11307	4.00E-156
356- 5:	transcript:Zm00001d026060_T003	transcript:EES12700	0
356- 6:	transcript:Zm00001d026061_T001	transcript:EES11304	4.00E-123
356- 7:	transcript:Zm00001d026062_T001	transcript:EES11302	0
356- 8:	transcript:Zm00001d026063_T001	transcript:OQU82190	0
356- 9:	transcript:Zm00001d026064_T005	transcript:KXG26962	0
356- 10:	transcript:Zm00001d026066_T001	transcript:EES12695	0
356- 11:	transcript:Zm00001d026067_T001	transcript:EES11294	0
356- 12:	transcript:Zm00001d026068_T001	transcript:EES12694	0
356- 13:	transcript:Zm00001d026069_T001	transcript:OQU82186	9.00E-37
356- 14:	transcript:Zm00001d026070_T002	transcript:OQU82184	0
356- 15:	transcript:Zm00001d026072_T001	transcript:KXG26952	7.00E-48
356- 16:	transcript:Zm00001d026076_T001	transcript:EES11285	0
356- 17:	transcript:Zm00001d026078_T001	transcript:EES11284	1.00E-138
356- 18:	transcript:Zm00001d026079_T001	transcript:KXG26943	0
356- 19:	transcript:Zm00001d026083_T001	transcript:EES12690	0
356- 20:	transcript:Zm00001d026084_T001	transcript:EES12687	0
356- 21:	transcript:Zm00001d026088_T002	transcript:KXG26935	0
356- 22:	transcript:Zm00001d026089_T001	transcript:EES11279	0
356- 23:	transcript:Zm00001d026091_T001	transcript:KXG26934	6.00E-177
356- 24:	transcript:Zm00001d026094_T001	transcript:OQU82174	6.00E-155
356- 25:	transcript:Zm00001d026095_T001	transcript:KXG26931	4.00E-122

356- 26: transcript:Zm00001d026096_T001 transcript:OQU82171 5.00E-125
356- 27: transcript:Zm00001d026097_T005 transcript:EES11275 0
356- 28: transcript:Zm00001d026102_T001 transcript:EES12682 0
356- 29: transcript:Zm00001d026104_T002 transcript:OQU82165 4.00E-61
356- 30: transcript:Zm00001d026106_T001 transcript:EES11274 1.00E-145
356- 31: transcript:Zm00001d026109_T001 transcript:EES11272 0
356- 32: transcript:Zm00001d026111_T008 transcript:OQU82149 0
Alignment 357: score=1104.0 e_value=1.3e-78 N=25 10&6 minus
357- 0: transcript:Zm00001d024897_T001 transcript:EES11750 2.00E-27
357- 1: transcript:Zm00001d024898_T001 transcript:EES11748 2.00E-167
357- 2: transcript:Zm00001d024902_T001 transcript:OQU81065 9.00E-49
357- 3: transcript:Zm00001d024903_T001 transcript:EES11741 0
357- 4: transcript:Zm00001d024904_T001 transcript:EES11739 0
357- 5: transcript:Zm00001d024905_T001 transcript:EES10353 2.00E-110
357- 6: transcript:Zm00001d024906_T001 transcript:EES10351 1.00E-156
357- 7: transcript:Zm00001d024908_T003 transcript:EES10350 0
357- 8: transcript:Zm00001d024909_T001 transcript:OQU81054 1.00E-94
357- 9: transcript:Zm00001d024913_T001 transcript:EES11737 1.00E-100
357- 10: transcript:Zm00001d024916_T001 transcript:EES10341 0
357- 11: transcript:Zm00001d024919_T001 transcript:EES10340 7.00E-120
357- 12: transcript:Zm00001d024925_T001 transcript:EES10337 0
357- 13: transcript:Zm00001d024926_T001 transcript:EES10336 3.00E-54
357- 14: transcript:Zm00001d024927_T002 transcript:EES10335 0
357- 15: transcript:Zm00001d024928_T003 transcript:EES10334 0
357- 16: transcript:Zm00001d024933_T003 transcript:EES11733 0
357- 17: transcript:Zm00001d024934_T001 transcript:OQU81046 0
357- 18: transcript:Zm00001d024935_T001 transcript:EES10332 3.00E-150
357- 19: transcript:Zm00001d024936_T002 transcript:EES11732 0
357- 20: transcript:Zm00001d024937_T001 transcript:KXG25757 0
357- 21: transcript:Zm00001d024938_T005 transcript:EES11731 1.00E-61
357- 22: transcript:Zm00001d024939_T002 transcript:KXG25754 1.00E-142
357- 23: transcript:Zm00001d024940_T001 transcript:OQU81039 0
357- 24: transcript:Zm00001d024941_T002 transcript:EES10326 3.00E-161
Alignment 358: score=1026.0 e_value=9.8e-68 N=23 10&6 minus
358- 0: transcript:EER88591 transcript:Zm00001d036925_T001 0
358- 1: transcript:EER89996 transcript:Zm00001d036922_T001 0
358- 2: transcript:EER88593 transcript:Zm00001d036919_T002 0
358- 3: transcript:KXG20284 transcript:Zm00001d036918_T001 1.00E-93
358- 4: transcript:KXG20286 transcript:Zm00001d036917_T009 0
358- 5: transcript:OQU76657 transcript:Zm00001d036912_T001 0
358- 6: transcript:EER90002 transcript:Zm00001d036905_T002 0
358- 7: transcript:KXG20293 transcript:Zm00001d036904_T005 0
358- 8: transcript:KXG20313 transcript:Zm00001d036903_T001 0
358- 9: transcript:EER88608 transcript:Zm00001d036902_T001 3.00E-64
358- 10: transcript:OQU76673 transcript:Zm00001d036900_T001 0
358- 11: transcript:KXG20315 transcript:Zm00001d036897_T001 0
358- 12: transcript:EER88614 transcript:Zm00001d036895_T001 0
358- 13: transcript:EER90009 transcript:Zm00001d036894_T001 8.00E-174
358- 14: transcript:EER90015 transcript:Zm00001d036893_T001 5.00E-86
358- 15: transcript:KXG20318 transcript:Zm00001d036892_T001 1.00E-159
358- 16: transcript:EER90018 transcript:Zm00001d036889_T001 1.00E-84
358- 17: transcript:EER90019 transcript:Zm00001d036888_T002 0
358- 18: transcript:KXG20323 transcript:Zm00001d036883_T001 4.00E-91
358- 19: transcript:EER90022 transcript:Zm00001d036880_T003 0

```
358- 20: transcript:EER90021            transcript:Zm00001d036879_T001          0
358- 21: transcript:KXG20324            transcript:Zm00001d036878_T007          0
358- 22: transcript:KXG20330            transcript:Zm00001d036877_T001 2.00E-169
## Alignment 359: score=960.0 e_value=6.1e-63 N=21 10&6 minus
359-  0: transcript:EER90355            transcript:Zm00001d036447_T003 5.00E-121
359-  1: transcript:KXG20822            transcript:Zm00001d036446_T001  3.00E-63
359-  2: transcript:EER88930            transcript:Zm00001d036443_T002 8.00E-128
359-  3: transcript:EER88931            transcript:Zm00001d036442_T001  4.00E-75
359-  4: transcript:EER88935            transcript:Zm00001d036441_T001  5.00E-69
359-  5: transcript:KXG20825            transcript:Zm00001d036440_T003          0
359-  6: transcript:KXG20827            transcript:Zm00001d036439_T001          0
359-  7: transcript:KXG20831            transcript:Zm00001d036438_T001  3.00E-17
359-  8: transcript:OQU77072            transcript:Zm00001d036436_T001  5.00E-31
359-  9: transcript:KXG20833            transcript:Zm00001d036435_T001 1.00E-100
359- 10: transcript:EER88944            transcript:Zm00001d036432_T003  8.00E-09
359- 11: transcript:KXG20844            transcript:Zm00001d036431_T001 9.00E-102
359- 12: transcript:EER88949            transcript:Zm00001d036430_T001 7.00E-115
359- 13: transcript:EER88950            transcript:Zm00001d036429_T006          0
359- 14: transcript:EER90369            transcript:Zm00001d036428_T006          0
359- 15: transcript:EER88951            transcript:Zm00001d036426_T001 6.00E-149
359- 16: transcript:EER90370            transcript:Zm00001d036425_T002 3.00E-153
359- 17: transcript:OQU77087            transcript:Zm00001d036423_T002          0
359- 18: transcript:KXG20848            transcript:Zm00001d036422_T003          0
359- 19: transcript:KXG20850            transcript:Zm00001d036418_T001 3.00E-126
359- 20: transcript:EER90374            transcript:Zm00001d036417_T004  8.00E-84
## Alignment 360: score=902.0 e_value=0 N=20 10&6 minus
360-  0: transcript:KXG20873            transcript:Zm00001d036416_T001          0
360-  1: transcript:EER90388            transcript:Zm00001d036415_T001  8.00E-52
360-  2: transcript:KXG20882            transcript:Zm00001d036410_T002          0
360-  3: transcript:EER88959            transcript:Zm00001d036409_T001  5.00E-53
360-  4: transcript:EER90393            transcript:Zm00001d036406_T001 6.00E-163
360-  5: transcript:EER88961            transcript:Zm00001d036403_T002 2.00E-165
360-  6: transcript:KXG20892            transcript:Zm00001d036402_T002          0
360-  7: transcript:KXG20895            transcript:Zm00001d036401_T001          0
360-  8: transcript:EER90398            transcript:Zm00001d036400_T001  6.00E-65
360-  9: transcript:KXG20897            transcript:Zm00001d036398_T001  1.00E-65
360- 10: transcript:KXG20902            transcript:Zm00001d036396_T001          0
360- 11: transcript:EER90402            transcript:Zm00001d036395_T016          0
360- 12: transcript:EER88970            transcript:Zm00001d036394_T001  7.00E-16
360- 13: transcript:EER90406            transcript:Zm00001d036392_T001  1.00E-98
360- 14: transcript:EER88975            transcript:Zm00001d036388_T002          0
360- 15: transcript:OQU77122            transcript:Zm00001d036387_T001          0
360- 16: transcript:OQU77124            transcript:Zm00001d036386_T001          0
360- 17: transcript:EER88977            transcript:Zm00001d036382_T001 5.00E-155
360- 18: transcript:EER88980            transcript:Zm00001d036376_T001  1.00E-10
360- 19: transcript:OQU77138            transcript:Zm00001d036373_T001  2.00E-27
## Alignment 361: score=869.0 e_value=9.9e-54 N=19 10&6 minus
361-  0: transcript:EER88892            transcript:Zm00001d036507_T004          0
361-  1: transcript:OQU76985            transcript:Zm00001d036506_T001          0
361-  2: transcript:EER88891            transcript:Zm00001d036499_T001  6.00E-56
361-  3: transcript:OQU76986            transcript:Zm00001d036496_T001          0
361-  4: transcript:EER90312            transcript:Zm00001d036495_T001          0
361-  5: transcript:EER88899            transcript:Zm00001d036494_T001 6.00E-173
361-  6: transcript:EER90313            transcript:Zm00001d036490_T001          0
```

```
361-  7: transcript:OQU76988           transcript:Zm00001d036489_T001          0
361-  8: transcript:KXG20763           transcript:Zm00001d036485_T003 7.00E-156
361-  9: transcript:KXG20764           transcript:Zm00001d036484_T001          0
361- 10: transcript:EER88901           transcript:Zm00001d036483_T006          0
361- 11: transcript:KXG20769           transcript:Zm00001d036482_T001          0
361- 12: transcript:EER88903           transcript:Zm00001d036481_T001 8.00E-135
361- 13: transcript:OQU76995           transcript:Zm00001d036480_T001          0
361- 14: transcript:KXG20771           transcript:Zm00001d036477_T002          0
361- 15: transcript:KXG20774           transcript:Zm00001d036475_T001          0
361- 16: transcript:EER90325           transcript:Zm00001d036465_T001  7.00E-82
361- 17: transcript:EER90326           transcript:Zm00001d036464_T001  1.00E-37
361- 18: transcript:OQU76999           transcript:Zm00001d036463_T001  2.00E-71
## Alignment 362: score=789.0 e_value=6.7e-54 N=18 10&6 minus
362-  0: transcript:OQU76881           transcript:Zm00001d036608_T003          0
362-  1: transcript:EER90214           transcript:Zm00001d036602_T003          0
362-  2: transcript:EER88811           transcript:Zm00001d036598_T006          0
362-  3: transcript:EER90217           transcript:Zm00001d036597_T001  2.00E-68
362-  4: transcript:EER88812           transcript:Zm00001d036594_T001          0
362-  5: transcript:EER90218           transcript:Zm00001d036593_T001          0
362-  6: transcript:KXG20626           transcript:Zm00001d036589_T001  8.00E-08
362-  7: transcript:EER88814           transcript:Zm00001d036588_T001 2.00E-156
362-  8: transcript:OQU76887           transcript:Zm00001d036579_T003          0
362-  9: transcript:EER90224           transcript:Zm00001d036577_T001 8.00E-144
362- 10: transcript:OQU76888           transcript:Zm00001d036574_T001  1.00E-52
362- 11: transcript:EER90225           transcript:Zm00001d036573_T002          0
362- 12: transcript:KXG20635           transcript:Zm00001d036571_T002          0
362- 13: transcript:KXG20637           transcript:Zm00001d036570_T001  1.00E-93
362- 14: transcript:KXG20642           transcript:Zm00001d036567_T002  7.00E-85
362- 15: transcript:KXG20650           transcript:Zm00001d036564_T001          0
362- 16: transcript:EER90236           transcript:Zm00001d036563_T001          0
362- 17: transcript:EER88830           transcript:Zm00001d036560_T001          0
## Alignment 363: score=700.0 e_value=1.4e-43 N=17 10&6 minus
363-  0: transcript:Zm00001d024314_T001 transcript:EES11410                    0
363-  1: transcript:Zm00001d024317_T001 transcript:EES11405            9.00E-126
363-  2: transcript:Zm00001d024324_T001 transcript:EES11400             1.00E-25
363-  3: transcript:Zm00001d024327_T001 transcript:OQU82282                    0
363-  4: transcript:Zm00001d024338_T008 transcript:EES12824                    0
363-  5: transcript:Zm00001d024339_T001 transcript:KXG27074                    0
363-  6: transcript:Zm00001d024342_T001 transcript:KXG27072                    0
363-  7: transcript:Zm00001d024348_T002 transcript:EES12818                    0
363-  8: transcript:Zm00001d024357_T001 transcript:KXG27066             4.00E-37
363-  9: transcript:Zm00001d024364_T001 transcript:EES12814                    0
363- 10: transcript:Zm00001d024371_T001 transcript:EES12810             3.00E-22
363- 11: transcript:Zm00001d024373_T001 transcript:KXG27000            2.00E-131
363- 12: transcript:Zm00001d024376_T001 transcript:EES11384             5.00E-70
363- 13: transcript:Zm00001d024392_T001 transcript:EES12801            8.00E-115
363- 14: transcript:Zm00001d024403_T001 transcript:EES12799             3.00E-28
363- 15: transcript:Zm00001d024406_T003 transcript:EES12797            3.00E-100
363- 16: transcript:Zm00001d024408_T005 transcript:KXG27049                    0
## Alignment 364: score=659.0 e_value=1e-43 N=16 10&6 minus
364-  0: transcript:EER89851           transcript:Zm00001d037041_T001          0
364-  1: transcript:KXG20093           transcript:Zm00001d037036_T001          0
364-  2: transcript:OQU76494           transcript:Zm00001d037035_T002          0
364-  3: transcript:EER88433           transcript:Zm00001d037034_T002          0
```

364- 4: transcript:OQU76497 transcript:Zm00001d037033_T001 7.00E-38
364- 5: transcript:OQU76499 transcript:Zm00001d037029_T001 2.00E-136
364- 6: transcript:EER89867 transcript:Zm00001d037025_T001 0
364- 7: transcript:EER89868 transcript:Zm00001d037024_T001 2.00E-23
364- 8: transcript:EER89870 transcript:Zm00001d037023_T001 2.00E-62
364- 9: transcript:KXG20125 transcript:Zm00001d037019_T001 5.00E-23
364- 10: transcript:EER89876 transcript:Zm00001d037018_T001 6.00E-86
364- 11: transcript:EER88476 transcript:Zm00001d037017_T001 0
364- 12: transcript:OQU76531 transcript:Zm00001d037015_T001 0
364- 13: transcript:EER89895 transcript:Zm00001d037013_T001 8.00E-162
364- 14: transcript:KXG20146 transcript:Zm00001d037009_T001 0
364- 15: transcript:EER89907 transcript:Zm00001d037008_T001 0
Alignment 365: score=620.0 e_value=2.2e-41 N=16 10&6 minus
365- 0: transcript:EER88189 transcript:Zm00001d037328_T001 5.00E-180
365- 1: transcript:EER88191 transcript:Zm00001d037327_T001 0
365- 2: transcript:KXG19738 transcript:Zm00001d037326_T002 0
365- 3: transcript:EER89571 transcript:Zm00001d037323_T001 5.00E-100
365- 4: transcript:KXG19752 transcript:Zm00001d037318_T001 0
365- 5: transcript:EER88199 transcript:Zm00001d037315_T016 0
365- 6: transcript:KXG19763 transcript:Zm00001d037313_T020 0
365- 7: transcript:KXG19770 transcript:Zm00001d037308_T001 0
365- 8: transcript:EER89586 transcript:Zm00001d037307_T001 0
365- 9: transcript:EER89587 transcript:Zm00001d037305_T001 0
365- 10: transcript:KXG19775 transcript:Zm00001d037301_T001 1.00E-134
365- 11: transcript:KXG19779 transcript:Zm00001d037300_T001 1.00E-83
365- 12: transcript:EER88218 transcript:Zm00001d037299_T001 4.00E-126
365- 13: transcript:EER89599 transcript:Zm00001d037298_T001 0
365- 14: transcript:EER88222 transcript:Zm00001d037297_T002 0
365- 15: transcript:OQU76225 transcript:Zm00001d037296_T001 0
Alignment 366: score=546.0 e_value=2.1e-31 N=13 10&6 minus
366- 0: transcript:KXG20201 transcript:Zm00001d036971_T004 0
366- 1: transcript:EER89955 transcript:Zm00001d036970_T001 1.00E-143
366- 2: transcript:OQU76591 transcript:Zm00001d036968_T015 0
366- 3: transcript:EER88542 transcript:Zm00001d036966_T001 2.00E-166
366- 4: transcript:KXG20214 transcript:Zm00001d036965_T001 2.00E-154
366- 5: transcript:OQU76596 transcript:Zm00001d036964_T001 1.00E-43
366- 6: transcript:OQU76598 transcript:Zm00001d036963_T001 9.00E-89
366- 7: transcript:OQU76599 transcript:Zm00001d036962_T001 0
366- 8: transcript:KXG20224 transcript:Zm00001d036961_T004 0
366- 9: transcript:EER89969 transcript:Zm00001d036959_T003 0
366- 10: transcript:EER89971 transcript:Zm00001d036956_T001 9.00E-145
366- 11: transcript:OQU76609 transcript:Zm00001d036955_T001 0
366- 12: transcript:EER88560 transcript:Zm00001d036949_T004 0
Alignment 367: score=491.0 e_value=7.9e-30 N=12 10&6 minus
367- 0: transcript:KXG20146 transcript:Zm00001d037005_T001 0
367- 1: transcript:EER89913 transcript:Zm00001d037004_T002 2.00E-100
367- 2: transcript:OQU76549 transcript:Zm00001d037003_T001 1.00E-34
367- 3: transcript:EER89920 transcript:Zm00001d037000_T002 2.00E-65
367- 4: transcript:KXG20160 transcript:Zm00001d036996_T001 3.00E-35
367- 5: transcript:KXG20161 transcript:Zm00001d036991_T001 2.00E-93
367- 6: transcript:EER89924 transcript:Zm00001d036989_T001 0
367- 7: transcript:KXG20162 transcript:Zm00001d036987_T001 0
367- 8: transcript:OQU76555 transcript:Zm00001d036986_T002 0
367- 9: transcript:EER88512 transcript:Zm00001d036985_T001 7.00E-91

367- 10: transcript:EER88513 transcript:Zm00001d036984_T001 0
367- 11: transcript:OQU76573 transcript:Zm00001d036977_T001 0
Alignment 368: score=483.0 e_value=6.2e-26 N=11 10&6 minus
368- 0: transcript:KXG20537 transcript:Zm00001d036668_T002 0
368- 1: transcript:OQU76827 transcript:Zm00001d036657_T003 6.00E-173
368- 2: transcript:KXG20556 transcript:Zm00001d036656_T003 0
368- 3: transcript:EER90170 transcript:Zm00001d036655_T003 2.00E-166
368- 4: transcript:EER88783 transcript:Zm00001d036654_T002 0
368- 5: transcript:OQU76836 transcript:Zm00001d036653_T001 0
368- 6: transcript:EER90182 transcript:Zm00001d036652_T001 0
368- 7: transcript:OQU76839 transcript:Zm00001d036650_T001 3.00E-63
368- 8: transcript:EER88787 transcript:Zm00001d036648_T001 2.00E-128
368- 9: transcript:KXG20560 transcript:Zm00001d036642_T001 4.00E-103
368- 10: transcript:OQU76843 transcript:Zm00001d036641_T001 2.00E-68
Alignment 369: score=441.0 e_value=4.3e-20 N=10 10&6 minus
369- 0: transcript:Zm00001d025031_T001 transcript:EES10473 0
369- 1: transcript:Zm00001d025033_T001 transcript:EES10469 4.00E-87
369- 2: transcript:Zm00001d025034_T002 transcript:KXG25874 0
369- 3: transcript:Zm00001d025035_T001 transcript:OQU81190 0
369- 4: transcript:Zm00001d025036_T003 transcript:EES11834 0
369- 5: transcript:Zm00001d025038_T001 transcript:OQU81187 0
369- 6: transcript:Zm00001d025040_T001 transcript:OQU81186 1.00E-33
369- 7: transcript:Zm00001d025041_T001 transcript:OQU81182 0
369- 8: transcript:Zm00001d025043_T004 transcript:EES11831 0
369- 9: transcript:Zm00001d025044_T029 transcript:EES11829 0
Alignment 370: score=409.0 e_value=4.8e-16 N=9 10&6 minus
370- 0: transcript:Zm00001d024875_T001 transcript:EES11763 0
370- 1: transcript:Zm00001d024878_T001 transcript:EES10379 0
370- 2: transcript:Zm00001d024879_T001 transcript:EES10378 7.00E-53
370- 3: transcript:Zm00001d024880_T001 transcript:EES11759 2.00E-41
370- 4: transcript:Zm00001d024883_T001 transcript:EES10376 0
370- 5: transcript:Zm00001d024885_T001 transcript:EES10373 0
370- 6: transcript:Zm00001d024886_T002 transcript:EES11757 0
370- 7: transcript:Zm00001d024889_T002 transcript:OQU81101 2.00E-137
370- 8: transcript:Zm00001d024890_T001 transcript:EES10370 7.00E-152
Alignment 371: score=402.0 e_value=6.9e-18 N=9 10&6 minus
371- 0: transcript:OQU77155 transcript:Zm00001d036371_T001 0
371- 1: transcript:KXG20942 transcript:Zm00001d036366_T001 5.00E-167
371- 2: transcript:KXG20943 transcript:Zm00001d036364_T001 1.00E-170
371- 3: transcript:OQU77158 transcript:Zm00001d036363_T001 3.00E-178
371- 4: transcript:EER90427 transcript:Zm00001d036361_T018 0
371- 5: transcript:KXG20945 transcript:Zm00001d036360_T002 0
371- 6: transcript:EER88999 transcript:Zm00001d036359_T001 0
371- 7: transcript:OQU77164 transcript:Zm00001d036357_T001 1.00E-175
371- 8: transcript:KXG20955 transcript:Zm00001d036355_T001 3.00E-29
Alignment 372: score=382.0 e_value=1.7e-20 N=9 10&6 minus
372- 0: transcript:OQU76916 transcript:Zm00001d036558_T001 0
372- 1: transcript:EER90248 transcript:Zm00001d036557_T002 0
372- 2: transcript:KXG20670 transcript:Zm00001d036550_T001 5.00E-51
372- 3: transcript:EER90263 transcript:Zm00001d036549_T001 1.00E-36
372- 4: transcript:KXG20671 transcript:Zm00001d036547_T001 3.00E-18
372- 5: transcript:OQU76933 transcript:Zm00001d036546_T007 0
372- 6: transcript:OQU76934 transcript:Zm00001d036543_T001 0
372- 7: transcript:KXG20680 transcript:Zm00001d036541_T002 6.00E-139

372- 8: transcript:EER90277 transcript:Zm00001d036540_T002 0
Alignment 373: score=377.0 e_value=8.4e-17 N=8 10&6 minus
373- 0: transcript:Zm00001d026034_T001 transcript:EES11345 5.00E-89
373- 1: transcript:Zm00001d026035_T001 transcript:EES12751 8.00E-156
373- 2: transcript:Zm00001d026036_T001 transcript:OQU82226 0
373- 3: transcript:Zm00001d026037_T001 transcript:EES11343 2.00E-49
373- 4: transcript:Zm00001d026038_T001 transcript:KXG27013 0
373- 5: transcript:Zm00001d026039_T002 transcript:KXG27012 0
373- 6: transcript:Zm00001d026040_T001 transcript:EES11341 0
373- 7: transcript:Zm00001d026042_T001 transcript:EES11332 1.00E-121
Alignment 374: score=347.0 e_value=3.5e-15 N=8 10&6 minus
374- 0: transcript:KXG20794 transcript:Zm00001d036459_T005 1.00E-166
374- 1: transcript:EER88921 transcript:Zm00001d036455_T001 0
374- 2: transcript:EER90343 transcript:Zm00001d036454_T001 4.00E-177
374- 3: transcript:OQU77036 transcript:Zm00001d036452_T001 5.00E-32
374- 4: transcript:KXG20810 transcript:Zm00001d036451_T002 2.00E-100
374- 5: transcript:EER88925 transcript:Zm00001d036450_T001 0
374- 6: transcript:EER90350 transcript:Zm00001d036449_T002 0
374- 7: transcript:EER88927 transcript:Zm00001d036448_T019 0
Alignment 375: score=331.0 e_value=5e-17 N=7 10&6 minus
375- 0: transcript:KXG19312 transcript:Zm00001d036202_T001 5.00E-104
375- 1: transcript:EER89207 transcript:Zm00001d036201_T001 2.00E-163
375- 2: transcript:EER89208 transcript:Zm00001d036199_T001 1.00E-99
375- 3: transcript:KXG19314 transcript:Zm00001d036198_T001 1.00E-21
375- 4: transcript:KXG19320 transcript:Zm00001d036196_T001 0
375- 5: transcript:EER89212 transcript:Zm00001d036195_T004 2.00E-104
375- 6: transcript:EER89215 transcript:Zm00001d036181_T001 0
Alignment 376: score=311.0 e_value=1.7e-12 N=7 10&6 minus
376- 0: transcript:Zm00001d025019_T001 transcript:EES11868 0
376- 1: transcript:Zm00001d025020_T001 transcript:OQU81217 6.00E-86
376- 2: transcript:Zm00001d025021_T008 transcript:KXG25899 0
376- 3: transcript:Zm00001d025022_T001 transcript:EES11864 0
376- 4: transcript:Zm00001d025023_T001 transcript:EES10492 0
376- 5: transcript:Zm00001d025024_T018 transcript:EES10487 0
376- 6: transcript:Zm00001d025025_T001 transcript:EES10488 3.00E-174
Alignment 377: score=298.0 e_value=6.6e-13 N=7 10&6 minus
377- 0: transcript:EER90280 transcript:Zm00001d036539_T001 5.00E-63
377- 1: transcript:EER88863 transcript:Zm00001d036536_T001 1.00E-82
377- 2: transcript:KXG20703 transcript:Zm00001d036535_T001 0
377- 3: transcript:EER88868 transcript:Zm00001d036534_T001 0
377- 4: transcript:OQU76954 transcript:Zm00001d036533_T002 0
377- 5: transcript:KXG20708 transcript:Zm00001d036532_T001 0
377- 6: transcript:KXG20719 transcript:Zm00001d036531_T002 6.00E-154
Alignment 378: score=290.0 e_value=9.6e-13 N=7 10&6 minus
378- 0: transcript:EER90442 transcript:Zm00001d036340_T001 0
378- 1: transcript:OQU77180 transcript:Zm00001d036339_T001 0
378- 2: transcript:EER89011 transcript:Zm00001d036338_T001 7.00E-154
378- 3: transcript:KXG20977 transcript:Zm00001d036337_T004 4.00E-139
378- 4: transcript:EER90445 transcript:Zm00001d036334_T003 0
378- 5: transcript:EER90448 transcript:Zm00001d036331_T002 0
378- 6: transcript:KXG20985 transcript:Zm00001d036330_T001 4.00E-81
Alignment 379: score=276.0 e_value=5.4e-10 N=6 10&6 minus
379- 0: transcript:KXG20722 transcript:Zm00001d036529_T001 0
379- 1: transcript:EER90290 transcript:Zm00001d036524_T001 0

```
379-  2: transcript:EER90291          transcript:Zm00001d036522_T001 7.00E-138
379-  3: transcript:KXG20732          transcript:Zm00001d036521_T001 1.00E-125
379-  4: transcript:KXG20736          transcript:Zm00001d036520_T001         0
379-  5: transcript:EER90296          transcript:Zm00001d036517_T001  6.00E-25
## Alignment 380: score=276.0 e_value=5.5e-10 N=6 10&6 minus
380-  0: transcript:OQU76184          transcript:Zm00001d037346_T007         0
380-  1: transcript:EER88180          transcript:Zm00001d037340_T001  8.00E-67
380-  2: transcript:EER89549          transcript:Zm00001d037338_T001         0
380-  3: transcript:EER89551          transcript:Zm00001d037337_T001         0
380-  4: transcript:EER88181          transcript:Zm00001d037336_T001         0
380-  5: transcript:OQU76190          transcript:Zm00001d037334_T001 4.00E-125
## Alignment 381: score=259.0 e_value=1.4e-09 N=6 10&6 minus
381-  0: transcript:OQU76459          transcript:Zm00001d037054_T001  6.00E-34
381-  1: transcript:EER89829          transcript:Zm00001d037052_T001  7.00E-71
381-  2: transcript:EER89830          transcript:Zm00001d037051_T001         0
381-  3: transcript:OQU76466          transcript:Zm00001d037050_T001         0
381-  4: transcript:EER88424          transcript:Zm00001d037044_T001  3.00E-40
381-  5: transcript:EER89844          transcript:Zm00001d037042_T001  2.00E-35
## Alignment 382: score=253.0 e_value=2.5e-09 N=6 10&6 minus
382-  0: transcript:Zm00001d025203_T001 transcript:EES11996                 0
382-  1: transcript:Zm00001d025205_T001 transcript:KXG26070         8.00E-162
382-  2: transcript:Zm00001d025206_T001 transcript:KXG26069                 0
382-  3: transcript:Zm00001d025210_T001 transcript:EES11991                 0
382-  4: transcript:Zm00001d025218_T001 transcript:OQU81347         3.00E-179
382-  5: transcript:Zm00001d025225_T001 transcript:KXG26045          8.00E-59
## Alignment 383: score=1145.0 e_value=1.8e-86 N=26 10&7 plus
383-  0: transcript:Zm00001d024300_T003 transcript:OQU80069                 0
383-  1: transcript:Zm00001d024301_T002 transcript:EES13516                 0
383-  2: transcript:Zm00001d024302_T001 transcript:OQU80070          7.00E-41
383-  3: transcript:Zm00001d024303_T002 transcript:OQU80072          7.00E-37
383-  4: transcript:Zm00001d024305_T001 transcript:EES13518          8.00E-88
383-  5: transcript:Zm00001d024307_T007 transcript:EES13519                 0
383-  6: transcript:Zm00001d024308_T001 transcript:EES14708         2.00E-172
383-  7: transcript:Zm00001d024310_T003 transcript:EES13521                 0
383-  8: transcript:Zm00001d024311_T001 transcript:KXG24710         7.00E-178
383-  9: transcript:Zm00001d024314_T001 transcript:OQU80077                 0
383- 10: transcript:Zm00001d024317_T001 transcript:KXG24714                 0
383- 11: transcript:Zm00001d024318_T009 transcript:EES14709                 0
383- 12: transcript:Zm00001d024319_T001 transcript:EES13525                 0
383- 13: transcript:Zm00001d024321_T001 transcript:OQU80084         2.00E-130
383- 14: transcript:Zm00001d024324_T001 transcript:EES14715          3.00E-32
383- 15: transcript:Zm00001d024327_T001 transcript:KXG24721                 0
383- 16: transcript:Zm00001d024333_T002 transcript:EES13536          5.00E-52
383- 17: transcript:Zm00001d024337_T001 transcript:EES13537                 0
383- 18: transcript:Zm00001d024338_T008 transcript:EES13540                 0
383- 19: transcript:Zm00001d024339_T001 transcript:EES14722                 0
383- 20: transcript:Zm00001d024342_T001 transcript:EES13543                 0
383- 21: transcript:Zm00001d024343_T001 transcript:KXG24731                 0
383- 22: transcript:Zm00001d024344_T001 transcript:KXG24732          6.00E-27
383- 23: transcript:Zm00001d024347_T001 transcript:EES14726         8.00E-122
383- 24: transcript:Zm00001d024348_T002 transcript:KXG24737                 0
383- 25: transcript:Zm00001d024351_T001 transcript:KXG24739          7.00E-51
## Alignment 384: score=628.0 e_value=2.8e-32 N=14 10&7 plus
384-  0: transcript:Zm00001d024392_T001 transcript:EES13579                 0
```

384- 1: transcript:Zm00001d024393_T001 transcript:EES14765 0
384- 2: transcript:Zm00001d024403_T001 transcript:EES13581 6.00E-42
384- 3: transcript:Zm00001d024406_T003 transcript:OQU80147 0
384- 4: transcript:Zm00001d024408_T005 transcript:EES14767 0
384- 5: transcript:Zm00001d024409_T001 transcript:EES13584 0
384- 6: transcript:Zm00001d024410_T001 transcript:KXG24798 6.00E-115
384- 7: transcript:Zm00001d024412_T002 transcript:EES13592 0
384- 8: transcript:Zm00001d024413_T001 transcript:KXG24804 3.00E-168
384- 9: transcript:Zm00001d024414_T001 transcript:EES14774 1.00E-82
384- 10: transcript:Zm00001d024415_T001 transcript:EES13595 5.00E-18
384- 11: transcript:Zm00001d024416_T001 transcript:EES14778 0
384- 12: transcript:Zm00001d024418_T004 transcript:EES14780 0
384- 13: transcript:Zm00001d024420_T007 transcript:KXG24818 0
Alignment 385: score=467.0 e_value=5.3e-23 N=10 10&7 plus
385- 0: transcript:Zm00001d024462_T006 transcript:KXG24534 1.00E-124
385- 1: transcript:Zm00001d024463_T009 transcript:KXG24537 0
385- 2: transcript:Zm00001d024464_T002 transcript:EES13425 1.00E-168
385- 3: transcript:Zm00001d024465_T001 transcript:OQU79969 7.00E-97
385- 4: transcript:Zm00001d024466_T001 transcript:EES14624 0
385- 5: transcript:Zm00001d024467_T004 transcript:EES13422 0
385- 6: transcript:Zm00001d024468_T003 transcript:KXG24541 1.00E-148
385- 7: transcript:Zm00001d024469_T001 transcript:EES14625 8.00E-171
385- 8: transcript:Zm00001d024470_T003 transcript:EES13423 0
385- 9: transcript:Zm00001d024471_T001 transcript:EES14627 2.00E-33
Alignment 386: score=458.0 e_value=9e-23 N=11 10&7 plus
386- 0: transcript:Zm00001d024349_T001 transcript:EES14737 8.00E-74
386- 1: transcript:Zm00001d024351_T001 transcript:OQU80122 0
386- 2: transcript:Zm00001d024353_T001 transcript:KXG24763 9.00E-10
386- 3: transcript:Zm00001d024354_T001 transcript:EES14747 4.00E-132
386- 4: transcript:Zm00001d024357_T001 transcript:EES13564 8.00E-82
386- 5: transcript:Zm00001d024364_T001 transcript:KXG24772 0
386- 6: transcript:Zm00001d024365_T001 transcript:EES14750 0
386- 7: transcript:Zm00001d024371_T001 transcript:KXG24780 8.00E-42
386- 8: transcript:Zm00001d024373_T001 transcript:OQU80130 2.00E-174
386- 9: transcript:Zm00001d024376_T001 transcript:EES14755 5.00E-126
386- 10: transcript:Zm00001d024378_T001 transcript:KXG24787 2.00E-123
Alignment 387: score=350.0 e_value=4e-14 N=8 10&7 plus
387- 0: transcript:Zm00001d024275_T013 transcript:OQU80053 7.00E-153
387- 1: transcript:Zm00001d024277_T001 transcript:OQU80058 3.00E-136
387- 2: transcript:Zm00001d024281_T001 transcript:OQU80059 0
387- 3: transcript:Zm00001d024286_T001 transcript:EES14697 0
387- 4: transcript:Zm00001d024291_T001 transcript:KXG24697 0
387- 5: transcript:Zm00001d024292_T002 transcript:EES14698 4.00E-180
387- 6: transcript:Zm00001d024294_T001 transcript:EES14699 0
387- 7: transcript:Zm00001d024298_T001 transcript:EES13508 0
Alignment 388: score=1751.0 e_value=3.7e-139 N=39 10&7 minus
388- 0: transcript:Zm00001d024615_T001 transcript:OQU79873 6.00E-11
388- 1: transcript:Zm00001d024624_T004 transcript:KXG24419 0
388- 2: transcript:Zm00001d024625_T002 transcript:KXG24418 0
388- 3: transcript:Zm00001d024627_T002 transcript:EES14540 0
388- 4: transcript:Zm00001d024630_T002 transcript:EES13346 0
388- 5: transcript:Zm00001d024631_T001 transcript:OQU79868 2.00E-112
388- 6: transcript:Zm00001d024632_T002 transcript:KXG24411 0
388- 7: transcript:Zm00001d024633_T001 transcript:KXG24413 0

388- 8: transcript:Zm00001d024634_T001 transcript:KXG24412 2.00E-123
388- 9: transcript:Zm00001d024637_T001 transcript:KXG24408 0
388- 10: transcript:Zm00001d024640_T003 transcript:EES14534 0
388- 11: transcript:Zm00001d024644_T001 transcript:EES13337 0
388- 12: transcript:Zm00001d024645_T001 transcript:OQU79861 0
388- 13: transcript:Zm00001d024646_T001 transcript:OQU79860 0
388- 14: transcript:Zm00001d024647_T001 transcript:EES14528 0
388- 15: transcript:Zm00001d024659_T001 transcript:KXG24393 7.00E-56
388- 16: transcript:Zm00001d024660_T001 transcript:EES14524 5.00E-29
388- 17: transcript:Zm00001d024661_T001 transcript:EES13332 6.00E-158
388- 18: transcript:Zm00001d024664_T001 transcript:EES13328 0
388- 19: transcript:Zm00001d024667_T002 transcript:OQU79853 2.00E-88
388- 20: transcript:Zm00001d024672_T001 transcript:OQU79851 0
388- 21: transcript:Zm00001d024674_T001 transcript:EES14516 6.00E-110
388- 22: transcript:Zm00001d024675_T001 transcript:EES14515 2.00E-74
388- 23: transcript:Zm00001d024676_T001 transcript:KXG24382 4.00E-34
388- 24: transcript:Zm00001d024677_T001 transcript:KXG24379 0
388- 25: transcript:Zm00001d024678_T001 transcript:KXG24378 0
388- 26: transcript:Zm00001d024679_T002 transcript:KXG24376 3.00E-177
388- 27: transcript:Zm00001d024680_T001 transcript:OQU79849 4.00E-28
388- 28: transcript:Zm00001d024687_T001 transcript:EES13321 0
388- 29: transcript:Zm00001d024693_T001 transcript:KXG24373 6.00E-98
388- 30: transcript:Zm00001d024698_T002 transcript:OQU79846 0
388- 31: transcript:Zm00001d024700_T001 transcript:EES13308 8.00E-63
388- 32: transcript:Zm00001d024701_T001 transcript:EES14500 0
388- 33: transcript:Zm00001d024702_T001 transcript:EES13315 0
388- 34: transcript:Zm00001d024703_T010 transcript:KXG24364 0
388- 35: transcript:Zm00001d024704_T004 transcript:EES13312 0
388- 36: transcript:Zm00001d024705_T001 transcript:EES13310 2.00E-35
388- 37: transcript:Zm00001d024708_T001 transcript:EES14504 0
388- 38: transcript:Zm00001d024709_T002 transcript:KXG24358 0
Alignment 389: score=618.0 e_value=4.7e-35 N=14 10&7 minus
389- 0: transcript:Zm00001d024567_T004 transcript:KXG24466 0
389- 1: transcript:Zm00001d024568_T001 transcript:EES13383 0
389- 2: transcript:Zm00001d024571_T001 transcript:EES13382 4.00E-80
389- 3: transcript:Zm00001d024572_T002 transcript:EES13381 0
389- 4: transcript:Zm00001d024573_T001 transcript:OQU79916 1.00E-97
389- 5: transcript:Zm00001d024574_T001 transcript:OQU79915 0
389- 6: transcript:Zm00001d024583_T003 transcript:EES13372 0
389- 7: transcript:Zm00001d024591_T001 transcript:EES13368 2.00E-23
389- 8: transcript:Zm00001d024594_T001 transcript:KXG24449 0
389- 9: transcript:Zm00001d024596_T002 transcript:EES13366 2.00E-158
389- 10: transcript:Zm00001d024597_T001 transcript:EES14559 4.00E-103
389- 11: transcript:Zm00001d024600_T002 transcript:OQU79902 0
389- 12: transcript:Zm00001d024601_T003 transcript:OQU79900 0
389- 13: transcript:Zm00001d024602_T001 transcript:EES14557 0
Alignment 390: score=588.0 e_value=2.8e-34 N=13 10&7 minus
390- 0: transcript:Zm00001d024421_T001 transcript:OQU79983 5.00E-178
390- 1: transcript:Zm00001d024423_T002 transcript:OQU79982 0
390- 2: transcript:Zm00001d024424_T001 transcript:EES13437 0
390- 3: transcript:Zm00001d024425_T003 transcript:KXG24568 0
390- 4: transcript:Zm00001d024427_T001 transcript:KXG24566 1.00E-85
390- 5: transcript:Zm00001d024429_T001 transcript:KXG24563 0
390- 6: transcript:Zm00001d024430_T002 transcript:EES13434 0

```
390-  7: transcript:Zm00001d024432_T001 transcript:OQU79976                 0
390-  8: transcript:Zm00001d024436_T001 transcript:KXG24553          7.00E-59
390-  9: transcript:Zm00001d024437_T001 transcript:KXG24552          7.00E-29
390- 10: transcript:Zm00001d024442_T001 transcript:KXG24550          5.00E-39
390- 11: transcript:Zm00001d024447_T001 transcript:KXG24548          7.00E-13
390- 12: transcript:Zm00001d024460_T001 transcript:KXG24546          6.00E-14
## Alignment 391: score=581.0 e_value=9e-34 N=13 10&7 minus
391-  0: transcript:Zm00001d024476_T001 transcript:OQU79965         3.00E-179
391-  1: transcript:Zm00001d024477_T001 transcript:KXG24530                 0
391-  2: transcript:Zm00001d024488_T001 transcript:EES13418          2.00E-29
391-  3: transcript:Zm00001d024489_T001 transcript:EES14615                 0
391-  4: transcript:Zm00001d024492_T001 transcript:EES14614          2.00E-57
391-  5: transcript:Zm00001d024494_T001 transcript:KXG24525          1.00E-43
391-  6: transcript:Zm00001d024497_T001 transcript:EES13415                 0
391-  7: transcript:Zm00001d024500_T001 transcript:EES14611         1.00E-102
391-  8: transcript:Zm00001d024507_T001 transcript:EES13412                 0
391-  9: transcript:Zm00001d024509_T002 transcript:EES14609          6.00E-35
391- 10: transcript:Zm00001d024510_T002 transcript:OQU79959         2.00E-159
391- 11: transcript:Zm00001d024511_T001 transcript:EES14608         4.00E-164
391- 12: transcript:Zm00001d024516_T001 transcript:EES13409                 0
## Alignment 392: score=515.0 e_value=1.2e-24 N=11 10&7 minus
392-  0: transcript:Zm00001d024525_T001 transcript:KXG24500                 0
392-  1: transcript:Zm00001d024527_T001 transcript:EES14600                 0
392-  2: transcript:Zm00001d024528_T001 transcript:EES14599                 0
392-  3: transcript:Zm00001d024530_T007 transcript:EES14598                 0
392-  4: transcript:Zm00001d024531_T001 transcript:EES14597                 0
392-  5: transcript:Zm00001d024532_T001 transcript:EES13398                 0
392-  6: transcript:Zm00001d024533_T001 transcript:EES14596                 0
392-  7: transcript:Zm00001d024534_T001 transcript:KXG24496                 0
392-  8: transcript:Zm00001d024537_T001 transcript:KXG24494         2.00E-120
392-  9: transcript:Zm00001d024538_T001 transcript:EES13396         7.00E-179
392- 10: transcript:Zm00001d024539_T001 transcript:EES14591                 0
## Alignment 393: score=320.0 e_value=3.2e-12 N=7 10&7 minus
393-  0: transcript:Zm00001d024540_T001 transcript:EES13390                 0
393-  1: transcript:Zm00001d024541_T001 transcript:EES14581         4.00E-158
393-  2: transcript:Zm00001d024543_T001 transcript:EES14580                 0
393-  3: transcript:Zm00001d024544_T002 transcript:KXG24478                 0
393-  4: transcript:Zm00001d024545_T001 transcript:EES13386         3.00E-120
393-  5: transcript:Zm00001d024546_T001 transcript:EES13385                 0
393-  6: transcript:Zm00001d024548_T001 transcript:KXG24475          2.00E-47
## Alignment 394: score=282.0 e_value=1.4e-10 N=6 10&7 minus
394-  0: transcript:Zm00001d024518_T001 transcript:EES14605          3.00E-53
394-  1: transcript:Zm00001d024519_T005 transcript:OQU79956         1.00E-170
394-  2: transcript:Zm00001d024520_T001 transcript:KXG24507         1.00E-115
394-  3: transcript:Zm00001d024521_T001 transcript:OQU79953                 0
394-  4: transcript:Zm00001d024522_T001 transcript:EES14602         1.00E-138
394-  5: transcript:Zm00001d024523_T003 transcript:KXG24503                 0
## Alignment 395: score=251.0 e_value=2.4e-08 N=6 10&7 minus
395-  0: transcript:Zm00001d026333_T001 transcript:KXG24659         1.00E-129
395-  1: transcript:Zm00001d026335_T001 transcript:EES13484          4.00E-79
395-  2: transcript:Zm00001d026336_T001 transcript:EES13481          3.00E-77
395-  3: transcript:Zm00001d026337_T011 transcript:KXG24650                 0
395-  4: transcript:Zm00001d026344_T001 transcript:OQU80028                 0
395-  5: transcript:Zm00001d026346_T002 transcript:KXG24662                 0
```

Alignment 396: score=915.0 e_value=1.3e-65 N=22 10&8 plus

396- 0:	transcript:Zm00001d023551_T001	transcript:KXG23070	3.00E-43
396- 1:	transcript:Zm00001d023554_T001	transcript:OQU78815	0
396- 2:	transcript:Zm00001d023559_T001	transcript:EES16731	0
396- 3:	transcript:Zm00001d023560_T002	transcript:EES16732	7.00E-150
396- 4:	transcript:Zm00001d023563_T001	transcript:EES15737	0
396- 5:	transcript:Zm00001d023564_T002	transcript:KXG23078	0
396- 6:	transcript:Zm00001d023566_T001	transcript:OQU78823	0
396- 7:	transcript:Zm00001d023568_T001	transcript:KXG23086	4.00E-25
396- 8:	transcript:Zm00001d023569_T001	transcript:KXG23087	4.00E-33
396- 9:	transcript:Zm00001d023570_T001	transcript:EES15744	3.00E-107
396- 10:	transcript:Zm00001d023576_T002	transcript:EES16745	0
396- 11:	transcript:Zm00001d023578_T001	transcript:EES15750	0
396- 12:	transcript:Zm00001d023579_T002	transcript:KXG23105	0
396- 13:	transcript:Zm00001d023580_T001	transcript:EES15756	0
396- 14:	transcript:Zm00001d023581_T001	transcript:EES16751	0
396- 15:	transcript:Zm00001d023582_T001	transcript:EES15758	0
396- 16:	transcript:Zm00001d023583_T005	transcript:EES16755	0
396- 17:	transcript:Zm00001d023585_T001	transcript:OQU78839	2.00E-100
396- 18:	transcript:Zm00001d023587_T002	transcript:EES15759	2.00E-46
396- 19:	transcript:Zm00001d023588_T001	transcript:OQU78841	0
396- 20:	transcript:Zm00001d023590_T005	transcript:EES16760	0
396- 21:	transcript:Zm00001d023592_T001	transcript:EES16761	0

Alignment 397: score=869.0 e_value=1.7e-67 N=21 10&8 plus

397- 0:	transcript:Zm00001d023394_T001	transcript:EES15668	0
397- 1:	transcript:Zm00001d023395_T001	transcript:EES16636	3.00E-44
397- 2:	transcript:Zm00001d023396_T001	transcript:OQU78711	5.00E-26
397- 3:	transcript:Zm00001d023398_T001	transcript:EES15672	0
397- 4:	transcript:Zm00001d023400_T002	transcript:EES16641	0
397- 5:	transcript:Zm00001d023401_T001	transcript:KXG22969	6.00E-42
397- 6:	transcript:Zm00001d023402_T001	transcript:EES15678	0
397- 7:	transcript:Zm00001d023404_T001	transcript:EES15679	0
397- 8:	transcript:Zm00001d023409_T001	transcript:KXG22974	2.00E-22
397- 9:	transcript:Zm00001d023419_T001	transcript:EES15680	1.00E-45
397- 10:	transcript:Zm00001d023420_T001	transcript:EES16646	6.00E-122
397- 11:	transcript:Zm00001d023422_T001	transcript:OQU78719	0
397- 12:	transcript:Zm00001d023423_T002	transcript:EES16649	3.00E-179
397- 13:	transcript:Zm00001d023424_T001	transcript:KXG22976	0
397- 14:	transcript:Zm00001d023425_T001	transcript:EES15682	0
397- 15:	transcript:Zm00001d023427_T001	transcript:KXG22978	7.00E-148
397- 16:	transcript:Zm00001d023426_T005	transcript:EES16654	0
397- 17:	transcript:Zm00001d023429_T001	transcript:EES16656	0
397- 18:	transcript:Zm00001d023431_T002	transcript:EES16662	0
397- 19:	transcript:Zm00001d023432_T001	transcript:OQU78730	0
397- 20:	transcript:Zm00001d023434_T002	transcript:EES16664	0

Alignment 398: score=845.0 e_value=6.5e-61 N=21 10&8 plus

398- 0:	transcript:Zm00001d023437_T001	transcript:EES15687	0
398- 1:	transcript:Zm00001d023439_T001	transcript:EES15689	0
398- 2:	transcript:Zm00001d023440_T001	transcript:EES15690	4.00E-73
398- 3:	transcript:Zm00001d023443_T001	transcript:EES15691	2.00E-54
398- 4:	transcript:Zm00001d023445_T001	transcript:KXG22985	0
398- 5:	transcript:Zm00001d023446_T001	transcript:KXG22988	2.00E-134
398- 6:	transcript:Zm00001d023450_T001	transcript:OQU78745	0
398- 7:	transcript:Zm00001d023452_T001	transcript:EES16672	0

398- 8: transcript:Zm00001d023453_T001 transcript:EES15696 4.00E-34
398- 9: transcript:Zm00001d023455_T001 transcript:OQU78754 3.00E-111
398- 10: transcript:Zm00001d023456_T001 transcript:EES16674 7.00E-75
398- 11: transcript:Zm00001d023459_T001 transcript:EES15698 2.00E-130
398- 12: transcript:Zm00001d023461_T001 transcript:EES16675 4.00E-77
398- 13: transcript:Zm00001d023465_T002 transcript:OQU78758 0
398- 14: transcript:Zm00001d023466_T001 transcript:EES16679 0
398- 15: transcript:Zm00001d023468_T001 transcript:EES16682 4.00E-124
398- 16: transcript:Zm00001d023472_T001 transcript:KXG23006 0
398- 17: transcript:Zm00001d023477_T001 transcript:EES15700 7.00E-21
398- 18: transcript:Zm00001d023478_T001 transcript:EES16684 1.00E-79
398- 19: transcript:Zm00001d023479_T006 transcript:EES15701 0
398- 20: transcript:Zm00001d023480_T001 transcript:KXG23010 3.00E-107
Alignment 399: score=600.0 e_value=1.2e-33 N=14 10&8 plus
399- 0: transcript:Zm00001d023365_T001 transcript:EES15653 0
399- 1: transcript:Zm00001d023366_T001 transcript:EES16622 8.00E-54
399- 2: transcript:Zm00001d023367_T002 transcript:OQU78687 0
399- 3: transcript:Zm00001d023368_T001 transcript:KXG22943 9.00E-106
399- 4: transcript:Zm00001d023371_T001 transcript:EES16626 4.00E-127
399- 5: transcript:Zm00001d023372_T001 transcript:OQU78689 2.00E-06
399- 6: transcript:Zm00001d023373_T004 transcript:EES16625 0
399- 7: transcript:Zm00001d023374_T001 transcript:OQU78692 1.00E-140
399- 8: transcript:Zm00001d023376_T002 transcript:KXG22950 1.00E-121
399- 9: transcript:Zm00001d023377_T001 transcript:EES16629 0
399- 10: transcript:Zm00001d023378_T001 transcript:EES15657 0
399- 11: transcript:Zm00001d023379_T001 transcript:KXG22956 0
399- 12: transcript:Zm00001d023384_T001 transcript:EES15665 3.00E-16
399- 13: transcript:Zm00001d023385_T001 transcript:EES15666 1.00E-20
Alignment 400: score=463.0 e_value=4.8e-20 N=10 10&8 plus
400- 0: transcript:Zm00001d023246_T001 transcript:EES16520 0
400- 1: transcript:Zm00001d023247_T001 transcript:EES16518 2.00E-161
400- 2: transcript:Zm00001d023249_T001 transcript:KXG22823 4.00E-35
400- 3: transcript:Zm00001d023252_T001 transcript:EES16527 4.00E-48
400- 4: transcript:Zm00001d023253_T001 transcript:KXG22829 0
400- 5: transcript:Zm00001d023254_T001 transcript:KXG22830 5.00E-122
400- 6: transcript:Zm00001d023258_T002 transcript:EES16530 0
400- 7: transcript:Zm00001d023259_T001 transcript:EES15558 2.00E-115
400- 8: transcript:Zm00001d023260_T001 transcript:EES16532 0
400- 9: transcript:Zm00001d023261_T001 transcript:EES16534 0
Alignment 401: score=397.0 e_value=5.7e-24 N=11 10&8 plus
401- 0: transcript:Zm00001d023330_T001 transcript:EES16599 0
401- 1: transcript:Zm00001d023331_T001 transcript:KXG22919 0
401- 2: transcript:Zm00001d023332_T001 transcript:EES15637 4.00E-91
401- 3: transcript:Zm00001d023333_T037 transcript:KXG22920 0
401- 4: transcript:Zm00001d023336_T001 transcript:OQU78673 8.00E-122
401- 5: transcript:Zm00001d023337_T010 transcript:KXG22925 0
401- 6: transcript:Zm00001d023340_T001 transcript:OQU78678 1.00E-113
401- 7: transcript:Zm00001d023341_T001 transcript:OQU78679 3.00E-70
401- 8: transcript:Zm00001d023345_T001 transcript:KXG22935 9.00E-24
401- 9: transcript:Zm00001d023349_T001 transcript:EES16619 0
401- 10: transcript:Zm00001d023353_T001 transcript:EES15653 0
Alignment 402: score=324.0 e_value=2.9e-11 N=7 10&8 plus
402- 0: transcript:Zm00001d023277_T001 transcript:EES15585 0
402- 1: transcript:Zm00001d023278_T002 transcript:EES15586 1.00E-170

402- 2: transcript:Zm00001d023279_T001 transcript:EES16550 0
402- 3: transcript:Zm00001d023280_T001 transcript:EES16559 1.00E-33
402- 4: transcript:Zm00001d023281_T004 transcript:EES15589 2.00E-122
402- 5: transcript:Zm00001d023282_T001 transcript:KXG22874 1.00E-143
402- 6: transcript:Zm00001d023283_T001 transcript:EES16562 0
Alignment 403: score=293.0 e_value=1.2e-13 N=7 10&8 plus
403- 0: transcript:Zm00001d023603_T001 transcript:EES16761 0
403- 1: transcript:Zm00001d023604_T001 transcript:KXG23124 2.00E-42
403- 2: transcript:Zm00001d023605_T001 transcript:EES16769 2.00E-75
403- 3: transcript:Zm00001d023606_T005 transcript:OQU78843 0
403- 4: transcript:Zm00001d023608_T001 transcript:KXG23128 0
403- 5: transcript:Zm00001d023611_T001 transcript:KXG23129 0
403- 6: transcript:Zm00001d023615_T001 transcript:EES15768 2.00E-108
Alignment 404: score=286.0 e_value=2.1e-10 N=7 10&8 plus
404- 0: transcript:Zm00001d023974_T001 transcript:OQU78949 0
404- 1: transcript:Zm00001d023978_T001 transcript:EES15848 8.00E-79
404- 2: transcript:Zm00001d023979_T002 transcript:KXG23275 0
404- 3: transcript:Zm00001d023984_T001 transcript:KXG23277 0
404- 4: transcript:Zm00001d023987_T001 transcript:EES16897 0
404- 5: transcript:Zm00001d023990_T001 transcript:EES16901 0
404- 6: transcript:Zm00001d023992_T001 transcript:OQU78964 9.00E-96
Alignment 405: score=284.0 e_value=1.6e-13 N=7 10&8 plus
405- 0: transcript:Zm00001d023305_T001 transcript:EES15617 2.00E-24
405- 1: transcript:Zm00001d023306_T001 transcript:KXG22907 0
405- 2: transcript:Zm00001d023309_T001 transcript:EES16591 0
405- 3: transcript:Zm00001d023311_T001 transcript:KXG22911 1.00E-40
405- 4: transcript:Zm00001d023312_T001 transcript:EES15627 5.00E-116
405- 5: transcript:Zm00001d023313_T001 transcript:EES16593 0
405- 6: transcript:Zm00001d023317_T002 transcript:KXG22916 2.00E-94
Alignment 406: score=272.0 e_value=3.4e-09 N=6 10&8 plus
406- 0: transcript:Zm00001d023284_T001 transcript:KXG22874 1.00E-71
406- 1: transcript:Zm00001d023286_T001 transcript:EES16563 1.00E-40
406- 2: transcript:Zm00001d023290_T001 transcript:EES16564 3.00E-62
406- 3: transcript:Zm00001d023291_T001 transcript:KXG22878 0
406- 4: transcript:Zm00001d023293_T001 transcript:EES15592 2.00E-96
406- 5: transcript:Zm00001d023294_T001 transcript:EES16571 2.00E-146
Alignment 407: score=265.0 e_value=4.7e-09 N=6 10&8 plus
407- 0: transcript:Zm00001d023298_T001 transcript:EES16574 3.00E-115
407- 1: transcript:Zm00001d023299_T003 transcript:KXG22886 3.00E-140
407- 2: transcript:Zm00001d023300_T002 transcript:KXG22889 1.00E-158
407- 3: transcript:Zm00001d023301_T002 transcript:EES15601 0
407- 4: transcript:Zm00001d023302_T001 transcript:KXG22891 0
407- 5: transcript:Zm00001d023303_T012 transcript:KXG22895 0
Alignment 408: score=261.0 e_value=1.3e-10 N=6 10&8 plus
408- 0: transcript:Zm00001d023943_T001 transcript:EES16855 0
408- 1: transcript:Zm00001d023944_T001 transcript:OQU78921 0
408- 2: transcript:Zm00001d023945_T003 transcript:EES15821 0
408- 3: transcript:Zm00001d023946_T001 transcript:EES16856 0
408- 4: transcript:Zm00001d023950_T001 transcript:EES16857 4.00E-175
408- 5: transcript:Zm00001d023953_T001 transcript:EES15823 2.00E-45
Alignment 409: score=261.0 e_value=2.7e-09 N=6 10&8 plus
409- 0: transcript:Zm00001d023707_T002 transcript:EES16784 4.00E-110
409- 1: transcript:Zm00001d023710_T005 transcript:KXG23153 0
409- 2: transcript:Zm00001d023713_T001 transcript:KXG23155 1.00E-76

409- 3: transcript:Zm00001d023715_T001 transcript:KXG23156 0
409- 4: transcript:Zm00001d023718_T002 transcript:OQU78863 0
409- 5: transcript:Zm00001d023719_T001 transcript:OQU78866 2.00E-34
Alignment 410: score=256.0 e_value=5.5e-09 N=6 10&8 plus
410- 0: transcript:Zm00001d023534_T002 transcript:OQU78801 0
410- 1: transcript:Zm00001d023535_T001 transcript:OQU78803 8.00E-87
410- 2: transcript:Zm00001d023536_T002 transcript:EES16721 0
410- 3: transcript:Zm00001d023537_T002 transcript:EES15725 0
410- 4: transcript:Zm00001d023538_T006 transcript:KXG23059 0
410- 5: transcript:Zm00001d023539_T001 transcript:OQU78806 0
Alignment 411: score=1580.0 e_value=9.3e-120 N=35 10&8 minus
411- 0: transcript:Zm00001d023859_T001 transcript:EES16102 0
411- 1: transcript:Zm00001d023863_T001 transcript:KXG23615 8.00E-175
411- 2: transcript:Zm00001d023865_T001 transcript:EES16100 2.00E-95
411- 3: transcript:Zm00001d023867_T005 transcript:EES17139 0
411- 4: transcript:Zm00001d023868_T004 transcript:KXG23612 0
411- 5: transcript:Zm00001d023869_T001 transcript:EES16096 2.00E-32
411- 6: transcript:Zm00001d023873_T001 transcript:EES16092 1.00E-67
411- 7: transcript:Zm00001d023874_T001 transcript:EES16091 0
411- 8: transcript:Zm00001d023877_T001 transcript:EES17134 3.00E-86
411- 9: transcript:Zm00001d023882_T001 transcript:KXG23976 6.00E-71
411- 10: transcript:Zm00001d023885_T002 transcript:EES16087 0
411- 11: transcript:Zm00001d023887_T001 transcript:EES17130 2.00E-87
411- 12: transcript:Zm00001d023888_T001 transcript:EES17129 1.00E-97
411- 13: transcript:Zm00001d023892_T001 transcript:KXG23603 0
411- 14: transcript:Zm00001d023895_T002 transcript:KXG23601 5.00E-130
411- 15: transcript:Zm00001d023896_T003 transcript:EES17120 2.00E-139
411- 16: transcript:Zm00001d023897_T003 transcript:EES17116 0
411- 17: transcript:Zm00001d023899_T001 transcript:EES17115 0
411- 18: transcript:Zm00001d023901_T001 transcript:EES17114 6.00E-89
411- 19: transcript:Zm00001d023903_T001 transcript:KXG23587 5.00E-44
411- 20: transcript:Zm00001d023908_T001 transcript:EES16076 4.00E-113
411- 21: transcript:Zm00001d023910_T001 transcript:EES16074 0
411- 22: transcript:Zm00001d023912_T001 transcript:KXG23585 0
411- 23: transcript:Zm00001d023914_T003 transcript:KXG23584 0
411- 24: transcript:Zm00001d023918_T001 transcript:KXG23582 0
411- 25: transcript:Zm00001d023919_T001 transcript:EES16070 0
411- 26: transcript:Zm00001d023922_T004 transcript:KXG23576 0
411- 27: transcript:Zm00001d023923_T001 transcript:OQU79215 4.00E-117
411- 28: transcript:Zm00001d023927_T001 transcript:KXG23574 0
411- 29: transcript:Zm00001d023929_T001 transcript:EES16067 0
411- 30: transcript:Zm00001d023931_T001 transcript:EES17106 6.00E-60
411- 31: transcript:Zm00001d023933_T001 transcript:EES17104 0
411- 32: transcript:Zm00001d023934_T001 transcript:EES16065 2.00E-81
411- 33: transcript:Zm00001d023936_T001 transcript:EES17099 2.00E-61
411- 34: transcript:Zm00001d023939_T001 transcript:KXG23568 4.00E-147
Alignment 412: score=595.0 e_value=2.4e-40 N=15 10&8 minus
412- 0: transcript:Zm00001d023646_T002 transcript:EES17033 0
412- 1: transcript:Zm00001d023648_T003 transcript:OQU79118 3.00E-139
412- 2: transcript:Zm00001d023650_T002 transcript:OQU79113 2.00E-65
412- 3: transcript:Zm00001d023651_T001 transcript:EES17023 0
412- 4: transcript:Zm00001d023652_T002 transcript:EES17021 0
412- 5: transcript:Zm00001d023653_T001 transcript:EES15972 0
412- 6: transcript:Zm00001d023654_T001 transcript:EES17020 4.00E-177

412- 7: transcript:Zm00001d023658_T002 transcript:EES15964 0
412- 8: transcript:Zm00001d023659_T007 transcript:KXG23609 0
412- 9: transcript:Zm00001d023661_T001 transcript:OQU79096 6.00E-10
412- 10: transcript:Zm00001d023664_T001 transcript:EES17007 2.00E-163
412- 11: transcript:Zm00001d023669_T001 transcript:EES17006 2.00E-121
412- 12: transcript:Zm00001d023671_T001 transcript:EES15958 2.00E-24
412- 13: transcript:Zm00001d023673_T001 transcript:OQU79094 0
412- 14: transcript:Zm00001d023677_T001 transcript:EES17001 0
Alignment 413: score=505.0 e_value=2.9e-27 N=12 10&8 minus
413- 0: transcript:Zm00001d023808_T001 transcript:EES16144 2.00E-133
413- 1: transcript:Zm00001d023810_T002 transcript:EES17177 0
413- 2: transcript:Zm00001d023815_T001 transcript:KXG23666 0
413- 3: transcript:Zm00001d023817_T001 transcript:EES16143 0
413- 4: transcript:Zm00001d023820_T001 transcript:EES17171 0
413- 5: transcript:Zm00001d023824_T002 transcript:EES16140 4.00E-54
413- 6: transcript:Zm00001d023825_T005 transcript:KXG23663 0
413- 7: transcript:Zm00001d023830_T001 transcript:OQU79284 3.00E-72
413- 8: transcript:Zm00001d023833_T001 transcript:EES16135 5.00E-143
413- 9: transcript:Zm00001d023838_T014 transcript:EES17168 0
413- 10: transcript:Zm00001d023839_T001 transcript:EES17167 0
413- 11: transcript:Zm00001d023841_T008 transcript:KXG23658 0
Alignment 414: score=385.0 e_value=3.3e-19 N=9 10&8 minus
414- 0: transcript:Zm00001d023728_T001 transcript:EES16019 0
414- 1: transcript:Zm00001d023729_T005 transcript:EES16018 0
414- 2: transcript:Zm00001d023732_T002 transcript:OQU79167 0
414- 3: transcript:Zm00001d023734_T003 transcript:EES16016 2.00E-66
414- 4: transcript:Zm00001d023735_T001 transcript:KXG23503 5.00E-29
414- 5: transcript:Zm00001d023736_T001 transcript:KXG23501 1.00E-62
414- 6: transcript:Zm00001d023737_T001 transcript:EES17056 3.00E-122
414- 7: transcript:Zm00001d023738_T001 transcript:EES16015 0
414- 8: transcript:Zm00001d023740_T001 transcript:OQU79162 2.00E-78
Alignment 415: score=303.0 e_value=2e-13 N=7 10&8 minus
415- 0: transcript:Zm00001d023749_T007 transcript:KXG23497 0
415- 1: transcript:Zm00001d023753_T001 transcript:KXG23494 8.00E-64
415- 2: transcript:Zm00001d023756_T001 transcript:EES17051 6.00E-104
415- 3: transcript:Zm00001d023757_T001 transcript:EES17048 4.00E-111
415- 4: transcript:Zm00001d023760_T001 transcript:EES17047 4.00E-158
415- 5: transcript:Zm00001d023762_T001 transcript:EES16005 0
415- 6: transcript:Zm00001d023767_T001 transcript:EES17040 8.00E-96
Alignment 416: score=265.0 e_value=6.7e-08 N=6 10&8 minus
416- 0: transcript:Zm00001d023384_T001 transcript:EES15666 1.00E-28
416- 1: transcript:Zm00001d023385_T001 transcript:EES15665 1.00E-18
416- 2: transcript:Zm00001d023387_T001 transcript:KXG22962 4.00E-79
416- 3: transcript:Zm00001d023390_T001 transcript:OQU78706 5.00E-162
416- 4: transcript:Zm00001d023391_T001 transcript:OQU78704 0
416- 5: transcript:Zm00001d023392_T002 transcript:OQU78697 0
Alignment 417: score=252.0 e_value=7.8e-10 N=6 10&8 minus
417- 0: transcript:Zm00001d023405_T001 transcript:KXG22974 5.00E-22
417- 1: transcript:Zm00001d023410_T001 transcript:EES15679 0
417- 2: transcript:Zm00001d023411_T001 transcript:EES15678 0
417- 3: transcript:Zm00001d023412_T001 transcript:KXG22969 1.00E-47
417- 4: transcript:Zm00001d023413_T001 transcript:EES16641 0
417- 5: transcript:Zm00001d023417_T001 transcript:EES15672 1.00E-178
Alignment 418: score=3014.0 e_value=2.2e-282 N=68 10&9 plus

418- 0:	transcript:Zm00001d024715_T001	transcript:EES18956	0
418- 1:	transcript:Zm00001d024717_T001	transcript:OQU77339	0
418- 2:	transcript:Zm00001d024718_T004	transcript:KXG21215	8.00E-130
418- 3:	transcript:Zm00001d024722_T002	transcript:EES17657	4.00E-104
418- 4:	transcript:Zm00001d024723_T001	transcript:KXG21217	0
418- 5:	transcript:Zm00001d024725_T001	transcript:KXG21220	2.00E-151
418- 6:	transcript:Zm00001d024729_T001	transcript:EES18967	0
418- 7:	transcript:Zm00001d024732_T001	transcript:EES17660	0
418- 8:	transcript:Zm00001d024733_T001	transcript:OQU77343	1.00E-112
418- 9:	transcript:Zm00001d024734_T001	transcript:EES17661	0
418- 10:	transcript:Zm00001d024738_T001	transcript:KXG21227	4.00E-136
418- 11:	transcript:Zm00001d024744_T012	transcript:OQU77347	0
418- 12:	transcript:Zm00001d024745_T001	transcript:OQU77354	0
418- 13:	transcript:Zm00001d024754_T001	transcript:EES17665	0
418- 14:	transcript:Zm00001d024755_T004	transcript:EES17666	0
418- 15:	transcript:Zm00001d024756_T004	transcript:EES18975	0
418- 16:	transcript:Zm00001d024757_T001	transcript:KXG21238	2.00E-134
418- 17:	transcript:Zm00001d024759_T001	transcript:EES18976	0
418- 18:	transcript:Zm00001d024762_T001	transcript:OQU77359	0
418- 19:	transcript:Zm00001d024763_T001	transcript:EES17668	0
418- 20:	transcript:Zm00001d024764_T001	transcript:EES17669	1.00E-127
418- 21:	transcript:Zm00001d024765_T001	transcript:EES17671	0
418- 22:	transcript:Zm00001d024767_T003	transcript:KXG21241	0
418- 23:	transcript:Zm00001d024768_T001	transcript:KXG21246	0
418- 24:	transcript:Zm00001d024770_T001	transcript:EES18981	3.00E-100
418- 25:	transcript:Zm00001d024772_T001	transcript:EES17673	5.00E-37
418- 26:	transcript:Zm00001d024777_T001	transcript:KXG21253	5.00E-149
418- 27:	transcript:Zm00001d024778_T001	transcript:EES18986	5.00E-29
418- 28:	transcript:Zm00001d024781_T001	transcript:OQU77368	2.00E-121
418- 29:	transcript:Zm00001d024783_T001	transcript:KXG21255	0
418- 30:	transcript:Zm00001d024784_T001	transcript:OQU77376	9.00E-66
418- 31:	transcript:Zm00001d024786_T001	transcript:OQU77377	7.00E-94
418- 32:	transcript:Zm00001d024787_T002	transcript:EES17679	1.00E-123
418- 33:	transcript:Zm00001d024788_T002	transcript:KXG21258	5.00E-143
418- 34:	transcript:Zm00001d024789_T001	transcript:EES18990	0
418- 35:	transcript:Zm00001d024795_T001	transcript:KXG21263	3.00E-84
418- 36:	transcript:Zm00001d024796_T001	transcript:EES17687	0
418- 37:	transcript:Zm00001d024798_T001	transcript:KXG21265	1.00E-28
418- 38:	transcript:Zm00001d024799_T001	transcript:EES18993	5.00E-44
418- 39:	transcript:Zm00001d024800_T001	transcript:KXG21266	1.00E-47
418- 40:	transcript:Zm00001d024802_T001	transcript:KXG21267	5.00E-30
418- 41:	transcript:Zm00001d024803_T001	transcript:KXG21270	1.00E-96
418- 42:	transcript:Zm00001d024804_T001	transcript:OQU77388	0
418- 43:	transcript:Zm00001d024805_T001	transcript:EES18994	2.00E-14
418- 44:	transcript:Zm00001d024807_T008	transcript:KXG21277	0
418- 45:	transcript:Zm00001d024809_T001	transcript:EES18995	7.00E-97
418- 46:	transcript:Zm00001d024813_T002	transcript:EES17692	0
418- 47:	transcript:Zm00001d024815_T005	transcript:KXG21286	0
418- 48:	transcript:Zm00001d024816_T007	transcript:EES19009	0
418- 49:	transcript:Zm00001d024819_T002	transcript:EES19010	0
418- 50:	transcript:Zm00001d024821_T002	transcript:OQU77395	0
418- 51:	transcript:Zm00001d024823_T001	transcript:EES17695	0
418- 52:	transcript:Zm00001d024824_T001	transcript:OQU77401	0
418- 53:	transcript:Zm00001d024825_T002	transcript:KXG21291	0

418- 54: transcript:Zm00001d024828_T003 transcript:EES17699 0
418- 55: transcript:Zm00001d024830_T001 transcript:EES17700 0
418- 56: transcript:Zm00001d024831_T001 transcript:EES19019 0
418- 57: transcript:Zm00001d024832_T002 transcript:KXG21297 0
418- 58: transcript:Zm00001d024833_T001 transcript:KXG21299 5.00E-163
418- 59: transcript:Zm00001d024835_T001 transcript:KXG21303 2.00E-103
418- 60: transcript:Zm00001d024839_T001 transcript:EES17708 2.00E-132
418- 61: transcript:Zm00001d024841_T001 transcript:OQU77406 0
418- 62: transcript:Zm00001d024843_T001 transcript:EES19027 0
418- 63: transcript:Zm00001d024854_T001 transcript:EES17717 0
418- 64: transcript:Zm00001d024855_T006 transcript:EES19028 0
418- 65: transcript:Zm00001d024857_T002 transcript:EES17718 0
418- 66: transcript:Zm00001d024858_T004 transcript:KXG21309 0
418- 67: transcript:Zm00001d024861_T007 transcript:KXG21311 0
Alignment 419: score=1841.0 e_value=1.6e-140 N=40 10&9 plus
419- 0: transcript:EER89844 transcript:Zm00001d046055_T001 4.00E-35
419- 1: transcript:KXG20071 transcript:Zm00001d046060_T001 0
419- 2: transcript:OQU76478 transcript:Zm00001d046068_T001 0
419- 3: transcript:EER88427 transcript:Zm00001d046072_T001 0
419- 4: transcript:KXG20081 transcript:Zm00001d046073_T002 0
419- 5: transcript:OQU76485 transcript:Zm00001d046089_T001 8.00E-82
419- 6: transcript:EER89851 transcript:Zm00001d046091_T002 0
419- 7: transcript:EER88428 transcript:Zm00001d046096_T001 0
419- 8: transcript:OQU76491 transcript:Zm00001d046097_T001 0
419- 9: transcript:EER88432 transcript:Zm00001d046112_T002 0
419- 10: transcript:OQU76497 transcript:Zm00001d046118_T001 2.00E-50
419- 11: transcript:EER88440 transcript:Zm00001d046120_T001 5.00E-55
419- 12: transcript:OQU76499 transcript:Zm00001d046126_T001 9.00E-141
419- 13: transcript:EER88443 transcript:Zm00001d046132_T001 1.00E-68
419- 14: transcript:EER88444 transcript:Zm00001d046134_T001 0
419- 15: transcript:EER89866 transcript:Zm00001d046135_T001 0
419- 16: transcript:EER89867 transcript:Zm00001d046136_T001 6.00E-145
419- 17: transcript:KXG20110 transcript:Zm00001d046137_T001 1.00E-38
419- 18: transcript:EER89869 transcript:Zm00001d046138_T001 2.00E-41
419- 19: transcript:OQU76504 transcript:Zm00001d046140_T001 6.00E-37
419- 20: transcript:KXG20113 transcript:Zm00001d046142_T001 1.00E-34
419- 21: transcript:OQU76505 transcript:Zm00001d046143_T001 5.00E-75
419- 22: transcript:EER88462 transcript:Zm00001d046148_T001 2.00E-141
419- 23: transcript:EER88463 transcript:Zm00001d046149_T001 7.00E-138
419- 24: transcript:KXG20125 transcript:Zm00001d046151_T001 2.00E-62
419- 25: transcript:OQU76515 transcript:Zm00001d046152_T001 2.00E-32
419- 26: transcript:OQU76517 transcript:Zm00001d046155_T001 3.00E-93
419- 27: transcript:KXG20131 transcript:Zm00001d046157_T001 1.00E-86
419- 28: transcript:EER89877 transcript:Zm00001d046158_T001 8.00E-57
419- 29: transcript:EER88482 transcript:Zm00001d046161_T001 2.00E-44
419- 30: transcript:EER88483 transcript:Zm00001d046166_T004 2.00E-176
419- 31: transcript:EER88484 transcript:Zm00001d046168_T001 0
419- 32: transcript:EER89887 transcript:Zm00001d046169_T001 3.00E-88
419- 33: transcript:EER89889 transcript:Zm00001d046170_T001 0
419- 34: transcript:OQU76531 transcript:Zm00001d046174_T001 0
419- 35: transcript:EER89894 transcript:Zm00001d046175_T001 0
419- 36: transcript:EER88491 transcript:Zm00001d046178_T001 0
419- 37: transcript:OQU76534 transcript:Zm00001d046182_T001 0
419- 38: transcript:EER89896 transcript:Zm00001d046183_T001 0

419- 39: transcript:EER88495 transcript:Zm00001d046184_T001 0
Alignment 420: score=1772.0 e_value=8.9e-136 N=39 10&9 plus
420- 0: transcript:KXG20536 transcript:Zm00001d046934_T002 1.00E-84
420- 1: transcript:EER90163 transcript:Zm00001d046935_T001 0
420- 2: transcript:KXG20537 transcript:Zm00001d046936_T002 0
420- 3: transcript:EER90165 transcript:Zm00001d046937_T001 2.00E-149
420- 4: transcript:EER90166 transcript:Zm00001d046938_T001 0
420- 5: transcript:KXG20556 transcript:Zm00001d046939_T001 0
420- 6: transcript:OQU76834 transcript:Zm00001d046940_T001 2.00E-110
420- 7: transcript:KXG20550 transcript:Zm00001d046941_T001 0
420- 8: transcript:EER90172 transcript:Zm00001d046942_T001 0
420- 9: transcript:KXG20551 transcript:Zm00001d046945_T001 0
420- 10: transcript:KXG20552 transcript:Zm00001d046947_T001 0
420- 11: transcript:EER88783 transcript:Zm00001d046948_T002 0
420- 12: transcript:EER90176 transcript:Zm00001d046949_T001 0
420- 13: transcript:OQU76836 transcript:Zm00001d046950_T001 0
420- 14: transcript:EER90179 transcript:Zm00001d046952_T001 0
420- 15: transcript:KXG20553 transcript:Zm00001d046953_T002 0
420- 16: transcript:EER90181 transcript:Zm00001d046958_T001 1.00E-140
420- 17: transcript:OQU76837 transcript:Zm00001d046959_T002 9.00E-94
420- 18: transcript:OQU76839 transcript:Zm00001d046961_T001 2.00E-67
420- 19: transcript:EER88786 transcript:Zm00001d046966_T001 1.00E-28
420- 20: transcript:EER88787 transcript:Zm00001d046967_T001 2.00E-138
420- 21: transcript:KXG20566 transcript:Zm00001d046968_T001 0
420- 22: transcript:OQU76853 transcript:Zm00001d046970_T002 0
420- 23: transcript:KXG20578 transcript:Zm00001d046971_T001 0
420- 24: transcript:EER90198 transcript:Zm00001d046972_T002 6.00E-153
420- 25: transcript:EER90199 transcript:Zm00001d046973_T001 0
420- 26: transcript:KXG20581 transcript:Zm00001d046974_T002 1.00E-115
420- 27: transcript:EER88792 transcript:Zm00001d046978_T002 3.00E-138
420- 28: transcript:KXG20587 transcript:Zm00001d046979_T001 0
420- 29: transcript:OQU76857 transcript:Zm00001d046981_T001 6.00E-89
420- 30: transcript:OQU76860 transcript:Zm00001d046986_T001 3.00E-59
420- 31: transcript:EER88799 transcript:Zm00001d046993_T001 6.00E-49
420- 32: transcript:EER88801 transcript:Zm00001d046995_T001 0
420- 33: transcript:EER90205 transcript:Zm00001d046996_T001 2.00E-165
420- 34: transcript:OQU76862 transcript:Zm00001d046998_T001 3.00E-128
420- 35: transcript:KXG20599 transcript:Zm00001d047000_T001 4.00E-12
420- 36: transcript:KXG20605 transcript:Zm00001d047006_T003 0
420- 37: transcript:KXG20612 transcript:Zm00001d047012_T001 1.00E-24
420- 38: transcript:KXG20615 transcript:Zm00001d047013_T001 0
Alignment 421: score=1256.0 e_value=1.9e-87 N=27 10&9 plus
421- 0: transcript:EER90008 transcript:Zm00001d046490_T001 0
421- 1: transcript:EER90009 transcript:Zm00001d046492_T001 3.00E-175
421- 2: transcript:EER90015 transcript:Zm00001d046496_T001 4.00E-88
421- 3: transcript:KXG20318 transcript:Zm00001d046499_T001 6.00E-178
421- 4: transcript:EER90017 transcript:Zm00001d046500_T001 0
421- 5: transcript:EER90018 transcript:Zm00001d046501_T001 2.00E-112
421- 6: transcript:KXG20319 transcript:Zm00001d046506_T002 0
421- 7: transcript:EER88618 transcript:Zm00001d046508_T001 0
421- 8: transcript:EER88619 transcript:Zm00001d046509_T002 0
421- 9: transcript:EER88620 transcript:Zm00001d046510_T001 2.00E-45
421- 10: transcript:KXG20322 transcript:Zm00001d046513_T001 2.00E-113
421- 11: transcript:KXG20323 transcript:Zm00001d046517_T001 1.00E-175

421- 12: transcript:EER88623 transcript:Zm00001d046530_T001 3.00E-120
421- 13: transcript:KXG20324 transcript:Zm00001d046531_T007 0
421- 14: transcript:EER88624 transcript:Zm00001d046533_T003 0
421- 15: transcript:EER88625 transcript:Zm00001d046534_T001 0
421- 16: transcript:KXG20330 transcript:Zm00001d046535_T001 8.00E-171
421- 17: transcript:OQU76682 transcript:Zm00001d046537_T002 0
421- 18: transcript:EER90026 transcript:Zm00001d046538_T003 0
421- 19: transcript:EER88627 transcript:Zm00001d046539_T002 0
421- 20: transcript:OQU76685 transcript:Zm00001d046540_T003 0
421- 21: transcript:EER88629 transcript:Zm00001d046542_T001 4.00E-39
421- 22: transcript:OQU76687 transcript:Zm00001d046545_T010 0
421- 23: transcript:EER90029 transcript:Zm00001d046549_T002 0
421- 24: transcript:EER88630 transcript:Zm00001d046552_T001 8.00E-142
421- 25: transcript:EER88631 transcript:Zm00001d046553_T003 1.00E-118
421- 26: transcript:KXG20338 transcript:Zm00001d046554_T001 0
Alignment 422: score=1062.0 e_value=3.7e-68 N=23 10&9 plus
422- 0: transcript:EER88547 transcript:Zm00001d046330_T002 0
422- 1: transcript:OQU76598 transcript:Zm00001d046332_T001 1.00E-124
422- 2: transcript:EER89960 transcript:Zm00001d046334_T001 0
422- 3: transcript:OQU76599 transcript:Zm00001d046335_T001 0
422- 4: transcript:KXG20224 transcript:Zm00001d046336_T001 0
422- 5: transcript:EER89963 transcript:Zm00001d046340_T001 1.00E-95
422- 6: transcript:EER89964 transcript:Zm00001d046342_T002 0
422- 7: transcript:EER88550 transcript:Zm00001d046346_T001 2.00E-158
422- 8: transcript:EER89968 transcript:Zm00001d046348_T003 0
422- 9: transcript:KXG20227 transcript:Zm00001d046350_T001 0
422- 10: transcript:EER89969 transcript:Zm00001d046352_T001 0
422- 11: transcript:EER88552 transcript:Zm00001d046354_T001 8.00E-177
422- 12: transcript:KXG20232 transcript:Zm00001d046356_T001 0
422- 13: transcript:EER88554 transcript:Zm00001d046357_T001 0
422- 14: transcript:OQU76607 transcript:Zm00001d046358_T001 0
422- 15: transcript:OQU76610 transcript:Zm00001d046362_T001 2.00E-168
422- 16: transcript:KXG20238 transcript:Zm00001d046363_T001 0
422- 17: transcript:EER89974 transcript:Zm00001d046364_T001 0
422- 18: transcript:OQU76614 transcript:Zm00001d046366_T001 0
422- 19: transcript:EER88560 transcript:Zm00001d046369_T002 0
422- 20: transcript:OQU76615 transcript:Zm00001d046370_T001 0
422- 21: transcript:OQU76618 transcript:Zm00001d046372_T005 0
422- 22: transcript:EER88566 transcript:Zm00001d046378_T001 2.00E-79
Alignment 423: score=890.0 e_value=7.9e-58 N=20 10&9 plus
423- 0: transcript:EER90117 transcript:Zm00001d046794_T001 4.00E-76
423- 1: transcript:KXG20495 transcript:Zm00001d046802_T001 0
423- 2: transcript:OQU76787 transcript:Zm00001d046803_T001 1.00E-107
423- 3: transcript:EER88729 transcript:Zm00001d046805_T001 1.00E-149
423- 4: transcript:KXG20499 transcript:Zm00001d046810_T001 0
423- 5: transcript:EER90127 transcript:Zm00001d046823_T001 0
423- 6: transcript:EER88739 transcript:Zm00001d046824_T001 6.00E-49
423- 7: transcript:OQU76796 transcript:Zm00001d046827_T002 0
423- 8: transcript:EER90135 transcript:Zm00001d046831_T002 0
423- 9: transcript:EER88740 transcript:Zm00001d046834_T001 5.00E-46
423- 10: transcript:EER88745 transcript:Zm00001d046835_T004 0
423- 11: transcript:KXG20506 transcript:Zm00001d046838_T005 0
423- 12: transcript:KXG20507 transcript:Zm00001d046850_T001 0
423- 13: transcript:EER90136 transcript:Zm00001d046852_T001 0

423- 14: transcript:EER90137 transcript:Zm00001d046855_T001 1.00E-149
423- 15: transcript:OQU76797 transcript:Zm00001d046859_T001 3.00E-56
423- 16: transcript:EER88750 transcript:Zm00001d046865_T001 0
423- 17: transcript:EER88751 transcript:Zm00001d046866_T001 0
423- 18: transcript:KXG20510 transcript:Zm00001d046867_T001 1.00E-48
423- 19: transcript:EER88753 transcript:Zm00001d046875_T002 0
Alignment 424: score=742.0 e_value=8.7e-51 N=18 10&9 plus
424- 0: transcript:KXG19733 transcript:Zm00001d045660_T001 7.00E-152
424- 1: transcript:EER88191 transcript:Zm00001d045661_T001 0
424- 2: transcript:KXG19738 transcript:Zm00001d045665_T003 0
424- 3: transcript:EER88193 transcript:Zm00001d045667_T001 0
424- 4: transcript:EER89569 transcript:Zm00001d045668_T001 2.00E-70
424- 5: transcript:EER89572 transcript:Zm00001d045673_T003 0
424- 6: transcript:EER89574 transcript:Zm00001d045675_T003 0
424- 7: transcript:KXG19752 transcript:Zm00001d045679_T001 0
424- 8: transcript:EER89582 transcript:Zm00001d045680_T001 6.00E-73
424- 9: transcript:KXG19762 transcript:Zm00001d045692_T010 0
424- 10: transcript:KXG19765 transcript:Zm00001d045695_T001 0
424- 11: transcript:EER89585 transcript:Zm00001d045696_T001 0
424- 12: transcript:EER89586 transcript:Zm00001d045703_T002 0
424- 13: transcript:KXG19772 transcript:Zm00001d045706_T001 0
424- 14: transcript:KXG19775 transcript:Zm00001d045708_T001 0
424- 15: transcript:OQU76222 transcript:Zm00001d045720_T001 0
424- 16: transcript:EER88219 transcript:Zm00001d045724_T001 2.00E-31
424- 17: transcript:OQU76224 transcript:Zm00001d045725_T002 0
Alignment 425: score=654.0 e_value=2.2e-40 N=15 10&9 plus
425- 0: transcript:EER88222 transcript:Zm00001d045729_T002 4.00E-65
425- 1: transcript:OQU76227 transcript:Zm00001d045731_T001 1.00E-63
425- 2: transcript:EER88227 transcript:Zm00001d045735_T002 0
425- 3: transcript:EER89601 transcript:Zm00001d045738_T001 1.00E-107
425- 4: transcript:KXG19790 transcript:Zm00001d045739_T001 0
425- 5: transcript:EER88229 transcript:Zm00001d045740_T001 0
425- 6: transcript:EER88230 transcript:Zm00001d045742_T001 3.00E-103
425- 7: transcript:EER88231 transcript:Zm00001d045744_T002 0
425- 8: transcript:OQU76234 transcript:Zm00001d045747_T004 2.00E-154
425- 9: transcript:EER88243 transcript:Zm00001d045755_T001 0
425- 10: transcript:OQU76241 transcript:Zm00001d045756_T001 0
425- 11: transcript:EER89609 transcript:Zm00001d045757_T001 0
425- 12: transcript:EER89610 transcript:Zm00001d045763_T001 0
425- 13: transcript:EER88246 transcript:Zm00001d045764_T002 0
425- 14: transcript:EER88247 transcript:Zm00001d045765_T001 1.00E-12
Alignment 426: score=478.0 e_value=9.8e-29 N=12 10&9 plus
426- 0: transcript:EER88334 transcript:Zm00001d045911_T002 0
426- 1: transcript:OQU76348 transcript:Zm00001d045913_T001 3.00E-54
426- 2: transcript:OQU76350 transcript:Zm00001d045914_T001 2.00E-57
426- 3: transcript:OQU76351 transcript:Zm00001d045919_T001 0
426- 4: transcript:EER88336 transcript:Zm00001d045926_T002 1.00E-151
426- 5: transcript:KXG19921 transcript:Zm00001d045927_T002 5.00E-180
426- 6: transcript:KXG19929 transcript:Zm00001d045928_T001 3.00E-153
426- 7: transcript:OQU76355 transcript:Zm00001d045931_T001 1.00E-69
426- 8: transcript:EER88344 transcript:Zm00001d045935_T001 2.00E-59
426- 9: transcript:KXG19933 transcript:Zm00001d045936_T001 1.00E-75
426- 10: transcript:OQU76359 transcript:Zm00001d045937_T001 6.00E-25
426- 11: transcript:KXG19937 transcript:Zm00001d045938_T005 0

Alignment 427: score=378.0 e_value=9.2e-23 N=10 10&9 plus
427- 0: transcript:KXG19894 transcript:Zm00001d045883_T001 0
427- 1: transcript:KXG19896 transcript:Zm00001d045884_T001 0
427- 2: transcript:EER88320 transcript:Zm00001d045885_T002 0
427- 3: transcript:KXG19898 transcript:Zm00001d045887_T004 0
427- 4: transcript:EER88321 transcript:Zm00001d045888_T001 2.00E-06
427- 5: transcript:EER89691 transcript:Zm00001d045893_T001 5.00E-78
427- 6: transcript:EER88322 transcript:Zm00001d045894_T001 0
427- 7: transcript:EER89693 transcript:Zm00001d045896_T001 4.00E-73
427- 8: transcript:OQU76337 transcript:Zm00001d045897_T003 0
427- 9: transcript:KXG19904 transcript:Zm00001d045905_T001 6.00E-12
Alignment 428: score=370.0 e_value=7.8e-15 N=8 10&9 plus
428- 0: transcript:KXG19307 transcript:Zm00001d045327_T001 0
428- 1: transcript:KXG19311 transcript:Zm00001d045328_T001 5.00E-31
428- 2: transcript:KXG19312 transcript:Zm00001d045334_T001 6.00E-61
428- 3: transcript:EER89208 transcript:Zm00001d045335_T003 1.00E-43
428- 4: transcript:KXG19314 transcript:Zm00001d045336_T001 0
428- 5: transcript:EER87855 transcript:Zm00001d045338_T001 0
428- 6: transcript:EER89212 transcript:Zm00001d045339_T002 2.00E-103
428- 7: transcript:OQU75837 transcript:Zm00001d045340_T004 1.00E-110
Alignment 429: score=314.0 e_value=4.6e-13 N=7 10&9 plus
429- 0: transcript:KXG20286 transcript:Zm00001d046438_T003 0
429- 1: transcript:OQU76660 transcript:Zm00001d046440_T001 0
429- 2: transcript:EER88599 transcript:Zm00001d046441_T002 0
429- 3: transcript:EER88600 transcript:Zm00001d046442_T002 0
429- 4: transcript:EER88601 transcript:Zm00001d046444_T003 0
429- 5: transcript:EER90002 transcript:Zm00001d046445_T004 0
429- 6: transcript:KXG20293 transcript:Zm00001d046449_T006 0
Alignment 430: score=311.0 e_value=8.1e-12 N=7 10&9 plus
430- 0: transcript:EER88513 transcript:Zm00001d046235_T002 0
430- 1: transcript:OQU76569 transcript:Zm00001d046236_T001 7.00E-55
430- 2: transcript:OQU76572 transcript:Zm00001d046238_T001 0
430- 3: transcript:KXG20192 transcript:Zm00001d046242_T001 1.00E-96
430- 4: transcript:KXG20193 transcript:Zm00001d046246_T001 0
430- 5: transcript:EER89945 transcript:Zm00001d046249_T001 3.00E-138
430- 6: transcript:EER88523 transcript:Zm00001d046254_T001 0
Alignment 431: score=308.0 e_value=4.2e-12 N=7 10&9 plus
431- 0: transcript:EER87676 transcript:Zm00001d045617_T001 0
431- 1: transcript:EER89024 transcript:Zm00001d045618_T001 7.00E-145
431- 2: transcript:EER87677 transcript:Zm00001d045620_T001 7.00E-81
431- 3: transcript:EER89027 transcript:Zm00001d045625_T001 1.00E-173
431- 4: transcript:KXG19082 transcript:Zm00001d045629_T002 0
431- 5: transcript:EER87680 transcript:Zm00001d045632_T004 0
431- 6: transcript:KXG19083 transcript:Zm00001d045635_T029 0
Alignment 432: score=255.0 e_value=3.5e-08 N=6 10&9 plus
432- 0: transcript:KXG19356 transcript:Zm00001d045235_T001 2.00E-59
432- 1: transcript:KXG19361 transcript:Zm00001d045244_T001 3.00E-66
432- 2: transcript:EER89245 transcript:Zm00001d045247_T001 5.00E-30
432- 3: transcript:EER87896 transcript:Zm00001d045251_T001 0
432- 4: transcript:KXG19372 transcript:Zm00001d045256_T001 7.00E-38
432- 5: transcript:KXG19378 transcript:Zm00001d045258_T001 7.00E-39
Alignment 433: score=253.0 e_value=6.9e-10 N=6 10&9 plus
433- 0: transcript:EER88257 transcript:Zm00001d045785_T001 0
433- 1: transcript:KXG19818 transcript:Zm00001d045792_T001 3.00E-150

```
433-  2: transcript:OQU76266          transcript:Zm00001d045793_T001  4.00E-55
433-  3: transcript:EER88270          transcript:Zm00001d045798_T001         0
433-  4: transcript:EER88272          transcript:Zm00001d045800_T001         0
433-  5: transcript:OQU76272          transcript:Zm00001d045804_T003         0
## Alignment 434: score=251.0 e_value=1.4e-08 N=6 10&9 plus
434-  0: transcript:EER88170          transcript:Zm00001d044725_T001  2.00E-53
434-  1: transcript:OQU76176          transcript:Zm00001d044726_T001         0
434-  2: transcript:EER89539          transcript:Zm00001d044728_T001  1.00E-87
434-  3: transcript:EER89540          transcript:Zm00001d044729_T001 6.00E-146
434-  4: transcript:OQU76180          transcript:Zm00001d044730_T001  2.00E-63
434-  5: transcript:OQU76181          transcript:Zm00001d044732_T001 1.00E-122
## Alignment 435: score=4063.0 e_value=0 N=89 10&9 minus
435-  0: transcript:EER88633          transcript:Zm00001d046793_T001         0
435-  1: transcript:EER90030          transcript:Zm00001d046792_T001 5.00E-150
435-  2: transcript:EER90031          transcript:Zm00001d046790_T001         0
435-  3: transcript:EER88636          transcript:Zm00001d046788_T001         0
435-  4: transcript:KXG20344          transcript:Zm00001d046786_T001         0
435-  5: transcript:OQU76690          transcript:Zm00001d046784_T001 6.00E-103
435-  6: transcript:OQU76691          transcript:Zm00001d046783_T001         0
435-  7: transcript:OQU76694          transcript:Zm00001d046781_T001         0
435-  8: transcript:KXG20349          transcript:Zm00001d046778_T001 6.00E-162
435-  9: transcript:EER88644          transcript:Zm00001d046774_T001         0
435- 10: transcript:KXG20351          transcript:Zm00001d046767_T002 1.00E-113
435- 11: transcript:EER90038          transcript:Zm00001d046765_T001         0
435- 12: transcript:KXG20354          transcript:Zm00001d046764_T001         0
435- 13: transcript:KXG20356          transcript:Zm00001d046761_T001         0
435- 14: transcript:KXG20357          transcript:Zm00001d046759_T001 4.00E-174
435- 15: transcript:EER88647          transcript:Zm00001d046758_T001         0
435- 16: transcript:KXG20361          transcript:Zm00001d046755_T001         0
435- 17: transcript:KXG20382          transcript:Zm00001d046751_T001  5.00E-84
435- 18: transcript:EER90047          transcript:Zm00001d046749_T001         0
435- 19: transcript:OQU76710          transcript:Zm00001d046748_T001         0
435- 20: transcript:EER88653          transcript:Zm00001d046743_T001  1.00E-14
435- 21: transcript:KXG20370          transcript:Zm00001d046742_T002         0
435- 22: transcript:KXG20371          transcript:Zm00001d046740_T001 9.00E-148
435- 23: transcript:EER88661          transcript:Zm00001d046729_T001  1.00E-21
435- 24: transcript:EER90053          transcript:Zm00001d046728_T001  3.00E-73
435- 25: transcript:EER88662          transcript:Zm00001d046723_T001 4.00E-173
435- 26: transcript:KXG20380          transcript:Zm00001d046722_T007         0
435- 27: transcript:KXG20383          transcript:Zm00001d046719_T006         0
435- 28: transcript:OQU76718          transcript:Zm00001d046718_T001 2.00E-179
435- 29: transcript:OQU76719          transcript:Zm00001d046717_T009         0
435- 30: transcript:EER90058          transcript:Zm00001d046716_T001 2.00E-158
435- 31: transcript:KXG20414          transcript:Zm00001d046714_T002 2.00E-128
435- 32: transcript:KXG20386          transcript:Zm00001d046713_T001  7.00E-30
435- 33: transcript:OQU76721          transcript:Zm00001d046711_T001  2.00E-52
435- 34: transcript:EER88668          transcript:Zm00001d046696_T001 2.00E-139
435- 35: transcript:OQU76726          transcript:Zm00001d046695_T001  1.00E-36
435- 36: transcript:KXG20397          transcript:Zm00001d046691_T005         0
435- 37: transcript:KXG20398          transcript:Zm00001d046690_T001         0
435- 38: transcript:EER88673          transcript:Zm00001d046687_T001  2.00E-64
435- 39: transcript:OQU76730          transcript:Zm00001d046684_T001         0
435- 40: transcript:OQU76732          transcript:Zm00001d046683_T002         0
435- 41: transcript:KXG20408          transcript:Zm00001d046682_T001 4.00E-136
```

```
435- 42: transcript:KXG20409            transcript:Zm00001d046681_T001         0
435- 43: transcript:OQU76738            transcript:Zm00001d046680_T001         0
435- 44: transcript:EER90068            transcript:Zm00001d046679_T002         0
435- 45: transcript:EER90070            transcript:Zm00001d046676_T001         0
435- 46: transcript:EER88680            transcript:Zm00001d046673_T001  5.00E-30
435- 47: transcript:EER88683            transcript:Zm00001d046667_T001  3.00E-31
435- 48: transcript:KXG20421            transcript:Zm00001d046665_T001  2.00E-47
435- 49: transcript:KXG20422            transcript:Zm00001d046664_T001  1.00E-25
435- 50: transcript:EER90075            transcript:Zm00001d046663_T001 4.00E-160
435- 51: transcript:EER88687            transcript:Zm00001d046661_T003         0
435- 52: transcript:OQU76743            transcript:Zm00001d046660_T001         0
435- 53: transcript:KXG20425            transcript:Zm00001d046659_T001  6.00E-88
435- 54: transcript:OQU76744            transcript:Zm00001d046656_T004 2.00E-108
435- 55: transcript:KXG20428            transcript:Zm00001d046655_T001         0
435- 56: transcript:EER90078            transcript:Zm00001d046654_T002         0
435- 57: transcript:EER90079            transcript:Zm00001d046652_T001         0
435- 58: transcript:OQU76746            transcript:Zm00001d046651_T001  3.00E-65
435- 59: transcript:OQU76747            transcript:Zm00001d046649_T001  4.00E-34
435- 60: transcript:KXG20435            transcript:Zm00001d046644_T001 6.00E-168
435- 61: transcript:EER90083            transcript:Zm00001d046643_T001         0
435- 62: transcript:EER90082            transcript:Zm00001d046642_T001         0
435- 63: transcript:EER88692            transcript:Zm00001d046641_T001  8.00E-43
435- 64: transcript:OQU76750            transcript:Zm00001d046634_T001  9.00E-29
435- 65: transcript:EER90085            transcript:Zm00001d046632_T001 1.00E-144
435- 66: transcript:OQU76753            transcript:Zm00001d046630_T003         0
435- 67: transcript:EER90086            transcript:Zm00001d046629_T001 1.00E-149
435- 68: transcript:EER88696            transcript:Zm00001d046628_T001  3.00E-98
435- 69: transcript:EER90087            transcript:Zm00001d046626_T001         0
435- 70: transcript:OQU76759            transcript:Zm00001d046625_T001 3.00E-132
435- 71: transcript:KXG20452            transcript:Zm00001d046624_T005         0
435- 72: transcript:KXG20453            transcript:Zm00001d046621_T001 3.00E-169
435- 73: transcript:KXG20459            transcript:Zm00001d046616_T001         0
435- 74: transcript:EER90094            transcript:Zm00001d046613_T007 2.00E-133
435- 75: transcript:EER90098            transcript:Zm00001d046604_T001         0
435- 76: transcript:EER88702            transcript:Zm00001d046602_T001  1.00E-38
435- 77: transcript:EER90100            transcript:Zm00001d046601_T005         0
435- 78: transcript:EER90101            transcript:Zm00001d046600_T001 1.00E-145
435- 79: transcript:EER88704            transcript:Zm00001d046599_T001 4.00E-149
435- 80: transcript:EER88705            transcript:Zm00001d046596_T001  3.00E-79
435- 81: transcript:OQU76768            transcript:Zm00001d046595_T002         0
435- 82: transcript:KXG20468            transcript:Zm00001d046593_T001         0
435- 83: transcript:EER88706            transcript:Zm00001d046592_T001 7.00E-127
435- 84: transcript:OQU76769            transcript:Zm00001d046591_T001         0
435- 85: transcript:EER88713            transcript:Zm00001d046590_T004         0
435- 86: transcript:EER88714            transcript:Zm00001d046587_T003         0
435- 87: transcript:EER90107            transcript:Zm00001d046586_T001         0
435- 88: transcript:EER88707            transcript:Zm00001d046583_T001 4.00E-105
## Alignment 436: score=3426.0 e_value=0 N=76 10&9 minus
436-  0: transcript:EER87942            transcript:Zm00001d045163_T001 2.00E-103
436-  1: transcript:EER89299            transcript:Zm00001d045162_T001 3.00E-153
436-  2: transcript:EER87945            transcript:Zm00001d045160_T002  3.00E-74
436-  3: transcript:KXG19432            transcript:Zm00001d045157_T002  6.00E-40
436-  4: transcript:OQU75939            transcript:Zm00001d045155_T001         0
436-  5: transcript:KXG19435            transcript:Zm00001d045154_T002         0
```

436- 6:	transcript:KXG19437	transcript:Zm00001d045153_T002	0
436- 7:	transcript:OQU75941	transcript:Zm00001d045149_T001	0
436- 8:	transcript:EER87951	transcript:Zm00001d045147_T015	0
436- 9:	transcript:KXG19439	transcript:Zm00001d045146_T002	0
436- 10:	transcript:EER87955	transcript:Zm00001d045145_T001	2.00E-98
436- 11:	transcript:EER87959	transcript:Zm00001d045144_T001	1.00E-112
436- 12:	transcript:EER89306	transcript:Zm00001d045141_T001	2.00E-64
436- 13:	transcript:KXG19445	transcript:Zm00001d045139_T002	0
436- 14:	transcript:KXG19454	transcript:Zm00001d045138_T001	0
436- 15:	transcript:OQU75953	transcript:Zm00001d045137_T001	1.00E-54
436- 16:	transcript:EER89314	transcript:Zm00001d045136_T001	0
436- 17:	transcript:KXG19459	transcript:Zm00001d045134_T001	4.00E-153
436- 18:	transcript:EER87965	transcript:Zm00001d045130_T001	0
436- 19:	transcript:KXG19463	transcript:Zm00001d045129_T001	2.00E-39
436- 20:	transcript:OQU75957	transcript:Zm00001d045128_T001	6.00E-179
436- 21:	transcript:EER89324	transcript:Zm00001d045127_T002	0
436- 22:	transcript:KXG19468	transcript:Zm00001d045125_T001	8.00E-102
436- 23:	transcript:OQU75963	transcript:Zm00001d045124_T012	0
436- 24:	transcript:OQU75965	transcript:Zm00001d045123_T001	0
436- 25:	transcript:OQU75967	transcript:Zm00001d045122_T001	0
436- 26:	transcript:OQU75968	transcript:Zm00001d045121_T001	2.00E-30
436- 27:	transcript:EER87969	transcript:Zm00001d045120_T001	4.00E-82
436- 28:	transcript:EER87970	transcript:Zm00001d045119_T001	1.00E-171
436- 29:	transcript:KXG19474	transcript:Zm00001d045118_T001	6.00E-102
436- 30:	transcript:KXG19478	transcript:Zm00001d045113_T001	0
436- 31:	transcript:KXG19480	transcript:Zm00001d045112_T001	0
436- 32:	transcript:EER89330	transcript:Zm00001d045111_T001	5.00E-169
436- 33:	transcript:KXG19483	transcript:Zm00001d045109_T016	0
436- 34:	transcript:EER89333	transcript:Zm00001d045108_T001	0
436- 35:	transcript:EER89334	transcript:Zm00001d045107_T001	2.00E-110
436- 36:	transcript:EER89335	transcript:Zm00001d045106_T001	1.00E-40
436- 37:	transcript:KXG19486	transcript:Zm00001d045105_T001	2.00E-162
436- 38:	transcript:EER89337	transcript:Zm00001d045104_T005	0
436- 39:	transcript:EER87977	transcript:Zm00001d045102_T001	0
436- 40:	transcript:EER89338	transcript:Zm00001d045101_T001	0
436- 41:	transcript:EER89339	transcript:Zm00001d045098_T001	0
436- 42:	transcript:EER89345	transcript:Zm00001d045097_T001	0
436- 43:	transcript:EER89340	transcript:Zm00001d045096_T001	1.00E-44
436- 44:	transcript:OQU75976	transcript:Zm00001d045094_T001	0
436- 45:	transcript:EER87979	transcript:Zm00001d045091_T001	0
436- 46:	transcript:EER87980	transcript:Zm00001d045090_T001	0
436- 47:	transcript:KXG19489	transcript:Zm00001d045089_T001	0
436- 48:	transcript:OQU75978	transcript:Zm00001d045086_T001	0
436- 49:	transcript:KXG19492	transcript:Zm00001d045085_T001	0
436- 50:	transcript:KXG19495	transcript:Zm00001d045084_T002	0
436- 51:	transcript:EER89351	transcript:Zm00001d045082_T002	0
436- 52:	transcript:OQU75979	transcript:Zm00001d045080_T001	0
436- 53:	transcript:OQU75980	transcript:Zm00001d045079_T001	5.00E-174
436- 54:	transcript:OQU75981	transcript:Zm00001d045078_T001	0
436- 55:	transcript:EER87986	transcript:Zm00001d045077_T001	0
436- 56:	transcript:EER87987	transcript:Zm00001d045076_T003	2.00E-92
436- 57:	transcript:EER87988	transcript:Zm00001d045075_T001	0
436- 58:	transcript:KXG19511	transcript:Zm00001d045074_T001	0
436- 59:	transcript:EER89358	transcript:Zm00001d045072_T035	0

436- 60:	transcript:KXG19510	transcript:Zm00001d045070_T001	6.00E-156
436- 61:	transcript:EER89359	transcript:Zm00001d045069_T001	2.00E-121
436- 62:	transcript:KXG19512	transcript:Zm00001d045065_T001	0
436- 63:	transcript:KXG19514	transcript:Zm00001d045064_T001	0
436- 64:	transcript:KXG19519	transcript:Zm00001d045056_T003	0
436- 65:	transcript:EER87993	transcript:Zm00001d045055_T001	0
436- 66:	transcript:EER89366	transcript:Zm00001d045053_T001	6.00E-38
436- 67:	transcript:OQU75990	transcript:Zm00001d045052_T001	5.00E-103
436- 68:	transcript:EER87996	transcript:Zm00001d045051_T001	1.00E-138
436- 69:	transcript:KXG19520	transcript:Zm00001d045050_T001	0
436- 70:	transcript:EER89368	transcript:Zm00001d045049_T001	3.00E-159
436- 71:	transcript:EER87997	transcript:Zm00001d045048_T001	0
436- 72:	transcript:EER88000	transcript:Zm00001d045046_T001	0
436- 73:	transcript:EER87998	transcript:Zm00001d045044_T001	2.00E-172
436- 74:	transcript:KXG19525	transcript:Zm00001d045043_T004	0
436- 75:	transcript:KXG19528	transcript:Zm00001d045042_T014	0

Alignment 437: score=1895.0 e_value=4.5e-158 N=44 10&9 minus

437- 0:	transcript:KXG19184	transcript:Zm00001d045491_T003	0
437- 1:	transcript:EER87769	transcript:Zm00001d045490_T001	0
437- 2:	transcript:EER89113	transcript:Zm00001d045488_T003	0
437- 3:	transcript:EER87768	transcript:Zm00001d045487_T001	1.00E-118
437- 4:	transcript:EER89116	transcript:Zm00001d045486_T001	3.00E-31
437- 5:	transcript:EER87771	transcript:Zm00001d045484_T001	0
437- 6:	transcript:EER87773	transcript:Zm00001d045483_T002	0
437- 7:	transcript:KXG19189	transcript:Zm00001d045482_T017	0
437- 8:	transcript:EER87774	transcript:Zm00001d045481_T004	0
437- 9:	transcript:OQU75735	transcript:Zm00001d045480_T003	0
437- 10:	transcript:EER87775	transcript:Zm00001d045479_T001	0
437- 11:	transcript:EER89121	transcript:Zm00001d045477_T001	0
437- 12:	transcript:EER87778	transcript:Zm00001d045472_T002	0
437- 13:	transcript:EER89125	transcript:Zm00001d045470_T001	0
437- 14:	transcript:EER87780	transcript:Zm00001d045468_T001	0
437- 15:	transcript:OQU75748	transcript:Zm00001d045465_T001	0
437- 16:	transcript:EER87784	transcript:Zm00001d045463_T001	2.00E-104
437- 17:	transcript:EER87785	transcript:Zm00001d045462_T001	0
437- 18:	transcript:EER87787	transcript:Zm00001d045461_T001	5.00E-27
437- 19:	transcript:EER87788	transcript:Zm00001d045459_T001	2.00E-12
437- 20:	transcript:EER87789	transcript:Zm00001d045458_T001	0
437- 21:	transcript:OQU75753	transcript:Zm00001d045454_T001	5.00E-89
437- 22:	transcript:KXG19207	transcript:Zm00001d045451_T001	0
437- 23:	transcript:EER87791	transcript:Zm00001d045450_T001	0
437- 24:	transcript:EER89131	transcript:Zm00001d045448_T001	7.00E-79
437- 25:	transcript:KXG19208	transcript:Zm00001d045447_T002	5.00E-110
437- 26:	transcript:EER89139	transcript:Zm00001d045445_T001	0
437- 27:	transcript:EER89136	transcript:Zm00001d045436_T001	0
437- 28:	transcript:EER89141	transcript:Zm00001d045435_T009	2.00E-154
437- 29:	transcript:OQU75763	transcript:Zm00001d045434_T004	0
437- 30:	transcript:EER87800	transcript:Zm00001d045432_T001	4.00E-63
437- 31:	transcript:OQU75768	transcript:Zm00001d045431_T001	0
437- 32:	transcript:KXG19237	transcript:Zm00001d045430_T002	4.00E-161
437- 33:	transcript:EER89149	transcript:Zm00001d045427_T001	6.00E-117
437- 34:	transcript:EER89151	transcript:Zm00001d045425_T001	0
437- 35:	transcript:KXG19242	transcript:Zm00001d045423_T001	1.00E-48
437- 36:	transcript:OQU75781	transcript:Zm00001d045421_T001	4.00E-121

```
437- 37: transcript:EER87803           transcript:Zm00001d045420_T001          0
437- 38: transcript:EER89155           transcript:Zm00001d045418_T001 1.00E-166
437- 39: transcript:OQU75788           transcript:Zm00001d045417_T002          0
437- 40: transcript:EER89158           transcript:Zm00001d045406_T001 1.00E-110
437- 41: transcript:OQU75790           transcript:Zm00001d045405_T001  3.00E-99
437- 42: transcript:EER87808           transcript:Zm00001d045404_T001 8.00E-161
437- 43: transcript:EER89160           transcript:Zm00001d045403_T001 4.00E-172
## Alignment 438: score=1488.0 e_value=5.9e-119 N=33 10&9 minus
438-  0: transcript:KXG19149           transcript:Zm00001d045546_T001  3.00E-09
438-  1: transcript:EER89080           transcript:Zm00001d045540_T001 1.00E-140
438-  2: transcript:EER89083           transcript:Zm00001d045539_T001  9.00E-57
438-  3: transcript:KXG19156           transcript:Zm00001d045538_T001          0
438-  4: transcript:EER87740           transcript:Zm00001d045537_T001          0
438-  5: transcript:EER89085           transcript:Zm00001d045535_T001  9.00E-88
438-  6: transcript:KXG19157           transcript:Zm00001d045534_T008          0
438-  7: transcript:EER89089           transcript:Zm00001d045533_T001 5.00E-168
438-  8: transcript:EER87742           transcript:Zm00001d045530_T001  3.00E-08
438-  9: transcript:EER87743           transcript:Zm00001d045528_T001 6.00E-130
438- 10: transcript:KXG19160           transcript:Zm00001d045522_T001 2.00E-144
438- 11: transcript:EER87745           transcript:Zm00001d045521_T001  8.00E-22
438- 12: transcript:EER87746           transcript:Zm00001d045520_T001  5.00E-16
438- 13: transcript:EER89102           transcript:Zm00001d045519_T002          0
438- 14: transcript:EER87749           transcript:Zm00001d045518_T001          0
438- 15: transcript:KXG19166           transcript:Zm00001d045517_T001 2.00E-125
438- 16: transcript:KXG19168           transcript:Zm00001d045516_T010          0
438- 17: transcript:EER89103           transcript:Zm00001d045515_T001          0
438- 18: transcript:EER89104           transcript:Zm00001d045514_T001  3.00E-86
438- 19: transcript:EER87751           transcript:Zm00001d045513_T001 3.00E-146
438- 20: transcript:EER87755           transcript:Zm00001d045509_T002 5.00E-158
438- 21: transcript:EER87757           transcript:Zm00001d045507_T001          0
438- 22: transcript:KXG19179           transcript:Zm00001d045505_T003          0
438- 23: transcript:EER87760           transcript:Zm00001d045501_T001          0
438- 24: transcript:EER87761           transcript:Zm00001d045500_T004  5.00E-85
438- 25: transcript:OQU75732           transcript:Zm00001d045499_T001 4.00E-179
438- 26: transcript:KXG19182           transcript:Zm00001d045498_T001  3.00E-86
438- 27: transcript:EER87763           transcript:Zm00001d045497_T001  8.00E-17
438- 28: transcript:KXG19183           transcript:Zm00001d045496_T001          0
438- 29: transcript:EER87765           transcript:Zm00001d045495_T001          0
438- 30: transcript:EER89111           transcript:Zm00001d045494_T001          0
438- 31: transcript:EER89112           transcript:Zm00001d045493_T002          0
438- 32: transcript:EER87766           transcript:Zm00001d045492_T002          0
## Alignment 439: score=1487.0 e_value=5.4e-114 N=33 10&9 minus
439-  0: transcript:EER89232           transcript:Zm00001d045272_T001 5.00E-140
439-  1: transcript:EER87885           transcript:Zm00001d045271_T001  1.00E-81
439-  2: transcript:OQU75869           transcript:Zm00001d045270_T001 3.00E-117
439-  3: transcript:KXG19351           transcript:Zm00001d045269_T021          0
439-  4: transcript:EER89236           transcript:Zm00001d045268_T001  2.00E-94
439-  5: transcript:EER89240           transcript:Zm00001d045264_T001          0
439-  6: transcript:EER89238           transcript:Zm00001d045263_T002 7.00E-119
439-  7: transcript:EER89242           transcript:Zm00001d045262_T001 4.00E-120
439-  8: transcript:KXG19354           transcript:Zm00001d045261_T006          0
439-  9: transcript:KXG19358           transcript:Zm00001d045256_T001  1.00E-40
439- 10: transcript:OQU75875           transcript:Zm00001d045254_T001          0
439- 11: transcript:EER87896           transcript:Zm00001d045251_T001          0
```

439- 12: transcript:KXG19373 transcript:Zm00001d045244_T001 6.00E-59
439- 13: transcript:KXG19378 transcript:Zm00001d045235_T001 2.00E-171
439- 14: transcript:EER87904 transcript:Zm00001d045233_T002 0
439- 15: transcript:KXG19379 transcript:Zm00001d045232_T001 0
439- 16: transcript:EER89250 transcript:Zm00001d045231_T002 2.00E-156
439- 17: transcript:EER89251 transcript:Zm00001d045230_T001 0
439- 18: transcript:OQU75886 transcript:Zm00001d045220_T003 3.00E-125
439- 19: transcript:EER89254 transcript:Zm00001d045219_T001 0
439- 20: transcript:EER87912 transcript:Zm00001d045218_T003 0
439- 21: transcript:EER87909 transcript:Zm00001d045217_T011 0
439- 22: transcript:EER89256 transcript:Zm00001d045216_T002 0
439- 23: transcript:OQU75889 transcript:Zm00001d045215_T001 0
439- 24: transcript:EER89259 transcript:Zm00001d045213_T002 0
439- 25: transcript:OQU75890 transcript:Zm00001d045212_T001 4.00E-154
439- 26: transcript:KXG19388 transcript:Zm00001d045207_T001 0
439- 27: transcript:EER87917 transcript:Zm00001d045206_T003 9.00E-167
439- 28: transcript:KXG19390 transcript:Zm00001d045205_T001 1.00E-92
439- 29: transcript:EER87919 transcript:Zm00001d045204_T001 9.00E-69
439- 30: transcript:KXG19391 transcript:Zm00001d045203_T001 7.00E-79
439- 31: transcript:EER87920 transcript:Zm00001d045202_T002 0
439- 32: transcript:KXG19392 transcript:Zm00001d045201_T001 1.00E-21
Alignment 440: score=1438.0 e_value=3.5e-94 N=30 10&9 minus
440- 0: transcript:EER88753 transcript:Zm00001d046933_T002 0
440- 1: transcript:EER90141 transcript:Zm00001d046930_T001 0
440- 2: transcript:EER90142 transcript:Zm00001d046929_T002 3.00E-43
440- 3: transcript:EER88755 transcript:Zm00001d046928_T002 0
440- 4: transcript:EER88756 transcript:Zm00001d046927_T001 2.00E-67
440- 5: transcript:EER88757 transcript:Zm00001d046926_T001 0
440- 6: transcript:EER90145 transcript:Zm00001d046925_T001 0
440- 7: transcript:EER90146 transcript:Zm00001d046923_T001 0
440- 8: transcript:EER90147 transcript:Zm00001d046921_T001 0
440- 9: transcript:EER90148 transcript:Zm00001d046919_T001 0
440- 10: transcript:EER88760 transcript:Zm00001d046916_T001 7.00E-90
440- 11: transcript:EER90150 transcript:Zm00001d046915_T001 0
440- 12: transcript:EER90151 transcript:Zm00001d046914_T001 4.00E-109
440- 13: transcript:EER88761 transcript:Zm00001d046913_T001 0
440- 14: transcript:EER90152 transcript:Zm00001d046912_T001 0
440- 15: transcript:KXG20523 transcript:Zm00001d046910_T001 0
440- 16: transcript:EER88764 transcript:Zm00001d046909_T002 0
440- 17: transcript:EER88765 transcript:Zm00001d046906_T001 2.00E-168
440- 18: transcript:KXG20526 transcript:Zm00001d046905_T001 0
440- 19: transcript:EER88767 transcript:Zm00001d046900_T003 1.00E-79
440- 20: transcript:EER88768 transcript:Zm00001d046898_T001 0
440- 21: transcript:EER90156 transcript:Zm00001d046897_T001 3.00E-64
440- 22: transcript:EER90158 transcript:Zm00001d046896_T001 5.00E-43
440- 23: transcript:EER88769 transcript:Zm00001d046893_T001 0
440- 24: transcript:EER90159 transcript:Zm00001d046891_T001 2.00E-40
440- 25: transcript:EER88770 transcript:Zm00001d046890_T001 0
440- 26: transcript:OQU76823 transcript:Zm00001d046889_T001 0
440- 27: transcript:OQU76824 transcript:Zm00001d046888_T001 9.00E-57
440- 28: transcript:KXG20535 transcript:Zm00001d046883_T001 3.00E-52
440- 29: transcript:OQU76825 transcript:Zm00001d046882_T001 0
Alignment 441: score=1075.0 e_value=1.7e-78 N=24 10&9 minus
441- 0: transcript:OQU75683 transcript:Zm00001d045597_T016 0

441- 1: transcript:OQU75684 transcript:Zm00001d045596_T001 3.00E-118
441- 2: transcript:EER87693 transcript:Zm00001d045590_T005 0
441- 3: transcript:EER87695 transcript:Zm00001d045589_T001 0
441- 4: transcript:EER87697 transcript:Zm00001d045583_T004 0
441- 5: transcript:OQU75688 transcript:Zm00001d045582_T002 7.00E-136
441- 6: transcript:EER89043 transcript:Zm00001d045581_T002 2.00E-152
441- 7: transcript:EER87703 transcript:Zm00001d045580_T001 1.00E-173
441- 8: transcript:EER87704 transcript:Zm00001d045579_T001 0
441- 9: transcript:EER89049 transcript:Zm00001d045577_T003 5.00E-137
441- 10: transcript:EER87705 transcript:Zm00001d045576_T001 0
441- 11: transcript:EER89050 transcript:Zm00001d045574_T003 1.00E-38
441- 12: transcript:KXG19106 transcript:Zm00001d045573_T006 6.00E-77
441- 13: transcript:KXG19110 transcript:Zm00001d045572_T001 0
441- 14: transcript:EER89054 transcript:Zm00001d045571_T001 2.00E-160
441- 15: transcript:OQU75689 transcript:Zm00001d045570_T001 5.00E-15
441- 16: transcript:KXG19113 transcript:Zm00001d045568_T001 2.00E-56
441- 17: transcript:OQU75691 transcript:Zm00001d045567_T001 0
441- 18: transcript:EER87717 transcript:Zm00001d045566_T001 0
441- 19: transcript:OQU75692 transcript:Zm00001d045564_T001 1.00E-86
441- 20: transcript:EER87721 transcript:Zm00001d045563_T001 0
441- 21: transcript:KXG19123 transcript:Zm00001d045560_T001 0
441- 22: transcript:EER89059 transcript:Zm00001d045559_T002 1.00E-168
441- 23: transcript:KXG19126 transcript:Zm00001d045558_T003 0
Alignment 442: score=1036.0 e_value=2.3e-69 N=23 10&9 minus
442- 0: transcript:EER89903 transcript:Zm00001d046323_T002 9.00E-173
442- 1: transcript:KXG20146 transcript:Zm00001d046318_T001 0
442- 2: transcript:KXG20149 transcript:Zm00001d046317_T001 2.00E-120
442- 3: transcript:OQU76539 transcript:Zm00001d046314_T001 1.00E-98
442- 4: transcript:EER89913 transcript:Zm00001d046313_T001 1.00E-157
442- 5: transcript:OQU76549 transcript:Zm00001d046311_T001 5.00E-33
442- 6: transcript:EER88499 transcript:Zm00001d046305_T001 0
442- 7: transcript:EER89914 transcript:Zm00001d046304_T001 0
442- 8: transcript:KXG20157 transcript:Zm00001d046303_T001 1.00E-138
442- 9: transcript:EER89918 transcript:Zm00001d046300_T001 5.00E-122
442- 10: transcript:EER89919 transcript:Zm00001d046299_T001 2.00E-137
442- 11: transcript:EER89920 transcript:Zm00001d046297_T001 2.00E-67
442- 12: transcript:KXG20160 transcript:Zm00001d046292_T001 2.00E-87
442- 13: transcript:EER88508 transcript:Zm00001d046289_T001 4.00E-158
442- 14: transcript:KXG20161 transcript:Zm00001d046288_T001 2.00E-115
442- 15: transcript:EER89924 transcript:Zm00001d046286_T001 6.00E-177
442- 16: transcript:EER89925 transcript:Zm00001d046281_T001 1.00E-136
442- 17: transcript:OQU76555 transcript:Zm00001d046277_T008 0
442- 18: transcript:KXG20175 transcript:Zm00001d046272_T001 1.00E-137
442- 19: transcript:EER88513 transcript:Zm00001d046263_T001 0
442- 20: transcript:EER89930 transcript:Zm00001d046261_T002 0
442- 21: transcript:OQU76573 transcript:Zm00001d046255_T035 0
442- 22: transcript:EER88523 transcript:Zm00001d046254_T001 0
Alignment 443: score=853.0 e_value=8.7e-53 N=19 10&9 minus
443- 0: transcript:EER88574 transcript:Zm00001d046489_T001 0
443- 1: transcript:EER88575 transcript:Zm00001d046488_T001 0
443- 2: transcript:EER89984 transcript:Zm00001d046487_T001 0
443- 3: transcript:EER89985 transcript:Zm00001d046485_T001 0
443- 4: transcript:EER88577 transcript:Zm00001d046483_T001 0
443- 5: transcript:EER89986 transcript:Zm00001d046482_T001 0

```
443-  6: transcript:EER89987          transcript:Zm00001d046480_T001          0
443-  7: transcript:KXG20265          transcript:Zm00001d046474_T001  1.00E-13
443-  8: transcript:OQU76640          transcript:Zm00001d046473_T001 8.00E-103
443-  9: transcript:EER89990          transcript:Zm00001d046472_T001 4.00E-125
443- 10: transcript:EER89991          transcript:Zm00001d046471_T001  5.00E-72
443- 11: transcript:OQU76642          transcript:Zm00001d046468_T003          0
443- 12: transcript:EER88586          transcript:Zm00001d046467_T001          0
443- 13: transcript:OQU76645          transcript:Zm00001d046460_T001 4.00E-113
443- 14: transcript:KXG20276          transcript:Zm00001d046456_T009          0
443- 15: transcript:EER89996          transcript:Zm00001d046455_T001 6.00E-114
443- 16: transcript:EER88593          transcript:Zm00001d046454_T003          0
443- 17: transcript:EER88596          transcript:Zm00001d046450_T001          0
443- 18: transcript:EER90002          transcript:Zm00001d046445_T004          0
## Alignment 444: score=850.0 e_value=1e-54 N=19 10&9 minus
444-  0: transcript:EER89197          transcript:Zm00001d045361_T001  6.00E-48
444-  1: transcript:OQU75818          transcript:Zm00001d045359_T002          0
444-  2: transcript:KXG19301          transcript:Zm00001d045358_T001  9.00E-94
444-  3: transcript:EER87839          transcript:Zm00001d045355_T001  1.00E-90
444-  4: transcript:EER89201          transcript:Zm00001d045354_T002          0
444-  5: transcript:OQU75822          transcript:Zm00001d045353_T003          0
444-  6: transcript:OQU75826          transcript:Zm00001d045352_T002          0
444-  7: transcript:EER87849          transcript:Zm00001d045351_T001  2.00E-59
444-  8: transcript:OQU75833          transcript:Zm00001d045344_T001  3.00E-68
444-  9: transcript:EER87855          transcript:Zm00001d045338_T001          0
444- 10: transcript:OQU75837          transcript:Zm00001d045327_T001  3.00E-87
444- 11: transcript:EER89214          transcript:Zm00001d045326_T004          0
444- 12: transcript:EER87859          transcript:Zm00001d045324_T001 2.00E-102
444- 13: transcript:KXG19322          transcript:Zm00001d045323_T004          0
444- 14: transcript:EER87861          transcript:Zm00001d045322_T001          0
444- 15: transcript:EER87862          transcript:Zm00001d045321_T001  6.00E-80
444- 16: transcript:EER89216          transcript:Zm00001d045320_T004  1.00E-39
444- 17: transcript:OQU75844          transcript:Zm00001d045319_T001          0
444- 18: transcript:EER87863          transcript:Zm00001d045318_T001  2.00E-72
## Alignment 445: score=823.0 e_value=1.3e-61 N=20 10&9 minus
445-  0: transcript:OQU75899          transcript:Zm00001d045201_T001 3.00E-127
445-  1: transcript:OQU75900          transcript:Zm00001d045199_T003          0
445-  2: transcript:KXG19397          transcript:Zm00001d045195_T012          0
445-  3: transcript:OQU75904          transcript:Zm00001d045194_T001          0
445-  4: transcript:OQU75905          transcript:Zm00001d045193_T002 2.00E-180
445-  5: transcript:EER89283          transcript:Zm00001d045192_T017          0
445-  6: transcript:OQU75907          transcript:Zm00001d045191_T001          0
445-  7: transcript:EER89287          transcript:Zm00001d045190_T001          0
445-  8: transcript:OQU75915          transcript:Zm00001d045185_T001  6.00E-77
445-  9: transcript:OQU75913          transcript:Zm00001d045184_T001 8.00E-132
445- 10: transcript:EER89290          transcript:Zm00001d045183_T001          0
445- 11: transcript:EER87930          transcript:Zm00001d045182_T001          0
445- 12: transcript:OQU75920          transcript:Zm00001d045181_T001          0
445- 13: transcript:EER87931          transcript:Zm00001d045180_T001 6.00E-131
445- 14: transcript:KXG19418          transcript:Zm00001d045179_T016          0
445- 15: transcript:EER87933          transcript:Zm00001d045176_T001          0
445- 16: transcript:EER87936          transcript:Zm00001d045174_T001  4.00E-37
445- 17: transcript:OQU75928          transcript:Zm00001d045171_T007          0
445- 18: transcript:EER87942          transcript:Zm00001d045169_T001  4.00E-61
445- 19: transcript:KXG19432          transcript:Zm00001d045164_T001  3.00E-22
```

Alignment 446: score=805.0 e_value=3.8e-56 N=19 10&9 minus
446- 0: transcript:EER89392 transcript:Zm00001d044976_T001 0
446- 1: transcript:EER89393 transcript:Zm00001d044973_T001 0
446- 2: transcript:KXG19571 transcript:Zm00001d044972_T001 2.00E-67
446- 3: transcript:EER88041 transcript:Zm00001d044971_T005 0
446- 4: transcript:EER89396 transcript:Zm00001d044970_T001 8.00E-141
446- 5: transcript:KXG19573 transcript:Zm00001d044966_T001 0
446- 6: transcript:EER89398 transcript:Zm00001d044954_T001 6.00E-166
446- 7: transcript:KXG19576 transcript:Zm00001d044953_T001 2.00E-145
446- 8: transcript:EER88045 transcript:Zm00001d044951_T001 0
446- 9: transcript:EER88048 transcript:Zm00001d044950_T001 2.00E-65
446- 10: transcript:EER88052 transcript:Zm00001d044947_T001 0
446- 11: transcript:EER88053 transcript:Zm00001d044945_T001 0
446- 12: transcript:EER89403 transcript:Zm00001d044943_T001 0
446- 13: transcript:KXG19586 transcript:Zm00001d044940_T004 0
446- 14: transcript:EER89404 transcript:Zm00001d044936_T001 3.00E-152
446- 15: transcript:OQU76044 transcript:Zm00001d044934_T001 1.00E-144
446- 16: transcript:EER89408 transcript:Zm00001d044931_T001 0
446- 17: transcript:KXG19588 transcript:Zm00001d044930_T001 3.00E-54
446- 18: transcript:OQU76046 transcript:Zm00001d044929_T002 0
Alignment 447: score=611.0 e_value=1.8e-42 N=16 10&9 minus
447- 0: transcript:EER88132 transcript:Zm00001d044785_T001 0
447- 1: transcript:KXG19671 transcript:Zm00001d044784_T001 6.00E-85
447- 2: transcript:KXG19676 transcript:Zm00001d044783_T001 5.00E-72
447- 3: transcript:EER88138 transcript:Zm00001d044781_T001 2.00E-130
447- 4: transcript:KXG19678 transcript:Zm00001d044780_T001 0
447- 5: transcript:OQU76129 transcript:Zm00001d044777_T001 9.00E-95
447- 6: transcript:EER89498 transcript:Zm00001d044776_T001 5.00E-168
447- 7: transcript:KXG19681 transcript:Zm00001d044774_T004 0
447- 8: transcript:KXG19688 transcript:Zm00001d044773_T001 0
447- 9: transcript:EER88144 transcript:Zm00001d044771_T001 0
447- 10: transcript:EER88145 transcript:Zm00001d044769_T001 6.00E-39
447- 11: transcript:EER89506 transcript:Zm00001d044768_T001 0
447- 12: transcript:EER88149 transcript:Zm00001d044766_T001 0
447- 13: transcript:OQU76149 transcript:Zm00001d044762_T001 3.00E-54
447- 14: transcript:KXG19701 transcript:Zm00001d044760_T001 0
447- 15: transcript:EER89512 transcript:Zm00001d044759_T001 0
Alignment 448: score=504.0 e_value=1.4e-27 N=12 10&9 minus
448- 0: transcript:EER89373 transcript:Zm00001d045041_T015 0
448- 1: transcript:KXG19530 transcript:Zm00001d045039_T001 5.00E-36
448- 2: transcript:EER88003 transcript:Zm00001d045036_T001 1.00E-120
448- 3: transcript:OQU76000 transcript:Zm00001d045030_T001 3.00E-48
448- 4: transcript:OQU76001 transcript:Zm00001d045029_T003 7.00E-118
448- 5: transcript:KXG19532 transcript:Zm00001d045028_T001 0
448- 6: transcript:EER89376 transcript:Zm00001d045027_T001 0
448- 7: transcript:KXG19536 transcript:Zm00001d045026_T002 0
448- 8: transcript:KXG19537 transcript:Zm00001d045025_T005 3.00E-165
448- 9: transcript:EER88010 transcript:Zm00001d045024_T001 7.00E-104
448- 10: transcript:OQU76007 transcript:Zm00001d045021_T004 7.00E-61
448- 11: transcript:EER89382 transcript:Zm00001d045017_T001 5.00E-56
Alignment 449: score=484.0 e_value=1.7e-27 N=12 10&9 minus
449- 0: transcript:OQU76060 transcript:Zm00001d044880_T001 1.00E-108
449- 1: transcript:KXG19604 transcript:Zm00001d044878_T002 0
449- 2: transcript:EER88090 transcript:Zm00001d044877_T002 0

```
449-  3: transcript:KXG19612            transcript:Zm00001d044875_T001  9.00E-50
449-  4: transcript:EER89430            transcript:Zm00001d044874_T001  3.00E-40
449-  5: transcript:EER88092            transcript:Zm00001d044869_T001 6.00E-113
449-  6: transcript:EER89435            transcript:Zm00001d044868_T001 6.00E-131
449-  7: transcript:EER89437            transcript:Zm00001d044866_T001         0
449-  8: transcript:KXG19617            transcript:Zm00001d044864_T001  7.00E-92
449-  9: transcript:EER88096            transcript:Zm00001d044861_T001  4.00E-20
449- 10: transcript:KXG19622            transcript:Zm00001d044860_T001         0
449- 11: transcript:KXG19625            transcript:Zm00001d044859_T001         0
## Alignment 450: score=479.0 e_value=1.7e-25 N=11 10&9 minus
450-  0: transcript:EER87812            transcript:Zm00001d045398_T001  4.00E-56
450-  1: transcript:KXG19254            transcript:Zm00001d045397_T001         0
450-  2: transcript:EER89165            transcript:Zm00001d045395_T001         0
450-  3: transcript:OQU75796            transcript:Zm00001d045394_T001         0
450-  4: transcript:EER89167            transcript:Zm00001d045393_T001  2.00E-95
450-  5: transcript:EER89174            transcript:Zm00001d045390_T002  6.00E-64
450-  6: transcript:EER87817            transcript:Zm00001d045389_T015         0
450-  7: transcript:KXG19268            transcript:Zm00001d045388_T001         0
450-  8: transcript:EER87823            transcript:Zm00001d045384_T003 1.00E-110
450-  9: transcript:EER87825            transcript:Zm00001d045382_T001         0
450- 10: transcript:EER87829            transcript:Zm00001d045381_T001         0
## Alignment 451: score=467.0 e_value=5.8e-23 N=10 10&9 minus
451-  0: transcript:KXG19328            transcript:Zm00001d045316_T002         0
451-  1: transcript:EER89220            transcript:Zm00001d045315_T003         0
451-  2: transcript:OQU75845            transcript:Zm00001d045314_T001 2.00E-109
451-  3: transcript:EER87866            transcript:Zm00001d045313_T001         0
451-  4: transcript:KXG19329            transcript:Zm00001d045311_T015         0
451-  5: transcript:EER89222            transcript:Zm00001d045310_T001         0
451-  6: transcript:EER87870            transcript:Zm00001d045309_T001         0
451-  7: transcript:EER89223            transcript:Zm00001d045308_T001  1.00E-94
451-  8: transcript:EER87873            transcript:Zm00001d045305_T001         0
451-  9: transcript:EER89224            transcript:Zm00001d045304_T001         0
## Alignment 452: score=437.0 e_value=9.3e-25 N=11 10&9 minus
452-  0: transcript:KXG19623            transcript:Zm00001d044854_T001         0
452-  1: transcript:EER88100            transcript:Zm00001d044851_T001  8.00E-93
452-  2: transcript:KXG19627            transcript:Zm00001d044850_T001 2.00E-135
452-  3: transcript:OQU76080            transcript:Zm00001d044849_T001  1.00E-67
452-  4: transcript:KXG19632            transcript:Zm00001d044845_T001         0
452-  5: transcript:EER88105            transcript:Zm00001d044844_T001  2.00E-57
452-  6: transcript:EER89465            transcript:Zm00001d044843_T001         0
452-  7: transcript:EER88108            transcript:Zm00001d044841_T001 2.00E-162
452-  8: transcript:EER88111            transcript:Zm00001d044839_T001  2.00E-52
452-  9: transcript:EER89468            transcript:Zm00001d044838_T002 2.00E-128
452- 10: transcript:EER88112            transcript:Zm00001d044836_T001 3.00E-123
## Alignment 453: score=429.0 e_value=2.6e-19 N=9 10&9 minus
453-  0: transcript:OQU76667            transcript:Zm00001d046423_T001         0
453-  1: transcript:OQU76668            transcript:Zm00001d046422_T001         0
453-  2: transcript:KXG20313            transcript:Zm00001d046420_T001         0
453-  3: transcript:EER88607            transcript:Zm00001d046405_T001         0
453-  4: transcript:EER88608            transcript:Zm00001d046404_T001  1.00E-60
453-  5: transcript:OQU76672            transcript:Zm00001d046402_T001  3.00E-57
453-  6: transcript:EER88613            transcript:Zm00001d046401_T001  7.00E-39
453-  7: transcript:EER90006            transcript:Zm00001d046400_T001         0
453-  8: transcript:EER88614            transcript:Zm00001d046399_T001         0
```

Alignment 454: score=374.0 e_value=1.9e-13 N=8 10&9 minus
454- 0: transcript:EER88061 transcript:Zm00001d044925_T001 2.00E-56
454- 1: transcript:OQU76048 transcript:Zm00001d044923_T001 1.00E-58
454- 2: transcript:EER88065 transcript:Zm00001d044922_T001 0
454- 3: transcript:EER89413 transcript:Zm00001d044921_T001 3.00E-140
454- 4: transcript:OQU76052 transcript:Zm00001d044920_T001 0
454- 5: transcript:EER88070 transcript:Zm00001d044918_T001 9.00E-159
454- 6: transcript:EER89414 transcript:Zm00001d044917_T001 9.00E-164
454- 7: transcript:EER89415 transcript:Zm00001d044916_T001 0
Alignment 455: score=368.0 e_value=5.8e-14 N=8 10&9 minus
455- 0: transcript:OQU76779 transcript:Zm00001d046574_T001 6.00E-27
455- 1: transcript:EER88721 transcript:Zm00001d046569_T002 0
455- 2: transcript:KXG20488 transcript:Zm00001d046568_T001 0
455- 3: transcript:EER90114 transcript:Zm00001d046564_T001 1.00E-100
455- 4: transcript:EER88722 transcript:Zm00001d046561_T001 0
455- 5: transcript:EER88725 transcript:Zm00001d046560_T001 0
455- 6: transcript:OQU76784 transcript:Zm00001d046558_T001 1.00E-108
455- 7: transcript:EER88723 transcript:Zm00001d046555_T001 3.00E-94
Alignment 456: score=326.0 e_value=5e-17 N=8 10&9 minus
456- 0: transcript:EER89471 transcript:Zm00001d044828_T001 0
456- 1: transcript:EER89472 transcript:Zm00001d044826_T001 0
456- 2: transcript:KXG19645 transcript:Zm00001d044825_T001 0
456- 3: transcript:EER89473 transcript:Zm00001d044824_T001 7.00E-69
456- 4: transcript:OQU76111 transcript:Zm00001d044823_T011 0
456- 5: transcript:EER89477 transcript:Zm00001d044822_T001 2.00E-112
456- 6: transcript:EER88121 transcript:Zm00001d044821_T001 0
456- 7: transcript:OQU76115 transcript:Zm00001d044819_T002 0
Alignment 457: score=318.0 e_value=1.1e-16 N=7 10&9 minus
457- 0: transcript:EER87677 transcript:Zm00001d045620_T001 7.00E-81
457- 1: transcript:EER89026 transcript:Zm00001d045621_T001 0
457- 2: transcript:EER89028 transcript:Zm00001d045611_T001 0
457- 3: transcript:EER89029 transcript:Zm00001d045610_T003 0
457- 4: transcript:EER89030 transcript:Zm00001d045607_T001 2.00E-112
457- 5: transcript:KXG19087 transcript:Zm00001d045606_T002 0
457- 6: transcript:EER87687 transcript:Zm00001d045604_T003 0
Alignment 458: score=296.0 e_value=3.7e-12 N=7 10&9 minus
458- 0: transcript:EER88177 transcript:Zm00001d045657_T001 7.00E-99
458- 1: transcript:KXG19719 transcript:Zm00001d045655_T013 0
458- 2: transcript:KXG19720 transcript:Zm00001d045651_T001 2.00E-47
458- 3: transcript:EER89550 transcript:Zm00001d045650_T001 2.00E-69
458- 4: transcript:EER89551 transcript:Zm00001d045649_T001 0
458- 5: transcript:EER88181 transcript:Zm00001d045647_T002 0
458- 6: transcript:EER89557 transcript:Zm00001d045644_T001 1.00E-166
Alignment 459: score=279.0 e_value=2.3e-10 N=6 10&9 minus
459- 0: transcript:EER89186 transcript:Zm00001d045378_T001 0
459- 1: transcript:KXG19285 transcript:Zm00001d045374_T001 2.00E-111
459- 2: transcript:OQU75807 transcript:Zm00001d045373_T001 0
459- 3: transcript:EER89188 transcript:Zm00001d045372_T002 2.00E-109
459- 4: transcript:EER89191 transcript:Zm00001d045370_T001 3.00E-43
459- 5: transcript:EER89192 transcript:Zm00001d045369_T001 8.00E-14
Alignment 460: score=276.0 e_value=3.4e-08 N=6 10&9 minus
460- 0: transcript:KXG20201 transcript:Zm00001d046221_T017 5.00E-160
460- 1: transcript:KXG20202 transcript:Zm00001d046218_T001 0
460- 2: transcript:KXG20204 transcript:Zm00001d046214_T002 0

```
460-  3: transcript:EER89952           transcript:Zm00001d046210_T001          0
460-  4: transcript:OQU76588           transcript:Zm00001d046207_T001          0
460-  5: transcript:KXG20211           transcript:Zm00001d046204_T001 6.00E-165
## Alignment 461: score=270.0 e_value=6.2e-09 N=6 10&9 minus
461-  0: transcript:EER87723           transcript:Zm00001d045557_T001          0
461-  1: transcript:EER87724           transcript:Zm00001d045556_T001 9.00E-102
461-  2: transcript:EER87725           transcript:Zm00001d045555_T002  2.00E-77
461-  3: transcript:OQU75697           transcript:Zm00001d045554_T002          0
461-  4: transcript:KXG19130           transcript:Zm00001d045553_T001          0
461-  5: transcript:OQU75698           transcript:Zm00001d045551_T032          0
## Alignment 462: score=3988.0 e_value=0 N=88 2&2 plus
462-  0: transcript:OQU89696           transcript:Zm00001d006034_T003 3.00E-173
462-  1: transcript:EER99104           transcript:Zm00001d006036_T001          0
462-  2: transcript:OQU89704           transcript:Zm00001d006037_T001 4.00E-152
462-  3: transcript:EER96980           transcript:Zm00001d006040_T001          0
462-  4: transcript:OQU89707           transcript:Zm00001d006041_T002 2.00E-128
462-  5: transcript:OQU89708           transcript:Zm00001d006042_T001 2.00E-155
462-  6: transcript:EER99114           transcript:Zm00001d006045_T001 1.00E-179
462-  7: transcript:KXG35939           transcript:Zm00001d006046_T002          0
462-  8: transcript:KXG35944           transcript:Zm00001d006049_T001 9.00E-156
462-  9: transcript:KXG35943           transcript:Zm00001d006050_T001          0
462- 10: transcript:KXG35950           transcript:Zm00001d006051_T001 2.00E-161
462- 11: transcript:OQU89720           transcript:Zm00001d006052_T004          0
462- 12: transcript:EER99125           transcript:Zm00001d006053_T001          0
462- 13: transcript:EER99119           transcript:Zm00001d006054_T001          0
462- 14: transcript:OQU89723           transcript:Zm00001d006055_T002          0
462- 15: transcript:EER99128           transcript:Zm00001d006057_T001          0
462- 16: transcript:EER99130           transcript:Zm00001d006059_T001 1.00E-118
462- 17: transcript:EER96997           transcript:Zm00001d006060_T001  4.00E-28
462- 18: transcript:KXG35958           transcript:Zm00001d006061_T001  4.00E-83
462- 19: transcript:KXG35959           transcript:Zm00001d006063_T001          0
462- 20: transcript:EER97002           transcript:Zm00001d006064_T002  9.00E-94
462- 21: transcript:OQU89735           transcript:Zm00001d006065_T002          0
462- 22: transcript:KXG35961           transcript:Zm00001d006066_T002          0
462- 23: transcript:EER97004           transcript:Zm00001d006069_T001          0
462- 24: transcript:EER99142           transcript:Zm00001d006070_T004 1.00E-109
462- 25: transcript:OQU89741           transcript:Zm00001d006071_T001 7.00E-101
462- 26: transcript:OQU89743           transcript:Zm00001d006078_T001  3.00E-63
462- 27: transcript:EER99145           transcript:Zm00001d006079_T001          0
462- 28: transcript:OQU89744           transcript:Zm00001d006080_T004          0
462- 29: transcript:KXG35972           transcript:Zm00001d006082_T001          0
462- 30: transcript:EER99151           transcript:Zm00001d006084_T003          0
462- 31: transcript:OQU89746           transcript:Zm00001d006085_T002 1.00E-126
462- 32: transcript:OQU89747           transcript:Zm00001d006089_T002          0
462- 33: transcript:OQU89749           transcript:Zm00001d006090_T002 8.00E-107
462- 34: transcript:EER97011           transcript:Zm00001d006091_T001          0
462- 35: transcript:EER97012           transcript:Zm00001d006094_T001 8.00E-177
462- 36: transcript:EER97013           transcript:Zm00001d006096_T001  5.00E-67
462- 37: transcript:OQU89751           transcript:Zm00001d006097_T001          0
462- 38: transcript:KXG35976           transcript:Zm00001d006098_T002          0
462- 39: transcript:EER99157           transcript:Zm00001d006099_T001  3.00E-59
462- 40: transcript:EER99160           transcript:Zm00001d006100_T001          0
462- 41: transcript:OQU89753           transcript:Zm00001d006101_T001  2.00E-68
462- 42: transcript:EER99164           transcript:Zm00001d006102_T002          0
```

462- 43: transcript:EER97018 transcript:Zm00001d006106_T001 6.00E-114
462- 44: transcript:EER97019 transcript:Zm00001d006107_T001 0
462- 45: transcript:EER97020 transcript:Zm00001d006108_T002 0
462- 46: transcript:EER99167 transcript:Zm00001d006109_T001 3.00E-12
462- 47: transcript:EER99171 transcript:Zm00001d006110_T002 6.00E-150
462- 48: transcript:EER99174 transcript:Zm00001d006111_T001 3.00E-121
462- 49: transcript:KXG35994 transcript:Zm00001d006112_T002 1.00E-145
462- 50: transcript:KXG35997 transcript:Zm00001d006113_T001 0
462- 51: transcript:EER99175 transcript:Zm00001d006115_T002 0
462- 52: transcript:KXG35998 transcript:Zm00001d006116_T001 7.00E-124
462- 53: transcript:OQU89769 transcript:Zm00001d006117_T001 8.00E-66
462- 54: transcript:EER97039 transcript:Zm00001d006118_T005 0
462- 55: transcript:EER99179 transcript:Zm00001d006119_T001 2.00E-107
462- 56: transcript:EER97043 transcript:Zm00001d006124_T001 0
462- 57: transcript:EER99181 transcript:Zm00001d006125_T001 0
462- 58: transcript:EER99178 transcript:Zm00001d006126_T001 3.00E-145
462- 59: transcript:EER99183 transcript:Zm00001d006127_T003 0
462- 60: transcript:EER99185 transcript:Zm00001d006128_T002 0
462- 61: transcript:EER99187 transcript:Zm00001d006130_T001 0
462- 62: transcript:EER99188 transcript:Zm00001d006131_T003 0
462- 63: transcript:EER97050 transcript:Zm00001d006132_T001 0
462- 64: transcript:EER99194 transcript:Zm00001d006133_T001 0
462- 65: transcript:EER97051 transcript:Zm00001d006137_T001 0
462- 66: transcript:EER99195 transcript:Zm00001d006145_T001 1.00E-100
462- 67: transcript:EER97054 transcript:Zm00001d006147_T006 0
462- 68: transcript:EER97057 transcript:Zm00001d006148_T001 4.00E-37
462- 69: transcript:OQU89785 transcript:Zm00001d006149_T001 0
462- 70: transcript:EER97058 transcript:Zm00001d006150_T001 3.00E-74
462- 71: transcript:EER97059 transcript:Zm00001d006153_T003 0
462- 72: transcript:OQU89788 transcript:Zm00001d006154_T001 0
462- 73: transcript:EER97061 transcript:Zm00001d006157_T001 0
462- 74: transcript:EER99204 transcript:Zm00001d006158_T001 1.00E-38
462- 75: transcript:EER99205 transcript:Zm00001d006159_T002 1.00E-80
462- 76: transcript:EER97062 transcript:Zm00001d006160_T001 0
462- 77: transcript:EER99200 transcript:Zm00001d006161_T001 0
462- 78: transcript:EER97064 transcript:Zm00001d006162_T001 3.00E-148
462- 79: transcript:EER99208 transcript:Zm00001d006165_T001 1.00E-158
462- 80: transcript:KXG36028 transcript:Zm00001d006166_T001 0
462- 81: transcript:EER99209 transcript:Zm00001d006167_T003 0
462- 82: transcript:KXG36029 transcript:Zm00001d006168_T002 0
462- 83: transcript:OQU89795 transcript:Zm00001d006169_T001 5.00E-41
462- 84: transcript:KXG36033 transcript:Zm00001d006173_T001 2.00E-129
462- 85: transcript:EER97068 transcript:Zm00001d006175_T001 3.00E-141
462- 86: transcript:EER99219 transcript:Zm00001d006176_T001 0
462- 87: transcript:KXG36036 transcript:Zm00001d006177_T002 1.00E-173
Alignment 463: score=3684.0 e_value=0 N=81 2&2 plus
463- 0: transcript:KXG35763 transcript:Zm00001d005881_T011 0
463- 1: transcript:EER96850 transcript:Zm00001d005884_T002 1.00E-113
463- 2: transcript:EER99009 transcript:Zm00001d005885_T001 0
463- 3: transcript:EER96851 transcript:Zm00001d005888_T002 2.00E-151
463- 4: transcript:EER99010 transcript:Zm00001d005889_T001 0
463- 5: transcript:EER96852 transcript:Zm00001d005890_T001 0
463- 6: transcript:EER96855 transcript:Zm00001d005892_T001 1.00E-114
463- 7: transcript:KXG35766 transcript:Zm00001d005893_T001 0

463- 8:	transcript:KXG35767	transcript:Zm00001d005894_T001	0
463- 9:	transcript:EER96861	transcript:Zm00001d005895_T001	2.00E-59
463- 10:	transcript:EER96862	transcript:Zm00001d005897_T001	4.00E-67
463- 11:	transcript:KXG35772	transcript:Zm00001d005899_T001	2.00E-77
463- 12:	transcript:EER96866	transcript:Zm00001d005901_T001	7.00E-124
463- 13:	transcript:EER96867	transcript:Zm00001d005902_T003	0
463- 14:	transcript:KXG35780	transcript:Zm00001d005905_T001	0
463- 15:	transcript:EER96865	transcript:Zm00001d005910_T001	6.00E-115
463- 16:	transcript:KXG35787	transcript:Zm00001d005911_T001	0
463- 17:	transcript:KXG35789	transcript:Zm00001d005912_T001	0
463- 18:	transcript:KXG35791	transcript:Zm00001d005913_T001	6.00E-180
463- 19:	transcript:KXG35796	transcript:Zm00001d005917_T003	0
463- 20:	transcript:OQU89629	transcript:Zm00001d005918_T001	4.00E-76
463- 21:	transcript:EER96881	transcript:Zm00001d005919_T001	8.00E-118
463- 22:	transcript:KXG35798	transcript:Zm00001d005920_T004	0
463- 23:	transcript:OQU89633	transcript:Zm00001d005923_T001	2.00E-151
463- 24:	transcript:EER96885	transcript:Zm00001d005924_T002	7.00E-149
463- 25:	transcript:EER99030	transcript:Zm00001d005925_T002	0
463- 26:	transcript:EER99031	transcript:Zm00001d005926_T001	9.00E-96
463- 27:	transcript:EER96886	transcript:Zm00001d005928_T002	0
463- 28:	transcript:EER96888	transcript:Zm00001d005931_T001	3.00E-101
463- 29:	transcript:KXG35809	transcript:Zm00001d005933_T001	0
463- 30:	transcript:EER99041	transcript:Zm00001d005936_T001	9.00E-173
463- 31:	transcript:EER96894	transcript:Zm00001d005939_T001	4.00E-150
463- 32:	transcript:EER99042	transcript:Zm00001d005940_T001	1.00E-155
463- 33:	transcript:EER96895	transcript:Zm00001d005944_T001	7.00E-99
463- 34:	transcript:KXG35819	transcript:Zm00001d005948_T001	7.00E-88
463- 35:	transcript:EER96900	transcript:Zm00001d005950_T001	0
463- 36:	transcript:EER96901	transcript:Zm00001d005951_T001	2.00E-128
463- 37:	transcript:EER96902	transcript:Zm00001d005957_T001	1.00E-51
463- 38:	transcript:EER99045	transcript:Zm00001d005958_T002	0
463- 39:	transcript:KXG35825	transcript:Zm00001d005959_T001	0
463- 40:	transcript:EER96911	transcript:Zm00001d005961_T001	1.00E-101
463- 41:	transcript:EER96905	transcript:Zm00001d005962_T001	5.00E-79
463- 42:	transcript:KXG35834	transcript:Zm00001d005964_T001	0
463- 43:	transcript:OQU89652	transcript:Zm00001d005965_T001	5.00E-42
463- 44:	transcript:EER99049	transcript:Zm00001d005966_T001	0
463- 45:	transcript:KXG35839	transcript:Zm00001d005969_T004	0
463- 46:	transcript:OQU89656	transcript:Zm00001d005970_T001	5.00E-87
463- 47:	transcript:KXG35845	transcript:Zm00001d005971_T001	1.00E-110
463- 48:	transcript:KXG35851	transcript:Zm00001d005973_T001	0
463- 49:	transcript:OQU89663	transcript:Zm00001d005975_T001	1.00E-100
463- 50:	transcript:KXG35854	transcript:Zm00001d005976_T012	0
463- 51:	transcript:EER99058	transcript:Zm00001d005977_T001	3.00E-16
463- 52:	transcript:OQU89666	transcript:Zm00001d005978_T001	5.00E-82
463- 53:	transcript:EER96927	transcript:Zm00001d005980_T001	0
463- 54:	transcript:KXG35867	transcript:Zm00001d005984_T001	4.00E-65
463- 55:	transcript:EER96944	transcript:Zm00001d005989_T001	1.00E-32
463- 56:	transcript:EER96945	transcript:Zm00001d005993_T001	3.00E-60
463- 57:	transcript:EER96946	transcript:Zm00001d005995_T008	0
463- 58:	transcript:EER96948	transcript:Zm00001d005996_T001	1.00E-88
463- 59:	transcript:EER96942	transcript:Zm00001d005997_T002	0
463- 60:	transcript:EER99070	transcript:Zm00001d005998_T003	4.00E-164
463- 61:	transcript:OQU89672	transcript:Zm00001d006000_T001	2.00E-106

463- 62:	transcript:KXG35872	transcript:Zm00001d006001_T006	0
463- 63:	transcript:EER99072	transcript:Zm00001d006002_T001	1.00E-79
463- 64:	transcript:EER99075	transcript:Zm00001d006008_T001	0
463- 65:	transcript:KXG35889	transcript:Zm00001d006009_T001	9.00E-132
463- 66:	transcript:KXG35895	transcript:Zm00001d006011_T001	0
463- 67:	transcript:EER96966	transcript:Zm00001d006013_T001	2.00E-74
463- 68:	transcript:OQU89687	transcript:Zm00001d006016_T001	9.00E-79
463- 69:	transcript:EER99080	transcript:Zm00001d006017_T001	0
463- 70:	transcript:KXG35901	transcript:Zm00001d006019_T001	5.00E-38
463- 71:	transcript:KXG35903	transcript:Zm00001d006022_T001	0
463- 72:	transcript:EER96974	transcript:Zm00001d006024_T001	0
463- 73:	transcript:EER99087	transcript:Zm00001d006025_T001	0
463- 74:	transcript:OQU89690	transcript:Zm00001d006026_T001	2.00E-70
463- 75:	transcript:KXG35908	transcript:Zm00001d006027_T005	0
463- 76:	transcript:EER99092	transcript:Zm00001d006028_T001	0
463- 77:	transcript:EER99093	transcript:Zm00001d006029_T001	5.00E-38
463- 78:	transcript:EER96978	transcript:Zm00001d006030_T001	0
463- 79:	transcript:EER99096	transcript:Zm00001d006031_T001	0
463- 80:	transcript:OQU89695	transcript:Zm00001d006032_T001	2.00E-31
## Alignment 464: score=3617.0 e_value=0 N=83 2&2 plus			
464- 0:	transcript:EER97253	transcript:Zm00001d006439_T001	2.00E-135
464- 1:	transcript:KXG36269	transcript:Zm00001d006440_T003	0
464- 2:	transcript:EER97255	transcript:Zm00001d006443_T001	0
464- 3:	transcript:KXG36274	transcript:Zm00001d006445_T001	7.00E-46
464- 4:	transcript:EER97258	transcript:Zm00001d006447_T001	1.00E-111
464- 5:	transcript:OQU89985	transcript:Zm00001d006449_T001	0
464- 6:	transcript:EER97257	transcript:Zm00001d006451_T001	2.00E-79
464- 7:	transcript:EER99425	transcript:Zm00001d006453_T001	2.00E-36
464- 8:	transcript:OQU89990	transcript:Zm00001d006454_T003	0
464- 9:	transcript:EER99427	transcript:Zm00001d006455_T001	0
464- 10:	transcript:KXG36286	transcript:Zm00001d006456_T001	0
464- 11:	transcript:KXG36287	transcript:Zm00001d006459_T003	0
464- 12:	transcript:KXG36288	transcript:Zm00001d006460_T002	2.00E-67
464- 13:	transcript:EER99434	transcript:Zm00001d006461_T001	0
464- 14:	transcript:EER97271	transcript:Zm00001d006463_T001	4.00E-132
464- 15:	transcript:KXG36291	transcript:Zm00001d006464_T001	8.00E-139
464- 16:	transcript:EER99436	transcript:Zm00001d006466_T001	0
464- 17:	transcript:EER99437	transcript:Zm00001d006467_T002	0
464- 18:	transcript:EER97274	transcript:Zm00001d006470_T003	0
464- 19:	transcript:EER99438	transcript:Zm00001d006471_T001	0
464- 20:	transcript:KXG36300	transcript:Zm00001d006472_T003	0
464- 21:	transcript:KXG36299	transcript:Zm00001d006473_T001	0
464- 22:	transcript:EER99442	transcript:Zm00001d006474_T002	5.00E-72
464- 23:	transcript:KXG36319	transcript:Zm00001d006475_T001	1.00E-122
464- 24:	transcript:EER97286	transcript:Zm00001d006476_T004	0
464- 25:	transcript:OQU90004	transcript:Zm00001d006478_T001	0
464- 26:	transcript:KXG36322	transcript:Zm00001d006479_T006	0
464- 27:	transcript:OQU90011	transcript:Zm00001d006480_T006	0
464- 28:	transcript:EER97289	transcript:Zm00001d006486_T001	0
464- 29:	transcript:KXG36331	transcript:Zm00001d006488_T001	4.00E-40
464- 30:	transcript:EER99449	transcript:Zm00001d006489_T001	1.00E-80
464- 31:	transcript:EER97294	transcript:Zm00001d006494_T004	0
464- 32:	transcript:KXG36335	transcript:Zm00001d006495_T001	1.00E-16
464- 33:	transcript:KXG36337	transcript:Zm00001d006496_T004	0

464- 34: transcript:EER99450 transcript:Zm00001d006497_T002 0
464- 35: transcript:EER97300 transcript:Zm00001d006499_T005 0
464- 36: transcript:KXG36347 transcript:Zm00001d006502_T002 1.00E-20
464- 37: transcript:OQU90022 transcript:Zm00001d006503_T001 0
464- 38: transcript:EER99458 transcript:Zm00001d006504_T005 0
464- 39: transcript:OQU90023 transcript:Zm00001d006505_T002 4.00E-73
464- 40: transcript:OQU90024 transcript:Zm00001d006507_T021 0
464- 41: transcript:EER97313 transcript:Zm00001d006508_T011 0
464- 42: transcript:KXG36361 transcript:Zm00001d006509_T001 1.00E-175
464- 43: transcript:OQU90027 transcript:Zm00001d006510_T001 7.00E-53
464- 44: transcript:OQU90029 transcript:Zm00001d006511_T002 2.00E-174
464- 45: transcript:KXG36366 transcript:Zm00001d006512_T001 0
464- 46: transcript:EER99462 transcript:Zm00001d006515_T001 4.00E-104
464- 47: transcript:EER97318 transcript:Zm00001d006517_T001 0
464- 48: transcript:OQU90040 transcript:Zm00001d006520_T001 0
464- 49: transcript:OQU90041 transcript:Zm00001d006521_T001 8.00E-29
464- 50: transcript:EER97325 transcript:Zm00001d006524_T001 2.00E-129
464- 51: transcript:OQU90044 transcript:Zm00001d006525_T001 3.00E-85
464- 52: transcript:OQU90045 transcript:Zm00001d006526_T001 2.00E-92
464- 53: transcript:EER99466 transcript:Zm00001d006527_T001 0
464- 54: transcript:KXG36397 transcript:Zm00001d006533_T007 0
464- 55: transcript:KXG36409 transcript:Zm00001d006534_T002 3.00E-13
464- 56: transcript:KXG36410 transcript:Zm00001d006535_T001 0
464- 57: transcript:EER99491 transcript:Zm00001d006538_T001 0
464- 58: transcript:EER97340 transcript:Zm00001d006539_T001 0
464- 59: transcript:EER97341 transcript:Zm00001d006540_T001 2.00E-110
464- 60: transcript:EER99496 transcript:Zm00001d006541_T002 4.00E-138
464- 61: transcript:EER97344 transcript:Zm00001d006547_T001 2.00E-89
464- 62: transcript:EER99497 transcript:Zm00001d006549_T002 0
464- 63: transcript:EER97346 transcript:Zm00001d006551_T001 0
464- 64: transcript:KXG36430 transcript:Zm00001d006553_T001 0
464- 65: transcript:EER99501 transcript:Zm00001d006555_T001 2.00E-39
464- 66: transcript:EER99506 transcript:Zm00001d006556_T001 5.00E-36
464- 67: transcript:EER97353 transcript:Zm00001d006561_T001 2.00E-111
464- 68: transcript:EER99510 transcript:Zm00001d006562_T001 9.00E-176
464- 69: transcript:OQU90065 transcript:Zm00001d006564_T002 2.00E-131
464- 70: transcript:EER97356 transcript:Zm00001d006566_T002 6.00E-43
464- 71: transcript:EER97358 transcript:Zm00001d006567_T002 0
464- 72: transcript:EER99514 transcript:Zm00001d006568_T001 2.00E-119
464- 73: transcript:EER99515 transcript:Zm00001d006570_T003 4.00E-85
464- 74: transcript:KXG36445 transcript:Zm00001d006571_T001 0
464- 75: transcript:KXG36457 transcript:Zm00001d006573_T023 0
464- 76: transcript:EER99518 transcript:Zm00001d006574_T001 2.00E-167
464- 77: transcript:EER97371 transcript:Zm00001d006580_T005 0
464- 78: transcript:EER99535 transcript:Zm00001d006581_T001 7.00E-153
464- 79: transcript:KXG36472 transcript:Zm00001d006582_T002 0
464- 80: transcript:EER99536 transcript:Zm00001d006585_T001 2.00E-116
464- 81: transcript:EER99537 transcript:Zm00001d006587_T001 0
464- 82: transcript:EER99538 transcript:Zm00001d006588_T001 0
Alignment 465: score=2937.0 e_value=1.9e-269 N=66 2&2 plus
465- 0: transcript:EER97704 transcript:Zm00001d007123_T001 2.00E-61
465- 1: transcript:KXG36916 transcript:Zm00001d007124_T001 1.00E-50
465- 2: transcript:EER99880 transcript:Zm00001d007125_T001 0
465- 3: transcript:OQU90456 transcript:Zm00001d007130_T001 2.00E-82

465- 4:	transcript:EER97694	transcript:Zm00001d007133_T002	0
465- 5:	transcript:EER97709	transcript:Zm00001d007135_T001	2.00E-34
465- 6:	transcript:OQU90457	transcript:Zm00001d007139_T007	0
465- 7:	transcript:EER99891	transcript:Zm00001d007143_T001	0
465- 8:	transcript:EER97711	transcript:Zm00001d007144_T001	0
465- 9:	transcript:KXG36925	transcript:Zm00001d007145_T001	0
465- 10:	transcript:KXG36926	transcript:Zm00001d007146_T001	0
465- 11:	transcript:KXG36922	transcript:Zm00001d007151_T005	0
465- 12:	transcript:EER97716	transcript:Zm00001d007152_T001	0
465- 13:	transcript:EER97717	transcript:Zm00001d007153_T001	2.00E-48
465- 14:	transcript:EER97718	transcript:Zm00001d007154_T001	2.00E-75
465- 15:	transcript:EER99899	transcript:Zm00001d007155_T001	0
465- 16:	transcript:OQU90463	transcript:Zm00001d007156_T001	0
465- 17:	transcript:EER97719	transcript:Zm00001d007157_T001	1.00E-84
465- 18:	transcript:OQU90466	transcript:Zm00001d007158_T014	0
465- 19:	transcript:EER99905	transcript:Zm00001d007159_T001	7.00E-52
465- 20:	transcript:EER97728	transcript:Zm00001d007160_T001	3.00E-135
465- 21:	transcript:EER97734	transcript:Zm00001d007164_T001	0
465- 22:	transcript:KXG36942	transcript:Zm00001d007166_T001	8.00E-107
465- 23:	transcript:OQU90474	transcript:Zm00001d007168_T001	2.00E-79
465- 24:	transcript:OQU90475	transcript:Zm00001d007169_T001	0
465- 25:	transcript:OQU90478	transcript:Zm00001d007172_T001	0
465- 26:	transcript:EER99914	transcript:Zm00001d007173_T003	0
465- 27:	transcript:EER99916	transcript:Zm00001d007174_T001	2.00E-23
465- 28:	transcript:KXG36951	transcript:Zm00001d007175_T001	0
465- 29:	transcript:KXG36952	transcript:Zm00001d007179_T001	2.00E-158
465- 30:	transcript:OQU90483	transcript:Zm00001d007180_T001	0
465- 31:	transcript:EER97743	transcript:Zm00001d007181_T001	3.00E-115
465- 32:	transcript:EER97744	transcript:Zm00001d007183_T002	0
465- 33:	transcript:EER97746	transcript:Zm00001d007184_T001	0
465- 34:	transcript:OQU90487	transcript:Zm00001d007185_T001	1.00E-165
465- 35:	transcript:EER97748	transcript:Zm00001d007186_T004	0
465- 36:	transcript:KXG36963	transcript:Zm00001d007187_T002	0
465- 37:	transcript:OQU90496	transcript:Zm00001d007188_T001	0
465- 38:	transcript:EER97757	transcript:Zm00001d007189_T003	6.00E-74
465- 39:	transcript:OQU90497	transcript:Zm00001d007190_T001	0
465- 40:	transcript:KXG36991	transcript:Zm00001d007191_T001	8.00E-172
465- 41:	transcript:EER97762	transcript:Zm00001d007192_T001	0
465- 42:	transcript:OQU90509	transcript:Zm00001d007193_T002	1.00E-91
465- 43:	transcript:KXG37006	transcript:Zm00001d007194_T001	7.00E-63
465- 44:	transcript:OQU90522	transcript:Zm00001d007195_T003	0
465- 45:	transcript:EER99942	transcript:Zm00001d007197_T001	0
465- 46:	transcript:OQU90528	transcript:Zm00001d007199_T001	7.00E-39
465- 47:	transcript:EER99929	transcript:Zm00001d007200_T003	0
465- 48:	transcript:KXG37007	transcript:Zm00001d007201_T001	3.00E-62
465- 49:	transcript:EER97776	transcript:Zm00001d007202_T003	0
465- 50:	transcript:EER99954	transcript:Zm00001d007204_T002	0
465- 51:	transcript:EER97779	transcript:Zm00001d007205_T001	0
465- 52:	transcript:EER97780	transcript:Zm00001d007207_T002	0
465- 53:	transcript:OQU90545	transcript:Zm00001d007208_T001	0
465- 54:	transcript:EER97781	transcript:Zm00001d007209_T001	8.00E-16
465- 55:	transcript:EER99943	transcript:Zm00001d007213_T001	0
465- 56:	transcript:EER99960	transcript:Zm00001d007214_T001	0
465- 57:	transcript:EER99961	transcript:Zm00001d007215_T001	0

465- 58: transcript:OQU90552 transcript:Zm00001d007216_T001 0
465- 59: transcript:KXG37031 transcript:Zm00001d007225_T019 0
465- 60: transcript:OQU90555 transcript:Zm00001d007226_T005 0
465- 61: transcript:OQU90559 transcript:Zm00001d007227_T002 1.00E-73
465- 62: transcript:EER99967 transcript:Zm00001d007229_T001 0
465- 63: transcript:EER99968 transcript:Zm00001d007231_T002 5.00E-162
465- 64: transcript:OQU90563 transcript:Zm00001d007232_T001 0
465- 65: transcript:EER99972 transcript:Zm00001d007234_T001 0
Alignment 466: score=2896.0 e_value=8.5e-263 N=65 2&2 plus
466- 0: transcript:EER99221 transcript:Zm00001d006179_T005 0
466- 1: transcript:EER99225 transcript:Zm00001d006180_T001 0
466- 2: transcript:KXG36043 transcript:Zm00001d006181_T003 0
466- 3: transcript:EER99228 transcript:Zm00001d006182_T001 0
466- 4: transcript:EER97069 transcript:Zm00001d006183_T001 6.00E-180
466- 5: transcript:KXG36047 transcript:Zm00001d006184_T001 3.00E-158
466- 6: transcript:EER97076 transcript:Zm00001d006185_T001 0
466- 7: transcript:EER97077 transcript:Zm00001d006192_T001 0
466- 8: transcript:EER99230 transcript:Zm00001d006193_T001 0
466- 9: transcript:EER97078 transcript:Zm00001d006194_T001 7.00E-129
466- 10: transcript:EER99231 transcript:Zm00001d006195_T001 1.00E-114
466- 11: transcript:EER99232 transcript:Zm00001d006196_T001 5.00E-46
466- 12: transcript:KXG36051 transcript:Zm00001d006197_T003 0
466- 13: transcript:EER97079 transcript:Zm00001d006198_T001 7.00E-94
466- 14: transcript:EER97082 transcript:Zm00001d006199_T001 0
466- 15: transcript:KXG36061 transcript:Zm00001d006204_T001 2.00E-126
466- 16: transcript:EER97086 transcript:Zm00001d006205_T001 0
466- 17: transcript:KXG36059 transcript:Zm00001d006206_T001 2.00E-19
466- 18: transcript:OQU89813 transcript:Zm00001d006207_T001 2.00E-105
466- 19: transcript:KXG36062 transcript:Zm00001d006209_T001 3.00E-150
466- 20: transcript:OQU89815 transcript:Zm00001d006210_T009 0
466- 21: transcript:EER99239 transcript:Zm00001d006211_T001 6.00E-155
466- 22: transcript:EER99234 transcript:Zm00001d006212_T002 0
466- 23: transcript:EER99246 transcript:Zm00001d006213_T001 5.00E-98
466- 24: transcript:EER99247 transcript:Zm00001d006214_T002 0
466- 25: transcript:EER99248 transcript:Zm00001d006217_T004 0
466- 26: transcript:KXG36074 transcript:Zm00001d006218_T001 2.00E-78
466- 27: transcript:EER97092 transcript:Zm00001d006219_T003 0
466- 28: transcript:EER97093 transcript:Zm00001d006220_T002 1.00E-126
466- 29: transcript:EER99254 transcript:Zm00001d006221_T001 0
466- 30: transcript:EER97094 transcript:Zm00001d006226_T001 8.00E-115
466- 31: transcript:OQU89823 transcript:Zm00001d006227_T003 0
466- 32: transcript:EER99259 transcript:Zm00001d006231_T001 0
466- 33: transcript:OQU89832 transcript:Zm00001d006232_T001 0
466- 34: transcript:EER97100 transcript:Zm00001d006235_T001 0
466- 35: transcript:EER99264 transcript:Zm00001d006236_T001 2.00E-148
466- 36: transcript:EER97101 transcript:Zm00001d006237_T001 0
466- 37: transcript:EER99265 transcript:Zm00001d006238_T003 0
466- 38: transcript:EER99266 transcript:Zm00001d006242_T001 0
466- 39: transcript:KXG36088 transcript:Zm00001d006243_T005 0
466- 40: transcript:EER99271 transcript:Zm00001d006244_T001 0
466- 41: transcript:EER97105 transcript:Zm00001d006246_T001 0
466- 42: transcript:EER97111 transcript:Zm00001d006247_T001 7.00E-45
466- 43: transcript:EER99275 transcript:Zm00001d006249_T003 3.00E-88
466- 44: transcript:EER99277 transcript:Zm00001d006250_T001 5.00E-131

```
466- 45: transcript:OQU89843          transcript:Zm00001d006251_T001 1.00E-153
466- 46: transcript:EER99281          transcript:Zm00001d006254_T001         0
466- 47: transcript:EER99282          transcript:Zm00001d006255_T001         0
466- 48: transcript:EER97114          transcript:Zm00001d006256_T001 5.00E-101
466- 49: transcript:KXG36094          transcript:Zm00001d006257_T006         0
466- 50: transcript:OQU89853          transcript:Zm00001d006260_T001         0
466- 51: transcript:EER97120          transcript:Zm00001d006267_T002         0
466- 52: transcript:EER99290          transcript:Zm00001d006268_T001 5.00E-156
466- 53: transcript:EER99291          transcript:Zm00001d006269_T001         0
466- 54: transcript:EER99285          transcript:Zm00001d006270_T001         0
466- 55: transcript:OQU89863          transcript:Zm00001d006275_T001  1.00E-48
466- 56: transcript:EER97129          transcript:Zm00001d006276_T001  2.00E-56
466- 57: transcript:OQU89866          transcript:Zm00001d006277_T001  1.00E-43
466- 58: transcript:EER97127          transcript:Zm00001d006279_T001  1.00E-59
466- 59: transcript:EER97130          transcript:Zm00001d006286_T001         0
466- 60: transcript:EER97137          transcript:Zm00001d006287_T001  9.00E-91
466- 61: transcript:EER97138          transcript:Zm00001d006291_T002 6.00E-176
466- 62: transcript:EER97139          transcript:Zm00001d006293_T024         0
466- 63: transcript:EER99307          transcript:Zm00001d006294_T001 5.00E-120
466- 64: transcript:EER99310          transcript:Zm00001d006295_T002         0
## Alignment 467: score=1979.0 e_value=4.8e-153 N=43 2&2 plus
467-  0: transcript:EER97420          transcript:Zm00001d006646_T001  2.00E-37
467-  1: transcript:EER99587          transcript:Zm00001d006649_T001         0
467-  2: transcript:KXG36544          transcript:Zm00001d006651_T001         0
467-  3: transcript:EER99585          transcript:Zm00001d006653_T001 1.00E-139
467-  4: transcript:EER99592          transcript:Zm00001d006654_T001 1.00E-112
467-  5: transcript:EER99597          transcript:Zm00001d006656_T001  2.00E-51
467-  6: transcript:OQU90150          transcript:Zm00001d006657_T001         0
467-  7: transcript:OQU90151          transcript:Zm00001d006658_T005         0
467-  8: transcript:EER97437          transcript:Zm00001d006659_T001         0
467-  9: transcript:KXG36551          transcript:Zm00001d006663_T001 1.00E-179
467- 10: transcript:KXG36553          transcript:Zm00001d006667_T003 4.00E-157
467- 11: transcript:OQU90154          transcript:Zm00001d006668_T001  9.00E-94
467- 12: transcript:EER99600          transcript:Zm00001d006669_T001         0
467- 13: transcript:EER99602          transcript:Zm00001d006670_T001  7.00E-84
467- 14: transcript:KXG36556          transcript:Zm00001d006673_T001         0
467- 15: transcript:EER97439          transcript:Zm00001d006676_T001 9.00E-171
467- 16: transcript:EER99604          transcript:Zm00001d006677_T001 2.00E-145
467- 17: transcript:EER99605          transcript:Zm00001d006678_T001         0
467- 18: transcript:EER99606          transcript:Zm00001d006679_T001         0
467- 19: transcript:EER97440          transcript:Zm00001d006680_T001         0
467- 20: transcript:EER97441          transcript:Zm00001d006681_T004         0
467- 21: transcript:EER97442          transcript:Zm00001d006682_T001 3.00E-178
467- 22: transcript:EER97443          transcript:Zm00001d006687_T001 2.00E-107
467- 23: transcript:EER97444          transcript:Zm00001d006688_T001         0
467- 24: transcript:OQU90161          transcript:Zm00001d006699_T001  9.00E-41
467- 25: transcript:EER97446          transcript:Zm00001d006700_T003         0
467- 26: transcript:EER97449          transcript:Zm00001d006701_T001         0
467- 27: transcript:KXG36569          transcript:Zm00001d006702_T001         0
467- 28: transcript:EER97452          transcript:Zm00001d006704_T005         0
467- 29: transcript:EER97453          transcript:Zm00001d006705_T001         0
467- 30: transcript:KXG36570          transcript:Zm00001d006708_T001         0
467- 31: transcript:OQU90170          transcript:Zm00001d006710_T004         0
467- 32: transcript:OQU90171          transcript:Zm00001d006711_T001  3.00E-44
```

467- 33: transcript:EER97457 transcript:Zm00001d006713_T001 5.00E-170
467- 34: transcript:EER99617 transcript:Zm00001d006714_T001 4.00E-110
467- 35: transcript:KXG36580 transcript:Zm00001d006715_T001 1.00E-44
467- 36: transcript:EER97461 transcript:Zm00001d006717_T008 0
467- 37: transcript:EER99623 transcript:Zm00001d006720_T001 5.00E-149
467- 38: transcript:EER97462 transcript:Zm00001d006721_T001 0
467- 39: transcript:EER99626 transcript:Zm00001d006722_T001 0
467- 40: transcript:KXG36586 transcript:Zm00001d006723_T001 2.00E-66
467- 41: transcript:EER99630 transcript:Zm00001d006725_T001 0
467- 42: transcript:EER97464 transcript:Zm00001d006726_T001 0
Alignment 468: score=1566.0 e_value=5e-120 N=35 2&2 plus
468- 0: transcript:EER97536 transcript:Zm00001d006865_T001 2.00E-111
468- 1: transcript:EER99702 transcript:Zm00001d006866_T003 0
468- 2: transcript:OQU90257 transcript:Zm00001d006868_T007 0
468- 3: transcript:KXG36687 transcript:Zm00001d006871_T001 2.00E-167
468- 4: transcript:EER97540 transcript:Zm00001d006872_T001 2.00E-64
468- 5: transcript:OQU90263 transcript:Zm00001d006874_T001 0
468- 6: transcript:EER99708 transcript:Zm00001d006875_T001 0
468- 7: transcript:KXG36696 transcript:Zm00001d006879_T001 1.00E-97
468- 8: transcript:KXG36703 transcript:Zm00001d006882_T001 7.00E-52
468- 9: transcript:EER97550 transcript:Zm00001d006883_T001 1.00E-133
468- 10: transcript:OQU90271 transcript:Zm00001d006884_T001 0
468- 11: transcript:EER99712 transcript:Zm00001d006885_T004 0
468- 12: transcript:EER97555 transcript:Zm00001d006886_T002 0
468- 13: transcript:OQU90278 transcript:Zm00001d006894_T001 0
468- 14: transcript:EER97560 transcript:Zm00001d006896_T001 7.00E-158
468- 15: transcript:OQU90282 transcript:Zm00001d006899_T001 3.00E-155
468- 16: transcript:EER99720 transcript:Zm00001d006900_T004 0
468- 17: transcript:OQU90285 transcript:Zm00001d006903_T001 1.00E-74
468- 18: transcript:EER99723 transcript:Zm00001d006904_T001 2.00E-90
468- 19: transcript:EER97564 transcript:Zm00001d006905_T001 2.00E-151
468- 20: transcript:KXG36734 transcript:Zm00001d006906_T010 1.00E-36
468- 21: transcript:EER99727 transcript:Zm00001d006907_T002 4.00E-85
468- 22: transcript:EER99728 transcript:Zm00001d006910_T003 0
468- 23: transcript:EER99729 transcript:Zm00001d006911_T001 6.00E-81
468- 24: transcript:OQU90293 transcript:Zm00001d006912_T001 6.00E-12
468- 25: transcript:EER97568 transcript:Zm00001d006913_T002 0
468- 26: transcript:OQU90298 transcript:Zm00001d006914_T001 2.00E-36
468- 27: transcript:EER99735 transcript:Zm00001d006915_T001 3.00E-48
468- 28: transcript:EER97576 transcript:Zm00001d006916_T001 0
468- 29: transcript:KXG36751 transcript:Zm00001d006917_T001 0
468- 30: transcript:OQU90305 transcript:Zm00001d006918_T001 0
468- 31: transcript:EER97587 transcript:Zm00001d006920_T001 0
468- 32: transcript:OQU90306 transcript:Zm00001d006921_T003 2.00E-18
468- 33: transcript:OQU90308 transcript:Zm00001d006922_T001 0
468- 34: transcript:OQU90309 transcript:Zm00001d006924_T001 2.00E-136
Alignment 469: score=1488.0 e_value=8.6e-116 N=34 2&2 plus
469- 0: transcript:KXG35672 transcript:Zm00001d005782_T001 0
469- 1: transcript:EER98942 transcript:Zm00001d005784_T001 6.00E-174
469- 2: transcript:EER96769 transcript:Zm00001d005785_T006 0
469- 3: transcript:EER98944 transcript:Zm00001d005786_T001 2.00E-61
469- 4: transcript:EER96770 transcript:Zm00001d005789_T001 6.00E-100
469- 5: transcript:OQU89547 transcript:Zm00001d005790_T001 2.00E-180
469- 6: transcript:EER98950 transcript:Zm00001d005792_T004 0

469- 7: transcript:EER96774 transcript:Zm00001d005793_T001 4.00E-38
469- 8: transcript:KXG35687 transcript:Zm00001d005794_T009 0
469- 9: transcript:EER98955 transcript:Zm00001d005795_T002 0
469- 10: transcript:KXG35689 transcript:Zm00001d005798_T001 3.00E-133
469- 11: transcript:KXG35699 transcript:Zm00001d005799_T001 1.00E-105
469- 12: transcript:OQU89560 transcript:Zm00001d005802_T001 2.00E-82
469- 13: transcript:EER96781 transcript:Zm00001d005803_T001 3.00E-81
469- 14: transcript:OQU89562 transcript:Zm00001d005804_T001 4.00E-18
469- 15: transcript:KXG35705 transcript:Zm00001d005807_T001 1.00E-51
469- 16: transcript:EER96789 transcript:Zm00001d005808_T007 0
469- 17: transcript:KXG35707 transcript:Zm00001d005811_T001 1.00E-105
469- 18: transcript:EER98967 transcript:Zm00001d005812_T002 5.00E-107
469- 19: transcript:EER98969 transcript:Zm00001d005813_T002 5.00E-98
469- 20: transcript:KXG35708 transcript:Zm00001d005814_T001 2.00E-175
469- 21: transcript:EER96791 transcript:Zm00001d005816_T001 0
469- 22: transcript:EER98970 transcript:Zm00001d005817_T006 0
469- 23: transcript:EER98972 transcript:Zm00001d005818_T001 0
469- 24: transcript:KXG35713 transcript:Zm00001d005819_T001 2.00E-82
469- 25: transcript:EER98974 transcript:Zm00001d005821_T002 0
469- 26: transcript:EER96796 transcript:Zm00001d005824_T001 5.00E-64
469- 27: transcript:EER96802 transcript:Zm00001d005826_T004 0
469- 28: transcript:OQU89583 transcript:Zm00001d005829_T001 0
469- 29: transcript:EER96807 transcript:Zm00001d005830_T001 3.00E-120
469- 30: transcript:EER98983 transcript:Zm00001d005831_T003 0
469- 31: transcript:EER96809 transcript:Zm00001d005833_T002 0
469- 32: transcript:KXG35731 transcript:Zm00001d005834_T001 0
469- 33: transcript:OQU89585 transcript:Zm00001d005837_T001 2.00E-121
Alignment 470: score=1344.0 e_value=2.1e-93 N=30 2&2 plus
470- 0: transcript:EER97465 transcript:Zm00001d006731_T003 0
470- 1: transcript:KXG36592 transcript:Zm00001d006732_T001 0
470- 2: transcript:EER99632 transcript:Zm00001d006733_T001 5.00E-156
470- 3: transcript:OQU90180 transcript:Zm00001d006735_T001 2.00E-36
470- 4: transcript:KXG36595 transcript:Zm00001d006737_T006 0
470- 5: transcript:KXG36600 transcript:Zm00001d006738_T001 0
470- 6: transcript:EER97470 transcript:Zm00001d006739_T001 4.00E-101
470- 7: transcript:EER97471 transcript:Zm00001d006744_T001 0
470- 8: transcript:OQU90181 transcript:Zm00001d006746_T001 6.00E-95
470- 9: transcript:EER99634 transcript:Zm00001d006749_T001 0
470- 10: transcript:EER97475 transcript:Zm00001d006750_T001 1.00E-44
470- 11: transcript:EER99637 transcript:Zm00001d006751_T001 0
470- 12: transcript:EER99638 transcript:Zm00001d006752_T003 0
470- 13: transcript:EER99639 transcript:Zm00001d006753_T001 0
470- 14: transcript:EER97478 transcript:Zm00001d006754_T001 1.00E-106
470- 15: transcript:EER97479 transcript:Zm00001d006756_T001 3.00E-52
470- 16: transcript:EER99646 transcript:Zm00001d006757_T003 0
470- 17: transcript:OQU90190 transcript:Zm00001d006758_T003 0
470- 18: transcript:EER99633 transcript:Zm00001d006759_T012 0
470- 19: transcript:KXG36613 transcript:Zm00001d006760_T001 5.00E-115
470- 20: transcript:EER97486 transcript:Zm00001d006761_T001 0
470- 21: transcript:KXG36619 transcript:Zm00001d006762_T004 0
470- 22: transcript:KXG36622 transcript:Zm00001d006763_T001 0
470- 23: transcript:EER99658 transcript:Zm00001d006766_T001 2.00E-47
470- 24: transcript:KXG36626 transcript:Zm00001d006768_T001 5.00E-163
470- 25: transcript:EER97491 transcript:Zm00001d006769_T003 0

470- 26: transcript:KXG36630 transcript:Zm00001d006771_T002 4.00E-170
470- 27: transcript:KXG36634 transcript:Zm00001d006774_T001 4.00E-27
470- 28: transcript:EER97492 transcript:Zm00001d006776_T001 2.00E-61
470- 29: transcript:KXG36639 transcript:Zm00001d006778_T004 0
Alignment 471: score=1267.0 e_value=1.1e-99 N=29 2&2 plus
471- 0: transcript:EER97214 transcript:Zm00001d006364_T001 0
471- 1: transcript:EER99386 transcript:Zm00001d006371_T001 0
471- 2: transcript:EER99387 transcript:Zm00001d006373_T002 0
471- 3: transcript:KXG36229 transcript:Zm00001d006374_T001 0
471- 4: transcript:OQU89957 transcript:Zm00001d006375_T001 0
471- 5: transcript:KXG36232 transcript:Zm00001d006377_T001 0
471- 6: transcript:EER99382 transcript:Zm00001d006382_T004 0
471- 7: transcript:EER97216 transcript:Zm00001d006384_T005 0
471- 8: transcript:EER97229 transcript:Zm00001d006388_T001 3.00E-148
471- 9: transcript:OQU89960 transcript:Zm00001d006389_T001 4.00E-151
471- 10: transcript:KXG36238 transcript:Zm00001d006396_T001 9.00E-78
471- 11: transcript:EER99395 transcript:Zm00001d006397_T001 0
471- 12: transcript:EER97233 transcript:Zm00001d006398_T001 7.00E-96
471- 13: transcript:KXG36242 transcript:Zm00001d006399_T001 1.00E-151
471- 14: transcript:KXG36243 transcript:Zm00001d006400_T001 1.00E-104
471- 15: transcript:KXG36246 transcript:Zm00001d006402_T004 4.00E-106
471- 16: transcript:EER97237 transcript:Zm00001d006408_T001 0
471- 17: transcript:KXG36249 transcript:Zm00001d006409_T002 2.00E-128
471- 18: transcript:KXG36254 transcript:Zm00001d006410_T013 0
471- 19: transcript:KXG36256 transcript:Zm00001d006415_T001 0
471- 20: transcript:EER97242 transcript:Zm00001d006416_T001 2.00E-165
471- 21: transcript:EER99396 transcript:Zm00001d006417_T002 0
471- 22: transcript:EER99406 transcript:Zm00001d006419_T002 0
471- 23: transcript:EER97238 transcript:Zm00001d006420_T002 0
471- 24: transcript:EER99408 transcript:Zm00001d006421_T002 2.00E-50
471- 25: transcript:EER99409 transcript:Zm00001d006422_T006 0
471- 26: transcript:KXG36264 transcript:Zm00001d006428_T026 0
471- 27: transcript:OQU89976 transcript:Zm00001d006430_T001 0
471- 28: transcript:EER97251 transcript:Zm00001d006433_T001 1.00E-152
Alignment 472: score=1234.0 e_value=2.1e-87 N=27 2&2 plus
472- 0: transcript:EER99339 transcript:Zm00001d006324_T005 0
472- 1: transcript:OQU89926 transcript:Zm00001d006327_T001 0
472- 2: transcript:EER97171 transcript:Zm00001d006328_T003 0
472- 3: transcript:EER97173 transcript:Zm00001d006329_T001 2.00E-43
472- 4: transcript:EER97174 transcript:Zm00001d006330_T001 0
472- 5: transcript:KXG36174 transcript:Zm00001d006331_T001 1.00E-170
472- 6: transcript:KXG36176 transcript:Zm00001d006332_T001 4.00E-162
472- 7: transcript:EER97179 transcript:Zm00001d006333_T001 0
472- 8: transcript:KXG36179 transcript:Zm00001d006335_T003 0
472- 9: transcript:EER99354 transcript:Zm00001d006336_T001 5.00E-55
472- 10: transcript:EER99355 transcript:Zm00001d006337_T001 4.00E-122
472- 11: transcript:KXG36186 transcript:Zm00001d006340_T002 0
472- 12: transcript:KXG36188 transcript:Zm00001d006343_T001 1.00E-138
472- 13: transcript:EER99357 transcript:Zm00001d006344_T007 0
472- 14: transcript:EER99360 transcript:Zm00001d006345_T001 0
472- 15: transcript:EER97188 transcript:Zm00001d006347_T002 0
472- 16: transcript:EER99361 transcript:Zm00001d006348_T001 2.00E-166
472- 17: transcript:EER97196 transcript:Zm00001d006350_T001 0
472- 18: transcript:EER97198 transcript:Zm00001d006351_T001 0

```
472- 19: transcript:OQU89942           transcript:Zm00001d006353_T001          0
472- 20: transcript:KXG36200           transcript:Zm00001d006354_T002          0
472- 21: transcript:EER99364           transcript:Zm00001d006355_T011          0
472- 22: transcript:KXG36201           transcript:Zm00001d006357_T001          0
472- 23: transcript:EER97204           transcript:Zm00001d006358_T001 3.00E-134
472- 24: transcript:EER99370           transcript:Zm00001d006360_T001 5.00E-168
472- 25: transcript:EER99372           transcript:Zm00001d006361_T001          0
472- 26: transcript:KXG36212           transcript:Zm00001d006368_T003 2.00E-150
## Alignment 473: score=1149.0 e_value=1.2e-81 N=26 2&2 plus
473-  0: transcript:KXG35518           transcript:Zm00001d005670_T001  4.00E-31
473-  1: transcript:KXG35519           transcript:Zm00001d005671_T002          0
473-  2: transcript:EER98819           transcript:Zm00001d005674_T001          0
473-  3: transcript:OQU89424           transcript:Zm00001d005675_T006  2.00E-14
473-  4: transcript:EER96673           transcript:Zm00001d005678_T001          0
473-  5: transcript:OQU89427           transcript:Zm00001d005681_T001 7.00E-126
473-  6: transcript:EER98823           transcript:Zm00001d005682_T001          0
473-  7: transcript:KXG35533           transcript:Zm00001d005684_T001  4.00E-94
473-  8: transcript:EER96679           transcript:Zm00001d005685_T001 5.00E-145
473-  9: transcript:EER98826           transcript:Zm00001d005687_T001          0
473- 10: transcript:KXG35541           transcript:Zm00001d005688_T001          0
473- 11: transcript:EER98832           transcript:Zm00001d005692_T001 9.00E-114
473- 12: transcript:OQU89440           transcript:Zm00001d005693_T001  1.00E-80
473- 13: transcript:EER98844           transcript:Zm00001d005694_T001 2.00E-103
473- 14: transcript:EER98845           transcript:Zm00001d005695_T001  8.00E-50
473- 15: transcript:EER98846           transcript:Zm00001d005696_T001          0
473- 16: transcript:OQU89449           transcript:Zm00001d005698_T001 2.00E-102
473- 17: transcript:OQU89450           transcript:Zm00001d005699_T001          0
473- 18: transcript:EER96695           transcript:Zm00001d005705_T001  5.00E-99
473- 19: transcript:EER98855           transcript:Zm00001d005707_T007          0
473- 20: transcript:KXG35573           transcript:Zm00001d005708_T001          0
473- 21: transcript:OQU89456           transcript:Zm00001d005711_T005          0
473- 22: transcript:EER96704           transcript:Zm00001d005713_T001          0
473- 23: transcript:EER98864           transcript:Zm00001d005714_T001  2.00E-77
473- 24: transcript:EER96705           transcript:Zm00001d005715_T002 4.00E-113
473- 25: transcript:KXG35585           transcript:Zm00001d005716_T001  5.00E-96
## Alignment 474: score=1039.0 e_value=4.8e-68 N=23 2&2 plus
474-  0: transcript:EER99788           transcript:Zm00001d007029_T001          0
474-  1: transcript:EER97627           transcript:Zm00001d007031_T002  2.00E-60
474-  2: transcript:EER99790           transcript:Zm00001d007032_T001  3.00E-64
474-  3: transcript:KXG36798           transcript:Zm00001d007034_T008          0
474-  4: transcript:KXG36802           transcript:Zm00001d007035_T001  8.00E-11
474-  5: transcript:OQU90351           transcript:Zm00001d007037_T002          0
474-  6: transcript:EER97635           transcript:Zm00001d007038_T001 3.00E-175
474-  7: transcript:KXG36804           transcript:Zm00001d007039_T001          0
474-  8: transcript:EER97638           transcript:Zm00001d007042_T001          0
474-  9: transcript:EER99801           transcript:Zm00001d007043_T002          0
474- 10: transcript:EER97640           transcript:Zm00001d007044_T001          0
474- 11: transcript:EER99803           transcript:Zm00001d007045_T001 1.00E-127
474- 12: transcript:EER97642           transcript:Zm00001d007047_T001  5.00E-84
474- 13: transcript:KXG36814           transcript:Zm00001d007048_T001          0
474- 14: transcript:OQU90369           transcript:Zm00001d007049_T001  9.00E-84
474- 15: transcript:OQU90373           transcript:Zm00001d007050_T001          0
474- 16: transcript:EER97654           transcript:Zm00001d007053_T001 3.00E-149
474- 17: transcript:KXG36822           transcript:Zm00001d007056_T001          0
```

```
474- 18: transcript:EER99810           transcript:Zm00001d007057_T001  5.00E-49
474- 19: transcript:EER97658           transcript:Zm00001d007058_T002         0
474- 20: transcript:OQU90377           transcript:Zm00001d007059_T001  1.00E-41
474- 21: transcript:EER99814           transcript:Zm00001d007060_T003 8.00E-115
474- 22: transcript:EER99817           transcript:Zm00001d007062_T001 2.00E-161
## Alignment 475: score=806.0 e_value=3.1e-54 N=19 2&2 plus
475-  0: transcript:EER99851           transcript:Zm00001d007090_T001         0
475-  1: transcript:OQU90415           transcript:Zm00001d007091_T001         0
475-  2: transcript:EER97680           transcript:Zm00001d007092_T005         0
475-  3: transcript:OQU90417           transcript:Zm00001d007093_T006         0
475-  4: transcript:EER99854           transcript:Zm00001d007095_T008         0
475-  5: transcript:OQU90419           transcript:Zm00001d007096_T007         0
475-  6: transcript:EER99855           transcript:Zm00001d007097_T001  1.00E-66
475-  7: transcript:EER99858           transcript:Zm00001d007102_T001 7.00E-133
475-  8: transcript:EER97684           transcript:Zm00001d007103_T001 4.00E-165
475-  9: transcript:OQU90429           transcript:Zm00001d007105_T001 1.00E-157
475- 10: transcript:EER97690           transcript:Zm00001d007106_T001 2.00E-149
475- 11: transcript:EER99873           transcript:Zm00001d007107_T001 3.00E-178
475- 12: transcript:KXG36887           transcript:Zm00001d007108_T001         0
475- 13: transcript:KXG36890           transcript:Zm00001d007111_T001         0
475- 14: transcript:KXG36897           transcript:Zm00001d007113_T001         0
475- 15: transcript:EER97695           transcript:Zm00001d007117_T001         0
475- 16: transcript:EER97697           transcript:Zm00001d007118_T003  2.00E-89
475- 17: transcript:KXG36907           transcript:Zm00001d007119_T001 3.00E-127
475- 18: transcript:OQU90452           transcript:Zm00001d007121_T024         0
## Alignment 476: score=786.0 e_value=1.1e-46 N=17 2&2 plus
476-  0: transcript:EER99554           transcript:Zm00001d006608_T001  2.00E-60
476-  1: transcript:EER97389           transcript:Zm00001d006610_T001         0
476-  2: transcript:EER99555           transcript:Zm00001d006611_T001         0
476-  3: transcript:EER99556           transcript:Zm00001d006612_T001  2.00E-48
476-  4: transcript:EER97390           transcript:Zm00001d006613_T004         0
476-  5: transcript:EER99559           transcript:Zm00001d006614_T001         0
476-  6: transcript:OQU90111           transcript:Zm00001d006616_T001  3.00E-13
476-  7: transcript:EER99561           transcript:Zm00001d006617_T001 3.00E-125
476-  8: transcript:OQU90112           transcript:Zm00001d006618_T001  4.00E-69
476-  9: transcript:EER97396           transcript:Zm00001d006619_T001         0
476- 10: transcript:EER99552           transcript:Zm00001d006620_T001 5.00E-180
476- 11: transcript:EER99568           transcript:Zm00001d006621_T004         0
476- 12: transcript:EER99569           transcript:Zm00001d006623_T001         0
476- 13: transcript:EER99570           transcript:Zm00001d006624_T001  3.00E-84
476- 14: transcript:EER97403           transcript:Zm00001d006625_T002         0
476- 15: transcript:EER99573           transcript:Zm00001d006626_T001 3.00E-122
476- 16: transcript:KXG36510           transcript:Zm00001d006627_T001         0
## Alignment 477: score=651.0 e_value=2.5e-37 N=15 2&2 plus
477-  0: transcript:KXG36142           transcript:Zm00001d006297_T001         0
477-  1: transcript:OQU89887           transcript:Zm00001d006298_T001  3.00E-37
477-  2: transcript:KXG36147           transcript:Zm00001d006307_T002         0
477-  3: transcript:EER99321           transcript:Zm00001d006308_T001         0
477-  4: transcript:KXG36157           transcript:Zm00001d006309_T007         0
477-  5: transcript:EER99325           transcript:Zm00001d006310_T005 5.00E-165
477-  6: transcript:EER97162           transcript:Zm00001d006311_T001 7.00E-101
477-  7: transcript:EER99326           transcript:Zm00001d006312_T001 3.00E-117
477-  8: transcript:EER99327           transcript:Zm00001d006315_T001 8.00E-103
477-  9: transcript:EER97156           transcript:Zm00001d006316_T001  4.00E-36
```

```
477- 10: transcript:EER99333           transcript:Zm00001d006317_T001          0
477- 11: transcript:OQU89911           transcript:Zm00001d006319_T001          0
477- 12: transcript:EER99336           transcript:Zm00001d006320_T004 3.00E-120
477- 13: transcript:KXG36164           transcript:Zm00001d006321_T001  1.00E-15
477- 14: transcript:EER99338           transcript:Zm00001d006322_T001          0
## Alignment 478: score=603.0 e_value=3.4e-40 N=15 2&2 plus
478-  0: transcript:EER97808           transcript:Zm00001d007921_T001 1.00E-160
478-  1: transcript:KXG34242           transcript:Zm00001d007927_T001          0
478-  2: transcript:EER95718           transcript:Zm00001d007929_T002          0
478-  3: transcript:EER97820           transcript:Zm00001d007930_T001          0
478-  4: transcript:EER95730           transcript:Zm00001d007931_T001          0
478-  5: transcript:EER97827           transcript:Zm00001d007932_T001          0
478-  6: transcript:EER95733           transcript:Zm00001d007934_T001          0
478-  7: transcript:EER97832           transcript:Zm00001d007935_T001  1.00E-69
478-  8: transcript:OQU88300           transcript:Zm00001d007936_T001          0
478-  9: transcript:KXG34265           transcript:Zm00001d007937_T001          0
478- 10: transcript:KXG34268           transcript:Zm00001d007938_T003          0
478- 11: transcript:KXG34269           transcript:Zm00001d007940_T001 6.00E-106
478- 12: transcript:EER97841           transcript:Zm00001d007941_T001          0
478- 13: transcript:OQU88311           transcript:Zm00001d007942_T002          0
478- 14: transcript:EER97849           transcript:Zm00001d007943_T009          0
## Alignment 479: score=589.0 e_value=7.4e-37 N=14 2&2 plus
479-  0: transcript:EER99684           transcript:Zm00001d006813_T001 6.00E-101
479-  1: transcript:EER97507           transcript:Zm00001d006833_T001 2.00E-108
479-  2: transcript:EER97521           transcript:Zm00001d006834_T002 1.00E-141
479-  3: transcript:EER99688           transcript:Zm00001d006835_T001          0
479-  4: transcript:EER97522           transcript:Zm00001d006836_T002          0
479-  5: transcript:EER99689           transcript:Zm00001d006838_T002  7.00E-58
479-  6: transcript:OQU90235           transcript:Zm00001d006839_T002          0
479-  7: transcript:OQU90236           transcript:Zm00001d006840_T001  5.00E-38
479-  8: transcript:EER99692           transcript:Zm00001d006841_T001          0
479-  9: transcript:EER97528           transcript:Zm00001d006845_T002          0
479- 10: transcript:KXG36675           transcript:Zm00001d006853_T001 9.00E-180
479- 11: transcript:EER97530           transcript:Zm00001d006855_T001          0
479- 12: transcript:OQU90253           transcript:Zm00001d006856_T001          0
479- 13: transcript:EER97533           transcript:Zm00001d006860_T001 2.00E-148
## Alignment 480: score=536.0 e_value=4.3e-28 N=13 2&2 plus
480-  0: transcript:EER96129           transcript:Zm00001d005459_T001 3.00E-131
480-  1: transcript:KXG34775           transcript:Zm00001d005460_T003          0
480-  2: transcript:EER96133           transcript:Zm00001d005461_T001          0
480-  3: transcript:KXG34779           transcript:Zm00001d005462_T001          0
480-  4: transcript:EER96136           transcript:Zm00001d005464_T001 4.00E-126
480-  5: transcript:EER96139           transcript:Zm00001d005466_T001          0
480-  6: transcript:KXG34786           transcript:Zm00001d005468_T001 3.00E-162
480-  7: transcript:EER96145           transcript:Zm00001d005470_T001  8.00E-84
480-  8: transcript:EER98287           transcript:Zm00001d005471_T001  1.00E-24
480-  9: transcript:EER98288           transcript:Zm00001d005472_T001 2.00E-137
480- 10: transcript:EER98290           transcript:Zm00001d005473_T001          0
480- 11: transcript:EER96156           transcript:Zm00001d005478_T009          0
480- 12: transcript:OQU88782           transcript:Zm00001d005479_T001          0
## Alignment 481: score=495.0 e_value=2.1e-24 N=11 2&2 plus
481-  0: transcript:EER97669           transcript:Zm00001d007076_T001          0
481-  1: transcript:EER97670           transcript:Zm00001d007077_T001 3.00E-106
481-  2: transcript:EER97659           transcript:Zm00001d007079_T001 3.00E-135
```

```
481-  3: transcript:KXG36846           transcript:Zm00001d007080_T002 6.00E-124
481-  4: transcript:EER97672           transcript:Zm00001d007081_T002         0
481-  5: transcript:EER99841           transcript:Zm00001d007082_T001         0
481-  6: transcript:KXG36850           transcript:Zm00001d007083_T001 2.00E-109
481-  7: transcript:EER97675           transcript:Zm00001d007084_T001  7.00E-76
481-  8: transcript:KXG36853           transcript:Zm00001d007085_T001 2.00E-172
481-  9: transcript:EER97676           transcript:Zm00001d007086_T001  6.00E-30
481- 10: transcript:KXG36860           transcript:Zm00001d007089_T003         0
## Alignment 482: score=495.0 e_value=7.2e-28 N=11 2&2 plus
482-  0: transcript:EER96587           transcript:Zm00001d005620_T001         0
482-  1: transcript:EER96589           transcript:Zm00001d005622_T001  7.00E-86
482-  2: transcript:EER96598           transcript:Zm00001d005624_T001         0
482-  3: transcript:KXG35450           transcript:Zm00001d005627_T002         0
482-  4: transcript:EER96601           transcript:Zm00001d005628_T001  1.00E-65
482-  5: transcript:KXG35452           transcript:Zm00001d005629_T001         0
482-  6: transcript:OQU89345           transcript:Zm00001d005630_T001 1.00E-114
482-  7: transcript:EER98775           transcript:Zm00001d005631_T001  8.00E-85
482-  8: transcript:EER98776           transcript:Zm00001d005632_T001         0
482-  9: transcript:EER96609           transcript:Zm00001d005636_T003 2.00E-133
482- 10: transcript:EER98780           transcript:Zm00001d005638_T001         0
## Alignment 483: score=457.0 e_value=7.8e-24 N=11 2&2 plus
483-  0: transcript:EER96252           transcript:Zm00001d005148_T002         0
483-  1: transcript:KXG34942           transcript:Zm00001d005149_T001         0
483-  2: transcript:KXG34945           transcript:Zm00001d005151_T004         0
483-  3: transcript:OQU88874           transcript:Zm00001d005154_T001         0
483-  4: transcript:EER98389           transcript:Zm00001d005156_T001         0
483-  5: transcript:EER98390           transcript:Zm00001d005157_T003         0
483-  6: transcript:EER98393           transcript:Zm00001d005158_T001  7.00E-98
483-  7: transcript:KXG34956           transcript:Zm00001d005159_T001         0
483-  8: transcript:EER98399           transcript:Zm00001d005160_T001  2.00E-31
483-  9: transcript:KXG34959           transcript:Zm00001d005161_T001         0
483- 10: transcript:EER98402           transcript:Zm00001d005164_T001         0
## Alignment 484: score=414.0 e_value=1.3e-17 N=9 2&2 plus
484-  0: transcript:OQU90128           transcript:Zm00001d006628_T005         0
484-  1: transcript:OQU90131           transcript:Zm00001d006631_T001         0
484-  2: transcript:EER97408           transcript:Zm00001d006633_T001  5.00E-75
484-  3: transcript:EER99582           transcript:Zm00001d006637_T001  7.00E-52
484-  4: transcript:EER97410           transcript:Zm00001d006638_T001         0
484-  5: transcript:EER97412           transcript:Zm00001d006639_T001  7.00E-31
484-  6: transcript:EER97415           transcript:Zm00001d006640_T001         0
484-  7: transcript:EER97416           transcript:Zm00001d006644_T002         0
484-  8: transcript:EER97419           transcript:Zm00001d006645_T001 4.00E-148
## Alignment 485: score=363.0 e_value=7.7e-14 N=8 2&2 plus
485-  0: transcript:OQU90088           transcript:Zm00001d006590_T002         0
485-  1: transcript:KXG36477           transcript:Zm00001d006591_T001  6.00E-84
485-  2: transcript:OQU90095           transcript:Zm00001d006592_T001  3.00E-53
485-  3: transcript:EER99532           transcript:Zm00001d006593_T009         0
485-  4: transcript:EER97378           transcript:Zm00001d006594_T001  1.00E-66
485-  5: transcript:EER97379           transcript:Zm00001d006595_T001         0
485-  6: transcript:EER97380           transcript:Zm00001d006596_T001         0
485-  7: transcript:KXG36480           transcript:Zm00001d006597_T003         0
## Alignment 486: score=354.0 e_value=6.3e-16 N=8 2&2 plus
486-  0: transcript:EER99823           transcript:Zm00001d007067_T003         0
486-  1: transcript:EER99824           transcript:Zm00001d007068_T001         0
```

```
486-  2: transcript:KXG36835           transcript:Zm00001d007069_T001  1.00E-70
486-  3: transcript:OQU90390           transcript:Zm00001d007070_T001         0
486-  4: transcript:OQU90393           transcript:Zm00001d007071_T001  3.00E-20
486-  5: transcript:EER97649           transcript:Zm00001d007072_T001 1.00E-146
486-  6: transcript:OQU90395           transcript:Zm00001d007073_T001         0
486-  7: transcript:KXG36842           transcript:Zm00001d007075_T007         0
## Alignment 487: score=352.0 e_value=1.6e-14 N=8 2&2 plus
487-  0: transcript:KXG35476           transcript:Zm00001d005641_T001         0
487-  1: transcript:EER98790           transcript:Zm00001d005642_T002  1.00E-73
487-  2: transcript:EER96625           transcript:Zm00001d005645_T001 7.00E-139
487-  3: transcript:EER96629           transcript:Zm00001d005647_T002         0
487-  4: transcript:EER96630           transcript:Zm00001d005648_T002         0
487-  5: transcript:EER96631           transcript:Zm00001d005649_T001  3.00E-67
487-  6: transcript:EER96634           transcript:Zm00001d005650_T004         0
487-  7: transcript:KXG35486           transcript:Zm00001d005653_T004         0
## Alignment 488: score=341.0 e_value=1.4e-13 N=7 2&2 plus
488-  0: transcript:EER96652           transcript:Zm00001d005657_T002  3.00E-75
488-  1: transcript:OQU89414           transcript:Zm00001d005658_T001  9.00E-37
488-  2: transcript:OQU89415           transcript:Zm00001d005659_T001         0
488-  3: transcript:EER96662           transcript:Zm00001d005661_T001 2.00E-180
488-  4: transcript:EER98811           transcript:Zm00001d005662_T001         0
488-  5: transcript:OQU89416           transcript:Zm00001d005663_T001         0
488-  6: transcript:KXG35511           transcript:Zm00001d005664_T001 3.00E-164
## Alignment 489: score=340.0 e_value=1.9e-15 N=8 2&2 plus
489-  0: transcript:EER95753           transcript:Zm00001d007949_T001 6.00E-147
489-  1: transcript:EER97857           transcript:Zm00001d007951_T001         0
489-  2: transcript:EER95758           transcript:Zm00001d007952_T001  7.00E-38
489-  3: transcript:EER95762           transcript:Zm00001d007953_T001         0
489-  4: transcript:EER97859           transcript:Zm00001d007954_T001  3.00E-85
489-  5: transcript:EER95764           transcript:Zm00001d007957_T001  2.00E-31
489-  6: transcript:EER95768           transcript:Zm00001d007958_T001  9.00E-44
489-  7: transcript:OQU88327           transcript:Zm00001d007959_T001 7.00E-165
## Alignment 490: score=315.0 e_value=7.4e-13 N=7 2&2 plus
490-  0: transcript:EER98909           transcript:Zm00001d005767_T001         0
490-  1: transcript:EER98911           transcript:Zm00001d005769_T001         0
490-  2: transcript:EER98913           transcript:Zm00001d005770_T001         0
490-  3: transcript:EER96747           transcript:Zm00001d005772_T001  8.00E-90
490-  4: transcript:EER98912           transcript:Zm00001d005773_T001 5.00E-114
490-  5: transcript:EER96750           transcript:Zm00001d005775_T005         0
490-  6: transcript:OQU89518           transcript:Zm00001d005776_T002         0
## Alignment 491: score=286.0 e_value=1.2e-11 N=6 2&2 plus
491-  0: transcript:EER99546           transcript:Zm00001d006601_T001 5.00E-165
491-  1: transcript:EER99548           transcript:Zm00001d006602_T001         0
491-  2: transcript:OQU90105           transcript:Zm00001d006603_T001  1.00E-46
491-  3: transcript:EER97386           transcript:Zm00001d006604_T001  2.00E-99
491-  4: transcript:EER99550           transcript:Zm00001d006605_T002         0
491-  5: transcript:EER99542           transcript:Zm00001d006606_T001         0
## Alignment 492: score=278.0 e_value=2.5e-11 N=6 2&2 plus
492-  0: transcript:EER96573           transcript:Zm00001d005593_T001  3.00E-25
492-  1: transcript:EER96576           transcript:Zm00001d005609_T001 1.00E-153
492-  2: transcript:OQU89311           transcript:Zm00001d005612_T001  7.00E-46
492-  3: transcript:EER96579           transcript:Zm00001d005614_T001         0
492-  4: transcript:EER98749           transcript:Zm00001d005615_T001  6.00E-63
492-  5: transcript:KXG35438           transcript:Zm00001d005616_T001         0
```

```
## Alignment 493: score=269.0 e_value=9.1e-09 N=6 2&2 plus
493-  0: transcript:EER98873            transcript:Zm00001d005726_T001 2.00E-180
493-  1: transcript:OQU89468            transcript:Zm00001d005727_T001 2.00E-146
493-  2: transcript:EER98878            transcript:Zm00001d005729_T001         0
493-  3: transcript:KXG35596            transcript:Zm00001d005732_T001         0
493-  4: transcript:EER96715            transcript:Zm00001d005735_T001         0
493-  5: transcript:KXG35610            transcript:Zm00001d005736_T003  1.00E-55
## Alignment 494: score=251.0 e_value=4.3e-09 N=6 2&2 plus
494-  0: transcript:KXG35333            transcript:Zm00001d005538_T001         0
494-  1: transcript:OQU89206            transcript:Zm00001d005539_T003         0
494-  2: transcript:OQU89208            transcript:Zm00001d005542_T001         0
494-  3: transcript:EER96516            transcript:Zm00001d005544_T001  3.00E-93
494-  4: transcript:EER98660            transcript:Zm00001d005545_T001 2.00E-109
494-  5: transcript:EER98661            transcript:Zm00001d005546_T002         0
## Alignment 495: score=1020.0 e_value=3e-64 N=22 2&2 minus
495-  0: transcript:KXG35737            transcript:Zm00001d005878_T001  9.00E-42
495-  1: transcript:EER98990            transcript:Zm00001d005875_T001 2.00E-118
495-  2: transcript:KXG35739            transcript:Zm00001d005874_T001 1.00E-127
495-  3: transcript:EER98992            transcript:Zm00001d005873_T001  5.00E-58
495-  4: transcript:EER96827            transcript:Zm00001d005871_T002         0
495-  5: transcript:EER96828            transcript:Zm00001d005869_T001         0
495-  6: transcript:OQU89603            transcript:Zm00001d005867_T002  7.00E-70
495-  7: transcript:EER96832            transcript:Zm00001d005866_T003         0
495-  8: transcript:EER96833            transcript:Zm00001d005865_T001         0
495-  9: transcript:EER98997            transcript:Zm00001d005864_T001 2.00E-130
495- 10: transcript:EER96835            transcript:Zm00001d005859_T001  7.00E-98
495- 11: transcript:EER98999            transcript:Zm00001d005858_T001  9.00E-98
495- 12: transcript:EER96837            transcript:Zm00001d005857_T001  2.00E-92
495- 13: transcript:KXG35750            transcript:Zm00001d005856_T003         0
495- 14: transcript:OQU89606            transcript:Zm00001d005851_T002         0
495- 15: transcript:KXG35752            transcript:Zm00001d005849_T001         0
495- 16: transcript:EER96841            transcript:Zm00001d005848_T003         0
495- 17: transcript:EER99004            transcript:Zm00001d005847_T001         0
495- 18: transcript:OQU89611            transcript:Zm00001d005846_T002         0
495- 19: transcript:KXG35754            transcript:Zm00001d005844_T002 6.00E-130
495- 20: transcript:EER96844            transcript:Zm00001d005843_T001 4.00E-149
495- 21: transcript:EER96848            transcript:Zm00001d005841_T001  9.00E-79
## Alignment 496: score=932.0 e_value=2.5e-65 N=21 2&2 minus
496-  0: transcript:EER97594            transcript:Zm00001d007027_T001  6.00E-74
496-  1: transcript:EER97595            transcript:Zm00001d007015_T001  1.00E-90
496-  2: transcript:EER97597            transcript:Zm00001d007012_T001 4.00E-156
496-  3: transcript:EER97598            transcript:Zm00001d007011_T001  2.00E-33
496-  4: transcript:EER97599            transcript:Zm00001d007009_T001         0
496-  5: transcript:EER97600            transcript:Zm00001d006951_T001 2.00E-166
496-  6: transcript:EER97605            transcript:Zm00001d006950_T001  1.00E-33
496-  7: transcript:EER97609            transcript:Zm00001d006947_T001         0
496-  8: transcript:EER97613            transcript:Zm00001d006945_T001         0
496-  9: transcript:EER97615            transcript:Zm00001d006944_T001         0
496- 10: transcript:OQU90338            transcript:Zm00001d006943_T001         0
496- 11: transcript:EER97616            transcript:Zm00001d006942_T007  2.00E-68
496- 12: transcript:EER99772            transcript:Zm00001d006940_T001  5.00E-45
496- 13: transcript:EER97617            transcript:Zm00001d006939_T001 2.00E-180
496- 14: transcript:OQU90340            transcript:Zm00001d006938_T001         0
496- 15: transcript:EER99776            transcript:Zm00001d006936_T002  2.00E-74
```

```
496- 16: transcript:KXG36791            transcript:Zm00001d006933_T001          0
496- 17: transcript:EER97624            transcript:Zm00001d006931_T002          0
496- 18: transcript:OQU90344            transcript:Zm00001d006930_T001 3.00E-115
496- 19: transcript:EER99786            transcript:Zm00001d006929_T001          0
496- 20: transcript:EER97625            transcript:Zm00001d006928_T001 2.00E-171
## Alignment 497: score=582.0 e_value=2.3e-32 N=14 2&2 minus
497-  0: transcript:EER96094            transcript:Zm00001d005205_T003          0
497-  1: transcript:KXG34718            transcript:Zm00001d005203_T001  2.00E-42
497-  2: transcript:EER98223            transcript:Zm00001d005200_T002  7.00E-98
497-  3: transcript:EER96096            transcript:Zm00001d005199_T002          0
497-  4: transcript:EER96097            transcript:Zm00001d005196_T001  2.00E-59
497-  5: transcript:OQU88735            transcript:Zm00001d005193_T001  4.00E-37
497-  6: transcript:EER96099            transcript:Zm00001d005190_T001          0
497-  7: transcript:OQU88740            transcript:Zm00001d005188_T001 1.00E-118
497-  8: transcript:OQU88744            transcript:Zm00001d005185_T001 3.00E-152
497-  9: transcript:EER96102            transcript:Zm00001d005182_T001 5.00E-156
497- 10: transcript:EER98238            transcript:Zm00001d005179_T001 2.00E-108
497- 11: transcript:EER98240            transcript:Zm00001d005178_T001  2.00E-33
497- 12: transcript:KXG34745            transcript:Zm00001d005177_T001  4.00E-99
497- 13: transcript:EER96106            transcript:Zm00001d005174_T001          0
## Alignment 498: score=578.0 e_value=2e-29 N=13 2&2 minus
498-  0: transcript:EER98893            transcript:Zm00001d005766_T001  5.00E-59
498-  1: transcript:KXG35617            transcript:Zm00001d005765_T004 2.00E-170
498-  2: transcript:OQU89487            transcript:Zm00001d005764_T001          0
498-  3: transcript:EER96729            transcript:Zm00001d005762_T002          0
498-  4: transcript:KXG35628            transcript:Zm00001d005760_T001 2.00E-167
498-  5: transcript:KXG35632            transcript:Zm00001d005757_T001 1.00E-151
498-  6: transcript:EER98900            transcript:Zm00001d005756_T002          0
498-  7: transcript:EER96734            transcript:Zm00001d005754_T001  7.00E-61
498-  8: transcript:KXG35635            transcript:Zm00001d005752_T001 2.00E-177
498-  9: transcript:EER98903            transcript:Zm00001d005751_T002 4.00E-153
498- 10: transcript:OQU89496            transcript:Zm00001d005750_T002 3.00E-159
498- 11: transcript:EER98905            transcript:Zm00001d005749_T001 2.00E-130
498- 12: transcript:EER98907            transcript:Zm00001d005748_T001          0
## Alignment 499: score=495.0 e_value=4.7e-27 N=12 2&2 minus
499-  0: transcript:OQU88465            transcript:Zm00001d007919_T001 2.00E-158
499-  1: transcript:EER95892            transcript:Zm00001d007918_T001          0
499-  2: transcript:EER98012            transcript:Zm00001d007915_T001          0
499-  3: transcript:EER98014            transcript:Zm00001d007912_T009          0
499-  4: transcript:KXG34439            transcript:Zm00001d007911_T001  2.00E-31
499-  5: transcript:EER98015            transcript:Zm00001d007910_T001  3.00E-93
499-  6: transcript:OQU88478            transcript:Zm00001d007908_T001  1.00E-95
499-  7: transcript:EER98022            transcript:Zm00001d007907_T003          0
499-  8: transcript:KXG34446            transcript:Zm00001d007905_T001          0
499-  9: transcript:KXG34449            transcript:Zm00001d007904_T003          0
499- 10: transcript:EER98026            transcript:Zm00001d007902_T001          0
499- 11: transcript:KXG34455            transcript:Zm00001d007901_T001  2.00E-20
## Alignment 500: score=465.0 e_value=2.6e-23 N=10 2&2 minus
500-  0: transcript:KXG34614            transcript:Zm00001d007773_T001  9.00E-23
500-  1: transcript:EER98147            transcript:Zm00001d007772_T001          0
500-  2: transcript:OQU88645            transcript:Zm00001d007770_T001          0
500-  3: transcript:EER98148            transcript:Zm00001d007769_T003          0
500-  4: transcript:OQU88647            transcript:Zm00001d007768_T001  8.00E-82
500-  5: transcript:EER96027            transcript:Zm00001d007767_T001 8.00E-166
```

500- 6: transcript:EER98151 transcript:Zm00001d007766_T001 1.00E-15
500- 7: transcript:EER98153 transcript:Zm00001d007765_T001 6.00E-165
500- 8: transcript:EER98157 transcript:Zm00001d007763_T001 8.00E-28
500- 9: transcript:KXG34620 transcript:Zm00001d007762_T001 0
Alignment 501: score=464.0 e_value=3.4e-21 N=10 2&2 minus
501- 0: transcript:KXG36646 transcript:Zm00001d006828_T001 0
501- 1: transcript:EER97496 transcript:Zm00001d006825_T003 0
501- 2: transcript:EER97499 transcript:Zm00001d006823_T001 0
501- 3: transcript:EER97500 transcript:Zm00001d006822_T001 0
501- 4: transcript:EER99681 transcript:Zm00001d006821_T001 6.00E-154
501- 5: transcript:EER97509 transcript:Zm00001d006820_T001 0
501- 6: transcript:OQU90223 transcript:Zm00001d006816_T001 2.00E-104
501- 7: transcript:OQU90224 transcript:Zm00001d006815_T001 2.00E-61
501- 8: transcript:KXG36663 transcript:Zm00001d006810_T001 1.00E-99
501- 9: transcript:EER99687 transcript:Zm00001d006808_T001 1.00E-98
Alignment 502: score=428.0 e_value=6.9e-26 N=11 2&2 minus
502- 0: transcript:KXG35085 transcript:Zm00001d005379_T001 2.00E-20
502- 1: transcript:EER98482 transcript:Zm00001d005375_T001 4.00E-62
502- 2: transcript:KXG35088 transcript:Zm00001d005373_T001 0
502- 3: transcript:OQU88996 transcript:Zm00001d005372_T002 0
502- 4: transcript:EER98485 transcript:Zm00001d005370_T004 0
502- 5: transcript:EER98488 transcript:Zm00001d005369_T001 1.00E-46
502- 6: transcript:KXG35092 transcript:Zm00001d005368_T002 0
502- 7: transcript:KXG35095 transcript:Zm00001d005361_T007 5.00E-144
502- 8: transcript:EER98490 transcript:Zm00001d005359_T001 1.00E-179
502- 9: transcript:KXG35104 transcript:Zm00001d005355_T001 0
502- 10: transcript:EER96348 transcript:Zm00001d005351_T001 0
Alignment 503: score=423.0 e_value=1.1e-20 N=10 2&2 minus
503- 0: transcript:EER96059 transcript:Zm00001d005250_T001 0
503- 1: transcript:EER98198 transcript:Zm00001d005248_T003 0
503- 2: transcript:OQU88693 transcript:Zm00001d005244_T002 2.00E-176
503- 3: transcript:KXG34686 transcript:Zm00001d005241_T001 0
503- 4: transcript:EER96065 transcript:Zm00001d005240_T004 0
503- 5: transcript:EER96067 transcript:Zm00001d005239_T001 0
503- 6: transcript:KXG34695 transcript:Zm00001d005238_T001 0
503- 7: transcript:EER96075 transcript:Zm00001d005231_T013 1.00E-132
503- 8: transcript:KXG34699 transcript:Zm00001d005230_T001 2.00E-17
503- 9: transcript:EER96079 transcript:Zm00001d005229_T001 1.00E-159
Alignment 504: score=375.0 e_value=5.2e-17 N=9 2&2 minus
504- 0: transcript:EER98531 transcript:Zm00001d005322_T001 8.00E-101
504- 1: transcript:OQU89045 transcript:Zm00001d005317_T001 0
504- 2: transcript:EER98534 transcript:Zm00001d005315_T001 6.00E-125
504- 3: transcript:OQU89052 transcript:Zm00001d005313_T001 0
504- 4: transcript:EER96388 transcript:Zm00001d005312_T001 0
504- 5: transcript:KXG35151 transcript:Zm00001d005309_T001 3.00E-63
504- 6: transcript:OQU89054 transcript:Zm00001d005308_T002 0
504- 7: transcript:KXG35154 transcript:Zm00001d005307_T001 1.00E-119
504- 8: transcript:OQU89056 transcript:Zm00001d005305_T001 8.00E-133
Alignment 505: score=364.0 e_value=1e-18 N=9 2&2 minus
505- 0: transcript:OQU89013 transcript:Zm00001d005350_T005 0
505- 1: transcript:OQU89015 transcript:Zm00001d005348_T001 0
505- 2: transcript:KXG35115 transcript:Zm00001d005347_T001 0
505- 3: transcript:OQU89025 transcript:Zm00001d005346_T001 0
505- 4: transcript:OQU89026 transcript:Zm00001d005344_T002 2.00E-100

```
505-  5: transcript:EER98509           transcript:Zm00001d005343_T003           0
505-  6: transcript:KXG35119           transcript:Zm00001d005338_T005           0
505-  7: transcript:EER96363           transcript:Zm00001d005334_T001  6.00E-92
505-  8: transcript:KXG35124           transcript:Zm00001d005333_T001  4.00E-60
## Alignment 506: score=346.0 e_value=2.1e-18 N=9 2&2 minus
506-  0: transcript:KXG34534           transcript:Zm00001d007824_T001           0
506-  1: transcript:EER95950           transcript:Zm00001d007823_T001           0
506-  2: transcript:OQU88569           transcript:Zm00001d007822_T001  2.00E-18
506-  3: transcript:EER98072           transcript:Zm00001d007820_T002           0
506-  4: transcript:EER98081           transcript:Zm00001d007810_T001  7.00E-59
506-  5: transcript:EER95967           transcript:Zm00001d007807_T006           0
506-  6: transcript:OQU88580           transcript:Zm00001d007806_T003 1.00E-132
506-  7: transcript:EER95968           transcript:Zm00001d007802_T001           0
506-  8: transcript:OQU88584           transcript:Zm00001d007800_T001  2.00E-68
## Alignment 507: score=279.0 e_value=1.9e-12 N=7 2&2 minus
507-  0: transcript:EER96488           transcript:Zm00001d005535_T002           0
507-  1: transcript:KXG35300           transcript:Zm00001d005534_T001           0
507-  2: transcript:EER98641           transcript:Zm00001d005532_T003 5.00E-107
507-  3: transcript:KXG35311           transcript:Zm00001d005528_T001           0
507-  4: transcript:OQU89186           transcript:Zm00001d005516_T001           0
507-  5: transcript:EER96498           transcript:Zm00001d005513_T001 1.00E-178
507-  6: transcript:EER98648           transcript:Zm00001d005503_T001  2.00E-69
## Alignment 508: score=262.0 e_value=1.6e-09 N=6 2&2 minus
508-  0: transcript:EER95909           transcript:Zm00001d007896_T001           0
508-  1: transcript:EER95912           transcript:Zm00001d007894_T001           0
508-  2: transcript:EER98038           transcript:Zm00001d007893_T001 5.00E-170
508-  3: transcript:EER98039           transcript:Zm00001d007892_T001           0
508-  4: transcript:KXG34475           transcript:Zm00001d007890_T002           0
508-  5: transcript:KXG34480           transcript:Zm00001d007889_T009           0
## Alignment 509: score=826.0 e_value=6.1e-54 N=20 2&4 plus
509-  0: transcript:Zm00001d002512_T001 transcript:EES05698            4.00E-71
509-  1: transcript:Zm00001d002515_T001 transcript:OQU85596            7.00E-58
509-  2: transcript:Zm00001d002517_T001 transcript:KXG30985            3.00E-42
509-  3: transcript:Zm00001d002519_T001 transcript:KXG30986            1.00E-94
509-  4: transcript:Zm00001d002520_T001 transcript:KXG30991            9.00E-18
509-  5: transcript:Zm00001d002523_T001 transcript:EES05711            1.00E-34
509-  6: transcript:Zm00001d002531_T001 transcript:OQU85613           3.00E-176
509-  7: transcript:Zm00001d002535_T001 transcript:EES05717            3.00E-59
509-  8: transcript:Zm00001d002540_T002 transcript:OQU85618                   0
509-  9: transcript:Zm00001d002542_T001 transcript:KXG30997                   0
509- 10: transcript:Zm00001d002544_T004 transcript:OQU85620                   0
509- 11: transcript:Zm00001d002546_T001 transcript:OQU85624            7.00E-67
509- 12: transcript:Zm00001d002549_T002 transcript:EES05720            1.00E-07
509- 13: transcript:Zm00001d002553_T001 transcript:EES05721                   0
509- 14: transcript:Zm00001d002562_T001 transcript:EES07445           4.00E-119
509- 15: transcript:Zm00001d002565_T001 transcript:EES05726            1.00E-10
509- 16: transcript:Zm00001d002573_T001 transcript:EES05727                   0
509- 17: transcript:Zm00001d002576_T001 transcript:EES05728            5.00E-66
509- 18: transcript:Zm00001d002579_T001 transcript:EES05729                   0
509- 19: transcript:Zm00001d002580_T004 transcript:KXG31007                   0
## Alignment 510: score=623.0 e_value=1.8e-35 N=14 2&4 plus
510-  0: transcript:Zm00001d002819_T002 transcript:EES05821           1.00E-170
510-  1: transcript:Zm00001d002820_T001 transcript:OQU85754            5.00E-65
510-  2: transcript:Zm00001d002823_T001 transcript:EES07540                   0
```

```
510-  3: transcript:Zm00001d002824_T001 transcript:EES05825                 0
510-  4: transcript:Zm00001d002825_T001 transcript:EES05819          8.00E-92
510-  5: transcript:Zm00001d002826_T001 transcript:EES07542          2.00E-41
510-  6: transcript:Zm00001d002828_T001 transcript:KXG31125         2.00E-153
510-  7: transcript:Zm00001d002829_T001 transcript:EES05828          2.00E-91
510-  8: transcript:Zm00001d002836_T006 transcript:EES07545                 0
510-  9: transcript:Zm00001d002837_T001 transcript:EES05833         2.00E-106
510- 10: transcript:Zm00001d002842_T001 transcript:KXG31130          3.00E-67
510- 11: transcript:Zm00001d002843_T001 transcript:OQU85763          1.00E-42
510- 12: transcript:Zm00001d002844_T003 transcript:EES05836         5.00E-105
510- 13: transcript:Zm00001d002845_T001 transcript:EES07550          8.00E-19
## Alignment 511: score=552.0 e_value=1.3e-33 N=14 2&4 plus
511-  0: transcript:EER97077            transcript:Zm00001d053200_T001         0
511-  1: transcript:EER99230            transcript:Zm00001d053202_T001         0
511-  2: transcript:KXG36062            transcript:Zm00001d053208_T001  1.00E-92
511-  3: transcript:KXG36070            transcript:Zm00001d053210_T001 2.00E-101
511-  4: transcript:EER97092            transcript:Zm00001d053211_T009 3.00E-118
511-  5: transcript:EER97094            transcript:Zm00001d053212_T002  1.00E-92
511-  6: transcript:OQU89832            transcript:Zm00001d053214_T001         0
511-  7: transcript:EER97098            transcript:Zm00001d053215_T001 1.00E-134
511-  8: transcript:EER97100            transcript:Zm00001d053217_T001 2.00E-177
511-  9: transcript:EER99264            transcript:Zm00001d053220_T001 4.00E-104
511- 10: transcript:KXG36088            transcript:Zm00001d053225_T005         0
511- 11: transcript:EER99269            transcript:Zm00001d053228_T002  3.00E-57
511- 12: transcript:EER97111            transcript:Zm00001d053229_T001  9.00E-15
511- 13: transcript:KXG36092            transcript:Zm00001d053236_T001  6.00E-90
## Alignment 512: score=375.0 e_value=1.2e-16 N=9 2&4 plus
512-  0: transcript:Zm00001d002679_T001 transcript:EES07479                 0
512-  1: transcript:Zm00001d002687_T001 transcript:OQU85673                 0
512-  2: transcript:Zm00001d002688_T002 transcript:EES05759                 0
512-  3: transcript:Zm00001d002690_T001 transcript:EES07484                 0
512-  4: transcript:Zm00001d002693_T001 transcript:OQU85674                 0
512-  5: transcript:Zm00001d002699_T001 transcript:KXG31053                 0
512-  6: transcript:Zm00001d002700_T001 transcript:KXG31055          7.00E-19
512-  7: transcript:Zm00001d002703_T001 transcript:EES07487          1.00E-26
512-  8: transcript:Zm00001d002704_T002 transcript:KXG31059          1.00E-73
## Alignment 513: score=353.0 e_value=6.3e-18 N=9 2&4 plus
513-  0: transcript:Zm00001d002743_T001 transcript:EES05783          7.00E-21
513-  1: transcript:Zm00001d002744_T001 transcript:EES07513          4.00E-55
513-  2: transcript:Zm00001d002756_T001 transcript:EES05789          1.00E-27
513-  3: transcript:Zm00001d002757_T001 transcript:EES05790         5.00E-158
513-  4: transcript:Zm00001d002758_T002 transcript:EES07522         2.00E-106
513-  5: transcript:Zm00001d002760_T001 transcript:KXG31094          1.00E-63
513-  6: transcript:Zm00001d002762_T001 transcript:EES05793          2.00E-91
513-  7: transcript:Zm00001d002772_T001 transcript:OQU85738          7.00E-52
513-  8: transcript:Zm00001d002776_T001 transcript:EES05807                 0
## Alignment 514: score=338.0 e_value=2.3e-13 N=8 2&4 plus
514-  0: transcript:Zm00001d002794_T001 transcript:KXG31104          5.00E-76
514-  1: transcript:Zm00001d002797_T001 transcript:EES05808                 0
514-  2: transcript:Zm00001d002799_T001 transcript:EES05814          2.00E-57
514-  3: transcript:Zm00001d002801_T001 transcript:EES05815         2.00E-130
514-  4: transcript:Zm00001d002802_T001 transcript:EES05816         1.00E-148
514-  5: transcript:Zm00001d002806_T001 transcript:EES07539          1.00E-83
514-  6: transcript:Zm00001d002811_T002 transcript:KXG31117          2.00E-91
```

```
514-  7: transcript:Zm00001d002817_T004 transcript:KXG31119               0
## Alignment 515: score=314.0 e_value=4.6e-15 N=8 2&4 plus
515-  0: transcript:EER97717            transcript:Zm00001d052333_T002  3.00E-15
515-  1: transcript:EER97728            transcript:Zm00001d052335_T003 8.00E-136
515-  2: transcript:KXG36940            transcript:Zm00001d052336_T001 3.00E-130
515-  3: transcript:KXG36943            transcript:Zm00001d052340_T001         0
515-  4: transcript:OQU90475            transcript:Zm00001d052344_T003  2.00E-94
515-  5: transcript:EER99915            transcript:Zm00001d052354_T005         0
515-  6: transcript:KXG36951            transcript:Zm00001d052361_T001  2.00E-17
515-  7: transcript:OQU90483            transcript:Zm00001d052367_T003 2.00E-133
## Alignment 516: score=306.0 e_value=9.7e-11 N=7 2&4 plus
516-  0: transcript:Zm00001d002485_T001 transcript:EES07420               0
516-  1: transcript:Zm00001d002488_T001 transcript:EES07422        7.00E-21
516-  2: transcript:Zm00001d002489_T001 transcript:KXG30975       1.00E-127
516-  3: transcript:Zm00001d002491_T001 transcript:EES05692       5.00E-114
516-  4: transcript:Zm00001d002492_T001 transcript:EES07423        2.00E-35
516-  5: transcript:Zm00001d002498_T001 transcript:EES07426       4.00E-172
516-  6: transcript:Zm00001d002500_T001 transcript:OQU85580        8.00E-14
## Alignment 517: score=297.0 e_value=3.5e-12 N=7 2&4 plus
517-  0: transcript:KXG35755            transcript:Zm00001d052155_T001  1.00E-64
517-  1: transcript:KXG35763            transcript:Zm00001d052157_T005 2.00E-104
517-  2: transcript:EER96855            transcript:Zm00001d052167_T001  2.00E-36
517-  3: transcript:EER99011            transcript:Zm00001d052170_T001 8.00E-154
517-  4: transcript:EER96861            transcript:Zm00001d052172_T001  2.00E-18
517-  5: transcript:EER96862            transcript:Zm00001d052173_T001  2.00E-30
517-  6: transcript:EER99015            transcript:Zm00001d052174_T001  2.00E-11
## Alignment 518: score=280.0 e_value=1.5e-11 N=7 2&4 plus
518-  0: transcript:OQU89720            transcript:Zm00001d049764_T001         0
518-  1: transcript:EER99119            transcript:Zm00001d049765_T001 2.00E-111
518-  2: transcript:EER99130            transcript:Zm00001d049768_T001  4.00E-46
518-  3: transcript:EER96997            transcript:Zm00001d049769_T001  3.00E-14
518-  4: transcript:KXG35959            transcript:Zm00001d049785_T003         0
518-  5: transcript:EER99135            transcript:Zm00001d049789_T001  2.00E-41
518-  6: transcript:EER97002            transcript:Zm00001d049790_T007  4.00E-94
## Alignment 519: score=277.0 e_value=7.7e-09 N=6 2&4 plus
519-  0: transcript:Zm00001d002618_T001 transcript:OQU85641        2.00E-80
519-  1: transcript:Zm00001d002620_T001 transcript:KXG31013        1.00E-58
519-  2: transcript:Zm00001d002621_T002 transcript:KXG31014        2.00E-63
519-  3: transcript:Zm00001d002623_T001 transcript:EES07463               0
519-  4: transcript:Zm00001d002625_T001 transcript:EES05735               0
519-  5: transcript:Zm00001d002626_T001 transcript:EES05736               0
## Alignment 520: score=276.0 e_value=1.6e-08 N=6 2&4 plus
520-  0: transcript:EER99087            transcript:Zm00001d052889_T001         0
520-  1: transcript:EER99092            transcript:Zm00001d052890_T001 5.00E-112
520-  2: transcript:EER99094            transcript:Zm00001d052892_T001  1.00E-83
520-  3: transcript:EER96978            transcript:Zm00001d052893_T002  2.00E-63
520-  4: transcript:EER99096            transcript:Zm00001d052895_T001 2.00E-103
520-  5: transcript:OQU89696            transcript:Zm00001d052901_T001 3.00E-116
## Alignment 521: score=263.0 e_value=3.3e-11 N=7 2&4 plus
521-  0: transcript:Zm00001d002418_T030 transcript:EES05664               0
521-  1: transcript:Zm00001d002422_T004 transcript:KXG30930       7.00E-119
521-  2: transcript:Zm00001d002424_T002 transcript:OQU85554        2.00E-20
521-  3: transcript:Zm00001d002429_T002 transcript:EES07405       3.00E-113
521-  4: transcript:Zm00001d002432_T001 transcript:OQU85560       2.00E-124
```

521- 5: transcript:Zm00001d002434_T001 transcript:EES05672 7.00E-151
521- 6: transcript:Zm00001d002439_T002 transcript:EES07407 2.00E-53
Alignment 522: score=815.0 e_value=5.4e-52 N=19 2&4 minus
522- 0: transcript:Zm00001d002956_T005 transcript:EES05482 0
522- 1: transcript:Zm00001d002958_T001 transcript:EES07199 3.00E-72
522- 2: transcript:Zm00001d002966_T001 transcript:EES05471 6.00E-147
522- 3: transcript:Zm00001d002969_T001 transcript:EES05469 9.00E-140
522- 4: transcript:Zm00001d002970_T001 transcript:EES07196 0
522- 5: transcript:Zm00001d002972_T001 transcript:OQU85362 0
522- 6: transcript:Zm00001d002982_T001 transcript:EES07188 1.00E-76
522- 7: transcript:Zm00001d002992_T002 transcript:OQU85355 2.00E-92
522- 8: transcript:Zm00001d002996_T001 transcript:OQU85352 1.00E-25
522- 9: transcript:Zm00001d002999_T001 transcript:KXG30679 4.00E-156
522- 10: transcript:Zm00001d003006_T001 transcript:EES05459 0
522- 11: transcript:Zm00001d003009_T001 transcript:EES07181 6.00E-20
522- 12: transcript:Zm00001d003011_T001 transcript:EES05458 3.00E-47
522- 13: transcript:Zm00001d003012_T001 transcript:KXG30670 7.00E-108
522- 14: transcript:Zm00001d003013_T001 transcript:EES05457 1.00E-134
522- 15: transcript:Zm00001d003015_T001 transcript:EES07175 0
522- 16: transcript:Zm00001d003016_T001 transcript:EES07174 0
522- 17: transcript:Zm00001d003017_T002 transcript:KXG30663 0
522- 18: transcript:Zm00001d003021_T001 transcript:KXG30662 0
Alignment 523: score=395.0 e_value=4.5e-19 N=9 2&4 minus
523- 0: transcript:Zm00001d003345_T001 transcript:EES07061 1.00E-73
523- 1: transcript:Zm00001d003347_T002 transcript:EES05332 1.00E-61
523- 2: transcript:Zm00001d003349_T001 transcript:KXG30524 0
523- 3: transcript:Zm00001d003353_T001 transcript:EES07056 1.00E-15
523- 4: transcript:Zm00001d003357_T001 transcript:KXG30519 2.00E-18
523- 5: transcript:Zm00001d003363_T001 transcript:EES07051 1.00E-08
523- 6: transcript:Zm00001d003364_T001 transcript:KXG30516 1.00E-10
523- 7: transcript:Zm00001d003369_T004 transcript:EES07046 1.00E-71
523- 8: transcript:Zm00001d003375_T001 transcript:EES05328 6.00E-19
Alignment 524: score=370.0 e_value=1.6e-13 N=8 2&4 minus
524- 0: transcript:Zm00001d002850_T001 transcript:EES05490 8.00E-69
524- 1: transcript:Zm00001d002854_T001 transcript:EES07212 3.00E-71
524- 2: transcript:Zm00001d002856_T001 transcript:EES07211 3.00E-72
524- 3: transcript:Zm00001d002860_T002 transcript:EES05488 1.00E-142
524- 4: transcript:Zm00001d002865_T002 transcript:EES05484 0
524- 5: transcript:Zm00001d002867_T001 transcript:OQU85375 6.00E-86
524- 6: transcript:Zm00001d002868_T001 transcript:KXG30703 4.00E-39
524- 7: transcript:Zm00001d002869_T001 transcript:EES07205 2.00E-129
Alignment 525: score=325.0 e_value=1.4e-16 N=8 2&4 minus
525- 0: transcript:Zm00001d003269_T001 transcript:OQU85247 4.00E-07
525- 1: transcript:Zm00001d003281_T001 transcript:EES05369 0
525- 2: transcript:Zm00001d003287_T001 transcript:EES07094 6.00E-74
525- 3: transcript:Zm00001d003288_T001 transcript:EES05367 3.00E-168
525- 4: transcript:Zm00001d003292_T001 transcript:KXG30544 0
525- 5: transcript:Zm00001d003293_T001 transcript:KXG30609 3.00E-91
525- 6: transcript:Zm00001d003297_T001 transcript:EES05363 0
525- 7: transcript:Zm00001d003300_T001 transcript:EES05348 2.00E-112
Alignment 526: score=315.0 e_value=2.1e-13 N=8 2&4 minus
526- 0: transcript:Zm00001d003492_T001 transcript:KXG30480 1.00E-68
526- 1: transcript:Zm00001d003493_T002 transcript:KXG30522 3.00E-91
526- 2: transcript:Zm00001d003497_T001 transcript:EES07019 3.00E-48

526- 3: transcript:Zm00001d003499_T001 transcript:EES05293 2.00E-74
526- 4: transcript:Zm00001d003505_T001 transcript:EES05291 5.00E-22
526- 5: transcript:Zm00001d003509_T001 transcript:EES07012 0
526- 6: transcript:Zm00001d003518_T003 transcript:EES05287 1.00E-116
526- 7: transcript:Zm00001d003520_T001 transcript:KXG30470 2.00E-166
Alignment 527: score=601.0 e_value=1.7e-34 N=14 2&5 plus
527- 0: transcript:Zm00001d007457_T003 transcript:EES08659 6.00E-176
527- 1: transcript:Zm00001d007464_T001 transcript:EES09937 0
527- 2: transcript:Zm00001d007466_T001 transcript:KXG28680 6.00E-39
527- 3: transcript:Zm00001d007468_T001 transcript:OQU83648 0
527- 4: transcript:Zm00001d007470_T001 transcript:KXG28687 6.00E-18
527- 5: transcript:Zm00001d007473_T001 transcript:EES09947 1.00E-111
527- 6: transcript:Zm00001d007474_T001 transcript:EES08678 0
527- 7: transcript:Zm00001d007477_T001 transcript:EES09952 0
527- 8: transcript:Zm00001d007478_T001 transcript:KXG28702 6.00E-17
527- 9: transcript:Zm00001d007479_T001 transcript:EES09955 0
527- 10: transcript:Zm00001d007481_T001 transcript:EES09958 0
527- 11: transcript:Zm00001d007485_T001 transcript:OQU83661 1.00E-32
527- 12: transcript:Zm00001d007486_T001 transcript:KXG28711 2.00E-113
527- 13: transcript:Zm00001d007490_T001 transcript:KXG28714 2.00E-77
Alignment 528: score=457.0 e_value=1.1e-25 N=11 2&5 plus
528- 0: transcript:Zm00001d007640_T001 transcript:EES08900 0
528- 1: transcript:Zm00001d007652_T001 transcript:OQU83913 3.00E-138
528- 2: transcript:Zm00001d007653_T003 transcript:EES08902 0
528- 3: transcript:Zm00001d007654_T001 transcript:OQU83918 1.00E-43
528- 4: transcript:Zm00001d007657_T001 transcript:KXG29014 1.00E-47
528- 5: transcript:Zm00001d007658_T001 transcript:EES10178 2.00E-17
528- 6: transcript:Zm00001d007665_T001 transcript:KXG29028 5.00E-104
528- 7: transcript:Zm00001d007672_T001 transcript:KXG29029 5.00E-92
528- 8: transcript:Zm00001d007677_T001 transcript:OQU83927 0
528- 9: transcript:Zm00001d007686_T001 transcript:OQU83937 3.00E-07
528- 10: transcript:Zm00001d007687_T001 transcript:EES10195 1.00E-133
Alignment 529: score=408.0 e_value=5.2e-24 N=10 2&5 plus
529- 0: transcript:Zm00001d007522_T023 transcript:EES08750 0
529- 1: transcript:Zm00001d007529_T001 transcript:KXG28797 0
529- 2: transcript:Zm00001d007531_T001 transcript:KXG28799 0
529- 3: transcript:Zm00001d007549_T003 transcript:KXG28800 1.00E-149
529- 4: transcript:Zm00001d007550_T001 transcript:EES10013 2.00E-122
529- 5: transcript:Zm00001d007553_T001 transcript:KXG28801 1.00E-60
529- 6: transcript:Zm00001d007571_T001 transcript:OQU83756 0
529- 7: transcript:Zm00001d007572_T001 transcript:OQU83761 0
529- 8: transcript:Zm00001d007573_T001 transcript:OQU83765 0
529- 9: transcript:Zm00001d007575_T001 transcript:KXG28822 7.00E-96
Alignment 530: score=372.0 e_value=3.6e-18 N=9 2&5 plus
530- 0: transcript:Zm00001d007407_T024 transcript:OQU83580 0
530- 1: transcript:Zm00001d007411_T001 transcript:EES08607 4.00E-82
530- 2: transcript:Zm00001d007417_T001 transcript:EES08608 0
530- 3: transcript:Zm00001d007418_T001 transcript:OQU83581 3.00E-77
530- 4: transcript:Zm00001d007419_T001 transcript:EES09869 0
530- 5: transcript:Zm00001d007420_T001 transcript:OQU83582 1.00E-58
530- 6: transcript:Zm00001d007421_T004 transcript:OQU83587 0
530- 7: transcript:Zm00001d007422_T008 transcript:EES08617 0
530- 8: transcript:Zm00001d007433_T001 transcript:KXG28588 3.00E-72
Alignment 531: score=286.0 e_value=4.3e-09 N=6 2&5 plus

531- 0: transcript:Zm00001d007331_T001 transcript:EES08514 4.00E-127
531- 1: transcript:Zm00001d007334_T001 transcript:OQU83467 0
531- 2: transcript:Zm00001d007339_T001 transcript:OQU83468 0
531- 3: transcript:Zm00001d007341_T001 transcript:EES08516 2.00E-75
531- 4: transcript:Zm00001d007345_T001 transcript:EES08518 0
531- 5: transcript:Zm00001d007347_T001 transcript:EES09765 8.00E-72
Alignment 532: score=268.0 e_value=2.2e-08 N=6 2&5 plus
532- 0: transcript:Zm00001d007388_T005 transcript:EES08581 0
532- 1: transcript:Zm00001d007390_T001 transcript:EES08586 2.00E-81
532- 2: transcript:Zm00001d007391_T005 transcript:EES09845 0
532- 3: transcript:Zm00001d007394_T001 transcript:EES09846 5.00E-73
532- 4: transcript:Zm00001d007395_T001 transcript:KXG28532 0
532- 5: transcript:Zm00001d007397_T003 transcript:KXG28538 0
Alignment 533: score=257.0 e_value=1.8e-09 N=6 2&5 plus
533- 0: transcript:Zm00001d007632_T003 transcript:KXG28995 0
533- 1: transcript:Zm00001d007633_T001 transcript:EES10163 4.00E-41
533- 2: transcript:Zm00001d007636_T002 transcript:EES10164 0
533- 3: transcript:Zm00001d007638_T001 transcript:EES10167 0
533- 4: transcript:Zm00001d007639_T003 transcript:KXG29006 0
533- 5: transcript:Zm00001d007645_T002 transcript:EES08899 1.00E-160
Alignment 534: score=665.0 e_value=1.4e-41 N=16 2&5 minus
534- 0: transcript:Zm00001d004992_T001 transcript:EES09210 0
534- 1: transcript:Zm00001d004993_T003 transcript:EES09209 0
534- 2: transcript:Zm00001d004994_T001 transcript:EES09206 4.00E-39
534- 3: transcript:Zm00001d005001_T001 transcript:EES09204 0
534- 4: transcript:Zm00001d005003_T002 transcript:EES09201 2.00E-118
534- 5: transcript:Zm00001d005004_T001 transcript:EES07926 0
534- 6: transcript:Zm00001d005005_T001 transcript:OQU82801 3.00E-42
534- 7: transcript:Zm00001d005006_T003 transcript:EES09188 0
534- 8: transcript:Zm00001d005007_T001 transcript:OQU82792 3.00E-19
534- 9: transcript:Zm00001d005008_T002 transcript:EES09186 0
534- 10: transcript:Zm00001d005010_T001 transcript:EES09185 4.00E-117
534- 11: transcript:Zm00001d005011_T001 transcript:EES07919 9.00E-35
534- 12: transcript:Zm00001d005012_T002 transcript:EES09184 0
534- 13: transcript:Zm00001d005015_T001 transcript:OQU82789 0
534- 14: transcript:Zm00001d005016_T005 transcript:EES09182 0
534- 15: transcript:Zm00001d005018_T001 transcript:EES09180 0
Alignment 535: score=601.0 e_value=1e-37 N=14 2&5 minus
535- 0: transcript:Zm00001d004924_T001 transcript:EES07975 8.00E-136
535- 1: transcript:Zm00001d004925_T001 transcript:KXG27729 0
535- 2: transcript:Zm00001d004927_T001 transcript:OQU82869 2.00E-103
535- 3: transcript:Zm00001d004929_T001 transcript:OQU82868 0
535- 4: transcript:Zm00001d004930_T001 transcript:EES07974 0
535- 5: transcript:Zm00001d004931_T001 transcript:EES07966 8.00E-39
535- 6: transcript:Zm00001d004933_T001 transcript:KXG27714 4.00E-157
535- 7: transcript:Zm00001d004934_T001 transcript:EES07962 0
535- 8: transcript:Zm00001d004936_T001 transcript:EES07961 4.00E-48
535- 9: transcript:Zm00001d004955_T001 transcript:OQU82859 0
535- 10: transcript:Zm00001d004956_T001 transcript:EES09238 1.00E-135
535- 11: transcript:Zm00001d004957_T001 transcript:OQU82855 0
535- 12: transcript:Zm00001d004959_T003 transcript:EES09236 2.00E-166
535- 13: transcript:Zm00001d004960_T002 transcript:EES07951 0
Alignment 536: score=526.0 e_value=2.5e-32 N=13 2&5 minus
536- 0: transcript:Zm00001d004889_T001 transcript:OQU82902 8.00E-124

```
536-  1: transcript:Zm00001d004894_T001 transcript:EES09292            1.00E-110
536-  2: transcript:Zm00001d004895_T001 transcript:KXG27770            4.00E-154
536-  3: transcript:Zm00001d004896_T002 transcript:OQU82899             7.00E-93
536-  4: transcript:Zm00001d004897_T001 transcript:KXG27769            2.00E-123
536-  5: transcript:Zm00001d004898_T002 transcript:EES08001                    0
536-  6: transcript:Zm00001d004903_T001 transcript:KXG27760            5.00E-129
536-  7: transcript:Zm00001d004908_T001 transcript:EES09276            2.00E-103
536-  8: transcript:Zm00001d004909_T001 transcript:KXG27755            2.00E-157
536-  9: transcript:Zm00001d004910_T001 transcript:EES09275             9.00E-38
536- 10: transcript:Zm00001d004912_T001 transcript:OQU82890             4.00E-18
536- 11: transcript:Zm00001d004913_T001 transcript:EES07996            1.00E-159
536- 12: transcript:Zm00001d004915_T001 transcript:KXG27750                    0
## Alignment 537: score=424.0 e_value=2.8e-22 N=10 2&5 minus
537-  0: transcript:Zm00001d004861_T002 transcript:KXG27795                    0
537-  1: transcript:Zm00001d004862_T001 transcript:KXG27794             5.00E-51
537-  2: transcript:Zm00001d004865_T001 transcript:EES09309             6.00E-38
537-  3: transcript:Zm00001d004868_T001 transcript:EES08020                    0
537-  4: transcript:Zm00001d004874_T001 transcript:EES08015             6.00E-20
537-  5: transcript:Zm00001d004875_T002 transcript:EES08014                    0
537-  6: transcript:Zm00001d004876_T001 transcript:OQU82910            1.00E-102
537-  7: transcript:Zm00001d004877_T003 transcript:KXG27779             8.00E-66
537-  8: transcript:Zm00001d004881_T001 transcript:EES09297             3.00E-78
537-  9: transcript:Zm00001d004882_T001 transcript:EES09295            7.00E-113
## Alignment 538: score=361.0 e_value=9.1e-19 N=9 2&5 minus
538-  0: transcript:EER96837            transcript:Zm00001d018194_T001  2.00E-66
538-  1: transcript:EER99004            transcript:Zm00001d018193_T001  1.00E-58
538-  2: transcript:KXG35755            transcript:Zm00001d018183_T001 2.00E-180
538-  3: transcript:EER96846            transcript:Zm00001d018182_T001         0
538-  4: transcript:KXG35763            transcript:Zm00001d018179_T015  1.00E-85
538-  5: transcript:EER96852            transcript:Zm00001d018159_T001         0
538-  6: transcript:EER96855            transcript:Zm00001d018158_T001  3.00E-34
538-  7: transcript:EER96862            transcript:Zm00001d018150_T001  5.00E-30
538-  8: transcript:EER99015            transcript:Zm00001d018149_T002  1.00E-11
## Alignment 539: score=355.0 e_value=1e-16 N=8 2&5 minus
539-  0: transcript:Zm00001d005075_T001 transcript:OQU82693             6.00E-32
539-  1: transcript:Zm00001d005080_T002 transcript:KXG27560                    0
539-  2: transcript:Zm00001d005081_T001 transcript:EES09094             4.00E-42
539-  3: transcript:Zm00001d005082_T001 transcript:EES07857                    0
539-  4: transcript:Zm00001d005083_T001 transcript:OQU82689                    0
539-  5: transcript:Zm00001d005085_T002 transcript:OQU82686                    0
539-  6: transcript:Zm00001d005087_T001 transcript:EES09087             2.00E-99
539-  7: transcript:Zm00001d005089_T005 transcript:EES09085            7.00E-138
## Alignment 540: score=311.0 e_value=1.6e-12 N=7 2&5 minus
540-  0: transcript:Zm00001d007351_T001 transcript:EES09797                    0
540-  1: transcript:Zm00001d007352_T001 transcript:KXG28474             7.00E-43
540-  2: transcript:Zm00001d007354_T001 transcript:OQU83503            6.00E-177
540-  3: transcript:Zm00001d007357_T003 transcript:EES09791                    0
540-  4: transcript:Zm00001d007361_T001 transcript:KXG28467             2.00E-55
540-  5: transcript:Zm00001d007363_T001 transcript:OQU83496            3.00E-118
540-  6: transcript:Zm00001d007364_T001 transcript:OQU83493            2.00E-107
## Alignment 541: score=310.0 e_value=5.1e-11 N=7 2&5 minus
541-  0: transcript:Zm00001d005029_T001 transcript:OQU82754                    0
541-  1: transcript:Zm00001d005032_T001 transcript:EES07900                    0
541-  2: transcript:Zm00001d005035_T001 transcript:EES09161                    0
```

541- 3: transcript:Zm00001d005036_T003 transcript:EES09159 6.00E-116
541- 4: transcript:Zm00001d005037_T001 transcript:EES09157 3.00E-50
541- 5: transcript:Zm00001d005038_T001 transcript:EES09154 1.00E-92
541- 6: transcript:Zm00001d005039_T001 transcript:EES07898 4.00E-111
Alignment 542: score=305.0 e_value=9.3e-11 N=7 2&5 minus
542- 0: transcript:Zm00001d004775_T004 transcript:OQU82997 0
542- 1: transcript:Zm00001d004779_T001 transcript:KXG27890 0
542- 2: transcript:Zm00001d004782_T005 transcript:EES08084 0
542- 3: transcript:Zm00001d004784_T001 transcript:EES09396 0
542- 4: transcript:Zm00001d004790_T008 transcript:EES09393 0
542- 5: transcript:Zm00001d004804_T001 transcript:EES09390 0
542- 6: transcript:Zm00001d004805_T001 transcript:OQU82990 1.00E-17
Alignment 543: score=252.0 e_value=7.2e-10 N=6 2&5 minus
543- 0: transcript:Zm00001d005052_T002 transcript:EES09143 0
543- 1: transcript:Zm00001d005053_T001 transcript:EES07894 2.00E-103
543- 2: transcript:Zm00001d005055_T001 transcript:EES07893 3.00E-76
543- 3: transcript:Zm00001d005056_T001 transcript:OQU82732 9.00E-20
543- 4: transcript:Zm00001d005057_T001 transcript:OQU82725 2.00E-129
543- 5: transcript:Zm00001d005060_T001 transcript:EES09132 2.00E-52
Alignment 544: score=1064.0 e_value=4.8e-76 N=24 2&6 plus
544- 0: transcript:Zm00001d002876_T001 transcript:EES13137 0
544- 1: transcript:Zm00001d002878_T001 transcript:OQU82621 1.00E-71
544- 2: transcript:Zm00001d002879_T001 transcript:KXG27486 3.00E-07
544- 3: transcript:Zm00001d002880_T001 transcript:EES11691 0
544- 4: transcript:Zm00001d002881_T001 transcript:KXG27487 0
544- 5: transcript:Zm00001d002882_T001 transcript:EES13145 0
544- 6: transcript:Zm00001d002884_T001 transcript:EES11692 3.00E-107
544- 7: transcript:Zm00001d002888_T001 transcript:EES11693 0
544- 8: transcript:Zm00001d002889_T002 transcript:KXG27493 3.00E-147
544- 9: transcript:Zm00001d002890_T001 transcript:KXG27495 9.00E-115
544- 10: transcript:Zm00001d002891_T001 transcript:EES13147 9.00E-97
544- 11: transcript:Zm00001d002893_T001 transcript:KXG27497 0
544- 12: transcript:Zm00001d002896_T001 transcript:OQU82627 0
544- 13: transcript:Zm00001d002897_T001 transcript:EES13148 0
544- 14: transcript:Zm00001d002904_T008 transcript:EES13154 0
544- 15: transcript:Zm00001d002905_T001 transcript:KXG27500 0
544- 16: transcript:Zm00001d002906_T001 transcript:KXG27501 2.00E-138
544- 17: transcript:Zm00001d002917_T001 transcript:EES11699 1.00E-127
544- 18: transcript:Zm00001d002919_T001 transcript:KXG27505 0
544- 19: transcript:Zm00001d002928_T001 transcript:KXG27507 3.00E-96
544- 20: transcript:Zm00001d002929_T001 transcript:EES13156 0
544- 21: transcript:Zm00001d002933_T014 transcript:EES13157 0
544- 22: transcript:Zm00001d002934_T001 transcript:EES13158 1.00E-177
544- 23: transcript:Zm00001d002936_T024 transcript:EES11709 0
Alignment 545: score=659.0 e_value=1.3e-32 N=14 2&6 plus
545- 0: transcript:Zm00001d003044_T001 transcript:OQU81967 0
545- 1: transcript:Zm00001d003047_T001 transcript:OQU81970 0
545- 2: transcript:Zm00001d003048_T001 transcript:EES12509 0
545- 3: transcript:Zm00001d003049_T003 transcript:KXG26713 0
545- 4: transcript:Zm00001d003050_T001 transcript:KXG26715 0
545- 5: transcript:Zm00001d003051_T001 transcript:EES11113 5.00E-67
545- 6: transcript:Zm00001d003052_T001 transcript:KXG26719 2.00E-154
545- 7: transcript:Zm00001d003058_T001 transcript:OQU81978 5.00E-22
545- 8: transcript:Zm00001d003060_T001 transcript:OQU81979 0

545- 9: transcript:Zm00001d003062_T001 transcript:EES11116 4.00E-120
545- 10: transcript:Zm00001d003064_T001 transcript:EES12516 2.00E-148
545- 11: transcript:Zm00001d003066_T001 transcript:EES12517 0
545- 12: transcript:Zm00001d003068_T001 transcript:EES11118 0
545- 13: transcript:Zm00001d003069_T001 transcript:OQU81982 0
Alignment 546: score=619.0 e_value=2.5e-35 N=14 2&6 plus
546- 0: transcript:Zm00001d002775_T002 transcript:EES11201 0
546- 1: transcript:Zm00001d002776_T001 transcript:EES12581 0
546- 2: transcript:Zm00001d002778_T001 transcript:OQU82059 2.00E-161
546- 3: transcript:Zm00001d002781_T003 transcript:KXG26818 0
546- 4: transcript:Zm00001d002782_T001 transcript:OQU82063 1.00E-99
546- 5: transcript:Zm00001d002783_T001 transcript:OQU82065 0
546- 6: transcript:Zm00001d002784_T001 transcript:OQU82069 1.00E-85
546- 7: transcript:Zm00001d002787_T001 transcript:EES12588 0
546- 8: transcript:Zm00001d002788_T001 transcript:EES12591 5.00E-115
546- 9: transcript:Zm00001d002789_T001 transcript:EES12592 0
546- 10: transcript:Zm00001d002790_T001 transcript:KXG26822 2.00E-159
546- 11: transcript:Zm00001d002791_T002 transcript:EES12594 0
546- 12: transcript:Zm00001d002794_T001 transcript:KXG26823 9.00E-134
546- 13: transcript:Zm00001d002796_T004 transcript:EES12597 9.00E-174
Alignment 547: score=511.0 e_value=5.4e-24 N=11 2&6 plus
547- 0: transcript:Zm00001d003024_T001 transcript:KXG26691 4.00E-71
547- 1: transcript:Zm00001d003025_T001 transcript:KXG26692 0
547- 2: transcript:Zm00001d003031_T002 transcript:OQU81941 0
547- 3: transcript:Zm00001d003033_T005 transcript:EES11106 0
547- 4: transcript:Zm00001d003034_T001 transcript:EES12483 0
547- 5: transcript:Zm00001d003037_T002 transcript:EES11108 4.00E-23
547- 6: transcript:Zm00001d003038_T001 transcript:OQU81947 0
547- 7: transcript:Zm00001d003039_T001 transcript:EES12487 0
547- 8: transcript:Zm00001d003040_T001 transcript:EES12490 0
547- 9: transcript:Zm00001d003041_T001 transcript:EES12491 1.00E-60
547- 10: transcript:Zm00001d003044_T001 transcript:OQU81951 0
Alignment 548: score=380.0 e_value=2.1e-19 N=9 2&6 plus
548- 0: transcript:Zm00001d004126_T001 transcript:EES10507 0
548- 1: transcript:Zm00001d004129_T001 transcript:KXG25916 7.00E-52
548- 2: transcript:Zm00001d004132_T001 transcript:EES10519 0
548- 3: transcript:Zm00001d004133_T001 transcript:EES11875 0
548- 4: transcript:Zm00001d004134_T001 transcript:OQU81236 1.00E-147
548- 5: transcript:Zm00001d004136_T001 transcript:EES10523 0
548- 6: transcript:Zm00001d004138_T001 transcript:EES11880 1.00E-105
548- 7: transcript:Zm00001d004139_T003 transcript:KXG25921 0
548- 8: transcript:Zm00001d004140_T001 transcript:OQU81241 0
Alignment 549: score=354.0 e_value=2.3e-19 N=9 2&6 plus
549- 0: transcript:Zm00001d002179_T001 transcript:OQU82382 9.00E-113
549- 1: transcript:Zm00001d002182_T001 transcript:OQU82383 7.00E-102
549- 2: transcript:Zm00001d002184_T003 transcript:OQU82385 2.00E-81
549- 3: transcript:Zm00001d002185_T003 transcript:KXG27171 0
549- 4: transcript:Zm00001d002186_T001 transcript:EES12911 0
549- 5: transcript:Zm00001d002190_T001 transcript:KXG27174 0
549- 6: transcript:Zm00001d002191_T001 transcript:EES11491 0
549- 7: transcript:Zm00001d002192_T003 transcript:EES11493 4.00E-36
549- 8: transcript:Zm00001d002198_T002 transcript:KXG27204 7.00E-127
Alignment 550: score=296.0 e_value=4.5e-13 N=7 2&6 plus
550- 0: transcript:Zm00001d002938_T001 transcript:EES13161 0

550- 1: transcript:Zm00001d002939_T001 transcript:OQU82647 0
550- 2: transcript:Zm00001d002940_T001 transcript:OQU82649 0
550- 3: transcript:Zm00001d002941_T001 transcript:EES13163 0
550- 4: transcript:Zm00001d002942_T002 transcript:EES13164 3.00E-159
550- 5: transcript:Zm00001d002943_T002 transcript:EES11713 0
550- 6: transcript:Zm00001d002945_T010 transcript:OQU82661 0
Alignment 551: score=291.0 e_value=1.9e-13 N=7 2&6 plus
551- 0: transcript:Zm00001d002734_T001 transcript:OQU82091 0
551- 1: transcript:Zm00001d002736_T001 transcript:OQU82092 0
551- 2: transcript:Zm00001d002738_T001 transcript:OQU82093 6.00E-62
551- 3: transcript:Zm00001d002739_T004 transcript:KXG26851 0
551- 4: transcript:Zm00001d002741_T001 transcript:KXG26853 0
551- 5: transcript:Zm00001d002742_T002 transcript:EES12618 0
551- 6: transcript:Zm00001d002743_T001 transcript:OQU82096 6.00E-32
Alignment 552: score=270.0 e_value=1.8e-08 N=6 2&6 plus
552- 0: transcript:Zm00001d002204_T001 transcript:EES12892 3.00E-151
552- 1: transcript:Zm00001d002209_T001 transcript:EES12893 1.00E-72
552- 2: transcript:Zm00001d002214_T001 transcript:EES12896 1.00E-64
552- 3: transcript:Zm00001d002220_T001 transcript:EES11475 8.00E-80
552- 4: transcript:Zm00001d002226_T001 transcript:EES12897 3.00E-25
552- 5: transcript:Zm00001d002231_T001 transcript:OQU82382 3.00E-112
Alignment 553: score=4426.0 e_value=0 N=97 2&6 minus
553- 0: transcript:Zm00001d003369_T004 transcript:OQU81742 0
553- 1: transcript:Zm00001d003370_T001 transcript:KXG26505 5.00E-74
553- 2: transcript:Zm00001d003372_T001 transcript:EES12317 0
553- 3: transcript:Zm00001d003373_T002 transcript:KXG26501 3.00E-112
553- 4: transcript:Zm00001d003375_T001 transcript:KXG26500 4.00E-36
553- 5: transcript:Zm00001d003377_T001 transcript:OQU81736 0
553- 6: transcript:Zm00001d003378_T001 transcript:EES10963 0
553- 7: transcript:Zm00001d003379_T001 transcript:EES10962 4.00E-100
553- 8: transcript:Zm00001d003380_T001 transcript:OQU81731 3.00E-50
553- 9: transcript:Zm00001d003381_T001 transcript:OQU81730 0
553- 10: transcript:Zm00001d003382_T002 transcript:EES10959 0
553- 11: transcript:Zm00001d003386_T001 transcript:KXG26490 1.00E-22
553- 12: transcript:Zm00001d003392_T001 transcript:OQU81721 0
553- 13: transcript:Zm00001d003393_T002 transcript:OQU81720 0
553- 14: transcript:Zm00001d003394_T001 transcript:EES12304 0
553- 15: transcript:Zm00001d003395_T001 transcript:KXG26484 1.00E-180
553- 16: transcript:Zm00001d003396_T001 transcript:EES10956 0
553- 17: transcript:Zm00001d003398_T001 transcript:OQU81717 5.00E-145
553- 18: transcript:Zm00001d003399_T001 transcript:KXG26481 0
553- 19: transcript:Zm00001d003400_T001 transcript:EES10953 5.00E-88
553- 20: transcript:Zm00001d003401_T003 transcript:EES10954 0
553- 21: transcript:Zm00001d003403_T001 transcript:KXG26478 0
553- 22: transcript:Zm00001d003404_T001 transcript:OQU81715 2.00E-146
553- 23: transcript:Zm00001d003405_T001 transcript:EES10952 5.00E-87
553- 24: transcript:Zm00001d003406_T002 transcript:EES12298 0
553- 25: transcript:Zm00001d003408_T001 transcript:OQU81712 2.00E-74
553- 26: transcript:Zm00001d003411_T001 transcript:EES10949 1.00E-88
553- 27: transcript:Zm00001d003412_T001 transcript:KXG26475 0
553- 28: transcript:Zm00001d003414_T001 transcript:EES10946 1.00E-162
553- 29: transcript:Zm00001d003415_T002 transcript:EES10945 2.00E-138
553- 30: transcript:Zm00001d003417_T001 transcript:KXG26473 0
553- 31: transcript:Zm00001d003418_T001 transcript:EES10944 7.00E-115

553- 32:	transcript:Zm00001d003420_T001	transcript:KXG26471	3.00E-26
553- 33:	transcript:Zm00001d003422_T001	transcript:EES10943	0
553- 34:	transcript:Zm00001d003423_T002	transcript:EES10942	1.00E-166
553- 35:	transcript:Zm00001d003425_T001	transcript:EES10941	2.00E-19
553- 36:	transcript:Zm00001d003426_T002	transcript:EES10940	0
553- 37:	transcript:Zm00001d003427_T001	transcript:KXG26469	0
553- 38:	transcript:Zm00001d003428_T001	transcript:OQU81707	5.00E-133
553- 39:	transcript:Zm00001d003429_T001	transcript:KXG26463	0
553- 40:	transcript:Zm00001d003430_T001	transcript:OQU81705	0
553- 41:	transcript:Zm00001d003431_T009	transcript:EES12286	0
553- 42:	transcript:Zm00001d003434_T002	transcript:EES10934	9.00E-81
553- 43:	transcript:Zm00001d003438_T001	transcript:EES10931	0
553- 44:	transcript:Zm00001d003446_T002	transcript:OQU81698	0
553- 45:	transcript:Zm00001d003447_T001	transcript:EES12282	6.00E-31
553- 46:	transcript:Zm00001d003448_T002	transcript:EES12281	0
553- 47:	transcript:Zm00001d003451_T001	transcript:KXG26452	0
553- 48:	transcript:Zm00001d003457_T001	transcript:EES10928	2.00E-100
553- 49:	transcript:Zm00001d003459_T001	transcript:EES12280	1.00E-97
553- 50:	transcript:Zm00001d003460_T001	transcript:OQU81690	0
553- 51:	transcript:Zm00001d003462_T001	transcript:EES12278	0
553- 52:	transcript:Zm00001d003463_T001	transcript:EES12277	1.00E-82
553- 53:	transcript:Zm00001d003464_T001	transcript:EES12276	0
553- 54:	transcript:Zm00001d003468_T001	transcript:EES10921	4.00E-145
553- 55:	transcript:Zm00001d003470_T001	transcript:EES12270	0
553- 56:	transcript:Zm00001d003476_T002	transcript:EES12269	0
553- 57:	transcript:Zm00001d003477_T001	transcript:KXG26438	0
553- 58:	transcript:Zm00001d003482_T001	transcript:EES10915	3.00E-08
553- 59:	transcript:Zm00001d003483_T001	transcript:EES12267	0
553- 60:	transcript:Zm00001d003491_T005	transcript:EES12266	0
553- 61:	transcript:Zm00001d003492_T001	transcript:KXG26433	0
553- 62:	transcript:Zm00001d003493_T002	transcript:KXG26432	1.00E-94
553- 63:	transcript:Zm00001d003494_T004	transcript:OQU81673	3.00E-81
553- 64:	transcript:Zm00001d003495_T008	transcript:OQU81672	0
553- 65:	transcript:Zm00001d003497_T001	transcript:OQU81670	2.00E-153
553- 66:	transcript:Zm00001d003499_T001	transcript:KXG26428	0
553- 67:	transcript:Zm00001d003500_T001	transcript:EES10908	0
553- 68:	transcript:Zm00001d003502_T003	transcript:EES10907	1.00E-85
553- 69:	transcript:Zm00001d003504_T002	transcript:KXG26427	1.00E-162
553- 70:	transcript:Zm00001d003505_T001	transcript:KXG26420	5.00E-43
553- 71:	transcript:Zm00001d003508_T001	transcript:EES12256	0
553- 72:	transcript:Zm00001d003509_T001	transcript:KXG26417	0
553- 73:	transcript:Zm00001d003510_T001	transcript:EES12255	0
553- 74:	transcript:Zm00001d003511_T001	transcript:KXG26414	0
553- 75:	transcript:Zm00001d003512_T001	transcript:EES12253	0
553- 76:	transcript:Zm00001d003514_T001	transcript:KXG26411	6.00E-38
553- 77:	transcript:Zm00001d003515_T001	transcript:EES10894	4.00E-70
553- 78:	transcript:Zm00001d003518_T003	transcript:EES10893	0
553- 79:	transcript:Zm00001d003520_T001	transcript:KXG26410	0
553- 80:	transcript:Zm00001d003521_T001	transcript:KXG26409	2.00E-94
553- 81:	transcript:Zm00001d003522_T002	transcript:EES10890	0
553- 82:	transcript:Zm00001d003530_T001	transcript:EES10885	1.00E-135
553- 83:	transcript:Zm00001d003531_T002	transcript:EES12248	0
553- 84:	transcript:Zm00001d003533_T001	transcript:EES10882	0
553- 85:	transcript:Zm00001d003534_T001	transcript:KXG26355	0

553- 86: transcript:Zm00001d003535_T001 transcript:EES10880 2.00E-46
553- 87: transcript:Zm00001d003538_T001 transcript:EES12247 1.00E-26
553- 88: transcript:Zm00001d003540_T003 transcript:EES10879 5.00E-125
553- 89: transcript:Zm00001d003543_T001 transcript:OQU81656 0
553- 90: transcript:Zm00001d003544_T003 transcript:EES10878 3.00E-139
553- 91: transcript:Zm00001d003545_T001 transcript:OQU81655 1.00E-38
553- 92: transcript:Zm00001d003546_T001 transcript:EES10876 7.00E-88
553- 93: transcript:Zm00001d003549_T001 transcript:EES12241 0
553- 94: transcript:Zm00001d003550_T001 transcript:EES10871 4.00E-88
553- 95: transcript:Zm00001d003552_T001 transcript:KXG26394 3.00E-67
553- 96: transcript:Zm00001d003553_T001 transcript:KXG26392 0
Alignment 554: score=3867.0 e_value=0 N=88 2&6 minus
554- 0: transcript:Zm00001d003088_T001 transcript:KXG26673 2.00E-150
554- 1: transcript:Zm00001d003089_T001 transcript:EES12465 0
554- 2: transcript:Zm00001d003091_T001 transcript:EES11087 0
554- 3: transcript:Zm00001d003099_T003 transcript:OQU81913 0
554- 4: transcript:Zm00001d003101_T013 transcript:KXG26669 0
554- 5: transcript:Zm00001d003102_T001 transcript:OQU81911 0
554- 6: transcript:Zm00001d003103_T001 transcript:KXG26667 0
554- 7: transcript:Zm00001d003106_T003 transcript:EES12460 0
554- 8: transcript:Zm00001d003107_T001 transcript:OQU81905 3.00E-80
554- 9: transcript:Zm00001d003108_T007 transcript:KXG26662 1.00E-125
554- 10: transcript:Zm00001d003109_T002 transcript:KXG26658 0
554- 11: transcript:Zm00001d003110_T002 transcript:OQU81903 0
554- 12: transcript:Zm00001d003112_T004 transcript:OQU81900 0
554- 13: transcript:Zm00001d003114_T006 transcript:KXG26657 0
554- 14: transcript:Zm00001d003116_T001 transcript:EES11079 0
554- 15: transcript:Zm00001d003118_T001 transcript:KXG26656 6.00E-82
554- 16: transcript:Zm00001d003119_T001 transcript:KXG26655 0
554- 17: transcript:Zm00001d003121_T001 transcript:EES12456 2.00E-68
554- 18: transcript:Zm00001d003123_T001 transcript:OQU81892 0
554- 19: transcript:Zm00001d003124_T001 transcript:EES11076 0
554- 20: transcript:Zm00001d003125_T001 transcript:EES11075 0
554- 21: transcript:Zm00001d003127_T003 transcript:KXG26651 3.00E-89
554- 22: transcript:Zm00001d003129_T001 transcript:EES11073 8.00E-89
554- 23: transcript:Zm00001d003139_T001 transcript:EES11072 5.00E-63
554- 24: transcript:Zm00001d003144_T001 transcript:EES11071 0
554- 25: transcript:Zm00001d003147_T001 transcript:EES12452 0
554- 26: transcript:Zm00001d003148_T002 transcript:EES12451 3.00E-62
554- 27: transcript:Zm00001d003149_T001 transcript:EES11070 0
554- 28: transcript:Zm00001d003153_T004 transcript:EES12450 0
554- 29: transcript:Zm00001d003157_T001 transcript:OQU81878 0
554- 30: transcript:Zm00001d003160_T001 transcript:OQU81872 0
554- 31: transcript:Zm00001d003161_T001 transcript:OQU81871 0
554- 32: transcript:Zm00001d003162_T001 transcript:EES12446 0
554- 33: transcript:Zm00001d003164_T001 transcript:KXG26638 0
554- 34: transcript:Zm00001d003166_T006 transcript:EES12441 0
554- 35: transcript:Zm00001d003167_T002 transcript:KXG26636 6.00E-59
554- 36: transcript:Zm00001d003172_T001 transcript:OQU81867 8.00E-134
554- 37: transcript:Zm00001d003173_T001 transcript:OQU81866 8.00E-155
554- 38: transcript:Zm00001d003175_T001 transcript:OQU81865 2.00E-88
554- 39: transcript:Zm00001d003176_T001 transcript:KXG26633 0
554- 40: transcript:Zm00001d003179_T001 transcript:KXG26632 1.00E-123
554- 41: transcript:Zm00001d003180_T001 transcript:EES12438 0

554- 42: transcript:Zm00001d003181_T005 transcript:EES11058 0
554- 43: transcript:Zm00001d003182_T001 transcript:EES11057 9.00E-91
554- 44: transcript:Zm00001d003184_T001 transcript:EES11054 3.00E-162
554- 45: transcript:Zm00001d003188_T001 transcript:OQU81860 1.00E-107
554- 46: transcript:Zm00001d003193_T001 transcript:KXG26623 2.00E-81
554- 47: transcript:Zm00001d003195_T001 transcript:EES12431 2.00E-32
554- 48: transcript:Zm00001d003196_T001 transcript:EES11049 0
554- 49: transcript:Zm00001d003197_T001 transcript:EES11048 3.00E-101
554- 50: transcript:Zm00001d003198_T010 transcript:KXG26622 0
554- 51: transcript:Zm00001d003200_T002 transcript:OQU81856 0
554- 52: transcript:Zm00001d003203_T014 transcript:EES11045 0
554- 53: transcript:Zm00001d003205_T001 transcript:EES11044 4.00E-110
554- 54: transcript:Zm00001d003207_T010 transcript:EES11042 0
554- 55: transcript:Zm00001d003208_T001 transcript:KXG26617 0
554- 56: transcript:Zm00001d003212_T001 transcript:EES11040 0
554- 57: transcript:Zm00001d003213_T001 transcript:EES11039 0
554- 58: transcript:Zm00001d003214_T002 transcript:EES11038 0
554- 59: transcript:Zm00001d003217_T001 transcript:OQU81845 1.00E-25
554- 60: transcript:Zm00001d003220_T001 transcript:EES11033 0
554- 61: transcript:Zm00001d003222_T016 transcript:EES12419 0
554- 62: transcript:Zm00001d003223_T001 transcript:EES12417 3.00E-116
554- 63: transcript:Zm00001d003226_T001 transcript:EES11032 1.00E-94
554- 64: transcript:Zm00001d003227_T002 transcript:OQU81835 0
554- 65: transcript:Zm00001d003228_T001 transcript:EES11030 0
554- 66: transcript:Zm00001d003229_T002 transcript:EES12415 0
554- 67: transcript:Zm00001d003232_T001 transcript:OQU81830 6.00E-21
554- 68: transcript:Zm00001d003234_T001 transcript:EES11027 1.00E-100
554- 69: transcript:Zm00001d003242_T001 transcript:KXG26596 2.00E-128
554- 70: transcript:Zm00001d003245_T001 transcript:EES11026 0
554- 71: transcript:Zm00001d003246_T001 transcript:EES11025 0
554- 72: transcript:Zm00001d003247_T002 transcript:KXG26594 0
554- 73: transcript:Zm00001d003248_T003 transcript:EES11024 2.00E-80
554- 74: transcript:Zm00001d003249_T001 transcript:EES12410 1.00E-114
554- 75: transcript:Zm00001d003250_T001 transcript:EES12408 0
554- 76: transcript:Zm00001d003251_T001 transcript:EES11020 0
554- 77: transcript:Zm00001d003252_T002 transcript:KXG26589 0
554- 78: transcript:Zm00001d003254_T008 transcript:EES11019 6.00E-142
554- 79: transcript:Zm00001d003256_T001 transcript:KXG26588 0
554- 80: transcript:Zm00001d003257_T002 transcript:KXG26586 0
554- 81: transcript:Zm00001d003258_T001 transcript:EES12405 0
554- 82: transcript:Zm00001d003259_T006 transcript:KXG26585 0
554- 83: transcript:Zm00001d003261_T003 transcript:OQU81824 0
554- 84: transcript:Zm00001d003262_T004 transcript:EES11014 0
554- 85: transcript:Zm00001d003264_T001 transcript:EES11012 3.00E-72
554- 86: transcript:Zm00001d003265_T003 transcript:EES12400 0
554- 87: transcript:Zm00001d003266_T003 transcript:KXG26579 0
Alignment 555: score=3219.0 e_value=5.5e-297 N=73 2&6 minus
555- 0: transcript:Zm00001d002512_T001 transcript:EES12746 4.00E-121
555- 1: transcript:Zm00001d002515_T001 transcript:EES11332 0
555- 2: transcript:Zm00001d002517_T001 transcript:EES11330 6.00E-60
555- 3: transcript:Zm00001d002519_T001 transcript:KXG27003 2.00E-168
555- 4: transcript:Zm00001d002520_T001 transcript:EES12739 9.00E-26
555- 5: transcript:Zm00001d002522_T001 transcript:EES12737 9.00E-15
555- 6: transcript:Zm00001d002523_T001 transcript:KXG27001 5.00E-19

555- 7:	transcript:Zm00001d002531_T001	transcript:OQU82212	0
555- 8:	transcript:Zm00001d002532_T003	transcript:KXG26996	0
555- 9:	transcript:Zm00001d002534_T001	transcript:EES12729	0
555- 10:	transcript:Zm00001d002535_T001	transcript:EES12728	4.00E-90
555- 11:	transcript:Zm00001d002536_T001	transcript:EES11324	0
555- 12:	transcript:Zm00001d002537_T002	transcript:EES12727	0
555- 13:	transcript:Zm00001d002538_T001	transcript:EES11323	0
555- 14:	transcript:Zm00001d002539_T001	transcript:KXG26994	0
555- 15:	transcript:Zm00001d002540_T002	transcript:EES11322	0
555- 16:	transcript:Zm00001d002541_T001	transcript:EES11319	0
555- 17:	transcript:Zm00001d002542_T001	transcript:KXG26992	0
555- 18:	transcript:Zm00001d002544_T004	transcript:KXG26989	0
555- 19:	transcript:Zm00001d002546_T001	transcript:EES12723	7.00E-67
555- 20:	transcript:Zm00001d002548_T001	transcript:EES11318	0
555- 21:	transcript:Zm00001d002549_T002	transcript:EES12722	2.00E-20
555- 22:	transcript:Zm00001d002550_T001	transcript:OQU82206	0
555- 23:	transcript:Zm00001d002551_T001	transcript:EES11273	4.00E-138
555- 24:	transcript:Zm00001d002556_T001	transcript:EES11315	1.00E-30
555- 25:	transcript:Zm00001d002558_T001	transcript:OQU82200	3.00E-69
555- 26:	transcript:Zm00001d002562_T001	transcript:EES12712	0
555- 27:	transcript:Zm00001d002564_T001	transcript:KXG26978	1.00E-129
555- 28:	transcript:Zm00001d002565_T001	transcript:KXG26977	6.00E-66
555- 29:	transcript:Zm00001d002568_T001	transcript:EES12708	4.00E-78
555- 30:	transcript:Zm00001d002569_T001	transcript:KXG26973	0
555- 31:	transcript:Zm00001d002572_T002	transcript:EES12704	2.00E-52
555- 32:	transcript:Zm00001d002573_T001	transcript:OQU82196	0
555- 33:	transcript:Zm00001d002576_T001	transcript:KXG26971	2.00E-100
555- 34:	transcript:Zm00001d002579_T001	transcript:EES12701	0
555- 35:	transcript:Zm00001d002580_T004	transcript:EES11308	0
555- 36:	transcript:Zm00001d002584_T001	transcript:KXG26970	3.00E-35
555- 37:	transcript:Zm00001d002589_T001	transcript:EES11307	0
555- 38:	transcript:Zm00001d002590_T001	transcript:EES11306	6.00E-60
555- 39:	transcript:Zm00001d002591_T001	transcript:EES11305	2.00E-149
555- 40:	transcript:Zm00001d002592_T002	transcript:EES12700	0
555- 41:	transcript:Zm00001d002593_T001	transcript:EES12699	3.00E-73
555- 42:	transcript:Zm00001d002594_T001	transcript:EES11304	1.00E-152
555- 43:	transcript:Zm00001d002595_T002	transcript:EES12698	6.00E-165
555- 44:	transcript:Zm00001d002596_T001	transcript:OQU82191	1.00E-51
555- 45:	transcript:Zm00001d002597_T002	transcript:EES11301	0
555- 46:	transcript:Zm00001d002598_T001	transcript:KXG26964	0
555- 47:	transcript:Zm00001d002599_T002	transcript:OQU82190	0
555- 48:	transcript:Zm00001d002600_T005	transcript:KXG26962	0
555- 49:	transcript:Zm00001d002601_T001	transcript:EES11298	9.00E-55
555- 50:	transcript:Zm00001d002602_T002	transcript:EES12697	0
555- 51:	transcript:Zm00001d002603_T001	transcript:KXG26959	0
555- 52:	transcript:Zm00001d002607_T001	transcript:OQU82187	0
555- 53:	transcript:Zm00001d002609_T001	transcript:EES12695	2.00E-168
555- 54:	transcript:Zm00001d002610_T001	transcript:EES11294	0
555- 55:	transcript:Zm00001d002611_T002	transcript:OQU82186	1.00E-174
555- 56:	transcript:Zm00001d002613_T002	transcript:KXG26955	0
555- 57:	transcript:Zm00001d002614_T001	transcript:OQU82184	0
555- 58:	transcript:Zm00001d002615_T003	transcript:KXG26948	0
555- 59:	transcript:Zm00001d002616_T001	transcript:KXG26947	2.00E-144
555- 60:	transcript:Zm00001d002617_T028	transcript:EES12692	0

555- 61: transcript:Zm00001d002618_T001 transcript:EES11288 4.00E-116
555- 62: transcript:Zm00001d002620_T001 transcript:EES11287 7.00E-114
555- 63: transcript:Zm00001d002621_T002 transcript:KXG26945 8.00E-117
555- 64: transcript:Zm00001d002623_T001 transcript:EES11285 0
555- 65: transcript:Zm00001d002624_T003 transcript:EES11283 2.00E-142
555- 66: transcript:Zm00001d002625_T001 transcript:KXG26943 0
555- 67: transcript:Zm00001d002626_T001 transcript:EES12690 0
555- 68: transcript:Zm00001d002630_T002 transcript:EES12687 0
555- 69: transcript:Zm00001d002631_T003 transcript:KXG26938 0
555- 70: transcript:Zm00001d002632_T001 transcript:EES11282 0
555- 71: transcript:Zm00001d002639_T001 transcript:KXG26934 0
555- 72: transcript:Zm00001d002640_T011 transcript:KXG26932 0
Alignment 556: score=2502.0 e_value=1.3e-216 N=54 2&6 minus
556- 0: transcript:Zm00001d003268_T001 transcript:OQU81813 3.00E-83
556- 1: transcript:Zm00001d003269_T001 transcript:EES12396 4.00E-24
556- 2: transcript:Zm00001d003271_T001 transcript:EES12389 7.00E-128
556- 3: transcript:Zm00001d003272_T001 transcript:KXG26570 0
556- 4: transcript:Zm00001d003274_T002 transcript:EES12384 8.00E-58
556- 5: transcript:Zm00001d003275_T001 transcript:KXG26569 3.00E-166
556- 6: transcript:Zm00001d003276_T001 transcript:EES11004 2.00E-171
556- 7: transcript:Zm00001d003279_T001 transcript:EES12382 2.00E-71
556- 8: transcript:Zm00001d003281_T001 transcript:EES11002 0
556- 9: transcript:Zm00001d003282_T001 transcript:EES11000 5.00E-40
556- 10: transcript:Zm00001d003283_T001 transcript:EES12375 0
556- 11: transcript:Zm00001d003284_T001 transcript:EES10999 0
556- 12: transcript:Zm00001d003287_T001 transcript:KXG26563 2.00E-107
556- 13: transcript:Zm00001d003288_T001 transcript:EES10998 0
556- 14: transcript:Zm00001d003291_T002 transcript:OQU81802 0
556- 15: transcript:Zm00001d003292_T001 transcript:EES12371 0
556- 16: transcript:Zm00001d003293_T001 transcript:OQU81795 2.00E-122
556- 17: transcript:Zm00001d003294_T001 transcript:OQU81794 0
556- 18: transcript:Zm00001d003296_T001 transcript:EES12366 3.00E-60
556- 19: transcript:Zm00001d003297_T001 transcript:EES10995 0
556- 20: transcript:Zm00001d003300_T001 transcript:KXG26553 0
556- 21: transcript:Zm00001d003301_T001 transcript:EES10990 0
556- 22: transcript:Zm00001d003302_T001 transcript:EES10991 0
556- 23: transcript:Zm00001d003308_T001 transcript:KXG26549 0
556- 24: transcript:Zm00001d003309_T002 transcript:EES12364 0
556- 25: transcript:Zm00001d003310_T001 transcript:OQU81792 2.00E-59
556- 26: transcript:Zm00001d003311_T001 transcript:EES12363 0
556- 27: transcript:Zm00001d003313_T001 transcript:EES10982 6.00E-152
556- 28: transcript:Zm00001d003314_T001 transcript:EES12360 6.00E-99
556- 29: transcript:Zm00001d003315_T001 transcript:EES12359 6.00E-101
556- 30: transcript:Zm00001d003317_T001 transcript:OQU81782 0
556- 31: transcript:Zm00001d003319_T001 transcript:KXG26533 0
556- 32: transcript:Zm00001d003320_T001 transcript:OQU81776 2.00E-43
556- 33: transcript:Zm00001d003321_T001 transcript:KXG26530 0
556- 34: transcript:Zm00001d003322_T002 transcript:KXG26529 7.00E-132
556- 35: transcript:Zm00001d003328_T001 transcript:EES12349 0
556- 36: transcript:Zm00001d003329_T003 transcript:EES10975 0
556- 37: transcript:Zm00001d003331_T001 transcript:KXG26525 0
556- 38: transcript:Zm00001d003333_T005 transcript:OQU81762 0
556- 39: transcript:Zm00001d003334_T001 transcript:OQU81760 0
556- 40: transcript:Zm00001d003335_T001 transcript:KXG26524 2.00E-159

556- 41: transcript:Zm00001d003343_T001 transcript:KXG26523 0
556- 42: transcript:Zm00001d003345_T001 transcript:KXG26522 6.00E-54
556- 43: transcript:Zm00001d003346_T001 transcript:EES12339 1.00E-21
556- 44: transcript:Zm00001d003347_T002 transcript:EES10970 9.00E-63
556- 45: transcript:Zm00001d003349_T001 transcript:EES10969 0
556- 46: transcript:Zm00001d003352_T003 transcript:EES12337 0
556- 47: transcript:Zm00001d003353_T001 transcript:EES12334 2.00E-33
556- 48: transcript:Zm00001d003354_T002 transcript:OQU81756 0
556- 49: transcript:Zm00001d003355_T001 transcript:EES12328 4.00E-13
556- 50: transcript:Zm00001d003357_T001 transcript:EES12322 1.00E-18
556- 51: transcript:Zm00001d003363_T001 transcript:EES12324 7.00E-09
556- 52: transcript:Zm00001d003364_T001 transcript:EES12323 5.00E-13
556- 53: transcript:Zm00001d003368_T001 transcript:OQU81751 0
Alignment 557: score=2037.0 e_value=4e-153 N=43 2&6 minus
557- 0: transcript:Zm00001d002952_T001 transcript:EES11105 2.00E-156
557- 1: transcript:Zm00001d002953_T001 transcript:EES11150 0
557- 2: transcript:Zm00001d002954_T002 transcript:EES11149 5.00E-78
557- 3: transcript:Zm00001d002955_T001 transcript:EES12541 5.00E-46
557- 4: transcript:Zm00001d002956_T005 transcript:KXG26761 0
557- 5: transcript:Zm00001d002958_T001 transcript:KXG26758 2.00E-68
557- 6: transcript:Zm00001d002960_T001 transcript:EES12537 2.00E-48
557- 7: transcript:Zm00001d002961_T002 transcript:EES11147 0
557- 8: transcript:Zm00001d002962_T001 transcript:EES11144 0
557- 9: transcript:Zm00001d002965_T001 transcript:EES12536 1.00E-128
557- 10: transcript:Zm00001d002966_T001 transcript:KXG26686 0
557- 11: transcript:Zm00001d002967_T015 transcript:KXG26754 0
557- 12: transcript:Zm00001d002968_T001 transcript:EES11141 0
557- 13: transcript:Zm00001d002969_T001 transcript:KXG26752 8.00E-178
557- 14: transcript:Zm00001d002970_T001 transcript:EES12534 0
557- 15: transcript:Zm00001d002971_T001 transcript:EES12533 1.00E-165
557- 16: transcript:Zm00001d002972_T001 transcript:KXG26749 0
557- 17: transcript:Zm00001d002979_T001 transcript:EES11135 0
557- 18: transcript:Zm00001d002980_T001 transcript:EES11134 0
557- 19: transcript:Zm00001d002982_T001 transcript:EES12531 9.00E-86
557- 20: transcript:Zm00001d002984_T001 transcript:KXG26678 4.00E-60
557- 21: transcript:Zm00001d002989_T001 transcript:EES11132 0
557- 22: transcript:Zm00001d002990_T001 transcript:KXG26740 3.00E-06
557- 23: transcript:Zm00001d002992_T002 transcript:EES12528 0
557- 24: transcript:Zm00001d002993_T001 transcript:KXG26739 9.00E-95
557- 25: transcript:Zm00001d002996_T001 transcript:OQU81997 6.00E-48
557- 26: transcript:Zm00001d002999_T001 transcript:KXG26736 0
557- 27: transcript:Zm00001d003002_T001 transcript:OQU81996 0
557- 28: transcript:Zm00001d003003_T001 transcript:OQU81995 2.00E-57
557- 29: transcript:Zm00001d003004_T001 transcript:KXG26733 0
557- 30: transcript:Zm00001d003005_T001 transcript:EES11125 1.00E-42
557- 31: transcript:Zm00001d003006_T001 transcript:EES11124 0
557- 32: transcript:Zm00001d003007_T001 transcript:EES11123 0
557- 33: transcript:Zm00001d003009_T001 transcript:KXG26730 4.00E-72
557- 34: transcript:Zm00001d003011_T001 transcript:KXG26729 6.00E-76
557- 35: transcript:Zm00001d003012_T001 transcript:EES11121 6.00E-116
557- 36: transcript:Zm00001d003013_T001 transcript:EES11120 1.00E-160
557- 37: transcript:Zm00001d003014_T002 transcript:KXG26728 3.00E-69
557- 38: transcript:Zm00001d003015_T001 transcript:OQU81984 0
557- 39: transcript:Zm00001d003016_T001 transcript:EES12521 0

557- 40: transcript:Zm00001d003017_T002 transcript:EES12520 0
557- 41: transcript:Zm00001d003018_T001 transcript:OQU81983 2.00E-65
557- 42: transcript:Zm00001d003019_T002 transcript:OQU81982 0
Alignment 558: score=1950.0 e_value=1.1e-144 N=41 2&6 minus
558- 0: transcript:Zm00001d002819_T002 transcript:EES12570 0
558- 1: transcript:Zm00001d002820_T001 transcript:EES12569 7.00E-123
558- 2: transcript:Zm00001d002821_T001 transcript:EES12567 1.00E-75
558- 3: transcript:Zm00001d002822_T001 transcript:EES12566 0
558- 4: transcript:Zm00001d002823_T001 transcript:EES11189 0
558- 5: transcript:Zm00001d002824_T001 transcript:EES12565 0
558- 6: transcript:Zm00001d002825_T001 transcript:EES11188 7.00E-156
558- 7: transcript:Zm00001d002826_T001 transcript:EES11187 2.00E-72
558- 8: transcript:Zm00001d002827_T010 transcript:OQU82027 0
558- 9: transcript:Zm00001d002828_T001 transcript:OQU82026 0
558- 10: transcript:Zm00001d002829_T001 transcript:EES12563 4.00E-135
558- 11: transcript:Zm00001d002830_T001 transcript:OQU82023 0
558- 12: transcript:Zm00001d002833_T001 transcript:EES12561 2.00E-121
558- 13: transcript:Zm00001d002834_T001 transcript:EES11184 6.00E-142
558- 14: transcript:Zm00001d002835_T003 transcript:KXG26785 0
558- 15: transcript:Zm00001d002836_T006 transcript:KXG26784 8.00E-168
558- 16: transcript:Zm00001d002837_T001 transcript:KXG26781 1.00E-19
558- 17: transcript:Zm00001d002842_T001 transcript:EES12557 8.00E-115
558- 18: transcript:Zm00001d002843_T001 transcript:EES11178 1.00E-157
558- 19: transcript:Zm00001d002844_T003 transcript:EES12556 2.00E-134
558- 20: transcript:Zm00001d002845_T001 transcript:KXG26779 4.00E-33
558- 21: transcript:Zm00001d002847_T001 transcript:EES11173 0
558- 22: transcript:Zm00001d002848_T001 transcript:OQU82017 1.00E-65
558- 23: transcript:Zm00001d002849_T001 transcript:EES12554 7.00E-36
558- 24: transcript:Zm00001d002850_T001 transcript:EES11168 1.00E-86
558- 25: transcript:Zm00001d002851_T001 transcript:EES11166 0
558- 26: transcript:Zm00001d002853_T001 transcript:EES12553 0
558- 27: transcript:Zm00001d002854_T001 transcript:EES12552 2.00E-109
558- 28: transcript:Zm00001d002856_T001 transcript:EES12550 1.00E-171
558- 29: transcript:Zm00001d002857_T001 transcript:KXG26776 9.00E-23
558- 30: transcript:Zm00001d002859_T001 transcript:EES11163 0
558- 31: transcript:Zm00001d002860_T002 transcript:OQU82013 2.00E-123
558- 32: transcript:Zm00001d002864_T001 transcript:OQU82012 0
558- 33: transcript:Zm00001d002865_T002 transcript:EES11157 0
558- 34: transcript:Zm00001d002867_T001 transcript:KXG26694 0
558- 35: transcript:Zm00001d002868_T001 transcript:KXG26766 8.00E-80
558- 36: transcript:Zm00001d002869_T001 transcript:EES12544 0
558- 37: transcript:Zm00001d002871_T001 transcript:OQU82009 0
558- 38: transcript:Zm00001d002872_T001 transcript:EES11154 7.00E-42
558- 39: transcript:Zm00001d002873_T004 transcript:KXG26764 4.00E-55
558- 40: transcript:Zm00001d002874_T001 transcript:EES12542 4.00E-178
Alignment 559: score=1895.0 e_value=2.3e-159 N=44 2&6 minus
559- 0: transcript:Zm00001d003554_T001 transcript:KXG26390 2.00E-107
559- 1: transcript:Zm00001d003555_T001 transcript:EES10868 0
559- 2: transcript:Zm00001d003556_T003 transcript:KXG26389 0
559- 3: transcript:Zm00001d003559_T001 transcript:EES10867 0
559- 4: transcript:Zm00001d003563_T001 transcript:EES12237 0
559- 5: transcript:Zm00001d003566_T001 transcript:OQU81648 0
559- 6: transcript:Zm00001d003569_T001 transcript:EES12235 2.00E-20
559- 7: transcript:Zm00001d003572_T001 transcript:EES10864 0

559- 8:	transcript:Zm00001d003573_T001	transcript:KXG26384	4.00E-59
559- 9:	transcript:Zm00001d003575_T001	transcript:EES10862	1.00E-96
559- 10:	transcript:Zm00001d003583_T001	transcript:EES12234	0
559- 11:	transcript:Zm00001d003584_T001	transcript:KXG26381	0
559- 12:	transcript:Zm00001d003588_T002	transcript:EES10859	6.00E-92
559- 13:	transcript:Zm00001d003589_T002	transcript:EES10858	0
559- 14:	transcript:Zm00001d003590_T001	transcript:KXG26376	0
559- 15:	transcript:Zm00001d003593_T001	transcript:OQU81644	8.00E-80
559- 16:	transcript:Zm00001d003594_T001	transcript:EES12223	1.00E-45
559- 17:	transcript:Zm00001d003598_T003	transcript:EES10856	1.00E-100
559- 18:	transcript:Zm00001d003599_T001	transcript:EES10855	3.00E-32
559- 19:	transcript:Zm00001d003600_T001	transcript:EES12221	2.00E-90
559- 20:	transcript:Zm00001d003601_T006	transcript:EES10850	0
559- 21:	transcript:Zm00001d003602_T002	transcript:EES12220	0
559- 22:	transcript:Zm00001d003603_T001	transcript:EES12219	0
559- 23:	transcript:Zm00001d003604_T001	transcript:EES10849	4.00E-160
559- 24:	transcript:Zm00001d003605_T001	transcript:EES12212	9.00E-80
559- 25:	transcript:Zm00001d003611_T003	transcript:KXG26361	0
559- 26:	transcript:Zm00001d003612_T001	transcript:EES12206	4.00E-176
559- 27:	transcript:Zm00001d003614_T001	transcript:EES12205	0
559- 28:	transcript:Zm00001d003615_T002	transcript:KXG26359	0
559- 29:	transcript:Zm00001d003616_T024	transcript:OQU81622	7.00E-180
559- 30:	transcript:Zm00001d003617_T001	transcript:EES10841	0
559- 31:	transcript:Zm00001d003618_T001	transcript:EES12200	2.00E-163
559- 32:	transcript:Zm00001d003621_T002	transcript:EES12199	0
559- 33:	transcript:Zm00001d003622_T001	transcript:OQU81615	7.00E-61
559- 34:	transcript:Zm00001d003624_T001	transcript:OQU81614	0
559- 35:	transcript:Zm00001d003626_T002	transcript:KXG26346	2.00E-112
559- 36:	transcript:Zm00001d003630_T001	transcript:OQU81612	7.00E-104
559- 37:	transcript:Zm00001d003631_T001	transcript:KXG26342	2.00E-33
559- 38:	transcript:Zm00001d003632_T003	transcript:EES10837	0
559- 39:	transcript:Zm00001d003640_T001	transcript:OQU81610	0
559- 40:	transcript:Zm00001d003643_T005	transcript:EES12190	0
559- 41:	transcript:Zm00001d003644_T002	transcript:EES12188	0
559- 42:	transcript:Zm00001d003645_T001	transcript:OQU81605	1.00E-30
559- 43:	transcript:Zm00001d003646_T002	transcript:EES12187	1.00E-105
## Alignment 560: score=1525.0 e_value=2.3e-117 N=34 2&6 minus			
560- 0:	transcript:Zm00001d001929_T003	transcript:OQU82526	1.00E-115
560- 1:	transcript:Zm00001d001931_T001	transcript:EES13045	3.00E-42
560- 2:	transcript:Zm00001d001932_T001	transcript:EES11611	0
560- 3:	transcript:Zm00001d001933_T001	transcript:EES11610	0
560- 4:	transcript:Zm00001d001934_T001	transcript:EES11609	4.00E-87
560- 5:	transcript:Zm00001d001935_T001	transcript:EES13044	0
560- 6:	transcript:Zm00001d001936_T001	transcript:OQU82518	0
560- 7:	transcript:Zm00001d001937_T003	transcript:KXG27277	0
560- 8:	transcript:Zm00001d001939_T003	transcript:EES13042	0
560- 9:	transcript:Zm00001d001940_T001	transcript:KXG27361	7.00E-22
560- 10:	transcript:Zm00001d001941_T001	transcript:EES13037	0
560- 11:	transcript:Zm00001d001945_T006	transcript:OQU82517	0
560- 12:	transcript:Zm00001d001947_T001	transcript:EES13036	0
560- 13:	transcript:Zm00001d001948_T001	transcript:OQU82514	2.00E-143
560- 14:	transcript:Zm00001d001949_T001	transcript:EES11607	5.00E-68
560- 15:	transcript:Zm00001d001951_T001	transcript:EES13034	8.00E-76
560- 16:	transcript:Zm00001d001952_T001	transcript:EES13033	0

560- 17: transcript:Zm00001d001953_T003 transcript:EES11606 0
560- 18: transcript:Zm00001d001959_T001 transcript:KXG27351 0
560- 19: transcript:Zm00001d001960_T001 transcript:EES13031 0
560- 20: transcript:Zm00001d001961_T001 transcript:EES13029 3.00E-46
560- 21: transcript:Zm00001d001962_T014 transcript:KXG27341 0
560- 22: transcript:Zm00001d001963_T001 transcript:OQU82502 2.00E-30
560- 23: transcript:Zm00001d001965_T001 transcript:EES13024 5.00E-24
560- 24: transcript:Zm00001d001966_T001 transcript:EES11600 0
560- 25: transcript:Zm00001d001967_T001 transcript:EES13023 7.00E-105
560- 26: transcript:Zm00001d001968_T001 transcript:OQU82499 0
560- 27: transcript:Zm00001d001970_T001 transcript:EES11599 2.00E-103
560- 28: transcript:Zm00001d001972_T001 transcript:OQU82498 9.00E-31
560- 29: transcript:Zm00001d001973_T001 transcript:EES11597 0
560- 30: transcript:Zm00001d001974_T001 transcript:KXG27325 0
560- 31: transcript:Zm00001d001976_T001 transcript:EES13016 0
560- 32: transcript:Zm00001d001977_T001 transcript:KXG27321 0
560- 33: transcript:Zm00001d001978_T012 transcript:EES13018 0
Alignment 561: score=1358.0 e_value=4.9e-106 N=31 2&6 minus
561- 0: transcript:Zm00001d004440_T001 transcript:KXG25806 0
561- 1: transcript:Zm00001d004442_T001 transcript:KXG25805 0
561- 2: transcript:Zm00001d004443_T001 transcript:EES10404 0
561- 3: transcript:Zm00001d004446_T001 transcript:EES11787 9.00E-58
561- 4: transcript:Zm00001d004448_T001 transcript:KXG25802 0
561- 5: transcript:Zm00001d004451_T001 transcript:EES10400 0
561- 6: transcript:Zm00001d004452_T001 transcript:EES11784 2.00E-81
561- 7: transcript:Zm00001d004457_T001 transcript:EES11783 4.00E-34
561- 8: transcript:Zm00001d004459_T001 transcript:OQU81125 2.00E-51
561- 9: transcript:Zm00001d004460_T004 transcript:EES10394 0
561- 10: transcript:Zm00001d004463_T006 transcript:KXG25794 0
561- 11: transcript:Zm00001d004465_T001 transcript:EES10392 2.00E-126
561- 12: transcript:Zm00001d004466_T010 transcript:EES10391 0
561- 13: transcript:Zm00001d004467_T001 transcript:EES11777 0
561- 14: transcript:Zm00001d004472_T002 transcript:EES11770 0
561- 15: transcript:Zm00001d004473_T001 transcript:EES10385 0
561- 16: transcript:Zm00001d004477_T005 transcript:EES11769 3.00E-119
561- 17: transcript:Zm00001d004478_T001 transcript:EES10383 0
561- 18: transcript:Zm00001d004480_T001 transcript:EES11768 4.00E-149
561- 19: transcript:Zm00001d004482_T001 transcript:EES11767 4.00E-61
561- 20: transcript:Zm00001d004483_T001 transcript:OQU81111 7.00E-110
561- 21: transcript:Zm00001d004484_T001 transcript:EES11762 0
561- 22: transcript:Zm00001d004495_T001 transcript:KXG25783 2.00E-131
561- 23: transcript:Zm00001d004496_T001 transcript:EES10378 4.00E-48
561- 24: transcript:Zm00001d004497_T001 transcript:EES10376 0
561- 25: transcript:Zm00001d004498_T001 transcript:OQU81108 2.00E-130
561- 26: transcript:Zm00001d004501_T001 transcript:EES11758 1.00E-14
561- 27: transcript:Zm00001d004512_T001 transcript:EES11756 0
561- 28: transcript:Zm00001d004513_T001 transcript:OQU81103 0
561- 29: transcript:Zm00001d004517_T005 transcript:OQU81101 6.00E-163
561- 30: transcript:Zm00001d004521_T001 transcript:EES10369 0
Alignment 562: score=1299.0 e_value=1.6e-94 N=29 2&6 minus
562- 0: transcript:Zm00001d002418_T030 transcript:KXG27048 0
562- 1: transcript:Zm00001d002419_T001 transcript:OQU82257 0
562- 2: transcript:Zm00001d002420_T001 transcript:EES12793 0
562- 3: transcript:Zm00001d002421_T001 transcript:EES11379 0

562- 4: transcript:Zm00001d002422_T004 transcript:EES11375 0
562- 5: transcript:Zm00001d002423_T001 transcript:EES12792 0
562- 6: transcript:Zm00001d002424_T002 transcript:EES12791 6.00E-155
562- 7: transcript:Zm00001d002425_T001 transcript:EES12790 3.00E-35
562- 8: transcript:Zm00001d002426_T003 transcript:KXG26982 0
562- 9: transcript:Zm00001d002427_T001 transcript:OQU82256 0
562- 10: transcript:Zm00001d002428_T001 transcript:EES12788 3.00E-120
562- 11: transcript:Zm00001d002429_T002 transcript:EES12787 6.00E-178
562- 12: transcript:Zm00001d002430_T001 transcript:KXG27043 1.00E-29
562- 13: transcript:Zm00001d002432_T001 transcript:OQU82255 0
562- 14: transcript:Zm00001d002433_T002 transcript:EES12784 0
562- 15: transcript:Zm00001d002434_T001 transcript:EES12783 0
562- 16: transcript:Zm00001d002439_T002 transcript:KXG27037 5.00E-76
562- 17: transcript:Zm00001d002440_T002 transcript:EES11372 0
562- 18: transcript:Zm00001d002441_T002 transcript:OQU82248 0
562- 19: transcript:Zm00001d002444_T003 transcript:KXG27033 5.00E-153
562- 20: transcript:Zm00001d002446_T001 transcript:KXG27032 4.00E-159
562- 21: transcript:Zm00001d002449_T001 transcript:EES11362 0
562- 22: transcript:Zm00001d002450_T001 transcript:EES11363 3.00E-113
562- 23: transcript:Zm00001d002451_T001 transcript:EES12779 1.00E-61
562- 24: transcript:Zm00001d002452_T001 transcript:KXG27029 0
562- 25: transcript:Zm00001d002454_T002 transcript:EES12777 0
562- 26: transcript:Zm00001d002455_T001 transcript:OQU82238 1.00E-29
562- 27: transcript:Zm00001d002456_T002 transcript:EES12775 1.00E-101
562- 28: transcript:Zm00001d002457_T002 transcript:OQU82234 2.00E-75
Alignment 563: score=1281.0 e_value=7.9e-98 N=30 2&6 minus
563- 0: transcript:Zm00001d001789_T002 transcript:EES11670 0
563- 1: transcript:Zm00001d001790_T004 transcript:KXG27461 0
563- 2: transcript:Zm00001d001791_T001 transcript:EES13119 0
563- 3: transcript:Zm00001d001797_T001 transcript:EES13118 3.00E-125
563- 4: transcript:Zm00001d001798_T002 transcript:EES13117 0
563- 5: transcript:Zm00001d001799_T001 transcript:KXG27371 1.00E-139
563- 6: transcript:Zm00001d001802_T001 transcript:KXG27455 2.00E-38
563- 7: transcript:Zm00001d001803_T001 transcript:EES13116 0
563- 8: transcript:Zm00001d001804_T005 transcript:OQU82585 0
563- 9: transcript:Zm00001d001806_T001 transcript:EES11666 0
563- 10: transcript:Zm00001d001807_T001 transcript:EES13115 8.00E-90
563- 11: transcript:Zm00001d001808_T005 transcript:KXG27452 0
563- 12: transcript:Zm00001d001809_T002 transcript:EES11663 0
563- 13: transcript:Zm00001d001811_T001 transcript:EES13114 2.00E-125
563- 14: transcript:Zm00001d001812_T001 transcript:EES13112 0
563- 15: transcript:Zm00001d001813_T001 transcript:KXG27447 0
563- 16: transcript:Zm00001d001814_T001 transcript:EES11661 6.00E-157
563- 17: transcript:Zm00001d001817_T001 transcript:KXG27443 8.00E-15
563- 18: transcript:Zm00001d001819_T001 transcript:OQU82575 0
563- 19: transcript:Zm00001d001820_T001 transcript:EES11656 0
563- 20: transcript:Zm00001d001824_T001 transcript:EES13107 1.00E-86
563- 21: transcript:Zm00001d001825_T001 transcript:EES11653 0
563- 22: transcript:Zm00001d001826_T002 transcript:EES11652 1.00E-11
563- 23: transcript:Zm00001d001827_T005 transcript:KXG27435 0
563- 24: transcript:Zm00001d001828_T033 transcript:OQU82573 0
563- 25: transcript:Zm00001d001829_T001 transcript:EES11651 8.00E-55
563- 26: transcript:Zm00001d001830_T001 transcript:EES11650 7.00E-105
563- 27: transcript:Zm00001d001831_T001 transcript:EES13103 0

563- 28: transcript:Zm00001d001832_T001 transcript:EES13101 2.00E-136
563- 29: transcript:Zm00001d001833_T001 transcript:OQU82570 0
Alignment 564: score=1145.0 e_value=4.3e-75 N=25 2&6 minus
564- 0: transcript:Zm00001d002474_T001 transcript:OQU82234 0
564- 1: transcript:Zm00001d002475_T001 transcript:EES12769 0
564- 2: transcript:Zm00001d002476_T001 transcript:EES12768 0
564- 3: transcript:Zm00001d002477_T001 transcript:KXG27022 1.00E-46
564- 4: transcript:Zm00001d002478_T001 transcript:EES11354 0
564- 5: transcript:Zm00001d002479_T001 transcript:EES11351 4.00E-41
564- 6: transcript:Zm00001d002482_T001 transcript:KXG27021 6.00E-126
564- 7: transcript:Zm00001d002483_T007 transcript:EES12762 0
564- 8: transcript:Zm00001d002485_T001 transcript:EES11350 0
564- 9: transcript:Zm00001d002488_T001 transcript:OQU82229 2.00E-67
564- 10: transcript:Zm00001d002489_T001 transcript:EES12755 8.00E-155
564- 11: transcript:Zm00001d002490_T001 transcript:OQU82227 3.00E-155
564- 12: transcript:Zm00001d002491_T001 transcript:EES12753 0
564- 13: transcript:Zm00001d002492_T001 transcript:KXG27015 9.00E-54
564- 14: transcript:Zm00001d002494_T001 transcript:EES11345 3.00E-79
564- 15: transcript:Zm00001d002495_T001 transcript:EES12751 0
564- 16: transcript:Zm00001d002496_T001 transcript:EES11344 1.00E-137
564- 17: transcript:Zm00001d002498_T001 transcript:EES11341 0
564- 18: transcript:Zm00001d002499_T003 transcript:EES11340 0
564- 19: transcript:Zm00001d002500_T001 transcript:EES11339 4.00E-41
564- 20: transcript:Zm00001d002501_T001 transcript:EES12747 1.00E-32
564- 21: transcript:Zm00001d002503_T001 transcript:KXG27008 0
564- 22: transcript:Zm00001d002504_T002 transcript:EES11337 0
564- 23: transcript:Zm00001d002505_T001 transcript:KXG27007 0
564- 24: transcript:Zm00001d002510_T001 transcript:KXG27006 0
Alignment 565: score=1105.0 e_value=1.2e-80 N=26 2&6 minus
565- 0: transcript:Zm00001d001979_T002 transcript:EES11595 0
565- 1: transcript:Zm00001d001980_T001 transcript:EES13017 0
565- 2: transcript:Zm00001d001982_T001 transcript:KXG27318 7.00E-79
565- 3: transcript:Zm00001d001983_T003 transcript:KXG27317 0
565- 4: transcript:Zm00001d001984_T001 transcript:EES11591 0
565- 5: transcript:Zm00001d001987_T002 transcript:EES11590 0
565- 6: transcript:Zm00001d001988_T002 transcript:EES13014 0
565- 7: transcript:Zm00001d001989_T001 transcript:EES11587 7.00E-41
565- 8: transcript:Zm00001d001990_T001 transcript:EES13012 6.00E-25
565- 9: transcript:Zm00001d001993_T001 transcript:EES11585 0
565- 10: transcript:Zm00001d001994_T001 transcript:OQU82490 0
565- 11: transcript:Zm00001d001995_T003 transcript:OQU82488 0
565- 12: transcript:Zm00001d001996_T001 transcript:KXG27310 6.00E-55
565- 13: transcript:Zm00001d001997_T001 transcript:EES11583 0
565- 14: transcript:Zm00001d001999_T009 transcript:EES11582 0
565- 15: transcript:Zm00001d002000_T003 transcript:KXG27309 0
565- 16: transcript:Zm00001d002001_T001 transcript:EES11581 3.00E-28
565- 17: transcript:Zm00001d002003_T001 transcript:KXG27305 0
565- 18: transcript:Zm00001d002004_T003 transcript:EES13003 0
565- 19: transcript:Zm00001d002005_T001 transcript:EES11579 0
565- 20: transcript:Zm00001d002006_T013 transcript:EES11577 0
565- 21: transcript:Zm00001d002011_T001 transcript:EES12999 3.00E-62
565- 22: transcript:Zm00001d002012_T002 transcript:OQU82476 0
565- 23: transcript:Zm00001d002014_T001 transcript:EES11575 4.00E-60
565- 24: transcript:Zm00001d002020_T003 transcript:KXG27294 0

565- 25: transcript:Zm00001d002021_T001 transcript:OQU82471 4.00E-26
Alignment 566: score=1064.0 e_value=3.2e-66 N=23 2&6 minus
566- 0: transcript:Zm00001d002332_T026 transcript:EES11416 0
566- 1: transcript:Zm00001d002333_T003 transcript:EES12852 0
566- 2: transcript:Zm00001d002334_T001 transcript:EES12845 0
566- 3: transcript:Zm00001d002338_T001 transcript:KXG27103 0
566- 4: transcript:Zm00001d002339_T001 transcript:EES11414 2.00E-114
566- 5: transcript:Zm00001d002340_T006 transcript:EES12846 0
566- 6: transcript:Zm00001d002341_T001 transcript:OQU82312 1.00E-124
566- 7: transcript:Zm00001d002342_T001 transcript:EES11412 0
566- 8: transcript:Zm00001d002343_T001 transcript:EES12842 0
566- 9: transcript:Zm00001d002344_T001 transcript:EES11411 1.00E-71
566- 10: transcript:Zm00001d002345_T001 transcript:KXG27099 0
566- 11: transcript:Zm00001d002346_T002 transcript:EES11410 0
566- 12: transcript:Zm00001d002347_T001 transcript:OQU82307 3.00E-50
566- 13: transcript:Zm00001d002348_T001 transcript:KXG27096 9.00E-68
566- 14: transcript:Zm00001d002350_T001 transcript:EES11408 0
566- 15: transcript:Zm00001d002352_T001 transcript:EES11406 2.00E-76
566- 16: transcript:Zm00001d002353_T001 transcript:EES11405 0
566- 17: transcript:Zm00001d002357_T007 transcript:KXG27092 0
566- 18: transcript:Zm00001d002358_T001 transcript:EES11403 0
566- 19: transcript:Zm00001d002359_T001 transcript:EES12835 0
566- 20: transcript:Zm00001d002360_T001 transcript:KXG27020 1.00E-163
566- 21: transcript:Zm00001d002362_T001 transcript:EES12833 3.00E-84
566- 22: transcript:Zm00001d002364_T001 transcript:EES11400 2.00E-76
Alignment 567: score=951.0 e_value=2.9e-64 N=21 2&6 minus
567- 0: transcript:Zm00001d003655_T006 transcript:KXG26331 0
567- 1: transcript:Zm00001d003657_T001 transcript:OQU81603 3.00E-151
567- 2: transcript:Zm00001d003658_T001 transcript:OQU81602 1.00E-175
567- 3: transcript:Zm00001d003659_T002 transcript:KXG26327 0
567- 4: transcript:Zm00001d003660_T001 transcript:OQU81598 1.00E-73
567- 5: transcript:Zm00001d003661_T001 transcript:EES10826 4.00E-117
567- 6: transcript:Zm00001d003663_T001 transcript:EES12177 0
567- 7: transcript:Zm00001d003664_T001 transcript:OQU81597 0
567- 8: transcript:Zm00001d003666_T001 transcript:EES10822 0
567- 9: transcript:Zm00001d003667_T002 transcript:EES12176 8.00E-116
567- 10: transcript:Zm00001d003668_T001 transcript:EES10821 0
567- 11: transcript:Zm00001d003669_T001 transcript:EES10820 1.00E-152
567- 12: transcript:Zm00001d003670_T005 transcript:KXG26320 0
567- 13: transcript:Zm00001d003671_T001 transcript:EES10817 0
567- 14: transcript:Zm00001d003672_T001 transcript:EES10818 9.00E-121
567- 15: transcript:Zm00001d003673_T001 transcript:EES10816 0
567- 16: transcript:Zm00001d003674_T001 transcript:KXG26318 0
567- 17: transcript:Zm00001d003676_T001 transcript:OQU81588 0
567- 18: transcript:Zm00001d003677_T003 transcript:KXG26314 0
567- 19: transcript:Zm00001d003678_T003 transcript:KXG26313 0
567- 20: transcript:Zm00001d003679_T006 transcript:EES10812 0
Alignment 568: score=920.0 e_value=2.4e-64 N=22 2&6 minus
568- 0: transcript:Zm00001d001879_T035 transcript:KXG27404 0
568- 1: transcript:Zm00001d001880_T001 transcript:EES13075 4.00E-39
568- 2: transcript:Zm00001d001881_T001 transcript:EES13073 0
568- 3: transcript:Zm00001d001883_T008 transcript:EES13070 0
568- 4: transcript:Zm00001d001884_T001 transcript:KXG27402 0
568- 5: transcript:Zm00001d001885_T001 transcript:OQU82545 1.00E-77

568- 6: transcript:Zm00001d001894_T001 transcript:KXG27398 2.00E-142
568- 7: transcript:Zm00001d001895_T002 transcript:EES11631 0
568- 8: transcript:Zm00001d001896_T002 transcript:KXG27303 1.00E-175
568- 9: transcript:Zm00001d001897_T001 transcript:KXG27394 0
568- 10: transcript:Zm00001d001898_T008 transcript:EES11627 0
568- 11: transcript:Zm00001d001899_T001 transcript:EES13065 6.00E-41
568- 12: transcript:Zm00001d001900_T002 transcript:EES13064 0
568- 13: transcript:Zm00001d001901_T002 transcript:EES11624 9.00E-109
568- 14: transcript:Zm00001d001902_T002 transcript:OQU82541 1.00E-153
568- 15: transcript:Zm00001d001905_T001 transcript:OQU82540 2.00E-62
568- 16: transcript:Zm00001d001906_T004 transcript:EES11620 0
568- 17: transcript:Zm00001d001907_T001 transcript:OQU82539 6.00E-67
568- 18: transcript:Zm00001d001909_T002 transcript:EES13056 0
568- 19: transcript:Zm00001d001910_T002 transcript:EES11619 3.00E-85
568- 20: transcript:Zm00001d001913_T004 transcript:EES13053 2.00E-108
568- 21: transcript:Zm00001d001914_T001 transcript:EES11615 0
Alignment 569: score=867.0 e_value=6.6e-57 N=20 2&6 minus
569- 0: transcript:Zm00001d002703_T001 transcript:OQU82115 0
569- 1: transcript:Zm00001d002704_T002 transcript:EES11249 7.00E-120
569- 2: transcript:Zm00001d002708_T001 transcript:EES11247 0
569- 3: transcript:Zm00001d002711_T001 transcript:KXG26863 0
569- 4: transcript:Zm00001d002713_T001 transcript:EES12629 6.00E-168
569- 5: transcript:Zm00001d002714_T003 transcript:EES12628 1.00E-40
569- 6: transcript:Zm00001d002715_T001 transcript:OQU82107 0
569- 7: transcript:Zm00001d002716_T001 transcript:OQU82106 0
569- 8: transcript:Zm00001d002718_T001 transcript:EES11245 0
569- 9: transcript:Zm00001d002720_T001 transcript:EES12624 1.00E-54
569- 10: transcript:Zm00001d002721_T001 transcript:EES11244 6.00E-51
569- 11: transcript:Zm00001d002723_T001 transcript:EES12623 2.00E-99
569- 12: transcript:Zm00001d002725_T001 transcript:OQU82099 0
569- 13: transcript:Zm00001d002726_T002 transcript:EES12621 0
569- 14: transcript:Zm00001d002729_T001 transcript:OQU82097 3.00E-118
569- 15: transcript:Zm00001d002730_T001 transcript:EES11239 0
569- 16: transcript:Zm00001d002731_T001 transcript:EES11238 3.00E-139
569- 17: transcript:Zm00001d002732_T001 transcript:EES11237 0
569- 18: transcript:Zm00001d002733_T001 transcript:EES11236 3.00E-108
569- 19: transcript:Zm00001d002736_T001 transcript:OQU82092 0
Alignment 570: score=829.0 e_value=3.6e-49 N=18 2&6 minus
570- 0: transcript:Zm00001d002742_T002 transcript:EES12618 0
570- 1: transcript:Zm00001d002744_T001 transcript:OQU82089 3.00E-82
570- 2: transcript:Zm00001d002747_T001 transcript:OQU82088 5.00E-110
570- 3: transcript:Zm00001d002748_T001 transcript:OQU82086 2.00E-32
570- 4: transcript:Zm00001d002750_T001 transcript:EES11224 2.00E-178
570- 5: transcript:Zm00001d002751_T001 transcript:OQU82083 0
570- 6: transcript:Zm00001d002754_T001 transcript:EES12613 3.00E-123
570- 7: transcript:Zm00001d002755_T001 transcript:KXG26838 0
570- 8: transcript:Zm00001d002756_T001 transcript:OQU82082 8.00E-123
570- 9: transcript:Zm00001d002757_T001 transcript:EES12610 0
570- 10: transcript:Zm00001d002758_T002 transcript:EES11216 1.00E-134
570- 11: transcript:Zm00001d002759_T001 transcript:EES11215 0
570- 12: transcript:Zm00001d002760_T001 transcript:EES11211 6.00E-101
570- 13: transcript:Zm00001d002762_T001 transcript:OQU82076 2.00E-129
570- 14: transcript:Zm00001d002768_T001 transcript:EES12607 5.00E-75
570- 15: transcript:Zm00001d002770_T006 transcript:OQU82075 0

```
570- 16: transcript:Zm00001d002772_T001 transcript:EES12600              7.00E-28
570- 17: transcript:Zm00001d002774_T001 transcript:KXG26767              4.00E-16
## Alignment 571: score=801.0 e_value=9.6e-54 N=19 2&6 minus
571-  0: transcript:Zm00001d002115_T001 transcript:EES11530                     0
571-  1: transcript:Zm00001d002119_T006 transcript:EES12952                     0
571-  2: transcript:Zm00001d002121_T001 transcript:EES12951              2.00E-45
571-  3: transcript:Zm00001d002122_T007 transcript:EES12950             3.00E-176
571-  4: transcript:Zm00001d002123_T001 transcript:OQU82428                     0
571-  5: transcript:Zm00001d002124_T001 transcript:EES11528             5.00E-143
571-  6: transcript:Zm00001d002125_T019 transcript:EES11527                     0
571-  7: transcript:Zm00001d002126_T001 transcript:KXG27238                     0
571-  8: transcript:Zm00001d002127_T001 transcript:EES11524              1.00E-54
571-  9: transcript:Zm00001d002128_T001 transcript:EES12946             1.00E-176
571- 10: transcript:Zm00001d002129_T001 transcript:OQU82423                     0
571- 11: transcript:Zm00001d002130_T002 transcript:OQU82421                     0
571- 12: transcript:Zm00001d002131_T001 transcript:EES11521             3.00E-133
571- 13: transcript:Zm00001d002132_T001 transcript:KXG27231              4.00E-11
571- 14: transcript:Zm00001d002135_T001 transcript:KXG27229                     0
571- 15: transcript:Zm00001d002136_T001 transcript:EES11515             7.00E-154
571- 16: transcript:Zm00001d002137_T001 transcript:EES12943              9.00E-17
571- 17: transcript:Zm00001d002139_T001 transcript:OQU82414                     0
571- 18: transcript:Zm00001d002140_T001 transcript:EES12937                     0
## Alignment 572: score=758.0 e_value=4.6e-45 N=17 2&6 minus
572-  0: transcript:Zm00001d001849_T001 transcript:EES13090                     0
572-  1: transcript:Zm00001d001850_T001 transcript:EES11642                     0
572-  2: transcript:Zm00001d001855_T002 transcript:EES13087                     0
572-  3: transcript:Zm00001d001856_T003 transcript:KXG27420             2.00E-177
572-  4: transcript:Zm00001d001857_T001 transcript:OQU82564             3.00E-149
572-  5: transcript:Zm00001d001858_T001 transcript:KXG27417                     0
572-  6: transcript:Zm00001d001859_T001 transcript:OQU82561                     0
572-  7: transcript:Zm00001d001860_T001 transcript:EES11638             4.00E-178
572-  8: transcript:Zm00001d001861_T002 transcript:EES13085             2.00E-145
572-  9: transcript:Zm00001d001862_T001 transcript:EES13084             6.00E-121
572- 10: transcript:Zm00001d001864_T002 transcript:KXG27412                     0
572- 11: transcript:Zm00001d001865_T001 transcript:EES11637              1.00E-67
572- 12: transcript:Zm00001d001866_T012 transcript:KXG27408                     0
572- 13: transcript:Zm00001d001868_T001 transcript:EES11636                     0
572- 14: transcript:Zm00001d001869_T001 transcript:OQU82554                     0
572- 15: transcript:Zm00001d001870_T001 transcript:EES11635             2.00E-154
572- 16: transcript:Zm00001d001877_T001 transcript:EES11633                     0
## Alignment 573: score=753.0 e_value=1.1e-47 N=18 2&6 minus
573-  0: transcript:Zm00001d002063_T001 transcript:OQU82456                     0
573-  1: transcript:Zm00001d002064_T001 transcript:EES12978                     0
573-  2: transcript:Zm00001d002065_T006 transcript:KXG27268                     0
573-  3: transcript:Zm00001d002066_T001 transcript:KXG27192                     0
573-  4: transcript:Zm00001d002068_T001 transcript:KXG27266                     0
573-  5: transcript:Zm00001d002071_T001 transcript:OQU82451              6.00E-45
573-  6: transcript:Zm00001d002072_T001 transcript:OQU82450              2.00E-22
573-  7: transcript:Zm00001d002075_T001 transcript:OQU82447             4.00E-135
573-  8: transcript:Zm00001d002079_T001 transcript:KXG27254              2.00E-92
573-  9: transcript:Zm00001d002080_T003 transcript:EES11547                     0
573- 10: transcript:Zm00001d002082_T004 transcript:OQU82441                     0
573- 11: transcript:Zm00001d002083_T001 transcript:EES11545              3.00E-81
573- 12: transcript:Zm00001d002084_T007 transcript:OQU82439                     0
```

573- 13: transcript:Zm00001d002085_T001 transcript:EES12965 0
573- 14: transcript:Zm00001d002086_T001 transcript:EES12962 0
573- 15: transcript:Zm00001d002087_T001 transcript:EES12961 0
573- 16: transcript:Zm00001d002088_T001 transcript:OQU82436 3.00E-57
573- 17: transcript:Zm00001d002089_T003 transcript:KXG27246 0
Alignment 574: score=720.0 e_value=2.8e-49 N=17 2&6 minus
574- 0: transcript:Zm00001d004301_T001 transcript:EES11845 0
574- 1: transcript:Zm00001d004309_T001 transcript:EES11842 5.00E-39
574- 2: transcript:Zm00001d004310_T001 transcript:OQU81203 2.00E-30
574- 3: transcript:Zm00001d004315_T001 transcript:KXG25890 1.00E-08
574- 4: transcript:Zm00001d004320_T001 transcript:KXG25889 6.00E-41
574- 5: transcript:Zm00001d004322_T001 transcript:EES10476 3.00E-26
574- 6: transcript:Zm00001d004328_T001 transcript:OQU81201 3.00E-49
574- 7: transcript:Zm00001d004330_T001 transcript:KXG25879 1.00E-16
574- 8: transcript:Zm00001d004335_T001 transcript:EES10473 0
574- 9: transcript:Zm00001d004337_T001 transcript:EES10472 3.00E-46
574- 10: transcript:Zm00001d004339_T003 transcript:KXG25874 0
574- 11: transcript:Zm00001d004340_T002 transcript:KXG25873 0
574- 12: transcript:Zm00001d004354_T001 transcript:KXG25870 8.00E-101
574- 13: transcript:Zm00001d004355_T001 transcript:OQU81188 2.00E-175
574- 14: transcript:Zm00001d004363_T001 transcript:EES10457 0
574- 15: transcript:Zm00001d004371_T001 transcript:OQU81187 2.00E-167
574- 16: transcript:Zm00001d004390_T001 transcript:OQU81178 4.00E-74
Alignment 575: score=708.0 e_value=6.3e-48 N=17 2&6 minus
575- 0: transcript:Zm00001d003745_T001 transcript:KXG26241 0
575- 1: transcript:Zm00001d003749_T001 transcript:KXG26278 0
575- 2: transcript:Zm00001d003750_T001 transcript:EES10780 0
575- 3: transcript:Zm00001d003751_T001 transcript:EES12138 8.00E-137
575- 4: transcript:Zm00001d003754_T003 transcript:EES10778 0
575- 5: transcript:Zm00001d003755_T001 transcript:OQU81542 0
575- 6: transcript:Zm00001d003756_T001 transcript:EES10775 2.00E-85
575- 7: transcript:Zm00001d003760_T001 transcript:EES10773 5.00E-60
575- 8: transcript:Zm00001d003761_T001 transcript:KXG26271 1.00E-40
575- 9: transcript:Zm00001d003762_T002 transcript:KXG26269 3.00E-162
575- 10: transcript:Zm00001d003763_T001 transcript:EES10769 1.00E-56
575- 11: transcript:Zm00001d003765_T001 transcript:KXG26268 0
575- 12: transcript:Zm00001d003767_T001 transcript:EES10765 3.00E-95
575- 13: transcript:Zm00001d003769_T001 transcript:EES12134 5.00E-38
575- 14: transcript:Zm00001d003770_T001 transcript:EES10764 1.00E-31
575- 15: transcript:Zm00001d003773_T005 transcript:KXG26266 0
575- 16: transcript:Zm00001d003774_T005 transcript:KXG26255 0
Alignment 576: score=672.0 e_value=5.3e-37 N=15 2&6 minus
576- 0: transcript:Zm00001d002034_T001 transcript:EES11563 2.00E-141
576- 1: transcript:Zm00001d002035_T001 transcript:EES11561 0
576- 2: transcript:Zm00001d002036_T001 transcript:KXG27281 0
576- 3: transcript:Zm00001d002037_T001 transcript:EES11559 0
576- 4: transcript:Zm00001d002038_T003 transcript:EES12990 0
576- 5: transcript:Zm00001d002039_T001 transcript:KXG27279 0
576- 6: transcript:Zm00001d002042_T001 transcript:OQU82465 0
576- 7: transcript:Zm00001d002046_T003 transcript:KXG27209 0
576- 8: transcript:Zm00001d002051_T001 transcript:EES12983 0
576- 9: transcript:Zm00001d002052_T001 transcript:EES11555 0
576- 10: transcript:Zm00001d002053_T001 transcript:OQU82463 0
576- 11: transcript:Zm00001d002054_T001 transcript:KXG27275 1.00E-180

576- 12: transcript:Zm00001d002055_T001 transcript:KXG27271 8.00E-126
576- 13: transcript:Zm00001d002056_T006 transcript:EES11553 0
576- 14: transcript:Zm00001d002058_T001 transcript:OQU82458 6.00E-112
Alignment 577: score=654.0 e_value=2e-37 N=15 2&6 minus
577- 0: transcript:Zm00001d002277_T001 transcript:EES11441 2.00E-152
577- 1: transcript:Zm00001d002278_T001 transcript:EES11440 2.00E-28
577- 2: transcript:Zm00001d002279_T001 transcript:OQU82350 6.00E-164
577- 3: transcript:Zm00001d002282_T001 transcript:EES12876 0
577- 4: transcript:Zm00001d002283_T002 transcript:EES12875 0
577- 5: transcript:Zm00001d002284_T003 transcript:KXG27134 0
577- 6: transcript:Zm00001d002285_T001 transcript:OQU82346 5.00E-166
577- 7: transcript:Zm00001d002286_T001 transcript:OQU82343 5.00E-137
577- 8: transcript:Zm00001d002287_T001 transcript:OQU82341 0
577- 9: transcript:Zm00001d002288_T002 transcript:EES11436 1.00E-120
577- 10: transcript:Zm00001d002292_T004 transcript:KXG27129 0
577- 11: transcript:Zm00001d002295_T001 transcript:EES12867 5.00E-141
577- 12: transcript:Zm00001d002296_T001 transcript:EES11434 1.00E-157
577- 13: transcript:Zm00001d002301_T001 transcript:OQU82335 1.00E-111
577- 14: transcript:Zm00001d002302_T001 transcript:KXG27121 7.00E-40
Alignment 578: score=644.0 e_value=1.1e-32 N=14 2&6 minus
578- 0: transcript:Zm00001d002679_T001 transcript:KXG26894 0
578- 1: transcript:Zm00001d002680_T003 transcript:EES11259 0
578- 2: transcript:Zm00001d002682_T001 transcript:EES12650 0
578- 3: transcript:Zm00001d002684_T001 transcript:EES12649 0
578- 4: transcript:Zm00001d002685_T001 transcript:EES11258 4.00E-92
578- 5: transcript:Zm00001d002687_T001 transcript:EES12648 0
578- 6: transcript:Zm00001d002688_T002 transcript:OQU82130 0
578- 7: transcript:Zm00001d002690_T001 transcript:EES11257 0
578- 8: transcript:Zm00001d002694_T004 transcript:OQU82128 1.00E-132
578- 9: transcript:Zm00001d002695_T009 transcript:KXG26891 0
578- 10: transcript:Zm00001d002696_T001 transcript:EES11256 0
578- 11: transcript:Zm00001d002698_T001 transcript:OQU82126 0
578- 12: transcript:Zm00001d002699_T001 transcript:EES11255 0
578- 13: transcript:Zm00001d002700_T001 transcript:EES12643 2.00E-50
Alignment 579: score=636.0 e_value=4.9e-37 N=14 2&6 minus
579- 0: transcript:Zm00001d002369_T003 transcript:EES12828 0
579- 1: transcript:Zm00001d002370_T001 transcript:EES11393 0
579- 2: transcript:Zm00001d002371_T002 transcript:EES12824 0
579- 3: transcript:Zm00001d002372_T002 transcript:KXG27074 0
579- 4: transcript:Zm00001d002373_T006 transcript:KXG27072 0
579- 5: transcript:Zm00001d002374_T001 transcript:OQU82277 1.00E-101
579- 6: transcript:Zm00001d002377_T001 transcript:OQU82276 3.00E-124
579- 7: transcript:Zm00001d002378_T001 transcript:OQU82274 0
579- 8: transcript:Zm00001d002382_T002 transcript:EES12818 0
579- 9: transcript:Zm00001d002384_T001 transcript:EES12817 2.00E-145
579- 10: transcript:Zm00001d002385_T002 transcript:OQU82265 2.00E-126
579- 11: transcript:Zm00001d002386_T003 transcript:EES11389 1.00E-154
579- 12: transcript:Zm00001d002387_T002 transcript:KXG27066 3.00E-72
579- 13: transcript:Zm00001d002388_T003 transcript:EES12812 0
Alignment 580: score=627.0 e_value=9.1e-37 N=14 2&6 minus
580- 0: transcript:Zm00001d002144_T001 transcript:KXG27223 9.00E-53
580- 1: transcript:Zm00001d002145_T001 transcript:EES12936 0
580- 2: transcript:Zm00001d002146_T001 transcript:EES11510 0
580- 3: transcript:Zm00001d002147_T002 transcript:KXG27213 0

580- 4: transcript:Zm00001d002148_T001 transcript:KXG27212 7.00E-166
580- 5: transcript:Zm00001d002150_T001 transcript:EES12873 0
580- 6: transcript:Zm00001d002149_T007 transcript:EES11508 0
580- 7: transcript:Zm00001d002153_T001 transcript:KXG27211 0
580- 8: transcript:Zm00001d002154_T003 transcript:EES12872 0
580- 9: transcript:Zm00001d002155_T001 transcript:EES12932 8.00E-64
580- 10: transcript:Zm00001d002156_T001 transcript:EES12930 5.00E-140
580- 11: transcript:Zm00001d002157_T001 transcript:EES11506 0
580- 12: transcript:Zm00001d002158_T001 transcript:EES12924 5.00E-19
580- 13: transcript:Zm00001d002163_T001 transcript:EES12920 0
Alignment 581: score=597.0 e_value=2.4e-31 N=13 2&6 minus
581- 0: transcript:Zm00001d002797_T001 transcript:OQU82052 0
581- 1: transcript:Zm00001d002798_T001 transcript:EES11200 7.00E-88
581- 2: transcript:Zm00001d002799_T001 transcript:EES11198 6.00E-118
581- 3: transcript:Zm00001d002801_T001 transcript:OQU82045 0
581- 4: transcript:Zm00001d002802_T001 transcript:OQU82043 0
581- 5: transcript:Zm00001d002803_T001 transcript:KXG26803 0
581- 6: transcript:Zm00001d002806_T001 transcript:EES11195 5.00E-151
581- 7: transcript:Zm00001d002810_T001 transcript:OQU82036 0
581- 8: transcript:Zm00001d002811_T002 transcript:EES11194 0
581- 9: transcript:Zm00001d002812_T002 transcript:KXG26792 2.00E-59
581- 10: transcript:Zm00001d002815_T001 transcript:EES11192 2.00E-126
581- 11: transcript:Zm00001d002816_T001 transcript:EES11191 7.00E-64
581- 12: transcript:Zm00001d002817_T004 transcript:KXG26789 0
Alignment 582: score=572.0 e_value=6.6e-27 N=12 2&6 minus
582- 0: transcript:Zm00001d001835_T001 transcript:OQU82570 0
582- 1: transcript:Zm00001d001837_T001 transcript:EES13099 0
582- 2: transcript:Zm00001d001838_T001 transcript:EES13098 0
582- 3: transcript:Zm00001d001839_T001 transcript:OQU82569 2.00E-81
582- 4: transcript:Zm00001d001841_T001 transcript:KXG27428 3.00E-84
582- 5: transcript:Zm00001d001842_T001 transcript:KXG27427 0
582- 6: transcript:Zm00001d001843_T001 transcript:EES11645 1.00E-105
582- 7: transcript:Zm00001d001844_T001 transcript:EES13095 0
582- 8: transcript:Zm00001d001845_T001 transcript:OQU82566 0
582- 9: transcript:Zm00001d001846_T001 transcript:EES13093 0
582- 10: transcript:Zm00001d001847_T001 transcript:KXG27423 0
582- 11: transcript:Zm00001d001848_T003 transcript:KXG27425 0
Alignment 583: score=563.0 e_value=2.2e-33 N=14 2&6 minus
583- 0: transcript:Zm00001d002246_T001 transcript:EES12890 0
583- 1: transcript:Zm00001d002247_T001 transcript:OQU82372 1.00E-54
583- 2: transcript:Zm00001d002250_T001 transcript:OQU82370 0
583- 3: transcript:Zm00001d002252_T001 transcript:EES12886 0
583- 4: transcript:Zm00001d002253_T001 transcript:EES11452 3.00E-93
583- 5: transcript:Zm00001d002255_T001 transcript:OQU82369 1.00E-49
583- 6: transcript:Zm00001d002256_T022 transcript:KXG27148 0
583- 7: transcript:Zm00001d002257_T006 transcript:KXG27145 5.00E-84
583- 8: transcript:Zm00001d002258_T001 transcript:EES11449 0
583- 9: transcript:Zm00001d002259_T003 transcript:OQU82368 2.00E-61
583- 10: transcript:Zm00001d002261_T003 transcript:EES11443 0
583- 11: transcript:Zm00001d002266_T002 transcript:EES12883 0
583- 12: transcript:Zm00001d002267_T001 transcript:OQU82367 4.00E-170
583- 13: transcript:Zm00001d002272_T007 transcript:OQU82356 0
Alignment 584: score=506.0 e_value=4.4e-23 N=11 2&6 minus
584- 0: transcript:Zm00001d003071_T003 transcript:OQU81934 5.00E-142

584- 1: transcript:Zm00001d003072_T001 transcript:KXG26687 0
584- 2: transcript:Zm00001d003076_T001 transcript:EES12476 0
584- 3: transcript:Zm00001d003079_T001 transcript:EES11097 0
584- 4: transcript:Zm00001d003080_T001 transcript:OQU81923 5.00E-165
584- 5: transcript:Zm00001d003081_T003 transcript:EES11094 1.00E-80
584- 6: transcript:Zm00001d003083_T002 transcript:EES11093 0
584- 7: transcript:Zm00001d003084_T001 transcript:EES11092 1.00E-41
584- 8: transcript:Zm00001d003085_T001 transcript:OQU81918 0
584- 9: transcript:Zm00001d003086_T001 transcript:KXG26675 9.00E-127
584- 10: transcript:Zm00001d003087_T002 transcript:KXG26674 0
Alignment 585: score=466.0 e_value=1.9e-21 N=10 2&6 minus
585- 0: transcript:Zm00001d002090_T001 transcript:EES11541 0
585- 1: transcript:Zm00001d002091_T002 transcript:EES11540 0
585- 2: transcript:Zm00001d002093_T001 transcript:EES12958 7.00E-55
585- 3: transcript:Zm00001d002094_T001 transcript:EES12957 2.00E-99
585- 4: transcript:Zm00001d002095_T001 transcript:EES11538 0
585- 5: transcript:Zm00001d002096_T001 transcript:EES11537 8.00E-88
585- 6: transcript:Zm00001d002097_T001 transcript:EES11535 3.00E-22
585- 7: transcript:Zm00001d002098_T001 transcript:EES11534 0
585- 8: transcript:Zm00001d002099_T003 transcript:EES11533 0
585- 9: transcript:Zm00001d002100_T001 transcript:EES11531 2.00E-132
Alignment 586: score=445.0 e_value=5.6e-28 N=11 2&6 minus
586- 0: transcript:Zm00001d004401_T001 transcript:EES11809 8.00E-161
586- 1: transcript:Zm00001d004405_T001 transcript:KXG25826 4.00E-14
586- 2: transcript:Zm00001d004409_T002 transcript:EES11806 4.00E-53
586- 3: transcript:Zm00001d004410_T001 transcript:EES10420 2.00E-76
586- 4: transcript:Zm00001d004413_T002 transcript:EES11801 0
586- 5: transcript:Zm00001d004415_T001 transcript:OQU81143 2.00E-129
586- 6: transcript:Zm00001d004417_T001 transcript:EES10417 1.00E-35
586- 7: transcript:Zm00001d004426_T001 transcript:EES10416 9.00E-81
586- 8: transcript:Zm00001d004437_T001 transcript:OQU81138 6.00E-153
586- 9: transcript:Zm00001d004438_T005 transcript:KXG25811 0
586- 10: transcript:Zm00001d004439_T001 transcript:EES10410 2.00E-110
Alignment 587: score=435.0 e_value=2.9e-22 N=10 2&6 minus
587- 0: transcript:Zm00001d004539_T001 transcript:EES10336 1.00E-50
587- 1: transcript:Zm00001d004541_T009 transcript:EES10334 0
587- 2: transcript:Zm00001d004543_T001 transcript:EES11733 0
587- 3: transcript:Zm00001d004545_T001 transcript:OQU81046 0
587- 4: transcript:Zm00001d004546_T001 transcript:EES10332 1.00E-151
587- 5: transcript:Zm00001d004547_T002 transcript:KXG25757 0
587- 6: transcript:Zm00001d004551_T007 transcript:EES11731 3.00E-60
587- 7: transcript:Zm00001d004552_T003 transcript:KXG25754 2.00E-176
587- 8: transcript:Zm00001d004553_T001 transcript:OQU81039 0
587- 9: transcript:Zm00001d004554_T004 transcript:EES10326 5.00E-162
Alignment 588: score=411.0 e_value=2.1e-20 N=10 2&6 minus
588- 0: transcript:Zm00001d002312_T004 transcript:KXG27116 0
588- 1: transcript:Zm00001d002314_T001 transcript:KXG27114 5.00E-134
588- 2: transcript:Zm00001d002315_T002 transcript:EES12856 0
588- 3: transcript:Zm00001d002316_T001 transcript:KXG27113 0
588- 4: transcript:Zm00001d002317_T002 transcript:KXG27108 0
588- 5: transcript:Zm00001d002319_T001 transcript:EES11423 0
588- 6: transcript:Zm00001d002323_T024 transcript:KXG27107 0
588- 7: transcript:Zm00001d002325_T002 transcript:EES11421 0
588- 8: transcript:Zm00001d002326_T003 transcript:EES11419 0

588- 9: transcript:Zm00001d002330_T006 transcript:EES12853 0
Alignment 589: score=370.0 e_value=1e-15 N=8 2&6 minus
589- 0: transcript:Zm00001d002225_T001 transcript:OQU82382 8.00E-110
589- 1: transcript:Zm00001d002226_T001 transcript:EES12897 3.00E-25
589- 2: transcript:Zm00001d002227_T001 transcript:EES11475 8.00E-80
589- 3: transcript:Zm00001d002228_T001 transcript:EES12896 6.00E-70
589- 4: transcript:Zm00001d002229_T001 transcript:EES12893 5.00E-72
589- 5: transcript:Zm00001d002230_T001 transcript:EES12892 3.00E-84
589- 6: transcript:Zm00001d002234_T001 transcript:EES11464 0
589- 7: transcript:Zm00001d002235_T002 transcript:EES11468 0
Alignment 590: score=366.0 e_value=2.9e-14 N=8 2&6 minus
590- 0: transcript:Zm00001d002399_T001 transcript:KXG27059 3.00E-145
590- 1: transcript:Zm00001d002403_T001 transcript:KXG27060 2.00E-96
590- 2: transcript:Zm00001d002405_T001 transcript:EES11384 8.00E-164
590- 3: transcript:Zm00001d002406_T001 transcript:OQU82260 0
590- 4: transcript:Zm00001d002412_T001 transcript:EES12801 0
590- 5: transcript:Zm00001d002413_T005 transcript:EES11381 0
590- 6: transcript:Zm00001d002415_T001 transcript:EES12799 1.00E-48
590- 7: transcript:Zm00001d002416_T002 transcript:EES12797 0
Alignment 591: score=323.0 e_value=4.2e-12 N=7 2&6 minus
591- 0: transcript:Zm00001d002668_T004 transcript:KXG26900 0
591- 1: transcript:Zm00001d002669_T002 transcript:KXG26898 0
591- 2: transcript:Zm00001d002673_T001 transcript:EES12656 0
591- 3: transcript:Zm00001d002675_T001 transcript:KXG26897 0
591- 4: transcript:Zm00001d002676_T001 transcript:KXG26896 0
591- 5: transcript:Zm00001d002677_T001 transcript:OQU82132 4.00E-156
591- 6: transcript:Zm00001d002678_T004 transcript:EES11262 0
Alignment 592: score=281.0 e_value=1.5e-09 N=6 2&6 minus
592- 0: transcript:Zm00001d003648_T029 transcript:EES12185 0
592- 1: transcript:Zm00001d003649_T005 transcript:KXG26334 0
592- 2: transcript:Zm00001d003650_T001 transcript:KXG26333 6.00E-38
592- 3: transcript:Zm00001d003652_T003 transcript:EES10830 0
592- 4: transcript:Zm00001d003653_T002 transcript:OQU81604 2.00E-68
592- 5: transcript:Zm00001d003654_T001 transcript:EES12182 0
Alignment 593: score=281.0 e_value=4.8e-11 N=6 2&6 minus
593- 0: transcript:Zm00001d002211_T001 transcript:OQU82382 2.00E-112
593- 1: transcript:Zm00001d002212_T001 transcript:EES12897 1.00E-25
593- 2: transcript:Zm00001d002220_T001 transcript:EES11475 8.00E-80
593- 3: transcript:Zm00001d002221_T001 transcript:EES12896 1.00E-63
593- 4: transcript:Zm00001d002223_T001 transcript:EES12893 1.00E-72
593- 5: transcript:Zm00001d002224_T001 transcript:EES12892 3.00E-151
Alignment 594: score=273.0 e_value=8.1e-14 N=7 2&6 minus
594- 0: transcript:Zm00001d001769_T001 transcript:EES13136 0
594- 1: transcript:Zm00001d001771_T001 transcript:EES11686 0
594- 2: transcript:Zm00001d001773_T001 transcript:KXG27475 0
594- 3: transcript:Zm00001d001774_T001 transcript:EES11684 2.00E-171
594- 4: transcript:Zm00001d001776_T001 transcript:EES11682 5.00E-41
594- 5: transcript:Zm00001d001779_T001 transcript:EES11679 4.00E-100
594- 6: transcript:Zm00001d001780_T002 transcript:EES11678 0
Alignment 595: score=257.0 e_value=1.6e-08 N=6 2&6 minus
595- 0: transcript:Zm00001d002167_T001 transcript:EES12922 0
595- 1: transcript:Zm00001d002168_T001 transcript:EES11501 0
595- 2: transcript:Zm00001d002172_T001 transcript:OQU82401 0
595- 3: transcript:Zm00001d002173_T001 transcript:OQU82396 0

```
595-  4: transcript:Zm00001d002175_T001 transcript:EES11497                 0
595-  5: transcript:Zm00001d002178_T002 transcript:KXG27189         3.00E-113
## Alignment 596: score=254.0 e_value=8.1e-11 N=6 2&6 minus
596-  0: transcript:Zm00001d003778_T001 transcript:OQU81523                 0
596-  1: transcript:Zm00001d003779_T001 transcript:EES10743                 0
596-  2: transcript:Zm00001d003780_T001 transcript:OQU81517         3.00E-108
596-  3: transcript:Zm00001d003781_T002 transcript:EES10742         1.00E-152
596-  4: transcript:Zm00001d003784_T001 transcript:OQU81515                 0
596-  5: transcript:Zm00001d003786_T002 transcript:KXG26230                 0
## Alignment 597: score=10086.0 e_value=0 N=219 2&7 plus
597-  0: transcript:OQU90181            transcript:Zm00001d022001_T001 9.00E-105
597-  1: transcript:EER97472            transcript:Zm00001d022002_T001  5.00E-77
597-  2: transcript:KXG36606            transcript:Zm00001d022003_T001         0
597-  3: transcript:EER99634            transcript:Zm00001d022006_T001         0
597-  4: transcript:EER97477            transcript:Zm00001d022009_T001         0
597-  5: transcript:EER99637            transcript:Zm00001d022010_T004         0
597-  6: transcript:EER99638            transcript:Zm00001d022016_T001         0
597-  7: transcript:EER99639            transcript:Zm00001d022017_T001         0
597-  8: transcript:KXG36608            transcript:Zm00001d022021_T001  5.00E-91
597-  9: transcript:KXG36609            transcript:Zm00001d022023_T001  3.00E-32
597- 10: transcript:EER97479            transcript:Zm00001d022025_T001  2.00E-55
597- 11: transcript:EER97480            transcript:Zm00001d022026_T001  2.00E-87
597- 12: transcript:EER99642            transcript:Zm00001d022027_T001  6.00E-87
597- 13: transcript:EER99643            transcript:Zm00001d022028_T005         0
597- 14: transcript:EER99645            transcript:Zm00001d022032_T001 3.00E-150
597- 15: transcript:EER97483            transcript:Zm00001d022037_T001         0
597- 16: transcript:EER99646            transcript:Zm00001d022038_T007         0
597- 17: transcript:OQU90190            transcript:Zm00001d022040_T002         0
597- 18: transcript:EER99633            transcript:Zm00001d022041_T001         0
597- 19: transcript:KXG36613            transcript:Zm00001d022042_T006 5.00E-115
597- 20: transcript:OQU90195            transcript:Zm00001d022043_T001         0
597- 21: transcript:EER99654            transcript:Zm00001d022044_T001         0
597- 22: transcript:OQU90200            transcript:Zm00001d022045_T001 2.00E-124
597- 23: transcript:KXG36617            transcript:Zm00001d022046_T004         0
597- 24: transcript:KXG36627            transcript:Zm00001d022048_T002         0
597- 25: transcript:EER97491            transcript:Zm00001d022049_T003         0
597- 26: transcript:KXG36630            transcript:Zm00001d022050_T002 6.00E-132
597- 27: transcript:EER99662            transcript:Zm00001d022052_T001         0
597- 28: transcript:KXG36634            transcript:Zm00001d022053_T001  1.00E-52
597- 29: transcript:OQU90210            transcript:Zm00001d022055_T001  5.00E-06
597- 30: transcript:EER99652            transcript:Zm00001d022058_T001         0
597- 31: transcript:EER97492            transcript:Zm00001d022059_T001  1.00E-63
597- 32: transcript:KXG36638            transcript:Zm00001d022060_T001         0
597- 33: transcript:EER97494            transcript:Zm00001d022062_T001  9.00E-19
597- 34: transcript:EER99673            transcript:Zm00001d022063_T001  1.00E-54
597- 35: transcript:KXG36643            transcript:Zm00001d022065_T001  2.00E-83
597- 36: transcript:OQU90213            transcript:Zm00001d022066_T001         0
597- 37: transcript:EER99675            transcript:Zm00001d022067_T001         0
597- 38: transcript:EER99676            transcript:Zm00001d022069_T001 3.00E-143
597- 39: transcript:OQU90215            transcript:Zm00001d022071_T001         0
597- 40: transcript:KXG36646            transcript:Zm00001d022072_T001         0
597- 41: transcript:EER97496            transcript:Zm00001d022073_T001         0
597- 42: transcript:EER97498            transcript:Zm00001d022075_T001         0
597- 43: transcript:EER97501            transcript:Zm00001d022078_T001         0
```

597- 44:	transcript:EER97506	transcript:Zm00001d022081_T001	0
597- 45:	transcript:EER99681	transcript:Zm00001d022083_T001	1.00E-140
597- 46:	transcript:OQU90222	transcript:Zm00001d022084_T001	1.00E-54
597- 47:	transcript:EER97509	transcript:Zm00001d022085_T001	0
597- 48:	transcript:EER97510	transcript:Zm00001d022088_T004	2.00E-161
597- 49:	transcript:EER97513	transcript:Zm00001d022089_T001	3.00E-16
597- 50:	transcript:EER99682	transcript:Zm00001d022092_T001	0
597- 51:	transcript:OQU90226	transcript:Zm00001d022097_T004	0
597- 52:	transcript:EER99684	transcript:Zm00001d022099_T001	8.00E-95
597- 53:	transcript:EER97518	transcript:Zm00001d022101_T001	1.00E-177
597- 54:	transcript:KXG36663	transcript:Zm00001d022102_T001	5.00E-115
597- 55:	transcript:EER97519	transcript:Zm00001d022103_T002	0
597- 56:	transcript:OQU90232	transcript:Zm00001d022104_T001	1.00E-134
597- 57:	transcript:OQU90234	transcript:Zm00001d022106_T001	0
597- 58:	transcript:EER97507	transcript:Zm00001d022107_T002	3.00E-116
597- 59:	transcript:EER97521	transcript:Zm00001d022108_T004	7.00E-149
597- 60:	transcript:EER99688	transcript:Zm00001d022109_T011	0
597- 61:	transcript:EER97523	transcript:Zm00001d022110_T002	3.00E-176
597- 62:	transcript:EER99689	transcript:Zm00001d022111_T001	4.00E-169
597- 63:	transcript:OQU90236	transcript:Zm00001d022112_T001	2.00E-159
597- 64:	transcript:KXG36666	transcript:Zm00001d022114_T004	0
597- 65:	transcript:KXG36669	transcript:Zm00001d022115_T002	0
597- 66:	transcript:EER97527	transcript:Zm00001d022116_T001	0
597- 67:	transcript:EER99692	transcript:Zm00001d022117_T002	2.00E-89
597- 68:	transcript:OQU90245	transcript:Zm00001d022119_T001	3.00E-136
597- 69:	transcript:OQU90247	transcript:Zm00001d022122_T001	0
597- 70:	transcript:EER99694	transcript:Zm00001d022124_T001	2.00E-101
597- 71:	transcript:EER97528	transcript:Zm00001d022125_T002	0
597- 72:	transcript:OQU90248	transcript:Zm00001d022126_T001	0
597- 73:	transcript:OQU90249	transcript:Zm00001d022127_T001	2.00E-25
597- 74:	transcript:OQU90250	transcript:Zm00001d022130_T001	4.00E-65
597- 75:	transcript:KXG36677	transcript:Zm00001d022131_T001	1.00E-127
597- 76:	transcript:EER97530	transcript:Zm00001d022132_T002	0
597- 77:	transcript:OQU90252	transcript:Zm00001d022133_T001	5.00E-120
597- 78:	transcript:EER99700	transcript:Zm00001d022134_T002	1.00E-130
597- 79:	transcript:KXG36681	transcript:Zm00001d022135_T001	1.00E-45
597- 80:	transcript:EER97533	transcript:Zm00001d022139_T001	2.00E-139
597- 81:	transcript:OQU90254	transcript:Zm00001d022141_T007	1.00E-73
597- 82:	transcript:OQU90255	transcript:Zm00001d022142_T001	0
597- 83:	transcript:KXG36684	transcript:Zm00001d022143_T010	0
597- 84:	transcript:EER99702	transcript:Zm00001d022144_T003	0
597- 85:	transcript:OQU90261	transcript:Zm00001d022148_T001	0
597- 86:	transcript:OQU90262	transcript:Zm00001d022149_T001	1.00E-72
597- 87:	transcript:OQU90263	transcript:Zm00001d022151_T001	0
597- 88:	transcript:EER99708	transcript:Zm00001d022152_T001	0
597- 89:	transcript:EER97543	transcript:Zm00001d022153_T004	0
597- 90:	transcript:KXG36696	transcript:Zm00001d022154_T001	2.00E-108
597- 91:	transcript:EER97544	transcript:Zm00001d022155_T001	0
597- 92:	transcript:EER97545	transcript:Zm00001d022159_T001	0
597- 93:	transcript:KXG36699	transcript:Zm00001d022160_T001	6.00E-121
597- 94:	transcript:KXG36701	transcript:Zm00001d022161_T001	0
597- 95:	transcript:EER97550	transcript:Zm00001d022163_T001	4.00E-108
597- 96:	transcript:EER99712	transcript:Zm00001d022166_T001	0
597- 97:	transcript:EER97552	transcript:Zm00001d022167_T001	9.00E-120

597- 98:	transcript:KXG36712	transcript:Zm00001d022168_T001	0
597- 99:	transcript:OQU90275	transcript:Zm00001d022169_T018	0
597-100:	transcript:EER97557	transcript:Zm00001d022171_T001	2.00E-19
597-101:	transcript:EER99714	transcript:Zm00001d022172_T001	1.00E-139
597-102:	transcript:OQU90276	transcript:Zm00001d022174_T001	1.00E-20
597-103:	transcript:EER97558	transcript:Zm00001d022175_T001	0
597-104:	transcript:OQU90279	transcript:Zm00001d022176_T002	0
597-105:	transcript:EER99718	transcript:Zm00001d022177_T001	3.00E-60
597-106:	transcript:EER97562	transcript:Zm00001d022179_T001	0
597-107:	transcript:OQU90282	transcript:Zm00001d022180_T009	1.00E-162
597-108:	transcript:EER99720	transcript:Zm00001d022181_T004	0
597-109:	transcript:KXG36727	transcript:Zm00001d022182_T001	0
597-110:	transcript:OQU90287	transcript:Zm00001d022184_T008	0
597-111:	transcript:KXG36731	transcript:Zm00001d022185_T003	0
597-112:	transcript:OQU90289	transcript:Zm00001d022188_T002	2.00E-51
597-113:	transcript:KXG36734	transcript:Zm00001d022189_T002	2.00E-12
597-114:	transcript:EER99728	transcript:Zm00001d022190_T003	5.00E-177
597-115:	transcript:OQU90293	transcript:Zm00001d022191_T001	2.00E-88
597-116:	transcript:EER97568	transcript:Zm00001d022192_T002	0
597-117:	transcript:KXG36743	transcript:Zm00001d022194_T001	1.00E-130
597-118:	transcript:EER99735	transcript:Zm00001d022195_T001	7.00E-70
597-119:	transcript:OQU90301	transcript:Zm00001d022196_T001	4.00E-17
597-120:	transcript:EER97576	transcript:Zm00001d022199_T003	0
597-121:	transcript:KXG36748	transcript:Zm00001d022200_T003	0
597-122:	transcript:KXG36751	transcript:Zm00001d022201_T002	0
597-123:	transcript:OQU90305	transcript:Zm00001d022202_T001	0
597-124:	transcript:EER99739	transcript:Zm00001d022203_T001	0
597-125:	transcript:EER97581	transcript:Zm00001d022204_T001	0
597-126:	transcript:KXG36753	transcript:Zm00001d022205_T001	0
597-127:	transcript:KXG36755	transcript:Zm00001d022206_T004	0
597-128:	transcript:EER97586	transcript:Zm00001d022209_T001	0
597-129:	transcript:EER99740	transcript:Zm00001d022210_T001	6.00E-62
597-130:	transcript:KXG36756	transcript:Zm00001d022211_T001	1.00E-60
597-131:	transcript:OQU90306	transcript:Zm00001d022212_T002	4.00E-22
597-132:	transcript:EER99744	transcript:Zm00001d022217_T001	6.00E-94
597-133:	transcript:OQU90309	transcript:Zm00001d022218_T001	1.00E-102
597-134:	transcript:OQU90310	transcript:Zm00001d022224_T001	0
597-135:	transcript:EER99751	transcript:Zm00001d022225_T001	8.00E-174
597-136:	transcript:EER99752	transcript:Zm00001d022226_T002	0
597-137:	transcript:OQU90312	transcript:Zm00001d022227_T001	0
597-138:	transcript:EER97591	transcript:Zm00001d022228_T001	2.00E-151
597-139:	transcript:OQU90313	transcript:Zm00001d022229_T001	0
597-140:	transcript:EER97593	transcript:Zm00001d022230_T001	0
597-141:	transcript:KXG36765	transcript:Zm00001d022231_T001	0
597-142:	transcript:KXG36766	transcript:Zm00001d022233_T001	5.00E-39
597-143:	transcript:EER97595	transcript:Zm00001d022234_T001	9.00E-100
597-144:	transcript:EER97596	transcript:Zm00001d022236_T001	0
597-145:	transcript:EER97597	transcript:Zm00001d022237_T001	1.00E-158
597-146:	transcript:EER97600	transcript:Zm00001d022238_T001	3.00E-168
597-147:	transcript:EER99756	transcript:Zm00001d022239_T001	0
597-148:	transcript:OQU90319	transcript:Zm00001d022240_T006	0
597-149:	transcript:OQU90320	transcript:Zm00001d022241_T001	5.00E-166
597-150:	transcript:EER99759	transcript:Zm00001d022242_T001	3.00E-97
597-151:	transcript:EER97602	transcript:Zm00001d022243_T002	8.00E-113

597-152:	transcript:EER99760	transcript:Zm00001d022244_T002	7.00E-80
597-153:	transcript:EER97603	transcript:Zm00001d022245_T001	0
597-154:	transcript:EER99761	transcript:Zm00001d022246_T001	0
597-155:	transcript:EER99762	transcript:Zm00001d022247_T001	0
597-156:	transcript:EER97604	transcript:Zm00001d022248_T001	3.00E-29
597-157:	transcript:EER97605	transcript:Zm00001d022249_T001	3.00E-34
597-158:	transcript:OQU90326	transcript:Zm00001d022250_T001	3.00E-164
597-159:	transcript:EER97606	transcript:Zm00001d022251_T002	7.00E-160
597-160:	transcript:EER97589	transcript:Zm00001d022252_T001	0
597-161:	transcript:EER99765	transcript:Zm00001d022254_T002	4.00E-168
597-162:	transcript:EER97607	transcript:Zm00001d022258_T002	0
597-163:	transcript:EER97608	transcript:Zm00001d022259_T002	1.00E-162
597-164:	transcript:EER97609	transcript:Zm00001d022262_T001	0
597-165:	transcript:OQU90331	transcript:Zm00001d022263_T001	0
597-166:	transcript:KXG36781	transcript:Zm00001d022264_T001	6.00E-32
597-167:	transcript:EER99769	transcript:Zm00001d022265_T001	0
597-168:	transcript:EER97613	transcript:Zm00001d022266_T001	0
597-169:	transcript:EER97614	transcript:Zm00001d022268_T001	2.00E-42
597-170:	transcript:KXG36787	transcript:Zm00001d022270_T002	6.00E-98
597-171:	transcript:EER97615	transcript:Zm00001d022272_T002	0
597-172:	transcript:EER99772	transcript:Zm00001d022273_T016	0
597-173:	transcript:KXG36786	transcript:Zm00001d022274_T001	0
597-174:	transcript:EER97617	transcript:Zm00001d022275_T001	0
597-175:	transcript:KXG36790	transcript:Zm00001d022276_T002	0
597-176:	transcript:OQU90341	transcript:Zm00001d022277_T001	0
597-177:	transcript:EER97619	transcript:Zm00001d022278_T001	6.00E-152
597-178:	transcript:EER97620	transcript:Zm00001d022279_T001	0
597-179:	transcript:EER99779	transcript:Zm00001d022286_T005	0
597-180:	transcript:EER99780	transcript:Zm00001d022289_T001	1.00E-94
597-181:	transcript:EER97624	transcript:Zm00001d022294_T002	5.00E-48
597-182:	transcript:EER99785	transcript:Zm00001d022295_T001	1.00E-168
597-183:	transcript:OQU90344	transcript:Zm00001d022296_T001	1.00E-97
597-184:	transcript:EER97626	transcript:Zm00001d022302_T001	8.00E-157
597-185:	transcript:EER99787	transcript:Zm00001d022303_T001	0
597-186:	transcript:EER99788	transcript:Zm00001d022305_T001	0
597-187:	transcript:OQU90346	transcript:Zm00001d022306_T001	1.00E-30
597-188:	transcript:EER97627	transcript:Zm00001d022307_T003	3.00E-62
597-189:	transcript:OQU90350	transcript:Zm00001d022309_T002	9.00E-80
597-190:	transcript:EER97632	transcript:Zm00001d022313_T001	0
597-191:	transcript:OQU90351	transcript:Zm00001d022314_T001	1.00E-165
597-192:	transcript:OQU90356	transcript:Zm00001d022315_T001	0
597-193:	transcript:EER97635	transcript:Zm00001d022316_T001	0
597-194:	transcript:OQU90360	transcript:Zm00001d022317_T001	0
597-195:	transcript:KXG36804	transcript:Zm00001d022322_T001	0
597-196:	transcript:EER99796	transcript:Zm00001d022324_T001	0
597-197:	transcript:EER99799	transcript:Zm00001d022330_T001	2.00E-109
597-198:	transcript:KXG36809	transcript:Zm00001d022332_T001	0
597-199:	transcript:OQU90363	transcript:Zm00001d022333_T001	4.00E-59
597-200:	transcript:EER97639	transcript:Zm00001d022334_T002	0
597-201:	transcript:EER99801	transcript:Zm00001d022335_T002	0
597-202:	transcript:KXG36812	transcript:Zm00001d022337_T002	3.00E-155
597-203:	transcript:KXG36813	transcript:Zm00001d022338_T002	0
597-204:	transcript:KXG36814	transcript:Zm00001d022341_T001	0
597-205:	transcript:EER99805	transcript:Zm00001d022342_T001	0

597-206: transcript:KXG36818 transcript:Zm00001d022344_T001 0
597-207: transcript:OQU90373 transcript:Zm00001d022347_T001 0
597-208: transcript:EER97657 transcript:Zm00001d022349_T001 0
597-209: transcript:EER97658 transcript:Zm00001d022350_T001 8.00E-24
597-210: transcript:OQU90374 transcript:Zm00001d022351_T001 0
597-211: transcript:OQU90375 transcript:Zm00001d022352_T001 1.00E-137
597-212: transcript:KXG36823 transcript:Zm00001d022353_T001 0
597-213: transcript:OQU90377 transcript:Zm00001d022354_T001 5.00E-44
597-214: transcript:KXG36825 transcript:Zm00001d022355_T001 0
597-215: transcript:EER97660 transcript:Zm00001d022360_T002 0
597-216: transcript:EER99816 transcript:Zm00001d022361_T001 0
597-217: transcript:EER99817 transcript:Zm00001d022364_T002 0
597-218: transcript:EER99824 transcript:Zm00001d022366_T001 0
Alignment 598: score=8219.0 e_value=0 N=177 2&7 plus
598- 0: transcript:EER96777 transcript:Zm00001d020603_T004 0
598- 1: transcript:OQU89560 transcript:Zm00001d020605_T001 2.00E-82
598- 2: transcript:EER96781 transcript:Zm00001d020606_T001 5.00E-93
598- 3: transcript:OQU89562 transcript:Zm00001d020607_T001 6.00E-21
598- 4: transcript:KXG35704 transcript:Zm00001d020609_T001 0
598- 5: transcript:KXG35705 transcript:Zm00001d020610_T001 8.00E-77
598- 6: transcript:EER96789 transcript:Zm00001d020612_T001 0
598- 7: transcript:KXG35707 transcript:Zm00001d020613_T001 3.00E-160
598- 8: transcript:EER98969 transcript:Zm00001d020614_T001 6.00E-96
598- 9: transcript:EER96790 transcript:Zm00001d020615_T001 0
598- 10: transcript:EER96791 transcript:Zm00001d020617_T001 0
598- 11: transcript:EER98970 transcript:Zm00001d020618_T003 0
598- 12: transcript:KXG35709 transcript:Zm00001d020620_T001 1.00E-51
598- 13: transcript:KXG35712 transcript:Zm00001d020622_T002 0
598- 14: transcript:KXG35713 transcript:Zm00001d020623_T001 8.00E-81
598- 15: transcript:OQU89566 transcript:Zm00001d020626_T001 1.00E-13
598- 16: transcript:EER96794 transcript:Zm00001d020627_T001 0
598- 17: transcript:EER98974 transcript:Zm00001d020628_T001 0
598- 18: transcript:EER96796 transcript:Zm00001d020629_T001 9.00E-21
598- 19: transcript:EER98978 transcript:Zm00001d020631_T001 0
598- 20: transcript:EER96801 transcript:Zm00001d020636_T001 0
598- 21: transcript:OQU89581 transcript:Zm00001d020638_T002 0
598- 22: transcript:EER96802 transcript:Zm00001d020639_T002 0
598- 23: transcript:KXG35723 transcript:Zm00001d020640_T002 1.00E-93
598- 24: transcript:EER96805 transcript:Zm00001d020643_T002 0
598- 25: transcript:KXG35726 transcript:Zm00001d020644_T001 8.00E-160
598- 26: transcript:EER96807 transcript:Zm00001d020646_T001 6.00E-102
598- 27: transcript:EER98983 transcript:Zm00001d020647_T002 0
598- 28: transcript:EER96809 transcript:Zm00001d020650_T001 7.00E-108
598- 29: transcript:EER98982 transcript:Zm00001d020651_T001 0
598- 30: transcript:OQU89585 transcript:Zm00001d020653_T001 3.00E-104
598- 31: transcript:EER96812 transcript:Zm00001d020655_T001 2.00E-55
598- 32: transcript:EER96813 transcript:Zm00001d020656_T001 0
598- 33: transcript:OQU89590 transcript:Zm00001d020657_T001 4.00E-108
598- 34: transcript:OQU89588 transcript:Zm00001d020658_T001 3.00E-45
598- 35: transcript:OQU89591 transcript:Zm00001d020659_T001 5.00E-68
598- 36: transcript:KXG35736 transcript:Zm00001d020664_T016 0
598- 37: transcript:OQU89595 transcript:Zm00001d020669_T001 0
598- 38: transcript:EER98990 transcript:Zm00001d020670_T001 2.00E-151
598- 39: transcript:OQU89596 transcript:Zm00001d020673_T001 0

598- 40:	transcript:EER96818	transcript:Zm00001d020674_T001	0
598- 41:	transcript:KXG35739	transcript:Zm00001d020675_T001	1.00E-154
598- 42:	transcript:KXG35742	transcript:Zm00001d020679_T001	4.00E-159
598- 43:	transcript:EER96823	transcript:Zm00001d020680_T001	0
598- 44:	transcript:EER98992	transcript:Zm00001d020681_T001	1.00E-76
598- 45:	transcript:EER96827	transcript:Zm00001d020683_T001	0
598- 46:	transcript:EER96829	transcript:Zm00001d020684_T001	4.00E-90
598- 47:	transcript:EER98994	transcript:Zm00001d020685_T002	0
598- 48:	transcript:EER98995	transcript:Zm00001d020686_T001	0
598- 49:	transcript:EER96832	transcript:Zm00001d020687_T002	0
598- 50:	transcript:EER96833	transcript:Zm00001d020688_T001	0
598- 51:	transcript:EER96834	transcript:Zm00001d020691_T001	0
598- 52:	transcript:KXG35748	transcript:Zm00001d020692_T002	0
598- 53:	transcript:EER99001	transcript:Zm00001d020694_T001	0
598- 54:	transcript:KXG35750	transcript:Zm00001d020695_T001	0
598- 55:	transcript:EER96840	transcript:Zm00001d020696_T001	0
598- 56:	transcript:KXG35752	transcript:Zm00001d020697_T001	0
598- 57:	transcript:KXG35755	transcript:Zm00001d020702_T001	0
598- 58:	transcript:EER96846	transcript:Zm00001d020703_T001	0
598- 59:	transcript:EER96848	transcript:Zm00001d020705_T001	8.00E-104
598- 60:	transcript:KXG35759	transcript:Zm00001d020706_T001	0
598- 61:	transcript:KXG35760	transcript:Zm00001d020707_T001	0
598- 62:	transcript:KXG35763	transcript:Zm00001d020708_T002	0
598- 63:	transcript:EER96850	transcript:Zm00001d020711_T001	3.00E-167
598- 64:	transcript:EER99009	transcript:Zm00001d020713_T001	0
598- 65:	transcript:EER96851	transcript:Zm00001d020714_T001	5.00E-149
598- 66:	transcript:EER99010	transcript:Zm00001d020717_T001	0
598- 67:	transcript:KXG35766	transcript:Zm00001d020719_T002	0
598- 68:	transcript:EER99011	transcript:Zm00001d020721_T001	0
598- 69:	transcript:EER96860	transcript:Zm00001d020722_T001	5.00E-113
598- 70:	transcript:EER96862	transcript:Zm00001d020723_T001	3.00E-62
598- 71:	transcript:OQU89614	transcript:Zm00001d020725_T001	1.00E-98
598- 72:	transcript:OQU89615	transcript:Zm00001d020726_T001	0
598- 73:	transcript:EER96863	transcript:Zm00001d020728_T005	0
598- 74:	transcript:EER96866	transcript:Zm00001d020731_T001	5.00E-110
598- 75:	transcript:OQU89623	transcript:Zm00001d020732_T001	0
598- 76:	transcript:KXG35780	transcript:Zm00001d020736_T001	0
598- 77:	transcript:KXG35782	transcript:Zm00001d020737_T001	2.00E-107
598- 78:	transcript:EER99019	transcript:Zm00001d020738_T001	7.00E-90
598- 79:	transcript:EER96865	transcript:Zm00001d020740_T001	2.00E-79
598- 80:	transcript:KXG35787	transcript:Zm00001d020743_T001	2.00E-15
598- 81:	transcript:KXG35788	transcript:Zm00001d020746_T001	6.00E-51
598- 82:	transcript:KXG35789	transcript:Zm00001d020748_T002	0
598- 83:	transcript:KXG35791	transcript:Zm00001d020750_T001	0
598- 84:	transcript:OQU89627	transcript:Zm00001d020752_T001	0
598- 85:	transcript:OQU89629	transcript:Zm00001d020757_T001	2.00E-144
598- 86:	transcript:KXG35797	transcript:Zm00001d020762_T001	0
598- 87:	transcript:EER96881	transcript:Zm00001d020763_T001	1.00E-113
598- 88:	transcript:KXG35798	transcript:Zm00001d020764_T004	0
598- 89:	transcript:OQU89633	transcript:Zm00001d020768_T001	3.00E-136
598- 90:	transcript:KXG35804	transcript:Zm00001d020769_T005	0
598- 91:	transcript:EER96885	transcript:Zm00001d020770_T002	2.00E-132
598- 92:	transcript:EER99030	transcript:Zm00001d020771_T003	0
598- 93:	transcript:EER96886	transcript:Zm00001d020772_T001	2.00E-102

598- 94:	transcript:EER96884	transcript:Zm00001d020773_T001	7.00E-152
598- 95:	transcript:EER96888	transcript:Zm00001d020774_T001	1.00E-111
598- 96:	transcript:EER99032	transcript:Zm00001d020775_T001	0
598- 97:	transcript:OQU89636	transcript:Zm00001d020776_T002	0
598- 98:	transcript:EER96889	transcript:Zm00001d020779_T001	8.00E-28
598- 99:	transcript:EER96890	transcript:Zm00001d020780_T001	8.00E-152
598-100:	transcript:KXG35809	transcript:Zm00001d020781_T003	3.00E-114
598-101:	transcript:EER99039	transcript:Zm00001d020782_T001	1.00E-53
598-102:	transcript:KXG35813	transcript:Zm00001d020783_T005	2.00E-125
598-103:	transcript:OQU89638	transcript:Zm00001d020787_T001	1.00E-161
598-104:	transcript:EER99041	transcript:Zm00001d020789_T001	0
598-105:	transcript:EER96894	transcript:Zm00001d020790_T001	4.00E-153
598-106:	transcript:EER99042	transcript:Zm00001d020791_T001	1.00E-152
598-107:	transcript:EER96896	transcript:Zm00001d020793_T001	5.00E-46
598-108:	transcript:KXG35819	transcript:Zm00001d020799_T003	0
598-109:	transcript:EER96900	transcript:Zm00001d020803_T001	6.00E-38
598-110:	transcript:EER96901	transcript:Zm00001d020804_T001	2.00E-157
598-111:	transcript:EER96902	transcript:Zm00001d020807_T003	0
598-112:	transcript:OQU89643	transcript:Zm00001d020808_T001	3.00E-125
598-113:	transcript:EER96904	transcript:Zm00001d020810_T002	0
598-114:	transcript:KXG35822	transcript:Zm00001d020811_T001	0
598-115:	transcript:OQU89644	transcript:Zm00001d020820_T001	4.00E-77
598-116:	transcript:OQU89646	transcript:Zm00001d020821_T005	0
598-117:	transcript:EER96905	transcript:Zm00001d020823_T001	4.00E-86
598-118:	transcript:KXG35833	transcript:Zm00001d020825_T001	0
598-119:	transcript:KXG35834	transcript:Zm00001d020826_T001	0
598-120:	transcript:EER99049	transcript:Zm00001d020828_T001	0
598-121:	transcript:KXG35839	transcript:Zm00001d020834_T004	4.00E-58
598-122:	transcript:KXG35843	transcript:Zm00001d020835_T022	0
598-123:	transcript:OQU89656	transcript:Zm00001d020837_T001	4.00E-83
598-124:	transcript:EER96917	transcript:Zm00001d020838_T001	9.00E-165
598-125:	transcript:EER96918	transcript:Zm00001d020839_T001	7.00E-80
598-126:	transcript:KXG35845	transcript:Zm00001d020840_T001	9.00E-113
598-127:	transcript:KXG35846	transcript:Zm00001d020843_T001	7.00E-21
598-128:	transcript:EER96920	transcript:Zm00001d020845_T001	2.00E-93
598-129:	transcript:KXG35849	transcript:Zm00001d020850_T001	8.00E-10
598-130:	transcript:KXG35850	transcript:Zm00001d020851_T001	0
598-131:	transcript:EER99055	transcript:Zm00001d020852_T001	0
598-132:	transcript:EER96924	transcript:Zm00001d020853_T004	0
598-133:	transcript:KXG35851	transcript:Zm00001d020857_T001	0
598-134:	transcript:KXG35854	transcript:Zm00001d020858_T001	0
598-135:	transcript:OQU89666	transcript:Zm00001d020861_T001	2.00E-81
598-136:	transcript:KXG35861	transcript:Zm00001d020862_T001	0
598-137:	transcript:OQU89668	transcript:Zm00001d020863_T001	2.00E-82
598-138:	transcript:KXG35865	transcript:Zm00001d020867_T001	0
598-139:	transcript:EER99067	transcript:Zm00001d020869_T001	1.00E-31
598-140:	transcript:EER96940	transcript:Zm00001d020870_T001	0
598-141:	transcript:KXG35867	transcript:Zm00001d020871_T001	2.00E-56
598-142:	transcript:EER96944	transcript:Zm00001d020872_T001	4.00E-34
598-143:	transcript:EER96945	transcript:Zm00001d020874_T001	3.00E-133
598-144:	transcript:EER96947	transcript:Zm00001d020876_T001	1.00E-95
598-145:	transcript:EER96948	transcript:Zm00001d020877_T001	1.00E-78
598-146:	transcript:EER99068	transcript:Zm00001d020878_T001	1.00E-115
598-147:	transcript:EER96942	transcript:Zm00001d020879_T001	8.00E-124

598-148:	transcript:KXG35872	transcript:Zm00001d020881_T002	0
598-149:	transcript:KXG35874	transcript:Zm00001d020882_T001	0
598-150:	transcript:EER96951	transcript:Zm00001d020886_T001	0
598-151:	transcript:EER99074	transcript:Zm00001d020888_T001	5.00E-51
598-152:	transcript:KXG35876	transcript:Zm00001d020892_T003	0
598-153:	transcript:OQU89678	transcript:Zm00001d020900_T001	0
598-154:	transcript:EER99078	transcript:Zm00001d020901_T001	0
598-155:	transcript:KXG35886	transcript:Zm00001d020902_T002	0
598-156:	transcript:KXG35889	transcript:Zm00001d020903_T001	4.00E-136
598-157:	transcript:KXG35890	transcript:Zm00001d020908_T001	0
598-158:	transcript:OQU89681	transcript:Zm00001d020909_T001	0
598-159:	transcript:KXG35895	transcript:Zm00001d020913_T001	0
598-160:	transcript:EER96962	transcript:Zm00001d020915_T002	0
598-161:	transcript:EER96963	transcript:Zm00001d020921_T001	0
598-162:	transcript:EER96965	transcript:Zm00001d020923_T001	1.00E-161
598-163:	transcript:OQU89684	transcript:Zm00001d020925_T001	5.00E-118
598-164:	transcript:OQU89687	transcript:Zm00001d020926_T001	2.00E-70
598-165:	transcript:EER99080	transcript:Zm00001d020927_T001	0
598-166:	transcript:EER99082	transcript:Zm00001d020929_T002	0
598-167:	transcript:EER99083	transcript:Zm00001d020930_T001	2.00E-78
598-168:	transcript:EER99085	transcript:Zm00001d020931_T001	0
598-169:	transcript:KXG35903	transcript:Zm00001d020932_T001	0
598-170:	transcript:KXG35906	transcript:Zm00001d020935_T002	0
598-171:	transcript:EER96975	transcript:Zm00001d020936_T001	0
598-172:	transcript:EER99087	transcript:Zm00001d020937_T001	0
598-173:	transcript:KXG35908	transcript:Zm00001d020938_T002	0
598-174:	transcript:EER99089	transcript:Zm00001d020939_T001	0
598-175:	transcript:OQU89693	transcript:Zm00001d020940_T001	5.00E-69
598-176:	transcript:EER99092	transcript:Zm00001d020941_T001	0

Alignment 599: score=6109.0 e_value=0 N=133 2&7 plus

599- 0:	transcript:EER98806	transcript:Zm00001d020260_T001	0
599- 1:	transcript:EER96652	transcript:Zm00001d020264_T001	8.00E-80
599- 2:	transcript:EER96655	transcript:Zm00001d020265_T001	2.00E-83
599- 3:	transcript:KXG35507	transcript:Zm00001d020267_T001	1.00E-150
599- 4:	transcript:EER96661	transcript:Zm00001d020272_T001	0
599- 5:	transcript:EER96662	transcript:Zm00001d020273_T001	8.00E-152
599- 6:	transcript:EER98811	transcript:Zm00001d020274_T001	8.00E-24
599- 7:	transcript:KXG35511	transcript:Zm00001d020275_T001	1.00E-112
599- 8:	transcript:OQU89417	transcript:Zm00001d020277_T001	0
599- 9:	transcript:EER98814	transcript:Zm00001d020279_T001	0
599- 10:	transcript:KXG35517	transcript:Zm00001d020283_T001	0
599- 11:	transcript:KXG35519	transcript:Zm00001d020296_T001	0
599- 12:	transcript:KXG35520	transcript:Zm00001d020297_T002	0
599- 13:	transcript:KXG35522	transcript:Zm00001d020303_T001	0
599- 14:	transcript:EER96671	transcript:Zm00001d020306_T001	0
599- 15:	transcript:EER98819	transcript:Zm00001d020309_T001	0
599- 16:	transcript:EER96675	transcript:Zm00001d020311_T001	2.00E-58
599- 17:	transcript:KXG35529	transcript:Zm00001d020312_T017	0
599- 18:	transcript:KXG35533	transcript:Zm00001d020315_T001	5.00E-92
599- 19:	transcript:OQU89431	transcript:Zm00001d020317_T001	4.00E-29
599- 20:	transcript:EER96678	transcript:Zm00001d020322_T001	0
599- 21:	transcript:EER96679	transcript:Zm00001d020323_T001	4.00E-163
599- 22:	transcript:EER98827	transcript:Zm00001d020325_T001	0
599- 23:	transcript:KXG35540	transcript:Zm00001d020332_T001	0

599- 24:	transcript:KXG35541	transcript:Zm00001d020337_T001	1.00E-109
599- 25:	transcript:KXG35543	transcript:Zm00001d020339_T001	0
599- 26:	transcript:KXG35545	transcript:Zm00001d020340_T001	0
599- 27:	transcript:KXG35546	transcript:Zm00001d020343_T002	0
599- 28:	transcript:OQU89437	transcript:Zm00001d020344_T001	2.00E-173
599- 29:	transcript:EER96683	transcript:Zm00001d020345_T001	0
599- 30:	transcript:OQU89439	transcript:Zm00001d020346_T001	6.00E-43
599- 31:	transcript:EER96686	transcript:Zm00001d020348_T001	5.00E-91
599- 32:	transcript:KXG35555	transcript:Zm00001d020350_T001	5.00E-145
599- 33:	transcript:OQU89444	transcript:Zm00001d020353_T001	9.00E-80
599- 34:	transcript:EER98845	transcript:Zm00001d020354_T001	1.00E-42
599- 35:	transcript:EER98846	transcript:Zm00001d020355_T002	0
599- 36:	transcript:KXG35557	transcript:Zm00001d020357_T003	0
599- 37:	transcript:OQU89450	transcript:Zm00001d020358_T001	0
599- 38:	transcript:EER98853	transcript:Zm00001d020359_T003	0
599- 39:	transcript:EER96695	transcript:Zm00001d020361_T001	2.00E-154
599- 40:	transcript:EER98854	transcript:Zm00001d020362_T001	9.00E-152
599- 41:	transcript:EER98855	transcript:Zm00001d020363_T002	0
599- 42:	transcript:KXG35568	transcript:Zm00001d020364_T001	2.00E-64
599- 43:	transcript:EER96700	transcript:Zm00001d020365_T001	0
599- 44:	transcript:KXG35570	transcript:Zm00001d020366_T001	3.00E-06
599- 45:	transcript:KXG35571	transcript:Zm00001d020367_T003	0
599- 46:	transcript:EER98860	transcript:Zm00001d020371_T001	0
599- 47:	transcript:KXG35575	transcript:Zm00001d020374_T003	0
599- 48:	transcript:OQU89459	transcript:Zm00001d020378_T001	0
599- 49:	transcript:EER98863	transcript:Zm00001d020380_T002	0
599- 50:	transcript:EER96704	transcript:Zm00001d020381_T008	0
599- 51:	transcript:KXG35581	transcript:Zm00001d020382_T001	1.00E-52
599- 52:	transcript:EER98866	transcript:Zm00001d020392_T002	0
599- 53:	transcript:EER96705	transcript:Zm00001d020395_T002	2.00E-172
599- 54:	transcript:KXG35582	transcript:Zm00001d020396_T004	0
599- 55:	transcript:KXG35583	transcript:Zm00001d020399_T007	0
599- 56:	transcript:EER98869	transcript:Zm00001d020400_T001	0
599- 57:	transcript:EER98872	transcript:Zm00001d020403_T001	0
599- 58:	transcript:EER96710	transcript:Zm00001d020405_T001	3.00E-147
599- 59:	transcript:EER98873	transcript:Zm00001d020409_T002	0
599- 60:	transcript:OQU89468	transcript:Zm00001d020411_T001	1.00E-142
599- 61:	transcript:EER98875	transcript:Zm00001d020412_T001	6.00E-125
599- 62:	transcript:OQU89470	transcript:Zm00001d020414_T002	0
599- 63:	transcript:KXG35595	transcript:Zm00001d020416_T002	0
599- 64:	transcript:KXG35593	transcript:Zm00001d020417_T001	2.00E-123
599- 65:	transcript:EER98878	transcript:Zm00001d020418_T001	0
599- 66:	transcript:KXG35596	transcript:Zm00001d020419_T003	4.00E-155
599- 67:	transcript:KXG35599	transcript:Zm00001d020420_T001	0
599- 68:	transcript:KXG35601	transcript:Zm00001d020424_T002	0
599- 69:	transcript:KXG35600	transcript:Zm00001d020425_T001	1.00E-53
599- 70:	transcript:OQU89472	transcript:Zm00001d020426_T001	0
599- 71:	transcript:EER96715	transcript:Zm00001d020429_T001	0
599- 72:	transcript:KXG35603	transcript:Zm00001d020430_T001	3.00E-56
599- 73:	transcript:KXG35610	transcript:Zm00001d020432_T001	0
599- 74:	transcript:EER96723	transcript:Zm00001d020433_T001	2.00E-122
599- 75:	transcript:EER98887	transcript:Zm00001d020434_T001	1.00E-95
599- 76:	transcript:OQU89478	transcript:Zm00001d020435_T001	0
599- 77:	transcript:EER96725	transcript:Zm00001d020436_T001	0

599- 78:	transcript:EER98889	transcript:Zm00001d020437_T001	0
599- 79:	transcript:EER98890	transcript:Zm00001d020438_T001	2.00E-76
599- 80:	transcript:EER98892	transcript:Zm00001d020441_T001	0
599- 81:	transcript:EER96726	transcript:Zm00001d020443_T001	7.00E-52
599- 82:	transcript:EER98894	transcript:Zm00001d020444_T003	0
599- 83:	transcript:EER96727	transcript:Zm00001d020445_T001	0
599- 84:	transcript:OQU89481	transcript:Zm00001d020446_T001	0
599- 85:	transcript:OQU89486	transcript:Zm00001d020448_T001	0
599- 86:	transcript:EER98897	transcript:Zm00001d020450_T001	9.00E-81
599- 87:	transcript:OQU89487	transcript:Zm00001d020454_T003	0
599- 88:	transcript:KXG35628	transcript:Zm00001d020457_T001	0
599- 89:	transcript:KXG35630	transcript:Zm00001d020459_T001	1.00E-61
599- 90:	transcript:KXG35632	transcript:Zm00001d020460_T001	1.00E-169
599- 91:	transcript:EER96732	transcript:Zm00001d020461_T001	0
599- 92:	transcript:EER96733	transcript:Zm00001d020463_T002	0
599- 93:	transcript:EER96734	transcript:Zm00001d020472_T001	7.00E-42
599- 94:	transcript:EER98903	transcript:Zm00001d020485_T001	8.00E-157
599- 95:	transcript:EER96736	transcript:Zm00001d020486_T001	0
599- 96:	transcript:EER96737	transcript:Zm00001d020487_T001	0
599- 97:	transcript:OQU89496	transcript:Zm00001d020490_T001	9.00E-94
599- 98:	transcript:EER98905	transcript:Zm00001d020492_T001	1.00E-159
599- 99:	transcript:EER98907	transcript:Zm00001d020496_T001	0
599-100:	transcript:EER96738	transcript:Zm00001d020497_T001	0
599-101:	transcript:OQU89500	transcript:Zm00001d020500_T001	9.00E-06
599-102:	transcript:OQU89502	transcript:Zm00001d020501_T001	0
599-103:	transcript:EER96744	transcript:Zm00001d020503_T001	3.00E-39
599-104:	transcript:EER98909	transcript:Zm00001d020505_T001	2.00E-131
599-105:	transcript:EER98910	transcript:Zm00001d020506_T001	1.00E-145
599-106:	transcript:KXG35644	transcript:Zm00001d020510_T003	7.00E-41
599-107:	transcript:EER96746	transcript:Zm00001d020511_T001	2.00E-78
599-108:	transcript:EER98911	transcript:Zm00001d020512_T001	0
599-109:	transcript:OQU89509	transcript:Zm00001d020515_T002	0
599-110:	transcript:EER96747	transcript:Zm00001d020519_T001	5.00E-120
599-111:	transcript:EER98912	transcript:Zm00001d020521_T001	0
599-112:	transcript:EER98918	transcript:Zm00001d020528_T001	0
599-113:	transcript:EER98920	transcript:Zm00001d020530_T001	0
599-114:	transcript:EER96750	transcript:Zm00001d020531_T002	0
599-115:	transcript:KXG35650	transcript:Zm00001d020532_T002	0
599-116:	transcript:OQU89518	transcript:Zm00001d020533_T001	0
599-117:	transcript:KXG35653	transcript:Zm00001d020535_T001	0
599-118:	transcript:EER96754	transcript:Zm00001d020536_T001	0
599-119:	transcript:EER98922	transcript:Zm00001d020537_T001	0
599-120:	transcript:KXG35655	transcript:Zm00001d020538_T001	8.00E-50
599-121:	transcript:OQU89519	transcript:Zm00001d020540_T002	0
599-122:	transcript:EER96758	transcript:Zm00001d020541_T003	0
599-123:	transcript:EER98924	transcript:Zm00001d020543_T001	0
599-124:	transcript:OQU89520	transcript:Zm00001d020544_T001	2.00E-20
599-125:	transcript:OQU89521	transcript:Zm00001d020549_T005	0
599-126:	transcript:EER96763	transcript:Zm00001d020552_T001	1.00E-178
599-127:	transcript:EER96762	transcript:Zm00001d020555_T001	0
599-128:	transcript:EER98930	transcript:Zm00001d020557_T001	0
599-129:	transcript:KXG35667	transcript:Zm00001d020558_T001	1.00E-33
599-130:	transcript:KXG35666	transcript:Zm00001d020560_T001	0
599-131:	transcript:OQU89525	transcript:Zm00001d020561_T002	0

599-132: transcript:OQU89527 transcript:Zm00001d020563_T001 0
Alignment 600: score=3085.0 e_value=1.1e-281 N=68 2&7 plus
600- 0: transcript:OQU89729 transcript:Zm00001d021001_T001 2.00E-86
600- 1: transcript:EER99130 transcript:Zm00001d021006_T001 3.00E-120
600- 2: transcript:EER96997 transcript:Zm00001d021010_T001 4.00E-30
600- 3: transcript:KXG35959 transcript:Zm00001d021014_T001 0
600- 4: transcript:EER96994 transcript:Zm00001d021015_T002 0
600- 5: transcript:EER99135 transcript:Zm00001d021016_T001 0
600- 6: transcript:EER97001 transcript:Zm00001d021017_T001 3.00E-94
600- 7: transcript:EER97002 transcript:Zm00001d021018_T002 9.00E-91
600- 8: transcript:OQU89735 transcript:Zm00001d021019_T001 0
600- 9: transcript:EER99137 transcript:Zm00001d021021_T001 6.00E-96
600- 10: transcript:KXG35961 transcript:Zm00001d021023_T001 0
600- 11: transcript:EER99139 transcript:Zm00001d021024_T001 0
600- 12: transcript:EER97004 transcript:Zm00001d021025_T001 3.00E-152
600- 13: transcript:EER99141 transcript:Zm00001d021026_T001 0
600- 14: transcript:EER97007 transcript:Zm00001d021031_T002 0
600- 15: transcript:EER99142 transcript:Zm00001d021032_T001 1.00E-107
600- 16: transcript:OQU89741 transcript:Zm00001d021035_T001 7.00E-13
600- 17: transcript:KXG35966 transcript:Zm00001d021036_T001 1.00E-155
600- 18: transcript:OQU89743 transcript:Zm00001d021038_T001 2.00E-84
600- 19: transcript:OQU89744 transcript:Zm00001d021046_T003 0
600- 20: transcript:EER99149 transcript:Zm00001d021047_T001 0
600- 21: transcript:EER99152 transcript:Zm00001d021050_T002 2.00E-160
600- 22: transcript:OQU89746 transcript:Zm00001d021051_T002 2.00E-128
600- 23: transcript:EER99154 transcript:Zm00001d021053_T001 0
600- 24: transcript:OQU89749 transcript:Zm00001d021054_T002 5.00E-87
600- 25: transcript:EER97011 transcript:Zm00001d021056_T001 0
600- 26: transcript:EER97012 transcript:Zm00001d021057_T001 1.00E-169
600- 27: transcript:EER97013 transcript:Zm00001d021058_T001 1.00E-72
600- 28: transcript:KXG35977 transcript:Zm00001d021059_T001 0
600- 29: transcript:EER99157 transcript:Zm00001d021060_T001 3.00E-93
600- 30: transcript:EER99160 transcript:Zm00001d021061_T001 0
600- 31: transcript:OQU89753 transcript:Zm00001d021062_T001 3.00E-67
600- 32: transcript:EER99162 transcript:Zm00001d021063_T001 1.00E-145
600- 33: transcript:OQU89754 transcript:Zm00001d021064_T001 5.00E-160
600- 34: transcript:EER99164 transcript:Zm00001d021065_T001 0
600- 35: transcript:EER97019 transcript:Zm00001d021070_T002 0
600- 36: transcript:EER97020 transcript:Zm00001d021071_T001 0
600- 37: transcript:KXG35983 transcript:Zm00001d021072_T001 0
600- 38: transcript:KXG35982 transcript:Zm00001d021073_T002 0
600- 39: transcript:EER97022 transcript:Zm00001d021085_T001 3.00E-51
600- 40: transcript:EER99171 transcript:Zm00001d021086_T001 4.00E-173
600- 41: transcript:EER99172 transcript:Zm00001d021087_T003 0
600- 42: transcript:EER97023 transcript:Zm00001d021089_T001 4.00E-88
600- 43: transcript:EER99165 transcript:Zm00001d021090_T001 0
600- 44: transcript:KXG35988 transcript:Zm00001d021093_T001 1.00E-99
600- 45: transcript:KXG35989 transcript:Zm00001d021119_T008 0
600- 46: transcript:EER97031 transcript:Zm00001d021120_T001 0
600- 47: transcript:EER97032 transcript:Zm00001d021122_T001 1.00E-101
600- 48: transcript:KXG35994 transcript:Zm00001d021123_T002 7.00E-147
600- 49: transcript:EER97033 transcript:Zm00001d021126_T002 0
600- 50: transcript:KXG35997 transcript:Zm00001d021127_T001 0
600- 51: transcript:EER97035 transcript:Zm00001d021128_T001 3.00E-49

600- 52: transcript:OQU89765 transcript:Zm00001d021131_T002 0
600- 53: transcript:KXG36001 transcript:Zm00001d021136_T001 0
600- 54: transcript:EER97037 transcript:Zm00001d021137_T002 0
600- 55: transcript:EER97038 transcript:Zm00001d021138_T003 0
600- 56: transcript:OQU89769 transcript:Zm00001d021139_T008 7.00E-67
600- 57: transcript:KXG36003 transcript:Zm00001d021140_T002 0
600- 58: transcript:EER99179 transcript:Zm00001d021141_T001 6.00E-90
600- 59: transcript:KXG36004 transcript:Zm00001d021142_T006 0
600- 60: transcript:EER97044 transcript:Zm00001d021145_T003 0
600- 61: transcript:EER99181 transcript:Zm00001d021147_T002 3.00E-165
600- 62: transcript:EER99178 transcript:Zm00001d021148_T001 2.00E-81
600- 63: transcript:KXG36008 transcript:Zm00001d021160_T001 1.00E-12
600- 64: transcript:EER97051 transcript:Zm00001d021167_T001 3.00E-145
600- 65: transcript:EER97054 transcript:Zm00001d021169_T002 0
600- 66: transcript:EER99199 transcript:Zm00001d021170_T016 0
600- 67: transcript:KXG36019 transcript:Zm00001d021171_T001 2.00E-51
Alignment 601: score=2986.0 e_value=1.6e-249 N=64 2&7 plus
601- 0: transcript:KXG36544 transcript:Zm00001d021891_T005 0
601- 1: transcript:EER99585 transcript:Zm00001d021892_T001 1.00E-133
601- 2: transcript:EER99592 transcript:Zm00001d021893_T002 0
601- 3: transcript:EER99593 transcript:Zm00001d021895_T001 0
601- 4: transcript:EER99594 transcript:Zm00001d021896_T001 0
601- 5: transcript:OQU90148 transcript:Zm00001d021897_T001 0
601- 6: transcript:EER99597 transcript:Zm00001d021898_T001 0
601- 7: transcript:EER97430 transcript:Zm00001d021899_T002 0
601- 8: transcript:EER97431 transcript:Zm00001d021900_T001 0
601- 9: transcript:EER99598 transcript:Zm00001d021901_T001 7.00E-149
601- 10: transcript:OQU90151 transcript:Zm00001d021903_T005 0
601- 11: transcript:EER97436 transcript:Zm00001d021904_T001 6.00E-109
601- 12: transcript:KXG36551 transcript:Zm00001d021906_T001 0
601- 13: transcript:KXG36553 transcript:Zm00001d021908_T005 4.00E-170
601- 14: transcript:EER99600 transcript:Zm00001d021912_T001 0
601- 15: transcript:EER99602 transcript:Zm00001d021913_T001 2.00E-80
601- 16: transcript:KXG36556 transcript:Zm00001d021915_T001 0
601- 17: transcript:KXG36558 transcript:Zm00001d021920_T001 1.00E-42
601- 18: transcript:EER99603 transcript:Zm00001d021926_T001 1.00E-55
601- 19: transcript:EER99604 transcript:Zm00001d021927_T001 6.00E-141
601- 20: transcript:EER99605 transcript:Zm00001d021929_T001 0
601- 21: transcript:EER99606 transcript:Zm00001d021930_T001 0
601- 22: transcript:EER97440 transcript:Zm00001d021931_T001 0
601- 23: transcript:EER97442 transcript:Zm00001d021932_T001 0
601- 24: transcript:EER97443 transcript:Zm00001d021934_T001 3.00E-108
601- 25: transcript:EER97444 transcript:Zm00001d021935_T001 0
601- 26: transcript:KXG36561 transcript:Zm00001d021943_T002 0
601- 27: transcript:KXG36562 transcript:Zm00001d021945_T001 2.00E-13
601- 28: transcript:EER97449 transcript:Zm00001d021946_T001 0
601- 29: transcript:KXG36569 transcript:Zm00001d021947_T002 0
601- 30: transcript:EER97450 transcript:Zm00001d021948_T001 0
601- 31: transcript:EER97451 transcript:Zm00001d021949_T002 0
601- 32: transcript:EER97452 transcript:Zm00001d021950_T007 0
601- 33: transcript:EER97453 transcript:Zm00001d021951_T001 0
601- 34: transcript:KXG36570 transcript:Zm00001d021952_T001 0
601- 35: transcript:OQU90170 transcript:Zm00001d021954_T001 0
601- 36: transcript:OQU90171 transcript:Zm00001d021955_T002 2.00E-41

601- 37:	transcript:EER99616	transcript:Zm00001d021956_T002	0
601- 38:	transcript:EER97457	transcript:Zm00001d021957_T001	9.00E-172
601- 39:	transcript:EER99617	transcript:Zm00001d021958_T002	7.00E-49
601- 40:	transcript:KXG36580	transcript:Zm00001d021959_T001	4.00E-127
601- 41:	transcript:KXG36581	transcript:Zm00001d021960_T001	7.00E-167
601- 42:	transcript:EER99620	transcript:Zm00001d021961_T001	0
601- 43:	transcript:KXG36582	transcript:Zm00001d021962_T001	0
601- 44:	transcript:EER97460	transcript:Zm00001d021965_T001	0
601- 45:	transcript:EER97461	transcript:Zm00001d021966_T002	0
601- 46:	transcript:OQU90174	transcript:Zm00001d021967_T001	2.00E-65
601- 47:	transcript:EER99622	transcript:Zm00001d021968_T001	0
601- 48:	transcript:EER99623	transcript:Zm00001d021971_T001	1.00E-164
601- 49:	transcript:KXG36585	transcript:Zm00001d021972_T002	0
601- 50:	transcript:EER97462	transcript:Zm00001d021973_T001	0
601- 51:	transcript:EER99626	transcript:Zm00001d021974_T002	0
601- 52:	transcript:KXG36586	transcript:Zm00001d021976_T001	0
601- 53:	transcript:EER99629	transcript:Zm00001d021977_T001	1.00E-27
601- 54:	transcript:EER97464	transcript:Zm00001d021978_T001	0
601- 55:	transcript:EER97465	transcript:Zm00001d021979_T001	0
601- 56:	transcript:OQU90179	transcript:Zm00001d021980_T001	0
601- 57:	transcript:EER97466	transcript:Zm00001d021985_T001	0
601- 58:	transcript:EER99632	transcript:Zm00001d021988_T001	1.00E-145
601- 59:	transcript:OQU90180	transcript:Zm00001d021990_T001	1.00E-65
601- 60:	transcript:KXG36595	transcript:Zm00001d021991_T002	0
601- 61:	transcript:EER97470	transcript:Zm00001d021995_T001	1.00E-115
601- 62:	transcript:EER97471	transcript:Zm00001d021998_T001	0
601- 63:	transcript:KXG36601	transcript:Zm00001d021999_T002	0
## Alignment 602: score=2284.0 e_value=8.5e-196 N=52 2&7 plus			
602- 0:	transcript:EER97253	transcript:Zm00001d021554_T001	2.00E-130
602- 1:	transcript:KXG36269	transcript:Zm00001d021557_T001	0
602- 2:	transcript:OQU89981	transcript:Zm00001d021558_T001	0
602- 3:	transcript:KXG36274	transcript:Zm00001d021562_T001	2.00E-168
602- 4:	transcript:OQU89982	transcript:Zm00001d021564_T001	7.00E-38
602- 5:	transcript:KXG36277	transcript:Zm00001d021565_T001	0
602- 6:	transcript:EER99420	transcript:Zm00001d021566_T005	0
602- 7:	transcript:KXG36279	transcript:Zm00001d021567_T001	0
602- 8:	transcript:EER99421	transcript:Zm00001d021569_T001	0
602- 9:	transcript:EER99422	transcript:Zm00001d021571_T001	0
602- 10:	transcript:EER97257	transcript:Zm00001d021573_T001	2.00E-43
602- 11:	transcript:EER99425	transcript:Zm00001d021574_T001	0
602- 12:	transcript:OQU89990	transcript:Zm00001d021579_T002	0
602- 13:	transcript:EER99427	transcript:Zm00001d021580_T001	1.00E-127
602- 14:	transcript:EER99428	transcript:Zm00001d021582_T001	0
602- 15:	transcript:KXG36286	transcript:Zm00001d021583_T001	0
602- 16:	transcript:EER99430	transcript:Zm00001d021584_T003	0
602- 17:	transcript:KXG36288	transcript:Zm00001d021587_T002	0
602- 18:	transcript:KXG36289	transcript:Zm00001d021588_T002	0
602- 19:	transcript:EER97271	transcript:Zm00001d021591_T001	9.00E-160
602- 20:	transcript:EER99435	transcript:Zm00001d021592_T001	0
602- 21:	transcript:EER99436	transcript:Zm00001d021595_T001	3.00E-166
602- 22:	transcript:EER99437	transcript:Zm00001d021596_T004	0
602- 23:	transcript:KXG36295	transcript:Zm00001d021598_T005	0
602- 24:	transcript:EER97278	transcript:Zm00001d021599_T002	0
602- 25:	transcript:OQU89996	transcript:Zm00001d021600_T002	0

```
602- 26: transcript:KXG36296            transcript:Zm00001d021605_T001  1.00E-13
602- 27: transcript:EER97282            transcript:Zm00001d021607_T001         0
602- 28: transcript:KXG36300            transcript:Zm00001d021609_T018         0
602- 29: transcript:KXG36299            transcript:Zm00001d021610_T003         0
602- 30: transcript:EER99440            transcript:Zm00001d021613_T001         0
602- 31: transcript:KXG36301            transcript:Zm00001d021614_T001         0
602- 32: transcript:KXG36303            transcript:Zm00001d021615_T002         0
602- 33: transcript:EER99443            transcript:Zm00001d021616_T001  2.00E-44
602- 34: transcript:KXG36308            transcript:Zm00001d021617_T001         0
602- 35: transcript:KXG36319            transcript:Zm00001d021620_T001         0
602- 36: transcript:KXG36318            transcript:Zm00001d021621_T010         0
602- 37: transcript:KXG36322            transcript:Zm00001d021623_T004         0
602- 38: transcript:KXG36329            transcript:Zm00001d021626_T002 2.00E-106
602- 39: transcript:EER99449            transcript:Zm00001d021627_T001  3.00E-80
602- 40: transcript:EER97292            transcript:Zm00001d021628_T001  7.00E-60
602- 41: transcript:KXG36335            transcript:Zm00001d021630_T001  3.00E-16
602- 42: transcript:KXG36337            transcript:Zm00001d021633_T006         0
602- 43: transcript:KXG36340            transcript:Zm00001d021634_T001  7.00E-77
602- 44: transcript:EER97300            transcript:Zm00001d021635_T011         0
602- 45: transcript:OQU90019            transcript:Zm00001d021636_T004         0
602- 46: transcript:KXG36343            transcript:Zm00001d021638_T001         0
602- 47: transcript:OQU90022            transcript:Zm00001d021639_T001         0
602- 48: transcript:EER99458            transcript:Zm00001d021641_T002         0
602- 49: transcript:EER97310            transcript:Zm00001d021645_T001 1.00E-123
602- 50: transcript:OQU90024            transcript:Zm00001d021646_T024         0
602- 51: transcript:KXG36359            transcript:Zm00001d021647_T003         0
## Alignment 603: score=2186.0 e_value=2.2e-171 N=47 2&7 plus
603-  0: transcript:KXG36963            transcript:Zm00001d022516_T001  2.00E-86
603-  1: transcript:EER97754            transcript:Zm00001d022517_T001 1.00E-153
603-  2: transcript:KXG36973            transcript:Zm00001d022518_T002 4.00E-152
603-  3: transcript:EER97739            transcript:Zm00001d022524_T001 1.00E-126
603-  4: transcript:OQU90492            transcript:Zm00001d022525_T001         0
603-  5: transcript:EER99928            transcript:Zm00001d022526_T001 4.00E-170
603-  6: transcript:OQU90493            transcript:Zm00001d022527_T005         0
603-  7: transcript:OQU90495            transcript:Zm00001d022529_T001         0
603-  8: transcript:OQU90496            transcript:Zm00001d022530_T001         0
603-  9: transcript:EER99933            transcript:Zm00001d022531_T001 3.00E-137
603- 10: transcript:OQU90498            transcript:Zm00001d022535_T004 7.00E-145
603- 11: transcript:KXG36990            transcript:Zm00001d022536_T008         0
603- 12: transcript:OQU90502            transcript:Zm00001d022537_T001         0
603- 13: transcript:EER97763            transcript:Zm00001d022538_T001 4.00E-150
603- 14: transcript:OQU90509            transcript:Zm00001d022541_T004  9.00E-95
603- 15: transcript:KXG36997            transcript:Zm00001d022542_T002         0
603- 16: transcript:OQU90514            transcript:Zm00001d022544_T002 5.00E-168
603- 17: transcript:OQU90515            transcript:Zm00001d022545_T001         0
603- 18: transcript:KXG37006            transcript:Zm00001d022546_T001         0
603- 19: transcript:KXG37001            transcript:Zm00001d022547_T001         0
603- 20: transcript:EER99941            transcript:Zm00001d022549_T001         0
603- 21: transcript:KXG37010            transcript:Zm00001d022550_T001 1.00E-119
603- 22: transcript:KXG37011            transcript:Zm00001d022551_T002         0
603- 23: transcript:EER99948            transcript:Zm00001d022552_T001  3.00E-73
603- 24: transcript:OQU90537            transcript:Zm00001d022553_T001  2.00E-11
603- 25: transcript:EER99950            transcript:Zm00001d022554_T003         0
603- 26: transcript:EER99952            transcript:Zm00001d022558_T001         0
```

603- 27: transcript:EER97776 transcript:Zm00001d022560_T001 0
603- 28: transcript:KXG37016 transcript:Zm00001d022561_T003 1.00E-44
603- 29: transcript:EER99954 transcript:Zm00001d022562_T002 0
603- 30: transcript:EER97779 transcript:Zm00001d022563_T001 0
603- 31: transcript:KXG37018 transcript:Zm00001d022564_T001 0
603- 32: transcript:EER97780 transcript:Zm00001d022565_T001 0
603- 33: transcript:EER97781 transcript:Zm00001d022567_T001 0
603- 34: transcript:KXG37021 transcript:Zm00001d022569_T001 7.00E-93
603- 35: transcript:KXG37022 transcript:Zm00001d022570_T001 2.00E-93
603- 36: transcript:EER99960 transcript:Zm00001d022572_T001 1.00E-81
603- 37: transcript:EER99961 transcript:Zm00001d022573_T001 0
603- 38: transcript:KXG37024 transcript:Zm00001d022574_T001 2.00E-76
603- 39: transcript:OQU90552 transcript:Zm00001d022575_T001 0
603- 40: transcript:KXG37026 transcript:Zm00001d022576_T001 0
603- 41: transcript:EER99949 transcript:Zm00001d022578_T001 0
603- 42: transcript:OQU90553 transcript:Zm00001d022579_T001 0
603- 43: transcript:EER97797 transcript:Zm00001d022581_T001 0
603- 44: transcript:EER99964 transcript:Zm00001d022582_T002 0
603- 45: transcript:OQU90556 transcript:Zm00001d022583_T001 0
603- 46: transcript:OQU90558 transcript:Zm00001d022584_T001 0
Alignment 604: score=2150.0 e_value=1.4e-181 N=48 2&7 plus
604- 0: transcript:KXG36361 transcript:Zm00001d021649_T001 4.00E-166
604- 1: transcript:OQU90027 transcript:Zm00001d021652_T001 7.00E-53
604- 2: transcript:OQU90029 transcript:Zm00001d021653_T001 2.00E-177
604- 3: transcript:EER97316 transcript:Zm00001d021654_T001 1.00E-105
604- 4: transcript:OQU90035 transcript:Zm00001d021655_T001 0
604- 5: transcript:EER97318 transcript:Zm00001d021659_T010 0
604- 6: transcript:KXG36369 transcript:Zm00001d021661_T002 0
604- 7: transcript:EER97319 transcript:Zm00001d021662_T002 0
604- 8: transcript:EER97312 transcript:Zm00001d021664_T001 2.00E-93
604- 9: transcript:EER99470 transcript:Zm00001d021665_T001 3.00E-36
604- 10: transcript:EER99471 transcript:Zm00001d021666_T001 0
604- 11: transcript:KXG36371 transcript:Zm00001d021667_T001 0
604- 12: transcript:EER97314 transcript:Zm00001d021668_T008 4.00E-81
604- 13: transcript:KXG36373 transcript:Zm00001d021669_T007 0
604- 14: transcript:OQU90040 transcript:Zm00001d021672_T001 0
604- 15: transcript:OQU90041 transcript:Zm00001d021673_T001 4.00E-30
604- 16: transcript:EER97325 transcript:Zm00001d021674_T001 5.00E-132
604- 17: transcript:OQU90043 transcript:Zm00001d021675_T001 0
604- 18: transcript:EER97326 transcript:Zm00001d021676_T001 0
604- 19: transcript:OQU90044 transcript:Zm00001d021677_T001 4.00E-81
604- 20: transcript:EER99466 transcript:Zm00001d021682_T001 7.00E-158
604- 21: transcript:KXG36397 transcript:Zm00001d021685_T022 0
604- 22: transcript:KXG36398 transcript:Zm00001d021686_T002 1.00E-13
604- 23: transcript:KXG36402 transcript:Zm00001d021687_T004 0
604- 24: transcript:KXG36403 transcript:Zm00001d021690_T001 0
604- 25: transcript:KXG36410 transcript:Zm00001d021692_T001 0
604- 26: transcript:EER99491 transcript:Zm00001d021695_T001 0
604- 27: transcript:KXG36419 transcript:Zm00001d021696_T003 0
604- 28: transcript:EER99493 transcript:Zm00001d021698_T001 0
604- 29: transcript:EER97339 transcript:Zm00001d021700_T001 8.00E-89
604- 30: transcript:EER99495 transcript:Zm00001d021701_T002 5.00E-158
604- 31: transcript:EER97340 transcript:Zm00001d021702_T001 0
604- 32: transcript:EER97341 transcript:Zm00001d021703_T001 1.00E-126

```
604- 33: transcript:EER99496            transcript:Zm00001d021704_T001 5.00E-147
604- 34: transcript:EER97342            transcript:Zm00001d021705_T002  9.00E-61
604- 35: transcript:EER97344            transcript:Zm00001d021706_T001  6.00E-92
604- 36: transcript:EER97347            transcript:Zm00001d021708_T003 2.00E-163
604- 37: transcript:EER97348            transcript:Zm00001d021709_T001 7.00E-175
604- 38: transcript:EER97349            transcript:Zm00001d021710_T004         0
604- 39: transcript:EER99499            transcript:Zm00001d021711_T001  1.00E-39
604- 40: transcript:KXG36430            transcript:Zm00001d021714_T002         0
604- 41: transcript:KXG36435            transcript:Zm00001d021715_T001         0
604- 42: transcript:EER99505            transcript:Zm00001d021716_T001         0
604- 43: transcript:EER97353            transcript:Zm00001d021718_T001 5.00E-131
604- 44: transcript:KXG36442            transcript:Zm00001d021719_T001 1.00E-141
604- 45: transcript:EER99509            transcript:Zm00001d021720_T001  5.00E-59
604- 46: transcript:OQU90065            transcript:Zm00001d021721_T001         0
604- 47: transcript:KXG36444            transcript:Zm00001d021726_T003         0
## Alignment 605: score=1884.0 e_value=3.1e-150 N=42 2&7 plus
605-  0: transcript:EER99377            transcript:Zm00001d021484_T004 2.00E-163
605-  1: transcript:EER99378            transcript:Zm00001d021486_T001  3.00E-52
605-  2: transcript:EER97215            transcript:Zm00001d021487_T001         0
605-  3: transcript:KXG36224            transcript:Zm00001d021488_T003 2.00E-122
605-  4: transcript:EER99384            transcript:Zm00001d021489_T001         0
605-  5: transcript:EER97217            transcript:Zm00001d021490_T002         0
605-  6: transcript:EER97218            transcript:Zm00001d021491_T001         0
605-  7: transcript:EER99386            transcript:Zm00001d021494_T001         0
605-  8: transcript:EER99387            transcript:Zm00001d021498_T008         0
605-  9: transcript:EER97223            transcript:Zm00001d021504_T001  4.00E-39
605- 10: transcript:EER99388            transcript:Zm00001d021506_T001         0
605- 11: transcript:KXG36232            transcript:Zm00001d021507_T002         0
605- 12: transcript:EER97229            transcript:Zm00001d021508_T001 3.00E-147
605- 13: transcript:OQU89960            transcript:Zm00001d021512_T002 1.00E-156
605- 14: transcript:EER97231            transcript:Zm00001d021513_T001         0
605- 15: transcript:KXG36238            transcript:Zm00001d021514_T001  5.00E-86
605- 16: transcript:EER97233            transcript:Zm00001d021515_T001 4.00E-115
605- 17: transcript:KXG36244            transcript:Zm00001d021516_T003         0
605- 18: transcript:KXG36246            transcript:Zm00001d021517_T001 6.00E-142
605- 19: transcript:OQU89965            transcript:Zm00001d021518_T001 5.00E-143
605- 20: transcript:EER97237            transcript:Zm00001d021519_T001         0
605- 21: transcript:KXG36249            transcript:Zm00001d021520_T002 1.00E-124
605- 22: transcript:OQU89966            transcript:Zm00001d021521_T001  2.00E-60
605- 23: transcript:KXG36254            transcript:Zm00001d021522_T002 2.00E-176
605- 24: transcript:KXG36256            transcript:Zm00001d021524_T003         0
605- 25: transcript:EER99394            transcript:Zm00001d021525_T002         0
605- 26: transcript:EER99399            transcript:Zm00001d021526_T001 2.00E-103
605- 27: transcript:KXG36261            transcript:Zm00001d021528_T001 9.00E-153
605- 28: transcript:EER99403            transcript:Zm00001d021529_T002 2.00E-100
605- 29: transcript:EER99396            transcript:Zm00001d021532_T001         0
605- 30: transcript:EER99405            transcript:Zm00001d021533_T001         0
605- 31: transcript:EER99406            transcript:Zm00001d021534_T001         0
605- 32: transcript:EER97238            transcript:Zm00001d021535_T001         0
605- 33: transcript:EER99408            transcript:Zm00001d021536_T003  7.00E-51
605- 34: transcript:EER97244            transcript:Zm00001d021537_T001  3.00E-98
605- 35: transcript:KXG36264            transcript:Zm00001d021541_T008         0
605- 36: transcript:OQU89976            transcript:Zm00001d021542_T001  9.00E-71
605- 37: transcript:EER99411            transcript:Zm00001d021544_T001         0
```

605- 38: transcript:KXG36267 transcript:Zm00001d021545_T005 0
605- 39: transcript:KXG36268 transcript:Zm00001d021546_T003 0
605- 40: transcript:EER97251 transcript:Zm00001d021551_T002 6.00E-142
605- 41: transcript:EER97252 transcript:Zm00001d021553_T004 0
Alignment 606: score=1511.0 e_value=2.2e-115 N=33 2&7 plus
606- 0: transcript:EER99094 transcript:Zm00001d020944_T001 0
606- 1: transcript:EER96978 transcript:Zm00001d020946_T005 0
606- 2: transcript:EER99095 transcript:Zm00001d020947_T001 0
606- 3: transcript:EER99096 transcript:Zm00001d020948_T001 0
606- 4: transcript:EER99097 transcript:Zm00001d020949_T001 1.00E-47
606- 5: transcript:OQU89695 transcript:Zm00001d020950_T001 3.00E-77
606- 6: transcript:OQU89696 transcript:Zm00001d020951_T002 5.00E-140
606- 7: transcript:EER99100 transcript:Zm00001d020953_T001 0
606- 8: transcript:KXG35928 transcript:Zm00001d020954_T001 0
606- 9: transcript:KXG35923 transcript:Zm00001d020955_T001 0
606- 10: transcript:EER99104 transcript:Zm00001d020956_T001 2.00E-51
606- 11: transcript:KXG35925 transcript:Zm00001d020957_T001 2.00E-173
606- 12: transcript:KXG35927 transcript:Zm00001d020960_T001 3.00E-56
606- 13: transcript:EER96980 transcript:Zm00001d020963_T001 0
606- 14: transcript:OQU89707 transcript:Zm00001d020965_T007 0
606- 15: transcript:OQU89708 transcript:Zm00001d020966_T001 4.00E-146
606- 16: transcript:OQU89709 transcript:Zm00001d020967_T001 6.00E-67
606- 17: transcript:KXG35935 transcript:Zm00001d020969_T013 0
606- 18: transcript:EER99113 transcript:Zm00001d020970_T001 0
606- 19: transcript:EER99115 transcript:Zm00001d020971_T002 1.00E-147
606- 20: transcript:OQU89712 transcript:Zm00001d020972_T010 0
606- 21: transcript:EER99120 transcript:Zm00001d020973_T002 0
606- 22: transcript:EER96985 transcript:Zm00001d020974_T001 0
606- 23: transcript:KXG35939 transcript:Zm00001d020975_T001 0
606- 24: transcript:OQU89716 transcript:Zm00001d020976_T001 5.00E-128
606- 25: transcript:EER96986 transcript:Zm00001d020977_T001 2.00E-151
606- 26: transcript:OQU89718 transcript:Zm00001d020978_T005 0
606- 27: transcript:EER96987 transcript:Zm00001d020980_T001 0
606- 28: transcript:EER99125 transcript:Zm00001d020982_T001 0
606- 29: transcript:EER96988 transcript:Zm00001d020983_T001 2.00E-92
606- 30: transcript:OQU89722 transcript:Zm00001d020984_T001 0
606- 31: transcript:EER99121 transcript:Zm00001d020985_T002 0
606- 32: transcript:EER99128 transcript:Zm00001d020986_T002 0
Alignment 607: score=1476.0 e_value=2.9e-103 N=32 2&7 plus
607- 0: transcript:EER97079 transcript:Zm00001d021278_T001 3.00E-141
607- 1: transcript:EER97080 transcript:Zm00001d021279_T002 3.00E-93
607- 2: transcript:OQU89810 transcript:Zm00001d021280_T004 0
607- 3: transcript:KXG36061 transcript:Zm00001d021282_T001 2.00E-133
607- 4: transcript:EER97086 transcript:Zm00001d021283_T001 0
607- 5: transcript:KXG36055 transcript:Zm00001d021284_T001 2.00E-33
607- 6: transcript:KXG36062 transcript:Zm00001d021285_T001 2.00E-152
607- 7: transcript:KXG36063 transcript:Zm00001d021286_T001 0
607- 8: transcript:OQU89815 transcript:Zm00001d021287_T001 0
607- 9: transcript:EER99239 transcript:Zm00001d021288_T001 3.00E-145
607- 10: transcript:EER99240 transcript:Zm00001d021289_T001 2.00E-112
607- 11: transcript:EER97088 transcript:Zm00001d021290_T001 9.00E-94
607- 12: transcript:EER99234 transcript:Zm00001d021291_T002 0
607- 13: transcript:EER97089 transcript:Zm00001d021294_T001 0
607- 14: transcript:KXG36065 transcript:Zm00001d021295_T006 0

607- 15: transcript:KXG36070 transcript:Zm00001d021296_T004 1.00E-165
607- 16: transcript:EER97090 transcript:Zm00001d021297_T006 0
607- 17: transcript:EER99245 transcript:Zm00001d021298_T001 0
607- 18: transcript:EER97091 transcript:Zm00001d021299_T001 0
607- 19: transcript:EER99246 transcript:Zm00001d021300_T001 3.00E-101
607- 20: transcript:EER99247 transcript:Zm00001d021301_T002 0
607- 21: transcript:EER99248 transcript:Zm00001d021303_T003 0
607- 22: transcript:EER99250 transcript:Zm00001d021304_T001 0
607- 23: transcript:EER99253 transcript:Zm00001d021305_T001 0
607- 24: transcript:EER97092 transcript:Zm00001d021306_T001 0
607- 25: transcript:EER97093 transcript:Zm00001d021309_T003 0
607- 26: transcript:EER99254 transcript:Zm00001d021310_T001 0
607- 27: transcript:EER99255 transcript:Zm00001d021313_T001 1.00E-174
607- 28: transcript:EER97094 transcript:Zm00001d021314_T007 2.00E-155
607- 29: transcript:EER97095 transcript:Zm00001d021315_T001 0
607- 30: transcript:EER99258 transcript:Zm00001d021316_T001 6.00E-65
607- 31: transcript:EER99259 transcript:Zm00001d021318_T003 0
Alignment 608: score=1285.0 e_value=5.4e-94 N=29 2&7 plus
608- 0: transcript:OQU88760 transcript:Zm00001d019266_T002 0
608- 1: transcript:EER96120 transcript:Zm00001d019268_T001 0
608- 2: transcript:EER98268 transcript:Zm00001d019269_T001 0
608- 3: transcript:KXG34769 transcript:Zm00001d019270_T001 3.00E-33
608- 4: transcript:EER96124 transcript:Zm00001d019277_T001 6.00E-33
608- 5: transcript:EER96129 transcript:Zm00001d019279_T001 1.00E-132
608- 6: transcript:EER96131 transcript:Zm00001d019282_T001 0
608- 7: transcript:KXG34775 transcript:Zm00001d019283_T005 0
608- 8: transcript:EER96133 transcript:Zm00001d019287_T001 0
608- 9: transcript:KXG34779 transcript:Zm00001d019288_T001 0
608- 10: transcript:EER96136 transcript:Zm00001d019290_T001 3.00E-131
608- 11: transcript:KXG34784 transcript:Zm00001d019294_T001 0
608- 12: transcript:OQU88765 transcript:Zm00001d019297_T001 0
608- 13: transcript:KXG34786 transcript:Zm00001d019298_T002 3.00E-156
608- 14: transcript:EER98280 transcript:Zm00001d019299_T001 0
608- 15: transcript:OQU88766 transcript:Zm00001d019300_T001 1.00E-20
608- 16: transcript:EER96145 transcript:Zm00001d019303_T001 2.00E-68
608- 17: transcript:EER98285 transcript:Zm00001d019305_T001 2.00E-47
608- 18: transcript:OQU88772 transcript:Zm00001d019306_T001 0
608- 19: transcript:EER98287 transcript:Zm00001d019312_T002 6.00E-121
608- 20: transcript:EER98290 transcript:Zm00001d019314_T003 0
608- 21: transcript:EER96156 transcript:Zm00001d019317_T002 0
608- 22: transcript:OQU88778 transcript:Zm00001d019320_T001 3.00E-47
608- 23: transcript:KXG34808 transcript:Zm00001d019324_T001 2.00E-33
608- 24: transcript:KXG34809 transcript:Zm00001d019325_T002 0
608- 25: transcript:OQU88779 transcript:Zm00001d019326_T001 2.00E-69
608- 26: transcript:OQU88782 transcript:Zm00001d019327_T001 0
608- 27: transcript:OQU88783 transcript:Zm00001d019328_T003 6.00E-159
608- 28: transcript:KXG34814 transcript:Zm00001d019329_T001 0
Alignment 609: score=1182.0 e_value=4.1e-79 N=25 2&7 plus
609- 0: transcript:EER99554 transcript:Zm00001d021812_T001 1.00E-65
609- 1: transcript:EER99556 transcript:Zm00001d021813_T001 1.00E-48
609- 2: transcript:EER97390 transcript:Zm00001d021815_T002 0
609- 3: transcript:KXG36496 transcript:Zm00001d021816_T001 3.00E-43
609- 4: transcript:EER99559 transcript:Zm00001d021817_T002 0
609- 5: transcript:OQU90111 transcript:Zm00001d021818_T001 0

609- 6: transcript:EER99561 transcript:Zm00001d021820_T001 7.00E-125
609- 7: transcript:KXG36499 transcript:Zm00001d021821_T001 0
609- 8: transcript:EER97384 transcript:Zm00001d021823_T002 0
609- 9: transcript:EER99563 transcript:Zm00001d021825_T001 6.00E-100
609- 10: transcript:KXG36498 transcript:Zm00001d021826_T001 0
609- 11: transcript:OQU90116 transcript:Zm00001d021827_T001 3.00E-36
609- 12: transcript:KXG36500 transcript:Zm00001d021828_T004 0
609- 13: transcript:EER97395 transcript:Zm00001d021829_T001 0
609- 14: transcript:EER99566 transcript:Zm00001d021830_T002 0
609- 15: transcript:EER97396 transcript:Zm00001d021831_T001 0
609- 16: transcript:EER99552 transcript:Zm00001d021834_T001 1.00E-177
609- 17: transcript:EER99568 transcript:Zm00001d021835_T007 0
609- 18: transcript:EER99569 transcript:Zm00001d021836_T001 0
609- 19: transcript:EER97403 transcript:Zm00001d021838_T001 0
609- 20: transcript:EER99573 transcript:Zm00001d021839_T001 9.00E-123
609- 21: transcript:EER97404 transcript:Zm00001d021840_T001 2.00E-135
609- 22: transcript:KXG36512 transcript:Zm00001d021841_T001 2.00E-78
609- 23: transcript:OQU90125 transcript:Zm00001d021842_T001 0
609- 24: transcript:EER97406 transcript:Zm00001d021843_T001 2.00E-29
Alignment 610: score=1108.0 e_value=4.6e-81 N=25 2&7 plus
610- 0: transcript:KXG35452 transcript:Zm00001d020170_T001 0
610- 1: transcript:KXG35455 transcript:Zm00001d020171_T002 0
610- 2: transcript:EER96609 transcript:Zm00001d020175_T002 5.00E-134
610- 3: transcript:OQU89349 transcript:Zm00001d020176_T001 0
610- 4: transcript:EER96611 transcript:Zm00001d020177_T001 0
610- 5: transcript:EER98780 transcript:Zm00001d020178_T002 0
610- 6: transcript:EER98781 transcript:Zm00001d020180_T002 3.00E-79
610- 7: transcript:EER96613 transcript:Zm00001d020182_T002 0
610- 8: transcript:OQU89354 transcript:Zm00001d020184_T001 2.00E-81
610- 9: transcript:KXG35475 transcript:Zm00001d020187_T001 1.00E-108
610- 10: transcript:EER98789 transcript:Zm00001d020188_T001 0
610- 11: transcript:KXG35476 transcript:Zm00001d020193_T001 3.00E-37
610- 12: transcript:OQU89358 transcript:Zm00001d020195_T001 2.00E-58
610- 13: transcript:EER96623 transcript:Zm00001d020198_T001 8.00E-86
610- 14: transcript:EER98790 transcript:Zm00001d020201_T005 9.00E-68
610- 15: transcript:EER96624 transcript:Zm00001d020204_T002 0
610- 16: transcript:EER96625 transcript:Zm00001d020205_T001 3.00E-134
610- 17: transcript:EER98791 transcript:Zm00001d020206_T001 0
610- 18: transcript:OQU89360 transcript:Zm00001d020208_T003 0
610- 19: transcript:EER96629 transcript:Zm00001d020213_T003 0
610- 20: transcript:EER98794 transcript:Zm00001d020214_T001 9.00E-56
610- 21: transcript:EER96631 transcript:Zm00001d020219_T004 0
610- 22: transcript:EER98793 transcript:Zm00001d020220_T001 0
610- 23: transcript:EER98795 transcript:Zm00001d020224_T002 1.00E-54
610- 24: transcript:KXG35481 transcript:Zm00001d020225_T002 0
Alignment 611: score=963.0 e_value=1.6e-66 N=22 2&7 plus
611- 0: transcript:EER96094 transcript:Zm00001d019215_T002 0
611- 1: transcript:KXG34718 transcript:Zm00001d019216_T001 2.00E-34
611- 2: transcript:EER98221 transcript:Zm00001d019217_T002 0
611- 3: transcript:KXG34719 transcript:Zm00001d019218_T001 2.00E-140
611- 4: transcript:EER98223 transcript:Zm00001d019219_T001 3.00E-97
611- 5: transcript:EER98229 transcript:Zm00001d019220_T001 0
611- 6: transcript:KXG34722 transcript:Zm00001d019221_T001 0
611- 7: transcript:EER96097 transcript:Zm00001d019222_T001 3.00E-48

611- 8:	transcript:OQU88735	transcript:Zm00001d019223_T001	3.00E-31
611- 9:	transcript:EER98235	transcript:Zm00001d019225_T001	0
611- 10:	transcript:EER96099	transcript:Zm00001d019226_T003	0
611- 11:	transcript:EER98236	transcript:Zm00001d019227_T001	7.00E-12
611- 12:	transcript:EER96101	transcript:Zm00001d019230_T002	0
611- 13:	transcript:EER96102	transcript:Zm00001d019233_T001	6.00E-157
611- 14:	transcript:EER98238	transcript:Zm00001d019234_T001	1.00E-121
611- 15:	transcript:KXG34739	transcript:Zm00001d019239_T001	2.00E-13
611- 16:	transcript:KXG34742	transcript:Zm00001d019240_T001	0
611- 17:	transcript:EER98240	transcript:Zm00001d019241_T002	1.00E-34
611- 18:	transcript:KXG34744	transcript:Zm00001d019248_T001	0
611- 19:	transcript:EER98239	transcript:Zm00001d019249_T001	0
611- 20:	transcript:EER96106	transcript:Zm00001d019250_T002	7.00E-145
611- 21:	transcript:EER98242	transcript:Zm00001d019251_T001	0
## Alignment 612: score=916.0 e_value=5.7e-58 N=20 2&7 plus			
612- 0:	transcript:EER96062	transcript:Zm00001d019163_T001	0
612- 1:	transcript:EER98198	transcript:Zm00001d019164_T002	0
612- 2:	transcript:EER98199	transcript:Zm00001d019165_T002	0
612- 3:	transcript:OQU88693	transcript:Zm00001d019166_T002	5.00E-169
612- 4:	transcript:EER96065	transcript:Zm00001d019169_T004	0
612- 5:	transcript:OQU88697	transcript:Zm00001d019170_T001	0
612- 6:	transcript:EER96067	transcript:Zm00001d019171_T001	3.00E-174
612- 7:	transcript:KXG34689	transcript:Zm00001d019172_T001	0
612- 8:	transcript:OQU88702	transcript:Zm00001d019173_T001	0
612- 9:	transcript:KXG34690	transcript:Zm00001d019177_T001	0
612- 10:	transcript:EER98202	transcript:Zm00001d019180_T001	7.00E-59
612- 11:	transcript:EER96069	transcript:Zm00001d019181_T001	1.00E-130
612- 12:	transcript:KXG34695	transcript:Zm00001d019182_T001	0
612- 13:	transcript:EER96072	transcript:Zm00001d019184_T001	2.00E-112
612- 14:	transcript:OQU88705	transcript:Zm00001d019185_T001	0
612- 15:	transcript:EER96074	transcript:Zm00001d019186_T001	6.00E-78
612- 16:	transcript:EER96075	transcript:Zm00001d019191_T007	9.00E-133
612- 17:	transcript:KXG34699	transcript:Zm00001d019192_T001	1.00E-56
612- 18:	transcript:EER96079	transcript:Zm00001d019194_T008	1.00E-174
612- 19:	transcript:EER96081	transcript:Zm00001d019195_T001	0
## Alignment 613: score=873.0 e_value=1.1e-68 N=22 2&7 plus			
613- 0:	transcript:EER98423	transcript:Zm00001d019515_T008	0
613- 1:	transcript:OQU88903	transcript:Zm00001d019518_T001	1.00E-77
613- 2:	transcript:EER96294	transcript:Zm00001d019520_T001	1.00E-167
613- 3:	transcript:EER98424	transcript:Zm00001d019522_T001	0
613- 4:	transcript:EER96297	transcript:Zm00001d019525_T001	0
613- 5:	transcript:OQU88910	transcript:Zm00001d019527_T005	7.00E-69
613- 6:	transcript:KXG35006	transcript:Zm00001d019531_T002	1.00E-167
613- 7:	transcript:EER96302	transcript:Zm00001d019534_T003	0
613- 8:	transcript:OQU88918	transcript:Zm00001d019536_T001	0
613- 9:	transcript:EER98432	transcript:Zm00001d019537_T001	0
613- 10:	transcript:OQU88922	transcript:Zm00001d019538_T002	0
613- 11:	transcript:OQU88923	transcript:Zm00001d019545_T001	8.00E-12
613- 12:	transcript:EER96307	transcript:Zm00001d019546_T001	1.00E-36
613- 13:	transcript:EER98436	transcript:Zm00001d019547_T002	0
613- 14:	transcript:KXG35021	transcript:Zm00001d019549_T002	2.00E-09
613- 15:	transcript:EER98440	transcript:Zm00001d019551_T001	0
613- 16:	transcript:OQU88930	transcript:Zm00001d019552_T002	0
613- 17:	transcript:KXG35023	transcript:Zm00001d019553_T001	9.00E-30

613- 18: transcript:EER96308 transcript:Zm00001d019554_T001 2.00E-103
613- 19: transcript:EER96309 transcript:Zm00001d019555_T002 1.00E-87
613- 20: transcript:EER98447 transcript:Zm00001d019557_T001 3.00E-132
613- 21: transcript:OQU88952 transcript:Zm00001d019561_T007 0
Alignment 614: score=869.0 e_value=5.5e-55 N=20 2&7 plus
614- 0: transcript:OQU90086 transcript:Zm00001d021772_T001 7.00E-16
614- 1: transcript:EER97374 transcript:Zm00001d021773_T001 9.00E-18
614- 2: transcript:EER99539 transcript:Zm00001d021774_T001 4.00E-172
614- 3: transcript:EER99540 transcript:Zm00001d021775_T001 0
614- 4: transcript:OQU90088 transcript:Zm00001d021777_T001 0
614- 5: transcript:OQU90092 transcript:Zm00001d021778_T001 5.00E-34
614- 6: transcript:KXG36477 transcript:Zm00001d021779_T001 5.00E-53
614- 7: transcript:EER97376 transcript:Zm00001d021781_T001 0
614- 8: transcript:OQU90095 transcript:Zm00001d021784_T001 0
614- 9: transcript:EER99532 transcript:Zm00001d021785_T002 0
614- 10: transcript:EER97379 transcript:Zm00001d021787_T003 0
614- 11: transcript:EER97380 transcript:Zm00001d021788_T001 0
614- 12: transcript:KXG36480 transcript:Zm00001d021790_T010 0
614- 13: transcript:EER99546 transcript:Zm00001d021791_T001 6.00E-177
614- 14: transcript:OQU90102 transcript:Zm00001d021799_T010 0
614- 15: transcript:EER99548 transcript:Zm00001d021802_T001 0
614- 16: transcript:OQU90105 transcript:Zm00001d021803_T001 7.00E-48
614- 17: transcript:EER97387 transcript:Zm00001d021804_T001 0
614- 18: transcript:EER99550 transcript:Zm00001d021805_T006 0
614- 19: transcript:KXG36486 transcript:Zm00001d021806_T019 0
Alignment 615: score=867.0 e_value=3.7e-56 N=19 2&7 plus
615- 0: transcript:EER99577 transcript:Zm00001d021850_T001 2.00E-83
615- 1: transcript:OQU90128 transcript:Zm00001d021858_T007 0
615- 2: transcript:EER99579 transcript:Zm00001d021861_T007 0
615- 3: transcript:KXG36527 transcript:Zm00001d021863_T001 7.00E-96
615- 4: transcript:EER99582 transcript:Zm00001d021864_T001 0
615- 5: transcript:KXG36525 transcript:Zm00001d021866_T001 0
615- 6: transcript:EER97411 transcript:Zm00001d021869_T001 0
615- 7: transcript:EER97412 transcript:Zm00001d021870_T001 3.00E-31
615- 8: transcript:KXG36530 transcript:Zm00001d021871_T002 0
615- 9: transcript:EER97413 transcript:Zm00001d021873_T001 6.00E-144
615- 10: transcript:EER97416 transcript:Zm00001d021875_T002 0
615- 11: transcript:EER97418 transcript:Zm00001d021876_T001 2.00E-40
615- 12: transcript:EER97419 transcript:Zm00001d021877_T001 0
615- 13: transcript:EER97421 transcript:Zm00001d021878_T003 0
615- 14: transcript:EER97422 transcript:Zm00001d021879_T001 0
615- 15: transcript:EER99588 transcript:Zm00001d021880_T001 0
615- 16: transcript:EER99589 transcript:Zm00001d021881_T001 1.00E-179
615- 17: transcript:EER99590 transcript:Zm00001d021883_T001 0
615- 18: transcript:KXG36540 transcript:Zm00001d021890_T007 0
Alignment 616: score=803.0 e_value=9.7e-55 N=19 2&7 plus
616- 0: transcript:EER98026 transcript:Zm00001d018867_T002 0
616- 1: transcript:KXG34455 transcript:Zm00001d018868_T001 2.00E-23
616- 2: transcript:EER98027 transcript:Zm00001d018869_T001 0
616- 3: transcript:EER95900 transcript:Zm00001d018870_T001 0
616- 4: transcript:OQU88487 transcript:Zm00001d018871_T001 0
616- 5: transcript:EER98028 transcript:Zm00001d018872_T009 0
616- 6: transcript:EER98031 transcript:Zm00001d018873_T001 0
616- 7: transcript:OQU88488 transcript:Zm00001d018879_T001 9.00E-28

616- 8: transcript:EER95905 transcript:Zm00001d018880_T001 4.00E-55
616- 9: transcript:EER98035 transcript:Zm00001d018881_T001 0
616- 10: transcript:KXG34463 transcript:Zm00001d018882_T001 1.00E-165
616- 11: transcript:OQU88494 transcript:Zm00001d018883_T001 0
616- 12: transcript:EER98036 transcript:Zm00001d018884_T002 8.00E-69
616- 13: transcript:KXG34465 transcript:Zm00001d018887_T002 0
616- 14: transcript:KXG34470 transcript:Zm00001d018890_T006 0
616- 15: transcript:KXG34469 transcript:Zm00001d018891_T001 0
616- 16: transcript:EER95910 transcript:Zm00001d018894_T009 2.00E-116
616- 17: transcript:EER95913 transcript:Zm00001d018895_T001 2.00E-172
616- 18: transcript:KXG34475 transcript:Zm00001d018896_T002 0
Alignment 617: score=776.0 e_value=0 N=17 2&7 plus
617- 0: transcript:KXG35672 transcript:Zm00001d020563_T001 0
617- 1: transcript:KXG35673 transcript:Zm00001d020568_T001 0
617- 2: transcript:EER98942 transcript:Zm00001d020569_T001 0
617- 3: transcript:EER96767 transcript:Zm00001d020571_T003 6.00E-106
617- 4: transcript:EER96769 transcript:Zm00001d020572_T001 2.00E-32
617- 5: transcript:EER98944 transcript:Zm00001d020573_T001 2.00E-60
617- 6: transcript:EER98945 transcript:Zm00001d020577_T001 1.00E-55
617- 7: transcript:EER98946 transcript:Zm00001d020578_T001 0
617- 8: transcript:EER96770 transcript:Zm00001d020580_T001 5.00E-99
617- 9: transcript:OQU89547 transcript:Zm00001d020583_T001 3.00E-136
617- 10: transcript:EER98950 transcript:Zm00001d020586_T001 0
617- 11: transcript:EER96772 transcript:Zm00001d020589_T001 6.00E-89
617- 12: transcript:KXG35682 transcript:Zm00001d020590_T009 0
617- 13: transcript:OQU89550 transcript:Zm00001d020591_T001 2.00E-09
617- 14: transcript:KXG35687 transcript:Zm00001d020593_T020 0
617- 15: transcript:KXG35689 transcript:Zm00001d020595_T001 3.00E-121
617- 16: transcript:EER98958 transcript:Zm00001d020602_T009 0
Alignment 618: score=760.0 e_value=3.6e-48 N=18 2&7 plus
618- 0: transcript:EER96010 transcript:Zm00001d019062_T003 0
618- 1: transcript:OQU88639 transcript:Zm00001d019064_T001 0
618- 2: transcript:EER98141 transcript:Zm00001d019067_T001 0
618- 3: transcript:KXG34607 transcript:Zm00001d019068_T001 4.00E-169
618- 4: transcript:EER96015 transcript:Zm00001d019069_T002 1.00E-41
618- 5: transcript:OQU88642 transcript:Zm00001d019070_T001 1.00E-162
618- 6: transcript:EER96017 transcript:Zm00001d019075_T001 0
618- 7: transcript:KXG34613 transcript:Zm00001d019078_T002 0
618- 8: transcript:EER96020 transcript:Zm00001d019079_T001 0
618- 9: transcript:EER98147 transcript:Zm00001d019084_T002 0
618- 10: transcript:OQU88645 transcript:Zm00001d019087_T015 0
618- 11: transcript:EER96025 transcript:Zm00001d019089_T001 0
618- 12: transcript:EER98148 transcript:Zm00001d019090_T003 0
618- 13: transcript:EER96027 transcript:Zm00001d019091_T002 1.00E-161
618- 14: transcript:OQU88648 transcript:Zm00001d019092_T001 7.00E-40
618- 15: transcript:EER98157 transcript:Zm00001d019093_T001 2.00E-80
618- 16: transcript:KXG34620 transcript:Zm00001d019094_T001 1.00E-110
618- 17: transcript:EER98158 transcript:Zm00001d019096_T001 2.00E-114
Alignment 619: score=749.0 e_value=6.8e-46 N=17 2&7 plus
619- 0: transcript:EER97828 transcript:Zm00001d018625_T106 0
619- 1: transcript:KXG34255 transcript:Zm00001d018626_T001 0
619- 2: transcript:EER97830 transcript:Zm00001d018627_T001 0
619- 3: transcript:OQU88297 transcript:Zm00001d018628_T001 2.00E-62
619- 4: transcript:EER97832 transcript:Zm00001d018631_T001 9.00E-100

619- 5: transcript:OQU88300 transcript:Zm00001d018632_T001 0
619- 6: transcript:EER97834 transcript:Zm00001d018634_T001 2.00E-170
619- 7: transcript:EER95739 transcript:Zm00001d018635_T001 0
619- 8: transcript:EER95740 transcript:Zm00001d018636_T001 0
619- 9: transcript:OQU88302 transcript:Zm00001d018637_T004 5.00E-103
619- 10: transcript:OQU88304 transcript:Zm00001d018638_T001 0
619- 11: transcript:KXG34265 transcript:Zm00001d018639_T002 0
619- 12: transcript:EER95743 transcript:Zm00001d018641_T001 3.00E-74
619- 13: transcript:EER97839 transcript:Zm00001d018642_T002 3.00E-169
619- 14: transcript:EER95744 transcript:Zm00001d018645_T001 3.00E-38
619- 15: transcript:EER95746 transcript:Zm00001d018647_T001 0
619- 16: transcript:EER95747 transcript:Zm00001d018648_T001 0
Alignment 620: score=743.0 e_value=4.6e-45 N=17 2&7 plus
620- 0: transcript:Zm00001d005867_T002 transcript:EES13984 1.00E-53
620- 1: transcript:Zm00001d005881_T011 transcript:KXG25311 3.00E-124
620- 2: transcript:Zm00001d005884_T002 transcript:OQU80630 9.00E-63
620- 3: transcript:Zm00001d005885_T001 transcript:OQU80634 9.00E-164
620- 4: transcript:Zm00001d005889_T001 transcript:EES15128 0
620- 5: transcript:Zm00001d005890_T001 transcript:EES14002 0
620- 6: transcript:Zm00001d005892_T001 transcript:OQU80635 6.00E-61
620- 7: transcript:Zm00001d005897_T001 transcript:EES14004 5.00E-25
620- 8: transcript:Zm00001d005902_T003 transcript:OQU80636 0
620- 9: transcript:Zm00001d005905_T001 transcript:OQU80637 3.00E-121
620- 10: transcript:Zm00001d005910_T001 transcript:KXG25323 6.00E-128
620- 11: transcript:Zm00001d005917_T003 transcript:KXG25324 5.00E-179
620- 12: transcript:Zm00001d005918_T001 transcript:EES14020 4.00E-67
620- 13: transcript:Zm00001d005919_T001 transcript:EES14023 5.00E-63
620- 14: transcript:Zm00001d005923_T001 transcript:KXG25333 7.00E-73
620- 15: transcript:Zm00001d005928_T002 transcript:EES14027 7.00E-121
620- 16: transcript:Zm00001d005931_T001 transcript:EES15144 4.00E-94
Alignment 621: score=705.0 e_value=1.3e-40 N=16 2&7 plus
621- 0: transcript:EER97669 transcript:Zm00001d022380_T001 0
621- 1: transcript:OQU90400 transcript:Zm00001d022381_T016 0
621- 2: transcript:KXG36846 transcript:Zm00001d022384_T002 2.00E-139
621- 3: transcript:OQU90402 transcript:Zm00001d022385_T001 8.00E-48
621- 4: transcript:EER99838 transcript:Zm00001d022386_T001 0
621- 5: transcript:EER97672 transcript:Zm00001d022387_T010 0
621- 6: transcript:KXG36849 transcript:Zm00001d022388_T004 0
621- 7: transcript:EER97673 transcript:Zm00001d022390_T001 0
621- 8: transcript:EER99841 transcript:Zm00001d022391_T001 0
621- 9: transcript:EER99842 transcript:Zm00001d022393_T001 4.00E-92
621- 10: transcript:EER99844 transcript:Zm00001d022394_T002 0
621- 11: transcript:KXG36853 transcript:Zm00001d022395_T001 0
621- 12: transcript:OQU90406 transcript:Zm00001d022396_T003 0
621- 13: transcript:EER97676 transcript:Zm00001d022400_T003 2.00E-175
621- 14: transcript:EER97677 transcript:Zm00001d022401_T002 0
621- 15: transcript:KXG36862 transcript:Zm00001d022403_T001 5.00E-119
Alignment 622: score=684.0 e_value=8.9e-42 N=15 2&7 plus
622- 0: transcript:EER99209 transcript:Zm00001d021203_T003 0
622- 1: transcript:KXG36029 transcript:Zm00001d021204_T005 0
622- 2: transcript:OQU89795 transcript:Zm00001d021205_T001 2.00E-43
622- 3: transcript:EER97067 transcript:Zm00001d021214_T001 9.00E-90
622- 4: transcript:EER99216 transcript:Zm00001d021219_T001 0
622- 5: transcript:KXG36033 transcript:Zm00001d021221_T001 3.00E-127

```
622-  6: transcript:EER97068           transcript:Zm00001d021223_T001  1.00E-18
622-  7: transcript:EER99217           transcript:Zm00001d021224_T001         0
622-  8: transcript:EER99218           transcript:Zm00001d021225_T005 1.00E-121
622-  9: transcript:OQU89797           transcript:Zm00001d021226_T001  5.00E-37
622- 10: transcript:EER99219           transcript:Zm00001d021227_T003         0
622- 11: transcript:EER99221           transcript:Zm00001d021231_T005         0
622- 12: transcript:OQU89800           transcript:Zm00001d021232_T001         0
622- 13: transcript:EER99222           transcript:Zm00001d021236_T004         0
622- 14: transcript:KXG36047           transcript:Zm00001d021241_T009  1.00E-57
## Alignment 623: score=659.0 e_value=1.1e-40 N=15 2&7 plus
623-  0: transcript:EER96576           transcript:Zm00001d020100_T001 1.00E-167
623-  1: transcript:EER98746           transcript:Zm00001d020101_T001         0
623-  2: transcript:OQU89311           transcript:Zm00001d020102_T001  6.00E-45
623-  3: transcript:OQU89313           transcript:Zm00001d020103_T003         0
623-  4: transcript:EER98751           transcript:Zm00001d020108_T002         0
623-  5: transcript:EER98749           transcript:Zm00001d020109_T001         0
623-  6: transcript:OQU89317           transcript:Zm00001d020112_T001         0
623-  7: transcript:EER96586           transcript:Zm00001d020122_T001         0
623-  8: transcript:OQU89331           transcript:Zm00001d020129_T003  9.00E-80
623-  9: transcript:EER96588           transcript:Zm00001d020133_T001  7.00E-40
623- 10: transcript:EER98758           transcript:Zm00001d020134_T003         0
623- 11: transcript:EER96589           transcript:Zm00001d020136_T001 7.00E-137
623- 12: transcript:OQU89339           transcript:Zm00001d020138_T001  1.00E-25
623- 13: transcript:KXG35447           transcript:Zm00001d020139_T001         0
623- 14: transcript:EER96598           transcript:Zm00001d020147_T001         0
## Alignment 624: score=649.0 e_value=1.4e-35 N=14 2&7 plus
624-  0: transcript:KXG34928           transcript:Zm00001d019399_T001  8.00E-34
624-  1: transcript:EER96244           transcript:Zm00001d019404_T001 5.00E-176
624-  2: transcript:EER98375           transcript:Zm00001d019405_T001         0
624-  3: transcript:OQU88871           transcript:Zm00001d019414_T001 2.00E-140
624-  4: transcript:EER96246           transcript:Zm00001d019420_T001         0
624-  5: transcript:EER96247           transcript:Zm00001d019423_T002         0
624-  6: transcript:KXG34933           transcript:Zm00001d019426_T001 6.00E-172
624-  7: transcript:EER96250           transcript:Zm00001d019430_T001 9.00E-170
624-  8: transcript:KXG34936           transcript:Zm00001d019432_T002         0
624-  9: transcript:KXG34937           transcript:Zm00001d019434_T001 4.00E-153
624- 10: transcript:EER98384           transcript:Zm00001d019439_T001 3.00E-158
624- 11: transcript:EER96251           transcript:Zm00001d019445_T001  3.00E-46
624- 12: transcript:EER98385           transcript:Zm00001d019446_T001 5.00E-149
624- 13: transcript:KXG34938           transcript:Zm00001d019447_T001  1.00E-71
## Alignment 625: score=647.0 e_value=3.1e-41 N=15 2&7 plus
625-  0: transcript:EER97976           transcript:Zm00001d018796_T001  1.00E-57
625-  1: transcript:EER95868           transcript:Zm00001d018797_T001  3.00E-84
625-  2: transcript:KXG34404           transcript:Zm00001d018798_T002         0
625-  3: transcript:OQU88438           transcript:Zm00001d018799_T001         0
625-  4: transcript:KXG34405           transcript:Zm00001d018801_T001  2.00E-37
625-  5: transcript:EER95872           transcript:Zm00001d018802_T001  1.00E-62
625-  6: transcript:OQU88440           transcript:Zm00001d018803_T001         0
625-  7: transcript:EER95875           transcript:Zm00001d018806_T001         0
625-  8: transcript:KXG34410           transcript:Zm00001d018807_T001         0
625-  9: transcript:EER95876           transcript:Zm00001d018809_T001  8.00E-69
625- 10: transcript:KXG34415           transcript:Zm00001d018810_T002         0
625- 11: transcript:EER97987           transcript:Zm00001d018811_T001  2.00E-42
625- 12: transcript:EER97990           transcript:Zm00001d018812_T001 6.00E-143
```

625- 13: transcript:EER97991 transcript:Zm00001d018813_T001 0
625- 14: transcript:EER97992 transcript:Zm00001d018816_T001 4.00E-69
Alignment 626: score=614.0 e_value=1.4e-38 N=15 2&7 plus
626- 0: transcript:EER95885 transcript:Zm00001d018826_T001 2.00E-95
626- 1: transcript:EER95886 transcript:Zm00001d018827_T001 1.00E-93
626- 2: transcript:OQU88462 transcript:Zm00001d018828_T002 0
626- 3: transcript:EER95888 transcript:Zm00001d018829_T002 1.00E-120
626- 4: transcript:EER95889 transcript:Zm00001d018831_T008 1.00E-116
626- 5: transcript:OQU88465 transcript:Zm00001d018832_T001 2.00E-100
626- 6: transcript:KXG34438 transcript:Zm00001d018837_T001 3.00E-149
626- 7: transcript:EER98009 transcript:Zm00001d018838_T002 2.00E-135
626- 8: transcript:EER95892 transcript:Zm00001d018839_T001 0
626- 9: transcript:EER98013 transcript:Zm00001d018842_T001 0
626- 10: transcript:KXG34439 transcript:Zm00001d018844_T001 7.00E-45
626- 11: transcript:EER98015 transcript:Zm00001d018846_T001 6.00E-122
626- 12: transcript:OQU88478 transcript:Zm00001d018848_T001 0
626- 13: transcript:EER95897 transcript:Zm00001d018853_T001 0
626- 14: transcript:KXG34449 transcript:Zm00001d018859_T010 0
Alignment 627: score=610.0 e_value=5.7e-39 N=15 2&7 plus
627- 0: transcript:KXG34887 transcript:Zm00001d019349_T008 0
627- 1: transcript:KXG34888 transcript:Zm00001d019354_T001 0
627- 2: transcript:EER98353 transcript:Zm00001d019357_T002 6.00E-177
627- 3: transcript:OQU88844 transcript:Zm00001d019358_T001 2.00E-34
627- 4: transcript:OQU88848 transcript:Zm00001d019363_T001 0
627- 5: transcript:EER96217 transcript:Zm00001d019364_T001 8.00E-104
627- 6: transcript:OQU88855 transcript:Zm00001d019365_T001 0
627- 7: transcript:EER96218 transcript:Zm00001d019370_T006 2.00E-152
627- 8: transcript:KXG34897 transcript:Zm00001d019372_T001 0
627- 9: transcript:KXG34905 transcript:Zm00001d019373_T001 3.00E-69
627- 10: transcript:OQU88862 transcript:Zm00001d019376_T004 0
627- 11: transcript:KXG34919 transcript:Zm00001d019377_T001 1.00E-161
627- 12: transcript:OQU88866 transcript:Zm00001d019380_T001 6.00E-95
627- 13: transcript:EER96237 transcript:Zm00001d019397_T006 0
627- 14: transcript:KXG34927 transcript:Zm00001d019398_T001 0
Alignment 628: score=605.0 e_value=3.7e-34 N=14 2&7 plus
628- 0: transcript:OQU88523 transcript:Zm00001d018930_T003 0
628- 1: transcript:OQU88522 transcript:Zm00001d018931_T001 1.00E-157
628- 2: transcript:KXG34507 transcript:Zm00001d018935_T003 0
628- 3: transcript:KXG34505 transcript:Zm00001d018936_T001 0
628- 4: transcript:KXG34512 transcript:Zm00001d018937_T012 0
628- 5: transcript:EER98056 transcript:Zm00001d018938_T001 7.00E-157
628- 6: transcript:EER95939 transcript:Zm00001d018939_T001 0
628- 7: transcript:OQU88531 transcript:Zm00001d018940_T001 4.00E-60
628- 8: transcript:OQU88534 transcript:Zm00001d018943_T003 0
628- 9: transcript:EER95944 transcript:Zm00001d018944_T002 0
628- 10: transcript:OQU88537 transcript:Zm00001d018946_T001 6.00E-126
628- 11: transcript:KXG34522 transcript:Zm00001d018947_T001 3.00E-41
628- 12: transcript:KXG34525 transcript:Zm00001d018950_T001 8.00E-103
628- 13: transcript:OQU88538 transcript:Zm00001d018951_T001 1.00E-35
Alignment 629: score=593.0 e_value=9.1e-33 N=14 2&7 plus
629- 0: transcript:EER96269 transcript:Zm00001d019473_T001 0
629- 1: transcript:EER96271 transcript:Zm00001d019474_T001 3.00E-67
629- 2: transcript:KXG34964 transcript:Zm00001d019475_T001 1.00E-78
629- 3: transcript:OQU88891 transcript:Zm00001d019477_T001 0

```
629-  4: transcript:EER98410            transcript:Zm00001d019479_T004          0
629-  5: transcript:EER98411            transcript:Zm00001d019480_T001          0
629-  6: transcript:OQU88892            transcript:Zm00001d019481_T001 6.00E-127
629-  7: transcript:EER98412            transcript:Zm00001d019483_T001          0
629-  8: transcript:EER96277            transcript:Zm00001d019487_T001  2.00E-62
629-  9: transcript:EER96279            transcript:Zm00001d019490_T001 7.00E-180
629- 10: transcript:EER96281            transcript:Zm00001d019492_T001          0
629- 11: transcript:KXG34973            transcript:Zm00001d019493_T001          0
629- 12: transcript:KXG34975            transcript:Zm00001d019495_T007          0
629- 13: transcript:KXG34976            transcript:Zm00001d019497_T004          0
## Alignment 630: score=588.0 e_value=2.2e-34 N=14 2&7 plus
630-  0: transcript:KXG34640            transcript:Zm00001d019115_T048          0
630-  1: transcript:KXG34641            transcript:Zm00001d019116_T001  5.00E-85
630-  2: transcript:EER96043            transcript:Zm00001d019117_T002  3.00E-71
630-  3: transcript:EER96044            transcript:Zm00001d019120_T001  6.00E-33
630-  4: transcript:EER98182            transcript:Zm00001d019123_T002          0
630-  5: transcript:KXG34650            transcript:Zm00001d019124_T003          0
630-  6: transcript:EER96048            transcript:Zm00001d019125_T003          0
630-  7: transcript:EER98184            transcript:Zm00001d019130_T005          0
630-  8: transcript:EER98187            transcript:Zm00001d019138_T001          0
630-  9: transcript:KXG34661            transcript:Zm00001d019139_T012          0
630- 10: transcript:EER98192            transcript:Zm00001d019142_T001  9.00E-86
630- 11: transcript:EER98193            transcript:Zm00001d019145_T001          0
630- 12: transcript:EER96052            transcript:Zm00001d019147_T001          0
630- 13: transcript:OQU88678            transcript:Zm00001d019148_T002          0
## Alignment 631: score=532.0 e_value=5.4e-33 N=13 2&7 plus
631-  0: transcript:EER95716            transcript:Zm00001d018605_T001 3.00E-117
631-  1: transcript:EER97816            transcript:Zm00001d018609_T001          0
631-  2: transcript:EER97817            transcript:Zm00001d018610_T001          0
631-  3: transcript:KXG34247            transcript:Zm00001d018611_T001 4.00E-156
631-  4: transcript:EER97821            transcript:Zm00001d018613_T004          0
631-  5: transcript:EER95720            transcript:Zm00001d018614_T003          0
631-  6: transcript:EER97822            transcript:Zm00001d018616_T001 5.00E-172
631-  7: transcript:EER95722            transcript:Zm00001d018617_T001          0
631-  8: transcript:EER95724            transcript:Zm00001d018618_T001 6.00E-127
631-  9: transcript:EER95727            transcript:Zm00001d018620_T001 6.00E-176
631- 10: transcript:EER95728            transcript:Zm00001d018621_T001  3.00E-74
631- 11: transcript:EER95729            transcript:Zm00001d018623_T001 2.00E-123
631- 12: transcript:OQU88295            transcript:Zm00001d018624_T001          0
## Alignment 632: score=517.0 e_value=3.1e-27 N=12 2&7 plus
632-  0: transcript:EER96508            transcript:Zm00001d020002_T002          0
632-  1: transcript:EER98657            transcript:Zm00001d020003_T002 2.00E-104
632-  2: transcript:OQU89208            transcript:Zm00001d020004_T003          0
632-  3: transcript:EER96517            transcript:Zm00001d020006_T001          0
632-  4: transcript:OQU89210            transcript:Zm00001d020008_T001          0
632-  5: transcript:EER96518            transcript:Zm00001d020011_T001          0
632-  6: transcript:EER98666            transcript:Zm00001d020013_T001          0
632-  7: transcript:OQU89219            transcript:Zm00001d020014_T001          0
632-  8: transcript:EER96520            transcript:Zm00001d020017_T001          0
632-  9: transcript:EER98668            transcript:Zm00001d020018_T001 5.00E-171
632- 10: transcript:EER96522            transcript:Zm00001d020019_T002 5.00E-116
632- 11: transcript:EER96523            transcript:Zm00001d020020_T003          0
## Alignment 633: score=482.0 e_value=2.1e-30 N=13 2&7 plus
633-  0: transcript:EER95753            transcript:Zm00001d018667_T003 1.00E-145
```

633- 1: transcript:OQU88318 transcript:Zm00001d018668_T002 2.00E-140
633- 2: transcript:EER97857 transcript:Zm00001d018669_T004 0
633- 3: transcript:EER95756 transcript:Zm00001d018670_T001 5.00E-140
633- 4: transcript:EER97859 transcript:Zm00001d018671_T001 0
633- 5: transcript:EER97861 transcript:Zm00001d018677_T001 0
633- 6: transcript:EER97863 transcript:Zm00001d018680_T001 2.00E-134
633- 7: transcript:EER95768 transcript:Zm00001d018681_T001 5.00E-159
633- 8: transcript:OQU88323 transcript:Zm00001d018682_T001 1.00E-79
633- 9: transcript:EER95777 transcript:Zm00001d018685_T001 0
633- 10: transcript:EER97876 transcript:Zm00001d018691_T001 1.00E-34
633- 11: transcript:EER97880 transcript:Zm00001d018694_T002 0
633- 12: transcript:OQU88335 transcript:Zm00001d018699_T002 3.00E-82
Alignment 634: score=439.0 e_value=1.1e-24 N=10 2&7 plus
634- 0: transcript:EER96634 transcript:Zm00001d020228_T002 0
634- 1: transcript:EER96635 transcript:Zm00001d020230_T006 0
634- 2: transcript:KXG35486 transcript:Zm00001d020233_T001 0
634- 3: transcript:EER98796 transcript:Zm00001d020234_T001 0
634- 4: transcript:OQU89390 transcript:Zm00001d020236_T001 2.00E-75
634- 5: transcript:OQU89389 transcript:Zm00001d020237_T001 0
634- 6: transcript:EER98797 transcript:Zm00001d020238_T002 0
634- 7: transcript:KXG35490 transcript:Zm00001d020242_T002 0
634- 8: transcript:OQU89394 transcript:Zm00001d020243_T001 2.00E-13
634- 9: transcript:EER96646 transcript:Zm00001d020250_T001 1.00E-60
Alignment 635: score=439.0 e_value=1.5e-19 N=10 2&7 plus
635- 0: transcript:EER96257 transcript:Zm00001d019449_T004 0
635- 1: transcript:EER98388 transcript:Zm00001d019450_T001 0
635- 2: transcript:KXG34948 transcript:Zm00001d019454_T001 2.00E-177
635- 3: transcript:EER98393 transcript:Zm00001d019457_T001 4.00E-95
635- 4: transcript:EER98394 transcript:Zm00001d019459_T002 7.00E-67
635- 5: transcript:EER98396 transcript:Zm00001d019461_T002 0
635- 6: transcript:EER98397 transcript:Zm00001d019462_T001 0
635- 7: transcript:OQU88881 transcript:Zm00001d019463_T001 0
635- 8: transcript:KXG34956 transcript:Zm00001d019467_T001 0
635- 9: transcript:EER96263 transcript:Zm00001d019472_T005 0
Alignment 636: score=427.0 e_value=1.7e-20 N=10 2&7 plus
636- 0: transcript:OQU88373 transcript:Zm00001d018714_T001 1.00E-20
636- 1: transcript:EER95817 transcript:Zm00001d018717_T001 5.00E-45
636- 2: transcript:EER97906 transcript:Zm00001d018718_T001 8.00E-142
636- 3: transcript:KXG34315 transcript:Zm00001d018719_T001 0
636- 4: transcript:EER97909 transcript:Zm00001d018724_T003 0
636- 5: transcript:EER97911 transcript:Zm00001d018725_T001 2.00E-25
636- 6: transcript:OQU88383 transcript:Zm00001d018727_T001 1.00E-18
636- 7: transcript:EER95819 transcript:Zm00001d018729_T001 3.00E-21
636- 8: transcript:EER95824 transcript:Zm00001d018730_T001 0
636- 9: transcript:KXG34322 transcript:Zm00001d018731_T001 0
Alignment 637: score=414.0 e_value=1.2e-20 N=10 2&7 plus
637- 0: transcript:EER95850 transcript:Zm00001d018770_T003 0
637- 1: transcript:KXG34371 transcript:Zm00001d018772_T001 2.00E-28
637- 2: transcript:EER95852 transcript:Zm00001d018773_T001 0
637- 3: transcript:EER97956 transcript:Zm00001d018775_T001 2.00E-115
637- 4: transcript:KXG34372 transcript:Zm00001d018776_T004 0
637- 5: transcript:EER97959 transcript:Zm00001d018779_T001 2.00E-159
637- 6: transcript:KXG34373 transcript:Zm00001d018780_T001 2.00E-71
637- 7: transcript:KXG34376 transcript:Zm00001d018781_T001 7.00E-156

```
637-  8: transcript:KXG34380            transcript:Zm00001d018786_T004         0
637-  9: transcript:EER97966            transcript:Zm00001d018787_T001         0
## Alignment 638: score=412.0 e_value=6.2e-21 N=9 2&7 plus
638-  0: transcript:EER96549            transcript:Zm00001d020051_T001         0
638-  1: transcript:EER98698            transcript:Zm00001d020052_T002         0
638-  2: transcript:EER98699            transcript:Zm00001d020053_T002         0
638-  3: transcript:EER96550            transcript:Zm00001d020054_T001  2.00E-54
638-  4: transcript:EER96552            transcript:Zm00001d020055_T001 1.00E-167
638-  5: transcript:EER98700            transcript:Zm00001d020057_T004         0
638-  6: transcript:EER96560            transcript:Zm00001d020062_T001         0
638-  7: transcript:OQU89271            transcript:Zm00001d020063_T001         0
638-  8: transcript:KXG35381            transcript:Zm00001d020064_T001 5.00E-117
## Alignment 639: score=406.0 e_value=1.5e-22 N=10 2&7 plus
639-  0: transcript:OQU88987            transcript:Zm00001d019629_T001 4.00E-126
639-  1: transcript:EER98483            transcript:Zm00001d019631_T003         0
639-  2: transcript:KXG35088            transcript:Zm00001d019637_T001         0
639-  3: transcript:OQU88990            transcript:Zm00001d019641_T002         0
639-  4: transcript:OQU88997            transcript:Zm00001d019643_T007 5.00E-163
639-  5: transcript:EER96343            transcript:Zm00001d019644_T003         0
639-  6: transcript:EER98486            transcript:Zm00001d019645_T001  5.00E-29
639-  7: transcript:OQU88998            transcript:Zm00001d019648_T002 9.00E-146
639-  8: transcript:KXG35092            transcript:Zm00001d019650_T001         0
639-  9: transcript:KXG35102            transcript:Zm00001d019656_T001         0
## Alignment 640: score=404.0 e_value=2.8e-18 N=9 2&7 plus
640-  0: transcript:EER97364            transcript:Zm00001d021744_T001  1.00E-79
640-  1: transcript:EER99528            transcript:Zm00001d021745_T001 3.00E-167
640-  2: transcript:EER99518            transcript:Zm00001d021746_T001         0
640-  3: transcript:OQU90076            transcript:Zm00001d021747_T001 2.00E-109
640-  4: transcript:EER97371            transcript:Zm00001d021754_T005         0
640-  5: transcript:EER99533            transcript:Zm00001d021755_T001         0
640-  6: transcript:EER99536            transcript:Zm00001d021761_T001 2.00E-170
640-  7: transcript:EER99537            transcript:Zm00001d021763_T001         0
640-  8: transcript:EER99538            transcript:Zm00001d021764_T002 4.00E-138
## Alignment 641: score=402.0 e_value=3.1e-20 N=10 2&7 plus
641-  0: transcript:OQU88550            transcript:Zm00001d018950_T001  6.00E-15
641-  1: transcript:EER95948            transcript:Zm00001d018954_T002 7.00E-121
641-  2: transcript:KXG34534            transcript:Zm00001d018957_T001         0
641-  3: transcript:EER95950            transcript:Zm00001d018959_T001         0
641-  4: transcript:EER95951            transcript:Zm00001d018961_T002         0
641-  5: transcript:EER98071            transcript:Zm00001d018964_T003         0
641-  6: transcript:EER98073            transcript:Zm00001d018965_T001 2.00E-107
641-  7: transcript:KXG34543            transcript:Zm00001d018972_T005         0
641-  8: transcript:EER98076            transcript:Zm00001d018973_T001 1.00E-111
641-  9: transcript:OQU88575            transcript:Zm00001d018976_T003         0
## Alignment 642: score=391.0 e_value=1.1e-15 N=8 2&7 plus
642-  0: transcript:OQU90392            transcript:Zm00001d022367_T002         0
642-  1: transcript:OQU90393            transcript:Zm00001d022368_T002 9.00E-119
642-  2: transcript:EER97663            transcript:Zm00001d022369_T001         0
642-  3: transcript:EER97649            transcript:Zm00001d022371_T001 7.00E-164
642-  4: transcript:EER97666            transcript:Zm00001d022373_T001  3.00E-61
642-  5: transcript:OQU90395            transcript:Zm00001d022374_T001         0
642-  6: transcript:EER99834            transcript:Zm00001d022376_T001 1.00E-132
642-  7: transcript:KXG36842            transcript:Zm00001d022378_T003         0
## Alignment 643: score=379.0 e_value=1.2e-16 N=8 2&7 plus
```

```
643-  0: transcript:KXG36444           transcript:Zm00001d021729_T001          0
643-  1: transcript:EER99514           transcript:Zm00001d021730_T001 4.00E-148
643-  2: transcript:KXG36445           transcript:Zm00001d021731_T001          0
643-  3: transcript:OQU90071           transcript:Zm00001d021737_T001          0
643-  4: transcript:EER97362           transcript:Zm00001d021738_T001  4.00E-17
643-  5: transcript:EER99527           transcript:Zm00001d021739_T001 6.00E-157
643-  6: transcript:EER97364           transcript:Zm00001d021740_T001          0
643-  7: transcript:KXG36457           transcript:Zm00001d021741_T001          0
## Alignment 644: score=352.0 e_value=3.1e-17 N=9 2&7 plus
644-  0: transcript:Zm00001d002333_T003 transcript:OQU80069                    0
644-  1: transcript:Zm00001d002334_T001 transcript:EES13516                    0
644-  2: transcript:Zm00001d002338_T001 transcript:EES13519                    0
644-  3: transcript:Zm00001d002343_T001 transcript:EES13520             2.00E-91
644-  4: transcript:Zm00001d002346_T002 transcript:OQU80077                    0
644-  5: transcript:Zm00001d002353_T001 transcript:KXG24714            2.00E-111
644-  6: transcript:Zm00001d002358_T001 transcript:EES14712                    0
644-  7: transcript:Zm00001d002362_T001 transcript:KXG24716             5.00E-19
644-  8: transcript:Zm00001d002364_T001 transcript:EES14717             1.00E-27
## Alignment 645: score=333.0 e_value=2.4e-13 N=8 2&7 plus
645-  0: transcript:Zm00001d005648_T002 transcript:KXG25127                    0
645-  1: transcript:Zm00001d005650_T004 transcript:OQU80395             3.00E-64
645-  2: transcript:Zm00001d005653_T004 transcript:EES13800            2.00E-132
645-  3: transcript:Zm00001d005654_T001 transcript:EES14962             8.00E-16
645-  4: transcript:Zm00001d005656_T001 transcript:EES13808             2.00E-56
645-  5: transcript:Zm00001d005657_T002 transcript:EES14968             3.00E-41
645-  6: transcript:Zm00001d005658_T001 transcript:EES13815                    0
645-  7: transcript:Zm00001d005664_T001 transcript:OQU80413             4.00E-42
## Alignment 646: score=329.0 e_value=3.7e-12 N=7 2&7 plus
646-  0: transcript:EER97061           transcript:Zm00001d021191_T001          0
646-  1: transcript:EER99204           transcript:Zm00001d021192_T001  2.00E-37
646-  2: transcript:KXG36025           transcript:Zm00001d021195_T002          0
646-  3: transcript:EER97062           transcript:Zm00001d021196_T004          0
646-  4: transcript:EER99200           transcript:Zm00001d021197_T001          0
646-  5: transcript:EER97064           transcript:Zm00001d021199_T002 3.00E-158
646-  6: transcript:KXG36028           transcript:Zm00001d021201_T001          0
## Alignment 647: score=315.0 e_value=4.6e-12 N=7 2&7 plus
647-  0: transcript:KXG36860           transcript:Zm00001d022405_T051          0
647-  1: transcript:EER97679           transcript:Zm00001d022406_T002          0
647-  2: transcript:EER99854           transcript:Zm00001d022407_T007          0
647-  3: transcript:OQU90419           transcript:Zm00001d022414_T001          0
647-  4: transcript:EER99855           transcript:Zm00001d022416_T001  3.00E-48
647-  5: transcript:KXG36868           transcript:Zm00001d022417_T003          0
647-  6: transcript:EER99861           transcript:Zm00001d022418_T001  8.00E-38
## Alignment 648: score=310.0 e_value=8.8e-11 N=7 2&7 plus
648-  0: transcript:EER98081           transcript:Zm00001d018979_T001 1.00E-108
648-  1: transcript:EER95966           transcript:Zm00001d018981_T002  2.00E-54
648-  2: transcript:OQU88579           transcript:Zm00001d018983_T001          0
648-  3: transcript:EER98085           transcript:Zm00001d018984_T001 7.00E-109
648-  4: transcript:EER95968           transcript:Zm00001d018986_T001          0
648-  5: transcript:EER95974           transcript:Zm00001d018987_T001          0
648-  6: transcript:KXG34554           transcript:Zm00001d018988_T002  6.00E-65
## Alignment 649: score=305.0 e_value=2.1e-11 N=7 2&7 plus
649-  0: transcript:KXG34485           transcript:Zm00001d018906_T001  3.00E-52
649-  1: transcript:KXG34488           transcript:Zm00001d018907_T003 1.00E-150
```

```
649-  2: transcript:EER95920           transcript:Zm00001d018908_T001  6.00E-69
649-  3: transcript:KXG34492           transcript:Zm00001d018911_T001         0
649-  4: transcript:EER95921           transcript:Zm00001d018912_T010         0
649-  5: transcript:EER95922           transcript:Zm00001d018913_T001  2.00E-30
649-  6: transcript:OQU88517           transcript:Zm00001d018915_T007  2.00E-19
## Alignment 650: score=298.0 e_value=1.4e-12 N=7 2&7 plus
650-  0: transcript:KXG35063           transcript:Zm00001d019579_T007         0
650-  1: transcript:OQU88970           transcript:Zm00001d019582_T001         0
650-  2: transcript:KXG35066           transcript:Zm00001d019586_T016         0
650-  3: transcript:OQU88974           transcript:Zm00001d019587_T002         0
650-  4: transcript:EER96325           transcript:Zm00001d019588_T001 2.00E-172
650-  5: transcript:OQU88975           transcript:Zm00001d019589_T001 2.00E-103
650-  6: transcript:KXG35071           transcript:Zm00001d019591_T006         0
## Alignment 651: score=291.0 e_value=9e-12 N=7 2&7 plus
651-  0: transcript:KXG34354           transcript:Zm00001d018750_T001  6.00E-30
651-  1: transcript:EER97947           transcript:Zm00001d018751_T001         0
651-  2: transcript:EER97948           transcript:Zm00001d018752_T001         0
651-  3: transcript:EER95842           transcript:Zm00001d018754_T001         0
651-  4: transcript:KXG34359           transcript:Zm00001d018755_T001 7.00E-175
651-  5: transcript:OQU88410           transcript:Zm00001d018756_T001         0
651-  6: transcript:KXG34367           transcript:Zm00001d018758_T001         0
## Alignment 652: score=286.0 e_value=5.9e-15 N=7 2&7 plus
652-  0: transcript:Zm00001d005696_T001 transcript:KXG25169                  0
652-  1: transcript:Zm00001d005699_T001 transcript:OQU80448          1.00E-119
652-  2: transcript:Zm00001d005705_T001 transcript:KXG25172           1.00E-86
652-  3: transcript:Zm00001d005713_T001 transcript:EES13861          1.00E-171
652-  4: transcript:Zm00001d005714_T001 transcript:EES15006           2.00E-44
652-  5: transcript:Zm00001d005726_T001 transcript:EES15016           5.00E-70
652-  6: transcript:Zm00001d005729_T001 transcript:OQU80479                  0
## Alignment 653: score=283.0 e_value=3.2e-09 N=6 2&7 plus
653-  0: transcript:EER98048           transcript:Zm00001d018917_T001  3.00E-59
653-  1: transcript:EER95927           transcript:Zm00001d018918_T001         0
653-  2: transcript:KXG34496           transcript:Zm00001d018925_T004         0
653-  3: transcript:OQU88519           transcript:Zm00001d018926_T001  6.00E-81
653-  4: transcript:KXG34499           transcript:Zm00001d018927_T002 8.00E-110
653-  5: transcript:EER98050           transcript:Zm00001d018929_T002         0
## Alignment 654: score=281.0 e_value=4.1e-09 N=6 2&7 plus
654-  0: transcript:KXG34391           transcript:Zm00001d018789_T001         0
654-  1: transcript:OQU88433           transcript:Zm00001d018790_T001 1.00E-169
654-  2: transcript:EER97970           transcript:Zm00001d018791_T001         0
654-  3: transcript:EER95864           transcript:Zm00001d018793_T001         0
654-  4: transcript:OQU88434           transcript:Zm00001d018792_T004         0
654-  5: transcript:OQU88435           transcript:Zm00001d018794_T003         0
## Alignment 655: score=279.0 e_value=9.1e-09 N=6 2&7 plus
655-  0: transcript:KXG36398           transcript:Zm00001d021688_T001  4.00E-12
655-  1: transcript:OQU90051           transcript:Zm00001d021689_T001  7.00E-07
655-  2: transcript:EER99485           transcript:Zm00001d021692_T001         0
655-  3: transcript:KXG36403           transcript:Zm00001d021695_T001         0
655-  4: transcript:OQU90052           transcript:Zm00001d021696_T003         0
655-  5: transcript:KXG36425           transcript:Zm00001d021698_T001 1.00E-101
## Alignment 656: score=274.0 e_value=1.4e-09 N=6 2&7 plus
656-  0: transcript:OQU88587           transcript:Zm00001d019039_T002 8.00E-145
656-  1: transcript:KXG34561           transcript:Zm00001d019040_T001         0
656-  2: transcript:EER95980           transcript:Zm00001d019041_T001         0
```

656- 3: transcript:KXG34566   transcript:Zm00001d019044_T001 3.00E-47
656- 4: transcript:EER95981   transcript:Zm00001d019045_T001 6.00E-68
656- 5: transcript:EER98097   transcript:Zm00001d019047_T001   0
Alignment 657: score=273.0 e_value=0 N=6 2&7 plus
657- 0: transcript:OQU88453   transcript:Zm00001d018819_T001   0
657- 1: transcript:EER95880   transcript:Zm00001d018820_T004   0
657- 2: transcript:EER98001   transcript:Zm00001d018821_T001   0
657- 3: transcript:EER95883   transcript:Zm00001d018822_T001   0
657- 4: transcript:OQU88460   transcript:Zm00001d018823_T001 1.00E-83
657- 5: transcript:OQU88461   transcript:Zm00001d018825_T001 8.00E-111
Alignment 658: score=3071.0 e_value=2.4e-274 N=67 2&7 minus
658- 0: transcript:KXG36142   transcript:Zm00001d021434_T001   0
658- 1: transcript:OQU89887   transcript:Zm00001d021433_T002 3.00E-55
658- 2: transcript:EER99311   transcript:Zm00001d021430_T013   0
658- 3: transcript:EER99320   transcript:Zm00001d021426_T002   0
658- 4: transcript:KXG36150   transcript:Zm00001d021425_T001   0
658- 5: transcript:KXG36156   transcript:Zm00001d021423_T001 3.00E-107
658- 6: transcript:KXG36157   transcript:Zm00001d021421_T003   0
658- 7: transcript:EER99325   transcript:Zm00001d021420_T003   0
658- 8: transcript:EER97162   transcript:Zm00001d021419_T001 1.00E-127
658- 9: transcript:EER99326   transcript:Zm00001d021418_T001 7.00E-135
658- 10: transcript:EER97163   transcript:Zm00001d021417_T001 4.00E-83
658- 11: transcript:OQU89905   transcript:Zm00001d021416_T001   0
658- 12: transcript:OQU89906   transcript:Zm00001d021415_T001 2.00E-167
658- 13: transcript:EER99328   transcript:Zm00001d021414_T001 3.00E-86
658- 14: transcript:KXG36159   transcript:Zm00001d021413_T001   0
658- 15: transcript:EER97165   transcript:Zm00001d021412_T001   0
658- 16: transcript:OQU89909   transcript:Zm00001d021411_T001   0
658- 17: transcript:EER99331   transcript:Zm00001d021410_T001   0
658- 18: transcript:OQU89910   transcript:Zm00001d021407_T001   0
658- 19: transcript:EER99333   transcript:Zm00001d021404_T001   0
658- 20: transcript:OQU89911   transcript:Zm00001d021403_T001   0
658- 21: transcript:EER99336   transcript:Zm00001d021401_T001 1.00E-91
658- 22: transcript:EER97166   transcript:Zm00001d021400_T001   0
658- 23: transcript:OQU89913   transcript:Zm00001d021396_T001 1.00E-178
658- 24: transcript:EER99339   transcript:Zm00001d021395_T004   0
658- 25: transcript:EER99342   transcript:Zm00001d021393_T001   0
658- 26: transcript:EER97171   transcript:Zm00001d021392_T002   0
658- 27: transcript:KXG36173   transcript:Zm00001d021391_T001 3.00E-100
658- 28: transcript:KXG36172   transcript:Zm00001d021390_T001   0
658- 29: transcript:KXG36174   transcript:Zm00001d021389_T001   0
658- 30: transcript:EER97175   transcript:Zm00001d021388_T001   0
658- 31: transcript:EER99337   transcript:Zm00001d021387_T001   0
658- 32: transcript:EER99346   transcript:Zm00001d021386_T001 9.00E-61
658- 33: transcript:EER99347   transcript:Zm00001d021385_T001   0
658- 34: transcript:EER97178   transcript:Zm00001d021384_T001   0
658- 35: transcript:EER97181   transcript:Zm00001d021379_T003   0
658- 36: transcript:EER99353   transcript:Zm00001d021378_T007   0
658- 37: transcript:OQU89932   transcript:Zm00001d021376_T001   0
658- 38: transcript:EER99354   transcript:Zm00001d021375_T003 8.00E-38
658- 39: transcript:EER97183   transcript:Zm00001d021372_T001   0
658- 40: transcript:EER97184   transcript:Zm00001d021371_T001   0
658- 41: transcript:EER97176   transcript:Zm00001d021370_T001 5.00E-90
658- 42: transcript:KXG36189   transcript:Zm00001d021368_T001   0

```
658- 43: transcript:KXG36191            transcript:Zm00001d021365_T001          0
658- 44: transcript:EER97188            transcript:Zm00001d021363_T001          0
658- 45: transcript:EER99361            transcript:Zm00001d021362_T001 1.00E-162
658- 46: transcript:EER97189            transcript:Zm00001d021360_T001  3.00E-67
658- 47: transcript:EER97191            transcript:Zm00001d021359_T001  2.00E-60
658- 48: transcript:KXG36195            transcript:Zm00001d021358_T001 2.00E-101
658- 49: transcript:EER97193            transcript:Zm00001d021357_T001  3.00E-60
658- 50: transcript:OQU89940            transcript:Zm00001d021356_T001  1.00E-42
658- 51: transcript:EER97185            transcript:Zm00001d021351_T003          0
658- 52: transcript:KXG36196            transcript:Zm00001d021350_T003 2.00E-167
658- 53: transcript:EER97196            transcript:Zm00001d021347_T004          0
658- 54: transcript:EER97197            transcript:Zm00001d021346_T002          0
658- 55: transcript:EER97198            transcript:Zm00001d021345_T001          0
658- 56: transcript:OQU89942            transcript:Zm00001d021344_T002          0
658- 57: transcript:KXG36199            transcript:Zm00001d021343_T001          0
658- 58: transcript:EER99364            transcript:Zm00001d021342_T017          0
658- 59: transcript:EER97204            transcript:Zm00001d021341_T001 6.00E-116
658- 60: transcript:EER99369            transcript:Zm00001d021340_T001 2.00E-131
658- 61: transcript:EER99371            transcript:Zm00001d021337_T001  2.00E-63
658- 62: transcript:EER99373            transcript:Zm00001d021336_T004          0
658- 63: transcript:EER97205            transcript:Zm00001d021335_T003 2.00E-126
658- 64: transcript:EER99374            transcript:Zm00001d021334_T001          0
658- 65: transcript:KXG36207            transcript:Zm00001d021332_T004 1.00E-168
658- 66: transcript:EER97212            transcript:Zm00001d021321_T001 1.00E-157
## Alignment 659: score=1538.0 e_value=1.2e-114 N=34 2&7 minus
659-  0: transcript:EER99891            transcript:Zm00001d022467_T001          0
659-  1: transcript:KXG36925            transcript:Zm00001d022466_T006          0
659-  2: transcript:KXG36926            transcript:Zm00001d022465_T007 4.00E-160
659-  3: transcript:EER99898            transcript:Zm00001d022464_T001  2.00E-37
659-  4: transcript:EER97718            transcript:Zm00001d022463_T001  4.00E-76
659-  5: transcript:EER97722            transcript:Zm00001d022462_T001 2.00E-132
659-  6: transcript:EER99901            transcript:Zm00001d022461_T001  5.00E-80
659-  7: transcript:EER99903            transcript:Zm00001d022460_T001          0
659-  8: transcript:EER97724            transcript:Zm00001d022459_T001 6.00E-131
659-  9: transcript:EER97728            transcript:Zm00001d022456_T001 2.00E-102
659- 10: transcript:KXG36942            transcript:Zm00001d022450_T001          0
659- 11: transcript:EER99908            transcript:Zm00001d022449_T002          0
659- 12: transcript:OQU90473            transcript:Zm00001d022448_T001  6.00E-77
659- 13: transcript:OQU90474            transcript:Zm00001d022446_T001  5.00E-97
659- 14: transcript:EER97737            transcript:Zm00001d022445_T002          0
659- 15: transcript:OQU90475            transcript:Zm00001d022444_T001          0
659- 16: transcript:OQU90477            transcript:Zm00001d022443_T001 2.00E-163
659- 17: transcript:OQU90478            transcript:Zm00001d022442_T002          0
659- 18: transcript:EER99914            transcript:Zm00001d022440_T005          0
659- 19: transcript:EER99915            transcript:Zm00001d022439_T001          0
659- 20: transcript:EER99916            transcript:Zm00001d022438_T001  8.00E-38
659- 21: transcript:EER99917            transcript:Zm00001d022437_T001 1.00E-109
659- 22: transcript:KXG36949            transcript:Zm00001d022436_T005          0
659- 23: transcript:KXG36951            transcript:Zm00001d022435_T001 7.00E-171
659- 24: transcript:EER97738            transcript:Zm00001d022434_T001          0
659- 25: transcript:KXG36952            transcript:Zm00001d022433_T001 2.00E-158
659- 26: transcript:KXG36955            transcript:Zm00001d022432_T001          0
659- 27: transcript:OQU90480            transcript:Zm00001d022431_T001 1.00E-115
659- 28: transcript:EER97743            transcript:Zm00001d022430_T001 9.00E-116
```

659- 29: transcript:EER97744 transcript:Zm00001d022429_T009 0
659- 30: transcript:EER97746 transcript:Zm00001d022428_T005 0
659- 31: transcript:OQU90486 transcript:Zm00001d022427_T003 0
659- 32: transcript:EER97748 transcript:Zm00001d022425_T002 0
659- 33: transcript:EER99924 transcript:Zm00001d022424_T001 1.00E-141
Alignment 660: score=1230.0 e_value=4.2e-86 N=27 2&7 minus
660- 0: transcript:EER97120 transcript:Zm00001d021473_T002 0
660- 1: transcript:OQU89855 transcript:Zm00001d021469_T002 0
660- 2: transcript:EER99292 transcript:Zm00001d021467_T001 0
660- 3: transcript:KXG36104 transcript:Zm00001d021465_T002 1.00E-105
660- 4: transcript:EER99291 transcript:Zm00001d021464_T005 0
660- 5: transcript:EER99285 transcript:Zm00001d021462_T001 2.00E-80
660- 6: transcript:OQU89856 transcript:Zm00001d021461_T002 0
660- 7: transcript:OQU89865 transcript:Zm00001d021457_T001 8.00E-64
660- 8: transcript:OQU89866 transcript:Zm00001d021456_T001 7.00E-47
660- 9: transcript:EER97128 transcript:Zm00001d021454_T001 2.00E-73
660- 10: transcript:EER99304 transcript:Zm00001d021453_T002 0
660- 11: transcript:EER97130 transcript:Zm00001d021452_T001 0
660- 12: transcript:EER97131 transcript:Zm00001d021450_T001 6.00E-158
660- 13: transcript:KXG36120 transcript:Zm00001d021449_T001 3.00E-22
660- 14: transcript:OQU89872 transcript:Zm00001d021448_T002 0
660- 15: transcript:EER97124 transcript:Zm00001d021447_T001 0
660- 16: transcript:KXG36122 transcript:Zm00001d021446_T002 0
660- 17: transcript:EER97137 transcript:Zm00001d021445_T001 3.00E-91
660- 18: transcript:OQU89874 transcript:Zm00001d021443_T001 6.00E-21
660- 19: transcript:EER97139 transcript:Zm00001d021442_T013 0
660- 20: transcript:EER99307 transcript:Zm00001d021441_T001 2.00E-122
660- 21: transcript:EER97140 transcript:Zm00001d021440_T001 1.00E-123
660- 22: transcript:OQU89879 transcript:Zm00001d021439_T001 1.00E-84
660- 23: transcript:EER99310 transcript:Zm00001d021438_T003 0
660- 24: transcript:KXG36129 transcript:Zm00001d021436_T002 2.00E-94
660- 25: transcript:EER97143 transcript:Zm00001d021435_T001 0
660- 26: transcript:KXG36131 transcript:Zm00001d021434_T001 0
Alignment 661: score=1093.0 e_value=2.1e-69 N=23 2&7 minus
661- 0: transcript:KXG36088 transcript:Zm00001d022621_T005 0
661- 1: transcript:EER97106 transcript:Zm00001d022619_T001 0
661- 2: transcript:EER99267 transcript:Zm00001d022618_T001 1.00E-79
661- 3: transcript:EER99268 transcript:Zm00001d022617_T001 6.00E-55
661- 4: transcript:EER99269 transcript:Zm00001d022616_T001 2.00E-82
661- 5: transcript:EER99270 transcript:Zm00001d022615_T001 0
661- 6: transcript:EER97107 transcript:Zm00001d022614_T002 0
661- 7: transcript:EER97108 transcript:Zm00001d022613_T001 3.00E-97
661- 8: transcript:EER97105 transcript:Zm00001d022611_T001 0
661- 9: transcript:KXG36090 transcript:Zm00001d022608_T001 3.00E-177
661- 10: transcript:EER99274 transcript:Zm00001d022607_T001 3.00E-86
661- 11: transcript:EER97111 transcript:Zm00001d022605_T001 2.00E-56
661- 12: transcript:EER99275 transcript:Zm00001d022604_T001 6.00E-95
661- 13: transcript:EER99276 transcript:Zm00001d022603_T002 6.00E-85
661- 14: transcript:EER97112 transcript:Zm00001d022602_T001 2.00E-123
661- 15: transcript:EER99278 transcript:Zm00001d022600_T001 0
661- 16: transcript:EER97113 transcript:Zm00001d022598_T001 0
661- 17: transcript:KXG36092 transcript:Zm00001d022597_T001 2.00E-125
661- 18: transcript:EER97114 transcript:Zm00001d022596_T001 1.00E-76
661- 19: transcript:KXG36093 transcript:Zm00001d022595_T001 3.00E-48

661- 20: transcript:KXG36094 transcript:Zm00001d022594_T002 0
661- 21: transcript:EER99286 transcript:Zm00001d022593_T001 0
661- 22: transcript:OQU89853 transcript:Zm00001d022592_T001 0
Alignment 662: score=977.0 e_value=9.5e-64 N=22 2&7 minus
662- 0: transcript:EER99862 transcript:Zm00001d022514_T001 0
662- 1: transcript:EER97683 transcript:Zm00001d022513_T001 5.00E-117
662- 2: transcript:OQU90424 transcript:Zm00001d022512_T001 0
662- 3: transcript:OQU90426 transcript:Zm00001d022510_T016 0
662- 4: transcript:OQU90427 transcript:Zm00001d022509_T001 7.00E-47
662- 5: transcript:EER97687 transcript:Zm00001d022506_T001 7.00E-108
662- 6: transcript:KXG36881 transcript:Zm00001d022505_T002 8.00E-91
662- 7: transcript:EER97689 transcript:Zm00001d022504_T001 0
662- 8: transcript:EER99853 transcript:Zm00001d022502_T003 0
662- 9: transcript:EER99872 transcript:Zm00001d022501_T001 0
662- 10: transcript:EER99873 transcript:Zm00001d022500_T001 6.00E-74
662- 11: transcript:KXG36890 transcript:Zm00001d022499_T001 0
662- 12: transcript:EER99875 transcript:Zm00001d022498_T007 0
662- 13: transcript:EER99876 transcript:Zm00001d022496_T001 0
662- 14: transcript:OQU90437 transcript:Zm00001d022494_T001 4.00E-99
662- 15: transcript:EER97693 transcript:Zm00001d022493_T001 0
662- 16: transcript:EER99879 transcript:Zm00001d022492_T001 0
662- 17: transcript:OQU90438 transcript:Zm00001d022491_T005 0
662- 18: transcript:KXG36897 transcript:Zm00001d022490_T002 0
662- 19: transcript:KXG36907 transcript:Zm00001d022488_T001 4.00E-145
662- 20: transcript:KXG36910 transcript:Zm00001d022487_T001 3.00E-67
662- 21: transcript:EER99886 transcript:Zm00001d022485_T001 0
Alignment 663: score=801.0 e_value=5e-55 N=20 2&7 minus
663- 0: transcript:Zm00001d006052_T004 transcript:KXG25593 6.00E-166
663- 1: transcript:Zm00001d006054_T001 transcript:EES15344 8.00E-132
663- 2: transcript:Zm00001d006059_T001 transcript:KXG25581 1.00E-53
663- 3: transcript:Zm00001d006060_T001 transcript:EES15334 2.00E-16
663- 4: transcript:Zm00001d006063_T001 transcript:EES15333 0
663- 5: transcript:Zm00001d006064_T002 transcript:EES15331 2.00E-91
663- 6: transcript:Zm00001d006065_T002 transcript:EES15330 2.00E-100
663- 7: transcript:Zm00001d006066_T002 transcript:OQU80851 2.00E-23
663- 8: transcript:Zm00001d006078_T001 transcript:OQU80845 1.00E-28
663- 9: transcript:Zm00001d006079_T001 transcript:EES14205 6.00E-36
663- 10: transcript:Zm00001d006082_T001 transcript:OQU80833 3.00E-64
663- 11: transcript:Zm00001d006085_T002 transcript:EES15319 5.00E-140
663- 12: transcript:Zm00001d006091_T001 transcript:KXG25534 2.00E-144
663- 13: transcript:Zm00001d006094_T001 transcript:EES15309 4.00E-123
663- 14: transcript:Zm00001d006097_T001 transcript:EES14190 3.00E-141
663- 15: transcript:Zm00001d006101_T001 transcript:KXG25521 6.00E-26
663- 16: transcript:Zm00001d006102_T002 transcript:EES14182 0
663- 17: transcript:Zm00001d006106_T001 transcript:OQU80816 3.00E-43
663- 18: transcript:Zm00001d006107_T001 transcript:KXG25507 0
663- 19: transcript:Zm00001d006110_T002 transcript:EES14175 9.00E-81
Alignment 664: score=620.0 e_value=4.1e-31 N=13 2&7 minus
664- 0: transcript:OQU89832 transcript:Zm00001d022647_T001 0
664- 1: transcript:EER97097 transcript:Zm00001d022643_T001 5.00E-177
664- 2: transcript:EER97098 transcript:Zm00001d022637_T001 0
664- 3: transcript:EER99261 transcript:Zm00001d022636_T002 0
664- 4: transcript:EER97099 transcript:Zm00001d022631_T002 3.00E-156
664- 5: transcript:EER99262 transcript:Zm00001d022630_T001 8.00E-54

```
664-  6: transcript:EER97100           transcript:Zm00001d022629_T001         0
664-  7: transcript:EER99264           transcript:Zm00001d022628_T001 3.00E-142
664-  8: transcript:EER97101           transcript:Zm00001d022627_T002         0
664-  9: transcript:KXG36086           transcript:Zm00001d022626_T001         0
664- 10: transcript:EER99265           transcript:Zm00001d022625_T006         0
664- 11: transcript:EER97103           transcript:Zm00001d022624_T001  3.00E-15
664- 12: transcript:EER99266           transcript:Zm00001d022622_T005         0
## Alignment 665: score=476.0 e_value=2e-22 N=11 2&7 minus
665-  0: transcript:Zm00001d006016_T001 transcript:OQU80949            1.00E-58
665-  1: transcript:Zm00001d006017_T001 transcript:EES15407           6.00E-159
665-  2: transcript:Zm00001d006019_T001 transcript:OQU80944            3.00E-26
665-  3: transcript:Zm00001d006022_T001 transcript:OQU80938           5.00E-117
665-  4: transcript:Zm00001d006025_T001 transcript:EES14272                   0
665-  5: transcript:Zm00001d006026_T001 transcript:OQU80933            3.00E-40
665-  6: transcript:Zm00001d006028_T001 transcript:EES14266            8.00E-98
665-  7: transcript:Zm00001d006029_T001 transcript:EES14265            2.00E-14
665-  8: transcript:Zm00001d006030_T001 transcript:EES15392            7.00E-55
665-  9: transcript:Zm00001d006031_T001 transcript:EES14262           1.00E-100
665- 10: transcript:Zm00001d006032_T001 transcript:EES14256            9.00E-28
## Alignment 666: score=458.0 e_value=1.2e-24 N=11 2&7 minus
666-  0: transcript:OQU89168           transcript:Zm00001d020001_T007 6.00E-117
666-  1: transcript:EER98634           transcript:Zm00001d019994_T001         0
666-  2: transcript:EER98635           transcript:Zm00001d019993_T001         0
666-  3: transcript:EER98637           transcript:Zm00001d019989_T001         0
666-  4: transcript:EER96488           transcript:Zm00001d019988_T001         0
666-  5: transcript:KXG35300           transcript:Zm00001d019987_T001         0
666-  6: transcript:EER96489           transcript:Zm00001d019983_T001         0
666-  7: transcript:EER98641           transcript:Zm00001d019981_T002 3.00E-109
666-  8: transcript:EER98643           transcript:Zm00001d019980_T001 7.00E-146
666-  9: transcript:OQU89186           transcript:Zm00001d019967_T001 7.00E-114
666- 10: transcript:KXG35317           transcript:Zm00001d019966_T001         0
## Alignment 667: score=439.0 e_value=4.7e-19 N=10 2&7 minus
667-  0: transcript:Zm00001d005841_T001 transcript:EES15124            1.00E-48
667-  1: transcript:Zm00001d005844_T002 transcript:EES15119            2.00E-54
667-  2: transcript:Zm00001d005849_T001 transcript:OQU80624           1.00E-154
667-  3: transcript:Zm00001d005857_T001 transcript:KXG25366            1.00E-63
667-  4: transcript:Zm00001d005859_T001 transcript:EES13987            1.00E-99
667-  5: transcript:Zm00001d005865_T001 transcript:KXG25358            1.00E-75
667-  6: transcript:Zm00001d005869_T001 transcript:EES13983           5.00E-113
667-  7: transcript:Zm00001d005871_T002 transcript:EES13982           6.00E-167
667-  8: transcript:Zm00001d005874_T001 transcript:KXG25300            7.00E-35
667-  9: transcript:Zm00001d005875_T001 transcript:KXG25294            1.00E-75
## Alignment 668: score=414.0 e_value=1.5e-17 N=9 2&7 minus
668-  0: transcript:OQU90452           transcript:Zm00001d022481_T004         0
668-  1: transcript:EER99887           transcript:Zm00001d022480_T001         0
668-  2: transcript:OQU90453           transcript:Zm00001d022479_T001  2.00E-50
668-  3: transcript:OQU90454           transcript:Zm00001d022478_T001 4.00E-165
668-  4: transcript:EER97704           transcript:Zm00001d022477_T001  2.00E-69
668-  5: transcript:OQU90456           transcript:Zm00001d022474_T001 8.00E-110
668-  6: transcript:EER99889           transcript:Zm00001d022473_T010 7.00E-101
668-  7: transcript:EER97707           transcript:Zm00001d022472_T001  5.00E-36
668-  8: transcript:EER97694           transcript:Zm00001d022469_T001         0
## Alignment 669: score=401.0 e_value=1.9e-20 N=9 2&7 minus
669-  0: transcript:KXG36038           transcript:Zm00001d021266_T001 3.00E-110
```

669- 1:	transcript:OQU89801	transcript:Zm00001d021263_T001	0
669- 2:	transcript:KXG36042	transcript:Zm00001d021249_T003	0
669- 3:	transcript:EER99225	transcript:Zm00001d021248_T002	0
669- 4:	transcript:EER97073	transcript:Zm00001d021246_T002	9.00E-151
669- 5:	transcript:OQU89803	transcript:Zm00001d021245_T001	2.00E-145
669- 6:	transcript:OQU89804	transcript:Zm00001d021243_T001	0
669- 7:	transcript:EER97075	transcript:Zm00001d021242_T001	0
669- 8:	transcript:EER97077	transcript:Zm00001d021240_T001	0
## Alignment 670: score=386.0 e_value=2.1e-17 N=9 2&7 minus			
670- 0:	transcript:Zm00001d006147_T006	transcript:EES15259	0
670- 1:	transcript:Zm00001d006150_T001	transcript:OQU80777	4.00E-54
670- 2:	transcript:Zm00001d006157_T001	transcript:EES15257	2.00E-93
670- 3:	transcript:Zm00001d006165_T001	transcript:OQU80770	2.00E-68
670- 4:	transcript:Zm00001d006166_T001	transcript:EES15253	0
670- 5:	transcript:Zm00001d006169_T001	transcript:EES14131	2.00E-51
670- 6:	transcript:Zm00001d006171_T001	transcript:EES14130	0
670- 7:	transcript:Zm00001d006173_T001	transcript:EES14129	8.00E-130
670- 8:	transcript:Zm00001d006176_T001	transcript:KXG25450	7.00E-177
## Alignment 671: score=386.0 e_value=2e-21 N=10 2&7 minus			
671- 0:	transcript:Zm00001d005976_T012	transcript:EES15458	0
671- 1:	transcript:Zm00001d005978_T001	transcript:EES14320	4.00E-38
671- 2:	transcript:Zm00001d005984_T001	transcript:OQU80994	4.00E-35
671- 3:	transcript:Zm00001d005993_T001	transcript:EES15444	2.00E-50
671- 4:	transcript:Zm00001d005995_T008	transcript:OQU80990	0
671- 5:	transcript:Zm00001d005997_T002	transcript:EES14314	3.00E-97
671- 6:	transcript:Zm00001d005998_T003	transcript:EES14310	1.00E-89
671- 7:	transcript:Zm00001d006001_T006	transcript:KXG25679	2.00E-90
671- 8:	transcript:Zm00001d006008_T001	transcript:EES14299	0
671- 9:	transcript:Zm00001d006009_T001	transcript:EES14287	6.00E-102
## Alignment 672: score=325.0 e_value=9e-11 N=7 2&7 minus			
672- 0:	transcript:Zm00001d006179_T005	transcript:OQU80759	3.00E-138
672- 1:	transcript:Zm00001d006180_T001	transcript:EES14126	7.00E-133
672- 2:	transcript:Zm00001d006181_T003	transcript:EES15247	5.00E-29
672- 3:	transcript:Zm00001d006185_T001	transcript:OQU80754	3.00E-155
672- 4:	transcript:Zm00001d006192_T001	transcript:EES15245	0
672- 5:	transcript:Zm00001d006193_T001	transcript:OQU80753	0
672- 6:	transcript:Zm00001d006197_T003	transcript:KXG25443	0
## Alignment 673: score=316.0 e_value=2.9e-12 N=7 2&7 minus			
673- 0:	transcript:EER99183	transcript:Zm00001d021189_T001	0
673- 1:	transcript:OQU89775	transcript:Zm00001d021188_T006	0
673- 2:	transcript:KXG36013	transcript:Zm00001d021187_T002	0
673- 3:	transcript:EER99187	transcript:Zm00001d021181_T005	0
673- 4:	transcript:EER99188	transcript:Zm00001d021180_T005	0
673- 5:	transcript:EER99190	transcript:Zm00001d021177_T001	2.00E-58
673- 6:	transcript:EER97050	transcript:Zm00001d021173_T001	0
## Alignment 674: score=305.0 e_value=2.8e-13 N=7 2&7 minus			
674- 0:	transcript:Zm00001d005958_T002	transcript:EES15476	1.00E-88
674- 1:	transcript:Zm00001d005961_T001	transcript:EES15473	2.00E-24
674- 2:	transcript:Zm00001d005962_T001	transcript:KXG25724	1.00E-26
674- 3:	transcript:Zm00001d005964_T001	transcript:OQU81018	1.00E-71
674- 4:	transcript:Zm00001d005969_T004	transcript:EES15468	2.00E-122
674- 5:	transcript:Zm00001d005970_T001	transcript:OQU81009	1.00E-52
674- 6:	transcript:Zm00001d005971_T001	transcript:EES15462	5.00E-80
## Alignment 675: score=301.0 e_value=9.3e-12 N=7 2&8 plus			

```
675-  0: transcript:Zm00001d004934_T001 transcript:KXG22815           1.00E-125
675-  1: transcript:Zm00001d004936_T001 transcript:EES16514            4.00E-48
675-  2: transcript:Zm00001d004955_T001 transcript:KXG22816                   0
675-  3: transcript:Zm00001d004956_T001 transcript:KXG22818            5.00E-33
675-  4: transcript:Zm00001d004960_T002 transcript:EES16519                   0
675-  5: transcript:Zm00001d004966_T001 transcript:EES16520                   0
675-  6: transcript:Zm00001d004972_T001 transcript:EES16521            4.00E-46
## Alignment 676: score=259.0 e_value=3e-10 N=6 2&8 plus
676-  0: transcript:Zm00001d005006_T003 transcript:EES15579                   0
676-  1: transcript:Zm00001d005007_T001 transcript:KXG22865            4.00E-19
676-  2: transcript:Zm00001d005010_T001 transcript:EES15581            2.00E-98
676-  3: transcript:Zm00001d005011_T001 transcript:OQU78649            2.00E-36
676-  4: transcript:Zm00001d005012_T002 transcript:EES16544           3.00E-161
676-  5: transcript:Zm00001d005018_T001 transcript:EES15585                   0
## Alignment 677: score=401.0 e_value=6.2e-20 N=9 2&8 minus
677-  0: transcript:Zm00001d005075_T001 transcript:KXG22778            2.00E-42
677-  1: transcript:Zm00001d005077_T001 transcript:OQU78594            2.00E-19
677-  2: transcript:Zm00001d005080_T002 transcript:KXG22777                   0
677-  3: transcript:Zm00001d005081_T001 transcript:EES16496            1.00E-41
677-  4: transcript:Zm00001d005082_T001 transcript:EES15514                   0
677-  5: transcript:Zm00001d005083_T001 transcript:KXG22773                   0
677-  6: transcript:Zm00001d005085_T002 transcript:EES16493                   0
677-  7: transcript:Zm00001d005087_T001 transcript:EES15503            1.00E-87
677-  8: transcript:Zm00001d005089_T005 transcript:KXG22765           2.00E-100
## Alignment 678: score=415.0 e_value=3.2e-21 N=10 2&9 plus
678-  0: transcript:EER97718            transcript:Zm00001d047500_T001  6.00E-56
678-  1: transcript:KXG36938            transcript:Zm00001d047512_T002  8.00E-79
678-  2: transcript:EER99906            transcript:Zm00001d047514_T001 2.00E-121
678-  3: transcript:EER97734            transcript:Zm00001d047516_T001 6.00E-180
678-  4: transcript:OQU90478            transcript:Zm00001d047519_T001 3.00E-150
678-  5: transcript:EER99915            transcript:Zm00001d047522_T002         0
678-  6: transcript:EER99917            transcript:Zm00001d047523_T001  1.00E-40
678-  7: transcript:KXG36951            transcript:Zm00001d047528_T001  1.00E-13
678-  8: transcript:EER97738            transcript:Zm00001d047531_T001         0
678-  9: transcript:EER97743            transcript:Zm00001d047535_T001  5.00E-78
## Alignment 679: score=397.0 e_value=6.2e-19 N=9 2&9 plus
679-  0: transcript:EER99759            transcript:Zm00001d047399_T001  4.00E-50
679-  1: transcript:EER97602            transcript:Zm00001d047401_T001  2.00E-67
679-  2: transcript:EER97603            transcript:Zm00001d047402_T001         0
679-  3: transcript:OQU90326            transcript:Zm00001d047403_T001 1.00E-117
679-  4: transcript:EER97589            transcript:Zm00001d047404_T001  9.00E-72
679-  5: transcript:EER99768            transcript:Zm00001d047417_T001 1.00E-164
679-  6: transcript:KXG36781            transcript:Zm00001d047419_T001  1.00E-20
679-  7: transcript:EER97613            transcript:Zm00001d047421_T001  4.00E-99
679-  8: transcript:KXG36786            transcript:Zm00001d047422_T002         0
## Alignment 680: score=3185.0 e_value=3.8e-287 N=71 3&3 plus
680-  0: transcript:EES00464            transcript:Zm00001d040196_T001 1.00E-139
680-  1: transcript:KXG32145            transcript:Zm00001d040197_T001  3.00E-83
680-  2: transcript:OQU86563            transcript:Zm00001d040201_T001         0
680-  3: transcript:EES00468            transcript:Zm00001d040202_T002 3.00E-163
680-  4: transcript:OQU86566            transcript:Zm00001d040203_T003         0
680-  5: transcript:EES02659            transcript:Zm00001d040204_T001         0
680-  6: transcript:KXG32148            transcript:Zm00001d040205_T001         0
680-  7: transcript:EES00472            transcript:Zm00001d040210_T001  2.00E-43
```

680- 8:	transcript:OQU86571	transcript:Zm00001d040213_T002	0
680- 9:	transcript:OQU86574	transcript:Zm00001d040214_T001	0
680- 10:	transcript:EES02664	transcript:Zm00001d040215_T001	1.00E-111
680- 11:	transcript:OQU86578	transcript:Zm00001d040218_T016	0
680- 12:	transcript:OQU86587	transcript:Zm00001d040220_T002	7.00E-176
680- 13:	transcript:KXG32153	transcript:Zm00001d040221_T002	0
680- 14:	transcript:OQU86588	transcript:Zm00001d040222_T001	0
680- 15:	transcript:EES00471	transcript:Zm00001d040223_T001	5.00E-114
680- 16:	transcript:EES00478	transcript:Zm00001d040228_T001	0
680- 17:	transcript:OQU86592	transcript:Zm00001d040232_T001	4.00E-144
680- 18:	transcript:OQU86594	transcript:Zm00001d040233_T004	0
680- 19:	transcript:KXG32159	transcript:Zm00001d040234_T001	0
680- 20:	transcript:EES02674	transcript:Zm00001d040235_T005	0
680- 21:	transcript:EES00480	transcript:Zm00001d040238_T002	0
680- 22:	transcript:KXG32163	transcript:Zm00001d040239_T001	0
680- 23:	transcript:KXG32164	transcript:Zm00001d040242_T001	1.00E-108
680- 24:	transcript:OQU86596	transcript:Zm00001d040243_T002	0
680- 25:	transcript:KXG32168	transcript:Zm00001d040244_T001	3.00E-153
680- 26:	transcript:KXG32170	transcript:Zm00001d040245_T001	6.00E-26
680- 27:	transcript:EES00486	transcript:Zm00001d040246_T001	5.00E-82
680- 28:	transcript:KXG32172	transcript:Zm00001d040247_T001	5.00E-56
680- 29:	transcript:EES02682	transcript:Zm00001d040248_T001	0
680- 30:	transcript:EES00488	transcript:Zm00001d040252_T001	5.00E-26
680- 31:	transcript:EES02686	transcript:Zm00001d040254_T001	0
680- 32:	transcript:EES00491	transcript:Zm00001d040257_T006	0
680- 33:	transcript:OQU86609	transcript:Zm00001d040259_T001	0
680- 34:	transcript:EES02687	transcript:Zm00001d040260_T001	8.00E-44
680- 35:	transcript:EES00498	transcript:Zm00001d040263_T010	0
680- 36:	transcript:EES02689	transcript:Zm00001d040264_T001	2.00E-131
680- 37:	transcript:KXG32181	transcript:Zm00001d040265_T001	0
680- 38:	transcript:KXG32183	transcript:Zm00001d040268_T002	0
680- 39:	transcript:OQU86611	transcript:Zm00001d040269_T001	0
680- 40:	transcript:EES02695	transcript:Zm00001d040270_T011	0
680- 41:	transcript:EES00501	transcript:Zm00001d040271_T001	1.00E-101
680- 42:	transcript:KXG32186	transcript:Zm00001d040274_T001	0
680- 43:	transcript:EES02696	transcript:Zm00001d040275_T001	2.00E-178
680- 44:	transcript:KXG32188	transcript:Zm00001d040276_T001	3.00E-49
680- 45:	transcript:EES02699	transcript:Zm00001d040278_T001	5.00E-142
680- 46:	transcript:KXG32191	transcript:Zm00001d040279_T023	0
680- 47:	transcript:EES02700	transcript:Zm00001d040281_T001	2.00E-21
680- 48:	transcript:KXG32194	transcript:Zm00001d040285_T001	4.00E-103
680- 49:	transcript:EES02705	transcript:Zm00001d040286_T003	4.00E-154
680- 50:	transcript:EES00506	transcript:Zm00001d040290_T001	0
680- 51:	transcript:EES00507	transcript:Zm00001d040291_T001	7.00E-78
680- 52:	transcript:EES02706	transcript:Zm00001d040292_T001	1.00E-20
680- 53:	transcript:KXG32197	transcript:Zm00001d040293_T001	0
680- 54:	transcript:EES02709	transcript:Zm00001d040294_T002	4.00E-94
680- 55:	transcript:EES02710	transcript:Zm00001d040297_T001	1.00E-59
680- 56:	transcript:EES02711	transcript:Zm00001d040298_T001	2.00E-70
680- 57:	transcript:EES02712	transcript:Zm00001d040302_T005	5.00E-160
680- 58:	transcript:EES00508	transcript:Zm00001d040303_T001	0
680- 59:	transcript:KXG32201	transcript:Zm00001d040304_T001	4.00E-09
680- 60:	transcript:OQU86626	transcript:Zm00001d040305_T014	0
680- 61:	transcript:EES02716	transcript:Zm00001d040308_T001	2.00E-104

680- 62:	transcript:EES00509	transcript:Zm00001d040309_T003	7.00E-133
680- 63:	transcript:KXG32203	transcript:Zm00001d040310_T001	0
680- 64:	transcript:EES02721	transcript:Zm00001d040311_T010	0
680- 65:	transcript:OQU86634	transcript:Zm00001d040312_T007	0
680- 66:	transcript:OQU86638	transcript:Zm00001d040313_T001	2.00E-143
680- 67:	transcript:EES00513	transcript:Zm00001d040314_T001	0
680- 68:	transcript:OQU86639	transcript:Zm00001d040315_T001	6.00E-45
680- 69:	transcript:OQU86641	transcript:Zm00001d040317_T005	7.00E-153
680- 70:	transcript:OQU86647	transcript:Zm00001d040318_T001	0

Alignment 681: score=3014.0 e_value=3.3e-282 N=66 3&3 plus

681- 0:	transcript:EES03176	transcript:Zm00001d044315_T009	0
681- 1:	transcript:EES03182	transcript:Zm00001d044316_T001	2.00E-35
681- 2:	transcript:KXG32796	transcript:Zm00001d044317_T001	4.00E-06
681- 3:	transcript:EES03185	transcript:Zm00001d044318_T001	0
681- 4:	transcript:EES03186	transcript:Zm00001d044323_T001	8.00E-65
681- 5:	transcript:EES03187	transcript:Zm00001d044324_T001	0
681- 6:	transcript:EES00980	transcript:Zm00001d044325_T001	5.00E-53
681- 7:	transcript:EES00981	transcript:Zm00001d044326_T001	1.00E-30
681- 8:	transcript:EES03190	transcript:Zm00001d044327_T001	9.00E-85
681- 9:	transcript:EES03191	transcript:Zm00001d044328_T001	0
681- 10:	transcript:OQU87082	transcript:Zm00001d044329_T003	8.00E-154
681- 11:	transcript:EES00984	transcript:Zm00001d044330_T002	1.00E-162
681- 12:	transcript:EES03192	transcript:Zm00001d044331_T001	0
681- 13:	transcript:EES03193	transcript:Zm00001d044332_T001	2.00E-136
681- 14:	transcript:EES00985	transcript:Zm00001d044333_T001	1.00E-138
681- 15:	transcript:EES00986	transcript:Zm00001d044335_T001	2.00E-101
681- 16:	transcript:EES03194	transcript:Zm00001d044336_T001	0
681- 17:	transcript:EES00987	transcript:Zm00001d044338_T003	0
681- 18:	transcript:KXG32803	transcript:Zm00001d044339_T003	0
681- 19:	transcript:OQU87085	transcript:Zm00001d044340_T001	0
681- 20:	transcript:EES00994	transcript:Zm00001d044355_T001	2.00E-140
681- 21:	transcript:KXG32804	transcript:Zm00001d044356_T001	2.00E-23
681- 22:	transcript:EES00995	transcript:Zm00001d044357_T001	2.00E-130
681- 23:	transcript:OQU87096	transcript:Zm00001d044358_T002	0
681- 24:	transcript:KXG32810	transcript:Zm00001d044364_T002	0
681- 25:	transcript:EES00998	transcript:Zm00001d044365_T001	6.00E-161
681- 26:	transcript:OQU87098	transcript:Zm00001d044367_T001	9.00E-104
681- 27:	transcript:EES01000	transcript:Zm00001d044373_T001	0
681- 28:	transcript:KXG32812	transcript:Zm00001d044374_T001	1.00E-110
681- 29:	transcript:KXG32814	transcript:Zm00001d044375_T001	3.00E-35
681- 30:	transcript:EES03209	transcript:Zm00001d044376_T001	1.00E-42
681- 31:	transcript:EES01003	transcript:Zm00001d044379_T006	0
681- 32:	transcript:EES01006	transcript:Zm00001d044382_T001	4.00E-166
681- 33:	transcript:OQU87108	transcript:Zm00001d044385_T001	1.00E-113
681- 34:	transcript:OQU87118	transcript:Zm00001d044387_T001	0
681- 35:	transcript:EES03220	transcript:Zm00001d044388_T001	0
681- 36:	transcript:KXG32826	transcript:Zm00001d044389_T001	6.00E-149
681- 37:	transcript:EES01017	transcript:Zm00001d044390_T001	0
681- 38:	transcript:OQU87121	transcript:Zm00001d044391_T002	4.00E-81
681- 39:	transcript:EES01019	transcript:Zm00001d044393_T002	0
681- 40:	transcript:EES01020	transcript:Zm00001d044394_T002	0
681- 41:	transcript:EES03224	transcript:Zm00001d044395_T001	7.00E-168
681- 42:	transcript:OQU87124	transcript:Zm00001d044396_T001	0
681- 43:	transcript:EES01025	transcript:Zm00001d044405_T002	0

681- 44: transcript:EES03232 transcript:Zm00001d044407_T001 4.00E-60
681- 45: transcript:EES01026 transcript:Zm00001d044409_T001 3.00E-132
681- 46: transcript:OQU87132 transcript:Zm00001d044410_T001 3.00E-71
681- 47: transcript:EES03239 transcript:Zm00001d044411_T001 1.00E-129
681- 48: transcript:EES03240 transcript:Zm00001d044412_T001 0
681- 49: transcript:EES01034 transcript:Zm00001d044416_T001 0
681- 50: transcript:EES03241 transcript:Zm00001d044415_T002 2.00E-98
681- 51: transcript:EES03242 transcript:Zm00001d044417_T001 2.00E-168
681- 52: transcript:OQU87134 transcript:Zm00001d044418_T001 0
681- 53: transcript:EES01036 transcript:Zm00001d044419_T001 0
681- 54: transcript:EES03243 transcript:Zm00001d044420_T001 0
681- 55: transcript:EES01039 transcript:Zm00001d044421_T001 2.00E-165
681- 56: transcript:OQU87136 transcript:Zm00001d044422_T002 2.00E-87
681- 57: transcript:KXG32844 transcript:Zm00001d044423_T001 2.00E-46
681- 58: transcript:EES03248 transcript:Zm00001d044425_T001 0
681- 59: transcript:EES01042 transcript:Zm00001d044426_T001 2.00E-100
681- 60: transcript:OQU87138 transcript:Zm00001d044427_T001 4.00E-19
681- 61: transcript:KXG32848 transcript:Zm00001d044428_T001 4.00E-48
681- 62: transcript:KXG32850 transcript:Zm00001d044429_T001 2.00E-99
681- 63: transcript:EES01047 transcript:Zm00001d044430_T001 5.00E-77
681- 64: transcript:OQU87139 transcript:Zm00001d044431_T001 0
681- 65: transcript:EES03249 transcript:Zm00001d044432_T010 0
Alignment 682: score=1438.0 e_value=5e-104 N=32 3&3 plus
682- 0: transcript:KXG31936 transcript:Zm00001d039920_T001 4.00E-149
682- 1: transcript:OQU86378 transcript:Zm00001d039924_T001 4.00E-24
682- 2: transcript:OQU86380 transcript:Zm00001d039926_T001 0
682- 3: transcript:KXG31949 transcript:Zm00001d039927_T001 9.00E-147
682- 4: transcript:EES00350 transcript:Zm00001d039930_T001 3.00E-70
682- 5: transcript:EES00357 transcript:Zm00001d039932_T001 2.00E-39
682- 6: transcript:EES02531 transcript:Zm00001d039941_T001 3.00E-27
682- 7: transcript:EES00360 transcript:Zm00001d039944_T001 3.00E-86
682- 8: transcript:EES00361 transcript:Zm00001d039946_T001 0
682- 9: transcript:OQU86384 transcript:Zm00001d039947_T002 1.00E-175
682- 10: transcript:KXG31953 transcript:Zm00001d039951_T001 2.00E-132
682- 11: transcript:EES00362 transcript:Zm00001d039958_T001 0
682- 12: transcript:OQU86387 transcript:Zm00001d039963_T001 0
682- 13: transcript:EES02540 transcript:Zm00001d039965_T001 0
682- 14: transcript:EES00364 transcript:Zm00001d039966_T001 0
682- 15: transcript:EES02541 transcript:Zm00001d039967_T001 0
682- 16: transcript:EES02542 transcript:Zm00001d039969_T001 0
682- 17: transcript:KXG31957 transcript:Zm00001d039971_T002 0
682- 18: transcript:KXG31959 transcript:Zm00001d039973_T002 0
682- 19: transcript:EES00365 transcript:Zm00001d039974_T001 1.00E-80
682- 20: transcript:KXG31963 transcript:Zm00001d039975_T001 0
682- 21: transcript:EES02547 transcript:Zm00001d039977_T001 5.00E-114
682- 22: transcript:OQU86393 transcript:Zm00001d039983_T001 5.00E-74
682- 23: transcript:EES00368 transcript:Zm00001d039987_T003 0
682- 24: transcript:OQU86401 transcript:Zm00001d039988_T001 6.00E-53
682- 25: transcript:OQU86404 transcript:Zm00001d039991_T001 7.00E-65
682- 26: transcript:OQU86408 transcript:Zm00001d039993_T001 7.00E-43
682- 27: transcript:EES02554 transcript:Zm00001d039994_T001 0
682- 28: transcript:KXG31973 transcript:Zm00001d039997_T001 3.00E-39
682- 29: transcript:KXG31975 transcript:Zm00001d040002_T001 0
682- 30: transcript:EES00374 transcript:Zm00001d040003_T001 5.00E-39

682- 31: transcript:EES02556 transcript:Zm00001d040004_T001 1.00E-160
Alignment 683: score=1217.0 e_value=2.5e-82 N=27 3&3 plus
683- 0: transcript:KXG32865 transcript:Zm00001d044442_T036 0
683- 1: transcript:EES01054 transcript:Zm00001d044445_T001 2.00E-117
683- 2: transcript:OQU87152 transcript:Zm00001d044446_T001 4.00E-124
683- 3: transcript:EES03263 transcript:Zm00001d044447_T007 0
683- 4: transcript:OQU87154 transcript:Zm00001d044451_T002 1.00E-41
683- 5: transcript:EES03266 transcript:Zm00001d044452_T001 5.00E-117
683- 6: transcript:OQU87155 transcript:Zm00001d044455_T001 0
683- 7: transcript:KXG32881 transcript:Zm00001d044456_T001 1.00E-31
683- 8: transcript:EES03269 transcript:Zm00001d044457_T001 0
683- 9: transcript:EES01062 transcript:Zm00001d044459_T002 7.00E-32
683- 10: transcript:KXG32884 transcript:Zm00001d044460_T001 8.00E-158
683- 11: transcript:KXG32886 transcript:Zm00001d044461_T001 7.00E-157
683- 12: transcript:OQU87160 transcript:Zm00001d044463_T001 0
683- 13: transcript:OQU87162 transcript:Zm00001d044464_T001 4.00E-65
683- 14: transcript:EES01067 transcript:Zm00001d044465_T001 0
683- 15: transcript:EES01068 transcript:Zm00001d044466_T001 0
683- 16: transcript:OQU87164 transcript:Zm00001d044467_T001 1.00E-104
683- 17: transcript:EES03271 transcript:Zm00001d044468_T002 0
683- 18: transcript:EES03272 transcript:Zm00001d044469_T001 0
683- 19: transcript:EES01071 transcript:Zm00001d044470_T006 0
683- 20: transcript:KXG32891 transcript:Zm00001d044475_T001 0
683- 21: transcript:EES03276 transcript:Zm00001d044476_T001 0
683- 22: transcript:EES01072 transcript:Zm00001d044478_T022 0
683- 23: transcript:EES01073 transcript:Zm00001d044479_T001 0
683- 24: transcript:EES01075 transcript:Zm00001d044480_T001 2.00E-152
683- 25: transcript:KXG32898 transcript:Zm00001d044481_T002 3.00E-120
683- 26: transcript:EES01079 transcript:Zm00001d044488_T003 0
Alignment 684: score=1015.0 e_value=4.1e-76 N=24 3&3 plus
684- 0: transcript:KXG31837 transcript:Zm00001d039818_T001 2.00E-42
684- 1: transcript:EES00304 transcript:Zm00001d039821_T001 1.00E-98
684- 2: transcript:KXG31852 transcript:Zm00001d039822_T001 0
684- 3: transcript:EES00306 transcript:Zm00001d039825_T001 6.00E-23
684- 4: transcript:EES00307 transcript:Zm00001d039827_T001 9.00E-39
684- 5: transcript:EES00303 transcript:Zm00001d039833_T001 0
684- 6: transcript:KXG31859 transcript:Zm00001d039837_T005 0
684- 7: transcript:EES02468 transcript:Zm00001d039841_T001 9.00E-115
684- 8: transcript:KXG31868 transcript:Zm00001d039842_T001 4.00E-66
684- 9: transcript:EES00313 transcript:Zm00001d039845_T001 2.00E-36
684- 10: transcript:EES00314 transcript:Zm00001d039847_T001 3.00E-45
684- 11: transcript:EES02479 transcript:Zm00001d039848_T002 0
684- 12: transcript:EES02480 transcript:Zm00001d039851_T002 0
684- 13: transcript:KXG31876 transcript:Zm00001d039852_T001 0
684- 14: transcript:EES02482 transcript:Zm00001d039854_T001 0
684- 15: transcript:KXG31877 transcript:Zm00001d039856_T002 6.00E-43
684- 16: transcript:KXG31883 transcript:Zm00001d039858_T001 0
684- 17: transcript:OQU86320 transcript:Zm00001d039859_T001 5.00E-22
684- 18: transcript:EES02483 transcript:Zm00001d039860_T003 0
684- 19: transcript:EES00320 transcript:Zm00001d039865_T002 2.00E-175
684- 20: transcript:KXG31889 transcript:Zm00001d039866_T001 0
684- 21: transcript:EES00322 transcript:Zm00001d039867_T007 0
684- 22: transcript:EES00318 transcript:Zm00001d039870_T002 0
684- 23: transcript:EES02488 transcript:Zm00001d039871_T002 0

Alignment 685: score=965.0 e_value=5e-67 N=22 3&3 plus

685- 0:	transcript:EES00272	transcript:Zm00001d039764_T001	9.00E-114
685- 1:	transcript:KXG31816	transcript:Zm00001d039766_T001	3.00E-21
685- 2:	transcript:OQU86270	transcript:Zm00001d039768_T003	0
685- 3:	transcript:EES00281	transcript:Zm00001d039769_T002	0
685- 4:	transcript:EES00282	transcript:Zm00001d039770_T001	6.00E-116
685- 5:	transcript:OQU86274	transcript:Zm00001d039771_T001	8.00E-120
685- 6:	transcript:EES02427	transcript:Zm00001d039772_T001	0
685- 7:	transcript:EES02430	transcript:Zm00001d039776_T001	3.00E-140
685- 8:	transcript:EES00289	transcript:Zm00001d039780_T001	8.00E-77
685- 9:	transcript:OQU86276	transcript:Zm00001d039781_T001	2.00E-89
685- 10:	transcript:EES02432	transcript:Zm00001d039782_T001	3.00E-126
685- 11:	transcript:OQU86277	transcript:Zm00001d039783_T001	8.00E-89
685- 12:	transcript:OQU86282	transcript:Zm00001d039785_T001	7.00E-132
685- 13:	transcript:OQU86283	transcript:Zm00001d039786_T001	4.00E-09
685- 14:	transcript:KXG31832	transcript:Zm00001d039787_T001	0
685- 15:	transcript:EES02439	transcript:Zm00001d039788_T001	2.00E-144
685- 16:	transcript:KXG31834	transcript:Zm00001d039791_T001	3.00E-40
685- 17:	transcript:EES00296	transcript:Zm00001d039795_T001	3.00E-104
685- 18:	transcript:EES00299	transcript:Zm00001d039796_T001	3.00E-61
685- 19:	transcript:KXG31841	transcript:Zm00001d039803_T001	3.00E-28
685- 20:	transcript:EES02445	transcript:Zm00001d039807_T001	4.00E-52
685- 21:	transcript:KXG31849	transcript:Zm00001d039808_T001	1.00E-12

Alignment 686: score=954.0 e_value=1.7e-61 N=21 3&3 plus

686- 0:	transcript:OQU86697	transcript:Zm00001d040435_T001	2.00E-81
686- 1:	transcript:EES00573	transcript:Zm00001d040438_T004	0
686- 2:	transcript:KXG32258	transcript:Zm00001d040440_T001	6.00E-92
686- 3:	transcript:KXG32259	transcript:Zm00001d040441_T001	3.00E-101
686- 4:	transcript:EES02765	transcript:Zm00001d040442_T001	0
686- 5:	transcript:EES00577	transcript:Zm00001d040445_T001	0
686- 6:	transcript:EES02767	transcript:Zm00001d040446_T001	0
686- 7:	transcript:KXG32260	transcript:Zm00001d040448_T001	5.00E-177
686- 8:	transcript:KXG32262	transcript:Zm00001d040450_T002	2.00E-41
686- 9:	transcript:EES00579	transcript:Zm00001d040455_T001	0
686- 10:	transcript:OQU86699	transcript:Zm00001d040456_T002	0
686- 11:	transcript:EES00580	transcript:Zm00001d040459_T006	4.00E-150
686- 12:	transcript:KXG32265	transcript:Zm00001d040461_T001	4.00E-57
686- 13:	transcript:EES02774	transcript:Zm00001d040462_T002	0
686- 14:	transcript:EES02775	transcript:Zm00001d040463_T001	0
686- 15:	transcript:EES00586	transcript:Zm00001d040464_T001	0
686- 16:	transcript:KXG32271	transcript:Zm00001d040465_T001	0
686- 17:	transcript:EES02777	transcript:Zm00001d040466_T001	0
686- 18:	transcript:EES02778	transcript:Zm00001d040467_T003	1.00E-50
686- 19:	transcript:EES02787	transcript:Zm00001d040468_T001	0
686- 20:	transcript:EES02789	transcript:Zm00001d040470_T001	0

Alignment 687: score=951.0 e_value=1.1e-60 N=21 3&3 plus

687- 0:	transcript:EES03566	transcript:Zm00001d043528_T001	5.00E-148
687- 1:	transcript:EES01419	transcript:Zm00001d043534_T002	0
687- 2:	transcript:EES01420	transcript:Zm00001d043535_T001	4.00E-135
687- 3:	transcript:KXG33312	transcript:Zm00001d043536_T001	2.00E-134
687- 4:	transcript:EES01424	transcript:Zm00001d043538_T001	0
687- 5:	transcript:EES03571	transcript:Zm00001d043539_T002	0
687- 6:	transcript:EES01426	transcript:Zm00001d043541_T001	9.00E-49
687- 7:	transcript:EES01429	transcript:Zm00001d043543_T002	0

687- 8: transcript:EES01430 transcript:Zm00001d043544_T002 0
687- 9: transcript:KXG33317 transcript:Zm00001d043548_T001 0
687- 10: transcript:EES01438 transcript:Zm00001d043549_T001 2.00E-144
687- 11: transcript:OQU87487 transcript:Zm00001d043550_T001 8.00E-110
687- 12: transcript:KXG33320 transcript:Zm00001d043551_T003 5.00E-169
687- 13: transcript:OQU87488 transcript:Zm00001d043552_T006 0
687- 14: transcript:KXG33323 transcript:Zm00001d043554_T001 4.00E-27
687- 15: transcript:OQU87491 transcript:Zm00001d043555_T002 0
687- 16: transcript:EES01445 transcript:Zm00001d043556_T001 0
687- 17: transcript:OQU87493 transcript:Zm00001d043558_T001 3.00E-47
687- 18: transcript:EES01448 transcript:Zm00001d043560_T006 0
687- 19: transcript:EES01447 transcript:Zm00001d043562_T008 0
687- 20: transcript:OQU87495 transcript:Zm00001d043563_T002 0
Alignment 688: score=907.0 e_value=3.1e-63 N=22 3&3 plus
688- 0: transcript:KXG32292 transcript:Zm00001d040503_T005 0
688- 1: transcript:EES02796 transcript:Zm00001d040504_T001 7.00E-117
688- 2: transcript:KXG32293 transcript:Zm00001d040505_T001 0
688- 3: transcript:OQU86731 transcript:Zm00001d040506_T001 5.00E-96
688- 4: transcript:KXG32297 transcript:Zm00001d040508_T001 3.00E-50
688- 5: transcript:EES02798 transcript:Zm00001d040513_T001 0
688- 6: transcript:EES00597 transcript:Zm00001d040515_T001 0
688- 7: transcript:KXG32299 transcript:Zm00001d040518_T001 7.00E-17
688- 8: transcript:EES02802 transcript:Zm00001d040519_T002 9.00E-131
688- 9: transcript:EES02803 transcript:Zm00001d040526_T001 5.00E-101
688- 10: transcript:OQU86737 transcript:Zm00001d040527_T001 1.00E-111
688- 11: transcript:EES00603 transcript:Zm00001d040535_T005 0
688- 12: transcript:KXG32305 transcript:Zm00001d040536_T005 0
688- 13: transcript:EES00604 transcript:Zm00001d040539_T004 4.00E-36
688- 14: transcript:EES00605 transcript:Zm00001d040541_T001 2.00E-93
688- 15: transcript:OQU86741 transcript:Zm00001d040542_T001 0
688- 16: transcript:KXG32309 transcript:Zm00001d040544_T001 2.00E-34
688- 17: transcript:EES02810 transcript:Zm00001d040545_T002 5.00E-13
688- 18: transcript:EES02817 transcript:Zm00001d040548_T001 6.00E-42
688- 19: transcript:EES00609 transcript:Zm00001d040554_T001 0
688- 20: transcript:KXG32327 transcript:Zm00001d040555_T001 0
688- 21: transcript:EES02821 transcript:Zm00001d040562_T002 0
Alignment 689: score=844.0 e_value=4.5e-52 N=19 3&3 plus
689- 0: transcript:EES02393 transcript:Zm00001d039700_T001 3.00E-110
689- 1: transcript:KXG31766 transcript:Zm00001d039701_T001 2.00E-149
689- 2: transcript:OQU86239 transcript:Zm00001d039702_T001 0
689- 3: transcript:EES02395 transcript:Zm00001d039703_T001 0
689- 4: transcript:KXG31768 transcript:Zm00001d039706_T001 0
689- 5: transcript:EES00240 transcript:Zm00001d039709_T001 0
689- 6: transcript:KXG31771 transcript:Zm00001d039710_T004 4.00E-93
689- 7: transcript:EES00241 transcript:Zm00001d039711_T001 0
689- 8: transcript:EES02394 transcript:Zm00001d039712_T005 0
689- 9: transcript:KXG31778 transcript:Zm00001d039714_T001 0
689- 10: transcript:EES00245 transcript:Zm00001d039715_T001 9.00E-46
689- 11: transcript:EES02399 transcript:Zm00001d039716_T001 8.00E-89
689- 12: transcript:KXG31782 transcript:Zm00001d039717_T005 0
689- 13: transcript:EES00246 transcript:Zm00001d039718_T001 0
689- 14: transcript:KXG31785 transcript:Zm00001d039719_T002 5.00E-99
689- 15: transcript:EES00247 transcript:Zm00001d039726_T001 4.00E-84
689- 16: transcript:EES02400 transcript:Zm00001d039727_T001 5.00E-118

```
689- 17: transcript:KXG31787            transcript:Zm00001d039728_T001  8.00E-38
689- 18: transcript:KXG31790            transcript:Zm00001d039729_T005         0
## Alignment 690: score=806.0 e_value=3.2e-46 N=17 3&3 plus
690-  0: transcript:EES01469            transcript:Zm00001d043444_T001 7.00E-164
690-  1: transcript:KXG33362            transcript:Zm00001d043445_T003         0
690-  2: transcript:KXG33363            transcript:Zm00001d043446_T001         0
690-  3: transcript:EES01471            transcript:Zm00001d043449_T001         0
690-  4: transcript:EES03601            transcript:Zm00001d043450_T001  1.00E-86
690-  5: transcript:EES03602            transcript:Zm00001d043451_T001         0
690-  6: transcript:EES03605            transcript:Zm00001d043452_T008         0
690-  7: transcript:OQU87536            transcript:Zm00001d043453_T001 7.00E-118
690-  8: transcript:OQU87538            transcript:Zm00001d043454_T001         0
690-  9: transcript:KXG33366            transcript:Zm00001d043455_T001  1.00E-53
690- 10: transcript:EES01473            transcript:Zm00001d043458_T001         0
690- 11: transcript:EES03608            transcript:Zm00001d043459_T005         0
690- 12: transcript:EES01474            transcript:Zm00001d043461_T001 4.00E-123
690- 13: transcript:EES01475            transcript:Zm00001d043462_T004         0
690- 14: transcript:OQU87541            transcript:Zm00001d043463_T001         0
690- 15: transcript:EES03609            transcript:Zm00001d043464_T001 8.00E-166
690- 16: transcript:EES01477            transcript:Zm00001d043465_T004         0
## Alignment 691: score=783.0 e_value=1.2e-50 N=18 3&3 plus
691-  0: transcript:KXG31610            transcript:Zm00001d039449_T001         0
691-  1: transcript:EES02299            transcript:Zm00001d039450_T001 1.00E-119
691-  2: transcript:KXG31613            transcript:Zm00001d039451_T001 2.00E-116
691-  3: transcript:KXG31614            transcript:Zm00001d039452_T002         0
691-  4: transcript:EES02301            transcript:Zm00001d039453_T001         0
691-  5: transcript:EES02302            transcript:Zm00001d039454_T001         0
691-  6: transcript:EES00126            transcript:Zm00001d039455_T001 9.00E-109
691-  7: transcript:EES02303            transcript:Zm00001d039456_T001         0
691-  8: transcript:KXG31616            transcript:Zm00001d039459_T001 3.00E-149
691-  9: transcript:EES00127            transcript:Zm00001d039460_T003 1.00E-131
691- 10: transcript:EES00128            transcript:Zm00001d039461_T001  1.00E-99
691- 11: transcript:KXG31618            transcript:Zm00001d039464_T001  3.00E-48
691- 12: transcript:OQU86148            transcript:Zm00001d039465_T001         0
691- 13: transcript:EES02307            transcript:Zm00001d039467_T001         0
691- 14: transcript:EES02308            transcript:Zm00001d039468_T001  2.00E-58
691- 15: transcript:EES00131            transcript:Zm00001d039469_T001         0
691- 16: transcript:KXG31622            transcript:Zm00001d039471_T001         0
691- 17: transcript:EES00132            transcript:Zm00001d039472_T001 2.00E-122
## Alignment 692: score=722.0 e_value=4.2e-49 N=18 3&3 plus
692-  0: transcript:EES00222            transcript:Zm00001d039664_T001  3.00E-19
692-  1: transcript:EES00223            transcript:Zm00001d039667_T001 2.00E-128
692-  2: transcript:KXG31745            transcript:Zm00001d039668_T001         0
692-  3: transcript:KXG31749            transcript:Zm00001d039670_T007         0
692-  4: transcript:EES00225            transcript:Zm00001d039673_T002         0
692-  5: transcript:KXG31751            transcript:Zm00001d039674_T001         0
692-  6: transcript:EES00226            transcript:Zm00001d039675_T002         0
692-  7: transcript:EES00227            transcript:Zm00001d039677_T004 7.00E-149
692-  8: transcript:EES02381            transcript:Zm00001d039678_T002         0
692-  9: transcript:OQU86232            transcript:Zm00001d039679_T001         0
692- 10: transcript:KXG31755            transcript:Zm00001d039681_T001  2.00E-94
692- 11: transcript:EES00230            transcript:Zm00001d039682_T003         0
692- 12: transcript:OQU86234            transcript:Zm00001d039683_T009         0
692- 13: transcript:EES00228            transcript:Zm00001d039685_T001         0
```

692- 14: transcript:KXG31759 transcript:Zm00001d039686_T001 0
692- 15: transcript:KXG31760 transcript:Zm00001d039687_T001 6.00E-119
692- 16: transcript:KXG31764 transcript:Zm00001d039691_T001 0
692- 17: transcript:EES02391 transcript:Zm00001d039693_T013 0
Alignment 693: score=685.0 e_value=3.1e-38 N=15 3&3 plus
693- 0: transcript:EES00939 transcript:Zm00001d044250_T001 0
693- 1: transcript:EES03151 transcript:Zm00001d044253_T001 0
693- 2: transcript:EES03152 transcript:Zm00001d044254_T001 2.00E-67
693- 3: transcript:EES00941 transcript:Zm00001d044255_T001 9.00E-84
693- 4: transcript:EES00942 transcript:Zm00001d044259_T001 0
693- 5: transcript:EES00943 transcript:Zm00001d044260_T002 0
693- 6: transcript:EES00944 transcript:Zm00001d044261_T001 0
693- 7: transcript:EES00946 transcript:Zm00001d044266_T012 0
693- 8: transcript:OQU87035 transcript:Zm00001d044267_T004 0
693- 9: transcript:OQU87034 transcript:Zm00001d044270_T001 4.00E-114
693- 10: transcript:EES00947 transcript:Zm00001d044271_T011 0
693- 11: transcript:EES00948 transcript:Zm00001d044272_T001 3.00E-172
693- 12: transcript:KXG32765 transcript:Zm00001d044273_T002 0
693- 13: transcript:OQU87037 transcript:Zm00001d044276_T001 0
693- 14: transcript:OQU87038 transcript:Zm00001d044277_T002 0
Alignment 694: score=681.0 e_value=1.7e-41 N=16 3&3 plus
694- 0: transcript:OQU86341 transcript:Zm00001d039891_T001 0
694- 1: transcript:OQU86343 transcript:Zm00001d039892_T001 2.00E-79
694- 2: transcript:KXG31919 transcript:Zm00001d039893_T002 3.00E-161
694- 3: transcript:EES02500 transcript:Zm00001d039895_T001 0
694- 4: transcript:KXG31922 transcript:Zm00001d039899_T002 3.00E-107
694- 5: transcript:OQU86345 transcript:Zm00001d039900_T002 0
694- 6: transcript:EES02509 transcript:Zm00001d039901_T003 0
694- 7: transcript:OQU86349 transcript:Zm00001d039903_T003 1.00E-166
694- 8: transcript:EES00346 transcript:Zm00001d039904_T006 0
694- 9: transcript:EES02511 transcript:Zm00001d039907_T001 0
694- 10: transcript:EES02512 transcript:Zm00001d039908_T001 3.00E-107
694- 11: transcript:EES02514 transcript:Zm00001d039910_T001 0
694- 12: transcript:EES00348 transcript:Zm00001d039916_T001 0
694- 13: transcript:OQU86363 transcript:Zm00001d039918_T001 0
694- 14: transcript:EES02517 transcript:Zm00001d039919_T002 5.00E-180
694- 15: transcript:OQU86365 transcript:Zm00001d039920_T001 0
Alignment 695: score=652.0 e_value=2.1e-36 N=14 3&3 plus
695- 0: transcript:EES02644 transcript:Zm00001d040169_T001 0
695- 1: transcript:KXG32127 transcript:Zm00001d040171_T001 1.00E-122
695- 2: transcript:EES00454 transcript:Zm00001d040172_T001 2.00E-94
695- 3: transcript:KXG32128 transcript:Zm00001d040173_T001 5.00E-155
695- 4: transcript:OQU86545 transcript:Zm00001d040178_T001 3.00E-138
695- 5: transcript:KXG32133 transcript:Zm00001d040183_T001 0
695- 6: transcript:OQU86549 transcript:Zm00001d040185_T002 1.00E-89
695- 7: transcript:KXG32134 transcript:Zm00001d040186_T001 0
695- 8: transcript:EES00462 transcript:Zm00001d040188_T001 0
695- 9: transcript:EES02651 transcript:Zm00001d040189_T001 0
695- 10: transcript:OQU86550 transcript:Zm00001d040190_T001 3.00E-139
695- 11: transcript:KXG32138 transcript:Zm00001d040191_T002 0
695- 12: transcript:EES00463 transcript:Zm00001d040192_T001 0
695- 13: transcript:OQU86557 transcript:Zm00001d040193_T005 0
Alignment 696: score=646.0 e_value=1.5e-35 N=15 3&3 plus
696- 0: transcript:KXG31977 transcript:Zm00001d040005_T001 0

696- 1: transcript:KXG31978 transcript:Zm00001d040006_T001 6.00E-94
696- 2: transcript:EES02558 transcript:Zm00001d040008_T005 0
696- 3: transcript:KXG31980 transcript:Zm00001d040010_T003 0
696- 4: transcript:OQU86410 transcript:Zm00001d040011_T001 8.00E-92
696- 5: transcript:KXG31983 transcript:Zm00001d040014_T002 4.00E-79
696- 6: transcript:EES00381 transcript:Zm00001d040019_T001 1.00E-145
696- 7: transcript:KXG31991 transcript:Zm00001d040020_T001 0
696- 8: transcript:KXG31993 transcript:Zm00001d040023_T001 1.00E-76
696- 9: transcript:EES02563 transcript:Zm00001d040027_T002 1.00E-38
696- 10: transcript:KXG31997 transcript:Zm00001d040033_T002 0
696- 11: transcript:EES02566 transcript:Zm00001d040034_T001 0
696- 12: transcript:EES02568 transcript:Zm00001d040036_T021 0
696- 13: transcript:KXG32003 transcript:Zm00001d040038_T001 0
696- 14: transcript:EES02570 transcript:Zm00001d040040_T001 5.00E-135
Alignment 697: score=631.0 e_value=3.4e-37 N=15 3&3 plus
697- 0: transcript:EES02356 transcript:Zm00001d039613_T004 1.00E-123
697- 1: transcript:EES02357 transcript:Zm00001d039614_T001 0
697- 2: transcript:EES00189 transcript:Zm00001d039615_T001 0
697- 3: transcript:EES00191 transcript:Zm00001d039616_T001 0
697- 4: transcript:KXG31700 transcript:Zm00001d039617_T006 0
697- 5: transcript:EES00192 transcript:Zm00001d039618_T001 0
697- 6: transcript:KXG31701 transcript:Zm00001d039619_T009 6.00E-51
697- 7: transcript:EES00194 transcript:Zm00001d039620_T003 2.00E-117
697- 8: transcript:KXG31703 transcript:Zm00001d039621_T001 2.00E-19
697- 9: transcript:KXG31704 transcript:Zm00001d039623_T001 0
697- 10: transcript:KXG31706 transcript:Zm00001d039624_T001 1.00E-103
697- 11: transcript:EES00199 transcript:Zm00001d039625_T001 0
697- 12: transcript:EES02363 transcript:Zm00001d039626_T002 0
697- 13: transcript:KXG31711 transcript:Zm00001d039628_T001 6.00E-142
697- 14: transcript:EES00201 transcript:Zm00001d039631_T002 0
Alignment 698: score=569.0 e_value=6.4e-31 N=12 3&3 plus
698- 0: transcript:EES03131 transcript:Zm00001d044224_T001 0
698- 1: transcript:OQU87019 transcript:Zm00001d044225_T001 0
698- 2: transcript:EES03132 transcript:Zm00001d044226_T001 8.00E-122
698- 3: transcript:EES03133 transcript:Zm00001d044227_T001 2.00E-76
698- 4: transcript:EES03134 transcript:Zm00001d044228_T001 0
698- 5: transcript:EES03135 transcript:Zm00001d044230_T001 9.00E-114
698- 6: transcript:EES00928 transcript:Zm00001d044231_T001 3.00E-51
698- 7: transcript:EES03137 transcript:Zm00001d044232_T003 0
698- 8: transcript:OQU87025 transcript:Zm00001d044242_T001 3.00E-106
698- 9: transcript:EES03139 transcript:Zm00001d044243_T001 0
698- 10: transcript:EES03140 transcript:Zm00001d044244_T001 2.00E-54
698- 11: transcript:EES03141 transcript:Zm00001d044245_T001 0
Alignment 699: score=531.0 e_value=1.3e-28 N=12 3&3 plus
699- 0: transcript:EES02339 transcript:Zm00001d039565_T001 1.00E-99
699- 1: transcript:EES00167 transcript:Zm00001d039566_T001 4.00E-107
699- 2: transcript:EES02341 transcript:Zm00001d039568_T001 0
699- 3: transcript:OQU86178 transcript:Zm00001d039574_T001 5.00E-55
699- 4: transcript:EES00168 transcript:Zm00001d039575_T001 0
699- 5: transcript:KXG31669 transcript:Zm00001d039576_T001 0
699- 6: transcript:EES00169 transcript:Zm00001d039578_T003 0
699- 7: transcript:EES00170 transcript:Zm00001d039579_T001 0
699- 8: transcript:KXG31673 transcript:Zm00001d039580_T001 1.00E-56
699- 9: transcript:EES02346 transcript:Zm00001d039581_T001 3.00E-66

699- 10: transcript:EES00172 transcript:Zm00001d039582_T002 0
699- 11: transcript:OQU86187 transcript:Zm00001d039584_T001 2.00E-131
Alignment 700: score=525.0 e_value=6.6e-23 N=11 3&3 plus
700- 0: transcript:EES01375 transcript:Zm00001d043612_T002 1.00E-100
700- 1: transcript:KXG33257 transcript:Zm00001d043613_T004 0
700- 2: transcript:KXG33264 transcript:Zm00001d043614_T001 0
700- 3: transcript:EES01377 transcript:Zm00001d043615_T001 3.00E-79
700- 4: transcript:OQU87433 transcript:Zm00001d043616_T001 0
700- 5: transcript:KXG33265 transcript:Zm00001d043617_T001 0
700- 6: transcript:EES03535 transcript:Zm00001d043618_T002 9.00E-60
700- 7: transcript:EES03536 transcript:Zm00001d043619_T001 1.00E-54
700- 8: transcript:EES03537 transcript:Zm00001d043621_T001 1.00E-166
700- 9: transcript:EES01382 transcript:Zm00001d043622_T001 1.00E-120
700- 10: transcript:EES03538 transcript:Zm00001d043625_T002 3.00E-146
Alignment 701: score=509.0 e_value=2.9e-28 N=12 3&3 plus
701- 0: transcript:EES00405 transcript:Zm00001d040056_T005 0
701- 1: transcript:OQU86466 transcript:Zm00001d040057_T001 9.00E-78
701- 2: transcript:KXG32043 transcript:Zm00001d040058_T001 2.00E-12
701- 3: transcript:KXG32050 transcript:Zm00001d040059_T002 0
701- 4: transcript:EES00412 transcript:Zm00001d040060_T001 0
701- 5: transcript:EES00407 transcript:Zm00001d040061_T002 0
701- 6: transcript:OQU86475 transcript:Zm00001d040071_T001 0
701- 7: transcript:EES02601 transcript:Zm00001d040076_T008 0
701- 8: transcript:KXG32067 transcript:Zm00001d040082_T003 0
701- 9: transcript:KXG32068 transcript:Zm00001d040084_T001 0
701- 10: transcript:EES02603 transcript:Zm00001d040089_T001 0
701- 11: transcript:OQU86477 transcript:Zm00001d040090_T002 0
Alignment 702: score=509.0 e_value=1.7e-28 N=12 3&3 plus
702- 0: transcript:EES00077 transcript:Zm00001d039387_T002 2.00E-133
702- 1: transcript:EES00078 transcript:Zm00001d039390_T001 0
702- 2: transcript:EES02268 transcript:Zm00001d039391_T001 6.00E-174
702- 3: transcript:EES00079 transcript:Zm00001d039392_T001 0
702- 4: transcript:EES00081 transcript:Zm00001d039394_T001 2.00E-140
702- 5: transcript:EES02270 transcript:Zm00001d039395_T001 5.00E-131
702- 6: transcript:KXG31585 transcript:Zm00001d039400_T002 0
702- 7: transcript:EES00083 transcript:Zm00001d039401_T001 0
702- 8: transcript:EES02271 transcript:Zm00001d039402_T001 4.00E-168
702- 9: transcript:EES02272 transcript:Zm00001d039403_T003 0
702- 10: transcript:EES00084 transcript:Zm00001d039404_T001 0
702- 11: transcript:EES00086 transcript:Zm00001d039406_T002 0
Alignment 703: score=474.0 e_value=1.3e-24 N=11 3&3 plus
703- 0: transcript:OQU86094 transcript:Zm00001d039341_T126 0
703- 1: transcript:KXG31540 transcript:Zm00001d039343_T001 0
703- 2: transcript:EES00046 transcript:Zm00001d039345_T001 0
703- 3: transcript:EES00047 transcript:Zm00001d039347_T001 1.00E-107
703- 4: transcript:EES02235 transcript:Zm00001d039348_T001 5.00E-80
703- 5: transcript:KXG31543 transcript:Zm00001d039349_T002 0
703- 6: transcript:EES02236 transcript:Zm00001d039350_T001 5.00E-162
703- 7: transcript:EES02238 transcript:Zm00001d039351_T001 2.00E-66
703- 8: transcript:EES02240 transcript:Zm00001d039352_T001 0
703- 9: transcript:KXG31546 transcript:Zm00001d039354_T001 2.00E-112
703- 10: transcript:KXG31547 transcript:Zm00001d039355_T010 0
Alignment 704: score=464.0 e_value=2.2e-22 N=10 3&3 plus
704- 0: transcript:KXG32078 transcript:Zm00001d040094_T001 0

704- 1: transcript:KXG32080 transcript:Zm00001d040097_T002 5.00E-11
704- 2: transcript:EES00428 transcript:Zm00001d040100_T001 9.00E-11
704- 3: transcript:EES00430 transcript:Zm00001d040107_T001 0
704- 4: transcript:EES00431 transcript:Zm00001d040108_T001 3.00E-156
704- 5: transcript:EES00432 transcript:Zm00001d040109_T001 0
704- 6: transcript:EES02611 transcript:Zm00001d040110_T001 1.00E-13
704- 7: transcript:EES02613 transcript:Zm00001d040112_T001 3.00E-18
704- 8: transcript:EES02614 transcript:Zm00001d040113_T003 7.00E-117
704- 9: transcript:EES00434 transcript:Zm00001d040116_T001 0
Alignment 705: score=446.0 e_value=1.4e-22 N=10 3&3 plus
705- 0: transcript:EES03161 transcript:Zm00001d044297_T002 0
705- 1: transcript:EES00964 transcript:Zm00001d044301_T001 0
705- 2: transcript:OQU87056 transcript:Zm00001d044302_T001 0
705- 3: transcript:KXG32782 transcript:Zm00001d044303_T003 0
705- 4: transcript:OQU87062 transcript:Zm00001d044304_T002 0
705- 5: transcript:EES00971 transcript:Zm00001d044307_T001 0
705- 6: transcript:OQU87065 transcript:Zm00001d044308_T001 3.00E-43
705- 7: transcript:OQU87067 transcript:Zm00001d044310_T001 2.00E-109
705- 8: transcript:EES00973 transcript:Zm00001d044312_T003 0
705- 9: transcript:EES00974 transcript:Zm00001d044313_T004 0
Alignment 706: score=441.0 e_value=1.1e-21 N=10 3&3 plus
706- 0: transcript:EES02224 transcript:Zm00001d039323_T001 0
706- 1: transcript:KXG31528 transcript:Zm00001d039324_T001 1.00E-166
706- 2: transcript:KXG31530 transcript:Zm00001d039325_T001 0
706- 3: transcript:EES02226 transcript:Zm00001d039326_T001 0
706- 4: transcript:EES02227 transcript:Zm00001d039327_T001 5.00E-120
706- 5: transcript:EES00037 transcript:Zm00001d039330_T001 2.00E-145
706- 6: transcript:KXG31533 transcript:Zm00001d039331_T001 0
706- 7: transcript:KXG31535 transcript:Zm00001d039337_T001 0
706- 8: transcript:KXG31537 transcript:Zm00001d039338_T001 4.00E-115
706- 9: transcript:EES02231 transcript:Zm00001d039339_T001 2.00E-35
Alignment 707: score=425.0 e_value=1.1e-23 N=10 3&3 plus
707- 0: transcript:EES00261 transcript:Zm00001d039739_T001 0
707- 1: transcript:EES02410 transcript:Zm00001d039740_T001 0
707- 2: transcript:KXG31802 transcript:Zm00001d039745_T001 0
707- 3: transcript:EES00264 transcript:Zm00001d039746_T001 0
707- 4: transcript:KXG31803 transcript:Zm00001d039747_T002 3.00E-164
707- 5: transcript:EES00265 transcript:Zm00001d039748_T001 7.00E-89
707- 6: transcript:KXG31806 transcript:Zm00001d039749_T002 1.00E-178
707- 7: transcript:EES02415 transcript:Zm00001d039750_T001 4.00E-143
707- 8: transcript:KXG31807 transcript:Zm00001d039758_T002 0
707- 9: transcript:EES00270 transcript:Zm00001d039763_T001 0
Alignment 708: score=421.0 e_value=1.7e-19 N=9 3&3 plus
708- 0: transcript:EES02495 transcript:Zm00001d039873_T002 2.00E-17
708- 1: transcript:EES02496 transcript:Zm00001d039874_T001 3.00E-118
708- 2: transcript:KXG31900 transcript:Zm00001d039875_T001 1.00E-121
708- 3: transcript:KXG31903 transcript:Zm00001d039878_T003 6.00E-161
708- 4: transcript:EES02497 transcript:Zm00001d039879_T001 3.00E-160
708- 5: transcript:EES00328 transcript:Zm00001d039880_T001 0
708- 6: transcript:EES00331 transcript:Zm00001d039881_T003 0
708- 7: transcript:EES00332 transcript:Zm00001d039882_T001 6.00E-31
708- 8: transcript:EES00336 transcript:Zm00001d039884_T002 0
Alignment 709: score=415.0 e_value=1.6e-17 N=9 3&3 plus
709- 0: transcript:EES03610 transcript:Zm00001d043469_T022 0

709- 1: transcript:EES01480 transcript:Zm00001d043470_T001 2.00E-124
709- 2: transcript:KXG33369 transcript:Zm00001d043473_T004 0
709- 3: transcript:EES03611 transcript:Zm00001d043474_T001 1.00E-161
709- 4: transcript:EES01481 transcript:Zm00001d043477_T001 0
709- 5: transcript:OQU87546 transcript:Zm00001d043478_T001 0
709- 6: transcript:KXG33371 transcript:Zm00001d043479_T001 4.00E-124
709- 7: transcript:KXG33373 transcript:Zm00001d043480_T001 0
709- 8: transcript:EES01482 transcript:Zm00001d043483_T001 0
Alignment 710: score=392.0 e_value=8.5e-23 N=10 3&3 plus
710- 0: transcript:EES00633 transcript:Zm00001d040611_T002 0
710- 1: transcript:EES00635 transcript:Zm00001d040612_T003 0
710- 2: transcript:KXG32368 transcript:Zm00001d040613_T001 2.00E-95
710- 3: transcript:KXG32370 transcript:Zm00001d040614_T002 9.00E-141
710- 4: transcript:EES00638 transcript:Zm00001d040619_T001 4.00E-84
710- 5: transcript:OQU86782 transcript:Zm00001d040621_T002 5.00E-153
710- 6: transcript:KXG32373 transcript:Zm00001d040622_T001 5.00E-150
710- 7: transcript:KXG32374 transcript:Zm00001d040623_T001 4.00E-24
710- 8: transcript:KXG32375 transcript:Zm00001d040624_T001 3.00E-113
710- 9: transcript:KXG32379 transcript:Zm00001d040625_T002 0
Alignment 711: score=374.0 e_value=1e-15 N=8 3&3 plus
711- 0: transcript:KXG33374 transcript:Zm00001d043486_T001 1.00E-53
711- 1: transcript:KXG33375 transcript:Zm00001d043489_T001 0
711- 2: transcript:EES03617 transcript:Zm00001d043490_T001 0
711- 3: transcript:KXG33377 transcript:Zm00001d043491_T001 2.00E-84
711- 4: transcript:KXG33379 transcript:Zm00001d043492_T001 0
711- 5: transcript:EES01486 transcript:Zm00001d043494_T005 0
711- 6: transcript:EES03620 transcript:Zm00001d043497_T001 4.00E-106
711- 7: transcript:KXG33380 transcript:Zm00001d043500_T008 0
Alignment 712: score=361.0 e_value=3.8e-17 N=9 3&3 plus
712- 0: transcript:EES02207 transcript:Zm00001d039309_T001 7.00E-152
712- 1: transcript:OQU86080 transcript:Zm00001d039311_T001 3.00E-166
712- 2: transcript:EES02213 transcript:Zm00001d039312_T001 0
712- 3: transcript:EES02214 transcript:Zm00001d039313_T001 7.00E-105
712- 4: transcript:KXG31512 transcript:Zm00001d039314_T001 0
712- 5: transcript:EES00021 transcript:Zm00001d039315_T003 0
712- 6: transcript:KXG31516 transcript:Zm00001d039316_T001 1.00E-175
712- 7: transcript:EES00022 transcript:Zm00001d039318_T001 4.00E-112
712- 8: transcript:EES02221 transcript:Zm00001d039319_T002 0
Alignment 713: score=350.0 e_value=2.8e-18 N=9 3&3 plus
713- 0: transcript:EES00139 transcript:Zm00001d039487_T001 0
713- 1: transcript:EES00140 transcript:Zm00001d039488_T002 0
713- 2: transcript:EES02317 transcript:Zm00001d039489_T002 4.00E-180
713- 3: transcript:EES00141 transcript:Zm00001d039492_T001 7.00E-140
713- 4: transcript:KXG31642 transcript:Zm00001d039495_T001 0
713- 5: transcript:EES00143 transcript:Zm00001d039496_T001 6.00E-130
713- 6: transcript:EES00144 transcript:Zm00001d039498_T001 0
713- 7: transcript:EES02318 transcript:Zm00001d039499_T002 0
713- 8: transcript:KXG31651 transcript:Zm00001d039503_T002 2.00E-110
Alignment 714: score=344.0 e_value=4e-19 N=9 3&3 plus
714- 0: transcript:KXG32338 transcript:Zm00001d040579_T001 3.00E-46
714- 1: transcript:EES00616 transcript:Zm00001d040581_T001 0
714- 2: transcript:OQU86763 transcript:Zm00001d040582_T002 2.00E-107
714- 3: transcript:EES00619 transcript:Zm00001d040588_T004 5.00E-147
714- 4: transcript:EES00620 transcript:Zm00001d040589_T001 0

714- 5: transcript:EES00623 transcript:Zm00001d040593_T001 0
714- 6: transcript:EES00624 transcript:Zm00001d040594_T001 3.00E-151
714- 7: transcript:EES00626 transcript:Zm00001d040596_T001 3.00E-69
714- 8: transcript:KXG32356 transcript:Zm00001d040597_T001 0
Alignment 715: score=310.0 e_value=3.7e-16 N=8 3&3 plus
715- 0: transcript:EES02869 transcript:Zm00001d040650_T001 2.00E-137
715- 1: transcript:EES00666 transcript:Zm00001d040651_T001 7.00E-105
715- 2: transcript:EES02873 transcript:Zm00001d040652_T001 0
715- 3: transcript:OQU86800 transcript:Zm00001d040656_T002 3.00E-135
715- 4: transcript:EES02876 transcript:Zm00001d040660_T001 8.00E-30
715- 5: transcript:KXG32398 transcript:Zm00001d040665_T001 0
715- 6: transcript:EES00668 transcript:Zm00001d040666_T003 0
715- 7: transcript:EES02878 transcript:Zm00001d040667_T001 0
Alignment 716: score=308.0 e_value=1.5e-11 N=7 3&3 plus
716- 0: transcript:EES02330 transcript:Zm00001d039529_T001 0
716- 1: transcript:KXG31655 transcript:Zm00001d039530_T001 0
716- 2: transcript:EES00157 transcript:Zm00001d039531_T001 2.00E-91
716- 3: transcript:EES02331 transcript:Zm00001d039532_T001 0
716- 4: transcript:KXG31656 transcript:Zm00001d039533_T001 5.00E-172
716- 5: transcript:KXG31658 transcript:Zm00001d039534_T001 0
716- 6: transcript:EES02334 transcript:Zm00001d039535_T001 0
Alignment 717: score=292.0 e_value=1e-11 N=7 3&3 plus
717- 0: transcript:KXG31735 transcript:Zm00001d039653_T005 0
717- 1: transcript:EES00213 transcript:Zm00001d039654_T001 0
717- 2: transcript:EES00217 transcript:Zm00001d039656_T003 0
717- 3: transcript:EES00218 transcript:Zm00001d039657_T001 2.00E-54
717- 4: transcript:EES00219 transcript:Zm00001d039658_T001 3.00E-62
717- 5: transcript:KXG31743 transcript:Zm00001d039660_T010 0
717- 6: transcript:EES02380 transcript:Zm00001d039661_T001 8.00E-68
Alignment 718: score=291.0 e_value=5.6e-12 N=7 3&3 plus
718- 0: transcript:KXG31683 transcript:Zm00001d039596_T004 0
718- 1: transcript:EES00181 transcript:Zm00001d039597_T001 1.00E-125
718- 2: transcript:OQU86191 transcript:Zm00001d039598_T001 0
718- 3: transcript:EES02350 transcript:Zm00001d039600_T001 0
718- 4: transcript:KXG31691 transcript:Zm00001d039606_T002 0
718- 5: transcript:EES00187 transcript:Zm00001d039607_T001 0
718- 6: transcript:KXG31694 transcript:Zm00001d039608_T007 0
Alignment 719: score=290.0 e_value=4.6e-14 N=7 3&3 plus
719- 0: transcript:EES00202 transcript:Zm00001d039634_T001 0
719- 1: transcript:OQU86218 transcript:Zm00001d039635_T001 3.00E-75
719- 2: transcript:EES02371 transcript:Zm00001d039636_T001 0
719- 3: transcript:EES02372 transcript:Zm00001d039637_T001 0
719- 4: transcript:EES00205 transcript:Zm00001d039638_T001 0
719- 5: transcript:EES02368 transcript:Zm00001d039639_T001 0
719- 6: transcript:KXG31729 transcript:Zm00001d039642_T001 0
Alignment 720: score=270.0 e_value=1e-08 N=6 3&3 plus
720- 0: transcript:EES02638 transcript:Zm00001d040158_T001 0
720- 1: transcript:EES00452 transcript:Zm00001d040160_T001 0
720- 2: transcript:KXG32119 transcript:Zm00001d040161_T002 0
720- 3: transcript:KXG32120 transcript:Zm00001d040163_T001 0
720- 4: transcript:EES02639 transcript:Zm00001d040164_T002 2.00E-86
720- 5: transcript:OQU86533 transcript:Zm00001d040166_T014 0
Alignment 721: score=10088.0 e_value=0 N=217 3&3 minus
721- 0: transcript:KXG33608 transcript:Zm00001d043091_T002 0

721- 1:	transcript:EES01652	transcript:Zm00001d043090_T001	0
721- 2:	transcript:OQU87751	transcript:Zm00001d043089_T001	2.00E-161
721- 3:	transcript:OQU87754	transcript:Zm00001d043088_T002	0
721- 4:	transcript:EES01655	transcript:Zm00001d043087_T001	8.00E-102
721- 5:	transcript:KXG33616	transcript:Zm00001d043086_T002	0
721- 6:	transcript:EES01656	transcript:Zm00001d043083_T003	0
721- 7:	transcript:EES01658	transcript:Zm00001d043082_T001	0
721- 8:	transcript:KXG33617	transcript:Zm00001d043081_T001	0
721- 9:	transcript:EES01659	transcript:Zm00001d043080_T001	0
721- 10:	transcript:EES01660	transcript:Zm00001d043076_T001	0
721- 11:	transcript:EES03800	transcript:Zm00001d043075_T002	0
721- 12:	transcript:EES01661	transcript:Zm00001d043074_T004	0
721- 13:	transcript:EES01662	transcript:Zm00001d043071_T002	0
721- 14:	transcript:EES01663	transcript:Zm00001d043070_T001	6.00E-108
721- 15:	transcript:EES01664	transcript:Zm00001d043069_T001	0
721- 16:	transcript:OQU87761	transcript:Zm00001d043068_T001	0
721- 17:	transcript:EES03802	transcript:Zm00001d043067_T002	0
721- 18:	transcript:EES01666	transcript:Zm00001d043066_T001	8.00E-151
721- 19:	transcript:EES01667	transcript:Zm00001d043063_T001	1.00E-144
721- 20:	transcript:EES01668	transcript:Zm00001d043062_T001	4.00E-23
721- 21:	transcript:EES01669	transcript:Zm00001d043060_T001	5.00E-132
721- 22:	transcript:KXG33619	transcript:Zm00001d043059_T002	0
721- 23:	transcript:EES03806	transcript:Zm00001d043058_T001	0
721- 24:	transcript:EES03807	transcript:Zm00001d043056_T002	0
721- 25:	transcript:EES03808	transcript:Zm00001d043052_T001	0
721- 26:	transcript:EES03810	transcript:Zm00001d043049_T001	2.00E-61
721- 27:	transcript:OQU87763	transcript:Zm00001d043047_T001	1.00E-180
721- 28:	transcript:KXG33624	transcript:Zm00001d043046_T001	3.00E-149
721- 29:	transcript:EES01674	transcript:Zm00001d043045_T001	1.00E-109
721- 30:	transcript:EES03812	transcript:Zm00001d043044_T001	0
721- 31:	transcript:KXG33626	transcript:Zm00001d043043_T001	0
721- 32:	transcript:EES01676	transcript:Zm00001d043039_T001	2.00E-68
721- 33:	transcript:KXG33627	transcript:Zm00001d043038_T001	0
721- 34:	transcript:KXG33629	transcript:Zm00001d043037_T001	2.00E-98
721- 35:	transcript:OQU87768	transcript:Zm00001d043036_T001	2.00E-90
721- 36:	transcript:EES03814	transcript:Zm00001d043031_T001	0
721- 37:	transcript:KXG33634	transcript:Zm00001d043029_T001	0
721- 38:	transcript:EES01683	transcript:Zm00001d043026_T001	0
721- 39:	transcript:KXG33637	transcript:Zm00001d043025_T001	0
721- 40:	transcript:EES03819	transcript:Zm00001d043024_T001	3.00E-90
721- 41:	transcript:EES03820	transcript:Zm00001d043023_T002	0
721- 42:	transcript:OQU87773	transcript:Zm00001d043022_T001	0
721- 43:	transcript:EES01686	transcript:Zm00001d043019_T003	0
721- 44:	transcript:EES03822	transcript:Zm00001d043018_T001	1.00E-137
721- 45:	transcript:OQU87774	transcript:Zm00001d043015_T001	0
721- 46:	transcript:OQU87775	transcript:Zm00001d043014_T001	4.00E-127
721- 47:	transcript:EES01692	transcript:Zm00001d043013_T001	5.00E-112
721- 48:	transcript:OQU87776	transcript:Zm00001d043012_T018	0
721- 49:	transcript:EES03824	transcript:Zm00001d043011_T001	1.00E-105
721- 50:	transcript:EES01697	transcript:Zm00001d043009_T002	0
721- 51:	transcript:KXG33640	transcript:Zm00001d043006_T001	6.00E-148
721- 52:	transcript:OQU87785	transcript:Zm00001d043001_T001	1.00E-158
721- 53:	transcript:EES03828	transcript:Zm00001d042998_T001	5.00E-110
721- 54:	transcript:EES03830	transcript:Zm00001d042997_T001	4.00E-91

721- 55: transcript:EES03831	transcript:Zm00001d042996_T001	0
721- 56: transcript:EES01702	transcript:Zm00001d042993_T001	0
721- 57: transcript:OQU87789	transcript:Zm00001d042988_T001	2.00E-76
721- 58: transcript:EES01703	transcript:Zm00001d042985_T001	0
721- 59: transcript:EES01705	transcript:Zm00001d042980_T002	0
721- 60: transcript:EES01706	transcript:Zm00001d042979_T007	0
721- 61: transcript:EES01707	transcript:Zm00001d042978_T001	0
721- 62: transcript:EES03838	transcript:Zm00001d042977_T001	0
721- 63: transcript:EES01708	transcript:Zm00001d042976_T001	1.00E-131
721- 64: transcript:EES03839	transcript:Zm00001d042975_T003	0
721- 65: transcript:EES01710	transcript:Zm00001d042974_T001	9.00E-148
721- 66: transcript:KXG33651	transcript:Zm00001d042973_T003	0
721- 67: transcript:KXG33654	transcript:Zm00001d042972_T002	0
721- 68: transcript:EES03840	transcript:Zm00001d042969_T002	5.00E-146
721- 69: transcript:OQU87808	transcript:Zm00001d042968_T002	1.00E-96
721- 70: transcript:EES03842	transcript:Zm00001d042966_T001	2.00E-100
721- 71: transcript:EES01713	transcript:Zm00001d042965_T001	0
721- 72: transcript:KXG33657	transcript:Zm00001d042964_T001	4.00E-44
721- 73: transcript:OQU87809	transcript:Zm00001d042963_T001	1.00E-25
721- 74: transcript:EES03843	transcript:Zm00001d042962_T002	0
721- 75: transcript:OQU87817	transcript:Zm00001d042961_T002	0
721- 76: transcript:OQU87816	transcript:Zm00001d042960_T001	5.00E-77
721- 77: transcript:EES03844	transcript:Zm00001d042958_T001	3.00E-142
721- 78: transcript:OQU87818	transcript:Zm00001d042955_T001	0
721- 79: transcript:EES01718	transcript:Zm00001d042953_T001	0
721- 80: transcript:EES01717	transcript:Zm00001d042950_T001	0
721- 81: transcript:EES01719	transcript:Zm00001d042949_T001	0
721- 82: transcript:EES03848	transcript:Zm00001d042948_T001	0
721- 83: transcript:EES01720	transcript:Zm00001d042946_T001	1.00E-168
721- 84: transcript:EES03847	transcript:Zm00001d042944_T001	0
721- 85: transcript:EES01723	transcript:Zm00001d042943_T005	0
721- 86: transcript:EES01724	transcript:Zm00001d042941_T001	3.00E-15
721- 87: transcript:EES01725	transcript:Zm00001d042940_T001	1.00E-61
721- 88: transcript:EES03850	transcript:Zm00001d042939_T002	0
721- 89: transcript:KXG33666	transcript:Zm00001d042938_T004	0
721- 90: transcript:EES03851	transcript:Zm00001d042936_T001	1.00E-52
721- 91: transcript:OQU87825	transcript:Zm00001d042933_T001	5.00E-70
721- 92: transcript:EES03854	transcript:Zm00001d042932_T001	0
721- 93: transcript:EES01728	transcript:Zm00001d042931_T001	0
721- 94: transcript:EES03855	transcript:Zm00001d042930_T001	7.00E-92
721- 95: transcript:EES03856	transcript:Zm00001d042929_T001	3.00E-119
721- 96: transcript:EES03857	transcript:Zm00001d042926_T001	1.00E-85
721- 97: transcript:EES01730	transcript:Zm00001d042923_T003	2.00E-82
721- 98: transcript:EES01731	transcript:Zm00001d042922_T007	0
721- 99: transcript:KXG33669	transcript:Zm00001d042921_T001	0
721-100: transcript:OQU87830	transcript:Zm00001d042920_T001	1.00E-93
721-101: transcript:OQU87835	transcript:Zm00001d042918_T001	7.00E-155
721-102: transcript:EES03863	transcript:Zm00001d042917_T001	2.00E-166
721-103: transcript:EES01733	transcript:Zm00001d042916_T003	0
721-104: transcript:EES01737	transcript:Zm00001d042911_T001	0
721-105: transcript:EES01738	transcript:Zm00001d042910_T010	0
721-106: transcript:EES01739	transcript:Zm00001d042909_T001	1.00E-106
721-107: transcript:OQU87839	transcript:Zm00001d042908_T002	0
721-108: transcript:EES01740	transcript:Zm00001d042907_T001	0

721-109:	transcript:OQU87842	transcript:Zm00001d042903_T001	1.00E-52
721-110:	transcript:KXG33680	transcript:Zm00001d042902_T001	9.00E-39
721-111:	transcript:EES01742	transcript:Zm00001d042901_T001	0
721-112:	transcript:KXG33681	transcript:Zm00001d042900_T001	2.00E-28
721-113:	transcript:EES03870	transcript:Zm00001d042899_T001	0
721-114:	transcript:EES03871	transcript:Zm00001d042898_T001	0
721-115:	transcript:KXG33682	transcript:Zm00001d042892_T001	6.00E-74
721-116:	transcript:EES03872	transcript:Zm00001d042887_T001	0
721-117:	transcript:EES01744	transcript:Zm00001d042886_T001	0
721-118:	transcript:KXG33686	transcript:Zm00001d042885_T006	0
721-119:	transcript:EES01747	transcript:Zm00001d042884_T003	0
721-120:	transcript:EES03874	transcript:Zm00001d042883_T001	0
721-121:	transcript:OQU87854	transcript:Zm00001d042880_T001	0
721-122:	transcript:OQU87855	transcript:Zm00001d042879_T003	0
721-123:	transcript:EES01750	transcript:Zm00001d042875_T001	5.00E-46
721-124:	transcript:EES01751	transcript:Zm00001d042874_T001	0
721-125:	transcript:EES01749	transcript:Zm00001d042872_T001	3.00E-94
721-126:	transcript:EES01752	transcript:Zm00001d042868_T001	1.00E-108
721-127:	transcript:EES03878	transcript:Zm00001d042867_T001	5.00E-27
721-128:	transcript:EES03880	transcript:Zm00001d042866_T001	3.00E-37
721-129:	transcript:EES03882	transcript:Zm00001d042864_T003	0
721-130:	transcript:OQU87858	transcript:Zm00001d042863_T001	2.00E-86
721-131:	transcript:EES03885	transcript:Zm00001d042862_T001	1.00E-75
721-132:	transcript:EES03886	transcript:Zm00001d042861_T001	0
721-133:	transcript:EES01756	transcript:Zm00001d042857_T001	1.00E-41
721-134:	transcript:EES03887	transcript:Zm00001d042856_T002	2.00E-09
721-135:	transcript:EES01757	transcript:Zm00001d042853_T001	0
721-136:	transcript:EES01758	transcript:Zm00001d042851_T003	0
721-137:	transcript:OQU87860	transcript:Zm00001d042850_T001	2.00E-47
721-138:	transcript:OQU87862	transcript:Zm00001d042849_T001	2.00E-65
721-139:	transcript:EES01759	transcript:Zm00001d042848_T001	0
721-140:	transcript:OQU87871	transcript:Zm00001d042847_T001	0
721-141:	transcript:EES03891	transcript:Zm00001d042846_T001	2.00E-138
721-142:	transcript:EES03892	transcript:Zm00001d042845_T001	0
721-143:	transcript:OQU87873	transcript:Zm00001d042843_T001	8.00E-158
721-144:	transcript:EES03893	transcript:Zm00001d042842_T003	0
721-145:	transcript:OQU87874	transcript:Zm00001d042841_T002	2.00E-129
721-146:	transcript:EES01767	transcript:Zm00001d042840_T001	0
721-147:	transcript:EES03894	transcript:Zm00001d042837_T001	0
721-148:	transcript:EES03898	transcript:Zm00001d042836_T001	3.00E-23
721-149:	transcript:EES01769	transcript:Zm00001d042833_T001	0
721-150:	transcript:KXG33716	transcript:Zm00001d042830_T002	2.00E-159
721-151:	transcript:EES01771	transcript:Zm00001d042826_T001	0
721-152:	transcript:EES01772	transcript:Zm00001d042822_T001	5.00E-61
721-153:	transcript:OQU87881	transcript:Zm00001d042821_T001	2.00E-123
721-154:	transcript:KXG33719	transcript:Zm00001d042820_T001	0
721-155:	transcript:EES03905	transcript:Zm00001d042817_T001	0
721-156:	transcript:OQU87885	transcript:Zm00001d042814_T001	0
721-157:	transcript:EES03906	transcript:Zm00001d042813_T001	0
721-158:	transcript:EES01775	transcript:Zm00001d042812_T001	0
721-159:	transcript:KXG33721	transcript:Zm00001d042811_T001	0
721-160:	transcript:EES01777	transcript:Zm00001d042810_T001	0
721-161:	transcript:EES01778	transcript:Zm00001d042809_T003	0
721-162:	transcript:KXG33724	transcript:Zm00001d042807_T001	0

721-163: transcript:KXG33725	transcript:Zm00001d042804_T001	0
721-164: transcript:EES03912	transcript:Zm00001d042802_T001	0
721-165: transcript:EES03913	transcript:Zm00001d042801_T001	0
721-166: transcript:OQU87888	transcript:Zm00001d042800_T001	0
721-167: transcript:KXG33729	transcript:Zm00001d042796_T001	4.00E-104
721-168: transcript:KXG33728	transcript:Zm00001d042794_T005	0
721-169: transcript:KXG33730	transcript:Zm00001d042793_T002	0
721-170: transcript:EES03919	transcript:Zm00001d042792_T002	0
721-171: transcript:OQU87889	transcript:Zm00001d042787_T001	0
721-172: transcript:EES03917	transcript:Zm00001d042786_T001	0
721-173: transcript:EES03920	transcript:Zm00001d042781_T001	6.00E-179
721-174: transcript:EES01786	transcript:Zm00001d042780_T001	0
721-175: transcript:KXG33733	transcript:Zm00001d042779_T001	1.00E-178
721-176: transcript:EES01788	transcript:Zm00001d042778_T001	0
721-177: transcript:KXG33737	transcript:Zm00001d042777_T006	0
721-178: transcript:EES03923	transcript:Zm00001d042774_T001	0
721-179: transcript:OQU87899	transcript:Zm00001d042772_T001	2.00E-136
721-180: transcript:EES01789	transcript:Zm00001d042769_T001	1.00E-164
721-181: transcript:EES03924	transcript:Zm00001d042768_T001	1.00E-129
721-182: transcript:EES01795	transcript:Zm00001d042767_T001	0
721-183: transcript:KXG33742	transcript:Zm00001d042766_T001	2.00E-109
721-184: transcript:EES03926	transcript:Zm00001d042765_T001	2.00E-59
721-185: transcript:EES01796	transcript:Zm00001d042764_T001	0
721-186: transcript:EES03928	transcript:Zm00001d042762_T001	0
721-187: transcript:EES03929	transcript:Zm00001d042761_T001	0
721-188: transcript:EES01798	transcript:Zm00001d042760_T001	1.00E-112
721-189: transcript:EES01799	transcript:Zm00001d042758_T001	3.00E-133
721-190: transcript:EES01800	transcript:Zm00001d042756_T001	6.00E-100
721-191: transcript:EES03930	transcript:Zm00001d042755_T001	9.00E-62
721-192: transcript:OQU87905	transcript:Zm00001d042754_T001	6.00E-43
721-193: transcript:OQU87906	transcript:Zm00001d042753_T001	8.00E-87
721-194: transcript:EES03931	transcript:Zm00001d042752_T001	4.00E-98
721-195: transcript:EES01803	transcript:Zm00001d042751_T001	5.00E-50
721-196: transcript:OQU87908	transcript:Zm00001d042748_T001	7.00E-22
721-197: transcript:KXG33746	transcript:Zm00001d042747_T003	0
721-198: transcript:KXG33747	transcript:Zm00001d042746_T003	2.00E-147
721-199: transcript:OQU87909	transcript:Zm00001d042739_T001	0
721-200: transcript:EES03937	transcript:Zm00001d042738_T001	0
721-201: transcript:EES03938	transcript:Zm00001d042736_T001	0
721-202: transcript:EES01809	transcript:Zm00001d042735_T001	7.00E-89
721-203: transcript:EES01810	transcript:Zm00001d042731_T001	0
721-204: transcript:OQU87919	transcript:Zm00001d042729_T003	9.00E-148
721-205: transcript:KXG33753	transcript:Zm00001d042727_T001	0
721-206: transcript:EES03940	transcript:Zm00001d042726_T001	2.00E-60
721-207: transcript:EES03941	transcript:Zm00001d042725_T001	3.00E-38
721-208: transcript:EES01812	transcript:Zm00001d042724_T001	0
721-209: transcript:KXG33757	transcript:Zm00001d042723_T001	0
721-210: transcript:EES01813	transcript:Zm00001d042722_T001	0
721-211: transcript:EES03943	transcript:Zm00001d042721_T001	6.00E-128
721-212: transcript:EES01814	transcript:Zm00001d042720_T005	0
721-213: transcript:EES01815	transcript:Zm00001d042719_T001	0
721-214: transcript:EES03944	transcript:Zm00001d042718_T001	4.00E-168
721-215: transcript:KXG33764	transcript:Zm00001d042717_T001	2.00E-126
721-216: transcript:OQU87936	transcript:Zm00001d042713_T005	0

Alignment 722: score=7322.0 e_value=0 N=159 3&3 minus

722- 0:	transcript:OQU87608	transcript:Zm00001d043347_T006	0
722- 1:	transcript:EES03672	transcript:Zm00001d043346_T001	4.00E-93
722- 2:	transcript:KXG33448	transcript:Zm00001d043339_T001	5.00E-98
722- 3:	transcript:OQU87610	transcript:Zm00001d043338_T001	2.00E-29
722- 4:	transcript:EES03676	transcript:Zm00001d043337_T001	1.00E-49
722- 5:	transcript:KXG33450	transcript:Zm00001d043336_T001	0
722- 6:	transcript:KXG33451	transcript:Zm00001d043335_T006	0
722- 7:	transcript:OQU87613	transcript:Zm00001d043333_T001	8.00E-174
722- 8:	transcript:OQU87616	transcript:Zm00001d043330_T001	0
722- 9:	transcript:EES01527	transcript:Zm00001d043329_T001	0
722- 10:	transcript:EES01528	transcript:Zm00001d043328_T003	0
722- 11:	transcript:EES01529	transcript:Zm00001d043327_T002	0
722- 12:	transcript:KXG33458	transcript:Zm00001d043325_T001	0
722- 13:	transcript:EES03683	transcript:Zm00001d043324_T001	0
722- 14:	transcript:KXG33461	transcript:Zm00001d043323_T001	4.00E-63
722- 15:	transcript:OQU87621	transcript:Zm00001d043320_T009	0
722- 16:	transcript:EES03690	transcript:Zm00001d043318_T001	1.00E-118
722- 17:	transcript:KXG33464	transcript:Zm00001d043317_T001	2.00E-149
722- 18:	transcript:EES01533	transcript:Zm00001d043314_T001	1.00E-120
722- 19:	transcript:KXG33468	transcript:Zm00001d043312_T001	0
722- 20:	transcript:EES01535	transcript:Zm00001d043311_T001	8.00E-109
722- 21:	transcript:KXG33474	transcript:Zm00001d043309_T004	0
722- 22:	transcript:EES01541	transcript:Zm00001d043307_T001	7.00E-172
722- 23:	transcript:OQU87632	transcript:Zm00001d043305_T001	0
722- 24:	transcript:EES01544	transcript:Zm00001d043303_T003	0
722- 25:	transcript:OQU87635	transcript:Zm00001d043302_T005	2.00E-169
722- 26:	transcript:KXG33478	transcript:Zm00001d043301_T006	0
722- 27:	transcript:KXG33481	transcript:Zm00001d043300_T001	0
722- 28:	transcript:EES03698	transcript:Zm00001d043299_T001	8.00E-54
722- 29:	transcript:EES03699	transcript:Zm00001d043298_T001	0
722- 30:	transcript:KXG33485	transcript:Zm00001d043296_T002	0
722- 31:	transcript:OQU87640	transcript:Zm00001d043295_T003	0
722- 32:	transcript:EES01550	transcript:Zm00001d043294_T006	0
722- 33:	transcript:EES03702	transcript:Zm00001d043293_T001	0
722- 34:	transcript:OQU87645	transcript:Zm00001d043292_T001	0
722- 35:	transcript:OQU87648	transcript:Zm00001d043291_T001	0
722- 36:	transcript:OQU87651	transcript:Zm00001d043289_T002	1.00E-81
722- 37:	transcript:EES01554	transcript:Zm00001d043288_T001	0
722- 38:	transcript:OQU87654	transcript:Zm00001d043287_T002	0
722- 39:	transcript:OQU87661	transcript:Zm00001d043283_T001	2.00E-112
722- 40:	transcript:OQU87663	transcript:Zm00001d043280_T001	0
722- 41:	transcript:EES01558	transcript:Zm00001d043278_T001	0
722- 42:	transcript:EES03711	transcript:Zm00001d043277_T013	0
722- 43:	transcript:EES03713	transcript:Zm00001d043276_T001	1.00E-38
722- 44:	transcript:KXG33509	transcript:Zm00001d043275_T002	0
722- 45:	transcript:EES01562	transcript:Zm00001d043274_T008	0
722- 46:	transcript:OQU87668	transcript:Zm00001d043273_T001	5.00E-122
722- 47:	transcript:OQU87671	transcript:Zm00001d043272_T002	0
722- 48:	transcript:EES03716	transcript:Zm00001d043271_T001	0
722- 49:	transcript:EES01567	transcript:Zm00001d043270_T001	2.00E-165
722- 50:	transcript:EES01566	transcript:Zm00001d043269_T001	4.00E-150
722- 51:	transcript:EES03719	transcript:Zm00001d043267_T002	0
722- 52:	transcript:EES01568	transcript:Zm00001d043266_T002	0

722- 53:	transcript:EES03721	transcript:Zm00001d043265_T001	8.00E-41
722- 54:	transcript:KXG33521	transcript:Zm00001d043264_T001	1.00E-72
722- 55:	transcript:EES03727	transcript:Zm00001d043263_T006	0
722- 56:	transcript:EES01573	transcript:Zm00001d043262_T001	5.00E-26
722- 57:	transcript:EES03729	transcript:Zm00001d043261_T001	2.00E-136
722- 58:	transcript:EES01574	transcript:Zm00001d043259_T001	5.00E-125
722- 59:	transcript:KXG33526	transcript:Zm00001d043258_T001	1.00E-50
722- 60:	transcript:OQU87691	transcript:Zm00001d043256_T001	6.00E-174
722- 61:	transcript:KXG33529	transcript:Zm00001d043253_T002	0
722- 62:	transcript:KXG33530	transcript:Zm00001d043252_T003	0
722- 63:	transcript:KXG33531	transcript:Zm00001d043249_T001	5.00E-141
722- 64:	transcript:KXG33534	transcript:Zm00001d043248_T001	0
722- 65:	transcript:EES01578	transcript:Zm00001d043244_T001	0
722- 66:	transcript:EES03733	transcript:Zm00001d043243_T001	0
722- 67:	transcript:EES03734	transcript:Zm00001d043242_T001	3.00E-98
722- 68:	transcript:OQU87698	transcript:Zm00001d043240_T002	0
722- 69:	transcript:KXG33537	transcript:Zm00001d043239_T004	0
722- 70:	transcript:EES03738	transcript:Zm00001d043238_T001	0
722- 71:	transcript:EES01583	transcript:Zm00001d043235_T001	6.00E-175
722- 72:	transcript:OQU87702	transcript:Zm00001d043234_T001	4.00E-82
722- 73:	transcript:EES03739	transcript:Zm00001d043233_T001	0
722- 74:	transcript:EES01586	transcript:Zm00001d043232_T001	7.00E-79
722- 75:	transcript:EES01587	transcript:Zm00001d043231_T001	0
722- 76:	transcript:EES03740	transcript:Zm00001d043230_T001	0
722- 77:	transcript:EES03741	transcript:Zm00001d043229_T001	2.00E-159
722- 78:	transcript:OQU87703	transcript:Zm00001d043228_T001	0
722- 79:	transcript:EES01588	transcript:Zm00001d043227_T002	0
722- 80:	transcript:EES03743	transcript:Zm00001d043226_T001	0
722- 81:	transcript:EES01589	transcript:Zm00001d043225_T001	0
722- 82:	transcript:EES03744	transcript:Zm00001d043224_T001	2.00E-72
722- 83:	transcript:EES01590	transcript:Zm00001d043223_T001	7.00E-45
722- 84:	transcript:EES03746	transcript:Zm00001d043222_T001	0
722- 85:	transcript:EES03747	transcript:Zm00001d043220_T001	4.00E-27
722- 86:	transcript:EES01592	transcript:Zm00001d043218_T001	0
722- 87:	transcript:EES03750	transcript:Zm00001d043217_T002	2.00E-70
722- 88:	transcript:EES01595	transcript:Zm00001d043211_T001	0
722- 89:	transcript:EES01596	transcript:Zm00001d043209_T001	2.00E-12
722- 90:	transcript:KXG33547	transcript:Zm00001d043207_T004	3.00E-151
722- 91:	transcript:EES03752	transcript:Zm00001d043206_T002	2.00E-107
722- 92:	transcript:OQU87713	transcript:Zm00001d043204_T001	5.00E-26
722- 93:	transcript:EES03753	transcript:Zm00001d043202_T001	0
722- 94:	transcript:KXG33551	transcript:Zm00001d043201_T001	3.00E-122
722- 95:	transcript:OQU87715	transcript:Zm00001d043200_T001	0
722- 96:	transcript:EES01602	transcript:Zm00001d043199_T001	0
722- 97:	transcript:EES03756	transcript:Zm00001d043198_T001	0
722- 98:	transcript:EES01603	transcript:Zm00001d043197_T001	7.00E-151
722- 99:	transcript:KXG33555	transcript:Zm00001d043196_T001	0
722-100:	transcript:EES01606	transcript:Zm00001d043195_T001	0
722-101:	transcript:OQU87717	transcript:Zm00001d043193_T001	0
722-102:	transcript:OQU87718	transcript:Zm00001d043191_T005	0
722-103:	transcript:EES01612	transcript:Zm00001d043190_T001	4.00E-131
722-104:	transcript:KXG33560	transcript:Zm00001d043188_T001	0
722-105:	transcript:EES03760	transcript:Zm00001d043187_T001	0
722-106:	transcript:EES03763	transcript:Zm00001d043185_T001	0

```
722-107: transcript:KXG33564            transcript:Zm00001d043183_T001          0
722-108: transcript:KXG33565            transcript:Zm00001d043182_T001          0
722-109: transcript:KXG33566            transcript:Zm00001d043181_T001  1.00E-71
722-110: transcript:EES01618            transcript:Zm00001d043180_T001 4.00E-162
722-111: transcript:EES03766            transcript:Zm00001d043179_T001          0
722-112: transcript:KXG33567            transcript:Zm00001d043178_T001          0
722-113: transcript:KXG33568            transcript:Zm00001d043175_T003  2.00E-64
722-114: transcript:EES03767            transcript:Zm00001d043174_T001          0
722-115: transcript:EES03768            transcript:Zm00001d043171_T001          0
722-116: transcript:KXG33570            transcript:Zm00001d043170_T001  3.00E-77
722-117: transcript:EES03769            transcript:Zm00001d043168_T001 4.00E-121
722-118: transcript:KXG33572            transcript:Zm00001d043167_T001 1.00E-171
722-119: transcript:KXG33573            transcript:Zm00001d043165_T002 2.00E-133
722-120: transcript:EES03772            transcript:Zm00001d043164_T002          0
722-121: transcript:EES03773            transcript:Zm00001d043160_T004          0
722-122: transcript:EES01623            transcript:Zm00001d043158_T002          0
722-123: transcript:EES03774            transcript:Zm00001d043157_T002          0
722-124: transcript:EES03775            transcript:Zm00001d043156_T001          0
722-125: transcript:EES03776            transcript:Zm00001d043155_T001          0
722-126: transcript:OQU87725            transcript:Zm00001d043152_T002          0
722-127: transcript:EES03778            transcript:Zm00001d043153_T001          0
722-128: transcript:EES01628            transcript:Zm00001d043150_T002          0
722-129: transcript:OQU87727            transcript:Zm00001d043149_T002 4.00E-150
722-130: transcript:EES01629            transcript:Zm00001d043147_T002          0
722-131: transcript:EES03780            transcript:Zm00001d043146_T002          0
722-132: transcript:OQU87729            transcript:Zm00001d043145_T001 3.00E-157
722-133: transcript:EES01631            transcript:Zm00001d043144_T001 2.00E-109
722-134: transcript:EES01630            transcript:Zm00001d043137_T004 3.00E-139
722-135: transcript:EES01635            transcript:Zm00001d043135_T003          0
722-136: transcript:EES03781            transcript:Zm00001d043134_T004          0
722-137: transcript:KXG33583            transcript:Zm00001d043131_T002          0
722-138: transcript:OQU87732            transcript:Zm00001d043128_T001 3.00E-127
722-139: transcript:EES01639            transcript:Zm00001d043125_T001          0
722-140: transcript:EES01640            transcript:Zm00001d043121_T001 9.00E-138
722-141: transcript:KXG33587            transcript:Zm00001d043119_T001          0
722-142: transcript:EES03784            transcript:Zm00001d043118_T002  1.00E-73
722-143: transcript:EES03785            transcript:Zm00001d043117_T002          0
722-144: transcript:KXG33588            transcript:Zm00001d043116_T002          0
722-145: transcript:OQU87737            transcript:Zm00001d043113_T001 5.00E-132
722-146: transcript:KXG33589            transcript:Zm00001d043112_T002          0
722-147: transcript:KXG33592            transcript:Zm00001d043110_T012          0
722-148: transcript:EES01643            transcript:Zm00001d043109_T004          0
722-149: transcript:EES03787            transcript:Zm00001d043108_T001          0
722-150: transcript:OQU87745            transcript:Zm00001d043107_T001          0
722-151: transcript:EES03789            transcript:Zm00001d043104_T001 4.00E-167
722-152: transcript:EES03790            transcript:Zm00001d043102_T001  2.00E-79
722-153: transcript:EES03791            transcript:Zm00001d043101_T001  1.00E-57
722-154: transcript:EES03792            transcript:Zm00001d043098_T002          0
722-155: transcript:EES03793            transcript:Zm00001d043097_T001 2.00E-140
722-156: transcript:EES03794            transcript:Zm00001d043096_T001  2.00E-35
722-157: transcript:EES01648            transcript:Zm00001d043095_T004          0
722-158: transcript:OQU87747            transcript:Zm00001d043094_T001          0
## Alignment 723: score=5438.0 e_value=0 N=119 3&3 minus
723-  0: transcript:OQU88089            transcript:Zm00001d042398_T001          0
```

723- 1:	transcript:KXG33965	transcript:Zm00001d042397_T001	0
723- 2:	transcript:KXG33967	transcript:Zm00001d042396_T003	0
723- 3:	transcript:EES04087	transcript:Zm00001d042395_T001	3.00E-78
723- 4:	transcript:EES01942	transcript:Zm00001d042394_T001	0
723- 5:	transcript:OQU88090	transcript:Zm00001d042393_T002	0
723- 6:	transcript:EES04089	transcript:Zm00001d042389_T001	0
723- 7:	transcript:OQU88093	transcript:Zm00001d042388_T001	7.00E-89
723- 8:	transcript:KXG33973	transcript:Zm00001d042386_T001	4.00E-69
723- 9:	transcript:EES04091	transcript:Zm00001d042383_T001	1.00E-19
723- 10:	transcript:EES01946	transcript:Zm00001d042382_T001	6.00E-44
723- 11:	transcript:OQU88098	transcript:Zm00001d042381_T001	2.00E-53
723- 12:	transcript:KXG33974	transcript:Zm00001d042380_T001	0
723- 13:	transcript:OQU88099	transcript:Zm00001d042378_T001	0
723- 14:	transcript:EES04096	transcript:Zm00001d042376_T001	4.00E-158
723- 15:	transcript:EES04098	transcript:Zm00001d042373_T002	0
723- 16:	transcript:EES04100	transcript:Zm00001d042372_T002	7.00E-108
723- 17:	transcript:EES04101	transcript:Zm00001d042371_T001	3.00E-41
723- 18:	transcript:EES04102	transcript:Zm00001d042370_T003	0
723- 19:	transcript:EES01953	transcript:Zm00001d042367_T002	0
723- 20:	transcript:EES01956	transcript:Zm00001d042366_T002	0
723- 21:	transcript:KXG33982	transcript:Zm00001d042365_T001	1.00E-62
723- 22:	transcript:KXG33985	transcript:Zm00001d042363_T007	0
723- 23:	transcript:EES01957	transcript:Zm00001d042362_T001	0
723- 24:	transcript:EES04106	transcript:Zm00001d042361_T002	8.00E-61
723- 25:	transcript:EES04107	transcript:Zm00001d042359_T001	6.00E-109
723- 26:	transcript:EES04108	transcript:Zm00001d042357_T002	5.00E-178
723- 27:	transcript:KXG33989	transcript:Zm00001d042355_T001	0
723- 28:	transcript:EES04111	transcript:Zm00001d042353_T003	0
723- 29:	transcript:EES01962	transcript:Zm00001d042350_T002	3.00E-124
723- 30:	transcript:EES01963	transcript:Zm00001d042349_T001	0
723- 31:	transcript:OQU88110	transcript:Zm00001d042346_T001	8.00E-119
723- 32:	transcript:OQU88108	transcript:Zm00001d042345_T001	9.00E-160
723- 33:	transcript:EES04113	transcript:Zm00001d042344_T002	0
723- 34:	transcript:EES04112	transcript:Zm00001d042343_T001	0
723- 35:	transcript:EES01966	transcript:Zm00001d042341_T001	7.00E-127
723- 36:	transcript:KXG34000	transcript:Zm00001d042340_T001	3.00E-62
723- 37:	transcript:EES04116	transcript:Zm00001d042338_T002	0
723- 38:	transcript:EES01968	transcript:Zm00001d042336_T001	3.00E-95
723- 39:	transcript:OQU88120	transcript:Zm00001d042335_T006	0
723- 40:	transcript:EES01975	transcript:Zm00001d042333_T001	0
723- 41:	transcript:EES01977	transcript:Zm00001d042329_T001	3.00E-137
723- 42:	transcript:KXG34006	transcript:Zm00001d042328_T001	0
723- 43:	transcript:EES04120	transcript:Zm00001d042325_T001	2.00E-152
723- 44:	transcript:EES01979	transcript:Zm00001d042319_T001	0
723- 45:	transcript:KXG34011	transcript:Zm00001d042317_T001	6.00E-23
723- 46:	transcript:EES01980	transcript:Zm00001d042315_T001	6.00E-36
723- 47:	transcript:EES01984	transcript:Zm00001d042314_T004	0
723- 48:	transcript:EES01985	transcript:Zm00001d042313_T004	0
723- 49:	transcript:KXG34012	transcript:Zm00001d042312_T012	0
723- 50:	transcript:EES01986	transcript:Zm00001d042310_T001	4.00E-47
723- 51:	transcript:EES04122	transcript:Zm00001d042309_T001	0
723- 52:	transcript:EES01990	transcript:Zm00001d042308_T001	9.00E-150
723- 53:	transcript:EES01991	transcript:Zm00001d042307_T001	9.00E-79
723- 54:	transcript:EES04123	transcript:Zm00001d042306_T004	0

723- 55:	transcript:EES01992	transcript:Zm00001d042305_T001	2.00E-134
723- 56:	transcript:EES01994	transcript:Zm00001d042303_T001	0
723- 57:	transcript:EES01993	transcript:Zm00001d042302_T002	0
723- 58:	transcript:EES04127	transcript:Zm00001d042292_T001	2.00E-70
723- 59:	transcript:EES04124	transcript:Zm00001d042291_T002	0
723- 60:	transcript:KXG34023	transcript:Zm00001d042290_T001	0
723- 61:	transcript:OQU88137	transcript:Zm00001d042289_T001	1.00E-167
723- 62:	transcript:EES01998	transcript:Zm00001d042288_T002	7.00E-105
723- 63:	transcript:KXG34025	transcript:Zm00001d042287_T001	0
723- 64:	transcript:EES01999	transcript:Zm00001d042286_T001	2.00E-87
723- 65:	transcript:EES04133	transcript:Zm00001d042285_T001	0
723- 66:	transcript:EES02001	transcript:Zm00001d042284_T003	8.00E-152
723- 67:	transcript:KXG34027	transcript:Zm00001d042282_T003	0
723- 68:	transcript:OQU88139	transcript:Zm00001d042279_T001	0
723- 69:	transcript:EES02002	transcript:Zm00001d042274_T001	3.00E-141
723- 70:	transcript:OQU88147	transcript:Zm00001d042272_T003	0
723- 71:	transcript:OQU88149	transcript:Zm00001d042271_T001	1.00E-162
723- 72:	transcript:EES02003	transcript:Zm00001d042270_T005	5.00E-76
723- 73:	transcript:KXG34037	transcript:Zm00001d042269_T001	0
723- 74:	transcript:EES02007	transcript:Zm00001d042268_T001	0
723- 75:	transcript:EES02008	transcript:Zm00001d042267_T003	0
723- 76:	transcript:KXG34040	transcript:Zm00001d042266_T001	0
723- 77:	transcript:OQU88150	transcript:Zm00001d042264_T006	0
723- 78:	transcript:KXG34042	transcript:Zm00001d042263_T001	0
723- 79:	transcript:EES04143	transcript:Zm00001d042262_T003	1.00E-73
723- 80:	transcript:KXG34044	transcript:Zm00001d042259_T002	0
723- 81:	transcript:OQU88151	transcript:Zm00001d042258_T001	3.00E-83
723- 82:	transcript:OQU88153	transcript:Zm00001d042257_T001	2.00E-135
723- 83:	transcript:KXG34046	transcript:Zm00001d042256_T010	0
723- 84:	transcript:OQU88154	transcript:Zm00001d042250_T001	0
723- 85:	transcript:EES02012	transcript:Zm00001d042245_T001	4.00E-48
723- 86:	transcript:EES02013	transcript:Zm00001d042244_T001	0
723- 87:	transcript:EES04150	transcript:Zm00001d042243_T001	3.00E-70
723- 88:	transcript:EES04154	transcript:Zm00001d042241_T001	2.00E-178
723- 89:	transcript:EES04155	transcript:Zm00001d042237_T001	2.00E-45
723- 90:	transcript:EES02018	transcript:Zm00001d042234_T004	0
723- 91:	transcript:KXG34059	transcript:Zm00001d042226_T001	2.00E-11
723- 92:	transcript:KXG34066	transcript:Zm00001d042225_T002	8.00E-139
723- 93:	transcript:EES04162	transcript:Zm00001d042217_T001	5.00E-73
723- 94:	transcript:KXG34068	transcript:Zm00001d042216_T001	6.00E-135
723- 95:	transcript:EES04163	transcript:Zm00001d042215_T002	0
723- 96:	transcript:EES04164	transcript:Zm00001d042214_T001	7.00E-124
723- 97:	transcript:KXG34069	transcript:Zm00001d042213_T001	0
723- 98:	transcript:OQU88162	transcript:Zm00001d042212_T007	0
723- 99:	transcript:EES02021	transcript:Zm00001d042211_T001	4.00E-164
723-100:	transcript:KXG34073	transcript:Zm00001d042207_T001	3.00E-73
723-101:	transcript:KXG34074	transcript:Zm00001d042204_T001	2.00E-45
723-102:	transcript:EES04172	transcript:Zm00001d042202_T001	0
723-103:	transcript:KXG34077	transcript:Zm00001d042200_T001	5.00E-70
723-104:	transcript:OQU88166	transcript:Zm00001d042196_T001	2.00E-41
723-105:	transcript:EES04173	transcript:Zm00001d042193_T001	0
723-106:	transcript:EES04177	transcript:Zm00001d042192_T001	0
723-107:	transcript:EES02024	transcript:Zm00001d042189_T004	0
723-108:	transcript:EES02025	transcript:Zm00001d042187_T001	1.00E-70

```
723-109: transcript:KXG34080          transcript:Zm00001d042184_T001 1.00E-154
723-110: transcript:EES04181          transcript:Zm00001d042183_T001         0
723-111: transcript:OQU88170          transcript:Zm00001d042182_T001 2.00E-116
723-112: transcript:EES04183          transcript:Zm00001d042180_T003         0
723-113: transcript:EES04184          transcript:Zm00001d042179_T001  2.00E-77
723-114: transcript:EES02031          transcript:Zm00001d042172_T001  2.00E-73
723-115: transcript:KXG34084          transcript:Zm00001d042169_T001  7.00E-45
723-116: transcript:EES04190          transcript:Zm00001d042168_T001         0
723-117: transcript:OQU88174          transcript:Zm00001d042164_T002         0
723-118: transcript:KXG34089          transcript:Zm00001d042158_T011  2.00E-51
## Alignment 724: score=4691.0 e_value=0 N=102 3&3 minus
724-  0: transcript:KXG33766          transcript:Zm00001d042712_T001         0
724-  1: transcript:EES03952          transcript:Zm00001d042709_T001         0
724-  2: transcript:KXG33767          transcript:Zm00001d042706_T002         0
724-  3: transcript:EES01823          transcript:Zm00001d042699_T001         0
724-  4: transcript:EES01824          transcript:Zm00001d042697_T002         0
724-  5: transcript:KXG33770          transcript:Zm00001d042695_T002         0
724-  6: transcript:KXG33773          transcript:Zm00001d042694_T002         0
724-  7: transcript:OQU87940          transcript:Zm00001d042691_T002 4.00E-125
724-  8: transcript:EES01827          transcript:Zm00001d042689_T002  7.00E-56
724-  9: transcript:KXG33778          transcript:Zm00001d042686_T001         0
724- 10: transcript:KXG33777          transcript:Zm00001d042685_T001         0
724- 11: transcript:EES01830          transcript:Zm00001d042683_T001 1.00E-169
724- 12: transcript:OQU87951          transcript:Zm00001d042680_T001 1.00E-138
724- 13: transcript:EES03961          transcript:Zm00001d042676_T001         0
724- 14: transcript:OQU87957          transcript:Zm00001d042673_T001         0
724- 15: transcript:EES01838          transcript:Zm00001d042672_T001  8.00E-70
724- 16: transcript:EES03964          transcript:Zm00001d042670_T025         0
724- 17: transcript:EES03963          transcript:Zm00001d042669_T002         0
724- 18: transcript:EES03967          transcript:Zm00001d042667_T001         0
724- 19: transcript:EES01839          transcript:Zm00001d042665_T001 4.00E-162
724- 20: transcript:KXG33790          transcript:Zm00001d042664_T001         0
724- 21: transcript:OQU87964          transcript:Zm00001d042663_T001         0
724- 22: transcript:EES01840          transcript:Zm00001d042662_T002         0
724- 23: transcript:EES01843          transcript:Zm00001d042661_T001         0
724- 24: transcript:KXG33794          transcript:Zm00001d042660_T001 7.00E-147
724- 25: transcript:EES01844          transcript:Zm00001d042658_T002 1.00E-135
724- 26: transcript:EES03970          transcript:Zm00001d042657_T001  2.00E-47
724- 27: transcript:EES01845          transcript:Zm00001d042654_T005         0
724- 28: transcript:EES01846          transcript:Zm00001d042653_T001         0
724- 29: transcript:EES03971          transcript:Zm00001d042652_T001         0
724- 30: transcript:EES01847          transcript:Zm00001d042651_T001         0
724- 31: transcript:OQU87972          transcript:Zm00001d042646_T001         0
724- 32: transcript:EES03974          transcript:Zm00001d042643_T001         0
724- 33: transcript:OQU87973          transcript:Zm00001d042642_T002  1.00E-86
724- 34: transcript:EES01849          transcript:Zm00001d042641_T013 3.00E-179
724- 35: transcript:OQU87974          transcript:Zm00001d042640_T001 1.00E-120
724- 36: transcript:KXG33802          transcript:Zm00001d042639_T004         0
724- 37: transcript:EES03975          transcript:Zm00001d042638_T002  4.00E-83
724- 38: transcript:EES01852          transcript:Zm00001d042637_T002         0
724- 39: transcript:OQU87977          transcript:Zm00001d042636_T002         0
724- 40: transcript:EES03978          transcript:Zm00001d042634_T001         0
724- 41: transcript:EES01853          transcript:Zm00001d042633_T001 1.00E-122
724- 42: transcript:EES01854          transcript:Zm00001d042632_T002         0
```

724- 43:	transcript:OQU87982	transcript:Zm00001d042631_T001	4.00E-91
724- 44:	transcript:EES01856	transcript:Zm00001d042627_T005	0
724- 45:	transcript:OQU87984	transcript:Zm00001d042626_T001	0
724- 46:	transcript:KXG33812	transcript:Zm00001d042624_T001	2.00E-160
724- 47:	transcript:OQU87990	transcript:Zm00001d042621_T005	0
724- 48:	transcript:OQU87991	transcript:Zm00001d042620_T001	1.00E-54
724- 49:	transcript:EES03989	transcript:Zm00001d042619_T002	0
724- 50:	transcript:EES01864	transcript:Zm00001d042618_T001	8.00E-147
724- 51:	transcript:EES01868	transcript:Zm00001d042617_T001	3.00E-77
724- 52:	transcript:EES01869	transcript:Zm00001d042615_T003	0
724- 53:	transcript:EES01867	transcript:Zm00001d042613_T001	9.00E-85
724- 54:	transcript:OQU87995	transcript:Zm00001d042611_T001	0
724- 55:	transcript:EES03991	transcript:Zm00001d042610_T001	2.00E-53
724- 56:	transcript:KXG33829	transcript:Zm00001d042609_T001	0
724- 57:	transcript:EES01872	transcript:Zm00001d042608_T002	0
724- 58:	transcript:KXG33831	transcript:Zm00001d042605_T001	0
724- 59:	transcript:KXG33833	transcript:Zm00001d042603_T001	8.00E-135
724- 60:	transcript:OQU88000	transcript:Zm00001d042602_T001	0
724- 61:	transcript:EES03995	transcript:Zm00001d042601_T001	0
724- 62:	transcript:EES03996	transcript:Zm00001d042600_T001	1.00E-78
724- 63:	transcript:EES03997	transcript:Zm00001d042599_T001	0
724- 64:	transcript:KXG33835	transcript:Zm00001d042598_T003	0
724- 65:	transcript:EES01874	transcript:Zm00001d042596_T001	0
724- 66:	transcript:KXG33837	transcript:Zm00001d042593_T001	1.00E-62
724- 67:	transcript:EES01878	transcript:Zm00001d042591_T002	2.00E-165
724- 68:	transcript:EES01879	transcript:Zm00001d042590_T001	0
724- 69:	transcript:OQU88003	transcript:Zm00001d042589_T001	1.00E-54
724- 70:	transcript:KXG33839	transcript:Zm00001d042588_T001	1.00E-133
724- 71:	transcript:KXG33843	transcript:Zm00001d042585_T004	0
724- 72:	transcript:EES01881	transcript:Zm00001d042584_T001	2.00E-179
724- 73:	transcript:EES04005	transcript:Zm00001d042583_T001	1.00E-145
724- 74:	transcript:KXG33846	transcript:Zm00001d042582_T001	0
724- 75:	transcript:EES04006	transcript:Zm00001d042581_T001	0
724- 76:	transcript:EES01882	transcript:Zm00001d042580_T001	0
724- 77:	transcript:KXG33848	transcript:Zm00001d042578_T001	0
724- 78:	transcript:KXG33850	transcript:Zm00001d042575_T002	0
724- 79:	transcript:KXG33851	transcript:Zm00001d042574_T001	2.00E-18
724- 80:	transcript:OQU88007	transcript:Zm00001d042573_T001	2.00E-22
724- 81:	transcript:OQU88008	transcript:Zm00001d042572_T001	1.00E-49
724- 82:	transcript:EES01884	transcript:Zm00001d042569_T001	0
724- 83:	transcript:OQU88009	transcript:Zm00001d042568_T001	9.00E-142
724- 84:	transcript:KXG33854	transcript:Zm00001d042567_T005	7.00E-131
724- 85:	transcript:OQU88012	transcript:Zm00001d042560_T001	0
724- 86:	transcript:KXG33862	transcript:Zm00001d042558_T002	0
724- 87:	transcript:EES01886	transcript:Zm00001d042555_T001	0
724- 88:	transcript:KXG33870	transcript:Zm00001d042553_T001	1.00E-150
724- 89:	transcript:KXG33869	transcript:Zm00001d042551_T009	0
724- 90:	transcript:EES01891	transcript:Zm00001d042549_T001	0
724- 91:	transcript:OQU88024	transcript:Zm00001d042543_T002	0
724- 92:	transcript:EES04025	transcript:Zm00001d042540_T008	0
724- 93:	transcript:EES04029	transcript:Zm00001d042536_T001	0
724- 94:	transcript:EES01893	transcript:Zm00001d042535_T001	7.00E-83
724- 95:	transcript:KXG33883	transcript:Zm00001d042534_T002	1.00E-102
724- 96:	transcript:KXG33884	transcript:Zm00001d042533_T002	3.00E-121

```
724- 97: transcript:EES01896            transcript:Zm00001d042530_T003          0
724- 98: transcript:KXG33892            transcript:Zm00001d042528_T007          0
724- 99: transcript:EES04034            transcript:Zm00001d042527_T001  3.00E-94
724-100: transcript:EES04035            transcript:Zm00001d042526_T001          0
724-101: transcript:KXG33893            transcript:Zm00001d042525_T003          0
## Alignment 725: score=4216.0 e_value=0 N=93 3&3 minus
725-  0: transcript:EES01272            transcript:Zm00001d043843_T001          0
725-  1: transcript:KXG33134            transcript:Zm00001d043842_T002          0
725-  2: transcript:KXG33136            transcript:Zm00001d043841_T003 1.00E-173
725-  3: transcript:EES01276            transcript:Zm00001d043840_T001          0
725-  4: transcript:EES01278            transcript:Zm00001d043839_T001 1.00E-151
725-  5: transcript:EES03448            transcript:Zm00001d043838_T002          0
725-  6: transcript:OQU87345            transcript:Zm00001d043837_T001 1.00E-164
725-  7: transcript:KXG33145            transcript:Zm00001d043835_T006          0
725-  8: transcript:KXG33146            transcript:Zm00001d043834_T001          0
725-  9: transcript:EES01283            transcript:Zm00001d043831_T001          0
725- 10: transcript:KXG33148            transcript:Zm00001d043830_T001  5.00E-90
725- 11: transcript:EES01285            transcript:Zm00001d043827_T001          0
725- 12: transcript:EES01287            transcript:Zm00001d043815_T005          0
725- 13: transcript:EES03453            transcript:Zm00001d043814_T002 1.00E-160
725- 14: transcript:KXG33152            transcript:Zm00001d043813_T004          0
725- 15: transcript:KXG33157            transcript:Zm00001d043812_T001          0
725- 16: transcript:OQU87355            transcript:Zm00001d043809_T014 1.00E-144
725- 17: transcript:EES03457            transcript:Zm00001d043808_T001  2.00E-91
725- 18: transcript:EES01291            transcript:Zm00001d043806_T001  3.00E-69
725- 19: transcript:EES03455            transcript:Zm00001d043805_T012          0
725- 20: transcript:EES03458            transcript:Zm00001d043803_T003  2.00E-58
725- 21: transcript:EES01292            transcript:Zm00001d043801_T002          0
725- 22: transcript:OQU87357            transcript:Zm00001d043800_T003          0
725- 23: transcript:EES01296            transcript:Zm00001d043799_T001 7.00E-114
725- 24: transcript:OQU87362            transcript:Zm00001d043798_T001  3.00E-97
725- 25: transcript:EES01302            transcript:Zm00001d043797_T020          0
725- 26: transcript:KXG33169            transcript:Zm00001d043796_T001  1.00E-20
725- 27: transcript:EES03468            transcript:Zm00001d043789_T010 4.00E-108
725- 28: transcript:EES01304            transcript:Zm00001d043786_T001          0
725- 29: transcript:EES01305            transcript:Zm00001d043785_T002          0
725- 30: transcript:OQU87370            transcript:Zm00001d043784_T002 6.00E-169
725- 31: transcript:EES03472            transcript:Zm00001d043782_T001          0
725- 32: transcript:OQU87373            transcript:Zm00001d043781_T001          0
725- 33: transcript:OQU87374            transcript:Zm00001d043780_T001          0
725- 34: transcript:EES03474            transcript:Zm00001d043779_T001 2.00E-161
725- 35: transcript:KXG33173            transcript:Zm00001d043778_T001          0
725- 36: transcript:EES01311            transcript:Zm00001d043776_T001          0
725- 37: transcript:OQU87377            transcript:Zm00001d043775_T006          0
725- 38: transcript:EES01312            transcript:Zm00001d043773_T003          0
725- 39: transcript:EES01313            transcript:Zm00001d043770_T001  2.00E-37
725- 40: transcript:KXG33181            transcript:Zm00001d043768_T001          0
725- 41: transcript:KXG33185            transcript:Zm00001d043767_T002 6.00E-171
725- 42: transcript:EES01319            transcript:Zm00001d043766_T005          0
725- 43: transcript:EES03480            transcript:Zm00001d043765_T001          0
725- 44: transcript:EES03481            transcript:Zm00001d043757_T001          0
725- 45: transcript:EES03482            transcript:Zm00001d043752_T001          0
725- 46: transcript:EES03483            transcript:Zm00001d043751_T001  2.00E-74
725- 47: transcript:EES01323            transcript:Zm00001d043745_T001  1.00E-78
```

725- 48: transcript:EES01324 transcript:Zm00001d043738_T001 1.00E-112
725- 49: transcript:EES01328 transcript:Zm00001d043737_T001 8.00E-103
725- 50: transcript:EES01330 transcript:Zm00001d043736_T002 8.00E-144
725- 51: transcript:EES01331 transcript:Zm00001d043735_T001 1.00E-151
725- 52: transcript:EES03488 transcript:Zm00001d043734_T001 0
725- 53: transcript:KXG33200 transcript:Zm00001d043733_T002 0
725- 54: transcript:KXG33202 transcript:Zm00001d043731_T001 0
725- 55: transcript:KXG33203 transcript:Zm00001d043730_T001 9.00E-65
725- 56: transcript:EES03490 transcript:Zm00001d043729_T001 1.00E-95
725- 57: transcript:OQU87393 transcript:Zm00001d043728_T001 3.00E-146
725- 58: transcript:EES01337 transcript:Zm00001d043727_T001 0
725- 59: transcript:EES01338 transcript:Zm00001d043726_T001 0
725- 60: transcript:EES03491 transcript:Zm00001d043725_T001 1.00E-107
725- 61: transcript:KXG33207 transcript:Zm00001d043724_T001 0
725- 62: transcript:OQU87398 transcript:Zm00001d043723_T001 1.00E-118
725- 63: transcript:EES03493 transcript:Zm00001d043722_T001 0
725- 64: transcript:KXG33208 transcript:Zm00001d043721_T001 0
725- 65: transcript:KXG33209 transcript:Zm00001d043713_T001 1.00E-37
725- 66: transcript:EES01342 transcript:Zm00001d043710_T001 2.00E-130
725- 67: transcript:KXG33211 transcript:Zm00001d043709_T003 3.00E-102
725- 68: transcript:OQU87399 transcript:Zm00001d043708_T001 3.00E-46
725- 69: transcript:KXG33213 transcript:Zm00001d043707_T002 1.00E-117
725- 70: transcript:EES01345 transcript:Zm00001d043706_T001 0
725- 71: transcript:EES01343 transcript:Zm00001d043705_T005 1.00E-49
725- 72: transcript:KXG33218 transcript:Zm00001d043704_T001 4.00E-47
725- 73: transcript:EES03497 transcript:Zm00001d043703_T007 0
725- 74: transcript:EES01349 transcript:Zm00001d043702_T001 2.00E-20
725- 75: transcript:EES03499 transcript:Zm00001d043701_T001 0
725- 76: transcript:EES01350 transcript:Zm00001d043700_T006 7.00E-119
725- 77: transcript:KXG33221 transcript:Zm00001d043699_T001 2.00E-178
725- 78: transcript:EES01351 transcript:Zm00001d043696_T004 2.00E-165
725- 79: transcript:EES01352 transcript:Zm00001d043695_T001 0
725- 80: transcript:EES01353 transcript:Zm00001d043694_T001 0
725- 81: transcript:EES01354 transcript:Zm00001d043693_T002 0
725- 82: transcript:OQU87402 transcript:Zm00001d043692_T001 2.00E-148
725- 83: transcript:EES03502 transcript:Zm00001d043691_T001 0
725- 84: transcript:EES03506 transcript:Zm00001d043687_T001 2.00E-47
725- 85: transcript:EES01356 transcript:Zm00001d043686_T009 0
725- 86: transcript:KXG33225 transcript:Zm00001d043684_T001 0
725- 87: transcript:EES01357 transcript:Zm00001d043682_T002 0
725- 88: transcript:EES03507 transcript:Zm00001d043681_T001 0
725- 89: transcript:OQU87408 transcript:Zm00001d043680_T001 0
725- 90: transcript:EES01361 transcript:Zm00001d043675_T001 2.00E-169
725- 91: transcript:EES03512 transcript:Zm00001d043674_T001 2.00E-168
725- 92: transcript:EES03516 transcript:Zm00001d043669_T004 0
Alignment 726: score=3686.0 e_value=0 N=81 3&3 minus
726- 0: transcript:EES01094 transcript:Zm00001d044185_T004 0
726- 1: transcript:EES03303 transcript:Zm00001d044184_T001 0
726- 2: transcript:EES01095 transcript:Zm00001d044182_T001 0
726- 3: transcript:EES03305 transcript:Zm00001d044181_T002 0
726- 4: transcript:EES01097 transcript:Zm00001d044179_T001 1.00E-106
726- 5: transcript:EES03307 transcript:Zm00001d044177_T001 2.00E-112
726- 6: transcript:EES01098 transcript:Zm00001d044176_T001 2.00E-91
726- 7: transcript:KXG32926 transcript:Zm00001d044175_T002 0

726- 8:	transcript:EES01100	transcript:Zm00001d044173_T002	2.00E-114
726- 9:	transcript:EES01101	transcript:Zm00001d044172_T002	0
726- 10:	transcript:EES03310	transcript:Zm00001d044171_T001	3.00E-154
726- 11:	transcript:EES01102	transcript:Zm00001d044170_T001	0
726- 12:	transcript:EES01103	transcript:Zm00001d044168_T001	3.00E-146
726- 13:	transcript:EES03313	transcript:Zm00001d044167_T001	4.00E-122
726- 14:	transcript:EES01104	transcript:Zm00001d044164_T002	0
726- 15:	transcript:EES01107	transcript:Zm00001d044162_T001	0
726- 16:	transcript:KXG32930	transcript:Zm00001d044157_T001	0
726- 17:	transcript:EES03321	transcript:Zm00001d044154_T001	0
726- 18:	transcript:EES01118	transcript:Zm00001d044146_T001	0
726- 19:	transcript:EES01122	transcript:Zm00001d044144_T008	0
726- 20:	transcript:OQU87197	transcript:Zm00001d044139_T001	0
726- 21:	transcript:KXG32942	transcript:Zm00001d044138_T001	0
726- 22:	transcript:EES01127	transcript:Zm00001d044136_T001	0
726- 23:	transcript:KXG32944	transcript:Zm00001d044132_T001	0
726- 24:	transcript:KXG32945	transcript:Zm00001d044131_T001	2.00E-73
726- 25:	transcript:EES03324	transcript:Zm00001d044130_T002	1.00E-91
726- 26:	transcript:OQU87201	transcript:Zm00001d044129_T001	0
726- 27:	transcript:EES01133	transcript:Zm00001d044127_T003	0
726- 28:	transcript:KXG32947	transcript:Zm00001d044124_T001	0
726- 29:	transcript:EES01135	transcript:Zm00001d044122_T001	0
726- 30:	transcript:OQU87203	transcript:Zm00001d044121_T002	0
726- 31:	transcript:KXG32950	transcript:Zm00001d044120_T001	0
726- 32:	transcript:EES01138	transcript:Zm00001d044119_T001	8.00E-138
726- 33:	transcript:KXG32953	transcript:Zm00001d044117_T001	4.00E-22
726- 34:	transcript:EES01141	transcript:Zm00001d044116_T001	0
726- 35:	transcript:KXG32956	transcript:Zm00001d044112_T001	4.00E-43
726- 36:	transcript:EES03331	transcript:Zm00001d044111_T001	0
726- 37:	transcript:EES01142	transcript:Zm00001d044110_T001	0
726- 38:	transcript:EES01143	transcript:Zm00001d044107_T001	3.00E-126
726- 39:	transcript:EES01144	transcript:Zm00001d044106_T001	0
726- 40:	transcript:EES01147	transcript:Zm00001d044105_T001	7.00E-72
726- 41:	transcript:OQU87208	transcript:Zm00001d044104_T001	7.00E-155
726- 42:	transcript:EES01149	transcript:Zm00001d044103_T004	2.00E-169
726- 43:	transcript:EES03335	transcript:Zm00001d044102_T002	0
726- 44:	transcript:EES01150	transcript:Zm00001d044101_T003	0
726- 45:	transcript:EES01152	transcript:Zm00001d044100_T001	2.00E-88
726- 46:	transcript:KXG32970	transcript:Zm00001d044099_T002	0
726- 47:	transcript:OQU87217	transcript:Zm00001d044094_T001	0
726- 48:	transcript:EES03337	transcript:Zm00001d044093_T001	2.00E-118
726- 49:	transcript:EES03338	transcript:Zm00001d044092_T001	5.00E-34
726- 50:	transcript:EES01155	transcript:Zm00001d044091_T003	0
726- 51:	transcript:EES01156	transcript:Zm00001d044090_T001	6.00E-174
726- 52:	transcript:EES03339	transcript:Zm00001d044089_T002	0
726- 53:	transcript:EES01158	transcript:Zm00001d044088_T001	0
726- 54:	transcript:EES03342	transcript:Zm00001d044086_T001	5.00E-138
726- 55:	transcript:EES01159	transcript:Zm00001d044083_T001	0
726- 56:	transcript:EES03343	transcript:Zm00001d044081_T001	1.00E-127
726- 57:	transcript:OQU87227	transcript:Zm00001d044080_T001	2.00E-79
726- 58:	transcript:EES03347	transcript:Zm00001d044079_T001	5.00E-57
726- 59:	transcript:OQU87230	transcript:Zm00001d044078_T001	1.00E-45
726- 60:	transcript:EES01162	transcript:Zm00001d044076_T001	1.00E-20
726- 61:	transcript:OQU87231	transcript:Zm00001d044074_T001	4.00E-170

```
726- 62: transcript:OQU87233           transcript:Zm00001d044069_T001          0
726- 63: transcript:KXG32993           transcript:Zm00001d044068_T001          0
726- 64: transcript:OQU87235           transcript:Zm00001d044065_T001 2.00E-166
726- 65: transcript:EES01166           transcript:Zm00001d044061_T001          0
726- 66: transcript:EES03353           transcript:Zm00001d044060_T001          0
726- 67: transcript:EES01168           transcript:Zm00001d044059_T001          0
726- 68: transcript:EES03354           transcript:Zm00001d044056_T003          0
726- 69: transcript:KXG32997           transcript:Zm00001d044054_T001 1.00E-163
726- 70: transcript:KXG33002           transcript:Zm00001d044042_T001          0
726- 71: transcript:EES01172           transcript:Zm00001d044040_T003  5.00E-99
726- 72: transcript:OQU87244           transcript:Zm00001d044039_T001 9.00E-165
726- 73: transcript:KXG33005           transcript:Zm00001d044038_T001          0
726- 74: transcript:OQU87247           transcript:Zm00001d044037_T001          0
726- 75: transcript:EES01184           transcript:Zm00001d044036_T001 1.00E-101
726- 76: transcript:EES01185           transcript:Zm00001d044035_T001          0
726- 77: transcript:EES01186           transcript:Zm00001d044034_T001  2.00E-80
726- 78: transcript:EES03360           transcript:Zm00001d044031_T001 2.00E-149
726- 79: transcript:EES01188           transcript:Zm00001d044030_T001          0
726- 80: transcript:KXG33008           transcript:Zm00001d044027_T001  2.00E-40
## Alignment 727: score=3144.0 e_value=6.5e-290 N=69 3&3 minus
727-  0: transcript:EES02060           transcript:Zm00001d042082_T001 1.00E-107
727-  1: transcript:KXG34126           transcript:Zm00001d042078_T001  2.00E-74
727-  2: transcript:KXG34127           transcript:Zm00001d042074_T001          0
727-  3: transcript:KXG34128           transcript:Zm00001d042066_T016          0
727-  4: transcript:EES02065           transcript:Zm00001d042064_T010          0
727-  5: transcript:OQU88207           transcript:Zm00001d042063_T001 9.00E-113
727-  6: transcript:KXG34132           transcript:Zm00001d042062_T001 7.00E-116
727-  7: transcript:KXG34135           transcript:Zm00001d042061_T001 5.00E-130
727-  8: transcript:OQU88209           transcript:Zm00001d042060_T001  5.00E-77
727-  9: transcript:EES02070           transcript:Zm00001d042059_T001  4.00E-21
727- 10: transcript:EES04236           transcript:Zm00001d042057_T001          0
727- 11: transcript:EES04237           transcript:Zm00001d042056_T001  2.00E-65
727- 12: transcript:EES02075           transcript:Zm00001d042055_T001 3.00E-135
727- 13: transcript:EES04241           transcript:Zm00001d042054_T001 2.00E-152
727- 14: transcript:KXG34152           transcript:Zm00001d042051_T001          0
727- 15: transcript:EES04251           transcript:Zm00001d042045_T001          0
727- 16: transcript:EES04254           transcript:Zm00001d042044_T001          0
727- 17: transcript:EES02081           transcript:Zm00001d042043_T001          0
727- 18: transcript:EES02078           transcript:Zm00001d042042_T001 3.00E-134
727- 19: transcript:EES02086           transcript:Zm00001d042040_T001 5.00E-111
727- 20: transcript:EES02087           transcript:Zm00001d042039_T001          0
727- 21: transcript:EES04259           transcript:Zm00001d042035_T001          0
727- 22: transcript:EES04260           transcript:Zm00001d042034_T002          0
727- 23: transcript:KXG34170           transcript:Zm00001d042033_T001          0
727- 24: transcript:EES02093           transcript:Zm00001d042030_T001  2.00E-23
727- 25: transcript:KXG34176           transcript:Zm00001d042028_T014          0
727- 26: transcript:EES02099           transcript:Zm00001d042027_T001 6.00E-129
727- 27: transcript:EES04263           transcript:Zm00001d042026_T001          0
727- 28: transcript:EES04268           transcript:Zm00001d042025_T001  6.00E-55
727- 29: transcript:EES04269           transcript:Zm00001d042024_T001          0
727- 30: transcript:EES04270           transcript:Zm00001d042023_T001 2.00E-102
727- 31: transcript:EES02102           transcript:Zm00001d042019_T002 1.00E-131
727- 32: transcript:EES04275           transcript:Zm00001d042018_T003 1.00E-158
727- 33: transcript:EES02107           transcript:Zm00001d042017_T002 2.00E-107
```

727- 34: transcript:EES02110 transcript:Zm00001d042016_T001 5.00E-57
727- 35: transcript:EES02112 transcript:Zm00001d042014_T001 9.00E-18
727- 36: transcript:EES04277 transcript:Zm00001d042013_T001 0
727- 37: transcript:EES02113 transcript:Zm00001d042011_T006 4.00E-73
727- 38: transcript:EES02115 transcript:Zm00001d042008_T001 5.00E-117
727- 39: transcript:KXG34193 transcript:Zm00001d042007_T001 0
727- 40: transcript:EES02118 transcript:Zm00001d042005_T001 0
727- 41: transcript:OQU88240 transcript:Zm00001d042003_T001 6.00E-148
727- 42: transcript:EES02122 transcript:Zm00001d041996_T001 0
727- 43: transcript:OQU88246 transcript:Zm00001d041995_T002 0
727- 44: transcript:EES04285 transcript:Zm00001d041994_T002 0
727- 45: transcript:EES02124 transcript:Zm00001d041993_T002 4.00E-77
727- 46: transcript:OQU88249 transcript:Zm00001d041991_T001 0
727- 47: transcript:EES04287 transcript:Zm00001d041990_T001 8.00E-165
727- 48: transcript:EES02126 transcript:Zm00001d041989_T001 0
727- 49: transcript:OQU88250 transcript:Zm00001d041988_T001 0
727- 50: transcript:OQU88251 transcript:Zm00001d041985_T001 0
727- 51: transcript:OQU88253 transcript:Zm00001d041983_T006 0
727- 52: transcript:EES02135 transcript:Zm00001d041982_T001 0
727- 53: transcript:EES02136 transcript:Zm00001d041981_T001 2.00E-113
727- 54: transcript:EES04290 transcript:Zm00001d041979_T002 2.00E-54
727- 55: transcript:EES02138 transcript:Zm00001d041973_T001 3.00E-155
727- 56: transcript:EES04292 transcript:Zm00001d041972_T002 0
727- 57: transcript:OQU88254 transcript:Zm00001d041969_T001 4.00E-75
727- 58: transcript:EES04293 transcript:Zm00001d041968_T001 0
727- 59: transcript:KXG34214 transcript:Zm00001d041965_T001 8.00E-113
727- 60: transcript:EES04294 transcript:Zm00001d041964_T001 0
727- 61: transcript:EES02143 transcript:Zm00001d041963_T001 9.00E-95
727- 62: transcript:OQU88264 transcript:Zm00001d041962_T002 0
727- 63: transcript:EES02141 transcript:Zm00001d041961_T001 2.00E-146
727- 64: transcript:EES04298 transcript:Zm00001d041959_T001 0
727- 65: transcript:EES04299 transcript:Zm00001d041958_T001 4.00E-121
727- 66: transcript:EES02145 transcript:Zm00001d041957_T004 0
727- 67: transcript:OQU88270 transcript:Zm00001d041956_T002 5.00E-147
727- 68: transcript:EES02148 transcript:Zm00001d041954_T002 0
Alignment 728: score=3125.0 e_value=6.1e-268 N=68 3&3 minus
728- 0: transcript:EES01208 transcript:Zm00001d043975_T001 3.00E-167
728- 1: transcript:EES03386 transcript:Zm00001d043974_T001 4.00E-98
728- 2: transcript:KXG33046 transcript:Zm00001d043973_T001 2.00E-64
728- 3: transcript:EES01209 transcript:Zm00001d043972_T001 1.00E-90
728- 4: transcript:EES03388 transcript:Zm00001d043971_T001 8.00E-110
728- 5: transcript:KXG33047 transcript:Zm00001d043969_T001 3.00E-113
728- 6: transcript:EES03391 transcript:Zm00001d043968_T001 3.00E-50
728- 7: transcript:EES01213 transcript:Zm00001d043965_T001 0
728- 8: transcript:KXG33050 transcript:Zm00001d043963_T001 1.00E-117
728- 9: transcript:KXG33051 transcript:Zm00001d043962_T001 0
728- 10: transcript:EES03392 transcript:Zm00001d043959_T003 0
728- 11: transcript:EES01219 transcript:Zm00001d043955_T001 0
728- 12: transcript:KXG33056 transcript:Zm00001d043954_T003 0
728- 13: transcript:KXG33060 transcript:Zm00001d043953_T001 0
728- 14: transcript:EES03393 transcript:Zm00001d043950_T001 6.00E-168
728- 15: transcript:EES01222 transcript:Zm00001d043948_T008 0
728- 16: transcript:KXG33063 transcript:Zm00001d043947_T001 1.00E-113
728- 17: transcript:OQU87294 transcript:Zm00001d043946_T001 3.00E-66

728- 18: transcript:EES01227 transcript:Zm00001d043944_T001 7.00E-97
728- 19: transcript:EES03398 transcript:Zm00001d043943_T001 7.00E-159
728- 20: transcript:EES03399 transcript:Zm00001d043942_T001 4.00E-133
728- 21: transcript:EES03401 transcript:Zm00001d043941_T001 0
728- 22: transcript:EES01229 transcript:Zm00001d043938_T001 0
728- 23: transcript:EES03403 transcript:Zm00001d043937_T001 3.00E-60
728- 24: transcript:OQU87300 transcript:Zm00001d043935_T001 3.00E-156
728- 25: transcript:EES03406 transcript:Zm00001d043932_T001 1.00E-55
728- 26: transcript:EES03407 transcript:Zm00001d043929_T001 6.00E-132
728- 27: transcript:OQU87303 transcript:Zm00001d043928_T001 0
728- 28: transcript:OQU87306 transcript:Zm00001d043923_T001 0
728- 29: transcript:OQU87307 transcript:Zm00001d043922_T001 0
728- 30: transcript:OQU87308 transcript:Zm00001d043921_T001 0
728- 31: transcript:OQU87310 transcript:Zm00001d043915_T002 0
728- 32: transcript:KXG33084 transcript:Zm00001d043912_T002 3.00E-104
728- 33: transcript:KXG33085 transcript:Zm00001d043911_T001 2.00E-162
728- 34: transcript:KXG33086 transcript:Zm00001d043909_T010 0
728- 35: transcript:OQU87312 transcript:Zm00001d043907_T003 0
728- 36: transcript:EES03419 transcript:Zm00001d043906_T002 0
728- 37: transcript:EES03421 transcript:Zm00001d043905_T003 2.00E-103
728- 38: transcript:OQU87316 transcript:Zm00001d043904_T001 2.00E-46
728- 39: transcript:KXG33097 transcript:Zm00001d043903_T004 0
728- 40: transcript:EES03423 transcript:Zm00001d043902_T001 2.00E-178
728- 41: transcript:EES03424 transcript:Zm00001d043900_T022 0
728- 42: transcript:EES01241 transcript:Zm00001d043898_T001 2.00E-42
728- 43: transcript:EES03425 transcript:Zm00001d043895_T003 0
728- 44: transcript:KXG33100 transcript:Zm00001d043890_T002 0
728- 45: transcript:KXG33101 transcript:Zm00001d043889_T001 0
728- 46: transcript:EES01246 transcript:Zm00001d043880_T001 4.00E-143
728- 47: transcript:EES03429 transcript:Zm00001d043879_T002 0
728- 48: transcript:KXG33104 transcript:Zm00001d043878_T001 1.00E-137
728- 49: transcript:OQU87320 transcript:Zm00001d043877_T001 5.00E-21
728- 50: transcript:EES01248 transcript:Zm00001d043875_T001 9.00E-144
728- 51: transcript:KXG33105 transcript:Zm00001d043874_T001 9.00E-78
728- 52: transcript:EES01250 transcript:Zm00001d043873_T001 0
728- 53: transcript:EES01251 transcript:Zm00001d043872_T001 0
728- 54: transcript:EES01253 transcript:Zm00001d043870_T001 0
728- 55: transcript:KXG33113 transcript:Zm00001d043868_T001 1.00E-24
728- 56: transcript:OQU87329 transcript:Zm00001d043864_T001 0
728- 57: transcript:EES01262 transcript:Zm00001d043863_T001 6.00E-67
728- 58: transcript:OQU87332 transcript:Zm00001d043860_T001 0
728- 59: transcript:EES01264 transcript:Zm00001d043858_T001 0
728- 60: transcript:EES01269 transcript:Zm00001d043857_T001 3.00E-56
728- 61: transcript:EES03442 transcript:Zm00001d043855_T001 0
728- 62: transcript:EES03440 transcript:Zm00001d043854_T001 0
728- 63: transcript:EES03441 transcript:Zm00001d043853_T001 4.00E-21
728- 64: transcript:EES01271 transcript:Zm00001d043851_T001 0
728- 65: transcript:KXG33127 transcript:Zm00001d043850_T001 0
728- 66: transcript:KXG33130 transcript:Zm00001d043849_T001 4.00E-30
728- 67: transcript:KXG33132 transcript:Zm00001d043847_T001 1.00E-19
Alignment 729: score=2580.0 e_value=1.8e-225 N=57 3&3 minus
729- 0: transcript:KXG33894 transcript:Zm00001d042523_T001 0
729- 1: transcript:KXG33896 transcript:Zm00001d042520_T001 0
729- 2: transcript:OQU88032 transcript:Zm00001d042519_T002 2.00E-157

729- 3:	transcript:OQU88033	transcript:Zm00001d042511_T005	3.00E-57
729- 4:	transcript:EES01903	transcript:Zm00001d042510_T002	3.00E-140
729- 5:	transcript:EES01904	transcript:Zm00001d042508_T001	0
729- 6:	transcript:EES01905	transcript:Zm00001d042507_T001	0
729- 7:	transcript:OQU88036	transcript:Zm00001d042506_T002	0
729- 8:	transcript:EES04041	transcript:Zm00001d042505_T005	0
729- 9:	transcript:OQU88040	transcript:Zm00001d042504_T001	0
729- 10:	transcript:EES01908	transcript:Zm00001d042503_T001	2.00E-80
729- 11:	transcript:OQU88043	transcript:Zm00001d042500_T001	0
729- 12:	transcript:OQU88046	transcript:Zm00001d042499_T001	0
729- 13:	transcript:EES04044	transcript:Zm00001d042498_T001	0
729- 14:	transcript:EES04045	transcript:Zm00001d042494_T004	0
729- 15:	transcript:EES04046	transcript:Zm00001d042493_T001	2.00E-168
729- 16:	transcript:KXG33917	transcript:Zm00001d042492_T001	0
729- 17:	transcript:KXG33919	transcript:Zm00001d042490_T001	0
729- 18:	transcript:OQU88047	transcript:Zm00001d042487_T002	0
729- 19:	transcript:OQU88051	transcript:Zm00001d042486_T001	1.00E-164
729- 20:	transcript:KXG33923	transcript:Zm00001d042482_T001	0
729- 21:	transcript:OQU88052	transcript:Zm00001d042481_T004	2.00E-103
729- 22:	transcript:EES04050	transcript:Zm00001d042480_T002	0
729- 23:	transcript:EES04051	transcript:Zm00001d042479_T001	0
729- 24:	transcript:KXG33926	transcript:Zm00001d042478_T003	0
729- 25:	transcript:EES04053	transcript:Zm00001d042476_T002	3.00E-116
729- 26:	transcript:KXG33931	transcript:Zm00001d042475_T001	0
729- 27:	transcript:OQU88056	transcript:Zm00001d042474_T001	0
729- 28:	transcript:KXG33937	transcript:Zm00001d042473_T002	0
729- 29:	transcript:EES01920	transcript:Zm00001d042472_T001	0
729- 30:	transcript:KXG33943	transcript:Zm00001d042470_T003	0
729- 31:	transcript:OQU88062	transcript:Zm00001d042469_T002	2.00E-59
729- 32:	transcript:EES04059	transcript:Zm00001d042468_T004	0
729- 33:	transcript:EES04060	transcript:Zm00001d042464_T002	0
729- 34:	transcript:EES04061	transcript:Zm00001d042463_T001	0
729- 35:	transcript:KXG33944	transcript:Zm00001d042461_T001	8.00E-114
729- 36:	transcript:KXG33945	transcript:Zm00001d042460_T002	0
729- 37:	transcript:KXG33947	transcript:Zm00001d042455_T001	1.00E-155
729- 38:	transcript:EES01923	transcript:Zm00001d042454_T001	2.00E-75
729- 39:	transcript:EES04064	transcript:Zm00001d042453_T001	0
729- 40:	transcript:EES04065	transcript:Zm00001d042452_T001	1.00E-37
729- 41:	transcript:KXG33950	transcript:Zm00001d042451_T003	0
729- 42:	transcript:EES04067	transcript:Zm00001d042450_T002	0
729- 43:	transcript:OQU88071	transcript:Zm00001d042449_T001	2.00E-93
729- 44:	transcript:EES01925	transcript:Zm00001d042448_T001	1.00E-47
729- 45:	transcript:KXG33953	transcript:Zm00001d042447_T002	0
729- 46:	transcript:EES01927	transcript:Zm00001d042446_T001	0
729- 47:	transcript:EES01930	transcript:Zm00001d042445_T001	0
729- 48:	transcript:KXG33955	transcript:Zm00001d042444_T001	2.00E-51
729- 49:	transcript:OQU88080	transcript:Zm00001d042443_T001	1.00E-111
729- 50:	transcript:EES04071	transcript:Zm00001d042441_T001	0
729- 51:	transcript:EES01933	transcript:Zm00001d042438_T001	0
729- 52:	transcript:EES04073	transcript:Zm00001d042437_T005	0
729- 53:	transcript:EES04074	transcript:Zm00001d042436_T001	0
729- 54:	transcript:KXG33958	transcript:Zm00001d042435_T002	0
729- 55:	transcript:KXG33960	transcript:Zm00001d042434_T001	0
729- 56:	transcript:KXG33961	transcript:Zm00001d042433_T001	2.00E-11

```
## Alignment 730: score=1927.0 e_value=3.1e-141 N=41 3&3 minus
730-  0: transcript:EES01494           transcript:Zm00001d043414_T001          0
730-  1: transcript:OQU87576           transcript:Zm00001d043413_T001  4.00E-08
730-  2: transcript:EES01498           transcript:Zm00001d043411_T001          0
730-  3: transcript:EES01499           transcript:Zm00001d043410_T002          0
730-  4: transcript:EES03634           transcript:Zm00001d043407_T001 4.00E-134
730-  5: transcript:EES03635           transcript:Zm00001d043406_T002 5.00E-177
730-  6: transcript:EES03636           transcript:Zm00001d043405_T001          0
730-  7: transcript:EES01500           transcript:Zm00001d043404_T001          0
730-  8: transcript:EES03638           transcript:Zm00001d043403_T001  6.00E-91
730-  9: transcript:KXG33408           transcript:Zm00001d043402_T001  8.00E-80
730- 10: transcript:EES03642           transcript:Zm00001d043401_T002          0
730- 11: transcript:EES03643           transcript:Zm00001d043399_T001  2.00E-93
730- 12: transcript:EES01501           transcript:Zm00001d043395_T002          0
730- 13: transcript:KXG33412           transcript:Zm00001d043392_T016 7.00E-179
730- 14: transcript:KXG33413           transcript:Zm00001d043391_T001 2.00E-111
730- 15: transcript:OQU87585           transcript:Zm00001d043390_T001 8.00E-153
730- 16: transcript:EES03646           transcript:Zm00001d043389_T003          0
730- 17: transcript:EES01504           transcript:Zm00001d043387_T002          0
730- 18: transcript:KXG33416           transcript:Zm00001d043386_T001  2.00E-62
730- 19: transcript:OQU87589           transcript:Zm00001d043383_T001          0
730- 20: transcript:KXG33418           transcript:Zm00001d043382_T001 3.00E-147
730- 21: transcript:OQU87593           transcript:Zm00001d043380_T001  9.00E-15
730- 22: transcript:KXG33425           transcript:Zm00001d043378_T001          0
730- 23: transcript:KXG33428           transcript:Zm00001d043376_T006          0
730- 24: transcript:EES03649           transcript:Zm00001d043374_T001 3.00E-148
730- 25: transcript:KXG33430           transcript:Zm00001d043371_T001  6.00E-42
730- 26: transcript:EES03656           transcript:Zm00001d043370_T002 1.00E-125
730- 27: transcript:EES03657           transcript:Zm00001d043368_T001 2.00E-147
730- 28: transcript:EES03658           transcript:Zm00001d043367_T001  2.00E-77
730- 29: transcript:KXG33433           transcript:Zm00001d043366_T001  3.00E-20
730- 30: transcript:KXG33434           transcript:Zm00001d043365_T001          0
730- 31: transcript:EES03659           transcript:Zm00001d043364_T001  3.00E-65
730- 32: transcript:OQU87600           transcript:Zm00001d043361_T001 3.00E-101
730- 33: transcript:OQU87601           transcript:Zm00001d043359_T001  7.00E-46
730- 34: transcript:EES01515           transcript:Zm00001d043358_T001          0
730- 35: transcript:KXG33437           transcript:Zm00001d043357_T001  1.00E-67
730- 36: transcript:EES03663           transcript:Zm00001d043356_T004 3.00E-176
730- 37: transcript:OQU87603           transcript:Zm00001d043353_T001  3.00E-55
730- 38: transcript:KXG33442           transcript:Zm00001d043352_T002 2.00E-109
730- 39: transcript:EES01517           transcript:Zm00001d043350_T001          0
730- 40: transcript:EES03665           transcript:Zm00001d043348_T020          0
## Alignment 731: score=1517.0 e_value=9e-116 N=33 3&3 minus
731-  0: transcript:EES02034           transcript:Zm00001d042152_T005          0
731-  1: transcript:EES02036           transcript:Zm00001d042150_T001  2.00E-72
731-  2: transcript:EES02037           transcript:Zm00001d042148_T001          0
731-  3: transcript:OQU88184           transcript:Zm00001d042146_T002          0
731-  4: transcript:EES02039           transcript:Zm00001d042144_T001  2.00E-65
731-  5: transcript:OQU88185           transcript:Zm00001d042142_T001          0
731-  6: transcript:EES04196           transcript:Zm00001d042141_T001          0
731-  7: transcript:EES04197           transcript:Zm00001d042138_T001 6.00E-137
731-  8: transcript:EES04195           transcript:Zm00001d042137_T002          0
731-  9: transcript:KXG34096           transcript:Zm00001d042136_T001          0
731- 10: transcript:KXG34100           transcript:Zm00001d042133_T003          0
```

731- 11: transcript:KXG34097 transcript:Zm00001d042132_T001 0
731- 12: transcript:EES04209 transcript:Zm00001d042131_T003 0
731- 13: transcript:KXG34104 transcript:Zm00001d042128_T005 5.00E-117
731- 14: transcript:EES02045 transcript:Zm00001d042127_T001 0
731- 15: transcript:EES02043 transcript:Zm00001d042122_T001 4.00E-163
731- 16: transcript:EES02047 transcript:Zm00001d042118_T001 9.00E-82
731- 17: transcript:EES04210 transcript:Zm00001d042117_T002 0
731- 18: transcript:KXG34107 transcript:Zm00001d042116_T001 0
731- 19: transcript:EES04212 transcript:Zm00001d042114_T001 2.00E-12
731- 20: transcript:OQU88196 transcript:Zm00001d042111_T001 0
731- 21: transcript:EES02048 transcript:Zm00001d042108_T001 1.00E-76
731- 22: transcript:EES04217 transcript:Zm00001d042107_T001 9.00E-112
731- 23: transcript:KXG34116 transcript:Zm00001d042098_T001 1.00E-73
731- 24: transcript:OQU88198 transcript:Zm00001d042096_T001 2.00E-93
731- 25: transcript:EES04219 transcript:Zm00001d042095_T001 4.00E-95
731- 26: transcript:EES02053 transcript:Zm00001d042094_T001 9.00E-115
731- 27: transcript:EES04224 transcript:Zm00001d042093_T001 2.00E-101
731- 28: transcript:OQU88202 transcript:Zm00001d042092_T001 5.00E-129
731- 29: transcript:EES04226 transcript:Zm00001d042091_T002 0
731- 30: transcript:KXG34124 transcript:Zm00001d042088_T001 0
731- 31: transcript:EES04227 transcript:Zm00001d042087_T001 0
731- 32: transcript:EES02059 transcript:Zm00001d042084_T060 0
Alignment 732: score=1352.0 e_value=5.8e-91 N=29 3&3 minus
732- 0: transcript:EES01384 transcript:Zm00001d043611_T001 2.00E-137
732- 1: transcript:EES01388 transcript:Zm00001d043610_T001 1.00E-49
732- 2: transcript:EES03540 transcript:Zm00001d043609_T001 0
732- 3: transcript:EES03543 transcript:Zm00001d043607_T001 0
732- 4: transcript:EES03542 transcript:Zm00001d043606_T001 3.00E-94
732- 5: transcript:KXG33287 transcript:Zm00001d043601_T003 0
732- 6: transcript:EES01390 transcript:Zm00001d043600_T001 2.00E-169
732- 7: transcript:EES03544 transcript:Zm00001d043599_T001 0
732- 8: transcript:EES01392 transcript:Zm00001d043598_T003 0
732- 9: transcript:OQU87450 transcript:Zm00001d043596_T001 4.00E-101
732- 10: transcript:OQU87451 transcript:Zm00001d043595_T001 0
732- 11: transcript:OQU87453 transcript:Zm00001d043592_T004 0
732- 12: transcript:EES03545 transcript:Zm00001d043590_T001 1.00E-13
732- 13: transcript:KXG33296 transcript:Zm00001d043589_T002 9.00E-113
732- 14: transcript:EES01397 transcript:Zm00001d043588_T001 4.00E-67
732- 15: transcript:KXG33297 transcript:Zm00001d043587_T001 0
732- 16: transcript:OQU87454 transcript:Zm00001d043586_T001 3.00E-11
732- 17: transcript:OQU87456 transcript:Zm00001d043581_T001 0
732- 18: transcript:EES01401 transcript:Zm00001d043580_T001 5.00E-99
732- 19: transcript:EES01402 transcript:Zm00001d043579_T001 0
732- 20: transcript:KXG33301 transcript:Zm00001d043578_T008 0
732- 21: transcript:EES03557 transcript:Zm00001d043574_T001 0
732- 22: transcript:EES01408 transcript:Zm00001d043573_T001 0
732- 23: transcript:KXG33303 transcript:Zm00001d043572_T001 1.00E-31
732- 24: transcript:EES01411 transcript:Zm00001d043571_T001 0
732- 25: transcript:KXG33305 transcript:Zm00001d043570_T001 5.00E-58
732- 26: transcript:EES03563 transcript:Zm00001d043569_T001 8.00E-126
732- 27: transcript:KXG33308 transcript:Zm00001d043566_T005 0
732- 28: transcript:EES03565 transcript:Zm00001d043565_T001 1.00E-68
Alignment 733: score=1095.0 e_value=4e-70 N=24 3&3 minus
733- 0: transcript:KXG33015 transcript:Zm00001d044023_T017 0

733- 1: transcript:EES03365   transcript:Zm00001d044022_T001 2.00E-43
733- 2: transcript:OQU87252   transcript:Zm00001d044021_T001 0
733- 3: transcript:OQU87254   transcript:Zm00001d044020_T001 2.00E-84
733- 4: transcript:OQU87255   transcript:Zm00001d044019_T004 0
733- 5: transcript:EES01191   transcript:Zm00001d044017_T001 0
733- 6: transcript:EES01192   transcript:Zm00001d044016_T001 0
733- 7: transcript:KXG33023   transcript:Zm00001d044015_T001 0
733- 8: transcript:OQU87261   transcript:Zm00001d044013_T001 9.00E-76
733- 9: transcript:EES03370   transcript:Zm00001d044010_T001 2.00E-139
733- 10: transcript:KXG33029   transcript:Zm00001d044008_T010 0
733- 11: transcript:OQU87262   transcript:Zm00001d044005_T001 7.00E-131
733- 12: transcript:EES03374   transcript:Zm00001d044004_T001 4.00E-148
733- 13: transcript:KXG33034   transcript:Zm00001d043998_T001 8.00E-104
733- 14: transcript:EES01199   transcript:Zm00001d043994_T001 1.00E-151
733- 15: transcript:EES03376   transcript:Zm00001d043993_T003 0
733- 16: transcript:OQU87266   transcript:Zm00001d043992_T001 0
733- 17: transcript:EES01200   transcript:Zm00001d043991_T001 3.00E-164
733- 18: transcript:EES01201   transcript:Zm00001d043990_T001 3.00E-163
733- 19: transcript:EES01202   transcript:Zm00001d043989_T001 0
733- 20: transcript:KXG33039   transcript:Zm00001d043988_T001 0
733- 21: transcript:EES03378   transcript:Zm00001d043986_T005 0
733- 22: transcript:EES03381   transcript:Zm00001d043985_T001 2.00E-54
733- 23: transcript:EES01207   transcript:Zm00001d043984_T001 6.00E-77
Alignment 734: score=959.0 e_value=1.9e-67 N=22 3&3 minus
734- 0: transcript:EES00519   transcript:Zm00001d040372_T002 0
734- 1: transcript:OQU86655   transcript:Zm00001d040371_T001 0
734- 2: transcript:KXG32214   transcript:Zm00001d040365_T001 4.00E-30
734- 3: transcript:EES02729   transcript:Zm00001d040364_T001 8.00E-134
734- 4: transcript:EES02730   transcript:Zm00001d040363_T001 0
734- 5: transcript:EES02731   transcript:Zm00001d040362_T001 0
734- 6: transcript:EES00528   transcript:Zm00001d040361_T001 5.00E-09
734- 7: transcript:EES02732   transcript:Zm00001d040360_T001 0
734- 8: transcript:EES00531   transcript:Zm00001d040357_T001 6.00E-170
734- 9: transcript:EES00532   transcript:Zm00001d040356_T001 0
734- 10: transcript:OQU86658   transcript:Zm00001d040351_T005 3.00E-117
734- 11: transcript:EES02735   transcript:Zm00001d040348_T002 2.00E-108
734- 12: transcript:EES02736   transcript:Zm00001d040344_T001 0
734- 13: transcript:KXG32223   transcript:Zm00001d040343_T001 2.00E-58
734- 14: transcript:EES02737   transcript:Zm00001d040342_T001 0
734- 15: transcript:OQU86665   transcript:Zm00001d040341_T001 2.00E-131
734- 16: transcript:KXG32230   transcript:Zm00001d040340_T005 0
734- 17: transcript:EES00543   transcript:Zm00001d040334_T001 0
734- 18: transcript:OQU86667   transcript:Zm00001d040333_T001 0
734- 19: transcript:OQU86672   transcript:Zm00001d040331_T001 0
734- 20: transcript:KXG32238   transcript:Zm00001d040324_T001 8.00E-161
734- 21: transcript:OQU86675   transcript:Zm00001d040323_T001 1.00E-76
Alignment 735: score=894.0 e_value=3.3e-60 N=21 3&3 minus
735- 0: transcript:EES03280   transcript:Zm00001d044222_T032 0
735- 1: transcript:KXG32903   transcript:Zm00001d044221_T001 1.00E-49
735- 2: transcript:EES03281   transcript:Zm00001d044219_T004 6.00E-107
735- 3: transcript:KXG32905   transcript:Zm00001d044217_T001 2.00E-171
735- 4: transcript:EES03283   transcript:Zm00001d044216_T002 0
735- 5: transcript:EES01080   transcript:Zm00001d044213_T001 0
735- 6: transcript:KXG32909   transcript:Zm00001d044212_T001 0

735- 7: transcript:EES03286 transcript:Zm00001d044211_T001 0
735- 8: transcript:EES01082 transcript:Zm00001d044208_T001 0
735- 9: transcript:KXG32911 transcript:Zm00001d044202_T001 0
735- 10: transcript:KXG32912 transcript:Zm00001d044201_T005 0
735- 11: transcript:EES03288 transcript:Zm00001d044197_T001 0
735- 12: transcript:EES03289 transcript:Zm00001d044195_T018 0
735- 13: transcript:EES03290 transcript:Zm00001d044194_T001 1.00E-46
735- 14: transcript:EES03291 transcript:Zm00001d044193_T002 0
735- 15: transcript:OQU87179 transcript:Zm00001d044192_T001 0
735- 16: transcript:EES03295 transcript:Zm00001d044191_T004 0
735- 17: transcript:EES01088 transcript:Zm00001d044190_T001 2.00E-34
735- 18: transcript:EES01092 transcript:Zm00001d044189_T001 0
735- 19: transcript:OQU87181 transcript:Zm00001d044188_T006 0
735- 20: transcript:OQU87185 transcript:Zm00001d044187_T001 1.00E-05
Alignment 736: score=891.0 e_value=5.7e-58 N=20 3&3 minus
736- 0: transcript:EES01449 transcript:Zm00001d043527_T002 0
736- 1: transcript:KXG33329 transcript:Zm00001d043526_T004 0
736- 2: transcript:EES03581 transcript:Zm00001d043525_T001 3.00E-75
736- 3: transcript:EES01451 transcript:Zm00001d043524_T001 0
736- 4: transcript:EES01452 transcript:Zm00001d043523_T001 3.00E-89
736- 5: transcript:EES01453 transcript:Zm00001d043520_T001 0
736- 6: transcript:OQU87502 transcript:Zm00001d043517_T002 0
736- 7: transcript:OQU87503 transcript:Zm00001d043516_T001 0
736- 8: transcript:KXG33335 transcript:Zm00001d043515_T002 4.00E-173
736- 9: transcript:OQU87508 transcript:Zm00001d043514_T001 0
736- 10: transcript:KXG33339 transcript:Zm00001d043512_T001 0
736- 11: transcript:EES03587 transcript:Zm00001d043511_T001 0
736- 12: transcript:KXG33341 transcript:Zm00001d043510_T002 0
736- 13: transcript:KXG33344 transcript:Zm00001d043509_T002 0
736- 14: transcript:EES03590 transcript:Zm00001d043508_T002 0
736- 15: transcript:KXG33345 transcript:Zm00001d043506_T004 0
736- 16: transcript:OQU87518 transcript:Zm00001d043505_T001 3.00E-91
736- 17: transcript:OQU87522 transcript:Zm00001d043504_T030 0
736- 18: transcript:EES01465 transcript:Zm00001d043503_T001 0
736- 19: transcript:EES03594 transcript:Zm00001d043502_T001 0
Alignment 737: score=672.0 e_value=5.5e-45 N=17 3&3 minus
737- 0: transcript:EES00098 transcript:Zm00001d039446_T001 0
737- 1: transcript:EES00099 transcript:Zm00001d039444_T001 0
737- 2: transcript:OQU86135 transcript:Zm00001d039441_T001 3.00E-115
737- 3: transcript:EES00101 transcript:Zm00001d039439_T001 0
737- 4: transcript:EES02283 transcript:Zm00001d039437_T001 0
737- 5: transcript:OQU86137 transcript:Zm00001d039435_T001 0
737- 6: transcript:EES00105 transcript:Zm00001d039434_T001 4.00E-164
737- 7: transcript:EES02285 transcript:Zm00001d039432_T004 3.00E-126
737- 8: transcript:EES00106 transcript:Zm00001d039430_T001 2.00E-78
737- 9: transcript:EES00107 transcript:Zm00001d039429_T001 1.00E-55
737- 10: transcript:EES00109 transcript:Zm00001d039428_T001 3.00E-71
737- 11: transcript:KXG31606 transcript:Zm00001d039427_T002 0
737- 12: transcript:EES02286 transcript:Zm00001d039426_T001 0
737- 13: transcript:EES02288 transcript:Zm00001d039425_T005 0
737- 14: transcript:EES00113 transcript:Zm00001d039424_T001 2.00E-19
737- 15: transcript:EES02291 transcript:Zm00001d039423_T001 3.00E-98
737- 16: transcript:EES02292 transcript:Zm00001d039422_T003 0
Alignment 738: score=659.0 e_value=1.3e-37 N=15 3&3 minus

```
738-  0: transcript:EES03092           transcript:Zm00001d044551_T003          0
738-  1: transcript:EES03093           transcript:Zm00001d044548_T001          0
738-  2: transcript:OQU86972           transcript:Zm00001d044547_T003          0
738-  3: transcript:EES00878           transcript:Zm00001d044546_T001  4.00E-50
738-  4: transcript:KXG32681           transcript:Zm00001d044542_T001 1.00E-142
738-  5: transcript:EES00882           transcript:Zm00001d044541_T002 4.00E-153
738-  6: transcript:EES00883           transcript:Zm00001d044540_T001          0
738-  7: transcript:EES03100           transcript:Zm00001d044538_T001  3.00E-71
738-  8: transcript:KXG32684           transcript:Zm00001d044537_T001          0
738-  9: transcript:EES03101           transcript:Zm00001d044535_T001 6.00E-132
738- 10: transcript:KXG32687           transcript:Zm00001d044534_T001          0
738- 11: transcript:OQU86992           transcript:Zm00001d044533_T003  3.00E-51
738- 12: transcript:OQU86994           transcript:Zm00001d044532_T001 7.00E-138
738- 13: transcript:KXG32695           transcript:Zm00001d044529_T001          0
738- 14: transcript:KXG32697           transcript:Zm00001d044528_T001          0
## Alignment 739: score=607.0 e_value=3e-32 N=13 3&3 minus
739-  0: transcript:EES00895           transcript:Zm00001d044527_T002 2.00E-153
739-  1: transcript:OQU87002           transcript:Zm00001d044526_T001  1.00E-84
739-  2: transcript:EES03112           transcript:Zm00001d044525_T001          0
739-  3: transcript:OQU87005           transcript:Zm00001d044520_T001  9.00E-80
739-  4: transcript:EES00900           transcript:Zm00001d044519_T001 8.00E-119
739-  5: transcript:KXG32705           transcript:Zm00001d044518_T001  2.00E-81
739-  6: transcript:OQU87009           transcript:Zm00001d044517_T001          0
739-  7: transcript:EES00901           transcript:Zm00001d044516_T001 6.00E-178
739-  8: transcript:EES03116           transcript:Zm00001d044515_T001          0
739-  9: transcript:EES00903           transcript:Zm00001d044514_T004          0
739- 10: transcript:KXG32707           transcript:Zm00001d044513_T009          0
739- 11: transcript:EES00905           transcript:Zm00001d044512_T001  8.00E-46
739- 12: transcript:EES00906           transcript:Zm00001d044511_T001          0
## Alignment 740: score=598.0 e_value=2.5e-30 N=13 3&3 minus
740-  0: transcript:OQU87414           transcript:Zm00001d043667_T003          0
740-  1: transcript:OQU87416           transcript:Zm00001d043666_T001          0
740-  2: transcript:KXG33243           transcript:Zm00001d043665_T001  6.00E-86
740-  3: transcript:KXG33244           transcript:Zm00001d043663_T001 7.00E-117
740-  4: transcript:KXG33246           transcript:Zm00001d043662_T001          0
740-  5: transcript:EES03523           transcript:Zm00001d043661_T001 3.00E-155
740-  6: transcript:EES03524           transcript:Zm00001d043660_T001          0
740-  7: transcript:OQU87425           transcript:Zm00001d043656_T001          0
740-  8: transcript:KXG33251           transcript:Zm00001d043655_T002          0
740-  9: transcript:EES01370           transcript:Zm00001d043654_T001 6.00E-180
740- 10: transcript:EES01371           transcript:Zm00001d043653_T003          0
740- 11: transcript:EES03529           transcript:Zm00001d043652_T003          0
740- 12: transcript:KXG33254           transcript:Zm00001d043650_T001          0
## Alignment 741: score=509.0 e_value=3.8e-27 N=11 3&3 minus
741-  0: transcript:EES00551           transcript:Zm00001d040411_T001          0
741-  1: transcript:OQU86680           transcript:Zm00001d040409_T001 3.00E-154
741-  2: transcript:KXG32246           transcript:Zm00001d040408_T003          0
741-  3: transcript:OQU86683           transcript:Zm00001d040399_T001          0
741-  4: transcript:EES00556           transcript:Zm00001d040398_T001          0
741-  5: transcript:EES02750           transcript:Zm00001d040393_T001          0
741-  6: transcript:EES00558           transcript:Zm00001d040392_T001 7.00E-155
741-  7: transcript:KXG32249           transcript:Zm00001d040390_T003 5.00E-110
741-  8: transcript:EES00559           transcript:Zm00001d040389_T002  7.00E-11
741-  9: transcript:EES00560           transcript:Zm00001d040383_T005          0
```

741- 10: transcript:EES02752 transcript:Zm00001d040382_T001 8.00E-95
Alignment 742: score=486.0 e_value=8.8e-21 N=10 3&3 minus
742- 0: transcript:KXG33388 transcript:Zm00001d043428_T005 0
742- 1: transcript:EES03626 transcript:Zm00001d043427_T001 2.00E-26
742- 2: transcript:KXG33391 transcript:Zm00001d043426_T001 0
742- 3: transcript:OQU87571 transcript:Zm00001d043425_T003 0
742- 4: transcript:KXG33398 transcript:Zm00001d043423_T001 0
742- 5: transcript:EES01492 transcript:Zm00001d043422_T001 8.00E-126
742- 6: transcript:KXG33401 transcript:Zm00001d043421_T001 0
742- 7: transcript:EES01493 transcript:Zm00001d043420_T001 2.00E-142
742- 8: transcript:OQU87574 transcript:Zm00001d043419_T001 1.00E-68
742- 9: transcript:KXG33403 transcript:Zm00001d043418_T136 0
Alignment 743: score=425.0 e_value=8.4e-20 N=10 3&3 minus
743- 0: transcript:KXG31557 transcript:Zm00001d039378_T001 1.00E-26
743- 1: transcript:KXG31561 transcript:Zm00001d039374_T001 1.00E-25
743- 2: transcript:EES00066 transcript:Zm00001d039372_T002 3.00E-25
743- 3: transcript:KXG31563 transcript:Zm00001d039371_T001 0
743- 4: transcript:EES00064 transcript:Zm00001d039370_T002 7.00E-130
743- 5: transcript:EES02258 transcript:Zm00001d039369_T004 0
743- 6: transcript:EES02260 transcript:Zm00001d039368_T003 0
743- 7: transcript:KXG31566 transcript:Zm00001d039367_T001 3.00E-16
743- 8: transcript:KXG31573 transcript:Zm00001d039366_T001 0
743- 9: transcript:EES00059 transcript:Zm00001d039365_T001 0
Alignment 744: score=420.0 e_value=3.3e-19 N=9 3&3 minus
744- 0: transcript:OQU88275 transcript:Zm00001d041953_T010 0
744- 1: transcript:EES02150 transcript:Zm00001d041951_T001 0
744- 2: transcript:EES04301 transcript:Zm00001d041950_T001 0
744- 3: transcript:EES02151 transcript:Zm00001d041949_T001 2.00E-37
744- 4: transcript:OQU88278 transcript:Zm00001d041948_T002 0
744- 5: transcript:KXG34231 transcript:Zm00001d041947_T001 0
744- 6: transcript:EES02152 transcript:Zm00001d041944_T001 0
744- 7: transcript:EES04305 transcript:Zm00001d041941_T001 0
744- 8: transcript:KXG34234 transcript:Zm00001d041940_T001 0
Alignment 745: score=418.0 e_value=1.2e-22 N=10 3&3 minus
745- 0: transcript:KXG32714 transcript:Zm00001d044510_T002 0
745- 1: transcript:EES00907 transcript:Zm00001d044506_T006 0
745- 2: transcript:OQU87011 transcript:Zm00001d044504_T001 0
745- 3: transcript:EES00914 transcript:Zm00001d044503_T004 0
745- 4: transcript:EES03122 transcript:Zm00001d044502_T001 2.00E-116
745- 5: transcript:OQU87012 transcript:Zm00001d044500_T001 1.00E-11
745- 6: transcript:KXG32731 transcript:Zm00001d044498_T003 0
745- 7: transcript:EES00918 transcript:Zm00001d044497_T002 1.00E-148
745- 8: transcript:EES00921 transcript:Zm00001d044496_T013 0
745- 9: transcript:EES00922 transcript:Zm00001d044495_T005 0
Alignment 746: score=319.0 e_value=7.2e-13 N=7 3&3 minus
746- 0: transcript:KXG33962 transcript:Zm00001d042432_T007 0
746- 1: transcript:EES01938 transcript:Zm00001d042427_T001 0
746- 2: transcript:OQU88086 transcript:Zm00001d042426_T001 5.00E-166
746- 3: transcript:EES04078 transcript:Zm00001d042425_T001 0
746- 4: transcript:EES01940 transcript:Zm00001d042421_T001 0
746- 5: transcript:EES04079 transcript:Zm00001d042420_T001 9.00E-59
746- 6: transcript:EES04084 transcript:Zm00001d042416_T002 0
Alignment 747: score=275.0 e_value=3.2e-10 N=6 3&5 plus
747- 0: transcript:Zm00001d044601_T006 transcript:KXG27541 0

747- 1: transcript:Zm00001d044602_T001 transcript:OQU82677 0
747- 2: transcript:Zm00001d044605_T001 transcript:EES09078 0
747- 3: transcript:Zm00001d044606_T001 transcript:EES09081 0
747- 4: transcript:Zm00001d044607_T001 transcript:KXG27548 2.00E-39
747- 5: transcript:Zm00001d044608_T001 transcript:EES09082 0
Alignment 748: score=837.0 e_value=1.1e-51 N=19 3&6 plus
748- 0: transcript:EES01425 transcript:Zm00001d038761_T001 7.00E-61
748- 1: transcript:EES01429 transcript:Zm00001d038762_T001 0
748- 2: transcript:EES01428 transcript:Zm00001d038763_T001 8.00E-130
748- 3: transcript:EES01437 transcript:Zm00001d038764_T001 2.00E-136
748- 4: transcript:OQU87487 transcript:Zm00001d038766_T001 3.00E-63
748- 5: transcript:KXG33320 transcript:Zm00001d038768_T001 6.00E-136
748- 6: transcript:OQU87495 transcript:Zm00001d038775_T002 0
748- 7: transcript:EES01449 transcript:Zm00001d038779_T001 2.00E-145
748- 8: transcript:EES03581 transcript:Zm00001d038780_T001 2.00E-35
748- 9: transcript:EES01452 transcript:Zm00001d038783_T001 1.00E-84
748- 10: transcript:KXG33335 transcript:Zm00001d038784_T001 6.00E-76
748- 11: transcript:KXG33339 transcript:Zm00001d038791_T001 0
748- 12: transcript:EES03587 transcript:Zm00001d038792_T001 0
748- 13: transcript:KXG33341 transcript:Zm00001d038793_T001 3.00E-65
748- 14: transcript:KXG33344 transcript:Zm00001d038794_T002 0
748- 15: transcript:KXG33345 transcript:Zm00001d038796_T001 0
748- 16: transcript:OQU87522 transcript:Zm00001d038797_T001 0
748- 17: transcript:EES03595 transcript:Zm00001d038801_T001 2.00E-105
748- 18: transcript:KXG33358 transcript:Zm00001d038804_T002 0
Alignment 749: score=381.0 e_value=1.5e-20 N=10 3&6 plus
749- 0: transcript:EES00189 transcript:Zm00001d037619_T001 0
749- 1: transcript:KXG31703 transcript:Zm00001d037623_T001 4.00E-14
749- 2: transcript:KXG31704 transcript:Zm00001d037624_T001 2.00E-158
749- 3: transcript:EES00199 transcript:Zm00001d037626_T001 0
749- 4: transcript:EES00202 transcript:Zm00001d037627_T001 3.00E-138
749- 5: transcript:KXG31729 transcript:Zm00001d037630_T001 2.00E-80
749- 6: transcript:EES00210 transcript:Zm00001d037631_T001 1.00E-23
749- 7: transcript:KXG31743 transcript:Zm00001d037636_T001 0
749- 8: transcript:EES02380 transcript:Zm00001d037637_T001 3.00E-29
749- 9: transcript:KXG31749 transcript:Zm00001d037643_T001 0
Alignment 750: score=311.0 e_value=0 N=7 3&6 plus
750- 0: transcript:KXG33770 transcript:Zm00001d038326_T003 0
750- 1: transcript:EES01827 transcript:Zm00001d038328_T001 0
750- 2: transcript:KXG33783 transcript:Zm00001d038331_T001 4.00E-41
750- 3: transcript:EES01830 transcript:Zm00001d038333_T001 0
750- 4: transcript:OQU87951 transcript:Zm00001d038334_T001 0
750- 5: transcript:EES03963 transcript:Zm00001d038336_T001 9.00E-142
750- 6: transcript:EES01839 transcript:Zm00001d038338_T001 4.00E-114
Alignment 751: score=292.0 e_value=7.7e-12 N=7 3&6 plus
751- 0: transcript:EES03545 transcript:Zm00001d038740_T002 1.00E-50
751- 1: transcript:EES01405 transcript:Zm00001d038742_T001 0
751- 2: transcript:EES01406 transcript:Zm00001d038745_T002 0
751- 3: transcript:EES01408 transcript:Zm00001d038747_T003 3.00E-172
751- 4: transcript:EES01416 transcript:Zm00001d038751_T001 2.00E-29
751- 5: transcript:KXG33308 transcript:Zm00001d038752_T001 0
751- 6: transcript:EES03565 transcript:Zm00001d038753_T002 2.00E-54
Alignment 752: score=272.0 e_value=2.2e-08 N=6 3&6 plus
752- 0: transcript:EES02301 transcript:Zm00001d037745_T001 0

752- 1: transcript:EES00126 transcript:Zm00001d037747_T001 1.00E-56
752- 2: transcript:KXG31616 transcript:Zm00001d037749_T001 3.00E-38
752- 3: transcript:EES00128 transcript:Zm00001d037751_T001 2.00E-97
752- 4: transcript:KXG31618 transcript:Zm00001d037753_T001 1.00E-09
752- 5: transcript:EES02308 transcript:Zm00001d037757_T001 4.00E-43
Alignment 753: score=257.0 e_value=5.1e-09 N=6 3&6 plus
753- 0: transcript:OQU87868 transcript:Zm00001d038263_T001 2.00E-157
753- 1: transcript:EES03895 transcript:Zm00001d038268_T001 1.00E-156
753- 2: transcript:EES03894 transcript:Zm00001d038269_T001 0
753- 3: transcript:KXG33716 transcript:Zm00001d038270_T001 4.00E-107
753- 4: transcript:EES01775 transcript:Zm00001d038274_T002 1.00E-150
753- 5: transcript:EES01778 transcript:Zm00001d038275_T002 0
Alignment 754: score=1139.0 e_value=3.1e-85 N=27 3&6 minus
754- 0: transcript:KXG33109 transcript:Zm00001d038972_T002 0
754- 1: transcript:OQU87329 transcript:Zm00001d038968_T001 2.00E-114
754- 2: transcript:EES01264 transcript:Zm00001d038965_T001 2.00E-141
754- 3: transcript:EES03442 transcript:Zm00001d038963_T001 5.00E-83
754- 4: transcript:EES03441 transcript:Zm00001d038959_T001 2.00E-24
754- 5: transcript:OQU87335 transcript:Zm00001d038955_T001 0
754- 6: transcript:KXG33132 transcript:Zm00001d038950_T001 5.00E-09
754- 7: transcript:EES03446 transcript:Zm00001d038948_T001 0
754- 8: transcript:EES03447 transcript:Zm00001d038944_T003 0
754- 9: transcript:OQU87345 transcript:Zm00001d038930_T001 2.00E-90
754- 10: transcript:KXG33146 transcript:Zm00001d038929_T001 0
754- 11: transcript:OQU87350 transcript:Zm00001d038926_T001 2.00E-67
754- 12: transcript:EES01286 transcript:Zm00001d038923_T001 0
754- 13: transcript:KXG33157 transcript:Zm00001d038921_T001 7.00E-136
754- 14: transcript:OQU87356 transcript:Zm00001d038920_T001 4.00E-30
754- 15: transcript:EES01291 transcript:Zm00001d038918_T001 4.00E-38
754- 16: transcript:EES03455 transcript:Zm00001d038916_T001 0
754- 17: transcript:OQU87357 transcript:Zm00001d038915_T001 0
754- 18: transcript:EES03464 transcript:Zm00001d038911_T001 2.00E-15
754- 19: transcript:KXG33168 transcript:Zm00001d038910_T001 3.00E-96
754- 20: transcript:OQU87370 transcript:Zm00001d038909_T001 1.00E-94
754- 21: transcript:KXG33171 transcript:Zm00001d038908_T004 7.00E-149
754- 22: transcript:EES03472 transcript:Zm00001d038907_T001 2.00E-171
754- 23: transcript:OQU87374 transcript:Zm00001d038904_T001 0
754- 24: transcript:EES03474 transcript:Zm00001d038903_T001 1.00E-113
754- 25: transcript:EES01312 transcript:Zm00001d038892_T001 0
754- 26: transcript:EES01313 transcript:Zm00001d038891_T001 0
Alignment 755: score=944.0 e_value=1.5e-63 N=22 3&6 minus
755- 0: transcript:EES01844 transcript:Zm00001d038251_T001 4.00E-93
755- 1: transcript:EES01846 transcript:Zm00001d038250_T001 8.00E-131
755- 2: transcript:OQU87973 transcript:Zm00001d038248_T002 3.00E-50
755- 3: transcript:EES03975 transcript:Zm00001d038239_T001 6.00E-40
755- 4: transcript:EES01856 transcript:Zm00001d038229_T001 0
755- 5: transcript:EES03982 transcript:Zm00001d038228_T001 3.00E-153
755- 6: transcript:KXG33819 transcript:Zm00001d038226_T001 8.00E-128
755- 7: transcript:KXG33820 transcript:Zm00001d038225_T003 0
755- 8: transcript:EES03986 transcript:Zm00001d038224_T003 0
755- 9: transcript:OQU87990 transcript:Zm00001d038222_T003 0
755- 10: transcript:KXG33829 transcript:Zm00001d038221_T001 5.00E-150
755- 11: transcript:EES03996 transcript:Zm00001d038218_T001 5.00E-73
755- 12: transcript:KXG33835 transcript:Zm00001d038217_T002 8.00E-99

755- 13: transcript:KXG33837 transcript:Zm00001d038216_T001 3.00E-39
755- 14: transcript:KXG33843 transcript:Zm00001d038209_T001 0
755- 15: transcript:KXG33846 transcript:Zm00001d038208_T001 3.00E-129
755- 16: transcript:EES01882 transcript:Zm00001d038207_T001 3.00E-112
755- 17: transcript:KXG33848 transcript:Zm00001d038205_T002 0
755- 18: transcript:OQU88008 transcript:Zm00001d038203_T001 6.00E-17
755- 19: transcript:OQU88012 transcript:Zm00001d038197_T001 1.00E-77
755- 20: transcript:KXG33864 transcript:Zm00001d038196_T001 0
755- 21: transcript:KXG33866 transcript:Zm00001d038195_T001 4.00E-84
Alignment 756: score=934.0 e_value=7.2e-68 N=23 3&6 minus
756- 0: transcript:EES03800 transcript:Zm00001d038494_T011 0
756- 1: transcript:EES01661 transcript:Zm00001d038489_T001 0
756- 2: transcript:EES01662 transcript:Zm00001d038487_T003 0
756- 3: transcript:EES01664 transcript:Zm00001d038483_T001 2.00E-63
756- 4: transcript:EES03806 transcript:Zm00001d038481_T001 0
756- 5: transcript:OQU87763 transcript:Zm00001d038476_T001 5.00E-156
756- 6: transcript:KXG33624 transcript:Zm00001d038473_T001 3.00E-80
756- 7: transcript:KXG33626 transcript:Zm00001d038471_T001 0
756- 8: transcript:EES01676 transcript:Zm00001d038469_T001 3.00E-31
756- 9: transcript:KXG33629 transcript:Zm00001d038466_T001 7.00E-36
756- 10: transcript:EES03814 transcript:Zm00001d038465_T001 0
756- 11: transcript:KXG33634 transcript:Zm00001d038459_T001 8.00E-179
756- 12: transcript:KXG33637 transcript:Zm00001d038451_T001 3.00E-141
756- 13: transcript:EES03819 transcript:Zm00001d038450_T001 3.00E-65
756- 14: transcript:OQU87773 transcript:Zm00001d038449_T001 6.00E-168
756- 15: transcript:OQU87774 transcript:Zm00001d038447_T001 0
756- 16: transcript:EES03824 transcript:Zm00001d038442_T001 1.00E-75
756- 17: transcript:KXG33643 transcript:Zm00001d038431_T001 3.00E-152
756- 18: transcript:EES03833 transcript:Zm00001d038420_T001 2.00E-41
756- 19: transcript:OQU87792 transcript:Zm00001d038412_T001 0
756- 20: transcript:EES01704 transcript:Zm00001d038409_T001 0
756- 21: transcript:EES01709 transcript:Zm00001d038408_T001 4.00E-57
756- 22: transcript:OQU87808 transcript:Zm00001d038397_T005 5.00E-86
Alignment 757: score=932.0 e_value=5.6e-69 N=23 3&6 minus
757- 0: transcript:EES03761 transcript:Zm00001d038577_T001 4.00E-157
757- 1: transcript:KXG33562 transcript:Zm00001d038574_T004 3.00E-83
757- 2: transcript:EES01618 transcript:Zm00001d038563_T001 2.00E-120
757- 3: transcript:KXG33567 transcript:Zm00001d038562_T001 0
757- 4: transcript:KXG33568 transcript:Zm00001d038558_T005 5.00E-33
757- 5: transcript:EES03767 transcript:Zm00001d038555_T001 0
757- 6: transcript:EES03768 transcript:Zm00001d038554_T001 0
757- 7: transcript:KXG33573 transcript:Zm00001d038553_T001 2.00E-131
757- 8: transcript:EES03777 transcript:Zm00001d038548_T001 0
757- 9: transcript:EES01631 transcript:Zm00001d038543_T004 8.00E-71
757- 10: transcript:EES01635 transcript:Zm00001d038541_T002 0
757- 11: transcript:EES01637 transcript:Zm00001d038538_T001 3.00E-102
757- 12: transcript:EES01639 transcript:Zm00001d038537_T001 0
757- 13: transcript:OQU87737 transcript:Zm00001d038533_T004 1.00E-129
757- 14: transcript:EES01645 transcript:Zm00001d038532_T001 3.00E-45
757- 15: transcript:OQU87743 transcript:Zm00001d038530_T003 0
757- 16: transcript:EES03792 transcript:Zm00001d038529_T003 2.00E-173
757- 17: transcript:EES03794 transcript:Zm00001d038526_T002 1.00E-09
757- 18: transcript:KXG33608 transcript:Zm00001d038522_T002 0
757- 19: transcript:EES01652 transcript:Zm00001d038521_T001 0

757- 20: transcript:EES01654 transcript:Zm00001d038517_T001 0
757- 21: transcript:EES01655 transcript:Zm00001d038514_T001 1.00E-80
757- 22: transcript:EES03800 transcript:Zm00001d038503_T001 0
Alignment 758: score=738.0 e_value=6.7e-44 N=17 3&6 minus
758- 0: transcript:KXG33049 transcript:Zm00001d039043_T011 0
758- 1: transcript:KXG33051 transcript:Zm00001d039041_T007 0
758- 2: transcript:KXG33053 transcript:Zm00001d039039_T002 3.00E-36
758- 3: transcript:EES01219 transcript:Zm00001d039038_T001 0
758- 4: transcript:KXG33056 transcript:Zm00001d039037_T001 0
758- 5: transcript:EES03393 transcript:Zm00001d039032_T001 3.00E-92
758- 6: transcript:OQU87291 transcript:Zm00001d039031_T001 3.00E-17
758- 7: transcript:EES03398 transcript:Zm00001d039021_T001 9.00E-90
758- 8: transcript:EES03399 transcript:Zm00001d039020_T001 4.00E-124
758- 9: transcript:EES03403 transcript:Zm00001d039017_T001 2.00E-39
758- 10: transcript:OQU87300 transcript:Zm00001d039016_T001 1.00E-122
758- 11: transcript:KXG33070 transcript:Zm00001d039015_T001 6.00E-82
758- 12: transcript:OQU87301 transcript:Zm00001d039014_T001 9.00E-153
758- 13: transcript:EES03406 transcript:Zm00001d039011_T001 3.00E-39
758- 14: transcript:EES03407 transcript:Zm00001d039010_T001 7.00E-36
758- 15: transcript:OQU87307 transcript:Zm00001d039006_T007 0
758- 16: transcript:OQU87308 transcript:Zm00001d039004_T002 2.00E-147
Alignment 759: score=583.0 e_value=2.5e-33 N=14 3&6 minus
759- 0: transcript:EES03483 transcript:Zm00001d038886_T001 2.00E-19
759- 1: transcript:EES01324 transcript:Zm00001d038883_T001 3.00E-133
759- 2: transcript:EES01332 transcript:Zm00001d038882_T002 0
759- 3: transcript:KXG33200 transcript:Zm00001d038880_T001 0
759- 4: transcript:EES01335 transcript:Zm00001d038879_T003 0
759- 5: transcript:EES03490 transcript:Zm00001d038878_T001 9.00E-109
759- 6: transcript:EES03491 transcript:Zm00001d038876_T001 2.00E-25
759- 7: transcript:KXG33209 transcript:Zm00001d038873_T001 3.00E-21
759- 8: transcript:KXG33211 transcript:Zm00001d038870_T001 2.00E-25
759- 9: transcript:EES01347 transcript:Zm00001d038865_T003 7.00E-89
759- 10: transcript:KXG33221 transcript:Zm00001d038863_T001 1.00E-54
759- 11: transcript:OQU87402 transcript:Zm00001d038862_T001 6.00E-119
759- 12: transcript:EES03502 transcript:Zm00001d038861_T001 5.00E-132
759- 13: transcript:OQU87407 transcript:Zm00001d038860_T001 3.00E-33
Alignment 760: score=516.0 e_value=8.7e-26 N=12 3&6 minus
760- 0: transcript:OQU87888 transcript:Zm00001d038301_T002 2.00E-95
760- 1: transcript:EES03915 transcript:Zm00001d038300_T002 0
760- 2: transcript:EES03920 transcript:Zm00001d038297_T001 7.00E-116
760- 3: transcript:KXG33737 transcript:Zm00001d038296_T001 5.00E-149
760- 4: transcript:EES01793 transcript:Zm00001d038291_T001 1.00E-54
760- 5: transcript:EES01799 transcript:Zm00001d038289_T001 3.00E-77
760- 6: transcript:EES01800 transcript:Zm00001d038288_T001 1.00E-73
760- 7: transcript:OQU87905 transcript:Zm00001d038287_T001 1.00E-26
760- 8: transcript:OQU87906 transcript:Zm00001d038284_T001 2.00E-44
760- 9: transcript:EES03931 transcript:Zm00001d038283_T001 2.00E-31
760- 10: transcript:KXG33746 transcript:Zm00001d038282_T001 0
760- 11: transcript:EES03938 transcript:Zm00001d038281_T001 5.00E-83
Alignment 761: score=400.0 e_value=1.8e-17 N=9 3&6 minus
761- 0: transcript:KXG32997 transcript:Zm00001d039090_T001 2.00E-153
761- 1: transcript:KXG33002 transcript:Zm00001d039089_T001 0
761- 2: transcript:KXG33004 transcript:Zm00001d039087_T001 3.00E-164
761- 3: transcript:KXG33005 transcript:Zm00001d039086_T001 0

```
761-  4: transcript:EES01185           transcript:Zm00001d039084_T001  7.00E-24
761-  5: transcript:EES01186           transcript:Zm00001d039083_T001  3.00E-71
761-  6: transcript:KXG33008           transcript:Zm00001d039082_T001  3.00E-06
761-  7: transcript:KXG33012           transcript:Zm00001d039081_T002         0
761-  8: transcript:OQU87252           transcript:Zm00001d039079_T002         0
## Alignment 762: score=399.0 e_value=2.4e-17 N=9 3&6 minus
762-  0: transcript:KXG33081           transcript:Zm00001d039002_T019         0
762-  1: transcript:KXG33085           transcript:Zm00001d038999_T001 1.00E-101
762-  2: transcript:EES03419           transcript:Zm00001d038998_T001 4.00E-112
762-  3: transcript:EES03421           transcript:Zm00001d038995_T001 3.00E-105
762-  4: transcript:EES03423           transcript:Zm00001d038994_T001  2.00E-93
762-  5: transcript:EES01241           transcript:Zm00001d038993_T001  4.00E-11
762-  6: transcript:KXG33100           transcript:Zm00001d038991_T001         0
762-  7: transcript:KXG33101           transcript:Zm00001d038989_T002 1.00E-177
762-  8: transcript:KXG33104           transcript:Zm00001d038987_T001  3.00E-32
## Alignment 763: score=386.0 e_value=2.9e-19 N=10 3&6 minus
763-  0: transcript:OQU87820           transcript:Zm00001d038393_T012         0
763-  1: transcript:EES01717           transcript:Zm00001d038392_T001         0
763-  2: transcript:EES01719           transcript:Zm00001d038388_T001  9.00E-99
763-  3: transcript:EES01725           transcript:Zm00001d038387_T001  2.00E-33
763-  4: transcript:EES03852           transcript:Zm00001d038379_T001  3.00E-38
763-  5: transcript:EES03856           transcript:Zm00001d038376_T001 2.00E-117
763-  6: transcript:EES03860           transcript:Zm00001d038374_T007  6.00E-56
763-  7: transcript:EES01733           transcript:Zm00001d038373_T001         0
763-  8: transcript:EES03867           transcript:Zm00001d038371_T002         0
763-  9: transcript:EES03870           transcript:Zm00001d038367_T002 1.00E-175
## Alignment 764: score=337.0 e_value=2.4e-16 N=8 3&6 minus
764-  0: transcript:EES01473           transcript:Zm00001d038726_T001 8.00E-167
764-  1: transcript:EES01474           transcript:Zm00001d038725_T001  6.00E-71
764-  2: transcript:EES01480           transcript:Zm00001d038714_T001  9.00E-46
764-  3: transcript:KXG33373           transcript:Zm00001d038708_T001 4.00E-178
764-  4: transcript:KXG33374           transcript:Zm00001d038706_T001  5.00E-12
764-  5: transcript:KXG33380           transcript:Zm00001d038704_T002         0
764-  6: transcript:KXG33383           transcript:Zm00001d038699_T001 1.00E-108
764-  7: transcript:OQU87551           transcript:Zm00001d038698_T001         0
## Alignment 765: score=327.0 e_value=7.9e-12 N=7 3&6 minus
765-  0: transcript:KXG33695           transcript:Zm00001d038358_T002         0
765-  1: transcript:EES03882           transcript:Zm00001d038355_T001         0
765-  2: transcript:OQU87858           transcript:Zm00001d038352_T001  1.00E-19
765-  3: transcript:EES03887           transcript:Zm00001d038351_T004  4.00E-47
765-  4: transcript:EES01757           transcript:Zm00001d038346_T002 2.00E-104
765-  5: transcript:EES01758           transcript:Zm00001d038343_T001         0
765-  6: transcript:OQU87860           transcript:Zm00001d038342_T002  1.00E-30
## Alignment 766: score=308.0 e_value=5.5e-15 N=8 3&6 minus
766-  0: transcript:Zm00001d042730_T001 transcript:EES10786            9.00E-93
766-  1: transcript:Zm00001d042735_T001 transcript:OQU81550            1.00E-27
766-  2: transcript:Zm00001d042738_T001 transcript:KXG26241            1.00E-77
766-  3: transcript:Zm00001d042750_T001 transcript:EES12138            1.00E-12
766-  4: transcript:Zm00001d042753_T001 transcript:KXG26269            1.00E-20
766-  5: transcript:Zm00001d042754_T001 transcript:EES10769            4.00E-12
766-  6: transcript:Zm00001d042760_T001 transcript:OQU81531            1.00E-18
766-  7: transcript:Zm00001d042767_T001 transcript:OQU81515           4.00E-146
## Alignment 767: score=3540.0 e_value=0 N=80 3&8 plus
767-  0: transcript:EES03446           transcript:Zm00001d011610_T063         0
```

767- 1:	transcript:EES01274	transcript:Zm00001d011611_T005	0
767- 2:	transcript:OQU87345	transcript:Zm00001d011614_T001	2.00E-163
767- 3:	transcript:EES01283	transcript:Zm00001d011615_T004	0
767- 4:	transcript:OQU87350	transcript:Zm00001d011616_T001	6.00E-152
767- 5:	transcript:KXG33157	transcript:Zm00001d011618_T001	0
767- 6:	transcript:EES01291	transcript:Zm00001d011620_T001	1.00E-63
767- 7:	transcript:EES03455	transcript:Zm00001d011622_T004	0
767- 8:	transcript:EES03458	transcript:Zm00001d011623_T001	2.00E-34
767- 9:	transcript:EES01292	transcript:Zm00001d011624_T001	0
767- 10:	transcript:OQU87357	transcript:Zm00001d011625_T001	0
767- 11:	transcript:EES01295	transcript:Zm00001d011626_T001	3.00E-91
767- 12:	transcript:EES01297	transcript:Zm00001d011627_T001	5.00E-17
767- 13:	transcript:EES03464	transcript:Zm00001d011630_T001	2.00E-45
767- 14:	transcript:KXG33168	transcript:Zm00001d011631_T001	1.00E-133
767- 15:	transcript:EES03465	transcript:Zm00001d011632_T002	0
767- 16:	transcript:EES01303	transcript:Zm00001d011634_T002	0
767- 17:	transcript:KXG33169	transcript:Zm00001d011635_T001	7.00E-37
767- 18:	transcript:OQU87370	transcript:Zm00001d011636_T001	4.00E-153
767- 19:	transcript:OQU87371	transcript:Zm00001d011637_T001	3.00E-97
767- 20:	transcript:KXG33171	transcript:Zm00001d011638_T002	0
767- 21:	transcript:EES03472	transcript:Zm00001d011639_T001	0
767- 22:	transcript:KXG33172	transcript:Zm00001d011641_T001	4.00E-103
767- 23:	transcript:EES01313	transcript:Zm00001d011642_T002	0
767- 24:	transcript:EES01319	transcript:Zm00001d011644_T002	0
767- 25:	transcript:KXG33190	transcript:Zm00001d011645_T001	7.00E-111
767- 26:	transcript:EES03481	transcript:Zm00001d011648_T001	0
767- 27:	transcript:EES03482	transcript:Zm00001d011649_T001	0
767- 28:	transcript:EES03483	transcript:Zm00001d011650_T001	3.00E-118
767- 29:	transcript:EES01323	transcript:Zm00001d011652_T001	0
767- 30:	transcript:EES01324	transcript:Zm00001d011654_T001	0
767- 31:	transcript:EES01328	transcript:Zm00001d011657_T002	2.00E-100
767- 32:	transcript:EES01330	transcript:Zm00001d011658_T001	2.00E-126
767- 33:	transcript:EES01332	transcript:Zm00001d011660_T003	0
767- 34:	transcript:KXG33200	transcript:Zm00001d011663_T003	0
767- 35:	transcript:EES01335	transcript:Zm00001d011668_T001	0
767- 36:	transcript:EES03490	transcript:Zm00001d011669_T001	2.00E-116
767- 37:	transcript:EES01337	transcript:Zm00001d011673_T001	0
767- 38:	transcript:EES01338	transcript:Zm00001d011676_T002	0
767- 39:	transcript:EES03491	transcript:Zm00001d011678_T002	3.00E-111
767- 40:	transcript:EES01339	transcript:Zm00001d011679_T001	0
767- 41:	transcript:OQU87399	transcript:Zm00001d011681_T001	7.00E-44
767- 42:	transcript:EES03498	transcript:Zm00001d011684_T002	0
767- 43:	transcript:EES03497	transcript:Zm00001d011685_T002	0
767- 44:	transcript:EES03499	transcript:Zm00001d011687_T001	0
767- 45:	transcript:EES01350	transcript:Zm00001d011688_T004	1.00E-158
767- 46:	transcript:EES01351	transcript:Zm00001d011691_T002	2.00E-159
767- 47:	transcript:EES01352	transcript:Zm00001d011692_T001	0
767- 48:	transcript:EES01354	transcript:Zm00001d011696_T002	0
767- 49:	transcript:EES01355	transcript:Zm00001d011697_T001	0
767- 50:	transcript:KXG33224	transcript:Zm00001d011698_T001	2.00E-39
767- 51:	transcript:EES01356	transcript:Zm00001d011699_T002	0
767- 52:	transcript:KXG33225	transcript:Zm00001d011700_T014	0
767- 53:	transcript:EES01357	transcript:Zm00001d011705_T002	0
767- 54:	transcript:EES01359	transcript:Zm00001d011706_T003	0

```
767- 55: transcript:KXG33230           transcript:Zm00001d011707_T001          0
767- 56: transcript:EES01361           transcript:Zm00001d011708_T001 2.00E-156
767- 57: transcript:OQU87411           transcript:Zm00001d011709_T003          0
767- 58: transcript:EES03512           transcript:Zm00001d011710_T001 6.00E-156
767- 59: transcript:KXG33236           transcript:Zm00001d011712_T001          0
767- 60: transcript:OQU87414           transcript:Zm00001d011713_T003          0
767- 61: transcript:OQU87416           transcript:Zm00001d011716_T001          0
767- 62: transcript:EES03521           transcript:Zm00001d011717_T001 5.00E-129
767- 63: transcript:OQU87421           transcript:Zm00001d011718_T002          0
767- 64: transcript:EES03528           transcript:Zm00001d011719_T008 1.00E-140
767- 65: transcript:KXG33253           transcript:Zm00001d011720_T002          0
767- 66: transcript:EES03532           transcript:Zm00001d011721_T001          0
767- 67: transcript:EES01374           transcript:Zm00001d011722_T001  3.00E-07
767- 68: transcript:OQU87429           transcript:Zm00001d011723_T002          0
767- 69: transcript:OQU87430           transcript:Zm00001d011730_T001 4.00E-155
767- 70: transcript:EES01377           transcript:Zm00001d011731_T001  1.00E-74
767- 71: transcript:EES03536           transcript:Zm00001d011732_T001 4.00E-147
767- 72: transcript:EES03537           transcript:Zm00001d011734_T001 7.00E-164
767- 73: transcript:OQU87436           transcript:Zm00001d011735_T001          0
767- 74: transcript:EES01382           transcript:Zm00001d011736_T001 1.00E-125
767- 75: transcript:OQU87437           transcript:Zm00001d011738_T002  1.00E-70
767- 76: transcript:EES01384           transcript:Zm00001d011739_T001 5.00E-105
767- 77: transcript:KXG33273           transcript:Zm00001d011740_T001          0
767- 78: transcript:EES03542           transcript:Zm00001d011741_T002  5.00E-94
767- 79: transcript:KXG33276           transcript:Zm00001d011742_T005          0
## Alignment 768: score=2460.0 e_value=1.3e-226 N=56 3&8 plus
768-  0: transcript:Zm00001d041343_T001 transcript:EES16256                 0
768-  1: transcript:Zm00001d041345_T001 transcript:EES17294                 0
768-  2: transcript:Zm00001d041346_T001 transcript:OQU79459                 0
768-  3: transcript:Zm00001d041351_T001 transcript:EES17296          1.00E-83
768-  4: transcript:Zm00001d041352_T001 transcript:EES17297         8.00E-125
768-  5: transcript:Zm00001d041353_T002 transcript:OQU79460                 0
768-  6: transcript:Zm00001d041363_T003 transcript:OQU79465                 0
768-  7: transcript:Zm00001d041365_T001 transcript:KXG23847          7.00E-98
768-  8: transcript:Zm00001d041374_T001 transcript:EES17304                 0
768-  9: transcript:Zm00001d041375_T001 transcript:OQU79477          8.00E-49
768- 10: transcript:Zm00001d041376_T001 transcript:OQU79478          2.00E-56
768- 11: transcript:Zm00001d041378_T002 transcript:KXG23863                 0
768- 12: transcript:Zm00001d041380_T001 transcript:EES16270                 0
768- 13: transcript:Zm00001d041381_T001 transcript:OQU79483         2.00E-170
768- 14: transcript:Zm00001d041382_T001 transcript:EES16277          4.00E-36
768- 15: transcript:Zm00001d041383_T002 transcript:OQU79488         9.00E-141
768- 16: transcript:Zm00001d041384_T001 transcript:EES16281          9.00E-33
768- 17: transcript:Zm00001d041387_T001 transcript:OQU79494         2.00E-136
768- 18: transcript:Zm00001d041390_T001 transcript:OQU79495         2.00E-151
768- 19: transcript:Zm00001d041392_T001 transcript:OQU79501         2.00E-115
768- 20: transcript:Zm00001d041395_T002 transcript:OQU79502                 0
768- 21: transcript:Zm00001d041397_T001 transcript:EES17321                 0
768- 22: transcript:Zm00001d041402_T001 transcript:OQU79511                 0
768- 23: transcript:Zm00001d041403_T001 transcript:OQU79513          1.00E-38
768- 24: transcript:Zm00001d041404_T001 transcript:EES17325          2.00E-48
768- 25: transcript:Zm00001d041405_T001 transcript:OQU79520                 0
768- 26: transcript:Zm00001d041407_T002 transcript:KXG23899                 0
768- 27: transcript:Zm00001d041410_T001 transcript:EES17331          5.00E-80
```

768- 28: transcript:Zm00001d041414_T002 transcript:OQU79521 0
768- 29: transcript:Zm00001d041415_T001 transcript:KXG23904 0
768- 30: transcript:Zm00001d041416_T001 transcript:EES17334 2.00E-119
768- 31: transcript:Zm00001d041418_T001 transcript:EES16315 4.00E-104
768- 32: transcript:Zm00001d041420_T025 transcript:EES17335 0
768- 33: transcript:Zm00001d041422_T001 transcript:EES17341 0
768- 34: transcript:Zm00001d041426_T001 transcript:EES17343 5.00E-177
768- 35: transcript:Zm00001d041430_T001 transcript:EES16322 1.00E-95
768- 36: transcript:Zm00001d041437_T001 transcript:OQU79535 3.00E-140
768- 37: transcript:Zm00001d041438_T001 transcript:OQU79536 0
768- 38: transcript:Zm00001d041439_T001 transcript:OQU79537 0
768- 39: transcript:Zm00001d041443_T012 transcript:EES17351 0
768- 40: transcript:Zm00001d041444_T001 transcript:EES17352 0
768- 41: transcript:Zm00001d041445_T006 transcript:KXG23930 0
768- 42: transcript:Zm00001d041455_T002 transcript:OQU79550 0
768- 43: transcript:Zm00001d041456_T001 transcript:EES17355 0
768- 44: transcript:Zm00001d041458_T001 transcript:OQU79557 5.00E-167
768- 45: transcript:Zm00001d041462_T001 transcript:EES17360 7.00E-54
768- 46: transcript:Zm00001d041464_T001 transcript:OQU79569 0
768- 47: transcript:Zm00001d041465_T001 transcript:KXG23954 2.00E-58
768- 48: transcript:Zm00001d041467_T001 transcript:EES17361 1.00E-81
768- 49: transcript:Zm00001d041471_T001 transcript:EES16348 0
768- 50: transcript:Zm00001d041472_T001 transcript:OQU79579 5.00E-165
768- 51: transcript:Zm00001d041474_T001 transcript:KXG23966 2.00E-134
768- 52: transcript:Zm00001d041475_T001 transcript:EES16349 0
768- 53: transcript:Zm00001d041476_T001 transcript:OQU79583 1.00E-25
768- 54: transcript:Zm00001d041480_T001 transcript:EES17375 0
768- 55: transcript:Zm00001d041481_T001 transcript:OQU79585 0
Alignment 769: score=1937.0 e_value=3.2e-154 N=43 3&8 plus
769- 0: transcript:KXG33012 transcript:Zm00001d011486_T003 0
769- 1: transcript:OQU87252 transcript:Zm00001d011487_T001 0
769- 2: transcript:OQU87254 transcript:Zm00001d011490_T003 0
769- 3: transcript:OQU87255 transcript:Zm00001d011492_T001 0
769- 4: transcript:KXG33023 transcript:Zm00001d011495_T001 2.00E-179
769- 5: transcript:EES03370 transcript:Zm00001d011496_T002 2.00E-168
769- 6: transcript:EES01196 transcript:Zm00001d011497_T001 1.00E-18
769- 7: transcript:EES03373 transcript:Zm00001d011498_T001 0
769- 8: transcript:EES03374 transcript:Zm00001d011499_T001 2.00E-143
769- 9: transcript:KXG33034 transcript:Zm00001d011500_T014 6.00E-78
769- 10: transcript:EES03375 transcript:Zm00001d011501_T001 2.00E-28
769- 11: transcript:EES01199 transcript:Zm00001d011503_T001 6.00E-141
769- 12: transcript:EES01200 transcript:Zm00001d011504_T003 6.00E-163
769- 13: transcript:EES01201 transcript:Zm00001d011506_T001 3.00E-60
769- 14: transcript:EES03381 transcript:Zm00001d011507_T001 5.00E-59
769- 15: transcript:EES01206 transcript:Zm00001d011510_T001 0
769- 16: transcript:EES01208 transcript:Zm00001d011511_T001 3.00E-24
769- 17: transcript:KXG33045 transcript:Zm00001d011512_T004 4.00E-143
769- 18: transcript:EES01209 transcript:Zm00001d011513_T001 1.00E-74
769- 19: transcript:EES03388 transcript:Zm00001d011514_T001 2.00E-88
769- 20: transcript:EES03391 transcript:Zm00001d011515_T001 5.00E-53
769- 21: transcript:KXG33051 transcript:Zm00001d011517_T005 0
769- 22: transcript:OQU87278 transcript:Zm00001d011518_T001 0
769- 23: transcript:EES01216 transcript:Zm00001d011519_T003 0
769- 24: transcript:EES03392 transcript:Zm00001d011520_T001 2.00E-163

769- 25: transcript:KXG33056 transcript:Zm00001d011525_T002 0
769- 26: transcript:EES01221 transcript:Zm00001d011526_T002 0
769- 27: transcript:EES03393 transcript:Zm00001d011527_T001 4.00E-136
769- 28: transcript:OQU87294 transcript:Zm00001d011528_T001 5.00E-45
769- 29: transcript:EES03396 transcript:Zm00001d011531_T001 7.00E-170
769- 30: transcript:KXG33067 transcript:Zm00001d011532_T002 0
769- 31: transcript:EES03399 transcript:Zm00001d011534_T004 3.00E-129
769- 32: transcript:EES03400 transcript:Zm00001d011536_T001 8.00E-109
769- 33: transcript:EES03403 transcript:Zm00001d011537_T001 2.00E-35
769- 34: transcript:OQU87300 transcript:Zm00001d011540_T001 2.00E-32
769- 35: transcript:KXG33070 transcript:Zm00001d011541_T004 0
769- 36: transcript:EES03406 transcript:Zm00001d011543_T001 5.00E-57
769- 37: transcript:EES03408 transcript:Zm00001d011550_T001 0
769- 38: transcript:KXG33074 transcript:Zm00001d011551_T001 0
769- 39: transcript:OQU87308 transcript:Zm00001d011555_T001 2.00E-170
769- 40: transcript:KXG33079 transcript:Zm00001d011558_T001 4.00E-144
769- 41: transcript:KXG33080 transcript:Zm00001d011559_T001 0
769- 42: transcript:KXG33081 transcript:Zm00001d011560_T012 0
Alignment 770: score=1626.0 e_value=2e-123 N=37 3&8 plus
770- 0: transcript:EES01073 transcript:Zm00001d011352_T001 0
770- 1: transcript:EES01075 transcript:Zm00001d011353_T008 8.00E-152
770- 2: transcript:KXG32898 transcript:Zm00001d011355_T003 1.00E-140
770- 3: transcript:EES01079 transcript:Zm00001d011357_T003 0
770- 4: transcript:EES03280 transcript:Zm00001d011359_T001 1.00E-47
770- 5: transcript:KXG32903 transcript:Zm00001d011360_T004 0
770- 6: transcript:KXG32905 transcript:Zm00001d011362_T005 6.00E-122
770- 7: transcript:EES03282 transcript:Zm00001d011363_T002 1.00E-144
770- 8: transcript:EES01081 transcript:Zm00001d011364_T001 0
770- 9: transcript:KXG32911 transcript:Zm00001d011365_T001 0
770- 10: transcript:KXG32912 transcript:Zm00001d011366_T002 0
770- 11: transcript:EES01085 transcript:Zm00001d011369_T001 0
770- 12: transcript:KXG32913 transcript:Zm00001d011370_T001 0
770- 13: transcript:EES03295 transcript:Zm00001d011373_T009 0
770- 14: transcript:EES03296 transcript:Zm00001d011377_T001 0
770- 15: transcript:EES01090 transcript:Zm00001d011378_T001 0
770- 16: transcript:OQU87181 transcript:Zm00001d011380_T001 9.00E-69
770- 17: transcript:EES03302 transcript:Zm00001d011386_T004 1.00E-158
770- 18: transcript:EES01094 transcript:Zm00001d011392_T003 0
770- 19: transcript:EES03305 transcript:Zm00001d011393_T004 0
770- 20: transcript:EES01097 transcript:Zm00001d011396_T001 2.00E-101
770- 21: transcript:EES01098 transcript:Zm00001d011398_T001 9.00E-104
770- 22: transcript:EES01100 transcript:Zm00001d011399_T001 4.00E-117
770- 23: transcript:EES01101 transcript:Zm00001d011401_T001 0
770- 24: transcript:EES03310 transcript:Zm00001d011403_T001 2.00E-158
770- 25: transcript:EES01102 transcript:Zm00001d011405_T001 0
770- 26: transcript:EES01103 transcript:Zm00001d011406_T001 7.00E-126
770- 27: transcript:EES03313 transcript:Zm00001d011410_T001 1.00E-123
770- 28: transcript:EES01104 transcript:Zm00001d011411_T002 0
770- 29: transcript:EES03314 transcript:Zm00001d011412_T001 2.00E-31
770- 30: transcript:EES01107 transcript:Zm00001d011413_T001 0
770- 31: transcript:KXG32930 transcript:Zm00001d011417_T001 0
770- 32: transcript:EES03321 transcript:Zm00001d011418_T002 0
770- 33: transcript:EES01116 transcript:Zm00001d011419_T002 0
770- 34: transcript:EES01118 transcript:Zm00001d011422_T001 0

770- 35: transcript:EES01122 transcript:Zm00001d011424_T008 0
770- 36: transcript:KXG32938 transcript:Zm00001d011426_T002 0
Alignment 771: score=1188.0 e_value=3.5e-85 N=27 3&8 plus
771- 0: transcript:OQU87197 transcript:Zm00001d011427_T035 0
771- 1: transcript:KXG32942 transcript:Zm00001d011428_T003 0
771- 2: transcript:EES01127 transcript:Zm00001d011430_T001 0
771- 3: transcript:EES01128 transcript:Zm00001d011431_T001 2.00E-119
771- 4: transcript:EES01129 transcript:Zm00001d011433_T002 0
771- 5: transcript:EES01130 transcript:Zm00001d011434_T001 1.00E-31
771- 6: transcript:KXG32945 transcript:Zm00001d011435_T001 1.00E-64
771- 7: transcript:EES01135 transcript:Zm00001d011438_T001 0
771- 8: transcript:KXG32952 transcript:Zm00001d011442_T001 4.00E-140
771- 9: transcript:OQU87205 transcript:Zm00001d011443_T001 0
771- 10: transcript:EES01142 transcript:Zm00001d011444_T002 0
771- 11: transcript:EES03332 transcript:Zm00001d011445_T009 3.00E-168
771- 12: transcript:OQU87208 transcript:Zm00001d011446_T001 7.00E-141
771- 13: transcript:OQU87213 transcript:Zm00001d011447_T001 0
771- 14: transcript:EES01150 transcript:Zm00001d011448_T001 0
771- 15: transcript:KXG32962 transcript:Zm00001d011450_T001 0
771- 16: transcript:EES01152 transcript:Zm00001d011451_T001 1.00E-89
771- 17: transcript:KXG32970 transcript:Zm00001d011454_T001 1.00E-128
771- 18: transcript:EES03337 transcript:Zm00001d011455_T001 6.00E-101
771- 19: transcript:EES03338 transcript:Zm00001d011457_T001 0
771- 20: transcript:EES01155 transcript:Zm00001d011458_T001 0
771- 21: transcript:EES01157 transcript:Zm00001d011459_T001 0
771- 22: transcript:EES03340 transcript:Zm00001d011461_T001 5.00E-29
771- 23: transcript:OQU87226 transcript:Zm00001d011465_T001 0
771- 24: transcript:EES01161 transcript:Zm00001d011466_T001 3.00E-46
771- 25: transcript:EES01162 transcript:Zm00001d011468_T001 3.00E-27
771- 26: transcript:OQU87231 transcript:Zm00001d011470_T001 1.00E-148
Alignment 772: score=1120.0 e_value=1.5e-77 N=25 3&8 plus
772- 0: transcript:OQU87075 transcript:Zm00001d011236_T001 4.00E-96
772- 1: transcript:EES03177 transcript:Zm00001d011237_T001 7.00E-109
772- 2: transcript:EES03178 transcript:Zm00001d011238_T001 2.00E-93
772- 3: transcript:EES03181 transcript:Zm00001d011239_T001 0
772- 4: transcript:EES03182 transcript:Zm00001d011241_T001 2.00E-29
772- 5: transcript:EES03185 transcript:Zm00001d011242_T002 0
772- 6: transcript:OQU87079 transcript:Zm00001d011243_T001 7.00E-30
772- 7: transcript:EES00981 transcript:Zm00001d011245_T001 0
772- 8: transcript:EES03190 transcript:Zm00001d011246_T001 9.00E-142
772- 9: transcript:EES00984 transcript:Zm00001d011252_T001 6.00E-160
772- 10: transcript:EES00985 transcript:Zm00001d011255_T001 2.00E-132
772- 11: transcript:EES00986 transcript:Zm00001d011256_T001 2.00E-97
772- 12: transcript:KXG32799 transcript:Zm00001d011257_T001 2.00E-17
772- 13: transcript:EES00987 transcript:Zm00001d011258_T001 0
772- 14: transcript:KXG32803 transcript:Zm00001d011259_T001 0
772- 15: transcript:KXG32804 transcript:Zm00001d011260_T001 6.00E-20
772- 16: transcript:EES03203 transcript:Zm00001d011263_T001 6.00E-167
772- 17: transcript:OQU87098 transcript:Zm00001d011266_T002 2.00E-103
772- 18: transcript:KXG32812 transcript:Zm00001d011268_T001 1.00E-53
772- 19: transcript:EES01001 transcript:Zm00001d011269_T001 0
772- 20: transcript:EES03209 transcript:Zm00001d011270_T001 3.00E-42
772- 21: transcript:EES01003 transcript:Zm00001d011272_T007 0
772- 22: transcript:OQU87104 transcript:Zm00001d011274_T001 4.00E-70

772- 23: transcript:EES01005 transcript:Zm00001d011275_T001 7.00E-78
772- 24: transcript:OQU87108 transcript:Zm00001d011276_T001 8.00E-167
Alignment 773: score=998.0 e_value=3e-73 N=23 3&8 plus
773- 0: transcript:EES03221 transcript:Zm00001d011276_T001 8.00E-86
773- 1: transcript:EES01018 transcript:Zm00001d011277_T001 0
773- 2: transcript:OQU87121 transcript:Zm00001d011278_T001 8.00E-83
773- 3: transcript:EES01019 transcript:Zm00001d011282_T001 0
773- 4: transcript:EES01020 transcript:Zm00001d011283_T002 0
773- 5: transcript:EES03224 transcript:Zm00001d011284_T001 9.00E-104
773- 6: transcript:OQU87124 transcript:Zm00001d011285_T001 0
773- 7: transcript:EES03227 transcript:Zm00001d011286_T002 6.00E-68
773- 8: transcript:EES01025 transcript:Zm00001d011288_T006 0
773- 9: transcript:EES01026 transcript:Zm00001d011297_T001 6.00E-129
773- 10: transcript:OQU87135 transcript:Zm00001d011298_T001 0
773- 11: transcript:EES01039 transcript:Zm00001d011299_T001 2.00E-159
773- 12: transcript:KXG32845 transcript:Zm00001d011300_T001 2.00E-41
773- 13: transcript:EES01041 transcript:Zm00001d011301_T001 0
773- 14: transcript:EES03248 transcript:Zm00001d011302_T001 0
773- 15: transcript:OQU87138 transcript:Zm00001d011303_T001 1.00E-25
773- 16: transcript:EES01047 transcript:Zm00001d011304_T001 0
773- 17: transcript:KXG32852 transcript:Zm00001d011308_T001 5.00E-147
773- 18: transcript:EES03249 transcript:Zm00001d011309_T009 0
773- 19: transcript:KXG32856 transcript:Zm00001d011311_T001 0
773- 20: transcript:EES03251 transcript:Zm00001d011312_T001 0
773- 21: transcript:EES01052 transcript:Zm00001d011314_T001 6.00E-41
773- 22: transcript:KXG32865 transcript:Zm00001d011315_T002 0
Alignment 774: score=919.0 e_value=1.7e-58 N=21 3&8 plus
774- 0: transcript:EES00904 transcript:Zm00001d011154_T001 2.00E-149
774- 1: transcript:KXG32714 transcript:Zm00001d011155_T012 0
774- 2: transcript:KXG32718 transcript:Zm00001d011156_T001 0
774- 3: transcript:EES00909 transcript:Zm00001d011157_T001 0
774- 4: transcript:KXG32723 transcript:Zm00001d011158_T005 0
774- 5: transcript:EES03122 transcript:Zm00001d011159_T001 3.00E-142
774- 6: transcript:OQU87012 transcript:Zm00001d011160_T002 3.00E-09
774- 7: transcript:EES03124 transcript:Zm00001d011162_T002 2.00E-151
774- 8: transcript:EES03125 transcript:Zm00001d011164_T001 3.00E-46
774- 9: transcript:EES00919 transcript:Zm00001d011167_T001 2.00E-154
774- 10: transcript:EES00920 transcript:Zm00001d011168_T007 3.00E-113
774- 11: transcript:EES00922 transcript:Zm00001d011174_T001 0
774- 12: transcript:EES03130 transcript:Zm00001d011177_T006 0
774- 13: transcript:EES03131 transcript:Zm00001d011178_T002 0
774- 14: transcript:EES00925 transcript:Zm00001d011179_T001 3.00E-151
774- 15: transcript:EES03132 transcript:Zm00001d011180_T004 4.00E-98
774- 16: transcript:EES03134 transcript:Zm00001d011183_T001 0
774- 17: transcript:EES00928 transcript:Zm00001d011185_T001 2.00E-50
774- 18: transcript:OQU87025 transcript:Zm00001d011187_T001 3.00E-121
774- 19: transcript:EES03139 transcript:Zm00001d011188_T001 0
774- 20: transcript:EES03140 transcript:Zm00001d011189_T001 2.00E-61
Alignment 775: score=907.0 e_value=2e-56 N=20 3&8 plus
775- 0: transcript:KXG33085 transcript:Zm00001d011561_T004 1.00E-165
775- 1: transcript:EES03419 transcript:Zm00001d011562_T003 0
775- 2: transcript:OQU87315 transcript:Zm00001d011563_T019 0
775- 3: transcript:EES03421 transcript:Zm00001d011565_T002 2.00E-100
775- 4: transcript:KXG33095 transcript:Zm00001d011569_T001 0

775- 5: transcript:KXG33097 transcript:Zm00001d011570_T002 0
775- 6: transcript:EES03423 transcript:Zm00001d011571_T001 7.00E-174
775- 7: transcript:EES03424 transcript:Zm00001d011572_T001 0
775- 8: transcript:EES01241 transcript:Zm00001d011576_T001 3.00E-44
775- 9: transcript:EES03425 transcript:Zm00001d011578_T003 0
775- 10: transcript:EES01242 transcript:Zm00001d011579_T001 0
775- 11: transcript:KXG33100 transcript:Zm00001d011580_T002 0
775- 12: transcript:EES03426 transcript:Zm00001d011581_T001 4.00E-114
775- 13: transcript:KXG33102 transcript:Zm00001d011582_T001 4.00E-103
775- 14: transcript:EES01246 transcript:Zm00001d011585_T001 3.00E-127
775- 15: transcript:KXG33104 transcript:Zm00001d011588_T003 1.00E-136
775- 16: transcript:OQU87320 transcript:Zm00001d011589_T001 4.00E-27
775- 17: transcript:KXG33105 transcript:Zm00001d011592_T001 2.00E-69
775- 18: transcript:OQU87322 transcript:Zm00001d011594_T001 6.00E-43
775- 19: transcript:KXG33111 transcript:Zm00001d011595_T007 0
Alignment 776: score=883.0 e_value=1e-53 N=19 3&8 plus
776- 0: transcript:EES01389 transcript:Zm00001d011745_T001 5.00E-16
776- 1: transcript:OQU87453 transcript:Zm00001d011746_T002 0
776- 2: transcript:EES03545 transcript:Zm00001d011747_T001 3.00E-87
776- 3: transcript:KXG33296 transcript:Zm00001d011748_T001 9.00E-124
776- 4: transcript:EES03551 transcript:Zm00001d011750_T004 1.00E-60
776- 5: transcript:OQU87454 transcript:Zm00001d011751_T001 6.00E-40
776- 6: transcript:OQU87455 transcript:Zm00001d011752_T001 1.00E-20
776- 7: transcript:EES03552 transcript:Zm00001d011753_T001 2.00E-106
776- 8: transcript:KXG33301 transcript:Zm00001d011754_T001 0
776- 9: transcript:EES03555 transcript:Zm00001d011755_T001 1.00E-173
776- 10: transcript:KXG33302 transcript:Zm00001d011756_T001 0
776- 11: transcript:EES01405 transcript:Zm00001d011757_T001 0
776- 12: transcript:EES01408 transcript:Zm00001d011758_T001 0
776- 13: transcript:EES01410 transcript:Zm00001d011759_T001 0
776- 14: transcript:EES01416 transcript:Zm00001d011760_T001 6.00E-36
776- 15: transcript:EES01417 transcript:Zm00001d011761_T001 1.00E-148
776- 16: transcript:KXG33308 transcript:Zm00001d011762_T001 0
776- 17: transcript:OQU87465 transcript:Zm00001d011763_T005 9.00E-69
776- 18: transcript:KXG33311 transcript:Zm00001d011764_T001 0
Alignment 777: score=676.0 e_value=6.4e-37 N=15 3&8 plus
777- 0: transcript:KXG32865 transcript:Zm00001d011319_T003 0
777- 1: transcript:EES01054 transcript:Zm00001d011321_T001 2.00E-117
777- 2: transcript:KXG32874 transcript:Zm00001d011323_T001 0
777- 3: transcript:EES03262 transcript:Zm00001d011325_T001 0
777- 4: transcript:EES03268 transcript:Zm00001d011326_T001 1.00E-155
777- 5: transcript:KXG32882 transcript:Zm00001d011329_T002 0
777- 6: transcript:EES01062 transcript:Zm00001d011330_T001 2.00E-114
777- 7: transcript:KXG32886 transcript:Zm00001d011331_T001 1.00E-171
777- 8: transcript:EES01067 transcript:Zm00001d011334_T003 0
777- 9: transcript:EES01070 transcript:Zm00001d011335_T001 0
777- 10: transcript:EES03272 transcript:Zm00001d011336_T006 0
777- 11: transcript:EES03274 transcript:Zm00001d011347_T005 0
777- 12: transcript:KXG32891 transcript:Zm00001d011348_T001 0
777- 13: transcript:EES03276 transcript:Zm00001d011350_T001 0
777- 14: transcript:EES01072 transcript:Zm00001d011351_T001 0
Alignment 778: score=511.0 e_value=1.5e-28 N=12 3&8 plus
778- 0: transcript:Zm00001d044683_T001 transcript:KXG22931 9.00E-124
778- 1: transcript:Zm00001d044685_T001 transcript:KXG22935 4.00E-54

778- 2: transcript:Zm00001d044689_T002 transcript:KXG22937 0
778- 3: transcript:Zm00001d044691_T001 transcript:EES16622 4.00E-50
778- 4: transcript:Zm00001d044692_T001 transcript:OQU78687 0
778- 5: transcript:Zm00001d044693_T001 transcript:EES15654 0
778- 6: transcript:Zm00001d044694_T002 transcript:EES16626 8.00E-128
778- 7: transcript:Zm00001d044695_T001 transcript:EES15657 0
778- 8: transcript:Zm00001d044696_T002 transcript:KXG22954 0
778- 9: transcript:Zm00001d044698_T003 transcript:EES15664 0
778- 10: transcript:Zm00001d044699_T001 transcript:OQU78707 0
778- 11: transcript:Zm00001d044703_T001 transcript:EES16641 0
Alignment 779: score=492.0 e_value=5.4e-25 N=11 3&8 plus
779- 0: transcript:KXG33777 transcript:Zm00001d010190_T002 0
779- 1: transcript:KXG33783 transcript:Zm00001d010191_T001 2.00E-37
779- 2: transcript:EES01830 transcript:Zm00001d010195_T001 0
779- 3: transcript:OQU87957 transcript:Zm00001d010197_T001 0
779- 4: transcript:EES01838 transcript:Zm00001d010198_T001 0
779- 5: transcript:EES03963 transcript:Zm00001d010199_T002 0
779- 6: transcript:EES03967 transcript:Zm00001d010200_T001 0
779- 7: transcript:EES01839 transcript:Zm00001d010201_T001 5.00E-86
779- 8: transcript:KXG33790 transcript:Zm00001d010205_T002 0
779- 9: transcript:EES01844 transcript:Zm00001d010211_T002 2.00E-84
779- 10: transcript:OQU87973 transcript:Zm00001d010213_T001 4.00E-49
Alignment 780: score=461.0 e_value=1.6e-22 N=10 3&8 plus
780- 0: transcript:Zm00001d044659_T001 transcript:EES16558 9.00E-80
780- 1: transcript:Zm00001d044660_T001 transcript:EES16559 4.00E-66
780- 2: transcript:Zm00001d044661_T001 transcript:EES16562 0
780- 3: transcript:Zm00001d044662_T001 transcript:EES16563 5.00E-40
780- 4: transcript:Zm00001d044663_T002 transcript:KXG22878 0
780- 5: transcript:Zm00001d044664_T001 transcript:EES15592 2.00E-100
780- 6: transcript:Zm00001d044665_T001 transcript:KXG22879 0
780- 7: transcript:Zm00001d044666_T001 transcript:EES16567 5.00E-84
780- 8: transcript:Zm00001d044667_T002 transcript:KXG22886 0
780- 9: transcript:Zm00001d044669_T001 transcript:KXG22890 1.00E-155
Alignment 781: score=454.0 e_value=5e-20 N=10 3&8 plus
781- 0: transcript:KXG31557 transcript:Zm00001d008912_T001 3.00E-33
781- 1: transcript:EES00069 transcript:Zm00001d008914_T001 6.00E-17
781- 2: transcript:EES00066 transcript:Zm00001d008916_T001 1.00E-57
781- 3: transcript:KXG31563 transcript:Zm00001d008919_T001 0
781- 4: transcript:EES02258 transcript:Zm00001d008922_T001 0
781- 5: transcript:EES02261 transcript:Zm00001d008923_T001 3.00E-20
781- 6: transcript:KXG31566 transcript:Zm00001d008924_T001 7.00E-28
781- 7: transcript:EES02259 transcript:Zm00001d008925_T002 0
781- 8: transcript:EES02257 transcript:Zm00001d008927_T001 2.00E-53
781- 9: transcript:KXG31572 transcript:Zm00001d008934_T001 8.00E-71
Alignment 782: score=436.0 e_value=5.8e-23 N=11 3&8 plus
782- 0: transcript:EES03982 transcript:Zm00001d010222_T001 0
782- 1: transcript:KXG33814 transcript:Zm00001d010228_T001 3.00E-166
782- 2: transcript:OQU87990 transcript:Zm00001d010230_T001 0
782- 3: transcript:EES03989 transcript:Zm00001d010231_T001 0
782- 4: transcript:EES01864 transcript:Zm00001d010233_T004 1.00E-102
782- 5: transcript:KXG33831 transcript:Zm00001d010235_T022 3.00E-163
782- 6: transcript:EES03996 transcript:Zm00001d010236_T001 1.00E-73
782- 7: transcript:KXG33843 transcript:Zm00001d010243_T002 0
782- 8: transcript:KXG33848 transcript:Zm00001d010249_T001 0

782- 9: transcript:OQU88008 transcript:Zm00001d010256_T001 2.00E-18
782- 10: transcript:OQU88012 transcript:Zm00001d010264_T001 2.00E-79
Alignment 783: score=410.0 e_value=2.4e-18 N=9 3&8 plus
783- 0: transcript:EES01750 transcript:Zm00001d010052_T001 5.00E-09
783- 1: transcript:EES01749 transcript:Zm00001d010053_T001 3.00E-67
783- 2: transcript:EES01752 transcript:Zm00001d010054_T001 4.00E-166
783- 3: transcript:EES03880 transcript:Zm00001d010055_T003 2.00E-138
783- 4: transcript:KXG33695 transcript:Zm00001d010056_T002 0
783- 5: transcript:EES03882 transcript:Zm00001d010060_T001 0
783- 6: transcript:OQU87858 transcript:Zm00001d010061_T001 2.00E-20
783- 7: transcript:EES03886 transcript:Zm00001d010062_T001 0
783- 8: transcript:EES01757 transcript:Zm00001d010066_T003 1.00E-101
Alignment 784: score=408.0 e_value=1.7e-16 N=9 3&8 plus
784- 0: transcript:EES04064 transcript:Zm00001d012103_T001 0
784- 1: transcript:EES04065 transcript:Zm00001d012108_T003 0
784- 2: transcript:OQU88071 transcript:Zm00001d012109_T001 2.00E-90
784- 3: transcript:EES01925 transcript:Zm00001d012118_T002 0
784- 4: transcript:KXG33953 transcript:Zm00001d012119_T001 0
784- 5: transcript:EES01927 transcript:Zm00001d012120_T001 0
784- 6: transcript:KXG33951 transcript:Zm00001d012121_T002 3.00E-140
784- 7: transcript:KXG33955 transcript:Zm00001d012126_T004 0
784- 8: transcript:EES04070 transcript:Zm00001d012127_T001 2.00E-38
Alignment 785: score=398.0 e_value=1.5e-19 N=9 3&8 plus
785- 0: transcript:EES03683 transcript:Zm00001d012644_T001 0
785- 1: transcript:OQU87620 transcript:Zm00001d012645_T001 1.00E-159
785- 2: transcript:OQU87623 transcript:Zm00001d012646_T001 3.00E-79
785- 3: transcript:EES01541 transcript:Zm00001d012647_T001 1.00E-178
785- 4: transcript:EES03695 transcript:Zm00001d012648_T001 0
785- 5: transcript:OQU87629 transcript:Zm00001d012651_T004 0
785- 6: transcript:EES01544 transcript:Zm00001d012652_T003 0
785- 7: transcript:KXG33478 transcript:Zm00001d012653_T002 0
785- 8: transcript:EES01546 transcript:Zm00001d012654_T004 0
Alignment 786: score=367.0 e_value=4.7e-15 N=8 3&8 plus
786- 0: transcript:KXG33566 transcript:Zm00001d010651_T001 3.00E-11
786- 1: transcript:EES01618 transcript:Zm00001d010652_T001 4.00E-124
786- 2: transcript:KXG33567 transcript:Zm00001d010654_T001 0
786- 3: transcript:EES03768 transcript:Zm00001d010655_T001 0
786- 4: transcript:EES03772 transcript:Zm00001d010656_T001 4.00E-143
786- 5: transcript:EES03778 transcript:Zm00001d010658_T001 1.00E-152
786- 6: transcript:EES01628 transcript:Zm00001d010659_T001 0
786- 7: transcript:EES01627 transcript:Zm00001d010661_T001 1.00E-20
Alignment 787: score=362.0 e_value=5.5e-15 N=8 3&8 plus
787- 0: transcript:EES00140 transcript:Zm00001d008805_T001 0
787- 1: transcript:EES00141 transcript:Zm00001d008808_T002 4.00E-133
787- 2: transcript:KXG31642 transcript:Zm00001d008812_T001 0
787- 3: transcript:EES00144 transcript:Zm00001d008815_T001 0
787- 4: transcript:EES02318 transcript:Zm00001d008816_T001 0
787- 5: transcript:EES00142 transcript:Zm00001d008817_T001 0
787- 6: transcript:EES02320 transcript:Zm00001d008819_T001 0
787- 7: transcript:KXG31653 transcript:Zm00001d008820_T002 0
Alignment 788: score=353.0 e_value=1.6e-14 N=8 3&8 plus
788- 0: transcript:EES03353 transcript:Zm00001d011471_T001 0
788- 1: transcript:EES03354 transcript:Zm00001d011473_T003 0
788- 2: transcript:KXG32997 transcript:Zm00001d011474_T001 2.00E-165

788- 3: transcript:EES01174 transcript:Zm00001d011475_T001 0
788- 4: transcript:EES01176 transcript:Zm00001d011476_T001 0
788- 5: transcript:KXG33004 transcript:Zm00001d011477_T001 4.00E-144
788- 6: transcript:EES01184 transcript:Zm00001d011478_T002 7.00E-103
788- 7: transcript:KXG33009 transcript:Zm00001d011483_T004 0
Alignment 789: score=351.0 e_value=3.9e-14 N=8 3&8 plus
789- 0: transcript:Zm00001d041578_T001 transcript:EES16171 3.00E-96
789- 1: transcript:Zm00001d041580_T001 transcript:EES17207 4.00E-19
789- 2: transcript:Zm00001d041582_T005 transcript:KXG23713 0
789- 3: transcript:Zm00001d041584_T001 transcript:KXG23714 0
789- 4: transcript:Zm00001d041590_T001 transcript:KXG23716 0
789- 5: transcript:Zm00001d041592_T002 transcript:KXG23720 0
789- 6: transcript:Zm00001d041593_T001 transcript:EES16175 0
789- 7: transcript:Zm00001d041594_T001 transcript:EES16176 2.00E-69
Alignment 790: score=350.0 e_value=9.1e-15 N=8 3&8 plus
790- 0: transcript:Zm00001d041484_T001 transcript:EES17383 0
790- 1: transcript:Zm00001d041488_T002 transcript:EES17384 0
790- 2: transcript:Zm00001d041489_T009 transcript:EES17386 0
790- 3: transcript:Zm00001d041491_T001 transcript:EES17387 7.00E-135
790- 4: transcript:Zm00001d041495_T005 transcript:KXG23990 0
790- 5: transcript:Zm00001d041496_T001 transcript:KXG23993 2.00E-159
790- 6: transcript:Zm00001d041497_T005 transcript:EES17392 0
790- 7: transcript:Zm00001d041504_T002 transcript:OQU79608 0
Alignment 791: score=337.0 e_value=5.8e-16 N=8 3&8 plus
791- 0: transcript:Zm00001d041796_T001 transcript:EES16783 3.00E-32
791- 1: transcript:Zm00001d041800_T001 transcript:EES15778 0
791- 2: transcript:Zm00001d041803_T037 transcript:KXG23150 0
791- 3: transcript:Zm00001d041804_T001 transcript:EES16784 2.00E-106
791- 4: transcript:Zm00001d041816_T001 transcript:KXG23153 0
791- 5: transcript:Zm00001d041818_T001 transcript:EES15780 1.00E-109
791- 6: transcript:Zm00001d041819_T001 transcript:KXG23155 6.00E-86
791- 7: transcript:Zm00001d041822_T001 transcript:KXG23158 3.00E-48
Alignment 792: score=324.0 e_value=5.4e-12 N=7 3&8 plus
792- 0: transcript:Zm00001d041702_T001 transcript:KXG23574 0
792- 1: transcript:Zm00001d041703_T001 transcript:EES16070 0
792- 2: transcript:Zm00001d041708_T001 transcript:KXG23584 0
792- 3: transcript:Zm00001d041709_T001 transcript:OQU79218 0
792- 4: transcript:Zm00001d041710_T001 transcript:KXG23586 0
792- 5: transcript:Zm00001d041711_T003 transcript:EES16076 2.00E-94
792- 6: transcript:Zm00001d041712_T001 transcript:KXG23587 7.00E-12
Alignment 793: score=310.0 e_value=6.8e-14 N=7 3&8 plus
793- 0: transcript:KXG32703 transcript:Zm00001d011140_T004 0
793- 1: transcript:EES00900 transcript:Zm00001d011142_T001 3.00E-135
793- 2: transcript:KXG32705 transcript:Zm00001d011143_T001 7.00E-80
793- 3: transcript:EES00901 transcript:Zm00001d011144_T002 5.00E-177
793- 4: transcript:EES03116 transcript:Zm00001d011145_T001 0
793- 5: transcript:KXG32707 transcript:Zm00001d011147_T011 0
793- 6: transcript:KXG32708 transcript:Zm00001d011149_T002 8.00E-61
Alignment 794: score=306.0 e_value=2.2e-12 N=7 3&8 plus
794- 0: transcript:Zm00001d044629_T001 transcript:OQU78622 0
794- 1: transcript:Zm00001d044630_T002 transcript:EES15540 0
794- 2: transcript:Zm00001d044631_T001 transcript:EES16515 0
794- 3: transcript:Zm00001d044632_T001 transcript:EES15549 1.00E-158
794- 4: transcript:Zm00001d044634_T002 transcript:KXG22823 6.00E-88

794- 5: transcript:Zm00001d044635_T001 transcript:EES16527 5.00E-48
794- 6: transcript:Zm00001d044639_T001 transcript:KXG22829 0
Alignment 795: score=291.0 e_value=9.6e-14 N=7 3&8 plus
795- 0: transcript:Zm00001d041826_T001 transcript:EES16801 0
795- 1: transcript:Zm00001d041827_T001 transcript:EES16804 0
795- 2: transcript:Zm00001d041829_T001 transcript:EES16808 0
795- 3: transcript:Zm00001d041830_T001 transcript:EES16809 5.00E-37
795- 4: transcript:Zm00001d041831_T002 transcript:KXG23197 0
795- 5: transcript:Zm00001d041833_T005 transcript:EES15802 0
795- 6: transcript:Zm00001d041836_T001 transcript:EES15803 5.00E-125
Alignment 796: score=288.0 e_value=3.4e-15 N=8 3&8 plus
796- 0: transcript:OQU86986 transcript:Zm00001d009395_T001 3.00E-71
796- 1: transcript:OQU86993 transcript:Zm00001d009397_T001 1.00E-39
796- 2: transcript:EES00891 transcript:Zm00001d009398_T003 3.00E-177
796- 3: transcript:KXG32695 transcript:Zm00001d009399_T001 0
796- 4: transcript:KXG32697 transcript:Zm00001d009401_T001 0
796- 5: transcript:EES00894 transcript:Zm00001d009402_T001 8.00E-117
796- 6: transcript:EES00895 transcript:Zm00001d009403_T002 9.00E-149
796- 7: transcript:EES00896 transcript:Zm00001d009404_T001 0
Alignment 797: score=281.0 e_value=3.2e-13 N=7 3&8 plus
797- 0: transcript:OQU86478 transcript:Zm00001d008461_T001 3.00E-55
797- 1: transcript:EES02605 transcript:Zm00001d008465_T001 0
797- 2: transcript:OQU86479 transcript:Zm00001d008466_T001 0
797- 3: transcript:OQU86482 transcript:Zm00001d008477_T003 0
797- 4: transcript:EES00429 transcript:Zm00001d008478_T001 2.00E-40
797- 5: transcript:OQU86487 transcript:Zm00001d008479_T001 2.00E-98
797- 6: transcript:KXG32078 transcript:Zm00001d008488_T006 0
Alignment 798: score=270.0 e_value=8.9e-09 N=6 3&8 plus
798- 0: transcript:EES01738 transcript:Zm00001d012404_T001 0
798- 1: transcript:KXG33675 transcript:Zm00001d012407_T005 3.00E-106
798- 2: transcript:EES03866 transcript:Zm00001d012408_T001 0
798- 3: transcript:OQU87842 transcript:Zm00001d012413_T001 5.00E-82
798- 4: transcript:KXG33681 transcript:Zm00001d012414_T001 8.00E-31
798- 5: transcript:EES03871 transcript:Zm00001d012415_T001 0
Alignment 799: score=255.0 e_value=7.8e-10 N=6 3&8 plus
799- 0: transcript:EES02887 transcript:Zm00001d009161_T002 2.00E-133
799- 1: transcript:EES02889 transcript:Zm00001d009163_T001 0
799- 2: transcript:KXG32426 transcript:Zm00001d009167_T001 0
799- 3: transcript:EES02892 transcript:Zm00001d009171_T001 0
799- 4: transcript:EES00704 transcript:Zm00001d009177_T001 0
799- 5: transcript:OQU86811 transcript:Zm00001d009178_T001 0
Alignment 800: score=255.0 e_value=3.9e-09 N=6 3&8 plus
800- 0: transcript:EES00151 transcript:Zm00001d008788_T003 0
800- 1: transcript:EES02326 transcript:Zm00001d008789_T018 0
800- 2: transcript:EES00154 transcript:Zm00001d008791_T001 0
800- 3: transcript:EES02329 transcript:Zm00001d008792_T001 9.00E-75
800- 4: transcript:EES00157 transcript:Zm00001d008793_T001 7.00E-89
800- 5: transcript:EES02331 transcript:Zm00001d008794_T001 0
Alignment 801: score=254.0 e_value=6.2e-09 N=6 3&8 plus
801- 0: transcript:Zm00001d041767_T002 transcript:OQU78932 5.00E-158
801- 1: transcript:Zm00001d041769_T001 transcript:KXG23240 0
801- 2: transcript:Zm00001d041772_T001 transcript:EES15835 5.00E-71
801- 3: transcript:Zm00001d041773_T002 transcript:EES15836 0
801- 4: transcript:Zm00001d041774_T001 transcript:KXG23250 6.00E-11

801- 5: transcript:Zm00001d041775_T001 transcript:KXG23253 0
Alignment 802: score=253.0 e_value=2.1e-12 N=6 3&8 plus
802- 0: transcript:EES02605 transcript:Zm00001d008457_T001 0
802- 1: transcript:OQU86479 transcript:Zm00001d008458_T001 0
802- 2: transcript:OQU86482 transcript:Zm00001d008461_T001 1.00E-44
802- 3: transcript:OQU86483 transcript:Zm00001d008466_T001 0
802- 4: transcript:EES00429 transcript:Zm00001d008468_T001 0
802- 5: transcript:KXG32078 transcript:Zm00001d008477_T003 0
Alignment 803: score=6424.0 e_value=0 N=141 3&8 minus
803- 0: transcript:KXG33605 transcript:Zm00001d012525_T012 0
803- 1: transcript:EES01652 transcript:Zm00001d012524_T001 0
803- 2: transcript:OQU87751 transcript:Zm00001d012522_T001 6.00E-164
803- 3: transcript:EES01654 transcript:Zm00001d012521_T001 0
803- 4: transcript:EES01655 transcript:Zm00001d012520_T001 4.00E-91
803- 5: transcript:KXG33616 transcript:Zm00001d012518_T003 0
803- 6: transcript:EES01659 transcript:Zm00001d012517_T001 0
803- 7: transcript:EES01660 transcript:Zm00001d012516_T001 4.00E-161
803- 8: transcript:EES01661 transcript:Zm00001d012515_T004 0
803- 9: transcript:EES01662 transcript:Zm00001d012514_T001 0
803- 10: transcript:EES01663 transcript:Zm00001d012513_T001 6.00E-108
803- 11: transcript:EES01664 transcript:Zm00001d012512_T001 0
803- 12: transcript:EES03802 transcript:Zm00001d012511_T010 0
803- 13: transcript:EES03803 transcript:Zm00001d012510_T001 0
803- 14: transcript:EES01666 transcript:Zm00001d012508_T001 8.00E-93
803- 15: transcript:EES01668 transcript:Zm00001d012507_T001 5.00E-125
803- 16: transcript:EES01669 transcript:Zm00001d012505_T001 2.00E-132
803- 17: transcript:KXG33619 transcript:Zm00001d012504_T001 1.00E-143
803- 18: transcript:KXG33620 transcript:Zm00001d012503_T003 0
803- 19: transcript:EES03808 transcript:Zm00001d012501_T002 0
803- 20: transcript:OQU87764 transcript:Zm00001d012499_T001 8.00E-144
803- 21: transcript:KXG33624 transcript:Zm00001d012498_T001 5.00E-169
803- 22: transcript:KXG33626 transcript:Zm00001d012494_T001 0
803- 23: transcript:EES03814 transcript:Zm00001d012488_T001 0
803- 24: transcript:KXG33633 transcript:Zm00001d012486_T001 0
803- 25: transcript:KXG33636 transcript:Zm00001d012485_T001 0
803- 26: transcript:EES01683 transcript:Zm00001d012484_T002 3.00E-31
803- 27: transcript:KXG33637 transcript:Zm00001d012482_T001 0
803- 28: transcript:EES03819 transcript:Zm00001d012480_T001 2.00E-94
803- 29: transcript:OQU87773 transcript:Zm00001d012479_T004 8.00E-18
803- 30: transcript:EES01686 transcript:Zm00001d012477_T001 0
803- 31: transcript:EES03823 transcript:Zm00001d012476_T001 0
803- 32: transcript:OQU87775 transcript:Zm00001d012475_T001 1.00E-135
803- 33: transcript:OQU87776 transcript:Zm00001d012474_T001 3.00E-133
803- 34: transcript:EES03824 transcript:Zm00001d012473_T001 1.00E-105
803- 35: transcript:EES03825 transcript:Zm00001d012472_T001 0
803- 36: transcript:KXG33640 transcript:Zm00001d012471_T001 7.00E-69
803- 37: transcript:KXG33641 transcript:Zm00001d012468_T001 3.00E-40
803- 38: transcript:EES01699 transcript:Zm00001d012467_T001 0
803- 39: transcript:OQU87786 transcript:Zm00001d012466_T001 0
803- 40: transcript:EES03828 transcript:Zm00001d012465_T001 1.00E-160
803- 41: transcript:EES01700 transcript:Zm00001d012464_T001 0
803- 42: transcript:KXG33643 transcript:Zm00001d012463_T001 0
803- 43: transcript:EES01703 transcript:Zm00001d012460_T001 4.00E-160
803- 44: transcript:OQU87790 transcript:Zm00001d012459_T001 0

803- 45:	transcript:EES03837	transcript:Zm00001d012458_T001	4.00E-129
803- 46:	transcript:EES01704	transcript:Zm00001d012457_T003	0
803- 47:	transcript:EES01705	transcript:Zm00001d012456_T004	0
803- 48:	transcript:EES01706	transcript:Zm00001d012452_T004	0
803- 49:	transcript:EES03838	transcript:Zm00001d012451_T004	0
803- 50:	transcript:EES01709	transcript:Zm00001d012450_T001	1.00E-73
803- 51:	transcript:EES03839	transcript:Zm00001d012449_T001	6.00E-35
803- 52:	transcript:EES01711	transcript:Zm00001d012448_T001	2.00E-46
803- 53:	transcript:KXG33654	transcript:Zm00001d012447_T005	0
803- 54:	transcript:EES03840	transcript:Zm00001d012446_T002	0
803- 55:	transcript:OQU87808	transcript:Zm00001d012445_T001	4.00E-60
803- 56:	transcript:KXG33657	transcript:Zm00001d012444_T001	3.00E-41
803- 57:	transcript:OQU87809	transcript:Zm00001d012443_T001	3.00E-18
803- 58:	transcript:EES03844	transcript:Zm00001d012441_T001	1.00E-135
803- 59:	transcript:OQU87818	transcript:Zm00001d012440_T002	0
803- 60:	transcript:EES00251	transcript:Zm00001d012439_T001	6.00E-67
803- 61:	transcript:EES01718	transcript:Zm00001d012438_T001	0
803- 62:	transcript:EES01717	transcript:Zm00001d012437_T001	0
803- 63:	transcript:EES01719	transcript:Zm00001d012435_T001	9.00E-177
803- 64:	transcript:EES03847	transcript:Zm00001d012434_T001	1.00E-46
803- 65:	transcript:EES01723	transcript:Zm00001d012433_T002	0
803- 66:	transcript:EES03849	transcript:Zm00001d012432_T001	4.00E-100
803- 67:	transcript:EES01725	transcript:Zm00001d012431_T001	2.00E-38
803- 68:	transcript:OQU87825	transcript:Zm00001d012427_T001	4.00E-64
803- 69:	transcript:EES03854	transcript:Zm00001d012426_T001	3.00E-171
803- 70:	transcript:EES01727	transcript:Zm00001d012425_T002	1.00E-100
803- 71:	transcript:EES01728	transcript:Zm00001d012424_T001	3.00E-57
803- 72:	transcript:EES03855	transcript:Zm00001d012423_T001	1.00E-91
803- 73:	transcript:EES03856	transcript:Zm00001d012421_T004	1.00E-123
803- 74:	transcript:EES01731	transcript:Zm00001d012420_T001	0
803- 75:	transcript:OQU87830	transcript:Zm00001d012419_T001	1.00E-74
803- 76:	transcript:OQU87835	transcript:Zm00001d012418_T001	3.00E-150
803- 77:	transcript:EES03863	transcript:Zm00001d012417_T001	0
803- 78:	transcript:OQU87842	transcript:Zm00001d012413_T001	5.00E-82
803- 79:	transcript:EES01742	transcript:Zm00001d012408_T001	0
803- 80:	transcript:EES03872	transcript:Zm00001d012402_T001	0
803- 81:	transcript:EES01744	transcript:Zm00001d012401_T001	0
803- 82:	transcript:EES03874	transcript:Zm00001d012399_T003	6.00E-171
803- 83:	transcript:OQU87854	transcript:Zm00001d012398_T002	0
803- 84:	transcript:OQU87855	transcript:Zm00001d012396_T007	0
803- 85:	transcript:EES01751	transcript:Zm00001d012395_T005	0
803- 86:	transcript:KXG33695	transcript:Zm00001d012391_T003	0
803- 87:	transcript:KXG33698	transcript:Zm00001d012387_T001	0
803- 88:	transcript:OQU87858	transcript:Zm00001d012383_T002	6.00E-81
803- 89:	transcript:EES03887	transcript:Zm00001d012382_T002	2.00E-89
803- 90:	transcript:EES01757	transcript:Zm00001d012381_T001	0
803- 91:	transcript:EES01758	transcript:Zm00001d012380_T002	0
803- 92:	transcript:OQU87862	transcript:Zm00001d012379_T001	0
803- 93:	transcript:OQU87867	transcript:Zm00001d012378_T001	8.00E-137
803- 94:	transcript:EES01763	transcript:Zm00001d012363_T001	5.00E-87
803- 95:	transcript:OQU87868	transcript:Zm00001d012362_T001	3.00E-177
803- 96:	transcript:EES03892	transcript:Zm00001d012361_T001	0
803- 97:	transcript:EES03894	transcript:Zm00001d012350_T001	0
803- 98:	transcript:EES03897	transcript:Zm00001d012349_T001	0

```
803- 99: transcript:OQU87880            transcript:Zm00001d012340_T001          0
803-100: transcript:EES03898            transcript:Zm00001d012339_T001 3.00E-175
803-101: transcript:EES01769            transcript:Zm00001d012338_T001          0
803-102: transcript:KXG33716            transcript:Zm00001d012337_T001 2.00E-122
803-103: transcript:KXG33717            transcript:Zm00001d012336_T001 9.00E-161
803-104: transcript:EES01771            transcript:Zm00001d012333_T001          0
803-105: transcript:EES01772            transcript:Zm00001d012332_T001  8.00E-42
803-106: transcript:OQU87881            transcript:Zm00001d012330_T001  4.00E-99
803-107: transcript:OQU87882            transcript:Zm00001d012327_T001          0
803-108: transcript:OQU87885            transcript:Zm00001d012326_T001          0
803-109: transcript:EES01775            transcript:Zm00001d012325_T001          0
803-110: transcript:EES01776            transcript:Zm00001d012322_T001          0
803-111: transcript:KXG33721            transcript:Zm00001d012321_T001          0
803-112: transcript:EES01777            transcript:Zm00001d012320_T005          0
803-113: transcript:KXG33722            transcript:Zm00001d012314_T005          0
803-114: transcript:EES01781            transcript:Zm00001d012313_T001          0
803-115: transcript:EES03911            transcript:Zm00001d012312_T001 7.00E-180
803-116: transcript:OQU87887            transcript:Zm00001d012307_T001          0
803-117: transcript:EES03915            transcript:Zm00001d012304_T001          0
803-118: transcript:EES03917            transcript:Zm00001d012302_T001          0
803-119: transcript:KXG33733            transcript:Zm00001d012296_T001          0
803-120: transcript:EES01788            transcript:Zm00001d012295_T003 5.00E-174
803-121: transcript:KXG33737            transcript:Zm00001d012294_T009          0
803-122: transcript:EES01793            transcript:Zm00001d012293_T001  1.00E-63
803-123: transcript:EES01795            transcript:Zm00001d012292_T001          0
803-124: transcript:KXG33742            transcript:Zm00001d012291_T001 1.00E-111
803-125: transcript:EES01796            transcript:Zm00001d012290_T001 5.00E-108
803-126: transcript:KXG33743            transcript:Zm00001d012289_T001          0
803-127: transcript:EES01797            transcript:Zm00001d012287_T001  1.00E-95
803-128: transcript:EES03929            transcript:Zm00001d012286_T001          0
803-129: transcript:EES01800            transcript:Zm00001d012285_T001  5.00E-94
803-130: transcript:EES03930            transcript:Zm00001d012284_T001          0
803-131: transcript:EES03931            transcript:Zm00001d012282_T001  2.00E-90
803-132: transcript:OQU87908            transcript:Zm00001d012281_T001  1.00E-30
803-133: transcript:EES03938            transcript:Zm00001d012280_T001 3.00E-142
803-134: transcript:EES01809            transcript:Zm00001d012279_T001  6.00E-86
803-135: transcript:EES01810            transcript:Zm00001d012277_T003          0
803-136: transcript:OQU87919            transcript:Zm00001d012275_T001 2.00E-152
803-137: transcript:KXG33757            transcript:Zm00001d012274_T001          0
803-138: transcript:EES03943            transcript:Zm00001d012273_T001  7.00E-97
803-139: transcript:EES01815            transcript:Zm00001d012270_T001 3.00E-105
803-140: transcript:EES03944            transcript:Zm00001d012269_T003          0
## Alignment 804: score=3035.0 e_value=1.6e-255 N=65 3&8 minus
804-  0: transcript:EES01573            transcript:Zm00001d012611_T001          0
804-  1: transcript:EES03729            transcript:Zm00001d012610_T001 2.00E-177
804-  2: transcript:OQU87691            transcript:Zm00001d012609_T001 1.00E-173
804-  3: transcript:KXG33531            transcript:Zm00001d012607_T001 7.00E-141
804-  4: transcript:EES01579            transcript:Zm00001d012606_T001          0
804-  5: transcript:EES03733            transcript:Zm00001d012605_T001          0
804-  6: transcript:KXG33538            transcript:Zm00001d012604_T001  2.00E-74
804-  7: transcript:EES01583            transcript:Zm00001d012603_T001 2.00E-149
804-  8: transcript:EES01586            transcript:Zm00001d012602_T001  2.00E-81
804-  9: transcript:EES03741            transcript:Zm00001d012601_T002 1.00E-157
804- 10: transcript:EES01588            transcript:Zm00001d012600_T001          0
```

804- 11:	transcript:EES03744	transcript:Zm00001d012599_T001	4.00E-70
804- 12:	transcript:EES01590	transcript:Zm00001d012598_T001	1.00E-56
804- 13:	transcript:OQU87705	transcript:Zm00001d012597_T003	1.00E-24
804- 14:	transcript:EES03749	transcript:Zm00001d012596_T001	1.00E-50
804- 15:	transcript:EES03746	transcript:Zm00001d012595_T001	0
804- 16:	transcript:EES03747	transcript:Zm00001d012594_T001	3.00E-19
804- 17:	transcript:EES01592	transcript:Zm00001d012593_T001	0
804- 18:	transcript:EES01593	transcript:Zm00001d012591_T001	0
804- 19:	transcript:EES01597	transcript:Zm00001d012590_T001	0
804- 20:	transcript:KXG33549	transcript:Zm00001d012586_T001	0
804- 21:	transcript:OQU87713	transcript:Zm00001d012585_T001	3.00E-112
804- 22:	transcript:EES01601	transcript:Zm00001d012584_T001	2.00E-66
804- 23:	transcript:EES03753	transcript:Zm00001d012581_T001	0
804- 24:	transcript:KXG33553	transcript:Zm00001d012580_T001	3.00E-87
804- 25:	transcript:KXG33555	transcript:Zm00001d012578_T002	0
804- 26:	transcript:KXG33556	transcript:Zm00001d012577_T001	1.00E-29
804- 27:	transcript:OQU87717	transcript:Zm00001d012576_T001	0
804- 28:	transcript:OQU87718	transcript:Zm00001d012575_T001	0
804- 29:	transcript:KXG33559	transcript:Zm00001d012573_T001	0
804- 30:	transcript:EES01610	transcript:Zm00001d012572_T001	2.00E-51
804- 31:	transcript:KXG33560	transcript:Zm00001d012571_T002	0
804- 32:	transcript:EES03760	transcript:Zm00001d012569_T001	0
804- 33:	transcript:EES03761	transcript:Zm00001d012568_T001	0
804- 34:	transcript:EES03762	transcript:Zm00001d012567_T002	0
804- 35:	transcript:EES03763	transcript:Zm00001d012566_T001	3.00E-51
804- 36:	transcript:KXG33564	transcript:Zm00001d012564_T001	6.00E-179
804- 37:	transcript:KXG33566	transcript:Zm00001d012563_T001	1.00E-46
804- 38:	transcript:KXG33567	transcript:Zm00001d012562_T001	0
804- 39:	transcript:KXG33568	transcript:Zm00001d012561_T001	1.00E-69
804- 40:	transcript:EES03772	transcript:Zm00001d012560_T002	0
804- 41:	transcript:EES03773	transcript:Zm00001d012559_T004	0
804- 42:	transcript:KXG33575	transcript:Zm00001d012557_T001	6.00E-117
804- 43:	transcript:EES01623	transcript:Zm00001d012556_T001	0
804- 44:	transcript:KXG33577	transcript:Zm00001d012555_T001	8.00E-136
804- 45:	transcript:EES03778	transcript:Zm00001d012553_T003	0
804- 46:	transcript:EES01628	transcript:Zm00001d012552_T005	0
804- 47:	transcript:EES01627	transcript:Zm00001d012551_T001	1.00E-80
804- 48:	transcript:OQU87727	transcript:Zm00001d012550_T001	4.00E-147
804- 49:	transcript:EES03779	transcript:Zm00001d012549_T001	4.00E-161
804- 50:	transcript:EES03780	transcript:Zm00001d012548_T001	4.00E-09
804- 51:	transcript:EES01630	transcript:Zm00001d012546_T002	2.00E-138
804- 52:	transcript:KXG33583	transcript:Zm00001d012544_T001	0
804- 53:	transcript:KXG33586	transcript:Zm00001d012543_T001	3.00E-19
804- 54:	transcript:KXG33587	transcript:Zm00001d012539_T001	3.00E-169
804- 55:	transcript:EES03785	transcript:Zm00001d012538_T001	1.00E-124
804- 56:	transcript:OQU87737	transcript:Zm00001d012537_T003	3.00E-97
804- 57:	transcript:KXG33589	transcript:Zm00001d012536_T002	0
804- 58:	transcript:EES01645	transcript:Zm00001d012535_T001	4.00E-82
804- 59:	transcript:OQU87743	transcript:Zm00001d012534_T001	0
804- 60:	transcript:EES03789	transcript:Zm00001d012532_T001	6.00E-158
804- 61:	transcript:EES03793	transcript:Zm00001d012530_T001	1.00E-101
804- 62:	transcript:EES01648	transcript:Zm00001d012529_T001	0
804- 63:	transcript:EES01649	transcript:Zm00001d012528_T001	0
804- 64:	transcript:EES03797	transcript:Zm00001d012527_T001	0

Alignment 805: score=3026.0 e_value=5.6e-273 N=67 3&8 minus

805- 0:	transcript:EES01819	transcript:Zm00001d012268_T001	2.00E-30
805- 1:	transcript:EES03952	transcript:Zm00001d012267_T001	0
805- 2:	transcript:EES01822	transcript:Zm00001d012266_T001	3.00E-60
805- 3:	transcript:EES01823	transcript:Zm00001d012265_T001	0
805- 4:	transcript:KXG33770	transcript:Zm00001d012263_T002	0
805- 5:	transcript:EES01826	transcript:Zm00001d012261_T001	0
805- 6:	transcript:KXG33778	transcript:Zm00001d012260_T001	0
805- 7:	transcript:EES01830	transcript:Zm00001d012259_T001	0
805- 8:	transcript:EES01835	transcript:Zm00001d012258_T001	0
805- 9:	transcript:EES03963	transcript:Zm00001d012257_T013	0
805- 10:	transcript:EES01839	transcript:Zm00001d012255_T001	5.00E-154
805- 11:	transcript:KXG33790	transcript:Zm00001d012254_T002	0
805- 12:	transcript:EES01841	transcript:Zm00001d012249_T001	0
805- 13:	transcript:OQU87964	transcript:Zm00001d012248_T001	1.00E-47
805- 14:	transcript:EES01843	transcript:Zm00001d012247_T004	0
805- 15:	transcript:KXG33794	transcript:Zm00001d012246_T001	3.00E-152
805- 16:	transcript:EES01844	transcript:Zm00001d012245_T002	2.00E-127
805- 17:	transcript:EES03970	transcript:Zm00001d012244_T001	0
805- 18:	transcript:EES01846	transcript:Zm00001d012242_T001	0
805- 19:	transcript:OQU87972	transcript:Zm00001d012241_T001	0
805- 20:	transcript:EES03974	transcript:Zm00001d012240_T001	0
805- 21:	transcript:OQU87973	transcript:Zm00001d012239_T003	0
805- 22:	transcript:EES01849	transcript:Zm00001d012238_T003	3.00E-173
805- 23:	transcript:OQU87974	transcript:Zm00001d012237_T001	1.00E-124
805- 24:	transcript:KXG33803	transcript:Zm00001d012234_T002	0
805- 25:	transcript:EES01851	transcript:Zm00001d012233_T001	0
805- 26:	transcript:OQU87977	transcript:Zm00001d012229_T001	0
805- 27:	transcript:EES03978	transcript:Zm00001d012228_T002	0
805- 28:	transcript:EES01853	transcript:Zm00001d012227_T001	2.00E-23
805- 29:	transcript:OQU87981	transcript:Zm00001d012226_T001	9.00E-157
805- 30:	transcript:EES01856	transcript:Zm00001d012224_T014	0
805- 31:	transcript:EES03982	transcript:Zm00001d012223_T001	0
805- 32:	transcript:EES01858	transcript:Zm00001d012221_T001	0
805- 33:	transcript:KXG33814	transcript:Zm00001d012220_T001	0
805- 34:	transcript:EES03984	transcript:Zm00001d012219_T001	0
805- 35:	transcript:EES01865	transcript:Zm00001d012217_T001	1.00E-132
805- 36:	transcript:EES03987	transcript:Zm00001d012216_T001	0
805- 37:	transcript:EES01869	transcript:Zm00001d012213_T003	1.00E-122
805- 38:	transcript:OQU87995	transcript:Zm00001d012212_T001	0
805- 39:	transcript:EES03991	transcript:Zm00001d012211_T010	0
805- 40:	transcript:KXG33833	transcript:Zm00001d012206_T001	1.00E-129
805- 41:	transcript:EES03995	transcript:Zm00001d012205_T001	0
805- 42:	transcript:EES03997	transcript:Zm00001d012204_T001	0
805- 43:	transcript:EES03999	transcript:Zm00001d012203_T001	3.00E-174
805- 44:	transcript:EES04002	transcript:Zm00001d012199_T002	0
805- 45:	transcript:KXG33843	transcript:Zm00001d012198_T001	0
805- 46:	transcript:EES01881	transcript:Zm00001d012197_T002	3.00E-153
805- 47:	transcript:KXG33846	transcript:Zm00001d012196_T005	1.00E-143
805- 48:	transcript:KXG33850	transcript:Zm00001d012195_T002	0
805- 49:	transcript:KXG33851	transcript:Zm00001d012194_T001	3.00E-19
805- 50:	transcript:EES04016	transcript:Zm00001d012184_T001	2.00E-36
805- 51:	transcript:OQU88010	transcript:Zm00001d012178_T001	0
805- 52:	transcript:KXG33860	transcript:Zm00001d012177_T001	0

```
805- 53: transcript:KXG33864            transcript:Zm00001d012176_T001          0
805- 54: transcript:KXG33870            transcript:Zm00001d012175_T004 1.00E-156
805- 55: transcript:EES04029            transcript:Zm00001d012173_T001          0
805- 56: transcript:KXG33883            transcript:Zm00001d012171_T001  3.00E-29
805- 57: transcript:EES01894            transcript:Zm00001d012170_T002          0
805- 58: transcript:EES04031            transcript:Zm00001d012169_T001 4.00E-180
805- 59: transcript:EES01896            transcript:Zm00001d012168_T002          0
805- 60: transcript:EES01898            transcript:Zm00001d012167_T001          0
805- 61: transcript:KXG33892            transcript:Zm00001d012166_T002          0
805- 62: transcript:EES04034            transcript:Zm00001d012165_T001  5.00E-95
805- 63: transcript:KXG33893            transcript:Zm00001d012161_T001          0
805- 64: transcript:EES01903            transcript:Zm00001d012160_T003 2.00E-130
805- 65: transcript:EES01905            transcript:Zm00001d012159_T006          0
805- 66: transcript:OQU88036            transcript:Zm00001d012158_T001          0
## Alignment 806: score=3014.0 e_value=7.8e-272 N=66 3&8 minus
806-  0: transcript:KXG34018            transcript:Zm00001d011981_T007          0
806-  1: transcript:EES01993            transcript:Zm00001d011979_T001          0
806-  2: transcript:EES04127            transcript:Zm00001d011978_T001  2.00E-59
806-  3: transcript:EES04124            transcript:Zm00001d011975_T002          0
806-  4: transcript:EES04130            transcript:Zm00001d011972_T001          0
806-  5: transcript:KXG34022            transcript:Zm00001d011971_T001          0
806-  6: transcript:KXG34023            transcript:Zm00001d011970_T001          0
806-  7: transcript:EES01998            transcript:Zm00001d011969_T001 4.00E-111
806-  8: transcript:EES01999            transcript:Zm00001d011968_T001  8.00E-62
806-  9: transcript:EES04133            transcript:Zm00001d011967_T001          0
806- 10: transcript:EES04134            transcript:Zm00001d011966_T003 1.00E-139
806- 11: transcript:KXG34027            transcript:Zm00001d011965_T001          0
806- 12: transcript:EES04135            transcript:Zm00001d011964_T001          0
806- 13: transcript:EES04136            transcript:Zm00001d011959_T001          0
806- 14: transcript:KXG34030            transcript:Zm00001d011958_T001          0
806- 15: transcript:OQU88147            transcript:Zm00001d011956_T002          0
806- 16: transcript:EES02003            transcript:Zm00001d011955_T002  4.00E-76
806- 17: transcript:EES02008            transcript:Zm00001d011953_T008          0
806- 18: transcript:KXG34040            transcript:Zm00001d011952_T001 9.00E-136
806- 19: transcript:KXG34044            transcript:Zm00001d011945_T001          0
806- 20: transcript:OQU88151            transcript:Zm00001d011944_T001 1.00E-114
806- 21: transcript:EES04145            transcript:Zm00001d011943_T001          0
806- 22: transcript:EES02009            transcript:Zm00001d011941_T003          0
806- 23: transcript:EES04149            transcript:Zm00001d011940_T001  9.00E-43
806- 24: transcript:EES02012            transcript:Zm00001d011938_T001  7.00E-69
806- 25: transcript:EES02015            transcript:Zm00001d011932_T001          0
806- 26: transcript:KXG34055            transcript:Zm00001d011930_T001 3.00E-152
806- 27: transcript:EES04152            transcript:Zm00001d011929_T002          0
806- 28: transcript:EES04153            transcript:Zm00001d011928_T001 2.00E-155
806- 29: transcript:KXG34058            transcript:Zm00001d011927_T001          0
806- 30: transcript:EES04155            transcript:Zm00001d011924_T001  3.00E-47
806- 31: transcript:EES02018            transcript:Zm00001d011923_T002          0
806- 32: transcript:KXG34061            transcript:Zm00001d011921_T001  5.00E-23
806- 33: transcript:EES04163            transcript:Zm00001d011920_T003          0
806- 34: transcript:EES04164            transcript:Zm00001d011919_T001 1.00E-139
806- 35: transcript:KXG34069            transcript:Zm00001d011918_T002          0
806- 36: transcript:KXG34073            transcript:Zm00001d011917_T001 2.00E-128
806- 37: transcript:OQU88166            transcript:Zm00001d011915_T001  6.00E-41
806- 38: transcript:EES02024            transcript:Zm00001d011913_T002          0
```

806- 39: transcript:EES04179 transcript:Zm00001d011912_T002 0
806- 40: transcript:EES04183 transcript:Zm00001d011904_T001 0
806- 41: transcript:EES04184 transcript:Zm00001d011903_T001 1.00E-70
806- 42: transcript:OQU88171 transcript:Zm00001d011902_T001 3.00E-29
806- 43: transcript:OQU88172 transcript:Zm00001d011901_T005 0
806- 44: transcript:KXG34085 transcript:Zm00001d011900_T001 0
806- 45: transcript:KXG34084 transcript:Zm00001d011899_T001 2.00E-50
806- 46: transcript:EES04190 transcript:Zm00001d011898_T001 0
806- 47: transcript:OQU88174 transcript:Zm00001d011896_T001 0
806- 48: transcript:OQU88178 transcript:Zm00001d011895_T001 0
806- 49: transcript:EES02036 transcript:Zm00001d011891_T001 0
806- 50: transcript:EES02037 transcript:Zm00001d011890_T001 0
806- 51: transcript:OQU88184 transcript:Zm00001d011889_T001 0
806- 52: transcript:EES02039 transcript:Zm00001d011887_T001 6.00E-52
806- 53: transcript:EES04199 transcript:Zm00001d011886_T001 2.00E-110
806- 54: transcript:KXG34100 transcript:Zm00001d011885_T005 0
806- 55: transcript:EES02045 transcript:Zm00001d011881_T001 0
806- 56: transcript:EES02047 transcript:Zm00001d011880_T002 4.00E-154
806- 57: transcript:EES04212 transcript:Zm00001d011879_T001 3.00E-10
806- 58: transcript:OQU88196 transcript:Zm00001d011878_T001 0
806- 59: transcript:KXG34115 transcript:Zm00001d011877_T003 7.00E-123
806- 60: transcript:OQU88197 transcript:Zm00001d011876_T001 0
806- 61: transcript:EES02053 transcript:Zm00001d011874_T002 1.00E-164
806- 62: transcript:EES04224 transcript:Zm00001d011873_T002 6.00E-136
806- 63: transcript:EES02054 transcript:Zm00001d011872_T003 0
806- 64: transcript:KXG34124 transcript:Zm00001d011855_T002 0
806- 65: transcript:EES02059 transcript:Zm00001d011854_T087 0
Alignment 807: score=2935.0 e_value=6e-256 N=66 3&8 minus
807- 0: transcript:EES03605 transcript:Zm00001d012751_T002 0
807- 1: transcript:EES01475 transcript:Zm00001d012750_T002 0
807- 2: transcript:EES01479 transcript:Zm00001d012749_T001 0
807- 3: transcript:EES03610 transcript:Zm00001d012748_T002 0
807- 4: transcript:KXG33369 transcript:Zm00001d012747_T007 0
807- 5: transcript:EES03611 transcript:Zm00001d012746_T001 3.00E-139
807- 6: transcript:EES01481 transcript:Zm00001d012744_T002 0
807- 7: transcript:KXG33373 transcript:Zm00001d012743_T002 0
807- 8: transcript:EES03615 transcript:Zm00001d012741_T001 2.00E-44
807- 9: transcript:KXG33374 transcript:Zm00001d012740_T001 1.00E-49
807- 10: transcript:KXG33375 transcript:Zm00001d012738_T001 0
807- 11: transcript:OQU87548 transcript:Zm00001d012737_T001 3.00E-115
807- 12: transcript:KXG33377 transcript:Zm00001d012736_T001 6.00E-46
807- 13: transcript:EES03620 transcript:Zm00001d012735_T003 7.00E-88
807- 14: transcript:KXG33380 transcript:Zm00001d012734_T002 0
807- 15: transcript:KXG33381 transcript:Zm00001d012732_T002 9.00E-107
807- 16: transcript:OQU87551 transcript:Zm00001d012731_T010 0
807- 17: transcript:OQU87569 transcript:Zm00001d012729_T009 9.00E-36
807- 18: transcript:EES03625 transcript:Zm00001d012728_T001 0
807- 19: transcript:KXG33392 transcript:Zm00001d012726_T001 3.00E-21
807- 20: transcript:KXG33398 transcript:Zm00001d012725_T002 0
807- 21: transcript:EES01492 transcript:Zm00001d012720_T001 4.00E-109
807- 22: transcript:EES01493 transcript:Zm00001d012719_T001 1.00E-136
807- 23: transcript:EES01494 transcript:Zm00001d012718_T004 0
807- 24: transcript:EES01496 transcript:Zm00001d012717_T002 0
807- 25: transcript:OQU87576 transcript:Zm00001d012714_T001 1.00E-06

```
807- 26: transcript:EES01498           transcript:Zm00001d012712_T002          0
807- 27: transcript:EES03634           transcript:Zm00001d012710_T001 6.00E-130
807- 28: transcript:EES03635           transcript:Zm00001d012709_T001 4.00E-165
807- 29: transcript:EES03636           transcript:Zm00001d012708_T001          0
807- 30: transcript:EES03641           transcript:Zm00001d012707_T003 2.00E-122
807- 31: transcript:KXG33408           transcript:Zm00001d012703_T001  9.00E-88
807- 32: transcript:KXG33409           transcript:Zm00001d012702_T008          0
807- 33: transcript:EES03642           transcript:Zm00001d012701_T004          0
807- 34: transcript:EES03643           transcript:Zm00001d012700_T001  2.00E-81
807- 35: transcript:KXG33412           transcript:Zm00001d012699_T005 1.00E-178
807- 36: transcript:OQU87587           transcript:Zm00001d012697_T009          0
807- 37: transcript:EES03646           transcript:Zm00001d012696_T001          0
807- 38: transcript:EES01504           transcript:Zm00001d012694_T002          0
807- 39: transcript:EES01505           transcript:Zm00001d012693_T002          0
807- 40: transcript:OQU87589           transcript:Zm00001d012691_T001 8.00E-150
807- 41: transcript:KXG33422           transcript:Zm00001d012690_T002  2.00E-98
807- 42: transcript:OQU87593           transcript:Zm00001d012689_T001  3.00E-76
807- 43: transcript:KXG33425           transcript:Zm00001d012688_T001          0
807- 44: transcript:EES03651           transcript:Zm00001d012687_T001          0
807- 45: transcript:KXG33429           transcript:Zm00001d012686_T001  2.00E-45
807- 46: transcript:EES03653           transcript:Zm00001d012685_T004          0
807- 47: transcript:KXG33430           transcript:Zm00001d012684_T001  8.00E-38
807- 48: transcript:EES03655           transcript:Zm00001d012683_T001  7.00E-90
807- 49: transcript:EES01510           transcript:Zm00001d012681_T003 2.00E-154
807- 50: transcript:EES03658           transcript:Zm00001d012679_T004  7.00E-71
807- 51: transcript:EES01511           transcript:Zm00001d012677_T012          0
807- 52: transcript:EES03659           transcript:Zm00001d012676_T001          0
807- 53: transcript:EES03661           transcript:Zm00001d012675_T001 2.00E-153
807- 54: transcript:EES03663           transcript:Zm00001d012674_T010          0
807- 55: transcript:KXG33439           transcript:Zm00001d012673_T002          0
807- 56: transcript:EES03667           transcript:Zm00001d012672_T001          0
807- 57: transcript:EES03668           transcript:Zm00001d012671_T001 7.00E-146
807- 58: transcript:KXG33446           transcript:Zm00001d012670_T002          0
807- 59: transcript:EES03678           transcript:Zm00001d012667_T009          0
807- 60: transcript:EES01521           transcript:Zm00001d012662_T001          0
807- 61: transcript:EES01523           transcript:Zm00001d012661_T002          0
807- 62: transcript:EES01524           transcript:Zm00001d012660_T002          0
807- 63: transcript:OQU87612           transcript:Zm00001d012659_T009          0
807- 64: transcript:EES01528           transcript:Zm00001d012656_T002          0
807- 65: transcript:EES01529           transcript:Zm00001d012655_T001          0
## Alignment 808: score=2525.0 e_value=2.5e-219 N=56 3&8 minus
808-  0: transcript:EES02060           transcript:Zm00001d011853_T001 6.00E-127
808-  1: transcript:KXG34127           transcript:Zm00001d011851_T001          0
808-  2: transcript:EES02063           transcript:Zm00001d011850_T002          0
808-  3: transcript:EES02064           transcript:Zm00001d011849_T002 9.00E-119
808-  4: transcript:KXG34132           transcript:Zm00001d011847_T001 1.00E-113
808-  5: transcript:KXG34134           transcript:Zm00001d011846_T001 8.00E-140
808-  6: transcript:EES02068           transcript:Zm00001d011845_T001 8.00E-107
808-  7: transcript:OQU88210           transcript:Zm00001d011844_T001  7.00E-14
808-  8: transcript:KXG34140           transcript:Zm00001d011843_T001 9.00E-147
808-  9: transcript:KXG34141           transcript:Zm00001d011842_T001          0
808- 10: transcript:EES04237           transcript:Zm00001d011841_T001  2.00E-42
808- 11: transcript:KXG34144           transcript:Zm00001d011840_T001 1.00E-104
808- 12: transcript:EES02075           transcript:Zm00001d011839_T001 4.00E-126
```

```
808- 13: transcript:EES04242          transcript:Zm00001d011838_T001          0
808- 14: transcript:KXG34153          transcript:Zm00001d011835_T003          0
808- 15: transcript:EES02080          transcript:Zm00001d011832_T001          0
808- 16: transcript:EES02083          transcript:Zm00001d011831_T001          0
808- 17: transcript:OQU88220          transcript:Zm00001d011830_T001          0
808- 18: transcript:EES02084          transcript:Zm00001d011829_T001          0
808- 19: transcript:EES02086          transcript:Zm00001d011828_T001 1.00E-100
808- 20: transcript:OQU88222          transcript:Zm00001d011827_T002          0
808- 21: transcript:EES02088          transcript:Zm00001d011826_T001  8.00E-83
808- 22: transcript:EES02089          transcript:Zm00001d011825_T001  3.00E-97
808- 23: transcript:KXG34164          transcript:Zm00001d011823_T002          0
808- 24: transcript:KXG34171          transcript:Zm00001d011821_T007          0
808- 25: transcript:EES04263          transcript:Zm00001d011819_T001          0
808- 26: transcript:EES02101          transcript:Zm00001d011817_T003          0
808- 27: transcript:EES04270          transcript:Zm00001d011816_T001  6.00E-95
808- 28: transcript:KXG34187          transcript:Zm00001d011813_T001  4.00E-72
808- 29: transcript:EES02110          transcript:Zm00001d011812_T001  4.00E-61
808- 30: transcript:EES04276          transcript:Zm00001d011811_T002  9.00E-89
808- 31: transcript:EES02112          transcript:Zm00001d011810_T001  3.00E-20
808- 32: transcript:KXG34192          transcript:Zm00001d011805_T001          0
808- 33: transcript:EES02118          transcript:Zm00001d011803_T003          0
808- 34: transcript:EES02119          transcript:Zm00001d011802_T001          0
808- 35: transcript:OQU88242          transcript:Zm00001d011800_T001  5.00E-21
808- 36: transcript:OQU88245          transcript:Zm00001d011799_T001  3.00E-81
808- 37: transcript:KXG34204          transcript:Zm00001d011797_T002 5.00E-107
808- 38: transcript:OQU88246          transcript:Zm00001d011796_T002 8.00E-170
808- 39: transcript:EES02124          transcript:Zm00001d011795_T001  2.00E-43
808- 40: transcript:EES02127          transcript:Zm00001d011793_T001 5.00E-161
808- 41: transcript:EES04291          transcript:Zm00001d011792_T003          0
808- 42: transcript:KXG34211          transcript:Zm00001d011790_T001          0
808- 43: transcript:EES02140          transcript:Zm00001d011789_T001  3.00E-43
808- 44: transcript:EES04293          transcript:Zm00001d011788_T001          0
808- 45: transcript:EES02142          transcript:Zm00001d011787_T001          0
808- 46: transcript:EES02143          transcript:Zm00001d011786_T001  3.00E-93
808- 47: transcript:KXG34215          transcript:Zm00001d011784_T001  2.00E-19
808- 48: transcript:EES02146          transcript:Zm00001d011782_T003          0
808- 49: transcript:OQU88270          transcript:Zm00001d011781_T001 2.00E-114
808- 50: transcript:OQU88272          transcript:Zm00001d011780_T002          0
808- 51: transcript:EES04303          transcript:Zm00001d011778_T001 1.00E-146
808- 52: transcript:EES02152          transcript:Zm00001d011774_T001          0
808- 53: transcript:EES04306          transcript:Zm00001d011773_T003 5.00E-139
808- 54: transcript:EES04307          transcript:Zm00001d011771_T001  3.00E-83
808- 55: transcript:KXG34234          transcript:Zm00001d011770_T001          0
## Alignment 809: score=2497.0 e_value=2.3e-228 N=58 3&8 minus
809-  0: transcript:KXG31682          transcript:Zm00001d008785_T001  2.00E-90
809-  1: transcript:EES00181          transcript:Zm00001d008782_T001 7.00E-121
809-  2: transcript:EES02350          transcript:Zm00001d008779_T001          0
809-  3: transcript:EES02351          transcript:Zm00001d008777_T008          0
809-  4: transcript:KXG31691          transcript:Zm00001d008769_T002          0
809-  5: transcript:EES00187          transcript:Zm00001d008764_T001          0
809-  6: transcript:KXG31694          transcript:Zm00001d008763_T014          0
809-  7: transcript:EES02354          transcript:Zm00001d008762_T002          0
809-  8: transcript:EES02356          transcript:Zm00001d008759_T002 4.00E-106
809-  9: transcript:EES02357          transcript:Zm00001d008758_T001          0
```

809- 10: transcript:EES00189 transcript:Zm00001d008756_T001 0
809- 11: transcript:EES00190 transcript:Zm00001d008753_T001 9.00E-67
809- 12: transcript:EES00193 transcript:Zm00001d008752_T003 0
809- 13: transcript:EES02362 transcript:Zm00001d008750_T001 0
809- 14: transcript:KXG31706 transcript:Zm00001d008749_T002 1.00E-94
809- 15: transcript:EES02364 transcript:Zm00001d008748_T001 0
809- 16: transcript:KXG31711 transcript:Zm00001d008744_T007 0
809- 17: transcript:EES02367 transcript:Zm00001d008743_T004 0
809- 18: transcript:KXG31722 transcript:Zm00001d008742_T010 0
809- 19: transcript:EES00204 transcript:Zm00001d008739_T001 0
809- 20: transcript:KXG31728 transcript:Zm00001d008738_T001 0
809- 21: transcript:KXG31735 transcript:Zm00001d008737_T001 6.00E-76
809- 22: transcript:KXG31737 transcript:Zm00001d008736_T001 2.00E-112
809- 23: transcript:EES00215 transcript:Zm00001d008735_T001 2.00E-92
809- 24: transcript:EES00219 transcript:Zm00001d008734_T001 1.00E-76
809- 25: transcript:KXG31741 transcript:Zm00001d008733_T001 0
809- 26: transcript:EES00222 transcript:Zm00001d008731_T001 7.00E-27
809- 27: transcript:EES02382 transcript:Zm00001d008730_T001 2.00E-87
809- 28: transcript:KXG31749 transcript:Zm00001d008727_T006 0
809- 29: transcript:EES00224 transcript:Zm00001d008726_T001 6.00E-95
809- 30: transcript:EES00225 transcript:Zm00001d008725_T004 0
809- 31: transcript:EES02381 transcript:Zm00001d008724_T005 0
809- 32: transcript:KXG31752 transcript:Zm00001d008722_T001 1.00E-20
809- 33: transcript:EES02389 transcript:Zm00001d008721_T001 9.00E-103
809- 34: transcript:KXG31754 transcript:Zm00001d008720_T002 9.00E-134
809- 35: transcript:EES00230 transcript:Zm00001d008716_T002 0
809- 36: transcript:EES02390 transcript:Zm00001d008715_T001 0
809- 37: transcript:KXG31759 transcript:Zm00001d008708_T001 0
809- 38: transcript:KXG31760 transcript:Zm00001d008706_T001 2.00E-124
809- 39: transcript:KXG31764 transcript:Zm00001d008700_T002 0
809- 40: transcript:KXG31765 transcript:Zm00001d008699_T001 1.00E-06
809- 41: transcript:EES02393 transcript:Zm00001d008695_T001 4.00E-46
809- 42: transcript:KXG31766 transcript:Zm00001d008693_T002 4.00E-158
809- 43: transcript:OQU86239 transcript:Zm00001d008692_T001 0
809- 44: transcript:EES02395 transcript:Zm00001d008691_T001 0
809- 45: transcript:KXG31768 transcript:Zm00001d008690_T001 0
809- 46: transcript:EES00240 transcript:Zm00001d008689_T003 0
809- 47: transcript:EES00245 transcript:Zm00001d008681_T001 2.00E-43
809- 48: transcript:EES02399 transcript:Zm00001d008680_T001 2.00E-72
809- 49: transcript:KXG31782 transcript:Zm00001d008679_T010 0
809- 50: transcript:EES00247 transcript:Zm00001d008676_T001 8.00E-76
809- 51: transcript:EES00250 transcript:Zm00001d008669_T001 6.00E-67
809- 52: transcript:KXG31795 transcript:Zm00001d008668_T001 1.00E-135
809- 53: transcript:KXG31798 transcript:Zm00001d008666_T003 0
809- 54: transcript:EES02409 transcript:Zm00001d008665_T001 9.00E-148
809- 55: transcript:EES00261 transcript:Zm00001d008664_T001 5.00E-178
809- 56: transcript:EES02410 transcript:Zm00001d008662_T001 1.00E-86
809- 57: transcript:KXG31803 transcript:Zm00001d008656_T002 5.00E-171
Alignment 810: score=1737.0 e_value=2.7e-131 N=37 3&8 minus
810- 0: transcript:KXG33959 transcript:Zm00001d012097_T001 0
810- 1: transcript:KXG33960 transcript:Zm00001d012096_T001 1.00E-170
810- 2: transcript:KXG33961 transcript:Zm00001d012092_T001 5.00E-24
810- 3: transcript:EES04079 transcript:Zm00001d012091_T001 5.00E-127
810- 4: transcript:KXG33963 transcript:Zm00001d012090_T001 1.00E-36

```
810-  5: transcript:EES04081            transcript:Zm00001d012088_T001  1.00E-31
810-  6: transcript:EES04083            transcript:Zm00001d012087_T005         0
810-  7: transcript:OQU88089            transcript:Zm00001d012086_T009         0
810-  8: transcript:EES01942            transcript:Zm00001d012085_T005         0
810-  9: transcript:EES01944            transcript:Zm00001d012084_T001         0
810- 10: transcript:EES01945            transcript:Zm00001d012083_T001 1.00E-169
810- 11: transcript:EES04091            transcript:Zm00001d012081_T001  5.00E-33
810- 12: transcript:EES01946            transcript:Zm00001d012080_T001  9.00E-44
810- 13: transcript:OQU88098            transcript:Zm00001d012079_T001  3.00E-53
810- 14: transcript:KXG33974            transcript:Zm00001d012078_T001  3.00E-35
810- 15: transcript:KXG33976            transcript:Zm00001d012074_T001         0
810- 16: transcript:EES04096            transcript:Zm00001d012069_T001 8.00E-143
810- 17: transcript:EES01950            transcript:Zm00001d012068_T001  2.00E-59
810- 18: transcript:EES04098            transcript:Zm00001d012067_T003         0
810- 19: transcript:EES04100            transcript:Zm00001d012064_T001  7.00E-83
810- 20: transcript:EES04101            transcript:Zm00001d012063_T001  3.00E-46
810- 21: transcript:EES04102            transcript:Zm00001d012061_T005         0
810- 22: transcript:EES04103            transcript:Zm00001d012060_T003         0
810- 23: transcript:EES01953            transcript:Zm00001d012059_T001  7.00E-51
810- 24: transcript:EES01954            transcript:Zm00001d012052_T001  6.00E-68
810- 25: transcript:EES01955            transcript:Zm00001d012050_T001  1.00E-51
810- 26: transcript:KXG33985            transcript:Zm00001d012049_T003         0
810- 27: transcript:EES01957            transcript:Zm00001d012048_T001         0
810- 28: transcript:EES04106            transcript:Zm00001d012047_T001  1.00E-58
810- 29: transcript:EES04107            transcript:Zm00001d012045_T001 3.00E-117
810- 30: transcript:EES04108            transcript:Zm00001d012043_T002  2.00E-33
810- 31: transcript:EES04109            transcript:Zm00001d012042_T002  6.00E-49
810- 32: transcript:KXG33988            transcript:Zm00001d012041_T001  1.00E-73
810- 33: transcript:KXG33990            transcript:Zm00001d012040_T003         0
810- 34: transcript:EES04111            transcript:Zm00001d012036_T001         0
810- 35: transcript:EES01963            transcript:Zm00001d012035_T001         0
810- 36: transcript:EES04113            transcript:Zm00001d012033_T002         0
## Alignment 811: score=1310.0 e_value=2.8e-101 N=30 3&8 minus
811-  0: transcript:OQU86083            transcript:Zm00001d008984_T001         0
811-  1: transcript:EES00021            transcript:Zm00001d008983_T001         0
811-  2: transcript:KXG31516            transcript:Zm00001d008982_T001 5.00E-171
811-  3: transcript:EES00022            transcript:Zm00001d008980_T001 1.00E-102
811-  4: transcript:KXG31519            transcript:Zm00001d008977_T002         0
811-  5: transcript:EES00024            transcript:Zm00001d008976_T001 2.00E-169
811-  6: transcript:KXG31524            transcript:Zm00001d008975_T002         0
811-  7: transcript:KXG31527            transcript:Zm00001d008974_T001         0
811-  8: transcript:EES00028            transcript:Zm00001d008971_T001  2.00E-32
811-  9: transcript:OQU86088            transcript:Zm00001d008970_T001  4.00E-95
811- 10: transcript:KXG31528            transcript:Zm00001d008968_T001 2.00E-122
811- 11: transcript:KXG31529            transcript:Zm00001d008966_T001         0
811- 12: transcript:KXG31530            transcript:Zm00001d008965_T001         0
811- 13: transcript:KXG31536            transcript:Zm00001d008963_T001         0
811- 14: transcript:KXG31537            transcript:Zm00001d008962_T001 2.00E-179
811- 15: transcript:EES02231            transcript:Zm00001d008961_T001  2.00E-41
811- 16: transcript:EES00042            transcript:Zm00001d008960_T001         0
811- 17: transcript:EES00046            transcript:Zm00001d008957_T001         0
811- 18: transcript:EES00049            transcript:Zm00001d008956_T001  5.00E-61
811- 19: transcript:KXG31543            transcript:Zm00001d008954_T002         0
811- 20: transcript:EES00050            transcript:Zm00001d008952_T001         0
```

811- 21: transcript:EES02236 transcript:Zm00001d008951_T002 7.00E-162
811- 22: transcript:EES02237 transcript:Zm00001d008950_T001 1.00E-58
811- 23: transcript:EES02238 transcript:Zm00001d008946_T001 9.00E-71
811- 24: transcript:EES02240 transcript:Zm00001d008945_T001 7.00E-76
811- 25: transcript:EES02242 transcript:Zm00001d008944_T002 1.00E-15
811- 26: transcript:KXG31547 transcript:Zm00001d008941_T002 0
811- 27: transcript:KXG31552 transcript:Zm00001d008940_T002 0
811- 28: transcript:KXG31553 transcript:Zm00001d008939_T001 2.00E-124
811- 29: transcript:EES02247 transcript:Zm00001d008930_T003 0
Alignment 812: score=1085.0 e_value=7.8e-73 N=24 3&8 minus
812- 0: transcript:EES01966 transcript:Zm00001d012031_T001 2.00E-128
812- 1: transcript:KXG34000 transcript:Zm00001d012030_T003 1.00E-74
812- 2: transcript:OQU88114 transcript:Zm00001d012029_T001 0
812- 3: transcript:EES04116 transcript:Zm00001d012027_T002 0
812- 4: transcript:EES01968 transcript:Zm00001d012022_T001 2.00E-56
812- 5: transcript:KXG34001 transcript:Zm00001d012021_T001 1.00E-96
812- 6: transcript:EES01976 transcript:Zm00001d012020_T001 0
812- 7: transcript:EES04118 transcript:Zm00001d012019_T001 0
812- 8: transcript:EES01977 transcript:Zm00001d012017_T001 8.00E-125
812- 9: transcript:KXG34006 transcript:Zm00001d012016_T001 0
812- 10: transcript:EES01979 transcript:Zm00001d012015_T001 0
812- 11: transcript:OQU88129 transcript:Zm00001d012010_T001 2.00E-45
812- 12: transcript:EES01984 transcript:Zm00001d012007_T006 0
812- 13: transcript:KXG34012 transcript:Zm00001d012005_T007 0
812- 14: transcript:EES01986 transcript:Zm00001d012003_T001 4.00E-170
812- 15: transcript:KXG34016 transcript:Zm00001d011997_T002 0
812- 16: transcript:KXG34017 transcript:Zm00001d011996_T002 0
812- 17: transcript:EES04122 transcript:Zm00001d011994_T001 0
812- 18: transcript:OQU88132 transcript:Zm00001d011993_T001 3.00E-118
812- 19: transcript:EES01990 transcript:Zm00001d011992_T001 1.00E-150
812- 20: transcript:EES01991 transcript:Zm00001d011987_T001 4.00E-74
812- 21: transcript:EES01992 transcript:Zm00001d011985_T001 4.00E-142
812- 22: transcript:EES01994 transcript:Zm00001d011984_T001 0
812- 23: transcript:KXG34018 transcript:Zm00001d011983_T031 0
Alignment 813: score=1004.0 e_value=2.9e-76 N=25 3&8 minus
813- 0: transcript:KXG33081 transcript:Zm00001d009562_T007 0
813- 1: transcript:EES03419 transcript:Zm00001d009555_T003 1.00E-116
813- 2: transcript:EES03421 transcript:Zm00001d009552_T002 6.00E-106
813- 3: transcript:KXG33101 transcript:Zm00001d009549_T002 8.00E-178
813- 4: transcript:EES03429 transcript:Zm00001d009539_T002 0
813- 5: transcript:KXG33104 transcript:Zm00001d009532_T001 4.00E-85
813- 6: transcript:KXG33105 transcript:Zm00001d009525_T002 1.00E-30
813- 7: transcript:KXG33109 transcript:Zm00001d009513_T003 5.00E-145
813- 8: transcript:EES01253 transcript:Zm00001d009511_T001 0
813- 9: transcript:OQU87329 transcript:Zm00001d009510_T001 4.00E-138
813- 10: transcript:OQU87331 transcript:Zm00001d009509_T001 9.00E-29
813- 11: transcript:EES03442 transcript:Zm00001d009508_T001 2.00E-87
813- 12: transcript:EES03440 transcript:Zm00001d009506_T001 0
813- 13: transcript:OQU87335 transcript:Zm00001d009504_T002 0
813- 14: transcript:KXG33132 transcript:Zm00001d009501_T001 3.00E-07
813- 15: transcript:EES03447 transcript:Zm00001d009500_T002 0
813- 16: transcript:EES01277 transcript:Zm00001d009496_T001 2.00E-57
813- 17: transcript:EES03448 transcript:Zm00001d009494_T001 0
813- 18: transcript:KXG33146 transcript:Zm00001d009488_T001 0

```
813- 19: transcript:KXG33148           transcript:Zm00001d009487_T001  5.00E-33
813- 20: transcript:EES01286           transcript:Zm00001d009480_T001         0
813- 21: transcript:EES01291           transcript:Zm00001d009475_T001  2.00E-44
813- 22: transcript:OQU87357           transcript:Zm00001d009473_T001         0
813- 23: transcript:EES01304           transcript:Zm00001d009469_T001  4.00E-33
813- 24: transcript:EES03472           transcript:Zm00001d009468_T001 4.00E-162
## Alignment 814: score=986.0 e_value=3e-61 N=22 3&8 minus
814-  0: transcript:EES01424           transcript:Zm00001d012791_T001 4.00E-161
814-  1: transcript:EES01425           transcript:Zm00001d012789_T001 2.00E-102
814-  2: transcript:EES01428           transcript:Zm00001d012788_T001 2.00E-158
814-  3: transcript:KXG33317           transcript:Zm00001d012787_T003         0
814-  4: transcript:EES01436           transcript:Zm00001d012782_T001 1.00E-144
814-  5: transcript:EES03574           transcript:Zm00001d012780_T001         0
814-  6: transcript:OQU87487           transcript:Zm00001d012779_T001  3.00E-96
814-  7: transcript:KXG33320           transcript:Zm00001d012776_T001 1.00E-162
814-  8: transcript:OQU87493           transcript:Zm00001d012775_T001  3.00E-42
814-  9: transcript:EES01447           transcript:Zm00001d012774_T001         0
814- 10: transcript:EES01452           transcript:Zm00001d012772_T001  4.00E-92
814- 11: transcript:OQU87505           transcript:Zm00001d012771_T001         0
814- 12: transcript:EES01455           transcript:Zm00001d012770_T001         0
814- 13: transcript:KXG33339           transcript:Zm00001d012769_T001         0
814- 14: transcript:KXG33341           transcript:Zm00001d012767_T001 5.00E-126
814- 15: transcript:KXG33344           transcript:Zm00001d012766_T001         0
814- 16: transcript:EES01459           transcript:Zm00001d012765_T005         0
814- 17: transcript:EES01460           transcript:Zm00001d012764_T001         0
814- 18: transcript:OQU87522           transcript:Zm00001d012763_T002         0
814- 19: transcript:OQU87524           transcript:Zm00001d012761_T002  4.00E-57
814- 20: transcript:EES03594           transcript:Zm00001d012760_T002         0
814- 21: transcript:EES03595           transcript:Zm00001d012757_T001 1.00E-159
## Alignment 815: score=961.0 e_value=2.8e-70 N=24 3&8 minus
815-  0: transcript:KXG32178           transcript:Zm00001d008363_T007         0
815-  1: transcript:EES00497           transcript:Zm00001d008360_T001         0
815-  2: transcript:EES02687           transcript:Zm00001d008359_T001  3.00E-29
815-  3: transcript:EES00498           transcript:Zm00001d008357_T002         0
815-  4: transcript:EES00499           transcript:Zm00001d008356_T005 1.00E-107
815-  5: transcript:KXG32180           transcript:Zm00001d008355_T001  2.00E-22
815-  6: transcript:EES02691           transcript:Zm00001d008354_T006         0
815-  7: transcript:OQU86611           transcript:Zm00001d008347_T001         0
815-  8: transcript:EES00501           transcript:Zm00001d008346_T001  2.00E-59
815-  9: transcript:KXG32186           transcript:Zm00001d008345_T003         0
815- 10: transcript:OQU86616           transcript:Zm00001d008343_T001  1.00E-33
815- 11: transcript:OQU86618           transcript:Zm00001d008340_T003         0
815- 12: transcript:KXG32191           transcript:Zm00001d008338_T001         0
815- 13: transcript:EES02701           transcript:Zm00001d008334_T001         0
815- 14: transcript:EES02705           transcript:Zm00001d008333_T003 3.00E-112
815- 15: transcript:KXG32196           transcript:Zm00001d008331_T001         0
815- 16: transcript:EES00507           transcript:Zm00001d008330_T001  9.00E-80
815- 17: transcript:EES02706           transcript:Zm00001d008329_T001  3.00E-17
815- 18: transcript:KXG32197           transcript:Zm00001d008327_T001         0
815- 19: transcript:KXG32198           transcript:Zm00001d008326_T001         0
815- 20: transcript:EES02711           transcript:Zm00001d008325_T001  6.00E-68
815- 21: transcript:EES02712           transcript:Zm00001d008322_T002 3.00E-154
815- 22: transcript:OQU86626           transcript:Zm00001d008321_T011         0
815- 23: transcript:EES02716           transcript:Zm00001d008320_T004         0
```

```
## Alignment 816: score=931.0 e_value=7.3e-63 N=21 3&8 minus
816-  0: transcript:Zm00001d041186_T005 transcript:KXG24158                0
816-  1: transcript:Zm00001d041198_T001 transcript:EES16463                0
816-  2: transcript:Zm00001d041201_T001 transcript:EES17499                0
816-  3: transcript:Zm00001d041203_T001 transcript:EES16459                0
816-  4: transcript:Zm00001d041204_T001 transcript:KXG24148                0
816-  5: transcript:Zm00001d041205_T001 transcript:OQU79734         1.00E-56
816-  6: transcript:Zm00001d041212_T001 transcript:EES17501         2.00E-56
816-  7: transcript:Zm00001d041214_T003 transcript:EES16456                0
816-  8: transcript:Zm00001d041215_T002 transcript:EES17498                0
816-  9: transcript:Zm00001d041216_T001 transcript:EES17494        5.00E-119
816- 10: transcript:Zm00001d041217_T002 transcript:EES17493                0
816- 11: transcript:Zm00001d041220_T001 transcript:KXG24127        2.00E-162
816- 12: transcript:Zm00001d041221_T001 transcript:KXG24124                0
816- 13: transcript:Zm00001d041226_T001 transcript:EES17483        2.00E-173
816- 14: transcript:Zm00001d041229_T002 transcript:OQU79722                0
816- 15: transcript:Zm00001d041230_T001 transcript:OQU79720        8.00E-113
816- 16: transcript:Zm00001d041232_T001 transcript:EES16439                0
816- 17: transcript:Zm00001d041243_T001 transcript:KXG24112                0
816- 18: transcript:Zm00001d041246_T001 transcript:EES16438         4.00E-55
816- 19: transcript:Zm00001d041255_T001 transcript:EES16437                0
816- 20: transcript:Zm00001d041258_T001 transcript:OQU79716        5.00E-167
## Alignment 817: score=880.0 e_value=1.5e-59 N=21 3&8 minus
817-  0: transcript:OQU86551            transcript:Zm00001d008399_T001  1.00E-84
817-  1: transcript:OQU86553            transcript:Zm00001d008398_T001         0
817-  2: transcript:EES02656            transcript:Zm00001d008397_T001  3.00E-60
817-  3: transcript:KXG32145            transcript:Zm00001d008396_T003 2.00E-110
817-  4: transcript:EES00469            transcript:Zm00001d008395_T001 4.00E-151
817-  5: transcript:EES02660            transcript:Zm00001d008394_T001 9.00E-113
817-  6: transcript:OQU86571            transcript:Zm00001d008393_T001         0
817-  7: transcript:EES02664            transcript:Zm00001d008392_T001 9.00E-102
817-  8: transcript:OQU86578            transcript:Zm00001d008390_T001         0
817-  9: transcript:EES02668            transcript:Zm00001d008389_T003         0
817- 10: transcript:EES02669            transcript:Zm00001d008388_T002         0
817- 11: transcript:OQU86591            transcript:Zm00001d008387_T002 2.00E-139
817- 12: transcript:EES02670            transcript:Zm00001d008386_T002         0
817- 13: transcript:OQU86592            transcript:Zm00001d008381_T001         0
817- 14: transcript:OQU86594            transcript:Zm00001d008380_T002         0
817- 15: transcript:EES02674            transcript:Zm00001d008379_T001         0
817- 16: transcript:EES02675            transcript:Zm00001d008377_T001         0
817- 17: transcript:EES00480            transcript:Zm00001d008376_T001         0
817- 18: transcript:OQU86596            transcript:Zm00001d008374_T001         0
817- 19: transcript:OQU86601            transcript:Zm00001d008370_T001 1.00E-177
817- 20: transcript:EES00491            transcript:Zm00001d008369_T001         0
## Alignment 818: score=857.0 e_value=2.1e-58 N=20 3&8 minus
818-  0: transcript:EES00105            transcript:Zm00001d008882_T001 1.00E-139
818-  1: transcript:EES00107            transcript:Zm00001d008881_T001  4.00E-56
818-  2: transcript:EES00109            transcript:Zm00001d008878_T001  1.00E-97
818-  3: transcript:KXG31606            transcript:Zm00001d008875_T001         0
818-  4: transcript:EES02288            transcript:Zm00001d008874_T002         0
818-  5: transcript:EES00113            transcript:Zm00001d008872_T001  2.00E-81
818-  6: transcript:EES02290            transcript:Zm00001d008871_T001         0
818-  7: transcript:EES00114            transcript:Zm00001d008870_T009 2.00E-159
818-  8: transcript:EES00115            transcript:Zm00001d008869_T001         0
```

```
818-  9: transcript:EES00119          transcript:Zm00001d008866_T001  7.00E-99
818- 10: transcript:EES02297          transcript:Zm00001d008865_T002         0
818- 11: transcript:EES00121          transcript:Zm00001d008863_T004         0
818- 12: transcript:EES00123          transcript:Zm00001d008862_T001         0
818- 13: transcript:EES00124          transcript:Zm00001d008859_T001         0
818- 14: transcript:KXG31611          transcript:Zm00001d008858_T001 7.00E-107
818- 15: transcript:KXG31616          transcript:Zm00001d008853_T001         0
818- 16: transcript:EES00127          transcript:Zm00001d008851_T002 2.00E-125
818- 17: transcript:EES00128          transcript:Zm00001d008850_T001 2.00E-106
818- 18: transcript:KXG31619          transcript:Zm00001d008849_T001         0
818- 19: transcript:EES02308          transcript:Zm00001d008843_T001  3.00E-52
## Alignment 819: score=843.0 e_value=2.7e-55 N=19 3&8 minus
819-  0: transcript:EER99976          transcript:Zm00001d009032_T001  7.00E-61
819-  1: transcript:EER99980          transcript:Zm00001d009031_T001  1.00E-73
819-  2: transcript:EES02157          transcript:Zm00001d009030_T002         0
819-  3: transcript:EES02162          transcript:Zm00001d009029_T001         0
819-  4: transcript:EER99986          transcript:Zm00001d009028_T001         0
819-  5: transcript:EES02163          transcript:Zm00001d009027_T004         0
819-  6: transcript:KXG31453          transcript:Zm00001d009025_T001         0
819-  7: transcript:EES02167          transcript:Zm00001d009023_T001 9.00E-100
819-  8: transcript:OQU86052          transcript:Zm00001d009020_T001  8.00E-66
819-  9: transcript:KXG31456          transcript:Zm00001d009017_T002         0
819- 10: transcript:EES02175          transcript:Zm00001d009014_T004         0
819- 11: transcript:EER99993          transcript:Zm00001d009013_T001         0
819- 12: transcript:OQU86056          transcript:Zm00001d009010_T003  1.00E-80
819- 13: transcript:EES00001          transcript:Zm00001d009009_T001  7.00E-90
819- 14: transcript:KXG31480          transcript:Zm00001d009008_T001         0
819- 15: transcript:OQU86057          transcript:Zm00001d009007_T001 2.00E-149
819- 16: transcript:KXG31482          transcript:Zm00001d009005_T002         0
819- 17: transcript:EES00004          transcript:Zm00001d009004_T002         0
819- 18: transcript:EES02188          transcript:Zm00001d009003_T001         0
## Alignment 820: score=837.0 e_value=3.4e-51 N=19 3&8 minus
820-  0: transcript:OQU86378          transcript:Zm00001d008586_T001  9.00E-23
820-  1: transcript:EES02523          transcript:Zm00001d008585_T001 3.00E-169
820-  2: transcript:EES00350          transcript:Zm00001d008582_T001  1.00E-41
820-  3: transcript:EES00357          transcript:Zm00001d008579_T001  4.00E-31
820-  4: transcript:EES02531          transcript:Zm00001d008577_T001  1.00E-81
820-  5: transcript:EES00360          transcript:Zm00001d008573_T001  4.00E-88
820-  6: transcript:OQU86387          transcript:Zm00001d008570_T001         0
820-  7: transcript:EES02540          transcript:Zm00001d008569_T001         0
820-  8: transcript:EES02542          transcript:Zm00001d008568_T001 3.00E-151
820-  9: transcript:KXG31959          transcript:Zm00001d008567_T008         0
820- 10: transcript:KXG31963          transcript:Zm00001d008565_T001         0
820- 11: transcript:EES02546          transcript:Zm00001d008564_T001 2.00E-105
820- 12: transcript:EES02547          transcript:Zm00001d008562_T001 2.00E-110
820- 13: transcript:EES02545          transcript:Zm00001d008559_T002 6.00E-178
820- 14: transcript:EES02550          transcript:Zm00001d008549_T002         0
820- 15: transcript:KXG31970          transcript:Zm00001d008548_T001  4.00E-52
820- 16: transcript:EES02552          transcript:Zm00001d008546_T001 6.00E-148
820- 17: transcript:OQU86404          transcript:Zm00001d008540_T001  9.00E-62
820- 18: transcript:OQU86408          transcript:Zm00001d008539_T001  3.00E-32
## Alignment 821: score=820.0 e_value=2.1e-52 N=19 3&8 minus
821-  0: transcript:KXG31902          transcript:Zm00001d008614_T001         0
821-  1: transcript:EES02497          transcript:Zm00001d008613_T001 3.00E-140
```

821- 2: transcript:EES00330 transcript:Zm00001d008612_T002 3.00E-105
821- 3: transcript:EES00331 transcript:Zm00001d008611_T003 0
821- 4: transcript:EES00334 transcript:Zm00001d008610_T001 5.00E-51
821- 5: transcript:KXG31909 transcript:Zm00001d008606_T003 5.00E-129
821- 6: transcript:KXG31914 transcript:Zm00001d008604_T011 0
821- 7: transcript:KXG31919 transcript:Zm00001d008601_T001 2.00E-55
821- 8: transcript:EES02505 transcript:Zm00001d008600_T001 0
821- 9: transcript:EES02509 transcript:Zm00001d008599_T001 7.00E-74
821- 10: transcript:EES00342 transcript:Zm00001d008597_T001 0
821- 11: transcript:EES00346 transcript:Zm00001d008596_T005 0
821- 12: transcript:EES02512 transcript:Zm00001d008594_T001 3.00E-109
821- 13: transcript:OQU86360 transcript:Zm00001d008592_T001 0
821- 14: transcript:EES00348 transcript:Zm00001d008591_T001 0
821- 15: transcript:EES02516 transcript:Zm00001d008590_T001 2.00E-34
821- 16: transcript:OQU86363 transcript:Zm00001d008588_T001 0
821- 17: transcript:KXG31931 transcript:Zm00001d008587_T001 0
821- 18: transcript:OQU86365 transcript:Zm00001d008585_T001 3.00E-154
Alignment 822: score=818.0 e_value=3.6e-49 N=18 3&8 minus
822- 0: transcript:EES01683 transcript:Zm00001d010607_T001 1.00E-107
822- 1: transcript:EES03820 transcript:Zm00001d010606_T004 0
822- 2: transcript:OQU87773 transcript:Zm00001d010604_T006 0
822- 3: transcript:EES03822 transcript:Zm00001d010603_T001 2.00E-106
822- 4: transcript:OQU87774 transcript:Zm00001d010602_T001 0
822- 5: transcript:OQU87776 transcript:Zm00001d010599_T002 0
822- 6: transcript:EES03824 transcript:Zm00001d010596_T001 8.00E-84
822- 7: transcript:KXG33640 transcript:Zm00001d010594_T001 6.00E-99
822- 8: transcript:KXG33641 transcript:Zm00001d010592_T001 9.00E-07
822- 9: transcript:OQU87785 transcript:Zm00001d010591_T001 6.00E-19
822- 10: transcript:EES01699 transcript:Zm00001d010590_T001 0
822- 11: transcript:KXG33643 transcript:Zm00001d010589_T001 2.00E-166
822- 12: transcript:EES03833 transcript:Zm00001d010587_T001 4.00E-37
822- 13: transcript:EES03837 transcript:Zm00001d010583_T001 2.00E-90
822- 14: transcript:EES01704 transcript:Zm00001d010579_T001 0
822- 15: transcript:EES01706 transcript:Zm00001d010578_T003 0
822- 16: transcript:EES03838 transcript:Zm00001d010576_T002 3.00E-113
822- 17: transcript:OQU87808 transcript:Zm00001d010574_T002 1.00E-82
Alignment 823: score=770.0 e_value=3.4e-49 N=18 3&8 minus
823- 0: transcript:Zm00001d041535_T001 transcript:KXG23778 4.00E-18
823- 1: transcript:Zm00001d041536_T001 transcript:EES16206 0
823- 2: transcript:Zm00001d041537_T001 transcript:EES16200 0
823- 3: transcript:Zm00001d041538_T001 transcript:KXG23762 9.00E-11
823- 4: transcript:Zm00001d041539_T001 transcript:EES17233 4.00E-122
823- 5: transcript:Zm00001d041544_T001 transcript:KXG23755 7.00E-73
823- 6: transcript:Zm00001d041548_T002 transcript:OQU79382 0
823- 7: transcript:Zm00001d041549_T001 transcript:OQU79380 3.00E-157
823- 8: transcript:Zm00001d041550_T006 transcript:EES16191 0
823- 9: transcript:Zm00001d041553_T001 transcript:EES17228 5.00E-94
823- 10: transcript:Zm00001d041556_T004 transcript:EES16185 0
823- 11: transcript:Zm00001d041567_T001 transcript:EES16184 6.00E-48
823- 12: transcript:Zm00001d041568_T001 transcript:KXG23748 0
823- 13: transcript:Zm00001d041569_T001 transcript:OQU79368 3.00E-116
823- 14: transcript:Zm00001d041570_T001 transcript:EES17225 0
823- 15: transcript:Zm00001d041573_T002 transcript:EES16180 6.00E-73
823- 16: transcript:Zm00001d041576_T001 transcript:KXG23733 2.00E-131

823- 17: transcript:Zm00001d041575_T008 transcript:KXG23730 3.00E-141
Alignment 824: score=742.0 e_value=3.3e-45 N=16 3&8 minus
824- 0: transcript:Zm00001d041595_T001 transcript:EES17205 0
824- 1: transcript:Zm00001d041596_T001 transcript:KXG23710 0
824- 2: transcript:Zm00001d041597_T001 transcript:EES16167 1.00E-130
824- 3: transcript:Zm00001d041598_T001 transcript:KXG23705 5.00E-30
824- 4: transcript:Zm00001d041599_T004 transcript:EES16163 0
824- 5: transcript:Zm00001d041600_T001 transcript:EES17198 9.00E-91
824- 6: transcript:Zm00001d041601_T001 transcript:EES17197 0
824- 7: transcript:Zm00001d041603_T001 transcript:KXG23702 0
824- 8: transcript:Zm00001d041604_T001 transcript:EES16161 9.00E-79
824- 9: transcript:Zm00001d041606_T002 transcript:KXG23699 0
824- 10: transcript:Zm00001d041607_T001 transcript:EES16160 1.00E-127
824- 11: transcript:Zm00001d041608_T001 transcript:KXG23698 0
824- 12: transcript:Zm00001d041609_T001 transcript:EES17194 1.00E-82
824- 13: transcript:Zm00001d041610_T001 transcript:KXG23694 0
824- 14: transcript:Zm00001d041612_T001 transcript:EES17190 1.00E-92
824- 15: transcript:Zm00001d041620_T001 transcript:EES17187 0
Alignment 825: score=725.0 e_value=0 N=17 3&8 minus
825- 0: transcript:OQU86478 transcript:Zm00001d008485_T001 0
825- 1: transcript:EES02605 transcript:Zm00001d008478_T001 1.00E-147
825- 2: transcript:OQU86482 transcript:Zm00001d008477_T003 0
825- 3: transcript:OQU86483 transcript:Zm00001d008466_T001 0
825- 4: transcript:EES00429 transcript:Zm00001d008465_T001 0
825- 5: transcript:KXG32078 transcript:Zm00001d008462_T002 0
825- 6: transcript:OQU86489 transcript:Zm00001d008458_T001 0
825- 7: transcript:EES00430 transcript:Zm00001d008455_T001 0
825- 8: transcript:EES00432 transcript:Zm00001d008454_T001 0
825- 9: transcript:EES02611 transcript:Zm00001d008453_T001 1.00E-17
825- 10: transcript:EES02613 transcript:Zm00001d008451_T001 3.00E-15
825- 11: transcript:EES00433 transcript:Zm00001d008449_T001 2.00E-78
825- 12: transcript:EES02614 transcript:Zm00001d008446_T006 8.00E-110
825- 13: transcript:EES00435 transcript:Zm00001d008444_T001 1.00E-128
825- 14: transcript:EES02615 transcript:Zm00001d008443_T002 0
825- 15: transcript:EES00436 transcript:Zm00001d008440_T001 3.00E-146
825- 16: transcript:KXG32095 transcript:Zm00001d008435_T007 0
Alignment 826: score=672.0 e_value=1.9e-45 N=17 3&8 minus
826- 0: transcript:EES02729 transcript:Zm00001d008299_T002 4.00E-129
826- 1: transcript:EES00529 transcript:Zm00001d008297_T001 2.00E-98
826- 2: transcript:OQU86656 transcript:Zm00001d008296_T001 0
826- 3: transcript:OQU86657 transcript:Zm00001d008295_T002 1.00E-131
826- 4: transcript:KXG32221 transcript:Zm00001d008294_T001 0
826- 5: transcript:KXG32223 transcript:Zm00001d008293_T002 1.00E-51
826- 6: transcript:EES00536 transcript:Zm00001d008289_T007 0
826- 7: transcript:EES00537 transcript:Zm00001d008288_T001 4.00E-32
826- 8: transcript:KXG32228 transcript:Zm00001d008287_T001 0
826- 9: transcript:KXG32230 transcript:Zm00001d008285_T005 0
826- 10: transcript:EES00543 transcript:Zm00001d008284_T004 0
826- 11: transcript:OQU86667 transcript:Zm00001d008283_T001 1.00E-140
826- 12: transcript:EES02742 transcript:Zm00001d008282_T002 5.00E-124
826- 13: transcript:OQU86673 transcript:Zm00001d008281_T001 0
826- 14: transcript:KXG32238 transcript:Zm00001d008279_T001 0
826- 15: transcript:OQU86675 transcript:Zm00001d008278_T001 3.00E-86
826- 16: transcript:KXG32241 transcript:Zm00001d008273_T001 0

Alignment 827: score=648.0 e_value=1.4e-39 N=16 3&8 minus
827- 0: transcript:Zm00001d041844_T021 transcript:KXG23110 0
827- 1: transcript:Zm00001d041846_T001 transcript:KXG23107 0
827- 2: transcript:Zm00001d041847_T002 transcript:EES15751 0
827- 3: transcript:Zm00001d041852_T002 transcript:EES16745 0
827- 4: transcript:Zm00001d041853_T001 transcript:OQU78829 5.00E-88
827- 5: transcript:Zm00001d041854_T002 transcript:OQU78826 6.00E-40
827- 6: transcript:Zm00001d041856_T001 transcript:KXG23087 2.00E-35
827- 7: transcript:Zm00001d041857_T001 transcript:OQU78822 0
827- 8: transcript:Zm00001d041864_T007 transcript:KXG23082 0
827- 9: transcript:Zm00001d041870_T001 transcript:OQU78817 3.00E-179
827- 10: transcript:Zm00001d041871_T001 transcript:EES16732 6.00E-116
827- 11: transcript:Zm00001d041873_T001 transcript:OQU78812 0
827- 12: transcript:Zm00001d041877_T002 transcript:KXG23068 0
827- 13: transcript:Zm00001d041880_T005 transcript:EES16726 0
827- 14: transcript:Zm00001d041882_T002 transcript:KXG23066 7.00E-108
827- 15: transcript:Zm00001d041883_T001 transcript:KXG23063 6.00E-37
Alignment 828: score=626.0 e_value=1.4e-41 N=15 3&8 minus
828- 0: transcript:KXG31565 transcript:Zm00001d008949_T001 2.00E-34
828- 1: transcript:EES02257 transcript:Zm00001d008934_T001 4.00E-48
828- 2: transcript:EES02254 transcript:Zm00001d008927_T001 1.00E-52
828- 3: transcript:KXG31580 transcript:Zm00001d008911_T001 7.00E-42
828- 4: transcript:EES00076 transcript:Zm00001d008910_T001 9.00E-56
828- 5: transcript:EES00081 transcript:Zm00001d008909_T002 4.00E-153
828- 6: transcript:EES00083 transcript:Zm00001d008907_T001 0
828- 7: transcript:KXG31587 transcript:Zm00001d008906_T003 0
828- 8: transcript:OQU86128 transcript:Zm00001d008905_T001 0
828- 9: transcript:EES00087 transcript:Zm00001d008903_T001 0
828- 10: transcript:EES00090 transcript:Zm00001d008902_T001 0
828- 11: transcript:EES00092 transcript:Zm00001d008901_T001 0
828- 12: transcript:EES02276 transcript:Zm00001d008900_T001 2.00E-20
828- 13: transcript:EES02274 transcript:Zm00001d008899_T001 0
828- 14: transcript:EES00088 transcript:Zm00001d008898_T001 0
Alignment 829: score=613.0 e_value=1.7e-35 N=14 3&8 minus
829- 0: transcript:EES04041 transcript:Zm00001d012156_T007 0
829- 1: transcript:OQU88038 transcript:Zm00001d012155_T021 0
829- 2: transcript:EES01908 transcript:Zm00001d012154_T001 3.00E-90
829- 3: transcript:KXG33907 transcript:Zm00001d012153_T002 0
829- 4: transcript:OQU88051 transcript:Zm00001d012147_T001 0
829- 5: transcript:EES04050 transcript:Zm00001d012146_T003 0
829- 6: transcript:EES04051 transcript:Zm00001d012145_T001 0
829- 7: transcript:EES01913 transcript:Zm00001d012144_T001 0
829- 8: transcript:EES04053 transcript:Zm00001d012142_T001 5.00E-110
829- 9: transcript:OQU88055 transcript:Zm00001d012141_T001 0
829- 10: transcript:OQU88056 transcript:Zm00001d012140_T001 0
829- 11: transcript:KXG33937 transcript:Zm00001d012138_T001 0
829- 12: transcript:KXG33943 transcript:Zm00001d012137_T010 0
829- 13: transcript:EES04061 transcript:Zm00001d012128_T001 0
Alignment 830: score=600.0 e_value=3.3e-32 N=13 3&8 minus
830- 0: transcript:EES01081 transcript:Zm00001d009719_T003 5.00E-149
830- 1: transcript:EES01085 transcript:Zm00001d009717_T001 0
830- 2: transcript:EES03295 transcript:Zm00001d009716_T001 0
830- 3: transcript:EES03296 transcript:Zm00001d009714_T002 0
830- 4: transcript:EES01090 transcript:Zm00001d009710_T001 0

830- 5: transcript:EES01092 transcript:Zm00001d009709_T001 0
830- 6: transcript:EES01094 transcript:Zm00001d009708_T001 0
830- 7: transcript:EES03303 transcript:Zm00001d009707_T001 4.00E-125
830- 8: transcript:EES03305 transcript:Zm00001d009705_T001 0
830- 9: transcript:KXG32925 transcript:Zm00001d009702_T001 0
830- 10: transcript:EES01098 transcript:Zm00001d009701_T001 2.00E-23
830- 11: transcript:EES01100 transcript:Zm00001d009700_T001 7.00E-80
830- 12: transcript:EES03310 transcript:Zm00001d009698_T001 4.00E-58
Alignment 831: score=568.0 e_value=5.1e-34 N=13 3&8 minus
831- 0: transcript:Zm00001d041719_T001 transcript:KXG23570 0
831- 1: transcript:Zm00001d041725_T001 transcript:EES16055 1.00E-139
831- 2: transcript:Zm00001d041726_T001 transcript:KXG23555 9.00E-133
831- 3: transcript:Zm00001d041727_T001 transcript:OQU79193 2.00E-37
831- 4: transcript:Zm00001d041730_T001 transcript:OQU79192 3.00E-111
831- 5: transcript:Zm00001d041732_T001 transcript:KXG23548 5.00E-70
831- 6: transcript:Zm00001d041733_T001 transcript:EES17080 2.00E-72
831- 7: transcript:Zm00001d041735_T001 transcript:EES17083 3.00E-67
831- 8: transcript:Zm00001d041740_T001 transcript:EES16036 0
831- 9: transcript:Zm00001d041741_T001 transcript:OQU79188 0
831- 10: transcript:Zm00001d041744_T001 transcript:EES16032 4.00E-79
831- 11: transcript:Zm00001d041746_T001 transcript:KXG23533 2.00E-90
831- 12: transcript:Zm00001d041748_T003 transcript:KXG23525 0
Alignment 832: score=557.0 e_value=9.7e-34 N=14 3&8 minus
832- 0: transcript:EES02802 transcript:Zm00001d008210_T002 6.00E-129
832- 1: transcript:EES00602 transcript:Zm00001d008209_T001 2.00E-162
832- 2: transcript:EES02803 transcript:Zm00001d008208_T001 1.00E-102
832- 3: transcript:OQU86737 transcript:Zm00001d008206_T001 4.00E-47
832- 4: transcript:KXG32305 transcript:Zm00001d008205_T001 0
832- 5: transcript:EES00604 transcript:Zm00001d008203_T001 0
832- 6: transcript:EES00605 transcript:Zm00001d008201_T001 8.00E-32
832- 7: transcript:KXG32309 transcript:Zm00001d008200_T001 2.00E-33
832- 8: transcript:EES02809 transcript:Zm00001d008199_T001 4.00E-178
832- 9: transcript:EES02810 transcript:Zm00001d008196_T001 1.00E-41
832- 10: transcript:EES02817 transcript:Zm00001d008194_T001 1.00E-29
832- 11: transcript:EES00609 transcript:Zm00001d008190_T001 0
832- 12: transcript:KXG32321 transcript:Zm00001d008189_T004 0
832- 13: transcript:KXG32327 transcript:Zm00001d008187_T001 0
Alignment 833: score=555.0 e_value=3.2e-29 N=12 3&8 minus
833- 0: transcript:EES00406 transcript:Zm00001d008503_T002 0
833- 1: transcript:EES02593 transcript:Zm00001d008502_T001 0
833- 2: transcript:OQU86466 transcript:Zm00001d008501_T001 7.00E-80
833- 3: transcript:KXG32043 transcript:Zm00001d008500_T001 5.00E-79
833- 4: transcript:KXG32044 transcript:Zm00001d008499_T001 2.00E-144
833- 5: transcript:KXG32046 transcript:Zm00001d008498_T001 3.00E-165
833- 6: transcript:OQU86468 transcript:Zm00001d008497_T002 0
833- 7: transcript:OQU86469 transcript:Zm00001d008496_T001 0
833- 8: transcript:KXG32050 transcript:Zm00001d008495_T001 0
833- 9: transcript:EES00414 transcript:Zm00001d008494_T001 0
833- 10: transcript:EES02601 transcript:Zm00001d008493_T001 0
833- 11: transcript:KXG32068 transcript:Zm00001d008491_T001 0
Alignment 834: score=537.0 e_value=1.1e-28 N=12 3&8 minus
834- 0: transcript:Zm00001d041268_T004 transcript:KXG24045 3.00E-177
834- 1: transcript:Zm00001d041269_T010 transcript:EES16404 0
834- 2: transcript:Zm00001d041277_T001 transcript:EES16403 1.00E-116

834- 3: transcript:Zm00001d041280_T001 transcript:OQU79660 0
834- 4: transcript:Zm00001d041287_T001 transcript:KXG24034 0
834- 5: transcript:Zm00001d041290_T002 transcript:EES17423 6.00E-118
834- 6: transcript:Zm00001d041291_T001 transcript:OQU79657 2.00E-41
834- 7: transcript:Zm00001d041298_T003 transcript:OQU79652 0
834- 8: transcript:Zm00001d041305_T001 transcript:EES17420 0
834- 9: transcript:Zm00001d041307_T002 transcript:KXG24021 0
834- 10: transcript:Zm00001d041308_T001 transcript:OQU79638 0
834- 11: transcript:Zm00001d041309_T001 transcript:EES16393 0
Alignment 835: score=536.0 e_value=5e-28 N=12 3&8 minus
835- 0: transcript:KXG32244 transcript:Zm00001d008271_T002 0
835- 1: transcript:KXG32246 transcript:Zm00001d008269_T001 0
835- 2: transcript:EES02749 transcript:Zm00001d008268_T001 4.00E-154
835- 3: transcript:OQU86683 transcript:Zm00001d008266_T001 0
835- 4: transcript:EES00556 transcript:Zm00001d008265_T001 0
835- 5: transcript:EES00557 transcript:Zm00001d008264_T003 7.00E-161
835- 6: transcript:EES00558 transcript:Zm00001d008262_T001 4.00E-139
835- 7: transcript:KXG32249 transcript:Zm00001d008260_T001 1.00E-123
835- 8: transcript:EES00559 transcript:Zm00001d008259_T001 6.00E-19
835- 9: transcript:KXG32250 transcript:Zm00001d008258_T001 0
835- 10: transcript:EES02755 transcript:Zm00001d008257_T001 5.00E-56
835- 11: transcript:EES02756 transcript:Zm00001d008256_T003 0
Alignment 836: score=531.0 e_value=5.8e-27 N=12 3&8 minus
836- 0: transcript:OQU86303 transcript:Zm00001d008632_T001 0
836- 1: transcript:OQU86311 transcript:Zm00001d008631_T001 0
836- 2: transcript:EES02481 transcript:Zm00001d008628_T001 0
836- 3: transcript:KXG31876 transcript:Zm00001d008625_T001 0
836- 4: transcript:EES02482 transcript:Zm00001d008624_T001 0
836- 5: transcript:KXG31877 transcript:Zm00001d008623_T001 1.00E-79
836- 6: transcript:EES02478 transcript:Zm00001d008622_T002 0
836- 7: transcript:KXG31883 transcript:Zm00001d008621_T001 0
836- 8: transcript:OQU86320 transcript:Zm00001d008620_T001 3.00E-22
836- 9: transcript:EES00320 transcript:Zm00001d008619_T001 1.00E-170
836- 10: transcript:EES00322 transcript:Zm00001d008618_T005 0
836- 11: transcript:EES02488 transcript:Zm00001d008617_T001 0
Alignment 837: score=516.0 e_value=1.7e-29 N=12 3&8 minus
837- 0: transcript:EES01547 transcript:Zm00001d012643_T007 0
837- 1: transcript:EES01548 transcript:Zm00001d012642_T001 3.00E-98
837- 2: transcript:EES03702 transcript:Zm00001d012641_T001 0
837- 3: transcript:KXG33492 transcript:Zm00001d012640_T010 0
837- 4: transcript:EES01553 transcript:Zm00001d012636_T001 0
837- 5: transcript:OQU87651 transcript:Zm00001d012635_T001 1.00E-83
837- 6: transcript:EES01554 transcript:Zm00001d012634_T002 0
837- 7: transcript:KXG33497 transcript:Zm00001d012633_T001 0
837- 8: transcript:EES01557 transcript:Zm00001d012632_T001 1.00E-40
837- 9: transcript:EES01562 transcript:Zm00001d012627_T001 3.00E-173
837- 10: transcript:OQU87666 transcript:Zm00001d012626_T001 7.00E-45
837- 11: transcript:EES01563 transcript:Zm00001d012625_T001 0
Alignment 838: score=513.0 e_value=5.5e-23 N=11 3&8 minus
838- 0: transcript:EES03151 transcript:Zm00001d011228_T001 0
838- 1: transcript:EES00940 transcript:Zm00001d011226_T002 0
838- 2: transcript:EES03152 transcript:Zm00001d011225_T003 3.00E-90
838- 3: transcript:EES00941 transcript:Zm00001d011224_T001 1.00E-85
838- 4: transcript:EES00944 transcript:Zm00001d011223_T002 0

838- 5: transcript:KXG32756 transcript:Zm00001d011222_T001 0
838- 6: transcript:EES00946 transcript:Zm00001d011220_T003 0
838- 7: transcript:OQU87034 transcript:Zm00001d011217_T001 4.00E-141
838- 8: transcript:EES00947 transcript:Zm00001d011213_T005 0
838- 9: transcript:EES00948 transcript:Zm00001d011212_T001 3.00E-151
838- 10: transcript:OQU87037 transcript:Zm00001d011211_T001 0
Alignment 839: score=497.0 e_value=1.3e-26 N=11 3&8 minus
839- 0: transcript:KXG32370 transcript:Zm00001d009119_T001 2.00E-40
839- 1: transcript:EES00637 transcript:Zm00001d009118_T001 2.00E-115
839- 2: transcript:EES00638 transcript:Zm00001d009116_T001 7.00E-75
839- 3: transcript:EES00640 transcript:Zm00001d009112_T001 4.00E-50
839- 4: transcript:OQU86782 transcript:Zm00001d009088_T001 8.00E-67
839- 5: transcript:KXG32375 transcript:Zm00001d009087_T001 2.00E-120
839- 6: transcript:OQU86785 transcript:Zm00001d009084_T001 3.00E-179
839- 7: transcript:EES02859 transcript:Zm00001d009082_T001 2.00E-64
839- 8: transcript:EES00645 transcript:Zm00001d009081_T001 4.00E-12
839- 9: transcript:EES00648 transcript:Zm00001d009077_T001 0
839- 10: transcript:KXG32383 transcript:Zm00001d009075_T001 0
Alignment 840: score=495.0 e_value=7.8e-26 N=11 3&8 minus
840- 0: transcript:Zm00001d041506_T001 transcript:KXG23800 0
840- 1: transcript:Zm00001d041510_T003 transcript:EES17264 0
840- 2: transcript:Zm00001d041511_T003 transcript:EES16223 0
840- 3: transcript:Zm00001d041514_T002 transcript:EES17261 0
840- 4: transcript:Zm00001d041515_T006 transcript:KXG23793 0
840- 5: transcript:Zm00001d041518_T001 transcript:EES16218 0
840- 6: transcript:Zm00001d041522_T001 transcript:KXG23788 9.00E-137
840- 7: transcript:Zm00001d041525_T001 transcript:KXG23785 0
840- 8: transcript:Zm00001d041530_T001 transcript:EES17247 0
840- 9: transcript:Zm00001d041534_T002 transcript:EES17245 0
840- 10: transcript:Zm00001d041535_T001 transcript:KXG23779 1.00E-23
Alignment 841: score=493.0 e_value=2e-28 N=12 3&8 minus
841- 0: transcript:EES03391 transcript:Zm00001d009599_T001 8.00E-35
841- 1: transcript:KXG33049 transcript:Zm00001d009594_T011 0
841- 2: transcript:KXG33051 transcript:Zm00001d009591_T006 0
841- 3: transcript:KXG33056 transcript:Zm00001d009587_T002 0
841- 4: transcript:OQU87291 transcript:Zm00001d009583_T001 3.00E-17
841- 5: transcript:EES03399 transcript:Zm00001d009579_T001 1.00E-129
841- 6: transcript:EES03400 transcript:Zm00001d009578_T001 3.00E-50
841- 7: transcript:OQU87300 transcript:Zm00001d009572_T001 8.00E-125
841- 8: transcript:KXG33070 transcript:Zm00001d009571_T002 5.00E-141
841- 9: transcript:OQU87301 transcript:Zm00001d009570_T001 3.00E-161
841- 10: transcript:EES03406 transcript:Zm00001d009568_T001 8.00E-45
841- 11: transcript:EES03407 transcript:Zm00001d009566_T001 3.00E-89
Alignment 842: score=470.0 e_value=8.2e-22 N=10 3&8 minus
842- 0: transcript:Zm00001d041624_T001 transcript:KXG23666 0
842- 1: transcript:Zm00001d041625_T008 transcript:EES16143 0
842- 2: transcript:Zm00001d041626_T001 transcript:EES17171 0
842- 3: transcript:Zm00001d041627_T001 transcript:EES16141 0
842- 4: transcript:Zm00001d041632_T002 transcript:KXG23665 0
842- 5: transcript:Zm00001d041634_T001 transcript:KXG23664 0
842- 6: transcript:Zm00001d041635_T002 transcript:KXG23663 0
842- 7: transcript:Zm00001d041638_T001 transcript:OQU79284 9.00E-91
842- 8: transcript:Zm00001d041645_T001 transcript:EES17169 7.00E-22
842- 9: transcript:Zm00001d041647_T002 transcript:EES17168 0

Alignment 843: score=447.0 e_value=1.6e-29 N=11 3&8 minus
843- 0: transcript:Zm00001d041671_T001 transcript:EES17150 1.00E-41
843- 1: transcript:Zm00001d041684_T001 transcript:OQU79251 1.00E-123
843- 2: transcript:Zm00001d041685_T001 transcript:EES16106 3.00E-48
843- 3: transcript:Zm00001d041690_T001 transcript:EES17142 2.00E-52
843- 4: transcript:Zm00001d041691_T002 transcript:EES16103 5.00E-106
843- 5: transcript:Zm00001d041693_T004 transcript:EES17139 0
843- 6: transcript:Zm00001d041696_T001 transcript:EES17134 5.00E-89
843- 7: transcript:Zm00001d041697_T001 transcript:EES17131 0
843- 8: transcript:Zm00001d041698_T001 transcript:EES16087 0
843- 9: transcript:Zm00001d041701_T002 transcript:EES17130 5.00E-86
843- 10: transcript:Zm00001d041714_T001 transcript:EES16082 0
Alignment 844: score=412.0 e_value=3.7e-21 N=10 3&8 minus
844- 0: transcript:EES03793 transcript:Zm00001d010633_T001 8.00E-21
844- 1: transcript:EES03794 transcript:Zm00001d010632_T002 2.00E-13
844- 2: transcript:EES01652 transcript:Zm00001d010630_T003 0
844- 3: transcript:EES01654 transcript:Zm00001d010629_T001 0
844- 4: transcript:EES01655 transcript:Zm00001d010627_T001 1.00E-74
844- 5: transcript:EES01659 transcript:Zm00001d010622_T002 8.00E-128
844- 6: transcript:EES01661 transcript:Zm00001d010620_T001 0
844- 7: transcript:EES01663 transcript:Zm00001d010618_T003 8.00E-108
844- 8: transcript:EES01669 transcript:Zm00001d010617_T001 1.00E-35
844- 9: transcript:EES01676 transcript:Zm00001d010613_T002 8.00E-17
Alignment 845: score=404.0 e_value=1.2e-17 N=9 3&8 minus
845- 0: transcript:OQU87667 transcript:Zm00001d012624_T006 0
845- 1: transcript:OQU87668 transcript:Zm00001d012621_T001 2.00E-127
845- 2: transcript:OQU87669 transcript:Zm00001d012620_T025 0
845- 3: transcript:EES01567 transcript:Zm00001d012619_T001 2.00E-126
845- 4: transcript:EES01566 transcript:Zm00001d012618_T001 6.00E-150
845- 5: transcript:EES01568 transcript:Zm00001d012615_T001 0
845- 6: transcript:EES03720 transcript:Zm00001d012614_T001 0
845- 7: transcript:EES03721 transcript:Zm00001d012613_T001 2.00E-23
845- 8: transcript:EES01570 transcript:Zm00001d012612_T001 0
Alignment 846: score=394.0 e_value=4.9e-21 N=10 3&8 minus
846- 0: transcript:EES01312 transcript:Zm00001d009452_T007 0
846- 1: transcript:EES01319 transcript:Zm00001d009447_T002 0
846- 2: transcript:EES03483 transcript:Zm00001d009446_T001 2.00E-23
846- 3: transcript:KXG33200 transcript:Zm00001d009439_T004 0
846- 4: transcript:EES01335 transcript:Zm00001d009436_T003 0
846- 5: transcript:EES03490 transcript:Zm00001d009435_T001 6.00E-98
846- 6: transcript:EES01337 transcript:Zm00001d009431_T001 0
846- 7: transcript:KXG33209 transcript:Zm00001d009429_T001 6.00E-18
846- 8: transcript:OQU87399 transcript:Zm00001d009425_T001 2.00E-14
846- 9: transcript:EES01350 transcript:Zm00001d009421_T001 7.00E-49
Alignment 847: score=379.0 e_value=5.9e-20 N=9 3&8 minus
847- 0: transcript:KXG32283 transcript:Zm00001d008224_T002 0
847- 1: transcript:EES02790 transcript:Zm00001d008223_T001 8.00E-31
847- 2: transcript:EES02792 transcript:Zm00001d008222_T002 6.00E-104
847- 3: transcript:EES02793 transcript:Zm00001d008221_T001 0
847- 4: transcript:KXG32287 transcript:Zm00001d008219_T001 2.00E-85
847- 5: transcript:KXG32292 transcript:Zm00001d008218_T002 0
847- 6: transcript:KXG32297 transcript:Zm00001d008216_T002 2.00E-164
847- 7: transcript:EES00597 transcript:Zm00001d008215_T002 0
847- 8: transcript:EES02800 transcript:Zm00001d008214_T001 4.00E-88

Alignment 848: score=369.0 e_value=3.1e-15 N=8 3&8 minus
848- 0: transcript:KXG31977 transcript:Zm00001d008535_T001 0
848- 1: transcript:KXG31978 transcript:Zm00001d008532_T001 4.00E-104
848- 2: transcript:KXG31980 transcript:Zm00001d008531_T001 0
848- 3: transcript:OQU86410 transcript:Zm00001d008530_T001 4.00E-95
848- 4: transcript:KXG31983 transcript:Zm00001d008529_T001 4.00E-150
848- 5: transcript:EES00381 transcript:Zm00001d008528_T001 7.00E-164
848- 6: transcript:OQU86414 transcript:Zm00001d008526_T001 2.00E-161
848- 7: transcript:KXG31991 transcript:Zm00001d008524_T001 9.00E-95
Alignment 849: score=356.0 e_value=1.9e-16 N=8 3&8 minus
849- 0: transcript:KXG32126 transcript:Zm00001d008411_T003 0
849- 1: transcript:EES02644 transcript:Zm00001d008409_T001 0
849- 2: transcript:KXG32127 transcript:Zm00001d008408_T001 2.00E-126
849- 3: transcript:OQU86545 transcript:Zm00001d008407_T001 5.00E-127
849- 4: transcript:KXG32133 transcript:Zm00001d008406_T002 0
849- 5: transcript:OQU86549 transcript:Zm00001d008405_T001 1.00E-102
849- 6: transcript:KXG32134 transcript:Zm00001d008404_T001 2.00E-135
849- 7: transcript:EES02651 transcript:Zm00001d008403_T001 2.00E-127
Alignment 850: score=356.0 e_value=1e-16 N=8 3&8 minus
850- 0: transcript:EES00626 transcript:Zm00001d009132_T001 1.00E-64
850- 1: transcript:OQU86772 transcript:Zm00001d009131_T001 1.00E-94
850- 2: transcript:KXG32361 transcript:Zm00001d009130_T001 1.00E-125
850- 3: transcript:KXG32363 transcript:Zm00001d009128_T001 2.00E-64
850- 4: transcript:KXG32365 transcript:Zm00001d009127_T001 0
850- 5: transcript:EES00628 transcript:Zm00001d009125_T008 0
850- 6: transcript:EES00630 transcript:Zm00001d009123_T001 0
850- 7: transcript:EES02848 transcript:Zm00001d009122_T001 0
Alignment 851: score=332.0 e_value=7.3e-15 N=8 3&8 minus
851- 0: transcript:KXG32260 transcript:Zm00001d008236_T004 0
851- 1: transcript:KXG32269 transcript:Zm00001d008235_T001 4.00E-128
851- 2: transcript:EES02774 transcript:Zm00001d008233_T001 0
851- 3: transcript:EES02775 transcript:Zm00001d008230_T001 0
851- 4: transcript:EES00586 transcript:Zm00001d008229_T001 0
851- 5: transcript:EES02778 transcript:Zm00001d008228_T001 8.00E-76
851- 6: transcript:KXG32275 transcript:Zm00001d008227_T001 0
851- 7: transcript:EES00589 transcript:Zm00001d008226_T001 0
Alignment 852: score=317.0 e_value=1.2e-15 N=8 3&8 minus
852- 0: transcript:OQU87235 transcript:Zm00001d009646_T001 4.00E-79
852- 1: transcript:KXG33002 transcript:Zm00001d009640_T002 0
852- 2: transcript:KXG33004 transcript:Zm00001d009639_T001 2.00E-126
852- 3: transcript:EES01185 transcript:Zm00001d009638_T001 0
852- 4: transcript:KXG33008 transcript:Zm00001d009637_T001 1.00E-07
852- 5: transcript:KXG33012 transcript:Zm00001d009631_T001 0
852- 6: transcript:KXG33023 transcript:Zm00001d009626_T001 6.00E-126
852- 7: transcript:EES03370 transcript:Zm00001d009619_T002 1.00E-42
Alignment 853: score=312.0 e_value=2.5e-11 N=7 3&8 minus
853- 0: transcript:EES00278 transcript:Zm00001d008651_T001 0
853- 1: transcript:KXG31816 transcript:Zm00001d008650_T001 3.00E-18
853- 2: transcript:EES00281 transcript:Zm00001d008649_T003 0
853- 3: transcript:EES00285 transcript:Zm00001d008648_T003 0
853- 4: transcript:KXG31820 transcript:Zm00001d008645_T002 5.00E-42
853- 5: transcript:EES02427 transcript:Zm00001d008642_T002 0
853- 6: transcript:EES02429 transcript:Zm00001d008640_T002 0
Alignment 854: score=311.0 e_value=3.3e-13 N=7 3&8 minus

854- 0: transcript:EES00958 transcript:Zm00001d011210_T001 4.00E-166
854- 1: transcript:EES00964 transcript:Zm00001d011202_T001 0
854- 2: transcript:EES00965 transcript:Zm00001d011199_T002 8.00E-179
854- 3: transcript:OQU87056 transcript:Zm00001d011198_T001 0
854- 4: transcript:KXG32782 transcript:Zm00001d011197_T007 0
854- 5: transcript:EES03167 transcript:Zm00001d011195_T001 0
854- 6: transcript:EES00971 transcript:Zm00001d011193_T001 0
Alignment 855: score=305.0 e_value=6e-13 N=7 3&8 minus
855- 0: transcript:KXG32436 transcript:Zm00001d009224_T001 2.00E-55
855- 1: transcript:EES00707 transcript:Zm00001d009222_T001 0
855- 2: transcript:EES02902 transcript:Zm00001d009220_T002 0
855- 3: transcript:KXG32441 transcript:Zm00001d009212_T004 0
855- 4: transcript:EES00709 transcript:Zm00001d009211_T001 2.00E-135
855- 5: transcript:KXG32445 transcript:Zm00001d009210_T001 1.00E-176
855- 6: transcript:EES00712 transcript:Zm00001d009205_T001 0
Alignment 856: score=302.0 e_value=3.6e-14 N=7 3&8 minus
856- 0: transcript:KXG32453 transcript:Zm00001d009193_T001 2.00E-66
856- 1: transcript:EES02912 transcript:Zm00001d009189_T001 3.00E-119
856- 2: transcript:KXG32456 transcript:Zm00001d009187_T001 2.00E-28
856- 3: transcript:EES00722 transcript:Zm00001d009186_T001 4.00E-21
856- 4: transcript:EES00724 transcript:Zm00001d009183_T008 0
856- 5: transcript:KXG32464 transcript:Zm00001d009182_T009 0
856- 6: transcript:EES02921 transcript:Zm00001d009181_T005 5.00E-147
Alignment 857: score=295.0 e_value=8.1e-11 N=7 3&8 minus
857- 0: transcript:KXG32256 transcript:Zm00001d008248_T002 0
857- 1: transcript:EES00573 transcript:Zm00001d008244_T003 0
857- 2: transcript:KXG32258 transcript:Zm00001d008242_T001 0
857- 3: transcript:EES00575 transcript:Zm00001d008241_T001 2.00E-24
857- 4: transcript:EES02765 transcript:Zm00001d008239_T001 0
857- 5: transcript:EES02766 transcript:Zm00001d008238_T001 0
857- 6: transcript:EES00577 transcript:Zm00001d008237_T001 0
Alignment 858: score=295.0 e_value=3.5e-12 N=7 3&8 minus
858- 0: transcript:KXG32411 transcript:Zm00001d009160_T001 0
858- 1: transcript:EES00676 transcript:Zm00001d009156_T001 0
858- 2: transcript:KXG32413 transcript:Zm00001d009152_T001 0
858- 3: transcript:EES02883 transcript:Zm00001d009150_T002 0
858- 4: transcript:EES00678 transcript:Zm00001d009147_T002 5.00E-131
858- 5: transcript:KXG32419 transcript:Zm00001d009146_T002 0
858- 6: transcript:EES00685 transcript:Zm00001d009138_T001 6.00E-124
Alignment 859: score=269.0 e_value=2.2e-09 N=6 3&8 minus
859- 0: transcript:EES03752 transcript:Zm00001d010678_T003 2.00E-173
859- 1: transcript:EES01601 transcript:Zm00001d010676_T001 5.00E-38
859- 2: transcript:EES01606 transcript:Zm00001d010673_T001 5.00E-166
859- 3: transcript:EES03761 transcript:Zm00001d010671_T001 6.00E-69
859- 4: transcript:EES03762 transcript:Zm00001d010670_T001 0
859- 5: transcript:KXG33562 transcript:Zm00001d010667_T001 2.00E-75
Alignment 860: score=268.0 e_value=9.6e-12 N=7 3&8 minus
860- 0: transcript:EES03856 transcript:Zm00001d010534_T001 2.00E-98
860- 1: transcript:EES01731 transcript:Zm00001d010529_T001 0
860- 2: transcript:EES03860 transcript:Zm00001d010528_T001 3.00E-60
860- 3: transcript:EES03863 transcript:Zm00001d010527_T001 0
860- 4: transcript:EES01733 transcript:Zm00001d010526_T002 0
860- 5: transcript:EES03868 transcript:Zm00001d010522_T001 0
860- 6: transcript:OQU87848 transcript:Zm00001d010515_T001 2.00E-22

```
## Alignment 861: score=256.0 e_value=5.4e-09 N=6 3&8 minus
861-  0: transcript:Zm00001d041908_T001 transcript:EES16704                  0
861-  1: transcript:Zm00001d041910_T001 transcript:KXG23038         2.00E-141
861-  2: transcript:Zm00001d041911_T011 transcript:KXG23037                  0
861-  3: transcript:Zm00001d041912_T006 transcript:EES16701                  0
861-  4: transcript:Zm00001d041913_T001 transcript:EES15714                  0
861-  5: transcript:Zm00001d041914_T001 transcript:KXG23035                  0
## Alignment 862: score=252.0 e_value=4.4e-09 N=6 3&8 minus
862-  0: transcript:EES03581           transcript:Zm00001d010798_T001  5.00E-33
862-  1: transcript:EES01452           transcript:Zm00001d010797_T001  6.00E-87
862-  2: transcript:EES03587           transcript:Zm00001d010796_T001         0
862-  3: transcript:KXG33341           transcript:Zm00001d010795_T001  1.00E-70
862-  4: transcript:KXG33344           transcript:Zm00001d010793_T002         0
862-  5: transcript:OQU87518           transcript:Zm00001d010791_T001  7.00E-71
## Alignment 863: score=1395.0 e_value=3.8e-107 N=33 3&9 plus
863-  0: transcript:Zm00001d043853_T001 transcript:EES18651                  0
863-  1: transcript:Zm00001d043854_T001 transcript:EES18654                  0
863-  2: transcript:Zm00001d043855_T001 transcript:KXG22515          2.00E-89
863-  3: transcript:Zm00001d043858_T001 transcript:OQU78406         9.00E-154
863-  4: transcript:Zm00001d043864_T001 transcript:OQU78407         2.00E-161
863-  5: transcript:Zm00001d043870_T001 transcript:EES19928                  0
863-  6: transcript:Zm00001d043874_T001 transcript:EES19934          1.00E-41
863-  7: transcript:Zm00001d043878_T001 transcript:OQU78411          1.00E-35
863-  8: transcript:Zm00001d043879_T002 transcript:EES18664                  0
863-  9: transcript:Zm00001d043889_T001 transcript:EES19937                  0
863- 10: transcript:Zm00001d043890_T002 transcript:KXG22525                  0
863- 11: transcript:Zm00001d043895_T003 transcript:OQU78419         7.00E-152
863- 12: transcript:Zm00001d043898_T001 transcript:KXG22531          7.00E-15
863- 13: transcript:Zm00001d043902_T001 transcript:EES18672          1.00E-95
863- 14: transcript:Zm00001d043906_T002 transcript:EES18674         3.00E-127
863- 15: transcript:Zm00001d043911_T001 transcript:EES19942         2.00E-110
863- 16: transcript:Zm00001d043921_T001 transcript:EES18675         5.00E-143
863- 17: transcript:Zm00001d043922_T001 transcript:EES18676                  0
863- 18: transcript:Zm00001d043929_T001 transcript:EES18677          5.00E-87
863- 19: transcript:Zm00001d043932_T001 transcript:OQU78423          4.00E-45
863- 20: transcript:Zm00001d043935_T001 transcript:EES19950         6.00E-123
863- 21: transcript:Zm00001d043937_T001 transcript:KXG22537          2.00E-12
863- 22: transcript:Zm00001d043942_T001 transcript:EES18687         2.00E-129
863- 23: transcript:Zm00001d043943_T001 transcript:EES18688          1.00E-85
863- 24: transcript:Zm00001d043950_T001 transcript:EES18690          3.00E-77
863- 25: transcript:Zm00001d043954_T003 transcript:EES19958                  0
863- 26: transcript:Zm00001d043955_T001 transcript:EES19959                  0
863- 27: transcript:Zm00001d043962_T001 transcript:KXG22544                  0
863- 28: transcript:Zm00001d043968_T001 transcript:KXG22549          1.00E-31
863- 29: transcript:Zm00001d043969_T001 transcript:EES19963          6.00E-50
863- 30: transcript:Zm00001d043971_T001 transcript:KXG22553          8.00E-82
863- 31: transcript:Zm00001d043973_T001 transcript:EES18692          2.00E-18
863- 32: transcript:Zm00001d043974_T001 transcript:EES18706          4.00E-11
## Alignment 864: score=1214.0 e_value=1e-86 N=28 3&9 plus
864-  0: transcript:Zm00001d043001_T001 transcript:EES18373          3.00E-25
864-  1: transcript:Zm00001d043006_T001 transcript:EES19677         1.00E-101
864-  2: transcript:Zm00001d043011_T001 transcript:EES18364          5.00E-85
864-  3: transcript:Zm00001d043014_T001 transcript:EES19683          5.00E-91
864-  4: transcript:Zm00001d043015_T001 transcript:EES19684                  0
```

864- 5: transcript:Zm00001d043018_T001 transcript:KXG22202 4.00E-107
864- 6: transcript:Zm00001d043022_T001 transcript:OQU78173 5.00E-167
864- 7: transcript:Zm00001d043023_T002 transcript:KXG22205 0
864- 8: transcript:Zm00001d043024_T001 transcript:EES18380 1.00E-55
864- 9: transcript:Zm00001d043025_T001 transcript:OQU78174 1.00E-153
864- 10: transcript:Zm00001d043026_T001 transcript:EES19686 1.00E-106
864- 11: transcript:Zm00001d043029_T001 transcript:KXG22212 0
864- 12: transcript:Zm00001d043031_T001 transcript:EES18382 0
864- 13: transcript:Zm00001d043037_T001 transcript:EES19689 2.00E-33
864- 14: transcript:Zm00001d043038_T001 transcript:EES19682 0
864- 15: transcript:Zm00001d043039_T001 transcript:KXG22215 1.00E-28
864- 16: transcript:Zm00001d043043_T001 transcript:OQU78182 0
864- 17: transcript:Zm00001d043046_T001 transcript:EES18387 6.00E-71
864- 18: transcript:Zm00001d043047_T001 transcript:EES19693 1.00E-158
864- 19: transcript:Zm00001d043050_T001 transcript:EES18392 2.00E-35
864- 20: transcript:Zm00001d043058_T001 transcript:EES18394 0
864- 21: transcript:Zm00001d043066_T001 transcript:EES19695 7.00E-21
864- 22: transcript:Zm00001d043069_T001 transcript:EES19696 7.00E-69
864- 23: transcript:Zm00001d043070_T001 transcript:EES19701 2.00E-107
864- 24: transcript:Zm00001d043071_T002 transcript:EES19702 0
864- 25: transcript:Zm00001d043074_T004 transcript:KXG22223 0
864- 26: transcript:Zm00001d043075_T002 transcript:EES18391 0
864- 27: transcript:Zm00001d043080_T001 transcript:OQU78195 1.00E-115
Alignment 865: score=1133.0 e_value=4.2e-84 N=26 3&9 plus
865- 0: transcript:Zm00001d043164_T002 transcript:EES18429 1.00E-153
865- 1: transcript:Zm00001d043165_T002 transcript:EES19738 2.00E-155
865- 2: transcript:Zm00001d043171_T001 transcript:OQU78229 0
865- 3: transcript:Zm00001d043174_T001 transcript:EES18431 0
865- 4: transcript:Zm00001d043175_T003 transcript:EES19739 9.00E-37
865- 5: transcript:Zm00001d043178_T001 transcript:KXG22255 0
865- 6: transcript:Zm00001d043180_T001 transcript:KXG22257 2.00E-125
865- 7: transcript:Zm00001d043181_T001 transcript:EES18433 5.00E-07
865- 8: transcript:Zm00001d043194_T001 transcript:EES19743 1.00E-177
865- 9: transcript:Zm00001d043195_T001 transcript:OQU78237 3.00E-173
865- 10: transcript:Zm00001d043204_T001 transcript:KXG22269 6.00E-36
865- 11: transcript:Zm00001d043206_T002 transcript:KXG22271 5.00E-64
865- 12: transcript:Zm00001d043217_T002 transcript:EES18447 3.00E-68
865- 13: transcript:Zm00001d043218_T001 transcript:EES19759 0
865- 14: transcript:Zm00001d043220_T001 transcript:KXG22279 3.00E-08
865- 15: transcript:Zm00001d043227_T002 transcript:EES19760 0
865- 16: transcript:Zm00001d043238_T001 transcript:EES18451 2.00E-121
865- 17: transcript:Zm00001d043244_T001 transcript:EES19769 0
865- 18: transcript:Zm00001d043248_T001 transcript:EES18457 4.00E-68
865- 19: transcript:Zm00001d043249_T001 transcript:KXG22286 4.00E-132
865- 20: transcript:Zm00001d043256_T001 transcript:OQU78259 0
865- 21: transcript:Zm00001d043261_T001 transcript:EES18459 1.00E-110
865- 22: transcript:Zm00001d043265_T001 transcript:KXG22290 7.00E-10
865- 23: transcript:Zm00001d043267_T002 transcript:EES18461 0
865- 24: transcript:Zm00001d043272_T002 transcript:EES19774 0
865- 25: transcript:Zm00001d043273_T001 transcript:OQU78261 3.00E-30
Alignment 866: score=833.0 e_value=1.5e-53 N=19 3&9 plus
866- 0: transcript:Zm00001d042800_T001 transcript:EES18312 5.00E-69
866- 1: transcript:Zm00001d042808_T001 transcript:EES19620 6.00E-87
866- 2: transcript:Zm00001d042809_T003 transcript:EES19621 0

866- 3:	transcript:Zm00001d042812_T001	transcript:KXG22120	2.00E-162
866- 4:	transcript:Zm00001d042813_T001	transcript:KXG22121	0
866- 5:	transcript:Zm00001d042826_T001	transcript:EES19625	0
866- 6:	transcript:Zm00001d042833_T001	transcript:EES18318	0
866- 7:	transcript:Zm00001d042837_T001	transcript:OQU78116	0
866- 8:	transcript:Zm00001d042841_T002	transcript:EES18320	2.00E-126
866- 9:	transcript:Zm00001d042850_T001	transcript:EES18331	3.00E-93
866- 10:	transcript:Zm00001d042851_T003	transcript:EES19630	0
866- 11:	transcript:Zm00001d042853_T001	transcript:KXG22131	5.00E-103
866- 12:	transcript:Zm00001d042861_T001	transcript:EES18335	0
866- 13:	transcript:Zm00001d042863_T001	transcript:EES19633	3.00E-15
866- 14:	transcript:Zm00001d042864_T003	transcript:EES18336	0
866- 15:	transcript:Zm00001d042866_T001	transcript:KXG22142	1.00E-33
866- 16:	transcript:Zm00001d042868_T001	transcript:KXG22143	1.00E-87
866- 17:	transcript:Zm00001d042872_T001	transcript:EES18339	2.00E-63
866- 18:	transcript:Zm00001d042875_T001	transcript:OQU78128	1.00E-09
## Alignment 867: score=819.0 e_value=3.7e-51 N=19 3&9 plus			
867- 0:	transcript:Zm00001d042714_T001	transcript:EES18293	8.00E-137
867- 1:	transcript:Zm00001d042717_T001	transcript:OQU78085	1.00E-38
867- 2:	transcript:Zm00001d042718_T001	transcript:EES18295	2.00E-149
867- 3:	transcript:Zm00001d042719_T001	transcript:EES19595	2.00E-111
867- 4:	transcript:Zm00001d042721_T001	transcript:EES18296	2.00E-65
867- 5:	transcript:Zm00001d042724_T001	transcript:EES19596	0
867- 6:	transcript:Zm00001d042727_T001	transcript:KXG22100	0
867- 7:	transcript:Zm00001d042731_T001	transcript:EES19603	0
867- 8:	transcript:Zm00001d042735_T001	transcript:KXG22101	2.00E-34
867- 9:	transcript:Zm00001d042736_T001	transcript:EES18302	3.00E-74
867- 10:	transcript:Zm00001d042747_T003	transcript:KXG22104	0
867- 11:	transcript:Zm00001d042752_T001	transcript:EES18304	1.00E-35
867- 12:	transcript:Zm00001d042753_T001	transcript:KXG22105	9.00E-40
867- 13:	transcript:Zm00001d042754_T001	transcript:EES19598	9.00E-26
867- 14:	transcript:Zm00001d042756_T001	transcript:EES19607	3.00E-72
867- 15:	transcript:Zm00001d042758_T001	transcript:KXG22106	1.00E-75
867- 16:	transcript:Zm00001d042767_T001	transcript:EES18301	0
867- 17:	transcript:Zm00001d042777_T006	transcript:OQU78097	0
867- 18:	transcript:Zm00001d042781_T001	transcript:EES18308	3.00E-123
## Alignment 868: score=776.0 e_value=6.5e-54 N=19 3&9 plus			
868- 0:	transcript:Zm00001d042560_T001	transcript:OQU78015	4.00E-95
868- 1:	transcript:Zm00001d042572_T001	transcript:EES18240	1.00E-12
868- 2:	transcript:Zm00001d042578_T001	transcript:EES19537	0
868- 3:	transcript:Zm00001d042580_T001	transcript:EES19541	8.00E-116
868- 4:	transcript:Zm00001d042582_T001	transcript:EES18243	4.00E-130
868- 5:	transcript:Zm00001d042585_T004	transcript:OQU78021	0
868- 6:	transcript:Zm00001d042589_T001	transcript:EES18244	1.00E-29
868- 7:	transcript:Zm00001d042590_T001	transcript:EES19544	1.00E-177
868- 8:	transcript:Zm00001d042593_T001	transcript:EES19546	5.00E-41
868- 9:	transcript:Zm00001d042598_T003	transcript:EES18249	4.00E-103
868- 10:	transcript:Zm00001d042600_T001	transcript:EES18250	7.00E-74
868- 11:	transcript:Zm00001d042605_T001	transcript:KXG22029	3.00E-66
868- 12:	transcript:Zm00001d042609_T001	transcript:EES18253	9.00E-144
868- 13:	transcript:Zm00001d042611_T001	transcript:OQU78027	2.00E-118
868- 14:	transcript:Zm00001d042618_T001	transcript:EES18254	2.00E-115
868- 15:	transcript:Zm00001d042619_T002	transcript:OQU78029	0
868- 16:	transcript:Zm00001d042621_T005	transcript:EES19550	0

868- 17: transcript:Zm00001d042627_T005 transcript:EES19558 0
868- 18: transcript:Zm00001d042638_T002 transcript:KXG22043 8.00E-30
Alignment 869: score=662.0 e_value=1.4e-39 N=16 3&9 plus
869- 0: transcript:Zm00001d043691_T001 transcript:KXG22437 1.00E-136
869- 1: transcript:Zm00001d043692_T001 transcript:EES18588 6.00E-115
869- 2: transcript:Zm00001d043699_T001 transcript:EES18589 4.00E-62
869- 3: transcript:Zm00001d043702_T001 transcript:EES19883 2.00E-16
869- 4: transcript:Zm00001d043708_T001 transcript:EES19885 1.00E-19
869- 5: transcript:Zm00001d043709_T003 transcript:KXG22444 3.00E-22
869- 6: transcript:Zm00001d043713_T001 transcript:OQU78377 1.00E-23
869- 7: transcript:Zm00001d043725_T001 transcript:EES18599 3.00E-30
869- 8: transcript:Zm00001d043727_T001 transcript:EES19888 0
869- 9: transcript:Zm00001d043729_T001 transcript:EES18602 4.00E-38
869- 10: transcript:Zm00001d043733_T002 transcript:KXG22453 0
869- 11: transcript:Zm00001d043738_T001 transcript:KXG22458 6.00E-120
869- 12: transcript:Zm00001d043751_T001 transcript:EES18606 4.00E-14
869- 13: transcript:Zm00001d043766_T005 transcript:OQU78384 0
869- 14: transcript:Zm00001d043770_T001 transcript:EES19896 2.00E-36
869- 15: transcript:Zm00001d043773_T003 transcript:EES19899 0
Alignment 870: score=474.0 e_value=7.8e-25 N=11 3&9 plus
870- 0: transcript:Zm00001d043504_T030 transcript:OQU78322 0
870- 1: transcript:Zm00001d043505_T001 transcript:EES19834 3.00E-75
870- 2: transcript:Zm00001d043506_T004 transcript:EES18517 1.00E-111
870- 3: transcript:Zm00001d043509_T002 transcript:OQU78325 0
870- 4: transcript:Zm00001d043510_T002 transcript:KXG22374 2.00E-61
870- 5: transcript:Zm00001d043511_T001 transcript:EES18534 0
870- 6: transcript:Zm00001d043512_T001 transcript:EES19836 0
870- 7: transcript:Zm00001d043515_T002 transcript:KXG22376 1.00E-92
870- 8: transcript:Zm00001d043523_T001 transcript:OQU78328 1.00E-52
870- 9: transcript:Zm00001d043525_T001 transcript:EES18536 5.00E-29
870- 10: transcript:Zm00001d043527_T002 transcript:EES19839 9.00E-142
Alignment 871: score=444.0 e_value=2.3e-22 N=11 3&9 plus
871- 0: transcript:Zm00001d044074_T001 transcript:EES18731 3.00E-87
871- 1: transcript:Zm00001d044080_T001 transcript:EES19991 1.00E-30
871- 2: transcript:Zm00001d044087_T001 transcript:OQU78484 2.00E-155
871- 3: transcript:Zm00001d044088_T001 transcript:OQU78486 0
871- 4: transcript:Zm00001d044091_T003 transcript:EES19998 0
871- 5: transcript:Zm00001d044100_T001 transcript:EES20000 5.00E-43
871- 6: transcript:Zm00001d044104_T001 transcript:EES18736 0
871- 7: transcript:Zm00001d044110_T001 transcript:EES20001 0
871- 8: transcript:Zm00001d044117_T001 transcript:EES20004 4.00E-41
871- 9: transcript:Zm00001d044121_T002 transcript:EES18741 1.00E-106
871- 10: transcript:Zm00001d044129_T001 transcript:OQU78492 0
Alignment 872: score=416.0 e_value=9.4e-21 N=10 3&9 plus
872- 0: transcript:Zm00001d042916_T003 transcript:OQU78135 0
872- 1: transcript:Zm00001d042917_T001 transcript:EES19637 1.00E-134
872- 2: transcript:Zm00001d042922_T007 transcript:EES19649 0
872- 3: transcript:Zm00001d042929_T001 transcript:KXG22163 9.00E-114
872- 4: transcript:Zm00001d042934_T001 transcript:EES18355 4.00E-37
872- 5: transcript:Zm00001d042936_T001 transcript:EES18357 6.00E-25
872- 6: transcript:Zm00001d042940_T001 transcript:EES19655 1.00E-33
872- 7: transcript:Zm00001d042949_T001 transcript:KXG22169 4.00E-102
872- 8: transcript:Zm00001d042950_T001 transcript:EES18358 0
872- 9: transcript:Zm00001d042963_T001 transcript:OQU78154 1.00E-08

Alignment 873: score=374.0 e_value=1e-16 N=9 3&9 plus
873- 0: transcript:Zm00001d043113_T001 transcript:EES19719 2.00E-131
873- 1: transcript:Zm00001d043117_T002 transcript:EES18419 1.00E-134
873- 2: transcript:Zm00001d043125_T001 transcript:EES19721 0
873- 3: transcript:Zm00001d043126_T007 transcript:EES19722 1.00E-97
873- 4: transcript:Zm00001d043135_T003 transcript:EES19725 0
873- 5: transcript:Zm00001d043144_T001 transcript:EES19728 4.00E-87
873- 6: transcript:Zm00001d043149_T002 transcript:EES19716 1.00E-83
873- 7: transcript:Zm00001d043150_T002 transcript:EES19734 0
873- 8: transcript:Zm00001d043153_T001 transcript:KXG22247 9.00E-134
Alignment 874: score=346.0 e_value=8.6e-14 N=8 3&9 plus
874- 0: transcript:Zm00001d042642_T002 transcript:KXG22055 3.00E-56
874- 1: transcript:Zm00001d042653_T001 transcript:EES19566 3.00E-124
874- 2: transcript:Zm00001d042656_T001 transcript:KXG22057 0
874- 3: transcript:Zm00001d042658_T002 transcript:EES19570 6.00E-94
874- 4: transcript:Zm00001d042664_T001 transcript:OQU78068 0
874- 5: transcript:Zm00001d042665_T001 transcript:KXG22070 4.00E-111
874- 6: transcript:Zm00001d042667_T001 transcript:OQU78069 0
874- 7: transcript:Zm00001d042669_T002 transcript:EES18280 0
Alignment 875: score=345.0 e_value=9.9e-15 N=8 3&9 plus
875- 0: transcript:Zm00001d044162_T001 transcript:OQU78504 1.00E-55
875- 1: transcript:Zm00001d044167_T001 transcript:OQU78505 1.00E-25
875- 2: transcript:Zm00001d044171_T001 transcript:KXG22622 7.00E-64
875- 3: transcript:Zm00001d044173_T002 transcript:EES20018 8.00E-75
875- 4: transcript:Zm00001d044176_T001 transcript:OQU78506 7.00E-17
875- 5: transcript:Zm00001d044181_T002 transcript:EES20021 0
875- 6: transcript:Zm00001d044184_T001 transcript:KXG22631 4.00E-116
875- 7: transcript:Zm00001d044185_T004 transcript:EES20022 0
Alignment 876: score=293.0 e_value=1.8e-13 N=7 3&9 plus
876- 0: transcript:EES03843 transcript:Zm00001d047762_T045 0
876- 1: transcript:EES01719 transcript:Zm00001d047771_T001 5.00E-12
876- 2: transcript:EES03848 transcript:Zm00001d047772_T002 0
876- 3: transcript:EES01723 transcript:Zm00001d047774_T002 2.00E-64
876- 4: transcript:EES03849 transcript:Zm00001d047775_T001 1.00E-15
876- 5: transcript:EES01725 transcript:Zm00001d047779_T001 6.00E-25
876- 6: transcript:OQU87825 transcript:Zm00001d047786_T001 3.00E-24
Alignment 877: score=258.0 e_value=4.2e-09 N=6 3&9 plus
877- 0: transcript:Zm00001d043652_T003 transcript:EES18569 0
877- 1: transcript:Zm00001d043653_T003 transcript:KXG22411 8.00E-130
877- 2: transcript:Zm00001d043655_T002 transcript:KXG22418 1.00E-168
877- 3: transcript:Zm00001d043661_T001 transcript:EES18574 3.00E-108
877- 4: transcript:Zm00001d043663_T001 transcript:EES18577 3.00E-62
877- 5: transcript:Zm00001d043667_T003 transcript:OQU78360 0
Alignment 878: score=251.0 e_value=1.8e-10 N=6 3&9 plus
878- 0: transcript:Zm00001d042672_T001 transcript:EES19582 2.00E-60
878- 1: transcript:Zm00001d042673_T001 transcript:EES18281 0
878- 2: transcript:Zm00001d042684_T001 transcript:OQU78070 3.00E-157
878- 3: transcript:Zm00001d042685_T001 transcript:EES19586 0
878- 4: transcript:Zm00001d042689_T002 transcript:EES19587 1.00E-28
878- 5: transcript:Zm00001d042695_T002 transcript:EES19589 0
Alignment 879: score=352.0 e_value=4.2e-15 N=8 3&9 minus
879- 0: transcript:Zm00001d043441_T002 transcript:EES19829 0
879- 1: transcript:Zm00001d043444_T001 transcript:EES19824 9.00E-103
879- 2: transcript:Zm00001d043450_T001 transcript:EES18519 3.00E-49

879- 3: transcript:Zm00001d043452_T008 transcript:OQU78312 7.00E-158
879- 4: transcript:Zm00001d043455_T001 transcript:KXG22361 1.00E-16
879- 5: transcript:Zm00001d043456_T001 transcript:KXG22360 2.00E-06
879- 6: transcript:Zm00001d043458_T001 transcript:EES19821 0
879- 7: transcript:Zm00001d043461_T001 transcript:KXG22359 7.00E-104
Alignment 880: score=317.0 e_value=4.2e-12 N=7 3&9 minus
880- 0: transcript:Zm00001d042954_T001 transcript:EES18362 7.00E-67
880- 1: transcript:Zm00001d042955_T001 transcript:KXG22182 0
880- 2: transcript:Zm00001d042958_T001 transcript:KXG22181 2.00E-67
880- 3: transcript:Zm00001d042962_T002 transcript:EES19666 0
880- 4: transcript:Zm00001d042966_T001 transcript:EES19663 2.00E-100
880- 5: transcript:Zm00001d042968_T002 transcript:KXG22176 2.00E-86
880- 6: transcript:Zm00001d042973_T003 transcript:OQU78148 1.00E-71
Alignment 881: score=284.0 e_value=3.2e-09 N=6 3&9 minus
881- 0: transcript:Zm00001d043541_T001 transcript:EES19851 4.00E-45
881- 1: transcript:Zm00001d043543_T002 transcript:KXG22389 0
881- 2: transcript:Zm00001d043547_T001 transcript:EES19844 6.00E-153
881- 3: transcript:Zm00001d043549_T001 transcript:EES19842 6.00E-78
881- 4: transcript:Zm00001d043550_T001 transcript:EES18545 5.00E-71
881- 5: transcript:Zm00001d043551_T003 transcript:EES19841 4.00E-147
Alignment 882: score=261.0 e_value=4.5e-09 N=6 3&9 minus
882- 0: transcript:Zm00001d044405_T002 transcript:EES18801 0
882- 1: transcript:Zm00001d044407_T001 transcript:EES18796 1.00E-18
882- 2: transcript:Zm00001d044409_T001 transcript:KXG22659 4.00E-46
882- 3: transcript:Zm00001d044412_T001 transcript:EES18791 7.00E-143
882- 4: transcript:Zm00001d044416_T001 transcript:EES20041 0
882- 5: transcript:Zm00001d044421_T001 transcript:EES20039 4.00E-112
Alignment 883: score=3468.0 e_value=0 N=79 4&4 plus
883- 0: transcript:OQU85082 transcript:Zm00001d050664_T002 2.00E-150
883- 1: transcript:EES06920 transcript:Zm00001d050666_T001 0
883- 2: transcript:EES06922 transcript:Zm00001d050669_T004 0
883- 3: transcript:KXG30353 transcript:Zm00001d050671_T001 3.00E-34
883- 4: transcript:EES05205 transcript:Zm00001d050689_T001 0
883- 5: transcript:EES05207 transcript:Zm00001d050694_T001 0
883- 6: transcript:EES06931 transcript:Zm00001d050696_T005 8.00E-46
883- 7: transcript:EES05210 transcript:Zm00001d050697_T001 2.00E-94
883- 8: transcript:EES05214 transcript:Zm00001d050698_T001 0
883- 9: transcript:EES06934 transcript:Zm00001d050705_T001 1.00E-64
883- 10: transcript:EES05216 transcript:Zm00001d050708_T002 0
883- 11: transcript:EES06938 transcript:Zm00001d050712_T001 0
883- 12: transcript:KXG30367 transcript:Zm00001d050714_T001 0
883- 13: transcript:EES06978 transcript:Zm00001d050715_T003 0
883- 14: transcript:EES06941 transcript:Zm00001d050716_T002 0
883- 15: transcript:EES06943 transcript:Zm00001d050723_T002 0
883- 16: transcript:EES05228 transcript:Zm00001d050730_T001 0
883- 17: transcript:EES05234 transcript:Zm00001d050735_T003 4.00E-105
883- 18: transcript:EES06956 transcript:Zm00001d050737_T003 0
883- 19: transcript:KXG30418 transcript:Zm00001d050741_T004 1.00E-116
883- 20: transcript:KXG30419 transcript:Zm00001d050747_T007 0
883- 21: transcript:EES06963 transcript:Zm00001d050748_T001 9.00E-106
883- 22: transcript:EES05242 transcript:Zm00001d050753_T001 0
883- 23: transcript:EES05244 transcript:Zm00001d050755_T003 0
883- 24: transcript:EES05247 transcript:Zm00001d050768_T003 5.00E-92
883- 25: transcript:EES06965 transcript:Zm00001d050775_T001 2.00E-106

883- 26:	transcript:EES06968	transcript:Zm00001d050781_T001	0
883- 27:	transcript:EES05255	transcript:Zm00001d050783_T001	2.00E-50
883- 28:	transcript:EES06981	transcript:Zm00001d050785_T001	0
883- 29:	transcript:KXG30429	transcript:Zm00001d050787_T001	2.00E-81
883- 30:	transcript:OQU85140	transcript:Zm00001d050790_T005	0
883- 31:	transcript:EES05262	transcript:Zm00001d050793_T001	0
883- 32:	transcript:EES06987	transcript:Zm00001d050798_T001	1.00E-74
883- 33:	transcript:OQU85147	transcript:Zm00001d050800_T001	0
883- 34:	transcript:OQU85157	transcript:Zm00001d050805_T001	0
883- 35:	transcript:EES05271	transcript:Zm00001d050810_T001	0
883- 36:	transcript:EES05272	transcript:Zm00001d050811_T002	3.00E-105
883- 37:	transcript:OQU85159	transcript:Zm00001d050815_T001	0
883- 38:	transcript:EES05274	transcript:Zm00001d050816_T002	2.00E-155
883- 39:	transcript:KXG30458	transcript:Zm00001d050822_T008	0
883- 40:	transcript:EES07001	transcript:Zm00001d050823_T002	2.00E-84
883- 41:	transcript:OQU85165	transcript:Zm00001d050824_T001	3.00E-19
883- 42:	transcript:KXG30465	transcript:Zm00001d050827_T003	9.00E-107
883- 43:	transcript:EES07003	transcript:Zm00001d050830_T001	5.00E-24
883- 44:	transcript:EES07004	transcript:Zm00001d050831_T001	7.00E-14
883- 45:	transcript:EES07005	transcript:Zm00001d050833_T002	0
883- 46:	transcript:OQU85171	transcript:Zm00001d050834_T001	1.00E-131
883- 47:	transcript:EES05288	transcript:Zm00001d050837_T001	0
883- 48:	transcript:EES05289	transcript:Zm00001d050838_T001	0
883- 49:	transcript:EES07019	transcript:Zm00001d050840_T001	2.00E-107
883- 50:	transcript:EES07020	transcript:Zm00001d050844_T005	0
883- 51:	transcript:KXG30480	transcript:Zm00001d050848_T001	0
883- 52:	transcript:KXG30485	transcript:Zm00001d050850_T001	0
883- 53:	transcript:OQU85189	transcript:Zm00001d050851_T009	3.00E-175
883- 54:	transcript:OQU85192	transcript:Zm00001d050860_T001	0
883- 55:	transcript:EES05301	transcript:Zm00001d050861_T001	0
883- 56:	transcript:EES05304	transcript:Zm00001d050862_T001	0
883- 57:	transcript:OQU85193	transcript:Zm00001d050864_T001	7.00E-11
883- 58:	transcript:EES07029	transcript:Zm00001d050865_T001	0
883- 59:	transcript:EES07030	transcript:Zm00001d050868_T004	0
883- 60:	transcript:EES05307	transcript:Zm00001d050872_T001	0
883- 61:	transcript:EES07034	transcript:Zm00001d050873_T001	0
883- 62:	transcript:EES07035	transcript:Zm00001d050874_T001	1.00E-148
883- 63:	transcript:KXG30492	transcript:Zm00001d050884_T004	6.00E-168
883- 64:	transcript:OQU85197	transcript:Zm00001d050885_T001	0
883- 65:	transcript:OQU85198	transcript:Zm00001d050886_T001	7.00E-167
883- 66:	transcript:EES07037	transcript:Zm00001d050889_T001	4.00E-88
883- 67:	transcript:EES05316	transcript:Zm00001d050893_T001	0
883- 68:	transcript:OQU85202	transcript:Zm00001d050897_T002	7.00E-88
883- 69:	transcript:EES07039	transcript:Zm00001d050899_T001	5.00E-90
883- 70:	transcript:OQU85206	transcript:Zm00001d050903_T001	7.00E-112
883- 71:	transcript:KXG30503	transcript:Zm00001d050904_T001	3.00E-60
883- 72:	transcript:EES07042	transcript:Zm00001d050905_T001	8.00E-107
883- 73:	transcript:OQU85212	transcript:Zm00001d050907_T001	0
883- 74:	transcript:EES05328	transcript:Zm00001d050908_T001	1.00E-43
883- 75:	transcript:EES07045	transcript:Zm00001d050910_T004	0
883- 76:	transcript:EES07046	transcript:Zm00001d050911_T001	2.00E-51
883- 77:	transcript:OQU85219	transcript:Zm00001d050913_T001	3.00E-134
883- 78:	transcript:EES07048	transcript:Zm00001d050914_T001	0

Alignment 884: score=2357.0 e_value=7.3e-195 N=52 4&4 plus

884- 0: transcript:KXG30709 transcript:Zm00001d052192_T001 5.00E-161
884- 1: transcript:OQU85383 transcript:Zm00001d052193_T012 0
884- 2: transcript:EES07215 transcript:Zm00001d052194_T001 5.00E-128
884- 3: transcript:KXG30711 transcript:Zm00001d052198_T002 1.00E-105
884- 4: transcript:KXG30712 transcript:Zm00001d052200_T005 0
884- 5: transcript:EES07217 transcript:Zm00001d052205_T001 0
884- 6: transcript:EES05501 transcript:Zm00001d052208_T002 5.00E-60
884- 7: transcript:EES07219 transcript:Zm00001d052209_T001 0
884- 8: transcript:KXG30724 transcript:Zm00001d052212_T001 2.00E-73
884- 9: transcript:KXG30728 transcript:Zm00001d052213_T002 0
884- 10: transcript:EES05510 transcript:Zm00001d052215_T001 0
884- 11: transcript:EES07227 transcript:Zm00001d052216_T001 6.00E-91
884- 12: transcript:OQU85395 transcript:Zm00001d052218_T001 0
884- 13: transcript:EES07228 transcript:Zm00001d052219_T002 0
884- 14: transcript:EES07230 transcript:Zm00001d052220_T001 2.00E-65
884- 15: transcript:KXG30730 transcript:Zm00001d052221_T001 0
884- 16: transcript:EES07231 transcript:Zm00001d052223_T001 3.00E-158
884- 17: transcript:EES07233 transcript:Zm00001d052225_T001 5.00E-152
884- 18: transcript:EES07234 transcript:Zm00001d052226_T001 4.00E-08
884- 19: transcript:EES07235 transcript:Zm00001d052229_T001 3.00E-166
884- 20: transcript:OQU85411 transcript:Zm00001d052230_T001 0
884- 21: transcript:EES05517 transcript:Zm00001d052231_T003 0
884- 22: transcript:KXG30738 transcript:Zm00001d052232_T002 0
884- 23: transcript:KXG30739 transcript:Zm00001d052233_T001 0
884- 24: transcript:KXG30742 transcript:Zm00001d052234_T001 2.00E-63
884- 25: transcript:KXG30744 transcript:Zm00001d052237_T001 0
884- 26: transcript:OQU85415 transcript:Zm00001d052238_T001 2.00E-73
884- 27: transcript:EES07241 transcript:Zm00001d052239_T001 2.00E-67
884- 28: transcript:KXG30749 transcript:Zm00001d052240_T001 0
884- 29: transcript:EES07249 transcript:Zm00001d052242_T001 8.00E-145
884- 30: transcript:OQU85421 transcript:Zm00001d052243_T001 0
884- 31: transcript:EES05522 transcript:Zm00001d052244_T001 2.00E-16
884- 32: transcript:KXG30755 transcript:Zm00001d052247_T001 0
884- 33: transcript:EES07254 transcript:Zm00001d052248_T009 0
884- 34: transcript:EES05526 transcript:Zm00001d052252_T001 0
884- 35: transcript:KXG30756 transcript:Zm00001d052253_T001 0
884- 36: transcript:EES05528 transcript:Zm00001d052254_T001 9.00E-49
884- 37: transcript:KXG30761 transcript:Zm00001d052255_T001 6.00E-83
884- 38: transcript:EES07255 transcript:Zm00001d052256_T001 7.00E-152
884- 39: transcript:EES07258 transcript:Zm00001d052258_T001 0
884- 40: transcript:KXG30762 transcript:Zm00001d052259_T001 0
884- 41: transcript:EES07260 transcript:Zm00001d052260_T001 0
884- 42: transcript:EES07262 transcript:Zm00001d052261_T001 7.00E-144
884- 43: transcript:EES05532 transcript:Zm00001d052263_T001 0
884- 44: transcript:KXG30767 transcript:Zm00001d052266_T001 0
884- 45: transcript:EES07268 transcript:Zm00001d052268_T001 5.00E-135
884- 46: transcript:EES05538 transcript:Zm00001d052269_T001 0
884- 47: transcript:EES07276 transcript:Zm00001d052270_T005 1.00E-176
884- 48: transcript:EES05543 transcript:Zm00001d052271_T003 0
884- 49: transcript:EES05544 transcript:Zm00001d052273_T004 1.00E-119
884- 50: transcript:EES05545 transcript:Zm00001d052276_T005 2.00E-120
884- 51: transcript:KXG30786 transcript:Zm00001d052277_T001 7.00E-85
Alignment 885: score=1412.0 e_value=1.2e-103 N=31 4&4 plus
885- 0: transcript:OQU85313 transcript:Zm00001d051110_T001 0

885- 1: transcript:OQU85315   transcript:Zm00001d051111_T001   0
885- 2: transcript:EES05435   transcript:Zm00001d051112_T001   0
885- 3: transcript:EES05438   transcript:Zm00001d051114_T001   0
885- 4: transcript:EES07156   transcript:Zm00001d051116_T001 1.00E-36
885- 5: transcript:EES07157   transcript:Zm00001d051117_T001 3.00E-112
885- 6: transcript:OQU85322   transcript:Zm00001d051118_T001 1.00E-69
885- 7: transcript:OQU85324   transcript:Zm00001d051119_T001   0
885- 8: transcript:EES05442   transcript:Zm00001d051120_T001 2.00E-28
885- 9: transcript:EES05443   transcript:Zm00001d051121_T001 2.00E-71
885- 10: transcript:KXG30647   transcript:Zm00001d051126_T004   0
885- 11: transcript:EES05446   transcript:Zm00001d051127_T001 2.00E-55
885- 12: transcript:EES07163   transcript:Zm00001d051128_T001 6.00E-71
885- 13: transcript:EES05447   transcript:Zm00001d051129_T002   0
885- 14: transcript:EES07164   transcript:Zm00001d051130_T001   0
885- 15: transcript:EES05449   transcript:Zm00001d051135_T005   0
885- 16: transcript:KXG30659   transcript:Zm00001d051136_T001   0
885- 17: transcript:EES05450   transcript:Zm00001d051138_T001 1.00E-137
885- 18: transcript:EES07168   transcript:Zm00001d051139_T002 7.00E-137
885- 19: transcript:EES05451   transcript:Zm00001d051140_T001 4.00E-128
885- 20: transcript:KXG30661   transcript:Zm00001d051143_T001 5.00E-97
885- 21: transcript:EES07173   transcript:Zm00001d051149_T001 3.00E-137
885- 22: transcript:EES05452   transcript:Zm00001d051156_T001 9.00E-177
885- 23: transcript:KXG30663   transcript:Zm00001d051157_T001   0
885- 24: transcript:KXG30665   transcript:Zm00001d051158_T001   0
885- 25: transcript:EES07174   transcript:Zm00001d051161_T003   0
885- 26: transcript:EES07176   transcript:Zm00001d051163_T001   0
885- 27: transcript:EES05458   transcript:Zm00001d051172_T001   0
885- 28: transcript:EES05459   transcript:Zm00001d051174_T001   0
885- 29: transcript:EES05462   transcript:Zm00001d051178_T001   0
885- 30: transcript:OQU85355   transcript:Zm00001d051180_T002 1.00E-132
Alignment 886: score=1405.0 e_value=1.9e-109 N=31 4&4 plus
886- 0: transcript:EES06208   transcript:Zm00001d053936_T001 4.00E-113
886- 1: transcript:KXG29345   transcript:Zm00001d053940_T001 3.00E-18
886- 2: transcript:EES06213   transcript:Zm00001d053941_T001 4.00E-164
886- 3: transcript:EES04425   transcript:Zm00001d053952_T001 2.00E-157
886- 4: transcript:EES04429   transcript:Zm00001d053953_T001 2.00E-131
886- 5: transcript:KXG29354   transcript:Zm00001d053954_T001   0
886- 6: transcript:KXG29356   transcript:Zm00001d053956_T001   0
886- 7: transcript:OQU84280   transcript:Zm00001d053957_T003   0
886- 8: transcript:EES04434   transcript:Zm00001d053961_T002 3.00E-43
886- 9: transcript:EES04435   transcript:Zm00001d053962_T002   0
886- 10: transcript:EES06217   transcript:Zm00001d053963_T001   0
886- 11: transcript:EES04436   transcript:Zm00001d053964_T001 2.00E-120
886- 12: transcript:EES04437   transcript:Zm00001d053965_T001 3.00E-85
886- 13: transcript:EES04438   transcript:Zm00001d053966_T001 1.00E-75
886- 14: transcript:EES04440   transcript:Zm00001d053967_T001 2.00E-174
886- 15: transcript:KXG29373   transcript:Zm00001d053969_T002   0
886- 16: transcript:OQU84288   transcript:Zm00001d053974_T001   0
886- 17: transcript:KXG29378   transcript:Zm00001d053975_T003   0
886- 18: transcript:EES06222   transcript:Zm00001d053976_T001 2.00E-152
886- 19: transcript:EES06223   transcript:Zm00001d053977_T001 4.00E-163
886- 20: transcript:EES06227   transcript:Zm00001d053978_T001   0
886- 21: transcript:EES06225   transcript:Zm00001d053979_T001 2.00E-57
886- 22: transcript:KXG29379   transcript:Zm00001d053981_T002 1.00E-124

```
886- 23: transcript:KXG29385           transcript:Zm00001d053982_T001          0
886- 24: transcript:EES06231           transcript:Zm00001d053983_T002 7.00E-153
886- 25: transcript:KXG29394           transcript:Zm00001d053984_T002          0
886- 26: transcript:KXG29398           transcript:Zm00001d053985_T001          0
886- 27: transcript:OQU84306           transcript:Zm00001d053986_T001          0
886- 28: transcript:OQU84307           transcript:Zm00001d053987_T003          0
886- 29: transcript:EES06238           transcript:Zm00001d053988_T001  2.00E-74
886- 30: transcript:EES04464           transcript:Zm00001d053989_T003          0
## Alignment 887: score=842.0 e_value=8.3e-53 N=19 4&4 plus
887-  0: transcript:EES05369           transcript:Zm00001d050974_T001          0
887-  1: transcript:EES07097           transcript:Zm00001d050977_T001          0
887-  2: transcript:EES07098           transcript:Zm00001d050978_T001 2.00E-147
887-  3: transcript:KXG30555           transcript:Zm00001d050981_T001          0
887-  4: transcript:EES07101           transcript:Zm00001d050985_T002          0
887-  5: transcript:EES05371           transcript:Zm00001d050988_T001  1.00E-73
887-  6: transcript:EES07102           transcript:Zm00001d050992_T001          0
887-  7: transcript:KXG30570           transcript:Zm00001d050993_T001          0
887-  8: transcript:EES05377           transcript:Zm00001d051001_T001          0
887-  9: transcript:EES05380           transcript:Zm00001d051005_T001 3.00E-159
887- 10: transcript:EES05382           transcript:Zm00001d051007_T001  3.00E-87
887- 11: transcript:EES05385           transcript:Zm00001d051009_T002  3.00E-87
887- 12: transcript:KXG30580           transcript:Zm00001d051012_T001          0
887- 13: transcript:KXG30587           transcript:Zm00001d051014_T001          0
887- 14: transcript:KXG30589           transcript:Zm00001d051015_T001 6.00E-177
887- 15: transcript:EES07118           transcript:Zm00001d051016_T002 3.00E-123
887- 16: transcript:KXG30633           transcript:Zm00001d051017_T007          0
887- 17: transcript:EES07119           transcript:Zm00001d051018_T002 1.00E-152
887- 18: transcript:KXG30593           transcript:Zm00001d051020_T002 1.00E-148
## Alignment 888: score=738.0 e_value=1.4e-46 N=17 4&4 plus
888-  0: transcript:EES05395           transcript:Zm00001d051039_T010          0
888-  1: transcript:KXG30597           transcript:Zm00001d051041_T001          0
888-  2: transcript:EES05399           transcript:Zm00001d051043_T001          0
888-  3: transcript:EES07131           transcript:Zm00001d051044_T001          0
888-  4: transcript:EES05406           transcript:Zm00001d051047_T001 3.00E-148
888-  5: transcript:KXG30615           transcript:Zm00001d051052_T001  9.00E-84
888-  6: transcript:OQU85291           transcript:Zm00001d051053_T001  1.00E-96
888-  7: transcript:KXG30617           transcript:Zm00001d051054_T001  4.00E-07
888-  8: transcript:OQU85293           transcript:Zm00001d051055_T003  5.00E-38
888-  9: transcript:EES05409           transcript:Zm00001d051056_T001          0
888- 10: transcript:KXG30618           transcript:Zm00001d051057_T002          0
888- 11: transcript:EES07137           transcript:Zm00001d051061_T001  1.00E-72
888- 12: transcript:OQU85297           transcript:Zm00001d051062_T001 1.00E-160
888- 13: transcript:EES05413           transcript:Zm00001d051063_T001 6.00E-111
888- 14: transcript:EES07139           transcript:Zm00001d051065_T001 3.00E-165
888- 15: transcript:EES07141           transcript:Zm00001d051067_T003          0
888- 16: transcript:EES05415           transcript:Zm00001d051069_T001          0
## Alignment 889: score=733.0 e_value=3.6e-41 N=16 4&4 plus
889-  0: transcript:EES05473           transcript:Zm00001d051214_T001          0
889-  1: transcript:EES05476           transcript:Zm00001d051216_T001  8.00E-85
889-  2: transcript:KXG30697           transcript:Zm00001d051219_T001          0
889-  3: transcript:EES07199           transcript:Zm00001d051223_T001 3.00E-141
889-  4: transcript:EES07202           transcript:Zm00001d051227_T001          0
889-  5: transcript:KXG30699           transcript:Zm00001d051229_T002          0
889-  6: transcript:EES05483           transcript:Zm00001d051232_T009 2.00E-180
```

889- 7: transcript:KXG30701 transcript:Zm00001d051233_T001 6.00E-61
889- 8: transcript:KXG30751 transcript:Zm00001d051234_T001 2.00E-89
889- 9: transcript:EES07205 transcript:Zm00001d051235_T001 0
889- 10: transcript:KXG30703 transcript:Zm00001d051238_T001 2.00E-63
889- 11: transcript:OQU85375 transcript:Zm00001d051239_T001 0
889- 12: transcript:EES05484 transcript:Zm00001d051241_T001 0
889- 13: transcript:EES05486 transcript:Zm00001d051242_T002 0
889- 14: transcript:OQU85378 transcript:Zm00001d051245_T001 0
889- 15: transcript:EES05490 transcript:Zm00001d051249_T003 5.00E-86
Alignment 890: score=719.0 e_value=2.4e-49 N=18 4&4 plus
890- 0: transcript:EES05109 transcript:Zm00001d050544_T001 3.00E-112
890- 1: transcript:KXG30258 transcript:Zm00001d050550_T001 0
890- 2: transcript:EES05114 transcript:Zm00001d050551_T007 0
890- 3: transcript:KXG30261 transcript:Zm00001d050552_T001 0
890- 4: transcript:EES06855 transcript:Zm00001d050553_T001 6.00E-91
890- 5: transcript:EES05119 transcript:Zm00001d050557_T001 4.00E-61
890- 6: transcript:OQU84998 transcript:Zm00001d050558_T001 3.00E-114
890- 7: transcript:EES05123 transcript:Zm00001d050563_T001 1.00E-102
890- 8: transcript:KXG30275 transcript:Zm00001d050565_T001 0
890- 9: transcript:OQU85003 transcript:Zm00001d050567_T001 0
890- 10: transcript:OQU85004 transcript:Zm00001d050572_T001 0
890- 11: transcript:EES05138 transcript:Zm00001d050575_T002 0
890- 12: transcript:EES06868 transcript:Zm00001d050577_T001 1.00E-156
890- 13: transcript:OQU85015 transcript:Zm00001d050580_T001 0
890- 14: transcript:KXG30289 transcript:Zm00001d050583_T001 6.00E-168
890- 15: transcript:EES05145 transcript:Zm00001d050584_T034 0
890- 16: transcript:KXG30300 transcript:Zm00001d050600_T002 4.00E-124
890- 17: transcript:KXG30309 transcript:Zm00001d050604_T008 0
Alignment 891: score=714.0 e_value=1.7e-42 N=16 4&4 plus
891- 0: transcript:EES05332 transcript:Zm00001d050915_T004 3.00E-134
891- 1: transcript:EES07061 transcript:Zm00001d050916_T002 4.00E-75
891- 2: transcript:EES05342 transcript:Zm00001d050917_T002 0
891- 3: transcript:EES07065 transcript:Zm00001d050918_T001 7.00E-30
891- 4: transcript:EES07070 transcript:Zm00001d050920_T002 1.00E-151
891- 5: transcript:EES07071 transcript:Zm00001d050923_T002 0
891- 6: transcript:EES07073 transcript:Zm00001d050925_T007 0
891- 7: transcript:OQU85235 transcript:Zm00001d050929_T002 0
891- 8: transcript:EES05347 transcript:Zm00001d050935_T001 8.00E-66
891- 9: transcript:KXG30534 transcript:Zm00001d050938_T001 0
891- 10: transcript:EES07079 transcript:Zm00001d050942_T001 0
891- 11: transcript:EES07081 transcript:Zm00001d050943_T001 0
891- 12: transcript:OQU85237 transcript:Zm00001d050944_T001 9.00E-64
891- 13: transcript:EES05353 transcript:Zm00001d050947_T007 0
891- 14: transcript:EES05354 transcript:Zm00001d050948_T001 2.00E-149
891- 15: transcript:EES05356 transcript:Zm00001d050955_T002 4.00E-166
Alignment 892: score=656.0 e_value=5.6e-35 N=14 4&4 plus
892- 0: transcript:EES06373 transcript:Zm00001d053729_T001 2.00E-32
892- 1: transcript:EES04626 transcript:Zm00001d053731_T001 2.00E-43
892- 2: transcript:EES06374 transcript:Zm00001d053732_T001 0
892- 3: transcript:OQU84487 transcript:Zm00001d053734_T001 2.00E-47
892- 4: transcript:KXG29620 transcript:Zm00001d053735_T002 0
892- 5: transcript:EES06376 transcript:Zm00001d053736_T001 3.00E-143
892- 6: transcript:EES04633 transcript:Zm00001d053738_T001 0
892- 7: transcript:KXG29623 transcript:Zm00001d053739_T001 0

```
892-  8: transcript:KXG29624            transcript:Zm00001d053740_T001          0
892-  9: transcript:EES06381            transcript:Zm00001d053741_T002          0
892- 10: transcript:EES04638            transcript:Zm00001d053743_T002 1.00E-121
892- 11: transcript:KXG29625            transcript:Zm00001d053745_T001          0
892- 12: transcript:EES04642            transcript:Zm00001d053746_T001 2.00E-147
892- 13: transcript:EES04645            transcript:Zm00001d053747_T002          0
## Alignment 893: score=570.0 e_value=2.7e-26 N=12 4&4 plus
893-  0: transcript:KXG29315            transcript:Zm00001d053895_T001          0
893-  1: transcript:EES06185            transcript:Zm00001d053896_T001          0
893-  2: transcript:KXG29316            transcript:Zm00001d053897_T001  2.00E-84
893-  3: transcript:EES06186            transcript:Zm00001d053899_T001 1.00E-120
893-  4: transcript:OQU84238            transcript:Zm00001d053900_T001  3.00E-90
893-  5: transcript:EES06189            transcript:Zm00001d053901_T001          0
893-  6: transcript:EES06188            transcript:Zm00001d053908_T001  8.00E-82
893-  7: transcript:EES06191            transcript:Zm00001d053909_T002          0
893-  8: transcript:EES06192            transcript:Zm00001d053910_T001  9.00E-40
893-  9: transcript:KXG29319            transcript:Zm00001d053911_T002          0
893- 10: transcript:EES04389            transcript:Zm00001d053912_T001  6.00E-74
893- 11: transcript:EES04390            transcript:Zm00001d053916_T002          0
## Alignment 894: score=542.0 e_value=1.4e-25 N=12 4&4 plus
894-  0: transcript:EES04394            transcript:Zm00001d053918_T001 5.00E-167
894-  1: transcript:EES06194            transcript:Zm00001d053919_T001  3.00E-86
894-  2: transcript:EES06196            transcript:Zm00001d053923_T003 4.00E-145
894-  3: transcript:EES06198            transcript:Zm00001d053925_T001 2.00E-133
894-  4: transcript:OQU84249            transcript:Zm00001d053926_T001  8.00E-11
894-  5: transcript:KXG29333            transcript:Zm00001d053927_T021          0
894-  6: transcript:EES06202            transcript:Zm00001d053928_T001          0
894-  7: transcript:EES04403            transcript:Zm00001d053930_T001          0
894-  8: transcript:EES04404            transcript:Zm00001d053931_T003          0
894-  9: transcript:EES06204            transcript:Zm00001d053932_T002          0
894- 10: transcript:KXG29335            transcript:Zm00001d053933_T001  1.00E-35
894- 11: transcript:KXG29336            transcript:Zm00001d053934_T001  2.00E-35
## Alignment 895: score=467.0 e_value=1.4e-19 N=10 4&4 plus
895-  0: transcript:EES04879            transcript:Zm00001d053352_T001 2.00E-110
895-  1: transcript:OQU84748            transcript:Zm00001d053359_T001 8.00E-137
895-  2: transcript:OQU84749            transcript:Zm00001d053367_T001  5.00E-22
895-  3: transcript:EES04883            transcript:Zm00001d053369_T001 3.00E-167
895-  4: transcript:EES06620            transcript:Zm00001d053371_T001 3.00E-136
895-  5: transcript:OQU84754            transcript:Zm00001d053372_T001          0
895-  6: transcript:EES06624            transcript:Zm00001d053373_T001 1.00E-106
895-  7: transcript:EES04890            transcript:Zm00001d053374_T001          0
895-  8: transcript:OQU84761            transcript:Zm00001d053375_T001 9.00E-112
895-  9: transcript:EES06629            transcript:Zm00001d053376_T003          0
## Alignment 896: score=426.0 e_value=2.6e-21 N=10 4&4 plus
896-  0: transcript:EES06546            transcript:Zm00001d053522_T001          0
896-  1: transcript:KXG29870            transcript:Zm00001d053524_T001          0
896-  2: transcript:EES04807            transcript:Zm00001d053541_T002          0
896-  3: transcript:OQU84681            transcript:Zm00001d053543_T001  9.00E-75
896-  4: transcript:EES04812            transcript:Zm00001d053544_T001          0
896-  5: transcript:EES04814            transcript:Zm00001d053545_T003          0
896-  6: transcript:EES04818            transcript:Zm00001d053547_T003 2.00E-130
896-  7: transcript:EES06566            transcript:Zm00001d053548_T001          0
896-  8: transcript:EES06567            transcript:Zm00001d053552_T001          0
896-  9: transcript:KXG29896            transcript:Zm00001d053554_T001          0
```

Alignment 897: score=414.0 e_value=6.9e-18 N=9 4&4 plus

897- 0:	transcript:EES07187	transcript:Zm00001d051183_T031	0
897- 1:	transcript:OQU85359	transcript:Zm00001d051187_T009	0
897- 2:	transcript:KXG30684	transcript:Zm00001d051189_T001	1.00E-44
897- 3:	transcript:OQU85362	transcript:Zm00001d051193_T004	0
897- 4:	transcript:EES07195	transcript:Zm00001d051199_T001	8.00E-70
897- 5:	transcript:EES05469	transcript:Zm00001d051203_T001	5.00E-176
897- 6:	transcript:EES05471	transcript:Zm00001d051206_T001	0
897- 7:	transcript:KXG30693	transcript:Zm00001d051207_T001	8.00E-39
897- 8:	transcript:EES05472	transcript:Zm00001d051211_T004	2.00E-159

Alignment 898: score=389.0 e_value=1e-14 N=8 4&4 plus

898- 0:	transcript:EES05363	transcript:Zm00001d050959_T001	0
898- 1:	transcript:KXG30609	transcript:Zm00001d050960_T001	0
898- 2:	transcript:KXG30544	transcript:Zm00001d050961_T001	0
898- 3:	transcript:KXG30547	transcript:Zm00001d050963_T001	1.00E-69
898- 4:	transcript:EES07092	transcript:Zm00001d050964_T001	6.00E-125
898- 5:	transcript:EES07093	transcript:Zm00001d050965_T001	7.00E-141
898- 6:	transcript:EES05366	transcript:Zm00001d050969_T001	0
898- 7:	transcript:KXG30548	transcript:Zm00001d050970_T003	0

Alignment 899: score=384.0 e_value=7.5e-15 N=8 4&4 plus

899- 0:	transcript:EES05709	transcript:Zm00001d051484_T001	1.00E-130
899- 1:	transcript:EES05711	transcript:Zm00001d051492_T001	7.00E-58
899- 2:	transcript:EES05712	transcript:Zm00001d051495_T001	2.00E-71
899- 3:	transcript:EES05714	transcript:Zm00001d051498_T001	0
899- 4:	transcript:EES07437	transcript:Zm00001d051499_T001	1.00E-101
899- 5:	transcript:OQU85613	transcript:Zm00001d051500_T001	0
899- 6:	transcript:EES05717	transcript:Zm00001d051501_T001	3.00E-97
899- 7:	transcript:OQU85618	transcript:Zm00001d051502_T001	0

Alignment 900: score=342.0 e_value=1.4e-13 N=7 4&4 plus

900- 0:	transcript:OQU85513	transcript:Zm00001d051653_T001	2.00E-16
900- 1:	transcript:OQU85514	transcript:Zm00001d051658_T001	3.00E-24
900- 2:	transcript:OQU85515	transcript:Zm00001d051660_T001	4.00E-114
900- 3:	transcript:EES05620	transcript:Zm00001d051661_T001	7.00E-138
900- 4:	transcript:EES05621	transcript:Zm00001d051662_T001	6.00E-67
900- 5:	transcript:EES07361	transcript:Zm00001d051663_T001	1.00E-104
900- 6:	transcript:KXG30878	transcript:Zm00001d051664_T001	0

Alignment 901: score=271.0 e_value=1.9e-10 N=6 4&4 plus

901- 0:	transcript:EES05675	transcript:Zm00001d051542_T047	0
901- 1:	transcript:EES07409	transcript:Zm00001d051543_T001	5.00E-113
901- 2:	transcript:KXG30941	transcript:Zm00001d051544_T004	0
901- 3:	transcript:EES07410	transcript:Zm00001d051545_T003	0
901- 4:	transcript:EES07411	transcript:Zm00001d051546_T001	3.00E-140
901- 5:	transcript:EES07413	transcript:Zm00001d051550_T001	0

Alignment 902: score=268.0 e_value=7.3e-10 N=6 4&4 plus

902- 0:	transcript:EES07147	transcript:Zm00001d051075_T002	0
902- 1:	transcript:EES05417	transcript:Zm00001d051080_T004	4.00E-68
902- 2:	transcript:EES05418	transcript:Zm00001d051081_T001	0
902- 3:	transcript:EES07148	transcript:Zm00001d051082_T001	5.00E-44
902- 4:	transcript:OQU85311	transcript:Zm00001d051093_T001	1.00E-133
902- 5:	transcript:EES05426	transcript:Zm00001d051102_T001	0

Alignment 903: score=267.0 e_value=1.5e-08 N=6 4&4 plus

903- 0:	transcript:KXG29407	transcript:Zm00001d053994_T008	0
903- 1:	transcript:KXG29411	transcript:Zm00001d053995_T001	2.00E-82
903- 2:	transcript:EES06244	transcript:Zm00001d053996_T001	9.00E-40

903- 3: transcript:EES06245   transcript:Zm00001d053997_T004   0
903- 4: transcript:KXG29416   transcript:Zm00001d053998_T001   0
903- 5: transcript:EES04470   transcript:Zm00001d054000_T001   0
Alignment 904: score=4840.0 e_value=0 N=108 4&4 minus
904- 0: transcript:OQU85586   transcript:Zm00001d051507_T007   0
904- 1: transcript:KXG30983   transcript:Zm00001d051506_T001 2.00E-66
904- 2: transcript:EES05698   transcript:Zm00001d051505_T001 1.00E-107
904- 3: transcript:OQU85596   transcript:Zm00001d051504_T001 1.00E-114
904- 4: transcript:KXG30985   transcript:Zm00001d051503_T001 2.00E-72
904- 5: transcript:EES05711   transcript:Zm00001d051492_T001 7.00E-58
904- 6: transcript:EES05718   transcript:Zm00001d051480_T001 9.00E-132
904- 7: transcript:OQU85620   transcript:Zm00001d051479_T014   0
904- 8: transcript:OQU85624   transcript:Zm00001d051478_T001 7.00E-67
904- 9: transcript:EES05720   transcript:Zm00001d051475_T002 1.00E-72
904- 10: transcript:EES07443   transcript:Zm00001d051474_T001   0
904- 11: transcript:OQU85625   transcript:Zm00001d051473_T001   0
904- 12: transcript:OQU85626   transcript:Zm00001d051472_T001 6.00E-94
904- 13: transcript:EES07445   transcript:Zm00001d051471_T001 1.00E-178
904- 14: transcript:EES05726   transcript:Zm00001d051468_T001 3.00E-68
904- 15: transcript:EES07448   transcript:Zm00001d051465_T002 1.00E-168
904- 16: transcript:EES05727   transcript:Zm00001d051461_T001   0
904- 17: transcript:EES05730   transcript:Zm00001d051459_T001 3.00E-171
904- 18: transcript:KXG31009   transcript:Zm00001d051458_T002 1.00E-43
904- 19: transcript:EES07452   transcript:Zm00001d051457_T001   0
904- 20: transcript:OQU85635   transcript:Zm00001d051456_T001 1.00E-55
904- 21: transcript:OQU85640   transcript:Zm00001d051453_T001 9.00E-145
904- 22: transcript:KXG31013   transcript:Zm00001d051451_T001 2.00E-79
904- 23: transcript:KXG31014   transcript:Zm00001d051448_T001 3.00E-135
904- 24: transcript:KXG31015   transcript:Zm00001d051447_T001   0
904- 25: transcript:EES05735   transcript:Zm00001d051442_T002   0
904- 26: transcript:KXG31018   transcript:Zm00001d051441_T001 5.00E-58
904- 27: transcript:EES05738   transcript:Zm00001d051440_T001 3.00E-49
904- 28: transcript:KXG31020   transcript:Zm00001d051439_T001 2.00E-151
904- 29: transcript:EES05742   transcript:Zm00001d051431_T002   0
904- 30: transcript:KXG31022   transcript:Zm00001d051430_T001 1.00E-74
904- 31: transcript:OQU85652   transcript:Zm00001d051429_T001   0
904- 32: transcript:OQU85654   transcript:Zm00001d051427_T002 6.00E-46
904- 33: transcript:EES05745   transcript:Zm00001d051424_T001   0
904- 34: transcript:EES07474   transcript:Zm00001d051422_T001 7.00E-66
904- 35: transcript:OQU85665   transcript:Zm00001d051421_T002 2.00E-89
904- 36: transcript:EES05747   transcript:Zm00001d051420_T001 4.00E-69
904- 37: transcript:KXG31039   transcript:Zm00001d051419_T002   0
904- 38: transcript:KXG31041   transcript:Zm00001d051418_T001   0
904- 39: transcript:KXG31045   transcript:Zm00001d051416_T001   0
904- 40: transcript:EES07479   transcript:Zm00001d051415_T001   0
904- 41: transcript:EES05753   transcript:Zm00001d051411_T001 2.00E-40
904- 42: transcript:EES05750   transcript:Zm00001d051410_T001 1.00E-39
904- 43: transcript:EES05755   transcript:Zm00001d051406_T001 8.00E-53
904- 44: transcript:EES05756   transcript:Zm00001d051405_T001 3.00E-122
904- 45: transcript:EES05759   transcript:Zm00001d051404_T004   0
904- 46: transcript:EES07484   transcript:Zm00001d051403_T001   0
904- 47: transcript:OQU85674   transcript:Zm00001d051397_T001 5.00E-160
904- 48: transcript:KXG31056   transcript:Zm00001d051396_T004   0
904- 49: transcript:OQU85677   transcript:Zm00001d051395_T001 1.00E-104

904- 50:	transcript:EES05761	transcript:Zm00001d051394_T001	9.00E-73
904- 51:	transcript:EES07487	transcript:Zm00001d051393_T002	0
904- 52:	transcript:KXG31059	transcript:Zm00001d051392_T001	3.00E-76
904- 53:	transcript:EES05763	transcript:Zm00001d051389_T001	0
904- 54:	transcript:EES05762	transcript:Zm00001d051388_T001	3.00E-96
904- 55:	transcript:EES05768	transcript:Zm00001d051387_T001	0
904- 56:	transcript:OQU85682	transcript:Zm00001d051386_T001	2.00E-32
904- 57:	transcript:KXG31075	transcript:Zm00001d051384_T001	2.00E-58
904- 58:	transcript:EES07504	transcript:Zm00001d051383_T001	3.00E-156
904- 59:	transcript:EES07505	transcript:Zm00001d051380_T001	5.00E-118
904- 60:	transcript:EES07500	transcript:Zm00001d051376_T001	4.00E-127
904- 61:	transcript:EES05775	transcript:Zm00001d051373_T001	2.00E-69
904- 62:	transcript:OQU85696	transcript:Zm00001d051371_T001	1.00E-116
904- 63:	transcript:OQU85697	transcript:Zm00001d051370_T001	7.00E-119
904- 64:	transcript:EES07509	transcript:Zm00001d051368_T001	3.00E-91
904- 65:	transcript:KXG31084	transcript:Zm00001d051367_T001	0
904- 66:	transcript:OQU85705	transcript:Zm00001d051366_T002	3.00E-19
904- 67:	transcript:EES05783	transcript:Zm00001d051365_T001	6.00E-44
904- 68:	transcript:KXG31086	transcript:Zm00001d051362_T001	2.00E-156
904- 69:	transcript:KXG31087	transcript:Zm00001d051361_T001	0
904- 70:	transcript:KXG31088	transcript:Zm00001d051360_T001	5.00E-137
904- 71:	transcript:EES07512	transcript:Zm00001d051359_T001	0
904- 72:	transcript:EES07513	transcript:Zm00001d051350_T001	6.00E-48
904- 73:	transcript:KXG31090	transcript:Zm00001d051345_T003	0
904- 74:	transcript:EES05789	transcript:Zm00001d051344_T001	9.00E-71
904- 75:	transcript:EES05790	transcript:Zm00001d051343_T001	0
904- 76:	transcript:KXG31094	transcript:Zm00001d051340_T001	2.00E-134
904- 77:	transcript:OQU85717	transcript:Zm00001d051339_T001	2.00E-144
904- 78:	transcript:KXG31098	transcript:Zm00001d051338_T002	0
904- 79:	transcript:EES07525	transcript:Zm00001d051337_T002	2.00E-156
904- 80:	transcript:EES07529	transcript:Zm00001d051335_T001	4.00E-23
904- 81:	transcript:EES05795	transcript:Zm00001d051334_T001	0
904- 82:	transcript:OQU85738	transcript:Zm00001d051333_T001	1.00E-77
904- 83:	transcript:KXG31102	transcript:Zm00001d051329_T002	9.00E-162
904- 84:	transcript:KXG31104	transcript:Zm00001d051328_T002	7.00E-115
904- 85:	transcript:KXG31163	transcript:Zm00001d051324_T001	0
904- 86:	transcript:EES05807	transcript:Zm00001d051323_T003	0
904- 87:	transcript:OQU85746	transcript:Zm00001d051321_T007	0
904- 88:	transcript:EES05813	transcript:Zm00001d051320_T001	0
904- 89:	transcript:EES05815	transcript:Zm00001d051316_T001	0
904- 90:	transcript:EES05816	transcript:Zm00001d051314_T001	0
904- 91:	transcript:OQU85751	transcript:Zm00001d051313_T001	0
904- 92:	transcript:EES07539	transcript:Zm00001d051309_T001	4.00E-110
904- 93:	transcript:KXG31119	transcript:Zm00001d051308_T009	0
904- 94:	transcript:EES05821	transcript:Zm00001d051307_T001	0
904- 95:	transcript:EES07540	transcript:Zm00001d051306_T001	0
904- 96:	transcript:EES05819	transcript:Zm00001d051305_T001	2.00E-134
904- 97:	transcript:EES07542	transcript:Zm00001d051302_T001	1.00E-71
904- 98:	transcript:KXG31125	transcript:Zm00001d051294_T001	4.00E-100
904- 99:	transcript:EES07541	transcript:Zm00001d051291_T003	0
904-100:	transcript:KXG31127	transcript:Zm00001d051288_T001	5.00E-135
904-101:	transcript:EES07545	transcript:Zm00001d051287_T001	0
904-102:	transcript:EES05833	transcript:Zm00001d051272_T001	7.00E-162
904-103:	transcript:OQU85763	transcript:Zm00001d051269_T001	6.00E-87

904-104: transcript:EES07547 transcript:Zm00001d051267_T001 5.00E-114
904-105: transcript:EES05836 transcript:Zm00001d051262_T001 5.00E-121
904-106: transcript:KXG31133 transcript:Zm00001d051251_T003 2.00E-114
904-107: transcript:OQU85770 transcript:Zm00001d051246_T001 2.00E-150
Alignment 905: score=4042.0 e_value=0 N=90 4&4 minus

905- 0:	transcript:EES07724	transcript:Zm00001d051958_T001	1.00E-142
905- 1:	transcript:EES07725	transcript:Zm00001d051956_T001	8.00E-147
905- 2:	transcript:KXG31323	transcript:Zm00001d051952_T001	3.00E-63
905- 3:	transcript:KXG31326	transcript:Zm00001d051951_T008	0
905- 4:	transcript:OQU85924	transcript:Zm00001d051950_T003	0
905- 5:	transcript:EES05988	transcript:Zm00001d051948_T001	0
905- 6:	transcript:KXG31328	transcript:Zm00001d051946_T001	0
905- 7:	transcript:KXG31389	transcript:Zm00001d051945_T001	0
905- 8:	transcript:KXG31329	transcript:Zm00001d051944_T001	5.00E-17
905- 9:	transcript:EES07730	transcript:Zm00001d051939_T001	0
905- 10:	transcript:EES05992	transcript:Zm00001d051938_T002	0
905- 11:	transcript:EES05994	transcript:Zm00001d051936_T001	3.00E-170
905- 12:	transcript:KXG31339	transcript:Zm00001d051934_T001	0
905- 13:	transcript:OQU85936	transcript:Zm00001d051932_T002	0
905- 14:	transcript:OQU85937	transcript:Zm00001d051930_T002	0
905- 15:	transcript:EES06002	transcript:Zm00001d051929_T001	1.00E-97
905- 16:	transcript:KXG31344	transcript:Zm00001d051928_T001	0
905- 17:	transcript:EES07746	transcript:Zm00001d051927_T001	7.00E-126
905- 18:	transcript:KXG31345	transcript:Zm00001d051926_T001	2.00E-106
905- 19:	transcript:OQU85939	transcript:Zm00001d051925_T001	0
905- 20:	transcript:EES06003	transcript:Zm00001d051924_T002	2.00E-159
905- 21:	transcript:EES07753	transcript:Zm00001d051923_T001	0
905- 22:	transcript:KXG31351	transcript:Zm00001d051917_T007	0
905- 23:	transcript:EES07755	transcript:Zm00001d051915_T001	0
905- 24:	transcript:EES06017	transcript:Zm00001d051914_T001	0
905- 25:	transcript:KXG31415	transcript:Zm00001d051913_T005	0
905- 26:	transcript:EES06018	transcript:Zm00001d051912_T001	0
905- 27:	transcript:EES07758	transcript:Zm00001d051911_T002	2.00E-161
905- 28:	transcript:KXG31417	transcript:Zm00001d051910_T001	0
905- 29:	transcript:KXG31364	transcript:Zm00001d051908_T001	3.00E-123
905- 30:	transcript:KXG31366	transcript:Zm00001d051906_T003	0
905- 31:	transcript:OQU85956	transcript:Zm00001d051905_T001	2.00E-121
905- 32:	transcript:EES06026	transcript:Zm00001d051902_T003	0
905- 33:	transcript:KXG31376	transcript:Zm00001d051901_T004	0
905- 34:	transcript:EES07767	transcript:Zm00001d051900_T001	0
905- 35:	transcript:KXG31383	transcript:Zm00001d051899_T001	0
905- 36:	transcript:KXG31384	transcript:Zm00001d051898_T001	0
905- 37:	transcript:EES06032	transcript:Zm00001d051896_T001	0
905- 38:	transcript:OQU85969	transcript:Zm00001d051894_T001	2.00E-81
905- 39:	transcript:EES07769	transcript:Zm00001d051893_T001	0
905- 40:	transcript:OQU85970	transcript:Zm00001d051892_T001	0
905- 41:	transcript:EES07771	transcript:Zm00001d051891_T001	5.00E-125
905- 42:	transcript:OQU85971	transcript:Zm00001d051890_T001	5.00E-20
905- 43:	transcript:OQU85974	transcript:Zm00001d051889_T002	0
905- 44:	transcript:EES07775	transcript:Zm00001d051887_T001	0
905- 45:	transcript:OQU85978	transcript:Zm00001d051886_T001	7.00E-175
905- 46:	transcript:EES06036	transcript:Zm00001d051885_T001	0
905- 47:	transcript:EES06038	transcript:Zm00001d051884_T002	0
905- 48:	transcript:EES06039	transcript:Zm00001d051883_T001	0

905- 49: transcript:KXG31391 transcript:Zm00001d051882_T001 8.00E-24
905- 50: transcript:EES06040 transcript:Zm00001d051881_T001 0
905- 51: transcript:EES06041 transcript:Zm00001d051879_T001 0
905- 52: transcript:EES06043 transcript:Zm00001d051877_T001 3.00E-149
905- 53: transcript:OQU85983 transcript:Zm00001d051876_T002 0
905- 54: transcript:OQU85984 transcript:Zm00001d051875_T001 0
905- 55: transcript:EES06047 transcript:Zm00001d051873_T002 0
905- 56: transcript:OQU85985 transcript:Zm00001d051872_T001 0
905- 57: transcript:EES06049 transcript:Zm00001d051871_T002 0
905- 58: transcript:EES06050 transcript:Zm00001d051870_T001 0
905- 59: transcript:EES07782 transcript:Zm00001d051869_T001 5.00E-48
905- 60: transcript:EES06044 transcript:Zm00001d051866_T004 0
905- 61: transcript:EES07783 transcript:Zm00001d051865_T001 4.00E-134
905- 62: transcript:OQU85991 transcript:Zm00001d051864_T003 0
905- 63: transcript:KXG31398 transcript:Zm00001d051863_T001 0
905- 64: transcript:OQU85994 transcript:Zm00001d051861_T001 0
905- 65: transcript:EES07787 transcript:Zm00001d051860_T001 2.00E-45
905- 66: transcript:EES06056 transcript:Zm00001d051859_T001 5.00E-33
905- 67: transcript:EES06057 transcript:Zm00001d051856_T002 0
905- 68: transcript:KXG31404 transcript:Zm00001d051854_T001 0
905- 69: transcript:EES07795 transcript:Zm00001d051851_T005 0
905- 70: transcript:EES07797 transcript:Zm00001d051850_T001 1.00E-97
905- 71: transcript:KXG31418 transcript:Zm00001d051849_T023 0
905- 72: transcript:OQU86011 transcript:Zm00001d051848_T002 0
905- 73: transcript:OQU86016 transcript:Zm00001d051847_T001 0
905- 74: transcript:EES07809 transcript:Zm00001d051846_T001 0
905- 75: transcript:EES07811 transcript:Zm00001d051843_T001 0
905- 76: transcript:EES07812 transcript:Zm00001d051842_T003 0
905- 77: transcript:EES07819 transcript:Zm00001d051840_T001 0
905- 78: transcript:KXG31426 transcript:Zm00001d051839_T004 0
905- 79: transcript:EES06072 transcript:Zm00001d051838_T001 0
905- 80: transcript:KXG31429 transcript:Zm00001d051837_T002 0
905- 81: transcript:OQU86028 transcript:Zm00001d051836_T001 1.00E-37
905- 82: transcript:OQU86029 transcript:Zm00001d051835_T001 6.00E-82
905- 83: transcript:EES07825 transcript:Zm00001d051833_T001 1.00E-92
905- 84: transcript:EES06080 transcript:Zm00001d051832_T001 0
905- 85: transcript:EES06081 transcript:Zm00001d051830_T001 1.00E-79
905- 86: transcript:EES07827 transcript:Zm00001d051829_T001 0
905- 87: transcript:EES06083 transcript:Zm00001d051824_T001 0
905- 88: transcript:OQU86039 transcript:Zm00001d051823_T001 0
905- 89: transcript:KXG31443 transcript:Zm00001d051819_T001 1.00E-37
Alignment 906: score=2151.0 e_value=9.2e-183 N=48 4&4 minus
906- 0: transcript:EES05623 transcript:Zm00001d051644_T004 0
906- 1: transcript:EES07363 transcript:Zm00001d051643_T001 1.00E-84
906- 2: transcript:EES05624 transcript:Zm00001d051637_T001 0
906- 3: transcript:EES05625 transcript:Zm00001d051636_T001 0
906- 4: transcript:EES07365 transcript:Zm00001d051635_T001 1.00E-26
906- 5: transcript:EES07369 transcript:Zm00001d051634_T001 0
906- 6: transcript:OQU85519 transcript:Zm00001d051633_T003 0
906- 7: transcript:KXG30886 transcript:Zm00001d051632_T002 0
906- 8: transcript:EES05627 transcript:Zm00001d051629_T003 0
906- 9: transcript:EES05628 transcript:Zm00001d051628_T002 0
906- 10: transcript:OQU85520 transcript:Zm00001d051627_T002 5.00E-158
906- 11: transcript:EES05629 transcript:Zm00001d051626_T003 0

906- 12:	transcript:EES05630	transcript:Zm00001d051620_T001	6.00E-134
906- 13:	transcript:KXG30889	transcript:Zm00001d051615_T001	0
906- 14:	transcript:EES05635	transcript:Zm00001d051611_T001	2.00E-96
906- 15:	transcript:KXG30894	transcript:Zm00001d051610_T001	4.00E-91
906- 16:	transcript:KXG30897	transcript:Zm00001d051609_T001	0
906- 17:	transcript:EES05638	transcript:Zm00001d051606_T001	2.00E-146
906- 18:	transcript:KXG30901	transcript:Zm00001d051600_T004	0
906- 19:	transcript:EES05641	transcript:Zm00001d051599_T004	0
906- 20:	transcript:EES07382	transcript:Zm00001d051598_T001	6.00E-152
906- 21:	transcript:EES07383	transcript:Zm00001d051596_T001	1.00E-46
906- 22:	transcript:EES05643	transcript:Zm00001d051595_T002	1.00E-147
906- 23:	transcript:EES05644	transcript:Zm00001d051594_T001	3.00E-67
906- 24:	transcript:OQU85528	transcript:Zm00001d051592_T001	2.00E-64
906- 25:	transcript:EES07386	transcript:Zm00001d051591_T001	8.00E-99
906- 26:	transcript:KXG30908	transcript:Zm00001d051590_T001	0
906- 27:	transcript:EES05647	transcript:Zm00001d051589_T004	0
906- 28:	transcript:OQU85541	transcript:Zm00001d051588_T002	1.00E-32
906- 29:	transcript:EES07390	transcript:Zm00001d051587_T001	2.00E-143
906- 30:	transcript:EES07391	transcript:Zm00001d051586_T001	0
906- 31:	transcript:EES05648	transcript:Zm00001d051584_T001	5.00E-131
906- 32:	transcript:EES05649	transcript:Zm00001d051577_T001	3.00E-155
906- 33:	transcript:OQU85543	transcript:Zm00001d051576_T001	0
906- 34:	transcript:KXG30914	transcript:Zm00001d051573_T001	1.00E-134
906- 35:	transcript:EES07394	transcript:Zm00001d051572_T001	0
906- 36:	transcript:EES05656	transcript:Zm00001d051569_T001	3.00E-176
906- 37:	transcript:EES07397	transcript:Zm00001d051568_T001	0
906- 38:	transcript:KXG30919	transcript:Zm00001d051567_T001	0
906- 39:	transcript:EES05655	transcript:Zm00001d051565_T009	0
906- 40:	transcript:KXG30922	transcript:Zm00001d051564_T001	3.00E-125
906- 41:	transcript:EES07398	transcript:Zm00001d051563_T001	0
906- 42:	transcript:OQU85551	transcript:Zm00001d051562_T001	0
906- 43:	transcript:OQU85552	transcript:Zm00001d051561_T001	4.00E-150
906- 44:	transcript:KXG30924	transcript:Zm00001d051556_T001	0
906- 45:	transcript:EES05661	transcript:Zm00001d051554_T002	0
906- 46:	transcript:EES05665	transcript:Zm00001d051553_T004	0
906- 47:	transcript:EES07404	transcript:Zm00001d051552_T001	0

Alignment 907: score=1606.0 e_value=1.4e-131 N=37 4&4 minus

907- 0:	transcript:KXG29487	transcript:Zm00001d053876_T011	0
907- 1:	transcript:KXG29488	transcript:Zm00001d053875_T001	0
907- 2:	transcript:EES04535	transcript:Zm00001d053873_T001	9.00E-137
907- 3:	transcript:OQU84386	transcript:Zm00001d053872_T021	0
907- 4:	transcript:EES06292	transcript:Zm00001d053868_T001	5.00E-65
907- 5:	transcript:KXG29501	transcript:Zm00001d053865_T007	0
907- 6:	transcript:EES06302	transcript:Zm00001d053864_T001	3.00E-100
907- 7:	transcript:EES04539	transcript:Zm00001d053863_T002	4.00E-60
907- 8:	transcript:KXG29509	transcript:Zm00001d053862_T002	5.00E-78
907- 9:	transcript:OQU84405	transcript:Zm00001d053861_T005	0
907- 10:	transcript:EES04542	transcript:Zm00001d053860_T001	4.00E-134
907- 11:	transcript:OQU84412	transcript:Zm00001d053858_T001	0
907- 12:	transcript:EES04545	transcript:Zm00001d053857_T007	0
907- 13:	transcript:EES06311	transcript:Zm00001d053856_T001	0
907- 14:	transcript:EES06312	transcript:Zm00001d053855_T001	2.00E-151
907- 15:	transcript:EES04550	transcript:Zm00001d053852_T001	0
907- 16:	transcript:KXG29512	transcript:Zm00001d053851_T001	4.00E-35

907- 17: transcript:OQU84417 transcript:Zm00001d053850_T001 6.00E-80
907- 18: transcript:EES04556 transcript:Zm00001d053848_T001 0
907- 19: transcript:EES04558 transcript:Zm00001d053846_T001 0
907- 20: transcript:EES06319 transcript:Zm00001d053843_T001 6.00E-37
907- 21: transcript:KXG29521 transcript:Zm00001d053841_T014 0
907- 22: transcript:EES06321 transcript:Zm00001d053839_T001 7.00E-109
907- 23: transcript:KXG29527 transcript:Zm00001d053838_T005 0
907- 24: transcript:KXG29532 transcript:Zm00001d053832_T001 1.00E-57
907- 25: transcript:EES04566 transcript:Zm00001d053831_T001 8.00E-67
907- 26: transcript:EES04567 transcript:Zm00001d053829_T001 4.00E-61
907- 27: transcript:EES04569 transcript:Zm00001d053828_T001 1.00E-29
907- 28: transcript:KXG29535 transcript:Zm00001d053826_T004 0
907- 29: transcript:EES04570 transcript:Zm00001d053825_T002 0
907- 30: transcript:OQU84426 transcript:Zm00001d053824_T001 1.00E-39
907- 31: transcript:EES04574 transcript:Zm00001d053822_T001 1.00E-133
907- 32: transcript:EES04575 transcript:Zm00001d053819_T002 0
907- 33: transcript:OQU84428 transcript:Zm00001d053818_T003 0
907- 34: transcript:EES04577 transcript:Zm00001d053817_T001 7.00E-121
907- 35: transcript:KXG29548 transcript:Zm00001d053816_T012 0
907- 36: transcript:OQU84433 transcript:Zm00001d053815_T001 1.00E-49
Alignment 908: score=1454.0 e_value=6.7e-114 N=32 4&4 minus
908- 0: transcript:EES05560 transcript:Zm00001d051815_T002 0
908- 1: transcript:EES07289 transcript:Zm00001d051814_T001 0
908- 2: transcript:OQU85445 transcript:Zm00001d051812_T003 0
908- 3: transcript:EES07294 transcript:Zm00001d051810_T001 2.00E-20
908- 4: transcript:KXG30803 transcript:Zm00001d051809_T001 0
908- 5: transcript:EES05568 transcript:Zm00001d051808_T002 0
908- 6: transcript:EES07298 transcript:Zm00001d051807_T001 0
908- 7: transcript:EES07299 transcript:Zm00001d051806_T001 0
908- 8: transcript:KXG30808 transcript:Zm00001d051804_T001 0
908- 9: transcript:EES07301 transcript:Zm00001d051803_T001 6.00E-92
908- 10: transcript:EES07303 transcript:Zm00001d051800_T002 1.00E-120
908- 11: transcript:OQU85454 transcript:Zm00001d051799_T001 1.00E-108
908- 12: transcript:EES05569 transcript:Zm00001d051798_T001 4.00E-38
908- 13: transcript:EES07306 transcript:Zm00001d051796_T003 0
908- 14: transcript:EES05570 transcript:Zm00001d051795_T001 3.00E-79
908- 15: transcript:EES05571 transcript:Zm00001d051793_T001 3.00E-78
908- 16: transcript:OQU85457 transcript:Zm00001d051790_T001 0
908- 17: transcript:KXG30816 transcript:Zm00001d051788_T001 8.00E-154
908- 18: transcript:KXG30818 transcript:Zm00001d051787_T001 0
908- 19: transcript:EES05582 transcript:Zm00001d051784_T001 3.00E-09
908- 20: transcript:EES05585 transcript:Zm00001d051761_T001 0
908- 21: transcript:EES05586 transcript:Zm00001d051760_T001 2.00E-106
908- 22: transcript:KXG30824 transcript:Zm00001d051759_T001 0
908- 23: transcript:KXG30825 transcript:Zm00001d051756_T004 0
908- 24: transcript:KXG30827 transcript:Zm00001d051754_T003 0
908- 25: transcript:EES07315 transcript:Zm00001d051753_T001 0
908- 26: transcript:KXG30829 transcript:Zm00001d051749_T001 9.00E-152
908- 27: transcript:EES05589 transcript:Zm00001d051746_T001 0
908- 28: transcript:EES05591 transcript:Zm00001d051741_T001 2.00E-77
908- 29: transcript:EES05594 transcript:Zm00001d051740_T005 0
908- 30: transcript:EES07324 transcript:Zm00001d051739_T001 1.00E-55
908- 31: transcript:OQU85475 transcript:Zm00001d051736_T001 1.00E-152
Alignment 909: score=1397.0 e_value=2.7e-115 N=33 4&4 minus

909- 0:	transcript:EES04327	transcript:Zm00001d054076_T010	0
909- 1:	transcript:OQU84152	transcript:Zm00001d054075_T001	5.00E-173
909- 2:	transcript:EES06107	transcript:Zm00001d054074_T001	2.00E-16
909- 3:	transcript:EES04335	transcript:Zm00001d054072_T002	0
909- 4:	transcript:OQU84157	transcript:Zm00001d054071_T001	0
909- 5:	transcript:KXG29267	transcript:Zm00001d054070_T002	0
909- 6:	transcript:EES06120	transcript:Zm00001d054069_T001	0
909- 7:	transcript:OQU84187	transcript:Zm00001d054067_T001	0
909- 8:	transcript:EES04344	transcript:Zm00001d054066_T001	0
909- 9:	transcript:EES04345	transcript:Zm00001d054065_T001	1.00E-62
909- 10:	transcript:EES04350	transcript:Zm00001d054057_T001	0
909- 11:	transcript:OQU84199	transcript:Zm00001d054056_T001	5.00E-52
909- 12:	transcript:EES06137	transcript:Zm00001d054055_T001	0
909- 13:	transcript:KXG29286	transcript:Zm00001d054052_T005	0
909- 14:	transcript:EES04356	transcript:Zm00001d054047_T001	0
909- 15:	transcript:OQU84207	transcript:Zm00001d054044_T001	0
909- 16:	transcript:KXG29291	transcript:Zm00001d054043_T003	0
909- 17:	transcript:EES06156	transcript:Zm00001d054042_T008	0
909- 18:	transcript:EES04360	transcript:Zm00001d054039_T001	0
909- 19:	transcript:KXG29295	transcript:Zm00001d054038_T003	1.00E-156
909- 20:	transcript:EES06157	transcript:Zm00001d054037_T002	0
909- 21:	transcript:EES04363	transcript:Zm00001d054034_T001	0
909- 22:	transcript:EES06160	transcript:Zm00001d054033_T001	1.00E-98
909- 23:	transcript:KXG29298	transcript:Zm00001d054017_T003	1.00E-47
909- 24:	transcript:OQU84212	transcript:Zm00001d054016_T004	0
909- 25:	transcript:EES04369	transcript:Zm00001d054015_T001	3.00E-131
909- 26:	transcript:EES04370	transcript:Zm00001d054014_T001	0
909- 27:	transcript:EES04378	transcript:Zm00001d054012_T001	0
909- 28:	transcript:EES04381	transcript:Zm00001d054011_T002	0
909- 29:	transcript:EES04382	transcript:Zm00001d054010_T001	0
909- 30:	transcript:EES04383	transcript:Zm00001d054009_T001	5.00E-24
909- 31:	transcript:OQU84235	transcript:Zm00001d054006_T001	7.00E-61
909- 32:	transcript:EES06180	transcript:Zm00001d054005_T006	0
## Alignment 910: score=1338.0 e_value=4.1e-106 N=32 4&4 minus			
910- 0:	transcript:OQU84616	transcript:Zm00001d053609_T011	0
910- 1:	transcript:OQU84621	transcript:Zm00001d053608_T002	3.00E-30
910- 2:	transcript:EES06503	transcript:Zm00001d053607_T001	1.00E-176
910- 3:	transcript:EES04736	transcript:Zm00001d053605_T001	7.00E-50
910- 4:	transcript:EES04733	transcript:Zm00001d053603_T005	0
910- 5:	transcript:KXG29788	transcript:Zm00001d053599_T001	6.00E-12
910- 6:	transcript:EES06507	transcript:Zm00001d053597_T006	4.00E-20
910- 7:	transcript:EES04741	transcript:Zm00001d053595_T004	0
910- 8:	transcript:EES06508	transcript:Zm00001d053594_T001	0
910- 9:	transcript:OQU84635	transcript:Zm00001d053593_T001	3.00E-41
910- 10:	transcript:EES04747	transcript:Zm00001d053591_T002	0
910- 11:	transcript:EES06511	transcript:Zm00001d053589_T001	6.00E-132
910- 12:	transcript:KXG29810	transcript:Zm00001d053588_T001	0
910- 13:	transcript:OQU84639	transcript:Zm00001d053587_T001	0
910- 14:	transcript:EES04752	transcript:Zm00001d053586_T001	0
910- 15:	transcript:EES06516	transcript:Zm00001d053585_T002	0
910- 16:	transcript:EES04754	transcript:Zm00001d053580_T002	0
910- 17:	transcript:EES06520	transcript:Zm00001d053578_T001	0
910- 18:	transcript:EES04758	transcript:Zm00001d053576_T001	0
910- 19:	transcript:KXG29817	transcript:Zm00001d053575_T005	0

```
910- 20: transcript:EES06526           transcript:Zm00001d053572_T002 2.00E-156
910- 21: transcript:EES06527           transcript:Zm00001d053569_T001         0
910- 22: transcript:OQU84657           transcript:Zm00001d053568_T001  2.00E-77
910- 23: transcript:EES04776           transcript:Zm00001d053566_T002 3.00E-173
910- 24: transcript:KXG29832           transcript:Zm00001d053565_T014         0
910- 25: transcript:EES04777           transcript:Zm00001d053564_T020         0
910- 26: transcript:EES04779           transcript:Zm00001d053563_T002         0
910- 27: transcript:EES06530           transcript:Zm00001d053562_T001 1.00E-132
910- 28: transcript:KXG29840           transcript:Zm00001d053561_T001         0
910- 29: transcript:KXG29853           transcript:Zm00001d053559_T005         0
910- 30: transcript:OQU84673           transcript:Zm00001d053556_T004  2.00E-46
910- 31: transcript:EES04794           transcript:Zm00001d053555_T001         0
## Alignment 911: score=1328.0 e_value=7.9e-103 N=30 4&4 minus
911-  0: transcript:EES05908           transcript:Zm00001d052072_T003         0
911-  1: transcript:KXG31235           transcript:Zm00001d052070_T032         0
911-  2: transcript:EES05913           transcript:Zm00001d052069_T001         0
911-  3: transcript:EES05917           transcript:Zm00001d052068_T001  7.00E-27
911-  4: transcript:OQU85839           transcript:Zm00001d052067_T004         0
911-  5: transcript:OQU85840           transcript:Zm00001d052066_T001         0
911-  6: transcript:KXG31244           transcript:Zm00001d052064_T001         0
911-  7: transcript:EES07655           transcript:Zm00001d052063_T003         0
911-  8: transcript:EES07659           transcript:Zm00001d052062_T002  3.00E-50
911-  9: transcript:EES05934           transcript:Zm00001d052061_T001         0
911- 10: transcript:EES05935           transcript:Zm00001d052060_T005         0
911- 11: transcript:EES07660           transcript:Zm00001d052059_T001         0
911- 12: transcript:EES07661           transcript:Zm00001d052058_T001 2.00E-162
911- 13: transcript:EES05937           transcript:Zm00001d052057_T013         0
911- 14: transcript:EES05938           transcript:Zm00001d052056_T005         0
911- 15: transcript:EES05939           transcript:Zm00001d052054_T001  1.00E-42
911- 16: transcript:EES05936           transcript:Zm00001d052051_T001         0
911- 17: transcript:OQU85861           transcript:Zm00001d052050_T001         0
911- 18: transcript:EES07666           transcript:Zm00001d052049_T002  9.00E-83
911- 19: transcript:KXG31260           transcript:Zm00001d052047_T001         0
911- 20: transcript:EES07669           transcript:Zm00001d052044_T002         0
911- 21: transcript:KXG31264           transcript:Zm00001d052043_T003         0
911- 22: transcript:EES07673           transcript:Zm00001d052042_T001         0
911- 23: transcript:EES05949           transcript:Zm00001d052041_T001 2.00E-170
911- 24: transcript:OQU85875           transcript:Zm00001d052040_T001  4.00E-30
911- 25: transcript:OQU85879           transcript:Zm00001d052039_T001 5.00E-130
911- 26: transcript:EES05953           transcript:Zm00001d052038_T001 3.00E-123
911- 27: transcript:EES07679           transcript:Zm00001d052036_T001  5.00E-47
911- 28: transcript:EES05957           transcript:Zm00001d052031_T001         0
911- 29: transcript:EES07683           transcript:Zm00001d052030_T001         0
## Alignment 912: score=1231.0 e_value=9e-84 N=26 4&4 minus
912-  0: transcript:OQU85775           transcript:Zm00001d052189_T001         0
912-  1: transcript:EES05842           transcript:Zm00001d052188_T001         0
912-  2: transcript:EES07559           transcript:Zm00001d052186_T001         0
912-  3: transcript:EES07560           transcript:Zm00001d052185_T001         0
912-  4: transcript:KXG31147           transcript:Zm00001d052184_T001 4.00E-108
912-  5: transcript:EES05876           transcript:Zm00001d052183_T001 1.00E-169
912-  6: transcript:KXG31153           transcript:Zm00001d052182_T001 2.00E-130
912-  7: transcript:KXG31156           transcript:Zm00001d052180_T001 2.00E-152
912-  8: transcript:EES07566           transcript:Zm00001d052179_T001         0
912-  9: transcript:EES07567           transcript:Zm00001d052177_T001  5.00E-68
```

912- 10: transcript:KXG31161 transcript:Zm00001d052176_T001 2.00E-20
912- 11: transcript:EES05848 transcript:Zm00001d052174_T001 8.00E-99
912- 12: transcript:OQU85783 transcript:Zm00001d052173_T001 8.00E-88
912- 13: transcript:KXG31165 transcript:Zm00001d052172_T001 7.00E-63
912- 14: transcript:EES05849 transcript:Zm00001d052171_T001 7.00E-116
912- 15: transcript:OQU85785 transcript:Zm00001d052170_T001 0
912- 16: transcript:KXG31168 transcript:Zm00001d052167_T001 9.00E-82
912- 17: transcript:EES07578 transcript:Zm00001d052164_T002 0
912- 18: transcript:EES07579 transcript:Zm00001d052163_T001 5.00E-151
912- 19: transcript:EES05858 transcript:Zm00001d052162_T001 5.00E-81
912- 20: transcript:KXG31170 transcript:Zm00001d052158_T001 0
912- 21: transcript:EES07582 transcript:Zm00001d052157_T005 0
912- 22: transcript:EES07586 transcript:Zm00001d052155_T001 1.00E-83
912- 23: transcript:KXG31176 transcript:Zm00001d052154_T001 2.00E-163
912- 24: transcript:EES07587 transcript:Zm00001d052153_T001 0
912- 25: transcript:EES07588 transcript:Zm00001d052152_T001 2.00E-107
Alignment 913: score=1166.0 e_value=1.8e-84 N=27 4&4 minus
913- 0: transcript:KXG29906 transcript:Zm00001d053448_T001 0
913- 1: transcript:EES06575 transcript:Zm00001d053447_T001 2.00E-104
913- 2: transcript:OQU84700 transcript:Zm00001d053446_T001 0
913- 3: transcript:KXG29909 transcript:Zm00001d053445_T001 1.00E-37
913- 4: transcript:EES04836 transcript:Zm00001d053442_T001 9.00E-129
913- 5: transcript:KXG29912 transcript:Zm00001d053438_T002 1.00E-19
913- 6: transcript:KXG29921 transcript:Zm00001d053435_T001 8.00E-23
913- 7: transcript:EES06590 transcript:Zm00001d053434_T001 9.00E-25
913- 8: transcript:KXG29926 transcript:Zm00001d053433_T001 3.00E-102
913- 9: transcript:EES04848 transcript:Zm00001d053429_T001 4.00E-144
913- 10: transcript:OQU84708 transcript:Zm00001d053427_T004 0
913- 11: transcript:EES04851 transcript:Zm00001d053425_T001 0
913- 12: transcript:EES06596 transcript:Zm00001d053424_T001 0
913- 13: transcript:EES04854 transcript:Zm00001d053416_T001 4.00E-119
913- 14: transcript:OQU84716 transcript:Zm00001d053415_T001 6.00E-33
913- 15: transcript:KXG29936 transcript:Zm00001d053412_T001 0
913- 16: transcript:KXG29938 transcript:Zm00001d053410_T001 4.00E-22
913- 17: transcript:EES04858 transcript:Zm00001d053409_T001 8.00E-96
913- 18: transcript:EES06601 transcript:Zm00001d053406_T001 2.00E-49
913- 19: transcript:EES06602 transcript:Zm00001d053404_T001 0
913- 20: transcript:KXG29948 transcript:Zm00001d053401_T014 0
913- 21: transcript:EES04862 transcript:Zm00001d053399_T001 0
913- 22: transcript:EES04863 transcript:Zm00001d053397_T001 5.00E-179
913- 23: transcript:EES04864 transcript:Zm00001d053396_T001 5.00E-135
913- 24: transcript:EES04867 transcript:Zm00001d053395_T001 0
913- 25: transcript:KXG29951 transcript:Zm00001d053394_T001 3.00E-87
913- 26: transcript:OQU84731 transcript:Zm00001d053393_T002 0
Alignment 914: score=1135.0 e_value=3.2e-79 N=25 4&4 minus
914- 0: transcript:KXG31179 transcript:Zm00001d052149_T015 0
914- 1: transcript:EES05864 transcript:Zm00001d052148_T001 2.00E-55
914- 2: transcript:OQU85800 transcript:Zm00001d052147_T001 0
914- 3: transcript:EES07597 transcript:Zm00001d052146_T001 0
914- 4: transcript:EES07598 transcript:Zm00001d052145_T001 0
914- 5: transcript:EES05867 transcript:Zm00001d052144_T001 0
914- 6: transcript:OQU85801 transcript:Zm00001d052143_T009 0
914- 7: transcript:EES07601 transcript:Zm00001d052139_T001 0
914- 8: transcript:KXG31183 transcript:Zm00001d052138_T004 0

914- 9: transcript:EES07604 transcript:Zm00001d052137_T001 1.00E-140
914- 10: transcript:EES05870 transcript:Zm00001d052136_T001 0
914- 11: transcript:EES05874 transcript:Zm00001d052133_T003 0
914- 12: transcript:EES05875 transcript:Zm00001d052131_T001 2.00E-75
914- 13: transcript:KXG31190 transcript:Zm00001d052130_T001 1.00E-56
914- 14: transcript:KXG31196 transcript:Zm00001d052125_T001 1.00E-73
914- 15: transcript:EES07614 transcript:Zm00001d052124_T002 0
914- 16: transcript:KXG31198 transcript:Zm00001d052123_T001 5.00E-102
914- 17: transcript:EES07615 transcript:Zm00001d052122_T001 2.00E-49
914- 18: transcript:KXG31200 transcript:Zm00001d052120_T001 2.00E-95
914- 19: transcript:EES07621 transcript:Zm00001d052118_T001 1.00E-98
914- 20: transcript:KXG31202 transcript:Zm00001d052117_T001 8.00E-24
914- 21: transcript:EES07625 transcript:Zm00001d052113_T002 0
914- 22: transcript:EES07627 transcript:Zm00001d052112_T001 0
914- 23: transcript:EES07628 transcript:Zm00001d052111_T007 0
914- 24: transcript:EES05882 transcript:Zm00001d052110_T044 0
Alignment 915: score=879.0 e_value=4.6e-57 N=20 4&4 minus
915- 0: transcript:EES07697 transcript:Zm00001d052010_T001 7.00E-117
915- 1: transcript:EES05969 transcript:Zm00001d052009_T002 9.00E-167
915- 2: transcript:KXG31293 transcript:Zm00001d052008_T003 0
915- 3: transcript:EES07702 transcript:Zm00001d052007_T001 1.00E-89
915- 4: transcript:EES07706 transcript:Zm00001d052003_T002 7.00E-36
915- 5: transcript:EES05975 transcript:Zm00001d052002_T001 3.00E-69
915- 6: transcript:KXG31305 transcript:Zm00001d052001_T002 0
915- 7: transcript:EES07710 transcript:Zm00001d051998_T003 0
915- 8: transcript:EES05978 transcript:Zm00001d051995_T001 0
915- 9: transcript:KXG31311 transcript:Zm00001d051990_T001 0
915- 10: transcript:OQU85910 transcript:Zm00001d051988_T001 0
915- 11: transcript:OQU85913 transcript:Zm00001d051987_T001 0
915- 12: transcript:EES07716 transcript:Zm00001d051986_T001 1.00E-64
915- 13: transcript:EES05983 transcript:Zm00001d051981_T005 0
915- 14: transcript:EES07720 transcript:Zm00001d051980_T004 0
915- 15: transcript:OQU85917 transcript:Zm00001d051977_T001 0
915- 16: transcript:EES06023 transcript:Zm00001d051976_T002 0
915- 17: transcript:KXG31320 transcript:Zm00001d051967_T001 0
915- 18: transcript:EES05986 transcript:Zm00001d051966_T001 1.00E-98
915- 19: transcript:EES07724 transcript:Zm00001d051965_T017 0
Alignment 916: score=874.0 e_value=9.1e-61 N=21 4&4 minus
916- 0: transcript:KXG29638 transcript:Zm00001d053728_T001 2.00E-13
916- 1: transcript:EES06395 transcript:Zm00001d053727_T003 0
916- 2: transcript:EES06399 transcript:Zm00001d053726_T001 0
916- 3: transcript:EES06400 transcript:Zm00001d053725_T002 0
916- 4: transcript:EES04658 transcript:Zm00001d053724_T001 8.00E-106
916- 5: transcript:OQU84518 transcript:Zm00001d053722_T001 3.00E-159
916- 6: transcript:OQU84522 transcript:Zm00001d053719_T002 0
916- 7: transcript:EES04660 transcript:Zm00001d053718_T001 0
916- 8: transcript:EES04667 transcript:Zm00001d053716_T001 5.00E-151
916- 9: transcript:OQU84527 transcript:Zm00001d053715_T001 0
916- 10: transcript:KXG29659 transcript:Zm00001d053714_T001 3.00E-19
916- 11: transcript:KXG29660 transcript:Zm00001d053713_T001 6.00E-81
916- 12: transcript:EES06417 transcript:Zm00001d053709_T001 3.00E-74
916- 13: transcript:EES06418 transcript:Zm00001d053707_T001 4.00E-72
916- 14: transcript:KXG29671 transcript:Zm00001d053706_T002 0
916- 15: transcript:EES06425 transcript:Zm00001d053705_T001 5.00E-107

```
916- 16: transcript:OQU84547           transcript:Zm00001d053704_T001 8.00E-146
916- 17: transcript:EES06428           transcript:Zm00001d053703_T001  8.00E-34
916- 18: transcript:EES06429           transcript:Zm00001d053702_T006         0
916- 19: transcript:KXG29681           transcript:Zm00001d053699_T001  5.00E-23
916- 20: transcript:EES06432           transcript:Zm00001d053698_T004 8.00E-113
## Alignment 917: score=832.0 e_value=5.3e-56 N=19 4&4 minus
917-  0: transcript:EES04674           transcript:Zm00001d053697_T007         0
917-  1: transcript:OQU84552           transcript:Zm00001d053696_T003         0
917-  2: transcript:EES04677           transcript:Zm00001d053695_T001         0
917-  3: transcript:EES04683           transcript:Zm00001d053694_T001  1.00E-37
917-  4: transcript:OQU84559           transcript:Zm00001d053689_T001  4.00E-35
917-  5: transcript:KXG29692           transcript:Zm00001d053688_T002         0
917-  6: transcript:EES04686           transcript:Zm00001d053687_T001         0
917-  7: transcript:EES04687           transcript:Zm00001d053686_T001         0
917-  8: transcript:KXG29696           transcript:Zm00001d053684_T004         0
917-  9: transcript:EES04688           transcript:Zm00001d053682_T001  4.00E-83
917- 10: transcript:EES06447           transcript:Zm00001d053680_T001         0
917- 11: transcript:KXG29699           transcript:Zm00001d053676_T002         0
917- 12: transcript:KXG29700           transcript:Zm00001d053675_T001         0
917- 13: transcript:EES06449           transcript:Zm00001d053674_T001         0
917- 14: transcript:EES04692           transcript:Zm00001d053672_T007         0
917- 15: transcript:KXG29703           transcript:Zm00001d053671_T001  2.00E-95
917- 16: transcript:EES04693           transcript:Zm00001d053667_T001         0
917- 17: transcript:OQU84569           transcript:Zm00001d053665_T002 2.00E-179
917- 18: transcript:EES06452           transcript:Zm00001d053664_T001         0
## Alignment 918: score=744.0 e_value=1.5e-39 N=16 4&4 minus
918-  0: transcript:EES07415           transcript:Zm00001d051535_T001         0
918-  1: transcript:OQU85568           transcript:Zm00001d051534_T001         0
918-  2: transcript:EES05679           transcript:Zm00001d051532_T002         0
918-  3: transcript:EES05680           transcript:Zm00001d051529_T001         0
918-  4: transcript:KXG30955           transcript:Zm00001d051528_T001         0
918-  5: transcript:KXG30956           transcript:Zm00001d051527_T001 2.00E-158
918-  6: transcript:EES07417           transcript:Zm00001d051526_T002 7.00E-137
918-  7: transcript:EES05682           transcript:Zm00001d051525_T001         0
918-  8: transcript:OQU85574           transcript:Zm00001d051524_T001         0
918-  9: transcript:EES05685           transcript:Zm00001d051520_T001 3.00E-171
918- 10: transcript:KXG30963           transcript:Zm00001d051518_T001         0
918- 11: transcript:EES05734           transcript:Zm00001d051516_T001         0
918- 12: transcript:EES05688           transcript:Zm00001d051514_T002         0
918- 13: transcript:OQU85579           transcript:Zm00001d051513_T001         0
918- 14: transcript:KXG30975           transcript:Zm00001d051511_T001 2.00E-146
918- 15: transcript:EES07423           transcript:Zm00001d051509_T001 7.00E-152
## Alignment 919: score=666.0 e_value=3e-38 N=15 4&4 minus
919-  0: transcript:EES04592           transcript:Zm00001d053801_T001         0
919-  1: transcript:OQU84444           transcript:Zm00001d053800_T001 5.00E-100
919-  2: transcript:OQU84446           transcript:Zm00001d053799_T005         0
919-  3: transcript:EES04595           transcript:Zm00001d053798_T004         0
919-  4: transcript:KXG29567           transcript:Zm00001d053792_T001  5.00E-42
919-  5: transcript:EES04600           transcript:Zm00001d053786_T002 7.00E-109
919-  6: transcript:EES06348           transcript:Zm00001d053783_T001         0
919-  7: transcript:OQU84456           transcript:Zm00001d053782_T006         0
919-  8: transcript:OQU84462           transcript:Zm00001d053781_T001  7.00E-24
919-  9: transcript:OQU84463           transcript:Zm00001d053779_T001  5.00E-31
919- 10: transcript:EES06351           transcript:Zm00001d053778_T001         0
```

919- 11: transcript:KXG29578 transcript:Zm00001d053776_T015 0
919- 12: transcript:EES04609 transcript:Zm00001d053775_T001 0
919- 13: transcript:EES06353 transcript:Zm00001d053770_T001 0
919- 14: transcript:EES04611 transcript:Zm00001d053769_T001 0
Alignment 920: score=593.0 e_value=6e-34 N=13 4&4 minus
920- 0: transcript:EES07686 transcript:Zm00001d052028_T007 0
920- 1: transcript:OQU85887 transcript:Zm00001d052027_T001 4.00E-84
920- 2: transcript:OQU85888 transcript:Zm00001d052026_T001 1.00E-80
920- 3: transcript:EES05958 transcript:Zm00001d052025_T001 1.00E-30
920- 4: transcript:EES05959 transcript:Zm00001d052022_T002 0
920- 5: transcript:EES07689 transcript:Zm00001d052021_T002 0
920- 6: transcript:EES05961 transcript:Zm00001d052020_T001 0
920- 7: transcript:KXG31281 transcript:Zm00001d052019_T001 0
920- 8: transcript:EES05962 transcript:Zm00001d052018_T001 6.00E-174
920- 9: transcript:KXG31285 transcript:Zm00001d052017_T001 9.00E-26
920- 10: transcript:EES05965 transcript:Zm00001d052016_T001 3.00E-32
920- 11: transcript:KXG31286 transcript:Zm00001d052015_T001 8.00E-54
920- 12: transcript:EES05967 transcript:Zm00001d052011_T003 3.00E-123
Alignment 921: score=579.0 e_value=3.5e-30 N=13 4&4 minus
921- 0: transcript:KXG29710 transcript:Zm00001d053663_T009 0
921- 1: transcript:EES06456 transcript:Zm00001d053661_T003 2.00E-33
921- 2: transcript:EES06457 transcript:Zm00001d053660_T001 0
921- 3: transcript:EES04701 transcript:Zm00001d053659_T001 4.00E-149
921- 4: transcript:EES04704 transcript:Zm00001d053658_T002 0
921- 5: transcript:OQU84580 transcript:Zm00001d053655_T001 1.00E-55
921- 6: transcript:OQU84582 transcript:Zm00001d053654_T001 0
921- 7: transcript:KXG29731 transcript:Zm00001d053652_T001 3.00E-111
921- 8: transcript:EES06466 transcript:Zm00001d053650_T001 0
921- 9: transcript:EES06471 transcript:Zm00001d053649_T002 0
921- 10: transcript:EES04709 transcript:Zm00001d053648_T005 6.00E-179
921- 11: transcript:EES04710 transcript:Zm00001d053643_T001 0
921- 12: transcript:EES06473 transcript:Zm00001d053642_T001 0
Alignment 922: score=541.0 e_value=1.3e-30 N=13 4&4 minus
922- 0: transcript:EES05888 transcript:Zm00001d052108_T003 2.00E-137
922- 1: transcript:EES05889 transcript:Zm00001d052107_T001 0
922- 2: transcript:EES05891 transcript:Zm00001d052106_T001 1.00E-99
922- 3: transcript:EES07654 transcript:Zm00001d052104_T001 0
922- 4: transcript:EES05901 transcript:Zm00001d052103_T001 0
922- 5: transcript:KXG31222 transcript:Zm00001d052102_T001 9.00E-131
922- 6: transcript:EES07636 transcript:Zm00001d052101_T001 3.00E-166
922- 7: transcript:EES07640 transcript:Zm00001d052098_T011 0
922- 8: transcript:EES07641 transcript:Zm00001d052093_T002 1.00E-150
922- 9: transcript:EES07642 transcript:Zm00001d052087_T001 0
922- 10: transcript:EES05907 transcript:Zm00001d052082_T001 0
922- 11: transcript:EES07645 transcript:Zm00001d052081_T002 0
922- 12: transcript:KXG31271 transcript:Zm00001d052079_T039 0
Alignment 923: score=448.0 e_value=7.7e-23 N=10 4&4 minus
923- 0: transcript:EES06475 transcript:Zm00001d053641_T001 2.00E-110
923- 1: transcript:KXG29743 transcript:Zm00001d053639_T001 1.00E-41
923- 2: transcript:EES04714 transcript:Zm00001d053628_T003 0
923- 3: transcript:OQU84590 transcript:Zm00001d053627_T001 0
923- 4: transcript:EES04719 transcript:Zm00001d053626_T001 2.00E-153
923- 5: transcript:EES06481 transcript:Zm00001d053625_T001 9.00E-68
923- 6: transcript:EES06482 transcript:Zm00001d053624_T001 0

```
923-  7: transcript:OQU84598            transcript:Zm00001d053623_T001          0
923-  8: transcript:KXG29751            transcript:Zm00001d053622_T003 6.00E-110
923-  9: transcript:KXG29753            transcript:Zm00001d053620_T043 3.00E-142
## Alignment 924: score=446.0 e_value=1.2e-21 N=10 4&4 minus
924-  0: transcript:EES04615            transcript:Zm00001d053765_T001          0
924-  1: transcript:EES04616            transcript:Zm00001d053763_T001          0
924-  2: transcript:EES06357            transcript:Zm00001d053762_T001          0
924-  3: transcript:OQU84478            transcript:Zm00001d053761_T001          0
924-  4: transcript:EES06360            transcript:Zm00001d053759_T001  2.00E-66
924-  5: transcript:KXG29601            transcript:Zm00001d053757_T002          0
924-  6: transcript:OQU84482            transcript:Zm00001d053756_T001 3.00E-118
924-  7: transcript:EES04622            transcript:Zm00001d053755_T001 1.00E-148
924-  8: transcript:EES06368            transcript:Zm00001d053754_T001  2.00E-56
924-  9: transcript:EES06372            transcript:Zm00001d053753_T003          0
## Alignment 925: score=417.0 e_value=2.5e-19 N=9 4&4 minus
925-  0: transcript:EES05596            transcript:Zm00001d051706_T001  9.00E-58
925-  1: transcript:OQU85481            transcript:Zm00001d051704_T002          0
925-  2: transcript:KXG30838            transcript:Zm00001d051703_T001          0
925-  3: transcript:EES07330            transcript:Zm00001d051702_T002 2.00E-140
925-  4: transcript:KXG30845            transcript:Zm00001d051700_T001  1.00E-82
925-  5: transcript:EES05601            transcript:Zm00001d051699_T001          0
925-  6: transcript:EES05602            transcript:Zm00001d051697_T001  3.00E-26
925-  7: transcript:KXG30849            transcript:Zm00001d051696_T001  7.00E-62
925-  8: transcript:EES07335            transcript:Zm00001d051692_T001 3.00E-125
## Alignment 926: score=386.0 e_value=6.4e-20 N=9 4&4 minus
926-  0: transcript:KXG29245            transcript:Zm00001d054104_T002  5.00E-27
926-  1: transcript:KXG29246            transcript:Zm00001d054095_T001 4.00E-174
926-  2: transcript:KXG29248            transcript:Zm00001d054094_T001 5.00E-149
926-  3: transcript:KXG29249            transcript:Zm00001d054089_T002          0
926-  4: transcript:EES06100            transcript:Zm00001d054086_T001  3.00E-09
926-  5: transcript:OQU84143            transcript:Zm00001d054084_T001  1.00E-95
926-  6: transcript:EES06104            transcript:Zm00001d054080_T001          0
926-  7: transcript:EES04324            transcript:Zm00001d054079_T002          0
926-  8: transcript:EES04326            transcript:Zm00001d054077_T003 5.00E-118
## Alignment 927: score=349.0 e_value=2.4e-15 N=8 4&4 minus
927-  0: transcript:EES07342            transcript:Zm00001d051684_T004          0
927-  1: transcript:OQU85499            transcript:Zm00001d051682_T001 3.00E-139
927-  2: transcript:EES07343            transcript:Zm00001d051681_T001          0
927-  3: transcript:EES05610            transcript:Zm00001d051678_T001          0
927-  4: transcript:EES07344            transcript:Zm00001d051677_T003          0
927-  5: transcript:EES05611            transcript:Zm00001d051676_T007          0
927-  6: transcript:KXG30863            transcript:Zm00001d051675_T001          0
927-  7: transcript:KXG30865            transcript:Zm00001d051674_T037          0
## Alignment 928: score=323.0 e_value=4.3e-11 N=7 4&4 minus
928-  0: transcript:EES05617            transcript:Zm00001d051692_T001 2.00E-132
928-  1: transcript:EES07354            transcript:Zm00001d051691_T001 6.00E-142
928-  2: transcript:KXG30873            transcript:Zm00001d051690_T001          0
928-  3: transcript:EES07356            transcript:Zm00001d051689_T001 4.00E-102
928-  4: transcript:EES07357            transcript:Zm00001d051688_T002          0
928-  5: transcript:OQU85511            transcript:Zm00001d051687_T001  1.00E-87
928-  6: transcript:KXG30875            transcript:Zm00001d051685_T001          0
## Alignment 929: score=255.0 e_value=4.7e-08 N=6 4&4 minus
929-  0: transcript:EES06570            transcript:Zm00001d053518_T001          0
929-  1: transcript:EES04829            transcript:Zm00001d053515_T002          0
```

929- 2: transcript:KXG29899 transcript:Zm00001d053513_T001 0
929- 3: transcript:EES04831 transcript:Zm00001d053453_T002 0
929- 4: transcript:KXG29904 transcript:Zm00001d053452_T002 5.00E-149
929- 5: transcript:OQU84698 transcript:Zm00001d053450_T082 0
Alignment 930: score=5592.0 e_value=0 N=123 4&5 plus
930- 0: transcript:EES06956 transcript:Zm00001d016822_T012 0
930- 1: transcript:EES06961 transcript:Zm00001d016824_T001 0
930- 2: transcript:KXG30418 transcript:Zm00001d016825_T008 3.00E-114
930- 3: transcript:EES06963 transcript:Zm00001d016826_T001 3.00E-107
930- 4: transcript:EES05242 transcript:Zm00001d016827_T001 0
930- 5: transcript:EES05244 transcript:Zm00001d016832_T001 0
930- 6: transcript:EES06965 transcript:Zm00001d016834_T001 2.00E-159
930- 7: transcript:KXG30422 transcript:Zm00001d016835_T002 0
930- 8: transcript:OQU85120 transcript:Zm00001d016836_T002 0
930- 9: transcript:EES05250 transcript:Zm00001d016837_T001 2.00E-133
930- 10: transcript:EES06968 transcript:Zm00001d016838_T001 0
930- 11: transcript:EES06981 transcript:Zm00001d016851_T001 1.00E-164
930- 12: transcript:OQU85147 transcript:Zm00001d016856_T009 0
930- 13: transcript:EES05269 transcript:Zm00001d016857_T001 2.00E-17
930- 14: transcript:KXG30444 transcript:Zm00001d016858_T001 0
930- 15: transcript:EES06996 transcript:Zm00001d016860_T001 3.00E-131
930- 16: transcript:EES05274 transcript:Zm00001d016861_T003 1.00E-157
930- 17: transcript:EES06997 transcript:Zm00001d016863_T001 0
930- 18: transcript:EES05275 transcript:Zm00001d016864_T001 1.00E-40
930- 19: transcript:KXG30452 transcript:Zm00001d016865_T001 0
930- 20: transcript:KXG30456 transcript:Zm00001d016866_T001 0
930- 21: transcript:EES07000 transcript:Zm00001d016867_T001 1.00E-144
930- 22: transcript:KXG30462 transcript:Zm00001d016873_T001 0
930- 23: transcript:KXG30464 transcript:Zm00001d016876_T001 0
930- 24: transcript:KXG30467 transcript:Zm00001d016878_T001 0
930- 25: transcript:EES07005 transcript:Zm00001d016879_T001 0
930- 26: transcript:EES05283 transcript:Zm00001d016882_T001 1.00E-64
930- 27: transcript:KXG30470 transcript:Zm00001d016884_T002 0
930- 28: transcript:EES05287 transcript:Zm00001d016890_T001 0
930- 29: transcript:OQU85173 transcript:Zm00001d016891_T001 0
930- 30: transcript:OQU85174 transcript:Zm00001d016892_T001 8.00E-81
930- 31: transcript:OQU85175 transcript:Zm00001d016893_T001 4.00E-101
930- 32: transcript:EES07012 transcript:Zm00001d016894_T005 0
930- 33: transcript:EES05288 transcript:Zm00001d016895_T001 0
930- 34: transcript:KXG30473 transcript:Zm00001d016896_T001 0
930- 35: transcript:EES05289 transcript:Zm00001d016897_T002 0
930- 36: transcript:EES07014 transcript:Zm00001d016898_T001 0
930- 37: transcript:EES05291 transcript:Zm00001d016899_T001 2.00E-18
930- 38: transcript:EES05293 transcript:Zm00001d016901_T001 5.00E-40
930- 39: transcript:EES07020 transcript:Zm00001d016902_T002 0
930- 40: transcript:KXG30522 transcript:Zm00001d016908_T001 9.00E-92
930- 41: transcript:KXG30480 transcript:Zm00001d016909_T001 5.00E-168
930- 42: transcript:OQU85186 transcript:Zm00001d016911_T001 0
930- 43: transcript:KXG30485 transcript:Zm00001d016915_T002 0
930- 44: transcript:OQU85189 transcript:Zm00001d016916_T004 0
930- 45: transcript:OQU85190 transcript:Zm00001d016918_T001 0
930- 46: transcript:OQU85192 transcript:Zm00001d016919_T001 0
930- 47: transcript:OQU85191 transcript:Zm00001d016922_T009 0
930- 48: transcript:EES05301 transcript:Zm00001d016924_T001 0

930- 49:	transcript:EES05306	transcript:Zm00001d016926_T001	0
930- 50:	transcript:EES07030	transcript:Zm00001d016928_T005	0
930- 51:	transcript:EES05307	transcript:Zm00001d016933_T001	0
930- 52:	transcript:EES05308	transcript:Zm00001d016934_T001	0
930- 53:	transcript:EES07034	transcript:Zm00001d016938_T001	0
930- 54:	transcript:KXG30492	transcript:Zm00001d016941_T003	1.00E-180
930- 55:	transcript:EES05311	transcript:Zm00001d016942_T001	0
930- 56:	transcript:EES07037	transcript:Zm00001d016943_T001	2.00E-98
930- 57:	transcript:EES05313	transcript:Zm00001d016946_T001	5.00E-33
930- 58:	transcript:EES05314	transcript:Zm00001d016947_T001	4.00E-10
930- 59:	transcript:EES05316	transcript:Zm00001d016950_T002	5.00E-168
930- 60:	transcript:OQU85201	transcript:Zm00001d016952_T001	3.00E-169
930- 61:	transcript:OQU85202	transcript:Zm00001d016957_T001	3.00E-170
930- 62:	transcript:OQU85205	transcript:Zm00001d016972_T001	0
930- 63:	transcript:OQU85206	transcript:Zm00001d016973_T001	1.00E-86
930- 64:	transcript:KXG30502	transcript:Zm00001d016974_T001	1.00E-134
930- 65:	transcript:EES07042	transcript:Zm00001d016977_T002	3.00E-95
930- 66:	transcript:EES07043	transcript:Zm00001d016979_T001	0
930- 67:	transcript:OQU85212	transcript:Zm00001d016980_T001	0
930- 68:	transcript:EES05323	transcript:Zm00001d016982_T001	0
930- 69:	transcript:EES07044	transcript:Zm00001d016987_T001	0
930- 70:	transcript:KXG30504	transcript:Zm00001d016988_T001	4.00E-92
930- 71:	transcript:EES05322	transcript:Zm00001d016990_T007	0
930- 72:	transcript:KXG30508	transcript:Zm00001d016991_T001	4.00E-26
930- 73:	transcript:EES05328	transcript:Zm00001d016992_T001	3.00E-35
930- 74:	transcript:EES07045	transcript:Zm00001d016993_T007	0
930- 75:	transcript:OQU85218	transcript:Zm00001d016995_T001	0
930- 76:	transcript:KXG30510	transcript:Zm00001d016997_T001	8.00E-41
930- 77:	transcript:EES07046	transcript:Zm00001d016998_T001	0
930- 78:	transcript:OQU85219	transcript:Zm00001d016999_T002	3.00E-72
930- 79:	transcript:EES07050	transcript:Zm00001d017000_T001	2.00E-152
930- 80:	transcript:KXG30516	transcript:Zm00001d017001_T001	2.00E-29
930- 81:	transcript:EES07051	transcript:Zm00001d017008_T001	4.00E-56
930- 82:	transcript:KXG30520	transcript:Zm00001d017009_T001	2.00E-46
930- 83:	transcript:EES07057	transcript:Zm00001d017021_T001	4.00E-75
930- 84:	transcript:OQU85223	transcript:Zm00001d017025_T001	0
930- 85:	transcript:EES05332	transcript:Zm00001d017026_T003	3.00E-152
930- 86:	transcript:EES05334	transcript:Zm00001d017028_T001	0
930- 87:	transcript:EES05335	transcript:Zm00001d017029_T004	0
930- 88:	transcript:EES05337	transcript:Zm00001d017030_T001	0
930- 89:	transcript:EES05338	transcript:Zm00001d017031_T001	1.00E-24
930- 90:	transcript:OQU85227	transcript:Zm00001d017032_T001	4.00E-29
930- 91:	transcript:EES07062	transcript:Zm00001d017034_T007	0
930- 92:	transcript:EES05342	transcript:Zm00001d017036_T002	0
930- 93:	transcript:EES05341	transcript:Zm00001d017037_T001	2.00E-74
930- 94:	transcript:EES07065	transcript:Zm00001d017041_T001	3.00E-26
930- 95:	transcript:EES07068	transcript:Zm00001d017042_T002	0
930- 96:	transcript:EES05344	transcript:Zm00001d017043_T002	6.00E-115
930- 97:	transcript:KXG30530	transcript:Zm00001d017046_T001	0
930- 98:	transcript:EES07070	transcript:Zm00001d017047_T001	6.00E-153
930- 99:	transcript:EES07071	transcript:Zm00001d017049_T001	0
930-100:	transcript:EES07073	transcript:Zm00001d017050_T002	0
930-101:	transcript:EES07075	transcript:Zm00001d017053_T003	0
930-102:	transcript:EES05347	transcript:Zm00001d017060_T001	3.00E-70

930-103: transcript:EES07079 transcript:Zm00001d017061_T001 3.00E-46
930-104: transcript:EES07080 transcript:Zm00001d017065_T001 1.00E-46
930-105: transcript:EES07081 transcript:Zm00001d017067_T001 0
930-106: transcript:EES05349 transcript:Zm00001d017069_T001 0
930-107: transcript:OQU85237 transcript:Zm00001d017070_T001 5.00E-63
930-108: transcript:EES05350 transcript:Zm00001d017071_T002 0
930-109: transcript:EES05348 transcript:Zm00001d017072_T002 0
930-110: transcript:EES05356 transcript:Zm00001d017077_T001 0
930-111: transcript:OQU85242 transcript:Zm00001d017078_T002 0
930-112: transcript:EES05363 transcript:Zm00001d017079_T001 0
930-113: transcript:KXG30609 transcript:Zm00001d017084_T001 0
930-114: transcript:KXG30544 transcript:Zm00001d017085_T002 0
930-115: transcript:KXG30545 transcript:Zm00001d017086_T001 6.00E-42
930-116: transcript:KXG30547 transcript:Zm00001d017089_T001 2.00E-74
930-117: transcript:EES07092 transcript:Zm00001d017090_T001 1.00E-127
930-118: transcript:EES07093 transcript:Zm00001d017091_T001 0
930-119: transcript:EES05366 transcript:Zm00001d017092_T001 0
930-120: transcript:EES05367 transcript:Zm00001d017094_T001 0
930-121: transcript:EES07094 transcript:Zm00001d017095_T001 5.00E-108
930-122: transcript:KXG30548 transcript:Zm00001d017096_T005 0
Alignment 931: score=3551.0 e_value=0 N=79 4&5 plus
931- 0: transcript:EES06208 transcript:Zm00001d015084_T001 0
931- 1: transcript:EES04420 transcript:Zm00001d015085_T002 3.00E-77
931- 2: transcript:KXG29349 transcript:Zm00001d015086_T003 0
931- 3: transcript:EES06213 transcript:Zm00001d015088_T002 0
931- 4: transcript:KXG29351 transcript:Zm00001d015090_T001 3.00E-138
931- 5: transcript:EES04425 transcript:Zm00001d015091_T003 3.00E-143
931- 6: transcript:OQU84271 transcript:Zm00001d015092_T001 2.00E-103
931- 7: transcript:KXG29352 transcript:Zm00001d015093_T001 0
931- 8: transcript:OQU84276 transcript:Zm00001d015094_T002 0
931- 9: transcript:EES04429 transcript:Zm00001d015095_T002 1.00E-138
931- 10: transcript:KXG29356 transcript:Zm00001d015096_T002 0
931- 11: transcript:KXG29361 transcript:Zm00001d015100_T002 0
931- 12: transcript:EES04430 transcript:Zm00001d015101_T001 0
931- 13: transcript:EES04432 transcript:Zm00001d015102_T001 2.00E-167
931- 14: transcript:OQU84280 transcript:Zm00001d015103_T003 5.00E-98
931- 15: transcript:EES04433 transcript:Zm00001d015114_T001 0
931- 16: transcript:EES06216 transcript:Zm00001d015115_T002 0
931- 17: transcript:EES04438 transcript:Zm00001d015116_T001 8.00E-73
931- 18: transcript:EES04440 transcript:Zm00001d015118_T001 2.00E-166
931- 19: transcript:EES06219 transcript:Zm00001d015121_T001 2.00E-141
931- 20: transcript:OQU84289 transcript:Zm00001d015124_T001 0
931- 21: transcript:EES06222 transcript:Zm00001d015125_T001 6.00E-152
931- 22: transcript:EES06224 transcript:Zm00001d015126_T001 4.00E-32
931- 23: transcript:EES06225 transcript:Zm00001d015127_T001 3.00E-52
931- 24: transcript:EES04449 transcript:Zm00001d015129_T001 0
931- 25: transcript:KXG29382 transcript:Zm00001d015130_T001 0
931- 26: transcript:KXG29383 transcript:Zm00001d015131_T003 2.00E-172
931- 27: transcript:KXG29386 transcript:Zm00001d015132_T001 2.00E-50
931- 28: transcript:KXG29392 transcript:Zm00001d015133_T001 0
931- 29: transcript:EES06231 transcript:Zm00001d015134_T001 1.00E-165
931- 30: transcript:KXG29394 transcript:Zm00001d015135_T002 0
931- 31: transcript:OQU84303 transcript:Zm00001d015138_T004 0
931- 32: transcript:EES06235 transcript:Zm00001d015140_T001 1.00E-66

931- 33: transcript:KXG29398 transcript:Zm00001d015141_T001 0
931- 34: transcript:OQU84305 transcript:Zm00001d015142_T001 2.00E-33
931- 35: transcript:OQU84307 transcript:Zm00001d015148_T002 0
931- 36: transcript:KXG29401 transcript:Zm00001d015151_T004 0
931- 37: transcript:EES04459 transcript:Zm00001d015152_T001 0
931- 38: transcript:EES06238 transcript:Zm00001d015153_T001 2.00E-80
931- 39: transcript:OQU84311 transcript:Zm00001d015156_T001 0
931- 40: transcript:KXG29404 transcript:Zm00001d015163_T001 0
931- 41: transcript:EES06241 transcript:Zm00001d015164_T001 0
931- 42: transcript:KXG29407 transcript:Zm00001d015174_T010 0
931- 43: transcript:EES04466 transcript:Zm00001d015180_T001 0
931- 44: transcript:KXG29410 transcript:Zm00001d015181_T001 0
931- 45: transcript:EES04468 transcript:Zm00001d015182_T001 6.00E-122
931- 46: transcript:EES04469 transcript:Zm00001d015183_T002 0
931- 47: transcript:EES06244 transcript:Zm00001d015184_T001 4.00E-91
931- 48: transcript:EES04470 transcript:Zm00001d015186_T001 0
931- 49: transcript:EES04471 transcript:Zm00001d015194_T001 0
931- 50: transcript:EES06247 transcript:Zm00001d015195_T001 0
931- 51: transcript:EES04472 transcript:Zm00001d015196_T003 0
931- 52: transcript:EES04478 transcript:Zm00001d015197_T002 1.00E-67
931- 53: transcript:KXG29420 transcript:Zm00001d015198_T001 1.00E-160
931- 54: transcript:KXG29421 transcript:Zm00001d015200_T003 0
931- 55: transcript:EES06248 transcript:Zm00001d015201_T001 0
931- 56: transcript:KXG29423 transcript:Zm00001d015202_T001 0
931- 57: transcript:KXG29425 transcript:Zm00001d015203_T005 0
931- 58: transcript:EES06249 transcript:Zm00001d015204_T001 4.00E-168
931- 59: transcript:EES04482 transcript:Zm00001d015205_T001 0
931- 60: transcript:EES04483 transcript:Zm00001d015208_T003 0
931- 61: transcript:EES04484 transcript:Zm00001d015209_T022 0
931- 62: transcript:EES04485 transcript:Zm00001d015210_T001 0
931- 63: transcript:EES04487 transcript:Zm00001d015211_T001 1.00E-80
931- 64: transcript:EES06253 transcript:Zm00001d015212_T008 0
931- 65: transcript:KXG29435 transcript:Zm00001d015213_T001 0
931- 66: transcript:KXG29433 transcript:Zm00001d015215_T001 3.00E-64
931- 67: transcript:KXG29437 transcript:Zm00001d015216_T001 4.00E-105
931- 68: transcript:EES06255 transcript:Zm00001d015225_T001 4.00E-177
931- 69: transcript:OQU84339 transcript:Zm00001d015226_T001 5.00E-169
931- 70: transcript:EES06256 transcript:Zm00001d015227_T004 3.00E-96
931- 71: transcript:EES06257 transcript:Zm00001d015228_T004 0
931- 72: transcript:KXG29442 transcript:Zm00001d015231_T002 0
931- 73: transcript:EES04495 transcript:Zm00001d015233_T001 0
931- 74: transcript:EES06258 transcript:Zm00001d015234_T002 0
931- 75: transcript:KXG29446 transcript:Zm00001d015239_T007 0
931- 76: transcript:EES04498 transcript:Zm00001d015242_T002 3.00E-92
931- 77: transcript:OQU84350 transcript:Zm00001d015243_T001 0
931- 78: transcript:KXG29455 transcript:Zm00001d015245_T002 0
Alignment 932: score=2889.0 e_value=9.4e-254 N=63 4&5 plus
932- 0: transcript:EES07095 transcript:Zm00001d017097_T001 2.00E-144
932- 1: transcript:KXG30552 transcript:Zm00001d017099_T001 0
932- 2: transcript:EES07098 transcript:Zm00001d017100_T006 5.00E-145
932- 3: transcript:EES05370 transcript:Zm00001d017101_T001 6.00E-169
932- 4: transcript:OQU85247 transcript:Zm00001d017102_T001 1.00E-38
932- 5: transcript:EES07100 transcript:Zm00001d017103_T003 2.00E-158
932- 6: transcript:KXG30555 transcript:Zm00001d017106_T001 0

932- 7:	transcript:EES05371	transcript:Zm00001d017107_T001	2.00E-82
932- 8:	transcript:KXG30556	transcript:Zm00001d017110_T002	0
932- 9:	transcript:EES07102	transcript:Zm00001d017111_T001	0
932- 10:	transcript:OQU85252	transcript:Zm00001d017112_T001	0
932- 11:	transcript:EES05373	transcript:Zm00001d017113_T002	0
932- 12:	transcript:KXG30559	transcript:Zm00001d017114_T001	0
932- 13:	transcript:OQU85258	transcript:Zm00001d017117_T001	0
932- 14:	transcript:KXG30566	transcript:Zm00001d017119_T003	0
932- 15:	transcript:KXG30570	transcript:Zm00001d017120_T002	0
932- 16:	transcript:EES05377	transcript:Zm00001d017121_T001	0
932- 17:	transcript:OQU85259	transcript:Zm00001d017124_T001	0
932- 18:	transcript:EES05379	transcript:Zm00001d017125_T005	0
932- 19:	transcript:EES07108	transcript:Zm00001d017126_T001	0
932- 20:	transcript:EES05381	transcript:Zm00001d017128_T002	0
932- 21:	transcript:KXG30576	transcript:Zm00001d017130_T001	0
932- 22:	transcript:EES07109	transcript:Zm00001d017131_T001	0
932- 23:	transcript:EES07110	transcript:Zm00001d017134_T001	1.00E-122
932- 24:	transcript:EES07111	transcript:Zm00001d017135_T001	0
932- 25:	transcript:EES05388	transcript:Zm00001d017138_T001	3.00E-152
932- 26:	transcript:EES07113	transcript:Zm00001d017139_T003	0
932- 27:	transcript:EES05386	transcript:Zm00001d017140_T001	0
932- 28:	transcript:KXG30583	transcript:Zm00001d017141_T001	9.00E-72
932- 29:	transcript:KXG30587	transcript:Zm00001d017144_T007	0
932- 30:	transcript:KXG30589	transcript:Zm00001d017145_T003	4.00E-176
932- 31:	transcript:EES07118	transcript:Zm00001d017146_T001	3.00E-141
932- 32:	transcript:EES05393	transcript:Zm00001d017150_T001	0
932- 33:	transcript:KXG30593	transcript:Zm00001d017151_T002	4.00E-142
932- 34:	transcript:EES05394	transcript:Zm00001d017152_T001	1.00E-170
932- 35:	transcript:EES07121	transcript:Zm00001d017153_T018	0
932- 36:	transcript:EES05392	transcript:Zm00001d017157_T001	0
932- 37:	transcript:OQU85270	transcript:Zm00001d017158_T001	0
932- 38:	transcript:EES07120	transcript:Zm00001d017160_T001	1.00E-56
932- 39:	transcript:KXG30598	transcript:Zm00001d017165_T002	0
932- 40:	transcript:EES07126	transcript:Zm00001d017166_T001	0
932- 41:	transcript:KXG30604	transcript:Zm00001d017167_T001	0
932- 42:	transcript:EES05399	transcript:Zm00001d017168_T002	0
932- 43:	transcript:OQU85274	transcript:Zm00001d017169_T001	6.00E-44
932- 44:	transcript:KXG30606	transcript:Zm00001d017170_T001	0
932- 45:	transcript:EES07130	transcript:Zm00001d017171_T001	0
932- 46:	transcript:OQU85286	transcript:Zm00001d017174_T001	2.00E-84
932- 47:	transcript:EES07133	transcript:Zm00001d017175_T003	0
932- 48:	transcript:EES05406	transcript:Zm00001d017176_T001	2.00E-179
932- 49:	transcript:KXG30615	transcript:Zm00001d017177_T007	1.00E-64
932- 50:	transcript:OQU85291	transcript:Zm00001d017178_T004	1.00E-78
932- 51:	transcript:EES05407	transcript:Zm00001d017179_T001	0
932- 52:	transcript:EES07132	transcript:Zm00001d017180_T001	3.00E-07
932- 53:	transcript:EES05410	transcript:Zm00001d017185_T001	0
932- 54:	transcript:EES05411	transcript:Zm00001d017186_T001	0
932- 55:	transcript:OQU85297	transcript:Zm00001d017187_T001	4.00E-161
932- 56:	transcript:EES07139	transcript:Zm00001d017193_T002	4.00E-173
932- 57:	transcript:EES07140	transcript:Zm00001d017195_T001	0
932- 58:	transcript:EES07141	transcript:Zm00001d017197_T015	0
932- 59:	transcript:EES07143	transcript:Zm00001d017199_T001	5.00E-138
932- 60:	transcript:EES05415	transcript:Zm00001d017201_T001	0

932- 61: transcript:KXG30624 transcript:Zm00001d017202_T001 0
932- 62: transcript:KXG30626 transcript:Zm00001d017203_T021 0
Alignment 933: score=2735.0 e_value=1.6e-232 N=60 4&5 plus
933- 0: transcript:EES05864 transcript:Zm00001d018200_T001 1.00E-52
933- 1: transcript:EES07595 transcript:Zm00001d018201_T001 0
933- 2: transcript:EES05866 transcript:Zm00001d018203_T001 0
933- 3: transcript:EES05867 transcript:Zm00001d018204_T001 0
933- 4: transcript:EES07601 transcript:Zm00001d018206_T001 0
933- 5: transcript:KXG31183 transcript:Zm00001d018207_T001 0
933- 6: transcript:EES07603 transcript:Zm00001d018208_T001 6.00E-138
933- 7: transcript:EES07604 transcript:Zm00001d018209_T001 5.00E-98
933- 8: transcript:EES05870 transcript:Zm00001d018211_T001 0
933- 9: transcript:EES07606 transcript:Zm00001d018214_T001 0
933- 10: transcript:EES07602 transcript:Zm00001d018217_T003 0
933- 11: transcript:EES05872 transcript:Zm00001d018218_T001 9.00E-171
933- 12: transcript:EES05874 transcript:Zm00001d018225_T001 0
933- 13: transcript:EES05875 transcript:Zm00001d018226_T002 0
933- 14: transcript:KXG31190 transcript:Zm00001d018227_T001 1.00E-49
933- 15: transcript:EES05878 transcript:Zm00001d018229_T001 2.00E-111
933- 16: transcript:KXG31193 transcript:Zm00001d018230_T001 0
933- 17: transcript:EES05879 transcript:Zm00001d018233_T011 0
933- 18: transcript:OQU85809 transcript:Zm00001d018234_T001 8.00E-64
933- 19: transcript:KXG31196 transcript:Zm00001d018235_T001 9.00E-55
933- 20: transcript:EES07614 transcript:Zm00001d018237_T001 0
933- 21: transcript:KXG31198 transcript:Zm00001d018238_T001 2.00E-119
933- 22: transcript:EES07616 transcript:Zm00001d018241_T001 3.00E-165
933- 23: transcript:KXG31199 transcript:Zm00001d018242_T001 0
933- 24: transcript:EES07617 transcript:Zm00001d018243_T001 0
933- 25: transcript:EES07618 transcript:Zm00001d018244_T001 1.00E-63
933- 26: transcript:KXG31200 transcript:Zm00001d018246_T015 0
933- 27: transcript:EES07621 transcript:Zm00001d018247_T001 6.00E-111
933- 28: transcript:EES07622 transcript:Zm00001d018251_T001 6.00E-42
933- 29: transcript:OQU85812 transcript:Zm00001d018254_T001 9.00E-34
933- 30: transcript:OQU85815 transcript:Zm00001d018255_T001 1.00E-152
933- 31: transcript:EES07620 transcript:Zm00001d018256_T001 1.00E-115
933- 32: transcript:KXG31206 transcript:Zm00001d018258_T012 0
933- 33: transcript:EES07626 transcript:Zm00001d018259_T001 5.00E-62
933- 34: transcript:EES07627 transcript:Zm00001d018260_T001 2.00E-157
933- 35: transcript:KXG31210 transcript:Zm00001d018261_T002 0
933- 36: transcript:OQU85819 transcript:Zm00001d018262_T001 0
933- 37: transcript:KXG31251 transcript:Zm00001d018274_T003 0
933- 38: transcript:EES05887 transcript:Zm00001d018275_T001 0
933- 39: transcript:EES05888 transcript:Zm00001d018277_T002 1.00E-145
933- 40: transcript:EES05889 transcript:Zm00001d018278_T001 0
933- 41: transcript:EES05890 transcript:Zm00001d018279_T011 8.00E-68
933- 42: transcript:EES05891 transcript:Zm00001d018280_T001 5.00E-134
933- 43: transcript:EES07654 transcript:Zm00001d018281_T001 0
933- 44: transcript:EES05899 transcript:Zm00001d018284_T001 1.00E-53
933- 45: transcript:EES05900 transcript:Zm00001d018286_T002 0
933- 46: transcript:OQU85823 transcript:Zm00001d018287_T001 0
933- 47: transcript:KXG31222 transcript:Zm00001d018288_T001 1.00E-116
933- 48: transcript:EES05902 transcript:Zm00001d018289_T001 0
933- 49: transcript:OQU85830 transcript:Zm00001d018290_T001 2.00E-97
933- 50: transcript:EES07638 transcript:Zm00001d018291_T002 0

933- 51: transcript:KXG31226 transcript:Zm00001d018292_T001 0
933- 52: transcript:KXG31228 transcript:Zm00001d018295_T001 0
933- 53: transcript:EES07641 transcript:Zm00001d018297_T002 2.00E-112
933- 54: transcript:EES05905 transcript:Zm00001d018298_T001 9.00E-94
933- 55: transcript:EES05906 transcript:Zm00001d018303_T010 0
933- 56: transcript:EES07642 transcript:Zm00001d018305_T001 7.00E-113
933- 57: transcript:KXG31271 transcript:Zm00001d018307_T001 6.00E-145
933- 58: transcript:EES07646 transcript:Zm00001d018309_T003 0
933- 59: transcript:EES05911 transcript:Zm00001d018312_T001 1.00E-72
Alignment 934: score=2595.0 e_value=7.7e-227 N=57 4&5 plus
934- 0: transcript:EES07698 transcript:Zm00001d018405_T002 0
934- 1: transcript:EES05968 transcript:Zm00001d018406_T001 0
934- 2: transcript:KXG31292 transcript:Zm00001d018407_T001 3.00E-179
934- 3: transcript:EES05972 transcript:Zm00001d018408_T001 1.00E-145
934- 4: transcript:KXG31297 transcript:Zm00001d018409_T007 0
934- 5: transcript:EES07706 transcript:Zm00001d018410_T001 7.00E-38
934- 6: transcript:EES05974 transcript:Zm00001d018411_T001 0
934- 7: transcript:EES07707 transcript:Zm00001d018412_T001 1.00E-52
934- 8: transcript:KXG31305 transcript:Zm00001d018413_T003 0
934- 9: transcript:KXG31308 transcript:Zm00001d018414_T001 1.00E-93
934- 10: transcript:EES05978 transcript:Zm00001d018415_T002 0
934- 11: transcript:KXG31310 transcript:Zm00001d018416_T001 8.00E-160
934- 12: transcript:OQU85913 transcript:Zm00001d018418_T002 0
934- 13: transcript:EES07716 transcript:Zm00001d018419_T001 6.00E-77
934- 14: transcript:KXG31315 transcript:Zm00001d018420_T008 0
934- 15: transcript:EES05983 transcript:Zm00001d018421_T004 0
934- 16: transcript:EES07718 transcript:Zm00001d018422_T001 0
934- 17: transcript:OQU85915 transcript:Zm00001d018423_T005 0
934- 18: transcript:OQU85917 transcript:Zm00001d018428_T001 0
934- 19: transcript:EES07722 transcript:Zm00001d018429_T001 0
934- 20: transcript:EES06023 transcript:Zm00001d018430_T008 2.00E-22
934- 21: transcript:KXG31318 transcript:Zm00001d018431_T001 0
934- 22: transcript:KXG31320 transcript:Zm00001d018432_T001 0
934- 23: transcript:EES05986 transcript:Zm00001d018433_T001 1.00E-172
934- 24: transcript:EES07725 transcript:Zm00001d018435_T001 1.00E-159
934- 25: transcript:KXG31323 transcript:Zm00001d018437_T001 7.00E-81
934- 26: transcript:EES07727 transcript:Zm00001d018438_T003 0
934- 27: transcript:KXG31325 transcript:Zm00001d018439_T003 0
934- 28: transcript:KXG31326 transcript:Zm00001d018440_T002 0
934- 29: transcript:OQU85924 transcript:Zm00001d018441_T008 0
934- 30: transcript:EES05988 transcript:Zm00001d018443_T001 0
934- 31: transcript:KXG31328 transcript:Zm00001d018444_T001 0
934- 32: transcript:KXG31389 transcript:Zm00001d018445_T004 0
934- 33: transcript:EES07730 transcript:Zm00001d018447_T001 0
934- 34: transcript:EES05993 transcript:Zm00001d018450_T009 0
934- 35: transcript:EES05994 transcript:Zm00001d018451_T001 1.00E-164
934- 36: transcript:OQU85929 transcript:Zm00001d018452_T001 5.00E-110
934- 37: transcript:KXG31334 transcript:Zm00001d018454_T004 0
934- 38: transcript:KXG31336 transcript:Zm00001d018455_T001 0
934- 39: transcript:EES07740 transcript:Zm00001d018460_T003 0
934- 40: transcript:EES07741 transcript:Zm00001d018461_T001 5.00E-178
934- 41: transcript:KXG31340 transcript:Zm00001d018462_T009 0
934- 42: transcript:EES06001 transcript:Zm00001d018464_T001 1.00E-89
934- 43: transcript:EES07735 transcript:Zm00001d018465_T003 0

934- 44: transcript:EES06005 transcript:Zm00001d018466_T001 3.00E-155
934- 45: transcript:EES06006 transcript:Zm00001d018467_T001 4.00E-32
934- 46: transcript:EES06007 transcript:Zm00001d018468_T001 1.00E-41
934- 47: transcript:EES07748 transcript:Zm00001d018469_T001 0
934- 48: transcript:KXG31346 transcript:Zm00001d018470_T001 0
934- 49: transcript:KXG31347 transcript:Zm00001d018471_T002 1.00E-90
934- 50: transcript:OQU85939 transcript:Zm00001d018472_T002 0
934- 51: transcript:EES07745 transcript:Zm00001d018473_T001 0
934- 52: transcript:EES07753 transcript:Zm00001d018474_T001 0
934- 53: transcript:EES06009 transcript:Zm00001d018475_T001 0
934- 54: transcript:KXG31351 transcript:Zm00001d018477_T006 0
934- 55: transcript:EES06012 transcript:Zm00001d018478_T001 7.00E-67
934- 56: transcript:EES06013 transcript:Zm00001d018479_T001 0
Alignment 935: score=2091.0 e_value=2.2e-165 N=46 4&5 plus
935- 0: transcript:KXG31235 transcript:Zm00001d018316_T028 0
935- 1: transcript:KXG31236 transcript:Zm00001d018318_T006 0
935- 2: transcript:EES05913 transcript:Zm00001d018319_T001 4.00E-59
935- 3: transcript:EES05916 transcript:Zm00001d018321_T001 4.00E-86
935- 4: transcript:EES05918 transcript:Zm00001d018323_T001 2.00E-118
935- 5: transcript:KXG31240 transcript:Zm00001d018325_T001 0
935- 6: transcript:OQU85840 transcript:Zm00001d018326_T001 0
935- 7: transcript:OQU85841 transcript:Zm00001d018328_T003 0
935- 8: transcript:EES07650 transcript:Zm00001d018330_T002 0
935- 9: transcript:EES05919 transcript:Zm00001d018331_T001 0
935- 10: transcript:KXG31244 transcript:Zm00001d018332_T001 0
935- 11: transcript:EES07651 transcript:Zm00001d018333_T001 0
935- 12: transcript:EES07653 transcript:Zm00001d018334_T001 9.00E-146
935- 13: transcript:KXG31250 transcript:Zm00001d018335_T001 0
935- 14: transcript:EES07655 transcript:Zm00001d018336_T002 0
935- 15: transcript:EES07656 transcript:Zm00001d018337_T001 4.00E-137
935- 16: transcript:EES07658 transcript:Zm00001d018339_T001 0
935- 17: transcript:EES07659 transcript:Zm00001d018341_T007 4.00E-59
935- 18: transcript:EES05935 transcript:Zm00001d018342_T002 0
935- 19: transcript:EES07660 transcript:Zm00001d018343_T001 0
935- 20: transcript:EES07661 transcript:Zm00001d018344_T001 2.00E-156
935- 21: transcript:EES07662 transcript:Zm00001d018348_T001 2.00E-99
935- 22: transcript:EES05938 transcript:Zm00001d018349_T001 0
935- 23: transcript:EES05940 transcript:Zm00001d018350_T001 0
935- 24: transcript:EES05936 transcript:Zm00001d018352_T001 0
935- 25: transcript:KXG31257 transcript:Zm00001d018355_T005 0
935- 26: transcript:EES05942 transcript:Zm00001d018358_T001 0
935- 27: transcript:EES07664 transcript:Zm00001d018359_T002 0
935- 28: transcript:EES07665 transcript:Zm00001d018360_T001 0
935- 29: transcript:OQU85861 transcript:Zm00001d018361_T001 1.00E-20
935- 30: transcript:KXG31258 transcript:Zm00001d018362_T002 0
935- 31: transcript:EES05944 transcript:Zm00001d018363_T001 2.00E-66
935- 32: transcript:OQU85864 transcript:Zm00001d018364_T001 7.00E-125
935- 33: transcript:KXG31264 transcript:Zm00001d018365_T004 0
935- 34: transcript:OQU85866 transcript:Zm00001d018366_T001 7.00E-173
935- 35: transcript:EES07672 transcript:Zm00001d018367_T001 0
935- 36: transcript:OQU85873 transcript:Zm00001d018369_T001 0
935- 37: transcript:EES07676 transcript:Zm00001d018370_T003 0
935- 38: transcript:OQU85876 transcript:Zm00001d018371_T006 0
935- 39: transcript:OQU85881 transcript:Zm00001d018372_T001 2.00E-98

935- 40:	transcript:EES05953	transcript:Zm00001d018374_T001	0
935- 41:	transcript:EES07679	transcript:Zm00001d018375_T006	0
935- 42:	transcript:EES05956	transcript:Zm00001d018377_T006	0
935- 43:	transcript:EES05957	transcript:Zm00001d018378_T001	1.00E-33
935- 44:	transcript:EES07684	transcript:Zm00001d018380_T001	0
935- 45:	transcript:EES07686	transcript:Zm00001d018382_T011	0

Alignment 936: score=2079.0 e_value=3.3e-181 N=47 4&5 plus

936- 0:	transcript:EES05094	transcript:Zm00001d016520_T001	0
936- 1:	transcript:KXG30244	transcript:Zm00001d016522_T001	1.00E-63
936- 2:	transcript:EES06843	transcript:Zm00001d016528_T001	0
936- 3:	transcript:EES05101	transcript:Zm00001d016530_T001	0
936- 4:	transcript:EES06845	transcript:Zm00001d016531_T001	0
936- 5:	transcript:KXG30250	transcript:Zm00001d016535_T002	1.00E-149
936- 6:	transcript:EES05102	transcript:Zm00001d016542_T001	2.00E-172
936- 7:	transcript:EES05105	transcript:Zm00001d016543_T005	2.00E-139
936- 8:	transcript:EES05106	transcript:Zm00001d016544_T008	0
936- 9:	transcript:EES05109	transcript:Zm00001d016545_T001	6.00E-112
936- 10:	transcript:EES05110	transcript:Zm00001d016549_T001	4.00E-80
936- 11:	transcript:EES06851	transcript:Zm00001d016550_T002	0
936- 12:	transcript:OQU84980	transcript:Zm00001d016551_T001	6.00E-106
936- 13:	transcript:EES05114	transcript:Zm00001d016552_T002	0
936- 14:	transcript:EES06854	transcript:Zm00001d016553_T001	0
936- 15:	transcript:KXG30265	transcript:Zm00001d016558_T001	0
936- 16:	transcript:KXG30267	transcript:Zm00001d016559_T001	3.00E-179
936- 17:	transcript:EES05119	transcript:Zm00001d016561_T001	4.00E-60
936- 18:	transcript:EES05120	transcript:Zm00001d016564_T001	3.00E-93
936- 19:	transcript:EES06863	transcript:Zm00001d016566_T001	0
936- 20:	transcript:EES06864	transcript:Zm00001d016567_T001	1.00E-40
936- 21:	transcript:EES05123	transcript:Zm00001d016570_T001	3.00E-90
936- 22:	transcript:OQU85003	transcript:Zm00001d016572_T001	0
936- 23:	transcript:EES05126	transcript:Zm00001d016577_T002	1.00E-144
936- 24:	transcript:EES05127	transcript:Zm00001d016580_T001	3.00E-76
936- 25:	transcript:OQU85005	transcript:Zm00001d016581_T004	0
936- 26:	transcript:KXG30278	transcript:Zm00001d016582_T001	1.00E-80
936- 27:	transcript:EES06868	transcript:Zm00001d016590_T001	7.00E-160
936- 28:	transcript:EES05143	transcript:Zm00001d016599_T002	2.00E-97
936- 29:	transcript:EES06876	transcript:Zm00001d016601_T004	0
936- 30:	transcript:EES06877	transcript:Zm00001d016602_T001	0
936- 31:	transcript:KXG30289	transcript:Zm00001d016604_T001	1.00E-163
936- 32:	transcript:EES05145	transcript:Zm00001d016606_T022	0
936- 33:	transcript:KXG30290	transcript:Zm00001d016607_T001	0
936- 34:	transcript:KXG30293	transcript:Zm00001d016615_T002	0
936- 35:	transcript:OQU85019	transcript:Zm00001d016616_T001	1.00E-60
936- 36:	transcript:KXG30297	transcript:Zm00001d016619_T001	3.00E-177
936- 37:	transcript:EES05147	transcript:Zm00001d016620_T001	5.00E-29
936- 38:	transcript:KXG30300	transcript:Zm00001d016621_T001	1.00E-127
936- 39:	transcript:KXG30301	transcript:Zm00001d016623_T001	4.00E-120
936- 40:	transcript:OQU85020	transcript:Zm00001d016625_T001	5.00E-20
936- 41:	transcript:EES05153	transcript:Zm00001d016632_T001	0
936- 42:	transcript:KXG30309	transcript:Zm00001d016634_T003	0
936- 43:	transcript:EES05156	transcript:Zm00001d016635_T001	2.00E-178
936- 44:	transcript:EES05158	transcript:Zm00001d016636_T001	6.00E-80
936- 45:	transcript:EES05159	transcript:Zm00001d016637_T001	6.00E-56
936- 46:	transcript:EES06884	transcript:Zm00001d016640_T003	0

```
## Alignment 937: score=1867.0 e_value=1.8e-140 N=40 4&5 plus
937-  0: transcript:OQU85775           transcript:Zm00001d018122_T001          0
937-  1: transcript:EES07558           transcript:Zm00001d018123_T001          0
937-  2: transcript:OQU85776           transcript:Zm00001d018125_T001          0
937-  3: transcript:EES05842           transcript:Zm00001d018126_T001          0
937-  4: transcript:EES07559           transcript:Zm00001d018127_T001          0
937-  5: transcript:EES07560           transcript:Zm00001d018128_T001          0
937-  6: transcript:KXG31147           transcript:Zm00001d018130_T001 1.00E-111
937-  7: transcript:EES07561           transcript:Zm00001d018131_T004  6.00E-84
937-  8: transcript:EES05876           transcript:Zm00001d018133_T013          0
937-  9: transcript:EES07562           transcript:Zm00001d018134_T001  5.00E-75
937- 10: transcript:EES07563           transcript:Zm00001d018135_T001 9.00E-113
937- 11: transcript:KXG31156           transcript:Zm00001d018142_T001 2.00E-154
937- 12: transcript:KXG31158           transcript:Zm00001d018144_T003          0
937- 13: transcript:KXG31160           transcript:Zm00001d018145_T001          0
937- 14: transcript:EES07569           transcript:Zm00001d018146_T008          0
937- 15: transcript:KXG31164           transcript:Zm00001d018147_T002          0
937- 16: transcript:EES07570           transcript:Zm00001d018148_T003          0
937- 17: transcript:EES05848           transcript:Zm00001d018149_T002 2.00E-109
937- 18: transcript:OQU85783           transcript:Zm00001d018150_T001  2.00E-82
937- 19: transcript:OQU85784           transcript:Zm00001d018151_T003          0
937- 20: transcript:EES05850           transcript:Zm00001d018155_T001 3.00E-154
937- 21: transcript:EES07574           transcript:Zm00001d018157_T001 1.00E-168
937- 22: transcript:KXG31168           transcript:Zm00001d018158_T001 6.00E-100
937- 23: transcript:EES07577           transcript:Zm00001d018159_T001          0
937- 24: transcript:EES07578           transcript:Zm00001d018161_T001          0
937- 25: transcript:EES07580           transcript:Zm00001d018178_T001          0
937- 26: transcript:EES07582           transcript:Zm00001d018179_T015          0
937- 27: transcript:EES05859           transcript:Zm00001d018180_T003          0
937- 28: transcript:OQU85792           transcript:Zm00001d018181_T001 3.00E-170
937- 29: transcript:EES07585           transcript:Zm00001d018182_T001          0
937- 30: transcript:EES07586           transcript:Zm00001d018183_T001          0
937- 31: transcript:KXG31176           transcript:Zm00001d018190_T001 4.00E-151
937- 32: transcript:EES07588           transcript:Zm00001d018191_T001  3.00E-98
937- 33: transcript:EES05861           transcript:Zm00001d018192_T001  4.00E-34
937- 34: transcript:EES07589           transcript:Zm00001d018193_T001          0
937- 35: transcript:EES07590           transcript:Zm00001d018194_T001 8.00E-103
937- 36: transcript:EES07591           transcript:Zm00001d018195_T001          0
937- 37: transcript:EES05862           transcript:Zm00001d018197_T001          0
937- 38: transcript:OQU85793           transcript:Zm00001d018198_T001  4.00E-60
937- 39: transcript:KXG31179           transcript:Zm00001d018199_T001          0
## Alignment 938: score=1464.0 e_value=2.1e-113 N=33 4&5 plus
938-  0: transcript:EES04635           transcript:Zm00001d015499_T003          0
938-  1: transcript:EES04637           transcript:Zm00001d015504_T004          0
938-  2: transcript:EES04638           transcript:Zm00001d015505_T001 2.00E-129
938-  3: transcript:KXG29625           transcript:Zm00001d015508_T001          0
938-  4: transcript:OQU84492           transcript:Zm00001d015509_T001 2.00E-148
938-  5: transcript:EES04640           transcript:Zm00001d015513_T001          0
938-  6: transcript:EES04642           transcript:Zm00001d015515_T001 1.00E-172
938-  7: transcript:EES04643           transcript:Zm00001d015517_T001 2.00E-115
938-  8: transcript:EES06385           transcript:Zm00001d015519_T001 8.00E-129
938-  9: transcript:EES06386           transcript:Zm00001d015520_T001          0
938- 10: transcript:EES06387           transcript:Zm00001d015521_T001          0
938- 11: transcript:OQU84499           transcript:Zm00001d015525_T001  2.00E-55
```

938- 12:	transcript:EES06388	transcript:Zm00001d015526_T002	2.00E-145
938- 13:	transcript:EES06389	transcript:Zm00001d015527_T001	0
938- 14:	transcript:EES04646	transcript:Zm00001d015546_T001	2.00E-123
938- 15:	transcript:OQU84501	transcript:Zm00001d015549_T001	0
938- 16:	transcript:EES06390	transcript:Zm00001d015550_T001	2.00E-42
938- 17:	transcript:KXG29634	transcript:Zm00001d015553_T001	4.00E-133
938- 18:	transcript:EES06393	transcript:Zm00001d015560_T001	3.00E-170
938- 19:	transcript:KXG29638	transcript:Zm00001d015568_T001	2.00E-32
938- 20:	transcript:EES06395	transcript:Zm00001d015569_T001	0
938- 21:	transcript:KXG29640	transcript:Zm00001d015570_T002	0
938- 22:	transcript:EES04652	transcript:Zm00001d015571_T001	5.00E-167
938- 23:	transcript:KXG29641	transcript:Zm00001d015578_T001	0
938- 24:	transcript:EES06400	transcript:Zm00001d015581_T001	0
938- 25:	transcript:OQU84514	transcript:Zm00001d015585_T001	0
938- 26:	transcript:EES04658	transcript:Zm00001d015586_T003	7.00E-121
938- 27:	transcript:EES06398	transcript:Zm00001d015589_T001	0
938- 28:	transcript:OQU84518	transcript:Zm00001d015599_T001	2.00E-159
938- 29:	transcript:OQU84522	transcript:Zm00001d015600_T002	0
938- 30:	transcript:EES04660	transcript:Zm00001d015603_T001	5.00E-109
938- 31:	transcript:OQU84524	transcript:Zm00001d015605_T001	0
938- 32:	transcript:KXG29647	transcript:Zm00001d015609_T003	0

Alignment 939: score=1462.0 e_value=7.1e-117 N=33 4&5 plus

939- 0:	transcript:OQU85994	transcript:Zm00001d018515_T003	0
939- 1:	transcript:OQU85998	transcript:Zm00001d018516_T002	0
939- 2:	transcript:EES06055	transcript:Zm00001d018517_T001	0
939- 3:	transcript:EES07790	transcript:Zm00001d018521_T001	1.00E-36
939- 4:	transcript:EES07791	transcript:Zm00001d018522_T001	0
939- 5:	transcript:EES07793	transcript:Zm00001d018523_T014	0
939- 6:	transcript:EES06057	transcript:Zm00001d018524_T003	0
939- 7:	transcript:EES07794	transcript:Zm00001d018525_T004	0
939- 8:	transcript:EES06058	transcript:Zm00001d018526_T001	0
939- 9:	transcript:EES06059	transcript:Zm00001d018527_T002	0
939- 10:	transcript:OQU86001	transcript:Zm00001d018528_T001	0
939- 11:	transcript:KXG31409	transcript:Zm00001d018529_T001	0
939- 12:	transcript:EES07800	transcript:Zm00001d018530_T001	6.00E-92
939- 13:	transcript:EES07806	transcript:Zm00001d018531_T004	7.00E-161
939- 14:	transcript:EES06066	transcript:Zm00001d018532_T001	0
939- 15:	transcript:EES07813	transcript:Zm00001d018533_T003	0
939- 16:	transcript:EES07814	transcript:Zm00001d018534_T001	0
939- 17:	transcript:KXG31424	transcript:Zm00001d018537_T001	0
939- 18:	transcript:KXG31425	transcript:Zm00001d018535_T003	0
939- 19:	transcript:EES07820	transcript:Zm00001d018541_T002	0
939- 20:	transcript:KXG31426	transcript:Zm00001d018543_T001	5.00E-75
939- 21:	transcript:OQU86025	transcript:Zm00001d018544_T001	3.00E-132
939- 22:	transcript:OQU86024	transcript:Zm00001d018545_T001	0
939- 23:	transcript:EES07822	transcript:Zm00001d018552_T002	8.00E-173
939- 24:	transcript:OQU86028	transcript:Zm00001d018554_T001	3.00E-23
939- 25:	transcript:EES06074	transcript:Zm00001d018555_T001	0
939- 26:	transcript:OQU86032	transcript:Zm00001d018557_T001	6.00E-47
939- 27:	transcript:OQU86033	transcript:Zm00001d018562_T006	0
939- 28:	transcript:EES06084	transcript:Zm00001d018571_T003	3.00E-52
939- 29:	transcript:KXG31438	transcript:Zm00001d018573_T002	7.00E-88
939- 30:	transcript:EES07831	transcript:Zm00001d018574_T001	2.00E-129
939- 31:	transcript:KXG31439	transcript:Zm00001d018575_T001	3.00E-130

939- 32: transcript:KXG31443 transcript:Zm00001d018579_T001 5.00E-38
Alignment 940: score=1403.0 e_value=5.1e-107 N=31 4&5 plus
940- 0: transcript:EES06920 transcript:Zm00001d016736_T001 0
940- 1: transcript:KXG30352 transcript:Zm00001d016746_T003 0
940- 2: transcript:EES05214 transcript:Zm00001d016755_T001 0
940- 3: transcript:EES05215 transcript:Zm00001d016756_T001 1.00E-43
940- 4: transcript:EES06934 transcript:Zm00001d016758_T001 8.00E-65
940- 5: transcript:EES06935 transcript:Zm00001d016760_T001 3.00E-29
940- 6: transcript:EES06936 transcript:Zm00001d016762_T002 0
940- 7: transcript:EES06938 transcript:Zm00001d016764_T001 0
940- 8: transcript:EES05221 transcript:Zm00001d016765_T001 0
940- 9: transcript:EES06978 transcript:Zm00001d016766_T002 0
940- 10: transcript:KXG30370 transcript:Zm00001d016767_T001 0
940- 11: transcript:EES06941 transcript:Zm00001d016768_T001 0
940- 12: transcript:KXG30376 transcript:Zm00001d016770_T002 0
940- 13: transcript:OQU85097 transcript:Zm00001d016771_T002 0
940- 14: transcript:KXG30379 transcript:Zm00001d016772_T001 0
940- 15: transcript:KXG30385 transcript:Zm00001d016773_T001 7.00E-174
940- 16: transcript:EES06943 transcript:Zm00001d016783_T002 0
940- 17: transcript:OQU85098 transcript:Zm00001d016786_T001 0
940- 18: transcript:OQU85099 transcript:Zm00001d016787_T001 1.00E-44
940- 19: transcript:OQU85100 transcript:Zm00001d016788_T001 8.00E-61
940- 20: transcript:KXG30389 transcript:Zm00001d016792_T004 0
940- 21: transcript:EES05228 transcript:Zm00001d016793_T002 0
940- 22: transcript:EES05229 transcript:Zm00001d016794_T001 0
940- 23: transcript:EES05231 transcript:Zm00001d016797_T004 0
940- 24: transcript:EES05232 transcript:Zm00001d016798_T001 0
940- 25: transcript:EES06948 transcript:Zm00001d016799_T001 2.00E-168
940- 26: transcript:OQU85104 transcript:Zm00001d016800_T001 6.00E-61
940- 27: transcript:KXG30399 transcript:Zm00001d016802_T001 0
940- 28: transcript:EES05235 transcript:Zm00001d016803_T001 5.00E-32
940- 29: transcript:OQU85110 transcript:Zm00001d016804_T002 0
940- 30: transcript:KXG30407 transcript:Zm00001d016814_T001 7.00E-119
Alignment 941: score=1362.0 e_value=1.4e-115 N=32 4&5 plus
941- 0: transcript:Zm00001d048598_T001 transcript:OQU84122 3.00E-18
941- 1: transcript:EES04365 transcript:Zm00001d015004_T001 0
941- 2: transcript:EES04366 transcript:Zm00001d015005_T002 2.00E-87
941- 3: transcript:OQU84212 transcript:Zm00001d015007_T004 0
941- 4: transcript:EES06161 transcript:Zm00001d015008_T001 0
941- 5: transcript:OQU84217 transcript:Zm00001d015009_T002 0
941- 6: transcript:EES06164 transcript:Zm00001d015010_T001 0
941- 7: transcript:EES04370 transcript:Zm00001d015012_T001 0
941- 8: transcript:EES04371 transcript:Zm00001d015014_T001 0
941- 9: transcript:EES06166 transcript:Zm00001d015018_T001 3.00E-45
941- 10: transcript:EES06170 transcript:Zm00001d015020_T001 0
941- 11: transcript:EES04377 transcript:Zm00001d015025_T001 0
941- 12: transcript:EES06171 transcript:Zm00001d015026_T001 0
941- 13: transcript:EES04384 transcript:Zm00001d015029_T002 0
941- 14: transcript:EES06175 transcript:Zm00001d015030_T001 0
941- 15: transcript:EES04386 transcript:Zm00001d015031_T001 3.00E-122
941- 16: transcript:EES06176 transcript:Zm00001d015032_T003 0
941- 17: transcript:OQU84227 transcript:Zm00001d015033_T001 0
941- 18: transcript:EES06186 transcript:Zm00001d015036_T007 6.00E-146
941- 19: transcript:EES06189 transcript:Zm00001d015037_T002 0

941- 20: transcript:EES06192 transcript:Zm00001d015040_T001 9.00E-15
941- 21: transcript:EES04394 transcript:Zm00001d015053_T001 0
941- 22: transcript:EES06194 transcript:Zm00001d015058_T003 3.00E-110
941- 23: transcript:OQU84248 transcript:Zm00001d015059_T001 0
941- 24: transcript:OQU84245 transcript:Zm00001d015060_T001 0
941- 25: transcript:EES06196 transcript:Zm00001d015061_T001 2.00E-135
941- 26: transcript:OQU84251 transcript:Zm00001d015064_T001 0
941- 27: transcript:EES06203 transcript:Zm00001d015065_T005 0
941- 28: transcript:EES04405 transcript:Zm00001d015067_T001 0
941- 29: transcript:EES04410 transcript:Zm00001d015068_T001 5.00E-17
941- 30: transcript:OQU84257 transcript:Zm00001d015076_T001 3.00E-33
941- 31: transcript:EES06209 transcript:Zm00001d015082_T001 0
Alignment 942: score=1259.0 e_value=4e-88 N=28 4&5 plus
942- 0: transcript:KXG31354 transcript:Zm00001d018482_T004 0
942- 1: transcript:EES06017 transcript:Zm00001d018483_T001 0
942- 2: transcript:KXG31415 transcript:Zm00001d018484_T003 0
942- 3: transcript:EES06018 transcript:Zm00001d018485_T003 0
942- 4: transcript:KXG31361 transcript:Zm00001d018487_T001 0
942- 5: transcript:KXG31365 transcript:Zm00001d018488_T001 0
942- 6: transcript:KXG31366 transcript:Zm00001d018489_T007 0
942- 7: transcript:EES06019 transcript:Zm00001d018490_T001 0
942- 8: transcript:KXG31367 transcript:Zm00001d018491_T001 4.00E-08
942- 9: transcript:EES06024 transcript:Zm00001d018494_T002 0
942- 10: transcript:OQU85961 transcript:Zm00001d018495_T001 2.00E-63
942- 11: transcript:EES06026 transcript:Zm00001d018496_T007 0
942- 12: transcript:KXG31376 transcript:Zm00001d018497_T002 0
942- 13: transcript:EES07765 transcript:Zm00001d018498_T002 0
942- 14: transcript:EES07767 transcript:Zm00001d018499_T003 0
942- 15: transcript:KXG31382 transcript:Zm00001d018500_T002 0
942- 16: transcript:EES07768 transcript:Zm00001d018501_T001 2.00E-103
942- 17: transcript:KXG31383 transcript:Zm00001d018502_T001 0
942- 18: transcript:KXG31384 transcript:Zm00001d018503_T002 6.00E-180
942- 19: transcript:EES07772 transcript:Zm00001d018504_T001 0
942- 20: transcript:OQU85971 transcript:Zm00001d018505_T001 1.00E-113
942- 21: transcript:KXG31387 transcript:Zm00001d018507_T002 7.00E-82
942- 22: transcript:OQU85972 transcript:Zm00001d018508_T001 9.00E-131
942- 23: transcript:OQU85973 transcript:Zm00001d018509_T001 2.00E-40
942- 24: transcript:EES07774 transcript:Zm00001d018510_T001 2.00E-66
942- 25: transcript:EES06029 transcript:Zm00001d018511_T003 4.00E-101
942- 26: transcript:KXG31391 transcript:Zm00001d018512_T001 0
942- 27: transcript:EES06040 transcript:Zm00001d018514_T003 0
Alignment 943: score=1236.0 e_value=3.4e-85 N=27 4&5 plus
943- 0: transcript:EES05467 transcript:Zm00001d017317_T001 0
943- 1: transcript:EES07193 transcript:Zm00001d017320_T001 0
943- 2: transcript:OQU85362 transcript:Zm00001d017323_T001 0
943- 3: transcript:KXG30689 transcript:Zm00001d017324_T001 1.00E-120
943- 4: transcript:EES07195 transcript:Zm00001d017325_T001 8.00E-142
943- 5: transcript:EES07196 transcript:Zm00001d017326_T001 0
943- 6: transcript:EES05469 transcript:Zm00001d017330_T001 1.00E-170
943- 7: transcript:EES05470 transcript:Zm00001d017331_T002 0
943- 8: transcript:EES05471 transcript:Zm00001d017333_T002 0
943- 9: transcript:KXG30693 transcript:Zm00001d017334_T006 5.00E-116
943- 10: transcript:EES05472 transcript:Zm00001d017336_T007 2.00E-171
943- 11: transcript:OQU85369 transcript:Zm00001d017345_T002 0

943- 12: transcript:EES07199 transcript:Zm00001d017353_T001 1.00E-69
943- 13: transcript:EES07201 transcript:Zm00001d017357_T001 2.00E-19
943- 14: transcript:EES05483 transcript:Zm00001d017360_T013 0
943- 15: transcript:KXG30751 transcript:Zm00001d017361_T001 1.00E-82
943- 16: transcript:EES07206 transcript:Zm00001d017363_T001 0
943- 17: transcript:KXG30703 transcript:Zm00001d017364_T001 2.00E-80
943- 18: transcript:OQU85374 transcript:Zm00001d017365_T001 0
943- 19: transcript:OQU85375 transcript:Zm00001d017366_T001 0
943- 20: transcript:EES05484 transcript:Zm00001d017371_T008 0
943- 21: transcript:EES05486 transcript:Zm00001d017373_T001 0
943- 22: transcript:KXG30707 transcript:Zm00001d017374_T002 0
943- 23: transcript:EES05487 transcript:Zm00001d017375_T001 0
943- 24: transcript:EES05488 transcript:Zm00001d017377_T001 2.00E-180
943- 25: transcript:EES07211 transcript:Zm00001d017378_T001 1.00E-122
943- 26: transcript:EES07212 transcript:Zm00001d017379_T001 2.00E-101
Alignment 944: score=1093.0 e_value=2.9e-71 N=24 4&5 plus
944- 0: transcript:OQU85331 transcript:Zm00001d017252_T002 0
944- 1: transcript:EES07163 transcript:Zm00001d017256_T001 6.00E-76
944- 2: transcript:EES05447 transcript:Zm00001d017258_T010 0
944- 3: transcript:EES05449 transcript:Zm00001d017261_T006 0
944- 4: transcript:KXG30661 transcript:Zm00001d017263_T001 5.00E-93
944- 5: transcript:KXG30662 transcript:Zm00001d017264_T001 0
944- 6: transcript:EES07173 transcript:Zm00001d017268_T001 5.00E-138
944- 7: transcript:EES05452 transcript:Zm00001d017270_T001 0
944- 8: transcript:KXG30663 transcript:Zm00001d017271_T005 0
944- 9: transcript:EES07174 transcript:Zm00001d017274_T001 0
944- 10: transcript:EES07175 transcript:Zm00001d017275_T001 0
944- 11: transcript:OQU85344 transcript:Zm00001d017280_T001 0
944- 12: transcript:EES05456 transcript:Zm00001d017281_T001 0
944- 13: transcript:KXG30670 transcript:Zm00001d017282_T001 0
944- 14: transcript:EES05458 transcript:Zm00001d017284_T001 0
944- 15: transcript:KXG30674 transcript:Zm00001d017285_T001 2.00E-44
944- 16: transcript:EES07181 transcript:Zm00001d017287_T001 1.00E-48
944- 17: transcript:EES05459 transcript:Zm00001d017288_T001 0
944- 18: transcript:KXG30676 transcript:Zm00001d017291_T001 0
944- 19: transcript:EES05460 transcript:Zm00001d017292_T003 1.00E-40
944- 20: transcript:KXG30679 transcript:Zm00001d017294_T001 0
944- 21: transcript:EES05462 transcript:Zm00001d017296_T001 0
944- 22: transcript:OQU85352 transcript:Zm00001d017297_T001 2.00E-18
944- 23: transcript:OQU85355 transcript:Zm00001d017298_T003 1.00E-132
Alignment 945: score=1090.0 e_value=2e-80 N=26 4&5 plus
945- 0: transcript:Zm00001d052472_T012 transcript:EES07951 0
945- 1: transcript:Zm00001d052473_T002 transcript:EES09236 9.00E-172
945- 2: transcript:Zm00001d052475_T001 transcript:OQU82855 0
945- 3: transcript:Zm00001d052476_T001 transcript:KXG27703 4.00E-48
945- 4: transcript:Zm00001d052478_T001 transcript:EES09243 0
945- 5: transcript:Zm00001d052479_T001 transcript:OQU82861 0
945- 6: transcript:Zm00001d052481_T001 transcript:EES07964 0
945- 7: transcript:Zm00001d052482_T001 transcript:EES09254 5.00E-33
945- 8: transcript:Zm00001d052485_T001 transcript:KXG27718 0
945- 9: transcript:Zm00001d052488_T001 transcript:EES07965 4.00E-156
945- 10: transcript:Zm00001d052489_T001 transcript:KXG27720 0
945- 11: transcript:Zm00001d052492_T001 transcript:OQU82866 0
945- 12: transcript:Zm00001d052493_T001 transcript:EES09256 7.00E-139

945- 13: transcript:Zm00001d052494_T001 transcript:EES09257 0
945- 14: transcript:Zm00001d052495_T001 transcript:EES07975 4.00E-146
945- 15: transcript:Zm00001d052500_T001 transcript:EES07977 2.00E-30
945- 16: transcript:Zm00001d052501_T001 transcript:EES07980 2.00E-14
945- 17: transcript:Zm00001d052509_T001 transcript:EES07983 0
945- 18: transcript:Zm00001d052518_T003 transcript:EES09261 2.00E-64
945- 19: transcript:Zm00001d052519_T004 transcript:EES07984 0
945- 20: transcript:Zm00001d052520_T001 transcript:KXG27737 5.00E-147
945- 21: transcript:Zm00001d052523_T001 transcript:EES07987 0
945- 22: transcript:Zm00001d052525_T001 transcript:EES09263 0
945- 23: transcript:Zm00001d052527_T001 transcript:KXG27740 0
945- 24: transcript:Zm00001d052530_T001 transcript:OQU82886 0
945- 25: transcript:Zm00001d052532_T001 transcript:KXG27743 0
Alignment 946: score=1077.0 e_value=1.7e-68 N=23 4&5 plus
946- 0: transcript:OQU84443 transcript:Zm00001d015377_T003 0
946- 1: transcript:OQU84444 transcript:Zm00001d015378_T001 2.00E-107
946- 2: transcript:EES06344 transcript:Zm00001d015379_T001 1.00E-74
946- 3: transcript:EES06346 transcript:Zm00001d015381_T001 5.00E-179
946- 4: transcript:EES06345 transcript:Zm00001d015382_T001 1.00E-28
946- 5: transcript:EES04595 transcript:Zm00001d015383_T001 0
946- 6: transcript:KXG29565 transcript:Zm00001d015384_T001 5.00E-44
946- 7: transcript:EES04597 transcript:Zm00001d015385_T001 2.00E-174
946- 8: transcript:OQU84450 transcript:Zm00001d015386_T001 6.00E-103
946- 9: transcript:EES04599 transcript:Zm00001d015389_T002 0
946- 10: transcript:EES04603 transcript:Zm00001d015394_T001 3.00E-147
946- 11: transcript:EES06349 transcript:Zm00001d015395_T001 1.00E-67
946- 12: transcript:OQU84462 transcript:Zm00001d015397_T001 5.00E-45
946- 13: transcript:EES04606 transcript:Zm00001d015399_T002 0
946- 14: transcript:EES04608 transcript:Zm00001d015400_T004 0
946- 15: transcript:EES06351 transcript:Zm00001d015401_T001 0
946- 16: transcript:KXG29578 transcript:Zm00001d015404_T002 4.00E-113
946- 17: transcript:EES06352 transcript:Zm00001d015407_T001 0
946- 18: transcript:EES04609 transcript:Zm00001d015410_T002 0
946- 19: transcript:KXG29583 transcript:Zm00001d015412_T001 1.00E-108
946- 20: transcript:EES04611 transcript:Zm00001d015414_T001 0
946- 21: transcript:EES06354 transcript:Zm00001d015415_T001 1.00E-75
946- 22: transcript:EES04613 transcript:Zm00001d015420_T001 4.00E-103
Alignment 947: score=1076.0 e_value=1.2e-72 N=23 4&5 plus
947- 0: transcript:EES05417 transcript:Zm00001d017204_T003 1.00E-140
947- 1: transcript:EES05418 transcript:Zm00001d017205_T001 0
947- 2: transcript:EES07148 transcript:Zm00001d017206_T001 7.00E-46
947- 3: transcript:KXG30667 transcript:Zm00001d017207_T001 0
947- 4: transcript:EES05420 transcript:Zm00001d017208_T001 1.00E-144
947- 5: transcript:OQU85309 transcript:Zm00001d017209_T001 0
947- 6: transcript:OQU85310 transcript:Zm00001d017210_T001 0
947- 7: transcript:EES05423 transcript:Zm00001d017212_T001 0
947- 8: transcript:EES07150 transcript:Zm00001d017213_T004 0
947- 9: transcript:EES07151 transcript:Zm00001d017214_T002 0
947- 10: transcript:KXG30638 transcript:Zm00001d017215_T001 6.00E-140
947- 11: transcript:OQU85319 transcript:Zm00001d017216_T001 0
947- 12: transcript:EES05435 transcript:Zm00001d017239_T001 0
947- 13: transcript:EES05437 transcript:Zm00001d017240_T001 2.00E-66
947- 14: transcript:EES05438 transcript:Zm00001d017241_T002 0
947- 15: transcript:EES07156 transcript:Zm00001d017242_T001 1.00E-38

947- 16:	transcript:EES07157	transcript:Zm00001d017243_T001	2.00E-154
947- 17:	transcript:EES05440	transcript:Zm00001d017246_T001	0
947- 18:	transcript:OQU85324	transcript:Zm00001d017247_T003	0
947- 19:	transcript:EES05443	transcript:Zm00001d017248_T001	9.00E-74
947- 20:	transcript:EES05444	transcript:Zm00001d017249_T001	0
947- 21:	transcript:OQU85328	transcript:Zm00001d017250_T001	0
947- 22:	transcript:EES07161	transcript:Zm00001d017251_T003	0

Alignment 948: score=1026.0 e_value=1.4e-77 N=23 4&5 plus

948- 0:	transcript:EES06406	transcript:Zm00001d015612_T001	9.00E-105
948- 1:	transcript:EES04667	transcript:Zm00001d015613_T001	3.00E-156
948- 2:	transcript:EES04668	transcript:Zm00001d015614_T001	1.00E-165
948- 3:	transcript:KXG29648	transcript:Zm00001d015618_T001	0
948- 4:	transcript:OQU84527	transcript:Zm00001d015623_T001	0
948- 5:	transcript:OQU84529	transcript:Zm00001d015626_T001	1.00E-22
948- 6:	transcript:KXG29652	transcript:Zm00001d015627_T002	7.00E-81
948- 7:	transcript:KXG29660	transcript:Zm00001d015628_T001	8.00E-79
948- 8:	transcript:EES06416	transcript:Zm00001d015635_T002	1.00E-77
948- 9:	transcript:EES04670	transcript:Zm00001d015636_T001	0
948- 10:	transcript:OQU84537	transcript:Zm00001d015637_T001	0
948- 11:	transcript:KXG29668	transcript:Zm00001d015638_T004	5.00E-128
948- 12:	transcript:EES06418	transcript:Zm00001d015639_T001	1.00E-77
948- 13:	transcript:EES04672	transcript:Zm00001d015649_T003	0
948- 14:	transcript:OQU84544	transcript:Zm00001d015651_T001	0
948- 15:	transcript:KXG29675	transcript:Zm00001d015654_T001	1.00E-39
948- 16:	transcript:KXG29676	transcript:Zm00001d015656_T001	0
948- 17:	transcript:EES06426	transcript:Zm00001d015657_T001	5.00E-83
948- 18:	transcript:KXG29679	transcript:Zm00001d015658_T003	0
948- 19:	transcript:EES06428	transcript:Zm00001d015664_T001	1.00E-67
948- 20:	transcript:EES06429	transcript:Zm00001d015665_T007	0
948- 21:	transcript:EES06430	transcript:Zm00001d015670_T001	8.00E-65
948- 22:	transcript:KXG29681	transcript:Zm00001d015686_T001	5.00E-25

Alignment 949: score=1014.0 e_value=2.7e-63 N=22 4&5 plus

949- 0:	transcript:KXG29596	transcript:Zm00001d015446_T016	0
949- 1:	transcript:KXG29599	transcript:Zm00001d015448_T001	1.00E-43
949- 2:	transcript:EES04620	transcript:Zm00001d015449_T001	0
949- 3:	transcript:EES06365	transcript:Zm00001d015450_T001	0
949- 4:	transcript:OQU84482	transcript:Zm00001d015451_T001	0
949- 5:	transcript:KXG29603	transcript:Zm00001d015457_T001	0
949- 6:	transcript:EES06368	transcript:Zm00001d015460_T001	5.00E-55
949- 7:	transcript:EES06371	transcript:Zm00001d015463_T001	8.00E-85
949- 8:	transcript:EES06372	transcript:Zm00001d015464_T004	0
949- 9:	transcript:OQU84486	transcript:Zm00001d015468_T001	1.00E-163
949- 10:	transcript:KXG29612	transcript:Zm00001d015469_T004	0
949- 11:	transcript:EES06373	transcript:Zm00001d015470_T001	5.00E-57
949- 12:	transcript:EES04626	transcript:Zm00001d015473_T003	2.00E-43
949- 13:	transcript:EES06374	transcript:Zm00001d015474_T001	0
949- 14:	transcript:OQU84487	transcript:Zm00001d015475_T001	3.00E-54
949- 15:	transcript:KXG29617	transcript:Zm00001d015476_T001	9.00E-65
949- 16:	transcript:EES04629	transcript:Zm00001d015477_T001	0
949- 17:	transcript:KXG29621	transcript:Zm00001d015484_T001	2.00E-160
949- 18:	transcript:OQU84489	transcript:Zm00001d015485_T001	0
949- 19:	transcript:KXG29623	transcript:Zm00001d015490_T001	3.00E-60
949- 20:	transcript:EES06381	transcript:Zm00001d015493_T001	0
949- 21:	transcript:EES04635	transcript:Zm00001d015495_T013	0

Alignment 950: score=964.0 e_value=2.8e-61 N=20 4&5 plus
950- 0: transcript:EES04577 transcript:Zm00001d015338_T001 2.00E-143
950- 1: transcript:EES04578 transcript:Zm00001d015339_T001 2.00E-31
950- 2: transcript:EES04579 transcript:Zm00001d015342_T001 2.00E-125
950- 3: transcript:EES04580 transcript:Zm00001d015344_T001 0
950- 4: transcript:KXG29548 transcript:Zm00001d015345_T001 3.00E-08
950- 5: transcript:OQU84432 transcript:Zm00001d015346_T001 0
950- 6: transcript:EES06333 transcript:Zm00001d015347_T001 2.00E-126
950- 7: transcript:EES04582 transcript:Zm00001d015348_T002 0
950- 8: transcript:OQU84433 transcript:Zm00001d015354_T001 1.00E-51
950- 9: transcript:OQU84436 transcript:Zm00001d015355_T001 3.00E-152
950- 10: transcript:EES06335 transcript:Zm00001d015356_T001 0
950- 11: transcript:EES04587 transcript:Zm00001d015361_T001 0
950- 12: transcript:KXG29552 transcript:Zm00001d015362_T001 0
950- 13: transcript:EES06336 transcript:Zm00001d015363_T001 0
950- 14: transcript:EES06337 transcript:Zm00001d015364_T003 3.00E-169
950- 15: transcript:OQU84439 transcript:Zm00001d015366_T001 0
950- 16: transcript:EES06339 transcript:Zm00001d015367_T001 5.00E-100
950- 17: transcript:EES06342 transcript:Zm00001d015374_T001 0
950- 18: transcript:EES06343 transcript:Zm00001d015375_T001 5.00E-97
950- 19: transcript:KXG29557 transcript:Zm00001d015376_T002 0
Alignment 951: score=804.0 e_value=2.8e-54 N=19 4&5 plus
951- 0: transcript:Zm00001d053094_T001 transcript:OQU83624 4.00E-122
951- 1: transcript:Zm00001d053095_T001 transcript:KXG28654 3.00E-26
951- 2: transcript:Zm00001d053100_T004 transcript:EES08655 0
951- 3: transcript:Zm00001d053101_T001 transcript:EES09918 0
951- 4: transcript:Zm00001d053102_T001 transcript:KXG28661 8.00E-90
951- 5: transcript:Zm00001d053104_T002 transcript:EES10304 9.00E-79
951- 6: transcript:Zm00001d053106_T001 transcript:KXG28673 9.00E-161
951- 7: transcript:Zm00001d053107_T001 transcript:KXG28677 0
951- 8: transcript:Zm00001d053109_T002 transcript:OQU83648 0
951- 9: transcript:Zm00001d053112_T001 transcript:EES09943 3.00E-29
951- 10: transcript:Zm00001d053113_T001 transcript:KXG28687 2.00E-27
951- 11: transcript:Zm00001d053117_T001 transcript:EES09947 2.00E-115
951- 12: transcript:Zm00001d053118_T001 transcript:EES09948 0
951- 13: transcript:Zm00001d053119_T002 transcript:EES09952 0
951- 14: transcript:Zm00001d053120_T001 transcript:KXG28704 0
951- 15: transcript:Zm00001d053122_T001 transcript:OQU83660 1.00E-159
951- 16: transcript:Zm00001d053123_T001 transcript:EES08689 1.00E-61
951- 17: transcript:Zm00001d053124_T001 transcript:EES09957 2.00E-166
951- 18: transcript:Zm00001d053127_T005 transcript:KXG28707 0
Alignment 952: score=766.0 e_value=2.7e-43 N=17 4&5 plus
952- 0: transcript:Zm00001d052367_T003 transcript:KXG27624 0
952- 1: transcript:Zm00001d052368_T001 transcript:EES09150 8.00E-27
952- 2: transcript:Zm00001d052371_T001 transcript:EES09152 1.00E-62
952- 3: transcript:Zm00001d052373_T001 transcript:EES09154 3.00E-117
952- 4: transcript:Zm00001d052375_T002 transcript:EES09157 1.00E-46
952- 5: transcript:Zm00001d052376_T006 transcript:EES09159 0
952- 6: transcript:Zm00001d052377_T001 transcript:EES09160 0
952- 7: transcript:Zm00001d052378_T001 transcript:EES09161 0
952- 8: transcript:Zm00001d052379_T001 transcript:EES09162 1.00E-102
952- 9: transcript:Zm00001d052380_T001 transcript:OQU82754 0
952- 10: transcript:Zm00001d052382_T002 transcript:OQU82755 2.00E-109
952- 11: transcript:Zm00001d052384_T001 transcript:OQU82759 3.00E-38

```
952- 12: transcript:Zm00001d052385_T001 transcript:OQU82760                  0
952- 13: transcript:Zm00001d052386_T001 transcript:EES07905                  0
952- 14: transcript:Zm00001d052389_T001 transcript:EES09166                  0
952- 15: transcript:Zm00001d052390_T001 transcript:EES07908         1.00E-114
952- 16: transcript:Zm00001d052391_T001 transcript:EES09167         4.00E-121
## Alignment 953: score=751.0 e_value=6e-44 N=17 4&5 plus
953-  0: transcript:Zm00001d053146_T001 transcript:EES08018         5.00E-118
953-  1: transcript:Zm00001d053149_T002 transcript:KXG27790                  0
953-  2: transcript:Zm00001d053150_T007 transcript:EES08020                  0
953-  3: transcript:Zm00001d053151_T003 transcript:EES09309                  0
953-  4: transcript:Zm00001d053155_T002 transcript:OQU82917                  0
953-  5: transcript:Zm00001d053156_T001 transcript:EES09313                  0
953-  6: transcript:Zm00001d053158_T001 transcript:OQU82924                  0
953-  7: transcript:Zm00001d053160_T001 transcript:OQU82926           2.00E-47
953-  8: transcript:Zm00001d053162_T003 transcript:EES08023                  0
953-  9: transcript:Zm00001d053163_T001 transcript:OQU82928                  0
953- 10: transcript:Zm00001d053168_T001 transcript:KXG27800                  0
953- 11: transcript:Zm00001d053173_T006 transcript:EES09317                  0
953- 12: transcript:Zm00001d053175_T002 transcript:KXG27802           2.00E-42
953- 13: transcript:Zm00001d053177_T001 transcript:EES08028                  0
953- 14: transcript:Zm00001d053178_T001 transcript:OQU82934                  0
953- 15: transcript:Zm00001d053179_T002 transcript:EES08030                  0
953- 16: transcript:Zm00001d053181_T001 transcript:KXG27804         9.00E-179
## Alignment 954: score=748.0 e_value=1.6e-39 N=16 4&5 plus
954-  0: transcript:EES05958            transcript:Zm00001d018383_T002  7.00E-31
954-  1: transcript:EES07689            transcript:Zm00001d018384_T002         0
954-  2: transcript:KXG31276            transcript:Zm00001d018386_T005         0
954-  3: transcript:OQU85889            transcript:Zm00001d018388_T001  1.00E-73
954-  4: transcript:KXG31278            transcript:Zm00001d018389_T001         0
954-  5: transcript:KXG31279            transcript:Zm00001d018390_T001  3.00E-63
954-  6: transcript:KXG31281            transcript:Zm00001d018391_T005         0
954-  7: transcript:OQU85891            transcript:Zm00001d018392_T004         0
954-  8: transcript:EES07692            transcript:Zm00001d018393_T001  4.00E-66
954-  9: transcript:EES05962            transcript:Zm00001d018394_T003         0
954- 10: transcript:KXG31285            transcript:Zm00001d018396_T001  4.00E-30
954- 11: transcript:EES07693            transcript:Zm00001d018399_T002         0
954- 12: transcript:EES05965            transcript:Zm00001d018400_T001  3.00E-30
954- 13: transcript:KXG31287            transcript:Zm00001d018401_T001         0
954- 14: transcript:KXG31289            transcript:Zm00001d018403_T001  2.00E-66
954- 15: transcript:EES05967            transcript:Zm00001d018404_T002         0
## Alignment 955: score=738.0 e_value=2.4e-44 N=16 4&5 plus
955-  0: transcript:EES05172            transcript:Zm00001d016686_T002 4.00E-176
955-  1: transcript:EES06903            transcript:Zm00001d016687_T001         0
955-  2: transcript:EES06904            transcript:Zm00001d016690_T001         0
955-  3: transcript:EES06905            transcript:Zm00001d016691_T001  2.00E-46
955-  4: transcript:KXG30328            transcript:Zm00001d016692_T001  8.00E-28
955-  5: transcript:OQU85055            transcript:Zm00001d016693_T001  1.00E-38
955-  6: transcript:KXG30330            transcript:Zm00001d016694_T003         0
955-  7: transcript:KXG30334            transcript:Zm00001d016695_T004         0
955-  8: transcript:EES05176            transcript:Zm00001d016696_T002         0
955-  9: transcript:EES06908            transcript:Zm00001d016697_T001 2.00E-137
955- 10: transcript:EES05177            transcript:Zm00001d016698_T001  4.00E-55
955- 11: transcript:EES06909            transcript:Zm00001d016702_T006         0
955- 12: transcript:EES06910            transcript:Zm00001d016705_T001  1.00E-32
```

955- 13: transcript:KXG30339 transcript:Zm00001d016708_T002 0
955- 14: transcript:EES06914 transcript:Zm00001d016714_T003 0
955- 15: transcript:OQU85062 transcript:Zm00001d016715_T001 1.00E-71
Alignment 956: score=713.0 e_value=9.6e-48 N=17 4&5 plus
956- 0: transcript:Zm00001d052392_T003 transcript:EES09169 0
956- 1: transcript:Zm00001d052393_T001 transcript:KXG27630 0
956- 2: transcript:Zm00001d052395_T001 transcript:EES07913 2.00E-39
956- 3: transcript:Zm00001d052396_T001 transcript:EES07915 0
956- 4: transcript:Zm00001d052397_T001 transcript:OQU82780 1.00E-142
956- 5: transcript:Zm00001d052399_T009 transcript:KXG27640 0
956- 6: transcript:Zm00001d052401_T001 transcript:KXG27642 0
956- 7: transcript:Zm00001d052402_T001 transcript:OQU82781 4.00E-26
956- 8: transcript:Zm00001d052403_T001 transcript:OQU82787 3.00E-136
956- 9: transcript:Zm00001d052404_T002 transcript:EES07918 0
956- 10: transcript:Zm00001d052405_T002 transcript:EES09182 0
956- 11: transcript:Zm00001d052407_T004 transcript:EES09184 0
956- 12: transcript:Zm00001d052409_T001 transcript:KXG27654 7.00E-133
956- 13: transcript:Zm00001d052410_T005 transcript:EES09187 8.00E-132
956- 14: transcript:Zm00001d052411_T002 transcript:EES09188 0
956- 15: transcript:Zm00001d052412_T001 transcript:OQU82801 3.00E-34
956- 16: transcript:Zm00001d052423_T002 transcript:EES07922 0
Alignment 957: score=625.0 e_value=1.1e-41 N=16 4&5 plus
957- 0: transcript:EES06097 transcript:Zm00001d014928_T002 0
957- 1: transcript:EES06100 transcript:Zm00001d014931_T001 8.00E-60
957- 2: transcript:OQU84145 transcript:Zm00001d014933_T001 0
957- 3: transcript:OQU84149 transcript:Zm00001d014937_T001 3.00E-125
957- 4: transcript:EES06104 transcript:Zm00001d014938_T001 0
957- 5: transcript:EES04327 transcript:Zm00001d014943_T002 0
957- 6: transcript:EES04329 transcript:Zm00001d014944_T002 0
957- 7: transcript:EES04331 transcript:Zm00001d014946_T001 0
957- 8: transcript:OQU84152 transcript:Zm00001d014947_T001 0
957- 9: transcript:EES04334 transcript:Zm00001d014948_T002 0
957- 10: transcript:EES06107 transcript:Zm00001d014949_T001 4.00E-54
957- 11: transcript:EES04335 transcript:Zm00001d014950_T002 0
957- 12: transcript:EES04336 transcript:Zm00001d014951_T002 0
957- 13: transcript:OQU84157 transcript:Zm00001d014952_T001 0
957- 14: transcript:EES06115 transcript:Zm00001d014953_T002 0
957- 15: transcript:OQU84170 transcript:Zm00001d014954_T002 4.00E-96
Alignment 958: score=596.0 e_value=2.2e-33 N=13 4&5 plus
958- 0: transcript:EES05160 transcript:Zm00001d016647_T001 0
958- 1: transcript:OQU85027 transcript:Zm00001d016648_T001 1.00E-39
958- 2: transcript:EES06890 transcript:Zm00001d016653_T001 0
958- 3: transcript:EES05163 transcript:Zm00001d016659_T001 0
958- 4: transcript:EES05164 transcript:Zm00001d016660_T001 5.00E-120
958- 5: transcript:OQU85040 transcript:Zm00001d016662_T003 0
958- 6: transcript:EES05165 transcript:Zm00001d016664_T001 0
958- 7: transcript:EES06894 transcript:Zm00001d016665_T001 6.00E-166
958- 8: transcript:EES05166 transcript:Zm00001d016666_T001 0
958- 9: transcript:OQU85043 transcript:Zm00001d016669_T001 6.00E-123
958- 10: transcript:OQU85044 transcript:Zm00001d016671_T001 1.00E-80
958- 11: transcript:EES06898 transcript:Zm00001d016674_T001 0
958- 12: transcript:KXG30326 transcript:Zm00001d016675_T013 0
Alignment 959: score=591.0 e_value=2.7e-31 N=13 4&5 plus
959- 0: transcript:Zm00001d052956_T001 transcript:OQU83729 1.00E-27

959- 1: transcript:Zm00001d052958_T001 transcript:EES08739 3.00E-110
959- 2: transcript:Zm00001d052961_T003 transcript:OQU83731 0
959- 3: transcript:Zm00001d052962_T001 transcript:EES08741 3.00E-66
959- 4: transcript:Zm00001d052963_T001 transcript:OQU83736 5.00E-77
959- 5: transcript:Zm00001d052964_T002 transcript:EES10004 3.00E-132
959- 6: transcript:Zm00001d052967_T001 transcript:EES10005 3.00E-45
959- 7: transcript:Zm00001d052969_T001 transcript:EES10007 0
959- 8: transcript:Zm00001d052971_T005 transcript:KXG28793 0
959- 9: transcript:Zm00001d052974_T002 transcript:EES08750 0
959- 10: transcript:Zm00001d052977_T001 transcript:EES10011 0
959- 11: transcript:Zm00001d052978_T001 transcript:EES08754 3.00E-133
959- 12: transcript:Zm00001d052981_T001 transcript:EES10014 0
Alignment 960: score=587.0 e_value=5.5e-33 N=13 4&5 plus
960- 0: transcript:EES06311 transcript:Zm00001d015306_T001 0
960- 1: transcript:OQU84413 transcript:Zm00001d015307_T001 0
960- 2: transcript:EES06314 transcript:Zm00001d015308_T001 4.00E-108
960- 3: transcript:EES04548 transcript:Zm00001d015309_T002 0
960- 4: transcript:EES04549 transcript:Zm00001d015310_T001 0
960- 5: transcript:EES06317 transcript:Zm00001d015313_T001 0
960- 6: transcript:KXG29518 transcript:Zm00001d015314_T003 0
960- 7: transcript:EES04556 transcript:Zm00001d015319_T001 0
960- 8: transcript:EES06320 transcript:Zm00001d015325_T001 0
960- 9: transcript:EES04559 transcript:Zm00001d015326_T003 0
960- 10: transcript:KXG29527 transcript:Zm00001d015327_T001 0
960- 11: transcript:EES06324 transcript:Zm00001d015329_T006 0
960- 12: transcript:EES04567 transcript:Zm00001d015331_T001 3.00E-47
Alignment 961: score=570.0 e_value=9.8e-35 N=14 4&5 plus
961- 0: transcript:OQU84929 transcript:Zm00001d016475_T001 0
961- 1: transcript:OQU84933 transcript:Zm00001d016476_T001 0
961- 2: transcript:OQU84935 transcript:Zm00001d016479_T001 0
961- 3: transcript:EES05054 transcript:Zm00001d016483_T002 0
961- 4: transcript:OQU84937 transcript:Zm00001d016486_T001 0
961- 5: transcript:EES05056 transcript:Zm00001d016490_T001 0
961- 6: transcript:OQU84939 transcript:Zm00001d016491_T001 2.00E-50
961- 7: transcript:EES05060 transcript:Zm00001d016492_T001 6.00E-112
961- 8: transcript:EES06799 transcript:Zm00001d016493_T001 3.00E-105
961- 9: transcript:OQU84946 transcript:Zm00001d016496_T004 0
961- 10: transcript:KXG30215 transcript:Zm00001d016500_T002 2.00E-38
961- 11: transcript:EES06804 transcript:Zm00001d016501_T001 0
961- 12: transcript:KXG30224 transcript:Zm00001d016505_T001 1.00E-124
961- 13: transcript:OQU84950 transcript:Zm00001d016506_T003 0
Alignment 962: score=535.0 e_value=4.1e-29 N=12 4&5 plus
962- 0: transcript:EES04356 transcript:Zm00001d014988_T001 0
962- 1: transcript:EES06150 transcript:Zm00001d014989_T001 3.00E-157
962- 2: transcript:OQU84205 transcript:Zm00001d014990_T001 0
962- 3: transcript:EES04357 transcript:Zm00001d014991_T004 0
962- 4: transcript:OQU84206 transcript:Zm00001d014992_T001 1.00E-35
962- 5: transcript:KXG29291 transcript:Zm00001d014993_T003 0
962- 6: transcript:EES04360 transcript:Zm00001d014994_T001 0
962- 7: transcript:KXG29295 transcript:Zm00001d014995_T001 9.00E-155
962- 8: transcript:EES04361 transcript:Zm00001d014996_T001 0
962- 9: transcript:OQU84210 transcript:Zm00001d014998_T001 6.00E-59
962- 10: transcript:Zm00001d048600_T001 transcript:OQU84105 0
962- 11: transcript:Zm00001d048601_T001 transcript:KXG29219 0

Alignment 963: score=514.0 e_value=3.7e-28 N=12 4&5 plus

963- 0:	transcript:Zm00001d052533_T001	transcript:OQU82887	3.00E-147
963- 1:	transcript:Zm00001d052534_T001	transcript:KXG27749	2.00E-17
963- 2:	transcript:Zm00001d052537_T001	transcript:EES09271	8.00E-116
963- 3:	transcript:Zm00001d052540_T001	transcript:EES09272	0
963- 4:	transcript:Zm00001d052542_T002	transcript:EES07996	1.00E-159
963- 5:	transcript:Zm00001d052543_T001	transcript:OQU82890	0
963- 6:	transcript:Zm00001d052545_T001	transcript:EES09275	8.00E-46
963- 7:	transcript:Zm00001d052551_T001	transcript:KXG27755	3.00E-160
963- 8:	transcript:Zm00001d052556_T001	transcript:KXG27759	0
963- 9:	transcript:Zm00001d052557_T001	transcript:KXG27763	1.00E-41
963- 10:	transcript:Zm00001d052570_T001	transcript:OQU82897	0
963- 11:	transcript:Zm00001d052572_T001	transcript:EES09286	1.00E-50

Alignment 964: score=488.0 e_value=2.2e-24 N=11 4&5 plus

964- 0:	transcript:EES04519	transcript:Zm00001d015274_T001	0
964- 1:	transcript:EES04520	transcript:Zm00001d015275_T001	5.00E-161
964- 2:	transcript:OQU84365	transcript:Zm00001d015276_T001	1.00E-75
964- 3:	transcript:EES06277	transcript:Zm00001d015277_T001	0
964- 4:	transcript:EES04525	transcript:Zm00001d015279_T003	0
964- 5:	transcript:KXG29475	transcript:Zm00001d015282_T001	1.00E-27
964- 6:	transcript:OQU84367	transcript:Zm00001d015284_T001	1.00E-110
964- 7:	transcript:EES04530	transcript:Zm00001d015289_T002	0
964- 8:	transcript:EES04532	transcript:Zm00001d015290_T001	0
964- 9:	transcript:KXG29486	transcript:Zm00001d015291_T003	0
964- 10:	transcript:EES06282	transcript:Zm00001d015292_T001	0

Alignment 965: score=472.0 e_value=5.6e-26 N=11 4&5 plus

965- 0:	transcript:EES04502	transcript:Zm00001d015246_T002	0
965- 1:	transcript:OQU84355	transcript:Zm00001d015247_T001	0
965- 2:	transcript:EES04510	transcript:Zm00001d015248_T018	0
965- 3:	transcript:EES04511	transcript:Zm00001d015249_T002	0
965- 4:	transcript:EES04512	transcript:Zm00001d015251_T001	0
965- 5:	transcript:EES04513	transcript:Zm00001d015259_T001	0
965- 6:	transcript:KXG29465	transcript:Zm00001d015260_T001	0
965- 7:	transcript:EES04515	transcript:Zm00001d015265_T003	0
965- 8:	transcript:EES04516	transcript:Zm00001d015268_T001	0
965- 9:	transcript:EES06273	transcript:Zm00001d015269_T001	0
965- 10:	transcript:EES04518	transcript:Zm00001d015272_T004	0

Alignment 966: score=458.0 e_value=3.3e-24 N=11 4&5 plus

966- 0:	transcript:Zm00001d053017_T001	transcript:EES09390	0
966- 1:	transcript:Zm00001d053018_T004	transcript:KXG27888	0
966- 2:	transcript:Zm00001d053022_T001	transcript:EES09393	0
966- 3:	transcript:Zm00001d053025_T001	transcript:EES09394	3.00E-53
966- 4:	transcript:Zm00001d053026_T001	transcript:KXG27887	0
966- 5:	transcript:Zm00001d053028_T001	transcript:EES09396	0
966- 6:	transcript:Zm00001d053038_T007	transcript:EES08084	0
966- 7:	transcript:Zm00001d053041_T002	transcript:KXG27890	0
966- 8:	transcript:Zm00001d053042_T001	transcript:EES09398	3.00E-26
966- 9:	transcript:Zm00001d053046_T001	transcript:OQU83005	2.00E-40
966- 10:	transcript:Zm00001d053049_T001	transcript:KXG27901	0

Alignment 967: score=450.0 e_value=7.5e-28 N=11 4&5 plus

967- 0:	transcript:Zm00001d052340_T001	transcript:EES09124	0
967- 1:	transcript:Zm00001d052341_T001	transcript:EES09125	8.00E-32
967- 2:	transcript:Zm00001d052343_T001	transcript:OQU82714	0
967- 3:	transcript:Zm00001d052344_T003	transcript:EES09127	0

```
967-  4: transcript:Zm00001d052353_T001 transcript:EES07890                    0
967-  5: transcript:Zm00001d052354_T005 transcript:EES09132                    0
967-  6: transcript:Zm00001d052355_T001 transcript:OQU82725           4.00E-132
967-  7: transcript:Zm00001d052358_T001 transcript:OQU82732           9.00E-119
967-  8: transcript:Zm00001d052361_T001 transcript:KXG27614                    0
967-  9: transcript:Zm00001d052363_T001 transcript:EES07894           3.00E-151
967- 10: transcript:Zm00001d052365_T001 transcript:OQU82736            1.00E-86
## Alignment 968: score=446.0 e_value=2.1e-22 N=11 4&5 plus
968-  0: transcript:Zm00001d049059_T001 transcript:KXG28254           2.00E-177
968-  1: transcript:Zm00001d049060_T001 transcript:KXG28256            6.00E-07
968-  2: transcript:Zm00001d049068_T001 transcript:KXG28257           2.00E-129
968-  3: transcript:Zm00001d049071_T001 transcript:EES08383                    0
968-  4: transcript:Zm00001d049073_T001 transcript:KXG28260                    0
968-  5: transcript:Zm00001d049076_T001 transcript:EES08386                    0
968-  6: transcript:Zm00001d049077_T001 transcript:EES08387                    0
968-  7: transcript:Zm00001d049079_T001 transcript:EES09660           5.00E-160
968-  8: transcript:Zm00001d049080_T001 transcript:KXG28265                    0
968-  9: transcript:Zm00001d049081_T001 transcript:KXG28268                    0
968- 10: transcript:Zm00001d049082_T001 transcript:EES08397           5.00E-177
## Alignment 969: score=440.0 e_value=2.5e-24 N=10 4&5 plus
969-  0: transcript:Zm00001d052911_T002 transcript:EES08709                    0
969-  1: transcript:Zm00001d052923_T001 transcript:OQU83677            2.00E-48
969-  2: transcript:Zm00001d052928_T001 transcript:KXG28732           3.00E-166
969-  3: transcript:Zm00001d052929_T002 transcript:EES08716                    0
969-  4: transcript:Zm00001d052930_T003 transcript:KXG28742                    0
969-  5: transcript:Zm00001d052932_T001 transcript:EES08717                    0
969-  6: transcript:Zm00001d052933_T001 transcript:EES09979                    0
969-  7: transcript:Zm00001d052935_T001 transcript:EES08718            8.00E-89
969-  8: transcript:Zm00001d052939_T001 transcript:EES09980            3.00E-94
969-  9: transcript:Zm00001d052940_T001 transcript:EES09981           3.00E-128
## Alignment 970: score=371.0 e_value=2.3e-15 N=8 4&5 plus
970-  0: transcript:EES04616            transcript:Zm00001d015429_T006         0
970-  1: transcript:EES06356            transcript:Zm00001d015430_T001         0
970-  2: transcript:EES04617            transcript:Zm00001d015431_T001  3.00E-61
970-  3: transcript:EES06357            transcript:Zm00001d015433_T001         0
970-  4: transcript:OQU84476            transcript:Zm00001d015434_T001  3.00E-51
970-  5: transcript:OQU84478            transcript:Zm00001d015435_T016         0
970-  6: transcript:EES06360            transcript:Zm00001d015440_T001  1.00E-35
970-  7: transcript:KXG29595            transcript:Zm00001d015442_T001  1.00E-33
## Alignment 971: score=349.0 e_value=6.9e-14 N=8 4&5 plus
971-  0: transcript:Zm00001d053055_T001 transcript:EES08092            5.00E-43
971-  1: transcript:Zm00001d053059_T007 transcript:EES09411                    0
971-  2: transcript:Zm00001d053060_T001 transcript:OQU83016           9.00E-151
971-  3: transcript:Zm00001d053062_T001 transcript:KXG27914                    0
971-  4: transcript:Zm00001d053065_T002 transcript:EES08096                    0
971-  5: transcript:Zm00001d053066_T001 transcript:EES08097           2.00E-133
971-  6: transcript:Zm00001d053067_T001 transcript:EES08101                    0
971-  7: transcript:Zm00001d053068_T001 transcript:EES09416            6.00E-78
## Alignment 972: score=327.0 e_value=6.4e-13 N=7 4&5 plus
972-  0: transcript:EES06689            transcript:Zm00001d015921_T001  3.00E-76
972-  1: transcript:OQU84819            transcript:Zm00001d015925_T001         0
972-  2: transcript:EES06690            transcript:Zm00001d015927_T001         0
972-  3: transcript:KXG30054            transcript:Zm00001d015929_T002         0
972-  4: transcript:OQU84821            transcript:Zm00001d015932_T002 2.00E-124
```

972- 5: transcript:KXG30058 transcript:Zm00001d015934_T001 5.00E-77
972- 6: transcript:EES04945 transcript:Zm00001d015945_T001 7.00E-75
Alignment 973: score=327.0 e_value=3.5e-12 N=7 4&5 plus
973- 0: transcript:Zm00001d052316_T001 transcript:KXG27559 2.00E-157
973- 1: transcript:Zm00001d052317_T003 transcript:EES07851 2.00E-152
973- 2: transcript:Zm00001d052318_T002 transcript:OQU82688 0
973- 3: transcript:Zm00001d052321_T002 transcript:EES07858 2.00E-66
973- 4: transcript:Zm00001d052322_T001 transcript:OQU82692 7.00E-82
973- 5: transcript:Zm00001d052323_T001 transcript:EES07860 0
973- 6: transcript:Zm00001d052324_T001 transcript:EES07861 0
Alignment 974: score=323.0 e_value=2.2e-12 N=7 4&5 plus
974- 0: transcript:OQU84171 transcript:Zm00001d014958_T001 0
974- 1: transcript:OQU84172 transcript:Zm00001d014960_T001 0
974- 2: transcript:EES06120 transcript:Zm00001d014961_T001 0
974- 3: transcript:OQU84173 transcript:Zm00001d014962_T003 0
974- 4: transcript:OQU84182 transcript:Zm00001d014963_T001 3.00E-53
974- 5: transcript:EES04344 transcript:Zm00001d014965_T001 0
974- 6: transcript:EES06126 transcript:Zm00001d014966_T001 0
Alignment 975: score=322.0 e_value=1e-12 N=7 4&5 plus
975- 0: transcript:KXG30353 transcript:Zm00001d016718_T001 2.00E-39
975- 1: transcript:EES05205 transcript:Zm00001d016719_T001 0
975- 2: transcript:EES05207 transcript:Zm00001d016720_T001 0
975- 3: transcript:OQU85090 transcript:Zm00001d016721_T001 1.00E-129
975- 4: transcript:EES06931 transcript:Zm00001d016722_T003 1.00E-44
975- 5: transcript:OQU85091 transcript:Zm00001d016723_T001 5.00E-94
975- 6: transcript:EES06938 transcript:Zm00001d016730_T001 1.00E-50
Alignment 976: score=320.0 e_value=5.4e-14 N=7 4&5 plus
976- 0: transcript:Zm00001d052434_T001 transcript:KXG27678 0
976- 1: transcript:Zm00001d052437_T001 transcript:EES07930 7.00E-178
976- 2: transcript:Zm00001d052438_T001 transcript:EES07931 0
976- 3: transcript:Zm00001d052439_T003 transcript:EES09209 0
976- 4: transcript:Zm00001d052442_T001 transcript:EES09211 0
976- 5: transcript:Zm00001d052444_T002 transcript:EES07933 0
976- 6: transcript:Zm00001d052445_T003 transcript:KXG27681 0
Alignment 977: score=319.0 e_value=2.5e-10 N=7 4&5 plus
977- 0: transcript:Zm00001d052586_T002 transcript:OQU82900 0
977- 1: transcript:Zm00001d052591_T001 transcript:EES08005 4.00E-134
977- 2: transcript:Zm00001d052592_T001 transcript:EES09291 0
977- 3: transcript:Zm00001d052594_T001 transcript:EES08006 2.00E-35
977- 4: transcript:Zm00001d052595_T001 transcript:EES09292 4.00E-110
977- 5: transcript:Zm00001d052598_T001 transcript:OQU82902 1.00E-122
977- 6: transcript:Zm00001d052601_T001 transcript:EES09293 3.00E-141
Alignment 978: score=313.0 e_value=1.2e-11 N=7 4&5 plus
978- 0: transcript:Zm00001d053008_T001 transcript:EES08069 0
978- 1: transcript:Zm00001d053009_T009 transcript:OQU82968 0
978- 2: transcript:Zm00001d053010_T001 transcript:EES09364 3.00E-111
978- 3: transcript:Zm00001d053011_T010 transcript:EES09367 0
978- 4: transcript:Zm00001d053014_T001 transcript:EES08070 0
978- 5: transcript:Zm00001d053015_T001 transcript:EES09371 0
978- 6: transcript:Zm00001d053016_T002 transcript:EES09373 0
Alignment 979: score=307.0 e_value=1.8e-11 N=7 4&5 plus
979- 0: transcript:Zm00001d049285_T001 transcript:EES09821 8.00E-85
979- 1: transcript:Zm00001d049286_T001 transcript:OQU83531 0
979- 2: transcript:Zm00001d049288_T001 transcript:KXG28516 4.00E-15

979- 3: transcript:Zm00001d049294_T001 transcript:EES08566 0
979- 4: transcript:Zm00001d049295_T001 transcript:OQU83539 0
979- 5: transcript:Zm00001d049297_T003 transcript:EES08568 0
979- 6: transcript:Zm00001d049299_T001 transcript:OQU83547 0
Alignment 980: score=301.0 e_value=1.8e-12 N=7 4&5 plus
980- 0: transcript:Zm00001d049092_T002 transcript:EES09475 0
980- 1: transcript:Zm00001d049093_T001 transcript:OQU83081 8.00E-43
980- 2: transcript:Zm00001d049094_T001 transcript:OQU83083 0
980- 3: transcript:Zm00001d049095_T002 transcript:EES08169 0
980- 4: transcript:Zm00001d049096_T001 transcript:OQU83086 2.00E-164
980- 5: transcript:Zm00001d049099_T005 transcript:KXG28002 0
980- 6: transcript:Zm00001d049100_T003 transcript:EES09482 0
Alignment 981: score=299.0 e_value=2.4e-13 N=7 4&5 plus
981- 0: transcript:Zm00001d052943_T023 transcript:EES09985 0
981- 1: transcript:Zm00001d052947_T001 transcript:OQU83709 0
981- 2: transcript:Zm00001d052948_T001 transcript:EES09994 0
981- 3: transcript:Zm00001d052951_T001 transcript:OQU83721 0
981- 4: transcript:Zm00001d052952_T001 transcript:EES08734 1.00E-115
981- 5: transcript:Zm00001d052953_T001 transcript:EES09997 0
981- 6: transcript:Zm00001d052955_T001 transcript:EES08735 0
Alignment 982: score=290.0 e_value=5.1e-08 N=6 4&5 plus
982- 0: transcript:Zm00001d052601_T001 transcript:EES09296 5.00E-147
982- 1: transcript:Zm00001d052605_T001 transcript:EES09295 1.00E-105
982- 2: transcript:Zm00001d052606_T001 transcript:EES09297 4.00E-71
982- 3: transcript:Zm00001d052609_T001 transcript:EES09299 8.00E-49
982- 4: transcript:Zm00001d052612_T001 transcript:EES08010 0
982- 5: transcript:Zm00001d052613_T001 transcript:OQU82905 0
Alignment 983: score=285.0 e_value=1.9e-10 N=6 4&5 plus
983- 0: transcript:Zm00001d052776_T001 transcript:EES08013 2.00E-132
983- 1: transcript:Zm00001d052777_T001 transcript:OQU82910 6.00E-98
983- 2: transcript:Zm00001d052779_T004 transcript:OQU82911 0
983- 3: transcript:Zm00001d052781_T005 transcript:EES08014 0
983- 4: transcript:Zm00001d052783_T001 transcript:EES08015 9.00E-38
983- 5: transcript:Zm00001d052784_T001 transcript:EES08016 0
Alignment 984: score=276.0 e_value=3.4e-08 N=6 4&5 plus
984- 0: transcript:EES04792 transcript:Zm00001d016271_T001 5.00E-97
984- 1: transcript:KXG29848 transcript:Zm00001d016273_T001 0
984- 2: transcript:KXG29849 transcript:Zm00001d016274_T006 0
984- 3: transcript:EES06539 transcript:Zm00001d016276_T001 5.00E-92
984- 4: transcript:OQU84670 transcript:Zm00001d016277_T002 3.00E-159
984- 5: transcript:EES06537 transcript:Zm00001d016285_T001 0
Alignment 985: score=268.0 e_value=5.1e-12 N=6 4&5 plus
985- 0: transcript:Zm00001d049189_T001 transcript:OQU83395 1.00E-114
985- 1: transcript:Zm00001d049190_T002 transcript:OQU83398 0
985- 2: transcript:Zm00001d049191_T001 transcript:OQU83400 0
985- 3: transcript:Zm00001d049192_T002 transcript:OQU83401 4.00E-138
985- 4: transcript:Zm00001d049196_T001 transcript:OQU83407 0
985- 5: transcript:Zm00001d049197_T002 transcript:OQU83404 1.00E-177
Alignment 986: score=266.0 e_value=6.8e-12 N=6 4&5 plus
986- 0: transcript:Zm00001d049038_T014 transcript:EES08355 0
986- 1: transcript:Zm00001d049042_T001 transcript:KXG28234 0
986- 2: transcript:Zm00001d049043_T001 transcript:EES08356 0
986- 3: transcript:Zm00001d049046_T002 transcript:KXG28238 0
986- 4: transcript:Zm00001d049048_T001 transcript:EES08362 2.00E-142

986- 5: transcript:Zm00001d049049_T001 transcript:EES09641 0
Alignment 987: score=262.0 e_value=1.1e-07 N=6 4&5 plus
987- 0: transcript:Zm00001d053182_T003 transcript:KXG27814 0
987- 1: transcript:Zm00001d053183_T001 transcript:KXG27818 0
987- 2: transcript:Zm00001d053184_T001 transcript:EES09328 0
987- 3: transcript:Zm00001d053186_T001 transcript:EES09329 8.00E-46
987- 4: transcript:Zm00001d053189_T004 transcript:EES08036 0
987- 5: transcript:Zm00001d053192_T001 transcript:EES08037 0
Alignment 988: score=3839.0 e_value=0 N=84 4&5 minus
988- 0: transcript:OQU85586 transcript:Zm00001d017660_T021 0
988- 1: transcript:KXG30983 transcript:Zm00001d017659_T001 1.00E-118
988- 2: transcript:OQU85592 transcript:Zm00001d017658_T001 3.00E-178
988- 3: transcript:EES05698 transcript:Zm00001d017657_T001 2.00E-138
988- 4: transcript:EES05704 transcript:Zm00001d017654_T002 0
988- 5: transcript:KXG30986 transcript:Zm00001d017653_T001 2.00E-166
988- 6: transcript:OQU85598 transcript:Zm00001d017651_T001 1.00E-175
988- 7: transcript:OQU85603 transcript:Zm00001d017650_T002 1.00E-34
988- 8: transcript:EES05709 transcript:Zm00001d017649_T001 5.00E-140
988- 9: transcript:KXG30991 transcript:Zm00001d017646_T001 2.00E-15
988- 10: transcript:EES05717 transcript:Zm00001d017645_T001 2.00E-108
988- 11: transcript:KXG30997 transcript:Zm00001d017643_T001 0
988- 12: transcript:EES05718 transcript:Zm00001d017642_T001 0
988- 13: transcript:OQU85620 transcript:Zm00001d017641_T004 0
988- 14: transcript:KXG30999 transcript:Zm00001d017640_T006 0
988- 15: transcript:EES07443 transcript:Zm00001d017625_T001 0
988- 16: transcript:EES07444 transcript:Zm00001d017621_T001 8.00E-104
988- 17: transcript:KXG31003 transcript:Zm00001d017620_T005 0
988- 18: transcript:OQU85628 transcript:Zm00001d017619_T001 3.00E-24
988- 19: transcript:EES07445 transcript:Zm00001d017618_T001 0
988- 20: transcript:EES05724 transcript:Zm00001d017617_T001 2.00E-41
988- 21: transcript:EES05725 transcript:Zm00001d017616_T001 0
988- 22: transcript:EES05726 transcript:Zm00001d017615_T001 6.00E-65
988- 23: transcript:EES07448 transcript:Zm00001d017614_T003 3.00E-135
988- 24: transcript:KXG31006 transcript:Zm00001d017613_T001 0
988- 25: transcript:EES05729 transcript:Zm00001d017611_T001 0
988- 26: transcript:KXG31009 transcript:Zm00001d017609_T011 0
988- 27: transcript:OQU85630 transcript:Zm00001d017608_T001 2.00E-151
988- 28: transcript:EES07451 transcript:Zm00001d017607_T001 0
988- 29: transcript:OQU85631 transcript:Zm00001d017606_T001 4.00E-116
988- 30: transcript:EES07454 transcript:Zm00001d017604_T001 0
988- 31: transcript:KXG31012 transcript:Zm00001d017603_T001 0
988- 32: transcript:OQU85635 transcript:Zm00001d017602_T002 4.00E-08
988- 33: transcript:EES07456 transcript:Zm00001d017601_T001 0
988- 34: transcript:OQU85636 transcript:Zm00001d017598_T001 0
988- 35: transcript:EES07458 transcript:Zm00001d017597_T001 0
988- 36: transcript:EES07459 transcript:Zm00001d017595_T002 0
988- 37: transcript:OQU85641 transcript:Zm00001d017592_T001 2.00E-113
988- 38: transcript:KXG31013 transcript:Zm00001d017591_T001 2.00E-89
988- 39: transcript:EES07463 transcript:Zm00001d017590_T001 0
988- 40: transcript:EES07464 transcript:Zm00001d017584_T002 0
988- 41: transcript:EES05735 transcript:Zm00001d017583_T002 3.00E-156
988- 42: transcript:EES05736 transcript:Zm00001d017582_T001 0
988- 43: transcript:KXG31020 transcript:Zm00001d017575_T001 2.00E-171
988- 44: transcript:EES05737 transcript:Zm00001d017574_T001 2.00E-112

988- 45: transcript:EES07467 transcript:Zm00001d017573_T003 0
988- 46: transcript:EES05741 transcript:Zm00001d017570_T001 0
988- 47: transcript:EES05742 transcript:Zm00001d017568_T002 0
988- 48: transcript:EES07469 transcript:Zm00001d017566_T001 0
988- 49: transcript:KXG31023 transcript:Zm00001d017564_T001 0
988- 50: transcript:KXG31025 transcript:Zm00001d017563_T002 2.00E-125
988- 51: transcript:EES07471 transcript:Zm00001d017560_T002 0
988- 52: transcript:EES07470 transcript:Zm00001d017559_T001 0
988- 53: transcript:OQU85652 transcript:Zm00001d017558_T001 0
988- 54: transcript:EES07473 transcript:Zm00001d017557_T001 0
988- 55: transcript:OQU85654 transcript:Zm00001d017555_T001 3.00E-51
988- 56: transcript:OQU85656 transcript:Zm00001d017553_T001 0
988- 57: transcript:KXG31037 transcript:Zm00001d017551_T001 2.00E-79
988- 58: transcript:OQU85665 transcript:Zm00001d017550_T009 5.00E-169
988- 59: transcript:EES05747 transcript:Zm00001d017547_T001 2.00E-83
988- 60: transcript:KXG31039 transcript:Zm00001d017546_T001 1.00E-39
988- 61: transcript:EES05749 transcript:Zm00001d017545_T001 2.00E-136
988- 62: transcript:EES07478 transcript:Zm00001d017544_T002 0
988- 63: transcript:KXG31044 transcript:Zm00001d017543_T001 1.00E-73
988- 64: transcript:KXG31045 transcript:Zm00001d017540_T001 0
988- 65: transcript:EES07479 transcript:Zm00001d017539_T002 0
988- 66: transcript:OQU85667 transcript:Zm00001d017537_T001 2.00E-124
988- 67: transcript:OQU85671 transcript:Zm00001d017536_T001 0
988- 68: transcript:EES05753 transcript:Zm00001d017535_T001 5.00E-47
988- 69: transcript:EES05750 transcript:Zm00001d017534_T001 2.00E-43
988- 70: transcript:EES07481 transcript:Zm00001d017532_T001 1.00E-71
988- 71: transcript:KXG31048 transcript:Zm00001d017531_T001 0
988- 72: transcript:EES07483 transcript:Zm00001d017530_T001 4.00E-119
988- 73: transcript:OQU85673 transcript:Zm00001d017528_T001 0
988- 74: transcript:EES07484 transcript:Zm00001d017526_T001 0
988- 75: transcript:KXG31053 transcript:Zm00001d017524_T001 0
988- 76: transcript:EES05761 transcript:Zm00001d017523_T001 2.00E-98
988- 77: transcript:EES07490 transcript:Zm00001d017522_T003 0
988- 78: transcript:EES05764 transcript:Zm00001d017518_T001 0
988- 79: transcript:EES05762 transcript:Zm00001d017516_T001 1.00E-94
988- 80: transcript:OQU85678 transcript:Zm00001d017514_T002 0
988- 81: transcript:EES05768 transcript:Zm00001d017513_T001 0
988- 82: transcript:OQU85684 transcript:Zm00001d017512_T003 2.00E-163
988- 83: transcript:EES07497 transcript:Zm00001d017508_T001 7.00E-39
Alignment 989: score=3196.0 e_value=7.6e-274 N=69 4&5 minus
989- 0: transcript:EES07504 transcript:Zm00001d017505_T003 4.00E-118
989- 1: transcript:KXG31078 transcript:Zm00001d017504_T001 7.00E-106
989- 2: transcript:EES05773 transcript:Zm00001d017503_T001 1.00E-102
989- 3: transcript:OQU85690 transcript:Zm00001d017502_T001 0
989- 4: transcript:EES05775 transcript:Zm00001d017501_T002 1.00E-173
989- 5: transcript:KXG31080 transcript:Zm00001d017499_T001 4.00E-108
989- 6: transcript:OQU85696 transcript:Zm00001d017497_T001 4.00E-109
989- 7: transcript:EES05780 transcript:Zm00001d017493_T001 4.00E-109
989- 8: transcript:EES05781 transcript:Zm00001d017492_T001 0
989- 9: transcript:OQU85705 transcript:Zm00001d017491_T001 3.00E-24
989- 10: transcript:EES05783 transcript:Zm00001d017486_T001 5.00E-51
989- 11: transcript:KXG31086 transcript:Zm00001d017485_T003 2.00E-162
989- 12: transcript:KXG31088 transcript:Zm00001d017482_T002 2.00E-157
989- 13: transcript:EES07513 transcript:Zm00001d017477_T001 5.00E-42

989- 14:	transcript:EES07516	transcript:Zm00001d017476_T001	0
989- 15:	transcript:OQU85714	transcript:Zm00001d017475_T001	0
989- 16:	transcript:EES05789	transcript:Zm00001d017474_T001	6.00E-140
989- 17:	transcript:EES05790	transcript:Zm00001d017473_T002	0
989- 18:	transcript:EES07518	transcript:Zm00001d017472_T002	0
989- 19:	transcript:EES05791	transcript:Zm00001d017470_T001	0
989- 20:	transcript:EES07521	transcript:Zm00001d017469_T001	0
989- 21:	transcript:EES07522	transcript:Zm00001d017468_T001	5.00E-146
989- 22:	transcript:EES05792	transcript:Zm00001d017467_T001	3.00E-158
989- 23:	transcript:KXG31094	transcript:Zm00001d017466_T001	6.00E-160
989- 24:	transcript:EES05793	transcript:Zm00001d017462_T001	4.00E-122
989- 25:	transcript:KXG31098	transcript:Zm00001d017461_T001	0
989- 26:	transcript:EES07525	transcript:Zm00001d017460_T003	2.00E-152
989- 27:	transcript:EES07526	transcript:Zm00001d017458_T003	3.00E-141
989- 28:	transcript:EES07528	transcript:Zm00001d017457_T001	0
989- 29:	transcript:EES07529	transcript:Zm00001d017456_T003	3.00E-144
989- 30:	transcript:OQU85738	transcript:Zm00001d017455_T001	2.00E-60
989- 31:	transcript:KXG31101	transcript:Zm00001d017453_T001	0
989- 32:	transcript:EES05797	transcript:Zm00001d017449_T001	2.00E-127
989- 33:	transcript:KXG31102	transcript:Zm00001d017448_T001	2.00E-136
989- 34:	transcript:EES05799	transcript:Zm00001d017445_T001	0
989- 35:	transcript:KXG31104	transcript:Zm00001d017444_T001	9.00E-128
989- 36:	transcript:OQU85741	transcript:Zm00001d017441_T001	4.00E-93
989- 37:	transcript:EES07533	transcript:Zm00001d017438_T001	3.00E-17
989- 38:	transcript:EES05803	transcript:Zm00001d017435_T001	0
989- 39:	transcript:KXG31106	transcript:Zm00001d017432_T001	4.00E-179
989- 40:	transcript:EES05808	transcript:Zm00001d017427_T002	0
989- 41:	transcript:OQU85747	transcript:Zm00001d017425_T001	5.00E-65
989- 42:	transcript:OQU85746	transcript:Zm00001d017424_T003	0
989- 43:	transcript:EES05814	transcript:Zm00001d017422_T001	2.00E-113
989- 44:	transcript:EES05815	transcript:Zm00001d017420_T001	0
989- 45:	transcript:EES05816	transcript:Zm00001d017419_T001	0
989- 46:	transcript:EES05817	transcript:Zm00001d017418_T001	0
989- 47:	transcript:EES07538	transcript:Zm00001d017415_T001	2.00E-17
989- 48:	transcript:EES07539	transcript:Zm00001d017412_T001	8.00E-68
989- 49:	transcript:KXG31117	transcript:Zm00001d017409_T001	1.00E-150
989- 50:	transcript:KXG31120	transcript:Zm00001d017404_T001	6.00E-62
989- 51:	transcript:EES05821	transcript:Zm00001d017403_T001	0
989- 52:	transcript:OQU85754	transcript:Zm00001d017402_T001	2.00E-83
989- 53:	transcript:EES07540	transcript:Zm00001d017401_T001	0
989- 54:	transcript:EES05825	transcript:Zm00001d017399_T001	0
989- 55:	transcript:KXG31122	transcript:Zm00001d017398_T005	0
989- 56:	transcript:EES07542	transcript:Zm00001d017397_T001	4.00E-78
989- 57:	transcript:KXG31125	transcript:Zm00001d017396_T001	2.00E-135
989- 58:	transcript:EES07544	transcript:Zm00001d017395_T002	9.00E-161
989- 59:	transcript:EES07541	transcript:Zm00001d017392_T009	0
989- 60:	transcript:EES05828	transcript:Zm00001d017391_T002	9.00E-145
989- 61:	transcript:KXG31127	transcript:Zm00001d017390_T001	3.00E-138
989- 62:	transcript:KXG31129	transcript:Zm00001d017387_T006	0
989- 63:	transcript:KXG31130	transcript:Zm00001d017386_T001	1.00E-75
989- 64:	transcript:OQU85763	transcript:Zm00001d017385_T001	9.00E-103
989- 65:	transcript:EES07547	transcript:Zm00001d017383_T001	4.00E-147
989- 66:	transcript:EES05836	transcript:Zm00001d017382_T001	7.00E-124
989- 67:	transcript:KXG31133	transcript:Zm00001d017381_T001	6.00E-110

```
989- 68: transcript:EES05839           transcript:Zm00001d017380_T001         0
## Alignment 990: score=2905.0 e_value=8.7e-270 N=67 4&5 minus
990-  0: transcript:OQU84616           transcript:Zm00001d016402_T012         0
990-  1: transcript:KXG29773           transcript:Zm00001d016400_T001  3.00E-38
990-  2: transcript:KXG29776           transcript:Zm00001d016397_T008         0
990-  3: transcript:EES04733           transcript:Zm00001d016396_T001         0
990-  4: transcript:KXG29778           transcript:Zm00001d016395_T001         0
990-  5: transcript:KXG29787           transcript:Zm00001d016394_T001         0
990-  6: transcript:KXG29797           transcript:Zm00001d016382_T004         0
990-  7: transcript:EES04741           transcript:Zm00001d016380_T001  5.00E-30
990-  8: transcript:OQU84635           transcript:Zm00001d016379_T001  2.00E-35
990-  9: transcript:EES06511           transcript:Zm00001d016378_T002         0
990- 10: transcript:KXG29808           transcript:Zm00001d016374_T002         0
990- 11: transcript:EES04750           transcript:Zm00001d016373_T003         0
990- 12: transcript:OQU84646           transcript:Zm00001d016371_T001  8.00E-74
990- 13: transcript:EES04754           transcript:Zm00001d016365_T002         0
990- 14: transcript:EES04755           transcript:Zm00001d016364_T001 2.00E-160
990- 15: transcript:EES06519           transcript:Zm00001d016363_T002  3.00E-91
990- 16: transcript:KXG29816           transcript:Zm00001d016361_T001 2.00E-152
990- 17: transcript:EES04758           transcript:Zm00001d016358_T006         0
990- 18: transcript:KXG29821           transcript:Zm00001d016354_T001         0
990- 19: transcript:KXG29825           transcript:Zm00001d016350_T001  4.00E-51
990- 20: transcript:EES06526           transcript:Zm00001d016349_T002 2.00E-166
990- 21: transcript:EES04771           transcript:Zm00001d016347_T002         0
990- 22: transcript:OQU84657           transcript:Zm00001d016342_T001  2.00E-99
990- 23: transcript:EES04776           transcript:Zm00001d016332_T001         0
990- 24: transcript:EES04777           transcript:Zm00001d016326_T001         0
990- 25: transcript:EES04778           transcript:Zm00001d016311_T002         0
990- 26: transcript:EES04779           transcript:Zm00001d016308_T003         0
990- 27: transcript:EES06530           transcript:Zm00001d016306_T001 8.00E-133
990- 28: transcript:EES06531           transcript:Zm00001d016305_T001 1.00E-141
990- 29: transcript:EES04782           transcript:Zm00001d016301_T001 1.00E-156
990- 30: transcript:EES04783           transcript:Zm00001d016299_T001         0
990- 31: transcript:EES04785           transcript:Zm00001d016298_T001         0
990- 32: transcript:EES06532           transcript:Zm00001d016294_T001 8.00E-119
990- 33: transcript:KXG29841           transcript:Zm00001d016287_T001  5.00E-80
990- 34: transcript:OQU84666           transcript:Zm00001d016281_T001  1.00E-79
990- 35: transcript:KXG29849           transcript:Zm00001d016274_T006         0
990- 36: transcript:KXG29854           transcript:Zm00001d016269_T001  2.00E-96
990- 37: transcript:KXG29857           transcript:Zm00001d016260_T001  3.00E-67
990- 38: transcript:EES06545           transcript:Zm00001d016259_T001         0
990- 39: transcript:EES04798           transcript:Zm00001d016257_T001  8.00E-97
990- 40: transcript:EES04799           transcript:Zm00001d016256_T001 1.00E-128
990- 41: transcript:KXG29862           transcript:Zm00001d016255_T001 5.00E-140
990- 42: transcript:KXG29864           transcript:Zm00001d016254_T001         0
990- 43: transcript:EES04803           transcript:Zm00001d016253_T001 1.00E-124
990- 44: transcript:EES04804           transcript:Zm00001d016248_T001         0
990- 45: transcript:KXG29872           transcript:Zm00001d016242_T002         0
990- 46: transcript:EES06549           transcript:Zm00001d016237_T001         0
990- 47: transcript:EES04807           transcript:Zm00001d016234_T001         0
990- 48: transcript:OQU84681           transcript:Zm00001d016231_T001 5.00E-126
990- 49: transcript:EES04810           transcript:Zm00001d016230_T001 2.00E-155
990- 50: transcript:OQU84683           transcript:Zm00001d016228_T001         0
990- 51: transcript:EES04812           transcript:Zm00001d016225_T001         0
```

990- 52: transcript:KXG29880 transcript:Zm00001d016223_T001 0
990- 53: transcript:EES04811 transcript:Zm00001d016219_T001 0
990- 54: transcript:KXG29882 transcript:Zm00001d016216_T001 3.00E-42
990- 55: transcript:EES04815 transcript:Zm00001d016203_T002 3.00E-123
990- 56: transcript:OQU84689 transcript:Zm00001d016199_T001 3.00E-152
990- 57: transcript:KXG29888 transcript:Zm00001d016198_T003 0
990- 58: transcript:EES06565 transcript:Zm00001d016197_T001 0
990- 59: transcript:EES06566 transcript:Zm00001d016188_T002 0
990- 60: transcript:EES04822 transcript:Zm00001d016185_T001 0
990- 61: transcript:KXG29892 transcript:Zm00001d016182_T001 0
990- 62: transcript:EES06567 transcript:Zm00001d016180_T001 0
990- 63: transcript:KXG29896 transcript:Zm00001d016177_T001 7.00E-159
990- 64: transcript:EES04827 transcript:Zm00001d016175_T001 0
990- 65: transcript:EES04830 transcript:Zm00001d016170_T001 0
990- 66: transcript:EES04831 transcript:Zm00001d016166_T001 0
Alignment 991: score=2904.0 e_value=2.9e-254 N=63 4&5 minus
991- 0: transcript:KXG30709 transcript:Zm00001d018119_T001 3.00E-156
991- 1: transcript:EES05493 transcript:Zm00001d018118_T001 2.00E-72
991- 2: transcript:EES05494 transcript:Zm00001d018117_T001 5.00E-64
991- 3: transcript:OQU85383 transcript:Zm00001d018113_T006 0
991- 4: transcript:KXG30711 transcript:Zm00001d018112_T004 1.00E-86
991- 5: transcript:KXG30712 transcript:Zm00001d018111_T002 0
991- 6: transcript:EES07217 transcript:Zm00001d018105_T001 6.00E-100
991- 7: transcript:EES05501 transcript:Zm00001d018104_T001 5.00E-64
991- 8: transcript:KXG30724 transcript:Zm00001d018103_T002 2.00E-173
991- 9: transcript:OQU85392 transcript:Zm00001d018102_T001 0
991- 10: transcript:EES05510 transcript:Zm00001d018099_T001 0
991- 11: transcript:KXG30729 transcript:Zm00001d018098_T005 0
991- 12: transcript:EES05511 transcript:Zm00001d018097_T001 0
991- 13: transcript:EES07227 transcript:Zm00001d018096_T001 5.00E-91
991- 14: transcript:OQU85397 transcript:Zm00001d018093_T001 0
991- 15: transcript:EES05513 transcript:Zm00001d018090_T002 0
991- 16: transcript:EES07230 transcript:Zm00001d018089_T001 2.00E-66
991- 17: transcript:KXG30730 transcript:Zm00001d018088_T001 0
991- 18: transcript:EES07231 transcript:Zm00001d018087_T001 6.00E-179
991- 19: transcript:EES07233 transcript:Zm00001d018085_T001 1.00E-53
991- 20: transcript:EES07234 transcript:Zm00001d018082_T001 0
991- 21: transcript:EES07235 transcript:Zm00001d018081_T001 4.00E-175
991- 22: transcript:EES05516 transcript:Zm00001d018080_T001 0
991- 23: transcript:KXG30738 transcript:Zm00001d018078_T002 0
991- 24: transcript:KXG30740 transcript:Zm00001d018077_T001 1.00E-122
991- 25: transcript:KXG30742 transcript:Zm00001d018076_T001 3.00E-70
991- 26: transcript:KXG30743 transcript:Zm00001d018075_T001 2.00E-83
991- 27: transcript:KXG30744 transcript:Zm00001d018074_T001 0
991- 28: transcript:OQU85415 transcript:Zm00001d018073_T001 6.00E-29
991- 29: transcript:EES07241 transcript:Zm00001d018072_T001 2.00E-68
991- 30: transcript:EES05520 transcript:Zm00001d018070_T001 0
991- 31: transcript:EES07249 transcript:Zm00001d018069_T001 1.00E-144
991- 32: transcript:EES07243 transcript:Zm00001d018066_T001 1.00E-56
991- 33: transcript:EES07252 transcript:Zm00001d018065_T001 3.00E-61
991- 34: transcript:OQU85421 transcript:Zm00001d018064_T004 0
991- 35: transcript:KXG30755 transcript:Zm00001d018061_T002 0
991- 36: transcript:EES05526 transcript:Zm00001d018058_T001 2.00E-174
991- 37: transcript:EES05527 transcript:Zm00001d018057_T001 0

```
991- 38: transcript:EES05528            transcript:Zm00001d018056_T001  2.00E-54
991- 39: transcript:KXG30761            transcript:Zm00001d018050_T003         0
991- 40: transcript:EES07256            transcript:Zm00001d018047_T002  8.00E-41
991- 41: transcript:EES07255            transcript:Zm00001d018045_T001 1.00E-160
991- 42: transcript:EES05529            transcript:Zm00001d018043_T001         0
991- 43: transcript:EES07257            transcript:Zm00001d018042_T001         0
991- 44: transcript:EES07258            transcript:Zm00001d018041_T001         0
991- 45: transcript:KXG30762            transcript:Zm00001d018040_T002         0
991- 46: transcript:EES07261            transcript:Zm00001d018038_T001         0
991- 47: transcript:EES07262            transcript:Zm00001d018037_T001         0
991- 48: transcript:EES05530            transcript:Zm00001d018036_T002         0
991- 49: transcript:OQU85426            transcript:Zm00001d018035_T001 1.00E-111
991- 50: transcript:EES07263            transcript:Zm00001d018034_T001         0
991- 51: transcript:EES05532            transcript:Zm00001d018033_T001         0
991- 52: transcript:KXG30765            transcript:Zm00001d018032_T001         0
991- 53: transcript:OQU85427            transcript:Zm00001d018031_T001         0
991- 54: transcript:EES07267            transcript:Zm00001d018030_T001  8.00E-74
991- 55: transcript:EES07268            transcript:Zm00001d018028_T001 4.00E-137
991- 56: transcript:EES07273            transcript:Zm00001d018027_T001  2.00E-68
991- 57: transcript:EES07274            transcript:Zm00001d018025_T001         0
991- 58: transcript:EES05538            transcript:Zm00001d018024_T001         0
991- 59: transcript:EES05539            transcript:Zm00001d018019_T001         0
991- 60: transcript:EES05540            transcript:Zm00001d018017_T001  1.00E-93
991- 61: transcript:EES07275            transcript:Zm00001d018016_T001         0
991- 62: transcript:KXG30782            transcript:Zm00001d018014_T030         0
## Alignment 992: score=2035.0 e_value=5.5e-157 N=44 4&5 minus
992-  0: transcript:KXG30782            transcript:Zm00001d018009_T026         0
992-  1: transcript:OQU85432            transcript:Zm00001d018006_T003         0
992-  2: transcript:EES05543            transcript:Zm00001d018005_T001         0
992-  3: transcript:EES05544            transcript:Zm00001d018004_T002 5.00E-138
992-  4: transcript:EES05546            transcript:Zm00001d018001_T001         0
992-  5: transcript:KXG30786            transcript:Zm00001d017998_T001 4.00E-106
992-  6: transcript:EES05547            transcript:Zm00001d017997_T010         0
992-  7: transcript:EES07282            transcript:Zm00001d017996_T001         0
992-  8: transcript:EES07283            transcript:Zm00001d017995_T003         0
992-  9: transcript:EES07284            transcript:Zm00001d017994_T001         0
992- 10: transcript:OQU85439            transcript:Zm00001d017992_T001 4.00E-159
992- 11: transcript:EES05550            transcript:Zm00001d017991_T001  2.00E-39
992- 12: transcript:EES07286            transcript:Zm00001d017990_T001  2.00E-65
992- 13: transcript:KXG30790            transcript:Zm00001d017989_T001         0
992- 14: transcript:KXG30792            transcript:Zm00001d017988_T002         0
992- 15: transcript:EES05553            transcript:Zm00001d017987_T003         0
992- 16: transcript:KXG30794            transcript:Zm00001d017986_T005         0
992- 17: transcript:EES05555            transcript:Zm00001d017985_T002         0
992- 18: transcript:EES05556            transcript:Zm00001d017984_T001         0
992- 19: transcript:EES05554            transcript:Zm00001d017983_T001         0
992- 20: transcript:OQU85441            transcript:Zm00001d017982_T001         0
992- 21: transcript:EES05560            transcript:Zm00001d017979_T002         0
992- 22: transcript:EES07289            transcript:Zm00001d017978_T001         0
992- 23: transcript:OQU85445            transcript:Zm00001d017977_T010         0
992- 24: transcript:EES05563            transcript:Zm00001d017976_T001         0
992- 25: transcript:KXG30800            transcript:Zm00001d017973_T001  3.00E-15
992- 26: transcript:EES07294            transcript:Zm00001d017969_T001  2.00E-35
992- 27: transcript:EES05564            transcript:Zm00001d017968_T001  1.00E-40
```

992- 28:	transcript:KXG30805	transcript:Zm00001d017967_T006	0
992- 29:	transcript:EES05565	transcript:Zm00001d017966_T001	0
992- 30:	transcript:EES05566	transcript:Zm00001d017965_T001	0
992- 31:	transcript:KXG30806	transcript:Zm00001d017962_T001	9.00E-105
992- 32:	transcript:EES07297	transcript:Zm00001d017961_T003	0
992- 33:	transcript:EES05568	transcript:Zm00001d017960_T001	0
992- 34:	transcript:EES07299	transcript:Zm00001d017959_T003	0
992- 35:	transcript:KXG30808	transcript:Zm00001d017958_T002	0
992- 36:	transcript:OQU85452	transcript:Zm00001d017957_T002	0
992- 37:	transcript:EES07301	transcript:Zm00001d017956_T001	2.00E-93
992- 38:	transcript:OQU85453	transcript:Zm00001d017955_T001	5.00E-120
992- 39:	transcript:EES07303	transcript:Zm00001d017952_T001	1.00E-68
992- 40:	transcript:OQU85454	transcript:Zm00001d017951_T001	1.00E-110
992- 41:	transcript:EES05570	transcript:Zm00001d017950_T001	2.00E-95
992- 42:	transcript:EES07307	transcript:Zm00001d017949_T001	0
992- 43:	transcript:EES05573	transcript:Zm00001d017947_T001	2.00E-94
## Alignment 993: score=2031.0 e_value=9.1e-165 N=45 4&5 minus			
993- 0:	transcript:EES05576	transcript:Zm00001d017946_T001	0
993- 1:	transcript:EES05578	transcript:Zm00001d017945_T002	2.00E-105
993- 2:	transcript:EES07309	transcript:Zm00001d017943_T001	1.00E-145
993- 3:	transcript:KXG30818	transcript:Zm00001d017941_T001	0
993- 4:	transcript:EES05581	transcript:Zm00001d017939_T001	0
993- 5:	transcript:EES05582	transcript:Zm00001d017937_T003	0
993- 6:	transcript:EES05583	transcript:Zm00001d017936_T001	1.00E-73
993- 7:	transcript:OQU85463	transcript:Zm00001d017932_T002	1.00E-144
993- 8:	transcript:EES05584	transcript:Zm00001d017931_T001	0
993- 9:	transcript:KXG30822	transcript:Zm00001d017930_T003	0
993- 10:	transcript:OQU85465	transcript:Zm00001d017928_T001	0
993- 11:	transcript:EES05586	transcript:Zm00001d017927_T001	4.00E-137
993- 12:	transcript:KXG30824	transcript:Zm00001d017926_T003	0
993- 13:	transcript:KXG30825	transcript:Zm00001d017925_T004	0
993- 14:	transcript:EES07316	transcript:Zm00001d017923_T001	4.00E-32
993- 15:	transcript:KXG30829	transcript:Zm00001d017920_T001	0
993- 16:	transcript:EES07319	transcript:Zm00001d017918_T001	0
993- 17:	transcript:EES07321	transcript:Zm00001d017917_T001	1.00E-139
993- 18:	transcript:KXG30834	transcript:Zm00001d017915_T001	0
993- 19:	transcript:EES05591	transcript:Zm00001d017914_T001	8.00E-102
993- 20:	transcript:EES05592	transcript:Zm00001d017913_T001	0
993- 21:	transcript:OQU85472	transcript:Zm00001d017912_T001	0
993- 22:	transcript:EES07324	transcript:Zm00001d017911_T001	5.00E-94
993- 23:	transcript:KXG30839	transcript:Zm00001d017908_T001	6.00E-171
993- 24:	transcript:KXG30840	transcript:Zm00001d017907_T001	2.00E-124
993- 25:	transcript:OQU85485	transcript:Zm00001d017906_T003	0
993- 26:	transcript:EES07330	transcript:Zm00001d017905_T001	4.00E-172
993- 27:	transcript:KXG30845	transcript:Zm00001d017900_T001	2.00E-110
993- 28:	transcript:EES05602	transcript:Zm00001d017898_T001	4.00E-25
993- 29:	transcript:EES07334	transcript:Zm00001d017897_T002	0
993- 30:	transcript:KXG30849	transcript:Zm00001d017896_T002	2.00E-90
993- 31:	transcript:EES05604	transcript:Zm00001d017895_T001	0
993- 32:	transcript:KXG30853	transcript:Zm00001d017894_T001	0
993- 33:	transcript:EES07339	transcript:Zm00001d017889_T001	0
993- 34:	transcript:KXG30856	transcript:Zm00001d017887_T002	0
993- 35:	transcript:OQU85498	transcript:Zm00001d017886_T001	0
993- 36:	transcript:EES07342	transcript:Zm00001d017885_T001	0

993- 37: transcript:EES05610 transcript:Zm00001d017883_T002 0
993- 38: transcript:EES07344 transcript:Zm00001d017882_T001 0
993- 39: transcript:EES05611 transcript:Zm00001d017881_T002 0
993- 40: transcript:EES07345 transcript:Zm00001d017880_T006 0
993- 41: transcript:OQU85503 transcript:Zm00001d017878_T001 0
993- 42: transcript:KXG30863 transcript:Zm00001d017877_T001 0
993- 43: transcript:KXG30865 transcript:Zm00001d017876_T018 0
993- 44: transcript:EES05613 transcript:Zm00001d017874_T001 3.00E-74
Alignment 994: score=1716.0 e_value=4.7e-130 N=37 4&5 minus
994- 0: transcript:EES05623 transcript:Zm00001d017848_T004 0
994- 1: transcript:EES07362 transcript:Zm00001d017847_T003 1.00E-104
994- 2: transcript:EES07364 transcript:Zm00001d017845_T001 4.00E-156
994- 3: transcript:EES05624 transcript:Zm00001d017842_T001 3.00E-117
994- 4: transcript:EES05625 transcript:Zm00001d017840_T001 0
994- 5: transcript:KXG30883 transcript:Zm00001d017838_T001 8.00E-104
994- 6: transcript:EES07366 transcript:Zm00001d017837_T001 6.00E-117
994- 7: transcript:EES07367 transcript:Zm00001d017836_T002 0
994- 8: transcript:EES07368 transcript:Zm00001d017834_T001 0
994- 9: transcript:EES07369 transcript:Zm00001d017833_T001 0
994- 10: transcript:OQU85519 transcript:Zm00001d017832_T006 0
994- 11: transcript:KXG30886 transcript:Zm00001d017831_T001 0
994- 12: transcript:EES05627 transcript:Zm00001d017830_T001 0
994- 13: transcript:OQU85520 transcript:Zm00001d017829_T002 1.00E-159
994- 14: transcript:EES05629 transcript:Zm00001d017826_T001 0
994- 15: transcript:EES05630 transcript:Zm00001d017825_T001 0
994- 16: transcript:EES07372 transcript:Zm00001d017823_T001 0
994- 17: transcript:EES07373 transcript:Zm00001d017822_T001 4.00E-49
994- 18: transcript:KXG30889 transcript:Zm00001d017821_T002 0
994- 19: transcript:EES05632 transcript:Zm00001d017820_T001 0
994- 20: transcript:KXG30891 transcript:Zm00001d017818_T001 0
994- 21: transcript:EES05636 transcript:Zm00001d017814_T001 7.00E-44
994- 22: transcript:OQU85522 transcript:Zm00001d017813_T001 2.00E-63
994- 23: transcript:KXG30892 transcript:Zm00001d017812_T002 4.00E-154
994- 24: transcript:KXG30894 transcript:Zm00001d017811_T001 0
994- 25: transcript:KXG30895 transcript:Zm00001d017810_T010 0
994- 26: transcript:KXG30897 transcript:Zm00001d017809_T002 0
994- 27: transcript:KXG30898 transcript:Zm00001d017808_T001 0
994- 28: transcript:EES05638 transcript:Zm00001d017807_T001 0
994- 29: transcript:EES07379 transcript:Zm00001d017806_T002 0
994- 30: transcript:OQU85525 transcript:Zm00001d017805_T002 0
994- 31: transcript:EES07381 transcript:Zm00001d017804_T001 5.00E-142
994- 32: transcript:EES05644 transcript:Zm00001d017803_T001 2.00E-55
994- 33: transcript:EES05645 transcript:Zm00001d017802_T001 0
994- 34: transcript:EES07384 transcript:Zm00001d017801_T001 3.00E-43
994- 35: transcript:OQU85528 transcript:Zm00001d017800_T001 5.00E-72
994- 36: transcript:EES07385 transcript:Zm00001d017799_T001 0
Alignment 995: score=1597.0 e_value=3.5e-117 N=35 4&5 minus
995- 0: transcript:KXG30908 transcript:Zm00001d017798_T002 0
995- 1: transcript:EES05647 transcript:Zm00001d017793_T002 0
995- 2: transcript:EES07390 transcript:Zm00001d017791_T002 0
995- 3: transcript:EES07391 transcript:Zm00001d017790_T002 0
995- 4: transcript:EES07392 transcript:Zm00001d017789_T001 2.00E-97
995- 5: transcript:EES05649 transcript:Zm00001d017788_T001 3.00E-179
995- 6: transcript:EES05650 transcript:Zm00001d017786_T003 0

995- 7:	transcript:KXG30913	transcript:Zm00001d017785_T002	3.00E-174
995- 8:	transcript:KXG30914	transcript:Zm00001d017784_T001	5.00E-126
995- 9:	transcript:EES07394	transcript:Zm00001d017783_T001	0
995- 10:	transcript:OQU85546	transcript:Zm00001d017782_T001	1.00E-36
995- 11:	transcript:EES07395	transcript:Zm00001d017780_T002	0
995- 12:	transcript:EES05656	transcript:Zm00001d017778_T001	0
995- 13:	transcript:KXG30917	transcript:Zm00001d017777_T003	3.00E-122
995- 14:	transcript:EES07397	transcript:Zm00001d017773_T001	0
995- 15:	transcript:EES05657	transcript:Zm00001d017771_T001	0
995- 16:	transcript:KXG30922	transcript:Zm00001d017770_T001	7.00E-138
995- 17:	transcript:EES07398	transcript:Zm00001d017769_T001	0
995- 18:	transcript:OQU85551	transcript:Zm00001d017768_T006	0
995- 19:	transcript:OQU85552	transcript:Zm00001d017767_T001	7.00E-157
995- 20:	transcript:KXG30924	transcript:Zm00001d017766_T001	0
995- 21:	transcript:KXG30925	transcript:Zm00001d017764_T001	4.00E-11
995- 22:	transcript:EES05661	transcript:Zm00001d017762_T003	0
995- 23:	transcript:EES07401	transcript:Zm00001d017761_T001	5.00E-121
995- 24:	transcript:EES05663	transcript:Zm00001d017756_T001	2.00E-140
995- 25:	transcript:EES05665	transcript:Zm00001d017755_T011	0
995- 26:	transcript:EES05664	transcript:Zm00001d017754_T031	0
995- 27:	transcript:KXG30930	transcript:Zm00001d017753_T002	0
995- 28:	transcript:OQU85554	transcript:Zm00001d017752_T001	7.00E-74
995- 29:	transcript:EES07403	transcript:Zm00001d017751_T001	0
995- 30:	transcript:EES07404	transcript:Zm00001d017748_T001	0
995- 31:	transcript:KXG30932	transcript:Zm00001d017746_T001	0
995- 32:	transcript:EES07405	transcript:Zm00001d017742_T001	0
995- 33:	transcript:OQU85560	transcript:Zm00001d017730_T001	0
995- 34:	transcript:OQU85557	transcript:Zm00001d017729_T029	0
## Alignment 996: score=1547.0 e_value=2.3e-108 N=33 4&5 minus			
996- 0:	transcript:EES05675	transcript:Zm00001d017720_T009	0
996- 1:	transcript:EES07409	transcript:Zm00001d017719_T001	3.00E-113
996- 2:	transcript:KXG30941	transcript:Zm00001d017718_T004	0
996- 3:	transcript:EES07411	transcript:Zm00001d017717_T001	2.00E-135
996- 4:	transcript:EES07413	transcript:Zm00001d017712_T001	0
996- 5:	transcript:KXG30948	transcript:Zm00001d017711_T001	0
996- 6:	transcript:KXG30949	transcript:Zm00001d017709_T002	7.00E-160
996- 7:	transcript:EES05677	transcript:Zm00001d017708_T001	3.00E-102
996- 8:	transcript:EES07415	transcript:Zm00001d017707_T003	0
996- 9:	transcript:EES05679	transcript:Zm00001d017706_T002	0
996- 10:	transcript:KXG30951	transcript:Zm00001d017704_T001	0
996- 11:	transcript:KXG30955	transcript:Zm00001d017703_T002	0
996- 12:	transcript:KXG30956	transcript:Zm00001d017702_T001	3.00E-168
996- 13:	transcript:KXG30958	transcript:Zm00001d017700_T020	0
996- 14:	transcript:EES07417	transcript:Zm00001d017699_T001	0
996- 15:	transcript:EES05682	transcript:Zm00001d017698_T003	0
996- 16:	transcript:OQU85576	transcript:Zm00001d017697_T001	4.00E-54
996- 17:	transcript:OQU85577	transcript:Zm00001d017696_T001	0
996- 18:	transcript:KXG30961	transcript:Zm00001d017693_T001	0
996- 19:	transcript:KXG30966	transcript:Zm00001d017692_T002	4.00E-89
996- 20:	transcript:KXG30968	transcript:Zm00001d017691_T001	0
996- 21:	transcript:EES05689	transcript:Zm00001d017689_T002	2.00E-166
996- 22:	transcript:OQU85579	transcript:Zm00001d017686_T003	0
996- 23:	transcript:EES05690	transcript:Zm00001d017685_T003	6.00E-73
996- 24:	transcript:EES07422	transcript:Zm00001d017684_T001	7.00E-67

996- 25: transcript:KXG30975 transcript:Zm00001d017682_T001 2.00E-155
996- 26: transcript:EES05692 transcript:Zm00001d017678_T001 0
996- 27: transcript:EES07423 transcript:Zm00001d017677_T001 1.00E-162
996- 28: transcript:EES07424 transcript:Zm00001d017676_T001 0
996- 29: transcript:EES07425 transcript:Zm00001d017675_T003 3.00E-169
996- 30: transcript:EES07426 transcript:Zm00001d017671_T001 0
996- 31: transcript:EES07427 transcript:Zm00001d017670_T001 0
996- 32: transcript:KXG30977 transcript:Zm00001d017666_T001 0
Alignment 997: score=994.0 e_value=1.2e-63 N=22 4&5 minus
997- 0: transcript:OQU84698 transcript:Zm00001d016165_T013 0
997- 1: transcript:KXG29906 transcript:Zm00001d016164_T003 0
997- 2: transcript:EES06575 transcript:Zm00001d016163_T001 8.00E-11
997- 3: transcript:OQU84700 transcript:Zm00001d016160_T001 0
997- 4: transcript:EES04832 transcript:Zm00001d016158_T001 1.00E-86
997- 5: transcript:KXG29910 transcript:Zm00001d016155_T001 0
997- 6: transcript:EES04836 transcript:Zm00001d016154_T001 7.00E-132
997- 7: transcript:KXG29911 transcript:Zm00001d016152_T002 0
997- 8: transcript:EES04838 transcript:Zm00001d016151_T001 0
997- 9: transcript:KXG29912 transcript:Zm00001d016149_T001 1.00E-19
997- 10: transcript:OQU84705 transcript:Zm00001d016145_T001 3.00E-71
997- 11: transcript:EES04844 transcript:Zm00001d016144_T002 2.00E-124
997- 12: transcript:EES06583 transcript:Zm00001d016143_T001 3.00E-52
997- 13: transcript:EES06586 transcript:Zm00001d016142_T001 3.00E-40
997- 14: transcript:EES06590 transcript:Zm00001d016137_T001 3.00E-22
997- 15: transcript:KXG29926 transcript:Zm00001d016136_T001 3.00E-98
997- 16: transcript:KXG29927 transcript:Zm00001d016135_T001 4.00E-40
997- 17: transcript:EES04848 transcript:Zm00001d016132_T001 1.00E-163
997- 18: transcript:OQU84708 transcript:Zm00001d016131_T006 0
997- 19: transcript:EES04851 transcript:Zm00001d016130_T001 0
997- 20: transcript:EES06598 transcript:Zm00001d016128_T001 0
997- 21: transcript:EES04857 transcript:Zm00001d016119_T001 0
Alignment 998: score=894.0 e_value=3.8e-59 N=20 4&5 minus
998- 0: transcript:EES04870 transcript:Zm00001d016100_T001 0
998- 1: transcript:EES06610 transcript:Zm00001d016095_T001 2.00E-163
998- 2: transcript:OQU84734 transcript:Zm00001d016084_T003 0
998- 3: transcript:EES04874 transcript:Zm00001d016083_T001 4.00E-45
998- 4: transcript:KXG29959 transcript:Zm00001d016082_T001 0
998- 5: transcript:KXG29960 transcript:Zm00001d016081_T002 0
998- 6: transcript:EES04875 transcript:Zm00001d016077_T005 0
998- 7: transcript:EES04876 transcript:Zm00001d016076_T001 3.00E-52
998- 8: transcript:EES06612 transcript:Zm00001d016075_T001 0
998- 9: transcript:EES06613 transcript:Zm00001d016072_T001 2.00E-140
998- 10: transcript:OQU84742 transcript:Zm00001d016070_T004 0
998- 11: transcript:KXG29968 transcript:Zm00001d016066_T001 0
998- 12: transcript:EES04878 transcript:Zm00001d016063_T006 0
998- 13: transcript:EES04879 transcript:Zm00001d016062_T001 2.00E-112
998- 14: transcript:OQU84748 transcript:Zm00001d016061_T001 1.00E-144
998- 15: transcript:EES06617 transcript:Zm00001d016060_T001 3.00E-143
998- 16: transcript:EES04883 transcript:Zm00001d016052_T001 6.00E-170
998- 17: transcript:EES06620 transcript:Zm00001d016049_T003 6.00E-92
998- 18: transcript:OQU84754 transcript:Zm00001d016044_T002 0
998- 19: transcript:OQU84758 transcript:Zm00001d016041_T001 4.00E-156
Alignment 999: score=842.0 e_value=1.6e-55 N=19 4&5 minus
999- 0: transcript:EES04677 transcript:Zm00001d015884_T001 0

999- 1: transcript:KXG29686 transcript:Zm00001d015868_T001 3.00E-85
999- 2: transcript:EES06442 transcript:Zm00001d015867_T001 2.00E-103
999- 3: transcript:OQU84558 transcript:Zm00001d015866_T001 2.00E-17
999- 4: transcript:KXG29690 transcript:Zm00001d015865_T002 6.00E-87
999- 5: transcript:EES04683 transcript:Zm00001d015863_T001 0
999- 6: transcript:OQU84559 transcript:Zm00001d015862_T001 9.00E-37
999- 7: transcript:EES04685 transcript:Zm00001d015861_T002 8.00E-147
999- 8: transcript:KXG29696 transcript:Zm00001d015856_T007 0
999- 9: transcript:EES06446 transcript:Zm00001d015853_T001 0
999- 10: transcript:KXG29700 transcript:Zm00001d015852_T004 0
999- 11: transcript:EES06449 transcript:Zm00001d015851_T001 0
999- 12: transcript:KXG29702 transcript:Zm00001d015845_T001 1.00E-148
999- 13: transcript:EES06450 transcript:Zm00001d015844_T001 0
999- 14: transcript:KXG29703 transcript:Zm00001d015842_T001 1.00E-118
999- 15: transcript:EES04693 transcript:Zm00001d015839_T001 0
999- 16: transcript:EES06452 transcript:Zm00001d015837_T001 0
999- 17: transcript:EES04697 transcript:Zm00001d015829_T001 0
999- 18: transcript:OQU84570 transcript:Zm00001d015825_T001 0
Alignment 1000: score=820.0 e_value=9.7e-50 N=18 4&5 minus
1000- 0:transcript:OQU84570 transcript:Zm00001d015820_T002 6.00E-82
1000- 1:transcript:KXG29714 transcript:Zm00001d015816_T003 0
1000- 2:transcript:KXG29713 transcript:Zm00001d015815_T001 0
1000- 3:transcript:KXG29717 transcript:Zm00001d015810_T001 3.00E-170
1000- 4:transcript:KXG29720 transcript:Zm00001d015806_T001 4.00E-29
1000- 5:transcript:KXG29722 transcript:Zm00001d015804_T013 0
1000- 6:transcript:KXG29725 transcript:Zm00001d015802_T001 0
1000- 7:transcript:OQU84578 transcript:Zm00001d015799_T001 3.00E-24
1000- 8:transcript:OQU84580 transcript:Zm00001d015798_T001 0
1000- 9:transcript:OQU84582 transcript:Zm00001d015796_T001 2.00E-115
1000- 10:transcript:OQU84581 transcript:Zm00001d015795_T001 6.00E-95
1000- 11:transcript:OQU84585 transcript:Zm00001d015791_T001 0
1000- 12:transcript:KXG29736 transcript:Zm00001d015789_T002 0
1000- 13:transcript:EES06471 transcript:Zm00001d015788_T001 0
1000- 14:transcript:EES04709 transcript:Zm00001d015785_T005 0
1000- 15:transcript:KXG29738 transcript:Zm00001d015783_T001 0
1000- 16:transcript:EES04710 transcript:Zm00001d015780_T002 0
1000- 17:transcript:EES06473 transcript:Zm00001d015778_T001 0
Alignment 1001: score=741.0 e_value=3.2e-42 N=16 4&5 minus
1001- 0:transcript:EES05614 transcript:Zm00001d017872_T001 0
1001- 1:transcript:KXG30910 transcript:Zm00001d017871_T002 0
1001- 2:transcript:EES07349 transcript:Zm00001d017866_T001 0
1001- 3:transcript:KXG30869 transcript:Zm00001d017865_T007 0
1001- 4:transcript:EES05617 transcript:Zm00001d017864_T001 1.00E-131
1001- 5:transcript:EES07355 transcript:Zm00001d017863_T001 0
1001- 6:transcript:KXG30873 transcript:Zm00001d017862_T001 0
1001- 7:transcript:EES07356 transcript:Zm00001d017861_T001 1.00E-100
1001- 8:transcript:EES07357 transcript:Zm00001d017860_T005 0
1001- 9:transcript:OQU85511 transcript:Zm00001d017859_T002 2.00E-97
1001- 10:transcript:KXG30875 transcript:Zm00001d017857_T002 0
1001- 11:transcript:EES05618 transcript:Zm00001d017853_T001 0
1001- 12:transcript:OQU85513 transcript:Zm00001d017852_T001 1.00E-18
1001- 13:transcript:OQU85514 transcript:Zm00001d017851_T001 2.00E-10
1001- 14:transcript:EES05621 transcript:Zm00001d017850_T001 1.00E-84
1001- 15:transcript:EES07359 transcript:Zm00001d017849_T001 0

Alignment 1002: score=568.0 e_value=1.3e-34 N=14 4&5 minus

1002- 0:	transcript:Zm00001d048794_T001	transcript:EES10152	0
1002- 1:	transcript:Zm00001d048795_T001	transcript:EES10151	4.00E-47
1002- 2:	transcript:Zm00001d048796_T003	transcript:KXG28983	5.00E-153
1002- 3:	transcript:Zm00001d048799_T001	transcript:EES08879	1.00E-90
1002- 4:	transcript:Zm00001d048801_T001	transcript:EES08878	8.00E-64
1002- 5:	transcript:Zm00001d048802_T003	transcript:EES10143	0
1002- 6:	transcript:Zm00001d048804_T001	transcript:EES10142	4.00E-156
1002- 7:	transcript:Zm00001d048806_T001	transcript:KXG28976	2.00E-81
1002- 8:	transcript:Zm00001d048816_T001	transcript:EES10132	5.00E-73
1002- 9:	transcript:Zm00001d048819_T001	transcript:OQU83877	3.00E-105
1002- 10:	transcript:Zm00001d048822_T001	transcript:EES08871	0
1002- 11:	transcript:Zm00001d048823_T007	transcript:EES08863	0
1002- 12:	transcript:Zm00001d048824_T004	transcript:EES08860	0
1002- 13:	transcript:Zm00001d048825_T001	transcript:KXG28966	9.00E-97

Alignment 1003: score=509.0 e_value=1.8e-24 N=11 4&5 minus

1003- 0:	transcript:OQU85125	transcript:Zm00001d016855_T002	0
1003- 1:	transcript:OQU85132	transcript:Zm00001d016854_T001	0
1003- 2:	transcript:EES06980	transcript:Zm00001d016853_T001	0
1003- 3:	transcript:EES05255	transcript:Zm00001d016850_T002	7.00E-54
1003- 4:	transcript:EES06976	transcript:Zm00001d016849_T002	0
1003- 5:	transcript:KXG30429	transcript:Zm00001d016848_T001	1.00E-68
1003- 6:	transcript:EES05258	transcript:Zm00001d016846_T001	0
1003- 7:	transcript:KXG30434	transcript:Zm00001d016845_T001	1.00E-43
1003- 8:	transcript:EES05261	transcript:Zm00001d016841_T002	6.00E-180
1003- 9:	transcript:EES06987	transcript:Zm00001d016840_T001	1.00E-63
1003- 10:	transcript:OQU85146	transcript:Zm00001d016839_T001	4.00E-167

Alignment 1004: score=501.0 e_value=3.5e-26 N=11 4&5 minus

1004- 0:	transcript:OQU85064	transcript:Zm00001d016745_T001	5.00E-166
1004- 1:	transcript:EES05191	transcript:Zm00001d016744_T001	3.00E-54
1004- 2:	transcript:EES05192	transcript:Zm00001d016743_T001	3.00E-46
1004- 3:	transcript:EES05193	transcript:Zm00001d016738_T001	5.00E-49
1004- 4:	transcript:OQU85082	transcript:Zm00001d016737_T002	0
1004- 5:	transcript:EES05200	transcript:Zm00001d016735_T001	0
1004- 6:	transcript:EES06921	transcript:Zm00001d016733_T001	3.00E-124
1004- 7:	transcript:EES06922	transcript:Zm00001d016732_T006	0
1004- 8:	transcript:EES05202	transcript:Zm00001d016731_T001	0
1004- 9:	transcript:OQU85086	transcript:Zm00001d016730_T001	0
1004- 10:	transcript:EES05207	transcript:Zm00001d016720_T001	0

Alignment 1005: score=489.0 e_value=7.6e-29 N=12 4&5 minus

1005- 0:	transcript:Zm00001d049161_T001	transcript:EES08518	0
1005- 1:	transcript:Zm00001d049162_T001	transcript:EES08514	8.00E-144
1005- 2:	transcript:Zm00001d049163_T001	transcript:EES08513	0
1005- 3:	transcript:Zm00001d049165_T001	transcript:EES09763	5.00E-176
1005- 4:	transcript:Zm00001d049166_T001	transcript:EES08512	6.00E-41
1005- 5:	transcript:Zm00001d049168_T003	transcript:OQU83463	0
1005- 6:	transcript:Zm00001d049169_T001	transcript:KXG28418	0
1005- 7:	transcript:Zm00001d049170_T001	transcript:KXG28402	4.00E-60
1005- 8:	transcript:Zm00001d049171_T001	transcript:OQU83448	0
1005- 9:	transcript:Zm00001d049173_T001	transcript:EES08501	2.00E-104
1005- 10:	transcript:Zm00001d049174_T001	transcript:EES08500	0
1005- 11:	transcript:Zm00001d049175_T001	transcript:OQU83444	2.00E-24

Alignment 1006: score=465.0 e_value=3.2e-25 N=11 4&5 minus

1006- 0:	transcript:Zm00001d048993_T001	transcript:KXG28118	0

1006- 1:	transcript:Zm00001d048994_T003	transcript:OQU83167	0
1006- 2:	transcript:Zm00001d048997_T001	transcript:EES09552	3.00E-57
1006- 3:	transcript:Zm00001d048998_T001	transcript:EES09551	0
1006- 4:	transcript:Zm00001d049000_T001	transcript:KXG28111	8.00E-161
1006- 5:	transcript:Zm00001d049002_T001	transcript:EES08262	0
1006- 6:	transcript:Zm00001d049006_T001	transcript:EES08261	1.00E-63
1006- 7:	transcript:Zm00001d049008_T001	transcript:OQU83157	3.00E-57
1006- 8:	transcript:Zm00001d049011_T001	transcript:EES08256	4.00E-156
1006- 9:	transcript:Zm00001d049015_T001	transcript:OQU83156	3.00E-159
1006- 10:	transcript:Zm00001d049016_T001	transcript:EES08254	0
## Alignment 1007: score=435.0 e_value=1.3e-23 N=10 4&5 minus			
1007- 0:	transcript:EES06492	transcript:Zm00001d016439_T003	0
1007- 1:	transcript:EES06493	transcript:Zm00001d016438_T001	0
1007- 2:	transcript:EES06494	transcript:Zm00001d016417_T001	3.00E-172
1007- 3:	transcript:OQU84608	transcript:Zm00001d016414_T001	3.00E-86
1007- 4:	transcript:KXG29763	transcript:Zm00001d016411_T001	0
1007- 5:	transcript:KXG29764	transcript:Zm00001d016410_T006	0
1007- 6:	transcript:EES04729	transcript:Zm00001d016409_T001	9.00E-86
1007- 7:	transcript:EES06497	transcript:Zm00001d016408_T001	5.00E-18
1007- 8:	transcript:EES04731	transcript:Zm00001d016407_T007	0
1007- 9:	transcript:KXG29768	transcript:Zm00001d016406_T015	0
## Alignment 1008: score=425.0 e_value=5.5e-23 N=10 4&5 minus			
1008- 0:	transcript:Zm00001d048715_T004	transcript:KXG29048	0
1008- 1:	transcript:Zm00001d048716_T001	transcript:KXG29046	7.00E-35
1008- 2:	transcript:Zm00001d048717_T004	transcript:EES08921	0
1008- 3:	transcript:Zm00001d048718_T010	transcript:OQU83951	0
1008- 4:	transcript:Zm00001d048719_T001	transcript:EES08918	2.00E-54
1008- 5:	transcript:Zm00001d048721_T001	transcript:EES08917	0
1008- 6:	transcript:Zm00001d048723_T001	transcript:EES08911	8.00E-09
1008- 7:	transcript:Zm00001d048726_T001	transcript:EES10195	4.00E-98
1008- 8:	transcript:Zm00001d048727_T001	transcript:EES10193	2.00E-39
1008- 9:	transcript:Zm00001d048729_T001	transcript:OQU83941	9.00E-11
## Alignment 1009: score=424.0 e_value=2.6e-23 N=10 4&5 minus			
1009- 0:	transcript:Zm00001d052636_T001	transcript:OQU83591	3.00E-122
1009- 1:	transcript:Zm00001d052651_T001	transcript:OQU83588	0
1009- 2:	transcript:Zm00001d052653_T003	transcript:OQU83587	0
1009- 3:	transcript:Zm00001d052654_T001	transcript:EES08612	1.00E-102
1009- 4:	transcript:Zm00001d052655_T001	transcript:EES08611	8.00E-164
1009- 5:	transcript:Zm00001d052660_T002	transcript:KXG28573	0
1009- 6:	transcript:Zm00001d052661_T001	transcript:OQU83582	7.00E-70
1009- 7:	transcript:Zm00001d052662_T001	transcript:EES08610	3.00E-124
1009- 8:	transcript:Zm00001d052663_T001	transcript:EES09869	0
1009- 9:	transcript:Zm00001d052666_T015	transcript:OQU83580	0
## Alignment 1010: score=399.0 e_value=3e-22 N=10 4&5 minus			
1010- 0:	transcript:Zm00001d048666_T001	transcript:EES10237	3.00E-17
1010- 1:	transcript:Zm00001d048669_T001	transcript:EES08954	0
1010- 2:	transcript:Zm00001d048670_T001	transcript:OQU83991	0
1010- 3:	transcript:Zm00001d048671_T001	transcript:OQU83990	4.00E-101
1010- 4:	transcript:Zm00001d048672_T001	transcript:EES10228	0
1010- 5:	transcript:Zm00001d048673_T003	transcript:KXG29090	1.00E-166
1010- 6:	transcript:Zm00001d048680_T001	transcript:EES08941	3.00E-147
1010- 7:	transcript:Zm00001d048681_T001	transcript:OQU83977	3.00E-133
1010- 8:	transcript:Zm00001d048683_T001	transcript:OQU83975	0
1010- 9:	transcript:Zm00001d048684_T001	transcript:EES10219	7.00E-66

```
## Alignment 1011: score=391.0 e_value=6.1e-19 N=9 4&5 minus
1011-  0:transcript:Zm00001d048763_T001 transcript:EES08897                0
1011-  1:transcript:Zm00001d048765_T001 transcript:OQU83910          6.00E-57
1011-  2:transcript:Zm00001d048766_T001 transcript:EES10167                0
1011-  3:transcript:Zm00001d048769_T002 transcript:OQU83902          2.00E-80
1011-  4:transcript:Zm00001d048770_T001 transcript:EES10163          2.00E-37
1011-  5:transcript:Zm00001d048771_T001 transcript:EES10162                0
1011-  6:transcript:Zm00001d048772_T001 transcript:OQU83898                0
1011-  7:transcript:Zm00001d048776_T001 transcript:KXG28997                0
1011-  8:transcript:Zm00001d048777_T004 transcript:KXG28995                0
## Alignment 1012: score=345.0 e_value=4.8e-16 N=8 4&5 minus
1012-  0:transcript:Zm00001d052415_T001 transcript:EES09201         6.00E-103
1012-  1:transcript:Zm00001d052416_T002 transcript:OQU82814                0
1012-  2:transcript:Zm00001d052418_T001 transcript:KXG27672                0
1012-  3:transcript:Zm00001d052420_T001 transcript:EES07926                0
1012-  4:transcript:Zm00001d052422_T001 transcript:EES07924         9.00E-107
1012-  5:transcript:Zm00001d052424_T001 transcript:OQU82802          5.00E-85
1012-  6:transcript:Zm00001d052425_T006 transcript:KXG27663                0
1012-  7:transcript:Zm00001d052430_T001 transcript:OQU82801          4.00E-35
## Alignment 1013: score=335.0 e_value=3.4e-13 N=7 4&5 minus
1013-  0:transcript:OQU85111            transcript:Zm00001d016817_T001  6.00E-52
1013-  1:transcript:EES06957            transcript:Zm00001d016816_T002         0
1013-  2:transcript:EES05239            transcript:Zm00001d016815_T001         0
1013-  3:transcript:OQU85115            transcript:Zm00001d016811_T001         0
1013-  4:transcript:EES06958            transcript:Zm00001d016810_T001         0
1013-  5:transcript:OQU85117            transcript:Zm00001d016807_T001 2.00E-160
1013-  6:transcript:KXG30413            transcript:Zm00001d016806_T001         0
## Alignment 1014: score=331.0 e_value=1.5e-14 N=8 4&5 minus
1014-  0:transcript:KXG30018            transcript:Zm00001d015997_T001  9.00E-72
1014-  1:transcript:EES04919            transcript:Zm00001d015992_T001         0
1014-  2:transcript:KXG30022            transcript:Zm00001d015990_T001         0
1014-  3:transcript:EES04922            transcript:Zm00001d015988_T003 3.00E-108
1014-  4:transcript:KXG30028            transcript:Zm00001d015986_T002 7.00E-115
1014-  5:transcript:OQU84803            transcript:Zm00001d015985_T003         0
1014-  6:transcript:EES06669            transcript:Zm00001d015984_T002         0
1014-  7:transcript:EES04926            transcript:Zm00001d015977_T001         0
## Alignment 1015: score=329.0 e_value=3.3e-14 N=8 4&5 minus
1015-  0:transcript:Zm00001d048608_T001 transcript:EES09049                0
1015-  1:transcript:Zm00001d048609_T001 transcript:EES10309         3.00E-133
1015-  2:transcript:Zm00001d048611_T001 transcript:EES09048          2.00E-19
1015-  3:transcript:Zm00001d048614_T001 transcript:EES09047                0
1015-  4:transcript:Zm00001d048615_T001 transcript:EES09046         3.00E-102
1015-  5:transcript:Zm00001d048616_T001 transcript:EES10302         4.00E-158
1015-  6:transcript:Zm00001d048617_T001 transcript:OQU84094          3.00E-66
1015-  7:transcript:Zm00001d048618_T001 transcript:EES09033          2.00E-82
## Alignment 1016: score=321.0 e_value=2e-15 N=8 4&5 minus
1016-  0:transcript:Zm00001d048590_T001 transcript:OQU84125         4.00E-144
1016-  1:transcript:Zm00001d048592_T001 transcript:EES10316                0
1016-  2:transcript:Zm00001d048594_T001 transcript:OQU84123          1.00E-96
1016-  3:transcript:Zm00001d048595_T001 transcript:EES10314         3.00E-156
1016-  4:transcript:Zm00001d048599_T002 transcript:OQU84121          1.00E-78
1016-  5:transcript:Zm00001d048600_T001 transcript:OQU84114          2.00E-88
1016-  6:transcript:Zm00001d048601_T001 transcript:OQU84111                0
1016-  7:transcript:Zm00001d048606_T001 transcript:OQU84103                0
```

Alignment 1017: score=315.0 e_value=2.5e-12 N=7 4&5 minus
1017- 0:transcript:KXG30283 transcript:Zm00001d016595_T014 6.00E-172
1017- 1:transcript:EES05136 transcript:Zm00001d016593_T001 2.00E-86
1017- 2:transcript:EES05138 transcript:Zm00001d016591_T002 0
1017- 3:transcript:EES05139 transcript:Zm00001d016588_T002 0
1017- 4:transcript:KXG30287 transcript:Zm00001d016587_T001 0
1017- 5:transcript:OQU85015 transcript:Zm00001d016586_T002 0
1017- 6:transcript:EES06873 transcript:Zm00001d016584_T001 1.00E-40
Alignment 1018: score=312.0 e_value=4.2e-14 N=7 4&5 minus
1018- 0:transcript:EES06474 transcript:Zm00001d015777_T001 9.00E-113
1018- 1:transcript:EES06475 transcript:Zm00001d015776_T001 1.00E-113
1018- 2:transcript:EES06476 transcript:Zm00001d015772_T001 0
1018- 3:transcript:KXG29743 transcript:Zm00001d015767_T002 2.00E-40
1018- 4:transcript:EES04713 transcript:Zm00001d015765_T004 0
1018- 5:transcript:KXG29745 transcript:Zm00001d015759_T001 1.00E-143
1018- 6:transcript:OQU84591 transcript:Zm00001d015758_T001 0
Alignment 1019: score=290.0 e_value=3.4e-11 N=7 4&5 minus
1019- 0:transcript:Zm00001d048896_T001 transcript:KXG28878 1.00E-31
1019- 1:transcript:Zm00001d048898_T001 transcript:OQU83795 0
1019- 2:transcript:Zm00001d048899_T001 transcript:EES08798 0
1019- 3:transcript:Zm00001d048901_T001 transcript:OQU83792 6.00E-109
1019- 4:transcript:Zm00001d048902_T004 transcript:EES08797 0
1019- 5:transcript:Zm00001d048903_T001 transcript:KXG28871 2.00E-135
1019- 6:transcript:Zm00001d048904_T001 transcript:KXG28867 5.00E-26
Alignment 1020: score=289.0 e_value=2.1e-09 N=6 4&5 minus
1020- 0:transcript:OQU85562 transcript:Zm00001d017728_T001 0
1020- 1:transcript:EES05672 transcript:Zm00001d017727_T001 0
1020- 2:transcript:EES07407 transcript:Zm00001d017726_T001 4.00E-175
1020- 3:transcript:KXG30937 transcript:Zm00001d017725_T001 4.00E-62
1020- 4:transcript:EES05673 transcript:Zm00001d017724_T001 0
1020- 5:transcript:EES05674 transcript:Zm00001d017721_T001 0
Alignment 1021: score=286.0 e_value=3.3e-15 N=8 4&5 minus
1021- 0:transcript:EES04486 transcript:Zm00001d014716_T002 0
1021- 1:transcript:KXG29433 transcript:Zm00001d014704_T002 7.00E-25
1021- 2:transcript:OQU84339 transcript:Zm00001d014701_T002 1.00E-114
1021- 3:transcript:EES04495 transcript:Zm00001d014698_T006 3.00E-128
1021- 4:transcript:EES06258 transcript:Zm00001d014695_T001 0
1021- 5:transcript:EES04498 transcript:Zm00001d014692_T001 1.00E-56
1021- 6:transcript:OQU84350 transcript:Zm00001d014690_T014 0
1021- 7:transcript:EES06260 transcript:Zm00001d014689_T006 0
Alignment 1022: score=283.0 e_value=3.2e-12 N=7 4&5 minus
1022- 0:transcript:Zm00001d048643_T002 transcript:EES10269 0
1022- 1:transcript:Zm00001d048644_T001 transcript:OQU84025 1.00E-155
1022- 2:transcript:Zm00001d048647_T001 transcript:EES10257 1.00E-136
1022- 3:transcript:Zm00001d048648_T006 transcript:OQU84022 0
1022- 4:transcript:Zm00001d048649_T001 transcript:EES08981 2.00E-90
1022- 5:transcript:Zm00001d048651_T001 transcript:OQU84013 1.00E-132
1022- 6:transcript:Zm00001d048655_T012 transcript:EES08974 0
Alignment 1023: score=277.0 e_value=2.6e-09 N=6 4&5 minus
1023- 0:transcript:EES06649 transcript:Zm00001d016008_T001 0
1023- 1:transcript:KXG30006 transcript:Zm00001d016006_T001 0
1023- 2:transcript:EES06653 transcript:Zm00001d016002_T002 2.00E-161
1023- 3:transcript:OQU84790 transcript:Zm00001d016001_T002 0
1023- 4:transcript:EES06654 transcript:Zm00001d016000_T006 9.00E-38

1023- 5:transcript:OQU84793 transcript:Zm00001d015999_T001 0
Alignment 1024: score=276.0 e_value=3.1e-09 N=6 4&5 minus
1024- 0:transcript:Zm00001d048829_T001 transcript:EES10116 0
1024- 1:transcript:Zm00001d048831_T001 transcript:EES08851 5.00E-76
1024- 2:transcript:Zm00001d048832_T001 transcript:EES08846 0
1024- 3:transcript:Zm00001d048833_T001 transcript:EES10114 2.00E-107
1024- 4:transcript:Zm00001d048834_T001 transcript:OQU83842 0
1024- 5:transcript:Zm00001d048835_T004 transcript:EES08844 0
Alignment 1025: score=263.0 e_value=3.8e-09 N=6 4&5 minus
1025- 0:transcript:EES06644 transcript:Zm00001d016031_T002 0
1025- 1:transcript:EES06645 transcript:Zm00001d016029_T001 2.00E-162
1025- 2:transcript:KXG29998 transcript:Zm00001d016022_T001 1.00E-51
1025- 3:transcript:OQU84787 transcript:Zm00001d016021_T001 0
1025- 4:transcript:KXG30001 transcript:Zm00001d016019_T001 3.00E-167
1025- 5:transcript:EES06648 transcript:Zm00001d016016_T001 1.00E-51
Alignment 1026: score=253.0 e_value=4e-09 N=6 4&5 minus
1026- 0:transcript:Zm00001d048691_T001 transcript:EES08930 0
1026- 1:transcript:Zm00001d048692_T003 transcript:EES10216 0
1026- 2:transcript:Zm00001d048693_T001 transcript:OQU83960 8.00E-160
1026- 3:transcript:Zm00001d048695_T001 transcript:KXG29052 0
1026- 4:transcript:Zm00001d048696_T001 transcript:KXG29050 0
1026- 5:transcript:Zm00001d048697_T003 transcript:KXG29049 4.00E-157
Alignment 1027: score=251.0 e_value=1e-09 N=6 4&5 minus
1027- 0:transcript:KXG30661 transcript:Zm00001d014032_T001 1.00E-80
1027- 1:transcript:EES07173 transcript:Zm00001d014029_T001 3.00E-82
1027- 2:transcript:EES05457 transcript:Zm00001d014016_T001 6.00E-118
1027- 3:transcript:OQU85349 transcript:Zm00001d014015_T001 8.00E-38
1027- 4:transcript:EES05458 transcript:Zm00001d014013_T002 0
1027- 5:transcript:EES07181 transcript:Zm00001d014007_T001 9.00E-11
Alignment 1028: score=980.0 e_value=3.8e-70 N=23 4&6 plus
1028- 0:transcript:Zm00001d051235_T001 transcript:EES12544 2.00E-132
1028- 1:transcript:Zm00001d051238_T001 transcript:KXG26766 9.00E-42
1028- 2:transcript:Zm00001d051239_T001 transcript:KXG26694 1.00E-83
1028- 3:transcript:Zm00001d051241_T001 transcript:EES11157 0
1028- 4:transcript:Zm00001d051242_T002 transcript:OQU82010 0
1028- 5:transcript:Zm00001d051249_T003 transcript:EES11168 2.00E-68
1028- 6:transcript:Zm00001d051262_T001 transcript:EES12556 2.00E-106
1028- 7:transcript:Zm00001d051267_T001 transcript:EES11177 4.00E-90
1028- 8:transcript:Zm00001d051269_T001 transcript:EES11178 6.00E-107
1028- 9:transcript:Zm00001d051272_T001 transcript:KXG26783 3.00E-123
1028- 10:transcript:Zm00001d051287_T001 transcript:KXG26784 2.00E-174
1028- 11:transcript:Zm00001d051288_T001 transcript:EES12558 4.00E-88
1028- 12:transcript:Zm00001d051294_T001 transcript:OQU82026 1.00E-93
1028- 13:transcript:Zm00001d051302_T001 transcript:EES11187 3.00E-41
1028- 14:transcript:Zm00001d051305_T001 transcript:EES11188 1.00E-90
1028- 15:transcript:Zm00001d051306_T001 transcript:EES11189 0
1028- 16:transcript:Zm00001d051307_T001 transcript:EES12570 4.00E-177
1028- 17:transcript:Zm00001d051308_T009 transcript:KXG26789 0
1028- 18:transcript:Zm00001d051309_T001 transcript:EES11195 3.00E-78
1028- 19:transcript:Zm00001d051314_T001 transcript:OQU82043 4.00E-149
1028- 20:transcript:Zm00001d051316_T001 transcript:OQU82048 3.00E-48
1028- 21:transcript:Zm00001d051322_T001 transcript:EES11201 7.00E-20
1028- 22:transcript:Zm00001d051323_T003 transcript:EES12581 0
Alignment 1029: score=933.0 e_value=4.6e-61 N=21 4&6 plus

1029- 0:transcript:Zm00001d051328_T002 transcript:KXG26823 4.00E-70
1029- 1:transcript:Zm00001d051333_T001 transcript:EES12600 7.00E-28
1029- 2:transcript:Zm00001d051334_T001 transcript:EES12602 9.00E-97
1029- 3:transcript:Zm00001d051337_T002 transcript:EES11210 3.00E-120
1029- 4:transcript:Zm00001d051340_T001 transcript:EES11211 6.00E-59
1029- 5:transcript:Zm00001d051343_T001 transcript:EES12610 2.00E-135
1029- 6:transcript:Zm00001d051344_T001 transcript:OQU82082 2.00E-14
1029- 7:transcript:Zm00001d051350_T001 transcript:OQU82086 7.00E-45
1029- 8:transcript:Zm00001d051359_T001 transcript:OQU82091 0
1029- 9:transcript:Zm00001d051360_T001 transcript:KXG26850 2.00E-64
1029- 10:transcript:Zm00001d051362_T001 transcript:OQU82093 9.00E-57
1029- 11:transcript:Zm00001d051365_T001 transcript:OQU82096 3.00E-35
1029- 12:transcript:Zm00001d051368_T001 transcript:EES11234 9.00E-73
1029- 13:transcript:Zm00001d051370_T001 transcript:EES11237 8.00E-49
1029- 14:transcript:Zm00001d051371_T001 transcript:EES11238 1.00E-59
1029- 15:transcript:Zm00001d051383_T001 transcript:KXG26858 1.00E-67
1029- 16:transcript:Zm00001d051384_T001 transcript:EES11241 2.00E-55
1029- 17:transcript:Zm00001d051387_T001 transcript:EES11245 0
1029- 18:transcript:Zm00001d051388_T001 transcript:EES12628 4.00E-87
1029- 19:transcript:Zm00001d051392_T001 transcript:EES11249 6.00E-117
1029- 20:transcript:Zm00001d051393_T002 transcript:OQU82115 5.00E-24
Alignment 1030: score=638.0 e_value=6e-36 N=15 4&6 plus
1030- 0:transcript:EES05526 transcript:Zm00001d037218_T001 1.00E-107
1030- 1:transcript:EES07256 transcript:Zm00001d037220_T001 8.00E-99
1030- 2:transcript:EES07255 transcript:Zm00001d037221_T001 1.00E-120
1030- 3:transcript:EES07258 transcript:Zm00001d037225_T001 4.00E-157
1030- 4:transcript:KXG30762 transcript:Zm00001d037227_T002 3.00E-88
1030- 5:transcript:EES07262 transcript:Zm00001d037228_T001 7.00E-173
1030- 6:transcript:EES05532 transcript:Zm00001d037234_T005 0
1030- 7:transcript:EES07268 transcript:Zm00001d037237_T002 3.00E-92
1030- 8:transcript:EES07273 transcript:Zm00001d037239_T001 8.00E-19
1030- 9:transcript:EES05540 transcript:Zm00001d037243_T001 5.00E-48
1030- 10:transcript:EES07275 transcript:Zm00001d037244_T001 4.00E-45
1030- 11:transcript:EES07276 transcript:Zm00001d037246_T002 2.00E-169
1030- 12:transcript:EES05543 transcript:Zm00001d037247_T007 0
1030- 13:transcript:KXG30786 transcript:Zm00001d037251_T001 6.00E-56
1030- 14:transcript:EES07283 transcript:Zm00001d037257_T001 0
Alignment 1031: score=598.0 e_value=3.2e-35 N=14 4&6 plus
1031- 0:transcript:Zm00001d051460_T001 transcript:KXG26971 2.00E-59
1031- 1:transcript:Zm00001d051461_T001 transcript:OQU82196 6.00E-145
1031- 2:transcript:Zm00001d051465_T002 transcript:KXG26975 6.00E-104
1031- 3:transcript:Zm00001d051468_T001 transcript:EES12708 5.00E-39
1031- 4:transcript:Zm00001d051471_T001 transcript:EES12712 3.00E-101
1031- 5:transcript:Zm00001d051475_T002 transcript:EES12722 1.00E-33
1031- 6:transcript:Zm00001d051478_T001 transcript:EES12723 7.00E-67
1031- 7:transcript:Zm00001d051479_T014 transcript:KXG26989 0
1031- 8:transcript:Zm00001d051480_T001 transcript:OQU82207 4.00E-55
1031- 9:transcript:Zm00001d051492_T001 transcript:KXG27001 9.00E-17
1031- 10:transcript:Zm00001d051495_T001 transcript:EES12737 2.00E-24
1031- 11:transcript:Zm00001d051503_T001 transcript:EES11330 2.00E-33
1031- 12:transcript:Zm00001d051504_T001 transcript:EES11332 3.00E-166
1031- 13:transcript:Zm00001d051505_T001 transcript:EES12746 2.00E-59
Alignment 1032: score=490.0 e_value=6e-23 N=11 4&6 plus
1032- 0:transcript:Zm00001d051135_T005 transcript:KXG26713 0

1032- 1:transcript:Zm00001d051140_T001 transcript:KXG26719 5.00E-93
1032- 2:transcript:Zm00001d051143_T001 transcript:EES11114 4.00E-97
1032- 3:transcript:Zm00001d051149_T001 transcript:EES12516 3.00E-93
1032- 4:transcript:Zm00001d051156_T001 transcript:EES11118 3.00E-148
1032- 5:transcript:Zm00001d051157_T001 transcript:EES12520 0
1032- 6:transcript:Zm00001d051161_T003 transcript:EES12521 0
1032- 7:transcript:Zm00001d051163_T001 transcript:OQU81984 0
1032- 8:transcript:Zm00001d051172_T001 transcript:KXG26729 0
1032- 9:transcript:Zm00001d051174_T001 transcript:EES11124 0
1032- 10:transcript:Zm00001d051180_T002 transcript:EES12528 6.00E-106
Alignment 1033: score=384.0 e_value=2.7e-18 N=9 4&6 plus
1033- 0:transcript:EES06356 transcript:Zm00001d036672_T005 0
1033- 1:transcript:OQU84476 transcript:Zm00001d036676_T001 3.00E-33
1033- 2:transcript:OQU84478 transcript:Zm00001d036678_T002 0
1033- 3:transcript:KXG29599 transcript:Zm00001d036690_T001 8.00E-13
1033- 4:transcript:OQU84482 transcript:Zm00001d036692_T001 9.00E-85
1033- 5:transcript:EES04622 transcript:Zm00001d036699_T001 2.00E-34
1033- 6:transcript:EES06371 transcript:Zm00001d036700_T001 5.00E-35
1033- 7:transcript:EES06373 transcript:Zm00001d036703_T001 2.00E-17
1033- 8:transcript:EES04626 transcript:Zm00001d036708_T001 1.00E-42
Alignment 1034: score=356.0 e_value=1.1e-17 N=9 4&6 plus
1034- 0:transcript:Zm00001d050747_T007 transcript:KXG26359 0
1034- 1:transcript:Zm00001d050753_T001 transcript:OQU81625 0
1034- 2:transcript:Zm00001d050755_T003 transcript:EES12170 3.00E-107
1034- 3:transcript:Zm00001d050768_T003 transcript:EES10856 3.00E-81
1034- 4:transcript:Zm00001d050787_T001 transcript:EES12227 2.00E-27
1034- 5:transcript:Zm00001d050798_T001 transcript:EES10862 2.00E-40
1034- 6:transcript:Zm00001d050800_T001 transcript:EES10864 0
1034- 7:transcript:Zm00001d050805_T001 transcript:EES12235 3.00E-17
1034- 8:transcript:Zm00001d050816_T002 transcript:KXG26388 6.00E-116
Alignment 1035: score=350.0 e_value=1.9e-15 N=8 4&6 plus
1035- 0:transcript:EES05493 transcript:Zm00001d037194_T001 3.00E-39
1035- 1:transcript:EES05494 transcript:Zm00001d037197_T001 9.00E-06
1035- 2:transcript:OQU85383 transcript:Zm00001d037198_T001 0
1035- 3:transcript:KXG30711 transcript:Zm00001d037200_T004 7.00E-116
1035- 4:transcript:EES07217 transcript:Zm00001d037204_T001 7.00E-98
1035- 5:transcript:KXG30713 transcript:Zm00001d037205_T001 3.00E-140
1035- 6:transcript:KXG30724 transcript:Zm00001d037209_T001 2.00E-73
1035- 7:transcript:EES05513 transcript:Zm00001d037211_T001 6.00E-175
Alignment 1036: score=273.0 e_value=1.4e-10 N=7 4&6 plus
1036- 0:transcript:EES06507 transcript:Zm00001d036945_T001 3.00E-173
1036- 1:transcript:OQU84632 transcript:Zm00001d036947_T001 0
1036- 2:transcript:EES06511 transcript:Zm00001d036949_T004 0
1036- 3:transcript:EES04755 transcript:Zm00001d036956_T001 5.00E-112
1036- 4:transcript:EES04758 transcript:Zm00001d036959_T003 0
1036- 5:transcript:OQU84653 transcript:Zm00001d036961_T004 0
1036- 6:transcript:EES06526 transcript:Zm00001d036966_T001 8.00E-124
Alignment 1037: score=265.0 e_value=2.4e-08 N=6 4&6 plus
1037- 0:transcript:Zm00001d051439_T001 transcript:OQU82171 6.00E-95
1037- 1:transcript:Zm00001d051441_T001 transcript:KXG26934 9.00E-141
1037- 2:transcript:Zm00001d051442_T002 transcript:KXG26943 0
1037- 3:transcript:Zm00001d051447_T001 transcript:EES11284 4.00E-146
1037- 4:transcript:Zm00001d051448_T001 transcript:KXG26945 1.00E-66
1037- 5:transcript:Zm00001d051451_T001 transcript:EES11287 1.00E-64

Alignment 1038: score=265.0 e_value=1.1e-08 N=6 4&6 plus
1038- 0:transcript:Zm00001d050874_T001 transcript:EES12291 5.00E-101
1038- 1:transcript:Zm00001d050893_T001 transcript:EES10946 3.00E-104
1038- 2:transcript:Zm00001d050897_T002 transcript:OQU81712 1.00E-54
1038- 3:transcript:Zm00001d050899_T001 transcript:EES12297 1.00E-59
1038- 4:transcript:Zm00001d050903_T001 transcript:EES10954 5.00E-21
1038- 5:transcript:Zm00001d050905_T001 transcript:EES10953 2.00E-76
Alignment 1039: score=260.0 e_value=1.8e-10 N=7 4&6 plus
1039- 0:transcript:Zm00001d051041_T001 transcript:OQU81866 3.00E-147
1039- 1:transcript:Zm00001d051047_T001 transcript:EES12446 7.00E-121
1039- 2:transcript:Zm00001d051056_T001 transcript:OQU81878 0
1039- 3:transcript:Zm00001d051061_T001 transcript:EES12451 3.00E-64
1039- 4:transcript:Zm00001d051067_T003 transcript:OQU81892 6.00E-172
1039- 5:transcript:Zm00001d051069_T001 transcript:KXG26657 0
1039- 6:transcript:Zm00001d051080_T004 transcript:KXG26662 3.00E-52
Alignment 1040: score=412.0 e_value=0 N=9 4&6 minus
1040- 0:transcript:KXG31165 transcript:Zm00001d037173_T001 2.00E-38
1040- 1:transcript:KXG31166 transcript:Zm00001d037172_T001 2.00E-169
1040- 2:transcript:OQU85787 transcript:Zm00001d037171_T001 1.00E-22
1040- 3:transcript:EES07580 transcript:Zm00001d037170_T001 7.00E-112
1040- 4:transcript:KXG31176 transcript:Zm00001d037167_T001 4.00E-74
1040- 5:transcript:EES07588 transcript:Zm00001d037165_T001 4.00E-53
1040- 6:transcript:EES07590 transcript:Zm00001d037163_T002 5.00E-119
1040- 7:transcript:EES07591 transcript:Zm00001d037160_T002 0
1040- 8:transcript:OQU85793 transcript:Zm00001d037158_T001 5.00E-52
Alignment 1041: score=271.0 e_value=1.2e-08 N=6 4&6 minus
1041- 0:transcript:Zm00001d050190_T001 transcript:KXG27066 8.00E-27
1041- 1:transcript:Zm00001d050191_T001 transcript:EES12814 0
1041- 2:transcript:Zm00001d050193_T001 transcript:KXG27000 1.00E-130
1041- 3:transcript:Zm00001d050195_T001 transcript:EES11384 4.00E-65
1041- 4:transcript:Zm00001d050201_T001 transcript:EES12801 2.00E-104
1041- 5:transcript:Zm00001d050208_T003 transcript:KXG27049 0
Alignment 1042: score=1611.0 e_value=7.2e-126 N=36 4&7 plus
1042- 0:transcript:Zm00001d050329_T003 transcript:KXG25169 0
1042- 1:transcript:Zm00001d050330_T003 transcript:KXG25172 7.00E-155
1042- 2:transcript:Zm00001d050333_T001 transcript:KXG25173 1.00E-127
1042- 3:transcript:Zm00001d050335_T001 transcript:EES15000 0
1042- 4:transcript:Zm00001d050336_T003 transcript:EES15001 0
1042- 5:transcript:Zm00001d050339_T001 transcript:EES13852 0
1042- 6:transcript:Zm00001d050340_T001 transcript:OQU80464 0
1042- 7:transcript:Zm00001d050341_T001 transcript:EES13856 1.00E-64
1042- 8:transcript:Zm00001d050346_T001 transcript:EES13861 0
1042- 9:transcript:Zm00001d050347_T002 transcript:EES15006 5.00E-97
1042- 10:transcript:Zm00001d050350_T001 transcript:KXG25180 6.00E-95
1042- 11:transcript:Zm00001d050352_T001 transcript:EES13865 1.00E-100
1042- 12:transcript:Zm00001d050355_T001 transcript:EES15012 0
1042- 13:transcript:Zm00001d050357_T005 transcript:EES15014 0
1042- 14:transcript:Zm00001d050359_T001 transcript:EES13867 0
1042- 15:transcript:Zm00001d050365_T006 transcript:EES15016 1.00E-162
1042- 16:transcript:Zm00001d050368_T001 transcript:EES15017 7.00E-100
1042- 17:transcript:Zm00001d050371_T001 transcript:OQU80479 0
1042- 18:transcript:Zm00001d050372_T002 transcript:EES15019 0
1042- 19:transcript:Zm00001d050375_T001 transcript:OQU80487 0
1042- 20:transcript:Zm00001d050380_T001 transcript:KXG25196 0

1042- 21:transcript:Zm00001d050382_T001 transcript:EES13877 0
1042- 22:transcript:Zm00001d050383_T001 transcript:EES15031 0
1042- 23:transcript:Zm00001d050384_T001 transcript:EES13880 4.00E-69
1042- 24:transcript:Zm00001d050386_T001 transcript:OQU80503 3.00E-29
1042- 25:transcript:Zm00001d050389_T001 transcript:EES13882 2.00E-102
1042- 26:transcript:Zm00001d050393_T001 transcript:EES15037 2.00E-104
1042- 27:transcript:Zm00001d050394_T001 transcript:EES15038 3.00E-144
1042- 28:transcript:Zm00001d050395_T002 transcript:EES15039 1.00E-153
1042- 29:transcript:Zm00001d050400_T001 transcript:OQU80505 0
1042- 30:transcript:Zm00001d050401_T003 transcript:EES13890 0
1042- 31:transcript:Zm00001d050403_T001 transcript:KXG25214 2.00E-171
1042- 32:transcript:Zm00001d050406_T008 transcript:KXG25219 0
1042- 33:transcript:Zm00001d050409_T001 transcript:OQU80513 0
1042- 34:transcript:Zm00001d050410_T001 transcript:EES13897 0
1042- 35:transcript:Zm00001d050411_T001 transcript:EES15047 6.00E-81
Alignment 1043: score=651.0 e_value=3.8e-39 N=15 4&7 plus
1043- 0:transcript:Zm00001d050259_T002 transcript:OQU80389 0
1043- 1:transcript:Zm00001d050265_T001 transcript:KXG25127 0
1043- 2:transcript:Zm00001d050267_T001 transcript:KXG25109 8.00E-10
1043- 3:transcript:Zm00001d050269_T001 transcript:EES13801 3.00E-138
1043- 4:transcript:Zm00001d050273_T001 transcript:EES13807 0
1043- 5:transcript:Zm00001d050277_T001 transcript:KXG25122 2.00E-61
1043- 6:transcript:Zm00001d050283_T001 transcript:OQU80410 0
1043- 7:transcript:Zm00001d050284_T005 transcript:EES13811 0
1043- 8:transcript:Zm00001d050285_T001 transcript:EES14968 2.00E-84
1043- 9:transcript:Zm00001d050294_T001 transcript:OQU80413 2.00E-137
1043- 10:transcript:Zm00001d050300_T001 transcript:OQU80418 4.00E-76
1043- 11:transcript:Zm00001d050302_T001 transcript:OQU80420 0
1043- 12:transcript:Zm00001d050303_T001 transcript:EES14976 0
1043- 13:transcript:Zm00001d050304_T002 transcript:EES13821 0
1043- 14:transcript:Zm00001d050307_T001 transcript:EES13822 6.00E-72
Alignment 1044: score=547.0 e_value=7.1e-28 N=12 4&7 plus
1044- 0:transcript:Zm00001d050241_T001 transcript:EES13765 0
1044- 1:transcript:Zm00001d050242_T002 transcript:EES13767 1.00E-125
1044- 2:transcript:Zm00001d050243_T001 transcript:EES14939 0
1044- 3:transcript:Zm00001d050244_T002 transcript:OQU80376 0
1044- 4:transcript:Zm00001d050245_T001 transcript:EES14941 6.00E-83
1044- 5:transcript:Zm00001d050247_T001 transcript:OQU80381 2.00E-175
1044- 6:transcript:Zm00001d050251_T002 transcript:EES13774 0
1044- 7:transcript:Zm00001d050253_T002 transcript:EES13775 0
1044- 8:transcript:Zm00001d050255_T001 transcript:OQU80383 0
1044- 9:transcript:Zm00001d050256_T001 transcript:KXG25094 2.00E-102
1044- 10:transcript:Zm00001d050258_T001 transcript:EES14951 2.00E-170
1044- 11:transcript:Zm00001d050259_T002 transcript:KXG25097 0
Alignment 1045: score=498.0 e_value=5e-27 N=11 4&7 plus
1045- 0:transcript:Zm00001d052753_T002 transcript:EES13174 0
1045- 1:transcript:Zm00001d052756_T001 transcript:EES13179 3.00E-142
1045- 2:transcript:Zm00001d052757_T001 transcript:EES13180 1.00E-100
1045- 3:transcript:Zm00001d052758_T001 transcript:EES14349 0
1045- 4:transcript:Zm00001d052759_T001 transcript:EES13181 4.00E-24
1045- 5:transcript:Zm00001d052760_T001 transcript:EES14354 3.00E-104
1045- 6:transcript:Zm00001d052763_T001 transcript:KXG24164 0
1045- 7:transcript:Zm00001d052765_T002 transcript:KXG24166 0
1045- 8:transcript:Zm00001d052766_T001 transcript:EES14356 0

```
1045-  9:transcript:Zm00001d052767_T002 transcript:EES14357                    0
1045- 10:transcript:Zm00001d052768_T001 transcript:EES14358            5.00E-127
## Alignment 1046: score=427.0 e_value=2.7e-21 N=10 4&7 plus
1046-  0:transcript:Zm00001d050201_T001 transcript:EES13579            1.00E-116
1046-  1:transcript:Zm00001d050202_T002 transcript:OQU80144                    0
1046-  2:transcript:Zm00001d050208_T003 transcript:EES14767                    0
1046-  3:transcript:Zm00001d050211_T001 transcript:EES13588                    0
1046-  4:transcript:Zm00001d050214_T001 transcript:EES13591                    0
1046-  5:transcript:Zm00001d050216_T003 transcript:KXG24799                    0
1046-  6:transcript:Zm00001d050218_T001 transcript:EES13592                    0
1046-  7:transcript:Zm00001d050219_T004 transcript:KXG24803             2.00E-96
1046-  8:transcript:Zm00001d050222_T001 transcript:EES13595             3.00E-41
1046-  9:transcript:Zm00001d050224_T002 transcript:EES13602                    0
## Alignment 1047: score=354.0 e_value=2.3e-14 N=8 4&7 plus
1047-  0:transcript:Zm00001d050308_T001 transcript:OQU80426                    0
1047-  1:transcript:Zm00001d050310_T001 transcript:KXG25139            2.00E-120
1047-  2:transcript:Zm00001d050315_T001 transcript:OQU80427                    0
1047-  3:transcript:Zm00001d050316_T035 transcript:KXG25141                    0
1047-  4:transcript:Zm00001d050319_T001 transcript:KXG25145            3.00E-161
1047-  5:transcript:Zm00001d050320_T001 transcript:EES14990                    0
1047-  6:transcript:Zm00001d050323_T001 transcript:OQU80433            3.00E-174
1047-  7:transcript:Zm00001d050324_T002 transcript:OQU80434             2.00E-06
## Alignment 1048: score=271.0 e_value=3.4e-11 N=7 4&7 plus
1048-  0:transcript:Zm00001d050097_T001 transcript:OQU80592                    0
1048-  1:transcript:Zm00001d050099_T001 transcript:OQU80595             2.00E-59
1048-  2:transcript:Zm00001d050100_T001 transcript:EES15092             3.00E-98
1048-  3:transcript:Zm00001d050103_T002 transcript:EES13969                    0
1048-  4:transcript:Zm00001d050104_T002 transcript:KXG25287                    0
1048-  5:transcript:Zm00001d050106_T001 transcript:KXG25288            2.00E-127
1048-  6:transcript:Zm00001d050107_T001 transcript:EES13971                    0
## Alignment 1049: score=254.0 e_value=5.6e-09 N=6 4&7 plus
1049-  0:transcript:Zm00001d049880_T001 transcript:EES15148                    0
1049-  1:transcript:Zm00001d049881_T001 transcript:KXG25347            4.00E-136
1049-  2:transcript:Zm00001d049883_T001 transcript:EES15149             3.00E-78
1049-  3:transcript:Zm00001d049884_T006 transcript:EES15150                    0
1049-  4:transcript:Zm00001d049886_T001 transcript:OQU80669             7.00E-67
1049-  5:transcript:Zm00001d049889_T001 transcript:OQU80673            2.00E-120
## Alignment 1050: score=2080.0 e_value=1.8e-177 N=47 4&7 minus
1050-  0:transcript:Zm00001d052794_T001 transcript:EES15481                    0
1050-  1:transcript:Zm00001d052795_T001 transcript:EES15477                    0
1050-  2:transcript:Zm00001d052796_T003 transcript:EES14338            2.00E-160
1050-  3:transcript:Zm00001d052797_T002 transcript:EES14335                    0
1050-  4:transcript:Zm00001d052798_T001 transcript:EES15479            3.00E-154
1050-  5:transcript:Zm00001d052799_T001 transcript:OQU81032            9.00E-177
1050-  6:transcript:Zm00001d052800_T002 transcript:EES15476                    0
1050-  7:transcript:Zm00001d052801_T004 transcript:OQU81022                    0
1050-  8:transcript:Zm00001d052803_T004 transcript:KXG25727             3.00E-90
1050-  9:transcript:Zm00001d052804_T001 transcript:EES14333            2.00E-137
1050- 10:transcript:Zm00001d052807_T001 transcript:KXG25724             2.00E-64
1050- 11:transcript:Zm00001d052809_T002 transcript:EES14330                    0
1050- 12:transcript:Zm00001d052810_T001 transcript:KXG25721                    0
1050- 13:transcript:Zm00001d052813_T001 transcript:EES14328                    0
1050- 14:transcript:Zm00001d052814_T001 transcript:EES15471             1.00E-48
1050- 15:transcript:Zm00001d052815_T001 transcript:EES14325             7.00E-93
```

1050- 16:	transcript:Zm00001d052816_T001	transcript:EES15461	4.00E-180
1050- 17:	transcript:Zm00001d052817_T001	transcript:EES15463	2.00E-56
1050- 18:	transcript:Zm00001d052818_T001	transcript:EES15462	9.00E-110
1050- 19:	transcript:Zm00001d052820_T001	transcript:OQU81002	2.00E-158
1050- 20:	transcript:Zm00001d052827_T001	transcript:EES15454	0
1050- 21:	transcript:Zm00001d052835_T001	transcript:EES15451	0
1050- 22:	transcript:Zm00001d052837_T001	transcript:EES15449	0
1050- 23:	transcript:Zm00001d052838_T001	transcript:EES15444	6.00E-83
1050- 24:	transcript:Zm00001d052840_T016	transcript:OQU80990	0
1050- 25:	transcript:Zm00001d052842_T001	transcript:EES14310	3.00E-102
1050- 26:	transcript:Zm00001d052843_T001	transcript:EES14309	3.00E-88
1050- 27:	transcript:Zm00001d052845_T009	transcript:EES15437	2.00E-157
1050- 28:	transcript:Zm00001d052847_T001	transcript:KXG25679	0
1050- 29:	transcript:Zm00001d052848_T004	transcript:EES14305	0
1050- 30:	transcript:Zm00001d052851_T002	transcript:EES15427	0
1050- 31:	transcript:Zm00001d052852_T001	transcript:KXG25674	0
1050- 32:	transcript:Zm00001d052853_T001	transcript:EES15429	0
1050- 33:	transcript:Zm00001d052854_T001	transcript:OQU80975	0
1050- 34:	transcript:Zm00001d052855_T001	transcript:EES14299	0
1050- 35:	transcript:Zm00001d052856_T001	transcript:EES15422	0
1050- 36:	transcript:Zm00001d052859_T001	transcript:KXG25664	0
1050- 37:	transcript:Zm00001d052864_T001	transcript:EES14287	4.00E-130
1050- 38:	transcript:Zm00001d052873_T001	transcript:KXG25654	0
1050- 39:	transcript:Zm00001d052874_T001	transcript:KXG25656	7.00E-112
1050- 40:	transcript:Zm00001d052875_T001	transcript:OQU80956	0
1050- 41:	transcript:Zm00001d052876_T001	transcript:EES14282	0
1050- 42:	transcript:Zm00001d052882_T001	transcript:EES15413	8.00E-46
1050- 43:	transcript:Zm00001d052883_T001	transcript:EES15412	0
1050- 44:	transcript:Zm00001d052885_T001	transcript:EES15406	5.00E-90
1050- 45:	transcript:Zm00001d052886_T001	transcript:KXG25647	4.00E-172
1050- 46:	transcript:Zm00001d052888_T004	transcript:EES15409	0
## Alignment 1051: score=1705.0 e_value=1.6e-142 N=40 4&7 minus			
1051- 0:	transcript:Zm00001d049762_T006	transcript:OQU80872	0
1051- 1:	transcript:Zm00001d049763_T002	transcript:EES14236	0
1051- 2:	transcript:Zm00001d049764_T001	transcript:KXG25593	0
1051- 3:	transcript:Zm00001d049765_T001	transcript:EES15344	3.00E-151
1051- 4:	transcript:Zm00001d049766_T001	transcript:EES14235	0
1051- 5:	transcript:Zm00001d049767_T001	transcript:EES15338	0
1051- 6:	transcript:Zm00001d049768_T001	transcript:KXG25581	1.00E-106
1051- 7:	transcript:Zm00001d049769_T001	transcript:EES15334	7.00E-31
1051- 8:	transcript:Zm00001d049784_T001	transcript:OQU80859	1.00E-55
1051- 9:	transcript:Zm00001d049785_T003	transcript:EES15333	0
1051- 10:	transcript:Zm00001d049786_T001	transcript:EES14216	0
1051- 11:	transcript:Zm00001d049789_T001	transcript:EES15332	8.00E-104
1051- 12:	transcript:Zm00001d049790_T007	transcript:EES15331	3.00E-95
1051- 13:	transcript:Zm00001d049795_T001	transcript:EES14208	4.00E-38
1051- 14:	transcript:Zm00001d049796_T001	transcript:OQU80855	0
1051- 15:	transcript:Zm00001d049804_T001	transcript:EES14210	0
1051- 16:	transcript:Zm00001d049805_T001	transcript:EES14200	0
1051- 17:	transcript:Zm00001d049806_T001	transcript:EES14205	3.00E-58
1051- 18:	transcript:Zm00001d049807_T001	transcript:OQU80836	1.00E-22
1051- 19:	transcript:Zm00001d049809_T001	transcript:EES14202	0
1051- 20:	transcript:Zm00001d049813_T001	transcript:OQU80829	2.00E-94
1051- 21:	transcript:Zm00001d049815_T005	transcript:OQU80827	4.00E-90

1051- 22:transcript:Zm00001d049816_T001 transcript:EES15312 2.00E-69
1051- 23:transcript:Zm00001d049817_T003 transcript:KXG25536 0
1051- 24:transcript:Zm00001d049821_T001 transcript:EES15311 1.00E-146
1051- 25:transcript:Zm00001d049822_T001 transcript:KXG25534 0
1051- 26:transcript:Zm00001d049823_T001 transcript:EES15308 4.00E-160
1051- 27:transcript:Zm00001d049826_T001 transcript:EES14191 7.00E-79
1051- 28:transcript:Zm00001d049830_T002 transcript:EES15306 7.00E-106
1051- 29:transcript:Zm00001d049831_T001 transcript:EES15304 0
1051- 30:transcript:Zm00001d049832_T001 transcript:EES15303 0
1051- 31:transcript:Zm00001d049834_T001 transcript:EES14189 9.00E-90
1051- 32:transcript:Zm00001d049835_T004 transcript:EES14188 6.00E-152
1051- 33:transcript:Zm00001d049851_T001 transcript:EES15301 2.00E-108
1051- 34:transcript:Zm00001d049853_T001 transcript:KXG25524 0
1051- 35:transcript:Zm00001d049855_T002 transcript:EES15299 3.00E-113
1051- 36:transcript:Zm00001d049856_T002 transcript:OQU80819 1.00E-55
1051- 37:transcript:Zm00001d049857_T002 transcript:EES14182 0
1051- 38:transcript:Zm00001d049858_T001 transcript:EES14180 1.00E-47
1051- 39:transcript:Zm00001d049860_T001 transcript:OQU80816 4.00E-106
Alignment 1052: score=1057.0 e_value=1.7e-65 N=23 4&7 minus
1052- 0:transcript:Zm00001d053196_T001 transcript:EES15248 0
1052- 1:transcript:Zm00001d053197_T006 transcript:EES15247 6.00E-83
1052- 2:transcript:Zm00001d053200_T001 transcript:EES15245 0
1052- 3:transcript:Zm00001d053202_T001 transcript:OQU80753 0
1052- 4:transcript:Zm00001d053207_T001 transcript:KXG25445 0
1052- 5:transcript:Zm00001d053208_T001 transcript:KXG25440 0
1052- 6:transcript:Zm00001d053210_T001 transcript:OQU80746 1.00E-164
1052- 7:transcript:Zm00001d053211_T009 transcript:EES15240 8.00E-128
1052- 8:transcript:Zm00001d053212_T002 transcript:OQU80744 6.00E-67
1052- 9:transcript:Zm00001d053214_T001 transcript:EES15234 0
1052- 10:transcript:Zm00001d053215_T001 transcript:EES15233 0
1052- 11:transcript:Zm00001d053216_T001 transcript:KXG25432 1.00E-164
1052- 12:transcript:Zm00001d053217_T001 transcript:EES15231 0
1052- 13:transcript:Zm00001d053220_T001 transcript:EES14116 4.00E-138
1052- 14:transcript:Zm00001d053223_T001 transcript:EES14115 4.00E-178
1052- 15:transcript:Zm00001d053225_T005 transcript:KXG25431 0
1052- 16:transcript:Zm00001d053228_T002 transcript:EES14114 3.00E-86
1052- 17:transcript:Zm00001d053229_T001 transcript:EES15227 6.00E-34
1052- 18:transcript:Zm00001d053230_T001 transcript:EES15226 3.00E-27
1052- 19:transcript:Zm00001d053232_T005 transcript:EES14113 0
1052- 20:transcript:Zm00001d053234_T001 transcript:EES15223 0
1052- 21:transcript:Zm00001d053236_T001 transcript:OQU80738 1.00E-118
1052- 22:transcript:Zm00001d053237_T001 transcript:EES15222 0
Alignment 1053: score=957.0 e_value=3e-63 N=22 4&7 minus
1053- 0:transcript:Zm00001d052689_T002 transcript:OQU80804 0
1053- 1:transcript:Zm00001d052690_T001 transcript:EES14167 0
1053- 2:transcript:Zm00001d052692_T001 transcript:EES15285 0
1053- 3:transcript:Zm00001d052695_T021 transcript:EES14166 0
1053- 4:transcript:Zm00001d052696_T001 transcript:EES14162 0
1053- 5:transcript:Zm00001d052697_T002 transcript:OQU80800 0
1053- 6:transcript:Zm00001d052698_T001 transcript:EES15274 2.00E-161
1053- 7:transcript:Zm00001d052700_T001 transcript:EES15272 0
1053- 8:transcript:Zm00001d052701_T001 transcript:EES14153 0
1053- 9:transcript:Zm00001d052702_T001 transcript:KXG25483 0
1053- 10:transcript:Zm00001d052709_T002 transcript:EES15269 0

1053- 11:transcript:Zm00001d052713_T002 transcript:OQU80791 1.00E-148
1053- 12:transcript:Zm00001d052715_T001 transcript:OQU80788 0
1053- 13:transcript:Zm00001d052716_T001 transcript:EES15266 1.00E-53
1053- 14:transcript:Zm00001d052717_T001 transcript:EES14151 0
1053- 15:transcript:Zm00001d052718_T002 transcript:OQU80786 3.00E-38
1053- 16:transcript:Zm00001d052719_T002 transcript:EES15262 5.00E-175
1053- 17:transcript:Zm00001d052720_T002 transcript:EES14146 0
1053- 18:transcript:Zm00001d052721_T001 transcript:KXG25467 0
1053- 19:transcript:Zm00001d052723_T017 transcript:EES14142 0
1053- 20:transcript:Zm00001d052725_T001 transcript:EES15257 0
1053- 21:transcript:Zm00001d052726_T008 transcript:OQU80771 0
Alignment 1054: score=941.0 e_value=4.7e-72 N=23 4&7 minus
1054- 0:transcript:Zm00001d049545_T001 transcript:EES14575 3.00E-86
1054- 1:transcript:Zm00001d049548_T002 transcript:KXG24466 0
1054- 2:transcript:Zm00001d049550_T001 transcript:KXG24464 0
1054- 3:transcript:Zm00001d049552_T007 transcript:EES13382 1.00E-75
1054- 4:transcript:Zm00001d049553_T001 transcript:KXG24453 5.00E-85
1054- 5:transcript:Zm00001d049556_T011 transcript:EES13369 0
1054- 6:transcript:Zm00001d049557_T002 transcript:EES13368 5.00E-64
1054- 7:transcript:Zm00001d049558_T002 transcript:EES13367 0
1054- 8:transcript:Zm00001d049559_T001 transcript:EES14564 1.00E-70
1054- 9:transcript:Zm00001d049563_T004 transcript:EES13357 0
1054- 10:transcript:Zm00001d049564_T001 transcript:EES14559 1.00E-150
1054- 11:transcript:Zm00001d049565_T001 transcript:OQU79902 0
1054- 12:transcript:Zm00001d049571_T001 transcript:EES14557 0
1054- 13:transcript:Zm00001d049572_T004 transcript:OQU79887 0
1054- 14:transcript:Zm00001d049573_T001 transcript:EES14551 0
1054- 15:transcript:Zm00001d049576_T001 transcript:KXG24421 3.00E-10
1054- 16:transcript:Zm00001d049578_T001 transcript:EES14542 1.00E-144
1054- 17:transcript:Zm00001d049579_T003 transcript:KXG24419 0
1054- 18:transcript:Zm00001d049581_T001 transcript:EES14540 0
1054- 19:transcript:Zm00001d049582_T001 transcript:EES13345 1.00E-96
1054- 20:transcript:Zm00001d049588_T001 transcript:EES13340 0
1054- 21:transcript:Zm00001d049592_T001 transcript:KXG24400 1.00E-50
1054- 22:transcript:Zm00001d049594_T001 transcript:EES13336 0
Alignment 1055: score=888.0 e_value=9.5e-66 N=21 4&7 minus
1055- 0:transcript:Zm00001d049708_T007 transcript:EES14396 0
1055- 1:transcript:Zm00001d049709_T001 transcript:EES14393 0
1055- 2:transcript:Zm00001d049712_T006 transcript:EES14389 0
1055- 3:transcript:Zm00001d049713_T001 transcript:EES13223 0
1055- 4:transcript:Zm00001d049714_T003 transcript:EES13225 0
1055- 5:transcript:Zm00001d049715_T001 transcript:EES13224 1.00E-127
1055- 6:transcript:Zm00001d049716_T001 transcript:KXG24208 0
1055- 7:transcript:Zm00001d049717_T001 transcript:EES14386 0
1055- 8:transcript:Zm00001d049718_T008 transcript:KXG24206 0
1055- 9:transcript:Zm00001d049721_T001 transcript:EES14382 1.00E-91
1055- 10:transcript:Zm00001d049722_T001 transcript:EES14378 3.00E-77
1055- 11:transcript:Zm00001d049723_T001 transcript:EES13219 3.00E-114
1055- 12:transcript:Zm00001d049724_T001 transcript:EES13218 0
1055- 13:transcript:Zm00001d049725_T002 transcript:KXG24196 0
1055- 14:transcript:Zm00001d049727_T001 transcript:KXG24193 0
1055- 15:transcript:Zm00001d049729_T008 transcript:KXG24185 0
1055- 16:transcript:Zm00001d049730_T004 transcript:KXG24181 5.00E-162
1055- 17:transcript:Zm00001d049732_T001 transcript:EES13198 1.00E-58

1055- 18:transcript:Zm00001d049734_T012 transcript:KXG24176 0
1055- 19:transcript:Zm00001d049736_T001 transcript:KXG24174 0
1055- 20:transcript:Zm00001d049737_T001 transcript:KXG24173 0
Alignment 1056: score=604.0 e_value=4.3e-39 N=15 4&7 minus
1056- 0:transcript:Zm00001d049596_T001 transcript:EES14524 2.00E-102
1056- 1:transcript:Zm00001d049597_T001 transcript:EES14522 0
1056- 2:transcript:Zm00001d049598_T001 transcript:EES13332 4.00E-158
1056- 3:transcript:Zm00001d049601_T001 transcript:EES13328 0
1056- 4:transcript:Zm00001d049602_T001 transcript:KXG24382 1.00E-47
1056- 5:transcript:Zm00001d049605_T036 transcript:KXG24379 0
1056- 6:transcript:Zm00001d049606_T003 transcript:EES13326 0
1056- 7:transcript:Zm00001d049607_T001 transcript:EES13321 0
1056- 8:transcript:Zm00001d049608_T001 transcript:OQU79846 0
1056- 9:transcript:Zm00001d049610_T001 transcript:EES13315 0
1056- 10:transcript:Zm00001d049612_T001 transcript:EES14506 2.00E-85
1056- 11:transcript:Zm00001d049614_T001 transcript:OQU79842 1.00E-59
1056- 12:transcript:Zm00001d049615_T001 transcript:EES14492 0
1056- 13:transcript:Zm00001d049617_T007 transcript:OQU79837 0
1056- 14:transcript:Zm00001d049619_T003 transcript:EES14495 0
Alignment 1057: score=580.0 e_value=1.2e-32 N=13 4&7 minus
1057- 0:transcript:Zm00001d049656_T001 transcript:EES14457 0
1057- 1:transcript:Zm00001d049657_T001 transcript:EES13278 3.00E-140
1057- 2:transcript:Zm00001d049659_T001 transcript:EES13275 5.00E-52
1057- 3:transcript:Zm00001d049660_T002 transcript:EES14442 0
1057- 4:transcript:Zm00001d049661_T001 transcript:KXG24283 0
1057- 5:transcript:Zm00001d049662_T001 transcript:KXG24282 0
1057- 6:transcript:Zm00001d049663_T001 transcript:OQU79802 0
1057- 7:transcript:Zm00001d049665_T001 transcript:OQU79799 2.00E-99
1057- 8:transcript:Zm00001d049666_T001 transcript:EES14427 7.00E-107
1057- 9:transcript:Zm00001d049674_T002 transcript:EES13261 3.00E-68
1057- 10:transcript:Zm00001d049676_T001 transcript:OQU79793 0
1057- 11:transcript:Zm00001d049678_T001 transcript:KXG24274 0
1057- 12:transcript:Zm00001d049680_T005 transcript:KXG24270 0
Alignment 1058: score=515.0 e_value=2.8e-30 N=12 4&7 minus
1058- 0:transcript:Zm00001d049741_T001 transcript:EES15376 0
1058- 1:transcript:Zm00001d049742_T001 transcript:KXG25612 0
1058- 2:transcript:Zm00001d049743_T001 transcript:EES14251 0
1058- 3:transcript:Zm00001d049744_T001 transcript:EES14249 0
1058- 4:transcript:Zm00001d049746_T005 transcript:OQU80887 4.00E-178
1058- 5:transcript:Zm00001d049748_T001 transcript:OQU80886 0
1058- 6:transcript:Zm00001d049750_T002 transcript:EES15369 0
1058- 7:transcript:Zm00001d049753_T001 transcript:KXG25605 0
1058- 8:transcript:Zm00001d049756_T001 transcript:EES15366 0
1058- 9:transcript:Zm00001d049759_T003 transcript:EES15356 0
1058- 10:transcript:Zm00001d049760_T001 transcript:KXG25602 0
1058- 11:transcript:Zm00001d049761_T009 transcript:KXG25600 0
Alignment 1059: score=503.0 e_value=2.8e-26 N=11 4&7 minus
1059- 0:transcript:Zm00001d049486_T001 transcript:OQU79964 1.00E-47
1059- 1:transcript:Zm00001d049490_T001 transcript:KXG24526 1.00E-109
1059- 2:transcript:Zm00001d049491_T001 transcript:EES14615 0
1059- 3:transcript:Zm00001d049493_T001 transcript:OQU79963 0
1059- 4:transcript:Zm00001d049494_T001 transcript:EES13412 0
1059- 5:transcript:Zm00001d049495_T001 transcript:OQU79960 0
1059- 6:transcript:Zm00001d049496_T001 transcript:OQU79959 5.00E-153

1059- 7:transcript:Zm00001d049499_T001	transcript:EES13410	0
1059- 8:transcript:Zm00001d049500_T001	transcript:EES14608	7.00E-159
1059- 9:transcript:Zm00001d049501_T001	transcript:KXG24513	1.00E-125
1059- 10:transcript:Zm00001d049502_T001	transcript:EES14606	2.00E-118

Alignment 1060: score=493.0 e_value=3.9e-23 N=11 4&7 minus

1060- 0:transcript:Zm00001d050412_T002	transcript:EES13917	3.00E-171
1060- 1:transcript:Zm00001d050417_T001	transcript:EES15061	0
1060- 2:transcript:Zm00001d050422_T001	transcript:EES13913	0
1060- 3:transcript:Zm00001d050428_T003	transcript:EES13911	0
1060- 4:transcript:Zm00001d050430_T003	transcript:EES15057	6.00E-141
1060- 5:transcript:Zm00001d050433_T001	transcript:EES13910	2.00E-177
1060- 6:transcript:Zm00001d050434_T002	transcript:EES13909	0
1060- 7:transcript:Zm00001d050436_T001	transcript:EES13906	0
1060- 8:transcript:Zm00001d050440_T004	transcript:EES13905	4.00E-153
1060- 9:transcript:Zm00001d050443_T001	transcript:EES15050	2.00E-125
1060- 10:transcript:Zm00001d050452_T001	transcript:KXG25227	2.00E-48

Alignment 1061: score=446.0 e_value=3e-21 N=10 4&7 minus

1061- 0:transcript:Zm00001d049418_T001	transcript:EES13470	7.00E-151
1061- 1:transcript:Zm00001d049420_T001	transcript:EES13467	4.00E-148
1061- 2:transcript:Zm00001d049431_T001	transcript:OQU80016	4.00E-148
1061- 3:transcript:Zm00001d049434_T001	transcript:OQU80015	3.00E-88
1061- 4:transcript:Zm00001d049439_T001	transcript:OQU80013	1.00E-83
1061- 5:transcript:Zm00001d049440_T004	transcript:EES14665	0
1061- 6:transcript:Zm00001d049441_T002	transcript:EES13461	0
1061- 7:transcript:Zm00001d049442_T009	transcript:OQU80002	0
1061- 8:transcript:Zm00001d049443_T003	transcript:EES13460	0
1061- 9:transcript:Zm00001d049449_T002	transcript:OQU80001	0

Alignment 1062: score=441.0 e_value=5.6e-21 N=10 4&7 minus

1062- 0:transcript:Zm00001d049623_T002	transcript:KXG24345	3.00E-83
1062- 1:transcript:Zm00001d049628_T001	transcript:EES14486	0
1062- 2:transcript:Zm00001d049629_T002	transcript:KXG24330	0
1062- 3:transcript:Zm00001d049630_T001	transcript:OQU79822	0
1062- 4:transcript:Zm00001d049632_T001	transcript:KXG24326	0
1062- 5:transcript:Zm00001d049636_T002	transcript:EES13297	0
1062- 6:transcript:Zm00001d049637_T001	transcript:EES14475	0
1062- 7:transcript:Zm00001d049638_T009	transcript:KXG24325	0
1062- 8:transcript:Zm00001d049640_T002	transcript:KXG24322	0
1062- 9:transcript:Zm00001d049641_T001	transcript:KXG24317	0

Alignment 1063: score=417.0 e_value=2.1e-18 N=9 4&7 minus

1063- 0:transcript:Zm00001d049505_T001	transcript:EES14599	0
1063- 1:transcript:Zm00001d049506_T001	transcript:EES13402	5.00E-179
1063- 2:transcript:Zm00001d049507_T001	transcript:KXG24499	1.00E-76
1063- 3:transcript:Zm00001d049510_T001	transcript:EES14597	0
1063- 4:transcript:Zm00001d049511_T001	transcript:EES13398	4.00E-179
1063- 5:transcript:Zm00001d049516_T001	transcript:KXG24496	3.00E-97
1063- 6:transcript:Zm00001d049521_T001	transcript:KXG24494	1.00E-120
1063- 7:transcript:Zm00001d049525_T004	transcript:EES14592	2.00E-48
1063- 8:transcript:Zm00001d049526_T002	transcript:EES13397	2.00E-144

Alignment 1064: score=382.0 e_value=8e-21 N=10 4&7 minus

1064- 0:transcript:OQU85783	transcript:Zm00001d020723_T001	2.00E-36
1064- 1:transcript:EES05850	transcript:Zm00001d020721_T001	3.00E-160
1064- 2:transcript:EES07580	transcript:Zm00001d020711_T001	9.00E-62
1064- 3:transcript:EES07582	transcript:Zm00001d020708_T002	2.00E-105
1064- 4:transcript:EES07585	transcript:Zm00001d020703_T001	0

1064- 5:transcript:EES07586 transcript:Zm00001d020702_T001 1.00E-172
1064- 6:transcript:EES07595 transcript:Zm00001d020691_T001 4.00E-119
1064- 7:transcript:EES07603 transcript:Zm00001d020688_T001 5.00E-13
1064- 8:transcript:EES05870 transcript:Zm00001d020686_T001 1.00E-169
1064- 9:transcript:KXG31190 transcript:Zm00001d020675_T001 3.00E-28
Alignment 1065: score=378.0 e_value=4.5e-16 N=9 4&7 minus
1065- 0:transcript:Zm00001d052889_T001 transcript:EES14272 0
1065- 1:transcript:Zm00001d052890_T001 transcript:EES14266 9.00E-169
1065- 2:transcript:Zm00001d052893_T002 transcript:EES15392 2.00E-164
1065- 3:transcript:Zm00001d052895_T001 transcript:EES14262 7.00E-179
1065- 4:transcript:Zm00001d052898_T001 transcript:EES14260 3.00E-64
1065- 5:transcript:Zm00001d052901_T001 transcript:KXG25625 0
1065- 6:transcript:Zm00001d052903_T001 transcript:EES15382 0
1065- 7:transcript:Zm00001d052905_T002 transcript:KXG25620 0
1065- 8:transcript:Zm00001d052906_T001 transcript:EES15376 4.00E-159
Alignment 1066: score=376.0 e_value=1.9e-19 N=9 4&7 minus
1066- 0:transcript:Zm00001d049363_T001 transcript:OQU80087 0
1066- 1:transcript:Zm00001d049364_T001 transcript:OQU80085 2.00E-38
1066- 2:transcript:Zm00001d049365_T001 transcript:KXG24716 0
1066- 3:transcript:Zm00001d049369_T002 transcript:OQU80084 3.00E-115
1066- 4:transcript:Zm00001d049370_T002 transcript:EES14709 0
1066- 5:transcript:Zm00001d049371_T002 transcript:KXG24710 0
1066- 6:transcript:Zm00001d049372_T002 transcript:EES13521 0
1066- 7:transcript:Zm00001d049375_T001 transcript:EES13520 4.00E-122
1066- 8:transcript:Zm00001d049376_T001 transcript:EES13518 0
Alignment 1067: score=367.0 e_value=5.1e-16 N=8 4&7 minus
1067- 0:transcript:Zm00001d049404_T001 transcript:KXG24654 1.00E-39
1067- 1:transcript:Zm00001d049405_T001 transcript:EES13484 7.00E-82
1067- 2:transcript:Zm00001d049406_T001 transcript:EES13483 5.00E-25
1067- 3:transcript:Zm00001d049407_T001 transcript:EES13481 0
1067- 4:transcript:Zm00001d049409_T001 transcript:EES14669 0
1067- 5:transcript:Zm00001d049413_T001 transcript:EES14668 7.00E-66
1067- 6:transcript:Zm00001d049416_T001 transcript:KXG24662 0
1067- 7:transcript:Zm00001d049419_T001 transcript:OQU80026 2.00E-128
Alignment 1068: score=339.0 e_value=1.8e-15 N=8 4&7 minus
1068- 0:transcript:Zm00001d049642_T001 transcript:EES14467 0
1068- 1:transcript:Zm00001d049643_T001 transcript:EES14466 0
1068- 2:transcript:Zm00001d049647_T001 transcript:EES13286 2.00E-63
1068- 3:transcript:Zm00001d049648_T001 transcript:EES13283 0
1068- 4:transcript:Zm00001d049649_T001 transcript:OQU79811 3.00E-138
1068- 5:transcript:Zm00001d049650_T001 transcript:EES14462 2.00E-53
1068- 6:transcript:Zm00001d049651_T001 transcript:OQU79810 1.00E-77
1068- 7:transcript:Zm00001d049652_T008 transcript:EES13281 0
Alignment 1069: score=319.0 e_value=7e-11 N=7 4&7 minus
1069- 0:transcript:Zm00001d049532_T001 transcript:OQU79941 8.00E-39
1069- 1:transcript:Zm00001d049533_T001 transcript:EES13390 0
1069- 2:transcript:Zm00001d049534_T001 transcript:EES14584 6.00E-31
1069- 3:transcript:Zm00001d049536_T001 transcript:EES14581 0
1069- 4:transcript:Zm00001d049540_T002 transcript:EES14580 0
1069- 5:transcript:Zm00001d049543_T016 transcript:EES13385 0
1069- 6:transcript:Zm00001d049544_T001 transcript:EES13384 0
Alignment 1070: score=308.0 e_value=3.3e-12 N=7 4&7 minus
1070- 0:transcript:Zm00001d049691_T002 transcript:KXG24248 8.00E-93
1070- 1:transcript:Zm00001d049692_T001 transcript:EES13239 6.00E-123

1070- 2:transcript:Zm00001d049694_T004 transcript:KXG24244 5.00E-180
1070- 3:transcript:Zm00001d049698_T003 transcript:KXG24243 0
1070- 4:transcript:Zm00001d049703_T001 transcript:EES14403 2.00E-129
1070- 5:transcript:Zm00001d049704_T001 transcript:KXG24238 0
1070- 6:transcript:Zm00001d049706_T009 transcript:KXG24228 0
Alignment 1071: score=291.0 e_value=7.6e-10 N=6 4&7 minus
1071- 0:transcript:Zm00001d052744_T001 transcript:EES13178 0
1071- 1:transcript:Zm00001d052747_T001 transcript:EES13177 0
1071- 2:transcript:Zm00001d052748_T001 transcript:EES14346 0
1071- 3:transcript:Zm00001d052749_T001 transcript:EES14345 2.00E-124
1071- 4:transcript:Zm00001d052750_T001 transcript:EES14344 0
1071- 5:transcript:Zm00001d052751_T005 transcript:EES13173 0
Alignment 1072: score=266.0 e_value=8.8e-08 N=6 4&7 minus
1072- 0:transcript:Zm00001d049457_T042 transcript:EES14647 0
1072- 1:transcript:Zm00001d049459_T001 transcript:EES13447 4.00E-153
1072- 2:transcript:Zm00001d049461_T001 transcript:KXG24583 3.00E-158
1072- 3:transcript:Zm00001d049464_T001 transcript:EES14650 7.00E-102
1072- 4:transcript:Zm00001d049467_T001 transcript:EES13444 0
1072- 5:transcript:Zm00001d049471_T001 transcript:OQU79987 0
Alignment 1073: score=253.0 e_value=2.2e-08 N=6 4&7 minus
1073- 0:transcript:Zm00001d052728_T001 transcript:KXG25462 1.00E-173
1073- 1:transcript:Zm00001d052731_T001 transcript:OQU80769 0
1073- 2:transcript:Zm00001d052733_T001 transcript:EES14129 9.00E-171
1073- 3:transcript:Zm00001d052735_T013 transcript:KXG25450 0
1073- 4:transcript:Zm00001d052738_T001 transcript:OQU80758 0
1073- 5:transcript:Zm00001d052741_T001 transcript:EES14126 4.00E-159
Alignment 1074: score=639.0 e_value=6.9e-40 N=15 4&8 plus
1074- 0:transcript:Zm00001d052493_T001 transcript:KXG22950 2.00E-91
1074- 1:transcript:Zm00001d052494_T001 transcript:KXG22956 0
1074- 2:transcript:Zm00001d052495_T001 transcript:EES15662 6.00E-140
1074- 3:transcript:Zm00001d052500_T001 transcript:EES15666 3.00E-17
1074- 4:transcript:Zm00001d052501_T001 transcript:KXG22963 1.00E-11
1074- 5:transcript:Zm00001d052509_T001 transcript:EES15668 0
1074- 6:transcript:Zm00001d052520_T001 transcript:KXG22966 7.00E-132
1074- 7:transcript:Zm00001d052525_T001 transcript:EES16640 0
1074- 8:transcript:Zm00001d052530_T001 transcript:EES16641 0
1074- 9:transcript:Zm00001d052534_T001 transcript:KXG22974 1.00E-14
1074- 10:transcript:Zm00001d052537_T001 transcript:EES16646 2.00E-112
1074- 11:transcript:Zm00001d052539_T001 transcript:EES15681 2.00E-30
1074- 12:transcript:Zm00001d052540_T001 transcript:OQU78719 0
1074- 13:transcript:Zm00001d052543_T001 transcript:KXG22976 0
1074- 14:transcript:Zm00001d052545_T001 transcript:EES16652 2.00E-29
Alignment 1075: score=324.0 e_value=1.2e-11 N=7 4&8 plus
1075- 0:transcript:Zm00001d053158_T001 transcript:OQU78778 6.00E-152
1075- 1:transcript:Zm00001d053162_T003 transcript:EES15709 4.00E-156
1075- 2:transcript:Zm00001d053163_T001 transcript:KXG23028 1.00E-125
1075- 3:transcript:Zm00001d053168_T001 transcript:EES16697 0
1075- 4:transcript:Zm00001d053175_T002 transcript:EES16699 2.00E-36
1075- 5:transcript:Zm00001d053177_T001 transcript:KXG23031 0
1075- 6:transcript:Zm00001d053178_T001 transcript:EES15712 0
Alignment 1076: score=257.0 e_value=2e-09 N=6 4&8 plus
1076- 0:transcript:Zm00001d052586_T002 transcript:OQU78745 0
1076- 1:transcript:Zm00001d052594_T001 transcript:EES15696 2.00E-25
1076- 2:transcript:Zm00001d052605_T001 transcript:OQU78754 2.00E-60

1076- 3:transcript:Zm00001d052606_T001 transcript:EES16674 3.00E-45
1076- 4:transcript:Zm00001d052609_T001 transcript:EES16675 1.00E-37
1076- 5:transcript:Zm00001d052612_T001 transcript:OQU78760 3.00E-163
Alignment 1077: score=250.0 e_value=1.5e-08 N=6 4&8 plus
1077- 0:transcript:Zm00001d053038_T007 transcript:KXG23082 0
1077- 1:transcript:Zm00001d053042_T001 transcript:KXG23086 4.00E-10
1077- 2:transcript:Zm00001d053046_T001 transcript:KXG23087 2.00E-09
1077- 3:transcript:Zm00001d053055_T001 transcript:EES15744 9.00E-51
1077- 4:transcript:Zm00001d053060_T001 transcript:OQU78829 5.00E-91
1077- 5:transcript:Zm00001d053066_T001 transcript:OQU78831 3.00E-80
Alignment 1078: score=381.0 e_value=1.8e-16 N=9 4&8 minus
1078- 0:transcript:Zm00001d052453_T014 transcript:EES16532 0
1078- 1:transcript:Zm00001d052457_T001 transcript:EES16530 0
1078- 2:transcript:Zm00001d052461_T001 transcript:KXG22823 3.00E-33
1078- 3:transcript:Zm00001d052463_T001 transcript:EES16518 3.00E-109
1078- 4:transcript:Zm00001d052468_T002 transcript:EES16520 0
1078- 5:transcript:Zm00001d052472_T012 transcript:EES16519 0
1078- 6:transcript:Zm00001d052476_T001 transcript:KXG22820 2.00E-38
1078- 7:transcript:Zm00001d052479_T001 transcript:EES16515 5.00E-148
1078- 8:transcript:Zm00001d052485_T001 transcript:EES15543 0
Alignment 1079: score=306.0 e_value=1.1e-11 N=7 4&8 minus
1079- 0:transcript:Zm00001d052294_T004 transcript:EES15502 4.00E-143
1079- 1:transcript:Zm00001d052295_T024 transcript:EES15499 0
1079- 2:transcript:Zm00001d052296_T001 transcript:OQU78570 1.00E-147
1079- 3:transcript:Zm00001d052297_T001 transcript:OQU78568 0
1079- 4:transcript:Zm00001d052303_T002 transcript:EES15494 6.00E-108
1079- 5:transcript:Zm00001d052306_T002 transcript:KXG22749 0
1079- 6:transcript:Zm00001d052307_T001 transcript:KXG22747 0
Alignment 1080: score=279.0 e_value=2.9e-11 N=7 4&8 minus
1080- 0:transcript:Zm00001d052375_T002 transcript:EES15614 8.00E-32
1080- 1:transcript:Zm00001d052376_T006 transcript:EES15612 0
1080- 2:transcript:Zm00001d052380_T001 transcript:KXG22896 0
1080- 3:transcript:Zm00001d052385_T001 transcript:KXG22886 0
1080- 4:transcript:Zm00001d052386_T001 transcript:OQU78658 1.00E-152
1080- 5:transcript:Zm00001d052395_T001 transcript:EES16563 3.00E-44
1080- 6:transcript:Zm00001d052397_T001 transcript:KXG22874 1.00E-117
Alignment 1081: score=536.0 e_value=1.1e-26 N=12 4&9 plus
1081- 0:transcript:EES04792 transcript:Zm00001d046281_T001 6.00E-49
1081- 1:transcript:KXG29854 transcript:Zm00001d046288_T001 1.00E-40
1081- 2:transcript:KXG29857 transcript:Zm00001d046292_T001 1.00E-39
1081- 3:transcript:EES04799 transcript:Zm00001d046297_T001 1.00E-43
1081- 4:transcript:KXG29862 transcript:Zm00001d046299_T001 5.00E-83
1081- 5:transcript:EES04803 transcript:Zm00001d046300_T001 9.00E-94
1081- 6:transcript:EES06549 transcript:Zm00001d046303_T001 1.00E-87
1081- 7:transcript:EES04807 transcript:Zm00001d046304_T001 0
1081- 8:transcript:OQU84681 transcript:Zm00001d046305_T001 7.00E-37
1081- 9:transcript:KXG29882 transcript:Zm00001d046311_T001 2.00E-15
1081- 10:transcript:EES04818 transcript:Zm00001d046317_T001 1.00E-130
1081- 11:transcript:OQU84689 transcript:Zm00001d046318_T001 3.00E-47
Alignment 1082: score=491.0 e_value=3.2e-25 N=11 4&9 plus
1082- 0:transcript:OQU84476 transcript:Zm00001d046888_T001 3.00E-18
1082- 1:transcript:OQU84478 transcript:Zm00001d046889_T001 0
1082- 2:transcript:KXG29595 transcript:Zm00001d046896_T001 3.00E-09
1082- 3:transcript:OQU84482 transcript:Zm00001d046906_T001 1.00E-81

1082- 4:transcript:KXG29603 transcript:Zm00001d046910_T001 8.00E-52
1082- 5:transcript:EES04622 transcript:Zm00001d046914_T001 7.00E-33
1082- 6:transcript:EES04623 transcript:Zm00001d046915_T001 0
1082- 7:transcript:EES06371 transcript:Zm00001d046916_T001 4.00E-35
1082- 8:transcript:OQU84486 transcript:Zm00001d046925_T001 1.00E-111
1082- 9:transcript:EES06373 transcript:Zm00001d046927_T001 5.00E-19
1082- 10:transcript:EES04626 transcript:Zm00001d046929_T002 2.00E-42
Alignment 1083: score=447.0 e_value=4.8e-22 N=11 4&9 plus
1083- 0:transcript:EES07566 transcript:Zm00001d044918_T001 2.00E-36
1083- 1:transcript:KXG31161 transcript:Zm00001d044922_T001 3.00E-167
1083- 2:transcript:EES05848 transcript:Zm00001d044927_T001 2.00E-22
1083- 3:transcript:KXG31165 transcript:Zm00001d044930_T001 9.00E-42
1083- 4:transcript:EES05850 transcript:Zm00001d044933_T001 0
1083- 5:transcript:KXG31166 transcript:Zm00001d044934_T001 3.00E-179
1083- 6:transcript:EES07580 transcript:Zm00001d044940_T004 3.00E-111
1083- 7:transcript:EES07583 transcript:Zm00001d044947_T001 0
1083- 8:transcript:EES07588 transcript:Zm00001d044950_T001 1.00E-45
1083- 9:transcript:EES07591 transcript:Zm00001d044951_T001 0
1083- 10:transcript:OQU85793 transcript:Zm00001d044953_T001 5.00E-55
Alignment 1084: score=351.0 e_value=3.5e-18 N=9 4&9 plus
1084- 0:transcript:EES07658 transcript:Zm00001d045075_T001 0
1084- 1:transcript:EES07659 transcript:Zm00001d045076_T003 2.00E-61
1084- 2:transcript:EES07660 transcript:Zm00001d045077_T001 3.00E-105
1084- 3:transcript:EES05940 transcript:Zm00001d045078_T001 0
1084- 4:transcript:KXG31260 transcript:Zm00001d045084_T002 9.00E-179
1084- 5:transcript:EES07672 transcript:Zm00001d045091_T001 3.00E-105
1084- 6:transcript:OQU85876 transcript:Zm00001d045102_T001 3.00E-131
1084- 7:transcript:OQU85879 transcript:Zm00001d045105_T001 2.00E-34
1084- 8:transcript:EES05953 transcript:Zm00001d045107_T001 1.00E-75
Alignment 1085: score=342.0 e_value=9.9e-17 N=8 4&9 plus
1085- 0:transcript:EES07684 transcript:Zm00001d045112_T001 0
1085- 1:transcript:OQU85888 transcript:Zm00001d045120_T001 4.00E-66
1085- 2:transcript:EES05958 transcript:Zm00001d045121_T001 7.00E-31
1085- 3:transcript:EES05959 transcript:Zm00001d045122_T001 0
1085- 4:transcript:KXG31279 transcript:Zm00001d045125_T001 1.00E-41
1085- 5:transcript:EES05962 transcript:Zm00001d045128_T001 0
1085- 6:transcript:KXG31285 transcript:Zm00001d045129_T001 1.00E-06
1085- 7:transcript:EES05967 transcript:Zm00001d045138_T001 0
Alignment 1086: score=309.0 e_value=4.9e-11 N=7 4&9 plus
1086- 0:transcript:EES04672 transcript:Zm00001d046655_T001 0
1086- 1:transcript:KXG29676 transcript:Zm00001d046660_T001 0
1086- 2:transcript:KXG29679 transcript:Zm00001d046661_T003 0
1086- 3:transcript:OQU84547 transcript:Zm00001d046663_T001 2.00E-27
1086- 4:transcript:EES06428 transcript:Zm00001d046664_T001 4.00E-14
1086- 5:transcript:EES06430 transcript:Zm00001d046667_T001 2.00E-16
1086- 6:transcript:KXG29681 transcript:Zm00001d046673_T001 2.00E-06
Alignment 1087: score=252.0 e_value=8.7e-13 N=7 4&9 plus
1087- 0:transcript:EES04688 transcript:Zm00001d046740_T001 2.00E-123
1087- 1:transcript:KXG29702 transcript:Zm00001d046751_T001 1.00E-125
1087- 2:transcript:EES04692 transcript:Zm00001d046765_T001 0
1087- 3:transcript:KXG29703 transcript:Zm00001d046767_T002 4.00E-91
1087- 4:transcript:KXG29713 transcript:Zm00001d046783_T001 2.00E-149
1087- 5:transcript:EES04701 transcript:Zm00001d046792_T001 8.00E-116
1087- 6:transcript:OQU84580 transcript:Zm00001d046793_T001 0

```
## Alignment 1088: score=418.0 e_value=7.3e-19 N=10 4&9 minus
1088-  0:transcript:OQU84635           transcript:Zm00001d046378_T001  3.00E-37
1088-  1:transcript:EES06511           transcript:Zm00001d046369_T002         0
1088-  2:transcript:OQU84639           transcript:Zm00001d046364_T001         0
1088-  3:transcript:EES06516           transcript:Zm00001d046357_T001         0
1088-  4:transcript:EES06520           transcript:Zm00001d046356_T001         0
1088-  5:transcript:KXG29816           transcript:Zm00001d046354_T001  2.00E-63
1088-  6:transcript:EES04758           transcript:Zm00001d046352_T001         0
1088-  7:transcript:KXG29820           transcript:Zm00001d046348_T003         0
1088-  8:transcript:KXG29821           transcript:Zm00001d046346_T001  7.00E-44
1088-  9:transcript:OQU84653           transcript:Zm00001d046336_T001         0
## Alignment 1089: score=300.0 e_value=3.8e-10 N=7 4&9 minus
1089-  0:transcript:OQU84706           transcript:Zm00001d046151_T001  2.00E-16
1089-  1:transcript:KXG29921           transcript:Zm00001d046143_T001  2.00E-27
1089-  2:transcript:KXG29926           transcript:Zm00001d046142_T001  3.00E-20
1089-  3:transcript:EES04847           transcript:Zm00001d046138_T001  2.00E-14
1089-  4:transcript:EES04851           transcript:Zm00001d046136_T001 1.00E-135
1089-  5:transcript:OQU84716           transcript:Zm00001d046132_T001  2.00E-17
1089-  6:transcript:EES04857           transcript:Zm00001d046126_T001 8.00E-139
## Alignment 1090: score=367.0 e_value=2.7e-13 N=8 4&B73V4_ctg107 plus
1090-  0:transcript:EES04522           transcript:Zm00001d000300_T001         0
1090-  1:transcript:EES06277           transcript:Zm00001d000301_T001         0
1090-  2:transcript:EES04525           transcript:Zm00001d000302_T002         0
1090-  3:transcript:EES04526           transcript:Zm00001d000303_T001  1.00E-85
1090-  4:transcript:OQU84367           transcript:Zm00001d000304_T005 4.00E-103
1090-  5:transcript:EES04528           transcript:Zm00001d000305_T003         0
1090-  6:transcript:EES04532           transcript:Zm00001d000306_T001  5.00E-62
1090-  7:transcript:EES06280           transcript:Zm00001d000307_T001 2.00E-157
## Alignment 1091: score=1277.0 e_value=1.6e-97 N=30 5&6 plus
1091-  0:transcript:Zm00001d017352_T004 transcript:EES12535           6.00E-146
1091-  1:transcript:Zm00001d017353_T001 transcript:KXG26758            1.00E-76
1091-  2:transcript:Zm00001d017357_T001 transcript:KXG26759            9.00E-17
1091-  3:transcript:Zm00001d017364_T001 transcript:KXG26766            2.00E-45
1091-  4:transcript:Zm00001d017366_T001 transcript:KXG26694            3.00E-93
1091-  5:transcript:Zm00001d017371_T008 transcript:EES11157                   0
1091-  6:transcript:Zm00001d017373_T001 transcript:OQU82010                   0
1091-  7:transcript:Zm00001d017377_T001 transcript:OQU82013           2.00E-104
1091-  8:transcript:Zm00001d017378_T001 transcript:EES12550            6.00E-74
1091-  9:transcript:Zm00001d017379_T001 transcript:EES12552            7.00E-72
1091- 10:transcript:Zm00001d017381_T001 transcript:KXG26779           3.00E-123
1091- 11:transcript:Zm00001d017382_T001 transcript:EES12556           3.00E-101
1091- 12:transcript:Zm00001d017383_T001 transcript:EES11177           2.00E-115
1091- 13:transcript:Zm00001d017385_T001 transcript:EES11178           4.00E-116
1091- 14:transcript:Zm00001d017386_T001 transcript:EES12557            2.00E-62
1091- 15:transcript:Zm00001d017390_T001 transcript:EES12558            1.00E-93
1091- 16:transcript:Zm00001d017391_T002 transcript:EES12563            7.00E-84
1091- 17:transcript:Zm00001d017396_T001 transcript:OQU82026            4.00E-94
1091- 18:transcript:Zm00001d017397_T001 transcript:EES11187            6.00E-43
1091- 19:transcript:Zm00001d017399_T001 transcript:EES12565                   0
1091- 20:transcript:Zm00001d017401_T001 transcript:EES11189                   0
1091- 21:transcript:Zm00001d017402_T001 transcript:EES12569            4.00E-69
1091- 22:transcript:Zm00001d017403_T001 transcript:EES12570           1.00E-164
1091- 23:transcript:Zm00001d017409_T001 transcript:EES11194            1.00E-89
1091- 24:transcript:Zm00001d017412_T001 transcript:EES11195            4.00E-60
```

1091- 25:transcript:Zm00001d017418_T001 transcript:OQU82042 0
1091- 26:transcript:Zm00001d017419_T001 transcript:OQU82043 6.00E-151
1091- 27:transcript:Zm00001d017420_T001 transcript:OQU82045 3.00E-144
1091- 28:transcript:Zm00001d017422_T001 transcript:EES11198 2.00E-69
1091- 29:transcript:Zm00001d017427_T002 transcript:OQU82052 0
Alignment 1092: score=1226.0 e_value=2.3e-94 N=29 5&6 plus
1092- 0:transcript:Zm00001d017570_T001 transcript:EES12682 0
1092- 1:transcript:Zm00001d017573_T003 transcript:EES11275 0
1092- 2:transcript:Zm00001d017574_T001 transcript:EES11276 1.00E-102
1092- 3:transcript:Zm00001d017575_T001 transcript:OQU82171 5.00E-106
1092- 4:transcript:Zm00001d017582_T001 transcript:EES12690 0
1092- 5:transcript:Zm00001d017583_T002 transcript:KXG26943 6.00E-132
1092- 6:transcript:Zm00001d017590_T001 transcript:EES11285 0
1092- 7:transcript:Zm00001d017591_T001 transcript:EES11287 4.00E-66
1092- 8:transcript:Zm00001d017592_T001 transcript:EES11288 9.00E-77
1092- 9:transcript:Zm00001d017597_T001 transcript:OQU82184 0
1092- 10:transcript:Zm00001d017598_T001 transcript:KXG26955 0
1092- 11:transcript:Zm00001d017604_T001 transcript:EES11301 0
1092- 12:transcript:Zm00001d017606_T001 transcript:EES11304 1.00E-30
1092- 13:transcript:Zm00001d017611_T001 transcript:EES12701 0
1092- 14:transcript:Zm00001d017612_T001 transcript:KXG26971 5.00E-57
1092- 15:transcript:Zm00001d017614_T003 transcript:KXG26975 4.00E-75
1092- 16:transcript:Zm00001d017615_T001 transcript:EES12708 3.00E-42
1092- 17:transcript:Zm00001d017618_T001 transcript:EES12712 3.00E-115
1092- 18:transcript:Zm00001d017619_T001 transcript:KXG26980 3.00E-24
1092- 19:transcript:Zm00001d017622_T001 transcript:EES12722 6.00E-35
1092- 20:transcript:Zm00001d017641_T004 transcript:KXG26989 0
1092- 21:transcript:Zm00001d017642_T001 transcript:OQU82207 1.00E-71
1092- 22:transcript:Zm00001d017643_T001 transcript:KXG26992 0
1092- 23:transcript:Zm00001d017645_T001 transcript:EES12728 4.00E-57
1092- 24:transcript:Zm00001d017646_T001 transcript:KXG27001 3.00E-22
1092- 25:transcript:Zm00001d017647_T001 transcript:EES12737 2.00E-23
1092- 26:transcript:Zm00001d017648_T001 transcript:EES12745 1.00E-18
1092- 27:transcript:Zm00001d017653_T001 transcript:KXG27003 4.00E-92
1092- 28:transcript:Zm00001d017657_T001 transcript:EES12746 5.00E-72
Alignment 1093: score=821.0 e_value=3.3e-51 N=19 5&6 plus
1093- 0:transcript:Zm00001d017441_T001 transcript:EES12591 8.00E-82
1093- 1:transcript:Zm00001d017444_T001 transcript:KXG26823 5.00E-78
1093- 2:transcript:Zm00001d017445_T001 transcript:EES12597 1.00E-154
1093- 3:transcript:Zm00001d017455_T001 transcript:EES12600 2.00E-26
1093- 4:transcript:Zm00001d017460_T003 transcript:EES11210 2.00E-114
1093- 5:transcript:Zm00001d017462_T001 transcript:OQU82076 2.00E-93
1093- 6:transcript:Zm00001d017468_T001 transcript:EES11216 8.00E-102
1093- 7:transcript:Zm00001d017473_T002 transcript:EES12610 3.00E-158
1093- 8:transcript:Zm00001d017474_T001 transcript:OQU82082 8.00E-26
1093- 9:transcript:Zm00001d017476_T001 transcript:OQU82085 0
1093- 10:transcript:Zm00001d017477_T001 transcript:OQU82086 9.00E-54
1093- 11:transcript:Zm00001d017480_T001 transcript:OQU82088 2.00E-41
1093- 12:transcript:Zm00001d017482_T002 transcript:KXG26850 3.00E-66
1093- 13:transcript:Zm00001d017485_T003 transcript:OQU82093 1.00E-59
1093- 14:transcript:Zm00001d017486_T001 transcript:OQU82096 2.00E-33
1093- 15:transcript:Zm00001d017493_T001 transcript:EES11234 5.00E-133
1093- 16:transcript:Zm00001d017497_T001 transcript:EES11238 2.00E-54
1093- 17:transcript:Zm00001d017499_T001 transcript:OQU82097 7.00E-50

1093- 18:transcript:Zm00001d017505_T003	transcript:KXG26858	6.00E-67
## Alignment 1094: score=674.0 e_value=2.5e-37 N=15 5&6 plus		
1094- 0:transcript:Zm00001d017261_T006	transcript:KXG26713	0
1094- 1:transcript:Zm00001d017263_T001	transcript:EES11114	3.00E-97
1094- 2:transcript:Zm00001d017268_T001	transcript:EES12516	2.00E-97
1094- 3:transcript:Zm00001d017270_T001	transcript:EES11118	3.00E-149
1094- 4:transcript:Zm00001d017271_T005	transcript:EES12520	0
1094- 5:transcript:Zm00001d017274_T001	transcript:EES12521	0
1094- 6:transcript:Zm00001d017275_T001	transcript:OQU81984	0
1094- 7:transcript:Zm00001d017282_T001	transcript:EES11121	0
1094- 8:transcript:Zm00001d017284_T001	transcript:KXG26729	0
1094- 9:transcript:Zm00001d017287_T001	transcript:KXG26730	3.00E-26
1094- 10:transcript:Zm00001d017288_T001	transcript:EES11124	0
1094- 11:transcript:Zm00001d017292_T003	transcript:EES11129	3.00E-26
1094- 12:transcript:Zm00001d017294_T001	transcript:KXG26736	1.00E-153
1094- 13:transcript:Zm00001d017297_T001	transcript:OQU81997	2.00E-12
1094- 14:transcript:Zm00001d017298_T003	transcript:EES12528	1.00E-106
## Alignment 1095: score=448.0 e_value=1.4e-24 N=11 5&6 plus		
1095- 0:transcript:Zm00001d016901_T001	transcript:KXG26428	9.00E-44
1095- 1:transcript:Zm00001d016908_T001	transcript:KXG26432	4.00E-95
1095- 2:transcript:Zm00001d016909_T001	transcript:OQU81680	4.00E-68
1095- 3:transcript:Zm00001d016910_T001	transcript:EES12267	1.00E-97
1095- 4:transcript:Zm00001d016915_T002	transcript:KXG26438	0
1095- 5:transcript:Zm00001d016918_T001	transcript:EES12269	0
1095- 6:transcript:Zm00001d016919_T001	transcript:EES10921	7.00E-162
1095- 7:transcript:Zm00001d016924_T001	transcript:KXG26452	5.00E-131
1095- 8:transcript:Zm00001d016928_T005	transcript:EES12285	0
1095- 9:transcript:Zm00001d016933_T001	transcript:EES10931	0
1095- 10:transcript:Zm00001d016934_T001	transcript:KXG26457	3.00E-153
## Alignment 1096: score=373.0 e_value=4.7e-15 N=8 5&6 plus		
1096- 0:transcript:Zm00001d017071_T002	transcript:EES10994	0
1096- 1:transcript:Zm00001d017072_T002	transcript:KXG26553	3.00E-149
1096- 2:transcript:Zm00001d017079_T001	transcript:EES10995	0
1096- 3:transcript:Zm00001d017084_T001	transcript:OQU81795	4.00E-26
1096- 4:transcript:Zm00001d017085_T002	transcript:EES12371	0
1096- 5:transcript:Zm00001d017091_T001	transcript:KXG26562	0
1096- 6:transcript:Zm00001d017094_T001	transcript:EES10998	0
1096- 7:transcript:Zm00001d017095_T001	transcript:KXG26563	1.00E-74
## Alignment 1097: score=356.0 e_value=2.3e-15 N=8 5&6 plus		
1097- 0:transcript:Zm00001d016876_T001	transcript:EES12241	7.00E-109
1097- 1:transcript:Zm00001d016878_T001	transcript:OQU81656	7.00E-58
1097- 2:transcript:Zm00001d016884_T002	transcript:KXG26410	2.00E-166
1097- 3:transcript:Zm00001d016890_T001	transcript:EES10893	1.00E-121
1097- 4:transcript:Zm00001d016894_T005	transcript:KXG26417	0
1097- 5:transcript:Zm00001d016895_T001	transcript:KXG26418	5.00E-120
1097- 6:transcript:Zm00001d016897_T002	transcript:EES10898	0
1097- 7:transcript:Zm00001d016899_T001	transcript:KXG26420	8.00E-09
## Alignment 1098: score=350.0 e_value=7.1e-14 N=8 5&6 plus		
1098- 0:transcript:Zm00001d017671_T001	transcript:EES11341	2.00E-173
1098- 1:transcript:Zm00001d017677_T001	transcript:KXG27015	1.00E-84
1098- 2:transcript:Zm00001d017678_T001	transcript:EES12753	1.00E-126
1098- 3:transcript:Zm00001d017682_T001	transcript:EES12755	4.00E-120
1098- 4:transcript:Zm00001d017684_T001	transcript:OQU82229	3.00E-30
1098- 5:transcript:Zm00001d017689_T002	transcript:EES12761	3.00E-139

1098- 6:transcript:Zm00001d017692_T002 transcript:EES11354 1.00E-39
1098- 7:transcript:Zm00001d017698_T003 transcript:EES11358 0
Alignment 1099: score=325.0 e_value=2.7e-11 N=7 5&6 plus
1099- 0:transcript:Zm00001d016655_T001 transcript:EES12088 2.00E-21
1099- 1:transcript:Zm00001d016659_T001 transcript:KXG26206 0
1099- 2:transcript:Zm00001d016662_T003 transcript:EES12091 0
1099- 3:transcript:Zm00001d016664_T001 transcript:OQU81478 0
1099- 4:transcript:Zm00001d016665_T001 transcript:EES12092 8.00E-133
1099- 5:transcript:Zm00001d016669_T001 transcript:OQU81480 1.00E-60
1099- 6:transcript:Zm00001d016671_T001 transcript:EES12096 6.00E-57
Alignment 1100: score=301.0 e_value=5.3e-15 N=8 5&6 plus
1100- 0:transcript:Zm00001d017186_T001 transcript:EES11073 0
1100- 1:transcript:Zm00001d017195_T001 transcript:OQU81891 0
1100- 2:transcript:Zm00001d017197_T015 transcript:OQU81892 0
1100- 3:transcript:Zm00001d017201_T001 transcript:KXG26657 0
1100- 4:transcript:Zm00001d017203_T021 transcript:OQU81900 0
1100- 5:transcript:Zm00001d017204_T003 transcript:KXG26662 2.00E-109
1100- 6:transcript:Zm00001d017209_T001 transcript:EES11085 7.00E-124
1100- 7:transcript:Zm00001d017213_T004 transcript:EES12465 0
Alignment 1101: score=300.0 e_value=4.2e-12 N=7 5&6 plus
1101- 0:transcript:Zm00001d017240_T001 transcript:EES11094 5.00E-56
1101- 1:transcript:Zm00001d017243_T001 transcript:KXG26684 3.00E-90
1101- 2:transcript:Zm00001d017246_T001 transcript:EES11097 0
1101- 3:transcript:Zm00001d017247_T003 transcript:OQU81932 0
1101- 4:transcript:Zm00001d017248_T001 transcript:KXG26691 1.00E-53
1101- 5:transcript:Zm00001d017249_T001 transcript:KXG26692 0
1101- 6:transcript:Zm00001d017251_T003 transcript:EES12488 0
Alignment 1102: score=299.0 e_value=5.9e-13 N=7 5&6 plus
1102- 0:transcript:Zm00001d016765_T001 transcript:EES10816 0
1102- 1:transcript:Zm00001d016766_T002 transcript:OQU81597 1.00E-49
1102- 2:transcript:Zm00001d016783_T002 transcript:KXG26327 0
1102- 3:transcript:Zm00001d016786_T001 transcript:EES12182 0
1102- 4:transcript:Zm00001d016787_T001 transcript:OQU81604 5.00E-47
1102- 5:transcript:Zm00001d016788_T001 transcript:KXG26333 8.00E-17
1102- 6:transcript:Zm00001d016792_T004 transcript:KXG26334 3.00E-171
Alignment 1103: score=256.0 e_value=9.1e-12 N=7 5&6 plus
1103- 0:transcript:Zm00001d016564_T001 transcript:EES12026 1.00E-44
1103- 1:transcript:Zm00001d016568_T001 transcript:EES10625 5.00E-19
1103- 2:transcript:Zm00001d016569_T001 transcript:EES12036 1.00E-20
1103- 3:transcript:Zm00001d016577_T002 transcript:KXG26117 1.00E-94
1103- 4:transcript:Zm00001d016580_T001 transcript:OQU81408 3.00E-27
1103- 5:transcript:Zm00001d016582_T001 transcript:EES10642 2.00E-24
1103- 6:transcript:Zm00001d016591_T002 transcript:EES10653 0
Alignment 1104: score=341.0 e_value=6.6e-14 N=8 5&6 minus
1104- 0:transcript:Zm00001d016721_T001 transcript:OQU81550 5.00E-74
1104- 1:transcript:Zm00001d016730_T001 transcript:KXG26241 7.00E-170
1104- 2:transcript:Zm00001d016731_T001 transcript:EES10780 0
1104- 3:transcript:Zm00001d016733_T001 transcript:EES12138 2.00E-87
1104- 4:transcript:Zm00001d016735_T001 transcript:EES10778 0
1104- 5:transcript:Zm00001d016737_T002 transcript:OQU81542 1.00E-137
1104- 6:transcript:Zm00001d016743_T001 transcript:EES10769 2.00E-24
1104- 7:transcript:Zm00001d016745_T001 transcript:EES12134 2.00E-128
Alignment 1105: score=304.0 e_value=1.5e-13 N=8 5&8 minus
1105- 0:transcript:Zm00001d013659_T001 transcript:OQU79668 6.00E-86

1105- 1:	transcript:Zm00001d013661_T001	transcript:EES16403	6.00E-19
1105- 2:	transcript:Zm00001d013667_T001	transcript:EES17423	2.00E-43
1105- 3:	transcript:Zm00001d013672_T001	transcript:EES17420	0
1105- 4:	transcript:Zm00001d013676_T001	transcript:OQU79642	4.00E-77
1105- 5:	transcript:Zm00001d013680_T002	transcript:KXG24021	0
1105- 6:	transcript:Zm00001d013688_T001	transcript:EES16393	0
1105- 7:	transcript:Zm00001d013692_T001	transcript:KXG24018	0
## Alignment 1106: score=272.0 e_value=1.2e-09 N=6 5&8 minus			
1106- 0:	transcript:Zm00001d013546_T003	transcript:EES17494	6.00E-89
1106- 1:	transcript:Zm00001d013547_T002	transcript:KXG24130	0
1106- 2:	transcript:Zm00001d013549_T001	transcript:EES16449	2.00E-110
1106- 3:	transcript:Zm00001d013550_T001	transcript:OQU79728	6.00E-06
1106- 4:	transcript:Zm00001d013551_T001	transcript:KXG24122	2.00E-18
1106- 5:	transcript:Zm00001d013554_T001	transcript:OQU79723	9.00E-28
## Alignment 1107: score=269.0 e_value=7.9e-09 N=6 5&8 minus			
1107- 0:	transcript:Zm00001d013626_T002	transcript:OQU79693	1.00E-21
1107- 1:	transcript:Zm00001d013627_T001	transcript:EES16411	5.00E-122
1107- 2:	transcript:Zm00001d013631_T001	transcript:EES16412	2.00E-121
1107- 3:	transcript:Zm00001d013638_T001	transcript:EES16410	1.00E-174
1107- 4:	transcript:Zm00001d013641_T001	transcript:OQU79684	2.00E-22
1107- 5:	transcript:Zm00001d013644_T001	transcript:KXG24060	0
## Alignment 1108: score=1436.0 e_value=3e-124 N=34 6&7 plus			
1108- 0:	transcript:Zm00001d035124_T001	transcript:EES13280	0
1108- 1:	transcript:Zm00001d035130_T001	transcript:EES13281	0
1108- 2:	transcript:Zm00001d035132_T001	transcript:KXG24301	8.00E-139
1108- 3:	transcript:Zm00001d035134_T001	transcript:OQU79810	8.00E-67
1108- 4:	transcript:Zm00001d035135_T001	transcript:EES14462	4.00E-51
1108- 5:	transcript:Zm00001d035136_T001	transcript:OQU79811	2.00E-148
1108- 6:	transcript:Zm00001d035139_T002	transcript:EES14463	0
1108- 7:	transcript:Zm00001d035140_T001	transcript:EES14467	0
1108- 8:	transcript:Zm00001d035153_T002	transcript:EES13285	0
1108- 9:	transcript:Zm00001d035154_T001	transcript:EES14471	6.00E-59
1108- 10:	transcript:Zm00001d035155_T001	transcript:KXG24316	8.00E-34
1108- 11:	transcript:Zm00001d035156_T003	transcript:KXG24317	0
1108- 12:	transcript:Zm00001d035157_T001	transcript:KXG24322	0
1108- 13:	transcript:Zm00001d035163_T001	transcript:EES13292	0
1108- 14:	transcript:Zm00001d035167_T009	transcript:KXG24325	0
1108- 15:	transcript:Zm00001d035168_T003	transcript:EES13296	3.00E-108
1108- 16:	transcript:Zm00001d035169_T001	transcript:EES14475	0
1108- 17:	transcript:Zm00001d035170_T002	transcript:EES13297	0
1108- 18:	transcript:Zm00001d035171_T001	transcript:EES14476	2.00E-45
1108- 19:	transcript:Zm00001d035184_T001	transcript:KXG24329	0
1108- 20:	transcript:Zm00001d035185_T001	transcript:OQU79824	0
1108- 21:	transcript:Zm00001d035186_T001	transcript:EES14486	0
1108- 22:	transcript:Zm00001d035191_T004	transcript:EES14488	0
1108- 23:	transcript:Zm00001d035194_T001	transcript:KXG24341	0
1108- 24:	transcript:Zm00001d035195_T001	transcript:EES14490	3.00E-89
1108- 25:	transcript:Zm00001d035197_T001	transcript:KXG24348	0
1108- 26:	transcript:Zm00001d035198_T001	transcript:EES13306	0
1108- 27:	transcript:Zm00001d035199_T001	transcript:EES14493	0
1108- 28:	transcript:Zm00001d035200_T001	transcript:EES14494	0
1108- 29:	transcript:Zm00001d035201_T001	transcript:EES14495	0
1108- 30:	transcript:Zm00001d035206_T003	transcript:EES13307	0
1108- 31:	transcript:Zm00001d035207_T004	transcript:KXG24353	6.00E-160

1108- 32:transcript:Zm00001d035208_T001	transcript:EES14497		0
1108- 33:transcript:Zm00001d035211_T001	transcript:EES13310		0
## Alignment 1109: score=820.0 e_value=2.3e-60 N=20 6&7 plus			
1109- 0:transcript:Zm00001d035080_T001	transcript:EES13253		3.00E-18
1109- 1:transcript:Zm00001d035081_T001	transcript:KXG24264		2.00E-161
1109- 2:transcript:Zm00001d035082_T001	transcript:OQU79790		0
1109- 3:transcript:Zm00001d035083_T001	transcript:EES14420		4.00E-140
1109- 4:transcript:Zm00001d035084_T001	transcript:KXG24274		0
1109- 5:transcript:Zm00001d035086_T001	transcript:OQU79793		0
1109- 6:transcript:Zm00001d035087_T001	transcript:EES13261		4.00E-69
1109- 7:transcript:Zm00001d035089_T003	transcript:EES13259		0
1109- 8:transcript:Zm00001d035090_T002	transcript:EES14424		0
1109- 9:transcript:Zm00001d035091_T001	transcript:KXG24277		0
1109- 10:transcript:Zm00001d035092_T001	transcript:EES13264		0
1109- 11:transcript:Zm00001d035093_T002	transcript:EES13267		0
1109- 12:transcript:Zm00001d035094_T001	transcript:EES14430		3.00E-105
1109- 13:transcript:Zm00001d035095_T001	transcript:OQU79799		2.00E-101
1109- 14:transcript:Zm00001d035097_T001	transcript:EES13268		0
1109- 15:transcript:Zm00001d035098_T002	transcript:KXG24282		0
1109- 16:transcript:Zm00001d035100_T001	transcript:EES14433		9.00E-155
1109- 17:transcript:Zm00001d035113_T001	transcript:KXG24287		0
1109- 18:transcript:Zm00001d035115_T004	transcript:OQU79805		0
1109- 19:transcript:Zm00001d035119_T001	transcript:EES14442		0
## Alignment 1110: score=659.0 e_value=1.8e-39 N=15 6&7 plus			
1110- 0:transcript:Zm00001d035014_T001	transcript:EES14371		0
1110- 1:transcript:Zm00001d035015_T001	transcript:KXG24196		0
1110- 2:transcript:Zm00001d035018_T001	transcript:EES14373		0
1110- 3:transcript:Zm00001d035019_T001	transcript:EES13219		4.00E-96
1110- 4:transcript:Zm00001d035020_T001	transcript:EES14378		5.00E-63
1110- 5:transcript:Zm00001d035023_T001	transcript:EES13221		0
1110- 6:transcript:Zm00001d035024_T001	transcript:KXG24202		2.00E-61
1110- 7:transcript:Zm00001d035027_T001	transcript:EES14382		2.00E-120
1110- 8:transcript:Zm00001d035028_T001	transcript:KXG24203		3.00E-140
1110- 9:transcript:Zm00001d035029_T006	transcript:KXG24206		0
1110- 10:transcript:Zm00001d035030_T002	transcript:EES13222		0
1110- 11:transcript:Zm00001d035031_T001	transcript:EES14386		0
1110- 12:transcript:Zm00001d035032_T002	transcript:KXG24208		0
1110- 13:transcript:Zm00001d035034_T002	transcript:EES14387		1.00E-167
1110- 14:transcript:Zm00001d035035_T001	transcript:EES13223		0
## Alignment 1111: score=491.0 e_value=1.5e-27 N=12 6&7 plus			
1111- 0:transcript:Zm00001d034964_T083	transcript:EES13173		0
1111- 1:transcript:Zm00001d034965_T002	transcript:EES14344		0
1111- 2:transcript:Zm00001d034966_T001	transcript:EES13176		7.00E-26
1111- 3:transcript:Zm00001d034968_T001	transcript:EES14347		0
1111- 4:transcript:Zm00001d034970_T002	transcript:EES14349		0
1111- 5:transcript:Zm00001d034973_T001	transcript:EES13182		7.00E-96
1111- 6:transcript:Zm00001d034976_T001	transcript:EES14352		8.00E-121
1111- 7:transcript:Zm00001d034977_T001	transcript:EES14353		2.00E-155
1111- 8:transcript:Zm00001d034978_T001	transcript:EES14354		2.00E-104
1111- 9:transcript:Zm00001d034979_T001	transcript:EES13183		4.00E-98
1111- 10:transcript:Zm00001d034984_T001	transcript:OQU79754		2.00E-115
1111- 11:transcript:Zm00001d034986_T001	transcript:OQU79755		9.00E-49
## Alignment 1112: score=421.0 e_value=1.3e-21 N=10 6&7 plus			
1112- 0:transcript:Zm00001d035046_T001	transcript:KXG24230		0

1112- 1:transcript:Zm00001d035047_T003 transcript:EES14400 0
1112- 2:transcript:Zm00001d035048_T003 transcript:EES14404 0
1112- 3:transcript:Zm00001d035050_T002 transcript:KXG24243 0
1112- 4:transcript:Zm00001d035051_T001 transcript:KXG24244 2.00E-170
1112- 5:transcript:Zm00001d035052_T001 transcript:EES13238 2.00E-98
1112- 6:transcript:Zm00001d035053_T002 transcript:EES13239 1.00E-113
1112- 7:transcript:Zm00001d035054_T002 transcript:KXG24248 6.00E-90
1112- 8:transcript:Zm00001d035055_T001 transcript:EES13242 0
1112- 9:transcript:Zm00001d035057_T001 transcript:EES13245 0
Alignment 1113: score=270.0 e_value=7e-09 N=6 6&7 plus
1113- 0:transcript:Zm00001d035066_T001 transcript:EES13246 0
1113- 1:transcript:Zm00001d035067_T001 transcript:KXG24256 0
1113- 2:transcript:Zm00001d035069_T001 transcript:EES13248 0
1113- 3:transcript:Zm00001d035072_T001 transcript:EES13250 0
1113- 4:transcript:Zm00001d035076_T001 transcript:EES13251 0
1113- 5:transcript:Zm00001d035079_T001 transcript:KXG24258 2.00E-09
Alignment 1114: score=631.0 e_value=2.3e-43 N=15 6&7 minus
1114- 0:transcript:Zm00001d034989_T001 transcript:EES13213 0
1114- 1:transcript:Zm00001d034990_T001 transcript:KXG24195 0
1114- 2:transcript:Zm00001d034991_T001 transcript:KXG24194 0
1114- 3:transcript:Zm00001d034992_T001 transcript:EES13205 1.00E-67
1114- 4:transcript:Zm00001d034995_T001 transcript:EES14365 1.00E-100
1114- 5:transcript:Zm00001d034996_T005 transcript:KXG24185 0
1114- 6:transcript:Zm00001d034998_T004 transcript:KXG24181 0
1114- 7:transcript:Zm00001d035000_T001 transcript:KXG24179 1.00E-44
1114- 8:transcript:Zm00001d035001_T001 transcript:EES13198 1.00E-71
1114- 9:transcript:Zm00001d035004_T002 transcript:KXG24176 0
1114- 10:transcript:Zm00001d035005_T001 transcript:KXG24175 0
1114- 11:transcript:Zm00001d035007_T001 transcript:EES13196 0
1114- 12:transcript:Zm00001d035008_T006 transcript:KXG24174 0
1114- 13:transcript:Zm00001d035012_T002 transcript:EES14361 0
1114- 14:transcript:Zm00001d035013_T001 transcript:OQU79755 6.00E-47
Alignment 1115: score=262.0 e_value=8.1e-09 N=6 6&7 minus
1115- 0:transcript:KXG26935 transcript:Zm00001d021236_T004 0
1115- 1:transcript:EES11282 transcript:Zm00001d021231_T005 2.00E-135
1115- 2:transcript:EES11283 transcript:Zm00001d021225_T005 3.00E-44
1115- 3:transcript:EES11284 transcript:Zm00001d021224_T001 5.00E-120
1115- 4:transcript:EES11285 transcript:Zm00001d021216_T001 1.00E-172
1115- 5:transcript:EES11288 transcript:Zm00001d021207_T001 3.00E-37
Alignment 1116: score=1044.0 e_value=9.2e-81 N=23 6&8 minus
1116- 0:transcript:Zm00001d035268_T001 transcript:EES17104 1.00E-25
1116- 1:transcript:Zm00001d035267_T003 transcript:EES16065 6.00E-131
1116- 2:transcript:Zm00001d035270_T011 transcript:EES17101 4.00E-74
1116- 3:transcript:Zm00001d035285_T003 transcript:KXG23570 0
1116- 4:transcript:Zm00001d035288_T002 transcript:EES17098 3.00E-42
1116- 5:transcript:Zm00001d035294_T001 transcript:EES16059 0
1116- 6:transcript:Zm00001d035297_T001 transcript:KXG23563 0
1116- 7:transcript:Zm00001d035298_T005 transcript:OQU79205 5.00E-121
1116- 8:transcript:Zm00001d035300_T001 transcript:KXG23561 0
1116- 9:transcript:Zm00001d035303_T001 transcript:KXG23560 0
1116- 10:transcript:Zm00001d035304_T001 transcript:EES16055 9.00E-141
1116- 11:transcript:Zm00001d035306_T001 transcript:OQU79203 3.00E-14
1116- 12:transcript:Zm00001d035309_T001 transcript:EES17091 1.00E-32
1116- 13:transcript:Zm00001d035310_T001 transcript:KXG23553 1.00E-28

1116- 14:	transcript:Zm00001d035312_T002	transcript:OQU79193	9.00E-21
1116- 15:	transcript:Zm00001d035313_T002	transcript:OQU79192	3.00E-64
1116- 16:	transcript:Zm00001d035317_T002	transcript:KXG23548	2.00E-93
1116- 17:	transcript:Zm00001d035318_T001	transcript:EES17080	1.00E-149
1116- 18:	transcript:Zm00001d035320_T001	transcript:EES17083	2.00E-65
1116- 19:	transcript:Zm00001d035319_T005	transcript:KXG23544	3.00E-19
1116- 20:	transcript:Zm00001d035321_T001	transcript:EES17082	4.00E-75
1116- 21:	transcript:Zm00001d035322_T001	transcript:KXG23542	6.00E-134
1116- 22:	transcript:Zm00001d035323_T001	transcript:EES16036	0
## Alignment 1117: score=8245.0 e_value=0 N=180 6&9 plus			
1117- 0:	transcript:Zm00001d038341_T001	transcript:EES18324	1.00E-68
1117- 1:	transcript:Zm00001d038342_T002	transcript:EES18331	3.00E-147
1117- 2:	transcript:Zm00001d038343_T001	transcript:EES19630	0
1117- 3:	transcript:Zm00001d038346_T002	transcript:KXG22131	0
1117- 4:	transcript:Zm00001d038348_T002	transcript:EES19631	0
1117- 5:	transcript:Zm00001d038351_T004	transcript:EES18332	1.00E-82
1117- 6:	transcript:Zm00001d038352_T001	transcript:EES19633	5.00E-72
1117- 7:	transcript:Zm00001d038355_T001	transcript:EES18336	0
1117- 8:	transcript:Zm00001d038357_T001	transcript:EES18337	2.00E-132
1117- 9:	transcript:Zm00001d038358_T002	transcript:KXG22138	0
1117- 10:	transcript:Zm00001d038361_T001	transcript:EES18338	3.00E-92
1117- 11:	transcript:Zm00001d038363_T001	transcript:KXG22143	3.00E-35
1117- 12:	transcript:Zm00001d038364_T001	transcript:KXG22144	2.00E-49
1117- 13:	transcript:Zm00001d038366_T001	transcript:EES18341	0
1117- 14:	transcript:Zm00001d038367_T002	transcript:KXG22154	3.00E-165
1117- 15:	transcript:Zm00001d038368_T002	transcript:EES19645	1.00E-127
1117- 16:	transcript:Zm00001d038371_T002	transcript:EES18346	0
1117- 17:	transcript:Zm00001d038372_T001	transcript:EES18347	5.00E-137
1117- 18:	transcript:Zm00001d038373_T001	transcript:OQU78135	0
1117- 19:	transcript:Zm00001d038374_T007	transcript:KXG22160	2.00E-64
1117- 20:	transcript:Zm00001d038375_T003	transcript:KXG22161	0
1117- 21:	transcript:Zm00001d038376_T001	transcript:KXG22163	1.00E-123
1117- 22:	transcript:Zm00001d038378_T001	transcript:KXG22165	1.00E-123
1117- 23:	transcript:Zm00001d038379_T001	transcript:EES18355	2.00E-81
1117- 24:	transcript:Zm00001d038380_T004	transcript:EES19652	3.00E-159
1117- 25:	transcript:Zm00001d038381_T001	transcript:EES19653	1.00E-75
1117- 26:	transcript:Zm00001d038382_T001	transcript:EES19654	2.00E-24
1117- 27:	transcript:Zm00001d038387_T001	transcript:EES19655	5.00E-56
1117- 28:	transcript:Zm00001d038388_T001	transcript:KXG22169	6.00E-152
1117- 29:	transcript:Zm00001d038389_T008	transcript:EES19657	0
1117- 30:	transcript:Zm00001d038392_T001	transcript:EES18358	0
1117- 31:	transcript:Zm00001d038393_T012	transcript:OQU78144	0
1117- 32:	transcript:Zm00001d038394_T001	transcript:EES19660	0
1117- 33:	transcript:Zm00001d038395_T005	transcript:OQU78148	0
1117- 34:	transcript:Zm00001d038396_T011	transcript:KXG22175	0
1117- 35:	transcript:Zm00001d038397_T005	transcript:KXG22176	2.00E-124
1117- 36:	transcript:Zm00001d038400_T001	transcript:EES19664	0
1117- 37:	transcript:Zm00001d038401_T001	transcript:EES19665	1.00E-29
1117- 38:	transcript:Zm00001d038403_T001	transcript:OQU78154	3.00E-20
1117- 39:	transcript:Zm00001d038404_T003	transcript:EES19666	0
1117- 40:	transcript:Zm00001d038407_T004	transcript:KXG22182	0
1117- 41:	transcript:Zm00001d038408_T001	transcript:EES19670	6.00E-90
1117- 42:	transcript:Zm00001d038409_T001	transcript:OQU78157	0
1117- 43:	transcript:Zm00001d038411_T001	transcript:OQU78161	0

1117- 44:	transcript:Zm00001d038412_T001	transcript:EES18369	0
1117- 45:	transcript:Zm00001d038420_T001	transcript:EES18370	3.00E-78
1117- 46:	transcript:Zm00001d038431_T001	transcript:EES18372	0
1117- 47:	transcript:Zm00001d038432_T006	transcript:KXG22192	0
1117- 48:	transcript:Zm00001d038440_T001	transcript:KXG22199	0
1117- 49:	transcript:Zm00001d038446_T001	transcript:EES18376	9.00E-88
1117- 50:	transcript:Zm00001d038447_T001	transcript:EES19684	0
1117- 51:	transcript:Zm00001d038449_T001	transcript:OQU78173	0
1117- 52:	transcript:Zm00001d038450_T001	transcript:EES18380	7.00E-90
1117- 53:	transcript:Zm00001d038451_T001	transcript:OQU78174	0
1117- 54:	transcript:Zm00001d038459_T001	transcript:KXG22212	0
1117- 55:	transcript:Zm00001d038460_T007	transcript:EES19688	0
1117- 56:	transcript:Zm00001d038465_T001	transcript:EES18382	0
1117- 57:	transcript:Zm00001d038466_T001	transcript:EES19689	6.00E-98
1117- 58:	transcript:Zm00001d038467_T001	transcript:OQU78181	0
1117- 59:	transcript:Zm00001d038469_T001	transcript:KXG22215	1.00E-52
1117- 60:	transcript:Zm00001d038471_T001	transcript:OQU78182	0
1117- 61:	transcript:Zm00001d038473_T001	transcript:EES18387	6.00E-179
1117- 62:	transcript:Zm00001d038475_T001	transcript:EES19687	0
1117- 63:	transcript:Zm00001d038476_T001	transcript:EES19693	3.00E-157
1117- 64:	transcript:Zm00001d038478_T001	transcript:EES18392	4.00E-63
1117- 65:	transcript:Zm00001d038479_T001	transcript:EES19694	1.00E-61
1117- 66:	transcript:Zm00001d038480_T001	transcript:EES18393	1.00E-127
1117- 67:	transcript:Zm00001d038481_T001	transcript:EES18394	0
1117- 68:	transcript:Zm00001d038483_T001	transcript:EES19696	0
1117- 69:	transcript:Zm00001d038484_T001	transcript:EES19691	1.00E-103
1117- 70:	transcript:Zm00001d038486_T002	transcript:EES19700	6.00E-54
1117- 71:	transcript:Zm00001d038487_T003	transcript:EES19702	0
1117- 72:	transcript:Zm00001d038489_T001	transcript:KXG22223	0
1117- 73:	transcript:Zm00001d038490_T002	transcript:KXG22226	6.00E-159
1117- 74:	transcript:Zm00001d038491_T002	transcript:KXG22227	2.00E-145
1117- 75:	transcript:Zm00001d038503_T001	transcript:EES18391	0
1117- 76:	transcript:Zm00001d038504_T001	transcript:EES19705	5.00E-127
1117- 77:	transcript:Zm00001d038506_T001	transcript:OQU78195	7.00E-117
1117- 78:	transcript:Zm00001d038508_T001	transcript:EES18401	6.00E-42
1117- 79:	transcript:Zm00001d038509_T001	transcript:OQU78197	0
1117- 80:	transcript:Zm00001d038510_T001	transcript:OQU78198	4.00E-163
1117- 81:	transcript:Zm00001d038511_T001	transcript:KXG22230	0
1117- 82:	transcript:Zm00001d038512_T001	transcript:EES18405	0
1117- 83:	transcript:Zm00001d038513_T002	transcript:EES18406	0
1117- 84:	transcript:Zm00001d038514_T001	transcript:EES19708	2.00E-118
1117- 85:	transcript:Zm00001d038517_T001	transcript:EES19709	0
1117- 86:	transcript:Zm00001d038518_T001	transcript:EES18409	0
1117- 87:	transcript:Zm00001d038521_T001	transcript:EES19711	0
1117- 88:	transcript:Zm00001d038522_T002	transcript:KXG22231	0
1117- 89:	transcript:Zm00001d038525_T004	transcript:KXG22232	0
1117- 90:	transcript:Zm00001d038526_T002	transcript:KXG22235	2.00E-24
1117- 91:	transcript:Zm00001d038527_T001	transcript:KXG22236	0
1117- 92:	transcript:Zm00001d038528_T001	transcript:KXG22238	0
1117- 93:	transcript:Zm00001d038529_T003	transcript:EES18413	0
1117- 94:	transcript:Zm00001d038530_T003	transcript:EES18414	0
1117- 95:	transcript:Zm00001d038531_T002	transcript:EES18415	0
1117- 96:	transcript:Zm00001d038532_T001	transcript:EES19718	4.00E-91
1117- 97:	transcript:Zm00001d038533_T004	transcript:EES19719	4.00E-132

1117- 98:	transcript:Zm00001d038534_T001	transcript:EES18420	4.00E-100
1117- 99:	transcript:Zm00001d038535_T001	transcript:EES18421	1.00E-68
1117-100:	transcript:Zm00001d038536_T003	transcript:EES18422	0
1117-101:	transcript:Zm00001d038537_T001	transcript:EES19721	0
1117-102:	transcript:Zm00001d038538_T001	transcript:EES19722	0
1117-103:	transcript:Zm00001d038539_T001	transcript:EES19723	3.00E-95
1117-104:	transcript:Zm00001d038540_T001	transcript:OQU78222	0
1117-105:	transcript:Zm00001d038541_T002	transcript:EES19725	0
1117-106:	transcript:Zm00001d038542_T001	transcript:EES19726	4.00E-83
1117-107:	transcript:Zm00001d038543_T004	transcript:OQU78227	2.00E-31
1117-108:	transcript:Zm00001d038546_T001	transcript:EES19732	0
1117-109:	transcript:Zm00001d038547_T001	transcript:KXG22248	0
1117-110:	transcript:Zm00001d038548_T001	transcript:KXG22249	0
1117-111:	transcript:Zm00001d038550_T001	transcript:EES19735	0
1117-112:	transcript:Zm00001d038552_T001	transcript:KXG22252	2.00E-46
1117-113:	transcript:Zm00001d038553_T001	transcript:EES19738	0
1117-114:	transcript:Zm00001d038554_T001	transcript:OQU78229	0
1117-115:	transcript:Zm00001d038555_T001	transcript:EES18431	0
1117-116:	transcript:Zm00001d038557_T005	transcript:OQU78230	2.00E-141
1117-117:	transcript:Zm00001d038558_T005	transcript:EES19739	3.00E-63
1117-118:	transcript:Zm00001d038562_T001	transcript:KXG22255	0
1117-119:	transcript:Zm00001d038563_T001	transcript:KXG22257	6.00E-154
1117-120:	transcript:Zm00001d038570_T005	transcript:KXG22258	0
1117-121:	transcript:Zm00001d038572_T001	transcript:EES18435	0
1117-122:	transcript:Zm00001d038574_T004	transcript:EES19742	1.00E-126
1117-123:	transcript:Zm00001d038576_T001	transcript:EES18436	0
1117-124:	transcript:Zm00001d038577_T001	transcript:OQU78235	0
1117-125:	transcript:Zm00001d038578_T001	transcript:EES18439	6.00E-68
1117-126:	transcript:Zm00001d038579_T001	transcript:EES19743	0
1117-127:	transcript:Zm00001d038581_T001	transcript:KXG22268	1.00E-69
1117-128:	transcript:Zm00001d038583_T001	transcript:EES18430	0
1117-129:	transcript:Zm00001d038584_T001	transcript:KXG22269	1.00E-84
1117-130:	transcript:Zm00001d038585_T001	transcript:EES19749	3.00E-70
1117-131:	transcript:Zm00001d038586_T001	transcript:OQU78239	3.00E-16
1117-132:	transcript:Zm00001d038587_T033	transcript:OQU78241	0
1117-133:	transcript:Zm00001d038589_T005	transcript:EES19752	0
1117-134:	transcript:Zm00001d038594_T002	transcript:EES18447	5.00E-70
1117-135:	transcript:Zm00001d038595_T001	transcript:EES19759	0
1117-136:	transcript:Zm00001d038596_T001	transcript:KXG22279	1.00E-36
1117-137:	transcript:Zm00001d038597_T001	transcript:EES19761	1.00E-81
1117-138:	transcript:Zm00001d038598_T001	transcript:EES18450	0
1117-139:	transcript:Zm00001d038600_T001	transcript:OQU78254	0
1117-140:	transcript:Zm00001d038604_T001	transcript:KXG22281	0
1117-141:	transcript:Zm00001d038606_T001	transcript:EES19766	0
1117-142:	transcript:Zm00001d038607_T001	transcript:EES19768	3.00E-56
1117-143:	transcript:Zm00001d038608_T001	transcript:EES18454	5.00E-122
1117-144:	transcript:Zm00001d038609_T001	transcript:EES18455	0
1117-145:	transcript:Zm00001d038610_T001	transcript:KXG22282	3.00E-123
1117-146:	transcript:Zm00001d038612_T001	transcript:EES18457	1.00E-94
1117-147:	transcript:Zm00001d038613_T001	transcript:KXG22286	2.00E-22
1117-148:	transcript:Zm00001d038616_T001	transcript:EES18459	0
1117-149:	transcript:Zm00001d038617_T001	transcript:EES19770	1.00E-141
1117-150:	transcript:Zm00001d038618_T001	transcript:KXG22291	0
1117-151:	transcript:Zm00001d038620_T006	transcript:EES18461	0

1117-152:	transcript:Zm00001d038622_T001	transcript:EES19773	0
1117-153:	transcript:Zm00001d038623_T001	transcript:EES19774	0
1117-154:	transcript:Zm00001d038624_T001	transcript:OQU78261	8.00E-104
1117-155:	transcript:Zm00001d038625_T001	transcript:EES19776	0
1117-156:	transcript:Zm00001d038626_T001	transcript:OQU78262	1.00E-14
1117-157:	transcript:Zm00001d038638_T001	transcript:KXG22297	2.00E-32
1117-158:	transcript:Zm00001d038641_T001	transcript:KXG22299	4.00E-35
1117-159:	transcript:Zm00001d038642_T001	transcript:EES18464	0
1117-160:	transcript:Zm00001d038643_T001	transcript:KXG22300	0
1117-161:	transcript:Zm00001d038644_T004	transcript:OQU78267	0
1117-162:	transcript:Zm00001d038646_T002	transcript:EES19780	0
1117-163:	transcript:Zm00001d038647_T001	transcript:EES18467	1.00E-106
1117-164:	transcript:Zm00001d038648_T005	transcript:EES18468	0
1117-165:	transcript:Zm00001d038650_T001	transcript:EES18460	0
1117-166:	transcript:Zm00001d038651_T004	transcript:KXG22302	0
1117-167:	transcript:Zm00001d038652_T003	transcript:OQU78269	7.00E-73
1117-168:	transcript:Zm00001d038654_T001	transcript:KXG22304	3.00E-11
1117-169:	transcript:Zm00001d038655_T003	transcript:KXG22306	0
1117-170:	transcript:Zm00001d038657_T005	transcript:EES18475	0
1117-171:	transcript:Zm00001d038658_T001	transcript:KXG22309	0
1117-172:	transcript:Zm00001d038660_T003	transcript:EES19781	0
1117-173:	transcript:Zm00001d038661_T002	transcript:EES19782	0
1117-174:	transcript:Zm00001d038665_T001	transcript:EES18469	0
1117-175:	transcript:Zm00001d038667_T001	transcript:EES18483	0
1117-176:	transcript:Zm00001d038668_T001	transcript:EES19787	7.00E-152
1117-177:	transcript:Zm00001d038671_T004	transcript:EES19790	0
1117-178:	transcript:Zm00001d038672_T001	transcript:EES18485	0
1117-179:	transcript:Zm00001d038673_T002	transcript:OQU78280	0
## Alignment 1118: score=5097.0 e_value=0 N=109 6&9 plus			
1118- 0:	transcript:Zm00001d038820_T001	transcript:KXG22407	7.00E-152
1118- 1:	transcript:Zm00001d038823_T001	transcript:EES18568	0
1118- 2:	transcript:Zm00001d038824_T002	transcript:EES18569	0
1118- 3:	transcript:Zm00001d038825_T001	transcript:EES19860	0
1118- 4:	transcript:Zm00001d038829_T001	transcript:KXG22409	0
1118- 5:	transcript:Zm00001d038830_T002	transcript:KXG22411	0
1118- 6:	transcript:Zm00001d038832_T003	transcript:EES19871	5.00E-131
1118- 7:	transcript:Zm00001d038833_T001	transcript:EES19872	0
1118- 8:	transcript:Zm00001d038835_T002	transcript:OQU78357	0
1118- 9:	transcript:Zm00001d038838_T001	transcript:KXG22418	0
1118- 10:	transcript:Zm00001d038839_T002	transcript:KXG22420	1.00E-52
1118- 11:	transcript:Zm00001d038840_T001	transcript:EES18574	0
1118- 12:	transcript:Zm00001d038841_T007	transcript:EES19875	0
1118- 13:	transcript:Zm00001d038842_T001	transcript:OQU78358	2.00E-129
1118- 14:	transcript:Zm00001d038843_T001	transcript:EES18577	6.00E-114
1118- 15:	transcript:Zm00001d038844_T001	transcript:OQU78360	0
1118- 16:	transcript:Zm00001d038846_T001	transcript:KXG22422	0
1118- 17:	transcript:Zm00001d038847_T001	transcript:KXG22427	8.00E-179
1118- 18:	transcript:Zm00001d038849_T005	transcript:OQU78363	1.00E-95
1118- 19:	transcript:Zm00001d038850_T001	transcript:KXG22429	0
1118- 20:	transcript:Zm00001d038851_T001	transcript:KXG22432	0
1118- 21:	transcript:Zm00001d038852_T001	transcript:KXG22433	2.00E-158
1118- 22:	transcript:Zm00001d038854_T006	transcript:EES18583	0
1118- 23:	transcript:Zm00001d038855_T001	transcript:OQU78366	0
1118- 24:	transcript:Zm00001d038856_T001	transcript:EES18584	1.00E-33

1118- 25:transcript:Zm00001d038857_T001	transcript:EES19881	0
1118- 26:transcript:Zm00001d038859_T007	transcript:EES18585	0
1118- 27:transcript:Zm00001d038860_T001	transcript:OQU78367	0
1118- 28:transcript:Zm00001d038861_T001	transcript:KXG22437	0
1118- 29:transcript:Zm00001d038862_T001	transcript:EES18588	7.00E-146
1118- 30:transcript:Zm00001d038863_T001	transcript:EES18589	3.00E-164
1118- 31:transcript:Zm00001d038864_T001	transcript:EES18592	1.00E-119
1118- 32:transcript:Zm00001d038865_T003	transcript:KXG22440	3.00E-96
1118- 33:transcript:Zm00001d038866_T002	transcript:KXG22443	0
1118- 34:transcript:Zm00001d038868_T003	transcript:EES18579	0
1118- 35:transcript:Zm00001d038870_T001	transcript:KXG22444	7.00E-76
1118- 36:transcript:Zm00001d038871_T001	transcript:EES18595	0
1118- 37:transcript:Zm00001d038873_T001	transcript:OQU78377	1.00E-48
1118- 38:transcript:Zm00001d038874_T002	transcript:OQU78378	0
1118- 39:transcript:Zm00001d038876_T001	transcript:EES18599	9.00E-91
1118- 40:transcript:Zm00001d038877_T001	transcript:KXG22451	2.00E-171
1118- 41:transcript:Zm00001d038878_T001	transcript:EES18602	3.00E-157
1118- 42:transcript:Zm00001d038879_T003	transcript:EES18587	0
1118- 43:transcript:Zm00001d038880_T001	transcript:KXG22453	0
1118- 44:transcript:Zm00001d038881_T001	transcript:EES19889	2.00E-128
1118- 45:transcript:Zm00001d038882_T002	transcript:OQU78381	0
1118- 46:transcript:Zm00001d038883_T001	transcript:KXG22458	0
1118- 47:transcript:Zm00001d038886_T001	transcript:EES18606	4.00E-92
1118- 48:transcript:Zm00001d038887_T001	transcript:EES19894	2.00E-142
1118- 49:transcript:Zm00001d038890_T002	transcript:EES19895	3.00E-75
1118- 50:transcript:Zm00001d038891_T001	transcript:EES19896	0
1118- 51:transcript:Zm00001d038892_T001	transcript:EES19899	0
1118- 52:transcript:Zm00001d038894_T002	transcript:KXG22467	0
1118- 53:transcript:Zm00001d038896_T001	transcript:KXG22471	3.00E-154
1118- 54:transcript:Zm00001d038897_T001	transcript:KXG22472	3.00E-105
1118- 55:transcript:Zm00001d038901_T001	transcript:KXG22473	4.00E-88
1118- 56:transcript:Zm00001d038903_T001	transcript:EES18612	4.00E-160
1118- 57:transcript:Zm00001d038904_T001	transcript:OQU78386	0
1118- 58:transcript:Zm00001d038905_T001	transcript:EES18614	0
1118- 59:transcript:Zm00001d038907_T001	transcript:KXG22484	0
1118- 60:transcript:Zm00001d038908_T004	transcript:EES19904	0
1118- 61:transcript:Zm00001d038909_T001	transcript:EES18624	8.00E-177
1118- 62:transcript:Zm00001d038910_T001	transcript:EES19907	1.00E-142
1118- 63:transcript:Zm00001d038911_T001	transcript:EES18625	3.00E-41
1118- 64:transcript:Zm00001d038915_T001	transcript:KXG22487	0
1118- 65:transcript:Zm00001d038916_T001	transcript:KXG22489	0
1118- 66:transcript:Zm00001d038917_T002	transcript:EES18629	0
1118- 67:transcript:Zm00001d038918_T001	transcript:EES19909	2.00E-59
1118- 68:transcript:Zm00001d038919_T001	transcript:KXG22490	8.00E-77
1118- 69:transcript:Zm00001d038921_T001	transcript:KXG22495	1.00E-179
1118- 70:transcript:Zm00001d038922_T001	transcript:KXG22496	0
1118- 71:transcript:Zm00001d038923_T001	transcript:EES18632	0
1118- 72:transcript:Zm00001d038924_T001	transcript:EES18633	0
1118- 73:transcript:Zm00001d038925_T003	transcript:EES18634	0
1118- 74:transcript:Zm00001d038926_T001	transcript:KXG22500	2.00E-134
1118- 75:transcript:Zm00001d038927_T001	transcript:EES19913	0
1118- 76:transcript:Zm00001d038928_T002	transcript:OQU78399	0
1118- 77:transcript:Zm00001d038929_T001	transcript:KXG22502	0
1118- 78:transcript:Zm00001d038930_T001	transcript:EES18637	2.00E-125

1118- 79:	transcript:Zm00001d038931_T002	transcript:EES18638	0
1118- 80:	transcript:Zm00001d038932_T001	transcript:EES18639	1.00E-134
1118- 81:	transcript:Zm00001d038933_T001	transcript:KXG22504	2.00E-24
1118- 82:	transcript:Zm00001d038936_T001	transcript:EES18641	0
1118- 83:	transcript:Zm00001d038937_T001	transcript:EES18642	0
1118- 84:	transcript:Zm00001d038944_T003	transcript:EES18643	0
1118- 85:	transcript:Zm00001d038946_T001	transcript:KXG22507	3.00E-96
1118- 86:	transcript:Zm00001d038948_T001	transcript:KXG22508	0
1118- 87:	transcript:Zm00001d038950_T001	transcript:EES18645	1.00E-43
1118- 88:	transcript:Zm00001d038954_T001	transcript:KXG22510	6.00E-62
1118- 89:	transcript:Zm00001d038955_T001	transcript:EES19921	0
1118- 90:	transcript:Zm00001d038959_T001	transcript:EES18651	0
1118- 91:	transcript:Zm00001d038960_T001	transcript:EES19922	0
1118- 92:	transcript:Zm00001d038961_T001	transcript:KXG22514	0
1118- 93:	transcript:Zm00001d038963_T001	transcript:KXG22515	3.00E-139
1118- 94:	transcript:Zm00001d038964_T001	transcript:EES19923	0
1118- 95:	transcript:Zm00001d038965_T001	transcript:OQU78406	0
1118- 96:	transcript:Zm00001d038968_T001	transcript:OQU78407	0
1118- 97:	transcript:Zm00001d038970_T004	transcript:EES18659	0
1118- 98:	transcript:Zm00001d038971_T001	transcript:EES19927	0
1118- 99:	transcript:Zm00001d038972_T002	transcript:EES19929	0
1118-100:	transcript:Zm00001d038973_T001	transcript:EES19931	1.00E-139
1118-101:	transcript:Zm00001d038974_T001	transcript:KXG22517	0
1118-102:	transcript:Zm00001d038975_T001	transcript:KXG22518	0
1118-103:	transcript:Zm00001d038976_T001	transcript:EES18660	2.00E-59
1118-104:	transcript:Zm00001d038978_T001	transcript:EES18661	4.00E-55
1118-105:	transcript:Zm00001d038979_T001	transcript:EES19935	2.00E-135
1118-106:	transcript:Zm00001d038980_T001	transcript:OQU78411	4.00E-83
1118-107:	transcript:Zm00001d038981_T001	transcript:EES18664	0
1118-108:	transcript:Zm00001d038982_T007	transcript:KXG22522	0
## Alignment 1119: score=3762.0 e_value=0 N=81 6&9 plus			
1119- 0:	transcript:Zm00001d039004_T002	transcript:EES18675	0
1119- 1:	transcript:Zm00001d039005_T003	transcript:EES19946	0
1119- 2:	transcript:Zm00001d039006_T007	transcript:EES18676	0
1119- 3:	transcript:Zm00001d039007_T001	transcript:EES19947	4.00E-162
1119- 4:	transcript:Zm00001d039010_T001	transcript:EES18677	2.00E-35
1119- 5:	transcript:Zm00001d039011_T001	transcript:OQU78423	2.00E-54
1119- 6:	transcript:Zm00001d039012_T001	transcript:EES19949	0
1119- 7:	transcript:Zm00001d039013_T001	transcript:EES18680	1.00E-146
1119- 8:	transcript:Zm00001d039014_T001	transcript:EES18681	0
1119- 9:	transcript:Zm00001d039015_T001	transcript:KXG22536	0
1119- 10:	transcript:Zm00001d039016_T001	transcript:EES19950	3.00E-156
1119- 11:	transcript:Zm00001d039017_T001	transcript:KXG22537	0
1119- 12:	transcript:Zm00001d039019_T001	transcript:KXG22538	1.00E-117
1119- 13:	transcript:Zm00001d039020_T001	transcript:EES18686	2.00E-100
1119- 14:	transcript:Zm00001d039021_T001	transcript:EES18688	7.00E-149
1119- 15:	transcript:Zm00001d039022_T001	transcript:OQU78429	4.00E-80
1119- 16:	transcript:Zm00001d039029_T001	transcript:KXG22542	0
1119- 17:	transcript:Zm00001d039031_T001	transcript:EES19955	8.00E-40
1119- 18:	transcript:Zm00001d039032_T001	transcript:EES18690	9.00E-154
1119- 19:	transcript:Zm00001d039036_T003	transcript:EES18691	0
1119- 20:	transcript:Zm00001d039037_T001	transcript:EES19958	0
1119- 21:	transcript:Zm00001d039038_T001	transcript:EES19959	0
1119- 22:	transcript:Zm00001d039039_T002	transcript:EES19960	4.00E-129

1119- 23:	transcript:Zm00001d039040_T001	transcript:EES18693	0
1119- 24:	transcript:Zm00001d039041_T007	transcript:KXG22544	0
1119- 25:	transcript:Zm00001d039043_T011	transcript:KXG22545	0
1119- 26:	transcript:Zm00001d039044_T001	transcript:KXG22547	0
1119- 27:	transcript:Zm00001d039046_T011	transcript:EES18696	0
1119- 28:	transcript:Zm00001d039047_T001	transcript:EES18697	0
1119- 29:	transcript:Zm00001d039049_T001	transcript:KXG22549	5.00E-45
1119- 30:	transcript:Zm00001d039051_T002	transcript:EES18699	0
1119- 31:	transcript:Zm00001d039053_T001	transcript:EES19964	0
1119- 32:	transcript:Zm00001d039055_T001	transcript:EES18704	1.00E-46
1119- 33:	transcript:Zm00001d039056_T001	transcript:EES18692	9.00E-38
1119- 34:	transcript:Zm00001d039057_T001	transcript:EES18706	2.00E-49
1119- 35:	transcript:Zm00001d039058_T001	transcript:EES18707	5.00E-82
1119- 36:	transcript:Zm00001d039059_T001	transcript:OQU78439	0
1119- 37:	transcript:Zm00001d039061_T001	transcript:EES18708	1.00E-122
1119- 38:	transcript:Zm00001d039062_T006	transcript:OQU78441	0
1119- 39:	transcript:Zm00001d039064_T001	transcript:EES18711	2.00E-74
1119- 40:	transcript:Zm00001d039065_T011	transcript:EES18712	0
1119- 41:	transcript:Zm00001d039066_T001	transcript:EES18713	0
1119- 42:	transcript:Zm00001d039070_T001	transcript:OQU78451	5.00E-67
1119- 43:	transcript:Zm00001d039071_T001	transcript:KXG22559	2.00E-87
1119- 44:	transcript:Zm00001d039072_T005	transcript:EES19971	0
1119- 45:	transcript:Zm00001d039076_T001	transcript:EES19972	1.00E-89
1119- 46:	transcript:Zm00001d039077_T001	transcript:EES19973	1.00E-125
1119- 47:	transcript:Zm00001d039078_T001	transcript:KXG22564	0
1119- 48:	transcript:Zm00001d039079_T002	transcript:OQU78460	0
1119- 49:	transcript:Zm00001d039080_T001	transcript:EES19977	8.00E-144
1119- 50:	transcript:Zm00001d039081_T002	transcript:OQU78463	2.00E-37
1119- 51:	transcript:Zm00001d039082_T001	transcript:OQU78467	8.00E-23
1119- 52:	transcript:Zm00001d039083_T001	transcript:EES19980	2.00E-86
1119- 53:	transcript:Zm00001d039084_T001	transcript:EES19981	2.00E-25
1119- 54:	transcript:Zm00001d039085_T001	transcript:KXG22574	4.00E-126
1119- 55:	transcript:Zm00001d039086_T001	transcript:KXG22577	0
1119- 56:	transcript:Zm00001d039087_T001	transcript:OQU78469	0
1119- 57:	transcript:Zm00001d039089_T001	transcript:EES19985	0
1119- 58:	transcript:Zm00001d039094_T001	transcript:EES19987	0
1119- 59:	transcript:Zm00001d039096_T001	transcript:OQU78471	0
1119- 60:	transcript:Zm00001d039098_T001	transcript:OQU78473	5.00E-57
1119- 61:	transcript:Zm00001d039100_T001	transcript:EES18730	2.00E-51
1119- 62:	transcript:Zm00001d039101_T001	transcript:EES18731	1.00E-160
1119- 63:	transcript:Zm00001d039102_T001	transcript:EES19989	0
1119- 64:	transcript:Zm00001d039103_T001	transcript:OQU78477	2.00E-67
1119- 65:	transcript:Zm00001d039104_T001	transcript:KXG22587	0
1119- 66:	transcript:Zm00001d039105_T001	transcript:EES18732	0
1119- 67:	transcript:Zm00001d039106_T003	transcript:EES19994	4.00E-95
1119- 68:	transcript:Zm00001d039108_T001	transcript:OQU78484	3.00E-175
1119- 69:	transcript:Zm00001d039109_T001	transcript:OQU78486	9.00E-44
1119- 70:	transcript:Zm00001d039110_T001	transcript:EES19997	6.00E-101
1119- 71:	transcript:Zm00001d039111_T002	transcript:EES18735	1.00E-117
1119- 72:	transcript:Zm00001d039112_T001	transcript:EES20000	7.00E-88
1119- 73:	transcript:Zm00001d039113_T001	transcript:EES20002	1.00E-90
1119- 74:	transcript:Zm00001d039115_T001	transcript:EES18737	0
1119- 75:	transcript:Zm00001d039116_T001	transcript:EES18738	0
1119- 76:	transcript:Zm00001d039118_T001	transcript:EES20004	4.00E-52

1119- 77:	transcript:Zm00001d039120_T002	transcript:EES18741	0
1119- 78:	transcript:Zm00001d039121_T001	transcript:EES18742	0
1119- 79:	transcript:Zm00001d039131_T007	transcript:OQU78492	0
1119- 80:	transcript:Zm00001d039132_T001	transcript:KXG22609	2.00E-129

Alignment 1120: score=2249.0 e_value=2.7e-193 N=51 6&9 plus

1120- 0:	transcript:Zm00001d038049_T001	transcript:KXG21874	0
1120- 1:	transcript:Zm00001d038051_T001	transcript:EES18140	0
1120- 2:	transcript:Zm00001d038054_T002	transcript:KXG21877	0
1120- 3:	transcript:Zm00001d038056_T001	transcript:OQU77894	2.00E-61
1120- 4:	transcript:Zm00001d038057_T002	transcript:EES19437	9.00E-72
1120- 5:	transcript:Zm00001d038059_T001	transcript:EES19439	0
1120- 6:	transcript:Zm00001d038060_T001	transcript:EES18142	0
1120- 7:	transcript:Zm00001d038061_T001	transcript:EES19444	5.00E-93
1120- 8:	transcript:Zm00001d038062_T001	transcript:OQU77915	0
1120- 9:	transcript:Zm00001d038063_T001	transcript:EES18146	0
1120- 10:	transcript:Zm00001d038064_T001	transcript:EES18148	0
1120- 11:	transcript:Zm00001d038065_T001	transcript:KXG21896	1.00E-162
1120- 12:	transcript:Zm00001d038066_T002	transcript:EES18149	0
1120- 13:	transcript:Zm00001d038068_T001	transcript:KXG21900	0
1120- 14:	transcript:Zm00001d038070_T001	transcript:EES18150	1.00E-133
1120- 15:	transcript:Zm00001d038076_T002	transcript:EES18154	0
1120- 16:	transcript:Zm00001d038084_T001	transcript:EES18162	0
1120- 17:	transcript:Zm00001d038085_T001	transcript:EES18163	0
1120- 18:	transcript:Zm00001d038087_T002	transcript:EES19464	0
1120- 19:	transcript:Zm00001d038088_T002	transcript:EES19467	1.00E-59
1120- 20:	transcript:Zm00001d038089_T001	transcript:EES18165	2.00E-127
1120- 21:	transcript:Zm00001d038091_T006	transcript:EES19469	0
1120- 22:	transcript:Zm00001d038092_T005	transcript:EES18166	0
1120- 23:	transcript:Zm00001d038097_T002	transcript:EES18169	0
1120- 24:	transcript:Zm00001d038098_T001	transcript:EES18170	8.00E-103
1120- 25:	transcript:Zm00001d038100_T002	transcript:EES18171	0
1120- 26:	transcript:Zm00001d038101_T002	transcript:EES19477	0
1120- 27:	transcript:Zm00001d038104_T003	transcript:EES18172	0
1120- 28:	transcript:Zm00001d038105_T002	transcript:EES19478	0
1120- 29:	transcript:Zm00001d038106_T002	transcript:KXG21921	0
1120- 30:	transcript:Zm00001d038107_T002	transcript:EES18174	0
1120- 31:	transcript:Zm00001d038108_T001	transcript:OQU77940	4.00E-73
1120- 32:	transcript:Zm00001d038109_T002	transcript:EES18176	0
1120- 33:	transcript:Zm00001d038113_T001	transcript:OQU77942	0
1120- 34:	transcript:Zm00001d038114_T001	transcript:EES19480	0
1120- 35:	transcript:Zm00001d038115_T002	transcript:OQU77944	2.00E-89
1120- 36:	transcript:Zm00001d038117_T001	transcript:KXG21927	2.00E-83
1120- 37:	transcript:Zm00001d038118_T001	transcript:EES18183	0
1120- 38:	transcript:Zm00001d038121_T001	transcript:OQU77947	0
1120- 39:	transcript:Zm00001d038122_T001	transcript:EES19484	0
1120- 40:	transcript:Zm00001d038123_T003	transcript:EES18187	0
1120- 41:	transcript:Zm00001d038125_T001	transcript:EES18180	0
1120- 42:	transcript:Zm00001d038127_T001	transcript:EES19487	2.00E-176
1120- 43:	transcript:Zm00001d038128_T004	transcript:KXG21937	0
1120- 44:	transcript:Zm00001d038130_T004	transcript:EES18189	2.00E-161
1120- 45:	transcript:Zm00001d038131_T004	transcript:KXG21940	0
1120- 46:	transcript:Zm00001d038133_T001	transcript:EES19489	4.00E-158
1120- 47:	transcript:Zm00001d038134_T001	transcript:EES19490	0
1120- 48:	transcript:Zm00001d038135_T001	transcript:EES18191	0

1120- 49:transcript:Zm00001d038136_T002 transcript:EES18192 2.00E-161
1120- 50:transcript:Zm00001d038139_T001 transcript:EES19493 1.00E-127
Alignment 1121: score=2011.0 e_value=1.9e-165 N=46 6&9 plus
1121- 0:transcript:Zm00001d038170_T001 transcript:EES19518 0
1121- 1:transcript:Zm00001d038171_T001 transcript:EES19519 0
1121- 2:transcript:Zm00001d038172_T001 transcript:OQU78005 0
1121- 3:transcript:Zm00001d038173_T001 transcript:EES18222 5.00E-99
1121- 4:transcript:Zm00001d038174_T002 transcript:KXG21994 0
1121- 5:transcript:Zm00001d038176_T001 transcript:EES18226 0
1121- 6:transcript:Zm00001d038177_T001 transcript:EES18227 0
1121- 7:transcript:Zm00001d038179_T001 transcript:EES18229 2.00E-180
1121- 8:transcript:Zm00001d038189_T010 transcript:KXG21996 0
1121- 9:transcript:Zm00001d038190_T012 transcript:KXG21997 0
1121- 10:transcript:Zm00001d038192_T001 transcript:EES19527 7.00E-155
1121- 11:transcript:Zm00001d038193_T005 transcript:OQU78012 0
1121- 12:transcript:Zm00001d038194_T001 transcript:EES19528 0
1121- 13:transcript:Zm00001d038195_T001 transcript:EES19530 0
1121- 14:transcript:Zm00001d038196_T001 transcript:KXG22005 0
1121- 15:transcript:Zm00001d038197_T001 transcript:OQU78015 4.00E-98
1121- 16:transcript:Zm00001d038198_T001 transcript:OQU78016 0
1121- 17:transcript:Zm00001d038203_T001 transcript:EES18240 2.00E-48
1121- 18:transcript:Zm00001d038205_T002 transcript:EES19537 0
1121- 19:transcript:Zm00001d038206_T001 transcript:KXG22024 0
1121- 20:transcript:Zm00001d038207_T001 transcript:EES19541 0
1121- 21:transcript:Zm00001d038208_T001 transcript:EES18243 0
1121- 22:transcript:Zm00001d038209_T001 transcript:OQU78021 0
1121- 23:transcript:Zm00001d038211_T001 transcript:EES18246 0
1121- 24:transcript:Zm00001d038216_T001 transcript:EES19546 4.00E-59
1121- 25:transcript:Zm00001d038217_T002 transcript:EES18249 0
1121- 26:transcript:Zm00001d038218_T001 transcript:EES18250 1.00E-77
1121- 27:transcript:Zm00001d038220_T001 transcript:EES18252 3.00E-69
1121- 28:transcript:Zm00001d038221_T001 transcript:EES18253 0
1121- 29:transcript:Zm00001d038222_T003 transcript:EES19550 0
1121- 30:transcript:Zm00001d038223_T001 transcript:EES19545 0
1121- 31:transcript:Zm00001d038224_T003 transcript:EES18256 0
1121- 32:transcript:Zm00001d038225_T003 transcript:EES18257 0
1121- 33:transcript:Zm00001d038226_T001 transcript:OQU78035 9.00E-124
1121- 34:transcript:Zm00001d038227_T002 transcript:KXG22035 0
1121- 35:transcript:Zm00001d038228_T001 transcript:KXG22037 0
1121- 36:transcript:Zm00001d038229_T001 transcript:EES19558 0
1121- 37:transcript:Zm00001d038239_T001 transcript:KXG22043 4.00E-69
1121- 38:transcript:Zm00001d038240_T001 transcript:OQU78045 0
1121- 39:transcript:Zm00001d038248_T002 transcript:KXG22055 1.00E-93
1121- 40:transcript:Zm00001d038250_T001 transcript:EES19566 0
1121- 41:transcript:Zm00001d038251_T001 transcript:EES19570 6.00E-128
1121- 42:transcript:Zm00001d038252_T001 transcript:KXG22060 0
1121- 43:transcript:Zm00001d038257_T018 transcript:KXG22066 0
1121- 44:transcript:Zm00001d038258_T001 transcript:EES19576 6.00E-139
1121- 45:transcript:Zm00001d038262_T001 transcript:OQU78068 0
Alignment 1122: score=1650.0 e_value=7.1e-127 N=36 6&9 plus
1122- 0:transcript:Zm00001d038676_T001 transcript:KXG22328 0
1122- 1:transcript:Zm00001d038678_T001 transcript:EES18491 0
1122- 2:transcript:Zm00001d038680_T001 transcript:EES19795 8.00E-42
1122- 3:transcript:Zm00001d038681_T002 transcript:OQU78288 3.00E-22

1122- 4:	transcript:Zm00001d038682_T001	transcript:EES18492	2.00E-76
1122- 5:	transcript:Zm00001d038683_T001	transcript:EES18493	4.00E-178
1122- 6:	transcript:Zm00001d038684_T003	transcript:KXG22332	0
1122- 7:	transcript:Zm00001d038685_T002	transcript:EES18495	0
1122- 8:	transcript:Zm00001d038687_T001	transcript:KXG22334	0
1122- 9:	transcript:Zm00001d038688_T002	transcript:EES18497	9.00E-153
1122- 10:	transcript:Zm00001d038689_T001	transcript:KXG22335	0
1122- 11:	transcript:Zm00001d038690_T001	transcript:EES18498	0
1122- 12:	transcript:Zm00001d038691_T001	transcript:EES18499	0
1122- 13:	transcript:Zm00001d038692_T001	transcript:OQU78297	3.00E-116
1122- 14:	transcript:Zm00001d038693_T001	transcript:OQU78298	6.00E-66
1122- 15:	transcript:Zm00001d038695_T001	transcript:EES19801	0
1122- 16:	transcript:Zm00001d038696_T001	transcript:KXG22340	8.00E-26
1122- 17:	transcript:Zm00001d038697_T001	transcript:KXG22341	2.00E-25
1122- 18:	transcript:Zm00001d038698_T001	transcript:EES18504	0
1122- 19:	transcript:Zm00001d038699_T001	transcript:EES19802	4.00E-166
1122- 20:	transcript:Zm00001d038700_T001	transcript:EES19804	4.00E-167
1122- 21:	transcript:Zm00001d038704_T002	transcript:EES18506	0
1122- 22:	transcript:Zm00001d038706_T001	transcript:KXG22353	3.00E-38
1122- 23:	transcript:Zm00001d038708_T001	transcript:KXG22354	0
1122- 24:	transcript:Zm00001d038709_T001	transcript:EES18509	7.00E-151
1122- 25:	transcript:Zm00001d038712_T001	transcript:EES18510	6.00E-54
1122- 26:	transcript:Zm00001d038714_T001	transcript:EES19813	5.00E-103
1122- 27:	transcript:Zm00001d038717_T001	transcript:KXG22356	2.00E-110
1122- 28:	transcript:Zm00001d038718_T001	transcript:OQU78310	5.00E-106
1122- 29:	transcript:Zm00001d038719_T001	transcript:EES18512	8.00E-30
1122- 30:	transcript:Zm00001d038724_T001	transcript:EES18513	2.00E-123
1122- 31:	transcript:Zm00001d038725_T001	transcript:KXG22359	2.00E-81
1122- 32:	transcript:Zm00001d038726_T001	transcript:EES19821	0
1122- 33:	transcript:Zm00001d038728_T003	transcript:EES18516	0
1122- 34:	transcript:Zm00001d038729_T001	transcript:KXG22360	1.00E-42
1122- 35:	transcript:Zm00001d038739_T001	transcript:OQU78316	0
## Alignment 1123: score=843.0 e_value=1.1e-50 N=19 6&9 plus			
1123- 0:	transcript:Zm00001d038280_T001	transcript:KXG22102	0
1123- 1:	transcript:Zm00001d038281_T001	transcript:EES18302	2.00E-154
1123- 2:	transcript:Zm00001d038282_T001	transcript:KXG22104	0
1123- 3:	transcript:Zm00001d038283_T001	transcript:EES18304	3.00E-64
1123- 4:	transcript:Zm00001d038284_T001	transcript:KXG22105	6.00E-111
1123- 5:	transcript:Zm00001d038287_T001	transcript:EES19598	9.00E-40
1123- 6:	transcript:Zm00001d038288_T001	transcript:EES19607	1.00E-102
1123- 7:	transcript:Zm00001d038289_T001	transcript:KXG22106	1.00E-135
1123- 8:	transcript:Zm00001d038290_T004	transcript:EES19609	0
1123- 9:	transcript:Zm00001d038291_T001	transcript:EES19611	1.00E-79
1123- 10:	transcript:Zm00001d038292_T004	transcript:EES19612	0
1123- 11:	transcript:Zm00001d038296_T001	transcript:OQU78097	0
1123- 12:	transcript:Zm00001d038297_T001	transcript:EES18308	8.00E-169
1123- 13:	transcript:Zm00001d038299_T003	transcript:EES18309	0
1123- 14:	transcript:Zm00001d038300_T002	transcript:EES19608	0
1123- 15:	transcript:Zm00001d038301_T002	transcript:EES18312	0
1123- 16:	transcript:Zm00001d038302_T041	transcript:KXG22114	0
1123- 17:	transcript:Zm00001d038304_T001	transcript:EES19619	0
1123- 18:	transcript:Zm00001d038306_T002	transcript:EES18315	4.00E-116
## Alignment 1124: score=620.0 e_value=1.6e-31 N=13 6&9 plus			
1124- 0:	transcript:Zm00001d038984_T001	transcript:EES18667	2.00E-60

1124- 1:transcript:Zm00001d038988_T005 transcript:KXG22524 0
1124- 2:transcript:Zm00001d038989_T002 transcript:EES19937 0
1124- 3:transcript:Zm00001d038990_T001 transcript:EES19938 3.00E-149
1124- 4:transcript:Zm00001d038991_T001 transcript:KXG22525 0
1124- 5:transcript:Zm00001d038992_T001 transcript:EES18670 0
1124- 6:transcript:Zm00001d038993_T001 transcript:KXG22531 2.00E-29
1124- 7:transcript:Zm00001d038994_T001 transcript:EES18672 3.00E-171
1124- 8:transcript:Zm00001d038996_T001 transcript:EES19940 0
1124- 9:transcript:Zm00001d038997_T001 transcript:KXG22532 3.00E-25
1124- 10:transcript:Zm00001d038998_T001 transcript:EES18674 0
1124- 11:transcript:Zm00001d038999_T001 transcript:EES19942 1.00E-152
1124- 12:transcript:Zm00001d039000_T001 transcript:EES19943 0
Alignment 1125: score=566.0 e_value=2.4e-28 N=12 6&9 plus
1125- 0:transcript:Zm00001d039135_T001 transcript:EES18751 9.00E-89
1125- 1:transcript:Zm00001d039138_T012 transcript:KXG22612 0
1125- 2:transcript:Zm00001d039139_T001 transcript:EES20007 2.00E-118
1125- 3:transcript:Zm00001d039140_T001 transcript:EES20008 1.00E-96
1125- 4:transcript:Zm00001d039141_T001 transcript:KXG22615 0
1125- 5:transcript:Zm00001d039142_T001 transcript:EES20010 0
1125- 6:transcript:Zm00001d039144_T002 transcript:EES18756 0
1125- 7:transcript:Zm00001d039145_T001 transcript:OQU78503 0
1125- 8:transcript:Zm00001d039146_T001 transcript:EES18757 0
1125- 9:transcript:Zm00001d039147_T003 transcript:KXG22620 0
1125- 10:transcript:Zm00001d039148_T001 transcript:KXG22624 1.00E-114
1125- 11:transcript:Zm00001d039150_T001 transcript:EES20021 0
Alignment 1126: score=460.0 e_value=2.8e-21 N=10 6&9 plus
1126- 0:transcript:Zm00001d037890_T001 transcript:OQU77874 0
1126- 1:transcript:Zm00001d037891_T001 transcript:OQU77876 0
1126- 2:transcript:Zm00001d037892_T001 transcript:EES19414 2.00E-99
1126- 3:transcript:Zm00001d037894_T001 transcript:EES18117 1.00E-47
1126- 4:transcript:Zm00001d037897_T002 transcript:OQU77881 3.00E-44
1126- 5:transcript:Zm00001d037898_T012 transcript:OQU77882 0
1126- 6:transcript:Zm00001d037899_T001 transcript:EES19420 5.00E-118
1126- 7:transcript:Zm00001d037900_T001 transcript:EES19421 0
1126- 8:transcript:Zm00001d037901_T001 transcript:OQU77884 6.00E-128
1126- 9:transcript:Zm00001d037902_T001 transcript:EES18130 3.00E-151
Alignment 1127: score=422.0 e_value=6.2e-21 N=10 6&9 plus
1127- 0:transcript:Zm00001d038142_T034 transcript:EES18196 0
1127- 1:transcript:Zm00001d038143_T002 transcript:KXG21943 8.00E-166
1127- 2:transcript:Zm00001d038146_T004 transcript:EES19495 0
1127- 3:transcript:Zm00001d038147_T001 transcript:KXG21947 0
1127- 4:transcript:Zm00001d038149_T004 transcript:KXG21948 0
1127- 5:transcript:Zm00001d038150_T001 transcript:KXG21950 0
1127- 6:transcript:Zm00001d038151_T001 transcript:OQU77960 0
1127- 7:transcript:Zm00001d038158_T001 transcript:OQU77961 1.00E-119
1127- 8:transcript:Zm00001d038161_T001 transcript:EES19501 2.00E-132
1127- 9:transcript:Zm00001d038163_T001 transcript:KXG21960 0
Alignment 1128: score=403.0 e_value=3e-18 N=9 6&9 plus
1128- 0:transcript:Zm00001d037593_T004 transcript:EES19050 0
1128- 1:transcript:Zm00001d037596_T001 transcript:EES19051 0
1128- 2:transcript:Zm00001d037599_T001 transcript:EES17741 0
1128- 3:transcript:Zm00001d037601_T001 transcript:OQU77459 0
1128- 4:transcript:Zm00001d037602_T001 transcript:EES17747 5.00E-177
1128- 5:transcript:Zm00001d037603_T002 transcript:EES17748 9.00E-123

1128- 6:transcript:Zm00001d037604_T001 transcript:EES19059 0
1128- 7:transcript:Zm00001d037605_T001 transcript:KXG21349 9.00E-56
1128- 8:transcript:Zm00001d037606_T001 transcript:EES19061 0
Alignment 1129: score=369.0 e_value=2.5e-14 N=8 6&9 plus
1129- 0:transcript:Zm00001d037703_T001 transcript:KXG21457 2.00E-53
1129- 1:transcript:Zm00001d037704_T001 transcript:EES19137 2.00E-174
1129- 2:transcript:Zm00001d037708_T001 transcript:EES19138 3.00E-155
1129- 3:transcript:Zm00001d037711_T001 transcript:OQU77549 0
1129- 4:transcript:Zm00001d037712_T001 transcript:KXG21461 0
1129- 5:transcript:Zm00001d037713_T001 transcript:EES19146 2.00E-132
1129- 6:transcript:Zm00001d037714_T001 transcript:EES17814 4.00E-62
1129- 7:transcript:Zm00001d037717_T002 transcript:EES19148 0
Alignment 1130: score=335.0 e_value=3.8e-13 N=7 6&9 plus
1130- 0:transcript:Zm00001d038012_T001 transcript:EES18032 3.00E-122
1130- 1:transcript:Zm00001d038013_T001 transcript:EES19343 3.00E-101
1130- 2:transcript:Zm00001d038016_T001 transcript:EES19345 3.00E-99
1130- 3:transcript:Zm00001d038018_T001 transcript:EES18035 1.00E-138
1130- 4:transcript:Zm00001d038020_T001 transcript:KXG21748 0
1130- 5:transcript:Zm00001d038021_T001 transcript:EES19347 0
1130- 6:transcript:Zm00001d038022_T001 transcript:OQU77788 0
Alignment 1131: score=269.0 e_value=8.4e-10 N=6 6&9 plus
1131- 0:transcript:Zm00001d038003_T001 transcript:EES19328 5.00E-80
1131- 1:transcript:Zm00001d038004_T001 transcript:EES19329 3.00E-127
1131- 2:transcript:Zm00001d038005_T001 transcript:EES18028 0
1131- 3:transcript:Zm00001d038006_T001 transcript:KXG21733 0
1131- 4:transcript:Zm00001d038007_T001 transcript:EES18029 0
1131- 5:transcript:Zm00001d038010_T001 transcript:OQU77780 8.00E-63
Alignment 1132: score=2373.0 e_value=9.8e-196 N=52 6&9 minus
1132- 0:transcript:Zm00001d038730_T001 transcript:KXG22403 3.00E-123
1132- 1:transcript:Zm00001d038732_T001 transcript:EES18564 1.00E-148
1132- 2:transcript:Zm00001d038733_T001 transcript:EES19865 0
1132- 3:transcript:Zm00001d038734_T003 transcript:KXG22401 0
1132- 4:transcript:Zm00001d038737_T001 transcript:EES19864 0
1132- 5:transcript:Zm00001d038739_T001 transcript:EES18556 0
1132- 6:transcript:Zm00001d038740_T002 transcript:EES18554 4.00E-15
1132- 7:transcript:Zm00001d038742_T001 transcript:EES19861 0
1132- 8:transcript:Zm00001d038745_T002 transcript:EES19859 0
1132- 9:transcript:Zm00001d038746_T001 transcript:EES18551 0
1132- 10:transcript:Zm00001d038747_T003 transcript:KXG22394 0
1132- 11:transcript:Zm00001d038748_T001 transcript:KXG22393 2.00E-17
1132- 12:transcript:Zm00001d038750_T003 transcript:EES19858 0
1132- 13:transcript:Zm00001d038751_T001 transcript:EES19857 0
1132- 14:transcript:Zm00001d038752_T001 transcript:EES19840 0
1132- 15:transcript:Zm00001d038753_T002 transcript:KXG22390 7.00E-64
1132- 16:transcript:Zm00001d038754_T002 transcript:EES19856 5.00E-108
1132- 17:transcript:Zm00001d038755_T001 transcript:EES18539 5.00E-153
1132- 18:transcript:Zm00001d038756_T001 transcript:EES19854 0
1132- 19:transcript:Zm00001d038761_T001 transcript:EES19853 1.00E-98
1132- 20:transcript:Zm00001d038762_T001 transcript:KXG22389 0
1132- 21:transcript:Zm00001d038763_T001 transcript:KXG22388 0
1132- 22:transcript:Zm00001d038764_T001 transcript:EES18546 1.00E-174
1132- 23:transcript:Zm00001d038766_T001 transcript:EES18545 2.00E-114
1132- 24:transcript:Zm00001d038768_T001 transcript:EES19841 0
1132- 25:transcript:Zm00001d038769_T003 transcript:KXG22385 0

1132- 26:transcript:Zm00001d038772_T001 transcript:OQU78336 1.00E-146
1132- 27:transcript:Zm00001d038773_T002 transcript:OQU78335 0
1132- 28:transcript:Zm00001d038775_T002 transcript:OQU78332 0
1132- 29:transcript:Zm00001d038777_T001 transcript:OQU78330 0
1132- 30:transcript:Zm00001d038779_T001 transcript:EES19839 0
1132- 31:transcript:Zm00001d038780_T001 transcript:EES18536 6.00E-64
1132- 32:transcript:Zm00001d038783_T001 transcript:OQU78328 3.00E-52
1132- 33:transcript:Zm00001d038784_T001 transcript:KXG22376 2.00E-154
1132- 34:transcript:Zm00001d038791_T001 transcript:EES19836 0
1132- 35:transcript:Zm00001d038792_T001 transcript:EES18534 0
1132- 36:transcript:Zm00001d038793_T001 transcript:KXG22374 4.00E-158
1132- 37:transcript:Zm00001d038794_T002 transcript:OQU78325 0
1132- 38:transcript:Zm00001d038795_T001 transcript:EES18531 4.00E-40
1132- 39:transcript:Zm00001d038796_T001 transcript:EES18517 0
1132- 40:transcript:Zm00001d038797_T001 transcript:OQU78322 0
1132- 41:transcript:Zm00001d038799_T001 transcript:EES18527 1.00E-102
1132- 42:transcript:Zm00001d038801_T001 transcript:EES18524 4.00E-153
1132- 43:transcript:Zm00001d038803_T001 transcript:EES19830 0
1132- 44:transcript:Zm00001d038804_T002 transcript:EES19829 0
1132- 45:transcript:Zm00001d038805_T002 transcript:EES18523 9.00E-137
1132- 46:transcript:Zm00001d038806_T001 transcript:EES19828 0
1132- 47:transcript:Zm00001d038807_T005 transcript:EES19826 0
1132- 48:transcript:Zm00001d038809_T008 transcript:KXG22364 0
1132- 49:transcript:Zm00001d038810_T001 transcript:EES18519 5.00E-86
1132- 50:transcript:Zm00001d038813_T001 transcript:OQU78312 0
1132- 51:transcript:Zm00001d038814_T001 transcript:KXG22361 8.00E-48
Alignment 1133: score=1695.0 e_value=4.6e-148 N=41 6&9 minus
1133- 0:transcript:Zm00001d037909_T001 transcript:EES18107 2.00E-10
1133- 1:transcript:Zm00001d037914_T001 transcript:EES18100 8.00E-44
1133- 2:transcript:Zm00001d037915_T001 transcript:OQU77870 6.00E-106
1133- 3:transcript:Zm00001d037916_T001 transcript:EES19407 3.00E-160
1133- 4:transcript:Zm00001d037917_T001 transcript:EES19406 2.00E-118
1133- 5:transcript:Zm00001d037918_T001 transcript:EES18092 0
1133- 6:transcript:Zm00001d037920_T001 transcript:EES18089 5.00E-46
1133- 7:transcript:Zm00001d037925_T006 transcript:KXG21829 4.00E-122
1133- 8:transcript:Zm00001d037932_T003 transcript:KXG21827 0
1133- 9:transcript:Zm00001d037934_T011 transcript:EES19402 0
1133- 10:transcript:Zm00001d037935_T001 transcript:KXG21817 0
1133- 11:transcript:Zm00001d037936_T001 transcript:EES19396 1.00E-55
1133- 12:transcript:Zm00001d037937_T001 transcript:OQU77848 2.00E-87
1133- 13:transcript:Zm00001d037939_T003 transcript:EES18080 0
1133- 14:transcript:Zm00001d037941_T001 transcript:EES18078 7.00E-72
1133- 15:transcript:Zm00001d037943_T001 transcript:OQU77841 3.00E-57
1133- 16:transcript:Zm00001d037946_T001 transcript:OQU77834 0
1133- 17:transcript:Zm00001d037949_T001 transcript:EES19385 0
1133- 18:transcript:Zm00001d037951_T001 transcript:OQU77830 0
1133- 19:transcript:Zm00001d037958_T001 transcript:EES19380 5.00E-68
1133- 20:transcript:Zm00001d037962_T003 transcript:KXG21798 0
1133- 21:transcript:Zm00001d037966_T006 transcript:OQU77822 2.00E-139
1133- 22:transcript:Zm00001d037967_T002 transcript:EES18066 0
1133- 23:transcript:Zm00001d037969_T001 transcript:KXG21784 2.00E-29
1133- 24:transcript:Zm00001d037970_T022 transcript:EES19365 0
1133- 25:transcript:Zm00001d037971_T002 transcript:KXG21772 0
1133- 26:transcript:Zm00001d037972_T001 transcript:EES19362 8.00E-37

1133- 27:transcript:Zm00001d037973_T001 transcript:KXG21771 0
1133- 28:transcript:Zm00001d037974_T001 transcript:OQU77810 2.00E-101
1133- 29:transcript:Zm00001d037975_T001 transcript:EES18052 0
1133- 30:transcript:Zm00001d037976_T001 transcript:KXG21769 0
1133- 31:transcript:Zm00001d037978_T001 transcript:EES19360 0
1133- 32:transcript:Zm00001d037979_T001 transcript:KXG21767 0
1133- 33:transcript:Zm00001d037982_T006 transcript:EES18046 0
1133- 34:transcript:Zm00001d037984_T004 transcript:OQU77805 0
1133- 35:transcript:Zm00001d037985_T001 transcript:KXG21764 6.00E-42
1133- 36:transcript:Zm00001d037992_T001 transcript:EES19357 0
1133- 37:transcript:Zm00001d037993_T001 transcript:EES19356 2.00E-112
1133- 38:transcript:Zm00001d037994_T001 transcript:EES18041 7.00E-89
1133- 39:transcript:Zm00001d038001_T001 transcript:KXG21759 5.00E-110
1133- 40:transcript:Zm00001d038002_T001 transcript:EES18037 1.00E-103
Alignment 1134: score=1278.0 e_value=1.6e-98 N=30 6&9 minus
1134- 0:transcript:Zm00001d037722_T001 transcript:EES17874 1.00E-44
1134- 1:transcript:Zm00001d037724_T002 transcript:OQU77607 1.00E-136
1134- 2:transcript:Zm00001d037725_T001 transcript:EES17872 0
1134- 3:transcript:Zm00001d037728_T001 transcript:EES19211 0
1134- 4:transcript:Zm00001d037729_T001 transcript:EES19209 0
1134- 5:transcript:Zm00001d037730_T001 transcript:KXG21527 7.00E-11
1134- 6:transcript:Zm00001d037732_T002 transcript:EES19208 2.00E-124
1134- 7:transcript:Zm00001d037733_T001 transcript:EES19204 0
1134- 8:transcript:Zm00001d037734_T011 transcript:EES17868 0
1134- 9:transcript:Zm00001d037735_T001 transcript:EES17867 0
1134- 10:transcript:Zm00001d037737_T001 transcript:KXG21521 2.00E-179
1134- 11:transcript:Zm00001d037738_T001 transcript:OQU77602 1.00E-57
1134- 12:transcript:Zm00001d037740_T001 transcript:EES17861 8.00E-148
1134- 13:transcript:Zm00001d037743_T001 transcript:EES19201 2.00E-114
1134- 14:transcript:Zm00001d037744_T001 transcript:EES19200 0
1134- 15:transcript:Zm00001d037745_T001 transcript:EES17857 0
1134- 16:transcript:Zm00001d037747_T001 transcript:OQU77594 3.00E-81
1134- 17:transcript:Zm00001d037748_T001 transcript:EES19197 1.00E-133
1134- 18:transcript:Zm00001d037749_T001 transcript:KXG21509 6.00E-52
1134- 19:transcript:Zm00001d037751_T001 transcript:EES19193 1.00E-148
1134- 20:transcript:Zm00001d037753_T001 transcript:KXG21508 1.00E-44
1134- 21:transcript:Zm00001d037756_T004 transcript:EES17852 0
1134- 22:transcript:Zm00001d037757_T001 transcript:EES17851 6.00E-66
1134- 23:transcript:Zm00001d037759_T002 transcript:EES19190 0
1134- 24:transcript:Zm00001d037760_T001 transcript:EES17849 0
1134- 25:transcript:Zm00001d037762_T001 transcript:KXG21504 6.00E-104
1134- 26:transcript:Zm00001d037766_T001 transcript:KXG21503 1.00E-176
1134- 27:transcript:Zm00001d037767_T001 transcript:EES17847 3.00E-29
1134- 28:transcript:Zm00001d037769_T001 transcript:EES19185 0
1134- 29:transcript:Zm00001d037770_T002 transcript:EES19183 0
Alignment 1135: score=1183.0 e_value=3.7e-89 N=28 6&9 minus
1135- 0:transcript:Zm00001d035562_T007 transcript:EES17610 1.00E-62
1135- 1:transcript:Zm00001d035569_T001 transcript:EES17609 6.00E-18
1135- 2:transcript:Zm00001d035574_T001 transcript:EES17607 0
1135- 3:transcript:Zm00001d035579_T007 transcript:EES17606 0
1135- 4:transcript:Zm00001d035585_T001 transcript:KXG21128 7.00E-53
1135- 5:transcript:Zm00001d035589_T007 transcript:OQU77259 7.00E-59
1135- 6:transcript:Zm00001d035593_T001 transcript:EES17598 0
1135- 7:transcript:Zm00001d035595_T003 transcript:OQU77257 2.00E-120

1135- 8:transcript:Zm00001d035597_T001	transcript:EES18910	0
1135- 9:transcript:Zm00001d035598_T001	transcript:EES18909	0
1135- 10:transcript:Zm00001d035599_T001	transcript:OQU77253	0
1135- 11:transcript:Zm00001d035601_T001	transcript:EES17597	0
1135- 12:transcript:Zm00001d035603_T001	transcript:EES17594	2.00E-124
1135- 13:transcript:Zm00001d035604_T001	transcript:EES17592	7.00E-59
1135- 14:transcript:Zm00001d035608_T001	transcript:OQU77248	0
1135- 15:transcript:Zm00001d035609_T001	transcript:KXG21109	1.00E-98
1135- 16:transcript:Zm00001d035610_T001	transcript:OQU77247	8.00E-44
1135- 17:transcript:Zm00001d035614_T001	transcript:EES18903	0
1135- 18:transcript:Zm00001d035616_T001	transcript:EES18902	4.00E-155
1135- 19:transcript:Zm00001d035624_T006	transcript:KXG21107	3.00E-85
1135- 20:transcript:Zm00001d035625_T001	transcript:EES17589	8.00E-88
1135- 21:transcript:Zm00001d035631_T001	transcript:KXG21102	0
1135- 22:transcript:Zm00001d035634_T001	transcript:OQU77242	0
1135- 23:transcript:Zm00001d035637_T001	transcript:EES18900	1.00E-33
1135- 24:transcript:Zm00001d035646_T001	transcript:EES18898	1.00E-135
1135- 25:transcript:Zm00001d035649_T001	transcript:EES18896	2.00E-108
1135- 26:transcript:Zm00001d035651_T001	transcript:KXG21095	2.00E-135
1135- 27:transcript:Zm00001d035652_T001	transcript:EES18894	0
## Alignment 1136: score=1153.0 e_value=8e-78 N=25 6&9 minus		
1136- 0:transcript:Zm00001d039165_T046	transcript:KXG22678	0
1136- 1:transcript:Zm00001d039166_T002	transcript:EES18806	2.00E-170
1136- 2:transcript:Zm00001d039167_T001	transcript:EES20050	0
1136- 3:transcript:Zm00001d039169_T001	transcript:EES18804	2.00E-98
1136- 4:transcript:Zm00001d039173_T002	transcript:KXG22672	0
1136- 5:transcript:Zm00001d039174_T001	transcript:KXG22670	1.00E-153
1136- 6:transcript:Zm00001d039175_T001	transcript:KXG22669	4.00E-99
1136- 7:transcript:Zm00001d039176_T001	transcript:EES18801	0
1136- 8:transcript:Zm00001d039177_T001	transcript:EES18800	0
1136- 9:transcript:Zm00001d039179_T001	transcript:EES18799	0
1136- 10:transcript:Zm00001d039180_T001	transcript:EES18798	0
1136- 11:transcript:Zm00001d039181_T001	transcript:EES18778	0
1136- 12:transcript:Zm00001d039182_T001	transcript:EES18791	0
1136- 13:transcript:Zm00001d039183_T001	transcript:EES20041	0
1136- 14:transcript:Zm00001d039185_T001	transcript:OQU78520	0
1136- 15:transcript:Zm00001d039188_T001	transcript:EES18787	0
1136- 16:transcript:Zm00001d039189_T001	transcript:KXG22647	0
1136- 17:transcript:Zm00001d039190_T001	transcript:KXG22644	2.00E-152
1136- 18:transcript:Zm00001d039192_T001	transcript:OQU78513	0
1136- 19:transcript:Zm00001d039193_T001	transcript:EES18784	0
1136- 20:transcript:Zm00001d039194_T001	transcript:EES18781	0
1136- 21:transcript:Zm00001d039195_T001	transcript:EES18780	0
1136- 22:transcript:Zm00001d039196_T001	transcript:EES18766	0
1136- 23:transcript:Zm00001d039197_T001	transcript:KXG22635	3.00E-147
1136- 24:transcript:Zm00001d039200_T001	transcript:EES20024	1.00E-30
## Alignment 1137: score=875.0 e_value=1.2e-53 N=19 6&9 minus		
1137- 0:transcript:Zm00001d038310_T003	transcript:EES19596	0
1137- 1:transcript:Zm00001d038311_T001	transcript:EES18296	1.00E-113
1137- 2:transcript:Zm00001d038312_T001	transcript:EES19595	2.00E-146
1137- 3:transcript:Zm00001d038316_T001	transcript:KXG22095	1.00E-62
1137- 4:transcript:Zm00001d038318_T002	transcript:EES19594	0
1137- 5:transcript:Zm00001d038319_T003	transcript:EES18295	2.00E-154
1137- 6:transcript:Zm00001d038320_T001	transcript:OQU78085	1.00E-73

```
1137-  7:transcript:Zm00001d038321_T001 transcript:EES18293                    0
1137-  8:transcript:Zm00001d038325_T001 transcript:KXG22077           7.00E-141
1137-  9:transcript:Zm00001d038326_T003 transcript:EES19589                    0
1137- 10:transcript:Zm00001d038327_T001 transcript:EES19588            1.00E-76
1137- 11:transcript:Zm00001d038328_T001 transcript:EES19587                    0
1137- 12:transcript:Zm00001d038329_T001 transcript:EES18284           9.00E-113
1137- 13:transcript:Zm00001d038330_T001 transcript:KXG22075                    0
1137- 14:transcript:Zm00001d038331_T001 transcript:KXG22074            1.00E-51
1137- 15:transcript:Zm00001d038335_T001 transcript:EES19584            4.00E-31
1137- 16:transcript:Zm00001d038337_T001 transcript:EES19581                    0
1137- 17:transcript:Zm00001d038338_T001 transcript:KXG22070           6.00E-122
1137- 18:transcript:Zm00001d038340_T001 transcript:EES19579                    0
## Alignment 1138: score=854.0 e_value=3.5e-54 N=19 6&9 minus
1138-  0:transcript:Zm00001d035439_T001 transcript:EES18954           8.00E-173
1138-  1:transcript:Zm00001d035440_T001 transcript:EES17653            1.00E-73
1138-  2:transcript:Zm00001d035442_T001 transcript:KXG21209                    0
1138-  3:transcript:Zm00001d035443_T001 transcript:EES17651                    0
1138-  4:transcript:Zm00001d035445_T001 transcript:EES17650           6.00E-171
1138-  5:transcript:Zm00001d035447_T001 transcript:KXG21208                    0
1138-  6:transcript:Zm00001d035450_T001 transcript:EES18951                    0
1138-  7:transcript:Zm00001d035454_T001 transcript:OQU77329           9.00E-133
1138-  8:transcript:Zm00001d035455_T001 transcript:OQU77325                    0
1138-  9:transcript:Zm00001d035456_T001 transcript:EES17646            8.00E-55
1138- 10:transcript:Zm00001d035457_T001 transcript:EES17645           2.00E-103
1138- 11:transcript:Zm00001d035462_T001 transcript:EES18946                    0
1138- 12:transcript:Zm00001d035467_T001 transcript:OQU77320                    0
1138- 13:transcript:Zm00001d035470_T004 transcript:OQU77310                    0
1138- 14:transcript:Zm00001d035473_T001 transcript:EES18943            1.00E-91
1138- 15:transcript:Zm00001d035475_T001 transcript:EES18942                    0
1138- 16:transcript:Zm00001d035476_T002 transcript:OQU77305                    0
1138- 17:transcript:Zm00001d035486_T001 transcript:OQU77304           2.00E-129
1138- 18:transcript:Zm00001d035487_T025 transcript:KXG21178                    0
## Alignment 1139: score=784.0 e_value=1.3e-49 N=18 6&9 minus
1139-  0:transcript:Zm00001d037509_T001 transcript:EES17781           1.00E-158
1139-  1:transcript:Zm00001d037512_T001 transcript:KXG21417                    0
1139-  2:transcript:Zm00001d037513_T001 transcript:EES17780           4.00E-141
1139-  3:transcript:Zm00001d037515_T001 transcript:EES19107            7.00E-78
1139-  4:transcript:Zm00001d037519_T004 transcript:KXG21405                    0
1139-  5:transcript:Zm00001d037521_T002 transcript:OQU77505                    0
1139-  6:transcript:Zm00001d037528_T001 transcript:EES19097            1.00E-93
1139-  7:transcript:Zm00001d037529_T001 transcript:KXG21396                    0
1139-  8:transcript:Zm00001d037530_T002 transcript:EES19096                    0
1139-  9:transcript:Zm00001d037532_T001 transcript:EES19095                    0
1139- 10:transcript:Zm00001d037533_T001 transcript:OQU77500            1.00E-77
1139- 11:transcript:Zm00001d037536_T006 transcript:EES19093                    0
1139- 12:transcript:Zm00001d037537_T001 transcript:OQU77499           3.00E-170
1139- 13:transcript:Zm00001d037538_T001 transcript:KXG21394                    0
1139- 14:transcript:Zm00001d037540_T001 transcript:EES19087                    0
1139- 15:transcript:Zm00001d037543_T001 transcript:KXG21387            2.00E-77
1139- 16:transcript:Zm00001d037544_T001 transcript:EES17770                    0
1139- 17:transcript:Zm00001d037546_T011 transcript:EES19084                    0
## Alignment 1140: score=761.0 e_value=2.2e-52 N=18 6&9 minus
1140-  0:transcript:Zm00001d035491_T001 transcript:EES18940           7.00E-171
1140-  1:transcript:Zm00001d035492_T003 transcript:KXG21175                    0
```

1140- 2:transcript:Zm00001d035494_T001 transcript:OQU77301 5.00E-91
1140- 3:transcript:Zm00001d035498_T001 transcript:EES18939 1.00E-104
1140- 4:transcript:Zm00001d035501_T001 transcript:EES17631 0
1140- 5:transcript:Zm00001d035503_T001 transcript:KXG21172 1.00E-163
1140- 6:transcript:Zm00001d035505_T001 transcript:EES18938 3.00E-29
1140- 7:transcript:Zm00001d035506_T003 transcript:OQU77296 0
1140- 8:transcript:Zm00001d035509_T001 transcript:EES17627 0
1140- 9:transcript:Zm00001d035512_T001 transcript:OQU77285 5.00E-136
1140- 10:transcript:Zm00001d035514_T001 transcript:EES17624 0
1140- 11:transcript:Zm00001d035515_T001 transcript:EES18931 0
1140- 12:transcript:Zm00001d035519_T001 transcript:EES17623 1.00E-117
1140- 13:transcript:Zm00001d035526_T010 transcript:EES18930 0
1140- 14:transcript:Zm00001d035527_T001 transcript:EES17622 0
1140- 15:transcript:Zm00001d035535_T001 transcript:OQU77281 1.00E-101
1140- 16:transcript:Zm00001d035540_T001 transcript:OQU77280 7.00E-32
1140- 17:transcript:Zm00001d035542_T001 transcript:EES17619 1.00E-37
Alignment 1141: score=502.0 e_value=1.3e-24 N=11 6&9 minus
1141- 0:transcript:Zm00001d037557_T065 transcript:EES17763 0
1141- 1:transcript:Zm00001d037565_T002 transcript:OQU77483 0
1141- 2:transcript:Zm00001d037567_T001 transcript:OQU77476 1.00E-82
1141- 3:transcript:Zm00001d037569_T001 transcript:KXG21369 8.00E-38
1141- 4:transcript:Zm00001d037573_T001 transcript:EES19072 3.00E-118
1141- 5:transcript:Zm00001d037574_T001 transcript:EES17755 2.00E-125
1141- 6:transcript:Zm00001d037576_T001 transcript:EES19071 0
1141- 7:transcript:Zm00001d037577_T001 transcript:KXG21365 5.00E-149
1141- 8:transcript:Zm00001d037582_T001 transcript:EES19069 0
1141- 9:transcript:Zm00001d037586_T001 transcript:EES17752 0
1141- 10:transcript:Zm00001d037590_T002 transcript:EES19066 0
Alignment 1142: score=493.0 e_value=3.6e-24 N=11 6&9 minus
1142- 0:transcript:Zm00001d037675_T001 transcript:OQU77586 0
1142- 1:transcript:Zm00001d037680_T001 transcript:EES19172 2.00E-97
1142- 2:transcript:Zm00001d037682_T001 transcript:EES17840 0
1142- 3:transcript:Zm00001d037684_T001 transcript:EES17836 3.00E-36
1142- 4:transcript:Zm00001d037687_T001 transcript:EES19170 7.00E-158
1142- 5:transcript:Zm00001d037688_T001 transcript:KXG21487 0
1142- 6:transcript:Zm00001d037689_T001 transcript:EES19165 0
1142- 7:transcript:Zm00001d037691_T003 transcript:EES17832 6.00E-103
1142- 8:transcript:Zm00001d037693_T003 transcript:EES19164 0
1142- 9:transcript:Zm00001d037694_T001 transcript:EES19163 2.00E-93
1142- 10:transcript:Zm00001d037695_T003 transcript:KXG21485 0
Alignment 1143: score=466.0 e_value=1.4e-25 N=11 6&9 minus
1143- 0:transcript:Zm00001d035682_T001 transcript:KXG21061 0
1143- 1:transcript:Zm00001d035685_T001 transcript:EES17559 5.00E-29
1143- 2:transcript:Zm00001d035689_T001 transcript:KXG21060 0
1143- 3:transcript:Zm00001d035693_T001 transcript:EES17556 6.00E-47
1143- 4:transcript:Zm00001d035694_T001 transcript:KXG21057 0
1143- 5:transcript:Zm00001d035695_T001 transcript:EES17554 0
1143- 6:transcript:Zm00001d035696_T001 transcript:KXG21055 0
1143- 7:transcript:Zm00001d035698_T001 transcript:OQU77211 0
1143- 8:transcript:Zm00001d035700_T001 transcript:EES18857 0
1143- 9:transcript:Zm00001d035701_T001 transcript:EES17549 0
1143- 10:transcript:Zm00001d035702_T001 transcript:KXG21044 2.00E-34
Alignment 1144: score=415.0 e_value=2.5e-16 N=9 6&9 minus
1144- 0:transcript:Zm00001d038263_T001 transcript:KXG22127 0

1144- 1:transcript:Zm00001d038267_T001 transcript:EES19629 9.00E-97
1144- 2:transcript:Zm00001d038268_T001 transcript:EES18320 0
1144- 3:transcript:Zm00001d038269_T001 transcript:OQU78116 0
1144- 4:transcript:Zm00001d038270_T001 transcript:EES19627 7.00E-180
1144- 5:transcript:Zm00001d038272_T006 transcript:KXG22123 0
1144- 6:transcript:Zm00001d038273_T001 transcript:EES18318 0
1144- 7:transcript:Zm00001d038274_T002 transcript:KXG22120 0
1144- 8:transcript:Zm00001d038275_T002 transcript:EES19621 0
Alignment 1145: score=406.0 e_value=2.9e-17 N=9 6&9 minus
1145- 0:transcript:Zm00001d037619_T001 transcript:OQU77548 0
1145- 1:transcript:Zm00001d037620_T002 transcript:EES17810 8.00E-149
1145- 2:transcript:Zm00001d037623_T001 transcript:EES17809 1.00E-26
1145- 3:transcript:Zm00001d037624_T001 transcript:KXG21455 0
1145- 4:transcript:Zm00001d037625_T001 transcript:EES17807 0
1145- 5:transcript:Zm00001d037626_T001 transcript:EES19134 0
1145- 6:transcript:Zm00001d037627_T001 transcript:KXG21453 0
1145- 7:transcript:Zm00001d037630_T001 transcript:OQU77541 0
1145- 8:transcript:Zm00001d037631_T001 transcript:EES19127 4.00E-36
Alignment 1146: score=365.0 e_value=2.1e-16 N=8 6&9 minus
1146- 0:transcript:Zm00001d039213_T001 transcript:OQU78555 7.00E-96
1146- 1:transcript:Zm00001d039214_T025 transcript:EES18816 0
1146- 2:transcript:Zm00001d039216_T001 transcript:EES18815 1.00E-172
1146- 3:transcript:Zm00001d039217_T001 transcript:KXG22731 0
1146- 4:transcript:Zm00001d039218_T002 transcript:EES20075 0
1146- 5:transcript:Zm00001d039219_T001 transcript:KXG22727 5.00E-171
1146- 6:transcript:Zm00001d039221_T001 transcript:KXG22725 3.00E-150
1146- 7:transcript:Zm00001d039222_T001 transcript:KXG22724 5.00E-151
Alignment 1147: score=324.0 e_value=1.4e-11 N=7 6&9 minus
1147- 0:transcript:Zm00001d037659_T001 transcript:EES17796 0
1147- 1:transcript:Zm00001d037663_T001 transcript:EES17794 1.00E-67
1147- 2:transcript:Zm00001d037664_T001 transcript:EES19118 4.00E-27
1147- 3:transcript:Zm00001d037666_T001 transcript:EES17792 0
1147- 4:transcript:Zm00001d037668_T001 transcript:EES19115 0
1147- 5:transcript:Zm00001d037672_T001 transcript:EES17789 0
1147- 6:transcript:Zm00001d037674_T001 transcript:OQU77514 0
Alignment 1148: score=306.0 e_value=1.3e-15 N=8 6&9 minus
1148- 0:transcript:Zm00001d035666_T002 transcript:EES17567 1.00E-42
1148- 1:transcript:Zm00001d035669_T002 transcript:EES18883 2.00E-82
1148- 2:transcript:Zm00001d035670_T001 transcript:EES18880 0
1148- 3:transcript:Zm00001d035672_T001 transcript:EES17563 7.00E-76
1148- 4:transcript:Zm00001d035677_T001 transcript:EES17560 9.00E-178
1148- 5:transcript:Zm00001d035679_T006 transcript:EES18877 0
1148- 6:transcript:Zm00001d035680_T001 transcript:EES18867 3.00E-26
1148- 7:transcript:Zm00001d035682_T001 transcript:KXG21068 0
Alignment 1149: score=279.0 e_value=2.4e-09 N=6 6&9 minus
1149- 0:transcript:Zm00001d037607_T001 transcript:EES19157 3.00E-105
1149- 1:transcript:Zm00001d037611_T001 transcript:EES19154 0
1149- 2:transcript:Zm00001d037613_T001 transcript:KXG21479 5.00E-175
1149- 3:transcript:Zm00001d037615_T002 transcript:EES17824 0
1149- 4:transcript:Zm00001d037616_T001 transcript:OQU77561 0
1149- 5:transcript:Zm00001d037617_T001 transcript:OQU77560 6.00E-153
Alignment 1150: score=271.0 e_value=1.4e-09 N=6 6&9 minus
1150- 0:transcript:Zm00001d038071_T002 transcript:EES19457 1.00E-162
1150- 1:transcript:Zm00001d038072_T001 transcript:EES18159 0

1150- 2:transcript:Zm00001d038075_T001 transcript:KXG21909 2.00E-11
1150- 3:transcript:Zm00001d038080_T002 transcript:EES18152 8.00E-114
1150- 4:transcript:Zm00001d038081_T001 transcript:EES19454 2.00E-130
1150- 5:transcript:Zm00001d038082_T002 transcript:OQU77923 0
Alignment 1151: score=263.0 e_value=9.3e-09 N=6 6&9 minus
1151- 0:transcript:Zm00001d038153_T001 transcript:EES18214 0
1151- 1:transcript:Zm00001d038155_T001 transcript:KXG21969 0
1151- 2:transcript:Zm00001d038156_T017 transcript:OQU77974 0
1151- 3:transcript:Zm00001d038158_T001 transcript:EES18211 0
1151- 4:transcript:Zm00001d038160_T001 transcript:OQU77971 1.00E-45
1151- 5:transcript:Zm00001d038161_T001 transcript:KXG21961 2.00E-23
Alignment 1152: score=269.0 e_value=4.2e-09 N=6 6&B73V4_ctg56 plus
1152- 0:transcript:KXG26203 transcript:Zm00001d000122_T006 0
1152- 1:transcript:EES10717 transcript:Zm00001d000123_T003 0
1152- 2:transcript:EES12087 transcript:Zm00001d000124_T010 0
1152- 3:transcript:EES12088 transcript:Zm00001d000126_T002 6.00E-24
1152- 4:transcript:EES12089 transcript:Zm00001d000128_T002 2.00E-86
1152- 5:transcript:KXG26206 transcript:Zm00001d000133_T009 0
Alignment 1153: score=610.0 e_value=2e-33 N=14 7&7 plus
1153- 0:transcript:KXG25294 transcript:Zm00001d020670_T001 3.00E-92
1153- 1:transcript:EES15110 transcript:Zm00001d020673_T001 4.00E-121
1153- 2:transcript:OQU80618 transcript:Zm00001d020674_T001 0
1153- 3:transcript:KXG25300 transcript:Zm00001d020675_T001 2.00E-36
1153- 4:transcript:EES13982 transcript:Zm00001d020683_T001 0
1153- 5:transcript:KXG25358 transcript:Zm00001d020688_T001 9.00E-77
1153- 6:transcript:OQU80624 transcript:Zm00001d020697_T001 3.00E-154
1153- 7:transcript:EES15124 transcript:Zm00001d020705_T001 1.00E-58
1153- 8:transcript:KXG25311 transcript:Zm00001d020708_T002 2.00E-167
1153- 9:transcript:OQU80630 transcript:Zm00001d020711_T001 3.00E-100
1153- 10:transcript:OQU80634 transcript:Zm00001d020713_T001 3.00E-162
1153- 11:transcript:EES15128 transcript:Zm00001d020717_T001 0
1153- 12:transcript:EES14004 transcript:Zm00001d020723_T001 6.00E-23
1153- 13:transcript:EES15129 transcript:Zm00001d020725_T001 6.00E-24
Alignment 1154: score=504.0 e_value=0 N=12 7&7 plus
1154- 0:transcript:KXG25161 transcript:Zm00001d020345_T001 0
1154- 1:transcript:OQU80443 transcript:Zm00001d020348_T001 1.00E-50
1154- 2:transcript:KXG25169 transcript:Zm00001d020355_T002 0
1154- 3:transcript:OQU80448 transcript:Zm00001d020358_T001 2.00E-130
1154- 4:transcript:KXG25172 transcript:Zm00001d020361_T001 4.00E-137
1154- 5:transcript:OQU80464 transcript:Zm00001d020365_T001 0
1154- 6:transcript:OQU80467 transcript:Zm00001d020367_T003 0
1154- 7:transcript:EES15002 transcript:Zm00001d020371_T001 0
1154- 8:transcript:EES15004 transcript:Zm00001d020378_T001 5.00E-145
1154- 9:transcript:EES13861 transcript:Zm00001d020381_T008 8.00E-170
1154- 10:transcript:KXG25180 transcript:Zm00001d020382_T001 2.00E-15
1154- 11:transcript:OQU80473 transcript:Zm00001d020384_T001 7.00E-86
Alignment 1155: score=489.0 e_value=5.3e-25 N=11 7&7 plus
1155- 0:transcript:OQU80639 transcript:Zm00001d020732_T001 8.00E-82
1155- 1:transcript:KXG25323 transcript:Zm00001d020742_T001 5.00E-72
1155- 2:transcript:EES14016 transcript:Zm00001d020746_T001 7.00E-11
1155- 3:transcript:EES14020 transcript:Zm00001d020757_T001 2.00E-107
1155- 4:transcript:EES14021 transcript:Zm00001d020762_T001 5.00E-116
1155- 5:transcript:EES14023 transcript:Zm00001d020763_T001 3.00E-63
1155- 6:transcript:KXG25333 transcript:Zm00001d020768_T001 4.00E-71

1155- 7:transcript:OQU80649 transcript:Zm00001d020769_T005 0
1155- 8:transcript:EES14027 transcript:Zm00001d020772_T001 6.00E-47
1155- 9:transcript:EES15144 transcript:Zm00001d020774_T001 6.00E-101
1155- 10:transcript:OQU80661 transcript:Zm00001d020776_T002 6.00E-141
Alignment 1156: score=450.0 e_value=4.4e-24 N=11 7&7 plus
1156- 0:transcript:EES13953 transcript:Zm00001d020605_T001 3.00E-59
1156- 1:transcript:KXG25271 transcript:Zm00001d020607_T001 6.00E-38
1156- 2:transcript:KXG25273 transcript:Zm00001d020609_T001 0
1156- 3:transcript:EES13957 transcript:Zm00001d020617_T001 0
1156- 4:transcript:OQU80592 transcript:Zm00001d020628_T001 0
1156- 5:transcript:EES13964 transcript:Zm00001d020629_T001 2.00E-13
1156- 6:transcript:KXG25279 transcript:Zm00001d020631_T001 0
1156- 7:transcript:KXG25280 transcript:Zm00001d020639_T002 0
1156- 8:transcript:EES13972 transcript:Zm00001d020650_T001 8.00E-68
1156- 9:transcript:OQU80608 transcript:Zm00001d020651_T001 2.00E-96
1156- 10:transcript:EES13973 transcript:Zm00001d020653_T001 6.00E-48
Alignment 1157: score=435.0 e_value=1.1e-19 N=10 7&7 plus
1157- 0:transcript:EES13800 transcript:Zm00001d020233_T001 5.00E-132
1157- 1:transcript:OQU80404 transcript:Zm00001d020238_T002 0
1157- 2:transcript:EES13801 transcript:Zm00001d020243_T001 2.00E-34
1157- 3:transcript:EES13805 transcript:Zm00001d020251_T001 1.00E-06
1157- 4:transcript:EES13808 transcript:Zm00001d020257_T001 5.00E-61
1157- 5:transcript:EES14968 transcript:Zm00001d020264_T001 6.00E-42
1157- 6:transcript:EES13814 transcript:Zm00001d020267_T001 2.00E-91
1157- 7:transcript:EES13815 transcript:Zm00001d020272_T001 0
1157- 8:transcript:OQU80413 transcript:Zm00001d020275_T001 3.00E-31
1157- 9:transcript:EES13816 transcript:Zm00001d020279_T001 3.00E-172
Alignment 1158: score=332.0 e_value=6.3e-10 N=7 7&7 plus
1158- 0:transcript:EES15012 transcript:Zm00001d020403_T001 0
1158- 1:transcript:KXG25181 transcript:Zm00001d020405_T001 2.00E-87
1158- 2:transcript:EES15016 transcript:Zm00001d020409_T002 5.00E-83
1158- 3:transcript:EES15017 transcript:Zm00001d020412_T001 2.00E-22
1158- 4:transcript:EES15018 transcript:Zm00001d020414_T002 0
1158- 5:transcript:OQU80478 transcript:Zm00001d020417_T001 2.00E-70
1158- 6:transcript:OQU80479 transcript:Zm00001d020418_T001 0
Alignment 1159: score=312.0 e_value=5.6e-13 N=7 7&7 plus
1159- 0:transcript:KXG25224 transcript:Zm00001d020445_T001 4.00E-153
1159- 1:transcript:EES13897 transcript:Zm00001d020446_T001 2.00E-137
1159- 2:transcript:EES15047 transcript:Zm00001d020450_T001 8.00E-79
1159- 3:transcript:EES13898 transcript:Zm00001d020457_T001 1.00E-134
1159- 4:transcript:KXG25227 transcript:Zm00001d020459_T001 1.00E-31
1159- 5:transcript:EES15050 transcript:Zm00001d020460_T001 7.00E-85
1159- 6:transcript:EES13906 transcript:Zm00001d020470_T001 7.00E-134
Alignment 1160: score=579.0 e_value=1.8e-34 N=14 7&7 minus
1160- 0:transcript:EES14266 transcript:Zm00001d020941_T001 2.00E-117
1160- 1:transcript:EES15398 transcript:Zm00001d020939_T001 0
1160- 2:transcript:EES14272 transcript:Zm00001d020937_T001 0
1160- 3:transcript:OQU80938 transcript:Zm00001d020932_T001 1.00E-123
1160- 4:transcript:EES14275 transcript:Zm00001d020930_T001 6.00E-63
1160- 5:transcript:EES14276 transcript:Zm00001d020929_T002 0
1160- 6:transcript:EES15407 transcript:Zm00001d020927_T001 2.00E-163
1160- 7:transcript:OQU80949 transcript:Zm00001d020926_T001 7.00E-57
1160- 8:transcript:EES15412 transcript:Zm00001d020923_T001 6.00E-139
1160- 9:transcript:OQU80954 transcript:Zm00001d020921_T001 0

1160- 10:transcript:EES14287 transcript:Zm00001d020903_T001 4.00E-99
1160- 11:transcript:EES15423 transcript:Zm00001d020899_T001 2.00E-21
1160- 12:transcript:EES14299 transcript:Zm00001d020898_T002 0
1160- 13:transcript:KXG25679 transcript:Zm00001d020881_T002 1.00E-84
Alignment 1161: score=565.0 e_value=1.6e-34 N=14 7&7 minus
1161- 0:transcript:EES15278 transcript:Zm00001d021093_T001 1.00E-15
1161- 1:transcript:OQU80802 transcript:Zm00001d021090_T001 0
1161- 2:transcript:OQU80805 transcript:Zm00001d021089_T001 7.00E-30
1161- 3:transcript:OQU80812 transcript:Zm00001d021087_T003 0
1161- 4:transcript:EES14175 transcript:Zm00001d021086_T001 2.00E-88
1161- 5:transcript:KXG25506 transcript:Zm00001d021072_T001 2.00E-116
1161- 6:transcript:KXG25507 transcript:Zm00001d021070_T002 0
1161- 7:transcript:EES14182 transcript:Zm00001d021065_T001 0
1161- 8:transcript:EES14183 transcript:Zm00001d021064_T001 8.00E-112
1161- 9:transcript:KXG25521 transcript:Zm00001d021062_T001 9.00E-27
1161- 10:transcript:EES15309 transcript:Zm00001d021057_T001 4.00E-120
1161- 11:transcript:KXG25534 transcript:Zm00001d021056_T001 1.00E-138
1161- 12:transcript:EES15319 transcript:Zm00001d021051_T002 1.00E-139
1161- 13:transcript:OQU80845 transcript:Zm00001d021038_T001 3.00E-29
Alignment 1162: score=442.0 e_value=3.2e-21 N=10 7&7 minus
1162- 0:transcript:EES14314 transcript:Zm00001d020879_T001 1.00E-54
1162- 1:transcript:OQU80992 transcript:Zm00001d020875_T001 2.00E-14
1162- 2:transcript:EES15444 transcript:Zm00001d020874_T001 8.00E-83
1162- 3:transcript:OQU80994 transcript:Zm00001d020871_T001 5.00E-36
1162- 4:transcript:EES15451 transcript:Zm00001d020862_T001 0
1162- 5:transcript:EES14320 transcript:Zm00001d020861_T001 1.00E-38
1162- 6:transcript:EES15458 transcript:Zm00001d020858_T001 0
1162- 7:transcript:OQU81003 transcript:Zm00001d020851_T001 3.00E-80
1162- 8:transcript:EES15462 transcript:Zm00001d020840_T001 3.00E-77
1162- 9:transcript:OQU81009 transcript:Zm00001d020837_T001 3.00E-55
Alignment 1163: score=437.0 e_value=7.3e-21 N=10 7&7 minus
1163- 0:transcript:EES14210 transcript:Zm00001d021026_T001 0
1163- 1:transcript:EES14212 transcript:Zm00001d021024_T001 0
1163- 2:transcript:OQU80851 transcript:Zm00001d021023_T001 4.00E-24
1163- 3:transcript:KXG25559 transcript:Zm00001d021021_T001 1.00E-27
1163- 4:transcript:EES15330 transcript:Zm00001d021019_T001 5.00E-111
1163- 5:transcript:EES15331 transcript:Zm00001d021018_T002 2.00E-88
1163- 6:transcript:EES15332 transcript:Zm00001d021016_T001 3.00E-43
1163- 7:transcript:EES15333 transcript:Zm00001d021014_T001 0
1163- 8:transcript:EES15334 transcript:Zm00001d021010_T001 9.00E-17
1163- 9:transcript:KXG25581 transcript:Zm00001d021006_T001 2.00E-45
Alignment 1164: score=368.0 e_value=1.9e-18 N=9 7&7 minus
1164- 0:transcript:EES15468 transcript:Zm00001d020834_T004 1.00E-19
1164- 1:transcript:EES14330 transcript:Zm00001d020827_T001 0
1164- 2:transcript:OQU81018 transcript:Zm00001d020826_T001 1.00E-78
1164- 3:transcript:KXG25724 transcript:Zm00001d020823_T001 1.00E-31
1164- 4:transcript:OQU81020 transcript:Zm00001d020820_T001 1.00E-14
1164- 5:transcript:KXG25732 transcript:Zm00001d020810_T002 0
1164- 6:transcript:OQU81036 transcript:Zm00001d020804_T001 1.00E-70
1164- 7:transcript:EES14336 transcript:Zm00001d020803_T001 1.00E-28
1164- 8:transcript:KXG25739 transcript:Zm00001d020794_T001 7.00E-32
Alignment 1165: score=311.0 e_value=8.7e-17 N=7 7&7 minus
1165- 0:transcript:EES15382 transcript:Zm00001d020963_T001 0
1165- 1:transcript:KXG25625 transcript:Zm00001d020951_T002 5.00E-99

```
1165-  2:transcript:EES14256          transcript:Zm00001d020950_T001  4.00E-63
1165-  3:transcript:EES14259          transcript:Zm00001d020949_T001  7.00E-08
1165-  4:transcript:EES14262          transcript:Zm00001d020948_T001  2.00E-99
1165-  5:transcript:EES14263          transcript:Zm00001d020947_T001  2.00E-98
1165-  6:transcript:EES15392          transcript:Zm00001d020946_T005  3.00E-60
## Alignment 1166: score=301.0 e_value=3.4e-13 N=7 7&7 minus
1166-  0:transcript:EES15245          transcript:Zm00001d021240_T001         0
1166-  1:transcript:OQU80759          transcript:Zm00001d021231_T005 3.00E-135
1166-  2:transcript:KXG25450          transcript:Zm00001d021227_T003 2.00E-170
1166-  3:transcript:EES15249          transcript:Zm00001d021226_T001  6.00E-12
1166-  4:transcript:EES14129          transcript:Zm00001d021221_T001 1.00E-143
1166-  5:transcript:EES14130          transcript:Zm00001d021216_T001         0
1166-  6:transcript:EES14131          transcript:Zm00001d021205_T001  1.00E-42
## Alignment 1167: score=262.0 e_value=1.6e-09 N=6 7&B73V4_ctg150 minus
1167-  0:transcript:OQU80002          transcript:Zm00001d000023_T001         0
1167-  1:transcript:EES13461          transcript:Zm00001d000021_T002         0
1167-  2:transcript:OQU80003          transcript:Zm00001d000018_T001 1.00E-111
1167-  3:transcript:EES13464          transcript:Zm00001d000012_T001 1.00E-154
1167-  4:transcript:OQU80009          transcript:Zm00001d000009_T001  2.00E-89
1167-  5:transcript:EES13478          transcript:Zm00001d000006_T001 1.00E-147
## Alignment 1168: score=3272.0 e_value=4.7e-304 N=71 8&9 plus
1168-  0:transcript:Zm00001d009407_T001 transcript:EES18582          1.00E-143
1168-  1:transcript:Zm00001d009409_T002 transcript:EES18583                  0
1168-  2:transcript:Zm00001d009410_T001 transcript:EES18584           3.00E-73
1168-  3:transcript:Zm00001d009411_T002 transcript:EES18585                  0
1168-  4:transcript:Zm00001d009413_T001 transcript:EES18592          8.00E-116
1168-  5:transcript:Zm00001d009415_T001 transcript:EES19883           3.00E-15
1168-  6:transcript:Zm00001d009416_T001 transcript:OQU78369          1.00E-107
1168-  7:transcript:Zm00001d009417_T006 transcript:KXG22440           8.00E-98
1168-  8:transcript:Zm00001d009418_T001 transcript:KXG22443                  0
1168-  9:transcript:Zm00001d009424_T005 transcript:EES18579                  0
1168- 10:transcript:Zm00001d009425_T001 transcript:EES19885           9.00E-31
1168- 11:transcript:Zm00001d009426_T005 transcript:EES18595          3.00E-144
1168- 12:transcript:Zm00001d009429_T001 transcript:OQU78377           4.00E-39
1168- 13:transcript:Zm00001d009430_T002 transcript:KXG22451          3.00E-167
1168- 14:transcript:Zm00001d009431_T001 transcript:EES19888                  0
1168- 15:transcript:Zm00001d009435_T001 transcript:EES18602          1.00E-123
1168- 16:transcript:Zm00001d009436_T003 transcript:EES18587                  0
1168- 17:transcript:Zm00001d009439_T004 transcript:KXG22453                  0
1168- 18:transcript:Zm00001d009440_T001 transcript:EES19889          6.00E-146
1168- 19:transcript:Zm00001d009445_T001 transcript:EES19893           2.00E-78
1168- 20:transcript:Zm00001d009446_T001 transcript:EES18606          9.00E-101
1168- 21:transcript:Zm00001d009448_T001 transcript:OQU78384                  0
1168- 22:transcript:Zm00001d009449_T001 transcript:EES18610                  0
1168- 23:transcript:Zm00001d009450_T002 transcript:EES19895           4.00E-86
1168- 24:transcript:Zm00001d009452_T007 transcript:EES19899                  0
1168- 25:transcript:Zm00001d009458_T001 transcript:KXG22473          3.00E-139
1168- 26:transcript:Zm00001d009465_T001 transcript:EES18614                  0
1168- 27:transcript:Zm00001d009468_T001 transcript:KXG22484                  0
1168- 28:transcript:Zm00001d009469_T001 transcript:EES19900                  0
1168- 29:transcript:Zm00001d009473_T001 transcript:KXG22487                  0
1168- 30:transcript:Zm00001d009474_T001 transcript:OQU78393                  0
1168- 31:transcript:Zm00001d009475_T001 transcript:EES19909           1.00E-83
1168- 32:transcript:Zm00001d009476_T001 transcript:KXG22494          8.00E-147
```

```
1168- 33:transcript:Zm00001d009480_T001 transcript:EES18632 0
1168- 34:transcript:Zm00001d009481_T001 transcript:EES18634 0
1168- 35:transcript:Zm00001d009484_T001 transcript:EES19914 0
1168- 36:transcript:Zm00001d009487_T001 transcript:EES19915 4.00E-61
1168- 37:transcript:Zm00001d009488_T001 transcript:KXG22502 0
1168- 38:transcript:Zm00001d009490_T001 transcript:EES18637 5.00E-26
1168- 39:transcript:Zm00001d009493_T001 transcript:EES18639 1.00E-131
1168- 40:transcript:Zm00001d009494_T001 transcript:OQU78400 0
1168- 41:transcript:Zm00001d009495_T001 transcript:KXG22504 9.00E-22
1168- 42:transcript:Zm00001d009496_T001 transcript:EES19917 2.00E-72
1168- 43:transcript:Zm00001d009497_T004 transcript:EES18641 0
1168- 44:transcript:Zm00001d009500_T002 transcript:EES18643 0
1168- 45:transcript:Zm00001d009501_T001 transcript:EES18645 2.00E-35
1168- 46:transcript:Zm00001d009503_T001 transcript:KXG22510 9.00E-43
1168- 47:transcript:Zm00001d009504_T002 transcript:EES19921 0
1168- 48:transcript:Zm00001d009506_T001 transcript:EES18654 0
1168- 49:transcript:Zm00001d009508_T001 transcript:KXG22515 6.00E-154
1168- 50:transcript:Zm00001d009509_T001 transcript:EES18658 2.00E-61
1168- 51:transcript:Zm00001d009510_T001 transcript:OQU78407 0
1168- 52:transcript:Zm00001d009511_T001 transcript:EES19928 0
1168- 53:transcript:Zm00001d009513_T003 transcript:EES19929 0
1168- 54:transcript:Zm00001d009517_T001 transcript:EES19931 2.00E-116
1168- 55:transcript:Zm00001d009520_T002 transcript:EES18661 0
1168- 56:transcript:Zm00001d009522_T005 transcript:EES19933 3.00E-171
1168- 57:transcript:Zm00001d009525_T002 transcript:EES19934 8.00E-91
1168- 58:transcript:Zm00001d009530_T001 transcript:EES19935 3.00E-126
1168- 59:transcript:Zm00001d009532_T001 transcript:OQU78411 3.00E-80
1168- 60:transcript:Zm00001d009539_T002 transcript:EES18664 0
1168- 61:transcript:Zm00001d009540_T001 transcript:EES18666 1.00E-23
1168- 62:transcript:Zm00001d009541_T002 transcript:KXG22522 2.00E-180
1168- 63:transcript:Zm00001d009543_T001 transcript:EES18667 3.00E-10
1168- 64:transcript:Zm00001d009548_T005 transcript:KXG22524 0
1168- 65:transcript:Zm00001d009549_T002 transcript:EES19937 0
1168- 66:transcript:Zm00001d009553_T001 transcript:KXG22533 4.00E-25
1168- 67:transcript:Zm00001d009554_T001 transcript:EES19941 0
1168- 68:transcript:Zm00001d009555_T003 transcript:EES18674 0
1168- 69:transcript:Zm00001d009556_T001 transcript:EES19943 0
1168- 70:transcript:Zm00001d009557_T001 transcript:EES19945 2.00E-133
## Alignment 1169: score=2957.0 e_value=1.3e-270 N=66 8&9 plus
1169-  0:transcript:Zm00001d010572_T013 transcript:KXG22177 0
1169-  1:transcript:Zm00001d010575_T001 transcript:EES18362 6.00E-67
1169-  2:transcript:Zm00001d010576_T002 transcript:EES18363 1.00E-160
1169-  3:transcript:Zm00001d010578_T003 transcript:OQU78157 0
1169-  4:transcript:Zm00001d010579_T001 transcript:EES19672 0
1169-  5:transcript:Zm00001d010583_T001 transcript:OQU78158 3.00E-128
1169-  6:transcript:Zm00001d010584_T001 transcript:OQU78161 0
1169-  7:transcript:Zm00001d010585_T002 transcript:KXG22191 0
1169-  8:transcript:Zm00001d010586_T001 transcript:EES19674 3.00E-103
1169-  9:transcript:Zm00001d010587_T001 transcript:EES18370 3.00E-66
1169- 10:transcript:Zm00001d010588_T001 transcript:EES18371 0
1169- 11:transcript:Zm00001d010589_T001 transcript:EES18372 0
1169- 12:transcript:Zm00001d010590_T001 transcript:EES19675 0
1169- 13:transcript:Zm00001d010591_T001 transcript:EES18373 3.00E-178
1169- 14:transcript:Zm00001d010592_T001 transcript:KXG22193 7.00E-36
```

1169- 15:transcript:Zm00001d010594_T001 transcript:EES19677 6.00E-132
1169- 16:transcript:Zm00001d010596_T001 transcript:EES18364 9.00E-92
1169- 17:transcript:Zm00001d010601_T001 transcript:EES18377 0
1169- 18:transcript:Zm00001d010602_T001 transcript:EES19684 0
1169- 19:transcript:Zm00001d010603_T001 transcript:KXG22202 2.00E-124
1169- 20:transcript:Zm00001d010604_T006 transcript:OQU78173 0
1169- 21:transcript:Zm00001d010606_T004 transcript:KXG22205 0
1169- 22:transcript:Zm00001d010607_T001 transcript:EES19686 0
1169- 23:transcript:Zm00001d010610_T001 transcript:OQU78181 0
1169- 24:transcript:Zm00001d010613_T002 transcript:KXG22215 2.00E-29
1169- 25:transcript:Zm00001d010614_T007 transcript:EES19692 1.00E-89
1169- 26:transcript:Zm00001d010615_T001 transcript:EES18393 3.00E-138
1169- 27:transcript:Zm00001d010616_T001 transcript:EES19695 1.00E-112
1169- 28:transcript:Zm00001d010618_T003 transcript:EES19701 3.00E-108
1169- 29:transcript:Zm00001d010620_T001 transcript:KXG22223 0
1169- 30:transcript:Zm00001d010622_T002 transcript:OQU78195 1.00E-147
1169- 31:transcript:Zm00001d010623_T001 transcript:OQU78197 0
1169- 32:transcript:Zm00001d010624_T001 transcript:EES18404 4.00E-20
1169- 33:transcript:Zm00001d010625_T001 transcript:EES18406 0
1169- 34:transcript:Zm00001d010627_T001 transcript:EES19708 5.00E-110
1169- 35:transcript:Zm00001d010629_T001 transcript:EES19709 0
1169- 36:transcript:Zm00001d010630_T003 transcript:EES19711 0
1169- 37:transcript:Zm00001d010631_T003 transcript:EES19715 1.00E-88
1169- 38:transcript:Zm00001d010632_T002 transcript:KXG22235 1.00E-31
1169- 39:transcript:Zm00001d010633_T001 transcript:OQU78215 4.00E-31
1169- 40:transcript:Zm00001d010634_T001 transcript:KXG22236 0
1169- 41:transcript:Zm00001d010635_T001 transcript:KXG22238 4.00E-158
1169- 42:transcript:Zm00001d010638_T001 transcript:EES18419 0
1169- 43:transcript:Zm00001d010639_T002 transcript:EES18422 0
1169- 44:transcript:Zm00001d010640_T001 transcript:EES19721 0
1169- 45:transcript:Zm00001d010646_T001 transcript:EES19725 0
1169- 46:transcript:Zm00001d010649_T001 transcript:OQU78227 4.00E-74
1169- 47:transcript:Zm00001d010650_T001 transcript:EES19728 3.00E-77
1169- 48:transcript:Zm00001d010658_T001 transcript:KXG22247 0
1169- 49:transcript:Zm00001d010667_T001 transcript:EES19742 5.00E-93
1169- 50:transcript:Zm00001d010670_T001 transcript:EES18437 0
1169- 51:transcript:Zm00001d010671_T001 transcript:OQU78235 6.00E-136
1169- 52:transcript:Zm00001d010672_T001 transcript:EES19743 1.00E-113
1169- 53:transcript:Zm00001d010673_T001 transcript:OQU78237 0
1169- 54:transcript:Zm00001d010676_T001 transcript:KXG22269 6.00E-93
1169- 55:transcript:Zm00001d010678_T003 transcript:KXG22271 0
1169- 56:transcript:Zm00001d010679_T001 transcript:EES18444 0
1169- 57:transcript:Zm00001d010684_T003 transcript:OQU78247 2.00E-57
1169- 58:transcript:Zm00001d010687_T004 transcript:EES19760 0
1169- 59:transcript:Zm00001d010689_T001 transcript:KXG22281 3.00E-171
1169- 60:transcript:Zm00001d010693_T001 transcript:EES18454 1.00E-112
1169- 61:transcript:Zm00001d010697_T001 transcript:EES19769 0
1169- 62:transcript:Zm00001d010698_T002 transcript:KXG22286 5.00E-142
1169- 63:transcript:Zm00001d010700_T002 transcript:OQU78259 0
1169- 64:transcript:Zm00001d010702_T001 transcript:EES19772 1.00E-129
1169- 65:transcript:Zm00001d010703_T002 transcript:EES19774 0
Alignment 1170: score=2422.0 e_value=3.2e-212 N=54 8&9 plus
1170- 0:transcript:Zm00001d009564_T001 transcript:EES19946 0
1170- 1:transcript:Zm00001d009566_T001 transcript:EES18677 2.00E-109

1170- 2:transcript:Zm00001d009568_T001	transcript:OQU78423	1.00E-54
1170- 3:transcript:Zm00001d009570_T001	transcript:EES18681	0
1170- 4:transcript:Zm00001d009571_T002	transcript:KXG22536	0
1170- 5:transcript:Zm00001d009572_T001	transcript:EES19950	2.00E-156
1170- 6:transcript:Zm00001d009573_T001	transcript:KXG22538	7.00E-120
1170- 7:transcript:Zm00001d009578_T001	transcript:EES18686	6.00E-78
1170- 8:transcript:Zm00001d009579_T001	transcript:EES18687	5.00E-132
1170- 9:transcript:Zm00001d009580_T001	transcript:KXG22540	0
1170- 10:transcript:Zm00001d009582_T001	transcript:EES19954	0
1170- 11:transcript:Zm00001d009583_T001	transcript:EES19955	5.00E-48
1170- 12:transcript:Zm00001d009587_T002	transcript:EES19958	0
1170- 13:transcript:Zm00001d009589_T001	transcript:EES18693	0
1170- 14:transcript:Zm00001d009591_T006	transcript:KXG22544	0
1170- 15:transcript:Zm00001d009594_T011	transcript:KXG22545	0
1170- 16:transcript:Zm00001d009595_T001	transcript:KXG22547	0
1170- 17:transcript:Zm00001d009596_T001	transcript:OQU78430	0
1170- 18:transcript:Zm00001d009597_T002	transcript:EES18697	0
1170- 19:transcript:Zm00001d009599_T001	transcript:KXG22549	1.00E-47
1170- 20:transcript:Zm00001d009600_T017	transcript:EES18699	1.00E-158
1170- 21:transcript:Zm00001d009604_T001	transcript:EES19963	5.00E-73
1170- 22:transcript:Zm00001d009608_T001	transcript:EES19964	0
1170- 23:transcript:Zm00001d009611_T001	transcript:EES18692	3.00E-10
1170- 24:transcript:Zm00001d009612_T001	transcript:EES18706	2.00E-07
1170- 25:transcript:Zm00001d009613_T001	transcript:OQU78439	0
1170- 26:transcript:Zm00001d009617_T017	transcript:EES19971	0
1170- 27:transcript:Zm00001d009618_T001	transcript:OQU78452	0
1170- 28:transcript:Zm00001d009619_T002	transcript:EES18716	4.00E-125
1170- 29:transcript:Zm00001d009622_T001	transcript:EES19973	2.00E-113
1170- 30:transcript:Zm00001d009626_T001	transcript:OQU78453	0
1170- 31:transcript:Zm00001d009627_T001	transcript:EES19976	8.00E-175
1170- 32:transcript:Zm00001d009628_T001	transcript:EES19977	4.00E-148
1170- 33:transcript:Zm00001d009631_T001	transcript:OQU78463	4.00E-36
1170- 34:transcript:Zm00001d009636_T001	transcript:OQU78466	8.00E-62
1170- 35:transcript:Zm00001d009637_T001	transcript:OQU78467	5.00E-26
1170- 36:transcript:Zm00001d009638_T001	transcript:EES19981	0
1170- 37:transcript:Zm00001d009639_T001	transcript:OQU78469	0
1170- 38:transcript:Zm00001d009640_T002	transcript:EES19985	0
1170- 39:transcript:Zm00001d009645_T001	transcript:OQU78470	2.00E-138
1170- 40:transcript:Zm00001d009647_T001	transcript:OQU78477	7.00E-35
1170- 41:transcript:Zm00001d009648_T001	transcript:EES19991	8.00E-74
1170- 42:transcript:Zm00001d009649_T001	transcript:KXG22587	0
1170- 43:transcript:Zm00001d009653_T008	transcript:OQU78483	7.00E-48
1170- 44:transcript:Zm00001d009658_T001	transcript:OQU78486	0
1170- 45:transcript:Zm00001d009660_T001	transcript:EES19998	0
1170- 46:transcript:Zm00001d009661_T002	transcript:EES19999	0
1170- 47:transcript:Zm00001d009666_T001	transcript:EES18736	0
1170- 48:transcript:Zm00001d009667_T001	transcript:EES20001	0
1170- 49:transcript:Zm00001d009668_T001	transcript:EES20002	4.00E-79
1170- 50:transcript:Zm00001d009669_T005	transcript:EES20003	0
1170- 51:transcript:Zm00001d009673_T001	transcript:EES18737	7.00E-171
1170- 52:transcript:Zm00001d009674_T001	transcript:OQU78488	1.00E-116
1170- 53:transcript:Zm00001d009681_T001	transcript:OQU78496	1.00E-64
## Alignment 1171: score=1676.0 e_value=8.5e-127 N=36 8&9 plus		
1171- 0:transcript:Zm00001d009677_T001	transcript:KXG22611	0

1171- 1:transcript:Zm00001d009683_T004 transcript:KXG22612 0
1171- 2:transcript:Zm00001d009686_T002 transcript:EES20007 4.00E-132
1171- 3:transcript:Zm00001d009687_T001 transcript:EES20008 4.00E-100
1171- 4:transcript:Zm00001d009688_T001 transcript:KXG22615 0
1171- 5:transcript:Zm00001d009690_T001 transcript:EES18755 0
1171- 6:transcript:Zm00001d009693_T001 transcript:EES18756 0
1171- 7:transcript:Zm00001d009694_T001 transcript:EES20013 0
1171- 8:transcript:Zm00001d009695_T001 transcript:OQU78503 0
1171- 9:transcript:Zm00001d009698_T001 transcript:KXG22622 3.00E-118
1171- 10:transcript:Zm00001d009700_T001 transcript:EES20018 4.00E-112
1171- 11:transcript:Zm00001d009701_T001 transcript:OQU78506 4.00E-22
1171- 12:transcript:Zm00001d009702_T001 transcript:EES20020 0
1171- 13:transcript:Zm00001d009705_T001 transcript:EES20021 0
1171- 14:transcript:Zm00001d009707_T001 transcript:KXG22631 0
1171- 15:transcript:Zm00001d009708_T001 transcript:EES20022 0
1171- 16:transcript:Zm00001d009709_T001 transcript:EES20023 0
1171- 17:transcript:Zm00001d009710_T001 transcript:KXG22633 0
1171- 18:transcript:Zm00001d009711_T001 transcript:EES20024 1.00E-33
1171- 19:transcript:Zm00001d009714_T002 transcript:EES18777 0
1171- 20:transcript:Zm00001d009715_T001 transcript:KXG22637 0
1171- 21:transcript:Zm00001d009716_T001 transcript:KXG22639 0
1171- 22:transcript:Zm00001d009718_T001 transcript:KXG22640 1.00E-112
1171- 23:transcript:Zm00001d009719_T003 transcript:OQU78513 0
1171- 24:transcript:Zm00001d009723_T001 transcript:EES18769 0
1171- 25:transcript:Zm00001d009724_T009 transcript:OQU78515 0
1171- 26:transcript:Zm00001d009726_T001 transcript:OQU78517 2.00E-167
1171- 27:transcript:Zm00001d009727_T006 transcript:KXG22648 3.00E-35
1171- 28:transcript:Zm00001d009728_T001 transcript:OQU78518 3.00E-120
1171- 29:transcript:Zm00001d009729_T001 transcript:KXG22652 2.00E-116
1171- 30:transcript:Zm00001d009731_T004 transcript:OQU78520 0
1171- 31:transcript:Zm00001d009734_T001 transcript:EES20041 4.00E-178
1171- 32:transcript:Zm00001d009735_T001 transcript:EES18791 2.00E-12
1171- 33:transcript:Zm00001d009740_T004 transcript:EES18796 7.00E-152
1171- 34:transcript:Zm00001d009741_T001 transcript:KXG22662 0
1171- 35:transcript:Zm00001d009743_T001 transcript:EES18799 0
Alignment 1172: score=1100.0 e_value=3.6e-73 N=24 8&9 plus
1172- 0:transcript:Zm00001d010791_T001 transcript:EES19834 3.00E-85
1172- 1:transcript:Zm00001d010792_T001 transcript:EES18531 7.00E-37
1172- 2:transcript:Zm00001d010793_T002 transcript:OQU78325 0
1172- 3:transcript:Zm00001d010795_T001 transcript:KXG22374 2.00E-148
1172- 4:transcript:Zm00001d010796_T001 transcript:EES18534 0
1172- 5:transcript:Zm00001d010797_T001 transcript:OQU78328 8.00E-55
1172- 6:transcript:Zm00001d010798_T001 transcript:EES18536 1.00E-50
1172- 7:transcript:Zm00001d010800_T001 transcript:OQU78336 1.00E-144
1172- 8:transcript:Zm00001d010802_T001 transcript:EES19844 0
1172- 9:transcript:Zm00001d010803_T001 transcript:EES19851 2.00E-86
1172- 10:transcript:Zm00001d010804_T003 transcript:EES19852 6.00E-90
1172- 11:transcript:Zm00001d010805_T001 transcript:EES19853 2.00E-105
1172- 12:transcript:Zm00001d010806_T001 transcript:EES19854 0
1172- 13:transcript:Zm00001d010808_T001 transcript:EES19856 2.00E-157
1172- 14:transcript:Zm00001d010812_T001 transcript:EES18551 0
1172- 15:transcript:Zm00001d010811_T007 transcript:EES19859 0
1172- 16:transcript:Zm00001d010813_T001 transcript:EES19861 0
1172- 17:transcript:Zm00001d010814_T001 transcript:EES18552 3.00E-07

1172- 18:transcript:Zm00001d010816_T001 transcript:EES18556 0
1172- 19:transcript:Zm00001d010818_T001 transcript:EES19862 0
1172- 20:transcript:Zm00001d010819_T001 transcript:EES19847 6.00E-45
1172- 21:transcript:Zm00001d010821_T002 transcript:EES18558 0
1172- 22:transcript:Zm00001d010822_T001 transcript:EES18564 1.00E-138
1172- 23:transcript:Zm00001d010823_T002 transcript:KXG22405 0
Alignment 1173: score=1029.0 e_value=4.8e-70 N=23 8&9 plus
1173- 0:transcript:Zm00001d010515_T001 transcript:EES18340 3.00E-42
1173- 1:transcript:Zm00001d010519_T002 transcript:EES19643 0
1173- 2:transcript:Zm00001d010520_T001 transcript:EES18341 0
1173- 3:transcript:Zm00001d010521_T001 transcript:EES18344 0
1173- 4:transcript:Zm00001d010522_T001 transcript:EES18345 0
1173- 5:transcript:Zm00001d010525_T001 transcript:EES18348 0
1173- 6:transcript:Zm00001d010526_T002 transcript:OQU78135 0
1173- 7:transcript:Zm00001d010527_T001 transcript:EES19637 0
1173- 8:transcript:Zm00001d010528_T001 transcript:KXG22160 1.00E-70
1173- 9:transcript:Zm00001d010529_T001 transcript:EES19649 0
1173- 10:transcript:Zm00001d010530_T002 transcript:KXG22161 0
1173- 11:transcript:Zm00001d010534_T001 transcript:KXG22163 6.00E-100
1173- 12:transcript:Zm00001d010535_T001 transcript:OQU78139 0
1173- 13:transcript:Zm00001d010536_T001 transcript:OQU78141 1.00E-142
1173- 14:transcript:Zm00001d010537_T001 transcript:KXG22165 1.00E-115
1173- 15:transcript:Zm00001d010538_T008 transcript:EES19652 3.00E-159
1173- 16:transcript:Zm00001d010540_T001 transcript:EES18357 1.00E-40
1173- 17:transcript:Zm00001d010543_T001 transcript:KXG22169 1.00E-142
1173- 18:transcript:Zm00001d010545_T001 transcript:EES18358 0
1173- 19:transcript:Zm00001d010546_T003 transcript:OQU78144 0
1173- 20:transcript:Zm00001d010548_T002 transcript:EES19660 0
1173- 21:transcript:Zm00001d010556_T001 transcript:KXG22175 0
1173- 22:transcript:Zm00001d010558_T001 transcript:KXG22182 0
Alignment 1174: score=1028.0 e_value=7.9e-77 N=25 8&9 plus
1174- 0:transcript:Zm00001d012263_T002 transcript:EES19589 0
1174- 1:transcript:Zm00001d012268_T001 transcript:KXG22088 0
1174- 2:transcript:Zm00001d012269_T003 transcript:EES18295 4.00E-167
1174- 3:transcript:Zm00001d012270_T001 transcript:EES19595 8.00E-58
1174- 4:transcript:Zm00001d012273_T001 transcript:EES18296 9.00E-38
1174- 5:transcript:Zm00001d012277_T003 transcript:EES19603 0
1174- 6:transcript:Zm00001d012279_T001 transcript:KXG22101 4.00E-34
1174- 7:transcript:Zm00001d012280_T001 transcript:EES18302 9.00E-70
1174- 8:transcript:Zm00001d012282_T001 transcript:EES18304 1.00E-38
1174- 9:transcript:Zm00001d012285_T001 transcript:EES19607 1.00E-69
1174- 10:transcript:Zm00001d012292_T001 transcript:EES18301 9.00E-162
1174- 11:transcript:Zm00001d012293_T001 transcript:EES19611 3.00E-57
1174- 12:transcript:Zm00001d012294_T009 transcript:OQU78097 5.00E-179
1174- 13:transcript:Zm00001d012304_T001 transcript:EES19608 0
1174- 14:transcript:Zm00001d012313_T001 transcript:EES19619 2.00E-57
1174- 15:transcript:Zm00001d012325_T001 transcript:KXG22120 6.00E-163
1174- 16:transcript:Zm00001d012333_T001 transcript:EES19625 0
1174- 17:transcript:Zm00001d012338_T001 transcript:EES18318 0
1174- 18:transcript:Zm00001d012350_T001 transcript:OQU78116 0
1174- 19:transcript:Zm00001d012362_T001 transcript:KXG22127 0
1174- 20:transcript:Zm00001d012380_T002 transcript:EES19630 0
1174- 21:transcript:Zm00001d012381_T001 transcript:KXG22131 9.00E-107
1174- 22:transcript:Zm00001d012382_T002 transcript:EES18332 3.00E-71

1174- 23:transcript:Zm00001d012383_T002 transcript:EES19633 1.00E-15
1174- 24:transcript:Zm00001d012391_T003 transcript:KXG22138 0
Alignment 1175: score=1012.0 e_value=1.1e-72 N=24 8&9 plus
1175- 0:transcript:Zm00001d012530_T001 transcript:OQU78215 1.00E-44
1175- 1:transcript:Zm00001d012534_T001 transcript:EES18414 0
1175- 2:transcript:Zm00001d012535_T001 transcript:EES19718 5.00E-51
1175- 3:transcript:Zm00001d012537_T003 transcript:EES19719 5.00E-94
1175- 4:transcript:Zm00001d012538_T001 transcript:EES18419 4.00E-86
1175- 5:transcript:Zm00001d012550_T001 transcript:EES19716 2.00E-80
1175- 6:transcript:Zm00001d012551_T001 transcript:OQU78228 4.00E-17
1175- 7:transcript:Zm00001d012552_T005 transcript:EES19734 0
1175- 8:transcript:Zm00001d012553_T003 transcript:KXG22247 3.00E-138
1175- 9:transcript:Zm00001d012560_T002 transcript:EES18429 5.00E-160
1175- 10:transcript:Zm00001d012561_T001 transcript:EES19739 2.00E-37
1175- 11:transcript:Zm00001d012562_T001 transcript:KXG22255 0
1175- 12:transcript:Zm00001d012567_T002 transcript:EES18437 0
1175- 13:transcript:Zm00001d012568_T001 transcript:OQU78235 4.00E-162
1175- 14:transcript:Zm00001d012584_T001 transcript:KXG22269 3.00E-37
1175- 15:transcript:Zm00001d012585_T001 transcript:EES19749 2.00E-68
1175- 16:transcript:Zm00001d012593_T001 transcript:EES19759 0
1175- 17:transcript:Zm00001d012594_T001 transcript:KXG22279 7.00E-07
1175- 18:transcript:Zm00001d012600_T001 transcript:EES19760 0
1175- 19:transcript:Zm00001d012607_T001 transcript:KXG22286 4.00E-127
1175- 20:transcript:Zm00001d012609_T001 transcript:OQU78259 0
1175- 21:transcript:Zm00001d012610_T001 transcript:EES18459 3.00E-110
1175- 22:transcript:Zm00001d012613_T001 transcript:KXG22290 1.00E-08
1175- 23:transcript:Zm00001d012614_T001 transcript:KXG22291 0
Alignment 1176: score=807.0 e_value=5.1e-49 N=18 8&9 plus
1176- 0:transcript:Zm00001d012450_T001 transcript:EES19670 1.00E-58
1176- 1:transcript:Zm00001d012451_T004 transcript:EES18363 2.00E-148
1176- 2:transcript:Zm00001d012452_T004 transcript:OQU78157 0
1176- 3:transcript:Zm00001d012457_T003 transcript:EES19672 0
1176- 4:transcript:Zm00001d012458_T001 transcript:OQU78158 2.00E-96
1176- 5:transcript:Zm00001d012463_T001 transcript:EES18372 6.00E-162
1176- 6:transcript:Zm00001d012467_T001 transcript:EES19675 0
1176- 7:transcript:Zm00001d012468_T001 transcript:KXG22193 6.00E-06
1176- 8:transcript:Zm00001d012471_T001 transcript:EES19677 1.00E-47
1176- 9:transcript:Zm00001d012473_T001 transcript:EES18364 4.00E-89
1176- 10:transcript:Zm00001d012475_T001 transcript:EES19683 4.00E-88
1176- 11:transcript:Zm00001d012479_T004 transcript:OQU78173 4.00E-09
1176- 12:transcript:Zm00001d012480_T001 transcript:EES18380 5.00E-60
1176- 13:transcript:Zm00001d012482_T001 transcript:OQU78174 8.00E-143
1176- 14:transcript:Zm00001d012488_T001 transcript:EES18382 0
1176- 15:transcript:Zm00001d012494_T001 transcript:OQU78182 0
1176- 16:transcript:Zm00001d012498_T001 transcript:EES18387 3.00E-73
1176- 17:transcript:Zm00001d012500_T001 transcript:EES18392 2.00E-33
Alignment 1177: score=698.0 e_value=3.4e-43 N=16 8&9 plus
1177- 0:transcript:Zm00001d010720_T001 transcript:EES18485 0
1177- 1:transcript:Zm00001d010722_T001 transcript:OQU78281 0
1177- 2:transcript:Zm00001d010723_T001 transcript:EES18486 0
1177- 3:transcript:Zm00001d010724_T001 transcript:KXG22329 0
1177- 4:transcript:Zm00001d010726_T001 transcript:EES18490 3.00E-49
1177- 5:transcript:Zm00001d010727_T001 transcript:KXG22330 2.00E-132
1177- 6:transcript:Zm00001d010728_T005 transcript:EES18492 1.00E-67

1177- 7:transcript:Zm00001d010730_T001 transcript:EES18493 4.00E-156
1177- 8:transcript:Zm00001d010731_T001 transcript:EES19788 3.00E-173
1177- 9:transcript:Zm00001d010732_T002 transcript:EES18497 5.00E-155
1177- 10:transcript:Zm00001d010736_T001 transcript:EES19804 7.00E-152
1177- 11:transcript:Zm00001d010744_T001 transcript:OQU78307 8.00E-96
1177- 12:transcript:Zm00001d010749_T001 transcript:KXG22356 7.00E-106
1177- 13:transcript:Zm00001d010752_T001 transcript:KXG22359 6.00E-122
1177- 14:transcript:Zm00001d010754_T001 transcript:EES18514 0
1177- 15:transcript:Zm00001d010755_T001 transcript:EES18516 0
Alignment 1178: score=555.0 e_value=2.7e-31 N=13 8&9 plus
1178- 0:transcript:Zm00001d009888_T001 transcript:KXG21801 0
1178- 1:transcript:Zm00001d009890_T001 transcript:KXG21805 0
1178- 2:transcript:Zm00001d009891_T001 transcript:KXG21810 6.00E-65
1178- 3:transcript:Zm00001d009894_T001 transcript:OQU77841 2.00E-26
1178- 4:transcript:Zm00001d009899_T001 transcript:EES19395 0
1178- 5:transcript:Zm00001d009902_T001 transcript:OQU77848 3.00E-84
1178- 6:transcript:Zm00001d009903_T001 transcript:KXG21823 0
1178- 7:transcript:Zm00001d009904_T001 transcript:OQU77852 0
1178- 8:transcript:Zm00001d009907_T001 transcript:EES18085 0
1178- 9:transcript:Zm00001d009908_T005 transcript:EES19402 0
1178- 10:transcript:Zm00001d009910_T001 transcript:EES19404 8.00E-69
1178- 11:transcript:Zm00001d009912_T001 transcript:KXG21831 0
1178- 12:transcript:Zm00001d009918_T001 transcript:EES18088 0
Alignment 1179: score=479.0 e_value=2.7e-22 N=11 8&9 plus
1179- 0:transcript:Zm00001d010461_T001 transcript:EES19467 1.00E-59
1179- 1:transcript:Zm00001d010462_T002 transcript:EES18166 0
1179- 2:transcript:Zm00001d010463_T001 transcript:EES18164 0
1179- 3:transcript:Zm00001d010465_T001 transcript:KXG21919 2.00E-145
1179- 4:transcript:Zm00001d010466_T001 transcript:EES19478 0
1179- 5:transcript:Zm00001d010470_T005 transcript:KXG21921 0
1179- 6:transcript:Zm00001d010476_T002 transcript:EES18176 0
1179- 7:transcript:Zm00001d010478_T003 transcript:EES19480 0
1179- 8:transcript:Zm00001d010480_T001 transcript:KXG21925 0
1179- 9:transcript:Zm00001d010481_T001 transcript:KXG21927 1.00E-77
1179- 10:transcript:Zm00001d010488_T008 transcript:EES18173 0
Alignment 1180: score=470.0 e_value=1.6e-23 N=11 8&9 plus
1180- 0:transcript:Zm00001d012757_T001 transcript:EES18524 2.00E-97
1180- 1:transcript:Zm00001d012763_T002 transcript:OQU78322 0
1180- 2:transcript:Zm00001d012766_T001 transcript:OQU78325 0
1180- 3:transcript:Zm00001d012767_T001 transcript:KXG22374 3.00E-41
1180- 4:transcript:Zm00001d012769_T001 transcript:EES19836 0
1180- 5:transcript:Zm00001d012772_T001 transcript:OQU78328 2.00E-53
1180- 6:transcript:Zm00001d012776_T001 transcript:EES19841 5.00E-144
1180- 7:transcript:Zm00001d012779_T001 transcript:EES18545 1.00E-69
1180- 8:transcript:Zm00001d012782_T001 transcript:EES18546 2.00E-155
1180- 9:transcript:Zm00001d012788_T001 transcript:KXG22388 2.00E-129
1180- 10:transcript:Zm00001d012789_T001 transcript:EES19853 3.00E-66
Alignment 1181: score=451.0 e_value=4.3e-26 N=10 8&9 plus
1181- 0:transcript:Zm00001d009750_T005 transcript:OQU78538 0
1181- 1:transcript:Zm00001d009753_T009 transcript:EES20060 0
1181- 2:transcript:Zm00001d009755_T001 transcript:EES18807 0
1181- 3:transcript:Zm00001d009756_T002 transcript:OQU78542 0
1181- 4:transcript:Zm00001d009759_T001 transcript:KXG22709 0
1181- 5:transcript:Zm00001d009760_T001 transcript:KXG22710 0

1181- 6:transcript:Zm00001d009761_T004 transcript:KXG22711 0
1181- 7:transcript:Zm00001d009762_T001 transcript:EES20067 3.00E-176
1181- 8:transcript:Zm00001d009763_T001 transcript:KXG22713 0
1181- 9:transcript:Zm00001d009765_T008 transcript:OQU78545 0
Alignment 1182: score=427.0 e_value=1.4e-20 N=10 8&9 plus
1182- 0:transcript:Zm00001d012401_T001 transcript:KXG22151 1.00E-152
1182- 1:transcript:Zm00001d012408_T001 transcript:EES18346 0
1182- 2:transcript:Zm00001d012417_T001 transcript:EES19637 0
1182- 3:transcript:Zm00001d012420_T001 transcript:EES19649 0
1182- 4:transcript:Zm00001d012421_T004 transcript:KXG22163 1.00E-117
1182- 5:transcript:Zm00001d012430_T001 transcript:EES18355 2.00E-34
1182- 6:transcript:Zm00001d012431_T001 transcript:EES19655 1.00E-29
1182- 7:transcript:Zm00001d012435_T001 transcript:KXG22169 3.00E-99
1182- 8:transcript:Zm00001d012437_T001 transcript:EES18358 0
1182- 9:transcript:Zm00001d012445_T001 transcript:KXG22176 2.00E-45
Alignment 1183: score=413.0 e_value=1.1e-21 N=10 8&9 plus
1183- 0:transcript:Zm00001d008666_T003 transcript:KXG21405 0
1183- 1:transcript:Zm00001d008679_T010 transcript:EES19110 0
1183- 2:transcript:Zm00001d008691_T001 transcript:KXG21420 2.00E-114
1183- 3:transcript:Zm00001d008700_T002 transcript:OQU77514 1.00E-163
1183- 4:transcript:Zm00001d008708_T001 transcript:EES19114 2.00E-176
1183- 5:transcript:Zm00001d008715_T001 transcript:EES17789 0
1183- 6:transcript:Zm00001d008716_T002 transcript:EES19115 0
1183- 7:transcript:Zm00001d008721_T001 transcript:KXG21431 7.00E-74
1183- 8:transcript:Zm00001d008722_T001 transcript:EES19118 3.00E-07
1183- 9:transcript:Zm00001d008724_T005 transcript:KXG21435 1.00E-17
Alignment 1184: score=373.0 e_value=7.3e-16 N=8 8&9 plus
1184- 0:transcript:Zm00001d009771_T003 transcript:EES18810 0
1184- 1:transcript:Zm00001d009772_T001 transcript:EES20073 2.00E-85
1184- 2:transcript:Zm00001d009773_T001 transcript:KXG22724 0
1184- 3:transcript:Zm00001d009774_T001 transcript:KXG22725 7.00E-159
1184- 4:transcript:Zm00001d009775_T001 transcript:KXG22731 0
1184- 5:transcript:Zm00001d009776_T001 transcript:EES20076 1.00E-85
1184- 6:transcript:Zm00001d009777_T001 transcript:EES18815 1.00E-171
1184- 7:transcript:Zm00001d009778_T001 transcript:KXG22733 7.00E-107
Alignment 1185: score=365.0 e_value=4.2e-15 N=8 8&9 plus
1185- 0:transcript:Zm00001d010067_T001 transcript:EES19620 1.00E-110
1185- 1:transcript:Zm00001d010068_T004 transcript:EES19623 2.00E-103
1185- 2:transcript:Zm00001d010075_T001 transcript:KXG22120 0
1185- 3:transcript:Zm00001d010078_T001 transcript:KXG22121 0
1185- 4:transcript:Zm00001d010079_T001 transcript:EES19625 0
1185- 5:transcript:Zm00001d010082_T002 transcript:EES18318 0
1185- 6:transcript:Zm00001d010087_T001 transcript:EES19627 3.00E-105
1185- 7:transcript:Zm00001d010108_T001 transcript:EES18324 3.00E-29
Alignment 1186: score=343.0 e_value=1.9e-14 N=8 8&9 plus
1186- 0:transcript:Zm00001d009837_T001 transcript:EES19051 0
1186- 1:transcript:Zm00001d009840_T001 transcript:EES17741 0
1186- 2:transcript:Zm00001d009844_T001 transcript:OQU77459 0
1186- 3:transcript:Zm00001d009847_T001 transcript:OQU77460 2.00E-81
1186- 4:transcript:Zm00001d009848_T001 transcript:EES17748 1.00E-131
1186- 5:transcript:Zm00001d009849_T001 transcript:EES17749 8.00E-180
1186- 6:transcript:Zm00001d009850_T002 transcript:EES19061 0
1186- 7:transcript:Zm00001d009856_T005 transcript:KXG21351 0
Alignment 1187: score=326.0 e_value=1.2e-14 N=8 8&9 plus

1187- 0:transcript:Zm00001d012234_T002 transcript:KXG22053 0
1187- 1:transcript:Zm00001d012239_T003 transcript:KXG22055 7.00E-57
1187- 2:transcript:Zm00001d012242_T001 transcript:EES19566 2.00E-139
1187- 3:transcript:Zm00001d012245_T002 transcript:EES19570 3.00E-94
1187- 4:transcript:Zm00001d012254_T002 transcript:OQU78068 0
1187- 5:transcript:Zm00001d012255_T001 transcript:KXG22070 2.00E-108
1187- 6:transcript:Zm00001d012257_T013 transcript:EES18280 0
1187- 7:transcript:Zm00001d012259_T001 transcript:KXG22072 0
Alignment 1188: score=321.0 e_value=3.2e-14 N=8 8&9 plus
1188- 0:transcript:Zm00001d010358_T001 transcript:EES19164 0
1188- 1:transcript:Zm00001d010360_T001 transcript:EES17832 2.00E-93
1188- 2:transcript:Zm00001d010366_T001 transcript:KXG21487 0
1188- 3:transcript:Zm00001d010368_T003 transcript:EES19170 1.00E-149
1188- 4:transcript:Zm00001d010373_T001 transcript:EES19172 4.00E-159
1188- 5:transcript:Zm00001d010375_T001 transcript:EES17845 0
1188- 6:transcript:Zm00001d010376_T001 transcript:EES19183 2.00E-42
1188- 7:transcript:Zm00001d010380_T001 transcript:EES19185 0
Alignment 1189: score=317.0 e_value=1.6e-11 N=7 8&9 plus
1189- 0:transcript:Zm00001d010779_T001 transcript:OQU78316 2.00E-86
1189- 1:transcript:Zm00001d010783_T001 transcript:EES19830 0
1189- 2:transcript:Zm00001d010784_T005 transcript:EES19831 4.00E-134
1189- 3:transcript:Zm00001d010785_T001 transcript:EES18524 4.00E-155
1189- 4:transcript:Zm00001d010787_T001 transcript:EES18525 2.00E-86
1189- 5:transcript:Zm00001d010788_T001 transcript:EES18527 7.00E-98
1189- 6:transcript:Zm00001d010790_T017 transcript:OQU78322 0
Alignment 1190: score=316.0 e_value=2.4e-11 N=7 8&9 plus
1190- 0:transcript:Zm00001d010008_T001 transcript:EES19205 1.00E-178
1190- 1:transcript:Zm00001d010009_T003 transcript:EES19208 3.00E-113
1190- 2:transcript:Zm00001d010010_T001 transcript:KXG21527 2.00E-25
1190- 3:transcript:Zm00001d010012_T001 transcript:EES19209 0
1190- 4:transcript:Zm00001d010016_T002 transcript:EES17871 5.00E-42
1190- 5:transcript:Zm00001d010018_T004 transcript:EES19211 0
1190- 6:transcript:Zm00001d010020_T001 transcript:EES17872 2.00E-83
Alignment 1191: score=314.0 e_value=7.6e-15 N=8 8&9 plus
1191- 0:transcript:Zm00001d012196_T005 transcript:EES18243 1.00E-121
1191- 1:transcript:Zm00001d012198_T001 transcript:OQU78021 0
1191- 2:transcript:Zm00001d012203_T001 transcript:EES18248 3.00E-115
1191- 3:transcript:Zm00001d012212_T001 transcript:OQU78027 2.00E-115
1191- 4:transcript:Zm00001d012216_T001 transcript:OQU78028 0
1191- 5:transcript:Zm00001d012220_T001 transcript:EES18258 2.00E-161
1191- 6:transcript:Zm00001d012223_T001 transcript:KXG22037 0
1191- 7:transcript:Zm00001d012224_T014 transcript:EES19558 0
Alignment 1192: score=288.0 e_value=3.7e-09 N=6 8&9 plus
1192- 0:transcript:Zm00001d010239_T001 transcript:EES19541 0
1192- 1:transcript:Zm00001d010240_T001 transcript:EES19542 0
1192- 2:transcript:Zm00001d010241_T002 transcript:EES18243 6.00E-116
1192- 3:transcript:Zm00001d010243_T002 transcript:OQU78021 0
1192- 4:transcript:Zm00001d010244_T001 transcript:OQU78023 4.00E-46
1192- 5:transcript:Zm00001d010248_T001 transcript:EES18248 0
Alignment 1193: score=282.0 e_value=7.8e-13 N=7 8&9 plus
1193- 0:transcript:Zm00001d009193_T001 transcript:KXG21349 8.00E-41
1193- 1:transcript:Zm00001d009196_T001 transcript:EES17751 1.00E-87
1193- 2:transcript:Zm00001d009210_T001 transcript:EES17752 1.00E-151
1193- 3:transcript:Zm00001d009212_T004 transcript:EES19070 0

1193- 4:transcript:Zm00001d009220_T002	transcript:KXG21365	2.00E-89
1193- 5:transcript:Zm00001d009222_T001	transcript:EES19071	0
1193- 6:transcript:Zm00001d009224_T001	transcript:KXG21369	3.00E-12
## Alignment 1194: score=264.0 e_value=4.5e-08 N=6 8&9 plus		
1194- 0:transcript:Zm00001d010858_T007	transcript:EES19028	0
1194- 1:transcript:Zm00001d010862_T002	transcript:EES17718	0
1194- 2:transcript:Zm00001d010863_T001	transcript:KXG21312	0
1194- 3:transcript:Zm00001d010866_T004	transcript:OQU77409	0
1194- 4:transcript:Zm00001d010867_T001	transcript:EES17721	0
1194- 5:transcript:Zm00001d010868_T001	transcript:OQU77415	0
## Alignment 1195: score=260.0 e_value=2.4e-09 N=6 8&9 plus		
1195- 0:transcript:Zm00001d010490_T001	transcript:OQU77880	0
1195- 1:transcript:Zm00001d010491_T002	transcript:EES19420	1.00E-116
1195- 2:transcript:Zm00001d010492_T010	transcript:EES18123	0
1195- 3:transcript:Zm00001d010493_T001	transcript:EES18130	3.00E-144
1195- 4:transcript:Zm00001d010494_T001	transcript:EES19422	3.00E-24
1195- 5:transcript:Zm00001d010495_T002	transcript:KXG21862	0
## Alignment 1196: score=2645.0 e_value=2.7e-239 N=60 8&9 minus		
1196- 0:transcript:Zm00001d010152_T002	transcript:EES19605	1.00E-155
1196- 1:transcript:Zm00001d010159_T002	transcript:EES19603	0
1196- 2:transcript:Zm00001d010170_T001	transcript:KXG22096	4.00E-37
1196- 3:transcript:Zm00001d010173_T003	transcript:EES19594	0
1196- 4:transcript:Zm00001d010174_T004	transcript:EES18295	2.00E-155
1196- 5:transcript:Zm00001d010175_T002	transcript:OQU78085	9.00E-102
1196- 6:transcript:Zm00001d010178_T001	transcript:KXG22089	0
1196- 7:transcript:Zm00001d010179_T001	transcript:KXG22088	0
1196- 8:transcript:Zm00001d010180_T002	transcript:KXG22087	4.00E-144
1196- 9:transcript:Zm00001d010183_T001	transcript:OQU78080	8.00E-66
1196- 10:transcript:Zm00001d010185_T001	transcript:KXG22077	2.00E-27
1196- 11:transcript:Zm00001d010189_T001	transcript:KXG22075	0
1196- 12:transcript:Zm00001d010190_T002	transcript:EES19586	0
1196- 13:transcript:Zm00001d010191_T001	transcript:KXG22074	2.00E-45
1196- 14:transcript:Zm00001d010193_T001	transcript:EES18282	1.00E-43
1196- 15:transcript:Zm00001d010195_T001	transcript:KXG22072	0
1196- 16:transcript:Zm00001d010197_T001	transcript:EES18281	0
1196- 17:transcript:Zm00001d010198_T001	transcript:EES19582	0
1196- 18:transcript:Zm00001d010199_T002	transcript:EES18280	0
1196- 19:transcript:Zm00001d010200_T001	transcript:OQU78069	0
1196- 20:transcript:Zm00001d010201_T001	transcript:KXG22070	6.00E-84
1196- 21:transcript:Zm00001d010202_T001	transcript:EES19579	0
1196- 22:transcript:Zm00001d010205_T002	transcript:OQU78068	0
1196- 23:transcript:Zm00001d010206_T001	transcript:EES19576	4.00E-156
1196- 24:transcript:Zm00001d010207_T001	transcript:KXG22066	3.00E-57
1196- 25:transcript:Zm00001d010209_T001	transcript:KXG22062	5.00E-74
1196- 26:transcript:Zm00001d010210_T001	transcript:KXG22060	0
1196- 27:transcript:Zm00001d010211_T002	transcript:EES19570	4.00E-125
1196- 28:transcript:Zm00001d010212_T002	transcript:KXG22057	0
1196- 29:transcript:Zm00001d010213_T001	transcript:KXG22055	7.00E-124
1196- 30:transcript:Zm00001d010214_T003	transcript:OQU78054	2.00E-179
1196- 31:transcript:Zm00001d010217_T001	transcript:EES18271	5.00E-58
1196- 32:transcript:Zm00001d010218_T001	transcript:EES18266	2.00E-172
1196- 33:transcript:Zm00001d010220_T002	transcript:EES18264	1.00E-95
1196- 34:transcript:Zm00001d010222_T001	transcript:KXG22037	0
1196- 35:transcript:Zm00001d010225_T001	transcript:OQU78038	0

```
1196- 36:transcript:Zm00001d010227_T004 transcript:KXG22035                0
1196- 37:transcript:Zm00001d010228_T001 transcript:EES18258                0
1196- 38:transcript:Zm00001d010230_T001 transcript:EES19550                0
1196- 39:transcript:Zm00001d010231_T001 transcript:OQU78029                0
1196- 40:transcript:Zm00001d010234_T001 transcript:OQU78028                0
1196- 41:transcript:Zm00001d010235_T022 transcript:KXG22029                0
1196- 42:transcript:Zm00001d010236_T001 transcript:EES18250         1.00E-78
1196- 43:transcript:Zm00001d010240_T001 transcript:EES19542                0
1196- 44:transcript:Zm00001d010249_T001 transcript:EES19537                0
1196- 45:transcript:Zm00001d010251_T003 transcript:KXG22017                0
1196- 46:transcript:Zm00001d010256_T001 transcript:EES18240         5.00E-46
1196- 47:transcript:Zm00001d010259_T001 transcript:EES19534        4.00E-168
1196- 48:transcript:Zm00001d010262_T001 transcript:KXG22009                0
1196- 49:transcript:Zm00001d010264_T001 transcript:OQU78015         2.00E-82
1196- 50:transcript:Zm00001d010266_T001 transcript:KXG22004        4.00E-103
1196- 51:transcript:Zm00001d010271_T003 transcript:EES19522                0
1196- 52:transcript:Zm00001d010274_T001 transcript:OQU78012                0
1196- 53:transcript:Zm00001d010282_T001 transcript:KXG21997                0
1196- 54:transcript:Zm00001d010284_T001 transcript:EES18227                0
1196- 55:transcript:Zm00001d010287_T002 transcript:EES19520                0
1196- 56:transcript:Zm00001d010290_T002 transcript:EES18222         2.00E-85
1196- 57:transcript:Zm00001d010292_T002 transcript:OQU78005        2.00E-176
1196- 58:transcript:Zm00001d010294_T001 transcript:EES19519                0
1196- 59:transcript:Zm00001d010295_T001 transcript:KXG21991        3.00E-126
## Alignment 1197: score=1485.0 e_value=9e-113 N=34 8&9 minus
1197-  0:transcript:Zm00001d010896_T001 transcript:OQU77381         2.00E-40
1197-  1:transcript:Zm00001d010901_T001 transcript:EES17679        3.00E-123
1197-  2:transcript:Zm00001d010907_T001 transcript:OQU77376                0
1197-  3:transcript:Zm00001d010908_T002 transcript:EES17675                0
1197-  4:transcript:Zm00001d010910_T002 transcript:EES18988        5.00E-178
1197-  5:transcript:Zm00001d010911_T001 transcript:EES17674        8.00E-171
1197-  6:transcript:Zm00001d010912_T001 transcript:EES17673         1.00E-34
1197-  7:transcript:Zm00001d010914_T001 transcript:EES17669                0
1197-  8:transcript:Zm00001d010915_T001 transcript:KXG21235                0
1197-  9:transcript:Zm00001d010918_T001 transcript:EES18975                0
1197- 10:transcript:Zm00001d010919_T001 transcript:EES17666                0
1197- 11:transcript:Zm00001d010920_T001 transcript:EES17665                0
1197- 12:transcript:Zm00001d010924_T001 transcript:EES18974                0
1197- 13:transcript:Zm00001d010925_T001 transcript:OQU77354                0
1197- 14:transcript:Zm00001d010926_T001 transcript:EES17661        7.00E-104
1197- 15:transcript:Zm00001d010927_T001 transcript:OQU77343                0
1197- 16:transcript:Zm00001d010928_T001 transcript:KXG21223                0
1197- 17:transcript:Zm00001d010933_T001 transcript:KXG21220        3.00E-129
1197- 18:transcript:Zm00001d010939_T004 transcript:EES18963                0
1197- 19:transcript:Zm00001d010940_T001 transcript:EES17657        3.00E-110
1197- 20:transcript:Zm00001d010945_T002 transcript:EES17654                0
1197- 21:transcript:Zm00001d010946_T004 transcript:EES18955                0
1197- 22:transcript:Zm00001d010948_T001 transcript:EES18954                0
1197- 23:transcript:Zm00001d010950_T003 transcript:EES17650                0
1197- 24:transcript:Zm00001d010954_T001 transcript:KXG21208                0
1197- 25:transcript:Zm00001d010956_T001 transcript:OQU77325                0
1197- 26:transcript:Zm00001d010958_T001 transcript:EES17644                0
1197- 27:transcript:Zm00001d010961_T001 transcript:OQU77320                0
1197- 28:transcript:Zm00001d010967_T001 transcript:OQU77310                0
```

1197- 29:transcript:Zm00001d010969_T001 transcript:EES17639 0
1197- 30:transcript:Zm00001d010970_T003 transcript:EES18942 1.00E-130
1197- 31:transcript:Zm00001d010971_T003 transcript:OQU77305 0
1197- 32:transcript:Zm00001d010972_T001 transcript:EES17636 0
1197- 33:transcript:Zm00001d010974_T008 transcript:KXG21178 0
Alignment 1198: score=1142.0 e_value=3.2e-83 N=26 8&9 minus
1198- 0:transcript:Zm00001d010975_T003 transcript:KXG21175 0
1198- 1:transcript:Zm00001d010976_T001 transcript:OQU77301 1.00E-137
1198- 2:transcript:Zm00001d010978_T001 transcript:EES18939 7.00E-98
1198- 3:transcript:Zm00001d010982_T001 transcript:EES17631 0
1198- 4:transcript:Zm00001d010984_T001 transcript:EES18937 0
1198- 5:transcript:Zm00001d010985_T001 transcript:EES18936 0
1198- 6:transcript:Zm00001d010986_T001 transcript:EES18935 0
1198- 7:transcript:Zm00001d010987_T001 transcript:OQU77285 5.00E-157
1198- 8:transcript:Zm00001d010994_T001 transcript:EES18932 2.00E-128
1198- 9:transcript:Zm00001d010995_T001 transcript:EES18931 0
1198- 10:transcript:Zm00001d010997_T001 transcript:EES17622 0
1198- 11:transcript:Zm00001d010998_T001 transcript:OQU77281 4.00E-52
1198- 12:transcript:Zm00001d011001_T001 transcript:OQU77279 9.00E-171
1198- 13:transcript:Zm00001d011003_T001 transcript:KXG21161 9.00E-11
1198- 14:transcript:Zm00001d011006_T001 transcript:EES17614 0
1198- 15:transcript:Zm00001d011012_T001 transcript:EES17611 3.00E-75
1198- 16:transcript:Zm00001d011013_T001 transcript:EES18919 0
1198- 17:transcript:Zm00001d011014_T001 transcript:OQU77263 4.00E-61
1198- 18:transcript:Zm00001d011034_T004 transcript:OQU77257 2.00E-122
1198- 19:transcript:Zm00001d011036_T001 transcript:EES18910 0
1198- 20:transcript:Zm00001d011037_T001 transcript:OQU77253 0
1198- 21:transcript:Zm00001d011038_T002 transcript:EES17597 0
1198- 22:transcript:Zm00001d011040_T001 transcript:KXG21113 0
1198- 23:transcript:Zm00001d011041_T001 transcript:EES17594 1.00E-135
1198- 24:transcript:Zm00001d011049_T001 transcript:EES17591 4.00E-83
1198- 25:transcript:Zm00001d011050_T001 transcript:OQU77247 4.00E-34
Alignment 1199: score=895.0 e_value=6.9e-63 N=22 8&9 minus
1199- 0:transcript:Zm00001d011610_T063 transcript:KXG22508 0
1199- 1:transcript:Zm00001d011614_T001 transcript:EES18637 3.00E-91
1199- 2:transcript:Zm00001d011616_T001 transcript:KXG22500 9.00E-73
1199- 3:transcript:Zm00001d011618_T001 transcript:KXG22495 9.00E-125
1199- 4:transcript:Zm00001d011620_T001 transcript:EES19909 1.00E-24
1199- 5:transcript:Zm00001d011622_T004 transcript:KXG22489 0
1199- 6:transcript:Zm00001d011625_T001 transcript:KXG22487 0
1199- 7:transcript:Zm00001d011630_T001 transcript:EES18625 5.00E-20
1199- 8:transcript:Zm00001d011631_T001 transcript:EES19907 2.00E-100
1199- 9:transcript:Zm00001d011636_T001 transcript:EES18624 1.00E-91
1199- 10:transcript:Zm00001d011637_T001 transcript:KXG22473 1.00E-52
1199- 11:transcript:Zm00001d011642_T002 transcript:EES19896 0
1199- 12:transcript:Zm00001d011644_T002 transcript:OQU78384 0
1199- 13:transcript:Zm00001d011650_T001 transcript:EES18606 3.00E-19
1199- 14:transcript:Zm00001d011654_T001 transcript:KXG22458 2.00E-114
1199- 15:transcript:Zm00001d011660_T003 transcript:OQU78381 0
1199- 16:transcript:Zm00001d011663_T003 transcript:KXG22453 0
1199- 17:transcript:Zm00001d011668_T001 transcript:EES18587 0
1199- 18:transcript:Zm00001d011669_T001 transcript:EES18602 1.00E-38
1199- 19:transcript:Zm00001d011673_T001 transcript:EES19888 0
1199- 20:transcript:Zm00001d011678_T002 transcript:EES18599 1.00E-28

1199- 21:transcript:Zm00001d011681_T001 transcript:EES19885 1.00E-19
Alignment 1200: score=719.0 e_value=1.4e-49 N=18 8&9 minus
1200- 0:transcript:Zm00001d010851_T001 transcript:EES17727 0
1200- 1:transcript:Zm00001d010852_T002 transcript:OQU77419 0
1200- 2:transcript:Zm00001d010855_T001 transcript:OQU77417 4.00E-142
1200- 3:transcript:Zm00001d010856_T001 transcript:EES17723 1.00E-74
1200- 4:transcript:Zm00001d010859_T001 transcript:EES19029 4.00E-176
1200- 5:transcript:Zm00001d010862_T002 transcript:EES17718 0
1200- 6:transcript:Zm00001d010869_T001 transcript:OQU77406 0
1200- 7:transcript:Zm00001d010870_T001 transcript:EES17708 3.00E-121
1200- 8:transcript:Zm00001d010871_T001 transcript:KXG21303 4.00E-100
1200- 9:transcript:Zm00001d010872_T001 transcript:EES19020 4.00E-68
1200- 10:transcript:Zm00001d010877_T003 transcript:KXG21291 0
1200- 11:transcript:Zm00001d010880_T001 transcript:KXG21288 0
1200- 12:transcript:Zm00001d010884_T001 transcript:EES17695 0
1200- 13:transcript:Zm00001d010885_T001 transcript:OQU77397 3.00E-28
1200- 14:transcript:Zm00001d010887_T002 transcript:EES17694 0
1200- 15:transcript:Zm00001d010892_T013 transcript:KXG21285 0
1200- 16:transcript:Zm00001d010894_T003 transcript:EES17692 0
1200- 17:transcript:Zm00001d010903_T001 transcript:EES18993 3.00E-28
Alignment 1201: score=609.0 e_value=3.6e-34 N=14 8&9 minus
1201- 0:transcript:Zm00001d010109_T001 transcript:EES18314 0
1201- 1:transcript:Zm00001d010112_T001 transcript:KXG22127 0
1201- 2:transcript:Zm00001d010115_T001 transcript:EES18320 0
1201- 3:transcript:Zm00001d010116_T001 transcript:OQU78116 0
1201- 4:transcript:Zm00001d010126_T004 transcript:EES19618 0
1201- 5:transcript:Zm00001d010127_T001 transcript:KXG22114 9.00E-65
1201- 6:transcript:Zm00001d010128_T003 transcript:KXG22113 7.00E-43
1201- 7:transcript:Zm00001d010129_T001 transcript:KXG22110 0
1201- 8:transcript:Zm00001d010131_T001 transcript:EES18308 3.00E-172
1201- 9:transcript:Zm00001d010135_T003 transcript:KXG22108 0
1201- 10:transcript:Zm00001d010136_T001 transcript:EES19611 9.00E-75
1201- 11:transcript:Zm00001d010137_T001 transcript:EES18301 0
1201- 12:transcript:Zm00001d010141_T005 transcript:EES19609 0
1201- 13:transcript:Zm00001d010144_T001 transcript:KXG22106 1.00E-114
Alignment 1202: score=563.0 e_value=4.9e-31 N=13 8&9 minus
1202- 0:transcript:Zm00001d011514_T001 transcript:KXG22553 8.00E-64
1202- 1:transcript:Zm00001d011515_T001 transcript:KXG22549 6.00E-31
1202- 2:transcript:Zm00001d011517_T005 transcript:KXG22544 0
1202- 3:transcript:Zm00001d011525_T002 transcript:EES19958 0
1202- 4:transcript:Zm00001d011527_T001 transcript:EES18690 2.00E-73
1202- 5:transcript:Zm00001d011534_T004 transcript:EES18687 8.00E-126
1202- 6:transcript:Zm00001d011536_T001 transcript:EES18686 7.00E-56
1202- 7:transcript:Zm00001d011537_T001 transcript:KXG22537 2.00E-09
1202- 8:transcript:Zm00001d011540_T001 transcript:EES19950 5.00E-22
1202- 9:transcript:Zm00001d011541_T004 transcript:KXG22536 1.00E-151
1202- 10:transcript:Zm00001d011543_T001 transcript:OQU78423 2.00E-47
1202- 11:transcript:Zm00001d011555_T001 transcript:EES18675 5.00E-136
1202- 12:transcript:Zm00001d011558_T001 transcript:EES19945 4.00E-94
Alignment 1203: score=551.0 e_value=8.1e-32 N=12 8&9 minus
1203- 0:transcript:Zm00001d011097_T001 transcript:KXG21012 0
1203- 1:transcript:Zm00001d011099_T012 transcript:EES18836 0
1203- 2:transcript:Zm00001d011101_T002 transcript:OQU77195 6.00E-100
1203- 3:transcript:Zm00001d011102_T001 transcript:EES18832 0

1203- 4:transcript:Zm00001d011103_T001 transcript:EES18831 2.00E-90
1203- 5:transcript:Zm00001d011104_T001 transcript:EES17522 5.00E-174
1203- 6:transcript:Zm00001d011108_T004 transcript:EES17520 0
1203- 7:transcript:Zm00001d011111_T001 transcript:EES18828 3.00E-94
1203- 8:transcript:Zm00001d011112_T001 transcript:EES17519 3.00E-147
1203- 9:transcript:Zm00001d011117_T001 transcript:OQU77192 6.00E-98
1203- 10:transcript:Zm00001d011118_T001 transcript:EES18824 1.00E-75
1203- 11:transcript:Zm00001d011119_T004 transcript:KXG20995 0
Alignment 1204: score=456.0 e_value=9.9e-22 N=11 8&9 minus
1204- 0:transcript:Zm00001d009939_T001 transcript:EES19157 2.00E-116
1204- 1:transcript:Zm00001d009941_T001 transcript:KXG21479 0
1204- 2:transcript:Zm00001d009943_T001 transcript:OQU77558 6.00E-65
1204- 3:transcript:Zm00001d009945_T001 transcript:KXG21468 3.00E-64
1204- 4:transcript:Zm00001d009946_T001 transcript:KXG21466 7.00E-77
1204- 5:transcript:Zm00001d009950_T002 transcript:EES19148 0
1204- 6:transcript:Zm00001d009952_T001 transcript:OQU77549 0
1204- 7:transcript:Zm00001d009954_T001 transcript:EES17812 3.00E-114
1204- 8:transcript:Zm00001d009957_T001 transcript:KXG21457 5.00E-36
1204- 9:transcript:Zm00001d009960_T001 transcript:OQU77548 0
1204- 10:transcript:Zm00001d009961_T001 transcript:EES17809 7.00E-22
Alignment 1205: score=453.0 e_value=3e-20 N=10 8&9 minus
1205- 0:transcript:Zm00001d010052_T001 transcript:OQU78128 1.00E-33
1205- 1:transcript:Zm00001d010053_T001 transcript:EES18339 1.00E-91
1205- 2:transcript:Zm00001d010054_T001 transcript:KXG22143 2.00E-160
1205- 3:transcript:Zm00001d010055_T003 transcript:KXG22142 2.00E-149
1205- 4:transcript:Zm00001d010056_T002 transcript:KXG22138 0
1205- 5:transcript:Zm00001d010060_T001 transcript:EES18336 0
1205- 6:transcript:Zm00001d010061_T001 transcript:EES19633 2.00E-66
1205- 7:transcript:Zm00001d010062_T001 transcript:EES18335 0
1205- 8:transcript:Zm00001d010063_T001 transcript:EES19631 1.00E-28
1205- 9:transcript:Zm00001d010066_T003 transcript:KXG22131 1.00E-171
Alignment 1206: score=396.0 e_value=3.6e-17 N=9 8&9 minus
1206- 0:transcript:Zm00001d009965_T002 transcript:EES18159 0
1206- 1:transcript:Zm00001d009967_T001 transcript:EES18158 0
1206- 2:transcript:Zm00001d009969_T001 transcript:EES18155 2.00E-95
1206- 3:transcript:Zm00001d009971_T001 transcript:EES19454 4.00E-132
1206- 4:transcript:Zm00001d009975_T001 transcript:EES18150 9.00E-137
1206- 5:transcript:Zm00001d009978_T001 transcript:EES19450 3.00E-100
1206- 6:transcript:Zm00001d009979_T002 transcript:EES18149 0
1206- 7:transcript:Zm00001d009983_T003 transcript:EES18143 0
1206- 8:transcript:Zm00001d009985_T001 transcript:EES19444 4.00E-93
Alignment 1207: score=384.0 e_value=7.8e-17 N=9 8&9 minus
1207- 0:transcript:Zm00001d011443_T001 transcript:EES18738 0
1207- 1:transcript:Zm00001d011444_T002 transcript:EES20001 0
1207- 2:transcript:Zm00001d011446_T001 transcript:EES18736 0
1207- 3:transcript:Zm00001d011451_T001 transcript:EES20000 1.00E-46
1207- 4:transcript:Zm00001d011458_T001 transcript:EES19998 0
1207- 5:transcript:Zm00001d011463_T001 transcript:OQU78484 8.00E-141
1207- 6:transcript:Zm00001d011465_T001 transcript:EES18732 0
1207- 7:transcript:Zm00001d011470_T001 transcript:EES18731 9.00E-85
1207- 8:transcript:Zm00001d011474_T001 transcript:KXG22580 1.00E-41
Alignment 1208: score=378.0 e_value=8.2e-15 N=8 8&9 minus
1208- 0:transcript:Zm00001d010651_T001 transcript:EES18433 1.00E-49
1208- 1:transcript:Zm00001d010652_T001 transcript:KXG22257 1.00E-160

1208- 2:transcript:Zm00001d010654_T001 transcript:KXG22255 0
1208- 3:transcript:Zm00001d010655_T001 transcript:OQU78229 0
1208- 4:transcript:Zm00001d010656_T001 transcript:EES18429 0
1208- 5:transcript:Zm00001d010659_T001 transcript:EES19734 0
1208- 6:transcript:Zm00001d010661_T001 transcript:OQU78228 2.00E-27
1208- 7:transcript:Zm00001d010662_T001 transcript:EES19730 1.00E-164
Alignment 1209: score=372.0 e_value=1.6e-15 N=8 8&9 minus
1209- 0:transcript:Zm00001d011051_T002 transcript:OQU77242 0
1209- 1:transcript:Zm00001d011055_T001 transcript:EES18900 6.00E-29
1209- 2:transcript:Zm00001d011056_T001 transcript:EES18897 6.00E-143
1209- 3:transcript:Zm00001d011058_T001 transcript:KXG21095 2.00E-113
1209- 4:transcript:Zm00001d011060_T001 transcript:OQU77236 0
1209- 5:transcript:Zm00001d011061_T005 transcript:EES17580 0
1209- 6:transcript:Zm00001d011062_T001 transcript:EES17579 0
1209- 7:transcript:Zm00001d011064_T003 transcript:KXG21091 3.00E-92
Alignment 1210: score=372.0 e_value=1.3e-14 N=8 8&9 minus
1210- 0:transcript:Zm00001d011130_T002 transcript:KXG22432 0
1210- 1:transcript:Zm00001d011131_T001 transcript:KXG22422 1.00E-154
1210- 2:transcript:Zm00001d011133_T001 transcript:EES18577 5.00E-87
1210- 3:transcript:Zm00001d011134_T001 transcript:EES19874 0
1210- 4:transcript:Zm00001d011135_T001 transcript:EES18574 8.00E-154
1210- 5:transcript:Zm00001d011136_T002 transcript:KXG22420 3.00E-46
1210- 6:transcript:Zm00001d011138_T003 transcript:KXG22418 0
1210- 7:transcript:Zm00001d011139_T005 transcript:EES19871 1.00E-78
Alignment 1211: score=368.0 e_value=5.1e-17 N=9 8&9 minus
1211- 0:transcript:Zm00001d010445_T001 transcript:EES17886 5.00E-139
1211- 1:transcript:Zm00001d010446_T001 transcript:EES19222 0
1211- 2:transcript:Zm00001d010447_T001 transcript:KXG21547 0
1211- 3:transcript:Zm00001d010448_T003 transcript:OQU77612 0
1211- 4:transcript:Zm00001d010449_T001 transcript:EES19219 3.00E-83
1211- 5:transcript:Zm00001d010450_T001 transcript:EES17880 4.00E-97
1211- 6:transcript:Zm00001d010451_T001 transcript:KXG21539 0
1211- 7:transcript:Zm00001d010452_T003 transcript:EES17873 5.00E-93
1211- 8:transcript:Zm00001d010454_T001 transcript:EES19212 0
Alignment 1212: score=349.0 e_value=1.6e-15 N=8 8&9 minus
1212- 0:transcript:Zm00001d009795_T001 transcript:EES19124 0
1212- 1:transcript:Zm00001d009797_T001 transcript:OQU77538 9.00E-48
1212- 2:transcript:Zm00001d009798_T001 transcript:EES19123 6.00E-59
1212- 3:transcript:Zm00001d009799_T001 transcript:EES17800 0
1212- 4:transcript:Zm00001d009800_T001 transcript:EES19121 8.00E-179
1212- 5:transcript:Zm00001d009801_T001 transcript:KXG21439 0
1212- 6:transcript:Zm00001d009803_T001 transcript:EES17794 2.00E-66
1212- 7:transcript:Zm00001d009810_T003 transcript:EES19116 4.00E-134
Alignment 1213: score=334.0 e_value=1.6e-16 N=8 8&9 minus
1213- 0:transcript:Zm00001d011083_T001 transcript:OQU77204 4.00E-102
1213- 1:transcript:Zm00001d011086_T005 transcript:KXG21030 0
1213- 2:transcript:Zm00001d011089_T001 transcript:EES18849 0
1213- 3:transcript:Zm00001d011091_T004 transcript:KXG21027 0
1213- 4:transcript:Zm00001d011092_T001 transcript:EES18845 2.00E-68
1213- 5:transcript:Zm00001d011093_T001 transcript:EES17536 2.00E-54
1213- 6:transcript:Zm00001d011095_T001 transcript:EES17533 2.00E-34
1213- 7:transcript:Zm00001d011096_T004 transcript:EES18841 0
Alignment 1214: score=325.0 e_value=1.9e-15 N=8 8&9 minus
1214- 0:transcript:Zm00001d011889_T001 transcript:OQU77891 0

1214- 1:transcript:Zm00001d011890_T001 transcript:EES18136 0
1214- 2:transcript:Zm00001d011898_T001 transcript:OQU77888 2.00E-128
1214- 3:transcript:Zm00001d011900_T001 transcript:EES18133 0
1214- 4:transcript:Zm00001d011904_T001 transcript:KXG21862 3.00E-106
1214- 5:transcript:Zm00001d011908_T001 transcript:EES18122 0
1214- 6:transcript:Zm00001d011912_T002 transcript:OQU77882 3.00E-146
1214- 7:transcript:Zm00001d011917_T001 transcript:KXG21852 2.00E-93
Alignment 1215: score=305.0 e_value=2.5e-11 N=7 8&9 minus
1215- 0:transcript:Zm00001d010705_T001 transcript:KXG22318 0
1215- 1:transcript:Zm00001d010710_T002 transcript:KXG22316 0
1215- 2:transcript:Zm00001d010711_T003 transcript:EES19783 7.00E-171
1215- 3:transcript:Zm00001d010713_T006 transcript:EES19781 0
1215- 4:transcript:Zm00001d010714_T005 transcript:KXG22310 0
1215- 5:transcript:Zm00001d010715_T001 transcript:KXG22308 1.00E-55
1215- 6:transcript:Zm00001d010716_T002 transcript:KXG22306 0
Alignment 1216: score=296.0 e_value=1.1e-11 N=7 8&9 minus
1216- 0:transcript:Zm00001d011392_T003 transcript:EES20022 0
1216- 1:transcript:Zm00001d011393_T004 transcript:EES20021 0
1216- 2:transcript:Zm00001d011398_T001 transcript:OQU78506 2.00E-21
1216- 3:transcript:Zm00001d011399_T001 transcript:EES20018 5.00E-75
1216- 4:transcript:Zm00001d011403_T001 transcript:KXG22622 1.00E-68
1216- 5:transcript:Zm00001d011410_T001 transcript:OQU78505 2.00E-26
1216- 6:transcript:Zm00001d011413_T001 transcript:OQU78504 1.00E-51
Alignment 1217: score=279.0 e_value=9.1e-08 N=6 8&9 minus
1217- 0:transcript:Zm00001d011747_T001 transcript:EES18554 2.00E-11
1217- 1:transcript:Zm00001d011751_T001 transcript:EES18552 2.00E-12
1217- 2:transcript:Zm00001d011757_T001 transcript:EES19861 0
1217- 3:transcript:Zm00001d011758_T001 transcript:KXG22394 0
1217- 4:transcript:Zm00001d011760_T001 transcript:EES19857 2.00E-165
1217- 5:transcript:Zm00001d011762_T001 transcript:EES19840 0
Alignment 1218: score=277.0 e_value=6.6e-09 N=6 8&9 minus
1218- 0:transcript:Zm00001d009811_T001 transcript:EES19111 0
1218- 1:transcript:Zm00001d009813_T001 transcript:EES19110 0
1218- 2:transcript:Zm00001d009814_T001 transcript:OQU77509 0
1218- 3:transcript:Zm00001d009820_T001 transcript:EES19107 9.00E-90
1218- 4:transcript:Zm00001d009823_T001 transcript:OQU77508 5.00E-93
1218- 5:transcript:Zm00001d009826_T001 transcript:EES17778 7.00E-82
Alignment 1219: score=275.0 e_value=2.3e-09 N=6 8&9 minus
1219- 0:transcript:Zm00001d011066_T004 transcript:EES18885 0
1219- 1:transcript:Zm00001d011067_T001 transcript:KXG21081 7.00E-176
1219- 2:transcript:Zm00001d011068_T001 transcript:EES18884 1.00E-116
1219- 3:transcript:Zm00001d011070_T001 transcript:KXG21079 0
1219- 4:transcript:Zm00001d011072_T001 transcript:KXG21078 1.00E-36
1219- 5:transcript:Zm00001d011073_T001 transcript:EES17562 4.00E-69
Alignment 1220: score=268.0 e_value=5.2e-08 N=6 8&9 minus
1220- 0:transcript:Zm00001d009927_T001 transcript:EES19495 0
1220- 1:transcript:Zm00001d009928_T001 transcript:EES18197 1.00E-61
1220- 2:transcript:Zm00001d009929_T001 transcript:EES19494 0
1220- 3:transcript:Zm00001d009932_T001 transcript:EES18195 1.00E-101
1220- 4:transcript:Zm00001d009936_T001 transcript:OQU77955 0
1220- 5:transcript:Zm00001d009938_T006 transcript:EES18192 3.00E-124
Alignment 1221: score=266.0 e_value=1.2e-08 N=6 8&9 minus
1221- 0:transcript:Zm00001d010733_T002 transcript:EES18509 5.00E-150
1221- 1:transcript:Zm00001d010735_T001 transcript:EES18507 3.00E-83

1221- 2:transcript:Zm00001d010736_T001 transcript:EES19802	1.00E-175
1221- 3:transcript:Zm00001d010737_T001 transcript:KXG22337	2.00E-139
1221- 4:transcript:Zm00001d010740_T001 transcript:OQU78298	7.00E-81
1221- 5:transcript:Zm00001d010743_T001 transcript:EES18498	0
## Alignment 1222: score=265.0 e_value=4.3e-09 N=6 8&9 minus	
1222- 0:transcript:Zm00001d011571_T001 transcript:EES18672	2.00E-94
1222- 1:transcript:Zm00001d011576_T001 transcript:KXG22531	1.00E-13
1222- 2:transcript:Zm00001d011578_T003 transcript:OQU78419	2.00E-170
1222- 3:transcript:Zm00001d011580_T002 transcript:KXG22525	0
1222- 4:transcript:Zm00001d011588_T003 transcript:OQU78411	8.00E-40
1222- 5:transcript:Zm00001d011592_T001 transcript:EES19934	1.00E-38
## Alignment 1223: score=260.0 e_value=2.6e-10 N=6 8&9 minus	
1223- 0:transcript:Zm00001d010304_T001 transcript:EES18214	0
1223- 1:transcript:Zm00001d010313_T001 transcript:KXG21968	0
1223- 2:transcript:Zm00001d010314_T001 transcript:KXG21969	0
1223- 3:transcript:Zm00001d010315_T012 transcript:OQU77974	0
1223- 4:transcript:Zm00001d010319_T001 transcript:EES19509	0
1223- 5:transcript:Zm00001d010321_T001 transcript:KXG21960	0
## Alignment 1224: score=259.0 e_value=4.1e-08 N=6 8&9 minus	
1224- 0:transcript:Zm00001d008803_T002 transcript:KXG21504	2.00E-06
1224- 1:transcript:Zm00001d008805_T001 transcript:KXG21503	0
1224- 2:transcript:Zm00001d008808_T002 transcript:EES19187	1.00E-74
1224- 3:transcript:Zm00001d008812_T001 transcript:EES19185	3.00E-157
1224- 4:transcript:Zm00001d008816_T001 transcript:EES17845	0
1224- 5:transcript:Zm00001d008817_T001 transcript:KXG21498	4.00E-87
## Alignment 1225: score=251.0 e_value=6.1e-11 N=6 8&9 minus	
1225- 0:transcript:Zm00001d010023_T004 transcript:EES18062	0
1225- 1:transcript:Zm00001d010029_T006 transcript:EES19365	0
1225- 2:transcript:Zm00001d010030_T001 transcript:EES19362	7.00E-36
1225- 3:transcript:Zm00001d010031_T002 transcript:OQU77810	9.00E-97
1225- 4:transcript:Zm00001d010032_T001 transcript:EES18052	0
1225- 5:transcript:Zm00001d010033_T002 transcript:EES19360	0
